# *De novo* transcriptome analysis of *Bagarius yarrelli *(Siluriformes: Sisoridae) and the search for potential SSR markers using RNA-Seq

**DOI:** 10.1371/journal.pone.0190343

**Published:** 2018-02-09

**Authors:** Min Du, Na Li, Baozhen Niu, Yanhong Liu, Dongjing You, Defu Jiang, Congquan Ruan, Zhengquan Qin, Taowen Song, Wentao Wang

**Affiliations:** 1 Key Lab for Quality, Efficient Cultivation and Security Control of Crops in Colleges and University of Yunnan Province, Honghe University, Mengzi, Yunnan Province, P.R. China; 2 College of Life Science and Technology, Honghe University, Mengzi, Yunnan Province, P.R. China; Youngstown State University, UNITED STATES

## Abstract

**Background:**

The yellow sisorid catfish (*Bagarius yarrelli*) is a carnivorous freshwater fish that inhabits the Honghe River, Lanchangjiang River and Nujiang River of southern China and other Southeast Asian countries. However, the publicly available genomic data for *B*. *yarrelli* are limited.

**Methodology and principal findings:**

Illumina Solexa paired-end technology produced 1,706,456 raw reads from muscle, liver and caudal fin tissues of *B*. *yarrelli*. Nearly 5 Gb of data were acquired, and *de novo* assembly generated 14,607 unigenes, with an N50 of 2006 bp. A total of 9093 unigenes showed significant similarities to known proteins in public databases: 4477 and 6391 of *B*. *yarrelli* unigenes were mapped to the Gene Ontology (GO) and Clusters of Orthologous Groups (COG) databases, respectively. Moreover, 9635 unigenes were assigned to 242 Kyoto Encyclopedia of Genes and Genomes (KEGG) pathways. In addition, 8568 microsatellites (simple sequence repeats, SSRs) were detected, and 31 pairs of polymorphic primers were characterized using wild populations of *B*. *yarrelli* from the Nujiang River, Yunnan Province, China.

**Conclusion/Significance:**

These sequences enrich the genomic resources for *B*. *yarrelli* and will benefit future investigations into the evolutionary and biological processes of this and related *Bagarius* species. The SSR markers developed in this study will facilitate construction of genetic maps, investigations of genetic structures and germplasm polymorphism assessments in *B*. *yarrelli*.

## Introduction

The yellow sisorid catfish (*Bagarius yarrelli*, Osteichthyes, Siluriformes, Sisoridae) [1] is distributed mainly in southwestern China, including the Nujiang, Lanchangjiang, and Yuanjiang Rivers, Southeast Asia, including Vietnam, Laos, Thailand and Burma, South Asia, and India [2]. Its common names are “yellow fish” in the Yuanjiang River region, “tiger fish” in the Nujiang River region, and “surface melon fish” in the Lanchangjiang River region. Though wild resources of *B*. *yarrelli* are currently under threat of population decline due to environmental changes and overfishing, the fish is consumed by locals, which has resulted in a high price. Previous investigations have largely addressed its biological characteristics [3], noting that *B*. *yarrelli* is a benthic carnivorous fish. Its physiology [4] results in a protein content that is higher than that of conventional fish, including *Cyprinus carpio*, *Carassius auratus*, *Ctenopharyngodon idellus*, and *Hypophthalmichthys molitrix*. Artificial propagation of *B*.*yarrelli* succeeded for the first time on 24 March, 2012 and the larvae were hatched 20 hours after fertilization at a water temperature of 27°C[5]. In addition, mitochondrial and nuclear DNA sequencing have been utilized to study the species’ genetic phylogeny, including 16S rRNA [6], cytochrome oxidase [7], ND6 [8] and rag1 and rag2 [9]. Random amplified polymorphic DNA (RAPD) and microsatellite (simple sequence repeat, SSR) marker technologies have also been applied to study the population genetic diversity of *B*. *yarrelli* [10–11]. However, the available gene sequences and molecular markers are extremely limited, and as of August 15, 2017, only 37 nucleotide sequences were available in the NCBI GenBank database.

In investigations of model and non-model fish species, next-generation sequencing techniques, such as 454 pyrosequencing technology and Illumina paired-end sequencing technology [12], have provided abundant data at a low cost. Recently, High-throughput sequencing technology has been broadly implemented for many species, for example, sesame [13], blunt snout bream [14], common carp [15], butterfly [16], lake sturgeon [17], and mud crab [18].

To acquire comprehensive gene sequences of *B*. *yarrelli*, next-generation sequencing technology was used to perform transcriptome sequencing with a pool of tissues (this fish originates from the Honghe River,Yunnan Province,China) and the first sequencing library was constructed. We then performed *de novo* assembly and gene annotation and utilized a set of SSR markers to assess the genetic diversity of a wild *B*. *yarrelli* population (these fish originate from the Nujiang River, Yunnan Province, China). These findings provide a very useful genomic resource for subsequent investigation of *B*. *yarrelli* and related species at the levels of biochemistry, molecular biology and genetics.

## Results

### Sequencing and *de novo* assembly

Approximately 1,706,456 raw reads were obtained from muscle, fin and liver tissues of *B*. *yarrelli* specimens obtained from the Honghe River in Hekou County, Yunnan Province, China. The entire length of the raw reads was 5.119 Gb. Raw reads were processed to eliminate low-quality reads and then *de novo* assembled into 151,911 contigs using Trinity software, with an average length of 644 bp. The resulting final assembly contained 14,607 unigenes, with an average length of 1216 bp and the N50 length was 2006bp (Table 1). N50 is an indicator of the assembly effect, which is calculated by sorting the assembly fragments from large to small and starting to add their length values; when the cumulative sum is 50 percent greater than the total length, the final cumulative length of the segment is the N50 value. The raw reads in this study were submitted to the NCBI Short Read Archive (SRA) database under accession number SRR5943894.

**Table 1:** Transcriptome statistics of *Bagarius yarrelli*.

Contig length	Number	Percentage (%)	Unigene length	Number	Percentage (%)
200–300	66,148	43.54	200–300	1846	12.63
300–400	23,827	15.68	300–400	2034	13.93
400–500	12,427	8.18	400–500	1422	9.74
500–600	7826	5.15	500–600	1053	7.21
600–700	5542	3.65	600–700	743	5.09
700–800	4262	2.81	700–800	644	4.41
800–900	3501	2.30	800–900	549	3.76
900–1000	2859	1.88	900–1000	478	3.27
1000–1100	2589	1.71	1000–1100	444	3.04
1100–1200	2239	1.47	1100–1200	386	2.64
1200–1300	2015	1.33	1200–1300	395	2.70
1300–1400	1751	1.15	1300–1400	337	2.31
1400–1500	1665	1.40	1400–1500	336	2.30
1500–2000	6156	4.05	1500–2000	1301	8.91
2000–3000	5692	3.75	2000–3000	1443	9.88
3000–4000	1963	1.29	3000–4000	585	4.01
4000–5000	749	0.49	4000–5000	276	1.89
5000–10,000	664	0.44	5000–10,000	322	2.20
>10,000	36	0.02	>10,000	13	0.09
Total contigs	151,911	100	Total contigs	14,607	100
Total length(bp)	97,832,254		Total length(bp)	17,768,104	
Range in length (bp)	200–18,629		Range in length (bp)	200–15,207	
(G+C)/(A+T+G+C)	0.457		(G+C)/(A+T+G+C)	0.471	
N50 length (bp)	1097		N50 length (bp)	2006	
Average length (bp)	644		Average length (bp)	1216	

### Gene identification and open reading frame (ORF) search

The numbers of contigs and unigenes in this study were 151,911 and 14,607, respectively. Approximately 9093 unigenes yielded BLASTX hits to known proteins in the NR database; 4477 matched to the Gene Ontology (GO) database, 6391 matched to the Clusters of Orthologous Groups (COG) database and 9635 matched to the Kyoto Encyclopedia of Genes and Genomes (KEGG) database. The final unigenes and annotation information are provided in S1 File and S1 Table. The predicted protein number using Transdecoder (http://transecoder.fourceforge.net/) in Trinity software, which was set at a length of greater than 80 aa, was 9310; the data are listed in S2 File.

### GO analysis

For functional analysis of *B*. *yarrelli* unigenes, 14607 unigenes were classified into three main categories namely, cellular components (3204, 21.93%), biological processes (5982, 40.95%) and molecular functions (5249, 35.93%) using Blast2GO software [19]. The GO classifications are shown in S1 Fig. Among nine cellular components, cell (GO: 0005623), cell part (GO: 0044464), and organelle (GO: 0043226) were the dominant terms. Among 19 biological process terms, cellular process (GO: 0009987) was the top category, followed by metabolic process (GO: 0008152) and biological regulation (GO: 0065007). Among 10 molecular function terms, binding (GO: 0005488) and catalytic activity (GO: 0003824) were highly represented.

### COG analysis

A total of 6391 unigenes were analyzed with the COG database (NCBI, http://www.ncbi.nlm.nih.gov/.structure/bwrpsb/bwrpsb.cgi, COG-4873PSSMs, E-Value = 0.01, maximum number of hits = 500) and were divided into 24 possible functional categories. Among them, the highest group was general function prediction (1433, 22.44%), the second group was signal transduction mechanisms (660, 10.33%), the third group was transcription (634, 9.92%), the fourth group was replication, recombination and repair (582, 9.11%), and finally, cell cycle control, cell division, and chromosome partitioning (574, 8.98%). Only a few unigenes were classified into nuclear structure and defense mechanisms (S2 Fig).

### KEGG pathway analysis

A total of 9635 unigenes were classified into 242 KEGG pathways (S2 Table). Metabolic pathways (KO:01100) was the most represented category, followed by pathways in cancer (KO:05200), focal adhesion (KO:04510), regulation of the actin cytoskeleton (KO:04810), endocytosis (KO:04144), the MAPK signaling pathway (KO:04010), tight junctions (KO:04530), the chemokine signaling pathway (KO:04062), biosynthesis of secondary metabolites (KO01110), and RNA transport (KO:03013), which included 474, 230, 188, 184, 183, 159, 142, 141, 136, and 120 unigenes, respectively (S3 File).

### Comparative analysis

The 14,607 unigenes were searched against NR BLAST data to assess the acquired *B*. *yarrelli* transcriptome. Although 204 unigenes were unmatched, 7524 unigenes of *B*. *yarrelli* showed significant hits with 10 species: *Danio rerio*, *Oreochromis niloticus*, *Oryzias latipes*, *Salmo salar*, *Takifugu rubripes*, *Ictalurus punctatus*, *Tetraodon nigroviridis*, *Homo sapiens*, *Ictalurus furcatus*, and *Mus musculus*. The matched numbers and percentages are denoted in Table 2.

**Table 2:** Distribution of *Bagarius yarrelli* unigenes with matched species in the NR database.

Matched species	Number	Percentage (%)
*Danio rerio*	4655	51.19
*Oreochromis niloticus*	921	10.13
*Oryzias latipes*	373	4.10
*Salmo salar*	370	4.07
*Takifugu rubripes*	347	3.82
*Ictalurus punctatus*	328	3.61
*Tetraodon nigroviridis*	192	2.11
*Homo sapiens*	144	1.58
*Ictalurus furcatus*	120	1.32
*Mus musculus*	74	0.81
Others	1365	15.01
Unmatched	204	2.24

### Identification of SSR markers

In total, 14,607 unigene sequences, including 5,119,369,800 bp, were obtained. Overall, 8568 SSRs, 58.66% of the unigene sequences, were examined (Table 3). In accordance with the repeat motif classification criteria reported by Weber [20], we obtained 7352 perfect repeats, 236 imperfect repeats and 980 compound repeats (Table 4).

**Table 3:** General statistics of the SSR searches for *B*. *yarrelli*.

Source	Number
Total number of sequences examined	14,607
Total size of examined sequences (bp)	17,782,710
Total number of identified SSRs	14,812
Number of SSR-containing sequences	8568
Number of sequences containing more than 1 SSR	3824
Number of SSRs present in compound formation	2241

**Table 4:** Repeat motif type distribution in genic SSRs of *B*. *yarrelli*.

Repeat motif type		Number	Frequency (%)
Perfect	Mono-	1531	17.87
	Di-	4699	54.84
	Tri-	849	9.91
	Tetra-	254	2.96
	Penta-	18	0.21
	Hexa-	1	0.01
	Total	7352	85.81
Imperfect		236	2.75
Compound		980	11.44

The obtained microsatellites include mono-, di-, tri-, tetra-, penta- and hexa-nucleotide repeats, with (AC/GT)n di-nucleotide repeat motifs as the most abundant, with 1901 perfect SSRs. The other main motif types were (A/T)n mono-nucleotide, (CTC/GAG)n tri-nucleotide, (AGAC/GTCT)n tetra-nucleotide, (AGAC/GTCT)n tetra-nucleotide, (ATTAG/CGCTG/CTTTT/ TTTTC)n penta-nucleotide and (TGTCTG)n hexa-nucleotide repeat sequences (Table 5).

**Table 5:** Number and frequency of repeat types in perfect genic SSRs of *B*. *yarrelli*.

Repeat motif type	Number of repeat motifs	Frequency (%)	Most abundant
Mono-	2	2.27	(A/T)n
Di-	8	9.09	(AC/GT)n
Tri-	27	30.68	(CTC/GAG)n
Tetra-	46	52.27	(AGAC/GTCG)n
Penta-	14	15.91	(ATTAG/CGCTG/CTTTT/TTTTC)n
Hexa-	1	1.14	(TGTCTG)n
Total	88	100	

Among the perfect SSR data, an in-depth study revealed that the copy numbers of different repeat sequences were non-uniformly distributed, ranging from 5 to 42 (Table 6); the (ATC/GAT)n tri-nucleotide repeat showed the greatest copy number. The frequencies of the top four copy numbers for *B*. *yarrelli* SSRs were 10 (21.15%), 9 (18.78%), 6 (13.38%), and 7 (10.12%). The longest SSR sequences among the six types were 24 bp for mono-nucleotides (A/T), 46 bp for di-nucleotides (AC/GT), 126 bp for tri-nucleotides (ATC/GAT), 80 bp for tetra-nucleotides (GATA/TATC), 125 bp for penta-nucleotides (ACGTG/CACGT), and 36 bp for hexa-nucleotides (CAGACA/TGTCTG).

**Table 6:** Distribution of different repeat sequences in perfect SSRs of *B*. *yarrelli*.

Number of repeat units	Mono-	Di-	Tri-	Tetra-	Penta-	Hexa-	Total	Frequency (%)
5	0	0	307	157	10	0	474	6.45
6	0	717	200	60	6	1	984	13.38
7	0	488	243	13	0	0	744	10.12
8	0	461	46	4	0	0	511	6.95
9	0	1351	24	6	0	0	1381	18.78
10	488	1055	11	1	0	0	1555	21.15
11	279	297	11	2	0	0	589	8.01
12	218	83	2	0	0	0	303	4.12
13	127	40	2	1	0	0	176	2.39
14	76	46	2	1	1	0	138	1.88
15	87	58	0	4	0	0	131	1.78
16	46	33	0	3	0	0	82	1.12
17	45	19	0	0	0	0	64	0.87
18	44	19	0	0	0	0	63	0.86
19	41	8	0	0	0	0	49	0.67
20	44	5	1	1	0	0	51	0.69
21	24	8	0	0	0	0	32	0.44
22	5	5	0	0	0	0	10	0.14
23	5	5	0	0	0	0	10	0.14
24	2	0	0	0	0	0	2	0.03
25	0	0	0	0	1	0	1	0.01
27	0	1	0	0	0	0	1	0.01
42	0	0	1	0	0	0	1	0.01
Total	1531	4699	850	253	18	1	7352	100
Frequency(%)	20.82	63.91	11.56	3.24	0.44	0.01	100	

### PCR amplification and polymorphisms of genic SSRs

Primer3 software [21] was used to design 3951 SSR primer pairs for 8568 SSR sequences and 90 primer pairs were randomly selected and synthesized by Sangon Biotech Co., Ltd. (Shanghai) to examine the amplification proportions. Of these, 31primer pairs are polymorphic in a wild population of 40 fish from Nujiang River, Yunnan Province, China. Altogether, 73 alleles were found, with an average of 2.4 for each locus. The parameters of these 31 polymorphic SSR sequences are shown in S4 File. The SSR primer names and locus, number of alleles, effective number alleles, expected heterozygosity (He), observed heterozygosity (Ho), PIC values, Index of Shannon (I) values, and P values are provided in S5 File. The PAGE histogram of the Microsatellite Baya297 locus obtained through EB staining of 40 *B*.*yarrelli* fish is denoted in S6 File.

## Materials and methods

### Ethics statement

All *B*. *yarrelli* fish used in this study were assessed according to relevant guidelines in China. This experiment was approved by the Key Lab for Quality, Efficient Cultivation and Security Control of Crops in Colleges and University of Yunnan Province, Honghe University. China does not require a specific license to catch wild *B*. *yarrelli* from rivers because it is not an endangered species.

### Samples, RNA isolation, and library preparation

The samples examined in this study were caught from Honghe River in Hekou County, Yunnan Province, China (103°56’21E, 22°31’51N) by professional fishers using a fishnet. The fish were killed using MS-222 (finquel) followed by a stick to their heads. The samples were dissected on ice using scissors. Muscle, liver, and caudal fin tissues were excised and rapidly frozen at -80°C. A wild population of 40 *B*. *yarrelli* individuals was obtained from the area downstream of the Nujiang River in Yunnan Province, China. These 40 individuals were used to test microsatellite polymorphisms. Total RNA was extracted using TRIzol (Invitrogen, USA) following the manufacturer’s protocol, and total RNA from tissues was pooled and purified to obtain mRNA using oligo (dT) magnetic beads. The quality results of *B*. *yarrelli* RNA are shown in S7 File. cDNA libraries were analyzed after preparation for Illumina sequencing.

### Sequence assembly

An Illumina TruSeq RNA Sample Prep Kit was applied to construct cDNA libraries in accordance with the manufacturer’s protocol. The libraries were then sequenced using the HiSeq 2500 sequencing platform (TruSeq SBS Kit v3-HS, Illumina). Low-quality sequences, trimming primers and adaptors were filtered. In other words, the reads with sequences containning the adaptor sequence, the paired reads with the amount of N contained in single-end sequencing reads exceeding 10% of the read length and the paired reads with a low-quality(less than 5) base number of the single-end sequencing reads greater than 50% of the read length were excluded. High-quality sequences (>Q20) were assembled using Trinity software (r20131110) [22]. The ending contigs were embedded in scaffolds based on paired-end information using TGI Clustering (TGICL) tools [23].

### Identification of microsatellite markers

To detect SSR markers, the MISA tool (http://pgrc.ipk-gatersleben.de/misa/) was used with the default settings: the smallest numbers of repeats were five for tri-, tetra-, penta- and hexa-nucleotides, six for di-nucleotides, and 10 for single nucleotides. Altogether, 90 pairs of specific primers were designed with Primer3 [21]. These SSR primer pairs were synthesized by Shanghai Sangon Biological Engineering Technology Service Co., Ltd. (Shanghai, China). Forty individuals from the wild population were used to screen these microsatellite loci through PCR. The PCR reaction system and the cycling parameters for PCR amplification were performed as described by M Du et al [12]. After staining with ethidium bromide, a 8% non-denaturing polyacrylamide (w/v) gel was used to identify the PCR products. POPgene 1.32 software was applied to perform the genetic analysis. All SSR markers examined in unigenes are listed in S4 File. Polymorphism microsatellite loci and genetic parameters are provided in S5 File.

### DNA extraction and electrophoresis

The normal phenol-chloroform method described previously [24] was used to extract genomic DNA from the caudal fin tissues of the 40 wild *B*. *yarrelli* individuals. The DNA quality and concentration were assessed by 1% agarose gel electrophoresis and then use of a Thermo Scientific NanoDrop 2000 spectrophotometer, respectively.

## Discussion

The next-generation sequencing technique is used to obtain large amounts of transcriptome data from a known or an unknown reference sequence organism [25] because it is inexpensive and rapid [26]. In this study, we use the high-throughput Illumina Solexa paired-end technique for *de novo* assembly of a transcriptome using clean reads for *B*.*yarrelli*,a species with limited sequence data in public databases. Here, the N50 length and the average length of unigenes of *B*.*yarrelli* were 2006bp and 1216bp, respectively, indicating that our assembly was effective and accurate. Similar results have been found for speices such as mud crab (*Scylla paramamosain*), in which the N50 length and the average length of contigs are 639bp and 606bp, respectively [18], sesame (*Sesamum indicum* L.), in which the N50 length and the average length of unigenes are 1901bp and 1127bp, respectively [13], whitefly (*Bemisia tabaci*), in which the average length of scaffolds is 266bp [27], and sweet potato (*Lpomoea batatas*), in which the N50 length and the average length of unigenes are 765bp and 481bp, respectively [28]. Illumina sequencing produced 1,706,456 raw reads for *B*.*yarrelli*.The 14607 unigenes yielded in the present study will facilitates further research on the physiology, biochemistry, and molecular genetics of *B*.*yarrelli* or related species.

SSR markers have been extensively used to perform genetic diversity research, gene mapping and population genetic analysis in many species [29–30]. NGS technology is a powerful tool for identifying microsatellites for plant or animal organisms [31–32]. Here, a total of 8568 microsatellites were identified based on the *B*.*yarrelli* dataset. Di-nucleotide repeats were the most frequent type, and mono-nucleotide repeats were the second most frequent type, followed by the tri-nucleotide type, which is somewhat similar to previous reports. For example, di-nucleotide repeats as the most common type in EST data was also found in mud crab (*S*. *paramamosain*) (N = 8161, 42.9%) [18] and blunt snout bream (*Megalobrama amblycephala*)(N = 3107,62.7%) [32]. The top di-, mono-, and tri- nucleotide motifs in this study were AC/GT, A/T and CTC/GAG in *B*.*yarrelli*, respectively.

In this study, 90 primer pairs were randomly designed to assess the successful amplification ratio of these SSRs by Primer 3. Thirty-one (33.3%) loci showed polymorphisms across panels of 40 individual *B*.*yarrelli* fish from the Nujiang River. The ratio (33.3%) of polymorphism microsatellites investigated in this study is lower than that of a previous study [11] (47%), in which the core motifs of the microsatellite loci only contained five bases, possibly because the core motifs of the microsatellite loci examined in this study are shorter than five bases. From a genetic perspective, the average observed heterozygosity (H_O_), average expected heterozygosity (H_E_), and the average polymorphic information content (PIC) can reflect the genetic diversity and inheritance patterns of a population from multiple angles [33]. We generally believe that PIC >0.5 denotes a high polymorphism rate, 0.25<PIC<0.5 denotes a moderate polymorphism rate, and PIC<0.25 denotes a low polymorphism rate [34]. In this study, the average observed heterozygosity (H_O_), average expected heterozygosity (H_E_), and average PIC value were 0.19, 0.3588 and 0.2954, respectively, indicating moderate genetic diversity among Nujiang River populations of *B*.*yarrelli*. Here, 8568 SSRs were defined in our dataset, and PCR primers could be designed for further research on the germplasm polymorphism [31], comparative genomics [35], and functional genomics of this fish.

## Conclusions

This study constitutes the first analysis of the transcriptome of *B*. *yarrelli* using HiSeq 2500 technology, generating 14,607 unigenes. A total of 8568 sequences contained SSR motifs. Thirty-one of 90 primer pairs were assessed in 40 wild *B*. *yarrelli* via PCR amplification, and the loci exhibited polymorphisms in the wild populations from Nujiang River of China. The large quantity of transcriptome and SSR data for *B*. *yarrelli* obtained in this study will facilitate future detailed genetic studies.

## Supporting information

S1 File Sequences of unigenes. (DOC)

S2 File Protein information. (DOC)

S3 File The top 20 KEGG pathways with the highest sequence numbers. (DOC)

S4 File Characteristics of *B*.*yarrelli* genic-SSR primers. (DOC)

S5 File Characteristics of 31 pairs of *B*. *yarrelli* genic-SSR primers screened from 40 individuals. (DOC)

S6 File PAGE histogram of the microsatellite Baya297 locus identified by EB staining of 40 *B*.*yarrelli* individuals. (DOC)

S7 File Quality of the *B*. *yarrelli* RNA results. (DOC)

S1 Fig Histogram of GO classifications for *Bagarius yarrelli*. (TIF)

S2 Fig Histogram of a cluster of orthologous groups of protein functional classes for *Bagarius yarrelli*. (TIF)

S1 Table Unigene annotation. (XLS)

S2 Table KEGG annotation. (XLS)
