# Supplementary material for: De novo transcriptome analysis of Bagarius yarrelli (Siluriformes: Sisoridae) and the search for potential SSR markers using RNA-Seq
Source: PLoS One. 2018 Feb 9;13(2):e0190343. doi: 10.1371/journal.pone.0190343 (PMC5806860; doi:10.1371/journal.pone.0190343)
Supplement: S2 File — (DOC) [file pone.0190343.s002.doc]

File S3 Protein information

>m.1 g.1 ORF g.1 m.1 type:5prime_partial len:2484 (+) Unigene000001:3-7454(+)

KPPSQKDASNKTQSQKKTSNNTQSQKDESAETQPILDQNANVDQSAKASAGPGTQKNQEV

YKSRMVFGPQSRTEVESLSSSVDPQPLETCQPVQSAREKGDALSSQKSPENRRKQKREER

DTKKDSTPERIPTSTQQVSASERLTIYFHAVLSKDFKFDPDEDRVFIRAGPQICDWETNL

VELSVTRDLNEHGLLVEGSLVSTKDKAEAVSIPYKYVVYKKKKNKYEYEYIYKLDTNKTT

NRCLFVKRLLLNAEGEWHQYDDIICQEPSKNVFKRLKDNFWPEQRKSVIEGREIAGRVML

ETIFDFLRSWTDINLRNFIFQLKQFHHIYGNPFVFEEKEEKWLNLNYTEKDVSKLLKNFI

MDHITPELHKDGLSKKSEYINVPLKAAIITLYLWKQYELRLEFGELNRLCSALCLPNLPK

NEFIPFWTNIRKSFFCLKNLPDTLVDLINTVKGQGMTRWIVAIPLLHLLKGISNPFEPIS

LTANTKNEQAWVGLQGLKSCSTKPVTSEGRRSMLNLMKTNHYLVEVDTLVVRSWMFLLSV

DELVEYCTNIHVELFDVLQLFYLKAPADISYSVPKNVSDMLTFIQSQLLEQKYRCFSDEY

RKSCLAASVRLLEKICKGARSSYSNTDIPVACLNLVASVSYFSQSFPKMEFQEEDTSQKP

IEESSKINEILSEALKIVRTWVEQTFKERLVYAYFLSATLTSKIEMWNNIISVSFESEEF

TKEWRDTLSRDFEGKFQQESSIDQIEVYCSRIEELNDTHPYVARSIEKCALQAVTSMCQM

KTEAKLFEKCKINWKFGKLISAIVQKSWPKTESGSYQEREELVLKHLLNWTAAKNVFQLY

GADEKLIDQLSEEAKERLTMATSLLTNISKQFIRGSITIKVLNHILEKKDTFIELVKIDC

FCENEKCKDTNTMKRLLQLRQDEVNAVYNDSELVKGLIAMCRNIQEYVTVDLDNLEEKQQ

VDIEEMELDKFMEVHLFDQISSPTAGYVTYFSLEDTVWHMAEIFHTFKESYVFKICWKSQ

AKDFAESASSECESDDEEDLRATIDLIYDEIFQPCYSKYKQIYESLKDGSMAFEEVDITF

KAYIGKYEELAEEATVMSKLDPTDDKRWIQRRIQQIEQYHELHLAVESAQAVMMVKQTLG

LQGDFQVLEKLLIVTELDFKKEPLNSIDNELMQAKTVLVDITEPRRLCLQELGLRKNFVM

WVKEALEDINELKVFVDLASISAGENDLDVDRVACFHDAVLGYSPIFYELKTDASFRVFK

EVLKKLWKALDNDPNLPKKLRDTARHLEWLKTVKDSHGSVELSSLSLASAINKKGIYFIS

AENQKKLSLDKVLKLRITEEHAEDNEVRCYSLDDLKELQNKLMLMSGKGDQGQNEVDRFA

EVFASVQRLGTAFMDLFAAGNPLFRLWEAIIHCNSQEACIVMDFNLDGVTSVVMLRGDVA

EQLLDVCKKMERCLSFWKDFMDKQRSQNYYLNYFTAEQIVYLCSQLNQTNLSHLEDQALI

MLSFIKLNCTTYNLRQSWHVLQYEMLTNPTKQNEDIDFQTFADVPNQIENEIDINSVPFS

DSGIENDTRRFDMIWNMYMRNMKTFLPKSLDVRSLGRLLEILSDSPSKTDVDDDDDDDNA

EKSTLENLHKNVQRVLPRGLVIGKPNLIVCPFTEIQTACISIYMSSEDEPLPTYDEVLLC

DSSTSYEQAELFLRRCLSAGYKGQKIYTMLYVDQLTYEVSYRVEQFFQRHKAQSRNDYRL

VLICSTDREHAYLPSVFSQFRLHMVPQEPLGRIQEYLTKHYTVPKGYFSAAAVFKTKQYV

GVVSSRRAGVGKSLYIRRLYEKVKLITTKSSQLKCIRLTKSKVDENAVLQSMLHGPQREL

TIFHFDITSSVQKGLHEFLFRLLVLGYLMDSEGRMWKCNSKHLYIIELLQSSANISRNLP

RSSESVQSAFQDVFPSVYCRTPKEVLDLEMRMSEDPTIIVNDPLMDDKEFRNEAYQRPYQ

YLKRFHHGVNLDGFMYKGIEGTHVECLQLLFLYCGVVDPSWAELRNFTWFLNLQLRDCET

SVFCDASFTGDTLLGFKNFVVDFMILMAKDFATPSLSISDQSPGRQQMDISGVSEEDLAP

FRVRKRWESEPHPYIFFNDDHVSMTFIGFHLRPNDQNLVDAIDPSTNRVIKKNIMTSELY

EGLKLQRVPFNINFDSLPRGEKIERICNVLGIQWPLDPDETYELTTDNILKILAIHMRFR

CGIPVIIMGETGCGKTRLIKFLCELRRSGVATENMKLVKVHGGTSDDMIYTKVREAESIA

CSNKQDYGFDSVLFFDEANTTEAISSIKEVLCDKTVKGEVMVPNTGLQIIAACNPYRKHS

DIMIKRLESAGLGYRVRAEQTDEKLGSIPLRQLVYRVQALPPSMIPLVWDFGQLNDQTEK

MYIQQIVQRVAEDHFFSVEYIKIFTDVLSASQKYMRTRKDECSFVSLRDVERCMQVFTWF

YQNNEMLLSELARYEINQKAQKK*

>m.4 g.4 ORF g.4 m.4 type:complete len:158 (+) Unigene000001:7507-7980(+)

MAIGVCYHACLEDKEKYRNKICGYFSKTYSPVNLMQEISVMQDLLLSGVPLGETIARNNA

LKENVFHDGNLHRVTNSSFPGWKAWKLQIIVQNPGSRCYARSGSSFRLVQKTKTDSSGVI

PVQSSFHTRRYHQYIQAMCTFSRGQKSKRVHLGGGTG*

>m.2 g.2 ORF g.2 m.2 type:complete len:1832 (+) Unigene000001:9216-14711(+)

MLWFAWIVQPFLKWRVSTFQECTLRTKSTAVWLTLSLVTFSRWSGTIPVLLRYSSIGEIN

KFQKEHDSRVFVYFITKLPRVEGGTSYVGFHGGPWKSVHIDDLRKSKDIISDIKALQCLT

ISQLFKETEDQSEVMETEDMYREEEETEKMEQDDNTSWKNVLDTTALVRSCVQSAVGMLR

DQGDVGCRSTKRVEILLTLLSDSEELQATFLKTMKHRLYSLLITHDEHTHSAKNWVFKEA

SNIDSLQEGGTFRNTLWKRVQAAIIPFLAQLVSVIDREHNLDLLLDENSGESVKKLWLDI

FGNVKLLNVPYTRVDSTSMTKTILVQNCIVVYKNTGCSMPFSWIIKDHLEELWLHALHRE

GHSLRQFEEFFWKTPLGRYIAKAEQEMQQEFFYRYLQDFISLTTTITTEVELKLLVKALS

CCVNELRSRQEAVQNEVTSLPWIHAAYHEFKNRLQNFSRMIIIEPQIAQVLLDNTQSRDG

KEMVLDVYAALACVEYLEPQGLDACINCLTWLRQVKKLQIPIELVCSEASIRHYGAMSKA

IIPRVQHGWRRIHAISLFVEHILLDIENIEKKLKPLVLEHTRKLAQVLDKNSDLKMKQPF

EAVIAILKTCKDGANEQIFRFGFQPCPVCMGDPHDPLSLPCDHVYCLACIKQWLIPGQMY

CPLCIQGVDDDFDLQPSDTIRTLIKQNSHFRKQCNAFFIDFVSTICFRDNSPPSKTIILH

LLSFLMVEAGSVPTIRGCERRFLTKALSPFDDSVDKNPVVRSVVLKLLLKYSFADVKGYL

QQHLTEVEQSNILEKADKTELYCLYINCLEDSMFERIQWHSVAEQQACLLEEKHFLVEYL

RSGAVATETVTIEHLQQVARTRLCLDMAADLLISRSSTADAEKESSAGVTAFLDSVQNLC

KYSKNDWYRVYLIRKLCSLSGVEYIQKLLQEDQLSWLFPQEVLQMRKTSSQIDQYLVCGV

EYKAIRDAVAKAMLEGKMETIEEACKICQCSPEKKNVYILLALFREVTTLYKANNPNIHP

TPEQLAALDDFTKNSKVFVVPESRDFAQALVHNQLPVLHLHPSLLANEFVILELTVHLAA

VLLCTNQPLLQPLIQLAFFPINMQKAFLPTMPDDMLAVAQQVMGQLQWYFCPNGHPCTVG

ECGRPMEKSHCVDCNAEIGGVAHNPVQGFQPIAVQGDRTQTGHLLGDPHRRDQPDMQDTK

NMSPGPFTLLRLLTHMAMLLGSSRQPQEVAAIIKPPVPNPATFLLAHLLKDIEQLTRAIG

KGADDTICTIHLTICILLEPHQPRPLPYDNLLSTKDARNNWEITVTTDIITCQLKVLESH

LANVNTFIRNDERVSSNPIMKVTFGEPRLFLRTLPQGSLIHSSSVWCCRQRVSLLGLSHI

LEQYGGRDRLPILWRFLQKEAELRLVKFLPDILALQRDLVKKFQNVIELQCGTIAQFLQN

LKAESLMTSYKKRINIFLSTWNQLRASLETNGEIKLPTQYCQDDLDLSADLQVLLPRRQG

LGLCSTALVSYLIALQNDLIYAVDKHTGEETSYTVSPADLTELHVIRYEVERDLLPLVLS

NCQYSMECGRETLSEYDLPKIQQQLLTRFLQGKPFIGLTGIPTLVNRHDRNYETIFRDVK

GKVQQESMTSLNQSTLCAELQCYSDVCEALSTVEIALGFLAMTGGEPNMQLSNYLKEELQ

MADQIPLHVLKALSRCCLKHCLALWQLLTSLKSETMLRLKRDPFERFSKDYKKTLGEQEK

RLLTGFFSKTSADSFLLEMHEFLLLVLKNANATDTYKPNWSVKDTILSYMEQKDMDVPPD

VVELFPDEILLAHYIEAWKFSILLKQERIQR*

>m.3 g.3 ORF g.3 m.3 type:complete len:564 (+) Unigene000001:7697-9388(+)

MFFMMVICIELRIPLFLVGKPGSSKSLSKTLVADAMQGQAAHSDLFRKLKQIHLVSFQCS

PHSTPEGIINTFKQCARFQEGKNLSEYISVVVLDEIGLAEDSPKMPLKTLHPLLEEGCIE

DQPLPHKRVGFIGISNWALDPAKMNRGIFVSRGDPDEKELIESAKGICSSDAMILEKVGD

FFQPFAKAYLKICSEGGKGFFGLRDYYSLIKMLFSIAKASDQKPSADQVVEAVLRNFSGK

DTVNAVTVFTNRLRLKPNLESIDTIELVRQNITAVAQDEECRYLLILTKNYAALQILQQT

FFSEQYQPEIIFGSSFPKDQQYTQICRNINRVKICMETGQVIVLLNLQNLYESLYDALNQ

YYVCLGGQKYVDLGLGTHRVKCRVHKDFRLIVIEEKDIVYKQFPIPLINRLEKHYLDINT

VLKNEQKQLVKILEEWVDHFIKCKHAPTPQAAKHCPTDVFIGYHSDTCASVIMQVIERQK

GRMEFSDPERQLLDEAKLILLNCATPDAVVRLDSSALSKVESEHLSRVYFEDQKHSCMAD

FVFGHIQQVEWNHSCFTEVLLHW*

>m.12 g.12 ORF g.12 m.12 type:complete len:4649 (+) Unigene000002:244-14190(+)

MHRALLLQTATILTFFLQCSAQGSGCEVGQFRCGTGTCILASWRCDGTADCTDDSDESGC

PQATCDADQFQCLSDGECIPVHWVCDDEEDCEDGSDERQHCPGRTCSSIQFSCTNGACIP

AEYRCDHLPDCSDGADERNCHYPECPELRCASGACYNTSQRCDQVLNCRDSSDEANCTLR

CISGQFQCNNGECIPRGYICDHDDDCGDMSDEQNCTYPTCKGTYFTCPSGRCIHQVWLCD

GEDDCGDNADERGCDNVQHECYPGEWPCPSSGICIRMEQLCDGTPHCPDGEDETNFTAGH

NCSIWRCASLSCEHQCVASPQGGTCSCPSGYVVSFNDSRSCIDYDDCQLWGVCDQLCEDR

IGTHRCSCKEGYVLEQHRYCRANTSSGVASLLFSNGRDILIGDIHGHSSRVLVHSENRGV

AVGVDFHYQLQRVFWTDTIQNKVFSVDMDGSHLQVILNVSVDYPENLAVDWVNNKLYIVE

ASVNRIDMVDFDGNNRVTLIAENLGNPRGLALDPTVGYLFFSDWDTLNGEPGLERAYMDG

SNRYSIIRSKLGWPAGITLDIDAKRVYWVDSRYDYIETTTYDGLHRKTVVHGGSLIPHPF

GISLFEHSVYYTDWTKMAIMKANKFTENNPHVLYPTAHTPYGVAVVHPFRQPYMSNPCGT

DQGGCEQICVLSHRTDNGGLGYRCRCRMGYDLHPDGKRCVAVKRFLLFSSQLAVRGIPFN

LSSQEDIILPVTGTPSFFVGVDFCAEDNSIFFSDTAKDIIFKQKIDGTDRKVLAANRVEG

VEDLAYDWISKNLYWTDPRYRSISVMKVADKSRRAIIRNLNNPRSIVVHPIVGFIFWTDW

YRPAKIMRSWCDGSHAQPIVNTTLGWPNGLAIDWSSMRLYWVDAFFDKIEHSTFDGQNRL

SLDRITQISHPFGLTIYGGYAYFTDWRLGGIVRVRKTDGGELTVIRRGISHIMHVKSFNA

DLQTGSNFCNRPSNPNGDCSHFCFPAPYSQRVCGCPYGMKLAPNQQTCVDDPSSEPPTLQ

CGSNSFSCSNGKCVPASYQCDGVNDCYDNSDEANCGTNNNTCSSSAFTCANQRCVPRNWR

CDGHNDCFDGSDERDCPTQTPGTCQADQFSCANQRCIPRTWLCDTDNDCGDGSDETNCDA

IGTCHPGQFQCPDHRCIDPAYVCDGDRDCVDGADEQGCVYNCTGYEFKCANGHQCVNSYY

RCDGVYDCNDRSDESGCPTRPPGMCHHESEFQCQSDGTCLPSAWECDGHADCEDGSDEHH

GCPPRTCPTSLFRCDNGNCVFRSWLCDGDNDCRDGSDERDCPTPPFRCPSWQFQCPGHTV

CINISKVCDNTPDCPNGADESPLCNQEICSDNNAGCTHGCIQGPFGAQCTCPVGYQLSND

SKTCEDIDECDPPGLCSQHCFNERGSFRCHCQDGYTLEGDGRTCKASGSREAILLVASRN

QIITDDIVSQPNVVRSLVRDGRNIVAVDFDSVTDRVYWSDTTQDKIWSAHKNGSDRTVIF

DSGVTVTESIAVDWVGRNLYWTDYILETVEVAKLDGSHRAVLLSENVTNPRGLVLDPRNG

FHLMFWTDWGRNPRIERASMDGKQRTTIITTKLYWPNGLTIDYPNNLLYFADAYLDFIDY

CDYEGNNRKQVLASDLVLQHPHAITIFEDFVYWTDRYINRVIRANKWHGENQTVLLYNVP

QPMGLVAMHPVRQPAGDNPCALNPCTHICLLSAVGPRFYSCACPSGWTLANDQFSCAKVD

DPFLVVVRDSIIYGISLNPDEKSNDAMVPIAGLQNGYDVDFDDAEQMIYWVEHPGEIHRV

KSDGTNRTEFAPAAILGSPVGLALDWMSQNLYYTNPSSQSIEVLRLKGEVQYRKTLITNN

GTPTGAGSPVGIAVDPARGKLYWTDQGTESGIPAKVACADMDGSNAVVLFTNNLEHVEFI

TIDIKENKLYWAVTGTGVIERGDPNGLNRITVVNGLSHPWGVAVHGPYLYFTDRDFEVIE

RVDKATGSSRVVLRNNVPGLRVLKVHSRETSVGSSNGCSNNVGACEQLCLPRPGGIFSCA

CATGFRLNADNRTCAPYQSYVVISMLTAIKGFSLEGEDHSEAMVPVAGRGRNVLHVDVHM

PSGFLYWCDFSSTVASQNGIRRIKPNGSGFRNIVTSGIGRNGIRGIAVDWAAGNLYFTNA

FLTETYIEVVRLNTTFRRVLLKTQVDMPRHIVVDPKNKFLFWADYGQNPKIERAFLDGTN

RTVLVTSGIITPRGLALDHGNGYIYWVDDSLDMIARVPPDGGETEIVRYGSRYPTPYGIT

VFENNIIWVDRNLKKVFQVSKEPGNTDQPMVIRDNINMLRDVTIFDQRMQPWSAQELNNN

PCLSSNGGCSHFCFALPGTQARNCACAFGNLAVDGMTCVVSRDDYLIYTTESTVRSLRLD

PDDHSLPFPVVNVARTSVALDYDRLDNRIYFTQSSGAGQSKISYISLSSPTSSATVVASD

LGAPDGIAYDWINKRIYYSDYVNQTINSMAVDGSQRTLIAHVPRPRAIMLDPCSGYMYWT

DWGTIAKIERATLGGNFRTEIVNSSLVWPNGLTLDYDDQRLYWADASLQKIERCSLTGTN

REVIVSTAIYPFAMTVYGQHIFWTDWNTRSIYRANKHDGSDQRVMIQNLPTRPMDIHVLS

NSKQQQCSSPCLQFNGGCSHICTPGPQGAECQCPSEGRWYLADNKYCIPDNGTRCQPSQF

TCMNGRCIRAAWKCDTDDDCGDGSDELERVCAFHTCEPTLYTCGNGRCVPYHYRCDHYND

CGDNSDEEGCLFRPCDPDTEFTCNNGRCIAREYVCNGINNCFDNGTSDERNCAERTCQPG

HTKCQSTNICIPRSYLCDGDNDCGDMSDESPTHCATSTCTQSEFRCTSGRCIPAHWYCDG

GTDCADGSDEPSTCTTIVRTCSSDQFRCDDGRCIASSWICDGDNDCGDMSDEDQRHNCAN

RTCSSLEFTCVNNRPPQRKCIPQDWVCDGDADCSDAYDEHQNCTRRSCSATEFTCSNGLC

IRSAYRCDRRNDCGDGSDEQSCTYQPCQQHQFTCQNGRCISQDFQCDGDNDCGDESDELP

HMCHTPAPTCPPGKFRCDNGNCVPSSQVCDHSDDCNDNSDEKGCGINECTDPSVHHCDHE

CTDTPTSFVCRCRPGYRLMSDKKSCDDVDECSETPSVCSQMCENTLGSYVCKCAPGFLRE

PDGRRCRQNSNISPYLIFSNRYYLRNLSADGAAYSLILQGLSSVVALDFDRVDKRLYWID

VSRRVVERMFFNGTGREVVLNGIMHGEGLAVDWVGRKLYWVDSFLDCLKVSELDGRFIRK

LAEHCVDVNNTYCFENPRAIVVHPKFGYVYWTDWGSKAFIGRVGMDGNNKSAIITSKIEW

PNGLTIDYTNDMLYWADAPLNYIESSNLDGPHRHTVYDGVLPHPFAITVFEDTVYWTDWN

TRTVEKGNKYDGTGRQALVNTTHRPFDIHVLHPYRQPIVTNPCGINNGGCSHLCLLKAGG

RGYTCQCPDHFLTVQIGGVARCLPSCSSTQYRCADNERCIPIWWKCDGQSDCRDGSDEPS

TCPLRHCRLGQFQCNDGNCTSSHFLCNSNRDCPDGSDEDTVLCATHQCESHQWQCSNKRC

IPEAWQCDGENDCGDGSDEEPSHCSSRTCRPGQFKCRNGRCIPQSWKCDVDDDCGDNSDE

PLDECMGPEYRCDNHTEFDCSTNYRCVPLWAVCNGQNDCRDNSDEQNCEEMTCDPAGDFR

CDNHKCIPLRWKCDGDSDCGDGSDERNCNPRACTESEYRCDNLRCIPERWVCDHDNDCED

NSDERDCELRTCHPGYFQCDSGHCIAERLKCDGSADCMDVTDETSCPTRYPNGTYCPPFL

FECKNHICVQQHWKCDGDNDCGDGSDEELHLCLDIPCEAPFRFRCDNNRCIYSHELCNSV

DDCGDGTDEKQEHCLTPTHGPCSSDEFKCGNSQCIPLQYVCDDYDDCGDQTDELGCNSGN

GRSCSENLCEHNCTELTGGGFICSCRQGYKAREDNRNSCEDVNECELYGTCPQLCKNTKG

SYECFCAEGFRSVGPQHRTECAAEGNPPVLLLPDNVRIRRYNLSSKQYLDYIDNAEHIHA

VDYIWDPEDQGLSIVYWTILGHGAQFGSIKRAYMTTFDDHGSNQMKDVDLNLRYISSPDG

IAVDWVGRHIYWTDAGTNRIEVAKLDGRYRKWLIYSNLDQPAAIVVNPAIGMMYWTDWGS

RPKIEAAWMDGQHRQVLLDEDLGWPTGLTLDYLNGNRIYWCDSKENIIESMKPDGTDRKI

VLSGDIGNPYSLDVFEGHVYWTTKVRGEVWKADKFGRGDKVKMLTINPWLTQVRIYHEHR

YNRSVLNPCKGVCSHLCLLRPGGYTCACPQGSSLVSFNENECDAASLPEVAMPVECGCIN

GGTCSTDKPGLPKCKCPYGYSGRHCEIGKSRGAPAGTAVAVLLAVIIILLSGALAVGLFL

NYKRTGSLIPSMPKLPSLSSLAKLSDAGNGVSFRSGDGSTVDPQTLGVSFIDRAMQLNEN

FTDAGREPVTFENPLYSTATGAAADAAVIHATQVTVNISGDSTENVFANPMYNEQQQAVD

MKNTPNEQAAKESKWNFFKKKLKPSTTFENPAYSEMRDKQSALTTEDISTSHPQPALPPL

KREKSSMYSPTEDSFQDTANLVKEDSDI*

>m.31 g.31 ORF g.31 m.31 type:3prime_partial len:140 (-) Unigene000003:2-421(-)

MDSLSSEIHELFRCSSHFNFTGGIIAAMIPLHSYTRSLTTNCQRGLLCTKNKRGIHISPQ

RCKIKNACPKKKKKKIDSTCFFSPFSFSYPFSSSSGWVESLAEDGKTLRNRATLLRYSVL

CFEVLWRGDVTGAHALPICL

>m.29 g.29 ORF g.29 m.29 type:complete len:4810 (+) Unigene000003:339-14768(+)

MAAMIPPVKLKWLEHLNSSWISEDSESISTREGVALLYSKLLANKEVVLLPQQVLCLKGP

QLPDFERECLSRDEQEHYLDALLASQLALAKTVCSDSPFASALRKRLLVLQRIFYALSSK

YHDSGKAKQQQHSGESALNSSELQAASERPRSSTDALIEMGVRTGLSLLFALLRQSWALP

TATPGLSLCNDVITTAIEVVGSLPPLSLANESKIPPMGLDCLVQVTAFLKGAIACQSGAD

VVGRRLACELLLGLAAQRGSLRYLLEWVEMALGASSGVSKLEQNQNQEGLMGYDCFMNIL

MQMRRSLGSSADRSQWREPTRTPDGLCSLYEAALCLFEEVCRMASDYSRTCASPDSIQTG

EAAMVSETCEVYVWGSNSSHQLVEGTQEKILQPKLAVSFSDAQTIEAGQYCTFVISSDGA

VRACGKGSYGRLGLGDSNNQSTLKKLTFEPHRAIKKVSSSKGSDGHTLAFTSDGEVFSWG

DGDYGKLGHGNSSTQKYPKLIQGPLQGKAVVSVSAGYRHSAAVSEDGELYTWGEGDFGRL

GHGDSNSRNIPMLVKDISNVGEVSCGSSHTIALSKDGRTVWSFGGGDNGKLGHGDTNRVY

KPKVVEALQGMFIRKVCAGSQSSLALTSTGQVYAWGCGACLGCGSSEATALRPKLIEELA

TTRVVDISIGDSHCLALSHDNEVYAWGNNSMGQCGQGNSTGPITKPKKVIGLDGVAIQQI

SAGTSHSLAWTALPRDRQVVAWHRPYCVDLEESTFAHLRFFLERYCEGINNKVPPLPFPS

SKEHHNFLKLCLKLLSNHLALALAGGVATSILGRQARPLRNLLFRLMDASVPDEIQEAVI

ETLSVGATMLLPPLRERMELLHSLLPQGPDRWESLSKGQRMQLDIILTSLQDHTHVASLL

GYSSPPDMSESLTLSSDSTHTPDTHPDTHLAEILMKTLLRNLGFYTDQAFGELEKNSDKH

LQGTSSSENSQPAHLHELLCSLQKQLLAFCHMNPVTEDSSSVALLHKHLQLLLPHATDIF

TRSAVLLKESSGNGSVREKLRDVMYVSAAGSMLCQIMTSLLLLPVWVARPLLSFLLDLLP

PLDRLNRLLPAAAPLEDQEMQWPVQGISELTQTECVPGPAQSWVWLMDLERTVALLIGRC

LGGMLQGSAPSLEEQHTAYWLKTPLFSNGLETEIPRLDSCISALLEAALSGNEEKKPFDC

ALRADISVLVELALGSTKEPTNSLWTNMVDYAISKDWDSATLSNEALLDTVSHFVLATLL

KHTGLLGHACGGGRYQPCKLLAEVYRSVYKVRNRLLACKNMELVQTHSPSRERRISENVD

SVEMDPQEHAFTRTIDEEAELEERAERERDREDGHQEQEDEEEREHEVMTAGKIFQCFLS

ARDVARGRERPRTSSVGVPDESVQPEQERRNSIPLQDRQDLYTTTCNSIIHRCAMLLMAV

SPTLPQTTNEQPALCGGVTQDGSGFLIRSESLSTESRSIQSGPSYRLTKSRSESDLSQPE

SDEEGYSLSGKRNADLDLAFTPKKREFAHSTPDSADWFGRHSGRERLYSSPQSYEESDLD

FSCTLHIHAVIDNIVSFISGDVGNTPGFKEPEDSMSTSPQATVVAMEQQQGRAELRLDAL

HQIVVLISGMEEKSSQGGAEAVAGCGPAAFHSASLLTSVRLQFLAGCFGLGAYNGGLNGE

SVQLHHYQDGIQAAKKRLQMNIQTAVHKIYQQLSITLERALQSNKHHIEAQQRLLLVTVF

ALSVRYQPVDVSLAISSGLLDVLSQLCGKETLLGHTLQLLHKPAGSQLSTALKVASTRLL

QILAISTGTYADRLSPKVVQALLDLLCSQLKTLLSQAAGSVFGTNSESEEKTEDSTGSMK

KDFRALLRKQHIAELHLGDFLVFLRRVVSSKAIQSKMASAKWTEVLLNIAAQKCCSGIPL

VGNLRTRLLALHVLEAVLPACESSVEDHQMSQVVERLFSLLSDCMWEAPVAQAKNTVHLK

EKEVKLQGEAEEEEENLPVQEVIFDPEKTQCCVVENGQGLTHGSGGKGYGLAATGISSGC

YQWKFYIVKENRGNEGTCVGVSRWPVNDFNHRTTADMWLYRAYSGNLYHNGEQTLALSSY

TQGDYITCVLDMEARTVSFGKNGEEPKLAFEDVDATELYPCVMFYSSNPGEKVKICDMQM

RGTPRDLLPGDPICSPVATVLAEAVSQLVRILHRSDSWTQPINRCMLQRLKQIRGCLHDG

ASPTGGARLRKSRSAQSREEQDEAKDEDRDEERGRMGRELTEAQLRTLCNLIWPVLAVIG

GVDGGLRVGGRCVHKQSGRHATLLGVVKEGSMSAKVQWDEAEITISDTPLYNLEPCEPLP

FDVARFRGLTAALLLELTALASLQDDGKFSPTACSLSSSSRRHERRHRHEPHEPEEGPGD

LRVSHSLDEVRSHAELQKPPVMAEVHAVQLSYLCLGAIKSLSVLLSCSKYAELLLIPKAV

HDNGHNADCGSPSIGVSQEEAEMRSALQFLMRHMVKRAVMRSPIKRVLGLAELERAQAMI

YKLVVNSVLEEQEGGKTKQYGECEELEGEQQFQTPVTTSPSASSSTSFMSSSLEDTTTAT

TPVTTATTPITDAETAPASESPGVMPLSLLRQMFSSYPTTTLLPARRAQTPPVSSLPTSP

SDEHGRRQSFTSPESQPARTTNRTALSDPSSRLSTSPPPPAIAVPLLEMGFSLRQIMKAL

EATGTRGEADAQNITVLAMWMIEHPGNEDTEERTLSSDCSGAAGGGKSAERSFLRSPGDI

PNADAAELEEGFSESPEGIDQDSASASNGPALRGRSAASRKHRFDLAARTLLARAAGLYH

SVQAHRSQVRREGSGLQQDSSSLGGVYDFNLDEELELELDQEAMEAMFTQELASDSDILG

MWIPERVCESEEREEVVVCELCDVSVSSFNQHMKKSHPGCGRSANRQGYRSNGSYVDGWF

GGECGSGNPYYLLCGSCREKYLGMKSKHKGAVSERYKGQAPDLLGKQDSVYEEDWDMLDV

DEAEKLTGEEEFELLSAPLGLTERKPVPEAVQFPDTDPLGASAAMVTATNSMEETLLQIG

CQSSVDKSTSGRMCLGEQAAGLQSSADRMVALRRVTAAAQVLVARTMVMRALSLLSVSGS

SCSLAAGLEALGLTDIRTLVRLMCLAAAGRAGLSTGLASGPGALERPRGTNKPTKPISCL

AYLSTAVGCLASNSPNAAKLLVQLCTQNLISAATGMNLTTVDDPIQRKFLPSFLRGVAEE

NKLITSPNFIVTQALVALLADKGARLRPGYDKAEMEKRGPLELANALAACCLSSRLSSQH

RQWAAQQLVRTLAAHDRDNQSRPQTFADMAGDLRKCSTIKLEAHQGRVISFGWCNKKGLL

ATSGSDGTVRVWNVNKNQYTLQQTCVFNKTDFTSEECSTGLGSPGEPSLAPVAWSVSGKL

VAAAMEKVVNIWQVNGGKALLDLQPHWVSSLVWPENEAGCLWAGESRELLLVGRIDGSLG

LLEILDSSNIQRTELLHCYRKDAVMNIAWYSEDRPFAVGYADGKLLIGCKEALENGSVVV

IDAHKESISSLRWAPGGQILLSCAKEETACLWAESGAGLGRSWVCLQSIAHPSVVNAVAW

CGLTGQGPKALNMLATCCQNGLVSVWTVPQDPTAFSQSGSSSTDAWWETDSKSKPRFTPQ

WHEAAVCVFQLRGHMTPVRTIAFSPDGLALVSGGVGGLLNIWSLRDGSVLQTVVVGSGAI

QNTVWIPDVGVAVCSNRSKDVLVVNCSSEFMTANQVLATCRTALKKQGVVGLNTAPCMRT

FLERLPIMLQEQYAYEKPHVVCGEQLIHSPYMQCLASLAVGLQLDSLLCRPAVPLHLSHC

LAQPVSSSSSSSQSSSSSGPSALMHSCGSSEWAWLHCFSTTIKTAEALARAHTFPESFNV

PDLEPVAKDKMAVLMDNSKWVPGMDEQLMAWATSRPEDWHLGGKCDVYLWGAGRHGQLAE

VGRNILVPTPAASFSQAQQVVCGQNCTFVIQANGTVSACGEGSYGRLGQGNSDDLHILTV

ISALQGFVVTQLVTSCGSDGHSMALTESGEVFSWGDGDYGKLGHGNSDRQRRPRQIEALQ

GEEVVQMSCGFKHSAVVTADGKLFCFGNGDYGRLGLGNTSNKKLPERVTALEGQQIGQVA

CGLNHTLVVSSDGSTVWAFGDGDYGKLGLGNSTAKSSPQKVDVLCGIGIKKVACGTQFSV

ALTKDGNVYTFGQDRLIGLPEGRARNHNRPQQLPALVGVIIKDVAVGAEHTLALASTGDV

YAWGSNSEGQLGLGHTNHVREPTLITALQGKNTQQISAGRCHSAAWTAPSVPPRAPGSSM

PLQLGLPVCVPPQYGSLRDISMEALRARLRLLYHFSDLMYSSWRLLNLSPNNQSCTSHYN

PGTWGIVQGQLRPLLAPRVYMLPMVRSIGKTMVQGKNYGPQITVKRISTRGRKCKPIFVQ

IARQVVKLNASDLRLPSRAWKVKLVGEGADDAGGVFDDTITEMCQELENGVVDLLIPSPN

ATAEVGYNRDRFLLNPSACLDEHLLQFKFLGILMGVAIRTKKPLDLHLAPMVWKQLCCIP

LTLEDLEEVDLLYVQTLNSIVHLEDSSITEQNFHEMIPLDSFVGQSADGKMVPIIPGGNS

IPLTFSNRKEYVERAIEYRLHELDRQVAAVREGMSWIVPVPLLSLLTARQLEQMVCGLAE

ISVEVLKKVVRYREVEEQQQLVQWFWQTLDEFSNEERMLFMRFVSGRSRLPANTADISQR

FQIMKVDRPYDSLPTSQTCFFQLRLPPYSSQSVMAERLRYAINNCRSIDMDNYMLSRNVD

NTEGSDTDY*

>m.49 g.49 ORF g.49 m.49 type:5prime_partial len:545 (+) Unigene000004:3-1637(+)

GKIGRASGRTEGLPAKDTENKLTKEFVDQKSIDGDMQIKGLVQTEMSKVSDISDVFEYIV

ESPPQEREDIRSVHVMQQRQTMENENLVTETSKNLVDVNRDTKDEMESQMQPIVTNDMSK

IQADNEIDFNKDYAPDEAEKKRKKKRKAKKRMMEKIGKESEESNADKLVQIPPVDNQPQS

DQFSAVEKVEMEQPQKSTQLEKEALLMKAKESILRKVFERGVSEKQAAEELEALRQGSTK

EQHTTAQEKAKLDERSVSLPADENRTVKYQATQTDRNINLSANILPKSDSIQLHFSQTDL

TDDNFYEKQDLSCTVSSDVSMDTSKQISKPIIILDKAETTVKEHSHLKSENLSETAVPKE

PYTSPTGENKDHQKSRQKPQNSFSALEQSEINLLEETSKPELEDTVSKEPMQATTTEYSS

NDDEFKDESSLEESSEARLSYLEEVPESDTTECWDEEDLEECQDDVDKKQAEDLVKKPRT

AAQISKSSLVRQECLEHDQQIVALLSMVRHTEVRLKQQQQQFMGKSLASLDDIIKQTEAC

SRAY*

>m.48 g.48 ORF g.48 m.48 type:complete len:4150 (+) Unigene000004:1594-14043(+)

MTSLNRQRLALELTDMEPQVNGEVEAAKQLLKSHTEDVPPQLFTALEKDQQSLSRTFTAA

KNLAESSLQGMRSHRDAQKEAVGSELESLTVRVDGLHAWLKDKEAQVEQDADSSEDGAIK

ENNRETLVKLTQKLQQVKDLQNCLTSRSSDVNAVAFDIQVFISERAQDLAPEQSRRLLRQ

LQQLQNAFHQASGRTHARAETLSVQKVREEEREQKEQEREQEEREKEKERQTAREREVVQ

QQKAESSQKLEGLSMWLAGAASLMAIQRPGAESEDVNVLQQKHSQLKEVQKDLQTKADGM

METIRSVEEFLAERGEDMSPEERAKLQAALTHIKEQYNVLTDTAQSSLTQLDSTINTTLQ

QNTQRAKAEEQLQETQGKIEVLLKELSSLDTSRRESLGETVPDGSMPVGSLNLHNDMLKA

ELQQLQAQQAHLLQLAQSTHSLLEQPDSNVSPEEKQRLRDRLDHLQSQHKERLQSCQERL

RRTEALQENLSKFMEEHGNLGTWLEQSERQLCSLREGETDAQGLKDKLEEHKKLADDVIC

HKADLRFVSISGQKVLDSIQGSQESGIAIDPVLQDTRQLVSDKLQDATQRYSSLQNKSGN

LGTHLSTLLDQYQQYQDEATALDTWLTAQEQNQSILKPSGEQTDTQTLQNTLNTVQHMQD

ELAERSVQLEKVKRAGKVLTSNQETPKLKTADINTTTDKIERRFDTLTECVSQRAKQLQT

AAAQSVSVQEGLKALVSWLDGLALDPEPVQPTTQAVQEALAQNQKLRQELLSRQGSVEAT

RDSVSKLLKSADALTVSGLQGALQDLNQRYTAAQTKQTENEAELRAVLPKLESFERLNSD

LHNFVQSRERVLSLGGLPDRSVEDYKHSIQEVQSDLGQETSHLKTFVELSTDLSKSKLFV

NAHSLVDSSKEVSDEFARLEASLNERLNAIQSCDQQLVQFRSHSDSILRWLQMAQEQLPA

KEPQQSTDSLQRRVQQLKDLLSEWESRGNQIQELNKNSSELESRIISITAPQSKTSAPML

NGAGGSSSLNGIHTCRDLTEIQVVVADVNSRYEQLGGDLRNRQSKQQAMLELCQKARQDS

EALHQWLALREQRLAQGQSASPSRPDVARAQAQENKALLSELTEHSGKVEDLKNTLKQLI

QENPDSPEAESWKQQLKDLDIRWEKASQVAAQRQAELETCADRLGNFASAANQLGPWLRE

KELMMSVLGPLSIDPNMLNTQKQQVQFMLREFETRHAQFDQLTQAAEGILSPVEEQGSGD

NQDVAEVKKELASITQQWEDLTSRLSGRFDQIERAQGTSESFQSLLKELSQSLSQLAEKL

DAQASLSAQPEALRHRLKETGEIRAELEQRKGQLAQAEQLCEDLSAIVVEPYLREELHKR

LESVSGPLNNLEERAADGLSQLQAALSSTQQFQQMFEELRSWLDGQAAAPSMPDPLPCQP

EALQVLVAQQEELQRCVAQQRGSYDLLQAEGASILASLPAGDERSALQAQLTKLRQDWEE

VNQRLTEKQAHIKETLGRAVLFRQHRDELDPWLKECEQKEAEIQPSLEPAALEEALQKAK

QLGLDLDRRRPLLEILNSAADQLLEQSRTGEEEVRDEKAKLNRRLDGLSERLHGRMAQLE

ELNSRLKEFEDGRLAVERRLEAARHQLEVQEALGPQACSNKSLERLRSQQDTLGSLQPQV

VYLHNLAKGLAQDAPQMTGGSGDGGQKLLQQAQTTEKEFGEVTEKIQQCCSSLESRLQGV

GEVQSHVRDIFSRLADLDDELDSLSPVGRDVDSLASQADAIRGFLGRLASLRAELEGHGS

ACTTMLKREGSSPDLLAIRRETEALNRQAAKLAERGQGRLALIEEAEGRVKEFYARLMEL

QRLLDQAEEGLNVQSVVGTEVDVIKRQLQEFKAVEREQVDSIQPKLHHVNAVGQGLIQSA

AKHTDTQALEHDLETTNLQWNSLNKRVAERIAQLQEALLHCGKFQDALEPLLSWLSDTEE

LIANQKPPSAEYRVVKAQIQEQKLLQRLLDDRLGTVEMIRAEGERIAATAETQDREKIQR

QLQCLGERWTDLLEKANARQRQLEELQVLALQFHESVDPLGEWLSATERRLSSAEPMGTQ

ASKISQQINRHKALQDEVLAREKEVDHLEALGQSLSPLSCAADRDWLSERVGAVRSGHTE

LRNWCFRRAAMLEQALANAQLFGEEEVEVLNWLAEVAQRLSDVSVQSYQPELLAEQHKYT

LSLNEEIVSRKKTVDQAIKNGQALLKQTTGEEVLLIQEKLDGIKSRYSEMTAGSSKALRN

LEQALQLATRFASAHEDLSQWLDSMEAELNNMEPDTTPAYQERQKDLKCVSAEKRLILDT

VNEVGSALLDLVPWRAREGLDRLVADANQRYRQADETITQRVQLVQAAIQRSQQYEEAVD

AELAWVGETERKLTSLGPLSLEPDVTVAQLQVQRAFNIDIIRHKDTVDQLLHTREDILES

CSDQQREALKVKTDSLSMRYEAVSQNHAERFSALEQAQVLVARFWETYEELDPWLGETET

LISQLPPPAIDTEALRQQQDQMRMLRESIAEHKPHIDKLLKIGPQLAELSSQEGATLRQR

YSEAERRYLAIKEDVKGRAAVLDEAFSQSAQFHDKMDPLLETLEGAVQRLRQPPPVAAEV

EKIREQLAEHRAAGLELDKLLPSFSTLCARGEELITRAAHDDPAAQAVRSRLLRLRSLWD

EIRQRAEEREGKLQDVLDLAGKFWADMAALLSTLRDSQEIVKELEDPGLDPSLIKQQIEA

AEAIKAETDGLREELEIVRTLGADLIFACGETEKPEVKKTIDEMNAAWEGLNRTWRERME

KLEEAMTASVQYQDALQGMFDYLDNAVIKLCDMQAVGTDLSTVKQQIEELKQFKVEVYQQ

QIDMEKLCHQGELLLKKVSDQADRDMIQEPLTELRHLWDNLGDKITVRQHKLEGALLALG

QFQHALSELQSWLSHTHATLDTQRPVNSDPKAIEIELAKHHVLRNDVLSHRATVETVNKA

GSELLESSPGDEASHLRDQLDELNRSWDSLLLKTDERQKLLETALQQAEGFHGELEEFLQ

WLRRTESQLSAAKPTGGLPETAREQLQQHMELQAQLTQRGEQYHRLLDQGESMLLARGAE

ENSPGTTQTQQNLALLQNKWASLNAKMDDRRAKLEEAVSLATGFQSSLQDTINWLTQAEQ

TLNMAQSPSLILDTVLFQIDEHKVFVNEVNTHREQVLALEKAGSQLRFASLKQDVVLIKN

LLLSVQARWDKLVQRSLDRGRHLDEARKRAKQFHEAWRKLTDWLEEAEKRLDAELEISNE

PDKIKVQLTKHKEFQKTLGSKQPVYDTTVRSGKAMRDKATLPADTQKLDNLLGEVRDKWD

TVCGKSVERQHKLEEALLFSGQFAEALQALVDWLYRVEPQLAEDQPVHGDLDLVSNLMDS

HKAFQKELGKRTSNIQALKRSARELMETGRDDTAWVKVQLQELSNRWETICALSVSKQTR

LQQALKQAEEFRTAVQMLLEWLSEAEQTLRFRGILPEEVETLQVLLHTHRNFMQTVEEKR

VDVNKAAGMGEAILAVCHPDCITTIKHWITIIRARFEEVLTWAKQHEQRLEAALAELLNN

ATLLEDLLSWLQWAETTLVQRDTEPLPQDITQLKTLITEHQVFMEEMTRKQPDVDKVTKT

YKRKPSETSSSLAERRGVRKQQQQQQQPAMQVSGGNPRLNQLCSRWQQVWLLALDRQRKL

HDGLDRLEELKEFANFDFDVWRKKYMRWMNHKKSRVMDFFRRIDKDQDGKITRQEFIDGI

LASKFPTSRLEMTAVADIFDRDGDGYIDYYEFVAALHPNKDAYKPTTDADKIEDEVTRQV

AQCKCAKRFQVEQIGENKYRFFLGNQFGDSQQLRLVRILRSTVMVRVGGGWMALDEFLVK

NDPCRVQHPGLRILRSDSSCSISSRIARGRTNLELREKFILPEGVSQGMAAFRSRGRRSK

PSSRTASPTRSSSSASQSAQSCASGPSAPATPTASARSSQSQSRGYAKPWLAHSKTPTPT

KCQSCTEHSHTPGHEGGSSSKLKRPPFHSSRGSLTGENGGTTHATKPVRSDNKRTPSSTS

GPTSRAGSRAGSRASSRRGSDASDASELMETRSACSDTSDTPRRPGTKPSKIPTISKKTP

SPKTPTTKK*

>m.61 g.61 ORF g.61 m.61 type:5prime_partial len:3717 (+) Unigene000005:2-11152(+)

APDRKKLWKALDNDPNLPKKLRDTARHLEWLKTVKDSHGSVELSSLSLASAINKKGIYFI

SAENQKKLSLDKVLKLRITEEHAEDNEVRCYSLDDLKELQNKLMLMSGKGDQGQNEVDRF

AEVFASVQRLGTAFMDLFAAGNPLFRLWEAIIHCNSQEACIVMDFNLDGVTSVVMLRGDV

AEQLLDVCKKMERCLSFWKDFMDKQRSQNYYLNYFTAEQIVYLCSQLNQTNLSHLEDQAL

IMLSFIKLNCTTYNLRQSWHVLQYEMLTNPTKQNEDIDFQTFADVPNQIENEIDINSVPF

SDSGIENDTRRFDMIWNMYMRNMKTFLPKSLDVRSLGRLLEILSDSPSKTDVDDDDDDDN

AEKSTLENLHKNVQRVLPRGLVIGKPNLIVCPFTEIQTACISIYMSSEDEPLPTYDEVLL

CDSSTSYEQAELFLRRCLSAGYKGQKIYTMLYVDQLTYEVSYRVEQFFQRHKAQSRNDYR

LVLICSTDREHAYLPSVFSQFRLHMVPQEPLGRIQEYLTKHYTVPKGYFSAAAVFKTKQY

VGVVSSRRAGVGKSLYIRRLYEKVKLITTKSSQLKCIRLTKSKVDENAVLQSMLHGPQRE

LTIFHFDITSSVQKGLHEFLFRLLVLGYLMDSEGRMWKCNSKHLYIIELLQSSANISRNL

PRSSESVQSAFQDVFPSVYCRTPKEVLDLEMRMSEDPTIIVNDPLMDDKEFRNEAYQRPY

QYLKRFHHGVNLDGFMYKGIEGTHVECLQLLFLYCGVVDPSWAELRNFTWFLNLQLRDCE

TSVFCDASFTGDTLLGFKNFVVDFMILMAKDFATPSLSISDQSPGRQQMDISGVSEEDLA

PFRVRKRWESEPHPYIFFNDDHVSMTFIGFHLRPNDQNLVDAIDPSTNRVIKKNIMTSEL

YEGLKLQRVPFNINFDSLPRGEKIERICNVLGIQWPLDPDETYELTTDNILKILAIHMRF

RCGIPVIIMGETGCGKTRLIKFLCELRRSGVATENMKLVKVHGGTSDDMIYTKVREAESI

ACSNKQDYGFDSVLFFDEANTTEAISSIKEVLCDKTVKGEVMVPNTGLQIIAACNPYRKH

SDIMIKRLESAGLGYRVRAEQTDEKLGSIPLRQLVYRVQALPPSMIPLVWDFGQLNDQTE

KMYIQQIVQRVAEDHFFSVEYIKIFTDVLSASQKYMRTRKDECSFVSLRDVERCMQVFTW

FYQNNEMLLSELARYEINQKAQKNEHNQRDNDVRDPILWSLVMAIGVCYHACLEDKEKYR

NKICGYFSKTYSPVNLMQEISVMQDLLLSGVPLGETIARNNALKENVFMMVICIELRIPL

FLVGKPGSSKSLSKTLVADAMQGQAAHSDLFRKLKQIHLVSFQCSPHSTPEGIINTFKQC

ARFQEGKNLSEYISVVVLDEIGLAEDSPKMPLKTLHPLLEEGCIEDQPLPHKRVGFIGIS

NWALDPAKMNRGIFVSRGDPDEKELIESAKGICSSDAMILEKVGDFFQPFAKAYLKICSE

GGKGFFGLRDYYSLIKMLFSIAKASDQKPSADQVVEAVLRNFSGKDTVNAVTVFTNRLRL

KPNLESIDTIELVRQNITAVAQDEECRYLLILTKNYAALQILQQTFFSEQYQPEIIFGSS

FPKDQQYTQICRNINRVKICMETGQVIVLLNLQNLYESLYDALNQYYVCLGGQKYVDLGL

GTHRVKCRVHKDFRLIVIEEKDIVYKQFPIPLINRLEKHYLDINTVLKNEQKQLVKILEE

WVDHFIKCKHAPTPQAAKHCPTDVFIGYHSDTCASVIMQVIERQKGRMEFSDPERQLLDE

AKLILLNCATPDAVVRLDSSALSKVESEHLSRVYFEDQKHSCMADFVFGHIQQVEWNHSC

FTEVTTFSRLLTASDIEQLQEVVPNIELLALQQFDTEHSFLKKIRNFLNCTSGNKMLIIQ

TEFDEAVQSYNLIASAKYSSIGEINKFQKEHDSRVFVYFITKLPRVEGGTSYVGFHGGPW

KSVHIDDLRKSKDIISDIKALQCLTISQLFKETEDQSEVMETEDMYREEEETEKMEQDDN

TSWKNVLDTTALVRSCVQSAVGMLRDQGDVGCRSTKRVEILLTLLSDSEELQATFLKTMK

HRLYSLLITHDEHTHSAKNWVFKEASNIDSLQEGGTFRNTLWKRVQAAIIPFLAQLVSVI

DREHNLDLLLDENSGESVKKLWLDIFGNVKLLNVPYTRVDSTSMTKTILVQNCIVVYKNT

GCSMPFSWIIKDHLEELWLHALHREGHSLRQFEEFFWKTPLGRYIAKAEQEMQQEFFYRY

LQDFISLTTTITTEVELKLLVKALSCCVNELRSRQEAVQNEVPSFPWIQAPSHDLKNRLQ

NFSRMIIIEPQIAQVLLDNTQSRDGKEMVLDVYAALACVEYLEPQGLDACINCLTWLRQV

KKLQIPIELVCSEASIRHYGAMSKAIIPRVQHGWRRIHAISLFVEHILLDIENIEKKLKP

LVLEHTRKLAQVLDKNSDLKMKQPFEAVIAILKTCKDGANEQIFRFGFQPCPVCMGDPHD

PLSLPCDHVYCLACIKQWLIPGQMYCPLCIQGVDDDFDLQPSDTIRTLIKQNSHFRKQCN

AFFIDFVSTICFRDNSPPSKTIILHLLSFLMVEAGSVPTIRGCERRFLTKALSPFDDSVD

KNPVVRSVVLKLLLKYSFADVKGYLQQHLTEVEQSNILEKADKTELYCLYINCLEDSMFE

RIQWHSVAEQQACLLEEKHFLVEYLRSGAVATETVTIEHLQQVARTRLCLDMAADLLISR

SSTADAEKESSAGVTAFLDSVQNLCKYSKNDWYRVYLIRKLCSLSGVEYIQKLLQEDQLS

WLFPQEVLQMRKTSSQIDQYLVCGVEYKAIRDAVAKAMLEGKMETIEEACKICQCSPEKK

NVYILLALFREVTTLYKANNPNIHPTPEQLAALDDFTKNSKVFVVPESRDFAQALVHNQL

PVLHLHPSLLANEFVILELTVHLAAVLLCTNQPLLQPLIQLAFFPINMQKAFLPTMPDDM

LAVAQQVMGQLQWYFCPNGHPCTVGECGRPMEKSHCVDCNAEIGGVAHNPVQGFQPIAVQ

GDRTQTGHLLGDPHRRDQPDMQDTKNMSPGPFTLLRLLTHMAMLLGSSRQPQEVAAIIKP

PVPNPATFLLAHLLKDIEQLTRAIGKGADDTICTIHLTICILLEPHQPRPLPYDNLLSTK

DARNNWEITVTTDIITCQLKVLESHLANVNTFIRNDERVSSNPIMKVTFGEPRLFLRTLP

QGSLIHSSSVWCCRQRVSLLGLSHILEQYGGRDRLPILWRFLQKEAELRLVKFLPDILAL

QRDLVKKFQNVIELQCGTIAQFLQNLKAESLMTSYKKRINIFLSTWNQLRASLETNGEIK

LPTQYCQDDLDLSADLQVLLPRRQGLGLCSTALVSYLIALQNDLIYAVDKHTGEETSYTV

SPADLTELHVIRYEVERDLLPLVLSNCQYSMECGRETLSEYDLPKIQQQLLTRFLQGKPF

IGLTGIPTLVNRHDRNYETIFRDVKGKVQQESMTSLNQSTLCAELQCYSDVCEALSTVEI

ALGFLAMTGGEPNMQLSNYLKEELQMADQIPLHVLKALSRCCLKHCLALWQLLTSLKSET

MLRLKRDPFERFSKDYKKTLGEQEKRLLTGFFSKTSADSFLLEMHEFLLLVLKNANATDT

YKPNWSVKDTILSYMEQKDMDVPPDVVELFPDEILLAHYIEAWKFSILLKQERIQR*

>m.69 g.69 ORF g.69 m.69 type:5prime_partial len:3640 (+) Unigene000006:1-10920(+)

VNTHTHTDSCISALLEAALSGNEEKKPFDCALRADISVLVELALGSTKEPTNSLWTNMVD

YAISKDWDSATLSNEALLDTVSHFVLATLLKHTGLLGHACGGGRYQPCKLLAEVYRSVYK

VRNRLLACKNMELVQTHSPSRERRISENVDSVEMDPQEHAFTRTIDEEAELEERAERERD

REDGHQEQEDEEEREHEVMTAGKIFQCFLSARDVARGRERPRTSSVGVPDESVQPEQERR

NSIPLQDRQDLYTTTCNSIIHRCAMLLMAVSPTLPQTTNEQPALCGGVTQDGSGFLIRSE

SLSTESRSIQSGPSYRLTKSRSESDLSQPESDEEGYSLSGKRNADLDLAFTPKKREFAHS

TPDSADWFGRHSGRERLYSSPQSYEESDLDFSCTLHIHAVIDNIVSFISGDVGNTPGFKE

PEDSMSTSPQATVVAMEQQQGRAELRLDALHQIVVLISGMEEKSSQGGAEAVAGCGPAAF

HSASLLTSVRLQFLAGCFGLGAYNGGLNGESVQLHHYQDGIQAAKKRLQMNIQTAVHKIY

QQLSITLERALQSNKHHIEAQQRLLLVTVFALSVRYQPVDVSLAISSGLLDVLSQLCGKE

TLLGHTLQLLHKPAGSQLSTALKVASTRLLQILAISTGTYADRLSPKVVQALLDLLCSQL

KTLLSQAAGSVFGTNSESEEKTEDSTGSMKKDFRALLRKQHIAELHLGDFLVFLRRVVSS

KAIQSKMASAKWTEVLLNIAAQKCCSGIPLVGNLRTRLLALHVLEAVLPACESSVEDHQM

SQVVERLFSLLSDCMWEAPVAQAKNTVHLKEKEVKLQGEAEEEEENLPVQEVIFDPEKTQ

CCVVENGQGLTHGSGGKGYGLAATGISSGCYQWKFYIVKENRGNEGTCVGVSRWPVNDFN

HRTTADMWLYRAYSGNLYHNGEQTLALSSYTQGDYITCVLDMEARTVSFGKNGEEPKLAF

EDVDATELYPCVMFYSSNPGEKVKICDMQMRGTPRDLLPGDPICSPVATVLAEAVSQLVR

ILHRSDSWTQPINRCMLQRLKQIRGCLHDGASPTGGARLRKSRSAQSREEQDEAKDEDRD

EERGRMGRELTEAQLRTLCNLIWPVLAVIGGVDGGLRVGGRCVHKQSGRHATLLGVVKEG

SMSAKVQWDEAEITISDTPLYNLEPCEPLPFDVARFRGLTAALLLELTALASLQDDGKFS

PTACSLSSSSRRHERRHRHEPHEPEEGPGDLRVSHSLDEVRSHAELQKPPVMAEVHAVQL

SYLCLGAIKSLSVLLSCSKYAELLLIPKAVHDNGHNADCGSPSIGVSQEEAEMRSALQFL

MRHMVKRAVMRSPIKRVLGLAELERAQAMIYKLVVNSVLEEQEGGKTKQYGECEELEGEQ

QFQTPVTTSPSASSSTSFMSSSLEDTTTATTPVTTATTPITDAETAPASESPGVMPLSLL

RQMFSSYPTTTLLPARRAQTPPVSSLPTSPSDEHGRRQSFTSPESQPARTTNRTALSDPS

SRLSTSPPPPAIAVPLLEMGFSLRQIMKALEATGTRGEADAQNITVLAMWMIEHPGNEDT

EERTLSSDCSGAAGGGKSAERSFLRSPGDIPNADAAELEEGFSESPEGIDQDSASASNGP

ALRGRSAASRKHRFDLAARTLLARAAGLYHSVQAHRSQVRREGSGLQQDSSSLGGVYDFN

LDEELELELDQEAMEAMFTQELASDSDILGMWIPERVCESEEREEVVVCELCDVSVSSFN

QHMKKSHPGCGRSANRQGYRSNGSYVDGWFGGECGSGNPYYLLCGSCREKYLGMKSKHKG

AVSERYKGQAPDLLGKQDSVYEEDWDMLDVDEAEKLTGEEEFELLSAPLGLTERKPVPEA

VQFPDTDPLGASAAMVTATNSMEETLLQIGCQSSVDKSTSGRMCLGEQAAGLQSSADRMV

ALRRVTAAAQVLVARTMVMRALSLLSVSGSSCSLAAGLEALGLTDIRTLVRLMCLAAAGR

AGLSTGLASGPGALERPRGTNKPTKPISCLAYLSTAVGCLASNSPNAAKLLVQLCTQNLI

SAATGMNLTTVDDPIQRKFLPSFLRGVAEENKLITSPNFIVTQALVALLADKGARLRPGY

DKAEMEKRGPLELANALAACCLSSRLSSQHRQWAAQQLVRTLAAHDRDNQSRPQTFADMA

GDLRKCSTIKLEAHQGRVISFGWCNKKGLLATSGSDGTVRVWNVNKNQYTLQQTCVFNKT

DFTSEECSTGLGSPGEPSLAPVAWSVSGKLVAAAMEKVVNIWQVNGGKALLDLQPHWVSS

LVWPENEAGCLWAGESRELLLVGRIDGSLGLLEILDSSNIQRTELLHCYRKDAVMNIAWY

SEDRPFAVGYADGKLLIGCKEALENGSVVVIDAHKESISSLRWAPGGQILLSCAKEETAC

LWAESGAGLGRSWVCLQSIAHPSVVNAVAWCGLTGQGPKALNMLATCCQNGLVSVWTVPQ

DPTAFSQSGSSSTDAWWETDSKSKPRFTPQWHEAAVCVFQLRGHMTPVRTIAFSPDGLAL

VSGGVGGLLNIWSLRDGSVLQTVVVGSGAIQNTVWIPDVGVAVCSNRSKDVLVVNCSSEF

MTANQVLATCRTALKKQGVVGLNTAPCMRTFLERLPIMLQEQYAYEKPHVVCGEQLIHSP

YMQCLASLAVGLQLDSLLCRPAVPLHLSHCLAQPVSSSSSSSQSSSSSGPSALMHSCGSS

EWAWLHCFSTTIKTAEALARAHTFPESFNVPDLEPVAKDKMAVLMDNSKWVPGMDEQLMA

WATSRPEDWHLGGKCDVYLWGAGRHGQLAEVGRNILVPTPAASFSQAQQVVCGQNCTFVI

QANGTVSACGEGSYGRLGQGNSDDLHILTVISALQGFVVTQLVTSCGSDGHSMALTESGE

VFSWGDGDYGKLGHGNSDRQRRPRQIEALQGEEVVQMSCGFKHSAVVTADGKLFCFGNGD

YGRLGLGNTSNKKLPERVTALEGQQIGQVACGLNHTLVVSSDGSTVWAFGDGDYGKLGLG

NSTAKSSPQKVDVLCGIGIKKVACGTQFSVALTKDGNVYTFGQDRLIGLPEGRARNHNRP

QQLPALVGVIIKDVAVGAEHTLALASTGDVYAWGSNSEGQLGLGHTNHVREPTLITALQG

KNTQQISAGRCHSAAWTAPSVPPRAPGSSMPLQLGLPVCVPPQYGSLRDISMEALRARLR

LLYHFSDLMYSSWRLLNLSPNNQSCTSHYNPGTWGIVQGQLRPLLAPRVYMLPMVRSIGK

TMVQGKNYGPQITVKRISTRGRKCKPIFVQIARQVVKLNASDLRLPSRAWKVKLVGEGAD

DAGGVFDDTITEMCQELENGVVDLLIPSPNATAEVGYNRDRFLLNPSACLDEHLLQFKFL

GILMGVAIRTKKPLDLHLAPMVWKQLCCIPLTLEDLEEVDLLYVQTLNSIVHLEDSSITE

QNFHEMIPLDSFVGQSADGKMVPIIPGGNSIPLTFSNRKEYVERAIEYRLHELDRQVAAV

REGMSWIVPVPLLSLLTARQLEQMVCGLAEISVEVLKKVVRYREVEEQQQLVQWFWQTLD

EFSNEERMLFMRFVSGRSRLPANTADISQRFQIMKVDRPYDSLPTSQTCFFQLRLPPYSS

QSVMAERLRYAINNCRSIDMDNYMLSRNVDNTEGSDTDY*

>m.84 g.84 ORF g.84 m.84 type:5prime_partial len:3627 (+) Unigene000007:1-10881(+)

SADRKSGNEEKKPFDCALRADISVLVELALGSTKEPTNSLWTNMVDYAISKDWDSATLSN

EALLDTVSHFVLATLLKHTGLLGHACGGGRYQPCKLLAEVYRSVYKVRNRLLACKNMELV

QTHSPSRERRISENVDSVEMDPQEHAFTRTIDEEAELEERAERERDREDGHQEQEDEEER

EHEVMTAGKIFQCFLSARDVARGRERPRTSSVGVPDESVQPEQERRNSIPLQDRQDLYTT

TCNSIIHRCAMLLMAVSPTLPQTTNEQPALCGGVTQDGSGFLIRSESLSTESRSIQSGPS

YRLTKSRSESDLSQPESDEEGYSLSGKRNADLDLAFTPKKREFAHSTPDSADWFGRHSGR

ERLYSSPQSYEESDLDFSCTLHIHAVIDNIVSFISGDVGNTPGFKEPEDSMSTSPQATVV

AMEQQQGRAELRLDALHQIVVLISGMEEKSSQGGAEAVAGCGPAAFHSASLLTSVRLQFL

AGCFGLGAYNGGLNGESVQLHHYQDGIQAAKKRLQMNIQTAVHKIYQQLSITLERALQSN

KHHIEAQQRLLLVTVFALSVRYQPVDVSLAISSGLLDVLSQLCGKETLLGHTLQLLHKPA

GSQLSTALKVASTRLLQILAISTGTYADRLSPKVVQALLDLLCSQLKTLLSQAAGSVFGT

NSESEEKTEDSTGSMKKDFRALLRKQHIAELHLGDFLVFLRRVVSSKAIQSKMASAKWTE

VLLNIAAQKCCSGIPLVGNLRTRLLALHVLEAVLPACESSVEDHQMSQVVERLFSLLSDC

MWEAPVAQAKNTVHLKEKEVKLQGEAEEEEENLPVQEVIFDPEKTQCCVVENGQGLTHGS

GGKGYGLAATGISSGCYQWKFYIVKENRGNEGTCVGVSRWPVNDFNHRTTADMWLYRAYS

GNLYHNGEQTLALSSYTQGDYITCVLDMEARTVSFGKNGEEPKLAFEDVDATELYPCVMF

YSSNPGEKVKICDMQMRGTPRDLLPGDPICSPVATVLAEAVSQLVRILHRSDSWTQPINR

CMLQRLKQIRGCLHDGASPTGGARLRKSRSAQSREEQDEAKDEDRDEERGRMGRELTEAQ

LRTLCNLIWPVLAVIGGVDGGLRVGGRCVHKQSGRHATLLGVVKEGSMSAKVQWDEAEIT

ISDTPLYNLEPCEPLPFDVARFRGLTAALLLELTALASLQDDGKFSPTACSLSSSSRRHE

RRHRHEPHEPEEGPGDLRVSHSLDEVRSHAELQKPPVMAEVHAVQLSYLCLGAIKSLSVL

LSCSKYAELLLIPKAVHDNGHNADCGSPSIGVSQEEAEMRSALQFLMRHMVKRAVMRSPI

KRVLGLAELERAQAMIYKLVVNSVLEEQEGGKTKQSDGECEELEGEQQFQTPVTTSPSAS

SSTSFMSSSLEDTTTATTPVTTATTPITDAETAPASESPGVMPLSLLRQMFSSYPTTTLL

PARRAQTPPVSSLPTSPSDEHGRRQSFTSPESQPARTTNRTALSDPSSRLSTSPPPPAIA

VPLLEMGFSLRQIMKALEATGTRGEADAQNITVLAMWMIEHPGNEDTEERTLSSDCSGAA

GGGKSAERSFLRSPGDIPNADAAELEEGFSESPEGIDQDSASASNGPALRGRSAASRKHR

FDLAARTLLARAAGLYHSVQAHRSQVRREGSGLQQDSSSLGGVYDFNLDEELELELDQEA

MEAMFTQELASDSDILGMWIPERVCESEEREEVVVCELCDVSVSSFNQHMKKSHPGCGRS

ANRQGYRSNGSYVDGWFGGECGSGNPYYLLCGSCREKYLGMKSKHKGAVSERYKGQAPDL

LGKQDSVYEEDWDMLDVDEAEKLTGEEEFELLSAPLGLTERKPVPEAVQFPDTDPLGASA

AMVTATNSMEETLLQIGCQSSVDKSTSGRMCLGEQAAGLQSSADRMVALRRVTAAAQVLV

ARTMVMRALSLLSVSGSSCSLAAGLEALGLTDIRTLVRLMCLAAAGRAGLSTGLASGPGA

LERPRGTNKPTKPISCLAYLSTAVGCLASNSPNAAKLLVQLCTQNLISAATGMNLTTVDD

PIQRKFLPSFLRGVAEENKLITSPNFIVTQALVALLADKGARLRPGYDKAEMEKRGPLEL

ANALAACCLSSRLSSQHRQWAAQQLVRTLAAHDRDNQSRPQTFADMAGDLRKCSTIKLEA

HQGRVISFGWCNKKGLLATSGSDGTVRVWNVNKNQYTLQQTCVFNKTDFTSEECSTGLGS

PGEPSLAPVAWSVSGKLVAAAMEKVVNIWQVNGGKALLDLQPHWVSSLVWPENEAGCLWA

GESRELLLVGRIDGSLGLLEILDSSNIQRTELLHCYRKDAVMNIAWYSEDRPFAVGYADG

KLLIGCKEALENGSVVVIDAHKESISSLRWAPGGQILLSCAKEETACLWAESGAGLGRSW

VCLQSIAHPSVVNAVAWCGLTGQGPKALNMLATCCQNGLVSVWTVPQDPTAFSQSGSSST

DAWWETDSKSKPRFTPQWHEAAVCVFQLRGHMTPVRTIAFSPDGLALVSGGVGGLLNIWS

LRDGSVLQTVVVGSGAIQNTVWIPDVGVAVCSNRSKDVLVVNCSSEFMTANQVLATCRTA

LKKQGVVGLNTAPCMRTFLERLPIMLQEQYAYEKPHVVCGEQLIHSPYMQCLASLAVGLQ

LDSLLCRPAVPLHLSHCLAQPVSSSSSSSQSSSSSGPSALMHSCGSSEWAWLHCFSTTIK

TAEALARAHTFPESFNVPDLEPVAKDKMAVLMDNSKWVPGMDEQLMAWATSRPEDWHLGG

KCDVYLWGAGRHGQLAEVGRNILVPTPAASFSQAQQVVCGQNCTFVIQANGTVSACGEGS

YGRLGQGNSDDLHILTVISALQGFVVTQLVTSCGSDGHSMALTESGEVFSWGDGDYGKLG

HGNSDRQRRPRQIEALQGEEVVQMSCGFKHSAVVTADGKLFCFGNGDYGRLGLGNTSNKK

LPERVTALEGQQIGQVACGLNHTLVVSSDGSTVWAFGDGDYGKLGLGNSTAKSSPQKVDV

LCGIGIKKVACGTQFSVALTKDGNVYTFGQDRLIGLPEGRARNHNRPQQLPALVGVIIKD

VAVGAEHTLALASTGDVYAWGSNSEGQLGLGHTNHVREPTLITALQGKNTQQISAGRCHS

AAWTAPSVPPRAPGSSMPLQLGLPVCVPPQYGSLRDISMEALRARLRLLYHFSDLMYSSW

RLLNLSPNNQSCTSHYNPGTWGIVQGQLRPLLAPRVYMLPMVRSIGKTMVQGKNYGPQIT

VKRISTRGRKCKPIFVQIARQVVKLNASDLRLPSRAWKVKLVGEGADDAGGVFDDTITEM

CQELENGVVDLLIPSPNATAEVGYNRDRFLLNPSACLDEHLLQFKFLGILMGVAIRTKKP

LDLHLAPMVWKQLCCIPLTLEDLEEVDLLYVQTLNSIVHLEDSSITEQNFHEMIPLDSFV

GQSADGKMVPIIPGGNSIPLTFSNRKEYVERAIEYRLHELDRQVAAVREGMSWIVPVPLL

SLLTARQLEQMVCGLAEISVEVLKKVVRYREVEEQQQLVQWFWQTLDEFSNEERMLFMRF

VSGRSRLPANTADISQRFQIMKVDRPYDSLPTSQTCFFQLRLPPYSSQSVMAERLRYAIN

NCRSIDMDNYMLSRNVDNTEGSDTDY*

>m.99 g.99 ORF g.99 m.99 type:5prime_partial len:3001 (+) Unigene000008:2-9004(+)

SFNTTSSELNQSLDLGKELLIQQCRDLTEQLEVKEKQLEVLQEEVRRSAEDLEEARERWS

KASEELEEAKWELESERDKRLQCEELLNEKIHEQDNLKNKPGFLENQIEKDQPTSNMEYD

EKSFSPEKLTEELELVLQEQLGQMDYREDRMENVQRLEKVRVDLESELTCLKENLSNMEE

ALRQGSAEKAAMEKRLQKLEEKNKSMEKALEMELENFELQLQAKDVAFEQMKEAREKAEE

ELMEKETTLQKEKNEHEEERRRLEEKHQQEMLDWSLRLEKELSDLRSDLEEEQKKQITLI

KQVHEREHEREMDELVAQQKAELERLKAELSEELRDSMEAAHQAELSQVQTQHTLELEAL

RLSLTNMHTAQLELTQSNLQKEKEAALSELQASLREKWAQESAMLQTRQQFELEKFREQG

REQERQTELQHQLAVDDLNCVWERRLSEERAALEQQLAGGIEDAKIRWIQDFKKEQAELQ

EALNKAEDRLAETKRKLEELQRSKEEEIKHLESELSQAWADRDSAARAVEELVASHRVVL

QEQQAHTQELEEKEKWLKQEVDRLLEQKSSSEQEITNLWSQLESMRTSRQELGELKEQLL

ARKSHVNDIEQLKQDFSQQRRELQEQNETELENLRVYFEQRLRASEENHREEIALLQLRL

VEGALEDSVLKTGEESFLSDGKSEEEKGDSLAEITEQLDKHKVELDALRLQLEEKHKQDL

EQLRCNMALSYREELLQARTELTDRYYSDMEQLKTKHALEIEQLRAKLSDSHLKEITRLR

LQSVADVARQVETELEEKSRLQTQEHQARLAQLASDTESILSLEKKLAALTLQHSRELER

ATEQHAEELRQLEERLKKNFSEELHTRLAEAQQEERTHVTEELLNRSSEERLQLREQLQA

QAEERVASLREELQQAASKDRQELEQKLVQAEELLAEEKQRLQALQVSLEREESPQLLAL

KQRLESQYAGELHTAKSTMAAEVKELNTLLQEQTESRLKEATCRHQEEQRQLAEKLVQQK

EEALQKLRQQHAQELQVQETLLEQNVSELQHLQKEYQELKSRHQEELDLLSSNHKTALDS

LAEKHKAELDKLQVVFQETNLAQLEAQEAELEERHKQEMEELETRMLCNMDTLESTYLKE

IQAVREEKEAALRDLGASLEQQQAEEIERLKQEELIVREELRKKLAQVHMDKFSVMATEL

SHAHQAELSAALASQKAALESEHHSALETLRQHVLDLEAQHGAALHEVTDMTTKENQQLK

KQLDALNAQHQQQLQELRATSSREIEALRRELEEEASRQRLHFLEEAELLKCQSEEQLQK

KLMELKEESDREKVSALEELERSLCEQHQQNELSYTEKMNHLTTQLQQLDTVVSQLRAEV

SGLQGELEGKRSEMDTLETLLQRRERESQEGANLLAMLRDDLNTATQQRQDLQLTHDRLQ

RLVLEMLRITIATEEEISRRLGISMEGDDITLNRKQSVNRDPQKTDAEVGDGAAAAVEAS

ANCSMFSSLMDEGLELSQRLCESLFTGPDGELDPEREELVLGACTHLRVAVDKLLEVVSD

STVQLEQLQALQALLDEKFSAGGEAKNALFLQHTQLLEQLDQEASVKSQLQLELHKADGL

IEGYVVEKAALEDALQQKESREQKLVKELESTRAQLQELGEEHALLQRQRDALSASLGEP

EKALLAEAERLGQERVDVQRQAEKDRVGLAGRLRLLEQTLEEHESRTQQQEEQHRTQTED

LQQHISALEKQLKHHRQFIDEQAVEREHERDEFQQEIKNLEAQLKTTSKGHAGEDTKGQR

VESLQALIKDKTEDYSVLLSLKEQCQRDLEERNEEIDKMASRIRELEQALLSSVEERRAA

AQLEQELQKARKNCQELSQDKEALQQQLYSNKLQISALQSKLDETRHRYPDPGADPNLRE

RLETLQQDLQNKEEQVEVLLERVEVLEKALAVKEEDVRQLSLQLELKTTESRAREEELRL

SVAELQDTASLLRRQRQEEADASALQLPSALLEEKNLEIDHLNQQLLHLQQELDASKENK

TEEKQADVEDLRAQVERLHGDQERLHQAKEEEVEQLHEIIHKLQEELSQLNPNRHEVSDP

NTDSPEPPDFPWSPRPQHGAEESLCHELSSQTLQSCRTKICELQADLERSVEEKVALERL

LLMQEEQYGQQLEALGCSLGEGKRKLALLEQEAGELKLQLDQKETKVEELQARIQTLEDG

ERSVNDLELRLIKAEETMKETQQEMTRLNDKARKHTEERERTESLIAELQQRYEDSERTA

TQARLQVAELKETIQQRKSDIAILETGKTELYTEKQALQKRERKLQEEIEKLKQEVSAKS

LQIQEVSLQLEERAADPDQSQEEVLTCAEETLAKAAAALREKEEQLAHLRSQHDALRAEL

AAVKEGLSSSTERAEKLQEEGQTKDRALADLEEHNQRLRAELRGLQDDLEVQEEELAYQQ

RELDELRQRCRLQEHTELDCTKPERRFLDGLSHNSTLSSPEVLRRLDCSEERPSHVHASH

LSELSGLRNTSLELMSKHSLMERNELKRPLVLPPETDPPSTHSPSASTHSLSISENLSVL

NSLDDDKVQVLENLDDVTPSRSPLSSTSPVSAPEWASDGYGSNVSSELEAKLKNELEHME

RLDAHFVEYLRSRDMAPAPCSDSAAGSLHHTYELLSPELQAMLNRVYRESYRVLSLSHRP

PPAGQPHSDAEEAPPASWQRERRALQETVLSLRELLCRMAEREPKVEGEVDWRTELLQAV

RSVFDSERKWLHAQLQDIITTNTGIDYTSLLKDLETLLQKQEDQQRRSLEQLLSADRNSL

LAEIRSLHKQLHTCTLQNQEQLQRSLGAVREEGAQTQQQLHRQVGELERQLQQEQSSSHD

LQRSLGQEQSRSTNLHQQLEIQQKLAVEMKLELEEGTRQLRASEKSREELQAQIHKLRLK

LESEEQEHQACVEAANEMRSGLSSFRRNFITRGSAANARRRSMRTCKSLSGPPCASMRLV

*

>m.100 g.100 ORF g.100 m.100 type:complete len:578 (+) Unigene000008:8956-10689(+)

MQESLRASLCEHATRLNELNAALEQERVAVSNLRAELQIEQSRCEALLAQERERTELTLS

RLDEERSRCAELSRSLSHQAQEHARRLEEEVRSQQAASADDRKFIQELRAQLEQERRQAE

DLAATVDNLQSQVLQSKRRQEEAAQQEAQRGQEEVKRLRSALDGLQAQRVEVNRTLETER

HRAAQLQTELDAVKEKMREVKEKERAKEEQREKQRWCEKQEQEDRERRQEQTKEKLLELE

SLRERDQQRLRQLQQTLADLEQQEKQLTSERLQRDCVAHTQDSSSSSSLLERVLKENSEL

AERLAALSEEKISLKHTVSCLERDLHNLRCTQQSWHKERMFSAPIASQKAEEEFTDSRNR

NRPITDLSNNKVQRLYEKYLRAESYRKSLVYQKRYLLLLLGGFQDCEQATLALIARMGAR

PSLITQTSPPISRFRSAVRAVIAVSRLKFLTRKWQRATRKSSGGGVIVNSAAIKTSILKP

QHSGVAAHQSPPTRDGILSQRTALSPLVPPLKSPFRLHNGGYANSTAAPSERTLTPSQDQ

ERSLADYIHHLESVQQRLGAVRPGSPATFSFLRKNDR*

>m.116 g.116 ORF g.116 m.116 type:5prime_partial len:274 (+) Unigene000009:1-822(+)

TGRSEVRRSAEDLEEARERWSKASEELEEAKWELESERDKRLQCEELLNEKIHEQDNLKN

KPGFLENQIEKDQPTSNMEYDEKSFSPEKLTEDKVELVLQEQLGQMDYREDRMENVQRLE

KVRVDLESELTCLKENLSNMEEALRQGSAEKAAMEKRLQKLEEKNKSMEKALEMELENFE

LQLQAKDVAFEQMKEAREKAEEELMEKETTLQKEKNEHEEERRRLEEKHQQEMLDWSLRL

EKELSDLRSDLEEEQKKQITLIKQVFISCVFYF*

>m.114 g.114 ORF g.114 m.114 type:complete len:2697 (+) Unigene000009:893-8983(+)

MDELVAQQKAELERLKAELSEELRDSMEAAHQAELSQVQTQHTLELEALRLSLTNMHTAQ

LELTQSNLQKEKEAALSELQASLREKWAQESAMLQTRQQFELEKFREQGREQERQTELQH

QLAVDDLNCVWERRLSEERAALEQQLAGGIEDAKIRWIQDFKKEQAELQEALNKAEDRLA

ETKRKLEELQRSKEEEIKHLESELSQAWADRDSAARAVEELVASHRVVLQEQQAHTQELE

EKEKWLKQEVDRLLEQKSSSEQEITNLWSQLESMRTSRQELGELKEQLLARKSHVNDIEQ

LKQDFSQQRRELQEQNETELENLRVYFEQRLRASEENHREEIALLQLRLVEGALEDSVLK

TGEESFLSDGKSEEEKGDSLAEITEQLDKHKVELDALRLQLEEKHKQDLEQLRCNMALSY

REELLQARTELTDRYYSDMEQLKTKHALEIEQLRAKLSDSHLKEITRLRLQSVADVARQV

ETELEEKSRLQTQEHQARLAQLASDTESILSLEKKLAALTLQHSRELERATEQHAEELRQ

LEERLKKNFSEELHTRLAEAQQEERTHVTEELLNRSSEERLQLREQLQAQAEERVASLRE

ELQQAASKDRQELEQKLVQAEELLAEEKQRLQALQVSLEREESPQLLALKQRLESQYAGE

LHTAKSTMAAEVKELNTLLQEQTESRLKEATCRHQEEQRQLAEKLVQQKEEALQKLRQQH

AQELQVQETLLEQNVSELQHLQKEYQELKSRHQEELDLLSSNHKTALDSLAEKHKAELDK

LQVVFQETNLAQLEAQEAELEERHKQEMEELETRMLCNMDTLESTYLKEIQAVREEKEAA

LRDLGASLEQQQAEEIERLKQEELIVREELRKKLAQVHMDKFSVMATELSHAHQAELSAA

LASQKAALESEHHSALETLRQHVLDLEAQHGAALHEVTDMTTKENQQLKKQLDALNAQHQ

QQLQELRATSSREIEALRRELEEEASRQRLHFLEEAELLKCQSEEQLQKKLMELKEESDR

EKVSALEELERSLCEQHQQNELSYTEKMNHLTTQLQQLDTVVSQLRAEVSGLQGELEGKR

SEMDTLETLLQRRERESQEGANLLAMLRDDLNTATQQRQDLQLTHDRLQRLVLEMLRITI

ATEEEISRRLGISMEGDDITLNRKQSVNRDPQKTDAEVGDGAAAAVEASANCSMFSSLMD

EGLELSQRLCESLFTGPDGELDPEREELVLGACTHLRVAVDKLLEVVSDSTVQLEQLQAL

QALLDEKFSAGGEAKNALFLQHTQLLEQLDQEASVKSQLQLELHKADGLIEGYVVEKAAL

EDALQQKESREQKLVKELESTRAQLQELGEEHALLQRQRDALSASLGEPEKALLAEAERL

GQERVDVQRQAEKDRVGLAGRLRLLEQTLEEHESRTQQQEEQHRTQTEDLQQHISALEKQ

LKHHRQFIDEQAVEREHERDEFQQEIKNLEAQLKTTSKGHAGEDTKGQRIEDLVIQVESL

QALIKDKTEDYSVLLSLKEQCQRDLEERNEEIDKMASRIRELEQALLSSVEERRAAAQLE

QELQKARKNCQELSQDKEALQQQLYSNKLQISALQSKLDETRHRYPDPGADPNLRERLET

LQQDLQNKEEQVEVLLERVEVLEKALAVKEEDVRQLSLQLELKTTESRAREEELRLSVAE

LQDTASLLRRQRQEEADASALQLPSALLEEKNLEIDHLNQQLLHLQQELDASKENKTEEK

QADVEDLRAQVERLHGDQERLHQAKEEEVEQLHEIIHKLQEELSQLNPNRHEVSDPNTDS

PEPPDFPWSPRPQHGAEESLCHELSSQTLQSCRTKICELQADLERSVEEKVALERLLLMQ

EEQYGQQLEALGCSLGEGKRKLALLEQEAGELKLQLDQKETKVEELQARIQTLEDGERSV

NDLELRLIKAEETMKETQQEMTRLNDKARKHTEERERTESLIAELQQRYEDSERTATQAR

LQVAELKETIQQRKSDIAILETGKTELYTEKQALQKRERKLQEEIEKLKQEVSAKSLQIQ

EVSLQLEERAADPDQSQEEVLTCAEETLAKAAAALREKEEQLAHLRSQHDALRAELAAVK

EGLSSSTERAEKLQEEGQTKDRALADLEEHNQRLRAELRGLQDDLEVQEEELAYQQRELD

ELRQRCRLQEHTELDCTKPERRFLDGLSHNSTLSSPEVLRRLDCSEERPSHVHASHLSEL

SGLRNTSLELMSKHSLMERNELKRPLVLPPETDPPSTHSPSASTHSLSISENLSVLNSLD

DDKVQVLENLDDVTPSRSPLSSTSPVSAPEWASDGYGSNVSSELEAKLKNELEHMERLDA

HFVEYLRSRDMAPAPCSDSAAGSLHHTYELLSPELQAMLNRVYRESYRVLSLSHRPPPAG

QPHSDAEEAPPASWQRERRALQETVLSLRELLCRMAEREPKVEGEVDWRTELLQAVRSVF

DSERKWLHAQLQDIITTNTGIDYTSLLKDLETLLQKQEDQQRRSLEQLLSADRNSLLAEI

RSLHKQLHTCTLQNQEQLQRSLGAVREEGAQTQQQLHRQVGELERQLQQEQSSSHDLQRS

LGQEQSRSTNLHQQLEIQQKLAVEMKLELEEGTRQLRASEKSREELQAQIHKLRLKLESE

EQEHQACVEAANEMRSGLSSFRRNFITRGSAANARRRSMRTCKSLSGPPCASMRLV*

>m.115 g.115 ORF g.115 m.115 type:complete len:578 (+) Unigene000009:8935-10668(+)

MQESLRASLCEHATRLNELNAALEQERVAVSNLRAELQIEQSRCEALLAQERERTELTLS

RLDEERSRCAELSRSLSHQAQEHARRLEEEVRSQQAASADDRKFIQELRAQLEQERRQAE

DLAATVDNLQSQVLQSKRRQEEAAQQEAQRGQEEVKRLRSALDGLQAQRVEVNRTLETER

HRAAQLQTELDAVKEKMREVKEKERAKEEQREKQRWCEKQEQEDRERRQEQTKEKLLELE

SLRERDQQRLRQLQQTLADLEQQEKQLTSERLQRDCVAHTQDSSSSSSLLERVLKENSEL

AERLAALSEEKISLKHTVSCLERDLHNLRCTQQSWHKERMFSAPIASQKAEEEFTDSRNR

NRPITDLSNNKVQRLYEKYLRAESYRKSLVYQKRYLLLLLGGFQDCEQATLALIARMGAR

PSLITQTSPPISRFRSAVRAVIAVSRLKFLTRKWQRATRKSSGGGVIVNSAAIKTSILKP

QHSGVAAHQSPPTRDGILSQRTALSPLVPPLKSPFRLHNGGYANSTAAPSERTLTPSQDQ

ERSLADYIHHLESVQQRLGAVRPGSPATFSFLRKNDR*

>m.130 g.130 ORF g.130 m.130 type:5prime_partial len:3558 (+) Unigene000010:1-10674(+)

THTRAHTRRHEVIFFFVCVRVRYQPCKLLAEVYRSVYKVRNRLLACKNMELVQTHSPSRE

RRISENVDSVEMDPQEHAFTRTIDEEAELEERAERERDREDGHQEQEDEEEREHEVMTAG

KIFQCFLSARDVARGRERPRTSSVGVPDESVQPEQERRNSIPLQDRQDLYTTTCNSIIHR

CAMLLMAVSPTLPQTTNEQPALCGGVTQDGSGFLIRSESLSTESRSIQSGPSYRLTKSRS

ESDLSQPESDEEGYSLSGKRNADLDLAFTPKKREFAHSTPDSADWFGRHSGRERLYSSPQ

SYEESDLDFSCTLHIHAVIDNIVSFISGDVGNTPGFKEPEDSMSTSPQATVVAMEQQQGR

AELRLDALHQIVVLISGMEEKSSQGGAEAVAGCGPAAFHSASLLTSVRLQFLAGCFGLGA

YNGGLNGESVQLHHYQDGIQAAKKRLQMNIQTAVHKIYQQLSITLERALQSNKHHIEAQQ

RLLLVTVFALSVRYQPVDVSLAISSGLLDVLSQLCGKETLLGHTLQLLHKPAGSQLSTAL

KVASTRLLQILAISTGTYADRLSPKVVQALLDLLCSQLKTLLSQAAGSVFGTNSESEEKT

EDSTGSMKKDFRALLRKQHIAELHLGDFLVFLRRVVSSKAIQSKMASAKWTEVLLNIAAQ

KCCSGIPLVGNLRTRLLALHVLEAVLPACESSVEDHQMSQVVERLFSLLSDCMWEAPVAQ

AKNTVHLKEKEVKLQGEAEEEEENLPVQEVIFDPEKTQCCVVENGQGLTHGSGGKGYGLA

ATGISSGCYQWKFYIVKENRGNEGTCVGVSRWPVNDFNHRTTADMWLYRAYSGNLYHNGE

QTLALSSYTQGDYITCVLDMEARTVSFGKNGEEPKLAFEDVDATELYPCVMFYSSNPGEK

VKICDMQMRGTPRDLLPGDPICSPVATVLAEAVSQLVRILHRSDSWTQPINRCMLQRLKQ

IRGCLHDGASPTGGARLRKSRSAQSREEQDEAKDEDRDEERGRMGRELTEAQLRTLCNLI

WPVLAVIGGVDGGLRVGGRCVHKQSGRHATLLGVVKEGSMSAKVQWDEAEITISDTPLYN

LEPCEPLPFDVARFRGLTAALLLELTALASLQDDGKFSPTACSLSSSSRRHERRHRHEPH

EPEEGPGDLRVSHSLDEVRSHAELQKPPVMAEVHAVQLSYLCLGAIKSLSVLLSCSKYAE

LLLIPKAVHDNGHNADCGSPSIGVSQEEAEMRSALQFLMRHMVKRAVMRSPIKRVLGLAE

LERAQAMIYKLVVNSVLEEQEGGKTKQYGECEELEGEQQFQTPVTTSPSASSSTSFMSSS

LEDTTTATTPVTTATTPITDAETAPASESPGVMPLSLLRQMFSSYPTTTLLPARRAQTPP

VSSLPTSPSDEHGRRQSFTSPESQPARTTNRTALSDPSSRLSTSPPPPAIAVPLLEMGFS

LRQIMKALEATGTRGEADAQNITVLAMWMIEHPGNEDTEERTLSSDCSGAAGGGKSAERS

FLRSPGDIPNADAAELEEGFSESPEGIDQDSASASNGPALRGRSAASRKHRFDLAARTLL

ARAAGLYHSVQAHRSQVRREGSGLQQDSSSLGGVYDFNLDEELELELDQEAMEAMFTQEL

ASDSDILGMWIPERVCESEEREEVVVCELCDVSVSSFNQHMKKSHPGCGRSANRQGYRSN

GSYVDGWFGGECGSGNPYYLLCGSCREKYLGMKSKHKGAVSERYKGQAPDLLGKQDSVYE

EDWDMLDVDEAEKLTGEEEFELLSAPLGLTERKPVPEAVQFPDTDPLGASAAMVTATNSM

EETLLQIGCQSSVDKSTSGRMCLGEQAAGLQSSADRMVALRRVTAAAQVLVARTMVMRAL

SLLSVSGSSCSLAAGLEALGLTDIRTLVRLMCLAAAGRAGLSTGLASGPGALERPRGTNK

PTKPISCLAYLSTAVGCLASNSPNAAKLLVQLCTQNLISAATGMNLTTVDDPIQRKFLPS

FLRGVAEENKLITSPNFIVTQALVALLADKGARLRPGYDKAEMEKRGPLELANALAACCL

SSRLSSQHRQWAAQQLVRTLAAHDRDNQSRPQTFADMAGDLRKCSTIKLEAHQGRVISFG

WCNKKGLLATSGSDGTVRVWNVNKNQYTLQQTCVFNKTDFTSEECSTGLGSPGEPSLAPV

AWSVSGKLVAAAMEKVVNIWQVNGGKALLDLQPHWVSSLVWPENEAGCLWAGESRELLLV

GRIDGSLGLLEILDSSNIQRTELLHCYRKDAVMNIAWYSEDRPFAVGYADGKLLIGCKEA

LENGSVVVIDAHKESISSLRWAPGGQILLSCAKEETACLWAESGAGLGRSWVCLQSIAHP

SVVNAVAWCGLTGQGPKALNMLATCCQNGLVSVWTVPQDPTAFSQSGSSSTDAWWETDSK

SKPRFTPQWHEAAVCVFQLRGHMTPVRTIAFSPDGLALVSGGVGGLLNIWSLRDGSVLQT

VVVGSGAIQNTVWIPDVGVAVCSNRSKDVLVVNCSSEFMTANQVLATCRTALKKQGVVGL

NTAPCMRTFLERLPIMLQEQYAYEKPHVVCGEQLIHSPYMQCLASLAVGLQLDSLLCRPA

VPLHLSHCLAQPVSSSSSSSQSSSSSGPSALMHSCGSSEWAWLHCFSTTIKTAEALARAH

TFPESFNVPDLEPVAKDKMAVLMDNSKWVPGMDEQLMAWATSRPEDWHLGGKCDVYLWGA

GRHGQLAEVGRNILVPTPAASFSQAQQVVCGQNCTFVIQANGTVSACGEGSYGRLGQGNS

DDLHILTVISALQGFVVTQLVTSCGSDGHSMALTESGEVFSWGDGDYGKLGHGNSDRQRR

PRQIEALQGEEVVQMSCGFKHSAVVTADGKLFCFGNGDYGRLGLGNTSNKKLPERVTALE

GQQIGQVACGLNHTLVVSSDGSTVWAFGDGDYGKLGLGNSTAKSSPQKVDVLCGIGIKKV

ACGTQFSVALTKDGNVYTFGQDRLIGLPEGRARNHNRPQQLPALVGVIIKDVAVGAEHTL

ALASTGDVYAWGSNSEGQLGLGHTNHVREPTLITALQGKNTQQISAGRCHSAAWTAPSVP

PRAPGSSMPLQLGLPVCVPPQYGSLRDISMEALRARLRLLYHFSDLMYSSWRLLNLSPNN

QSCTSHYNPGTWGIVQGQLRPLLAPRVYMLPMVRSIGKTMVQGKNYGPQITVKRISTRGR

KCKPIFVQIARQVVKLNASDLRLPSRAWKVKLVGEGADDAGGVFDDTITEMCQELENGVV

DLLIPSPNATAEVGYNRDRFLLNPSACLDEHLLQFKFLGILMGVAIRTKKPLDLHLAPMV

WKQLCCIPLTLEDLEEVDLLYVQTLNSIVHLEDSSITEQNFHEMIPLDSFVGQSADGKMV

PIIPGGNSIPLTFSNRKEYVERAIEYRLHELDRQVAAVREGMSWIVPVPLLSLLTARQLE

QMVCGLAEISVEVLKKVVRYREVEEQQQLVQWFWQTLDEFSNEERMLFMRFVSGRSRLPA

NTADISQRFQIMKVDRPYDSLPTSQTCFFQLRLPPYSSQSVMAERLRYAINNCRSIDMDN

YMLSRNVDNTEGSDTDY*

>m.145 g.145 ORF g.145 m.145 type:complete len:206 (+) Unigene000011:10119-10736(+)

MDYWETVYALLEEETLNLFNDQNAAEENCTRWPPISMAGADCRDNPYYRRKNYTFKLILN

DGSRYMFAAPSHEEQQKWLEEMQNCTGQREVSENTGIHKEKKTETTKQVDSELSEKLHVS

TSELEKDSTKSLEKVKEPPPKPPHTYYNKHRYPDVGDPRDSVTLRGLRKNQPPSFPPPLP

PQNQQESEGKDKSKNKNVFKKLFKI*

>m.144 g.144 ORF g.144 m.144 type:5prime_partial len:3235 (+) Unigene000011:2-9706(+)

SKQEVISRQMSISKKWQSLLQQLQQNRDSVGDVVNTLGVLKDTELIALDLKELQVQAASS

DLGKHLPEVVDLLQKQELLDTKIISLGESLSAISSNALRGKHRDVSQVQRTVKELNSQYT

SLLALSQNRRRALEGQLKLFEFFHDCEEMEGWIYEKWLLVQAASLGRDLSQIEQAIQNHK

MLEAEVQSQESLCSKVLSRGQELGRGGHPNESDILKWIRTLQKQWQQLRDQVTNRKNRLH

AASVIKQYFADAAEANSWLNDHKPFLTDENYGKDEMSTISLLQRYQRLEKEIGAYASEVK

RLSEQSQSAAQLAPLTTEPQQTIMVHYSDSSGDERETVKEKTGTSVKIRAPSKTLPQMQE

LNAKIRFKYRGMKLTWDRGEMVTIISKEKEDKMLVRDSKGNEQLVSSTYITELSSTQAPP

APTDVVPESQIRKVSRPRRTRSMRRGTAEIITTLLPDPQFQKDMIENTQSSLEKDYNSLC

QLLQYKRRILDETAQLHRFYNGCEEFESWMEDKENILTTFSSNSENTEQARYENFLTELA

SGKGQLDDITKLGDELVKKQHSKKREIRSRYAQVTQRWDHIQELKDEKAQELLKSSDIKS

FLHTCEYAKSQLLEKLHQLDTSGVGSSASALQAEEQSQTQAEREIEGLERKIEYLKSVSK

TKRDCSPAESAAIMKEVYALEELLCQVKGQAAKRQNLLEQARHLQLFRKESRDLLLWAEE

VEERLLEEGNSYNVESAQGLLNDNQELKLEIEQQRARLKSIERLAKSLEASSGEKFTKNI

QQTLDQLNQEWSRLDELWSNQNRRLEQALQLQKWNREADRIDATIAGHEARLKVNDLGDS

VDGVYSLLGRQEELEGLLVALDQNISFLQEKSQELINKNHFASKQHRQRSWAIEERNQKL

KDSCKKRNHDLFASKSYQEFYRDAEELQIWMDEKFKIAEDESYRDPNNILRKLKRHEAAE

KEMQANHIRRDRLIQAGEEMVSKDHYNSQSIRSKTRQIQSRWSELETKMAERGDKLRQAG

QQEQLMELLQDAKVKIEKIQKMLQHAPKGYDLRSSRLLLKEHKQVEQEAQELADKINSII

THAKHLATNHFDSQRILHETDMYLKLFKSLEKPLNHRRDQLEEEVTLYSFFYDVELELSW

IAEHEPKADTSSYTKSLAGAISLLQKHKELQAEVNAHRQYFQRVLDRGRAMGKREVYQRC

RDLSAAWEKLEDACEKRFSHLSKAVMREQVLLDCAELEVCLSETSALVSTDYGKNELATQ

SLIKQHHIVEGQIEVLSAQVDELKSRVDQAVHTWGLEEVTRNYNHIRTQLTELQHLAALR

EQKLQDALHLHEFKRESSELEEWISQQQLIASSDDYGSDYERVLHMQRRFAVFLKQLDVG

RERMRSCQELAQRLIKSNHPESRFIQETLGLLRESWNELQHLANSRKENLQKSEECYKVY

SDLTDALEQTEDRYKCIPEDIAKDLKGVELQLRKHEAQVNELVGNELQMQELLDIADSVL

DECSLGLREKIQERQQLLVEQWEKLRMAMEQREHELRLAKQRYIFLKRVQDYSLWCSQIL

SGIKAEESIRDMATCDLQLFQHQQLWAEIVAHEETYSQAVTIGQHLHEQDVSNPQEVQDK

LKALKDERERLTANWESKQKWLENTYQEQVFYRDIEHMERLTYSQEVQLKNSDLGSTVDE

TDTLIKRHEAFEKLLSAQEEKMIALQKSVEHLNKGEVTKGKGNYKNTLNSLRARRDRIRD

LSMKRREDLELSRLFCIFTRDSSEAEDWVSERMQKMQEDSKMDLSNFQAKMKLLQKHQVF

EAEILAHGKIINSVLETGEELISLRHSKSREIKQSISNLISHWEALKEAISTRGKVLEGH

RDFLEFLQKVEQVEVWIRQKEVMINVGDLGKDYEHCLELLRKLSEFRSSSPGDMTVDDAH

IKTINNLAARLEKQNCDEMITVKKRRQQLNERWNSFHGNLTSYKRKLKEAKEVHSLIRDL

EEVRDRASEKMLLLQGQGYGEDVESVENLIRRHEEMEREVRVIHERRAVLDQETKIQLRT

HSDLSDKLSKKQQEINNTLIKLDKAMKLRKEHLQESHQLQLFKGNQHLLLDWTIKQTTEM

GKKRLPKSKAQAESLIVEHHDWKVEIDARGERIDSVKNFGQSLVKSGHSKTAEIKKALSK

LEDAKVSLIQAWEERKITLDQALELQIFLGNVDQSESLLSNLEAFLANDDVGSSLSDVEA

LQRKHALFENSLEAQIQEVEEVERYAQQLIQRQHYDSDNISKRSKAIQLRKNRLLETSKA

RQKALAESVQLQKFLEDSYEVCLWLNEKKLVAQDESWREPINLQAKMQKHQSFEAEIIAN

RNSVEVLTKEGEKMLLTGHPAKDKIKSRIMDLNEGWEQLLIRCKEKKSRLQEAYQALQFL

HSLDDVESWLDSVEAELSQTDCGEDLASVSRLLKALEGLEEVMDGHLEKVQNLVDTAKSL

SSQGNFQAREIQQQVWHMANRYNSLAEPLQARRETLESWQLLFQFYQDIDDEMIWIQDKL

QATALNDCGTSFESTQAVIKKHQVVIQEITARTPLVQAVLEAGHNLVKGRHFASQEIYAR

LEELKDNHETLKKESENKSQLLKEALKIQTFLSEVSDIELWMEELRPTLESTDYGKTEDA

TEALLRKLDAVDLELVNHHRKVKSLQETGAEMEKCGHPNSYLVSKSLTTMKKQYETLLQL

SSDYRISLEDQYNLYVFEREARDLKNWLISNQILAESDDFGQDLEDVETLQKKFEDFTSE

VNTLGQNKLNTVQNLKQEVKSPEAQQRENDLLNLWGELQRALKTRAENLQSAHEVHQFDH

DLDELKSWISEKEIMLDSEDRKDDLISVKACIRQHEGLERDLVVIEEELLRIKKEGRSLV

RRCPQVRDSLSERMQEVEENWDSLLEKACQCKHRLQQVQVVQKYLTDWRELMTWLRESLS

LVTGEGLRGEVKDLSQLIKRHDEYHKQIERQLDKSEIVKNEGRRLMQEGNFMIEKLEKHL

EELQDLEVLVLQGWADRKVLYQEELELQQLQRELEQAEHWLNTYENVLTAQDYGDSVSDV

LELMKKQEDLEAMIQAQSDRFNTLHNRKTQKEQKLQKSFEEDNGSIKKPFLMTSLKKNPS

DHLDSVSQPSVAKHVLRRNSSGRKDGIKLTTAPLRRHSSGKGEGPSTVPRLASMLKRNTS

NSANSIDSSMPSVKSFADSSQTQYTSDSSLVYRQIRNSDHTAMYKDEWRDRGTP*

>m.146 g.146 ORF g.146 m.146 type:complete len:124 (+) Unigene000011:9678-10049(+)

MSGGIEEPPESTHLKSNEEIEEPFISTPPSPPVAMERELAAISTASPVSDSPPDFSTLQP

PSHPQYHPPSSPSLRKSTEETKDFTEELPKLPSLADNVCCLNLTFIYPFISGIFLSVKCI

CAL*

>m.152 g.152 ORF g.152 m.152 type:complete len:768 (+) Unigene000012:8310-10613(+)

MALTESGEVFSWGDGDYGKLGHGNSDRQRRPRQIEALQGEEVVQMSCGFKHSAVVTADGK

LFCFGNGDYGRLGLGNTSNKKLPERVTALEGQQIGQVACGLNHTLVVSSDGSTVWAFGDG

DYGKLGLGNSTAKSSPQKVDVLCGIGIKKVACGTQFSVALTKDGNVYTFGQDRLIGLPEG

RARNHNRPQQLPALVGVIIKDVAVGAEHTLALASTGDVYAWGSNSEGQLGLGHTNHVREP

TLITALQGKNTQQISAGRCHSAAWTAPSVPPRAPGSSMPLQLGLPVCVPPQYGSLRDISM

EALRARLRLLYHFSDLMYSSWRLLNLSPNNQSCTSHYNPGTWGIVQGQLRPLLAPRVYML

PMVRSIGKTMVQGKNYGPQITVKRISTRGRKCKPIFVQIARQVVKLNASDLRLPSRAWKV

KLVGEGADDAGGVFDDTITEMCQELENGVVDLLIPSPNATAEVGYNRDRFLLNPSACLDE

HLLQFKFLGILMGVAIRTKKPLDLHLAPMVWKQLCCIPLTLEDLEEVDLLYVQTLNSIVH

LEDSSITEQNFHEMIPLDSFVGQSADGKMVPIIPGGNSIPLTFSNRKEYVERAIEYRLHE

LDRQVAAVREGMSWIVPVPLLSLLTARQLEQMVCGLAEISVEVLKKVVRYREVEEQQQLV

QWFWQTLDEFSNEERMLFMRFVSGRSRLPANTADISQRFQIMKVDRPYDSLPTSQTCFFQ

LRLPPYSSQSVMAERLRYAINNCRSIDMDNYMLSRNVDNTEGSDTDY*

>m.151 g.151 ORF g.151 m.151 type:5prime_partial len:2647 (+) Unigene000012:3-7943(+)

VCVCVLQISENVDSVEMDPQEHAFTRTIDEEAELEERAERERDREDGHQEQEDEEEREHE

VMTAGKIFQCFLSARDVARGRERPRTSSVGVPDESVQPEQERRNSIPLQDRQDLYTTTCN

SIIHRCAMLLMAVSPTLPQTTNEQPALCGGVTQDGSGFLIRSESLSTESRSIQSGPSYRL

TKSRSESDLSQPESDEEGYSLSGKRNADLDLAFTPKKREFAHSTPDSADWFGRHSGRERL

YSSPQSYEESDLDFSCTLHIHAVIDNIVSFISGDVGNTPGFKEPEDSMSTSPQATVVAME

QQQGRAELRLDALHQIVVLISGMEEKSSQGGAEAVAGCGPAAFHSASLLTSVRLQFLAGC

FGLGAYNGGLNGESVQLHHYQDGIQAAKKRLQMNIQTAVHKIYQQLSITLERALQSNKHH

IEAQQRLLLVTVFALSVRYQPVDVSLAISSGLLDVLSQLCGKETLLGHTLQLLHKPAGSQ

LSTALKVASTRLLQILAISTGTYADRLSPKVVQALLDLLCSQLKTLLSQAAGSVFGTNSE

SEEKTEDSTGSMKKDFRALLRKQHIAELHLGDFLVFLRRVVSSKAIQSKMASAKWTEVLL

NIAAQKCCSGIPLVGNLRTRLLALHVLEAVLPACESSVEDHQMSQVVERLFSLLSDCMWE

APVAQAKNTVHLKEKEVKLQGEAEEEEENLPVQEVIFDPEKTQCCVVENGQGLTHGSGGK

GYGLAATGISSGCYQWKFYIVKENRGNEGTCVGVSRWPVNDFNHRTTADMWLYRAYSGNL

YHNGEQTLALSSYTQGDYITCVLDMEARTVSFGKNGEEPKLAFEDVDATELYPCVMFYSS

NPGEKVKICDMQMRGTPRDLLPGDPICSPVATVLAEAVSQLVRILHRSDSWTQPINRCML

QRLKQIRGCLHDGASPTGGARLRKSRSAQSREEQDEAKDEDRDEERGRMGRELTEAQLRT

LCNLIWPVLAVIGGVDGGLRVGGRCVHKQSGRHATLLGVVKEGSMSAKVQWDEAEITISD

TPLYNLEPCEPLPFDVARFRGLTAALLLELTALASLQDDGKFSPTACSLSSSSRRHERRH

RHEPHEPEEGPGDLRVSHSLDEVRSHAELQKPPVMAEVHAVQLSYLCLGAIKSLSVLLSC

SKYAELLLIPKAVHDNGHNADCGSPSIGVSQEEAEMRSALQFLMRHMVKRAVMRSPIKRV

LGLAELERAQAMIYKLVVNSVLEEQEGGKTKQSDGECEELEGEQQFQTPVTTSPSASSST

SFMSSSLEDTTTATTPVTTATTPITDAETAPASESPGVMPLSLLRQMFSSYPTTTLLPAR

RAQTPPVSSLPTSPSDEHGRRQSFTSPESQPARTTNRTALSDPSSRLSTSPPPPAIAVPL

LEMGFSLRQIMKALEATGTRGEADAQNITVLAMWMIEHPGNEDTEERTLSSDCSGAAGGG

KSAERSFLRSPGDIPNADAAELEEGFSESPEGIDQDSASASNGPALRGRSAASRKHRFDL

AARTLLARAAGLYHSVQAHRSQVRREGSGLQQDSSSLGGVYDFNLDEELELELDQEAMEA

MFTQELASDSDILGMWIPERVCESEEREEVVVCELCDVSVSSFNQHMKKSHPGCGRSANR

QGYRSNGSYVDGWFGGECGSGNPYYLLCGSCREKYLGMKSKHKGAVSERYKGQAPDLLGK

QDSVYEEDWDMLDVDEAEKLTGEEEFELLSAPLGLTERKPVPEAVQFPDTDPLGASAAMV

TATNSMEETLLQIGCQSSVDKSTSGRMCLGEQAAGLQSSADRMVALRRVTAAAQVLVART

MVMRALSLLSVSGSSCSLAAGLEALGLTDIRTLVRLMCLAAAGRAGLSTGLASGPGALER

PRGTNKPTKPISCLAYLSTAVGCLASNSPNAAKLLVQLCTQNLISAATGMNLTTVDDPIQ

RKFLPSFLRGVAEENKLITSPNFIVTQALVALLADKGARLRPGYDKAEMEKRGPLELANA

LAACCLSSRLSSQHRQWAAQQLVRTLAAHDRDNQSRPQTFADMAGDLRKCSTIKLEAHQG

RVISFGWCNKKGLLATSGSDGTVRVWNVNKNQYTLQQTCVFNKTDFTSEECSTGLGSPGE

PSLAPVAWSVSGKLVAAAMEKVVNIWQVNGGKALLDLQPHWVSSLVWPENEAGCLWAGES

RELLLVGRIDGSLGLLEILDSSNIQRTELLHCYRKDVAVMNIAWYSEDRPFAVGYADGKL

LIGCKEALENGSVVVIDAHKESISSLRWAPGGQILLSCAKEETACLWAESGAGLGRSWVC

LQSIAHPSVVNAVAWCGLTGQGPKALNMLATCCQNGLVSVWTVPQDPTAFSQSGSSSTDA

WWETDSKSKPRFTPQWHEAAVCVFQLRGHMTPVRTIAFSPDGLALVSGGVGGLLNIWSLR

DGSVLQTVVVGSGAIQNTVWIPDVGVAVCSNRSKDVLVVNCSSEFMTANQVLATCRTALK

KQGVVGLNTAPCMRTFLERLPIMLQEQYAYEKPHVVCGEQLIHSPYMQCLASLAVGLQLD

SLLCRPAVPLHLSHCLAQPVSSSSSSSQSSSSSGPSALMHSCGSSEWAWLHCFSTTIKTA

EALARAHTFPESFNVPDLEPVAKDKMAVLMDNSKWVPGMDEQLMAWATSRPEVLISPVII

SDSLII*

>m.166 g.166 ORF g.166 m.166 type:5prime_partial len:557 (+) Unigene000013:3-1673(+)

TRSEERVSQCPPNEFECQGTDVCIHLSKVCNGISDCLDGRDEGHHCLEFVHNCTVMGCHD

SCAITPSGPKCYCGSGFEVGEDGKACRDFNECAVYGTCSQTCTNTEGSYTCSCVEGYLPQ

PDNRSCKAKNDPVDRPPYLLIANSQNIQATSLNGANPISVSTRKTTAMDFIYAEETVCWI

HVGDAPSATMLKCAKFPNAKGFNEERTINISLSLHHVEQMAIDWLTGNFYFVDDVDERIF

VCTKDGATCVILLDLELYNPKGIVLDPTMGKMFFTDYGQIPKVERCDMDGQNRTKLVDSK

IVFPHGITLDLVNRLVYWADAYLDYIEVVDYEGKNRHTIIQGLLVEHLYSLTVFENYLYA

TNSDTGNIQPKTSVIRVNRFNSSDFQVVTRVDKGGALHVYHQRRQPAVRSHACAPDQFGK

PGGCSDICLLANSHKIRTCRCRSGFSLGSDGKSCKKPEHELFLVYGKGRPGVIRGMDLHA

RVYDEHIIPIENLNNPRALDFHAETEFIYFSDAAMYTIGRQKIDGTERDIIVKSGEIFFA

VTSNVQNNGDINVLEF*

>m.164 g.164 ORF g.164 m.164 type:3prime_partial len:1618 (+) Unigene000013:5397-10253(+)

MGDKLWWADQGTDQVGTCDKKDGGSWKVLRNGTSPVMHMKIYNETVQQKGTNLCTMNNGD

CSQLCLPTSPTSRACMCTAGYNLKSGQQSCEGMGSFLLYSVHEGIRGIPLDPLDKSDALV

PVSGTSLAVGIDFHAENDTIYWVDMGLSTISRAKRDQTWREDVVTNGIGRVEGIAVDWIA

GNIYWTDQGFDMIEVARLNGSFRYVVISHGLDKPRAIAVHPEKGYLFWTEWGQYPRIERS

RLDGSERVVLVNVSISWPNGISIDYEEGLLYWCDARTDKIERINLETGGNRELVLAVHNM

DMFAVSVFENYIYWSDRTHANGSIKRGNKNNATDMVYLRKGIGVQLKDIKVFNRARQQGT

NICKDKNGGCEQLCLFRGNGARTCACAHGMLAEDGRTCRDYDGYLLYSERTILKSIHLSD

ETNLNAPIKPFEDPDHMRNVIALTFDHQGGDGKGANRIFFSDIHFGNIQQINSDGSARKT

VVENVGSVEGLAYHRGWDTLYWTSYTTSTITRHTVDQTLSGAFNRETVVSMSGDDHPRAF

ALDECQGLMFWTNWNELAPSIMRSSLAGSNVLVIVGNNIRTPNGLAIDHRSEKLYFSDAT

LDKIERCEYDGTNRFVVLKNEPVHPFGLAVYGEYIFWTDWVRRAVLRADKFGRDMKVLRA

DIPQQPMGIIAVAKDTNSCEFSPCHTNNGGCQDLCLLTSEGRVNCSCRGDRKLVEGNVCV

AENTTCHSVDEFECGNGDCINYSLTCDGRAHCKDKSDEKPSYCSNRGCKKGYRRCLNGRC

IKHSSWCDGTDDCGDRSDERPCNMTLCSASEFQCKDGSCITNTSRCNQVVDCEDASDEMN

CSPTDCHSFYLLGVKGVIFQKCEFTTLCYTPIWQCDGSNDCGDFSDERNCPEKRQLKCPV

NFFACPSGRCIPMSWTCDKENDCENGTDETHCDKFCSPTQFECGNHHCISSSWVCDGTDD

CGDGTDEGSRCSSKTCSPEAFQCPGSHCVPQRWKCDGDNDCPDGADEGVQAGCTNNKTCD

DTEFQCQNKQCIPKHFVCDHDLDCRDGSDESPECEYPTCGPNDFRCENGQCLKQKSWECD

GEFDCRDHSDEAPKNLHCTEPEKRCNDSAYLCNNGKCVSEDSLCDWKDDCGDGSDEHNCF

VNECLNNKLSGCTQHCEDLKIGYKCRCDPGFRLKNDGKTCVDVDECTTTYPCSQRCFNSY

GSFHCFCIEGYVAYANDWTSCKSSSEEEAYLIFANRYYLRKLNLDGSNYTLIKTDLNNAV

ALDYHYAEQMIYWTDVTTQGSMIRRMLMNGSNVEVLHRTSLSNPDGLAVDWVGGNLYWCD

KGRDTIEVSKLNGAYRSMLVNSGLREPRAVAVDVRNGYLYWSDWGDVPHIGRIGMDGTDR

SIIIKDKITWPNGLTLDFINDRIYWADAREDYIAFASLDGTNRHIVLNQDIPHIFAMSLF

EDYIYWTDWETKSINRAHKTLGTNKTMLISTLHRPMDIHIYHPYRQPAVENHPCQVDNGG

CSNLCLLSPGGGYKCACPTNFYLAADGKQCLSNCTASQFVCKNDKCIPFWWKCDTEDDCG

DRSDEPADCPEFKCRPGQFQCGTGICTNPAYICDGDNDCQDNSDEANCDIHVCLPSQF

>m.165 g.165 ORF g.165 m.165 type:complete len:1225 (+) Unigene000013:1768-5442(+)

MDISVCSSVTNTVHTYLIIGIHTVEGIAVDWMGQNLYWTDDGPEKTISVAKLEKASQTRK

TLLEGKMTHPRAIVVDPSHGMMYWSDWQEDSRDANRGSIRKAWMDGSKSGILITSKNMLW

PNGLSLDIEQNVLYWVDAYYDRIEMVLLNTTERRTVYEGQELNHPFGLCHYKNFLFWNEY

RIGGIYKLDMTSKKVTLLRSERPPIYEIRTYDAQQQQIPGHNPCRGNNGGCSSLCLLTPA

GRTCACADDQILDFDNKTCKANPSYVPPPQCQPGEFACKNNRCIQERWKCDGDNDCLDNS

DEVPELCHQHTCPADRFKCQNNRCIPMRWLCDGDNDCGSNEDESNTTCSARTCPPNQHPC

ASGRCIPKTWICDLDDDCGDRSDEPASCAYPTCFPQTQFTCANGRCINVNWRCDNDNDCG

DNSDEAGCSHSCSSVQFKCNSGRCIPEYWTCDGDNDCGDNSDEKSANCTNQATRPPGGCH

SDEFQCRLDGLCIPMRWRCDGENDCIDDTDEKHCEGVTHTCNPSAQFACRDSARCISKAW

VCDGDSDCEDNSDEDNCEALLCKLSHHTCAQNDSICLPAEKLCDGKDDCPDGSDEKLCDL

CSLENGGCSHNCTIAPGEGILCSCPTGMELGSDNKTCQIQSFCAKHLKCSQRCIQEKATV

KCACYEGWALEPDNESCKSTDPFKPFIIFSNRHEIRRIDLYKGEFSVLVPSLRNTIALDF

HLNQSSLYWTDVVEDKIYRGKLSENGALTSFEVVIQYGLATPEGLAVDWIAGNIYWVESN

LDQIEVAKLDGTMRTTLLAGEVEHPRAIALDPRDGILFWTDWDASSPRIEAASMSGDGRR

TIHRETGSGGWPNGLTVDYLERRILWIDARSDAIYSAAYDGSGLIEVLRGHEYLSHPFAV

TMYGGEVYWTDWRTNTLAKANKWTGHNVTVVQRTNTQPFDLQVYHPSRQPQAPNPCAAND

GRGPCSHLCLINYNQTFSCACPHLMKLQADKHTCYESRQFLLYARQIEIRGVDIDNPYYN

YIISFTVPVIDNVTAVDYDAQEQRIYWSDVRTQMIKRAFINGTGVETVVSADVPNAQGLA

VDWVSRNLFWTSYDTSKKQINVARLDGSFKNSVVHGLDKPHCLVVHPMLGKLYWTDGDNV

SMANTDGSNQSLLFTSQKGPVGLSIDFDEGKLYWISSGNSTINRCNLDGTGLEVIESVKG

NSPEPPLSPSWGTSCGGRTRAQTR*

>m.185 g.185 ORF g.185 m.185 type:5prime_partial len:3241 (+) Unigene000014:1-9723(+)

TSRSEELSEELRDSMEAAHQAELSQVQTQHTLELEALRLSLTNMHTAQLELTQSNLQKEK

EAALSELQASLREKWAQESAMLQTRQQFELEKFREQGREQERQTELQHQLAVDDLNCVWE

RRLSEERAALEQQLAGGIEDAKIRWIQDFKKEQAELQEALNKAEDRLAETKRKLEELQRS

KEEEIKHLESELSQAWADRDSAARAVEELVASHRVVLQEQQAHTQELEEKEKWLKQEVDR

LLEQKSSSEQEITNLWSQLESMRTSRQELGELKEQLLARKSHVNDIEQLKQDFSQQRREL

QEQNETELENLRVYFEQRLRASEENHREEIALLQLRLVEGALEDSVLKTGEESFLSDGKS

EEEKGDSLAEITEQLDKHKVELDALRLQLEEKHKQDLEQLRCNMALSYREELLQARTELT

DRYYSDMEQLKTKHALEIEQLRAKLSDSHLKEITRLRLQSVADVARQVETELEEKSRLQT

QEHQARLAQLASDTESILSLEKKLAALTLQHSRELERATEQHAEELRQLEERLKKNFSEE

LHTRLAEAQQEERTHVTEELLNRSSEERLQLREQLQAQAEERVASLREELQQAASKDRQE

LEQKLVQAEELLAEEKQRLQALQVSLEREESPQLLALKQRLESQYAGELHTAKSTMAAEV

KELNTLLQEQTESRLKEATCRHQEEQRQLAEKLVQQKEEALQKLRQQHAQELQVQETLLE

QNVSELQHLQKEYQELKSRHQEELDLLSSNHKTALDSLAEKHKAELDKLQVVFQETNLAQ

LEAQEAELEERHKQEMEELETRMLCNMDTLESTYLKEIQAVREEKEAALRDLGASLEQQQ

AEEIERLKQEELIVREELRKKLAQVHMDKFSVMATELSHAHQAELSAALASQKAALESEH

HSALETLRQHVLDLEAQHGAALHEVTDMTTKENQQLKKQLDALNAQHQQQLQELRATSSR

EIEALRRELEEEASRQRLHFLEEAELLKCQSEEQLQKKLMELKEESDREKVSALEELERS

LCEQHQQNELSYTEKMNHLTTQLQQLDTVVSQLRAEVSGLQGELEGKRSEMDTLETLLQR

RERESQEGANLLAMLRDDLNTATQQRQDLQLTHDRLQRLVLEMLRITIATEEEISRRLGI

SMEGDDITLNRKQSVNRDPQKTDAEVGDGAAAAVEASANCSMFSSLMDEGLELSQRLCES

LFTGPDGELDPEREELVLGACTHLRVAVDKLLEVVSDSTVQLEQLQALQALLDEKFSAGG

EAKNALFLQHTQLLEQLDQEASVKSQLQLELHKADGLIEGYVVEKAALEDALQQKESREQ

KLVKELESTRAQLQELGEEHALLQRQRDALSASLGEPEKALLAEAERLGQERVDVQRQAE

KDRVGLAGRLRLLEQTLEEHESRTQQQEEQHRTQTEDLQQHISALEKQLKHHRQFIDEQA

VEREHERDEFQQEIKNLEAQLKTTSKGHAGEDTKGQRVESLQALIKDKTEDYSVLLSLKE

QCQRDLEERNEEIDKMASRIRELEQALLSSVEERRAAAQLEQELQKARKNCQELSQDKEA

LQQQLYSNKLQISALQSKLDETRHRYPDPGADPNLRERLETLQQDLQNKEEQVEVLLERV

EVLEKALAVKEEDVRQLSLQLELKTTESRAREEELRLSVAELQDTASLLRRQRQEEADAS

ALQLPSALLEEKNLEIDHLNQQLLHLQQELDASKENKTEEKQADVEDLRAQVERLHGDQE

RLHQAKEEEVEQLHEIIHKLQEELSQLNPNRHEVSDPNTDSPEPPDFPWSPRPQHGAEES

LCHELSSQTLQSCRTKICELQADLERSVEEKVALERLLLMQEEQYGQQLEALGCSLGEGK

RKLALLEQEAGELKLQLDQKETKVEELQARIQTLEDGERSVNDLELRLIKAEETMKETQQ

EMTRLNDKARKHTEERERTESLIAELQQRYEDSERTATQARLQVAELKETIQQRKSDIAI

LETGKTELYTEKQALQKRERKLQEEIEKLKQEVSAKSLQIQEVSLQLEERAADPDQSQEE

VLTCAEETLAKAAAALREKEEQLAHLRSQHDALRAELAAVKEGLSSSTERAEKLQEEGQT

KDRALADLEEHNQRLRAELRGLQDDLEVQEEELAYQQRELDELRQRCRLQEHTELDCTKP

ERRFLDGLSHNSTLSSPEVLRRLDCSEERPSHVHASHLSELSGLRNTSLELMSKHSLMER

NELKRPLVLPPETDPPSTHSPSASTHSLSISENLSVLNSLDDDKVQVLENLDDVTPSRSP

LSSTSPVSAPEWASDGYGSNVSSELEAKLKNELEHMERLDAHFVEYLRSRDMAPAPCSDS

AAGSLHHTYELLSPELQAMLNRVYRESYRVLSLSHRPPPAGQPHSDAEEAPPASWQRERR

ALQETVLSLRELLCRMAEREPKVEGEVDWRTELLQAVRSVFDSERKWLHAQLQDIITTNT

GIDYTSLLKDLETLLQKQEDQQRRSLEQLLSADRNSLLAEIRSLHKQLHTCTLQNQEQLQ

RSLGAVREEGAQTQQQLHRQVGELERQLQQEQSSSHDLQRSLGQEQSRSTNLHQQLEIQQ

KLAVEMKLELEEGTRQLRASEKSREELQAQIHKLRLKLESEEQEHQACVEAAKRDEVRVK

QLQEELHHERLSSKRAAEEHAHMQESLRASLCEHATRLNELNAALEQERVAVSNLRAELQ

IEQSRCEALLAQERERTELTLSRLDEERSRCAELSRSLSHQAQEHARRLEEEVRSQQAAS

ADDRKFIQELRAQLEQERRQAEDLAATVDNLQSQVLQSKRRQEEAAQQEAQRGQEEVKRL

RSALDGLQAQRVEVNRTLETERHRAAQLQTELDAVKEKMREVKEKERAKEEQREKQRWCE

KQEQEDRERRQEQTKEKLLELESLRERDQQRLRQLQQTLADLEQQEKQLTSERLQRDCVA

HTQDSSSSSSLLERVLKENSELAERLAALSEEKISLKHTVSCLERDLHNLRCTQQQSWHK

ERMFSAPIASQKAEEEFTDSRNRNRPITDLSNNKVQRLYEKYLRAESYRKSLVYQKRYLL

LLLGGFQDCEQATLALIARMGARPSLITQTSPPISRFRSAVRAVIAVSRLKFLTRKWQRA

TRKSSGGGVIVNSAAIKTSILKPQHSGVAAHQSPPTRDGILSQRTALSPLVPPLKSPFRL

HNGGYANSTAAPSERTLTPSQDQERSLADYIHHLESVQQRLGAVRPGSPATFSFLRKNDR

*

>m.199 g.199 ORF g.199 m.199 type:3prime_partial len:1648 (+) Unigene000015:4859-9805(+)

MTAGSSKALRNLEQALQLATRFASAHEDLSQWLDSMEAELNNMEPDTTPAYQERQKDLKC

VSAEKRLILDTVNEVGSALLDLVPWRAREGLDRLVADANQRYRQADETITQRVQLVQAAI

QRSQQYEEAVDAELAWVGETERKLTSLGPLSLEPDVTVAQLQVQRAFNIDIIRHKDTVDQ

LLHTREDILESCSDQQREALKVKTDSLSMRYEAVSQNHAERFSALEQAQVLVARFWETYE

ELDPWLGETETLISQLPPPAIDTEALRQQQDQMRMLRESIAEHKPHIDKLLKIGPQLAEL

SSQEGATLRQRYSEAERRYLAIKEDVKGRAAVLDEAFSQSAQFHDKMDPLLETLEGAVQR

LRQPPPVAAEVEKIREQLAEHRAAGLELDKLLPSFSTLCARGEELITRAAHDDPAAQAVR

SRLLRLRSLWDEIRQRAEEREGKLQDVLDLAGKFWADMAALLSTLRDSQEIVKELEDPGL

DPSLIKQQIEAAEAIKAETDGLREELEIVRTLGADLIFACGETEKPEVKKTIDEMNAAWE

GLNRTWRERMEKLEEAMTASVQYQDALQGMFDYLDNAVIKLCDMQAVGTDLSTVKQQIEE

LKQFKVEVYQQQIDMEKLCHQGELLLKKVSDQADRDMIQEPLTELRHLWDNLGDKITVRQ

HKLEGALLALGQFQHALSELQSWLSHTHATLDTQRPVNSDPKAIEIELAKHHVLRNDVLS

HRATVETVNKAGSELLESSPGDEASHLRDQLDELNRSWDSLLLKTDERQKLLETALQQAE

GFHGELEEFLQWLRRTESQLSAAKPTGGLPETAREQLQQHMELQAQLTQRGEQYHRLLDQ

GESMLLARGAEENSPGTTQTQQNLALLQNKWASLNAKMDDRRAKLEEAVSLATGFQSSLQ

DTINWLTQAEQTLNMAQSPSLILDTVLFQIDEHKVFVNEVNTHREQVLALEKAGSQLRFA

SLKQDVVLIKNLLLSVQARWDKLVQRSLDRGRHLDEARKRAKQFHEAWRKLTDWLEEAEK

RLDAELEISNEPDKIKVQLTKHKEFQKTLGSKQPVYDTTVRSGKAMRDKATLPADTQKLD

NLLGEVRDKWDTVCGKSVERQHKLEEALLFSGQFAEALQALVDWLYRVEPQLAEDQPVHG

DLDLVSNLMDSHKAFQKELGKRTSNIQALKRSARELMETGRDDTAWVKVQLQELSNRWET

ICALSVSKQTRLQQALKQAEEFRTAVQMLLEWLSEAEQTLRFRGILPEEVETLQVLLHTH

RNFMQTVEEKRVDVNKAAGMGEAILAVCHPDCITTIKHWITIIRARFEEVLTWAKQHEQR

LEAALAELLNNATLLEDLLSWLQWAETTLVQRDTEPLPQDITQLKTLITEHQVFMEEMTR

KQPDVDKVTKTYKRKPSETSSSLAERRGVRKQQQQQQQPAMQVSGGNPRLNQLCSRWQQV

WLLALDRQRKLHDGLDRLEELKEFANFDFDVWRKKYMRWMNHKKSRVMDFFRRIDKDQDG

KITRQEFIDGILASKFPTSRLEMTAVADIFDRDGDGYIDYYEFVAALHPNKDAYKPTTDA

DKIEDEVTRQVAQCKCAKRFQVEQIGENKYRFFLGNQFGDSQQLRLVRILRSTVMVRVGG

GWMALDEFLVKNDPCRGKAYLHKHTIIH

>m.200 g.200 ORF g.200 m.200 type:5prime_partial len:1545 (+) Unigene000015:2-4636(+)

ELDRKSVDTLTECVSQRAKQLQTAAAQSVSVQEGLKALVSWLDGLALDPEPVQPTTQAVQ

EALAQNQKLRQELLSRQGSVEATRDSVSKLLKSADALTVSGLQGALQDLNQRYTAAQTKQ

TENEAELRAVLPKLESFERLNSDLHNFVQSRERVLSLGGLPDRSVEDYKHSIQEVQSDLG

QETSHLKTFVELSTDLSKSKLFVNAHSLVDSSKEVSDEFARLEASLNERLNAIQSCDQQL

VQFRSHSDSILRWLQMAQEQLPAKEPQQSTDSLQRRVQQLKDLLSEWESRGNQIQELNKN

SSELESRIISITAPQSKTSAPMLNGAGGSSSLNGIHTCRDLTEIQVVVADVNSRYEQLGG

DLRNRQSKQQAMLELCQKARQDSEALHQWLALREQRLAQGQSASPSRPDVARAQAQENKA

LLSELTEHSGKVEDLKNTLKQLIQENPDSPEAESWKQQLKDLDIRWEKASQVAAQRQAEL

ETCADRLGNFASAANQLGPWLREKELMMSVLGPLSIDPNMLNTQKQQVQFMLREFETRHA

QFDQLTQAAEGILSPVEEQGSGDNQDVAEVKKELASITQQWEDLTSRLSGRFDQIERAQG

TSESFQSLLKELSQSLSQLAEKLDAQASLSAQPEALRHRLKETGEIRAELEQRKGQLAQA

EQLCEDLSAIVVEPYLREELHKRLESVSGPLNNLEERAADGLSQLQAALSSTQQFQQMFE

ELRSWLDGQAAAPSMPDPLPCQPEALQVLVAQQEELQRCVAQQRGSYDLLQAEGASILAS

LPAGDERSALQAQLTKLRQDWEEVNQRLTEKQAHIKETLGRAVLFRQHRDELDPWLKECE

QKEAEIQPSLEPAALEEALQKAKQLGLDLDRRRPLLEILNSAADQLLEQSRTGEEEVRDE

KAKLNRRLDGLSERLHGRMAQLEELNSRLKEFEDGRLAVERRLEAARHQLEVQEALGPQA

CSNKSLERLRSQQDTLGSLQPQVVYLHNLAKGLAQDAPQMTGGSGDGGQKLLQQAQTTEK

EFGEVTEKIQQCCSSLESRLQGVGEVQSHVRDIFSRLADLDDELDSLSPVGRDVDSLASQ

ADAIRGFLGRLASLRAELEGHGSACTTMLKREGSSPDLLAIRRETEALNRQAAKLAERGQ

GRLALIEEAEGRVKEFYARLMELQRLLDQAEEGLNVQSVVGTEVDVIKRQLQEFKAVERE

QVDSIQPKLHHVNAVGQGLIQSAAKHTDTQALEHDLETTNLQWNSLNKRVAERIAQLQEA

LLHCGKFQDALEPLLSWLSDTEELIANQKPPSAEYRVVKAQIQEQKLLQRLLDDRLGTVE

MIRAEGERIAATAETQDREKIQRQLQCLGERWTDLLEKANARQRQLEELQVLALQFHESV

DPLGEWLSATERRLSSAEPMGTQASKISQQINRHKALQDEVLAREKEVDHLEALGQSLSP

LSCAADRDWLSERVGAVRSGHTELRNWCFRRAAMLEQALANAQLFGEEEVEVLNWLAEVA

QRLSDVSVQSYQPELLAEQHKYTLVGHRRQFTDAVDLATFKRRH*

>m.211 g.211 ORF g.211 m.211 type:complete len:1609 (+) Unigene000016:4111-8937(+)

MGNSESQFRTQGSRSSSFTLSNRQEPYPVRCRTFKEDSLTPHCRWRTGSSLKSKAEKRCG

LSQHRANQSSLLRHCDYITKGGEGKRNISAQWNNGHFEENRRDCNGHALNGYKTPERKPK

LEDLDDHSSPKVVIKSDGSVRVEFSHMSKNSTLPDENGGSVQLLKFSPTSHSVSTPESAL

VQNSPINRTSKRNSLSSEGSWYDSPWGPGIELNYDGVCALPNRLSETLPSVRIEYFDEPC

IPHLLYRDPVMAATFSTAEDLKLANELSFKHRTPFLHVMEEPVLAESSGARQYSSYTLPC

PQTKPTAENSGKKEIIKNRMRRFSDWTGSLTRRKKKSKELRYKDSIDCFDSGIDGLTADT

SSPSQVSSLLGYHKVQCRGRSQSQVLPSCSGGSFSALHPATDGLRQNIYDNFMRELETSQ

TTTGSGERRGENSAEDTESSSQETVGSLEQLDLLCEKEQDVVRRAGWLTFKPLLTLHKDR

KLELVARRKWKQYWVTLKGCTLLFYETYGKGSPEQEASPSYALLAEDGIVQAVPEHPKKE

NVFCLSNAFGDVYLFQATSQTDLENWVTAIHSASASLFAKRHGKEDTPLLLRGQIRSLLQ

KIDMDGKMKKMAELQMSIVTDSKSRKAIENQIQQWDQNLERFNIDLFRMRCYLSSLQGGE

LPNPKSLLATASRPSKAVLGRLGVFSVSSFHALICSRDEATLRKRSLSLPRRSRGKRGMF

SSHKALDSLTKRSRDRRPCTSQVFDGAAGDHSGLVPSYSRERPDAPADTYSQAPPDGSQW

ERSVTERLVWVYMPDHQVVTVTLSGQHTVEELLNTVCKMQNLDPSAHCLRLRRCVSDRME

VWTPGPHKLFQDLLYDELEVSALNVYRLHMTKPTYAGDFGFAISGSVDSLNQSRIFVTQV

FSGGIAFSEGLRPGDEILVLNGCQVSRLDLELIQTLFSENTLHLTIRREPTAMTHTHTGQ

QPMRDLLYRHHRAKSATDVSCVSEGKAGVETRQHHRLSLRAVQHSKSAETVFALYHSCQD

SSVRLMEVQTETAPPGGTLLRACPRHMSVSERLRKVVQELVDTEKSYVKDLSCLLEIYLK

PLQKETFLTQDEMESLFGSLPEMLNFQKVFLQTLEERIASTPDFGTLQTPVQFRKLLFSL

GGSFLYYADHFKLYSGFCANHIKVQKVLERAKTDRAFKDFLDARNPTEQHSTTLESYLIK

PVQRVLKYPLLLRELVSLTDTDSEEHYHLTEALKAMEKVASHINEMQKIHEDYGAVFDQL

VAEQSGSEREVTEISIGEFLTHSSVMWLNPFPSLGRMKKEPELTVFVFKKAVILIYRENK

QLKKKTGDQDLVKFRWLIPRSALQVRLGNTADSNCMWDLIHTKSQLEGRPETLFQLCSSS

PESRVKTLKTIRAILRENTRKGALGSERPAERSSKEQLAPLTGSLPSSAKFGSSRAFWLV

QKPDSASESSGDPPTAPSSEAPPTNSTLGRERMRESDILSDDGWFSERSATESMESRFYQ

LRLSDETRVEPEGGEINAHETQPQLRRAHFSAARRRLARSDTRRSQAALLSLRQNSCSLD

GQTDTAVVSADLNMLLERDFSVHSLTSVINEDCFYQNTTTRSPTTTLS*

>m.212 g.212 ORF g.212 m.212 type:complete len:657 (+) Unigene000016:168-2138(+)

MEAVKAFNGELYSLNEYKPPISKAKMTQITKSAIKAIKFYKHVVQSVEKFIQKCKPEYKV

PGLYVIDSIVRQSRHQFGQEKDVFAPRFSKNIISTFQNLYRCPSDDKSKIVRVLNLWQKN

SVFKSDIIQPLLDMAAGIPPPSITPVSASTALPVNNATPGTPATPATPANIMPSLPDWAS

QISNSDTVAAVAQILQSPQGQQLQQLVQSLQMQQQKPQPSLLQALDAGLVVQLQALTAQL

TAAAAANTLPLEQRVSSFNKKLLGQFDFVGESEPSEDSKKDTAASQLPMVSESINNSLFH

QLAEQLQQQNLEQFQKQILEHQQKSMTLESQENIFCPENSAPPSQSSVQSQLQEQDVKLD

DSIDNQQQDMDLDEGQDTVDEDMFESDEKKTSGTRSRTHSRSRSRSPKRRRSRSRSGSRK

RKHRKRSRSRSRDRKRKSSRSYSSERRAREREKERQKKGLPPIRSRFLSVCSTTLWVGQV

DKKATQQDLTNLFEEFGQIESINMIPPRGCAYICMVHRQDAYRARQKLSTGSYKIGAKGI

KIAWALNKGVKQEYKQFWDVDLGVTYIPWEKVKLDDLDGFAEGGMIDQETVNSEWESQKN

VEAPKDLQVQQVSAESSAASNSQNETYTQPVAMMPVQVFVHLLLLGGCCYFYNLQY*

>m.213 g.213 ORF g.213 m.213 type:complete len:587 (+) Unigene000016:2234-3994(+)

MAMPPPGFGPPPPFLRAGFNTAQPPPGFMAPSGVAPSAVGAALTSGPTAHNQDGNKDSTF

GAILPPSSTIPASFMPSGMPGAVFNSVGAQAQQASTDKTSVSGESVDTPNELTLQGMQNA

VRSGMGLLGMHPSAALSHPLSGQRMPGLLPIDMRPNLLQGAGARFSMLMQQGLAQQANSL

LDASLQTRARAPFPQMEHFNCADAPFVRAPNPAAPAPDSIDKSSNNITATATDSRSQQED

QDYRFPPPEKQSTGLLRTPPPEMRTEPTAVRAPLLDRPLRAGMHMEGREGPGRREPMPAP

FRAETRWGAPRGDLDERDMRGMSAGPPKSFPEDPGNPNFQNRFEMRGGGAMGGAGPGWTR

GGGGGGPFNMNMHQDFDDRRRPWERQKDRDDRDFRRDINDNRHRDRDRERERDRERGRDR

NRERERDRDRDNDKERERERGGWAPVQPLLPLPRPSQPLLPLPTPLLNLPPTQPESHPKP

QPASRPESQPDGKQPQPEKQNSGEREKTHVFSDIAHGPDTEHETVTEPTAESSRSSSPAP

TAELQHQTQTPEVSASTGGRQTPLKEEAETGTEPEPELVKTDTEET*

>m.224 g.224 ORF g.224 m.224 type:5prime_partial len:2735 (+) Unigene000017:2-8206(+)

PCVLKDTELIALDLKELQVQAASSDLGKHLPEVVDLLQKQELLDTKIISLGESLSAISSN

ALRGKHRDVSQVQRTVKELNSQYTSLLALSQNRRRALEGQLKLFEFFHDCEEMEGWIYEK

WLLVQAASLGRDLSQIEQAIQNHKMLEAEVQSQESLCSKVLSRGQELGRGGHPNESDILK

WIRTLQKQWQQLRDQVTNRKNRLHAASVIKQYFADAAEANSWLNDHKPFLTDENYGKDEM

STISLLQRYQRLEKEIGAYASEVKRLSEQSQSAAQLAPLTTEPQQTIMVHYSDSSGDERE

TVKEKTGTSVKIRAPSKTLPQMQELNAKIRFKYRGMKLTWDRGEMVTIISKEKEDKMLVR

DSKGNEQLVSSTYITELSSTQAPPAPTDVVPESQIRKVSRPRRTRSMRRGTAEIITTLLP

DPQFQKDMIENTQSSLEKDYNSLCQLLQYKRRILDETAQLHRFYNGCEEFESWMEDKENI

LTTFSSNSENTEQARYENFLTELASGKGQLDDITKLGDELVKKQHSKKREIRSRYAQVTQ

RWDHIQELKDEKAQELLKSSDIKSFLHTCEYAKSQLLEKLHQLDTSGVGSSASALQAEEQ

SQTQAEREIEGLERKIEYLKSVSKTKRDCSPAESAAIMKEVYALEELLCQVKGQAAKRQN

LLEQARHLQLFRKESRDLLLWAEEVEERLLEEGNSYNVESAQGLLNDNQELKLEIEQQRA

RLKSIERLAKSLEASSGEKFTKNIQQTLDQLNQEWSRLDELWSNQNRRLEQALQLQKWNR

EADRIDATIAGHEARLKVNDLGDSVDGVYSLLGRQEELEGLLVALDQNISFLQEKSQELI

NKNHFASKQHRQRSWAIEERNQKLKDSCKKRNHDLFASKSYQEFYRDAEELQIWMDEKFK

IAEDESYRDPNNILRKLKRHEAAEKEMQANHIRRDRLIQAGEEMVSKDHYNSQSIRSKTR

QIQSRWSELETKMAERGDKLRQAGQQEQLMELLQDAKVKIEKIQKMLQHAPKGYDLRSSR

LLLKEHKQVEQEAQELADKINSIITHAKHLATNHFDSQRILHETDMYLKLFKSLEKPLNH

RRDQLEEEVTLYSFFYDVELELSWIAEHEPKADTSSYTKSLAGAISLLQKHKELQAEVNA

HRQYFQRVLDRGRAMGKREVYQRCRDLSAAWEKLEDACEKRFSHLSKAVMREQVLLDCAE

LEVCLSETSALVSTDYGKNELATQSLIKQHHIVEGQIEVLSAQVDELKSRVDQAVHTWGL

EEVTRNYNHIRTQLTELQHLAALREQKLQDALHLHEFKRESSELEEWISQQQLIASSDDY

GSDYERVLHMQRRFAVFLKQLDVGRERMRSCQELAQRLIKSNHPESRFIQETLGLLRESW

NELQHLANSRKENLQKSEECYKVYSDLTDALEQTEDRYKCIPEDIAKDLKGVELQLRKHE

AQVNELVGNELQMQELLDIADSVLDECSLGLREKIQERQQLLVEQWEKLRMAMEQREHEL

RLAKQRYIFLKRVQDYSLWCSQILSGIKAEESIRDMATCDLQLFQHQQLWAEIVAHEETY

SQAVTIGQHLHEQDVSNPQEVQDKLKALKDERERLTANWESKQKWLENTYQEQVFYRDIE

HMERLTYSQEVQLKNSDLGSTVDETDTLIKRHEAFEKLLSAQEEKMIALQKSVEHLNKGE

VTKGKGNYKNTLNSLRARRDRIRDLSMKRREDLELSRLFCIFTRDSSEAEDWVSERMQKM

QEDSKMDLSNFQAKMKLLQKHQVFEAEILAHGKIINSVLETGEELISLRHSKSREIKQSI

SNLISHWEALKEAISTRGKVLEGHRDFLEFLQKVEQVEVWIRQKEVMINVGDLGKDYEHC

LELLRKLSEFRSSSPGDMTVDDAHIKTINNLAARLEKQNCDEMITVKKRRQQLNERWNSF

HGNLTSYKRKLKEAKEVHSLIRDLEEVRDRASEKMLLLQGQGYGEDVESVENLIRRHEEM

EREVRVIHERRAVLDQETKIQLRTHSDLSDKLSKKQQEINNTLIKLDKAMKLRKEHLQES

HQLQLFKGNQHLLLDWTIKQTTEMGKKRLPKSKAQAESLIVEHHDWKVEIDARGERIDSV

KNFGQSLVKSGHSKTAEIKKALSKLEDAKVSLIQAWEERKITLDQALELQIFLGNVDQSE

SLLSNLEAFLANDDVGSSLSDVEALQRKHALFENSLEAQIQEVEEVERYAQQLIQRQHYD

SDNISKRSKAIQLRKNRLLETSKARQKALAESVQLQKFLEDSYEVCLWLNEKKLVAQDES

WREPINLQAKMQKHQSFEAEIIANRNSVEVLTKEGEKMLLTGHPAKDKIKSRIMDLNEGW

EQLLIRCKEKKSRLQEAYQALQFLHSLDDVESWLDSVEAELSQTDCGEDLASVSRLLKAL

EGLEEVMDGHLEKVQNLVDTAKSLSSQGNFQAREIQQQVWHMANRYNSLAEPLQARRETL

ESWQLLFQFYQDIDDEMIWIQDKLQATALNDCGTSFESTQAVIKKHQVVIQEITARTPLV

QAVLEAGHNLVKGRHFASQEIYARLEELKDNHETLKKESENKSQLLKEALKIQTFLSEVS

DIELWMEELRPTLESTDYGKTEDATEALLRKLDAVDLELVNHHRKVKSLQETGAEMEKCG

HPNSYLVSKSLTTMKKQYETLLQLSSDYRISLEDQYNLYVFEREARDLKNWLISNQILAE

SDDFGQDLEDVEVPLHVIIIYKMSTVLRDLAEKI*

>m.225 g.225 ORF g.225 m.225 type:3prime_partial len:271 (+) Unigene000017:8445-9260(+)

MLDSEDRKDDLISVKACIRQHEGLERDLVVIEEELLRIKKEGRSLVRRCPQVRDSLSERM

QEVEENWDSLLEKACQCKHRLQQVQVVQKYLTDWRELMTWLRESLSLVTGEGLRGEVKDL

SQLIKRHDEYHKQIERQLDKSEIVKNEGRRLMQEGNFMIEKLEKHLEELQDLEVLVLQGW

ADRKVLYQEELELQQLQRELEQAEHWLNTYENVLTAQDYGDSVSDVLELMKKQEDLEAMI

QAQSDRFNTLHNRKTQVKKQEAKLGSQVLVR

>m.229 g.229 ORF g.229 m.229 type:5prime_partial len:2773 (-) Unigene000018:928-9246(-)

GGKEPWLERLYHSCQRLDKREGAVVPRSLLKTEAVTWQWAVWEAAQFTVLSKLRTPLGRA

QDTFQTIEGMIRSLAAHSLNSDQELSQWSGGESDEGHHTNQLRLALLLQFLENLEKLMYN

AYEGCANALTAPPKGIRTFFYTNRQTCQDWLTRIRLALMRVGLMSGQPAVTVRHGFDLLT

EISSAQGPEMEVPITMLVEALCELRCPEAIQGLAAWSLAHMGKSVAWVASVALQAEGKFE

KAALEYQEQLCAVTGVDCSIKGCNTPLLKLTNTPTGSSSSPKHTGNGDVKKTVLLKSSEC

SPEVLNFLANKACECYVALSDWASVQEWQSSMMALKKNSNSSASVTLKTDFNYIKALSRF

EEGDFAECRAQLELLPGEDYGLLNTKDKLDLKRLLPAVLSPDPSELQKAIEVQLLRSAVG

AITSNPEQDQKVPPSSDTLVKYLKQTSRIALGPLRLSTLTLCDSLPTLGTLQLHCSSALE

TSLCSQHPSEECLIPLYSDALSTCKQQDVQPWLHALRYSTFQRQLFLKLRGSSGPVESHL

VELCLTAVKFARKQGNIALASRLLAQCNKPSTEDSDTSDLVQSFRLLSLEGAVAEKWGPE

LKIERAKVLFTAGQSVAAMEMLSSCALSYCHSGKCERAACRSILTLCKWLLADWKELTPQ

LKQVVKRNSSGAPSNLSKNIAALLELPVEEQSILRITTETTVSVGVGEADFVLGQLYQLS

ASQAPEVAKSWAALASWAYRWGRKVVDNASQGEGVPLLPGEKKEIEELLPSGTTDEDKEA

IFSILGQAMCRPAGIQDEDMALQNEEDDDDMVDVIWRQLLSSCPWLAEVEDGVTEGLIRV

WRRVVDRIFSLYRVSCRAYFTFLKLNAGQTPIDEDDPKLLLNPQSSKQSSDDMIVMATLR

LLRLLVKHAGELREGLELGLASTPTAPWRGIIPQLFSRLNHPEAYIRQSICSLLCRVAQD

SPHLILYPAIVGLISLGAEAQTTGIKLPSALPTLLGTMPGEPLCVGESEGGSPPASQESG

RGDELGLCSVEDQAMMQDCYSKIVDTLSSANPTMVQQVQLLVGELRRVTVLWDELWLGVL

QQQHMHVLRRIQQLEDEVKRVQNNNTLRKEEKVAIMREKHSALMRPVVFALDHARSITAA

AAETPHETWFQETYGEGINNALERLKNPQNPANPANSWVPFKQIMLSLQQRAQKRASYLL

RLEEISPRLGSMTHTEMALPGEVSATDAITIHNVGNTITILPTKTKPKKLYFLGSDGKNY

PYLFKGLEDLHLDERIMQFLSIVNTMFTKVNQQESPRFHARHYSVTPLGTRSGLIQWVDG

ATPLFGLYKRWQQREAVLQAQKAQDSIQQPQNPPMVPRPSELYYSKIGPALKAVGLSLDV

SRRDWPLSVMRDVLRELMEATPPNLLAKELWCSCTTPSEWWRVTQSYARSTAVMSMVGYI

IGLGDRHLDNVLIDMTTGEVVHIDYNVCFEKGKSLRVPEKVPFRMTHNIETALGVTGVEG

IFRLSCEQVIQMMRRGRETLLTLLEAFVYDPLVDWTAGGEVGFAGAVYGGGGQQAESKQS

KREMERDITRSLFSSRVAEIKVNWFKNRDEMQAVLPQVEMAVEEYLNLQEQLSQVDKAQG

KLLEEMEFLEGADSRVDHSIHTLEHRYSEHTQLQSRQRTVQDDIQSKLSDLDQWISQYQA

AFGSLEATQLASLLQEISSPIDLGPPSYVPATAFLQNAGQAHLISQCEALEAEVSALLQQ

RRSILRACLEHLHSYATVALLYPRAVLHRHRVYTWKQWMEELMCDMTVENCQAIYHHYEM

QFAPQPPTATCQFLSSVELALQHHAAETNTRLLRQMERLKAEGASVPVCEEQLQEIERCI

KVFLHEDAELASFSLAGIIISALCTLTRRNLVMEGAAASAGEQLVELTSRDGAWFLEELC

SMSGNVTCLVQLLKECQLQSHELDILCLEDTSQAVYLANGVYTCLQELNTNFRQIIFPEA

LRCMLKGESTLESMLSELDSLIEQCADGVSLQGLAEALQTHLRNASMGLQEDVDAHYLQV

TRVLRVQYSELIQPRNMEGSIQDTPKMSAGQMLLVAFDGMFAQLETAFGLLIEKLNSMDI

PTAWRKVDVIWEARATQVHFFDTVQTRQVLEEIFFLKRLQTIRDFFRLCASFAQTLSGTC

PPPNDEPPPSNGPVSLVKPIYRTSTVVNEDQMTRPIKAFTADFVRQMLMGLPTQALGLAL

CSALSALGSDLIAQVEAKDFGAEGKVSLDDLCKKAVEQGVQAGRISQLLLNRATLLASSY

DTAWKKLDLVRRLELSIDACKVSLQRAQLHIAMFQWQHEDVLGTRPQPMTVSPPPRSIIL

SNMKKKLYKLSQDDAAIASVQEKLASLEGSIEQRLKWAGGANPALAPVLQDFESTITERR

ALVVKESQRTTQVTFLCSTVLNFEGLRTRTSEALSMDAALFDLVKRCQATCSYAAQFSST

VSTLELQLLHRLSPVMEQSIGTPEWLACAQKHLAQEMANQRAAQEEREQQLDSVTETLQL

LVDTIKGTLSNHNRLLADVKHLLRAMAKDEESALAEGEEVMYEGSVRQFLCEYKGWQDNV

QIVLFTVVQATGQPRSLEQVELLQEIPPTLKELKGQSQRVYNGLVGFAFPLVTDRGGDCA

SPTATVQTSFAAAVRCSGVKTQPDSMSQNARKALPRNLGTPADTPPSTLLITSKSLMPSP

KRTVRDPKTGRAVQERNSYAVSVWKRVKAKLEGRDVDPNRRMSVTEQVDYVIKEATNLDN

LAQLYEGWTAWV*

>m.237 g.237 ORF g.237 m.237 type:complete len:2493 (+) Unigene000019:315-7793(+)

MADVDPDTLLEWLQMGQGDERDMQLIALEQLCMLLLMSDNVDRCFETCPPRTFLPALCKI

FLDESAPDNVLEVTARAITYYLDVSAECTRRIVGVDGAIKALCNRLVVVELNNRTSRDLA

EQCVKVLELICTRESGAVFEAGGLNCVLSFIRDSGHLVHKDTLHSAMAVVSRLCSKMEPQ

DSSLETCVESLSSLLKHEDHQVSDGALRCFASLADRFTRRGVDPAPLAKHGLSEELLSRM

AAAGGSAAGPSSTCKPGRTSTGGAPSAPDSKLSNQVSTIVSLLSTLCRGSPLVTHDLLRS

ELPDSMERALQGDERCVLDTMRLVDLLLVLLFEGRKALPKSTAGSGGRIPGLRRLDSSGE

RSHRQLIDCIRSKDTDALIDAIDTGAFEVNFMDDVGQTLLNWASAFGTQEMVEFLCERGA

DVNRGQRSSSLHYAACFGRPQVAKTLLRHGANPDLRDEDGKTPLDKARERGHSEVVAILQ

SPGDWMCPVNKGDDKKKKDINRDEEEGSEPKGDPEMAPLYLKRLLPVFAQTFQHTMLPSI

RKASLALIRKMVHYSSEVLLKEVCDSDAGHNLPTILVEITATVLDQEDDDDGHLLALQII

RDLVDKGGDVFLDQLARLGVINKVSTLAGPTSDDENEEEAKPEKEDELQEDAKEVQQGKP

YHWRDWSIIRGRDCLYIWSDAAALELSNGSNGWFRFILDGKLATMYSSGSPEGGSDSSES

RSEFLEKLQRARSQVKPVTASQPILSTVGPTKLTVGNWSLTCLKESEIAIHNSDGQQATI

LKEDLPGFVFESNRGTKHSFTAETSLGSEFVTGWTGKRGRKLKSKLEKTKQKVKTMARDL

YDDHFKAVESMPRGVVVTLRNIATQLESAWELHTNRQCIEGENTWRDLMKTALENLIVVL

KDENTISPYEMCSSGLVQALFTVLNNSVDLDMKPDCKQLVERINVFKTAFSENEDDESRP

AVALIRKLIAVLESIERLPLHLYDTPGSAYNLQILTRRLRFRLERAPGETALIDRTGRML

KMEPLATVESLEQYLLKMVAKQWYDFDRSSFIFVKKLREGQSFIFRHQHDFDENGIVYWI

GTNAKTAYEWVNPAAYGLVVVTSSEGRNLPYGRLEDILSRDSSALNCHTNDDKNAWFAVD

LGLWVIPSAYTLRHARGYGRSALRNWVFQVSKDGQNWMTLYTHVDDGSLNEPGSTATWPL

DPSKDEKHGWRHIRIKQMGKNASGQTHYLSLSGLEIYGTVTGVCEDQLGKAVKEAEANLR

RQRRLFRSQVMKYIVPGARVVRGIDWKWRDQDGNPSGEGTVTGEAHNGWIDVTWDAGGSN

SYRMGAEGKFDLKLAPGYDPESAPSPKTVSCAVAGPPTPWSSLAKNNCPDRGGPSSVSSG

RKGSSSSACSDVGLGSARRLEGLSEQGTLEVGGPLGTEGQEPLVVLSTAEGGSASNESAE

NKSESTAISVGPVSVSSPDVSSVSDSSGKAAVSQRPLGPGTGARLSVSSLLAAGAPMSSS

ASVPNLSSREASLMESFVRRAPNMARTNATNNMNLSRSSSDNNTNTIGRNVAAASASPLM

GAQSFPNLTTTGTTSTVTMSTSIVTSSNNVATATTTGLSVGQLLSNTLTTSLTSTSSESD

TGQEAEFSLYDFLDSCRANTLLAELDDEEDLPEPDDDDDENEDDNQEDQEYEEVLVRSRV

NLGYHVHIHREEEEYEIKGGRRRTWDDDFVLKRQFSALVPAFDPRPGRTNVQQTTDLEIP

IPGTPRSEVLEEVECAPSPHLALILKVAGLGTTREVELPLNNYKSTIFYYVQKLLQLSCN

GSIKSDKLRRIWEPTYTIMYRELKDSDKEKESGKLDFCERGCRSSGLGSSSLAATPTCDI

LSGAHEQPQAKAGSAQSACGVEDVLQLLRILYIIGGEPVVGHTLQEDLDYLQFNASPEEF

TSKKITTKILQQIEEPLALASGALPDWCEQLTSKCPFLIPFETRQLFFTCTAFGASRAIV

WLQNRREATMERTRPSTTVRRDEPGEFRVGRLKHERVKVPRGESMMEWAESVMHIHADRK

SVLEVEFQGEEGTGLGPTLEFYALVAAEFQRTSLGIWLCDDDFPDDESRQVDLGGGLKPP

GYYVQRSCGLFPAPFPQDSDELERITKLVHFLGIFLAKCIQDNRLVDLPVSQPFFKLLCM

GDIKSNMSKLLYERRSDSDRRFSDIHSEASTEEEPYLMGSFDEDSKSEFILDPPKPKPPA

WYHGILTWEDFELVNPHRARFLKEIKELAVKRRQILGNKSISEDEKNTRLQGLMLKNPVG

SGPPLSVEDLGLNFQFCPSSKVHGFSAVDLKPNGEDEMVTLDNAEEYVELLFDFCMHTGI

QKQMEAFREGFNRVFPMEKLSSFSHKEVQMILCGNQSPSWTAEDIVNYTEPKLGYTRDSP

GFLRFVRVMCGMCSDERKAFLQFTTGCSTLPPGGLANLHPRLTIVRKVDATDASYPSVNT

CVHYLKLPEYSSEEIMRERLLAATMEKGFHLN*

>m.243 g.243 ORF g.243 m.243 type:5prime_partial len:110 (-) Unigene000019:8842-9171(-)

AKACLFRKKDECSFVEYKDYKLVYRQYAALFILVGITDNENELSIYELVQNFIEVLDKYF

SRVSELDILFNLDKVHIILDEMIQNGHIVETNKNRILAPLLALDKMAEG*

>m.253 g.253 ORF g.253 m.253 type:5prime_partial len:313 (+) Unigene000020:2-940(+)

SFNTTSSELNQSLDLGKELLIQQCRDLTEQLEVKEKQLEVLQEEVRRSAEDLEEARERWS

KASEELEEAKWELESERDKRLQCEELLNEKIHEQDNLKNKPGFLENQIEKDQPTSNMEYD

EKSFSPEKLTEDKVELVLQEQLGQMDYREDRMENVQRLEKVRVDLESELTCLKENLSNME

EALRQGSAEKAAMEKRLQKLEEKNKSMEKALEMELENFELQLQAKDVAFEQMKEAREKAE

EELMEKETTLQKEKNEHEEERRRLEEKHQQEMLDWSLRLEKELSDLRSDLEEEQKKQITL

IKQVFISCVFYF*

>m.252 g.252 ORF g.252 m.252 type:complete len:2681 (+) Unigene000020:1011-9053(+)

MDELVAQQKAELERLKAELSEELRDSMEAAHQAELSQVQTQHTLELEALRLSLTNMHTAQ

LELTQSNLQKEKEAALSELQASLREKWAQESAMLQTRQQFELEKFREQGREQERQTELQH

QLAVDDLNCVWERRLSEERAALEQQLAGGIEDAKIRWIQDFKKEQAELQEALNKAEDRLA

ETKRKLEELQRSKEEEIKHLESELSQAWADRDSAARAVEELVASHRVVLQEQQAHTQELE

EKEKWLKQEVDRLLEQKSSSEQEITNLWSQLESMRTSRQELGELKEQLLARKSHVNDIEQ

LKQDFSQQRRELQEQNETELENLRVYFEQRLRASEENHREEIALLQLRLVEGALEDSVLK

TGEESFLSDGKSEEEKGDSLAEITEQLDKHKVELDALRLQLEEKHKQDLEQLRCNMALSY

REELLQARTELTDRYYSDMEQLKTKHALEIEQLRAKLSDSHLKEITRLRLQSVADVARQV

ETELEEKSRLQTQEHQARLAQLASDTESILSLEKKLAALTLQHSRELERATEQHAEELRQ

LEERLKKNFSEELHTRLAEAQQEERTHVTEELLNRSSEERLQLREQLQAQAEERVASLRE

ELQQAASKDRQELEQKLVQAEELLAEEKQRLQALQVSLEREESPQLLALKQRLESQYAGE

LHTAKSTMAAEVKELNTLLQEQTESRLKEATCRHQEEQRQLAEKLVQQKEEALQKLRQQH

AQELQVQETLLEQNVSELQHLQKEYQELKSRHQEELDLLSSNHKTALDSLAEKHKAELDK

LQVVFQETNLAQLEAQEAELEERHKQEMEELETRMLCNMDTLESTYLKEIQAVREEKEAA

LRDLGASLEQQQAEEIERLKQEELIVREELRKKLAQVHMDKFSVMATELSHAHQAELSAA

LASQKAALESEHHSALETLRQHVLDLEAQHGAALHEVTDMTTKENQQLKKQLDALNAQHQ

QQLQELRATSSREIEALRRELEEEASRQRLHFLEEAELLKCQSEEQLQKKLMELKEESDR

EKVSALEELERSLCEQHQQNELSYTEKMNHLTTQLQQLDTVVSQLRAEVSGLQGELEGKR

SEMDTLETLLQRRERESQEGANLLAMLRDDLNTATQQRQDLQLTHDRLQRLVLEMLRITI

ATEEEISRRLGISMEGDDITLNRKQSVNRDPQKTDAEVGDGAAAAVEASANCSMFSSLMD

EGLELSQRLCESLFTGPDGELDPEREELVLGACTHLRVAVDKLLEVVSDSTVQLEQLQAL

QALLDEKFSAGGEAKNALFLQHTQLLEQLDQEASVKSQLQLELHKADGLIEGYVVEKAAL

EDALQQKESREQKLVKELESTRAQLQELGEEHALLQRQRDALSASLGEPEKALLAEAERL

GQERVDVQRQAEKDRVGLAGRLRLLEQTLEEHESRTQQQEEQHRTQTEDLQQHISALEKQ

LKHHRQFIDEQAVEREHERDEFQQEIKNLEAQLKTTSKGHAGEDTKGQRVESLQALIKDK

TEDYSVLLSLKEQCQRDLEERNEEIDKMASRIRELEQALLSSVEERRAAAQLEQELQKAR

KNCQELSQDKEALQQQLYSNKLQISALQSKLDETRHRYPDPGADPNLRERLETLQQDLQN

KEEQVEVLLERVEVLEKALAVKEEDVRQLSLQLELKTTESRAREEELRLSVAELQDTASL

LRRQRQEEADASALQLPSALLEEKNLEIDHLNQQLLHLQQELDASKENKTEEKQADVEDL

RAQVERLHGDQERLHQAKEEEVEQLHEIIHKLQEELSQLNPNRHEVSDPNTDSPEPPDFP

WSPRPQHGAEESLCHELSSQTLQSCRTKICELQADLERSVEEKVALERLLLMQEEQYGQQ

LEALGCSLGEGKRKLALLEQEAGELKLQLDQKETKVEELQARIQTLEDGERSVNDLELRL

IKAEETMKETQQEMTRLNDKARKHTEERERTESLIAELQQRYEDSERTATQARLQVAELK

ETIQQRKSDIAILETGKTELYTEKQALQKRERKLQEEIEKLKQEVSAKSLQIQEVSLQLE

ERAADPDQSQEEVLTCAEETLAKAAAALREKEEQLAHLRSQHDALRAELAAVKEGLSSST

ERAEKLQEEGQTKDRALADLEEHNQRLRAELRGLQDDLEVQEEELAYQQRELDELRQRCR

LQEHTELDCTKPERRFLDGLSHNSTLSSPEVLRRLDCSEERPSHVHASHLSELSGLRNTS

LELMSKHSLMERNELKRPLVLPPETDPPSTHSPSASTHSLSISENLSVLNSLDDDKVQVL

ENLDDVTPSRSPLSSTSPVSAPEWASDGYGSNVSSELEAKLKNELEHMERLDAHFVEYLR

SRDMAPAPCSDSAAGSLHHTYELLSPELQAMLNRVYRESYRVLSLSHRPPPAGQPHSDAE

EAPPASWQRERRALQETVLSLRELLCRMAEREPKVEGEVDWRTELLQAVRSVFDSERKWL

HAQLQDIITTNTGIDYTSLLKDLETLLQKQEDQQRRSLEQLLSADRNSLLAEIRSLHKQL

HTCTLQNQEQLQRSLGAVREEGAQTQQQLHRQVGELERQLQQEQSSSHDLQRSLGQEQSR

STNLHQQLEIQQKLAVEMKLELEEGTRQLRASEKSREELQAQIHKLRLKLESEEQEHQAC

VEAAKRDEVRVKQLQEELHHERLSSKRAAEEHAHMQEVLE*

>m.266 g.266 ORF g.266 m.266 type:internal len:3041 (+) Unigene000021:1-9126(+)

RVLPENIYGIGEPCETTDSVLVCEIPSVPQDLQVIDITKSTVILAWDKPFYDGGSKLTGY

LIEACKKDTDRWMVVANVRASVLRHTIDSLNENEQYLFRIRAENNRGVSEPRDLMTPITI

QEQRVMPKIDLSSIPQKIVNVHAGKPIVFNIPITGRPAPTCSWFFGGIKMKDKLDRIKIE

TTAKYTKLTVRETTVDDTGDYTLEVKNITGVASEIIKVIILDKPGTPAGQMRIDEIDANS

ITCSWQQPEKDGGANISGYVVEQRDAHRPGWLTISESVTRPTFKFTRLTEGTEYVFRVAA

TNRFGIGAFIQSEVVECKSAKTAPGAPSMPEIFDVSHDGMTLTWKPPENDGGSSLAGYII

ERKETRSDRWVRINKNPVTMTRYRSTGLIEGLEYEYRVTAMNSRGTGKPSLSSKPVIALD

PIEPPGIPSNPRVTDTTRTSVSLAWSPPEEEGGAIVTGYLIEMQKVDQIEWTKCNTTPTK

ICEYTLTHMPQGAEYRFRVMACNAGGPGEPAEIPGVVKVTEMLEYPDYELDTKYQEGYIV

RQGGVVRLSVPIKGKPYPTCKWTKEGRDISHRAMVATSEERTELVIKEAHRDDTGTYDLV

LENKCGKKAVYIKVKVIGRPDPPEGPLEFDDIQARSVRVSWRPPSDDGGSDILGYIVERR

EVPKAAWYTVDSRVVETSLVVKGLKENVEYHFRVSAENQFGISKSLKSDESVIPKTPLCP

PEPPSFPPEIMDVTKTTIGLSWSRPKDDGGSRVTGYYIERREMSTEKWVRHNKTHITTTM

YTLTGLIPDAEYQFRVIAQNDIGQSEPGPVSEAVVCKDPFDKPSQPGEIDIISVTKDCIT

IHWLRPESDGGKEILGYWIEFRQAGESAWKKCNKERSKDRQFTMGGLMEATEYEFRIFAE

NETGLSRPRRTAMGIKTKLSVGEAPSLKEEIKDTTTKLGESGTLTCQIMGRPLPEIKWYR

YGKELTQSRKYKMSSDGRNHSLTVLTDEQEDEGLYTCRAINEAGEIETSGKLLLQAAPQF

HPGFPLKEKYYAGCGTSLRLHVVYIGRPIPQIMWFYGKKPLNPSENVVIENTENYTHLVV

RNVQRKTNAGRYKVQLSNKFGTIDTVLRVEIQDKPLIPEGPIIVEALLKNSVIISWKLPK

DDGGSMITNYIVEKREAKEGEQWHLVSSCVPGTTCRIPNLIESAGYYFRVSAQNQYGISE

SLEIPAVVIIKSPYEKPGIPQQPVVTSVTKDSCVVTWKPPSSDGGAKIKTYYLEKREKKQ

NKWIAVTTEEIHETYYTVKGLIEGFEYEFRVKCENIGGESDWSMISEPVIPMSDTAPRAP

FFKEELRDMCVKYKANATFVTKVIGYPKPTVKWYKNGKEVLPDGEKIKIQEFKGGYYQLL

ISSADENDVAVYQIRATNQEGSISTTITLDVEVPAKVHLPLHLQGMGAVHAVRGEVVTIK

IPISGKPEPAVTWQKGQELLSNTAYHQVIVTRSFTSLVFQKGVQRKDSGYYIICAKNRFG

VDKQTVELDVADVPDPPKDVKVSDVGRDTITLTWTPPNDGGSDIIRYIVEKCPLSGDRWI

RVAQTSESQYTVMSLFGKTKYQFRVIAENKFGLSNPSVPTEPVTAREDKLAIRNYDEEVD

TQREVTKEEAPHSKVKNLPSLYKISEELARCGHFGIVHRCVEISSKKTFMAKLIKVKGAD

RELVAREIGTLNIARHKNLLYLHESFDSLEEYVLIYEFISGIDIFERLGTNFDLTEREIV

LYLRQVCQALKFLHGLNYGHFDIRPENIVYATRNSSTVKIIEMGQARLLTPGENIRIQFT

APEYYAPEIHNSDYVTTATDMWSVGVLAYVLLSGLNPFAAESTHKMIENISNAEYIFDRE

AFKHTSLEGMDFIDRLLTKECKHRLTASEALEHPWLKTKIENISSKVIKTLRHRRYYQSI

IKKEWTTVISAARVAYGGGYRNQRNVSIGRVKIGHPEQGLRAGPVMHGSAEPGGHVRFMC

TIENYDKTTEVTWYFGSRKLIASHKYEISYANGVASIYVKDIEESDDGLYRCKVVSEDRE

ENGYAELFVESVRSYREYFLGRSLKKPKRRIDKTKLLQRPPEFTLPLYNRTAYVGEDVRF

GVTITVHPEPQVTWLKAGHRIKPNPSKYTFTTDKGLYQLMIHNVEPDDDCEYTVVANNRF

GEDSCTARLTVTHHPVAEDTMRPIFRRLLANIDCVEGDSVHFELRVSGTPPPALKWEKDG

KPLEFRPQVEIVQEDVGYYVLYIRETLIEDSGIYRVTATNSAGSESCQATLKVERLTYIR

RKFKSEEERHVYVQNQIEKTLKMAQEFSTAEVTTLNPAAQEALKEAAELYKPAVSTKNVQ

GEFDISDHEDKEIKRIKEESRKIRMPYEIPEPRVHDPTVLEEDMNIKHFVPLSDMKWYKK

LRDQYEISERMERVVQKRQKRIRLSRWEQFYVMPLPRITDQYRPRWRIPKLTLDDLETVR

PARRSPSPESDIFIQRRRSLGDISDEDFLQLKEDYLTKRRIEEEKQQLEEELELGFSASP

PSGSPVHFELYALRRASPRIMQKAEVKHAEETKLAVKQIKSTTSPPPISHFMRRRRSLSP

TYIELMRPVSELIRPSRAPVEYVTEAVERRSPTPERTRPRSPSPVSPERRSSRSSSRFER

SARFDIMSRYEARKAALKSERKYQIVTQQPFSLDHAPRITVRMRSHRVPCGQDTKFTLNV

QSRPEGEIQWFHNGQQIQESSKYHFTNMSGVLTLQITDCQGDDSGTYRAVCTNSKGEASD

YATLDVSGGAFTTYSSRRKDEDAPTAFVPDVLKTDYYHTSSIRASSASRTHIEIKETKTK

LTERHEASGFEKYESERLASSPIRYASTEYLSSASYSTSERHASSEYKTKFVDSTSTASI

TAKKVKATLSAKILTKPHSLTVSLGESARFTCDIDGEPAPNVTWMHGQHVLVTSHRVRVT

TTQYKSTFEISFVEYSDEGSYTLVVENSQGRQEAEFTLNIHKPQTKEEALAPSRLVKSSE

PEVKSPEPPVKSPELLVKSQEAVVKSQEAVIKSLEPAVKSP

>m.271 g.271 ORF g.271 m.271 type:internal len:3031 (+) Unigene000022:1-9096(+)

RVLPENIYGIGEPCETTDSVLVCEIPSVPQDLQVIDITKSTVILAWDKPFYDGGSKLTGY

LIEACKKDTDRWMVVANVRASVLRHTIDSLNENEQYLFRIRAENNRGVSEPRDLMTPITI

QEQRVMPKIDLSSIPQKIVNVHAGKPIVFNIPITGRPAPTCSWFFGGIKMKDKLDRIKIE

TTAKYTKLTVRETTVDDTGDYTLEVKNITGVASEIIKVIILDKPGTPAGQMRIDEIDANS

ITCSWQQPEKDGGANISGYVVEQRDAHRPGWLTISESVTRPTFKFTRLTEGTEYVFRVAA

TNRFGIGAFIQSEVVECKSAKTAPGAPSMPEIFDVSHDGMTLTWKPPENDGGSSLAGYII

ERKETRSDRWVRINKNPVTMTRYRSTGLIEGLEYEYRVTAMNSRGTGKPSLSSKPVIALD

PIEPPGIPSNPRVTDTTRTSVSLAWSPPEEEGGAIVTGYLIEMQKVDQIEWTKCNTTPTK

ICEYTLTHMPQGAEYRFRVMACNAGGPGEPAEIPGVVKVTEMLEYPDYELDTKYQEGYIV

RQGGVVRLSVPIKGKPYPTCKWTKEGRDISHRAMVATSEERTELVIKEAHRDDTGTYDLV

LENKCGKKAVYIKVKVIGRPDPPEGPLEFDDIQARSVRVSWRPPSDDGGSDILGYIVERR

EVPKAAWYTVDSRVVETSLVVKGLKENVEYHFRVSAENQFGISKSLKSDESVIPKTPLCP

PEPPSFPPEIMDVTKTTIGLSWSRPKDDGGSRVTGYYIERREMSTEKWVRHNKTHITTTM

YTLTGLIPDAEYQFRVIAQNDIGQSEPGPVSEAVVCKDPFDKPSQPGEIDIISVTKDCIT

IHWLRPESDGGKEILGYWIEFRQAGESAWKKCNKERSKDRQFTMGGLMEATEYEFRIFAE

NETGLSRPRRTAMGIKTKLSVGEAPSLKEEIKDTTTKLGESGTLTCQIMGRPLPEIKWYR

YGKELTQSRKYKMSSDGRNHSLTVLTDEQEDEGLYTCRAINEAGEIETSGKLLLQAAPQF

HPGFPLKEKYYAGCGTSLRLHVVYIGRPIPQIMWFYGKKPLNPSENVVIENTENYTHLVV

RNVQRKTNAGRYKVQLSNKFGTIDTVLRVEIQDKPLIPEGPIIVEALLKNSVIISWKLPK

DDGGSMITNYIVEKREAKEGEQWHLVSSCVPGTTCRIPNLIESAGYYFRVSAQNQYGISE

SLEIPAVVIIKSPYEKPGIPQQPVVTSVTKDSCVVTWKPPSSDGGAKIKTYYLEKREKKQ

NKWIAVTTEEIHETYYTVKGLIEGFEYEFRVKCENIGGESDWSMISEPVIPMSDTAPRAP

FFKEELRDMCVKYKANATFVTKVIGYPKPTVKWYKNGKEVLPDGEKIKIQEFKGGYYQLL

ISSADENDVAVYQIRATNQEGSISTTITLDVEVPAKVHLPLHLQGMGAVHAVRGEVVTIK

IPISGKPEPAVTWQKGQELLSNTAYHQVIVTRSFTSLVFQKGVQRKDSGYYIICAKNRFG

VDKQTVELDVADVPDPPKDVKVSDVGRDTITLTWTPPNDGGSDIIRYIVEKCPLSGDRWI

RVAQTSESQYTVMSLFGKTKYQFRVIAENKFGLSNPSVPTEPVTAREDKLAIRNYDEEVD

TQREVTKEEAPHSKVKNLPSLYKISEELARCGHFGIVHRCVEISSKKTFMAKLIKVKGAD

RELVAREIGTLNIARHKNLLYLHESFDSLEEYVLIYEFISGIDIFERLGTNFDLTEREIV

LYLRQVCQALKFLHGLNYGHFDIRPENIVYATRNSSTVKIIEMGQARLLTPGENIRIQFT

APEYYAPEIHNSDYVTTATDMWSVGVLAYVLLSGLNPFAAESTHKMIENISNAEYIFDRE

AFKHTSLEGMDFIDRLLTKECKHRLTASEALEHPWLKTKIENISSKVIKTLRHRRYYQSI

IKKEWTTVISAARVAYGGGYRNQRNVSIGRVKIGHPEQGLRAGPVMHGSAEPGGHVRFMC

TIENYDKTTEVTWYFGSRKLIASHKYEISYANGVASIYVKDIEESDDGLYRCKVVSEDRE

ENGYAELFVESVRSYREYFLGRSLKKPKRRIDKTKLLQRPPEFTLPLYNRTAYVGEDVRF

GVTITVHPEPQVTWLKAGHRIKPNPSKYTFTTDKGLYQLMIHNVEPDDDCEYTVVANNRF

GEDSCTARLTVTHHPVAEDTMRPIFRRLLANIDCVEGDSVHFELRVSGTPPPALKWEKDG

KPLEFRPQVEIVQEDVGYYVLYIRETLIEDSGIYRVTATNSAGSESCQATLKVERLTYIR

RKFKSEEERHVYVQNQIEKTLKMAQEFSTAEVTTLNPAAQEALKEAAELYKPAVSTKNVQ

GEFDISDHEDKEIKRIKEESRKIRMPYEIPEPRVHDPTVLEEDMNIKHFVPLSDMKWYKK

LRDQYEISERMERVVQKRQKRIRLSRWEQFYVMPLPRITDQYRPRWRIPKLTLDDLETVR

PARRSPSPESDIFIQRRRSLGDISDEDFLQLKEDYLTKRRIEEEKQQLEEELELGFSASP

PSGSPVHFELYALRRASPRIMQKAEVKHAEETKLAVKQIKSTTSPPPISHFMRRRRSLSP

TYIELMRPVSELIRPSRAPVEYVTEAVERRSPTPERTRPRSPSPVSPERRSSRSSSRFER

SARFDIMSRYEARKAALKSERKYQIVTQQPFSLDHAPRITVRMRSHRVPCGQDTKFTLNV

QSRPEGEIQWFHNGQQIQESSKYHFTNMSGVLTLQITDCQGDDSGTYRAVCTNSKGEASD

YATLDVSGGAFTTYSSRRKDEDAPTAFVPDVLKTDYYHTSSIRASSASRTHIEIKETKTK

LTERHEASGFEKYESERLASSPIRYASTEYLSSASYSTSERHASSKYKTKFVVSTSTASI

TAKKVKATLSAKILTKPHSLTVSLGESARFTCDIDGEPAPNVTWMHGQHVLVTSHRVRVT

TTQYNSDEGSYTLVVENSQGRQEAEFTLNIHKPQTKEEALAPSRLVKSSEPEVKSPEPPV

KSPELLVKSQEAVVKSQEAVIKSLEPAVKSP

>m.288 g.288 ORF g.288 m.288 type:complete len:411 (+) Unigene000024:7318-8550(+)

MLAQSACRRQARINTLPLNLFDQLDKLRPFVEVVNDGVPLVQAVVPRNQTHSFIIPGSDV

LVTVSAKGQIGTHSWLPYDKNIANYFTFTKDPTINNPKTQRFLSGPFSPGVDMGAQVLVV

SNDARLLFSGGHWDCSLRVTTLAKAKLVGRICRHIDVVTCLALDLCGIYLISGSRDTTCM

VWQVLQQGGFSSGLSPRPVQVLCGHDAEVTCVSISTELDMAVSGSKDGTVIVHSIRRGQY

LRTLRPPSEGCVSVRVNQLQVGMEGHIVAQSVLEGCSARKEKCVLHVYSVNGTLLASTVM

EEPVSALYMVSDYLILGTLQGNLHIKDLFSLEQAAKPLALMLPIRIVSVTKENSHILVGL

EDGKLIVVGAGKPEELRSGQFPRRLWGSTRRISQVSAGETEYNPTENAGK*

>m.287 g.287 ORF g.287 m.287 type:complete len:1804 (+) Unigene000024:292-5703(+)

MDWPQTEHQLKDVSYLKQCLEDFVASFKKVIDVQSLEPRRLEEWNAEVPAVPRDFLVFLE

TQLSHSVLHLSGQEPNNSMPHPLLLIKFFIIICRNMENLDLDKTPGFVLETIKLLTYCLN

QIKEKPEEQSSLQSVVQHCLLLCENLFDPYQTWRRRQAGEEVSMLERSKYKFHPLTLPEE

LPAFFHDCLQETERIPNALLLRLVHLQGAVTSGCKKNGLLSITPEAVEDLMSVLRSWCLR

PAEEDQSKDPQVLRLTLRCLTAMIHLLHCSSPAERQVEMKTVLNNYFQLLNWNRPPTSEQ

ENSLAWEESIITLQTHMLNAIPEILQCSDRPVLQAIFLNNNCFEHILRLIQNSKVWEKGS

DCITVHAIGVLTAIMSNSPSAKEVFKERIGYSQLFDVLKSQGQPTKRLLQELMNMAVEGE

HCQAVPLGISNVHPLLLLLQWLPELGSRSLQLLVCEWLLAVCRGSLACRSVAVEAGLVDA

VLDVLNQGPERLERQCADSLLGLVQELGSLSLRPHQLKSLLRLLRTNPGSPPHPYCTRTI

RALAAMATHAHTGISALQYFDLTPPMAGIMVPSINRWPGNGFAFHAWLCLNSNFPPPVHQ

HHSESHLSIPDNTLRMTKGPRRKQLYSFFTASGTGFEAFFTMEGVLVVAVCTKKEYMAVS

LPELPLNDGAWHSLAIVHIAGRRPFGQNLVTIHVDGNLCKTAQLRFPTLSEPFTSCCIGS

AGHRTTTTTTSPTLLPPVHSSEMAFAMSSGPPALTRSQSFPASFAAGGGGRWGLGRDVPV

YTIPAGLQDTEWGSPSSLDGLLATTFICHEALQPAQTRALYMAGPHSVPLFKGDGELSEL

CSKLLLYYTPQAFKNQICLDLSPNHMYDGRLTGHRVVNWDIKDVLNCVGGMGALLPLLEQ

VCVLEQGETAGQGTSDLLGPDLTSSRGPAGMLLPLGKSCESRLEKNSVAAFLLMIRNMLR

NHPVNQESLLQCYGPAIIGAMLNKVPSTMMDMSVLMACQLLLEQVSIEGNSALLSQLYQY

LLFDFRIWSHCHFAVCLGHVQYLSSVLKEGKQRTRRKYGVQYILDSIRTHYSVEKNGSAL

SDEKKTVQISLLELLKDFLKSPTTKDLHSILAYTVVVQDQQQAIMLLDILYTVLKNNPQA

LSVPLEWGVEQFLCLLLKPSYNDEAREKVFRIMYKLLKSERVPERSKQRVKLRETTYMGL

VCFLEEVPVTMTIIRCLCEQVLATDPSPNFRDLLSVVYLSHRADLSVRLDICRKLFSLID

SNEDCVKQLARQPGWQDILIKLYVKESFESHKTSVSSPYPSLEPIPTRTLLRKDDSIDGP

RSDPFNPYSSQREEAEEDECDEEEEASRDISERFIGLSQSPPARGQLKGFSDSMNFKSFD

SVEQSSRSSSLSNAVDVPSTQLLEEEEESLYNPLSPLGMSPDLELGGQRASLTPDTPPST

EHNKLFLGPRARKSSSLSNVLDETSSCTEPPTADTISNNSNPQQAPEEELCNLLTYIVFR

VLWSGTEGAEDAVWRERGQVFSALTKLGSSWQLVRHPDDIKRSLLEMMLESSLSDLRDSQ

GLSLPHIPSLLRLLRLLHDFLFAEGTDNQTLWSEKIFEGVVNLLDRLQAWHTTSATAGAT

ELKRMAQIGLWIITGYIQQQNSQVCEMACVKLHSLLQTVLCLSWEEVYFLLGRLGATLWP

AEGMSLTSPAGTEAVLRPMVPVVRTLLDQHADPTKLQQSLPNLPPTNGSATFAQDLLSYC

NTVEWQLFYCNHVRPTMQQYEQDTFGKSHDLLSNFWNSCFDDLMSTAEKKNKDKAEFKAK

FQS*

>m.289 g.289 ORF g.289 m.289 type:complete len:399 (+) Unigene000024:5823-7019(+)

MTSERATWADRDQPEMKWKLSSAETYSKMRLKLVPNYNFDSHSEASALRDNMGAESPRNS

TEPLPLAVAKEAKVSDMEDDQLEEEDLLAMDSQAEEVEDESQKEKLVLSEDCELITIEAV

VMGRLEVTTHHIYFYDASSEKEETEEGIGFDFKRPLSQLREVHLRRYNLRRSALELFFID

QAHYFINFKKKVRNKVYSRILGLRPPNLFYFGSRSPQELLKASNLTQKWVCREISNFEYL

MQLNTISGRTYNDLSQYPVFPWVLCDYTSNVLDLEDPSVFRDLSKPIGVVTPRHAQDVRE

KYESFEDPTGTVDKFHYGTHYSNAAGVMHYMIRMEPFTRLHIQLQSGKFDCADRQFHSVA

AAWQARMESPADVKELIPEFFYFPEFLQNINGQSGAGE*

>m.300 g.300 ORF g.300 m.300 type:complete len:2480 (+) Unigene000025:189-7628(+)

MVGERRNGNLLVQLGPKQQAYPEELIRQRRTHDGQTEYLIRWSLQAVEDGSSSGSSNEGG

SNRSGSSGVGSSSGCTSGESKTENILMWMSTEDVYANCPTLLGKRPPQEEIARPSGQFPS

DVTFNEVDLRDMKEDVKNLVERARKQMAKNSDFAISLTHTIHVLSAYASIGSLVGVFKET

GALDLLMELLGNKERQTRRSAGKMLRALASHDAGSRAYVLLSLSQQDGIEQHMDFDNRYT

LLELFAETTSSEEHGISFEGIHLPQIPGKLLFSLVKRYLCVTSLMDKLNTSGSELSTERQ

EPEPSSSTASHQSDQSRLQRQFEFTMAMANLISELVRVMGWDRNRQPSDASPNTARGAEE

TVAEVNRHTLRSIFQPRFSASTVITATVTSAPAPPKKKTNGFRTRTDFSTRSAYVEYVQE

TLQSGMHVRMLEDYEDVSSGDEGEFRYSNDGSPPVQVYWNSISRTYWVHWHMIEIVGSGS

SVQEEKKTHEKAFSLTETLKHNAVSQMFFLKPPGGLYSLPYLTDSLQDDAGTLTRAEWWE

VLFFIKKLEPKQQQEINNILQQNNVKQMAELDESTLIQMSVSSELACKILHYLKQTLQSS

CLNDLLCSHAFVKHYLRRGAASLEDEEMFNECSLNDSGLGGQGSSSSLLATPKASTSSAS

FSINGCVSKKPKKEFPIDKGSCNSDTESELPAEDETKYPEDLEEKMKLFNNPKVQSKKTA

LEKIGEVVDIMKKSSSDSGQLLAGMKVICKILDEEGPQEHSTLRSDLAQSTRDKVLKLLV

VMVGSQQKPNVIMALLLTRSLMLKYEWRVSFATEGGVKAILSCMQEYPTCTQVQQIALAT

LKVITGASKHDLRSVGSCLPLSESGTQMMLEIFASIGSATPEGSKGLLEAIPSAIDLMLN

TAGGGLSIRNGLLVIIMLISNHKSLAEQLVACEVTTVLRKCLSGHTSESMLAIIALNHIS

NVHKLEKRESEEMLDFKDTELKMLVVGLKEMTTTKEVIQTLEQLLCDESSQLEDERNEVL

HSRDTFQDLVRLMDQHRVDRTVQLSILRILNKFLDNYQEDLLPWHESIEPCISSLTACIN

DREAVQQFIHFLYRLASQNKDCTVVMCRLGTKEALVKALDKHSTNLLLVTELRDLINDCE

KYASLYKKMTTSVLAGCIQMVLGQIEEHRRNHQPINIPFFDVFLRNLCQGSSVELKEDKC

WEKVEVSSNHHRANKLTDKNPKTFWESNGCTGSHFINVYMHKGVVIRQLVLLVASDDSSY

MPARIIVLGGDDPTSIKTELNTVNVAPSASRVILLENMTRFWPIIQIGIKRCQQGGIDTR

VHGFEILGPKPTFWPVFKEQLCCRTYLFYTTKAHTWCQEILGDKKQLLQLFNKLNSALRH

EQMFADRFLPDAEAAEALGRTCWEALITPIVQSITLSESHVLSPLAWLLGEYLENSESLK

CYKSRAAIFNSRVRRLTHLLVHVDTSRVDIEELKPPVKCKGINRSKDVKNGKEGKNKGAS

GVTSPSSTAKPKIKNSSSIAGVALCWQSVVQGQMKKFLDLTYNLPEFVECYKNMYLRLKN

AMEELFGQQTAFVLALRQGFSAALLQLSILTSMHVSERFAQYIDHMIRQSGVDSGNVETL

NKLQKFLEPMLFLSGLELANTFEHFYRYYLGDRLLSQGNLWLESAVIEQICTCFPERFPQ

QMLNNLSESDELQQEFHLYRLQQLDKSLQDMNEEMADEQMLDPEDEGEVKVLVLSPRCWA

VSAPCYLENPTKYFPQNLCSYLEEFADFYSNSQCIYQLGNSMLRRLQWTWLGHAEVCYGS

LTLYVSTLQMYILLQFNHQEEVSLQALQQATGLSLPVLIQALTPLISEKGILSHEGLNQD

LQKGLLRLNKMSLAQSSEKQRYCNLLPKQTYLNVDEDAALTLERKRNHLYCLIVQIMKNE

KEMHIDNLVFKVLDTCQKQEAASNTGRFSCSTTDVLSCIMHVINKGYIRRNEDNPHIVEY

IVEDPSTPQKSQAHFFSRVDLNKCKGSTKADTSLGGSILVPQQVEDGALETMLFSMGRTM

TQEEVKRLMRCTIQQLADTLSLEEEPAEHLLMHCKWNIDMLIQRYTDDPETLMLAAGLKI

RNPHSLASPMSLCPSCWQEYLTARIEQNLVMNCNCPITDCPAQPTPKFFYSILTDKDTIA

KYENALLRGYVECCSNLTWCTNPQGCDQILCKENIGSMGTCSKCCWSSCFSCNFPEAHYP

ASCSHMSQWMDDGGYYEGMSMEAQSKHLAKLISKRCPSCQAQIEKNEGCLHMTCAKCNHG

FCWRCLKPWKPTHKDYYNCSAMVSKAARQEKKFQDYNERCTFHNQAKEFAINLENKVSSI

NEALQMKSLTFVIDACKILAQGRKVLAYSCVYSYYNQDTEKMDVMEQQTEALDLHTNALQ

ILLEETLLQCTDLASCVRLLKPEHLNTGLELIRRIQERLVAILQHSTQDFRVGYQSKTGP

DHETAQALNIANNTEQNKV*

>m.305 g.305 ORF g.305 m.305 type:5prime_partial len:2880 (+) Unigene000026:2-8641(+)

SPPQPKMGHPFDPFGQYDPQGGRMIANGPGPGPGSANGMSSPHSRYHGGNGTATGHHAYP

GAQGDGRGGGGAAAGQSFQDGSGAATVNNWGPQGMQLPDQNRAQFPQSQQQQQVHGHQVA

PYIGRTDFGGMQGHNQTQAPPRMNHFPQGLPQEANRFMGHMGANMTSSTMRHPSQTPQHQ

QAGQQFLHHHTDSQFPQSPSKQQQMLSGRGHQNMGFSLNNQQVANSSSGQYAAYPQYSGL

NQGLANASGMSLNTSSNPNASVQCYPTPAGPQCYPVNQLPPQNMHPKLPQQQQQPPPQQQ

HLVPPPQGSYATPPSMSPMRNLGGNPAGTPPPQRGQGPISHPQQHPQMYGTLSPNQRAMN

APNHTEVLPQQLQTSTLQFQQVRTPPTLSVSQQPPGSSPPTPLPQGNWSSNTHSSPPANH

QQVPHMQPSPSDASQTLSSTHNSTPSSTLQLGSCDTQPLDSDSKPKKKKKVKSEEKDEDM

GVCGELGGAGVSGETSSPEKKRKKKPKEKQAKDTKEPKTPKTPKAPKTPKEPKEKKVKNT

TPKPRSTKKNSKQEECESSSKKEGKRKRESSITLDPEKTPPLSPEEEEEDDGVQKRRSCR

QVKRKRYTEDLEFRISDDDDEDSTGPKSPSTTSEPTEVLDAEGPVVEKIMGLRTVKKQLE

SGQEVEVEEFYVKFKSFSYLHCRWADLEELEKDKRIQQKVKRFKAKQAHTNFLSEMDDEP

FNPDYVEVDRVLDVSESTDENGEVVTLYLVKWCSLPYEDSTWELKVDVDAGKIEEFEQVM

SREPQLKRVERPPASDWQKSQSSRQYRNNNALREYQLEGVNWLLFNWYNTRNCILADEMG

LGKTVQSITFLYEIYLKGVHGPFLVIAPLSTIPNWEREFRTWTDLNVVVYHGSQASRRTI

QTYEMNYRDTQGRVIKGAYKFHAIITTFEMILTDCPELRGVPWRCVIIDEAHRLKNRNCK

LLEGLKLMDMEHKVLLTGTPLQNTVEELFSLLNFLEPERFPSESTFMQEFGDLKTEEQVQ

KLQGILKPMMLRRLKEDVEKNLAPKEETIIEVELTNIQKKYYRAILEKNFTFLSKSGGGG

GGGGSNVPNLLNTMMELRKCCNHPYLINGAEEKIMEEFRETHPADLPDFHLQSMIQAAGK

LVLIDKLLPKLKAGGHRVLVFSQMVRCLDILEDYLIQRRYPYERIDGRVRGNLRQAAIDR

FSRPDSDRFVFLLCTRAGGLGINLTAADTCIIFDSDWNPQNDLQAQARCHRIGQSKAVKI

YRLITRNSYEREMFDKASLKLGLDKAVLQSMSGRENAANGVQQLSKKEIEDLLRKGAYGA

LMDEEDEGSKFCEEDIDQILQRRTQTITIESEGKGSTFAKASFVSSGNRTDISLEDPDFW

QKWAKKAELDLDAINGRNTLVIDTPRVRKQTRHYSSIKEDEMMEYSELESDSEEKPVQKS

RRPQDRTQGYPRSECFRVEKNLLVYGWGRWKDILCHGRFKRPLYERDVELICRALLAYCL

LHYRGDENIKSFIWDLITPTEDGQSKTLTNHSGLSAPVPRGRKGKKGKLPVTQMNMPQAD

WLAECNPDVLLQEDSYKRHLKHHCNKVLLRVRMLYYLRQEVIGDLADRILEGADSSELDI

WIPQPLHAEVPTDWWDVEADKSLLIGVFKHGYEKYNSMRADSALCFLERVGMPDAKAIAA

EQRGTDMVADGEDEDPEYKPLRMPFKDDLDDFTNSPLDEKEEVMDVEAGDAPKESEVNGG

KAGNLYWPTASAFTARLRRLITAYQRCHKRQETLIKPDGRRRRRHRDDHLLAMATEGGAY

SLEPSRVTPTAYLTEGASFKTSPFLHESAALLADGTAFFKERRQRWTRREEADFYRVIST

FGVVYDVQTQRFDWSQFRAFARLDKKTDESLEKYYYAFVAMCKRVCRMQVKDSELADPTL

IIDPITEERASRTLYRIELLRRIREQVLPHPKLEERLHLCQPNPELPAWWEAGKHDRDLL

HGAAKHGVSRTDYHIQNDPELSFLQAQRKFIQNRGSDTALTSDPPPTGDLIKDEELRNDP

AENTGMEDMKEENTSSPKQEVKPEEEEKTANEKMESENEAEVKSEENRSVPQMTEEKVTE

SEDTPTPHVTVHQDTEKEEPENPKELEKNSEEEEEEEEKMDEDDKSEKSSQAEAGASSDR

KNFDEESNASLSTARDETRDYFGLDDVEPSVSQMFGERAPFSFWPKDRVMINRLDSICEA

VIKGKWPSNRKQFFDMPGLLPGYVAMATDSPVPRGMGDFSIMNQSSYSGSDDITLSPQVN

KVQDEALSLSVPRQRRRRRRKVEIEAERAAKRRNLMEMVAQLRESHATESRTRVMDLTKA

LHGLAPSSSTSSSSAVFPGTVASPMELLHAAGASRANGAMLEAEIPTRRRRGRRKNVEGL

ELLFMGNKSSQLNTEDSNGTKGFTDGAQMSSRTQTHLQTPAQKDEGDHLRGGDDGTSTKD

LQEWLRQHPTYTMDVPGYIPKNEELLLSQLGGKPKQKRHRCRNPNKIDINTLTGEERVPV

VNKRNGRKMGGAMAPPMKDLPRWLAENPEFSIAPDWTDIVKQSGFLPESMFDRLLTGPVV

REEGVRRRGRRPKSEMAKAAAAQASLSAASSASASGINPFLLNGLFGGMDLSSLQNLQNL

QLLMGLSPSLSAGAADSKNSATMLPLMLPAMGALPNVFGLGGLFGGNMAAAAANLNTNSN

SAATSESEETKKDGEPKEEAEEKKVEVEKPAESSDANTAAAATGLSTNPLAFNPFLLSTM

APGLFYPSMFLPPGLGGLGLPGFSPAALAELQSVMSGALGGAGEEKEVSKETEQRQEAVK

PSSAVENSDSEEGENKTHLSAAAEELDSIEAGEEEDDDDDEDEDDEEDVGEDDDEDKSD*

>m.321 g.321 ORF g.321 m.321 type:5prime_partial len:98 (-) Unigene000027:8649-8942(-)

GYSDYIQCSGFDRAITMVTGVTKVTVWASVPGSVFMMPSVEVVVEEDDAARCHASNDTSL

VSGYGHGANSSIAAWISEELFEIMNLPHTKNTSLSST*

>m.316 g.316 ORF g.316 m.316 type:complete len:2818 (+) Unigene000027:405-8858(+)

MAEEKAPPGGEGITPIVPIRGIRMKFAVLEGLIEAGEVTNRDIVETVFNLLVGGQFDLEL

NFIIQEPESIPCTVDLLEKCEVTCQAEVWSMFTAVLKKSVRNLQACTDVGLITLVLQRID

RAHSMIADLLVDMLGVLASYSITVKELKLYFSKLQGEKRQWPPHAVKLLSVLKSMAQRSG

PDAFFTFPGKSAAAIALPPIAKWPYQNGFTFHTWLRLDPINNINIDKDKPYLYCFRTSKG

MGYSAHFVGGCLIVTALKAKGKGFQHCVKFDFKPQKWYMVTIVHVYHRWKNSEISCYVNG

ELASFGEITWFVNTSDTFDKCFLGSAETADANRVFCGQMGAVYLFNDALNAAQIFAIYQL

GSAYKGVFKYKAESDLLFADHHKILLYDGKLSSTIAFTYNPRATDAQLCLESSPRDNTSI

FIHSPHALMLQDVRAVITHSVQSAVHSIGGVQVLFPLFTQLDFVQPNSEEPDISVSCTLL

SFLLDLLKSSVAMQEQMLTCKGFLVIGYSLEKSSKVHVTRPVLDIVLSFARYLSNLHNGI

LLLKQLCDHILFNPAIWIHTPAKQVLYTYLATEFISTVTIYNAVRRVGTVLQVMHTLKYY

YWVVNPQDRSGVMPKGLDGPRPSQKEIHSLRAFLLLFVKQLILKDTGVKEDELQSILNYL

LTMHEDDNLMDVLQLLVALMSEHPSSMVQAFDQRNGIRVIYKLLASKSEGIRVQALKVLG

YFLRHLQPKRKSEVMLSHSLFSLLTERLLLHSNQFSMTTYNVLFEILTEQICTQVIHKQH

PEPDSALKIQNPQILKVIAALFKNNSCPESMEIRRVFLSDMIKLFNSSRENRRSLLQCSV

WQEWMLSLCFINPNSSEELKITEMVYTVFRILLYHAIKHEWGGWRVWVDTLSITHSKVTF

EKHKENLSRVFQEYQRMGSTDDTEIRTVSGSSRNSELTAQPSETVVEPPSSIIEVEEPIC

GEHDEHEEKAQVIISNILAGVEEEDTKEKEEKATKDEGNVQEALEEKDTVSEVVEEKKDC

NKAMDKDGTEGGDHKNLEEENNQKVESDQQANAVKISENEKDKPATSDIDEEEKESEKAE

GQKENEMKPPPETPVQGDGEEKKQKEDKNSDLSEVSPGAEKGSDMEVQTRVEASTDVQAS

PDQETNLGLEKVSSTQQGPSSETSPSDQESPKAETSPSLQEVPSVPESPKAETSPSLQEV

PSVQESPDALMSPSKEESPSMQELSSVEKSPSVEEDQSTQIPKESPSTQEASGVPESLNV

ETSIKSKGVKIARLDVSSVAIDTERLELKDNTAADGGQSQAAAGTSTQNQDGSSSSPRPS

MFRIPEFKWSHMHQRLLTDLLFSIETDVQMWRSHSTKTVMDFVNSSENVVFVHNTVHLIS

QVVDNLIMASGGILPLLSAATSSTHELEAIDASQGLEVETSLSFLQRLVSLVDVLVLSSS

LNFTEIEAEKNMTSGGVLRQCLRLVCTMAVRSCLECQLHSLYKAGTEKTQRGLVGNAQFL

PAQSPVDMLTGGIAPIRDMERLLQDMDINRLRAIVFRDIEDSKQAQFLALAVVYFISVLM

VSKYRDILEPHNDKKINKNSSRRTESVSSDRGNSSPLQRLDSAPGSEDSPVPQHNAESRS

APDAVSEALSTLSSEVRGHENMDKDKKGKSMKVKDILRSLVSAAPADDVIVDASLLPPTF

LGKQEIHGQFSSFDRSVVVGSRKVGGAPAAGSSPSFTAATTGGTPVVSVSVVSTDDPAPS

DAAESGGTTEVTSNLPSVPLSPTTTLSISERLEHALEKAAPLLREIFVDFAPFLSRTLLG

SHGQELLIEGTSLVCMKSSSSVVELVMLLCSQEWQNSIQKNAGLAFIELVNEGRLLSHTM

KDHLVRVANEAEFILSRQRAEDIHKHADFESHCAQYAADKREEEKMCDHLIRAAKYRDHV

TAAQLIQKIINILTDKHGAWGSSTHSKQCFWRLDFWEDDLRRRRRMVRNPFGSSHSEAAL

KSAAEHAPEDDVLKGKQLGKEPARNQSSESESSAEGDDETISSLEEKELDNLTGTVSFST

VAELVSPALVMKGTLSITAHELYFEVDEEEASFKGIDPKLLVYADGLHGKWMFSEMRAIF

SRRYLLQNTALEIFMANRTSVMFNFVDAATVKKVVHALPRVGVGTNFGLPQTRRISLATP

KQLLKASNMTQRWQKREISNFEYLMFLNTIAGRTFNDLNQYPVFPWIISNYDSEELDLTL

PSNYRDLSKPIGALNPKRAAFFSERYESWEDEQVPKFHYGTHYSTASFTMMWLLRIEPFT

TFFLNFQGGKFDHADRTFSSVARAWRNCQRDTSDVKELIPEFFYLPEMFINANNYNLGVM

DDGTVVSDVELPPWAKSPEDFVRFNRMALESEFVSCQLHQWVDLIFGYKQRGPEAVRALN

IYYYLTYEGAVNLSSITDPLLREAVESQIRSFGQTPSQLLIEPHPPRSSAMQVTPLMFTE

QMQQDVIMVLKFPSNSPVVYVSAHTQAGLSNPSIVTVTANRIFSINKWHGLTGHQSSSNT

EQQYQLPVEIDPVIVANLMHRRQVCDLLDQSVEVNSHCFLVSSDNRFLFIAGFWDKSFRI

YNTDTGKLMQIVFGHRDVVTCVFRSESYIGGDCYILSGSRDATLLLWYWSGKHCCIGDTQ

NDFVTPRAILTGHDCEVTCASVCAELGVVISGSKDGPCLLHSVSGELLRVFENSQRPMLV

QTSSEGHCVIYYSSGQVCVYSINGKLLCDTHIEDNIKAMLLSKDGQYLLCGGERGVLSVW

QVHDLKQLFTYPGCDAAIRSMAITADQRCIITGMASGSIVLFYNDFNRWHHEYRTRY*

>m.325 g.325 ORF g.325 m.325 type:3prime_partial len:82 (-) Unigene000027:3-248(-)

MILSFSLSLAAKNTPKQNTNSPAVDSSALCELTVLLLGVFFLFLERHLFLNRLALGRRRH

DSLQVGERALRMRSSGLRGAVR

>m.327 g.327 ORF g.327 m.327 type:complete len:2379 (+) Unigene000028:266-7402(+)

MTATTRGSPMGGNESQEQGQAPDGQSQPPLPQNQTSTPTSSNENSPVSPPDEQVQGDCTG

QLEEEEEPAFPHTDLAKLDDMINRPRWVVPVLPKGELEVLLEAAIDLCKKGLDVKCEACQ

RFFRDGLTISFTKILTDEAVSGWKFEIHRCIINNTHRLIELCVTKLSQDWFPLLELLAMA

TNPHCKFHIYNGTRPSETVPAGVQLAEDELFARPPDPRSPKGWLVDLINKFGTLNGFQIL

HDRFMSGQALNVQIIAALIKPFGQCYEFLTLHTVKKYFLPVIEMVPQFLENLTDEELKKE

AKNEAKNDALSMIIKSLKNLASRVPGQEETVKNMEIFRLKMILRLLQISSFNGKMNALNE

VNKVISSVSYYTHRHGNPEEAEWLTAERMAEWIQQNNILSIVLRDSLHQPQYVEKLEKIL

RFVIKEKALTLQDLDNIWAAQAGKHEAIVKNVHDLLAKLAWDFSPEQLDHLFDCFKESWT

NASKKQREKLLELIRRLAEDDKDGVMAHKVLNLLWNLAHSDDVPVDIMDQALSAHIKILD

YSCSQDRDTQKIQWIDRFIEELRSNDKWVIPALKQIREICSLFGEAPQNLSQTQRSPHVF

YRHDLINQLQHNHALVTLVAENLSAYMENMRQFSKAEHTDFDPQTVRPGSRYSHVQEVQE

RLNFLRFLLKDGQLWLCAPQAKQIWKCLAENAVFLCDREACFKWYSKLMGDEPDLDPDIN

KDFFENNVLQLDPSLLTENGMKCFERFFKAVNCREGKLVAKRRVYMMDDLELIGLDYLWR

VVIQGSDDIASRAIDLLKEIYTNLGPKLQANQVEIHEDFIQSCFDRLKASYDTLCVLDGD

KDSINCARQEAIRMVRVLTVLREYITECDSDYHEERTILPMSRAFRGKHITLIVRFPNQG

RQVDDLDIWSHTNDTIGSVRRCILNRIKANSAHTKIELFIGGDIIDPADDRKLIGQLNLK

DKTLITAKLTQVSANMPSSPDSSSDSSTGSPGNHGNHYSDGPNPEVESCLPGVIMSLHLR

YISFLWQVADLGCNLNMPLLRDGARVLMKLMPPDNTTVGNLRAICLDHAKLGESSLSPTL

DSRFFGPSPSQVLYLIEVVYALLMPASGTLGEDASDFQYNFLRSGGLPLVLSMLTRNNFL

PNADMETRRGAYLNALKIAKLLLTAVGFGHVKAVAEACQPVAEGNIPVSPINQATHDQAL

VLQSALQNIPNPSAECMLRNVAIRLAQQISDENFFQASKYIPDIGVIRAVQKIVWASGCG

SIQLVFSSIEEISNIYEKTNAGNEPDSEDEQVCCEALEVMTLCFALIPTALDALSKEKAW

QTFIIDLLLHCQSKSVRQMAQEQFFLMATRCCMGHRPLLFFITLLFTVLGSTAKERAKHA

GDYFTLLRHLLNYAYNSNINLPNAEVLLNNEIDWLKRIKDEVKRTGETGVEETILEGHIG

VTKELLAFQTAEKKYYIGCEKGGANLIKELIDDFIFPASNVYLQYMKSGEFPPEQAIPVC

STPATITAGFELLVALAVGCMRNLRQIVDTLTDMYYSGCEALTEWEYLPPVGPRPTKGFV

GLKNAGATCYMNSVIQQLYMIPAIRNGILAIEGTGSDVDDDVPGDEKQDNESNVDPRDEV

FSYHHQFDDKPSLSKSEDRKEYNIGVLRHLQVIFGHLASSRLQYYVPRGFWKQFRLWGEP

VNLREQHDALEFFNSLVDSLDEALKALGHPPMLSKVLGGSFADQKICQGCPHRYECEESF

TTLNVDIRNHQNLLDSMEQYVKGDLLEGANAYHCEKCNKKVDTVKRLLIKKLPPVLAIQL

KRFDYDWERECAIKFNDYFEFPRELDMEPYTVAGVAKLEGSDVHPENQVIQQNEPSEPEA

PCSSRYRLVGVLVHSGQASGGHYYSYIIQRNGSGSEGETNRWYKFDDGDVTECKMDDDEE

MKNQCFGGEYMGEVFDHMMKRMSYRRQKRWWNAYILFYERIDSMDKDGELVKYISELTVS

SKPHQIKMPPAIECSVRKQNVQFMHSRMQYSLEYFQFIKKLLTCNSVYLNPPPGQDHLLP

EAEEMAKISIQLAARFLFSTGFHTKKAVRGPASDWYDALCILLRHSKNVRCWFAHNALFS

YPNRFSEYLLECPSAEVRGAFSKLIVFIAHFSLQDGPYLSPVASPGPSTQGCDNLSLSDH

LLRAVLNLLRREVSEHGRHLQQYFNLFVMYANLGLAEKTQLLKLGVPATFMLVALDEGPG

PPIKYQYAELGKLYTVVSQLVRCCDVTSRMQSSINGNPPLANPYGDPSLTQPIMSLHQLV

AEILFVRTSYVKKIIEDCSNSEETIKLLRFCCWENPQFSSTVLSELLWQVAYSYTYELRP

YLDLLLQILLVEDSWQTHRSAYAFLASRLIPATLKLHF*

>m.329 g.329 ORF g.329 m.329 type:complete len:201 (+) Unigene000028:7538-8140(+)

MVALFSNCSVAYQILQSNGDLKRKWTWAVEWLGDELERRPYTGNAQYTYNNWSPPVQSNE

TSNGYFLERSHSARMTLAKACELCPEECHLTKHEVVSEEDAGRKLSSPQQLLPGEVTGQQ

QHTEQDEQEAPDDQDSSPPEDTSLYPHSPGTQYQPQNNLPHGQPYTGPAAQHMNNPQRPG

PRAQENWEPPEEVPPAQTKE*

>m.334 g.334 ORF g.334 m.334 type:5prime_partial len:2866 (+) Unigene000029:2-8599(+)

DRKSVVSEIGFSSVADFIGSLNEHLVVKNGTIFHRNHLPQTQDTATENKVAQSLPEAFQN

ADNDKISQEVLELIQEHPEGIPINQLATFYNIRYRKNLIVSNLGFASLSSFVKSMSKDLL

VKNGSILHKMHRVVSERPALQKETLSTVKANSKMSDGTACTRKEGEMSKVELLKKVKEVI

QVYPDAATSITQLMNGYFLHFGSILPLGLYISLYDSQTSTQTATVGQAQVIAGHKTGSPP

TSIKMFTSATASPQHAPVTEEIMRSKSGSPVWRLTATPPPSDSSLTSSIDFPPLTTRPSR

AEDRRHGQEGSKLVFREAHHAQLREVHGANVRAVEALEINETTDRRKTRPSLEEVNNLAE

DAIQDLAAQGEHVTVEKVTNKVCRLLQVPSLNALGIRSYWQLPALKELERTIKEINLFVQ

SIEATTSMCTLYELGHAIASIKNKKRFEELQLGPLSKIPIIHRLFTIDSNTKDDDIHQIE

TVDLLRSLRNFRRKQNKPKVDLAEFLQYLADQYNCDSPYALGIRIQSVGLPISTLSKAYG

SENASLERARETIQKEIEEEVQTRLWKIKKTLLEPAQGSPLYSSYGSLELRKKYTTLTAS

EAVLEVFKNSVGIFNQRMTKRVQEFLTNISEDRLARTLFQLAICGGSLAIPNDLAPKEKP

QKQTQEQSASEKKATEMLPTEDAVKQYFLDTVSSFMGTVTLSYLNRLERTIAKHFRFKEF

SQLHQGSFLEFLVKNMQILQDAAGGAVAIANQEVRANGFRPSKQDVYEFIKQCGETDPSK

LPFIEAAIRSHYKVQDSRELGYGKLHTLADFARHQKELCDGVASTFVFYECPLFHKEPED

MLVNSEGLLGDVSREQAVASLLSAPLLEDLAEWSQWELVFQSNHGPLKHFIDKYCGNTDL

LALEVSPGVLLRITSVTGDKFFSQASQALDPAGTAGHLVSIVVRDGILNTPTALLANLME

SSLAAAAAKEDPSLGEDRCSFSIVAKFVLDCVSRMPTRICRELLQQVFLEPLSKVLGQAR

SKIVLLDTARSEARYLNKLHQLGIMLGITEWVKDFHNKLILPPTPELPQQNLAAESVSDL

SSVLSLADSEDLLETFQQDINDCIKDNVEDDSEDDAEDEDEDLFELASEKNEKVNGSVES

KEEDEETNDVDSIPKGHAEEDETFSEQLSQQMAIIEDIRKNEFGVGVELNEAGQNLMKKQ

QARLGRSLERLSTELYSKDTHFVLELIQNADDNSYPLHRKEEPALAFIVQKDCITILNNE

FGFEEKNVRAICDVGKSTKGKHTCGYIGQKGIGFKSVFKVTDCPEIHSNDFHIQFDKASG

PMGYILPHWVEEERHVPPVAKEIAEKSWTTKIFLPLRSESYQTKNLFHDVHPSLLLFLHR

LRSITIYSEAERKVVSMMRRDLNHNVLEVEHTGGVERWLVVKKILYPEKVKVDVESTELA

LAFQLNNGSEGKVKVQPRQQPVFAFLPLRSFGFRFVIQGDFDIPSSREDVDRDSSWNQWL

RSEIPQLFLQAMDVFTTHPEFSGLQGLCHFLQYIPQPSEILDFFNPVAKQIIQLLKGKPC

LPAKMDSNGGVQFKLPSQVAVCQDPLIQDVIGGEELCKHLNLSYLHPELQSSLSSSLLSA

LGVQRLKAANITTVICAMAKSLVQEGDIYTDSNLKKLAKLLACNFRALESEYNEVDSLLQ

ALRDIPMIPLADGRVVSLSAEGVFFPLSEETHKGLNALYKDLSIVEPRLLECLDKLGNSQ

VRELLCRLEVHELEPKEVLQNHIYPILKQAAWKEKPEKITVSYLVFIKEHSQEQDYRALR

EAIPVLTNKGFLCPGQCKVQFSKEYGNIDLPNKLPGVDWVLLDSCYLCDGDLSGWRDFLS

DLGVRDLLIFKKERRNLKDTELVSSPWAAEAELWPKPTDKLYVIEDQQCEELHSLITADQ

LPPTIKLQQRQALINLLQNNWDTGEKYSQYLSAQVLDNQGRTLKDTRSSFYYHLTQLAWM

PSYKPTQDEKNSVEYLIPNRVYLFSNKVHSLLGSHVFYVNLDPSEFSRAVGMRHTVVVDE

MISYLKRWCTKESDSGEAEGADFITTAQHIHTVYLYLQAECSHTQLRDLFQHSPAVYIEY

DRKDEWSSGRFYHLKDVCWRDPTDMFVRYKELIRKPDSRVQEPKVLAPFYSKLPQMEELF

KSLNVYPSPSMKQYVDLLETVCESSPLPTGEVLQDVSIIFARLADKCKNPTNVGQDQDIE

MNQCYCMTLKEMVSRLKVFPTKTQGWVTLARKPMIADSPSLEKIFKSYKEVCLLNLPSAQ

KKVQRHKPNQKAKLHDEKLAFNEGDRELFLEICGVKKLAQCVTTEPTTELYRPCPAMQNL

VRNLIPYIQRFIYHHDELGEIYDDLKENGIAQEIKSLSFGQVGMLYINYRLEQPDESPIV

ESEDIICLLKDRKELYIQKDHLSSKMAICRELVKLFTTEKSFVEELKHFLEELVIRISDP

DSLKRLLDGKNIQELPQDEERWEVPAPLETFEPRTFTISSSGIVSAEEEEINTEEDTLHS

WPPKASISKTGGRSTGKGSQAVEAVMKMWPPPAPLTSSAQSVSPAPSAHWSSGAERSPSN

LNSQERTTELQMHREPGSLNIPGNQEPRERAGIPRAEAQTSNADVPVVQGVEAPNEAHAG

SNGAPDEHTEVCVDKPENSTLSPAEEQQPTLQPTEQVNKPVHPPEVMESQFQGSVIYRPP

MPLDSAVWTKPTVAGVLEDLALDFSLPNTLQVPCAYDDSDDIGRWGEQLVYSFLTRWRES

GEGPREISWSNEKGESGQPYDFKLIFPTGCNTTREVFVEVKTTVKQERHFIHMSANEIDF

ALKEKEKYHIYRVYGAGDSQHTRLCRIKNLAQHLHSKTLELFLFV*

>m.340 g.340 ORF g.340 m.340 type:5prime_partial len:2839 (+) Unigene000030:1-8517(+)

SEERRGGNGTATGHHAYPGAQGDGRGGGGAAAGQSFQDGSGAATVNNWGPQGMQLPDQNR

AQFPQSQQQQQVHGHQVAPYIGRTDFGGMQGHNQTQAPPRMNHFPQGLPQEANRFMGHMG

ANMTSSTMRHPSQTPQHQQAGQQFLHHHTDSQFPQSPSKQQQMLSGRGHQNMGFSLNNQQ

VANSSSGQYAAYPQYSGLNQGLANASGMSLNTSSNPNASVQCYPTPAGPQCYPVNQLPPQ

NMHPKLPQQQQQPPPQQQHLVPPPQGSYATPPSMSPMRNLGGNPAGTPPPQRGQGPISHP

QQHPQMYGTLSPNQRAMNAPNHTEVLPQQLQTSTLQFQQVRTPPTLSVSQQPPGSSPPTP

LPQGNWSSNTHSSPPANHQQVPHMQPSPSDASQTLSSTHNSTPSSTLQLGSCDTQPLDSD

SKPKKKKKVKSEEKDEDMGVCGELGGAGVSGETSSPEKKRKKKPKEKQAKDTKEPKTPKT

PKAPKTPKEPKEKKVKNTTPKPRSTKKNSSKQEECESSSKKEGKRKRESSITLDPEKTPP

LSPEEEEEDDGVQKRRSCRQVKRKRYTEDLEFRISDDDDEDSTGPKSPSTTSEPTEVLDA

EGPVVEKIMGLRTVKKQLESGQEVEVEEFYVKFKSFSYLHCRWADLEELEKDKRIQQKVK

RFKAKQAHTNFLSEMDDEPFNPDYVEVDRVLDVSESTDENGEVVTLYLVKWCSLPYEDST

WELKVDVDAGKIEEFEQVMSREPQLKRVERPPASDWQKSQSSRQYRNNNALREYQLEGVN

WLLFNWYNTRNCILADEMGLGKTVQSITFLYEIYLKGVHGPFLVIAPLSTIPNWEREFRT

WTDLNVVVYHGSQASRRTIQTYEMNYRDTQGRVIKGAYKFHAIITTFEMILTDCPELRGV

PWRCVIIDEAHRLKNRNCKLLEGLKLMDMEHKVLLTGTPLQNTVEELFSLLNFLEPERFP

SESTFMQEFGDLKTEEQVQKLQGILKPMMLRRLKEDVEKNLAPKEETIIEVELTNIQKKY

YRAILEKNFTFLSKSGGGGGGGGSNVPNLLNTMMELRKCCNHPYLINGAEEKIMEEFRET

HPADLPDFHLQSMIQAAGKLVLIDKLLPKLKAGGHRVLVFSQMVRCLDILEDYLIQRRYP

YERIDGRVRGNLRQAAIDRFSRPDSDRFVFLLCTRAGGLGINLTAADTCIIFDSDWNPQN

DLQAQARCHRIGQSKAVKIYRLITRNSYEREMFDKASLKLGLDKAVLQSMSGRENAANGV

QQLSKKEIEDLLRKGAYGALMDEEDEGSKFCEEDIDQILQRRTQTITIESEGKGSTFAKA

SFVSSGNRTDISLEDPDFWQKWAKKAELDLDAINGRNTLVIDTPRVRKQTRHYSSIKEDE

MMEYSELESDSEEKPVQKSRRPQDRTQGYPRSECFRVEKNLLVYGWGRWKDILCHGRFKR

PLYERDVELICRALLAYCLLHYRGDENIKSFIWDLITPTEDGQSKTLTNHSGLSAPVPRG

RKGKKGKLPVTQMNMPQADWLAECNPDVLLQEDSYKRHLKHHCNKVLLRVRMLYYLRQEV

IGDLADRILEGADSSELDIWIPQPLHAEVPTDWWDVEADKSLLIGVFKHGYEKYNSMRAD

SALCFLERVGMPDAKAIAAEQRGTDMVADGEDEDPEYKPLRMPFKDDLDDFTNSPLDEKE

EVMDVEAGDAPKESEVNGGKAGNLYWPTASAFTARLRRLITAYQRCHKRQETLIKPDGRR

RRRHRDDHLLAMATEGGAYSLEPSRVTPTAYLTEGASFKTSPFLHESAALLADGTAFFKE

RRQRWTRREEADFYRVISTFGVVYDVQTQRFDWSQFRAFARLDKKTDESLEKYYYAFVAM

CKRVCRMQVKDSELADPTLIIDPITEERASRTLYRIELLRRIREQVLPHPKLEERLHLCQ

PNPELPAWWEAGKHDRDLLHGAAKHGVSRTDYHIQNDPELSFLQAQRKFIQNRGSDTALT

SDPPPTGDLIKDEELRNDPAENTGMEDMKEENTSSPKQEVKPEEEEKTANEKMESENEAE

VKSEENRSVPQMTEEKVTESEDTPTPHVTVHQDTEKEEPENPKELEKNSEEEEEEEEKMD

EDDKSEKSSQAEAGASSDRKNFDEESNASLSTARDETRDYFGLDDVEPSVSQMFGERAPF

SFWPKDRVMINRLDSICEAVIKGKWPSNRKQFFDMPGLLPGYVAMATDSPVPRGMGDFSI

MNQSSYSGSDDITLSPQVNKVQDEALSLSVPRQRRRRRRKVEIEAERAAKRRNLMEMVAQ

LRESHATESRTRVMDLTKALHGLAPSSSTSSSSAVFPGTVASPMELLHAAGASRANGAML

EAEIPTRRRRGRRKNVEGLELLFMGNKSSQLNTEDSNGTKGFTDGAQMSSRTQTHLQTPA

QKDEGDHLRGGDDGTSTKDLQEWLRQHPTYTMDVPGYIPKNEELLLSQLGGKPKQKRHRC

RNPNKIDINTLTGEERVPVVNKRNGRKMGGAMAPPMKDLPRWLAENPEFSIAPDWTDIVK

QSGFLPESMFDRLLTGPVVREEGVRRRGRRPKSEMAKAAAAQASLSAASSASASGINPFL

LNGLFGGMDLSSLQNLQNLQLLMGLSPSLSAGAADSKNSATMLPLMLPAMGALPNVFGLG

GLFGGNMAAAAANLNTNSNSAATSESEETKKDGEPKEEAEEKKVEVEKPAESSDANTAAA

ATGLSTNPLAFNPFLLSTMAPGLFYPSMFLPPGLGGLGLPGFSPAALAELQSVMSGALGG

AGEEKEVSKETEQRQEAVKPSSAVENSDSEEGENKTHLSAAAEELDSIEAGEEEDDDDDE

DEDDEEDVGEDDDEDKSD*

>m.365 g.365 ORF g.365 m.365 type:3prime_partial len:80 (-) Unigene000031:3-242(-)

MSPVLLSPNLNVLYNLNHKKVSLLIVFFFTPAPLRATRLRSEPQNGKFSLKHVKCCKKNN

NKSCSEPLGLTSPSATSTTR

>m.360 g.360 ORF g.360 m.360 type:complete len:96 (-) Unigene000031:3273-3560(-)

MFSLPPSSFINKLSPKSENPKQKRLIAHFPANQKGSGCKYQRSHRVMFKLKKKKKMKIKV

TSGTGSLQQPLRCEIRSLMSEGKSWMSSHSHSNSA*

>m.351 g.351 ORF g.351 m.351 type:complete len:982 (+) Unigene000031:241-3186(+)

MATQVETLLHTTINTTTAALLQDDDLQSPVGHGELSPVPSSKQAGNENSPQENMEEKKEF

KEAPPPKVNPWTKKLNSVTVNGQTAPEQTGPAKVVRAGNPRPRRGGKVGDFGDVTNWPTP

GEIATKEVQGCKKASVKKDSREKRDSEECKENQKAKSDDSGEEKNREEDLHKNNAHRKKG

NKHKWVPLMIEVKSEGPRERSASRNNARQGETPRMHHSNRNDLRDWHSGKYERDQFRDDH

DEVSSVKSEGAPFRGGFRGRGRGRGRGRGRGRGGRGQYDYPYSYKCEGKDGVYDPKYTQS

VTYYYDSMSSAELYSVDQDLLKEYIKRQIEYYFSMENLQRDFFLRRKMDEGGFLPVALIA

SFHRVQALTTDIQLIMEALKDSKVVEIIDMKIRCKEEPAKWPLPGLTLSEHTHTDFSQLI

NCPEFIPRNTSDTPHTGSPRVSSAAEQKLEEVCNLKTMPKGLSASLPDLDSECWIEVKKR

PRPSPARPKKLEDLRSALTPGDQDEPEELDFLFDEEMEAMDGRRNTFTEWTDDESDGEID

DHDVNKILIVTQTPPYLRKHPGGDRTGNHVSRSKLTNELVKVINDGLFYYEQDLWHDTYE

PEYATIKQEVENFKKVHLISREQFDCLTPEPPIDPNQEVPPCPPRPQHIPTDDLANKLFG

APERSVMARSLPTAVPDSPAYRAPHTPHTPRTPRCKHNTPRFYPVIKDGRPVDAKTPRKR

KTRHSSNPPMECHVGWVMDSREHRSRTASVSSNASPSEGTAALGNIGCTPQSLPKFQHPS

HELLKENGFTQHVYHKYRRRCLNERKRLGIGQSQEMNTLFRFWSFFLRDHFNKKMYEEFR

QLGVEDAKEGYRYGLECLFRYYSYGLERKFRAEIFKDFQEETIKDYEAGQLYGLEKFWAF

LKYSKAKNLEIDSRLQEYLCSFRRLEDFRVDPPMDEGRKRHSSSGDGRRRHPSQSSARHT

HTSDHNLDPKPGPAPTADCSK*

>m.352 g.352 ORF g.352 m.352 type:complete len:666 (+) Unigene000031:5801-7798(+)

MHYKEAIRISPTFADAYSNMGNTLKEMQDVQGALQCYTRAIQINPAFADAHSNLASIHKD

SGNIPEAIASYRTALKLKPDFPDAYCNLAHCLQIVCDWTDYDERMKKLVSIVADQLEKNR

LPSVHPHHSMLYPLSHGFRKAIAERHGNLCLDKINALHKPAFEHPKDLKASGGRLRVGYV

SSDFGNHPTSHLMQSIPGMHNSEKFEVFCYALSPDDGTNFRVKVMAEAHHFTDLSQIPCN

GKAADRIHQDGIHILVNMNGYTKGARNELFALRPAPIQAMWLGYPGTSGAPFMDYIITDK

ETSPAELAEQYSEKMAYMPNTFFIGDHANMFPHLKKKAVIDFKSNGHIFDNRIVLNGIDL

KAFLDSLPDVKVIKMEGDGQESTDINGALSMPVIPMNTAAEAIINMINQGQIQVTINSFT

VSNGLATTQINNKAATGEEVPRTIVVTTRSQYGLPEDSIVYCNFNQLYKIDPPTLQMWAN

ILKRVPNSILWLLRFPAVGEPNIQQYAQNMGLPTSRIVFSPVAPKEEHVRRGQLADVCLD

TPLCNGHTTGMDVLWAGTPMVTMPGETLASRVAASQLTCLGCPELIAQSRQEYEDVAVKL

GTDMEFLKKVRGRVWKQRICSPLFNTKQYTVELERLYLQMWEHHAGGSKPEHLVKMQLLD

SSESA*

>m.367 g.367 ORF g.367 m.367 type:3prime_partial len:1618 (+) Unigene000032:3957-8813(+)

MGDKLWWADQGTDQVGTCDKKDGGSWKVLRNGTSPVMHMKIYNETVQQKGTNLCTMNNGD

CSQLCLPTSPTSRACMCTAGYNLKSGQQSCEGMGSFLLYSVHEGIRGIPLDPLDKSDALV

PVSGTSLAVGIDFHAENDTIYWVDMGLSTISRAKRDQTWREDVVTNGIGRVEGIAVDWIA

GNIYWTDQGFDMIEVARLNGSFRYVVISHGLDKPRAIAVHPEKGYLFWTEWGQYPRIERS

RLDGSERVVLVNVSISWPNGISIDYEEGLLYWCDARTDKIERINLETGGNRELVLAVHNM

DMFAVSVFENYIYWSDRTHANGSIKRGNKNNATDMVYLRKGIGVQLKDIKVFNRARQQGT

NICKDKNGGCEQLCLFRGNGARTCACAHGMLAEDGRTCRDYDGYLLYSERTILKSIHLSD

ETNLNAPIKPFEDPDHMRNVIALTFDHQGGDGKGANRIFFSDIHFGNIQQINSDGSARKT

VVENVGSVEGLAYHRGWDTLYWTSYTTSTITRHTVDQTLSGAFNRETVVSMSGDDHPRAF

ALDECQGLMFWTNWNELAPSIMRSSLAGSNVLVIVGNNIRTPNGLAIDHRSEKLYFSDAT

LDKIERCEYDGTNRFVVLKNEPVHPFGLAVYGEYIFWTDWVRRAVLRADKFGRDMKVLRA

DIPQQPMGIIAVAKDTNSCEFSPCHTNNGGCQDLCLLTSEGRVNCSCRGDRKLVEGNVCV

AENTTCHSVDEFECGNGDCINYSLTCDGRAHCKDKSDEKPSYCSNRGCKKGYRRCLNGRC

IKHSSWCDGTDDCGDRSDERPCNMTLCSASEFQCKDGSCITNTSRCNQVVDCEDASDEMN

CSPTDCHSFYLLGVKGVIFQKCEFTTLCYTPIWQCDGSNDCGDFSDERNCPEKRQLKCPV

NFFACPSGRCIPMSWTCDKENDCENGTDETHCDKFCSPTQFECGNHHCISSSWVCDGTDD

CGDGTDEGSRCSSKTCSPEAFQCPGSHCVPQRWKCDGDNDCPDGADEGVQAGCTNNKTCD

DTEFQCQNKQCIPKHFVCDHDLDCRDGSDESPECEYPTCGPNDFRCENGQCLKQKSWECD

GEFDCRDHSDEAPKNLHCTEPEKRCNDSAYLCNNGKCVSEDSLCDWKDDCGDGSDEHNCF

VNECLNNKLSGCTQHCEDLKIGYKCRCDPGFRLKNDGKTCVDVDECTTTYPCSQRCFNSY

GSFHCFCIEGYVAYANDWTSCKSSSEEEAYLIFANRYYLRKLNLDGSNYTLIKTDLNNAV

ALDYHYAEQMIYWTDVTTQGSMIRRMLMNGSNVEVLHRTSLSNPDGLAVDWVGGNLYWCD

KGRDTIEVSKLNGAYRSMLVNSGLREPRAVAVDVRNGYLYWSDWGDVPHIGRIGMDGTDR

SIIIKDKITWPNGLTLDFINDRIYWADAREDYIAFASLDGTNRHIVLNQDIPHIFAMSLF

EDYIYWTDWETKSINRAHKTLGTNKTMLISTLHRPMDIHIYHPYRQPAVENHPCQVDNGG

CSNLCLLSPGGGYKCACPTNFYLAADGKQCLSNCTASQFVCKNDKCIPFWWKCDTEDDCG

DRSDEPADCPEFKCRPGQFQCGTGICTNPAYICDGDNDCQDNSDEANCDIHVCLPSQF

>m.368 g.368 ORF g.368 m.368 type:5prime_partial len:1334 (+) Unigene000032:1-4002(+)

AVRSHACAPDQFGKPGGCSDICLLANSHKIRTCRCRSGFSLGSDGKSCKKPEHELFLVYG

KGRPGVIRGMDLHARVYDEHIIPIENLNNPRALDFHAETEFIYFSDAAMYTIGRQKIDGT

ERDIIVKSGIHTVEGIAVDWMGQNLYWTDDGPEKTISVAKLEKASQTRKTLLEGKMTHPR

AIVVDPSHGMMYWSDWQEDSRDANRGSIRKAWMDGSKSGILITSKNMLWPNGLSLDIEQN

VLYWVDAYYDRIEMVLLNTTERRTVYEGQELNHPFGLCHYKNFLFWNEYRIGGIYKLDMT

SKKVTLLRSERPPIYEIRTYDAQQQQIPGHNPCRGNNGGCSSLCLLTPAGRTCACADDQI

LDFDNKTCKANPSYVPPPQCQPGEFACKNNRCIQERWKCDGDNDCLDNSDEVPELCHQHT

CPADRFKCQNNRCIPMRWLCDGDNDCGSNEDESNTTCSARTCPPNQHPCASGRCIPKTWI

CDLDDDCGDRSDEPASCAYPTCFPQTQFTCANGRCINVNWRCDNDNDCGDNSDEAGCSHS

CSSVQFKCNSGRCIPEYWTCDGDNDCGDNSDEKSANCTNQATRPPGGCHSDEFQCRLDGL

CIPMRWRCDGENDCIDDTDEKHCEGVTHTCNPSAQFACRDSARCISKAWVCDGDSDCEDN

SDEDNCEALLCKLSHHTCAQNDSICLPAEKLCDGKDDCPDGSDEKLCDLCSLENGGCSHN

CTIAPGEGILCSCPTGMELGSDNKTCQIQSFCAKHLKCSQRCIQEKATVKCACYEGWALE

PDNESCKSTDPFKPFIIFSNRHEIRRIDLYKGEFSVLVPSLRNTIALDFHLNQSSLYWTD

VVEDKIYRGKLSENGALTSFEVVIQYGLATPEGLAVDWIAGNIYWVESNLDQIEVAKLDG

TMRTTLLAGEVEHPRAIALDPRDGILFWTDWDASSPRIEAASMSGDGRRTIHRETGSGGW

PNGLTVDYLERRILWIDARSDAIYSAAYDGSGLIEVLRGHEYLSHPFAVTMYGGEVYWTD

WRTNTLAKANKWTGHNVTVVQRTNTQPFDLQVYHPSRQPQAPNPCAANDGRGPCSHLCLI

NYNQTFSCACPHLMKLQADKHTCYESRQFLLYARQIEIRGVDIDNPYYNYIISFTVPVID

NVTAVDYDAQEQRIYWSDVRTQMIKRAFINGTGVETVVSADVPNAQGLAVDWVSRNLFWT

SYDTSKKQINVARLDGSFKNSVVHGLDKPHCLVVHPMLGKLYWTDGDNVSMANTDGSNQS

LLFTSQKGPVGLSIDFDEGKLYWISSGNSTINRCNLDGTGLEVIESVKGNSPEPPLSPSW

GTSCGGRTRAQTR*

>m.385 g.385 ORF g.385 m.385 type:complete len:2493 (+) Unigene000033:767-8245(+)

MDRNYPGAGFGDLGAGAGWSYERSAKASLVYGSSRSSHPESELLHRQAYATPHPLQGYAT

NHHPGSSGQGGAWGAAGRSLGLSGLFDAGLHHASPSGPDPSVMNLISALESRGPQPPPSA

SSLLSQFRTPSWQTAMHTPAPAELFISGALPGSGSFPSSSALSAYQHPASFSGRSFPGVT

PSLSLQDTPTFSPTSNGLLSPHDPLLHIKTPSQSSLGFDRLLSSQGPAAAYRGSHDPSGS

SGVTSAQASSAASASARHLQSHQFNLLSSQLQDQSSQLYSASVFSSAPPQPASQERTVPR

QDSVIKHYQRPSPAQAPSSTPHPLQHYLSCGVSGYQQAPHSRHAGLSCSPLGEHSPSSDH

KPSPRTEQYRPIIQPSYGSSSSSSSGGPGKAAKSSSSSGYSSSGSGSSSRTPHTPPSASS

ASSSSSSSSSSSASANARQSNSIPSSTSASASSRQQPPTQSVPPSQTHSQPAPSTNSSQQ

TLTKACLSGYGSPAAPVKSSNSLTGQTPPQQQPQSFSPNQPPSHLSQPYAGFSSPQTHDL

PSARTAGVAKGYGNLGSQSFSAESVYGTDSGYGSLPSSLGGAGSPSMGYAASGHSPALLR

SGASGGTAGGSSNGGSTSTGGGNNVTGGGGSYHIPDSSPSPSGNSGIIRPGLHSPAPSRP

AQSPVGTGSNKYLSSVLSPSFLPSPQGYPDTRGPRSQTYHPSAPPKTKSDTSLLGVTESR

PQQDDDDDDFLIQHLLQAQSPTPQASHHHPPQSHHSSQSTQQPPVLPNQDGSKGLSYEIG

KSSEERYHLQSVIRTHSATTNAAVSGTGNGAGLDSQLELSLKKQQHQQQHQTQHQQQQQQ

HKHQQQKSSRSVTDGGGRGNPDQAHSHSHHHHDSMSSVVHYGRGDPYSQHSISQHPSSHH

QHTPHTPTHSHLHSHPHMDLQKKPQESADMAYIRKTPDLQQHHHQQQSGHHQSHQQQHQQ

QQQQSQTLMESPTDQSRQPPHLLQSVLSHTTRNKIETQQQQHPASQQALIEATGGVTAAE

THSQPQASQLQLQLQTQALETASHYSHGSQQQDQNHQKSNTVSSLDMLERSLSRTSSQEG

GMVDDRPGNEGGRGNSAAGTGGPVERHRSQDQQRLPSHHPTQHHPPELHTYLSEPDLGLS

TPSHGHHLSHHHHQSQQPPPAQQPNSHHHAHHQHQNPQSHHHLASQSASATAQQQEQSHS

SHSSQITQAQLDQQHQGDHHFDSSGSAVKSNANQNQSHHNQRFVPLTSICFPDSLLPDEE

RSFFPGMEDMFCPEEYKSSCSGGTGQGQDGVSQAQTGQEGMEGIKSTAEGGEDDTGASYD

MLGHPGDQDYGQYCHDLTEHDNGTMHLDLDSLKTHELPSTVNTEQLGLIQSQPTNLGIGS

SANVGDGAGAKMVGSGGSVSGSGGLTSPIFCSSRPKKLLKSSSFHLLKERPDPNSIPKKS

YAQEYEFEDDEDKADVPADIRLNSRRLPDLIPDLVSSCRKAGGGTMSPLMSDLDFGYPSL

GPPPQLLPNDGPKKRGRKPTKPKREGPPRPRGRPRIRPLPEPHHSRAMMGECGTGYIPER

GRGRGRGRGRGRRDESMMDSKDQSQLYQQHMQQQQMHHLPQQHQEPIKPIKIKLPIGSMS

SSDALLRTDSLSGTDPALSDGSVGSAPSLGLSPGTPGVPDMTRPLDKNKAKTQDMEEKDS

EKTGFVASFLDFLKSGKRPGSSSGNGDSSPGKSGGIHPISPQPPPPPAPAGSSGYGDSEG

DGGLSLGGCPSPCKRLDEELKRNLETLPSFSSDEEDSVGKNQDLQKSISSAISALYDTPQ

LSTSSMQPPSLPPPPPPVAPTPLTPTQHPPSLSPQTPTHMHTQPSSHTHPQSIESTILSR

DEQGDEVEEEIINDEDKEEEQDKGVEEKVTEMELLSVPKVGDSPKEASQAVAPHSLLPLF

PSSPASSSSPSPPPLPPLSLPSPLPELEENPSPPQKPSAQLSSPPASDLATTLPVLSPPH

PLTVASPPPLETPTPSSPPPTQQTVPAPPSPEETPATQILPLHLAQKQSGAAIAGETDDD

ESESGGEGIFRERDEFVVRIEDIKTLKLALQTGREPPPIWRVQKALLQKFAPEIKDGQRQ

FCATSNYLGYFGDAKKRYQRLYVKFLENVNKKDYVRVCSCKPWHRPSVTLRRQAQSHPVS

KVNPSPNNQTLPRVVRDEKDKDREKGRNKEQRDSRDKNNIKVKEQQEKEKVVKIKEREEK

KDKEKKMPPPPAPQRAGKQTAVTEPGRKEEKRGVERKIERTTKQQPLKVKAEPPPKKRRK

WLKEVPSSSDSDSSASDDEISVRAGLNTRAMREMYRSYVEMLVSTALDPDMIQALEDTED

ELYLPPMRKIDSILSEHKRKLLKRINMNPQHQEALHLFPQITAEPLDSGAVRVKLGGECY

NRKTLNRVKKSISKPQDIKLSTETCRLYSFYHSLHHYKYHTFLICKKETNTIEQASEDPG

QEEVVQQCMANQSWLDTLFNAFLELMTLSAKA*

>m.397 g.397 ORF g.397 m.397 type:complete len:2592 (+) Unigene000034:266-8041(+)

MTATTRGSPMGGNESQEQGQAPDGQSQPPLPQNQTSTPTSSNENSPVSPPDEQVQGDCTG

QLEEEEEPAFPHTDLAKLDDMINRPRWVVPVLPKGELEVLLEAAIDLCKKGLDVKCEACQ

RFFRDGLTISFTKILTDEAVSGWKFEIHRCIINNTHRLIELCVTKLSQDWFPLLELLAMA

TNPHCKFHIYNGTRPSETVPAGVQLAEDELFARPPDPRSPKGWLVDLINKFGTLNGFQIL

HDRFMSGQALNVQIIAALIKPFGQCYEFLTLHTVKKYFLPVIEMVPQFLENLTDEELKKE

AKNEAKNDALSMIIKSLKNLASRVPGQEETVKNMEIFRLKMILRLLQISSFNGKMNALNE

VNKVISSVSYYTHRHGNPEEAEWLTAERMAEWIQQNNILSIVLRDSLHQPQYVEKLEKIL

RFVIKEKALTLQDLDNIWAAQAGKHEAIVKNVHDLLAKLAWDFSPEQLDHLFDCFKESWT

NASKKQREKLLELIRRLAEDDKDGVMAHKVLNLLWNLAHSDDVPVDIMDQALSAHIKILD

YSCSQDRDTQKIQWIDRFIEELRSNDKWVIPALKQIREICSLFGEAPQNLSQTQRSPHVF

YRHDLINQLQHNHALVTLVAENLSAYMENMRQFSKAEHTDFDPQTVRPGSRYSHVQEVQE

RLNFLRFLLKDGQLWLCAPQAKQIWKCLAENAVFLCDREACFKWYSKLMGDEPDLDPDIN

KDFFENNVLQLDPSLLTENGMKCFERFFKAVNCREGKLVAKRRVYMMDDLELIGLDYLWR

VVIQGSDDIASRAIDLLKEIYTNLGPKLQANQVEIHEDFIQSCFDRLKASYDTLCVLDGD

KDSINCARQEAIRMVRVLTVLREYITECDSDYHEERTILPMSRAFRGKHITLIVRFPNQG

RQVDDLDIWSHTNDTIGSVRRCILNRIKANSAHTKIELFIGGDIIDPADDRKLIGQLNLK

DKTLITAKLTQVSANMPSSPDSSSDSSTGSPGNHGNHYSDGPNPEVESCLPGVIMSLHLR

YISFLWQVADLGCNLNMPLLRDGARVLMKLMPPDNTTVGNLRAICLDHAKLGESSLSPTL

DSRFFGPSPSQVLYLIEVVYALLMPASGTLGEDASDFQYNFLRSGGLPLVLSMLTRNNFL

PNADMETRRGAYLNALKIAKLLLTAVGFGHVKAVAEACQPVAEGNIPVSPINQATHDQAL

VLQSALQNIPNPSAECMLRNVAIRLAQQISDEASKYIPDIGVIRAVQKIVWASGCGSIQL

VFSSIEEISNIYEKTNAGNEPDSEDEQVCCEALEVMTLCFALIPTALDALSKEKAWQTFI

IDLLLHCQSKSVRQMAQEQFFLMATRCCMGHRPLLFFITLLFTVLGSTAKERAKHAGDYF

TLLRHLLNYAYNSNINLPNAEVLLNNEIDWLKRIKDEVKRTGETGVEETILEGHIGVTKE

LLAFQTAEKKYYIGCEKGGANLIKELIDDFIFPASNVYLQYMKSGEFPPEQAIPVCSTPA

TITAGFELLVALAVGCMRNLRQIVDTLTDMYYSGCEALTEWEYLPPVGPRPTKGFVGLKN

AGATCYMNSVIQQLYMIPAIRNGILAIEGTGSDVDDDVPGDEKQDNESNVDPRDEVFSYH

HQFDDKPSLSKSEDRKEYNIGVLRHLQVIFGHLASSRLQYYVPRGFWKQFRLWGEPVNLR

EQHDALEFFNSLVDSLDEALKALGHPPMLSKVLGGSFADQKICQGCPHRYECEESFTTLN

VDIRNHQNLLDSMEQYVKGDLLEGANAYHCEKCNKKVDTVKRLLIKKLPPVLAIQLKRFD

YDWERECAIKFNDYFEFPRELDMEPYTVAGVAKLEGSDVHPENQVIQQNEPSEPEAPCSS

RYRLVGVLVHSGQASGGHYYSYIIQRNGSGSEGETNRWYKFDDGDVTECKMDDDEEMKNQ

CFGGEYMGEVFDHMMKRMSYRRQKRWWNAYILFYERIDSMDKDGELVKYISELTVSSKPH

QIKMPPAIECSVRKQNVQFMHSRMQYSLEYFQFIKKLLTCNSVYLNPPPGQDHLLPEAEE

MAKISIQLAARFLFSTGFHTKKAVRGPASDWYDALCILLRHSKNVRCWFAHNALFSYPNR

FSEYLLECPSAEVRGAFSKLIVFIAHFSLQDGPYLSPVASPGPSTQGCDNLSLSDHLLRA

VLNLLRREVSEHGRHLQQYFNLFVMYANLGLAEKTQLLKLGVPATFMLVALDEGPGPPIK

YQYAELGKLYTVVSQLVRCCDVTSRMQSSINGNPPLANPYGDPSLTQPIMSLHQLVAEIL

FVRTSYVKKIIEDCSNSEETIKLLRFCCWENPQFSSTVLSELLWQVAYSYTYELRPYLDL

LLQILLVEDSWQTHRIHNVLKGIPDDRDGLFDTIQRSKNHYQKRAYQCIKCMVALFSNCS

VAYQILQSNGDLKRKWTWAVEWLGDELERRPYTGNAQYTYNNWSPPVQSNETSNGYFLER

SHSARMTLAKACELCPEECHLTKHEVVSEEDAGRKLSSPQQLLPGEVTGQQQHTEQDEQE

APDDQDSSPPEDTSLYPHSPGTQYQPQNNLPHGQPYTGPAAQHMNNPQRPGPRAQENWEP

PEEVPPAQTKE*

>m.403 g.403 ORF g.403 m.403 type:3prime_partial len:2797 (+) Unigene000035:405-8798(+)

MAEEKAPPGGEGITPIVPIRGIRMKFAVLEGLIEAGEVTNRDIVETVFNLLVGGQFDLEL

NFIIQEPESIPCTVDLLEKCEVTCQAEVWSMFTAVLKKSVRNLQACTDVGLITLVLQRID

RAHSMIADLLVDMLGVLASYSITVKELKLYFSKLQGEKRQWPPHAVKLLSVLKSMAQRSG

PDAFFTFPGKSAAAIALPPIAKWPYQNGFTFHTWLRLDPINNINIDKDKPYLYCFRTSKG

MGYSAHFVGGCLIVTALKAKGKGFQHCVKFDFKPQKWYMVTIVHVYHRWKNSEISCYVNG

ELASFGEITWFVNTSDTFDKCFLGSAETADANRVFCGQMGAVYLFNDALNAAQIFAIYQL

GSAYKGVFKYKAESDLLFADHHKILLYDGKLSSTIAFTYNPRATDAQLCLESSPRDNTSI

FIHSPHALMLQDVRAVITHSVQSAVHSIGGVQVLFPLFTQLDFVQPNSEEPDISVSCTLL

SFLLDLLKSSVAMQEQMLTCKGFLVIGYSLEKSSKVHVTRPVLDIVLSFARYLSNLHNGI

LLLKQLCDHILFNPAIWIHTPAKQVLYTYLATEFISTVTIYNAVRRVGTVLQVMHTLKYY

YWVVNPQDRSGVMPKGLDGPRPSQKEIHSLRAFLLLFVKQLILKDTGVKEDELQSILNYL

LTMHEDDNLMDVLQLLVALMSEHPSSMVQAFDQRNGIRVIYKLLASKSEGIRVQALKVLG

YFLRHLQPKRKSEVMLSHSLFSLLTERLLLHSNQFSMTTYNVLFEILTEQICTQVIHKQH

PEPDSALKIQNPQILKVIAALFKNNSCPESMEIRRVFLSDMIKLFNSSRENRRSLLQCSV

WQEWMLSLCFINPNSSEELKITEMVYTVFRILLYHAIKHEWGGWRVWVDTLSITHSKVTF

EKHKENLSRVFQEYQRMGSTDDTEIRTVSGSSRNSELTAQPSETVVEPPSSIIEVEEPIC

GEHDEHEEKAQVIISNILAGVEEEDTKEKEEKATKDEGNVQEALEEKDTVSEVVEEKKDC

NKAMDKDGTEGGDHKNLEEENNQKVESDQQANAVKISENEKDKPATSDIDEEEKESEKAE

GQKENEMKPPPETPVQGDGEEKKQKEDKNSDLSEVSPGAEKGSDMEVQTRVEASTDVQAS

PDQETNLGLEKVSSTQQGPSSETSPSDQESPKAETSPSLQEVPSVPESPKAETSPSLQEV

PSVQESPDALMSPSKEESPSMQELSSVEKSPSVEEDQSTQIPKESPSTQEASGVPESLNV

ETSIKSKGVKIARLDVSSVAIDTERLELKDNTAADGGQSQAAAGTSTQNQDGSSSSPRPS

MFRIPEFKWSHMHQRLLTDLLFSIETDVQMWRSHSTKTVMDFVNSSENVVFVHNTVHLIS

QVVDNLIMASGGILPLLSAATSSTHELEAIDASQGLEVETSLSFLQRLVSLVDVLVLSSS

LNFTEIEAEKNMTSGGVLRQCLRLVCTMAVRSCLECQLHSLYKAGTEKTQRGLVGNAQFL

PAQSPVDMLTGGIAPIRDMERLLQDMDINRLRAIVFRDIEDSKQAQFLALAVVYFISVLM

VSKYRDILEPHNDKKINKNSSRRTESVSSDRGNSSPLQRLDSAPGSEDSPVPQHNAESRS

APDAVSEALSTLSSEVRGHENMDKDKKGKSMKVKDILRSLVSAAPADDVIVDASLLPPTF

LGKQEIHGQFSSFDRSVVVGSRKVGGAPAAGSSPSFTAATTGGTPVVSVSVVSTDDPAPS

DAAESGGTTEVTSNLPSVPLSPTTTLSISERLEHALEKAAPLLREIFVDFAPFLSRTLLG

SHGQELLIEGTSLVCMKSSSSVVELVMLLCSQEWQNSIQKNAGLAFIELVNEGRLLSHTM

KDHLVRVANEAEFILSRQRAEDIHKHADFESHCAQYAADKREEEKMCDHLIRAAKYRDHV

TAAQLIQKIINILTDKHGAWGSSTHSKQCFWRLDFWEDDLRRRRRMVRNPFGSSHSEAAL

KSAAEHAPEDDVLKGKQLGKEPARNQSSESESSAEGDDETISSLEEKELDNLTGTVSFST

VAELVSPALVMKGTLSITAHELYFEVDEEEASFKGIDPKLLVYADGLHGKWMFSEMRAIF

SRRYLLQNTALEIFMANRTSVMFNFVDAATVKKVVHALPRVGVGTNFGLPQTRRISLATP

KQLLKASNMTQRWQKREISNFEYLMFLNTIAGRTFNDLNQYPVFPWIISNYDSEELDLTL

PSNYRDLSKPIGALNPKRAAFFSERYESWEDEQVPKFHYGTHYSTASFTMMWLLRIEPFT

TFFLNFQGGKFDHADRTFSSVARAWRNCQRDTSDVKELIPEFFYLPEMFINANNYNLGVM

DDGTVVSDVELPPWAKSPEDFVRFNRMALESEFVSCQLHQWVDLIFGYKQRGPEAVRALN

IYYYLTYEGAVNLSSITDPLLREAVESQIRSFGQTPSQLLIEPHPPRSSAMQVTPLMFTE

QMQQDVIMVLKFPSNSPVVYVSAHTQAGLSNPSIVTVTANRIFSINKWHGLTGHQSSSNT

EQQYQLPVEIDPVIVANLMHRRQVCDLLDQSVEVNSHCFLVSSDNRFLFIAGFWDKSFRI

YNTDTGKLMQIVFGHRDVVTCVFRSESYIGGDCYILSGSRDATLLLWYWSGKHCCIGDTQ

NDFVTPRAILTGHDCEVTCASVCAELGVVISGSKDGPCLLHSVSGELLRVFENSQRPMLV

QTSSEGHCVIYYSSGQVCVYSINGKLLCDTHIEDNIKAMLLSKDGQYLLCGGERGVLSVW

QVHDLKQLFTYPGCDAAIRSMAITADQRYIHTYYIYT

>m.411 g.411 ORF g.411 m.411 type:3prime_partial len:82 (-) Unigene000035:3-248(-)

MILSFSLSLAAKNTPKQNTNSPAVDSSALCELTVLLLGVFFLFLERHLFLNRLALGRRRH

DSLQVGERALRMRSSGLRGAVR

>m.416 g.416 ORF g.416 m.416 type:complete len:115 (-) Unigene000036:319-663(-)

MQRERDMAPSPTARTPSLPLEFIFSFCFYTSQHNNLLPIRMTSSVKKTARVQGMLGLVQP

ALKDVKKKEEERSSSRRRRVESAGRSRCKSPASKQVACKATRPQVAEFVTPCRP*

>m.413 g.413 ORF g.413 m.413 type:complete len:2367 (+) Unigene000036:615-7715(+)

MEYGRLEREPCLSPAAFVNQVQYSNILEGRFKQLQDEREAVQKKTFTKWVNSHLGRVTCR

INDLYTDLRDGRMLIRLLEVLSSEQLPKPTKGRMRIHCLENVDKALQFLKEQKVHLENMG

SHDIVDGNHRLTLGLIWTIILRFQIQDISVETEDNKEKKSAKDALLLWCQMKTAGYPNVN

VHNFTTSWRDGLAFNAIVHKHRPDLIEFDTLKRSNAHYNLQNAFNIAEKEMGLTKLLDPE

DVNVDQPDEKSIITYVATYYHYFSKMKALAVEGKRIGKVLDYAIEADQLIEKYETLASEL

LQWIEQTIETLNDRQLANSLSGVQNQLQAFNTYRTVEKPPKFTEKGNLEVLLFTIQSKMR

ANNQKVYMPREGKLISDINKAWERLEKAEHERELALRNELIRQEKLEMLAARFDRKAAMR

ETWLSENQRLVSQDNFGFDLSAVEAATRKHEAIETDIVAYGERVAAVEAVARELDAERYH

DVRRILARRDNVLRLWEYLKELLAARRERLVAHRDLQRLLQEMSYIMDWMEDMKGRLQSQ

DSGKHLHDVEDLLQKHTLVEADISAQAERIRAVQAAANRFTSEEITYKPCEPALVEEKVA

LLGQTYEELGQLVVERRAQLEDSRRLWQLLWELGEEAAWMREQEQIMATEECGKDLSSAL

RLLSKHEAFREEMAARYGPLGNSIAAGEQLVKEGHCGAPEITERIKEIKAQWGRLEEASR

LREERLKESVTFHQFQTDACDMEAWLQEALRQVSSQEVGHDEYSTQTLARKHKEVEEEIQ

SHRSLIDSLHEQAQSLPEEYAQSPQVEGRLPAIEQRYEELKALSSARRDALEGALALYRM

YSEADACKLWIGEKEQWLLTMEIPSKLEDLEVVQQRFETLEPEMNNIGSRVSDVNQVAEQ

LLKSDNCNKEQIHQTQDQLNDRWHEFQRLANERKQAMESALNLQNYHLECNELQSWMKEK

TKVIESTQGLGNDLAGVMALQRKLTGMERDLEAIQGKLGGQRAEAEKLASEHPEQAEEIQ

ARLTDMEDVWEELCGTMKKREESLGEASKLQGFLRHLDDFQAWLSRTQTAVASEDTPTSL

AEAEQLLAQHEAIKNEVDNYRDDYERIKATGAEVTQGQTDAQYMFLAQRLQALDTGWQDL

RRMWESRQCLLAQAFDFQTFLRDAKQAESFLNSQEYVLSHTEMPSSLEGALEAIKKHEDF

LTTMEASEEKINGVVESGRRLVSDGNANADKIQEKADSIEERHQKNKQAANELLGKLKDN

RELQHFLQDGQELSLWINEKMLTAQDMTYDEARNLHSKWQKHQAFMAELASNKDWLDKIA

KEGQALVKEKPELEETVSETLRELQKQWDLLETTTQTKAQCLFDANRAELFTQSCSALDT

WLQNLSSQLQSDDFGKDLTSVNILLKKHQMLEHQMEVREKEVQSLQSQALALSQEDARIV

EVDGQQRRVIDTFAQLQDPLRDRLQHLLASKEAHQFNRDLEDEILWVKERMPLATSVDHG

KDLPSVQLLIKKNQTLQKEIQGHQPRIDDLQAHGRNMAPGGGAEMKGRRDEPLNELGDLW

TWLIAETEQRHKRLVEAHRSQQFYADAAEAEAWMGEQELHMMSEEKAKDEQSALVMVKKH

QILEQALEDYAQTIHQLANSSRLMVDSEHPESERIALRQAQVDKLYAGLKDLAEERRGKL

QERLRLTQLKREVDDLEQWIAEREVVAGSHELGQDYEHVTMLRDRFREFARDTSAIGQER

VDTVNAQADALIESGHPENASVAEWKDGLNEAWADLLELMDTRTQMLAASYELQRFQQDA

REALARIRNKREALNSAELGRDLNTVQHLHRQHTTYEHDVHALSGQVTQVQDDAARLQKA

YAGEKADDIHRHERAVTEAWEELLSATQARRLLLLDTVEKFRFFNMVRDLMLWMDGINLQ

IQSHDSPRDVSSAGLVIANHQDIKAEIEARSGSFTACTDMGHALINNNHYASDEIREKLE

QLQEKRDEINQKWQDKMDHLQIVLEVLQFGRDASVAESWLVSQEPLVRAAELGSNVDEVE

SLIKRHEAFEKLAAGWEERFTLLEKLTTLEEQEFQRRKEEEERARRPPTPPPVEEVVPSE

VEYESGARTSLDQTTLNQSVSVNGVHSDQDTSQSLSVSGSEFPNTEPKPLIKPVSNPVPK

PYKAHQRGSEAESVNGPGQDSGLDTASRHEPSGTLPSRIEASAEAMEGTLCRKQEMESQG

KKAANRSWQNVYCVLRKGSLGFYKDNKSASNGIPYHGEVPISLGEATCEVAHDYKKRKHV

FKLKLGDGKEFLFQAKDEAEMSSWIRAIHSSMPPNNIDDLSPAPPLTRAMTMPPMSPSEG

EGVTMRNKEGKEKDREKRFSFFGKKK*

>m.428 g.428 ORF g.428 m.428 type:5prime_partial len:2549 (+) Unigene000037:3-7649(+)

RKSVLYWCDFSSTVASQNGIRRIKPNGSGFRNIVTSGIGRNGIRGIAVDWAAGNLYFTNA

FLTETYIEVVRLNTTFRRVLLKTQVDMPRHIVVDPKNKFLFWADYGQNPKIERAFLDGTN

RTVLVTSGIITPRGLALDHGNGYIYWVDDSLDMIARVPPDGGETEIVRYGSRYPTPYGIT

VFENNIIWVDRNLKKVFQVSKEPGNTDQPMVIRDNINMLRDVTIFDQRMQPWSAQELNNN

PCLSSNGGCSHFCFALPGTQARNCACAFGNLAVDGMTCVVSRDDYLIYTTESTVRSLRLD

PDDHSLPFPVVNVARTSVALDYDRLDNRIYFTQSSGAGQSKISYISLSSPTSSATVVASD

LGAPDGIAYDWINKRIYYSDYVNQTINSMAVDGSQRTLIAHVPRPRAIMLDPCSGYMYWT

DWGTIAKIERATLGGNFRTEIVNSSLVWPNGLTLDYDDQRLYWADASLQKIERCSLTGTN

REVIVSTAIYPFAMTVYGQHIFWTDWNTRSIYRANKHDGSDQRVMIQNLPTRPMDIHVLS

NSKQQQCSSPCLQFNGGCSHICTPGPQGAECQCPSEGRWYLADNKYCIPDNGTRCQPSQF

TCMNGRCIRAAWKCDTDDDCGDGSDELERVCAFHTCEPTLYTCGNGRCVPYHYRCDHYND

CGDNSDEEGCLFRPCDPDTEFTCNNGRCIAREYVCNGINNCFDNGTSDERNCAERTCQPG

HTKCQSTNICIPRSYLCDGDNDCGDMSDESPTHCATSTCTQSEFRCTSGRCIPAHWYCDG

GTDCADGSDEPSTCTTIVRTCSSDQFRCDDGRCIASSWICDGDNDCGDMSDEDQRHNCAN

RTCSSLEFTCVNNRPPQRKCIPQDWVCDGDADCSDAYDEHQNCTRRSCSATEFTCSNGLC

IRSAYRCDRRNDCGDGSDEQSCTYQPCQQHQFTCQNGRCISQDFQCDGDNDCGDESDELP

HMCHTPAPTCPPGKFRCDNGNCVPSSQVCDHSDDCNDNSDEKGCGINECTDPSVHHCDHE

CTDTPTSFVCRCRPGYRLMSDKKSCDDVDECSETPSVCSQMCENTLGSYVCKCAPGFLRE

PDGRRCRQNSNISPYLIFSNRYYLRNLSADGAAYSLILQGLSSVVALDFDRVDKRLYWID

VSRRVVERMFFNGTGREVVLNGIMHGEGLAVDWVGRKLYWVDSFLDCLKVSELDGRFIRK

LAEHCVDVNNTYCFENPRAIVVHPKFGYVYWTDWGSKAFIGRVGMDGNNKSAIITSKIEW

PNGLTIDYTNDMLYWADAHLNYIEYSNLDGHHRHTVYDGVLPHPFAITVFEDTVYWTDWN

TRTVEKGNKYDGTGRQALVNTTHRPFDIHVLHPYRQPIVTNPCGINNGGCSHLCLLKAGG

RGYTCQCPDHFLTVQIGGVARCLPSCSSTQYRCADNERCIPIWWKCDGQSDCRDGSDEPS

TCPLRHCRLGQFQCNDGNCTSSHFLCNSNRDCPDGSDEDTVLCATHQCESHQWQCSNKRC

IPEAWQCDGENDCGDGSDEEPSHCSSRTCRPGQFKCRNGRCIPQSWKCDVDDDCGDNSDE

PLDECMGPEYRCDNHTEFDCSTNYRCVPLWAVCNGQNDCRDNSDEQNCEEMTCDPAGDFR

CDNHKCIPLRWKCDGDSDCGDGSDERNCNPRACTESEYRCDNLRCIPERWVCDHDNDCED

NSDERDCELRTCHPGYFQCDSGHCIAERLKCDGSADCMDVTDETSCPTRYPNGTYCPPFL

FECKNHICVQQHWKCDGDNDCGDGSDEELHLCLDIPCEAPFRFRCDNNRCIYSHELCNSV

DDCGDGTDEKQEHCLTPTHGPCSSDEFKCGNSQCIPLQYVCDDYDDCGDQTDELGCNSGN

GRSCSENLCEHNCTELTGGGFICSCRQGYKAREDNRNSCEDVNECELYGTCPQLCKNTKG

SYECFCAEGFRSVGPQHRTECAAEGNPPVLLLPDNVRIRRYNLSSKQYLDYIDNAEHIHA

VDYIWDPEDQGLSIVYWTILGHGAQFGSIKRAYMTTFDDHGSNQMKDVDLNLRYISSPDG

IAVDWVGRHIYWTDAGTNRIEVAKLDGRYRKWLIYSNLDQPAAIVVNPAIGMMYWTDWGS

RPKIEAAWMDGQHRQVLLDEDLGWPTGLTLDYLNGNRIYWCDSKENIIESMKPDGTDRKI

VLSGDIGNPYSLDVFEGHVYWTTKVRGEVWKADKFGRGDKVKMLTINPWLTQVRIYHEHR

YNRSVLNPCKGVCSHLCLLRPGGYTCACPQGSSLVSFNENECDAASLPEVAMPVECGCIN

GGTCSTDKPGLPKCKCPYGYSGRHCEIGKSRGAPAGTAVAVLLAVIIILLSGALAVGLFL

NYKRTGSLIPSMPKLPSLSSLAKLSDAGNGVSFRSGDGSTVDPQTLGVSFIDRAMQLNEN

FTDAGREPVTFENPLYSTATGAAADAAVIHATQVTVNISGDSTENVFANPMYNEQQQAVD

MKNTPNEQAAKESKWNFFKKKLKPSTTFENPAYSEMRDKQSALTTEDISTSHPQPALPPL

KREKSSMYSPTEDSFQDTANLVKEDSDI*

>m.438 g.438 ORF g.438 m.438 type:3prime_partial len:1836 (+) Unigene000038:3055-8565(+)

MDNVTASMFPGQKMSPSKAMTMKDYENQITALKKENFNLKLRIYFLEERVQQKCDDSTEE

IYKTNIELKVEVESMKRELAEKQELLVSASKALESLANRDSEAGHRERAQRDMDALREAF

NARIRDLEESLRCAEDEVEKMASIAEQEKLRSIELEKELEAVCQSGPLHDADSARGPDHK

LQQALQEKERLINQLRESIKKQDALIGELQERDQDQPIREQMLQLNKLIGEKDAEVQALK

EELDRERGKAEKESQVCMITQDELKTLHQKTRQLTEELNSTRSSGQKLKHRLEEMEGENK

ILSGQLEERESELAGEKKNALKRDKTIQGLSILLKEKERQVDELYGNLEEKERTLTKARE

ALHKAQLQKYQGGEEQQALLQSQNAELSRLQAECHSSLLELQRLQRALSSRDAQLELLQQ

DKVQLENELELLQQHKRKGDKTINDLQNQLKKLSGELAERENLLEHQRLAQQEETRTTEQ

KLQSTIQQLTTNITKKEQQLQEYMSMMQHLEQNQASAGSDVMLAKLREKLREKEKALEKA

LDEKFVAVEEKENEIHLLQLSMREKDRDLERLNHLLTHNEETVNSFDALLKEKDMELQQL

LNSFKNLQRSKQESEENLQRALREKDSIIQQLQYALQLKTKDTEDGATTGLTQSDSQEYN

SAEQMSQRLKVTEAMLIEEVKHRERLVCENQTVVDNLLAIISSKDQLLKESAERHSQALG

DQTAEVQDLRRQLADTERRVSNGQNLSAGLNEKDVLINKLLDHTPERDLEVSPPQVVELR

QTINILQQRLEEKEDELSQRRSEENSEKLRAANKKPVVILKKELAQKTEALNSALKRENQ

LQMSLAELQSSVSELEGRLEGQTANIESLKSIISTKDEIITELQQHLSEMGLRRSTVDHH

SQAAEELSFRSLPQRERTVIGGDRQQQQETLSLGDLCSEQAELNHILREEQQLYTKLIHA

VKEQDSVQQLQSLQLELSSVTLLRQQLEDGVRNNNKLREQLHQEIQRVNQREGVDPAELK

SMRDALEDAQRWNASLQARLGQIQSRGGGVGQANDTDDSLSLIGEQTSYMSICLGEGQEE

ELQQLSIEQLRLKVSELQAKNAELQKQLAQPERDVMSNSEREPQGQRDLIGFSPPCTQPQ

RPVTPLAVNANERQTASSVKKTADDSLEVQRRMASSPSLLPKKDNGSVSYSPGQQSENEQ

EELAALLRDCGAESISQLREQVNKLRSEMSKLGVLLKEETVAESKESTESSGENDSHTNL

HQTVQTLRSEARSHRKIVRLLKEQLLRDRKPKTGGEPGLDSERVDSMARKMERLRKEHEA

TQRHAAILEERLKEVQSRERNEDREQEEKDSKKKGRQEHSKKLRHAAYSKSRLPVAIRPA

KPKDRHRMTHHDPEKSPEPLSTSDSEQLSPKSEGSCGAVLQRSSENSFKTLRGSESRQNR

EVRKKSKNVSDIPQSELLSQLELLNQECQEKTELISQLQLQMQNWEKLQAELQEKERLNS

QYLEALHAAESTIAYLTACNLDSESGLGQPKPGSDSSLQQQCSELQRAVQEKDQVSIQLL

ECLNKAEAAMASLTSANLPESFFSAQKDPHALSERFDFLLSQIKTLQEQKNACTSKELQQ

NRLNAELQEKLRAAEETITKLSAQDDTTTGQKLIYSHSCTSPQKEMVDEYEEGQQRLAAS

FSECIFAVEQAVGSLAEYGLRSDRLLHGVETSTNAELEQNLDRLQKVLLERDRFWNSIST

DISHSTIVGPFHQPVHHNLQILDAAQQKTSTPISQEQQGQQKISETCDPDRSGSQHLNLY

KNLTLLVQIFKQHALKVRELEEALKVKPGQDLKANR

>m.440 g.440 ORF g.440 m.440 type:complete len:441 (+) Unigene000038:1628-2950(+)

MDSQKVQLESVHTSISSDLLMEHLLEVRALRQRLEETIHTNERLRQQLERKLQQAEKDPD

THIFIAGSDDPSQLNTDIRYLWAQNHTLKEQLNQAARDKQKENDSLRKTLARRTAKLELS

RKECEMLREEQTNLQNSLYRLQCEQNLQKQQLADTQQLLQSVQVELQVHEQIRNSVRTHT

DEERGGSESSDVDLKELLGEVRSLRLQLEKSIQTNTALRHKLEQQLLTQSDPPSTININY

LLSQTDDGGKSEMLRQHSHTDPSSAAYDSGSSEHGGSPALSRLVPGHRLWADRHGQHVLG

LVEDYSALSKQIREAKRITSAMDTQLQDRFVECASFSGSVSTMQQMLEEAGRLLKLLWKV

SLPTGDLTNTQQNVLRSEITQLKSRLSQQERMLTGAVKRLRSTNQLKEGMERIIIEQLSL

THGVLKKARGNLEEIPLNAQ*

>m.439 g.439 ORF g.439 m.439 type:complete len:446 (+) Unigene000038:151-1488(+)

MDSVVGEDQTLPFDINGALKVKPGQDLKANREDVVKSLQKALKENQKMCQKLEKKLASAQ

SIIALQNSSRKDKDSERHKAPCSEQEDKGVQVDAQDLGYETSGKSETDVEKEEGSTTDGG

VCLDPGVSGFTSADNLNTTASSPSYPSSPDLSSPRPSDPSHELLQLRSLVKHQHKVIAQL

QQLLPKNCLTAEVLTCNPASCEEEEEDRTALKARLFQLTPEPEKEQTIDRSNESASPSRM

ESLVQSQARELCELREQIRVSRTLGAGQRRQLLELRGALEELMAPNDGQSALSPTIRKHL

DQSLSLLDKLEQDSCVEKAELPGLELFQRLTAELQEKETLIQQLKNRLFGKVSGTQQGFE

SETSETSSNDGNVSVQGSLNDLHTRTTRGATEGLKSSSGESAPDSESHPSSVCVAAGSTG

SQGEEGSVFTLQRDNSRLQEQLKKE*

>m.445 g.445 ORF g.445 m.445 type:complete len:1246 (+) Unigene000039:166-3903(+)

MAPVYEGMASQVQVFSPHTLQSSAFFSVKKLKVEQSCNWDMTGYGTHSKVYGQNSKQSVS

AAPVGLNAASLQVSNSSLPYEQALLFPASSGHIVVASASSTSGVAGQLLGSTGSSSGSGS

GGHNLTRRSTVSLLDTYQRCGLKRKSEELDNNNGSGSSVHVVEELQQPPAAQMLQNNGQS

GPTGTVATASTTATSKTSGANSEGDYQLLQHEVLCSMTNTYEVLEFLGRGTFGQVVKCWK

RGTSEIVAIKILKNHPSYARQGQIEVSILARLSTESADDYNFVRAYECFQHKNHTCLVFE

MLEQNLYDFLKQNKFSPLPLKYIRPILQQVATALMKLKSLGLIHADLKPENIMLVDPSRQ

PYRVKVIDFGSASHVSKAVCSTYLQSRYYRAPEIILGLPFCEAIDMWSLGCVIAELFLGW

PLYPGASEYDQIRYISQTQGLPAEYLLSAGTKTTRFFNRDPDSTYPLWRLKTPEDHEGET

GIKSKEARKYIFNCLDDMAQVNVSTELEGSDMLAEKADRREFIDLLTKMLTIDADKRITP

IETLNHPFVTMAHLLDFPHSTHVKSCFQNMEICKRRVNMYDAVNQSKTPFITHVAPSTST

NLTMTFNNQLNTVHSQATNLAPSSTSATLSLANPDVSILNYQSALYQPSAAPMAAVAPRT

MPLQPGAPQLCATRPDPFQQALIVCPPTFQGLQASPSKHSGYSVRMENAVPIVTQAPGGQ

PLQIQPGLLTQAWPSGTQQILLPPAWQQLTHTSVQHATVIPDSMGTSQPLPNWRNPHPHG

SHYNPIMQQPALLASHVSLPSQQPLNVGVAHVMRQPVNNSTASKKSKPQQMSNRNVSAYE

VSSSQAMLSPQRSKRVKENTPPRCAVVQNGPSVPCALSQACGGGGGGWGEEQASSTTREH

HGPHNQHLQHNQHNPPRQTIVIPDTPSPAVSIITISSDTDEEDEHKQPGNTRSAGTSSKQ

RKNVISCVTVHDSPESDSSNISPYAVESRSNCPNANNYDSKSTVLDNYNNGNPRTIIIPP

LKTQTSESLSECDRLMPDTMNHQNPAYKFNSSNGGLSTNNHSSVGIPGGGAYRQQRSGPH

PFQQQPLNLSQAQQHMAVDRTRGHRRQQAYITPTMTAQAPYSFHHNSPSHSTNVHPHLAA

THLSGQPHLYTYTTPAALGSTGTVAHLVASQGSAHHAVQHAGYPPSIVHQVPVSMGHRVL

PSPTLHHGQYQPQFAHQTYISASPASTVYTGYPLSPTKMNQYPYL*

>m.450 g.450 ORF g.450 m.450 type:complete len:2333 (+) Unigene000040:615-7613(+)

MEYGRLEREPCLSPAAFVNQVQYSNILEGRFKQLQDEREAVQKKTFTKWVNSHLGRVTCR

INDLYTDLRDGRMLIRLLEVLSSEQLPKPTKGRMRIHCLENVDKALQFLKEQKVHLENMG

SHDIVDGNHRLTLGLIWTIILRFQIQDISVETEDNKEKKSAKDALLLWCQMKTAGYPNVN

VHNFTTSWRDGLAFNAIVHKHRPDLIEFDTLKRSNAHYNLQNAFNIAEKEMGLTKLLDPE

DVNVDQPDEKSIITYVATYYHYFSKMKALAVEGKRIGKVLDYAIEADQLIEKYETLASEL

LQWIEQTIETLNDRQLANSLSGVQNQLQAFNTYRTVEKPPKFTEKGNLEVLLFTIQSKMR

ANNQKVYMPREGKLISDINKAWERLEKAEHERELALRNELIRQEKLEMLAARFDRKAAMR

ETWLSENQRLVSQDNFGFDLSAVEAATRKHEAIETDIVAYGERVAAVEAVARELDAERYH

DVRRILARRDNVLRLWEYLKELLAARRERLVAHRDLQRLLQEMSYIMDWMEDMKGRLQSQ

DSGKHLHDVEDLLQKHTLVEADISAQAERIRAVQAAANRFTSEEITYKPCEPALVEEKVA

LLGQTYEELGQLVVERRAQLEDSRRLWQLLWELGEEAAWMREQEQIMATEECGKDLSSAL

RLLSKHEAFREEMAARYGPLGNSIAAGEQLVKEGHCGAPEITERIKEIKAQWGRLEEASR

LREERLKESVTFHQFQTDACDMEAWLQEALRQVSSQEVGHDEYSTQTLARKHKEVEEEIQ

SHRSLIDSLHEQAQSLPEEYAQSPQVEGRLPAIEQRYEELKALSSARRDALEGALALYRM

YSEADACKLWIGEKEQWLLTMEIPSKLEDLEVVQQRFETLEPEMNNIGSRVSDVNQVAEQ

LLKSDNCNKEQIHQTQDQLNDRWHEFQRLANERKQAMESALNLQNYHLECNELQSWMKEK

TKVIESTQGLGNDLAGVMALQRKLTGMERDLEAIQGKLGGQRAEAEKLASEHPEQAEEIQ

ARLTDMEDVWEELCGTMKKREESLGEASKLQGFLRHLDDFQAWLSRTQTAVASEDTPTSL

AEAEQLLAQHEAIKNEVDNYRDDYERIKATGAEVTQGQTDAQYMFLAQRLQALDTGWQDL

RRMWESRQCLLAQAFDFQTFLRDAKQAESFLNSQEYVLSHTEMPSSLEGALEAIKKHEDF

LTTMEASEEKINGVVESGRRLVSDGNANADKIQEKADSIEERHQKNKQAANELLGKLKDN

RELQHFLQDGQELSLWINEKMLTAQDMTYDEARNLHSKWQKHQAFMAELASNKDWLDKIA

KEGQALVKEKPELEETVSETLRELQKQWDLLETTTQTKAQCLFDANRAELFTQSCSALDT

WLQNLSSQLQSDDFGKDLTSVNILLKKHQMLEHQMEVREKEVQSLQSQALALSQEDARIV

EVDGQQRRVIDTFAQLQDPLRDRLQHLLASKEAHQFNRDLEDEILWVKERMPLATSVDHG

KDLPSVQLLIKKNQTLQKEIQGHQPRIDDLQAHGRNMAPGGGAEMKGQRDEPLNELGDLW

TWLIAETEQRHKRLVEAHRSQQFYADAAEAEAWMGEQELHMMSEEKAKDEQSALVMVKKH

QILEQALEDYAQTIHQLANSSRLMVDSEHPESERIALRQAQVDKLYAGLKDLAEERRGKL

QERLRLTQLKREVDDLEQWIAEREVVAGSHELGQDYEHVTMLRDRFREFARDTSAIGQER

VDTVNAQADALIESGHPENASVAEWKDGLNEAWADLLELMDTRTQMLAASYELQRFQQDA

REALARIRNKREALNSAELGRDLNTVQHLHRQHTTYEHDVHALSGQVTQVQDDAARLQKA

YAGEKADDIHRHERAVTEAWEELLSATQARRLLLLDTVEKFRFFNMVRDLMLWMDGINLQ

IQSHDSPRDVSSAGLVIANHQDIKAEIEARSGSFTACTDMGHALINNNHYASDEIREKLE

QLQEKRDEINQKWQDKMDHLQIVLEVLQFGRDASVAESWLVSQEPLVRAAELGSNVDEVE

SLIKRHEAFEKLAAGWEERFTLLEKLTTLEEQEFQRRKEEEERARRPPTPPPVEEVVPSE

VEYESGARTSLDQTTLNQSVSVNGVHSDQDTSQGSEAESVNGPGQDSGLDTASRHEPSGT

LPSRIEASAEAMEGTLCRKQEMESQGKKAANRSWQNVYCVLRKGSLGFYKDNKSASNGIP

YHGEVPISLGEATCEVAHDYKKRKHVFKLKLGDGKEFLFQAKDEAEMSSWIRAIHSSMPP

NNIDDLSPAPPLTRAMTMPPMSPSEGEGVTMRNKEGKEKDREKRFSFFGKKK*

>m.453 g.453 ORF g.453 m.453 type:complete len:115 (-) Unigene000040:319-663(-)

MQRERDMAPSPTARTPSLPLEFIFSFCFYTSQHNNLLPIRMTSSVKKTARVQGMLGLVQP

ALKDVKKKEEERSSSRRRRVESAGRSRCKSPASKQVACKATRPQVAEFVTPCRP*

>m.465 g.465 ORF g.465 m.465 type:complete len:1866 (+) Unigene000041:232-5829(+)

MEIDHGCLEKGMINQELSNGLRQHGEETNGLGGATLARLSHQQKKTLACLPQTQGWGPGN

LKGSSTHQQQEAGWSDQSTTSSGNSVDIENARTLVAFSASAGSLPPCSVPQPTHNPNTHL

YEKFNQEMSNRGDSAKQKTWSRDDQHCTSEDLNTLQMALNQARHGHKPPNCDCDGPDCPD

YLEWLEKKIKMAGNSHEKGFCKLSGASQKEQQQYQSEPQPQPNSANTPQSQNPANQPPAP

RPYGPLPPIPCSPSVLSIAKERNVSLQTAIAIEALTQLSGTGGDSTFGNTNYQNLPQPLS

QTQDGMRFTAASPASLSSSPASTINQPSTSGHFPQKNSASWEQHRTESPDPAHQSTLYKP

PNSPFSHSSTTSPSPHQWQQSTAASCEKSSPRNPWILKSENQACFPQQSHGSSDPMSELK

QLLGDSSGKYTSTTFKFPAPPQNLKDNQVMPHVKPEVNSGDFPCDMAAAMGQYRINNQQQ

FQGQQISQSIRYSTQAALQQHLHHKRNLFTGPQTFDLGAPMTCQTLRKWWPQSTPESALP

IKQEPREPKKKKNTQSSPLLKQHVGGLLGSLGPPLPKPKQIIIKKTKQKASQPLFLPQIQ

ITIQKSSMSTSIPSLTQPTPSLPVMEAALPPCLPSYNSSQVVVSQAAPVQSQESFLNTGS

TPISGNTPLDASTLNVPMPPAQDKLTSSDPTIVNEGSLATTTSTSQTTTSHSTPPLCGLN

SMDPKFEELIRQFEEEFGDTSPVTDTTEQGFSDVVTKTQLNPNLGQGRPRSPEKLNNLPV

TTNEPEPNLKQAEGGNEKFMSSNQLSAIKQEVEEYEVDVKPSDQLLALATEAYLQQQHHK

IVVNPFTTTCSPPNKRMKIESSGGVTVLSTTASLSTAGENLDTPTKECYPSSPSLKGFLE

SPLHYLDTPTKNLLDTPTKNAQTEFPTCDCVEQIIEKDEGPYYNHLGSGPTVASIRELME

NRYGEKGDSIRIEKVVYTGREGKSSQGCPIAKWVIRRGSEKEKLLCLVRKRAGHHCANAV

IIVVIIAWEGIPKALGDKLYREITDTLIKYGNPTSRRCGLNEDRTCACQGKDPETCGASF

SFGCSWSMYFNGCKYARSKTPRKFRLQGEHPKEEEILRDNFQELATKVAPVYRKLAPQAY

QNQCATEKMAPDCRLGLQEGRPFSGITACMDFCAHAHKDQHNLQNGCTVVCTLTKEDNRA

VGVIPEDEQLHVLPLYKISLTDEFGSEENQRLKMQTGAIQVLSKFRREVRKLPEPAKSCR

QRRLEAKKAASEKKNKKQQLSETPEKTNKKEVSLHGNKAILKQEVKPNIKKELVDHFQAI

NGALDGFPPTGNTKMCPDPYSMKDAYSSCPAPYARGSLPSISQSSTHSPINGFHPNIQGM

PYNYYNQAKKGLIPPEVLGCVNHNGTSHKAQVEEQKPSIQSLQARLVPNHSDHHHQQGVN

MVNHNYPHQSELLESTAPLKRAPSAPPDQMHCVTPNIKQEPMEVPLYDRRTDSQFRSCPV

TPSVTPQPDTWPVHTPNGSLAPKGWDGNFRPGQVHSPFTPEKQRLHKQLHNQLQHSIYVQ

QQQQWRSHPGTPVASPSPAPAFNTGLSPAPSPHPVTPQHWGSPAPSPQPKTWGQPGSLGY

GHDGLGQGTPIGAFPDKMLAEAAEIRGSTPLGLQEKAWKSGGASAAGSNPSPAPEGRLFP

DTLQKVNGQACWDSEVESEREPEEEEVWSDSEHNFLDPNIGGVAVAPAHGSILIECARRE

LHATTPLKKPDRSHPTRISLVFYQHKNLNQPCHGLALWEAKMKLLAERARQRQQEAALLG

LSQEDMKAYTKKRKWADGLVSSASEPMKDKREVVTRLAPTQHTTNMVTVSPYAFTRVTGP

YSCFM*

>m.472 g.472 ORF g.472 m.472 type:5prime_partial len:2551 (+) Unigene000042:1-7653(+)

LLRSEERTGEEEVRDEKAKLNRRLDGLSERLHGRMAQLEELNSRLKEFEDGRLAVERRLE

AARHQLEVQEALGPQACSNKSLERLRSQQDTLGSLQPQVVYLHNLAKGLAQDAPQMTGGS

GDGGQKLLQQAQTTEKEFGEVTEKIQQCCSSLESRLQGVGEVQSHVRDIFSRLADLDDEL

DSLSPVGRDVDSLASQADAIRGFLGRLASLRAELEGHGSACTTMLKREGSSPDLLAIRRE

TEALNRQAAKLAERGQGRLALIEEAEGRVKEFYARLMELQRLLDQAEEGLNVQSVVGTEV

DVIKRQLQEFKAVEREQVDSIQPKLHHVNAVGQGLIQSAAKHTDTQALEHDLETTNLQWN

SLNKRVAERIAQLQEALLHCGKFQDALEPLLSWLSDTEELIANQKPPSAEYRVVKAQIQE

QKLLQRLLDDRLGTVEMIRAEGERIAATAETQDREKIQRQLQCLGERWTDLLEKANARQR

QLEELQVLALQFHESVDPLGEWLSATERRLSSAEPMGTQASKISQQINRHKALQDEVLAR

EKEVDHLEALGQSLSPLSCAADRDWLSERVGAVRSGHTELRNWCFRRAAMLEQALANAQL

FGEEEVEVLNWLAEVAQRLSDVSVQSYQPELLAEQHKYTLSLNEEIVSRKKTVDQAIKNG

QALLKQTTGEEVLLIQEKLDGIKSRYSEMTAGSSKALRNLEQALQLATRFASAHEDLSQW

LDSMEAELNNMEPDTTPAYQERQKDLKCVSAEKRLILDTVNEVGSALLDLVPWRAREGLD

RLVADANQRYRQADETITQRVQLVQAAIQRSQQYEEAVDAELAWVGETERKLTSLGPLSL

EPDVTVAQLQVQRAFNIDIIRHKDTVDQLLHTREDILESCSDQQREALKVKTDSLSMRYE

AVSQNHAERFSALEQAQVLVARFWETYEELDPWLGETETLISQLPPPAIDTEALRQQQDQ

MRMLRESIAEHKPHIDKLLKIGPQLAELSSQEGATLRQRYSEAERRYLAIKEDVKGRAAV

LDEAFSQSAQLIEFHDKMDPLLETLEGAVQRLRQPPPVAAEVEKIREQLAEHRAAGLELD

KLLPSFSTLCARGEELITRAAHDDPAAQAVRSRLLRLRSLWDEIRQRAEEREGKLQDVLD

LAGKFWADMAALLSTLRDSQEIVKELEDPGLDPSLIKQQIEAAEAIKAETDGLREELEIV

RTLGADLIFACGETEKPEVKKTIDEMNAAWEGLNRTWRERMEKLEEAMTASVQYQDALQG

MFDYLDNAVIKLCDMQAVGTDLSTVKQQIEELKQFKVEVYQQQIDMEKLCHQGELLLKKV

SDQADRDMIQEPLTELRHLWDNLGDKITVRQHKLEGALLALGQFQHALSELQSWLSHTHA

TLDTQRPVNSDPKAIEIELAKHHVLRNDVLSHRATVETVNKAGSELLESSPGDEASHLRD

QLDELNRSWDSLLLKTDERQKLLETALQQAEGFHGELEEFLQWLRRTESQLSAAKPTGGL

PETAREQLQQHMELQAQLTQRGEQYHRLLDQGESMLLARGAEENSPGTTQTQQNLALLQN

KWASLNAKMDDRRAKLEEAVSLATGFQSSLQDTINWLTQAEQTLNMAQSPSLILDTVLFQ

IDEHKVFVNEVNTHREQVLALEKAGSQLRFASLKQDVVLIKNLLLSVQARWDKLVQRSLD

RGRHLDEARKRAKQFHEAWRKLTDWLEEAEKRLDAELEISNEPDKIKVQLTKHKEFQKTL

GSKQPVYDTTVRSGKAMRDKATLPADTQKLDNLLGEVRDKWDTVCGKSVERQHKLEEALL

FSGQFAEALQALVDWLYRVEPQLAEDQPVHGDLDLVSNLMDSHKAFQKELGKRTSNIQAL

KRSARELMETGRDDTAWVKVQLQELSNRWETICALSVSKQTRLQQALKQAEEFRTAVQML

LEWLSEAEQTLRFRGILPEEVETLQVLLHTHRNFMQTVEEKRVDVNKAAGMGEAILAVCH

PDCITTIKHWITIIRARFEEVLTWAKQHEQRLEAALAELLNNATLLEDLLSWLQWAETTL

VQRDTEPLPQDITQLKTLITEHQVFMEEMTRKQPDVDKVTKTYKRKPSETSSSLAERRGV

RKQQQQQQQPAMQVSGGNPRLNQLCSRWQQVWLLALDRQRKLHDGLDRLEELKEFANFDF

DVWRKKYMRWMNHKKSRVMDFFRRIDKDQDGKITRQEFIDGILASKFPTSRLEMTAVADI

FDRDGDGYIDYYEFVAALHPNKDAYKPTTDADKIEDEVTRQVAQCKCAKRFQVEQIGENK

YRFFLGNQFGDSQQLRLVRILRSTVMVRVGGGWMALDEFLVKNDPCRARGRTNLELREKF

ILPEGVSQGMAAFRSRGRRSKPSSRTASPTRSSSSASQSAQSCASGPSAPATPTASARSS

QSQSRGYAKPWLAHSKTPTPTKCQSCTEHSHTPGHEGGSSSKLKRPPFHSSRGSLTGENG

GTTHATKPVRSDNKRTPSSTSGPTSRAGSRAGSRASSRRGSDASDASELMETRSACSDTS

DTPRRPGTKPSKIPTISKKTPSPKTPTTKK*

>m.481 g.481 ORF g.481 m.481 type:3prime_partial len:1618 (+) Unigene000043:3625-8481(+)

MGDKLWWADQGTDQVGTCDKKDGGSWKVLRNGTSPVMHMKIYNETVQQKGTNLCTMNNGD

CSQLCLPTSPTSRACMCTAGYNLKSGQQSCEGMGSFLLYSVHEGIRGIPLDPLDKSDALV

PVSGTSLAVGIDFHAENDTIYWVDMGLSTISRAKRDQTWREDVVTNGIGRVEGIAVDWIA

GNIYWTDQGFDMIEVARLNGSFRYVVISHGLDKPRAIAVHPEKGYLFWTEWGQYPRIERS

RLDGSERVVLVNVSISWPNGISIDYEEGLLYWCDARTDKIERINLETGGNRELVLAVHNM

DMFAVSVFENYIYWSDRTHANGSIKRGNKNNATDMVYLRKGIGVQLKDIKVFNRARQQGT

NICKDKNGGCEQLCLFRGNGARTCACAHGMLAEDGRTCRDYDGYLLYSERTILKSIHLSD

ETNLNAPIKPFEDPDHMRNVIALTFDHQGGDGKGANRIFFSDIHFGNIQQINSDGSARKT

VVENVGSVEGLAYHRGWDTLYWTSYTTSTITRHTVDQTLSGAFNRETVVSMSGDDHPRAF

ALDECQGLMFWTNWNELAPSIMRSSLAGSNVLVIVGNNIRTPNGLAIDHRSEKLYFSDAT

LDKIERCEYDGTNRFVVLKNEPVHPFGLAVYGEYIFWTDWVRRAVLRADKFGRDMKVLRA

DIPQQPMGIIAVAKDTNSCEFSPCHTNNGGCQDLCLLTSEGRVNCSCRGDRKLVEGNVCV

AENTTCHSVDEFECGNGDCINYSLTCDGRAHCKDKSDEKPSYCSNRGCKKGYRRCLNGRC

IKHSSWCDGTDDCGDRSDERPCNMTLCSASEFQCKDGSCITNTSRCNQVVDCEDASDEMN

CSPTDCHSFYLLGVKGVIFQKCEFTTLCYTPIWQCDGSNDCGDFSDERNCPEKRQLKCPV

NFFACPSGRCIPMSWTCDKENDCENGTDETHCDKFCSPTQFECGNHHCISSSWVCDGTDD

CGDGTDEGSRCSSKTCSPEAFQCPGSHCVPQRWKCDGDNDCPDGADEGVQAGCTNNKTCD

DTEFQCQNKQCIPKHFVCDHDLDCRDGSDESPECEYPTCGPNDFRCENGQCLKQKSWECD

GEFDCRDHSDEAPKNLHCTEPEKRCNDSAYLCNNGKCVSEDSLCDWKDDCGDGSDEHNCF

VNECLNNKLSGCTQHCEDLKIGYKCRCDPGFRLKNDGKTCVDVDECTTTYPCSQRCFNSY

GSFHCFCIEGYVAYANDWTSCKSSSEEEAYLIFANRYYLRKLNLDGSNYTLIKTDLNNAV

ALDYHYAEQMIYWTDVTTQGSMIRRMLMNGSNVEVLHRTSLSNPDGLAVDWVGGNLYWCD

KGRDTIEVSKLNGAYRSMLVNSGLREPRAVAVDVRNGYLYWSDWGDVPHIGRIGMDGTDR

SIIIKDKITWPNGLTLDFINDRIYWADAREDYIAFASLDGTNRHIVLNQDIPHIFAMSLF

EDYIYWTDWETKSINRAHKTLGTNKTMLISTLHRPMDIHIYHPYRQPAVENHPCQVDNGG

CSNLCLLSPGGGYKCACPTNFYLAADGKQCLSNCTASQFVCKNDKCIPFWWKCDTEDDCG

DRSDEPADCPEFKCRPGQFQCGTGICTNPAYICDGDNDCQDNSDEANCDIHVCLPSQF

>m.482 g.482 ORF g.482 m.482 type:5prime_partial len:1223 (+) Unigene000043:2-3670(+)

REDRKIDGTERDIIVKSGIHTVEGIAVDWMGQNLYWTDDGPEKTISVAKLEKASQTRKTL

LEGKMTHPRAIVVDPSHGMMYWSDWQEDSRDANRGSIRKAWMDGSKSGILITSKNMLWPN

GLSLDIEQNVLYWVDAYYDRIEMVLLNTTERRTVYEGQELNHPFGLCHYKNFLFWNEYRI

GGIYKLDMTSKKVTLLRSERPPIYEIRTYDAQQQQIPGHNPCRGNNGGCSSLCLLTPAGR

TCACADDQILDFDNKTCKANPSYVPPPQCQPGEFACKNNRCIQERWKCDGDNDCLDNSDE

VPELCHQHTCPADRFKCQNNRCIPMRWLCDGDNDCGSNEDESNTTCSARTCPPNQHPCAS

GRCIPKTWICDLDDDCGDRSDEPASCAYPTCFPQTQFTCANGRCINVNWRCDNDNDCGDN

SDEAGCSHSCSSVQFKCNSGRCIPEYWTCDGDNDCGDNSDEKSANCTNQATRPPGGCHSD

EFQCRLDGLCIPMRWRCDGENDCIDDTDEKHCEGVTHTCNPSAQFACRDSARCISKAWVC

DGDSDCEDNSDEDNCEALLCKLSHHTCAQNDSICLPAEKLCDGKDDCPDGSDEKLCDLCS

LENGGCSHNCTIAPGEGILCSCPTGMELGSDNKTCQIQSFCAKHLKCSQRCIQEKATVKC

ACYEGWALEPDNESCKSTDPFKPFIIFSNRHEIRRIDLYKGEFSVLVPSLRNTIALDFHL

NQSSLYWTDVVEDKIYRGKLSENGALTSFEVVIQYGLATPEGLAVDWIAGNIYWVESNLD

QIEVAKLDGTMRTTLLAGEVEHPRAIALDPRDGILFWTDWDASSPRIEAASMSGDGRRTI

HRETGSGGWPNGLTVDYLERRILWIDARSDAIYSAAYDGSGLIEVLRGHEYLSHPFAVTM

YGGEVYWTDWRTNTLAKANKWTGHNVTVVQRTNTQPFDLQVYHPSRQPQAPNPCAANDGR

GPCSHLCLINYNQTFSCACPHLMKLQADKHTCYESRQFLLYARQIEIRGVDIDNPYYNYI

ISFTVPVIDNVTAVDYDAQEQRIYWSDVRTQMIKRAFINGTGVETVVSADVPNAQGLAVD

WVSRNLFWTSYDTSKKQINVARLDGSFKNSVVHGLDKPHCLVVHPMLGKLYWTDGDNVSM

ANTDGSNQSLLFTSQKGPVGLSIDFDEGKLYWISSGNSTINRCNLDGTGLEVIESVKGNS

PEPPLSPSWGTSCGGRTRAQTR*

>m.499 g.499 ORF g.499 m.499 type:complete len:2549 (+) Unigene000044:148-7794(+)

MWSQSEGKKPSAMEPRMADYELSSAWRNLNHNKAALRHIENQLEAAVGSGVVITSLNTEK

SHKHKRRGRRSSDGDDATVKPRPQSRRSPDKSSRSPLRTSTQDNTHTSSPADASLSRCVY

DRDRRPVHSADADSTPSSALDATNVRVLNDSFALQRSEVTMGASEVSPGSARLEKLRQRQ

TDEKLEKLKERIRRQREQLEETAGRGGAIEAGHGNSAHQHLTKVRKVAAAPPAPNYKGFN

SSEVKIQSSDSRTWGDTESRLKPENYLTDALSERVKRKPVEKDKQQERKLIKPVRKIHRS

TSAPEPKPAISASSWREGQKVVQMMLGPLPREPWAQTAGRERLAAASSRSSSVPRLESRH

RSRSSGHRLSSTEKPTVTRPSRPPAGTRERKTVTTQSSRPIEGPGALSADLELLPAEFRS

ILDDLHLEDRGVKSHDPGRVDRKRAGRSPSKPRPDNTEGALPRKRHYDADEVRRYIAKQQ

EERKRKQVEERRSQREEQERRSHRLQELYRKQREGVIKPQVPPPNLLNPHLRETYTKMLL

KHKLQEAQPVYQPSGESDKENKKMEPLSPSTSELSEHTPSPLCRADLVPPAGEFFSHLLS

VEPECVNSTPHVRDTHTEDAHWVGKGWVDTHTISRLNRVEALKACAASLSNRIESEARKL

ITYSDHDNQDGQNHQSKPPSPPEREQPHSTSRNHDYLRELPGVGNLHGFITREGWGEIPK

PRPLTASTALPFTVQHEEHDSSGGSISEGVLSDASLSDHADHPDDRITLFRKEAELYVPY

RPVVTSQPPWEELSKGSPHSVINIFAKNLNKYNNVMDMEENGATYEDDFVSSRSSSQSAS

KRTASGVSRSSAAADRFPHSSASVLLSSPSSTHSVSKRGSDRSLERSLVDGQRSMPLIEP

RRKIDSPRGSHRSAEGNGTPGGASVRSSHSMVSEFPHFPRSPADSPRISGSSSSSPASAR

DSSPKVETGPAPNLVSSPGPAHSITERHFPPDVLQQRLNAELNFLDAVEESVRQLSDVER

VRAVSLAQQESVSLAQIIKAQQQRQERELQLLKLKAEQETLETQRHVQESRERAAQAHVE

LCMNLVQSHQKALMELQENSSKINHQHTQTPQHLREMTEMSRPQLPSVSSVTPVRQPHPQ

TDSSRSEVTTEKTPTAHTPQSITAELESFSFTQSNGGGVDRSGSGGEEGKTPADDSLQTD

KDSVSINYSSKFDESVTEDELEKSFRSVLPSESHWRGSVERKRQSDEEPHADKISTKDGS

VPVFSADQYSFSEFTMAMVKQCMQEEEVRSQHQSSLLRLRHRALKEKTRAELEWLELQKR

HLRDKGEDDKMPPIRKKQRGLLLKLQQEQAEIRRLQEVNRAARRERKLLLKQQEEIERIQ

HTTQKLREKLRNAEDTNQRSPVSGVSEDLAIPTKVDIEFRSPSPLSVSGSETSSVMKKLK

KMNYHTDERFLTQRELRLVHWRQQVEAELRRSDGAEPQPKGRALNAERSTQQKHGTDELK

RDRQEERKTGHEVRSDCDSSSALSVSSIHTVQECDNTHSREFSLVPASSSSKVTSHSSTR

KRDLPLSAPTSEAASDQSDIEGRVLALREELRRRKAEVQLLKREQRLRHKERLKAQEASL

LKQLESCNNFIQKTKAELSNADVATTSTKPQIKTPTSDSHKSGTESFTTDSSGTEKSREE

KSHESESERSNRIHTQSPALSDEDPSTVTPTPVLGSPNLLSPRELQHVEQTPQPESPGRP

GKDASTDLSTASSPGSDVIEELESLRSDPTRSEHSHPLLNLKLQDSDGNSSKNQPEIEVK

FSHVEEFTNSEAVKSSSIIVTKLEPEEPKLPERSLTLDVSDLEDKPPESLSEHSYNDDFE

SAIKEETGSHSLSSEDPEEVSYKFSFSSEDEIEEKISLQSGNTSRTFEQERPPDLHFLGR

CTAESIHEDVPSLPKSSKDEMMDEMPDYVIGERVLVSNLQPGILRFKGQTSFADGFWGGV

ELDASEGSNDGTYNGIVYFQCKEKHGIFAPPGKISHLPETHEDHTRSSEEKDSSFKDQTC

RKTNKSESSLQNKRVESDPPSETEELSGKPVVLDASVTKDLMIDHVALNGETFENLNGEK

NLLALESFKDIVLEFKNVSKHEEGRDQTPDILDLLIRDESSTATEDLQKSSVVSPEKTNE

DNVDGSVVTEHKPLTALADTLIERFMSDALKQFQQIKKNKEQKILAANQLVFFGEDEEPS

ENDVFRFGTSASCRVDFDKDQEEVSSPEMCNRLESPVLGQKELVQRLEELELNREFLHVL

GDEPDWFDEDFGLSSRRQTQNKNKLRQDELTPTSAAKLPHQRQQDEAVAPAAMLVPHKAE

EVEKLVSAALLEIWMSCGMGRGKASLTGVAKPQASESLLGDQSGEACVRSYKQAVFDLVW

ELMQEIYAEDPNTYRPLWIKPHRIHSPYIHRIDNPTDLHKVQAFVTGEVLKLYNLNKEQN

HRTDWQKMLKFGRKKRDKVDHILVQELHEEEALWVNYDEDELLVKMQLADGILEALLKDT

VDVLNKIHQSRSSRALTPSSSTHLLLQP*

>m.513 g.513 ORF g.513 m.513 type:complete len:1213 (+) Unigene000045:166-3804(+)

MAPVYEGMASQVQVFSPHTLQSSAFFSVKKLKVEQSCNWDMTGYGTHSKVYGQNSKQSVS

AAPVGLNAASLQVSNSSLPYEQALLFPASSGHIVVASASSTSGVAGQLLGSTGSSSGSGS

GGHNLTRRSTVSLLDTYQRCGLKRKSEELDNNNGSGSSVHVVEELQQPPAAQMLQNNGQS

GPTGTVATASTTATSKTSGANSEGDYQLLQHEVLCSMTNTYEVLEFLGRGTFGQVVKCWK

RGTSEIVAIKILKNHPSYARQGQIEVSILARLSTESADDYNFVRAYECFQHKNHTCLVFE

MLEQNLYDFLKQNKFSPLPLKYIRPILQQVATALMKLKSLGLIHADLKPENIMLVDPSRQ

PYRVKVIDFGSASHVSKAVCSTYLQSRYYRAPEIILGLPFCEAIDMWSLGCVIAELFLGW

PLYPGASEYDQIRYISQTQGLPAEYLLSAGTKTTRFFNRDPDSTYPLWRLKTPEDHEGET

GIKSKEARKYIFNCLDDMAQVNVSTELEGSDMLAEKADRREFIDLLTKMLTIDADKRITP

IETLNHPFVTMAHLLDFPHSTHVKSCFQNMEICKRRVNMYDAVNQSKTPFITHVAPSTST

NLTMTFNNQLNTVHSQPSAAPMAAVAPRTMPLQPGAPQLCATRPDPFQQALIVCPPTFQG

LQASPSKHSGYSVRMENAVPIVTQAPGGQPLQIQPGLLTQQAWPSGTQQILLPPAWQQLT

HTSVQHATVIPDSMGTSQPLPNWRNPHPHGSHYNPIMQQPALLASHVSLPSQQPLNVGVA

HVMRQPVNNSTASKKSKPQQMSNRNVSAYEVSSSQAMLSPQRSKRVKENTPPRCAVVQNG

PSVPCALSQACGGGGGGWGEEQASSTTREHHGPHNQHLQHNQHNPPRQTIVIPDTPSPAV

SIITISSDTDEEDEHKQPGNTSTSSKQRKNVISCVTVHDSPESDSSNISPYAVESRSNCP

NANNYDSKSTVLDNYNNGNPRTIIIPPLKTQTSESLSECDRLMPDTMNHQNPAYKFNSSN

GGLSTNNHSSVGIPGGGAYRQQRSGPHPFQQQPLNLSQAQQHMAVDRTRGHRRQQAYITP

TMTAQAPYSFHHNSPSHSTNVHPHLAATHLSGQPHLYTYTTPAALGSTGTVAHLVASQGS

AHHAVQHAGYPPSIVHQVPVSMGHRVLPSPTLHHGQYQPQFAHQTYISASPASTVYTGYP

LSPTKMNQYPYL*

>m.519 g.519 ORF g.519 m.519 type:complete len:241 (+) Unigene000046:7600-8322(+)

MQESLRASLCEHATRLNELNAALEQERVAVSNLRAELQIEQSRCEALLAQERERTELTLS

RLDEERSRCAELSRSLSHQAQEHARRLEEEVRSQQAASADDRKFIQELRAQLEQERRQAE

DLAATVDNLQSQVLQSKRRQEEAAQQEAQRGQEEVKRLRSALDGLQAQRVEVNRTLETER

HRAAQLQTELDAVKEKMREVKEKERAKEEQREKQRWCEKQEQEDRERRQEQTKEKLVLQR

*

>m.518 g.518 ORF g.518 m.518 type:5prime_partial len:2549 (+) Unigene000046:2-7648(+)

SEERRAGGIEDAKIRWIQDFKKEQAELQEALNKAEDRLAETKRKLEELQRSKEEEIKHLE

SELSQAWADRDSAARAVEELVASHRVVLQEQQAHTQELEEKEKWLKQEVDRLLEQKSSSE

QEITNLWSQLESMRTSRQELGELKEQLLARKSHVNDIEQLKQDFSQQRRELQEQNETELE

NLRVYFEQRLRASEENHREEIALLQLRLVEGALEDSVLKTGEESFLSDGKSEEEKGDSLA

EITEQLDKHKVELDALRLQLEEKHKQDLEQLRCNMALSYREELLQARTELTDRYYSDMEQ

LKTKHALEIEQLRAKLSDSHLKEITRLRLQSVADVARQVETELEEKSRLQTQEHQARLAQ

LASDTESILSLEKKLAALTLQHSRELERATEQHAEELRQLEERLKKNFSEELHTRLAEAQ

QEERTHVTEELLNRSSEERLQLREQLQAQAEERVASLREELQQAASKDRQELEQKLVQAE

ELLAEEKQRLQALQVSLEREESPQLLALKQRLESQYAGELHTAKSTMAAEVKELNTLLQE

QTESRLKEATCRHQEEQRQLAEKLVQQKEEALQKLRQQHAQELQVQETLLEQNVSELQHL

QKEYQELKSRHQEELDLLSSNHKTALDSLAEKHKAELDKLQVVFQETNLAQLEAQEAELE

ERHKQEMEELETRMLCNMDTLESTYLKEIQAVREEKEAALRDLGASLEQQQAEEIERLKQ

EELIVREELRKKLAQVHMDKFSVMATELSHAHQAELSAALASQKAALESEHHSALETLRQ

HVLDLEAQHGAALHEVTDMTTKENQQLKKQLDALNAQHQQQLQELRATSSREIEALRREL

EEEASRQRLHFLEEAELLKCQSEEQLQKKLMELKEESDREKVSALEELERSLCEQHQQNE

LSYTEKMNHLTTQLQQLDTVVSQLRAEVSGLQGELEGKRSEMDTLETLLQRRERESQEGA

NLLAMLRDDLNTATQQRQDLQLTHDRLQRLVLEMLRITIATEEEISRRLGISMEGDDITL

NRKQSVNRDPQKTDAEVGDGAAAAVEASANCSMFSSLMDEGLELSQRLCESLFTGPDGEL

DPEREELVLGACTHLRVAVDKLLEVVSDSTVQLEQLQALQALLDEKFSAGGEAKNALFLQ

HTQLLEQLDQEASVKSQLQLELHKADGLIEGYVVEKAALEDALQQKESREQKLVKELEST

RAQLQELGEEHALLQRQRDALSASLGEPEKALLAEAERLGQERVDVQRQAEKDRVGLAGR

LRLLEQTLEEHESRTQQQEEQHRTQTEDLQQHISALEKQLKHHRQFIDEQAVEREHERDE

FQQEIKNLEAQLKTTSKGHAGEDTKGQRVESLQALIKDKTEDYSVLLSLKEQCQRDLEER

NEEIDKMASRIRELEQALLSSVEERRAAAQLEQELQKARKNCQELSQDKEALQQQLYSNK

LQISALQSKLDETRHRYPDPGADPNLRERLETLQQDLQNKEEQVEVLLERVEVLEKALAV

KEEDVRQLSLQLELKTTESRAREEELRLSVAELQDTASLLRRQRQEEADASALQLPSALL

EEKNLEIDHLNQQLLHLQQELDASKENKTEEKQADVEDLRAQVERLHGDQERLHQAKEEE

VEQLHEIIHKLQEELSQLNPNRHEVSDPNTDSPEPPDFPWSPRPQHGAEESLCHELSSQT

LQSCRTKICELQADLERSVEEKVALERLLLMQEEQYGQQLEALGCSLGEGKRKLALLEQE

AGELKLQLDQKETKVEELQARIQTLEDGERSVNDLELRLIKAEETMKETQQEMTRLNDKA

RKHTEERERTESLIAELQQRYEDSERTATQARLQVAELKETIQQRKSDIAILETGKTELY

TEKQALQKRERKLQEEIEKLKQEVSAKSLQIQEVSLQLEERAADPDQSQEEVLTCAEETL

AKAAAALREKEEQLAHLRSQHDALRAELAAVKEGLSSSTERAEKLQEEGQTKDRALADLE

EHNQRLRAELRGLQDDLEVQEEELAYQQRELDELRQRCRLQEHTELDCTKPERRFLDGLS

HNSTLSSPEVLRRLDCSEERPSHVHASHLSELSGLRNTSLELMSKHSLMERNELKRPLVL

PPETDPPSTHSPSASTHSLSISENLSVLNSLDDDKVQVLENLDDVTPSRSPLSSTSPVSA

PEWASDGYGSNVSSELEAKLKNELEHMERLDAHFVEYLRSRDMAPAPCSDSAAGSLHHTY

ELLSPELQAMLNRVYRESYRVLSLSHRPPPAGQPHSDAEEAPPASWQRERRALQETVLSL

RELLCRMAEREPKVEGEVDWRTELLQAVRSVFDSERKWLHAQLQDIITTNTGIDYTSLLK

DLETLLQKQEDQQRRSLEQLLSADRNSLLAEIRSLHKQLHTCTLQNQEQLQRSLGAVREE

GAQTQQQLHRQVGELERQLQQEQSSSHDLQRSLGQEQSRSTNLHQQLEIQQKLAVEMKLE

LEEGTRQLRASEKSREELQAQIHKLRLKLESEEQEHQACVEAANEMRSGLSSFRRNFITR

GSAANARRRSMRTCKSLSGPPCASMRLV*

>m.531 g.531 ORF g.531 m.531 type:complete len:2607 (+) Unigene000047:76-7896(+)

MPATEKDLAEDAPWKKIQQNTFTRWCNEHLKCVNKRIADLQMDLNDGLRLIALLEVLSQK

KMYRKYHARPTFRQMKLENVSVALEFLDRENIRLVSIDSKAIVDGNLKLILGLVWTLILH

YSISMPVWEEDEDEESKKATPKQRLLTWVQNKVPDLPISNFSQDWRDGRALGALVDSCAP

GLCPDWETWDPVKPVENATEAMQLADDWLGIPQVIAPEEIIDPSVDEQSVMTYLSQFPKA

KLKPGAPLKPKLNPKKARAYGPGIEPVGNRVMQPAVFTVDTFSAGQGHVTVYVEDPEGRR

EEVKAVYNESKKTYSVTYIPQVMGPHKVTVLFAGQEIPKSPFEVNVDKAQGDPTKVTAKG

PGLEPVGIIANKPTYFDIYTAGAGVGDVTAVIKDPQGNKNTVEAMMEDKGDSVFRCTYRP

VQAGPHTVSIMFGGIAIPKSPYAVSVGPASTPSACRATGRGLQPRGVRVNQVADFKVDTR

NAGSGDLKVTVKGPKGLEEQVKLKEAMDGVYAYEYYPNTQGKYTVSVTWGGQHIPKSPFE

VQVSPEAGPQKIRAWGPGLEGGIVGKSADFVVESVGTDVGVLGFAIEGPSQARIECEDQN

DGSCDVKYWPTEPGEYAVHIMCDDEDIEESPFMAYIIPDNKSSNPDQVKAYGPGLEKSGC

IINKPAEFTVEAKDAGKGPLKISAQNADGEPIDVKVKSKGDGVYACSYTPTSAVKHTVVV

TWGGISVPNSPFRVQVGKGSHPHKVKVFGPGVERTGLKAHEPTHFTVDCTDAGEGDVSVG

IKCDANIISDKEEDVDFGIIPNANDTITVKYIPPGAGRLTIKVLFTDQEIPASPFRVKVD

PSHDASKVKAEGPGLAQAGVESGKPTHFTVYTKGAGKAPLDVQFSGPSRTQPVQDFEIID

NYDYSYTVKYTPVQQGEMVITVTFGGDPISKSPFAVGVAAPLDLSKIEVDGLESRVEVGQ

NQEFAVDTKEAGGQGKLEARITSPTGKSVPCVVQPQRGKGASLVKYVPKEEGAYSVELLY

DSNPVPGSPFTVDAMLPPDPSKVKAYGPGLKGGLVGNPAQFTIDTNGAGTGGLGLTVEGP

TEAKIECSDNGDCTCSVSYLPTEPGEYLVNILFEGVHIPGSPFHADVEYPFDPTKVVVSG

TGLKRGKVGETSVLNVDCAKAGPGQLVLEAVSDAGSKAKTEVLDNKDGTYTVTYVPLTAG

MYTLLLKYGGKTVQNFPAKVVVDPAVDTSKVKVFGPGVAGEGVFREATTDFTVDARALTK

VGGQHIKAQIINPSGASTSCSISDQNDGTYKVEYTPFENGVHAVEVLYDDTPLPKSPFQV

GVSEGCDPTRVLAKGPGLDEALTDKPNKFSIITRGAGIGGLGITVEGPSESKMSCKDNKD

GSCDVEYIPYAPGLYDVNITYGGEHIPGSPFRVPVKDVVDSSKVKLSGPGVSSGVRAKIP

QTFTVDCSKAGIAPLSVAVTGPKGKAEPVQIMDNGDGTHTVAYTPSVEGPYAVAVKYAEE

EIPRSPFKLRVLPTHDASKVRASGPGLTTSLPASLPVAFTIDAKDAGEGQLSVEITDKDG

KPKKISIHDNQDGTYDVSYVPDKVGRYTIVIKYGGDEIPTSPYRVRATTTGDASKCTVTG

PGIGPTIGIGEEVGFVVNTKGAGKGKVSCVVVTPDGTEVDAEVIENEDGTFDIFYTAPKP

GTYIIYVRFGGENIPHSPFKVMATDEPPLLQQQQQSVQQNRLAPGAGFQPWVTDGAYIPV

SSMNGAGFRPFDMVIPFTLSKGEITGEVHMPSGKTAQPEIIDNKDGTVTVKYAPTELGLH

EMHIKYNGAHIPESPLQFYVNSANSPNVTAYGPGLVYGVANQPATFTICTEDASEGGLDL

AVEGPSKAEIGCVDNKDGTCTVTYLPTLPGDYNILVRYNDKHIAGSPFTARITEDNKRRS

QVKLGSAADFALDITETDLSQLTASIKAPSGRDEPCLLKRQPNNHIGISFIPREVGEHLV

SIKKNGHHVANSPISIMVVQSEIGDASRVKVYGPGLLEGRTFQMADFIVDTRDAGYGGLA

LSIEGPSKVDIQTEDLEDGTCGVAYCPTEPGTYMVSIRFAEEHVPGSPFSVKVTGEGRIR

ESISRRQRAASVATVGSACDLNLRIPEIDIHDLSAQVTSPSGVTEPAEIGAVGNHTYCVR

FVPHEMGVHTVNVKYRGQHVPGSPFQFTVGPLGEGGAQKVRAGGPGLEKAEIGVPAEFSV

WTREAGAGGLSIAVEGPSRAELSFEDRKDGSCGVSYVAQEPGDYEVSVKFNEQHIPDSPF

LVPVCAPPHDARCLTVTSLQESGLKLNQPASFAVRLNGAQGVIAAKVHSPSGALEDCVVS

ELEHDKYAIRFIPRENGIHTIDVKFNDTHIPGSPFQVRVGEPGQGGDAGLVSAYGPGLEK

GTTGNQSEFTINTSKAGPGSLAVTVEGPSKVKLECQECPEGFKVHYTPMAPGNYLISIKY

GGPNHITGSPFKAKVTGQRLVNVTNASETSTLLLDPTVRAVSAGSTNAMPRAASDASKVT

SRGPGLSKAYVGQKASFSVDCSKAGKNMLLVGVLGPQTPCEEIMVKHMGSMQYNVSYVLK

ERGNYVLAVKWGEDHIPGSPFQITVP*

>m.545 g.545 ORF g.545 m.545 type:5prime_partial len:2536 (+) Unigene000048:2-7609(+)

CVCVCVYAELSSAWRNLNHNKAALRHIENQLEAAVGSGVVITSLNTEKSHKHKRRGRRSS

DGDDATVKPRPQSRRSPDKSSRSPLRTSTQDNTHTSSPADASLSRCVYDRDRRPVHSADA

DSTPSSALDATNVRVLNDSFALQRSEVTMGASEVSPGSARLEKLRQRQTDEKLEKLKERI

RRQREQLEETAGRGGAIEAGHGNSAHQHLTKVRKVAAAPPAPNYKGFNSSEVKIQSSDSR

TWGDTESRLKPENYLTDALSERVKRKPVEKDKQQERKLIKPVRKIHRSTSAPEPKPAISA

SSWREGQKVVQMMLGPLPREPWAQTAGRERLAAASSRSSSVPRLESRHRSRSSGHRLSST

EKPTVTRPSRPPAGTRERKTVTTQSSRPIEGPGALSADLELLPAEFRSILDDLHLEDRGV

KSHDPGRVDRKRAGRSPSKPRPDNTEGALPRKRHYDADEVRRYIAKQQEERKRKQVEERR

SQREEQERRSHRLQELYRKQREGVIKPQVPPPNLLNPHLRETYTKMLLKHKLQEAQPVYQ

PSGESDKENKKMEPLSPSTSELSEHTPSPLCRADLVPPAGEFFSHLLSVEPECVNSTPHV

RDTHTEDAHWVGKGWVDTHTISRLNRVEALKACAASLSNRIESEARKLITYSDHDNQDGQ

NHQSKPPSPPEREQPHSTSRNHDYLRELPGVGNLHGFITREGWGEIPKPRPLTASTALPF

TVQHEEHDSSGGSISEGVLSDASLSDHADHPDDRITLFRKEAELYVPYRPVVTSQPPWEE

LSKGSPHSVINIFAKNLNKYNNVMDMEENGATYEDDFVSSRSSSQSASKRTASGVSRSSA

AADRFPHSSASVLLSSPSSTHSVSKRGSDRSLERSLVDGQRSMPLIEPRRKIDSPRGSHR

SAEGNGTPGGASVRSSHSMVSEFPHFPRSPADSPRISGSSSSSPASARDSSPKVETGPAP

NLVSSPGPAHSITERHFPPDVLQQRLNAELNFLDAVEESVRQLSDVERVRAVSLAQQESV

SLAQIIKAQQQRQERELQLLKLKAEQETLETQRHVQESRERAAQAHVELCMNLVQSHQKA

LMELQENSSKINHQHTQTPQHLREMTEMSRPQLPSVSSVTPVRQPHPQTDSRSEVTTEKT

PTAHTPQSITAELESFSFTQSNGGGVDRSGSVEEEVNTAADDSLQTDKDSVSINYSSKFD

ESVTEDELEKSFRSVLPSESHWRGSVERKRQSDEEPHADKISTKDGSVPVFSADQYSFSE

FTMAMVKQCMQEEEVRSQHQSSLLRLRHRALKEKTRAELEWLELQKRHLRDKGEDDKMPP

IRKKQRGLLLKLQQEQAEIRRLQEVNRAARRERKLLLKQQEEIERIQHTTQKLREKLRNA

EDTNQRSPVSGVSEDLAIPTKVDIEFRSPSPLSVSGSETSSVMKKLKKMNYHTDERFLTQ

RELRLVHWRQQVEAELRRSDGAEPQPKGRALNAERSTQQKHGTDELKRDRQEERKTGHEV

RSDCDSSSALSVSSIHTVQECDNTHSREFSLVPASSSSKVTSHSSTRKRDLPLSAPTSEA

ASDQSDIEGRVLALREELRRRKAEVQLLKREQRLRHKERLKAQEASLLKQLESCNNFIQK

TKAELSNADVATTSTKPQIKTPTSDSHKSGTESFTTDSSGTEKSREEKSHESESERSNRI

HTQSPALSDEDPSTVTPTPVLGSPNLLSPRELQHVEQTPQPESPGRPGKDASTDLSTASS

PGSDVIEELESLRSDPTRSEHSHPLLNLKLQDSDGNSSKNQPEIEVKFSHVEEFTNSEAV

KSSSIIVTKLEPEEPKLPERSLTLDVSDLEDKPPESLSEHSYNDDFESAIKEETGSHSLS

SEDPEEVSYKFSFSSEDEIEEKISLQSGNTSRTFEQERPPDLHFLGRCTAESIHEDVPSL

PKSSKDEMMDEMPDYVIGERVLVSNLQPGILRFKGQTSFADGFWGGVELDASEGSNDGTY

NGIVYFQCKEKHGIFAPPGKISHLPETHEDHTRSSEEKDSSFKDQTCRKTNKSESSLQNK

RVESDPPSETEELSGKPVVLDASVTKDLMIDHVALNGETFENLNGEKNLLALESFKDIVL

EFKNVSKHEEGRDQTPDILDLLIRDESSTATEDLQKSSVVSPEKTNEDNVDGSVVTEHKP

LTALADTLIERFMSDALKQFQQIKKNKEQKILAANQLVFFGEDEEPSENDVFRFGTSASC

RVDFDKDQEEVSSPEMCNRLESPVLGQKELVQRLEELELNREFLHVLGDEPDWFDEDFGL

SSRRQTQNKNKLRQDELTPTSAAKLPHQRQQDEAVAPAAMLVPHKAEEVEKLVSAALLEI

WMSCGMGRGKASLTGVAKPQASESLLGDQSGEACVRSYKQAVFDLVWELMQEIYAEDPNT

YRPLWIKPHRIHSPYIHRIDNPTDLHKVQAFVTGEVLKLYNLNKEQNHRTDWQKMLKFGR

KKRDKVDHILVQELHEEEALWVNYDEDELLVKMQLADGILEALLKDTVDVLNKIHQSRSS

RALTPSSSTHLLLQP*

>m.559 g.559 ORF g.559 m.559 type:5prime_partial len:2698 (+) Unigene000049:3-8096(+)

THTHTHFGIFLCWTFIFSEKRKQRAATMSAHGTPTSRFPSMSRGPSMRVNEQMFMSGNGY

QSDLSENIMQQRYSTNTYSVKSQGSMMPLQQNSQVVYNRCMRNLEKAQRQLELVLQGAPR

SEFDQLMAMAEMDIKQLHQLAIEGKNFGQYPEFIIRGLEECNATYAELSAGAGKISVRQN

RNSTSREEYSLTLTDAYAWIKQQKRLIETAPFGDDCEAIEQQIANHNRFHSSIQRSAEVQ

RARDELMQLGDKPNMHALEQEWDSLQKMSHDRLTHLRDMQNILTDISQAIMWVNDKEEEE

LMFDWGDKNIDTYIPKKQESYSRLMSELEMKEKELNKLKSRVDQLLKNNHPASDKIEAYM

DTLQTQWSWMLQITNCIDTHLKENAAYSQFFKEANETYTRLQKDDENLRKKFTCNKSAPL

ESLTELLRNMEKEKERLMENKRQVQTLVNKSKNIVCLKPRNPEHKSNNRVIVKALCDFKQ

DQRGIYKGNEAILLDNAERSKWQVTGPGGLNMFIPSVCLIIPPPNPLCIDLANKNEKYFE

AVMGIWNQLFINNKSLIAWHYCLRDINQINSLTISMLSKMKPEEYRSIIKSLEYHYEEFR

RLSQQSSLFEEADQRKMAVDYNSAQTHYDTILTKVQPGFDASQRPKAPQHTHNLLTLTHT

LRRRLEAAESGLSQHLHVPLRENSVQECTQRLQLLQGVHHDLGSIQDEYLHLRELVLKQE

VNADPEQAKFLRAELDLINQKLSSLRSYSNSYLNRLTALRALMQNLTRAEDIIKVHEARL

TEKETSSLDQGELQDYHKTLKNMKVDLDQRKEVLSAMEADLDQAYHWNGQIDQNFHQCDV

DLSKFSDLVKQMISRWNRITSQINTRMGDLNKQEKQLSQYKHNSSNTNQWIDQTSNRLDK

LQNYKLNTLQDLMDHLNQHKTLHSEIKGKKDKVDDVQKDASTCSASIKDYELELASYSAG

LETLLNIPIKRTMLQSPATILSQEAADVQTRYIELLTRSTDYYKFLTEMQKNMDELKIRN

TRIDLLEEELRRLKDDLQDRSQKNRSLEEMLARLKIDLSQSKEQLISMEQVKSTEAMRYN

AARENLDTTQNQLKDLNDQVARLTLLIDEEKRKRRLAEERYSSQQQEYEGAIRKRQKELD

ELNWTKIDFEKAVKDKEREIERLKVQLEDEASRRRTAESETAKVRNQFNQDLTSLKQTYE

SEIHVTKTTVLKDMQQKEEDTAALKMQLEKLMNEKKDLEEELRRLQMSIRQMEEARKRAE

QEVHQQRSSGTEETRRRKELEIHVQTVTRQRTELESRFNEELVQANNKLQEKARQISVLT

QNLEDETRKKRALEQENHRLRQSEADLQAKHSSSLELINKLKITEKETKLICVELEKQAS

EKGKVEQGAARMQTRIIDLQNMLEKLEFELEKEKKSNQDELTRRKRIETELERVNQVCRE

YTTTINSLRAHKEEESATSRRQEQDLHRVQQDLERCRKEYNVTSENLARLTAELKALQQQ

LIQEQARVREVNLRNESLYKTIEEKSRVLNESMSEIEKLKGLTQNLTKERLKLEEELRSV

RQEREDLRNNKNNNESEHMAQLSAIQLQLQASNKRGLELQGHISDLTKERENLKTEIAKI

QKQYTETSLMVHESQMHYKEILQERDSLLAKLKLLEMDKGKHQRSEEELSRIKMSLESEL

RQKQRLQEEIDKIRKDFNYWKSQYEIKDGQVKQCHADKDKSERDRSSLQSEITRLTAELR

SLDERYRTRLQSSEFEVSELTRKREALERELRRLQQRPSAFTKQTQTDENVPMIDPSKLV

FDGIRKKVTAHQLCDCGIIDKSTLDKLLKGQIAVSDISAKIQPYLKGTGVVAGVQKDSHG

RMPISEAKSKNLLSPQSAIMLLEAQAATGYIIDPKFNERMPVDTACSRGIVDTEDRDVLV

TAEAACTGFKDPFSGKVLSVGQACKKGQLDKEKALRLLQAQETVGGIIDPVLSVFLPKDI

ALDRGLIDEELYRALNKKPACYINPANSEKISYSDLRRKCVTEPTNGLLLFPVNENSYYV

KGIRGQVSLTELVNSSLITEGDVDKVKQGLLTTKDIENRLKTYLSGSGCIAGIYDEARDR

VLPFYQAMKENLLRPGTTLELLEAQAASGFMIDPVNNVYLTVEDAWRRGLVGQEFKGKLL

SAEKAVTGYHDPSTGQLISLFQAIEKELIEKGHGIRLLESQIASGGIIDPKESHRIDVEV

AYRRGYFDREMNEILSYEGDDTKGFFDPNTQENLTYLQLQKRCMKDPVTGLLLLPLKDKN

KQKQQLSQKNTLRKRRVVIVDPDTGKEMSVREAYHRELIDYDTFLNLSEQECEWEEITIT

DSAGKTRLVIVDRKTGTQYDVQDFLDKGLIRKSDLDQYRSGSLTLTQFANLFSTKATSAE

LSICTSIPEDVTNCGSPTEVRPSSPTVRKRFASVSITLSPPSEILDDQSPVAAIFDTETL

EKITITEALRRGIVDAITAQRLLEAQACTGGIINPANGKKLSLQDAVHQSIIDEEMANKL

KPAQKAYIGYEDVKTRRKMSAAEAIKEKWLPYEAGQRFLEFQYLTGGLLEPESGKRISIE

EAIRRGWLDGKGAQKLQDTRSYLKSLTCPKTKLKISYKEAMDNCMVEENNGMKMLQATSV

STKGISSPYNVSSGPSSRSGSRAGSRGSSRSGSRRGSVDYSTYTYSSTTSSSYTPFP*

>m.568 g.568 ORF g.568 m.568 type:complete len:2157 (+) Unigene000050:232-6702(+)

MSVSTQLGLLLWKNFTYRRRQTFQLLIEIIWPLFIFFILIAVRLNYPPYEQHECHFPNKA

MPSAGTLPWIQGIVCNANNPCFRHPTPGESPGVVGNFNDSIISRLFSDAKKILLYSQNNK

SVDGFKEFISAVKHMQNNSSGFRLKDFLNDNETLSAFLMQNASLTDNVVEDILRAEINLE

KVLTRGYGVHLRNMCNTSVLEQFVKFPNRTVADLTHKMICSSPQHWLNKAEKHFLANLDF

FRPLRSDVRSERNDTHHVAKATDNLLESLGSVAVELASMRSWRDLRNEILFLTQNATGSP

GQMYQAVSRIVCGHPEGGGLKIKSLNWYEDSNFKAMFGNYNSSDEEPVSVYDNTTTPYCN

NLMKNIETSPISRMIWRAIKPLLMGKILYTPQTPATQKIIQEVNRTFQELGILRDLGGMW

EEARPKVWNFMENSEEVDLVRMLLQNNVTAYFFSAQLSGTQWSVQDLSSFLTKHSEDTRP

PGTVFTWRNVFNETDQAIMSISRFMECVNLDKLEAVSTEEKLVNESLSLLDNRKFWAGLV

FPDIDINSSELPPHVSYKIRMDIDNVERTNKIKDAYWDPGPRADPFEDMRYVWGGFSYLQ

DVIEQSIIRAVTGTKEKTGVYIQQMPYPCYVDDIFLRVMSRSMPLFMTLAWMYSVAIIIK

SVVYEKEARLKETMRIMGLDNGILWFSWFISSLIPLLMSAGLLVFALKMGNLLPYSDPGV

VFLFLGSFAVVTIMQCFLISTAFSRANLAAACGGIIYFTLYLPYVLCVAWEEYAGLPAKI

IASILSPVAFGFGCEYFALFEEQGVGIQWSNLISSPMEGDDYSLTTSIILMYFDAFLYGV

MTWYIEAVFPGQYGIPRPWYFPFTKSYWFGEEEQKQTPIIQGKKANAGAVCIEEEPGHLE

LGVYIKNLVKVYRHGKKLAVDDLTLGFYEGQITSFLGHNGAGKTTTMSILTGLFPPTSGT

AYIMGKDIQSELSAIRQSLGVCPQHNVLFSMLTVEEHIWFYARLKGLSEEEVKDEMDQIL

YDTGLPHKRKSKTSELSGGMQRKLSVALAFVGGSKVVILDEPTAGVDPYARRGIWDLLLK

YRAGRTIILSTHHMDEADILGDRIAIISHGKLCCVGSSLFLKTHLGTGYYLTLVKKDQEA

SLSSSRNSTTTESYIKKDEDDSESSSDAGLGSDQESDAATAIGTSWNDSPVVPADVSLIL

GLILKHVPNARMVEDLGHEITFVLPYESAKDGAFVDLFHDLDDHLSALGISSYGVSDTTL

EEIFLKVAEDSGVDTEMMSGDGTLPVRRRRRHAFGADHQSCLKPLTEDENFDCNESEVEP

DTKETDHLNSTDGKGSYQVQGWSLMRQQFVALLWKRFLYARRSRKGFFAQIVLPAVFVCI

ALVFSLIVPPFGKYPSLALQPWMYEDQVTFISNDAPEDASTQKLLSALLDDPGFGTRCMK

GEPVPDVPCTMGDEDWSIPEVPDNVWEVFKNENWTMENPSPECECSCDGNKKMLPECPAS

AGGLPPPQIKISERDTLQNLTGRNISDYLVKTYAQIIGKSLNNKVWVNEFRYGGFSLGAR

STQVLPPAQEIDDTISRVREIFHLHKNSGTAADRFLKSLSAFINGLDTKNNVKIWFNNKG

WHSVVSFLNVMNNGMLRANLPPGKDPSKYGITAYNHPLNLTKEQISQVALVTTSVDVLVS

ICVIFAMSFVPASFVVFLIQERVSKAKHMQFISGVQPYLYWLANFIWDMCNYIVPATLVI

IIFMCFQQKAYVSSTNLPVLALLLLLYGWSITPLMYPASFLFNIPSTAYVVLTSVNILIG

INGSVSTFVLELFGGDEVGGINDILKNVLLIFPHFCLGRGLIDMVKNQAMADALERFGEN

RFRSPLEWDMVGKNLFAMAVEGVVFFIITVLIQYRFFIKPKAVRPKLSPIGEEDEDVARE

RQKIMSGAGHGDILELRELTKVYERKEKPAVDRLCVGIPPGECFGLLGVNGAGKTSTFKM

LTGDCAVTSGEAFLARKSILREIDEVHQNMGYCPQFDAINDLLTGREHLEFYAILRGVPE

GEVCEVAEWGIRKLGLIKYADKAAGSYSGGNMRKLSTAMALIGGPPVVFLDEPTTGMDPK

ARRALWNCILSIIKEGRSVVLTSHSMEECEALCTRMAIMVNGRFRCLGSVQHLKNR*

>m.571 g.571 ORF g.571 m.571 type:complete len:107 (+) Unigene000050:6864-7184(+)

MELIERELPGSTLKEKHRNMLQFQLPSSLSSLARIFSILSKNKERLHIEDYSVSQTTLDQ

VFVNFAKDQSDEDHIKDISMNKSDAVVDFSQLDAFLTDAKAQETVV*

>m.573 g.573 ORF g.573 m.573 type:complete len:2307 (+) Unigene000051:741-7661(+)

MQPAVFTVDTFSAGQGHVTVYVEDPEGRREEVKAVYNESKKTYSVTYIPQVMGPHKVTVL

FAGQEIPKSPFEVNVDKAQGDPTKVTAKGPGLEPVGIIANKPTYFDIYTAGAGVGDVTAV

IKDPQGNKNTVEAMMEDKGDSVFRCTYRPVQAGPHTVSIMFGGIAIPKSPYAVSVGPAST

PSACRATGRGLQPRGVRVNQVADFKVDTRNAGSGDLKVTVKGPKGLEEQVKLKEAMDGVY

AYEYYPNTQGKYTVSVTWGGQHIPKSPFEVQVSPEAGPQKIRAWGPGLEGGIVGKSADFV

VESVGTDVGVLGFAIEGPSQARIECEDQNDGSCDVKYWPTEPGEYAVHIMCDDEDIEESP

FMAYIIPDNKSSNPDQVKAYGPGLEKSGCIINKPAEFTVEAKDAGKGPLKISAQNADGEP

IDVKVKSKGDGVYACSYTPTSAVKHTVVVTWGGISVPNSPFRVQVGKGSHPHKVKVFGPG

VERTGLKAHEPTHFTVDCTDAGEGDVSVGIKCDANIISDKEEDVDFGIIPNANDTITVKY

IPPGAGRLTIKVLFTDQEIPASPFRVKVDPSHDASKVKAEGPGLAQAGVESGKPTHFTVY

TKGAGKAPLDVQFSGPSRTQPVQDFEIIDNYDYSYTVKYTPVQQGEMVITVTFGGDPISK

SPFAVGVAAPLDLSKIEVDGLESRVEVGQNQEFAVDTKEAGGQGKLEARITSPTGKSVPC

VVQPQRGKGASLVKYVPKEEGAYSVELLYDSNPVPGSPFTVDAMLPPDPSKVKAYGPGLK

GGLVGNPAQFTIDTNGAGTGGLGLTVEGPTEAKIECSDNGDCTCSVSYLPTEPGEYLVNI

LFEGVHIPGSPFHADVEYPFDPTKVVVSGTGLKRGKVGETSVLNVDCAKAGPGQLVLEAV

SDAGSKAKTEVLDNKDGTYTVTYVPLTAGMYTLLLKYGGKTVQNFPAKVVVDPAVDTSKV

KVFGPGVAGEGVFREATTDFTVDARALTKVGGQHIKAQIINPSGASTSCSISDQNDGTYK

VEYTPFENGVHAVEVLYDDTPLPKSPFQVGVSEGCDPTRVLAKGPGLDEALTDKPNKFSI

ITRGAGIGGLGITVEGPSESKMSCKDNKDGSCDVEYIPYAPGLYDVNITYGGEHIPGSPF

RVPVKDVVDSSKVKLSGPGVSSGVRAKIPQTFTVDCSKAGIAPLSVAVTGPKGKAEPVQI

MDNGDGTHTVAYTPSVEGPYAVAVKYAEEEIPRSPFKLRVLPTHDASKVRASGPGLTTSL

PASLPVAFTIDAKDAGEGQLSVEITDKDGKPKKISIHDNQDGTYDVSYVPDKVGRYTIVI

KYGGDEIPTSPYRVRATTTGDASKCTVTGPGIGPTIGIGEEVGFVVNTKGAGKGKVSCVV

VTPDGTEVDAEVIENEDGTFDIFYTAPKPGTYIIYVRFGGENIPHSPFKVMVTDGAYIPV

SSMNGAGFRPFDMVIPFTLSKGEITGEVHMPSGKTAQPEIIDNKDGTVTVKYAPTELGLH

EMHIKYNGAHIPESPLQFYVNSANSPNVTAYGPGLVYGVANQPATFTICTEDASEGGLDL

AVEGPSKAEIGCVDNKDGTCTVTYLPTLPGDYNILVRYNDKHIAGSPFTARITEDNKRRS

QVKLGSAADFALDITETDLSQLTASIKAPSGRDEPCLLKRQPNNHIGISFIPREVGEHLV

SIKKNGHHVANSPISIMVVQSEIGDASRVKVYGPGLLEGRTFQMADFIVDTRDAGYGGLA

LSIEGPSKVDIQTEDLEDGTCGVAYCPTEPGTYMVSIRFAEEHVPGSPFSVKVTGEGRIR

ESISRRQRAASVATVGSACDLNLRIPEIDIHDLSAQVTSPSGVTEPAEIGAVGNHTYCVR

FVPHEMGVHTVNVKYRGQHVPGSPFQFTVGPLGEGGAQKVRAGGPGLEKAEIGVPAEFSV

WTREAGAGGLSIAVEGPSRAELSFEDRKDGSCGVSYVAQEPGDYEVSVKFNEQHIPDSPF

LVPVCAPPHDARCLTVTSLQESGLKLNQPASFAVRLNGAQGVIAAKVHSPSGALEDCVVS

ELEHDKYAIRFIPRENGIHTIDVKFNDTHIPGSPFQVRVGEPGQGGDAGLVSAYGPGLEK

GTTGNQSEFTINTSKAGPGSLAVTVEGPSKVKLECQECPEGFKVHYTPMAPGNYLISIKY

GGPNHITGSPFKAKVTGQRLVNVTNASETSTLLLDPTVRAVSAGSTNAMPRAASDASKVT

SRGPGLSKAYVGQKASFSVDCSKAGKNMLLVGVLGPQTPCEEIMVKHMGSMQYNVSYVLK

ERGNYVLAVKWGEDHIPGSPFQITVP*

>m.575 g.575 ORF g.575 m.575 type:complete len:222 (+) Unigene000051:76-741(+)

MPATEKDLAEDAPWKKIQQNTFTRWCNEHLKCVNKRIADLQMDLNDGLRLIALLEVLSQK

KMYRKYHARPTFRQMKLENVSVALEFLDRENIRLVSIDSKAIVDGNLKLILGLVWTLILH

YSISMPVWEEDEDEESKKATPKQRLLTWVQNKVPDLPISNFSQDWRDGRALGALVDSCAP

GLCPDWETWDPVKPVENATEAMQLADDWLGIPQVLSQWVTV*

>m.574 g.574 ORF g.574 m.574 type:complete len:358 (-) Unigene000051:711-1784(-)

MDGVLARLRGPILDVTGSVILILALDPGLRWTLDSKAQHSYIRANRLHNKISRLAHNAPL

QTRTPSSDLLWSCFRADLDLKRTLGNVLATPCDAHCVFALRVGIILVGVDAVHRFLQLHL

LLQAFRSLHCDLQVSTSCISGVHLEVGHLIHTHSSGLQAPTRCSASTRGGRWTNADGVRT

LGNSDASEHDADRVRTGLYWSVRAAEHAVALVLHHGLHSVLIPLGVFNYRRDVAHTSACC

VDVEVGGFVCDYAHWLQPRTFSRDLGGVTLSLVHVDFERAFGNFLSGKQHCHLVRSHDLW

NVCYAVGLLAFIVNGLHFFPSAFGILHVDGDVSLSCAECVHGEHGWLHHTVTHWLNT*

>m.587 g.587 ORF g.587 m.587 type:complete len:2541 (+) Unigene000052:289-7911(+)

MVALSLKIGVGNVVKTMQFEPSTMVYDACRIIRERVPEAQLGQPNDYGLFLSDEDPKKGI

WLEAGKALDYYMLRNGDTLEYKKKQRPLKIRMLDGTVKTVMVDDSKIVTDLLMTICARIG

ITNYDEYSLVRDIGEEKKEETTGTLKRDKTLLRDDKKMEKLKQKLHTDDELNWLDHGRTL

REQGVEETEMLLLRRKFFYSDQNVDSRDPVQLNLLYVQARDDILNGSHPVSFDKACEFAG

YQCQIQFGDHNESKHKPGFLDLKEFLPKEYVKNKGEKKIFQAHKNCQNMTEIEAKVSYVK

LARSLKTYGVSFFLVKEKMKGKNKLVPRLLGITKESVMRVDEKTKEVMQEWSLTNIKRWA

ASPKSFTLDFGDYQDGYYSVQTTEGEQIAQLIAGYIDIILKKKKSKDHFGLEGDEESTML

EDSVSPKKSTVLQQQFNKVARVETGSVALPVIMRPGAGGPESIYTGSMPKAKQQITTGQM

HTGHMPPLTSAQQALTGTINSSMQAVQAAQASLTDFDSLPPLGTDAASQAWRKNKMDESK

HEIHSQVDAITAGTASMVNLTAGDPAETDYTAVGCAVTTISSNLTEMSKGVKLLAALMED

EGGNGQQLLGAARNLACAVSDMLKTAQPASAEPRQNLLQAAGNVGQASGELLQQIGESDA

DQQFQDMLMKLAKAVATAAATLVLKAKNVAQKTEDSAQQNRVIAAATQCALSTSQLVACT

RVVAPTISSPVCQEQLIEAGKLVAKSVEGCVEASQGATNDEQLLKQVGVAATGVTQALNE

LLQHIKQYASGGQPIGRHGEATDRILDVTENIFSSMGDAGEMVRQARILAQATSDLVNAI

KADAEGETDLENSRKLLSAAKLLADATAKMVEAAKGAAANPDSEEQQQKLREAAEGLRMA

TNAAAQNAIKKRLVNKLENAAKQAAAAATQIIAAAQHAASSNKNPAAQQQLVQSCKVVAD

QIPQLVQGVRGSQAQPDSPSAQLALIGASQNFLQPGAKMVTAAKATVPTIGDQASAMQLS

QCAKNLASALAELRTAAQKAQEACGPLEIDNALNAVRDLEKDMQEAKASAAEGKLRPLPG

ETLEKCSQDLGSNTKAVSSAVAQLLSEATQGNENYTGMAARDVAQALRSLASAARGVAAT

TDDPQACSAMLDCAGDVLDKSGNLIEETKRAIAKPGDPESQQRLAQVAKAVAQALNRCVN

CLPGQRDVDNAIRTVGEASKKLLSDQFPASGKTFQEAQANLNQAAAGLNQSANELVQASR

GTTQDLAKASGKFGQDFSHFLEAGVDMAGQSQSKEDQTQVVTNLKTISMSSSKLLLAAKA

LSTDPNSPNLKNQLAAAARAVTDSINQLITMCTQQAPGQKECDNALRELETVRGMLENPT

EAVNDMGYFDCIDSVMENSKVLGEAMAGISHNAKNSNLPEFGDAVSSASKALCGLTEAAA

QGAYLVGVSDPNSHAGQKGLVDPSQFAKAKQSIQMACQNLVDPACTQSQVLSAATIVAKH

TSALCNACRLASSKTSNPVAKRQFVQSAKEVANSTANLVKSIKALDGAFNQENRQKCKAA

TGPLIEAVDNLTAFASNPEFASVPAQISPEGLAAMEPIVAAAKTMLESSTGLIQTARSLA

VNPKDPPKWSVLAGHSRTVSDSIKKLITNMRDKAPGQRECDEAIEVLNNCIREVDQASLA

AISQHLTPRDDISHEALHEQMAASVQEISHLIDPVAIAARSDASQLGHKVSQMASYFEPL

IMAAIGTASKILNSQQQMSVLDQTKTLAESALQMLYTAKEAGGNPKAAHTQEALEESVQM

MKEAVDDLGGTMAETASAAGAVGGMVDSITQALNKLEDPGVEPEGTFVDYQTTMVKTAKA

IAVTVQEMVTKSNTNPDELGGLANQLTTEFGDLASEAKCAAITAENAEIGSHIKKQVTEL

GYGCTGLVTKAGALQCSPNDSITKKELIDAARKVSEKVSHVLAALQAGNRGTQACITAAS

AVAGIIADLDTTIMFATAGTLNRENAETFADHRENILKTAKVLVEDTKLLVSGAGASQEK

LAQAAQSSVNTITKLADVVKLGAASLGSEDPETQVVLINAVKDVAKALGDLIRTTKAAAG

KPHDDPAMLQLKSSAKVMVTNVTSLLKTVKAVEDEATKGTRALEATIEHIKQELAVFSSP

DPPPKTATPEEFIRMTKGITQATAKAVAAGNSCRQEDIIATANLSRRAIAEMLHSCKQAA

YHPEVNLEVRTRALRFGKECANGYLGLLEHVLVIIQKPTHDLKQQLATFSKRVAGSVTEL

IQAAEAMKGTEWVDPEDPTVIAENELLGAAAAIEAAAKKLEQLKPRAKPKEADESLNFEE

QILEAAKSIAAATSALVKAASAAQRELVAQGKVGAIPANAVDDGQWSQGLISAARMVAAA

TNNLCEAANSAVQGHASEEKLISSAKQVAASTAQLLVACKVKADQDSQTMKRLQAAGNAV

KKASDNLVKAAQKAAFDAQDDQAVMVKSRMVGGIAQIIAAQEEMLRKERELDEARRKLAQ

IRQQQYKFLPSELREDGHEQ*

>m.596 g.596 ORF g.596 m.596 type:complete len:741 (+) Unigene000053:1584-3806(+)

MIPPRGCAYICMVHRQDAYRARQKLSTGSYKIGSKVIKIAWALNKGVKQEYKQFWDVDLG

VTYIPWEKVKLDDLDGFAEGGMIDQETVNSEWESQKNVEAPKDLQVQQVSAESSAASNSQ

NETYTQPVAMMPVQIPVAQTVSAVGLVPPTFPVTMAMPPPGFGPPPPFLRAGFNTAQPPP

GFMAPSGVAPSAVGAALTSGPTAHNQDGNKDSTFGAILPPSSTIPASFMPSGMPGAVFNS

VGAQAQQASTDKTSVSGESVDTPNELTLQGMQNAVRSGMGLLGMHPSAALSHPLSGQRMP

GLLPIDMRPNLLQGAGARFSMLMQQGLAQQANSLLDASLQTRARAPFPQMEHFNCADAPF

VRAPNPAAPAPDSIDKSSNNITATATDSRSQQEDQDYRFPPPEKQSTGLLRTPPPEMRTE

PTAVRAPLLDRPLRAGMHMEGREGPGRREPMPAPFRAETRWGAPRGDLDERDMRGMSAGP

PKSFPEDPGNPNFQNRFEMRGGGAMGGAGPGWTRGGGGGGPFNMNMHQDFDDRRRPWERQ

KDRDDRDFRRDINDNRHRDRDRERERDRERGRDRNRERERDRDRDNDKERERERGGWAPV

QPLLPLPRPSQPLLPLPTPLLNLPPTQPESHPKPQPASRPESQPDGKQPQPEKQNSGERE

KTHVFSDIAHGPDTEHETVTEPTAESSRSSSPAPTAELQHQTQTPEVSASTGGRQTPLKE

EAETGTEPEPELVKTDTEET*

>m.597 g.597 ORF g.597 m.597 type:5prime_partial len:514 (+) Unigene000053:1-1542(+)

CVCVCVFQLYSLNEYKPPISKAKMTQITKSAIKAIKFYKHVVQSVEKFIQKCKPEYKVPG

LYVIDSIVRQSRHQFGQEKDVFAPRFSKNIISTFQNLYRCPSDDKSKIVRVLNLWQKNSV

FKSDIIQPLLDMAAGIPPPSITPVSASTALPVNNATPGTPATPATPANIMPSLPDWASQI

SNSDTVAAVAQILQSPQGQQLQQLVQSLQMQQQKPQPSLLQALDAGLVVQLQALTAQLTA

AAAANTLPLEQRVSSFNKKLLGQFDFVGESEPSEDSKKDTAASQLPMVSESINNSLFHQL

AEQLQQQNLEQFQKQILEHQQKSMTLESQENIFCPENSAPPSQSSVQSQLQEQDVKLDDS

IDNQQQDMDLDEGQDTVDEDMFESDEKKTSGTRSRTHSRSRSRLDTYSCSNRLHIIPHCT

SHLISLPTAGPQREGGHGHARGLESANTGNGPAHGPETGRGNPPVRTRASAERGKEKRSG

RKKVFLQSAPDFSACVARHCGWAKLIRKPRSRT*

>m.595 g.595 ORF g.595 m.595 type:complete len:1294 (+) Unigene000053:3923-7804(+)

MGNSESQFRTQGSRSSSFTLSNRQEPYPVRCRTFKEDSLTPHCRWRTGSSLKSKAEKRCG

LSQHRANQSSLLRHCDYITKGGEGKRNISAQWNNGHFEENRRDCNGHALNGYKTPERKPK

LEDLDDHSSPKVVIKSDGSVRVEFSHMSKNSTLPDENGGSVQLLKFSPTSHSVSTPESAL

VQNSPINRTSKRNSLSSEGSWYDSPWGPGIELNYDGVCALPNRLSETLPSVRIEYFDEPC

IPHLLYRDPVMAATFSTAEDLKLANELSFKHRTPFLHVMEEPVLAESSGARQYSSYTLPC

PQTKPTAENSGKKEIIKNRMRRFSDWTGSLTRRKKKSKELRYKDSIDCFDSGIDGLTADT

SSPSQVSSLLGYHKVQCRGRSQSQVLPSCSGGSFSALHPATDGLRQNIYDNFMRELETSQ

TTTGSGERRGENSAEDTESSSQETVGSLEQLDLLCEKEQDVVRRAGWLTFKPLLTLHKDR

KLELVARRKWKQYWVTLKGCTLLFYETYGKGSPEQEASPSYALLAEDGIVQAVPEHPKKE

NVFCLSNAFGDVYLFQATSQTDLENWVTAIHSASASLFAKRHGKEDTPLLLRGQIRSLLQ

KIDMDGKMKKMAELQMSIVTDSKSRKAIENQIQQWDQNLERFNIDLFRMRCYLSSLQGGE

LPNPKSLLATASRPSKAVLGRLGVFSVSSFHALICSRDEATLRKRSLSLPRRSRGKRGMF

SSHKALDSLTKRSRDRRPCTSQDCISVSSCSNPEVFDGAAGDHSGLVPSYSRERPDAPAD

TYSQAPPDGSQWERSVTERLVWVYMPDHQVVTVTLSGQHTVEELLNTVCKMQNLDPSAHC

LRLRRCVSDRMEVWTPGPHKLFQDLLYDELEVSALNVYRLHMTKPTYAGDFGFAISGSVD

SLNQSRIFVTQVFSGGIAFSEGLRPGDEILVLNGCQVSRLDLELIQTLFSENTLHLTIRR

EPTAMTHTHTGQQPMRDLLYRHHRAKSATDVSCVSEGKAGVETRQHHRLSLRAVQHSKSA

ETVFALYHSCQDSSVRLMEVQTETAPPGGTLLRACPRHMSVSERLRKVVQELVDTEKSYV

KDLSCLLEIYLKPLQKETFLTQDEMESLFGSLPEMLNFQKVFLQTLEERIASTPDFGTLQ

TPVQFRKLLFSLGGSFLYYADHFKLYSGFCANHIKVQKVLERAKTDRAFKDFLDARNPTE

QHSTTLESYLIKPVQRVLKYPLLLRELVSLTDTDSEEHYHLTEALKAMEKVASHINEMQK

IHEDYGAVFDQLVAEQSGSEREVLYHTVSRFFC*

>m.606 g.606 ORF g.606 m.606 type:5prime_partial len:2511 (+) Unigene000054:1-7533(+)

VSAHLCVRSENVGDVLERLSLSCVELLLSIAEDFPDGLWEQFQSSIQMTHELLQANAIPQ

LHLLSAVTQERGMWSNCTLQSLLRNEMPPEAKVHEFLMREGPVLLQLRVKYLLKVNCMEK

AALLAKACADVQEFGGSRGHFTQCFLVCVCSCAPQETLMEELSKVDCRDALEMICNLEAE

GDERGAFTLCSGFLTRQLLQEDSYCAWELTLFWSKLLKRLEPSEQSFLEKCQKMSLLAKT

VFHLLFFIKVIQSELDKIGLPACIEMCIRALRMENCEGANKATICKTISCLLPSDLEVKR

ACQLTEFLLDPTVDSYYAVETLYNEPDQKLEEETLLIPTSLRCDLFLVFKTQWSFDPEFW

DWKTLKRHCLGLMGEEASIVSSIDELNDDRFLEIERDELDSAQEEFKDVFECFLDTTKEL

KEISDHRQKNREVKKLREKGFVSARFRNWQAYMQYCVLCDKEFLGHRIVRHAQTHFTDGY

YSCPICTETFETRETLDPHVASHVKLSCKERLAAMKTTRKLTGAKNSASNDAPLDNKSGE

NQARKAKAKHCNGESDDVYNMGSCIEDAGVRVHRKEFNVCPVSSCRKVFKFFRNLLTHVK

DHGDVEEAKRFLELQATKIVCQYCRRHFVSVGHLNDHLQVHCGAKPYTCIQLNCKASFDT

NAELLVHSKRHPCFKAKCMFPGCGKIFNEAYKLYDHEAQHYKTFTCKVPGCGKVFHAQSQ

LDMHKESHNPPKLETQPAGSVSPLGEPQSHLSSEPAQQDAHSGPPADSGCMISSEDQSTG

LVKVKHSIENMLSVAGQGLSQEFGHVNCKAEPSVPNLVQQPPMPHMVNTEQIQPQLTPSM

PSLIEPRSEESLLDALMNDPMLTSSCSLPPASSYQSLLEDFQQAPSGDVLQAQMESAVSQ

GHTQSLYSNENTEYGQYNIKPETPISYPPPYDNTVPLAQNGHNDPPVLPVSQRLPPQLTM

FSSNRPVQALQANTLSSVVEEKDRYKCAYETCSRDYSSYRSLTKHMKAAHCEFYTQWKLA

KRSNKVSPNTCSASSINGKCMSAEPSQNQLSQRTPAPQNPVQKPPAQTHSPASCLSSHYP

SGSSHLTGPNGQTFPNQMDSILNPIVLSHLGSIANQSNVVSHSSWNSISVNTSLRQQTCH

SQAMTSSSGQFPSQLDTVPQTQLSDSYNRVMAYPSLQHQNSPSCRFIQTRPCAPQTINSP

AKISHPGDSSLPQYVQQPKTSSKSTLKHTDCVVKLERNPILIPSHSNLLQQSNPPTAMKP

ETCQKDISGFAHDGDVKKRKRSSRTKWPAIVKDGKFICCRCFREFQSPKSLGGHLSKRTV

CKPYDEAVLSADLPSSFLELLNSPHPSETPQSSPSTQVTSSKAWPSLTKGPLDPKLFPNV

TFLNTKNSTYNSGTIHQNSPSLEKGVTGEQTFPESSGSYHQKSKNSVIQHTGNIRDKCIY

DLMNYTQPCNTLPSDTSSSEQLLSHMLSENKTLDSNLSRSPNNYVDRILQAETLNKIREI

KEEPTRTNISGLSNDGLLAAMASLAQNLVSEKSVKERLREQILAGDFQKRSSLGQGQGVE

NSNSQSPMTDVPLGLQTAVQSKEHIVRRASELGELYTSVVPDETLTNISLAGASNNRETN

NAFSNSLEQEDDKDLSEILKSFERLDLNKEIEKNEQTTSNLLEAERVTKASEATCTLMLR

GYSCDNDGCGYRAMTKDALFKHLVKQHNYTEDMMNQCRKEQSNLAPFNCQLCPKTFTRNS

NLRTHYQAVHNYSHEQVVKLGITRQYSRKSIEADQGLCTTLNISELASPVFAGQNAESGA

GITALVSGVRSIKSEFSVQPFEQQHPGASFDSCPVSSTILRAQTTPHHLQSQTTVHLSVI

KATPSLLTSTSVEQQLNRPNEFVPESLVPSQHTQGFLSGGLSSSGAPLLAPISLASAQLG

SLSSDTKPKFGKPKMAKVKEVTKKPKEKKLDMVDVFSPYRPYRCVHQGCVAAFTIQHNLI

LHYKAVHQSALPKFEVNEDEEPIEEEHNENQEEIGMPDSEVNEEFRCQVKDCSRIFQVVT

DLLQHYLQLHKLGLDKAGIMMSGMNLGRFRCDQPDCHVTFTGFWKYVNHIDNEHKHVKLS

RTEPVNGMFQCEVEGCSCVYATRSNLLRHFMKKHPELYKLRLLNSGGVKLGRPPKNRFVS

LEKENREINKKTFQKGGAKKKRRQKNLWTKYGNPILKSKEEASSMCTKRIQLQYPCMLKG

CETVAESERKLLKHYVQHGLPKQYLEEQRSNYIFCKKMPRSKYKRIASRSDDTDKSDGSS

IEASENEDAADTGPSESEFSKPTSEKESTEDTEVSDAKLSSDLSSDISVVVKRKRGRPRK

GEQVKRTQLARKRMTRLRAAQTRSINYADMNSDSTSSSVTLPQEPNTSLSSFKPMGFEVS

FLKFLEESSQSPGKRKSCSLMDLLPHRKFPTVRLKTASVVCSRINPDYHHCRDVLKHVEF

KNPQKLTSLSRVTFEVHRGFSNVYELLLKQLHDMRPAVVIQKEGVSETRA*

>m.613 g.613 ORF g.613 m.613 type:3prime_partial len:2612 (+) Unigene000055:118-7956(+)

MKVDRSKLKKTPTEAPADCRALIEKLKGCSDEQLLVELQHIKTWNIGKCELYHWVDLLDR

FDGILCDAGQTVENMSWMLVCDRPDNGQLKALLLAVLNFTALLIEYSFSRHLYSSIEHLT

TLLASCDMQVVLSVLNLLYVFSKRSNYITRLGSDKRSPLLARLQHLAESWGGKENGFGLA

ECCRDLPMTKYPPSATTLHFEFYAEPSSEVKVEKKSSSNTLHYIHIEQLDKISESPSEIM

ESLTVMYNIPKDKQTLLFTHIRLAHGFSNHKKRLQAVQARLHAISILVYSNALQESANSI

LYNGLIEELVDVLQITDKQLVDIKAASLRTLTSIVHLERTPKLSNIIDCTGTASYHGFLP

VLVRNCIQAMIDPIMEPYPHQFATALFSFLYHLASYDAGGEALVSCGMMEALLKVIKFLG

DEQDQITFVTRAVRVVDLITNLDMAAFQSHSGLSIFICRLEHEVDLSRKECPFVIKPKIQ

RPSTTAETEDMDTDMDVSEVAMESSPGPSTSTGSKSDPDPRTQTSTVSAPRTGVQCIPQR

AALLKSMLNFLKKAIQDPAFSDGIRHVMDGSLPTSLKHIISNAEYYGPSLFLLATEVVTV

FVFQEPSLLSSLQDNGLTDVMLYALLIKDFLSLQVPATREVLGSLPNVFSALCLNARGLH

SFVQCQPFERLFKVLLSPDYLPAMRRRRSSDPLGDTASNLGSAVDELMRHQPTLKTDATT

AIIKLLEEICNLGRAPEYICQKPSIQKADGTVTVPPARSNHAAEEASSEDEEEEEALHTF

TQQQGEPESNRQVVGTEERIPIPLMDYILNVMKFVESILSNNTTDDHCQEFVNQKGLLPL

VSILGLPNLPIDFPTSAACQAVAGVCKSILTLSHEPKVLQEGLCQLDSILSALEPLHRPI

EEPGGSVLLRELASAGHVTDATLSARATPLLHALTAAHAYILMFVHTCRVGQSEIRAISV

NQWGSQLGLSVLNKLSQLYCSLVWESTVLLSLCTPNSLPPGCEFGQVDMQKLVPKEEKPS

GSTVTSGGRRTGKKTLLHAETESLSVGVDPSAQGLLEGMGLDGDSLAPMETDEPTTADPK

AKSKLTPAMATRIKQIKPLLSASSRLGRALAELFGLLVKLCVGSPVRQRRSHHTTNTGTA

PTPAARSTASSLTKLLTKGLSWQPPPYTPTPRFRLTFFICSVGFTSPMLFDERKYPYHLM

LQKFFCSGGHDALFETFNWALSMGGKVPVSEGLEHPELPDGTGEFLDAWLMLVEKMVNPS

TVLDSPHSLPAKVPGTTTTTPQFSAPRFLIVTQKAAFNCIRNLWNRKPLKVYGGRMAESM

LAILCHILRGEPVIQERLSKEREGTARAEEDGASVVGGASGGAAAGAVGESASGSGGSAS

TAGGTADDASATTRREPQVNQAQLTQLIDMGFSREHAMEALLNTTTMEQATEYLLTHPPP

LLSAAIREFTMSEEDQMMRAIAMSLGQEVSMEQRSDSPEEAARRREEEERRARERAEEEE

ARCLERFLEAEPLDSSELHAFTDTMLPGCFHLLDELPDTVYRLCDLLMTAIKRNGPEYRD

LILRQVVNQVWEAADVLIKAAVPLTTSDTKTVSEWTKQMATLPQASNLATRILLLTLLFE

ELKLSCAQIVESSGVLSVLIKLLEVVQPCLQAAKEQKDIQTPKWITPVLLLIDFYEKMAV

SSKRRAQMNKYLQPNGNNWRWFDDRSGRWCSYSASNNSTIDSAWRAGETSVRFTAGRRRY

TVQFNTMVQVNEETGNRRPVMLTVQRVPRMPKPNKSGNVADPEREEDQRAKTEETQVLAD

PASVAVEMVAPKEEPELKVSSAPASAAPAASQDSSSGSNSIVVQGLTDEMTTVLIRACVS

MISVPVDPDTLHATLRLCLRLTRVHHYAMMFAELKSTRMILGLTQSSGFNGFTPLVTLLF

RHIIEDPATLRHTMEKVVRSAVTSGAGSTTSGVVSGSLGSREINYILRVLGPAACRNPEI

FTETANSCVRIALPAPRGAGTASDDEFENFRIKGPNAVQLVKTTPLKLSPLPQIPETIKE

VIYDMLNALAAYHAPEEAEKGEERLATVPGSQDLCQILQDVGDDVYQQYRLTRQGSDFDS

QSAFHINTQVFAADGAVAESSQSGTPQGEASTPEEMREDKKEQEGDKAGSSEENKAAMAK

ASKPLMPTSTILRLLAELVRSYVGIATLIATYNYTAGQSELIKEDCSVLAFVLDHLLPHT

QNSEDKDTPALARLFLASLAAAGTGTEAQVTLVNEVKAALSRALAMAEGTEKHARLQAVM

CIISTIMESCPSTSSFYSTATAKTQHNGMNNIIRLFLKKGLVNDLARVPHSLDLSSPNMA

NTVNAALKPLETLSRIVNQPSSLFGGKGGSSKSKTEHDSVGTARDSNSNTQDQGEAGEAE

PVENSHRGQATDGDLMDGEAEGDTVVIAGQPEVLSTQAMQVENELVDLIDELLERDAGTV

NSTIIVGRSGDEESQEDVLMDEAPSNMSQASTLQANREDSMNILEPEDEEHTQEEDSSGS

NEDEDSQDEEEEEEEEEEDPDDEEGDEDDDDEGSEMELDEDFPDINAAPHIRFERFDRDD

DLIIEFDNMFSNSADIPPSPGNIPSSHPLMVR

>m.622 g.622 ORF g.622 m.622 type:complete len:1648 (+) Unigene000056:2911-7854(+)

MHNLHTALPRMTGPWQPGAAPHSVHWVRALMMMMRMRMMMIISFSEFVFFCSFALSVGSP

LLLFANRRDVRLVDVDSVRADSAVLVGDLEDAAAVDFLFSESLVFWTDVSEEAIKQTYYN

QSSNAVKEAVLGSGQKVVVSGLDSPDGLACDWLGRKLYWTDSETNRIEVANLDGTSRKVL

FWQDLDQPRAIALNPAQRYMYWTDWGEEPRIERSGMDGSSRKVIIDADIYWPNGLTIDLD

EQKLYWADAKLSFIHRANLDGTARETVVEGTLTHPFALTLFDETLYWTDWQTRSIHSCNK

HTGNNRREILNGIYSPMDIQVLEQRRQPYIHTPCSDGNGGCSHLCLLSPAPPYYSCSCPT

GVKLREDGRMCRAGAEEVLLLARRTDLRRISLDLPDFTDVVLQVGDVRHAIAIDYDPVDG

YVYWTDDEVRAIRRSRLDGSEAHTLVSAEVEHPDGLAIDWIARNLYWTDTGTDRIEVTRL

NGSSRRILISEGLSEPRAIVLDPVNGYMYWTDWGQKPKIERANLDGSERVVLVNTSLGWP

NGLALDHAAGKLYWGDAKTDKIEVINVDGSNRATLLDDKLPHIFGFTLLGDYIYWSDWQR

RSIERVHTTKITREVIVEQLPDLMGLKATKVTEIFGTNGCTDYNGGCSHLCFNGPRGVTC

ACPTGLELLSDLRTCVVPEAFLLFTNRADIRSISLGTNSNDVAIPLSGVKEASALDFDFA

ENRIFWTDVSTKTISRAFINGSGVEAVIEFGLDYPEGMAVDWMGRNIYWADTGTNRIEVA

RLDGQYRQVLVCKDLDNPRSLALDPANGYMYWTEWGGRPRIASAHMDGTNIITLVDKVGR

ANGLTIDYMDQRLYWTDLDTCMIESTNMQGLQREIVADDLPHPFGLTQYRDFIYWTDWNL

RSIERADKRSGQNRTLVQGQLEYVMDILVFHSSRQDGSNPCSHYNGYCAHLCLASPSGAQ

CRCASHYTLQPDGHNCSSPASFLLFSQKSSISRMVLGEQSPDMILPIHVFRNLRAISFDP

LKRFIYWIDGKQSVRKARDDGSQAATVVSPGPTQPLKQPHDLSVDPFSRTVYWTCENTNT

INVQRMDGQNIGEVLRDEVDKPRAIVLNAEKGYMYFTTLQERSAKIEGASLDGTERESLF

TTGLIRPVALALDNKLGKLFWVDADLKRIESSDLSGANRIVLQDSNILQPIGLTVLGDHL

YWIDKQHQMIERVDKRSGDRRTRIQGRIQSLTGIHAVELMETDEFSSHPCSRDNGGCSHI

CIAKGDGTPRCSCPMHLVLLQNLLSCGDPPTCSADHFTCATGEIDCIPMTWRCDGIAECA

DHSDEMNCPVCSEQQFQCHAGQCVDAKLRCNGEQDCTDGSDELDCHTICMPNQFRCKNNK

CISKKQQCDSYPDCSDDSDELFCDFSPPSSEERHTNTIGPVIGIILSVFVLGGICFVCQR

VVCRRYKGSSGAFSHDYITGTAHVPLNFISTNNTQHANCTGISCGKSVMSSVSLMGSSSS

GAPLYDRNHVTGASSSSSSSTKAAFYPQILNPPPSPATDRSLYNAEVFYSSNSPSTTRSY

RPYVVRGGAPPTTPCSTDVCDSDYTPSRWKSNKYYMEVNWDSEPCPPPPTPRSHYMSAEE

SCPPSPSTERSYFHLCPPPPSPCTDSS*

>m.623 g.623 ORF g.623 m.623 type:complete len:972 (+) Unigene000056:192-3107(+)

MATGGAPFDDEQELHNWTISNGSLDDRLNNMDWGVQQKKANRSSEKNRKKLSTVSESRLT

NDISPESTPGAGRRRARTPHSFPHVKYTTQMSVPDQAELDRLRQRINFTDLDERSIGSDS

QGRVTAANNQRQLSSEPKKPFNFLSLHVNTNKSRESSASTPPATTAGGKETKKQSPGKEL

FVPVPVVPVKEPRRDERTSGGPPVDGRQEPAIDSSQVVSKLVQIREYISRASSMRDDLLE

KNDVPANVERLSHLITHLKEQEKSYLRFLQEMLARQNMEEEAEGATVDSAVCSGSMAKST

SLNLEPRSEAADSTCYRVGGQHSEELENLRKQHDLLQKMLQQQEELRELQNRQATLLSMQ

NRSEHAMEDPVVTETTGSVSGVSITSELNDELNDLIRRFHNQLHDTRSQAVPDNRRQAES

LSLSREVSRSRSTHSPRGQQSPGTDAAVSASPASAKITKLQELQDKKQTMDKILQELHSL

RDQTLNNSSSRGASQRVSERPSASGRDGDSVCVARQESSSYDDGGNSAEKLRKLKEVHKR

LNELRELVQYYEQTSDMMVDTVNENVNEDEEDETDDGSLFEAMFDSEQENREPITNIRNP

AQPQSTSNWMDMNTLTNACSSSNNRVSQLNSQCEANNRSAANLRSFNGPSAIECQYNRDG

SYNGVKHGNGDGDEDEEDDDDDGAEGAAQARQRRRESSGSGSSRQSSIADDAVFAQKVHR

LQTAKQKLQQLRELVAMVQSDDTDTTTANEDEGLQQQPNNTRATAPKAKRELALSDKARE

KLYEEKLRQQQEELKQLHEERQRLMEIQGKIQDLQWACPDLQSSVSSAASGMVKKIPAAV

STPAVVPSTSAKCNTTVLKHTTEPAPVTITDNELWSEMRRHQVLREELRQRRKHLESLMA

EQQRRSDAQPSHSFTQDDRTMATWGSSTQCPLGEGVDDDDEDEDDDDYFLLGVCLFLLFC

AECRFSSAALC*

>m.634 g.634 ORF g.634 m.634 type:5prime_partial len:2566 (+) Unigene000057:1-7698(+)

GPRLDRNPQKEDMCVKDKVIEFRMNSVYGRGMFLLLIFVGTELQSTFAQNRCNKNTQVMT

KSECHLCAVSDLKNCPAGYSKKTPGKGQQNCRYQLKTSFSSDLYLRGCSHECFKEVIEER

CCSGYWGYDCLECPGSASTPCNNNGVCTDGIGGSGNCTCKEGFVGTACEKCEKNRYGSTC

SNVCTCKHGLCNSGLKGNGQCTCFSGYTGPDCDQELPGCAVLHCGPDARCFEDMNAGKLV

CKCQPGYFGDGVQCTSINPCQKRVCHEHAVCTHTGPNQHTCTCTEGYQGDGLVCLPIDPC

QTDYGGCQSNSSRCVYDGPGKAHCECLEGFEKLVVGAGCSLRDVCKPDSCHLQAYCITVA

PNTVECKCKDGFLGNGNVCFGNIIQRLQELNADPKSRSYGQLSSAISLFEGVMSWPLTSL

GPFTVFVPKNKAFKSTQPVKSLLADQAKARYLVKMHMIPGEVNYDMLKNGILYYTLTGKS

AESITESGQVKVRIHGSRKKAALLETDIISSNGMIHITDKLLDSVPSTVISDSQENLMKI

LSDNGKFSIFKSLIEKTSMVDILNKDGPYTLFAPTNTAFYLMKDNQLNYLKSEEGKPKLL

EFLRNHVVSSAQMKASYIVSISQIVSMANQVLTINVTAAGQILIGGVAVAEADVEAKNGC

LYSLLDVLIPSSIEPILPQRCDLTESNIYKSPCVSCTEVSKASCPTGVSLNTTSTGCVYR

KRSLVIDVPVLGCSLFCNETRTTPQCCKGFFGADCSPCPGGFTTPCSSNGKCLDGIEGNG

TCQCEPKFSGWRCQYCSDPNKYGPFCNQTCRCVRGICDNRPESNGACKPQSCQEGITGEL

CNKHALPCGPNQICHIHANCILEQAGEQTCVCKPGYMGDGFQCTHEDPCTWSPNGGCSDN

ARCVQLGKGRHKCECLTGWKEDGDECQPVNNCLEPSRGGCHPNATCVFLGPGQNDCVCKR

NFRGTGTDCEPVNQCVNIMGGCHQLASCVMMYPGKFECVCPADYIGDGRICYGTLGQELE

SNLDLSGFNTWVTKADLRQMMHEKENITLFVPSLSAIMTMSKDDQHFWLSSSNIQSLVMG

HVVPGHYRLSDLRSSTSPQLVSFLRGTLPVSWTNETTTIGGATVTLGDIVTKNGLIHLID

KVLIPDRKLSTGLIGVLDKTPEFSLFKNALITYNLSTEIEEATGFTVFAPTDSAIREYLR

NTGQDSLDLNVTQYHIVLDETLKQSDLQDGLYKDTMLGFSYQLGIFLNNSNWFVNKAWMN

ATDIETDNGVIHGLSAVLKIPKNRCYTASSTVFLGKCMDCFHPSESRCPAGTKQVKVSLK

RCMFSVRKFGRFIGCEASCEKLTITKKCCEGFYGTECEPCPGPEGQSCFGNGVCMDGING

TGVCQCNPGFNGTACENCQPGKYGIHCDQDCKCVNGRCNDGLEGDGSCQCDVGWRGIYCS

IAIKTDLCGGKCHTSANCLLKIPDNSYYCSCAAGYEGNGTHCTVMNACAKNNGGCSPNAV

CKRTLPGRRQCLCNPGYSGDGQVCVSINPCLDGSGGCSNNSECVHTGPNKSACVCLPGYT

GNGKICSPINLCKKKNGGCYVFAKCTMTGPGERNCTCPADQIGDGIKCKSKLMPEIRKKG

LTNFGVALQRADIYYLQSQGPFTVFAPNNNAFHKLGNQETVDKEKNAAILLYHIVPCRLL

RPEELQQPRNLTTLTGEILTISYSEGTIQINNNAKVVYSDEESRNGIFYEIDTVLIPNSF

YKMEETSKKNTSLKDVVDLYGFKTFYKLLEDTEVIKQIQEPLHQPVTLFMPTDTTMEALP

QAQKDFLYGLHNRAQLVEYLGYHIIQSKKLAEVMHLDSVKTLQGSGISVSCVGEDDIGSI

YVNDKSCRIIKRNLNFNEGLVYGIDCLLIPPSLGGRCDKKDTVDITMPCRYCGSVRSSDC

PEESKPKTTQPCHLARHSLYVNSGCQSVCSMIVWKPRCCPGYYGRDCLSCPGGPGAPCSN

HGQCDEGLLGNGTCTCNAGFQGVACELCTDGHFGPECKECNCTEHGTCNEGREGTGSCFC

DEGWAGLQCEHKLADGPVCLPACHKKAVCTENNTCVCKPFYEGDGITCKVTDMCKFWNGG

CSKNAKCTQKGEKVNCTCLIDFSGDGYVCSPVDPCIGENNGGCDEHALCTMTGPGKRKCA

CKTNYVGDGLTCELKVLPVNRCMLDNGQCHFDAQCTDLHYEDKTVGVFHYRSPLGAYKLN

YTSAQEACKMAGGTIATYTQLSYAQQAGYAMCSAGWLAQNRVAYPMSYSNPNCGFGHVGI

VDYGTQTDLSRTWDTFCYQVKDVKCECKIGYIGDGYSCTGNILQVLTTKPDFSNFLSQIM

NYSSAAAKGQEFIRRLSNITTKSTLFVPDNNGLYENQTLSFRDLEHHLLDGRALVLQDLI

NISHVKSHLGNTLNVKPVPSLQNPQTETSSGYVNDRYIIESDILASNGVIHVLQGPLKAP

PPPPSTLPPAHTAGLGIGVILLVILIVTAGFVAYYFYNRQTKPFQFHYFREDEKDENSSP

DSTLYISNPMYESSVAPGTTDLPVQDKDDKHQVVDGGSYDLLQNS*

>m.644 g.644 ORF g.644 m.644 type:complete len:2560 (+) Unigene000058:141-7820(+)

MRYLFLLGFVVLHSLQIKATSNRCDEPRTVRYFTVCTSCAAAPAISCPKGYIKTSTDAEC

RYNVQIGDRLLELPGCSRSCEKVTLIKRCCPGFFGSLCSPCPSLSGKVCNWHGTCMDGES

GNGTCVCEKGYTGFLCNKCPNGTYGERCSSVCDCVHGECNPDPDGDGQCYCQPPYSGPKC

DKVTSSCADCSPYSYCAGEGQNARCECLPSFAKVGRFCVGVCTKDVCDINADCSYKGAGQ

FQCTCKTGFEGDGKFCTPINSCAKNNGGCPTDSTICEITGSGQSICVCKSGMEGEDPRAG

CRLKSACKENTCHRSARCETGQDGTARCVCGSEQISDGWRCYSDIMERVLELDREGSQAG

NLTGSIALFEKGCKLSLSKHGPFTAFIPVDTSQISSGKLNQARTAEAICKHHLILGQHLY

KDLEGHDLWTYGGELMRFKPNKKFISKNDPDTVFTIINSDIPASNGIIHIVDKVFTFATV

DTYDSAEFSSKTIGDILAEDTRFNRFVSLVDNCGAPMPLQGPGPLTLFVPTNKAIDRSRD

GSIIYMLKEAKHKLQELLRHHMFSQAAVTMDQVASMTEIHTMANQAITVSISDDGRVLLT

EKKISFDMKDIIASNGIIHIVDGLLLPPSIIPIMPHRCDVNETTIITSPCVMCKFISESK

CPPGSEKQTDRFQFCEPPPDPRWSEFTTVRGCARYCKAKRARAECCKGFYGPDCKPCIGG

FQHPCYGKGTCSDGIYGDGSCKCKPDFMGIGCHICSNPMKHGENCDEDCRCVHGVCDNRP

DSQGVCRRNSCLSGYTGELCDLTAMLCDSDGASEHCHIHAYCTRVNNINMCVCNPGYEGD

GYSCTEANLCLRADRGGCHIGAQCLYAGPGNISCICNEGWTGDGFVCVEINNCLTANRGG

CHEQAECIYIGPGQSECACKKGFMGDGIMCQIVNPCLTDNGGCHATATCSFNNSGIHQCT

CPEDHAGDGFKCYGNILMELEGNSDFYTFNRYLLRHPVITAESNVTALVPSKDAFKNLSD

ADQVFWTDYYHLPYLMQAHFLDGIYSYDDLSQQVNKTVSTKSKTKWKITFKNGELMIDNA

IVLVSDLQATNGFIHIINTVLKPPISDIPPPPPDLMEVLHNTPSFSLFREAALLYNLTES

ISKKDFTIFIPSDSAVKEYLLKTNSTQLDENIVKYHVITKEQLFPEHLTDGTLKTTLLGN

EYQIMIHLNSENKTLINDVELDGNMTEIRHGVIISISRVLVIHKNYCSKDIYMKTFGRCG

DCNSSPKCLFGSKPIKDEFPLNMKSNCRYRMRVGKRRKSVPGCMIDCLKNVKDHSCCPGY

FGRDCFKCPGTVDNWCSNNGKCQDGLFGNGECLCSEGFHGTACEMCEPGRYGKDCKSECH

CDHGKCLDGIDGNGQCICYKGWKGVNCSVEIVNDECGGICDENANCLSPSSGVKPSCVCS

AGYHGNGTFCQETELCAINNGGCSEHATCTKISPGERSCTCKKDYTGDGTVCLELDPCLV

NNGGCSENADCIKTGPNTAACVCKSGFATIRNYCIPINLCLKDNNGGCSVNAICQNTGPG

ERNCTCRFGYKGNGIECMGTVSRELLHNSAANWFRKSLSQSRVRDLYSKGPFTVFVPHTD

YIGNYSIDLWESKNRIPDLLRYHIVSCEEISGSQLKSMTHVVSASGHVLNFSVRDGAVYI

NGDTKIITSDYECSNGVIHFINKVLIPYELKNETKGIEEMLNITNAVEAYGYTVFGKLLQ

KANLMATLQHTAFQPFTMFWPTDKAFNSLPQEQKNWLYSEDHLDKLQAYLKFHIIRDQRT

IAIALPTEKTVRTLYGSWLTFSCSQDLTGDILINGNDAKIVDRNLQFLTGMAHGIDKVLE

PPNIGAHCDDFSSIEVKGRCGSCLFPPPCPYGTVYTNKTNVCSRPWNSYRRHYFYDDFEF

PSPYSFKPFGCNRMCSKMSWVSKCCKNHYGRNCQVCSGGLEAPCGEHGDCDDGRYGTGTC

KCHTGFNGTACELCEKNHYGSNCTACTCTANGKCDDGIDGDGSCFCQEGWTGVMCESKIG

VKPVCSPECHSKAVCLPENQCQCEPAYVGNGYNCTAPDLCSEYNGGCHEQADCTQSEINV

ICTCKSGYSGDGNVCSPINACVEETNGGCSDFANCIFIGPNERRCECQQGYVGNGLQCFE

KVVPPVDRCLENNGDCHANAICKDLHFHTKTAGVFHLRSPAGKYMMNYSTAGAACKAEGA

TLATLNQLSDAQQLGMHLCVAGWIDGKKVGYPIRFPSVKCGDNQVGIILYKDPDVNTPYD

AYCYRIREVKCECGPGYVGDGDFCNGNLASVIATHSNFSIFYSMLVKTAEAAEEGKKLLD

FLSTSSTNLTLFVPHNAGFSPNETLSLRDLEYHVSSNNSLHYYEDLKHNTAIPSRLGFNL

KVIIKTDNQTQVEDSSPVKLVNKQTVLIWDIPATNGLIHIIEGPLKAPPIKVTPSTPSAV

RSQSSAPTVTAVLLVIFIGGIIAGVAYYFLRNKNDAFSFKYFKNDDEDGNSTKTSANPAI

MSIPNPLYSGYRAFVEPFGEEEQCESPASVAANTPNILD*

>m.651 g.651 ORF g.651 m.651 type:complete len:380 (+) Unigene000059:4949-6088(+)

MTAPQFSRTLPRPAREPTPPSRNHSPPQNQDPEQVKALVPQMPKLLKSLFPAREDKKDLR

PSPQSQQHMPRIMMQTSSSSDDGRVRGEPVPAVGRSSGSEKSSDTPDASISSMQDSHELP

LSPISEASSGYFSTSVSTATLSDVSAVSADLTSGSNANDAPRQAADECGGNSVPADTGRK

SSEEIPPQNLNLKDLQSMPQNGVPCSRTEAKLITVPSLVSKSEYAFSLQRVKPSNLKSFS

PILPTDEKEKGETGIVEGSYKGAGKDAELPHWLRAGESVIVANSKCGRVRYVGHVDFSDG

IWVGVELDTPAGKNDGSVGGRQYFRCNPNHGLLVRPDRVSLRDTSRRHMENRHSKEFLQV

KAGSGLTNQKAENRKSWSN*

>m.656 g.656 ORF g.656 m.656 type:complete len:90 (-) Unigene000059:6150-6419(-)

MEGSLARVRGDLMGNKKKKRHVLEINQALNTLKRRVIKRTRENQGRIKATVPPLRTGFYW

SLDSHSLFSVVGRYAGVKVRGRGRCSSSV*

>m.650 g.650 ORF g.650 m.650 type:complete len:1395 (+) Unigene000059:670-4854(+)

MGETSLNDSNVKVAVRVRPMNRREKDLNTKCVIEMEGNQTFLYPASGNLGKSDSRSQPKV

FAYDYCFWSMDESEKEKFAGQDVVFQCLGESLLHNAFQGYNACIFAYGQTGSGKSYTMMG

SADQPGLIPRLCSSLFERTVQHQREEESFTVEVSYMEIYNEKVRDLLDPKGSRQALKVRE

HKVLGPYVDGLSRLAVASYKDIESLMSEGNKSRTVAATNMNEESSRSHAVFNIILTHTLR

DLQSGTSGEKVSKLSLVDLAGSERAAKTGAAGERLKEGSNINKSLTTLGLVISALADQGA

GKNKSKFVPYRDSVLTWLLKDSLGGNSRTAMVATVSPAADNYDETLSTLRYADRAKSIVN

HAVVNEDPNARIIRELREEVEKLRDQLTQAESMKAPELKERLEESEKLIQEMTVTWEEKL

RKTEEIAQERQKQLESLGISLQSSGIRVGEDKCFLVNLNADPALNELLVYYLKEHTMVGS

ADSQDIQLCGMGIQAEHCVINITADGAVFLNPYKNSRTCVNGSPVTSQQQLQHGDRILWG

NNHFFRINLPKRRLRPAAEEEEGEGENMKNSNSSEQLDADGDTASEVSSEVSFSYEFAQT

EVMMKALGSNDPMQAVLQSLERQHEEEKRTALERQRLMYEQELQQLRRRLTPERQSTSSQ

QHYRSMERLSLGGTSSSSRLRQWSEEREAMLTRSLRKLREQVVKANLLVQEANFIAEEMD

KKTEYRVTLQIPAANLNANRKRDAVLSEPAIQVRRKGKGKQIWALERMENRLVDMRELYQ

EWKDHDEDNPVMRSYFKRADPFFDEQVNHSLIGVANVFLSCLFYDVKLQYAVPIINQKGE

VAGRLHVEVVRVAGGVEDSMAGGEDGDYSIEGDSTERKLVCMIKILQATGLPQYLSNFVF

CQYSFWDQAEPIIVAPEVDPSASSPSSKDPHCMVVFDSCKELAVTVTEDFIEYLTEGALA

IEVFGHRQADPGRNLALWDLSIIQAKTRTLRDRWSEVTRKLELWVQMLELNENGEYTPVE

VVAAGDVRTGGIFQLRQGQSRRLQVEVRSVQDSGTMPLIAEIILGVSIGCVEIRQTRPAK

SSDSQQLEGDEMDSYQERDLERLRRQWLAALTKRQQYLDEHLQSLVGKTDKSEDDVEREA

QLLECRLTLTEERNAVMVPSAGSGIPGAPAEWVPVPGMETHIPVLFLDLSADDFSSQENL

ESPEPGGWDAMLSGEDEDDFFDLQIVKHYDEEVKAEASWDSTVHECPQLSRGGAQDQRVY

LTIRAVVQLSHPAEMQLVLRKRICVNLSGRQGFAQNFLKRMSTRSTVPGCGVTFEVVSNI

PGDAQSSEDREMLARMAANTENPKASGNEAAIEKYLRSVLAVENILTLDRLRQEVAVKEH

LAGKGREQTESQLA*

>m.661 g.661 ORF g.661 m.661 type:complete len:2433 (+) Unigene000060:163-7461(+)

MDHSECGSSSFNSLYHHESIVLFLYATSTTDQFFPSGLSVSQSFLLILCWILIYLFKETV

ERSTTRTLPAGYPHKPVFSTRSYQAWSAAPVTIPGQTKCGLGSLSSSPSNTPSASPLKSV

WSITSASPLKSTLGTTNASPVKSVSDIASPIRTYRSISSPIKTVVQQGQYPASVSSGLMS

PSFKTGTDPVSIKGLAASLSSRTISVPPSGSILDKTFTTVTPPESPKSVANLYTSALSLR

SAYTSAPTTATPTFKTLTSLSSMKTLDRGTMSNGSILSPYTSPAGPAVASAETVLLNGSM

SPVKYPSYSSVSPRLLTEGAASLQERIQATTQAAASNVSAAFCEAEKTLIGYSTSGYDTI

KSMSSPLRSNVSNSLYSSYRAPGPSSAQLSTTAMTVPVFSVVNVLPEPQEKKLPDTVSTV

PRTTTQSTSFTHRSKPSKPQLYIPSAAFKSATSTQMSSSQDILRDVADMKEGLIKMSAIL

QTDVSNTKNYQSQATREAKMEDEEPFSLVEKVKEDLVKVSEILSKDVLSDTKVFTKDKAS

EDEWEEFSKDEIEEAQQSFLKSLPTFETTHPVRPQSVQDKDLNLAKVVDYLMNDIGASSL

SKIAGEKNKYIEMKEGEEKQKRLFKSEHKLKMPPPGMRTSPSEKDLCKITDSYSGTDAIL

ESPDDFSHDQDKSPLSDSGFETRSEKTPSAPQSAESTGPKPLFSDVPIPPVITETRTEVV

HVIRSYEHPEEACEPLLEEAAALPSPACIEPESHAGSIKDKVKAFQIKVSSEDDSVMSKS

MCLKEETHITTTTRMVYHKPQLKDVGSDRIEETMSVRDIMKAFQSGRDPSKELAGLFEHK

ANKDSIKGDELTPRFLDKDTKPKVERIIEVHIEKGNTTDPTEVIIRETKKHPEYVCAGSR

GLRELSNRVEGDFGALQEDELFTAEETVPSFLETSRVNTPVPQGEESRPSSAQLIADDTY

KAFKLLSQQSIEYHDDELSELRGESYRFAEKMLLSEQLDTSQSDTEDLVINQGHGRTPEG

FHSPKKESVYRSARDTSDNFFHKDQYDKLTVLHYTTEPGSPKHTVRMRFTQDRQDRNREK

LIYEDRIDQTVKEAEEKLSEVSQFFRDKTEKLNDELQSPEKKLPMKDFRETRSGPSSVCS

SPQKTKVKTEVSSEEWDKGKQKLFGSMNERKSASLPSSPERHVQSRLNEDKSKQENNVWQ

AKASDISSPVQTSRVSSVRLKFETEAQNQDKNMQSIQSKPPVKKLQESKLPVYQFFAGSK

ASNVDSTEEGKPLNKSADSDTSKVTSGQNSPFSKFSNAACMSETDDTIFSKPQFEVGKPK

DDTDEKPDFSRDRGSESSKEKVCNFFKNANTVDLASKKNSTDSDDTQIKTLKKKITESQI

PVRISSTLLDADQGKKAKQDTVGKQLEKNLSHIPTLAKTRVQGRSEVSREEAGKQSFEGS

VEGGGIRDPDSGNFKETMEKVTTVTTRESFKGLKTLPVYVSVQVGKQGEKDLAGGTNGSV

RKIVGSENRTIYTVKQRQPTSPQRSPDDDTLEQISFLDSSGKSPMTPETPSSEEVSYDLT

AKHPECFTTFIPGGAGPLAEVSEESEDSEQARAFTSKDPAPEKVIDTPAVNTCQDTGQKS

KDKRIAYIEFPPPPPLDSDSSQTEKKGSTPSLEMDTDMMEVNLQEEHDKHLLAEPIIRVQ

PPSPVPSGANDSDSSEDESVFQPIAVKKYTFKIDEQGQEKESKPKKHNRNGNYKEPGING

NGKAEDNDYEQNGNDQSITDCSIATTAEFSHDTDATEIDSLDGYDFQDEDDGLHEQDVKT

CGFSNDNRKDIWGSESILRPNDRTFSQSKLEVIEEESPTEEYKKVQTKSEPSVKKTENEK

DNENEKDKKQSKEEGLSDTYFSYKLDEEFNTPFKTVATKGLDFDPWSSKGGDDEVVDARI

KDDEPKPFGLAIDEKSQATTPDTTPARTPTDESTPTSEPNPFPFHEGKMFEMTRSGAIDM

SKRDFVEERLQFFQIGEHLYGRRPGNKREGGTNPSVVTSQPQQVEHAQVKMDDSSVISYN

IIQICADRSDTPSHDNIMEPPSYSEVSSNLCSSFKIGVKDTSGENKDLVCMDWDSVTQKP

SLASEGCKSETSTESHNEQRTDLSKTEQKCILIQNFNNNNNLQASSGANKYMQYSTIEQH

QQSHYEASGFNPISEDLLKTNSDSVRVDSNYSNKVKIILQCPEQSAVHKTNIIPQCKDVK

KKLLQSRLPILEHRSTKRYCSAGIRLELSNKEIKQVPRERLDPFPIGKPKSRIPMLKVKK

DSNSSSIGKGRTTTLVKEKKRTQVFIGKNKSKTDSSSTSKSKTSQKQVQAAGKIANSKAS

ETVKLVDCHANEDKKMQGDDKDEERSTSRNTSLSEASQPSTTSRSSRDVRAEVEQAGRDR

RRRRRSKRTSGSEGSQGAGSQVAPGVEIEPSL*

>m.668 g.668 ORF g.668 m.668 type:5prime_partial len:2427 (+) Unigene000061:3-7283(+)

RLFLVCASVHEFLMREGPVLLQLRVKYLLKVNCMEKAALLAKACADVQEFGGSRGHFTQC

FLVCVCSCAPQETLMEELSKVDCRDALEMICNLEAEGDERGAFTLCSGFLTRQLLQEDSY

CAWELTLFWSKLLKRLEPSEQSFLEKCQKMSLLAKTVFHLLFFIKVIQSELDKIGLPACI

EMCIRALRMENCEGANKATICKTISCLLPSDLEVKRACQLTEFLLDPTVDSYYAVETLYN

EPDQKLEEETLLIPTSLRCDLFLVFKTQWSFDPEFWDWKTLKRHCLGLMGEEASIVSSID

ELNDDRFLEIERDELDSAQEEFKDVFECFLDTTKELKEISDHRQKNREVKKLREKGFVSA

RFRNWQAYMQYCVLCDKEFLGHRIVRHAQTHFTDGYYSCPICTETFETRETLDPHVASHV

KLSCKERLAAMKTTRKLTGAKNSASNDAPLDNKSGENQARKAKAKHCNGESDDVYNMGSC

IEDAGVRVHRKEFNVCPVSSCRKVFKFFRNLLTHVKDHGDVEEAKRFLELQATKIVCQYC

RRHFVSVGHLNDHLQVHCGAKPYTCIQLNCKASFDTNAELLVHSKRHPCFKAKCMFPGCG

KIFNEAYKLYDHEAQHYKTFTCKVPGCGKVFHAQSQLDMHKESHNPPKLETQPAGSVSPL

GEPQSHLSSEPAQQDAHSGPPADSGCMISSEDQSTGLVKVKHSIENMLSVAGQGLSQEFG

HVNCKAEPSVPNLVQQPPMPHMVNTEQIQPQLTPSMPSLIEPRSEESLLDALMNDPMLTS

SCSLPPASSYQSLLEDFQQAPSGDVLQAQMESAVSQGHTQSLYSNENTEYGQYNIKPETP

ISYPPPYDNTVPLAQNGHNDPPVLPVSQRLPPQLTMFSSNRPVQALQANTLSSVVEEKDR

YKCAYETCSRDYSSYRSLTKHMKAAHCEFYTQWKLAKRSNKVSPNTCSASSINGKCMSAE

PSQNQLSQRTPAPQNPVQKPPAQTHSPASCLSSHYPSGSSHLTGPNGQTFPNQMDSILNP

IVLSHLGSIANQSNVVSHSSWNSISVNTSLRQQTCHSQAMTSSSGQFPSQLDTVPQTQLS

DSYNRVMAYPSLQHQNSPSCRFIQTRPCAPQTINSPAKISHPGDSSLPQYVQQPKTSSKS

TLKHTDCVVKLERNPILIPSHSNLLQQSNPPTAMKPETCQKDISGFAHDGDVKKRKRSSR

TKWPAIVKDGKFICCRCFREFQSPKSLGGHLSKRTVCKPYDEAVLSADLPSSFLELLNSP

HPSETPQSSPSTQVTSSKAWPSLTKGPLDPKLFPNVTFLNTKNSTYNSGTIHQNSPSLEK

GVTGEQTFPESSGSYHQKSKNSVIQHTGNIRDKCIYDLMNYTQPCNTLPSDTSSSEQLLS

HMLSENKTLDSNLSRSPNNYVDRILQAETLNKIREIKEEPTRTNISGLSNDGLLAAMASL

AQNLVSEKSVKERLREQILAGDFQKRSSLGQGQGVENSNSQSPMTDVPLGLQTAVQSKEH

IVRRASELGELYTSVVPDETLTNISLAGASNNRETNNAFSNSLEQEDDKDLSEILKSFER

LDLNKEIEKNEQTTSNLLEAERVTKASEATCTLMLRGYSCDNDGCGYRAMTKDALFKHLV

KQHNYTEDMMNQCRKEQSNLAPFNCQLCPKTFTRNSNLRTHYQAVHNYSHEQVVKLGITR

QYSRKSIEADQGLCTTLNISELASPVFAGQNAESGAGITALVSGVRSIKSEFSVQPFEQQ

HPGASFDSCPVSSTILRAQTTPHHLQSQTTVHLSVIKATPSLLTSTSVEQQLNRPNEFVP

ESLVPSQHTQGFLSGGLSSSGAPLLAPISLASAQLGSLSSDTKPKFGKPKMAKVKEVTKK

PKEKKLDMVDVFSPYRPYRCVHQGCVAAFTIQHNLILHYKAVHQSALPKFEVNEDEEPIE

EEHNENQEEIGMPDSEVNEEFRCQVKDCSRIFQVVTDLLQHYLQLHKLGLDKAGIMMSGM

NLGRFRCDQPDCHVTFTGFWKYVNHIDNEHKHVKLSRTEPVNGMFQCEVEGCSCVYATRS

NLLRHFMKKHPELYKLRLLNSGGVKLGRPPKNRFVSLEKENREINKKTFQKGGAKKKRRQ

KNLWTKYGNPILKSKEEASSMCTKRIQLQYPCMLKGCETVAESERKLLKHYVQHGLPKQY

LEEQRSNYIFCKKMPRSKYKRIASRSDDTDKSDGSSIEASENEDAADTGPSESEFSKPTS

EKESTEDTEVSDAKLSSDLSSDISVVVKRKRGRPRKGEQVKRTQLARKRMTRLRAAQTRS

INYADMNSDSTSSSVTLPQEPNTSLSSFKPMGFEVSFLKFLEESSQSPGKRKSCSLMDLL

PHRKFPTVRLKTASVVCSRINPDYHHCRDVLKHVEFKNPQKLTSLSRVTFEVHRGFSNVY

ELLLKQLHDMRPAVVIQKEGVSETRA*

>m.675 g.675 ORF g.675 m.675 type:complete len:2087 (+) Unigene000062:117-6377(+)

MASLPERRAFALKINRHSSAEIRKHFTGFGYGTAQSGLHQRHSSSTSASICLNIPQTYDA

VDPLDVEEFLMTQLRSGDAELMQELGEFPDDDLEVDFVDRECRTAYHSVPEEGVELDPHV

RDCVLGYTQPWLLVGRRCQGDGWSAYSERTGFHKLLSKQIFESDVKPEKQEPSVKSPSLA

VLCDNDSARSTITSSDFDLRALQPDPRLDSLLQFSNFEDLDRFNFESRKNSRHPELFSLY

PPPDEEDAVEIRPIPDSPKEHMGLRILVRCQAIKFEIEIEPIFATMALYDLKEKKKISEN

FHFDLNTDQMRSLLRSHTPNIDSSTLAKAAIFSITYPSPDIYLVIKLEKVLQQGEIGDCA

EPYIGIIDNAKNKDKLEKLRSQTENFCQRLGQYRMPFAFGTVNIMNVISAATLDRDTTDS

DSINGKNNVDKKAYLPRRNSERFTTLEDQSNLSSFKPAIINITTIFKQEGDRLSDEDLFK

FLADIKRTSTIQRRIKIIPGFLRLEVNPVPDTVMHCLSPELIPIKPVSEKNARPVKELLE

FPSNDVYVPHCIYRNLLFVYPQRFNFGNRGLTSLRNITIKIQFMNGDEPLKVIFGKSSGQ

EFLSEIYTPVTYHNKSPDFYEEVKIRLPARLTERHHLLFSFFHISCQQKQNQSGSMETLI

GYSWLPILNNERLQMGAFCLPLVLERLPVNYSLHSPEKLPIQVPPVKWIDGHKGIFSLEI

HAVSSVHTQESYLERFFTLCHALEGKATFPIRVGDEKVPENKLEHELKLSIISLSSSKLE

PLVLFLHLVLDKLFRLIMQPMVIAGQTANLAQIAFESVVSVVNILHNSQELAKDHQGRNC

LLATYLYYVFRLPDTLLEVINTGAPVQESRYSTMGRATATSVGNMLLYSRVRSSSNPDIP

NPQSPEDAEVNSILVEKGLNHPGSRMSTFIDVTNRYQNSASSTRPSNKKQFHEELALQMV

VSTGVCREMVYKYAWFFFELLVKSMAQHVSQMDKQEVSRKNRFSDRFKDDITTIVNVVTA

EIGTILVKQQKEQEQAEKMNVSLAFFLYDLMSLMDRGFVFNLVRNYCNQMSAKSVSMFTL

ISMRLEFLRVLCSHEHYLNLSLYFSSPSSAPTSPSPSVTSQTSSSCSLPDNKIVQMFDLT

QEFKQQHYLTGLLLTELSAALDMESEGSKVQRKAINAICSLLCAHDLDSRCSRLEVRTKI

ASLYLPLVGIIIDSLNYLDFTVSESRGNKAKSSGPEDDFDNIPPISQSVAMAIAGNPFNT

LARNALVSMASVANRNCSTLSAETSRNLLVCFLWIMKNADQSLIQRWIVDIAPSQLHRLL

ELLTICISCFDYRGKSSDKVSVQALQKSQQAKARLEEALLGGLGARGEMMKRVGANDRTM

GQRENLRWRKDLTQWRQTNDRQDKSKAELDQEAIISGNLATEANVIVLDLLEIIVQTVPL

VDYKDSAVGGVLRVLLHSLTCNQSTMYLSHCFSTIRALIVKFGDMLFEEEAEQCADLCQK

VLQYCSSCVDSNRNQACATLYLIMRYSFSSASNFSRVKMQVTMSLASLVGKSSDVHEEYL

RRSFRTILAYAEEDTEMQSTQLPSQVDELLRNLNSILSDTVKMKEFQEDPEMLMDLMYRI

AKGYQTSPDLRLTWLQNMAEKHNSRKCFTESAMCLVHAAALVAEYLSMLEDHKYLPIGSV

TFQNISPNVLEESAVSDDILSPDEDGVCSGRYFTEIGLVGLLEQAAELFSNGGLYEAVNE

VYKVIVPILEAHRDFRKLATTHDNLKRAFENVVQKGSKRMFGTYFRVAFFGSKFGDLDER

EFIYKEPGITHLPEISHRLENFYRQCLGDGVLEMIKDSTPVDRNKLNPNKAYIQITFVEP

YFDDYEMKDRMTIFEKNFNLRRFMYTTPFTKSGRPRGELNEQYKRKTILTTQHAFPYVKT

RINVIQKEEFDLTPIEVAIEDMQNKTRELAIATHKEKPDPKMLQMVLQGSVTATVNQGPL

EVAQVFLNEIPADPKLYRHHNKLRLCFKEFILRCGEAVEKNKHLITPDQREYQQELKKNY

NKLRENLRPMLERKIPELYKPIVKPRLENRDSTKRHSFRRIPEDSS*

>m.682 g.682 ORF g.682 m.682 type:complete len:2320 (+) Unigene000063:147-7106(+)

MRRFVLLALCACSAVVCLPKSDRNKRQGQQQQVLDIPVDELAYARQAGCTENGQFYKVND

QWERPYMDSTLLCTCRGSAGVQCESKPAAEEICFDKINARSYRVGETYERPKDGMIWDCT

CIGSGRGKISCTIANRCHEGGRSYRIGETWTRPHDTGDYMLECMCLGNGKGEWTCKPIAE

RCYDTSLGTSYVVGQTWEKPYQDWMIVDCTCLGEGNGRITCTSRNRCNDQDMRKAYRIGE

TWTKVDSQGRQQQCVCTGNGRGEWKCESHSSAQNALGSGSALSTEVRPVTHQLAILPELV

EIGACQTRSGTTYYNGMRWVQTQGSQQMICTCVNGGISCEEWEGQGHVYGGNSNGQPCVF

PFVFGGKTHYSCISEGRTDGQLWCSTTSDYDTDRQYSFCTQRNLMVTTRGGNSNGALCYF

PFLYNDRNYTDCTADGRRDGMKWCGTTANYDNERHYGFCPMAAHEEICTVNDVMYRLGDE

WDKRHDTMGHMMRCKCLGNGRGEWSCVAYSQLRDQCIVDGLTYEVDQKFDKRHREGYMMN

CTCFGQGRGRWKCDPIDQCQDAETKVFYQIGETWDKVNHGVQYRCTCYGNGIGEQACEPL

QSRLPVRVTITETGNQPNSHPIQWNPPASSHITQYILKWKVKNTRTPWKEVVIPGHVNSY

TISGLKPGLTYEGQLISILSYGHREVTRFDFTTSYGSLIPTEGVTRNNIRVVDASESITE

ITSSSFVVSWTSASETISGFRVQYELSEEGAEPTVIDLPRTSTSVNIDRLLPGRTYQVQV

YEVEPEGNMNLILTTTQTTAPDTTTEHRVTDVGETSIVISWTKPQAPITGYRVVYTPSLE

GSSTELILPETVTSVTLVDLQPGQSYNVSIFAVEGNLESEPVVLQVHTAGEAQPEEVQAP

TELQFYEVTDVKITITWKGPPSEVSGYRVTYEPVGSDGRATQRPLQLPVTPNAYAEITHL

QPGTLYRIYVYSVYGGTESQPLVGEKLTRPDAPTELRFPDVTEDTVLVAWSAPQAPITGY

RLIITAEDTTPPKQWRVRPEETQYTIQDLRPDTMYTITLHSEQGSTLSEGVSGTVTTSVP

VGNAPRFSTDVTDTSIIISWTPVPRFSYKMSVKPSQGGEAPRVVTSDSGSIYISGLTPGV

EYTYSLQPIFSGRRPGSPITRNVVTPLSPPSHLNLVSNPDTGNLNVYWQPTTTPDITGYR

VTCTPTTGQRGNSLEEFVRDGETSCTLENLSPGVEYNISVYAVKNHMESEPISSVITQAV

PAPTNLKFGEVGPDSMRLSWTRPSVRQSEISRFVIRYHPSNDDDNVQEVNVGGATSTYLL

QNLLPVTEYMVSVSCVYSERESIPITGRQTTILDAPTALIFSDVLTHSFTANWRAPRAQI

TGYRLVYEATSGGRRHEERLPPSRTHYALNNLQPDTLYTLHIYAISGRQESQPLTGTQST

ISDAPTDLEVTLSTPTSITISWDAPAIPVRYYRIKYGQSGERGPGQEFTVPGTDSTATIS

DLQPERDYTITIYAVTGRGDSPASSTPTIITHRTGSHGAPSTTDLDVSDVQDQAIIVRWT

PARGPVAGYRVTGKPKKGVGPTFSKEVGPDKTELRITGLVPTVEYVISVYAISRDGESTP

VVQKATKIETPDRPTDMRFPDVDSSGLTDRSSTPESEGTHTTAGVPSPTNIQFGDVGPSS

FVVLWRPPSVRLNGYRILVTPKNNFAHPKELNVAPDAMQATVTGLLVSTLYEVHVYALKD

SASSPPLIGEISTTEDISPPRRSRINEVKDTSITISWRSKIEPMTGFLIEAKPLSGESPT

IRKEIPAEQRSVVITGLQPATTYSINIYTLNGDRRSAPFTLIAKTIGSSLPPPTDLQFLA

LTPTSISFKWQPPTSRITGYYVTYEEEGKSPRELTPRPHAGANYASITDLKPATVYIIKI

IALQNTMRSPALVGRATTQESDFTLPSRSHSSLGPLDVPETEIVVNVMGPTVQPRPDKHG

QGMEYTEYNNQPTLPHSGQRPNPYVPGTGQTLIYVPAPGSDGSRVPKVVQLSERNAHNFL

FPENKTGTPQEAQTQTTISWKPFRQSKAYIVSCQPVTNRNEKMFQMQLPATSTSATLIGL

TSGASYRVLVEALKDALKYKILDEVITAGNTDPARVPASDDSCYDTITAAHYNVGDEWER

MSETGFKLWCRCLGLGSGHFRCDSSKWCHDNGNNYRIGERWERQAENGHMMSCTCLGNGK

GEFKCEPHESTCYDDGKTYQVGNQWQKEYLGAICTCTCYGGQQGWRCENCKKPGTEINTQ

MLRPVRYGDGIAKVNIHCPIECLRPDILADAVANPNPRE*

>m.688 g.688 ORF g.688 m.688 type:complete len:2314 (+) Unigene000064:147-7088(+)

MRRFVLLALCACSAVVCLPKSDRNKRQGQQQQVLDIPVDELAYARQAGCTENGQFYKVND

QWERPYMDSTLLCTCRGSAGVQCESKPAAEEICFDKINARSYRVGETYERPKDGMIWDCT

CIGSGRGKISCTIANRCHEGGRSYRIGETWTRPHDTGDYMLECMCLGNGKGEWTCKPIAE

RCYDTSLGTSYVVGQTWEKPYQDWMIVDCTCLGEGNGRITCTSRNRCNDQDMRKAYRIGE

TWTKVDSQGRQQQCVCTGNGRGEWKCESHSSAQNALGSGSALSTEVRPVTHQLAILPELV

EIGACQTRSGTTYYNGMRWVQTQGSQQMICTCVNGGISCEEWEGQGHVYGGNSNGQPCVF

PFVFGGKTHYSCISEGRTDGQLWCSTTSDYDTDRQYSFCTQRNLMVTTRGGNSNGALCYF

PFLYNDRNYTDCTADGRRDGMKWCGTTANYDNERHYGFCPMAAHEEICTVNDVMYRLGDE

WDKRHDTMGHMMRCKCLGNGRGEWSCVAYSQLRDQCIVDGLTYEVDQKFDKRHREGYMMN

CTCFGQGRGRWKCDPIDQCQDAETKVFYQIGETWDKVNHGVQYRCTCYGNGIGEQACEPL

QSRLPVRVTITETGNQPNSHPIQWNPPASSHITQYILKWKVKNTRTPWKEVVIPGHVNSY

TISGLKPGLTYEGQLISILSYGHREVTRFDFTTSYGSLIPTEGVTRNNIRVVDASESITE

ITSSSFVVSWTSASETISGFRVQYELSEEGAEPTVIDLPRTSTSVNIDRLLPGRTYQVQV

YEVEPEGNMNLILTTTQTTAPDTTTEHRVTDVGETSIVISWTKPQAPITGYRVVYTPSLE

GSSTELILPETVTSVTLVDLQPGQSYNVSIFAVEGNLESEPVVLQVHTAGEAQPEEVQAP

TELQFYEVTDVKITITWKGPPSEVSGYRVTYEPVGSDGRATQRPLQLPVTPNAYAEITHL

QPGTLYRIYVYSVYGGTESQPLVGEKLTRPDAPTELRFPDVTEDTVLVAWSAPQAPITGY

RLIITAEDTTPPKQWRVRPEETQYTIQDLRPDTMYTITLHSEQGSTLSEGVSGTVTTSVP

VGNAPRFSTDVTDTSIIISWTPVPRFSYKMSVKPSQGGEAPRVVTSDSGSIYISGLTPGV

EYTYSLQPIFSGRRPGSPITRNVVTPLSPPSHLNLVSNPDTGNLNVYWQPTTTPDITGYR

VTCTPTTGQRGNSLEEFVRDGETSCTLENLSPGVEYNISVYAVKNHMESEPISSVITQAV

PAPTNLKFGEVGPDSMRLSWTRPSVRQSEISRFVIRYHPSNDDDNVQEVNVGGATSTYLL

QNLLPVTEYMVSVSCVYSERESIPITGRQTTILDAPTALIFSDVLTHSFTANWRAPRAQI

TGYRLVYEATSGGRRHEERLPPSRTHYALNNLQPDTLYTLHIYAISGRQESQPLTGTQST

ISDAPTDLEVTLSTPTSITISWDAPAIPVRYYRIKYGQSGERGPGQEFTVPGTDSTATIS

DLQPERDYTITIYAVTGRGDSPASSTPTIITHRTGSHDLDVSDVQDQAIIVRWTPARGPV

AGYRVTGKPKKGVGPTFSKEVGPDKTELRITGLVPTVEYVISVYAISRDGESTPVVQKAT

KIETPDRPTDMRFPDVDSSGLTDRSSTPESEGTHTTAGVPSPTNIQFGDVGPSSFVVLWR

PPSVRLNGYRILVTPKNNFAHPKELNVAPDAMQATVTGLLVSTLYEVHVYALKDSASSPP

LIGEISTTEDISPPRRSRINEVKDTSITISWRSKIEPMTGFLIEAKPLSGESPTIRKEIP

AEQRSVVITGLQPATTYSINIYTLNGDRRSAPFTLIAKTIGSSLPPPTDLQFLALTPTSI

SFKWQPPTSRITGYYVTYEEEGKSPRELTPRPHAGANYASITDLKPATVYIIKIIALQNT

MRSPALVGRATTQESDFTLPSRSHSSLGPLDVPETEIVVNVMGPTVQPRPDKHGQGMEYT

EYNNQPTLPHSGQRPNPYVPGTGQTLIYVPAPGSDGSRVPKVVQLSERNAHNFLFPENKT

GTPQEAQTQTTISWKPFRQSKAYIVSCQPVTNRNEKMFQMQLPATSTSATLIGLTSGASY

RVLVEALKDALKYKILDEVITAGNTDPARVPASDDSCYDTITAAHYNVGDEWERMSETGF

KLWCRCLGLGSGHFRCDSSKWCHDNGNNYRIGERWERQAENGHMMSCTCLGNGKGEFKCE

PHESTCYDDGKTYQVGNQWQKEYLGAICTCTCYGGQQGWRCENCKKPGTEINTQMLRPVR

YGDGIAKVNIHCPIECLRPDILADAVANPNPRE*

>m.694 g.694 ORF g.694 m.694 type:complete len:2450 (+) Unigene000065:278-7627(+)

MLDGTVKTVMVDDSKIVTDLLMTICARIGITNYDEYSLVRDIGEEKKEETTGTLKRDKTL

LRDDKKMEKLKQKLHTDDELNWLDHGRTLREQGVEETEMLLLRRKFFYSDQNVDSRDPVQ

LNLLYVQARDDILNGSHPVSFDKACEFAGYQCQIQFGDHNESKHKPGFLDLKEFLPKEYV

KNKGEKKIFQAHKNCQNMTEIEAKVSYVKLARSLKTYGVSFFLVKEKMKGKNKLVPRLLG

ITKESVMRVDEKTKEVMQEWSLTNIKRWAASPKSFTLDFGDYQDGYYSVQTTEGEQIAQL

IAGYIDIILKKKKSKDHFGLEGDEESTMLEDSVSPKKSTVLQQQFNKVARVETGSVALPV

IMRPGAGGPESIYTGSMPKAKQQITTGQMHTGHMPPLTSAQQALTGTINSSMQAVQAAQA

SLTDFDSLPPLGTDAASQAWRKNKMDESKHEIHSQVDAITAGTASMVNLTAGDPAETDYT

AVGCAVTTISSNLTEMSKGVKLLAALMEDEGGNGQQLLGAARNLACAVSDMLKTAQPASA

EPRQNLLQAAGNVGQASGELLQQIGESDADQQFQDMLMKLAKAVATAAATLVLKAKNVAQ

KTEDSAQQNRVIAAATQCALSTSQLVACTRVVAPTISSPVCQEQLIEAGKLVAKSVEGCV

EASQGATNDEQLLKQVGVAATGVTQALNELLQHIKQYASGGQPIGRHGEATDRILDVTEN

IFSSMGDAGEMVRQARILAQATSDLVNAIKADAEGETDLENSRKLLSAAKLLADATAKMV

EAAKGAAANPDSEEQQQKLREAAEGLRMATNAAAQNAIKKRLVNKLENAAKQAAAAATQI

IAAAQHAASSNKNPAAQQQLVQSCKVVADQIPQLVQGVRGSQAQPDSPSAQLALIGASQN

FLQPGAKMVTAAKATVPTIGDQASAMQLSQCAKNLASALAELRTAAQKAQEACGPLEIDN

ALNAVRDLEKDMQEAKASAAEGKLRPLPGETLEKCSQDLGSNTKAVSSAVAQLLSEATQG

NENYTGMAARDVAQALRSLASAARGVAATTDDPQACSAMLDCAGDVLDKSGNLIEETKRA

IAKPGDPESQQRLAQVAKAVAQALNRCVNCLPGQRDVDNAIRTVGEASKKLLSDQFPASG

KTFQEAQANLNQAAAGLNQSANELVQASRGTTQDLAKASGKFGQDFSHFLEAGVDMAGQS

QSKEDQTQVVTNLKTISMSSSKLLLAAKALSTDPNSPNLKNQLAAAARAVTDSINQLITM

CTQQAPGQKECDNALRELETVRGMLENPTEAVNDMGYFDCIDSVMENSKVLGEAMAGISH

NAKNSNLPEFGDAVSSASKALCGLTEAAAQGAYLVGVSDPNSHAGQKGLVDPSQFAKAKQ

SIQMACQNLVDPACTQSQVLSAATIVAKHTSALCNACRLASSKTSNPVAKRQFVQSAKEV

ANSTANLVKSIKALDGAFNQENRQKCKAATGPLIEAVDNLTAFASNPEFASVPAQISPEG

LAAMEPIVAAAKTMLESSTGLIQTARSLAVNPKDPPKWSVLAGHSRTVSDSIKKLITNMR

DKAPGQRECDEAIEVLNNCIREVDQASLAAISQHLTPRDDISHEALHEQMAASVQEISHL

IDPVAIAARSDASQLGHKVSQMASYFEPLIMAAIGTASKILNSQQQMSVLDQTKTLAESA

LQMLYTAKEAGGNPKAAHTQEALEESVQMMKEAVDDLGGTMAETASAAGAVGGMVDSITQ

ALNKLEDPGVEPEGTFVDYQTTMVKTAKAIAVTVQEMVTKSNTNPDELGGLANQLTTEFG

DLASEAKCAAITAENAEIGSHIKKQVTELGYGCTGLVTKAGALQCSPNDSITKKELIDAA

RKVSEKVSHVLAALQAGNRGTQACITAASAVAGIIADLDTTIMFATAGTLNRENAETFAD

HRENILKTAKVLVEDTKLLVSGAGASQEKLAQAAQSSVNTITKLADVVKLGAASLGSEDP

ETQVVLINAVKDVAKALGDLIRTTKAAAGKPHDDPAMLQLKSSAKVMVTNVTSLLKTVKA

VEDEATKGTRALEATIEHIKQELAVFSSPDPPPKTATPEEFIRMTKGITQATAKAVAAGN

SCRQEDIIATANLSRRAIAEMLHSCKQAAYHPEVNLEVRTRALRFGKECANGYLGLLEHV

LVIIQKPTHDLKQQLATFSKRVAGSVTELIQAAEAMKGTEWVDPEDPTVIAENELLGAAA

AIEAAAKKLEQLKPRAKPKEADESLNFEEQILEAAKSIAAATSALVKAASAAQRELVAQG

KVGAIPANAVDDGQWSQGLISAARMVAAATNNLCEAANSAVQGHASEEKLISSAKQVAAS

TAQLLVACKVKADQDSQTMKRLQAAGNAVKKASDNLVKAAQKAAFDAQDDQAVMVKSRMV

GGIAQIIAAQEEMLRKERELDEARRKLAQIRQQQYKFLPSELREDGHEQ*

>m.702 g.702 ORF g.702 m.702 type:complete len:277 (+) Unigene000066:6312-7142(+)

MSNMACQNNTWRSREATTYSAKKMPRSKYKRIASRSDDTDKSDGSSIEASENEDAADTGP

SESEFSKPTSEKESTEDTEVSDAKLSSDLSSDISVVVKRKRGRPRKGEQVKRTQLARKRM

TRLRAAQTRSINYADMNSDSTSSSVTLPQEPNTSLSSFKPMGFEVSFLKFLEESSQSPGK

RKSCSLMDLLPHRKFPTVRLKTASVVCSRINPDYHHCRDVLKHVEFKNPQKLTSLSRVTF

EVHRGFSNVYELLLKQLHDMRPAVVIQKEGVSETRA*

>m.701 g.701 ORF g.701 m.701 type:complete len:2092 (+) Unigene000066:125-6400(+)

MICNLEAEGDERGAFTLCSGFLTRQLLQEDSYCAWELTLFWSKLLKRLEPSEQSFLEKCQ

KMSLLAKTVFHLLFFIKVIQSELDKIGLPACIEMCIRALRMENCEGANKATICKTISCLL

PSDLEVKRACQLTEFLLDPTVDSYYAVETLYNEPDQKLEEETLLIPTSLRCDLFLVFKTQ

WSFDPEFWDWKTLKRHCLGLMGEEASIVSSIDELNDDRFLEIERDELDSAQEEFKDVFEC

FLDTTKELKEISDHRQKNREVKKLREKGFVSARFRNWQAYMQYCVLCDKEFLGHRIVRHA

QTHFTDGYYSCPICTETFETRETLDPHVASHVKLSCKERLAAMKTTRKLTGAKNSASNDA

PLDNKSGENQARKAKAKHCNGESDDVYNMGSCIEDAGVRVHRKEFNVCPVSSCRKVFKFF

RNLLTHVKDHGDVEEAKRFLELQATKIVCQYCRRHFVSVGHLNDHLQVHCGAKPYTCIQL

NCKASFDTNAELLVHSKRHPCFKAKCMFPGCGKIFNEAYKLYDHEAQHYKTFTCKVPGCG

KVFHAQSQLDMHKESHNPPKLETQPAGSVSPLGEPQSHLSSEPAQQDAHSGPPADSGCMI

SSEDQSTGLVKVKHSIENMLSVAGQGLSQEFGHVNCKAEPSVPNLVQQPPMPHMVNTEQI

QPQLTPSMPSLIEPRSEESLLDALMNDPMLTSSCSLPPASSYQSLLEDFQQAPSGDVLQA

QMESAVSQGHTQSLYSNENTEYGQYNIKPETPISYPPPYDNTVPLAQNGHNDPPVLPVSQ

RLPPQLTMFSSNRPVQALQANTLSSVVEEKDRYKCAYETCSRDYSSYRSLTKHMKAAHCE

FYTQWKLAKRSNKVSPNTCSASSINGKCMSAEPSQNQLSQRTPAPQNPVQKPPAQTHSPA

SCLSSHYPSGSSHLTGPNGQTFPNQMDSILNPIVLSHLGSIANQSNVVSHSSWNSISVNT

SLRQQTCHSQAMTSSSGQFPSQLDTVPQTQLSDSYNRVMAYPSLQHQNSPSCRFIQTRPC

APQTINSPAKISHPGDSSLPQYVQQPKTSSKSTLKHTDCVVKLERNPILIPSHSNLLQQS

NPPTAMKPETCQKDISGFAHDGDVKKRKRSSRTKWPAIVKDGKFICCRCFREFQSPKSLG

GHLSKRTVCKPYDEAVLSADLPSSFLELLNSPHPSETPQSSPSTQVTSSKAWPSLTKGPL

DPKLFPNVTFLNTKNSTYNSGTIHQNSPSLEKGVTGEQTFPESSGSYHQKSKNSVIQHTG

NIRDKCIYDLMNYTQPCNTLPSDTSSSEQLLSHMLSENKTLDSNLSRSPNNYVDRILQAE

TLNKIREIKEEPTRTNISGLSNDGLLAAMASLAQNLVSEKSVKERLREQILAGDFQKRSS

LGQGQGVENSNSQSPMTDVPLGLQTAVQSKEHIVRRASELGELYTSVVPDETLTNISLAG

ASNNRETNNAFSNSLEQEDDKDLSEILKSFERLDLNKEIEKNEQTTSNLLEAERVTKASE

ATCTLMLRGYSCDNDGCGYRAMTKDALFKHLVKQHNYTEDMMNQCRKEQSNLAPFNCQLC

PKTFTRNSNLRTHYQAVHNYSHEQVVKLGITRQYSRKSIEADQGLCTTLNISELASPVFA

GQNAESGAGITALVSGVRSIKSEFSVQPFEQQHPGASFDSCPVSSTILRAQTTPHHLQSQ

TTVHLSVIKATPSLLTSTSVEQQLNRPNEFVPESLVPSQHTQGFLSGGLSSSGAPLLAPI

SLASAQLGSLSSDTKPKFGKPKMAKVKEVTKKPKEKKLDMVDVFSPYRPYRCVHQGCVAA

FTIQHNLILHYKAVHQSALPKFEVNEDEEPIEEEHNENQEEIGMPDSEVNEEFRCQVKDC

SRIFQVVTDLLQHYLQLHKLGLDKAGIMMSGMNLGRFRCDQPDCHVTFTGFWKYVNHIDN

EHKHVKLSRTEPVNGMFQCEVEGCSCVYATRSNLLRHFMKKHPELYKLRLLNSGGVKLGR

PPKNRFVSLEKENREINKKTFQKGGAKKKRRQKNLWTKYGNPILKSKEEASSMCTKRIQL

QYPCMLKGCETVAESERKLLKHYVQHGLPKQYLEEQRSNYIFCKKNASLKV*

>m.708 g.708 ORF g.708 m.708 type:complete len:2099 (+) Unigene000067:209-6505(+)

MWMIEHPGNEDTEERTLSSDCSGAAGGGKSAERSFLRSPGDIPNADAAELEEGFSESPEG

IDQDSASASNGPALRGRSAASRKHRFDLAARTLLARAAGLYHSVQAHRSQVRREGSGLQQ

DSSSLGGVYDFNLDEELELELDQEAMEAMFTQELASDSDILGMWIPEVLDWPTWRVCESE

EREEVVVCELCDVSVSSFNQHMKKSHPGCGRSANRQGYRSNGSYVDGWFGGECGSGNPYY

LLCGSCREKYLGMKSKHKGAVSERYKGQAPDLLGKQDSVYEEDWDMLDVDEAEKLTGEEE

FELLSAPLGLTERKPVPEAVQFPDTDPLGASAAMVTATNSMEETLLQIGCQSSVDKSTSG

RMCLGEQAAGLQSSADRMVALRRVTAAAQVLVARTMVMRALSLLSVSGSSCSLAAGLEAL

GLTDIRTLVRLMCLAAAGRAGLSTGLASGPGALERPRGTNKPTKPISCLAYLSTAVGCLA

SNSPNAAKLLVQLCTQNLISAATGMNLTTVDDPIQRKFLPSFLRGVAEENKLITSPNFIV

TQALVALLADKGARLRPGYDKAEMEKRGPLELANALAACCLSSRLSSQHRQWAAQQLVRT

LAAHDRDNQSRPQTFADMAGDLRKCSTIKLEAHQGRVISFGWCNKKGLLATSGSDGTVRV

WNVNKNQYTLQQTCVFNKTDFTSEECSTGLGSPGEPSLAPVAWSVSGKLVAAAMEKVVNI

WQVNGGKALLDLQPHWVSSLVWPENEAGCLWAGESRELLLVGRIDGSLGLLEILDSSNIQ

RTELLHCYRKDAVMNIAWYSEDRPFAVGYADGKLLIGCKEALENGSVVVIDAHKESISSL

RWAPGGQILLSCAKEETACLWAESGAGLGRSWVCLQSIAHPSVVNAVAWCGLTGQGPKAL

NMLATCCQNGLVSVWTVPQDPTAFSQSGSSSTDAWWETDSKSKPRFTPQWHEAAVCVFQL

RGHMTPVRTIAFSPDGLALVSGGVGGLLNIWSLRDGSVLQTVVVGSGAIQNTVWIPDVGV

AVCSNRSKDVLVVNCSSEFMTANQVLATCRTALKKQGVVGLNTAPCMRTFLERLPIMLQE

QYAYEKPHVVCGEQLIHSPYMQCLASLAVGLQLDSLLCRPAVPLHLSHCLAQPVSSSSSS

SQSSSSSGPSALMHSCGSSEWAWLHCFSTTIKTAEALARAHTFPESFNVPDLEPVAKDKM

AVLMDNSKWVPGMDEQLMAWATSRPEDWHLGGKCDVYLWGAGRHGQLAEVGRNILVPTPA

ASFSQAQQVVCGQNCTFVIQANGTVSACGEGSYGRLGQGNSDDLHILTVISALQGFVVTQ

LVTSCGSDGHSMALTESGEVFSWGDGDYGKLGHGNSDRQRRPRQIEALQGEEVVQMSCGF

KHSAVVTADGKLFCFGNGDYGRLGLGNTSNKKLPERVTALEGQQIGQVACGLNHTLVVSS

DGSTVWAFGDGDYGKLGLGNSTAKSSPQKVDVLCGIGIKKVACGTQFSVALTKDGNVYTF

GQDRLIGLPEGRARNHNRPQQLPALVGVIIKDVAVGAEHTLALASTGDVYAWGSNSEGQL

GLGHTNHVREPTLITALQGKNTQQISAGRCHSAAWTAPSVPPRAPGSSMPLQLGLPVCVP

PQYGSLRDISMEALRARLRLLYHFSDLMYSSWRLLNLSPNNQSCTSHYNPGTWGIVQGQL

RPLLAPRVYMLPMVRSIGKTMVQGKNYGPQITVKRISTRGRKCKPIFVQIARQVVKLNAS

DLRLPSRAWKVKLVGEGADDAGGVFDDTITEMCQELENGVVDLLIPSPNATAEVGYNRDR

FLLNPSACLDEHLLQFKFLGILMGVAIRTKKPLDLHLAPMVWKQLCCIPLTLEDLEEVDL

LYVQTLNSIVHLEDSSITEQNFHEMIPLDSFVGQSADGKMVPIIPGGNSIPLTFSNRKEY

VERAIEYRLHELDRQVAAVREGMSWIVPVPLLSLLTARQLEQMVCGLAEISVEVLKKVVR

YREVEEQQQLVQWFWQTLDEFSNEERMLFMRFVSGRSRLPANTADISQRFQIMKVDRPYD

SLPTSQTCFFQLRLPPYSSQSVMAERLRYAINNCRSIDMDNYMLSRNVDNTEGSDTDY*

>m.716 g.716 ORF g.716 m.716 type:5prime_partial len:2320 (+) Unigene000068:2-6961(+)

GHHIGALSIQDDPLLMLALNSAKIASDALYKKDFNKSKTKFHLPVDMLAFELAKKCQVQV

NDANYRTYLHNWTCLPDSNDVVQARQTYELQSDAVYRADLKWLKGLGWIPIGSLDVEKAK

KAGEALSERKYRQPPSNFKFTSTTDDMPIVLAKANNNIMNKRSYIQAWESDKTKIHIMPD

AMEVLHAKQNKINYSEKLYKLANELAKKKGYDMRGDAISILAARASTTIASDYKYKTGYR

KQVGHHIGALSVRDDPLLMLALNSAKIASDVLYKKDFNKSKTKFHLPVDMLAFELAKKNQ

IQVNDDNYRTYLHNWTCLPDQNDVIQARKAYDLASDNIYKTDLEWIRGCGWVAADSVDHV

KVRKAQQILNDRMYKKDAKDNFAKFTNIVDRPEVLLAKVNAFNLSDLKYKESFNLEKGHY

IGSDDTPQLAHSREAAKIISEKLYKLGWDEAKATGYQLNHEYLPLVSAKKVGSIVSEAKY

HDAHEKAKGHYLANTLVDFPAVVHSSQMEKMKNMRTYQKDYHTSKTKLHIPHDMISHVVA

KKCQEILSDVLYRSYLHQWTCHPEQNDAIRARKANEILSDVFYKDDLNWMKGIGCYAWDT

PEIVRAKKSYELQSDIKYKAEGKKEFNNYSIVTDTPVYVTAILGHTWASELNYREAYHKE

KHLYTTVLDTYDYARCYNLKQLYSSKTYSALWDRIKAKSYTIPIDANALVHAKQQKVLLS

KVKYKEDYEKFKSLYSLPKCLADDPATARCIKAGKLNLDRLYKDKWEKTKAKIHIPPDML

DVVAARNTQKMISEVDYRKYLHQWICLPDMQVYVHARKVNEQLSDIFYKEDMTWLKGVGC

YAWDTPEILRCKQAMNLQSENLYRAKGVEHFKNFSVVTDTPVYETCKQSAHNLSDLNYHH

DYVTNVRGKNTAPATTVDTERARLANYIQSDHIYKEASKSNMSTGYSLPFDTPLALQAKA

NNVITSNVKYKEAYENTKARSYKLDPNGVNFVTIRKANQVTNQRLYRALYEKEKDKVHSV

YDTPEIKQVKATQEAISDVCYKEKYYNSRGTLLPMPFTPQLMHCAHVNQINSDLKYKEDL

QWLRGIGCFVFDSPEMVRIREINKFRTSYDIDAKKNFSNFSVVLDTPEYKRVSELKTHLS

ERVYKAAGNIQRTQCSTTGDAMEFKRAKWAQTLTNKWLYTDLASKERAFYTPELHTPLLD

HARSMKVVYSENKYKEQYEKMKHKYTPVHDTPILIRAKKSYLNSSDLRYKETFELAKGHY

HTTKDALDIICAKRVRDDISEVKYREKYINSLGTWKSIPDRPEFFHSKVVRDCISDYKYK

EDLEWIKGVGCFVWDTPLLKQAEHNKALYSERLYKASYEKTKCNFKYTCDTPFFQAVKNA

SALSNDRSYRASYEKTKDKYTIVVDDPRNLLAKEVNNMSQWKYRSQYERLKSKYTSVLET

PEHKVHKLRKQISDKIYTMEYNKNKAKGYTLGHDTPLNLHMKKVKEITSNLKYKEVYEKN

KAQINIAPDAFDIRAAKEAYKNISNLDYKKKYEATKNKWIWTVDRPDFVHAAKINYQQSD

VEYKYDKEMLKGCVMPVTDDKYTLLAKQNTELSSDIKYKQKYEEARGHYHVVHDTPQILH

AKSVSGLVSESKYKLESKKARQSGSFTTLPETRDTAHSKAMTKITSSKVYKQKFEKDKGK

SIYNNMTVPPDVKHAMDVAKSQSNINYKQEAKAKLHYTPVADRPDILKATQAAKLISEVG

YRDKARQEASRGGSLVNRPDINLATEVSKLTSLVKYKEKFDKEMKGKRPQYDLKNSKIYQ

TLKDANTLASEVKYKGDLKKIHKPVTDMSESLAMQHNLSTSKLASSVKYKEKYERERGKA

MLDFETPTYVSAKEAQHMQSQREYKKALEQEIKGKGMLALANDTPDFMRARNATEILSQS

KYKHNAEQDRASYTTVIDTPDIIHAQQIRNIVSQKKYKEEAEKTMSHYNAVLDTPEMQRV

RENQRNFSTIKYKDMLGQGTAISDLPEVKRVRETQKHISSVLYKEPSGKGTSVIFTPEME

RVKRNQEHISSVLYSDSFRKQVQGKAAFVLDTPEMRRVKQTQRIISGVQYHQDFEKSKGS

FTPTTTDLVTERVKKNTQDFSDISYRGIQRRVVEMERRRAIEHDQETITDLRVWRTNPGS

VFDYDPAEDNIQSRSLHMMSVQAQRRSKEHSRSTSAMSGLGEEKSEPSESVDHHMSVYSN

GFQTHTTGYQQAKTVELQQRSSSVVTQQTTVSSVPSHPSTTGKTVRAMYDYAAADNDEVS

FKDGDVIVNVQSIDEGWMYGTVQRTGKTGMLPANYVEAV*

>m.734 g.734 ORF g.734 m.734 type:complete len:96 (-) Unigene000069:3264-3551(-)

MFSLPPSSFINKLSPKSENPKQKRLIAHFPANQKGSGCKYQRSHRVMFKLKKKKKMKIKV

TSGTGSLQQPLRCEIRSLMSEGKSWMSSHSHSNSA*

>m.724 g.724 ORF g.724 m.724 type:complete len:979 (+) Unigene000069:241-3177(+)

MATQVETLLHTTINTTTAALLQDDDLQSPVGHGELSPVPSSKQAGNENSPQENMEEKKEF

KEAPPPKVNPWTKKLNSVTVNGQTAPEQTGPAKVVRAGNPRPRRGGKVGDFGDVTNWPTP

GEIATKEVQGCKKASVKKDSREKRDSEECKENQKAKSDDSGEEKNREEDLHKNNAHRNKH

KWVPLMIEVKSEGPRERSASRNNARQGETPRMHHSNRNDLRDWHSGKYERDQFRDDHDEV

SSVKSEGAPFRGGFRGRGRGRGRGRGRGRGGRGQYDYPYSYKCEGKDGVYDPKYTQSVTY

YYDSMSSAELYSVDQDLLKEYIKRQIEYYFSMENLQRDFFLRRKMDEGGFLPVALIASFH

RVQALTTDIQLIMEALKDSKVVEIIDMKIRCKEEPAKWPLPGLTLSEHTHTDFSQLINCP

EFIPRNTSDTPHTGSPRVSSAAEQKLEEVCNLKTMPKGLSASLPDLDSECWIEVKKRPRP

SPARPKKLEDLRSALTPGDQDEPEELDFLFDEEMEAMDGRRNTFTEWTDDESDGEIDDHD

VNKILIVTQTPPYLRKHPGGDRTGNHVSRSKLTNELVKVINDGLFYYEQDLWHDTYEPEY

ATIKQEVENFKKVHLISREQFDCLTPEPPIDPNQEVPPCPPRPQHIPTDDLANKLFGAPE

RSVMARSLPTAVPDSPAYRAPHTPHTPRTPRCKHNTPRFYPVIKDGRPVDAKTPRKRKTR

HSSNPPMECHVGWVMDSREHRSRTASVSSNASPSEGTAALGNIGCTPQSLPKFQHPSHEL

LKENGFTQHVYHKYRRRCLNERKRLGIGQSQEMNTLFRFWSFFLRDHFNKKMYEEFRQLG

VEDAKEGYRYGLECLFRYYSYGLERKFRAEIFKDFQEETIKDYEAGQLYGLEKFWAFLKY

SKAKNLEIDSRLQEYLCSFRRLEDFRVDPPMDEGRKRHSSSGDGRRRHPSQSSARHTHTS

DHNLDPKPGPAPTADCSK*

>m.738 g.738 ORF g.738 m.738 type:3prime_partial len:80 (-) Unigene000069:3-242(-)

MSPVLLSPNLNVLYNLNHKKVSLLIVFFFTPAPLRATRLRSEPQNGKFSLKHVKCCKKNN

NKSCSEPLGLTSPSATSTTR

>m.725 g.725 ORF g.725 m.725 type:3prime_partial len:574 (+) Unigene000069:5792-7516(+)

MHYKEAIRISPTFADAYSNMGNTLKEMQDVQGALQCYTRAIQINPAFADAHSNLASIHKD

SGNIPEAIASYRTALKLKPDFPDAYCNLAHCLQIVCDWTDYDERMKKLVSIVADQLEKNR

LPSVHPHHSMLYPLSHGFRKAIAERHGNLCLDKINALHKPAFEHPKDLKASGGRLRVGYV

SSDFGNHPTSHLMQSIPGMHNSEKFEVFCYALSPDDGTNFRVKVMAEAHHFTDLSQIPCN

GKAADRIHQDGIHILVNMNGYTKGARNELFALRPAPIQAMWLGYPGTSGAPFMDYIITDK

ETSPAELAEQYSEKMAYMPNTFFIGDHANMFPHLKKKAVIDFKSNGHIFDNRIVLNGIDL

KAFLDSLPDVKVIKMEGDGQESTDINGALSMPVIPMNTAAEAIINMINQGQIQVTINSFT

VSNGLATTQINNKAATGEEVPRTIVVTTRSQYGLPEDSIVYCNFNQLYKIDPPTLQMWAN

ILKRVPNSILWLLRFPAVGEPNIQQYAQNMGLPTSRIVFSPVAPKEEHVRRGQLADVCLD

TPLCNGHTTGMDVLWAGTPMVTMPGLGNRFTTNT

>m.740 g.740 ORF g.740 m.740 type:5prime_partial len:2175 (+) Unigene000070:2-6526(+)

DRKSVERAKVLFTAGQSVAAMEMLSSCALSYCHSGKCERAACRSILTLCKWLLADWKELT

PQLKQVVKRNSSGAPSNLSKNIAALLELPVEEQSILRITTETTVSVGVGEADFVLGQLYQ

LSASQAPEVAKSWAALASWAYRWGRKVVDNASQGEGVPLLPGEKKEIEELLPSGTTDEDK

EAIFSILGQAMCRPAGIQDEDMALQNEEDDDDMVDVIWRQLLSSCPWLAEVEDGVTEGLI

RVWRRVVDRIFSLYRVSCRAYFTFLKLNAGQTPIDEDDPKLLLNPQSSKQSSDDMIVMAT

LRLLRLLVKHAGELREGLELGLASTPTAPWRGIIPQLFSRLNHPEAYIRQSICSLLCRVA

QDSPHLILYPAIVGLISLGAEAQTTGIKLPSALPTLLGTMPGEPLCVGESEGGSPPASQE

SGRGDELGLCSVEDQAMMQDCYSKIVDTLSSANPTMVQQVQLLVGELRRVTVLWDELWLG

VLQQQHMHVLRRIQQLEDEVKRVQNNNTLRKEEKVAIMREKHSALMRPVVFALDHARSIT

AAAAETPHETWFQETYGEGINNALERLKNPQNPANPANSWVPFKQIMLSLQQRAQKRASY

LLRLEEISPRLGSMTHTEMALPGEVSATDAITIHNVGNTITILPTKTKPKKLYFLGSDGK

NYPYLFKGLEDLHLDERIMQFLSIVNTMFTKVNQQESPRFHARHYSVTPLGTRSGLIQWV

DGATPLFGLYKRWQQREAVLQAQKAQDSIQQPQNPPMVPRPSELYYSKIGPALKAVGLSL

DVSRRDWPLSVMRDVLRELMEATPPNLLAKELWCSCTTPSEWWRVTQSYARSTAVMSMVG

YIIGLGDRHLDNVLIDMTTGEVVHIDYNVCFEKGKSLRVPEKVPFRMTHNIETALGVTGV

EGIFRLSCEQVIQMMRRGRETLLTLLEAFVYDPLVDWTAGGEVGFAGAVYGGGGQQAESK

QSKREMERDITRSLFSSRVAEIKVNWFKNRDEMQAVLPQVEMAVEEYLNLQEQLSQVDKA

QGKLLEEMEFLEGADSRVDHSIHTLEHRYSEHTQLQSRQRTVQDDIQSKLSDLDQWISQY

QAAFGSLEATQLASLLQEISSPIDLGPPSYVPATAFLQNAGQAHLISQCEALEAEVSALL

QQRRSILRACLEHLHSYATVALLYPRAVLHRHRVYTWKQWMEELMCDMTVENCQAIYHHY

EMQFAPQPPTATCQFLSSVELALQHHAAETNTRLLRQMERLKAEGASVPVCEEQLQEIER

CIKVFLHEDAELASFSLAGIIISALCTLTRRNLVMEGAAASAGEQLVELTSRDGAWFLEE

LCSMSGNVTCLVQLLKECQLQSHELDILCLEDTSQAVYLANGVYTCLQELNTNFRQIIFP

EALRCMLKGESTLESMLSELDSLIEQCADGVSLQGLAEALQTHLRNASMGLQEDVDAHYL

QVTRVLRVQYSELIQPRNMEGSIQDTPKMSAGQMLLVAFDGMFAQLETAFGLLIEKLNSM

DIPTAWRKVDVIWEARATQVHFFDTVQTRQVLEEIFFLKRLQTIRDFFRLCASFAQTLSG

TCPPPNDEPPPSNGPVSLVKPIYRTSTVVNEDQMTRPIKAFTADFVRQMLMGLPTQALGL

ALCSALSALGSDLIAQVEAKDFGAEGKVSLDDLCKKAVEQGVQAGRISQLLLNRATLLAS

SYDTAWKKLDLVRRLELSIDACKVSLQRAQLHIAMFQWQHEDVLGTRPQPMTVSPPPRSI

ILSNMKKKLYKLSQDDAAIASVQEKLASLEGSIEQRLKWAGGANPALAPVLQDFESTITE

RRALVVKESQRTTQVTFLCSTVLNFEGLRTRTSEALSMDAALFDLVKRCQATCSYAAQFS

STVSTLELQLLHRLSPVMEQSIGTPEWLACAQKHLAQEMANQRAAQEEREQQLDSVTETL

QLLVDTIKGTLSNHNRLLADVKHLLRAMAKDEESALAEGEEVMYEGSVRQFLCEYKGWQD

NVQIVLFTVVQATGQPRSLEQVELLQEIPPTLKELKGQSQRVYNGLVGFAFPLVTDRGGD

CASPTATVQTSFAAAVRCSGVKTQPDSMSQNARKALPRNLGTPADTPPSTLLITSKSLMP

SPKRTVRDPKTGRAVQERNSYAVSVWKRVKAKLEGRDVDPNRRMSVTEQVDYVIKEATNL

DNLAQLYEGWTAWV*

>m.748 g.748 ORF g.748 m.748 type:5prime_partial len:2317 (+) Unigene000071:2-6952(+)

RKSVVQEDSYCAWELTLFWSKLLKRLEPSEQSFLEKCQKMSLLAKTVFHLLFFIKVIQSE

LDKIGLPACIEMCIRALRMENCEGANKATICKTISCLLPSDLEVKRACQLTEFLLDPTVD

SYYAVETLYNEPDQKLEEETLLIPTSLRCDLFLVFKTQWSFDPEFWDWKTLKRHCLGLMG

EEASIVSSIDELNDDRFLEIERDELDSAQEEFKDVFECFLDTTKELKEISDHRQKNREVK

KLREKGFVSARFRNWQAYMQYCVLCDKEFLGHRIVRHAQTHFTDGYYSCPICTETFETRE

TLDPHVASHVKLSCKERLAAMKTTRKLTGAKNSASNDAPLDNKSGENQARKAKAKHCNGE

SDDVYNMGSCIEDAGVRVHRKEFNVCPVSSCRKVFKFFRNLLTHVKDHGDVEEAKRFLEL

QATKIVCQYCRRHFVSVGHLNDHLQVHCGAKPYTCIQLNCKASFDTNAELLVHSKRHPCF

KAKCMFPGCGKIFNEAYKLYDHEAQHYKTFTCKVPGCGKVFHAQSQLDMHKESHNPPKLE

TQPAGSVSPLGEPQSHLSSEPAQQDAHSGPPADSGCMISSEDQSTGLVKVKHSIENMLSV

AGQGLSQEFGHVNCKAEPSVPNLVQQPPMPHMVNTEQIQPQLTPSMPSLIEPRSEESLLD

ALMNDPMLTSSCSLPPASSYQSLLEDFQQAPSGDVLQAQMESAVSQGHTQSLYSNENTEY

GQYNIKPETPISYPPPYDNTVPLAQNGHNDPPVLPVSQRLPPQLTMFSSNRPVQALQANT

LSSVVEEKDRYKCAYETCSRDYSSYRSLTKHMKAAHCEFYTQWKLAKRSNKVSPNTCSAS

SINGKCMSAEPSQNQLSQRTPAPQNPVQKPPAQTHSPASCLSSHYPSGSSHLTGPNGQTF

PNQMDSILNPIVLSHLGSIANQSNVVSHSSWNSISVNTSLRQQTCHSQAMTSSSGQFPSQ

LDTVPQTQLSDSYNRVMAYPSLQHQNSPSCRFIQTRPCAPQTINSPAKISHPSDSSLPQY

VQQPKTSSKSTLKHTDCVVKLERNPILIPSHSNLLQQSNPPTAMKPETCQKDISGFAHDG

DVKKRKRSSRTKWPAIVKDGKFICCRCFREFQSPKSLGGHLSKRTVCKPYDEAVLSADLP

SSFLELLNSPHPSETPQSSPSTQVTSSKAWPSLTKGPLDPKLFPNVTFLNTKNSTYNSGT

IHQNSPSLEKGVTGEQTFPESSGSYHQKSKNSVIQHTGNIRDKCIYDLMNYTQPCNTLPS

DTSSSEQLLSHMLSENKTLDSNLSRSPNNYVDRILQAETLNKIREIKEEPTRTNISGLSN

DGLLAAMASLAQNLVSEKSVKERLREQILAGDFQKRSSLGQGQGVENSNSQSPMTDVPLG

LQTAVQSKEHIVRRASELGELYTSVVPDETLTNISLAGASNNRETNNAFSNSLEQEDDKD

LSEILKSFERLDLNKEIEKNEQTTSNLLEAERVTKASEATCTLMLRGYSCDNDGCGYRAM

TKDALFKHLVKQHNYTEDMMNQCRKEQSNLAPFNCQLCPKTFTRNSNLRTHYQAVHNYSH

EQVVKLGITRQYSRKSIEADQGLCTTLNISELASPVFAGQNAESGAGITALVSGVRSIKS

EFSVQPFEQQHPGASFDSCPVSSTILRAQTTPHHLQSQTTVHLSVIKATPSLLTSTSVEQ

QLNRPNEFVPESLVPSQHTQGFLSGGLSSSGAPLLAPISLASAQLGSLSSDTKPKFGKPK

MAKVKEVTKKPKEKKLDMVDVFSPYRPYRCVHQGCVAAFTIQHNLILHYKAVHQSALPKF

EVNEDEEPIEEEHNENQEEIGMPDSEVNEEFRCQVKDCSRIFQVVTDLLQHYLQLHKLGL

DKAGIMMSGMNLGRFRCDQPDCHVTFTGFWKYVNHIDNEHKHVKLSRTEPVNGMFQCEVE

GCSCVYATRSNLLRHFMKKHPELYKLRLLNSGGVKLGRPPKNRFVSLEKENREINKKTFQ

KGGAKKKRRQKNLWTKYGNPILKSKEEASSMCTKRIQLQYPCMLKGCETVAESERKLLKH

YVQHGLPKQYLEEQRSNYIFCKKMPRSKYKRIASRSDDTDKSDGSSIEASENEDAADTGP

SESEFSKPTSEKESTEDTEVSDAKLSSDLSSDISVVVKRKRGRPRKGEQVKRTQLARKRM

TRLRAAQTRSINYADMNSDSTSSSVTLPQEPNTSLSSFKPMGFEVSFLKFLEESSQSPGK

RKSCSLMDLLPHRKFPTVRLKTASVVCSRINPDYHHCRDVLKHVEFKNPQKLTSLSRVTF

EVHRGFSNVYELLLKQLHDMRPAVVIQKEGVSETRA*

>m.755 g.755 ORF g.755 m.755 type:complete len:2308 (+) Unigene000072:356-7279(+)

MDQRTQGGPTTPPPLPANALPVECEKEGGGGKKDDEDEKKKREKERDAVPVVSCNSESAA

GDQQQQQSPQFSVKETSYSEGNVKLKIGLQAKRMKKPPKILENYVCRPAFRATVRHGSRG

GGRGSRRVEPPNVNTPCLPHIKDAEKDPNENLGVSQSSAVLPNNIPTPSLPSSTSTNPEI

HVNANVPAKRGPPKLACKSEEIVDVKSIAFSDKSLKSHCPLSDSKSHSSGKRSLAPQTGP

PAPLLQTPTSNDSPHDPKIDRVTKPLQYTYTDSQREQERGAPNWGTPTVTEKLAQLIATC

PPTKCPKPKARKVSPAPSHSSSPPLISNLQRPERAMANRNTYSRVAHLAPPPPVSRPPGR

PYGSRNKDSILDKLSGIPRKEETSGLSKEITSSKSICSNISPVPYNSRLTNNDSVDYDNN

NELKSQSRLESHLSTPSLFSPALERIGSGISHQIMPASLGLRITASPSQTEEPVDIEKQR

ERDIFNVREMNKGSSTAVLGKQDSKEKGSILTSGERRKSLSPAKRNPAQESHACEELGAV

ESTRRTASPSISTGTSSSFPTLCDDHDPASPGSPAEQESKPLKKRRGRRPRWTKAVSRTH

RAIDDASPSQTKITTLPTDSSSSQIIRRPVGRPPNANRMLPVVSELYSQPLKKRGRPKSK

MPTLDAPARGRLPLKATPPKVYLMSKEEQDPPVLHPEVDLNPPKPMPRKRGRPKRLPPTL

PQETQPPTLAPEPGDTVAGDKGFQNKGNGQLIMKTIIGKINKMKTVKRKRFLSQILLGSG

SGTEPEVTKSTGMVGPVASTSQSLSSLAATFGGKLGPQINVSKKGTIYMGKRRGRKPKAP

VNNSPGAPTPPPEPFLTPNTSSPHHSHQSLASQNQIPPSDIFPSPSLSQSSGGQSPISEA

SFVEPGSGHLISHSHFSHHSFPFTPPTLTAPSPRTLGGSTSSHSSQKKATCRGHHYHHHH

YRQHYHCRKLSPPRPLLPTSPAPLSELKEATPSPVSESHSEETVPSDSGIGTDNNSTSDR

GEKAGNACSLAGLGVSQSLTNGLLMPGLMGSSVGIGLGHNFRGSRRHSSTVLLDCPSPTP

SPLGERSTANPRRPHPLAQASALVGHKEKHKHKCKRRGHGCPGYDKLKRQKRKRKKKYLQ

LRSRRQDPDFLAELDDICVRLCGIRISHRTPALRLGSSLGLSAGTAGTSHGAGLTGSRAG

SSIGSGTSTTPHHYLHRDLLPTIFRINFSGYYSPHPAYPCDPLHYVRKPDLKKKRGRPPK

LRESMSEVPFVPGLGFPLTSGGFYHPSYSVPYSSAPLGLGYYRGYPPASALYPHPHHQAT

HSGPSHHSHHSSSFPPPPPPSYVHHHHPPHLLLNPSKFHKKKHKLLRQEYLSGGRSAVLY

PPMSSELSFSWHHKHKHRHKHRDRCGEEEGDDGGGVSGGGGRNGGEGIGTGRGERARRGG

SLESLKHCGYGPDGSADSSANKQTAATSGNSPSSSSSSSAERYKQKEASMSCLVPSRLSL

SQGSRGQHPADSWFKIGSYDVDYSKLSHTQALGQGGFSDGHLHNSVVGCSDSEDEDHTSP

NEEQESGSHHTNLFASALTRTSLRCNQGRKGGRLEGPSLTRLDRSLRKELSTSAERRDSG

NCGIQTRYSQFSTPEVASHRHHHTPLPAPHSSSCLSTCGLEQPGESPSARSSQPACCLDS

SPSCPSQCSAPLTPKNNLHHVNKILRAKKLQRQARTGNNMVKKRGPGRPRKNPLPSPSPV

PPTPSPPPELEPEKHIEEWQEDTVTDAIESVIQRQKRKGRKRRYWEADRDENAEDEDETM

HEREEEEEEEARVEDREEQLVTQGPLTGGKGRLAHEEMPSFQSAVEGKSDGHSSLECAGT

TPTVQAPPTSMTTQREKKAARPPKKKFQKAGLYSDVYKTDDPRGQLLQLKKEKLEYIPGE

HEYGLLPAPIHVGKYLRQKRIDFQLPYDILWLWKHDQLHKRPDVPLYKKIRSNVYVDVKP

LSGYEATTCNCRCPEDCNAKGCSDDCLNRMIFAECSPNACPCGEQCDNQRIQRHEWVQCL

ERFRTEGKGWGIRTKESLRAGQFIIEYLGEVVSEQEFRSRMMEQYFSHSGQYCLNLDSGM

VIDSYRMGNEARFINHSCEPNCEMQKWSVNGVYRIGLFALKDMDSGTELTYDYNFHSFNT

EEQQVCMCGSEGCRGIIGGKSQRINGLPGKAGGSGSGARRLGRLKEKRKSKHHLKKREEE

SGDSSKFFQHLLMKPMSNRERNFVLKHRVFLVRNWEKMRDKRELSKRDGEREREKESSLS

QYARWGGVIRDDGNIKSGMCMFAWLPV*

>m.764 g.764 ORF g.764 m.764 type:internal len:2440 (+) Unigene000073:1-7323(+)

ACFLISSQSWGGKENGFGLAECCRDLPMTKYPPSATTLHFEFYAEPSSEVKVEKKSSSNT

LHYIHIEQLDKISESPSEIMESLTVMYNIPKDKQTLLFTHIRLAHGFSNHKKRLQAVQAR

LHAISILVYSNALQESANSILYNGLIEELVDVLQITDKQLVDIKAASLRTLTSIVHLERT

PKLSNIIDCTGTASYHGFLPVLVRNCIQAMIDPIMEPYPHQFATALFSFLYHLASYDAGG

EALVSCGMMEALLKVIKFLGDEQDQITFVTRAVRVVDLITNLDMAAFQSHSGLSIFICRL

EHEVDLSRKECPFVIKPKIQRPSTTAETEDMDTDMDVSEVAMESSPGPSTSTGSKSDPDP

RTQTSTVSAPRTGVQCIPQRAALLKSMLNFLKKAIQDPAFSDGIRHVMDGSLPTSLKHII

SNAEYYGPSLFLLATEVVTVFVFQEPSLLSSLQDNGLTDVMLYALLIKDVPATREVLGSL

PNVFSALCLNARGLHSFVQCQPFERLFKVLLSPDYLPAMRRRRSSDPLGDTASNLGSAVD

ELMRHQPTLKTDATTAIIKLLEEICNLGRAPEYICQKPSIQKADGTVTVPPARSNHAAEE

ASSEDEEEEEALHTFTQQQGEPESNRQVVGTEERIPIPLMDYILNVMKFVESILSNNTTD

DHCQEFVNQKGLLPLVSILGLPNLPIDFPTSAACQAVAGVCKSILTLSHEPKVLQEGLCQ

LDSILSALEPLHRPIEEPGGSVLLRELASAGHVTDATLSARATPLLHALTAAHAYILMFV

HTCRVGQSEIRAISVNQWGSQLGLSVLNKLSQLYCSLVWESTVLLSLCTPNSLPPGCEFG

QVDMQKLVPKEEKPSGSTVTSGGRRTAETESLSVGVDPSAQGLLEGMGLDGDSLAPMETD

EPTTADPKAKSKLTPAMATRIKQIKPLLSASSRLGRALAELFGLLVKLCVGSPVRQRRSH

HTTNTGTAPTPAARSTASSLTKLLTKGLSWQPPPYTPTPRFRLTFFICSVGFTSPMLFDE

RKYPYHLMLQKFFCSGGHDALFETFNWALSMGGKVPVSEGLEHPELPDGTGEFLDAWLML

VEKMVNPSTVLDSPHSLPAKVPGTTTTTPQFSAPRFLIVTQKAAFNCIRNLWNRKPLKVY

GGRMAESMLAILCHILRGEPVIQERLSKEREGTARAEEDGASVVGGASGGAAAGAVGESA

SGSGGSASTAGGTADDASATTRREPQVNQAQLTQLIDMGFSREHAMEALLNTTTMEQATE

YLLTHPPPLLSAAIREFTMSEEDQMMRAIAMSLGQEVSMEQRSDSPEEAARRREEEERRA

RERAEEEEARCLERFLEAEPLDSSELHAFTDTMLPGCFHLLDELPDTVYRLCDLLMTAIK

RNGPEYRDLILRQVVNQVWEAADVLIKAAVPLTTSDTKTVSEWTKQMATLPQASNLATRI

LLLTLLFEELKLSCAQIVESSGVLSVLIKLLEVVQPCLQAAKEQKDIQTPKWITPVLLLI

DFYEKMAVSSKRRAQMNKYLQPNGNNWRWFDDRSGRWCSYSASNNSTIDSAWRAGETSVR

FTAGRRRYTVQFNTMVQVNEETGNRRPVMLTVQRVPRMPKPNKSGNVADPEREEDQRAKT

EETQVLADPASVAVEMVAPKEEPELKVSSAPASAAPAASQDSSSGSNSIVVQGLTDEMTT

VLIRACVSMISVPVDPDTLHATLRLCLRLTRVHHYAMMFAELKSTRMILGLTQSSGFNGF

TPLVTLLFRHIIEDPATLRHTMEKVVRSAVTSGAGSTTSGVVSGSLGSREINYILRVLGP

AACRNPEIFTETANSCVRIALPAPRGAGTASDDEFENFRIKGPNAVQLVKTTPLKLSPLP

QIPETIKEVIYDMLNALAAYHAPEEAEKGEERLATVPGSQDLCQILQDVGDDVYQQYRLT

RQGSDFDSQSAFHINTQVFAADGAVAESSQSGTPQGEASTPEEMREDKKEQEGDKAGSSE

ENKAAMAKASKPLMPTSTILRLLAELVRSYVGIATLIATYNYTAGQSELIKEDCSVLAFV

LDHLLPHTQNSEDKDTPALARLFLASLAAAGTGTEAQVTLVNEVKAALSRALAMAEGTEK

HARLQAVMCIISTIMESCPSTSSFYSTATAKTQHNGMNNIIRLFLKKGLVNDLARVPHSL

DLSSPNMANTVNAALKPLETLSRIVNQPSSLFGGKGGSSKSKTEHDSVGTARDSNSNTQD

QGEAGEAEPVENSHRGQATDGDLMDGEAEGDTVVIAGQPEVLSTQAMQVENELVDLIDEL

LERDAGTVNSTIIVGRSGDEESQEDVLMDEAPSNMSQASTLQANREDSMNILEPEDEEHT

QEEDSSGSNEDEDSQDEEEEEEEEEEDPDDEEGDEDDDDEGSEMELDEDFPDINAAPHIR

FERFDRDDDLIIEFDNMFSNSADIPPSPGNIPSSHPLMVR

>m.772 g.772 ORF g.772 m.772 type:5prime_partial len:2237 (+) Unigene000074:1-6711(+)

KKNQIQVNDANYRTYLHNWTCLPDSNDVVQARQTYDIQSDAVYRADLKWLKGLGWIPIGS

LDVEKAKKAGEALSERKYRQPPSNFKFTSTTDDMPIVLAKANNNIMNKKAYVQAWDNEKS

NIHIMPDAMDVVLAKQNKVNYSEKQYKLANELSKKKGYDLRGDAISIIAAKASRDIASDY

KYKTGYRKQVGHHIGALSVRDDPLLMLALNSAKIASDVLYKKDFNKSKTKFHLPVDMLSF

ELAKKNQIQVNDDNYRTYLHNWTCLPDQNDVIQARKAYDLASDNIYKTDLEWIRGCGWVA

ADSVDHVKVRKAQQILNDRMYKKDAKDNFAKFTNIVDRPEVLLAKVNAFNLSDLKYKESF

NLEKGHYIGSDDTPQLAHSREAAKIISEKLYKLGWDEAKATGYQLNHEYLPLVSAKKVGS

IVSEAKYHDAHEKAKGHYLANTLVDFPAVVHSSQMEKMKNMRTYQKDYHTSKTKLHIPHD

MISHVVAKKCQEILSDVLYRSYLHQWTCHPEQNDAIRARKANEILSDVFYKDDLNWMKGI

GCYAWDTPEIVRAKKSYELQSDIKYKAEGKKEFNNYSIVTDTPVYVTAILGHTWASELNY

REAYHKEKHLYTTVLDTYDYARCYNLKQLYSSKTYSALWDRIKAKSYTIPIDANALVHAK

QQKVLLSKVKYKEDYEKFKSLYSLPKCLADDPATARCIKAGKLNLDRLYKDKWEKTKAKI

HIPPDMLDVVAARNTQKMISEVDYRKYLHQWICLPDMQVYVHARKVNEQLSDIFYKEDMT

WLKGVGCYAWDTPEILRCKQAMNLQSENLYRAKGVEHFKNFSVVTDTPVYETCKQSAHNL

SDLNYHHDYVTNVRGKNTAPATTVDTERARLANYIQSDHIYKEASKSNMSTGYSLPFDTP

LALQAKANNVITSNVKYKEAYENTKARSYKLDPNGVNFVTIRKANQVTNQRLYRALYEKE

KDKVHSVYDTPEIKQVKATQEAISDVCYKEKYYNSRGTLLPMPFTPQLMHCAHVNQINSD

LKYKEDLQWLRGIGCFVFDSPEMVRIREINKFRTSYDIDAKKNFSNFSVVLDTPEYKRVS

ELKTHLSERVYKAAGNIQRTQCSTTGDAMEFKRAKWAQTLTNKWLYTDLASKERAFYTPE

LHTPLLDHARSMKVVYSENKYKEQYEKMKHKYTPVHDTPILIRAKKSYLNSSDLRYKETF

ELAKGHYHTTKDALDIICAKRVRDDISEVKYREKYINSLGTWKSIPDRPEFFHSKVVRDC

ISDYKYKEDLEWIKGVGCFVWDTPLLKQAEHNKALYSERLYKASYEKTKCNFKYTCDTPF

FQAVKNASALSNDFKYRIRAKELLKSGCNELRRPDLLTAIYNTGMWSKWKYRSQYERLKS

KYTSVLETPEHKVHKLRKQISDKIYTMEYNKNKAKGYTLGHDTPLNLHMKKVKEITSNLK

YKEVYEKNKAQINIAPDAFDIRAAKEAYKNISNLDYKKKYEATKNKWIWTVDRPDFVHAA

KINYQQSDVEYKYDKEMLKGCVMPVTDDKYTLLAKQNTELSSDIKYKQKYEEARGHYHVV

HDTPQILHAKSVSGLVSESKYKLESKKARQSGSFTTLPETRDTAHSKAMTKITSSKVYKQ

KFEKDKGKSIYNNMTVPPDVKHAMDVAKSQSNINYKQEAKAKLHYTPVADRPDILKATQA

AKLISEVGYRDKARQEASRGGSLVNRPDINLATEVSKLTSLVKYKEKFDKEMKGKRPQYD

LKNSKIYQTLKDANTLASEVKYKGDLKKIHKPVTDMSESLAMQHNLSTSKLASSVKYKEK

YERERGKAMLDFETPTYVSAKEAQHMQSQREYKKALEQEIKGKGMLALANDTPDFMRARN

ATEILSQSKYKHNAEQDRASYTTVIDTPDIIHAQQIRNIVSQKKYKEEAEKTMSHYNAVL

DTPEMQRVRENQRNFSTVLYKEPSGKGTSVIFTPEMERVKRNQEHISSVLYSDSFRKQVQ

GKAAFVLDTPEMRRVKQTQRIISGVQYHQDFEKSKGSFTPTTTDLVTERVKKNTQDFSDI

SYRGIQRRVVEMERRRAIEHDQETITDLRVWRTNPGSVFDYDPAEDNIQSRSLHMMSVQA

QRRSKEHSRSTSAMSGLGEEKSEPSESVDHHMSVYSNGFQTHTTGYQQAKTVELQQRSSS

VVTQQTTVSSVPSHPSTTGKTVRAMYDYAAADNDEVSFKDGDVIVNVQSIDEGWMYGTVQ

RTGKTGMLPANYVEAV*

>m.779 g.779 ORF g.779 m.779 type:complete len:2299 (+) Unigene000075:305-7201(+)

MDESSILRRRGLQKELSLPRRGSFCRTGNRKSLIGSGQSSGLPRPHSPFSSHTGTSPQDS

PRNFSPSTSAHFSFARRTDGRRWSLASLPSSGYGTNTPSSTISSSCSSQEKLHQLPFQPT

ADELHFLSKHFCTESIAGDDHRRAASMRPRSRSLSPGRSPSCCDHEIIMMNHVYKERFPK

ATAQMEERIQIIISSSSPENVLPLADGVLSFAHHQIIELARDCLEKSRLGLITSRYFCEL

TDKLERLFQESTERSESAEVTFIKELVKKILIVIARPARLLECLEFNPEEFYHLLEAAEG

HAKEGQGIKTDIPRYIISQLGLTRDPLEEIAQLTSYDSGIAETPDTDDSVSSQSLSAPPQ

PRRKPCEIDFEMIKLISNGAYGAVYLVRHKETNQRFAMKKINKQNLMLRNQIQQAFVERD

ILTFAENPFVVSMYCSFETRRHLCMVMEYVEGGDCATLLKYMGPLPVDMARMYFAETVLA

LEYLHNYGIVHRDLKPDNLLVTSMGHIKLTDFGLSKVGLMNMTTNLYEGHIEKDAREFSD

KQVCGTPEYIAPEVILRQGYGKPVDWWAMGIILYEFLVGCVPFFGDTPEELFGQVISDEI

NWPEGEDAPPADSQELITLLLRQNPLERLGTAGGAYEVKHHQFFHSLDWNSLLRQKAEFI

PQLESEDDTSYFDTRSERYHHLETEEEEDTNDEDFNVELRQFSSSSHRFSKVYSSLDLSR

GQLEEKGEQPEKKSESPLTVDSLSWTPDFTEIPSLSHSSDLESSTNSLSRNSGLLPKFAI

SAEADSCDSSLSLDKASKVAFSVGELPQTEPDAITPSSPVSNATLSGSFSEHLDQLISRP

EGADSLDPPNKPSTDTVSHLTSLRPATSIPKSSSTSALSLMIPADVFGTSPLASPMSPHS

LSSNPSSRDSSPSRDSSLSAANSRQPIIIHSSGKKFGFTLRAIRVYACDSDVYTVYHMVW

NVEDSGPAQKAGLKAGDLITHVNGETVHGLVHTEVVELLLKSGSKVAISTTPFENTSIKT

GPARRNSYRSKMIRRTKKPKKEKTQERRRSVFRRFAMQPSPLLHTSRSFTSLNRSLSSGE

SLPGSPTHSLSPHSPTTPFRPTTELNQSGGTSSQSSSPSSSAPNSPAGSGHVRPNTLHGL

GPKLPGQRLRQSRRKSAGSIPLSPLARTPSPTPQPTSPQRSPSPLLAHSVGSSKTTQTFS

AKMHSPPTIVRHMVRPKSAEPPRSPLLKRVQSEEKLSPSYTGDKKHLCSRKQSLEVTQEE

VQTGFASGGEHTLQCVEESSCELPTITRVRPAEQGCLKRPVTRKVGRQEMMEDLDKDKLK

SNVVAKRQDWHERRESLQKQDAIQEAESTQSLTGCCSVDNKVVTATVKDVLYKKLNSRAC

ESIADSMSSTGDSASVFNESIRSSPAQSDRQLARQIKEGSKPDRHDFKAPNMEFARKRQS

FEEREDCLCRITSGVHESLHFNATRSKSLQLDSAGIATPGITTDSLSPKIFSGRGESAVE

KLQIISSTEGSIRKTSSEYKLEARLVSSLKPLKGTLDIGLLSGPRISKTDTCLSKIASSQ

ADSGGQVGAMQNQSEKQPLMLTHKPTQAALEIKSPGASSSKNAGKDEPTCSTDYVFGLFS

HEGTHDKNKQGEGIKFQTVASKMEPKDLSTGGKTENKLKTAQEMRPARHSNHFSCGKTPS

IREVSNEDQDDEMENPVEKPTLQNNTVETKTPMTHDQVITQNTSLPSGPCSVDKDKNVRE

ILISAQKSEQAFANTNKITMAKDRVLNSLGLTVVTHEVYQCKTVQDDASGSRSSEALVQT

LPPKPDRTVIVQKTGSDVKAGIVAIDEKKTLSTTGKSTDKKHEQITASSTPVLHAKTEPF

HSEGLVKEKVSVPRSSTVTTSSPSAASIVPDIKLPPNLPLQTKQDKNNVAPKGTPVKATS

AVVPQLPQHVHKSPQSFAEIQSDSTAINQSQQTTLKTPTGADSGNGQKRLNSISRTDDQN

AEVENGKRSSSKCQETCGVKTQIVDKDHKTCMVPEGSIDKSKITSSKDKKAQNIAVSKEE

VKCIMTNEPRSPTDLKTDGAKCVLATAHTPPSKSNQYSGVKGSEKNSNTVQVKSVNILEE

KLKDKNTPDVSCQKVTKPVLDVCKKEVVTATKKQPQLTGTSSNGKQGLSSSKSKAKLEPS

PATNQSTKATEGRGTQEGKAPVQVIIDHTNLKPKEQREVTLPTLKTQSQSENLPQTTSPQ

VGSVPVPTVKVHTPSGSPIGKGTGSTVETSKSPDSMQKGNRKDCQKSSGLAKDGAAPEKD

SPRTKQSKDLPRGSNNKK*

>m.791 g.791 ORF g.791 m.791 type:complete len:96 (+) Unigene000076:6063-6350(+)

MEATPFKWFMEMCVRGYLAVRPYMDAVVSLVTLMLDTGLPCFRGQTIKLLKQRFNPNMSE

KEAAAFMIKVIERCFLSSRSKTYDMLQYYQNQIPY*

>m.787 g.787 ORF g.787 m.787 type:complete len:2022 (+) Unigene000076:58-6123(+)

MAAKGGTRGFYFNTVLSLARSLAAHRPAPIDKVQKLQCMCPVDFRGVYQLDERRRDAVIA

LGIFLVESELQHKDPIVPYLLSLLKGLPRVQWIEESSERKGRETLPVAENFSFCLVTMLS

DVAQRDETLRIQILEAVMDIMQVLLDLCRNPEDQDKEYLCRYTLPCLLGVVRASGRYSNT

DEALLSKLFPRTSTQPVIASEEPESVRRRSFNDFRSILPSSLLTVCQSDTLRRKTSSVSS

ISQQASPERTGLPPSSPSDPSAPYFEAGSYLPDGSAVDPDYYFSTISSSFSVSPLFTGCS

TRGFGVPLDMLRRLLQMIEQFVAESFLKKLDQTMAEFLESNPGHHLFYKTFSDPLYVAIF

KMLRDTLYYMKDLQTSFVKEVHDFVLEQFSSSQSELQRILHEVESLHSELSPLKLRCQAN

AACVDLMVWAVTEEQGAENLCTKLSEKLQSKTSSKVIIAHMPLLICCLQGLGRLCERFPV

VAHSVTMSLRDFLVVPSPVLVKLYKYHSQYASGTGEIKIHVTNEHSQSTFSAHASKKSQP

SMYEQLRDISIDNICRCLKSGLTMDSVIVEAFLASLSNRLYISQENDKDAHLIPDHTIRA

LGHIAVALRDTPRVMEPILQILQQKFCQPPSQLDVLIIDQLGCMVITGNQYIYQEVWNLF

QQISVKASSMVYSTKDYKDHGYRHCSLAVINALANIAANLQAEQKVDELLVNLLELFVQL

GLEGKRASERASEKGPALKASSSAGNLGVLIPVIAVLTRRLPPIKEAKPRLQKLFRDFWL

YSVVMGFAVEGSGLWPEEWYEGVCEIATKSPLLTFPSGEPLRSELQYNSALKNDTVTAPE

LTDLRSTIINLLDPSPEVAALINKLDFAMSTYLLSVYRLEYMRMLRSLDSDRFQVMFRYF

EDRAIQKDKSGMMQCVIAVSDKVFDVFLQMMADKPKTKAHEEELERHAQFLLVNFNHIHK

RIRRVADKYLSGLAETFPHLLWSGRVLKTMLDILQTLSLSLSADIHKDQPYYDIPDTPYR

ITVPDTHEARESIVKDFAARCGEILKEAMKWAPSVTKSHLQEYLNKHQNWVSGLSQHTGL

AMATESILHFAGYNRQSTALGTNQLTERPACVKKDYSNFMASLNLRNRYAGEVAGMIHFL

EATSSTSDLNKLMIRQLNEALERQQPEEFTEAMFKMAALLITSKDCDPQLLHHLCWSPLK

MFTENGMETAIACWEWLLAARTSLEVPFMREMAGAWQMTVEQKIGLFSEAEVEADPLAAS

EESQPKPCAPNAIPHYIWIEFLFQRFEIAKYCSADQVEIFSSLLQRSLSLTVGGSNSSLN

RHVAAIGPRFRLLTLGLSLLHSDVVSNATIRNVLREKIYSTAFDYFSVASKFPTQGDKRL

REDISVMIKFYASILSDKKYLAASQLVPLDNQDPSLNSLSVMTAADPRNTMDMSVGTRQQ

SAQGWINTYPLSSGMSTTSKKSGISKKSNRGTQLHKYYMKRRTLLLALLASEIERLTTWY

NPLSAQELAISTEQSVETSIANWRSKYISLSEKQWKDNVNLAWSISPYLALHLPSRFKHT

EAIVAEVTRLVRLDPGAVSDVPEAVKFLVTWHTIDADSPELSHILCWAPADPPTGLSYFS

SMYPPHPLTAQYGVKVLRSFPPDAILFYIPQIVQALRYDKMGYVREYILWAAQKSQLLAH

QFIWNMKTNIYLDEEGHQKDPDIGELLEQMMEEITSSLSGPAKDFYQREFDFFNKITNVS

AIIKPVPKGEERKRACLKALSDIRVQPGCYLPSNPEAIVLDIDYESGTPMQSAAKAPYLA

KFKVKRCGVSELEKEGLRCPSDSLEEGGENSEAAQKVCWQAAIFKVGDDCRQDMLALQII

GLFKNIFELVGLDLFVFPYRVVATAPGCGVIECIPDCKSRDQLGRQTDFGMYDYFRNKYG

DESTLAFQKARYNFIRSMAAYSLLLFLLQIKDRHNGNIMLDSKGHLIHIDFGFMFESSPG

GNLGWEPDIKLTDEMVMIMGVKWKPLRLSGSWKCVCVDISL*

>m.793 g.793 ORF g.793 m.793 type:complete len:2290 (+) Unigene000077:305-7174(+)

MDESSILRRRGLQKELSLPRRGSFCRTGNRKSLIGSGQSSGLPRPHSPFSSHTGTSPQDS

PRNFSPSTSAHFSFARRTDGRRWSLASLPSSGYGTNTPSSTISEKLHQLPFQPTADELHF

LSKHFCTESIAGDDHRRAASMRPRSRSLSPGRSPSCCDHEIIMMNHVYKERFPKATAQME

ERIQIIISSSSPENVLPLADGVLSFAHHQIIELARDCLEKSRLGLITSRYFCELTDKLER

LFQESTERSESAEVTFIKELVKKILIVIARPARLLECLEFNPEEFYHLLEAAEGHAKEGQ

GIKTDIPRYIISQLGLTRDPLEEIAQLTSYDSGIAETPDTDDSSQSLSAPPQPRRKPCEI

DFEMIKLISNGAYGAVYLVRHKETNQRFAMKKINKQNLMLRNQIQQAFVERDILTFAENP

FVVSMYCSFETRRHLCMVMEYVEGGDCATLLKYMGPLPVDMARMYFAETVLALEYLHNYG

IVHRDLKPDNLLVTSMGHIKLTDFGLSKVGLMNMTTNLYEGHIEKDAREFSDKQVCGTPE

YIAPEVILRQGYGKPVDWWAMGIILYEFLVGCVPFFGDTPEELFGQVISDEINWPEGEDA

PPADSQELITLLLRQNPLERLGTGGAYEVKHHQFFHSLDWNSLLRQKAEFIPQLESEDDT

SYFDTRSERYHHLETEEEEDTNDEDFNVELRQFSSSSHRFSKVYSSLDLSRGQLEEKGEQ

PEKKSESPLTVDSLSWTPDFTEIPSLSHSSDLESSTNSLSRNSGLLPKFAISAEADSCDS

SLSLDKASKVAFSVGELPQTEPDAITPSSPVSNATLSGSFSEHLDQLISRPEGADSLDPP

NKPSTDTVSHLTSLRPATSIPKSSSTSALSLMIPADVFGTSPLASPMSPHSLSSNPSSRD

SSPSRDSSLSAANSRQPIIIHSSGKKFGFTLRAIRVYACDSDVYTVYHMVWNVEDSGPAQ

KAGLKAGDLITHVNGETVHGLVHTEVVELLLKSGSKVAISTTPFENTSIKTGPARRNSYR

SKMIRRTKKPKKEKTQERRRSVFRRFAMQPSPLLHTSRSFTSLNRSLSSGESLPGSPTHS

LSPHSPTTPFRPTTELNQSGGTSSQSSSPSSSAPNSPAGSGHVRPNTLHGLGPKLPGQRL

RQSRRKSAGSIPLSPLARTPSPTPQPTSPQRSPSPLLAHSVGSSKTTQTFSAKMHSPPTI

VRHMVRPKSAEPPRSPLLKRVQSEEKLSPSYTGDKKHLCSRKQSLEVTQEEVQTGFASGG

EHTLQCVEESSCELPTITRVRPAEQGCLKRPVTRKVGRQEMMEDLDKDKLKSNVVAKRQD

WHERRESLQKQDAIQEAESTQSLTGCCSVDNKVVTATVKDVLYKKLNSRACESIADSMSS

TGDSASVFNESIRSSPAQSDRQLARQIKEGSKPDRHDFKAPNMEFARKRQSFEEREDCLC

RITSGVHESLHFNATRSKSLQLDSAGIATPGITTDSLSPKIFSGRGESAVEKLQIISSTE

GSIRKTSSEYKLEARLVSSLKPLKGTLDIGLLSGPRISKTDTCLSKIASSQADSGGQVGA

MQNQSEKQPLMLTHKPTQAALEIKSPGASSSKNAGKDEPTCSTDYVFGLFSHEGTHDKNK

QGEGIKFQTVASKMEPKDLSTGGKTENKLKTAQEMRPARHSNHFSCGKTPSIREVSNEDQ

DDEMENPVEKPTLQNNTVETKTPMTHDQVITQNTSLPSGPCSVDKDKNVREILISAQKSE

QAFANTNKITMAKDRVLNSLGLTVVTHEVYQCKTVQDDASGSRSSEALVQTLPPKPDRTV

IVQKTGSDVKAGIVAIDEKKTLSTTGKSTDKKHEQITASSTPVLHAKTEPFHSEGLVKEK

VSVPRSSTVTTSSPSAASIVPDIKLPPNLPLQTKQDKNNVAPKGTPVKATSAVVPQLPQH

VHKSPQSFAEIQSDSTAINQSQQTTLKTPTGADSGNGQKRLNSISRTDDQNAEVENGKRS

SSKCQETCGVKTQIVDKDHKTCMVPEGSIDKSKITSSKDKKAQNIAVSKEEVKCIMTNEP

RSPTDLKTDGAKCVLATAHTPPSKSNQYSGVKGSEKNSNTVQVKSVNILEEKLKDKNTPD

VSCQKVTKPVLDVCKKEVVTATKKQPQLTGTSSNGKQGLSSSKSKAKLEPSPATNQSTKA

TEGRGTQEGKAPVQVIIDHTNLKPKEQREVTLPTLKTQSQSENLPQTTSPQVGSVPVPTV

KVHTPSGSPIGKGTGSTVETSKSPDSMQKGNRKDCQKSSGLAKDGAAPEKDSPRTKQSKD

LPRGSNNKK*

>m.801 g.801 ORF g.801 m.801 type:complete len:2237 (+) Unigene000078:260-6970(+)

MESGERLASPSSTPVSVHTSSSSSSSSVSPASNAKSSLNHGAAASLAACGPLFGVTGSEQ

PFSVTSVTSVPSAFPVMAHPAFGLLSPGTARPEFGGLGALGVTAALAAHPQLGAFTEWWR

AAEAHGRSPAAFFPPFLGLPPLFAPPLQNHEATPYTSKTLSKSSQGPKGVNGAMNGSVVS

PSTTKSGGSVASSAALNTSGGKPRARKASQNSNSAGELQEKLSQKPKEKKPRKKQTEGSG

MSDSESGSSLDSDIEGVSSSDLDDLGEEDDDDDDDDQSEESDSEKEPHKKKKAKVVASNL

RPGKKEHSRTAEVWDLREGNEPPGACVSKSRDRPAQPTSVIQSTGLAVSTNPLALIGQSQ

HDSSPQRLSSSSPRPIASHQPLPLSLCSSPKPLSVPSPPKPLPLSSSPKPPSLSPSPRAW

GSAHKSQNSLSRRKHLESSLSHIADYRLKQSLLAHDQEFPLQLKKQQDLYKTSKNSGVRS

SSSSSSTSASSILPSKSTAGRTKAPGAQAVTGSPSLMLTQTLLGLGHTNGVIQNSTQDTP

LALTTKPRADLPVNLSTGGRKDTSLHTSSLAPLPALVPASVLPARPRASRKNKTPRALEV

PKDVSQKHLVKSLVDLFHHAAGEQETPSKKDSDESGEDDEDDEDDDVDDDEDDEDEDDSL

SESDSNSDSELNGSTRRNMDTTETETDGERTPLKLSKNLPLLASASASYSLADCSPLNLQ

VIKPSGMATPTIVSGSGALTYHSSPSSSYSVGTPPGSGKRKRVMNEEDLKTPLEIGWRRE

TRIKSAGGRLQGDVAYYAPCGKRLRQYPDVVKGLQWCPLSEDEVIPRIRAMEGRRGRPPN

SERQHGAGNGEGSGARRRKGRPPNVGHTEFPSPSEAKLLRKLEAQEIARQAAQMKLMRKL

EKQALARAAKEARKQQAIMAAEERRKQKEQIKILKQQEKIKRIQQIRMEKELRAQQILEA

KRKKREEAANAKIMEAEKRLKEREMRRQQAVILKHQELERHRLDMERERRRQHVMLMKAV

EARKKAEERERLKQEKRDEKRLNKERKLELRRLELEMLREMKKPNEDMCLTDHKALPEFS

RMAGLVLPGQVFSDCLMVVQFLRSFGKVLGMEQSEVPTLGVLQEGLLNLGNSMGQVQDLL

VRLLSSAVCDPGLPPGHRAKSILGDHLTNVGLNRDNVSEVLQQYMESHCAQTELADVALS

LKTKAFQAHTPAQKASILAFLVNELACSRSVVSEIDKSLDQMNVLRKDECIVEGKLKKLK

NIHAKRTGRREAGGGDESHATGTPSAGQKRKRKTNDSDDDEEEDDDSDDAVDDDDEDDEE

EVKKSRRVETCDEDEGDQATSVEELEKHIDKISKQQNLIRRKLFESSHALRSMCYGQDRY

RRRYWVLPQCGGVFIEGVESGEGPEELEKEQERLRNFKPVRIKEEPQEEGDEEEEEEEAQ

VEDAQQQQPEGKTQPQVKQEEEQRAEQEVKKEDEKPCSPPNKDTPLSDSASCPHKEPSKP

LGDLATLCCTPDVSMPSVTTVTASASSPPTSKPPVLCSAATDSVALVAQFGAPPPQFGIP

LSLQHHQHLLANDQLLRVLTERSGHWFSLLPRSPCDESSLIQPTTSNVQPRSPPARSCSP

HQHLQPPSASSSPSNAAHDGQGNPTVSPLQVKPGGALLSLPMCAWSGGMISPNLPICSSP

MPICPLAEGNASPLMAPSVSTSKSGSPPPPGDKPCALSPAIDLPRNHDQPQPQPIPEDML

LGWWKMIDMEELQVLMKTLHSRGIRERALHKQLQKSIELVSHTFNKNKEVAVMEVSELDE

GQVSVETLQEWCVEEQAMETDIALLQQVEELERRVTSASLQVKGWMHPEPQSEREDLLYY

EHKPLPKVCPGAELHEERGSEKGLRRQPSNPLDIAVMRLAELERHIERRYLRSPLGTTIQ

IRLDNVGTVTVPAPAPSTSAGGEGGEEEIAPGMKVWRKALSEVRNSSQLAMCLQQLQKSI

AWERSIMKVYCQICRKGDNEDLLLLCDGCDKGCHTYCHKPKINAIPEGDWYCPACISKAS

SQSPKNKKPQSRVQSGGGGKKSAETTKKSKKQQEACEEDEAVGGSSTSNSSPKKTATASS

QAKKSSPVPSAAKPESPACVKRAKTARDNNRDLGLCRILLAELERHQDAWPFLTPVNLKS

VPGYRKVIKKPMDFSTIREKLVSSQYQNLETFIIDVNLVFDNCEKFNEDNSDIGRAGHNM

RKFFEKRWTELLKQTN*

>m.813 g.813 ORF g.813 m.813 type:complete len:877 (+) Unigene000079:893-3523(+)

MSKINDKKGDETSNHSNASSSGSEESNHSGSELASQSESECGSEHEQSQHSESNNTSESE

TCFESGSESAGSKSRQTVAEIKEKRVRKRDLADVKKMWEEHPDIYGVRRSNRSRQEPARL

NVGAEVSSESGSESHKRKNSRQKKKENTWKDDCHDEDDCEEEVSSSEIEQEGEKNRSRRL

PARRPQAKFYTTKQNLSQKGRKLRMQETSDEDDEDDIVEEENTPKRQTRRRAATKVSYKE

DQHDFETDSDDLIEMAEGTEDVRDDDSETIEKVLDTRIGKKGATGAATTVYAVEANGDPM

ADFSPEKEGGETQFLIKWKGWSYIHNTWESLDSLSQQKAKGMKKMENFKKKNEELSAWLE

KASPEDLEYYNCQQELTNDLNKQFQIIERVIAMKTGKAQVPSDFPSHKTMSDEPEYLCKW

MGLPYAECTWEDGALIGKKFQHCIDCFNNRNVSKTIPSKDCKVLKQRPRFVPLKKQPSYI

GDENLELRDYQLDGLNWLVHSWCRCNSVILADEMGLGKTIQTISFLSYLFHQHQLYGPFL

LVVPLSTLTSWQREFSTWAPDMNVVVYLGDVLSRKTIRDYEWIHQQTKRIKFNTLLTTYE

ILLKDKGVLGNINWAFLGVDEAHRLKNDDSLLYKTLIDFRSNHRLLITGTPLQNSLKELW

SLLHFLMPDKFELWEDFEEEHSMGRDNGYQSLHKVLEPFLLRRVKKDVEKSLPAKVEQIL

RVDMSAQQKQFYKWILTRNYKALSKGTKGSSTGFLNIVMELKKCCNHGFLIKQPDEAENE

NSQEHLQAVIRGSGKLVLLDKLLTRLKERGSRVLIFSQMVRMLDILADYLAMKRYPFQRL

DGSIKGELRKQALDHFNAEGSEVSTITIYHGITQFL*

>m.815 g.815 ORF g.815 m.815 type:complete len:335 (+) Unigene000079:5636-6640(+)

MSKPGAGIYGYLCRNLPSLELRNFTGYTKWLKKSAPKKRRKSTKRKMNHSKRRHFVQNLL

DQVETPLAHNLHRKHAFHIITQQDFHLNHSTGSITTNIRDTSLMIIGEIGTGKENIPTQE

TVTSHGKVTDITLMTSTVIRSITTVTDGLTQTCTTVLASETRVQLVNECMTSTAMIETTV

FTVNIMTGIQNQRGDAQVTITLQTATKEEVEYVTTRTSGGCLIIRVSQRLITTGRLTQTN

PFLCWTHVLLRPRNPHLSPYHLWRKPLSRNPVQILNGTESDLILNDSGTYRKTPVLDKAT

SPHHLFLNHDPIAAAQASMYLSPLDLLGLNMKPS*

>m.814 g.814 ORF g.814 m.814 type:complete len:863 (+) Unigene000079:3889-6477(+)

MVLDHLVIQRMDTTGRTILDNNSGNSNSNPFNKEELAAILKFGAEDLFKEPEGEESEPQE

MDIDEILRLAETRESDQGSSATDELLSQFKVANFTMDEITPELEEKPMRNWDEIIPEDQR

RQLEEEKKQKEMEDIYMLPRSRSLNKRALANDSDSDIGSKLKLKSHSSGSESETDDSEDD

KKPKRRGRPRARKNNVVGFSDAEIRRFIKAYKKFGAPLERLEAIARDSELVDKSIADLKR

LGELVHTTCVAAVQEHEEQIKENPTEAKGPGKRRGINIKISGVQVNAKSIIQHEEDFEPL

HRAVPSSSIERSKFQLGCRVKMPHFDVEWDVQDDAHLLLGIYEHGYGNWDQIKTDSELKL

IDKILPDNSDKKPQAKQLQMRADYLLKILKKKQEGEDASRAKEESQVKKRKPRVKKEKSQ

KDELASEISSPHLSEHTSEEGEVKDDKAEKFPTKVKQKKDNKENKEKQGTAKKDKERDRE

KKRTKLKKEKTKLGSKAKKTQGPVHITAGSEPVPIGEEDDELDQDTFSICKERMRPVKKA

LKQLDKPDEGLSVQEQLQHTRSCLLKIGDRITECLKAYSDPEHVKAWRRNLWIFVSKFTE

FGAKKLHRLYKMAQKKRSQEEAKEHKKKDEPFKKKTFRPEPSGSSRDSTGTQPSSKARFP

HNHPTGFSSQSQHREHYNQHKRHFPNDYRGDWHRERKYTYPGNSNQPWQGDRYNPYDQHS

YKEHHYSNRRAHTDLYHSTGFRNPGSARKRMYDQYSNDRDHRVHREYYDRHSEPKRRRSG

DYHSANCNQGGGGICYNQDFRRMPDHQGIPETDHYRSFNPDKPLPLLDPRSPQAQKSPLE

SLSPLEKTTEPKPSPDLKWNRK*

>m.819 g.819 ORF g.819 m.819 type:complete len:1281 (+) Unigene000080:2555-6397(+)

MCNLELKSQHGSDLQQQMPSNSTAKLTKLLEASFSDLHGVYEGWEGGSFPCIRVQKDSNT

QNPDSTPKIAVHVSGAADSDGKGGVGVCLNRDLGFGFVSMENPCELREKNSSPFSSNRTM

FETEEMVIPADPEEMKEFQAQCVAQSQCVVDIILPVAYILLPSKEAFQSIYNRINNDLLM

WEPPPPPQPPPNISPSPMQQRRSEGDEFQLCKSAFRLDSDSEEDEPQFHSASEPRKKAQP

EPQSNHALSLLSLSVQVNKGRLQFLTDSKNEAGRRMEQCHGELLLDFEGGKIFSVTQHLN

NPNLNFLTIEGKKVELYHKAEVPDSAVPTRLEMPKFTPPNHLDPTIYPTEVGVSSVGRRG

TGGQMLSTAIKITLDLQRNVKEFLVALRLQGATLRHHMALTNHSWHEQLVDFLDVIDDDI

LGYTPPAIITVLHTHLATCAVDYRPLYLPLRVLFTAESFSLSSNIIVDTATFHLRFILDD

SALYLTDKCDSNSVDLKRDYVCVLDIDLLELAITTWKGNNTGKLTQPLFELRCSNNVVHV

HTCADSCAALVNMLQYLISQGDLHPPPRHTSPTEIAGQKLPLSEAPTSLPPCPPAETAEI

NQCDLTDALIDTDRTLHEEDKEFAGSPSLVKRASPVSVYLFPGEGPKAHSSLLEGVESEL

DQLVVTAPEAHVDMMSEECSEGSTDNEDFCILEAPGIGIPPKDGEPVVKVLCQTPIKVRD

GYYSRPRGSSDLLRAPARFAMPQSRVVLREVSVVWHLYGGKDFGGKPISAHTQHSHRGRP

APAGARGSPSRSAGSSRPQNSWRWVGGSGRQHSLLMEIQLTKVSFQHESYPVVVPPVVSG

QDMEGAGLGEQPLSRQVFIVQELEVRDRLASSQINKFLYLYTSESMPRRAHSNMLTVKAL

QVLPESGLGGPECCLRISLLPLRLNIDQDALFFLKDFFSNMAAGINPYFPVDPAAEVKAD

ITQKPSEEVESGTGLGPELTASIETTYSEQSSSSAGSSSSSDQPIYFREFRFTSEVPIWL

DYHGKHVAIEQGTFAGILIGLAQLNCSELKLKRLCCRHGLLGVDKVIQYAVNEWLIDIRK

NQLPGILGGVGPMHSVVQLFHGVRDLFWMPIEQYRKDGRIIRGLQKGAASFGTSTASAAL

ELSNRLVQAIQATAETVYDILSPTPPLTRCITEGRPASRPRRTPQPADLREGVAKAYDTV

REGVIDTAQTLCDVASRGHEQKGLPGAVGGVLRQIPPTVVRPLIVASEATSNLLGGMRNQ

IKPDARKEDFLKWRTEDGQE*

>m.820 g.820 ORF g.820 m.820 type:complete len:705 (+) Unigene000080:384-2498(+)

MSRWLFPWSGSIKKRACRYLLQHYLGHFLEERLSLDQLSLDLYNGSGVIKEINLDVWAVN

ELLESLAAPLEIVEGFVQSIKVTIPWAALVTDHCTLEITGLQITCRPKDRTSGTWDSQGW

SSCMTSSMQLAQECLKDPPEASEEPPAPLEGLEMFAQTIETVLRRIKVTFLDTIVRLEHH

PLDSKNGVALEMQIKRLDYCDEAVRDSNQTVPVDIHQPPAFLHKILQLNSVQLLYETLGE

PQSFVPEHTEVEDEEKDSAPKPLRMHPGPLVIGRCSGFMETTVKIKQNDMLPGPKLELDG

KVGCLHLLLSPNQITNLTDLLTALCIETEGSSNSRGGGGAPSRPLVSDDLRLIEEDLSKQ

LSVDTVDKELELNAEPYIYSLENGEMFYSMGPGGMSSSVTSIRSGSELSDSEMESSIHSQ

SSLVAPTHPLSPQGILNCPRRYPVPGTFSSLPQCSRPSRRSTHTGQSESAKPDCLLRLSL

GGMTLTLLEQEPVEDRSSMAKVSQVFFHELGFFKDSIFSERDFENLRGSFAKACPHSHLR

LTGAAVQIACEMRSAGQSARSITSDLSFSRLELLECLWEPGKPQYTELLQFKNAGHFTVG

ATARPCAQIHYSLTEKQQRKSGSKQRIVRRESSLHIELGELTTEVDLDIVGRLDSIIQAL

SHCPSQPKQPANNQTPLLELHSSFVLLSPFAVLKLRFPSPTFDL*

>m.824 g.824 ORF g.824 m.824 type:complete len:1657 (+) Unigene000081:384-5354(+)

MSRWLFPWSGSIKKRACRYLLQHYLGHFLEERLSLDQLSLDLYNGSGVIKEINLDVWAVN

ELLESLAAPLEIVEGFVQSIKVTIPWAALVTDHCTLEITGLQITCRPKDRTSGTWDSQGW

SSCMTSSMQLAQECLKDPPEASEEPPAPLEGLEMFAQTIETVLRRIKVTFLDTIVRLEHH

PLDSKNGVALEMQIKRLDYCDEAVRDSNQTVPVDIHQPPAFLHKILQLNSVQLLYETLGE

PQSFVPEHTEVEDEEKDSAPKPLRMHPGPLVIGRCSGFMETTVKIKQNDMLPGPKLELDE

GSSNSRGGGGAPSRPLVSDDLRLIEEDLSKQLSVDTVDKELELNAEPYIYSLENGEMFYS

MGPGGMSSSVTSIRSGSELSDSEMESSIHSQSSLVAPTHPLSPQGILNCPRRYPVPGTFS

SLPQCSRPSRRSTHTGQSESAKPDCLLRLSLGGMTLTLLEQEPVEDRSSMAKVSQVFFHE

LGFFKDSIFSERDFENLRGSFAKACPHSHLRLTGAAVQIACEMRSAGQSARSITSDLSFS

RLELLECLWEPGKPQYTELLQFKNAGHFTVGATARPCAQIHYSLTEKQQRKSGSKQRIVR

RESSLHIELGELTTEVDLDIVGRLDSIIQALSHCPSQPKQPANNQTPLLELHSSFVLLSP

FAVLKLRFPIPDLRPLNERRPVSERAVRNEILSLEMCNLELKSQHGSDLQQQMPSNSTAK

LTKLLEASFSDLHGVYEGWEGGSFPCIRVQKDSNTQNPDSTPKIAVHVSGAADSDGKGGV

GVCLNRDLGFGFVSMENPCELREKNSSPFSSNRTMFETEEMVIPADPEEMKEFQAQCVAQ

SQCVVDIILPVAYILLPSKEAFQSIYNRINNDLLMWEPPPPPQPPPNISPSPMQQRRSEG

DEFQLCKSAFRLDSDSEEDEPQFHSASEPRKKAQPEPQSNHALSLLSLSVQVNKGRLQFL

TDSKNEAGRRMEQCHGELLLDFEGGKIFSVTQHLNNPNLNFLTIEGKKVELYHKAEVPDS

AVPTRLEMPKFTPPNHLDPTIYPTEVGVSSVGRRGTGGQMLSTAIKITLDLQRNVKEFLV

ALRLQGATLRHHMALTNHSWHEQLVDFLDVIDDDILGYTPPAIITVLHTHLATCAVDYRP

LYLPLRVLFTAESFSLSSNIIVDTATFHLRFILDDSALYLTDKCDSNSVDLKRDYVCVLD

IDLLELAITTWKGNNTGKLTQPLFELRCSNNVVHVHTCADSCAALVNMLQYLISQGDLHP

PPRHTSPTEIAGQKLPLSEAPTSLPPCPPAETAEINQCDLTDALIDTDRTLHEEDKEFAG

SPSLVKRASPVSVYLFPGEGPKAHSSLLEGVESELDQLVVTAPEAHVDMMSEECSEGSTD

NEDFCILEAPGIGIPPKDGEPVVKVLCQTPIKVRDGYYSRPRGSSDLLRAPARFAMPQSR

VVLREVSVVWHLYGGKDFGGKPISAHTQHSHRGRPAPAGARGSPSRSAGSSRPQNSWRWV

GGSGRQHSLLMEIQLTKVSFQHESYPVVVPPVVSGQDMEGAGLGEQPLSRQVFIVQELEV

RDRLASSQINKFLYLYTSESMPRRAHSNMLTVKALQVLPESGLGGPECCLRISLLPLRLN

IDQDALFFLKDFFSNMAAGINPYFPVDPAAEGKLNG*

>m.825 g.825 ORF g.825 m.825 type:complete len:189 (+) Unigene000081:5830-6396(+)

MHSVVQLFHGVRDLFWMPIEQYRKDGRIIRGLQKGAASFGTSTASAALELSNRLVQAIQA

TAETVYDILSPTPPLTRCITEGRPASRPRRTPQPADLREGVAKAYDTVREGVIDTAQTLC

DVASRGHEQKGLPGAVGGVLRQIPPTVVRPLIVASEATSNLLGGMRNQIKPDARKEDFLK

WRTEDGQE*

>m.829 g.829 ORF g.829 m.829 type:complete len:1811 (+) Unigene000082:851-6283(+)

MLGAMDNRISEEGMKVSCTHFQCAAGAFAYLRDHFSHNYSVDMSHQILNLNINLMLGQAQ

ECLLEKSMLDNRKSFLVARISAQVVEYYKEACRALENSDTASMLGKIQKDWKKLVQMKIY

YFAAVAHLHMGKQAEEQQKYGERLAYLQSSLDKLNEAIKLAKGQPDSVQEALRFTMDVIG

GKFNSAKKDNDFIYHETVPCLETLPSVKGAPLVKALPVNPTDPSVTGPDIFSKLVPMAAH

EASSLYSEEKAKLLRDVMTKIDSKNETLEQFMDSLNLDPDSVDNMDRYNSIPSVLMEKCA

ALSVRPDTVKSLIQSMQVLSGVFTDVEASLREIRLVLDDDEASDRKLEEVAGKQCVPAQP

ATIAEIRRDLEKYVEAHEKASFTNTELHRAMNLHISNLRLLGGPLDTLREALPRPQLSED

EVAGLQCMKRILGKVQEMKDQRNSLEKQLRELIQQDDITAALVTTERADMKRLFEEQLKK

YEQVKVYIDQNLAAQENILKALTDANVQYATVRKGLAETEHKWNSTVQMLVASYEAYEDL

MKKSQEGKEFYEDLEAKSSRLLERAKSICKTRDEERQAILEKELQKKVPQRPTAPKPAQK

KAPDMDLSGIEDPELAKINAAILALGGDLPEDLRSLPPELGRPSIPEAFHPGAAALYARP

LFPPNLPPPELLAQISRFTLPPTQGFNRNPTTGPLPHQHQVPQLPNPQIAQLPSVPGYGL

PSSQTQPAASGPPTAIGMSTTTVDSVHTPIPSYTSAPGHPVISTTPGHPMATSVGYNAPS

AVASQYFQQPGVPVSQPPQGVQVPQPKPPPSQSFPQVRRQGQPQPSPPQTFYQAVPAQQH

IAAPVTGPQLPQQTQPYSLHHMAQPRPPNIQQQANNIHSTPSALSHQVYPAVSFVPGCQP

APNQLPYHSMQPQMPPNSQPMPQVSMSHLPNVKQIPPCSQPYMPPLNQQLHTIPQTPLST

QQAVPASHRQMPPTPQQIPPLSQPQMLPSSAQISPPTHLQMPAVSQPGLHASHSPVSVSQ

SLPRSSHPPLHSVSQPLPHPSQVHPSYQQVFPGTTPRVPVPVQPQLPPASLPAQFPVHTN

APPSQPHSLPSQLPHGSLSSVPSQISLPQHHLPPQSTPMGYPGGIPLVPHQSTVPQQPQA

LPPQVPSGAQASYSGPHAGIHPGPIQNSGPVITPGIQSLPQGMIPPSSAAPGATPSNVLA

TPSASPSPGPSSLGIAPQRPSPALTPVPGAALPQTVVTPLPSSTHLFQHQNSSTDDLLSS

SPESQHGSSKDTANVLLPMKADPQDEQLRKKSEGLKIIQGDPYQAPERVSQLCAELERFR

STVQSLERPSGEGSLSELDARWKELQDQQEKDSRQLSIAIARCYTMKNRHQDVMPYDCNR

VVLDSGKDDYINASFIEELSPYCPRLIATQAPLTGTAADFWLMVYEQKVSLIVMLVSEQE

LEKQKVLRYFPSERGQQITQGSITLSLTTQKNTATHVERMIGLQYRDQSLKRTVIHLQFT

SWPELGLPESKSNLIRFIQEVHGHYLLQRPLHTPVVVHCSSGVGRTGAFSLLYAALQELE

AGNGIPDLPQLVRKMRQQRKNMLQEKLHLKFCYEAVLKHAEQVLQRRGIVTASSQKSTNS

AAVKQYSQPEAQDIVLGGDMPISSIQATVARLSIRPPSVESDQDPNQDPVACVAAGLEPE

LALVQDLQAFSPSDTIPASLSPPISCPTSPVKMLSPSPPNYQGNGFEGSTIMPVSNNHAA

LEATLPAPSSLDLLASLTPEAFTLDSGCRGKQRISKQSFFQAQEGQGLQGPSNQDDPLST

LDPLWTLNKS*

>m.836 g.836 ORF g.836 m.836 type:complete len:2206 (+) Unigene000083:260-6877(+)

MESGERLASPSSTPVSVHTSSSSSSSSVSPASNAKSSLNHGAAASLAACGPLFGVTGSEQ

PFSVTSVTSVPSAFPVMAHPAFGLLSPGTARPEFGGLGALGVTAALAAHPQLGAFTEWWR

AAEAHGRSPAAFFPPFLGLPPLFAPPLQNHEATPYTSKTLSKSSQGPKGVNGAMNGSVVS

PSTTKSGGSVASSAALNTSGGKPRARKASQNSNSAGELQEKLSQKPKEKKPRKKQTEGSG

MSDSESGSSLDSDIEGVSSSDLDDLGEEDDDDDDDDQSEESDSEKEPHKKKKAKVVASNL

RPGKKEHSRTAEVWDLREGNEPPGACVSKSRDRPAQPTSVIQSTGLAVSTNPLALIGQSQ

HDSSPQRLSSSSPRPIASHQPLPLSLCSSPKPLSVPSPPKPLPLSSSPKPPSLSPSPRAW

GSAHKSQNSLSRRKHLESSLSHIADYRLKQSLLAHDQEFPLQLKKQQDLYKTSKNSGVRS

SSSSSSTSASSILPSKSTAGRTKAPGAQAVTGSPSLMLTQTLLGLGHTNGVIQNSTQDTP

LALTTKPRADLPVNLSTGGRKDTSLHTSSLAPLPALVPASVLPARPRASRKNKTPRALEV

PKDVSQKHLVKSLVDLFHHAAGEQETPSKKDSDESGEDDEDDEDDDVDDDEDDEDEDDSL

SESDSNSDSELNGSTRRNMDTTETETDGERTPLKLSKNLPLLASASASYSLADCSPLNLQ

VIKPSGMATPTIVSGSGALTYHSSPSSSYSVGTPPGSGKRKRVMNEEDLKTPLEIGWRRE

TRIKSAGGRLQGDVAYYAPCGKRLRQYPDVVKGLQWCPLSEDEVIPRIRAMEGRRGRPPN

SERQHGAGNGEGSGARRRKGRPPNVGHTEFPSPSEAKLLRKLEAQEIARQAAQMKLMRKL

EKQALARAAKEARKQQAIMAAEERRKQKEQIKILKQQAKRKKREEAANAKIMEAEKRLKE

REMRRQQAVILKHQERERRRQHVMLMKAVEARKKAEERERLKQEKRDEKRLNKERKLELR

RLELEMLREMKKPNEDMCLTDHKALPEFSRMAGLVLPGQVFSDCLMVVQFLRSFGKVLGM

EQSEVPTLGVLQEGLLNLGNSMGQVQDLLVRLLSSAVCDPGLPPGHRAKSILGDHLTNVG

LNRDNVSEVLQQYMESHCAQTELADVALSLKTKAFQAHTPAQKASILAFLVNELACSRSV

VSEIDKSLDQMNVLRKDECIVEGKLKKLKNIHAKRTGRREAGGGDESHATGTPSAGQKRK

RKTNDSDDDEEEDDDSDDAVDDDDEDDEEEVKKSRRVETCDEDEGDQATSVEELEKHIDK

ISKQQNLIRRKLFESSHALRSMCYGQDRYRRRYWVLPQCGGVFIEGVESGEGPEELEKEQ

ERLRNFKPVRIKEEPQEEGDEEEEEEEAQVEDAQQQQPEGKTQPQVKQEEEQRAEQEVKK

EDEKPCSPPNKDTPLSDSASCPHKEPSKPLGDLATLCCTPDVSMPSVTTVTASASSPPTS

KPPVLCSAATDSVALVAQFGAPPPQFGIPLSLQHHQHLLANDQLLRVLTERSGHWFSLLP

RSPCDESSLIQPTTSNVQPRSPPARSCSPHQHLQPPSASSSPSNAAHDGQGNPTVSPLQV

KPGGALLSLPMCAWSGGMISPNLPICSSPMPICPLAEGNASPLMAPSVSTSKSGSPPPPG

DKPCALSPAIDLPRNHDQPQPQPIPEDMLLGWWKMIDMEELQVLMKTLHSRGIRERALHK

QLQKSIELVSHTFNKNKEVAVMEVSELDEGQVSVETLQEWCVEEQAMETDIALLQQVEEL

ERRVTSASLQVKGWMHPEPQSEREDLLYYEHKPLPKVCPGAELHEERGSEKGLRRQPSNP

LDIAVMRLAELERHIERRYLRSPLGTTIQIRLDNVGTVTVPAPAPSTSAGGEGGEEEIAP

GMKVWRKALSEVRNSSQLAMCLQQLQKSIAWERSIMKVYCQICRKGDNEDLLLLCDGCDK

GCHTYCHKPKINAIPEGDWYCPACISKASSQSPKNKKPQSRVQSGGGGKKSAETTKKSKK

QQEACEEDEAVGGSSTSNSSPKKTATASSQAKKSSPVPSAAKPESPACVKRAKTARDNNR

DLGLCRILLAELERHQDAWPFLTPVNLKSVPGYRKVIKKPMDFSTIREKLVSSQYQNLET

FIIDVNLVFDNCEKFNEDNSDIGRAGHNMRKFFEKRWTELLKQTN*

>m.849 g.849 ORF g.849 m.849 type:complete len:271 (+) Unigene000084:5963-6775(+)

MIVVIRVYEGQEKPSLVLSVSTVLCHYQFCQLCQTGDNEELLLLCDGCDKGCHTYCHNPK

ITAIPEGDWFCPACIAKTSEPPQRSRKQVSRNSGGGEKLSETKRSRKACVSGEGSENSTV

STSVSSTAKKGGKESRKRKPEDNLYNTTTTKQERDRSKTTTDNSKDLELCRLLLAELQTH

QDAWPFMTPVNPKSVPGYRKVIKKPMDFSTIQDKLSNSQYLNLETFIIDVNLVFENCEKF

NKDDSDIGRAGHSMRRFFQRRWTELLKQVN*

>m.848 g.848 ORF g.848 m.848 type:complete len:1907 (+) Unigene000084:176-5896(+)

MESEERLSSSSHSQASISSSATPPASSANITSESGHLFQLVSDPSFRASFPLLSHPAFRL

YAPVSGCSDFRGLGILGLQGGLTAHPHLGAFPDWWRVYEAQLHGAAALFPPFRGLPPLFT

PPSLSQSHTPKKSIRGSAKGLNGAVKAKSASSGASSTSSSPSPAYTTSEQTSTLKSGLNP

QNKTTKSNLNYCKEKQSATNPSNWNRVQKSKMGQVQERTGNKAKENPCMTVAEISSTSDS

QSRSSSDSSSDTLSSLGSVDLENEDDDEDLSVCSMDSDTQSDSSHIDQAAGCFSSGPNMC

SMDSLKHSQAAPLLFGSPSIREEHRKHTSVIQTTGAAGIAKTFSLPLQPNQDALSKPLCL

TMSPKPSSVSSSPKPLSVSSSPKQHSVSASPKPLLYTRSPNPASLSFSPNITSSPKPTSI

TNARNSPDGSRLHGNESNQDKQAKEHQESTKSLKKNVSSSSPSPQISTSLNPQHCNLFLT

SNITKNSSHLNGVVQGAVQDAPLALITKPRLDSSNTPDKTLLATTSPCFNTPINLSTGTR

HSYPGPPTSGKAFTHQESHRAREGSQPQRKTFREIESDIPSSRDSDDSGEYEDEEDLSDS

LSDSGSNLDTDSDNDEEDNKDEEMNTDAESHTDSMPLKLTKSSTSLADPSTGRSNSSSSL

NLQIYESPNILQTLNGSWALTYQSPPSSSCVVPPPAKRRRVTDEQALRRPLEHGWKRETR

ISNIGGRVQGEVAYYAPCGKKLKQYPDIMKYLARNGINEITRDHFSFSAKIRVGNFYEAQ

EGPEGLQWCLLKEDEVVSCISAMDSHRGRPKSLELQPTDDAFHSRKRKRPPSVSESEMAN

ATLIKLQQKLVAQEIARQAAQIKMMRKLEKRALAQAAKDAKRQKALLEAEEKRKQKEQLK

ILKQQEKVRRLEQIRAEKELRAQQILEAKRKRKEESINAKTLEAEKRLKEKELRRQQALI

LKQQELERHRLEMVWERERRKQHMILVKVTEARKKAEERERLKQEKRDEKRLNKERKLEL

RRLELAMVKELNKPNEDLCLPDQKALPDLSRIPGLLLPDSCFGDCLMVMQFLRCFGKVLG

LDRNIKLPTLHMLQAGLLNLSPSAAQLQNLVIGLLSAAVRDPGFPPGSMAQTTLGQHVSS

VEINQENMSEILQIYMAAHGSQTDLSPLVESLKTKGFMAHTPTQKASIMGFLVNELASSR

SVIMEIDKSIEHMTNLRREKCVIESSLRKLRSSYVKRTGKRDFSLGGEESQALETPTTGH

KRRRKVGGIEEEDDEDDDSNDHGEEDEDEEEDGGKKQRKTETCEEEDDEDQSASVEELVK

NIEKLTKHQLLIRHKLFESSYSLQSMMLGQDRYKRRYWALPQCGGVFVESTVSKEGPQGV

NQESGRMHSAQLTSVKKEPAEVPDGEPYDGPQTKLENSSLDLQQENGSFNLFLQKPSSFS

KLSNLLEVPKTSSSDSQSKNSTAAPFSTNALTVLPNSLSTNLNQNIKYEVSSQLLNPTHT

GNQQHPLHNDQLYTTQAEKHSAWFSLLPRSPCDEFSLTTSCTNPSSCRAKASSPAPATSN

SMTQSTSTFSSNSQNITSPVSPSATAPVSNFLGKELQMKHTMNPNMSFKDMFTKAADPLP

STSVSTSKIGSPVLLSDKSPVLEMAKRKDLPFPQPVPQEMLTGWWKVTSSEELSRIVSAC

HPRGIRERVLQKQIQKHMDYLTQVCAKNKNAAVIDVCELKQSQVCEETVQSWCVEEHAMD

RDIAVLQQVEELERKVISASLHVKSWMPTKPQSEREDLLYYEHKVLSHSESTDIVRQADN

PLDIAVARLYELERNIERRYLKSPLSTTIQVPLDNRGTVSILAPATPANTKCDSDVEDLV

PGLKLWRKALSKVRSGAQLSVCLQHLHNSIAWEKSILKVVRKIHIH*

>m.856 g.856 ORF g.856 m.856 type:complete len:118 (+) Unigene000085:4037-4390(+)

MKDKTGTTRDVVKIEAFIHYCETYTLEKKSTNTKLGYTVVKSEAPEEKQETFTGIQLDDT

AFKAKIHREKKEMSTGTPLGDTVVTAKVQQEKQGTLISTQLNDNVVTAKSLRSKTHS*

>m.855 g.855 ORF g.855 m.855 type:complete len:418 (+) Unigene000085:383-1636(+)

MASHGQGRKRTPNRNGITPEEDALNVIAREAEAQLTAKRVARAEAREIRMKELERQQKEK

YNGLENLDNKCWDIEQWMEDSEQYTHHPQQHVLVLGNEEGTSAGSKRSVQGSSRGPTLST

ETSLSGASSSKGSGGSSVTADTETSLRDIKEIHELKDQIRDVEINYMQILKVTKDSLSEM

EMKYRKAMVSNAQLDNEKSNLVYEMDSLKDSLMELEELLSETRRECEKKNNDLEREKHAH

SILHFQFSNRKETLKQSEELLSEIRQLHLKQEGFTREISDLQETVKWKDKKIGALEKQKE

FSDAIRNERDELRDEVVQLKDTLKKHGIALGSELRTNGEVGVNVSGGSELCTNREVGVNV

SGGSELCTNREVGVNVSGGSVCTELKHGSSVLGKVFFSNTSRFALALCLLKHLDWYF*

>m.859 g.859 ORF g.859 m.859 type:complete len:90 (+) Unigene000085:3348-3617(+)

MKGIGKDQQESYVKRSKTGKEGKHDFTHMFTCEISEKDINPHFDETVFCNSQIKMATDSS

PLAKTDTTFNSQSSNVSVKKKKKKKQKQK*

>m.861 g.861 ORF g.861 m.861 type:complete len:2163 (+) Unigene000086:338-6826(+)

MAHPAFGLLSPGTARPEFGGLGALGVTAALAAHPQLGAFTEWWRAAEAHGRSPAAFFPPF

LGLPPLFAPPLQNHEATPYTSKTLSKSSQGPKGVNGAMNGSVVSPSTTKSGGSVASSAAL

NTSGGKPRARKASQNSNSAGELQEKLSQKPKEKKPRKKQTEGSGMSDSESGSSLDSDIEG

VSSSDLDDLGEEDDDDDDDDQSEESDSEKEPHKKKKAKVVASNLRPGKKEHSRTAEVWDL

REGNEPPGACVSKSRDRPAQPTSVIQSTGLAVSTNPLALIGQSQHDSSPQRLSSSSPRPI

ASHQPLPLSLCSSPKPLSVPSPPKPLPLSSSPKPPSLSPSPRAWGSAHKSQNSLSRRKHL

ESSLSHIADYRLKQSLLAHDQEFPLQLKKQQDLYKTSKNSGVRSSSSSSSTSASSILPSK

STAGRTKAPGAQAVTGSPSLMLTQTLLGLGHTNGVIQNSTQDTPLALTTKPRADLPVNLS

TGGRKDTSLHTSSLAPLPALVPASVLPARPRASRKNKTPRALEVPKDVSQKHLVKSLVDL

FHHAAGEQETPSKKDSDESGEDDEDDEDDDVDDDEDDEDEDDSLSESDSNSDSELNGSTR

RNMDTTETETDGERTPLKLSKNLPLLASASASYSLADCSPLNLQVIKPSGMATPTIVSGS

GALTYHSSPSSSYSVGTPPGSGKRKRVMNEEDLKTPLEIGWRRETRIKSAGGRLQGDVAY

YAPCGKRLRQYPDVVKGLQWCPLSEDEVIPRIRAMEGRRGRPPNSERQHGAGNGEGSGAR

RRKGRPPNVGHTEFPSPSEAKLLRKLEAQEIARQAAQMKLMRKLEKQALARAAKEARKQQ

AIMAAEERRKQKEQIKILKQQEKIKRIQQIRMEKELRAQQILEAKRKKREEAANAKIMEA

EKRLKEREMRRQQAVILKHQELERHRLDMVWERERRRQHVMLMKAVEARKKAEERERLKQ

EKRDEKRLNKERKLELRRLELEMLREMKKPNEDMCLTDHKALPEFSRMAGLVLPGQVFSD

CLMVVQFLRSFGKVLGMEQSEVPTLGVLQEGLLNLGNSMGQVQDLLVRLLSSAVCDPGLP

PGHRAKSILGDHLTNVGLNRDNVSEVLQQYMESHCAQTELADVALSLKTKAFQAHTPAQK

ASILAFLVNELACSRSVVSEIDKSLDQMNVLRKDECIVEGKLKKLKNIHAKRTGRREAGG

GDESHATGTPSAGQKRKRKTNDSDDDEEEDDDSDDAVDDDDEDDEEEVKKSRRVETCDED

EGDQATSVEELEKHIDKISKQQNLIRRKLFESSHALRSMCYGQDRYRRRYWVLPQCGGVF

IEGVESGEGPEELEKEQERLRNFKPVRIKEEPQEEGDEEEEEEEAQVEDAQQQQPEGKTQ

PQVKQEEEQRAEQEVKKEDEKPCSPPNKDTPLSDSASCPHKEPSKPLGDLATLCCTPDVS

MPSVTTVTASASSPPTSKPPVLCSAATDSVALVAQFGAPPPQFGIPLSLQHHQHLLANDQ

LLRVLTERSGHWFSLLPRSPCDESSLIQPTTSNVQPRSPPARSCSPHQHLQPPSASSSPS

NAAHDGQGNPTVSPLQVKPGGALLSLPMCAWSGGMISPNLPICSSPMPICPLAEGNASPL

MAPSVSTSKSGSPPPPGDKPCALSPAIDLPRNHDQPQPQPIPEDMLLGWWKMIDMEELQV

LMKTLHSRGIRERALHKQLQKSIELVSHTFNKNKEVAVMEVSELDEGQVSVETLQEWCVE

EQAMETDIALLQQVEELERRVTSASLQVKGWMHPEPQSEREDLLYYEHKPLPKVCPGAEL

HEERGSEKGLRRQPSNPLDIAVMRLAELERHIERRYLRSPLGTTIQIRLDNVGTVTVPAP

APSTSAGGEGGEEEIAPGMKVWRKALSEVRNSSQLAMCLQQLQKSIAWERSIMKVYCQIC

RKGDNEDLLLLCDGCDKGCHTYCHKPKINAIPEGDWYCPACISKASSQSPKNKKPQSRVQ

SGGGGKKSAETTKKSKKQQEACEEDEAVGGSSTSNSSPKKTATASSQAKKSSPVPSAAKP

ESPACVKRAKTARDNNRDLGLCRILLAELERHQDAWPFLTPVNLKSVPGYRKVIKKPMDF

STIREKLVSSQYQNLETFIIDVNLVFDNCEKFNEDNSDIGRAGHNMRKFFEKRWTELLKQ

TN*

>m.873 g.873 ORF g.873 m.873 type:complete len:402 (+) Unigene000087:383-1588(+)

MASHGQGRKRTPNRNGITPEEDALNVIAREAEAQLTAKRVARAEAREIRMKELERQQKEK

YNGLENLDNKCWDIEQWMEDSEQYTHHPQQHVLVLGNEEGTSAGSKRSVQGSSRGPTLST

ETSLSGASSSKGSGGSSVTADTETSLRDIKEIHELKDQIRDVEINYMQILKVTKDSLSEM

EMKYRKAMVSNAQLDNEKSNLVYEMDSLKDSLMELEELLSETRRECEKKNNDLEREKHAH

SILHFQFSNRKETLKQSEELLSEIRQLHLKQEGFTREISDLQETVKWKDKKIGALEKQKE

FSDAIRNERDELRDEVVQLKDTLKKHGIALGSELRTNGEVGVNVSGGSELCTNREVGVNV

SGGSVCTELKHGSSVLGKVFFSNTSRFALALCLLKHLDWYF*

>m.874 g.874 ORF g.874 m.874 type:complete len:118 (+) Unigene000087:3989-4342(+)

MKDKTGTTRDVVKIEAFIHYCETYTLEKKSTNTKLGYTVVKSEAPEEKQETFTGIQLDDT

AFKAKIHREKKEMSTGTPLGDTVVTAKVQQEKQGTLISTQLNDNVVTAKSLRSKTHS*

>m.878 g.878 ORF g.878 m.878 type:complete len:90 (+) Unigene000087:3300-3569(+)

MKGIGKDQQESYVKRSKTGKEGKHDFTHMFTCEISEKDINPHFDETVFCNSQIKMATDSS

PLAKTDTTFNSQSSNVSVKKKKKKKQKQK*

>m.879 g.879 ORF g.879 m.879 type:complete len:1250 (+) Unigene000088:180-3929(+)

MDKRGTISLLESELYYLISRFLSSGPCQRAAEVLVSELEEHQLLPRRLDWEGNVHPRSYE

DLIAANKHIAPDHLLQICKQIGPILDKQVPSGLPGVHSLLGTGRHSLLRAAKDCGHSRWK

SSTFAALHRGRPPERPLSCRDAPNLVEVCRGRELTGTQRFSFVHPVGIYQNIKMHRRILG

HLSAVYCIAFDRTGSRIFTGSDDALVKIWSSFDGRLHSTLRGHHAEISDLAVNFENTLIA

AGSCDKTIRVWCLRTCAPVAVLQGHSGSITSLQFSPFAKGSKRYMVSTGTDATVCFWQWD

VMNTTFNDRPVKFTERPRPGVQMVCSSFSPGGVFLATGSTDDVIRIYYLGSGLPEKIAEL

NSHTDKVDSIQFCNTGERFVSGSRDGTARIWKLHQRHQWRSILLEMSATLPGTVPAEEES

FFKPKVTMVSWDRHDNTVITAVNNYLLKVWNSYTGQLLHILKGHEAEVFVLEPHPYDPRV

MLSAGHDGNIFIWDLIRGTKTHHYFNMIEGQGHGAVFDCKFTPDGHRFACTDSHGHLVIF

GFGSSKPYEKLPDQMFFHTDYRPLIRDSNGFVLDEQTQQAPHLMPPPFLVDVDGNPHPPK

YQRLVPGRENFTDEHLVPQLGYVATSDGEVVEQVISQQPVESEDPQARRSLLDDAIRQLQ

EQQDQQAGRQQGTPRRVSLSERMEVQSPPNVGLRRSGQVEGVRQMHQNAPRSQMATERDL

QAWRRRVVVPELTSSGYMDQESFRIAKGEEEMVLYNAKKRRIAQSSLRDDSDDEPVCTKR

EQLRKRHRNLESTSSYAESLIEFSCEEGEETASSEENEIEASEVDSSNEEEEEEWKSDSS

SRSSSEHSDWTADAGISAQAAMSSRRRARRQISSDEEEEDNSAEEEQTEQTQDEDVQSSS

PHKSKKKAKTQKSPKLRPSLNREISNEFRPSAWITDVIPRKSPFVPQMGDEVIYFRQGHE

AYVEAVCRSNLYPINLDKQPWKKMELRDQEFVKITGIKYEVCPPTLCCLKLTQIDTGTGK

ITTKSFSVKYHDMADVIDFLVLRQSYDEARSRNWRPNDAFRSVIDDAWWFGTIVCQEPYQ

PEYPDSHFQCFKVKWDNGEIEKLSPWDIEAIPENAQRPETVGASVQVTDSERADILYKPQ

ADEWGGKSREEECARIAAGIDQLITIDIAAPFSGPVDLTQYPTYCTVIAYPTDLGSIRMR

LKHNFYRRLSALIWDTRYIYFNARTFNEPRSKIAQSAKLITDVLLKFIE*

>m.880 g.880 ORF g.880 m.880 type:complete len:962 (+) Unigene000088:3992-6877(+)

MCVLFCSKPHCTDIMEIYNAVENMEYSEDEDMEDVDAPGTSTGRRANQLEQIPDRDAWKD

ACKQLLDFMFEREDSEPFRQPVDQDEYQNYSDIIDTPMDLETVRRNLEEDRYENPNELCK

DTRLIFTNAKVYTPNKRSKIYSMTLRLSAFFEESIRNIISDYKTAVKSSLKLRRSQRFKR

RTQQQEPLFPPPPVESTSQKRAAIKTQEQVEAATTKSTSAKTSTAEHRRSKKRRRRRSTN

TSMEDSNKSASSLSESENDSELSSNKSKAEEFSSSSQPFRRETRASRRAMQNREARRKIV

SQSGSEIDCSTEDDDDEDDDYSGKSSSLKRLQSSRNKSRTSRLTRNSEKRKLEGQVNGLS

SRSSRAERRRVQRSSGSDPEFSKEEENTGKEEDPEQSRQLRKTAMVAVSKMKLLDASEEE

DTSDHKARSRSSKRATVIQSSSESEEHDQQESNESEDCASEDEKSEASPDSDFSGSQQNG

ESKSGNRGTSRRRVVKEEGSLRVNGHKKQSQEESEDNSEDNEGESVSESVDEEEDSISRR

KLLRKTARVAVCKIKQMNDSDEEEEDHCRTRRQKNHPNNANNFKTSDEETETPQAEKHPH

QNGRKLNTQDIKYSEKMNHMTSKSQELVSVSKACAALKSAVKDSSALGRKIRSEDKEEEI

NESNSGEEKITNKRCSVARKNKCSRPAKSRSLDEDTNTSDSESETPSYQGRKKKESDSSE

EWRQSDESKKDKKRFSRRPTLRALPKKKYITDSEEDLDSDGEKQSGNGRRSSDSVEDGNT

HRQKSSEGSKRKHVSTSESDNKEENGSATDESDCDKPPGRRGKRFKKLSGSESDSGSREK

RTNGPSKRHRSEDELPEGASCPGKRQAGKRKHSKSYSSNDGTDSESQAESSNESQVSSSG

SQSNSERYGKGRLRARRRGTAHRKLSTSDIDSDTSYGKPQRATRIRTRNLGKRTVNYRDS

E*

>m.889 g.889 ORF g.889 m.889 type:complete len:2106 (+) Unigene000089:550-6867(+)

MGRCFLPTMSWCGTDCGHSRWKSSTFAALHRGRPPERPLSCRDAPNLVEVCRGRELTGTQ

RFSFVHPVGIYQNIKMHRRILGHLSAVYCIAFDRTGSRIFTGSDDALVKIWSSFDGRLHS

TLRGHHAEISDLAVNFENTLIAAGSCDKTIRVWCLRTCAPVAVLQGHSGSITSLQFSPFA

KGSKRYMVSTGTDATVCFWQWDVMNTTFNDRPVKFTERPRPGVQMVCSSFSPGGVFLATG

STDDVIRIYYLGSGLPEKIAELNSHTDKVDSIQFCNTGERFVSGSRDGTARIWKLHQRHQ

WRSILLEMSATLPGTVPAEEESFFKPKVTMVSWDRHDNTVITAVNNYLLKVWNSYTGQLL

HILKGHEAEVFVLEPHPYDPRVMLSAGHDGNIFIWDLIRGTKTHHYFNMIEGQGHGAVFD

CKFTPDGHRFACTDSHGHLVIFGFGSSKPYEKLPDQMFFHTDYRPLIRDSNGFVLDEQTQ

QAPHLMPPPFLVDVDGNPHPPKYQRLVPGRENFTDEHLVPQLGYVATSDGEVVEQVISQQ

PVESEDPQARRSLLDDAIRQLQEQQDQQAGRQQGTPRRVSLSERMEVQSPPNVGLRRSGQ

VEGVRQMHQNAPRSQMATERDLQAWRRRVVVPELTSSGYMDQESFRIAKGEEEMVLYNAK

KRRIAQSSLRDDSDDEPVCTKREQLRKRHRNLESTSSYAESLIEFSCEEGEETASSEENE

IEASEVDSSNEEEEEEWKSDSSSRSSSEHSDWTADAGISAQAAMSSRRRARRQISSDEEE

EDNSAEEEQTEQTQDEDVQSSSPHKSKKKAKTQKSPKLRPSLNREISNEFRPSAWITDVI

PRKSPFVPQMGDEVIYFRQGHEAYVEAVCRSNLYPINLDKQPWKKMELRDQEFVKITGIK

YEVCPPTLCCLKLTQIDTGTGKITTKSFSVKYHDMADVIDFLVLRQSYDEARSRNWRPND

AFRSVIDDAWWFGTIVCQEPYQPEYPDSHFQCFKVKWDNGEIEKLSPWDIEAIPENAQRP

ETVGASVQVTDSERADILYKPQADEWGGKSREEECARIAAGIDQLITIDIAAPFSGPVDL

TQYPTYCTVIAYPTDLGSIRMRLKHNFYRRLSALIWDTRYIYFNARTFNEPRSKIAQSAK

LITDVLLKFIDKPHCTDIMEIYNAVENMEYSEDEDMEDVDAPGTSTGRRANQLEQIPDRD

AWKDACKQLLDFMFEREDSEPFRQPVDQDEYQNYSDIIDTPMDLETVRRNLEEDRYENPN

ELCKDTRLIFTNAKVYTPNKRSKIYSMTLRLSAFFEESIRNIISDYKTAVKSSLKLRRSQ

RFKRRTQQQEPLFPPPPVESTSQKRAAIKTQEQVEAATTKSTSAKTSTAEHRRSKKRRRR

RSTNTSMEDSNKSASSLSESENDSELSSNKSKAEEFSSSSQPFRRETRASRRAMQNREAR

RKIVSQSGSEIDCSTEDDDDEDDDYSGKSSSLKRLQSSRNKSRTSRLTRNSEKRKLEGQV

NGLSSRSSRAERRRVQRSSGSDPEFSKEEENTGKEEDPEQSRQLRKTAMVAVSKMKLLDA

SEEEDTSDHKARSRSSKRATVIQSSSESEEHDQQESNESEDCASEDEKSEASPDSDFSGS

QQNGESKSGNRGTSRRRVVKEEGSLRVNGHKKQSQEESEDNSEDNEGESVSESVDEEEDS

ISRRKLLRKTARVAVCKIKQMNDSDEEEEDHCRTRRQKNHPNNANNFKTSDEETETPQAE

KHPHQNGRKLNTQDIKYSEKMNHMTSKSQELVSVSKACAALKSAVKDSSALGRKIRSEDK

EEEINESNSGEEKITNKRCSVARKNKCSRPAKSRSLDEDTNTSDSESETPSYQGRKKKES

DSSEEWRQSDESKKDKKRFSRRPTLRALPKKKYITDSEEDLDSDGEKQSGNGRRSSDSVE

DGNTHRQKSSEGSKRKHVSTSESDNKEENGSATDESDCDKPPGRRGKRFKKLSGSESDSG

SREKRTNGPSKRHRSEDELPEGASCPGKRQAGKRKHSKSYSSNDGTDSESQAESSNESQV

SSSGSQSNSERYGKGRLRARRRGTAHRKLSTSDIDSDTSYGKPQRATRIRTRNLGKRTVN

YRDSE*

>m.899 g.899 ORF g.899 m.899 type:5prime_partial len:2026 (+) Unigene000090:2-6079(+)

FLLFLSFFFCPISPHLFLIHPWAIHQALQDEVLAREKEVDHLEALGQSLSPLSCAADRDW

LSERVGAVRSGHTELRNWCFRRAAMLEQALANAQLFGEEEVEVLNWLAEVAQRLSDVSVQ

SYQPELLAEQHKYTLSLNEEIVSRKKTVDQAIKNGQALLKQTTGEEVLLIQEKLDGIKSR

YSEMTAGSSKALRNLEQALQLATRFASAHEDLSQWLDSMEAELNNMEPDTTPAYQERQKD

LKCVSAEKRLILDTVNEVGSALLDLVPWRAREGLDRLVADANQRYRQADETITQRVQLVQ

AAIQRSQQYEEAVDAELAWVGETERKLTSLGPLSLEPDVTVAQLQVQRAFNIDIIRHKDT

VDQLLHTREDILESCSDQQREALKVKTDSLSMRYEAVSQNHAERFSALEQAQVLVARFWE

TYEELDPWLGETETLISQLPPPAIDTEALRQQQDQMRMLRESIAEHKPHIDKLLKIGPQL

AELSSQEGATLRQRYSEAERRYLAIKEDVKGRAAVLDEAFSQSAQFHDKMDPLLETLEGA

VQRLRQPPPVAAEVEKIREQLAEHRAAGLELDKLLPSFSTLCARGEELITRAAHDDPAAQ

AVRSRLLRLRSLWDEIRQRAEEREGKLQDVLDLAGKFWADMAALLSTLRDSQEIVKELED

PGLDPSLIKQQIEAAEAIKAETDGLREELEIVRTLGADLIFACGETEKPEVKKTIDEMNA

AWEGLNRTWRERMEKLEEAMTASVQYQDALQGMFDYLDNAVIKLCDMQAVGTDLSTVKQQ

IEELKQFKVEVYQQQIDMEKLCHQGELLLKKVSDQADRDMIQEPLTELRHLWDNLGDKIT

VRQHKLEGALLALGQFQHALSELQSWLSHTHATLDTQRPVNSDPKAIEIELAKHHVLRND

VLSHRATVETVNKAGSELLESSPGDEASHLRDQLDELNRSWDSLLLKTDERQKLLETALQ

QAEGFHGELEEFLQWLRRTESQLSAAKPTGGLPETAREQLQQHMELQAQLTQRGEQYHRL

LDQGESMLLARGAEENSPGTTQTQQNLALLQNKWASLNAKMDDRRAKLEEAVSLATGFQS

SLQDTINWLTQAEQTLNMAQSPSLILDTVLFQIDEHKVFVNEVNTHREQVLALEKAGSQL

RFASLKQDVVLIKNLLLSVQARWDKLVQRSLDRGRHLDEARKRAKQFHEAWRKLTDWLEE

AEKRLDAELEISNEPDKIKVQLTKHKEFQKTLGSKQPVYDTTVRSGKAMRDKATLPADTQ

KLDNLLGEVRDKWDTVCGKSVERQHKLEEALLFSGQFAEALQALVDWLYRVEPQLAEDQP

VHGDLDLVSNLMDSHKAFQKELGKRTSNIQALKRSARELMETGRDDTAWVKVQLQELSNR

WETICALSVSKQTRLQQALKQAEEFRTAVQMLLEWLSEAEQTLRFRGILPEEVETLQVLL

HTHRNFMQTVEEKRVDVNKAAGMGEAILAVCHPDCITTIKHWITIIRARFEEVLTWAKQH

EQRLEAALAELLNNATLLEDLLSWLQWAETTLVQRDTEPLPQDITQLKTLITEHQVFMEE

MTRKQPDVDKVTKTYKRKPSETSSSLAERRGVRKQQQQQQQPAMQVSGGNPRLNQLCSRW

QQVWLLALDRQRKLHDGLDRLEELKEFANFDFDVWRKKYMRWMNHKKSRVMDFFRRIDKD

QDGKITRQEFIDGILASKFPTSRLEMTAVADIFDRDGDGYIDYYEFVAALHPNKDAYKPT

TDADKIEDEVTRQVAQCKCAKRFQVEQIGENKYRFFLGNQFGDSQQLRLVRILRSTVMVR

VGGGWMALDEFLVKNDPCRVQHPGLRILRSDSSCSISSRIARGRTNLELREKFILPEGVS

QGMAAFRSRGRRSKPSSRTASPTRSSSSASQSAQSCASGPSAPATPTASARGGSSSKLKR

PPFHSSRGSLTGENGGTTHATKPVRSDNKRTPSSTSGPTSRAGSRAGSRASSRRGSDASD

ASELMETRSACSDTSDTPRRPGTKPSKIPTISKKTPSPKTPTTKK*

>m.907 g.907 ORF g.907 m.907 type:internal len:2307 (+) Unigene000091:3-6926(+)

TRAARSPCRDSARCISKAWVCDGDSDCEDNSDEDNCEALLCKLSHHTCAQNDSICLPAEK

LCDGKDDCPDGSDEKLCDLCSLENGGCSHNCTIAPGEGILCSCPTGMELGSDNKTCQIQS

FCAKHLKCSQRCIQEKATVKCACYEGWALEPDNESCKSTDPFKPFIIFSNRHEIRRIDLY

KGEFSVLVPSLRNTIALDFHLNQSSLYWTDVVEDKIYRGKLSENGALTSFEVVIQYGLAT

PEGLAVDWIAGNIYWVESNLDQIEVAKLDGTMRTTLLAGEVEHPRAIALDPRDGILFWTD

WDASSPRIEAASMSGDGRRTIHRETGSGGWPNGLTVDYLERRILWIDARSDAIYSAAYDG

SGLIEVLRGHEYLSHPFAVTMYGGEVYWTDWRTNTLAKANKWTGHNVTVVQRTNTQPFDL

QVYHPSRQPQAPNPCAANDGRGPCSHLCLINYNQTFSCACPHLMKLQADKHTCYESRQFL

LYARQIEIRGVDIDNPYYNYIISFTVPVIDNVTAVDYDAQEQRIYWSDVRTQMIKRAFIN

GTGVETVVSADVPNAQGLAVDWVSRNLFWTSYDTSKKQINVARLDGSFKNSVVHGLDKPH

CLVVHPMLGKLYWTDGDNVSMANTDGSNQSLLFTSQKGPVGLSIDFDEGKLYWISSGNST

INRCNLDGTGLEVIESVKGKLTRATALAIMGDKLWWADQGTDQVGTCDKKDGGSWKVLRN

GTSPVMHMKIYNETVQQKGTNLCTMNNGDCSQLCLPTSPTSRACMCTAGYNLKSGQQSCE

GMGSFLLYSVHEGIRGIPLDPLDKSDALVPVSGTSLAVGIDFHAENDTIYWVDMGLSTIS

RAKRDQTWREDVVTNGIGRVEGIAVDWIAGNIYWTDQGFDMIEVARLNGSFRYVVISHGL

DKPRAIAVHPEKGYLFWTEWGQYPRIERSRLDGSERVVLVNVSISWPNGISIDYEEGLLY

WCDARTDKIERINLETGGNRELVLAVHNMDMFAVSVFENYIYWSDRTHANGSIKRGNKNN

ATDMVYLRKGIGVQLKDIKVFNRARQQGTNICKDKNGGCEQLCLFRGNGARTCACAHGML

AEDGRTCRDYDGYLLYSERTILKSIHLSDETNLNAPIKPFEDPDHMRNVIALTFDHQGGD

GKGANRIFFSDIHFGNIQQINSDGSARKTVVENVGSVEGLAYHRGWDTLYWTSYTTSTIT

RHTVDQTLSGAFNRETVVSMSGDDHPRAFALDECQGLMFWTNWNELAPSIMRSSLAGSNV

LVIVGNNIRTPNGLAIDHRSEKLYFSDATLDKIERCEYDGTNRFVVLKNEPVHPFGLAVY

GEYIFWTDWVRRAVLRADKFGRDMKVLRADIPQQPMGIIAVAKDTNSCEFSPCHTNNGGC

QDLCLLTSEGRVNCSCRGDRKLVEGNVCVAENTTCHSVDEFECGNGDCINYSLTCDGRAH

CKDKSDEKPSYCSNRGCKKGYRRCLNGRCIKHSSWCDGTDDCGDRSDERPCNMTLCSASE

FQCKDGSCITNTSRCNQVVDCEDASDEMNCSPTDCHSFYLLGVKGVIFQKCEFTTLCYTP

IWQCDGSNDCGDFSDERNCPEKRQLKCPVNFFACPSGRCIPMSWTCDKENDCENGTDETH

CDKFCSPTQFECGNHHCISSSWVCDGTDDCGDGTDEGSRCSSKTCSPEAFQCPGSHCVPQ

RWKCDGDNDCPDGADEGVQAGCTNNKTCDDTEFQCQNKQCIPKHFVCDHDLDCRDGSDES

PECEYPTCGPNDFRCENGQCLKQKSWECDGEFDCRDHSDEAPKNLHCTEPEKRCNDSAYL

CNNGKCVSEDSLCDWKDDCGDGSDEHNCFVNECLNNKLSGCTQHCEDLKIGYKCRCDPGF

RLKNDGKTCVDVDECTTTYPCSQRCFNSYGSFHCFCIEGYVAYANDWTSCKSSSEEEAYL

IFANRYYLRKLNLDGSNYTLIKTDLNNAVALDYHYAEQMIYWTDVTTQGSMIRRMLMNGS

NVEVLHRTSLSNPDGLAVDWVGGNLYWCDKGRDTIEVSKLNGAYRSMLVNSGLREPRAVA

VDVRNGYLYWSDWGDVPHIGRIGMDGTDRSIIIKDKITWPNGLTLDFINDRIYWADARED

YIAFASLDGTNRHIVLNQDIPHIFAMSLFEDYIYWTDWETKSINRAHKTLGTNKTMLIST

LHRPMDIHIYHPYRQPAVENHPCQVDNGGCSNLCLLSPGGGYKCACPTNFYLAADGKQCL

SNCTASQFVCKNDKCIPFWWKCDTEDDCGDRSDEPADCPEFKCRPGQFQCGTGICTNPAY

ICDGDNDCQDNSDEANCDIHVCLPSQF

>m.920 g.920 ORF g.920 m.920 type:complete len:1374 (-) Unigene000092:1760-5881(-)

MCSPFQAGIKGEQGPPGLPGKIGKDGENGAKGDKGTLGIPGFPGFKGQKGEKGPPGYGDG

APGPPGPLGSPGPLGEIGPPGYPGEPGPPGRNIVGPPGERGLPGEIGQKGDKGVDGESLR

GFNGADGPPGPPGPPGRLSDECDFDRGEPGPPGPPGLQGEVGQKGDKGDTCIQCSASGPP

GLPGSPGPKGNHGFPGQPGGKGEKGLPGPTGLPGFPGNDGSQGLMGSPGSKGEPGDIYIA

PDLKGEKGLPGLPGSRGLPGIDGLSGKDGRPGLPGLKGEPAREGIKGDRGLDGDPGIMGP

IGERGPPGVPGFGQTGEPGEKGSPGSPGVPGKPGIPGPKGEPGKGISTPGPPGPPGPRGE

TGLRGLQGERGSPGDQGWPGTPGEKGSPGLPGIGLPGPPGPKGHSGLPGAPGFPGDPGNP

GQAGSPGSPGTSGQKGEPGIGLRGPKGSMGQPGMQGFPGEKGNVGMPGVPGFEGKTGPPG

PQGVKGDAGPPGFPGLSGPPGPPGLGDPGPPGLIGPPGESGPAGRIGEKGEKGPQGFPGP

AMPGPQGEKGTPGFPGTPGPKGLPGEPGNPGLDGFPGERGIKGDMGIMGIPGSPGHPGQT

GGPGAIGPRGDPGLIGPRGVTGEQGPKGERGDPGLQGPPGNMTEVEMEHMKGEKGDIGNK

GEIGPTGQKGIIGLSGDPGLPGKDGMPGSPGKPGEKGDSGLPGQPGQIGAPGVKGSIGEM

GLPGPFGPKGSKGVAGQPGHHGFKGDPGGKGEKGLAGLPGIGIPGLPGEKGEMGPPGFPG

ESGQKGVKGSMGIPGIPGTPGQKGDIGQIGYPGSPGVPGEKGTPGLPGLTGEAGLKGRPG

EPGLQGSPGPQGKKGEAGKDGFPGVSGERGEPGLPGRGFPGTPGNAGEKGEKGNPGPPGM

SGSPGVPGSKGEKGLPGFQGVQGRTGERGLPGPSMEGPKGDKGVQGEPGEPGQPGAPGPA

GVPGGAGLKGEKGDKGFPGIQGTQGLKGDKGFTGLPGEPGTPGYKGLKGEMGPPGPPGFQ

GIKGEGGLPGFKGEIGDRGFPGAKGNDGLPGPPGPHTFIKGDIGFPGPQGPQGLPGPQGY

FGQKGQQGLIGIQGIKGEDGAPGFNGLPGNKGEPGPTGPPGSRGYPGPPGPDGVPGQVGP

PGPSSMDHGFLVTRHSQSVEVPLCPQGTTMIFDGYSLLYVQGNERSHGQDLGTAGSCLRK

FSPMPFLFCNINNVCNFASRNDYSYWLTSPEPMPMSMAPITGESIKPFISRCAVCEAPAM

VIAVHSQTIAIPSCPQGWISLWIGYSFVMHTSAGAEGSGQALASPGSCLEEFRSAPFIEC

HGRGTCNYYANSYSFWLATIEDNEMFSKPVPTTLKAGNLRTHISRCQVCMKRT*

>m.922 g.922 ORF g.922 m.922 type:complete len:132 (-) Unigene000092:6302-6697(-)

MRVLLILVAAVLLCARPSRAEGCSGSSCGKCDCSGVKGSKGERGLPGLQGTMGFPGMQGH

EGPQGPMGPKGDRGDAGAQGMKGIRGPSGLPGIPGIPGQPGIPGQDGPQGLPGIPGCNGT

KANIEIFYFCY*

>m.932 g.932 ORF g.932 m.932 type:complete len:1078 (+) Unigene000093:189-3422(+)

MWEEARPKVWNFMENSEEVDLVRMLLQNNVTAYFFSAQLSGTQWSVQDLSSFLTKHSEDT

RPPGTVFTWRNVFNETDQAIMSISRFMECVNLDKLEAVSTEEKLVNESLSLLDNRKFWAG

LVFPDIDINSSELPPHVSYKIRMDIDNVERTNKIKDAYWDPGPRADPFEDMRYVWGGFSY

LQDVIEQSIIRAVTGTKEKTGVYIQQMPYPCYVDDIFLRVMSRSMPLFMTLAWMYSVAII

IKSVVYEKEARLKETMRIMGLDNGILWFSWFISSLIPLLMSAGLLVFALKMGNLLPYSDP

GVVFLFLGSFAVVTIMQCFLISTAFSRANLAAACGGIIYFTLYLPYVLCVAWEEYAGLPA

KIIASILSPVAFGFGCEYFALFEEQGVGIQWSNLISSPMEGDDYSLTTSIILMYFDAFLY

GVMTWYIEAVFPGQYGIPRPWYFPFTKSYWFGEEEQKQTPIIQGKKANAGAVCIEEEPGH

LELGVYIKNLVKVYRHGKKLAVDDLTLGFYEGQITSFLGHNGAGKTTTMSILTGLFPPTS

GTAYIMGKDIRTDLSAIRQNLGVCPQHNVLFSMLTVEEHIWFYARLKGLSEERVKSEIHQ

IVTDLGLPHKRKSRTSQLSGGMQRKLSVALAFVGGSKVVILDEPTAGVDPYARRGIWDLL

LKYRQGRTIILSTHHMDEADILGDRIAIISHGKLCCVGSSLFLKTQLGTGYYLTLVKRDF

DLSLTSCTNSTNIVSYSKNSLKKDDSISESSSDGGLGSDQESETTTIDISLISNVIFKHV

PSARLVEDLGHEITYVLPYEAAKDGAFVELFHEIDDRLIDLGISSYGISDTTLEEIFLKV

ADENGVDAVTSDGTVLARRHRRHAFGDHQSCLKPFTEDDSFDFNDSEESRETDWLGVSDG

KGSYQVKGWRLKQQQFVALLWKRFLYARRSRKGFFAQIVLPAVFVCIALVFSLIVPPFGK

YPSLELEPSMYEEQYTFISNDVPEDLHTNKLLGSLTDSLAHRGQCAEDRTTEDGLCPVRV

SEWTIPVVPYSVSDMFQRGNWSMENPSPLCECSCKKKKKMLPDCPPGAGGLPPPQVN*

>m.933 g.933 ORF g.933 m.933 type:complete len:578 (+) Unigene000093:3711-5444(+)

MRARFNLERGTAADRFLRGLSSFIHGMDTKNNVKIWFNNKGWHSVVSFLNVMNNGMLRAN

LPPGKDPSKYGITAYNHPLNLTKEQISQVALVTTSVDVLVSICVIFAMSFVPASFVVFLI

QERVSKAKHMQFISGVQPYLYWLANFIWDMCNYIVPATLVIIIFMCFQQKAYVSSTNLPV

LALLLLLYGWSITPLMYPASFLFNIPSTAYVVLTSVNILIGINGSVSTFVLELFGGDEVG

GINDILKNVLLIFPHFCLGRGLIDMVKNQAMADALERFGENRFRSPLEWDMVGKNLFAMA

VEGVVFFIITVLIQYRFFIKPKAVRPKLSPIGEEDEDVARERQKIMSGAGHGDILELREL

TKVYERKEKPAVDRLCVGIPPGECFGLLGVNGAGKTSTFKMLTGDCAVTSGEAFLARKSI

LREIDEVHQNMGYCPQFDAINDLLTGREHLEFYAILRGVPEGEVCEVAEWGIRKLGLIKY

ADKAAGSYSGGNMRKLSTAMALIGGPPVVFLDEPTTGMDPKARRALWNCILSIIKEGRSV

VLTSHSMEECEALCTRMAIMVNGRFRCLGSVQHLKNR*

>m.936 g.936 ORF g.936 m.936 type:complete len:107 (+) Unigene000093:5606-5926(+)

MELIERELPGSTLKEKHRNMLQFQLPSSLSSLARIFSILSKNKERLHIEDYSVSQTTLDQ

VFVNFAKDQSDEDHIKDISMNKSDAVVDFSQLDAFLTDAKAQETVV*

>m.938 g.938 ORF g.938 m.938 type:complete len:1635 (-) Unigene000094:1760-6664(-)

MRVLLILVAAVLLCARPSRAEGCSGSSCGKCDCSGVKGSKGERGLPGLQGTMGFPGMQGH

EGPQGPMGPKGDRGDAGAQGMKGIRGPSGLPGIPGIPGQPGIPGQDGPQGLPGIPGCNGT

KGDRGRDGIPGIPGNSGPPGFPGEPGIKGDPGGIIGGFIPQKGERGFPGSPGLLGPPGNP

GLPGPVGPPGLDGIRGFPGEPGLPGPPGDKLSFVGPKGDKGEKGLRGPPGPPGTAGEFGE

NTLTYIPGPEGDKGDTGEKGDMCSPFQAGIKGEQGPPGLPGKIGKDGENGAKGDKGTLGI

PGFPGFKGQKGEKGPPGYGDGAPGPPGPLGSPGPLGEIGPPGYPGEPGPPGRNIVGPPGE

RGLPGEIGQKGDKGVDGESLRGFNGADGPPGPPGPPGRLSDECDFDRGEPGPPGPPGLQG

EVGQKGDKGDTCIQCSASGPPGLPGSPGPKGNHGFPGQPGGKGEKGLPGPTGLPGFPGND

GSQGLMGSPGSKGEPGDIYIAPDLKGEKGLPGLPGSRGLPGIDGLSGKDGRPGLPGLKGE

PAREGIKGDRGLDGDPGIMGPIGERGPPGVPGFGQTGEPGEKGSPGSPGVPGKPGIPGPK

GEPGKGISTPGPPGPPGPRGETGLRGLQGERGSPGDQGWPGTPGEKGSPGLPGIGLPGPP

GPKGHSGLPGAPGFPGDPGNPGQAGSPGSPGTSGQKGEPGIGLRGPKGSMGQPGMQGFPG

EKGNVGMPGVPGFEGKTGPPGPQGVKGDAGPPGFPGLSGPPGPPGLGDPGPPGLIGPPGE

SGPAGRIGEKGEKGPQGFPGPAMPGPQGEKGTPGFPGTPGPKGLPGEPGNPGLDGFPGER

GIKGDMGIMGIPGSPGHPGQTGGPGAIGPRGDPGLIGPRGVTGEQGPKGERGDPGLQGPP

GNMTEVEMEHMKGEKGDIGNKGEIGPTGQKGIIGLSGDPGLPGKDGMPGSPGKPGEKGDS

GLPGQPGQIGAPGVKGSIGEMGLPGPFGPKGSKGVAGQPGHHGFKGDPGGKGEKGLAGLP

GIGIPGLPGEKGEMGPPGFPGESGQKGVKGSMGIPGIPGTPGQKGDIGQIGYPGSPGVPG

EKGTPGLPGLTGEAGLKGRPGEPGLQGSPGPQGKKGEAGKDGFPGVSGERGEPGLPGRGF

PGTPGNAGEKGEKGNPGPPGMSGSPGVPGSKGEKGLPGFQGVQGRTGERGLPGPSMEGPK

GDKGVQGEPGEPGQPGAPGPAGVPGGAGLKGEKGDKGFPGIQGTQGLKGDKGFTGLPGEP

GTPGYKGLKGEMGPPGPPGFQGIKGEGGLPGFKGEIGDRGFPGAKGNDGLPGPPGPHTFI

KGDIGFPGPQGPQGLPGPQGYFGQKGQQGLIGIQGIKGEDGAPGFNGLPGNKGEPGPTGP

PGSRGYPGPPGPDGVPGQVGPPGPSSMDHGFLVTRHSQSVEVPLCPQGTTMIFDGYSLLY

VQGNERSHGQDLGTAGSCLRKFSPMPFLFCNINNVCNFASRNDYSYWLTSPEPMPMSMAP

ITGESIKPFISRCAVCEAPAMVIAVHSQTIAIPSCPQGWISLWIGYSFVMHTSAGAEGSG

QALASPGSCLEEFRSAPFIECHGRGTCNYYANSYSFWLATIEDNEMFSKPVPTTLKAGNL

RTHISRCQVCMKRT*

>m.948 g.948 ORF g.948 m.948 type:complete len:1124 (+) Unigene000095:383-3754(+)

MSGSEDDRDDFGPTEDLSMMQDDDLEEDLSENEAPKVKKKKKAKKSSRENKSSKRQRSRR

EDIPISSPEPVEGRDVDEVEDDGPGGRSDSEGSDYTPGRKKKKRASTSRDKKRSSTGADR

SSSLAVSKRKEPEEEDDEDDDDSSEPKTSSQLLDTWGMEDIDHVFTEEDYRSLTNYKAFS

QFVRPLIAAKNPKIAVSKMMMVLGAKWREFSTNNPLRGSASANAALAAANVAAAVESMVA

AETPATAPTPAPLAEPQPPPAPPLRKAKTKEGKGPNARRKSKPTPKPQEKKVKTKKVAPL

KIKLGGFNSKRKRSSSEEEDMDVDSDFDDGSINSVSVSDSSNSRSSRSKKKPKPKKKKKV

DEDADGYETDHQDYCEVCQQGGEIILCDTCPRAYHMVCLDPDMEKAPEGTWSCPHCEKEG

IQWEAREEASDGEEENDAGGGEAEEDDHHMEFCRVCKDGGELLCCDTCPSSYHMHCLNPP

LPDIPNGQWICPRCRCPPLKGKVQKVLTWRWGEAPPPIPVPRPPDLPVNAPDPPPLVGRK

EREFFVKWCNMSYWHCSWVQELQLELNCQVMFRNFQRKTDMEEPPSVDASECDDNKSMRR

KSKDPLYARMDHQFGRYGVKMEWLLIQRILNHRYTLITKHREITHSTNCLSVCLSLSVDK

KGNVHYLLKWRDLPYDQSAWESEDMDIPDYDTYKQQYWNHREMMIGDEGRPGKKLKKVKL

RKIEKPPVNPVVDPTVKFDRQPEYLDMTGGTLHPYQLEGLNWLRFSWSQGTDTILADEMG

LGKTVQTAVFLYSLFKEGHSKGPFLVSAPLSTIINWEREFEMWAPDMYVVTYVGDKDSRA

VIRENEFSFENHAIRGGKKAFKMKEAAVKFHVLLTSYELITIDQAVLGSIDWACLVVDEA

HRLKNNQSKFFRVLNNYPLQHKLLLTGTPLQNNLEELFHLLNFLTPERFNNLEGFLEEFA

DIAKEDQIKKLHDMLGPHMLRRLKADVFKHMPSKTELIVRVELSIMQKKYYKYILTRNFE

ALNTRGGGNQVSLLNVVMDLKKCCNHPYLFPTAAIEAPKMPNGMYEGGALTKSSGKLMLL

CKMLKKLKEGGHRVLIFSQVHTLLLTHFLATYNRIILLYTKCV*

>m.949 g.949 ORF g.949 m.949 type:complete len:942 (+) Unigene000095:3754-6579(+)

MQMTKMLDLLEDFLENEGYKYERIDGGVTGVMRQEAIDRFNAPGASQFVFLLSTRAGGLG

INLATADTVIIYDSDWNPHNDIQAFSRAHRIGQNRKVMIYRFVTKASVEERITQVAKKKM

MLTHLVVRPGLGSKAGSMSKQELDDILKFGTEELFKDDAGEGDNKEEDSSVIHYDDKAID

KLLDRNQDATDDTELQSMNEYLSSFKVAQYVVKDEDEEEVDREIIKQEENVDPDYWEKLL

RHHYEQQQEVLASHLGKGKRPRKPVNYNDCSQEEQADWHDDQSDNQSDYSVASEEGDEDF

DERSEANNRRSSRRGLRNDKEKPLPPLLARVGGNIEVLGFNSRQRKAFLNAVMRYGMPPQ

DAFTTQWLVRDLRGKSEKEFKAYVSLFMRHLCEPCADGAETFADGVPREGLSRQHVLTRI

GVMSLIRKKVQEFEHVNGQWSMPWMKELEENKRAVAVAMGDDPKTPSTGTPGDTQPNTPA

PEDLSRGEESERETDDKMEGEGEKDRKSTQLQDDEVIEIPDDGETPLSPEKTGPGQEMEK

DSTAPSLEKDGEIKDIAGEKDKEKGGAKEESTTCSLKIEEVKGERSEVTSAGDSSQPKEE

CSEEKSEKTDSNLTGEEKKEVKEEKDGVRAEESRINGEDGGVDNEEKKKHVKQRFMFNIA

DGGFTELHSLWQNEERAATVTKKTYDIWHRRHDYWLLAGIIQHGYARWQDVQNDPRFSIL

NQPFKGEMSRGNFLEIKNKFLARRFKLLEQALVIEEQLRRAAYLNMTQDPTHPAMALNAR

FAEVECLAESHQHLSKESLAGNKPANAVLHKVLNQLEELLSDMKADVTRLPATLSRVPPI

AARLQMSERSILSRLANKGNETHTPPPIPPGPYATPQNYGPPFTPAPPAMALAAANYSQM

PPGSFISVLNGPPMSVKKEREAELMMGRREQRSGEVICIDD*

>m.961 g.961 ORF g.961 m.961 type:complete len:2163 (+) Unigene000096:267-6755(+)

MSDRLGQITKSKDGKSKYSSLSLFDKYKGKSIETQKPTVVARHGLQSLGKVAAARRMPPP

AHLPSLKSENRGNDPNVIIVPKDGTGWANKQEQPDQKISVASTAQPPESQPPLPLQKSVS

NLQKPSAAANQESTSNGGPKQWAQLNGKAPDQDGLRASNRLQPFSHEEFPTLKAAGEQDK

GGKEKSAFDPSYGPGPSLRPQNVTSWREGGGRYLQPPSLSSALSSDSEGKTSASPEATTP

LPPPSSSSTSAASSSANAGTTASPDPKDPSLRPAQPTRRLTPGLQYQLHHSSNTTYHDML

PAFMCPKETRDGPASSEPGSAAAVAPMRFDARPTFRPTYETINGDLRRENRFTRVPPRPS

PRPIRRPGDRPSRPAIINPDDLKDLDDLDNDCEDGWAGLHEEVDYSEKLKFSDDEEDHSP

SDKSKICLVVYRGEWESRRERQSSMSSDGQEDSYHHQHDPLPSRKTNSRYGSTDLHQKSN

GTSESVEELDEPQRSAAAPPRGKFVPPDVSEAVERARRRREEEERRAREERLAACAEKLK

KLDEKFGKIEKPALTGDGHGAENKELLLSPGREAGKTQPETWQHVKDVSEQKSDSLHSQE

YRDDFSYRNEDDCSEYTQRHQKAVAPRFQKQQQDQVYKMQQWQQSGSGPAQRGYYPSHVL

GFDPRWMMMPPYMDPRMTQGRSTVDYYSSNVHSSVKPAGQQDHLHSPGSTSDEGCHPTMH

LDKKVLSADTYSVWSQEGYPPARSFTPPYQRQHESGERSQTDDRSERSGSQQDSYEDRGI

ERAESPLEDHLHQGYRPNRGPDISLGSQREDASHPLGHNEYFKPENQKEVISNRHHKDSL

DSHDEVCEGKKSFDSGFWRKEDSMRKDKDATQWSDPGSGGNGGVGQPSEPMSRTLRRTGP

IKKPVLKALKVEDKENEKPKSEPEEKSVPYRLEKEVLTNVYDLKKDNQPIISRQLPPPVT

CQEEKQTFRPKVEKLSNPTEESIKENGWDGSNSQLRDNSESREPPAPRRNNWIFIDEEQA

FAGVRGAGRGRGRGFREFNSRGGGRGGRGDNNRGMYNTGNIQRGGRGRGSREYVKTEDFQ

RGKPRRRNVSETLSEASEYEELPKRRRQKNGENGEAAYSTAGDTKRADRDSWRSNKVYNN

DQSTSGEAREKPKMRSFSRSLPPRLNTGYNRGFGSKEFSTWRGRGGQFGSGSMQENGYNF

TNDTFSRKNAEGDSLKYPSKFASSFTENGVEDREGDYHLDKDNLDNRPLRRRRPPRQDKP

PRFRRLRQERETGGQWNRDEYVNGDFTNPWSSRSKTSEDHWPGQYPGNRQQDFAPQAEDW

ETGSENSDLSDWREKRGEAPGEPGHGEPGSEKRELSKRSFSSQRPIVDRQNRKTDILESS

KLPRSTDTPSSSSSSNRNDSWQNGVRRSPEDSTSGLNAGSVYGVEQSEDGLSTSNSETSV

KKLDKDLKPRSMKGDMSESLSQYDLNSYPIESDSGVSIPSPDGFQDALNKKQRRPQEDER

RRKEQGATVKNRAITSKMPPRFAKKQGGISIDQSEETLSTSNLGTEIWEASSAALPVQSS

GGDSWTKQVSYNASEPNSEDSDAGPEHSKEHKPGPIGNERSLKNRKGSEGVERLEGPITP

VNGVDIHVENVLPVPPIEFGVSAKDSDFSLPPGSAPVVVSNPVTKLQDALASNAGLAQGI

PMLRRDHLQPAINLNPISYPTTDLTLKMESARKAWENSQSLPEQGSPGGAGSVTQPTCSS

SSAVSYSSFGGVSMPPMPVASVAPSVSLQGNHIPPLYLDGHVFPSQPRLVPPTMTQQQSY

QQAAAAQQIPISLHTSMQAQAQLGLRGGLPVSQSQEMFSSIPTFRSQVYMQPSLSHPSAM

VLSGAGPLKGPYSAFPGMQPSELVKPQSSSHFQPINGSQALVYDNAMNQAAGLGTSQLMD

SQLIQVTMPLPGSQLRYGSAQQHLILPQSIQLQQGQSLSVGTPRRILSHGSQPPVMTASR

EPSQLEMKGFQFPDKASHSPGIPAGSYRPSSASPSGKPSGPGAPPTVGPLPGHYAQQVPP

PQGNMVMHMRTPTNGPFPNPIQRPVMQVNKAVIMRSTPYTSPGRDPTHCTPPTNPESLNK

GTEEGTKVKLLHEARHVVSDLKTSSSSSVISGKLQDVLTGAPKPNRTGAIKPQSVKMEES

KA*

>m.973 g.973 ORF g.973 m.973 type:5prime_partial len:2248 (+) Unigene000097:1-6744(+)

KEQMDLQEEKHRQEIERLRSYFSEQIREMEERYTKEVLHLQDRLKDLKPMETLHSMAGIS

AEEQHLQMRQYEMEEEIARVIVQMSVEFARQSEMSRVSVQEETRDRAEPERGIQMTDESW

GGGVEWWNPSMQCSSTRTEQLSRSESEDEERKRKDKQCEKEEVTVTSQLTSTETQERVHQ

VLMDLLKMAALTEETVRDESILSAADEPETGKLSLLSGATDTDEGLDVCWEAGLAVERAG

SLWGVCSRLQKTVEKLIITLTSTHTQLDHARLTQTELMKEKFTYDQQITDLLNRQEELQR

RLEEERTAREKLTLQLHHAEGLIDGYSDERRALEEQVREGAGRQQQLEAELQVTNSRLQE

LHQERKEVTRQKDLLLRQQDAVIGGATEAELSLVEAAVDDAPAAGLLEETEKLLQEKVDV

QRQAQKDHTELLEQVKHLEAELNEQVRSTVKMEETHQSVTSDLHQQIHALEKQLENNRRF

LDEQAADREQERDVFQQEIQKLEQQLKSSSKTQAEHREVERLSVALQEKADWCSELSLRV

QQLQRDVQERNEEIERQEERVRVLEEVLVSHDNHHITSAVEDSKQYASMEGGADATLESL

LQTEREALDRKEKEIVNLEEQLEQFRDELQNKSEEVQQLHMQLEIQKKEISTQQQDLQTH

TRLKTEVEVKNQLVCDLESQVEYLRGEQDRLKRGSEEEVEQLNSVIEKLQQELSNIEHKQ

SVQEEDNDEEDQKDELKLKIDQIQRELDTLKEEHFLLVSKYGLLQEERETKEKKEKKNEM

LVHELEDMLRDKMAALVVAQVQIQALGESANSIVSGLTQRVEQLQTCLEEREDELRECRS

KGDEAKSEAGTLRLKVSQLEEKLKQKVAILSQMVNQKHCETDIQTKCEIRSRTSLDDELE

IKHAETLSDSIGGKEMDLTQQYQENDGKEWKFKVISEDKKEINLIKPDSALIASGSEMQV

KQCAVNAMENVLFLIKKLRKLEVDLCSMPKDQELQKHLLASSEEDIKEYEKMLTKLMKIL

DQMKSTPSSPAGTESGHVTETEKLHQKLQQARNQSAAANEHLDSCKIQNLQEQIQFKDEL

LEKIREDLRRASERSSEDSTVIVALHQELHEVKCQSEATEKELNSCKELSQRLQEQIEEQ

EKMISMLKDQMHKTTDEAERSELIQELQHIKLQTAANEEELNNYKEQNLKLQEQIQVCDA

SVVQLMEELQHHRTALNKSGEEKHSRSHRNTGQNDGKIMSDRAARQQDSLSPSSSLPAVP

SADRAQCISTEEVGTQVEGAELGAGLEEVIGEYTQRIGQIRELHAAEIMDMENRHITESE

SLKNENRRLEQECVTLREGIDQLHPLQVMRPDHSTFSVYRDGYTSDSSSDVGTEYRSTPE

GARPDDAHLPDRLKSLLREVHQEGAELGAGLEEVIGEYTQRIGQIRELHAAEIMDMENRH

ITESESLKNENRRLEQECVTLREGIDQLHPLQVMRPDHSTFSVYRDGYTSDSSSDVGTEY

RSTPEGARPDDAHLPDRLKSLLREVHQEGMQVLSLSELSVCDESADHSSLLAQITHLQAQ

LTQLRQDTHTNQQLHTTPTETDRQVEQCDRLRAELAQVRLELKTSLKTQHTHKRELDTLR

AEVCVKVSELDAVSERLAEEQRRARELQWEVERERSRTDRRTEGQREEQEDMKLALEEER

LVLAQKETELAKYTGLVKQLQVQLESQNSRLKEMRSDLQKEKELNAELLKHAQHPLQVGD

GVPSVADSMLQSLQVQLEKKQTTLMELRAQAEEKMLQELEQKQQWQDERRHLQRQVDELQ

SLLEGEKERGKATERERMRLEGRVSQMMERSSTSEQVSMQDSEQKSYKSVSLDSTGTPVL

NIAGVTCSRDTESVVAKLQKMSSKISRFAVEGADDESFSWLLKSIQEVISFLQKYSSVPP

VGPESAAQLTGGVSSVLSERLLHQNAELTGFVSRLTEEKNEQRNQILRLEEELRRQRHLK

AHNSLGFGSEVPGGVLEVQKELWMREKSKMEKSLLQAEAEISRLRADIRADVVRDLNTSD

ADNATLKRIYGKYLRSESFRKALIYQKKYLLLLLGGFQECEEATLSLIAGMASPHVHTHT

TFLECISQRRRGYSRFRSAARACIALFRMRFLVRRWQKVTGGSTTIIRNGVGQSQVIEMR

TDSPYLQPRGTTRGRTGRESPHTTHTTHHRYGGIVTDGGVVCSHLQSYDPDRALSDYITR

LEALQRRLGSVQSGSSSYTQMHFGLRR*

>m.979 g.979 ORF g.979 m.979 type:complete len:1937 (+) Unigene000098:227-6037(+)

MEAVPRMPMIWLDLKEAGEFQFSSTVKQFILKNYGEDPDTYNESLKKLEQLRQSAVNVTR

DFEGCSTLRKYFGQLHYLQSRVPLGPGQEAAVPVSWTEIFSGKTVTHDDISYEQACILYN

LGALHSMLGAMDNRISEEGMKVSCTHFQCAAGAFAYLRDHFSHNYSVDMSHQILNLNINL

MLGQAQECLLEKSMLDNRKSFLVARISAQVVEYYKEACRALENSDTASMLGKIQKDWKKL

VQMKIYYFAAVAHLHMGKQAEEQQKYGERLAYLQSSLDKLNEAIKLAKGQPDSVQEALRF

TMDVIGGKFNSAKKDNDFIYHETVPCLETLPSVKGAPLVKALPVNPTDPSVTGPDIFSKL

VPMAAHEASSLYSEEKAKLLRDVMTKIDSKNETLEQFMDSLNLDPDSVDNMDRYNSIPSV

LMEKCAALSVRPDTVKSLIQSMQVLSGVFTDVEASLREIRLVLDDDEASDRKLEEVAGKQ

CVPAQPATIAEIRRDLEKYVEAHEKASFTNTELHRAMNLHISNLRLLGGPLDTLREALPR

PQLSEDEVAGLQCMKRILGKVQEMKDQRNSLEKQLRELIQQDDITAALVTTERADMKRLF

EEQLKKYEQVKVYIDQNLAAQENILKALTDANVQYATVRKGLAETEHKWNSTVQMLVASY

EAYEDLMKKSQEGKEFYEDLEAKSSRLLERAKSICKTRDEERQAILEKELQKKVPQRPTA

PKPAQKKAPDMDLSGIEDPELAKINAAILALGGDLPEDLRSLPPELGRPSIPEAFHPGAA

ALYARPLFPPNLPPPELLAQISRFTLPPTQGFNRNPTTGPLPHQHQVPQLPNPQIAQLPS

VPGYGLPSSQTQPAASGPPTAIGMSTTTVDSVHTPIPSYTSAPGHPVISTTPGHPMATSV

GYNAPSAVASQYFQQPGVPVSQPPQGVQVPQPKPPPSQSFPQVRRQGQPQPSPPQTFYQA

VPAQQHIAAPVTGPQLPQQTQPYSLHHMAQPRPPNIQQQANNIHSTPSALSHQVYPAVSF

VPGCQPAPNQLPYHSMQPQMPPNSQPMPQVSMSHLPNVKQIPPCSQPYMPPLNQQLHTIP

QTPLSTQQAVPASHRQMPPTPQQIPPLSQPQMLPSSAQISPPTHLQMPAVSQPGLHASHS

PVSVSQSLPRSSHPPLHSVSQPLPHPSQVHPSYQQVFPGTTPRVPVPVQPQLPPASLPAQ

FPVHTNAPPSQPHSLPSQLPHGSLSSVPSQISLPQHHLPPQSTPMGYPGGIPLVPHQSTV

PQQPQALPPQVPSGAQASYSGPHAGIHPGPIQNSGPVITPGIQSLPQGMIPPSSAAPGAT

PSNVLATPSASPSPGPSSLGIAPQRPSPALTPVPGAALPQTVVTPLPSSTHLFQHQNSST

DDLLSSSPESQHGSSKDTANVLLPMKADPQDEQLRKKSEGLKIIQGDPYQAPERVSQLCA

ELERFRSTVQSLERPSGEGSLSELDARWKELQDQQEKDSRQLSIAIARCYTMKNRHQDVM

PYDCNRVVLDSGKDDYINASFIEELSPYCPRLIATQAPLTGTAADFWLMVYEQKVSLIVM

LVSEQELEKQKVLRYFPSERGQQITQGSITLSLTTQKNTATHVERMIGLQYRDQSLKRTV

IHLQFTSWPELGLPESKSNLIRFIQEVHGHYLLQRPLHTPVVVHCSSGVGRTGAFSLLYA

ALQELEAGNGIPDLPQLVRKMRQQRKNMLQEKLHLKFCYEAVLKHAEQVLQRRGIVTASS

QKSTNSAAVKQYSQPEAQDIVLGGDMPISSIQATVARLSIRPPSVESDQDPNQDPVACVA

AGLEPELALVQDLQAFSPSDTIPASLSPPISCPTSPVKMLSPSPPNYQGNGFEGSTIMPV

SNNHAALEATLPAPSSLDLLASLTPEAFTLDSGCRGKQRISKQSFFQAQEGQGLQGPSNQ

DDPLSTLDPLWTLNKS*

>m.985 g.985 ORF g.985 m.985 type:complete len:1965 (+) Unigene000099:197-6091(+)

MSAADRYLYVDRSLVNNPLAQADWASKKLVWIPSEKLGFESGSVKEETGDECTVELTDSG

KKIKVNKDDIQKMNPPKFNKVEDMAELTCLNEASVLHNLKDRYYSGLIYTYSGLFCVVIN

PYKNLPIYSEEIIDMYKGKKRHEMPPHIYAITDTAYRSMMQDREDQSILCTGESGAGKTE

NTKKVIQYLASVASSTKTKKDPTSTISSLSHGELEKQLLQANPILEAFGNAKTVKNDNSS

RFGKFIRINFDVNGYIVGANIETYLLEKSRAIRQAKEERAFHIFYYLLSGAGDKQRSELC

LEDYGKYRFLSNGKVTIAGQQDRDLYTETMDAFRIMSIPEDEQTGLLKVVSAVLHLGNII

FKKERNSDQASMPDDTAAQKVCHLLGMNVTDFVRAILSPRIKVGRDFVQKAQTQEQAEFA

VEALAKATYERLFRWLVMRINKALDKTKRQGASFLGILDIAGFEIFELNSFEQLCINYTN

EKLQQLFNHTMFILEQEEYQREGIEWNFIDFGLDLQPCIELIERPNNPPGILALLDEECW

FPKATDVSFVEKLTNQHANHLKFAKPKQLKDKTEFSVLHYAGRVDYNAVAWLTKNMDPLN

DNVTALLNNSSSVFVQDLWKDADRVVGLETIAKMSDSSMPSASKTKKGMFRTVGQLYKES

LAKLMTTLHNTQPNFVRCIIPNHEKRAGKLDAHLVLEQLRCNGVLEGIRICRQGFPNRIV

FQEFRQRYEILAANAIPKGFMDGKQACILMIKHLDLDPNLYRIGQSKIFFRTGVLAQLEE

ERDLKITVIIIAFQAQARGFLARKSFAKRQQQLTAMRVIQRNCAAYLKLRNWQWWRLFTK

VKPLLQVTRQEEELSVKEEELQKAKDSAQKYETELKDITLKHTQLVEERNALQEQLQAET

ELYAEAEEMRVRLVAKKQELEEILHEMEARLEEEEERSATLQVDRKKMQQQIQELEEHLE

EEEDARQKLQLEKVTCEGKIKKLEDDLLVIEDQNNKLLKEKKLLEERVADFSANLAEEEE

KSKNLTKLKNKHESMISDLEVRLKKEEKTRQELDKIKRKLEAESNDLQEQIADLQAQIAE

LKAQLAKKEEELQAALARLEDETAQKNNALKKIRELEGIISDLQDDLDSERVARNKAEKT

KRDLAEELEALKSELEDTLDTTATQQELRAKREQEVTLLKKAIEEESRNHEAQVQEMRQK

HTQAVEELTEHLEQAKRVKTNLEKAKQTLEKETSELTVEIRTLSQAKQEVEHKRKKVEGQ

LADLQSRFNDSEKQKAELGDRVSKMTVELESVTNLLNEAEGKNIKLNKDVASLTSQVQDT

QELLAEETRQKLQFSTKLRQMEDERNSLQDQLEEETEAKRNVERHVSTLNIQLSDFKKKL

DDVSGNVELLEESKKRLQKDLESSNSQLEEKSSAYEKLEKTKNRLQQELEDILMDLDNQR

QLVSNLEKKQKKFDQMLAEEKSVSCKYADERDRAEAEAREKETKALSLARALEEAQDTRE

ELERANKALRAEMEDLISSKDDVGKNVHELEKSKRGLEQQVEEMKTQLEELEDELQAAED

AKLRLEVNMQALKAQFERDLQARDELGEEKRKQLMKQVHEMEAELENERKQRSLALAVKK

KLELDLSDLEAQIDQAIKARDEAQRQLKKLQAQVKDQLRDMDELRAARDASLNSAKENER

KLKSLEAENMQLHEDLSAADRSRKQIQQERDDLHDEMTSCNSKMSVLSDEKRKSEARIAQ

LEEELDEEQCSTELVNDQLKKANAQVDTLGSELAGERSAVQKSETARQQLERQNKDLKSK

LQELEGSVKSKFKSTIAALEAKILQLEEQLEQEAKDKQQTSKSMRRTEKRLKEVLLQVED

ERRNSEQFKDQMEKANSRMKQLKRQLEEAEEEATRANASRRKLQRELEDATEASETMNRE

VNTLKSKLRRGGDFTLNVRRVVRGGLDSEEDADLTSETSEATPE*

>m.991 g.991 ORF g.991 m.991 type:complete len:1961 (+) Unigene000100:197-6079(+)

MSAADRYLYVDRSLVNNPLAQADWASKKLVWVPSERLGFEAGSLREEHGDEVVVELADSG

KKIRINKDDVQKMNPPKFNKVEDMAELTCLNEASVLHNLKERYYSGLIYTYSGLFCVVIN

PYKNLPIYSEEIVEMYKGKKRHEMPPHIYAITDTAYRSMMQDREDQSILCTGESGAGKTE

NTKKVIQYLAHVASSHKTKKDQSSSVLSHGELEKQLLQANPILEAFGNAKTVKNDNSSRF

GKFIRINFDVNGYIVGANIETYLLEKSRAIRQAREERTFHTFYYLLTGAGDKLRNELCLE

GYNKYRFLSNGNVTIPGQQDKDMFVETMEAMRIMGIPEDEQIGLLRVVSSVLQLGNMSFK

KERHSDQASMPDDTAAQKVCHLMGMNVTDFTRAILSPRIKVGRDYVQKAQTQEQAEFAVE

ALAKATYERMFRWLVMRINKALDKTKRQGASFIGILDIAGFEIFELNSFEQLCINYTNEK

LQQLFNHTMFILEQEEYQREGIEWSFIDFGLDLQPCIDLIEKPASPPGILALLDEECWFP

KATDKSFVEKVLQEQGTHPKFFKPKKLKDEADFCIIHYAGKVDYKADEWLMKNMDPLNDN

VASLLNQSTDKFVSELWKDVDRIVGLDKVAGMSEMPGAFKTRKGMFRTVGQLYKEQLSKL

MATLRNTNPNFVRCIIPNHEKKAGKLDPHLVLDQLRCNGVLEGIRICRQGFPNRIVFQEF

RQRYEILTPNAIPKGFMDGKQACVLMIKALELDPNLYRIGQSKVFFRAGVLAHLEEERDM

KITDVIISFQAWCRGYVARKAFAKRQQQLTAMRVIQRNCAAYLKLRNWQWWRLFTKVKPL

LQVSRQEEEMQAKDEELTKVREKQVVAEKMIEEMEVKHQQLNAEKMALQEQLQAEMELCA

EAEEMRSRLATRQQELEEILHDLENRVEEEEERVTQLQNEKKKMQQNITDLEQQLDEEEA

ARQKLQLEKVTTDAKLKKLEEDVMVLDDQNAKLNKEKKQLEDRISEFNINLAEEEEKSKS

LQKLKNKHEAMITDLEDRLKKEEKTRQELEKNRRKLESDFTELNDHIADLQSQIAELRAQ

LAKKEEELQAALARVEQETSLKNTAQKRVRELEAQVTELQEDLELERAARNKAEKQRRDL

GEELEALKSELEDTLDSTAAQQELRAKRESEVAQLKKTLEDEVRSHEQLLAEMRQKHTQA

FEELNEQLEQAKRSKASLEKVKHAAESERSELQIELKSITQSKNESEQRRKKAETQLQEL

QLKYNESEKQKKDMADKMSKMQVEVENLSSALSEVESKCIKATKDSSSVESQLQDTQGLL

QEETRQKLALSSRLRQVEDEQNRLKELLEEEEEGKKSVEKQLHASQAQLTELRKKMEQEA

STLETLEEGKRKLHREIENLTQRLEEKSSAYDKLEKTKVRLQQELDDMLVGQDHLRQNVQ

ELERKQKKFDQMLAEEKSISTRYGEERDRAEAEAREKETKALTLARELEALQEQRNELER

ANKLLKTEMEDLVSSKDDVGKSVHELERAKRAMEQQLEEMKLQVEELEDELQLTEDAKLR

LEVNLQAMKAQFERDLQARDELGEEKRKQLMKQVHEMEAELENERKQRSLALAVKKKLEL

DLSDLEAQIDQAIKARDEAQRQLKKLQAQVKDQLRDMDELRAARDASLNSAKENERKLKS

LEAENMQLHEDLSAADRSRKQIQQERDDLHDEMTSCNSKMSVLSDEKRKSEARIAQLEEE

LDEEQCSTELVNDQLKKANAQVDTLGSELAGERSAVQKSETARQQLERQNKDLKSKLQEL

EGSVKSKFKSTIAALEAKILQLEEQLEQEAKDKQQTSKSMRRTEKRLKEVLLQVEDERRN

SEQFKDQMEKANSRMKQLKRQLEEAEEEATRANASRRKLQRELEDATEASETMNREVNTL

KSKLRRGGDFTLNVRRVVRGGLDSEEDADLTSETSEATPE*

>m.997 g.997 ORF g.997 m.997 type:5prime_partial len:2255 (+) Unigene000101:1-6765(+)

GLRNNYSLSVRSCGNVTHVVFRCVCGSEQISDGWRCYSDIMERVLELDREGSQAGNLTGS

IALFEKGCKLSLSKHGPFTAFIPVDTSQISSGKLNQARTAEAICKHHLILGQHLYKDLEG

HDLWTYGGELMRFKPNKKFISKNDPDTVFTIINSDIPASNGIIHIVDKVFTFATVDTYDS

AEFSSKTIGDILAEDTRFNRFVSLVDNCGAPMPLQGPGPLTLFVPTNKAIDRSRDGSIIY

MLKEAKHKLQELLRHHMFSQAAVTMDQVASMTEIHTMANQAITVSISDDGRVLLTEKKIS

FDMKDIIASNGIIHIVDGLLLPPSIIPIMPHRCDVNETTIITSPCVMCKFISESKCPPGS

EKQTDRFQFCEPPPDPRWSEFTTVRGCARYCKAKRARAECCKGFYGPDCKPCIGGFQHPC

YGKGTCSDGIYGDGSCKCKPDFMGIGCHICSNPMKHGENCDEDCRCVHGVCDNRPDSQGV

CRRNSCLSGYTGELCDLTAMLCDSDGASEHCHIHAYCTRVNNINMCVCNPGYEGDGYSCT

EANLCLRADRGGCHIGAQCLYAGPGNISCICNEGWTGDGFVCVEINNCLTANRGGCHEQA

ECIYIGPGQSECACKKGFMGDGIMCQIVNPCLTDNGGCHATATCSFNNSGIHQCTCPEDH

AGDGFKCYGNILMELEGNSDFYTFNRYLLRHPVITAESNVTALVPSKDAFKNLSDADQVF

WTDYYHLPYLMQAHFLDGIYSYDDLSQQVNKTVSTKSKTKWKITFKNGELMIDNAIVLVS

DLQATNGFIHIINTVLKPPISDIPPPPPDLMEVLHNTPSFSLFREAALLYNLTESISKKD

FTIFIPSDSAVKEYLLKTNSTQLDENIVKYHVITKEQLFPEHLTDGTLKTTLLGNEYQIM

IHLNSENKTLINDVELDGNMTEIRHGVIISISRVLVIHKNYCSKDIYMKTFGRCGDCNSS

PKCLFGSKPIKDEFPLNMKSNCRYRMRVGKRRKSVPGCMIDCLKNVKDHSCCPGYFGRDC

FKCPGTVDNWCSNNGKCQDGLFGNGECLCSEGFHGTACEMCEPGRYGKDCKSECHCDHGK

CLDGIDGNGQCICYKGWKGVNCSVEIVNDECGGICDENANCLSPSSGVKPSCVCSAGYHG

NGTFCQETELCAINNGGCSEHATCTKISPGERSCTCKKDYTGDGTVCLELDPCLVNNGGC

SENADCIKTGPNTAACVCKSGFATIRNYCIPINLCLKDNNGGCSVNAICQNTGPGERNCT

CRFGYKGNGIECMGTVSRELLHNSAANWFRKSLSQSRVRDLYSKGPFTVFVPHTDYIGNY

SIDLWESKNRIPDLLRYHIVSCEEISGSQLKSMTHVVSASGHVLNFSVRDGAVYINGDTK

IITSDYECSNGVIHFINKVLIPYELKNETKGIEEMLNITNAVEAYGYTVFGKLLQKANLM

ATLQHTAFQPFTMFWPTDKAFNSLPQEQKNWLYSEDHLDKLQAYLKFHIIRDQRTIAIAL

PTEKTVRTLYGSWLTFSCSQDLTGDILINGNDAKIVDRNLQFLTGMAHGIDKVLEPPNIG

AHCDDFSSIEVKGRCGSCLFPPPCPYGTVYTNKTNVCSRPWNSYRRHYFYDDFEFPSPYS

FKPFGCNRMCSKMSWVSKCCKNHYGRNCQVCSGGLEAPCGEHGDCDDGRYGTGTCKCHTG

FNGTACELCEKNHYGSNCTACTCTANGKCDDGIDGDGSCFCQEGWTGVMCESKIGVKPVC

SPECHSKAVCLPENQCQCEPAYVGNGYNCTAPDLCSEYNGGCHEQADCTQSEINVICTCK

SGYSGDGNVCSPINACVEETNGGCSDFANCIFIGPNERRCECQQGYVGNGLQCFEKVVPP

VDRCLENNGDCHANAICKDLHFHTKTAGVFHLRSPAGKYMMNYSTAGAACKAEGATLATL

NQLSDAQQLGMHLCVAGWIDGKKVGYPIRFPSVKCGDNQVGIILYKDPDVNTPYDAYCYR

IREVKCECGPGYVGDGDFCNGNLASVIATHSNFSIFYSMLVKTAEAAEEGKKLLDFLSTS

STNLTLFVPHNAGFSPNETLSLRDLEYHVSSNNSLHYYEDLKHNTAIPSRLGFNLKVIIK

TDNQTQVEDSSPVKLVNKQTVLIWDIPATNGLIHIIEGPLKAPPIKVTPSTPSAVRSQSS

APTVTAVLLVIFIGGIIAGVAYYFLRNKNDAFSFKYFKNDDEDGNSTKTSANPAIMSIPN

PLYSGYRAFVEPFGEEEQCESPASVAANTPNILD*

>m.1002 g.1002 ORF g.1002 m.1002 type:5prime_partial len:1938 (+) Unigene000102:3-5816(+)

GSDELERVCVPHLVAFISLSAFHTCEPTLYTCGNGRCVPYHYRCDHYNDCGDNSDEEGCL

FRPCDPDTEFTCNNGRCIAREYVCNGINNCFDNGTSDERNCAERTCQPGHTKCQSTNICI

PRSYLCDGDNDCGDMSDESPTHCATSTCTQSEFRCTSGRCIPAHWYCDGGTDCADGSDEP

STCTTIVRTCSSDQFRCDDGRCIASSWICDGDNDCGDMSDEDQRHNCANRTCSSLEFTCV

NNRPPQRKCIPQDWVCDGDADCSDAYDEHQNCTRRSCSATEFTCSNGLCIRSAYRCDRRN

DCGDGSDEQSCTYQPCQQHQFTCQNGRCISQDFQCDGDNDCGDESDELPHMCHTPAPTCP

PGKFRCDNGNCVPSSQVCDHSDDCNDNSDEKGCGINECTDPSVHHCDHECTDTPTSFVCR

CRPGYRLMSDKKSCDDVDECSETPSVCSQMCENTLGSYVCKCAPGFLREPDGRRCRQNSN

ISPYLIFSNRYYLRNLSADGAAYSLILQGLSSVVALDFDRVDKRLYWIDVSRRVVERMFF

NGTGREVVLNGIMHGEGLAVDWVGRKLYWVDSFLDCLKVSELDGRFIRKLAEHCVDVNNT

YCFENPRAIVVHPKFGYVYWTDWGSKAFIGRVGMDGNNKSAIITSKIEWPNGLTIDYTND

MLYWADAPLNYIKSSNLEAPHRHTVYDGVLPHPFAITVFEDTVYWTDWNTRTVEKGNKYD

GTGRQALVNTTHRPFDIHVLHPYRQPIVTNPCGINNGGCSHLCLLKAGGRGYTCQCPDHF

LTVQIGGVARCLPSCSSTQYRCADNERCIPIWWKCDGQSDCRDGSDEPSTCPLRHCRLGQ

FQCNDGNCTSSHFLCNSNRDCPDGSDEDTVLCATHQCESHQWQCSNKRCIPEAWQCDGEN

DCGDGSDEEPSHCSSRTCRPGQFKCRNGRCIPQSWKCDVDDDCGDNSDEPLDECMGPEYR

CDNHTEFDCSTNYRCVPLWAVCNGQNDCRDNSDEQNCEEMTCDPAGDFRCDNHKCIPLRW

KCDGDSDCGDGSDERNCNPRACTESEYRCDNLRCIPERWVCDHDNDCEDNSDERDCELRT

CHPGYFQCDSGHCIAERLKCDGSADCMDVTDETSCPTRYPNGTYCPPFLFECKNHICVQQ

HWKCDGDNDCGDGSDEELHLCLDIPCEAPFRFRCDNNRCIYSHELCNSVDDCGDGTDEKQ

EHCLTPTHGPCSSDEFKCGNSQCIPLQYVCDDYDDCGDQTDELGCNSGNGRSCSENLCEH

NCTELTGGGFICSCRQGYKAREDNRNSCEDVNECELYGTCPQLCKNTKGSYECFCAEGFR

SVGPQHRTECAAEGNPPVLLLPDNVRIRRYNLSSKQYLDYIDNAEHIHAVDYIWDPEDQG

LSIVYWTILGHGAQFGSIKRAYMTTFDDHGSNQMKDVDLNLRYISSPDGIAVDWVGRHIY

WTDAGTNRIEVAKLDGRYRKWLIYSNLDQPAAIVVNPAIGMMYWTDWGSRPKIEAAWMDG

QHRQVLLDEDLGWPTGLTLDYLNGNRIYWCDSKENIIESMKPDGTDRKIVLSGDIGNPYS

LDVFEGHVYWTTKVRGEVWKADKFGRGDKVKMLTINPWLTQVRIYHEHRYNRSVLNPCKG

VCSHLCLLRPGGYTCACPQGSSLVSFNENECDAASLPEVAMPVECGCINGGTCSTDKPGL

PKCKCPYGYSGRHCEIGKSRGAPAGTAVAVLLAVIIILLSGALAVGLFLNYKRTGSLIPS

MPKLPSLSSLAKLSDAGNGVSFRSGDGSTVDPQTLGVSFIDRAMQLNENFTDAGREPVTF

ENPLYSTATGAAADAAVIHATQVTVNISGDSTENVFANPMYNEQQQAVDMKNTPNEQAAK

ESKWNFFKKKLKPSTTFENPAYSEMRDKQSALTTEDISTSHPQPALPPLKREKSSMYSPT

EDSFQDTANLVKEDSDI*

>m.1013 g.1013 ORF g.1013 m.1013 type:3prime_partial len:507 (+) Unigene000103:5224-6747(+)

MRYIGREVEVDGGVRLVMESALSARDRVGVQDFLLLDNYTSEAAFIENLRRRYKENLIYT

YIGSVLVSVNPYKELGIYSKTSMERYRGVNFYEISPHIYGLADNSYRALRTSRKDQCILI

SGESGAGKTEASKKILQYYTTTCPTRNNTPSIRERLLQSIPVLEAFGNAKTLRNDNSSRF

GKYMDVQFDYKGAPIGGHILNYLLEKSRVVHQNHGERNFHIFYQLLESGDSSLLMRLGLD

TTNPQQYRYLVKGNCPRVSSISDKNSWKAVSTGLTIIGFNEKEVEELLKVVASVLHLGNT

QFGEDEYGETHFTTETQLTYLTELLGVEVSALSEALTHKKIVAKGEEMIGPLTLEQALSA

RDALAKAIYGRTFTWLVQKINQSLAFQDEVYYTSKCSSVIGLLDIYGFEVFQNNSFEQFC

INYCNEKLQQLFIELTLKSEQEEYEAEGIGWESVEYFNNKIICDLVEEKFKGIIAILDEE

CLRPGDATDTTFLEKLEDRLGGHAHFV

>m.1012 g.1012 ORF g.1012 m.1012 type:5prime_partial len:564 (+) Unigene000103:2-1693(+)

SKDRKSVLEKLEDRLGGHAHFVTHKLANGKIRKAVGREEFRLLHYAGEVNYSVNGFLDKN

NDLLYRNLKEVMCQSKNYIIKQCFHPNELTDQRRPETAATQFKLSLAKLMEILMSKEPSY

IRCIKPNDAKQPGRFEEVLVRHQVKYLGLMENLRVRRAGFAYRRSFEAFLQRYKPLCPDT

WPNFNGKLSDGVSTLVKHLDYKPEEYKLGRSKIFIRFPKTLFRTEDALENVKPTIVIILQ

KCWRGYREWAKYQGIRHAAITIQSWWRGVKGRRRAKRRRQAADTIRTFIKGFILRQEPRC

PDNEYFLDHVRFSYLMTIKRNLPKSVLDRTWPVPPPSLEEASVYIHRLCIRNLVNDYCRK

IQPEWKNQLEQKVVASGIFRGQKDNYPRSVPRLFVGTRLENEEINLKVRQTLGTDNKLKY

GVPVIKYDRHGFRARPRQLLMTGSSVVLVQESKIKQRIDYTSLLGISVSSLSDGFFVLHV

PTADSKQKGDLVLQSDHVIEAVTKLAILSDKIHNVNVSQDSICFAITRGKEGIIDFTSGA

ELRVMKAKNGHLAVTAPYINSSA*

>m.1011 g.1011 ORF g.1011 m.1011 type:complete len:760 (+) Unigene000103:2510-4789(+)

MRYIGREVEVDGGVRLVMESALSARDRVGVQDFLLLDNYTSEAAFIENLRRRYKENLIYT

YIGSVLVSVNPYKELGIYSKTSMERYRGVNFYEISPHIYGLADNSYRALRTSRKDQCILI

SGESGAGKTEASKKILQYYTTTCPTRNNTPSIRERLLQSIPVLEAFGNAKTLRNDNSSRF

GKYMDVQFDYKGAPIGGHILNYLLEKSRVVHQNHGERNFHIFYQLLESGDSSLLMRLGLD

TTNPQQYRYLVKGNCPRVSSISDKNSWKAVSTGLTIIGFNEKEVEELLKVVASVLHLGNT

QFGEDEYGETHFTTETQLTYLTELLGVEVSALSEALTHKKIVAKGEEMIGPLTLEQALSA

RDALAKAIYGRTFTWLVQKINQSLAFQDEVYYTSKCSSVIGLLDIYGFEVFQNNSFEQFC

INYCNEKLQQLFIELTLKSEQEEYEAEGIGWESVEYFNNKIICDLVEEKFKGIIAILDEE

CLRPGDATDTTFLEKLEDRLGGHAHFVTHKLANGKIRKAVGREEFRLLHYAGEVNYSVNG

FLDKNNDLLYRNLKEVMCQSKNYIIKQCFHPNELTDQRRPETAATQFKLSLAKLMEILMS

KEPSYIRCIKPNDAKQPGRFEEVLVRHQVKYLGLMENLRVRRAGFAYRRSFEAFLQRYKP

LCPDTWPNFNGKLSDGVSTLVKHLDYKPEEYKLGRSKIFIRFPKTLFRTEDALENVKPTI

GEGVTRGGVLLRMIVTGWSMWCQGVHDGNRGSMMVTGSL*

>m.1015 g.1015 ORF g.1015 m.1015 type:complete len:2059 (+) Unigene000104:145-6321(+)

MADVTARSLQYEYKANSNLVLQADRSLIDRTRRDEPTGEVLSLVGKLEGTKMGDKSQRTK

PQMLEERRAKRRKRDEDRHDINKMKGFTLLSEGIDDMVGIVYKPKTKETRETYEILLSFI

QAALGDQPRDILCGAADEVLAVLKNDKMRDKERRREVEQLLGPADDTRYHVLVNLGKKIT

DYGGDKELQNMDDNIDETYGVNVQFESDEEEGDEDQFGEVRDEGSDDSEGEEADVGCTLS

ANLGSAGDVMTNKKKDLHPRDIDAFWLQRQLSRFYDDAIVSQKKADEVLEILKTASDDRE

CENQLVILLGFNTFDFIKVLRQHRRMILYCTMLASAQSDSEKERIMSKMEADQDLSQVLY

QLQETEKEDIIREERSRRERVRKSRVDSDLDSINIDHGQSMPARHLLDLEDLAFTQGSHF

MANKRCQLPDGSFRKQRKGYEEVHVPALKPKPFSEDEALVAIEKMPKYSQAAFEGFKTLN

RIQSKLFKTTMETDGNLLVCAPTGAGKTNVALMAMLREIGKHINMDGTINVDDFKMIYIA

PMRSLVQEMVGSFGKRLASYGITVSELTGDHQLCKEEINASQIIVCTPEKWDIITRKGGE

RTYTQLVRLIIIDEIHLLHDDRGPVLESLIARTIRNVELTQEDVRLIGLSATLPNYEDVA

TCLRVDPAKGLFYFDNSFRPVPLEQTYVGITEKKAIKRFQIMNEIVYEKIMEHAGKNQVL

VFVHSRKETGKTARAIRDMCLEKDTLGLFLREGSASTEVLRTEAEQCKNLELKDLLPYGF

AIHHAGMTRVDRTLVEDLFADRHIQVLVSTATLAWGVNLPAHTVIIKGTQVYSPEKGRWT

ELGALDILQMLGRAGRPQYDSKGEGILITSHGELQYYLSLLNQQLPIESQMLAKLPDMLN

AEIVLGNVQNAKDAVNWMGYTYLYVRMLRNPTLYGVSHDERSTDPLLEMRRMDLVHTAAT

VLEKSNLVKYDKRSGTFQVTDLGRIASHFYITHDSIMTYNQLLKPTLSEIELFRVFSLSS

EFRNITVREEEKLELQKLLERVPIPVKESIEEPSAKINVLLQAYISQLKLEGFALMADMV

YVTQSAGRLMRAIFEIVLSRGWAQLSDKTMNLCKMIDKRMWQSMSPLRQFRKLPEEVIKK

IEKKSFPFERLYDLNHNEIGELIRMPKIGKTIHKYVHQFPKLDLAVHLQPITRSTLKVEL

TITPDFQWDEKIHGSSEAFWILVEDVDSEVILHHEYFLLKAKYAQDEHLVTFFVPVFEPL

PPQYFIRVASDRWLSCETQLPVSFRHLILPEKYPPPTELLDLQPLPVSALRNSAFECLYQ

NMFPFFNPIQTQVFNAVYNSDDNVFVGAPTGSGKTICAEFAILRMLLHNSEGRCIYITPM

ESLAEQVFVDWHQKFQGLLNKKVVMLTGETSTDLKLLGKGDIIISTPEKWDILSRRWKQR

KNVQNVSLFIVDEVHLIGGENGPVLEVICSRMRYISSQIERPIRIVALSSSLSNAKDVAH

WLGCSTTATFNFHPNVRPVPLELHIQGFNVSHTQTRLLSMAKPVYHTIMKHSPSKPSLVF

VPSRRQTRLTAIDILTFCAADVVPQRFLHCSEKELAPFLDKLSDCALKETLSNGVGYLHE

GLSMTERRTVEQLFNSGVIQVVVASRSLCWGTNISSHLVIVMDTQYYNGKIHAYVDYPIY

DVLQMVGKANRPIQDDEGCCVIMCQGSKKDFFKKFLYEPLPVESHLDHRLHDHFNAEIVT

KTVENKQDAVDYLTWTFLYRRMTQNPNYYNLQGMSHRHLSDHLSELVENTLHDLEQSKCI

SIEDEMDVAPLNLGMIAAYYYINYTTIELFSMSLNAKTKIRGLIEIISNAAEYKNIPIRH

HEDALLHQLAQKVPHKLNSPKFNDPHVKTNLLLQAHLSRMQLSAELQSDTEEILSKAVRL

IQACVDVLSSNGWLSPALAAMELAQMVTQAMWSKDSYLKQLPHFTTEHIKRCTEKGVESI

FDIMEMEDEDRINLLQLSDAQMADVARFCNRYPNIELSYEVADKEDIKSGSPVVVQVQLE

REEEVTGPVIAPLFPQKV*

>m.1023 g.1023 ORF g.1023 m.1023 type:complete len:2077 (+) Unigene000105:412-6642(+)

MSIRINHTAGGSLRRSQRNTLATRLQSEQTKQKTNSPPENKRSYNKASKAPPTPAAGSSR

GHAPRRNSLSLASLSQSASEPDSEAAGSSGQREQREVSNNRALKRSSATEHNNASYSPSP

AKRPKAPPPESTEQLEAADPTADSAPELPPSSRSKKRRQTDEHPTSRTVNKKSSACPASS

SSHKRKKADNSFCSSSSSSFSSSSSFLSGSSSEQASRSSKPTKLASKSAASAKSVTESAS

SSSASSSSFTSSSSSSSVVVAGASGCPPPSQGARLKQGKDQSKSRRSRSASSPSPRRSSR

DKEHSKSSTAASKFDWTSRFSPRVNLPKPRLALPGSSKPETPSGSSKPGPSGLQAKLASL

RKSSKKRSESPPAELPSFRRSTRQKTTGSCASTSRRGSGLGKRGAAETRRQDKMAENDNS

QDGANSSRTEETPQGASASSSVAGAVGMTTSGESESDDSEMGRLQALLEARGLPPHLFGP

RMSQLFHRTIGTGASSKAQQLLQGLQATGDESQQLQAAIEMCQLLVMGNEETLGGFPVKS

VVPALITLLQMEHNFDIMNHASRALTYMMEALPRSSAVVVDAIPVFLEKLQVIQFIDVAE

QALTALEMLSRRHSKAILQAGGLADCLLYLEFFSINAQRNALAIAANCCQSITPDEFHFV

ADSLPLLTQRLTHQDKKSVESTCLCFARLVDNFQHEENLLQQVASRDLLTNIQQLLVVTP

PVLSSGMFIMVVRMFSLMCSNCPNLAVQLMKQNIAETLRFLLCGASNGSCQEQIDLIPRS

PQELYELTSLICELMPCLPREGIFAVDSMLKKGSAHTTEAAVWQWRDDRGLWHPYNRIDS

RIIETAHQNGEDEISLSTLGRVYTIDFNSMQQINEDTGTARGIQRKPNPLINPNTGHVDV

RGEDARAELMREEAELARCFIKTLFGVLYEVYSSSAGPAVRHKCLRAILRIIYFSDSELL

KDVLRNHAVSSHIASMLSSQDLKIVVGSLQMAEILMQKLPDVFSVYFRREGVMHQVKNLA

ESESFLPSPPKACTSGITAATTTITTATTPAASSVAPDLGSPSFHNNMEDSLDLSPQGRL

SDVLKRKRLPRRVPRRPKYSPPRDDDKVDNQAKSPTTTASPKSSFLASLNPKTWGKLSAQ

NNSTNSEPPRTAGVSGLARTANKDSVSNNRDKIKAWIKEQASKFVERYFNSENVDGSNPA

LNVLQRLCTATEQLSLQVDGGLECLVEICSIVSESDVSSFEIQHSGLVKQLLIYLTSNSE

RDLISRDQRIKRFLHIFCGSPLPGQEPLFRLEPTANGALLALVHKMNSCLSQMEQFPVKV

HDFPSGNGNGSRGSQALKFFNTHQLKCQLQRHPDCTNVKQWKGGPVKIDPLALVQAIERY

LVVRGYGRIREEDEDSDDDGSDDEIDESLAAQFLNSGSVRHRLQFYIADHLLPYNMTVYQ

AVRQFSLQPEEERESTDDEANPLGRAGIWTKTHTIWYKPVREDEDGSKDSVGGKRGRAQT

APTKTSPRNAKKQDELWHDGVCPSVSNPLESYLISEPPEGITFDDPSLEVILLLRVLHSI

SRYWFYLYENAVCKEIIPTSEFINSKLTAKANRQLQDPLVIMTGNIPPWLTELGKTCPFF

FPFDTRQMLFYVTAFDRDRAMQRLLDTNPEINQSDSQDSRVAPRLDRKKRTINRDELLKQ

AESVMQDLGSSRAMLEIQYENEVGTGLGPTLEFYALVSQELQRADLGLWRGEEVTLANPK

GSQEGTKYMFSSRGLFAVPFGRSTKPAHIAKIKMKFRFLGKLMAKAIMDFRLLDLPLGLP

FYKWMLRNESSISSHDLVNIDPGVAKSVQHLEDIIRQKRRLEQDKSYTRETLQQALESLN

MNGCSVEDLGLDFTLPGFPNIELKKGGKDVPVTIHNLEEYLRLVVYWTLNEGVSRQFESF

REGFESVFPLHHLQYFYPEELDQLLCGSKSETWDVKTLMECCRPDHGYTHDSVCVCSRAV

RFLFEVLSSFDAEQQRLFLQFVTGSPRLPVGGFRSLNPPLTIVRKTFESTENPDDFLPSV

MTCVNYLKLPDYSSIEIMREKLLMAAREGQQSFHLS*

>m.1031 g.1031 ORF g.1031 m.1031 type:complete len:335 (+) Unigene000106:691-1695(+)

MTKIGINGFGRIGRNVFRAALNNSEVEVVAINDLTDAKTLAHLLKYDTVHGTLNAEVSAN

ENSIVVNGKEIKVIAERDPAQLPWSDYGVEVVVESTGRFTKKSDAEKHLGGSVKKVIISA

PASDEDITVVMGVNHEQYDAANHNVVSNASCTTNCLAPFAKVLNEKFGVKRGMMTTIHSY

TNDQQILDLPHKDLRRARAAAENMIPTSTGAAKAVALVLPELKGKLNGGAVRVPTANVSL

VDLVVELDKEVTVEEVNAAFKAAAEGELKGILGYSEEPLVSIDYNGCTASSTIDALSTMV

MEGNMVKVLSWYDNETGYSNRVVDLAAYMTSKGL*

>m.1030 g.1030 ORF g.1030 m.1030 type:complete len:395 (+) Unigene000106:1835-3019(+)

MNKKSIRDVDLKGKRVFCRVDFNVPMKEGKITDETRIRAALPTIQYLVEQGAKVILASHL

GRPKGQVVEELRLTPVAARLGELLGKDVKKADEAFGPVAQEMVAAMNEGDVLVLENVRFY

AGEEKNDAELAKEFAALADIFVNDAFGAAHRAHASTAGIADYLPAVSGLLMEKELEVLGK

ALSNPERPFTAIIGGAKVKDKIGVIRHLLDKVDNLIIGGGLAYTFVKALGHEIGLSLCED

DKIELAKEFMQLAKEKGVNFYMPVDVVITEEFSETATTKIVGIDSIPSNWEGVDIGPKTR

EIYADVIKNSKLVVWNGPMGVFEMTPFSQGTKAVGQALADAEGTYSVIGGGDSAAAVEKF

GMADKMSHISTGGGASLEFMEGKELPGVVCLNDK*

>m.1029 g.1029 ORF g.1029 m.1029 type:complete len:432 (+) Unigene000106:5364-6659(+)

MSTIIDVYAREVLDSRGNPTVEVEVYTESGAFGRAIVPSGASTGEHEAVELRDGDKSRYL

GKGVMNAVNNVNEAIAPEIVGFDVTDQAGIDRAMIELDGTPNKGKLGANAILGVSMAVAH

AAADFVGLPLYRYLGGFNAKQLPTPMMNIINGGSHADNNVDFQEFMILPVGAPTFKESIR

MGAEVFHALKAVLHDKGLNTAVGDEGGFAPNLGSNREALEVIIEAIEKAGYKAGENVFLG

MDVASSEFYNKETGKYDLAGEGRTGLTSAEMVDFYEELCKDFPIISIEDGLDENDWDGHK

LLTERIGDKVQLVGDDLFVTNTQKLAEGIEKGISNSILIKVNQIGTLTETFEAIEMAKRA

GYTAVVSHRSGETEDATIADIAVATNAGQIKTGSMSRTDRIAKYNQLLRIEDELGEIAVY

DGIKSFYNIKR*

>m.1028 g.1028 ORF g.1028 m.1028 type:complete len:510 (+) Unigene000106:3804-5333(+)

MRKPTALIILDGFGLREETYGNAVAQAKKPNFDGYWNKFPHTTLTACGEAVGLPEGQMGN

SEVGHLNIGAGRIVYQSLTRVNVAIREGEFDKNETFQSAIKSVKEKGTALHLFGLLSDGG

VHSHMNHMFALLRLAAKEGVEKVYIHAFLDGRDVGPKTAQSYIDATNEVIKETGVGQFAT

ISGRYYSMDRDKRWDRVEKCYRAMVNGEGPTYKSAEECVEDSYANGIYDEFVLPSVIVNE

DNTPVATINDDDAVIFYNFRPDRAIQIARVFTNEDFREFDRGEKVPHIPEFVCMTHFSET

VDGYVAFKPMNLDNTLGEVVAQAGLKQLRIAETEKYPHVTFFFSGGREAEFPGEERILIN

SPKVATYDLKPEMSIYEVTDALVNEIENDKHDVIILNFANCDMVGHSGMMEPTIKAVEAT

DECLGKVVEAILAKDGVALITADHGNADEELTSEGEPMTAHTTNPVPFIVTKNDVELRED

GILGDIAPTMLTLLGVEQPKEMTGKTIIK*

>m.1036 g.1036 ORF g.1036 m.1036 type:5prime_partial len:121 (-) Unigene000107:6303-6665(-)

ERERERQKECVCVCSFSLNKWYNLSSLHLYKKNKNFRIKYIFYKERQFFFLFFLVPFLFF

FLHFSYFQKKKKNYHFSLDFGLKTGLRHEPAEEAEEKTERRKKRKRLLTKISSRLQNLAE

*

>m.1034 g.1034 ORF g.1034 m.1034 type:complete len:1956 (+) Unigene000107:197-6064(+)

MSAADRYLYVDRSLVNNPLAQADWASKKLVWVPSERLGFEAGSLREEHGDEVVVELADSG

KKIRINKDDVQKMNPPKFSKVEDMAELTCLNEASVLHNLRERYYSGLIYTYSGLFCVVVN

PYKMLPIYSEKIIDMYKGKKRHEVPPHIYSITDNAYRNMMQDREDQSILCTGESGAGKTE

NTKKVIQYLAVVASSHKGKKDTSAGELEKQLLQANPILEAFGNAKTIKNDNSSRFGKFIR

INFDVTGYIVGANIETYLLEKSRCIRQAKTERAFHIFYYMVAGARDKLREELLLENFNNY

RFLVAGHVQVPGNQDDEMYDETMEAMNIMGFTDEERIGIQKVVSTVLQLGNIEFKKERNQ

EQATMPDNTAAQKVCHLSGINVTDFTRAILTPRIKVGREVVQKAQTKEQADFAVEALAKA

TYERLFRWILARVNKALDKTKRQGASFLGILDIAGFEIFEDNSFEQLCINYTNEKLQQLF

NHTMFILEQEEYQREGIEWSFIDFGLDLQPCIDLIERPANPPGVLALLDEECWFPKATDK

SFVEKVLQEQGTHPKFFKPKKLKDEADFCIIHYAGKVDYKADEWLMKNMDPLNDNVASLL

NQSTDKFVSELWKDVDRIVGLDKVAGMSEMPGAFKTRKGMFRTVGQLYKEQLSKLMATLR

NTNPNFVRCIIPNHEKKAGKLDPHLVLDQLRCNGVLEGIRICRQGFPNRIVFQEFRQRYE

ILTPNAIPKGFMDGKQACVLMIKALELDPNLYRIGQSKVFFRAGVLAHLEEERDMKITDV

IISFQAWCRGYVARKAFAKRQQQLTAMRVIQRNCAAYLKLRNWQWWRLFTKVKPLLQVSR

QEEEMQAKDEELTKVREKQVVAEKMIEEMEVKHQQLNAEKMALQEQLQAEMDLCAEADEM

RNRLVAKKQELEEILHDLEARVEEEEERANQLQMEKKKMQQNITDLEQQLDEEEAARQKL

QLERVTMEAKLKKIEEDVMVLEDQNTKLNKEKKLMEERISEFTTNLAEEEEKSKSLQKLK

NKHEAMITDLEDRLRKEEKMRQELEKNRRKLEGDSTELHDQIAELQAQIAELRAQLAKKE

EELQEALARIEEEAAQKNLAHKKIRELEAQLSELQEDLELERAARTKAEKHRRDLGEELE

ALKTELEDTLDTTAAQEALRTKRETEVTHLKKSLEDEAKNHEQIVAEMRQKHGQAFDELN

EQLEQSKRNKASVEKMKQTLESERNELQIELQGLMQSKGESEHRRKKAEAQVQELQIKHS

ESEKQRAELMEKVSKLQAELDSVNSVLSDVEGKSIKAVKDCSAVESQLQDVQELLQEETR

QKLSLNTRMRQLEDEQHNLREQLEEEEEAKKNMEKQLIAAQSQLAEMRKKIEQEASSLES

TEEVKKRVQRDLESVSQRLDERNLAFDKLDKTKTRLQQELDDLLVDQDHLRQIVSNLEKK

QKKFDQMLAEEKTISARYAEERDRAEAEAREKETRALALTRELETLTDIKNEMERANKAL

RAEMEDLVSSKDDVGKSVHELEKSKRGMEQQLEEMRTQLEELEDELQATEDAKLRLEVNM

QAMKAQYERDLAGRDEMGEEKKRQLVKQVREMEMELEDERKQRSVAMAARKKLELDLKEL

EAAIDQANKNREEALKQLKKLQAQMKDVLRELDETRLSREEILALSKENEKKFKSMEAEM

IQIQEELAAAERAKRQAQQERDELLDEINNQNSKNALTVEERRRLEARIAQLEEELEEEQ

NNSELTNDRLKRTMLQVDQVNVELTAERSTAQRLEGARSQMERQNKEMKLKLTELEGAVK

SKYKASIGALEAKIAQLEEQLDIETRERQAATKLVRRSEKKLKEVILQVDDERRNADQYK

DQAEKMNSRMKQLKRQLEEAEEEAQRANANRRKLQRELEDATESADAMNREVSSLKSKLR

RGDLPFTMRRIVSRAGIESDEEPESKGETPEPKPE*

>m.1043 g.1043 ORF g.1043 m.1043 type:complete len:2077 (+) Unigene000108:377-6607(+)

MSIRINHTAGGSLRRSQRNTLATRLQSEQTKQKTNSPPENKRSYNKASKAPPTPAAGSSR

GHAPRRNSLSLASLSQSASEPDSEAAGSSGQREQREVSNNRALKRSSATEHNNASYSPSP

AKRPKAPPPESTEQLEAADPTADSAPELPPSSRSKKRRQTDEHPTSRTVNKKSSACPASS

SSHKRKKADNSFCSSSSSSFSSSSSFLSGSSSEQASRSSKPTKLASKSAASAKSVTESAS

SSSASSSSFTSSSSSSSVVVAGASGCPPPSQGARLKQGKDQSKSRRSRSASSPSPRRSSR

DKEHSKSSTAASKFDWTSRFSPRVNLPKPRLALPGSSKPETPSGSSKPGPSGLQAKLASL

RKSSKKRSESPPAELPSFRRSTRQKTTGSCASTSRRGSGLGKRGAAETRRQDKMAENDNS

QDGANSSRTEETPQGASASSSVAGAVGMTTSGESESDDSEMGRLQALLEARGLPPHLFGP

RMSQLFHRTIGTGASSKAQQLLQGLQATGDESQQLQAAIEMCQLLVMGNEETLGGFPVKS

VVPALITLLQMEHNFDIMNHASRALTYMMEALPRSSAVVVDAIPVFLEKLQVIQFIDVAE

QALTALEMLSRRHSKAILQAGGLADCLLYLEFFSINAQRNALAIAANCCQSITPDEFHFV

ADSLPLLTQRLTHQDKKSVESTCLCFARLVDNFQHEENLLQQVASRDLLTNIQQLLVVTP

PVLSSGMFIMVVRMFSLMCSNCPNLAVQLMKQNIAETLRFLLCGASNGSCQEQIDLIPRS

PQELYELTSLICELMPCLPREGIFAVDSMLKKGSAHTTEAAVWQWRDDRGLWHPYNRIDS

RIIETAHQNGEDEISLSTLGRVYTIDFNSMQQINEDTGTARGIQRKPNPLINPNTGHVDV

RGEDARAELMREEAELARCFIKTLFGVLYEVYSSSAGPAVRHKCLRAILRIIYFSDSELL

KDVLRNHAVSSHIASMLSSQDLKIVVGSLQMAEILMQKLPDVFSVYFRREGVMHQVKNLA

ESESFLPSPPKACTSGITAATTTITTATTPAASSVAPDLGSPSFHNNMEDSLDLSPQGRL

SDVLKRKRLPRRVPRRPKYSPPRDDDKVDNQAKSPTTTASPKSSFLASLNPKTWGKLSAQ

NNSTNSEPPRTAGVSGLARTANKDSVSNNRDKIKAWIKEQASKFVERYFNSENVDGSNPA

LNVLQRLCTATEQLSLQVDGGLECLVEICSIVSESDVSSFEIQHSGLVKQLLIYLTSNSE

RDLISRDQRIKRFLHIFCGSPLPGQEPLFRLEPTANGALLALVHKMNSCLSQMEQFPVKV

HDFPSGNGNGSRGSQALKFFNTHQLKCQLQRHPDCTNVKQWKGGPVKIDPLALVQAIERY

LVVRGYGRIREEDEDSDDDGSDDEIDESLAAQFLNSGSVRHRLQFYIADHLLPYNMTVYQ

AVRQFSLQPEEERESTDDEANPLGRAGIWTKTHTIWYKPVREDEDGSKDSVGGKRGRAQT

APTKTSPRNAKKQDELWHDGVCPSVSNPLESYLISEPPEGITFDDPSLEVILLLRVLHSI

SRYWFYLYENAVCKEIIPTSEFINSKLTAKANRQLQDPLVIMTGNIPPWLTELGKTCPFF

FPFDTRQMLFYVTAFDRDRAMQRLLDTNPEINQSDSQDSRVAPRLDRKKRTINRDELLKQ

AESVMQDLGSSRAMLEIQYENEVGTGLGPTLEFYALVSQELQRADLGLWRGEEVTLANPK

GSQEGTKYMFSSRGLFAVPFGRSTKPAHIAKIKMKFRFLGKLMAKAIMDFRLLDLPLGLP

FYKWMLRNESSISSHDLVNIDPGVAKSVQHLEDIIRQKRRLEQDKSYTRETLQQALESLN

MNGCSVEDLGLDFTLPGFPNIELKKGGKDVPVTIHNLEEYLRLVVYWTLNEGVSRQFESF

REGFESVFPLHHLQYFYPEELDQLLCGSKSETWDVKTLMECCRPDHGYTHDSVCVCSRAV

RFLFEVLSSFDAEQQRLFLQFVTGSPRLPVGGFRSLNPPLTIVRKTFESTENPDDFLPSV

MTCVNYLKLPDYSSIEIMREKLLMAAREGQQSFHLS*

>m.1048 g.1048 ORF g.1048 m.1048 type:5prime_partial len:2023 (+) Unigene000109:3-6071(+)

TLTHTHSSQQTLTKACLSGYGSPAAPVKSSNSLTGQTPPQQQPQSFSPNQPPSHLSQPYA

GFSSPQTHDLPSARTAGVAKGYGNLGSQSFSAESVYGTDSGYGSLPSSLGGAGSPSMGYA

ASGHSPALLRSGASGGTAGGSSNGGSTSTGGGNNVTGGGGSYHIPDSSPSPSGNSGIIRP

GLHSPAPSRPAQSPVGTGSNKYLSSVLSPSFLPSPQGYPDTRGPRSQTYHPSAPPKTKSD

TSLLGVTESRPQQDDDDDDFLIQHLLQAQSPTPQASHHHPPQSHHSSQSTQQPPVLPNQD

GSKGLSYEIGKSSEERYHLQSVIRTHSATTNAAVSGTGNGAGLDSQLELSLKKQQHQQQH

QTQHQQQQQQHKHQQQKSSRSVTDGGGRGNPDQAHSHSHHHHDSMSSVVHYGRGDPYSQH

SISQHPSSHHQHTPHTPTHSHLHSHPHMDLQKKPQESADMAYIRKTPDLQQHHHQQQSGH

HQSHQQQHQQQQQQSQTLMESPTDQSRQPPHLLQSVLSHTTRNKIETQQQQHPASQQALI

EATGGVTAAETHSQPQASQLQLQLQTQALETASHYSHGSQQQDQNHQKSNTVSSLDMLER

SLSRTSSQEGGMVDDRPGNEGGRGNSAAGTGGPVERHRSQDQQRLPSHHPTQHHPPELHT

YLSEPDLGLSTPSHGHHLSHHHHQSQQPPPAQQPNSHHHAHHQHQNPQSHHHLASQSASA

TAQQQEQSHSSHSSQITQAQLDQQHQGDHHFDSSGSAVKSNANQNQSHHNQRFVPLTSIC

FPDSLLPDEERSFFPGMEDMFCPEEYKSSCSGGTGQGQDGVSQAQTGQEGMEGIKSTAEG

GEDDTGASYDMLGHPGDQDYGQYCHDLTEHDNGTMHLDLDSLKTHELPSTVNTEQLGLIQ

SQPTNLGIGSSANVGDGAGAKMVGSGGSVSGSGGLTSPIFCSSRPKKLLKSSSFHLLKER

PDPNSIPKKSYAQEYEFEDDEDKADVPADIRLNSRRLPDLIPDLVSSCRKAGGGTMSPLM

SDLDFGYPSLGPPPQLLPNDGPKKRGRKPTKPKREGPPRPRGRPRIRPLPEPHHSRAMMG

ECGTGYIPERGRGRGRGRGRGRRDESMMDSKDQSQLYQQHMQQQQMHHLPQQHQEPIKPI

KIKLPIGSMSSSDALLRTDSLSGTDPALSDGSVGSAPSLGLSPGTPGVPDMTRPLDKNKA

KTQDMEEKDSEKTGFVASFLDFLKSGKRPGSSSGNGDSSPGKSGGIHPISPQPPPPPAPA

GSSGYGDSEGDGGLSLGGCPSPCKRLDEELKRNLETLPSFSSDEEDSVGKNQDLQKSISS

AISALYDTPQLSTSSMQPPSLPPPPPPVAPTPLTPTQHPPSLSPQTPTHMHTQPSSHTHP

QSIESTILSRDEQGDEVEEEIINDEDKEEEQDKGVEEKVTEMELLSVPKVGDSPKEASQA

VAPHSLLPLFPSSPASSSSPSPPPLPPLSLPSPLPELEENPSPPQKPSAQLSSPPASDLA

TTLPVLSPPHPLTVASPPPLETPTPSSPPPTQQTVPAPPSPEETPATQILPLHLAQKQSG

AAIAGETDDDESESGGEGIFRERDEFVVRIEDIKTLKLALQTGREPPPIWRVQKALLQKF

APEIKDGQRQFCATSNYLGYFGDAKKRYQRLYVKFLENVNKKDYVRVCSCKPWHRPSVTL

RRQAQSHPVSKVNPSPNNQTLPRVVRDEKDKDREKGRNKEQRDSRDKNNIKVKEQQEKEK

VVKIKEREEKKDKEKKMPPPPAPQRAGKQTAVTEPGRKEEKRGVERKIERTTKQQPLKVK

AEPPPKKRRKWLKEVPSSSDSDSSASDDEISVRAGLNTRAMREMYRSYVEMLVSTALDPD

MIQALEDTEDELYLPPMRKIDSILSEHKRKLLKRINMNPQHQEALHLFPQITAEPLDSGA

VRVKLGGECYNRKTLNRVKKSISKPQDIKLSTETCRLYSFYHSLHHYKYHTFLICKKETN

TIEQASEDPGQEEVVQQCMANQSWLDTLFNAFLELMTLSAKA*

>m.1057 g.1057 ORF g.1057 m.1057 type:complete len:2072 (+) Unigene000110:370-6585(+)

MSIRINHTAGGSLRRSQRNTLATRLQSEQTKQKTNSPPENKRSYNKASKAPPTPAAGSSR

GHAPRRNSLSLASLSQSASEPDSEAAGSSGQREQREVSNNRALKRSSATEHNNASYSPSP

AKRPKAPPPESTEQLEAADPTADSAPELPPSSRSKKRRQTDEHPTSRTVNKKSSACPASS

SSHKRKKADNSFCSSSSSSFSSSSSFLSGSSSEQASRSSKPTKLASKSAASAKSVTESAS

SSSASSSSFTSSSSSSSVVVAGASGCPPPSQGARLKQGKDQSKSRRSRSASSPSPRRSSR

DKEHSKSSTAASKFDWTSRFSPRVNLPKPRLALPGSSKPETPSGSSKPGPSGLQAKLASL

RKSSKKRSESPPAELPSFRRSTRQKTTGSCASTSRRGSGLGKRGAAETRRQDKMAENDNS

QDGANSSRTEETPQGASASSSVAGAVGMTTSGESESDDSEMGRLQALLEARGLPPHLFGP

RMSQLFHRTIGTGASSKAQQLLQGLQATGDESQQLQAAIEMCQLLVMGNEETLGGFPVKS

VVPALITLLQMEHNFDIMNHASRALTYMMEALPRSSAVVVDAIPVFLEKLQVIQFIDVAE

QALTALEMLSRRHSKAILQAGGLADCLLYLEFFSINAQRNALAIAANCCQSITPDEFHFV

ADSLPLLTQRLTHQDKKSVESTCLCFARLVDNFQHEENLLQQVASRDLLTNIQQLLVVTP

PVLSSGMFIMVVRMFSLMCSNCPNLAVQLMKQNIAETLRFLLCGASNGSCQEQIDLIPRS

PQELYELTSLICELMPCLPREGIFAVDSMLKKGSAHTTEAAVWQWRDDRGLWHPYNRIDS

RIIETAHQNGEDEISLSTLGRVYTIDFNSMQQINEDTGTARGIQRKPNPLINPNTGHVDV

RGEDARAELMREEAELARCFIKTLFGVLYEVYSSSAGPAVRHKCLRAILRIIYFSDSELL

KDVLRNHAVSSHIASMLSSQDLKIVVGSLQMAEILMQKLPDVFSVYFRREGVMHQVKNLA

ESESFLPSPPKACTSGITAATTTITTATTPAASSVAPDLGSPSFHNNMEDSLDLSPQGRL

SDVLKRKRLPRRVPRRPKYSPPRDDDKVDNQAKSPTTTASPKSSFLASLNPKTWGKLSAQ

NNSTNSEPPRTAGVSGLARTANKDSVSNNRDKIKAWIKEQASKFVERYFNSENVDGSNPA

LNVLQRLCTATEQLSLQVDGGLECLVEICSIVSESDVSSFEIQHSGLVKQLLIYLTSNSE

RDLISRDQRIKRFLHIFCGSPLPGQEPLFRLEPTANGALLALVHKMNSCLSQMEQFPVKV

HDFPSGNGNGSRGSQALKFFNTHQLKCQLQRHPDCTNVKQWKGGPVKIDPLALVQAIERY

LVVRGYGRIREEDEDSDDDGSDDEIDESLAAQFLNSGSVRHRLQFYIADHLLPYNMTVYQ

AVRQFSLQPEEERESTDDEANPLGRAGIWTKTHTIWYKPVREDEDGSKDSVGGKRGRAQT

APTKTSPRNAKKQDELWHDGVCPSVSNPLESYLISEPPEGITFDDPSLEVILLLRVLHSI

SRYWFYLYENAVCKEIIPTSEFINSKLTAKANRQLQDPLVIMTGNIPPWLTELGKTCPFF

FPFDTRQMLFYVTAFDRDRAMQRLLDTNPEINQSDSQDSRVAPRLDRKKRTINRDELLKQ

AESVMQDLGSSRAMLEIQYENEVGTGLGPTLEFYALVSQELQRADLGLWRGEEVTLANPK

GSQEGTKYMFSSRGLFAVPFGRSTKPAHIAKIKMKFRFLGKLMAKAIMDFRLLDLPLGLP

FYKWMLRNESSISSHDLVNIDPGVAKSVQHLEDIIRQKRRLEQDKSYTRETLQQALESLN

MNGCSVEDLGLDFTLPGFPNIELKKGGKDVPVTIHNLEEYLRLVVYWTLNEGVSRQFESF

REGFESVFPLHHLQYFYPEELDQLLCGSKSETWDVKTLMECCRPDHGYTHDSRAVRFLFE

VLSSFDAEQQRLFLQFVTGSPRLPVGGFRSLNPPLTIVRKTFESTENPDDFLPSVMTCVN

YLKLPDYSSIEIMREKLLMAAREGQQSFHLS*

>m.1062 g.1062 ORF g.1062 m.1062 type:complete len:1394 (+) Unigene000111:2046-6227(+)

MAKSCQADQFSVQIEEQRKKYADEIASLGERHQQEVEKLKMEHAEALNQQHCTDVEALEQ

KHQTETDVMMKDKEREFQEHVKEMNQKMLEKLEIKQAKLEALSSELSEMQKCKEQLEEHK

ESVRMELEVRLKDEQVKFQKEVDDLKQKHEEMLEGLEKHQKEEINQLQLVLEEKNKELEQ

YVEKERILQEDTNKAKQDVETMLKEIEDLRCVAQSEKEKLAEAAAQTTLLKEELTKSQSR

VQDLEKLLEESRNECQHKEEHLQQKSHENFELQQRIQQASNDLAETTSSHAQTHQAMQEE

QDGLKKQLEEERSSSEKRVESFRKEMESKLKSQETKMEKLRQKAKEMQEKFKKKLQEQEE

NAKAEMAKKEKELQQKEQQVKEKILEIAQANSEGLSSALSDLEANHKEQFEKIQKVHKLE

QDDLLRIWQEKLSQQEEELQEKHALTMQEKMQELEDISQQLLTHREEKRQMDQEIKNLKE

ELSMRETTVQKLQTELREAAGKLDSLSKVEELLKTQVETVEKNLNQALNERNIFQDQLTA

AEETSKEKILALSEELANACKKLTILEASKSKEGENLQKSLEEKTFELQNKESKFLEQIH

NVGEELEQHCHTAQTIINDFSARLCERVETKVNELQSRVMFNQKKVCHLKNLILTKNHRI

CCLEQELQQVVEENQKLKNSLEETTLQLHANSENLKALNVEKETLQNDAKNHSQLLSEKD

LSIEKTHEENKNLSENLKANILHISNLENVIDDLKKQLSCSITEKEEAISLLNQQHQEEK

QSVQNQMEQAENEKGLALKQVETLRNKLADFKKKTDFKISQNQNTVRSLQRQIEDMEKQI

AEKDEQLQKLTASIDNQCISKSELDQVLSEKEQKVSALNVELDDCTKKISELEQRLKSQS

MEQEKKEAELQNHVTSSEMEKTELIQQLEQTRQQCSLQQDLVSKTQEQLHALENEFQAVK

QKLESQQEEFEREKSKEEALKAAEGKVADLKKKAEQKISLIRKQLTSQIEQKEQTIKELQ

AQLEDIMQTQNEKEQQVRNLEENGRKMEEAINNLMEEHKKHLEQVLSDGQVEKECMDKED

LVAAAAAAATATVNDREEETWSRIRDLEAKLLQSEEQNTSCRAEINQLKEELCEQTSLVQ

ELQKSLEVQVKEKETNVIQMDKCFVEKSNNLLGMEHVQYVGDQNTKSHQDIWETEKELLV

KDYEMKLQDLHKRLEEKENVQLEHSLEQKLLDAENEKQKIHKDYTRLQKDLRSLRREHEK

DLEYLRKEIAEESEKKLKSDVEDLEMKHNSTLKQMMREFNTQIALKDKELETSVREAVEK

AQMVEAELMGIHKEEANQLQQIISQKDDDLKKNHPTLRATPSESRRRNGQSSVGGAAGAG

GAAAEESNRSSGC*

>m.1063 g.1063 ORF g.1063 m.1063 type:complete len:540 (+) Unigene000111:176-1795(+)

MFKKLKQKINEEQSPQRNVLSPQQAQVSSGGRAVKSPLLQPDTASSSSERENFSKGAVSP

QSGSVNGDGAVSPLREEAPSLAQKLQQRVSSVESLFRGSGRAEGLFRSGSRDSLVRSASR

ESLTPLGENEAPGVPSFDPPSDIESEAEDSPGNAEALSKDQLVYRLHRVEKSLANYRGKY

SELVTAYRTVQRDKEKTQVVLSQSQDKALRRIGELREELQMDQQAKKHLQEEFDAALEEK

DQMITVLQTQVALLKKRLHGSPGSLLTSEAGDSQTAETTETIADPQSVTQAEIEELIQAG

GSSEPENTADSEALQKRVRRQETLLQRCNELMKTNKERSAQLGSENDVLQQQLQERLQEL

EKMKELHTSEKTKLITQLRDAKNLIEQLEQDKGMVIAETKRQMHETLEMKEEEIAQLRTR

IQQTLTQKEELQEQREKAEKAAFEELERALGVAQRAEEARRQLQERMEKEVKQVEQTSEE

ERRSLQQELTRVKQEVISIMKKSTEERIAEMEQQHLEDLANKDLQMSVQIQEAVVELLS*

>m.1064 g.1064 ORF g.1064 m.1064 type:3prime_partial len:140 (+) Unigene000111:6148-6570(+)

MGSRVWEVQQELEELQQRSQTGPQGVDELQVQLAEKTTLLSETRLKEQEYQDRIHTLEDM

IRKAYKNSVVTHLGKDVSHHTAESLSEPTELEYLRKVMFEYMMGRETKTMAKVITSVLKF

PEDQAQKILEREDSRLMTWL

>m.1069 g.1069 ORF g.1069 m.1069 type:complete len:722 (+) Unigene000112:434-2599(+)

MKRLDDEPVHSGNRTSLSDNLTNGDSVTQRNPVALLQGLHLCQEAWNPGSPWDREGAHAA

LWGRPPGSFLVVSESSSENKLLCVSVSDEDKKVEDFPIIHTGSTQRLNTSHLAFADLLQL

VIFYNFSRDVLPFCLRIPSWVCGLTEQPKHLVSELGPKAWLCPPTDLLPDTMSPETADIQ

DTVMCTIQLTADNGALCFINPLYLQEHGDDWLTHSQVSANLANHLFTSKRDRRLSTTRPW

AGAGLKNQTKSVGDSAVMEISGSPVHGLTSPVTGVILRRASSTSDPQWRMSAESISSPIP

QSPHRVSWIEDKVWMSPPAPSSLLHPPCLEFDSLSMSSIEEEPEVEPAVSSCQVQNSPRL

PLADKVKNRLSAVGQALGGFIKPQKRLSKRVQEMSERKGSPFAEALKGFVEQTLKLRASC

HVTSTEMLQEVRSSLTALREMLYDSAEIQSIIDTLGDVPDFELDTMMEQALHKVALKPLC

SHLYECMKTARQQDGSLQRLQANQNTLKCRRLEELEGTAGAGVPDAAMLEKIQQRWATMH

QQYSPQMKVELLLKVCKNVYHSMTVNAKSGVVFGADDFLPCLTWVILRSDVVTLQIDTDY

MMELLDPSQLQGEGGYYLTSLYASLYYISSFQSRLAARQLSAEAQKSLSQWHRRRTLHCN

QSRRSRNRRTIRRHRNNEKEDGESGELDIRNMSKEKDWTHTDDVTETLPSVNEISGEEKE

E*

>m.1075 g.1075 ORF g.1075 m.1075 type:3prime_partial len:2152 (+) Unigene000113:108-6566(+)

MAAFGMLSYEHRPLKRPRLGPPDVYPQDPKQKEDELTALNVKQGFNNQPAVSGDEHGSAK

NVNFNPSKISSNFSSIIAEKLRCNTFPDTGKRKPQVNQKDNFWLVTARSQSSINNWFTDL

AGTKPLTQLAKKVPIFSKKEEVFGYLAKYSVPVMRSAWMIKMTCAYHAAITETKVKKRHV

IDPCIEWTQIITKYLSEQLQKVAEFYRQSPSQGCGSPLPAPPAEVETAMKQWEYNEKLAM

FMFQDGMLDRHEFLTWVLECFEKIRPGEDELLKLLLPLLLQYSGEFVQSAYLSRRLAYFC

TRRLNLLLSDGSIGPSSGGHPPHSITSQPGNALPTPPTSQPAGTTQPQTQFTDYYICPQH

RPVVFGLSCMLQSIVLCCPSALVWHYSLTDSRNKTGSPLDLLPIAPSNLPMPGANSTFNQ

QVRAKLREIEEQIKERGQAVEFRWSFDKCQETTAGFTIGRVLHTLEVLDNHSFEKSDFSN

SLDSLYNRIFGSGQSKDGHELSPDDDAVVTLLCEWAVCCKRSGRHRAMVVAKLLEKRQTE

IEAERCGESEVVDEKGSVSSGSLSAATLPVFQDVLLQFLDTQAPVLTEPGNESERVEFSN

LVLLFCELIRHDVFSHNIYMCTLISRGDLASDSHLSRPRSPSDEPSDESERKEQDAGSGV

KMEDTGLSEPMEIDNNSSANFDEMFSPPMHCEAKGSPSPEKNAPEQDGKSTAKEKGLDPT

FPQVYDQPRHIQYATHFPIPQEESASHECNQRLVVLYGVGKQRDEARHAIKKITKDILKV

LNRKSTAETGGEEGQKRKRSKPEAFPTAEDIFSKFQHLSHFDQHQVTSQVSRNVLEQITS

FALGMSYHLPLVQHIQFIFDLMEYSLNISGLIDFAIQLLNELSLVEAELLLKSSSLAGSY

TTSLCLCIVAVLRRYHSCLILNPDQTAQVFDGLRIVVKSGVNPADCSSAERCILAYLYDL

YTSCSHLKSKFGEIFSEFCSKVKNSIYWNIDPSDSNMLWDQVFMIDAIANPSAHNINHSM

VGKILNDSPANRYSFVCNVLMDVCVDHRDPDRVNDIGILCAELTAYCRSLSAEWLGVLKA

LCCSSNNGNCGFNDLLCNVDVSDLSFHDSLATFVAILIARQCLLLEDLVRCVAIPSLLNA

ACSEQDSEPGARLTCRILLHLFRTPQRNPCPQDSKTDKATVGIRSSCDRHLLAASQNSIV

VGAVFAVLKAVFMLGDAELKGSGFSHPAGLDDIGEDDIGSKKSGGRNISIETASLDVYAK

YVLKSICQQEWVGERCLKSLSEDSSALQDPVLVNIQAQRLLQLICYPHRQLDSEEGENPQ

RQRIKRILQNLDQWTMRQSSLELQLMIKQSSNNELYSLLENIAKATIEVFQKSAEMNSSN

PSWNGSAASSSSVSNSNNTSKLKPVLSSSERSGVWLVAPLIAKLPTSVQGHVLKAAGEEL

EKGQHLGSSSRKERDRQKQKSMSLLSQQPFLSLVLTCLKGQDEQREGLLTSLYSQVHQIV

TNWREDQYQDDCKAKQMMHEALKLRLNLVGGMFDTVQRSTQQTTEWAVLLLDIISSGTVD

MQSNNELFTTVLDMLSVLINGTLAADMSSISQGGMEENKRAYMNLVKKLRKELGDRQSES

LEKVRQLLPLPKQTRDVITCEPQGSLIDTKGNKIAGFEKEGLQVSTKQKISPWDVFEGLK

HSAPLSWGWFGTVRVDRKVTRFEEQQRLLLYHTHLKPKPRSYYLEPLPLPPEEEEPPTPV

QQEPEKKMVEAVKTDKSVPSVGPDTVKKKSKKKKTSSVNKEDYTARNPGVMTFHGPGMNP

DLMNMSQPNHSYRAMPYNQTPIMYTQNQPLPPGGPGLEPPYRPRVPMNNHQKIMPMRPNY

PNMMSNLQGGMGMVGMDNKQYPIGFKPQPAIPQGQIRQQLQVRLNQSMIGQQMRQMAPNQ

QYTPMQQSQGYTSYSSHVGMQAHPSQSGGIVPSSYTNQGFPGAHPGTNPAVVDPLRQMQQ

RPSGYVHQQAPGAYTANMQNTPRFTHQPMQQTPMMHGLGQGHMQAQGMHPNMRTNQMLDQ

QQQQQQAQQQQFLRQQALRQQAQQQQQQQQQVQVQQQQVPTQQVQQQQVGTQQPPGQNQS

LGMQPLPPQQPMFPRQGQGMQQTQQQQQTAALVRQLQQQLSSSRRSRSPHSR

>m.1081 g.1081 ORF g.1081 m.1081 type:internal len:2183 (+) Unigene000114:3-6554(+)

QLQGSRAKQIQWPALTFKPLVGKSVLGSITAVEFFSDRQLDFLTDDGACQPYQDEVDSLS

PVLRDNPQLHEEVKSWVKDQKVQEIFMQGPYSLNGYRVRVYRQDSATQWFTGIITHHDLF

SRTMVVMNDQVLEPQNVDPSMVQMTFLDNVVHSLLKGENIGITSRRRSRSSQNNNTAHGH

YTRAQANSPRPVMNSSGPATKQGSQAQQQQPQHAQQQVSQQQQQQPQVSPGQQRGSRSTR

RKGSDSSIPDDEKISDEKTDAGGGKGDVSKNKSKQVMNKRRKAEDEEKKAGLKRLKMEAS

DLSESSDSENSNKRLLDSSSEPSSENELKNKGILKASEEEEKSQSCKAMEEPDANSRMSP

WEEASTVDQIVKPTAMEVMPSTKSENTGDMRSLAQPSPLPPPSSCAQPRSLVPAEVQGCI

MEIKSTVKTSPKDHYSTGAPRTQTPKCVIDITEDSSSHPTTRENSETVPATLASQTRETY

VPESRHLVLNPLASECRKAEGELQQQLGQSLGSKIEFAHSEVIRPVVSVSELAAVAERER

EKVQQQYPSIMPCIKNASLAEDVRKPQKLSSSPDVAKSKSNPSPDAFKPKCNPSPDAMKS

KTHSVLEAMKPKPNTSPEVTKHKEIIEITSSSVARSAVKSRSQEIPRSSFKPVPARSTPS

ESIKSALIVDKNEHFTVYRDPALVRPEADTNHVTYLPPHLHPLHSSSHATCLTPSSHHHS

HLLPASSLSPHPSVHHPLLPTVLPAMPPSSLLGGHPRLDSGGLSHLALAHHHPHQQQQFL

QQQPPPPLLAQTHGGASYNQLGLYPIIWQYHNGTQHSYPSGISLPGSKWVHPENAVNSDG

SLSRNTSSPWLHQPTPVSSADSLGILSHVPGRPASADPHRPLKISSHSSPPLSKTVDHHK

GELESKVFVDPMRGLVTAHLKQEPDQSRTPNSRDLQRLYADSSHVKQQALPPRLPLDTPD

RAVKYKEENRRILQESIEVAPFTAKIRSGEPEREPYIRIPSLPISGPPKENEHSPSDLYK

YKHSAAQSLPQSNYFTTISNSVVNEPPRLYPSKELSSLNVSSPLTLGSYPNSGGNTKSLS

KPPPLIKHHPDGEGLVGKISEQLSHQVALNPLSTPVASCEPRSPAVSPSNQLRSMPALHR

APVFHPPTQQTLDRKESGYGRLSPPTLTPIQPVSVAGKVSEQQKPPTLLPELREVKGTVE

LSSSEPWRPSSNLQGYDKTVQWQLDKSQGKPQVATASVIVRSRTCIKYDSSPGSKPGARE

TSNSKPHTGKYQLDCSKLAETREPGRVIQQNTNKEDIFLQYKNNFVRVSQGSFPSSAVAV

VNSVCNNSTAVTTSASAASTHNVLSRGTAELPYSTSANPSSSNINRLEGAAPKCRTPTSL

ELQECNARTASPIGPLPQPGSALAPQPYSGNFIHLKKHKAALAAAQSRGSSSASESEGSS

RSSQESPTIITQDRASPGNPSSKVSPLPNGQPSLMNQPNYHKLKKAWLTRHSEEDRNTNK

VEMTSNAVSEIIRPCTVNLIASTSSDTEISKDGKCLEDKLSPEDRKPRRVPSKRPHESGS

DSGDDSDGSDSKHEQRAKRQPKPTFKKKQNDMQKKKGENDKEEEDVKPNGIFRSAKEKTK

LKLASSNGIPRSVLKDWRKVKKLKQTGESFLQDDSCSEIGPNLQKCRECRSIRTKKGEEP

THSPVFCRFYYFRRLSYSKNGVIRIDGFSSPDQHDEEALSLWAPDAYEENDLDLETSKYI

LSCIGDKFCQLVMSEKTAATWIKKDAKIAWKRAVRGVRESCDACEATLFNIHWVCQKCGF

VVCLDCYKAKEKKSSKDKDLYTWLKCVKGQPHDHKHLMPTQIIPGTVLTDLVNAMHMLRE

KFGIKAHCVCANKHNILNKLPSTNGVSQVLQNVLNHSNKLSLCKPEAGQHNLGQKVEANG

GSSPASDTSTDCKLPPPESQSPLHFLADLAEQKSREEKKENKESVVGKVKEESTDALETL

HCKTSSLVANSTEQGSTLRDLLTTTAGKLRLGSTDAGIAFAPVYSTASQTGKSGRSMPNI

LDDIIASVVENKIPADRSTKQSPKAKPQDEVKAERRKQAEDVPEQHTDIPHCWLYDHRLL

WLKDHRKSSNWKLFRECWKQGQPVLVSGVHKKLNASLWKAEAFSQEFADHQGDLLNCKDG

VVSNSGIKEFWDGFEDLTKRLKS

>m.1087 g.1087 ORF g.1087 m.1087 type:complete len:1706 (+) Unigene000115:340-5457(+)

MEDKSPRVADYLVVAGLTESPEPFEDDITSNENCQRSSVEAGAPITDVAVVFRSQGEEVP

QGYTCIEFTPSGLSAELNSGSIMGPQIFLCYRRGLDKPPLTDLGVLYEWRETLKPGCHII

QTTPSGRPANINSSSSQRIYITYRRAPEFHSHAMLAVTDISVIIPGKGEAPPYAFCKVDK

NLNSSMWGSSVYLCYKKSLAKTNTLAFKAGLLSRYPEEDYESFPLPESVPLFCLPMGASI

ECWPSRTKYSLPVFSTFVLTGASGEKVYGAAIQFYEAYPEERLTERQRTQLGLQASGLKS

KECMTVHTNKSICLLSHWPFFDAFRTYLTFLYRYSISGPHTLPIEKHICHFMHKVPFPSS

QRPRILVQLSPHDSLMLSQPVSSPLPLSGGSLSTLLLNLGPKNAVTLLVLAVTEHKILVH

SLRPAVLTSVTEALVSMIFPFHWPCPYIPLCPLALADVLSAPCPFIVGVDSRYFDLYEPP

ADISCIDLDTNTISNKEDRRALTWKILPRKACKRLINTLSNLHHQLVQDYLLSHEEGQID

VSMSERDPGSGEKSLQTLELEIQEAFLRFMAAILKGYRSYLLPITQAPSEKATDASSLFD

LQGFLKSRDRSHQKFYSLMTKTQMFIRFIEECSFVSDKDASLAFFDDCVDKVDSERIEDT

RLIELDKSHRSEHTVFITPPELPFVPEGEEPSILYSYSGFPVLNAELCEPQEGPGIPIAT

TNSRHTSPGSPAAIFRRSKQEIKSAQRMAKTYSSIPHMWSKCLLRHCHGLWFICLPAYVA

ACQSKVRALRAAYDVLRMMQDKKLQPPDEVCYRILMQLCGQYGQPVLAVRVLFEMKKAGV

QPNAITYGYYNRAVLESTWPSSTRGGHFLWGKLRNVVRGVVQFKQVWRRQASHAKEPQLS

DASDLDSVSHGSLDSTNDSAERASIDTDFTKMDSSDDGSSTGGQSDQGYDSLSKEEVQVS

SPHMKEKKERDSLPLAKTGASIKNDSSAALKPLNPSNVRDNAAVPIRPNSLDIFGKNTVR

PKTLELLLISASCKQKQRYSDKAMVEEETGTTNPLKPAAERSTSCTVAMETSTSDASCSV

SRSVSFSGTLGRLPQRTGIESGFDPLSLLAAESKVPDEQGETDDAPSTRRHLAEEIQLHL

EHLSSPVSQRLFSTDIRSLQSPSPSSSPCQTAIPGSPSTQLQPQPRSRLFSSPSLPLGCP

RKSKEARPTSFVSPSSPTPSASSFSMESLLTPTLDVFRSSFMSAGKGVAEKASRLYSRLS

SQTSIAQDLNSDRVSVSSLGSVEPDCSSVFEGDLCLDSEGLSSPQRDASVSVTPFKRSPN

RNSRCLESPSAPPRLFRQASLLGSSLSVVKAPQTPDVCPDISLTPGQQNYDIEVRMSSCS

RCNTCECLAYDEEIMAGWTADDSNLNSTCPFCGSAFLPLLNVNIQDLNNQDRIPFQDLPS

SQAETNDASENETKTCIETTDNGSNSSSHSAEPITVPYLSPLVLWKELESLLENEGEQII

SSPVIVDHHPIVFWNLVWFFRRLELPSSLPALILSSKHCSQNMQPFQCSASEDSKNVLVT

ILWDNPKLHQDPIPPCYILWNAYCSNTNVGVSSLLEDKQPVSLELLQSVVRSIQKNDVYQ

PMSQTLQLLGTRLGFIRQRSLYRDILFLTLVALGRNSINIDAFDREYKLAYDRLTPGQVK

LTHNCDRPPGAGAMECRRTFGLPSL*

>m.1092 g.1092 ORF g.1092 m.1092 type:internal len:2183 (+) Unigene000116:1-6552(+)

RTRHMTGGSGDGGQKLLQQAQTTEKEFGEVTEKIQQCCSSLESRLQGVGEVQSHVRDIFS

RLADLDDELDSLSPVGRDVDSLASQADAIRGFLGRLASLRAELEGHGSACTTMLKREGSS

PDLLAIRRETEALNRQAAKLAERGQGRLALIEEAEGRVKEFYARLMELQRLLDQAEEGLN

VQSVVGTEVDVIKRQLQEFKAVEREQVDSIQPKLHHVNAVGQGLIQSAAKHTDTQALEHD

LETTNLQWNSLNKRVAERIAQLQEALLHCGKFQDALEPLLSWLSDTEELIANQKPPSAEY

RVVKAQIQEQKLLQRLLDDRLGTVEMIRAEGERIAATAETQDREKIQRQLQCLGERWTDL

LEKANARQRQLEELQVLALQFHESVDPLGEWLSATERRLSSAEPMGTQASKISQQINRHK

ALQDEVLAREKEVDHLEALGQSLSPLSCAADRDWLSERVGAVRSGHTELRNWCFRRAAML

EQALANAQLFGEEEVEVLNWLAEVAQRLSDVSVQSYQPELLAEQHKYTLSLNEEIVSRKK

TVDQAIKNGQALLKQTTGEEVLLIQEKLDGIKSRYSEMTAGSSKALRNLEQALQLATRFA

SAHEDLSQWLDSMEAELNNMEPDTTPAYQERQKDLKCVSAEKRLILDTVNEVGSALLDLV

PWRAREGLDRLVADANQRYRQADETITQRVQLVQAAIQRSQQYEEAVDAELAWVGETERK

LTSLGPLSLEPDVTVAQLQVQRAFNIDIIRHKDTVDQLLHTREDILESCSDQQREALKVK

TDSLSMRYEAVSQNHAERFSALEQAQVLVARFWETYEELDPWLGETETLISQLPPPAIDT

EALRQQQDQMRMLRESIAEHKPHIDKLLKIGPQLAELSSQEGATLRQRYSEAERRYLAIK

EDVKGRAAVLDEAFSQSAQFHDKMDPLLETLEGAVQRLRQPPPVAAEVEKIREQLAEHRA

AGLELDKLLPSFSTLCARGEELITRAAHDDPAAQAVRSRLLRLRSLWDEIRQRAEEREGK

LQDVLDLAGKFWADMAALLSTLRDSQEIVKELEDPGLDPSLIKQQIEAAEAIKAETDGLR

EELEIVRTLGADLIFACGETEKPEVKKTIDEMNAAWEGLNRTWRERMEKLEEAMTASVQY

QDALQGMFDYLDNAVIKLCDMQAVGTDLSTVKQQIEELKQFKVEVYQQQIDMEKLCHQGE

LLLKKVSDQADRDMIQEPLTELRHLWDNLGDKITVRQHKLEGALLALGQFQHALSELQSW

LSHTHATLDTQRPVNSDPKAIEIELAKHHVLRNDVLSHRATVETVNKAGSELLESSPGDE

ASHLRDQLDELNRSWDSLLLKTDERQKLLETALQQAEGFHGELEEFLQWLRRTESQLSAA

KPTGGLPETAREQLQQHMELQAQLTQRGEQYHRLLDQGESMLLARGAEENSPGTTQTQQN

LALLQNKWASLNAKMDDRRAKLEEAVSLATGFQSSLQDTINWLTQAEQTLNMAQSPSLIL

DTVLFQIDEHKVFVNEVNTHREQVLALEKAGSQLRFASLKQDVVLIKNLLLSVQARWDKL

VQRSLDRGRHLDEARKRAKQFHEAWRKLTDWLEEAEKRLDAELEISNEPDKIKVQLTKHK

EFQKTLGSKQPVYDTTVRSGKAMRDKATLPADTQKLDNLLGEVRDKWDTVCGKSVERQHK

LEEALLFSGQFAEALQALVDWLYRVEPQLAEDQPVHGDLDLVSNLMDSHKAFQKELGKRT

SNIQALKRSARELMETGRDDTAWVKVQLQELSNRWETICALSVSKQTRLQQALKQAEEFR

TAVQMLLEWLSEAEQTLRFRGILPEEVETLQVLLHTHRNFMQTVEEKRVDVNKAAGMGEA

ILAVCHPDCITTIKHWITIIRARFEEVLTWAKQHEQRLEAALAELLNNATLLEDLLSWLQ

WAETTLVQRDTEPLPQDITQLKTLITEHQVFMEEMTRKQPDVDKVTKTYKRKPSETSSSL

AERRGVRMFAFSFTGKQQQQQQQPAMQVSGGNPRLNQLCSRWQQVWLLALDRQRKLHDGL

DRLEELKEFANFDFDVWRKKYMRWMNHKKSRVMDFFRRIDKDQDGKITRQEFIDGILASK

FPTSRLEMTAVADIFDRDGDGYIDYYEFVAALHPNKDAYKPTTDADKIEDEVTRQVAQCK

CAKRFQVEQIGENKYRVSSELVQ

>m.1099 g.1099 ORF g.1099 m.1099 type:complete len:2041 (+) Unigene000117:176-6298(+)

MESEERLSSSSHSQASISSSATPPASSANITSESGHLFQLVSDPSFRASFPLLSHPAFRL

YAPVSGCSDFRGLGILGLQGGLTAHPHLGAFPGLNGAVKAKSASSGASSTSSSPSPAYTT

SEQTSTLKSGLNPQNKTTKSNLNYCKEKQSATNPSNWNRVQKSKMGQVQERTGNKAKENP

CMTVAEISSTSDSQSRSSSDSSSDTLSSLGSVDLENEDDDEDLSVCSMDSDTQSVSRKVK

DSSHIDQAAGCFSSGPNMCSMDSLKHSQAAPLLFGSPSIREEHRKHTSVIQTTGAAGIAK

TFSLPLQPNQDALSKPLCLTMSPKPSSVSSSPKPLSVSSSPKQHSVSASPKPLLYTRSPN

PASLSFSPNITSSPKPTSITNARNSPDGSRLHGNESNQDKQAKEHQESTKSLKKNVSSSS

PSPQISTSLNPQHCNLFLTSNITKNSSHLNGVVQGAVQDAPLALITKPRLDSSNTPDKTL

LATTSPCFNTPINLSTGTRHSYPGPPTSGKAFTHQESHRAREGSQPQRKTFREIESDIPS

SRDSDDSGEYEDEEDLSDSLSDSGSNLDTDSDNDEEDNKDEEMNTDAESHTDSMPLKLTK

SSTSLADPSTGRSNSSSSLNLQIYESPNILQTLNGSWALTYQSPPSSSCVVPPPAKRRRV

TDEQALRRPLEHGWKRETRISNIGGRVQGEVAYYAPCGKKLKQYPDIMKYLARNGINEIT

RDHFSFSAKIRVGNFYEAQEGPEGLQWCLLKEDEVVSCISAMDSHRGRPKSLELQPTDDA

FHSRKRKRPPSVSESEMANATLIKLQQKLVAQEIARQAAQIKMMRKLEKRALAQAAKDAK

RQKALLEAEEKRKQKEQLKILKQQEKVRRLEQIRAEKELRAQQILEAKRKRKEESINAKT

LEAEKRLKEKELRRQQALILKQQERERRKQHMILVKVTEARKKAEERERLKQEKRDEKRL

NKERKLELRRLELAMVKELNKPNEDLCLPDQKALPDLSRIPGLLLPDSCFGDCLMVMQFL

RCFGKVLGLDRNIKLPTLHMLQAGLLNLSPSAAQLQNLVIGLLSAAVRDPGFPPGSMAQT

TLGQHVSSVEINQENMSEILQIYMAAHGSQTDLSPLVESLKTKGFMAHTPTQKASIMGFL

VNELASSRSVIMEIDKSIEHMTNLRREKCVIESSLRKLRSSYVKRTGKRDFSLGGEESQA

LETPTTGHKRRRKVGGIEEEDDEDDDSNDHGEEDEDEEEDGGKKQRKTETCEEEDDEDQS

ASVEELVKNIEKLTKHQLLIRHKLFESSYSLQSMMLGQDRYKRRYWALPQCGGVFVESTV

SKEGPQGVNQESGRMHSAQLTSVKKEPAEVPDGEPYDGPQTKLENSSLDLQQENGSFNLF

LQKPSSFSKLSNLLEVPKTSSSDSQSKNSTAAPFSTNALTVLPNSLSTNLNQNIKYEVSS

QLLNPTHTGNQQHPLHNDQLYTTQAEKHSAWFSLLPRSPCDEFSLTTSCTNPSSCRAKAS

SPAPATSNSMTQSTSTFSSNSQNITSPVSPSATAPVSNFLGKELQMKHTMNPNMSFKDMF

TKAADPLPSTSVSTSKIGSPVLLSDKSPVLEMAKRKDLPFPQPVPQEMLTGWWKVTSSEE

LSRIVSACHPRGIRERVLQKQIQKHMDYLTQVCAKNKNAAVIDVCELKQSQVCEETVQSW

CVEEHAMDRDIAVLQQVEELERKVISASLHVKSWMPTKPQSEREDLLYYEHKVLSHSEST

DIVRQADNPLDIAVARLYELERNIERRYLKSPLSTTIQVPLDNRGTVSILAPATPANTKC

DSDVEDLVPGLKLWRKALSKVRSGAQLSVCLQHLHNSIAWEKSILKVTSEPPQRSRKQVS

RNSGGGEKLSETKRSRKACVSGEGSENSTVSTSVSSTAKKGGKESRKRKPEDNLYNTTTT

KQERDRSKTTTDNSKDLELCRLLLAELQTHQDAWPFMTPVNPKSVPGYRKVIKKPMDFST

IQDKLSNSQYLNLETFIIDVNLVFENCEKFNKDDSDIGRAGHSMRRFFQRRWTELLKQVN

*

>m.1104 g.1104 ORF g.1104 m.1104 type:complete len:2014 (+) Unigene000118:124-6165(+)

MGDDSEWMKLPIDQKCEHKVWKARLNGYEEALKLFQRIGDEKSPEWGKYLGLIKKFVTES

NAVAQLKGLEAALIFIENAHVAGKTTGEVVSGVVSKVFNQPKARAKELGIDICLMYVEIE

KAEIVQDELIKGLDNKNPKIIVACIETLRKALSEFGSKIITLKPVVKVLPKLFESREKAV

RDEAKLLAVEIYKWIRDALRTPLQNINSVQLKELEEEWVKLPTGVPKQSRFLRSQQDLKA

KFEQQQAAGGDEADGDDDDVAETQVDPYELLEAVDILSKIPKDFYEKIEAKKWQERKEAL

EAVEALAKNPKLEGGDYGDMVRALKKVIGKDANVMLVTLAAKCLAGLAAGLRKKFGTYAG

HVVPTILEKFKEKKPQVVQALQEAIDAVFLTTNLQNISEDVLAVMDNKNPSIKQQASLFL

ARSFRHCTPTTLPKSVLKPLCAALLKQVNDSAPEVRDAAFEALGTAMKVVGERAVNPFLA

DVDKLKLDKIKECADKVELVGGKKGEGGGGQKKEKPAAKPPPVEEPPAKPAGPPKKAPAA

KAAGPSKKSKPAPAASSKSKKAAETKEVNETELSLEVCEERAAAVLPASCMQLLDSANWK

ERLASMEEFQRAVEQMDKTEMPCQALVRMLAKKPGWKETNFQVMQMKLHIVRLIAEKGSF

SKTSALVVLDGLVDKIGDVKCGNNAKEALTAIGEACSLPWAAEQVVSLAFAQKNPKNQAE

TLNWLANAMKEFGFAGINVKAFINNVKTALGATNPAVRTSAITLLGVMFLYMGAPLRMFF

EDEKPALLSQIDTEFEKMQGQSPPAPTRGLSKKPAKDDREEVDEDEADGGAGDIMDLLPR

TDISDKISSDMVAKISDKNWKIRKEGLDEVTAVISEAKFIQANLGELPMALKGRLSDSNK

LLVQQTLNILQQIATAMGPALKQHVKNLGFTIITVLGDSKPNVRAAALTTLNAWVEQTGL

KEWLEGEDLAEELKRENPFLRQEILGWLAERLPNMRSVPADLMLCVPLLYTCLEDRSGDV

RKKAQDALPMFMMHLGYEKMCKATSKLKPASKDQVVAMLEKARAVMPAKPAAPAKAAPSK

AAQSTPPAKSSPAPAKSQPAVDDYSPPEPKQDTKKPKTTGPAQKKGVLGKKAPVKAANKD

DEDKTGPIFILVPNGKEQRMKEEKALKILKWNFITPRDEYVEQLKTQMATCLAKWLQDEL

FHYDFQHHVKAINAMIEHLDGESDAAIGCLDLILKWFTLRFFDTNTSVLMKAMEFLKLLF

TMLSRENHHLSEYEASSFIPYLILKVGESKDVVRKDVRTILSMLCNVYSPSKMFPFLMEG

TKSKNAKQRSECLEELGCLIENNGMNVCQPTPAKALKDIAVHIGDRDTSVRNAALNTVVA

AYNVCGEQVFKLIGNLSEKDMSMLEERIKRSAKKAPAPVSTKQEKAQREQPNNPNATFLR

KPAQEEVPNKLNQARSQNAHEHTAPSIPKEFQLDLDMIENDHTRVSDFPDLVQHKLDELL

EPVMIPEPKIRSISPHFDDLHNSTASTINFVISQVASGDINTSIQALAQIDEVLRQEDKA

EAMSGHIDQFLIATFMQLRLIYNTHMADERLDKKDIFKLYSCIIGNMLSLFSMESLAREA

SMGVLKDLMHGLITLMLDSRVEDIEDGQQLIRSVNLLVVRVLEKSDQTNILSALLVLLQD

SLITTAGSPMFSELVMKCLWRMIRFLPETINSINLDRILLDVHNFMKVFPKEKLKQLKSD

VPHRTLKTLLHTLCKLTGAKILDHLSMIDNRNESELEAHLRRVVKHSGNLSGLKSDQHSE

KMALRSDDKVIKAKVSDILSEIFKKIGSKENTKEGLTELYEYKQKYTDADLEPFLRNTSQ

FFQSYVERGLRMIESEREGKSRLQSSSVIPQHSVDSAYPSNNSSMSVSSNGEDLKPAVYY

ERLKILRQRHGLENAKQQQQQEDERPLSNLLSRPPLASSTDMLHSKLSQLKETRESQLQL

EQTRSHSPARASSPASNLDDLKKRLERIKSNRQ*

>m.1111 g.1111 ORF g.1111 m.1111 type:complete len:624 (+) Unigene000119:434-2305(+)

MKRLDDEPVHSGNRTSLSDNLTNGDSVTQRNPVALLQGLHLCQEAWNPGSPWDREGAHAA

LWGRPPGSFLVVSESSSENKLLCVSVSDEDKKVEDFPIIHTGSTQRLNTSHLAFADLLQL

VIFYNFSRDVLPFCLRIPSWVCGLTEQPKHLVSELGPKAWLCPPTDLLPDTMSPETADIQ

DTVMCTIQLTADNGALCFINPLYLQEHGDDWLTHSQVSANLANHLFTSKRDRRLSTTRPW

AGAGLKNQTKSVGDSAVMEISGSPVHGLTSPVTGVILRRASSTSDPQWRMSAESISSPIP

QSPHRVSWIEDKVWMSPPAPSSLLHPPCLEFDSLSMSSIEEEPEVEPAVSSCQVQNSPRL

PLADKVKNRLSAVGQALGGFIKPQKRLSKRVQEMSERKGSPFAEALKGFVEQTLKLRASC

HVTSTEMLQEVRSSLTALREMLYDSAEIQSIIDTLGDVPDFELDTMMEQALHKVALKPLC

SHLYECMKTARQQDGSLQRLQANQNTLKCRRLEELEGTAGAGVPDAAMLEKIQQRWATMH

QQYSPQMKVELLLKVCKNVYHSMTVNAKSGVVFGADDFLPCLTWVILRSDVVTLQIDTDY

MMELLDPSQLQGEAFSLVWQRGS*

>m.1117 g.1117 ORF g.1117 m.1117 type:complete len:1804 (+) Unigene000120:157-5568(+)

MSDESPTHCATSTCTQSEFRCTSGRCIPAHWYCDGGTDCADGSDEPSTCTTIVRTCSSDQ

FRCDDGRCIASSWICDGDNDCGDMSDEDQRHNCANRTCSSLEFTCVNNRPPQRKCIPQDW

VCDGDADCSDAYDEHQNCTRRSCSATEFTCSNGLCIRSAYRCDRRNDCGDGSDEQSCTYQ

PCQQHQFTCQNGRCISQDFQCDGDNDCGDESDELPHMCHTPAPTCPPGKFRCDNGNCVPS

SQVCDHSDDCNDNSDEKGCGINECTDPSVHHCDHECTDTPTSFVCRCRPGYRLMSDKKSC

DDVDECSETPSVCSQMCENTLGSYVCKCAPGFLREPDGRRCRQNSNISPYLIFSNRYYLR

NLSADGAAYSLILQGLSSVVALDFDRVDKRLYWIDVSRRVVERMFFNGTGREVVLNGIMH

GEGLAVDWVGRKLYWVDSFLDCLKVSELDGRFIRKLAEHCVDVNNTYCFENPRAIVVHPK

FGYVYWTDWGSKAFIGRVGMDGNNKSAIITSKIEWPNGLTIDYTNDMLYWADAPLNYIES

SNLEAPHRHTVYDGVLPHPFAITVFEDTVYWTDWNTRTVEKGNKYDGTGRQALVNTTHRP

FDIHVLHPYRQPIVTNPCGINNGGCSHLCLLKAGGRGYTCQCPDHFLTVQIGGVARCLPS

CSSTQYRCADNERCIPIWWKCDGQSDCRDGSDEPSTCPLRHCRLGQFQCNDGNCTSSHFL

CNSNRDCPDGSDEDTVLCATHQCESHQWQCSNKRCIPEAWQCDGENDCGDGSDEEPSHCS

SRTCRPGQFKCRNGRCIPQSWKCDVDDDCGDNSDEPLDECMGPEYRCDNHTEFDCSTNYR

CVPLWAVCNGQNDCRDNSDEQNCEEMTCDPAGDFRCDNHKCIPLRWKCDGDSDCGDGSDE

RNCNPRACTESEYRCDNLRCIPERWVCDHDNDCEDNSDERDCELRTCHPGYFQCDSGHCI

AERLKCDGSADCMDVTDETSCPTRYPNGTYCPPFLFECKNHICVQQHWKCDGDNDCGDGS

DEELHLCLDIPCEAPFRFRCDNNRCIYSHELCNSVDDCGDGTDEKQEHCLTPTHGPCSSD

EFKCGNSQCIPLQYVCDDYDDCGDQTDELGCNSGNGRSCSENLCEHNCTELTGGGFICSC

RQGYKAREDNRNSCEDVNECELYGTCPQLCKNTKGSYECFCAEGFRSVGPQHRTECAAEG

NPPVLLLPDNVRIRRYNLSSKQYLDYIDNAEHIHAVDYIWDPEDQGLSIVYWTILGHGAQ

FGSIKRAYMTTFDDHGSNQMKDVDLNLRYISSPDGIAVDWVGRHIYWTDAGTNRIEVAKL

DGRYRKWLIYSNLDQPAAIVVNPAIGMMYWTDWGSRPKIEAAWMDGQHRQVLLDEDLGWP

TGLTLDYLNGNRIYWCDSKENIIESMKPDGTDRKIVLSGDIGNPYSLDVFEGHVYWTTKV

RGEVWKADKFGRGDKVKMLTINPWLTQVRIYHEHRYNRSVLNPCKGVCSHLCLLRPGGYT

CACPQGSSLVSFNENECDAASLPEVAMPVECGCINGGTCSTDKPGLPKCKCPYGYSGRHC

EIGKSRGAPAGTAVAVLLAVIIILLSGALAVGLFLNYKRTGSLIPSMPKLPSLSSLAKLS

DAGNGVSFRSGDGSTVDPQTLGVSFIDRAMQLNENFTDAGREPVTFENPLYSTATGAAAD

AAVIHATQVTVNISGDSTENVFANPMYNEQQQAVDMKNTPNEQAAKESKWNFFKKKLKPS

TTFENPAYSEMRDKQSALTTEDISTSHPQPALPPLKREKSSMYSPTEDSFQDTANLVKED

SDI*

>m.1125 g.1125 ORF g.1125 m.1125 type:3prime_partial len:2101 (+) Unigene000121:176-6481(+)

MFKKLKQKINEEQSPQRNVLSPQQAQVSSGGRAVKSPLLQPDTASSSSERENFSKGAVSP

QSGSVNGDGAVSPLREEAPSLAQKLQQRVSSVESLFRGSGRAEGLFRSGSRDSLVRSASR

ESLTPLGENEAPGVPSFDPPSDIESEAEDSPGNAEALSKDQLVYRLHRVEKSLANYRGKY

SELVTAYRTVQRDKEKTQVVLSQSQDKALRRIGELREELQMDQQAKKHLQEEFDAALEEK

DQMITVLQTQVALLKKRLHGSPGSLLTSEAGDSQTAETTETIADPQSVTQAEIEELIQAG

GSSEPENTADSEALQKRVRRQETLLQRCNELMKTNKERSAQLGSENDVLQQQLQERLQEL

EKMKELHTSEKTKLITQLRDAKNLIEQLEQDKGMVIAETKRQMHETLEMKEEEIAQLRTR

IQQTLTQKEELQEQREKAEKAAFEELERALGVAQRAEEARRQLQERMEKEVKQVEQTSEE

ERRSLQQELTRVKQEVISIMKKSTEERIAEMEQQHLEDLANKDLQMSVQIQEAVENCRKE

LLEAAQEKEQQASLAVEEAELQKSAVQAEGEAKTRELQLELESARTRILELESSMAKSCQ

ADQFSVQIEEQRKKYADEIASLGERHQQEVEKLKMEHAEALNQQHCTDVEALEQKHQTET

DVMMKDKEREFQEHVKEMNQKMLEKLEIKQAKLEALSSELSEMQKCKEQLEEHKESVRME

LEVRLKDEQVKFQKEVDDLKQKHEEMLEGLEKHQKEEINQLQLVLEEKNKELEQYVEKER

ILQEDTNKAKQDVETMLKEIEDLRCVAQSEKEKLAEAAAQTTLLKEELTKSQSRVQDLEK

LLEESRNECQHKEEHLQQKSHENFELQQRIQQASNDLAETTSSHAQTHQAMQEEQDGLKK

QLEEERSSSEKRVESFRKEMESKLKSQETKMEKLRQKAKEMQEKFKKKLQEQEENAKAEM

AKKEKELQQKEQQVKEKILEIAQANSEGLSSALSDLEANHKEQFEKIQKVHKLEQDDLLR

IWQEKLSQQEEELQEKHALTMQEKMQELEDISQQLLTHREEKRQMDQEIKNLKEELSMRE

TTVQKLQTELREAAGKLDSLSKVEELLKTQVETVEKNLNQALNERNIFQDQLTAAEETSK

EKILALSEELANACKKLTILEASKSKEGENLQKSLEEKTFELQNKESKFLEQIHNVGEEL

EQHCHTAQTIINDFSARLCERVETKVNELQSRVMFNQKKVCHLKNLILTKNHRICCLEQE

LQQVVEENQKLKNSLEETTLQLHANSENLKALNVEKETLQNDAKNHSQLLSEKDLSIEKT

HEENKNLSENLKANILHISNLENVIDDLKKQLSCSITEKEEAISLLNQQHQEEKQSVQNQ

MEQAENEKGLALKQVETLRNKLADFKKKTDFKISQNQNTVRSLQRQIEDMEKQIAEKDEQ

LQKLTASIDNQCISKSELDQVLSEKEQKVSALNVELDDCTKKISELEQRLKSQSMEQEKK

EAELQNHVTSSEMEKTELIQQLEQTRQQCSLQQDLVSKTQEQLHALENEFQAVKQKLESQ

QEEFEREKSKEEALKAAEGKVADLKKKAEQKISLIRKQLTSQIEQKEQTIKELQAQLEDI

MQTQNEKEQQVRNLEENGRKMEEAINNLMEEHKKHLEQVLSDGQVEKECMDKEDLVAAAA

AAATATVNDREEETWSRIRDLEAKLLQSEEQNTSCRAEINQLKEELCEQTSLVQELQKSL

EVQVKEKETNVIQMDKCFVEKSNNLLGMEHVQYVGDQNTKSHQDIWETEKELLVKDYEMK

LQDLHKRLEEKENVQLEHSLEQKLLDAENEKQKIHKDYTRLQKDLRSLRREHEKDLEYLR

KEIAEESEKKLKSDVEDLEMKHNSTLKQMMREFNTQIALKDKELETSVREAVEKAQMVEA

ELMGIHKEEANQLQQIISQKDDDLKKTIQRYEQLLQSREEEMGSRVWEVQQELEELQQRS

QTGPQGVDELQVQLAEKTTLLSETRLKEQEYQDRIHTLEDMIRKAYKNSVVTHLGKDVSH

HTAESLSEPTELEYLRKVMFEYMMGRETKTMAKVITSVLKFPEDQAQKILEREDSRLMTW

L

>m.1138 g.1138 ORF g.1138 m.1138 type:5prime_partial len:87 (-) Unigene000122:6177-6437(-)

HTHTHTLHYFSLSLSLKLSHRRFTTKEVGYKVQRYRVKTSFPPSVSFYLLQPNLESRSKV

TLSLDGVNVASSLSHSVQRKGSELVK*

>m.1130 g.1130 ORF g.1130 m.1130 type:complete len:1943 (+) Unigene000122:280-6108(+)

MSGSEDDRDDFGPTEDLSMMQDDDLEEDLSENEAPKVKKKKKAKKSSRENKSSKRQRSRR

EDIPISSPEPVEGRDVDEVEDDGPGGRSDSEGSDYTPGRKKKKRASTSRDKKRSSTGADR

SSSLAVSKRKEPEEEDDEDDDDSSEPKTSSQLLDTWGMEDIDHVFTEEDYRSLTNYKAFS

QFVRPLIAAKNPKIAVSKMMMVLGAKWREFSTNNPLRGSASANAALAAANVAAAVESMVA

AETPATAPTPAPLAEPQPPPAPPLRKAKTKEGKGPNARRKSKPTPKPQEKKVKTKKVAPL

KIKLGGFNSKRKRSSSEEEDMDVDSDFDDGSINSVSVSDSSNSRSSRSKKKPKPKKKKKV

DEDADGYETDHQDYCEVCQQGGEIILCDTCPRAYHMVCLDPDMEKAPEGTWSCPHCEKEG

IQWEAREEASDGEEENDAGGGEAEEDDHHMEFCRVCKDGGELLCCDTCPSSYHMHCLNPP

LPDIPNGQWICPRCRCPPLKGKVQKVLTWRWGEAPPPIPVPRPPDLPVNAPDPPPLVGRK

EREFFVKWCNMSYWHCSWVQELQLELNCQVMFRNFQRKTDMEEPPSVDASECDDNKSMRR

KSKDPLYARMDHQFGRYGVKMEWLLIQRILNHSVDKKGNVHYLLKWRDLPYDQSAWESED

MDIPDYDTYKQQYWNHREMMIGDEGRPGKKLKKVKLRKIEKPPVNPVVDPTVKFDRQPEY

LDMTGGTLHPYQLEGLNWLRFSWSQGTDTILADEMGLGKTVQTAVFLYSLFKEGHSKGPF

LVSAPLSTIINWEREFEMWAPDMYVVTYVGDKDSRAVIRENEFSFENHAIRGGKKAFKMK

KEAAVKFHVLLTSYELITIDQAVLGSIDWACLVVDEAHRLKNNQSKFFRVLNNYPLQHKL

LLTGTPLQNNLEELFHLLNFLTPERFNNLEGFLEEFADIAKEDQIKKLHDMLGPHMLRRL

KADVFKHMPSKTELIVRVELSIMQKKYYKYILTRNFEALNTRGGGNQVSLLNVVMDLKKC

CNHPYLFPTAAIEAPKMPNGMYEGGALTKSSGKLMLLCKMLKKLKEGGHRVLIFSQMTKM

LDLLEDFLENEGYKYERIDGGVTGVMRQEAIDRFNAPGASQFVFLLSTRAGGLGINLATA

DTVIIYDSDWNPHNDIQAFSRAHRIGQNRKVMIYRFVTKASVEERITQVAKKKMMLTHLV

VRPGLGSKAGSMSKQELDDILKFGTEELFKDDAGEGDNKEEDSSVIHYDDKAIDKLLDRN

QDATDDTELQSMNEYLSSFKVAQYVVKDEDEEEVDREIIKQEENVDPDYWEKLLRHHYEQ

QQEVLASHLGKGKRPRKPVNYNDCSQEEQDWHDDQSDNQSDYSVASEEGDEDFDERSEAN

NRRSSRRGLRNDKEKPLPPLLARVGGNIEVLGFNSRQRKAFLNAVMRYGMPPQDAFTTQW

LVRDLRGKSEKEFKAYVSLFMRHLCEPCADGAETFADGVPREGLSRQHVLTRIGVMSLIR

KKVQEFEHVNGQWSMPWMKELEENKRAVAVAMGDDPKTPSTGTPGDTQPNTPAPEDLSRG

EESERETDDKMEGEGEKDRKSTQLQDDEVIEIPDDGETPLSPEKTGPGQEMEKDSTAPSL

EKDGEIKDIAGEKDKEKGGAKEESTTCSLKIEEVKGERSEVTSAGDSSQPKEECSEEKSE

KTDSNLTGEEKKEVKEEKDGVRAEESRINGEDGGVDNEEKKKHVKQRFMFNIADGGFTEL

HSLWQNEERAATVTKKTYDIWHRRHDYWLLAGIIQHGYARWQDVQNDPRFSILNQPFKGE

MSRGNFLEIKNKFLARRFKLLEQALVIEEQLRRAAYLNMTEDPSHPSMTLNTRFSQVECL

AESHQHLSKESMMGNKPANAVLHKVLKQLEELLSDMKADVTRLPATIARIPPVAVRLQMS

ERSILSRLASRGSEVSQTQTPH*

>m.1142 g.1142 ORF g.1142 m.1142 type:complete len:2073 (+) Unigene000123:162-6380(+)

MSIRINHTAGGSLRRSQRNTLATRLQSEQTKQKTNSPPENKRSYNKASKAPPTPAAGSSR

GHAPRRNSLSLASLSQSASEPDSEAAGSSGQREQREVSNNRALKRSSATEHNNASYSPSP

AKRPKAPPPESTEQLEAADPTADSAPELPPSSRSKKRRQTDEHPTSRTVNKKSSACPASS

SSHKRKKADNSFCSSSSSSFSSSSSFLSGSSSEQASRSSKPTKLASKSAASAKSVTESAS

SSSASSSSFTSSSSSSSVVVAGASGCPPPSQGARLKQGKDQSKSRRSRSASSPSPRRSSR

DKEHSKSSTAASKFDWTSRFSPRVNLPKPRLALPGSSKPETPSGSSKPGPSGLQAKLASL

RKSSKKRSESPPAELPSFRRSTRQKTTGSCASTSRRGSGLGKRGAAETRRQDKMAENDNS

QDGANSSRTEETPQGASAASSSVAGAVGMTTSGESESDDSEMGRLQALLEARGLPPHLFG

PRMSQLFHRTIGTGASSKAQQLLQGLQATGDESQQLQAAIEMCQLLVMGNEETLGGFPVK

SVVPALITLLQMEHNFDIMNHASRALTYMMEALPRSSAVVVDAIPVFLEKLQVIQFIDVA

EQALTALEMLSRRHSKAILQAGGLADCLLYLEFFSINAQRNALAIAANCCQSITPDEFHF

VADSLPLLTQRLTHQDKKSVESTCLCFARLVDNFQHEENLLQQVASRDLLTNIQQLLVVT

PPVLSSGMFIMVVRMFSLMCSNCPNLAVQLMKQNIAETLRFLLCGASNGSCQEQIDLIPR

SPQELYELTSLICELMPCLPREGIFAVDSMLKKGSAHTTEAAVWQWRDDRGLWHPYNRID

SRIIETAHQNGEDEISLSTLGRVYTIDFNSMQQINEDTGTARGIQRKPNPLINPNTGHVD

VRGEDARAELMREEAELARCFIKTLFGVLYEVYSSSAGPAVRHKCLRAILRIIYFSDSEL

LKDVLRNHAVSSHIASMLSSQDLKIVVGSLQMAEILMQKLPDVFSVYFRREGVMHQVKNL

AESESFLPSPPKACTSGITAATTTITTATTPAASSVAPDLGSPSFHNNMEDSLDLSPQGR

LSDVLKRKRLPRRVPRRPKYSPPRDDDKVDNQAKSPTTTASPKSSFLASLNPKTWGKLSA

QNNSTNSEPPRTAGVSGLARTANKDSVSNNRDKIKAWIKEQASKFVERYFNSENVDGSNP

ALNVLQRLCTATEQLSLQVDGGLECLVEICSIVSESDVSSFEIQHSGLVKQLLIYLTSNS

ERDLISRDQRIKRFLHIFCGSPLPGQEPLFRLEPTANGALLALVHKMNSCLSQMEQFPVK

VHDFPSGNGNGSRGSQALKFFNTHQLKCQLQRHPDCTNVKQWKGGPVKIDPLALVQAIER

YLVVRGYGRIREEDEDSDDDGSDDEIDESLAAQFLNSGSVRHRLQFYIADHLLPYNMTVY

QAVRQFSLQPEEERESTDDEANPLGRAGIWTKTHTIWYKPVREDEDGSKDSVGGKRGRAQ

TAPTKTSPRNAKKQDELWHDGVCPSVSNPLESYLISEPPEGITFDDPSLEVILLLRVLHS

ISRYWFYLYENAVCKEIIPTSEFINSKLTAKANRQLQDPLVIMTGNIPPWLTELGKTCPF

FFPFDTRQMLFYVTAFDRDRAMQRLLDTNPEINQSDSQDSRVAPRLDRKKRTINRDELLK

QAESVMQDLGSSRAMLEIQYENEVGTGLGPTLEFYALVSQELQRADLGLWRGEEVTLANP

KGSQEGTKYMFSSRGLFAVPFGRSTKPAHIAKIKMKFRFLGKLMAKAIMDFRLLDLPLGL

PFYKWMLRNESSISSHDLVNIDPGVAKSVQHLEDIIRQKRRLEQDKSYTRETLQQALESL

NMNGCSVEDLGLDFTLPGFPNIELKKGGKDVPVTIHNLEEYLRLVVYWTLNEGVSRQFES

FREGFESVFPLHHLQYFYPEELDQLLCGSKSETWDVKTLMECCRPDHGYTHDSRAVRFLF

EVLSSFDAEQQRLFLQFVTGSPRLPVGGFRSLNPPLTIVRKTFESTENPDDFLPSVMTCV

NYLKLPDYSSIEIMREKLLMAAREGQQSFHLS*

>m.1148 g.1148 ORF g.1148 m.1148 type:complete len:497 (+) Unigene000124:128-1618(+)

MDDDFEWRREQRRQKREEMRLEAERMAYRRTEEDEEEAARERRRRARQERIRNKENEEIA

NPAESEGLNSHSVTATKTVSSSELNTGQAGEGDEEEQALLDRLAKREERRQKRMKEALDR

QKQLDSVDDPSYSTYSVESTSTTSYQAFSNQEDEPALNSKEEVKEELVPQMELVEEKPRR

SYLREQMEDEDAGGKIKQPNGGVYEDSKHKKVERTQSRGSVHSFEGPAAEEGDDSEQTRL

EAERKLEELKRRRDETENEEFERMKQRQQEAEVELEELKRKREDRRKVLEEEERLKKQEE

AERKEEKRRMKEEIERRRAEAAERRQKVGEEVDGDAGKPFKCVSPRGSSLKIGERAEFLN

RSAQKSAVKTSHSPVVSKIDNRLEQYTTAVQGNKEVRSPRGAAIDFPGGSDGVRNIKSMW

EKGNVFSSTISSPSSNKDAAGIKVGVAGRINDWLNKTPEASKTPGGRPVDLKPGDVTGKR

NLWENKSSSATKTSSC*

>m.1147 g.1147 ORF g.1147 m.1147 type:complete len:604 (+) Unigene000124:2022-3833(+)

MTAEDSTSAVTMSNSSHSSKSNPANNHAVLHGSITEGVAGAPNEAALMALMERTGYKMVQ

ENGQRKYGPPPGWQGASPPRGCEIFVGKIPRDVYEDELVPVFESVGRIYEMRLMMDFDGK

NRGYAFVMYTQKHEAKRAVRELNNYEVRPGRLLGVCSSVDNCRLFIGGIPKTKKREEILE

EVSKVTEGVLDVIVYASAADKMKNRGFAFVEYESHRAAAMARRKLMPGRIQLWGHQIAVD

WAEPEIDVDEDIMETVKILYVRNLMIETSEETLRKIFSQFNPGCVERVKKIRDYAFVHFT

SRDDAVVAMDNLNNTEIEGSFIEVTLAKPVDKEQYTRYQKASKGTMTSSDSTQQNFVYQC

DPYTLAYYSYPYNTLIGPNRDYFIKAGTARGRGRAGATNRGPGPRGSYLGGYSAGRGIYS

RYHEGKTKVPDKPYELMSSLELAAVNPVGIKPGTMTLPGLGAQYPAMFSAAPAATKLVEE

GKIHPVEHLINPLGLPHDHPAAPSSAAVIPAVSTPPPFQGRPITPVYAVAHNVPRIPAAA

AAAGLYGASFVPIAAHTNTAALAALQKNAAVAAAAYGGYAGYMPQAFPAATFQVPIHEMY

PTY*

>m.1154 g.1154 ORF g.1154 m.1154 type:complete len:2034 (+) Unigene000125:238-6339(+)

MADVTARSLQYEYKANSNLVLQADRSLIDRTRRDEPTGEVLSLVGKLEGTKMGDKSQRTK

PQMLEERRAKRRKRDEDRHDINKMKGFTLLSEGIDDMVGIVYKPKTKETRETYEILLSFI

QAALGDQPRDILCGAADEVLAVLKNDKMRDKERRREVEQLLGPADDTRYHVLVNLGKKIT

DYGGDKELQNMDDNIDETYGVNVQFESDEEEGDEDQFGEVRDEGSDDSEGEEADVGCTLS

ANLGSAGDVMTNKKKDLHPRDIDAFWLQRQLSRFYDDAIVSQKKADEVLEILKTASDDRE

CENQLVILLGFNTFDFIKVLRQHRRMILYCTMLASAQSDSEKERIMSKMEADQDLSQVLY

QLQETEKEDIIREERSRRERVRKSRVDSDLDSINIDHGQSMPARHLLDLEDLAFTQGSHF

MANKRCQLPDGSFRKQRKGYEEVHVPALKPKPFSEDEALVAIEKMPKYSQAAFEGFKTLN

RIQSKLFKTTMETDGNLLVCAPTGAGKTNVALMAMLREIGKHINMDGTINVDDFKMIYIA

PMRSLVQEMVGSFGKRLASYGITVSELTGDHQLCKEEINASQIIVCTPEKWDIITRKGGE

RTYTQLVRLIIIDEIHLLHDDRGPVLESLIARTIRNVELTQEDVRLIGLSATLPNYEDVA

TCLRVDPAKGLFYFDNSFRPVPLEQTYVGITEKKAIKRFQIMNEIVYEKIMEHAGKNQVL

VFVHSRKETGKTARAIRDMCLEKDTLGLFLREGSASTEVLRTEAEQCKNLELKDLLPYGF

AIHHAGMTRVDRTLVEDLFADRHIQVLVSTATLAWGVNLPAHTVIIKGTQVYSPEKGRWT

ELGALDILQMLGRAGRPQYDSKGEGILITSHGELQYYLSLLNQQLPIESQMLAKLPDMLN

AEIVLGNVQNAKDAVNWMGYTYLYVRMLRNPTLYGVSHDERSTDPLLEMRRMDLVHTAAT

VLEKSNLVKYDKRSGTFQVTDLGRIASHFYITHDSIMTYNQLLKPTLSEIELFRVFSLSS

EFRNITVREEEKLELQKLLERVPIPVKESIEEPSAKINVLLQAYISQLKLEGFALMADMV

YVTQSAGRLMRAIFEIVLSRGWAQLSDKTMNLCKMIDKRMWQSMSPLRQFRKLPEEVIKK

IEKKSFPFERLYDLNHNEIGELIRMPKIGKTIHKYVHQFPKLDLAVHLQPITRSTLKVEL

TITPDFQWDEKIHGSSEAFWILVEDVDSEVILHHEYFLLKAKYAQDEHLVTFFVPVFEPL

PPQYFIRVASDRWLSCETQLPVSFRHLILPEKYPPPTELLDLQPLPVSALRNSAFECLYQ

NMFPFFNPIQTQVFNAVYNSDDNVFVGAPTGSGKTICAEFAILRMLLHNSEGRCIYITPM

ESLAEQVFVDWHQKFQGLLNKKVVMLTGETSTDLKLLGKGDIIISTPEKWDILSRRWKQR

KNVQNVSLFIVDEVHLIGGENGPVLEVICSRMRYISSQIERPIRIVALSSSLSNAKDVAH

WLGCSTTATFNFHPNVRPVPLELHIQGFNVSHTQTRLLSMAKPVYHTIMKHSPSKPSLVF

VPSRRQTRLTAIDILTFCAADVVPQRFLHCSEKELAPFLDKLSDCALKETLSNGVGYLHE

GLSMTERRTVEQLFNSGVIQVVVASRSLCWGTNISSHLVIVMDTQYYNGKIHAYVDYPIY

DVLQMVGKANRPIQDDEGCCVIMCQGSKKDFFKKFLYEPLPVESHLDHRLHDHFNAEIVT

KTVENKQDAVDYLTWTFLYRRMTQNPNYYNLQGMSHRHLSDHLSELVENTLHDLEQSKCI

SIEDEMDVAPLNLGMIAAYYYINYTTIELFSMSLNAKTKIRGLIEIISNAAEYKNIPIRH

HEDALLHQLAQKVPHKLNSPKFNDPHVKTNLLLQAHLSRMQLSAELQSDTEEILSKAVRL

IQACVDVLSSNGWLSPALAAMELAQMVTQAMWSKDSYLKQLPHFTTEHIKRCTEKGVESI

FDIMEMEDEDRINLLQLSDAQMADVARFCNRYPNIELSYEVADKEDIKRFGYS*

>m.1162 g.1162 ORF g.1162 m.1162 type:complete len:2047 (+) Unigene000126:90-6230(+)

MVTKHFSKMASNDSNINEELMEKPGCSTEIPKATSRSKRKPAVVTKHTAVNEPESADECE

QDCTADDPNDTTIEMEEIPEDVVIVKPEPVSNEGKDEFKGPEFRNKGGSKVKEEGQRRRP

DEHLQEIRCSRVNCTACGQQVNHFQRDSVYQHPILKVLLCKSCFKYYMSDDISKDEDGMD

EQCRWCAEGGNLLCCDYCSNAFCKTCILRNLGRKELSGIMNNEDEKWHCYVCHPEPLEDH

VAACEKVLKNLECMKKPKAEQDKPKRDGIKHKQSKVKNSALNGKDVNSDGSGTLTFSYKT

LNVPKELLKKTKKLIETTNGLNSTFVQFIQQEPVYLDENIVRHRHLKAFRSVLADLKKAH

ATLEQALEEEFKALEVVNGDGNGLADNPPVAVTVPDSEPAEQSKDDQVVDQTVQDSTTDQ

VSAASEDEVDEDEVNAVQADVEMAEDVVPAAENPEEKLTENEANAEVVQSKPDCEDDASA

AGEASLDKDIVSIPPSVPDELFEMVESLSDCVKQESEKDSVDTDSMSSKNSESDQKPKSG

KRSAKVSKKLVVKLTPIPLKITIKKDESKSKSQEKEGALSPDESRRSPRMKTTPLRKSSE

RRSKSQSEDTRANDDECDASETPVETGDPDSDSDEVPEVLRQVEDEEATSDEPDAEPNGK

EAGNTSPKKSAEQSSKKTAKRKLLASSSDSDNTKSRKAKNAAKKKRRESDSSNHDSDLEK

ELKSLSRLRTTKKSKPGKKQTTEDEEEEEKEGKKTTKKKKEEETKKEGKKGPKRSFERKR

RSQKGKEKATKDSSSSSDEEEEEEQEADSGEESGDQQKIKPILESALSHMDAFQQSSGDE

AQVKAEPSQMLDDDDDPENRIAKKMLLAQIKANYSSGDDSSSEDDDSKTNKEERSPTKAK

NDASSEGEEEEDSDSDSGSEVDVKKAGRRHKLLRKKLTLSEGESDTEKPAKSKKEVKRRG

RRKAVNSDDSADSDFKRSGSGSRSESAMSDFVSEDDEDNKSNKRKTRSSKKADKDGERSY

QKEKKKKRRRIKVQDDSSSNKSAEEGDEDKEEEDEEGDDKGTPKGRKKIRKILKDDKLRS

ETQNALKEEEERRRRIAEKERLREKLRETIVVEESSQITCPITTKLVLDEDEETKEPLVQ

VHRNMVTKLKPHQVDGVQFIWDCCCESVKKTKNSSGSGCILAHCMGLGKTLQVVTFLHTV

LLCEKLNFSTALVVCPLNTVLNWLNEFEKWQEGLKDEESLEVTELATVKRPQERAYALQR

WQEDGGIMIIGYEMYRNLTQGRNIKSKKLKETFQKTLVNPGPDFVICDEGHVLKNEASAV

SKAMNSIKTRRRVILTGTPLQNNLIEYHCMVNFIKENLLGSVKEFRNRFINPIQNGQCAD

STMVDVRVMKKRAHILYEMLAGCVQRRDYTALTKFLPPKHEYVLAVRITPVQCKLYRYYL

EHFTGVGAALEGGRGRAGTKLFQDFQMLSRIWTHPWCLQLDYISKENKGYFDEDSMDDFI

ASETEESSMSLTSEDEKAKRKKKRGKGKGDSSDKSDSDDVEVIKEWNTSSRGGNPEGRNR

AEPVEEVRASSSGPGSPSPDWYKEFVTEADAEILEHSGKIVLLFEILRMAEDVEDKVLVF

SQSLISLDLIEDFLELAGRAKEEGKPSPYKGEGKWFKNIDYYRLDGSTNAMTRKKWAEDF

NDTSNVRGRLFLISTRAGSLGINLVAANRVIIFDASWNPSYDIQSIFRVYRFGQLKTVYV

YRFLAQGTMEEKIYDRQVTKQSLSFRVVDQQQIERHFTMNELTELYTFEPDQLDDPSEKK

SKRSTPMLPKDPVLAELLHNFKDHIVGYHEHDSLLDHKEEEALSEEDRKAAWAEYEAEKK

GLSMRINQPAYASVGIGSNYLPFNVAALASMSNQQLEDLINQGRQKVMEATNALRTFPRE

PLEDIISQVWKENPNLSESQVQGMALARQAAIDMELKHREATYRDALNKQQTLMMYVQKV

INNRKVQEQQLALARHGMLMNQMAMQNGIAGGMSQIDLLGLYQQLGALQGLPTSQIGKNP

GPSKGL*

>m.1170 g.1170 ORF g.1170 m.1170 type:complete len:1440 (+) Unigene000127:574-4893(+)

MELLLKHGASIQAVTESGLTPIHVAAFMGHENIVTQLMNHGASPNTTNVRGETALHMAAR

AGQAGVVKYLVQNGTHVDAKSKDDQTALHISSRLGKPEIVQQLLQHGAAPDSTTTSGYTP

LHLAAREGHKDVAAILLDQGASLSITTKKGFTPLHVAAKYGKIEVANLLLQKRAPPDASG

KSGLTPLHVAAHYDNQKVALLLLDQGASPHAAAKNGYTPLHIAAKKNQMEIATTLLEYGA

DTNAVTRQGISPLHLAAQEGNVDMVTLLLARDATINLGNKSGLTPLHLVAQEDKVNVAEV

LVNQSAAVDPETKMGYTPLHVACHYGNIKMAHFLLKNQAKVNAKTKNGYTPLHQAAQQGH

THIINLLLQHGASPNELTLNGNTALSIARRLGYISVVDTLKVVTEETLTTMTVTEKHKIN

VPETMNEVLDMSDDEVRRANVPEMLNEDYISDVEEGEDVMMGDTDKYLGPQDLRELGDDS

LPQEGYVGFSIGARSGSLRSFSSDRSNTLNRSSFTRDSMMIEEILAPSKDTHLTVAKDYD

TDSLRRYSWTADALDNVNLVSSPVHSGFLVSFMVDARGGSMRGSRHNGMRIIIPPRKCTA

PTRITCRLVKRHKLASPPPMVEGEGLASRLVEVGPAGAQFLGPVIVEIPHFGSMRGKERE

LIILRSDNGETWKEHQYDCKTEALNELLNGMDEELDCIEDLEKKRICRIITKDFPQYFAV

VSRIKQETNHMGPEGGVLSSTTVRKVQASFPEGALTKKIRVGLQTQPVPDETVKKIIGNR

ATFSPIVTVEPRRRKFHKPITMTIPVPPRSGEGMNGYKGDTTPCLRLLCSITGGTSPAQW

EDITGTTPLTFTNDCVSFTTNVSARFWLADCHQIPETVSLATQLYRELICVPYMAKFVVF

AKMNDPVESHLRCFCMTDDKVDKTLEQQENFEEVARSKDIEVLEGKPIYVDCYGNLTPLI

KAGQQLVLNFYAFKENRLPFCVKVRDNSQEPCGRLSFLREIKNAKGLPQVAVCNLNITLP

ALKKTEKPERRHTFASLALRKRYSYLTEPGLSPQSPCERTDLRMAIVADHLGLSWTELAR

EMDFSVDEINHIRVENPNSLTAQSFMLLKKWVNRDGKNATTDALTAALTKINRMDIVTLL

EGPIFDYGYHNISLELQSPSELSYEPPTPLRQDDFFSEDGGIVVSPSRTPVRPNELSLPT

TSRDALSPNAAPPTVVAEDSSIGGRGSYSREGGSTEDDLDAHDSDEMPKHPEVFMQSTYQ

EQEVPSNGAHRARESGRSGFDEEEEEEMTQEKSCFKSINLEEGLEIVEIPQEKVQEIRSQ

VEQAKKEMCSLAGLQEGPSSVSQEISASGLHTDTQLQDRSQKPTERQNGEHPELQGRAGS

LLEPQEPSSKMKGKGSRDSLDDTAAGPSTMDEDTVEPKAKVGEGDKKKEGRQRGKKGQS*

>m.1177 g.1177 ORF g.1177 m.1177 type:complete len:1656 (+) Unigene000128:200-5167(+)

MSVSDEADGPRPRYGSVLDGVERLTAEEMDERRQQNMAYEYLCHLEEAKRWMEACLDEEL

PPTTELEEGLRNGVYLAKLGNFFAPQVVSLKRIYDREQTRYKATGLHFRHTDNVIQWLNA

MADKGLPKIFYPETTDIYDRKNMPRCIYCIHALSLYLFKLGLAPQITDLYGKVAFTEEEI

NNMKIELEKYNIQMPAFSKIGGILANELSVDEAALHAAVIAINEAIDHGDPAGTLAAMKN

PNAMLLNLDESAAQHYQDTLYQAKAEKVCNARKRIGENVNAERDVYEELLTHAEIQGNVN

KVNSSNSLASVEQALFSGDEDKLYKALSSPALGLQSLKPKNKGWYLKQLVADREAKEQNN

PGESLTKEELQNGVEAANSVAESYQKMLQAVQRINAAIRKGVAEETVTELMNPEAQLPAV

YPRAADLYQRELFSLQQQSPEGSLTHPELLVAVEMLSSVVLINEALDVGDRGAVWKQLSS

SVTGLSNVEGEYAQRYIDELMRLKAAAREEGSDYLTWNDIQACVDQVNTTIQEEHERIAA

IGLINEALHEGDPKKTLEALQHPAAKLTDVDPSIAQHYFDKLLEARREKAHETRDPSAVL

WLDEIQEGILQANKDTEEALQFSQAIQAINEAVDSGDASQTLAALRAPGASLYGVTPECA

QTYQNDLTKIKNDKTEQGDNGSKWMKHWVKGGYNYYYNLNTKAGTWDEPAGFVQNNTQLN

KDDIQSVVSGVTAAYNREQLWLANESLIAKLQARCRGFLVRKNLRERLDFLRSQDPAIRS

IQAHWKGYKERKTFNERKQFLKDHTEEAVKIQSMVRMHQARKKYRDRLKYFRDHINEVVK

IQAFIRANKARDDYKTLINADDPPMAVVRKFVHLLDSSDQDFQEELELMRLREEVITNIR

SNQQLENDLNLMDIKIGLLVKNKITLQEVVSHSKKLTKKNKGELSNLMMMNKQKGGLKAL

SKEKRDKLEAYQYLFYLLQTNPTYLAKLIFQMPQNKSTKFMDSVIFTLYNYASNQREEYL

LLKLFKTALQEEIKSKVDQIQEIVTGNPTVIKMVVSFNRGARGQNALRQILAPVVKEIME

DKTLNIKTDPVDIYKAWVNQMESQTGEASKLPYDVTPEQAMSHEEVRNRLEASIKNMRTV

TDKFLSAIIVSVDKIPYGMRFTAKVLKDTLKEKFPDATEDELLKIVGNLLYYRYMNPAIV

APDAFDIIDMSAGGQLTTDQRRNLGSIAKMLQHAASNKMFLGDNAHLNPINEYLTNSYQK

FRRFFLAACDVPSLEDKFNVDQYSDLITVSKPVIYISIGEIINTHTLLLDHQDAIAPEHN

DPIHELLEDLGEVPTIESLIGENPLPPDDPNKEMMGKTEVSLTLTNKFDVPGEANAETDA

KTLLLNTKRLIVDVIRFQPGETLSEILDSSATPEQEIEYQRAMQRRAIRDAKTPEKMKRA

KPVVDDSLTLQGKKDKIKSNLQRLAELGKVQPENRYQDLINDIAKDIRNQRRYRQRRKAE

LVKLQQTNSALNTKTSFYNVQIDYYNQYIKTCMDNLASKGKLSKKPGDNKAKKSKQASQK

YTAARLHEKGVLIEIEDLQTNQFKNVIFEISPSENVGVFEIKAKFMGVHLETLLLEYQDL

LQLQYEGVAVMKLFDRATVNVNLLIFLLNKKFYGK*

>m.1186 g.1186 ORF g.1186 m.1186 type:complete len:513 (+) Unigene000129:133-1671(+)

MDDDFEWRREQRRQKREEMRLEAERMAYRRTEEDEEEAARERRRRARQERIRNKENEEIA

NPAESEGLNSHSVTATKTVSSSELNTGQAGEGDEEEQALLDRLAKREERRQKRMKEALDR

QKQLDSVDDPSYSTYSVESTSTTSYQAFSNQEDEPALNSKEEVKEELVPQMELVEEKPRR

SYLREQMEDEDAGGKIKQPNGGVYEDSKHKKVERTQSRGSVHSFEGPAAEEGDDSEQTRL

EAERKLEELKRRRDETENEEFERMKQRQQEAEVELEELKRKREDRRKVLEEEERLKKQEE

AERKAREEEEKRRMKEEIERRRAEAAERRQKVGEEVDGDAGKPFKCVSPRGSSLKIGERA

EFLNRSAQKSAVKTSHSPVVSKIDNRLEQYTTAVQGNKEVRSPRGAAIDFPGGSDGVRNI

KSMWEKGNVFSSTISSPSSNKDAAGIKVGVAGRINDWLNKTPEASKTPGGRPVDLKPGDV

TGKRNLWENKSSSATKVTSRGESKSVANGTGP*

>m.1185 g.1185 ORF g.1185 m.1185 type:complete len:604 (+) Unigene000129:1996-3807(+)

MTAEDSTSAVTMSNSSHSSKSNPANNHAVLHGSITEGVAGAPNEAALMALMERTGYKMVQ

ENGQRKYGPPPGWQGASPPRGCEIFVGKIPRDVYEDELVPVFESVGRIYEMRLMMDFDGK

NRGYAFVMYTQKHEAKRAVRELNNYEVRPGRLLGVCSSVDNCRLFIGGIPKTKKREEILE

EVSKVTEGVLDVIVYASAADKMKNRGFAFVEYESHRAAAMARRKLMPGRIQLWGHQIAVD

WAEPEIDVDEDIMETVKILYVRNLMIETSEETLRKIFSQFNPGCVERVKKIRDYAFVHFT

SRDDAVVAMDNLNNTEIEGSFIEVTLAKPVDKEQYTRYQKASKGTMTSSDSTQQNFVYQC

DPYTLAYYSYPYNTLIGPNRDYFIKAGTARGRGRAGATNRGPGPRGSYLGGYSAGRGIYS

RYHEGKTKVPDKPYELMSSLELAAVNPVGIKPGTMTLPGLGAQYPAMFSAAPAATKLVEE

GKIHPVEHLINPLGLPHDHPAAPSSAAVIPAVSTPPPFQGRPITPVYAVAHNVPRIPAAA

AAAGLYGASFVPIAAHTNTAALAALQKNAAVAAAAYGGYAGYMPQAFPAATFQVPIHEMY

PTY*

>m.1192 g.1192 ORF g.1192 m.1192 type:5prime_partial len:2060 (+) Unigene000130:2-6181(+)

KKLCIPEKAEILAVNVDSKGVHAVLKTGSWVRYCIFDLATGKAEQENHFPTSNLAFLGQS

ERNVAIFTAGQESPIILRDGNGTIYPMAKDCMGGIRDPDWLDLPPIASLGIGVHSLTNLP

NNSTIKKKAAVIIMAVEKQTLMQHVLRCDYEACRQYLVGLEQAVLLEQNPHALGALLGHR

CDGNRNLLHACVSVCFPVSNKETKEEEEAERSERNTFAERLSAVEAIANAISVVSSNSSG

NRTGSSSSRGLRLREMMRRSLRAATMSRHESGPSSSDHQDPVSPPIAPPSWVPDPPPMDP

DGDIDFILAPAVGSLTTASSGPSQGPSTSTIPGPSSEASVVESKDRKANAHLILKLMCDS

IVLRPHLRDLLSAKDARGMTPFMLAVSGRAYPAAITVLEAAQKIAKAGETNIGDKDDVNS

VFLEMICPAGTNPDDSPLYVLCCNDTCSFTWTGAEHINQDIFECRTCGLLESLCCCTECA

RVCHKGHDCKLKRTSPTAYCDCWEKCKCKTLIAGQKAARLDLLYRLLTTTNLVTSPNSRG

EHILLFLVQTVARQSVEHCQYRPPRIREDRNRKAVNAEDSDMPDHDLEPPRFAQLALERV

LQDWNALKCMILFGSQENKDPLSASSRIAHLLPEEQMYLNQQSGTIRLDCFTHCLIVKCA

PDLTFIDTLLGTLVKELQNKYTPGRREEAVCVTRRFLRSVARVFVILSVEMASSKKKNNF

VPQPIGKCRRVFQALLPYAVEELCNVAESLIVPVRMGIARPTAPFTLASTSIDAVQGSEE

LFSVEPLPPRPSPDQSNNSSQSASSYIIRNPQPRRSSQSQTTRGRDEEQDDIVSADVEEV

EVVEGVAGEEDHHDDQEEQGEESAEAEGQHDEHDEDGSDMELDLLAAAETESDSESNHSN

QDNASGRRSVVTAATAGSEAGASSVPAFFSEDDSQSNDSSDSDSSSSQSDDADQETYLLD

EPLERTTGSAHANSAAQAPRSMQWAVRTTPSQRTAGGAPASSSTPAASSAGLIYIDPSNL

RRSSAISTSAAAAAAALEASNSSSYLTSASSLARAYSIVIRQISDLMSLIPKYNHLVYSQ

YPAAVKLTYQDAVNLQNYVEEKLVPTWNWMVSIMDSTEAQLRYGSALSSAGDPGHPSHPL

HASQHSGRRERMTAREEASLRTLEGRRRAATLLTARQGMMSARGDFLNYALSLMRAHNDE

HSDVLPVLDVCSLKHVAYVFQALIYWIKAMNLQTTLDTSQMDRKRSRDPLELALDNEDSE

HENDDDTNQSSTLQDKEEESVSAETGQHHPFFRRSDSMTFLGCIPPNPFEVPVAEAIPLA

DQPHLLQPNARKEDLFGRPSQGLYSTSYMASKGLSDLTVDMNCLQILPTKMSYSANMKNV

MSMESRQRGGEEQQATAQDMDSSKPGPSPHDLAAQLKSSLLAEIGLTESEGPPLPTFIPH

CSFMGMVISHDMLLGRWRLSLELFGRVFMEDVGAEPGSILTELGGFEVKESKFRREMEKL

RNLQSRDLALEVDRDRDQLIQQTMRQLNTHFGRRCTSTPMAVHRVKVTFKDEPGEGSGVA

RSFYTAIAQAFLSNDKLPNLDCVQSVSKGMQASNLMQRLRNRDRERERRSGGLRTGSRRD

RDRDSRRQLSIDTRPFRPASEGNPSDEPDPLPAHRQALGERLYPRVHAMQPAFASKITGM

LLELSPAQLLLLLASEDSLRARVEEAMELLITHGRENGADSILDLGLLDTQEKAQENRKR

HASTRSVVDMELDDTDDGDDNAPLFYQPGKRGFYSPRPGKSSEARLNCFRNIGRILGLCL

LQNELCPITLNRHVIKVLLGRKVNWHDFAFFDPVMYESLRQLIRHSQTSEAEAVFAAMDL

AFAIDLCKEEGSGQVELLAGGGSIPVTPQNVYEYVRKYAEHRMLVVAEQPLHAMRKGLLD

VLPKNALEDLTAEDFRLLVNGCGEVNVQMLISFTSFNDESGENAEKLLQFKRWFWSIVEK

MSMTERQDLVYFWTSSPSLPASEEGFQPMPSITIRPPDDQHLPTANTCISRLYVPLYSSK

QILKQKLLLAIKTKNFGFV*

>m.1204 g.1204 ORF g.1204 m.1204 type:3prime_partial len:1763 (+) Unigene000131:1087-6378(+)

MMLAINSARIASDALYKKDFNKSKTKFHLPVDMLSLELAKKCQIQVNDFNYRTSLHNWTC

LPDQNDVIQARKAYDLASDNIYKTDLEWIRGCGWVAADSVDHVKVRKAQQILNDRMYKKD

AKDNFAKFTNIVDRPEVLLAKVNAFNLSDLKYKESFNLEKGHYIGSDDTPQLAHSREAAK

IISEKLYKLGWDEAKATGYQLNHEYLPLVSAKKVGSIVSEAKYHDAHEKAKGHYLANTLV

DFPAVVHSSQMEKMKNMRTYQKDYHTSKTKLHIPHDMISHVVAKKCQEILSDVLYRSYLH

QWTCHPEQNDAIRARKANEILSDVFYKDDLNWMKGIGCYAWDTPEIVRAKKSYELQSDIK

YKAEGKKEFNNYSIVTDTPVYVTAILGHTWASELNYREAYHKEKHLYTTVLDTYDYARCY

NLKQLYSSKTYSALWDRIKAKSYTIPIDANALVHAKQQKVLLSKVKYKEDYEKFKSLYSL

PKCLADDPATARCIKAGKLNLDRLYKDKWEKTKAKIHIPPDMLDVVAARNTQKMISEVDY

RKYLHQWICLPDMQVYVHARKVNEQLSDIFYKEDMTWLKGVGCYAWDTPEILRCKQAMNL

QSENLYRAKGVEHFKNFSVVTDTPVYETCKQSAHNLSDLNYHHDYVTNVRGKNTAPATTV

DTERARLANYIQSDHIYKEASKSNMSTGYSLPFDTPLALQAKANNVITSNVKYKEAYENT

KARSYKLDPNGVNFVTIRKANQVTNQRLYRALYEKEKDKVHSVYDTPEIKQVKATQEAIS

DVCYKEKYYNSRGTLLPMPFTPQLMHCAHVNQINSDLKYKEDLQWLRGIGCFVFDSPEMV

RIREINKFRTSYDIDAKKNFSNFSVVLDTPEYKRVSELKTHLSERVYKAAGNIQRTQCST

TGDAMEFKRAKWAQTLTNKWLYTDLASKERAFYTPELHTPLLDHARSMKVVYSENKYKEQ

YEKMKHKYTPVHDTPILIRAKKSYLNSSDLRYKETFELAKGHYHTTKDALDIICAKRVRD

DISEVKYREKYINSLGTWKSIPDRPEFFHSKVVRDCISDYKYKEDLEWIKGVGCFVWDTP

LLKQAEHNKALYSERLYKASYEKTKCNFKYTCDTPFFQAVKNASALSNDRSYRASYEKTK

DKYTIVVDDPRNLLAKEVNNMSQWKYRSQYERLKSKYTSVLETPEHKVHKLRKQISDKIY

TMEYNKNKAKGYTLGHDTPLNLHMKKVKEITSNLKYKEVYEKNKAQINIAPDAFDIRAAK

EAYKNISNLDYKKKYEATKNKWIWTVDRPDFVHAAKINYQQSDVEYKYDKEMLKGCVMPV

TDDKYTLLAKQNTELSSDIKYKQKYEEARGHYHVVHDTPQILHAKSVSGLVSESKYKLES

KKARQSGSFTTLPETRDTAHSKAMTKITSSKVYKQKFEKDKGKSIYNNMTVPPDVKHAMD

VAKSQSNINYKQEAKAKLHYTPVADRPDILKATQAAKLISEVGYRDKARQEASRGGSLVN

RPDINLATEVSKLTSLVKYKEKFDKEMKGKRPQYDLKNSKIYQTLKDANTLASEVKYKGD

LKKIHKPVTDMSESLAMQHNLSTSKLASSVKYKEKYERERGKAMLDFETPTYVSAKEAQH

MQSQREYKKALEQEIKGKGMLALANDTPDFMRARNATEILSQSKYKHNAEQDRASYTTVI

DTPDIIHAQQIRNIVSQKKYKEEAEKTMSHYNAVLDTPEMQRVRENQRNFSTVLYKEPSG

KGTSVIFTPEMERVKRNQEHISS

>m.1207 g.1207 ORF g.1207 m.1207 type:5prime_partial len:223 (+) Unigene000131:2-670(+)

GHHIGALSIQDDPLLMLALNSAKIASDALYKKDFNKSKTKFHLPVDMLAFELAKKCQVQV

NDANYRTYLHNWTCLPDSNDVVQARQTYELQSDAVYRADLKWLKGLGWIPIGSLDVEKAK

KAGEALSERKYRQPPSNFKFTSTTDDMPIVLAKANNNIMNKRSYIQAWESDKTKIHIMPD

AMEVLHAKQNKIKLMFKPGIMKSPISTSCQMQWMWSLPNKIK*

>m.1209 g.1209 ORF g.1209 m.1209 type:complete len:141 (+) Unigene000131:625-1047(+)

MPDAMDVVLAKQNKVNYSEKQYKLANELSKKKGYDLRGDAISIIAAKASRDIASDYKYKT

GYRRQVGHHIGARSVQDDPLIMLAINSAKIASDVLYKKDFNKSKTKFHLPVDMLSFELAK

KCQIQVNDDNYRTYGYRKQV*

>m.1213 g.1213 ORF g.1213 m.1213 type:complete len:1212 (+) Unigene000132:1082-4717(+)

MQSDDLFIRKFRRQSPRPTPVFEPKRTAVAEWPPRRDGEGAEGESLTTPSRAGLNQRAVA

RGHIMQRSNSDVTLGDLESAAKAGAKAVRSASEKTGQVGQGDSSIVLHREYGSLSSLEKQ

IQPPELCMDEQNMLSPNALRFKDPFLLLGLQETPPEPDGFFRALECPKPAKPPKPDALNK

KSKPSPLQVPSVPCDLTCGGAWVRNFAHYDVQSILFDLSEAATSRDSVGRKKNITSGASA

ASQLRPLSQAPPASPSQSLGSGTVGTASTEEPEPCLLDEGDGNDNDLLLSCPHFRNEMGG

EERAGLGRNQCCSGRWRSLQNPNDAVSVLEEPRESHVQQQGKSNYFIEHADLGARYYHKY

FYNKDHQNFFGMDERLGPVAISFRREEKEGTSGAQYMYRIIFRTTELKTLRGSILEESVP

SAARHTTPRGLSPKRLLEFIFPDLNLQCLRLASTSPKVRDTLLKLDEQGLNFQRKVGVMY

CRAGQSTEEDMYNNESAGPAFEEFLDLLGDRVRLKGWEKYRAQLDTKTDSTGTHSLYTRY

QDYEIMFHVSTMLPYTANNTQQLLRKRHIGNDIVTIVFQEPGAMPFTPKAIRSHFQHVFI

IVRVHEPCTENTYYRVAVTRSKDIPLFGPLFPKRARFPRSQAFRDFLLAKALNAENAAEK

SEKFRSMATRTRQEYLKDLAENYVTTTPIDSSTKFPLLSLGGKRKDKFKGAKGAELHSAG

ALVWAVCARQDDDGHKLACLLGVSAEFVVLIERCSKRVVFNCSCRDVIGWKAVTETGIKG

GPCLDIFYERGEAISVTMSESQIEDIKEVVQRLELVTRGCEALEITPLRDNTGQPGFLMS

AEGFVIEMQRFCYAESGGLQLSARVVRLCGHTLVHLSQEERTRLLRTAPKIHITVIPPDE

NGKPRRSFSELYQKSIQDAESKSGEGHSSEAWVLNEHDEDEDDEEEDERGGEREQGEGEE

SDSMNQSDTREEEESGSPSLSGLLSPPSLPLLRATSLQDNPPTQTLEITSSALTRSYSLE

RTLPCQDICDGHVYDNVGAKVNRHHIYENLGELIDATPDLILAVKPKVPLQEEGQVGLDV

LGEERASGSEAPPQSEHIERNSRAERNSRALSIHNSITKILSETTDSSEEEWQSISDLAT

ACRNILEALSHEDRKAGDGTQAGGDTGTQTDARIKDMKDSDSSSHLEEKVSQLEAMLKRL

QDDLQKGEQRR*

>m.1219 g.1219 ORF g.1219 m.1219 type:5prime_partial len:1869 (+) Unigene000133:2-5608(+)

SKVTVRIEKYTCKTSDCMFVQFLSHTHIHTLCVCVSEEVTFPDSRNVCVFVCVGALHSML

GAMDNRISEEGMKVSCTHFQCAAGAFAYLRDHFSHNYSVDMSHQILNLNINLMLGQAQEC

LLEKSMLDNRKSFLVARISAQVVEYYKEACRALENSDTASMLGKIQKDWKKLVQMKIYYF

AAVAHLHMGKQAEEQQKYGERLAYLQSSLDKLNEAIKLAKGQPDSVQEALRFTMDVIGGK

FNSAKKDNDFIYHETVPCLETLPSVKGAPLVKALPVNPTDPSVTGPDIFSKLVPMAAHEA

SSLYSEEKAKLLRDVMTKIDSKNETLEQFMDSLNLDPDSVDNMDRYNSIPSVLMEKCAAL

SVRPDTVKSLIQSMQVLSGVFTDVEASLREIRLVLDDDEASDRKLEEVAGKQCVPAQPAT

IAEIRRDLEKYVEAHEKASFTNTELHRAMNLHISNLRLLGGPLDTLREALPRPQLSEDEV

AGLQCMKRILGKVQEMKDQRNSLEKQLRELIQQDDITAALVTTERADMKRLFEEQLKKYE

QVKVYIDQNLAAQENILKALTDANVQYATVRKGLAETEHKWNSTVQMLVASYEAYEDLMK

KSQEGKEFYEDLEAKSSRLLERAKSICKTRDEERQAILEKELQKKVPQRPTAPKPAQKKA

PDMDLSGIEDPELAKINAAILALGGDLPEDLRSLPPELGRPSIPEAFHPGAAALYARPLF

PPNLPPPELLAQISRFTLPPTQGFNRNPTTGPLPHQHQVPQLPNPQIAQLPSVPGYGLPS

SQTQPAASGPPTAIGMSTTTVDSVHTPIPSYTSAPGHPVISTTPGHPMATSVGYNAPSAV

ASQYFQQPGVPVSQPPQGVQVPQPKPPPSQSFPQVRRQGQPQPSPPQTFYQAVPAQQHIA

APVTGPQLPQQTQPYSLHHMAQPRPPNIQQQANNIHSTPSALSHQVYPAVSFVPGCQPAP

NQLPYHSMQPQMPPNSQPMPQVSMSHLPNVKQIPPCSQPYMPPLNQQLHTIPQTPLSTQQ

AVPASHRQMPPTPQQIPPLSQPQMLPSSAQISPPTHLQMPAVSQPGLHASHSPVSVSQSL

PRSSHPPLHSVSQPLPHPSQVHPSYQQVFPGTTPRVPVPVQPQLPPASLPAQFPVHTNAP

PSQPHSLPSQLPHGSLSSVPSQISLPQHHLPPQSTPMGYPGGIPLVPHQSTVPQQPQALP

PQVPSGAQASYSGPHAGIHPGPIQNSGPVITPGIQSLPQGMIPPSSAAPGATPSNVLATP

SASPSPGPSSLGIAPQRPSPALTPVPGAALPQTVVTPLPSSTHLFQHQNSSTDDLLSSSP

ESQHGSSKDTANVLLPMKADPQDEQLRKKSEGLKIIQGDPYQAPERVSQLCAELERFRST

VQSLERPSGEGSLSELDARWKELQDQQEKDSRQLSIAIARCYTMKNRHQDVMPYDCNRVV

LDSGKDDYINASFIEELSPYCPRLIATQAPLTGTAADFWLMVYEQKVSLIVMLVSEQELE

KQKVLRYFPSERGQQITQGSITLSLTTQKNTATHVERMIGLQYRDQSLKRTVIHLQFTSW

PELGLPESKSNLIRFIQEVHGHYLLQRPLHTPVVVHCSSGVGRTGAFSLLYAALQELEAG

NGIPDLPQLVRKMRQQRKNMLQEKLHLKFCYEAVLKHAEQVLQRRGIVTASSQKSTNSAA

VKQYSQPEAQDIVLGGDMPISSIQATVARLSIRPPSVESDQDPNQDPVACVAAGLEPELA

LVQDLQAFSPSDTIPASLSPPISCPTSPVKMLSPSPPNYQGNGFEGSTIMPVSNNHAALE

ATLPAPSSLDLLASLTPEAFTLDSGCRGKQRISKQSFFQAQEGQGLQGPSNQDDPLSTLD

PLWTLNKS*

>m.1225 g.1225 ORF g.1225 m.1225 type:5prime_partial len:2058 (+) Unigene000134:1-6174(+)

RSEERQCVGFFFFFSNAGVHAVLKTGSWVRYCIFDLATGKAEQENHFPTSNLAFLGQSER

NVAIFTAGQESPIILRDGNGTIYPMAKDCMGGIRDPDWLDLPPIASLGIGVHSLTNLPNN

STIKKKAAVIIMAVEKQTLMQHVLRCDYEACRQYLVGLEQAVLLEQNPHALGALLGHRCD

GNRNLLHACVSVCFPVSNKETKEEEEAERSERNTFAERLSAVEAIANAISVVSSNSSGNR

TGSSSSRGLRLREMMRRSLRAATMSRHESGPSSSDHQDPVSPPIAPPSWVPDPPPMDPDG

DIDFILAPAVGSLTTASSGPSQGPSTSTIPGPSSEASVVESKDRKANAHLILKLMCDSIV

LRPHLRDLLSAKDARGMTPFMLAVSGRAYPAAITVLEAAQKIAKGETNIGDKDDVNSVFL

EMICPAGTNPDDSPLYVLCCNDTCSFTWTGAEHINQDIFECRTCGLLESLCCCTECARVC

HKGHDCKLKRTSPTAYCDCWEKCKCKTLIAGQKAARLDLLYRLLTTTNLVTSPNSRGEHI

LLFLVQTVARQSVEHCQYRPPRIREDRNRKAVNAEDSDMPDHDLEPPRFAQLALERVLQD

WNALKCMILFGSQENKDPLSASSRIAHLLPEEQMYLNQQSGTIRLDCFTHCLIVKCAPDL

TFIDTLLGTLVKELQNKYTPGRREEAVCVTRRFLRSVARVFVILSVEMASSKKKNNFVPQ

PIGKCRRVFQALLPYAVEELCNVAESLIVPVRMGIARPTAPFTLASTSIDAVQGSEELFS

VEPLPPRPSPDQSNNSSQSASSYIIRNPQPRRSSQSQTTRGRDEEQDDIVSADVEEVEVV

EGVAGEEDHHDDQEEQGEESAEAEGQHDEHDEDGSDMELDLLAAAETESDSESNHSNQDN

ASGRRSVVTAATAGSEAGASSVPAFFSEDDSQSNDSSDSDSSSSQSDDADQETYLLDEPL

ERTTGSAHANSAAQAPRSMQWAVRTTPSQRTAGGAPASSSTPAASSAGLIYIDPSNLRRS

SAISTSAAAAAAALEASNSSSYLTSASSLARAYSIVIRQISDLMSLIPKYNHLVYSQYPA

AVKLTYQDAVNLQNYVEEKLVPTWNWMVSIMDSTEAQLRYGSALSSAGDPGHPSHPLHAS

QHSGRRERMTAREEASLRTLEGRRRAATLLTARQGMMSARGDFLNYALSLMRAHNDEHSD

VLPVLDVCSLKHVAYVFQALIYWIKAMNLQTTLDTSQMDRKRSRDPLELALDNEDSEHEN

DDDTNQSSTLQDKEEESVSAETGQHHPFFRRSDSMTFLGCIPPNPFEVPVAEAIPLADQP

HLLQPNARKEDLFGRPSQGLYSTSYMASKGLSDLTVDMNCLQILPTKMSYSANMKNVMSM

ESRQRGGEEQQATAQDMDSSKPGPSPHDLAAQLKSSLLAEIGLTESEGPPLPTFIPHCSF

MGMVISHDMLLGRWRLSLELFGRVFMEDVGAEPGSILTELGGFEVKESKFRREMEKLRNL

QSRDLALEVDRDRDQLIQQTMRQLNTHFGRRCTSTPMAVHRVKVTFKDEPGEGSGVARSF

YTAIAQAFLSNDKLPNLDCVQSVSKGMQASNLMQRLRNRDRERERRSGGLRTGSRRDRDR

DSRRQLSIDTRPFRPASEGNPSDEPDPLPAHRQALGERLYPRVHAMQPAFASKITGMLLE

LSPAQLLLLLASEDSLRARVEEAMELLITHGRENGADSILDLGLLDTQEKAQQENRKRHA

STRSVVDMELDDTDDGDDNAPLFYQPGKRGFYSPRPGKSSEARLNCFRNIGRILGLCLLQ

NELCPITLNRHVIKVLLGRKVNWHDFAFFDPVMYESLRQLIRHSQTSEAEAVFAAMDLAF

AIDLCKEEGSGQVELLAGGGSIPVTPQNVYEYVRKYAEHRMLVVAEQPLHAMRKGLLDVL

PKNALEDLTAEDFRLLVNGCGEVNVQMLISFTSFNDESGENAEKLLQFKRWFWSIVEKMS

MTERQDLVYFWTSSPSLPASEEGFQPMPSITIRPPDDQHLPTANTCISRLYVPLYSSKQI

LKQKLLLAIKTKNFGFV*

>m.1239 g.1239 ORF g.1239 m.1239 type:complete len:342 (+) Unigene000135:5252-6277(+)

MLFMNEFLAEHWEDMRTFLQKVSNPDSELEMARFDGYVDLPLRLAVLHNLLVDIISPMKL

EVINNLQPLPSILNQITEHLGPDIPRITCSRSTCETEKPTYVPPRDLGKYSPLNKSLPHL

PTDPRPPQGKGKHRKQVARTQSVPASERHHRRPLKRTSSNAELSETAHIHHEESPERQME

ISLPQQEFFNKSAPAPVPWIKEHVQDETNILEKHAQELYELRSGVDQVTDRELEMAKRLE

DFIAVSQEQQAQLQAELQEVRNVLARREEQLASATFRLGVIEEEREEDERKLSVALAAAE

RVNVLEEQFVGLLKDMQQLNTVYNDVLSTSALNRDTETLRQ*

>m.1237 g.1237 ORF g.1237 m.1237 type:complete len:763 (+) Unigene000135:2553-4841(+)

MGTDKASETETHAKMEKEEVRGEEESGEERAEQDGNLLTSYTWQTGGARADPSEKSAGNK

WSRMQSWKKALSEDTPEKSATPTPGPRVGRKNPFRRALSEPPGSLLSSILSPSSSSPSAA

ETSAGADTQQRGKLRKYLQQVSQRLKRPRAQNRNNTSTTQQQQQREDVPCVEGADLVQFS

WVPQDVPVWDINNCILQDGQIFISREEEPLLRMRNRASSCLSSVSLQNLTESHSNLECSP

DPTSVTAPKPKTQDGGVRMKIKRRLQGGAHRKLSNTQLNVVGLSSAAWFGSRDSLSVPLS

AAESLNVSSDCTTVIRPVHSSILGEKYCFEVITSDSNHCFGCSSAAERDRWIENLRRAAR

PNKDNCERMENSLSLWVNEAKGLPPKKRYYCEVHLDGTLFARTSSRAVGKASSVPVSDSG

SSAVGVSSGGSASTGGCQLFWGEYFELDNLPPVTQITLHLFRDDDPKKKRHSKDENILYP

LGSVALSLANIKGRVFQEKWYPIVPYKPPGTGGSKEQLGPQACIRVKARFQNLKVLPIER

YKEFAESVTLGYMKMCSNLEPLLNVRDKEELAGALVHVLQSIGKAKDFLIELGHAEVQRS

GENEALLFRENTLATKAIDEYMKLVGQKYLIDTLGDFINRLYTTSESCEVDPQKCSASEL

LVNQRHLKDACGEVVRQITERHSSFPVELNEIFSSWVSDCEERGRADIGHRLISASLFLR

FLCPAILSPSLFGLIQPYPEHNTLRTLTLTAKGLQNLANFTL*

>m.1238 g.1238 ORF g.1238 m.1238 type:complete len:526 (+) Unigene000135:483-2060(+)

MAAVVNYSPPWWVNLLHRLPHFNLRFEQISSEFQPEDPSYQQSILLLGAVALACLALDLL

FLLFYSFWLCCRRKKSSEQPNADCCCTAWCVIIATLVCSAGIAVGFYGNGETCDGVNRLT

YSLRHANRTIAGVQKLVSDSTTSLNLTVDDNLQHLESQYTKHADYLSIVQKVQGQLDELL

KQMVEIPFWSNSRISLEALASKIELYDWYRWLGYLGLLLFDVLICLLVLFGLIRNSKRTL

IGVCLLGVLALVISWGSLGLELAVSSSSSDFCVAPDMYVAEVAERYGVINRDILQYYLHC

SVGQNNPFQQKLSGSHKALVEMQDDVLELLRAAVREYPQTRTNLEQIQGILNTTEISLHQ

LTALVDCRSLHMDYVQAVTGLCYDGLEGLIYLVLFSFVTALMFSSIICSVPHTWQGRRSS

EDTEEDSAASGGRQNHDNLYRVHMPSLYSCGSSYGSETSIPAAAHTVSNAPVTEYMTQNA

NFQNPRCENTPLIGRESPPPSYTSSMRAKYLANARPDPNSSTAAH*

>m.1246 g.1246 ORF g.1246 m.1246 type:complete len:2026 (+) Unigene000136:224-6301(+)

MVQMTFLDNVVHSLLKGENIGITSRRRSRSSQNNNTAHTTGGRPSGNTGSSQGHYTRAQA

NSPRPVMNSSGPATKQGSQAQQQQPQHAQQQVSQQQQQQPQVSPGQQRGSRSTRRKGSDS

SIPDDEKISDEKTDAGGGKGDVSKNKSKQVMNKRRKAEDEEKKAGLKRLKMEASDLSESS

DSENSNKRLLDSSSEPSSENELKNKGILKASEEEEKSQSCKAMEEPDANSRMSPWEEAST

VDQIVKPTAMEVMPSTKSENTGDMRSLAQPSPLPPPSSCAQPRSLVPAEVQGCIMEIKST

VKTSPKDHYSTGAPRTQTPKCVIDITEDSSSHPTTRENSETVPATLASQTRETYVPESRH

LVLNPLASECRKAEGELQQQLGQSLGSKIEFAHSEVIRPVVSVSELAAVAEREREKVQQQ

YPSIMPCIKNASLAEDVRKPQKLSSSPDVAKSKSNPSPDAFKPKCNPSPDAMKSKTHSVL

EAMKPKPNTSPEVTKHKEIIEITSSSVARSAVKSRSQEIPRSSFKPVPARSTPSESIKSA

LIVDKNEHFTVYRDPALVRPEADTNHVTYLPPHLHPLHSSSHATCLTPSSHHHSHLLPAS

SLSPHPSVHHPLLPTVLPAMPPSSLLGGHPRLDSGGLSHLALAHHHPHQQQQFLQQQPPP

PLLAQTHGGASYNQLGLYPIIWQYHNGTQHSYPSGISLPGSKWVHPENAVNSDGSLSRNT

SSPWLHQPTPVSSADSLGILSHVPGRPASADPHRPLKISSHSSPPLSKTVDHHKGELESK

VFVDPMRGLVTAHLKQEPDQSRTPNSRDLQRLYADSSHVKQQALPPRLPLDTPDRAVKYK

EENRRILQESIEVAPFTAKIRSGEPEREPYIRIPSLPISGPPKENEHSPSDLYKYKHSAA

QSLPQSNYFTTISNSVVNEPPRLYPSKELSSLNVSSPLTLGSYPNSGGNTKSLSKPPPLI

KHHPDGEGLVGKISEQLSHQVALNPLSTPVASCEPRSPAVSPSNQLRSMPALHRAPVFHP

PTQQTLDRKESGYGRLSPPTLTPIQPVSVAGKVSEQQKPPTLLPELREVKGTVELSSSEP

WRPSSNLQGYDKTVQWQLDKSQGKPQVATASVIVRSRTCIKYDSSPGSKPGARETSNSKP

HTGKYQLDCSKLAETREPGRVIQQNTNKEDIFLQYKNNFVRVSQGSFPSSAVAVVNSVCN

NSTAVTTSASAASTHNVLSRGTAELPYSTSANPSSSNINRLEGAAPKCRTPTSLELQECN

ARTASPIGPLPQPGSALAPQPYSGNFIHLKKHKAALAAAQSRGSSSASESEGSSRSSQES

PTIITQDRASPGNPSSKVSPLPNGQPSLMNQPNYHKLKKAWLTRHSEEDRNTNKVEMTSN

AVSEIIRPCTVNLIASTSSDTEISKDGKCLEDKLSPEDRKPRRVPSKRPHESGSDSGDDS

DGSDSKHEQRAKRQPKPTFKKKQNDMQKKKGENDKEEEDVKPNGIFRSAKEKTKLKLASS

NGIPRSVLKDWRKVKKLKQTGESFLQDDSCSEIGPNLQKCRECRSIRTKKGEEPTHSPVF

CRFYYFRRLSYSKNGVIRIDGFSSPDQHDEEALSLWAPDAYEENDLDLETSKYILSCIGD

KFCQLVMSEKTAATWIKKDAKIAWKRAVRGVRESCDACEATLFNIHWVCQKCGFVVCLDC

YKAKEKKSSKDKDLYTWLKCVKGQPHDHKHLMPTQIIPGTVLTDLVNAMHMLREKFGIKA

HCVCANKHNILNKLPSTNGVSQVLQNVLNHSNKLSLCKPEAGQHNLGQKVEANGGSSPAS

DTSTDCKLPPPESQSPLHFLADLAEQKSREEKKENKESVVGKVKEESTDALETLHCKTSS

LVANSTEQGSTLRDLLTTTAGKLRLGSTDAGIAFAPVYSTASQTGKSGRSMPNILDDIIA

SVVENKIPADRSTKQSPKAKPQDEVKAERRKQAEDVPEQHTDIPHCWLYDHRLLWLKDHR

KSSNWKLFRECWKQGQVSHSTTNHPAQPDQAFYMHTKKKKSMSEK*

>m.1252 g.1252 ORF g.1252 m.1252 type:complete len:510 (+) Unigene000137:3480-5009(+)

MRKPTALIILDGFGLREETYGNAVAQAKKPNFDGYWNKFPHTTLTACGEAVGLPEGQMGN

SEVGHLNIGAGRIVYQSLTRVNVAIREGEFDKNETFQSAIKSVKEKGTALHLFGLLSDGG

VHSHMNHMFALLRLAAKEGVEKVYIHAFLDGRDVGPKTAQSYIDATNEVIKETGVGQFAT

ISGRYYSMDRDKRWDRVEKCYRAMVNGEGPTYKSAEECVEDSYANGIYDEFVLPSVIVNE

DNTPVATINDDDAVIFYNFRPDRAIQIARVFTNEDFREFDRGEKVPHIPEFVCMTHFSET

VDGYVAFKPMNLDNTLGEVVAQAGLKQLRIAETEKYPHVTFFFSGGREAEFPGEERILIN

SPKVATYDLKPEMSIYEVTDALVNEIENDKHDVIILNFANCDMVGHSGMMEPTIKAVEAT

DECLGKVVEAILAKDGVALITADHGNADEELTSEGEPMTAHTTNPVPFIVTKNDVELRED

GILGDIAPTMLTLLGVEQPKEMTGKTIIK*

>m.1255 g.1255 ORF g.1255 m.1255 type:complete len:335 (+) Unigene000137:367-1371(+)

MTKIGINGFGRIGRNVFRAALNNSEVEVVAINDLTDAKTLAHLLKYDTVHGTLNAEVSAN

ENSIVVNGKEIKVIAERDPAQLPWSDYGVEVVVESTGRFTKKSDAEKHLGGSVKKVIISA

PASDEDITVVMGVNHEQYDAANHNVVSNASCTTNCLAPFAKVLNEKFGVKRGMMTTIHSY

TNDQQILDLPHKDLRRARAAAENMIPTSTGAAKAVALVLPELKGKLNGGAVRVPTANVSL

VDLVVELDKEVTVEEVNAAFKAAAEGELKGILGYSEEPLVSIDYNGCTASSTIDALSTMV

MEGNMVKVLSWYDNETGYSNRVVDLAAYMTSKGL*

>m.1254 g.1254 ORF g.1254 m.1254 type:complete len:395 (+) Unigene000137:1511-2695(+)

MNKKSIRDVDLKGKRVFCRVDFNVPMKEGKITDETRIRAALPTIQYLVEQGAKVILASHL

GRPKGQVVEELRLTPVAARLGELLGKDVKKADEAFGPVAQEMVAAMNEGDVLVLENVRFY

AGEEKNDAELAKEFAALADIFVNDAFGAAHRAHASTAGIADYLPAVSGLLMEKELEVLGK

ALSNPERPFTAIIGGAKVKDKIGVIRHLLDKVDNLIIGGGLAYTFVKALGHEIGLSLCED

DKIELAKEFMQLAKEKGVNFYMPVDVVITEEFSETATTKIVGIDSIPSNWEGVDIGPKTR

EIYADVIKNSKLVVWNGPMGVFEMTPFSQGTKAVGQALADAEGTYSVIGGGDSAAAVEKF

GMADKMSHISTGGGASLEFMEGKELPGVVCLNDK*

>m.1253 g.1253 ORF g.1253 m.1253 type:complete len:432 (+) Unigene000137:5040-6335(+)

MSTIIDVYAREVLDSRGNPTVEVEVYTESGAFGRAIVPSGASTGEHEAVELRDGDKSRYL

GKGVMNAVNNVNEAIAPEIVGFDVTDQAGIDRAMIELDGTPNKGKLGANAILGVSMAVAH

AAADFVGLPLYRYLGGFNAKQLPTPMMNIINGGSHADNNVDFQEFMILPVGAPTFKESIR

MGAEVFHALKAVLHDKGLNTAVGDEGGFAPNLGSNREALEVIIEAIEKAGYKAGENVFLG

MDVASSEFYNKETGKYDLAGEGRTGLTSAEMVDFYEELCKDFPIISIEDGLDENDWDGHK

LLTERIGDKVQLVGDDLFVTNTQKLAEGIEKGISNSILIKVNQIGTLTETFEAIEMAKRA

GYTAVVSHRSGETEDATIADIAVATNAGQIKTGSMSRTDRIAKYNQLLRIEDELGEIAVY

DGIKSFYNIKR*

>m.1256 g.1256 ORF g.1256 m.1256 type:complete len:252 (+) Unigene000137:2728-3483(+)

MRKPIIAGNWKMNKTLSEAVSFVEEVKGQIPAASAVDAVVCSPALFLERLVAATEGTDLQ

VGAQNMHFEKNGAFTGEISPVALSDLKVGYVVLGHSERREMFAETDESVNKKTIAAFEHG

LTPIVCCGETLEERESGKTFDLVAGQVTKALAGLTEEQVKATVIAYEPIWAIGTGKSSSS

ADANEVCAHIRKVVAEAVSPEAAEAVRIQYGGSVKPENIKEYMAQSDIDGALVGGASLEP

ASFLGLLGAVK*

>m.1258 g.1258 ORF g.1258 m.1258 type:complete len:1994 (+) Unigene000138:237-6218(+)

MAKPGTDRDGAMVEKTTGKKSKDKISPFTKTPKLDRSEIMGKEGKPKSSMKRKLSFSVSP

PRNGERDSDTDKDGPDKKRVKKESGGKKSTPVNILFGYPLSERKQMALLMQMTARDNSPD

TTPSHPSQAPAVQKKTPNSSTSRQKDKVNKRNERGETPLHMAAIRGDVKHVKELIGLGAD

VNVKDFAGWTPLHEACNLGYYDVAKVLIGAGAEVNTQGLDDDTPLHDASSSGHKDIVKLL

LQHGGNAFQANKRGERPVDVADSQEVEQLLKGEIPLSDPDDSSSDSEDLPSINPSSVDDN

MEYSDTEKDSDGKSTTAKATSSVSGVDEYEFKDEEEEEDLSKALNDRHILRREVRQKEEQ

NHYTPRQSNKSDQTVSSCKLKKPKSSRVLYCSSDSSDNELEAPSERKCSPTRSLCNDTHK

TESRTKRDSLTQTSTEKGKANKRNRSQSKNKENQEVQEDGKENSKSLLFSTATASDNSDK

SVREEDSFKMSFSPKDDSSVHLFHLSAVKSPKLSYGQPDKQITPLKQENAKTFVSIGDGS

CTMDGVKYNHYTDSDYTEGSSSKSCKHKDKSKHHYREHILDGDDRSSSPFKDASLSNSMD

SSEGAFRKSDKDGKVVKKHKLKHKEKDKYRKDYESERNRHRQRELSKDGHRNLEFDREFW

KENFFKSDESEELKGKCETPGSCSPQKSIDSSPVKEDKTTLKDKHSVCNSTKERKQKDER

EKDKPIKKEKKEVAYKDERGAKDSKGAENDRSDCPNLIRKPEDSNSCIKEETEDKPLTGN

LFDHNQLETTEKSSREKNDKRLPTKDRESEKGEKKQAEKEKKTKSDHLSDKPDLHNCTDR

WKERDKIMTLSNSSGERIHKESDKLKPVAPMKKHEENKKSKDKSERRSEKERQEREHFSS

DHRDRDKMGFEKKGKAPERSSDHSKIDRIKEKERDKDFDKKKKEKLKETMISSSGSNLKF

LLEEKKGYTSESNKTVTLKLKEEVIKTPEKDRDRRERDRDLERHRDKDRERHKEKDKERQ

HRDSKPSKTRPVEIESDRSKSKASPAQKETRPKEKRLVNDDLMQTSFERMLSLKDQEIEQ

WHKKHLEKIKQKERERLKQRPGSDSGKPKNKDKAKISTSSEPSANKELLRSKSSEIPDAS

GREKILKDATSGRTLSLDAKNLLCFGKSVPVIENSLSRSPRPDSERSAIMSRSVSMISVA

SSEDSCQATMLTPRPTEYDSDLNLEMSDSQPPFLQSSHIVQSSRSPIFNNKEINSLPDVA

SGSRTPLSSGLASSQSRTVHNEDDKASLAEPDRLTEESQKVGDVANSSEVPGKIHHTEAC

GAVTQKSLTINSSLSVHLHCAESEVQPPVGVEDANRSSSNQLACAPNSEDLPGHQTSFPR

SIGLTTTTVEVSKLNTDTLPEQSGQNQPDSFDTEMADGSNKNSLLASVPQVASMVSSEQS

FTKTPTQTGNLKWNSVFDPNNDHPARTDIDLTEEKSLRSLSSGPESLMEDRCMEKVSAGS

RMDLTTSQASSISSSRISSEESDKMLQCTVSVFRDSHQVTEQISVDDCKASHFGENISVA

DAHLGKKEYGLAPPSVSNVLHCTSPEQTSEELMEVSGEKSDHNQVIAETQVEKLLSSEDG

AQTQPFPDTKANVDVEMPDQLTVEEQQAKQGTPSDGEQNLGCTGVQLDFQSEQLSGATSG

GSSPLSVADRDSDLTAARTKVKNPDDEVDIQVTHPRKRKMPKSSLSTPAGLTAQQIKEKT

QQSLAAIVDSLKLEEIQPYQTERANPYYEFLHIRKKIEEKRKVLCSVIPQAPQYYDEYVT

FNGSYLLDGNPLSKLCIPTITPPPSLPEPLKEMFKQQEIVRMKLRLQHSIEREKLIVSNE

QEVLRVHYRAARTLANQTLPFSACTVLLDAEVYNMPQDVQGDDGKTSVRDRFNARQFMSW

LQDVDDKFDKLKTCLLMRQQHEAAALNAVQRLEWQLKLQELEPATYKSTSIFEIPEFHIP

LVEVNDDFDLTPI*

>m.1275 g.1275 ORF g.1275 m.1275 type:3prime_partial len:1895 (+) Unigene000140:665-6352(+)

MGKKTTSRRRSRKLSSGSQEPTSPSKPLSPEAENESALALPSPVQIHKAVWVETHLEEEG

SESSSLGQVTPAQQSPVLGFRASLIAQEAQDSGSDSSIYQDAVEGKTDEEADTKTEKRRS

VKLSTSEKFFAKRVWLNSQSSLDGEQEDTSVSSNEKQKSEVRILPSLKNVCVEIKKPDQA

SYDSISESNDAFVKEEAQTKLRLDNDVESPSGLPNNITDPGEDLPDMSGYKRVTSSGVRN

QQTAGTSSEDKASSGTNGVSAGKGPAPPVPLKTKASMARITSHTEARKEESTKKLAQNGT

EKKLSKSPTKDMPHVFTKPADKSKIPKKPAPELLPKPIKKPNTSMLPDTPVSSPVQTREP

ALSERSKPSSSNSVTKPQSPTSQPDQSQTKSPIRKEAAVASVDVDLSREPKPSQNKNPKK

GKDSKYIVEKTEPPSSPSGLEETKETSDPKEVTKSKPQTPTEKTPKSKTGKLLVSKSSKV

NKAEVDNKPHTLTEASQPENISTKDKTTNEMRVIGSKNPKKPKAEGSPTGSRLPRLTPPS

TPKQPTEDEFDYREDYSPKQPSFSDPRPTTDDSSLVNGPLSPGKSAECDQAAENAVVKDS

SENKPLTPLKHSTKLKSKEEEETLKVKPPVDLQNEVSDQNLEKAKKKQNVNVETKKNISE

LETSAQVPEEHKDKVRVAKVKDQTVNLEKETQPVTLTTTEPTADLKTSPRDLKERLNVSK

EAQNMSLQTDEHTVDLKSLSKVSQKEQDQTEEHSKTSVSEQKIDRPESVPCVDDKIKENK

TNLKLTHKVEQEMVKGEPDASVDDKTKDLKTKFKLTQEAEQKPPKASEKSTHLKNPPEVF

KKETQAQVVQNEKDTTEITSLSQVSESSKIKTDKMQSIDKKRVPNTDQVSEQKSDKAEKS

PKATVENKADLKAQPELSKEAKKGENVSGILDLPPSQASSLEGDKSDKLREGHCKPKDHT

QDLTALHQVLNQASSEKTAVEIKPETKANKTQVTETLNVGTSQVKPELSNKGAPPDTDKE

KVTLDTVDKISVKPTASVHETKEINQQSNTAQVTPDATKFIGKEQNNEINRLKLNKLSKT

TEEAGPREAAKDTKAFKSIDTIVPALKVSEFAKKDIKTKSGNKMELEPLAYGNLCTNQHP

VVKKAEQPKVTGQVSSHLEMTTEVKIPSNAIQKHKTDPTKSQKLPIIEKPPPCNTTAKMT

AQDFLLASKKLSKNESPSSWLDVDQGFEKKQKKMERKMDCSASNSNLQDTSDEPEDFIRK

IKELCSPFSFPPKKHAKSRTFVPPFAMPAIIEDHFEKTFDPEEFKFGIRKSTRPKDPSPA

MLIKKKSEDVRNKQLPKRKAAEDSMVFKALVSRQGQDKTDEEKTTESKENGEDQCNSEGP

GKVASRLERMSILSNLLNSRKPKTQPEGVSNGVTSPTVSTLGDTTNLTTLKSEIAPPGQI

DVKLQHLVIDPGVSGDSLKSPLTPPPLPNFSEIKLPDFLGKFLKMDQESPALGVSQNLEA

PSKLPAIQTEVSAGVVPDINTGLKTFPEPLKPIFPPKPPEQQTKSPSPTHTQITTARGFH

KRPGKMVIFQQAQFGGEAYEVFRDVDDATSLQLSPVISLKVVRGCWLLYEKPGFQGRTIA

LEEGPTELVNMWAEPDPDQEVGPDEIPIPSKPMVIGSIRLAVRDYSLPKIEIYSEPNGMG

RLSTFCDDIIELGTFGRPHSAGSIRVHSGVWLVFSDPDFQGLLSVLPEGEYPCPESWGFP

SPFVGSLRPLKMGGIKVENPHEVRAVLYEAPLFQGAWVEIDSDVYDVLEADEEEENDEEE

QNLVRRKKPTIIGSIKILSGLWVGYAEPGFEGRQYVLEEGEYADCSDWGGLEDELCSLRP

LQSTFVSPHLKMFSELEFNDRGLSVDLLVPVMAMG

>m.1282 g.1282 ORF g.1282 m.1282 type:3prime_partial len:1763 (+) Unigene000141:1059-6350(+)

MMLAINSARIASDALYKKDFNKSKTKFHLPVDMLSLELAKKCQIQVNDFNYRTSLHNWTC

LPDQNDVIQARKAYDLASDNIYKTDLEWIRGCGWVAADSVDHVKVRKAQQILNDRMYKKD

AKDNFAKFTNIVDRPEVLLAKVNAFNLSDLKYKESFNLEKGHYIGSDDTPQLAHSREAAK

IISEKLYKLGWDEAKATGYQLNHEYLPLVSAKKVGSIVSEAKYHDAHEKAKGHYLANTLV

DFPAVVHSSQMEKMKNMRTYQKDYHTSKTKLHIPHDMISHVVAKKCQEILSDVLYRSYLH

QWTCHPEQNDAIRARKANEILSDVFYKDDLNWMKGIGCYAWDTPEIVRAKKSYELQSDIK

YKAEGKKEFNNYSIVTDTPVYVTAILGHTWASELNYREAYHKEKHLYTTVLDTYDYARCY

NLKQLYSSKTYSALWDRIKAKSYTIPIDANALVHAKQQKVLLSKVKYKEDYEKFKSLYSL

PKCLADDPATARCIKAGKLNLDRLYKDKWEKTKAKIHIPPDMLDVVAARNTQKMISEVDY

RKYLHQWICLPDMQVYVHARKVNEQLSDIFYKEDMTWLKGVGCYAWDTPEILRCKQAMNL

QSENLYRAKGVEHFKNFSVVTDTPVYETCKQSAHNLSDLNYHHDYVTNVRGKNTAPATTV

DTERARLANYIQSDHIYKEASKSNMSTGYSLPFDTPLALQAKANNVITSNVKYKEAYENT

KARSYKLDPNGVNFVTIRKANQVTNQRLYRALYEKEKDKVHSVYDTPEIKQVKATQEAIS

DVCYKEKYYNSRGTLLPMPFTPQLMHCAHVNQINSDLKYKEDLQWLRGIGCFVFDSPEMV

RIREINKFRTSYDIDAKKNFSNFSVVLDTPEYKRVSELKTHLSERVYKAAGNIQRTQCST

TGDAMEFKRAKWAQTLTNKWLYTDLASKERAFYTPELHTPLLDHARSMKVVYSENKYKEQ

YEKMKHKYTPVHDTPILIRAKKSYLNSSDLRYKETFELAKGHYHTTKDALDIICAKRVRD

DISEVKYREKYINSLGTWKSIPDRPEFFHSKVVRDCISDYKYKEDLEWIKGVGCFVWDTP

LLKQAEHNKALYSERLYKASYEKTKCNFKYTCDTPFFQAVKNASALSNDRSYRASYEKTK

DKYTIVVDDPRNLLAKEVNNMSQWKYRSQYERLKSKYTSVLETPEHKVHKLRKQISDKIY

TMEYNKNKAKGYTLGHDTPLNLHMKKVKEITSNLKYKEVYEKNKAQINIAPDAFDIRAAK

EAYKNISNLDYKKKYEATKNKWIWTVDRPDFVHAAKINYQQSDVEYKYDKEMLKGCVMPV

TDDKYTLLAKQNTELSSDIKYKQKYEEARGHYHVVHDTPQILHAKSVSGLVSESKYKLES

KKARQSGSFTTLPETRDTAHSKAMTKITSSKVYKQKFEKDKGKSIYNNMTVPPDVKHAMD

VAKSQSNINYKQEAKAKLHYTPVADRPDILKATQAAKLISEVGYRDKARQEASRGGSLVN

RPDINLATEVSKLTSLVKYKEKFDKEMKGKRPQYDLKNSKIYQTLKDANTLASEVKYKGD

LKKIHKPVTDMSESLAMQHNLSTSKLASSVKYKEKYERERGKAMLDFETPTYVSAKEAQH

MQSQREYKKALEQEIKGKGMLALANDTPDFMRARNATEILSQSKYKHNAEQDRASYTTVI

DTPDIIHAQQIRNIVSQKKYKEEAEKTMSHYNAVLDTPEMQRVRENQRNFSTVLYKEPSG

KGTSVIFTPEMERVKRNQEHISS

>m.1284 g.1284 ORF g.1284 m.1284 type:5prime_partial len:339 (+) Unigene000141:3-1019(+)

AKIASDALYKKDFNKSKTKFHLPVDMLAFELAKKCQVQVNDANYRTYLHNWTCLPDSNDV

VQARQTYELQSDAVYRADLKWLKGLGWIPIGSLDVEKAKKAGEALSERKYRQPPSNFKFT

STTEDMPIVLAKANNNIMNKKAYVQAWDNEKSNIHIMPDAMDVVLAKQNKVNYSEKQYKL

ANELSKKKGYDLRGDAISIIAAKASRDKRYKLDNEESKKKGYDLRSDAIAIKAAKASRDI

ISEYKYKTGYRKQVGHHIGARWQVGHHIGALSIQDDPLLMLALNSAKIASDVLYKKDFNK

SKTKFHLPVDMLAFELAKKCQIQVNDANYRTYGYRKQV*

>m.1293 g.1293 ORF g.1293 m.1293 type:complete len:1798 (+) Unigene000142:207-5600(+)

MFNKSFGTPFGGGTGGFGTSSTFGQQNTGFGAAGGFGASAFGTANNTGGLFGATQNKPGG

LFGSNTFSQPVTSSTSSGFGFGATSGGTSNSLFGSTNTGGGGLFSQQGNAFGANKPASFG

TFGTSTSSGGLFGTTNTTSNPFGGTPSMFGSSGFTATQPGTTIKFNPPTGSDTMVKSGVT

TSINTKHQCITAMKEYENKSLEELRLEDYQAGRKGPSNTGGLFGATATATPSTATGLFGP

SGTGFSFNQPKPSFSAGTSGFGATPGGLFGQPPQTSSLFKSFGQPTTTQNTSFSFGATNT

MGPTNTSSMGLFGNTAPAQAGGLFGNTSNTSTATGFGTATGLFGQPNAGFGNNLFGNKPA

GFGTTTTSAPSFGTGTGLFGNKPTLTLGTNTNTFGFGANPAGGSLFGNKPATGTLGTGLG

TGFGAGVGTGGTSLFGNNQNKIGSTLGTVGAFGGTSFNTGANALNFGAPQQPVALTDPNA

AAQQAILQQQLSALNYSPFGDSPLFRNPLSDPKKKEERLKPTNPAAQKALTTPTHYKLTP

RPATRVRPKALTSSGSGKSQLFDGLDDDEPSLTNGAFMPRRSIKKLVLKNLNGSSLYSSP

LNRDADDLNSPSEYPENGLSLRSDAEQTQDVEAEGGAEDDLQVTKFYTNPIAKPIPQAQP

SPSLQDTISEFTMRRVSSVRNGLDLSSEEISLGDDSVQEERDEELEVQKPPHPAGIVLTR

VGYYTIPSMEELGKMLNENGECIVENFTVGRRGYGSIFFRGEVNLTNLNLDEIVHFRRKE

VIVYPDDENKPCIGEGLNRRGEVTLDGVWPIDKTTRSQIKSPQRLSEMNYEERLENASRK

QGARFLEYRPETGSWVFEVAHFSKYGLQDSDEEDDVPPAKTEVKKMKTAIPAGLQQIPLS

QQHQQMAPQAQSTAVLELLSRVPELDSDMADITQEHLADGVLFKEDDSMLEDDTRMARRP

ETPAEPEPISASSQIASSLGINPHALQIMKASLFAADDDCDLFYEQSPPKLLEELSSPRV

LLGKTPARLSVGGLLQSKFSSAGGLYSQLPEVSFGAISQKPSKPAVPEAPWPSLGTSVIL

PPPVPEVTLRTVGARRLGGPVPLDNSVTLGKGHLLMDAALYMGRSFRVGWGPNWTLVHCG

SPLSTIDESKEQTKDMMGYGFLPRPTKNRQITDSPFKVHVEHVVGMEPKETTQSQSVYHK

PLEIGLKNSTISTDDLCPFIKPDKGVNALHDYAEWIVEVLKEAASEDVVLNHWQQVWTLC

EALWGRLGERNLDPETGGGYREQHARRHSFSRWLSESAAQRIQDEVCQSQSHADAIFSYL

TGNCISKACRLAQKNGDHRLSLLLSQAVGSQLTRDLSALQLADWNRMQTDSFIQDERLHI

FALLAGKPVWEATDCCINVCARLDWKRCLAVHLWYMLPPTASVADALAKYEAAFQGLEEG

RKYACAPLPPYMDDDYEQEAFEEMEDTESKKPLYDICFHLLKLYSDKHYSLQQLLDPTTV

TAEQLDYRLSWHLWNMLQSLNYTHLAVPRQNLLHISYAAQLESAGLWEMAVFVLLHIPDS

VHREHAVREILNQHCTLEETEESAEKEHFLTHKLLIPMQWLHEAKAVRARRDGDRHGEAL

HLFRAGHWNRCHQLVIQDLASDCIINDNHEYLKEFLEGFEAPERSVQIQGWDTSGRVYLN

YIRVIQTLKAIQRMDSPGYQLERLHTDVMSLCSRIEHLPCFKAKDRLAQSEMAKRLANIL

RAVLSLQQGGEGTPDPRHIPLCHLAPHIGRLPMPEDYALEELRSLTQSYLHKYVVIH*

>m.1302 g.1302 ORF g.1302 m.1302 type:complete len:1462 (+) Unigene000143:433-4818(+)

MVHVCNGFTPLHIACKKNRIKVMELLLKHGASIQAVTESGLTPIHVAAFMGHENIVTQLM

NHGASPNTTNVRGETALHMAARAGQAGVVKYLVQNGTHVDAKSKDDQTALHISSRLGKPE

IVQQLLQHGAAPDSTTTSGYTPLHLAAREGHKDVAAILLDQGASLSITTKKGFTPLHVAA

KYGKIEVANLLLQKRAPPDASGKSGLTPLHVAAHYDNQKVALLLLDQGASPHAAAKNGYT

PLHIAAKKNQMEIATTLLEYGADTNAVTRQGISPLHLAAQEGNVDMVTLLLARDATINLG

NKSGLTPLHLVAQEDKVNVAEVLVNQSAAVDPETKMGYTPLHVACHYGNIKMAHFLLKNQ

AKVNAKTKNGYTPLHQAAQQGHTHIINLLLQHGASPNELTLNGNTALSIARRLGYISVVD

TLKVVTEETLTTMTVTEKHKINVPETMNEVLDMSDDEVRRANVPEMLNEDYISDVEEGED

VMMGDTDKYLGPQDLRELGDDSLPQEGYVGFSIGARSGSLRSFSSDRSNTLNRSSFTRDS

MMIEEILAPSKDTHLTVAKDYDTDSLRRYSWTADALDNVNLVSSPVHSGFLVSFMVDARG

GSMRGSRHNGMRIIIPPRKCTAPTRITCRLVKRHKLASPPPMVEGEGLASRLVEVGPAGA

QFLGPVIVEIPHFGSMRGKERELIILRSDNGETWKEHQYDCKTEALNELLNGMDEELDCI

EDLEKKRICRIITKDFPQYFAVVSRIKQETNHMGPEGGVLSSTTVRKVQASFPEGALTKK

IRVGLQTQPVPDETVKKIIGNRATFSPIVTVEPRRRKFHKPITMTIPVPPRSGEGMNGYK

GDTTPCLRLLCSITGGTSPAQWEDITGTTPLTFTNDCVSFTTNVSARFWLADCHQIPETV

SLATQLYRELICVPYMAKFVVFAKMNDPVESHLRCFCMTDDKVDKTLEQQENFEEVARSK

DIEVLEGKPIYVDCYGNLTPLIKAGQQLVLNFYAFKENRLPFCVKVRDNSQEPCGRLSFL

REIKNAKGLPQVAVCNLNITLPALKKTEKPERRHTFASLALRKRYSYLTEPGLSPQSPCE

RTDLRMAIVADHLGLSWTELAREMDFSVDEINHIRVENPNSLTAQSFMLLKKWVNRDGKN

ATTDALTAALTKINRMDIVTLLEGPIFDYGYHNISLELQSPSELSYEPPTPLRQDDFFSE

DGGIVVSPSRTPVRPNELSLPTTSRDALSPNAAPPTVVAEDSSIGGRGSYSREGGSTEDD

LDAHDSDEMPKHPEVFMQSTYQEQEVPSNGAHRARESGRSGFDEEEEEEMTQEKSCFKSI

NLEEGLEIVEIPQEKVQEIRSQVEQAKKEMCSLAGLQEGPSSVSQEISASGLHTDTQLQD

RSQKPTERQNGEHPELQGRAGSLLEPQEPSSKMKGKGSRDSLDDTAAGPSTMDEDTVEPK

AKVGEGDKKKEGRQRGKKGQS*

>m.1309 g.1309 ORF g.1309 m.1309 type:complete len:1676 (+) Unigene000144:268-5295(+)

MKCTLRMEGVMQSQHRGLVTIPRFLLICLVVLVLTSEVNAGGKKHSGICGGRDCSGGCQC

YPEKGARGQPGPLGPQGPTGPPGRAGEPGLQGLKGEKGNSGEAGIIGPKGAQGMIGSPGF

PGADGIPGHPGQQGPRGKPGADGCNGTRGDAGIPGLEGYSGTMGPKGLPGKKGEKGDPLE

ISVYMERFRGEPGIPGYTGPIGPPGKRGYQGPPGLSGVHGEKGFRGPPGLKGSATAIHPG

ERGEQGEVGDPGPPGTDRVVFIGSFTGPKGEKGMKGLPGDLGFNNKGETGIKGFPGDRGF

PGNAGLPGKKGEAGLAGEHGHPGSKGEPGEAGELIYTDGYSGEGPPGPPGERGPPGPVGS

IGIKGLPGPPGPHAQEQQIELFGSFGPKGAKGVKGDAGEPGIPASEPGPPGFDGLPGPPG

PPGPKGSWSVFFRGYEGPHGRPGNAGAKGQKGDQGHCECTLQTSPPGTPGPAGDQGDPGV

LGEWGHRGDQGDPGDRGRTGLHGFPGLQGLPGPKGLKGDTVEVKDKGTSGDPGDPGRPGE

QGTQGRPGTSGIIGAPGPHGLPGEGPVGESGEKGYPGRPGPSGLPGPAGVPGQVIGGTRG

IKGIAGDAGLDGYDGPSGLPGTSGDCILQGEGGEWITAGDCAAISGPPGEMGPPGLTGLE

GMPGQPGPKGFEGDNGKIGLKGEIGDQGIGGPPGKTGFTGTRGDIGTPGLKGHKGSPGIP

GRPGLDGDFGQKGPNGEALGASSGPPGERGPPGFQGIKGASGNAGTSGYPGMEGMPGMIG

DKGESGPLGLQGETGRPGPAGIFGFPGKPGYAGPPGPLGQTGPPGPRGETGYRGDSGPPG

HAGVKGVRGEPGSDAGPGDYGQHGDPGAPGPVGQTGLPGLKGEKGSQGMTGFPGMLGNSG

VNGFQGPKGLVGITGLEGVKGGMGQQGHKGERGVMGPPGKMSVVEMTHMKEVMKGEKGSC

GFEGDSGFTGPRGTKGLPGIPGEEGYHGLPGEPSNIKGLQGQTGAQGLPGQKGMPGPTGL

PGIQGFFGPTGTRGEKGSSGAYGAKGSFGIKGLKGDQGDVINFPGPTGTRGETGLPGPLG

HKGFLGVSGERGSPGFDGIKGNKGVQGELGEEGLSGADGLQGAPGNQGSKGWPGVPGAAG

TPGKPGHPGQIGFPGLDGISGLHGLKGQKGLQGVAGLDNWGLSGEDGEKGEIGEPGIPNT

EIGIPGTHGLKGSTGNPGDQGEPGPPGDQGPPGAHGIPGRRGLSGDTGLPGQKGLQGFPG

ATGLPGFPAPSGAKGIFGSEGDFGTPGPKGLLGDIGDKGFPGQFGVPGEKGIKGSMGGMG

VPGPYGLQGDRGLPGAKGGTGPPGPPGSPGARGLPPVPVKMPGERGPPGPPGDQGLQGLR

GNFGSMGLPGDAGYIGPKGQKGMPGLSGIHGTPGIRGDLGQTGHPGLQGLEGNRGRPGTP

GPTGMPGRSVSVGYLLVKHSQSDQTPMCPVGMSKLWDGYSLLYFEGQEKAHNQDLGLAGS

CLPRFNTMPFLYCTPGDVCYYASRNDKSFWLSTTAPIPMMPVEESEIKPYISRCSVCEAP

SVAIAVHSQDITIPQCPAGWRSLWIGYSFLMHTAAGDEGGGQSLVSPGSCLEDFRTTPFI

ECNGAKGTCHYFSNKHSFWLTSIEQTFHSSPESETLKAGQLLSRISRCQVCMKNL*

>m.1313 g.1313 ORF g.1313 m.1313 type:complete len:1830 (+) Unigene000145:99-5588(+)

MYKGKKRHEMPPHIYAITDTAYRSMMQDREDQSILCTGESGAGKTENTKKVIQYLASVAS

STKTKKDPTSTISSLSHGELEKQLLQANPILEAFGNAKTVKNDNSSRFGKFIRINFDVNG

YIVGANIETYLLEKSRAIRQAKEERAFHIFYYLLSGAGDKQRSELCLEDYGKYRFLSNGK

VTIAGQQDRDLYTETMDAFRIMSIPEDEQTGLLKVVSAVLHLGNIIFKKERNSDQASMPD

DTAAQKVCHLLGMNVTDFVRAILSPRIKVGRDFVQKAQTQEQAEFAVEALAKATYERLFR

WLVMRINKALDKTKRQGASFLGILDIAGFEIFELNSFEQLCINYTNEKLQQLFNHTMFIL

EQEEYQREGIEWSFIDFGLDLQPCIDLIERPANPPGVLALLDEECWFPKATDKTFVDKLV

QEQGNHPKFQKPKKLKDDADFCILHYAGKVDYKAVEWLMKNMDPLNDNVTTMLNQSSDRF

VSELWKDVDRIVGLDKVAGMAESAHGAFKTRKGMFRTVGQLYKEQLANLMTTLRNTNPNF

VRCIIPNHEKKAGKLDPHLVLDQLKCNGVLEGIRICRQGFPNRIIFQEFRQRYELLTPNA

IPKGFMDGKQACVLMIKALELDPNLYRIGQSKVFFRAGVLAHLEEERDMKITDVIISFQA

WCRGYVARKAFAKRQQQLTAMRVIQRNCAAYLKLRNWQWWRLFTKVKPLLQVSRQEEEMQ

AKDEELTKVREKQVVAEKMIEEMEVKHQQLNAEKMALQEQLQAEMDLCAEADEMRNRLVA

KKQELEEILHDLEARVEEEEERANQLQMEKKKMQQNITDLEQQLDEEEAARQKLQLERVT

MEAKLKKIEEDVMVLEDQNTKLNKEKKLMEERISEFTTNLAEEEEKSKSLQKLKNKHEAM

ITDLEDRLRKEEKMRQELEKNRRKLEGDSTELHDQIAELQAQIAELRAQLAKKEEELQEA

LARIEEEAAQKNLAHKKIRELEAQLSELQEDLELERAARTKAEKHRRDLGEELEALKTEL

EDTLDTTAAQEALRTKRETEVTHLKKSLEDEAKNHEQIVAEMRQKHGQAFDELNEQLEQS

KRNKASVEKMKQTLESERNELQIELQGLMQSKGESEHRRKKAEAQVQELQIKHSESEKQR

AELMEKVSKLQAELDSVNSVLSDVEGKSIKAVKDCSAVESQLQDVQELLQEETRQKLSLN

TRMRQLEDEQHNLREQLEEEEEAKKNMEKQLIAAQSQLAEMRKKIEQEASSLESTEEVKK

RVQRDLESVSQRLDERNLAFDKLDKTKTRLQQELDDLLVDQDHLRQIVSNLEKKQKKFDQ

MLAEEKTISARYAEERDRAEAEAREKETRALALTRELETLTDIKNEMERANKALRAEMED

LVSSKDDVGKSVHELEKSKRGMEQQLEEMRTQLEELEDELQATEDAKLRLEVNMQAMKAQ

YERDLAGRDEMGEEKKRQLVKQVREMEMELEDERKQRSVAMAARKKLELDLKELEAAIDQ

ANKNREEALKQLKKLQAQMKDVLRELDETRLSREEILALSKENEKKFKSMEAEMIQIQEE

LAAAERAKRQAQQERDELLDEINNQNSKNALTVEERRRLEARIAQLEEELDEEQCSTELV

NDQLKKANAQVDTLGSELAGERSAVQKSETARQQLERQNKDLKSKLQELEGSVKSKFKST

IAALEAKILQLEEQLEQEAKDKQQTSKSMRRTEKRLKEVLLQVEDERRNSEQFKDQMEKA

NSRMKQLKRQLEEAEEEATRANASRRKLQRELEDATEASETMNREVNTLKSKLRRGGDFT

LNVRRVVRGGLDSEEDADLTSETSEATPE*

>m.1321 g.1321 ORF g.1321 m.1321 type:complete len:1942 (+) Unigene000146:377-6202(+)

MHHTVEKFSARSSRDPFPLDGFNRGPWAPMGGRAWPPPPRCSPGGQPQFLPHLPPNHMTG

LNHPNKYYNSGALTRGGEPLPVLHRDIRVPPAPLRPWDPLGQHYEPLPSDDHVRLHNRYN

AGPSAHLTARANQLLKYGPPPLQHGSRPMPPIADMWEQSHQQQQQPPRAPFSRVGQLKRT

APLLGEQSVIQQTSLALNRPNEDCPSPSKRKKSSDSEQLIIGTHQYSGSIGSSLPLQQPQ

SHYPPLQKDTATWTPVEHKADRNEFQELRRQDMGDCNYKQFSSSKPSPISPPPITSASSY

EHGHGPPLKSLKPQSPHSLGSFSQNVQLHHTTFPFPKNKASAQNSIEHQVISIHKMHSSG

VRTTGSQVPLPQPLSLVDREHPVPTPAHQTSVPYSHPKFQPHPGLVSTTSPSSNSGPHAP

ESQCNPPKSWRNEIKHDNQALILGPYHRSVDPQLSTSSLQQQQKKTGQNQDEGTSLARAP

VITFTMQPAAPPCSAGCYSGVNTMSTLSSVPSSLSSTVSTIKNWKTAHPTTLNSNPSSIP

SSFPHQDYQRAQSGPESHLDHKQKVQIQHQELHLPQQGQDTPLYTPKVSSAPSSLSSGFQ

RSGNSVITSKAIDNLLQPAHSPLRQMQPAVPITVNTTPQQSNSLHHKLPHQVTPVTSQVP

PAETYERPCPQPAISNPQSIEEALKKLDAELQSHMQAEERRKEKAEEERKHNIGNNKVKK

HKVSEDSATEDMESLLSNSPTDPPPPCLSPAITPSTVLPPSQTSPPFPWLSRGGVPTRQL

APGANSVERSRPPPLTPQTDYAREKQRQRDQWNASVPPTLQNTSGIPIYSSKHIPTEFSN

KPIQTCSTEVSTTEKSSPNIKAMGPVLNTSNLREPPKLYQAFPRDTQSSLQKETSGGLSS

LGSSASSSSMDSDSAQFEEEPSELLPDGLANIMKMLDESIKKEEELQSVQSGGQSDPDIS

FSLTVAPIKTYTFAPDLMPALKQPLTVDYSTETHASPPVLSRQGSLASPCSRTSSLEEEE

DTLKIIDTYDTSVDKDGGVGSYRHSDLAKLYGLSEGAKSECDEEDEEAEQDEDTRSCSPP

PTRPHLHQTGVNSMFQNLASMLESQKYAYRGGPFGRPPPSALVGVKYSSSLSLGPDICRQ

QSTSPTSGSTKHPGFNHKDQASVNSRSEQTRSLSPSRSTPETKDLVGCQSDLSGCEEEEG

VRKGLMNEDASCIVNNFTEVERKPKLTTISESSLAELGRSCEVMLSRHNLPSIASDHNKK

QMPHITEKDRKRDRDYYQEKKHKRSSSSKKNEERKEKKKKHREKQENMLSNSSRRHKDSK

PHKEKRGHIDSHREKEKERDGEKKKGKEKEEWVCRNKEKKSGSSSVDHSSSSPSLGSADF

QKLKALTDGPPKELKIRLIKVESGDRETFIASEVEEKRIPLGEITIKNTASEIIRSCKGA

RVKGKFKESYLLPSFSLKPVVTMEQPIPREKLNPPTPSIYLESKRDAFSPVLLQFCTDPK

NPITVIRGLAGSLRLNLGLFSTKSLVEANAEHAVEVRTQVQQPADENWDPSGTGQIWLCE

SSRSHTTIAKYAQYQASSFQESLQEEKGSDDEEEDEDEKKPVTNLDSTGSNSLSSSLEQK

PVGKIIKFGTNIDLSDPKRWKPQLQELQKLPAFMRVSSSGNMLSHVGHTILGMNTVQLYM

KVPGSRTPGHQENNNFCSVNINIGPGDCEWFAVHDNYWESISNICEKHGVDYLTGSWWPV

LEDLYNANIPVYRFIQRPGDLVWINAGTVHWVQAVGWCNNIAWNVGPLNAYQYQLALERF

EWNEVKKVKSIVPMIHVSWNVARTVKVTDPDTYKMIKHCLLQSLKHIQILRDQLVAAGKK

ISYQSRVKDEPAYYCNECDVEVFNLLFVTSENGSKKSYVVHCEDCARQRSPNLSNVVVLE

QYRIEDLMNTYDTFSLASSSR*

>m.1327 g.1327 ORF g.1327 m.1327 type:complete len:1994 (+) Unigene000147:110-6091(+)

MAKPGTDRDGAMVEKTTGKKSKDKISPFTKTPKLDRSEIMGKEGKPKSSMKRKLSFSVSP

PRNGERDSDTDKDGPDKKRVKKESGGKKSTPVNILFGYPLSERKQMALLMQMTARDNSPD

TTPSHPSQAPAVQKKTPNSSTSRQKDKVNKRNERGETPLHMAAIRGDVKHVKELIGLGAD

VNVKDFAGWTPLHEACNLGYYDVAKVLIGAGAEVNTQGLDDDTPLHDASSSGHKDIVKLL

LQHGGNAFQANKRGERPVDVADSQEVEQLLKGEIPLSDPDDSSSDSEDLPSINPSSVDDN

MEYSDTEKDSDGKSTTAKATSSVSGVDEYEFKDEEEEEDLSKALNDRHILRREVRQKEEQ

NHYTPRQSNKSDQTVSSCKLKKPKSSRVLYCSSDSSDNELEAPSERKCSPTRSLCNDTHK

TESRTKRDSLTQTSTEKGKANKRNRSQSKNKENQEVQEDGKENSKSLLFSTATASDNSDK

SVREEDSFKMSFSPKDDSSVHLFHLSAVKSPKLSYGQPDKQITPLKQENAKTFVSIGDGS

CTMDGVKYNHYTDSDYTEGSSSKSCKHKDKSKHHYREHILDGDDRSSSPFKDASLSNSMD

SSEGAFRKSDKDGKVVKKHKLKHKEKDKYRKDYESERNRHRQRELSKDGHRNLEFDREFW

KENFFKSDESEELKGKCETPGSCSPQKSIDSSPVKEDKTTLKDKHSVCNSTKERKQKDER

EKDKPIKKEKKEVAYKDERGAKDSKGAENDRSDCPNLIRKPEDSNSCIKEETEDKPLTGN

LFDHNQLETTEKSSREKNDKRLPTKDRESEKGEKKQAEKEKKTKSDHLSDKPDLHNCTDR

WKERDKIMTLSNSSGERIHKESDKLKPVAPMKKHEENKKSKDKSERRSEKERQEREHFSS

DHRDRDKMGFEKKGKAPERSSDHSKIDRIKEKERDKDFDKKKKEKLKETMISSSGSNLKF

LLEEKKGYTSESNKTVTLKLKEEVIKTPEKDRDRRERDRDLERHRDKDRERHKEKDKERQ

HRDSKPSKTRPVEIESDRSKSKASPAQKETRPKEKRLVNDDLMQTSFERMLSLKDQEIEQ

WHKKHLEKIKQKERERLKQRPGSDSGKPKNKDKAKISTSSEPSANKELLRSKSSEIPDAS

GREKILKDATSGRTLSLDAKNLLCFGKSVPVIENSLSRSPRPDSERSAIMSRSVSMISVA

SSEDSCQATMLTPRPTEYDSDLNLEMSDSQPPFLQSSHIVQSSRSPIFNNKEINSLPDVA

SGSRTPLSSGLASSQSRTVHNEDDKASLAEPDRLTEESQKVGDVANSSEVPGKIHHTEAC

GAVTQKSLTINSSLSVHLHCAESEVQPPVGVEDANRSSSNQLACAPNSEDLPGHQTSFPR

SIGLTTTTVEVSKLNTDTLPEQSGQNQPDSFDTEMADGSNKNSLLASVPQVASMVSSEQS

FTKTPTQTGNLKWNSVFDPNNDHPARTDIDLTEEKSLRSLSSGPESLMEDRCMEKVSAGS

RMDLTTSQASSISSSRISSEESDKMLQCTVSVFRDSHQVTEQISVDDCKASHFGENISVA

DAHLGKKEYGLAPPSVSNVLHCTSPEQTSEELMEVSGEKSDHNQVIAETQVEKLLSSEDG

AQTQPFPDTKANVDVEMPDQLTVEEQQAKQGTPSDGEQNLGCTGVQLDFQSEQLSGATSG

GSSPLSVADRDSDLTAARTKVKNPDDEVDIQVTHPRKRKMPKSSLSTPAGLTAQQIKEKT

QQSLAAIVDSLKLEEIQPYQTERANPYYEFLHIRKKIEEKRKVLCSVIPQAPQYYDEYVT

FNGSYLLDGNPLSKLCIPTITPPPSLPEPLKEMFKQQEIVRMKLRLQHSIEREKLIVSNE

QEVLRVHYRAARTLANQTLPFSACTVLLDAEVYNMPQDVQGDDGKTSVRDRFNARQFMSW

LQDVDDKFDKLKTCLLMRQQHEAAALNAVQRLEWQLKLQELEPATYKSTSIFEIPEFHIP

LVEVNDDFDLTPI*

>m.1338 g.1338 ORF g.1338 m.1338 type:complete len:513 (+) Unigene000148:151-1689(+)

MDDDFEWRREQRRQKREEMRLEAERMAYRRTEEDEEEAARERRRRARQERIRNKENEEIA

NPAESEGLNSHSVTATKTVSSSELNTGQAGEGDEEEQALLDRLAKREERRQKRMKEALDR

QKQLDSVDDPSYSTYSVESTSTTSYQAFSNQEDEPALNSKEEVKEELVPQMELVEEKPRR

SYLREQMEDEDAGGKIKQPNGGVYEDSKHKKVERTQSRGSVHSFEGPAAEEGDDSEQTRL

EAERKLEELKRRRDETENEEFERMKQRQQEAEVELEELKRKREDRRKVLEEEERLKKQEE

AERKAREEEEKRRMKEEIERRRAEAAERRQKVGEEVDGDAGKPFKCVSPRGSSLKIGERA

EFLNRSAQKSAVKTSHSPVVSKIDNRLEQYTTAVQGNKEVRSPRGAAIDFPGGSDGVRNI

KSMWEKGNVFSSTISSPSSNKDAAGIKVGVAGRINDWLNKTPEASKTPGGRPVDLKPGDV

TGKRNLWENKSSSATKVTSRGESKSVANGTGP*

>m.1337 g.1337 ORF g.1337 m.1337 type:complete len:535 (+) Unigene000148:2014-3618(+)

MTAEDSTSAVTMSNSSHSSKSNPANNHAVLHGSITEGVAGAPNEAALMALMERTGYKMVQ

ENGQRKYGPPPGWQGASPPRGCEIFVGKIPRDVYEDELVPVFESVGRIYEMRLMMDFDGK

NRGYAFVMYTQKHEAKRAVRELNNYEVRPGRLLGVCSSVDNCRLFIGGIPKTKKREEILE

EVSKVTEGVLDVIVYASAADKMKNRGFAFVEYESHRAAAMARRKLMPGRIQLWGHQIAVD

WAEPEIDVDEDIMETVKILYVRNLMIETSEETLRKIFSQFNPGCVERVKKIRDYAFVHFT

SRDDAVVAMDNLNNTEIEGSFIEVTLAKPVDKEQYTRYQKASKGTMTSSDSTQQNFVYQC

DPYTLAYYSYPYNTLIGPNRDYFIKVTLPGLGAQYPAMFSAAPAATKLVEEGKIHPVEHL

INPLGLPHDHPAAPSSAAVIPAVSTPPPFQGRPITPVYAVAHNVPRIPAAAAAAGLYGAS

FVPIAAHTNTAALAALQKNAAVAAAAYGGYAGYMPQAFPAATFQVPIHEMYPTY*

>m.1343 g.1343 ORF g.1343 m.1343 type:5prime_partial len:1806 (+) Unigene000149:1-5418(+)

FRERLGVKTVKALKRNNNGLTHAAIDMLCALMCPMHDDYDLRQEQLNKASLLSSKKFLES

LLDKFIINVEHGTGALVVSALLDFLTFALCAPYSETSESQQFDMLLEMVASSGRTLFKLF

QHPSMAIVKGAGLVMKAIIEEGDKEIATKMQELALSEGALLRHLHTSMFTVSTDQRMLTN

RQLSRHLVGLWIAENPVAMTLLKRMLPTGLLAYLDSSDPVPERDMDRIHIRDNLKIASDQ

FGHTKVPEWQRIAGKAAKEVERFAKEKSDLMLMHWRDKMGIAQKEQDRNNLNTNQKPVVL

RKRRQRIKIEANWDLFYHKFSIDQARSNLIWNMKTREELRDALEGEMRSFSIDRELGSNS

VISWNHQEFEVRYECLYDEIRIGDYYLRLLLEENENEESGVIKRSYEFFNELYHRFLLTP

KVAMKCLCLQALTIVYDKCCKEIGPFTDTKYIVGMLDKSTDKLERDRLILFLNKLILNKK

NVKDIMDANGVRLLVDLLTLAHLHTSRATVPLQSNVLEAAPNMKRESEKEWYFGNADKER

RGPFSFEEMQEFWKSGTLTAKTRCWAQGMDGWRPLQAIPQLKWCTLASGQAVMNETDLAT

LILNMLITMCSYFPSRDQDNAVIRPLPKVKRLISDNACLPHIVQLLLTFDPILVEKVAIL

LYLVMQDNPNLPRLYLTGVFFFIMMYTGSNVLPVARFLKYTHLKQAFRSEEAKSQDIVQR

SVLGPVLPEAMVCYLENYEAELFSEIFLGDFDTPEAIWNSEMRRMMIEKIAAHLADFTPR

LESNTRALYQYCPIPVLNFPQLENELFCNIYYLRHLCDLQRFPEWPIRDPVKMLRDTLEA

WKKEVGKKPPSMSVDDAYDVLNLPAGQGQYEESKIRKAYFRLAQKYHPDKNPEGRETFEK

VNKAYEFLCTKSARFVDGPDPENIILILKAQSILFSQHKQELEPYKYAGYPMLIKTITME

TSDRQLFSKTSPLLPAASELTFHTVNCSALNAEELRRENGFEVLNEALSRCVAVLTGSSK

IGDMSVQVCGHICRCFSVAAQFEECREKIIKLPNIIRNICHILYYSNALSRLASLAVECV

SSFSVDFFLQTQLYQAGALWPLLLNLFNFDFTLEESGVQASQETNQQHVINSLAKNSLLA

IARLGGYRTRTEADGNHQIGGEPTPENPAVRKSVAAMLTPYIAYKLSTNSPAEVLKLVNC

NSETPYLIWNNATRAELLEFLEAQQEGNMKRGECDESFGAEFAFSEHNKELIVGEIFVRV

YNEQPTFPLEYPKVFAGSLLDYVGSQAQYLHTLLALTQTNKVESQQHAQRLLWVEMALEA

LCNVIKNNPGSETECIGHFKLLFSLLRVHGAGKVQQLVLEVVNTVTSNQECISSIAESLV

LANLLLLLHSLPSSRQLVLETLYALTSNTKIVKEAMNKGALIFLLDLFCNSTHPQVRTQT

AELLSKMTSDKLVGPKVRLILMRFLPGVFIDAMRDNAEAAVHIFEGTHENPELIWNDASR

EIVSTTVRELMLEHFKQQKDNPDVNWKLSEEFMVPYAAGQGELEVGGVFLRIFIAQPGWV

LRKPREFLVSLLDALTELLERNHPNDEALETVTTAAVCLFSTQTQLADQIPPLGHLPRIL

AALNHKNSAVLKSSIRLIHVLSENELCVRSMAALETIGPLMSGMKVRADMAGLACEALNR

MFQREQTDLVAQALRVELVPYLLKLLEGIGLETLDNPSATKAQIVKALKSMTGSLHYGEQ

VNEILSRSTVWSAFKDQKHDLFISESQLAGFLSGPAVAGYITAGTGATVMSTIPPPVDNN

IGDQC*

>m.1346 g.1346 ORF g.1346 m.1346 type:3prime_partial len:436 (+) Unigene000150:4898-6208(+)

MPAASLQVTRPHGPQHQAHLGLLVQPVLTFPLQVEPCLQIIPQRLRHTSRVPLEATPHRA

LATLLHLRRTRPPLRPTLLPAQTTAQHPHLIPPLLRLTLPHPHPIPRPPRATRPRRPLTR

QRLRLIPPPPHRTLRRHHHILQLHRLILRRLPATAQRPLHIHPLPRRTRPPPHHTRQPLL

RIPRLHHLIPLRLPVTARPPQITRQLRPATRPPLRLTAPPRRRTRPRLRITRPPARIILR

RLLRTPPPLRLTPHPAHGTRPNRPHTPRVRLLTARAPHLTPQRLRNTPPPLRPTAPALRN

TPRLHPNTLRPHPSTHPRLPNTVRRLQLTRPLHPNTAPLRRHTPQRLLPTPQPAPSTPPL

PPRILRHLQNTPRLHPLTRLLVLKARLTAQPHRGTAQRPRPTVQPSAPTTAMRKTTKAGD

PWISSAVSSHPSRKTQ

>m.1345 g.1345 ORF g.1345 m.1345 type:complete len:1967 (+) Unigene000150:247-6147(+)

MHGPPSSDSACPLRLIKRVQFGVLSPDELKRMSVTEGGIKYPETTEGGRPKLGGLMDPRQ

GVIERSGRCQTCAGNMTECPGHFGHIELAKPVFHVGFITKIMKVLRCVCFFCSKLLVDAN

NPKIKDILTKSKGQPRKRLMHVYDLCKGKNICEGGEEMDNKFGVEQQENDEDLTKEKGHG

GCGRYQPRIRRTGLELYAEWKHVNEDSQEKKILLSPERVHEIFKRISDEEDMILGMDPKF

ARPEWMIVTVLPVPPLAVRPAVVMQGSARNQDDLTHKLADIVKINNQLRRNEQSGAAAHV

IAEDVKLLQFHVATMVDNELPGLPRAMQKSGRPLKSIKQRLKGKEGRVRGNLMGKRVDFS

ARTVITPDPNLQIDQVGVPRSIAANMTFPEIVTPFNIDRLQELVRRGNSQYPGAKYIIRD

NGDRIDLRFHPKPSDLHLQIGYKVERHMCDGDIVIFNRQPTLHKMSMMGHRVRILPWSTF

RLNLSVTTPYNADFDGDEMNLHLPQSLETRAEIQELAMVPRMIVTPQSNRPVMGIVQDTL

TAVRKFTKRDVFLERGEVMNLLMFLSSWDGKVPQPAILKPRPLWTGKQIFSLIIPGHINV

IRTHSTHPDDEDSGPYKHISPGDTKVIVENGELIMGILCKKSLGTSAGSLVHISYLEMGH

DITRLFYSNIQTVVNNWLLIEGHSIGIGDSIADAKTYLDIQNTIKKAKQDVIEVIEKAHN

NELEPTPGNTLRQTFENQVNRILNDARDKTGSSAQKSLSEYNNFKSMVVAGSKGSKINIS

QVIAVVGQQNVEGKRIPFGFKHRTLPHFIKDDYGPESRGFVENSYLAGLTPTEFFFHAMG

GREGLIDTAVKTAETGYIQRRLIKSMESVMVKYDATVRNSINQVVQLRYGEDGLAGEAVE

FQNMATLKPSHKAFEKKFKFEYTNERALRRTLQEDVLKEVMTNAHVQSALEREFEKMKED

REILRAIFPNGDSKVVLPCNLARMIWNAQKIFRINPRTPTDLNPLRVVEGVHELSRKLVI

VNGDDPLSRQAQQNATLLFNIHLRSTLCSRRMTEEFRLSSEAFEWLLGEIETKFNQSIAH

PGEMVGALAAQSLGEPATQMTLNTFHYAGVSAKNVTLGVPRLKELINISKRPKTPSLTVF

LLGQAARDAERAKDILCRLEHTTLRKVTANTAIYYDPNPQSTVVSEDQEWVNVYYEMPDF

DVTRISPWLLRIELDRKHMTDRKLSMEQIAEKINAGFGDDLNCIFNDDNAEKLVLRIRIM

NSDENKFQEDEEVVDKMDDDVFLRCIESNMLTDMTLQGIEQISKVYMHLPQTENKKKTII

TEEGEFKALQEWILETDGVSLMRVLSEKDVDPVRTTSNDIVEIFTVLGIEAVRKALEREL

YHVISFDGSYVNYRHLALLCDTMTCRGHLMAITRHGINRQDTGPLMKCSFEETVDVLMEA

SSHGECDPMKGVSENIMLGQLAPAGTGCFDLLLDAEKCKYGMEIPTNIPGISVAGPTGMF

FGSAPSPMSGMSPAMTPWNTGATPAYGAWSPSVGSGMTPGAAGFSPSAASDASGFSPGYS

PAWSPTPGSPGSPGPASPYIPSPGGAMSPNYSPTSPAYEPRSPGGYTPQSPGYSPTSPSY

SPTSPSYSPTSPNYSPTSPSYSPTSPSYSPTSPSYSPTSPSYSPTSPSYSPTSPSYSPTS

PSYSPTSPSYSPTSPSYSPTSPSYSPTSPSYSPTSPSYSPTSPSYSPTSPSYSPTSPSYS

PTSPSYSPTSPNYTPTSPSYSPTSPSYSPTSPSYSPTSPNYTPTSPNYSPTSPSYSPTSP

SYSPSSPRYTPQSPTYTPSSPSYSPSSPSYSPTSPKYTPTSPSYSPSSPEYTPTSPKYSP

TSPKYSPTSPKYSPTSPTYSPTTPKYSPTSPTYSPTSPTYTPTSPKYSPTSPTYSPTSPK

YSPTSPTYSPTSPKGSTYSPTSPGYSPTSPTYSPAISPDDSDEENN*

>m.1356 g.1356 ORF g.1356 m.1356 type:3prime_partial len:1991 (+) Unigene000151:218-6193(+)

MNSSGPATKQGSQAQQQQPQHAQQQVSQQQQQQPQVSPGQQRGSRSTRRKGSDSSIPDDE

KISDEKTDAGGGKGDVSKNKSKQVMNKRRKAEDEEKKAGLKRLKMEASDLSESSDSENSN

KRLLDSSSEPSSENELKNKGILKASEEEEKSQSCKAMEEPDANSRMSPWEEASTVDQIVK

PTAMEVMPSTKSENTGDMRSLAQPSPLPPPSSCAQPRSLVPAEVQGCIMEIKSTVKTSPK

DHYSTGAPRTQTPKCVIDITEDSSSHPTTRENSETVPATLASQTRETYVPESRHLVLNPL

ASECRKAEGELQQQLGQSLGSKIEFAHSEVIRPVVSVSELAAVAEREREKVQQQYPSIMP

CIKNASLAEDVRKPQKLSSSPDVAKSKSNPSPDAFKPKCNPSPDAMKSKTHSVLEAMKPK

PNTSPEVTKHKEIIEITSSSVARSAVKSRSQEIPRSSFKPVPARSTPSESIKSALIVDKN

EHFTVYRDPALVRPEADTNHVTYLPPHLHPLHSSSHATCLTPSSHHHSHLLPASSLSPHP

SVHHPLLPTVLPAMPPSSLLGGHPRLDSGGLSHLALAHHHPHQQQQFLQQQPPPPLLAQT

HGGASYNQLGLYPIIWQYHNGTQHSYPSGISLPGSKWVHPENAVNSDGSLSRNTSSPWLH

QPTPVSSADSLGILSHVPGRPASADPHRPLKISSHSSPPLSKTVDHHKGELESKVFVDPM

RGLVTAHLKQEPDQSRTPNSRDLQRLYADSSHVKQQALPPRLPLDTPDRAVKYKEENRRI

LQESIEVAPFTAKIRSGEPEREPYIRIPSLPISGPPKENEHSPSDLYKYKHSAAQSLPQS

NYFTTISNSVVNEPPRLYPSKELSSLNVSSPLTLGSYPNSGGNTKSLSKPPPLIKHHPDG

EGLVGKISEQLSHQVALNPLSTPVASCEPRSPAVSPSNQLRSMPALHRAPVFHPPTQQTL

DRKESGYGRLSPPTLTPIQPVSVAGKVSEQQKPPTLLPELREVKGTVELSSSEPWRPSSN

LQGYDKTVQWQLDKSQGKPQVATASVIVRSRTCIKYDSSPGSKPGARETSNSKPHTGKYQ

LDCSKLAETREPGRVIQQNTNKEDIFLQYKNNFVRVSQGSFPSSAVAVVNSVCNNSTAVT

TSASAASTHNVLSRGTAELPYSTSANPSSSNINRLEGAAPKCRTPTSLELQECNARTASP

IGPLPQPGSALAPQPYSGNFIHLKKHKAALAAAQSRGSSSASESEGSSRSSQESPTIITQ

DRASPGNPSSKVSPLPNGQPSLMNQPNYHKLKKAWLTRHSEEDRNTNKVEMTSNAVSEII

RPCTVNLIASTSSDTEISKDGKCLEDKLSPEDRKPRRVPSKRPHESGSDSGDDSDGSDSK

HEQRAKRQPKPTFKKKQNDMQKKKGENDKEEEDVKPNGIFRSAKEKTKLKLASSNGIPRS

VLKDWRKVKKLKQTGESFLQDDSCSEIGPNLQKCRECRSIRTKKGEEPTHSPVFCRFYYF

RRLSYSKNGVIRIDGFSSPDQHDEEALSLWAPDAYEENDLDLETSKYILSCIGDKFCQLV

MSEKTAATWIKKDAKIAWKRAVRGVRESCDACEATLFNIHWVCQKCGFVVCLDCYKAKEK

KSSKDKDLYTWLKCVKGQPHDHKHLMPTQIIPGTVLTDLVNAMHMLREKFGIKAHCVCAN

KHNILNKLPSTNGVSQVLQNVLNHSNKLSLCKPEAGQHNLGQKVEANGGSSPASDTSTDC

KLPPPESQSPLHFLADLAEQKSREEKKENKESVVGKVKEESTDALETLHCKTSSLVANST

EQGSTLRDLLTTTAGKLRLGSTDAGIAFAPVYSTASQTGKSGRSMPNILDDIIASVVENK

IPADRSTKQSPKAKPQDEVKAERRKQAEDVPEQHTDIPHCWLYDHRLLWLKDHRKSSNWK

LFRECWKQGQPVLVSGVHKKLNASLWKAEAFSQEFADHQGDLLNCKDGVVSNSGIKEFWD

GFEDLTKRLKS

>m.1363 g.1363 ORF g.1363 m.1363 type:5prime_partial len:1881 (+) Unigene000152:3-5645(+)

QNFAGVPRALTRGGEPLPVLHRDIRVPPAPLRPWDPLGQHYEPLPSDDHVRLHNRYNAGP

SAHLTARANQLLKYGPPPLQHGSRPMPPIADMWEQSHQQQQQPPRAPFSRVGQLKRTAPL

LGEQSVIQQTSLALNRPNEDCPSPSKRKKSSDSEQLIIGTHQYSGSIGSSLPLQQPQSHY

PPLQKDTATWTPVEHKADRNEFQELRRQDMGDCNYKQFSSSKPSPISPPPITSASSYEHG

HGPPLKSLKPQSPHSLGSFSQNVQLHHTTFPFPKNKASAQNSIEHQVISIHKMHSSGVRT

TGSQVPLPQPLSLVDREHPVPTPAHQTSVPYSHPKFQPHPGLVSTTSPSSNSGPHAPESQ

CNPPKSWRNEIKHDNQALILGPYHRSVDPQLSTSSLQQQQKKTGQNQDEGTSLARAPVIT

FTMQPAAPPCSAGCYSGVNTMSTLSSVPSSLSSTVSTIKNWKTAHPTTLNSNPSSIPSSF

PHQDYQRAQSGPESHLDHKQKVQIQHQELHLPQQGQDTPLYTPKVSSAPSSLSSGFQRSG

NSVITSKAIDNLLQPAHSPLRQMQPAVPITVNTTPQQSNSLHHKLPHQVTPVTSQVPPAE

TYERPCPQPAISNPQSIEEALKKLDAELQSHMQAEERRKEKAEEERKHNIGNNKVKKHKV

SEDSATEDMESLLSNSPTDPPPPCLSPAITPSTVLPPSQTSPPFPWLSRGGVPTRQLAPG

ANSVERSRPPPLTPQTDYAREKQRQRDQWNASVPPTLQNTSGIPIYSSKHIPTEFSNKPI

QTCSTEVSTTEKSSPNIKAMGPVLNTSNLREPPKLYQAFPRDTQSSLQKETSGGLSSLGS

SASSSSMDSDSAQFEEEPSELLPDGLANIMKMLDESIKKEEELQSVQSGGQSDPDISFSL

TVAPIKTYTFAPDLMPALKQPLTVDYSTETHASPPVLSRQGSLASPCSRTSSLEEEEDTL

KIIDTYDTSVDKDGGVGSYRHSDLAKLYGLSEGAKSECDEEDEEAEQDEDTRSCSPPPTR

PHLHQTGVNSMFQNLASMLESQKYAYRGGPFGRPPPSALVGVKYSSSLSLGPDICRQQST

SPTSGSTKHPGFNHKDQASVNSRSEQTRSLSPSRSTPETKDLVGCQSDLSGCEEEEGVRK

GLMNEDASCIVNNFTEVERKPKLTTISESSLAELGRSCEVMLSRHNLPSIASDHNKKQMP

HITEKDRKRDRDYYQEKKHKRSSSSKKNEERKEKKKKHREKQENMLSNSSRRHKDSKPHK

EKRGHIDSHREKEKERDGEKKKGKEKEEWVCRNKEKKSGSSSVDHSSSSPSLGSADFQKL

KALTDGPPKELKIRLIKVESGDRETFIASEVEEKRIPLGEITIKNTASEIIRSCKGARVK

GKFKESYLLPSFSLKPVVTMEQPIPREKLNPPTPSIYLESKRDAFSPVLLQFCTDPKNPI

TVIRGLAGSLRLNLGLFSTKSLVEANAEHAVEVRTQVQQPADENWDPSGTGQIWLCESSR

SHTTIAKYAQYQASSFQESLQEEKDSEDEEDEGEKKTELISETLSSNLPSTLSSEQKTGG

KIIKFGTNIDLSDPKRWKLQLQELLKLPVFMRVSSTENMLSHVGHTILGMNTVQLYMKVP

GSRTPGHQENNNFCSVNINIGPGDCEWFAVHEHYWEAISHFCEKNGVDYLTGSWWPVLED

LYRSNIPVYRFIQRPGDLVWINAGTVHWVQAVGWCNNIAWNVGPLNSYQYQLALERFEWN

EVKKVKSIVPMIHVSWNVARTIKITDPSTYKMIRYCLLQSIKHIQILREQLVAEGKKICY

QSRVKDEPAYYCNECDVEVFNLLFVTSESSNRKTYVVHCEDCARRRNSNLSGVVVLEQYR

VEELMNTYDNFNLAPVPCVK*

>m.1370 g.1370 ORF g.1370 m.1370 type:3prime_partial len:2027 (+) Unigene000153:95-6178(+)

MPSQSSTIISVFVCVSSSACSQGHYTRAQANSPRPVMNSSGPATKQGSQAQQQQPQHAQQ

QVSQQQQQQPQVSPGQQRGSRSTRRKGSDSSIPDDEKISDEKTDAGGGKGDVSKNKSKQV

MNKRRKAEDEEKKAGLKRLKMEASDLSESSDSENSNKRLLDSSSEPSSENELKNKGILKA

SEEEEKSQSCKAMEEPDANSRMSPWEEASTVDQIVKPTAMEVMPSTKSENTGDMRSLAQP

SPLPPPSSCAQPRSLVPAEVQGCIMEIKSTVKTSPKDHYSTGAPRTQTPKCVIDITEDSS

SHPTTRENSETVPATLASQTRETYVPESRHLVLNPLASECRKAEGELQQQLGQSLGSKIE

FAHSEVIRPVVSVSELAAVAEREREKVQQQYPSIMPCIKNASLAEDVRKPQKLSSSPDVA

KSKSNPSPDAFKPKCNPSPDAMKSKTHSVLEAMKPKPNTSPEVTKHKEIIEITSSSVARS

AVKSRSQEIPRSSFKPVPARSTPSESIKSALIVDKNEHFTVYRDPALVRPEADTNHVTYL

PPHLHPLHSSSHATCLTPSSHHHSHLLPASSLSPHPSVHHPLLPTVLPAMPPSSLLGGHP

RLDSGGLSHLALAHHHPHQQQQFLQQQPPPPLLAQTHGGASYNQLGLYPIIWQYHNGTQH

SYPSGISLPGSKWVHPENAVNSDGSLSRNTSSPWLHQPTPVSSADSLGILSHVPGRPASA

DPHRPLKISSHSSPPLSKTVDHHKGELESKVFVDPMRGLVTAHLKQEPDQSRTPNSRDLQ

RLYADSSHVKQQALPPRLPLDTPDRAVKYKEENRRILQESIEVAPFTAKIRSGEPEREPY

IRIPSLPISGPPKENEHSPSDLYKYKHSAAQSLPQSNYFTTISNSVVNEPPRLYPSKELS

SLNVSSPLTLGSYPNSGGNTKSLSKPPPLIKHHPDGEGLVGKISEQLSHQVALNPLSTPV

ASCEPRSPAVSPSNQLRSMPALHRAPVFHPPTQQTLDRKESGYGRLSPPTLTPIQPVSVA

GKVSEQQKPPTLLPELREVKGTVELSSSEPWRPSSNLQGYDKTVQWQLDKSQGKPQVATA

SVIVRSRTCIKYDSSPGSKPGARETSNSKPHTGKYQLDCSKLAETREPGRVIQQNTNKED

IFLQYKNNFVRVSQGSFPSSAVAVVNSVCNNSTAVTTSASAASTHNVLSRGTAELPYSTS

ANPSSSNINRLEGAAPKCRTPTSLELQECNARTASPIGPLPQPGSALAPQPYSGNFIHLK

KHKAALAAAQSRGSSSASESEGSSRSSQESPTIITQDRASPGNPSSKVSPLPNGQPSLMN

QPNYHKLKKAWLTRHSEEDRNTNKVEMTSNAVSEIIRPCTVNLIASTSSDTEISKDGKCL

EDKLSPEDRKPRRVPSKRPHESGSDSGDDSDGSDSKHEQRAKRQPKPTFKKKQNDMQKKK

GENDKEEEDVKPNGIFRSAKEKTKLKLASSNGIPRSVLKDWRKVKKLKQTGESFLQDDSC

SEIGPNLQKCRECRSIRTKKGEEPTHSPVFCRFYYFRRLSYSKNGVIRIDGFSSPDQHDE

EALSLWAPDAYEENDLDLETSKYILSCIGDKFCQLVMSEKTAATWIKKDAKIAWKRAVRG

VRESCDACEATLFNIHWVCQKCGFVVCLDCYKAKEKKSSKDKDLYTWLKCVKGQPHDHKH

LMPTQIIPGTVLTDLVNAMHMLREKFGIKAHCVCANKHNILNKLPSTNGVSQVLQNVLNH

SNKLSLCKPEAGQHNLGQKVEANGGSSPASDTSTDCKLPPPESQSPLHFLADLAEQKSRE

EKKENKESVVGKVKEESTDALETLHCKTSSLVANSTEQGSTLRDLLTTTAGKLRLGSTDA

GIAFAPVYSTASQTGKSGRSMPNILDDIIASVVENKIPADRSTKQSPKAKPQDEVKAERR

KQAEDVPEQHTDIPHCWLYDHRLLWLKDHRKSSNWKLFRECWKQGQPVLVSGVHKKLNAS

LWKAEAFSQEFADHQGDLLNCKDGVVSNSGIKEFWDGFEDLTKRLKS

>m.1376 g.1376 ORF g.1376 m.1376 type:complete len:1627 (+) Unigene000154:190-5070(+)

MDAVDHHPGFSVRDHTLLSDPDRTLTPNGHGAADGHEAAEVWRLVCVSLYGGAPWGFTLR

GGQEHREPLIITKVEDGSKAAAVRLQVGDELVNINDVSLSGYRQEAICLVKGSHKTLTLV

VKRKNEPMSRPHSWHSTKFNENQSDTAKTQSTPSPVWQTRYDASAFTSDFTTGWDQPNLR

RVSDQFSSLGSMDSLEHSSCAYPPGRLSPSKSNGNSVEHLVCGKRDSAYSSFSTSSGTPD

YTLSRSNAASTENMLCKVNQWDAGSRASNSRHSQCLIEGVRQDDKIGYLQPPSVSSGRES

PRTEEQPGYRHSASGRSSIAPVWHVPDMKKAVAPSPPPPAPPTRSDSFAATKIHEKTLVP

SNTENPSTHTQSKIQGKGLKAATEVFEGNQRIYLAAESGRETSQNYSLQPKSGLINPYVA

GDGQQISLSNSNKKYSPSGMEPSYTHFAYHKRQYSDETNFYGPSRTPSSGKSQNCYSSMQ

ELPTNSYAPLCSQHQIRRHIASQSTTAIDQNADSQNSNRYYFVKSQPASQGGSQTSLFRV

EDRNANSVMEITQSGGDKTSVSSQGQLKDRFTTSQVLLHPNSKDSNGYYRQVDSQHRGYT

AINRSTFGDATTKNLEVRAPQKQNTVTNINQFITHYEQGKISESHRLGESSKENFPSKTE

TMLCPQKTPLLHSLSHDGNRLIDVQSEVVSGAGQQDIVDPQNGRQFHRTDRFATTLRNEI

QIRRAQLQKSRSTAGLSGVADPQEEPSVGRSTDIASPSSDGSFTSAYKDHLKEAQARVLK

ATSFRRRDLEPVLLEHPGAEFSEVLANKPASGTNHVHRICGRKRFPNDKKVRSFSEPEKI

HEVGVEERAPQDNDLTLVSRRKVSEATGKPTYPKPMLKPNSQISLDCRSDKPGELSQSGQ

NDVTKRNTKSKAIDSGPNSLQRLGTFAEYEATWSTQKKTDPRTSGRYHSADNILDSAVDE

RHKPSYFHERSRSSPSEDFYGQNIPAQGRKSAESHLPERKVSDHCNSTSRLSEEGHTTLL

PNQNPASLLTGEKWESNFSDPPYTGKSPSSPADPFQHFLTDPSCHEKNEGSNSLRPPSTC

PDKYKCPEAATSINTSQGGTGVSQPPIKDTVAKDIPVHTPVSWKDLEHIKSLKREEETQS

ETLSFPPPPPESPSHLTPPSMEEQRSPSPHFAPQRLTDKPPVSVSQQDEGTGRMEQVIDD

SSTVKKVPIKIVHSESSTEKENRQYLYPSTETPRNSQPAVLSLSSLGAQEQSSSPFCTYT

RQREQEDEAIRPQKEPEPSEDVSGPPKPQKKPCVDQSTNGDSSGIYSASFYSEEDQKRDI

LARDIIDKDKSLAEIFDQSKRRTTMDLMEGIFPEGEQLVEEAHQRRRAAPKLPSRNTEER

REEDSMTTAATAAAATLVTSSAYYSTSAPKAELLIKMKDMQEKSVELESEEELEEDNDAE

LANKKHELIKSLSKKLKVLREARESLQEDMQDNNALGEEVETTVQAICKPNELEKFRMFV

GDLDKVVSLLLSLSGRLARVENALNSLEEDAPLEERRTLMEKRKLLIRQHEDAKELKDNL

DRREKVVYDILTSYLKEENLADYEHFVKMKSALIIEQRKLEDKIKLGEEQLKCLTDSLPP

EQRSLF*

>m.1381 g.1381 ORF g.1381 m.1381 type:complete len:1988 (+) Unigene000155:96-6059(+)

MFTRRGYGDVKKSTQKVLDPKKDVLTRLKHLRSLLDFTERQELKIFFEANCSQIYFIFYE

NFVTLESNLKQKGNKSQREELDSILFIFEKILQLLPEKINSRWQFHSIGSILKKLLHTAN

SFKIRCEGMRLFLLWLQALQTNCSEEQLLIFACLVPGFPAVPSSRGPCTLDTIIYSPFSN

PPDAKIVPEEITPLVPAVPGEKAIDDQTCYILQTVLKFMVIQASSLEWYRKEKQDSGFRF

LFALFKKYYLPHIFPSFTKLTNLYKPLLDLPHHRPKPLYVPVSRNNESTFCTRDQYLAPR

VAFITWLVNFFLEKKYVSSASTSTKNGGEVFPKIIQTVTAGSTSQEKSNELEPNGPAEQE

KNHSNSSTLSDRKGSNSSLCSIEEEHRSVYDMVHSILLSTRDNVNFVNEVFHQAFLLPSC

EASATRKVVKVYRKWILQERPTFMTEPEKTDHDEEAEEPQQPVEIDSTQHTQEVHGHKRS

SSWGRTYSFSSAISRGCLTEQQNHDVKAGIQPTLQVFLTNSSNVFLLEPCHDVPKLLENQ

VEVCRAVLSIYRHMIMEHNMNRQTWEQMLQVLLRITEAVMKRSQDDQRKDMFSQSLASVL

FRTIIVAWVRANLSVFISRELWDDLLAVLSSLSHWEELVIEWGSIMDSLTVVLARCVYGL

DLNNLPLDKLSEQKEKKQRGRGVMQDSQKSSVISRTFSLSWRNAGGQPGTQEPMRIRSAT

TTGAPAVEKARNNVRQKASDVEECQLSECGGEGEAGDGDSPLPRSSSTSDITQLTESIPG

QNREDSPSASFSDGKSSVCDNSSVGKRETPTILVRRSSTPGDIDYPGEAGTLTARTTIRE

KSESLSSETSNSYLTDTDVNLLTWQAVEEEQDSSSANDVSVSVNLGLTTDTESQRSLLLS

QSEALAGSECVHSLHLAPLSPCPSVSPTLLLPQGYGDCPQLLEDAMHMPHDLDSSEFLAD

DISIIAGGCLTGWHADSAFVIWRRILGILGDVNNIRCPRIHAKVFSYLYELWHKLAKIRD

NLGINMDNQSVLPKTLFIPPLRMLASWLFKATMLPAEFKSGKLQAYRLICEMMTKHQDVP

PNSDFLVHLYHVMHKGFTSDDQDVLNTMIRWCSPRFFFLGMPGFTMLVRDCISAAARVLN

SDNSESPRIEAQTVLGSLVCFPNLYQQMPSLQSAAKSQDITVGREDLKDHLVNILLRTAR

NEPSEGARCVAICSLGLWVCEELGQQNIHPQVNDAINVLGVTLKFGNKVVAHVACDVFQL

LICHWQHLQKLEQSLPKRIIEIFVATIAFLLPSAEHSTVEADKKLIVSLLLCLLDWCMTV

PLSILLEPITMPMLDDPASHKTPLLDYIYRVLHCCVSGSNLHTQQSHYLLSLADLSSDYD

PFLTLGQVKNSEPPPMHNSTGEFGNLLTVAEEKKRRNMELIPLTARMIMTHLVNHLGHQP

LSGGPALLHSMVNENHDNPYVESSELSSEVFKSPNLQLFVFNDSTLISYLQIPSDPTSPS

SPDSKENSSEVRVIVRDISGKYSWDGGVLYKTFDRTNLDCKGAGDEPSHHYHSSTSSKHS

SSQASRCPSELEVEDGVDVLDQLLEELGNSSPECLPQPQQRLTQPAPAPVGMNPEMEGSI

MEAIYSQAQLEEEELTCRRNEDPSILASVSQRRPIYQEPKAPFYFCRLLLNELGMNSWDR

RKSFHLLKKNSKLLRELKNLDSRQCRETHKIAVFYIGEGQEDKCSILSNTEGSQAYEDFV

SGLGWEVDLATHCGFMGGLQRNGSTGNTAPYYATSNVEVIFHVSTRMPSDSDDSITKKLR

HLGNDEVHIVWSEHTRDYRRGIIPTDFGDVLIIIYPMKNHMFFIQVMKKPQVPFFGPLFD

GAIVTGTLLPSLVRSTCINASRAVKSRLTLYQSFYEERALYLEAIILNHKESLTFEDFAA

QVFSPFPTYYPVSGTGSLTGSTSAELNNTVTTSESTEQASPTMPRATKSRVSGKLRRSAS

AISKSSN*

>m.1386 g.1386 ORF g.1386 m.1386 type:complete len:1922 (+) Unigene000156:271-6036(+)

MSDSDSDEDQDRPFHLTGFLFGNINEDGQLEDDTVLDTESKKHLAGLGSLGLGALITEIT

ATEEDAEQSTDADGWVKSTDDAVDYSDISEVAEDETRKYKQAMSSMQLSHKSDDDDDDYD

ADSEDIDAKLMPPPPPPTQSITAKKEDSLNQSSSVTDEGDGIILPSIIAPSSAGEKAEFS

SSSDSESECDRPTPAGGGGLQSSLTLPLAGIMQKDTAKTLPQVTELFPEFRPGQVLRFLR

LFGPGKNMPSVWRSARRKRKKKHKDTHSDTPNADSESNATEGGDKKKSGWDYEYAPPPPP

EQCLSDDEISMMAPVECKSVSVVCEGEGVCDSRPRVAEWRYGPAQLWYDMLGVPEDGTGF

HYGLKLQQDNTDADTQTDTHKETHTDTDTNTETHTGTHTDTHADTHTDTNTPDAEQSSEE

GKQQSELEDELFLMVTQLQWEDDIIWNGEDVKHKGTKTQRASLAGWLPSSMTRNANAYNA

QQVSHEGDTPWYSIFPIDNEELVYGRWEDNIIWDDQNMNCLLCPPVLTLDPNDENLILEI

PSEKEERTSNSPSKENKKESSLKKSRILLGKTGVIKEEPQQNMSQPEVKDPWNLSNDEFY

YPKQQGLRGTFGGNIIQHSIPALELRQPFFPTHMGPMKLRQFHRPPLKKYSFGALSQPGP

HAVQPLLKHIKKKAKMREQERQASGGGDMFFMRTNQDLTGKDGDLILAEYSEEYPPLIHQ

VGMATKIKNYYKRKPGKDSGPPDYKYGETVYCHTSPFLGSLHPGQLLQAFENNLFRAPIY

LHKVPETDFLIIRTRQGYFIREVLDIFAVGQECPLYEVPGPNSKRANTHIRDFLQVFIYR

LFWKSKDRPRRIRMEDIKKAFPSHSESSIRKRLKLCADFKRTGMDSNWWVLKPDFRLPTE

EEIRAVVSPEQCCAYYSMLVAEQRLKDAGYGEKSFFAPEEENEEDFQMKIDDEVRTAPWN

TTRAFIAAMKGKCLLEVSGVADPTGCGEGFSYVKVPNKPTQQKDEREPQPAKKTVTGTDA

DLRRLSLKNAKQLLRKFGVPEEEIKKLSRWEVIDVVRTMSTEQARSGETPMSKFARGSRF

SVAEHQERYKEECQRIFDLQNKVLESTEVLSTDTDSSSAEDSDFEEMGKNIENMLQNKKT

SSQLSREREEEERKELQRMLMGDEGGERDKGKKDRRKGLSSALSTGSHKDDDASSVTSLN

SSATGRRLKIYRTFRDEDGKEYVRCETVRKPAVIDAYLRIRSTKDDDFIRKFALFDEQHR

EEMRKERRRIQEQLRRLKRNQEKDKFRGPPEKRPKKAKERPDLKLKCGACGAIGHMRTNK

FCPLYYQTNAPPSNPVAMTEEQEEELEKTVIHNDNEELIKVEGTKIVLGKQLIESADEVR

RKSLVLKFPKQQLPPKKKRRVGSTVHCDYLNRPHKSIHRRRTDPMVTLSSILESIINDMR

DHPNTYPFHTPVNSKLVKDYYKIITRPMDLQTLRENVRKRLYPSREEFRESVELIVKNSA

TYNGAKHPLTQVAQSILDLCDEKLKEKEERLVRLEKAINPLLDDDDQVAFSFILDNIVTQ

KMMAVPESWPFHHPVNKKFVPDYYKVITNPMDLENLRKNISKHKYQNREVFLADVNLIHT

NSVKYNGPDSSYTKTALEIVNVCKQTLAEYDEHLTQLEKDICTAKEAALDAADLESLDPL

TPGPYTPQGRGRGRLGLGEEESDVDIEGFEDDDDGKPKTPAPAEEGDLDDEDEDDDEMLL

PGRGHLEDEEDDEGSSRPVQSSVLYQDLLMSEGEDECSDEEGDNPFSSIHLSESGSDSDR

EVGHQDSTRMGMEHEESMMSYDGEGPDEDTHLEDSNVSYGSYDDGDSRQQGRGFSPEGEG

HEQGIGPGDGVSDEEEEEEEDESEEQRRGPSVLTRVQLSEDEEDSEDFQSIGGDSDMDSD

N*

>m.1394 g.1394 ORF g.1394 m.1394 type:complete len:1704 (+) Unigene000157:33-5144(+)

MMDLFFLLCVIHVLSVTEAQQRTYLITAPKVLRVDASETVLVHLYGYDQETIVNLYLKDN

LAHEGRIYASSTLKLNDRNNYQAETTLRVFPNIEQKVTHLHLQAVSSSFTNVKKIPVIHA

NGFLFIQTDKPLYTPEQQVEVRVYSLNEELRKTSRPVLLTFKDPDGIKVEIIEMNDIFNG

VKPLLPPFKIPLKPKFGIWKIEAAYTINFTTTAVAEFEVKEYVLPSISLRIQPEANYVSI

ANYEAFKLKIFAKYISGKAVRSADVFTRFGYINNHDVVMIPQTLSQYKMDDGEMEVVLNI

KHDLFSVNSGPQQLWDMNNHFLRVVVLLKESTGGLSQEAVLSNVKFVHAPFTLSLIATPP

FIKPALPYQIRVLVKDPLGEIARGVPVKARVTITYNNNEQEELHGYRNEMRQTSRNDGVA

YFVCNIPDNVARAELFFETDDPKYPPESQTQLKLSTLAYKSVNQRYLYISLPAHASAFEV

DDHASININFHYRDFLPLKTFSYQILSKGKVVQFATEQRISHSVQSINFKVTSNMVPSAR

LVVYYILFGEQTAELVADSVWFDVKAKCVNNLDVNLSAPSKSYKPKDRLMLSVKSRSANE

KFLVTLSAVDTALYNLRGSNKDPLNKVLQRFEHSDLGCGGGGGRNNADVFSRAGLTILTN

ANVEASDADETCTAVVRPKRSTLSDTEKQKYEFQANSVRRKRADLSLNEKLEAKAKEYRN

FHQCCTAGTDSSPTLETCRERTRKLKTEHVQCKFAFRDCCFYAEKLRAESGGVSLARSEI

EFLLEVKSEQIRSYFPESWLWEEYENERSGLLQVTRGLPHSLTIWEIRAVGVFNNGICMA

DPLQVSVQQAVSVDVPLPYSIVRGEQIELKGSVYNEYDTVTTYSVTLSASEGVCVFRGTL

LTEDGDQQSNKGTIKGHSVALVQFFIMALEVGNHKLSFTLTTRWGRETVVKTLRVVPEGI

RKEIYVGRRIDPSGIYGTSVTRIELRNSLPPKIVPKSTVERLLTVNGEVLGELLSIIINP

KGIMQLTNLPRGSGEAELVGLLPIFYVYDYIEQSEQWGKISKFGNTILLKRKLKQGITSV

MSFKSKWEDSFSLWKNKEPSTWLTALVVKTLASVNKYVTVDHNQLSSTVYWLITKCQTAD

GAFKEISSYKPMRLMGAGGDVNEQSVFLTSFVVIAIKNALTVPKSNLEMYHHAVEKATHY

LTLHLTKVSSLYVKAIAAYALTVVDFNNHHAVTLYENLKKKAKIKGNPVTVRFWEENEAP

QDPLKPNRATAQSVETTVYMLLNTLFRGDTAYSKPIIQWLTDDQRYGGGFHSTQDSILTL

EALTKYSNLVRRANLQMEVDVSYRRKGSIDRVTLTQDKPVGKPIEVKYDDDVILKTGYST

GVSFANLRTIYYETSDDNNNCNFDITVDVHPRDPNSQDPITLSPRIVACARYKPRKNEVE

NEAGHTVMEINLPTGMSPIQEDLKRFRDGLESRISDYEIIDNQVVLQIDSIPSEEFYCVG

FRIQEVFRTGMNSASVFKVYEYHDPDSQCTKFYYSQSHKLLRLCDEDQCQCMAAECCNFR

ANIDPTLTVEKKLHDVCKANIKYALKVKITAVDTEGDFLTYRANVEGVIEKGLLDITRSG

TSEVSFVKKATCTSANLEVGKQYLIMGTEVMQLRINRSYKYKFPLDLQASVDWWPSECQT

SACQQYTEVLNNFEFDYLSSSCS*

>m.1397 g.1397 ORF g.1397 m.1397 type:3prime_partial len:583 (+) Unigene000158:4375-6126(+)

MLQSQMQNLRENLGKVTTERDNLKKELEARTLDIQEKLRTITQVKKVGRRYKSQYEELKV

EHDKVVAAAASAPVQDQEAQQTSVQELQSLKDSLSLSEARTQELEGQLETLNKTIGEHET

GTRTAQDQVSHLQSELMRLRQELVEKSAQEDRLRQQFAEKEDRTRKAILGAKQKINHLVD

VKGQLVKENEELKQQREELEVRINALRSQYEGRLSRQEKELRELREQERHVEQRDEPLEQ

GPSKTQEQQRTTEQRQISLKSTPVAERGSASNSEPPTANIKPTPLVATPSKPPVIPGNKP

TPRASIKPMITPAAVPTPTPTATVMPTTQQESQEAMSSESPLDHVTVFGSTTGSVRSTSP

NIQTTLAQPMLSVQQSQTTAFVQPTQQQSLPLTEPVSQEPASATMMMEATPSNSQMERPS

TSTTVFGTVSATPGGTLTKRPREEELDSITVELEPQEEPTEPPISKKLRMQRVELEMEML

TENSTEAESVVPEGSQEAAVHELENYPTLEVDEESGVSQSVPVEFLLSQVSNIQSQEESD

EPVIVIVSDSESEEAPEEETEEEEQGFEEDEDEAEDDDDDDDD

>m.1396 g.1396 ORF g.1396 m.1396 type:5prime_partial len:1412 (+) Unigene000158:2-4237(+)

EEEGERTARNRRKMEVVLQQILDEDELSKVPRELQEKLEKYLAEQQSEADYYKAQYEQLR

VDSEQQISNLEKRYTESQDQCVSITKDHQKLQEEFTSLDEELKSLRVKTKEQESSYVKLA

TEQKELSKLRDELEAEKQELVRRLERTSQEVEGQSEDLKRISNRLVDVNATNMKLRLKVD

ELETLEVSIKYKEKRLEQEKDLLQGQVTWLSEELKTKSEEFLSLLREKSSEILELKCRIN

NKEDEYSRVQDQLTSLKSSNENLQKQAEDLIIKLKEAEEQQAVSEEKFRNELNANIKLSN

LYKAAAMDAEAKSEELTRALEELHKLLKEAGEAHKAMEIKLSEVESCKHKDVAELKGRIS

VLEKELDNANDLLSDSTRRVGTIFAPALPDEQLGTMSSTAAAVSKIKPSMKLTEIYTAYV

ESQEQLQLEKLENKRVNKYLDDIVQEVEAKAPILKKQREELERAQKSVASLSAKLEHAVQ

EVQCLQKEADEAKKHSSMLKRDNQRFGVQLSDMAHQVRVLLIELEEARGNHVVRDEDDLC

SADVSSTSEVISQHLVSFRSVEELQQQNQRLLVALRDLGEEQEKGEMEAESTKLAEMQNS

LENAQIELEQLREQRAHQMQLVESIVRQRDMYRVLLAQATGVSFPQQDEFSLTSTPCRSP

AATPTAATPTGLVATAIESVETVEAKAALKQLQEVFGVYKKEKAESEKTLTEQCEKLQEQ

VSEMRSKNTKISTQLEFTSKRYEMLQDNVEGYRKEIASLREKTQKQTTTLQQSEQTVNTL

AQDLRAANEKLSMIEAQAESLRKEKDMLKMVELSLSQEKESLLTQQRNQNLLLNNLKSIQ

AMLERSETETRQRLTAQVEKQEREISLLQKKLEHEVEQRHALGRINDKQLLDAKKQLEVQ

CALHQRTREQLSSTQQELSSLQQKPNTREGRVSSPATHTGTGLPGDEENMEELRKHLNEA

NSRAEELTERLDSTNASLEQYRFMCLSLEEALQKEKQVTEQVKVSVQSQVSEAHEQLQQM

ENNLLEAEKEKNKIQQEKQKTAAAIEQQVSELQRNLRRLQTEHQETVQKAALASAQMQQA

LLDSQEQARLATEAQEKYEQEMLLHAADMEALQAAKVKEQHASQLRQQLEEKIQTVSAQL

FEARVSWEEQEKILKEEQTRLESHCQDLQRQNELLHEQIQAMSGKMLTQMQQKAGDGSLN

VSFTEEGKSQEQLLEILSFVRREKEIAESRYEVAQGESLRYKLQVVHLERELKEVQDSLS

AEREKLQVTAKTLLQHDELMKKTETMNVLMETNKMLREEKEKLEQELQQTQAKVSKLQSD

ISPMQESNTELSEKSGMLQAEKNLLEEEIKRWKARTQVLVSQQKDTDPEEYKRLHLEKET

HLKRIQQLTEETSRLKAEATRYKDLLFTIKL*

>m.1406 g.1406 ORF g.1406 m.1406 type:5prime_partial len:1838 (+) Unigene000159:1-5514(+)

PLSDIGVMYEGKDRLIQGCEVIQATPYGRCANVNNSSATSQRIFITFRRAPLVQPRNSLA

VTDICVIVTSKGETPPHTFLKLDKNLNCGMWGSSVYLCYKKSVSSTNSIAYKAGLIFRYP

EEDYESFPLSESVPLFCLPMGAKIECWAPNTQYPLPVFSTFVLTNSSGEKVYGAAIQFYE

LYPSDNLTEKQKIQLGLFTTVDKKPIPNRTVNTNKCICLLSRWPFFQAFKDFLRFLYKLS

VNSVNPLPIEKHISHFMHNVPFPSPQRPRILVQLSAHDSLMLSQPVCTPLPLSGADYSTL

LTNLGPENCATLLHFILLESKILLHSLRPAVLTGVAEAVVAMIFPFQWQCPYIPLCPLSL

AGVLNAPCPFIVGVDSRYFDLYDPPPDVVCVDLDTNTIYLSDEKRNSNWKNLPKKPCKSL

INMLVNLHHQLNIVRRTSPGLSAVEMTPMEADFTWHKKMTALEMEIQEAFLRFMASILKG

YRYYLKPITEAPSEKATAADSLYDLQGFLKSRDRAHQKFYSQLTKTQLFIRFIEECTFVS

DKDTGLAFFDYCIEKLFPSDKGTDKSTKVEGELSEDTRLVELDESQKSEHTVFILPPEPP

TDDSPDPPPKYSYKSFPRLKLELFDRPAQLKPALSTRAAGASVSSSPALLAKRTKQEVKL

AYKMAKRFYSNPQLWAKCLLSHCYSLWFICLPAGMRLAHSKPRAMQQALDVLLKMRSSEV

EVLDEVCYRVVMQLCGLWEMPVMAVLVLMEMKKAGVEANAITYGYYNKAVLESPWPSRNR

SGRFMWTKIRNVVRGVAQFKQALRGEAQSKRAPPITAVVSLDAIADGDGDRLSHCSADSS

SEANGEEHTLFSHRLIVGDKNGSQFSTGGQSDQGYGSKDELHQDSTDACKATAFPHSDND

LANKSKTMKTQQNALVDIAGSVDSAMVVSEPPSPAETTPPPASIVKQSSGSFDKAMCRKD

SGSKLFTAHSKTEDIVLPEDEGATHPDATQSSQQQRAKAFTERSCSFSTESRAGMLRKKS

SLELSVDHMGADAKILAAAFSGGRTPPPATASASIFSDLGVAPIASSGLEEDTDEKMELI

TFEEKTDEGKGQKKETSEEVMSKESTEGMGRSISTSSSSSKAAEREDVEVGADPLSLLAS

ESDEAASIQSQESARLVPPVVSRNLADEIEMYMNLKSPLGAKASSMELHQVVQSKVDQAD

SPACKQSLGRRSSLPAGPVKAPDTLETPKRSPAVTRSKTFAIKSLKTPSSEGQRSSSLTA

LVKTSQSGSLGSVINSISGIKMDALLSGPKVDMLKSGMKQAANVASKVWGAVASAYSYSD

DEEEQSQPGDSFPARLEEQLMGGDGTGDTVPRGLMPNLASNGLTHSNTSLGSSSGSSETG

RGQHIASMTPGRSVRGVDSEQGSSHHASTSSIYQNCALEVLMSSCSQCRSCEVLVYDEEI

MAGWTADDSNLNTTCPFCNNSFLPLLHVEFHDLRPPSGFYVPSNSGDSIHSGSTHPPASS

SKVKVGSPSDPADLISFFESPDSKMEPEGNLTYQTGTQTALVPEPVISDPLGLLEKRSTS

LTRSNSVGGPLQSLDSIQRPNHGVSTTSLPNSLQEVVDTLGQKRPNPMPVSVPYLSPLVL

RKELESLLENEGDQVIYTQKFLTQHPIIFWNMVWYFRRLDLPSILPGLILTSEHCNNGVQ

LPQTSLPQDSKQVYVQLLWDNVNLHQEPTEPLYQLWRTFLKKKGSLAPTDHQEIRFLLNS

IVRNIQTNDVYGPINLLLREIKRHPEVKRQRSIYRQILFLSLVALGRENIDVEAFDREYQ

LAFEKLSPEQLKVLSSLDRPPSSSVQWCLKCFGTSFI*

>m.1410 g.1410 ORF g.1410 m.1410 type:complete len:259 (-) Unigene000160:2786-3562(-)

MGTDKASETETHAKMEKEEVRGEEESGEERAEQDGNLLTSYTWQTGGARADPSEKSAGNK

WSRMQSWKKALSEDTPEKSATPTPGPRVGRKNPFRRALSEPPGSLLSSILSPSSSSPSAA

ETSAGADTQQRGKLRKYLQQVSQRLKRPRAQNRNNTSTTQQQQQREDVPCVEGADLVQFS

WVPQDVPVWDINNCILQDGQIFISREEEPLLRMRNRASSCLSSVSLQNLTESHSNLECSP

DPTSVTAPKPKTQDGGVR*

>m.1409 g.1409 ORF g.1409 m.1409 type:complete len:526 (-) Unigene000160:4055-5632(-)

MAAVVNYSPPWWVNLLHRLPHFNLRFEQISSEFQPEDPSYQQSILLLGAVALACLALDLL

FLLFYSFWLCCRRKKSSEQPNADCCCTAWCVIIATLVCSAGIAVGFYGNGETCDGVNRLT

YSLRHANRTIAGVQKLVSDSTTSLNLTVDDNLQHLESQYTKHADYLSIVQKVQGQLDELL

KQMVEIPFWSNSRISLEALASKIELYDWYRWLGYLGLLLFDVLICLLVLFGLIRNSKRTL

IGVCLLGVLALVISWGSLGLELAVSSSSSDFCVAPDMYVAEVAERYGVINRDILQYYLHC

SVGQNNPFQQKLSGSHKALVEMQDDVLELLRAAVREYPQTRTNLEQIQGILNTTEISLHQ

LTALVDCRSLHMDYVQAVTGLCYDGLEGLIYLVLFSFVTALMFSSIICSVPHTWQGRRSS

EDTEEDSAASGGRQNHDNLYRVHMPSLYSCGSSYGSETSIPAAAHTVSNAPVTEYMTQNA

NFQNPRCENTPLIGRESPPPSYTSSMRAKYLANARPDPNSSTAAH*

>m.1408 g.1408 ORF g.1408 m.1408 type:complete len:743 (-) Unigene000160:91-2319(-)

MENSLSLWVNEAKGLPPKKRYYCEVHLDGTLFARTSSRAVGKASSVPVSDSGSSAVGVSS

GGSASTGGCQLFWGEYFELDNLPPVTQITLHLFRDDDPKKKRHSKDENILYPLGSVALSL

ANIKGRVFQEKWYPIVPYKPPGTGGSKEQLGPQACIRVKARFQNLKVLPIERYKEFAESV

TLGYMKMCSNLEPLLNVRDKEELAGALVHVLQSIGKAKDFLIELGHAEVQRSGENEALLF

RENTLATKAIDEYMKLVGQKYLIDTLGDFINRLYTTSESCEVDPQKCSASELLVNQRHLK

DACGEVVRQITERHSSFPVELNEIFSSWVSDCEERGRADIGHRLISASLFLRFLCPAILS

PSLFGLIQPYPEHNTLRTLTLTAKGLQNLANFTLFGEKEEYMLFMNEFLAEHWEDMRTFL

QKVSNPDSELEMARFDGYVDLPLRLAVLHNLLVDIISPMKLEVINNLQPLPSILNQITEH

LGPDIPRITCSRSTCETEKPTYVPPRDLGKYSPLNKSLPHLPTDPRPPQGKGKHRKQVAR

TQSVPASERHHRRPLKRTSSNAELSETAHIHHEESPERQMEISLPQQEFFNKSAPAPVPW

IKEHVQDETNILEKHAQELYELRSGVDQVTDRELEMAKRLEDFIAVSQEQQAQLQAELQE

VRNVLARREEQLASATFRLGVIEEEREEDERKLSVALAAAERVNVLEEQFVGLLKDMQQL

NTVYNDVLSTSALNRDTETLRQ*

>m.1417 g.1417 ORF g.1417 m.1417 type:5prime_partial len:1976 (+) Unigene000161:1-5928(+)

TLFPYKTLLCKNNEGLIVKDVDDNECGEEPEHDQDAFEEISKPMEIDPHDLEETTLNVPK

EPKDDYEEEVYESAKYFLLNCATPDSVLRLKNSGLGNHEVERLQRLYFHQQDHHSLRTFM

DSHLKRADKESKRFIEVTTFSSLLTKVDVRNLSKALGVSMDGLLLLSLHQFDTEVSFCSK

IRGFLKTAQLSLRILLVQIDMEESLCKNELIASAKYCTMNELLASQSEDPSCYIVFITKL

SRIASGNKYIGFQGGAWISIHIDDLRETEDMSLDLSVFCGTPISKLLSMPAVPAAMEVGD

QEKNTIINAQDESAYLHSLSLVKSCTQKAVSLLRDSNKKAPRSMERMNILLSLLQNDLGP

DGAHFQEVLLKRLVVALTQKEENIHNSGDWVNREIMKREALQEGGTLRHTLWRYLQGVLT

PILARILEVLDRDCNLDLLYSAGLGEGLVQFWLDIFDDGQILNLSVPQNLSISDQEIEVQ

CNLMVGEEACPCSAPFSWLIRQYCYNLWEEAQFVQGIEQSSHQRIKQFVSAVTSSRLCGY

MEKLSERARIELGQRYLNDFVILSFKIKTEDGVRVFSAAVSGCVAALQKDIGVTPDLSPA

WIMAATQHFSPRLDTLSHILQIQPYLAPLIILKQSTRKDQPDMCEDILALGICVEEMKLQ

SFSSLSQCSDFLGRVELLQPCLQRVFSPTYCSLCSPGYLRHLYAIKSAWQGMLVVAAFIE

QVVVKVNSCDKKLVALTLKHCSQLQRLMEECSNLQSKACLQQLIRILNEYYEESISEELC

FGLKCPVCLEELAEPCVLQCKHVFCLSCMRRCMQKEEVKCPKCRAQIPPNYQPVISTSID

LALKQHKELRRLWNSFFLEVVSHFCFSKEQKPEDDMVELLFSLLISAQGDVYKTRELTPF

LECVDQCPVVRSVLPKLLMQYSFDQVKGHIQRYLQNLEDKLLDSKDRMELYRLFVNCFQD

SLLCVDLSEALEAKELQRRQQEDITFLGRLARKQTPSREEHPAEFLLSMARLRMCLDSAA

RILSRAVSQQSGGCVQWEQRLLEEVEVVFKYAKDDWYKIYLLRTLNRQAGTDCVQAAMNS

ASYEWVFPAELLRQQRLIPVEVDRFLCCGGQYRAIRNGVGQVLLEGNANALKAALQNISS

SESAKCVLLALALFKQVTCRIDLPDSAMHPGEQKLDMLKHIVRENTAWSGFLRELLTSLI

SNPAEGLMSHLHVRANNSTQRRLLLELLVHAVAVFHCGSHIFNPFHLIASRPQSMRDSFL

PTMQDDDTSMVIQGLREQNLKRYYCKNGHLSLVGECGKPTALGKCDTCGVPIGGLNHKAV

AGFNPGQSSVGDQTQPGHILNDASRRSEAPNRGLSMAQSCVLRLFLHLAMLHGSFLNQQG

ISAMIHPTVYNVKEFLWRHLEKDIEVLGKTLNLNWDNAAITVHLILNTSAQLPTVRVQQG

ATGWASRNAREQWEKQVCETVINPILENLNRHLSNAQKQVEADDKLSNSALMKILKGDPR

SLIPLPSDCPSHHALFWTPPDTLSVEHLSVLIGQKRAQKQVPLLSLFLNKVQYIRHLTCL

PELAALLSDLIRIMPLDSDTNSTPIATLLQNISGGLQRNTLKKKLQTFFRVWNHLRMELA

NNTSVVVDPELCAKDITLDSPGQFLCLSRHGPGSCLHALIKKLSETHNSLVREAEKLSHQ

ENCDYSVPVGELSESQLAVCHPERELLPLVLAHCQYTLVKSQQIASDYNLLAIEKQLYRR

FLAGKPRIQTDTEKYLKRHHQDFSVVLKDVRSKIPQKPLKGSVCSSIRTVLRSFTDVCDA

VYALEIGLRFLGKTGGDPESQLLSYLLDSLKMKQHISSSVAKSLADIRLDQCTATWQLMS

CWKSELRLRRGQDPIPRLSQDYREAVTADVQKELNKFLDTTDVELFTLELHEILLLKTDN

NSEDRYDPQWDIQNTLESHLDGKGAPALPGLDNLSEEICLSNGAEVWRLAVDFKR*

>m.1422 g.1422 ORF g.1422 m.1422 type:complete len:1875 (+) Unigene000162:312-5936(+)

MLMKLAKAVATAAATLVLKAKNVAQKTEDSAQQNRVIAAATQCALSTSQLVACTRVVAPT

ISSPVCQEQLIEAGKLVAKSVEGCVEASQGATNDEQLLKQVGVAATGVTQALNELLQHIK

QYASGGQPIGRHGEATDRILDVTENIFSSMGDAGEMVRQARILAQATSDLVNAIKADAEG

ETDLENSRKLLSAAKLLADATAKMVEAAKGAAANPDSEEQQQKLREAAEGLRMATNAAAQ

NAIKKRLVNKLENAAKQAAAAATQIIAAAQHAASSNKNPAAQQQLVQSCKVVADQIPQLV

QGVRGSQAQPDSPSAQLALIGASQNFLQPGAKMVTAAKATVPTIGDQASAMQLSQCAKNL

ASALAELRTAAQKAQEACGPLEIDNALNAVRDLEKDMQEAKASAAEGKLRPLPGETLEKC

SQDLGSNTKAVSSAVAQLLSEATQGNENYTGMAARDVAQALRSLASAARGVAATTDDPQA

CSAMLDCAGDVLDKSGNLIEETKRAIAKPGDPESQQRLAQVAKAVAQALNRCVNCLPGQR

DVDNAIRTVGEASKKLLSDQFPASGKTFQEAQANLNQAAAGLNQSANELVQASRGTTQDL

AKASGKFGQDFSHFLEAGVDMAGQSQSKEDQTQVVTNLKTISMSSSKLLLAAKALSTDPN

SPNLKNQLAAAARAVTDSINQLITMCTQQAPGQKECDNALRELETVRGMLENPTEAVNDM

GYFDCIDSVMENSKVLGEAMAGISHNAKNSNLPEFGDAVSSASKALCGLTEAAAQGAYLV

GVSDPNSHAGQKGLVDPSQFAKAKQSIQMACQNLVDPACTQSQVLSAATIVAKHTSALCN

ACRLASSKTSNPVAKRQFVQSAKEVANSTANLVKSIKALDGAFNQENRQKCKAATGPLIE

AVDNLTAFASNPEFASVPAQISPEGLAAMEPIVAAAKTMLESSTGLIQTARSLAVNPKDP

PKWSVLAGHSRTVSDSIKKLITNMRDKAPGQRECDEAIEVLNNCIREVDQASLAAISQHL

TPRDDISHEALHEQMAASVQEISHLIDPVAIAARSDASQLGHKVSQMASYFEPLIMAAIG

TASKILNSQQQMSVLDQTKTLAESALQMLYTAKEAGGNPKAAHTQEALEESVQMMKEAVD

DLGGTMAETASAAGAVGGMVDSITQALNKLEDPGVEPEGTFVDYQTTMVKTAKAIAVTVQ

EMVTKSNTNPDELGGLANQLTTEFGDLASEAKCAAITAENAEIGSHIKKQVTELGYGCTG

LVTKAGALQCSPNDSITKKELIDAARKVSEKVSHVLAALQAGNRGTQACITAASAVAGII

ADLDTTIMFATAGTLNRENAETFADHRENILKTAKVLVEDTKLLVSGAGASQEKLAQAAQ

SSVNTITKLADVVKLGAASLGSEDPETQVVLINAVKDVAKALGDLIRTTKAAAGKPHDDP

AMLQLKSSAKVMVTNVTSLLKTVKAVEDEATKGTRALEATIEHIKQELAVFSSPDPPPKT

ATPEEFIRMTKGITQATAKAVAAGNSCRQEDIIATANLSRRAIAEMLHSCKQAAYHPEVN

LEVRTRALRFGKECANGYLGLLEHVLVIIQKPTHDLKQQLATFSKRVAGSVTELIQAAEA

MKGTEWVDPEDPTVIAENELLGAAAAIEAAAKKLEQLKPRAKPKEADESLNFEEQILEAA

KSIAAATSALVKAASAAQRELVAQGKVGAIPANAVDDGQWSQGLISAARMVAAATNNLCE

AANSAVQGHASEEKLISSAKQVAASTAQLLVACKVKADQDSQTMKRLQAAGNAVKKASDN

LVKAAQKAAFDAQDDQAVMVKSRMVGGIAQIIAAQEEMLRKERELDEARRKLAQIRQQQY

KFLPSELREDGHEQ*

>m.1427 g.1427 ORF g.1427 m.1427 type:complete len:1238 (+) Unigene000163:86-3799(+)

MSLTSWFLVSSGGTRHRLPREMIFVGRDDCELMLQSRSVDKQHAVINYEPASDEHKVKDL

GSLNGTFVNDVRIQEQMYITLKIDDKLRFGYDTNLFTVVRGELHVPEEALKHEKFTSQLQ

LGKKPVCTESAKTPKSPTASGAESKVSVPTAEPSTKVVEPCKTEDKMQGDTAALHRGTPL

YGQPSWWGDGDADDENSFKQENKAAGKKQEPHGTDTKDTRRTDKAKENGVSTAGPEPSYF

EIPSKDGQMAEDSIHEVPTKDTESSAAAAAAAAAAKAAQANTSFTIEFDNTSPGKVTIKD

HVSRPRSKKSQPGSKELSSLQAAIMASESKVADWLAHNNPPMVRRESTEEDSKSIKSDVP

VLLKRLKGSKHEDGTQSDSENGLGLRFGSRRHAALEERLKAAGLTHAKVTGAEGSGSSSR

SAFTIEFFDEENPRKRRSYSFSQTAPLLGGEGLCPAPPSHPKGTSLTLATVAPTAARLLQ

KKRSEEKTTTVGDETQKQDDDQSDKGTYTIELENHNPEEEEARRMIDKVFGVENPQNPSR

SVQQTTKDSKKVSSTKAEGLLDESVAVGSPHWVSQWATLAANHTRTDPEGSGAEPTNFVH

QEREADAFESGVSLRSTSSATSSQAERKRRTLPQLPCEEKSRLGKVSSTSGLRSEIGEKQ

DTELQEKQHKGDGECASTLIKKDTTSGIANQVQTATTKPPVPPVGGVEKQSEEFRRRRAE

DRNKGSDSERSGKPLVRQGSFTIDKPSTNVPLELIPRINQRQSGNRERSDSAGSMDTTTL

LKDTEAVMAFLEAKLRDEKKLDPSPPAGYPLGRSFSPELDTDAIKAAGRAEVSQKRRSLS

SIHREKSNMSSVGKSTTNARERLERKVKPKTSDGRLDTRRSTHTSQRTRQPSMDLTDDEQ

TSSLPVSDILSDDQENGRSPFTFPDDLLLSKLDSSKGAKVNKASRTPQATTASIGKHQLP

QPRPTRASLLRRARLGDTSDTDLADADRVSVASEVSTTSSTSRPPSGRKAPSRIDMLAQP

RRTRVGTMSARSDSEAPMSRSTTSRISAETALRLGLRPTNPADSKLNPRLRANSVSKLTD

TKTKTTASISSAPAASSRWRRLNTEYGSTSEDEFGSNRNSPKAGRLRPGTALRTSKLSSS

GMVTTSPGSITIKHRMREQEEYIRDWTAHSEEIARISQDLAKDLAILAREIHDVAGEIDS

VSSSGTAPSTTVSTAATTPGSAIDTKEEVQPASLKKP*

>m.1429 g.1429 ORF g.1429 m.1429 type:complete len:180 (+) Unigene000163:4038-4577(+)

MNGRPVELRPRAPDSLDPQNALRRRTWNRDEVRPAVLESLLLSSVSQLSSKIRQSVDKTA
[truncated: 4,262,988 more chars]
