# Supplementary material for: De novo transcriptome analysis of Bagarius yarrelli (Siluriformes: Sisoridae) and the search for potential SSR markers using RNA-Seq
Source: PLoS One. 2018 Feb 9;13(2):e0190343. doi: 10.1371/journal.pone.0190343 (PMC5806860; doi:10.1371/journal.pone.0190343)
Supplement: S3 File — (DOC) [file pone.0190343.s003.doc]

File S5 The top 20 pathways with the highest sequence numbers.

| Number | Pathway | All genes with pathway annotation(9635) | Pathway ID |
| --- | --- | --- | --- |
| 1  2  3  4 | Metabolic pathways  Pathways in cancer  Focal adhesion  Regulation of actin cytoskeleton | 474  230  188  184 | ko01100  ko05200  ko04510  ko04810 |
| 5 | Endocytosis | 183 | ko04144 |
| 6 | MAPK signaling pathway | 159 | ko04010 |
| 7 | Tight junction | 142 | ko04530 |
| 8 | Chemokine signaling pathway | 141 | ko04062 |
| 9  10  11  12  13  14  15  16  17  18  19  20 | Biosynthesis of secondary metabolites  RNA transport  Influenza A  Ubiquitin mediated proteolysis  Measles  Tuberculosis  Protein processing in endoplasmic reticulum  Neurotrophin signaling pathway  Jak-STAT signaling pathway  Phagosome  T cell receptor signaling pathway  Leukocyte transendothelial migration | 136  120  119  118  116  114  111  107  106  104  100  100 | ko01110  ko03013  ko05164  ko04120  ko05162  ko05152  ko04141  ko04722  ko04630  ko04145  ko04660  ko04670 |
