# Supplementary material for: De novo transcriptome analysis of Bagarius yarrelli (Siluriformes: Sisoridae) and the search for potential SSR markers using RNA-Seq
Source: PLoS One. 2018 Feb 9;13(2):e0190343. doi: 10.1371/journal.pone.0190343 (PMC5806860; doi:10.1371/journal.pone.0190343)
Supplement: S4 File — (DOC) [file pone.0190343.s004.doc]

**File S6 Characteristics of *B.yarrelli* genic-SSR primers screened in this study**

| SSR primer name | Product length | Primer sequence | Annealing temperature | Repeat motif |  |  |
| --- | --- | --- | --- | --- | --- | --- |
| Baya268 180-201 F: CCAGACACCACACCAGATTG 51.8 (TCTG)5  R: GATCCAGAGCAGAACCTCCA  Baya281 217-238 F:CTCCTGGTGGCTGTTTGTTT 51.8 (TCCA)10  R: GTGGATGAATGGATGGATGG  Baya297 123-217 F:AAGTTTACCCACGGCAAATG 53.2 (CTTT)6  R: CGTAAATTGTGAATGCCCCT  Baya332 242-309 F:TTAATACCAGCAGCAACCCC 54.2 (CAG)5  R: GATGAGAGATGGGACCCTGA  Baya381 242-309 F:GGCGTATACCTGTCGGTGTC 54.2 (GAT)6ga  R: GGAAAAAGCACAAAGCAAGGG gaactgt cgtg  gctgtc(ACT)5  Baya378 242-309 F:AAGATCGGCGAGTACCATTG 54.2 (CTC)7  R: GGACCGAAAACATTCTGAGC  Baya401 201-217 F:GTGCAGCGGAAGTGAGTGTA 54.2 (GAG)6  R: CTCTAAATGCTCCCTCGCTG  Baya267 201-217 F:CAAACATTGAGAGGGAACCAG 51.8 (ATAG)5  R: ATCCATTCATCCATCCGTGT  Baya277 147-180 F:CCCCGTTAGGAAGATGAGTG 51.8 (CTGT)5  R: CCACAATTCCAACACTGCAC  Baya218 242-309 F:GCGCAGTGGCTTCAGTAGAT 55.2 (ATGT)6  R: TTCGCCAAGTATGTTCAGACA  Baya254 180-190 F:CAGAAACAGGGTGCAGGTTT 55.2 (AGAC)5  R: TGCTGGAAGTTATGTGGGAA  Baya322 242-309 F:CTGTCCATCCATGTAGTCATCC 52.8 (ATCC)5  R: TCCCTTGTTCCTGTTTTGATG  Baya355 190-201 F:TAGCAGCTATCTTGGGGGTG 52.8 (CAC)6  R: AGAGGCAGTTTCCCATTCCT  Baya269 242-309 F:ACAGGCAGAGAGGCTCTTCA 51.8 (TGAA)6  R: CGTTAGAAACAGACCAGCCA  Baya271 123-160 F:TTGGGTAATTTTCTGTTTTGCAT 51.8 (TTTA)5  R: CGGATGATGCAAGAAATCCT  Baya287 217-238 F:CGTGACCCTGAATAAGGGAA 51.8 (ATGA)6  R: TCCTTCCATCTGGTGTCTCC  Baya389 160-180 F:GCAACTTTCAATTTCAATGCC 51.8 (TAT)6  R: AGAGATGAAATGTGGGGGT  Baya316 201-217 F:ATGGATAAAATCCTGCGACG 51.2 (ATTC)6  R: CTGGGATAGACTCCAGCAGC  Baya315 201-217 F:ACCAAAAGTCTTGTCCAGGC 51.2 (TTCA)7  R: TGAGGGAGTAAGTGGGTTGG  Baya299 123-147 F:TGTCTGTCTTTCTTCCTTTCTTTTC 51.8 (TCTT)5  R: CCGCAAGAAGGATTAGGATG  Baya282 217-242 F:CGAGGGTTGAAATAAGAGCG 51.8 (ACAG)7  R: AGAGACGGCACTCTGGATGT  Baya225 217-238 F:CAGTGCAAACTGACTGGACG 51.8 (TTGA)14  R: GCGAACCTGCTAAACACCAT  Baya295 238-309 F:ATTTCAGCCCACGTCAGAAG 50.8 (AGAA)6  R: AAGCAGTGCTGTTGCTCTCA  Baya25 123-160 F:GGAACCCGAATTAAAAGGGA 55.8 (A)10  R: AAAAGCGCTAACTGGGGAAT  Baya76 180-217 F:AGAACAGCGGCCAAAGTAAA 55.8 (TCA)6  R: ACGTTTTGATGTTGCACGAG  Baya81 238-309 F:TGTGCCAGTGAACAGAGGTC 59.9 (AC)6  R: CCTTTCAGCTGAGCCAGAAC  Baya85 201-217 F:CATTGATCAGAGGCTGGGAG 59.9 (T)10  R: CGGGGAACTATTGGTGTCTG  Baya3 160-217 F:CCACAGCCCTACAGGGTAAA 58.0 (AC)8  R:: TACGGATGCGTGTGTGTTTC  Baya19 201-238 F:CTCCCCTCACACTTGCTCTC 58.0 (T)10  R: GCGTGTGTGTGTATTGGGTC  Baya83 242-309 F:CACAGCAGCGCAAACTTAAA 59.2 (A)10  R: TGTCAGGTTGGGATAGAGGG  Baya94 90-110 F:GTGGGCTCTTATGTGTCCGT 59.2 (GT)6  R: GATCGTTGGTGGGAACTGAT | | | | | | |
