# Supplementary material for: De novo transcriptome analysis of Bagarius yarrelli (Siluriformes: Sisoridae) and the search for potential SSR markers using RNA-Seq
Source: PLoS One. 2018 Feb 9;13(2):e0190343. doi: 10.1371/journal.pone.0190343 (PMC5806860; doi:10.1371/journal.pone.0190343)
Supplement: S5 File — (DOC) [file pone.0190343.s005.doc]

**File S7 Characteristics of 31 pairs of *B.yarrelli* genic-SSR primers screened from 40 individuals of the downstream of Nujiang River in this study**

| Locus | No of alleles | effective number of alleles | Ho | H_E_ | PIC | Index of Shannon（I） | P value HWE |
| --- | --- | --- | --- | --- | --- | --- | --- |
|
| Baya268  Baya281 | 3.0000  2.0000 | 1.0000  4.0000 | 0.0750  0.0510 | 0.4850  0.0980 | 0.3850  0.0920 | 0.7450  0.2030 | 0.0030  0.0000 |
| Baya297 | 6.0000 | 1.6000 | 0.5500 | 0.7620 | 0.7120 | 1.5190 | 0.0000 |
| Baya332 | 2.0000 | 1.0000 | 0.1750 | 0.3920 | 0.3120 | 0.5750 | 0.3250 |
| Baya381 | 1.0000 | 1.7000 | 0.0000 | 0.0000 | 0.0000 | 0.0000 |  |
| Baya378 | 1.0000 | 2.3000 | 0.0000 | 0.0000 | 0.0000 | 0.0000 |  |
| Baya401 | 2.0000 | 2.6000 | 0.0000 | 0.4440 | 0.3420 | 0.6300 | 0.6180 |
| Baya267 | 4.0000 | 1.0000 | 0.5520 | 0.5860 | 0.5110 | 1.0320 | 0.0000 |
| Baya277 | 3.0000 | 1.6000 | 0.3230 | 0.6320 | 0.5490 | 1.0230 | 0.0000 |
| Baya218 | 2.0000 | 1.1000 | 0.0000 | 0.0670 | 0.0640 | 0.1500 | 0.0000 |
| Baya254 | 2.0000 | 1.0000 | 0.0000 | 0.4280 | 0.3040 | 0.5620 | 0.0000 |
| Baya322 | 2.0000 | 1.9000 | 0.0000 | 0.0490 | 0.0470 | 0.1160 | 0.0070 |
| Baya355 | 1.0000 | 1.8000 | 0.0000 | 0.0000 | 0.0000 | 0.0000 |  |
| Baya269 | 2.0000 | 1.7000 | 0.2360 | 0.5060 | 0.3740 | 0.6920 | 0.0010 |
| Baya271 | 2.0000 | 2.6000 | 0.0780 | 0.4780 | 0.3600 | 0.6640 | 0.0000 |
| Baya287 | 2.0000 | 1.0000 | 0.0250 | 0.4350 | 0.3240 | 0.6210 | 0.0050 |
| Baya389 | 3.0000 | 1.9000 | 0.1280 | 0.6330 | 0.5540 | 1.0390 | 0.0000 |
| Baya316 | 1.0000 | 1.8000 | 0.0000 | 0.0000 | 0.0000 | 0.0000 |  |
| Baya315 | 3.0000 | 1.4000 | 0.4250 | 0.4900 | 0.3780 | 0.7220 | 0.0000 |
| Baya299 | 2.0000 | 1.8000 | 0.0000 | 0.5330 | 0.2210 | 0.6360 | 0.0000 |
| Baya282 | 2.0000 | 2.2000 | 0.3500 | 0.3240 | 0.2680 | 0.5000 | 0.1000 |
| Baya225 | 3.0000 | 1.0000 | 0.0760 | 0.4580 | 0.3610 | 0.6900 | 0.6900 |
| Baya295 | 3.0000 | 2.9000 | 0.1500 | 0.5700 | 0.4910 | 0.9390 | 0.0200 |
| Baya25 | 1.0000 | 1.0000 | 0.0000 | 0.0000 | 0.0000 | 0.0000 |  |
| Baya76 | 2.0000 | 1.9000 | 0.9410 | 0.5050 | 0.3740 | 0.6910 | 0.6020 |
| Baya81 | 4.0000 | 1.0000 | 0.8960 | 0.6690 | 0.5910 | 0.1160 | 0.0000 |
| Baya85 | 1.0000 | 1.7000 | 0.0000 | 0.0000 | 0.0000 | 0.0000 |  |
| Baya3 | 7.0000 | 1.0000 | 0.2890 | 0.5020 | 0.6580 | 1.1750 | 0.0000 |
| Baya19 | 1.0000 | 1.0000 | 0.0000 | 0.0000 | 0.0000 | 0.0000 | 0.0000 |
| Baya83 | 2.0000 | 1.0000 | 0.0000 | 0.4310 | 0.3350 | 1.1260 | 0.0000 |
| Bya94 | 1.0000 | 1.0000 | 0.0000 | 0.0000 | 0.0000 | 0.0000 | 0.0000 |
| (Mean) | 2.4643 |  | 0.1900 | 0.3588 | 0.2954 | 0.5371 |  |
