# Supplementary material for: De novo transcriptome analysis of Bagarius yarrelli (Siluriformes: Sisoridae) and the search for potential SSR markers using RNA-Seq
Source: PLoS One. 2018 Feb 9;13(2):e0190343. doi: 10.1371/journal.pone.0190343 (PMC5806860; doi:10.1371/journal.pone.0190343)
Supplement: S6 File — (DOC) [file pone.0190343.s006.doc]

File S8


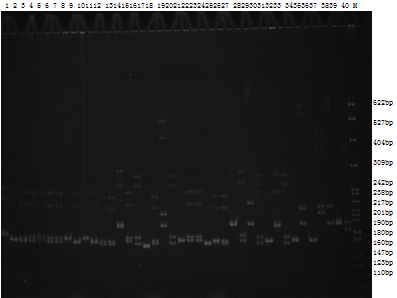


PAGE histogram of the Microsatellite Baya297 locus identified by EB staining of 40 *B.yarrelli*. individuals
