# Supplementary material for: De novo transcriptome analysis of Bagarius yarrelli (Siluriformes: Sisoridae) and the search for potential SSR markers using RNA-Seq
Source: PLoS One. 2018 Feb 9;13(2):e0190343. doi: 10.1371/journal.pone.0190343 (PMC5806860; doi:10.1371/journal.pone.0190343)
Supplement: S7 File — (DOC) [file pone.0190343.s007.doc]

File S9 Quality of the *Bagarius yarrelli* RNA results

| Sample | Concentration  (ug/ul) | Volume  (ul) | Total content  (ug) | OD_260/280_ | OD_260/230_ | Conclusion |
| --- | --- | --- | --- | --- | --- | --- |
| *Bagarius yarrelli* | 0.425 | 50 | 22.5 | 2.08 | 1.29 | Qualified |
