# Supplementary figures and images for: De novo transcriptome analysis of Bagarius yarrelli (Siluriformes: Sisoridae) and the search for potential SSR markers using RNA-Seq
Source: PLoS One. 2018 Feb 9;13(2):e0190343. doi: 10.1371/journal.pone.0190343 (PMC5806860; doi:10.1371/journal.pone.0190343)

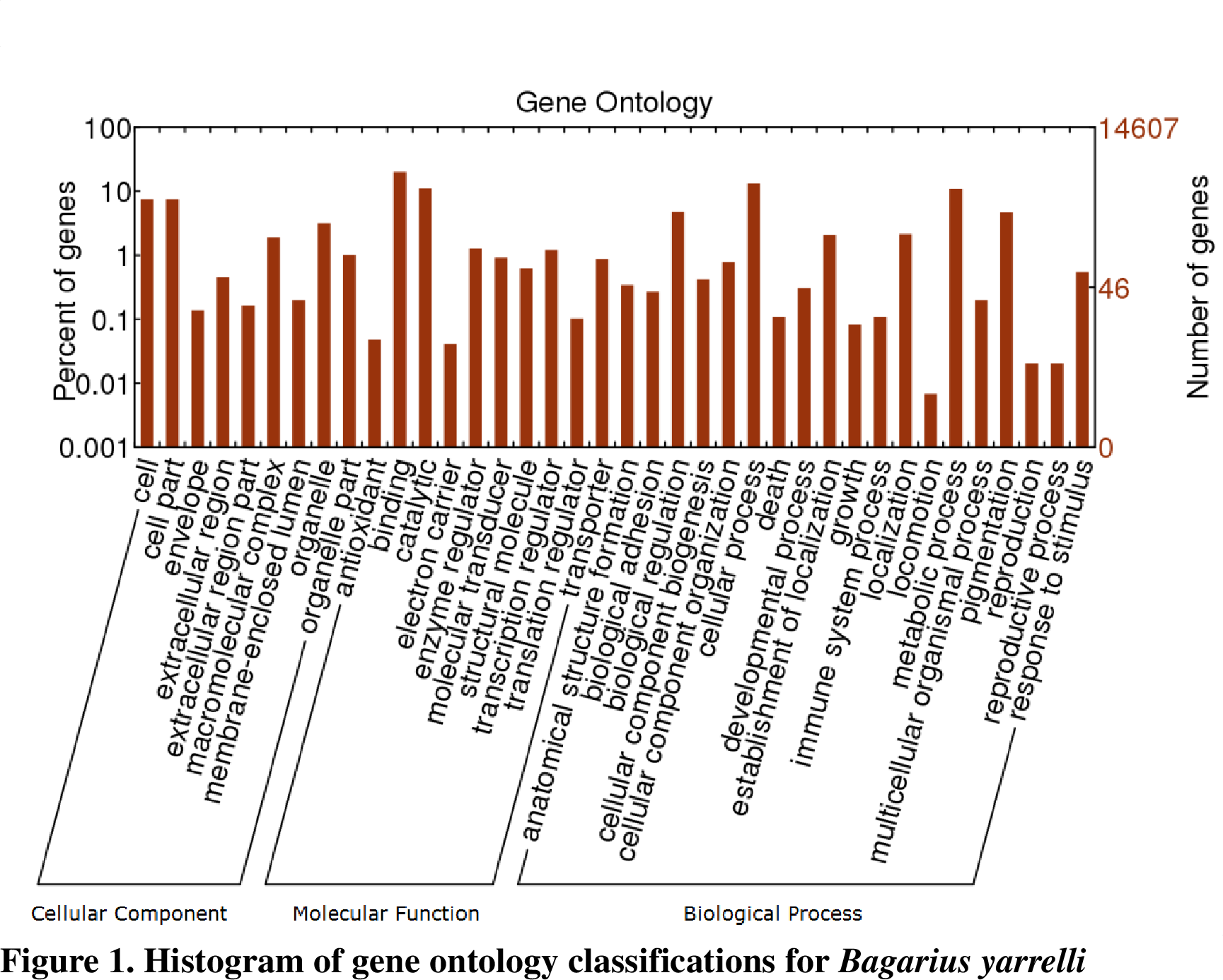

Supplement: S1 Fig — (TIF) [file pone.0190343.s008.tif]

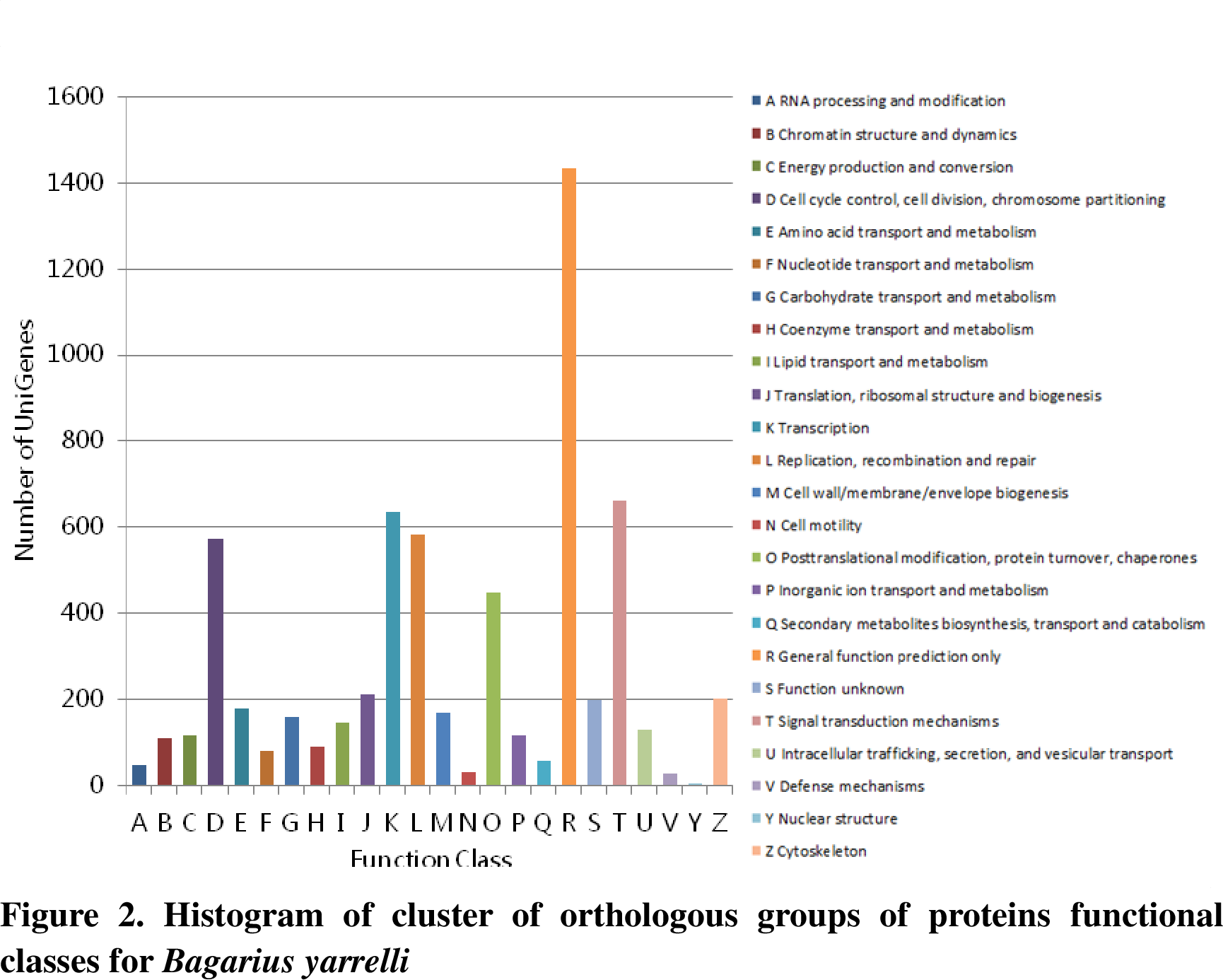

Supplement: S2 Fig — (TIF) [file pone.0190343.s009.tif]
