# Supplementary material for: De novo transcriptome analysis of Bagarius yarrelli (Siluriformes: Sisoridae) and the search for potential SSR markers using RNA-Seq
Source: PLoS One. 2018 Feb 9;13(2):e0190343. doi: 10.1371/journal.pone.0190343 (PMC5806860; doi:10.1371/journal.pone.0190343)
Supplement: S1 File — (DOC) [file pone.0190343.s001.doc]

>Unigene000001

AAAAACCACCAAGTCAGAAAGATGCATCTAACAAGACACAAAGCCAGAAAAAAACATCTA

ACAATACACAAAGCCAGAAAGATGAATCTGCTGAAACACAGCCCATTTTGGATCAGAATG

CCAATGTTGATCAATCTGCAAAAGCCTCAGCTGGACCTGGCACACAGAAAAACCAGGAAG

TATACAAATCTAGAATGGTGTTTGGACCACAGAGCAGAACTGAGGTGGAAAGTCTAAGCT

CCAGTGTTGATCCACAGCCACTGGAAACGTGTCAGCCTGTGCAGTCTGCACGTGAGAAAG

GAGATGCTTTAAGTAGCCAGAAAAGTCCAGAGAATAGAAGAAAACAAAAAAGAGAGGAGA

GAGATACCAAAAAAGATTCTACTCCAGAGAGAATTCCCACTTCAACTCAGCAGGTTTCCG

CCAGTGAGAGACTCACCATTTACTTTCATGCAGTCTTGTCAAAGGATTTTAAGTTCGACC

CTGATGAAGACCGCGTATTTATTAGGGCTGGTCCTCAAATTTGTGACTGGGAAACTAACC

TAGTGGAGCTGAGTGTGACACGGGATCTTAATGAACATGGACTTCTAGTGGAAGGAAGTC

TGGTGTCTACCAAAGACAAAGCGGAAGCTGTCTCAATACCCTACAAGTACGTGGTGTACA

AGAAAAAAAAGAACAAATATGAGTATGAGTATATCTACAAATTGGATACAAATAAAACCA

CAAACAGATGTCTATTTGTTAAAAGACTTCTACTTAATGCAGAAGGAGAATGGCATCAGT

ATGATGACATCATCTGCCAAGAACCATCTAAAAACGTGTTTAAACGTTTGAAAGATAACT

TTTGGCCTGAACAAAGGAAAAGTGTAATTGAAGGAAGGGAGATTGCAGGGCGAGTTATGC

TTGAGACAATCTTTGATTTTCTCAGAAGCTGGACTGACATCAATCTCAGAAACTTTATCT

TCCAGCTTAAGCAGTTCCATCACATCTATGGAAACCCCTTTGTGTTTGAGGAGAAGGAAG

AAAAGTGGTTAAATTTGAATTACACTGAGAAAGATGTTAGTAAACTTCTAAAGAACTTTA

TCATGGATCACATCACACCAGAACTGCACAAAGATGGCTTATCCAAAAAAAGTGAATATA

TTAATGTACCCCTGAAGGCTGCCATCATTACATTGTACTTGTGGAAGCAATATGAACTTC

GCCTGGAATTTGGGGAACTTAATCGTCTTTGCTCTGCCCTTTGCCTGCCGAATTTGCCTA

AAAATGAGTTTATACCCTTTTGGACCAACATTCGGAAGTCATTTTTTTGCTTAAAAAACT

TGCCTGACACCCTGGTGGATCTAATTAACACAGTGAAAGGACAAGGAATGACTCGATGGA

TTGTTGCTATTCCACTTCTGCATCTGCTTAAAGGAATTTCAAATCCATTTGAACCCATTT

CACTTACAGCAAACACTAAAAATGAACAGGCATGGGTTGGATTACAAGGACTAAAATCGT

GCTCTACAAAGCCGGTAACTTCAGAAGGCAGAAGATCCATGCTGAATCTCATGAAAACGA

ATCATTATCTCGTGGAAGTGGACACCCTGGTGGTTCGATCATGGATGTTCCTGTTGTCTG

TTGATGAGCTGGTGGAATACTGCACTAACATTCATGTGGAGCTCTTTGATGTGTTGCAGC

TTTTCTATCTCAAAGCTCCAGCTGATATTTCCTACAGTGTTCCCAAAAATGTGTCAGATA

TGCTGACATTCATCCAAAGCCAGCTTCTTGAACAAAAATACAGGTGCTTCTCTGATGAGT

ATAGGAAAAGTTGCTTAGCTGCATCTGTGAGGCTGCTGGAGAAGATATGCAAAGGAGCGA

GGTCTTCCTACAGCAATACAGACATTCCAGTTGCTTGCTTGAACCTTGTGGCTTCAGTTT

CATACTTTTCTCAGTCGTTCCCCAAAATGGAGTTTCAAGAGGAAGACACGAGTCAAAAAC

CCATAGAAGAAAGCTCCAAGATAAATGAAATTTTGTCAGAAGCGTTGAAAATTGTCAGAA

CGTGGGTAGAACAAACCTTTAAAGAGAGACTGGTGTATGCCTACTTTTTGAGTGCCACCC

TCACCTCAAAGATTGAGATGTGGAATAACATTATCTCTGTGAGCTTTGAAAGTGAGGAAT

TTACAAAGGAGTGGAGAGACACATTGTCTAGAGATTTTGAGGGAAAATTTCAGCAGGAGT

CCTCGATAGACCAAATTGAAGTGTACTGCAGTAGAATTGAAGAGCTTAATGATACCCATC

CTTATGTGGCAAGAAGTATTGAGAAGTGTGCCCTACAAGCAGTTACCTCAATGTGCCAGA

TGAAGACCGAAGCAAAACTTTTTGAGAAATGCAAAATAAACTGGAAGTTCGGAAAGCTCA

TTTCTGCAATTGTTCAGAAATCTTGGCCAAAAACTGAGAGTGGGTCATACCAAGAAAGGG

AGGAATTGGTTCTCAAGCACCTTCTCAACTGGACAGCAGCCAAGAATGTTTTCCAACTTT

ATGGTGCTGATGAAAAACTGATTGATCAGCTGTCTGAAGAGGCTAAAGAAAGACTTACTA

TGGCCACATCATTATTAACAAATATTTCCAAACAGTTCATCAGAGGAAGTATAACGATTA

AAGTTCTTAATCACATCTTGGAGAAGAAGGATACTTTCATTGAACTGGTAAAAATAGACT

GTTTCTGTGAAAATGAGAAATGCAAAGATACCAATACTATGAAGAGACTACTCCAGTTGA

GACAGGATGAGGTGAATGCCGTCTATAATGACAGTGAGCTTGTCAAAGGGCTCATTGCAA

TGTGCCGGAATATTCAGGAGTATGTAACAGTTGATCTTGATAATCTCGAAGAAAAACAAC

AGGTTGATATCGAAGAGATGGAACTGGATAAGTTCATGGAGGTTCATTTATTTGACCAGA

TCTCATCCCCAACCGCTGGCTATGTCACATACTTTTCTCTTGAGGACACTGTTTGGCACA

TGGCTGAAATTTTCCACACATTTAAAGAGAGCTATGTTTTTAAGATCTGCTGGAAGAGTC

AAGCTAAAGACTTCGCTGAAAGTGCTAGTAGCGAATGTGAGTCAGACGATGAGGAAGACT

TGCGGGCTACAATTGATTTGATTTATGATGAGATCTTTCAACCATGCTACAGTAAATACA

AGCAGATATATGAGAGCTTGAAAGATGGCAGCATGGCCTTTGAAGAAGTTGATATAACAT

TTAAAGCCTACATTGGAAAATATGAGGAACTAGCGGAAGAAGCGACAGTTATGTCAAAAC

TGGACCCGACAGACGACAAGCGGTGGATTCAGAGAAGGATTCAACAGATAGAACAGTATC

ACGAACTTCACTTAGCAGTGGAGTCAGCTCAAGCGGTCATGATGGTGAAGCAGACACTTG

GTCTTCAAGGAGACTTCCAAGTTTTGGAAAAGCTACTTATAGTTACAGAGTTGGACTTCA

AGAAGGAGCCTCTTAATAGTATTGACAATGAGCTAATGCAGGCCAAAACTGTACTTGTGG

ACATCACAGAGCCACGAAGGCTTTGTCTCCAGGAGCTGGGCTTAAGAAAGAACTTTGTTA

TGTGGGTCAAAGAAGCTCTTGAAGATATCAATGAGCTAAAAGTATTTGTGGACCTAGCTT

CGATCTCTGCGGGTGAAAATGATCTGGATGTAGACAGGGTGGCCTGTTTTCATGATGCAG

TTCTTGGCTACTCCCCAATATTTTATGAACTAAAGACAGATGCAAGCTTTAGAGTCTTCA

AGGAGGTATTGAAGAAGCTCTGGAAAGCACTCGATAATGATCCCAATCTTCCAAAGAAAC

TGCGTGACACTGCAAGACATCTTGAATGGTTGAAGACAGTTAAGGATAGCCATGGATCTG

TAGAACTTTCATCTCTGTCATTAGCTTCTGCTATCAATAAAAAAGGAATCTACTTTATCA

GTGCAGAGAACCAAAAGAAGTTGAGTCTGGATAAAGTCTTGAAGCTCCGGATTACAGAGG

AGCATGCCGAAGACAATGAGGTACGATGCTACTCCCTTGATGACCTTAAGGAACTTCAAA

ATAAGCTTATGCTTATGTCTGGAAAAGGGGACCAAGGCCAAAATGAAGTTGATCGATTTG

CAGAGGTTTTTGCCAGCGTACAGAGATTAGGTACTGCATTTATGGACCTATTTGCAGCAG

GAAATCCATTGTTCCGACTTTGGGAGGCAATTATTCACTGTAATAGTCAAGAGGCATGCA

TAGTTATGGACTTTAACTTGGATGGTGTCACCAGTGTTGTCATGTTAAGGGGTGATGTGG

CAGAGCAACTCCTAGATGTCTGTAAGAAAATGGAGCGATGCCTTAGTTTTTGGAAAGATT

TTATGGACAAGCAACGATCTCAGAATTACTATCTCAACTATTTTACAGCAGAACAGATTG

TCTATCTGTGCAGTCAGTTGAACCAGACAAACTTGTCACATCTGGAAGATCAAGCACTTA

TTATGCTCTCTTTTATCAAACTAAACTGTACTACCTACAACTTGAGGCAAAGCTGGCATG

TTCTTCAGTATGAGATGCTAACAAATCCTACAAAGCAAAATGAAGACATTGACTTTCAAA

CTTTTGCTGATGTTCCAAATCAAATTGAAAATGAGATAGATATTAACAGTGTCCCTTTTT

CTGATTCTGGTATAGAAAATGATACTAGAAGGTTTGACATGATTTGGAATATGTACATGA

GAAATATGAAAACATTTCTTCCAAAATCACTTGATGTCAGAAGTCTAGGAAGACTTTTGG

AGATTTTGTCTGATAGCCCCAGTAAAACTGATGTAGATGATGATGATGATGATGATAATG

CGGAAAAAAGTACTCTTGAAAATCTTCACAAAAATGTACAAAGAGTGCTTCCTAGGGGAC

TGGTTATTGGAAAACCCAATCTTATTGTCTGCCCATTCACAGAGATTCAAACTGCTTGCA

TCTCCATTTACATGAGTAGTGAAGATGAGCCACTGCCCACATATGATGAAGTGCTTCTCT

GTGATTCTTCCACCTCATATGAACAAGCAGAGTTGTTCCTTAGGCGCTGTCTTTCTGCTG

GTTACAAAGGACAGAAGATATACACCATGCTATATGTGGACCAGCTAACCTACGAAGTGA

GCTACAGAGTAGAACAGTTTTTCCAACGCCACAAAGCTCAGAGCAGAAATGACTACAGAC

TGGTTTTGATCTGTAGTACTGATCGAGAACATGCCTACCTTCCCTCAGTCTTCAGTCAGT

TTAGGCTACACATGGTACCCCAAGAACCTCTTGGGAGAATTCAAGAATATCTTACCAAGC

ACTACACAGTCCCCAAAGGCTATTTCAGCGCTGCTGCTGTTTTTAAAACAAAACAATATG

TAGGAGTTGTATCCTCAAGAAGAGCAGGTGTTGGCAAATCACTTTATATACGTCGGCTGT

ATGAAAAAGTAAAGTTGATTACCACGAAATCCTCACAGCTTAAGTGTATCCGGTTGACAA

AGTCAAAAGTGGATGAAAATGCTGTTCTTCAATCAATGCTTCATGGCCCCCAGAGAGAGC

TAACCATTTTTCATTTTGATATTACATCTTCGGTACAGAAAGGTCTCCATGAATTTCTCT

TTCGGCTTCTGGTCTTGGGTTACCTAATGGACTCAGAAGGAAGAATGTGGAAATGCAACA

GTAAACACCTATACATCATTGAACTCCTTCAGTCTAGTGCAAATATTTCTAGAAATCTTC

CAAGATCTAGTGAAAGTGTTCAGTCAGCCTTTCAGGATGTGTTTCCAAGTGTGTACTGTC

GAACTCCAAAAGAGGTCCTTGATCTAGAAATGAGAATGTCAGAAGACCCAACCATAATTG

TGAATGACCCCCTTATGGATGATAAAGAATTCAGAAATGAGGCTTATCAGCGTCCATACC

AGTATCTAAAGCGATTCCATCATGGAGTTAACTTGGATGGATTTATGTACAAAGGTATTG

AGGGAACCCATGTGGAATGCCTACAGTTGCTGTTTCTGTATTGTGGTGTTGTGGACCCTT

CCTGGGCAGAGTTGAGAAATTTTACATGGTTCTTGAATCTGCAGTTACGAGACTGTGAGA

CATCTGTATTCTGTGATGCATCATTTACAGGAGACACTTTGCTTGGATTTAAAAACTTTG

TGGTTGATTTTATGATACTCATGGCTAAGGACTTTGCTACACCCTCTTTGAGTATATCTG

ATCAGAGCCCAGGAAGGCAACAAATGGACATTTCCGGTGTTAGTGAAGAAGACCTTGCTC

CATTTAGGGTCAGAAAAAGATGGGAATCAGAGCCTCATCCTTACATTTTCTTTAATGATG

ACCATGTGTCCATGACATTCATTGGCTTTCATCTACGGCCAAACGATCAGAATTTGGTAG

ACGCAATTGATCCATCAACCAATAGAGTGATCAAAAAGAACATAATGACTAGTGAGCTGT

ATGAGGGTCTCAAGTTGCAGAGAGTTCCTTTCAACATTAATTTTGATAGTCTCCCAAGAG

GTGAGAAAATTGAGCGAATTTGCAATGTTCTGGGAATACAGTGGCCCCTTGACCCAGATG

AAACTTATGAGCTAACAACTGACAACATACTAAAAATCTTGGCTATACACATGCGCTTCA

GATGTGGCATTCCAGTTATCATCATGGGTGAGACTGGGTGTGGGAAGACCAGGTTAATAA

AATTTTTGTGTGAGCTGCGACGTAGTGGAGTTGCCACAGAGAATATGAAACTTGTCAAAG

TCCATGGAGGAACAAGCGATGACATGATTTATACCAAAGTGAGAGAGGCCGAGTCCATAG

CATGCAGCAATAAACAAGATTATGGTTTTGACTCTGTACTCTTTTTTGACGAGGCAAACA

CAACAGAGGCCATCAGCAGCATTAAAGAAGTACTATGTGATAAAACAGTAAAAGGTGAGG

TAATGGTGCCAAACACTGGCTTGCAAATTATTGCTGCATGTAATCCATACAGGAAACACT

CAGATATAATGATTAAGAGATTGGAGTCAGCTGGTTTGGGATACAGAGTGCGAGCTGAAC

AAACGGATGAAAAGCTTGGATCCATTCCCCTTCGGCAACTTGTGTACAGGGTGCAGGCCT

TGCCTCCGAGCATGATTCCATTAGTTTGGGACTTTGGACAACTTAATGACCAAACAGAGA

AAATGTACATTCAGCAAATAGTTCAGAGGGTGGCTGAAGACCACTTTTTCAGTGTGGAAT

ACATCAAGATTTTTACAGATGTCTTATCAGCTTCACAAAAATACATGAGGACAAGAAAAG

ATGAATGTAGTTTTGTAAGCTTGAGGGATGTTGAACGTTGCATGCAAGTTTTTACATGGT

TCTACCAAAATAATGAAATGTTGCTGTCAGAACTTGCCAGGTACGAAATAAATCAGAAAG

CACAAAAAAAATGAACACAATCAAAGAGACAATGATGTAAGAGACCCTATTCTGTGGTCA

TTAGTGATGGCTATTGGTGTGTGTTACCATGCCTGCTTGGAGGACAAAGAGAAGTACAGA

AACAAAATATGTGGCTACTTCTCAAAAACATACAGCCCCGTGAATTTAATGCAAGAAATT

TCAGTAATGCAAGATCTTCTTCTCAGTGGAGTTCCACTAGGTGAAACAATTGCAAGGAAT

AATGCTTTAAAGGAGAATGTTTTTCATGATGGTAATTTGCATCGAGTTACGAATTCCTCT

TTTCCTGGTTGGAAAGCCTGGAAGCTCCAAATCATTGTCCAAAACCCTGGTAGCAGATGC

TATGCAAGGTCAGGCAGCTCATTCAGACTTGTTCAGAAAACTAAAACAGATTCATCTGGT

GTCATTCCAGTGCAGTCCTCATTCCACACCAGAAGGTATCATCAATACATTCAAGCAATG

TGCACGTTTTCAAGAGGGCAAAAATCTAAGCGAGTACATCTCGGTGGTGGTACTGGATGA

AATTGGTCTAGCTGAGGATTCTCCTAAAATGCCTCTTAAAACTCTTCACCCTCTTCTTGA

GGAAGGGTGCATAGAAGATCAACCCCTGCCTCACAAGAGGGTTGGATTCATTGGTATATC

AAATTGGGCTTTAGACCCAGCGAAGATGAACAGAGGAATATTTGTTTCTCGAGGTGATCC

TGATGAGAAGGAGTTGATTGAGAGTGCCAAGGGCATTTGCTCCTCTGATGCAATGATCCT

TGAGAAGGTTGGGGATTTTTTTCAGCCTTTTGCCAAAGCTTACCTCAAAATCTGCAGCGA

AGGAGGAAAAGGGTTTTTTGGCTTGCGTGACTATTACAGCTTGATTAAGATGTTGTTTTC

TATTGCCAAAGCATCAGATCAAAAGCCAAGTGCAGATCAGGTTGTGGAGGCTGTGTTAAG

AAACTTTAGTGGGAAAGACACTGTGAATGCAGTTACTGTTTTTACCAACAGGCTTAGGTT

AAAACCTAACCTTGAAAGCATCGATACCATTGAGCTTGTGAGACAAAATATCACAGCAGT

TGCTCAAGATGAGGAGTGCCGCTACCTGCTGATTCTAACAAAAAATTATGCGGCCCTACA

GATACTTCAGCAAACATTCTTTTCAGAACAGTACCAACCTGAAATCATCTTTGGCTCAAG

CTTCCCTAAAGACCAACAGTACACCCAGATTTGCCGTAATATTAATCGTGTTAAGATTTG

CATGGAAACTGGTCAAGTCATTGTGCTTCTGAATTTGCAGAACTTGTATGAAAGCTTATA

TGATGCACTCAATCAGTACTATGTTTGCCTTGGAGGTCAGAAGTATGTGGATTTGGGCCT

CGGAACACACCGTGTCAAGTGTAGAGTACACAAGGACTTCCGGCTCATAGTGATTGAAGA

GAAGGACATTGTCTACAAACAGTTTCCCATTCCTCTAATTAACAGGTTGGAGAAACACTA

TCTGGACATTAACACTGTGCTGAAGAATGAACAAAAGCAGCTTGTGAAAATTCTTGAAGA

ATGGGTTGACCATTTTATTAAATGTAAGCATGCGCCTACTCCCCAGGCAGCAAAACACTG

CCCAACCGATGTTTTCATTGGATATCACTCTGATACCTGTGCTTCAGTCATCATGCAAGT

TATAGAAAGACAGAAAGGAAGGATGGAATTTTCAGACCCCGAGCGACAGCTGTTGGACGA

GGCCAAACTAATTCTACTGAACTGTGCTACACCAGATGCTGTGGTTCGCTTGGATAGTTC

AGCCCTTTCTAAAGTGGAGAGTGAGCACCTTTCAAGAGTGTACTTTGAGGACCAAAAGCA

CAGCTGTATGGCTGACTTTGTCTTTGGTCACATTCAGCAGGTGGAGTGGAACCATTCCTG

TTTTACTGAGGTACTCCTCCATTGGTGAAATCAACAAATTCCAAAAGGAGCATGACAGTA

GGGTGTTTGTTTACTTTATTACCAAACTACCAAGAGTGGAAGGTGGAACTTCTTATGTTG

GATTTCATGGAGGTCCCTGGAAGTCAGTCCACATTGATGACCTTAGAAAATCAAAAGATA

TTATCTCTGATATTAAAGCCTTGCAGTGTCTGACCATCAGTCAGCTGTTTAAGGAAACGG

AAGATCAGTCTGAGGTTATGGAAACAGAGGATATGTATAGAGAAGAAGAAGAAACTGAAA

AAATGGAGCAGGATGACAATACAAGCTGGAAAAATGTTCTAGATACCACAGCCCTGGTGC

GTAGCTGTGTTCAGAGTGCAGTCGGCATGCTGCGAGATCAGGGGGATGTGGGTTGTCGCA

GCACCAAGAGAGTAGAAATTCTCTTAACTCTCTTGTCTGATAGTGAAGAACTTCAAGCAA

CATTTTTAAAAACTATGAAGCATCGTCTTTATTCTCTCTTGATAACCCATGATGAGCACA

CACACTCAGCAAAGAATTGGGTTTTCAAAGAAGCATCCAATATTGACTCTTTACAAGAGG

GTGGAACATTCAGAAACACCTTATGGAAGCGGGTTCAAGCTGCTATTATTCCTTTCCTGG

CACAGCTGGTCTCGGTTATTGATCGCGAGCACAATCTCGACCTCTTGCTAGATGAAAACT

CTGGTGAATCAGTAAAAAAATTGTGGCTGGACATCTTTGGAAATGTCAAGTTATTAAATG

TTCCATATACAAGAGTGGATTCCACCTCAATGACAAAGACAATTCTTGTCCAGAACTGTA

TTGTGGTTTATAAGAATACAGGCTGCTCTATGCCGTTCAGCTGGATAATCAAAGATCATT

TAGAGGAACTCTGGTTACATGCATTGCACAGAGAAGGCCACAGTCTGAGACAGTTTGAGG

AATTCTTTTGGAAGACTCCACTGGGTAGATATATTGCCAAAGCTGAGCAGGAAATGCAGC

AGGAGTTTTTCTACAGATATCTTCAGGATTTTATCTCTTTGACAACAACAATAACAACCG

AGGTTGAACTAAAGCTCCTAGTGAAGGCTTTGTCCTGCTGCGTTAATGAATTGAGAAGCC

GTCAGGAGGCAGTGCAAAATGAAGTGACCTCTTTACCCTGGATCCATGCAGCCTACCATG

AGTTTAAAAACCGCCTGCAAAATTTCTCCAGAATGATAATCATTGAACCCCAGATTGCTC

AAGTGCTACTGGACAACACACAGTCCAGAGACGGCAAAGAAATGGTGCTGGATGTGTATG

CTGCCCTGGCCTGTGTTGAGTATCTGGAGCCCCAAGGCCTGGATGCTTGTATAAATTGTC

TGACTTGGCTTCGACAGGTGAAGAAGTTACAAATCCCGATTGAGTTAGTCTGCTCAGAGG

CAAGCATTCGACACTATGGAGCAATGAGCAAAGCCATTATTCCCCGTGTCCAACATGGGT

GGAGACGTATCCATGCCATTTCGCTATTTGTTGAGCACATACTTCTGGATATAGAGAACA

TAGAGAAAAAACTCAAGCCATTGGTCCTTGAGCACACACGAAAGTTGGCTCAAGTCTTGG

ACAAGAACTCTGACCTTAAAATGAAACAACCATTTGAGGCAGTTATTGCCATTCTCAAAA

CTTGCAAGGATGGAGCAAATGAGCAGATTTTTAGGTTTGGTTTCCAGCCATGTCCAGTGT

GTATGGGGGATCCTCATGATCCTCTGAGTCTTCCTTGTGATCATGTCTATTGTCTGGCCT

GCATCAAGCAGTGGCTGATTCCGGGACAGATGTACTGTCCACTCTGCATCCAAGGAGTAG

ATGATGACTTTGATCTACAGCCATCTGACACCATTCGGACCCTGATCAAACAAAATAGCC

ACTTCAGAAAGCAGTGCAATGCCTTCTTTATAGACTTTGTCTCCACCATTTGCTTCAGAG

ACAACTCACCACCCAGTAAAACAATCATCCTCCACCTGCTCTCCTTCCTCATGGTGGAGG

CAGGGTCTGTACCTACTATCAGAGGCTGTGAGAGACGTTTTTTGACCAAGGCTTTGTCTC

CTTTTGATGACTCTGTGGACAAAAATCCTGTTGTACGGTCTGTTGTGCTCAAACTGCTGC

TCAAGTACAGCTTTGCTGATGTAAAAGGCTACCTTCAACAACACCTCACAGAAGTGGAGC

AAAGTAACATACTAGAGAAAGCTGACAAAACTGAGCTTTATTGTCTGTACATAAACTGCC

TTGAGGACTCTATGTTTGAGCGGATCCAGTGGCATTCTGTGGCTGAACAGCAGGCCTGTC

TGCTGGAGGAGAAGCATTTTCTGGTGGAATACTTGCGTTCCGGAGCCGTGGCTACTGAAA

CTGTGACAATAGAACACCTACAGCAGGTGGCAAGAACTCGTCTATGCCTAGACATGGCAG

CAGATCTACTAATCAGCAGAAGCTCAACTGCAGATGCTGAAAAGGAAAGCTCAGCTGGGG

TGACTGCATTCTTGGATTCAGTGCAGAATCTTTGCAAGTACAGTAAGAATGACTGGTACA

GGGTCTACCTCATTAGAAAACTATGTAGTCTCAGTGGAGTAGAATACATCCAAAAGCTCC

TACAGGAAGATCAGCTAAGCTGGCTGTTTCCGCAAGAGGTTCTCCAGATGAGAAAAACCA

GTAGTCAGATTGACCAGTATTTGGTGTGTGGTGTGGAGTACAAGGCTATTCGAGATGCTG

TTGCCAAGGCCATGTTAGAAGGGAAAATGGAAACAATCGAAGAAGCATGCAAGATTTGCC

AGTGTTCACCTGAGAAGAAGAATGTGTACATTTTATTAGCTCTATTTAGAGAGGTCACTA

CTTTGTATAAAGCTAACAATCCTAACATCCACCCTACACCTGAGCAATTGGCAGCACTTG

ATGACTTCACCAAAAACTCTAAAGTCTTTGTCGTGCCAGAATCTAGGGACTTTGCTCAGG

CTCTAGTGCATAACCAACTGCCTGTCCTGCATTTACATCCCAGTTTATTGGCTAATGAGT

TTGTGATTCTGGAGCTGACTGTTCACCTGGCAGCAGTGCTTCTCTGTACAAACCAACCCT

TGTTGCAGCCTCTCATACAGCTTGCATTCTTTCCCATCAATATGCAGAAAGCCTTTTTAC

CCACTATGCCAGATGACATGCTTGCTGTTGCTCAGCAGGTCATGGGGCAGCTGCAGTGGT

ATTTCTGTCCAAATGGTCACCCATGCACTGTTGGTGAGTGTGGCCGGCCAATGGAGAAGA

GTCACTGTGTAGACTGCAACGCAGAGATAGGCGGTGTTGCGCACAATCCTGTTCAAGGCT

TCCAGCCAATTGCAGTACAAGGTGACCGCACACAAACAGGGCACCTCCTGGGGGATCCAC

ATCGCAGAGATCAGCCAGATATGCAGGACACGAAGAACATGTCTCCTGGTCCATTTACCC

TGCTCAGGTTGCTGACACATATGGCCATGCTGTTAGGATCCTCCAGGCAGCCCCAGGAAG

TGGCTGCAATCATTAAACCCCCTGTGCCGAATCCTGCAACATTTCTACTGGCTCACCTGC

TGAAAGATATTGAGCAGCTTACAAGAGCAATAGGGAAGGGGGCAGATGACACAATTTGCA

CCATCCACCTGACCATCTGCATTCTTCTGGAACCTCATCAGCCCAGGCCTCTACCTTATG

ATAATCTGCTCTCAACAAAAGATGCCAGAAATAACTGGGAGATCACAGTGACCACTGATA

TTATCACATGCCAGCTAAAGGTTTTGGAGAGTCACCTGGCAAATGTAAACACATTTATCA

GAAATGATGAGAGAGTTTCATCCAACCCAATAATGAAGGTGACCTTCGGAGAACCTCGGC

TGTTCCTGCGCACTTTACCTCAAGGTTCCTTGATTCATAGCTCATCTGTGTGGTGCTGCA

GACAGCGTGTCTCTCTCTTGGGTCTTAGCCACATTTTGGAGCAATATGGAGGCAGGGACA

GACTTCCCATCCTTTGGAGGTTTCTACAAAAGGAGGCAGAGTTAAGGTTGGTGAAGTTTC

TCCCAGACATTCTTGCTCTCCAGAGGGATCTGGTAAAAAAGTTCCAGAATGTGATAGAGC

TTCAATGTGGCACTATAGCACAGTTCCTGCAGAACCTGAAAGCAGAATCTCTAATGACTT

CATATAAAAAACGGATCAATATCTTTCTGTCCACATGGAACCAACTAAGAGCATCTCTGG

AAACAAATGGAGAGATCAAACTCCCAACGCAATACTGTCAAGACGATCTAGACCTTAGTG

CTGACCTGCAAGTGCTTCTGCCACGAAGACAGGGTCTGGGCCTGTGTTCCACCGCTCTAG

TCAGCTATCTAATTGCTTTGCAGAATGATTTAATTTATGCAGTGGACAAGCATACAGGAG

AAGAGACTAGTTACACAGTGAGTCCAGCTGACCTAACAGAGCTCCATGTCATCAGATACG

AGGTAGAGCGAGATCTGCTACCTCTTGTGCTTTCAAACTGCCAGTATAGCATGGAGTGTG

GCCGTGAGACCCTGTCCGAATATGACCTGCCCAAGATCCAGCAGCAGCTTCTCACACGCT

TTCTGCAGGGCAAACCATTCATTGGCCTCACTGGAATTCCAACTCTTGTAAACAGACATG

ACCGAAATTATGAAACAATCTTCCGGGATGTGAAAGGCAAAGTTCAGCAAGAATCTATGA

CTTCACTGAACCAGTCTACACTGTGTGCCGAGCTGCAGTGCTATAGTGACGTGTGTGAGG

CGTTGTCCACTGTGGAAATAGCCCTGGGATTTTTAGCCATGACTGGAGGTGAACCCAACA

TGCAACTGAGCAATTACCTGAAGGAAGAACTCCAAATGGCTGACCAAATACCTTTGCATG

TACTTAAGGCTCTGAGTAGGTGCTGTCTGAAACACTGTTTGGCTCTGTGGCAGCTACTCA

CCTCTCTCAAATCGGAGACAATGCTACGCCTTAAGAGGGACCCATTTGAAAGGTTTAGTA

AAGACTATAAGAAAACATTGGGGGAGCAGGAGAAGAGACTGCTTACTGGCTTCTTCTCAA

AGACCAGTGCTGACTCATTCTTGCTGGAAATGCATGAGTTTTTACTGTTGGTATTGAAGA

ACGCTAATGCCACAGATACATACAAGCCCAACTGGAGTGTGAAAGACACTATCTTGTCCT

ACATGGAGCAAAAAGACATGGATGTCCCACCAGATGTGGTGGAGCTGTTCCCTGATGAAA

TACTTCTAGCTCACTACATTGAGGCTTGGAAGTTTTCCATTCTCCTCAAGCAAGAGCGAA

TACAAAGATAAAGCTAAATTTAAAGATTTCTCATCATTATCATTTTTCCACTCCATACAT

CAGTGTACTTATCTTAAGATTTGCGTTTTTATTACAATCATCACTGCACATTCATATTAG

CTGGATGTGGGGTAAAAAGTGCCACTTGGAGGTTTAAACTTGACAATGTCTGTATTTGTT

GCTGCATAAATCTAATAGCACAGAAAATATAATCAAGCTACAGATTGCACAAGAGCAGTG

AATCCTTTTGTTTCGGCTGGCAATCAAATATAAGTTAATACAATCAGTGATTATTTCATT

TGTCATCATACAAATTTGTGGGATTATTCCATTATATAGCGTTACTGTAAATCTGTTTAC

ATTTACTTCTTTAGGTTTTGTTTGAGGAATATCTCCTATATATGCACTAATCAGGGTACA

ATAAAATAGTCCAAAATTATACTCAGGGACATTTTAAAAATCACATCAACTGGGTGAAAA

AAAAGATTGTTGATCCCCTGGGACCAA

>Unigene000002

CCGCGGCCTGTAGGATTCCGGTTAAAGTATTTGGCCCGGTGAGTGGATGTGTATAGACTA

TACGTGTACGTGCCTCTGTGTGTGTGTGTGTTTGGGGGTGGGGGTCGTGTCTAAGTCTTT

TAGTGCACGCCCTTTTTCATCAGCCCGGGACGAGTGTGTGAGAGGCGTGTGAGCATCCAT

CACCTTCAGTCCGCGGAGCTCCGTGTGTTCGGCTTGACCAGGAGCAGCCGCGCGCGCATC

ACCATGCACCGAGCACTGCTCCTCCAAACTGCGACTATACTCACGTTTTTCCTTCAGTGC

AGCGCTCAGGGTTCAGGTTGTGAAGTTGGTCAGTTCCGCTGTGGCACAGGCACATGTATC

CTAGCAAGCTGGCGCTGTGATGGCACTGCAGATTGCACAGATGATTCAGATGAGAGTGGC

TGCCCCCAAGCGACCTGTGATGCAGACCAGTTCCAGTGTCTGAGCGACGGGGAGTGTATT

CCAGTTCACTGGGTATGTGATGATGAAGAGGACTGTGAAGATGGCTCAGACGAGAGACAG

CACTGCCCTGGCAGGACCTGTTCCAGTATACAATTCAGCTGCACAAATGGGGCATGTATC

CCAGCAGAGTACAGGTGTGACCATCTGCCTGACTGCTCAGACGGAGCGGATGAGAGGAAC

TGCCACTATCCAGAGTGCCCAGAGTTGAGGTGTGCCAGTGGGGCATGTTATAACACGAGT

CAGCGCTGTGACCAAGTGCTCAACTGCCGAGACAGCTCAGACGAGGCCAACTGTACTCTG

CGATGCATAAGTGGCCAGTTTCAATGCAATAATGGCGAGTGTATTCCACGTGGATACATC

TGTGACCATGATGATGACTGTGGAGACATGAGTGATGAACAGAACTGCACTTACCCTACA

TGTAAGGGAACTTACTTCACTTGCCCCAGTGGTCGCTGTATACATCAGGTATGGTTGTGT

GATGGAGAGGACGACTGTGGAGATAATGCGGATGAGCGAGGCTGTGATAATGTCCAGCAT

GAGTGTTACCCTGGCGAATGGCCTTGCCCCTCATCTGGCATTTGCATCCGCATGGAGCAG

CTCTGTGATGGGACACCTCACTGTCCTGATGGGGAAGATGAGACCAATTTTACTGCTGGA

CACAACTGCAGTATCTGGAGGTGTGCATCTCTGAGCTGTGAGCATCAATGTGTGGCATCT

CCTCAGGGTGGTACCTGCTCCTGTCCGTCTGGCTATGTGGTCAGCTTTAATGACAGCCGC

TCTTGTATCGATTATGATGACTGCCAGTTGTGGGGTGTTTGTGATCAGCTGTGTGAAGAT

CGGATCGGTACACATCGCTGCAGCTGTAAGGAAGGCTACGTGTTAGAGCAGCACCGATAC

TGCAGAGCCAACACTTCCTCTGGAGTAGCATCTTTACTCTTCTCTAATGGACGAGACATT

CTGATCGGAGATATCCATGGCCACAGCTCTCGGGTCCTGGTTCACTCTGAGAACAGAGGA

GTGGCTGTGGGAGTGGACTTTCATTACCAGCTGCAAAGGGTCTTCTGGACTGACACCATT

CAAAACAAGGTATTTTCGGTGGACATGGATGGATCACATTTGCAGGTGATTCTAAATGTA

TCAGTTGATTACCCTGAAAATCTGGCTGTGGACTGGGTGAACAATAAACTTTATATAGTG

GAGGCCAGTGTAAACAGAATTGACATGGTGGACTTTGATGGAAATAATCGTGTCACACTC

ATTGCTGAGAACCTAGGAAATCCAAGGGGACTGGCACTAGATCCAACCGTTGGATATCTA

TTCTTCTCTGACTGGGACACTCTAAATGGAGAGCCTGGCCTGGAAAGAGCCTACATGGAT

GGTTCCAACCGCTACAGTATCATCAGGTCCAAGCTGGGTTGGCCTGCTGGAATCACGCTG

GACATTGATGCCAAGAGGGTGTATTGGGTGGACAGTCGCTATGACTACATAGAAACTACT

ACATATGATGGGCTGCACAGGAAAACAGTGGTGCATGGAGGTTCATTGATTCCTCACCCG

TTTGGCATAAGTCTGTTTGAACACTCTGTCTACTACACTGATTGGACCAAGATGGCCATC

ATGAAAGCAAACAAATTCACTGAAAACAACCCACATGTTCTCTACCCAACCGCTCATACA

CCATATGGCGTAGCTGTGGTACATCCCTTCAGGCAGCCCTATATGAGTAACCCATGTGGG

ACGGACCAAGGTGGCTGTGAGCAGATCTGTGTACTGAGTCACAGGACAGATAACGGTGGA

CTGGGATATCGATGCCGTTGCCGCATGGGCTATGACCTACACCCTGATGGCAAGAGATGT

GTGGCTGTTAAGAGGTTCTTGCTTTTCTCCAGTCAACTGGCAGTGAGAGGAATTCCGTTT

AACCTTTCATCTCAGGAGGACATCATTCTTCCTGTCACTGGCACACCATCATTTTTCGTT

GGTGTGGATTTCTGCGCAGAGGACAATTCTATATTTTTCTCTGATACCGCCAAAGATATT

ATTTTCAAGCAGAAAATTGATGGGACTGACAGAAAGGTGCTTGCAGCCAATCGCGTAGAG

GGTGTAGAAGATCTGGCTTATGATTGGATATCAAAAAACCTGTACTGGACAGACCCACGC

TATCGGAGTATTTCAGTTATGAAAGTAGCAGATAAATCCAGGAGAGCCATCATCCGCAAC

CTGAACAACCCCAGATCTATAGTGGTGCATCCAATAGTCGGCTTTATTTTCTGGACCGAC

TGGTACCGCCCGGCCAAAATAATGCGATCTTGGTGTGATGGGTCTCATGCCCAGCCGATT

GTTAACACTACACTGGGCTGGCCTAATGGCCTTGCCATAGACTGGAGCTCCATGCGTTTG

TATTGGGTTGATGCTTTTTTTGATAAGATCGAGCACAGTACTTTCGATGGGCAGAACAGA

TTGTCATTGGATAGGATTACACAGATATCTCATCCATTTGGACTTACTATTTATGGTGGT

TATGCTTACTTTACGGATTGGCGTCTCGGAGGAATAGTCCGTGTTAGGAAAACAGATGGT

GGAGAGCTGACGGTTATCCGGCGTGGAATCAGCCATATTATGCATGTCAAATCTTTTAAT

GCTGACTTGCAGACTGGTTCCAACTTCTGCAACAGACCATCAAATCCTAATGGAGACTGT

AGTCACTTTTGCTTTCCTGCACCTTACTCCCAGCGTGTGTGTGGATGCCCTTACGGCATG

AAGCTTGCACCCAATCAACAGACCTGTGTGGATGATCCATCCAGTGAGCCACCGACGCTG

CAGTGTGGCTCCAACTCGTTCTCTTGCTCCAATGGGAAGTGTGTCCCTGCCAGCTACCAG

TGTGATGGTGTGAACGACTGCTATGACAACAGTGATGAGGCCAACTGTGGAACTAACAAC

AATACCTGCTCCTCCTCCGCCTTCACCTGTGCCAACCAGCGCTGTGTGCCCAGGAACTGG

CGCTGTGATGGGCACAATGACTGTTTTGATGGCAGTGATGAAAGAGACTGTCCAACCCAG

ACCCCAGGAACATGTCAAGCTGACCAGTTTAGTTGTGCCAACCAACGGTGCATCCCGCGC

ACTTGGCTCTGTGACACAGATAATGACTGTGGTGATGGCTCTGATGAAACCAACTGTGAT

GCTATAGGCACATGCCACCCAGGTCAGTTTCAGTGTCCTGATCACCGTTGTATTGATCCA

GCCTATGTTTGCGATGGCGACAGGGACTGTGTGGATGGGGCAGATGAGCAGGGTTGTGTT

TACAATTGTACTGGCTATGAGTTCAAGTGTGCCAACGGTCACCAGTGCGTAAACTCCTAC

TACCGCTGTGATGGGGTGTATGATTGCAACGATCGCTCTGATGAATCTGGCTGCCCCACC

AGACCACCAGGCATGTGCCACCACGAGAGTGAGTTCCAGTGCCAGTCAGATGGAACCTGC

TTGCCCTCTGCATGGGAGTGTGATGGTCACGCAGACTGTGAGGACGGCTCAGATGAGCAT

CACGGGTGTCCCCCACGCACATGTCCTACCTCACTCTTCCGCTGCGACAACGGCAATTGT

GTGTTTCGCAGCTGGCTGTGTGATGGGGATAATGACTGCCGTGATGGCAGTGATGAGCGT

GACTGTCCTACTCCACCATTCCGCTGCCCCAGCTGGCAGTTTCAGTGTCCTGGACACACA

GTTTGTATCAATATCAGCAAAGTGTGTGATAACACACCTGACTGCCCAAACGGAGCTGAC

GAGTCCCCACTTTGCAACCAAGAAATTTGCTCAGACAACAATGCAGGCTGTACTCATGGC

TGTATCCAGGGTCCTTTCGGGGCACAATGCACATGTCCAGTGGGCTATCAGTTAAGCAAT

GACTCAAAGACATGTGAGGACATAGACGAATGTGATCCCCCTGGTCTGTGCAGCCAGCAT

TGTTTTAATGAACGAGGCTCCTTCCGCTGTCACTGCCAAGATGGATACACACTCGAAGGG

GATGGACGAACCTGCAAAGCCTCGGGATCTCGGGAGGCCATTCTGCTGGTGGCCAGTCGT

AATCAGATAATAACTGATGACATCGTCTCACAGCCAAATGTTGTGCGATCTCTTGTACGT

GATGGCCGGAACATTGTGGCAGTGGATTTTGATTCGGTGACAGACCGTGTGTATTGGTCA

GACACTACCCAAGACAAGATATGGAGTGCACATAAAAATGGCAGTGATAGAACAGTGATC

TTCGATAGTGGTGTGACAGTCACTGAGAGTATTGCAGTTGATTGGGTGGGTAGAAACTTA

TACTGGACAGACTACATCCTTGAGACAGTAGAAGTGGCCAAACTGGACGGCTCCCATAGA

GCAGTGCTCCTCAGTGAGAATGTCACGAATCCTAGAGGCCTTGTGCTAGACCCCAGGAAC

GGTTTTCACCTTATGTTCTGGACAGACTGGGGAAGAAACCCTCGCATTGAGAGAGCCAGC

ATGGATGGCAAGCAGAGGACCACCATCATTACCACCAAGCTTTACTGGCCCAATGGCCTG

ACCATAGACTACCCCAACAACCTACTCTACTTCGCAGATGCCTACCTGGATTTCATTGAC

TACTGTGATTACGAGGGAAACAATCGTAAACAAGTTCTAGCCAGTGATCTGGTACTACAA

CATCCACATGCAATTACCATTTTTGAGGATTTTGTGTATTGGACTGACAGATACATCAAT

CGTGTGATCCGGGCCAACAAGTGGCACGGAGAGAACCAGACAGTGTTGCTGTATAATGTC

CCTCAACCCATGGGTCTGGTGGCAATGCACCCAGTCAGACAGCCGGCAGGTGATAATCCA

TGTGCATTAAACCCCTGCACACATATCTGCCTTCTGTCTGCGGTTGGTCCCAGGTTTTAC

TCGTGTGCTTGTCCTTCAGGCTGGACACTGGCTAATGACCAGTTCTCCTGTGCCAAAGTG

GACGATCCTTTCCTGGTGGTTGTTAGGGACAGCATTATTTATGGTATTTCTCTCAACCCT

GATGAAAAGAGCAATGATGCCATGGTTCCAATCGCTGGCCTGCAGAACGGATATGATGTC

GATTTTGATGATGCTGAGCAGATGATCTACTGGGTTGAGCACCCTGGTGAGATCCACAGG

GTGAAGTCAGATGGCACTAACAGGACTGAATTTGCCCCGGCAGCCATCTTGGGGTCTCCA

GTCGGTTTGGCCCTTGATTGGATGAGTCAAAACTTGTACTACACCAACCCATCCTCACAA

TCCATAGAGGTTCTGAGGCTGAAAGGAGAGGTGCAGTACAGAAAAACTCTCATTACCAAC

AACGGCACCCCGACTGGTGCAGGCTCACCGGTTGGCATAGCAGTGGACCCGGCACGTGGG

AAACTGTACTGGACCGATCAAGGGACAGAGAGCGGCATTCCAGCCAAGGTGGCATGCGCA

GACATGGACGGGTCTAATGCTGTTGTACTATTCACTAACAACTTGGAGCATGTGGAGTTT

ATCACTATAGACATCAAAGAGAACAAACTTTACTGGGCTGTAACCGGGACCGGTGTGATC

GAGAGAGGAGATCCGAATGGTTTAAACCGCATCACGGTGGTTAATGGCTTGTCTCACCCA

TGGGGTGTGGCTGTGCACGGGCCCTACCTCTACTTTACTGATAGAGACTTTGAGGTGATT

GAACGTGTGGATAAAGCCACAGGATCCAGCAGGGTTGTGCTGAGGAACAATGTGCCTGGA

CTCAGAGTTCTTAAGGTGCACAGCAGAGAAACCTCTGTTGGCTCCTCTAATGGCTGTAGT

AATAATGTCGGTGCATGTGAGCAACTGTGTCTACCCCGACCCGGCGGCATTTTCTCCTGT

GCCTGTGCTACAGGCTTCAGACTCAATGCTGATAATAGGACCTGTGCGCCATATCAGTCC

TATGTGGTCATCTCAATGCTTACTGCCATCAAGGGATTTAGTCTCGAGGGAGAAGATCAC

TCTGAGGCCATGGTGCCTGTGGCAGGACGAGGTCGAAATGTCCTGCATGTGGATGTGCAC

ATGCCCTCCGGGTTCCTGTATTGGTGTGACTTTAGCAGCACTGTTGCCTCTCAGAATGGT

ATCCGCCGGATAAAGCCCAATGGATCCGGATTCCGTAATATTGTTACTTCTGGGATTGGA

CGCAATGGGATTCGAGGAATTGCTGTGGACTGGGCAGCAGGGAACCTATACTTCACAAAT

GCCTTCCTGACCGAAACCTACATCGAAGTTGTTCGACTTAACACCACTTTTCGGCGTGTT

CTCCTAAAGACCCAGGTGGATATGCCACGTCACATCGTAGTTGACCCAAAAAACAAATTC

CTTTTCTGGGCTGACTATGGTCAGAACCCCAAGATTGAAAGAGCTTTCTTAGACGGTACC

AACCGAACAGTGCTGGTGACATCTGGTATTATTACACCCAGAGGGCTTGCTCTGGACCAT

GGGAATGGGTATATCTACTGGGTGGATGATTCACTGGACATGATTGCAAGGGTTCCCCCC

GATGGAGGTGAGACCGAGATTGTACGGTACGGGAGTCGCTACCCCACTCCCTACGGTATT

ACTGTGTTTGAAAATAACATCATTTGGGTAGACAGGAACCTGAAAAAAGTTTTCCAGGTC

AGTAAAGAGCCAGGAAATACTGACCAACCAATGGTGATCAGAGACAACATTAACATGCTG

AGAGATGTCACCATTTTTGACCAGAGAATGCAGCCCTGGTCAGCACAGGAGCTTAATAAC

AATCCCTGTTTATCATCTAATGGAGGCTGTTCTCACTTTTGCTTTGCATTGCCTGGCACT

CAAGCAAGAAACTGTGCATGTGCCTTTGGGAACCTGGCCGTGGATGGTATGACCTGTGTG

GTATCCCGTGATGATTACCTCATATACACTACAGAAAGTACAGTGCGCTCACTACGCCTT

GATCCTGATGATCATTCCCTGCCTTTCCCTGTGGTCAATGTGGCCCGGACCTCTGTTGCA

CTGGATTATGACAGGCTGGACAACAGGATTTATTTCACCCAAAGCTCCGGGGCGGGACAA

AGCAAAATCAGCTACATTAGTTTATCATCTCCTACCTCATCCGCTACCGTAGTGGCTTCA

GATCTGGGTGCTCCTGATGGCATTGCCTATGACTGGATCAACAAAAGGATCTATTACAGC

GACTATGTTAATCAGACCATCAACTCGATGGCTGTGGATGGATCACAGAGGACTCTTATC

GCTCATGTGCCCAGACCACGAGCTATCATGCTGGATCCTTGCAGTGGATACATGTACTGG

ACTGACTGGGGTACCATTGCTAAGATTGAGCGTGCAACTCTGGGTGGAAATTTCCGGACT

GAGATTGTTAACAGCAGTTTGGTGTGGCCTAATGGTCTCACCTTGGATTATGATGATCAG

AGGCTCTACTGGGCCGATGCCAGCTTACAGAAGATTGAGCGCTGTTCTCTGACGGGCACT

AACCGTGAGGTTATTGTAAGCACAGCTATTTACCCGTTTGCCATGACTGTGTATGGTCAG

CACATTTTCTGGACAGACTGGAACACACGCAGCATCTACAGAGCGAACAAGCACGATGGC

TCGGATCAGAGGGTTATGATCCAGAACCTTCCAACCCGTCCTATGGACATCCATGTGCTG

TCAAACAGCAAACAGCAGCAATGCAGCAGCCCTTGCCTGCAGTTTAATGGAGGCTGTAGC

CATATCTGCACCCCAGGTCCTCAGGGGGCAGAGTGCCAGTGTCCATCAGAAGGTCGTTGG

TACCTGGCTGATAACAAATACTGCATCCCTGATAATGGCACTCGCTGCCAGCCAAGTCAG

TTCACCTGCATGAACGGTCGCTGCATCCGAGCAGCATGGAAATGTGACACCGATGATGAC

TGTGGTGATGGCAGTGACGAGCTGGAGAGAGTGTGCGCTTTTCACACCTGCGAGCCCACC

CTGTATACCTGTGGAAATGGCCGCTGTGTGCCGTACCACTACCGCTGTGATCACTATAAC

GACTGTGGTGATAACAGTGATGAGGAAGGCTGTTTGTTTCGGCCCTGCGACCCAGACACA

GAGTTCACCTGCAACAATGGCCGCTGCATTGCTCGCGAGTATGTGTGCAATGGCATCAAT

AACTGCTTCGACAACGGAACCTCGGATGAACGCAATTGCGCTGAAAGGACTTGTCAGCCT

GGTCACACCAAATGCCAAAGTACAAACATCTGCATTCCACGATCATACCTGTGTGATGGA

GATAACGACTGCGGAGACATGAGTGATGAGAGCCCCACACACTGCGCAACATCTACATGT

ACCCAGAGTGAGTTTCGCTGCACAAGCGGGCGATGTATCCCTGCACACTGGTACTGTGAT

GGTGGTACTGACTGTGCAGATGGGTCAGACGAACCCTCCACCTGTACCACCATAGTGAGA

ACCTGTAGCTCAGACCAGTTCCGCTGTGATGATGGAAGGTGCATCGCATCCTCCTGGATC

TGTGATGGAGACAATGACTGTGGGGACATGAGTGATGAGGACCAGAGGCACAACTGTGCT

AATCGAACCTGCTCTTCACTGGAGTTCACCTGTGTGAATAATCGGCCCCCACAGAGGAAG

TGCATCCCTCAGGACTGGGTGTGTGATGGTGATGCAGACTGCTCAGATGCATATGATGAA

CACCAGAACTGTACACGCAGGTCCTGTTCTGCAACTGAGTTTACATGCAGCAACGGCCTC

TGTATCCGCAGTGCATACAGGTGTGACCGGCGGAATGACTGTGGAGACGGTAGTGATGAG

CAAAGCTGTACCTACCAGCCGTGTCAACAGCACCAGTTCACCTGTCAGAATGGACGTTGT

ATTTCTCAGGACTTCCAGTGCGATGGCGATAATGACTGTGGTGATGAATCAGATGAGCTG

CCGCACATGTGCCACACTCCAGCGCCTACCTGCCCTCCTGGGAAGTTCAGGTGTGACAAT

GGCAACTGTGTGCCGTCCAGCCAAGTCTGTGACCACAGTGATGACTGCAATGATAACAGT

GATGAGAAAGGCTGTGGCATTAATGAATGTACAGACCCATCCGTGCACCATTGTGACCAC

GAGTGCACTGATACCCCCACCAGCTTTGTGTGCAGATGCCGTCCTGGCTACCGCCTTATG

TCTGATAAGAAGAGCTGTGATGATGTAGATGAGTGTTCAGAGACCCCCAGTGTATGCAGT

CAGATGTGTGAGAACACACTAGGCTCATATGTGTGTAAATGTGCACCAGGTTTCCTTCGC

GAGCCAGACGGGCGTCGTTGTCGGCAAAACAGCAACATCAGTCCTTACTTGATTTTTAGC

AACCGCTACTACCTGCGTAACTTATCTGCAGACGGAGCAGCCTACTCGCTCATCCTTCAG

GGCCTTAGCAGTGTTGTGGCTCTAGACTTCGATCGCGTCGACAAGCGTCTTTACTGGATC

GATGTGAGCCGTCGTGTGGTGGAGAGAATGTTTTTTAATGGCACAGGCAGAGAGGTGGTC

CTGAATGGCATTATGCACGGAGAAGGCCTGGCTGTGGACTGGGTGGGACGGAAGCTTTAT

TGGGTAGACAGCTTTTTAGACTGTCTGAAGGTGTCTGAGCTAGATGGACGCTTTATTAGG

AAGCTGGCTGAGCACTGTGTGGATGTTAACAACACCTACTGCTTTGAGAATCCAAGAGCC

ATTGTGGTGCATCCAAAGTTTGGATATGTGTACTGGACTGACTGGGGAAGTAAAGCATTT

ATAGGTCGTGTTGGGATGGACGGTAACAACAAGTCTGCCATTATTACCTCTAAGATCGAG

TGGCCCAATGGTCTTACCATCGACTACACCAATGATATGCTCTATTGGGCTGATGCTCCC

CTTAACTACATTGAATCCTCTAACTTAGATGGCCCCCACCGCCACACTGTTTATGATGGT

GTTCTTCCTCACCCGTTTGCTATAACTGTGTTTGAGGACACTGTGTACTGGACAGACTGG

AACACTCGCACTGTAGAAAAAGGCAATAAGTACGACGGCACTGGGCGCCAAGCTCTTGTC

AACACTACTCACAGGCCCTTTGATATCCACGTGCTTCACCCGTATCGTCAGCCCATAGTA

ACCAATCCCTGTGGAATAAACAACGGCGGCTGTTCCCACCTGTGTCTCTTAAAAGCTGGA

GGACGGGGATACACTTGTCAGTGCCCAGACCACTTTCTGACTGTGCAGATAGGGGGCGTA

GCCCGCTGCCTACCCAGCTGCTCCAGCACACAGTATAGATGCGCTGACAACGAGCGTTGC

ATCCCTATCTGGTGGAAGTGTGATGGTCAATCTGATTGCAGGGACGGTTCCGACGAGCCG

TCCACATGCCCCCTGCGCCATTGTAGGTTGGGCCAATTCCAATGTAATGACGGAAACTGT

ACCAGCTCACATTTTCTGTGTAACTCAAATCGCGACTGCCCTGATGGATCTGATGAGGAC

ACTGTGCTGTGTGCAACACACCAGTGTGAGTCCCACCAATGGCAGTGCAGTAATAAGCGG

TGTATCCCGGAGGCGTGGCAGTGCGACGGGGAGAATGACTGTGGTGACGGCTCGGATGAG

GAACCCTCACACTGCTCCAGCAGAACGTGTCGTCCTGGCCAGTTCAAGTGCAGGAATGGC

CGCTGCATCCCTCAGAGCTGGAAATGTGATGTAGATGATGATTGTGGAGATAACTCGGAT

GAGCCCTTGGATGAGTGCATGGGACCAGAATATCGATGTGATAACCACACTGAGTTTGAC

TGCAGTACAAACTACCGCTGTGTTCCTTTGTGGGCCGTGTGTAATGGACAAAACGACTGC

AGAGACAACAGTGATGAGCAGAACTGTGAGGAGATGACCTGTGACCCTGCTGGAGATTTC

CGCTGTGATAACCACAAGTGTATCCCCCTGCGCTGGAAGTGTGATGGAGACAGTGACTGT

GGAGATGGATCAGATGAACGCAACTGCAACCCTCGAGCCTGCACAGAGAGCGAGTATCGC

TGTGACAACCTGCGCTGCATTCCTGAACGCTGGGTGTGTGACCATGACAACGACTGTGAG

GATAACTCGGATGAAAGGGACTGTGAGTTGCGGACCTGCCATCCTGGATACTTTCAGTGT

GATAGTGGTCACTGCATTGCTGAGCGCTTAAAATGCGATGGCAGTGCAGATTGTATGGAC

GTCACTGATGAGACATCCTGCCCAACACGTTATCCAAATGGCACATACTGTCCTCCCTTC

CTGTTCGAGTGCAAAAATCATATATGTGTTCAGCAGCACTGGAAATGTGATGGAGACAAC

GACTGTGGAGATGGATCGGATGAAGAGCTGCATCTCTGCTTGGATATACCGTGTGAAGCC

CCTTTCCGCTTCCGCTGTGACAATAACCGCTGCATCTACAGCCACGAACTCTGCAACTCA

GTAGATGACTGTGGAGACGGAACGGATGAAAAGCAGGAGCACTGCTTGACTCCCACTCAT

GGACCCTGTTCCTCTGATGAGTTTAAATGTGGGAACAGCCAGTGCATTCCTCTGCAGTAT

GTCTGTGATGATTATGATGATTGTGGAGACCAGACTGATGAGTTGGGCTGTAACAGTGGA

AATGGACGCTCTTGCAGTGAGAATTTGTGTGAACATAACTGCACCGAACTGACGGGTGGA

GGCTTTATATGCTCCTGCAGACAGGGCTACAAAGCCCGAGAGGACAACAGAAACTCCTGT

GAAGATGTAAATGAGTGTGAGCTCTATGGCACTTGTCCCCAGCTGTGCAAAAACACTAAG

GGGAGTTACGAGTGCTTCTGTGCTGAGGGATTCCGCTCTGTGGGACCGCAACACAGGACT

GAATGCGCTGCTGAAGGCAATCCTCCAGTGCTACTGTTGCCTGATAATGTGCGTATCCGT

CGCTATAACCTGTCGTCTAAGCAGTATTTAGACTATATCGATAATGCTGAACACATTCAT

GCTGTGGACTACATTTGGGACCCAGAGGACCAGGGGCTCAGCATTGTATACTGGACCATT

TTAGGTCATGGTGCTCAGTTCGGATCCATAAAGAGGGCATATATGACCACATTTGATGAC

CATGGAAGTAACCAGATGAAAGATGTAGATCTGAACCTTAGATACATATCCAGTCCTGAT

GGAATTGCTGTGGACTGGGTTGGGCGACACATTTATTGGACAGATGCTGGAACCAATCGT

ATTGAAGTGGCAAAACTGGATGGGCGGTACAGGAAGTGGTTAATCTATTCCAATCTGGAT

CAACCAGCTGCAATAGTTGTCAACCCTGCTATTGGTATGATGTACTGGACTGACTGGGGT

AGCCGACCAAAGATTGAAGCAGCATGGATGGATGGTCAGCACAGACAGGTGCTGCTGGAT

GAGGATCTGGGCTGGCCTACAGGACTCACTCTAGATTACCTTAATGGAAACAGGATTTAT

TGGTGTGACTCAAAAGAGAACATTATTGAGTCTATGAAACCAGATGGAACAGACAGGAAG

ATCGTCCTGTCAGGAGATATTGGAAACCCCTACAGTCTGGATGTGTTTGAAGGACATGTG

TACTGGACAACAAAGGTCAGGGGTGAAGTATGGAAGGCTGACAAGTTTGGAAGGGGGGAT

AAAGTCAAGATGTTGACCATAAACCCTTGGTTGACTCAAGTCCGCATCTATCATGAGCAC

AGATACAATCGCTCAGTGTTAAACCCCTGTAAAGGTGTGTGTAGTCATCTGTGTCTGCTG

CGACCTGGTGGATACACCTGTGCCTGTCCACAAGGCTCTTCTCTGGTCAGCTTTAATGAA

AACGAGTGTGATGCTGCCAGCCTACCCGAGGTTGCTATGCCGGTTGAATGCGGATGTATT

AATGGTGGAACCTGCTCCACTGACAAGCCTGGCCTGCCAAAATGCAAGTGTCCTTATGGT

TATTCTGGAAGGCACTGTGAAATAGGAAAGTCCCGAGGAGCACCTGCTGGTACAGCAGTG

GCTGTGCTGTTGGCAGTGATTATTATTCTCTTAAGTGGGGCTCTCGCTGTTGGGCTTTTC

CTGAACTACAAACGCACAGGCTCACTCATCCCTTCAATGCCTAAGCTACCCAGCCTGAGC

AGCCTGGCTAAGTTAAGTGATGCAGGAAATGGTGTGTCCTTCCGCTCAGGGGATGGCAGT

ACTGTGGATCCTCAGACTCTTGGGGTATCGTTTATTGATAGAGCCATGCAGCTGAATGAA

AACTTTACAGATGCTGGGCGGGAACCTGTCACATTCGAGAATCCTCTTTATTCCACTGCA

ACAGGTGCAGCTGCTGATGCTGCGGTCATACATGCCACGCAGGTGACTGTGAATATAAGT

GGAGATTCAACAGAGAACGTATTCGCAAATCCGATGTACAATGAACAGCAGCAGGCAGTG

GACATGAAAAATACACCAAATGAACAAGCAGCAAAGGAGTCCAAGTGGAACTTTTTCAAG

AAAAAATTAAAACCTAGTACAACCTTTGAAAACCCAGCATACTCAGAGATGCGAGACAAA

CAGTCCGCTTTGACTACGGAGGACATTTCAACTAGCCATCCACAGCCAGCTCTTCCACCA

CTAAAGCGAGAGAAATCCAGCATGTACTCACCGACAGAAGACTCCTTCCAAGACACGGCC

AATCTGGTCAAAGAGGACAGCGACATATAACGCTTACTTTCTTTTTCAATCTTTCCTACA

CACAAACGCAGATACAACGGGAAAAAAGACACACTCTGTTCCTCTTTGCACAGAATCTAT

TTTTATATGCAGATTTTGTACAAAAAAAAAAAGAAATCTTAAGATTATTTTAAGAACACT

TAATAAGATGTTTTTATAACAGAAAATTAAAAGGTCTATGGAGACTATGCCTTCTGCTGT

TTCTTATGCCAATTCATGTCTATAATTGTTTTTATATTGTGATGTACTATATGTACTGTA

TATCTTTGCACTGATACTACTGATGGTAAAAATGTTGTACATTTGTAAAAAATTTTTAGA

AGATTTCGATGGTAACTGGAAATGATGTGCAGCAGATCCTGCGTTCTATTAAAGATAAAT

AAAATGACCGCTGAACATGGTAGTGTGTAGGTTAATGCACGCTGCTCAGTGATTTATTCC

TTTTTGTTGCTTCTTTGCATAGAAATTTACACTGATAATTTGTAAGTGGACTAAAAAAAC

ACATCTCATCCTGAACATTTATATACCTCATGTGTGGACTGGCCAGCACAGGAAAAAAAG

AGCCAATTTCTGTTTTGGTCCATGATTCACAATATGTTCTTAAGCAGTATCTTTGTTCAA

ATTAATATTACATTACCTTAAATACAGTATATATTAGACATTTATAGATTTATGAACTGC

TATAGATTATTTGTTGTTACCCAAGTTTTAATTGTAGATATGGTAATTACCAGAAATCAA

AATAACTGATGATATTTTTTGCCTTGATATCTACCAGGCTAATTTATACGGCGTGTCTTG

TAAAAACATTTACTGTATTGTTGCAGTAGGCAAATATTAAAGACTGCTGTTATTTAAAAG

AAGGGACTGATTTAAAAAAAAAAAAAAGCTAATAACCTATTTTCATTCATAATTTATATT

ACGAATGTCACTAAT

>Unigene000003

TTAAACAGATCGGAAGAGCGTGTGCTCCTGTAACGTCTCCTCTCCACAGCACCTCAAAGC

AAAGAACGCTGTAACGAAGAAGCGTTGCCCTGTTTCTGAGGGTTTTGCCATCCTCAGCGA

GTGACTCCACCCATCCAGACGACGAACTGAAAGGATACGAGAAGGAAAAGGGAGAAAAAA

AACACGTGGAGTCAATTTTTTTTTTTTTTTTTTTTGGACATGCGTTTTTTATTTTGCACC

TTTGTGGAGAAATATGAATCCCACGCTTGTTTTTAGTGCACAGGAGACCCCTTTGACAGT

TTGTGGTGAGACTCCGTGTGTATGAATGAAGCGGTATCATGGCTGCGATGATTCCGCCGG

TGAAGTTAAAATGGCTGGAGCATCTGAACAGCTCGTGGATCTCCGAGGATAGCGAGTCCA

TCTCCACACGAGAGGGTGTGGCTCTGCTCTACTCCAAACTCCTGGCTAATAAAGAGGTGG

TGCTGCTTCCTCAGCAGGTTTTGTGCCTCAAAGGTCCACAGCTGCCTGATTTCGAGCGTG

AATGTCTGTCTCGTGATGAACAGGAGCACTACCTGGATGCGCTGCTGGCCAGTCAGCTGG

CTTTGGCCAAAACCGTGTGTTCGGACTCTCCGTTCGCTAGCGCTCTACGCAAACGCCTGC

TGGTGCTGCAGCGAATCTTTTACGCTCTCTCCAGCAAGTACCACGACAGTGGCAAAGCGA

AGCAGCAGCAGCACTCGGGCGAGAGCGCTCTGAATTCCTCAGAGCTGCAGGCGGCGAGCG

AGCGGCCGCGCTCTAGTACGGACGCGCTCATCGAAATGGGCGTACGCACAGGGTTGAGCT

TACTCTTCGCTCTCCTGCGTCAGAGTTGGGCCCTGCCCACCGCCACACCCGGCCTCAGCC

TCTGCAACGACGTCATCACCACGGCGATCGAGGTAGTAGGCTCGCTGCCACCCCTGTCTC

TGGCCAACGAGAGCAAGATCCCGCCGATGGGTTTAGACTGTCTGGTGCAGGTCACCGCCT

TCCTAAAGGGCGCCATCGCCTGTCAGTCTGGTGCGGACGTAGTGGGACGGAGGTTGGCCT

GCGAGCTCCTCCTGGGTTTAGCGGCGCAGAGAGGATCGCTCAGGTATCTGCTGGAGTGGG

TGGAGATGGCGCTGGGCGCCTCATCCGGTGTCAGCAAACTGGAGCAGAACCAGAACCAGG

AAGGCCTGATGGGATACGACTGTTTCATGAACATCCTCATGCAGATGAGACGATCATTGG

GTTCTTCGGCTGACAGGAGTCAATGGAGAGAACCGACTCGCACTCCAGACGGTCTGTGCT

CTCTATACGAAGCAGCTCTCTGTCTGTTCGAAGAGGTCTGTCGCATGGCGTCGGATTACT

CGAGGACATGCGCCAGTCCAGACAGCATCCAGACGGGTGAGGCAGCTATGGTGTCTGAGA

CCTGTGAGGTGTACGTCTGGGGCAGTAACAGCAGTCACCAGCTGGTGGAGGGAACGCAGG

AGAAAATCCTTCAGCCCAAACTGGCAGTCAGCTTCAGCGACGCGCAGACAATTGAAGCTG

GTCAGTATTGCACATTCGTCATCTCGTCAGACGGCGCCGTTCGGGCTTGTGGCAAAGGAA

GTTATGGCCGACTTGGTCTGGGCGACTCCAACAACCAGTCCACGCTGAAGAAGCTGACCT

TCGAACCTCATCGTGCAATTAAAAAGGTTTCATCCTCCAAAGGCTCGGACGGCCACACGC

TAGCATTCACCAGCGATGGAGAAGTGTTCAGCTGGGGTGACGGAGACTACGGCAAACTGG

GACATGGCAACAGCTCCACCCAGAAATACCCCAAACTAATACAGGGGCCCCTGCAGGGAA

AGGCGGTGGTGTCGGTGTCGGCTGGCTACAGGCACAGTGCGGCGGTTAGCGAGGACGGAG

AGCTGTACACGTGGGGAGAAGGCGACTTTGGAAGACTGGGACATGGTGATAGCAACAGCC

GCAACATCCCCATGCTTGTGAAAGACATCAGTAACGTGGGTGAGGTGTCCTGCGGAAGCT

CTCACACCATCGCGCTGTCGAAAGACGGACGCACCGTTTGGTCCTTCGGAGGCGGAGACA

ACGGTAAGCTCGGTCATGGTGACACCAACCGTGTGTACAAACCCAAAGTGGTGGAGGCTC

TGCAGGGAATGTTCATCCGTAAAGTGTGTGCCGGAAGCCAGTCCTCTCTGGCCCTCACCT

CCACCGGCCAGGTATACGCGTGGGGTTGCGGCGCCTGTCTGGGCTGCGGATCCTCTGAAG

CCACCGCTCTTAGACCAAAACTAATCGAGGAACTGGCCACCACACGTGTCGTAGATATTT

CTATAGGAGACAGTCACTGCCTGGCATTGTCACATGATAATGAAGTGTACGCCTGGGGTA

ATAACTCCATGGGTCAGTGTGGTCAAGGAAACTCAACCGGTCCAATCACTAAACCCAAGA

AGGTGATTGGTTTAGACGGCGTGGCTATCCAGCAGATTTCTGCAGGCACCTCTCACAGTC

TGGCCTGGACCGCCCTTCCCAGAGACAGGCAAGTGGTGGCATGGCACAGGCCGTACTGTG

TAGACCTGGAGGAGAGCACTTTTGCTCATCTGCGTTTCTTCCTGGAAAGATATTGTGAAG

GCATCAATAACAAAGTCCCCCCTCTGCCCTTTCCTTCCTCCAAAGAGCACCATAACTTTC

TGAAGCTGTGCCTAAAGCTCTTATCCAATCACCTGGCTCTGGCGCTGGCAGGGGGCGTGG

CCACAAGCATCCTAGGACGACAGGCGCGGCCTCTTCGCAACTTGTTGTTCAGATTGATGG

ACGCTTCAGTGCCTGATGAGATTCAAGAGGCAGTCATTGAAACTTTGTCTGTTGGTGCGA

CCATGTTGCTGCCGCCTCTGAGGGAAAGGATGGAGCTTCTGCACTCACTCCTTCCTCAAG

GACCTGACCGCTGGGAAAGCCTCTCCAAAGGACAGAGGATGCAGCTGGACATCATTCTCA

CCAGTCTCCAGGATCACACCCATGTCGCATCTCTGCTGGGTTACAGTTCCCCTCCAGATA

TGTCCGAGTCTCTGACGCTGTCTTCAGATTCCACACACACACCAGACACGCACCCCGACA

CACACCTCGCCGAGATCCTCATGAAGACTTTATTAAGGAACCTGGGCTTCTACACCGATC

AGGCTTTTGGAGAGCTGGAAAAGAACAGCGATAAGCATTTACAGGGTACATCATCGTCTG

AGAACAGTCAACCTGCTCATCTCCACGAGCTTCTCTGTTCTCTACAGAAACAACTGCTGG

CTTTCTGCCACATGAATCCTGTTACCGAGGACTCTAGCAGCGTGGCCCTTCTCCATAAAC

ACCTGCAGCTTCTGCTGCCTCACGCTACTGACATTTTCACTCGCTCAGCAGTGCTTTTGA

AGGAGAGTTCCGGAAATGGCAGCGTGCGTGAAAAACTACGAGATGTGATGTATGTGTCAG

CAGCAGGAAGCATGCTGTGTCAAATTATGACGTCATTGCTGTTACTGCCCGTATGGGTCG

CTCGTCCACTGCTCAGTTTCCTGTTGGACCTCCTTCCTCCGTTGGACCGCCTCAACAGAC

TCCTGCCCGCTGCTGCACCGTTAGAGGACCAGGAGATGCAGTGGCCAGTTCAGGGAATCT

CAGAGCTTACGCAGACGGAGTGTGTGCCTGGTCCTGCGCAGTCCTGGGTGTGGCTCATGG

ATTTGGAGCGCACGGTGGCTCTGCTGATTGGCCGATGCCTTGGGGGAATGCTGCAAGGCT

CCGCCCCCTCGCTAGAGGAACAGCACACGGCATACTGGCTAAAAACTCCGCTGTTTAGCA

ACGGCCTGGAGACGGAAATACCACGCCTAGACTCCTGTATCAGCGCTCTCCTGGAAGCGG

CTCTGAGTGGAAATGAGGAAAAGAAGCCATTCGACTGTGCACTTCGAGCTGACATAAGTG

TGTTAGTGGAGCTGGCACTCGGATCCACAAAGGAGCCAACAAACAGCCTGTGGACCAACA

TGGTGGACTATGCCATCAGCAAGGACTGGGACAGTGCAACTCTAAGTAACGAGGCTTTGT

TGGACACCGTGTCTCACTTTGTTCTGGCAACGCTGCTGAAGCACACAGGGCTGCTGGGTC

ATGCCTGCGGAGGGGGCAGGTATCAGCCGTGTAAACTGTTGGCCGAAGTGTACCGGAGTG

TGTATAAGGTGCGGAATCGTTTGCTTGCCTGCAAGAACATGGAACTGGTCCAGACACACT

CGCCATCCAGAGAGCGCAGGATCTCTGAGAACGTGGACTCTGTGGAAATGGACCCACAGG

AACACGCTTTCACCCGCACCATCGACGAGGAGGCCGAACTTGAGGAACGAGCTGAACGAG

AGAGAGACCGAGAGGACGGCCATCAGGAGCAGGAGGACGAGGAGGAACGCGAGCATGAGG

TCATGACAGCAGGAAAAATCTTCCAGTGTTTTCTCTCGGCGCGGGACGTTGCGCGTGGTA

GAGAGCGACCTCGCACGAGCAGCGTCGGCGTCCCAGACGAGTCTGTTCAACCTGAACAGG

AGCGCAGAAACAGCATCCCGTTACAGGACAGACAAGACCTCTACACCACCACCTGCAACA

GCATCATCCACCGCTGCGCCATGCTCCTGATGGCAGTCAGCCCCACACTCCCGCAGACGA

CCAACGAGCAGCCAGCTTTGTGTGGTGGGGTCACGCAAGACGGAAGCGGATTTTTGATCC

GAAGCGAGAGTTTGTCAACTGAGAGTCGATCCATCCAGAGCGGTCCGAGTTACAGGCTCA

CTAAATCTAGGAGTGAATCTGATCTCTCCCAGCCCGAGTCTGATGAAGAAGGATACTCGC

TGAGTGGAAAAAGGAACGCAGACCTGGATTTGGCGTTCACTCCTAAAAAGAGAGAATTTG

CACATAGCACCCCTGACTCTGCGGACTGGTTCGGGCGGCATTCTGGTCGCGAGCGTCTGT

ACAGCTCCCCTCAGTCTTATGAGGAGTCGGACTTGGACTTCAGCTGCACGCTGCACATCC

ATGCAGTCATCGACAACATCGTCAGCTTCATCAGTGGGGATGTGGGAAACACGCCCGGAT

TCAAGGAACCAGAGGACAGCATGTCAACTAGTCCTCAAGCTACAGTTGTAGCCATGGAGC

AACAGCAGGGCAGAGCGGAGTTGCGGTTAGACGCCCTTCATCAGATTGTGGTTTTGATCT

CTGGAATGGAGGAGAAAAGCAGTCAGGGTGGAGCAGAGGCAGTGGCTGGGTGTGGTCCCG

CTGCCTTTCACTCCGCATCCTTGCTGACCTCAGTCAGACTGCAGTTCTTGGCAGGATGTT

TTGGCCTTGGCGCCTATAACGGGGGGCTAAACGGTGAGAGCGTCCAGCTGCACCACTACC

AGGACGGCATTCAAGCTGCTAAAAAGAGGTTACAGATGAACATTCAGACAGCAGTGCACA

AAATCTACCAGCAGCTTTCCATCACACTGGAGAGAGCGCTTCAGTCTAATAAACACCATA

TAGAGGCTCAGCAGCGGCTGCTGTTAGTGACCGTTTTTGCCCTGAGTGTTCGTTACCAGC

CTGTGGACGTTTCGTTAGCCATCTCCAGCGGTTTGCTAGATGTTCTCTCTCAACTGTGCG

GGAAAGAAACCCTGCTGGGACACACCCTTCAGCTCCTGCACAAACCCGCCGGCTCGCAAC

TCAGCACAGCACTCAAGGTTGCCAGCACCCGTCTGCTGCAGATCCTTGCCATTAGCACCG

GGACGTACGCAGATCGCCTGAGCCCTAAAGTGGTTCAAGCTCTGTTGGACCTGCTCTGTA

GTCAGCTGAAGACTCTGCTGTCGCAGGCTGCAGGAAGCGTCTTCGGCACAAATTCAGAGT

CAGAGGAAAAGACAGAGGACTCAACAGGCAGCATGAAAAAAGACTTCAGGGCCTTACTGC

GCAAACAGCACATTGCGGAACTACATCTCGGAGACTTTCTGGTGTTTCTCAGAAGAGTCG

TATCATCTAAAGCCATTCAGTCCAAGATGGCGTCTGCCAAATGGACCGAAGTGCTGCTTA

ACATTGCGGCACAGAAATGCTGTTCAGGTATCCCTCTGGTGGGGAATTTAAGGACACGTC

TGCTGGCACTGCATGTCCTAGAGGCTGTACTTCCTGCCTGCGAAAGTAGCGTGGAGGATC

ATCAAATGAGTCAGGTGGTTGAAAGGTTGTTCTCCTTGTTGTCGGACTGTATGTGGGAGG

CTCCTGTCGCTCAGGCCAAAAACACCGTCCACCTGAAGGAAAAGGAGGTCAAACTCCAGG

GTGAGGCAGAGGAGGAGGAAGAGAACCTGCCTGTCCAGGAAGTGATCTTCGATCCTGAAA

AGACACAGTGCTGCGTGGTGGAAAACGGACAGGGTTTGACGCACGGTAGCGGTGGAAAAG

GCTACGGTCTGGCTGCTACCGGCATCTCCTCAGGCTGTTACCAGTGGAAGTTCTACATCG

TGAAGGAGAATCGTGGGAATGAAGGGACGTGTGTGGGGGTTTCCCGTTGGCCTGTAAACG

ACTTTAACCACCGCACCACGGCCGACATGTGGCTGTACCGGGCTTACAGTGGCAACCTGT

ACCACAATGGCGAGCAGACGCTCGCGCTGTCCAGCTACACACAGGGCGATTACATCACCT

GCGTGCTGGACATGGAGGCTCGCACAGTTTCCTTCGGGAAGAACGGAGAGGAGCCCAAAC

TGGCTTTCGAAGACGTGGATGCGACAGAGCTTTATCCATGCGTCATGTTCTACAGCAGCA

ACCCAGGAGAAAAGGTCAAAATCTGTGACATGCAGATGCGAGGGACGCCACGTGACCTTT

TACCCGGCGACCCCATCTGCAGTCCCGTGGCCACCGTCCTTGCCGAAGCGGTGTCTCAGC

TGGTCAGGATCCTGCACCGGTCAGACAGCTGGACGCAACCTATCAACCGGTGCATGCTTC

AGCGGCTGAAGCAAATCCGGGGGTGCTTGCACGACGGCGCTTCGCCTACGGGTGGTGCGA

GACTCCGGAAGAGCCGCTCGGCTCAGAGCCGCGAGGAACAGGATGAGGCGAAAGACGAGG

ACAGGGATGAGGAGCGAGGCAGAATGGGGCGGGAACTGACTGAGGCGCAGCTGCGTACAC

TTTGTAACCTGATCTGGCCTGTGCTGGCGGTGATTGGAGGGGTGGACGGAGGTCTGAGAG

TCGGAGGTCGCTGCGTGCACAAGCAAAGCGGGAGACATGCAACCCTGCTGGGGGTGGTGA

AGGAGGGCAGCATGTCGGCCAAGGTGCAGTGGGACGAGGCAGAGATCACTATCAGTGACA

CGCCGCTATATAACCTGGAGCCCTGCGAGCCGCTGCCGTTTGACGTGGCACGTTTCCGGG

GACTGACGGCCGCGCTGCTTTTGGAACTGACCGCTCTGGCTTCGCTGCAGGACGACGGCA

AGTTCTCTCCTACGGCCTGCTCGTTATCATCTTCCTCGAGACGCCACGAGCGCCGCCACA

GACACGAGCCTCACGAGCCCGAAGAAGGCCCCGGTGACCTGCGTGTCTCTCACAGCCTGG

ATGAAGTGCGTTCCCACGCCGAACTGCAAAAGCCTCCTGTAATGGCCGAGGTTCATGCTG

TGCAACTCTCCTACCTCTGCCTTGGTGCCATAAAGAGCCTAAGCGTCCTTCTAAGCTGCA

GTAAGTATGCCGAGCTCCTGCTCATACCCAAGGCGGTGCACGACAACGGCCACAACGCAG

ACTGCGGCAGCCCCAGCATCGGCGTCTCACAGGAAGAGGCAGAGATGCGCTCAGCGCTGC

AGTTCCTTATGAGACACATGGTAAAGCGAGCAGTGATGAGGTCACCTATCAAACGCGTAC

TGGGTTTAGCTGAACTGGAGCGGGCGCAGGCCATGATTTACAAACTCGTCGTCAATAGTG

TGCTAGAAGAACAGGAGGGCGGGAAAACCAAACAATACGGCGAGTGTGAGGAGCTGGAAG

GCGAACAGCAGTTCCAGACGCCGGTTACCACCTCACCCTCTGCGTCCAGTAGCACCTCAT

TCATGAGCTCCTCCCTCGAGGACACCACCACAGCAACCACACCTGTTACCACGGCAACTA

CACCTATAACTGATGCTGAGACTGCGCCAGCCTCAGAATCGCCAGGTGTTATGCCTCTCA

GTCTGCTCAGACAGATGTTCTCCAGCTACCCCACCACAACGCTGCTGCCCGCTCGCCGAG

CTCAGACACCTCCAGTCTCGTCTCTGCCCACGTCTCCTTCAGACGAACACGGCCGAAGGC

AAAGTTTTACCTCTCCTGAATCTCAGCCTGCTAGAACTACCAACAGGACAGCGCTGTCGG

ACCCCAGCAGCCGTCTCTCCACATCTCCCCCTCCCCCTGCCATCGCTGTTCCCCTTCTCG

AGATGGGGTTCTCCCTCAGACAGATCATGAAGGCTCTGGAAGCTACAGGTACAAGAGGTG

AGGCTGACGCGCAAAACATCACGGTTCTGGCCATGTGGATGATCGAGCACCCGGGCAACG

AGGACACAGAGGAGCGGACCCTGTCCTCCGACTGCTCGGGGGCAGCAGGAGGGGGCAAAA

GTGCGGAGCGGAGCTTCCTGCGTTCACCTGGAGATATTCCCAACGCTGATGCCGCAGAGC

TTGAAGAGGGATTTAGCGAGAGCCCTGAAGGAATAGATCAAGACTCCGCCTCTGCATCCA

ACGGCCCCGCCCTCCGGGGGCGCTCAGCCGCCAGTAGGAAGCATCGCTTCGATCTGGCAG

CACGTACTCTATTGGCCCGTGCAGCTGGACTGTACCATTCGGTGCAGGCCCACCGCTCTC

AGGTGCGTAGGGAGGGCTCAGGGCTGCAGCAGGACTCCAGCTCACTGGGAGGAGTTTACG

ACTTTAACCTGGATGAGGAGCTGGAGCTAGAGCTGGACCAAGAGGCCATGGAGGCCATGT

TCACTCAGGAGCTTGCATCAGACTCGGATATTTTGGGAATGTGGATTCCGGAGCGTGTGT

GTGAGTCAGAAGAGCGCGAGGAGGTGGTGGTCTGTGAGCTCTGCGACGTCAGCGTGTCCA

GTTTTAACCAGCACATGAAGAAAAGTCATCCTGGCTGCGGACGGAGCGCAAATCGGCAGG

GTTACCGCAGTAACGGGTCCTACGTGGACGGGTGGTTTGGGGGAGAGTGTGGGAGCGGGA

ACCCCTATTACTTACTGTGCGGAAGCTGCAGGGAAAAGTACCTGGGCATGAAGAGCAAGC

ACAAAGGAGCAGTGTCGGAGAGATACAAGGGCCAGGCTCCAGACCTGCTGGGAAAACAGG

ACAGTGTCTATGAAGAAGACTGGGACATGCTGGACGTGGATGAGGCGGAGAAGCTGACAG

GCGAGGAGGAGTTTGAGCTGCTTTCAGCACCGCTGGGACTCACGGAACGGAAACCCGTCC

CCGAGGCTGTTCAGTTCCCTGACACAGACCCCCTCGGGGCCTCCGCTGCCATGGTTACCG

CCACCAATAGCATGGAGGAGACCCTGCTACAGATAGGGTGTCAGAGTTCGGTGGATAAAA

GCACATCAGGTCGCATGTGTTTGGGTGAGCAAGCAGCCGGTCTCCAGAGTTCTGCTGATC

GCATGGTTGCTTTGAGGAGAGTCACAGCAGCTGCTCAAGTCCTGGTGGCAAGAACCATGG

TGATGAGGGCGCTGTCTTTGCTGTCTGTCAGCGGTTCCAGCTGCAGTCTCGCTGCTGGTC

TGGAAGCTCTGGGTCTGACGGACATTAGGACCCTGGTGCGTTTGATGTGTCTGGCTGCGG

CCGGTCGAGCAGGTCTTTCTACCGGTCTCGCTTCAGGCCCCGGCGCTTTAGAGCGGCCAC

GTGGCACGAACAAGCCCACCAAACCCATCTCCTGTCTGGCCTACCTCAGCACAGCAGTGG

GCTGCCTCGCCTCCAACAGCCCAAACGCCGCCAAGCTTCTGGTCCAGCTGTGCACTCAGA

ACCTGATTTCAGCAGCTACAGGAATGAATCTGACCACTGTGGATGACCCAATTCAGAGGA

AGTTCCTGCCCAGTTTCCTGCGGGGCGTGGCTGAGGAAAACAAACTCATCACCTCGCCAA

ATTTCATAGTGACTCAGGCATTGGTGGCGCTCTTAGCCGATAAAGGAGCCAGACTGAGAC

CCGGGTACGACAAGGCCGAAATGGAGAAGAGAGGCCCGTTGGAGCTGGCCAATGCGCTGG

CGGCGTGCTGCCTCTCATCCCGCCTCTCCTCACAGCACCGCCAGTGGGCTGCGCAGCAGC

TGGTGCGGACACTTGCCGCCCATGACCGCGACAACCAGAGTCGACCGCAGACCTTCGCCG

ACATGGCCGGTGACTTACGCAAGTGTTCTACCATCAAGCTGGAGGCTCATCAGGGCAGAG

TGATCAGCTTCGGGTGGTGTAACAAGAAAGGTCTTTTGGCGACCAGCGGTAGTGATGGAA

CAGTTCGAGTCTGGAACGTGAACAAGAACCAATACACTCTGCAGCAGACCTGCGTCTTTA

ACAAAACAGACTTTACCTCTGAGGAGTGTTCCACCGGACTGGGGTCTCCTGGTGAACCGA

GCTTGGCGCCCGTGGCCTGGAGTGTGAGCGGGAAATTAGTTGCAGCTGCCATGGAGAAAG

TGGTGAACATCTGGCAGGTCAATGGCGGTAAGGCCTTGTTGGACCTGCAGCCTCACTGGG

TGTCGTCGCTGGTGTGGCCGGAGAACGAGGCAGGGTGTCTTTGGGCCGGAGAGTCTCGAG

AGCTCCTATTGGTCGGTCGCATAGATGGAAGTTTGGGCTTGCTGGAAATTTTAGATAGCT

CAAACATACAGAGAACCGAACTTCTGCACTGTTACAGGAAAGACGCTGTGATGAACATCG

CCTGGTACAGCGAGGATCGACCGTTTGCTGTCGGCTACGCTGACGGAAAGCTTCTGATTG

GCTGCAAAGAAGCGCTGGAGAACGGATCCGTGGTCGTCATCGATGCTCACAAGGAGAGCA

TCAGTTCTCTGCGCTGGGCTCCAGGAGGGCAAATTTTATTAAGCTGTGCCAAAGAGGAGA

CTGCCTGCCTTTGGGCGGAGTCTGGGGCGGGGCTTGGGCGGAGTTGGGTGTGTCTCCAGA

GCATCGCTCACCCGAGTGTTGTAAATGCTGTCGCCTGGTGTGGTCTCACCGGACAGGGAC

CTAAAGCACTAAACATGCTGGCCACGTGTTGTCAGAATGGCTTGGTGTCTGTGTGGACCG

TGCCTCAGGACCCCACAGCCTTCTCTCAGTCTGGCTCATCCAGCACTGATGCATGGTGGG

AAACGGACTCCAAGTCAAAACCAAGGTTCACTCCACAGTGGCACGAGGCCGCAGTGTGCG

TCTTCCAGCTAAGAGGTCACATGACTCCGGTTAGGACGATAGCCTTTAGTCCTGACGGTC

TTGCTCTGGTTTCTGGTGGTGTTGGTGGTCTCCTAAACATCTGGTCTCTCCGAGATGGCT

CTGTGCTGCAGACGGTGGTTGTCGGCTCAGGAGCGATTCAGAACACTGTGTGGATTCCAG

ACGTCGGGGTGGCCGTCTGTTCCAACCGCTCAAAGGACGTTCTGGTTGTGAACTGCTCCT

CGGAGTTTATGACCGCTAATCAGGTGTTAGCAACCTGTCGCACCGCGCTGAAGAAGCAGG

GGGTTGTGGGATTGAACACGGCTCCCTGTATGAGGACTTTTCTCGAGAGACTTCCCATAA

TGCTTCAGGAGCAGTACGCCTACGAGAAGCCCCATGTAGTGTGTGGAGAGCAGCTGATCC

ACAGTCCCTACATGCAGTGTTTGGCATCTCTGGCCGTCGGGTTGCAGTTGGACTCGTTAC

TATGTCGTCCCGCAGTGCCTCTTCACCTGTCTCATTGCCTCGCCCAACCTGTCTCTTCAT

CATCGTCATCATCACAATCATCCTCCTCATCCGGTCCTTCAGCACTCATGCACTCATGTG

GCAGCAGCGAGTGGGCGTGGCTTCACTGCTTCTCCACCACCATAAAGACGGCTGAGGCGC

TCGCCCGCGCTCACACTTTCCCAGAATCCTTTAACGTGCCTGATCTGGAGCCGGTGGCCA

AAGACAAGATGGCGGTGCTGATGGACAACAGTAAATGGGTTCCTGGGATGGATGAGCAGT

TGATGGCTTGGGCCACCTCCAGACCCGAGGACTGGCACCTGGGTGGGAAGTGTGACGTGT

ATCTGTGGGGCGCCGGTCGCCACGGTCAGCTCGCCGAGGTCGGCCGGAACATTCTGGTGC

CGACGCCTGCTGCGTCTTTCTCTCAGGCTCAACAGGTTGTATGTGGGCAGAACTGCACAT

TTGTGATTCAGGCGAACGGCACAGTGTCTGCGTGTGGGGAGGGCAGCTACGGTCGACTGG

GTCAGGGAAACTCTGATGACCTGCACATTCTTACGGTCATCTCAGCACTTCAAGGGTTTG

TTGTTACCCAGCTGGTCACGTCGTGTGGCTCTGATGGACACTCCATGGCTCTGACTGAGA

GCGGTGAGGTGTTCAGCTGGGGAGATGGCGACTACGGCAAACTGGGCCACGGCAACAGTG

ACCGCCAACGCCGGCCGCGCCAGATAGAGGCTTTACAGGGAGAGGAGGTGGTGCAGATGT

CCTGTGGGTTTAAACACTCTGCCGTCGTCACAGCAGACGGTAAACTCTTCTGCTTCGGCA

ATGGAGACTACGGGAGACTCGGACTGGGAAACACATCCAATAAGAAACTTCCAGAACGAG

TCACAGCTTTGGAGGGACAACAGATAGGCCAGGTGGCGTGCGGGCTAAATCACACACTGG

TGGTGTCATCTGACGGGTCGACCGTCTGGGCGTTTGGAGACGGAGATTATGGAAAACTTG

GCTTGGGAAACTCGACTGCTAAATCATCACCGCAGAAAGTGGATGTTCTGTGTGGAATTG

GAATCAAAAAGGTTGCATGTGGAACCCAGTTCTCTGTGGCTTTAACCAAAGATGGAAATG

TTTACACTTTTGGGCAAGATCGCCTGATCGGTCTCCCAGAGGGGCGTGCCCGCAATCACA

ACCGCCCGCAGCAGCTCCCCGCTCTGGTGGGCGTGATTATCAAAGATGTCGCTGTAGGGG

CGGAGCATACACTCGCGCTGGCGTCTACAGGAGATGTTTATGCTTGGGGGAGCAACTCTG

AGGGACAGCTCGGTTTGGGCCACACCAACCATGTACGAGAGCCGACTCTAATCACAGCTC

TGCAGGGTAAAAACACCCAGCAGATTTCAGCAGGACGCTGCCACAGTGCCGCATGGACCG

CGCCCTCAGTACCACCCAGAGCACCAGGCTCGTCCATGCCGCTGCAGCTGGGTTTGCCCG

TGTGTGTTCCTCCGCAGTACGGCAGTCTGAGAGACATCAGCATGGAGGCACTAAGAGCTC

GGCTACGTCTGCTCTACCACTTCTCTGACCTCATGTACTCCTCCTGGAGACTGCTTAACC

TCAGCCCTAACAACCAGAGCTGCACGTCTCATTATAACCCTGGGACGTGGGGTATCGTAC

AGGGGCAGCTGCGCCCTCTACTGGCTCCACGTGTGTATATGCTCCCGATGGTACGCTCCA

TCGGCAAGACCATGGTGCAGGGCAAGAATTACGGACCTCAGATCACCGTCAAGAGAATCT

CCACTCGTGGGAGGAAGTGTAAGCCCATTTTCGTGCAGATCGCTCGGCAGGTAGTGAAGC

TGAATGCGTCTGATCTCCGGCTGCCGTCTCGGGCGTGGAAGGTGAAGCTGGTGGGGGAGG

GAGCGGACGACGCTGGAGGAGTTTTTGATGACACCATTACAGAAATGTGTCAGGAGCTCG

AGAACGGGGTGGTGGATCTGCTCATCCCTTCTCCTAATGCCACTGCGGAAGTGGGCTACA

ACAGAGACAGGTTTCTGCTGAATCCCTCGGCTTGCCTGGACGAACATTTGCTGCAGTTCA

AGTTCCTGGGCATCCTGATGGGCGTGGCCATCCGCACCAAGAAACCTCTGGACCTGCATC

TGGCTCCGATGGTGTGGAAACAGCTGTGCTGCATCCCGCTGACGCTGGAGGACCTGGAGG

AGGTGGACCTGCTGTATGTCCAGACACTCAACAGCATTGTGCACCTGGAGGACAGCAGCA

TCACAGAGCAGAATTTCCACGAGATGATTCCTCTGGATTCATTTGTGGGTCAGAGCGCTG

ATGGGAAAATGGTGCCCATCATTCCGGGAGGAAACAGTATTCCACTCACCTTCTCCAACA

GGAAGGAGTACGTAGAACGCGCCATAGAGTACCGGCTACATGAACTGGATCGTCAGGTGG

CGGCCGTGCGTGAGGGCATGTCATGGATTGTACCGGTTCCTTTACTCTCGCTGCTGACCG

CTCGGCAGTTGGAGCAGATGGTGTGTGGTCTGGCGGAGATCAGTGTGGAGGTGTTGAAGA

AGGTGGTTCGTTATCGGGAGGTGGAGGAGCAGCAGCAGTTGGTGCAGTGGTTCTGGCAGA

CGCTTGACGAGTTCTCCAACGAGGAACGAATGCTGTTCATGCGCTTCGTCTCCGGACGCT

CGCGTCTCCCTGCTAACACAGCCGATATCTCGCAGCGCTTCCAGATCATGAAGGTGGACC

GGCCGTACGACAGCCTTCCCACGTCTCAGACCTGTTTCTTCCAGCTCCGCCTCCCGCCCT

ACTCCAGCCAATCGGTGATGGCAGAGCGGCTACGCTACGCCATTAATAACTGTCGTTCTA

TCGACATGGACAATTACATGCTGTCACGGAATGTGGACAATACTGAGGGTTCGGACACGG

ACTACTGACCGAGAGTTAAAGCACAAAGACACTCGCTGAACTTTAAACCGTCAACACAAA

AATCACCACCTTTAATTCTTCTCTTCTTTTATTAATCATTAAAGCAGTCAGACGGCGTTA

ACCAGGTACAGATAAATATAACCATTTCTGTTATTCACAATCCTCTCAACTCCCCACACA

CACACACACACACAC

>Unigene000004

GGGGAAAGATCGGAAGAGCGTCCGGTAGAACAGAGGGTTTGCCAGCCAAGGACACAGAGA

ACAAGCTTACAAAAGAATTTGTTGATCAAAAATCCATTGATGGAGATATGCAAATTAAGG

GACTAGTCCAGACAGAGATGTCCAAAGTTTCAGATATAAGTGATGTTTTTGAGTATATAG

TAGAGAGTCCACCTCAGGAAAGAGAGGATATAAGGAGTGTACACGTAATGCAGCAGCGAC

AAACAATGGAAAATGAAAATCTGGTAACTGAAACATCAAAAAATTTGGTAGATGTCAATA

GAGACACAAAAGATGAAATGGAGAGTCAAATGCAGCCCATAGTGACAAATGATATGTCAA

AAATACAGGCTGACAATGAAATAGATTTTAATAAGGATTATGCTCCAGATGAAGCAGAGA

AAAAGAGGAAGAAAAAAAGAAAAGCCAAAAAGAGAATGATGGAAAAGATCGGAAAGGAGT

CAGAGGAAAGTAACGCTGATAAACTAGTTCAAATACCACCAGTCGATAACCAACCTCAAA

GTGATCAATTCAGTGCAGTAGAAAAAGTTGAAATGGAACAACCTCAAAAATCGACACAGC

TGGAGAAAGAAGCTCTTCTGATGAAAGCCAAAGAAAGCATTCTTCGGAAAGTCTTTGAGA

GAGGTGTCAGTGAAAAGCAGGCTGCAGAGGAGTTAGAAGCATTACGTCAAGGGTCAACTA

AAGAACAGCATACGACAGCTCAGGAGAAAGCCAAGCTAGATGAGAGAAGTGTCTCACTCC

CTGCAGACGAAAATAGGACTGTGAAGTATCAGGCAACGCAGACAGACAGAAATATAAACC

TTTCAGCAAATATTTTACCTAAGTCAGACTCCATACAACTTCATTTCAGCCAGACAGACC

TGACTGATGATAACTTTTACGAAAAGCAGGACTTGAGCTGTACTGTGAGCTCAGATGTTT

CTATGGACACCAGTAAGCAAATATCAAAGCCCATCATTATTTTAGACAAAGCTGAGACAA

CTGTGAAAGAACATTCACATCTGAAAAGTGAAAATCTATCAGAAACTGCAGTCCCCAAAG

AACCATATACGAGTCCTACAGGGGAAAATAAAGACCACCAAAAGTCTCGGCAAAAACCAC

AGAACAGTTTCAGTGCTCTAGAACAGAGTGAAATAAATTTGTTGGAAGAAACCTCCAAAC

CAGAGCTGGAGGATACAGTTAGCAAGGAACCAATGCAGGCCACGACTACGGAATACTCTT

CTAATGATGATGAATTTAAAGACGAATCATCCCTGGAAGAGTCCTCGGAGGCAAGACTGT

CTTATTTAGAAGAGGTCCCTGAGTCTGATACTACAGAGTGCTGGGATGAGGAGGACTTGG

AAGAATGTCAAGATGATGTTGATAAAAAACAGGCTGAAGACCTGGTCAAAAAACCAAGGA

CCGCCGCACAGATTAGCAAGTCCTCGTTGGTCCGTCAAGAATGCCTGGAACATGATCAAC

AAATTGTAGCACTGCTCTCTATGGTGCGACACACTGAAGTCCGTCTCAAGCAGCAGCAGC

AACAATTCATGGGGAAGAGTCTTGCCTCACTTGATGACATCATTAAACAGACAGAGGCTT

GCTCTAGAGCTTACTGATATGGAACCCCAAGTAAATGGGGAGGTTGAAGCAGCCAAGCAG

CTCCTGAAGTCACACACTGAAGATGTCCCACCACAGTTATTTACAGCTCTGGAGAAGGAC

CAGCAAAGTTTGTCCCGTACGTTTACTGCTGCAAAAAATCTGGCAGAGAGCTCTCTGCAA

GGGATGCGATCCCATCGTGATGCACAGAAAGAAGCCGTTGGTAGTGAGTTGGAGTCTCTG

ACGGTGCGAGTGGACGGTCTTCATGCCTGGCTCAAAGACAAAGAAGCTCAGGTGGAACAA

GATGCAGATTCATCTGAGGATGGAGCAATTAAAGAGAATAATAGAGAAACATTAGTAAAG

CTCACTCAAAAACTGCAACAAGTTAAGGATCTACAGAACTGTCTCACTTCCCGCTCCAGT

GATGTCAATGCTGTGGCATTTGACATCCAGGTGTTCATCTCAGAGCGCGCACAGGATTTG

GCACCTGAGCAAAGCAGGCGTCTGCTGAGACAGCTGCAGCAGCTCCAGAATGCCTTTCAC

CAGGCCTCAGGCCGCACCCATGCCAGGGCTGAGACCCTCTCAGTCCAAAAAGTCAGAGAA

GAAGAGAGAGAACAGAAGGAACAGGAGAGGGAACAGGAGGAACGAGAGAAAGAAAAAGAG

AGACAGACTGCTAGGGAAAGAGAGGTAGTGCAGCAGCAGAAAGCAGAGTCCTCACAGAAG

CTTGAAGGGCTCAGTATGTGGTTGGCTGGTGCAGCGAGCCTAATGGCCATTCAGAGACCA

GGAGCTGAGTCAGAGGATGTAAATGTGCTTCAGCAAAAACACAGTCAGCTCAAGGAGGTG

CAGAAAGATCTTCAAACCAAAGCTGATGGCATGATGGAGACGATCCGGTCAGTTGAGGAG

TTCCTGGCTGAGCGTGGCGAGGATATGAGTCCAGAGGAAAGGGCTAAACTGCAGGCAGCA

CTGACGCATATAAAAGAGCAATACAACGTCCTCACAGACACAGCACAGTCCTCACTGACT

CAGCTAGACTCAACCATCAACACAACACTCCAGCAAAACACACAAAGGGCTAAAGCAGAG

GAGCAGCTGCAGGAGACTCAGGGAAAAATAGAAGTGCTTCTTAAGGAGTTATCATCACTG

GACACCTCTAGGAGGGAGTCACTTGGTGAGACAGTTCCAGATGGCAGCATGCCAGTGGGG

TCACTGAACTTGCATAACGACATGCTGAAGGCTGAATTACAGCAGTTACAAGCCCAGCAG

GCTCACCTCTTACAGCTGGCCCAGTCCACACACTCTCTCCTGGAGCAGCCAGACTCCAAT

GTGTCACCGGAAGAGAAGCAGCGTCTACGTGACAGACTGGACCACCTCCAAAGCCAGCAC

AAGGAACGTCTCCAGAGCTGCCAGGAGAGGCTAAGGCGGACTGAAGCACTTCAGGAAAAT

CTTTCGAAATTCATGGAAGAACATGGCAACTTGGGTACATGGCTGGAGCAAAGTGAAAGG

CAGCTGTGTTCTCTAAGGGAAGGAGAAACTGATGCACAGGGGCTAAAGGACAAGCTGGAG

GAGCACAAAAAGCTGGCAGATGATGTGATCTGCCACAAGGCAGATTTGCGCTTCGTGTCC

ATTTCTGGACAGAAGGTGCTGGATTCTATACAAGGGTCTCAGGAAAGTGGGATTGCTATT

GACCCAGTTCTACAGGACACAAGACAGCTTGTCAGTGATAAGCTACAAGATGCAACACAA

CGCTACAGTTCTCTACAGAACAAGTCTGGCAATCTTGGTACCCATCTGTCCACTTTACTG

GATCAGTACCAGCAGTACCAGGATGAGGCTACAGCTCTGGATACATGGCTTACTGCTCAG

GAACAGAACCAGAGCATCCTGAAACCCAGTGGAGAACAGACCGATACACAGACACTACAA

AATACACTGAATACTGTGCAGCACATGCAGGATGAGCTTGCTGAGCGCTCAGTACAATTA

GAGAAAGTCAAGAGAGCCGGAAAAGTTCTTACCAGCAACCAGGAGACCCCTAAACTTAAA

ACGGCAGACATCAACACCACCACAGATAAAATTGAGAGGAGGTTCGATACTCTCACAGAG

TGTGTGTCTCAGAGAGCTAAGCAGCTCCAGACTGCTGCTGCTCAGTCTGTCAGTGTTCAG

GAGGGACTCAAGGCACTGGTGTCCTGGTTGGATGGTCTAGCATTAGACCCTGAGCCAGTC

CAACCGACAACCCAAGCAGTACAGGAGGCGCTGGCGCAAAATCAGAAACTGCGTCAGGAA

TTATTGTCACGACAGGGAAGTGTAGAGGCCACTCGCGACTCCGTGTCCAAGCTGCTGAAA

TCGGCTGATGCATTAACAGTCTCTGGGCTGCAGGGGGCACTGCAAGACCTGAATCAGCGC

TACACTGCGGCCCAGACCAAGCAAACTGAAAATGAAGCAGAACTGCGGGCAGTTTTGCCT

AAACTAGAGAGTTTCGAGCGCCTAAACTCAGACCTTCACAACTTTGTTCAAAGCCGCGAG

AGAGTTCTTAGCCTTGGGGGGCTGCCGGATCGCAGTGTAGAGGATTATAAGCACAGCATA

CAGGAGGTACAATCAGATCTTGGTCAAGAAACAAGTCATCTGAAAACATTTGTGGAACTT

AGTACAGACCTGAGCAAGTCAAAGCTGTTTGTAAATGCACACAGCCTCGTAGACTCGTCT

AAAGAGGTGTCTGATGAATTTGCACGGCTAGAGGCCAGCCTCAATGAAAGGCTAAATGCC

ATCCAGAGCTGTGACCAGCAGCTGGTGCAGTTCCGCTCGCATTCCGACTCCATCCTCAGG

TGGCTGCAAATGGCACAAGAGCAGCTGCCTGCTAAAGAACCCCAGCAGAGCACAGACAGC

CTGCAGAGAAGAGTACAGCAGCTCAAGGATCTGCTGAGTGAGTGGGAGTCTCGAGGCAAT

CAGATTCAGGAGCTCAACAAGAACAGCTCCGAGCTGGAGAGTCGGATCATCAGCATTACT

GCTCCCCAAAGCAAGACCAGTGCCCCCATGCTCAATGGTGCCGGTGGCTCCAGCAGTCTT

AATGGTATTCACACCTGCAGAGACCTGACTGAGATCCAGGTGGTGGTGGCGGACGTGAAC

AGTAGGTATGAGCAGCTGGGTGGGGATCTTCGTAATAGGCAGAGCAAACAGCAGGCTATG

CTGGAGCTGTGCCAGAAGGCTAGGCAGGACTCTGAGGCTCTGCACCAGTGGCTAGCTCTT

CGGGAACAGAGGTTGGCACAGGGGCAGTCTGCATCACCTTCACGACCTGACGTGGCGCGT

GCCCAGGCCCAAGAGAACAAGGCCTTGCTGTCTGAACTCACAGAACACTCTGGGAAAGTA

GAAGACCTGAAAAACACCCTAAAGCAGCTGATACAAGAAAATCCTGACTCCCCTGAAGCA

GAGAGCTGGAAACAACAATTAAAAGACCTTGATATTCGATGGGAGAAAGCCAGTCAGGTC

GCGGCACAAAGACAGGCTGAACTGGAGACGTGCGCTGACAGACTGGGCAACTTTGCATCA

GCAGCCAATCAGCTTGGTCCCTGGCTACGAGAGAAAGAGCTGATGATGAGTGTTCTTGGG

CCCCTGAGCATTGACCCTAACATGCTTAACACTCAGAAGCAACAAGTGCAGTTTATGCTG

CGTGAATTTGAGACACGGCATGCCCAGTTTGATCAGCTGACCCAGGCAGCTGAAGGTATC

CTCTCACCTGTTGAAGAGCAGGGCTCTGGTGACAATCAAGATGTAGCTGAGGTGAAGAAG

GAGCTAGCCTCCATCACCCAGCAGTGGGAGGACCTAACTTCTCGTCTGTCTGGACGTTTT

GATCAAATTGAGCGTGCCCAGGGCACTAGTGAGAGTTTTCAGTCTCTGCTGAAAGAGCTT

TCACAAAGTTTGTCCCAATTGGCGGAAAAGCTGGATGCCCAAGCCTCACTTAGTGCCCAG

CCGGAGGCCCTGCGCCATAGACTAAAGGAGACAGGAGAGATCCGTGCAGAGCTCGAGCAG

AGGAAAGGCCAGCTGGCCCAGGCTGAGCAGTTGTGTGAGGATCTCAGTGCTATTGTGGTG

GAGCCTTACCTTCGAGAAGAGCTGCATAAGAGGCTAGAGAGTGTCAGTGGCCCACTTAAC

AACCTGGAGGAAAGAGCAGCGGATGGCCTGTCCCAGCTGCAGGCTGCTCTCTCAAGCACA

CAGCAGTTTCAGCAGATGTTCGAGGAGCTCCGTAGCTGGTTAGATGGGCAGGCTGCTGCA

CCCAGCATGCCTGATCCTCTCCCCTGCCAACCGGAAGCTCTGCAAGTCCTTGTTGCCCAA

CAAGAAGAACTTCAACGTTGTGTAGCACAACAGCGAGGATCATACGACCTCCTTCAAGCT

GAGGGAGCATCTATTCTGGCATCACTTCCTGCTGGAGATGAGCGCTCAGCTCTGCAAGCT

CAACTCACCAAGCTGCGTCAGGACTGGGAGGAAGTAAACCAACGCCTTACAGAAAAGCAA

GCACATATCAAAGAGACCCTGGGCCGTGCTGTACTATTCAGACAGCATCGTGATGAACTA

GATCCCTGGCTAAAGGAGTGTGAGCAAAAAGAGGCAGAGATCCAGCCTTCTTTGGAGCCT

GCTGCCCTTGAAGAAGCCCTGCAGAAAGCAAAGCAGTTAGGCCTGGACTTAGATCGCCGT

CGCCCCCTGTTGGAGATTCTGAATTCCGCAGCTGACCAGCTCCTGGAGCAGAGCCGGACT

GGAGAGGAGGAGGTGAGGGATGAGAAAGCCAAGCTGAACCGCAGGCTGGATGGCCTCTCT

GAGAGATTACATGGACGCATGGCTCAGCTGGAGGAGCTGAACAGCAGACTGAAAGAGTTT

GAAGATGGTAGACTAGCAGTAGAGAGAAGACTTGAGGCAGCAAGGCACCAGTTAGAGGTG

CAAGAGGCGTTGGGCCCTCAGGCATGCAGTAACAAGAGTCTGGAGCGCCTGAGAAGCCAG

CAGGACACACTCGGCTCCTTACAGCCACAAGTTGTATATCTGCACAATCTGGCCAAGGGG

CTGGCACAGGACGCGCCACAGATGACAGGGGGCAGTGGAGATGGGGGACAGAAGCTGTTG

CAACAGGCTCAGACTACAGAGAAGGAGTTTGGAGAAGTAACTGAAAAGATCCAACAATGC

TGCTCGTCTCTCGAGTCACGGCTGCAGGGTGTAGGAGAAGTCCAATCACACGTCCGTGAT

ATTTTCTCTCGGCTGGCGGACCTGGACGACGAGCTGGACAGCCTGAGCCCTGTAGGACGC

GATGTGGACTCCCTTGCCTCACAGGCAGATGCAATAAGGGGATTCCTGGGGCGTCTGGCC

TCTCTGCGGGCTGAACTTGAAGGCCATGGCAGTGCCTGCACCACCATGCTGAAGCGAGAA

GGTAGCTCCCCTGATCTCCTGGCTATTAGAAGGGAGACGGAAGCTCTAAATCGGCAGGCA

GCCAAGCTGGCCGAGCGGGGTCAGGGTCGTCTAGCACTCATAGAAGAAGCCGAGGGGCGA

GTAAAGGAGTTCTATGCCCGCCTCATGGAGCTACAGAGGCTACTTGACCAGGCAGAGGAA

GGGCTGAACGTACAAAGTGTTGTGGGAACCGAGGTGGATGTCATCAAACGACAGCTCCAG

GAATTTAAGGCTGTGGAGCGAGAGCAGGTAGACAGCATCCAGCCTAAACTGCATCACGTG

AACGCTGTTGGCCAGGGGCTAATCCAAAGTGCAGCTAAACATACCGACACGCAGGCCCTG

GAGCATGACCTGGAAACCACCAACCTGCAGTGGAACTCCCTCAACAAAAGGGTGGCCGAG

AGAATTGCTCAGCTCCAGGAAGCCCTTTTGCACTGCGGGAAGTTTCAAGACGCTCTGGAA

CCTCTTTTAAGCTGGTTGAGTGACACAGAGGAGCTGATCGCTAATCAGAAGCCTCCATCA

GCTGAATACCGTGTGGTTAAAGCACAAATCCAGGAACAGAAGCTGCTGCAGCGGTTGTTA

GATGACCGGCTTGGGACTGTAGAGATGATCCGGGCTGAAGGTGAGCGCATCGCAGCCACA

GCAGAGACGCAAGACCGAGAAAAGATCCAGAGACAGCTGCAATGTTTGGGAGAACGATGG

ACAGATCTGCTTGAAAAGGCCAATGCCAGGCAACGTCAGCTGGAGGAGCTGCAGGTACTT

GCGCTGCAGTTCCATGAGTCAGTGGATCCTTTGGGAGAGTGGCTGAGTGCTACAGAGAGG

CGACTAAGCTCTGCTGAACCCATGGGAACTCAGGCTTCCAAAATTAGCCAGCAAATCAAT

CGGCACAAGGCCCTCCAAGATGAGGTGTTGGCTCGGGAAAAAGAGGTGGACCATCTAGAG

GCCCTTGGCCAATCTCTCTCCCCTCTCAGCTGTGCGGCAGACCGTGATTGGCTTAGTGAG

CGAGTGGGAGCAGTGCGCAGCGGGCACACAGAGCTCCGGAATTGGTGCTTTCGGCGAGCA

GCTATGTTGGAGCAGGCTCTGGCCAATGCACAGCTGTTTGGGGAGGAGGAGGTGGAGGTG

CTGAACTGGCTGGCTGAGGTGGCCCAGCGCCTGTCTGACGTCTCAGTGCAGAGCTACCAG

CCCGAGCTTTTGGCTGAGCAGCACAAGTACACTCTGTCTCTGAACGAGGAGATTGTGAGC

CGGAAGAAGACAGTAGACCAGGCCATCAAGAATGGACAAGCATTGCTGAAACAGACCACT

GGTGAAGAAGTGCTGCTTATCCAGGAGAAGCTGGATGGAATAAAGAGCCGATATTCCGAG

ATGACAGCAGGTAGCAGCAAGGCCCTTCGTAACCTGGAGCAGGCACTGCAGCTCGCTACC

CGATTTGCCAGCGCCCACGAGGACCTCAGCCAGTGGCTTGACTCTATGGAGGCGGAGCTT

AATAACATGGAACCAGACACAACTCCAGCCTATCAGGAGAGACAAAAGGACCTGAAGTGT

GTGTCAGCTGAGAAGCGTCTCATTCTGGATACAGTTAATGAGGTGGGCAGTGCACTGCTG

GACCTGGTGCCCTGGCGAGCACGTGAAGGTCTGGATCGTCTAGTGGCCGATGCTAACCAA

CGCTACAGACAAGCCGATGAGACCATTACGCAAAGAGTGCAGCTAGTGCAGGCAGCCATC

CAGAGGTCACAGCAGTATGAAGAGGCCGTGGATGCGGAACTGGCCTGGGTGGGGGAGACA

GAGAGGAAGTTGACCTCCCTGGGGCCTCTAAGTCTGGAACCTGACGTGACAGTGGCACAG

CTCCAAGTCCAGAGAGCCTTCAACATTGACATTATCCGCCACAAGGACACGGTGGACCAG

CTCCTCCACACCCGAGAGGATATCCTGGAGAGCTGCAGTGACCAGCAAAGAGAAGCACTG

AAGGTAAAGACAGATTCATTAAGTATGCGGTATGAGGCAGTGAGTCAGAACCATGCTGAA

AGATTTTCAGCACTGGAACAAGCACAGGTGTTGGTAGCCCGCTTCTGGGAGACTTACGAG

GAGCTTGACCCATGGCTGGGTGAGACAGAGACCCTGATATCCCAGCTGCCTCCTCCTGCC

ATTGACACTGAGGCTCTACGACAACAGCAGGACCAGATGAGGATGCTGAGAGAGTCTATA

GCTGAGCACAAACCCCACATTGACAAGCTGCTGAAGATTGGCCCACAGTTGGCTGAGTTA

AGTTCCCAGGAGGGGGCGACACTGAGACAACGCTACAGTGAGGCTGAAAGGCGTTACCTC

GCCATCAAAGAGGACGTTAAAGGTCGTGCTGCAGTACTGGATGAGGCCTTCTCTCAGTCT

GCACAGTTCCATGATAAGATGGACCCCCTGTTAGAAACTCTGGAGGGAGCAGTGCAGCGT

TTGCGGCAGCCTCCTCCCGTGGCTGCTGAGGTAGAGAAAATCCGTGAACAGCTGGCGGAG

CACCGAGCAGCCGGGCTGGAACTGGACAAATTGTTGCCGTCCTTCAGCACTCTTTGTGCC

CGTGGGGAGGAGCTCATCACCCGTGCTGCACATGATGACCCTGCGGCTCAGGCAGTGCGT

TCTCGGCTTCTAAGACTCCGTTCCCTGTGGGATGAGATCAGACAGAGAGCAGAAGAAAGA

GAGGGAAAACTGCAGGATGTACTGGACTTGGCTGGAAAGTTCTGGGCTGACATGGCAGCT

TTGCTTAGCACCCTGCGAGACTCTCAGGAAATTGTAAAAGAACTGGAAGACCCTGGATTG

GATCCATCACTCATCAAACAGCAAATAGAGGCAGCAGAAGCTATCAAAGCAGAGACAGAC

GGTCTGCGTGAAGAACTGGAGATCGTCCGCACACTGGGAGCTGATCTAATTTTTGCTTGT

GGTGAAACCGAGAAACCAGAGGTCAAGAAAACCATCGATGAGATGAATGCTGCGTGGGAA

GGCTTGAACCGCACATGGAGAGAGAGAATGGAGAAGCTGGAGGAGGCTATGACAGCCTCT

GTGCAGTATCAGGATGCACTGCAGGGTATGTTTGACTACCTAGACAACGCTGTCATTAAA

CTGTGTGACATGCAAGCCGTAGGAACTGACCTCAGCACCGTCAAACAACAGATAGAAGAG

CTCAAGCAATTTAAGGTGGAAGTGTATCAGCAACAGATAGACATGGAGAAGCTCTGCCAC

CAGGGGGAGCTACTGCTCAAGAAGGTTTCTGACCAAGCGGACAGAGACATGATCCAGGAA

CCTCTCACTGAGCTCAGGCACCTGTGGGACAATCTGGGAGACAAGATCACTGTCAGACAG

CACAAGCTGGAAGGAGCTCTTCTAGCCCTGGGTCAGTTTCAGCATGCCCTCTCTGAACTG

CAGTCCTGGCTAAGCCACACTCATGCTACCCTGGACACCCAGAGACCTGTCAACTCTGAC

CCCAAAGCCATAGAGATCGAGCTGGCCAAACACCATGTATTGCGCAACGACGTCCTGTCC

CATCGAGCCACAGTAGAGACTGTGAACAAGGCAGGCTCAGAGCTGCTGGAGTCCAGTCCC

GGAGACGAGGCCAGCCACCTGAGAGACCAGCTGGATGAGCTGAACCGCAGCTGGGACAGC

TTACTTCTGAAAACAGACGAAAGACAGAAGCTACTAGAGACGGCACTGCAGCAGGCGGAG

GGTTTCCATGGTGAGCTGGAGGAGTTCCTGCAGTGGCTGAGACGCACAGAGAGTCAGCTA

TCAGCAGCCAAACCCACAGGTGGCCTTCCAGAGACCGCCAGGGAGCAGTTGCAGCAGCAC

ATGGAGCTGCAGGCTCAGTTGACCCAGCGCGGTGAACAGTACCACCGACTGCTAGATCAG

GGAGAGTCCATGCTGTTGGCTAGAGGGGCAGAGGAAAACAGCCCTGGAACCACTCAAACT

CAGCAGAACCTGGCCCTGCTCCAAAACAAGTGGGCCAGTTTGAATGCCAAGATGGATGAC

AGGAGGGCTAAGCTGGAGGAAGCAGTATCACTGGCAACAGGATTCCAGTCGTCACTGCAG

GACACCATCAACTGGCTGACGCAGGCAGAGCAGACACTAAACATGGCTCAGTCACCGAGC

CTCATCCTCGACACTGTCCTCTTCCAGATAGATGAGCACAAGGTGTTTGTAAATGAGGTG

AACACACACAGGGAGCAGGTGCTGGCTCTAGAGAAGGCCGGGTCTCAGCTGCGCTTTGCC

AGCCTTAAACAGGACGTGGTGCTCATCAAGAACCTTCTGTTGAGCGTGCAAGCTCGCTGG

GACAAGCTGGTGCAGAGGTCTCTGGACAGGGGACGGCACCTGGACGAGGCCCGCAAAAGA

GCCAAACAGTTCCATGAGGCCTGGAGGAAGCTGACAGACTGGCTGGAGGAGGCTGAGAAA

CGGCTGGATGCTGAGCTGGAAATCTCCAATGAGCCAGATAAAATTAAAGTGCAGCTTACT

AAGCATAAGGAGTTTCAGAAAACTCTTGGTTCTAAGCAGCCAGTATATGACACCACAGTG

CGCTCTGGGAAGGCCATGAGAGACAAAGCTACACTGCCAGCCGACACACAGAAACTGGAC

AACCTCCTCGGGGAGGTCAGAGACAAATGGGACACTGTGTGTGGAAAGAGTGTTGAAAGG

CAGCACAAGTTGGAGGAGGCCCTGCTGTTCTCGGGTCAGTTTGCTGAGGCACTGCAGGCT

CTTGTGGACTGGTTGTACCGGGTAGAGCCACAGTTGGCTGAAGACCAGCCTGTCCATGGT

GACCTGGACCTTGTGTCTAATCTCATGGATTCTCATAAGGCCTTTCAAAAAGAGCTGGGG

AAAAGGACCAGTAACATACAGGCACTGAAACGTTCTGCTCGGGAGCTAATGGAGACAGGC

AGGGATGACACCGCCTGGGTCAAAGTTCAGCTGCAAGAGCTCAGCAACCGCTGGGAGACG

ATCTGCGCACTGTCTGTCTCCAAACAGACCCGTCTGCAGCAGGCTCTCAAACAGGCTGAG

GAGTTCCGCACAGCAGTGCAGATGTTATTGGAGTGGCTCTCAGAAGCAGAGCAAACTCTA

CGTTTTCGAGGCATCCTGCCAGAGGAGGTGGAGACCTTGCAGGTTTTATTACACACCCAT

CGAAATTTTATGCAGACCGTAGAAGAGAAGCGAGTGGATGTGAACAAGGCAGCTGGCATG

GGGGAGGCCATACTGGCAGTGTGCCATCCTGACTGTATCACCACCATCAAACACTGGATC

ACCATCATCAGAGCCCGCTTTGAGGAGGTTTTAACCTGGGCGAAGCAGCATGAGCAGCGT

CTGGAGGCAGCACTTGCCGAACTGCTCAACAATGCAACACTGCTGGAAGACCTGTTATCA

TGGCTACAGTGGGCGGAGACCACATTAGTGCAACGAGACACTGAGCCCCTCCCCCAGGAT

ATCACTCAGCTTAAAACACTGATCACAGAGCATCAGGTTTTCATGGAGGAAATGACACGT

AAACAACCTGACGTCGATAAAGTGACAAAAACCTACAAGAGAAAACCATCAGAAACGTCT

AGCAGTCTTGCTGAGAGACGGGGTGTCCGTAAGCAGCAGCAACAGCAGCAGCAGCCTGCT

ATGCAGGTAAGTGGAGGAAACCCTCGTCTTAACCAGCTCTGCTCACGATGGCAACAAGTT

TGGCTACTAGCACTGGACCGCCAACGCAAGCTCCATGATGGCCTTGACAGGCTTGAAGAG

CTGAAAGAATTTGCCAACTTTGACTTTGATGTGTGGAGGAAGAAGTACATGCGCTGGATG

AATCATAAGAAGTCCCGCGTCATGGACTTTTTCAGACGCATTGACAAGGACCAGGATGGC

AAAATCACCCGGCAGGAATTTATCGACGGCATTCTGGCTTCAAAGTTCCCCACCAGCAGG

CTGGAGATGACTGCAGTGGCTGATATCTTTGACCGAGATGGTGACGGTTATATTGATTAC

TATGAGTTTGTGGCAGCTCTGCATCCTAATAAAGATGCCTACAAACCCACAACAGATGCA

GACAAGATTGAAGACGAGGTCACCAGACAAGTCGCCCAGTGCAAGTGTGCTAAGAGATTC

CAGGTTGAGCAGATTGGCGAGAACAAGTACAGGTTCTTCCTTGGGAACCAGTTTGGCGAC

TCTCAGCAGCTGCGTCTGGTGCGGATTTTGCGTAGCACAGTTATGGTACGAGTGGGAGGG

GGATGGATGGCGCTAGACGAGTTCCTGGTCAAAAACGACCCTTGCAGAGTGCAACATCCA

GGACTTAGAATTCTTCGCTCTGACTCCAGCTGCTCCATATCATCTCGTATAGCACGCGGA

CGTACCAACCTGGAGCTACGAGAAAAATTCATTCTTCCTGAAGGGGTTTCACAAGGTATG

GCGGCCTTCCGTTCCCGTGGTCGACGATCTAAACCTTCATCACGCACAGCCTCGCCAACA

CGCTCATCCTCCTCAGCCTCGCAGAGTGCCCAGAGCTGTGCCTCTGGGCCATCTGCCCCT

GCCACACCCACTGCTTCTGCTAGGTCCTCCCAATCTCAATCGCGGGGCTATGCCAAGCCC

TGGCTGGCACACAGTAAAACTCCAACTCCAACCAAGTGCCAGTCCTGCACAGAGCACAGC

CACACCCCTGGGCACGAGGGGGGCTCCTCATCCAAGCTTAAAAGGCCACCTTTCCATTCC

AGCCGTGGATCCCTGACAGGTGAAAATGGCGGAACCACACATGCTACTAAACCTGTGCGC

TCTGACAATAAGCGCACGCCGTCCTCAACCAGCGGCCCGACTAGCCGCGCAGGCAGCCGT

GCAGGAAGCAGAGCCAGCAGCCGAAGGGGCAGTGACGCCTCCGATGCATCCGAGCTGATG

GAGACGCGCTCTGCCTGCTCTGACACTTCAGACACACCACGCCGCCCAGGCACTAAACCC

TCCAAAATCCCCACCATCTCCAAGAAAACACCCAGTCCCAAAACCCCCACCACTAAGAAG

TAGCAGGTTTTCAGCATGGACAGATGGAGACGGGGGATTTTTTTTTCTCTAGTGCATGCC

ACTCAACCTTCCTGTCCACCAGCACAGGCATGTGGGATTGATGAGAATAGAGAACTGAAA

AATCTCTCTTTTAAAAACACCTGGGCAGTGCTGACCCCACATCTGCCCCTGCAACAAACC

TAATAGTGCTGCCTTATTTGTGTTACAATATGACTGACTTTATAGTTTCTTGAACATGTG

ACTGACCTGGAAAGCAACTACCACGTACGCACATAGACACGTGGACTGGCACCACCACCG

GACCTGGGATTTTATCTCAGCCTTGTTGATATGCAATGGCCAGGCGCTCTTCAGTGCTGA

AGCTGACAGATGGTACAGCCACCATTCCAATACTGACTCTCTTAGGCACAAAGATTTTTT

TGTGAAACAAACAGTGGCAATGATTCTAAAGTTTCATCCACTGTATGCAAAGCTACTTGA

GTATTCTGCATCCATGTGGTGTGTGTTTTAAGTGCTAGTTTCTTTTTTTTCGGTGGACAA

ACGGAAAACATTCTAAGGAAGTGGTGAGCGATTTATCATCTCAGCCTTGCTAGTGAGTCA

GAAGCATGGTCATGTGACCGCAGCTTCACTTATCTTTCTCAAGAAAGCATGCACCAACAC

TCACTGATGGCTTCTGATGTGATGTGTGTATGTATCGCCAGTCAGCAAATGAAGAAAGTC

CTAATTCCTGAGAGCACTTAAGTGACTCGTAGAGAAGAATTTCACATGTGCACTGACATT

TCCTTTCCTTTCAAACCTGATGCTAACATTAGGCACGCTTACCGCTGTGGTTTAACCATG

ATTAGTTACACGCATCATGTTACAG

>Unigene000005

GGCCCCAGATCGGAAGAAGCTCTGGAAAGCACTCGATAATGATCCCAATCTTCCAAAGAA

ACTGCGTGACACTGCAAGACATCTTGAATGGTTGAAGACAGTTAAGGATAGCCATGGATC

TGTAGAACTTTCATCTCTGTCATTAGCTTCTGCTATCAATAAAAAAGGAATCTACTTTAT

CAGTGCAGAGAACCAAAAGAAGTTGAGTCTGGATAAAGTCTTGAAGCTCCGGATTACAGA

GGAGCATGCCGAAGACAATGAGGTACGATGCTACTCCCTTGATGACCTTAAGGAACTTCA

AAATAAGCTTATGCTTATGTCTGGAAAAGGGGACCAAGGCCAAAATGAAGTTGATCGATT

TGCAGAGGTTTTTGCCAGCGTACAGAGATTAGGTACTGCATTTATGGACCTATTTGCAGC

AGGAAATCCATTGTTCCGACTTTGGGAGGCAATTATTCACTGTAATAGTCAAGAGGCATG

CATAGTTATGGACTTTAACTTGGATGGTGTCACCAGTGTTGTCATGTTAAGGGGTGATGT

GGCAGAGCAACTCCTAGATGTCTGTAAGAAAATGGAGCGATGCCTTAGTTTTTGGAAAGA

TTTTATGGACAAGCAACGATCTCAGAATTACTATCTCAACTATTTTACAGCAGAACAGAT

TGTCTATCTGTGCAGTCAGTTGAACCAGACAAACTTGTCACATCTGGAAGATCAAGCACT

TATTATGCTCTCTTTTATCAAACTAAACTGTACTACCTACAACTTGAGGCAAAGCTGGCA

TGTTCTTCAGTATGAGATGCTAACAAATCCTACAAAGCAAAATGAAGACATTGACTTTCA

AACTTTTGCTGATGTTCCAAATCAAATTGAAAATGAGATAGATATTAACAGTGTCCCTTT

TTCTGATTCTGGTATAGAAAATGATACTAGAAGGTTTGACATGATTTGGAATATGTACAT

GAGAAATATGAAAACATTTCTTCCAAAATCACTTGATGTCAGAAGTCTAGGAAGACTTTT

GGAGATTTTGTCTGATAGCCCCAGTAAAACTGATGTAGATGATGATGATGATGATGATAA

TGCGGAAAAAAGTACTCTTGAAAATCTTCACAAAAATGTACAAAGAGTGCTTCCTAGGGG

ACTGGTTATTGGAAAACCCAATCTTATTGTCTGCCCATTCACAGAGATTCAAACTGCTTG

CATCTCCATTTACATGAGTAGTGAAGATGAGCCACTGCCCACATATGATGAAGTGCTTCT

CTGTGATTCTTCCACCTCATATGAACAAGCAGAGTTGTTCCTTAGGCGCTGTCTTTCTGC

TGGTTACAAAGGACAGAAGATATACACCATGCTATATGTGGACCAGCTAACCTACGAAGT

GAGCTACAGAGTAGAACAGTTTTTCCAACGCCACAAAGCTCAGAGCAGAAATGACTACAG

ACTGGTTTTGATCTGTAGTACTGATCGAGAACATGCCTACCTTCCCTCAGTCTTCAGTCA

GTTTAGGCTACACATGGTACCCCAAGAACCTCTTGGGAGAATTCAAGAATATCTTACCAA

GCACTACACAGTCCCCAAAGGCTATTTCAGCGCTGCTGCTGTTTTTAAAACAAAACAATA

TGTAGGAGTTGTATCCTCAAGAAGAGCAGGTGTTGGCAAATCACTTTATATACGTCGGCT

GTATGAAAAAGTAAAGTTGATTACCACGAAATCCTCACAGCTTAAGTGTATCCGGTTGAC

AAAGTCAAAAGTGGATGAAAATGCTGTTCTTCAATCAATGCTTCATGGCCCCCAGAGAGA

GCTAACCATTTTTCATTTTGATATTACATCTTCGGTACAGAAAGGTCTCCATGAATTTCT

CTTTCGGCTTCTGGTCTTGGGTTACCTAATGGACTCAGAAGGAAGAATGTGGAAATGCAA

CAGTAAACACCTATACATCATTGAACTCCTTCAGTCTAGTGCAAATATTTCTAGAAATCT

TCCAAGATCTAGTGAAAGTGTTCAGTCAGCCTTTCAGGATGTGTTTCCAAGTGTGTACTG

TCGAACTCCAAAAGAGGTCCTTGATCTAGAAATGAGAATGTCAGAAGACCCAACCATAAT

TGTGAATGACCCCCTTATGGATGATAAAGAATTCAGAAATGAGGCTTATCAGCGTCCATA

CCAGTATCTAAAGCGATTCCATCATGGAGTTAACTTGGATGGATTTATGTACAAAGGTAT

TGAGGGAACCCATGTGGAATGCCTACAGTTGCTGTTTCTGTATTGTGGTGTTGTGGACCC

TTCCTGGGCAGAGTTGAGAAATTTTACATGGTTCTTGAATCTGCAGTTACGAGACTGTGA

GACATCTGTATTCTGTGATGCATCATTTACAGGAGACACTTTGCTTGGATTTAAAAACTT

TGTGGTTGATTTTATGATACTCATGGCTAAGGACTTTGCTACACCCTCTTTGAGTATATC

TGATCAGAGCCCAGGAAGGCAACAAATGGACATTTCCGGTGTTAGTGAAGAAGACCTTGC

TCCATTTAGGGTCAGAAAAAGATGGGAATCAGAGCCTCATCCTTACATTTTCTTTAATGA

TGACCATGTGTCCATGACATTCATTGGCTTTCATCTACGGCCAAACGATCAGAATTTGGT

AGACGCAATTGATCCATCAACCAATAGAGTGATCAAAAAGAACATAATGACTAGTGAGCT

GTATGAGGGTCTCAAGTTGCAGAGAGTTCCTTTCAACATTAATTTTGATAGTCTCCCAAG

AGGTGAGAAAATTGAGCGAATTTGCAATGTTCTGGGAATACAGTGGCCCCTTGACCCAGA

TGAAACTTATGAGCTAACAACTGACAACATACTAAAAATCTTGGCTATACACATGCGCTT

CAGATGTGGCATTCCAGTTATCATCATGGGTGAGACTGGGTGTGGGAAGACCAGGTTAAT

AAAATTTTTGTGTGAGCTGCGACGTAGTGGAGTTGCCACAGAGAATATGAAACTTGTCAA

AGTCCATGGAGGAACAAGCGATGACATGATTTATACCAAAGTGAGAGAGGCCGAGTCCAT

AGCATGCAGCAATAAACAAGATTATGGTTTTGACTCTGTACTCTTTTTTGACGAGGCAAA

CACAACAGAGGCCATCAGCAGCATTAAAGAAGTACTATGTGATAAAACAGTAAAAGGTGA

GGTAATGGTGCCAAACACTGGCTTGCAAATTATTGCTGCATGTAATCCATACAGGAAACA

CTCAGATATAATGATTAAGAGATTGGAGTCAGCTGGTTTGGGATACAGAGTGCGAGCTGA

ACAAACGGATGAAAAGCTTGGATCCATTCCCCTTCGGCAACTTGTGTACAGGGTGCAGGC

CTTGCCTCCGAGCATGATTCCATTAGTTTGGGACTTTGGACAACTTAATGACCAAACAGA

GAAAATGTACATTCAGCAAATAGTTCAGAGGGTGGCTGAAGACCACTTTTTCAGTGTGGA

ATACATCAAGATTTTTACAGATGTCTTATCAGCTTCACAAAAATACATGAGGACAAGAAA

AGATGAATGTAGTTTTGTAAGCTTGAGGGATGTTGAACGTTGCATGCAAGTTTTTACATG

GTTCTACCAAAATAATGAAATGTTGCTGTCAGAACTTGCCAGGTACGAAATAAATCAGAA

AGCACAAAAAAATGAACACAATCAAAGAGACAATGATGTAAGAGACCCTATTCTGTGGTC

ATTAGTGATGGCTATTGGTGTGTGTTACCATGCCTGCTTGGAGGACAAAGAGAAGTACAG

AAACAAAATATGTGGCTACTTCTCAAAAACATACAGCCCCGTGAATTTAATGCAAGAAAT

TTCAGTAATGCAAGATCTTCTTCTCAGTGGAGTTCCACTAGGTGAAACAATTGCAAGGAA

TAATGCTTTAAAGGAGAATGTTTTCATGATGGTAATTTGCATCGAGTTACGAATTCCTCT

TTTCCTGGTTGGAAAGCCTGGAAGCTCCAAATCATTGTCCAAAACCCTGGTAGCAGATGC

TATGCAAGGTCAGGCAGCTCATTCAGACTTGTTCAGAAAACTAAAACAGATTCATCTGGT

GTCATTCCAGTGCAGTCCTCATTCCACACCAGAAGGTATCATCAATACATTCAAGCAATG

TGCACGTTTTCAAGAGGGCAAAAATCTAAGCGAGTACATCTCGGTGGTGGTACTGGATGA

AATTGGTCTAGCTGAGGATTCTCCTAAAATGCCTCTTAAAACTCTTCACCCTCTTCTTGA

GGAAGGGTGCATAGAAGATCAACCCCTGCCTCACAAGAGGGTTGGATTCATTGGTATATC

AAATTGGGCTTTAGACCCAGCGAAGATGAACAGAGGAATATTTGTTTCTCGAGGTGATCC

TGATGAGAAGGAGTTGATTGAGAGTGCCAAGGGCATTTGCTCCTCTGATGCAATGATCCT

TGAGAAGGTTGGGGATTTTTTTCAGCCTTTTGCCAAAGCTTACCTCAAAATCTGCAGCGA

AGGAGGAAAAGGGTTTTTTGGCTTGCGTGACTATTACAGCTTGATTAAGATGTTGTTTTC

TATTGCCAAAGCATCAGATCAAAAGCCAAGTGCAGATCAGGTTGTGGAGGCTGTGTTAAG

AAACTTTAGTGGGAAAGACACTGTGAATGCAGTTACTGTTTTTACCAACAGGCTTAGGTT

AAAACCTAACCTTGAAAGCATCGATACCATTGAGCTTGTGAGACAAAATATCACAGCAGT

TGCTCAAGATGAGGAGTGCCGCTACCTGCTGATTCTAACAAAAAATTATGCGGCCCTACA

GATACTTCAGCAAACATTCTTTTCAGAACAGTACCAACCTGAAATCATCTTTGGCTCAAG

CTTCCCTAAAGACCAACAGTACACCCAGATTTGCCGTAATATTAATCGTGTTAAGATTTG

CATGGAAACTGGTCAAGTCATTGTGCTTCTGAATTTGCAGAACTTGTATGAAAGCTTATA

TGATGCACTCAATCAGTACTATGTTTGCCTTGGAGGTCAGAAGTATGTGGATTTGGGCCT

CGGAACACACCGTGTCAAGTGTAGAGTACACAAGGACTTCCGGCTCATAGTGATTGAAGA

GAAGGACATTGTCTACAAACAGTTTCCCATTCCTCTAATTAACAGGTTGGAGAAACACTA

TCTGGACATTAACACTGTGCTGAAGAATGAACAAAAGCAGCTTGTGAAAATTCTTGAAGA

ATGGGTTGACCATTTTATTAAATGTAAGCATGCGCCTACTCCCCAGGCAGCAAAACACTG

CCCAACCGATGTTTTCATTGGATATCACTCTGATACCTGTGCTTCAGTCATCATGCAAGT

TATAGAAAGACAGAAAGGAAGGATGGAATTTTCAGACCCCGAGCGACAGCTGTTGGACGA

GGCCAAACTAATTCTACTGAACTGTGCTACACCAGATGCTGTGGTTCGCTTGGATAGTTC

AGCCCTTTCTAAAGTGGAGAGTGAGCACCTTTCAAGAGTGTACTTTGAGGACCAAAAGCA

CAGCTGTATGGCTGACTTTGTCTTTGGTCACATTCAGCAGGTGGAGTGGAACCATTCCTG

TTTTACTGAGGTAACTACCTTCTCCAGACTTTTAACAGCATCAGACATTGAACAGCTACA

GGAGGTAGTTCCAAACATCGAACTTCTTGCATTGCAACAGTTTGATACAGAGCATTCTTT

CCTCAAGAAAATCAGGAACTTTTTAAACTGTACATCTGGAAACAAGATGCTAATTATCCA

GACCGAATTTGATGAAGCCGTACAAAGCTACAACCTTATTGCTTCAGCAAAGTACTCCTC

CATTGGTGAAATCAACAAATTCCAAAAGGAGCATGACAGTAGGGTGTTTGTTTACTTTAT

TACCAAACTACCAAGAGTGGAAGGTGGAACTTCTTATGTTGGATTTCATGGAGGTCCCTG

GAAGTCAGTCCACATTGATGACCTTAGAAAATCAAAAGATATTATCTCTGATATTAAAGC

CTTGCAGTGTCTGACCATCAGTCAGCTGTTTAAGGAAACGGAAGATCAGTCTGAGGTTAT

GGAAACAGAGGATATGTATAGAGAAGAAGAAGAAACTGAAAAAATGGAGCAGGATGACAA

TACAAGCTGGAAAAATGTTCTAGATACCACAGCCCTGGTGCGTAGCTGTGTTCAGAGTGC

AGTCGGCATGCTGCGAGATCAGGGGGATGTGGGTTGTCGCAGCACCAAGAGAGTAGAAAT

TCTCTTAACTCTCTTGTCTGATAGTGAAGAACTTCAAGCAACATTTTTAAAAACTATGAA

GCATCGTCTTTATTCTCTCTTGATAACCCATGATGAGCACACACACTCAGCAAAGAATTG

GGTTTTCAAAGAAGCATCCAATATTGACTCTTTACAAGAGGGTGGAACATTCAGAAACAC

CTTATGGAAGCGGGTTCAAGCTGCTATTATTCCTTTCCTGGCACAGCTGGTCTCGGTTAT

TGATCGCGAGCACAATCTCGACCTCTTGCTAGATGAAAACTCTGGTGAATCAGTAAAAAA

ATTGTGGCTGGACATCTTTGGAAATGTCAAGTTATTAAATGTTCCATATACAAGAGTGGA

TTCCACCTCAATGACAAAGACAATTCTTGTCCAGAACTGTATTGTGGTTTATAAGAATAC

AGGCTGCTCTATGCCGTTCAGCTGGATAATCAAAGATCATTTAGAGGAACTCTGGTTACA

TGCATTGCACAGAGAAGGCCACAGTCTGAGACAGTTTGAGGAATTCTTTTGGAAGACTCC

ACTGGGTAGATATATTGCCAAAGCTGAGCAGGAAATGCAGCAGGAGTTTTTCTACAGATA

TCTTCAGGATTTTATCTCTTTGACAACAACAATAACAACCGAGGTTGAACTAAAGCTCCT

AGTGAAGGCTTTGTCCTGCTGCGTTAATGAATTGAGAAGCCGTCAGGAGGCAGTGCAAAA

TGAAGTGCCCTCTTTCCCCTGGATCCAGGCACCCTCCCATGATTTAAAAAACCGCCTGCA

AAATTTCTCCAGAATGATAATCATTGAACCCCAGATTGCTCAAGTGCTACTGGACAACAC

ACAGTCCAGAGACGGCAAAGAAATGGTGCTGGATGTGTATGCTGCCCTGGCCTGTGTTGA

GTATCTGGAGCCCCAAGGCCTGGATGCTTGTATAAATTGTCTGACTTGGCTTCGACAGGT

GAAGAAGTTACAAATCCCGATTGAGTTAGTCTGCTCAGAGGCAAGCATTCGACACTATGG

AGCAATGAGCAAAGCCATTATTCCCCGTGTCCAACATGGGTGGAGACGTATCCATGCCAT

TTCGCTATTTGTTGAGCACATACTTCTGGATATAGAGAACATAGAGAAAAAACTCAAGCC

ATTGGTCCTTGAGCACACACGAAAGTTGGCTCAAGTCTTGGACAAGAACTCTGACCTTAA

AATGAAACAACCATTTGAGGCAGTTATTGCCATTCTCAAAACTTGCAAGGATGGAGCAAA

TGAGCAGATTTTTAGGTTTGGTTTCCAGCCATGTCCAGTGTGTATGGGGGATCCTCATGA

TCCTCTGAGTCTTCCTTGTGATCATGTCTATTGTCTGGCCTGCATCAAGCAGTGGCTGAT

TCCGGGACAGATGTACTGTCCACTCTGCATCCAAGGAGTAGATGATGACTTTGATCTACA

GCCATCTGACACCATTCGGACCCTGATCAAACAAAATAGCCACTTCAGAAAGCAGTGCAA

TGCCTTCTTTATAGACTTTGTCTCCACCATTTGCTTCAGAGACAACTCACCACCCAGTAA

AACAATCATCCTCCACCTGCTCTCCTTCCTCATGGTGGAGGCAGGGTCTGTACCTACTAT

CAGAGGCTGTGAGAGACGTTTTTTGACCAAGGCTTTGTCTCCTTTTGATGACTCTGTGGA

CAAAAATCCTGTTGTACGGTCTGTTGTGCTCAAACTGCTGCTCAAGTACAGCTTTGCTGA

TGTAAAAGGCTACCTTCAACAACACCTCACAGAAGTGGAGCAAAGTAACATACTAGAGAA

AGCTGACAAAACTGAGCTTTATTGTCTGTACATAAACTGCCTTGAGGACTCTATGTTTGA

GCGGATCCAGTGGCATTCTGTGGCTGAACAGCAGGCCTGTCTGCTGGAGGAGAAGCATTT

TCTGGTGGAATACTTGCGTTCCGGAGCCGTGGCTACTGAAACTGTGACAATAGAACACCT

ACAGCAGGTGGCAAGAACTCGTCTATGCCTAGACATGGCAGCAGATCTACTAATCAGCAG

AAGCTCAACTGCAGATGCTGAAAAGGAAAGCTCAGCTGGGGTGACTGCATTCTTGGATTC

AGTGCAGAATCTTTGCAAGTACAGTAAGAATGACTGGTACAGGGTCTACCTCATTAGAAA

ACTATGTAGTCTCAGTGGAGTAGAATACATCCAAAAGCTCCTACAGGAAGATCAGCTAAG

CTGGCTGTTTCCGCAAGAGGTTCTCCAGATGAGAAAAACCAGTAGTCAGATTGACCAGTA

TTTGGTGTGTGGTGTGGAGTACAAGGCTATTCGAGATGCTGTTGCCAAGGCCATGTTAGA

AGGGAAAATGGAAACAATCGAAGAAGCATGCAAGATTTGCCAGTGTTCACCTGAGAAGAA

GAATGTGTACATTTTATTAGCTCTATTTAGAGAGGTCACTACTTTGTATAAAGCTAACAA

TCCTAACATCCACCCTACACCTGAGCAATTGGCAGCACTTGATGACTTCACCAAAAACTC

TAAAGTCTTTGTCGTGCCAGAATCTAGGGACTTTGCTCAGGCTCTAGTGCATAACCAACT

GCCTGTCCTGCATTTACATCCCAGTTTATTGGCTAATGAGTTTGTGATTCTGGAGCTGAC

TGTTCACCTGGCAGCAGTGCTTCTCTGTACAAACCAACCCTTGTTGCAGCCTCTCATACA

GCTTGCATTCTTTCCCATCAATATGCAGAAAGCCTTTTTACCCACTATGCCAGATGACAT

GCTTGCTGTTGCTCAGCAGGTCATGGGGCAGCTGCAGTGGTATTTCTGTCCAAATGGTCA

CCCATGCACTGTTGGTGAGTGTGGCCGGCCAATGGAGAAGAGTCACTGTGTAGACTGCAA

CGCAGAGATAGGCGGTGTTGCGCACAATCCTGTTCAAGGCTTCCAGCCAATTGCAGTACA

AGGTGACCGCACACAAACAGGGCACCTCCTGGGGGATCCACATCGCAGAGATCAGCCAGA

TATGCAGGACACGAAGAACATGTCTCCTGGTCCATTTACCCTGCTCAGGTTGCTGACACA

TATGGCCATGCTGTTAGGATCCTCCAGGCAGCCCCAGGAAGTGGCTGCAATCATTAAACC

CCCTGTGCCGAATCCTGCAACATTTCTACTGGCTCACCTGCTGAAAGATATTGAGCAGCT

TACAAGAGCAATAGGGAAGGGGGCAGATGACACAATTTGCACCATCCACCTGACCATCTG

CATTCTTCTGGAACCTCATCAGCCCAGGCCTCTACCTTATGATAATCTGCTCTCAACAAA

AGATGCCAGAAATAACTGGGAGATCACAGTGACCACTGATATTATCACATGCCAGCTAAA

GGTTTTGGAGAGTCACCTGGCAAATGTAAACACATTTATCAGAAATGATGAGAGAGTTTC

ATCCAACCCAATAATGAAGGTGACCTTCGGAGAACCTCGGCTGTTCCTGCGCACTTTACC

TCAAGGTTCCTTGATTCATAGCTCATCTGTGTGGTGCTGCAGACAGCGTGTCTCTCTCTT

GGGTCTTAGCCACATTTTGGAGCAATATGGAGGCAGGGACAGACTTCCCATCCTTTGGAG

GTTTCTACAAAAGGAGGCAGAGTTAAGGTTGGTGAAGTTTCTCCCAGACATTCTTGCTCT

CCAGAGGGATCTGGTAAAAAAGTTCCAGAATGTGATAGAGCTTCAATGTGGCACTATAGC

ACAGTTCCTGCAGAACCTGAAAGCAGAATCTCTAATGACTTCATATAAAAAACGGATCAA

TATCTTTCTGTCCACATGGAACCAACTAAGAGCATCTCTGGAAACAAATGGAGAGATCAA

ACTCCCAACGCAATACTGTCAAGACGATCTAGACCTTAGTGCTGACCTGCAAGTGCTTCT

GCCACGAAGACAGGGTCTGGGCCTGTGTTCCACCGCTCTAGTCAGCTATCTAATTGCTTT

GCAGAATGATTTAATTTATGCAGTGGACAAGCATACAGGAGAAGAGACTAGTTACACAGT

GAGTCCAGCTGACCTAACAGAGCTCCATGTCATCAGATACGAGGTAGAGCGAGATCTGCT

ACCTCTTGTGCTTTCAAACTGCCAGTATAGCATGGAGTGTGGCCGTGAGACCCTGTCCGA

ATATGACCTGCCCAAGATCCAGCAGCAGCTTCTCACACGCTTTCTGCAGGGCAAACCATT

CATTGGCCTCACTGGAATTCCAACTCTTGTAAACAGACATGACCGAAATTATGAAACAAT

CTTCCGGGATGTGAAAGGCAAAGTTCAGCAAGAATCTATGACTTCACTGAACCAGTCTAC

ACTGTGTGCCGAGCTGCAGTGCTATAGTGACGTGTGTGAGGCGTTGTCCACTGTGGAAAT

AGCCCTGGGATTTTTAGCCATGACTGGAGGTGAACCCAACATGCAACTGAGCAATTACCT

GAAGGAAGAACTCCAAATGGCTGACCAAATACCTTTGCATGTACTTAAGGCTCTGAGTAG

GTGCTGTCTGAAACACTGTTTGGCTCTGTGGCAGCTACTCACCTCTCTCAAATCGGAGAC

AATGCTACGCCTTAAGAGGGACCCATTTGAAAGGTTTAGTAAAGACTATAAGAAAACATT

GGGGGAGCAGGAGAAGAGACTGCTTACTGGCTTCTTCTCAAAGACCAGTGCTGACTCATT

CTTGCTGGAAATGCATGAGTTTTTACTGTTGGTATTGAAGAACGCTAATGCCACAGATAC

ATACAAGCCCAACTGGAGTGTGAAAGACACTATCTTGTCCTACATGGAGCAAAAAGACAT

GGATGTCCCACCAGATGTGGTGGAGCTGTTCCCTGATGAAATACTTCTAGCTCACTACAT

TGAGGCTTGGAAGTTTTCCATTCTCCTCAAGCAAGAGCGAATACAAAGATAAAGCTAAAT

TTAAAGATTTCTCATCATTATCATTTTTCCACTCCATACATCAGTGTACTTATCTTAAGA

TTTGCGTTTTTATTACAATCATCACTGCACATTCATATTAGCTGGATGTGGGGTAAAAAG

TGCCACTTGGAGGTTTAAACTTGACAATGTCTGTATTTGTTGCTGCATAAATCTAATAGC

ACAGAAAATATAATCAAGCTACAGATTGCACAAGAGCAGTGAATCCTTTTGTTTCGGCTG

GCAATCAAATATAAGTTAATACAATCAGTGATTATTTCATTTGTCATCATACAAATTTGT

GGGATTATTCCATTATATAGCGTTACTGTAAATCTGTTTACATTTACTTCTTTAGGTTTT

GTTTGAGGAATATCTCCTATATATGCACTAATCAGGGTACAATAAAATAGTCCAAAATTA

TACTCAGGGACATTTTAAAAATCACATCAACTGGGTGAAAAAAAAGATTGTTGATCCCCT

GGGACCAA

>Unigene000006

GTAAACACACACACACACACAGACTCCTGTATCAGCGCTCTCCTGGAAGCGGCTCTGAGT

GGAAATGAGGAAAAGAAGCCATTCGACTGTGCACTTCGAGCTGACATAAGTGTGTTAGTG

GAGCTGGCACTCGGATCCACAAAGGAGCCAACAAACAGCCTGTGGACCAACATGGTGGAC

TATGCCATCAGCAAGGACTGGGACAGTGCAACTCTAAGTAACGAGGCTTTGTTGGACACC

GTGTCTCACTTTGTTCTGGCAACGCTGCTGAAGCACACAGGGCTGCTGGGTCATGCCTGC

GGAGGGGGCAGGTATCAGCCGTGTAAACTGTTGGCCGAAGTGTACCGGAGTGTGTATAAG

GTGCGGAATCGTTTGCTTGCCTGCAAGAACATGGAACTGGTCCAGACACACTCGCCATCC

AGAGAGCGCAGGATCTCTGAGAACGTGGACTCTGTGGAAATGGACCCACAGGAACACGCT

TTCACCCGCACCATCGACGAGGAGGCCGAACTTGAGGAACGAGCTGAACGAGAGAGAGAC

CGAGAGGACGGCCATCAGGAGCAGGAGGACGAGGAGGAACGCGAGCATGAGGTCATGACA

GCAGGAAAAATCTTCCAGTGTTTTCTCTCGGCGCGGGACGTTGCGCGTGGTAGAGAGCGA

CCTCGCACGAGCAGCGTCGGCGTCCCAGACGAGTCTGTTCAACCTGAACAGGAGCGCAGA

AACAGCATCCCGTTACAGGACAGACAAGACCTCTACACCACCACCTGCAACAGCATCATC

CACCGCTGCGCCATGCTCCTGATGGCAGTCAGCCCCACACTCCCGCAGACGACCAACGAG

CAGCCAGCTTTGTGTGGTGGGGTCACGCAAGACGGAAGCGGATTTTTGATCCGAAGCGAG

AGTTTGTCAACTGAGAGTCGATCCATCCAGAGCGGTCCGAGTTACAGGCTCACTAAATCT

AGGAGTGAATCTGATCTCTCCCAGCCCGAGTCTGATGAAGAAGGATACTCGCTGAGTGGA

AAAAGGAACGCAGACCTGGATTTGGCGTTCACTCCTAAAAAGAGAGAATTTGCACATAGC

ACCCCTGACTCTGCGGACTGGTTCGGGCGGCATTCTGGTCGCGAGCGTCTGTACAGCTCC

CCTCAGTCTTATGAGGAGTCGGACTTGGACTTCAGCTGCACGCTGCACATCCATGCAGTC

ATCGACAACATCGTCAGCTTCATCAGTGGGGATGTGGGAAACACGCCCGGATTCAAGGAA

CCAGAGGACAGCATGTCAACTAGTCCTCAAGCTACAGTTGTAGCCATGGAGCAACAGCAG

GGCAGAGCGGAGTTGCGGTTAGACGCCCTTCATCAGATTGTGGTTTTGATCTCTGGAATG

GAGGAGAAAAGCAGTCAGGGTGGAGCAGAGGCAGTGGCTGGGTGTGGTCCCGCTGCCTTT

CACTCCGCATCCTTGCTGACCTCAGTCAGACTGCAGTTCTTGGCAGGATGTTTTGGCCTT

GGCGCCTATAACGGGGGGCTAAACGGTGAGAGCGTCCAGCTGCACCACTACCAGGACGGC

ATTCAAGCTGCTAAAAAGAGGTTACAGATGAACATTCAGACAGCAGTGCACAAAATCTAC

CAGCAGCTTTCCATCACACTGGAGAGAGCGCTTCAGTCTAATAAACACCATATAGAGGCT

CAGCAGCGGCTGCTGTTAGTGACCGTTTTTGCCCTGAGTGTTCGTTACCAGCCTGTGGAC

GTTTCGTTAGCCATCTCCAGCGGTTTGCTAGATGTTCTCTCTCAACTGTGCGGGAAAGAA

ACCCTGCTGGGACACACCCTTCAGCTCCTGCACAAACCCGCCGGCTCGCAACTCAGCACA

GCACTCAAGGTTGCCAGCACCCGTCTGCTGCAGATCCTTGCCATTAGCACCGGGACGTAC

GCAGATCGCCTGAGCCCTAAAGTGGTTCAAGCTCTGTTGGACCTGCTCTGTAGTCAGCTG

AAGACTCTGCTGTCGCAGGCTGCAGGAAGCGTCTTCGGCACAAATTCAGAGTCAGAGGAA

AAGACAGAGGACTCAACAGGCAGCATGAAAAAAGACTTCAGGGCCTTACTGCGCAAACAG

CACATTGCGGAACTACATCTCGGAGACTTTCTGGTGTTTCTCAGAAGAGTCGTATCATCT

AAAGCCATTCAGTCCAAGATGGCGTCTGCCAAATGGACCGAAGTGCTGCTTAACATTGCG

GCACAGAAATGCTGTTCAGGTATCCCTCTGGTGGGGAATTTAAGGACACGTCTGCTGGCA

CTGCATGTCCTAGAGGCTGTACTTCCTGCCTGCGAAAGTAGCGTGGAGGATCATCAAATG

AGTCAGGTGGTTGAAAGGTTGTTCTCCTTGTTGTCGGACTGTATGTGGGAGGCTCCTGTC

GCTCAGGCCAAAAACACCGTCCACCTGAAGGAAAAGGAGGTCAAACTCCAGGGTGAGGCA

GAGGAGGAGGAAGAGAACCTGCCTGTCCAGGAAGTGATCTTCGATCCTGAAAAGACACAG

TGCTGCGTGGTGGAAAACGGACAGGGTTTGACGCACGGTAGCGGTGGAAAAGGCTACGGT

CTGGCTGCTACCGGCATCTCCTCAGGCTGTTACCAGTGGAAGTTCTACATCGTGAAGGAG

AATCGTGGGAATGAAGGGACGTGTGTGGGGGTTTCCCGTTGGCCTGTAAACGACTTTAAC

CACCGCACCACGGCCGACATGTGGCTGTACCGGGCTTACAGTGGCAACCTGTACCACAAT

GGCGAGCAGACGCTCGCGCTGTCCAGCTACACACAGGGCGATTACATCACCTGCGTGCTG

GACATGGAGGCTCGCACAGTTTCCTTCGGGAAGAACGGAGAGGAGCCCAAACTGGCTTTC

GAAGACGTGGATGCGACAGAGCTTTATCCATGCGTCATGTTCTACAGCAGCAACCCAGGA

GAAAAGGTCAAAATCTGTGACATGCAGATGCGAGGGACGCCACGTGACCTTTTACCCGGC

GACCCCATCTGCAGTCCCGTGGCCACCGTCCTTGCCGAAGCGGTGTCTCAGCTGGTCAGG

ATCCTGCACCGGTCAGACAGCTGGACGCAACCTATCAACCGGTGCATGCTTCAGCGGCTG

AAGCAAATCCGGGGGTGCTTGCACGACGGCGCTTCGCCTACGGGTGGTGCGAGACTCCGG

AAGAGCCGCTCGGCTCAGAGCCGCGAGGAACAGGATGAGGCGAAAGACGAGGACAGGGAT

GAGGAGCGAGGCAGAATGGGGCGGGAACTGACTGAGGCGCAGCTGCGTACACTTTGTAAC

CTGATCTGGCCTGTGCTGGCGGTGATTGGAGGGGTGGACGGAGGTCTGAGAGTCGGAGGT

CGCTGCGTGCACAAGCAAAGCGGGAGACATGCAACCCTGCTGGGGGTGGTGAAGGAGGGC

AGCATGTCGGCCAAGGTGCAGTGGGACGAGGCAGAGATCACTATCAGTGACACGCCGCTA

TATAACCTGGAGCCCTGCGAGCCGCTGCCGTTTGACGTGGCACGTTTCCGGGGACTGACG

GCCGCGCTGCTTTTGGAACTGACCGCTCTGGCTTCGCTGCAGGACGACGGCAAGTTCTCT

CCTACGGCCTGCTCGTTATCATCTTCCTCGAGACGCCACGAGCGCCGCCACAGACACGAG

CCTCACGAGCCCGAAGAAGGCCCCGGTGACCTGCGTGTCTCTCACAGCCTGGATGAAGTG

CGTTCCCACGCCGAACTGCAAAAGCCTCCTGTAATGGCCGAGGTTCATGCTGTGCAACTC

TCCTACCTCTGCCTTGGTGCCATAAAGAGCCTAAGCGTCCTTCTAAGCTGCAGTAAGTAT

GCCGAGCTCCTGCTCATACCCAAGGCGGTGCACGACAACGGCCACAACGCAGACTGCGGC

AGCCCCAGCATCGGCGTCTCACAGGAAGAGGCAGAGATGCGCTCAGCGCTGCAGTTCCTT

ATGAGACACATGGTAAAGCGAGCAGTGATGAGGTCACCTATCAAACGCGTACTGGGTTTA

GCTGAACTGGAGCGGGCGCAGGCCATGATTTACAAACTCGTCGTCAATAGTGTGCTAGAA

GAACAGGAGGGCGGGAAAACCAAACAATACGGCGAGTGTGAGGAGCTGGAAGGCGAACAG

CAGTTCCAGACGCCGGTTACCACCTCACCCTCTGCGTCCAGTAGCACCTCATTCATGAGC

TCCTCCCTCGAGGACACCACCACAGCAACCACACCTGTTACCACGGCAACTACACCTATA

ACTGATGCTGAGACTGCGCCAGCCTCAGAATCGCCAGGTGTTATGCCTCTCAGTCTGCTC

AGACAGATGTTCTCCAGCTACCCCACCACAACGCTGCTGCCCGCTCGCCGAGCTCAGACA

CCTCCAGTCTCGTCTCTGCCCACGTCTCCTTCAGACGAACACGGCCGAAGGCAAAGTTTT

ACCTCTCCTGAATCTCAGCCTGCTAGAACTACCAACAGGACAGCGCTGTCGGACCCCAGC

AGCCGTCTCTCCACATCTCCCCCTCCCCCTGCCATCGCTGTTCCCCTTCTCGAGATGGGG

TTCTCCCTCAGACAGATCATGAAGGCTCTGGAAGCTACAGGTACAAGAGGTGAGGCTGAC

GCGCAAAACATCACGGTTCTGGCCATGTGGATGATCGAGCACCCGGGCAACGAGGACACA

GAGGAGCGGACCCTGTCCTCCGACTGCTCGGGGGCAGCAGGAGGGGGCAAAAGTGCGGAG

CGGAGCTTCCTGCGTTCACCTGGAGATATTCCCAACGCTGATGCCGCAGAGCTTGAAGAG

GGATTTAGCGAGAGCCCTGAAGGAATAGATCAAGACTCCGCCTCTGCATCCAACGGCCCC

GCCCTCCGGGGGCGCTCAGCCGCCAGTAGGAAGCATCGCTTCGATCTGGCAGCACGTACT

CTATTGGCCCGTGCAGCTGGACTGTACCATTCGGTGCAGGCCCACCGCTCTCAGGTGCGT

AGGGAGGGCTCAGGGCTGCAGCAGGACTCCAGCTCACTGGGAGGAGTTTACGACTTTAAC

CTGGATGAGGAGCTGGAGCTAGAGCTGGACCAAGAGGCCATGGAGGCCATGTTCACTCAG

GAGCTTGCATCAGACTCGGATATTTTGGGAATGTGGATTCCGGAGCGTGTGTGTGAGTCA

GAAGAGCGCGAGGAGGTGGTGGTCTGTGAGCTCTGCGACGTCAGCGTGTCCAGTTTTAAC

CAGCACATGAAGAAAAGTCATCCTGGCTGCGGACGGAGCGCAAATCGGCAGGGTTACCGC

AGTAACGGGTCCTACGTGGACGGGTGGTTTGGGGGAGAGTGTGGGAGCGGGAACCCCTAT

TACTTACTGTGCGGAAGCTGCAGGGAAAAGTACCTGGGCATGAAGAGCAAGCACAAAGGA

GCAGTGTCGGAGAGATACAAGGGCCAGGCTCCAGACCTGCTGGGAAAACAGGACAGTGTC

TATGAAGAAGACTGGGACATGCTGGACGTGGATGAGGCGGAGAAGCTGACAGGCGAGGAG

GAGTTTGAGCTGCTTTCAGCACCGCTGGGACTCACGGAACGGAAACCCGTCCCCGAGGCT

GTTCAGTTCCCTGACACAGACCCCCTCGGGGCCTCCGCTGCCATGGTTACCGCCACCAAT

AGCATGGAGGAGACCCTGCTACAGATAGGGTGTCAGAGTTCGGTGGATAAAAGCACATCA

GGTCGCATGTGTTTGGGTGAGCAAGCAGCCGGTCTCCAGAGTTCTGCTGATCGCATGGTT

GCTTTGAGGAGAGTCACAGCAGCTGCTCAAGTCCTGGTGGCAAGAACCATGGTGATGAGG

GCGCTGTCTTTGCTGTCTGTCAGCGGTTCCAGCTGCAGTCTCGCTGCTGGTCTGGAAGCT

CTGGGTCTGACGGACATTAGGACCCTGGTGCGTTTGATGTGTCTGGCTGCGGCCGGTCGA

GCAGGTCTTTCTACCGGTCTCGCTTCAGGCCCCGGCGCTTTAGAGCGGCCACGTGGCACG

AACAAGCCCACCAAACCCATCTCCTGTCTGGCCTACCTCAGCACAGCAGTGGGCTGCCTC

GCCTCCAACAGCCCAAACGCCGCCAAGCTTCTGGTCCAGCTGTGCACTCAGAACCTGATT

TCAGCAGCTACAGGAATGAATCTGACCACTGTGGATGACCCAATTCAGAGGAAGTTCCTG

CCCAGTTTCCTGCGGGGCGTGGCTGAGGAAAACAAACTCATCACCTCGCCAAATTTCATA

GTGACTCAGGCATTGGTGGCGCTCTTAGCCGATAAAGGAGCCAGACTGAGACCCGGGTAC

GACAAGGCCGAAATGGAGAAGAGAGGCCCGTTGGAGCTGGCCAATGCGCTGGCGGCGTGC

TGCCTCTCATCCCGCCTCTCCTCACAGCACCGCCAGTGGGCTGCGCAGCAGCTGGTGCGG

ACACTTGCCGCCCATGACCGCGACAACCAGAGTCGACCGCAGACCTTCGCCGACATGGCC

GGTGACTTACGCAAGTGTTCTACCATCAAGCTGGAGGCTCATCAGGGCAGAGTGATCAGC

TTCGGGTGGTGTAACAAGAAAGGTCTTTTGGCGACCAGCGGTAGTGATGGAACAGTTCGA

GTCTGGAACGTGAACAAGAACCAATACACTCTGCAGCAGACCTGCGTCTTTAACAAAACA

GACTTTACCTCTGAGGAGTGTTCCACCGGACTGGGGTCTCCTGGTGAACCGAGCTTGGCG

CCCGTGGCCTGGAGTGTGAGCGGGAAATTAGTTGCAGCTGCCATGGAGAAAGTGGTGAAC

ATCTGGCAGGTCAATGGCGGTAAGGCCTTGTTGGACCTGCAGCCTCACTGGGTGTCGTCG

CTGGTGTGGCCGGAGAACGAGGCAGGGTGTCTTTGGGCCGGAGAGTCTCGAGAGCTCCTA

TTGGTCGGTCGCATAGATGGAAGTTTGGGCTTGCTGGAAATTTTAGATAGCTCAAACATA

CAGAGAACCGAACTTCTGCACTGTTACAGGAAAGACGCTGTGATGAACATCGCCTGGTAC

AGCGAGGATCGACCGTTTGCTGTCGGCTACGCTGACGGAAAGCTTCTGATTGGCTGCAAA

GAAGCGCTGGAGAACGGATCCGTGGTCGTCATCGATGCTCACAAGGAGAGCATCAGTTCT

CTGCGCTGGGCTCCAGGAGGGCAAATTTTATTAAGCTGTGCCAAAGAGGAGACTGCCTGC

CTTTGGGCGGAGTCTGGGGCGGGGCTTGGGCGGAGTTGGGTGTGTCTCCAGAGCATCGCT

CACCCGAGTGTTGTAAATGCTGTCGCCTGGTGTGGTCTCACCGGACAGGGACCTAAAGCA

CTAAACATGCTGGCCACGTGTTGTCAGAATGGCTTGGTGTCTGTGTGGACCGTGCCTCAG

GACCCCACAGCCTTCTCTCAGTCTGGCTCATCCAGCACTGATGCATGGTGGGAAACGGAC

TCCAAGTCAAAACCAAGGTTCACTCCACAGTGGCACGAGGCCGCAGTGTGCGTCTTCCAG

CTAAGAGGTCACATGACTCCGGTTAGGACGATAGCCTTTAGTCCTGACGGTCTTGCTCTG

GTTTCTGGTGGTGTTGGTGGTCTCCTAAACATCTGGTCTCTCCGAGATGGCTCTGTGCTG

CAGACGGTGGTTGTCGGCTCAGGAGCGATTCAGAACACTGTGTGGATTCCAGACGTCGGG

GTGGCCGTCTGTTCCAACCGCTCAAAGGACGTTCTGGTTGTGAACTGCTCCTCGGAGTTT

ATGACCGCTAATCAGGTGTTAGCAACCTGTCGCACCGCGCTGAAGAAGCAGGGGGTTGTG

GGATTGAACACGGCTCCCTGTATGAGGACTTTTCTCGAGAGACTTCCCATAATGCTTCAG

GAGCAGTACGCCTACGAGAAGCCCCATGTAGTGTGTGGAGAGCAGCTGATCCACAGTCCC

TACATGCAGTGTTTGGCATCTCTGGCCGTCGGGTTGCAGTTGGACTCGTTACTATGTCGT

CCCGCAGTGCCTCTTCACCTGTCTCATTGCCTCGCCCAACCTGTCTCTTCATCATCGTCA

TCATCACAATCATCCTCCTCATCCGGTCCTTCAGCACTCATGCACTCATGTGGCAGCAGC

GAGTGGGCGTGGCTTCACTGCTTCTCCACCACCATAAAGACGGCTGAGGCGCTCGCCCGC

GCTCACACTTTCCCAGAATCCTTTAACGTGCCTGATCTGGAGCCGGTGGCCAAAGACAAG

ATGGCGGTGCTGATGGACAACAGTAAATGGGTTCCTGGGATGGATGAGCAGTTGATGGCT

TGGGCCACCTCCAGACCCGAGGACTGGCACCTGGGTGGGAAGTGTGACGTGTATCTGTGG

GGCGCCGGTCGCCACGGTCAGCTCGCCGAGGTCGGCCGGAACATTCTGGTGCCGACGCCT

GCTGCGTCTTTCTCTCAGGCTCAACAGGTTGTATGTGGGCAGAACTGCACATTTGTGATT

CAGGCGAACGGCACAGTGTCTGCGTGTGGGGAGGGCAGCTACGGTCGACTGGGTCAGGGA

AACTCTGATGACCTGCACATTCTTACGGTCATCTCAGCACTTCAAGGGTTTGTTGTTACC

CAGCTGGTCACGTCGTGTGGCTCTGATGGACACTCCATGGCTCTGACTGAGAGCGGTGAG

GTGTTCAGCTGGGGAGATGGCGACTACGGCAAACTGGGCCACGGCAACAGTGACCGCCAA

CGCCGGCCGCGCCAGATAGAGGCTTTACAGGGAGAGGAGGTGGTGCAGATGTCCTGTGGG

TTTAAACACTCTGCCGTCGTCACAGCAGACGGTAAACTCTTCTGCTTCGGCAATGGAGAC

TACGGGAGACTCGGACTGGGAAACACATCCAATAAGAAACTTCCAGAACGAGTCACAGCT

TTGGAGGGACAACAGATAGGCCAGGTGGCGTGCGGGCTAAATCACACACTGGTGGTGTCA

TCTGACGGGTCGACCGTCTGGGCGTTTGGAGACGGAGATTATGGAAAACTTGGCTTGGGA

AACTCGACTGCTAAATCATCACCGCAGAAAGTGGATGTTCTGTGTGGAATTGGAATCAAA

AAGGTTGCATGTGGAACCCAGTTCTCTGTGGCTTTAACCAAAGATGGAAATGTTTACACT

TTTGGGCAAGATCGCCTGATCGGTCTCCCAGAGGGGCGTGCCCGCAATCACAACCGCCCG

CAGCAGCTCCCCGCTCTGGTGGGCGTGATTATCAAAGATGTCGCTGTAGGGGCGGAGCAT

ACACTCGCGCTGGCGTCTACAGGAGATGTTTATGCTTGGGGGAGCAACTCTGAGGGACAG

CTCGGTTTGGGCCACACCAACCATGTACGAGAGCCGACTCTAATCACAGCTCTGCAGGGT

AAAAACACCCAGCAGATTTCAGCAGGACGCTGCCACAGTGCCGCATGGACCGCGCCCTCA

GTACCACCCAGAGCACCAGGCTCGTCCATGCCGCTGCAGCTGGGTTTGCCCGTGTGTGTT

CCTCCGCAGTACGGCAGTCTGAGAGACATCAGCATGGAGGCACTAAGAGCTCGGCTACGT

CTGCTCTACCACTTCTCTGACCTCATGTACTCCTCCTGGAGACTGCTTAACCTCAGCCCT

AACAACCAGAGCTGCACGTCTCATTATAACCCTGGGACGTGGGGTATCGTACAGGGGCAG

CTGCGCCCTCTACTGGCTCCACGTGTGTATATGCTCCCGATGGTACGCTCCATCGGCAAG

ACCATGGTGCAGGGCAAGAATTACGGACCTCAGATCACCGTCAAGAGAATCTCCACTCGT

GGGAGGAAGTGTAAGCCCATTTTCGTGCAGATCGCTCGGCAGGTAGTGAAGCTGAATGCG

TCTGATCTCCGGCTGCCGTCTCGGGCGTGGAAGGTGAAGCTGGTGGGGGAGGGAGCGGAC

GACGCTGGAGGAGTTTTTGATGACACCATTACAGAAATGTGTCAGGAGCTCGAGAACGGG

GTGGTGGATCTGCTCATCCCTTCTCCTAATGCCACTGCGGAAGTGGGCTACAACAGAGAC

AGGTTTCTGCTGAATCCCTCGGCTTGCCTGGACGAACATTTGCTGCAGTTCAAGTTCCTG

GGCATCCTGATGGGCGTGGCCATCCGCACCAAGAAACCTCTGGACCTGCATCTGGCTCCG

ATGGTGTGGAAACAGCTGTGCTGCATCCCGCTGACGCTGGAGGACCTGGAGGAGGTGGAC

CTGCTGTATGTCCAGACACTCAACAGCATTGTGCACCTGGAGGACAGCAGCATCACAGAG

CAGAATTTCCACGAGATGATTCCTCTGGATTCATTTGTGGGTCAGAGCGCTGATGGGAAA

ATGGTGCCCATCATTCCGGGAGGAAACAGTATTCCACTCACCTTCTCCAACAGGAAGGAG

TACGTAGAACGCGCCATAGAGTACCGGCTACATGAACTGGATCGTCAGGTGGCGGCCGTG

CGTGAGGGCATGTCATGGATTGTACCGGTTCCTTTACTCTCGCTGCTGACCGCTCGGCAG

TTGGAGCAGATGGTGTGTGGTCTGGCGGAGATCAGTGTGGAGGTGTTGAAGAAGGTGGTT

CGTTATCGGGAGGTGGAGGAGCAGCAGCAGTTGGTGCAGTGGTTCTGGCAGACGCTTGAC

GAGTTCTCCAACGAGGAACGAATGCTGTTCATGCGCTTCGTCTCCGGACGCTCGCGTCTC

CCTGCTAACACAGCCGATATCTCGCAGCGCTTCCAGATCATGAAGGTGGACCGGCCGTAC

GACAGCCTTCCCACGTCTCAGACCTGTTTCTTCCAGCTCCGCCTCCCGCCCTACTCCAGC

CAATCGGTGATGGCAGAGCGGCTACGCTACGCCATTAATAACTGTCGTTCTATCGACATG

GACAATTACATGCTGTCACGGAATGTGGACAATACTGAGGGTTCGGACACGGACTACTGA

CCGAGAGTTAAAGCACAAAGACACTCGCTGAACTTTAAACCGTCAACACAAAAATCACCA

CCTTTAATTCTTCTCTTCTTTTATTAATCATTAAAGCAGTCAGACGGCGTTAACCAGGTA

CAGATAAATATAACCATTTCTGTTATTCACAATCCTCTCAACTCCCCACACACACACACA

CACACACCGTTTTTTTTAAAAGGTTTTTTGTATTTCTTAATCCTGGG

>Unigene000007

TCAGCAGATCGGAAGAGTGGAAATGAGGAAAAGAAGCCATTCGACTGTGCACTTCGAGCT

GACATAAGTGTGTTAGTGGAGCTGGCACTCGGATCCACAAAGGAGCCAACAAACAGCCTG

TGGACCAACATGGTGGACTATGCCATCAGCAAGGACTGGGACAGTGCAACTCTAAGTAAC

GAGGCTTTGTTGGACACCGTGTCTCACTTTGTTCTGGCAACGCTGCTGAAGCACACAGGG

CTGCTGGGTCATGCCTGCGGAGGGGGCAGGTATCAGCCGTGTAAACTGTTGGCCGAAGTG

TACCGGAGTGTGTATAAGGTGCGGAATCGTTTGCTTGCCTGCAAGAACATGGAACTGGTC

CAGACACACTCGCCATCCAGAGAGCGCAGGATCTCTGAGAACGTGGACTCTGTGGAAATG

GACCCACAGGAACACGCTTTCACCCGCACCATCGACGAGGAGGCCGAACTTGAGGAACGA

GCTGAACGAGAGAGAGACCGAGAGGACGGCCATCAGGAGCAGGAGGACGAGGAGGAACGC

GAGCATGAGGTCATGACAGCAGGAAAAATCTTCCAGTGTTTTCTCTCGGCGCGGGACGTT

GCGCGTGGTAGAGAGCGACCTCGCACGAGCAGCGTCGGCGTCCCAGACGAGTCTGTTCAA

CCTGAACAGGAGCGCAGAAACAGCATCCCGTTACAGGACAGACAAGACCTCTACACCACC

ACCTGCAACAGCATCATCCACCGCTGCGCCATGCTCCTGATGGCAGTCAGCCCCACACTC

CCGCAGACGACCAACGAGCAGCCAGCTTTGTGTGGTGGGGTCACGCAAGACGGAAGCGGA

TTTTTGATCCGAAGCGAGAGTTTGTCAACTGAGAGTCGATCCATCCAGAGCGGTCCGAGT

TACAGGCTCACTAAATCTAGGAGTGAATCTGATCTCTCCCAGCCCGAGTCTGATGAAGAA

GGATACTCGCTGAGTGGAAAAAGGAACGCAGACCTGGATTTGGCGTTCACTCCTAAAAAG

AGAGAATTTGCACATAGCACCCCTGACTCTGCGGACTGGTTCGGGCGGCATTCTGGTCGC

GAGCGTCTGTACAGCTCCCCTCAGTCTTATGAGGAGTCGGACTTGGACTTCAGCTGCACG

CTGCACATCCATGCAGTCATCGACAACATCGTCAGCTTCATCAGTGGGGATGTGGGAAAC

ACGCCCGGATTCAAGGAACCAGAGGACAGCATGTCAACTAGTCCTCAAGCTACAGTTGTA

GCCATGGAGCAACAGCAGGGCAGAGCGGAGTTGCGGTTAGACGCCCTTCATCAGATTGTG

GTTTTGATCTCTGGAATGGAGGAGAAAAGCAGTCAGGGTGGAGCAGAGGCAGTGGCTGGG

TGTGGTCCCGCTGCCTTTCACTCCGCATCCTTGCTGACCTCAGTCAGACTGCAGTTCTTG

GCAGGATGTTTTGGCCTTGGCGCCTATAACGGGGGGCTAAACGGTGAGAGCGTCCAGCTG

CACCACTACCAGGACGGCATTCAAGCTGCTAAAAAGAGGTTACAGATGAACATTCAGACA

GCAGTGCACAAAATCTACCAGCAGCTTTCCATCACACTGGAGAGAGCGCTTCAGTCTAAT

AAACACCATATAGAGGCTCAGCAGCGGCTGCTGTTAGTGACCGTTTTTGCCCTGAGTGTT

CGTTACCAGCCTGTGGACGTTTCGTTAGCCATCTCCAGCGGTTTGCTAGATGTTCTCTCT

CAACTGTGCGGGAAAGAAACCCTGCTGGGACACACCCTTCAGCTCCTGCACAAACCCGCC

GGCTCGCAACTCAGCACAGCACTCAAGGTTGCCAGCACCCGTCTGCTGCAGATCCTTGCC

ATTAGCACCGGGACGTACGCAGATCGCCTGAGCCCTAAAGTGGTTCAAGCTCTGTTGGAC

CTGCTCTGTAGTCAGCTGAAGACTCTGCTGTCGCAGGCTGCAGGAAGCGTCTTCGGCACA

AATTCAGAGTCAGAGGAAAAGACAGAGGACTCAACAGGCAGCATGAAAAAAGACTTCAGG

GCCTTACTGCGCAAACAGCACATTGCGGAACTACATCTCGGAGACTTTCTGGTGTTTCTC

AGAAGAGTCGTATCATCTAAAGCCATTCAGTCCAAGATGGCGTCTGCCAAATGGACCGAA

GTGCTGCTTAACATTGCGGCACAGAAATGCTGTTCAGGTATCCCTCTGGTGGGGAATTTA

AGGACACGTCTGCTGGCACTGCATGTCCTAGAGGCTGTACTTCCTGCCTGCGAAAGTAGC

GTGGAGGATCATCAAATGAGTCAGGTGGTTGAAAGGTTGTTCTCCTTGTTGTCGGACTGT

ATGTGGGAGGCTCCTGTCGCTCAGGCCAAAAACACCGTCCACCTGAAGGAAAAGGAGGTC

AAACTCCAGGGTGAGGCAGAGGAGGAGGAAGAGAACCTGCCTGTCCAGGAAGTGATCTTC

GATCCTGAAAAGACACAGTGCTGCGTGGTGGAAAACGGACAGGGTTTGACGCACGGTAGC

GGTGGAAAAGGCTACGGTCTGGCTGCTACCGGCATCTCCTCAGGCTGTTACCAGTGGAAG

TTCTACATCGTGAAGGAGAATCGTGGGAATGAAGGGACGTGTGTGGGGGTTTCCCGTTGG

CCTGTAAACGACTTTAACCACCGCACCACGGCCGACATGTGGCTGTACCGGGCTTACAGT

GGCAACCTGTACCACAATGGCGAGCAGACGCTCGCGCTGTCCAGCTACACACAGGGCGAT

TACATCACCTGCGTGCTGGACATGGAGGCTCGCACAGTTTCCTTCGGGAAGAACGGAGAG

GAGCCCAAACTGGCTTTCGAAGACGTGGATGCGACAGAGCTTTATCCATGCGTCATGTTC

TACAGCAGCAACCCAGGAGAAAAGGTCAAAATCTGTGACATGCAGATGCGAGGGACGCCA

CGTGACCTTTTACCCGGCGACCCCATCTGCAGTCCCGTGGCCACCGTCCTTGCCGAAGCG

GTGTCTCAGCTGGTCAGGATCCTGCACCGGTCAGACAGCTGGACGCAACCTATCAACCGG

TGCATGCTTCAGCGGCTGAAGCAAATCCGGGGGTGCTTGCACGACGGCGCTTCGCCTACG

GGTGGTGCGAGACTCCGGAAGAGCCGCTCGGCTCAGAGCCGCGAGGAACAGGATGAGGCG

AAAGACGAGGACAGGGATGAGGAGCGAGGCAGAATGGGGCGGGAACTGACTGAGGCGCAG

CTGCGTACACTTTGTAACCTGATCTGGCCTGTGCTGGCGGTGATTGGAGGGGTGGACGGA

GGTCTGAGAGTCGGAGGTCGCTGCGTGCACAAGCAAAGCGGGAGACATGCAACCCTGCTG

GGGGTGGTGAAGGAGGGCAGCATGTCGGCCAAGGTGCAGTGGGACGAGGCAGAGATCACT

ATCAGTGACACGCCGCTATATAACCTGGAGCCCTGCGAGCCGCTGCCGTTTGACGTGGCA

CGTTTCCGGGGACTGACGGCCGCGCTGCTTTTGGAACTGACCGCTCTGGCTTCGCTGCAG

GACGACGGCAAGTTCTCTCCTACGGCCTGCTCGTTATCATCTTCCTCGAGACGCCACGAG

CGCCGCCACAGACACGAGCCTCACGAGCCCGAAGAAGGCCCCGGTGACCTGCGTGTCTCT

CACAGCCTGGATGAAGTGCGTTCCCACGCCGAACTGCAAAAGCCTCCTGTAATGGCCGAG

GTTCATGCTGTGCAACTCTCCTACCTCTGCCTTGGTGCCATAAAGAGCCTAAGCGTCCTT

CTAAGCTGCAGTAAGTATGCCGAGCTCCTGCTCATACCCAAGGCGGTGCACGACAACGGC

CACAACGCAGACTGCGGCAGCCCCAGCATCGGCGTCTCACAGGAAGAGGCAGAGATGCGC

TCAGCGCTGCAGTTCCTTATGAGACACATGGTAAAGCGAGCAGTGATGAGGTCACCTATC

AAACGCGTACTGGGTTTAGCTGAACTGGAGCGGGCGCAGGCCATGATTTACAAACTCGTC

GTCAATAGTGTGCTAGAAGAACAGGAGGGCGGGAAAACCAAACAATCAGACGGCGAGTGT

GAGGAGCTGGAAGGCGAACAGCAGTTCCAGACGCCGGTTACCACCTCACCCTCTGCGTCC

AGTAGCACCTCATTCATGAGCTCCTCCCTCGAGGACACCACCACAGCAACCACACCTGTT

ACCACGGCAACTACACCTATAACTGATGCTGAGACTGCGCCAGCCTCAGAATCGCCAGGT

GTTATGCCTCTCAGTCTGCTCAGACAGATGTTCTCCAGCTACCCCACCACAACGCTGCTG

CCCGCTCGCCGAGCTCAGACACCTCCAGTCTCGTCTCTGCCCACGTCTCCTTCAGACGAA

CACGGCCGAAGGCAAAGTTTTACCTCTCCTGAATCTCAGCCTGCTAGAACTACCAACAGG

ACAGCGCTGTCGGACCCCAGCAGCCGTCTCTCCACATCTCCCCCTCCCCCTGCCATCGCT

GTTCCCCTTCTCGAGATGGGGTTCTCCCTCAGACAGATCATGAAGGCTCTGGAAGCTACA

GGTACAAGAGGTGAGGCTGACGCGCAAAACATCACGGTTCTGGCCATGTGGATGATCGAG

CACCCGGGCAACGAGGACACAGAGGAGCGGACCCTGTCCTCCGACTGCTCGGGGGCAGCA

GGAGGGGGCAAAAGTGCGGAGCGGAGCTTCCTGCGTTCACCTGGAGATATTCCCAACGCT

GATGCCGCAGAGCTTGAAGAGGGATTTAGCGAGAGCCCTGAAGGAATAGATCAAGACTCC

GCCTCTGCATCCAACGGCCCCGCCCTCCGGGGGCGCTCAGCCGCCAGTAGGAAGCATCGC

TTCGATCTGGCAGCACGTACTCTATTGGCCCGTGCAGCTGGACTGTACCATTCGGTGCAG

GCCCACCGCTCTCAGGTGCGTAGGGAGGGCTCAGGGCTGCAGCAGGACTCCAGCTCACTG

GGAGGAGTTTACGACTTTAACCTGGATGAGGAGCTGGAGCTAGAGCTGGACCAAGAGGCC

ATGGAGGCCATGTTCACTCAGGAGCTTGCATCAGACTCGGATATTTTGGGAATGTGGATT

CCGGAGCGTGTGTGTGAGTCAGAAGAGCGCGAGGAGGTGGTGGTCTGTGAGCTCTGCGAC

GTCAGCGTGTCCAGTTTTAACCAGCACATGAAGAAAAGTCATCCTGGCTGCGGACGGAGC

GCAAATCGGCAGGGTTACCGCAGTAACGGGTCCTACGTGGACGGGTGGTTTGGGGGAGAG

TGTGGGAGCGGGAACCCCTATTACTTACTGTGCGGAAGCTGCAGGGAAAAGTACCTGGGC

ATGAAGAGCAAGCACAAAGGAGCAGTGTCGGAGAGATACAAGGGCCAGGCTCCAGACCTG

CTGGGAAAACAGGACAGTGTCTATGAAGAAGACTGGGACATGCTGGACGTGGATGAGGCG

GAGAAGCTGACAGGCGAGGAGGAGTTTGAGCTGCTTTCAGCACCGCTGGGACTCACGGAA

CGGAAACCCGTCCCCGAGGCTGTTCAGTTCCCTGACACAGACCCCCTCGGGGCCTCCGCT

GCCATGGTTACCGCCACCAATAGCATGGAGGAGACCCTGCTACAGATAGGGTGTCAGAGT

TCGGTGGATAAAAGCACATCAGGTCGCATGTGTTTGGGTGAGCAAGCAGCCGGTCTCCAG

AGTTCTGCTGATCGCATGGTTGCTTTGAGGAGAGTCACAGCAGCTGCTCAAGTCCTGGTG

GCAAGAACCATGGTGATGAGGGCGCTGTCTTTGCTGTCTGTCAGCGGTTCCAGCTGCAGT

CTCGCTGCTGGTCTGGAAGCTCTGGGTCTGACGGACATTAGGACCCTGGTGCGTTTGATG

TGTCTGGCTGCGGCCGGTCGAGCAGGTCTTTCTACCGGTCTCGCTTCAGGCCCCGGCGCT

TTAGAGCGGCCACGTGGCACGAACAAGCCCACCAAACCCATCTCCTGTCTGGCCTACCTC

AGCACAGCAGTGGGCTGCCTCGCCTCCAACAGCCCAAACGCCGCCAAGCTTCTGGTCCAG

CTGTGCACTCAGAACCTGATTTCAGCAGCTACAGGAATGAATCTGACCACTGTGGATGAC

CCAATTCAGAGGAAGTTCCTGCCCAGTTTCCTGCGGGGCGTGGCTGAGGAAAACAAACTC

ATCACCTCGCCAAATTTCATAGTGACTCAGGCATTGGTGGCGCTCTTAGCCGATAAAGGA

GCCAGACTGAGACCCGGGTACGACAAGGCCGAAATGGAGAAGAGAGGCCCGTTGGAGCTG

GCCAATGCGCTGGCGGCGTGCTGCCTCTCATCCCGCCTCTCCTCACAGCACCGCCAGTGG

GCTGCGCAGCAGCTGGTGCGGACACTTGCCGCCCATGACCGCGACAACCAGAGTCGACCG

CAGACCTTCGCCGACATGGCCGGTGACTTACGCAAGTGTTCTACCATCAAGCTGGAGGCT

CATCAGGGCAGAGTGATCAGCTTCGGGTGGTGTAACAAGAAAGGTCTTTTGGCGACCAGC

GGTAGTGATGGAACAGTTCGAGTCTGGAACGTGAACAAGAACCAATACACTCTGCAGCAG

ACCTGCGTCTTTAACAAAACAGACTTTACCTCTGAGGAGTGTTCCACCGGACTGGGGTCT

CCTGGTGAACCGAGCTTGGCGCCCGTGGCCTGGAGTGTGAGCGGGAAATTAGTTGCAGCT

GCCATGGAGAAAGTGGTGAACATCTGGCAGGTCAATGGCGGTAAGGCCTTGTTGGACCTG

CAGCCTCACTGGGTGTCGTCGCTGGTGTGGCCGGAGAACGAGGCAGGGTGTCTTTGGGCC

GGAGAGTCTCGAGAGCTCCTATTGGTCGGTCGCATAGATGGAAGTTTGGGCTTGCTGGAA

ATTTTAGATAGCTCAAACATACAGAGAACCGAACTTCTGCACTGTTACAGGAAAGACGCT

GTGATGAACATCGCCTGGTACAGCGAGGATCGACCGTTTGCTGTCGGCTACGCTGACGGA

AAGCTTCTGATTGGCTGCAAAGAAGCGCTGGAGAACGGATCCGTGGTCGTCATCGATGCT

CACAAGGAGAGCATCAGTTCTCTGCGCTGGGCTCCAGGAGGGCAAATTTTATTAAGCTGT

GCCAAAGAGGAGACTGCCTGCCTTTGGGCGGAGTCTGGGGCGGGGCTTGGGCGGAGTTGG

GTGTGTCTCCAGAGCATCGCTCACCCGAGTGTTGTAAATGCTGTCGCCTGGTGTGGTCTC

ACCGGACAGGGACCTAAAGCACTAAACATGCTGGCCACGTGTTGTCAGAATGGCTTGGTG

TCTGTGTGGACCGTGCCTCAGGACCCCACAGCCTTCTCTCAGTCTGGCTCATCCAGCACT

GATGCATGGTGGGAAACGGACTCCAAGTCAAAACCAAGGTTCACTCCACAGTGGCACGAG

GCCGCAGTGTGCGTCTTCCAGCTAAGAGGTCACATGACTCCGGTTAGGACGATAGCCTTT

AGTCCTGACGGTCTTGCTCTGGTTTCTGGTGGTGTTGGTGGTCTCCTAAACATCTGGTCT

CTCCGAGATGGCTCTGTGCTGCAGACGGTGGTTGTCGGCTCAGGAGCGATTCAGAACACT

GTGTGGATTCCAGACGTCGGGGTGGCCGTCTGTTCCAACCGCTCAAAGGACGTTCTGGTT

GTGAACTGCTCCTCGGAGTTTATGACCGCTAATCAGGTGTTAGCAACCTGTCGCACCGCG

CTGAAGAAGCAGGGGGTTGTGGGATTGAACACGGCTCCCTGTATGAGGACTTTTCTCGAG

AGACTTCCCATAATGCTTCAGGAGCAGTACGCCTACGAGAAGCCCCATGTAGTGTGTGGA

GAGCAGCTGATCCACAGTCCCTACATGCAGTGTTTGGCATCTCTGGCCGTCGGGTTGCAG

TTGGACTCGTTACTATGTCGTCCCGCAGTGCCTCTTCACCTGTCTCATTGCCTCGCCCAA

CCTGTCTCTTCATCATCGTCATCATCACAATCATCCTCCTCATCCGGTCCTTCAGCACTC

ATGCACTCATGTGGCAGCAGCGAGTGGGCGTGGCTTCACTGCTTCTCCACCACCATAAAG

ACGGCTGAGGCGCTCGCCCGCGCTCACACTTTCCCAGAATCCTTTAACGTGCCTGATCTG

GAGCCGGTGGCCAAAGACAAGATGGCGGTGCTGATGGACAACAGTAAATGGGTTCCTGGG

ATGGATGAGCAGTTGATGGCTTGGGCCACCTCCAGACCCGAGGACTGGCACCTGGGTGGG

AAGTGTGACGTGTATCTGTGGGGCGCCGGTCGCCACGGTCAGCTCGCCGAGGTCGGCCGG

AACATTCTGGTGCCGACGCCTGCTGCGTCTTTCTCTCAGGCTCAACAGGTTGTATGTGGG

CAGAACTGCACATTTGTGATTCAGGCGAACGGCACAGTGTCTGCGTGTGGGGAGGGCAGC

TACGGTCGACTGGGTCAGGGAAACTCTGATGACCTGCACATTCTTACGGTCATCTCAGCA

CTTCAAGGGTTTGTTGTTACCCAGCTGGTCACGTCGTGTGGCTCTGATGGACACTCCATG

GCTCTGACTGAGAGCGGTGAGGTGTTCAGCTGGGGAGATGGCGACTACGGCAAACTGGGC

CACGGCAACAGTGACCGCCAACGCCGGCCGCGCCAGATAGAGGCTTTACAGGGAGAGGAG

GTGGTGCAGATGTCCTGTGGGTTTAAACACTCTGCCGTCGTCACAGCAGACGGTAAACTC

TTCTGCTTCGGCAATGGAGACTACGGGAGACTCGGACTGGGAAACACATCCAATAAGAAA

CTTCCAGAACGAGTCACAGCTTTGGAGGGACAACAGATAGGCCAGGTGGCGTGCGGGCTA

AATCACACACTGGTGGTGTCATCTGACGGGTCGACCGTCTGGGCGTTTGGAGACGGAGAT

TATGGAAAACTTGGCTTGGGAAACTCGACTGCTAAATCATCACCGCAGAAAGTGGATGTT

CTGTGTGGAATTGGAATCAAAAAGGTTGCATGTGGAACCCAGTTCTCTGTGGCTTTAACC

AAAGATGGAAATGTTTACACTTTTGGGCAAGATCGCCTGATCGGTCTCCCAGAGGGGCGT

GCCCGCAATCACAACCGCCCGCAGCAGCTCCCCGCTCTGGTGGGCGTGATTATCAAAGAT

GTCGCTGTAGGGGCGGAGCATACACTCGCGCTGGCGTCTACAGGAGATGTTTATGCTTGG

GGGAGCAACTCTGAGGGACAGCTCGGTTTGGGCCACACCAACCATGTACGAGAGCCGACT

CTAATCACAGCTCTGCAGGGTAAAAACACCCAGCAGATTTCAGCAGGACGCTGCCACAGT

GCCGCATGGACCGCGCCCTCAGTACCACCCAGAGCACCAGGCTCGTCCATGCCGCTGCAG

CTGGGTTTGCCCGTGTGTGTTCCTCCGCAGTACGGCAGTCTGAGAGACATCAGCATGGAG

GCACTAAGAGCTCGGCTACGTCTGCTCTACCACTTCTCTGACCTCATGTACTCCTCCTGG

AGACTGCTTAACCTCAGCCCTAACAACCAGAGCTGCACGTCTCATTATAACCCTGGGACG

TGGGGTATCGTACAGGGGCAGCTGCGCCCTCTACTGGCTCCACGTGTGTATATGCTCCCG

ATGGTACGCTCCATCGGCAAGACCATGGTGCAGGGCAAGAATTACGGACCTCAGATCACC

GTCAAGAGAATCTCCACTCGTGGGAGGAAGTGTAAGCCCATTTTCGTGCAGATCGCTCGG

CAGGTAGTGAAGCTGAATGCGTCTGATCTCCGGCTGCCGTCTCGGGCGTGGAAGGTGAAG

CTGGTGGGGGAGGGAGCGGACGACGCTGGAGGAGTTTTTGATGACACCATTACAGAAATG

TGTCAGGAGCTCGAGAACGGGGTGGTGGATCTGCTCATCCCTTCTCCTAATGCCACTGCG

GAAGTGGGCTACAACAGAGACAGGTTTCTGCTGAATCCCTCGGCTTGCCTGGACGAACAT

TTGCTGCAGTTCAAGTTCCTGGGCATCCTGATGGGCGTGGCCATCCGCACCAAGAAACCT

CTGGACCTGCATCTGGCTCCGATGGTGTGGAAACAGCTGTGCTGCATCCCGCTGACGCTG

GAGGACCTGGAGGAGGTGGACCTGCTGTATGTCCAGACACTCAACAGCATTGTGCACCTG

GAGGACAGCAGCATCACAGAGCAGAATTTCCACGAGATGATTCCTCTGGATTCATTTGTG

GGTCAGAGCGCTGATGGGAAAATGGTGCCCATCATTCCGGGAGGAAACAGTATTCCACTC

ACCTTCTCCAACAGGAAGGAGTACGTAGAACGCGCCATAGAGTACCGGCTACATGAACTG

GATCGTCAGGTGGCGGCCGTGCGTGAGGGCATGTCATGGATTGTACCGGTTCCTTTACTC

TCGCTGCTGACCGCTCGGCAGTTGGAGCAGATGGTGTGTGGTCTGGCGGAGATCAGTGTG

GAGGTGTTGAAGAAGGTGGTTCGTTATCGGGAGGTGGAGGAGCAGCAGCAGTTGGTGCAG

TGGTTCTGGCAGACGCTTGACGAGTTCTCCAACGAGGAACGAATGCTGTTCATGCGCTTC

GTCTCCGGACGCTCGCGTCTCCCTGCTAACACAGCCGATATCTCGCAGCGCTTCCAGATC

ATGAAGGTGGACCGGCCGTACGACAGCCTTCCCACGTCTCAGACCTGTTTCTTCCAGCTC

CGCCTCCCGCCCTACTCCAGCCAATCGGTGATGGCAGAGCGGCTACGCTACGCCATTAAT

AACTGTCGTTCTATCGACATGGACAATTACATGCTGTCACGGAATGTGGACAATACTGAG

GGTTCGGACACGGACTACTGACCGAGAGTTAAAGCACAAAGACACTCGCTGAACTTTAAA

CCGTCAACACAAAAATCACCACCTTTAATTCTTCTCTTCTTTTATTAATCATTAAAGCAG

TCAGACGGCGTTAACCAGGTACAGATAAATATAACCATTTCTGTTATTCACAACTCCCCA

CACACACACACACACACAC

>Unigene000008

GAGCTTCAATACAACCTCGAGTGAGTTGAACCAAAGTCTGGATCTGGGCAAGGAGCTTCT

GATCCAGCAGTGCAGAGACCTCACAGAGCAGCTAGAAGTAAAAGAAAAACAGCTGGAAGT

TCTTCAGGAAGAAGTGCGCCGCTCAGCCGAAGATTTGGAAGAGGCGCGAGAGCGTTGGTC

TAAAGCATCAGAGGAGCTTGAGGAAGCAAAGTGGGAGCTGGAGTCAGAACGAGACAAAAG

GCTTCAATGTGAGGAGCTCCTCAATGAGAAAATCCATGAGCAGGACAACCTAAAGAACAA

ACCCGGCTTCTTGGAGAACCAGATAGAGAAAGATCAGCCAACATCCAACATGGAGTATGA

CGAGAAAAGCTTTTCTCCAGAGAAACTAACTGAAGAACTCGAACTGGTTCTCCAAGAGCA

GCTAGGACAGATGGACTACAGGGAGGACAGAATGGAGAACGTGCAGAGGCTGGAAAAGGT

CCGAGTGGACCTGGAGTCTGAGCTGACGTGTCTAAAGGAGAACCTGAGTAACATGGAAGA

AGCTTTACGTCAAGGTTCTGCAGAGAAAGCGGCTATGGAGAAGCGACTGCAGAAGCTGGA

AGAAAAGAACAAGAGCATGGAAAAGGCTCTGGAAATGGAACTGGAGAACTTTGAGCTTCA

GCTTCAAGCGAAGGATGTGGCCTTTGAGCAGATGAAAGAAGCCAGAGAGAAGGCGGAAGA

GGAGCTCATGGAAAAGGAGACCACTCTGCAGAAAGAGAAGAATGAACATGAAGAGGAGAG

GAGACGACTGGAGGAGAAACATCAACAGGAGATGCTGGACTGGAGTCTGCGTTTGGAGAA

GGAGCTCTCTGATCTGCGGTCTGATCTGGAAGAGGAGCAGAAGAAACAGATCACACTGAT

TAAACAGGTACACGAACGAGAGCATGAGAGAGAGATGGACGAACTGGTGGCCCAACAAAA

AGCTGAACTGGAGCGGCTGAAGGCTGAGCTCTCTGAGGAGCTGAGGGACAGTATGGAGGC

TGCACATCAAGCCGAGCTTTCGCAAGTCCAGACGCAGCACACTCTGGAGCTGGAGGCTCT

CCGTCTGAGCCTGACCAACATGCACACGGCGCAGCTGGAGCTCACGCAATCCAACCTGCA

GAAGGAGAAGGAGGCGGCGCTGAGCGAGCTGCAGGCCAGTCTGAGGGAGAAGTGGGCTCA

GGAGAGCGCCATGCTGCAGACACGACAGCAGTTTGAGCTGGAGAAGTTCCGAGAACAGGG

CAGGGAGCAGGAGAGACAAACCGAGCTCCAGCACCAACTAGCCGTCGATGATCTAAACTG

TGTGTGGGAAAGGCGTCTTTCAGAAGAACGAGCCGCGCTGGAGCAACAGCTTGCTGGTGG

AATAGAGGATGCAAAAATTCGGTGGATACAAGACTTTAAAAAAGAACAGGCGGAGCTTCA

GGAAGCCCTGAACAAAGCCGAGGACCGTTTAGCCGAGACGAAACGCAAACTGGAGGAGCT

TCAGAGATCCAAAGAGGAGGAAATCAAACATCTGGAGTCGGAATTGAGTCAGGCGTGGGC

CGACCGAGACTCTGCTGCTCGTGCTGTTGAAGAACTGGTCGCTAGTCACAGGGTGGTGCT

ACAGGAGCAGCAGGCTCACACACAAGAGCTGGAGGAAAAGGAGAAATGGCTGAAGCAAGA

GGTGGATCGTCTGCTTGAACAGAAATCCAGCTCCGAGCAGGAGATCACAAACCTCTGGTC

TCAGCTGGAGAGCATGAGGACCAGCAGGCAGGAACTAGGAGAGTTGAAGGAGCAGTTGCT

AGCACGCAAGTCACATGTTAATGACATCGAGCAGTTGAAGCAGGACTTCTCGCAGCAGCG

GCGAGAGCTCCAGGAGCAAAACGAGACCGAGCTGGAGAACCTGCGTGTGTACTTCGAACA

GCGGCTCCGGGCCAGCGAAGAGAACCACCGGGAGGAAATCGCTCTGCTGCAGCTACGCCT

CGTAGAGGGAGCTTTGGAGGACTCGGTTCTCAAGACCGGAGAGGAAAGCTTCCTCTCTGA

TGGAAAGTCTGAGGAGGAGAAGGGAGACTCGTTGGCAGAGATTACAGAACAACTGGATAA

ACACAAGGTGGAGTTGGATGCACTGCGGCTGCAGCTGGAGGAGAAACACAAGCAGGACCT

GGAGCAGCTGCGCTGTAACATGGCTCTATCCTATAGAGAGGAGTTACTGCAAGCTCGTAC

CGAGCTCACCGACCGCTACTACAGCGACATGGAGCAGCTCAAAACTAAACACGCCTTGGA

GATCGAACAGCTGCGGGCCAAGCTCTCCGACAGCCACCTCAAAGAAATAACAAGGCTGCG

GCTGCAGTCAGTAGCAGATGTAGCGCGTCAGGTGGAGACTGAGCTGGAAGAGAAGAGCCG

GCTCCAAACCCAGGAGCACCAAGCCCGACTCGCTCAGCTCGCCTCAGACACAGAGAGCAT

ACTGAGCCTGGAGAAGAAGCTCGCCGCGCTCACCCTGCAGCACAGCCGAGAGCTGGAGAG

AGCAACTGAGCAGCATGCGGAGGAGCTCCGACAGCTGGAGGAGAGGCTGAAGAAAAACTT

TTCAGAAGAACTGCACACACGTCTCGCAGAGGCTCAACAGGAAGAAAGGACCCATGTGAC

CGAAGAGCTGCTCAACAGGAGCAGTGAGGAACGGCTCCAGCTCCGGGAACAGCTTCAAGC

ACAGGCTGAGGAGCGCGTGGCTTCCCTAAGAGAGGAGCTCCAACAAGCAGCCAGTAAGGA

CAGACAAGAGTTGGAGCAGAAGCTTGTTCAGGCTGAGGAGCTCCTGGCTGAGGAAAAGCA

GAGGCTACAAGCTTTACAGGTGAGTCTGGAGAGGGAGGAGAGCCCTCAGCTGCTGGCCCT

GAAGCAGAGGCTGGAATCTCAATACGCTGGCGAGTTGCACACAGCAAAGAGCACCATGGC

AGCTGAGGTAAAGGAGCTAAACACTTTGCTGCAGGAGCAGACGGAGAGCAGACTGAAGGA

GGCCACGTGCAGACATCAAGAGGAACAGCGGCAGCTAGCAGAGAAACTCGTGCAACAGAA

AGAGGAGGCTCTTCAGAAGCTGAGGCAACAACATGCTCAGGAGCTGCAGGTCCAGGAAAC

TCTCCTGGAACAGAACGTTTCAGAACTCCAACACCTCCAGAAGGAGTACCAGGAACTCAA

ATCCCGACACCAGGAGGAGCTCGACCTGCTCTCTTCCAATCACAAGACAGCGCTGGACTC

CCTCGCTGAGAAACACAAGGCTGAGCTGGATAAGCTGCAGGTGGTATTTCAGGAGACCAA

CTTGGCACAGCTCGAAGCTCAGGAGGCGGAGCTTGAAGAGCGTCACAAGCAGGAAATGGA

AGAACTGGAGACCCGGATGTTGTGCAACATGGACACTCTGGAGAGCACATACCTGAAGGA

GATCCAGGCTGTGCGCGAGGAGAAGGAGGCGGCGCTCCGGGATCTCGGGGCCTCCCTGGA

GCAACAGCAGGCTGAAGAGATTGAAAGACTGAAGCAGGAGGAGCTGATTGTCAGAGAAGA

GCTGAGAAAAAAACTGGCACAGGTGCACATGGACAAGTTTAGTGTCATGGCGACTGAACT

CAGCCACGCCCATCAGGCAGAGCTGTCTGCGGCGCTCGCTTCTCAGAAAGCGGCTCTGGA

GTCAGAGCACCACTCCGCGTTGGAGACCCTGCGACAGCACGTGCTCGATCTGGAGGCGCA

ACACGGCGCCGCCCTCCACGAGGTCACCGACATGACTACCAAGGAGAATCAGCAGCTGAA

GAAACAGCTGGATGCACTGAATGCTCAGCACCAGCAACAACTCCAGGAACTGAGGGCAAC

ATCCTCCAGGGAAATCGAAGCTCTACGCAGGGAGCTGGAAGAAGAAGCATCAAGACAGCG

GCTCCATTTCCTGGAAGAGGCGGAGCTTCTAAAGTGCCAGTCAGAGGAACAGCTACAGAA

AAAGCTAATGGAGTTAAAGGAGGAAAGTGATCGAGAAAAGGTCTCTGCTCTAGAGGAACT

GGAGAGAAGTCTGTGTGAGCAGCACCAGCAGAACGAGCTCTCCTACACTGAAAAAATGAA

CCATCTGACCACACAGCTGCAACAGCTGGACACCGTGGTCTCTCAGCTGCGTGCAGAGGT

GAGCGGTTTGCAAGGGGAGCTGGAGGGTAAGCGCTCGGAGATGGACACGCTGGAGACTCT

GCTGCAGCGACGCGAGCGCGAGAGTCAGGAAGGAGCCAACCTACTCGCCATGCTGCGTGA

TGACCTCAACACCGCCACCCAGCAGAGACAAGATCTTCAACTGACTCATGACCGACTCCA

GCGGCTTGTCCTGGAGATGCTTCGGATTACAATTGCGACAGAGGAGGAGATCAGTCGCAG

GTTGGGGATATCCATGGAAGGTGATGACATCACTCTGAACAGGAAGCAAAGTGTCAACCG

TGACCCGCAGAAAACGGACGCTGAGGTGGGCGACGGTGCTGCTGCAGCTGTAGAAGCCAG

TGCTAACTGCAGCATGTTCTCGTCACTGATGGATGAAGGTCTGGAGCTGTCTCAGCGACT

GTGTGAAAGTTTGTTTACAGGACCTGATGGAGAGCTGGACCCTGAAAGAGAGGAGCTGGT

ACTGGGGGCCTGCACTCATCTACGTGTCGCAGTGGACAAATTATTGGAGGTAGTTTCCGA

CTCCACAGTGCAGCTGGAGCAGCTGCAAGCTCTGCAGGCCCTGCTGGATGAAAAGTTCAG

TGCTGGTGGAGAAGCGAAGAACGCGCTCTTCCTGCAGCACACTCAGCTTCTGGAGCAACT

AGACCAGGAGGCCAGCGTGAAGAGTCAGCTGCAGCTAGAACTGCACAAGGCTGATGGTCT

GATCGAGGGTTATGTGGTGGAGAAAGCTGCTTTGGAGGATGCACTGCAGCAGAAGGAGTC

CCGTGAGCAGAAGCTGGTCAAAGAGCTGGAGAGCACTCGGGCTCAGCTGCAGGAACTTGG

TGAAGAACACGCACTCCTCCAGCGTCAACGGGATGCTCTCTCTGCAAGCCTCGGAGAACC

TGAGAAAGCACTGCTGGCCGAGGCGGAGCGTTTGGGGCAGGAGCGTGTCGACGTGCAGCG

GCAGGCTGAGAAGGACCGCGTCGGACTTGCAGGTCGGCTGCGTTTGCTGGAACAGACGCT

GGAGGAACACGAGAGCCGAACGCAGCAGCAGGAGGAGCAGCACCGCACCCAAACCGAGGA

CCTGCAGCAGCACATCAGCGCGCTGGAGAAACAGCTCAAACACCACCGCCAGTTTATAGA

CGAGCAAGCTGTGGAGCGGGAGCACGAGCGAGATGAGTTCCAGCAGGAGATTAAAAACCT

GGAAGCACAGCTGAAAACCACCTCTAAAGGACATGCTGGAGAGGACACCAAGGGTCAAAG

GGTGGAAAGCCTACAAGCTTTAATCAAAGACAAAACCGAGGACTACAGCGTTCTGCTCTC

GCTGAAGGAACAGTGCCAGCGTGACCTGGAGGAACGCAACGAAGAGATCGATAAGATGGC

ATCTCGCATCCGAGAGCTGGAGCAAGCTCTCCTGAGCAGCGTGGAGGAGCGACGGGCCGC

CGCGCAGCTGGAACAGGAGCTGCAGAAAGCGCGCAAAAACTGCCAGGAACTTTCTCAGGA

CAAGGAAGCCCTGCAGCAGCAGCTGTACTCTAATAAACTCCAGATCTCAGCTCTGCAGTC

CAAACTGGATGAAACGAGGCACCGCTACCCCGACCCAGGTGCTGACCCCAACCTCAGAGA

GCGGCTCGAGACGCTACAACAGGACCTGCAGAACAAAGAGGAGCAGGTGGAGGTGTTGCT

GGAGCGAGTGGAGGTGTTGGAGAAAGCTCTGGCAGTGAAGGAGGAGGACGTCAGGCAGCT

CTCTCTGCAGCTGGAGCTCAAAACTACCGAAAGCCGAGCGAGAGAAGAGGAGCTACGGCT

GAGCGTCGCCGAGCTGCAGGATACAGCGAGTCTGTTACGCAGACAGCGGCAGGAAGAAGC

CGATGCCTCGGCGCTACAGCTTCCTTCTGCTCTGCTGGAGGAGAAGAACCTGGAGATCGA

CCACCTGAACCAGCAGCTACTCCATCTGCAGCAGGAGCTGGACGCATCCAAAGAGAACAA

GACAGAGGAGAAGCAAGCAGACGTTGAAGATCTACGTGCTCAGGTGGAGCGACTCCATGG

CGATCAGGAACGTCTGCATCAAGCTAAAGAGGAGGAGGTGGAACAGCTGCATGAGATCAT

CCACAAGCTGCAGGAGGAGCTGAGCCAGCTCAATCCCAATCGGCACGAGGTCAGCGACCC

GAACACGGACAGCCCTGAACCTCCTGATTTCCCCTGGTCGCCCCGGCCTCAGCACGGTGC

CGAGGAGAGCCTGTGTCACGAGCTGAGCAGCCAGACCCTTCAGAGCTGTCGCACCAAGAT

CTGTGAGCTCCAGGCGGACTTGGAGCGCAGTGTTGAGGAGAAGGTGGCTCTCGAGAGACT

GCTCCTGATGCAGGAAGAACAGTACGGACAGCAGCTGGAGGCACTGGGTTGCAGTCTGGG

AGAGGGAAAAAGAAAGCTGGCGCTCCTGGAGCAAGAGGCCGGAGAGCTGAAGCTCCAGTT

AGACCAGAAGGAGACGAAGGTGGAGGAGCTGCAAGCTCGCATTCAAACGCTGGAGGATGG

AGAGAGGAGTGTAAATGATTTGGAACTCCGGCTGATTAAAGCAGAGGAAACGATGAAGGA

GACTCAGCAGGAGATGACCAGGTTGAACGACAAGGCACGAAAGCACACAGAAGAGCGAGA

GAGGACAGAGAGTCTGATTGCTGAGCTGCAGCAGAGGTATGAAGACAGCGAGAGAACTGC

GACGCAGGCGAGGCTGCAGGTTGCCGAGTTGAAGGAGACGATTCAGCAGCGGAAATCCGA

CATCGCCATCTTGGAGACGGGCAAGACGGAGCTGTACACGGAGAAACAGGCTCTTCAAAA

ACGCGAGAGAAAACTTCAAGAGGAAATCGAGAAGCTGAAACAGGAAGTGTCTGCTAAGAG

CCTTCAGATACAGGAAGTGAGTTTGCAGCTGGAGGAGAGAGCAGCTGACCCGGATCAGAG

TCAGGAAGAAGTTCTGACGTGTGCTGAGGAGACCCTGGCTAAAGCTGCAGCAGCTTTGCG

AGAAAAGGAGGAGCAGTTGGCTCATCTGAGGTCGCAGCACGATGCATTGCGAGCCGAACT

CGCTGCGGTGAAGGAAGGCCTGAGCAGCAGTACAGAGAGAGCTGAGAAACTACAGGAGGA

GGGGCAGACTAAGGACCGTGCTCTGGCTGATCTAGAGGAACATAACCAGCGTCTTCGGGC

CGAGCTGCGTGGCTTGCAGGACGATCTGGAGGTGCAGGAGGAGGAACTGGCCTACCAGCA

AAGGGAGTTGGATGAGCTGCGGCAGCGCTGCCGCCTTCAGGAGCACACCGAGCTCGACTG

CACCAAACCCGAACGCCGCTTCCTGGATGGCCTGTCGCATAACTCCACCCTTTCTTCTCC

CGAGGTGCTACGGCGGCTCGACTGCAGCGAAGAGCGACCGTCTCATGTACACGCGTCTCA

CCTGTCAGAACTGAGCGGCCTGCGCAATACCAGCCTCGAGCTGATGAGCAAACACTCGCT

TATGGAGAGAAACGAGTTAAAGCGACCGCTCGTCCTGCCGCCAGAGACCGACCCGCCCAG

CACACACTCGCCATCTGCTTCAACACATTCACTCTCCATCTCCGAGAACCTCTCTGTGCT

CAACTCACTGGACGATGACAAGGTTCAAGTGCTGGAGAATCTGGATGATGTGACTCCGTC

TCGCTCTCCTCTCTCTTCTACCTCCCCGGTGTCTGCACCAGAGTGGGCCAGCGATGGATA

CGGTAGCAACGTGAGCTCCGAGCTGGAGGCTAAACTAAAGAACGAGCTGGAGCATATGGA

GCGGCTTGACGCTCACTTTGTTGAGTACCTACGCAGCAGGGACATGGCACCCGCTCCATG

CTCGGACAGCGCCGCAGGCAGCCTGCATCACACTTACGAGCTGCTCTCACCTGAGTTACA

GGCCATGCTGAACCGCGTGTACCGCGAAAGCTACAGAGTGCTGTCTCTGTCTCATCGCCC

TCCCCCTGCCGGACAGCCTCATTCAGACGCCGAGGAGGCTCCACCCGCATCCTGGCAGAG

AGAGAGGCGAGCCTTGCAGGAAACGGTGCTGTCACTCAGGGAGCTCCTGTGCAGGATGGC

TGAGCGTGAACCCAAGGTGGAGGGTGAAGTAGACTGGCGCACAGAGCTGTTGCAGGCCGT

GCGGAGTGTGTTTGACAGTGAGAGGAAGTGGCTTCATGCACAGCTCCAGGACATCATCAC

AACCAACACTGGCATAGACTACACGTCTCTCCTGAAGGATTTAGAGACGCTTCTGCAAAA

ACAGGAGGATCAGCAGAGGAGGTCTTTGGAGCAGTTGCTAAGTGCAGACCGAAACAGTCT

CCTGGCTGAAATCCGCAGTCTGCATAAACAACTGCACACCTGCACGCTGCAGAACCAGGA

GCAGCTGCAGCGCTCGCTCGGTGCTGTTCGTGAGGAAGGTGCGCAGACGCAACAGCAGCT

GCACAGACAGGTTGGTGAGCTGGAGAGGCAGCTACAGCAGGAGCAGTCGTCGTCACATGA

CCTGCAGCGGTCGCTGGGACAGGAACAGTCCAGATCAACAAATCTACATCAGCAGCTGGA

GATCCAGCAGAAGCTGGCGGTAGAGATGAAGTTGGAGCTGGAGGAAGGAACCCGGCAGCT

TCGTGCGTCTGAGAAAAGCAGAGAGGAGCTGCAGGCTCAGATTCACAAACTCCGGCTCAA

GCTGGAGAGTGAGGAGCAGGAACATCAAGCTTGTGTGGAGGCTGCAAACGAGATGAGGTC

AGGGTTAAGCAGCTTCAGGAGGAACTTCATCACGAGAGGCTCAGCAGCAAACGCGCGGCG

GAGGAGCATGCGCACATGCAAGAGTCTCTCCGGGCCTCCCTGTGCGAGCATGCGACTCGT

CTGAATGAGCTGAACGCTGCACTGGAGCAGGAGCGTGTAGCAGTCAGTAACCTGCGTGCC

GAGCTGCAGATCGAACAATCCCGCTGTGAAGCCCTGCTGGCGCAGGAACGTGAGCGCACG

GAGCTAACCCTCTCGCGCCTGGACGAGGAGCGCTCGCGCTGTGCCGAACTTTCTCGCTCA

CTTTCGCACCAGGCTCAGGAACATGCTCGCCGCCTGGAGGAGGAGGTCCGCTCCCAACAG

GCTGCGAGCGCCGACGATAGGAAGTTCATCCAGGAGCTACGGGCACAGCTGGAGCAAGAG

CGGCGCCAGGCCGAGGACCTCGCCGCCACAGTGGACAATCTGCAGTCCCAGGTACTGCAA

AGCAAACGCAGGCAGGAGGAAGCAGCGCAGCAGGAAGCGCAAAGAGGACAGGAGGAGGTG

AAGAGGCTGCGCTCTGCTCTGGATGGTCTGCAGGCACAGCGGGTAGAGGTGAACCGCACC

CTGGAGACGGAGAGACACAGAGCTGCTCAGCTTCAGACAGAACTGGATGCCGTGAAGGAG

AAGATGAGGGAGGTGAAGGAAAAGGAGAGAGCGAAGGAGGAGCAAAGGGAAAAGCAAAGG

TGGTGCGAGAAACAGGAGCAGGAGGACAGGGAGCGGCGGCAGGAGCAAACCAAAGAAAAA

CTGTTGGAGCTGGAGTCGTTGCGCGAGCGAGATCAGCAGCGGTTGAGACAGCTGCAGCAG

ACTCTGGCTGACCTGGAGCAGCAGGAGAAACAGTTGACTTCAGAACGTCTGCAAAGGGAC

TGCGTAGCTCACACACAGGACTCTTCATCCTCCTCGTCTCTGTTAGAGCGAGTACTAAAG

GAGAACTCTGAACTGGCGGAACGTCTGGCAGCTTTAAGCGAGGAGAAGATCTCGCTTAAA

CACACCGTCTCCTGTCTGGAGAGAGATCTGCACAATCTCAGGTGCACTCAACAGTCCTGG

CACAAGGAGAGGATGTTCTCGGCGCCGATCGCTTCACAGAAAGCTGAGGAAGAATTTACA

GATTCCAGGAACCGAAACCGACCAATCACAGATCTTTCCAATAATAAGGTGCAGAGACTG

TATGAGAAGTACCTGAGGGCGGAGAGCTACCGGAAGTCTCTGGTGTATCAGAAGCGCTAC

TTGCTGCTTCTGCTCGGAGGGTTTCAGGACTGTGAGCAGGCCACGCTGGCGCTGATAGCA

CGGATGGGGGCACGGCCATCGCTGATCACGCAAACCTCCCCGCCAATCAGCCGCTTCAGG

AGCGCCGTGAGGGCAGTTATCGCTGTCTCCAGGCTTAAGTTCCTCACCAGGAAGTGGCAA

AGAGCGACGAGAAAAAGTTCAGGGGGAGGTGTGATTGTTAACAGTGCAGCTATAAAGACT

TCGATATTAAAGCCGCAGCACTCCGGCGTCGCCGCACATCAGTCTCCACCCACCCGGGAT

GGCATTCTCAGCCAGCGGACCGCGCTCTCGCCTCTGGTGCCGCCACTCAAATCTCCTTTC

AGACTGCACAACGGAGGTTACGCCAACAGCACGGCGGCCCCGTCTGAGCGCACGCTCACC

CCCTCCCAGGATCAGGAGCGCTCCCTCGCTGACTACATTCATCACCTGGAGAGCGTCCAG

CAGCGCCTAGGAGCTGTCCGTCCAGGTTCTCCTGCGACCTTCTCTTTTCTACGGAAAAAT

GACCGATGAAAAGACGACGACTTCTCTGCCCCGAGCCGGTTCGGTGCTCCTTCTTCACCA

GGGGGTTCAAGACACTTGATCATTTTGTAATAAATTATTGTAAATCTACTTTTACAATCC

GTCAGATTTCCCGCCTGATTGTGTTCCAGGTCAGCGGTGATGTTTCACTTCAGTCCGTCT

GTAAATTTACTCACAGTTGTTCGGTTTTGTGTAGCTTTTGTATTTTTAACCAACGTTTGC

ACTCGAATCCGTCGGCTCAGC

>Unigene000009

ACAGGAAGATCGGAAGTGCGCCGCTCAGCCGAAGATTTGGAAGAGGCGCGAGAGCGTTGG

TCTAAAGCATCAGAGGAGCTTGAGGAAGCAAAGTGGGAGCTGGAGTCAGAACGAGACAAA

AGGCTTCAATGTGAGGAGCTCCTCAATGAGAAAATCCATGAGCAGGACAACCTAAAGAAC

AAACCCGGCTTCTTGGAGAACCAGATAGAGAAAGATCAGCCAACATCCAACATGGAGTAT

GACGAGAAAAGCTTTTCTCCAGAGAAACTAACTGAAGACAAAGTCGAACTGGTTCTCCAA

GAGCAGCTAGGACAGATGGACTACAGGGAGGACAGAATGGAGAACGTGCAGAGGCTGGAA

AAGGTCCGAGTGGACCTGGAGTCTGAGCTGACGTGTCTAAAGGAGAACCTGAGTAACATG

GAAGAAGCTTTACGTCAAGGTTCTGCAGAGAAAGCGGCTATGGAGAAGCGACTGCAGAAG

CTGGAAGAAAAGAACAAGAGCATGGAAAAGGCTCTGGAAATGGAACTGGAGAACTTTGAG

CTTCAGCTTCAAGCGAAGGATGTGGCCTTTGAGCAGATGAAAGAAGCCAGAGAGAAGGCG

GAAGAGGAGCTCATGGAAAAGGAGACCACTCTGCAGAAAGAGAAGAATGAACATGAAGAG

GAGAGGAGACGACTGGAGGAGAAACATCAACAGGAGATGCTGGACTGGAGTCTGCGTTTG

GAGAAGGAGCTCTCTGATCTGCGGTCTGATCTGGAAGAGGAGCAGAAGAAACAGATCACA

CTGATTAAACAGGTATTTATTTCCTGTGTATTTTATTTTTAAAAAATTCCCTCTGGTTGT

ACATTTTTTTTTTTTTTCCACGTAGGTACACGAACGAGAGCATGAGAGAGAGATGGACGA

ACTGGTGGCCCAACAAAAAGCTGAACTGGAGCGGCTGAAGGCTGAGCTCTCTGAGGAGCT

GAGGGACAGTATGGAGGCTGCACATCAAGCCGAGCTTTCGCAAGTCCAGACGCAGCACAC

TCTGGAGCTGGAGGCTCTCCGTCTGAGCCTGACCAACATGCACACGGCGCAGCTGGAGCT

CACGCAATCCAACCTGCAGAAGGAGAAGGAGGCGGCGCTGAGCGAGCTGCAGGCCAGTCT

GAGGGAGAAGTGGGCTCAGGAGAGCGCCATGCTGCAGACACGACAGCAGTTTGAGCTGGA

GAAGTTCCGAGAACAGGGCAGGGAGCAGGAGAGACAAACCGAGCTCCAGCACCAACTAGC

CGTCGATGATCTAAACTGTGTGTGGGAAAGGCGTCTTTCAGAAGAACGAGCCGCGCTGGA

GCAACAGCTTGCTGGTGGAATAGAGGATGCAAAAATTCGGTGGATACAAGACTTTAAAAA

AGAACAGGCGGAGCTTCAGGAAGCCCTGAACAAAGCCGAGGACCGTTTAGCCGAGACGAA

ACGCAAACTGGAGGAGCTTCAGAGATCCAAAGAGGAGGAAATCAAACATCTGGAGTCGGA

ATTGAGTCAGGCGTGGGCCGACCGAGACTCTGCTGCTCGTGCTGTTGAAGAACTGGTCGC

TAGTCACAGGGTGGTGCTACAGGAGCAGCAGGCTCACACACAAGAGCTGGAGGAAAAGGA

GAAATGGCTGAAGCAAGAGGTGGATCGTCTGCTTGAACAGAAATCCAGCTCCGAGCAGGA

GATCACAAACCTCTGGTCTCAGCTGGAGAGCATGAGGACCAGCAGGCAGGAACTAGGAGA

GTTGAAGGAGCAGTTGCTAGCACGCAAGTCACATGTTAATGACATCGAGCAGTTGAAGCA

GGACTTCTCGCAGCAGCGGCGAGAGCTCCAGGAGCAAAACGAGACCGAGCTGGAGAACCT

GCGTGTGTACTTCGAACAGCGGCTCCGGGCCAGCGAAGAGAACCACCGGGAGGAAATCGC

TCTGCTGCAGCTACGCCTCGTAGAGGGAGCTTTGGAGGACTCGGTTCTCAAGACCGGAGA

GGAAAGCTTCCTCTCTGATGGAAAGTCTGAGGAGGAGAAGGGAGACTCGTTGGCAGAGAT

TACAGAACAACTGGATAAACACAAGGTGGAGTTGGATGCACTGCGGCTGCAGCTGGAGGA

GAAACACAAGCAGGACCTGGAGCAGCTGCGCTGTAACATGGCTCTATCCTATAGAGAGGA

GTTACTGCAAGCTCGTACCGAGCTCACCGACCGCTACTACAGCGACATGGAGCAGCTCAA

AACTAAACACGCCTTGGAGATCGAACAGCTGCGGGCCAAGCTCTCCGACAGCCACCTCAA

AGAAATAACAAGGCTGCGGCTGCAGTCAGTAGCAGATGTAGCGCGTCAGGTGGAGACTGA

GCTGGAAGAGAAGAGCCGGCTCCAAACCCAGGAGCACCAAGCCCGACTCGCTCAGCTCGC

CTCAGACACAGAGAGCATACTGAGCCTGGAGAAGAAGCTCGCCGCGCTCACCCTGCAGCA

CAGCCGAGAGCTGGAGAGAGCAACTGAGCAGCATGCGGAGGAGCTCCGACAGCTGGAGGA

GAGGCTGAAGAAAAACTTTTCAGAAGAACTGCACACACGTCTCGCAGAGGCTCAACAGGA

AGAAAGGACCCATGTGACCGAAGAGCTGCTCAACAGGAGCAGTGAGGAACGGCTCCAGCT

CCGGGAACAGCTTCAAGCACAGGCTGAGGAGCGCGTGGCTTCCCTAAGAGAGGAGCTCCA

ACAAGCAGCCAGTAAGGACAGACAAGAGTTGGAGCAGAAGCTTGTTCAGGCTGAGGAGCT

CCTGGCTGAGGAAAAGCAGAGGCTACAAGCTTTACAGGTGAGTCTGGAGAGGGAGGAGAG

CCCTCAGCTGCTGGCCCTGAAGCAGAGGCTGGAATCTCAATACGCTGGCGAGTTGCACAC

AGCAAAGAGCACCATGGCAGCTGAGGTAAAGGAGCTAAACACTTTGCTGCAGGAGCAGAC

GGAGAGCAGACTGAAGGAGGCCACGTGCAGACATCAAGAGGAACAGCGGCAGCTAGCAGA

GAAACTCGTGCAACAGAAAGAGGAGGCTCTTCAGAAGCTGAGGCAACAACATGCTCAGGA

GCTGCAGGTCCAGGAAACTCTCCTGGAACAGAACGTTTCAGAACTCCAACACCTCCAGAA

GGAGTACCAGGAACTCAAATCCCGACACCAGGAGGAGCTCGACCTGCTCTCTTCCAATCA

CAAGACAGCGCTGGACTCCCTCGCTGAGAAACACAAGGCTGAGCTGGATAAGCTGCAGGT

GGTATTTCAGGAGACCAACTTGGCACAGCTCGAAGCTCAGGAGGCGGAGCTTGAAGAGCG

TCACAAGCAGGAAATGGAAGAACTGGAGACCCGGATGTTGTGCAACATGGACACTCTGGA

GAGCACATACCTGAAGGAGATCCAGGCTGTGCGCGAGGAGAAGGAGGCGGCGCTCCGGGA

TCTCGGGGCCTCCCTGGAGCAACAGCAGGCTGAAGAGATTGAAAGACTGAAGCAGGAGGA

GCTGATTGTCAGAGAAGAGCTGAGAAAAAAACTGGCACAGGTGCACATGGACAAGTTTAG

TGTCATGGCGACTGAACTCAGCCACGCCCATCAGGCAGAGCTGTCTGCGGCGCTCGCTTC

TCAGAAAGCGGCTCTGGAGTCAGAGCACCACTCCGCGTTGGAGACCCTGCGACAGCACGT

GCTCGATCTGGAGGCGCAACACGGCGCCGCCCTCCACGAGGTCACCGACATGACTACCAA

GGAGAATCAGCAGCTGAAGAAACAGCTGGATGCACTGAATGCTCAGCACCAGCAACAACT

CCAGGAACTGAGGGCAACATCCTCCAGGGAAATCGAAGCTCTACGCAGGGAGCTGGAAGA

AGAAGCATCAAGACAGCGGCTCCATTTCCTGGAAGAGGCGGAGCTTCTAAAGTGCCAGTC

AGAGGAACAGCTACAGAAAAAGCTAATGGAGTTAAAGGAGGAAAGTGATCGAGAAAAGGT

CTCTGCTCTAGAGGAACTGGAGAGAAGTCTGTGTGAGCAGCACCAGCAGAACGAGCTCTC

CTACACTGAAAAAATGAACCATCTGACCACACAGCTGCAACAGCTGGACACCGTGGTCTC

TCAGCTGCGTGCAGAGGTGAGCGGTTTGCAAGGGGAGCTGGAGGGTAAGCGCTCGGAGAT

GGACACGCTGGAGACTCTGCTGCAGCGACGCGAGCGCGAGAGTCAGGAAGGAGCCAACCT

ACTCGCCATGCTGCGTGATGACCTCAACACCGCCACCCAGCAGAGACAAGATCTTCAACT

GACTCATGACCGACTCCAGCGGCTTGTCCTGGAGATGCTTCGGATTACAATTGCGACAGA

GGAGGAGATCAGTCGCAGGTTGGGGATATCCATGGAAGGTGATGACATCACTCTGAACAG

GAAGCAAAGTGTCAACCGTGACCCGCAGAAAACGGACGCTGAGGTGGGCGACGGTGCTGC

TGCAGCTGTAGAAGCCAGTGCTAACTGCAGCATGTTCTCGTCACTGATGGATGAAGGTCT

GGAGCTGTCTCAGCGACTGTGTGAAAGTTTGTTTACAGGACCTGATGGAGAGCTGGACCC

TGAAAGAGAGGAGCTGGTACTGGGGGCCTGCACTCATCTACGTGTCGCAGTGGACAAATT

ATTGGAGGTAGTTTCCGACTCCACAGTGCAGCTGGAGCAGCTGCAAGCTCTGCAGGCCCT

GCTGGATGAAAAGTTCAGTGCTGGTGGAGAAGCGAAGAACGCGCTCTTCCTGCAGCACAC

TCAGCTTCTGGAGCAACTAGACCAGGAGGCCAGCGTGAAGAGTCAGCTGCAGCTAGAACT

GCACAAGGCTGATGGTCTGATCGAGGGTTATGTGGTGGAGAAAGCTGCTTTGGAGGATGC

ACTGCAGCAGAAGGAGTCCCGTGAGCAGAAGCTGGTCAAAGAGCTGGAGAGCACTCGGGC

TCAGCTGCAGGAACTTGGTGAAGAACACGCACTCCTCCAGCGTCAACGGGATGCTCTCTC

TGCAAGCCTCGGAGAACCTGAGAAAGCACTGCTGGCCGAGGCGGAGCGTTTGGGGCAGGA

GCGTGTCGACGTGCAGCGGCAGGCTGAGAAGGACCGCGTCGGACTTGCAGGTCGGCTGCG

TTTGCTGGAACAGACGCTGGAGGAACACGAGAGCCGAACGCAGCAGCAGGAGGAGCAGCA

CCGCACCCAAACCGAGGACCTGCAGCAGCACATCAGCGCGCTGGAGAAACAGCTCAAACA

CCACCGCCAGTTTATAGACGAGCAAGCTGTGGAGCGGGAGCACGAGCGAGATGAGTTCCA

GCAGGAGATTAAAAACCTGGAAGCACAGCTGAAAACCACCTCTAAAGGACATGCTGGAGA

GGACACCAAGGGTCAAAGGATTGAAGACTTGGTTATTCAGGTGGAAAGCCTACAAGCTTT

AATCAAAGACAAAACCGAGGACTACAGCGTTCTGCTCTCGCTGAAGGAACAGTGCCAGCG

TGACCTGGAGGAACGCAACGAAGAGATCGATAAGATGGCATCTCGCATCCGAGAGCTGGA

GCAAGCTCTCCTGAGCAGCGTGGAGGAGCGACGGGCCGCCGCGCAGCTGGAACAGGAGCT

GCAGAAAGCGCGCAAAAACTGCCAGGAACTTTCTCAGGACAAGGAAGCCCTGCAGCAGCA

GCTGTACTCTAATAAACTCCAGATCTCAGCTCTGCAGTCCAAACTGGATGAAACGAGGCA

CCGCTACCCCGACCCAGGTGCTGACCCCAACCTCAGAGAGCGGCTCGAGACGCTACAACA

GGACCTGCAGAACAAAGAGGAGCAGGTGGAGGTGTTGCTGGAGCGAGTGGAGGTGTTGGA

GAAAGCTCTGGCAGTGAAGGAGGAGGACGTCAGGCAGCTCTCTCTGCAGCTGGAGCTCAA

AACTACCGAAAGCCGAGCGAGAGAAGAGGAGCTACGGCTGAGCGTCGCCGAGCTGCAGGA

TACAGCGAGTCTGTTACGCAGACAGCGGCAGGAAGAAGCCGATGCCTCGGCGCTACAGCT

TCCTTCTGCTCTGCTGGAGGAGAAGAACCTGGAGATCGACCACCTGAACCAGCAGCTACT

CCATCTGCAGCAGGAGCTGGACGCATCCAAAGAGAACAAGACAGAGGAGAAGCAAGCAGA

CGTTGAAGATCTACGTGCTCAGGTGGAGCGACTCCATGGCGATCAGGAACGTCTGCATCA

AGCTAAAGAGGAGGAGGTGGAACAGCTGCATGAGATCATCCACAAGCTGCAGGAGGAGCT

GAGCCAGCTCAATCCCAATCGGCACGAGGTCAGCGACCCGAACACGGACAGCCCTGAACC

TCCTGATTTCCCCTGGTCGCCCCGGCCTCAGCACGGTGCCGAGGAGAGCCTGTGTCACGA

GCTGAGCAGCCAGACCCTTCAGAGCTGTCGCACCAAGATCTGTGAGCTCCAGGCGGACTT

GGAGCGCAGTGTTGAGGAGAAGGTGGCTCTCGAGAGACTGCTCCTGATGCAGGAAGAACA

GTACGGACAGCAGCTGGAGGCACTGGGTTGCAGTCTGGGAGAGGGAAAAAGAAAGCTGGC

GCTCCTGGAGCAAGAGGCCGGAGAGCTGAAGCTCCAGTTAGACCAGAAGGAGACGAAGGT

GGAGGAGCTGCAAGCTCGCATTCAAACGCTGGAGGATGGAGAGAGGAGTGTAAATGATTT

GGAACTCCGGCTGATTAAAGCAGAGGAAACGATGAAGGAGACTCAGCAGGAGATGACCAG

GTTGAACGACAAGGCACGAAAGCACACAGAAGAGCGAGAGAGGACAGAGAGTCTGATTGC

TGAGCTGCAGCAGAGGTATGAAGACAGCGAGAGAACTGCGACGCAGGCGAGGCTGCAGGT

TGCCGAGTTGAAGGAGACGATTCAGCAGCGGAAATCCGACATCGCCATCTTGGAGACGGG

CAAGACGGAGCTGTACACGGAGAAACAGGCTCTTCAAAAACGCGAGAGAAAACTTCAAGA

GGAAATCGAGAAGCTGAAACAGGAAGTGTCTGCTAAGAGCCTTCAGATACAGGAAGTGAG

TTTGCAGCTGGAGGAGAGAGCAGCTGACCCGGATCAGAGTCAGGAAGAAGTTCTGACGTG

TGCTGAGGAGACCCTGGCTAAAGCTGCAGCAGCTTTGCGAGAAAAGGAGGAGCAGTTGGC

TCATCTGAGGTCGCAGCACGATGCATTGCGAGCCGAACTCGCTGCGGTGAAGGAAGGCCT

GAGCAGCAGTACAGAGAGAGCTGAGAAACTACAGGAGGAGGGGCAGACTAAGGACCGTGC

TCTGGCTGATCTAGAGGAACATAACCAGCGTCTTCGGGCCGAGCTGCGTGGCTTGCAGGA

CGATCTGGAGGTGCAGGAGGAGGAACTGGCCTACCAGCAAAGGGAGTTGGATGAGCTGCG

GCAGCGCTGCCGCCTTCAGGAGCACACCGAGCTCGACTGCACCAAACCCGAACGCCGCTT

CCTGGATGGCCTGTCGCATAACTCCACCCTTTCTTCTCCCGAGGTGCTACGGCGGCTCGA

CTGCAGCGAAGAGCGACCGTCTCATGTACACGCGTCTCACCTGTCAGAACTGAGCGGCCT

GCGCAATACCAGCCTCGAGCTGATGAGCAAACACTCGCTTATGGAGAGAAACGAGTTAAA

GCGACCGCTCGTCCTGCCGCCAGAGACCGACCCGCCCAGCACACACTCGCCATCTGCTTC

AACACATTCACTCTCCATCTCCGAGAACCTCTCTGTGCTCAACTCACTGGACGATGACAA

GGTTCAAGTGCTGGAGAATCTGGATGATGTGACTCCGTCTCGCTCTCCTCTCTCTTCTAC

CTCCCCGGTGTCTGCACCAGAGTGGGCCAGCGATGGATACGGTAGCAACGTGAGCTCCGA

GCTGGAGGCTAAACTAAAGAACGAGCTGGAGCATATGGAGCGGCTTGACGCTCACTTTGT

TGAGTACCTACGCAGCAGGGACATGGCACCCGCTCCATGCTCGGACAGCGCCGCAGGCAG

CCTGCATCACACTTACGAGCTGCTCTCACCTGAGTTACAGGCCATGCTGAACCGCGTGTA

CCGCGAAAGCTACAGAGTGCTGTCTCTGTCTCATCGCCCTCCCCCTGCCGGACAGCCTCA

TTCAGACGCCGAGGAGGCTCCACCCGCATCCTGGCAGAGAGAGAGGCGAGCCTTGCAGGA

AACGGTGCTGTCACTCAGGGAGCTCCTGTGCAGGATGGCTGAGCGTGAACCCAAGGTGGA

GGGTGAAGTAGACTGGCGCACAGAGCTGTTGCAGGCCGTGCGGAGTGTGTTTGACAGTGA

GAGGAAGTGGCTTCATGCACAGCTCCAGGACATCATCACAACCAACACTGGCATAGACTA

CACGTCTCTCCTGAAGGATTTAGAGACGCTTCTGCAAAAACAGGAGGATCAGCAGAGGAG

GTCTTTGGAGCAGTTGCTAAGTGCAGACCGAAACAGTCTCCTGGCTGAAATCCGCAGTCT

GCATAAACAACTGCACACCTGCACGCTGCAGAACCAGGAGCAGCTGCAGCGCTCGCTCGG

TGCTGTTCGTGAGGAAGGTGCGCAGACGCAACAGCAGCTGCACAGACAGGTTGGTGAGCT

GGAGAGGCAGCTACAGCAGGAGCAGTCGTCGTCACATGACCTGCAGCGGTCGCTGGGACA

GGAACAGTCCAGATCAACAAATCTACATCAGCAGCTGGAGATCCAGCAGAAGCTGGCGGT

AGAGATGAAGTTGGAGCTGGAGGAAGGAACCCGGCAGCTTCGTGCGTCTGAGAAAAGCAG

AGAGGAGCTGCAGGCTCAGATTCACAAACTCCGGCTCAAGCTGGAGAGTGAGGAGCAGGA

ACATCAAGCTTGTGTGGAGGCTGCAAACGAGATGAGGTCAGGGTTAAGCAGCTTCAGGAG

GAACTTCATCACGAGAGGCTCAGCAGCAAACGCGCGGCGGAGGAGCATGCGCACATGCAA

GAGTCTCTCCGGGCCTCCCTGTGCGAGCATGCGACTCGTCTGAATGAGCTGAACGCTGCA

CTGGAGCAGGAGCGTGTAGCAGTCAGTAACCTGCGTGCCGAGCTGCAGATCGAACAATCC

CGCTGTGAAGCCCTGCTGGCGCAGGAACGTGAGCGCACGGAGCTAACCCTCTCGCGCCTG

GACGAGGAGCGCTCGCGCTGTGCCGAACTTTCTCGCTCACTTTCGCACCAGGCTCAGGAA

CATGCTCGCCGCCTGGAGGAGGAGGTCCGCTCCCAACAGGCTGCGAGCGCCGACGATAGG

AAGTTCATCCAGGAGCTACGGGCACAGCTGGAGCAAGAGCGGCGCCAGGCCGAGGACCTC

GCCGCCACAGTGGACAATCTGCAGTCCCAGGTACTGCAAAGCAAACGCAGGCAGGAGGAA

GCAGCGCAGCAGGAAGCGCAAAGAGGACAGGAGGAGGTGAAGAGGCTGCGCTCTGCTCTG

GATGGTCTGCAGGCACAGCGGGTAGAGGTGAACCGCACCCTGGAGACGGAGAGACACAGA

GCTGCTCAGCTTCAGACAGAACTGGATGCCGTGAAGGAGAAGATGAGGGAGGTGAAGGAA

AAGGAGAGAGCGAAGGAGGAGCAAAGGGAAAAGCAAAGGTGGTGCGAGAAACAGGAGCAG

GAGGACAGGGAGCGGCGGCAGGAGCAAACCAAAGAAAAACTGTTGGAGCTGGAGTCGTTG

CGCGAGCGAGATCAGCAGCGGTTGAGACAGCTGCAGCAGACTCTGGCTGACCTGGAGCAG

CAGGAGAAACAGTTGACTTCAGAACGTCTGCAAAGGGACTGCGTAGCTCACACACAGGAC

TCTTCATCCTCCTCGTCTCTGTTAGAGCGAGTACTAAAGGAGAACTCTGAACTGGCGGAA

CGTCTGGCAGCTTTAAGCGAGGAGAAGATCTCGCTTAAACACACCGTCTCCTGTCTGGAG

AGAGATCTGCACAATCTCAGGTGCACTCAACAGTCCTGGCACAAGGAGAGGATGTTCTCG

GCGCCGATCGCTTCACAGAAAGCTGAGGAAGAATTTACAGATTCCAGGAACCGAAACCGA

CCAATCACAGATCTTTCCAATAATAAGGTGCAGAGACTGTATGAGAAGTACCTGAGGGCG

GAGAGCTACCGGAAGTCTCTGGTGTATCAGAAGCGCTACTTGCTGCTTCTGCTCGGAGGG

TTTCAGGACTGTGAGCAGGCCACGCTGGCGCTGATAGCACGGATGGGGGCACGGCCATCG

CTGATCACGCAAACCTCCCCGCCAATCAGCCGCTTCAGGAGCGCCGTGAGGGCAGTTATC

GCTGTCTCCAGGCTTAAGTTCCTCACCAGGAAGTGGCAAAGAGCGACGAGAAAAAGTTCA

GGGGGAGGTGTGATTGTTAACAGTGCAGCTATAAAGACTTCGATATTAAAGCCGCAGCAC

TCCGGCGTCGCCGCACATCAGTCTCCACCCACCCGGGATGGCATTCTCAGCCAGCGGACC

GCGCTCTCGCCTCTGGTGCCGCCACTCAAATCTCCTTTCAGACTGCACAACGGAGGTTAC

GCCAACAGCACGGCGGCCCCGTCTGAGCGCACGCTCACCCCCTCCCAGGATCAGGAGCGC

TCCCTCGCTGACTACATTCATCACCTGGAGAGCGTCCAGCAGCGCCTAGGAGCTGTCCGT

CCAGGTTCTCCTGCGACCTTCTCTTTTCTACGGAAAAATGACCGATGAAAAGACGACGAC

TTCTCTGCCCCGAGCCGGTTCGGTGCTCCTTCTTCACCAGGGGGTTCAAGACACTTGATC

ATTTTGTAATAAATTATTGTAAATCTACTTTTACAATCCGTCAGATTTCCCGCCTGATTG

TGTTCCAGGTCAGCGGTGATGTTTCACTTCAGTCCGTCTGTAAATTTACTCACAGTTGTT

CGGTTTTGTGTAGCTTTTGTATTTTTAACCAACGTTTGCACTCGAATCCGTCGGCTCAGC

>Unigene000010

ACACACACACGCGCACACACACGAAGACATGAGGTGATCTTTTTTTTTGTGTGTGTGCGT

GTAAGGTATCAGCCGTGTAAACTGTTGGCCGAAGTGTACCGGAGTGTGTATAAGGTGCGG

AATCGTTTGCTTGCCTGCAAGAACATGGAACTGGTCCAGACACACTCGCCATCCAGAGAG

CGCAGGATCTCTGAGAACGTGGACTCTGTGGAAATGGACCCACAGGAACACGCTTTCACC

CGCACCATCGACGAGGAGGCCGAACTTGAGGAACGAGCTGAACGAGAGAGAGACCGAGAG

GACGGCCATCAGGAGCAGGAGGACGAGGAGGAACGCGAGCATGAGGTCATGACAGCAGGA

AAAATCTTCCAGTGTTTTCTCTCGGCGCGGGACGTTGCGCGTGGTAGAGAGCGACCTCGC

ACGAGCAGCGTCGGCGTCCCAGACGAGTCTGTTCAACCTGAACAGGAGCGCAGAAACAGC

ATCCCGTTACAGGACAGACAAGACCTCTACACCACCACCTGCAACAGCATCATCCACCGC

TGCGCCATGCTCCTGATGGCAGTCAGCCCCACACTCCCGCAGACGACCAACGAGCAGCCA

GCTTTGTGTGGTGGGGTCACGCAAGACGGAAGCGGATTTTTGATCCGAAGCGAGAGTTTG

TCAACTGAGAGTCGATCCATCCAGAGCGGTCCGAGTTACAGGCTCACTAAATCTAGGAGT

GAATCTGATCTCTCCCAGCCCGAGTCTGATGAAGAAGGATACTCGCTGAGTGGAAAAAGG

AACGCAGACCTGGATTTGGCGTTCACTCCTAAAAAGAGAGAATTTGCACATAGCACCCCT

GACTCTGCGGACTGGTTCGGGCGGCATTCTGGTCGCGAGCGTCTGTACAGCTCCCCTCAG

TCTTATGAGGAGTCGGACTTGGACTTCAGCTGCACGCTGCACATCCATGCAGTCATCGAC

AACATCGTCAGCTTCATCAGTGGGGATGTGGGAAACACGCCCGGATTCAAGGAACCAGAG

GACAGCATGTCAACTAGTCCTCAAGCTACAGTTGTAGCCATGGAGCAACAGCAGGGCAGA

GCGGAGTTGCGGTTAGACGCCCTTCATCAGATTGTGGTTTTGATCTCTGGAATGGAGGAG

AAAAGCAGTCAGGGTGGAGCAGAGGCAGTGGCTGGGTGTGGTCCCGCTGCCTTTCACTCC

GCATCCTTGCTGACCTCAGTCAGACTGCAGTTCTTGGCAGGATGTTTTGGCCTTGGCGCC

TATAACGGGGGGCTAAACGGTGAGAGCGTCCAGCTGCACCACTACCAGGACGGCATTCAA

GCTGCTAAAAAGAGGTTACAGATGAACATTCAGACAGCAGTGCACAAAATCTACCAGCAG

CTTTCCATCACACTGGAGAGAGCGCTTCAGTCTAATAAACACCATATAGAGGCTCAGCAG

CGGCTGCTGTTAGTGACCGTTTTTGCCCTGAGTGTTCGTTACCAGCCTGTGGACGTTTCG

TTAGCCATCTCCAGCGGTTTGCTAGATGTTCTCTCTCAACTGTGCGGGAAAGAAACCCTG

CTGGGACACACCCTTCAGCTCCTGCACAAACCCGCCGGCTCGCAACTCAGCACAGCACTC

AAGGTTGCCAGCACCCGTCTGCTGCAGATCCTTGCCATTAGCACCGGGACGTACGCAGAT

CGCCTGAGCCCTAAAGTGGTTCAAGCTCTGTTGGACCTGCTCTGTAGTCAGCTGAAGACT

CTGCTGTCGCAGGCTGCAGGAAGCGTCTTCGGCACAAATTCAGAGTCAGAGGAAAAGACA

GAGGACTCAACAGGCAGCATGAAAAAAGACTTCAGGGCCTTACTGCGCAAACAGCACATT

GCGGAACTACATCTCGGAGACTTTCTGGTGTTTCTCAGAAGAGTCGTATCATCTAAAGCC

ATTCAGTCCAAGATGGCGTCTGCCAAATGGACCGAAGTGCTGCTTAACATTGCGGCACAG

AAATGCTGTTCAGGTATCCCTCTGGTGGGGAATTTAAGGACACGTCTGCTGGCACTGCAT

GTCCTAGAGGCTGTACTTCCTGCCTGCGAAAGTAGCGTGGAGGATCATCAAATGAGTCAG

GTGGTTGAAAGGTTGTTCTCCTTGTTGTCGGACTGTATGTGGGAGGCTCCTGTCGCTCAG

GCCAAAAACACCGTCCACCTGAAGGAAAAGGAGGTCAAACTCCAGGGTGAGGCAGAGGAG

GAGGAAGAGAACCTGCCTGTCCAGGAAGTGATCTTCGATCCTGAAAAGACACAGTGCTGC

GTGGTGGAAAACGGACAGGGTTTGACGCACGGTAGCGGTGGAAAAGGCTACGGTCTGGCT

GCTACCGGCATCTCCTCAGGCTGTTACCAGTGGAAGTTCTACATCGTGAAGGAGAATCGT

GGGAATGAAGGGACGTGTGTGGGGGTTTCCCGTTGGCCTGTAAACGACTTTAACCACCGC

ACCACGGCCGACATGTGGCTGTACCGGGCTTACAGTGGCAACCTGTACCACAATGGCGAG

CAGACGCTCGCGCTGTCCAGCTACACACAGGGCGATTACATCACCTGCGTGCTGGACATG

GAGGCTCGCACAGTTTCCTTCGGGAAGAACGGAGAGGAGCCCAAACTGGCTTTCGAAGAC

GTGGATGCGACAGAGCTTTATCCATGCGTCATGTTCTACAGCAGCAACCCAGGAGAAAAG

GTCAAAATCTGTGACATGCAGATGCGAGGGACGCCACGTGACCTTTTACCCGGCGACCCC

ATCTGCAGTCCCGTGGCCACCGTCCTTGCCGAAGCGGTGTCTCAGCTGGTCAGGATCCTG

CACCGGTCAGACAGCTGGACGCAACCTATCAACCGGTGCATGCTTCAGCGGCTGAAGCAA

ATCCGGGGGTGCTTGCACGACGGCGCTTCGCCTACGGGTGGTGCGAGACTCCGGAAGAGC

CGCTCGGCTCAGAGCCGCGAGGAACAGGATGAGGCGAAAGACGAGGACAGGGATGAGGAG

CGAGGCAGAATGGGGCGGGAACTGACTGAGGCGCAGCTGCGTACACTTTGTAACCTGATC

TGGCCTGTGCTGGCGGTGATTGGAGGGGTGGACGGAGGTCTGAGAGTCGGAGGTCGCTGC

GTGCACAAGCAAAGCGGGAGACATGCAACCCTGCTGGGGGTGGTGAAGGAGGGCAGCATG

TCGGCCAAGGTGCAGTGGGACGAGGCAGAGATCACTATCAGTGACACGCCGCTATATAAC

CTGGAGCCCTGCGAGCCGCTGCCGTTTGACGTGGCACGTTTCCGGGGACTGACGGCCGCG

CTGCTTTTGGAACTGACCGCTCTGGCTTCGCTGCAGGACGACGGCAAGTTCTCTCCTACG

GCCTGCTCGTTATCATCTTCCTCGAGACGCCACGAGCGCCGCCACAGACACGAGCCTCAC

GAGCCCGAAGAAGGCCCCGGTGACCTGCGTGTCTCTCACAGCCTGGATGAAGTGCGTTCC

CACGCCGAACTGCAAAAGCCTCCTGTAATGGCCGAGGTTCATGCTGTGCAACTCTCCTAC

CTCTGCCTTGGTGCCATAAAGAGCCTAAGCGTCCTTCTAAGCTGCAGTAAGTATGCCGAG

CTCCTGCTCATACCCAAGGCGGTGCACGACAACGGCCACAACGCAGACTGCGGCAGCCCC

AGCATCGGCGTCTCACAGGAAGAGGCAGAGATGCGCTCAGCGCTGCAGTTCCTTATGAGA

CACATGGTAAAGCGAGCAGTGATGAGGTCACCTATCAAACGCGTACTGGGTTTAGCTGAA

CTGGAGCGGGCGCAGGCCATGATTTACAAACTCGTCGTCAATAGTGTGCTAGAAGAACAG

GAGGGCGGGAAAACCAAACAATACGGCGAGTGTGAGGAGCTGGAAGGCGAACAGCAGTTC

CAGACGCCGGTTACCACCTCACCCTCTGCGTCCAGTAGCACCTCATTCATGAGCTCCTCC

CTCGAGGACACCACCACAGCAACCACACCTGTTACCACGGCAACTACACCTATAACTGAT

GCTGAGACTGCGCCAGCCTCAGAATCGCCAGGTGTTATGCCTCTCAGTCTGCTCAGACAG

ATGTTCTCCAGCTACCCCACCACAACGCTGCTGCCCGCTCGCCGAGCTCAGACACCTCCA

GTCTCGTCTCTGCCCACGTCTCCTTCAGACGAACACGGCCGAAGGCAAAGTTTTACCTCT

CCTGAATCTCAGCCTGCTAGAACTACCAACAGGACAGCGCTGTCGGACCCCAGCAGCCGT

CTCTCCACATCTCCCCCTCCCCCTGCCATCGCTGTTCCCCTTCTCGAGATGGGGTTCTCC

CTCAGACAGATCATGAAGGCTCTGGAAGCTACAGGTACAAGAGGTGAGGCTGACGCGCAA

AACATCACGGTTCTGGCCATGTGGATGATCGAGCACCCGGGCAACGAGGACACAGAGGAG

CGGACCCTGTCCTCCGACTGCTCGGGGGCAGCAGGAGGGGGCAAAAGTGCGGAGCGGAGC

TTCCTGCGTTCACCTGGAGATATTCCCAACGCTGATGCCGCAGAGCTTGAAGAGGGATTT

AGCGAGAGCCCTGAAGGAATAGATCAAGACTCCGCCTCTGCATCCAACGGCCCCGCCCTC

CGGGGGCGCTCAGCCGCCAGTAGGAAGCATCGCTTCGATCTGGCAGCACGTACTCTATTG

GCCCGTGCAGCTGGACTGTACCATTCGGTGCAGGCCCACCGCTCTCAGGTGCGTAGGGAG

GGCTCAGGGCTGCAGCAGGACTCCAGCTCACTGGGAGGAGTTTACGACTTTAACCTGGAT

GAGGAGCTGGAGCTAGAGCTGGACCAAGAGGCCATGGAGGCCATGTTCACTCAGGAGCTT

GCATCAGACTCGGATATTTTGGGAATGTGGATTCCGGAGCGTGTGTGTGAGTCAGAAGAG

CGCGAGGAGGTGGTGGTCTGTGAGCTCTGCGACGTCAGCGTGTCCAGTTTTAACCAGCAC

ATGAAGAAAAGTCATCCTGGCTGCGGACGGAGCGCAAATCGGCAGGGTTACCGCAGTAAC

GGGTCCTACGTGGACGGGTGGTTTGGGGGAGAGTGTGGGAGCGGGAACCCCTATTACTTA

CTGTGCGGAAGCTGCAGGGAAAAGTACCTGGGCATGAAGAGCAAGCACAAAGGAGCAGTG

TCGGAGAGATACAAGGGCCAGGCTCCAGACCTGCTGGGAAAACAGGACAGTGTCTATGAA

GAAGACTGGGACATGCTGGACGTGGATGAGGCGGAGAAGCTGACAGGCGAGGAGGAGTTT

GAGCTGCTTTCAGCACCGCTGGGACTCACGGAACGGAAACCCGTCCCCGAGGCTGTTCAG

TTCCCTGACACAGACCCCCTCGGGGCCTCCGCTGCCATGGTTACCGCCACCAATAGCATG

GAGGAGACCCTGCTACAGATAGGGTGTCAGAGTTCGGTGGATAAAAGCACATCAGGTCGC

ATGTGTTTGGGTGAGCAAGCAGCCGGTCTCCAGAGTTCTGCTGATCGCATGGTTGCTTTG

AGGAGAGTCACAGCAGCTGCTCAAGTCCTGGTGGCAAGAACCATGGTGATGAGGGCGCTG

TCTTTGCTGTCTGTCAGCGGTTCCAGCTGCAGTCTCGCTGCTGGTCTGGAAGCTCTGGGT

CTGACGGACATTAGGACCCTGGTGCGTTTGATGTGTCTGGCTGCGGCCGGTCGAGCAGGT

CTTTCTACCGGTCTCGCTTCAGGCCCCGGCGCTTTAGAGCGGCCACGTGGCACGAACAAG

CCCACCAAACCCATCTCCTGTCTGGCCTACCTCAGCACAGCAGTGGGCTGCCTCGCCTCC

AACAGCCCAAACGCCGCCAAGCTTCTGGTCCAGCTGTGCACTCAGAACCTGATTTCAGCA

GCTACAGGAATGAATCTGACCACTGTGGATGACCCAATTCAGAGGAAGTTCCTGCCCAGT

TTCCTGCGGGGCGTGGCTGAGGAAAACAAACTCATCACCTCGCCAAATTTCATAGTGACT

CAGGCATTGGTGGCGCTCTTAGCCGATAAAGGAGCCAGACTGAGACCCGGGTACGACAAG

GCCGAAATGGAGAAGAGAGGCCCGTTGGAGCTGGCCAATGCGCTGGCGGCGTGCTGCCTC

TCATCCCGCCTCTCCTCACAGCACCGCCAGTGGGCTGCGCAGCAGCTGGTGCGGACACTT

GCCGCCCATGACCGCGACAACCAGAGTCGACCGCAGACCTTCGCCGACATGGCCGGTGAC

TTACGCAAGTGTTCTACCATCAAGCTGGAGGCTCATCAGGGCAGAGTGATCAGCTTCGGG

TGGTGTAACAAGAAAGGTCTTTTGGCGACCAGCGGTAGTGATGGAACAGTTCGAGTCTGG

AACGTGAACAAGAACCAATACACTCTGCAGCAGACCTGCGTCTTTAACAAAACAGACTTT

ACCTCTGAGGAGTGTTCCACCGGACTGGGGTCTCCTGGTGAACCGAGCTTGGCGCCCGTG

GCCTGGAGTGTGAGCGGGAAATTAGTTGCAGCTGCCATGGAGAAAGTGGTGAACATCTGG

CAGGTCAATGGCGGTAAGGCCTTGTTGGACCTGCAGCCTCACTGGGTGTCGTCGCTGGTG

TGGCCGGAGAACGAGGCAGGGTGTCTTTGGGCCGGAGAGTCTCGAGAGCTCCTATTGGTC

GGTCGCATAGATGGAAGTTTGGGCTTGCTGGAAATTTTAGATAGCTCAAACATACAGAGA

ACCGAACTTCTGCACTGTTACAGGAAAGACGCTGTGATGAACATCGCCTGGTACAGCGAG

GATCGACCGTTTGCTGTCGGCTACGCTGACGGAAAGCTTCTGATTGGCTGCAAAGAAGCG

CTGGAGAACGGATCCGTGGTCGTCATCGATGCTCACAAGGAGAGCATCAGTTCTCTGCGC

TGGGCTCCAGGAGGGCAAATTTTATTAAGCTGTGCCAAAGAGGAGACTGCCTGCCTTTGG

GCGGAGTCTGGGGCGGGGCTTGGGCGGAGTTGGGTGTGTCTCCAGAGCATCGCTCACCCG

AGTGTTGTAAATGCTGTCGCCTGGTGTGGTCTCACCGGACAGGGACCTAAAGCACTAAAC

ATGCTGGCCACGTGTTGTCAGAATGGCTTGGTGTCTGTGTGGACCGTGCCTCAGGACCCC

ACAGCCTTCTCTCAGTCTGGCTCATCCAGCACTGATGCATGGTGGGAAACGGACTCCAAG

TCAAAACCAAGGTTCACTCCACAGTGGCACGAGGCCGCAGTGTGCGTCTTCCAGCTAAGA

GGTCACATGACTCCGGTTAGGACGATAGCCTTTAGTCCTGACGGTCTTGCTCTGGTTTCT

GGTGGTGTTGGTGGTCTCCTAAACATCTGGTCTCTCCGAGATGGCTCTGTGCTGCAGACG

GTGGTTGTCGGCTCAGGAGCGATTCAGAACACTGTGTGGATTCCAGACGTCGGGGTGGCC

GTCTGTTCCAACCGCTCAAAGGACGTTCTGGTTGTGAACTGCTCCTCGGAGTTTATGACC

GCTAATCAGGTGTTAGCAACCTGTCGCACCGCGCTGAAGAAGCAGGGGGTTGTGGGATTG

AACACGGCTCCCTGTATGAGGACTTTTCTCGAGAGACTTCCCATAATGCTTCAGGAGCAG

TACGCCTACGAGAAGCCCCATGTAGTGTGTGGAGAGCAGCTGATCCACAGTCCCTACATG

CAGTGTTTGGCATCTCTGGCCGTCGGGTTGCAGTTGGACTCGTTACTATGTCGTCCCGCA

GTGCCTCTTCACCTGTCTCATTGCCTCGCCCAACCTGTCTCTTCATCATCGTCATCATCA

CAATCATCCTCCTCATCCGGTCCTTCAGCACTCATGCACTCATGTGGCAGCAGCGAGTGG

GCGTGGCTTCACTGCTTCTCCACCACCATAAAGACGGCTGAGGCGCTCGCCCGCGCTCAC

ACTTTCCCAGAATCCTTTAACGTGCCTGATCTGGAGCCGGTGGCCAAAGACAAGATGGCG

GTGCTGATGGACAACAGTAAATGGGTTCCTGGGATGGATGAGCAGTTGATGGCTTGGGCC

ACCTCCAGACCCGAGGACTGGCACCTGGGTGGGAAGTGTGACGTGTATCTGTGGGGCGCC

GGTCGCCACGGTCAGCTCGCCGAGGTCGGCCGGAACATTCTGGTGCCGACGCCTGCTGCG

TCTTTCTCTCAGGCTCAACAGGTTGTATGTGGGCAGAACTGCACATTTGTGATTCAGGCG

AACGGCACAGTGTCTGCGTGTGGGGAGGGCAGCTACGGTCGACTGGGTCAGGGAAACTCT

GATGACCTGCACATTCTTACGGTCATCTCAGCACTTCAAGGGTTTGTTGTTACCCAGCTG

GTCACGTCGTGTGGCTCTGATGGACACTCCATGGCTCTGACTGAGAGCGGTGAGGTGTTC

AGCTGGGGAGATGGCGACTACGGCAAACTGGGCCACGGCAACAGTGACCGCCAACGCCGG

CCGCGCCAGATAGAGGCTTTACAGGGAGAGGAGGTGGTGCAGATGTCCTGTGGGTTTAAA

CACTCTGCCGTCGTCACAGCAGACGGTAAACTCTTCTGCTTCGGCAATGGAGACTACGGG

AGACTCGGACTGGGAAACACATCCAATAAGAAACTTCCAGAACGAGTCACAGCTTTGGAG

GGACAACAGATAGGCCAGGTGGCGTGCGGGCTAAATCACACACTGGTGGTGTCATCTGAC

GGGTCGACCGTCTGGGCGTTTGGAGACGGAGATTATGGAAAACTTGGCTTGGGAAACTCG

ACTGCTAAATCATCACCGCAGAAAGTGGATGTTCTGTGTGGAATTGGAATCAAAAAGGTT

GCATGTGGAACCCAGTTCTCTGTGGCTTTAACCAAAGATGGAAATGTTTACACTTTTGGG

CAAGATCGCCTGATCGGTCTCCCAGAGGGGCGTGCCCGCAATCACAACCGCCCGCAGCAG

CTCCCCGCTCTGGTGGGCGTGATTATCAAAGATGTCGCTGTAGGGGCGGAGCATACACTC

GCGCTGGCGTCTACAGGAGATGTTTATGCTTGGGGGAGCAACTCTGAGGGACAGCTCGGT

TTGGGCCACACCAACCATGTACGAGAGCCGACTCTAATCACAGCTCTGCAGGGTAAAAAC

ACCCAGCAGATTTCAGCAGGACGCTGCCACAGTGCCGCATGGACCGCGCCCTCAGTACCA

CCCAGAGCACCAGGCTCGTCCATGCCGCTGCAGCTGGGTTTGCCCGTGTGTGTTCCTCCG

CAGTACGGCAGTCTGAGAGACATCAGCATGGAGGCACTAAGAGCTCGGCTACGTCTGCTC

TACCACTTCTCTGACCTCATGTACTCCTCCTGGAGACTGCTTAACCTCAGCCCTAACAAC

CAGAGCTGCACGTCTCATTATAACCCTGGGACGTGGGGTATCGTACAGGGGCAGCTGCGC

CCTCTACTGGCTCCACGTGTGTATATGCTCCCGATGGTACGCTCCATCGGCAAGACCATG

GTGCAGGGCAAGAATTACGGACCTCAGATCACCGTCAAGAGAATCTCCACTCGTGGGAGG

AAGTGTAAGCCCATTTTCGTGCAGATCGCTCGGCAGGTAGTGAAGCTGAATGCGTCTGAT

CTCCGGCTGCCGTCTCGGGCGTGGAAGGTGAAGCTGGTGGGGGAGGGAGCGGACGACGCT

GGAGGAGTTTTTGATGACACCATTACAGAAATGTGTCAGGAGCTCGAGAACGGGGTGGTG

GATCTGCTCATCCCTTCTCCTAATGCCACTGCGGAAGTGGGCTACAACAGAGACAGGTTT

CTGCTGAATCCCTCGGCTTGCCTGGACGAACATTTGCTGCAGTTCAAGTTCCTGGGCATC

CTGATGGGCGTGGCCATCCGCACCAAGAAACCTCTGGACCTGCATCTGGCTCCGATGGTG

TGGAAACAGCTGTGCTGCATCCCGCTGACGCTGGAGGACCTGGAGGAGGTGGACCTGCTG

TATGTCCAGACACTCAACAGCATTGTGCACCTGGAGGACAGCAGCATCACAGAGCAGAAT

TTCCACGAGATGATTCCTCTGGATTCATTTGTGGGTCAGAGCGCTGATGGGAAAATGGTG

CCCATCATTCCGGGAGGAAACAGTATTCCACTCACCTTCTCCAACAGGAAGGAGTACGTA

GAACGCGCCATAGAGTACCGGCTACATGAACTGGATCGTCAGGTGGCGGCCGTGCGTGAG

GGCATGTCATGGATTGTACCGGTTCCTTTACTCTCGCTGCTGACCGCTCGGCAGTTGGAG

CAGATGGTGTGTGGTCTGGCGGAGATCAGTGTGGAGGTGTTGAAGAAGGTGGTTCGTTAT

CGGGAGGTGGAGGAGCAGCAGCAGTTGGTGCAGTGGTTCTGGCAGACGCTTGACGAGTTC

TCCAACGAGGAACGAATGCTGTTCATGCGCTTCGTCTCCGGACGCTCGCGTCTCCCTGCT

AACACAGCCGATATCTCGCAGCGCTTCCAGATCATGAAGGTGGACCGGCCGTACGACAGC

CTTCCCACGTCTCAGACCTGTTTCTTCCAGCTCCGCCTCCCGCCCTACTCCAGCCAATCG

GTGATGGCAGAGCGGCTACGCTACGCCATTAATAACTGTCGTTCTATCGACATGGACAAT

TACATGCTGTCACGGAATGTGGACAATACTGAGGGTTCGGACACGGACTACTGACCGAGA

GTTAAAGCACAAAGACACTCGCTGAACTTTAAACCGTCAACACAAAAATCACCACCTTTA

ATTCTTCTCTTCTTTTATTAATCATTAAAGCAGTCAGACGGCGTTAACCAGGTACAGATA

AATATAACCATTTCTGTTATTCACAACTCCCCACACACACACACACACACAC

>Unigene000011

CAGCAAGCAAGAAGTCATCTCTAGGCAAATGAGTATCTCCAAAAAATGGCAGTCTTTGCT

CCAGCAGTTGCAGCAGAACAGAGATTCTGTTGGAGATGTGGTGAACACACTTGGTGTTCT

CAAGGACACAGAGTTAATTGCACTGGACCTCAAGGAACTACAGGTTCAGGCAGCGTCCTC

TGATCTGGGAAAACATTTACCAGAGGTGGTAGATCTTCTCCAGAAACAAGAACTGTTGGA

CACTAAGATCATCTCTCTTGGAGAATCACTGAGTGCCATTAGCAGTAATGCCTTAAGGGG

AAAACACAGGGATGTTTCACAGGTTCAGAGAACAGTAAAAGAGCTTAATAGCCAGTACAC

CTCCCTCCTGGCACTTAGCCAGAACAGGAGGAGAGCACTGGAAGGACAGCTGAAGCTCTT

TGAGTTTTTCCATGACTGTGAAGAAATGGAAGGTTGGATTTATGAAAAGTGGCTTCTGGT

GCAAGCAGCGTCTTTGGGCAGAGACCTCAGTCAGATTGAACAAGCCATTCAAAACCATAA

GATGTTGGAAGCAGAAGTGCAGTCACAAGAGTCTCTTTGCTCCAAGGTTTTATCCAGGGG

TCAAGAACTAGGTAGAGGGGGACACCCTAATGAGAGCGATATACTGAAATGGATCCGGAC

CTTACAGAAACAATGGCAGCAGCTTAGGGATCAAGTTACAAACCGCAAAAACCGACTGCA

TGCAGCCAGTGTTATTAAACAGTACTTTGCTGATGCAGCAGAAGCAAACTCCTGGCTTAA

TGACCACAAGCCCTTTTTAACTGATGAAAATTATGGCAAGGATGAGATGAGCACAATTTC

TTTGCTACAGCGTTACCAACGACTGGAGAAGGAAATAGGGGCATATGCTTCAGAGGTAAA

GCGGCTCAGTGAGCAATCCCAAAGTGCTGCCCAGCTTGCTCCACTCACAACAGAACCTCA

GCAGACCATTATGGTCCACTACAGTGATTCATCAGGGGATGAAAGAGAGACCGTAAAAGA

AAAAACAGGAACCTCAGTGAAAATCAGAGCACCCTCCAAGACCTTACCTCAAATGCAGGA

ACTTAATGCCAAAATTAGGTTTAAATACAGAGGAATGAAATTAACCTGGGATCGTGGAGA

AATGGTGACTATCATCAGCAAAGAGAAAGAAGATAAAATGCTTGTGCGAGATAGCAAAGG

AAATGAACAACTGGTGTCTTCTACCTACATTACAGAACTTTCTTCAACTCAAGCCCCACC

TGCACCTACTGATGTAGTTCCAGAGAGCCAGATTCGGAAAGTGAGCCGGCCACGTCGCAC

ACGCTCCATGCGCCGTGGCACTGCTGAAATTATAACCACCCTCCTGCCAGACCCCCAGTT

CCAGAAAGACATGATAGAAAATACACAGAGCAGCTTAGAGAAAGACTACAACAGTCTCTG

TCAACTACTACAGTATAAGAGAAGAATCTTGGATGAGACTGCTCAGTTACATCGGTTTTA

TAATGGCTGTGAGGAGTTTGAGTCATGGATGGAGGACAAAGAGAACATTCTTACCACTTT

CAGCTCCAATTCTGAAAATACTGAGCAGGCCAGATATGAGAATTTTCTGACAGAGTTGGC

CAGTGGAAAGGGGCAGCTTGATGACATTACTAAATTGGGTGATGAACTTGTAAAAAAGCA

GCACAGCAAGAAGAGAGAGATTCGGTCCAGATATGCTCAGGTTACACAAAGGTGGGACCA

TATACAGGAACTTAAGGATGAAAAAGCTCAGGAGCTGCTCAAATCATCTGATATAAAATC

CTTTTTGCACACTTGTGAATATGCCAAATCCCAGCTCCTGGAAAAACTACACCAGCTGGA

TACATCTGGTGTGGGTAGCTCAGCATCAGCCCTGCAAGCTGAGGAACAGAGTCAAACCCA

GGCTGAAAGGGAAATCGAGGGCCTTGAAAGGAAGATAGAATACCTAAAGAGTGTGTCCAA

AACCAAGCGTGACTGCAGCCCTGCTGAGAGTGCTGCTATCATGAAAGAGGTCTATGCTTT

GGAAGAGCTTCTGTGCCAGGTTAAGGGTCAGGCTGCAAAAAGGCAAAATTTATTAGAGCA

GGCTCGACATCTGCAGCTCTTTCGTAAAGAGTCCAGAGACTTGCTGTTGTGGGCCGAGGA

AGTGGAAGAGCGTTTGCTTGAAGAAGGGAATAGCTATAATGTTGAATCTGCTCAAGGCTT

GTTAAATGATAATCAGGAACTGAAGCTGGAGATTGAGCAGCAAAGAGCAAGGTTGAAAAG

CATTGAAAGGCTAGCCAAGTCTTTGGAAGCTTCATCTGGAGAGAAATTCACTAAGAATAT

TCAACAAACACTAGATCAGTTAAACCAGGAATGGAGCAGGCTCGATGAACTTTGGTCTAA

TCAAAACAGGCGATTAGAGCAAGCACTTCAACTCCAGAAATGGAACAGAGAAGCTGATCG

AATAGATGCTACAATAGCTGGTCATGAAGCCAGGTTAAAGGTCAATGACCTAGGGGACTC

TGTGGATGGTGTTTATAGTTTGCTTGGCCGCCAAGAGGAGTTAGAAGGTTTGCTAGTAGC

TCTTGACCAAAATATAAGTTTTCTTCAAGAGAAGAGCCAGGAGCTCATTAATAAAAACCA

CTTTGCATCAAAGCAGCATCGGCAGCGATCCTGGGCCATTGAAGAACGCAACCAGAAGCT

TAAAGACAGCTGCAAAAAAAGAAACCATGATTTATTTGCTTCCAAATCTTACCAGGAATT

TTACAGAGATGCAGAGGAGCTACAAATTTGGATGGACGAAAAATTTAAGATAGCTGAAGA

TGAATCTTACAGAGATCCAAACAACATCTTGCGCAAACTTAAAAGGCATGAGGCAGCAGA

GAAGGAGATGCAAGCCAATCACATCCGCCGAGACAGACTGATTCAGGCAGGTGAAGAAAT

GGTGTCTAAAGACCACTATAACAGTCAGAGCATCCGGAGCAAAACTCGGCAAATCCAGAG

TAGATGGAGTGAACTGGAAACAAAAATGGCGGAAAGAGGGGACAAGCTAAGACAGGCTGG

CCAACAGGAACAACTCATGGAGCTGCTTCAGGATGCAAAAGTTAAAATTGAGAAAATTCA

GAAGATGCTACAGCATGCACCTAAAGGCTATGACCTTCGCTCCAGTCGATTACTCCTAAA

AGAACACAAGCAGGTTGAGCAGGAAGCTCAAGAACTGGCTGACAAAATTAACTCCATCAT

CACCCATGCCAAACACCTGGCTACAAACCATTTTGACTCTCAGAGAATTCTACATGAGAC

AGATATGTACCTGAAACTGTTTAAGTCTCTAGAAAAGCCTCTGAATCATCGCAGGGACCA

ATTAGAAGAGGAAGTGACACTTTACAGCTTTTTTTATGATGTTGAGTTGGAACTTAGTTG

GATTGCTGAACATGAGCCTAAAGCAGATACTAGTAGCTATACCAAATCCTTGGCTGGTGC

CATCAGCCTCCTACAAAAGCACAAGGAACTGCAAGCAGAGGTTAATGCCCACAGGCAGTA

TTTTCAGCGTGTACTGGACAGAGGCAGGGCCATGGGCAAGAGGGAAGTGTACCAGAGGTG

CAGGGATTTGTCTGCAGCCTGGGAAAAACTTGAGGATGCTTGTGAAAAACGCTTTTCCCA

CCTAAGCAAAGCAGTCATGCGGGAGCAGGTCTTGCTTGACTGTGCTGAATTAGAAGTGTG

CTTATCTGAAACGTCAGCTTTAGTCAGCACTGACTATGGCAAAAATGAGTTAGCCACACA

AAGCCTGATAAAGCAGCATCATATTGTAGAGGGGCAGATCGAGGTACTTTCAGCTCAGGT

GGATGAATTAAAGTCCAGGGTGGACCAGGCTGTGCATACATGGGGTTTGGAGGAAGTTAC

AAGAAATTACAACCACATTAGAACTCAGTTAACTGAATTACAGCACTTGGCAGCATTAAG

AGAGCAAAAGCTCCAGGATGCCCTCCACTTACATGAGTTTAAAAGAGAGTCTTCAGAATT

GGAAGAGTGGATATCACAGCAGCAGCTTATTGCCTCATCTGATGACTATGGCTCTGATTA

TGAGCGTGTCCTGCACATGCAGAGAAGGTTTGCAGTCTTCCTGAAACAGCTGGATGTGGG

TCGGGAGAGGATGCGTTCATGCCAAGAGCTGGCTCAAAGACTAATAAAGAGCAACCATCC

TGAAAGTCGGTTTATTCAAGAAACCCTGGGGTTACTGAGAGAATCATGGAATGAGCTGCA

GCACTTGGCCAACAGCCGTAAAGAGAATTTGCAAAAATCTGAGGAATGTTACAAGGTCTA

CAGCGACTTAACTGACGCTCTTGAACAAACAGAGGACAGGTACAAGTGCATTCCTGAGGA

TATTGCCAAGGACTTGAAAGGGGTTGAGTTGCAGCTAAGAAAGCATGAAGCACAAGTAAA

CGAGCTAGTGGGAAATGAACTACAGATGCAGGAGCTATTAGACATTGCAGACTCCGTTTT

AGATGAGTGCTCCCTGGGACTTAGAGAAAAGATACAGGAGAGACAGCAACTGTTGGTGGA

ACAATGGGAGAAGCTCCGCATGGCTATGGAGCAGAGGGAGCATGAGCTACGTCTTGCCAA

ACAGCGTTACATTTTTCTAAAGAGAGTACAGGATTATTCTTTGTGGTGTTCTCAAATTTT

AAGTGGCATAAAAGCAGAGGAGTCTATTCGTGATATGGCGACATGTGACCTGCAGCTTTT

CCAGCATCAGCAGCTGTGGGCTGAGATCGTAGCCCATGAGGAGACATATTCTCAGGCTGT

CACAATCGGTCAACACCTACATGAGCAAGATGTGTCCAATCCTCAGGAAGTCCAAGACAA

ACTAAAAGCTTTAAAGGATGAAAGAGAAAGACTGACAGCTAACTGGGAGTCTAAGCAGAA

ATGGCTTGAAAACACGTATCAGGAACAAGTGTTTTACAGAGACATTGAACACATGGAGAG

GCTCACTTACTCCCAAGAGGTCCAGCTGAAAAACAGTGATTTGGGGTCAACGGTGGATGA

GACAGACACTCTGATCAAACGCCATGAAGCCTTTGAAAAACTTCTTAGCGCTCAGGAAGA

AAAAATGATTGCTCTTCAGAAATCAGTTGAACATTTAAATAAAGGAGAAGTAACCAAGGG

AAAAGGCAACTACAAAAACACATTGAACTCTCTGAGAGCACGAAGGGACCGCATTAGAGA

TCTGTCCATGAAACGCAGGGAAGATCTTGAGCTCTCACGACTTTTTTGCATCTTCACTCG

GGATTCCTCTGAGGCTGAGGATTGGGTGTCAGAGCGCATGCAGAAGATGCAGGAGGATTC

CAAAATGGACCTGAGCAACTTTCAAGCCAAAATGAAACTATTGCAGAAGCACCAGGTTTT

TGAGGCAGAGATTCTGGCTCATGGCAAAATAATCAATTCTGTCTTGGAGACAGGCGAAGA

GCTGATCTCTCTGCGACATTCGAAGTCCAGGGAGATAAAACAAAGCATCTCTAATTTGAT

CTCTCACTGGGAGGCTCTGAAAGAAGCTATCTCCACCCGGGGAAAAGTTCTAGAAGGGCA

CCGTGACTTTCTTGAGTTCCTTCAAAAAGTGGAGCAGGTAGAAGTTTGGATCAGACAAAA

GGAGGTCATGATTAATGTTGGGGATCTCGGAAAGGATTATGAACATTGTCTAGAACTACT

CAGGAAACTAAGTGAGTTCAGAAGTTCCAGCCCTGGGGATATGACTGTTGATGATGCCCA

CATCAAAACCATCAACAACCTGGCGGCTCGGCTTGAGAAACAGAATTGTGATGAGATGAT

CACTGTAAAAAAGCGCAGACAGCAATTGAATGAGAGATGGAATTCTTTCCATGGAAACCT

AACCAGCTATAAGAGAAAGTTGAAAGAAGCAAAAGAGGTTCACTCTCTGATCCGTGACCT

TGAGGAAGTCAGGGACAGGGCTAGTGAAAAGATGCTGTTACTGCAAGGACAGGGTTATGG

AGAGGATGTGGAGAGTGTCGAGAACCTTATTCGCAGACATGAGGAGATGGAAAGAGAAGT

TCGAGTTATCCATGAGAGAAGAGCTGTGCTAGACCAAGAGACCAAAATCCAATTGAGAAC

TCACAGTGACCTTTCCGACAAGCTAAGTAAGAAGCAGCAAGAAATAAACAACACTTTAAT

AAAGCTGGACAAGGCAATGAAACTGAGGAAAGAACATTTGCAGGAGTCACACCAGCTTCA

GCTGTTTAAAGGTAATCAGCATCTGTTGCTGGACTGGACAATAAAGCAGACTACAGAAAT

GGGAAAGAAGAGATTGCCAAAAAGTAAAGCTCAAGCAGAAAGTCTTATAGTGGAGCACCA

TGACTGGAAGGTAGAGATTGATGCACGTGGTGAACGCATTGATTCAGTAAAGAATTTTGG

GCAGAGCCTAGTGAAGTCTGGACATAGTAAAACAGCAGAGATTAAAAAAGCTCTAAGCAA

ACTTGAAGATGCTAAAGTCAGCCTTATTCAAGCATGGGAAGAAAGAAAAATAACATTGGA

CCAAGCTCTGGAGCTTCAGATATTCCTTGGCAATGTGGACCAAAGTGAGAGCTTGTTGAG

CAACTTAGAAGCTTTTCTGGCTAATGACGATGTCGGGAGCTCTTTGTCAGACGTGGAGGC

TCTGCAAAGGAAACACGCTTTGTTTGAAAACTCCCTAGAAGCTCAAATACAAGAGGTGGA

AGAAGTGGAGAGATATGCTCAACAGCTGATCCAGAGACAGCATTATGACTCAGACAACAT

CTCAAAGAGAAGCAAAGCAATTCAGCTAAGGAAGAACAGATTGTTGGAGACAAGTAAAGC

CCGGCAAAAGGCTTTGGCAGAGTCAGTTCAGCTACAAAAGTTCTTGGAAGACAGTTATGA

GGTGTGCTTATGGTTGAATGAAAAGAAATTGGTGGCTCAAGATGAGAGCTGGAGAGAACC

CATCAATTTGCAGGCCAAGATGCAGAAACACCAGAGCTTCGAGGCTGAAATTATCGCCAA

CCGCAACAGTGTAGAAGTCCTAACAAAGGAAGGTGAGAAAATGCTACTTACTGGTCATCC

AGCCAAAGATAAAATAAAATCCAGAATAATGGATCTCAATGAAGGCTGGGAGCAGCTCTT

AATCCGCTGCAAGGAGAAGAAATCACGTTTACAGGAAGCATACCAGGCCTTGCAGTTCCT

ACACTCATTAGATGATGTTGAGTCATGGCTAGACTCAGTTGAAGCTGAGCTGAGTCAGAC

AGACTGTGGGGAGGACCTGGCCTCTGTCAGCAGACTGCTAAAGGCTCTAGAAGGCTTGGA

GGAAGTGATGGATGGACATCTGGAAAAAGTTCAGAATCTGGTGGACACAGCTAAAAGTCT

CAGCTCTCAAGGCAATTTTCAGGCTAGAGAAATCCAGCAACAGGTTTGGCATATGGCCAA

CAGGTATAATAGTCTGGCTGAGCCTCTGCAGGCTCGTAGAGAAACTCTGGAATCATGGCA

GCTGCTCTTTCAGTTTTACCAGGACATTGACGATGAAATGATATGGATTCAAGATAAGCT

GCAGGCAACAGCTTTAAATGACTGTGGAACATCATTTGAAAGCACACAAGCAGTGATCAA

AAAGCATCAAGTGGTGATACAGGAAATCACAGCTCGCACACCTTTGGTGCAGGCAGTACT

AGAAGCAGGACATAACTTGGTAAAAGGAAGGCACTTTGCCTCACAGGAGATATATGCAAG

ACTGGAGGAACTAAAGGACAACCATGAAACTCTGAAAAAAGAGTCGGAGAATAAAAGCCA

GCTGTTAAAAGAAGCCCTAAAAATCCAGACCTTCCTCTCAGAGGTCTCTGACATTGAGTT

GTGGATGGAGGAGTTGAGGCCAACCCTGGAGTCCACAGACTATGGAAAAACTGAAGATGC

TACAGAAGCACTCCTAAGAAAGTTAGATGCAGTGGATTTAGAGCTAGTCAACCACCATAG

AAAAGTCAAGTCTCTGCAGGAGACTGGGGCTGAAATGGAGAAGTGTGGCCACCCCAACAG

TTATTTGGTGAGCAAGAGTTTGACCACCATGAAAAAGCAGTATGAAACTCTGCTACAGCT

GTCTTCAGATTATCGAATATCATTGGAGGACCAATATAATCTCTATGTATTTGAAAGAGA

GGCCAGAGACCTCAAAAACTGGCTCATCTCCAACCAGATCCTCGCAGAGTCAGATGATTT

TGGCCAAGATCTTGAAGATGTGGAGACCTTGCAGAAAAAATTTGAGGATTTTACCAGTGA

AGTCAACACTCTGGGTCAGAACAAACTGAATACAGTACAAAACCTGAAACAAGAAGTAAA

ATCACCAGAAGCCCAGCAGAGAGAGAATGATCTACTAAATTTATGGGGAGAGTTACAGAG

GGCTTTGAAAACACGAGCAGAGAATTTACAATCAGCCCATGAAGTTCACCAGTTTGACCA

TGACTTAGACGAGTTGAAGAGCTGGATAAGTGAGAAGGAAATTATGTTGGACTCGGAGGA

CAGAAAAGATGACCTCATTAGCGTTAAAGCTTGTATTCGACAGCATGAAGGCCTTGAGAG

GGATTTGGTGGTAATAGAAGAGGAACTACTACGCATAAAAAAAGAGGGTAGGTCTCTTGT

TAGACGGTGTCCCCAGGTGAGAGACAGTCTGTCTGAAAGGATGCAAGAAGTGGAGGAAAA

CTGGGATAGTCTCCTGGAGAAAGCTTGCCAGTGCAAACACAGACTTCAGCAGGTGCAGGT

TGTCCAGAAATATCTTACTGACTGGAGAGAGCTTATGACATGGCTTAGGGAGTCCCTGTC

TTTAGTAACAGGAGAAGGATTGAGGGGTGAGGTCAAAGATCTGTCACAGCTAATTAAACG

GCATGACGAGTATCATAAACAGATTGAGCGGCAACTTGACAAATCAGAAATAGTGAAAAA

TGAAGGACGGCGTTTAATGCAAGAAGGAAACTTCATGATTGAAAAGCTGGAGAAACATCT

AGAAGAACTTCAGGATCTGGAAGTCCTGGTTCTGCAGGGCTGGGCTGACAGAAAAGTGCT

GTATCAGGAGGAGCTAGAGCTGCAGCAGTTGCAGAGAGAGCTGGAGCAAGCTGAACATTG

GCTAAACACATATGAGAATGTGCTTACTGCCCAAGATTATGGGGACTCTGTTTCAGATGT

TCTAGAGCTTATGAAAAAACAGGAGGATCTGGAGGCAATGATACAAGCTCAGAGTGACCG

ATTTAACACCCTACACAACAGGAAAACTCAGAAGGAACAGAAGTTGCAAAAAAGTTTTGA

AGAAGATAATGGTTCCATAAAGAAGCCTTTCCTTATGACATCGCTAAAGAAAAACCCTTC

TGATCATCTTGACTCAGTTTCTCAGCCTTCTGTAGCCAAACACGTCCTAAGAAGAAACAG

CAGTGGGAGAAAAGATGGTATAAAACTGACCACAGCACCGCTACGGAGACACAGCAGTGG

CAAGGGTGAAGGTCCTTCTACTGTTCCTCGATTAGCTTCTATGCTCAAGAGGAACACCAG

TAATAGTGCCAATAGCATAGACTCCAGTATGCCCTCTGTCAAGTCTTTTGCTGATTCTTC

TCAGACACAATACACATCTGATTCAAGCCTTGTTTACAGACAAATACGAAATTCAGACCA

CACAGCCATGTATAAAGATGAGTGGAGGGATAGAGGAACCCCCTGAATCCACACATTTAA

AATCAAATGAAGAGATTGAGGAACCTTTCATATCTACACCTCCAAGCCCTCCTGTAGCCA

TGGAAAGAGAACTTGCAGCAATCAGTACTGCTTCACCAGTAAGTGACTCCCCTCCAGATT

TCTCCACCTTGCAACCACCATCACATCCACAATATCATCCACCATCATCACCATCACTGA

GAAAATCTACAGAAGAAACGAAGGATTTTACTGAGGAACTTCCTAAGCTACCTTCTCTAG

CTGACAATGTATGTTGTCTAAATCTCACTTTTATTTATCCGTTTATTTCTGGTATATTTT

TGTCTGTAAAATGTATTTGTGCTCTCTAGGACAGAGCAGGCCAGGTCAAGATTGAGGGTC

CACTGGATATTAAACTAAAACAAGGGGGATTTAAAGGTATGGATTATTGGGAAACTGTCT

ATGCACTACTGGAAGAAGAGACTCTGAATTTGTTTAATGATCAAAATGCTGCTGAAGAGA

ACTGCACCCGATGGCCACCTATCTCTATGGCTGGAGCAGATTGTAGAGATAACCCATACT

ACAGAAGAAAGAACTACACCTTCAAATTGATACTAAATGATGGCAGTCGCTACATGTTTG

CTGCACCCTCTCATGAGGAACAACAGAAGTGGTTGGAGGAGATGCAGAATTGCACCGGAC

AGAGAGAGGTCTCAGAAAACACTGGGATACACAAAGAAAAAAAGACAGAGACAACAAAGC

AGGTGGACTCAGAATTATCAGAAAAACTGCATGTTTCAACCTCTGAGCTGGAAAAAGATT

CAACAAAGAGTTTAGAGAAAGTGAAGGAACCACCTCCCAAGCCGCCGCACACATACTACA

ACAAACATCGCTACCCAGATGTGGGAGACCCCAGAGACTCAGTGACTTTAAGAGGTCTGA

GGAAAAACCAACCCCCTTCTTTCCCTCCTCCTCTTCCCCCACAAAACCAGCAGGAGAGTG

AGGGCAAAGACAAGTCCAAAAACAAGAATGTCTTTAAAAAATTATTTAAAATATAATTTA

TATTGTTAAATCACTTGTTAAAAATGATCTGCTAAAATTCTACTCTACTCACTTGATTAA

GGTGGAGATGTTTCAGTGATTGCC

>Unigene000012

GTGTGTGTGTGTGTGTGTTACAGATCTCTGAGAACGTGGACTCTGTGGAAATGGACCCAC

AGGAACACGCTTTCACCCGCACCATCGACGAGGAGGCCGAACTTGAGGAACGAGCTGAAC

GAGAGAGAGACCGAGAGGACGGCCATCAGGAGCAGGAGGACGAGGAGGAACGCGAGCATG

AGGTCATGACAGCAGGAAAAATCTTCCAGTGTTTTCTCTCGGCGCGGGACGTTGCGCGTG

GTAGAGAGCGACCTCGCACGAGCAGCGTCGGCGTCCCAGACGAGTCTGTTCAACCTGAAC

AGGAGCGCAGAAACAGCATCCCGTTACAGGACAGACAAGACCTCTACACCACCACCTGCA

ACAGCATCATCCACCGCTGCGCCATGCTCCTGATGGCAGTCAGCCCCACACTCCCGCAGA

CGACCAACGAGCAGCCAGCTTTGTGTGGTGGGGTCACGCAAGACGGAAGCGGATTTTTGA

TCCGAAGCGAGAGTTTGTCAACTGAGAGTCGATCCATCCAGAGCGGTCCGAGTTACAGGC

TCACTAAATCTAGGAGTGAATCTGATCTCTCCCAGCCCGAGTCTGATGAAGAAGGATACT

CGCTGAGTGGAAAAAGGAACGCAGACCTGGATTTGGCGTTCACTCCTAAAAAGAGAGAAT

TTGCACATAGCACCCCTGACTCTGCGGACTGGTTCGGGCGGCATTCTGGTCGCGAGCGTC

TGTACAGCTCCCCTCAGTCTTATGAGGAGTCGGACTTGGACTTCAGCTGCACGCTGCACA

TCCATGCAGTCATCGACAACATCGTCAGCTTCATCAGTGGGGATGTGGGAAACACGCCCG

GATTCAAGGAACCAGAGGACAGCATGTCAACTAGTCCTCAAGCTACAGTTGTAGCCATGG

AGCAACAGCAGGGCAGAGCGGAGTTGCGGTTAGACGCCCTTCATCAGATTGTGGTTTTGA

TCTCTGGAATGGAGGAGAAAAGCAGTCAGGGTGGAGCAGAGGCAGTGGCTGGGTGTGGTC

CCGCTGCCTTTCACTCCGCATCCTTGCTGACCTCAGTCAGACTGCAGTTCTTGGCAGGAT

GTTTTGGCCTTGGCGCCTATAACGGGGGGCTAAACGGTGAGAGCGTCCAGCTGCACCACT

ACCAGGACGGCATTCAAGCTGCTAAAAAGAGGTTACAGATGAACATTCAGACAGCAGTGC

ACAAAATCTACCAGCAGCTTTCCATCACACTGGAGAGAGCGCTTCAGTCTAATAAACACC

ATATAGAGGCTCAGCAGCGGCTGCTGTTAGTGACCGTTTTTGCCCTGAGTGTTCGTTACC

AGCCTGTGGACGTTTCGTTAGCCATCTCCAGCGGTTTGCTAGATGTTCTCTCTCAACTGT

GCGGGAAAGAAACCCTGCTGGGACACACCCTTCAGCTCCTGCACAAACCCGCCGGCTCGC

AACTCAGCACAGCACTCAAGGTTGCCAGCACCCGTCTGCTGCAGATCCTTGCCATTAGCA

CCGGGACGTACGCAGATCGCCTGAGCCCTAAAGTGGTTCAAGCTCTGTTGGACCTGCTCT

GTAGTCAGCTGAAGACTCTGCTGTCGCAGGCTGCAGGAAGCGTCTTCGGCACAAATTCAG

AGTCAGAGGAAAAGACAGAGGACTCAACAGGCAGCATGAAAAAAGACTTCAGGGCCTTAC

TGCGCAAACAGCACATTGCGGAACTACATCTCGGAGACTTTCTGGTGTTTCTCAGAAGAG

TCGTATCATCTAAAGCCATTCAGTCCAAGATGGCGTCTGCCAAATGGACCGAAGTGCTGC

TTAACATTGCGGCACAGAAATGCTGTTCAGGTATCCCTCTGGTGGGGAATTTAAGGACAC

GTCTGCTGGCACTGCATGTCCTAGAGGCTGTACTTCCTGCCTGCGAAAGTAGCGTGGAGG

ATCATCAAATGAGTCAGGTGGTTGAAAGGTTGTTCTCCTTGTTGTCGGACTGTATGTGGG

AGGCTCCTGTCGCTCAGGCCAAAAACACCGTCCACCTGAAGGAAAAGGAGGTCAAACTCC

AGGGTGAGGCAGAGGAGGAGGAAGAGAACCTGCCTGTCCAGGAAGTGATCTTCGATCCTG

AAAAGACACAGTGCTGCGTGGTGGAAAACGGACAGGGTTTGACGCACGGTAGCGGTGGAA

AAGGCTACGGTCTGGCTGCTACCGGCATCTCCTCAGGCTGTTACCAGTGGAAGTTCTACA

TCGTGAAGGAGAATCGTGGGAATGAAGGGACGTGTGTGGGGGTTTCCCGTTGGCCTGTAA

ACGACTTTAACCACCGCACCACGGCCGACATGTGGCTGTACCGGGCTTACAGTGGCAACC

TGTACCACAATGGCGAGCAGACGCTCGCGCTGTCCAGCTACACACAGGGCGATTACATCA

CCTGCGTGCTGGACATGGAGGCTCGCACAGTTTCCTTCGGGAAGAACGGAGAGGAGCCCA

AACTGGCTTTCGAAGACGTGGATGCGACAGAGCTTTATCCATGCGTCATGTTCTACAGCA

GCAACCCAGGAGAAAAGGTCAAAATCTGTGACATGCAGATGCGAGGGACGCCACGTGACC

TTTTACCCGGCGACCCCATCTGCAGTCCCGTGGCCACCGTCCTTGCCGAAGCGGTGTCTC

AGCTGGTCAGGATCCTGCACCGGTCAGACAGCTGGACGCAACCTATCAACCGGTGCATGC

TTCAGCGGCTGAAGCAAATCCGGGGGTGCTTGCACGACGGCGCTTCGCCTACGGGTGGTG

CGAGACTCCGGAAGAGCCGCTCGGCTCAGAGCCGCGAGGAACAGGATGAGGCGAAAGACG

AGGACAGGGATGAGGAGCGAGGCAGAATGGGGCGGGAACTGACTGAGGCGCAGCTGCGTA

CACTTTGTAACCTGATCTGGCCTGTGCTGGCGGTGATTGGAGGGGTGGACGGAGGTCTGA

GAGTCGGAGGTCGCTGCGTGCACAAGCAAAGCGGGAGACATGCAACCCTGCTGGGGGTGG

TGAAGGAGGGCAGCATGTCGGCCAAGGTGCAGTGGGACGAGGCAGAGATCACTATCAGTG

ACACGCCGCTATATAACCTGGAGCCCTGCGAGCCGCTGCCGTTTGACGTGGCACGTTTCC

GGGGACTGACGGCCGCGCTGCTTTTGGAACTGACCGCTCTGGCTTCGCTGCAGGACGACG

GCAAGTTCTCTCCTACGGCCTGCTCGTTATCATCTTCCTCGAGACGCCACGAGCGCCGCC

ACAGACACGAGCCTCACGAGCCCGAAGAAGGCCCCGGTGACCTGCGTGTCTCTCACAGCC

TGGATGAAGTGCGTTCCCACGCCGAACTGCAAAAGCCTCCTGTAATGGCCGAGGTTCATG

CTGTGCAACTCTCCTACCTCTGCCTTGGTGCCATAAAGAGCCTAAGCGTCCTTCTAAGCT

GCAGTAAGTATGCCGAGCTCCTGCTCATACCCAAGGCGGTGCACGACAACGGCCACAACG

CAGACTGCGGCAGCCCCAGCATCGGCGTCTCACAGGAAGAGGCAGAGATGCGCTCAGCGC

TGCAGTTCCTTATGAGACACATGGTAAAGCGAGCAGTGATGAGGTCACCTATCAAACGCG

TACTGGGTTTAGCTGAACTGGAGCGGGCGCAGGCCATGATTTACAAACTCGTCGTCAATA

GTGTGCTAGAAGAACAGGAGGGCGGGAAAACCAAACAATCAGACGGCGAGTGTGAGGAGC

TGGAAGGCGAACAGCAGTTCCAGACGCCGGTTACCACCTCACCCTCTGCGTCCAGTAGCA

CCTCATTCATGAGCTCCTCCCTCGAGGACACCACCACAGCAACCACACCTGTTACCACGG

CAACTACACCTATAACTGATGCTGAGACTGCGCCAGCCTCAGAATCGCCAGGTGTTATGC

CTCTCAGTCTGCTCAGACAGATGTTCTCCAGCTACCCCACCACAACGCTGCTGCCCGCTC

GCCGAGCTCAGACACCTCCAGTCTCGTCTCTGCCCACGTCTCCTTCAGACGAACACGGCC

GAAGGCAAAGTTTTACCTCTCCTGAATCTCAGCCTGCTAGAACTACCAACAGGACAGCGC

TGTCGGACCCCAGCAGCCGTCTCTCCACATCTCCCCCTCCCCCTGCCATCGCTGTTCCCC

TTCTCGAGATGGGGTTCTCCCTCAGACAGATCATGAAGGCTCTGGAAGCTACAGGTACAA

GAGGTGAGGCTGACGCGCAAAACATCACGGTTCTGGCCATGTGGATGATCGAGCACCCGG

GCAACGAGGACACAGAGGAGCGGACCCTGTCCTCCGACTGCTCGGGGGCAGCAGGAGGGG

GCAAAAGTGCGGAGCGGAGCTTCCTGCGTTCACCTGGAGATATTCCCAACGCTGATGCCG

CAGAGCTTGAAGAGGGATTTAGCGAGAGCCCTGAAGGAATAGATCAAGACTCCGCCTCTG

CATCCAACGGCCCCGCCCTCCGGGGGCGCTCAGCCGCCAGTAGGAAGCATCGCTTCGATC

TGGCAGCACGTACTCTATTGGCCCGTGCAGCTGGACTGTACCATTCGGTGCAGGCCCACC

GCTCTCAGGTGCGTAGGGAGGGCTCAGGGCTGCAGCAGGACTCCAGCTCACTGGGAGGAG

TTTACGACTTTAACCTGGATGAGGAGCTGGAGCTAGAGCTGGACCAAGAGGCCATGGAGG

CCATGTTCACTCAGGAGCTTGCATCAGACTCGGATATTTTGGGAATGTGGATTCCGGAGC

GTGTGTGTGAGTCAGAAGAGCGCGAGGAGGTGGTGGTCTGTGAGCTCTGCGACGTCAGCG

TGTCCAGTTTTAACCAGCACATGAAGAAAAGTCATCCTGGCTGCGGACGGAGCGCAAATC

GGCAGGGTTACCGCAGTAACGGGTCCTACGTGGACGGGTGGTTTGGGGGAGAGTGTGGGA

GCGGGAACCCCTATTACTTACTGTGCGGAAGCTGCAGGGAAAAGTACCTGGGCATGAAGA

GCAAGCACAAAGGAGCAGTGTCGGAGAGATACAAGGGCCAGGCTCCAGACCTGCTGGGAA

AACAGGACAGTGTCTATGAAGAAGACTGGGACATGCTGGACGTGGATGAGGCGGAGAAGC

TGACAGGCGAGGAGGAGTTTGAGCTGCTTTCAGCACCGCTGGGACTCACGGAACGGAAAC

CCGTCCCCGAGGCTGTTCAGTTCCCTGACACAGACCCCCTCGGGGCCTCCGCTGCCATGG

TTACCGCCACCAATAGCATGGAGGAGACCCTGCTACAGATAGGGTGTCAGAGTTCGGTGG

ATAAAAGCACATCAGGTCGCATGTGTTTGGGTGAGCAAGCAGCCGGTCTCCAGAGTTCTG

CTGATCGCATGGTTGCTTTGAGGAGAGTCACAGCAGCTGCTCAAGTCCTGGTGGCAAGAA

CCATGGTGATGAGGGCGCTGTCTTTGCTGTCTGTCAGCGGTTCCAGCTGCAGTCTCGCTG

CTGGTCTGGAAGCTCTGGGTCTGACGGACATTAGGACCCTGGTGCGTTTGATGTGTCTGG

CTGCGGCCGGTCGAGCAGGTCTTTCTACCGGTCTCGCTTCAGGCCCCGGCGCTTTAGAGC

GGCCACGTGGCACGAACAAGCCCACCAAACCCATCTCCTGTCTGGCCTACCTCAGCACAG

CAGTGGGCTGCCTCGCCTCCAACAGCCCAAACGCCGCCAAGCTTCTGGTCCAGCTGTGCA

CTCAGAACCTGATTTCAGCAGCTACAGGAATGAATCTGACCACTGTGGATGACCCAATTC

AGAGGAAGTTCCTGCCCAGTTTCCTGCGGGGCGTGGCTGAGGAAAACAAACTCATCACCT

CGCCAAATTTCATAGTGACTCAGGCATTGGTGGCGCTCTTAGCCGATAAAGGAGCCAGAC

TGAGACCCGGGTACGACAAGGCCGAAATGGAGAAGAGAGGCCCGTTGGAGCTGGCCAATG

CGCTGGCGGCGTGCTGCCTCTCATCCCGCCTCTCCTCACAGCACCGCCAGTGGGCTGCGC

AGCAGCTGGTGCGGACACTTGCCGCCCATGACCGCGACAACCAGAGTCGACCGCAGACCT

TCGCCGACATGGCCGGTGACTTACGCAAGTGTTCTACCATCAAGCTGGAGGCTCATCAGG

GCAGAGTGATCAGCTTCGGGTGGTGTAACAAGAAAGGTCTTTTGGCGACCAGCGGTAGTG

ATGGAACAGTTCGAGTCTGGAACGTGAACAAGAACCAATACACTCTGCAGCAGACCTGCG

TCTTTAACAAAACAGACTTTACCTCTGAGGAGTGTTCCACCGGACTGGGGTCTCCTGGTG

AACCGAGCTTGGCGCCCGTGGCCTGGAGTGTGAGCGGGAAATTAGTTGCAGCTGCCATGG

AGAAAGTGGTGAACATCTGGCAGGTCAATGGCGGTAAGGCCTTGTTGGACCTGCAGCCTC

ACTGGGTGTCGTCGCTGGTGTGGCCGGAGAACGAGGCAGGGTGTCTTTGGGCCGGAGAGT

CTCGAGAGCTCCTATTGGTCGGTCGCATAGATGGAAGTTTGGGCTTGCTGGAAATTTTAG

ATAGCTCAAACATACAGAGAACCGAACTTCTGCACTGTTACAGGAAAGACGTAGCTGTGA

TGAACATCGCCTGGTACAGCGAGGATCGACCGTTTGCTGTCGGCTACGCTGACGGAAAGC

TTCTGATTGGCTGCAAAGAAGCGCTGGAGAACGGATCCGTGGTCGTCATCGATGCTCACA

AGGAGAGCATCAGTTCTCTGCGCTGGGCTCCAGGAGGGCAAATTTTATTAAGCTGTGCCA

AAGAGGAGACTGCCTGCCTTTGGGCGGAGTCTGGGGCGGGGCTTGGGCGGAGTTGGGTGT

GTCTCCAGAGCATCGCTCACCCGAGTGTTGTAAATGCTGTCGCCTGGTGTGGTCTCACCG

GACAGGGACCTAAAGCACTAAACATGCTGGCCACGTGTTGTCAGAATGGCTTGGTGTCTG

TGTGGACCGTGCCTCAGGACCCCACAGCCTTCTCTCAGTCTGGCTCATCCAGCACTGATG

CATGGTGGGAAACGGACTCCAAGTCAAAACCAAGGTTCACTCCACAGTGGCACGAGGCCG

CAGTGTGCGTCTTCCAGCTAAGAGGTCACATGACTCCGGTTAGGACGATAGCCTTTAGTC

CTGACGGTCTTGCTCTGGTTTCTGGTGGTGTTGGTGGTCTCCTAAACATCTGGTCTCTCC

GAGATGGCTCTGTGCTGCAGACGGTGGTTGTCGGCTCAGGAGCGATTCAGAACACTGTGT

GGATTCCAGACGTCGGGGTGGCCGTCTGTTCCAACCGCTCAAAGGACGTTCTGGTTGTGA

ACTGCTCCTCGGAGTTTATGACCGCTAATCAGGTGTTAGCAACCTGTCGCACCGCGCTGA

AGAAGCAGGGGGTTGTGGGATTGAACACGGCTCCCTGTATGAGGACTTTTCTCGAGAGAC

TTCCCATAATGCTTCAGGAGCAGTACGCCTACGAGAAGCCCCATGTAGTGTGTGGAGAGC

AGCTGATCCACAGTCCCTACATGCAGTGTTTGGCATCTCTGGCCGTCGGGTTGCAGTTGG

ACTCGTTACTATGTCGTCCCGCAGTGCCTCTTCACCTGTCTCATTGCCTCGCCCAACCTG

TCTCTTCATCATCGTCATCATCACAATCATCCTCCTCATCCGGTCCTTCAGCACTCATGC

ACTCATGTGGCAGCAGCGAGTGGGCGTGGCTTCACTGCTTCTCCACCACCATAAAGACGG

CTGAGGCGCTCGCCCGCGCTCACACTTTCCCAGAATCCTTTAACGTGCCTGATCTGGAGC

CGGTGGCCAAAGACAAGATGGCGGTGCTGATGGACAACAGTAAATGGGTTCCTGGGATGG

ATGAGCAGTTGATGGCTTGGGCCACCTCCAGACCCGAGGTACTGATATCACCAGTTATTA

TTTCAGACTCCCTGATTATTTAATTTTGATCTGTTTGTAATTTAGAGACGTTTGTGTGTG

TGGAATATTAACAGGACTGGCACCTGGGTGGGAAGTGTGACGTGTATCTGTGGGGCGCCG

GTCGCCACGGTCAGCTCGCCGAGGTCGGCCGGAACATTCTGGTGCCGACGCCTGCTGCGT

CTTTCTCTCAGGCTCAACAGGTTGTATGTGGGCAGAACTGCACATTTGTGATTCAGGCGA

ACGGCACAGTGTCTGCGTGTGGGGAGGGCAGCTACGGTCGACTGGGTCAGGGAAACTCTG

ATGACCTGCACATTCTTACGGTCATCTCAGCACTTCAAGGGTTTGTTGTTACCCAGCTGG

TCACGTCGTGTGGCTCTGATGGACACTCCATGGCTCTGACTGAGAGCGGTGAGGTGTTCA

GCTGGGGAGATGGCGACTACGGCAAACTGGGCCACGGCAACAGTGACCGCCAACGCCGGC

CGCGCCAGATAGAGGCTTTACAGGGAGAGGAGGTGGTGCAGATGTCCTGTGGGTTTAAAC

ACTCTGCCGTCGTCACAGCAGACGGTAAACTCTTCTGCTTCGGCAATGGAGACTACGGGA

GACTCGGACTGGGAAACACATCCAATAAGAAACTTCCAGAACGAGTCACAGCTTTGGAGG

GACAACAGATAGGCCAGGTGGCGTGCGGGCTAAATCACACACTGGTGGTGTCATCTGACG

GGTCGACCGTCTGGGCGTTTGGAGACGGAGATTATGGAAAACTTGGCTTGGGAAACTCGA

CTGCTAAATCATCACCGCAGAAAGTGGATGTTCTGTGTGGAATTGGAATCAAAAAGGTTG

CATGTGGAACCCAGTTCTCTGTGGCTTTAACCAAAGATGGAAATGTTTACACTTTTGGGC

AAGATCGCCTGATCGGTCTCCCAGAGGGGCGTGCCCGCAATCACAACCGCCCGCAGCAGC

TCCCCGCTCTGGTGGGCGTGATTATCAAAGATGTCGCTGTAGGGGCGGAGCATACACTCG

CGCTGGCGTCTACAGGAGATGTTTATGCTTGGGGGAGCAACTCTGAGGGACAGCTCGGTT

TGGGCCACACCAACCATGTACGAGAGCCGACTCTAATCACAGCTCTGCAGGGTAAAAACA

CCCAGCAGATTTCAGCAGGACGCTGCCACAGTGCCGCATGGACCGCGCCCTCAGTACCAC

CCAGAGCACCAGGCTCGTCCATGCCGCTGCAGCTGGGTTTGCCCGTGTGTGTTCCTCCGC

AGTACGGCAGTCTGAGAGACATCAGCATGGAGGCACTAAGAGCTCGGCTACGTCTGCTCT

ACCACTTCTCTGACCTCATGTACTCCTCCTGGAGACTGCTTAACCTCAGCCCTAACAACC

AGAGCTGCACGTCTCATTATAACCCTGGGACGTGGGGTATCGTACAGGGGCAGCTGCGCC

CTCTACTGGCTCCACGTGTGTATATGCTCCCGATGGTACGCTCCATCGGCAAGACCATGG

TGCAGGGCAAGAATTACGGACCTCAGATCACCGTCAAGAGAATCTCCACTCGTGGGAGGA

AGTGTAAGCCCATTTTCGTGCAGATCGCTCGGCAGGTAGTGAAGCTGAATGCGTCTGATC

TCCGGCTGCCGTCTCGGGCGTGGAAGGTGAAGCTGGTGGGGGAGGGAGCGGACGACGCTG

GAGGAGTTTTTGATGACACCATTACAGAAATGTGTCAGGAGCTCGAGAACGGGGTGGTGG

ATCTGCTCATCCCTTCTCCTAATGCCACTGCGGAAGTGGGCTACAACAGAGACAGGTTTC

TGCTGAATCCCTCGGCTTGCCTGGACGAACATTTGCTGCAGTTCAAGTTCCTGGGCATCC

TGATGGGCGTGGCCATCCGCACCAAGAAACCTCTGGACCTGCATCTGGCTCCGATGGTGT

GGAAACAGCTGTGCTGCATCCCGCTGACGCTGGAGGACCTGGAGGAGGTGGACCTGCTGT

ATGTCCAGACACTCAACAGCATTGTGCACCTGGAGGACAGCAGCATCACAGAGCAGAATT

TCCACGAGATGATTCCTCTGGATTCATTTGTGGGTCAGAGCGCTGATGGGAAAATGGTGC

CCATCATTCCGGGAGGAAACAGTATTCCACTCACCTTCTCCAACAGGAAGGAGTACGTAG

AACGCGCCATAGAGTACCGGCTACATGAACTGGATCGTCAGGTGGCGGCCGTGCGTGAGG

GCATGTCATGGATTGTACCGGTTCCTTTACTCTCGCTGCTGACCGCTCGGCAGTTGGAGC

AGATGGTGTGTGGTCTGGCGGAGATCAGTGTGGAGGTGTTGAAGAAGGTGGTTCGTTATC

GGGAGGTGGAGGAGCAGCAGCAGTTGGTGCAGTGGTTCTGGCAGACGCTTGACGAGTTCT

CCAACGAGGAACGAATGCTGTTCATGCGCTTCGTCTCCGGACGCTCGCGTCTCCCTGCTA

ACACAGCCGATATCTCGCAGCGCTTCCAGATCATGAAGGTGGACCGGCCGTACGACAGCC

TTCCCACGTCTCAGACCTGTTTCTTCCAGCTCCGCCTCCCGCCCTACTCCAGCCAATCGG

TGATGGCAGAGCGGCTACGCTACGCCATTAATAACTGTCGTTCTATCGACATGGACAATT

ACATGCTGTCACGGAATGTGGACAATACTGAGGGTTCGGACACGGACTACTGACCGAGAG

TTAAAGCACAAAGACACTCGCTGAACTTTAAACCGTCAACACAAAAATCACCACCTTTAA

TTCTTCTCTTCTTTTATTAATCATTAAAGCAGTCAGACGGCGTTAACCAGGTACAGATAA

ATATAACCATTTCTGTTATTCACAACTCCCCACACACACACACACACACAC

>Unigene000013

GCACTAGATCGGAAGAGCGTGTGTCCCAGTGCCCACCCAACGAGTTTGAGTGTCAGGGCA

CAGATGTGTGTATCCACCTGAGCAAAGTGTGTAACGGCATCTCAGACTGCCTGGACGGAC

GCGATGAAGGCCATCACTGTCTGGAATTTGTGCATAACTGCACCGTTATGGGCTGTCACG

ACAGCTGTGCCATCACCCCCTCTGGACCCAAGTGTTACTGCGGTAGCGGCTTTGAAGTCG

GAGAGGACGGCAAGGCGTGCCGAGATTTTAACGAGTGTGCAGTGTACGGAACGTGCAGTC

AGACCTGCACTAATACAGAAGGCTCCTACACCTGCAGCTGCGTAGAGGGCTACCTGCCTC

AGCCGGACAACCGCTCCTGCAAAGCCAAAAATGACCCCGTGGACCGCCCCCCATACCTCC

TGATCGCCAACTCGCAGAATATCCAGGCCACGTCTTTGAACGGTGCCAACCCCATCTCTG

TAAGCACACGCAAGACCACAGCTATGGACTTCATCTATGCTGAGGAAACTGTGTGCTGGA

TCCACGTAGGCGATGCGCCAAGTGCCACCATGCTCAAGTGTGCCAAGTTCCCCAACGCCA

AGGGCTTTAACGAAGAGAGAACGATTAACATCTCCTTGAGTCTGCACCATGTTGAGCAGA

TGGCCATAGACTGGCTAACAGGAAACTTCTACTTTGTAGACGATGTGGATGAACGCATCT

TTGTGTGCACCAAAGATGGCGCCACTTGCGTCATTCTGCTGGATCTTGAGCTGTACAACC

CCAAGGGCATTGTGCTGGACCCGACCATGGGGAAGATGTTCTTCACCGACTATGGGCAAA

TTCCCAAGGTGGAGCGCTGCGACATGGACGGGCAGAACCGCACCAAGCTGGTGGACAGCA

AGATTGTCTTTCCTCATGGCATCACACTGGATCTAGTCAACAGGCTGGTGTACTGGGCCG

ACGCGTACCTGGACTACATCGAAGTGGTTGACTACGAGGGCAAGAACCGACACACTATCA

TTCAAGGGCTGCTGGTCGAACACTTGTACAGCCTGACCGTGTTTGAGAACTACCTCTACG

CCACCAACTCGGACACTGGGAACATTCAACCCAAGACCAGCGTCATCCGCGTCAACCGTT

TCAACAGCTCCGACTTCCAGGTGGTGACACGAGTGGATAAAGGTGGAGCCCTGCACGTGT

ACCACCAGAGACGCCAGCCTGCAGTTCGCAGTCACGCCTGCGCACCTGATCAGTTTGGCA

AACCCGGAGGTTGTTCCGATATCTGTCTGCTAGCCAACAGCCACAAGATCCGCACCTGCC

GCTGCCGCTCCGGTTTCAGCCTGGGCAGCGACGGCAAATCCTGCAAGAAACCAGAGCATG

AGCTCTTCCTGGTGTATGGGAAAGGTCGGCCTGGGGTCATCCGAGGCATGGACCTGCACG

CCCGTGTGTATGACGAGCACATCATTCCTATCGAGAACCTGAACAACCCCAGGGCTTTGG

ACTTTCACGCCGAGACGGAGTTTATCTACTTTAGCGACGCCGCCATGTACACCATCGGAC

GGCAGAAAATCGACGGCACAGAAAGGGACATCATTGTGAAGAGCGGTGAGATTTTTTTCG

CAGTTACCTCGAATGTACAGAATAATGGTGATATTAATGTATTAGAATTTTAATCTTCAG

GATAGTCTTTACAAAGTTCTTGTTTCATCCAGGCGACAACAATTTCCAGGCTCAGTATGT

GGACATTCAAAAGTGAAATTCTTCAGTATGGACATTTCAGTCTGTTCTTCTGTCACCAAC

ACAGTCCACACTTATCTCATTATAGGTATCCACACTGTTGAGGGCATAGCGGTGGACTGG

ATGGGTCAGAACCTGTACTGGACAGATGATGGACCAGAGAAAACAATCAGTGTGGCCAAG

CTGGAGAAAGCCTCCCAGACACGCAAGACTCTCCTTGAGGGAAAGATGACTCACCCTCGA

GCTATAGTGGTGGACCCGTCACACGGGATGATGTACTGGAGTGACTGGCAGGAAGACTCG

AGAGATGCCAACCGTGGCAGCATTAGGAAGGCGTGGATGGATGGCTCCAAATCTGGCATC

CTCATCACCTCCAAAAACATGCTCTGGCCCAACGGTCTGAGTCTGGACATCGAACAGAAC

GTGCTGTACTGGGTGGACGCCTACTACGACCGCATCGAGATGGTGCTTCTTAACACCACC

GAGCGCAGGACCGTGTACGAGGGGCAGGAGCTGAACCACCCTTTTGGGCTTTGTCACTAT

AAGAACTTCTTGTTCTGGAACGAGTACCGAATCGGAGGCATTTACAAGCTGGACATGACG

TCCAAGAAGGTGACGCTGCTCAGGAGCGAGCGGCCGCCCATCTACGAGATCCGCACCTAC

GACGCCCAGCAGCAGCAAATTCCAGGCCATAATCCATGCAGGGGCAATAACGGTGGCTGC

AGCAGTCTGTGCTTGCTCACACCGGCAGGTCGTACCTGCGCTTGTGCAGATGACCAGATT

TTGGACTTTGACAATAAGACCTGTAAAGCCAATCCGTCCTACGTGCCCCCGCCTCAGTGC

CAGCCAGGGGAGTTTGCCTGCAAGAACAACCGCTGCATCCAGGAACGTTGGAAGTGCGAC

GGAGACAACGACTGCTTGGACAATAGCGATGAGGTGCCGGAGCTCTGCCATCAGCACACC

TGCCCTGCCGATCGCTTCAAGTGCCAGAACAACCGCTGCATTCCCATGCGCTGGCTGTGT

GACGGAGACAACGACTGCGGCAGCAACGAGGACGAATCCAATACCACCTGCTCAGCTCGT

ACATGTCCTCCTAACCAGCACCCGTGTGCCAGTGGACGCTGCATTCCTAAAACCTGGATA

TGTGACCTGGACGATGACTGTGGGGACCGCTCAGACGAACCAGCTTCCTGTGCCTACCCG

ACTTGTTTCCCACAAACCCAGTTCACGTGTGCCAACGGGCGCTGCATCAACGTTAACTGG

CGCTGTGATAATGATAATGACTGTGGAGACAACAGCGACGAAGCAGGCTGCAGTCACTCC

TGTTCCAGCGTGCAGTTCAAATGCAACAGCGGCCGCTGCATCCCAGAGTACTGGACCTGC

GACGGGGACAACGACTGCGGCGACAACAGCGACGAGAAAAGCGCCAACTGCACTAATCAG

GCTACGCGTCCCCCTGGTGGCTGCCACTCGGATGAGTTTCAGTGCCGCTTGGACGGTCTG

TGTATCCCCATGCGCTGGCGCTGTGACGGGGAGAACGACTGCATAGACGACACGGATGAG

AAGCACTGCGAGGGCGTCACCCACACGTGCAACCCGTCTGCGCAGTTCGCCTGCCGTGAC

TCGGCAAGGTGTATCAGTAAGGCGTGGGTGTGTGATGGAGACAGCGACTGTGAGGATAAC

TCTGATGAAGATAATTGTGAAGCGTTGTTGTGTAAACTTTCGCACCATACATGCGCCCAG

AACGATTCCATCTGCCTTCCTGCCGAAAAGCTGTGCGACGGTAAGGACGACTGTCCCGAC

GGCTCGGACGAGAAACTCTGCGACCTGTGCTCGCTAGAGAACGGCGGCTGCAGCCATAAC

TGCACCATTGCCCCCGGCGAGGGCATCCTGTGCTCCTGCCCGACTGGCATGGAACTCGGC

TCTGATAACAAGACCTGCCAGATCCAGAGCTTTTGCGCCAAACATCTGAAGTGCAGTCAA

CGCTGCATCCAGGAGAAAGCCACCGTCAAGTGTGCCTGCTACGAGGGCTGGGCTCTGGAA

CCGGACAACGAGAGCTGCAAGAGCACCGACCCTTTCAAGCCTTTCATCATTTTCTCCAAC

CGGCACGAAATTCGACGTATCGACCTGTACAAGGGCGAGTTCAGTGTCCTGGTGCCCAGC

CTGAGGAACACCATCGCCCTGGACTTCCACCTCAATCAGAGCTCCCTCTACTGGACCGAC

GTGGTGGAGGACAAGATCTATCGTGGCAAACTGTCTGAAAACGGAGCCCTGACCAGTTTT

GAGGTGGTGATCCAGTATGGTCTGGCCACTCCAGAGGGTTTGGCTGTGGACTGGATTGCT

GGAAACATCTACTGGGTGGAGAGCAACTTGGACCAGATCGAGGTGGCCAAGCTAGACGGA

ACCATGCGCACGACACTGCTCGCCGGAGAGGTGGAGCATCCACGAGCTATAGCCCTCGAT

CCACGTGACGGTATTTTGTTTTGGACAGACTGGGATGCCAGTTCTCCCCGAATCGAGGCT

GCCTCCATGAGCGGCGATGGCCGGCGTACCATTCACAGAGAAACAGGAAGTGGAGGCTGG

CCTAATGGGCTCACCGTTGACTACTTAGAGCGCCGTATCCTCTGGATCGATGCTCGGTCT

GATGCCATCTACTCTGCTGCTTACGACGGTTCGGGCCTGATCGAGGTGCTGCGTGGACAC

GAATACCTGTCGCACCCGTTCGCCGTCACCATGTACGGCGGCGAGGTTTACTGGACAGAC

TGGCGCACTAACACACTGGCTAAAGCCAACAAATGGACAGGACACAACGTTACAGTTGTA

CAGAGGACCAACACACAGCCGTTCGACCTGCAGGTCTATCATCCATCCAGACAGCCTCAG

GCTCCAAACCCTTGTGCTGCTAACGATGGAAGGGGTCCGTGCTCTCACCTCTGCCTCATC

AACTACAACCAGACCTTCTCCTGCGCCTGCCCCCACCTTATGAAGCTGCAGGCAGACAAA

CACACCTGCTACGAATCTCGTCAGTTCTTACTCTACGCTCGTCAGATCGAGATCAGAGGA

GTGGACATCGACAATCCATATTACAACTACATCATCTCCTTCACTGTTCCTGTGATCGAT

AATGTAACAGCGGTGGATTACGACGCCCAGGAACAGCGCATCTACTGGTCTGACGTTCGC

ACGCAAATGATCAAACGAGCCTTCATTAACGGCACGGGGGTGGAGACAGTGGTGTCGGCC

GATGTACCCAACGCTCAAGGCTTGGCTGTGGACTGGGTGTCCAGGAACCTGTTCTGGACT

AGCTACGATACCAGCAAGAAGCAGATCAACGTAGCCCGATTGGACGGCTCCTTTAAGAAC

TCGGTCGTCCACGGACTGGACAAACCACACTGCCTGGTGGTACATCCCATGCTTGGAAAG

CTCTACTGGACAGACGGGGACAACGTCAGCATGGCCAACACAGATGGCAGCAACCAGTCG

TTACTCTTCACCAGTCAGAAAGGCCCCGTGGGACTGTCTATTGACTTCGATGAGGGGAAG

CTGTACTGGATCAGCTCAGGCAACAGCACGATAAATCGCTGCAATCTGGACGGCACCGGC

CTGGAGGTCATTGAAAGTGTAAAAGGAAACTCACCAGAGCCACCGCTCTCGCCATCATGG

GGGACAAGCTGTGGTGGGCGGACCAGGGCACAGACCAGGTAGGCACCTGCGACAAGAAGG

ATGGCGGCAGCTGGAAGGTTCTTAGGAACGGCACGTCTCCGGTGATGCATATGAAGATCT

ACAACGAGACTGTCCAACAAAAAGGTACGAACCTGTGCACAATGAACAACGGGGACTGTT

CTCAGCTTTGCTTGCCCACCTCACCCACCAGCAGAGCCTGCATGTGTACGGCAGGCTACA

ACCTGAAGAGCGGCCAGCAGTCCTGTGAAGGTATGGGCTCGTTCTTGCTGTACTCGGTGC

ATGAGGGCATCCGCGGAATTCCTCTGGACCCGCTGGACAAATCTGACGCTCTGGTGCCAG

TTTCTGGAACCTCTTTGGCAGTGGGCATCGACTTTCACGCCGAAAACGACACCATTTACT

GGGTGGACATGGGTCTTAGCACCATCAGCCGAGCCAAAAGAGACCAGACATGGAGAGAAG

ATGTAGTTACTAACGGCATTGGACGTGTGGAAGGCATTGCTGTCGACTGGATTGCAGGAA

ACATCTACTGGACTGACCAAGGCTTCGACATGATTGAGGTGGCCAGACTCAACGGCTCCT

TCCGCTACGTGGTCATCTCTCACGGGCTGGACAAACCCCGCGCCATCGCCGTCCATCCAG

AGAAAGGGTACCTTTTCTGGACCGAGTGGGGTCAGTACCCACGCATCGAAAGGTCTCGAC

TGGACGGGTCAGAAAGAGTGGTTCTGGTTAATGTCAGTATTAGCTGGCCCAATGGCATCT

CCATCGATTATGAGGAAGGGCTGCTCTACTGGTGTGACGCTCGCACAGACAAAATCGAGC

GTATTAATCTGGAGACCGGTGGGAACCGAGAACTTGTCCTGGCAGTTCATAACATGGATA

TGTTCGCCGTGTCTGTATTCGAAAACTACATCTACTGGAGTGACAGAACCCATGCCAATG

GCTCCATTAAGAGGGGTAATAAGAACAATGCCACTGATATGGTTTATCTAAGAAAAGGCA

TTGGTGTGCAGCTCAAGGATATTAAAGTTTTCAACCGAGCCCGGCAGCAAGGCACCAACA

TCTGTAAAGACAAAAACGGTGGATGTGAGCAACTATGTCTATTCCGTGGCAACGGAGCAC

GCACCTGCGCCTGTGCCCATGGCATGCTAGCCGAAGACGGGCGCACTTGCCGCGACTACG

ACGGCTACCTTCTTTACTCCGAGCGTACAATACTGAAAAGCATACACTTATCAGACGAAA

CCAACCTCAACGCCCCGATCAAACCTTTCGAGGATCCAGATCATATGAGAAATGTCATCG

CTCTCACCTTCGACCACCAAGGTGGCGACGGCAAAGGAGCTAACCGCATCTTCTTCAGCG

ATATACACTTCGGCAACATCCAGCAGATCAACAGTGATGGCTCAGCGCGCAAGACCGTTG

TTGAAAATGTGGGCTCGGTGGAAGGCTTGGCGTATCACAGGGGTTGGGACACGCTTTACT

GGACCAGCTACACCACCTCCACCATTACGCGCCACACGGTGGACCAGACACTCAGCGGCG

CCTTTAACAGAGAGACGGTGGTCAGCATGTCTGGAGATGATCACCCCAGAGCCTTTGCGT

TAGATGAGTGCCAAGGGTTAATGTTCTGGACTAATTGGAATGAGTTGGCTCCCAGCATCA

TGCGCTCATCTCTAGCAGGCTCTAATGTCCTTGTGATCGTGGGTAACAATATCCGCACCC

CAAATGGCCTCGCTATAGACCATCGCTCCGAGAAGCTTTATTTCTCTGATGCCACGTTGG

ATAAGATTGAACGCTGCGAGTATGACGGCACCAATCGCTTTGTGGTTTTGAAGAACGAGC

CGGTCCACCCGTTTGGCTTGGCTGTCTACGGCGAGTACATCTTCTGGACGGACTGGGTGC

GCAGAGCCGTGCTGAGAGCGGATAAATTCGGCAGAGACATGAAAGTACTGCGTGCTGACA

TCCCCCAGCAGCCCATGGGCATCATAGCCGTTGCCAAAGACACCAACAGCTGTGAGTTCT

CTCCATGTCACACCAATAACGGCGGCTGTCAGGATCTGTGTCTTCTCACGTCCGAGGGCC

GGGTGAACTGCAGCTGCCGCGGAGATCGCAAACTGGTGGAGGGCAACGTGTGTGTTGCCG

AGAACACTACATGCCACAGCGTGGACGAGTTCGAATGCGGAAATGGTGACTGCATCAACT

ACAGCCTGACATGCGACGGACGCGCTCACTGCAAAGACAAATCAGACGAGAAACCGTCCT

ACTGCTCTAATCGAGGCTGTAAGAAGGGATACAGACGCTGCCTTAACGGACGCTGCATCA

AACACAGCTCCTGGTGCGACGGCACAGACGACTGCGGAGACCGATCAGACGAACGACCCT

GCAACATGACCTTGTGTAGTGCATCAGAGTTCCAATGTAAGGACGGTTCCTGTATCACCA

ACACGTCCCGCTGTAATCAGGTGGTAGACTGTGAGGATGCCAGTGATGAAATGAACTGCA

GTCCAACAGATTGCCACAGCTTCTACCTGTTAGGAGTTAAAGGTGTGATCTTCCAGAAAT

GTGAATTTACTACGCTGTGCTACACCCCTATCTGGCAGTGCGACGGCTCGAACGACTGTG

GAGACTTTTCGGATGAGAGGAACTGCCCAGAGAAGAGGCAGCTGAAATGTCCTGTGAACT

TTTTCGCCTGTCCCAGCGGTCGCTGTATTCCCATGAGCTGGACTTGCGACAAGGAGAACG

ACTGTGAAAACGGAACAGACGAGACTCACTGTGATAAGTTCTGCTCACCAACACAGTTTG

AGTGCGGGAATCACCACTGTATCTCCAGCTCCTGGGTGTGTGACGGCACCGATGACTGTG

GAGACGGCACCGATGAGGGCAGCCGATGCAGTTCAAAGACATGCAGTCCCGAAGCCTTTC

AATGCCCTGGCTCTCACTGCGTGCCCCAGCGCTGGAAGTGTGATGGGGACAACGACTGTC

CAGATGGTGCTGATGAGGGTGTGCAGGCTGGCTGCACAAACAATAAGACCTGCGACGACA

CGGAGTTCCAGTGCCAGAATAAGCAGTGCATCCCCAAGCATTTCGTCTGCGATCACGATT

TGGACTGCCGTGACGGCTCTGACGAGTCTCCCGAATGCGAGTACCCAACGTGTGGTCCCA

ATGATTTCCGCTGTGAAAACGGGCAGTGCCTTAAGCAGAAGAGCTGGGAGTGTGACGGCG

AATTTGACTGTCGTGACCACTCGGACGAAGCGCCCAAGAACCTCCACTGTACCGAGCCAG

AGAAGCGCTGTAACGACTCGGCATACCTGTGCAACAACGGGAAGTGTGTGAGCGAGGACT

CGCTGTGCGACTGGAAGGACGACTGCGGCGACGGCTCCGACGAGCACAACTGCTTCGTCA

ACGAGTGTCTTAACAACAAGCTCAGCGGCTGCACACAGCACTGCGAGGACCTAAAGATTG

GATACAAGTGCCGCTGTGATCCCGGCTTCCGTCTGAAAAACGACGGCAAGACATGCGTGG

ATGTGGATGAGTGCACCACCACTTACCCTTGTTCACAGCGATGTTTCAACAGCTACGGCA

GCTTCCACTGCTTCTGCATAGAGGGTTACGTGGCCTATGCGAACGACTGGACAAGCTGCA

AGTCCTCTTCTGAAGAGGAGGCTTATCTAATCTTTGCCAACCGTTACTACCTGAGGAAGC

TCAATCTTGATGGCAGCAATTACACCCTCATCAAAACGGATTTGAACAACGCTGTGGCTC

TGGATTATCACTACGCAGAGCAGATGATCTACTGGACAGACGTGACCACGCAGGGCAGCA

TGATCCGCCGCATGCTTATGAACGGCAGTAACGTCGAGGTGCTGCACCGGACGTCTCTGA

GCAATCCGGACGGTCTGGCGGTGGACTGGGTCGGGGGCAACCTGTACTGGTGCGATAAGG

GTCGAGACACCATCGAGGTGTCCAAGTTGAACGGGGCTTACAGGAGCATGCTGGTCAACA

GCGGTCTGAGGGAGCCTCGGGCGGTGGCTGTGGACGTCCGCAATGGTTATCTGTACTGGT

CAGACTGGGGAGATGTGCCTCACATCGGCCGTATTGGAATGGATGGAACAGACCGCAGCA

TCATCATCAAGGATAAGATCACATGGCCTAACGGCCTCACGCTGGACTTCATCAACGACC

GCATCTACTGGGCCGACGCTCGCGAGGACTACATCGCCTTCGCCAGTCTCGATGGCACCA

ACCGACACATCGTTCTGAATCAGGACATCCCACACATCTTCGCCATGTCTCTCTTCGAGG

ATTACATCTACTGGACCGACTGGGAGACCAAGTCTATCAATCGAGCTCACAAAACACTGG

GAACCAACAAGACCATGCTGATCAGCACGCTGCACAGACCTATGGATATTCACATCTACC

ATCCATACAGACAGCCTGCAGTTGAAAATCATCCCTGTCAGGTAGATAACGGAGGCTGCA

GTAACCTGTGCTTGTTGTCCCCTGGAGGAGGTTACAAGTGCGCCTGCCCCACCAACTTCT

ACCTGGCTGCAGATGGGAAGCAGTGCCTGTCTAACTGTACAGCGAGTCAGTTTGTGTGTA

AGAATGATAAGTGCATCCCCTTCTGGTGGAAGTGCGACACTGAAGATGACTGTGGAGATC

GCTCCGATGAACCTGCTGACTGCCCGGAATTTAAATGCCGTCCGGGGCAGTTCCAGTGCG

GCACAGGCATCTGCACAAACCCCGCCTACATCTGTGATGGCGACAATGACTGCCAGGACA

ACTCGGATGAAGCTAACTGCGACATCCACGTGTGCCTGCCGAGCCAGTTCAA

>Unigene000014

ACTTCTAGATCGGAAGAGCTCTCTGAGGAGCTGAGGGACAGTATGGAGGCTGCACATCAA

GCCGAGCTTTCGCAAGTCCAGACGCAGCACACTCTGGAGCTGGAGGCTCTCCGTCTGAGC

CTGACCAACATGCACACGGCGCAGCTGGAGCTCACGCAATCCAACCTGCAGAAGGAGAAG

GAGGCGGCGCTGAGCGAGCTGCAGGCCAGTCTGAGGGAGAAGTGGGCTCAGGAGAGCGCC

ATGCTGCAGACACGACAGCAGTTTGAGCTGGAGAAGTTCCGAGAACAGGGCAGGGAGCAG

GAGAGACAAACCGAGCTCCAGCACCAACTAGCCGTCGATGATCTAAACTGTGTGTGGGAA

AGGCGTCTTTCAGAAGAACGAGCCGCGCTGGAGCAACAGCTTGCTGGTGGAATAGAGGAT

GCAAAAATTCGGTGGATACAAGACTTTAAAAAAGAACAGGCGGAGCTTCAGGAAGCCCTG

AACAAAGCCGAGGACCGTTTAGCCGAGACGAAACGCAAACTGGAGGAGCTTCAGAGATCC

AAAGAGGAGGAAATCAAACATCTGGAGTCGGAATTGAGTCAGGCGTGGGCCGACCGAGAC

TCTGCTGCTCGTGCTGTTGAAGAACTGGTCGCTAGTCACAGGGTGGTGCTACAGGAGCAG

CAGGCTCACACACAAGAGCTGGAGGAAAAGGAGAAATGGCTGAAGCAAGAGGTGGATCGT

CTGCTTGAACAGAAATCCAGCTCCGAGCAGGAGATCACAAACCTCTGGTCTCAGCTGGAG

AGCATGAGGACCAGCAGGCAGGAACTAGGAGAGTTGAAGGAGCAGTTGCTAGCACGCAAG

TCACATGTTAATGACATCGAGCAGTTGAAGCAGGACTTCTCGCAGCAGCGGCGAGAGCTC

CAGGAGCAAAACGAGACCGAGCTGGAGAACCTGCGTGTGTACTTCGAACAGCGGCTCCGG

GCCAGCGAAGAGAACCACCGGGAGGAAATCGCTCTGCTGCAGCTACGCCTCGTAGAGGGA

GCTTTGGAGGACTCGGTTCTCAAGACCGGAGAGGAAAGCTTCCTCTCTGATGGAAAGTCT

GAGGAGGAGAAGGGAGACTCGTTGGCAGAGATTACAGAACAACTGGATAAACACAAGGTG

GAGTTGGATGCACTGCGGCTGCAGCTGGAGGAGAAACACAAGCAGGACCTGGAGCAGCTG

CGCTGTAACATGGCTCTATCCTATAGAGAGGAGTTACTGCAAGCTCGTACCGAGCTCACC

GACCGCTACTACAGCGACATGGAGCAGCTCAAAACTAAACACGCCTTGGAGATCGAACAG

CTGCGGGCCAAGCTCTCCGACAGCCACCTCAAAGAAATAACAAGGCTGCGGCTGCAGTCA

GTAGCAGATGTAGCGCGTCAGGTGGAGACTGAGCTGGAAGAGAAGAGCCGGCTCCAAACC

CAGGAGCACCAAGCCCGACTCGCTCAGCTCGCCTCAGACACAGAGAGCATACTGAGCCTG

GAGAAGAAGCTCGCCGCGCTCACCCTGCAGCACAGCCGAGAGCTGGAGAGAGCAACTGAG

CAGCATGCGGAGGAGCTCCGACAGCTGGAGGAGAGGCTGAAGAAAAACTTTTCAGAAGAA

CTGCACACACGTCTCGCAGAGGCTCAACAGGAAGAAAGGACCCATGTGACCGAAGAGCTG

CTCAACAGGAGCAGTGAGGAACGGCTCCAGCTCCGGGAACAGCTTCAAGCACAGGCTGAG

GAGCGCGTGGCTTCCCTAAGAGAGGAGCTCCAACAAGCAGCCAGTAAGGACAGACAAGAG

TTGGAGCAGAAGCTTGTTCAGGCTGAGGAGCTCCTGGCTGAGGAAAAGCAGAGGCTACAA

GCTTTACAGGTGAGTCTGGAGAGGGAGGAGAGCCCTCAGCTGCTGGCCCTGAAGCAGAGG

CTGGAATCTCAATACGCTGGCGAGTTGCACACAGCAAAGAGCACCATGGCAGCTGAGGTA

AAGGAGCTAAACACTTTGCTGCAGGAGCAGACGGAGAGCAGACTGAAGGAGGCCACGTGC

AGACATCAAGAGGAACAGCGGCAGCTAGCAGAGAAACTCGTGCAACAGAAAGAGGAGGCT

CTTCAGAAGCTGAGGCAACAACATGCTCAGGAGCTGCAGGTCCAGGAAACTCTCCTGGAA

CAGAACGTTTCAGAACTCCAACACCTCCAGAAGGAGTACCAGGAACTCAAATCCCGACAC

CAGGAGGAGCTCGACCTGCTCTCTTCCAATCACAAGACAGCGCTGGACTCCCTCGCTGAG

AAACACAAGGCTGAGCTGGATAAGCTGCAGGTGGTATTTCAGGAGACCAACTTGGCACAG

CTCGAAGCTCAGGAGGCGGAGCTTGAAGAGCGTCACAAGCAGGAAATGGAAGAACTGGAG

ACCCGGATGTTGTGCAACATGGACACTCTGGAGAGCACATACCTGAAGGAGATCCAGGCT

GTGCGCGAGGAGAAGGAGGCGGCGCTCCGGGATCTCGGGGCCTCCCTGGAGCAACAGCAG

GCTGAAGAGATTGAAAGACTGAAGCAGGAGGAGCTGATTGTCAGAGAAGAGCTGAGAAAA

AAACTGGCACAGGTGCACATGGACAAGTTTAGTGTCATGGCGACTGAACTCAGCCACGCC

CATCAGGCAGAGCTGTCTGCGGCGCTCGCTTCTCAGAAAGCGGCTCTGGAGTCAGAGCAC

CACTCCGCGTTGGAGACCCTGCGACAGCACGTGCTCGATCTGGAGGCGCAACACGGCGCC

GCCCTCCACGAGGTCACCGACATGACTACCAAGGAGAATCAGCAGCTGAAGAAACAGCTG

GATGCACTGAATGCTCAGCACCAGCAACAACTCCAGGAACTGAGGGCAACATCCTCCAGG

GAAATCGAAGCTCTACGCAGGGAGCTGGAAGAAGAAGCATCAAGACAGCGGCTCCATTTC

CTGGAAGAGGCGGAGCTTCTAAAGTGCCAGTCAGAGGAACAGCTACAGAAAAAGCTAATG

GAGTTAAAGGAGGAAAGTGATCGAGAAAAGGTCTCTGCTCTAGAGGAACTGGAGAGAAGT

CTGTGTGAGCAGCACCAGCAGAACGAGCTCTCCTACACTGAAAAAATGAACCATCTGACC

ACACAGCTGCAACAGCTGGACACCGTGGTCTCTCAGCTGCGTGCAGAGGTGAGCGGTTTG

CAAGGGGAGCTGGAGGGTAAGCGCTCGGAGATGGACACGCTGGAGACTCTGCTGCAGCGA

CGCGAGCGCGAGAGTCAGGAAGGAGCCAACCTACTCGCCATGCTGCGTGATGACCTCAAC

ACCGCCACCCAGCAGAGACAAGATCTTCAACTGACTCATGACCGACTCCAGCGGCTTGTC

CTGGAGATGCTTCGGATTACAATTGCGACAGAGGAGGAGATCAGTCGCAGGTTGGGGATA

TCCATGGAAGGTGATGACATCACTCTGAACAGGAAGCAAAGTGTCAACCGTGACCCGCAG

AAAACGGACGCTGAGGTGGGCGACGGTGCTGCTGCAGCTGTAGAAGCCAGTGCTAACTGC

AGCATGTTCTCGTCACTGATGGATGAAGGTCTGGAGCTGTCTCAGCGACTGTGTGAAAGT

TTGTTTACAGGACCTGATGGAGAGCTGGACCCTGAAAGAGAGGAGCTGGTACTGGGGGCC

TGCACTCATCTACGTGTCGCAGTGGACAAATTATTGGAGGTAGTTTCCGACTCCACAGTG

CAGCTGGAGCAGCTGCAAGCTCTGCAGGCCCTGCTGGATGAAAAGTTCAGTGCTGGTGGA

GAAGCGAAGAACGCGCTCTTCCTGCAGCACACTCAGCTTCTGGAGCAACTAGACCAGGAG

GCCAGCGTGAAGAGTCAGCTGCAGCTAGAACTGCACAAGGCTGATGGTCTGATCGAGGGT

TATGTGGTGGAGAAAGCTGCTTTGGAGGATGCACTGCAGCAGAAGGAGTCCCGTGAGCAG

AAGCTGGTCAAAGAGCTGGAGAGCACTCGGGCTCAGCTGCAGGAACTTGGTGAAGAACAC

GCACTCCTCCAGCGTCAACGGGATGCTCTCTCTGCAAGCCTCGGAGAACCTGAGAAAGCA

CTGCTGGCCGAGGCGGAGCGTTTGGGGCAGGAGCGTGTCGACGTGCAGCGGCAGGCTGAG

AAGGACCGCGTCGGACTTGCAGGTCGGCTGCGTTTGCTGGAACAGACGCTGGAGGAACAC

GAGAGCCGAACGCAGCAGCAGGAGGAGCAGCACCGCACCCAAACCGAGGACCTGCAGCAG

CACATCAGCGCGCTGGAGAAACAGCTCAAACACCACCGCCAGTTTATAGACGAGCAAGCT

GTGGAGCGGGAGCACGAGCGAGATGAGTTCCAGCAGGAGATTAAAAACCTGGAAGCACAG

CTGAAAACCACCTCTAAAGGACATGCTGGAGAGGACACCAAGGGTCAAAGGGTGGAAAGC

CTACAAGCTTTAATCAAAGACAAAACCGAGGACTACAGCGTTCTGCTCTCGCTGAAGGAA

CAGTGCCAGCGTGACCTGGAGGAACGCAACGAAGAGATCGATAAGATGGCATCTCGCATC

CGAGAGCTGGAGCAAGCTCTCCTGAGCAGCGTGGAGGAGCGACGGGCCGCCGCGCAGCTG

GAACAGGAGCTGCAGAAAGCGCGCAAAAACTGCCAGGAACTTTCTCAGGACAAGGAAGCC

CTGCAGCAGCAGCTGTACTCTAATAAACTCCAGATCTCAGCTCTGCAGTCCAAACTGGAT

GAAACGAGGCACCGCTACCCCGACCCAGGTGCTGACCCCAACCTCAGAGAGCGGCTCGAG

ACGCTACAACAGGACCTGCAGAACAAAGAGGAGCAGGTGGAGGTGTTGCTGGAGCGAGTG

GAGGTGTTGGAGAAAGCTCTGGCAGTGAAGGAGGAGGACGTCAGGCAGCTCTCTCTGCAG

CTGGAGCTCAAAACTACCGAAAGCCGAGCGAGAGAAGAGGAGCTACGGCTGAGCGTCGCC

GAGCTGCAGGATACAGCGAGTCTGTTACGCAGACAGCGGCAGGAAGAAGCCGATGCCTCG

GCGCTACAGCTTCCTTCTGCTCTGCTGGAGGAGAAGAACCTGGAGATCGACCACCTGAAC

CAGCAGCTACTCCATCTGCAGCAGGAGCTGGACGCATCCAAAGAGAACAAGACAGAGGAG

AAGCAAGCAGACGTTGAAGATCTACGTGCTCAGGTGGAGCGACTCCATGGCGATCAGGAA

CGTCTGCATCAAGCTAAAGAGGAGGAGGTGGAACAGCTGCATGAGATCATCCACAAGCTG

CAGGAGGAGCTGAGCCAGCTCAATCCCAATCGGCACGAGGTCAGCGACCCGAACACGGAC

AGCCCTGAACCTCCTGATTTCCCCTGGTCGCCCCGGCCTCAGCACGGTGCCGAGGAGAGC

CTGTGTCACGAGCTGAGCAGCCAGACCCTTCAGAGCTGTCGCACCAAGATCTGTGAGCTC

CAGGCGGACTTGGAGCGCAGTGTTGAGGAGAAGGTGGCTCTCGAGAGACTGCTCCTGATG

CAGGAAGAACAGTACGGACAGCAGCTGGAGGCACTGGGTTGCAGTCTGGGAGAGGGAAAA

AGAAAGCTGGCGCTCCTGGAGCAAGAGGCCGGAGAGCTGAAGCTCCAGTTAGACCAGAAG

GAGACGAAGGTGGAGGAGCTGCAAGCTCGCATTCAAACGCTGGAGGATGGAGAGAGGAGT

GTAAATGATTTGGAACTCCGGCTGATTAAAGCAGAGGAAACGATGAAGGAGACTCAGCAG

GAGATGACCAGGTTGAACGACAAGGCACGAAAGCACACAGAAGAGCGAGAGAGGACAGAG

AGTCTGATTGCTGAGCTGCAGCAGAGGTATGAAGACAGCGAGAGAACTGCGACGCAGGCG

AGGCTGCAGGTTGCCGAGTTGAAGGAGACGATTCAGCAGCGGAAATCCGACATCGCCATC

TTGGAGACGGGCAAGACGGAGCTGTACACGGAGAAACAGGCTCTTCAAAAACGCGAGAGA

AAACTTCAAGAGGAAATCGAGAAGCTGAAACAGGAAGTGTCTGCTAAGAGCCTTCAGATA

CAGGAAGTGAGTTTGCAGCTGGAGGAGAGAGCAGCTGACCCGGATCAGAGTCAGGAAGAA

GTTCTGACGTGTGCTGAGGAGACCCTGGCTAAAGCTGCAGCAGCTTTGCGAGAAAAGGAG

GAGCAGTTGGCTCATCTGAGGTCGCAGCACGATGCATTGCGAGCCGAACTCGCTGCGGTG

AAGGAAGGCCTGAGCAGCAGTACAGAGAGAGCTGAGAAACTACAGGAGGAGGGGCAGACT

AAGGACCGTGCTCTGGCTGATCTAGAGGAACATAACCAGCGTCTTCGGGCCGAGCTGCGT

GGCTTGCAGGACGATCTGGAGGTGCAGGAGGAGGAACTGGCCTACCAGCAAAGGGAGTTG

GATGAGCTGCGGCAGCGCTGCCGCCTTCAGGAGCACACCGAGCTCGACTGCACCAAACCC

GAACGCCGCTTCCTGGATGGCCTGTCGCATAACTCCACCCTTTCTTCTCCCGAGGTGCTA

CGGCGGCTCGACTGCAGCGAAGAGCGACCGTCTCATGTACACGCGTCTCACCTGTCAGAA

CTGAGCGGCCTGCGCAATACCAGCCTCGAGCTGATGAGCAAACACTCGCTTATGGAGAGA

AACGAGTTAAAGCGACCGCTCGTCCTGCCGCCAGAGACCGACCCGCCCAGCACACACTCG

CCATCTGCTTCAACACATTCACTCTCCATCTCCGAGAACCTCTCTGTGCTCAACTCACTG

GACGATGACAAGGTTCAAGTGCTGGAGAATCTGGATGATGTGACTCCGTCTCGCTCTCCT

CTCTCTTCTACCTCCCCGGTGTCTGCACCAGAGTGGGCCAGCGATGGATACGGTAGCAAC

GTGAGCTCCGAGCTGGAGGCTAAACTAAAGAACGAGCTGGAGCATATGGAGCGGCTTGAC

GCTCACTTTGTTGAGTACCTACGCAGCAGGGACATGGCACCCGCTCCATGCTCGGACAGC

GCCGCAGGCAGCCTGCATCACACTTACGAGCTGCTCTCACCTGAGTTACAGGCCATGCTG

AACCGCGTGTACCGCGAAAGCTACAGAGTGCTGTCTCTGTCTCATCGCCCTCCCCCTGCC

GGACAGCCTCATTCAGACGCCGAGGAGGCTCCACCCGCATCCTGGCAGAGAGAGAGGCGA

GCCTTGCAGGAAACGGTGCTGTCACTCAGGGAGCTCCTGTGCAGGATGGCTGAGCGTGAA

CCCAAGGTGGAGGGTGAAGTAGACTGGCGCACAGAGCTGTTGCAGGCCGTGCGGAGTGTG

TTTGACAGTGAGAGGAAGTGGCTTCATGCACAGCTCCAGGACATCATCACAACCAACACT

GGCATAGACTACACGTCTCTCCTGAAGGATTTAGAGACGCTTCTGCAAAAACAGGAGGAT

CAGCAGAGGAGGTCTTTGGAGCAGTTGCTAAGTGCAGACCGAAACAGTCTCCTGGCTGAA

ATCCGCAGTCTGCATAAACAACTGCACACCTGCACGCTGCAGAACCAGGAGCAGCTGCAG

CGCTCGCTCGGTGCTGTTCGTGAGGAAGGTGCGCAGACGCAACAGCAGCTGCACAGACAG

GTTGGTGAGCTGGAGAGGCAGCTACAGCAGGAGCAGTCGTCGTCACATGACCTGCAGCGG

TCGCTGGGACAGGAACAGTCCAGATCAACAAATCTACATCAGCAGCTGGAGATCCAGCAG

AAGCTGGCGGTAGAGATGAAGTTGGAGCTGGAGGAAGGAACCCGGCAGCTTCGTGCGTCT

GAGAAAAGCAGAGAGGAGCTGCAGGCTCAGATTCACAAACTCCGGCTCAAGCTGGAGAGT

GAGGAGCAGGAACATCAAGCTTGTGTGGAGGCTGCAAAACGAGATGAGGTCAGGGTTAAG

CAGCTTCAGGAGGAACTTCATCACGAGAGGCTCAGCAGCAAACGCGCGGCGGAGGAGCAT

GCGCACATGCAAGAGTCTCTCCGGGCCTCCCTGTGCGAGCATGCGACTCGTCTGAATGAG

CTGAACGCTGCACTGGAGCAGGAGCGTGTAGCAGTCAGTAACCTGCGTGCCGAGCTGCAG

ATCGAACAATCCCGCTGTGAAGCCCTGCTGGCGCAGGAACGTGAGCGCACGGAGCTAACC

CTCTCGCGCCTGGACGAGGAGCGCTCGCGCTGTGCCGAACTTTCTCGCTCACTTTCGCAC

CAGGCTCAGGAACATGCTCGCCGCCTGGAGGAGGAGGTCCGCTCCCAACAGGCTGCGAGC

GCCGACGATAGGAAGTTCATCCAGGAGCTACGGGCACAGCTGGAGCAAGAGCGGCGCCAG

GCCGAGGACCTCGCCGCCACAGTGGACAATCTGCAGTCCCAGGTACTGCAAAGCAAACGC

AGGCAGGAGGAAGCAGCGCAGCAGGAAGCGCAAAGAGGACAGGAGGAGGTGAAGAGGCTG

CGCTCTGCTCTGGATGGTCTGCAGGCACAGCGGGTAGAGGTGAACCGCACCCTGGAGACG

GAGAGACACAGAGCTGCTCAGCTTCAGACAGAACTGGATGCCGTGAAGGAGAAGATGAGG

GAGGTGAAGGAAAAGGAGAGAGCGAAGGAGGAGCAAAGGGAAAAGCAAAGGTGGTGCGAG

AAACAGGAGCAGGAGGACAGGGAGCGGCGGCAGGAGCAAACCAAAGAAAAACTGTTGGAG

CTGGAGTCGTTGCGCGAGCGAGATCAGCAGCGGTTGAGACAGCTGCAGCAGACTCTGGCT

GACCTGGAGCAGCAGGAGAAACAGTTGACTTCAGAACGTCTGCAAAGGGACTGCGTAGCT

CACACACAGGACTCTTCATCCTCCTCGTCTCTGTTAGAGCGAGTACTAAAGGAGAACTCT

GAACTGGCGGAACGTCTGGCAGCTTTAAGCGAGGAGAAGATCTCGCTTAAACACACCGTC

TCCTGTCTGGAGAGAGATCTGCACAATCTCAGGTGCACTCAACAGCAGTCCTGGCACAAG

GAGAGGATGTTCTCGGCGCCGATCGCTTCACAGAAAGCTGAGGAAGAATTTACAGATTCC

AGGAACCGAAACCGACCAATCACAGATCTTTCCAATAATAAGGTGCAGAGACTGTATGAG

AAGTACCTGAGGGCGGAGAGCTACCGGAAGTCTCTGGTGTATCAGAAGCGCTACTTGCTG

CTTCTGCTCGGAGGGTTTCAGGACTGTGAGCAGGCCACGCTGGCGCTGATAGCACGGATG

GGGGCACGGCCATCGCTGATCACGCAAACCTCCCCGCCAATCAGCCGCTTCAGGAGCGCC

GTGAGGGCAGTTATCGCTGTCTCCAGGCTTAAGTTCCTCACCAGGAAGTGGCAAAGAGCG

ACGAGAAAAAGTTCAGGGGGAGGTGTGATTGTTAACAGTGCAGCTATAAAGACTTCGATA

TTAAAGCCGCAGCACTCCGGCGTCGCCGCACATCAGTCTCCACCCACCCGGGATGGCATT

CTCAGCCAGCGGACCGCGCTCTCGCCTCTGGTGCCGCCACTCAAATCTCCTTTCAGACTG

CACAACGGAGGTTACGCCAACAGCACGGCGGCCCCGTCTGAGCGCACGCTCACCCCCTCC

CAGGATCAGGAGCGCTCCCTCGCTGACTACATTCATCACCTGGAGAGCGTCCAGCAGCGC

CTAGGAGCTGTCCGTCCAGGTTCTCCTGCGACCTTCTCTTTTCTACGGAAAAATGACCGA

TGAAAAGACGACGACTTCTCTGCCCCGAGCCGGTTCGGTGCTCCTTCTTCACCAGGGGGT

TCAAGACACTTGATCATTTTGTAATAAATTATTGTAAATCTACTTTTACAATCCGTCAGA

TTTCCCGCCTGATTGTGTTCCAGGTCAGCGGTGATGTTTCACTTCAGTCCGTCTGTAAAT

TTACTCACAGTTGTTCGGTTTTGTGTAGCTTTTGTATTTTTAACCAACGTTTGCACTCGA

ATCCGTCGGCTCAGC

>Unigene000015

CGAACTAGATCGGAAGAGCGTCGATACTCTCACAGAGTGTGTGTCTCAGAGAGCTAAGCA

GCTCCAGACTGCTGCTGCTCAGTCTGTCAGTGTTCAGGAGGGACTCAAGGCACTGGTGTC

CTGGTTGGATGGTCTAGCATTAGACCCTGAGCCAGTCCAACCGACAACCCAAGCAGTACA

GGAGGCGCTGGCGCAAAATCAGAAACTGCGTCAGGAATTATTGTCACGACAGGGAAGTGT

AGAGGCCACTCGCGACTCCGTGTCCAAGCTGCTGAAATCGGCTGATGCATTAACAGTCTC

TGGGCTGCAGGGGGCACTGCAAGACCTGAATCAGCGCTACACTGCGGCCCAGACCAAGCA

AACTGAAAATGAAGCAGAACTGCGGGCAGTTTTGCCTAAACTAGAGAGTTTCGAGCGCCT

AAACTCAGACCTTCACAACTTTGTTCAAAGCCGCGAGAGAGTTCTTAGCCTTGGGGGGCT

GCCGGATCGCAGTGTAGAGGATTATAAGCACAGCATACAGGAGGTACAATCAGATCTTGG

TCAAGAAACAAGTCATCTGAAAACATTTGTGGAACTTAGTACAGACCTGAGCAAGTCAAA

GCTGTTTGTAAATGCACACAGCCTCGTAGACTCGTCTAAAGAGGTGTCTGATGAATTTGC

ACGGCTAGAGGCCAGCCTCAATGAAAGGCTAAATGCCATCCAGAGCTGTGACCAGCAGCT

GGTGCAGTTCCGCTCGCATTCCGACTCCATCCTCAGGTGGCTGCAAATGGCACAAGAGCA

GCTGCCTGCTAAAGAACCCCAGCAGAGCACAGACAGCCTGCAGAGAAGAGTACAGCAGCT

CAAGGATCTGCTGAGTGAGTGGGAGTCTCGAGGCAATCAGATTCAGGAGCTCAACAAGAA

CAGCTCCGAGCTGGAGAGTCGGATCATCAGCATTACTGCTCCCCAAAGCAAGACCAGTGC

CCCCATGCTCAATGGTGCCGGTGGCTCCAGCAGTCTTAATGGTATTCACACCTGCAGAGA

CCTGACTGAGATCCAGGTGGTGGTGGCGGACGTGAACAGTAGGTATGAGCAGCTGGGTGG

GGATCTTCGTAATAGGCAGAGCAAACAGCAGGCTATGCTGGAGCTGTGCCAGAAGGCTAG

GCAGGACTCTGAGGCTCTGCACCAGTGGCTAGCTCTTCGGGAACAGAGGTTGGCACAGGG

GCAGTCTGCATCACCTTCACGACCTGACGTGGCGCGTGCCCAGGCCCAAGAGAACAAGGC

CTTGCTGTCTGAACTCACAGAACACTCTGGGAAAGTAGAAGACCTGAAAAACACCCTAAA

GCAGCTGATACAAGAAAATCCTGACTCCCCTGAAGCAGAGAGCTGGAAACAACAATTAAA

AGACCTTGATATTCGATGGGAGAAAGCCAGTCAGGTCGCGGCACAAAGACAGGCTGAACT

GGAGACGTGCGCTGACAGACTGGGCAACTTTGCATCAGCAGCCAATCAGCTTGGTCCCTG

GCTACGAGAGAAAGAGCTGATGATGAGTGTTCTTGGGCCCCTGAGCATTGACCCTAACAT

GCTTAACACTCAGAAGCAACAAGTGCAGTTTATGCTGCGTGAATTTGAGACACGGCATGC

CCAGTTTGATCAGCTGACCCAGGCAGCTGAAGGTATCCTCTCACCTGTTGAAGAGCAGGG

CTCTGGTGACAATCAAGATGTAGCTGAGGTGAAGAAGGAGCTAGCCTCCATCACCCAGCA

GTGGGAGGACCTAACTTCTCGTCTGTCTGGACGTTTTGATCAAATTGAGCGTGCCCAGGG

CACTAGTGAGAGTTTTCAGTCTCTGCTGAAAGAGCTTTCACAAAGTTTGTCCCAATTGGC

GGAAAAGCTGGATGCCCAAGCCTCACTTAGTGCCCAGCCGGAGGCCCTGCGCCATAGACT

AAAGGAGACAGGAGAGATCCGTGCAGAGCTCGAGCAGAGGAAAGGCCAGCTGGCCCAGGC

TGAGCAGTTGTGTGAGGATCTCAGTGCTATTGTGGTGGAGCCTTACCTTCGAGAAGAGCT

GCATAAGAGGCTAGAGAGTGTCAGTGGCCCACTTAACAACCTGGAGGAAAGAGCAGCGGA

TGGCCTGTCCCAGCTGCAGGCTGCTCTCTCAAGCACACAGCAGTTTCAGCAGATGTTCGA

GGAGCTCCGTAGCTGGTTAGATGGGCAGGCTGCTGCACCCAGCATGCCTGATCCTCTCCC

CTGCCAACCGGAAGCTCTGCAAGTCCTTGTTGCCCAACAAGAAGAACTTCAACGTTGTGT

AGCACAACAGCGAGGATCATACGACCTCCTTCAAGCTGAGGGAGCATCTATTCTGGCATC

ACTTCCTGCTGGAGATGAGCGCTCAGCTCTGCAAGCTCAACTCACCAAGCTGCGTCAGGA

CTGGGAGGAAGTAAACCAACGCCTTACAGAAAAGCAAGCACATATCAAAGAGACCCTGGG

CCGTGCTGTACTATTCAGACAGCATCGTGATGAACTAGATCCCTGGCTAAAGGAGTGTGA

GCAAAAAGAGGCAGAGATCCAGCCTTCTTTGGAGCCTGCTGCCCTTGAAGAAGCCCTGCA

GAAAGCAAAGCAGTTAGGCCTGGACTTAGATCGCCGTCGCCCCCTGTTGGAGATTCTGAA

TTCCGCAGCTGACCAGCTCCTGGAGCAGAGCCGGACTGGAGAGGAGGAGGTGAGGGATGA

GAAAGCCAAGCTGAACCGCAGGCTGGATGGCCTCTCTGAGAGATTACATGGACGCATGGC

TCAGCTGGAGGAGCTGAACAGCAGACTGAAAGAGTTTGAAGATGGTAGACTAGCAGTAGA

GAGAAGACTTGAGGCAGCAAGGCACCAGTTAGAGGTGCAAGAGGCGTTGGGCCCTCAGGC

ATGCAGTAACAAGAGTCTGGAGCGCCTGAGAAGCCAGCAGGACACACTCGGCTCCTTACA

GCCACAAGTTGTATATCTGCACAATCTGGCCAAGGGGCTGGCACAGGACGCGCCACAGAT

GACAGGGGGCAGTGGAGATGGGGGACAGAAGCTGTTGCAACAGGCTCAGACTACAGAGAA

GGAGTTTGGAGAAGTAACTGAAAAGATCCAACAATGCTGCTCGTCTCTCGAGTCACGGCT

GCAGGGTGTAGGAGAAGTCCAATCACACGTCCGTGATATTTTCTCTCGGCTGGCGGACCT

GGACGACGAGCTGGACAGCCTGAGCCCTGTAGGACGCGATGTGGACTCCCTTGCCTCACA

GGCAGATGCAATAAGGGGATTCCTGGGGCGTCTGGCCTCTCTGCGGGCTGAACTTGAAGG

CCATGGCAGTGCCTGCACCACCATGCTGAAGCGAGAAGGTAGCTCCCCTGATCTCCTGGC

TATTAGAAGGGAGACGGAAGCTCTAAATCGGCAGGCAGCCAAGCTGGCCGAGCGGGGTCA

GGGTCGTCTAGCACTCATAGAAGAAGCCGAGGGGCGAGTAAAGGAGTTCTATGCCCGCCT

CATGGAGCTACAGAGGCTACTTGACCAGGCAGAGGAAGGGCTGAACGTACAAAGTGTTGT

GGGAACCGAGGTGGATGTCATCAAACGACAGCTCCAGGAATTTAAGGCTGTGGAGCGAGA

GCAGGTAGACAGCATCCAGCCTAAACTGCATCACGTGAACGCTGTTGGCCAGGGGCTAAT

CCAAAGTGCAGCTAAACATACCGACACGCAGGCCCTGGAGCATGACCTGGAAACCACCAA

CCTGCAGTGGAACTCCCTCAACAAAAGGGTGGCCGAGAGAATTGCTCAGCTCCAGGAAGC

CCTTTTGCACTGCGGGAAGTTTCAAGACGCTCTGGAACCTCTTTTAAGCTGGTTGAGTGA

CACAGAGGAGCTGATCGCTAATCAGAAGCCTCCATCAGCTGAATACCGTGTGGTTAAAGC

ACAAATCCAGGAACAGAAGCTGCTGCAGCGGTTGTTAGATGACCGGCTTGGGACTGTAGA

GATGATCCGGGCTGAAGGTGAGCGCATCGCAGCCACAGCAGAGACGCAAGACCGAGAAAA

GATCCAGAGACAGCTGCAATGTTTGGGAGAACGATGGACAGATCTGCTTGAAAAGGCCAA

TGCCAGGCAACGTCAGCTGGAGGAGCTGCAGGTACTTGCGCTGCAGTTCCATGAGTCAGT

GGATCCTTTGGGAGAGTGGCTGAGTGCTACAGAGAGGCGACTAAGCTCTGCTGAACCCAT

GGGAACTCAGGCTTCCAAAATTAGCCAGCAAATCAATCGGCACAAGGCCCTCCAAGATGA

GGTGTTGGCTCGGGAAAAAGAGGTGGACCATCTAGAGGCCCTTGGCCAATCTCTCTCCCC

TCTCAGCTGTGCGGCAGACCGTGATTGGCTTAGTGAGCGAGTGGGAGCAGTGCGCAGCGG

GCACACAGAGCTCCGGAATTGGTGCTTTCGGCGAGCAGCTATGTTGGAGCAGGCTCTGGC

CAATGCACAGCTGTTTGGGGAGGAGGAGGTGGAGGTGCTGAACTGGCTGGCTGAGGTGGC

CCAGCGCCTGTCTGACGTCTCAGTGCAGAGCTACCAGCCCGAGCTTTTGGCTGAGCAGCA

CAAGTACACTCTGGTAGGACACAGGAGGCAGTTTACAGATGCAGTAGATTTGGCCACTTT

TAAAAGAAGGCATTAGGGAAAAAATCTGGTCTTTTTACAAGTAACATTTTACAAGGTAAA

TTTTGGATTAATAACATTTTATTATCTCTCGTAGTCTCTGAACGAGGAGATTGTGAGCCG

GAAGAAGACAGTAGACCAGGCCATCAAGAATGGACAAGCATTGCTGAAACAGACCACTGG

TGAAGAAGTGCTGCTTATCCAGGAGAAGCTGGATGGAATAAAGAGCCGATATTCCGAGAT

GACAGCAGGTAGCAGCAAGGCCCTTCGTAACCTGGAGCAGGCACTGCAGCTCGCTACCCG

ATTTGCCAGCGCCCACGAGGACCTCAGCCAGTGGCTTGACTCTATGGAGGCGGAGCTTAA

TAACATGGAACCAGACACAACTCCAGCCTATCAGGAGAGACAAAAGGACCTGAAGTGTGT

GTCAGCTGAGAAGCGTCTCATTCTGGATACAGTTAATGAGGTGGGCAGTGCACTGCTGGA

CCTGGTGCCCTGGCGAGCACGTGAAGGTCTGGATCGTCTAGTGGCCGATGCTAACCAACG

CTACAGACAAGCCGATGAGACCATTACGCAAAGAGTGCAGCTAGTGCAGGCAGCCATCCA

GAGGTCACAGCAGTATGAAGAGGCCGTGGATGCGGAACTGGCCTGGGTGGGGGAGACAGA

GAGGAAGTTGACCTCCCTGGGGCCTCTAAGTCTGGAACCTGACGTGACAGTGGCACAGCT

CCAAGTCCAGAGAGCCTTCAACATTGACATTATCCGCCACAAGGACACGGTGGACCAGCT

CCTCCACACCCGAGAGGATATCCTGGAGAGCTGCAGTGACCAGCAAAGAGAAGCACTGAA

GGTAAAGACAGATTCATTAAGTATGCGGTATGAGGCAGTGAGTCAGAACCATGCTGAAAG

ATTTTCAGCACTGGAACAAGCACAGGTGTTGGTAGCCCGCTTCTGGGAGACTTACGAGGA

GCTTGACCCATGGCTGGGTGAGACAGAGACCCTGATATCCCAGCTGCCTCCTCCTGCCAT

TGACACTGAGGCTCTACGACAACAGCAGGACCAGATGAGGATGCTGAGAGAGTCTATAGC

TGAGCACAAACCCCACATTGACAAGCTGCTGAAGATTGGCCCACAGTTGGCTGAGTTAAG

TTCCCAGGAGGGGGCGACACTGAGACAACGCTACAGTGAGGCTGAAAGGCGTTACCTCGC

CATCAAAGAGGACGTTAAAGGTCGTGCTGCAGTACTGGATGAGGCCTTCTCTCAGTCTGC

ACAGTTCCATGATAAGATGGACCCCCTGTTAGAAACTCTGGAGGGAGCAGTGCAGCGTTT

GCGGCAGCCTCCTCCCGTGGCTGCTGAGGTAGAGAAAATCCGTGAACAGCTGGCGGAGCA

CCGAGCAGCCGGGCTGGAACTGGACAAATTGTTGCCGTCCTTCAGCACTCTTTGTGCCCG

TGGGGAGGAGCTCATCACCCGTGCTGCACATGATGACCCTGCGGCTCAGGCAGTGCGTTC

TCGGCTTCTAAGACTCCGTTCCCTGTGGGATGAGATCAGACAGAGAGCAGAAGAAAGAGA

GGGAAAACTGCAGGATGTACTGGACTTGGCTGGAAAGTTCTGGGCTGACATGGCAGCTTT

GCTTAGCACCCTGCGAGACTCTCAGGAAATTGTAAAAGAACTGGAAGACCCTGGATTGGA

TCCATCACTCATCAAACAGCAAATAGAGGCAGCAGAAGCTATCAAAGCAGAGACAGACGG

TCTGCGTGAAGAACTGGAGATCGTCCGCACACTGGGAGCTGATCTAATTTTTGCTTGTGG

TGAAACCGAGAAACCAGAGGTCAAGAAAACCATCGATGAGATGAATGCTGCGTGGGAAGG

CTTGAACCGCACATGGAGAGAGAGAATGGAGAAGCTGGAGGAGGCTATGACAGCCTCTGT

GCAGTATCAGGATGCACTGCAGGGTATGTTTGACTACCTAGACAACGCTGTCATTAAACT

GTGTGACATGCAAGCCGTAGGAACTGACCTCAGCACCGTCAAACAACAGATAGAAGAGCT

CAAGCAATTTAAGGTGGAAGTGTATCAGCAACAGATAGACATGGAGAAGCTCTGCCACCA

GGGGGAGCTACTGCTCAAGAAGGTTTCTGACCAAGCGGACAGAGACATGATCCAGGAACC

TCTCACTGAGCTCAGGCACCTGTGGGACAATCTGGGAGACAAGATCACTGTCAGACAGCA

CAAGCTGGAAGGAGCTCTTCTAGCCCTGGGTCAGTTTCAGCATGCCCTCTCTGAACTGCA

GTCCTGGCTAAGCCACACTCATGCTACCCTGGACACCCAGAGACCTGTCAACTCTGACCC

CAAAGCCATAGAGATCGAGCTGGCCAAACACCATGTATTGCGCAACGACGTCCTGTCCCA

TCGAGCCACAGTAGAGACTGTGAACAAGGCAGGCTCAGAGCTGCTGGAGTCCAGTCCCGG

AGACGAGGCCAGCCACCTGAGAGACCAGCTGGATGAGCTGAACCGCAGCTGGGACAGCTT

ACTTCTGAAAACAGACGAAAGACAGAAGCTACTAGAGACGGCACTGCAGCAGGCGGAGGG

TTTCCATGGTGAGCTGGAGGAGTTCCTGCAGTGGCTGAGACGCACAGAGAGTCAGCTATC

AGCAGCCAAACCCACAGGTGGCCTTCCAGAGACCGCCAGGGAGCAGTTGCAGCAGCACAT

GGAGCTGCAGGCTCAGTTGACCCAGCGCGGTGAACAGTACCACCGACTGCTAGATCAGGG

AGAGTCCATGCTGTTGGCTAGAGGGGCAGAGGAAAACAGCCCTGGAACCACTCAAACTCA

GCAGAACCTGGCCCTGCTCCAAAACAAGTGGGCCAGTTTGAATGCCAAGATGGATGACAG

GAGGGCTAAGCTGGAGGAAGCAGTATCACTGGCAACAGGATTCCAGTCGTCACTGCAGGA

CACCATCAACTGGCTGACGCAGGCAGAGCAGACACTAAACATGGCTCAGTCACCGAGCCT

CATCCTCGACACTGTCCTCTTCCAGATAGATGAGCACAAGGTGTTTGTAAATGAGGTGAA

CACACACAGGGAGCAGGTGCTGGCTCTAGAGAAGGCCGGGTCTCAGCTGCGCTTTGCCAG

CCTTAAACAGGACGTGGTGCTCATCAAGAACCTTCTGTTGAGCGTGCAAGCTCGCTGGGA

CAAGCTGGTGCAGAGGTCTCTGGACAGGGGACGGCACCTGGACGAGGCCCGCAAAAGAGC

CAAACAGTTCCATGAGGCCTGGAGGAAGCTGACAGACTGGCTGGAGGAGGCTGAGAAACG

GCTGGATGCTGAGCTGGAAATCTCCAATGAGCCAGATAAAATTAAAGTGCAGCTTACTAA

GCATAAGGAGTTTCAGAAAACTCTTGGTTCTAAGCAGCCAGTATATGACACCACAGTGCG

CTCTGGGAAGGCCATGAGAGACAAAGCTACACTGCCAGCCGACACACAGAAACTGGACAA

CCTCCTCGGGGAGGTCAGAGACAAATGGGACACTGTGTGTGGAAAGAGTGTTGAAAGGCA

GCACAAGTTGGAGGAGGCCCTGCTGTTCTCGGGTCAGTTTGCTGAGGCACTGCAGGCTCT

TGTGGACTGGTTGTACCGGGTAGAGCCACAGTTGGCTGAAGACCAGCCTGTCCATGGTGA

CCTGGACCTTGTGTCTAATCTCATGGATTCTCATAAGGCCTTTCAAAAAGAGCTGGGGAA

AAGGACCAGTAACATACAGGCACTGAAACGTTCTGCTCGGGAGCTAATGGAGACAGGCAG

GGATGACACCGCCTGGGTCAAAGTTCAGCTGCAAGAGCTCAGCAACCGCTGGGAGACGAT

CTGCGCACTGTCTGTCTCCAAACAGACCCGTCTGCAGCAGGCTCTCAAACAGGCTGAGGA

GTTCCGCACAGCAGTGCAGATGTTATTGGAGTGGCTCTCAGAAGCAGAGCAAACTCTACG

TTTTCGAGGCATCCTGCCAGAGGAGGTGGAGACCTTGCAGGTTTTATTACACACCCATCG

AAATTTTATGCAGACCGTAGAAGAGAAGCGAGTGGATGTGAACAAGGCAGCTGGCATGGG

GGAGGCCATACTGGCAGTGTGCCATCCTGACTGTATCACCACCATCAAACACTGGATCAC

CATCATCAGAGCCCGCTTTGAGGAGGTTTTAACCTGGGCGAAGCAGCATGAGCAGCGTCT

GGAGGCAGCACTTGCCGAACTGCTCAACAATGCAACACTGCTGGAAGACCTGTTATCATG

GCTACAGTGGGCGGAGACCACATTAGTGCAACGAGACACTGAGCCCCTCCCCCAGGATAT

CACTCAGCTTAAAACACTGATCACAGAGCATCAGGTTTTCATGGAGGAAATGACACGTAA

ACAACCTGACGTCGATAAAGTGACAAAAACCTACAAGAGAAAACCATCAGAAACGTCTAG

CAGTCTTGCTGAGAGACGGGGTGTCCGTAAGCAGCAGCAACAGCAGCAGCAGCCTGCTAT

GCAGGTAAGTGGAGGAAACCCTCGTCTTAACCAGCTCTGCTCACGATGGCAACAAGTTTG

GCTACTAGCACTGGACCGCCAACGCAAGCTCCATGATGGCCTTGACAGGCTTGAAGAGCT

GAAAGAATTTGCCAACTTTGACTTTGATGTGTGGAGGAAGAAGTACATGCGCTGGATGAA

TCATAAGAAGTCCCGCGTCATGGACTTTTTCAGACGCATTGACAAGGACCAGGATGGCAA

AATCACCCGGCAGGAATTTATCGACGGCATTCTGGCTTCAAAGTTCCCCACCAGCAGGCT

GGAGATGACTGCAGTGGCTGATATCTTTGACCGAGATGGTGACGGTTATATTGATTACTA

TGAGTTTGTGGCAGCTCTGCATCCTAATAAAGATGCCTACAAACCCACAACAGATGCAGA

CAAGATTGAAGACGAGGTCACCAGACAAGTCGCCCAGTGCAAGTGTGCTAAGAGATTCCA

GGTTGAGCAGATTGGCGAGAACAAGTACAGGTTCTTCCTTGGGAACCAGTTTGGCGACTC

TCAGCAGCTGCGTCTGGTGCGGATTTTGCGTAGCACAGTTATGGTACGAGTGGGAGGGGG

ATGGATGGCGCTAGACGAGTTCCTGGTCAAAAACGACCCTTGCAGAGGTAAGGCTTATTT

ACACAAGCACACAATTATACAC

>Unigene000016

GCACTGCTGCTGCTCTCAGTGCCATGCAGCCGCTTTCACCTCCACCTCCAGCTCCAGTTC

CAGCTCCACCTCCAGCTCCGTCCCGCGGGCCGTAACGAGCAGAAGGCGAGAAAAAAACTC

CCTAATATTCGCTCTGGTACAGAAACAGAAGAAAGACCGAGTGAATCATGGAGGCTGTGA

AGGCGTTTAACGGAGAGCTGTACTCCCTGAACGAATACAAGCCCCCGATCTCTAAGGCGA

AGATGACCCAGATCACCAAATCAGCCATTAAAGCGATAAAGTTCTACAAACATGTGGTCC

AGAGTGTTGAGAAGTTCATTCAGAAGTGCAAACCCGAGTATAAAGTTCCCGGTCTGTACG

TCATCGACTCCATCGTGCGCCAGTCGCGACACCAGTTCGGTCAGGAGAAGGACGTGTTCG

CACCCCGCTTCAGCAAGAACATCATCAGCACCTTTCAGAACCTCTACAGATGCCCCTCTG

ATGATAAGAGCAAAATCGTCCGCGTGCTCAACCTGTGGCAGAAGAACAGCGTGTTTAAAA

GTGACATCATCCAGCCGTTATTGGACATGGCAGCAGGAATCCCGCCCCCAAGTATCACAC

CTGTCTCTGCCAGCACCGCACTTCCTGTTAACAACGCCACTCCTGGAACTCCTGCCACTC

CGGCTACTCCAGCCAACATCATGCCCAGTTTACCAGACTGGGCTTCTCAGATCTCCAACT

CGGACACCGTGGCAGCTGTAGCACAGATCCTACAGAGTCCTCAAGGACAGCAGTTGCAGC

AGTTGGTACAGAGTCTACAGATGCAGCAGCAGAAGCCTCAGCCCTCTCTCCTGCAGGCTC

TGGATGCTGGACTGGTCGTCCAACTGCAGGCCCTAACCGCTCAGCTGACAGCGGCCGCAG

CGGCCAACACACTCCCGCTCGAGCAGAGAGTCTCCTCTTTCAACAAGAAACTCTTGGGAC

AGTTTGACTTTGTTGGCGAGTCTGAACCCAGTGAGGATTCTAAAAAGGACACTGCAGCAT

CTCAGCTGCCGATGGTTTCGGAGTCCATTAACAACTCCCTCTTCCATCAGTTAGCTGAGC

AGCTCCAGCAGCAGAACCTGGAACAGTTTCAGAAACAAATTCTAGAACATCAGCAGAAGT

CCATGACTCTGGAGAGTCAGGAGAACATTTTCTGTCCCGAGAACTCGGCACCTCCGTCAC

AGAGTAGCGTCCAATCACAGCTTCAAGAACAGGACGTCAAACTGGACGATTCTATCGACA

ACCAACAGCAGGACATGGACCTGGATGAGGGACAGGACACTGTGGATGAGGATATGTTTG

AATCGGACGAGAAGAAGACCAGCGGTACTCGTTCCAGAACACACTCTCGATCCCGCTCCA

GGTCCCCAAAGAGAAGGAGGTCACGGTCACGCTCGGGGTCTCGAAAGCGCAAACACCGGA

AACGGTCCCGCTCACGGTCCAGAGACCGGAAGAGGAAATCCTCCCGTTCGTACTCGAGCG

AGCGCCGAGCGCGGGAAAGAGAAAAGGAGCGGCAGAAAAAAGGTCTTCCTCCAATCCGCT

CCAGATTTCTCAGCGTGTGTAGCACGACATTGTGGGTGGGCCAAGTTGATAAGAAAGCCA

CGCAGCAGGACCTGACCAACCTTTTTGAAGAATTCGGACAGATTGAATCCATCAATATGA

TTCCTCCCCGAGGATGTGCTTATATCTGCATGGTTCATCGGCAGGACGCTTATCGCGCAC

GGCAGAAATTAAGCACCGGCTCGTATAAAATCGGGGCAAAGGGTATTAAGATTGCCTGGG

CCCTTAATAAAGGTGTGAAACAGGAGTATAAACAGTTCTGGGACGTGGATCTCGGAGTGA

CGTATATCCCCTGGGAGAAGGTCAAACTGGACGACCTGGACGGATTTGCCGAAGGAGGAA

TGATTGACCAGGAAACAGTGAACAGTGAATGGGAATCTCAGAAGAATGTAGAGGCACCAA

AGGATCTCCAGGTTCAGCAGGTGTCTGCTGAGAGCAGTGCAGCCAGCAACTCACAGAATG

AAACCTACACACAGCCGGTGGCCATGATGCCTGTGCAGGTGTTTGTCCATTTGTTGTTGT

TAGGTGGATGTTGTTATTTTTATAACTTACAGTATTAAAGTTATTAAACACGCAATTACC

TTCTGTTTGTAAGATCCCAGTGGCGCAGACTGTCTCTGCTGTGGGCCTGGTCCCGCCCAC

TTTCCCCGTCACCATGGCAATGCCTCCACCAGGTTTCGGTCCCCCACCTCCCTTTCTCCG

GGCAGGATTTAACACTGCACAGCCTCCACCAGGTTTCATGGCTCCGTCCGGTGTGGCACC

GTCTGCCGTTGGAGCTGCTCTCACCTCAGGACCAACAGCTCACAATCAAGACGGAAACAA

GGACTCGACGTTTGGAGCGATTCTTCCTCCGAGCAGCACTATTCCAGCCAGCTTCATGCC

CTCTGGAATGCCGGGAGCGGTATTCAACTCTGTTGGAGCACAAGCACAGCAGGCCAGTAC

TGACAAGACGAGTGTGTCTGGAGAGAGCGTGGACACACCGAACGAATTAACACTACAGGG

AATGCAGAATGCAGTGCGCAGCGGAATGGGTCTACTGGGCATGCACCCTTCTGCAGCTCT

CTCTCACCCCCTCTCTGGTCAGAGGATGCCCGGGCTGCTGCCCATCGACATGCGTCCGAA

CCTCCTTCAGGGAGCTGGGGCTCGGTTCTCTATGCTCATGCAGCAAGGCCTGGCGCAGCA

AGCTAACAGCCTCCTGGACGCGTCTCTTCAGACACGCGCTCGAGCCCCTTTCCCTCAGAT

GGAGCACTTTAACTGCGCAGACGCTCCGTTTGTCAGAGCCCCCAATCCTGCAGCTCCTGC

ACCAGACAGTATAGACAAATCTAGTAACAATATCACAGCAACTGCTACAGACAGCAGGAG

CCAACAAGAAGATCAGGACTATCGCTTTCCACCGCCGGAGAAACAGAGCACGGGTCTGCT

GAGGACGCCTCCTCCTGAGATGAGGACGGAACCGACAGCTGTAAGAGCACCGCTGCTGGA

CAGACCTCTCAGAGCAGGCATGCACATGGAGGGCCGAGAGGGACCCGGGAGACGGGAACC

CATGCCGGCCCCTTTCAGAGCAGAGACACGCTGGGGTGCTCCGCGTGGGGACCTGGATGA

GCGGGACATGAGAGGAATGTCAGCAGGACCGCCGAAAAGCTTTCCGGAGGATCCAGGGAA

TCCGAACTTCCAAAACCGGTTTGAGATGCGAGGCGGAGGAGCGATGGGTGGGGCAGGACC

CGGTTGGACTCGTGGAGGAGGAGGGGGCGGACCCTTTAACATGAACATGCATCAGGACTT

TGATGATCGACGTCGACCCTGGGAAAGGCAGAAAGACCGAGACGACAGAGACTTCCGCAG

AGATATAAACGACAACCGGCACAGGGATCGAGACAGAGAACGGGAACGAGACCGCGAACG

AGGTCGGGATCGGAATCGGGAGCGAGAGAGAGACAGAGACCGAGATAACGATAAGGAAAG

AGAGAGAGAGCGCGGAGGCTGGGCACCTGTTCAACCTCTCCTCCCTCTCCCGAGACCGTC

TCAACCACTGCTCCCTCTACCGACACCACTGCTTAACCTTCCTCCAACTCAGCCCGAATC

GCATCCCAAACCACAACCCGCATCCAGACCGGAGTCGCAGCCCGACGGGAAGCAGCCGCA

GCCTGAAAAACAGAACTCAGGCGAGCGAGAGAAGACGCACGTCTTTAGTGACATCGCTCA

CGGCCCGGACACTGAACACGAAACGGTGACAGAACCTACAGCCGAGTCGTCTCGCTCCTC

TTCACCTGCACCCACCGCCGAACTCCAACACCAAACACAAACCCCAGAAGTGTCCGCTTC

CACAGGCGGCAGACAGACCCCGCTGAAGGAAGAAGCCGAGACTGGGACTGAGCCTGAGCC

TGAGCTTGTGAAAACGGACACTGAGGAAACATAATCGTCTCTCAGTAGACCAATTTGGTG

GATTGCTAGCTGACCAAAAGACGATGATGACTGCGGGGCTCTAACACTGTGGTGCTCTTG

ATCTGTCCTGATTACTAAATCTGGTCAGAGATGGGAAACTCAGAGAGCCAGTTCAGAACC

CAGGGATCTCGAAGCAGTAGCTTCACACTCTCAAACAGACAAGAGCCCTATCCAGTTAGG

TGTCGTACTTTCAAAGAAGACAGTTTGACCCCTCATTGTCGCTGGAGGACTGGATCCAGT

TTGAAATCCAAGGCGGAGAAGCGTTGCGGTTTGTCGCAGCACAGGGCCAATCAGTCGTCC

TTGTTAAGGCATTGTGACTACATTACTAAAGGAGGAGAAGGAAAGAGAAATATCAGCGCC

CAGTGGAACAACGGACACTTTGAGGAAAACAGGCGGGATTGTAACGGTCATGCATTAAAT

GGCTACAAGACCCCTGAGAGGAAGCCGAAATTAGAGGATTTGGATGATCACAGCAGCCCA

AAAGTCGTCATCAAGAGCGACGGCAGCGTTCGGGTCGAGTTCAGTCACATGTCTAAAAAC

TCAACACTCCCGGATGAGAACGGAGGTTCCGTCCAGCTGCTGAAGTTTTCCCCCACATCA

CACTCCGTGTCCACCCCTGAGTCTGCCCTGGTACAGAACAGCCCTATCAACAGGACCAGC

AAGAGAAACTCGCTGAGCTCAGAAGGCAGCTGGTACGACTCACCCTGGGGGCCCGGAATC

GAGTTGAACTACGACGGCGTCTGCGCCCTGCCCAACAGGCTATCAGAAACACTTCCCTCT

GTACGCATCGAGTACTTCGACGAGCCATGCATTCCGCATCTACTTTACCGGGACCCTGTC

ATGGCTGCCACTTTTTCCACTGCTGAAGATCTAAAGCTTGCAAATGAGCTGTCTTTTAAA

CACAGGACTCCGTTTCTGCATGTGATGGAGGAACCTGTTTTAGCAGAGTCCTCTGGGGCA

AGGCAGTACTCTTCATACACTTTACCGTGCCCCCAAACCAAACCGACCGCCGAGAACTCT

GGAAAGAAGGAAATCATCAAAAACAGGATGAGGCGTTTTAGTGACTGGACCGGGAGCTTA

ACCAGACGGAAGAAGAAGTCAAAGGAGTTGAGGTATAAGGATTCCATCGATTGTTTTGAC

AGCGGTATAGATGGACTGACTGCCGACACCAGCTCTCCTTCTCAGGTGTCCAGCCTGTTG

GGGTACCACAAGGTTCAGTGTCGGGGTCGCTCCCAGTCCCAGGTGCTGCCATCCTGCAGC

GGCGGCTCTTTCAGTGCCCTTCACCCGGCCACAGATGGTCTGCGGCAGAACATCTATGAC

AACTTCATGCGGGAGCTGGAGACAAGCCAGACAACGACAGGAAGCGGCGAGAGGAGAGGC

GAGAACTCTGCAGAGGACACTGAGAGCAGCAGCCAGGAGACGGTGGGCTCGCTGGAACAG

CTAGACCTTCTGTGTGAGAAGGAACAGGACGTGGTCCGAAGAGCCGGCTGGTTGACCTTT

AAACCGCTGCTCACACTGCACAAAGACCGTAAGCTGGAATTGGTTGCCAGGCGCAAGTGG

AAACAGTACTGGGTGACTCTTAAAGGCTGCACTCTCCTGTTTTACGAGACCTATGGCAAA

GGTTCTCCAGAGCAGGAGGCTTCACCCAGCTATGCTCTGCTCGCTGAAGACGGAATTGTC

CAGGCCGTCCCCGAACACCCGAAGAAGGAGAACGTGTTCTGCCTCAGCAACGCCTTCGGT

GATGTCTACCTCTTTCAGGCCACCAGCCAAACGGATTTGGAGAATTGGGTGACCGCAATC

CATTCGGCCAGCGCGTCTCTCTTCGCTAAACGCCACGGCAAGGAGGACACGCCGCTCCTG

CTCCGGGGACAGATCCGCAGCCTACTGCAGAAGATCGACATGGACGGAAAGATGAAGAAG

ATGGCAGAGCTCCAGATGTCCATCGTAACTGATTCCAAAAGCAGGAAGGCCATCGAGAAC

CAGATTCAGCAGTGGGATCAGAATCTGGAGCGGTTCAACATTGATTTGTTCCGTATGCGC

TGTTACCTGTCCAGCCTGCAGGGGGGGGAGCTGCCCAACCCCAAGAGCCTCCTGGCCACC

GCAAGCCGGCCCTCCAAAGCGGTCCTCGGACGTCTGGGCGTCTTCTCCGTCTCCTCTTTC

CACGCACTGATCTGCTCCCGAGACGAGGCCACACTGAGAAAGCGTTCTCTGTCTTTGCCC

CGGAGAAGCCGCGGTAAAAGAGGCATGTTTTCGTCACACAAAGCTCTGGACAGTTTGACC

AAACGCAGTCGAGACAGAAGACCCTGCACCTCACAGGTGTTTGATGGCGCTGCAGGCGAT

CACTCCGGTTTGGTTCCTTCGTACAGCAGAGAGAGACCGGACGCTCCAGCTGACACTTAC

TCACAGGCGCCACCCGATGGCAGTCAGTGGGAGCGCAGCGTGACTGAGAGGCTGGTGTGG

GTTTACATGCCTGACCACCAGGTGGTTACAGTGACACTGAGCGGACAGCACACAGTCGAG

GAGCTCCTGAACACCGTCTGCAAGATGCAGAATCTGGACCCCAGCGCACACTGCCTGCGT

CTGCGCAGATGCGTCAGCGATCGGATGGAGGTCTGGACTCCAGGACCGCACAAGCTTTTT

CAGGACCTGTTGTATGATGAGTTAGAAGTCAGCGCTCTCAATGTGTACAGGCTCCACATG

ACCAAGCCCACATACGCAGGGGATTTTGGCTTTGCTATTTCAGGAAGTGTGGACAGTCTC

AACCAAAGCCGCATCTTCGTCACTCAGGTTTTCTCTGGAGGAATCGCGTTCAGTGAAGGT

CTGAGGCCGGGTGATGAGATCCTGGTTCTGAACGGATGTCAGGTGTCGCGTCTGGACCTG

GAACTCATTCAGACTCTGTTTAGTGAGAACACACTGCACCTGACCATCAGACGAGAGCCG

ACCGCCATGACACACACACACACCGGCCAGCAGCCAATGAGGGACCTCCTGTACCGCCAT

CACAGAGCCAAGTCTGCTACAGACGTGTCGTGTGTGTCTGAGGGTAAAGCCGGTGTAGAG

ACGAGGCAACATCATCGTCTGAGTCTCAGAGCCGTACAGCACAGCAAGAGTGCGGAGACG

GTGTTTGCTCTCTATCACTCCTGCCAGGACTCCTCCGTACGACTGATGGAGGTTCAGACG

GAGACGGCGCCCCCGGGCGGGACCCTGCTCAGAGCCTGTCCACGGCACATGTCCGTCAGC

GAGAGACTACGCAAAGTGGTGCAGGAGTTAGTGGACACGGAGAAGTCCTACGTCAAGGAT

TTGAGCTGTTTGTTGGAGATCTACCTGAAGCCCCTGCAGAAGGAGACGTTTCTCACACAG

GATGAGATGGAGAGTTTGTTTGGGAGTCTCCCTGAGATGCTGAACTTCCAGAAGGTGTTT

CTGCAGACGTTGGAGGAGAGAATCGCTTCGACTCCAGACTTCGGCACGCTGCAGACGCCG

GTGCAGTTCAGGAAGTTGCTGTTTTCTCTCGGTGGCTCCTTCCTCTACTACGCCGATCAC

TTCAAACTCTACAGTGGATTTTGTGCCAATCACATCAAAGTCCAGAAGGTTCTTGAAAGA

GCTAAAACGGACCGCGCCTTTAAGGACTTCCTGGATGCCAGAAATCCCACGGAGCAGCAC

TCGACTACGCTGGAGTCTTACCTCATTAAACCGGTGCAGAGAGTGCTGAAGTACCCGCTA

CTACTGCGAGAACTGGTGTCTCTGACCGACACGGACAGCGAGGAGCACTATCACCTCACA

GAGGCACTGAAGGCGATGGAGAAGGTGGCCAGTCATATCAACGAGATGCAGAAGATCCAC

GAGGATTACGGCGCCGTGTTCGACCAGCTGGTGGCGGAACAAAGTGGCTCAGAAAGAGAG

GTCACTGAGATTTCTATAGGTGAGTTTCTCACACATTCCTCAGTCATGTGGTTGAACCCG

TTTCCTTCACTGGGCCGCATGAAGAAGGAGCCCGAATTAACTGTGTTCGTGTTTAAGAAA

GCAGTGATTCTCATCTACAGAGAAAACAAACAACTTAAAAAGAAAACTGGCGATCAGGAC

CTTGTGAAATTTCGCTGGTTAATTCCTCGCTCTGCGCTGCAGGTCAGACTGGGCAATACA

GCAGACAGTAACTGCATGTGGGATCTGATCCACACCAAATCACAGCTGGAGGGACGACCC

GAGACGCTGTTCCAGCTGTGCAGCAGTTCACCTGAGAGCAGAGTGAAGACGCTGAAGACC

ATTCGCGCGATTCTGCGGGAAAACACGCGCAAGGGGGCGCTAGGGAGCGAACGGCCGGCG

GAGAGAAGCAGCAAAGAACAGCTCGCACCTCTGACAGGAAGTTTACCATCATCAGCTAAA

TTTGGTTCCTCTCGTGCATTCTGGTTGGTTCAGAAGCCTGATTCGGCGAGTGAGAGCAGC

GGAGACCCGCCCACTGCACCATCATCAGAAGCCCCGCCCACTAACTCCACCCTCGGACGT

GAGCGTATGAGAGAGTCGGACATTCTCAGTGACGACGGCTGGTTCAGCGAGCGTTCGGCC

ACCGAATCCATGGAGTCCCGGTTCTACCAGCTGCGGCTGAGTGACGAGACCCGAGTGGAA

CCCGAAGGTGGTGAAATAAACGCTCACGAGACGCAGCCGCAGCTGAGACGGGCGCACTTC

AGCGCAGCAAGGCGCCGATTAGCCAGAAGTGACACGCGACGCAGCCAGGCGGCGCTGCTG

AGTCTCCGGCAGAACAGTTGCTCTCTGGACGGACAGACGGACACGGCGGTGGTCAGCGCA

GACCTGAACATGCTGCTGGAACGAGATTTCAGCGTCCACAGCCTCACATCAGTCATCAAC

GAGGACTGTTTCTACCAAAACACCACCACCCGCAGCCCCACCACTACCCTGTCCTAATCA

CCCGGCGGATCAACATCTGGAAGTGGACATTACGGTTGGCGTAGGTGAGGTTAAAGGTTA

ATTAATGTGGCCCTTTCCTAAGCCACGCCCCCACATTGATTTCCAAAGAAAAGAAGAAAA

ATGATTGACAGGTGAGGAGAGGCACTAACCCAGGAAAAAAAAAAAATCACTTACTGCACC

TGTAATAACCTGCGCTTCTCTTCAGGAACAAACCATCCAGATGTTGCAGCTGTTTGTTTT

TTCTGCTCTCTGCTTTTATTTATACATGCCAGTCTTTTAATTTTTATATCTTTTTTATTA

TTTATGAAGAACTTTATTCCTTTACAATAAGAAGTCAAACCTCGGATTCAGACTTGATGT

GTTTTTTTATTATCTTAAATTAAAGCGAGTTTCGGGACTGACCTGCGGCAGG

>Unigene000017

CCCCTGTGTTCTCAAGGACACAGAGTTAATTGCACTGGACCTCAAGGAACTACAGGTTCA

GGCAGCGTCCTCTGATCTGGGAAAACATTTACCAGAGGTGGTAGATCTTCTCCAGAAACA

AGAACTGTTGGACACTAAGATCATCTCTCTTGGAGAATCACTGAGTGCCATTAGCAGTAA

TGCCTTAAGGGGAAAACACAGGGATGTTTCACAGGTTCAGAGAACAGTAAAAGAGCTTAA

TAGCCAGTACACCTCCCTCCTGGCACTTAGCCAGAACAGGAGGAGAGCACTGGAAGGACA

GCTGAAGCTCTTTGAGTTTTTCCATGACTGTGAAGAAATGGAAGGTTGGATTTATGAAAA

GTGGCTTCTGGTGCAAGCAGCGTCTTTGGGCAGAGACCTCAGTCAGATTGAACAAGCCAT

TCAAAACCATAAGATGTTGGAAGCAGAAGTGCAGTCACAAGAGTCTCTTTGCTCCAAGGT

TTTATCCAGGGGTCAAGAACTAGGTAGAGGGGGACACCCTAATGAGAGCGATATACTGAA

ATGGATCCGGACCTTACAGAAACAATGGCAGCAGCTTAGGGATCAAGTTACAAACCGCAA

AAACCGACTGCATGCAGCCAGTGTTATTAAACAGTACTTTGCTGATGCAGCAGAAGCAAA

CTCCTGGCTTAATGACCACAAGCCCTTTTTAACTGATGAAAATTATGGCAAGGATGAGAT

GAGCACAATTTCTTTGCTACAGCGTTACCAACGACTGGAGAAGGAAATAGGGGCATATGC

TTCAGAGGTAAAGCGGCTCAGTGAGCAATCCCAAAGTGCTGCCCAGCTTGCTCCACTCAC

AACAGAACCTCAGCAGACCATTATGGTCCACTACAGTGATTCATCAGGGGATGAAAGAGA

GACCGTAAAAGAAAAAACAGGAACCTCAGTGAAAATCAGAGCACCCTCCAAGACCTTACC

TCAAATGCAGGAACTTAATGCCAAAATTAGGTTTAAATACAGAGGAATGAAATTAACCTG

GGATCGTGGAGAAATGGTGACTATCATCAGCAAAGAGAAAGAAGATAAAATGCTTGTGCG

AGATAGCAAAGGAAATGAACAACTGGTGTCTTCTACCTACATTACAGAACTTTCTTCAAC

TCAAGCCCCACCTGCACCTACTGATGTAGTTCCAGAGAGCCAGATTCGGAAAGTGAGCCG

GCCACGTCGCACACGCTCCATGCGCCGTGGCACTGCTGAAATTATAACCACCCTCCTGCC

AGACCCCCAGTTCCAGAAAGACATGATAGAAAATACACAGAGCAGCTTAGAGAAAGACTA

CAACAGTCTCTGTCAACTACTACAGTATAAGAGAAGAATCTTGGATGAGACTGCTCAGTT

ACATCGGTTTTATAATGGCTGTGAGGAGTTTGAGTCATGGATGGAGGACAAAGAGAACAT

TCTTACCACTTTCAGCTCCAATTCTGAAAATACTGAGCAGGCCAGATATGAGAATTTTCT

GACAGAGTTGGCCAGTGGAAAGGGGCAGCTTGATGACATTACTAAATTGGGTGATGAACT

TGTAAAAAAGCAGCACAGCAAGAAGAGAGAGATTCGGTCCAGATATGCTCAGGTTACACA

AAGGTGGGACCATATACAGGAACTTAAGGATGAAAAAGCTCAGGAGCTGCTCAAATCATC

TGATATAAAATCCTTTTTGCACACTTGTGAATATGCCAAATCCCAGCTCCTGGAAAAACT

ACACCAGCTGGATACATCTGGTGTGGGTAGCTCAGCATCAGCCCTGCAAGCTGAGGAACA

GAGTCAAACCCAGGCTGAAAGGGAAATCGAGGGCCTTGAAAGGAAGATAGAATACCTAAA

GAGTGTGTCCAAAACCAAGCGTGACTGCAGCCCTGCTGAGAGTGCTGCTATCATGAAAGA

GGTCTATGCTTTGGAAGAGCTTCTGTGCCAGGTTAAGGGTCAGGCTGCAAAAAGGCAAAA

TTTATTAGAGCAGGCTCGACATCTGCAGCTCTTTCGTAAAGAGTCCAGAGACTTGCTGTT

GTGGGCCGAGGAAGTGGAAGAGCGTTTGCTTGAAGAAGGGAATAGCTATAATGTTGAATC

TGCTCAAGGCTTGTTAAATGATAATCAGGAACTGAAGCTGGAGATTGAGCAGCAAAGAGC

AAGGTTGAAAAGCATTGAAAGGCTAGCCAAGTCTTTGGAAGCTTCATCTGGAGAGAAATT

CACTAAGAATATTCAACAAACACTAGATCAGTTAAACCAGGAATGGAGCAGGCTCGATGA

ACTTTGGTCTAATCAAAACAGGCGATTAGAGCAAGCACTTCAACTCCAGAAATGGAACAG

AGAAGCTGATCGAATAGATGCTACAATAGCTGGTCATGAAGCCAGGTTAAAGGTCAATGA

CCTAGGGGACTCTGTGGATGGTGTTTATAGTTTGCTTGGCCGCCAAGAGGAGTTAGAAGG

TTTGCTAGTAGCTCTTGACCAAAATATAAGTTTTCTTCAAGAGAAGAGCCAGGAGCTCAT

TAATAAAAACCACTTTGCATCAAAGCAGCATCGGCAGCGATCCTGGGCCATTGAAGAACG

CAACCAGAAGCTTAAAGACAGCTGCAAAAAAAGAAACCATGATTTATTTGCTTCCAAATC

TTACCAGGAATTTTACAGAGATGCAGAGGAGCTACAAATTTGGATGGACGAAAAATTTAA

GATAGCTGAAGATGAATCTTACAGAGATCCAAACAACATCTTGCGCAAACTTAAAAGGCA

TGAGGCAGCAGAGAAGGAGATGCAAGCCAATCACATCCGCCGAGACAGACTGATTCAGGC

AGGTGAAGAAATGGTGTCTAAAGACCACTATAACAGTCAGAGCATCCGGAGCAAAACTCG

GCAAATCCAGAGTAGATGGAGTGAACTGGAAACAAAAATGGCGGAAAGAGGGGACAAGCT

AAGACAGGCTGGCCAACAGGAACAACTCATGGAGCTGCTTCAGGATGCAAAAGTTAAAAT

TGAGAAAATTCAGAAGATGCTACAGCATGCACCTAAAGGCTATGACCTTCGCTCCAGTCG

ATTACTCCTAAAAGAACACAAGCAGGTTGAGCAGGAAGCTCAAGAACTGGCTGACAAAAT

TAACTCCATCATCACCCATGCCAAACACCTGGCTACAAACCATTTTGACTCTCAGAGAAT

TCTACATGAGACAGATATGTACCTGAAACTGTTTAAGTCTCTAGAAAAGCCTCTGAATCA

TCGCAGGGACCAATTAGAAGAGGAAGTGACACTTTACAGCTTTTTTTATGATGTTGAGTT

GGAACTTAGTTGGATTGCTGAACATGAGCCTAAAGCAGATACTAGTAGCTATACCAAATC

CTTGGCTGGTGCCATCAGCCTCCTACAAAAGCACAAGGAACTGCAAGCAGAGGTTAATGC

CCACAGGCAGTATTTTCAGCGTGTACTGGACAGAGGCAGGGCCATGGGCAAGAGGGAAGT

GTACCAGAGGTGCAGGGATTTGTCTGCAGCCTGGGAAAAACTTGAGGATGCTTGTGAAAA

ACGCTTTTCCCACCTAAGCAAAGCAGTCATGCGGGAGCAGGTCTTGCTTGACTGTGCTGA

ATTAGAAGTGTGCTTATCTGAAACGTCAGCTTTAGTCAGCACTGACTATGGCAAAAATGA

GTTAGCCACACAAAGCCTGATAAAGCAGCATCATATTGTAGAGGGGCAGATCGAGGTACT

TTCAGCTCAGGTGGATGAATTAAAGTCCAGGGTGGACCAGGCTGTGCATACATGGGGTTT

GGAGGAAGTTACAAGAAATTACAACCACATTAGAACTCAGTTAACTGAATTACAGCACTT

GGCAGCATTAAGAGAGCAAAAGCTCCAGGATGCCCTCCACTTACATGAGTTTAAAAGAGA

GTCTTCAGAATTGGAAGAGTGGATATCACAGCAGCAGCTTATTGCCTCATCTGATGACTA

TGGCTCTGATTATGAGCGTGTCCTGCACATGCAGAGAAGGTTTGCAGTCTTCCTGAAACA

GCTGGATGTGGGTCGGGAGAGGATGCGTTCATGCCAAGAGCTGGCTCAAAGACTAATAAA

GAGCAACCATCCTGAAAGTCGGTTTATTCAAGAAACCCTGGGGTTACTGAGAGAATCATG

GAATGAGCTGCAGCACTTGGCCAACAGCCGTAAAGAGAATTTGCAAAAATCTGAGGAATG

TTACAAGGTCTACAGCGACTTAACTGACGCTCTTGAACAAACAGAGGACAGGTACAAGTG

CATTCCTGAGGATATTGCCAAGGACTTGAAAGGGGTTGAGTTGCAGCTAAGAAAGCATGA

AGCACAAGTAAACGAGCTAGTGGGAAATGAACTACAGATGCAGGAGCTATTAGACATTGC

AGACTCCGTTTTAGATGAGTGCTCCCTGGGACTTAGAGAAAAGATACAGGAGAGACAGCA

ACTGTTGGTGGAACAATGGGAGAAGCTCCGCATGGCTATGGAGCAGAGGGAGCATGAGCT

ACGTCTTGCCAAACAGCGTTACATTTTTCTAAAGAGAGTACAGGATTATTCTTTGTGGTG

TTCTCAAATTTTAAGTGGCATAAAAGCAGAGGAGTCTATTCGTGATATGGCGACATGTGA

CCTGCAGCTTTTCCAGCATCAGCAGCTGTGGGCTGAGATCGTAGCCCATGAGGAGACATA

TTCTCAGGCTGTCACAATCGGTCAACACCTACATGAGCAAGATGTGTCCAATCCTCAGGA

AGTCCAAGACAAACTAAAAGCTTTAAAGGATGAAAGAGAAAGACTGACAGCTAACTGGGA

GTCTAAGCAGAAATGGCTTGAAAACACGTATCAGGAACAAGTGTTTTACAGAGACATTGA

ACACATGGAGAGGCTCACTTACTCCCAAGAGGTCCAGCTGAAAAACAGTGATTTGGGGTC

AACGGTGGATGAGACAGACACTCTGATCAAACGCCATGAAGCCTTTGAAAAACTTCTTAG

CGCTCAGGAAGAAAAAATGATTGCTCTTCAGAAATCAGTTGAACATTTAAATAAAGGAGA

AGTAACCAAGGGAAAAGGCAACTACAAAAACACATTGAACTCTCTGAGAGCACGAAGGGA

CCGCATTAGAGATCTGTCCATGAAACGCAGGGAAGATCTTGAGCTCTCACGACTTTTTTG

CATCTTCACTCGGGATTCCTCTGAGGCTGAGGATTGGGTGTCAGAGCGCATGCAGAAGAT

GCAGGAGGATTCCAAAATGGACCTGAGCAACTTTCAAGCCAAAATGAAACTATTGCAGAA

GCACCAGGTTTTTGAGGCAGAGATTCTGGCTCATGGCAAAATAATCAATTCTGTCTTGGA

GACAGGCGAAGAGCTGATCTCTCTGCGACATTCGAAGTCCAGGGAGATAAAACAAAGCAT

CTCTAATTTGATCTCTCACTGGGAGGCTCTGAAAGAAGCTATCTCCACCCGGGGAAAAGT

TCTAGAAGGGCACCGTGACTTTCTTGAGTTCCTTCAAAAAGTGGAGCAGGTAGAAGTTTG

GATCAGACAAAAGGAGGTCATGATTAATGTTGGGGATCTCGGAAAGGATTATGAACATTG

TCTAGAACTACTCAGGAAACTAAGTGAGTTCAGAAGTTCCAGCCCTGGGGATATGACTGT

TGATGATGCCCACATCAAAACCATCAACAACCTGGCGGCTCGGCTTGAGAAACAGAATTG

TGATGAGATGATCACTGTAAAAAAGCGCAGACAGCAATTGAATGAGAGATGGAATTCTTT

CCATGGAAACCTAACCAGCTATAAGAGAAAGTTGAAAGAAGCAAAAGAGGTTCACTCTCT

GATCCGTGACCTTGAGGAAGTCAGGGACAGGGCTAGTGAAAAGATGCTGTTACTGCAAGG

ACAGGGTTATGGAGAGGATGTGGAGAGTGTCGAGAACCTTATTCGCAGACATGAGGAGAT

GGAAAGAGAAGTTCGAGTTATCCATGAGAGAAGAGCTGTGCTAGACCAAGAGACCAAAAT

CCAATTGAGAACTCACAGTGACCTTTCCGACAAGCTAAGTAAGAAGCAGCAAGAAATAAA

CAACACTTTAATAAAGCTGGACAAGGCAATGAAACTGAGGAAAGAACATTTGCAGGAGTC

ACACCAGCTTCAGCTGTTTAAAGGTAATCAGCATCTGTTGCTGGACTGGACAATAAAGCA

GACTACAGAAATGGGAAAGAAGAGATTGCCAAAAAGTAAAGCTCAAGCAGAAAGTCTTAT

AGTGGAGCACCATGACTGGAAGGTAGAGATTGATGCACGTGGTGAACGCATTGATTCAGT

AAAGAATTTTGGGCAGAGCCTAGTGAAGTCTGGACATAGTAAAACAGCAGAGATTAAAAA

AGCTCTAAGCAAACTTGAAGATGCTAAAGTCAGCCTTATTCAAGCATGGGAAGAAAGAAA

AATAACATTGGACCAAGCTCTGGAGCTTCAGATATTCCTTGGCAATGTGGACCAAAGTGA

GAGCTTGTTGAGCAACTTAGAAGCTTTTCTGGCTAATGACGATGTCGGGAGCTCTTTGTC

AGACGTGGAGGCTCTGCAAAGGAAACACGCTTTGTTTGAAAACTCCCTAGAAGCTCAAAT

ACAAGAGGTGGAAGAAGTGGAGAGATATGCTCAACAGCTGATCCAGAGACAGCATTATGA

CTCAGACAACATCTCAAAGAGAAGCAAAGCAATTCAGCTAAGGAAGAACAGATTGTTGGA

GACAAGTAAAGCCCGGCAAAAGGCTTTGGCAGAGTCAGTTCAGCTACAAAAGTTCTTGGA

AGACAGTTATGAGGTGTGCTTATGGTTGAATGAAAAGAAATTGGTGGCTCAAGATGAGAG

CTGGAGAGAACCCATCAATTTGCAGGCCAAGATGCAGAAACACCAGAGCTTCGAGGCTGA

AATTATCGCCAACCGCAACAGTGTAGAAGTCCTAACAAAGGAAGGTGAGAAAATGCTACT

TACTGGTCATCCAGCCAAAGATAAAATAAAATCCAGAATAATGGATCTCAATGAAGGCTG

GGAGCAGCTCTTAATCCGCTGCAAGGAGAAGAAATCACGTTTACAGGAAGCATACCAGGC

CTTGCAGTTCCTACACTCATTAGATGATGTTGAGTCATGGCTAGACTCAGTTGAAGCTGA

GCTGAGTCAGACAGACTGTGGGGAGGACCTGGCCTCTGTCAGCAGACTGCTAAAGGCTCT

AGAAGGCTTGGAGGAAGTGATGGATGGACATCTGGAAAAAGTTCAGAATCTGGTGGACAC

AGCTAAAAGTCTCAGCTCTCAAGGCAATTTTCAGGCTAGAGAAATCCAGCAACAGGTTTG

GCATATGGCCAACAGGTATAATAGTCTGGCTGAGCCTCTGCAGGCTCGTAGAGAAACTCT

GGAATCATGGCAGCTGCTCTTTCAGTTTTACCAGGACATTGACGATGAAATGATATGGAT

TCAAGATAAGCTGCAGGCAACAGCTTTAAATGACTGTGGAACATCATTTGAAAGCACACA

AGCAGTGATCAAAAAGCATCAAGTGGTGATACAGGAAATCACAGCTCGCACACCTTTGGT

GCAGGCAGTACTAGAAGCAGGACATAACTTGGTAAAAGGAAGGCACTTTGCCTCACAGGA

GATATATGCAAGACTGGAGGAACTAAAGGACAACCATGAAACTCTGAAAAAAGAGTCGGA

GAATAAAAGCCAGCTGTTAAAAGAAGCCCTAAAAATCCAGACCTTCCTCTCAGAGGTCTC

TGACATTGAGTTGTGGATGGAGGAGTTGAGGCCAACCCTGGAGTCCACAGACTATGGAAA

AACTGAAGATGCTACAGAAGCACTCCTAAGAAAGTTAGATGCAGTGGATTTAGAGCTAGT

CAACCACCATAGAAAAGTCAAGTCTCTGCAGGAGACTGGGGCTGAAATGGAGAAGTGTGG

CCACCCCAACAGTTATTTGGTGAGCAAGAGTTTGACCACCATGAAAAAGCAGTATGAAAC

TCTGCTACAGCTGTCTTCAGATTATCGAATATCATTGGAGGACCAATATAATCTCTATGT

ATTTGAAAGAGAGGCCAGAGACCTCAAAAACTGGCTCATCTCCAACCAGATCCTCGCAGA

GTCAGATGATTTTGGCCAAGATCTTGAAGATGTGGAGGTACCTTTGCATGTGATTATAAT

CTATAAAATGTCAACAGTGCTCAGAGACCTTGCAGAAAAAATTTGAGGATTTTACCAGTG

AAGTCAACACTCTGGGTCAGAACAAACTGAATACAGTACAAAACCTGAAACAAGAAGTAA

AATCACCAGAAGCCCAGCAGAGAGAGAATGATCTACTAAATTTATGGGGAGAGTTACAGA

GGGCTTTGAAAACACGAGCAGAGAATTTACAATCAGCCCATGAAGTTCACCAGTTTGACC

ATGACTTAGACGAGTTGAAGAGCTGGATAAGTGAGAAGGAAATTATGTTGGACTCGGAGG

ACAGAAAAGATGACCTCATTAGCGTTAAAGCTTGTATTCGACAGCATGAAGGCCTTGAGA

GGGATTTGGTGGTAATAGAAGAGGAACTACTACGCATAAAAAAAGAGGGTAGGTCTCTTG

TTAGACGGTGTCCCCAGGTGAGAGACAGTCTGTCTGAAAGGATGCAAGAAGTGGAGGAAA

ACTGGGATAGTCTCCTGGAGAAAGCTTGCCAGTGCAAACACAGACTTCAGCAGGTGCAGG

TTGTCCAGAAATATCTTACTGACTGGAGAGAGCTTATGACATGGCTTAGGGAGTCCCTGT

CTTTAGTAACAGGAGAAGGATTGAGGGGTGAGGTCAAAGATCTGTCACAGCTAATTAAAC

GGCATGACGAGTATCATAAACAGATTGAGCGGCAACTTGACAAATCAGAAATAGTGAAAA

ATGAAGGACGGCGTTTAATGCAAGAAGGAAACTTCATGATTGAAAAGCTGGAGAAACATC

TAGAAGAACTTCAGGATCTGGAAGTCCTGGTTCTGCAGGGCTGGGCTGACAGAAAAGTGC

TGTATCAGGAGGAGCTAGAGCTGCAGCAGTTGCAGAGAGAGCTGGAGCAAGCTGAACATT

GGCTAAACACATATGAGAATGTGCTTACTGCCCAAGATTATGGGGACTCTGTTTCAGATG

TTCTAGAGCTTATGAAAAAACAGGAGGATCTGGAGGCAATGATACAAGCTCAGAGTGACC

GATTTAACACCCTACACAACAGGAAAACTCAGGTAAAAAAACAAGAAGCAAAACTCGGAT

CTCAAGTATTAGTCAGGTC

>Unigene000018

GAGAGAGAGAGAGAGAGAGAAAAATGGGAGGAGGCAAAGAATCAGAAAAACAATGACTAT

TCTGGAAACAGGCCAGCAGTCATAAGGATCGGAGTGGAATGCTTCAGAAAGTGTGGAATC

CTAAAGGAGGCTAAAAAAAAAAAATCAAAAAAAAATCCACAAGCTCACGTCCTCACGACC

AATACTAAATGAAAATTTCCCCAAAACAGATTTTTTTTAAATGTCCGCTCAAGTGTGAAA

GTGGCGGAGGTGTGTAAAGTAAGATTCAGGGGAAAATGTTTGACTAAAGAGAAAGGGGGG

AAAAAGGAAAGATTTTGAGAAAAAGTTTAAGCACTCGCAAATCCTCTAGCGGCAGTCTGA

CTTTGAACAGAGTTCCATTTTTAAAGCCTGGCACACCTGCCCCGGTGAACGCCAGCCTCA

ACTGCCCCACCCTTGATAAAGCCATCCAAAAAAACAGAAGGCCATAGGATGTTGGGAGGC

GCCCAGCCCCCATCTCCTGAGAGAAGTCCAGAGGTGCAACGCACCTGCGCTAAAACACAA

GCTTCATTATCGTTGACAGACGTGGCGTTAATATCGCTGATGAAGCAGGGGTTGGTTTGT

GGGGAGGGGGATGTTAAAGCTGGAAGGGCCGAATCATCATCATCCTCACCCCCCACACCC

CCCTCCCCTGTCCTCAGTGGGTTCCCCCCTTTGCACGTTTACAGGGAGACGGATGCAGAC

CAGAGAATCCTAGTGTGGGTGTTAGCTGGATCTGCTGCTGTGCTACGGAGGGGAGGGAGG

ATCCCGTGTGGGCGCACGGGAGGGCCTCGACGCCCCACCCAAACCCCGTTTATTTGACCG

CATGCAGCTCTGTGGCTTCCATGCCGCAGACCTGGTGGATTTTTGACCCTCGCTGGAACC

GAAGTGAACCGACGCGGAAATTCCTGTTCACACCCACGCCGTCCAGCCCTCGTACAGCTG

TGCCAGGTTGTCCAGATTTGTGGCTTCCTTGATGACATAATCCACCTGCTCTGTAACGCT

CATTCTGCGGTTCGGGTCCACATCCCTTCCCTCCAGCTTTGCCTTCACTCTTTTCCAAAC

ACTCACAGCATATGAGTTTCTCTCTTGCACAGCTCTCCCGGTTTTGGGGTCCCTGACGGT

ACGTTTGGGGCTCGGCATCAGGCTTTTGCTGGTGATAAGCAGGGTGCTGGGTGGTGTGTC

TGCCGGGGTCCCCAGGTTGCGAGGCAGGGCTTTGCGTGCATTCTGAGACATGCTGTCAGG

CTGTGTCTTTACGCCACTGCATCGCACCGCGGCAGCAAAGCTGGTCTGAACAGTTGCTGT

GGGGCTTGCACAATCACCTCCTCTGTCGGTGACTAGGGGGAACGCAAATCCAACCAGCCC

GTTATACACTCTCTGACTTTGTCCTTTGAGCTCTTTCAGGGTGGGGGGAATCTCCTGCAG

CAGCTCCACCTGCTCCAGGCTGCGTGGCTGCCCAGTCGCCTGCACCACCGTGAACAGCAC

GATCTGCACATTATCCTGCCACCCTTTGTACTCGCACAGGAACTGACGCACACTGCCCTC

GTACATTACTTCCTCTCCTTCTGCAAGAGCGCTTTCCTCATCCTTGGCCATAGCACGTAG

CAGGTGCTTGACGTCAGCCAGGAGTCGGTTGTGATTAGACAGGGTGCCTTTAATGGTGTC

CACAAGCAGCTGCAGGGTTTCAGTAACTGAGTCCAGCTGCTGCTCTCGTTCCTCCTGCGC

TGCCCTCTGATTCGCCATCTCCTGAGCCAGATGCTTCTGAGCGCATGCCAGCCACTCAGG

GGTGCCGATAGACTGCTCCATTACAGGACTCAGTCTGTGGAGAAGCTGAAGCTCTAAAGT

GGAGACAGTGGAGCTGAACTGGGCGGCGTAGGAGCACGTGGCCTGGCAACGCTTCACCAG

ATCAAACAGAGCGGCGTCCATGCTCAGAGCTTCTGAGGTACGTGTCCGAAGGCCCTCGAA

GTTCAAAACAGTGCTGCACAGGAAGGTGACCTGAGTGGTGCGCTGACTCTCCTTCACCAC

TAGGGCGCGCCGCTCAGTTATGGTGCTCTCGAAATCCTGCAGTACAGGCGCCAGAGCCGG

GTTTGCCCCGCCCGCCCACTTCAACCTCTGTTCGATGCTGCCCTCAAGTGATGCCAGTTT

CTCCTGAACACTGGCGATAGCTGCGTCATCCTGGCTGAGCTTGTACAGCTTCTTCTTCAT

GTTGCTGAGTATGATAGAGCGAGGTGGAGGGCTGACAGTCATGGGCTGAGGGCGAGTCCC

AAGAACATCCTCATGCTGCCACTGAAACATGGCGATGTGTAGCTGCGCTCTCTGCAGGCT

GACCTTGCAAGCGTCGATGCTGAGCTCCAGGCGGCGCACCAGGTCCAGCTTTTTCCAGGC

CGTGTCATACGAGCTGGCCAACAGTGTGGCTCTGTTTAGCAGCAGCTGGGAGATACGTCC

TGCCTGGACACCCTGTTCCACCGCCTTCTTACACAGGTCGTCCAGAGACACTTTCCCCTC

TGCCCCAAAGTCTTTAGCCTCAACCTGAGCGATGAGGTCTGAGCCGAGAGCACTGAGGGC

ACTGCAGAGAGCCAGCCCAAGAGCCTGAGTGGGGAGACCCATCAACATTTGCCGCACAAA

GTCTGCCGTAAAAGCCTTGATGGGCCGCGTCATCTGGTCCTCATTGACTACAGTGGAGGT

ACGGTATATGGGCTTCACTAACGAAACAGGGCCGTTAGACGGTGGGGGCTCGTCGTTGGG

CGGAGGACAGGTGCCTGACAGTGTCTGAGCAAATGAGGCACACAGCCGGAAGAAGTCACG

AATCGTTTGGAGCCTCTTAAGGAAAAAGATCTCCTCCAGGACCTGCCGCGTCTGAACCGT

GTCAAAAAAATGCACCTGTGTGGCTCGAGCCTCCCAAATCACATCCACCTTCCTCCAGGC

AGTCGGAATATCCATGGAGTTCAACTTCTCTATCAAGAGTCCAAAAGCAGTCTCCAGCTG

AGCAAACATCCCATCAAAGGCCACTAGCAGCATCTGTCCAGCGCTCATTTTGGGCGTGTC

CTGTATGGAGCCCTCCATGTTCCGAGGCTGAATCAGCTCTGAGTACTGAACACGAAGAAC

CCTGGTGACCTGGAGGTAGTGTGCATCCACATCCTCTTGCAGCCCCATGGAAGCGTTGCG

GAGGTGAGTCTGTAGCGCCTCTGCCAGGCCCTGCAGGGACACACCGTCTGCACACTGCTC

TATCAGAGAGTCCAGCTCCGACAGCATGCTCTCCAGAGTGCTCTCTCCCTTCAGCATGCA

GCGCAGAGCCTCTGGGAAGATAATCTGACGGAAGTTTGTGTTCAGCTCCTGTAGGCAGGT

GTAGACCCCATTGGCCAGGTAGACAGCCTGGGAGGTGTCCTCGAGGCAAAGGATGTCCAG

TTCGTGGGACTGGAGCTGGCATTCTTTCAACAGCTGCACCAGACAAGTGACGTTTCCACT

CATGCTGCAGAGCTCCTCCAAAAACCAGGCTCCGTCCCGGGACGTCAGCTCCACCAGCTG

CTCTCCTGCACTGGCTGCCGCACCCTCCATCACCAGGTTACGCCTGGTAAGGGTGCAGAG

AGCGGAGATTATAATCCCAGCCAGGCTGAAGGAGGCGAGCTCGGCATCCTCATGCAGGAA

GACCTTGATGCAGCGTTCAATCTCCTGCAGCTGCTCCTCGCACACAGGCACGCTCGCTCC

CTCTGCCTTCAGCCTTTCCATCTGACGCAGAAGCCGTGTGTTTGTCTCCGCAGCGTGGTG

CTGCAGCGCCAGCTCCACACTTGACAAGAACTGACACGTAGCAGTCGGTGGCTGGGGTGC

GAACTGCATTTCATAATGGTGATAGATGGCCTGGCAGTTCTCCACAGTCATATCACACAT

CAGCTCTTCCATCCACTGCTTCCAAGTGTAGACTCTGTGGCGGTGCAGGACAGCACGCGG

GTACAGCAGGGCCACGGTGGCGTAACTGTGCAAGTGCTCGAGGCAGGCGCGCAGTATAGA

GCGGCGCTGCTGCAAGAGGGCGCTCACCTCCGCCTCCAGAGCTTCACACTGGCTGATGAG

ATGAGCCTGACCTGCGTTCTGCAGAAACGCTGTAGCAGGGACATAACTGGGAGGACCGAG

ATCTATGGGGCTGCTGATCTCCTGCAGTAGGCTAGCAAGCTGCGTGGCCTCCAGACTTCC

AAACGCAGCCTGGTACTGAGAGATCCACTGGTCTAGATCAGACAGCTTGCTCTGGATGTC

GTCCTGTACAGTCCTCTGCCTCGACTGCAGCTGCGTGTGCTCGGAATATCTGTGCTCCAG

TGTATGAATTGAGTGGTCCACTCTGCTGTCTGCTCCCTCCAGGAACTCCATCTCCTCCAG

CAGCTTGCCCTGCGCCTTGTCCACCTGAGAGAGCTGTTCCTGAAGGTTCAGGTACTCCTC

CACAGCCATCTCCACCTGCGGCAGCACCGCCTGCATCTCGTCACGGTTCTTAAACCAGTT

AACCTTGATCTCGGCTACCCTTGAGGAGAACAGGCTGCGTGTGATGTCTCGCTCCATCTC

CCTCTTGCTTTGCTTGCTCTCGGCCTGCTGTCCTCCTCCTCCGTACACAGCTCCGGCGAA

GCCCACCTCTCCCCCTGCTGTCCAGTCCACCAGAGGGTCATACACAAATGCCTCCAGCAG

GGTCAGAAGAGTTTCTCTGCCTCTACGCATCATCTGGATCACCTGTTCACAGGAAAGTCT

AAAGATTCCCTCCACTCCCGTGACTCCTAACGCTGTCTCGATGTTGTGAGTCATACGGAA

AGGAACCTTCTCAGGAACCCTCAGACTTTTTCCTTTCTCAAAGCAGACGTTGTAGTCGAT

GTGCACCACCTCACCGGTCGTCATGTCAATCAACACGTTGTCCAGGTGACGATCACCTAA

ACCAATAATGTAACCCACCATCGACATCACGGCAGTGGACCTGGCGTATGACTGCGTGAC

TCTCCACCACTCACTCGGTGTGGTACACGAGCACCACAGCTCCTTAGCCAGCAGGTTGGG

GGGCGTGGCCTCCATCAGCTCCCGAAGCACGTCTCTCATGACGCTGAGCGGCCAATCACG

GCGTGACACGTCTAGGCTGAGTCCCACGGCTTTCAGAGCAGGCCCGATCTTACTGTAGTA

CAGCTCACTGGGCCGCGGAACCATGGGCGGGTTCTGTGGCTGCTGGATTGAGTCCTGAGC

CTTCTGAGCCTGAAGCACAGCCTCACGCTGCTGCCACCGTTTATAGAGTCCAAACAGGGG

CGTGGCTCCGTCTACCCACTGGATGAGACCCGAGCGAGTGCCCAGAGGCGTGACGGAGTA

GTGGCGGGCATGAAAGCGTGGACTCTCCTGCTGGTTGACTTTAGTAAACATTGTGTTGAC

AATAGACAAAAACTGCATGATCCTCTCATCTAAATGCAAATCCTCCAAACCTTTAAAGAG

GTATGGGTAGTTCTTGCCGTCTGAGCCGAGAAAGTAGAGTTTTTTGGGCTTGGTTTTGGT

GGGCAGGATAGTGATAGTGTTGCCGACGTTGTGGATGGTAATGGCGTCGGTGGCGGAAAC

CTCTCCCGGAAGAGCCATCTCTGTGTGTGTCATAGAGCCCAGACGAGGACTGATTTCCTC

CAGACGCAGGAGATAACTTGCTCGCTTCTGAGCTCGCTGCTGCAGACTTAACATGATCTG

TTTAAAGGGAACCCAGCTATTGGCAGGGTTGGCTGGGTTTTGTGGGTTCTTCAGTCGCTC

GAGAGCATTGTTGATGCCTTCTCCGTATGTCTCCTGGAACCATGTTTCGTGAGGCGTCTC

CGCTGCGGCGGCGGTGATGCTACGTGCGTGATCCAGAGCAAAGACTACCGGCCGCATAAG

AGCCGAGTGCTTCTCCCTCATAATGGCCACCTTCTCCTCCTTGCGCAGCGTGTTGTTGTT

CTGGACTCTCTTTACTTCGTCCTCGAGCTGCTGGATCCTGCGCAGGACGTGCATGTGTTG

CTGCTGCAGGACACCCAACCACAGCTCATCCCAAAGTACAGTGACCCTGCGCAGTTCCCC

AACCAGCAACTGCACCTGTTGGACCATGGTGGGGTTAGCTGAAGAAAGTGTGTCCACAAT

CTTGCTGTAGCAGTCCTGCATCATGGCCTGGTCCTCGACCGAACACAGACCCAGTTCGTC

TCCTCGGCCGCTCTCCTGAGACGCAGGGGGACTGCCGCCCTCACTTTCGCCCACACACAG

AGGTTCGCCCGGCATAGTGCCCAACAGAGTAGGCAGAGCAGAGGGCAGCTTGATACCTGT

GGTCTGAGCCTCCGCTCCAAGTGAGATTAAACCCACGATGGCAGGGTAGAGGATGAGGTG

GGGCGAGTCCTGGGCTACGCGACACAACAGACTGCAGATGCTCTGGCGGATGTAGGCTTC

GGGGTGGTTCAGTCTGGAGAACAGCTGCGGGATGATGCCTCTCCAGGGTGCGGTGGGTGT

GGAAGCCAGACCAAGCTCCAGTCCCTCCCTCAGCTCTCCAGCGTGCTTCACTAGCAGCCT

CAGCAGACGCAGCGTTGCCATCACGATCATGTCATCGCTGCTTTGCTTGCTGCTCTGAGG

GTTCAGCAGAAGTTTGGGGTCGTCCTCATCGATAGGCGTCTGTCCAGCGTTGAGCTTCAG

GAAGGTGAAATAAGCCCTGCAGGAGACGCGGTAGAGACTGAAGATGCGGTCCACAACCCG

CCTCCACACCCGGATCAGACCCTCAGTCACACCATCCTCCACTTCTGCCAGCCACGGGCA

CGAGCTTAACAGCTGCCTCCAGATCACATCCACCATGTCATCATCATCCTCTTCATTCTG

CAGAGCCATGTCCTCATCCTGAATCCCAGCAGGTCGACACATGGCCTGGCCCAGAATGCT

AAAAATGGCCTCCTTGTCCTCATCGGTGGTGCCAGATGGCAATAACTCCTCAATCTCCTT

CTTTTCTCCTGGCAGCAGAGGAACCCCTTCTCCTTGACTGGCATTGTCCACAACTTTCCG

GCCCCATCTGTACGCCCAGCTTGCTAAAGCTGCCCAGGATTTGGCCACCTCAGGTGCCTG

GCTAGCAGAAAGCTGGTAGAGCTGACCCAGGACAAAGTCTGCTTCTCCGACTCCTACACT

CACCGTCGTCTCCGTGGTAATGCGCAGTATGCTCTGCTCCTCCACGGGCAGCTCCAGCAG

GGCGGCGATGTTCTTGCTGAGGTTGGACGGTGCTCCGCTGCTGTTTCTCTTCACTACCTG

TTTGAGCTGAGGCGTCAGTTCCTTCCAGTCGGCCAGCAGCCACTTACACAGTGTGAGGAT

AGAGCGGCAGGCGGCGCGCTCGCACTTCCCCGAGTGGCAGTAGGATAGCGCACATGAGCT

CAGCATCTCCATAGCAGCCACCGACTGACCTGCAGTAAACAGGACTTTAGCCCTCTCGAT

CTTCAGCTCTGGTCCCCATTTTTCAGCCACGGCGCCTTCTAGAGACAGCAGCCTGAAGCT

TTGGACCAGGTCTGAGGTATCAGAGTCTTCGGTGGACGGTTTGTTGCACTGAGCAAGAAG

GCGTGAAGCCAAAGCGATGTTCCCCTGCTTACGAGCGAACTTCACAGCGGTCAGACACAA

CTCCACCAGGTGAGACTCCACAGGACCAGACGAGCCTCTGAGCTTTAGGAAGAGCTGCCT

CTGGAACGTGCTGTATCTAAGCGCGTGTAGCCACGGCTGAACATCCTGCTGCTTGCATGT

GCTCAAAGCGTCACTGTAGAGCGGGATAAGACACTCCTCAGATGGGTGCTGACTGCACAG

AGAGGTCTCCAACGCGCTGGAACAGTGGAGCTGCAGCGTTCCGAGGGTGGGCAGAGAGTC

ACACAGGGTGAGTGTAGACAGACGCAGAGGTCCCAGAGCGATCCGGCTCGTCTGCTTCAG

GTACTTCACCAGAGTGTCTGATGAAGGTGGAACTTTTTGGTCCTGCTCTGGATTAGATGT

GATGGCTCCTACAGCGCTCCTCAGCAGCTGGACCTCAATGGCCTTCTGCAGCTCTGAAGG

ATCAGGACTGAGTACGGCGGGAAGGAGACGCTTCAGGTCCAGTTTATCCTTGGTGTTAAG

AAGACCATAATCTTCACCAGGAAGCAGCTCTAACTGAGCACGGCACTCTGCGAAATCTCC

TTCCTCAAACCTGCTCAGAGCTTTGATGTAGTTGAAGTCAGTTTTGAGGGTAACAGAGGC

CGAGCTGTTGCTGTTCTTTTTCAGTGCCATCATGCTGCTCTGCCACTCCTGCACTGACGC

CCAGTCACTCAGAGCCACGTAACACTCGCACGCCTTATTGGCCAGGAAATTCAGGACCTC

GGGAGAACACTCACTGGACTTTAACAGGACAGTCTTCTTAACGTCTCCGTTTCCTGTATG

TTTTGGACTGGAGCTGCTGCCTGTGGGGGTGTTAGTGAGCTTTAATAAAGGTGTGTTACA

GCCTTTGATCGAGCAGTCGACCCCTGTGACGGCACACAGCTGTTCCTGATACTCCAGAGC

TGCTTTCTCAAACTTCCCCTCGGCCTGCAGAGCGACAGACGCTACCCAAGCCACGCTTTT

CCCCATGTGGGCCAAACTCCATGCTGCCAAACCCTGGATAGCTTCAGGACAGCGTAGCTC

ACACAGAGCCTCCACCAGCATAGTGATGGGGACCTCCATCTCGGGGCCCTGCGCGCTGCT

GATCTCTGTGAGCAGGTCGAAGCCGTGGCGGACGGTCACTGCGGGCTGGCCGGACATGAG

CCCCACCCTCATTAGAGCCAACCGGATCCTCGTCAGCCAATCCTGACACGTCTGACGATT

GGTGTAGAAGAAGGTTCTAATACCCTTAGGCGGGGCAGTGAGGGCGTTGGCACAGCCTTC

GTACGCGTTGTACATGAGCTTCTCGAGGTTCTCCAGGAACTGCAGCAGCAGAGCGAGACG

CAGCTGGTTGGTGTGGTGACCTTCATCACTCTCTCCTCCACTCCACTGACTCAACTCCTG

ATCACTGTTCAGACTGTGAGCTGCCAGACTGCGAATCATCCCTTCGATGGTTTGGAAGGT

GTCCTGAGCTCGTCCCAGTGGAGTGCGCAGTTTGGACAGGACGGTGAATTGAGCGGCTTC

CCACACGGCCCACTGCCATGTCACCGCCTCCGTCTTTAACAGAGACCGCGGCACCACCGC

ACCTTCGCGCTTATCCAAGCGCTGACAGCTGTGATACAACCGCTCCAACCACGGCTCCTT

CCCCCCGC

>Unigene000019

ATGCGGAGATCGGAAGAGCGTTGTGCCGCGTGATGCGGAGCCTCGCGCTGCTTCCCCTCG

ACGGCTCCATTTCGCTCGGCTGTTTCTCCGTGTTCACTTTGACCTTTAGTTTTGTGTCTC

TATAAAAGAAGCGGGATGAAGAAACATTAACGTTTTGGTTCTCTGACGGTCATATTTACG

GTCCGGTCTGACAGCCTTTTGGTTTCTTGGCGTCTCTGCTGAAAAACGGCAAAGACTTCA

TGAATGTGCAGAAGGACCAGTGACTTCCCAAACGTTTTCTCTTGTCTGACCGGCAAACGC

TGTGACTGCGTGTCATGGCTGATGTAGACCCGGACACACTGCTGGAATGGCTGCAGATGG

GGCAGGGTGATGAGCGTGACATGCAGCTCATTGCCCTGGAGCAGCTCTGCATGCTACTGC

TCATGTCGGATAATGTGGACCGCTGCTTCGAGACGTGTCCTCCAAGAACCTTTCTTCCAG

CTCTTTGTAAGATCTTCCTGGATGAAAGTGCCCCAGACAATGTTCTGGAAGTAACAGCCA

GGGCCATCACCTATTACCTCGATGTGTCCGCTGAGTGCACACGAAGGATAGTTGGAGTGG

ACGGTGCCATCAAGGCCCTCTGCAACCGACTGGTGGTGGTAGAGCTCAACAACAGGACTA

GCAGAGACCTGGCAGAGCAATGTGTAAAGGTGCTGGAGCTGATCTGCACACGAGAGTCTG

GTGCTGTGTTTGAGGCTGGAGGGCTAAACTGTGTTTTGAGTTTTATCAGGGACAGCGGCC

ACCTGGTGCATAAAGATACACTGCACTCAGCCATGGCAGTGGTATCTCGACTCTGCAGTA

AGATGGAGCCACAAGACTCGTCTCTTGAAACATGCGTGGAGTCCTTGTCCAGTTTGCTCA

AGCATGAAGACCACCAGGTTTCCGATGGAGCCCTGCGCTGCTTTGCATCTCTGGCTGATC

GTTTCACACGCCGTGGGGTTGATCCAGCTCCGCTGGCCAAGCACGGTCTAAGTGAGGAGC

TTCTATCACGCATGGCTGCTGCAGGAGGTTCAGCCGCTGGCCCTTCCTCCACCTGCAAAC

CTGGCAGAACATCCACAGGAGGTGCCCCTTCTGCCCCTGATTCCAAACTTAGCAATCAGG

TGTCCACTATCGTCAGCCTGCTGTCCACCCTCTGCAGGGGCTCACCGCTTGTCACCCACG

ACCTGCTGCGCTCAGAGCTGCCTGACTCAATGGAGCGGGCACTGCAGGGGGATGAGCGCT

GTGTGCTGGATACAATGCGTTTGGTAGACTTGTTGTTGGTCCTGCTGTTCGAAGGCCGGA

AAGCACTGCCCAAATCTACAGCTGGGTCAGGCGGGCGTATCCCAGGCTTACGGCGCCTTG

ACAGCTCCGGCGAACGCTCACATCGGCAGCTCATTGACTGTATTCGTAGCAAAGACACAG

ATGCACTCATTGATGCCATCGATACCGGAGCTTTTGAAGTAAATTTTATGGACGATGTGG

GCCAGACCTTATTGAACTGGGCCTCTGCGTTTGGTACACAGGAAATGGTGGAGTTCTTGT

GTGAGAGAGGTGCAGATGTTAACAGAGGCCAAAGATCTTCATCCCTGCATTATGCTGCTT

GTTTTGGTCGACCTCAAGTAGCCAAGACTTTACTGCGGCATGGAGCTAATCCTGATCTGA

GGGATGAAGATGGCAAAACACCTTTGGACAAGGCAAGGGAGAGGGGTCACAGTGAGGTTG

TAGCAATACTCCAGTCTCCTGGAGACTGGATGTGTCCTGTGAACAAGGGGGATGACAAGA

AAAAGAAGGATATAAACAGGGATGAGGAGGAGGGCAGCGAACCGAAGGGAGACCCTGAGA

TGGCACCCTTATATTTGAAGCGGCTGCTTCCAGTATTTGCACAAACCTTTCAGCACACCA

TGCTGCCTTCAATAAGGAAAGCTAGTTTAGCACTGATCAGGAAGATGGTTCATTACAGTT

CAGAAGTGCTGCTTAAGGAAGTCTGTGACTCTGATGCTGGACACAATCTCCCCACCATCC

TGGTGGAAATCACTGCTACTGTGCTGGATCAGGAGGATGATGATGATGGTCACTTGCTGG

CACTGCAGATCATAAGAGATCTGGTTGATAAGGGAGGTGATGTGTTCTTGGACCAGTTGG

CCAGACTAGGTGTCATTAATAAGGTGTCGACTCTGGCTGGACCTACTTCAGATGATGAGA

ATGAGGAAGAAGCCAAACCAGAAAAGGAGGATGAACTCCAGGAAGACGCCAAAGAGGTTC

AACAGGGAAAGCCCTACCATTGGCGGGACTGGTCCATTATCAGAGGCAGGGACTGTTTGT

ATATATGGAGTGATGCTGCTGCTCTTGAACTTTCCAATGGTAGCAATGGCTGGTTTCGCT

TCATCTTGGATGGCAAATTGGCTACTATGTACTCTAGTGGCAGTCCAGAGGGAGGCTCAG

ACAGTTCAGAAAGTCGAAGTGAGTTTTTGGAGAAACTTCAGCGTGCCAGAAGTCAGGTGA

AGCCGGTCACAGCTAGCCAGCCCATTCTGTCAACAGTGGGACCAACAAAACTCACAGTGG

GCAACTGGTCGCTTACTTGCCTAAAGGAAAGTGAAATAGCAATTCATAACTCAGATGGCC

AGCAGGCCACCATCCTCAAAGAAGATTTACCAGGCTTTGTGTTCGAATCCAATCGTGGCA

CCAAGCACTCTTTTACCGCTGAAACATCTCTTGGATCAGAATTTGTAACTGGCTGGACTG

GAAAACGAGGACGGAAGCTTAAATCTAAATTGGAGAAAACAAAACAGAAGGTGAAGACAA

TGGCTAGAGATTTGTATGATGATCATTTTAAGGCTGTAGAAAGTATGCCTAGGGGAGTAG

TTGTCACCTTACGAAACATTGCCACTCAGCTTGAGTCTGCATGGGAGCTTCATACCAACA

GACAGTGTATCGAAGGAGAGAACACTTGGCGAGACCTCATGAAAACTGCTTTAGAAAATT

TGATAGTGGTTCTCAAGGATGAGAACACGATCTCCCCCTATGAAATGTGCAGCAGTGGCC

TTGTGCAAGCACTTTTTACTGTCCTGAATAATAGTGTAGACCTCGATATGAAGCCTGATT

GTAAGCAATTAGTGGAGAGGATAAATGTGTTTAAAACTGCATTCAGTGAAAATGAAGATG

ATGAAAGCCGACCTGCAGTTGCCTTAATCCGCAAATTAATAGCAGTACTGGAGTCTATAG

AAAGGTTGCCGCTTCACTTGTATGACACACCTGGCTCAGCATACAATTTACAGATTCTCA

CCAGAAGGCTGCGTTTTCGGTTGGAGCGGGCTCCAGGTGAGACAGCCCTAATCGATCGAA

CTGGCAGAATGCTTAAGATGGAACCTCTTGCTACTGTGGAATCTCTGGAGCAGTATCTGC

TTAAAATGGTGGCTAAGCAGTGGTATGACTTTGATAGGTCCTCCTTCATCTTTGTTAAAA

AATTAAGAGAAGGCCAAAGTTTCATCTTTAGACACCAGCATGATTTTGATGAGAACGGCA

TTGTGTACTGGATCGGCACAAATGCAAAGACTGCATATGAGTGGGTAAACCCTGCAGCAT

ATGGGCTGGTGGTGGTAACCTCGTCTGAGGGCAGGAATCTTCCTTACGGGCGTCTAGAAG

ACATTCTAAGCAGAGACAGTTCTGCCCTCAACTGTCACACCAATGATGACAAAAATGCCT

GGTTTGCTGTGGATCTGGGTCTCTGGGTTATTCCCTCAGCTTACACACTACGGCATGCCC

GTGGATATGGTCGCTCTGCTCTCCGCAATTGGGTTTTTCAGGTGTCTAAAGATGGGCAGA

ACTGGATGACTCTTTATACCCATGTAGATGATGGCAGCCTCAATGAGCCAGGGTCAACTG

CCACATGGCCTTTGGACCCATCCAAAGATGAGAAGCACGGCTGGAGACACATTCGTATTA

AACAGATGGGGAAAAATGCAAGTGGACAAACGCACTACCTCTCACTGTCTGGACTGGAAA

TCTATGGCACAGTCACTGGTGTGTGTGAGGATCAGCTTGGTAAAGCTGTGAAGGAGGCAG

AAGCAAACCTGCGCAGACAACGGCGCCTCTTTCGTTCTCAGGTAATGAAGTACATAGTCC

CAGGGGCTCGGGTGGTACGCGGCATTGACTGGAAGTGGAGGGATCAGGATGGCAACCCTT

CAGGAGAGGGTACTGTGACTGGAGAAGCTCATAATGGCTGGATTGATGTCACCTGGGATG

CTGGTGGTTCGAACTCCTACCGCATGGGTGCGGAGGGAAAATTTGACCTGAAGTTAGCCC

CGGGGTATGACCCCGAATCAGCGCCATCACCCAAAACTGTTTCATGCGCTGTTGCAGGCC

CACCGACTCCTTGGAGCAGCCTGGCCAAAAACAACTGTCCGGACAGAGGTGGCCCGTCAT

CTGTATCTTCTGGTCGCAAGGGCAGCAGCAGCTCAGCATGCAGCGATGTCGGCCTTGGCT

CTGCCCGCAGACTTGAGGGGCTGTCTGAACAAGGCACACTTGAGGTGGGTGGCCCCCTGG

GTACAGAGGGACAGGAGCCCCTTGTAGTACTCTCCACAGCCGAGGGTGGTTCTGCATCAA

ACGAAAGTGCTGAGAACAAGAGTGAATCAACAGCCATCTCTGTTGGGCCAGTTAGTGTCA

GTTCACCTGATGTCAGCTCCGTCTCTGATTCATCTGGCAAGGCTGCGGTGTCTCAGAGGC

CCCTTGGTCCAGGCACTGGTGCTCGCCTTTCAGTCAGCTCGCTGCTTGCAGCTGGTGCAC

CTATGAGTTCCAGTGCCAGTGTGCCCAACCTGTCATCTCGTGAGGCCAGTCTTATGGAGT

CGTTTGTGCGCCGTGCTCCCAACATGGCGCGCACCAACGCCACCAACAACATGAACCTGA

GCCGCAGCAGCAGTGACAACAATACCAACACAATTGGCCGCAATGTTGCAGCTGCGTCTG

CTTCTCCTCTTATGGGTGCACAGAGCTTTCCTAACCTTACCACCACTGGTACCACCTCCA

CTGTTACAATGTCCACCTCCATAGTAACCAGTAGCAATAATGTAGCCACAGCCACTACCA

CAGGTTTGTCCGTTGGTCAGTTGCTGAGTAACACGCTGACGACCAGCCTGACCTCTACAT

CTAGTGAGAGCGACACAGGTCAGGAGGCTGAGTTTTCACTCTATGATTTCTTGGATAGCT

GTCGGGCAAACACGTTGCTTGCAGAGCTGGATGATGAGGAAGATTTGCCCGAACCAGATG

ATGACGATGATGAGAACGAGGACGACAATCAGGAGGACCAGGAGTATGAGGAAGTTCTGG

TACGGTCCAGGGTCAACCTTGGTTACCACGTTCATATACATAGGGAAGAGGAGGAATATG

AAATCAAAGGAGGCCGGCGTAGAACCTGGGATGATGATTTTGTCCTGAAACGGCAGTTTT

CTGCTTTAGTCCCTGCATTTGACCCCAGACCAGGTCGAACAAATGTCCAGCAAACCACAG

ATCTGGAAATCCCTATACCAGGTACACCTCGTTCAGAGGTGCTGGAGGAAGTGGAATGTG

CTCCTTCTCCCCATCTGGCTCTTATCTTGAAGGTGGCAGGCCTGGGTACCACACGGGAAG

TTGAACTACCTCTCAACAACTACAAATCTACCATTTTCTATTATGTCCAAAAGCTGCTGC

AGCTCTCCTGTAACGGCAGCATCAAATCCGACAAACTTCGACGCATTTGGGAGCCGACTT

ACACGATAATGTACAGAGAGTTGAAGGACTCCGACAAAGAGAAAGAAAGTGGGAAACTGG

ATTTCTGTGAACGAGGCTGCAGGTCGTCTGGACTTGGGTCAAGCTCTTTGGCAGCCACAC

CGACTTGTGACATCTTGAGTGGCGCACATGAGCAACCACAGGCCAAAGCTGGCTCAGCAC

AGAGCGCCTGTGGTGTTGAGGATGTGTTGCAGCTGCTGCGCATCCTCTACATTATTGGAG

GCGAGCCAGTGGTTGGCCATACACTGCAGGAAGATTTGGATTATCTACAGTTCAATGCAT

CACCTGAGGAGTTCACCAGCAAAAAAATTACAACCAAAATTCTGCAACAAATTGAGGAGC

CTTTGGCCTTGGCCAGTGGAGCGCTTCCAGACTGGTGTGAACAGTTGACCAGCAAATGTC

CGTTCCTAATTCCTTTTGAAACCCGACAGCTCTTTTTTACTTGCACTGCATTTGGAGCGT

CCAGGGCAATAGTGTGGCTCCAAAACCGAAGAGAAGCAACCATGGAACGCACACGTCCAT

CCACTACTGTGAGACGAGATGAACCAGGCGAGTTCCGCGTTGGCCGCCTCAAACACGAGC

GTGTGAAAGTTCCACGGGGAGAGAGCATGATGGAGTGGGCGGAAAGTGTCATGCACATTC

ATGCTGACAGAAAGTCAGTGCTTGAGGTGGAGTTTCAGGGAGAAGAAGGCACTGGCTTGG

GTCCCACGCTAGAGTTCTATGCCCTTGTGGCAGCTGAATTCCAGCGCACCTCACTGGGGA

TTTGGCTCTGTGATGATGATTTTCCAGATGATGAGTCACGCCAAGTGGATCTTGGTGGCG

GCCTTAAACCACCAGGCTATTATGTACAACGCTCGTGTGGCCTTTTCCCTGCCCCCTTCC

CTCAAGATAGTGATGAGCTGGAGCGAATTACCAAGCTGGTCCACTTTTTAGGCATATTCC

TGGCTAAGTGCATACAGGACAACCGGCTGGTAGACCTGCCTGTCTCACAACCTTTCTTCA

AGCTGCTCTGCATGGGTGACATCAAGAGCAACATGAGCAAACTTTTGTATGAGAGACGTT

CAGATTCAGATCGCCGCTTCTCTGACATCCACTCGGAGGCATCCACTGAGGAAGAACCAT

ATTTGATGGGCAGCTTTGATGAAGACTCTAAATCTGAGTTCATCTTGGACCCACCAAAGC

CCAAACCACCTGCCTGGTATCATGGCATCCTAACCTGGGAGGACTTTGAGCTAGTAAATC

CTCACAGAGCTCGCTTCCTCAAGGAGATAAAGGAGCTGGCAGTAAAGAGAAGGCAGATTC

TAGGCAATAAGAGCATTTCGGAGGATGAAAAGAACACACGACTACAGGGCCTCATGCTGA

AGAACCCTGTGGGCTCAGGCCCACCGCTGAGTGTGGAAGATCTTGGATTAAATTTTCAGT

TCTGTCCCTCGTCCAAAGTGCACGGCTTCTCAGCAGTGGACCTTAAACCCAATGGAGAGG

ATGAGATGGTGACTCTGGACAATGCAGAAGAGTATGTGGAGTTATTGTTCGACTTCTGTA

TGCACACTGGGATACAAAAGCAAATGGAGGCTTTTAGGGAGGGCTTTAACAGAGTTTTCC

CCATGGAGAAGCTGAGCTCATTTAGTCATAAAGAGGTCCAGATGATCCTGTGTGGGAACC

AGTCTCCATCCTGGACAGCTGAGGACATTGTCAACTACACCGAGCCCAAACTGGGATACA

CTCGGGACAGTCCTGGTTTCTTGCGCTTTGTGCGAGTGATGTGTGGAATGTGCTCGGATG

AGAGGAAAGCCTTTCTCCAGTTCACAACAGGTTGTTCAACTCTGCCCCCTGGTGGTCTCG

CCAATTTGCACCCCCGACTTACAATTGTTCGCAAGGTGGATGCGACGGATGCCAGCTACC

CCTCAGTGAACACGTGCGTACACTATCTGAAGTTGCCCGAGTATTCCTCTGAAGAGATCA

TGAGAGAGCGCCTCCTTGCTGCTACCATGGAAAAGGGCTTCCACCTGAACTGAGCATGTT

CCCTGCACCGCTGACCAATTCCATGTATTGATGAAGCCTGTCGTGTTTTGTTGCAAGAAA

AAAAAAAAAATACAAAAGCTGTGCTCGCCCATGATTTCTCTCCTTTATCAAAGAGCCTGC

GTTTACATCTGTAAATGAAAGGAAGCCCATGTCAGCGCATCACCTGCTGAAACAGATTAA

GGTCATGTCTGCAGCCCATGTTACCCCCACCTCAATCTCCTGGTGGGACTGCACTGCATA

AAGGGCTTGAGCCATTAGTAGCCAAGGGCAGTTCTCCTCTGTTTACTCACACCCCAGCGC

TGAGTGTGTGTGTGTAAAGTTCAGCATAGGTTTTTCTTTTCATTGTACACACATACCAGC

CCTGTTTCCTCGAAAGGGGGCACATAAGCACCTGTAGAGCTAACTGTCGAGTGTTTGGAC

TTTTTTTTCTGGTTTTAGGTTTACTTGTGCCTGCATTGTTTAAAATATTAGTCCCCTTCA

TTTTGTTTTGCATTCAGCGTCAATAACACACAACATGCTGTTTTCTCTTATTTTATTTCT

CTTCTCTCCTATGTGTTCGTGTACGTTTCAAATGTTCAGCACCCTGCTAAAAGATCAAAT

TTTCTGAGTGTGTCATTTACAATAAAGCTACAAAGACAAAAACACTGGTTGTGTGCAAGG

CACATTCGGGGGGGAGAGGTCTGCTTCTTGTATTTTCTTTGTATATTATAAACATTTATT

TAACTCAAGTTGCAGTTGGAAGTAAACAAATATTTTCAAATGTGTATGCTCCAAAATAAT

CATTAAAATGTTTGCAAAGTATAATTACTGCAGTGTGTTCCATTCGGCTATCTGTGTTTT

AGTCTTAATCTGTGCGTGGATAAATGCAGCAGGCGGTTTCACAGACATGATAAATATGAC

TAACTGTGTAATAGCAGCATTATGTAGCTATAGTGACAGTTTATAAACGTGAAGAGATAA

ACGGGGCTGCTGACTGAATGGATGGAATGTAGGAGTCCTTACACAATACACAGCAACAGC

TTCTCACTGTAACCGTTTCATTCATCCTTCTGCCATCTTGTCTAAAGCCAGCAGTGGTGC

CAGAATGCGGTTCTTGTTGGTTTCCACAATATGTCCATTCTGGATCATCTCATCGAGAAT

GATGTGAACTTTATCCAAATTAAACAATATATCTAGTTCACTCACACGACTGAAGTATTT

ATCGAGAACCTCAATGAAGTTTTGGACGAGCTCATAAATGGAAAGTTCGTTTTCATTGTC

TGTGATGCCGACTAGAATAAAAAGAGCTGCATACTGACGATACACCAGCTTGTAGTCTTT

GTACTCCACAAATGAGCACTCGTCTTTCTTCCTGAAGAGACAGGCCTTGGC

>Unigene000020

GAGCTTCAATACAACCTCGAGTGAGTTGAACCAAAGTCTGGATCTGGGCAAGGAGCTTCT

GATCCAGCAGTGCAGAGACCTCACAGAGCAGCTAGAAGTAAAAGAAAAACAGCTGGAAGT

TCTTCAGGAAGAAGTGCGCCGCTCAGCCGAAGATTTGGAAGAGGCGCGAGAGCGTTGGTC

TAAAGCATCAGAGGAGCTTGAGGAAGCAAAGTGGGAGCTGGAGTCAGAACGAGACAAAAG

GCTTCAATGTGAGGAGCTCCTCAATGAGAAAATCCATGAGCAGGACAACCTAAAGAACAA

ACCCGGCTTCTTGGAGAACCAGATAGAGAAAGATCAGCCAACATCCAACATGGAGTATGA

CGAGAAAAGCTTTTCTCCAGAGAAACTAACTGAAGACAAAGTCGAACTGGTTCTCCAAGA

GCAGCTAGGACAGATGGACTACAGGGAGGACAGAATGGAGAACGTGCAGAGGCTGGAAAA

GGTCCGAGTGGACCTGGAGTCTGAGCTGACGTGTCTAAAGGAGAACCTGAGTAACATGGA

AGAAGCTTTACGTCAAGGTTCTGCAGAGAAAGCGGCTATGGAGAAGCGACTGCAGAAGCT

GGAAGAAAAGAACAAGAGCATGGAAAAGGCTCTGGAAATGGAACTGGAGAACTTTGAGCT

TCAGCTTCAAGCGAAGGATGTGGCCTTTGAGCAGATGAAAGAAGCCAGAGAGAAGGCGGA

AGAGGAGCTCATGGAAAAGGAGACCACTCTGCAGAAAGAGAAGAATGAACATGAAGAGGA

GAGGAGACGACTGGAGGAGAAACATCAACAGGAGATGCTGGACTGGAGTCTGCGTTTGGA

GAAGGAGCTCTCTGATCTGCGGTCTGATCTGGAAGAGGAGCAGAAGAAACAGATCACACT

GATTAAACAGGTATTTATTTCCTGTGTATTTTATTTTTAAAAAATTCCCTCTGGTTGTAC

ATTTTTTTTTTTTTTCCACGTAGGTACACGAACGAGAGCATGAGAGAGAGATGGACGAAC

TGGTGGCCCAACAAAAAGCTGAACTGGAGCGGCTGAAGGCTGAGCTCTCTGAGGAGCTGA

GGGACAGTATGGAGGCTGCACATCAAGCCGAGCTTTCGCAAGTCCAGACGCAGCACACTC

TGGAGCTGGAGGCTCTCCGTCTGAGCCTGACCAACATGCACACGGCGCAGCTGGAGCTCA

CGCAATCCAACCTGCAGAAGGAGAAGGAGGCGGCGCTGAGCGAGCTGCAGGCCAGTCTGA

GGGAGAAGTGGGCTCAGGAGAGCGCCATGCTGCAGACACGACAGCAGTTTGAGCTGGAGA

AGTTCCGAGAACAGGGCAGGGAGCAGGAGAGACAAACCGAGCTCCAGCACCAACTAGCCG

TCGATGATCTAAACTGTGTGTGGGAAAGGCGTCTTTCAGAAGAACGAGCCGCGCTGGAGC

AACAGCTTGCTGGTGGAATAGAGGATGCAAAAATTCGGTGGATACAAGACTTTAAAAAAG

AACAGGCGGAGCTTCAGGAAGCCCTGAACAAAGCCGAGGACCGTTTAGCCGAGACGAAAC

GCAAACTGGAGGAGCTTCAGAGATCCAAAGAGGAGGAAATCAAACATCTGGAGTCGGAAT

TGAGTCAGGCGTGGGCCGACCGAGACTCTGCTGCTCGTGCTGTTGAAGAACTGGTCGCTA

GTCACAGGGTGGTGCTACAGGAGCAGCAGGCTCACACACAAGAGCTGGAGGAAAAGGAGA

AATGGCTGAAGCAAGAGGTGGATCGTCTGCTTGAACAGAAATCCAGCTCCGAGCAGGAGA

TCACAAACCTCTGGTCTCAGCTGGAGAGCATGAGGACCAGCAGGCAGGAACTAGGAGAGT

TGAAGGAGCAGTTGCTAGCACGCAAGTCACATGTTAATGACATCGAGCAGTTGAAGCAGG

ACTTCTCGCAGCAGCGGCGAGAGCTCCAGGAGCAAAACGAGACCGAGCTGGAGAACCTGC

GTGTGTACTTCGAACAGCGGCTCCGGGCCAGCGAAGAGAACCACCGGGAGGAAATCGCTC

TGCTGCAGCTACGCCTCGTAGAGGGAGCTTTGGAGGACTCGGTTCTCAAGACCGGAGAGG

AAAGCTTCCTCTCTGATGGAAAGTCTGAGGAGGAGAAGGGAGACTCGTTGGCAGAGATTA

CAGAACAACTGGATAAACACAAGGTGGAGTTGGATGCACTGCGGCTGCAGCTGGAGGAGA

AACACAAGCAGGACCTGGAGCAGCTGCGCTGTAACATGGCTCTATCCTATAGAGAGGAGT

TACTGCAAGCTCGTACCGAGCTCACCGACCGCTACTACAGCGACATGGAGCAGCTCAAAA

CTAAACACGCCTTGGAGATCGAACAGCTGCGGGCCAAGCTCTCCGACAGCCACCTCAAAG

AAATAACAAGGCTGCGGCTGCAGTCAGTAGCAGATGTAGCGCGTCAGGTGGAGACTGAGC

TGGAAGAGAAGAGCCGGCTCCAAACCCAGGAGCACCAAGCCCGACTCGCTCAGCTCGCCT

CAGACACAGAGAGCATACTGAGCCTGGAGAAGAAGCTCGCCGCGCTCACCCTGCAGCACA

GCCGAGAGCTGGAGAGAGCAACTGAGCAGCATGCGGAGGAGCTCCGACAGCTGGAGGAGA

GGCTGAAGAAAAACTTTTCAGAAGAACTGCACACACGTCTCGCAGAGGCTCAACAGGAAG

AAAGGACCCATGTGACCGAAGAGCTGCTCAACAGGAGCAGTGAGGAACGGCTCCAGCTCC

GGGAACAGCTTCAAGCACAGGCTGAGGAGCGCGTGGCTTCCCTAAGAGAGGAGCTCCAAC

AAGCAGCCAGTAAGGACAGACAAGAGTTGGAGCAGAAGCTTGTTCAGGCTGAGGAGCTCC

TGGCTGAGGAAAAGCAGAGGCTACAAGCTTTACAGGTGAGTCTGGAGAGGGAGGAGAGCC

CTCAGCTGCTGGCCCTGAAGCAGAGGCTGGAATCTCAATACGCTGGCGAGTTGCACACAG

CAAAGAGCACCATGGCAGCTGAGGTAAAGGAGCTAAACACTTTGCTGCAGGAGCAGACGG

AGAGCAGACTGAAGGAGGCCACGTGCAGACATCAAGAGGAACAGCGGCAGCTAGCAGAGA

AACTCGTGCAACAGAAAGAGGAGGCTCTTCAGAAGCTGAGGCAACAACATGCTCAGGAGC

TGCAGGTCCAGGAAACTCTCCTGGAACAGAACGTTTCAGAACTCCAACACCTCCAGAAGG

AGTACCAGGAACTCAAATCCCGACACCAGGAGGAGCTCGACCTGCTCTCTTCCAATCACA

AGACAGCGCTGGACTCCCTCGCTGAGAAACACAAGGCTGAGCTGGATAAGCTGCAGGTGG

TATTTCAGGAGACCAACTTGGCACAGCTCGAAGCTCAGGAGGCGGAGCTTGAAGAGCGTC

ACAAGCAGGAAATGGAAGAACTGGAGACCCGGATGTTGTGCAACATGGACACTCTGGAGA

GCACATACCTGAAGGAGATCCAGGCTGTGCGCGAGGAGAAGGAGGCGGCGCTCCGGGATC

TCGGGGCCTCCCTGGAGCAACAGCAGGCTGAAGAGATTGAAAGACTGAAGCAGGAGGAGC

TGATTGTCAGAGAAGAGCTGAGAAAAAAACTGGCACAGGTGCACATGGACAAGTTTAGTG

TCATGGCGACTGAACTCAGCCACGCCCATCAGGCAGAGCTGTCTGCGGCGCTCGCTTCTC

AGAAAGCGGCTCTGGAGTCAGAGCACCACTCCGCGTTGGAGACCCTGCGACAGCACGTGC

TCGATCTGGAGGCGCAACACGGCGCCGCCCTCCACGAGGTCACCGACATGACTACCAAGG

AGAATCAGCAGCTGAAGAAACAGCTGGATGCACTGAATGCTCAGCACCAGCAACAACTCC

AGGAACTGAGGGCAACATCCTCCAGGGAAATCGAAGCTCTACGCAGGGAGCTGGAAGAAG

AAGCATCAAGACAGCGGCTCCATTTCCTGGAAGAGGCGGAGCTTCTAAAGTGCCAGTCAG

AGGAACAGCTACAGAAAAAGCTAATGGAGTTAAAGGAGGAAAGTGATCGAGAAAAGGTCT

CTGCTCTAGAGGAACTGGAGAGAAGTCTGTGTGAGCAGCACCAGCAGAACGAGCTCTCCT

ACACTGAAAAAATGAACCATCTGACCACACAGCTGCAACAGCTGGACACCGTGGTCTCTC

AGCTGCGTGCAGAGGTGAGCGGTTTGCAAGGGGAGCTGGAGGGTAAGCGCTCGGAGATGG

ACACGCTGGAGACTCTGCTGCAGCGACGCGAGCGCGAGAGTCAGGAAGGAGCCAACCTAC

TCGCCATGCTGCGTGATGACCTCAACACCGCCACCCAGCAGAGACAAGATCTTCAACTGA

CTCATGACCGACTCCAGCGGCTTGTCCTGGAGATGCTTCGGATTACAATTGCGACAGAGG

AGGAGATCAGTCGCAGGTTGGGGATATCCATGGAAGGTGATGACATCACTCTGAACAGGA

AGCAAAGTGTCAACCGTGACCCGCAGAAAACGGACGCTGAGGTGGGCGACGGTGCTGCTG

CAGCTGTAGAAGCCAGTGCTAACTGCAGCATGTTCTCGTCACTGATGGATGAAGGTCTGG

AGCTGTCTCAGCGACTGTGTGAAAGTTTGTTTACAGGACCTGATGGAGAGCTGGACCCTG

AAAGAGAGGAGCTGGTACTGGGGGCCTGCACTCATCTACGTGTCGCAGTGGACAAATTAT

TGGAGGTAGTTTCCGACTCCACAGTGCAGCTGGAGCAGCTGCAAGCTCTGCAGGCCCTGC

TGGATGAAAAGTTCAGTGCTGGTGGAGAAGCGAAGAACGCGCTCTTCCTGCAGCACACTC

AGCTTCTGGAGCAACTAGACCAGGAGGCCAGCGTGAAGAGTCAGCTGCAGCTAGAACTGC

ACAAGGCTGATGGTCTGATCGAGGGTTATGTGGTGGAGAAAGCTGCTTTGGAGGATGCAC

TGCAGCAGAAGGAGTCCCGTGAGCAGAAGCTGGTCAAAGAGCTGGAGAGCACTCGGGCTC

AGCTGCAGGAACTTGGTGAAGAACACGCACTCCTCCAGCGTCAACGGGATGCTCTCTCTG

CAAGCCTCGGAGAACCTGAGAAAGCACTGCTGGCCGAGGCGGAGCGTTTGGGGCAGGAGC

GTGTCGACGTGCAGCGGCAGGCTGAGAAGGACCGCGTCGGACTTGCAGGTCGGCTGCGTT

TGCTGGAACAGACGCTGGAGGAACACGAGAGCCGAACGCAGCAGCAGGAGGAGCAGCACC

GCACCCAAACCGAGGACCTGCAGCAGCACATCAGCGCGCTGGAGAAACAGCTCAAACACC

ACCGCCAGTTTATAGACGAGCAAGCTGTGGAGCGGGAGCACGAGCGAGATGAGTTCCAGC

AGGAGATTAAAAACCTGGAAGCACAGCTGAAAACCACCTCTAAAGGACATGCTGGAGAGG

ACACCAAGGGTCAAAGGGTGGAAAGCCTACAAGCTTTAATCAAAGACAAAACCGAGGACT

ACAGCGTTCTGCTCTCGCTGAAGGAACAGTGCCAGCGTGACCTGGAGGAACGCAACGAAG

AGATCGATAAGATGGCATCTCGCATCCGAGAGCTGGAGCAAGCTCTCCTGAGCAGCGTGG

AGGAGCGACGGGCCGCCGCGCAGCTGGAACAGGAGCTGCAGAAAGCGCGCAAAAACTGCC

AGGAACTTTCTCAGGACAAGGAAGCCCTGCAGCAGCAGCTGTACTCTAATAAACTCCAGA

TCTCAGCTCTGCAGTCCAAACTGGATGAAACGAGGCACCGCTACCCCGACCCAGGTGCTG

ACCCCAACCTCAGAGAGCGGCTCGAGACGCTACAACAGGACCTGCAGAACAAAGAGGAGC

AGGTGGAGGTGTTGCTGGAGCGAGTGGAGGTGTTGGAGAAAGCTCTGGCAGTGAAGGAGG

AGGACGTCAGGCAGCTCTCTCTGCAGCTGGAGCTCAAAACTACCGAAAGCCGAGCGAGAG

AAGAGGAGCTACGGCTGAGCGTCGCCGAGCTGCAGGATACAGCGAGTCTGTTACGCAGAC

AGCGGCAGGAAGAAGCCGATGCCTCGGCGCTACAGCTTCCTTCTGCTCTGCTGGAGGAGA

AGAACCTGGAGATCGACCACCTGAACCAGCAGCTACTCCATCTGCAGCAGGAGCTGGACG

CATCCAAAGAGAACAAGACAGAGGAGAAGCAAGCAGACGTTGAAGATCTACGTGCTCAGG

TGGAGCGACTCCATGGCGATCAGGAACGTCTGCATCAAGCTAAAGAGGAGGAGGTGGAAC

AGCTGCATGAGATCATCCACAAGCTGCAGGAGGAGCTGAGCCAGCTCAATCCCAATCGGC

ACGAGGTCAGCGACCCGAACACGGACAGCCCTGAACCTCCTGATTTCCCCTGGTCGCCCC

GGCCTCAGCACGGTGCCGAGGAGAGCCTGTGTCACGAGCTGAGCAGCCAGACCCTTCAGA

GCTGTCGCACCAAGATCTGTGAGCTCCAGGCGGACTTGGAGCGCAGTGTTGAGGAGAAGG

TGGCTCTCGAGAGACTGCTCCTGATGCAGGAAGAACAGTACGGACAGCAGCTGGAGGCAC

TGGGTTGCAGTCTGGGAGAGGGAAAAAGAAAGCTGGCGCTCCTGGAGCAAGAGGCCGGAG

AGCTGAAGCTCCAGTTAGACCAGAAGGAGACGAAGGTGGAGGAGCTGCAAGCTCGCATTC

AAACGCTGGAGGATGGAGAGAGGAGTGTAAATGATTTGGAACTCCGGCTGATTAAAGCAG

AGGAAACGATGAAGGAGACTCAGCAGGAGATGACCAGGTTGAACGACAAGGCACGAAAGC

ACACAGAAGAGCGAGAGAGGACAGAGAGTCTGATTGCTGAGCTGCAGCAGAGGTATGAAG

ACAGCGAGAGAACTGCGACGCAGGCGAGGCTGCAGGTTGCCGAGTTGAAGGAGACGATTC

AGCAGCGGAAATCCGACATCGCCATCTTGGAGACGGGCAAGACGGAGCTGTACACGGAGA

AACAGGCTCTTCAAAAACGCGAGAGAAAACTTCAAGAGGAAATCGAGAAGCTGAAACAGG

AAGTGTCTGCTAAGAGCCTTCAGATACAGGAAGTGAGTTTGCAGCTGGAGGAGAGAGCAG

CTGACCCGGATCAGAGTCAGGAAGAAGTTCTGACGTGTGCTGAGGAGACCCTGGCTAAAG

CTGCAGCAGCTTTGCGAGAAAAGGAGGAGCAGTTGGCTCATCTGAGGTCGCAGCACGATG

CATTGCGAGCCGAACTCGCTGCGGTGAAGGAAGGCCTGAGCAGCAGTACAGAGAGAGCTG

AGAAACTACAGGAGGAGGGGCAGACTAAGGACCGTGCTCTGGCTGATCTAGAGGAACATA

ACCAGCGTCTTCGGGCCGAGCTGCGTGGCTTGCAGGACGATCTGGAGGTGCAGGAGGAGG

AACTGGCCTACCAGCAAAGGGAGTTGGATGAGCTGCGGCAGCGCTGCCGCCTTCAGGAGC

ACACCGAGCTCGACTGCACCAAACCCGAACGCCGCTTCCTGGATGGCCTGTCGCATAACT

CCACCCTTTCTTCTCCCGAGGTGCTACGGCGGCTCGACTGCAGCGAAGAGCGACCGTCTC

ATGTACACGCGTCTCACCTGTCAGAACTGAGCGGCCTGCGCAATACCAGCCTCGAGCTGA

TGAGCAAACACTCGCTTATGGAGAGAAACGAGTTAAAGCGACCGCTCGTCCTGCCGCCAG

AGACCGACCCGCCCAGCACACACTCGCCATCTGCTTCAACACATTCACTCTCCATCTCCG

AGAACCTCTCTGTGCTCAACTCACTGGACGATGACAAGGTTCAAGTGCTGGAGAATCTGG

ATGATGTGACTCCGTCTCGCTCTCCTCTCTCTTCTACCTCCCCGGTGTCTGCACCAGAGT

GGGCCAGCGATGGATACGGTAGCAACGTGAGCTCCGAGCTGGAGGCTAAACTAAAGAACG

AGCTGGAGCATATGGAGCGGCTTGACGCTCACTTTGTTGAGTACCTACGCAGCAGGGACA

TGGCACCCGCTCCATGCTCGGACAGCGCCGCAGGCAGCCTGCATCACACTTACGAGCTGC

TCTCACCTGAGTTACAGGCCATGCTGAACCGCGTGTACCGCGAAAGCTACAGAGTGCTGT

CTCTGTCTCATCGCCCTCCCCCTGCCGGACAGCCTCATTCAGACGCCGAGGAGGCTCCAC

CCGCATCCTGGCAGAGAGAGAGGCGAGCCTTGCAGGAAACGGTGCTGTCACTCAGGGAGC

TCCTGTGCAGGATGGCTGAGCGTGAACCCAAGGTGGAGGGTGAAGTAGACTGGCGCACAG

AGCTGTTGCAGGCCGTGCGGAGTGTGTTTGACAGTGAGAGGAAGTGGCTTCATGCACAGC

TCCAGGACATCATCACAACCAACACTGGCATAGACTACACGTCTCTCCTGAAGGATTTAG

AGACGCTTCTGCAAAAACAGGAGGATCAGCAGAGGAGGTCTTTGGAGCAGTTGCTAAGTG

CAGACCGAAACAGTCTCCTGGCTGAAATCCGCAGTCTGCATAAACAACTGCACACCTGCA

CGCTGCAGAACCAGGAGCAGCTGCAGCGCTCGCTCGGTGCTGTTCGTGAGGAAGGTGCGC

AGACGCAACAGCAGCTGCACAGACAGGTTGGTGAGCTGGAGAGGCAGCTACAGCAGGAGC

AGTCGTCGTCACATGACCTGCAGCGGTCGCTGGGACAGGAACAGTCCAGATCAACAAATC

TACATCAGCAGCTGGAGATCCAGCAGAAGCTGGCGGTAGAGATGAAGTTGGAGCTGGAGG

AAGGAACCCGGCAGCTTCGTGCGTCTGAGAAAAGCAGAGAGGAGCTGCAGGCTCAGATTC

ACAAACTCCGGCTCAAGCTGGAGAGTGAGGAGCAGGAACATCAAGCTTGTGTGGAGGCTG

CAAAACGAGATGAGGTCAGGGTTAAGCAGCTTCAGGAGGAACTTCATCACGAGAGGCTCA

GCAGCAAACGCGCGGCGGAGGAGCATGCGCACATGCAAGAGGTTCTTGAGTAGTTTAACA

GTAGAACAGTTGCCAACTTGTATTACTTAAAGTTAGAAATAAAATGCTTTAGTAAAAGTA

ACTCCACATCCCACCACCC

>Unigene000021

AGAGTACTGCCTGAAAACATATATGGCATTGGAGAACCTTGTGAAACCACTGATTCTGTG

CTAGTATGTGAAATTCCTTCTGTTCCTCAAGATTTGCAAGTCATTGACATTACAAAGTCC

ACTGTCATCCTTGCCTGGGATAAACCATTTTATGATGGTGGCAGCAAACTTACTGGATAC

CTTATTGAAGCCTGTAAGAAAGATACTGACCGATGGATGGTTGTCGCTAATGTGAGAGCA

TCAGTACTACGACACACCATAGATTCTCTGAATGAGAATGAGCAATACTTATTTAGAATC

AGAGCTGAAAACAACAGGGGTGTGAGTGAGCCCAGAGACCTGATGACACCTATAACTATT

CAAGAACAGAGAGTTATGCCAAAGATTGATCTGTCCAGCATTCCTCAGAAGATTGTAAAT

GTTCATGCTGGCAAACCAATTGTGTTCAACATTCCTATCACTGGAAGACCTGCACCAACT

TGCTCATGGTTCTTTGGTGGCATTAAAATGAAAGACAAGCTGGACCGTATTAAAATTGAG

ACAACTGCTAAGTACACAAAATTAACGGTTCGTGAAACTACTGTTGATGATACCGGTGAC

TATACTCTTGAAGTAAAGAATATAACAGGAGTAGCCTCAGAAATCATCAAGGTCATCATT

CTTGATAAACCTGGCACTCCAGCTGGGCAAATGAGAATCGATGAAATTGATGCCAATTCT

ATAACATGCAGCTGGCAGCAACCAGAAAAAGATGGAGGTGCCAATATCAGTGGCTATGTA

GTTGAGCAACGAGATGCTCATCGTCCTGGTTGGCTGACTATTTCTGAATCTGTTACAAGA

CCCACATTCAAATTTACTAGACTTACTGAGGGAACAGAATATGTGTTCCGTGTGGCTGCG

ACAAATCGATTTGGCATTGGTGCATTCATTCAGTCTGAAGTTGTTGAATGCAAGAGTGCC

AAAACTGCTCCTGGTGCACCAAGCATGCCGGAAATATTTGATGTATCCCATGATGGTATG

ACACTCACCTGGAAACCCCCAGAAAATGATGGTGGCTCCTCATTAGCTGGCTACATCATT

GAGCGCAAAGAAACCAGATCTGACAGATGGGTGCGCATTAATAAGAACCCAGTAACAATG

ACGAGATACAGATCAACTGGACTAATAGAAGGTCTTGAATACGAGTACAGAGTGACAGCT

ATGAACTCTAGAGGCACTGGCAAACCAAGTCTAAGCTCAAAACCAGTGATTGCATTGGAT

CCAATTGAACCTCCAGGCATACCTTCGAATCCAAGAGTCACAGATACAACCAGGACATCT

GTTTCATTGGCATGGTCTCCCCCAGAGGAAGAGGGAGGTGCTATTGTCACTGGTTACCTA

ATAGAGATGCAGAAAGTTGACCAGATTGAATGGACCAAATGTAATACAACTCCAACAAAG

ATTTGCGAATACACACTAACGCATATGCCTCAAGGTGCTGAGTATAGGTTCCGCGTGATG

GCATGTAATGCTGGTGGTCCTGGAGAACCAGCTGAGATTCCAGGAGTAGTTAAAGTCACA

GAAATGCTTGAATATCCTGATTATGAGCTGGACACAAAGTACCAGGAGGGTTATATCGTC

AGACAAGGTGGAGTTGTCAGACTTTCTGTGCCAATTAAGGGAAAACCATATCCCACCTGC

AAGTGGACAAAGGAAGGCCGTGATATTTCTCACAGAGCTATGGTTGCTACTAGTGAAGAA

CGCACAGAACTGGTAATTAAAGAAGCTCACAGGGATGACACTGGAACCTATGACCTTGTG

TTAGAAAACAAATGTGGAAAGAAAGCAGTGTACATCAAGGTTAAAGTCATTGGTCGCCCA

GATCCACCTGAAGGCCCTCTAGAATTTGATGATATTCAAGCTCGATCTGTGAGAGTAAGC

TGGAGACCACCCTCAGATGATGGTGGCTCCGACATTCTTGGTTACATTGTGGAAAGACGC

GAGGTACCAAAGGCAGCATGGTACACTGTAGACTCTCGAGTGGTTGAAACTTCATTAGTG

GTCAAAGGACTGAAAGAAAATGTTGAGTACCACTTTAGAGTTTCAGCAGAAAACCAGTTT

GGCATTAGTAAATCTCTGAAATCAGATGAATCAGTTATACCAAAGACACCACTCTGTCCA

CCCGAGCCTCCCAGTTTCCCTCCAGAAATTATGGATGTTACAAAAACCACTATCGGATTG

TCTTGGTCAAGACCAAAAGATGATGGTGGCTCTCGTGTTACTGGATACTATATTGAAAGA

AGGGAGATGTCAACTGAAAAATGGGTGCGCCATAACAAGACTCACATCACAACCACAATG

TATACTCTTACTGGTCTTATCCCAGATGCAGAATATCAGTTCCGTGTGATTGCCCAAAAC

GACATTGGTCAAAGTGAGCCTGGACCTGTATCTGAAGCAGTTGTGTGCAAGGATCCATTT

GATAAACCAAGCCAACCGGGTGAAATTGACATCATTTCAGTTACCAAGGATTGCATCACC

ATCCATTGGCTACGCCCAGAATCTGATGGTGGCAAAGAAATTCTTGGATATTGGATTGAG

TTTAGACAAGCAGGTGAAAGTGCCTGGAAAAAATGCAACAAGGAACGTTCAAAAGACAGG

CAATTTACCATGGGTGGACTTATGGAGGCAACCGAATATGAATTTAGAATTTTCGCTGAA

AATGAAACTGGATTGAGCAGACCACGTAGAACTGCCATGGGCATCAAAACCAAACTGAGT

GTTGGAGAGGCACCAAGTCTAAAAGAAGAAATAAAAGATACTACTACTAAACTGGGGGAA

TCTGGAACACTAACTTGTCAGATTATGGGAAGACCCCTTCCAGAAATAAAGTGGTATAGA

TATGGAAAGGAACTGACTCAGAGTCGCAAATACAAAATGAGCTCTGATGGCAGAAATCAT

TCTCTTACAGTTTTAACAGATGAACAAGAAGATGAGGGGCTGTACACTTGCCGGGCCATA

AATGAAGCCGGTGAGATTGAAACAAGTGGCAAGCTTCTTTTGCAGGCTGCACCTCAGTTC

CATCCCGGATTTCCTTTGAAGGAGAAATACTATGCAGGCTGTGGAACAAGCCTACGTCTT

CATGTGGTTTATATTGGACGTCCAATTCCACAAATCATGTGGTTTTATGGCAAGAAACCT

CTAAATCCATCAGAGAATGTTGTTATTGAAAACACAGAGAATTACACTCATTTGGTTGTT

AGAAATGTTCAGCGCAAAACAAATGCTGGCAGGTACAAGGTGCAACTGAGCAACAAATTT

GGCACAATTGATACAGTACTTCGTGTTGAAATTCAAGATAAGCCTTTGATTCCTGAAGGA

CCAATTATTGTTGAAGCTCTCTTGAAGAACTCTGTAATCATAAGTTGGAAACTGCCCAAA

GATGATGGTGGTTCAATGATTACAAACTATATTGTAGAAAAACGTGAAGCAAAGGAGGGA

GAACAGTGGCATCTTGTGTCATCATGTGTGCCAGGTACCACATGCCGCATTCCGAATCTA

ATTGAGAGTGCCGGATATTATTTCCGTGTTTCTGCCCAGAATCAATATGGCATTAGTGAG

TCATTAGAGATCCCTGCTGTTGTTATAATCAAGAGTCCATATGAAAAACCTGGAATTCCA

CAACAGCCAGTGGTTACTTCTGTCACAAAAGACTCCTGTGTTGTAACATGGAAACCTCCA

TCCAGTGATGGTGGTGCCAAAATTAAGACTTATTATCTGGAAAAGAGAGAGAAGAAACAG

AACAAATGGATTGCGGTCACAACTGAAGAAATTCATGAGACATACTACACTGTTAAAGGC

CTTATTGAAGGCTTTGAATACGAATTCCGTGTCAAATGCGAGAACATTGGTGGGGAGAGT

GATTGGAGTATGATTTCGGAACCTGTCATTCCGATGTCAGACACAGCTCCTCGCGCTCCC

TTCTTTAAAGAGGAACTGAGAGATATGTGTGTTAAATATAAAGCCAATGCAACCTTTGTT

ACCAAGGTCATTGGGTATCCTAAGCCAACAGTGAAATGGTACAAGAATGGCAAAGAAGTT

TTACCAGATGGTGAGAAAATCAAGATTCAAGAATTCAAAGGAGGCTACTACCAACTTCTT

ATCAGTAGTGCAGATGAGAATGATGTGGCTGTGTACCAGATTAGAGCTACAAATCAGGAA

GGCTCAATCTCAACTACTATAACACTTGATGTTGAAGTTCCTGCCAAGGTTCATTTGCCA

TTACATCTACAAGGAATGGGAGCTGTTCATGCTGTCCGTGGTGAGGTGGTCACCATCAAG

ATTCCAATCAGTGGTAAACCTGAACCTGCAGTCACATGGCAGAAGGGACAAGAATTACTA

AGTAATACAGCATATCATCAAGTGATTGTAACAAGATCCTTCACATCCCTTGTGTTCCAA

AAGGGGGTGCAAAGAAAAGATAGTGGCTACTATATAATTTGTGCCAAAAACAGATTTGGT

GTTGATAAACAGACAGTTGAACTAGATGTTGCGGATGTACCTGATCCACCTAAGGATGTT

AAAGTGAGTGATGTTGGAAGAGATACCATCACCCTCACATGGACTCCTCCAAATGATGGT

GGAAGTGACATCATCAGGTACATAGTAGAGAAGTGCCCATTATCTGGTGATAGGTGGATT

CGTGTTGCCCAGACATCTGAATCACAGTACACTGTAATGAGCCTATTTGGTAAGACCAAA

TATCAGTTCCGTGTTATTGCAGAAAATAAATTTGGTCTAAGTAACCCATCTGTACCAACT

GAGCCTGTAACAGCAAGAGAGGACAAATTAGCTATAAGAAATTATGATGAAGAAGTAGAT

ACACAAAGAGAAGTTACTAAAGAAGAAGCGCCTCATTCCAAAGTTAAGAATTTGCCATCT

CTTTACAAAATCTCTGAGGAACTAGCACGATGTGGGCACTTTGGTATTGTGCATCGTTGT

GTTGAAATTAGCTCCAAAAAGACTTTTATGGCTAAACTCATTAAGGTGAAAGGGGCTGAT

AGAGAGCTAGTTGCAAGAGAGATTGGAACACTCAATATTGCAAGGCACAAAAACCTACTC

TATCTTCATGAGTCTTTTGATAGCTTGGAGGAATACGTTCTGATATATGAATTCATATCA

GGGATAGACATATTTGAACGTCTTGGAACAAATTTTGACCTTACTGAGAGGGAAATTGTT

CTATACCTGAGACAAGTTTGCCAGGCTCTCAAGTTCCTACATGGCCTCAACTACGGTCAT

TTTGATATAAGACCTGAAAACATTGTCTATGCTACAAGAAACAGCAGCACAGTCAAGATA

ATTGAAATGGGCCAAGCCCGTCTGCTGACACCTGGAGAAAATATCAGAATTCAGTTTACA

GCACCTGAGTATTATGCACCTGAGATCCACAATAGTGACTATGTTACTACTGCTACAGAC

ATGTGGTCAGTTGGAGTTCTTGCATATGTGCTCCTTAGTGGCCTTAATCCTTTCGCTGCT

GAATCAACTCATAAAATGATCGAAAATATTTCCAATGCCGAGTACATTTTTGATAGAGAG

GCATTTAAACACACCAGTCTTGAAGGCATGGACTTCATTGACAGGCTTCTTACCAAGGAA

TGCAAACATCGTTTGACTGCATCTGAGGCTCTGGAACATCCTTGGCTCAAAACGAAGATT

GAAAATATTAGCTCCAAAGTCATTAAAACTTTAAGGCATAGGCGATATTATCAATCAATT

ATTAAGAAGGAGTGGACCACTGTTATTTCAGCAGCTCGTGTTGCATATGGAGGTGGTTAC

AGAAACCAGAGAAATGTTTCCATTGGCAGAGTAAAAATTGGACATCCAGAACAAGGCCTA

AGAGCAGGTCCAGTAATGCATGGCTCAGCTGAACCAGGTGGCCATGTACGATTTATGTGC

ACTATTGAAAACTATGACAAGACCACAGAGGTTACATGGTACTTTGGCTCTCGTAAATTG

ATAGCAAGTCATAAGTATGAAATCAGCTATGCCAATGGAGTTGCAAGCATATATGTAAAA

GACATTGAAGAAAGCGATGATGGACTATACAGATGCAAAGTTGTTAGTGAAGACAGGGAA

GAAAATGGCTATGCAGAACTATTTGTTGAGAGTGTAAGGTCCTATCGAGAATACTTCCTG

GGACGTTCACTAAAGAAACCTAAGAGAAGAATTGATAAAACCAAACTTCTGCAAAGACCA

CCTGAGTTTACACTGCCACTGTACAATCGTACAGCATATGTTGGAGAGGATGTACGCTTT

GGAGTAACAATAACTGTTCATCCAGAGCCACAGGTTACTTGGCTAAAGGCAGGTCACAGA

ATTAAACCTAACCCTTCAAAATATACATTTACAACAGACAAGGGTCTTTATCAGCTAATG

ATTCACAACGTTGAGCCTGATGATGATTGTGAATATACCGTGGTGGCAAATAACCGGTTT

GGTGAAGACAGCTGTACGGCACGCCTAACAGTAACACATCACCCTGTTGCTGAGGACACA

ATGAGACCAATTTTCAGACGCCTCTTAGCAAATATTGACTGTGTTGAGGGCGACAGTGTT

CATTTTGAGTTGAGAGTGTCAGGAACACCCCCACCTGCTCTGAAGTGGGAAAAAGATGGC

AAACCTCTTGAATTTAGACCACAAGTTGAAATTGTTCAAGAAGATGTTGGTTATTATGTC

CTGTACATTAGAGAAACACTGATTGAGGATTCAGGCATCTACAGAGTTACTGCAACAAAC

TCTGCTGGTTCAGAAAGTTGCCAAGCCACACTCAAGGTTGAACGTCTAACATACATTAGA

AGAAAGTTCAAGAGTGAGGAAGAGAGGCACGTGTATGTTCAAAATCAAATAGAAAAGACC

CTAAAAATGGCACAAGAATTCTCAACAGCAGAAGTGACTACACTCAATCCTGCAGCACAA

GAGGCACTAAAAGAAGCTGCTGAACTGTACAAACCTGCAGTGAGTACCAAAAATGTTCAG

GGTGAGTTTGATATCTCTGACCATGAGGATAAGGAAATTAAGAGAATTAAAGAAGAGAGT

AGGAAAATAAGAATGCCTTATGAAATTCCAGAACCTCGTGTACATGATCCAACCGTTCTC

GAGGAAGATATGAACATTAAACATTTTGTGCCTTTATCTGACATGAAGTGGTACAAAAAA

CTTCGTGATCAATATGAAATTTCTGAACGCATGGAGAGAGTTGTGCAGAAAAGACAGAAA

CGTATTCGATTATCCAGATGGGAACAGTTCTATGTCATGCCACTTCCTAGAATCACTGAT

CAATATAGGCCAAGATGGCGTATTCCAAAGCTTACGCTAGATGATTTGGAAACTGTAAGA

CCAGCTAGACGCTCACCATCTCCCGAGTCAGACATCTTTATACAGAGAAGGCGTTCCCTG

GGTGATATTTCAGATGAAGATTTTCTCCAACTCAAAGAAGATTATCTGACAAAGAGGAGA

ATTGAAGAAGAGAAGCAACAGCTTGAGGAGGAACTTGAACTTGGCTTCTCTGCCTCTCCT

CCAAGTGGTAGCCCTGTTCACTTCGAACTGTATGCATTACGCCGTGCATCACCTAGGATT

ATGCAAAAAGCTGAAGTGAAGCACGCAGAGGAAACAAAACTTGCAGTGAAACAGATAAAA

TCAACTACTTCTCCACCCCCCATTTCTCATTTCATGAGAAGGCGGCGATCTCTTTCTCCA

ACGTATATTGAATTGATGCGTCCTGTCTCTGAACTGATAAGACCATCCCGGGCACCAGTG

GAATATGTAACTGAGGCAGTAGAAAGGAGGTCCCCCACTCCTGAGAGGACACGACCACGT

TCCCCCAGCCCTGTGTCACCTGAAAGACGATCATCGAGATCTTCCTCTAGGTTTGAACGA

TCTGCAAGGTTCGACATTATGTCTAGATATGAGGCAAGAAAGGCTGCTTTAAAGTCTGAG

CGCAAGTATCAAATTGTAACACAACAACCATTTAGCCTTGATCATGCTCCGCGGATAACA

GTTAGAATGCGTTCACACCGAGTACCATGTGGTCAGGACACAAAATTCACATTGAATGTC

CAATCAAGGCCAGAAGGTGAGATTCAGTGGTTCCACAATGGACAACAGATCCAAGAAAGC

AGCAAATACCACTTTACAAACATGAGTGGTGTCTTAACCCTCCAGATTACTGATTGCCAG

GGGGATGATAGTGGCACATACCGTGCTGTCTGTACCAACTCTAAGGGAGAAGCATCTGAC

TATGCAACTTTAGATGTATCAGGTGGAGCATTTACTACATATTCCTCAAGACGCAAAGAT

GAGGATGCTCCAACAGCATTTGTACCTGATGTGCTAAAGACAGATTATTACCATACATCA

TCAATTAGGGCATCTTCTGCTTCAAGAACACACATAGAGATCAAAGAAACCAAGACAAAG

TTAACTGAGCGACATGAAGCTTCTGGGTTTGAAAAATATGAGTCTGAAAGACTTGCATCA

TCTCCTATCAGATATGCATCTACTGAATACCTGTCATCTGCTTCATATTCAACTTCAGAA

CGACATGCATCATCTGAATATAAGACAAAGTTTGTTGATTCTACATCTACAGCAAGCATA

ACCGCCAAGAAAGTCAAAGCCACTCTTTCAGCAAAAATACTCACCAAACCACACTCTTTA

ACAGTATCATTGGGGGAGTCTGCAAGATTTACTTGTGATATTGATGGTGAACCAGCACCA

AATGTTACGTGGATGCATGGACAACATGTCCTTGTCACTTCTCATCGTGTACGTGTGACA

ACAACACAATACAAATCTACATTTGAAATATCTTTTGTAGAGTACTCTGATGAAGGCAGC

TACACATTAGTGGTAGAAAATTCACAGGGAAGGCAAGAGGCAGAGTTTACATTAAACATC

CATAAGCCACAAACAAAAGAGGAGGCTCTCGCACCATCCCGTCTGGTTAAATCTTCTGAG

CCAGAAGTGAAGTCTCCAGAGCCACCAGTAAAATCTCCAGAGCTATTAGTTAAATCTCAG

GAGGCTGTAGTTAAGTCTCAAGAGGCAGTAATTAAGTCTCTAGAGCCAGCAGTCAAGTCT

CCAGA

>Unigene000022

AGAGTACTGCCTGAAAACATATATGGCATTGGAGAACCTTGTGAAACCACTGATTCTGTG

CTAGTATGTGAAATTCCTTCTGTTCCTCAAGATTTGCAAGTCATTGACATTACAAAGTCC

ACTGTCATCCTTGCCTGGGATAAACCATTTTATGATGGTGGCAGCAAACTTACTGGATAC

CTTATTGAAGCCTGTAAGAAAGATACTGACCGATGGATGGTTGTCGCTAATGTGAGAGCA

TCAGTACTACGACACACCATAGATTCTCTGAATGAGAATGAGCAATACTTATTTAGAATC

AGAGCTGAAAACAACAGGGGTGTGAGTGAGCCCAGAGACCTGATGACACCTATAACTATT

CAAGAACAGAGAGTTATGCCAAAGATTGATCTGTCCAGCATTCCTCAGAAGATTGTAAAT

GTTCATGCTGGCAAACCAATTGTGTTCAACATTCCTATCACTGGAAGACCTGCACCAACT

TGCTCATGGTTCTTTGGTGGCATTAAAATGAAAGACAAGCTGGACCGTATTAAAATTGAG

ACAACTGCTAAGTACACAAAATTAACGGTTCGTGAAACTACTGTTGATGATACCGGTGAC

TATACTCTTGAAGTAAAGAATATAACAGGAGTAGCCTCAGAAATCATCAAGGTCATCATT

CTTGATAAACCTGGCACTCCAGCTGGGCAAATGAGAATCGATGAAATTGATGCCAATTCT

ATAACATGCAGCTGGCAGCAACCAGAAAAAGATGGAGGTGCCAATATCAGTGGCTATGTA

GTTGAGCAACGAGATGCTCATCGTCCTGGTTGGCTGACTATTTCTGAATCTGTTACAAGA

CCCACATTCAAATTTACTAGACTTACTGAGGGAACAGAATATGTGTTCCGTGTGGCTGCG

ACAAATCGATTTGGCATTGGTGCATTCATTCAGTCTGAAGTTGTTGAATGCAAGAGTGCC

AAAACTGCTCCTGGTGCACCAAGCATGCCGGAAATATTTGATGTATCCCATGATGGTATG

ACACTCACCTGGAAACCCCCAGAAAATGATGGTGGCTCCTCATTAGCTGGCTACATCATT

GAGCGCAAAGAAACCAGATCTGACAGATGGGTGCGCATTAATAAGAACCCAGTAACAATG

ACGAGATACAGATCAACTGGACTAATAGAAGGTCTTGAATACGAGTACAGAGTGACAGCT

ATGAACTCTAGAGGCACTGGCAAACCAAGTCTAAGCTCAAAACCAGTGATTGCATTGGAT

CCAATTGAACCTCCAGGCATACCTTCGAATCCAAGAGTCACAGATACAACCAGGACATCT

GTTTCATTGGCATGGTCTCCCCCAGAGGAAGAGGGAGGTGCTATTGTCACTGGTTACCTA

ATAGAGATGCAGAAAGTTGACCAGATTGAATGGACCAAATGTAATACAACTCCAACAAAG

ATTTGCGAATACACACTAACGCATATGCCTCAAGGTGCTGAGTATAGGTTCCGCGTGATG

GCATGTAATGCTGGTGGTCCTGGAGAACCAGCTGAGATTCCAGGAGTAGTTAAAGTCACA

GAAATGCTTGAATATCCTGATTATGAGCTGGACACAAAGTACCAGGAGGGTTATATCGTC

AGACAAGGTGGAGTTGTCAGACTTTCTGTGCCAATTAAGGGAAAACCATATCCCACCTGC

AAGTGGACAAAGGAAGGCCGTGATATTTCTCACAGAGCTATGGTTGCTACTAGTGAAGAA

CGCACAGAACTGGTAATTAAAGAAGCTCACAGGGATGACACTGGAACCTATGACCTTGTG

TTAGAAAACAAATGTGGAAAGAAAGCAGTGTACATCAAGGTTAAAGTCATTGGTCGCCCA

GATCCACCTGAAGGCCCTCTAGAATTTGATGATATTCAAGCTCGATCTGTGAGAGTAAGC

TGGAGACCACCCTCAGATGATGGTGGCTCCGACATTCTTGGTTACATTGTGGAAAGACGC

GAGGTACCAAAGGCAGCATGGTACACTGTAGACTCTCGAGTGGTTGAAACTTCATTAGTG

GTCAAAGGACTGAAAGAAAATGTTGAGTACCACTTTAGAGTTTCAGCAGAAAACCAGTTT

GGCATTAGTAAATCTCTGAAATCAGATGAATCAGTTATACCAAAGACACCACTCTGTCCA

CCCGAGCCTCCCAGTTTCCCTCCAGAAATTATGGATGTTACAAAAACCACTATCGGATTG

TCTTGGTCAAGACCAAAAGATGATGGTGGCTCTCGTGTTACTGGATACTATATTGAAAGA

AGGGAGATGTCAACTGAAAAATGGGTGCGCCATAACAAGACTCACATCACAACCACAATG

TATACTCTTACTGGTCTTATCCCAGATGCAGAATATCAGTTCCGTGTGATTGCCCAAAAC

GACATTGGTCAAAGTGAGCCTGGACCTGTATCTGAAGCAGTTGTGTGCAAGGATCCATTT

GATAAACCAAGCCAACCGGGTGAAATTGACATCATTTCAGTTACCAAGGATTGCATCACC

ATCCATTGGCTACGCCCAGAATCTGATGGTGGCAAAGAAATTCTTGGATATTGGATTGAG

TTTAGACAAGCAGGTGAAAGTGCCTGGAAAAAATGCAACAAGGAACGTTCAAAAGACAGG

CAATTTACCATGGGTGGACTTATGGAGGCAACCGAATATGAATTTAGAATTTTCGCTGAA

AATGAAACTGGATTGAGCAGACCACGTAGAACTGCCATGGGCATCAAAACCAAACTGAGT

GTTGGAGAGGCACCAAGTCTAAAAGAAGAAATAAAAGATACTACTACTAAACTGGGGGAA

TCTGGAACACTAACTTGTCAGATTATGGGAAGACCCCTTCCAGAAATAAAGTGGTATAGA

TATGGAAAGGAACTGACTCAGAGTCGCAAATACAAAATGAGCTCTGATGGCAGAAATCAT

TCTCTTACAGTTTTAACAGATGAACAAGAAGATGAGGGGCTGTACACTTGCCGGGCCATA

AATGAAGCCGGTGAGATTGAAACAAGTGGCAAGCTTCTTTTGCAGGCTGCACCTCAGTTC

CATCCCGGATTTCCTTTGAAGGAGAAATACTATGCAGGCTGTGGAACAAGCCTACGTCTT

CATGTGGTTTATATTGGACGTCCAATTCCACAAATCATGTGGTTTTATGGCAAGAAACCT

CTAAATCCATCAGAGAATGTTGTTATTGAAAACACAGAGAATTACACTCATTTGGTTGTT

AGAAATGTTCAGCGCAAAACAAATGCTGGCAGGTACAAGGTGCAACTGAGCAACAAATTT

GGCACAATTGATACAGTACTTCGTGTTGAAATTCAAGATAAGCCTTTGATTCCTGAAGGA

CCAATTATTGTTGAAGCTCTCTTGAAGAACTCTGTAATCATAAGTTGGAAACTGCCCAAA

GATGATGGTGGTTCAATGATTACAAACTATATTGTAGAAAAACGTGAAGCAAAGGAGGGA

GAACAGTGGCATCTTGTGTCATCATGTGTGCCAGGTACCACATGCCGCATTCCGAATCTA

ATTGAGAGTGCCGGATATTATTTCCGTGTTTCTGCCCAGAATCAATATGGCATTAGTGAG

TCATTAGAGATCCCTGCTGTTGTTATAATCAAGAGTCCATATGAAAAACCTGGAATTCCA

CAACAGCCAGTGGTTACTTCTGTCACAAAAGACTCCTGTGTTGTAACATGGAAACCTCCA

TCCAGTGATGGTGGTGCCAAAATTAAGACTTATTATCTGGAAAAGAGAGAGAAGAAACAG

AACAAATGGATTGCGGTCACAACTGAAGAAATTCATGAGACATACTACACTGTTAAAGGC

CTTATTGAAGGCTTTGAATACGAATTCCGTGTCAAATGCGAGAACATTGGTGGGGAGAGT

GATTGGAGTATGATTTCGGAACCTGTCATTCCGATGTCAGACACAGCTCCTCGCGCTCCC

TTCTTTAAAGAGGAACTGAGAGATATGTGTGTTAAATATAAAGCCAATGCAACCTTTGTT

ACCAAGGTCATTGGGTATCCTAAGCCAACAGTGAAATGGTACAAGAATGGCAAAGAAGTT

TTACCAGATGGTGAGAAAATCAAGATTCAAGAATTCAAAGGAGGCTACTACCAACTTCTT

ATCAGTAGTGCAGATGAGAATGATGTGGCTGTGTACCAGATTAGAGCTACAAATCAGGAA

GGCTCAATCTCAACTACTATAACACTTGATGTTGAAGTTCCTGCCAAGGTTCATTTGCCA

TTACATCTACAAGGAATGGGAGCTGTTCATGCTGTCCGTGGTGAGGTGGTCACCATCAAG

ATTCCAATCAGTGGTAAACCTGAACCTGCAGTCACATGGCAGAAGGGACAAGAATTACTA

AGTAATACAGCATATCATCAAGTGATTGTAACAAGATCCTTCACATCCCTTGTGTTCCAA

AAGGGGGTGCAAAGAAAAGATAGTGGCTACTATATAATTTGTGCCAAAAACAGATTTGGT

GTTGATAAACAGACAGTTGAACTAGATGTTGCGGATGTACCTGATCCACCTAAGGATGTT

AAAGTGAGTGATGTTGGAAGAGATACCATCACCCTCACATGGACTCCTCCAAATGATGGT

GGAAGTGACATCATCAGGTACATAGTAGAGAAGTGCCCATTATCTGGTGATAGGTGGATT

CGTGTTGCCCAGACATCTGAATCACAGTACACTGTAATGAGCCTATTTGGTAAGACCAAA

TATCAGTTCCGTGTTATTGCAGAAAATAAATTTGGTCTAAGTAACCCATCTGTACCAACT

GAGCCTGTAACAGCAAGAGAGGACAAATTAGCTATAAGAAATTATGATGAAGAAGTAGAT

ACACAAAGAGAAGTTACTAAAGAAGAAGCGCCTCATTCCAAAGTTAAGAATTTGCCATCT

CTTTACAAAATCTCTGAGGAACTAGCACGATGTGGGCACTTTGGTATTGTGCATCGTTGT

GTTGAAATTAGCTCCAAAAAGACTTTTATGGCTAAACTCATTAAGGTGAAAGGGGCTGAT

AGAGAGCTAGTTGCAAGAGAGATTGGAACACTCAATATTGCAAGGCACAAAAACCTACTC

TATCTTCATGAGTCTTTTGATAGCTTGGAGGAATACGTTCTGATATATGAATTCATATCA

GGGATAGACATATTTGAACGTCTTGGAACAAATTTTGACCTTACTGAGAGGGAAATTGTT

CTATACCTGAGACAAGTTTGCCAGGCTCTCAAGTTCCTACATGGCCTCAACTACGGTCAT

TTTGATATAAGACCTGAAAACATTGTCTATGCTACAAGAAACAGCAGCACAGTCAAGATA

ATTGAAATGGGCCAAGCCCGTCTGCTGACACCTGGAGAAAATATCAGAATTCAGTTTACA

GCACCTGAGTATTATGCACCTGAGATCCACAATAGTGACTATGTTACTACTGCTACAGAC

ATGTGGTCAGTTGGAGTTCTTGCATATGTGCTCCTTAGTGGCCTTAATCCTTTCGCTGCT

GAATCAACTCATAAAATGATCGAAAATATTTCCAATGCCGAGTACATTTTTGATAGAGAG

GCATTTAAACACACCAGTCTTGAAGGCATGGACTTCATTGACAGGCTTCTTACCAAGGAA

TGCAAACATCGTTTGACTGCATCTGAGGCTCTGGAACATCCTTGGCTCAAAACGAAGATT

GAAAATATTAGCTCCAAAGTCATTAAAACTTTAAGGCATAGGCGATATTATCAATCAATT

ATTAAGAAGGAGTGGACCACTGTTATTTCAGCAGCTCGTGTTGCATATGGAGGTGGTTAC

AGAAACCAGAGAAATGTTTCCATTGGCAGAGTAAAAATTGGACATCCAGAACAAGGCCTA

AGAGCAGGTCCAGTAATGCATGGCTCAGCTGAACCAGGTGGCCATGTACGATTTATGTGC

ACTATTGAAAACTATGACAAGACCACAGAGGTTACATGGTACTTTGGCTCTCGTAAATTG

ATAGCAAGTCATAAGTATGAAATCAGCTATGCCAATGGAGTTGCAAGCATATATGTAAAA

GACATTGAAGAAAGCGATGATGGACTATACAGATGCAAAGTTGTTAGTGAAGACAGGGAA

GAAAATGGCTATGCAGAACTATTTGTTGAGAGTGTAAGGTCCTATCGAGAATACTTCCTG

GGACGTTCACTAAAGAAACCTAAGAGAAGAATTGATAAAACCAAACTTCTGCAAAGACCA

CCTGAGTTTACACTGCCACTGTACAATCGTACAGCATATGTTGGAGAGGATGTACGCTTT

GGAGTAACAATAACTGTTCATCCAGAGCCACAGGTTACTTGGCTAAAGGCAGGTCACAGA

ATTAAACCTAACCCTTCAAAATATACATTTACAACAGACAAGGGTCTTTATCAGCTAATG

ATTCACAACGTTGAGCCTGATGATGATTGTGAATATACCGTGGTGGCAAATAACCGGTTT

GGTGAAGACAGCTGTACGGCACGCCTAACAGTAACACATCACCCTGTTGCTGAGGACACA

ATGAGACCAATTTTCAGACGCCTCTTAGCAAATATTGACTGTGTTGAGGGCGACAGTGTT

CATTTTGAGTTGAGAGTGTCAGGAACACCCCCACCTGCTCTGAAGTGGGAAAAAGATGGC

AAACCTCTTGAATTTAGACCACAAGTTGAAATTGTTCAAGAAGATGTTGGTTATTATGTC

CTGTACATTAGAGAAACACTGATTGAGGATTCAGGCATCTACAGAGTTACTGCAACAAAC

TCTGCTGGTTCAGAAAGTTGCCAAGCCACACTCAAGGTTGAACGTCTAACATACATTAGA

AGAAAGTTCAAGAGTGAGGAAGAGAGGCACGTGTATGTTCAAAATCAAATAGAAAAGACC

CTAAAAATGGCACAAGAATTCTCAACAGCAGAAGTGACTACACTCAATCCTGCAGCACAA

GAGGCACTAAAAGAAGCTGCTGAACTGTACAAACCTGCAGTGAGTACCAAAAATGTTCAG

GGTGAGTTTGATATCTCTGACCATGAGGATAAGGAAATTAAGAGAATTAAAGAAGAGAGT

AGGAAAATAAGAATGCCTTATGAAATTCCAGAACCTCGTGTACATGATCCAACCGTTCTC

GAGGAAGATATGAACATTAAACATTTTGTGCCTTTATCTGACATGAAGTGGTACAAAAAA

CTTCGTGATCAATATGAAATTTCTGAACGCATGGAGAGAGTTGTGCAGAAAAGACAGAAA

CGTATTCGATTATCCAGATGGGAACAGTTCTATGTCATGCCACTTCCTAGAATCACTGAT

CAATATAGGCCAAGATGGCGTATTCCAAAGCTTACGCTAGATGATTTGGAAACTGTAAGA

CCAGCTAGACGCTCACCATCTCCCGAGTCAGACATCTTTATACAGAGAAGGCGTTCCCTG

GGTGATATTTCAGATGAAGATTTTCTCCAACTCAAAGAAGATTATCTGACAAAGAGGAGA

ATTGAAGAAGAGAAGCAACAGCTTGAGGAGGAACTTGAACTTGGCTTCTCTGCCTCTCCT

CCAAGTGGTAGCCCTGTTCACTTCGAACTGTATGCATTACGCCGTGCATCACCTAGGATT

ATGCAAAAAGCTGAAGTGAAGCACGCAGAGGAAACAAAACTTGCAGTGAAACAGATAAAA

TCAACTACTTCTCCACCCCCCATTTCTCATTTCATGAGAAGGCGGCGATCTCTTTCTCCA

ACGTATATTGAATTGATGCGTCCTGTCTCTGAACTGATAAGACCATCCCGGGCACCAGTG

GAATATGTAACTGAGGCAGTAGAAAGGAGGTCCCCCACTCCTGAGAGGACACGACCACGT

TCCCCCAGCCCTGTGTCACCTGAAAGACGATCATCGAGATCTTCCTCTAGGTTTGAACGA

TCTGCAAGGTTCGACATTATGTCTAGATATGAGGCAAGAAAGGCTGCTTTAAAGTCTGAG

CGCAAGTATCAAATTGTAACACAACAACCATTTAGCCTTGATCATGCTCCGCGGATAACA

GTTAGAATGCGTTCACACCGAGTACCATGTGGTCAGGACACAAAATTCACATTGAATGTC

CAATCAAGGCCAGAAGGTGAGATTCAGTGGTTCCACAATGGACAACAGATCCAAGAAAGC

AGCAAATACCACTTTACAAACATGAGTGGTGTCTTAACCCTCCAGATTACTGATTGCCAG

GGGGATGATAGTGGCACATACCGTGCTGTCTGTACCAACTCTAAGGGAGAAGCATCTGAC

TATGCAACTTTAGATGTATCAGGTGGAGCATTTACTACATATTCCTCAAGACGCAAAGAT

GAGGATGCTCCAACAGCATTTGTACCTGATGTGCTAAAGACAGATTATTACCATACATCA

TCAATTAGGGCATCTTCTGCTTCAAGAACACACATAGAGATCAAAGAAACCAAGACAAAG

TTAACTGAGCGACATGAAGCTTCTGGGTTTGAAAAATATGAGTCTGAAAGACTTGCATCA

TCTCCTATCAGATATGCATCTACTGAATACCTGTCATCTGCTTCATATTCAACTTCAGAA

CGACATGCATCATCTAAATATAAGACAAAGTTTGTTGTTTCTACATCTACAGCAAGCATA

ACCGCCAAGAAAGTCAAAGCCACTCTTTCAGCAAAAATACTCACCAAACCACACTCTTTA

ACAGTATCATTGGGGGAGTCTGCAAGATTTACTTGTGATATTGATGGTGAACCAGCACCA

AATGTTACGTGGATGCATGGACAACATGTCCTTGTCACTTCTCATCGTGTACGTGTGACA

ACAACACAATACAACTCTGATGAAGGCAGCTACACATTAGTGGTAGAAAATTCACAGGGA

AGGCAAGAGGCAGAGTTTACATTAAACATCCATAAGCCACAAACAAAAGAGGAGGCTCTC

GCACCATCCCGTCTGGTTAAATCTTCTGAGCCAGAAGTGAAGTCTCCAGAGCCACCAGTA

AAATCTCCAGAGCTATTAGTTAAATCTCAGGAGGCTGTAGTTAAGTCTCAAGAGGCAGTA

ATTAAGTCTCTAGAGCCAGCAGTCAAGTCTCCAGA

>Unigene000023

CCTCGATCCTACAAACTCCAAGTTAACAGCTAGGCGCTCAAACCAGCGAGCATTCATCTA

CTTTCCCCGCCCCCCTTCAAAAAGGCGGGGAAAGCCCCGGCGGGCCATTAACCTGCTTCT

TCGGGTTTGCAATCCGAAATGTTATACACCACGGGACTTGATAGGAAAAGGACTTAAACC

TTTGTTCATGGAGCTACAATCCACCGCCTAAACTCTCGGCCACCCTACCTGTGACAATCA

CACGCTGATTATTCTCAACCAACCATAAAGACATTGGCACCCTTTATTTAGTATTTGGTG

CTTGAGCCGGAATAGTTGGCACAGCTCTTAGCCTCCTAATTCGGGCAGAGCTAGCCCAAC

CTGGCGCTCTTCTAGGCGATGACCAAATTTATAATGTCATTGTTACTGCTCACGCCTTTG

TTATAATTTTCTTTATAGTGATGCCAATTATGATCGGTGGGTTCGGCAATTGACTTGTAC

CCTTAATGATCGGGGCCCCCGACATAGCATTCCCTCGAATAAATAACATAAGCTTCTGAC

TGCTACCCCCATCCCTTCTATTACTACTTACCTCTTCTGGTGTAGAGGCGGGGGCAGGAA

CAGGATGAACCGTATACCCCCCACTTGCAGGAAACCTTGCACATGCGGGAGCATCCGTGG

ATCTAACTATTTTTTCACTGCACCTCGCAGGAATCTCATCAATTCTAGGGGCCATCAATT

TTATCACAACTATTATTAACATAAAACCCCCAGCAATCTCCCAATACCAAACACCTTTAT

TTGTATGAGCTGTCCTCATTACAGCAGTACTTCTCCTGCTATCCCTACCAGTACTTGCCG

CAGGCATCACAATACTATTAACAGACCGGAACCTAAATACCACTTTCTTTGACCCAGCAG

GGGGAGGTGATCCAATTCTATATCAACATCTTTTCTGATTCTTTGGCCACCCAGAAGTAT

ATATTTTAATCCTCCCAGGATTTGGCATAATTTCCCACATCGTAGCTTACTATGCCGGTA

AGAAAGAACCATTTGGATACATAGGAATAGTGTGAGCTATAATGGCCATCGGCCTACTGG

GATTTATTGTCTGAGCTCACCACATGTTTACTGTAGGAATAGATGTAGACACCCGAGCAT

ACTTCACATCTGCAACAATAATTATTGCAATCCCCACTGGAGTAAAAGTATTTAGTTGAC

TCGCAACACTCCATGGAGGATCCATTAAATGAGAAACACCCATATTATGGGCCCTGGGGT

TTATTTTCCTTTTCACAGTTGGAGGACTAACTGGCATCGTACTAGCTAATTCATCCCTAG

ACATTGTACTACACGATACCTATTATGTGGTGGCCCACTTCCACTATGTCCTGTCAATAG

GAGCAGTATTTGCCATTATGGGGGCCTTCGTCCACTGATTCCCGCTATTTACAGGGTATT

CAATGCATGATACATGAACAAAAATTCACTTCGGCACAATATTCGTAGGAGTTAATCTTA

CCTTCTTCCCACAGCACTTTTTAGGATTAGCAGGCATGCCCCGACGATACTCGGACTACC

CAGATGCCTATGCACTATGAAACATTATTTCATCAATTGGCTCCCTAATCTCTTTAGTGG

CAGTAATTATATTCCTCTATATCTTATGGGAAGCTTTCACCGCCAAACGAGAAGTACTTT

CCGTAGAACTTGCCCACACAAATGTTGAATGACTACACGGATGCCCACCCCCCTATCATA

CATTTGAAGAGCCGGCCTTCGTGCAAGTTCAATCAAATTAACGAGAAAGGAGGGAATTGA

ACCCCCATAAACTAGTTTCAAGCCAGCTACATAACCACTCTGTCACTTTCTTCAATAAGA

TACTAGTAAAATGAGTATTACACTGCCTTGTCAAGGCAAAATTGTAGGTTTAACTCCTGC

GCATCTTAAGCCCTCTGGGCCTAATGGCACACCCATCTCAACTAGGATTTCAAGACGCAG

CCTCACCCGTAATAGAAGAACTTCTGCACTTCCACGACCATGCCTTAATAATCGTTTTCT

TAATTAGCACCTTAGTACTATATATTATTGTTGTAATAGTCACCACTAAACTCACTAATA

AATATATTCTAGACTCACAAGAAATTGAAATCGTCTGAACTATTCTACCAGCAGTAATCC

TTGTTTTAATTGCTCTTCCCTCCCTACGAATTCTCTATCTAATAGATGAAGTCAACGACC

CCCACCTAACTGTAAAAGCCATAGGCCATCAATGATACTGAAGCTATGAATACACTGACT

ACGAAAACCTGGCCTTCGACTCCTACATGGTCCCAACACAAGACCTACTACCAGGCCAGT

TTCGACTACTAGAAACAGACCACCGAATAGTAATTCCGATAGAATCACCTGTTCGAGTGT

TAGTTTCAGCTGAAGACGTACTTCACTCCTGAGCTGTACCAGCATTAGGAGTTAAAATAG

ACGCGGTCCCAGGACGATTAAATCAAACATCCTTCATCGCCTCCCGGCCCGGAGTATTTT

ACGGACAATGCTCCGAAATCTGCGGAGCAAACCACAGCTTCATACCTATCGTTGTAGAAT

CCGTCCCCTTAGAACACTTTGAAAACTGGTCTTCCCTAATACTTGAAGACGCCTCACTAG

AAAGCTAAATATGGGCCTAGCGTTAGCCTTTTAAGCTAAAGATTGGTGACCCCCGACCAC

CTCTAGTGACATGCCACAATTAAACCCAGCCCCATGATTTGCAATCTTACTATTCTCGTG

ATTAATCTTCTTAACACTAATCCCGAACAAAGTACTAAACCACACATTTACAAATGAAAT

TACAGCCCTAAGCGCTAAAAACCTTAAATCAAAAACCTGAAACTGACCATGGCACTAAAC

CTATTTGACCAATTTATAAGCCCCACACACTTAGGAGTTCCCTTAATTGCCATTGCACTT

ACCCTGCCCTGAATTCTAATCCCCACCCCAACTAATCGGTATCAAAACAACCGCCTTATT

TCCCTCCAAAGTTGATTCATTAAGTCCTTTACACAACAATTACTGCTGCCACTAAATCAA

AGCGGACATAAGTGAGCCATAATCCTGGCCTCCCTAATAATCTTTATCCTCACACTTAAT

ATACTAGGACTTCTCCCTTATACATTTACCCCGACTACCCAGCTATCTCTAAATATAGGC

CTAGCCGTCCCACTATGATTAGCAACCGTAATCATTGGTTTACGAAACCAACCTACAGCA

GCCCTAGGACATCTACTACCAGAAGGCACCCCGGCCCCTCTAATCCCAGTCTTAATTGTT

ATCGAAACAATTAGCCTATTTATTCGACCTTTAGCCCTCGGAGTACGACTAACTGCTAAC

TTAACAGCCGGCCACCTATTAATTCAGCTAATCTCAACCGCAACCATTACACTGCTACCA

ATAATAACTACAGTTGCAACCCTCACTGCTATCCTACTAGTAATACTTACTCTACTAGAA

ATTGCAGTAGCTATTATTCAAGCTTATGTATTTGTTCTACTACTAAGCTTATATTTACAA

GAAAACGTATAATGGCCCACCAAGCACATGCATATCATATGGTAGATCCAAGCCCATGGC

CCCTGACCGGCGCAGCAGCTGCTCTTCTAATAACTTCAGGACTAGCAATCTGATTTCATT

TCCACTCAGTAACACTTATATCTCTAGGTCTAATATTATTAATTTTAACAATAGCTCAAT

GATGACGAGACATTGTACGAGAAGGCACATTTCAAGGCCACCATACTCCTCAAGTACAAA

AAGGGTTACGATACGGAATAATTCTTTTCATTACCTCAGAAGTATTTTTCTTCCTGGGGT

TCTTCTGAGCCTTTTACCACTCCAGCCTTGCCCCCACCCCTGAATTAGGAGGATGCTGAC

CCCCCACAGGAATCACAACTCTAGATCCATTCGAAGTGCCACTCCTTAACACCGCAGTAC

TACTAGCCTCAGGTGTAACAGTAACTTGAGCGCACCATAGCCTAATGGAAGGAGAACGAA

AACAAGCAATTCAATCCCTGGCCCTAACAATTCTACTAGGCTTCTATTTTACTGCCCTCC

AGGCCATAGAATACTATGAGGCCCCCTTCACTATTGCAGACGGAGTGTACGGCTCAACAT

TCTTTGTAGCAACAGGCTTTCACGGCCTACACGTTATCATTGGCTCTACCTTCCTGGCCG

TCTGCCTACTCCGACAAATCAAACATCATTTTACCTCAGAACACCACTTCGGATTCGAAG

CAGCTGCCTGATATTGACACTTTGTAGATGTTGTATGACTATTCCTTTATGTCTCTATTT

ACTGATGAGGCTCATATCTTTCTAGTATTTAATGTTAGTACGAGTGACTTCCAATTACCT

GGTCTTGGTTAAAATCCAAGGAAAGATAATGAACTTAGTCTTAATAATAATCATCTCAGC

CATCCTATCAATTGCCCTGGCCCTCGTATCATTTTGACTCCCACAAATAAGCCCCGACAC

AGAAAAACTCTCCCCTTACGAGTGTGGTTTTGATCCTCTAGGATCAGCACGTCTCCCCTT

CTCTCTCCGATTTTTTTTAGTTGCCATCCTATTTCTATTATTTGATCTAGAAATCGCGCT

ACTACTACCACTACCATGAGGTAATCAACTACCAGACCCAGCCCACACTCTAATATGAGC

TGCAGCTGTCCTTGTACTACTCACGCTAGGCCTCATTTATGAGTGACTACAAGGAGGCCT

AGAATGAGCTGAATAGGGGGCTAGTCCAAATACAAGACCTCTGATTTCGGCTCAGACAAT

CCCGGTTTAAATCCGTGGCCCCCTTATGACGCCAGTATACCTTAGCTTCACCTCAGCATT

TACACTAGGCCTGACAGGACTGGCCCTCCACCGCTCCCATCTGATGTCTGCCTTACTATG

TTTAGAAGGTATAATACTCTCCCTATTTATTGCCCTTGCCATTTGAACCCTCCAACTAGA

ATCAACCACCTTTTCCGCAGCCCCCATCTTCCTGCTAGCCTTCTCAGCCTGCGAAGCCAG

TGCAGGATTAGCTTTACTAGTTGCTACAGCCCGGACCCACGGAACAGATCACCTACAAGC

CTTAAACCTACTACAATGCTAAAAATCCTAATTCCAACCATTATGCTATTCCCAACAATC

TGGCTTTCTACCCCTAAGTGGCTATGAACGTCTACAACCACACAAAGCTTAATTATTGCA

TTACTAAGCCTCACTTGAATTAAACTGCCCCTAGAAATCAGCTGATCAACCTCCAACCCA

TACATGGCTACAGATCCCTTATCAACCCCCTTATTAATCTTAACCTGCTGACTCCTCCCA

CTCATAATTTTAGCCAGCCAAAATCACATTAAAACAGAGCCCATTAACCGCCAACGAAGT

TACATTACTCTAATAGCCTTACTACAAACTTTTCTAATCCTAGCGTTTAGTGCTACCGAA

ATTATTATATTCTACGTAATATTTGAAGCAACTCTAATCCCAACCCTTATTATCATTACC

CGATGAGGAAACCAAGCTGAACGGCTAAGCGCCGGAACATATTTCCTATTTTATACACTA

GCCGGATCGCTACCACTCTTAGTAGCGCTCCTATTACTTCACCGTGACACCGGGACCCTA

TCAATATTTATTATTCAATATACACAGCCGCTAACCCTTAATTCTTGAGGAGATAAAATC

TGATGGGCGGCCTGCCTAATCGCTTTCTTAGTAAAAATGCCCTTATATGGTGTACATCTC

TGACTGCCAAAAGCCCATGTAGAAGCCCCTGTAGCTGGGTCTATAGTACTAGCAGCAGTC

CTACTAAAGCTAGGAGGCTATGGTATAATACGAATAATAGTAATCCTCGACCCCCTATCA

AAAGATCTAGTATATCCTATTCTTGCTCTGGCCCTATGAGGGGTAATCATAACCGGTTCT

ATTTGCCTACGACAAACAGACCTAAAATCACTAATCGCCTACTCATCCGTCAGCCACATA

GGCCTGGTAGCAGGGGGAATCCTAATCCAGACCCCATGAGGCTTTACTGGAGCATTAGTA

CTCATAATTGCCCACGGCCTAGTCTCCTCCGCCCTATTCTGCCTAGCCAACACCACATAT

GAACGAACCCACAGCCGAACCATACTCCTAGCTCGAGGAATACAAATTATCTTTCCACTA

ACCGCTGTTTGATGATTTATCTCTAACCTAGCAAACTTGGCATTACCCCCTATACCTAAC

CTAATAGGAGAATTAATAATCATCACAGCCATATTTAACTGATCCCCATGAACCCTTGTA

ATAACAGGAACTGGGACCCTAATCACAGCTGCCTATTCACTATACCTATTTCTAATAACA

CAACGAGGCCCGCTTCCACAACATATTATTAACCTACAACCCTTCCACACACGAGAACAT

CTATTAATAGCACTGCACCTCATTCCAGTTATCCTCTTAATCATCAAACCAGAGATTATA

TGGGGATGATGATATTGTAGATATAGTTTAACATAAAATATTAGATTGTGGTTCTAAAGA

TAGAGGTTAAACTCCCCTTATCCACCGAAAGAGGCCCAGAGGCAGTAAGGTCTGCTAACC

CTTTACCTCCGCAGTTAAATTCCGCGGCTCATTCGCCCCGCTTCTAAAGGATAATAGTCC

ATCCATTGGTCTTAGGAACCAAAAACTCTTGGTGCAACTCCAAGTAGAAGCTATGCTAAG

TGCCATCATACCTTCCTCCTTACTCCTTACCTTAACAATCCTTACACTACCCTTAATTAC

AACTCTCTCCCCAAAACCTCTTAACCAAAACTGAGCTAACAATGTAAAAAATGCAGTAAG

TACTGCATTCTTCATCAGTACTATTCCCTTAGCAATTTTTATAGATCAAGGAACAGAGAC

CGTAATCACAACATACCAATGAATTAATATCATAAACTTTGACATCAATATTAGCCTTAA

ATTTGATCACTACTCATTAATCTTTACTCCAATTGCCCTTTATGTTACATGATCCATTCT

AGAATTTGCACTATGATATATACATTCGGACCCTAACTTAAACCGATTTCTCAAATACTT

ACTTATATTTCTAGTAGCCATAATTACTCTTGTCACCGCTAATAATCTATTTCAACTATT

CATTGGGTGAGAGGGAGTAGGAATCATATCCTTCCTACTAATTGGATGATGACATGGCCG

GGCTGACGCCAATACAGCAGCCCTACAGGCAGTACTATACAATCGAGTAGGAGACATCGG

ATTAATTCTAGCTATTGCCTGAATCGCAATAAATCTCAATTCATGAGAACTACCCCAAAT

TTTTTTACTAGCCAAAGACTTCGACCTAACATTGCCAATACTAGGAATCATTCTAGCAGC

CACCGGAAAATCCGCCCAATTTGGCCTACATCCATGACTACCATCAGCAATAGAAGGCCC

AACCCCAGTCTCCGCTCTATTGCACTCAAGCACAATAGTGGTCGCAGGAATTTTCCTATT

AATTCGACTACACCCTTTAATAGAAAATAACCAGTTAATCTTAACTACCTGCCTATGTTT

GGGGGCCCTAACAACTTTTTTTACTGCCACATGTGCACTCACACAAAATGATATCAAAAA

AATCGTTGCATTTTCAACATCAAGCCAACTAGGCTTAATAATAGTCACTATTGGCCTTAA

CCAGCCCCAACTAGCCTTCCTCCACATCTGCACTCATGCCTTCTTTAAGGCCATACTATT

CCTTTGTTCGGGGTCAATTATTCACAGCCTAAACAACGAACAAGACATTCGAAAAATAGG

AGGACTATACAAATTAATACCCCTCACCTCCTCCTGTCTTACCATTGGAAGCCTTGCACT

CACAGGCATGCCCTTCCTAGCAGGCTTCTTCTCGAAAGACGCAATCATCGAAGCCCTCAA

CACCTCATACTTAAACACCTGAGCCTTAACCCTTACACTAATTGCCACATCATTCACTGC

CGTATACAGCCTGCGAATCATTTTCTTTGTAGTCATGGGCACTCCACGATTCTCAACCTT

CTCCCCTATTAATGAAAACCATAAAACAGTAATTATACCAATCGAACGCTTAGCCTGAGG

CAGCATTATTGCGGGTCTTATTATTACCTCAAACTTCCTTCCACTAAAAATTCCAACTAT

AACTATACCCCCAACCCTAAAACTGGCTGCACTAATTATTACAATTATAGGAACTATTAC

TGCGCTAGCACTGGCCACTGCCTCCCACAAACAAATTAAAATAAATCCTACCCTTACATT

ACATAACTTCTCCAACATACTGGGATTTTTCCCCCACATCACTCATCGACTAGCGCCCAA

AACTAACCTAATGCTTGGAAAAATAGCCACCAAAATCGACCAACAATGACTAGAAATCGT

ACCTAAAAACCTACATTCACTACACCAATACCTAACCTCACTATTTGACAGCAATACAAG

CACTATTAAAATATACTTAACAATCTACCTTATCTCCACAGCCATAGCTCTAATCTTCCT

GGCCACCATCTAAACAGCCCGAAGCGCCCCACGACTTAGCCCTCGAGTAAGCTCCAACAC

CACAAAAAGACATAATAACAGAACTCAAGCACATACAATTAACATACCCCCACCAACAGA

GTATATAAAAGAAACACCTCCAATATCCCCACGCACTACAAAAAACTCTTTAAGCTCATC

TACAACAATTCAATAGCCTCCGGACCCTGCCCAAAACCACCAAACCCCAACCCCTACCCC

CAACAAATATATTAAGACATAGACCTTAACCGACCCAGCCCCCCATGTTTCTGGGAAAGG

CTCAGCCGCCAAAGCTGCCGAATACGCAAAAACCACCAACATTCCCCCTAAATAAATTAA

AAACAATATAAAACCAATTAACGACCCACCATGACCCACTAAAACACCACACCCCACCCC

AGCCGCTACCGCTAACCCCAACGCTGCAAAATAGGGGGCAGGATTAGAGGCAACTCCAAC

TAAACCGACCACCAAACTTAACAAAAGCAAGTCAAAAAAATACATAATTCTCACCAGGAT

TTTAACCC

>Unigene000024

AGACAGCAGCAGTGGCAAGAACGGAAGCAGTGATGAGAGGAAATGTGCTTCACTTTAAGT

GACATGGCATTTCCTGGTTGCTTAACAAAAGCAGAGAGACAAAAAAGAAAGTCAAAGAAG

AGCGAGCGACAGAGAGAGAGACAGAGGAGTTAAAGTTTGTATGAGGTAGAACAGAGGAAT

GTGCCGGAAGACATGAGTAGGAATAAATGTTCCTGAGAGTCAGAGCTGTAGTGCGTCTGA

AGAGACCGGGGTTCCAGTGCCCCCTTCAACCTTCTTCAACTGCCATGAGACATGGACTGG

CCCCAAACAGAACACCAACTGAAAGATGTTAGCTACCTGAAGCAGTGCCTTGAAGATTTT

GTGGCGTCCTTCAAGAAGGTCATCGATGTCCAGTCGCTGGAGCCACGCCGTCTGGAGGAA

TGGAATGCCGAGGTGCCTGCGGTGCCACGAGATTTTCTCGTGTTCCTGGAAACACAGCTG

TCACACAGCGTCCTGCACCTGTCCGGTCAGGAGCCCAACAACAGCATGCCCCACCCATTG

CTCCTCATCAAGTTCTTTATCATCATCTGCAGGAACATGGAGAATCTCGACCTGGACAAG

ACCCCCGGCTTTGTGCTTGAAACCATCAAGCTGTTAACCTACTGTCTGAATCAGATAAAG

GAAAAGCCTGAGGAGCAAAGCAGTCTGCAGTCTGTTGTTCAGCACTGCCTTCTCCTTTGT

GAAAACCTCTTTGACCCTTATCAGACATGGAGGAGACGGCAGGCAGGTGAGGAGGTCAGT

ATGCTAGAAAGGAGCAAATATAAGTTCCACCCTCTCACCCTGCCAGAAGAGCTACCAGCA

TTCTTTCATGATTGCCTGCAGGAGACCGAGCGAATTCCCAATGCTCTGCTGCTGCGTCTG

GTGCATCTACAGGGTGCTGTCACCAGCGGGTGCAAAAAAAATGGCTTGCTCTCGATCACA

CCCGAAGCCGTGGAGGATCTTATGTCAGTCCTGCGCTCCTGGTGCCTCCGTCCTGCTGAA

GAAGATCAGTCCAAGGACCCGCAAGTTTTACGCCTGACTCTCCGCTGCCTGACCGCCATG

ATTCACCTTCTGCATTGCAGCAGCCCTGCTGAACGGCAGGTCGAAATGAAGACCGTGCTC

AACAATTATTTCCAGCTGCTTAATTGGAACAGACCGCCAACGAGCGAGCAGGAAAACAGT

CTAGCCTGGGAGGAGAGCATCATCACCCTGCAAACACACATGCTAAACGCCATCCCAGAG

ATCCTGCAGTGTTCAGACCGACCTGTGCTACAGGCCATTTTTCTGAACAATAACTGCTTC

GAGCACATTCTCCGGCTCATCCAAAACAGCAAGGTCTGGGAAAAAGGTTCCGACTGCATT

ACAGTACATGCTATTGGAGTCCTTACTGCCATCATGAGCAACTCTCCGTCTGCCAAGGAG

GTCTTCAAGGAGCGCATTGGGTATTCGCAGCTCTTTGATGTTTTAAAAAGTCAAGGGCAG

CCAACCAAAAGGCTCCTCCAAGAGCTCATGAACATGGCAGTAGAAGGTGAGCACTGCCAG

GCCGTCCCGCTGGGAATCAGTAACGTGCATCCCCTGCTTCTCCTTCTGCAGTGGCTTCCC

GAGCTAGGCTCTCGCTCGCTGCAGCTGCTGGTGTGTGAATGGTTGTTGGCCGTGTGCCGC

GGATCTTTGGCATGCCGCTCTGTGGCTGTGGAAGCTGGCTTGGTGGATGCAGTGCTGGAT

GTGCTGAACCAGGGACCCGAGCGGCTAGAGCGACAGTGTGCAGACTCTCTGTTGGGCCTG

GTGCAGGAGCTTGGCTCACTCTCACTGCGGCCTCATCAGCTGAAGAGCCTACTTAGACTG

CTGCGCACAAACCCGGGCTCGCCGCCTCATCCCTACTGCACTCGCACCATAAGGGCACTT

GCAGCAATGGCCACCCACGCCCACACAGGAATCAGTGCTCTGCAGTACTTTGACCTTACT

CCGCCCATGGCTGGTATCATGGTGCCATCTATTAACCGCTGGCCGGGGAATGGTTTCGCC

TTTCACGCCTGGCTATGCCTCAACTCCAACTTTCCTCCTCCGGTGCATCAACACCACTCT

GAGTCGCATCTTTCTATCCCTGACAATACGCTCCGCATGACCAAGGGCCCGCGCCGCAAA

CAGCTTTACAGCTTCTTCACGGCAAGTGGAACAGGGTTCGAGGCATTCTTCACCATGGAA

GGCGTGCTGGTGGTGGCAGTGTGCACCAAGAAGGAGTACATGGCTGTTTCACTGCCAGAA

CTCCCCCTCAACGATGGTGCCTGGCACTCTTTAGCCATAGTGCACATCGCAGGACGAAGG

CCGTTCGGACAAAACCTGGTCACAATCCACGTGGATGGAAACTTGTGTAAGACGGCACAG

TTGCGCTTCCCGACACTCAGTGAGCCTTTTACATCCTGCTGTATCGGTTCAGCGGGTCAC

CGCACCACCACTACCACCACTTCGCCGACACTGCTTCCCCCGGTACACTCATCTGAGATG

GCGTTTGCTATGTCCTCCGGCCCTCCAGCTCTCACCCGCTCTCAGTCTTTCCCAGCCTCG

TTTGCTGCAGGAGGAGGAGGACGCTGGGGACTGGGCAGAGATGTCCCTGTCTACACTATT

CCCGCAGGACTTCAAGATACAGAGTGGGGCTCGCCATCCTCGCTGGATGGCCTGTTGGCC

ACCACCTTCATTTGCCACGAAGCCCTGCAGCCTGCACAGACACGAGCACTCTATATGGCT

GGTCCTCATAGTGTGCCTCTGTTTAAAGGGGATGGGGAACTCTCAGAGCTCTGCAGTAAA

CTCCTGTTGTACTACACACCACAGGCCTTTAAAAACCAAATCTGTCTGGATCTGTCTCCC

AACCATATGTACGACGGCAGACTAACTGGACACAGAGTTGTCAACTGGGACATCAAGGAC

GTGCTGAACTGTGTTGGAGGAATGGGCGCTCTTTTACCCCTTCTTGAGCAGGTATGTGTA

CTGGAACAGGGTGAGACTGCTGGGCAGGGGACCTCTGACCTACTGGGACCAGATCTGACC

TCTTCCAGGGGCCCTGCAGGCATGCTGCTTCCTCTAGGAAAATCCTGTGAGAGCCGTTTG

GAAAAAAACAGTGTGGCCGCTTTCCTCCTTATGATTAGGAACATGCTGCGTAATCACCCA

GTCAATCAAGAGAGTCTGCTCCAGTGCTACGGGCCGGCCATTATAGGAGCTATGCTTAAC

AAGGTACCCAGCACTATGATGGACATGAGTGTGTTGATGGCATGCCAGCTGTTGTTGGAG

CAGGTCTCCATCGAGGGCAACAGCGCTCTGTTGTCTCAACTTTACCAGTACCTCCTGTTT

GACTTCCGCATCTGGAGCCACTGTCATTTTGCAGTCTGTCTCGGCCATGTGCAGTACCTG

TCCTCTGTCTTAAAGGAGGGCAAACAGAGAACACGCAGAAAGTATGGGGTTCAGTATATC

CTGGACTCTATTCGTACACACTACAGTGTGGAGAAGAATGGCAGTGCTCTATCGGATGAG

AAGAAAACAGTACAGATATCACTCTTAGAGCTACTGAAAGACTTCCTGAAGTCTCCCACT

ACCAAGGATCTGCACAGTATTCTGGCTTATACTGTCGTGGTGCAGGACCAGCAGCAGGCG

ATCATGCTGCTGGATATTTTGTATACAGTGCTAAAAAACAATCCTCAGGCTCTGTCTGTG

CCGCTGGAATGGGGGGTGGAGCAGTTTCTCTGTCTGCTGCTGAAGCCCAGCTATAACGAT

GAAGCCCGTGAAAAAGTCTTTAGGATCATGTACAAACTGCTAAAGAGCGAGCGAGTCCCA

GAACGTAGCAAACAGCGGGTCAAGCTGCGAGAGACAACCTACATGGGCCTGGTCTGCTTC

TTGGAGGAGGTCCCAGTCACAATGACCATCATCCGCTGTCTGTGTGAGCAGGTGCTGGCT

ACAGATCCCTCACCTAACTTCAGAGACCTCTTGTCAGTTGTGTATTTGTCCCATCGGGCT

GATCTCTCTGTCCGACTGGACATATGTCGCAAGCTTTTCAGTCTGATTGACTCCAATGAA

GACTGCGTTAAGCAGCTGGCCCGGCAGCCAGGCTGGCAGGACATTCTCATAAAACTCTAT

GTCAAAGAGTCCTTTGAGTCTCATAAGACCAGTGTATCCAGCCCTTATCCCTCCCTGGAG

CCTATTCCCACCAGAACTCTGCTCCGAAAGGATGACAGCATAGATGGTCCTCGCTCTGAC

CCCTTCAATCCCTATAGCTCCCAGCGTGAGGAAGCAGAAGAGGATGAATGTGATGAAGAG

GAAGAAGCATCTCGTGACATTTCAGAGCGTTTTATTGGATTGTCTCAGTCGCCGCCTGCA

AGAGGTCAGCTGAAGGGCTTTAGTGACTCGATGAACTTCAAGTCTTTCGACTCGGTGGAG

CAAAGCAGCCGCTCGTCATCCCTGTCCAACGCCGTGGACGTGCCATCCACACAGCTGTTG

GAGGAAGAAGAAGAGAGTCTATATAATCCACTCTCACCCTTAGGCATGTCACCTGATCTA

GAGCTGGGAGGGCAGAGAGCATCTCTGACTCCAGACACGCCGCCTTCTACAGAACATAAC

AAACTTTTTCTTGGCCCACGAGCTCGCAAGAGTTCCAGCTTGTCTAATGTGTTGGATGAA

ACCAGTTCCTGCACAGAGCCCCCAACCGCCGACACCATCTCTAACAACTCCAACCCACAG

CAGGCCCCAGAGGAGGAGTTGTGCAACTTGCTGACTTACATTGTGTTCCGAGTGCTGTGG

TCTGGCACAGAAGGAGCAGAAGATGCAGTGTGGCGGGAGAGAGGGCAAGTTTTCTCGGCC

CTCACCAAACTGGGATCTTCCTGGCAGCTTGTGCGTCACCCGGATGATATCAAACGCAGT

CTTCTGGAGATGATGCTGGAGTCGTCTCTTTCGGATCTGCGGGACTCTCAGGGCTTGTCA

TTGCCTCATATTCCCTCCTTACTTCGTCTCCTCAGACTGCTACACGATTTCCTGTTTGCA

GAAGGCACTGACAATCAAACACTGTGGAGTGAAAAGATCTTTGAAGGAGTGGTGAATTTG

CTCGACAGGCTGCAGGCGTGGCACACCACCTCTGCAACAGCCGGCGCAACAGAGCTCAAA

CGGATGGCTCAGATTGGCTTGTGGATCATAACAGGATACATACAGCAGCAGAACTCGCAG

GTGTGTGAGATGGCGTGTGTAAAGCTGCACAGTCTGCTGCAGACGGTCTTGTGTCTCTCA

TGGGAGGAGGTATACTTTCTGTTGGGCCGCCTGGGAGCCACACTGTGGCCTGCAGAAGGA

ATGTCTTTGACGAGCCCAGCTGGCACAGAGGCAGTCTTGAGGCCTATGGTACCAGTAGTG

CGCACACTCCTAGACCAGCACGCTGACCCTACCAAACTGCAGCAGTCTCTACCCAACCTG

CCCCCCACCAATGGCAGTGCCACCTTTGCCCAGGACCTACTGTCGTACTGCAACACAGTC

GAGTGGCAGCTTTTCTACTGCAATCATGTGAGACCCACCATGCAGCAGTATGAGCAGGAC

ACTTTTGGGAAAAGCCACGATTTACTCTCCAACTTTTGGAACTCGTGTTTCGATGATCTG

ATGAGCACCGCTGAAAAGAAGAACAAGGACAAAGCAGAATTCAAAGCCAAGTTTCAGAGT

TGATTGTGGACCCATATTTGAAGCGTGTACGCGTTGAGAACAGTCGCTATGTGAGTGGAC

AGAAGCTGAATAACAGCCAGCAGGAGGTGGTGTGGAAGCACTGGAAATGCCAGCGCAGAC

TGATGACCAGTGAACGAGCAACTTGGGCAGACAGGGATCAGCCAGAAATGAAGTGGAAGC

TGTCCAGCGCAGAAACATACTCCAAGATGCGTTTGAAGCTCGTGCCCAACTACAACTTCG

ACTCCCACAGCGAGGCCAGTGCACTCCGGGATAACATGGGAGCAGAGAGCCCTCGCAATT

CCACCGAGCCCCTCCCACTGGCTGTAGCTAAGGAAGCTAAAGTGAGTGACATGGAAGATG

ACCAACTGGAGGAAGAAGACCTTCTCGCCATGGACAGCCAAGCAGAGGAAGTGGAGGACG

AGAGCCAAAAGGAGAAGCTGGTACTCTCTGAGGACTGTGAGCTCATCACTATAGAAGCTG

TGGTCATGGGCCGGCTAGAAGTTACTACACATCACATTTACTTCTATGATGCCAGCAGTG

AGAAGGAGGAGACAGAGGAAGGGATCGGCTTCGATTTCAAGCGGCCACTGAGTCAGCTGA

GAGAAGTTCATCTCAGACGCTATAACCTGCGCCGCTCGGCCCTGGAGCTCTTCTTCATTG

ATCAGGCTCACTACTTTATCAACTTTAAGAAGAAGGTGCGGAACAAAGTCTACTCCAGGA

TTCTCGGTCTTCGACCACCTAATTTGTTTTATTTTGGTTCCCGCTCTCCTCAAGAACTCC

TCAAAGCCTCCAATCTTACACAGAAATGGGTGTGCAGAGAGATCTCAAACTTTGAGTATC

TTATGCAGCTCAACACCATCTCAGGTCGCACTTACAACGATCTGTCGCAGTACCCTGTGT

TCCCTTGGGTGCTGTGTGACTACACATCTAATGTATTGGATTTGGAAGACCCGTCTGTTT

TCAGAGATCTGTCTAAGCCTATCGGAGTGGTAACCCCCCGCCATGCCCAGGATGTCCGGG

AAAAGTACGAGAGCTTTGAAGACCCCACAGGCACCGTTGATAAGTTCCACTACGGCACAC

ACTACTCCAACGCGGCAGGCGTCATGCACTACATGATCCGCATGGAGCCTTTCACCAGGC

TGCACATCCAGCTGCAGAGCGGCAAGTTCGACTGTGCAGACAGACAGTTCCACTCAGTAG

CAGCAGCATGGCAGGCTCGTATGGAGAGCCCAGCTGATGTGAAAGAGCTCATCCCTGAGT

TCTTTTACTTCCCAGAATTCCTGCAGAATATCAACGGTCAATCAGGAGCAGGTGAATAAT

GTGGTGCTGCCACGCTGGGCTTCTTCCAGAGAAGATTTTATTAGAAAACACCGCCAGGCA

CTGGAGAGTGAGTATGTGTCTGCTCACCTACATGAATGGATCGATCTAATATTTGGCTAT

AAACAGCGCGGGCCAGAGGCTGTGCAGGCACTCAATGTGTTTTATTACTGCACATATGAG

GGTGCTGTGGATCTGGATGCCATAGCCAATGAGACTGAACGTAAGGCTCTGGAGGGCATC

ATCAGTAACTTTGGCCAAACACCCTGTCAGCTTCTCAAGGAGCCGCATCCTCCCCGTATG

TTAGCACAGAGCGCGTGCAGGCGGCAGGCACGTATCAATACTCTGCCTCTCAACCTCTTC

GACCAGCTGGACAAACTGAGACCCTTCGTTGAGGTGGTAAATGATGGTGTGCCTCTAGTG

CAGGCTGTGGTGCCCAGAAATCAGACACACTCCTTCATCATTCCAGGGTCTGATGTGCTG

GTGACTGTGAGTGCTAAAGGACAGATAGGGACACACAGCTGGCTGCCCTATGACAAGAAC

ATTGCCAATTATTTTACTTTTACTAAAGACCCCACTATCAACAATCCAAAAACACAACGG

TTCCTGAGCGGCCCCTTCTCCCCAGGGGTGGACATGGGGGCCCAGGTGCTGGTGGTGTCC

AATGACGCTCGCTTGCTGTTTAGTGGGGGACACTGGGACTGCAGCCTACGCGTCACCACG

CTGGCCAAAGCTAAACTAGTGGGCAGAATCTGCCGTCACATAGATGTGGTAACGTGTCTG

GCTTTGGATCTATGTGGTATCTACCTCATCTCAGGCTCTCGTGACACCACTTGCATGGTG

TGGCAGGTTCTCCAGCAGGGTGGTTTCTCCAGTGGTCTGTCCCCCCGACCAGTACAGGTG

CTATGTGGACATGATGCAGAGGTTACCTGTGTCTCCATTAGCACTGAGTTGGACATGGCT

GTTTCAGGATCAAAAGACGGGACAGTGATTGTACACAGCATCCGGCGAGGTCAGTATCTC

CGTACGCTGCGTCCTCCCAGTGAGGGCTGCGTTTCTGTACGGGTGAACCAGCTGCAGGTG

GGAATGGAGGGTCACATCGTTGCACAAAGCGTCCTAGAGGGCTGTTCTGCCAGAAAGGAA

AAGTGTGTGCTGCATGTGTACTCTGTGAACGGCACACTGTTGGCGTCCACGGTGATGGAG

GAGCCTGTGTCAGCACTCTATATGGTTTCAGACTACCTCATTCTGGGCACACTACAGGGC

AACCTGCACATCAAAGACCTGTTCAGCTTAGAGCAGGCTGCTAAGCCCTTGGCGCTGATG

CTCCCTATTCGCATTGTATCAGTCACCAAGGAGAACAGTCACATTCTGGTGGGATTGGAA

GACGGGAAACTCATTGTTGTGGGAGCTGGAAAACCAGAGGAGTTGCGTTCAGGTCAGTTC

CCACGGCGTCTCTGGGGATCAACACGCCGAATCTCTCAGGTGTCTGCAGGGGAGACTGAG

TACAACCCTACAGAGAATGCTGGGAAATAAGCATCATACACACTCCACATGCCTTTATAT

AACAGGCCTCTTCACATACACATACATACATGTACACGAACATGACCTGAGATGTTGACC

CGGAACACAGGGTGACTTAATGTACTGTGGACTGTTTCTCTGGTTTAATGGAAAAACACA

GAGTCGTCCAAAGCTGCACTTTTTTTACTTCTTCTTTTTTAAAGTTTCTATTTTAGACCG

TTTTTAAGCTGAAATCATTCCAAAAATTCCAAACATGAACTGGGATGTGAGAGTGGAAGT

CAATCTCAGAGTCAGAACTAAATGTGGATAACACACACGCCTGATGCTGAGAGCTGCTGG

CTGAAGTGTGTCGTCATGGTCGACGCTCGCTTTTGATCTGCCGTGCACAACACAACTTGA

CTAAAAAACACACACTGGAACAACAAGAGTTCTATTTTTAAAGTGTTTGGGTGAACTTGT

AGACGTGAAGAAAAAACATGGGAAAAAAAAGAGAG

>Unigene000025

TAAAAGTAATACGTCTTCAGGTGAAAGCGTATCTGGAAATATGGTGAATAGTTGTGTTGG

GAAAACATCTAGCTAATTTCAAAAACGTTTTCACGCTGGGCAACGATACGTTATCAAGAA

GAAGGAAATATCATCTAGAAAGAAAGACGGTGGTGAAGCCGCGAAAGTCCTTTAACACCG

GAGCGGTCATGGTGGGTGAGCGGCGCAACGGGAACCTGCTGGTTCAACTTGGCCCAAAAC

AGCAGGCTTACCCAGAGGAGCTGATCCGTCAAAGACGTACCCACGATGGCCAAACGGAGT

ATCTGATCCGCTGGAGCCTGCAAGCAGTGGAGGATGGCTCCAGCTCAGGCAGCAGCAATG

AAGGAGGCAGCAATCGAAGTGGAAGCTCCGGCGTAGGAAGCTCCAGTGGCTGCACTTCAG

GAGAGAGTAAAACAGAGAATATCCTGATGTGGATGTCTACTGAAGATGTGTACGCTAACT

GCCCTACGTTACTCGGAAAGCGACCACCACAAGAAGAAATAGCAAGACCCAGTGGCCAAT

TCCCCTCCGATGTAACCTTCAACGAAGTGGACCTCCGTGATATGAAGGAGGACGTCAAAA

ACCTTGTTGAGCGAGCACGGAAGCAGATGGCCAAGAACAGCGATTTTGCCATAAGCCTCA

CTCACACCATTCATGTACTGAGTGCTTATGCAAGCATCGGGTCGCTGGTGGGAGTTTTTA

AGGAGACTGGGGCGCTAGACCTGCTCATGGAGCTGCTGGGAAACAAAGAGCGACAGACCC

GGCGCAGTGCTGGCAAGATGCTGCGTGCTCTGGCCTCTCATGATGCAGGAAGTCGTGCCT

ATGTGCTGCTGTCCCTAAGCCAACAGGATGGCATTGAGCAACACATGGACTTTGACAACC

GCTACACTCTACTAGAGCTGTTTGCAGAGACCACGTCTTCAGAGGAGCATGGCATCTCTT

TTGAGGGCATCCACCTCCCACAAATCCCTGGCAAGCTGCTGTTTTCTCTGGTCAAGCGTT

ACCTTTGTGTGACCTCACTAATGGACAAGCTCAACACGTCTGGGTCGGAGTTAAGCACAG

AGCGGCAGGAGCCTGAGCCCTCCTCTTCCACGGCATCCCATCAATCAGATCAATCCCGAC

TGCAGCGACAGTTTGAGTTCACCATGGCAATGGCAAACCTCATCTCAGAGCTGGTACGAG

TGATGGGCTGGGATCGTAACCGCCAGCCATCCGATGCCTCCCCCAACACAGCCAGGGGAG

CAGAGGAAACCGTGGCCGAAGTAAATAGACATACCCTACGCTCCATCTTTCAGCCCCGTT

TTTCTGCTTCTACCGTTATAACTGCAACAGTAACATCAGCACCGGCTCCACCCAAGAAGA

AGACAAATGGCTTCCGAACTCGCACAGACTTTTCTACACGTTCAGCCTATGTGGAGTATG

TGCAAGAAACCCTGCAAAGCGGCATGCATGTGCGCATGCTCGAGGACTATGAGGATGTCA

GTTCTGGCGACGAGGGAGAGTTTCGCTACAGTAATGATGGCTCACCACCAGTCCAAGTGT

ACTGGAACTCGATCTCCAGAACCTACTGGGTGCACTGGCACATGATCGAGATTGTGGGTA

GTGGGAGCAGTGTACAAGAAGAAAAGAAAACTCATGAAAAGGCCTTTTCTCTAACTGAAA

CACTTAAACATAATGCAGTGAGTCAGATGTTCTTCTTGAAGCCACCTGGTGGTTTATACT

CCTTACCTTACCTAACTGACAGCCTTCAGGATGATGCAGGGACACTTACCCGTGCTGAGT

GGTGGGAGGTGCTTTTTTTCATTAAAAAGCTGGAGCCTAAGCAGCAACAAGAGATCAACA

ACATCCTTCAACAGAATAATGTTAAACAGATGGCTGAGCTGGATGAATCCACTTTAATTC

AGATGTCGGTCTCAAGCGAGCTCGCCTGTAAGATCCTGCATTACCTGAAACAGACCCTGC

AGTCCTCCTGCTTAAATGACCTGCTTTGTTCACATGCCTTTGTTAAGCACTACTTGCGCC

GTGGTGCGGCCAGTCTGGAGGATGAGGAAATGTTCAATGAATGCTCCCTTAACGATTCAG

GGCTTGGTGGACAGGGATCCTCATCATCTTTATTAGCGACCCCTAAAGCTTCGACCTCTT

CTGCATCCTTCTCCATTAATGGCTGTGTTTCTAAAAAACCTAAAAAAGAGTTTCCTATAG

ACAAGGGCAGCTGTAATAGTGACACAGAGAGTGAGCTTCCGGCAGAAGATGAAACTAAGT

ATCCTGAAGATCTGGAGGAAAAGATGAAATTATTTAATAACCCAAAAGTACAAAGTAAAA

AGACAGCCTTAGAAAAAATTGGAGAGGTTGTGGACATTATGAAAAAGAGTAGCTCTGACT

CTGGACAACTGCTGGCTGGCATGAAGGTCATCTGTAAAATCCTGGACGAGGAGGGACCTC

AGGAGCATAGCACTCTCAGATCAGACTTAGCCCAAAGCACCCGGGACAAAGTCTTAAAGC

TACTAGTTGTGATGGTTGGTAGCCAACAAAAACCAAATGTCATCATGGCCTTACTTCTAA

CTCGCAGTTTAATGCTTAAATATGAGTGGCGGGTTTCCTTTGCTACCGAGGGTGGAGTTA

AAGCCATTTTGTCCTGCATGCAGGAGTATCCCACCTGCACACAAGTGCAGCAGATTGCAC

TAGCAACCTTAAAAGTGATAACCGGTGCAAGTAAGCATGACCTGCGCAGTGTAGGAAGCT

GTTTGCCCCTCTCTGAATCTGGTACGCAAATGATGCTGGAAATCTTTGCTAGCATTGGCT

CAGCAACACCTGAAGGCTCAAAGGGGCTTCTTGAAGCAATTCCATCTGCAATTGATCTCA

TGCTCAACACTGCTGGTGGTGGCCTGTCTATACGGAATGGTCTGTTGGTGATAATAATGC

TGATCTCCAACCACAAGAGCCTAGCAGAGCAACTGGTGGCTTGTGAAGTCACCACTGTCC

TCAGAAAATGCCTTTCTGGTCACACCTCTGAAAGCATGCTGGCAATAATTGCCCTCAACC

ATATATCCAATGTTCATAAACTAGAGAAGAGAGAGTCTGAAGAGATGCTTGACTTCAAGG

ACACAGAGCTGAAGATGTTAGTTGTAGGTCTAAAGGAGATGACCACCACCAAGGAGGTGA

TTCAAACCTTGGAGCAGTTGCTGTGTGATGAATCGTCTCAGCTGGAGGATGAGAGAAATG

AGGTGCTGCATAGCAGAGACACGTTTCAGGACCTGGTGCGCCTCATGGACCAGCATAGAG

TGGATCGCACAGTGCAACTCTCTATTCTCAGAATCTTGAACAAGTTCTTGGACAATTACC

AGGAGGATTTGCTACCTTGGCATGAGAGTATCGAACCTTGCATATCTTCTTTGACAGCTT

GTATTAATGACAGAGAGGCTGTGCAGCAGTTTATACACTTTCTTTATCGACTGGCTTCTC

AGAACAAAGACTGCACTGTAGTTATGTGTCGGCTGGGCACTAAGGAAGCACTTGTCAAAG

CTTTGGACAAGCATAGCACCAATCTGCTACTGGTCACAGAGCTACGAGATCTCATTAACG

ATTGTGAAAAGTATGCCAGCCTTTACAAGAAGATGACCACTAGTGTGTTAGCTGGATGCA

TACAGATGGTCTTAGGCCAGATTGAAGAACATCGACGCAATCATCAGCCCATCAACATTC

CTTTCTTTGATGTGTTTTTACGCAACTTGTGCCAAGGTTCCAGTGTGGAGCTGAAAGAGG

ATAAATGCTGGGAGAAAGTGGAAGTCTCTTCTAACCATCACCGAGCTAACAAGTTAACTG

ACAAAAATCCCAAGACATTCTGGGAATCCAATGGCTGCACAGGCTCTCATTTTATTAATG

TTTACATGCACAAGGGAGTAGTTATAAGGCAACTGGTATTGCTGGTAGCTAGTGACGATT

CCAGTTACATGCCAGCACGTATAATAGTACTGGGTGGAGATGATCCCACAAGCATCAAAA

CTGAACTCAATACTGTAAATGTGGCACCTTCTGCTAGCCGTGTAATTCTACTGGAGAATA

TGACCCGCTTCTGGCCCATTATTCAAATCGGGATAAAGAGGTGTCAGCAGGGTGGGATTG

ATACTCGAGTACATGGCTTTGAAATTCTGGGGCCAAAGCCAACATTTTGGCCAGTGTTCA

AGGAGCAGCTGTGTTGCCGCACCTACCTCTTTTACACAACCAAAGCTCACACCTGGTGCC

AGGAGATCCTAGGGGACAAGAAACAACTGCTTCAGCTTTTCAATAAATTGAACAGTGCTC

TGCGTCATGAACAGATGTTTGCGGATCGTTTCCTGCCTGATGCTGAGGCTGCAGAGGCTC

TTGGTCGAACTTGTTGGGAGGCTCTGATCACTCCTATTGTACAGAGCATCACTCTCTCTG

AGAGCCACGTTCTAAGCCCATTGGCCTGGTTGCTTGGTGAATACCTAGAGAATTCAGAAT

CTTTAAAATGCTATAAGAGCAGGGCTGCTATTTTTAACTCCCGTGTGCGCCGCCTCACCC

ACTTACTGGTTCATGTGGACACCAGCCGTGTGGATATAGAAGAGCTTAAACCTCCTGTCA

AATGTAAAGGTATCAACAGAAGCAAAGATGTTAAGAATGGAAAGGAAGGAAAAAATAAGG

GCGCATCTGGAGTGACCTCTCCTTCATCTACTGCTAAGCCCAAAATAAAGAACAGCAGCA

GCATTGCTGGCGTTGCACTTTGCTGGCAGAGTGTTGTACAGGGGCAGATGAAGAAGTTTC

TGGACTTAACATACAATCTTCCAGAATTTGTGGAATGCTACAAGAACATGTACTTGCGCC

TGAAGAATGCCATGGAGGAGCTTTTTGGCCAGCAAACAGCCTTTGTGCTGGCACTGCGTC

AGGGGTTTTCTGCAGCACTGTTGCAGCTCTCCATCCTTACTTCTATGCATGTAAGTGAAC

GGTTTGCTCAATACATCGACCACATGATCCGGCAGAGTGGGGTAGATTCTGGCAATGTCG

AGACATTAAACAAGCTGCAGAAGTTCCTGGAGCCCATGCTCTTCCTCTCAGGACTAGAGT

TGGCCAACACTTTCGAGCACTTTTACAGGTACTACCTAGGGGATCGTCTGCTGAGCCAAG

GGAATCTGTGGCTGGAGAGTGCAGTAATCGAGCAGATTTGTACCTGTTTCCCTGAACGTT

TTCCCCAGCAGATGCTAAACAACCTGAGTGAATCAGATGAACTTCAGCAGGAATTTCACC

TCTACCGACTGCAGCAGCTGGACAAGAGTCTGCAAGATATGAATGAAGAGATGGCAGATG

AGCAAATGTTGGACCCAGAGGATGAGGGTGAAGTAAAGGTGCTTGTTTTATCGCCACGTT

GTTGGGCAGTTTCTGCTCCCTGCTACCTAGAAAATCCCACAAAGTATTTCCCTCAGAATC

TCTGCAGCTACCTAGAGGAGTTTGCTGACTTCTATTCCAACAGTCAGTGCATATATCAAC

TTGGAAACAGTATGCTGCGGCGGTTGCAGTGGACCTGGCTTGGCCATGCAGAAGTGTGTT

ACGGCTCCTTAACGCTATATGTCTCCACACTGCAGATGTACATCCTGCTTCAGTTCAATC

ACCAAGAGGAAGTGAGTTTGCAGGCTCTACAGCAGGCCACTGGCCTGTCCTTGCCTGTAT

TAATACAAGCTCTCACACCTTTAATATCAGAGAAAGGCATTCTGTCTCATGAAGGCTTGA

ATCAGGATCTTCAGAAGGGGCTTCTCAGGTTGAATAAGATGTCTTTGGCACAAAGCTCAG

AGAAGCAAAGGTATTGCAATCTACTACCAAAACAGACCTACCTAAATGTGGACGAAGATG

CAGCTCTCACACTGGAAAGGAAACGAAACCATTTGTACTGCCTCATTGTGCAAATCATGA

AGAATGAAAAGGAGATGCATATAGACAACTTGGTGTTTAAGGTGCTGGACACTTGTCAAA

AACAGGAGGCTGCTTCCAACACTGGGCGCTTTAGCTGCAGCACTACAGATGTACTATCCT

GCATCATGCACGTTATCAACAAGGGCTACATCCGCCGTAACGAGGACAACCCTCACATAG

TAGAGTACATAGTTGAAGACCCCTCCACCCCCCAGAAAAGCCAGGCACACTTCTTCAGCA

GGGTGGACCTAAACAAGTGCAAGGGCAGCACTAAAGCCGACACCAGCCTTGGAGGCTCCA

TTCTGGTCCCTCAGCAGGTGGAGGATGGGGCATTGGAGACTATGCTCTTTTCCATGGGTC

GCACAATGACCCAGGAGGAAGTGAAGCGTCTGATGCGGTGCACCATCCAGCAGTTGGCTG

ACACACTCAGCTTGGAGGAGGAACCAGCTGAGCACCTGCTCATGCACTGCAAGTGGAACA

TTGACATGTTAATCCAGCGCTACACAGATGACCCAGAGACACTGATGCTAGCTGCCGGTC

TCAAGATACGCAACCCTCATTCACTTGCCAGTCCCATGAGTCTCTGCCCGTCCTGCTGGC

AGGAGTATCTGACTGCCCGGATCGAACAGAACCTGGTGATGAACTGTAACTGTCCCATCA

CAGACTGTCCTGCTCAGCCTACTCCTAAGTTCTTCTATAGTATTTTGACTGACAAGGATA

CTATTGCAAAGTACGAGAATGCACTCCTGCGAGGATATGTTGAGTGCTGTTCCAACCTGA

CCTGGTGTACCAATCCTCAGGGCTGTGACCAGATCCTGTGCAAAGAGAACATCGGCAGCA

TGGGCACCTGCTCAAAGTGCTGCTGGTCATCCTGCTTCAGCTGTAACTTCCCTGAGGCTC

ACTATCCAGCTAGCTGTAGTCATATGTCCCAGTGGATGGATGATGGCGGCTATTATGAGG

GAATGAGTATGGAGGCCCAAAGTAAGCATTTGGCCAAACTAATCTCCAAGCGCTGTCCAA

GCTGCCAGGCACAAATTGAGAAAAATGAAGGCTGTCTTCATATGACCTGTGCCAAATGTA

ACCATGGATTTTGCTGGAGATGCCTTAAACCCTGGAAACCTACTCATAAGGATTATTACA

ACTGCTCAGCCATGGTCAGCAAAGCTGCAAGGCAGGAGAAAAAATTTCAGGACTATAATG

AGAGGTGTACTTTTCATAACCAGGCCAAGGAATTTGCAATCAATCTGGAAAACAAAGTGT

CCTCCATCAATGAAGCCTTGCAGATGAAATCTTTGACATTTGTTATTGATGCTTGTAAGA

TTCTGGCCCAAGGTCGAAAGGTGTTGGCCTATTCATGTGTGTACAGCTATTACAACCAAG

ACACAGAGAAGATGGATGTCATGGAGCAACAAACTGAGGCGCTGGACCTTCACACTAACG

CCTTACAGATCCTACTTGAGGAGACACTGTTGCAGTGCACAGACTTGGCATCTTGTGTGC

GACTGCTGAAGCCAGAACACCTTAACACTGGCCTGGAACTTATTCGCCGCATTCAGGAGC

GGCTGGTGGCAATTCTGCAGCACTCTACCCAGGATTTTCGTGTAGGATATCAGTCCAAGA

CTGGACCAGACCACGAGACTGCACAAGCATTAAACATTGCTAACAACACAGAGCAGAACA

AAGTGTGAGTTGTACAAAAATGAGTAAATGGCACTTCTCGTAAACTTGCAAAATTTGCTG

AGATCCAATTGTCTAGCACTTTTACTGCACTAGCTCCTACACACACAATGCTATGACTAA

TTCACAACTAACTGAAGATCATCATAAAAAGCAAGGATAATGATTTTTTGATTTATTTTG

CTTCCAATTATTAAGATTTAGTATATTGTGTAATTGTCTTGGAAAAAAAGAAAACAGATT

GTCCTACAGTATGTTTTACCAGTGTTCTTGCCAGTGCACAGTGTGCATCAGATTCCCAGA

AATATTTACTTATTAAATGACCAAAGGTCTTTGCACCTGGAAGTCCGAAAGGTGTTTTTT

TGCATATTCTAAAAATTTCTCAGACATTCAGTTTCTTTTAGGATGTCAGTGACCGTTTGA

TACTTTGCTAGCAATACTGGAGTCCCATATTAAAACAGAAGACCACTACTAGATGTATGT

CTAAGATATGGGCCTGCACAACACAACACCAGACAGGTGATTGCTGGGCATTGAAACTGT

GTGCAAGTACCTTAAAACTGCATTGGAAAAAGACCTCTCTTTTTCTCTGTGAGGATCGCC

TTAGACTCCATATTCATTAAGCCAAAAGCCAGAGTGGATGAAATCAGTTACCCTAGATTG

TGTAAATAAACTATGATTCACAAGCTGTTTTACTGCTCATATGGTCCTGTTAGTAAATCA

TCCTGCAAATTATTTTTACAGGTGCTAAAGATTTTTTTTTTCAGAAACAAGGAAACCAGA

GCCACAGCATACACCATATATAATATACTCTTTAATCCTTTCTTTTAATGCCTAAAAATA

GAGGAGTTGATCTAAACGCTTTCTGTAAGACCTAATGCAAAGAGCTAATTGGTTAATATT

TTTACAAACACTTGAACTATTTTCAGGAACAAATCAGACAGAGGCTCTGAGACCGGAGAG

TCTGACAACAACAATAACTTCAACGAAAATAACGGAGATGAAGCTGAGGACGATGATGAT

GAATATGATGACGAGTATGTCCCCGAATGGCACGAAGACTACGACGAAGAGGAAATAGAC

GAGGACGACTTTTTCTCTGACGATGATGAATCTGAAAACCTAGAGAGGGACTTTGTTCCA

TTCGACTGAGTTTAAAAAGACTGACCAGTCACTGTGCCTAGTAAGATGCTTCAGTTCTGG

AGGTCTCCCTATGCTGTAGAGCAGTGTTTTTCCTCTTGTTACATACATATTCACTAGTTC

AGCAGGTGTGTGAGGAAAACACTGAAGTGTAAAGCGCTCTAAACCCATGTGACTGAAAAC

ATTGGTTTCAAAACAGTAAGCAATCAAAAGCAACATTAAAAAAGTTAAAC

>Unigene000026

GTCCCCCCCTCAGCCCAAAATGGGACACCCCTTTGACCCCTTCGGGCAGTATGACCCTCA

AGGAGGCCGAATGATAGCCAATGGTCCTGGTCCTGGTCCTGGCTCCGCTAACGGGATGTC

GTCTCCTCATTCACGCTATCATGGTGGTAATGGCACAGCCACTGGACATCACGCTTATCC

GGGAGCACAGGGTGATGGTCGAGGAGGTGGAGGAGCGGCCGCAGGACAGTCGTTTCAGGA

TGGAAGCGGCGCTGCAACTGTGAATAACTGGGGTCCTCAAGGGATGCAGCTGCCTGACCA

AAACCGAGCCCAGTTTCCACAGTCGCAACAGCAGCAGCAAGTTCACGGTCATCAAGTGGC

TCCTTATATAGGACGCACTGACTTTGGTGGAATGCAAGGGCACAACCAGACCCAAGCACC

GCCAAGAATGAACCATTTCCCTCAGGGTTTGCCTCAGGAAGCAAACCGTTTTATGGGTCA

CATGGGAGCCAATATGACATCGTCCACCATGCGCCACCCGTCTCAGACGCCCCAGCATCA

GCAAGCTGGTCAGCAGTTTCTGCATCACCACACAGATTCACAGTTTCCGCAAAGTCCTTC

AAAGCAGCAGCAGATGCTGAGTGGGCGAGGACACCAGAACATGGGTTTCAGCTTGAACAA

CCAGCAGGTCGCTAACAGCAGCTCAGGTCAATATGCTGCTTATCCTCAATATAGCGGTCT

CAATCAGGGATTAGCCAATGCTTCAGGGATGAGCCTTAATACCAGCAGCAACCCCAATGC

TTCTGTGCAGTGTTACCCTACCCCTGCAGGGCCACAGTGTTACCCGGTGAACCAGTTACC

ACCTCAGAATATGCATCCCAAACTTCCCCAGCAGCAGCAGCAGCCGCCACCGCAGCAGCA

GCACCTTGTTCCACCTCCTCAGGGCTCTTATGCTACTCCTCCCTCCATGTCACCCATGAG

GAACCTGGGAGGCAACCCTGCAGGAACGCCACCCCCTCAAAGGGGTCAGGGTCCCATCTC

TCATCCCCAGCAGCACCCCCAGATGTATGGCACCTTGTCGCCCAATCAGAGAGCAATGAA

TGCGCCTAACCACACAGAGGTTCTCCCACAGCAGCTGCAAACCTCCACGCTGCAATTCCA

GCAGGTGAGAACTCCGCCCACACTCTCCGTGTCTCAGCAGCCCCCGGGCTCGTCTCCACC

AACACCGCTGCCACAGGGGAACTGGTCCTCCAACACACACTCGTCACCACCAGCTAATCA

CCAACAAGTGCCTCACATGCAGCCTTCTCCATCAGACGCTTCCCAAACCCTGTCCTCCAC

TCACAACTCCACCCCGAGCTCCACCCTGCAGCTGGGCAGCTGTGACACTCAACCACTGGA

CAGCGATTCCAAACCCAAGAAAAAGAAGAAAGTGAAGTCTGAGGAGAAGGACGAAGATAT

GGGTGTGTGTGGAGAGTTGGGAGGTGCAGGAGTTAGTGGAGAAACGTCTTCACCTGAGAA

AAAACGCAAAAAAAAACCAAAAGAGAAACAGGCCAAGGACACAAAAGAGCCCAAAACACC

TAAAACGCCTAAAGCCCCCAAAACACCCAAAGAACCCAAGGAGAAGAAAGTGAAGAACAC

CACACCTAAACCCAGGAGCACCAAGAAGAACAGTAAACAGGAGGAGTGTGAAAGTAGCAG

TAAGAAAGAGGGGAAGAGGAAGAGGGAGTCGTCCATCACCTTGGATCCAGAGAAAACTCC

TCCCCTTTCTCCGGAGGAGGAGGAGGAGGATGATGGGGTGCAGAAGAGGCGTTCGTGTCG

ACAGGTGAAGAGAAAGCGGTACACAGAAGATTTAGAGTTCAGGATATCTGATGATGATGA

TGAGGATTCTACAGGACCCAAATCTCCTTCTACCACGTCTGAACCAACTGAGGTGCTTGA

TGCTGAAGGTCCAGTGGTGGAGAAGATCATGGGATTACGTACAGTTAAGAAACAGCTGGA

GTCGGGACAGGAAGTGGAGGTCGAGGAATTTTACGTGAAGTTCAAGAGTTTCTCATATCT

GCACTGTCGCTGGGCTGATCTAGAGGAACTGGAGAAAGATAAAAGAATCCAGCAGAAAGT

CAAGCGCTTTAAAGCCAAACAGGCGCATACCAACTTCCTCAGTGAGATGGATGATGAGCC

GTTTAACCCGGATTATGTGGAGGTGGATCGAGTTCTGGATGTTTCTGAAAGTACAGATGA

AAATGGAGAGGTGGTGACTCTGTATCTGGTGAAATGGTGTTCTCTGCCCTATGAGGACAG

CACCTGGGAGCTGAAAGTCGATGTTGATGCAGGAAAAATTGAGGAGTTTGAGCAAGTGAT

GAGTCGTGAACCTCAGCTGAAACGTGTGGAGCGACCTCCGGCGAGCGACTGGCAGAAATC

CCAGAGTTCCCGCCAATATCGGAATAACAACGCACTCAGGGAATACCAGCTGGAGGGAGT

CAACTGGCTACTGTTCAACTGGTACAACACACGGAATTGTATCCTGGCAGATGAGATGGG

TTTGGGGAAGACGGTGCAGTCCATCACGTTCCTGTATGAGATTTATCTGAAAGGTGTCCA

TGGGCCGTTCCTGGTCATCGCTCCGCTCTCCACCATCCCCAACTGGGAGCGCGAGTTCAG

GACATGGACTGACCTCAATGTAGTGGTGTATCACGGCAGTCAGGCCAGTCGCAGGACCAT

CCAAACCTACGAGATGAACTACAGAGACACTCAGGGTCGTGTGATTAAAGGAGCCTATAA

ATTCCATGCCATCATCACGACGTTTGAGATGATCCTAACGGATTGTCCGGAGCTGCGCGG

TGTCCCCTGGCGCTGTGTGATTATCGATGAAGCTCACAGACTGAAGAACAGGAACTGTAA

ACTGCTCGAGGGACTCAAACTCATGGACATGGAACACAAAGTTCTCCTGACAGGAACTCC

ACTGCAGAACACGGTGGAGGAACTCTTTAGTCTCCTCAACTTCCTGGAACCTGAACGCTT

TCCCTCAGAGTCCACCTTCATGCAGGAGTTCGGAGACCTTAAAACAGAGGAACAGGTTCA

GAAACTGCAGGGAATCCTGAAGCCCATGATGTTGCGGAGACTGAAGGAAGATGTGGAGAA

GAATTTGGCCCCTAAAGAGGAAACGATCATTGAAGTGGAGCTCACAAACATCCAGAAGAA

ATATTACAGAGCAATTCTGGAGAAGAATTTCACCTTCCTGTCCAAGAGCGGCGGAGGGGG

AGGGGGAGGGGGATCTAATGTCCCTAATCTACTAAACACCATGATGGAGTTACGCAAGTG

CTGTAACCACCCCTACCTCATCAACGGAGCAGAGGAGAAGATCATGGAGGAGTTTAGGGA

GACTCACCCAGCTGATCTGCCGGATTTCCACCTGCAGTCGATGATCCAGGCGGCGGGGAA

GCTGGTGCTCATTGATAAGCTCCTCCCCAAACTGAAGGCTGGTGGTCACCGTGTCCTCGT

CTTCTCCCAGATGGTGCGCTGCCTCGATATCCTCGAGGATTACCTCATCCAGAGACGGTA

TCCCTATGAGCGTATTGATGGTAGAGTGAGAGGGAATCTGCGTCAGGCTGCGATTGATCG

ATTCTCTCGCCCGGATTCGGACCGTTTCGTCTTCCTGTTATGCACTCGTGCCGGAGGACT

GGGTATTAACCTGACCGCCGCAGACACCTGCATCATCTTCGACTCTGACTGGAACCCACA

GAACGACCTGCAGGCTCAGGCTCGCTGTCACCGGATTGGTCAGAGTAAAGCGGTGAAGAT

TTATCGTCTGATCACCAGGAACTCGTACGAGAGAGAGATGTTTGACAAAGCCAGTTTAAA

GCTCGGGCTGGATAAAGCAGTGCTACAGTCCATGAGTGGGAGAGAGAATGCTGCTAACGG

GGTTCAGCAGTTGTCTAAGAAGGAGATTGAGGACCTGCTGCGTAAAGGAGCTTATGGAGC

TCTGATGGACGAGGAGGACGAGGGCTCCAAATTCTGTGAGGAGGACATTGATCAAATCCT

ACAGAGACGAACACAAACCATCACTATCGAGTCTGAGGGCAAGGGGTCCACCTTCGCCAA

GGCCAGTTTCGTATCGTCGGGGAACAGAACAGATATTTCACTGGAGGATCCAGACTTCTG

GCAGAAATGGGCAAAGAAAGCTGAGCTGGACCTGGACGCCATTAATGGCAGGAACACTCT

GGTGATTGATACGCCGCGGGTGAGGAAACAGACGCGACACTACAGCTCCATTAAAGAGGA

TGAGATGATGGAGTACTCTGAGCTGGAGAGCGACTCAGAAGAGAAACCTGTTCAGAAATC

ACGACGGCCACAGGACCGAACTCAGGGGTACCCCCGCAGCGAGTGTTTCAGAGTGGAGAA

GAACCTGCTGGTCTACGGCTGGGGTCGGTGGAAGGACATCCTGTGTCATGGGCGGTTTAA

GCGTCCTCTGTATGAGCGGGATGTGGAACTGATCTGCAGAGCACTGCTGGCTTACTGCCT

GCTTCACTACAGAGGAGACGAAAACATCAAAAGCTTCATCTGGGACCTGATCACCCCTAC

TGAGGACGGGCAGAGTAAAACACTCACCAACCACTCGGGTCTGTCTGCTCCTGTACCGCG

AGGTCGTAAGGGGAAGAAGGGGAAGTTACCAGTGACTCAGATGAACATGCCTCAAGCTGA

TTGGTTGGCTGAGTGCAATCCAGATGTGCTGCTGCAGGAGGACAGCTACAAACGCCATCT

TAAACACCACTGCAACAAAGTGTTACTGCGGGTGCGAATGCTCTACTATCTGAGACAGGA

AGTGATTGGAGACCTGGCAGACCGCATCCTGGAGGGAGCTGATTCTAGTGAGCTGGATAT

CTGGATCCCGCAGCCCCTGCATGCTGAAGTTCCCACTGATTGGTGGGATGTGGAGGCTGA

TAAATCTCTCCTCATCGGTGTGTTTAAACATGGTTATGAGAAGTATAACTCCATGCGAGC

TGACTCGGCGCTGTGTTTTTTGGAGCGAGTGGGAATGCCGGATGCCAAAGCCATTGCGGC

GGAGCAGAGGGGAACGGACATGGTGGCAGATGGTGAGGATGAAGACCCTGAGTATAAACC

GCTGCGTATGCCCTTCAAAGACGATCTGGATGATTTCACTAACTCTCCCCTGGACGAGAA

GGAGGAGGTGATGGATGTGGAAGCTGGAGATGCCCCTAAAGAAAGTGAGGTAAACGGGGG

TAAAGCTGGGAATCTGTACTGGCCCACAGCTTCAGCGTTCACTGCACGCCTGCGTCGCCT

CATTACCGCGTACCAGCGCTGCCACAAGAGACAGGAAACACTGATTAAACCAGATGGACG

CAGGCGCCGGCGTCACCGTGACGACCACCTCCTCGCTATGGCCACTGAGGGCGGGGCTTA

CAGTCTGGAACCCAGTAGGGTAACTCCCACGGCATATCTGACTGAAGGAGCTTCTTTCAA

GACGTCACCTTTCCTGCACGAGAGCGCCGCCCTTCTGGCAGATGGCACTGCTTTCTTTAA

AGAGAGACGTCAGAGATGGACGCGGCGTGAGGAGGCTGATTTTTACCGTGTCATTTCTAC

CTTTGGGGTTGTTTATGATGTCCAGACGCAGCGTTTTGATTGGTCGCAGTTCCGGGCATT

TGCTCGTCTTGATAAAAAGACAGATGAGAGTCTGGAGAAATATTACTATGCCTTTGTGGC

CATGTGCAAGCGTGTGTGTCGGATGCAGGTCAAAGACTCGGAACTGGCTGACCCGACTCT

GATCATCGACCCCATCACAGAGGAACGAGCCTCCCGTACTCTGTACCGTATCGAGTTACT

GCGCCGGATCCGGGAGCAGGTTCTGCCCCATCCCAAATTGGAGGAGAGACTCCACCTCTG

TCAGCCCAATCCGGAGCTTCCGGCCTGGTGGGAGGCAGGGAAGCACGACCGGGACCTTCT

CCACGGCGCTGCGAAGCACGGAGTAAGTCGCACAGACTACCACATCCAAAACGACCCTGA

GCTCAGCTTTCTGCAGGCGCAACGCAAGTTCATCCAAAACCGAGGGTCTGACACGGCGCT

GACCTCCGACCCTCCCCCCACCGGGGATCTGATTAAAGATGAGGAGCTGAGGAACGACCC

TGCAGAAAACACAGGAATGGAGGACATGAAAGAGGAAAATACATCATCACCCAAACAGGA

AGTAAAACCTGAAGAGGAAGAGAAAACTGCCAATGAGAAGATGGAGAGTGAAAATGAAGC

CGAAGTCAAGAGTGAAGAAAATCGCAGCGTTCCTCAGATGACCGAGGAGAAGGTCACAGA

GTCTGAGGACACGCCCACACCTCATGTTACTGTCCACCAAGACACAGAAAAAGAGGAACC

GGAGAACCCAAAAGAACTGGAGAAAAATTCAGAGGAGGAAGAAGAGGAGGAAGAAAAGAT

GGATGAAGACGACAAATCAGAGAAATCGTCCCAGGCTGAAGCTGGTGCTTCTTCAGACAG

GAAGAACTTTGATGAAGAGAGTAACGCCTCCCTGAGCACCGCCCGGGACGAGACACGGGA

TTATTTTGGTCTGGATGATGTCGAACCTTCGGTGTCTCAGATGTTCGGTGAACGAGCTCC

GTTCTCTTTCTGGCCAAAGGATCGGGTGATGATCAATCGACTGGACAGCATCTGTGAGGC

GGTCATAAAGGGCAAGTGGCCATCCAATAGGAAGCAGTTCTTTGACATGCCTGGCCTGTT

GCCTGGATATGTTGCCATGGCAACCGACAGCCCTGTGCCCAGGGGTATGGGAGACTTCTC

CATAATGAATCAGAGCAGCTACAGTGGAAGTGATGACATCACATTGTCACCGCAGGTTAA

TAAGGTTCAGGACGAGGCTCTAAGTCTGTCTGTTCCACGTCAGCGCAGACGCAGGAGAAG

GAAAGTGGAGATCGAGGCAGAACGAGCTGCTAAGCGGCGCAATTTGATGGAGATGGTTGC

GCAGCTGCGTGAGTCTCATGCAACAGAGTCTCGGACTCGAGTAATGGATCTCACTAAAGC

TCTTCACGGTCTGGCTCCTTCCTCCTCCACCTCCTCTTCCTCTGCGGTGTTCCCCGGCAC

CGTGGCTTCCCCAATGGAGCTGCTGCACGCCGCCGGAGCCTCCAGAGCCAACGGAGCCAT

GCTGGAGGCTGAAATCCCCACACGCAGGAGGAGAGGACGCAGGAAAAACGTCGAGGGACT

CGAACTGCTCTTTATGGGCAATAAGAGTTCACAGCTCAACACGGAAGATTCAAATGGGAC

CAAAGGTTTTACAGATGGAGCACAGATGTCATCACGGACACAGACACACCTTCAGACTCC

TGCACAGAAAGACGAGGGAGACCATCTCAGAGGAGGAGATGATGGAACCAGCACAAAAGA

CCTGCAGGAGTGGCTTAGACAACATCCCACCTACACCATGGACGTGCCTGGATACATACC

AAAGAATGAGGAGCTTCTCCTCTCACAGCTGGGCGGGAAACCCAAGCAGAAGAGACATCG

CTGTCGAAACCCTAACAAGATCGACATCAACACACTGACCGGAGAGGAACGAGTGCCTGT

GGTCAACAAGAGGAACGGGCGAAAGATGGGGGGCGCTATGGCTCCACCAATGAAGGATCT

GCCGCGGTGGCTGGCTGAGAATCCGGAGTTCTCCATCGCTCCAGACTGGACAGACATCGT

CAAACAATCAGGATTCCTCCCCGAGTCCATGTTTGACCGTCTCCTGACCGGTCCGGTGGT

CAGAGAAGAAGGTGTTCGGCGCCGAGGACGAAGACCAAAATCTGAGATGGCTAAAGCAGC

AGCAGCTCAAGCTTCTCTTTCTGCAGCGTCTTCAGCCTCCGCCTCGGGCATTAACCCCTT

CCTGCTCAATGGCCTGTTCGGAGGAATGGACCTGAGCAGCTTGCAGAACCTCCAGAACCT

GCAGCTTCTCATGGGCTTGTCTCCTTCTCTAAGTGCCGGAGCTGCAGACAGCAAGAACTC

CGCCACCATGCTGCCCCTCATGCTACCTGCCATGGGGGCTCTGCCCAATGTCTTTGGCCT

CGGTGGTCTTTTTGGTGGCAACATGGCGGCTGCAGCTGCAAACTTGAACACCAATTCAAA

CAGCGCAGCCACTTCCGAGTCTGAGGAGACAAAGAAAGATGGAGAACCCAAAGAGGAAGC

TGAGGAGAAGAAGGTGGAGGTGGAGAAACCCGCAGAGTCCAGTGACGCAAACACGGCTGC

CGCAGCTACCGGTCTTTCCACTAATCCTCTGGCGTTTAACCCCTTCCTGCTCTCCACTAT

GGCTCCTGGACTGTTTTACCCCTCCATGTTCCTGCCCCCAGGCTTAGGTGGTTTGGGACT

GCCTGGCTTTTCCCCAGCGGCTCTTGCTGAGCTCCAGAGCGTGATGAGCGGGGCTTTAGG

GGGTGCTGGAGAGGAGAAGGAGGTTAGCAAGGAGACGGAACAGAGGCAAGAGGCAGTAAA

ACCCAGCAGTGCTGTGGAGAACAGCGACTCTGAGGAGGGTGAGAACAAAACCCACCTGAG

CGCCGCGGCCGAAGAACTCGACTCCATAGAGGCAGGAGAAGAAGAGGACGACGATGACGA

TGAGGATGAGGACGATGAGGAAGATGTCGGAGAAGATGATGACGAAGACAAAAGTGACTG

AAGAGGCAAATTCACCTGACTGACCTCCTGCCTTTTACCCTGAGGGCTTTTAAACAACTC

TACTGATACCCCAGACACAAGTTTTATATTATTTTCGTTTGAATTTTGTTTTTGACGTGT

AATTAATCTTATGATGGAGGAACACGAGACATCTTTGCAGGACACACACGCTGCACGCTG

TAGTGTGTGTATGTGGTGTGTGTTGTGCTGTAGGAGGCGCTGATGATGACTGAAGCGTTC

TAAATGTTAGGAATGAGAAGGTTCTGATAGCGTGCTGCTTTTTTTGTGTTTGATTATTTT

CTTTCTCTACTGTGGATGTTGTA

>Unigene000027

CCCCGCACAGCTCCTCTCAGACCTGAACTGCGCATGCGCAGCGCACGCTCACCCACTTGC

AAACTGTCATGGCGGCGGCGACCCAGAGCCAAGCGATTCAGGAAGAGATGGCGCTCTAAA

AAAAGGAAAAAAACTCCTAGAAGAAGAACTGTGAGCTCGCACAGAGCACTCGAATCAACA

GCAGGAGAATTTGTGTTCTGTTTCGGAGTGTTTTTAGCTGCCAGAGAAAGAGAGAAGGAG

AGAATCATTATTCCGCTCTGAAGAAGAAGAACAAGAAGAAGAAGAGAACCCCCAGCACTT

GATGGAGATTAGGTTCTGAAGCTTCTCTTTAATACACCGAGCTTAATCCGAGGTGCTACT

TTAAACCTGAGTCACCTTGAAGAGGAACAGGTGTCTCACCAGGTATGGCGGAGGAAAAAG

CGCCCCCTGGTGGAGAGGGCATCACACCCATCGTGCCCATCAGAGGAATCCGCATGAAGT

TTGCTGTGCTGGAGGGCCTCATCGAGGCTGGAGAGGTCACCAACCGAGACATTGTGGAGA

CCGTGTTCAACCTGCTGGTGGGCGGTCAGTTTGACCTGGAGCTGAACTTCATCATCCAGG

AGCCGGAGAGCATCCCGTGTACGGTGGATCTGTTGGAGAAGTGTGAGGTGACGTGTCAGG

CGGAGGTGTGGAGCATGTTCACGGCTGTGCTGAAGAAGAGCGTGAGGAACCTGCAGGCCT

GCACTGACGTCGGCCTTATCACACTCGTCCTGCAGAGGATCGACAGAGCCCACAGCATGA

TCGCAGACCTGCTGGTGGACATGCTGGGGGTTTTGGCCAGTTACAGCATCACAGTGAAGG

AGCTGAAGCTTTATTTCAGTAAACTACAGGGAGAGAAGAGACAGTGGCCTCCTCATGCTG

TGAAGCTGCTGTCGGTGTTGAAGTCCATGGCCCAGCGCTCCGGCCCGGACGCCTTCTTCA

CTTTCCCTGGAAAGAGTGCAGCAGCCATAGCACTTCCTCCGATCGCTAAGTGGCCATACC

AGAACGGTTTCACCTTCCACACCTGGCTGCGTCTGGATCCCATTAACAACATAAATATAG

ATAAAGACAAGCCTTACCTCTACTGTTTCCGGACGAGTAAAGGGATGGGTTATTCGGCTC

ATTTTGTCGGCGGATGTCTGATTGTCACTGCCTTGAAGGCCAAAGGAAAAGGATTCCAGC

ACTGTGTCAAGTTCGACTTTAAACCTCAGAAGTGGTACATGGTGACCATCGTGCATGTCT

ACCACCGCTGGAAGAACAGTGAAATCAGCTGCTATGTGAATGGCGAGCTGGCGTCTTTCG

GAGAGATCACGTGGTTCGTCAACACCAGTGATACATTCGATAAATGTTTTCTGGGCTCGG

CGGAGACGGCAGATGCGAACCGCGTGTTTTGTGGTCAGATGGGGGCGGTGTATCTTTTTA

ACGATGCCCTCAATGCCGCTCAAATCTTTGCCATCTATCAGCTAGGCTCCGCCTACAAGG

GTGTGTTTAAATACAAAGCAGAAAGCGACCTACTGTTTGCAGATCATCATAAAATTCTGC

TGTATGACGGTAAACTAAGCAGTACCATCGCCTTCACCTACAACCCCCGAGCTACAGACG

CCCAGCTGTGCCTGGAGTCCTCACCCAGAGACAACACGTCCATCTTCATACACTCCCCTC

ACGCCCTGATGCTGCAGGATGTTAGGGCGGTGATCACTCACTCGGTGCAGAGTGCTGTAC

ACTCCATCGGAGGAGTGCAGGTTCTCTTCCCGCTCTTCACTCAGCTGGATTTTGTGCAAC

CGAACAGCGAAGAGCCGGACATCTCTGTCTCCTGCACTCTGCTGTCCTTCCTGTTGGACT

TGTTGAAGTCGTCTGTGGCCATGCAGGAGCAGATGCTCACCTGTAAGGGCTTCCTGGTTA

TCGGATACTCTCTGGAGAAGTCTTCCAAAGTCCACGTAACAAGGCCCGTGCTGGACATTG

TTTTGTCCTTCGCTCGTTATCTCAGCAATCTGCACAACGGCATCTTACTGCTCAAACAGC

TGTGTGATCACATCCTATTTAACCCCGCCATCTGGATACACACACCAGCCAAGCAGGTCT

TGTACACATATTTGGCCACAGAGTTTATTAGCACGGTAACGATCTACAACGCCGTGCGGC

GGGTGGGAACCGTGCTGCAGGTTATGCACACGCTTAAATATTATTACTGGGTGGTGAATC

CACAGGACCGCAGCGGAGTGATGCCTAAAGGACTCGATGGTCCAAGGCCCAGTCAGAAGG

AGATCCATTCTCTTCGAGCGTTCCTGCTTCTCTTTGTCAAACAGCTCATCCTGAAGGACA

CGGGAGTGAAGGAAGATGAGCTTCAGAGCATCCTAAACTACCTGCTCACCATGCACGAGG

ATGATAACCTGATGGACGTCCTGCAGCTGCTTGTGGCGTTGATGTCTGAACACCCCAGTT

CAATGGTGCAAGCGTTCGACCAGCGCAACGGCATCCGGGTCATTTACAAACTGTTGGCAT

CCAAGAGTGAAGGGATTCGAGTGCAGGCTCTGAAGGTCTTGGGTTATTTCCTCCGCCATT

TACAGCCCAAGAGAAAGTCGGAGGTCATGCTGAGTCACAGTTTATTTTCCCTCCTGACGG

AACGCCTGCTTCTGCACTCCAACCAGTTCAGCATGACAACCTACAACGTCCTGTTTGAGA

TTCTGACTGAGCAGATCTGCACACAGGTGATCCACAAGCAGCATCCTGAACCCGACTCCG

CCCTCAAAATCCAAAACCCACAGATCCTAAAGGTGATTGCAGCTCTGTTCAAGAACAACT

CGTGTCCGGAGAGCATGGAGATTCGCAGAGTCTTTCTGTCTGACATGATCAAACTGTTTA

ACAGCAGCCGGGAGAACAGGAGGAGTCTACTACAGTGCTCGGTGTGGCAGGAGTGGATGC

TCTCACTCTGTTTTATAAACCCGAACAGCAGCGAGGAGCTGAAGATCACAGAGATGGTCT

ACACCGTCTTCCGCATCCTCCTCTATCACGCCATCAAGCACGAGTGGGGAGGCTGGCGCG

TGTGGGTCGACACGCTCTCCATCACACACTCCAAGGTAACCTTCGAGAAGCACAAAGAGA

ACTTATCGCGAGTTTTTCAGGAATACCAGCGGATGGGTTCCACTGATGACACTGAGATCC

GAACCGTTTCCGGATCCAGCAGGAACAGTGAACTTACTGCACAGCCATCTGAAACGGTGG

TGGAACCTCCATCTTCCATTATCGAGGTGGAAGAACCAATCTGTGGAGAACATGATGAAC

ATGAGGAGAAAGCACAAGTCATCATCTCAAACATTTTAGCCGGAGTTGAGGAGGAGGACA

CGAAAGAAAAGGAAGAAAAAGCAACAAAGGATGAAGGGAACGTTCAGGAAGCTTTGGAAG

AAAAGGATACAGTAAGTGAGGTTGTAGAGGAAAAGAAGGACTGTAATAAAGCAATGGACA

AAGACGGAACTGAAGGAGGTGATCATAAAAACTTAGAAGAAGAAAACAATCAGAAAGTAG

AATCTGATCAACAAGCCAATGCGGTGAAAATCTCAGAAAATGAAAAGGATAAACCCGCAA

CCTCAGACATTGATGAAGAAGAAAAAGAGTCAGAAAAAGCTGAGGGTCAGAAGGAAAATG

AGATGAAACCTCCTCCTGAGACCCCAGTTCAAGGGGACGGTGAAGAGAAAAAACAGAAAG

AGGACAAGAATTCTGATCTTTCTGAGGTGAGCCCCGGTGCAGAGAAGGGTTCAGACATGG

AGGTGCAGACCCGAGTGGAGGCCAGCACTGATGTCCAGGCAAGTCCAGATCAAGAGACAA

ATCTTGGTTTGGAGAAGGTTTCAAGTACGCAACAGGGCCCCAGTTCAGAGACGAGCCCCA

GTGACCAGGAGAGCCCTAAAGCAGAGACGAGCCCCAGTTTGCAGGAAGTCCCCAGTGTTC

CGGAAAGTCCTAAAGCAGAGACGAGCCCCAGTTTGCAGGAAGTTCCTAGTGTCCAGGAGA

GCCCTGATGCACTGATGAGCCCCAGCAAGGAGGAGAGTCCAAGTATGCAAGAGCTCTCCA

GTGTGGAGAAAAGCCCCAGTGTGGAGGAGGACCAGAGTACCCAAATTCCAAAGGAAAGCC

CCAGCACACAAGAGGCTTCTGGTGTCCCAGAGAGCCTCAATGTGGAGACAAGCATCAAGT

CCAAAGGTGTTAAAATTGCCAGACTGGATGTCTCTAGTGTTGCTATAGACACAGAACGAC

TGGAGCTAAAAGACAACACAGCTGCTGACGGAGGCCAATCCCAAGCTGCAGCCGGAACCT

CCACACAGAACCAAGACGGTTCCTCGTCCTCACCTCGTCCCAGCATGTTCCGTATCCCAG

AATTCAAATGGTCTCACATGCACCAGCGCCTGCTGACCGACTTGCTGTTCTCTATCGAGA

CGGATGTGCAGATGTGGAGGAGCCACTCTACTAAGACAGTGATGGACTTTGTAAACAGCA

GCGAGAATGTGGTGTTTGTGCACAACACGGTTCACCTGATCTCTCAGGTGGTGGATAATC

TGATCATGGCGTCGGGAGGAATCCTGCCTTTGCTCTCTGCAGCCACCTCATCTACGCATG

AGTTGGAGGCGATCGACGCGTCTCAGGGTCTGGAAGTGGAAACGTCTCTGTCTTTCCTGC

AACGTCTAGTCTCTCTGGTGGACGTCCTCGTCCTGTCCAGCTCTCTCAACTTTACGGAGA

TCGAGGCGGAGAAGAACATGACGTCAGGAGGGGTGCTGAGACAGTGTCTGCGCCTCGTGT

GCACTATGGCTGTAAGAAGTTGTTTGGAATGTCAGCTTCACTCTCTGTATAAAGCTGGAA

CAGAGAAAACACAGCGAGGCCTTGTGGGAAATGCTCAGTTTCTTCCAGCTCAGAGTCCCG

TGGACATGCTAACAGGAGGAATTGCTCCAATCCGAGATATGGAACGTCTGCTGCAGGACA

TGGACATCAACCGTCTTCGAGCCATCGTCTTCAGAGACATTGAGGATAGTAAGCAGGCTC

AGTTTTTGGCTCTGGCTGTTGTTTACTTCATCTCTGTTCTCATGGTGTCCAAATACCGAG

ACATTCTGGAGCCTCATAACGACAAAAAAATCAACAAAAACTCCTCTCGACGCACAGAAA

GCGTCAGTTCCGACCGGGGAAACTCCTCCCCCCTCCAACGTCTTGACTCCGCCCCCGGGA

GCGAGGACTCACCTGTTCCCCAGCACAATGCGGAATCCCGCTCCGCACCTGACGCCGTCT

CTGAGGCCTTGTCCACGTTGTCGTCTGAGGTCAGGGGTCACGAGAACATGGACAAGGATA

AAAAGGGAAAGAGCATGAAGGTGAAGGACATCCTGCGCAGCCTGGTAAGCGCCGCCCCTG

CTGATGATGTCATCGTGGATGCAAGTCTCTTACCACCAACGTTTTTGGGAAAGCAGGAAA

TTCACGGCCAGTTCAGCTCATTCGACAGAAGCGTCGTTGTGGGATCTAGGAAAGTGGGCG

GGGCTCCAGCCGCAGGCTCCTCCCCCTCCTTCACCGCTGCCACCACAGGTGGAACTCCGG

TCGTCAGTGTTTCTGTGGTTTCCACAGATGACCCCGCCCCAAGCGACGCTGCAGAGTCAG

GCGGAACAACAGAAGTCACTTCAAACCTGCCATCTGTCCCCCTCTCACCTACCACCACCC

TCAGTATCTCTGAGCGACTGGAACACGCTCTGGAGAAAGCGGCTCCGTTACTGCGGGAGA

TTTTTGTGGATTTTGCGCCGTTCCTTTCCCGCACTTTATTGGGAAGTCACGGACAGGAGC

TGCTCATCGAGGGAACGAGTTTGGTGTGTATGAAGTCCAGCAGCTCTGTAGTCGAGCTCG

TCATGCTGCTATGTTCTCAGGAATGGCAGAACTCCATCCAGAAGAATGCAGGTTTGGCTT

TCATCGAGCTCGTCAATGAGGGAAGGTTACTGAGCCACACGATGAAGGATCACCTGGTCC

GCGTTGCAAACGAAGCCGAGTTTATTCTGAGTCGACAAAGAGCTGAGGACATTCACAAAC

ACGCCGATTTCGAGTCTCACTGTGCTCAGTATGCTGCTGATAAGCGTGAAGAGGAGAAGA

TGTGTGATCACCTGATCCGAGCCGCAAAATACCGTGATCATGTGACCGCGGCGCAGCTCA

TCCAAAAGATCATCAACATCCTTACTGACAAACATGGAGCCTGGGGAAGCTCCACCCACA

GTAAGCAGTGTTTTTGGCGTTTGGATTTTTGGGAGGACGATTTGAGGAGACGTCGTCGTA

TGGTCAGGAATCCATTCGGGTCGTCACACTCAGAGGCCGCACTCAAATCTGCAGCTGAGC

ATGCTCCAGAGGACGACGTGCTGAAGGGAAAACAGCTCGGGAAAGAACCGGCCAGGAATC

AGAGCTCAGAGAGCGAGAGTTCAGCGGAGGGAGACGATGAGACCATTTCATCATTGGAGG

AAAAAGAGCTGGACAATCTGACAGGCACAGTGTCTTTCAGCACAGTAGCTGAGCTGGTCT

CTCCTGCACTTGTCATGAAAGGAACTCTGTCCATCACGGCTCATGAGCTTTACTTTGAGG

TGGACGAAGAGGAGGCGAGTTTCAAGGGCATCGATCCCAAGTTGTTGGTCTATGCAGATG

GACTGCACGGGAAGTGGATGTTCTCTGAGATGCGAGCGATCTTCTCGCGCCGGTACCTCC

TGCAGAACACAGCGCTGGAGATCTTTATGGCCAATCGGACTTCTGTGATGTTCAACTTTG

TTGATGCTGCAACGGTTAAGAAAGTCGTTCACGCTCTGCCTAGAGTCGGAGTCGGAACTA

ACTTCGGCCTCCCACAGACCAGGCGAATTTCTCTGGCGACTCCCAAGCAGCTACTGAAAG

CCTCAAACATGACTCAGCGCTGGCAGAAGCGCGAAATCTCCAACTTCGAGTACCTCATGT

TCCTCAACACAATTGCAGGCCGCACGTTTAACGACCTGAATCAGTATCCAGTGTTTCCCT

GGATCATCAGCAACTACGACAGCGAGGAGCTCGACCTCACACTGCCCAGCAACTACAGAG

ACCTGAGCAAGCCAATTGGAGCCTTGAACCCAAAGAGAGCAGCTTTCTTCTCCGAACGTT

ATGAATCCTGGGAGGACGAGCAGGTCCCCAAATTCCACTACGGCACACACTATTCCACAG

CCAGCTTCACTATGATGTGGCTGCTGAGAATTGAGCCCTTCACTACCTTCTTTCTCAACT

TCCAAGGAGGAAAGTTTGATCATGCAGACCGAACCTTCTCCTCAGTCGCCAGAGCCTGGA

GGAACTGTCAGAGAGACACATCTGATGTCAAGGAGCTGATACCAGAGTTCTTCTACCTGC

CAGAGATGTTCATTAACGCTAACAACTATAATCTGGGAGTGATGGATGATGGGACGGTGG

TGTCTGATGTCGAGCTGCCGCCCTGGGCAAAGAGTCCGGAGGATTTCGTTCGCTTTAACC

GCATGGCCCTGGAAAGTGAGTTTGTGTCGTGTCAGCTCCATCAATGGGTGGATCTAATCT

TCGGCTATAAGCAGCGAGGACCTGAAGCTGTGCGCGCACTTAACATCTACTACTACCTCA

CCTACGAGGGGGCAGTGAACCTCAGCTCCATTACTGACCCGCTACTGAGAGAGGCTGTAG

AGTCCCAAATTCGAAGTTTTGGTCAGACTCCATCTCAGCTGCTGATCGAGCCTCATCCAC

CACGGAGCTCTGCCATGCAGGTGACCCCACTGATGTTTACGGAGCAAATGCAGCAGGATG

TGATCATGGTGCTGAAGTTTCCCTCCAACTCTCCGGTGGTGTACGTGTCGGCTCACACTC

AGGCCGGTCTGTCCAATCCCTCCATCGTCACGGTCACAGCCAATCGAATCTTCTCCATCA

ATAAGTGGCACGGCCTCACCGGTCACCAGAGCTCCTCAAACACAGAGCAGCAGTACCAGC

TGCCGGTGGAAATCGACCCTGTGATAGTGGCTAACCTGATGCACCGCAGACAGGTGTGTG

ATTTATTGGATCAGAGTGTGGAGGTGAACTCACACTGTTTCCTCGTCTCGTCTGATAATC

GCTTCCTCTTCATCGCCGGCTTCTGGGACAAGAGTTTCCGCATCTACAACACAGACACAG

GGAAGCTGATGCAGATAGTGTTCGGTCACAGAGATGTGGTGACATGTGTGTTTCGGTCCG

AGTCGTACATCGGAGGAGACTGCTACATCCTGAGCGGCTCGAGAGACGCCACGCTGCTTC

TGTGGTACTGGAGCGGAAAACACTGCTGCATCGGAGACACACAGAATGATTTTGTGACTC

CTCGAGCAATTCTGACAGGCCATGACTGTGAAGTGACCTGTGCAAGTGTGTGTGCGGAGC

TCGGAGTGGTCATCAGCGGTTCTAAAGACGGCCCGTGTCTCTTACACTCGGTAAGCGGAG

AGCTGCTGCGTGTGTTTGAGAACAGTCAGAGGCCGATGCTGGTGCAGACGAGTTCAGAGG

GACACTGTGTTATCTACTACAGCTCCGGACAGGTGTGTGTGTACAGCATCAACGGCAAAC

TGCTGTGTGACACACACATCGAGGACAACATAAAGGCTATGTTATTAAGTAAGGATGGTC

AGTACCTGCTATGTGGAGGAGAGAGAGGTGTTCTTAGTGTGTGGCAGGTTCATGATCTCA

AACAGCTCTTCACTTATCCAGGCTGCGATGCTGCTATTCGCTCCATGGCCATAACCGCTG

ACCAAAGATGTATCATTACTGGCATGGCATCTGGCAGCATCGTCCTCTTCTACAACGACT

TCAACAGATGGCATCATGAATACCGAACCCGGTACTGATGCCCACACCGTGACCTTGGTG

ACCCCGGTGACCATGGTGATAGCCCTGTCGAACCCTGAACATTGAATGTAATCAGAATAG

CCC

>Unigene000028

CACACACACACACACACACACAACAAACGCACACATTCACACAACCGAATGACACATTTG

CGTTTTGGAAGTGCGTAATAAATCGCGTCTTGGGTCAGCTGGAAGAAGGTCCGATTGAAT

TTTTGGTTATGCAATGGTCTCTGTATCAGCGCTAGCACTTTCAGCTGTGTGCCACTTCTT

GATCAGTCCAGAGGAGGCAGTGATTTCATCTCGCGCGCCAGTGACATAAGCAGACAAGAT

TGCAAAGATCTGTCCTGTTTCGAGTATGACTGCCACGACCCGTGGCTCCCCCATGGGGGG

GAATGAAAGTCAGGAGCAGGGCCAAGCTCCGGACGGTCAATCTCAGCCCCCACTGCCCCA

AAACCAGACGTCCACTCCCACATCGTCCAACGAGAACTCTCCAGTGAGTCCTCCAGATGA

GCAGGTCCAGGGAGACTGTACAGGCCAGTTGGAGGAGGAGGAGGAGCCAGCTTTCCCTCA

CACAGACCTGGCCAAACTGGATGACATGATAAACAGACCCCGCTGGGTTGTTCCAGTTTT

GCCAAAGGGTGAACTAGAAGTTCTTCTTGAAGCTGCTATAGATCTTTGTAAAAAAGGACT

TGATGTCAAGTGTGAAGCCTGCCAGAGATTTTTCCGTGATGGTTTGACAATATCGTTCAC

TAAAATACTGACTGATGAGGCTGTCAGTGGATGGAAATTTGAAATCCATAGATGCATAAT

CAACAACACTCATCGCCTCATAGAACTGTGTGTGACCAAGCTCTCTCAGGACTGGTTTCC

TCTTCTAGAGCTCTTGGCCATGGCCACCAACCCCCACTGCAAGTTCCACATTTACAATGG

TACCCGGCCCTCAGAGACGGTGCCTGCTGGGGTGCAACTGGCTGAAGATGAGCTTTTCGC

TCGTCCACCTGACCCTCGCTCTCCTAAGGGCTGGCTGGTGGATTTAATAAACAAATTTGG

CACATTAAACGGGTTTCAAATTCTGCACGATCGATTCATGAGTGGTCAAGCTCTGAACGT

TCAAATCATCGCCGCACTCATAAAGCCTTTTGGGCAGTGCTACGAGTTTCTCACTTTGCA

CACGGTGAAGAAGTACTTCCTTCCCGTTATAGAAATGGTTCCCCAGTTTTTAGAAAACCT

CACTGATGAGGAGCTAAAAAAGGAAGCCAAGAATGAAGCCAAAAATGATGCTCTGTCTAT

GATAATCAAGTCGTTGAAGAACCTGGCTTCACGAGTACCAGGGCAAGAGGAAACCGTGAA

GAACATGGAGATATTTAGGTTAAAAATGATCCTTAGGTTATTGCAAATTTCTTCTTTTAA

TGGCAAAATGAATGCACTAAATGAAGTAAATAAGGTGATCTCAAGTGTCTCATACTACAC

ACACCGTCATGGCAACCCAGAAGAGGCAGAATGGCTCACGGCAGAGCGCATGGCGGAGTG

GATTCAGCAAAACAACATTTTGTCTATTGTGCTGAGGGATAGCCTTCATCAGCCTCAGTA

TGTTGAGAAGCTAGAGAAGATTCTGCGCTTTGTTATTAAGGAAAAAGCTCTTACCCTTCA

GGACTTGGACAACATTTGGGCTGCCCAGGCTGGGAAACATGAGGCTATTGTCAAGAATGT

TCATGATCTCCTTGCAAAATTGGCATGGGATTTTTCACCTGAACAACTAGACCATCTTTT

TGACTGTTTTAAGGAAAGCTGGACAAATGCGAGTAAAAAGCAACGAGAGAAGTTGCTGGA

ACTAATTCGTAGGCTGGCAGAAGACGATAAAGATGGCGTGATGGCACACAAAGTGCTTAA

TCTGCTTTGGAACTTGGCCCATAGTGATGATGTTCCTGTGGATATTATGGACCAGGCCCT

CAGTGCTCATATCAAGATCTTGGACTACAGCTGTTCCCAGGACAGGGACACCCAAAAGAT

CCAGTGGATAGATCGCTTCATTGAAGAGTTGCGATCAAATGATAAATGGGTAATCCCTGC

ACTGAAGCAAATTCGAGAGATCTGCAGTCTGTTCGGAGAGGCTCCTCAGAATCTCAGTCA

AACACAAAGGAGTCCACATGTTTTTTATCGCCATGACCTTATTAACCAACTTCAGCACAA

TCATGCGCTGGTCACTCTAGTAGCTGAGAATCTGTCTGCATACATGGAGAATATGAGGCA

GTTTTCCAAAGCAGAGCACACAGACTTTGACCCGCAGACGGTCCGGCCTGGAAGTCGATA

CAGTCATGTGCAGGAAGTGCAGGAGCGTCTAAACTTTTTAAGGTTTTTGTTAAAGGATGG

GCAGCTTTGGCTGTGTGCTCCTCAGGCCAAACAGATTTGGAAGTGCCTGGCAGAGAATGC

AGTGTTCCTGTGTGATCGTGAAGCCTGCTTTAAATGGTACTCCAAACTGATGGGGGACGA

GCCTGACCTTGATCCTGATATCAACAAGGACTTTTTCGAGAACAATGTGCTCCAGCTTGA

TCCTTCTCTGCTGACTGAAAACGGCATGAAATGCTTTGAGCGCTTCTTTAAAGCAGTAAA

CTGCCGAGAGGGCAAGCTGGTGGCCAAGCGCCGAGTGTATATGATGGATGACTTGGAACT

GATTGGACTGGATTATCTGTGGAGGGTTGTGATCCAAGGCAGTGATGACATCGCTAGCCG

AGCAATTGATTTACTTAAAGAGATTTATACAAACCTTGGACCAAAATTACAAGCCAATCA

GGTGGAGATCCATGAAGATTTTATTCAATCTTGCTTTGACCGACTCAAAGCCTCCTATGA

CACGCTGTGTGTGCTGGATGGAGATAAGGACAGCATTAACTGTGCCAGGCAGGAGGCCAT

CCGCATGGTGCGCGTTCTCACTGTGCTAAGAGAGTACATTACAGAATGTGACAGTGACTA

CCATGAGGAGCGGACCATCTTGCCGATGTCCAGGGCATTTCGTGGGAAGCACATCACCCT

CATTGTGCGATTTCCAAACCAAGGTCGCCAGGTGGATGATCTGGACATTTGGTCCCATAC

TAACGACACCATTGGTTCTGTGAGACGCTGCATTCTCAATCGAATAAAGGCTAACAGCGC

GCACACCAAGATTGAGCTCTTTATTGGCGGGGACATTATTGATCCTGCTGACGACAGGAA

ATTGATTGGACAGCTTAATCTCAAAGACAAAACTCTTATCACAGCCAAGCTTACCCAGGT

CAGTGCCAATATGCCTTCGAGCCCAGACAGTTCCTCTGACTCCTCCACCGGCTCCCCTGG

GAACCATGGCAACCACTATAGCGATGGACCAAACCCTGAAGTTGAGAGCTGCCTTCCTGG

AGTGATCATGTCCCTGCACTTGCGCTACATCTCTTTCTTGTGGCAAGTTGCTGACCTGGG

TTGTAATCTCAACATGCCTCTTCTGCGTGATGGTGCTCGGGTTCTTATGAAGCTCATGCC

TCCAGATAACACTACAGTGGGAAACCTGCGAGCAATCTGCCTGGATCATGCCAAACTTGG

AGAAAGTAGCCTTAGCCCCACTTTAGATTCCCGCTTTTTTGGTCCATCACCTTCACAAGT

CCTTTACTTAATAGAGGTGGTTTATGCCTTACTGATGCCAGCTAGTGGCACACTGGGAGA

GGATGCTAGTGACTTCCAGTACAACTTTTTGAGGAGTGGCGGCCTACCTTTGGTCTTGAG

CATGCTGACCCGAAATAATTTTCTTCCAAATGCTGACATGGAGACGCGCCGAGGAGCCTA

TCTCAACGCACTAAAAATAGCCAAGCTCCTGCTTACAGCTGTTGGCTTTGGACATGTAAA

GGCTGTGGCTGAAGCTTGCCAGCCTGTAGCAGAGGGAAACATCCCAGTGTCACCTATAAA

CCAGGCCACTCATGACCAAGCATTAGTGCTTCAAAGCGCCCTGCAAAACATCCCCAATCC

CTCGGCTGAGTGCATGCTTCGCAACGTGGCAATCCGTCTGGCTCAGCAGATCTCTGATGA

GAATTTTTTTCAGGCTTCTAAGTATATCCCAGACATCGGTGTGATCAGAGCAGTCCAGAA

GATTGTCTGGGCCTCTGGTTGTGGTTCTATTCAACTTGTTTTTAGCTCGATTGAGGAGAT

CAGCAACATCTATGAGAAGACTAATGCAGGGAATGAGCCCGATTCAGAGGATGAGCAGGT

GTGCTGTGAGGCCCTGGAGGTTATGACTCTTTGCTTCGCCCTTATTCCGACTGCACTTGA

TGCACTTAGCAAAGAGAAGGCCTGGCAGACCTTTATCATTGATTTACTTTTGCACTGTCA

GAGCAAGTCTGTTCGCCAGATGGCCCAGGAACAGTTCTTTCTCATGGCCACTAGGTGTTG

CATGGGTCACAGGCCTCTCCTGTTCTTTATTACTCTACTCTTCACAGTTTTAGGTAGCAC

TGCAAAAGAACGGGCCAAGCATGCTGGTGATTATTTTACACTTTTGAGGCACTTGCTCAA

CTACGCATATAATAGCAATATCAACCTCCCTAATGCTGAAGTTCTTCTCAACAATGAGAT

TGATTGGTTAAAAAGAATTAAGGATGAAGTTAAGCGAACTGGCGAGACTGGGGTGGAAGA

AACCATATTGGAGGGTCATATCGGTGTCACAAAGGAGCTACTGGCTTTTCAGACAGCAGA

AAAGAAATACTATATAGGATGTGAAAAGGGTGGAGCCAACCTGATCAAGGAGCTGATTGA

TGACTTCATCTTCCCAGCCTCAAATGTGTACCTGCAGTACATGAAGAGTGGAGAGTTTCC

TCCTGAGCAAGCCATACCTGTGTGTAGCACTCCTGCTACAATTACAGCTGGATTTGAGCT

CCTGGTTGCACTTGCTGTTGGATGTATGCGCAACCTCAGACAGATTGTGGACACCCTAAC

TGATATGTACTACTCGGGTTGTGAGGCACTTACAGAATGGGAGTACCTGCCACCAGTCGG

TCCAAGGCCCACTAAAGGCTTTGTTGGCCTGAAGAATGCAGGTGCCACTTGTTATATGAA

CTCAGTAATTCAACAGCTTTACATGATTCCAGCCATCAGAAACGGCATCTTGGCTATTGA

GGGCACGGGCAGTGATGTGGATGATGATGTGCCTGGGGATGAGAAACAAGATAATGAGAG

TAATGTCGATCCACGTGATGAGGTATTCAGTTATCACCACCAGTTTGATGACAAACCATC

GCTCAGCAAATCAGAGGACAGGAAAGAGTACAACATCGGGGTTCTGCGCCACCTTCAGGT

GATCTTTGGTCATCTCGCTTCCTCCAGGCTGCAGTACTATGTTCCCAGGGGATTTTGGAA

ACAGTTTAGGTTGTGGGGTGAGCCAGTAAATCTGAGAGAACAACATGATGCTCTGGAGTT

CTTCAACTCGCTCGTAGACAGTTTAGATGAGGCTCTTAAAGCACTGGGCCATCCTCCCAT

GCTAAGTAAAGTCCTTGGTGGTTCGTTTGCTGATCAGAAGATCTGTCAGGGGTGCCCTCA

TAGATATGAATGTGAAGAATCCTTTACAACACTGAATGTAGATATCCGAAATCATCAGAA

TCTGCTTGATTCTATGGAGCAGTATGTGAAGGGTGACTTGCTTGAGGGTGCTAATGCCTA

CCACTGTGAAAAATGCAACAAGAAAGTGGACACAGTAAAGCGCTTGCTTATTAAGAAACT

GCCTCCTGTACTGGCCATTCAGTTAAAACGATTTGATTATGACTGGGAACGTGAGTGTGC

TATCAAATTTAACGATTACTTTGAGTTTCCTCGGGAGCTGGACATGGAACCCTATACAGT

AGCAGGAGTAGCCAAGCTAGAAGGCTCTGATGTCCACCCTGAAAATCAGGTCATCCAACA

GAACGAACCCTCAGAGCCTGAGGCACCATGCAGTTCACGCTATAGACTTGTGGGAGTGCT

AGTGCACTCAGGCCAGGCCAGCGGTGGTCACTACTACTCTTACATCATCCAGAGGAATGG

CAGTGGAAGTGAGGGTGAAACCAATCGCTGGTACAAGTTTGATGACGGTGATGTCACCGA

GTGCAAGATGGACGACGACGAAGAGATGAAGAATCAGTGCTTTGGGGGAGAGTACATGGG

TGAAGTGTTTGACCACATGATGAAACGCATGTCCTACCGGAGACAGAAACGCTGGTGGAA

TGCCTACATCCTGTTCTATGAGCGCATAGACTCAATGGACAAGGATGGTGAGCTGGTGAA

GTACATCTCCGAGCTGACAGTAAGCAGCAAGCCACACCAGATCAAGATGCCACCAGCCAT

CGAGTGCAGCGTACGCAAGCAGAATGTGCAGTTCATGCACAGCCGCATGCAGTACAGTCT

GGAGTACTTTCAATTCATCAAGAAACTCCTGACCTGTAATAGTGTCTATCTTAACCCACC

ACCAGGACAAGACCATCTTTTGCCTGAAGCAGAGGAAATGGCTAAGATTAGCATTCAGCT

TGCTGCTCGATTCCTCTTCAGCACAGGATTTCATACCAAGAAAGCAGTCCGTGGCCCAGC

CAGCGATTGGTATGATGCCCTGTGCATCCTCCTTCGACACAGCAAGAATGTACGCTGCTG

GTTTGCACACAACGCCCTATTTTCCTACCCAAACCGCTTCTCTGAGTACCTGCTCGAGTG

CCCTAGTGCTGAGGTGCGGGGGGCCTTCTCCAAGCTTATTGTATTTATTGCACATTTCTC

TCTGCAAGATGGACCTTACCTATCACCTGTTGCCTCACCAGGCCCATCCACTCAGGGCTG

TGATAATCTGAGCCTAAGTGACCACTTGTTACGTGCTGTGCTCAACCTGTTACGGAGAGA

AGTGTCCGAGCATGGCCGTCACCTGCAGCAGTACTTCAACCTATTTGTCATGTATGCTAA

CCTAGGTTTGGCAGAGAAGACACAACTGTTGAAACTTGGTGTGCCAGCTACATTCATGCT

GGTGGCTTTGGATGAGGGCCCAGGCCCACCCATTAAGTACCAGTATGCTGAGCTGGGAAA

GCTGTATACAGTGGTGTCGCAGTTGGTTCGTTGCTGCGATGTAACTTCTCGTATGCAGTC

ATCAATCAATGGTAACCCTCCTCTTGCTAACCCTTATGGCGATCCCAGCTTGACTCAGCC

CATCATGTCTCTGCATCAGCTAGTGGCTGAGATCTTGTTTGTGCGCACCAGTTATGTGAA

GAAGATTATTGAGGATTGTAGTAACTCTGAGGAGACCATCAAACTCTTGCGCTTCTGCTG

CTGGGAAAACCCACAGTTTTCCTCCACTGTGCTCAGTGAACTGCTGTGGCAGGTTGCTTA

CTCCTACACTTATGAGCTAAGGCCCTACCTAGACTTGCTGCTACAGATCTTGCTTGTTGA

AGACTCTTGGCAAACTCACAGGTCAGCCTATGCTTTTCTGGCTAGCAGACTAATACCAGC

AACACTTAAATTGCATTTCTAAACATTTCCTTTTTTTTGATCCATCAGGATCCACAATGT

GCTGAAGGGGATTCCAGATGACCGTGATGGATTGTTTGATACCATCCAGCGCTCTAAAAA

TCACTACCAGAAGAGAGCTTACCAGTGCATCAAGTGCATGGTGGCACTGTTCAGCAATTG

CTCAGTTGCCTATCAGATTCTTCAGAGTAATGGAGACCTGAAGAGAAAGTGGACATGGGC

TGTAGAGTGGCTTGGGGATGAACTAGAACGCAGGCCATATACTGGCAATGCCCAGTACAC

ATATAACAACTGGTCACCTCCTGTTCAGAGTAATGAGACCTCCAATGGCTACTTCCTGGA

GCGTTCCCACAGTGCCCGCATGACCCTAGCCAAGGCCTGTGAGCTTTGCCCAGAAGAGTG

TCACTTAACGAAGCACGAGGTGGTGTCTGAAGAGGACGCCGGCCGGAAGCTGTCCTCGCC

ACAGCAGCTTTTGCCAGGGGAAGTGACAGGCCAGCAGCAACACACTGAGCAGGATGAGCA

GGAGGCCCCAGATGATCAGGACTCCTCTCCACCAGAAGATACTTCTCTGTATCCCCACTC

TCCAGGGACACAGTATCAACCGCAAAACAACCTTCCGCACGGTCAGCCGTACACCGGCCC

GGCAGCACAGCATATGAACAATCCCCAGCGTCCAGGTCCACGAGCACAAGAGAACTGGGA

GCCCCCAGAAGAGGTGCCACCTGCCCAAACAAAAGAGTAGCACCCTGCTTTCATCCTGTC

CTCCATCCGAGTACAAAACTTTCAAGTAAAGAGCGAGGCCCCAGGCCCCACCATGCAGCG

TCACTCCACAAACTGTGTCTCCAGAAAATTTTGCTCCCTGTCCTCCTCACCCTGGGGTGG

AGTATCACTGTCTGCCCTGCAGTATTACAACCTGCCTGCAGTGGATGGTGTATGGTGGCA

TAACAAGCCAGGCCTGATGCACTTGTCTCATGTGACAGTGGTGTACATATTCTAAAATAG

TTTTTGACTGTTCGAAACCATCAGCACCACTTGCTGGTTGGAGACTTGCGGATTTACAAA

ACTTTAAAAGTACAAAAATGCAAAAAAAAAAATGATGTAAAAAAAAAAAAAAAGCCCTGT

TTAATCACCTGTGCTCAGTGGTGCAGTTTGATCTGCCTTTTGGCCCTTTCGGTCATTCAG

CTCTATATTATTTTATCCATGTCCACTCTTGGTTAATCTCTCTCACTTCTGGAGCTGAGG

ATGGCTGTACTGTAGGCATGATTAAATGGTCCTGATTCTAGGTTGATGTAAGAGGTGATA

ACATTTGTTCTGAAAACTTTCATTGCCTTTCCTCTGTTTTGGTATTAACTGAATCATTTG

AAAGCTCTAGTTGATATGCTGTAATGTTTTAGCATGGCTTCACACCGAACTAGTGTAGCC

AAAGGGAAGTTATGTGATCCTAACCACCGTAGTTCCTAATTGACAGCTATTTTGACAAGC

CGTTCCAACCCCCCCACCCCAA

>Unigene000029

AGATCGGAAGAGCGTCGTGTCAGAGATTGGCTTTTCCAGCGTTGCTGATTTCATTGGCTC

TTTAAACGAGCACCTAGTGGTGAAAAATGGGACAATTTTCCACAGAAACCACTTACCTCA

GACCCAGGACACTGCAACGGAGAATAAAGTGGCTCAAAGCCTACCAGAAGCTTTTCAAAA

TGCAGACAATGACAAAATATCACAAGAGGTGTTGGAATTGATTCAGGAGCACCCGGAAGG

CATTCCTATCAATCAACTAGCTACGTTCTACAATATTCGCTACAGGAAAAACCTGATCGT

TTCAAACCTAGGCTTCGCTTCACTCAGCAGTTTTGTCAAATCTATGTCAAAGGATTTGCT

GGTGAAAAATGGCAGCATCCTTCACAAGATGCACAGGGTTGTTTCTGAGCGTCCAGCTTT

GCAGAAAGAAACCCTTTCTACAGTGAAGGCTAACTCAAAAATGTCTGATGGTACCGCTTG

TACACGAAAGGAAGGTGAGATGAGCAAAGTGGAACTGCTTAAGAAGGTTAAGGAGGTTAT

CCAGGTCTACCCAGATGCTGCAACTTCTATTACACAGCTGATGAATGGCTACTTTTTACA

TTTTGGGTCTATACTGCCACTGGGGCTGTACATTTCATTATACGACAGTCAGACCAGCAC

ACAGACGGCAACTGTTGGCCAGGCACAAGTTATTGCAGGACATAAAACAGGAAGCCCTCC

TACATCTATAAAGATGTTCACTTCAGCCACTGCAAGCCCGCAGCATGCTCCGGTGACTGA

GGAAATAATGCGTTCCAAGAGTGGCTCCCCTGTATGGCGTCTCACTGCAACTCCACCTCC

TTCAGATTCCAGTCTAACATCATCAATAGACTTCCCACCCCTTACTACACGACCCTCTAG

AGCTGAGGACAGGAGACATGGCCAAGAGGGAAGCAAGTTGGTGTTCCGTGAAGCCCATCA

TGCCCAGCTGCGAGAGGTGCACGGAGCTAACGTTCGTGCAGTAGAAGCCTTAGAGATAAA

TGAGACCACAGATAGGCGCAAAACTAGACCGAGTCTAGAGGAGGTCAACAACCTCGCTGA

GGACGCTATCCAAGACCTTGCTGCCCAGGGGGAACACGTCACTGTGGAGAAGGTGACAAA

TAAAGTGTGCAGGCTTTTACAAGTGCCATCCTTGAATGCATTGGGAATCAGATCTTATTG

GCAGCTGCCTGCTCTAAAGGAACTCGAGCGCACTATTAAAGAGATCAACCTCTTTGTTCA

GTCGATAGAAGCGACCACTTCCATGTGCACTCTGTACGAGCTGGGTCATGCTATTGCCAG

CATCAAGAACAAGAAACGATTTGAAGAGCTCCAGCTAGGCCCTCTCTCTAAAATACCAAT

TATTCATCGGCTGTTCACCATAGACAGCAACACAAAAGATGATGATATACACCAGATCGA

GACTGTGGATCTTCTTAGGAGTCTTCGTAACTTTAGGAGAAAGCAAAACAAGCCCAAGGT

AGATCTTGCTGAGTTCCTGCAGTATCTTGCAGACCAGTACAACTGTGATTCACCATATGC

TCTTGGAATCCGGATCCAGAGTGTTGGACTTCCCATTTCGACATTGTCGAAGGCATATGG

GAGTGAGAATGCTTCCTTGGAGAGAGCCAGAGAAACCATTCAGAAGGAGATTGAAGAGGA

GGTACAGACCAGGCTGTGGAAGATTAAAAAGACTTTATTAGAGCCAGCACAGGGATCGCC

GCTCTATTCCTCCTATGGCAGCCTGGAACTCAGAAAGAAATACACCACCTTGACGGCTTC

AGAGGCCGTCCTTGAGGTCTTTAAAAACTCTGTGGGCATTTTCAATCAAAGGATGACTAA

GCGAGTTCAGGAGTTCCTAACGAACATATCAGAAGACAGGCTGGCTCGGACCTTGTTTCA

GCTGGCTATCTGCGGCGGCTCTCTGGCCATCCCAAATGATCTAGCTCCCAAGGAGAAACC

CCAAAAACAAACCCAGGAGCAGAGCGCATCTGAGAAGAAGGCAACCGAGATGCTTCCCAC

TGAAGATGCTGTAAAGCAGTACTTCCTGGACACTGTATCCAGCTTTATGGGCACCGTGAC

GCTATCATATCTCAACCGGCTGGAGAGAACAATAGCCAAGCATTTCAGGTTTAAGGAGTT

TAGCCAGCTACATCAGGGCTCCTTCCTTGAATTCCTGGTCAAAAACATGCAGATTCTACA

AGATGCTGCAGGAGGCGCTGTGGCTATCGCCAACCAGGAAGTTCGAGCCAACGGCTTCAG

ACCCAGCAAGCAAGATGTGTATGAGTTTATCAAGCAATGTGGAGAAACAGATCCTTCAAA

GCTGCCATTTATCGAGGCTGCCATCAGGAGTCACTACAAGGTTCAGGACAGTCGAGAGCT

GGGCTATGGAAAACTTCATACACTCGCAGATTTCGCCAGGCACCAGAAGGAGCTGTGTGA

TGGGGTCGCGTCCACTTTTGTGTTCTATGAATGCCCTCTGTTCCATAAAGAGCCAGAGGA

CATGCTGGTGAATTCAGAGGGCCTGCTGGGGGACGTTAGCCGGGAACAGGCAGTGGCGAG

TCTTCTTTCTGCTCCATTGCTGGAGGATTTGGCTGAATGGAGTCAGTGGGAGCTGGTCTT

TCAGTCTAATCATGGACCTCTCAAACACTTTATCGACAAATACTGTGGTAACACAGACCT

GCTGGCTTTGGAAGTGTCTCCTGGAGTGCTGTTGAGGATCACCTCTGTCACTGGTGATAA

ATTCTTCTCTCAGGCCAGCCAGGCCCTTGACCCTGCAGGCACCGCCGGTCACCTGGTGTC

CATCGTAGTGCGTGATGGGATTTTAAACACCCCCACAGCACTGCTCGCCAATCTCATGGA

AAGTTCGCTAGCAGCTGCAGCTGCTAAAGAAGATCCATCCCTGGGTGAAGACAGATGCTC

CTTTAGCATAGTGGCCAAGTTTGTGCTCGATTGTGTAAGCCGGATGCCTACACGAATTTG

TAGAGAGCTGCTGCAACAGGTGTTCCTGGAGCCTCTTTCAAAAGTGCTTGGTCAGGCCCG

GTCTAAAATTGTCCTTTTGGACACAGCCAGGTCAGAGGCAAGATACCTCAACAAGCTTCA

TCAGCTGGGTATCATGCTGGGCATCACAGAGTGGGTGAAGGACTTCCACAACAAACTCAT

CCTGCCTCCCACACCTGAACTGCCTCAACAGAACCTGGCAGCTGAATCTGTAAGTGACTT

ATCATCTGTGTTGAGTCTTGCTGACAGTGAAGATTTGTTGGAAACCTTTCAGCAAGATAT

TAATGACTGCATTAAGGACAATGTTGAGGATGATTCAGAGGATGATGCAGAGGATGAGGA

TGAGGATCTCTTTGAACTGGCATCAGAGAAGAACGAGAAAGTGAACGGCAGTGTTGAGAG

TAAGGAAGAGGATGAAGAGACTAATGACGTAGACAGTATTCCTAAAGGCCATGCAGAAGA

AGATGAAACTTTCAGTGAGCAGCTCAGCCAGCAGATGGCTATCATCGAGGACATCAGGAA

AAATGAATTCGGTGTGGGAGTGGAGCTGAACGAAGCAGGCCAGAACCTCATGAAGAAGCA

ACAGGCTCGTCTTGGCCGCAGTCTCGAACGCCTCTCCACTGAACTCTACAGCAAAGACAC

ACACTTTGTTCTGGAGCTCATCCAGAATGCTGACGATAACAGCTACCCGCTACATAGAAA

GGAGGAGCCTGCTTTGGCCTTTATAGTGCAGAAAGACTGCATCACCATTCTAAACAACGA

GTTCGGCTTCGAAGAGAAGAACGTGCGAGCTATCTGTGACGTGGGAAAAAGCACCAAAGG

AAAACATACTTGTGGCTACATCGGTCAGAAAGGCATTGGATTTAAGTCAGTTTTTAAAGT

CACAGACTGTCCTGAGATCCACTCCAATGACTTCCATATTCAGTTTGACAAGGCCAGCGG

CCCTATGGGATACATCCTGCCTCACTGGGTGGAAGAAGAGAGGCATGTGCCCCCTGTTGC

CAAGGAGATAGCAGAAAAAAGCTGGACAACAAAGATTTTCCTGCCTCTTCGATCAGAGAG

CTACCAGACCAAAAACCTTTTTCACGACGTACACCCATCTCTGCTTCTCTTCCTTCACCG

TCTCCGCTCCATCACTATCTACAGCGAGGCCGAGAGAAAAGTGGTGTCAATGATGAGACG

AGATCTAAATCACAACGTGCTGGAGGTAGAGCACACCGGCGGTGTGGAGCGCTGGTTGGT

TGTCAAGAAGATACTCTACCCTGAGAAAGTTAAAGTGGATGTGGAATCTACCGAACTAGC

GCTTGCCTTCCAGCTCAATAATGGCTCAGAGGGCAAAGTAAAAGTTCAGCCTCGGCAGCA

GCCCGTCTTCGCCTTCCTTCCCCTGCGAAGCTTTGGCTTCCGCTTCGTCATCCAAGGAGA

TTTTGACATTCCTTCTTCTAGAGAGGATGTTGATAGAGACAGTTCCTGGAATCAGTGGCT

CCGCTCTGAAATCCCCCAACTCTTCTTGCAAGCCATGGATGTTTTCACTACTCACCCTGA

ATTCAGTGGACTGCAAGGACTCTGCCACTTCCTGCAGTATATCCCTCAGCCTAGTGAAAT

CCTGGACTTTTTCAACCCGGTGGCCAAACAGATTATCCAGCTGCTGAAGGGGAAACCCTG

CCTTCCTGCTAAGATGGACAGCAATGGAGGCGTGCAGTTCAAGCTGCCTTCGCAGGTGGC

TGTGTGCCAGGACCCCTTAATCCAGGATGTGATCGGAGGAGAAGAGCTGTGTAAGCACCT

GAACCTGTCCTACCTTCACCCTGAATTACAGTCATCACTGTCATCTTCTTTGCTCTCTGC

TTTGGGTGTCCAGCGTCTGAAGGCTGCCAACATCACCACCGTAATCTGTGCAATGGCCAA

GAGTCTTGTCCAGGAGGGTGATATTTACACAGATTCCAACCTGAAAAAACTGGCCAAGCT

GCTGGCTTGTAATTTCCGTGCTCTGGAATCTGAGTACAATGAGGTGGATAGCCTCCTGCA

GGCTCTCCGGGACATTCCCATGATTCCTCTGGCGGATGGCCGTGTGGTGTCTCTCAGCGC

AGAGGGTGTCTTTTTTCCTCTCAGTGAGGAGACTCACAAAGGCCTGAACGCTTTGTACAA

GGACCTGAGCATCGTAGAGCCGCGACTGCTAGAGTGTCTGGATAAATTGGGTAACTCTCA

GGTGCGTGAGCTGCTCTGTAGGCTGGAGGTTCATGAACTGGAGCCCAAAGAGGTGCTGCA

GAATCACATCTACCCCATACTGAAGCAAGCTGCTTGGAAGGAAAAGCCGGAGAAAATCAC

TGTCAGTTACCTGGTGTTTATCAAGGAGCATTCACAGGAGCAAGACTACAGGGCGCTGAG

GGAAGCTATCCCCGTACTCACCAACAAGGGTTTCCTCTGCCCCGGCCAGTGCAAAGTGCA

GTTCTCCAAAGAGTACGGCAACATTGATCTGCCCAACAAGCTGCCTGGTGTCGACTGGGT

TCTTTTGGATTCTTGCTACCTGTGTGATGGCGATTTATCAGGATGGAGGGATTTTTTGAG

TGATCTCGGAGTAAGAGATCTCCTAATTTTCAAGAAGGAGCGACGCAACCTCAAAGACAC

AGAGCTGGTATCGAGCCCATGGGCAGCGGAGGCAGAGCTGTGGCCCAAACCTACAGATAA

ACTGTATGTGATAGAGGATCAGCAGTGTGAAGAGCTTCACTCACTAATAACGGCAGATCA

ACTTCCCCCTACCATCAAACTTCAGCAAAGACAAGCTCTAATCAACCTTCTACAGAACAA

CTGGGACACTGGAGAGAAATATTCTCAGTACCTCAGTGCTCAGGTACTGGACAACCAGGG

CCGCACTCTAAAAGACACCAGGTCCTCGTTTTACTACCATCTCACCCAGTTGGCATGGAT

GCCTTCCTACAAGCCCACGCAGGACGAGAAAAACTCGGTGGAGTACTTGATTCCAAACAG

GGTGTATCTCTTTTCTAACAAAGTGCACAGTCTATTGGGATCCCACGTCTTTTATGTCAA

TCTGGATCCCAGCGAATTCTCCAGAGCTGTAGGAATGAGACACACTGTTGTGGTAGACGA

GATGATCAGCTACCTGAAACGCTGGTGTACGAAGGAGTCCGATAGCGGCGAGGCAGAAGG

AGCTGATTTTATCACCACCGCGCAGCACATCCACACCGTGTACCTCTACTTGCAAGCCGA

GTGCTCGCACACACAGCTTAGGGACCTCTTCCAGCACTCACCTGCTGTTTACATAGAGTA

TGACAGAAAGGATGAGTGGTCTTCAGGACGCTTCTATCATCTCAAAGACGTGTGCTGGAG

AGATCCAACAGACATGTTTGTCAGATACAAGGAGCTGATCCGCAAACCAGACAGCAGGGT

CCAGGAACCCAAAGTACTGGCTCCATTCTACAGCAAATTGCCCCAAATGGAAGAACTGTT

TAAGTCTCTAAACGTTTACCCCAGTCCATCCATGAAGCAGTACGTGGACCTGTTGGAGAC

TGTATGTGAGTCGTCACCACTTCCCACAGGAGAGGTGCTACAGGATGTCTCCATCATTTT

TGCCAGACTGGCGGACAAATGTAAAAACCCTACCAACGTAGGGCAAGATCAAGACATCGA

AATGAACCAGTGCTACTGCATGACCCTAAAAGAGATGGTGTCTCGTCTGAAGGTGTTTCC

TACCAAGACACAGGGTTGGGTCACACTGGCACGGAAACCTATGATAGCAGACAGCCCAAG

CCTGGAGAAGATCTTCAAATCTTACAAAGAAGTCTGCCTCCTCAACCTGCCCTCGGCTCA

GAAGAAAGTACAAAGACACAAACCGAACCAAAAAGCCAAGCTGCATGATGAGAAGCTGGC

CTTCAATGAAGGAGACCGAGAGCTCTTTCTGGAGATCTGCGGCGTAAAGAAACTCGCCCA

GTGTGTCACCACCGAACCTACAACGGAGCTCTACAGGCCGTGTCCAGCTATGCAAAACCT

GGTCCGTAATCTCATTCCTTACATCCAGAGGTTTATCTATCACCACGATGAGCTTGGAGA

AATATACGATGACCTAAAAGAAAATGGAATTGCCCAGGAGATCAAATCTCTCAGCTTCGG

TCAGGTGGGAATGCTGTACATTAATTACCGACTGGAACAGCCAGATGAGAGCCCCATTGT

AGAAAGTGAGGACATCATTTGTCTTCTGAAAGACAGAAAAGAGTTGTACATTCAGAAAGA

CCATCTTTCATCCAAAATGGCCATCTGCAGAGAACTGGTGAAACTGTTCACCACAGAGAA

GAGCTTTGTAGAGGAACTGAAACACTTTCTCGAGGAGCTTGTTATCCGTATCAGTGATCC

GGATTCTTTAAAGAGATTACTGGATGGTAAAAACATACAGGAGCTTCCTCAGGATGAAGA

GCGGTGGGAGGTTCCGGCACCGCTGGAGACATTCGAACCCAGGACCTTTACTATCAGCTC

ATCAGGGATTGTGAGTGCAGAAGAAGAGGAAATAAACACAGAAGAAGATACACTGCACAG

CTGGCCTCCTAAAGCATCCATCAGCAAAACAGGAGGCAGAAGCACAGGAAAAGGAAGCCA

GGCAGTAGAGGCAGTAATGAAGATGTGGCCTCCTCCAGCACCTCTGACCTCTTCAGCTCA

GTCTGTTTCTCCTGCACCTTCAGCTCACTGGAGTTCAGGAGCTGAGAGGAGTCCTTCCAA

TCTGAACTCTCAGGAACGCACGACTGAACTTCAGATGCACCGAGAGCCTGGCAGTTTAAA

TATTCCAGGGAATCAGGAGCCCAGGGAACGTGCTGGCATCCCACGTGCGGAAGCACAAAC

TAGTAACGCCGATGTGCCGGTGGTCCAGGGTGTCGAAGCTCCTAATGAAGCCCATGCTGG

GTCCAATGGAGCTCCTGATGAGCACACTGAGGTGTGTGTGGACAAGCCAGAGAACAGCAC

TCTATCTCCAGCAGAGGAACAACAACCCACACTACAGCCCACGGAACAGGTTAATAAACC

AGTTCACCCTCCAGAAGTCATGGAAAGCCAGTTCCAGGGAAGTGTTATTTACCGTCCACC

CATGCCACTGGACAGCGCAGTATGGACCAAACCCACAGTCGCTGGAGTGCTGGAGGACCT

TGCGCTGGACTTCAGTTTACCCAACACCCTGCAGGTGCCGTGTGCTTACGATGACAGCGA

TGATATCGGGCGATGGGGCGAGCAGCTGGTCTACTCCTTCCTCACCCGCTGGCGAGAAAG

TGGAGAAGGACCGCGGGAGATCAGCTGGTCCAACGAAAAGGGTGAGAGCGGCCAGCCGTA

CGATTTTAAACTGATCTTCCCGACTGGCTGTAACACCACGCGGGAAGTGTTTGTGGAAGT

CAAGACAACAGTGAAGCAAGAGAGGCATTTTATTCACATGTCAGCCAACGAGATCGATTT

TGCGCTCAAGGAGAAGGAAAAGTACCACATATACAGAGTGTACGGCGCTGGAGATTCCCA

ACATACCCGTCTCTGTCGCATCAAGAACCTTGCCCAACATCTGCATTCCAAAACACTGGA

ACTGTTTTTATTCGTGTAGGGAAAAAAAAATATTTTAATCTTTTTAGCAAGTGTAACTTT

TGAACTTTATAATTTTATTTCTGCACTAGTAACGTCATGCACAAGTTTAGTTATTTTATA

CCTCTCTGTCATCTGTTTAAATCCTTTATATGTTTAAAATTAAATACACTAAATTATTAC

TTATTTTTTTATTTTAGATGTTTTAAAGTTAAAATGATGTATATTAACTTTTATTTGAAA

AAAAAAAAAACATTAAACCAGAAATAACATGTCGGTCATTTAATCATTATTCATTCATCC

TTATAAAGCC

>Unigene000030

TCGGAAGAGCGTCGTGGTGGTAATGGCACAGCCACTGGACATCACGCTTATCCGGGAGCA

CAGGGTGATGGTCGAGGAGGTGGAGGAGCGGCCGCAGGACAGTCGTTTCAGGATGGAAGC

GGCGCTGCAACTGTGAATAACTGGGGTCCTCAAGGGATGCAGCTGCCTGACCAAAACCGA

GCCCAGTTTCCACAGTCGCAACAGCAGCAGCAAGTTCACGGTCATCAAGTGGCTCCTTAT

ATAGGACGCACTGACTTTGGTGGAATGCAAGGGCACAACCAGACCCAAGCACCGCCAAGA

ATGAACCATTTCCCTCAGGGTTTGCCTCAGGAAGCAAACCGTTTTATGGGTCACATGGGA

GCCAATATGACATCGTCCACCATGCGCCACCCGTCTCAGACGCCCCAGCATCAGCAAGCT

GGTCAGCAGTTTCTGCATCACCACACAGATTCACAGTTTCCGCAAAGTCCTTCAAAGCAG

CAGCAGATGCTGAGTGGGCGAGGACACCAGAACATGGGTTTCAGCTTGAACAACCAGCAG

GTCGCTAACAGCAGCTCAGGTCAATATGCTGCTTATCCTCAATATAGCGGTCTCAATCAG

GGATTAGCCAATGCTTCAGGGATGAGCCTTAATACCAGCAGCAACCCCAATGCTTCTGTG

CAGTGTTACCCTACCCCTGCAGGGCCACAGTGTTACCCGGTGAACCAGTTACCACCTCAG

AATATGCATCCCAAACTTCCCCAGCAGCAGCAGCAGCCGCCACCGCAGCAGCAGCACCTT

GTTCCACCTCCTCAGGGCTCTTATGCTACTCCTCCCTCCATGTCACCCATGAGGAACCTG

GGAGGCAACCCTGCAGGAACGCCACCCCCTCAAAGGGGTCAGGGTCCCATCTCTCATCCC

CAGCAGCACCCCCAGATGTATGGCACCTTGTCGCCCAATCAGAGAGCAATGAATGCGCCT

AACCACACAGAGGTTCTCCCACAGCAGCTGCAAACCTCCACGCTGCAATTCCAGCAGGTG

AGAACTCCGCCCACACTCTCCGTGTCTCAGCAGCCCCCGGGCTCGTCTCCACCAACACCG

CTGCCACAGGGGAACTGGTCCTCCAACACACACTCGTCACCACCAGCTAATCACCAACAA

GTGCCTCACATGCAGCCTTCTCCATCAGACGCTTCCCAAACCCTGTCCTCCACTCACAAC

TCCACCCCGAGCTCCACCCTGCAGCTGGGCAGCTGTGACACTCAACCACTGGACAGCGAT

TCCAAACCCAAGAAAAAGAAGAAAGTGAAGTCTGAGGAGAAGGACGAAGATATGGGTGTG

TGTGGAGAGTTGGGAGGTGCAGGAGTTAGTGGAGAAACGTCTTCACCTGAGAAAAAACGC

AAAAAAAAACCAAAAGAGAAACAGGCCAAGGACACAAAAGAGCCCAAAACACCTAAAACG

CCTAAAGCCCCCAAAACACCCAAAGAACCCAAGGAGAAGAAAGTGAAGAACACCACACCT

AAACCCAGGAGCACCAAGAAGAACAGCAGTAAACAGGAGGAGTGTGAAAGTAGCAGTAAG

AAAGAGGGGAAGAGGAAGAGGGAGTCGTCCATCACCTTGGATCCAGAGAAAACTCCTCCC

CTTTCTCCGGAGGAGGAGGAGGAGGATGATGGGGTGCAGAAGAGGCGTTCGTGTCGACAG

GTGAAGAGAAAGCGGTACACAGAAGATTTAGAGTTCAGGATATCTGATGATGATGATGAG

GATTCTACAGGACCCAAATCTCCTTCTACCACGTCTGAACCAACTGAGGTGCTTGATGCT

GAAGGTCCAGTGGTGGAGAAGATCATGGGATTACGTACAGTTAAGAAACAGCTGGAGTCG

GGACAGGAAGTGGAGGTCGAGGAATTTTACGTGAAGTTCAAGAGTTTCTCATATCTGCAC

TGTCGCTGGGCTGATCTAGAGGAACTGGAGAAAGATAAAAGAATCCAGCAGAAAGTCAAG

CGCTTTAAAGCCAAACAGGCGCATACCAACTTCCTCAGTGAGATGGATGATGAGCCGTTT

AACCCGGATTATGTGGAGGTGGATCGAGTTCTGGATGTTTCTGAAAGTACAGATGAAAAT

GGAGAGGTGGTGACTCTGTATCTGGTGAAATGGTGTTCTCTGCCCTATGAGGACAGCACC

TGGGAGCTGAAAGTCGATGTTGATGCAGGAAAAATTGAGGAGTTTGAGCAAGTGATGAGT

CGTGAACCTCAGCTGAAACGTGTGGAGCGACCTCCGGCGAGCGACTGGCAGAAATCCCAG

AGTTCCCGCCAATATCGGAATAACAACGCACTCAGGGAATACCAGCTGGAGGGAGTCAAC

TGGCTACTGTTCAACTGGTACAACACACGGAATTGTATCCTGGCAGATGAGATGGGTTTG

GGGAAGACGGTGCAGTCCATCACGTTCCTGTATGAGATTTATCTGAAAGGTGTCCATGGG

CCGTTCCTGGTCATCGCTCCGCTCTCCACCATCCCCAACTGGGAGCGCGAGTTCAGGACA

TGGACTGACCTCAATGTAGTGGTGTATCACGGCAGTCAGGCCAGTCGCAGGACCATCCAA

ACCTACGAGATGAACTACAGAGACACTCAGGGTCGTGTGATTAAAGGAGCCTATAAATTC

CATGCCATCATCACGACGTTTGAGATGATCCTAACGGATTGTCCGGAGCTGCGCGGTGTC

CCCTGGCGCTGTGTGATTATCGATGAAGCTCACAGACTGAAGAACAGGAACTGTAAACTG

CTCGAGGGACTCAAACTCATGGACATGGAACACAAAGTTCTCCTGACAGGAACTCCACTG

CAGAACACGGTGGAGGAACTCTTTAGTCTCCTCAACTTCCTGGAACCTGAACGCTTTCCC

TCAGAGTCCACCTTCATGCAGGAGTTCGGAGACCTTAAAACAGAGGAACAGGTTCAGAAA

CTGCAGGGAATCCTGAAGCCCATGATGTTGCGGAGACTGAAGGAAGATGTGGAGAAGAAT

TTGGCCCCTAAAGAGGAAACGATCATTGAAGTGGAGCTCACAAACATCCAGAAGAAATAT

TACAGAGCAATTCTGGAGAAGAATTTCACCTTCCTGTCCAAGAGCGGCGGAGGGGGAGGG

GGAGGGGGATCTAATGTCCCTAATCTACTAAACACCATGATGGAGTTACGCAAGTGCTGT

AACCACCCCTACCTCATCAACGGAGCAGAGGAGAAGATCATGGAGGAGTTTAGGGAGACT

CACCCAGCTGATCTGCCGGATTTCCACCTGCAGTCGATGATCCAGGCGGCGGGGAAGCTG

GTGCTCATTGATAAGCTCCTCCCCAAACTGAAGGCTGGTGGTCACCGTGTCCTCGTCTTC

TCCCAGATGGTGCGCTGCCTCGATATCCTCGAGGATTACCTCATCCAGAGACGGTATCCC

TATGAGCGTATTGATGGTAGAGTGAGAGGGAATCTGCGTCAGGCTGCGATTGATCGATTC

TCTCGCCCGGATTCGGACCGTTTCGTCTTCCTGTTATGCACTCGTGCCGGAGGACTGGGT

ATTAACCTGACCGCCGCAGACACCTGCATCATCTTCGACTCTGACTGGAACCCACAGAAC

GACCTGCAGGCTCAGGCTCGCTGTCACCGGATTGGTCAGAGTAAAGCGGTGAAGATTTAT

CGTCTGATCACCAGGAACTCGTACGAGAGAGAGATGTTTGACAAAGCCAGTTTAAAGCTC

GGGCTGGATAAAGCAGTGCTACAGTCCATGAGTGGGAGAGAGAATGCTGCTAACGGGGTT

CAGCAGTTGTCTAAGAAGGAGATTGAGGACCTGCTGCGTAAAGGAGCTTATGGAGCTCTG

ATGGACGAGGAGGACGAGGGCTCCAAATTCTGTGAGGAGGACATTGATCAAATCCTACAG

AGACGAACACAAACCATCACTATCGAGTCTGAGGGCAAGGGGTCCACCTTCGCCAAGGCC

AGTTTCGTATCGTCGGGGAACAGAACAGATATTTCACTGGAGGATCCAGACTTCTGGCAG

AAATGGGCAAAGAAAGCTGAGCTGGACCTGGACGCCATTAATGGCAGGAACACTCTGGTG

ATTGATACGCCGCGGGTGAGGAAACAGACGCGACACTACAGCTCCATTAAAGAGGATGAG

ATGATGGAGTACTCTGAGCTGGAGAGCGACTCAGAAGAGAAACCTGTTCAGAAATCACGA

CGGCCACAGGACCGAACTCAGGGGTACCCCCGCAGCGAGTGTTTCAGAGTGGAGAAGAAC

CTGCTGGTCTACGGCTGGGGTCGGTGGAAGGACATCCTGTGTCATGGGCGGTTTAAGCGT

CCTCTGTATGAGCGGGATGTGGAACTGATCTGCAGAGCACTGCTGGCTTACTGCCTGCTT

CACTACAGAGGAGACGAAAACATCAAAAGCTTCATCTGGGACCTGATCACCCCTACTGAG

GACGGGCAGAGTAAAACACTCACCAACCACTCGGGTCTGTCTGCTCCTGTACCGCGAGGT

CGTAAGGGGAAGAAGGGGAAGTTACCAGTGACTCAGATGAACATGCCTCAAGCTGATTGG

TTGGCTGAGTGCAATCCAGATGTGCTGCTGCAGGAGGACAGCTACAAACGCCATCTTAAA

CACCACTGCAACAAAGTGTTACTGCGGGTGCGAATGCTCTACTATCTGAGACAGGAAGTG

ATTGGAGACCTGGCAGACCGCATCCTGGAGGGAGCTGATTCTAGTGAGCTGGATATCTGG

ATCCCGCAGCCCCTGCATGCTGAAGTTCCCACTGATTGGTGGGATGTGGAGGCTGATAAA

TCTCTCCTCATCGGTGTGTTTAAACATGGTTATGAGAAGTATAACTCCATGCGAGCTGAC

TCGGCGCTGTGTTTTTTGGAGCGAGTGGGAATGCCGGATGCCAAAGCCATTGCGGCGGAG

CAGAGGGGAACGGACATGGTGGCAGATGGTGAGGATGAAGACCCTGAGTATAAACCGCTG

CGTATGCCCTTCAAAGACGATCTGGATGATTTCACTAACTCTCCCCTGGACGAGAAGGAG

GAGGTGATGGATGTGGAAGCTGGAGATGCCCCTAAAGAAAGTGAGGTAAACGGGGGTAAA

GCTGGGAATCTGTACTGGCCCACAGCTTCAGCGTTCACTGCACGCCTGCGTCGCCTCATT

ACCGCGTACCAGCGCTGCCACAAGAGACAGGAAACACTGATTAAACCAGATGGACGCAGG

CGCCGGCGTCACCGTGACGACCACCTCCTCGCTATGGCCACTGAGGGCGGGGCTTACAGT

CTGGAACCCAGTAGGGTAACTCCCACGGCATATCTGACTGAAGGAGCTTCTTTCAAGACG

TCACCTTTCCTGCACGAGAGCGCCGCCCTTCTGGCAGATGGCACTGCTTTCTTTAAAGAG

AGACGTCAGAGATGGACGCGGCGTGAGGAGGCTGATTTTTACCGTGTCATTTCTACCTTT

GGGGTTGTTTATGATGTCCAGACGCAGCGTTTTGATTGGTCGCAGTTCCGGGCATTTGCT

CGTCTTGATAAAAAGACAGATGAGAGTCTGGAGAAATATTACTATGCCTTTGTGGCCATG

TGCAAGCGTGTGTGTCGGATGCAGGTCAAAGACTCGGAACTGGCTGACCCGACTCTGATC

ATCGACCCCATCACAGAGGAACGAGCCTCCCGTACTCTGTACCGTATCGAGTTACTGCGC

CGGATCCGGGAGCAGGTTCTGCCCCATCCCAAATTGGAGGAGAGACTCCACCTCTGTCAG

CCCAATCCGGAGCTTCCGGCCTGGTGGGAGGCAGGGAAGCACGACCGGGACCTTCTCCAC

GGCGCTGCGAAGCACGGAGTAAGTCGCACAGACTACCACATCCAAAACGACCCTGAGCTC

AGCTTTCTGCAGGCGCAACGCAAGTTCATCCAAAACCGAGGGTCTGACACGGCGCTGACC

TCCGACCCTCCCCCCACCGGGGATCTGATTAAAGATGAGGAGCTGAGGAACGACCCTGCA

GAAAACACAGGAATGGAGGACATGAAAGAGGAAAATACATCATCACCCAAACAGGAAGTA

AAACCTGAAGAGGAAGAGAAAACTGCCAATGAGAAGATGGAGAGTGAAAATGAAGCCGAA

GTCAAGAGTGAAGAAAATCGCAGCGTTCCTCAGATGACCGAGGAGAAGGTCACAGAGTCT

GAGGACACGCCCACACCTCATGTTACTGTCCACCAAGACACAGAAAAAGAGGAACCGGAG

AACCCAAAAGAACTGGAGAAAAATTCAGAGGAGGAAGAAGAGGAGGAAGAAAAGATGGAT

GAAGACGACAAATCAGAGAAATCGTCCCAGGCTGAAGCTGGTGCTTCTTCAGACAGGAAG

AACTTTGATGAAGAGAGTAACGCCTCCCTGAGCACCGCCCGGGACGAGACACGGGATTAT

TTTGGTCTGGATGATGTCGAACCTTCGGTGTCTCAGATGTTCGGTGAACGAGCTCCGTTC

TCTTTCTGGCCAAAGGATCGGGTGATGATCAATCGACTGGACAGCATCTGTGAGGCGGTC

ATAAAGGGCAAGTGGCCATCCAATAGGAAGCAGTTCTTTGACATGCCTGGCCTGTTGCCT

GGATATGTTGCCATGGCAACCGACAGCCCTGTGCCCAGGGGTATGGGAGACTTCTCCATA

ATGAATCAGAGCAGCTACAGTGGAAGTGATGACATCACATTGTCACCGCAGGTTAATAAG

GTTCAGGACGAGGCTCTAAGTCTGTCTGTTCCACGTCAGCGCAGACGCAGGAGAAGGAAA

GTGGAGATCGAGGCAGAACGAGCTGCTAAGCGGCGCAATTTGATGGAGATGGTTGCGCAG

CTGCGTGAGTCTCATGCAACAGAGTCTCGGACTCGAGTAATGGATCTCACTAAAGCTCTT

CACGGTCTGGCTCCTTCCTCCTCCACCTCCTCTTCCTCTGCGGTGTTCCCCGGCACCGTG

GCTTCCCCAATGGAGCTGCTGCACGCCGCCGGAGCCTCCAGAGCCAACGGAGCCATGCTG

GAGGCTGAAATCCCCACACGCAGGAGGAGAGGACGCAGGAAAAACGTCGAGGGACTCGAA

CTGCTCTTTATGGGCAATAAGAGTTCACAGCTCAACACGGAAGATTCAAATGGGACCAAA

GGTTTTACAGATGGAGCACAGATGTCATCACGGACACAGACACACCTTCAGACTCCTGCA

CAGAAAGACGAGGGAGACCATCTCAGAGGAGGAGATGATGGAACCAGCACAAAAGACCTG

CAGGAGTGGCTTAGACAACATCCCACCTACACCATGGACGTGCCTGGATACATACCAAAG

AATGAGGAGCTTCTCCTCTCACAGCTGGGCGGGAAACCCAAGCAGAAGAGACATCGCTGT

CGAAACCCTAACAAGATCGACATCAACACACTGACCGGAGAGGAACGAGTGCCTGTGGTC

AACAAGAGGAACGGGCGAAAGATGGGGGGCGCTATGGCTCCACCAATGAAGGATCTGCCG

CGGTGGCTGGCTGAGAATCCGGAGTTCTCCATCGCTCCAGACTGGACAGACATCGTCAAA

CAATCAGGATTCCTCCCCGAGTCCATGTTTGACCGTCTCCTGACCGGTCCGGTGGTCAGA

GAAGAAGGTGTTCGGCGCCGAGGACGAAGACCAAAATCTGAGATGGCTAAAGCAGCAGCA

GCTCAAGCTTCTCTTTCTGCAGCGTCTTCAGCCTCCGCCTCGGGCATTAACCCCTTCCTG

CTCAATGGCCTGTTCGGAGGAATGGACCTGAGCAGCTTGCAGAACCTCCAGAACCTGCAG

CTTCTCATGGGCTTGTCTCCTTCTCTAAGTGCCGGAGCTGCAGACAGCAAGAACTCCGCC

ACCATGCTGCCCCTCATGCTACCTGCCATGGGGGCTCTGCCCAATGTCTTTGGCCTCGGT

GGTCTTTTTGGTGGCAACATGGCGGCTGCAGCTGCAAACTTGAACACCAATTCAAACAGC

GCAGCCACTTCCGAGTCTGAGGAGACAAAGAAAGATGGAGAACCCAAAGAGGAAGCTGAG

GAGAAGAAGGTGGAGGTGGAGAAACCCGCAGAGTCCAGTGACGCAAACACGGCTGCCGCA

GCTACCGGTCTTTCCACTAATCCTCTGGCGTTTAACCCCTTCCTGCTCTCCACTATGGCT

CCTGGACTGTTTTACCCCTCCATGTTCCTGCCCCCAGGCTTAGGTGGTTTGGGACTGCCT

GGCTTTTCCCCAGCGGCTCTTGCTGAGCTCCAGAGCGTGATGAGCGGGGCTTTAGGGGGT

GCTGGAGAGGAGAAGGAGGTTAGCAAGGAGACGGAACAGAGGCAAGAGGCAGTAAAACCC

AGCAGTGCTGTGGAGAACAGCGACTCTGAGGAGGGTGAGAACAAAACCCACCTGAGCGCC

GCGGCCGAAGAACTCGACTCCATAGAGGCAGGAGAAGAAGAGGACGACGATGACGATGAG

GATGAGGACGATGAGGAAGATGTCGGAGAAGATGATGACGAAGACAAAAGTGACTGAAGA

GGCAAATTCACCTGACTGACCTCCTGCCTTTTACCCTGAGGGCTTTTAAACAACTCTACT

GATACCCCAGACACAAGTTTTATATTATTTTCGTTTGAATTTTGTTTTTGACGTGTAATT

AATCTTATGATGGAGGAACACGAGACATCTTTGCAGGACACACACGCTGCACGCTGTAGT

GTGTGTATGTGGTGTGTGTTGTGCTGTAGGAGGCGCTGATGATGACTGAAGCGTTCTAAA

TGTTAGGAATGAGAAGGTTCTGATAGCGTGCTGCTTTTTTTGTGTTTGATTATTTTCTTT

CTCTACTGTGGATGTTGTA

>Unigene000031

GGTCGCGTGGTGCTGGTCGCTGAGGGAGAAGTCAGGCCGAGAGGTTCACTGCAGGATTTA

TTATTATTTTTTTTACAACATTTTACATGTTTTAACGAGAATTTGCCATTCTGCGGCTCA

GATCGAAGCCGGGTAGCGCGGAGAGGAGCAGGAGTGAAGAAAAAGACAATTAAAAGAGAG

ACTTTCTTGTGGTTTAAGTTGTAAAGCACGTTCAGGTTGGGGGAAAGGAGGACCGGAGAC

ATGGCCACTCAGGTGGAGACTCTCCTGCATACCACCATCAACACCACCACCGCCGCGCTC

CTGCAGGACGATGACCTCCAGTCGCCTGTAGGACATGGAGAGCTCTCCCCGGTGCCGAGC

TCGAAGCAGGCCGGGAATGAGAACAGCCCGCAGGAGAACATGGAGGAAAAGAAAGAGTTC

AAAGAAGCGCCTCCGCCCAAAGTCAATCCCTGGACCAAGAAACTCAACTCGGTGACTGTG

AATGGTCAGACCGCGCCCGAGCAAACTGGACCAGCAAAAGTGGTACGAGCAGGAAACCCC

CGACCAAGGAGAGGAGGAAAGGTTGGAGATTTTGGAGATGTGACCAACTGGCCGACACCT

GGAGAGATTGCCACCAAGGAAGTGCAGGGCTGTAAGAAGGCGTCAGTAAAAAAGGACTCG

AGAGAGAAGCGGGACAGTGAGGAATGTAAAGAGAACCAGAAGGCCAAATCAGATGATTCT

GGAGAGGAGAAGAACCGAGAGGAAGACCTGCACAAGAACAACGCTCACCGTAAGAAAGGA

AATAAACATAAATGGGTTCCTCTGATGATCGAGGTGAAGTCCGAAGGACCTCGGGAGCGT

TCGGCTTCCAGAAACAATGCACGACAGGGGGAAACTCCCAGAATGCACCACAGCAACAGG

AATGACCTGAGAGACTGGCACAGTGGAAAATACGAGCGTGATCAGTTCCGAGACGATCAT

GACGAGGTGTCGAGTGTGAAGAGTGAGGGAGCGCCGTTCAGAGGAGGCTTCAGAGGACGA

GGAAGAGGACGCGGTCGAGGAAGAGGACGGGGACGGGGAGGACGAGGTCAGTATGACTAT

CCATACAGCTACAAGTGTGAGGGAAAAGACGGTGTGTATGACCCCAAATACACACAGTCA

GTGACCTATTACTATGACAGCATGAGCAGCGCTGAGCTGTACAGCGTGGACCAGGACCTG

CTTAAAGAGTATATCAAGCGACAGATTGAGTACTACTTTAGCATGGAGAATCTGCAGCGG

GACTTTTTTCTTCGGCGAAAGATGGATGAGGGTGGATTCCTGCCTGTAGCTCTGATCGCT

AGCTTCCACCGTGTTCAAGCTCTCACCACTGACATTCAGCTCATCATGGAGGCTCTAAAG

GACAGTAAGGTGGTGGAGATTATTGACATGAAGATTCGATGTAAAGAGGAACCGGCGAAA

TGGCCTCTTCCTGGATTAACTCTTTCTGAACACACACACACGGACTTCTCCCAACTCATC

AACTGTCCTGAGTTCATCCCCCGCAACACTTCAGACACGCCTCACACAGGTTCACCTCGT

GTGTCGTCTGCTGCTGAGCAGAAGTTGGAGGAAGTGTGTAACCTCAAGACTATGCCAAAG

GGTTTATCAGCGAGTCTCCCTGATCTTGACTCGGAATGCTGGATAGAAGTGAAGAAACGG

CCACGCCCCTCACCAGCCAGACCCAAAAAGCTGGAGGACCTTCGGAGTGCCCTTACTCCA

GGGGATCAGGACGAACCAGAGGAGCTGGACTTCTTGTTTGATGAGGAGATGGAGGCGATG

GATGGCCGGAGGAACACTTTCACGGAGTGGACGGATGACGAGTCGGACGGAGAGATCGAC

GACCACGACGTCAACAAGATCCTTATTGTGACTCAGACACCACCGTACCTGCGCAAACAC

CCAGGGGGCGACCGGACTGGCAACCACGTCTCCCGTTCCAAACTCACTAACGAGCTTGTT

AAGGTTATTAACGACGGCCTGTTCTACTATGAACAGGATCTGTGGCACGACACGTATGAG

CCTGAGTACGCCACCATCAAGCAAGAGGTAGAAAACTTTAAGAAGGTTCATCTTATTAGC

CGTGAGCAGTTTGACTGCTTGACCCCTGAACCCCCCATCGACCCCAACCAGGAAGTGCCG

CCCTGCCCGCCCCGCCCACAACACATTCCCACGGATGATCTGGCGAATAAACTGTTTGGT

GCTCCGGAGCGTTCTGTGATGGCACGCTCTCTCCCCACCGCTGTGCCTGACTCGCCGGCA

TACAGAGCACCGCACACACCTCACACACCCCGCACACCCCGATGCAAACACAACACACCT

CGGTTTTATCCTGTCATAAAGGACGGCAGGCCTGTGGATGCCAAGACACCACGGAAAAGA

AAAACACGTCACAGCTCAAACCCCCCAATGGAGTGTCATGTCGGTTGGGTGATGGACTCC

AGAGAGCACAGGTCACGGACTGCCTCTGTCAGCTCTAACGCAAGTCCATCTGAAGGCACA

GCGGCGCTGGGAAACATCGGCTGCACTCCTCAGTCTTTACCTAAATTCCAGCATCCGTCA

CATGAGCTGCTAAAGGAAAACGGCTTCACACAGCACGTCTACCACAAATACAGACGACGC

TGTCTCAATGAGCGTAAGCGTTTGGGAATCGGTCAATCTCAGGAGATGAACACACTCTTC

CGTTTCTGGTCATTCTTCCTGCGTGATCACTTTAACAAAAAAATGTATGAAGAGTTCAGA

CAGCTGGGAGTCGAGGATGCTAAGGAGGGTTACAGGTATGGCTTGGAGTGTCTGTTCCGG

TATTACAGTTACGGTCTGGAGAGAAAGTTCAGAGCTGAAATCTTTAAAGATTTTCAGGAG

GAAACAATCAAAGATTATGAAGCAGGTCAGCTGTATGGTCTGGAGAAGTTCTGGGCTTTT

CTTAAATACTCTAAAGCGAAGAATCTGGAGATCGACTCCAGACTGCAGGAGTATTTGTGC

AGCTTCAGACGTCTGGAGGACTTCAGAGTTGATCCTCCTATGGATGAGGGTCGTAAACGT

CACTCCTCTAGTGGTGATGGACGTCGCCGTCACCCGTCTCAGTCCTCTGCTCGCCACACC

CATACCAGTGACCACAACCTAGACCCTAAGCCTGGTCCGGCTCCCACTGCTGACTGTAGC

AAGTGACTGACTAAGTGAAACCCACACCGCCGTCTTCACCGTTACCCGCCCATTTATCAT

CCCCCTCTGGGGTCCAGTCGTTCCCGCACGCATCATGCGCTGTTCGAGTGGCTGTGCGAG

GACATCCAGCTCTTTCCTTCAGACATTAGTGATCTAATTTCACACCTAAGAGGCTGCTGA

AGGGAACCTGTTCCACTCGTCACTTTTATTTTCATCTTTTTTTTTTTTTTTAATTTGAAC

ATCACTCGGTGTGACCTTTGATATTTACAGCCTGAACCCTTCTGATTGGCTGGGAAATGG

GCAATGAGGCGTTTTTGTTTTGGGTTTTCTGATTTGGGACTCAGTTTGTTGATAAATGAT

GAGGGAGGAAGACTGAACATTATAGCCGAACCCTCAGGACATTTCACATCGCTTTTCCCC

CCCACAGTTGCCTTATTTTACGGAGGTTTTTTCTGCTTATCGTGCGCCGGACACCTTAAA

CCCCCCCCCCCCTTTCCCCATCCACATCACCTCTTATTTGTAGCTGAAAACACACACAGC

TGTTGTAAGCAGACGCTTCCCCTCGTTTCTGCGTCTTCCATGTGCTCCGTTCTGGCCGTT

ATCGTCTGTTGCCGTGTGTTTGTCTCTGTGTCGTCCCCCAGTGTCCAAACTTAATCACAA

TTACTTGAAGGAGAGAAGCAGGTAAAAACATCTGATCATGCTGCAGGTGCTCAAGGACTT

GAAACTCATGCACAAGATGTAGAAATGGGGAAAAAAAAAAAAAAAAACCAAAAAAAAAAA

AAAAAAAAAAATTGTCTTGTGGCTACCTTCTGAGGAACAAAAAAAATGTAAAGGAAAAAA

CAGAGGAAAAAAAAGTATTTGACAAAAATAAGTTTTATTTAAACTTCCTCTGTACAACTT

AACATCGCCCATCACTGTCATTAAAAAAAAGAAATATGGATTGGATGCAGTAAGCAAACA

AACACAGTAAGAACCCTCTAACTCTTACTTGTTAATTATTTTTTTCCTGTTGCTTTAGTT

TATCATTTTGTTTTTCCTCTTGATTTGGAGTTGCAGCGTTCTGCTGAGCCGATGATGCGC

TGTGAGTCTGCAGTGACGCATGGGTTGCGCAGCGACAAAAACGCTCTCTATTGCGATTCA

ACAGCGCCTGCGTGCGACAAATGCTCCCTTTTCCCTTGCCCTCCCCTTTCGGCCTGCGGA

CCACAGATCTGCCCGTCTAGCCCGCTAGTATGCCACCCAGCGGACGCCATGCCCAGTACA

GGACACTATTGGCAAGCAGTTTAATTGGTTCGTGTTGTGCTTACAATACATTCTAAAAGT

CCAAACAGCAGTCAAACCGAACTTGTGTTCCTGAGGGTTGGTTGGATCCAGAGAGTGTGT

CAGTCTGGTGGGCCCAGGTCTACCTGCGGCTTTAGGTCTGTGGTTTGTTCACTGGCCGAC

GTAAGCGCAGCGAAAAGCGACAAGCAAGCACAGTGCGACCGCGGCGTGGCAAAACACAGT

GGCCGCCCGTGTGGCTATTTTGGCGCCTGCATTACAGGGACCTTCACCTACACTCTGCAC

AGGCATTTAAGGTTGTGACTATAGTCTAGCTAAATCTGTTGGGGCTTTTGGCTTTGAAAT

TTCTTTTCATGTGTAGCCGCAGCCTGGGTTTCTGTAGATTTTAATGCGTCTCAACGGTTT

TCTTCCAATAATGAGACCATAACTGAAAAATGTCCGTCAGGGTGAAGGTGCTGGTCAGAT

TTGCGCAGCGCAGGAATAAGCATGCAGCTTAGCGCGGCAGTATTCTGCGCATGCGAGCCC

ACGCTGTAGACTCACTTTTTGCTTTATGGAGTACGGGTTCTAACTCTGGTTTTCTCTGCC

CTTTTTGTTTTCTTCCTTCTTTCAACCTCTTATTTTTTTTTTTTTCTTTCAACCCCACAT

CTCTCTCTCTCTCTTTCTCACTCTCTTGCTCCTCTATTACTCTTTTTCCTCTCTTCCTTC

TGTTGTTTTCATCGTGAACCTATTAAAGGCTTGCTACCTGAAGGCAATCGAGACTCAGCC

AAACTTTGCGGTGGCATGGAGCAACCTGGGCTGCGTGTTCAATGCTCAGGGCGAGATCTG

GCTGGCCATTCATCATTTCGAGAAGGCGGTAACATTGGATCCAAACTTTTTGGATGCTTA

CATAAACTTGGGCAATGTGCTTAAAGAAGCCCGCATCTTCGACAGAGCTGTTGCTGGCTA

TCTGCGAGCCCTCAGTCTGAGCCCCAACCATGCTGTTGTCCATGGCAACTTGGCCTGCGT

GTACTATGAACAGGGTCTTATCGATTTGGCCATCGACACCTATCGACGTGCTATTGAGCT

GCAGCCTCACTTCCCTGATGCTTACTGCAACCTTGCTAATGCGCTTAAGGAAAAGGGCAA

TGTGTCTGAGGCTGAGGAATGCTACAACACAGCGCTGCGTCTTTGCCCCACACATGCTGA

CTCGCTTAATAACCTGGCTAACATCAAACGGGAGCAAGGCAACATTGAAGAGGCTGTGCA

GCTCTACAGGAAAGCTCTTGAGGTGTTCCCAGAGTTTGCTGCAGCCCACTCCAACCTTGC

CAGTGTCCTTCAACAGCAGGGCAAGCTGCAGGAAGCCCTCATGCATTACAAAGAAGCTAT

CAGAATCAGCCCCACCTTTGCTGATGCCTACTCTAACATGGGAAACACACTGAAGGAGAT

GCAGGATGTGCAGGGGGCGCTGCAGTGCTACACACGCGCTATTCAGATCAACCCTGCGTT

CGCAGATGCTCACAGCAACCTGGCTTCAATACACAAGGACTCTGGGAATATTCCAGAAGC

TATCGCATCTTACCGCACGGCATTGAAGCTCAAACCTGACTTCCCGGACGCTTACTGCAA

TCTTGCACACTGTCTGCAGATTGTGTGTGACTGGACCGACTATGATGAGCGCATGAAGAA

GTTGGTTAGCATTGTGGCTGACCAGCTGGAAAAGAACCGCCTGCCCTCAGTCCATCCTCA

TCACAGCATGCTGTACCCTCTTTCTCATGGGTTCCGCAAAGCCATTGCTGAAAGGCATGG

CAACCTCTGCTTGGATAAGATCAATGCTCTCCATAAACCTGCATTTGAGCATCCTAAGGA

CCTGAAAGCCAGTGGGGGGCGTTTGCGCGTTGGCTATGTCAGCTCAGATTTTGGCAACCA

CCCCACCTCCCATCTCATGCAGTCCATACCTGGCATGCATAACTCTGAAAAGTTTGAGGT

GTTCTGCTATGCCCTCAGTCCTGATGATGGCACAAACTTCAGAGTGAAAGTTATGGCTGA

GGCTCATCACTTTACTGATCTTTCCCAGATTCCCTGCAATGGAAAAGCTGCAGACAGGAT

CCATCAAGATGGAATTCACATCCTGGTCAACATGAATGGCTATACCAAAGGTGCTCGCAA

TGAGCTGTTCGCCTTGCGCCCAGCTCCTATTCAGGCCATGTGGCTTGGCTACCCGGGCAC

AAGTGGAGCTCCTTTTATGGACTACATCATTACCGATAAGGAGACGTCTCCAGCTGAGCT

GGCTGAGCAGTATTCTGAGAAGATGGCCTACATGCCTAATACATTCTTCATTGGGGATCA

TGCTAACATGTTCCCACATCTTAAGAAAAAGGCTGTGATTGACTTCAAGTCCAATGGACA

CATTTTTGACAATCGCATCGTGCTGAATGGAATTGATCTCAAAGCCTTTTTAGACAGCCT

ACCCGACGTCAAGGTCATCAAGATGGAGGGTGATGGCCAGGAGTCCACAGATATTAACGG

AGCCCTCTCCATGCCAGTCATTCCAATGAACACAGCCGCTGAGGCCATTATAAACATGAT

CAATCAAGGCCAGATCCAAGTCACTATCAATAGTTTTACAGTTAGCAATGGCTTGGCTAC

CACTCAGATTAATAACAAAGCTGCCACAGGAGAGGAGGTTCCTAGAACAATAGTTGTGAC

CACTCGCTCCCAGTATGGTCTGCCTGAGGATTCCATTGTCTACTGTAACTTTAACCAACT

CTACAAGATTGACCCTCCGACTCTGCAGATGTGGGCTAATATTCTAAAGCGTGTGCCAAA

CAGTATACTCTGGCTGTTGCGCTTCCCAGCTGTGGGTGAACCCAACATCCAGCAGTATGC

CCAGAACATGGGATTGCCTACTAGCCGCATCGTCTTCTCACCTGTTGCCCCGAAAGAGGA

ACATGTAAGGCGTGGCCAGCTGGCTGATGTCTGCCTTGACACACCTCTCTGCAATGGCCA

CACTACTGGCATGGATGTGCTCTGGGCTGGCACGCCAATGGTCACAATGCCAGGTGAAAC

ACTTGCCTCCCGTGTTGCTGCCTCTCAGCTGACTTGCCTAGGCTGCCCTGAGCTCATTGC

ACAGAGTCGGCAGGAGTATGAAGATGTGGCTGTCAAGCTCGGCACAGACATGGAGTTCTT

GAAGAAGGTTCGTGGCCGTGTTTGGAAGCAGCGTATCTGCAGCCCGCTCTTCAACACCAA

GCAGTACACCGTGGAGTTAGAGCGCCTCTACCTGCAGATGTGGGAGCACCACGCAGGGGG

TTCCAAACCTGAACACCTGGTGAAGATGCAGCTGCTGGACAGCAGCGAAAGCGCCTGAGC

TGCCCCCGTTTTAACCTCCAACCCCCACGCGTCTTCATTTTTATCAGTGTCAGAGACTCT

ACAGAGCCTGCTGTTGAGTACCATCACCATAATCTTACCCTTTAGCCACAGATTGAGTTT

CTCTGAGCCAGCTAGGAGAGTGTGATGATCGAACCCTCTTCACCGAGACTCCTTTTTCGT

CCGTATTGGCTTCACTTCCGCTGCTTCTCTCTTCTGCTTGGGTGTAGACTTGAAAACTGA

GCAACCTTTTGTCAAATTTGTGACCTTTGTCTTTAGATTATTATTTTTTTTGCTTAAGCA

TCAAAAAGGACATGTTGGTATGTTGCTAAAAGTAATGATGGCTTTAACATGCTAATGTGA

CTGTTTGCCTTTTAAGTTAGCTGATCTGTTTATGTATTTTAGTCTGTTCATTATGACCTG

GAATAGATCCCTGAACTGTAATAATTTGCCCGTGATTTATTTTCATGCTTTCCGTTTTAT

TGTTTGGTGAAACCTGCCCAGAATGACTATGGAGAAGCTAAAATGACTTTCTCACTGCAA

ATCTGATCATTGCCAACAGTCCACCCTAATCGTCATCCTACACGCTAATTTGTTTTATTT

TATTTTTTGCCAGACAGGTACAAAGGTAAGTATTTTCTAGGTTTGTCATTCTTTTCCACT

GTCTTCCTGTCAATCATTTTATTTATGCTGTTACTGACATTACAAGGCTCTTATATAAGC

TAAAATAAAGAAATGTGGCAAACTCCCGTAGTTTGTGTTAATATTAACTGACCTGATTAC

TGATTTTAATTAGTGACTTGTTCTATTGCTGCTGTTTATGATATGAAACTTGCTGTGGAA

TTTGGTGCAAATGTTATAGCGCAGTGGTTAAGATGTTAGGCTGATGATCGGAAGGTCAGA

GTTCGATCTCCTGCCGAAATTATTGAGTTGTTAGCACATGATCTTGTCATGGAGGGGCAT

CAAATTATGTACATGATTGGGAAATTGTATTTTATTAAAAATGGTTTAAGCAGAAAAAAA

TTACAAATAAAGAAC

>Unigene000032

GCAGTTCGCAGTCACGCCTGCGCCCCTGATCAGTTTGGCAAACCCGGAGGTTGTTCCGAT

ATCTGTCTGCTAGCCAACAGCCACAAGATCCGCACCTGCCGCTGCCGCTCCGGTTTCAGC

CTGGGCAGCGACGGCAAATCCTGCAAGAAACCAGAGCATGAGCTCTTCCTGGTGTATGGG

AAAGGTCGGCCTGGGGTCATCCGAGGCATGGACCTGCACGCCCGTGTGTATGACGAGCAC

ATCATTCCTATCGAGAACCTGAACAACCCCAGGGCTTTGGACTTTCACGCCGAGACGGAG

TTTATCTACTTTAGCGACGCCGCCATGTACACCATCGGACGGCAGAAAATCGACGGCACA

GAAAGGGACATCATTGTGAAGAGCGGTATCCACACTGTTGAGGGCATAGCGGTGGACTGG

ATGGGTCAGAACCTGTACTGGACAGATGATGGACCAGAGAAAACAATCAGTGTGGCCAAG

CTGGAGAAAGCCTCCCAGACACGCAAGACTCTCCTTGAGGGAAAGATGACTCACCCTCGA

GCTATAGTGGTGGACCCGTCACACGGGATGATGTACTGGAGTGACTGGCAGGAAGACTCG

AGAGATGCCAACCGTGGCAGCATTAGGAAGGCGTGGATGGATGGCTCCAAATCTGGCATC

CTCATCACCTCCAAAAACATGCTCTGGCCCAACGGTCTGAGTCTGGACATCGAACAGAAC

GTGCTGTACTGGGTGGACGCCTACTACGACCGCATCGAGATGGTGCTTCTTAACACCACC

GAGCGCAGGACCGTGTACGAGGGGCAGGAGCTGAACCACCCTTTTGGGCTTTGTCACTAT

AAGAACTTCTTGTTCTGGAACGAGTACCGAATCGGAGGCATTTACAAGCTGGACATGACG

TCCAAGAAGGTGACGCTGCTCAGGAGCGAGCGGCCGCCCATCTACGAGATCCGCACCTAC

GACGCCCAGCAGCAGCAAATTCCAGGCCATAATCCATGCAGGGGCAATAACGGTGGCTGC

AGCAGTCTGTGCTTGCTCACACCGGCAGGTCGTACCTGCGCTTGTGCAGATGACCAGATT

TTGGACTTTGACAATAAGACCTGTAAAGCCAATCCGTCCTACGTGCCCCCGCCTCAGTGC

CAGCCAGGGGAGTTTGCCTGCAAGAACAACCGCTGCATCCAGGAACGTTGGAAGTGCGAC

GGAGACAACGACTGCTTGGACAATAGCGATGAGGTGCCGGAGCTCTGCCATCAGCACACC

TGCCCTGCCGATCGCTTCAAGTGCCAGAACAACCGCTGCATTCCCATGCGCTGGCTGTGT

GACGGAGACAACGACTGCGGCAGCAACGAGGACGAATCCAATACCACCTGCTCAGCTCGT

ACATGTCCTCCTAACCAGCACCCGTGTGCCAGTGGACGCTGCATTCCTAAAACCTGGATA

TGTGACCTGGACGATGACTGTGGGGACCGCTCAGACGAACCAGCTTCCTGTGCCTACCCG

ACTTGTTTCCCACAAACCCAGTTCACGTGTGCCAACGGGCGCTGCATCAACGTTAACTGG

CGCTGTGATAATGATAATGACTGTGGAGACAACAGCGACGAAGCAGGCTGCAGTCACTCC

TGTTCCAGCGTGCAGTTCAAATGCAACAGCGGCCGCTGCATCCCAGAGTACTGGACCTGC

GACGGGGACAACGACTGCGGCGACAACAGCGACGAGAAAAGCGCCAACTGCACTAATCAG

GCTACGCGTCCCCCTGGTGGCTGCCACTCGGATGAGTTTCAGTGCCGCTTGGACGGTCTG

TGTATCCCCATGCGCTGGCGCTGTGACGGGGAGAACGACTGCATAGACGACACGGATGAG

AAGCACTGCGAGGGCGTCACCCACACGTGCAACCCGTCTGCGCAGTTCGCCTGCCGTGAC

TCGGCAAGGTGTATCAGTAAGGCGTGGGTGTGTGATGGAGACAGCGACTGTGAGGATAAC

TCTGATGAAGATAATTGTGAAGCGTTGTTGTGTAAACTTTCGCACCATACATGCGCCCAG

AACGATTCCATCTGCCTTCCTGCCGAAAAGCTGTGCGACGGTAAGGACGACTGTCCCGAC

GGCTCGGACGAGAAACTCTGCGACCTGTGCTCGCTAGAGAACGGCGGCTGCAGCCATAAC

TGCACCATTGCCCCCGGCGAGGGCATCCTGTGCTCCTGCCCGACTGGCATGGAACTCGGC

TCTGATAACAAGACCTGCCAGATCCAGAGCTTTTGCGCCAAACATCTGAAGTGCAGTCAA

CGCTGCATCCAGGAGAAAGCCACCGTCAAGTGTGCCTGCTACGAGGGCTGGGCTCTGGAA

CCGGACAACGAGAGCTGCAAGAGCACCGACCCTTTCAAGCCTTTCATCATTTTCTCCAAC

CGGCACGAAATTCGACGTATCGACCTGTACAAGGGCGAGTTCAGTGTCCTGGTGCCCAGC

CTGAGGAACACCATCGCCCTGGACTTCCACCTCAATCAGAGCTCCCTCTACTGGACCGAC

GTGGTGGAGGACAAGATCTATCGTGGCAAACTGTCTGAAAACGGAGCCCTGACCAGTTTT

GAGGTGGTGATCCAGTATGGTCTGGCCACTCCAGAGGGTTTGGCTGTGGACTGGATTGCT

GGAAACATCTACTGGGTGGAGAGCAACTTGGACCAGATCGAGGTGGCCAAGCTAGACGGA

ACCATGCGCACGACACTGCTCGCCGGAGAGGTGGAGCATCCACGAGCTATAGCCCTCGAT

CCACGTGACGGTATTTTGTTTTGGACAGACTGGGATGCCAGTTCTCCCCGAATCGAGGCT

GCCTCCATGAGCGGCGATGGCCGGCGTACCATTCACAGAGAAACAGGAAGTGGAGGCTGG

CCTAATGGGCTCACCGTTGACTACTTAGAGCGCCGTATCCTCTGGATCGATGCTCGGTCT

GATGCCATCTACTCTGCTGCTTACGACGGTTCGGGCCTGATCGAGGTGCTGCGTGGACAC

GAATACCTGTCGCACCCGTTCGCCGTCACCATGTACGGCGGCGAGGTTTACTGGACAGAC

TGGCGCACTAACACACTGGCTAAAGCCAACAAATGGACAGGACACAACGTTACAGTTGTA

CAGAGGACCAACACACAGCCGTTCGACCTGCAGGTCTATCATCCATCCAGACAGCCTCAG

GCTCCAAACCCTTGTGCTGCTAACGATGGAAGGGGTCCGTGCTCTCACCTCTGCCTCATC

AACTACAACCAGACCTTCTCCTGCGCCTGCCCCCACCTTATGAAGCTGCAGGCAGACAAA

CACACCTGCTACGAATCTCGTCAGTTCTTACTCTACGCTCGTCAGATCGAGATCAGAGGA

GTGGACATCGACAATCCATATTACAACTACATCATCTCCTTCACTGTTCCTGTGATCGAT

AATGTAACAGCGGTGGATTACGACGCCCAGGAACAGCGCATCTACTGGTCTGACGTTCGC

ACGCAAATGATCAAACGAGCCTTCATTAACGGCACGGGGGTGGAGACAGTGGTGTCGGCC

GATGTACCCAACGCTCAAGGCTTGGCTGTGGACTGGGTGTCCAGGAACCTGTTCTGGACT

AGCTACGATACCAGCAAGAAGCAGATCAACGTAGCCCGATTGGACGGCTCCTTTAAGAAC

TCGGTCGTCCACGGACTGGACAAACCACACTGCCTGGTGGTACATCCCATGCTTGGAAAG

CTCTACTGGACAGACGGGGACAACGTCAGCATGGCCAACACAGATGGCAGCAACCAGTCG

TTACTCTTCACCAGTCAGAAAGGCCCCGTGGGACTGTCTATTGACTTCGATGAGGGGAAG

CTGTACTGGATCAGCTCAGGCAACAGCACGATAAATCGCTGCAATCTGGACGGCACCGGC

CTGGAGGTCATTGAAAGTGTAAAAGGAAACTCACCAGAGCCACCGCTCTCGCCATCATGG

GGGACAAGCTGTGGTGGGCGGACCAGGGCACAGACCAGGTAGGCACCTGCGACAAGAAGG

ATGGCGGCAGCTGGAAGGTTCTTAGGAACGGCACGTCTCCGGTGATGCATATGAAGATCT

ACAACGAGACTGTCCAACAAAAAGGTACGAACCTGTGCACAATGAACAACGGGGACTGTT

CTCAGCTTTGCTTGCCCACCTCACCCACCAGCAGAGCCTGCATGTGTACGGCAGGCTACA

ACCTGAAGAGCGGCCAGCAGTCCTGTGAAGGTATGGGCTCGTTCTTGCTGTACTCGGTGC

ATGAGGGCATCCGCGGAATTCCTCTGGACCCGCTGGACAAATCTGACGCTCTGGTGCCAG

TTTCTGGAACCTCTTTGGCAGTGGGCATCGACTTTCACGCCGAAAACGACACCATTTACT

GGGTGGACATGGGTCTTAGCACCATCAGCCGAGCCAAAAGAGACCAGACATGGAGAGAAG

ATGTAGTTACTAACGGCATTGGACGTGTGGAAGGCATTGCTGTCGACTGGATTGCAGGAA

ACATCTACTGGACTGACCAAGGCTTCGACATGATTGAGGTGGCCAGACTCAACGGCTCCT

TCCGCTACGTGGTCATCTCTCACGGGCTGGACAAACCCCGCGCCATCGCCGTCCATCCAG

AGAAAGGGTACCTTTTCTGGACCGAGTGGGGTCAGTACCCACGCATCGAAAGGTCTCGAC

TGGACGGGTCAGAAAGAGTGGTTCTGGTTAATGTCAGTATTAGCTGGCCCAATGGCATCT

CCATCGATTATGAGGAAGGGCTGCTCTACTGGTGTGACGCTCGCACAGACAAAATCGAGC

GTATTAATCTGGAGACCGGTGGGAACCGAGAACTTGTCCTGGCAGTTCATAACATGGATA

TGTTCGCCGTGTCTGTATTCGAAAACTACATCTACTGGAGTGACAGAACCCATGCCAATG

GCTCCATTAAGAGGGGTAATAAGAACAATGCCACTGATATGGTTTATCTAAGAAAAGGCA

TTGGTGTGCAGCTCAAGGATATTAAAGTTTTCAACCGAGCCCGGCAGCAAGGCACCAACA

TCTGTAAAGACAAAAACGGTGGATGTGAGCAACTATGTCTATTCCGTGGCAACGGAGCAC

GCACCTGCGCCTGTGCCCATGGCATGCTAGCCGAAGACGGGCGCACTTGCCGCGACTACG

ACGGCTACCTTCTTTACTCCGAGCGTACAATACTGAAAAGCATACACTTATCAGACGAAA

CCAACCTCAACGCCCCGATCAAACCTTTCGAGGATCCAGATCATATGAGAAATGTCATCG

CTCTCACCTTCGACCACCAAGGTGGCGACGGCAAAGGAGCTAACCGCATCTTCTTCAGCG

ATATACACTTCGGCAACATCCAGCAGATCAACAGTGATGGCTCAGCGCGCAAGACCGTTG

TTGAAAATGTGGGCTCGGTGGAAGGCTTGGCGTATCACAGGGGTTGGGACACGCTTTACT

GGACCAGCTACACCACCTCCACCATTACGCGCCACACGGTGGACCAGACACTCAGCGGCG

CCTTTAACAGAGAGACGGTGGTCAGCATGTCTGGAGATGATCACCCCAGAGCCTTTGCGT

TAGATGAGTGCCAAGGGTTAATGTTCTGGACTAATTGGAATGAGTTGGCTCCCAGCATCA

TGCGCTCATCTCTAGCAGGCTCTAATGTCCTTGTGATCGTGGGTAACAATATCCGCACCC

CAAATGGCCTCGCTATAGACCATCGCTCCGAGAAGCTTTATTTCTCTGATGCCACGTTGG

ATAAGATTGAACGCTGCGAGTATGACGGCACCAATCGCTTTGTGGTTTTGAAGAACGAGC

CGGTCCACCCGTTTGGCTTGGCTGTCTACGGCGAGTACATCTTCTGGACGGACTGGGTGC

GCAGAGCCGTGCTGAGAGCGGATAAATTCGGCAGAGACATGAAAGTACTGCGTGCTGACA

TCCCCCAGCAGCCCATGGGCATCATAGCCGTTGCCAAAGACACCAACAGCTGTGAGTTCT

CTCCATGTCACACCAATAACGGCGGCTGTCAGGATCTGTGTCTTCTCACGTCCGAGGGCC

GGGTGAACTGCAGCTGCCGCGGAGATCGCAAACTGGTGGAGGGCAACGTGTGTGTTGCCG

AGAACACTACATGCCACAGCGTGGACGAGTTCGAATGCGGAAATGGTGACTGCATCAACT

ACAGCCTGACATGCGACGGACGCGCTCACTGCAAAGACAAATCAGACGAGAAACCGTCCT

ACTGCTCTAATCGAGGCTGTAAGAAGGGATACAGACGCTGCCTTAACGGACGCTGCATCA

AACACAGCTCCTGGTGCGACGGCACAGACGACTGCGGAGACCGATCAGACGAACGACCCT

GCAACATGACCTTGTGTAGTGCATCAGAGTTCCAATGTAAGGACGGTTCCTGTATCACCA

ACACGTCCCGCTGTAATCAGGTGGTAGACTGTGAGGATGCCAGTGATGAAATGAACTGCA

GTCCAACAGATTGCCACAGCTTCTACCTGTTAGGAGTTAAAGGTGTGATCTTCCAGAAAT

GTGAATTTACTACGCTGTGCTACACCCCTATCTGGCAGTGCGACGGCTCGAACGACTGTG

GAGACTTTTCGGATGAGAGGAACTGCCCAGAGAAGAGGCAGCTGAAATGTCCTGTGAACT

TTTTCGCCTGTCCCAGCGGTCGCTGTATTCCCATGAGCTGGACTTGCGACAAGGAGAACG

ACTGTGAAAACGGAACAGACGAGACTCACTGTGATAAGTTCTGCTCACCAACACAGTTTG

AGTGCGGGAATCACCACTGTATCTCCAGCTCCTGGGTGTGTGACGGCACCGATGACTGTG

GAGACGGCACCGATGAGGGCAGCCGATGCAGTTCAAAGACATGCAGTCCCGAAGCCTTTC

AATGCCCTGGCTCTCACTGCGTGCCCCAGCGCTGGAAGTGTGATGGGGACAACGACTGTC

CAGATGGTGCTGATGAGGGTGTGCAGGCTGGCTGCACAAACAATAAGACCTGCGACGACA

CGGAGTTCCAGTGCCAGAATAAGCAGTGCATCCCCAAGCATTTCGTCTGCGATCACGATT

TGGACTGCCGTGACGGCTCTGACGAGTCTCCCGAATGCGAGTACCCAACGTGTGGTCCCA

ATGATTTCCGCTGTGAAAACGGGCAGTGCCTTAAGCAGAAGAGCTGGGAGTGTGACGGCG

AATTTGACTGTCGTGACCACTCGGACGAAGCGCCCAAGAACCTCCACTGTACCGAGCCAG

AGAAGCGCTGTAACGACTCGGCATACCTGTGCAACAACGGGAAGTGTGTGAGCGAGGACT

CGCTGTGCGACTGGAAGGACGACTGCGGCGACGGCTCCGACGAGCACAACTGCTTCGTCA

ACGAGTGTCTTAACAACAAGCTCAGCGGCTGCACACAGCACTGCGAGGACCTAAAGATTG

GATACAAGTGCCGCTGTGATCCCGGCTTCCGTCTGAAAAACGACGGCAAGACATGCGTGG

ATGTGGATGAGTGCACCACCACTTACCCTTGTTCACAGCGATGTTTCAACAGCTACGGCA

GCTTCCACTGCTTCTGCATAGAGGGTTACGTGGCCTATGCGAACGACTGGACAAGCTGCA

AGTCCTCTTCTGAAGAGGAGGCTTATCTAATCTTTGCCAACCGTTACTACCTGAGGAAGC

TCAATCTTGATGGCAGCAATTACACCCTCATCAAAACGGATTTGAACAACGCTGTGGCTC

TGGATTATCACTACGCAGAGCAGATGATCTACTGGACAGACGTGACCACGCAGGGCAGCA

TGATCCGCCGCATGCTTATGAACGGCAGTAACGTCGAGGTGCTGCACCGGACGTCTCTGA

GCAATCCGGACGGTCTGGCGGTGGACTGGGTCGGGGGCAACCTGTACTGGTGCGATAAGG

GTCGAGACACCATCGAGGTGTCCAAGTTGAACGGGGCTTACAGGAGCATGCTGGTCAACA

GCGGTCTGAGGGAGCCTCGGGCGGTGGCTGTGGACGTCCGCAATGGTTATCTGTACTGGT

CAGACTGGGGAGATGTGCCTCACATCGGCCGTATTGGAATGGATGGAACAGACCGCAGCA

TCATCATCAAGGATAAGATCACATGGCCTAACGGCCTCACGCTGGACTTCATCAACGACC

GCATCTACTGGGCCGACGCTCGCGAGGACTACATCGCCTTCGCCAGTCTCGATGGCACCA

ACCGACACATCGTTCTGAATCAGGACATCCCACACATCTTCGCCATGTCTCTCTTCGAGG

ATTACATCTACTGGACCGACTGGGAGACCAAGTCTATCAATCGAGCTCACAAAACACTGG

GAACCAACAAGACCATGCTGATCAGCACGCTGCACAGACCTATGGATATTCACATCTACC

ATCCATACAGACAGCCTGCAGTTGAAAATCATCCCTGTCAGGTAGATAACGGAGGCTGCA

GTAACCTGTGCTTGTTGTCCCCTGGAGGAGGTTACAAGTGCGCCTGCCCCACCAACTTCT

ACCTGGCTGCAGATGGGAAGCAGTGCCTGTCTAACTGTACAGCGAGTCAGTTTGTGTGTA

AGAATGATAAGTGCATCCCCTTCTGGTGGAAGTGCGACACTGAAGATGACTGTGGAGATC

GCTCCGATGAACCTGCTGACTGCCCGGAATTTAAATGCCGTCCGGGGCAGTTCCAGTGCG

GCACAGGCATCTGCACAAACCCCGCCTACATCTGTGATGGCGACAATGACTGCCAGGACA

ACTCGGATGAAGCTAACTGCGACATCCACGTGTGCCTGCCGAGCCAGTTCAA

>Unigene000033

CGTTTTTTTTTTTTTTTTTTATTGTTAAAATATTCAGTCCAAAGACAAAGAATCAAATCA

GATAAAAAGGAAGGTTAACATAAACAAAGACAATTAAACTAAACGAGTCTTTTTTTTTAT

TTTTAATTAAGAAAAATGTTCTTGAGGACTGAGAAGAAAAAAAATAGGTTTTAATCCTAA

ATTGGTTAATTAAACCAACAATTGTCTTTACTAATCTTGTTTTCTGGCCGTTTGTGAGGA

TCAGGACTTTTTTTTTTTTCTTTTTTATTACGTGATTATTTTCGAAGCGCCGATAATCCT

AAAACCAGCAGGTGAACCCATCTTTACTTAAAATTGTGCCCTAGAACAGTGTTTACCATT

TATTTTGGGCCACAGTGGCACCAAAGACCGACGTGTGTGTGTGTGTCGCTTATTGGTTCT

GTTTGTTCTCTTGAGGGTTCTGAAATTAAGTCATTAAAACACTGTGTGTGTGTGTGTGCG

TGCGTGTGTGTGGGTTTTTTTCGGAGGAGGTTTGGTGAACACCCCCTGTATCACGCACTC

TCACTCTCCTCCCCTCCATTTCCCTCTCTGTCTCTCTCAATATGCTATTTTTTCTCTCGT

ATCCACATAGTCGCTTCTCATCAAGGTAGAATGATCGCAGATTAGCAGCGATCGCGTCGT

TGCGCGTTCACTTTACACATCCGGAGCTGCCCCCCTCCTCCCCCTTTAATTTATTGTAAT

TTTCTTGTTCTTCGTTCGATCCTCCATCCGTTCATCCGTGTCAGAAATGGACAGGAACTA

CCCGGGTGCAGGTTTCGGGGATTTGGGTGCAGGAGCCGGATGGAGCTACGAGCGATCTGC

TAAAGCAAGTTTGGTGTATGGTAGCTCCAGATCTTCCCATCCAGAGTCTGAACTCCTCCA

CAGGCAGGCCTATGCCACTCCTCATCCGCTGCAAGGTTATGCAACCAATCACCATCCAGG

CAGCTCTGGACAGGGTGGCGCGTGGGGTGCAGCTGGAAGGAGTCTGGGCTTATCTGGGCT

TTTTGATGCTGGGCTTCACCATGCGAGTCCGTCAGGCCCTGATCCCTCTGTAATGAACTT

GATATCAGCTCTGGAGTCCCGAGGACCACAGCCACCACCTTCTGCTTCTTCTCTTCTGTC

CCAGTTCCGCACTCCATCTTGGCAGACTGCAATGCACACACCTGCTCCTGCAGAGCTTTT

CATCTCCGGAGCCCTTCCTGGTTCTGGGTCGTTCCCATCATCCTCTGCCTTATCAGCTTA

CCAGCACCCAGCTTCATTTTCTGGACGCTCTTTTCCAGGAGTGACCCCCTCGCTCTCTTT

ACAGGACACTCCTACATTCAGCCCCACATCGAATGGCCTCTTGTCCCCTCATGATCCTCT

GCTGCACATTAAGACTCCCTCTCAGTCAAGTCTGGGCTTTGATCGTTTGTTGTCATCTCA

GGGTCCTGCTGCAGCCTACCGTGGAAGCCATGATCCTAGTGGTAGTAGTGGTGTTACTTC

TGCTCAGGCATCCTCAGCAGCTTCAGCTTCTGCACGTCATTTGCAGTCTCACCAGTTTAA

TCTGCTGTCCTCACAGCTACAGGACCAGTCCTCACAGTTATATAGTGCGTCTGTGTTCTC

ATCTGCTCCACCTCAGCCAGCCTCCCAAGAGCGAACTGTACCACGACAAGACAGTGTCAT

TAAGCACTACCAGCGTCCCTCGCCAGCACAAGCTCCATCCTCCACACCTCATCCTCTGCA

GCATTACCTAAGTTGTGGGGTGTCTGGGTATCAGCAGGCCCCTCATTCCCGTCATGCTGG

TCTCTCCTGCAGTCCATTAGGAGAGCACAGCCCCTCCTCTGACCATAAGCCCTCCCCCAG

GACAGAGCAGTACAGACCCATTATCCAACCTTCATATGGTTCCTCTTCCTCATCTTCCTC

TGGTGGACCTGGAAAGGCAGCAAAAAGCAGTTCCAGTAGTGGTTACTCTTCCTCTGGCTC

TGGATCATCCTCCAGAACTCCCCACACACCACCTTCTGCATCTTCTGCTTCCTCATCTTC

CTCATCATCTTCTTCCTCCTCAGCATCTGCCAATGCGCGCCAGTCTAACTCTATTCCAAG

CTCTACTTCTGCATCTGCATCTTCCCGACAGCAGCCACCAACTCAGTCTGTACCTCCTTC

ACAAACACATTCCCAGCCTGCTCCCAGCACTAACTCCTCTCAACAGACCCTTACTAAAGC

TTGTCTTTCAGGCTATGGCTCTCCAGCGGCTCCAGTAAAATCATCTAATAGTCTCACAGG

ACAGACCCCACCCCAACAGCAGCCTCAATCATTCTCTCCCAACCAGCCTCCATCACACTT

ATCTCAGCCATATGCAGGGTTTAGTTCTCCACAAACACATGATCTTCCTTCAGCCAGGAC

GGCGGGAGTAGCAAAAGGATACGGAAATTTGGGAAGCCAGTCATTTTCTGCAGAATCAGT

ATATGGGACAGATTCAGGTTATGGCTCACTGCCTTCATCACTAGGGGGAGCTGGAAGCCC

CTCCATGGGCTATGCTGCATCAGGACATTCTCCTGCACTTCTTCGTTCAGGGGCAAGCGG

GGGAACAGCTGGTGGAAGCAGCAATGGAGGAAGTACAAGTACAGGAGGCGGAAATAATGT

CACTGGCGGAGGTGGCTCTTACCACATTCCTGATTCCAGTCCATCACCATCTGGAAACTC

TGGAATCATTCGGCCAGGTCTTCATTCTCCAGCTCCTTCACGACCTGCTCAGTCACCTGT

TGGAACAGGATCCAACAAATACCTATCATCTGTTCTCTCGCCTTCCTTCTTACCCTCCCC

ACAAGGCTACCCAGACACCCGTGGACCACGATCACAAACGTATCATCCCTCTGCCCCTCC

CAAAACCAAGTCTGACACAAGCCTTCTTGGTGTGACTGAGTCTCGACCACAACAGGATGA

TGACGATGACGATTTCCTCATCCAGCATTTACTTCAGGCTCAAAGTCCTACTCCCCAAGC

ATCCCATCATCATCCACCACAGAGTCATCATTCCTCACAGTCTACACAACAGCCACCAGT

TCTTCCGAATCAAGATGGAAGCAAGGGTTTATCCTATGAGATTGGAAAGAGTTCAGAAGA

GCGATATCATCTTCAGAGTGTTATTCGAACTCACAGTGCAACCACAAATGCTGCAGTATC

TGGAACAGGAAATGGAGCAGGCCTGGACAGTCAGTTGGAATTGTCTTTAAAAAAGCAGCA

GCATCAGCAACAACATCAAACACAGCATCAACAACAGCAACAACAACACAAACATCAACA

GCAGAAAAGCAGCAGATCTGTCACAGATGGAGGAGGACGAGGAAACCCAGATCAAGCTCA

TTCACATTCACATCATCACCATGATTCAATGAGCTCAGTGGTACACTATGGTCGAGGTGA

TCCTTATTCTCAACATTCTATTTCACAGCATCCATCTAGTCACCATCAACACACTCCCCA

CACACCTACACATTCTCACTTGCACTCTCACCCCCATATGGATCTTCAGAAGAAGCCCCA

AGAGTCAGCAGATATGGCCTACATTCGCAAGACACCTGATCTGCAGCAGCACCACCACCA

ACAGCAATCAGGACATCATCAGTCTCATCAGCAACAACACCAACAGCAGCAGCAGCAATC

TCAAACATTAATGGAATCTCCAACTGACCAGTCACGTCAGCCTCCTCATTTGTTACAATC

TGTTCTCTCCCATACAACACGAAACAAAATAGAAACTCAGCAACAGCAACACCCTGCAAG

CCAGCAGGCTTTAATAGAAGCAACAGGTGGGGTTACAGCAGCAGAAACCCATTCCCAGCC

TCAGGCATCTCAACTACAGCTTCAACTCCAGACTCAAGCTTTGGAAACTGCTAGTCACTA

TAGCCATGGTTCTCAGCAGCAGGATCAGAACCATCAAAAATCTAATACTGTCTCTTCTCT

GGACATGTTGGAGCGCTCCCTTTCTCGAACCTCAAGCCAGGAAGGAGGAATGGTGGATGA

TAGACCAGGAAATGAGGGCGGCAGAGGAAATTCGGCAGCTGGGACCGGAGGACCTGTTGA

GAGGCACAGATCACAGGATCAGCAGAGACTTCCATCCCATCATCCTACACAACACCATCC

CCCTGAGTTACATACTTATCTTTCTGAGCCTGATTTGGGATTATCTACCCCGTCTCATGG

GCACCACCTTTCCCATCACCACCATCAGTCACAGCAACCACCTCCAGCTCAACAACCTAA

CTCACACCACCATGCTCACCATCAGCATCAAAATCCACAGAGCCATCACCATCTTGCTTC

ACAGTCAGCCTCTGCAACAGCACAGCAACAAGAGCAGTCTCACTCCTCCCATTCCTCTCA

AATCACTCAAGCTCAGCTTGACCAGCAGCATCAAGGCGATCATCACTTTGATAGTAGTGG

CTCAGCAGTAAAGTCAAATGCAAATCAGAATCAGTCCCATCACAACCAGAGATTTGTTCC

TTTAACATCTATTTGTTTTCCAGACTCTCTTCTTCCAGATGAAGAACGTTCTTTCTTTCC

TGGGATGGAGGACATGTTCTGCCCCGAAGAGTACAAGTCAAGCTGCTCTGGAGGAACCGG

GCAGGGTCAAGATGGAGTATCCCAAGCTCAGACAGGACAAGAAGGTATGGAGGGCATAAA

ATCAACTGCTGAGGGTGGAGAGGATGATACAGGTGCAAGCTATGACATGCTTGGTCACCC

TGGCGATCAGGATTATGGGCAGTATTGCCATGATCTCACAGAGCATGATAATGGAACCAT

GCATCTTGACCTTGACTCTTTAAAAACCCATGAACTCCCATCAACAGTCAATACAGAGCA

ACTTGGACTTATTCAGTCTCAGCCAACTAACCTTGGAATAGGTAGCAGTGCAAATGTCGG

GGATGGAGCTGGTGCTAAGATGGTAGGTTCTGGAGGTTCTGTTTCTGGATCAGGAGGACT

TACCTCTCCAATCTTCTGTTCATCCCGACCCAAAAAGCTTCTGAAATCCAGCTCTTTTCA

CCTGCTTAAAGAAAGACCTGATCCAAATTCCATTCCCAAAAAGAGCTATGCACAGGAGTA

TGAATTTGAAGATGATGAAGATAAGGCTGATGTTCCTGCTGACATTCGACTAAATAGCCG

AAGACTTCCTGACCTGATTCCTGACCTTGTATCCAGCTGTCGGAAAGCCGGTGGTGGAAC

TATGAGTCCTTTAATGAGCGACTTGGATTTTGGATATCCCTCTCTTGGTCCGCCACCACA

ATTACTTCCCAATGATGGACCAAAAAAGAGAGGCAGGAAGCCTACCAAGCCCAAACGGGA

GGGCCCCCCAAGGCCTCGTGGCCGTCCACGAATACGCCCTTTGCCGGAGCCCCACCACTC

CAGGGCCATGATGGGAGAGTGTGGAACAGGATATATTCCTGAGAGAGGCAGGGGTAGAGG

TAGAGGCAGAGGAAGAGGCAGGCGAGATGAATCTATGATGGACAGCAAAGATCAGAGTCA

GCTGTATCAACAACACATGCAGCAACAGCAAATGCATCACCTTCCACAACAGCACCAAGA

GCCAATCAAACCTATTAAGATCAAGCTTCCTATCGGTTCCATGTCATCTTCTGATGCCCT

CCTGCGGACAGATTCTTTGTCTGGTACTGACCCAGCCCTCTCAGATGGTTCTGTTGGCTC

TGCCCCTTCTTTGGGGTTAAGTCCAGGGACACCAGGAGTGCCTGATATGACCAGACCATT

AGACAAGAATAAGGCTAAAACTCAAGATATGGAAGAGAAAGATTCTGAAAAGACTGGCTT

TGTGGCATCTTTTTTGGATTTTCTGAAATCCGGGAAAAGACCAGGTAGCAGCTCTGGGAA

TGGTGATTCATCTCCAGGAAAAAGTGGAGGTATACATCCCATATCTCCTCAACCACCACC

TCCACCAGCACCTGCAGGATCATCTGGTTATGGGGATAGTGAAGGCGATGGAGGGCTGTC

TTTGGGTGGATGCCCAAGTCCTTGTAAGCGCTTAGACGAAGAACTGAAAAGGAATCTGGA

AACACTTCCATCATTTTCTTCTGATGAAGAAGACTCCGTGGGAAAAAACCAGGACCTCCA

GAAGAGTATTTCCTCAGCCATCTCTGCTCTGTATGACACTCCACAGCTAAGCACCTCTTC

AATGCAGCCTCCCTCTCTCCCTCCGCCGCCTCCTCCAGTTGCTCCTACACCATTGACACC

AACACAACATCCACCTTCCCTTAGCCCACAAACCCCAACACACATGCACACACAGCCTTC

TTCACATACACATCCACAGTCTATTGAGTCTACCATATTATCAAGAGATGAGCAAGGGGA

TGAGGTTGAGGAGGAAATAATTAATGATGAAGACAAAGAAGAGGAACAGGACAAAGGAGT

AGAAGAGAAAGTCACAGAAATGGAGTTGCTAAGTGTACCAAAAGTAGGAGACTCTCCTAA

AGAAGCTTCTCAAGCAGTAGCTCCGCATTCACTTCTACCTCTTTTCCCATCATCCCCTGC

ATCTTCATCTTCACCCTCTCCACCACCTCTTCCTCCACTTTCTCTTCCTTCACCCCTTCC

TGAGCTGGAAGAGAACCCATCTCCACCACAGAAACCTTCAGCTCAGCTGTCTTCACCTCC

AGCCAGCGATCTTGCCACTACATTACCTGTCCTTTCTCCTCCACATCCTCTGACTGTAGC

CTCTCCTCCCCCCTTGGAGACACCCACACCATCAAGCCCTCCTCCAACTCAGCAGACTGT

TCCAGCTCCTCCATCCCCAGAAGAAACCCCAGCAACTCAAATTTTGCCTTTACATCTGGC

ACAGAAACAGAGTGGTGCTGCTATTGCAGGAGAGACTGATGATGATGAGAGTGAAAGTGG

AGGAGAGGGTATCTTTAGGGAAAGGGATGAATTTGTGGTACGGATAGAAGACATCAAAAC

TCTTAAGCTGGCCCTGCAGACTGGTCGGGAGCCTCCACCCATTTGGCGTGTACAAAAAGC

TCTGCTCCAGAAGTTTGCGCCAGAAATTAAAGATGGACAGAGGCAGTTCTGTGCCACTAG

CAATTATCTGGGTTACTTTGGTGACGCTAAAAAACGGTACCAACGACTATATGTGAAATT

CCTAGAAAATGTGAATAAGAAGGATTATGTAAGAGTTTGCTCTTGCAAGCCCTGGCACAG

GCCTTCTGTGACACTCAGACGACAGGCACAGTCTCACCCGGTCTCAAAGGTGAACCCATC

TCCTAATAATCAGACACTGCCTCGTGTTGTAAGAGATGAGAAAGATAAAGACAGAGAAAA

AGGACGAAATAAGGAGCAGCGTGACAGCAGAGATAAGAACAACATCAAGGTGAAAGAGCA

ACAAGAAAAGGAAAAGGTGGTAAAAATTAAGGAGCGAGAAGAAAAGAAAGACAAAGAAAA

GAAAATGCCACCACCTCCTGCACCTCAAAGGGCAGGGAAACAAACAGCTGTGACAGAACC

TGGACGAAAGGAGGAGAAAAGAGGAGTGGAAAGAAAGATAGAACGAACTACAAAACAGCA

GCCACTAAAAGTAAAGGCTGAGCCTCCACCAAAAAAGAGAAGAAAGTGGCTGAAAGAAGT

TCCGTCATCATCCGATTCCGATTCTTCGGCCAGTGATGATGAAATTTCTGTGAGAGCAGG

CCTGAATACACGTGCCATGCGAGAGATGTACAGAAGCTATGTGGAAATGCTGGTCAGTAC

AGCTCTGGACCCTGACATGATACAAGCACTGGAGGATACAGAAGATGAATTGTACTTGCC

TCCAATGCGGAAGATTGACAGCATTCTTAGTGAGCATAAAAGAAAGCTGCTAAAACGGAT

CAACATGAACCCCCAACACCAGGAGGCCCTACACCTTTTTCCTCAGATCACAGCAGAGCC

CTTGGACTCAGGAGCTGTGAGGGTCAAACTTGGTGGGGAGTGCTATAATAGAAAAACCCT

GAACCGTGTCAAGAAGAGTATCTCCAAACCACAGGACATCAAGTTGTCAACAGAGACTTG

TCGCCTCTACAGCTTTTACCATTCTCTGCATCATTATAAATACCACACCTTCCTAATCTG

CAAAAAAGAGACCAACACTATAGAACAAGCGTCAGAAGATCCTGGGCAAGAGGAGGTGGT

TCAGCAGTGCATGGCCAATCAGAGCTGGCTGGACACACTCTTTAATGCCTTCTTAGAACT

GATGACCCTCAGTGCCAAAGCCTGACAGAATAAAAGCGTTCAAGAGGGGGGGGGCAGCGA

GCGACAAAGGACAAGCCCTGAGGATGCAGCAGAAGGCTGTTGTGGAACAAAAGAAATGGA

TTTGTTTTCACAGTGTCCCCCCTGCAGTCCTGTTCTATTGTTTTTCTTAAGGCATTTCTT

AAGATGATTGTGATACATGGCCAGGCAATAGAGCCGCCTCCCAGTAGGTAGCCTTTGCCG

AGTAAACTAATCAGCACTTCATTACTCAAGTCAATTAGAGCAAAGTTTTCATGCTGAATG

GATTCAAGGAAAAATGGGGACCATATTCACTAATTTTTTGTGTAAGGGGATGAGTGGATG

GATTGTCCCCTTTCGCTGTGCTGAATTTGCTAACAGACAGAAGAACAGAGAATGAATATG

ACTGAAGAATTCCGTACAAATTTTAAGGGTAGATTTGGGGGAAAAAACAAACTTTGAACA

ACTAGAATTTAAAGACATTGCAAGAAAAAAGTCTGTTACAATTTGAATTTTACACAGATT

ATTTTTAAGGATTACAAACTTTTATTCTGCTTTTTTTCCTTGAGTGGAAG

>Unigene000034

CACACACACACACACACACACAACAAACGCACACATTCACACAACCGAATGACACATTTG

CGTTTTGGAAGTGCGTAATAAATCGCGTCTTGGGTCAGCTGGAAGAAGGTCCGATTGAAT

TTTTGGTTATGCAATGGTCTCTGTATCAGCGCTAGCACTTTCAGCTGTGTGCCACTTCTT

GATCAGTCCAGAGGAGGCAGTGATTTCATCTCGCGCGCCAGTGACATAAGCAGACAAGAT

TGCAAAGATCTGTCCTGTTTCGAGTATGACTGCCACGACCCGTGGCTCCCCCATGGGGGG

GAATGAAAGTCAGGAGCAGGGCCAAGCTCCGGACGGTCAATCTCAGCCCCCACTGCCCCA

AAACCAGACGTCCACTCCCACATCGTCCAACGAGAACTCTCCAGTGAGTCCTCCAGATGA

GCAGGTCCAGGGAGACTGTACAGGCCAGTTGGAGGAGGAGGAGGAGCCAGCTTTCCCTCA

CACAGACCTGGCCAAACTGGATGACATGATAAACAGACCCCGCTGGGTTGTTCCAGTTTT

GCCAAAGGGTGAACTAGAAGTTCTTCTTGAAGCTGCTATAGATCTTTGTAAAAAAGGACT

TGATGTCAAGTGTGAAGCCTGCCAGAGATTTTTCCGTGATGGTTTGACAATATCGTTCAC

TAAAATACTGACTGATGAGGCTGTCAGTGGATGGAAATTTGAAATCCATAGATGCATAAT

CAACAACACTCATCGCCTCATAGAACTGTGTGTGACCAAGCTCTCTCAGGACTGGTTTCC

TCTTCTAGAGCTCTTGGCCATGGCCACCAACCCCCACTGCAAGTTCCACATTTACAATGG

TACCCGGCCCTCAGAGACGGTGCCTGCTGGGGTGCAACTGGCTGAAGATGAGCTTTTCGC

TCGTCCACCTGACCCTCGCTCTCCTAAGGGCTGGCTGGTGGATTTAATAAACAAATTTGG

CACATTAAACGGGTTTCAAATTCTGCACGATCGATTCATGAGTGGTCAAGCTCTGAACGT

TCAAATCATCGCCGCACTCATAAAGCCTTTTGGGCAGTGCTACGAGTTTCTCACTTTGCA

CACGGTGAAGAAGTACTTCCTTCCCGTTATAGAAATGGTTCCCCAGTTTTTAGAAAACCT

CACTGATGAGGAGCTAAAAAAGGAAGCCAAGAATGAAGCCAAAAATGATGCTCTGTCTAT

GATAATCAAGTCGTTGAAGAACCTGGCTTCACGAGTACCAGGGCAAGAGGAAACCGTGAA

GAACATGGAGATATTTAGGTTAAAAATGATCCTTAGGTTATTGCAAATTTCTTCTTTTAA

TGGCAAAATGAATGCACTAAATGAAGTAAATAAGGTGATCTCAAGTGTCTCATACTACAC

ACACCGTCATGGCAACCCAGAAGAGGCAGAATGGCTCACGGCAGAGCGCATGGCGGAGTG

GATTCAGCAAAACAACATTTTGTCTATTGTGCTGAGGGATAGCCTTCATCAGCCTCAGTA

TGTTGAGAAGCTAGAGAAGATTCTGCGCTTTGTTATTAAGGAAAAAGCTCTTACCCTTCA

GGACTTGGACAACATTTGGGCTGCCCAGGCTGGGAAACATGAGGCTATTGTCAAGAATGT

TCATGATCTCCTTGCAAAATTGGCATGGGATTTTTCACCTGAACAACTAGACCATCTTTT

TGACTGTTTTAAGGAAAGCTGGACAAATGCGAGTAAAAAGCAACGAGAGAAGTTGCTGGA

ACTAATTCGTAGGCTGGCAGAAGACGATAAAGATGGCGTGATGGCACACAAAGTGCTTAA

TCTGCTTTGGAACTTGGCCCATAGTGATGATGTTCCTGTGGATATTATGGACCAGGCCCT

CAGTGCTCATATCAAGATCTTGGACTACAGCTGTTCCCAGGACAGGGACACCCAAAAGAT

CCAGTGGATAGATCGCTTCATTGAAGAGTTGCGATCAAATGATAAATGGGTAATCCCTGC

ACTGAAGCAAATTCGAGAGATCTGCAGTCTGTTCGGAGAGGCTCCTCAGAATCTCAGTCA

AACACAAAGGAGTCCACATGTTTTTTATCGCCATGACCTTATTAACCAACTTCAGCACAA

TCATGCGCTGGTCACTCTAGTAGCTGAGAATCTGTCTGCATACATGGAGAATATGAGGCA

GTTTTCCAAAGCAGAGCACACAGACTTTGACCCGCAGACGGTCCGGCCTGGAAGTCGATA

CAGTCATGTGCAGGAAGTGCAGGAGCGTCTAAACTTTTTAAGGTTTTTGTTAAAGGATGG

GCAGCTTTGGCTGTGTGCTCCTCAGGCCAAACAGATTTGGAAGTGCCTGGCAGAGAATGC

AGTGTTCCTGTGTGATCGTGAAGCCTGCTTTAAATGGTACTCCAAACTGATGGGGGACGA

GCCTGACCTTGATCCTGATATCAACAAGGACTTTTTCGAGAACAATGTGCTCCAGCTTGA

TCCTTCTCTGCTGACTGAAAACGGCATGAAATGCTTTGAGCGCTTCTTTAAAGCAGTAAA

CTGCCGAGAGGGCAAGCTGGTGGCCAAGCGCCGAGTGTATATGATGGATGACTTGGAACT

GATTGGACTGGATTATCTGTGGAGGGTTGTGATCCAAGGCAGTGATGACATCGCTAGCCG

AGCAATTGATTTACTTAAAGAGATTTATACAAACCTTGGACCAAAATTACAAGCCAATCA

GGTGGAGATCCATGAAGATTTTATTCAATCTTGCTTTGACCGACTCAAAGCCTCCTATGA

CACGCTGTGTGTGCTGGATGGAGATAAGGACAGCATTAACTGTGCCAGGCAGGAGGCCAT

CCGCATGGTGCGCGTTCTCACTGTGCTAAGAGAGTACATTACAGAATGTGACAGTGACTA

CCATGAGGAGCGGACCATCTTGCCGATGTCCAGGGCATTTCGTGGGAAGCACATCACCCT

CATTGTGCGATTTCCAAACCAAGGTCGCCAGGTGGATGATCTGGACATTTGGTCCCATAC

TAACGACACCATTGGTTCTGTGAGACGCTGCATTCTCAATCGAATAAAGGCTAACAGCGC

GCACACCAAGATTGAGCTCTTTATTGGCGGGGACATTATTGATCCTGCTGACGACAGGAA

ATTGATTGGACAGCTTAATCTCAAAGACAAAACTCTTATCACAGCCAAGCTTACCCAGGT

CAGTGCCAATATGCCTTCGAGCCCAGACAGTTCCTCTGACTCCTCCACCGGCTCCCCTGG

GAACCATGGCAACCACTATAGCGATGGACCAAACCCTGAAGTTGAGAGCTGCCTTCCTGG

AGTGATCATGTCCCTGCACTTGCGCTACATCTCTTTCTTGTGGCAAGTTGCTGACCTGGG

TTGTAATCTCAACATGCCTCTTCTGCGTGATGGTGCTCGGGTTCTTATGAAGCTCATGCC

TCCAGATAACACTACAGTGGGAAACCTGCGAGCAATCTGCCTGGATCATGCCAAACTTGG

AGAAAGTAGCCTTAGCCCCACTTTAGATTCCCGCTTTTTTGGTCCATCACCTTCACAAGT

CCTTTACTTAATAGAGGTGGTTTATGCCTTACTGATGCCAGCTAGTGGCACACTGGGAGA

GGATGCTAGTGACTTCCAGTACAACTTTTTGAGGAGTGGCGGCCTACCTTTGGTCTTGAG

CATGCTGACCCGAAATAATTTTCTTCCAAATGCTGACATGGAGACGCGCCGAGGAGCCTA

TCTCAACGCACTAAAAATAGCCAAGCTCCTGCTTACAGCTGTTGGCTTTGGACATGTAAA

GGCTGTGGCTGAAGCTTGCCAGCCTGTAGCAGAGGGAAACATCCCAGTGTCACCTATAAA

CCAGGCCACTCATGACCAAGCATTAGTGCTTCAAAGCGCCCTGCAAAACATCCCCAATCC

CTCGGCTGAGTGCATGCTTCGCAACGTGGCAATCCGTCTGGCTCAGCAGATCTCTGATGA

GGCTTCTAAGTATATCCCAGACATCGGTGTGATCAGAGCAGTCCAGAAGATTGTCTGGGC

CTCTGGTTGTGGTTCTATTCAACTTGTTTTTAGCTCGATTGAGGAGATCAGCAACATCTA

TGAGAAGACTAATGCAGGGAATGAGCCCGATTCAGAGGATGAGCAGGTGTGCTGTGAGGC

CCTGGAGGTTATGACTCTTTGCTTCGCCCTTATTCCGACTGCACTTGATGCACTTAGCAA

AGAGAAGGCCTGGCAGACCTTTATCATTGATTTACTTTTGCACTGTCAGAGCAAGTCTGT

TCGCCAGATGGCCCAGGAACAGTTCTTTCTCATGGCCACTAGGTGTTGCATGGGTCACAG

GCCTCTCCTGTTCTTTATTACTCTACTCTTCACAGTTTTAGGTAGCACTGCAAAAGAACG

GGCCAAGCATGCTGGTGATTATTTTACACTTTTGAGGCACTTGCTCAACTACGCATATAA

TAGCAATATCAACCTCCCTAATGCTGAAGTTCTTCTCAACAATGAGATTGATTGGTTAAA

AAGAATTAAGGATGAAGTTAAGCGAACTGGCGAGACTGGGGTGGAAGAAACCATATTGGA

GGGTCATATCGGTGTCACAAAGGAGCTACTGGCTTTTCAGACAGCAGAAAAGAAATACTA

TATAGGATGTGAAAAGGGTGGAGCCAACCTGATCAAGGAGCTGATTGATGACTTCATCTT

CCCAGCCTCAAATGTGTACCTGCAGTACATGAAGAGTGGAGAGTTTCCTCCTGAGCAAGC

CATACCTGTGTGTAGCACTCCTGCTACAATTACAGCTGGATTTGAGCTCCTGGTTGCACT

TGCTGTTGGATGTATGCGCAACCTCAGACAGATTGTGGACACCCTAACTGATATGTACTA

CTCGGGTTGTGAGGCACTTACAGAATGGGAGTACCTGCCACCAGTCGGTCCAAGGCCCAC

TAAAGGCTTTGTTGGCCTGAAGAATGCAGGTGCCACTTGTTATATGAACTCAGTAATTCA

ACAGCTTTACATGATTCCAGCCATCAGAAACGGCATCTTGGCTATTGAGGGCACGGGCAG

TGATGTGGATGATGATGTGCCTGGGGATGAGAAACAAGATAATGAGAGTAATGTCGATCC

ACGTGATGAGGTATTCAGTTATCACCACCAGTTTGATGACAAACCATCGCTCAGCAAATC

AGAGGACAGGAAAGAGTACAACATCGGGGTTCTGCGCCACCTTCAGGTGATCTTTGGTCA

TCTCGCTTCCTCCAGGCTGCAGTACTATGTTCCCAGGGGATTTTGGAAACAGTTTAGGTT

GTGGGGTGAGCCAGTAAATCTGAGAGAACAACATGATGCTCTGGAGTTCTTCAACTCGCT

CGTAGACAGTTTAGATGAGGCTCTTAAAGCACTGGGCCATCCTCCCATGCTAAGTAAAGT

CCTTGGTGGTTCGTTTGCTGATCAGAAGATCTGTCAGGGGTGCCCTCATAGATATGAATG

TGAAGAATCCTTTACAACACTGAATGTAGATATCCGAAATCATCAGAATCTGCTTGATTC

TATGGAGCAGTATGTGAAGGGTGACTTGCTTGAGGGTGCTAATGCCTACCACTGTGAAAA

ATGCAACAAGAAAGTGGACACAGTAAAGCGCTTGCTTATTAAGAAACTGCCTCCTGTACT

GGCCATTCAGTTAAAACGATTTGATTATGACTGGGAACGTGAGTGTGCTATCAAATTTAA

CGATTACTTTGAGTTTCCTCGGGAGCTGGACATGGAACCCTATACAGTAGCAGGAGTAGC

CAAGCTAGAAGGCTCTGATGTCCACCCTGAAAATCAGGTCATCCAACAGAACGAACCCTC

AGAGCCTGAGGCACCATGCAGTTCACGCTATAGACTTGTGGGAGTGCTAGTGCACTCAGG

CCAGGCCAGCGGTGGTCACTACTACTCTTACATCATCCAGAGGAATGGCAGTGGAAGTGA

GGGTGAAACCAATCGCTGGTACAAGTTTGATGACGGTGATGTCACCGAGTGCAAGATGGA

CGACGACGAAGAGATGAAGAATCAGTGCTTTGGGGGAGAGTACATGGGTGAAGTGTTTGA

CCACATGATGAAACGCATGTCCTACCGGAGACAGAAACGCTGGTGGAATGCCTACATCCT

GTTCTATGAGCGCATAGACTCAATGGACAAGGATGGTGAGCTGGTGAAGTACATCTCCGA

GCTGACAGTAAGCAGCAAGCCACACCAGATCAAGATGCCACCAGCCATCGAGTGCAGCGT

ACGCAAGCAGAATGTGCAGTTCATGCACAGCCGCATGCAGTACAGTCTGGAGTACTTTCA

ATTCATCAAGAAACTCCTGACCTGTAATAGTGTCTATCTTAACCCACCACCAGGACAAGA

CCATCTTTTGCCTGAAGCAGAGGAAATGGCTAAGATTAGCATTCAGCTTGCTGCTCGATT

CCTCTTCAGCACAGGATTTCATACCAAGAAAGCAGTCCGTGGCCCAGCCAGCGATTGGTA

TGATGCCCTGTGCATCCTCCTTCGACACAGCAAGAATGTACGCTGCTGGTTTGCACACAA

CGCCCTATTTTCCTACCCAAACCGCTTCTCTGAGTACCTGCTCGAGTGCCCTAGTGCTGA

GGTGCGGGGGGCCTTCTCCAAGCTTATTGTATTTATTGCACATTTCTCTCTGCAAGATGG

ACCTTACCTATCACCTGTTGCCTCACCAGGCCCATCCACTCAGGGCTGTGATAATCTGAG

CCTAAGTGACCACTTGTTACGTGCTGTGCTCAACCTGTTACGGAGAGAAGTGTCCGAGCA

TGGCCGTCACCTGCAGCAGTACTTCAACCTATTTGTCATGTATGCTAACCTAGGTTTGGC

AGAGAAGACACAACTGTTGAAACTTGGTGTGCCAGCTACATTCATGCTGGTGGCTTTGGA

TGAGGGCCCAGGCCCACCCATTAAGTACCAGTATGCTGAGCTGGGAAAGCTGTATACAGT

GGTGTCGCAGTTGGTTCGTTGCTGCGATGTAACTTCTCGTATGCAGTCATCAATCAATGG

TAACCCTCCTCTTGCTAACCCTTATGGCGATCCCAGCTTGACTCAGCCCATCATGTCTCT

GCATCAGCTAGTGGCTGAGATCTTGTTTGTGCGCACCAGTTATGTGAAGAAGATTATTGA

GGATTGTAGTAACTCTGAGGAGACCATCAAACTCTTGCGCTTCTGCTGCTGGGAAAACCC

ACAGTTTTCCTCCACTGTGCTCAGTGAACTGCTGTGGCAGGTTGCTTACTCCTACACTTA

TGAGCTAAGGCCCTACCTAGACTTGCTGCTACAGATCTTGCTTGTTGAAGACTCTTGGCA

AACTCACAGGATCCACAATGTGCTGAAGGGGATTCCAGATGACCGTGATGGATTGTTTGA

TACCATCCAGCGCTCTAAAAATCACTACCAGAAGAGAGCTTACCAGTGCATCAAGTGCAT

GGTGGCACTGTTCAGCAATTGCTCAGTTGCCTATCAGATTCTTCAGAGTAATGGAGACCT

GAAGAGAAAGTGGACATGGGCTGTAGAGTGGCTTGGGGATGAACTAGAACGCAGGCCATA

TACTGGCAATGCCCAGTACACATATAACAACTGGTCACCTCCTGTTCAGAGTAATGAGAC

CTCCAATGGCTACTTCCTGGAGCGTTCCCACAGTGCCCGCATGACCCTAGCCAAGGCCTG

TGAGCTTTGCCCAGAAGAGTGTCACTTAACGAAGCACGAGGTGGTGTCTGAAGAGGACGC

CGGCCGGAAGCTGTCCTCGCCACAGCAGCTTTTGCCAGGGGAAGTGACAGGCCAGCAGCA

ACACACTGAGCAGGATGAGCAGGAGGCCCCAGATGATCAGGACTCCTCTCCACCAGAAGA

TACTTCTCTGTATCCCCACTCTCCAGGGACACAGTATCAACCGCAAAACAACCTTCCGCA

CGGTCAGCCGTACACCGGCCCGGCAGCACAGCATATGAACAATCCCCAGCGTCCAGGTCC

ACGAGCACAAGAGAACTGGGAGCCCCCAGAAGAGGTGCCACCTGCCCAAACAAAAGAGTA

GCACCCTGCTTTCATCCTGTCCTCCATCCGAGTACAAAACTTTCAAGTAAAGAGCGAGGC

CCCAGGCCCCACCATGCAGCGTCACTCCACAAACTGTGTCTCCAGAAAATTTTGCTCCCT

GTCCTCCTCACCCTGGGGTGGAGTATCACTGTCTGCCCTGCAGTATTACAACCTGCCTGC

AGTGGATGGTGTATGGTGGCATAACAAGCCAGGCCTGATGCACTTGTCTCATGTGACAGT

GGTGTACATATTCTAAAATAGTTTTTGACTGTTCGAAACCATCAGCACCACTTGCTGGTT

GGAGACTTGCGGATTTACAAAACTTTAAAAGTACAAAAATGCAAAAAAAAAAATGATGTA

AAAAAAAAAAAAAAGCCCTGTTTAATCACCTGTGCTCAGTGGTGCAGTTTGATCTGCCTT

TTGGCCCTTTCGGTCATTCAGCTCTATATTATTTTATCCATGTCCACTCTTGGTTAATCT

CTCTCACTTCTGGAGCTGAGGATGGCTGTACTGTAGGCATGATTAAATGGTCCTGATTCT

AGGTTGATGTAAGAGGTGATAACATTTGTTCTGAAAACTTTCATTGCCTTTCCTCTGTTT

TGGTATTAACTGAATCATTTGAAAGCTCTAGTTGATATGCTGTAATGTTTTAGCATGGCT

TCACACCGAACTAGTGTAGCCAAAGGGAAGTTATGTGATCCTAACCACCGTAGTTCCTAA

TTGACAGCTATTTTGACAAGCCGTTCCAACCCCCCCACCCCAA

>Unigene000035

CCCCGCACAGCTCCTCTCAGACCTGAACTGCGCATGCGCAGCGCACGCTCACCCACTTGC

AAACTGTCATGGCGGCGGCGACCCAGAGCCAAGCGATTCAGGAAGAGATGGCGCTCTAAA

AAAAGGAAAAAAACTCCTAGAAGAAGAACTGTGAGCTCGCACAGAGCACTCGAATCAACA

GCAGGAGAATTTGTGTTCTGTTTCGGAGTGTTTTTAGCTGCCAGAGAAAGAGAGAAGGAG

AGAATCATTATTCCGCTCTGAAGAAGAAGAACAAGAAGAAGAAGAGAACCCCCAGCACTT

GATGGAGATTAGGTTCTGAAGCTTCTCTTTAATACACCGAGCTTAATCCGAGGTGCTACT

TTAAACCTGAGTCACCTTGAAGAGGAACAGGTGTCTCACCAGGTATGGCGGAGGAAAAAG

CGCCCCCTGGTGGAGAGGGCATCACACCCATCGTGCCCATCAGAGGAATCCGCATGAAGT

TTGCTGTGCTGGAGGGCCTCATCGAGGCTGGAGAGGTCACCAACCGAGACATTGTGGAGA

CCGTGTTCAACCTGCTGGTGGGCGGTCAGTTTGACCTGGAGCTGAACTTCATCATCCAGG

AGCCGGAGAGCATCCCGTGTACGGTGGATCTGTTGGAGAAGTGTGAGGTGACGTGTCAGG

CGGAGGTGTGGAGCATGTTCACGGCTGTGCTGAAGAAGAGCGTGAGGAACCTGCAGGCCT

GCACTGACGTCGGCCTTATCACACTCGTCCTGCAGAGGATCGACAGAGCCCACAGCATGA

TCGCAGACCTGCTGGTGGACATGCTGGGGGTTTTGGCCAGTTACAGCATCACAGTGAAGG

AGCTGAAGCTTTATTTCAGTAAACTACAGGGAGAGAAGAGACAGTGGCCTCCTCATGCTG

TGAAGCTGCTGTCGGTGTTGAAGTCCATGGCCCAGCGCTCCGGCCCGGACGCCTTCTTCA

CTTTCCCTGGAAAGAGTGCAGCAGCCATAGCACTTCCTCCGATCGCTAAGTGGCCATACC

AGAACGGTTTCACCTTCCACACCTGGCTGCGTCTGGATCCCATTAACAACATAAATATAG

ATAAAGACAAGCCTTACCTCTACTGTTTCCGGACGAGTAAAGGGATGGGTTATTCGGCTC

ATTTTGTCGGCGGATGTCTGATTGTCACTGCCTTGAAGGCCAAAGGAAAAGGATTCCAGC

ACTGTGTCAAGTTCGACTTTAAACCTCAGAAGTGGTACATGGTGACCATCGTGCATGTCT

ACCACCGCTGGAAGAACAGTGAAATCAGCTGCTATGTGAATGGCGAGCTGGCGTCTTTCG

GAGAGATCACGTGGTTCGTCAACACCAGTGATACATTCGATAAATGTTTTCTGGGCTCGG

CGGAGACGGCAGATGCGAACCGCGTGTTTTGTGGTCAGATGGGGGCGGTGTATCTTTTTA

ACGATGCCCTCAATGCCGCTCAAATCTTTGCCATCTATCAGCTAGGCTCCGCCTACAAGG

GTGTGTTTAAATACAAAGCAGAAAGCGACCTACTGTTTGCAGATCATCATAAAATTCTGC

TGTATGACGGTAAACTAAGCAGTACCATCGCCTTCACCTACAACCCCCGAGCTACAGACG

CCCAGCTGTGCCTGGAGTCCTCACCCAGAGACAACACGTCCATCTTCATACACTCCCCTC

ACGCCCTGATGCTGCAGGATGTTAGGGCGGTGATCACTCACTCGGTGCAGAGTGCTGTAC

ACTCCATCGGAGGAGTGCAGGTTCTCTTCCCGCTCTTCACTCAGCTGGATTTTGTGCAAC

CGAACAGCGAAGAGCCGGACATCTCTGTCTCCTGCACTCTGCTGTCCTTCCTGTTGGACT

TGTTGAAGTCGTCTGTGGCCATGCAGGAGCAGATGCTCACCTGTAAGGGCTTCCTGGTTA

TCGGATACTCTCTGGAGAAGTCTTCCAAAGTCCACGTAACAAGGCCCGTGCTGGACATTG

TTTTGTCCTTCGCTCGTTATCTCAGCAATCTGCACAACGGCATCTTACTGCTCAAACAGC

TGTGTGATCACATCCTATTTAACCCCGCCATCTGGATACACACACCAGCCAAGCAGGTCT

TGTACACATATTTGGCCACAGAGTTTATTAGCACGGTAACGATCTACAACGCCGTGCGGC

GGGTGGGAACCGTGCTGCAGGTTATGCACACGCTTAAATATTATTACTGGGTGGTGAATC

CACAGGACCGCAGCGGAGTGATGCCTAAAGGACTCGATGGTCCAAGGCCCAGTCAGAAGG

AGATCCATTCTCTTCGAGCGTTCCTGCTTCTCTTTGTCAAACAGCTCATCCTGAAGGACA

CGGGAGTGAAGGAAGATGAGCTTCAGAGCATCCTAAACTACCTGCTCACCATGCACGAGG

ATGATAACCTGATGGACGTCCTGCAGCTGCTTGTGGCGTTGATGTCTGAACACCCCAGTT

CAATGGTGCAAGCGTTCGACCAGCGCAACGGCATCCGGGTCATTTACAAACTGTTGGCAT

CCAAGAGTGAAGGGATTCGAGTGCAGGCTCTGAAGGTCTTGGGTTATTTCCTCCGCCATT

TACAGCCCAAGAGAAAGTCGGAGGTCATGCTGAGTCACAGTTTATTTTCCCTCCTGACGG

AACGCCTGCTTCTGCACTCCAACCAGTTCAGCATGACAACCTACAACGTCCTGTTTGAGA

TTCTGACTGAGCAGATCTGCACACAGGTGATCCACAAGCAGCATCCTGAACCCGACTCCG

CCCTCAAAATCCAAAACCCACAGATCCTAAAGGTGATTGCAGCTCTGTTCAAGAACAACT

CGTGTCCGGAGAGCATGGAGATTCGCAGAGTCTTTCTGTCTGACATGATCAAACTGTTTA

ACAGCAGCCGGGAGAACAGGAGGAGTCTACTACAGTGCTCGGTGTGGCAGGAGTGGATGC

TCTCACTCTGTTTTATAAACCCGAACAGCAGCGAGGAGCTGAAGATCACAGAGATGGTCT

ACACCGTCTTCCGCATCCTCCTCTATCACGCCATCAAGCACGAGTGGGGAGGCTGGCGCG

TGTGGGTCGACACGCTCTCCATCACACACTCCAAGGTAACCTTCGAGAAGCACAAAGAGA

ACTTATCGCGAGTTTTTCAGGAATACCAGCGGATGGGTTCCACTGATGACACTGAGATCC

GAACCGTTTCCGGATCCAGCAGGAACAGTGAACTTACTGCACAGCCATCTGAAACGGTGG

TGGAACCTCCATCTTCCATTATCGAGGTGGAAGAACCAATCTGTGGAGAACATGATGAAC

ATGAGGAGAAAGCACAAGTCATCATCTCAAACATTTTAGCCGGAGTTGAGGAGGAGGACA

CGAAAGAAAAGGAAGAAAAAGCAACAAAGGATGAAGGGAACGTTCAGGAAGCTTTGGAAG

AAAAGGATACAGTAAGTGAGGTTGTAGAGGAAAAGAAGGACTGTAATAAAGCAATGGACA

AAGACGGAACTGAAGGAGGTGATCATAAAAACTTAGAAGAAGAAAACAATCAGAAAGTAG

AATCTGATCAACAAGCCAATGCGGTGAAAATCTCAGAAAATGAAAAGGATAAACCCGCAA

CCTCAGACATTGATGAAGAAGAAAAAGAGTCAGAAAAAGCTGAGGGTCAGAAGGAAAATG

AGATGAAACCTCCTCCTGAGACCCCAGTTCAAGGGGACGGTGAAGAGAAAAAACAGAAAG

AGGACAAGAATTCTGATCTTTCTGAGGTGAGCCCCGGTGCAGAGAAGGGTTCAGACATGG

AGGTGCAGACCCGAGTGGAGGCCAGCACTGATGTCCAGGCAAGTCCAGATCAAGAGACAA

ATCTTGGTTTGGAGAAGGTTTCAAGTACGCAACAGGGCCCCAGTTCAGAGACGAGCCCCA

GTGACCAGGAGAGCCCTAAAGCAGAGACGAGCCCCAGTTTGCAGGAAGTCCCCAGTGTTC

CGGAAAGTCCTAAAGCAGAGACGAGCCCCAGTTTGCAGGAAGTTCCTAGTGTCCAGGAGA

GCCCTGATGCACTGATGAGCCCCAGCAAGGAGGAGAGTCCAAGTATGCAAGAGCTCTCCA

GTGTGGAGAAAAGCCCCAGTGTGGAGGAGGACCAGAGTACCCAAATTCCAAAGGAAAGCC

CCAGCACACAAGAGGCTTCTGGTGTCCCAGAGAGCCTCAATGTGGAGACAAGCATCAAGT

CCAAAGGTGTTAAAATTGCCAGACTGGATGTCTCTAGTGTTGCTATAGACACAGAACGAC

TGGAGCTAAAAGACAACACAGCTGCTGACGGAGGCCAATCCCAAGCTGCAGCCGGAACCT

CCACACAGAACCAAGACGGTTCCTCGTCCTCACCTCGTCCCAGCATGTTCCGTATCCCAG

AATTCAAATGGTCTCACATGCACCAGCGCCTGCTGACCGACTTGCTGTTCTCTATCGAGA

CGGATGTGCAGATGTGGAGGAGCCACTCTACTAAGACAGTGATGGACTTTGTAAACAGCA

GCGAGAATGTGGTGTTTGTGCACAACACGGTTCACCTGATCTCTCAGGTGGTGGATAATC

TGATCATGGCGTCGGGAGGAATCCTGCCTTTGCTCTCTGCAGCCACCTCATCTACGCATG

AGTTGGAGGCGATCGACGCGTCTCAGGGTCTGGAAGTGGAAACGTCTCTGTCTTTCCTGC

AACGTCTAGTCTCTCTGGTGGACGTCCTCGTCCTGTCCAGCTCTCTCAACTTTACGGAGA

TCGAGGCGGAGAAGAACATGACGTCAGGAGGGGTGCTGAGACAGTGTCTGCGCCTCGTGT

GCACTATGGCTGTAAGAAGTTGTTTGGAATGTCAGCTTCACTCTCTGTATAAAGCTGGAA

CAGAGAAAACACAGCGAGGCCTTGTGGGAAATGCTCAGTTTCTTCCAGCTCAGAGTCCCG

TGGACATGCTAACAGGAGGAATTGCTCCAATCCGAGATATGGAACGTCTGCTGCAGGACA

TGGACATCAACCGTCTTCGAGCCATCGTCTTCAGAGACATTGAGGATAGTAAGCAGGCTC

AGTTTTTGGCTCTGGCTGTTGTTTACTTCATCTCTGTTCTCATGGTGTCCAAATACCGAG

ACATTCTGGAGCCTCATAACGACAAAAAAATCAACAAAAACTCCTCTCGACGCACAGAAA

GCGTCAGTTCCGACCGGGGAAACTCCTCCCCCCTCCAACGTCTTGACTCCGCCCCCGGGA

GCGAGGACTCACCTGTTCCCCAGCACAATGCGGAATCCCGCTCCGCACCTGACGCCGTCT

CTGAGGCCTTGTCCACGTTGTCGTCTGAGGTCAGGGGTCACGAGAACATGGACAAGGATA

AAAAGGGAAAGAGCATGAAGGTGAAGGACATCCTGCGCAGCCTGGTAAGCGCCGCCCCTG

CTGATGATGTCATCGTGGATGCAAGTCTCTTACCACCAACGTTTTTGGGAAAGCAGGAAA

TTCACGGCCAGTTCAGCTCATTCGACAGAAGCGTCGTTGTGGGATCTAGGAAAGTGGGCG

GGGCTCCAGCCGCAGGCTCCTCCCCCTCCTTCACCGCTGCCACCACAGGTGGAACTCCGG

TCGTCAGTGTTTCTGTGGTTTCCACAGATGACCCCGCCCCAAGCGACGCTGCAGAGTCAG

GCGGAACAACAGAAGTCACTTCAAACCTGCCATCTGTCCCCCTCTCACCTACCACCACCC

TCAGTATCTCTGAGCGACTGGAACACGCTCTGGAGAAAGCGGCTCCGTTACTGCGGGAGA

TTTTTGTGGATTTTGCGCCGTTCCTTTCCCGCACTTTATTGGGAAGTCACGGACAGGAGC

TGCTCATCGAGGGAACGAGTTTGGTGTGTATGAAGTCCAGCAGCTCTGTAGTCGAGCTCG

TCATGCTGCTATGTTCTCAGGAATGGCAGAACTCCATCCAGAAGAATGCAGGTTTGGCTT

TCATCGAGCTCGTCAATGAGGGAAGGTTACTGAGCCACACGATGAAGGATCACCTGGTCC

GCGTTGCAAACGAAGCCGAGTTTATTCTGAGTCGACAAAGAGCTGAGGACATTCACAAAC

ACGCCGATTTCGAGTCTCACTGTGCTCAGTATGCTGCTGATAAGCGTGAAGAGGAGAAGA

TGTGTGATCACCTGATCCGAGCCGCAAAATACCGTGATCATGTGACCGCGGCGCAGCTCA

TCCAAAAGATCATCAACATCCTTACTGACAAACATGGAGCCTGGGGAAGCTCCACCCACA

GTAAGCAGTGTTTTTGGCGTTTGGATTTTTGGGAGGACGATTTGAGGAGACGTCGTCGTA

TGGTCAGGAATCCATTCGGGTCGTCACACTCAGAGGCCGCACTCAAATCTGCAGCTGAGC

ATGCTCCAGAGGACGACGTGCTGAAGGGAAAACAGCTCGGGAAAGAACCGGCCAGGAATC

AGAGCTCAGAGAGCGAGAGTTCAGCGGAGGGAGACGATGAGACCATTTCATCATTGGAGG

AAAAAGAGCTGGACAATCTGACAGGCACAGTGTCTTTCAGCACAGTAGCTGAGCTGGTCT

CTCCTGCACTTGTCATGAAAGGAACTCTGTCCATCACGGCTCATGAGCTTTACTTTGAGG

TGGACGAAGAGGAGGCGAGTTTCAAGGGCATCGATCCCAAGTTGTTGGTCTATGCAGATG

GACTGCACGGGAAGTGGATGTTCTCTGAGATGCGAGCGATCTTCTCGCGCCGGTACCTCC

TGCAGAACACAGCGCTGGAGATCTTTATGGCCAATCGGACTTCTGTGATGTTCAACTTTG

TTGATGCTGCAACGGTTAAGAAAGTCGTTCACGCTCTGCCTAGAGTCGGAGTCGGAACTA

ACTTCGGCCTCCCACAGACCAGGCGAATTTCTCTGGCGACTCCCAAGCAGCTACTGAAAG

CCTCAAACATGACTCAGCGCTGGCAGAAGCGCGAAATCTCCAACTTCGAGTACCTCATGT

TCCTCAACACAATTGCAGGCCGCACGTTTAACGACCTGAATCAGTATCCAGTGTTTCCCT

GGATCATCAGCAACTACGACAGCGAGGAGCTCGACCTCACACTGCCCAGCAACTACAGAG

ACCTGAGCAAGCCAATTGGAGCCTTGAACCCAAAGAGAGCAGCTTTCTTCTCCGAACGTT

ATGAATCCTGGGAGGACGAGCAGGTCCCCAAATTCCACTACGGCACACACTATTCCACAG

CCAGCTTCACTATGATGTGGCTGCTGAGAATTGAGCCCTTCACTACCTTCTTTCTCAACT

TCCAAGGAGGAAAGTTTGATCATGCAGACCGAACCTTCTCCTCAGTCGCCAGAGCCTGGA

GGAACTGTCAGAGAGACACATCTGATGTCAAGGAGCTGATACCAGAGTTCTTCTACCTGC

CAGAGATGTTCATTAACGCTAACAACTATAATCTGGGAGTGATGGATGATGGGACGGTGG

TGTCTGATGTCGAGCTGCCGCCCTGGGCAAAGAGTCCGGAGGATTTCGTTCGCTTTAACC

GCATGGCCCTGGAAAGTGAGTTTGTGTCGTGTCAGCTCCATCAATGGGTGGATCTAATCT

TCGGCTATAAGCAGCGAGGACCTGAAGCTGTGCGCGCACTTAACATCTACTACTACCTCA

CCTACGAGGGGGCAGTGAACCTCAGCTCCATTACTGACCCGCTACTGAGAGAGGCTGTAG

AGTCCCAAATTCGAAGTTTTGGTCAGACTCCATCTCAGCTGCTGATCGAGCCTCATCCAC

CACGGAGCTCTGCCATGCAGGTGACCCCACTGATGTTTACGGAGCAAATGCAGCAGGATG

TGATCATGGTGCTGAAGTTTCCCTCCAACTCTCCGGTGGTGTACGTGTCGGCTCACACTC

AGGCCGGTCTGTCCAATCCCTCCATCGTCACGGTCACAGCCAATCGAATCTTCTCCATCA

ATAAGTGGCACGGCCTCACCGGTCACCAGAGCTCCTCAAACACAGAGCAGCAGTACCAGC

TGCCGGTGGAAATCGACCCTGTGATAGTGGCTAACCTGATGCACCGCAGACAGGTGTGTG

ATTTATTGGATCAGAGTGTGGAGGTGAACTCACACTGTTTCCTCGTCTCGTCTGATAATC

GCTTCCTCTTCATCGCCGGCTTCTGGGACAAGAGTTTCCGCATCTACAACACAGACACAG

GGAAGCTGATGCAGATAGTGTTCGGTCACAGAGATGTGGTGACATGTGTGTTTCGGTCCG

AGTCGTACATCGGAGGAGACTGCTACATCCTGAGCGGCTCGAGAGACGCCACGCTGCTTC

TGTGGTACTGGAGCGGAAAACACTGCTGCATCGGAGACACACAGAATGATTTTGTGACTC

CTCGAGCAATTCTGACAGGCCATGACTGTGAAGTGACCTGTGCAAGTGTGTGTGCGGAGC

TCGGAGTGGTCATCAGCGGTTCTAAAGACGGCCCGTGTCTCTTACACTCGGTAAGCGGAG

AGCTGCTGCGTGTGTTTGAGAACAGTCAGAGGCCGATGCTGGTGCAGACGAGTTCAGAGG

GACACTGTGTTATCTACTACAGCTCCGGACAGGTGTGTGTGTACAGCATCAACGGCAAAC

TGCTGTGTGACACACACATCGAGGACAACATAAAGGCTATGTTATTAAGTAAGGATGGTC

AGTACCTGCTATGTGGAGGAGAGAGAGGTGTTCTTAGTGTGTGGCAGGTTCATGATCTCA

AACAGCTCTTCACTTATCCAGGCTGCGATGCTGCTATTCGCTCCATGGCCATAACCGCTG

ACCAAAGGTACATACACACATACTACATCTACACACA

>Unigene000036

AGAGAGAGAGAGAGAGAGAGACTGTGTTCTGAGACAGAGGAAGAGACCAGAAGGCTTTGT

TGACTTATGCAGCCCACCATGCCGAGCTCAAACAAGGTGTGAGTGTGTACGCTCAACACA

CACCCACCTACACACACTCATTTTGGAGGGAAGGCTGAAGACCGAGAGCGTTGAACTTTG

CTCATCAGAGAAGAAAGGAAAGTCGGCAGGTCCGTACAGGAGGAAGGAGCAAAAGAAGTG

GAACAAGCTGTTCTTTCTTTATCCTTTTTGTTCTTAATTCATCCACTGGGTTTCTACATT

GAAGAAAGGAGGGGGTTTTTAGGGACGGCATGGCGTGACAAACTCTGCTACCTGTGGACG

TGTCGCTTTGCACGCAACTTGCTTCGAAGCCGGACTCTTGCACCTGGACCGACCTGCTGA

TTCCACTCTTCTTCTTCTGCTACTGCTTCTTTCTTCTTCTTTTTTTTTTACATCTTTTAA

CGCTGGTTGGACGAGTCCTAGCATGCCCTGAACCCTCGCTGTCTTTTTAACGGAGCTGGT

CATTCGTATCGGGAGCAGGTTATTGTGTTGGCTGGTGTAAAAGCAAAAGGAAAAAATAAA

CTCGAGAGGTAGAGATGGAGTACGGGCGGTTGGAGAGGGAGCCATGTCTCTCTCCCGCTG

CATTCGTCAACCAGGTGCAGTATTCCAACATCCTGGAGGGCCGTTTCAAACAGCTGCAAG

ATGAGCGAGAAGCCGTACAGAAGAAGACCTTCACTAAGTGGGTGAACTCTCACCTGGGCA

GGGTGACATGCAGGATCAACGATCTGTACACGGACCTGCGGGATGGACGCATGCTCATCC

GCCTGCTGGAGGTTCTTTCCTCCGAACAGCTGCCGAAGCCGACAAAAGGACGGATGAGAA

TCCACTGTCTGGAGAACGTGGATAAAGCGCTGCAGTTCCTCAAGGAGCAGAAGGTCCATT

TGGAAAACATGGGTTCTCACGACATCGTCGACGGCAACCACCGCCTCACGCTCGGCCTCA

TCTGGACCATCATCCTGCGCTTCCAGATTCAGGATATCAGCGTGGAGACGGAGGACAACA

AGGAGAAAAAATCCGCCAAAGATGCTTTACTGCTGTGGTGCCAGATGAAGACTGCTGGGT

ACCCGAATGTCAATGTTCACAATTTCACCACTAGCTGGAGAGACGGCCTGGCGTTCAACG

CTATCGTGCACAAACACAGACCGGATCTAATTGAGTTTGACACTCTGAAGCGATCCAACG

CTCACTACAACCTACAGAACGCCTTCAACATTGCCGAGAAGGAGATGGGGCTCACCAAAC

TGCTGGATCCTGAGGATGTAAACGTGGATCAGCCAGACGAGAAGTCGATCATCACCTATG

TGGCCACGTACTACCACTACTTCAGCAAGATGAAGGCGCTCGCCGTGGAGGGCAAAAGAA

TCGGCAAGGTGCTGGACTATGCCATCGAGGCAGACCAGCTGATCGAGAAGTATGAGACGT

TGGCGTCGGAGCTGCTGCAGTGGATCGAACAGACCATCGAGACGCTCAACGACCGGCAGC

TCGCCAACTCACTGAGCGGCGTGCAGAACCAGCTGCAGGCTTTCAACACCTATCGCACAG

TGGAGAAACCACCCAAGTTTACAGAGAAAGGCAATTTGGAGGTTCTTCTCTTCACCATCC

AGAGCAAAATGAGAGCCAACAACCAGAAAGTCTACATGCCTCGAGAGGGAAAGCTCATTT

CCGACATAAACAAGGCGTGGGAGCGTCTGGAGAAGGCGGAGCATGAACGTGAACTGGCTC

TGAGAAATGAGTTGATTCGTCAAGAGAAGCTAGAGATGCTGGCAGCGCGATTTGACCGCA

AAGCTGCCATGAGGGAGACGTGGCTCAGTGAGAACCAGCGTCTCGTCTCGCAGGACAACT

TCGGTTTTGATCTCAGCGCCGTGGAGGCTGCGACACGCAAGCATGAGGCCATCGAGACGG

ACATTGTCGCGTATGGAGAGCGTGTGGCTGCTGTAGAAGCCGTTGCCCGTGAGCTTGATG

CGGAGCGATACCATGACGTCCGGCGCATCCTGGCACGCCGGGATAACGTGCTACGCCTCT

GGGAGTACCTAAAGGAGCTGCTTGCAGCCAGGAGGGAAAGACTTGTGGCACACCGCGACC

TACAAAGACTTCTGCAGGAGATGAGCTACATCATGGACTGGATGGAAGACATGAAGGGTC

GTCTCCAGTCCCAGGACAGTGGGAAACATCTTCACGACGTGGAGGACTTACTTCAGAAAC

ACACTCTAGTAGAGGCGGACATATCGGCGCAGGCAGAGAGGATCCGAGCGGTGCAGGCAG

CAGCCAATCGCTTTACCTCGGAAGAAATAACCTATAAGCCCTGCGAGCCAGCGCTGGTGG

AGGAGAAGGTGGCGCTTCTGGGCCAGACGTATGAAGAGCTGGGTCAGCTGGTAGTCGAGC

GACGGGCACAGCTTGAGGACTCGCGGCGTCTCTGGCAGCTGCTGTGGGAGCTCGGGGAGG

AGGCAGCGTGGATGCGGGAACAGGAGCAGATCATGGCAACAGAGGAGTGCGGGAAGGATC

TGTCCTCTGCACTGCGTCTCCTTTCTAAACATGAGGCATTTCGAGAAGAGATGGCAGCAC

GCTATGGCCCGCTGGGAAACAGCATCGCTGCTGGGGAGCAACTGGTGAAGGAGGGGCACT

GCGGAGCACCCGAGATTACAGAGAGGATCAAAGAGATCAAAGCTCAGTGGGGCAGACTAG

AGGAGGCATCTCGTTTGCGAGAGGAGCGTCTAAAGGAGTCTGTGACTTTCCACCAGTTCC

AGACTGACGCCTGCGATATGGAGGCCTGGCTTCAGGAAGCTCTTAGACAGGTGTCCAGTC

AGGAGGTCGGCCATGATGAGTATTCTACACAGACGCTGGCACGGAAACACAAAGAGGTAG

AGGAGGAGATCCAGAGCCACCGCTCTCTCATAGACTCGCTGCATGAGCAGGCTCAATCAC

TGCCCGAAGAATACGCCCAGTCTCCACAGGTGGAGGGTCGTCTTCCTGCTATAGAGCAGC

GCTATGAAGAGTTGAAAGCTCTGTCCTCTGCACGACGCGACGCCCTGGAGGGCGCACTAG

CCCTTTACCGGATGTACAGTGAAGCCGACGCCTGCAAGCTGTGGATTGGAGAAAAGGAGC

AGTGGCTCCTCACTATGGAAATCCCTTCCAAACTCGAGGACCTGGAGGTGGTCCAGCAGA

GGTTCGAGACACTGGAGCCGGAAATGAATAATATTGGCTCTCGCGTCTCTGACGTCAACC

AGGTCGCGGAACAGCTGCTTAAATCCGACAACTGCAACAAGGAGCAAATTCACCAGACTC

AAGACCAGCTCAATGACCGGTGGCATGAGTTCCAGCGGCTTGCTAATGAGAGGAAACAGG

CTATGGAATCAGCTCTAAATCTCCAGAACTACCATCTAGAGTGCAACGAGCTGCAGAGCT

GGATGAAGGAAAAAACCAAAGTGATCGAGTCTACACAAGGGCTCGGCAATGATCTGGCTG

GGGTCATGGCACTGCAGCGCAAGCTCACGGGCATGGAGCGAGACCTAGAGGCCATACAGG

GGAAGCTGGGCGGCCAGCGTGCCGAGGCCGAAAAGTTGGCGTCCGAGCATCCGGAGCAGG

CTGAGGAGATCCAGGCACGGTTAACTGACATGGAGGATGTGTGGGAGGAGCTGTGTGGCA

CCATGAAGAAGCGTGAGGAGAGTCTGGGCGAGGCCTCCAAACTCCAGGGCTTCCTGCGTC

ACCTCGACGACTTCCAAGCCTGGCTCTCGCGCACGCAGACCGCCGTGGCTTCTGAGGACA

CACCCACCTCACTTGCTGAGGCGGAGCAACTCCTGGCTCAGCATGAAGCCATCAAAAATG

AGGTGGACAACTATCGCGACGACTACGAAAGGATCAAGGCCACCGGTGCCGAGGTGACCC

AGGGCCAAACGGATGCCCAGTACATGTTCCTGGCTCAGCGGCTGCAGGCACTGGACACGG

GCTGGCAAGATTTGCGCAGGATGTGGGAGAGCAGGCAGTGCCTCCTGGCCCAGGCATTCG

ACTTCCAGACGTTCCTTAGAGACGCCAAGCAGGCCGAGAGCTTCCTCAACAGCCAGGAGT

ATGTTCTCTCGCACACCGAGATGCCCTCGAGCCTGGAGGGGGCGCTGGAGGCTATCAAAA

AGCACGAGGACTTCCTCACCACCATGGAGGCCAGTGAGGAGAAGATCAACGGTGTGGTGG

AGTCAGGGCGACGCCTTGTTTCTGACGGCAACGCCAACGCCGACAAGATCCAGGAGAAAG

CCGACTCCATTGAGGAGCGGCACCAAAAGAACAAGCAGGCTGCTAATGAGTTGCTGGGTA

AACTGAAGGATAACAGAGAGTTGCAGCACTTCCTCCAGGATGGACAGGAGCTGAGTCTGT

GGATTAATGAGAAGATGTTGACAGCTCAGGACATGACATACGACGAGGCCAGAAACTTGC

ACAGTAAGTGGCAGAAACACCAAGCTTTCATGGCCGAGCTGGCCTCCAACAAGGACTGGC

TGGACAAAATCGCTAAGGAGGGTCAGGCTCTGGTGAAGGAGAAACCCGAGCTGGAGGAGA

CAGTGTCCGAGACCCTGCGTGAGCTGCAGAAGCAGTGGGATTTGTTGGAGACCACCACAC

AGACCAAAGCGCAGTGTCTGTTTGACGCCAACCGCGCTGAGCTTTTCACCCAGAGCTGCT

CGGCTCTCGACACATGGCTGCAGAACCTCTCCTCACAGCTGCAGAGCGACGACTTCGGCA

AGGACCTGACCAGCGTCAACATCCTGCTCAAGAAACACCAGATGCTGGAGCATCAGATGG

AAGTGCGAGAGAAGGAGGTGCAGTCTCTGCAGTCACAGGCTCTGGCCCTGTCTCAGGAGG

ACGCCAGGATCGTCGAGGTAGACGGTCAGCAGAGGAGAGTGATAGACACCTTCGCTCAGC

TCCAGGATCCTCTTAGAGACAGACTACAACACTTACTAGCATCGAAAGAGGCCCACCAGT

TCAACAGAGACCTGGAGGATGAGATACTGTGGGTAAAGGAAAGAATGCCACTGGCTACAT

CGGTAGATCACGGCAAAGACCTGCCCAGTGTTCAGCTGCTCATAAAAAAGAACCAGACGC

TTCAAAAAGAAATCCAGGGCCACCAGCCTCGCATCGATGACCTGCAAGCCCACGGGCGCA

ACATGGCCCCCGGGGGGGGGGCAGAGATGAAGGGGCGGCGGGACGAGCCTCTGAACGAGC

TTGGGGATCTGTGGACGTGGCTCATCGCAGAGACGGAGCAGAGACACAAACGCCTAGTTG

AGGCCCATCGCTCTCAGCAGTTCTATGCAGACGCGGCCGAGGCTGAGGCCTGGATGGGAG

AGCAGGAGCTGCACATGATGTCTGAAGAGAAGGCGAAGGACGAGCAGAGCGCTTTGGTGA

TGGTAAAGAAGCACCAGATCCTAGAGCAGGCGTTGGAGGATTATGCTCAGACCATCCACC

AGCTGGCCAATAGCAGCAGACTTATGGTGGACAGTGAACATCCTGAGAGTGAGAGGATCG

CGCTGAGGCAGGCTCAGGTGGATAAGCTGTACGCCGGGCTGAAGGACCTGGCTGAGGAGA

GGCGGGGCAAACTCCAGGAGCGACTGCGTCTCACACAGCTTAAGCGAGAGGTGGACGATC

TGGAGCAGTGGATTGCTGAGAGGGAAGTGGTGGCAGGATCTCACGAGCTGGGCCAGGACT

ACGAGCACGTCACTATGCTCCGGGATCGTTTCCGGGAGTTTGCACGGGACACGAGCGCGA

TTGGACAGGAGCGCGTGGACACTGTGAACGCCCAGGCGGACGCTCTGATTGAGTCAGGAC

ACCCGGAGAACGCCAGCGTGGCCGAGTGGAAGGACGGCCTGAACGAGGCTTGGGCCGATC

TGCTGGAGCTCATGGACACGCGCACGCAGATGCTAGCCGCTTCGTACGAGCTGCAGCGCT

TCCAACAGGACGCTCGCGAGGCGCTAGCACGCATCCGCAACAAAAGGGAGGCGCTAAACT

CGGCTGAGCTGGGCCGCGACCTCAACACCGTGCAGCACCTCCACCGGCAGCACACAACCT

ACGAGCATGACGTGCACGCTCTCAGTGGACAGGTGACACAGGTGCAGGACGACGCGGCAC

GTCTCCAGAAAGCTTATGCAGGAGAAAAGGCGGACGACATCCATCGGCACGAGCGCGCAG

TCACCGAGGCGTGGGAGGAGCTACTGTCTGCCACTCAAGCCAGGCGGCTGCTGCTATTGG

ACACGGTGGAGAAGTTCCGGTTCTTCAACATGGTCCGAGACCTGATGCTATGGATGGATG

GCATTAATCTACAAATCCAGTCTCACGACAGCCCTAGGGACGTGTCATCAGCGGGTCTTG

TCATTGCAAATCATCAAGACATTAAGGCTGAGATCGAGGCCCGATCCGGCAGCTTCACCG

CCTGCACTGACATGGGACATGCCCTCATCAACAACAACCATTACGCCTCAGACGAGATCC

GGGAAAAACTGGAGCAGCTTCAAGAGAAAAGAGACGAAATCAACCAGAAGTGGCAGGACA

AAATGGACCACTTGCAGATTGTCCTGGAGGTGCTGCAGTTCGGCAGGGACGCCTCCGTAG

CTGAGTCATGGTTGGTCAGCCAGGAGCCGTTGGTGCGGGCGGCCGAACTTGGCTCCAATG

TGGACGAGGTGGAGAGCCTCATCAAACGACACGAAGCCTTCGAGAAACTGGCTGCTGGTT

GGGAGGAACGTTTCACTTTGCTCGAGAAGCTCACTACGCTCGAAGAACAGGAATTTCAGA

GAAGGAAGGAGGAAGAAGAAAGAGCGCGTAGACCCCCTACGCCTCCCCCCGTAGAGGAAG

TGGTACCGTCTGAGGTTGAATACGAGTCTGGAGCGAGGACGAGTTTGGATCAGACCACAC

TGAATCAGTCGGTGTCTGTAAATGGGGTCCACAGTGACCAGGACACATCACAGTCTTTAT

CCGTCTCGGGATCCGAATTCCCGAACACCGAACCTAAACCTCTTATCAAACCGGTCTCTA

ATCCTGTGCCCAAACCCTACAAGGCACATCAGCGTGGCTCGGAGGCTGAGTCAGTGAACG

GTCCCGGGCAGGACAGCGGCTTGGACACGGCGTCGCGCCACGAACCGTCAGGAACACTGC

CTAGCCGAATAGAGGCGTCTGCCGAGGCCATGGAGGGAACTCTGTGCAGAAAACAGGAAA

TGGAAAGTCAGGGCAAGAAAGCGGCCAACAGATCGTGGCAGAACGTCTACTGTGTGTTGA

GGAAGGGAAGTCTGGGGTTCTATAAGGACAACAAGAGCGCGTCGAACGGCATCCCCTACC

ATGGAGAAGTTCCCATCAGCCTCGGCGAGGCCACCTGCGAGGTCGCCCATGACTACAAGA

AGAGGAAACATGTCTTCAAACTGAAGCTTGGAGACGGAAAAGAGTTCCTGTTTCAAGCCA

AGGACGAGGCTGAGATGAGCTCCTGGATTCGGGCAATCCACTCCTCTATGCCGCCTAACA

ACATCGACGACCTTTCCCCCGCTCCGCCTCTGACCCGGGCCATGACTATGCCCCCTATGT

CCCCCAGCGAGGGCGAAGGTGTGACCATGCGCAACAAAGAGGGCAAGGAGAAAGATCGAG

AGAAACGCTTCAGCTTCTTCGGCAAGAAAAAGTAACCAGGAAAAGAAGGAGGGACGTTCT

GTCAAAAGCGCAGGAACTGTCCTAGAGAAAGACGGCGCCCTCTACTGTCTCCTAACTCCA

CACCTTTATTTTCCCTGTCTCGCTGTCTCGCGCTGTCTTGCTCACTGCCTTCCGCCTTGA

CCGTAGCCCCGCCTCCCACAGGACGCTGGCTCAACTGCAATTGGACGGTCGGTCAGACGC

CTTTATTTTAACACTTTGTCCTTGAGTTTTAAGGCCCGACAGCGTTTCCCATAATGCAAC

GCTTCTAAATCTTTCTTCATTTATTCTTTCCATTTATTCTGTTTGACGTTGTGAAGAAGC

AAATTGTTTTTTTCTTCCTAGGAGTACGAATCTTTACGTTCTTAGTTAATATATCTGATT

GGTTTTATTTAACTTTTTGCCATTTGCAATGATTAATATTTTAAAAAGAGAAAAAAAAAA

GTTTTAACAGGGGATAAAAAGAAAAAAAATGAGAGCCAGAAGGAGAGAGAATGAGAGCGT

TTTCCACACAGAACCCAACTACATCTAGAACCTGAAGCTCCAGAATGAAACCCAGGGTTC

TCGATTCTGGTTTTTCTTTTTCCCCTTCTTTTATTTTAAAGTTTGCAGAGAATATTTTAA

TAACCGAATACTAAAAAAAACATTCTGAAAACTTTCCATCCTTTTTCTTTCCCAAAGCTG

AATTTAAAAAAAAAAATGTTTTTATTTGTGTTTAATGTTTTGACCTTAACCTTATTTCCC

TTCTTTAAATCAGATCACAAACTCACACTAAAGTCTTGTGGGATGGCGCGCTATGTTTTT

TTTTTTTTTGTTGTTTTTTGCCAAATTGCTGAGTCATTTACAGAAAAATAGGGCGGGGCT

AAAATTAGCTATACGATTTTCGGCATGTATATGCCTTTCACATGCAGTGTGTGTGTGTGT

GTGTGTGT

>Unigene000037

ATCGGAAGAGCGTCCTGTATTGGTGTGACTTTAGCAGCACTGTTGCCTCTCAGAATGGTA

TCCGCCGGATAAAGCCCAATGGATCCGGATTCCGTAATATTGTTACTTCTGGGATTGGAC

GCAATGGGATTCGAGGAATTGCTGTGGACTGGGCAGCAGGGAACCTATACTTCACAAATG

CCTTCCTGACCGAAACCTACATCGAAGTTGTTCGACTTAACACCACTTTTCGGCGTGTTC

TCCTAAAGACCCAGGTGGATATGCCACGTCACATCGTAGTTGACCCAAAAAACAAATTCC

TTTTCTGGGCTGACTATGGTCAGAACCCCAAGATTGAAAGAGCTTTCTTAGACGGTACCA

ACCGAACAGTGCTGGTGACATCTGGTATTATTACACCCAGAGGGCTTGCTCTGGACCATG

GGAATGGGTATATCTACTGGGTGGATGATTCACTGGACATGATTGCAAGGGTTCCCCCCG

ATGGAGGTGAGACCGAGATTGTACGGTACGGGAGTCGCTACCCCACTCCCTACGGTATTA

CTGTGTTTGAAAATAACATCATTTGGGTAGACAGGAACCTGAAAAAAGTTTTCCAGGTCA

GTAAAGAGCCAGGAAATACTGACCAACCAATGGTGATCAGAGACAACATTAACATGCTGA

GAGATGTCACCATTTTTGACCAGAGAATGCAGCCCTGGTCAGCACAGGAGCTTAATAACA

ATCCCTGTTTATCATCTAATGGAGGCTGTTCTCACTTTTGCTTTGCATTGCCTGGCACTC

AAGCAAGAAACTGTGCATGTGCCTTTGGGAACCTGGCCGTGGATGGTATGACCTGTGTGG

TATCCCGTGATGATTACCTCATATACACTACAGAAAGTACAGTGCGCTCACTACGCCTTG

ATCCTGATGATCATTCCCTGCCTTTCCCTGTGGTCAATGTGGCCCGGACCTCTGTTGCAC

TGGATTATGACAGGCTGGACAACAGGATTTATTTCACCCAAAGCTCCGGGGCGGGACAAA

GCAAAATCAGCTACATTAGTTTATCATCTCCTACCTCATCCGCTACCGTAGTGGCTTCAG

ATCTGGGTGCTCCTGATGGCATTGCCTATGACTGGATCAACAAAAGGATCTATTACAGCG

ACTATGTTAATCAGACCATCAACTCGATGGCTGTGGATGGATCACAGAGGACTCTTATCG

CTCATGTGCCCAGACCACGAGCTATCATGCTGGATCCTTGCAGTGGATACATGTACTGGA

CTGACTGGGGTACCATTGCTAAGATTGAGCGTGCAACTCTGGGTGGAAATTTCCGGACTG

AGATTGTTAACAGCAGTTTGGTGTGGCCTAATGGTCTCACCTTGGATTATGATGATCAGA

GGCTCTACTGGGCCGATGCCAGCTTACAGAAGATTGAGCGCTGTTCTCTGACGGGCACTA

ACCGTGAGGTTATTGTAAGCACAGCTATTTACCCGTTTGCCATGACTGTGTATGGTCAGC

ACATTTTCTGGACAGACTGGAACACACGCAGCATCTACAGAGCGAACAAGCACGATGGCT

CGGATCAGAGGGTTATGATCCAGAACCTTCCAACCCGTCCTATGGACATCCATGTGCTGT

CAAACAGCAAACAGCAGCAATGCAGCAGCCCTTGCCTGCAGTTTAATGGAGGCTGTAGCC

ATATCTGCACCCCAGGTCCTCAGGGGGCAGAGTGCCAGTGTCCATCAGAAGGTCGTTGGT

ACCTGGCTGATAACAAATACTGCATCCCTGATAATGGCACTCGCTGCCAGCCAAGTCAGT

TCACCTGCATGAACGGTCGCTGCATCCGAGCAGCATGGAAATGTGACACCGATGATGACT

GTGGTGATGGCAGTGACGAGCTGGAGAGAGTGTGCGCTTTTCACACCTGCGAGCCCACCC

TGTATACCTGTGGAAATGGCCGCTGTGTGCCGTACCACTACCGCTGTGATCACTATAACG

ACTGTGGTGATAACAGTGATGAGGAAGGCTGTTTGTTTCGGCCCTGCGACCCAGACACAG

AGTTCACCTGCAACAATGGCCGCTGCATTGCTCGCGAGTATGTGTGCAATGGCATCAATA

ACTGCTTCGACAACGGAACCTCGGATGAACGCAATTGCGCTGAAAGGACTTGTCAGCCTG

GTCACACCAAATGCCAAAGTACAAACATCTGCATTCCACGATCATACCTGTGTGATGGAG

ATAACGACTGCGGAGACATGAGTGATGAGAGCCCCACACACTGCGCAACATCTACATGTA

CCCAGAGTGAGTTTCGCTGCACAAGCGGGCGATGTATCCCTGCACACTGGTACTGTGATG

GTGGTACTGACTGTGCAGATGGGTCAGACGAACCCTCCACCTGTACCACCATAGTGAGAA

CCTGTAGCTCAGACCAGTTCCGCTGTGATGATGGAAGGTGCATCGCATCCTCCTGGATCT

GTGATGGAGACAATGACTGTGGGGACATGAGTGATGAGGACCAGAGGCACAACTGTGCTA

ATCGAACCTGCTCTTCACTGGAGTTCACCTGTGTGAATAATCGGCCCCCACAGAGGAAGT

GCATCCCTCAGGACTGGGTGTGTGATGGTGATGCAGACTGCTCAGATGCATATGATGAAC

ACCAGAACTGTACACGCAGGTCCTGTTCTGCAACTGAGTTTACATGCAGCAACGGCCTCT

GTATCCGCAGTGCATACAGGTGTGACCGGCGGAATGACTGTGGAGACGGTAGTGATGAGC

AAAGCTGTACCTACCAGCCGTGTCAACAGCACCAGTTCACCTGTCAGAATGGACGTTGTA

TTTCTCAGGACTTCCAGTGCGATGGCGATAATGACTGTGGTGATGAATCAGATGAGCTGC

CGCACATGTGCCACACTCCAGCGCCTACCTGCCCTCCTGGGAAGTTCAGGTGTGACAATG

GCAACTGTGTGCCGTCCAGCCAAGTCTGTGACCACAGTGATGACTGCAATGATAACAGTG

ATGAGAAAGGCTGTGGCATTAATGAATGTACAGACCCATCCGTGCACCATTGTGACCACG

AGTGCACTGATACCCCCACCAGCTTTGTGTGCAGATGCCGTCCTGGCTACCGCCTTATGT

CTGATAAGAAGAGCTGTGATGATGTAGATGAGTGTTCAGAGACCCCCAGTGTATGCAGTC

AGATGTGTGAGAACACACTAGGCTCATATGTGTGTAAATGTGCACCAGGTTTCCTTCGCG

AGCCAGACGGGCGTCGTTGTCGGCAAAACAGCAACATCAGTCCTTACTTGATTTTTAGCA

ACCGCTACTACCTGCGTAACTTATCTGCAGACGGAGCAGCCTACTCGCTCATCCTTCAGG

GCCTTAGCAGTGTTGTGGCTCTAGACTTCGATCGCGTCGACAAGCGTCTTTACTGGATCG

ATGTGAGCCGTCGTGTGGTGGAGAGAATGTTTTTTAATGGCACAGGCAGAGAGGTGGTCC

TGAATGGCATTATGCACGGAGAAGGCCTGGCTGTGGACTGGGTGGGACGGAAGCTTTATT

GGGTAGACAGCTTTTTAGACTGTCTGAAGGTGTCTGAGCTAGATGGACGCTTTATTAGGA

AGCTGGCTGAGCACTGTGTGGATGTTAACAACACCTACTGCTTTGAGAATCCAAGAGCCA

TTGTGGTGCATCCAAAGTTTGGATATGTGTACTGGACTGACTGGGGAAGTAAAGCATTTA

TAGGTCGTGTTGGGATGGACGGTAACAACAAGTCTGCCATTATTACCTCTAAGATCGAGT

GGCCCAATGGTCTTACCATCGACTACACCAATGATATGCTCTATTGGGCTGATGCTCACC

TTAACTACATTGAATACTCTAACTTAGATGGCCACCACCGCCACACTGTTTATGATGGTG

TTCTTCCTCACCCGTTTGCTATAACTGTGTTTGAGGACACTGTGTACTGGACAGACTGGA

ACACTCGCACTGTAGAAAAAGGCAATAAGTACGACGGCACTGGGCGCCAAGCTCTTGTCA

ACACTACTCACAGGCCCTTTGATATCCACGTGCTTCACCCGTATCGTCAGCCCATAGTAA

CCAATCCCTGTGGAATAAACAACGGCGGCTGTTCCCACCTGTGTCTCTTAAAAGCTGGAG

GACGGGGATACACTTGTCAGTGCCCAGACCACTTTCTGACTGTGCAGATAGGGGGCGTAG

CCCGCTGCCTACCCAGCTGCTCCAGCACACAGTATAGATGCGCTGACAACGAGCGTTGCA

TCCCTATCTGGTGGAAGTGTGATGGTCAATCTGATTGCAGGGACGGTTCCGACGAGCCGT

CCACATGCCCCCTGCGCCATTGTAGGTTGGGCCAATTCCAATGTAATGACGGAAACTGTA

CCAGCTCACATTTTCTGTGTAACTCAAATCGCGACTGCCCTGATGGATCTGATGAGGACA

CTGTGCTGTGTGCAACACACCAGTGTGAGTCCCACCAATGGCAGTGCAGTAATAAGCGGT

GTATCCCGGAGGCGTGGCAGTGCGACGGGGAGAATGACTGTGGTGACGGCTCGGATGAGG

AACCCTCACACTGCTCCAGCAGAACGTGTCGTCCTGGCCAGTTCAAGTGCAGGAATGGCC

GCTGCATCCCTCAGAGCTGGAAATGTGATGTAGATGATGATTGTGGAGATAACTCGGATG

AGCCCTTGGATGAGTGCATGGGACCAGAATATCGATGTGATAACCACACTGAGTTTGACT

GCAGTACAAACTACCGCTGTGTTCCTTTGTGGGCCGTGTGTAATGGACAAAACGACTGCA

GAGACAACAGTGATGAGCAGAACTGTGAGGAGATGACCTGTGACCCTGCTGGAGATTTCC

GCTGTGATAACCACAAGTGTATCCCCCTGCGCTGGAAGTGTGATGGAGACAGTGACTGTG

GAGATGGATCAGATGAACGCAACTGCAACCCTCGAGCCTGCACAGAGAGCGAGTATCGCT

GTGACAACCTGCGCTGCATTCCTGAACGCTGGGTGTGTGACCATGACAACGACTGTGAGG

ATAACTCGGATGAAAGGGACTGTGAGTTGCGGACCTGCCATCCTGGATACTTTCAGTGTG

ATAGTGGTCACTGCATTGCTGAGCGCTTAAAATGCGATGGCAGTGCAGATTGTATGGACG

TCACTGATGAGACATCCTGCCCAACACGTTATCCAAATGGCACATACTGTCCTCCCTTCC

TGTTCGAGTGCAAAAATCATATATGTGTTCAGCAGCACTGGAAATGTGATGGAGACAACG

ACTGTGGAGATGGATCGGATGAAGAGCTGCATCTCTGCTTGGATATACCGTGTGAAGCCC

CTTTCCGCTTCCGCTGTGACAATAACCGCTGCATCTACAGCCACGAACTCTGCAACTCAG

TAGATGACTGTGGAGACGGAACGGATGAAAAGCAGGAGCACTGCTTGACTCCCACTCATG

GACCCTGTTCCTCTGATGAGTTTAAATGTGGGAACAGCCAGTGCATTCCTCTGCAGTATG

TCTGTGATGATTATGATGATTGTGGAGACCAGACTGATGAGTTGGGCTGTAACAGTGGAA

ATGGACGCTCTTGCAGTGAGAATTTGTGTGAACATAACTGCACCGAACTGACGGGTGGAG

GCTTTATATGCTCCTGCAGACAGGGCTACAAAGCCCGAGAGGACAACAGAAACTCCTGTG

AAGATGTAAATGAGTGTGAGCTCTATGGCACTTGTCCCCAGCTGTGCAAAAACACTAAGG

GGAGTTACGAGTGCTTCTGTGCTGAGGGATTCCGCTCTGTGGGACCGCAACACAGGACTG

AATGCGCTGCTGAAGGCAATCCTCCAGTGCTACTGTTGCCTGATAATGTGCGTATCCGTC

GCTATAACCTGTCGTCTAAGCAGTATTTAGACTATATCGATAATGCTGAACACATTCATG

CTGTGGACTACATTTGGGACCCAGAGGACCAGGGGCTCAGCATTGTATACTGGACCATTT

TAGGTCATGGTGCTCAGTTCGGATCCATAAAGAGGGCATATATGACCACATTTGATGACC

ATGGAAGTAACCAGATGAAAGATGTAGATCTGAACCTTAGATACATATCCAGTCCTGATG

GAATTGCTGTGGACTGGGTTGGGCGACACATTTATTGGACAGATGCTGGAACCAATCGTA

TTGAAGTGGCAAAACTGGATGGGCGGTACAGGAAGTGGTTAATCTATTCCAATCTGGATC

AACCAGCTGCAATAGTTGTCAACCCTGCTATTGGTATGATGTACTGGACTGACTGGGGTA

GCCGACCAAAGATTGAAGCAGCATGGATGGATGGTCAGCACAGACAGGTGCTGCTGGATG

AGGATCTGGGCTGGCCTACAGGACTCACTCTAGATTACCTTAATGGAAACAGGATTTATT

GGTGTGACTCAAAAGAGAACATTATTGAGTCTATGAAACCAGATGGAACAGACAGGAAGA

TCGTCCTGTCAGGAGATATTGGAAACCCCTACAGTCTGGATGTGTTTGAAGGACATGTGT

ACTGGACAACAAAGGTCAGGGGTGAAGTATGGAAGGCTGACAAGTTTGGAAGGGGGGATA

AAGTCAAGATGTTGACCATAAACCCTTGGTTGACTCAAGTCCGCATCTATCATGAGCACA

GATACAATCGCTCAGTGTTAAACCCCTGTAAAGGTGTGTGTAGTCATCTGTGTCTGCTGC

GACCTGGTGGATACACCTGTGCCTGTCCACAAGGCTCTTCTCTGGTCAGCTTTAATGAAA

ACGAGTGTGATGCTGCCAGCCTACCCGAGGTTGCTATGCCGGTTGAATGCGGATGTATTA

ATGGTGGAACCTGCTCCACTGACAAGCCTGGCCTGCCAAAATGCAAGTGTCCTTATGGTT

ATTCTGGAAGGCACTGTGAAATAGGAAAGTCCCGAGGAGCACCTGCTGGTACAGCAGTGG

CTGTGCTGTTGGCAGTGATTATTATTCTCTTAAGTGGGGCTCTCGCTGTTGGGCTTTTCC

TGAACTACAAACGCACAGGCTCACTCATCCCTTCAATGCCTAAGCTACCCAGCCTGAGCA

GCCTGGCTAAGTTAAGTGATGCAGGAAATGGTGTGTCCTTCCGCTCAGGGGATGGCAGTA

CTGTGGATCCTCAGACTCTTGGGGTATCGTTTATTGATAGAGCCATGCAGCTGAATGAAA

ACTTTACAGATGCTGGGCGGGAACCTGTCACATTCGAGAATCCTCTTTATTCCACTGCAA

CAGGTGCAGCTGCTGATGCTGCGGTCATACATGCCACGCAGGTGACTGTGAATATAAGTG

GAGATTCAACAGAGAACGTATTCGCAAATCCGATGTACAATGAACAGCAGCAGGCAGTGG

ACATGAAAAATACACCAAATGAACAAGCAGCAAAGGAGTCCAAGTGGAACTTTTTCAAGA

AAAAATTAAAACCTAGTACAACCTTTGAAAACCCAGCATACTCAGAGATGCGAGACAAAC

AGTCCGCTTTGACTACGGAGGACATTTCAACTAGCCATCCACAGCCAGCTCTTCCACCAC

TAAAGCGAGAGAAATCCAGCATGTACTCACCGACAGAAGACTCCTTCCAAGACACGGCCA

ATCTGGTCAAAGAGGACAGCGACATATAACGCTTACTTTCTTTTTCAATCTTTCCTACAC

ACAAACGCAGATACAACGGGAAAAAAGACACACTCTGTTCCTCTTTGCACAGAATCTATT

TTTATATGCAGATTTTGTACAAAAAAAAAAAGAAATCTTAAGATTATTTTAAGAACACTT

AATAAGATGTTTTTATAACAGAAAATTAAAAGGTCTATGGAGACTATGCCTTCTGCTGTT

TCTTATGCCAATTCATGTCTATAATTGTTTTTATATTGTGATGTACTATATGTACTGTAT

ATCTTTGCACTGATACTACTGATGGTAAAAATGTTGTACATTTGTAAAAAATTTTTAGAA

GATTTCGATGGTAACTGGAAATGATGTGCAGCAGATCCTGCGTTCTATTAAAGATAAATA

AAATGACCGCTGAACATGGTAGTGTGTAGGTTAATGCACGCTGCTCAGTGATTTATTCCT

TTTTGTTGCTTCTTTGCATAGAAATTTACACTGATAATTTGTAAGTGGACTAAAAAAAAC

ACATCTCATCCTGAACATTTATATACCTCATGTGTGGACTGGCCAGCACAGGAAAAAAAG

AGCCAATTTCTGTTTTGGTCCATGATTCACAATATGTTCTTAAGCAGTATCTTTGTTCAA

ATTAATATTACATTACCTTAAATACAGTATATATTAGACATTTATAGATTTATGAACTGC

TATAGATTATTTGTTGTTACCCAAGTTTTAATTGTAGATATGGTAATTACCAGAAATCAA

AATAACTGATGATATTTTTTGCCTTGATATCTACCAGGCTAATTTATACGGCGTGTCTTG

TAAAAACATTTACTGTATTGTTGCAGTAGGCAAATATTAAAGACTGCTGTTATTTAAAAG

AAGGGACTGATTTAAAAAAAAAAAAAAGCTAATAACCTATTTTCATTCATAATTTATATT

ACGAATGTCACTAAT

>Unigene000038

GGCCGTCGCGGGCGGTTAGTGACGTCACCGTTTCTAAAATGGCGGTACTAAGTCTGCTTT

GAAATGTTCTTTTGCTGCTTGGATTTACAATATAAAAATGCAGAGAATGTTCAGGTTTTA

GAAGAATATATAAGAGTATAATTAATAAAAATGGATTCGGTGGTAGGGGAGGACCAGACG

CTGCCTTTCGACATCAATGGGGCACTTAAAGTCAAACCAGGGCAGGACCTAAAAGCAAAC

AGAGAGGATGTTGTTAAGAGCTTGCAGAAGGCTTTGAAGGAAAATCAGAAAATGTGTCAG

AAGCTGGAGAAGAAACTTGCTTCTGCTCAATCCATCATTGCTCTCCAGAATTCCTCCAGA

AAGGATAAAGACTCTGAGAGACACAAAGCACCTTGTAGTGAACAGGAAGATAAAGGCGTA

CAGGTGGATGCACAGGACCTTGGCTACGAGACAAGTGGGAAAAGTGAGACGGATGTGGAA

AAGGAGGAGGGCAGTACCACAGATGGTGGTGTGTGTTTGGACCCTGGTGTCTCCGGATTC

ACCTCGGCAGACAATTTAAACACTACAGCATCTAGCCCGTCTTACCCGAGCTCACCTGAT

CTCAGCTCTCCCCGTCCATCTGACCCCTCTCATGAGCTCCTGCAACTCAGGTCACTGGTC

AAACATCAGCACAAGGTCATTGCTCAACTCCAGCAACTTCTGCCCAAGAACTGTCTGACT

GCTGAGGTTTTGACCTGTAACCCAGCAAGCTGTGAAGAGGAAGAGGAGGACCGTACAGCA

CTGAAGGCACGGCTATTCCAGCTAACGCCTGAACCGGAGAAAGAACAAACGATCGACCGG

AGTAATGAATCAGCTTCTCCATCTCGGATGGAGTCTCTGGTGCAGTCTCAGGCCCGAGAG

TTGTGCGAGTTGCGTGAACAAATTAGAGTCTCAAGGACACTCGGTGCTGGACAGCGCAGG

CAGCTGCTGGAACTCCGGGGGGCACTAGAGGAGCTGATGGCACCTAACGACGGGCAGAGT

GCCCTGAGCCCAACCATCCGAAAACATCTGGACCAAAGTCTTAGCCTGTTGGACAAACTG

GAGCAAGACTCTTGTGTAGAAAAAGCTGAGTTGCCAGGACTTGAGCTGTTTCAGAGATTA

ACAGCAGAGCTCCAGGAAAAGGAGACGCTCATTCAGCAGCTGAAGAATCGGCTGTTTGGC

AAAGTCTCAGGTACCCAGCAGGGGTTCGAATCTGAGACGAGCGAGACAAGCTCTAACGAC

GGTAACGTTTCTGTTCAAGGCAGCCTAAATGACCTTCACACTCGCACCACCAGAGGAGCA

ACCGAAGGCTTAAAGTCAAGCAGCGGCGAAAGTGCTCCCGACTCCGAATCTCACCCTTCA

TCCGTTTGTGTGGCTGCTGGTTCGACTGGATCTCAGGGTGAAGAGGGAAGTGTGTTTACT

CTACAGAGGGATAACAGCCGTCTGCAGGAACAGCTAAAAAAAGAGTGAAGAGCTTAATGC

AACACTGAGAAGTGAACTAGATCTCACTCACTCCATCCTTACACACACACAGAGTCAGAA

AACCCCACTGACCAAACAGACACACAGCAGCTCAACAATCCCACACACACAACTCCAGAG

CCAAGATATGGACAGCCAGAAGGTTCAGCTGGAGTCAGTCCACACAAGCATCAGCTCAGA

TTTGTTAATGGAACACCTGCTAGAGGTTCGAGCTCTACGTCAGCGCTTAGAGGAAACAAT

CCACACTAACGAGAGACTGCGACAGCAGCTGGAGAGAAAACTTCAGCAGGCTGAAAAAGA

CCCAGATACTCACATCTTTATTGCTGGATCGGACGATCCGAGTCAACTGAACACTGATAT

TCGATATCTTTGGGCTCAGAATCACACACTTAAAGAACAACTTAACCAGGCAGCTAGAGA

TAAACAGAAAGAGAACGACAGTCTGAGGAAGACTTTGGCGAGGCGCACGGCTAAGCTAGA

GCTCAGCAGAAAAGAGTGTGAGATGCTGCGGGAGGAACAAACTAACTTGCAAAACAGCCT

CTACAGGTTGCAGTGTGAGCAAAATCTTCAAAAGCAGCAGTTGGCCGACACTCAGCAACT

TCTGCAATCAGTGCAAGTCGAACTTCAGGTGCACGAACAGATCCGGAACTCAGTACGCAC

CCACACAGACGAGGAGCGTGGTGGTTCGGAATCCTCTGACGTGGATTTGAAGGAGTTGTT

GGGTGAAGTACGGAGTCTCCGTCTGCAACTTGAGAAAAGTATCCAGACCAACACGGCACT

GAGGCACAAGCTGGAACAACAACTACTGACCCAAAGTGACCCTCCATCTACCATCAACAT

CAACTATCTGCTCTCTCAAACAGATGATGGAGGTAAATCAGAGATGCTCCGCCAGCACTC

ACACACCGACCCCTCCAGCGCTGCATACGACAGTGGTAGCTCTGAGCACGGTGGTTCTCC

GGCTTTGTCCCGACTGGTTCCAGGTCACAGACTGTGGGCAGACCGACATGGGCAGCATGT

TCTAGGTCTCGTAGAGGATTACAGCGCTCTCAGCAAACAGATCAGAGAAGCCAAAAGAAT

CACTTCAGCTATGGATACACAGCTACAGGACAGGTTTGTGGAGTGTGCCAGTTTTTCGGG

AAGTGTGAGCACCATGCAGCAGATGCTGGAGGAAGCCGGGCGACTGCTAAAGCTTCTCTG

GAAAGTATCGCTGCCCACAGGAGACCTTACAAACACTCAGCAGAATGTTCTAAGGAGTGA

GATCACTCAGCTGAAGAGCAGACTGTCTCAGCAGGAGCGAATGCTGACTGGAGCAGTCAA

ACGCCTGCGATCCACCAATCAGCTTAAAGAGGGCATGGAGCGCATCATCATCGAGCAATT

GTCACTGACTCATGGAGTGTTAAAGAAAGCACGAGGAAACCTGGAGGAAATTCCACTGAA

TGCCCAGTGATAAGTCAGCTGACTGACATCCCACCGTAACGCTAACATGACCCTCCTTCC

GCCCCAAGTTGCAGCATATCCAGACTACCAGACTCTCTAAACACAGACATCAGCATGGAC

AATGTGACAGCCTCCATGTTCCCCGGGCAGAAGATGTCTCCATCCAAAGCGATGACAATG

AAAGACTACGAGAATCAAATTACAGCTCTGAAAAAAGAGAACTTTAACCTCAAGCTGAGG

ATCTACTTCCTGGAGGAGCGCGTGCAGCAAAAATGCGACGATTCTACCGAAGAAATTTAC

AAAACGAACATTGAGTTAAAGGTGGAGGTGGAATCTATGAAGAGGGAACTTGCTGAAAAA

CAGGAGCTGCTTGTGTCGGCATCGAAAGCGCTAGAGAGTTTAGCTAACAGAGATTCCGAG

GCGGGTCACCGGGAACGGGCTCAGAGGGACATGGACGCACTCCGAGAAGCATTTAATGCC

AGAATCAGAGACCTGGAAGAGTCTTTGCGCTGTGCAGAAGACGAAGTAGAAAAAATGGCT

TCCATTGCCGAGCAGGAGAAGCTGAGGAGTATAGAGTTGGAGAAAGAACTTGAAGCTGTC

TGCCAGTCTGGACCATTGCACGATGCTGACTCTGCCCGGGGACCGGACCACAAACTCCAG

CAGGCTCTGCAGGAAAAAGAGCGTTTAATCAATCAACTTCGGGAGTCCATCAAAAAACAA

GATGCCCTGATAGGAGAACTTCAGGAGCGAGATCAGGATCAGCCAATCAGAGAGCAGATG

CTCCAGCTCAACAAACTGATTGGTGAAAAGGATGCTGAAGTACAGGCTTTAAAAGAAGAG

TTAGACCGGGAGAGAGGAAAAGCCGAGAAGGAAAGTCAGGTGTGTATGATAACACAGGAC

GAGCTGAAGACGCTACATCAGAAAACCCGACAGCTGACTGAAGAACTGAACTCCACCAGG

AGCAGCGGTCAAAAACTGAAACACAGACTGGAGGAGATGGAAGGAGAGAACAAGATTCTC

TCTGGACAACTGGAGGAGAGGGAGAGTGAACTGGCGGGAGAAAAGAAAAACGCCCTGAAG

CGAGACAAGACCATTCAGGGACTGAGCATCCTGCTTAAAGAGAAAGAACGACAGGTGGAT

GAGTTGTATGGAAATCTGGAGGAGAAAGAACGGACCTTAACCAAAGCCAGAGAAGCTCTT

CACAAAGCTCAGCTACAGAAATATCAGGGTGGTGAGGAACAACAGGCCCTGCTACAGTCG

CAGAATGCTGAGCTGTCTCGTCTACAGGCAGAGTGTCATTCGTCTCTGCTGGAGCTTCAG

AGGTTACAGCGTGCACTCAGCAGCAGAGATGCACAACTGGAGCTACTGCAGCAAGATAAA

GTGCAGCTGGAGAACGAGCTGGAGCTGCTTCAGCAACACAAAAGAAAAGGAGACAAGACC

ATTAATGATCTGCAGAACCAGTTAAAAAAGCTGAGTGGAGAGCTGGCCGAGCGTGAGAAC

CTTCTGGAACATCAGCGTTTAGCTCAGCAGGAGGAAACCCGCACAACAGAACAGAAACTG

CAAAGCACAATACAACAGTTAACAACCAACATCACAAAAAAAGAACAACAGCTACAGGAA

TACATGAGCATGATGCAACATCTGGAACAGAATCAAGCTTCAGCAGGAAGTGATGTAATG

CTGGCCAAACTCAGAGAGAAGCTGAGGGAAAAGGAAAAGGCTTTAGAGAAGGCACTGGAT

GAAAAGTTTGTGGCAGTGGAAGAGAAGGAGAATGAAATTCATCTGCTGCAGCTGAGCATG

AGAGAAAAGGACAGAGATCTGGAGCGTCTCAATCACCTGCTCACACACAACGAGGAGACC

GTTAACAGCTTTGATGCGTTACTGAAGGAGAAAGACATGGAATTGCAACAGCTGCTGAAT

TCCTTTAAAAACCTGCAGCGCTCCAAACAGGAGAGTGAAGAGAACCTGCAAAGAGCTCTA

AGGGAGAAAGATTCCATCATCCAGCAGCTCCAATATGCACTGCAACTCAAAACTAAAGAC

ACCGAGGACGGCGCTACTACGGGTCTCACACAGTCGGATTCTCAAGAGTATAATTCTGCC

GAGCAAATGAGTCAAAGGTTAAAGGTCACAGAGGCTATGCTAATCGAGGAAGTGAAGCAC

AGGGAAAGGCTGGTGTGTGAAAACCAGACCGTTGTGGACAACCTGCTTGCCATTATCAGT

AGTAAAGACCAGCTACTTAAGGAAAGTGCAGAGCGACATTCGCAGGCACTCGGTGATCAG

ACAGCAGAGGTGCAGGATCTGCGCAGACAGCTCGCAGACACAGAGCGGCGAGTTAGCAAC

GGCCAGAATCTCAGTGCTGGTCTGAATGAGAAGGATGTTCTAATAAATAAGCTGTTGGAT

CACACTCCAGAAAGAGATCTGGAGGTTTCGCCCCCTCAAGTGGTAGAGCTTAGACAGACC

ATTAATATACTGCAACAGAGACTGGAGGAGAAAGAGGATGAACTGTCTCAGCGGCGCAGC

GAGGAAAACAGCGAGAAATTGCGTGCAGCCAATAAAAAACCCGTAGTAATACTGAAGAAA

GAACTCGCCCAGAAGACAGAGGCACTCAACAGTGCTCTGAAGAGAGAAAATCAGCTCCAG

ATGTCTCTTGCGGAACTTCAGTCCTCTGTGTCCGAGCTGGAAGGTCGACTAGAGGGCCAG

ACAGCCAACATCGAGTCCCTCAAATCCATCATAAGCACTAAAGATGAAATTATTACTGAG

CTACAGCAACATTTGTCAGAAATGGGATTAAGACGGTCGACTGTTGATCACCACTCCCAA

GCCGCAGAGGAGCTCAGCTTCCGCTCTCTTCCTCAGAGAGAGAGAACCGTCATCGGGGGA

GACAGACAGCAGCAGCAGGAGACGTTGTCTCTGGGTGACCTTTGCTCTGAACAGGCTGAA

CTAAACCACATTCTTAGGGAAGAGCAGCAGCTTTACACCAAGCTTATTCATGCTGTAAAA

GAGCAAGACAGTGTTCAGCAGCTGCAGTCCTTACAGCTGGAGCTGAGCTCCGTAACACTC

CTCAGGCAGCAGTTGGAAGATGGCGTGCGTAACAACAATAAACTCCGTGAGCAGCTGCAC

CAAGAGATCCAGAGAGTTAATCAGAGAGAAGGTGTGGACCCTGCAGAGCTGAAAAGTATG

CGAGATGCTCTAGAAGATGCTCAGCGCTGGAACGCATCACTGCAAGCACGACTTGGGCAG

ATCCAAAGCAGAGGAGGGGGAGTAGGACAGGCTAATGACACAGATGATTCGCTGAGTCTG

ATTGGCGAGCAGACCTCCTATATGAGTATATGTCTAGGGGAGGGGCAGGAGGAGGAGCTA

CAGCAGCTCTCTATAGAACAACTCAGACTGAAGGTCTCGGAGCTACAGGCCAAAAATGCA

GAGCTGCAGAAACAGTTGGCGCAGCCAGAGAGAGACGTTATGAGCAATTCTGAAAGAGAG

CCACAGGGACAACGGGACCTTATTGGGTTTTCACCTCCATGCACGCAGCCACAGAGACCG

GTCACGCCACTAGCTGTTAATGCTAACGAGAGACAGACCGCTTCCAGTGTCAAGAAGACA

GCTGATGATAGTCTTGAAGTCCAAAGACGGATGGCAAGTTCCCCTTCACTTTTGCCTAAG

AAAGATAATGGAAGTGTCTCATATTCTCCCGGACAGCAGAGTGAGAATGAACAGGAGGAA

CTCGCAGCCTTGCTTAGAGACTGTGGAGCGGAATCCATTTCCCAACTCAGGGAGCAGGTA

AATAAACTTCGCTCAGAAATGTCAAAGCTCGGTGTGCTTTTGAAGGAAGAAACTGTGGCC

GAGTCCAAGGAAAGCACCGAAAGTTCCGGAGAAAACGACAGCCATACAAACCTGCATCAG

ACGGTGCAGACGCTCCGCTCCGAGGCTCGAAGTCACCGCAAAATTGTTCGACTGTTGAAG

GAACAGCTGCTGCGCGATAGAAAACCAAAAACAGGCGGCGAGCCAGGACTCGACTCGGAA

CGGGTTGACAGCATGGCTCGCAAGATGGAGCGTCTGAGAAAGGAGCATGAAGCCACTCAG

AGACATGCTGCCATCCTGGAGGAAAGGCTAAAGGAGGTACAAAGCAGAGAAAGAAATGAG

GACCGAGAACAAGAAGAGAAAGACAGTAAAAAGAAAGGCAGACAAGAACATAGCAAAAAG

TTGAGACATGCGGCATACAGCAAATCACGGCTGCCAGTAGCAATAAGACCTGCAAAGCCG

AAAGACAGACATAGAATGACTCATCATGATCCAGAGAAAAGTCCTGAGCCGCTTAGTACA

TCAGATTCTGAGCAGCTCAGCCCAAAAAGCGAAGGTTCTTGTGGGGCGGTTCTTCAAAGG

TCCTCTGAAAACAGCTTTAAAACCCTTCGGGGTTCTGAGTCTAGACAAAACAGAGAAGTC

AGAAAAAAATCCAAAAATGTGTCTGACATTCCCCAGTCGGAGCTTTTATCTCAGCTGGAG

TTACTGAACCAGGAATGCCAGGAGAAAACGGAGCTTATCTCACAACTTCAGCTTCAGATG

CAGAACTGGGAGAAGCTCCAAGCCGAGCTCCAAGAGAAGGAGCGGTTAAATAGTCAGTAT

CTGGAAGCCTTGCATGCTGCAGAGTCTACAATTGCATACCTTACAGCCTGTAACCTGGAT

TCGGAAAGTGGCCTGGGTCAGCCCAAACCGGGATCTGATTCATCACTGCAACAGCAGTGC

AGCGAGCTTCAACGAGCCGTGCAGGAAAAAGATCAAGTCAGCATTCAGCTGCTTGAGTGC

TTAAACAAAGCCGAAGCTGCCATGGCATCACTGACTTCGGCTAATCTGCCTGAAAGCTTT

TTCTCTGCCCAGAAAGACCCACATGCCTTAAGTGAGAGGTTTGACTTCCTTCTCAGCCAA

ATAAAGACCTTACAGGAGCAAAAAAATGCTTGCACTTCTAAGGAGTTGCAGCAGAACAGG

CTGAATGCTGAGTTACAGGAAAAACTCCGGGCCGCAGAAGAAACTATTACCAAACTATCT

GCTCAAGACGATACTACCACTGGTCAGAAGCTGATTTACAGCCACAGTTGCACAAGTCCA

CAAAAGGAGATGGTGGATGAGTATGAAGAGGGACAGCAGAGACTGGCTGCAAGTTTTTCA

GAGTGTATTTTTGCAGTAGAACAAGCAGTAGGGTCTCTTGCTGAATATGGCTTAAGGTCT

GATCGGCTTTTGCATGGTGTTGAAACCAGCACCAACGCAGAGCTTGAACAGAACTTGGAC

AGGCTTCAAAAGGTGCTCCTGGAAAGAGACAGGTTTTGGAACTCCATCAGTACAGATATA

AGCCACAGCACTATTGTGGGTCCGTTCCACCAACCAGTACATCACAATCTACAGATTTTA

GACGCTGCACAGCAGAAGACAAGCACACCAATTTCTCAAGAACAGCAGGGGCAACAGAAA

ATATCCGAAACATGCGATCCTGATCGATCCGGAAGTCAGCATCTAAACCTTTACAAAAAC

CTAACTCTGCTTGTCCAGATCTTTAAACAACATGCTCTGAAGGTCCGGGAGCTAGAGGAG

GCACTTAAAGTCAAACCAGGGCAGGACCTAAAAGCAAACAGAGA

>Unigene000039

TCTCTCTCTCTCTCTCTCTCTTCTTTTCTCTCCCCCGATGAACTGCTTCAGTAATGTGGG

AGACGGCGATTCTGTCAGCGGCTCAAGATGGCAGATTTTTCCCTGAGTTCTGAACTGAAG

CATCTCTTCACATTTTTGCCATGAATAGCAGGAACCCTTGCACCGATGGCCCCTGTATAC

GAAGGTATGGCCTCGCAAGTGCAAGTCTTTTCCCCTCACACTCTCCAGTCAAGTGCCTTC

TTTAGCGTGAAGAAACTGAAGGTGGAGCAAAGTTGCAACTGGGATATGACAGGGTACGGC

ACGCACAGCAAGGTCTACGGTCAGAACAGCAAGCAGAGCGTGTCCGCTGCCCCAGTGGGC

CTTAACGCCGCTTCGCTCCAGGTCTCCAACTCCTCCCTGCCATACGAGCAAGCTCTCCTC

TTCCCTGCCAGCTCTGGCCATATCGTAGTTGCCTCAGCGAGTAGCACGTCTGGGGTGGCC

GGACAACTTTTGGGGAGTACGGGCAGCAGTAGTGGCAGTGGCAGCGGAGGGCACAACCTG

ACGCGCCGCAGCACTGTGAGCTTGCTGGATACGTACCAGCGATGCGGGCTTAAGCGCAAG

AGTGAGGAGTTAGATAACAACAACGGCAGTGGAAGCAGTGTGCACGTGGTGGAGGAACTG

CAGCAGCCTCCAGCAGCGCAGATGCTCCAAAACAATGGCCAGAGCGGCCCGACAGGCACA

GTGGCCACCGCATCCACAACTGCCACATCCAAGACCAGTGGCGCCAACAGCGAAGGTGAC

TATCAACTCCTGCAGCATGAAGTTCTGTGCTCCATGACCAACACATATGAGGTGCTGGAG

TTCTTAGGTCGAGGTACCTTCGGGCAGGTGGTGAAGTGCTGGAAGCGTGGCACTAGTGAG

ATTGTGGCCATCAAGATCCTGAAAAACCACCCATCATATGCGCGCCAGGGCCAGATCGAG

GTCAGCATCCTGGCACGCCTTAGCACAGAGAGTGCAGATGACTACAACTTTGTGCGGGCA

TACGAGTGCTTTCAGCACAAGAACCACACGTGCCTGGTCTTTGAGATGTTGGAGCAGAAC

CTGTACGACTTTCTCAAGCAGAACAAGTTCAGCCCACTGCCCCTCAAGTACATTCGGCCT

ATTCTGCAGCAGGTGGCTACAGCGCTGATGAAGCTGAAAAGTCTGGGTCTGATTCATGCC

GACCTGAAGCCTGAGAACATCATGCTGGTGGATCCTTCTCGGCAACCCTACAGAGTCAAA

GTCATTGACTTTGGCTCTGCAAGCCATGTGTCCAAAGCTGTGTGCTCTACTTACCTCCAG

TCCAGATACTACAGAGCTCCAGAGATCATCCTTGGCCTGCCCTTCTGCGAGGCTATAGAC

ATGTGGTCCTTGGGCTGCGTCATCGCTGAGCTCTTCCTGGGCTGGCCGCTTTACCCTGGC

GCGTCCGAGTACGATCAAATTCGGTACATTTCTCAAACCCAGGGGCTGCCTGCCGAGTAT

CTGCTGAGTGCTGGAACAAAAACCACCAGGTTCTTTAACCGAGACCCAGACTCCACCTAT

CCACTGTGGAGATTAAAGACACCAGAGGATCATGAGGGTGAGACAGGGATCAAGTCTAAG

GAAGCCCGCAAGTACATTTTCAACTGCCTGGATGATATGGCACAGGTGAATGTGAGCACA

GAGCTGGAGGGCAGTGACATGCTGGCGGAGAAAGCAGACCGGAGAGAGTTCATCGACCTG

CTGACCAAGATGCTGACCATCGACGCAGATAAAAGGATCACGCCCATTGAGACGCTCAAC

CATCCATTTGTTACTATGGCTCACCTGCTGGATTTCCCCCACAGCACACATGTCAAATCC

TGCTTCCAAAACATGGAGATCTGTAAGCGCCGTGTGAACATGTACGACGCGGTCAACCAG

AGCAAGACCCCGTTCATCACTCACGTAGCCCCCAGCACTTCCACCAACCTCACCATGACC

TTTAACAACCAACTCAACACTGTGCACAGCCAGGCCACCAACCTGGCTCCCTCCTCCACC

AGTGCCACCCTTTCTCTTGCCAATCCTGATGTCTCCATACTAAACTACCAATCTGCACTC

TACCAGCCTTCTGCAGCACCCATGGCTGCCGTGGCACCCAGGACTATGCCCCTGCAGCCT

GGCGCCCCTCAGCTCTGCGCCACACGGCCTGACCCCTTCCAGCAGGCGCTTATTGTTTGC

CCGCCCACCTTTCAAGGGTTGCAGGCGTCCCCTTCCAAGCACAGCGGGTATTCCGTACGG

ATGGAGAATGCTGTTCCCATAGTGACTCAGGCCCCCGGTGGTCAACCCCTGCAGATTCAA

CCCGGGCTCCTTACACAGGCCTGGCCCTCTGGTACCCAGCAGATCTTACTTCCCCCAGCA

TGGCAGCAGCTCACCCACACCTCTGTACAGCACGCTACCGTCATCCCAGACTCCATGGGC

ACCTCTCAGCCTTTGCCCAACTGGAGGAATCCCCATCCCCATGGCAGCCATTACAATCCC

ATCATGCAACAGCCAGCCTTGCTTGCCAGTCACGTGTCATTGCCTTCCCAACAACCATTG

AATGTGGGCGTGGCTCATGTCATGAGGCAGCCAGTAAACAACAGTACAGCTTCTAAAAAG

AGTAAGCCACAGCAGATGAGCAACAGGAACGTGTCGGCCTACGAGGTGTCCTCCTCCCAG

GCGATGTTGTCTCCACAGCGTTCAAAGCGTGTCAAGGAGAACACACCGCCACGCTGCGCT

GTGGTGCAAAACGGACCCTCTGTTCCATGCGCCCTGTCCCAAGCTTGTGGAGGAGGTGGA

GGAGGATGGGGAGAGGAGCAAGCTTCCAGCACAACGCGAGAGCACCACGGACCACACAAC

CAACACCTGCAACACAATCAACACAACCCCCCAAGACAGACCATCGTCATCCCCGACACC

CCGAGTCCAGCAGTCAGCATCATCACCATCAGCAGCGACACAGACGAGGAAGATGAGCAC

AAGCAGCCCGGTAACACCAGGTCAGCAGGCACATCATCCAAGCAGCGGAAGAACGTCATC

AGCTGTGTCACTGTCCACGACTCCCCAGAATCCGATTCCAGCAATATCAGCCCCTACGCT

GTCGAGTCCCGAAGCAACTGCCCCAACGCCAACAACTACGACTCGAAGAGCACAGTGCTA

GACAACTACAACAACGGCAACCCCCGAACCATTATCATCCCTCCCCTGAAGACCCAGACC

AGCGAGAGCCTAAGCGAGTGTGATCGACTGATGCCTGACACAATGAACCACCAGAATCCT

GCCTACAAGTTTAATTCCTCCAATGGAGGCCTGTCAACTAACAATCACTCATCAGTAGGC

ATACCCGGAGGCGGAGCATATCGCCAACAACGTTCCGGGCCACATCCTTTCCAGCAGCAG

CCGCTTAATCTCAGCCAGGCTCAGCAGCACATGGCAGTGGACAGAACCAGAGGGCACAGG

AGACAGCAAGCCTACATCACACCTACAATGACTGCTCAAGCTCCCTATTCCTTTCACCAC

AACAGTCCCTCCCACAGCACTAATGTGCACCCCCACCTAGCTGCCACCCACTTATCTGGC

CAACCCCATCTTTACACATACACCACCCCCGCAGCCCTGGGATCCACTGGCACTGTGGCA

CATCTGGTGGCGTCGCAGGGTTCAGCTCATCACGCGGTCCAACACGCTGGCTACCCACCA

AGCATCGTGCACCAGGTACCTGTCAGCATGGGCCACCGTGTGCTCCCATCCCCCACGCTC

CACCACGGCCAGTACCAGCCCCAGTTCGCCCACCAAACCTACATCAGCGCTTCGCCTGCT

TCCACTGTCTACACTGGATATCCCCTGAGCCCCACAAAGATGAACCAGTACCCGTATCTG

TAGATCAGAAAACACTGGAGCCGAGCCCAACGCTGCTTTTTGATCTCTTCCTACTAGACA

CTGAAGAGTCAGTGAGACACGCCCTAGCTTGACAGCCCCTTAAACCTGTCCTCTCCCTGT

CCTCGTCACACTTAAACTTGAAGACAATGAAGACACTGATGGAGAAAGAAAGAGGGATGG

AATGAATAAGAAAATTTTTTTTTTTCATATGAGTTTCTTTTTAGAGGGCCAGTTCTGATT

CTTTTAGGTTTTCCAATATTTTTATAATGGAAATCATATATATGTGTTTTTTTTTTTCAT

TTAATCATTTTTACAGCATGCTAACGCTACTGCTGGCATTTTGGGAGTGACTGGGAATTT

AAATTAGATCGAGTCTTGTTTCTTTCTCTTTTTTTTTTGTTTCACCGACTGTCGAGCCGA

GGTCACGTGGTGGATGAGAACGTTCTATTAGGTGAAATACGGATATCTGCTGCTCTACAA

AGAAAAAAAAAATAACGATGGCCTTGAACTGTCTTAAACATTTCCGAATCAGTTGGTGAT

GGGTGCTGCAGGTACTGCGCTGCGCTGCACTGCACGTACTCCGCCGTTCACTCCAAACAA

CAGCCGGAGGAATCTTCTGTTATATTACAAGGAAAAAAAAAAGACAAAAGACTTTCTAGT

GTATTTATATATTTTTTGTGTTTCGTTTGTGTTCACACTTAGATGACAAATTTGAGTTTC

GACCCCCTCTCCTACCCCCCCACACACCTACGGTCCCCTTTCTGATTTTTTTTTCTAAAA

TGTAGCAAATTTCACCTGCTTATTTTATGAAGAACCTTTAAGATGCCAATAGAATTTTTG

TTCCTCTTTAAATGTGATTGCACAACGTTGTTGTAATAACATAAAGCATGTATAACCTAA

TAGTCTGTGCACGTGTGTGCTGCTGTCGGCCATGTTTGTGGTCCACCTTCCTCTGCGCCC

TTGTGCGTTGTACTCCTTCACGGTCTTAGGATTATCGTTGTTCCCTCGTAGTGCCTTACA

GTACACATATTACAATTTTTTTCCGAAACCAACAGGAACTAGTTTGCAGTTTGGCATCTA

GGTTGGACATCTCTGTCTGAGATGTTTTGCTCCTAACTTCCAGGTGCAAGCATAGTTCTA

AATGTAAGTAGCTAGATTATTATACATGACACCATTTTGTATAAAAAAAAAAAAACAACA

ACACAACAACGCTTCTTGTTTAGCCTGCATGAACTTATTTAGAGCGAGATTCTTCCTTGA

AATAATGAACCAAAGTTGTCATTCAGTAAAGAGGAGTAACATTTTATTACTGTTTTATAG

GATGTTTAGTAGCTGTGTGTCTTAGAAACCTGTACGGTGGCCCGGAGCTGTTTGTTTTGT

TAAAACTTGTTAGAAATAAAGCGCTACAAGTAATTCATTTCGAATACCCGCACTAATGCG

TCGGATTAACCGTCATAAAACTTTAAGGTGAAGGGCAAAGTGTTCTAGTATCTGCTAATG

AGGTCCAGCTGCAACACATCTGGGAGGGATTTTTGAAGGAAGGTCGAAAGAGCAGGAGTG

TTTGCCGAGCGAGCAGCATTGGTGCAGAAACGATGTTGAAACAGTGAACCTGTGAGGGTG

AGCGAGCGGGTCGAGGTAGGGACCGATTAAGGTGCTAAAAGATGGCACTGGATGGGTTTT

TTGGATGGGAATTTTAGAAAGCAGAGAGAGTGAGAAAGAGAGACAGAGATATGAAATTCC

ACATCCTAATGAGAATAGCATGTTGGTTTTGAGCATGTCAGTAAGATCTGGAGGAATCTG

CGCATAAAAAAACAACACCTTTTGATCCAAAGGGTAAGTTTAAAGAACCAGGCTGGGTTT

TGAAAGCTTTGGACACAGTGAAGATTGTTCACCGGGAACATGTGTTGTTACTCTTTTGTC

GGAATTACAGGAAGAACTTGTGCTAGATGATACAGGGAATAAAATATGGGCCAATGTTTA

GCTTTTATTTTATTTTCGTTGCCCGAGTTGATCAAGGCTTGTTTTTTCTCCGATGATTGA

CTGTCTTGATGAGGGCACGTGCATGTGAGTTTCTGAACACATCCATTTTTCCCCTCTCAC

CTCACGCCATAGTACTTGCATTTAAAGTGATTTTATTTTTGCTGGGGTTTTTTTAACATG

TCATGTTTAAATATATTTTGTCAGTTCGTGATACCTTTATTGTAATTGTTTTGCTAGATT

GTGTTAATTATCCGTTGTTTATTTGTATCTGTTATGGTCAATAAGTTAAGTGTATTCTTA

GGGGCATCTAGTAAGTAGGAAGTGTTGATTCTGATTGGGATACCTTTAACCCAAACATTG

TTTTATTCGTTCGGATCACAACGATGTGAGGATGTGTGTTGATTTTTGAGTTGTATTTGG

CTTGTTTTGGCTCTGTTATGGACTCACACTGGCTCCAGGACGTTGAAACATAGAGGTTCC

CATGTGGATTCGATTGCATCTAACATTCCACATAAGAAACTGTTATCCTGTCTCACTGCA

CTGTCTATGAGCTTTGCTCCTGTCTTACCTCACGTAGACCGGCTGTGTATGACCGCATGG

GGATATGGTGTCATTTTACACTTTTTTTCCACCAAAGAAATTTGTTGTCGAAGAGGCACA

GATTGTCCATGGAAGGTAATTGCAGGCCCAAGGCATAGGCCAACTAAGCGGCTGCTTATG

GCACCTCATGGCCACCAGGGGGCCCCCAAGAGTTTATGAAATGACATCATGTTTTTTGTT

GTTGTTTTTTATTTATATTTTAGTGAACAGTAGTAATTATACAAAAAAATGCAATATTAA

ACAAAGAAAAACAACGCAATATTGGGATGTAAGCAACACAGCGAAAGCAAATAAAATTGA

TAATGGGTGTGTTAAGATTTATAGTGTGCAAATAATTAAGGATTGACTATATTGGCAGCA

CTGAAGGGCCGCTAAATAAAAATACTGCCTAGGGACCCTAAAGGTTTATGCGGCCCTGAG

TTACTAAAATGACAGATGGTATCATATTAGAAAATACTCCAGAGCACAACATAAAATAAA

TTGACATCATTTTTGCTAAATCATCATCAAATCCCATTGATACACAAACGTGAGAAAGTA

CGACCACTTGTACTTTTTACCTCATTACATTTCTGTTACAAGCTCTTCCAGCTCTCCACA

TGTGAATCGTGAATTAGTTGCAAACCAATGAAAGAGGCAGGACTCCATAGTAACTGCCAG

TAATGATCACTAGCACGTACTCCGATATAATTGTATAATGTCAGACTAAGCAATTCATAT

CTATACATACATATACAGAGAGTTAAACAGCCATCTTTTTATTGTAAGTACATTTTTTCT

TTAAAAAAAAAATTAGAATGCAATATGTCTCACAACTCGGTTACGCTTAACATCCAGAAA

AGAATTATTATTCAAGTAGTATTCAAGTAATGTTGTGGTTTATTTTATTTAATTTAGCTT

ATCAAATTTTGACCTTATTTGTACCTGAATGTCTGAAAACTGGGCATCTTTTGTATAAGT

GACATATTTCGAAGATTTTTTAAGTTTTGATTTTAGTTCTCACCACCTGAAAATTCGTCC

ACATGTCAAATTGTTGCCTCGCAAACTACAAACTCAAAGCTTTAAGCAAAGGTCATAATT

GACCCTTTTCTGTCTTTTAAACATTTTTTTTTTTTTTGCTTTTTATCTATACACGTCTTC

TGTCCCCTTCAGTTTGAAAGGCGTTTCCAGCACCAGATGTCCTTCTGATGTTCCGAAGCG

TTTTTAAAAATTCCAGACAAGTTGTAAATGCTGTTTATTACCCCAAACAGTCTAATCAGT

GTACGAGCGCTTTGTTAAGCACACGCTGTTGAACATCGTCGGCGTCGATGCTGACAGGTG

TTTTATCGCTTCTTTTCGGTGGCAAAATGACAGCACTTCAACAATGTTTAAACTTTTTGT

GTTTAAGTGTTTGTGAAAATGTGTTTTTTTGTGTGTTTTATTACTGTTGGACGAGGGTTT

AAGCAGTGCGGTTGCAGGTTTTGCTGGAGTCATTGATCTGTGCTGAGAGGCCAAACGTTA

CAAGCGGCTATAGACAACTGTATACTTTTAAAGAACCAAAAACAAAATGTCAAAAAGTCT

TTAACCCTGATTCCAAAAGACGGTGCAGATCTAAAAGGCCCTCGCTTTACATCCGTCATG

AAGGTGTCTTAGCGACCGTAAATCCTGCTCTCCACATGGACGGTGCTGCAGTCGCACTCC

ATCACAAAAAAAACCTGTCAGACGCAACCAGATGAGACATTTCCTCATCGCTAATCATGC

TAATCTGTTGTGTAGCTGTGATGCGCTGTAGCCATCCACGTGTGCAGCGATTTATGACCA

GGACGTTTTTTCTATTTGCACAGTCAAATGTAATCGAAATATTTTTCTATAATATCTCAA

GACCTAAAAAAAAAAAAAAAAAACATCGCACCCTGACCCTGATTTTGTGTCTATCGCTGT

ATGAATTCTTGATGGCGTGTCTCATTAGCTGTTGTCAAAAGCTATTGTGTGCTACAGTAT

TTTCATGTCTTTGTTCATGTCTTCTTTTCTTTTTTTTTTTAGTCTTTATTGTCATTTTTC

TTTATCAGAATTCGAAGAAGAAGAAGAAA

>Unigene000040

AGAGAGAGAGAGAGAGAGAGACTGTGTTCTGAGACAGAGGAAGAGACCAGAAGGCTTTGT

TGACTTATGCAGCCCACCATGCCGAGCTCAAACAAGGTGTGAGTGTGTACGCTCAACACA

CACCCACCTACACACACTCATTTTGGAGGGAAGGCTGAAGACCGAGAGCGTTGAACTTTG

CTCATCAGAGAAGAAAGGAAAGTCGGCAGGTCCGTACAGGAGGAAGGAGCAAAAGAAGTG

GAACAAGCTGTTCTTTCTTTATCCTTTTTGTTCTTAATTCATCCACTGGGTTTCTACATT

GAAGAAAGGAGGGGGTTTTTAGGGACGGCATGGCGTGACAAACTCTGCTACCTGTGGACG

TGTCGCTTTGCACGCAACTTGCTTCGAAGCCGGACTCTTGCACCTGGACCGACCTGCTGA

TTCCACTCTTCTTCTTCTGCTACTGCTTCTTTCTTCTTCTTTTTTTTTTACATCTTTTAA

CGCTGGTTGGACGAGTCCTAGCATGCCCTGAACCCTCGCTGTCTTTTTAACGGAGCTGGT

CATTCGTATCGGGAGCAGGTTATTGTGTTGGCTGGTGTAAAAGCAAAAGGAAAAAATAAA

CTCGAGAGGTAGAGATGGAGTACGGGCGGTTGGAGAGGGAGCCATGTCTCTCTCCCGCTG

CATTCGTCAACCAGGTGCAGTATTCCAACATCCTGGAGGGCCGTTTCAAACAGCTGCAAG

ATGAGCGAGAAGCCGTACAGAAGAAGACCTTCACTAAGTGGGTGAACTCTCACCTGGGCA

GGGTGACATGCAGGATCAACGATCTGTACACGGACCTGCGGGATGGACGCATGCTCATCC

GCCTGCTGGAGGTTCTTTCCTCCGAACAGCTGCCGAAGCCGACAAAAGGACGGATGAGAA

TCCACTGTCTGGAGAACGTGGATAAAGCGCTGCAGTTCCTCAAGGAGCAGAAGGTCCATT

TGGAAAACATGGGTTCTCACGACATCGTCGACGGCAACCACCGCCTCACGCTCGGCCTCA

TCTGGACCATCATCCTGCGCTTCCAGATTCAGGATATCAGCGTGGAGACGGAGGACAACA

AGGAGAAAAAATCCGCCAAAGATGCTTTACTGCTGTGGTGCCAGATGAAGACTGCTGGGT

ACCCGAATGTCAATGTTCACAATTTCACCACTAGCTGGAGAGACGGCCTGGCGTTCAACG

CTATCGTGCACAAACACAGACCGGATCTAATTGAGTTTGACACTCTGAAGCGATCCAACG

CTCACTACAACCTACAGAACGCCTTCAACATTGCCGAGAAGGAGATGGGGCTCACCAAAC

TGCTGGATCCTGAGGATGTAAACGTGGATCAGCCAGACGAGAAGTCGATCATCACCTATG

TGGCCACGTACTACCACTACTTCAGCAAGATGAAGGCGCTCGCCGTGGAGGGCAAAAGAA

TCGGCAAGGTGCTGGACTATGCCATCGAGGCAGACCAGCTGATCGAGAAGTATGAGACGT

TGGCGTCGGAGCTGCTGCAGTGGATCGAACAGACCATCGAGACGCTCAACGACCGGCAGC

TCGCCAACTCACTGAGCGGCGTGCAGAACCAGCTGCAGGCTTTCAACACCTATCGCACAG

TGGAGAAACCACCCAAGTTTACAGAGAAAGGCAATTTGGAGGTTCTTCTCTTCACCATCC

AGAGCAAAATGAGAGCCAACAACCAGAAAGTCTACATGCCTCGAGAGGGAAAGCTCATTT

CCGACATAAACAAGGCGTGGGAGCGTCTGGAGAAGGCGGAGCATGAACGTGAACTGGCTC

TGAGAAATGAGTTGATTCGTCAAGAGAAGCTAGAGATGCTGGCAGCGCGATTTGACCGCA

AAGCTGCCATGAGGGAGACGTGGCTCAGTGAGAACCAGCGTCTCGTCTCGCAGGACAACT

TCGGTTTTGATCTCAGCGCCGTGGAGGCTGCGACACGCAAGCATGAGGCCATCGAGACGG

ACATTGTCGCGTATGGAGAGCGTGTGGCTGCTGTAGAAGCCGTTGCCCGTGAGCTTGATG

CGGAGCGATACCATGACGTCCGGCGCATCCTGGCACGCCGGGATAACGTGCTACGCCTCT

GGGAGTACCTAAAGGAGCTGCTTGCAGCCAGGAGGGAAAGACTTGTGGCACACCGCGACC

TACAAAGACTTCTGCAGGAGATGAGCTACATCATGGACTGGATGGAAGACATGAAGGGTC

GTCTCCAGTCCCAGGACAGTGGGAAACATCTTCACGACGTGGAGGACTTACTTCAGAAAC

ACACTCTAGTAGAGGCGGACATATCGGCGCAGGCAGAGAGGATCCGAGCGGTGCAGGCAG

CAGCCAATCGCTTTACCTCGGAAGAAATAACCTATAAGCCCTGCGAGCCAGCGCTGGTGG

AGGAGAAGGTGGCGCTTCTGGGCCAGACGTATGAAGAGCTGGGTCAGCTGGTAGTCGAGC

GACGGGCACAGCTTGAGGACTCGCGGCGTCTCTGGCAGCTGCTGTGGGAGCTCGGGGAGG

AGGCAGCGTGGATGCGGGAACAGGAGCAGATCATGGCAACAGAGGAGTGCGGGAAGGATC

TGTCCTCTGCACTGCGTCTCCTTTCTAAACATGAGGCATTTCGAGAAGAGATGGCAGCAC

GCTATGGCCCGCTGGGAAACAGCATCGCTGCTGGGGAGCAACTGGTGAAGGAGGGGCACT

GCGGAGCACCCGAGATTACAGAGAGGATCAAAGAGATCAAAGCTCAGTGGGGCAGACTAG

AGGAGGCATCTCGTTTGCGAGAGGAGCGTCTAAAGGAGTCTGTGACTTTCCACCAGTTCC

AGACTGACGCCTGCGATATGGAGGCCTGGCTTCAGGAAGCTCTTAGACAGGTGTCCAGTC

AGGAGGTCGGCCATGATGAGTATTCTACACAGACGCTGGCACGGAAACACAAAGAGGTAG

AGGAGGAGATCCAGAGCCACCGCTCTCTCATAGACTCGCTGCATGAGCAGGCTCAATCAC

TGCCCGAAGAATACGCCCAGTCTCCACAGGTGGAGGGTCGTCTTCCTGCTATAGAGCAGC

GCTATGAAGAGTTGAAAGCTCTGTCCTCTGCACGACGCGACGCCCTGGAGGGCGCACTAG

CCCTTTACCGGATGTACAGTGAAGCCGACGCCTGCAAGCTGTGGATTGGAGAAAAGGAGC

AGTGGCTCCTCACTATGGAAATCCCTTCCAAACTCGAGGACCTGGAGGTGGTCCAGCAGA

GGTTCGAGACACTGGAGCCGGAAATGAATAATATTGGCTCTCGCGTCTCTGACGTCAACC

AGGTCGCGGAACAGCTGCTTAAATCCGACAACTGCAACAAGGAGCAAATTCACCAGACTC

AAGACCAGCTCAATGACCGGTGGCATGAGTTCCAGCGGCTTGCTAATGAGAGGAAACAGG

CTATGGAATCAGCTCTAAATCTCCAGAACTACCATCTAGAGTGCAACGAGCTGCAGAGCT

GGATGAAGGAAAAAACCAAAGTGATCGAGTCTACACAAGGGCTCGGCAATGATCTGGCTG

GGGTCATGGCACTGCAGCGCAAGCTCACGGGCATGGAGCGAGACCTAGAGGCCATACAGG

GGAAGCTGGGCGGCCAGCGTGCCGAGGCCGAAAAGTTGGCGTCCGAGCATCCGGAGCAGG

CTGAGGAGATCCAGGCACGGTTAACTGACATGGAGGATGTGTGGGAGGAGCTGTGTGGCA

CCATGAAGAAGCGTGAGGAGAGTCTGGGCGAGGCCTCCAAACTCCAGGGCTTCCTGCGTC

ACCTCGACGACTTCCAAGCCTGGCTCTCGCGCACGCAGACCGCCGTGGCTTCTGAGGACA

CACCCACCTCACTTGCTGAGGCGGAGCAACTCCTGGCTCAGCATGAAGCCATCAAAAATG

AGGTGGACAACTATCGCGACGACTACGAAAGGATCAAGGCCACCGGTGCCGAGGTGACCC

AGGGCCAAACGGATGCCCAGTACATGTTCCTGGCTCAGCGGCTGCAGGCACTGGACACGG

GCTGGCAAGATTTGCGCAGGATGTGGGAGAGCAGGCAGTGCCTCCTGGCCCAGGCATTCG

ACTTCCAGACGTTCCTTAGAGACGCCAAGCAGGCCGAGAGCTTCCTCAACAGCCAGGAGT

ATGTTCTCTCGCACACCGAGATGCCCTCGAGCCTGGAGGGGGCGCTGGAGGCTATCAAAA

AGCACGAGGACTTCCTCACCACCATGGAGGCCAGTGAGGAGAAGATCAACGGTGTGGTGG

AGTCAGGGCGACGCCTTGTTTCTGACGGCAACGCCAACGCCGACAAGATCCAGGAGAAAG

CCGACTCCATTGAGGAGCGGCACCAAAAGAACAAGCAGGCTGCTAATGAGTTGCTGGGTA

AACTGAAGGATAACAGAGAGTTGCAGCACTTCCTCCAGGATGGACAGGAGCTGAGTCTGT

GGATTAATGAGAAGATGTTGACAGCTCAGGACATGACATACGACGAGGCCAGAAACTTGC

ACAGTAAGTGGCAGAAACACCAAGCTTTCATGGCCGAGCTGGCCTCCAACAAGGACTGGC

TGGACAAAATCGCTAAGGAGGGTCAGGCTCTGGTGAAGGAGAAACCCGAGCTGGAGGAGA

CAGTGTCCGAGACCCTGCGTGAGCTGCAGAAGCAGTGGGATTTGTTGGAGACCACCACAC

AGACCAAAGCGCAGTGTCTGTTTGACGCCAACCGCGCTGAGCTTTTCACCCAGAGCTGCT

CGGCTCTCGACACATGGCTGCAGAACCTCTCCTCACAGCTGCAGAGCGACGACTTCGGCA

AGGACCTGACCAGCGTCAACATCCTGCTCAAGAAACACCAGATGCTGGAGCATCAGATGG

AAGTGCGAGAGAAGGAGGTGCAGTCTCTGCAGTCACAGGCTCTGGCCCTGTCTCAGGAGG

ACGCCAGGATCGTCGAGGTAGACGGTCAGCAGAGGAGAGTGATAGACACCTTCGCTCAGC

TCCAGGATCCTCTTAGAGACAGACTACAACACTTACTAGCATCGAAAGAGGCCCACCAGT

TCAACAGAGACCTGGAGGATGAGATACTGTGGGTAAAGGAAAGAATGCCACTGGCTACAT

CGGTAGATCACGGCAAAGACCTGCCCAGTGTTCAGCTGCTCATAAAAAAGAACCAGACGC

TTCAAAAAGAAATCCAGGGCCACCAGCCTCGCATCGATGACCTGCAAGCCCACGGGCGCA

ACATGGCCCCCGGGGGGGGGGCAGAGATGAAGGGGCAGCGGGACGAGCCTCTGAACGAGC

TTGGGGATCTGTGGACGTGGCTCATCGCAGAGACGGAGCAGAGACACAAACGCCTAGTTG

AGGCCCATCGCTCTCAGCAGTTCTATGCAGACGCGGCCGAGGCTGAGGCCTGGATGGGAG

AGCAGGAGCTGCACATGATGTCTGAAGAGAAGGCGAAGGACGAGCAGAGCGCTTTGGTGA

TGGTAAAGAAGCACCAGATCCTAGAGCAGGCGTTGGAGGATTATGCTCAGACCATCCACC

AGCTGGCCAATAGCAGCAGACTTATGGTGGACAGTGAACATCCTGAGAGTGAGAGGATCG

CGCTGAGGCAGGCTCAGGTGGATAAGCTGTACGCCGGGCTGAAGGACCTGGCTGAGGAGA

GGCGGGGCAAACTCCAGGAGCGACTGCGTCTCACACAGCTTAAGCGAGAGGTGGACGATC

TGGAGCAGTGGATTGCTGAGAGGGAAGTGGTGGCAGGATCTCACGAGCTGGGCCAGGACT

ACGAGCACGTCACTATGCTCCGGGATCGTTTCCGGGAGTTTGCACGGGACACGAGCGCGA

TTGGACAGGAGCGCGTGGACACTGTGAACGCCCAGGCGGACGCTCTGATTGAGTCAGGAC

ACCCGGAGAACGCCAGCGTGGCCGAGTGGAAGGACGGCCTGAACGAGGCTTGGGCCGATC

TGCTGGAGCTCATGGACACGCGCACGCAGATGCTAGCCGCTTCGTACGAGCTGCAGCGCT

TCCAACAGGACGCTCGCGAGGCGCTAGCACGCATCCGCAACAAAAGGGAGGCGCTAAACT

CGGCTGAGCTGGGCCGCGACCTCAACACCGTGCAGCACCTCCACCGGCAGCACACAACCT

ACGAGCATGACGTGCACGCTCTCAGTGGACAGGTGACACAGGTGCAGGACGACGCGGCAC

GTCTCCAGAAAGCTTATGCAGGAGAAAAGGCGGACGACATCCATCGGCACGAGCGCGCAG

TCACCGAGGCGTGGGAGGAGCTACTGTCTGCCACTCAAGCCAGGCGGCTGCTGCTATTGG

ACACGGTGGAGAAGTTCCGGTTCTTCAACATGGTCCGAGACCTGATGCTATGGATGGATG

GCATTAATCTACAAATCCAGTCTCACGACAGCCCTAGGGACGTGTCATCAGCGGGTCTTG

TCATTGCAAATCATCAAGACATTAAGGCTGAGATCGAGGCCCGATCCGGCAGCTTCACCG

CCTGCACTGACATGGGACATGCCCTCATCAACAACAACCATTACGCCTCAGACGAGATCC

GGGAAAAACTGGAGCAGCTTCAAGAGAAAAGAGACGAAATCAACCAGAAGTGGCAGGACA

AAATGGACCACTTGCAGATTGTCCTGGAGGTGCTGCAGTTCGGCAGGGACGCCTCCGTAG

CTGAGTCATGGTTGGTCAGCCAGGAGCCGTTGGTGCGGGCGGCCGAACTTGGCTCCAATG

TGGACGAGGTGGAGAGCCTCATCAAACGACACGAAGCCTTCGAGAAACTGGCTGCTGGTT

GGGAGGAACGTTTCACTTTGCTCGAGAAGCTCACTACGCTCGAAGAACAGGAATTTCAGA

GAAGGAAGGAGGAAGAAGAAAGAGCGCGTAGACCCCCTACGCCTCCCCCCGTAGAGGAAG

TGGTACCGTCTGAGGTTGAATACGAGTCTGGAGCGAGGACGAGTTTGGATCAGACCACAC

TGAATCAGTCGGTGTCTGTAAATGGGGTCCACAGTGACCAGGACACATCACAGGGCTCGG

AGGCTGAGTCAGTGAACGGTCCCGGGCAGGACAGCGGCTTGGACACGGCGTCGCGCCACG

AACCGTCAGGAACACTGCCTAGCCGAATAGAGGCGTCTGCCGAGGCCATGGAGGGAACTC

TGTGCAGAAAACAGGAAATGGAAAGTCAGGGCAAGAAAGCGGCCAACAGATCGTGGCAGA

ACGTCTACTGTGTGTTGAGGAAGGGAAGTCTGGGGTTCTATAAGGACAACAAGAGCGCGT

CGAACGGCATCCCCTACCATGGAGAAGTTCCCATCAGCCTCGGCGAGGCCACCTGCGAGG

TCGCCCATGACTACAAGAAGAGGAAACATGTCTTCAAACTGAAGCTTGGAGACGGAAAAG

AGTTCCTGTTTCAAGCCAAGGACGAGGCTGAGATGAGCTCCTGGATTCGGGCAATCCACT

CCTCTATGCCGCCTAACAACATCGACGACCTTTCCCCCGCTCCGCCTCTGACCCGGGCCA

TGACTATGCCCCCTATGTCCCCCAGCGAGGGCGAAGGTGTGACCATGCGCAACAAAGAGG

GCAAGGAGAAAGATCGAGAGAAACGCTTCAGCTTCTTCGGCAAGAAAAAGTAACCAGGAA

AAGAAGGAGGGACGTTCTGTCAAAAGCGCAGGAACTGTCCTAGAGAAAGACGGCGCCCTC

TACTGTCTCCTAACTCCACACCTTTATTTTCCCTGTCTCGCTGTCTCGCGCTGTCTTGCT

CACTGCCTTCCGCCTTGACCGTAGCCCCGCCTCCCACAGGACGCTGGCTCAACTGCAATT

GGACGGTCGGTCAGACGCCTTTATTTTAACACTTTGTCCTTGAGTTTTAAGGCCCGACAG

CGTTTCCCATAATGCAACGCTTCTAAATCTTTCTTCATTTATTCTTTCCATTTATTCTGT

TTGACGTTGTGAAGAAGCAAATTGTTTTTTTCTTCCTAGGAGTACGAATCTTTACGTTCT

TAGTTAATATATCTGATTGGTTTTATTTAACTTTTTGCCATTTGCAATGATTAATATTTT

AAAAAGAGAAAAAAAAAAGTTTTAACAGGGGATAAAAAGAAAAAAAATGAGAGCCAGAAG

GAGAGAGAATGAGAGCGTTTTCCACACAGAACCCAACTACATCTAGAACCTGAAGCTCCA

GAATGAAACCCAGGGTTCTCGATTCTGGTTTTTCTTTTTCCCCTTCTTTTATTTTAAAGT

TTGCAGAGAATATTTTAATAACCGAATACTAAAAAAAACATTCTGAAAACTTTCCATCCT

TTTTCTTTCCCAAAGCTGAATTTAAAAAAAAAAATGTTTTTATTTGTGTTTAATGTTTTG

ACCTTAACCTTATTTCCCTTCTTTAAATCAGATCACAAACTCACACTAAAGTCTTGTGGG

ATGGCGCGCTATGTTTTTTTTTTTTTTGTTGTTTTTTGCCAAATTGCTGAGTCATTTACA

GAAAAATAGGGCGGGGCTAAAATTAGCTATACGATTTTCGGCATGTATATGCCTTTCACA

TGCAGTGTGTGTGTGTGTGTGTGTGT

>Unigene000041

GAGAGAGAGAGAGAGAGAGAGGAGCAGAAAGACAGAAAAGCGATAGATAGCTGAAAGGCT

TTTGATGGGGATTCGACTAGCTGGTGGTGAGAAGAAGAAAGCAGCAGCAGGACTGAGAGG

ACAGTGGACCATTCTGACGACGGTATAAGAGCACACACCTTAACCCCCACCCTGGCCACA

GAGACTGTTTCCGTGGTGGGTTCAGTGGATGGCCCCAGCAGAAGTGGCCAGATGGAAATT

GACCATGGATGTCTGGAGAAAGGCATGATAAACCAGGAGCTGTCCAATGGGCTACGTCAG

CATGGAGAGGAAACAAATGGTCTTGGAGGTGCAACTTTGGCAAGGCTTAGCCATCAGCAG

AAGAAGACCTTAGCATGCCTTCCCCAGACCCAGGGATGGGGGCCTGGAAATCTAAAAGGA

TCCTCTACCCACCAGCAACAGGAAGCAGGCTGGAGTGACCAGAGCACAACTAGCTCGGGA

AACAGTGTAGACATTGAGAATGCTAGAACTCTGGTGGCCTTTTCAGCTAGTGCAGGTTCC

CTACCTCCTTGCAGTGTCCCTCAACCTACTCATAACCCTAATACACATCTGTATGAGAAA

TTCAACCAGGAAATGTCAAATCGAGGGGATAGCGCTAAGCAAAAAACATGGAGTAGAGAT

GATCAGCATTGCACTTCAGAAGATCTAAACACCCTCCAGATGGCTTTAAATCAAGCCCGT

CATGGTCACAAGCCACCAAATTGTGACTGTGATGGACCTGACTGTCCTGATTACCTTGAG

TGGCTGGAAAAGAAGATCAAAATGGCAGGCAATAGCCACGAAAAGGGTTTTTGCAAGTTA

TCAGGTGCTTCTCAAAAAGAGCAACAACAATATCAGTCTGAGCCACAACCTCAACCCAAT

AGTGCAAACACACCGCAGTCACAAAACCCTGCTAACCAACCCCCTGCCCCAAGACCTTAT

GGCCCATTACCACCTATTCCCTGTTCTCCCAGTGTGCTATCCATTGCTAAGGAGAGAAAT

GTCAGTTTGCAGACAGCTATTGCCATTGAGGCCCTGACCCAACTATCAGGCACAGGTGGA

GACTCAACATTTGGAAACACCAATTACCAAAATTTACCTCAGCCCCTCTCTCAAACCCAA

GATGGGATGCGATTCACTGCTGCATCACCTGCATCCCTTTCATCTTCACCTGCTTCCACT

ATCAACCAACCTTCTACATCTGGACATTTTCCTCAAAAGAATTCTGCTTCATGGGAGCAA

CACAGGACTGAGTCACCAGATCCTGCACATCAATCCACCCTATACAAGCCCCCTAACTCC

CCCTTTTCACACTCATCCACCACAAGTCCATCACCCCACCAGTGGCAACAAAGCACTGCA

GCAAGCTGTGAGAAAAGCTCTCCTAGAAATCCATGGATATTAAAATCTGAAAATCAGGCC

TGTTTTCCACAACAGTCCCATGGCAGCAGTGACCCAATGTCTGAACTTAAACAACTCCTG

GGAGACAGCAGTGGAAAGTACACAAGCACTACCTTTAAATTTCCTGCCCCACCTCAAAAC

CTAAAAGACAACCAAGTGATGCCTCATGTTAAACCGGAGGTCAACAGTGGGGACTTTCCA

TGTGATATGGCTGCAGCCATGGGCCAGTATAGGATAAATAATCAGCAGCAGTTTCAAGGA

CAGCAGATTTCTCAGTCTATTAGATACTCAACTCAGGCAGCACTGCAACAGCACCTTCAT

CATAAACGTAACCTCTTTACTGGTCCTCAAACCTTCGATCTAGGAGCGCCAATGACATGC

CAGACTCTCCGGAAATGGTGGCCACAGTCTACACCAGAGAGCGCTCTGCCCATCAAACAG

GAACCCAGGGAACCCAAGAAGAAAAAGAACACACAGAGTTCTCCTCTTCTCAAGCAGCAT

GTTGGAGGACTTCTTGGATCATTGGGTCCACCGCTCCCTAAGCCTAAACAAATCATAATT

AAAAAGACCAAGCAAAAGGCTTCTCAGCCGCTTTTTCTCCCACAGATTCAGATCACAATA

CAGAAATCAAGCATGTCAACAAGTATACCATCCCTTACCCAACCTACACCATCTCTACCA

GTCATGGAGGCTGCACTTCCCCCTTGTCTACCTTCATATAACTCCTCTCAGGTGGTAGTG

AGCCAAGCAGCTCCGGTCCAATCTCAGGAATCATTTTTAAACACCGGTAGTACCCCTATC

TCTGGAAACACCCCCCTGGATGCCAGCACACTTAATGTCCCTATGCCCCCTGCCCAGGAT

AAACTTACTAGCTCAGACCCCACCATAGTAAATGAAGGTTCCTTGGCCACCACCACTTCC

ACAAGTCAGACAACTACATCTCACTCCACTCCACCGCTTTGTGGCCTAAATTCTATGGAT

CCTAAATTTGAAGAGCTGATTCGTCAGTTTGAAGAAGAATTCGGGGACACTAGCCCAGTC

ACAGATACAACTGAGCAGGGCTTTTCTGATGTGGTAACCAAAACACAATTAAATCCTAAC

TTGGGACAAGGTAGGCCCCGCTCACCAGAAAAACTCAACAACCTTCCTGTCACAACAAAT

GAGCCTGAACCCAACCTGAAGCAAGCTGAGGGAGGAAATGAGAAATTCATGTCCTCAAAC

CAACTTTCTGCCATTAAACAAGAGGTCGAAGAGTATGAAGTAGATGTTAAGCCATCAGAT

CAGCTATTGGCTCTGGCAACGGAGGCTTATCTGCAGCAGCAACATCACAAGATTGTTGTT

AACCCCTTCACCACAACTTGCTCTCCCCCAAACAAAAGGATGAAGATTGAATCTTCTGGT

GGTGTAACTGTGCTATCCACCACTGCCAGTCTCTCAACTGCAGGGGAGAATCTAGATACT

CCTACAAAAGAGTGTTATCCCTCATCCCCTTCATTAAAGGGTTTTCTGGAATCTCCATTA

CACTACTTAGACACCCCAACCAAGAATCTTTTGGACACACCTACAAAGAATGCTCAGACA

GAGTTCCCTACCTGCGACTGTGTGGAGCAAATCATTGAGAAAGATGAAGGCCCATACTAC

AATCATTTGGGATCAGGGCCAACCGTGGCTTCCATAAGAGAGCTCATGGAAAACAGGTAT

GGTGAGAAGGGTGACTCTATCCGTATAGAGAAGGTGGTGTACACGGGAAGGGAAGGCAAG

AGCTCTCAGGGATGCCCTATTGCTAAATGGGTGATTCGACGTGGCAGCGAGAAAGAGAAG

TTGCTTTGCCTGGTGCGTAAAAGGGCTGGGCACCACTGTGCCAACGCTGTCATTATTGTA

GTCATCATAGCGTGGGAGGGCATCCCAAAGGCTCTTGGTGATAAACTTTATCGAGAGATC

ACTGACACACTCATTAAATACGGCAATCCCACCAGTCGCCGTTGTGGCCTCAATGAAGAC

CGTACTTGTGCGTGCCAAGGAAAAGACCCCGAGACCTGTGGTGCTTCGTTCTCTTTCGGG

TGTTCTTGGAGCATGTATTTTAACGGGTGTAAATATGCCCGCAGCAAGACCCCTCGTAAA

TTCAGACTGCAAGGAGAACACCCCAAAGAGGAAGAAATTCTCAGGGATAACTTCCAGGAA

CTGGCCACTAAAGTGGCACCTGTATATAGGAAGCTGGCCCCTCAGGCATACCAAAACCAG

TGTGCAACTGAAAAAATGGCACCGGATTGTCGACTGGGACTCCAAGAAGGACGACCCTTT

TCTGGAATCACGGCATGCATGGACTTCTGTGCCCATGCTCACAAAGACCAACATAACCTC

CAGAACGGCTGCACTGTGGTTTGCACTCTTACAAAAGAGGACAATCGGGCAGTGGGGGTA

ATTCCAGAAGATGAGCAGCTTCATGTGCTTCCGCTTTATAAGATATCTTTGACCGACGAG

TTTGGCAGTGAAGAGAACCAGCGGCTAAAGATGCAAACCGGTGCAATTCAGGTGCTCAGC

AAGTTCCGCCGGGAGGTCCGGAAACTTCCAGAGCCTGCCAAGTCCTGCCGGCAACGGCGG

CTTGAGGCAAAGAAAGCTGCGTCTGAGAAAAAGAACAAGAAGCAGCAGCTGTCTGAAACA

CCCGAGAAGACGAACAAGAAAGAAGTTTCTTTGCATGGCAATAAAGCAATTCTAAAACAA

GAGGTAAAGCCCAACATCAAAAAAGAACTGGTCGACCATTTTCAGGCTATCAATGGAGCT

TTGGATGGCTTTCCACCTACTGGAAACACCAAAATGTGTCCAGATCCTTACAGTATGAAA

GATGCCTACTCTTCCTGTCCTGCTCCCTATGCAAGAGGTAGTCTGCCTTCAATCAGCCAG

TCTTCTACACATAGTCCCATCAATGGCTTCCATCCAAACATCCAAGGGATGCCCTACAAT

TACTACAACCAAGCTAAAAAAGGACTCATTCCCCCTGAAGTCTTAGGTTGTGTAAATCAT

AATGGGACTAGTCACAAGGCACAAGTAGAAGAACAGAAACCCAGTATCCAAAGTCTTCAG

GCCAGGCTGGTACCAAACCATTCTGATCATCACCACCAACAAGGGGTCAACATGGTCAAC

CATAACTACCCACACCAGTCTGAGTTATTAGAGTCTACAGCACCACTAAAACGTGCTCCA

TCTGCACCTCCAGATCAGATGCACTGTGTCACACCTAACATCAAGCAGGAGCCCATGGAG

GTACCTCTATATGACAGAAGAACTGATAGCCAATTCAGAAGTTGCCCAGTAACTCCCAGT

GTCACCCCACAACCCGACACCTGGCCGGTGCACACACCCAATGGCAGCTTAGCACCCAAG

GGCTGGGATGGAAATTTTAGACCAGGCCAAGTTCATTCACCCTTCACTCCAGAGAAGCAG

CGGCTTCACAAGCAGCTTCACAACCAACTTCAGCACTCTATATATGTCCAACAGCAGCAA

CAATGGCGCTCACACCCTGGTACCCCAGTAGCCTCACCTAGTCCAGCTCCAGCCTTTAAT

ACAGGGCTGTCTCCAGCTCCTTCCCCTCACCCAGTTACCCCACAACACTGGGGTAGCCCT

GCCCCCAGTCCACAGCCCAAAACTTGGGGTCAACCCGGGTCCTTGGGTTATGGGCATGAT

GGACTTGGACAGGGAACACCTATTGGGGCTTTTCCAGACAAGATGCTGGCTGAGGCAGCA

GAGATTCGAGGCTCCACTCCCCTCGGCCTCCAGGAGAAGGCATGGAAATCTGGAGGTGCC

TCTGCAGCAGGAAGCAATCCATCCCCTGCTCCAGAAGGTCGTCTGTTTCCTGACACACTG

CAGAAGGTGAATGGACAGGCATGCTGGGATAGTGAGGTGGAAAGTGAAAGAGAGCCCGAA

GAAGAGGAAGTGTGGTCAGACAGTGAACACAACTTCCTGGATCCCAACATTGGAGGAGTA

GCAGTAGCTCCTGCACATGGGTCTATTCTAATTGAATGTGCCAGACGGGAACTTCATGCT

ACCACACCCTTGAAAAAACCAGATCGCAGTCACCCGACCCGCATCTCTCTGGTATTCTAC

CAACATAAAAATTTAAATCAGCCCTGTCATGGACTGGCACTGTGGGAAGCCAAAATGAAG

CTGTTGGCTGAAAGAGCAAGGCAAAGGCAACAGGAGGCAGCTCTTCTAGGCCTTTCACAG

GAGGACATGAAAGCTTATACAAAGAAACGCAAGTGGGCTGATGGACTAGTAAGTTCCGCC

TCTGAGCCAATGAAGGACAAGCGGGAAGTGGTGACACGGCTGGCCCCCACACAGCACACC

ACCAACATGGTCACAGTGTCACCCTATGCCTTTACTCGAGTCACAGGGCCATACAGTTGC

TTTATGTGACCTCTGTAATAATGGCAGCAGGAAACAGAATTTTAGGATGAAGAAAAAACA

GCATCTCTTCCAATTCTGTATCCCCCTCAGACCAACCAGAGTATTGGTGGAAAATGAGAC

ACTTTTTTATTATTAATATTATTATTGATTTTTTTTTACCTCATATTTTGGTGGCAGTTT

GGGAGTCCCCCAAATGAACATGCTGCCTATGGTGCTCTTGTTCTCTCTAAAATGGGAAGG

GATTTCATATTTTGCCCTCCCTATGTGAATTTAATGATTCTAATATTTCTATTATCAATA

TTATGATTGCTATTGTTACCGTTGATGTTATTCCAGGTATTGCTATATTATGAAGATGAC

TATTTTTATTTTAATTGTATTGGTTTTCCCTCAATGTCATTATTATTATTATTATTTTAA

GCCCCCACCCTCTTGATTAGACACAAGCTCATCATCGTCACCCTTCCTTGTTTGATATGA

ATGTGTAAACAAGAAATGAATTTAGCTTTTCTGGCCTTTTAACCCACAACCTCTTCATAA

AACAGCATCTTCACTGTAAGCCAGGTGTCTACCACATATGCAAGACACTTTGGAGAAGGA

CAATGTTTCTAAGTCGGCAGGTTTCATTCTATGGTGACTTTGAATAAGTGACAAATGCTA

TGGCACCAATGCTGGCTTTTCTGGCAAATGCCACTTAACTTGTCCATGGGTGCTTTTTTC

CATTAGTGCTGTCATTGCCCACATCAAGACAGCTGCCACTGGACCAAGGTGCTCTTCAAC

AGCAAAAAAAAAAAGTCAAGTTAAGCAACCAGCTGATCTGATTAGATGTCATTGCCTAAA

ACATCATCAACCAGAAGGTGCTGACTAATTTCTTTATGGTGCAGAATCTAGTTTTTATCC

ATGAGACAGCAGTTAGTCAATTGTCTCTGAGGTGCTAATGCAGTTTGGCCCACCATAACC

TAGGGTGGACCCTGTGCTCTGTAGCACAAATTAATTGACTGCTCATTAATTAACTTGCAA

ACTGCTGAAGTTGTAGAATTCTCTACAAGTGAGGTAGTTAGCTGACAGGCTACAGCTTCA

GTGAAAGAAAAAACATGAAAGGGGATTGGTGCTACTCACTAGTCTTTCCATGCCTCTTTG

CCTGCTATTGCCCGGACAGCTAGTCTCCCCCTAACACATGGAATTTAGGTGCTTCTGTTT

GACACTCTTGTTCATGTGTAAAAACTGGGGGTCCTTAATAGCTACAGTTGGTTTACAAAA

GGCAGTTGGCTTCTTGGTGCTTACAGATTACAGATAGTCTGGTGCTTTTAAAGCTGTCAG

TTAGCTCTTTACCTCATGTTAAGACGTTGCACCCAATCACAACATGCTACTAATACATGG

GCTCTAAAGACTGTATGTCTTTAGCCTACATTCACATACCTATCTACTTTTTTTTAATTA

TTATTTATTACATTATGATGATTATTGTTATTGTGCTTGCTGTAATTTTGTTTCATTTTA

AACAAATTGTAAATAGAAAAAAAAAGATTTTATAAAAGCACTTTTAATCTTGATTTTAAG

GAAGAAAAATGATAATGGAATAATGACAACTTGAATCCTAATTTTTTTAATGGAATTTTA

TTGGGAGAAATAGAAATGTAAATAATTAAATATTTAGGTAAGATTATTTAAAACTGGTAA

GAATTAAATCATGAATGATTCCTTTAATTTTTAGTTGTCAGCATGTTACGCCCTCGATAC

TAAAAGAAGAGTGCTGGTCCTGGTCAGTACTCTTTCCCTGACACTTATGAACTTATGTGT

ATCCTCTGTACTTAGTGTACTGCTGTTCTTTTTAAAAAGTACTGTACTTAGACTGATACC

TCTGAACAAGCATCTATAGCAGCTTCCTTTTTCAACACATGATCCAGACAGACTTGAGAA

CAAAACCTACAATACCACAAAATTAGAGGATTCACAGGGTAATAATCTTGATATGATATT

ATAATCCTGTACAAGAAAATCCTGTATAATTCTTTGGTGACAGATTAAAAAACAAATCGA

ATGGATTTCCTACTTAAATGCGCTTGTTCAAGTGAAAGACTGTGCATGTGTCTTGCACTG

TAACAAATTTTCAATAGGAAAGTGACTTTACACCTTAGCAATCTTCTTAAGCTGCAAACT

CTTTTTAGATTGTTGTGTTTGTTTTTTTTGTTTCCTCGAAGCCACTTGATTTCATATCTG

ACAAAAGTAATTTTTTAACCTGTAGAGGTCACTGTAGAAATTAAGAGCAAAGCAAAAGCT

ACGTCGCTCTCGAAATCTTTACATGAGCCATTGTCTTGGGTTTGAGCGTTTTAGAACCAT

CTCTGGTGCTGAATGTGTTTTCATATTTTCCTTACCTGATTATGTTCATGATTTTAAAAG

CACTCAGATGTTTTGGCAGTTCTCTCTCTATCTCATTTTATCCCTAATCCCAAACTAAAT

ATGGTACTTGAAGCAGATATTAAGAAATCACTGATATACAGGTAAAAAACTTGTATCCTA

TGAAGATGAACTTATCATTCTGTTGATTATACCCAAACATTCCTTTCTTTCTGTCTCACA

CACACACACACTTAAGCTCAAATGCTTATTGTCCCAATCTCTTGGCACTCGGCATCAAAA

TCATGATCATGCCCAGTTTTACTTTTTTAATAATGACAACAAAAAAAAAAAAACCACATA

CCTGGCA

>Unigene000042

TTGTTGAGATCGGAAGAGCGGACTGGAGAGGAGGAGGTGAGGGATGAGAAAGCCAAGCTG

AACCGCAGGCTGGATGGCCTCTCTGAGAGATTACATGGACGCATGGCTCAGCTGGAGGAG

CTGAACAGCAGACTGAAAGAGTTTGAAGATGGTAGACTAGCAGTAGAGAGAAGACTTGAG

GCAGCAAGGCACCAGTTAGAGGTGCAAGAGGCGTTGGGCCCTCAGGCATGCAGTAACAAG

AGTCTGGAGCGCCTGAGAAGCCAGCAGGACACACTCGGCTCCTTACAGCCACAAGTTGTA

TATCTGCACAATCTGGCCAAGGGGCTGGCACAGGACGCGCCACAGATGACAGGGGGCAGT

GGAGATGGGGGACAGAAGCTGTTGCAACAGGCTCAGACTACAGAGAAGGAGTTTGGAGAA

GTAACTGAAAAGATCCAACAATGCTGCTCGTCTCTCGAGTCACGGCTGCAGGGTGTAGGA

GAAGTCCAATCACACGTCCGTGATATTTTCTCTCGGCTGGCGGACCTGGACGACGAGCTG

GACAGCCTGAGCCCTGTAGGACGCGATGTGGACTCCCTTGCCTCACAGGCAGATGCAATA

AGGGGATTCCTGGGGCGTCTGGCCTCTCTGCGGGCTGAACTTGAAGGCCATGGCAGTGCC

TGCACCACCATGCTGAAGCGAGAAGGTAGCTCCCCTGATCTCCTGGCTATTAGAAGGGAG

ACGGAAGCTCTAAATCGGCAGGCAGCCAAGCTGGCCGAGCGGGGTCAGGGTCGTCTAGCA

CTCATAGAAGAAGCCGAGGGGCGAGTAAAGGAGTTCTATGCCCGCCTCATGGAGCTACAG

AGGCTACTTGACCAGGCAGAGGAAGGGCTGAACGTACAAAGTGTTGTGGGAACCGAGGTG

GATGTCATCAAACGACAGCTCCAGGAATTTAAGGCTGTGGAGCGAGAGCAGGTAGACAGC

ATCCAGCCTAAACTGCATCACGTGAACGCTGTTGGCCAGGGGCTAATCCAAAGTGCAGCT

AAACATACCGACACGCAGGCCCTGGAGCATGACCTGGAAACCACCAACCTGCAGTGGAAC

TCCCTCAACAAAAGGGTGGCCGAGAGAATTGCTCAGCTCCAGGAAGCCCTTTTGCACTGC

GGGAAGTTTCAAGACGCTCTGGAACCTCTTTTAAGCTGGTTGAGTGACACAGAGGAGCTG

ATCGCTAATCAGAAGCCTCCATCAGCTGAATACCGTGTGGTTAAAGCACAAATCCAGGAA

CAGAAGCTGCTGCAGCGGTTGTTAGATGACCGGCTTGGGACTGTAGAGATGATCCGGGCT

GAAGGTGAGCGCATCGCAGCCACAGCAGAGACGCAAGACCGAGAAAAGATCCAGAGACAG

CTGCAATGTTTGGGAGAACGATGGACAGATCTGCTTGAAAAGGCCAATGCCAGGCAACGT

CAGCTGGAGGAGCTGCAGGTACTTGCGCTGCAGTTCCATGAGTCAGTGGATCCTTTGGGA

GAGTGGCTGAGTGCTACAGAGAGGCGACTAAGCTCTGCTGAACCCATGGGAACTCAGGCT

TCCAAAATTAGCCAGCAAATCAATCGGCACAAGGCCCTCCAAGATGAGGTGTTGGCTCGG

GAAAAAGAGGTGGACCATCTAGAGGCCCTTGGCCAATCTCTCTCCCCTCTCAGCTGTGCG

GCAGACCGTGATTGGCTTAGTGAGCGAGTGGGAGCAGTGCGCAGCGGGCACACAGAGCTC

CGGAATTGGTGCTTTCGGCGAGCAGCTATGTTGGAGCAGGCTCTGGCCAATGCACAGCTG

TTTGGGGAGGAGGAGGTGGAGGTGCTGAACTGGCTGGCTGAGGTGGCCCAGCGCCTGTCT

GACGTCTCAGTGCAGAGCTACCAGCCCGAGCTTTTGGCTGAGCAGCACAAGTACACTCTG

TCTCTGAACGAGGAGATTGTGAGCCGGAAGAAGACAGTAGACCAGGCCATCAAGAATGGA

CAAGCATTGCTGAAACAGACCACTGGTGAAGAAGTGCTGCTTATCCAGGAGAAGCTGGAT

GGAATAAAGAGCCGATATTCCGAGATGACAGCAGGTAGCAGCAAGGCCCTTCGTAACCTG

GAGCAGGCACTGCAGCTCGCTACCCGATTTGCCAGCGCCCACGAGGACCTCAGCCAGTGG

CTTGACTCTATGGAGGCGGAGCTTAATAACATGGAACCAGACACAACTCCAGCCTATCAG

GAGAGACAAAAGGACCTGAAGTGTGTGTCAGCTGAGAAGCGTCTCATTCTGGATACAGTT

AATGAGGTGGGCAGTGCACTGCTGGACCTGGTGCCCTGGCGAGCACGTGAAGGTCTGGAT

CGTCTAGTGGCCGATGCTAACCAACGCTACAGACAAGCCGATGAGACCATTACGCAAAGA

GTGCAGCTAGTGCAGGCAGCCATCCAGAGGTCACAGCAGTATGAAGAGGCCGTGGATGCG

GAACTGGCCTGGGTGGGGGAGACAGAGAGGAAGTTGACCTCCCTGGGGCCTCTAAGTCTG

GAACCTGACGTGACAGTGGCACAGCTCCAAGTCCAGAGAGCCTTCAACATTGACATTATC

CGCCACAAGGACACGGTGGACCAGCTCCTCCACACCCGAGAGGATATCCTGGAGAGCTGC

AGTGACCAGCAAAGAGAAGCACTGAAGGTAAAGACAGATTCATTAAGTATGCGGTATGAG

GCAGTGAGTCAGAACCATGCTGAAAGATTTTCAGCACTGGAACAAGCACAGGTGTTGGTA

GCCCGCTTCTGGGAGACTTACGAGGAGCTTGACCCATGGCTGGGTGAGACAGAGACCCTG

ATATCCCAGCTGCCTCCTCCTGCCATTGACACTGAGGCTCTACGACAACAGCAGGACCAG

ATGAGGATGCTGAGAGAGTCTATAGCTGAGCACAAACCCCACATTGACAAGCTGCTGAAG

ATTGGCCCACAGTTGGCTGAGTTAAGTTCCCAGGAGGGGGCGACACTGAGACAACGCTAC

AGTGAGGCTGAAAGGCGTTACCTCGCCATCAAAGAGGACGTTAAAGGTCGTGCTGCAGTA

CTGGATGAGGCCTTCTCTCAGTCTGCACAGCTGATAGAGTTCCATGATAAGATGGACCCC

CTGTTAGAAACTCTGGAGGGAGCAGTGCAGCGTTTGCGGCAGCCTCCTCCCGTGGCTGCT

GAGGTAGAGAAAATCCGTGAACAGCTGGCGGAGCACCGAGCAGCCGGGCTGGAACTGGAC

AAATTGTTGCCGTCCTTCAGCACTCTTTGTGCCCGTGGGGAGGAGCTCATCACCCGTGCT

GCACATGATGACCCTGCGGCTCAGGCAGTGCGTTCTCGGCTTCTAAGACTCCGTTCCCTG

TGGGATGAGATCAGACAGAGAGCAGAAGAAAGAGAGGGAAAACTGCAGGATGTACTGGAC

TTGGCTGGAAAGTTCTGGGCTGACATGGCAGCTTTGCTTAGCACCCTGCGAGACTCTCAG

GAAATTGTAAAAGAACTGGAAGACCCTGGATTGGATCCATCACTCATCAAACAGCAAATA

GAGGCAGCAGAAGCTATCAAAGCAGAGACAGACGGTCTGCGTGAAGAACTGGAGATCGTC

CGCACACTGGGAGCTGATCTAATTTTTGCTTGTGGTGAAACCGAGAAACCAGAGGTCAAG

AAAACCATCGATGAGATGAATGCTGCGTGGGAAGGCTTGAACCGCACATGGAGAGAGAGA

ATGGAGAAGCTGGAGGAGGCTATGACAGCCTCTGTGCAGTATCAGGATGCACTGCAGGGT

ATGTTTGACTACCTAGACAACGCTGTCATTAAACTGTGTGACATGCAAGCCGTAGGAACT

GACCTCAGCACCGTCAAACAACAGATAGAAGAGCTCAAGCAATTTAAGGTGGAAGTGTAT

CAGCAACAGATAGACATGGAGAAGCTCTGCCACCAGGGGGAGCTACTGCTCAAGAAGGTT

TCTGACCAAGCGGACAGAGACATGATCCAGGAACCTCTCACTGAGCTCAGGCACCTGTGG

GACAATCTGGGAGACAAGATCACTGTCAGACAGCACAAGCTGGAAGGAGCTCTTCTAGCC

CTGGGTCAGTTTCAGCATGCCCTCTCTGAACTGCAGTCCTGGCTAAGCCACACTCATGCT

ACCCTGGACACCCAGAGACCTGTCAACTCTGACCCCAAAGCCATAGAGATCGAGCTGGCC

AAACACCATGTATTGCGCAACGACGTCCTGTCCCATCGAGCCACAGTAGAGACTGTGAAC

AAGGCAGGCTCAGAGCTGCTGGAGTCCAGTCCCGGAGACGAGGCCAGCCACCTGAGAGAC

CAGCTGGATGAGCTGAACCGCAGCTGGGACAGCTTACTTCTGAAAACAGACGAAAGACAG

AAGCTACTAGAGACGGCACTGCAGCAGGCGGAGGGTTTCCATGGTGAGCTGGAGGAGTTC

CTGCAGTGGCTGAGACGCACAGAGAGTCAGCTATCAGCAGCCAAACCCACAGGTGGCCTT

CCAGAGACCGCCAGGGAGCAGTTGCAGCAGCACATGGAGCTGCAGGCTCAGTTGACCCAG

CGCGGTGAACAGTACCACCGACTGCTAGATCAGGGAGAGTCCATGCTGTTGGCTAGAGGG

GCAGAGGAAAACAGCCCTGGAACCACTCAAACTCAGCAGAACCTGGCCCTGCTCCAAAAC

AAGTGGGCCAGTTTGAATGCCAAGATGGATGACAGGAGGGCTAAGCTGGAGGAAGCAGTA

TCACTGGCAACAGGATTCCAGTCGTCACTGCAGGACACCATCAACTGGCTGACGCAGGCA

GAGCAGACACTAAACATGGCTCAGTCACCGAGCCTCATCCTCGACACTGTCCTCTTCCAG

ATAGATGAGCACAAGGTGTTTGTAAATGAGGTGAACACACACAGGGAGCAGGTGCTGGCT

CTAGAGAAGGCCGGGTCTCAGCTGCGCTTTGCCAGCCTTAAACAGGACGTGGTGCTCATC

AAGAACCTTCTGTTGAGCGTGCAAGCTCGCTGGGACAAGCTGGTGCAGAGGTCTCTGGAC

AGGGGACGGCACCTGGACGAGGCCCGCAAAAGAGCCAAACAGTTCCATGAGGCCTGGAGG

AAGCTGACAGACTGGCTGGAGGAGGCTGAGAAACGGCTGGATGCTGAGCTGGAAATCTCC

AATGAGCCAGATAAAATTAAAGTGCAGCTTACTAAGCATAAGGAGTTTCAGAAAACTCTT

GGTTCTAAGCAGCCAGTATATGACACCACAGTGCGCTCTGGGAAGGCCATGAGAGACAAA

GCTACACTGCCAGCCGACACACAGAAACTGGACAACCTCCTCGGGGAGGTCAGAGACAAA

TGGGACACTGTGTGTGGAAAGAGTGTTGAAAGGCAGCACAAGTTGGAGGAGGCCCTGCTG

TTCTCGGGTCAGTTTGCTGAGGCACTGCAGGCTCTTGTGGACTGGTTGTACCGGGTAGAG

CCACAGTTGGCTGAAGACCAGCCTGTCCATGGTGACCTGGACCTTGTGTCTAATCTCATG

GATTCTCATAAGGCCTTTCAAAAAGAGCTGGGGAAAAGGACCAGTAACATACAGGCACTG

AAACGTTCTGCTCGGGAGCTAATGGAGACAGGCAGGGATGACACCGCCTGGGTCAAAGTT

CAGCTGCAAGAGCTCAGCAACCGCTGGGAGACGATCTGCGCACTGTCTGTCTCCAAACAG

ACCCGTCTGCAGCAGGCTCTCAAACAGGCTGAGGAGTTCCGCACAGCAGTGCAGATGTTA

TTGGAGTGGCTCTCAGAAGCAGAGCAAACTCTACGTTTTCGAGGCATCCTGCCAGAGGAG

GTGGAGACCTTGCAGGTTTTATTACACACCCATCGAAATTTTATGCAGACCGTAGAAGAG

AAGCGAGTGGATGTGAACAAGGCAGCTGGCATGGGGGAGGCCATACTGGCAGTGTGCCAT

CCTGACTGTATCACCACCATCAAACACTGGATCACCATCATCAGAGCCCGCTTTGAGGAG

GTTTTAACCTGGGCGAAGCAGCATGAGCAGCGTCTGGAGGCAGCACTTGCCGAACTGCTC

AACAATGCAACACTGCTGGAAGACCTGTTATCATGGCTACAGTGGGCGGAGACCACATTA

GTGCAACGAGACACTGAGCCCCTCCCCCAGGATATCACTCAGCTTAAAACACTGATCACA

GAGCATCAGGTTTTCATGGAGGAAATGACACGTAAACAACCTGACGTCGATAAAGTGACA

AAAACCTACAAGAGAAAACCATCAGAAACGTCTAGCAGTCTTGCTGAGAGACGGGGTGTC

CGTAAGCAGCAGCAACAGCAGCAGCAGCCTGCTATGCAGGTAAGTGGAGGAAACCCTCGT

CTTAACCAGCTCTGCTCACGATGGCAACAAGTTTGGCTACTAGCACTGGACCGCCAACGC

AAGCTCCATGATGGCCTTGACAGGCTTGAAGAGCTGAAAGAATTTGCCAACTTTGACTTT

GATGTGTGGAGGAAGAAGTACATGCGCTGGATGAATCATAAGAAGTCCCGCGTCATGGAC

TTTTTCAGACGCATTGACAAGGACCAGGATGGCAAAATCACCCGGCAGGAATTTATCGAC

GGCATTCTGGCTTCAAAGTTCCCCACCAGCAGGCTGGAGATGACTGCAGTGGCTGATATC

TTTGACCGAGATGGTGACGGTTATATTGATTACTATGAGTTTGTGGCAGCTCTGCATCCT

AATAAAGATGCCTACAAACCCACAACAGATGCAGACAAGATTGAAGACGAGGTCACCAGA

CAAGTCGCCCAGTGCAAGTGTGCTAAGAGATTCCAGGTTGAGCAGATTGGCGAGAACAAG

TACAGGTTCTTCCTTGGGAACCAGTTTGGCGACTCTCAGCAGCTGCGTCTGGTGCGGATT

TTGCGTAGCACAGTTATGGTACGAGTGGGAGGGGGATGGATGGCGCTAGACGAGTTCCTG

GTCAAAAACGACCCTTGCAGAGCACGCGGACGTACCAACCTGGAGCTACGAGAAAAATTC

ATTCTTCCTGAAGGGGTTTCACAAGGTATGGCGGCCTTCCGTTCCCGTGGTCGACGATCT

AAACCTTCATCACGCACAGCCTCGCCAACACGCTCATCCTCCTCAGCCTCGCAGAGTGCC

CAGAGCTGTGCCTCTGGGCCATCTGCCCCTGCCACACCCACTGCTTCTGCTAGGTCCTCC

CAATCTCAATCGCGGGGCTATGCCAAGCCCTGGCTGGCACACAGTAAAACTCCAACTCCA

ACCAAGTGCCAGTCCTGCACAGAGCACAGCCACACCCCTGGGCACGAGGGGGGCTCCTCA

TCCAAGCTTAAAAGGCCACCTTTCCATTCCAGCCGTGGATCCCTGACAGGTGAAAATGGC

GGAACCACACATGCTACTAAACCTGTGCGCTCTGACAATAAGCGCACGCCGTCCTCAACC

AGCGGCCCGACTAGCCGCGCAGGCAGCCGTGCAGGAAGCAGAGCCAGCAGCCGAAGGGGC

AGTGACGCCTCCGATGCATCCGAGCTGATGGAGACGCGCTCTGCCTGCTCTGACACTTCA

GACACACCACGCCGCCCAGGCACTAAACCCTCCAAAATCCCCACCATCTCCAAGAAAACA

CCCAGTCCCAAAACCCCCACCACTAAGAAGTAGCAGGTTTTCAGCATGGACAGATGGAGA

CGGGGGATTTTTTTTCTCTAGTGCATGCCACTCAACCTTCCTGTCCACCAGCACAGGCAT

GTGGGATTGATGAGAATAGAGAACTGAAAAATCTCTCTTTTAAAAACACCTGGGCAGTGC

TGACCCCACATCTGCCCCTGCAACAAACCTAATAGTGCTGCCTTATTTGTGTTACAATAT

GACTGACTTTATAGTTTCTTGAACATGTGACTGACCTGGAAAGCAACTACCACGTACGCA

CATAGACACGTGGACTGGCACCACCACCGGACCTGGGATTTTATCTCAGCCTTGTTGATA

TGCAATGGCCAGGCGCTCTTCAGTGCTGAAGCTGACAGATGGTACAGCCACCATTCCAAT

ACTGACTCTCTTAGGCACAAAGATTTTTTTGTGAAACAAACAGTGGCAATGATTCTAAAG

TTTCATCCACTGTATGCAAAGCTACTTGAGTATTCTGCATCCATGTGGTGTGTGTTTTAA

GTGCTAGTTTCTTTTTTTTCGGTGGACAAACGGAAAACATTCTAAGGAAGTGGTGAGCGA

TTTATCATCTCAGCCTTGCTAGTGAGTCAGAAGCATGGTCATGTGACCGCAGCTTCACTT

ATCTTTCTCAAGAAAGCATGCACCAACACTCACTGATGGCTTCTGATGTGATGTGTGTAT

GTATCGCCAGTCAGCAAATGAAGAAAGTCCTAATTCCTGAGAGCACTTAAGTGACTCGTA

GAGAAGAATTTCACATGTGCACTGACATTTCCTTTCCTTTCAAACCTGATGCTAACATTA

GGCACGCTTACCGCTGTGGTTTAACCATGATTAGTTACACGCATCATGTTACAG

>Unigene000043

GCGTGAAGATCGGAAAATCGACGGCACAGAAAGGGACATCATTGTGAAGAGCGGTATCCA

CACTGTTGAGGGCATAGCGGTGGACTGGATGGGTCAGAACCTGTACTGGACAGATGATGG

ACCAGAGAAAACAATCAGTGTGGCCAAGCTGGAGAAAGCCTCCCAGACACGCAAGACTCT

CCTTGAGGGAAAGATGACTCACCCTCGAGCTATAGTGGTGGACCCGTCACACGGGATGAT

GTACTGGAGTGACTGGCAGGAAGACTCGAGAGATGCCAACCGTGGCAGCATTAGGAAGGC

GTGGATGGATGGCTCCAAATCTGGCATCCTCATCACCTCCAAAAACATGCTCTGGCCCAA

CGGTCTGAGTCTGGACATCGAACAGAACGTGCTGTACTGGGTGGACGCCTACTACGACCG

CATCGAGATGGTGCTTCTTAACACCACCGAGCGCAGGACCGTGTACGAGGGGCAGGAGCT

GAACCACCCTTTTGGGCTTTGTCACTATAAGAACTTCTTGTTCTGGAACGAGTACCGAAT

CGGAGGCATTTACAAGCTGGACATGACGTCCAAGAAGGTGACGCTGCTCAGGAGCGAGCG

GCCGCCCATCTACGAGATCCGCACCTACGACGCCCAGCAGCAGCAAATTCCAGGCCATAA

TCCATGCAGGGGCAATAACGGTGGCTGCAGCAGTCTGTGCTTGCTCACACCGGCAGGTCG

TACCTGCGCTTGTGCAGATGACCAGATTTTGGACTTTGACAATAAGACCTGTAAAGCCAA

TCCGTCCTACGTGCCCCCGCCTCAGTGCCAGCCAGGGGAGTTTGCCTGCAAGAACAACCG

CTGCATCCAGGAACGTTGGAAGTGCGACGGAGACAACGACTGCTTGGACAATAGCGATGA

GGTGCCGGAGCTCTGCCATCAGCACACCTGCCCTGCCGATCGCTTCAAGTGCCAGAACAA

CCGCTGCATTCCCATGCGCTGGCTGTGTGACGGAGACAACGACTGCGGCAGCAACGAGGA

CGAATCCAATACCACCTGCTCAGCTCGTACATGTCCTCCTAACCAGCACCCGTGTGCCAG

TGGACGCTGCATTCCTAAAACCTGGATATGTGACCTGGACGATGACTGTGGGGACCGCTC

AGACGAACCAGCTTCCTGTGCCTACCCGACTTGTTTCCCACAAACCCAGTTCACGTGTGC

CAACGGGCGCTGCATCAACGTTAACTGGCGCTGTGATAATGATAATGACTGTGGAGACAA

CAGCGACGAAGCAGGCTGCAGTCACTCCTGTTCCAGCGTGCAGTTCAAATGCAACAGCGG

CCGCTGCATCCCAGAGTACTGGACCTGCGACGGGGACAACGACTGCGGCGACAACAGCGA

CGAGAAAAGCGCCAACTGCACTAATCAGGCTACGCGTCCCCCTGGTGGCTGCCACTCGGA

TGAGTTTCAGTGCCGCTTGGACGGTCTGTGTATCCCCATGCGCTGGCGCTGTGACGGGGA

GAACGACTGCATAGACGACACGGATGAGAAGCACTGCGAGGGCGTCACCCACACGTGCAA

CCCGTCTGCGCAGTTCGCCTGCCGTGACTCGGCAAGGTGTATCAGTAAGGCGTGGGTGTG

TGATGGAGACAGCGACTGTGAGGATAACTCTGATGAAGATAATTGTGAAGCGTTGTTGTG

TAAACTTTCGCACCATACATGCGCCCAGAACGATTCCATCTGCCTTCCTGCCGAAAAGCT

GTGCGACGGTAAGGACGACTGTCCCGACGGCTCGGACGAGAAACTCTGCGACCTGTGCTC

GCTAGAGAACGGCGGCTGCAGCCATAACTGCACCATTGCCCCCGGCGAGGGCATCCTGTG

CTCCTGCCCGACTGGCATGGAACTCGGCTCTGATAACAAGACCTGCCAGATCCAGAGCTT

TTGCGCCAAACATCTGAAGTGCAGTCAACGCTGCATCCAGGAGAAAGCCACCGTCAAGTG

TGCCTGCTACGAGGGCTGGGCTCTGGAACCGGACAACGAGAGCTGCAAGAGCACCGACCC

TTTCAAGCCTTTCATCATTTTCTCCAACCGGCACGAAATTCGACGTATCGACCTGTACAA

GGGCGAGTTCAGTGTCCTGGTGCCCAGCCTGAGGAACACCATCGCCCTGGACTTCCACCT

CAATCAGAGCTCCCTCTACTGGACCGACGTGGTGGAGGACAAGATCTATCGTGGCAAACT

GTCTGAAAACGGAGCCCTGACCAGTTTTGAGGTGGTGATCCAGTATGGTCTGGCCACTCC

AGAGGGTTTGGCTGTGGACTGGATTGCTGGAAACATCTACTGGGTGGAGAGCAACTTGGA

CCAGATCGAGGTGGCCAAGCTAGACGGAACCATGCGCACGACACTGCTCGCCGGAGAGGT

GGAGCATCCACGAGCTATAGCCCTCGATCCACGTGACGGTATTTTGTTTTGGACAGACTG

GGATGCCAGTTCTCCCCGAATCGAGGCTGCCTCCATGAGCGGCGATGGCCGGCGTACCAT

TCACAGAGAAACAGGAAGTGGAGGCTGGCCTAATGGGCTCACCGTTGACTACTTAGAGCG

CCGTATCCTCTGGATCGATGCTCGGTCTGATGCCATCTACTCTGCTGCTTACGACGGTTC

GGGCCTGATCGAGGTGCTGCGTGGACACGAATACCTGTCGCACCCGTTCGCCGTCACCAT

GTACGGCGGCGAGGTTTACTGGACAGACTGGCGCACTAACACACTGGCTAAAGCCAACAA

ATGGACAGGACACAACGTTACAGTTGTACAGAGGACCAACACACAGCCGTTCGACCTGCA

GGTCTATCATCCATCCAGACAGCCTCAGGCTCCAAACCCTTGTGCTGCTAACGATGGAAG

GGGTCCGTGCTCTCACCTCTGCCTCATCAACTACAACCAGACCTTCTCCTGCGCCTGCCC

CCACCTTATGAAGCTGCAGGCAGACAAACACACCTGCTACGAATCTCGTCAGTTCTTACT

CTACGCTCGTCAGATCGAGATCAGAGGAGTGGACATCGACAATCCATATTACAACTACAT

CATCTCCTTCACTGTTCCTGTGATCGATAATGTAACAGCGGTGGATTACGACGCCCAGGA

ACAGCGCATCTACTGGTCTGACGTTCGCACGCAAATGATCAAACGAGCCTTCATTAACGG

CACGGGGGTGGAGACAGTGGTGTCGGCCGATGTACCCAACGCTCAAGGCTTGGCTGTGGA

CTGGGTGTCCAGGAACCTGTTCTGGACTAGCTACGATACCAGCAAGAAGCAGATCAACGT

AGCCCGATTGGACGGCTCCTTTAAGAACTCGGTCGTCCACGGACTGGACAAACCACACTG

CCTGGTGGTACATCCCATGCTTGGAAAGCTCTACTGGACAGACGGGGACAACGTCAGCAT

GGCCAACACAGATGGCAGCAACCAGTCGTTACTCTTCACCAGTCAGAAAGGCCCCGTGGG

ACTGTCTATTGACTTCGATGAGGGGAAGCTGTACTGGATCAGCTCAGGCAACAGCACGAT

AAATCGCTGCAATCTGGACGGCACCGGCCTGGAGGTCATTGAAAGTGTAAAAGGAAACTC

ACCAGAGCCACCGCTCTCGCCATCATGGGGGACAAGCTGTGGTGGGCGGACCAGGGCACA

GACCAGGTAGGCACCTGCGACAAGAAGGATGGCGGCAGCTGGAAGGTTCTTAGGAACGGC

ACGTCTCCGGTGATGCATATGAAGATCTACAACGAGACTGTCCAACAAAAAGGTACGAAC

CTGTGCACAATGAACAACGGGGACTGTTCTCAGCTTTGCTTGCCCACCTCACCCACCAGC

AGAGCCTGCATGTGTACGGCAGGCTACAACCTGAAGAGCGGCCAGCAGTCCTGTGAAGGT

ATGGGCTCGTTCTTGCTGTACTCGGTGCATGAGGGCATCCGCGGAATTCCTCTGGACCCG

CTGGACAAATCTGACGCTCTGGTGCCAGTTTCTGGAACCTCTTTGGCAGTGGGCATCGAC

TTTCACGCCGAAAACGACACCATTTACTGGGTGGACATGGGTCTTAGCACCATCAGCCGA

GCCAAAAGAGACCAGACATGGAGAGAAGATGTAGTTACTAACGGCATTGGACGTGTGGAA

GGCATTGCTGTCGACTGGATTGCAGGAAACATCTACTGGACTGACCAAGGCTTCGACATG

ATTGAGGTGGCCAGACTCAACGGCTCCTTCCGCTACGTGGTCATCTCTCACGGGCTGGAC

AAACCCCGCGCCATCGCCGTCCATCCAGAGAAAGGGTACCTTTTCTGGACCGAGTGGGGT

CAGTACCCACGCATCGAAAGGTCTCGACTGGACGGGTCAGAAAGAGTGGTTCTGGTTAAT

GTCAGTATTAGCTGGCCCAATGGCATCTCCATCGATTATGAGGAAGGGCTGCTCTACTGG

TGTGACGCTCGCACAGACAAAATCGAGCGTATTAATCTGGAGACCGGTGGGAACCGAGAA

CTTGTCCTGGCAGTTCATAACATGGATATGTTCGCCGTGTCTGTATTCGAAAACTACATC

TACTGGAGTGACAGAACCCATGCCAATGGCTCCATTAAGAGGGGTAATAAGAACAATGCC

ACTGATATGGTTTATCTAAGAAAAGGCATTGGTGTGCAGCTCAAGGATATTAAAGTTTTC

AACCGAGCCCGGCAGCAAGGCACCAACATCTGTAAAGACAAAAACGGTGGATGTGAGCAA

CTATGTCTATTCCGTGGCAACGGAGCACGCACCTGCGCCTGTGCCCATGGCATGCTAGCC

GAAGACGGGCGCACTTGCCGCGACTACGACGGCTACCTTCTTTACTCCGAGCGTACAATA

CTGAAAAGCATACACTTATCAGACGAAACCAACCTCAACGCCCCGATCAAACCTTTCGAG

GATCCAGATCATATGAGAAATGTCATCGCTCTCACCTTCGACCACCAAGGTGGCGACGGC

AAAGGAGCTAACCGCATCTTCTTCAGCGATATACACTTCGGCAACATCCAGCAGATCAAC

AGTGATGGCTCAGCGCGCAAGACCGTTGTTGAAAATGTGGGCTCGGTGGAAGGCTTGGCG

TATCACAGGGGTTGGGACACGCTTTACTGGACCAGCTACACCACCTCCACCATTACGCGC

CACACGGTGGACCAGACACTCAGCGGCGCCTTTAACAGAGAGACGGTGGTCAGCATGTCT

GGAGATGATCACCCCAGAGCCTTTGCGTTAGATGAGTGCCAAGGGTTAATGTTCTGGACT

AATTGGAATGAGTTGGCTCCCAGCATCATGCGCTCATCTCTAGCAGGCTCTAATGTCCTT

GTGATCGTGGGTAACAATATCCGCACCCCAAATGGCCTCGCTATAGACCATCGCTCCGAG

AAGCTTTATTTCTCTGATGCCACGTTGGATAAGATTGAACGCTGCGAGTATGACGGCACC

AATCGCTTTGTGGTTTTGAAGAACGAGCCGGTCCACCCGTTTGGCTTGGCTGTCTACGGC

GAGTACATCTTCTGGACGGACTGGGTGCGCAGAGCCGTGCTGAGAGCGGATAAATTCGGC

AGAGACATGAAAGTACTGCGTGCTGACATCCCCCAGCAGCCCATGGGCATCATAGCCGTT

GCCAAAGACACCAACAGCTGTGAGTTCTCTCCATGTCACACCAATAACGGCGGCTGTCAG

GATCTGTGTCTTCTCACGTCCGAGGGCCGGGTGAACTGCAGCTGCCGCGGAGATCGCAAA

CTGGTGGAGGGCAACGTGTGTGTTGCCGAGAACACTACATGCCACAGCGTGGACGAGTTC

GAATGCGGAAATGGTGACTGCATCAACTACAGCCTGACATGCGACGGACGCGCTCACTGC

AAAGACAAATCAGACGAGAAACCGTCCTACTGCTCTAATCGAGGCTGTAAGAAGGGATAC

AGACGCTGCCTTAACGGACGCTGCATCAAACACAGCTCCTGGTGCGACGGCACAGACGAC

TGCGGAGACCGATCAGACGAACGACCCTGCAACATGACCTTGTGTAGTGCATCAGAGTTC

CAATGTAAGGACGGTTCCTGTATCACCAACACGTCCCGCTGTAATCAGGTGGTAGACTGT

GAGGATGCCAGTGATGAAATGAACTGCAGTCCAACAGATTGCCACAGCTTCTACCTGTTA

GGAGTTAAAGGTGTGATCTTCCAGAAATGTGAATTTACTACGCTGTGCTACACCCCTATC

TGGCAGTGCGACGGCTCGAACGACTGTGGAGACTTTTCGGATGAGAGGAACTGCCCAGAG

AAGAGGCAGCTGAAATGTCCTGTGAACTTTTTCGCCTGTCCCAGCGGTCGCTGTATTCCC

ATGAGCTGGACTTGCGACAAGGAGAACGACTGTGAAAACGGAACAGACGAGACTCACTGT

GATAAGTTCTGCTCACCAACACAGTTTGAGTGCGGGAATCACCACTGTATCTCCAGCTCC

TGGGTGTGTGACGGCACCGATGACTGTGGAGACGGCACCGATGAGGGCAGCCGATGCAGT

TCAAAGACATGCAGTCCCGAAGCCTTTCAATGCCCTGGCTCTCACTGCGTGCCCCAGCGC

TGGAAGTGTGATGGGGACAACGACTGTCCAGATGGTGCTGATGAGGGTGTGCAGGCTGGC

TGCACAAACAATAAGACCTGCGACGACACGGAGTTCCAGTGCCAGAATAAGCAGTGCATC

CCCAAGCATTTCGTCTGCGATCACGATTTGGACTGCCGTGACGGCTCTGACGAGTCTCCC

GAATGCGAGTACCCAACGTGTGGTCCCAATGATTTCCGCTGTGAAAACGGGCAGTGCCTT

AAGCAGAAGAGCTGGGAGTGTGACGGCGAATTTGACTGTCGTGACCACTCGGACGAAGCG

CCCAAGAACCTCCACTGTACCGAGCCAGAGAAGCGCTGTAACGACTCGGCATACCTGTGC

AACAACGGGAAGTGTGTGAGCGAGGACTCGCTGTGCGACTGGAAGGACGACTGCGGCGAC

GGCTCCGACGAGCACAACTGCTTCGTCAACGAGTGTCTTAACAACAAGCTCAGCGGCTGC

ACACAGCACTGCGAGGACCTAAAGATTGGATACAAGTGCCGCTGTGATCCCGGCTTCCGT

CTGAAAAACGACGGCAAGACATGCGTGGATGTGGATGAGTGCACCACCACTTACCCTTGT

TCACAGCGATGTTTCAACAGCTACGGCAGCTTCCACTGCTTCTGCATAGAGGGTTACGTG

GCCTATGCGAACGACTGGACAAGCTGCAAGTCCTCTTCTGAAGAGGAGGCTTATCTAATC

TTTGCCAACCGTTACTACCTGAGGAAGCTCAATCTTGATGGCAGCAATTACACCCTCATC

AAAACGGATTTGAACAACGCTGTGGCTCTGGATTATCACTACGCAGAGCAGATGATCTAC

TGGACAGACGTGACCACGCAGGGCAGCATGATCCGCCGCATGCTTATGAACGGCAGTAAC

GTCGAGGTGCTGCACCGGACGTCTCTGAGCAATCCGGACGGTCTGGCGGTGGACTGGGTC

GGGGGCAACCTGTACTGGTGCGATAAGGGTCGAGACACCATCGAGGTGTCCAAGTTGAAC

GGGGCTTACAGGAGCATGCTGGTCAACAGCGGTCTGAGGGAGCCTCGGGCGGTGGCTGTG

GACGTCCGCAATGGTTATCTGTACTGGTCAGACTGGGGAGATGTGCCTCACATCGGCCGT

ATTGGAATGGATGGAACAGACCGCAGCATCATCATCAAGGATAAGATCACATGGCCTAAC

GGCCTCACGCTGGACTTCATCAACGACCGCATCTACTGGGCCGACGCTCGCGAGGACTAC

ATCGCCTTCGCCAGTCTCGATGGCACCAACCGACACATCGTTCTGAATCAGGACATCCCA

CACATCTTCGCCATGTCTCTCTTCGAGGATTACATCTACTGGACCGACTGGGAGACCAAG

TCTATCAATCGAGCTCACAAAACACTGGGAACCAACAAGACCATGCTGATCAGCACGCTG

CACAGACCTATGGATATTCACATCTACCATCCATACAGACAGCCTGCAGTTGAAAATCAT

CCCTGTCAGGTAGATAACGGAGGCTGCAGTAACCTGTGCTTGTTGTCCCCTGGAGGAGGT

TACAAGTGCGCCTGCCCCACCAACTTCTACCTGGCTGCAGATGGGAAGCAGTGCCTGTCT

AACTGTACAGCGAGTCAGTTTGTGTGTAAGAATGATAAGTGCATCCCCTTCTGGTGGAAG

TGCGACACTGAAGATGACTGTGGAGATCGCTCCGATGAACCTGCTGACTGCCCGGAATTT

AAATGCCGTCCGGGGCAGTTCCAGTGCGGCACAGGCATCTGCACAAACCCCGCCTACATC

TGTGATGGCGACAATGACTGCCAGGACAACTCGGATGAAGCTAACTGCGACATCCACGTG

TGCCTGCCGAGCCAGTTCAA

>Unigene000044

ATAACGTCACCAACTTCCTGCAATAGTTGTTTGGATAATGGCGGTGCGTCAGCTAACATC

GCTTCTAACCTTCCATTAAAACCGGAGAAAAACCTGAGAAACGACGGGAATCGAGCACAA

TAACCGATTTCAAAGGGCGGAGTTTCGATGTGGAGCCAATCAGAAGGAAAGAAGCCTTCA

GCGATGGAGCCACGCATGGCGGATTACGAGCTGTCATCTGCATGGAGGAATCTGAACCAC

AACAAAGCAGCTCTGCGTCACATCGAGAATCAGCTGGAGGCGGCGGTTGGTTCAGGAGTT

GTCATTACCTCGTTGAACACTGAGAAGTCTCACAAACACAAACGCAGAGGTAGGCGAAGC

TCTGATGGTGACGATGCAACAGTTAAGCCCCGCCCACAAAGCCGTCGCAGCCCTGATAAG

TCCTCCCGCAGCCCGCTGAGGACCAGCACACAGGACAACACACACACGTCCTCTCCCGCT

GACGCATCACTGAGTCGCTGCGTTTACGATCGAGACCGTCGTCCTGTCCACTCAGCTGAC

GCAGACAGCACGCCATCCTCAGCGCTGGACGCCACGAACGTACGTGTCCTCAACGACTCC

TTCGCTCTGCAGAGGTCAGAGGTCACGATGGGGGCATCGGAGGTCAGCCCAGGGTCAGCG

CGGCTGGAGAAGTTACGTCAGCGCCAGACGGACGAGAAGCTGGAGAAGCTTAAGGAGAGA

ATCCGCAGGCAGAGGGAGCAGCTGGAGGAGACAGCAGGGAGGGGCGGGGCAATAGAGGCG

GGGCATGGAAACTCCGCCCACCAGCACCTGACCAAAGTGCGCAAAGTGGCAGCTGCTCCA

CCTGCGCCGAACTATAAAGGGTTTAACAGCAGTGAGGTGAAGATCCAGTCCTCAGACAGC

AGAACGTGGGGAGACACCGAGTCTCGCTTAAAGCCTGAGAATTATCTCACTGACGCTCTC

TCAGAACGAGTGAAACGTAAACCGGTGGAAAAGGATAAGCAGCAAGAGAGGAAGCTGATC

AAACCCGTGAGGAAGATCCACCGATCTACATCTGCTCCAGAACCCAAACCAGCCATCAGC

GCGTCGTCATGGCGAGAGGGCCAGAAAGTGGTGCAGATGATGCTGGGGCCGCTGCCACGG

GAACCTTGGGCTCAGACTGCGGGGAGAGAGAGACTGGCAGCTGCTTCATCGCGCTCCAGT

TCTGTTCCTCGGCTGGAGTCGAGGCACCGCTCAAGATCATCAGGTCATCGCTTGAGCAGC

ACAGAGAAGCCTACAGTAACTCGGCCCTCCAGACCACCAGCAGGCACCAGAGAGCGAAAA

ACCGTAACAACTCAGTCATCTAGACCAATAGAGGGCCCCGGCGCTCTTTCTGCCGATCTG

GAGCTTCTTCCTGCAGAGTTTCGCAGCATCCTAGATGATCTGCATCTGGAGGACCGCGGA

GTCAAGTCACATGACCCAGGTCGAGTCGACAGAAAGAGGGCGGGACGAAGCCCCTCTAAA

CCCCGCCCAGATAACACAGAGGGAGCGCTGCCACGGAAACGGCACTACGACGCAGACGAG

GTGCGACGCTACATTGCTAAGCAGCAGGAGGAGAGGAAGAGGAAACAGGTGGAGGAGAGG

CGGAGTCAGAGGGAGGAGCAAGAGAGGCGGAGTCACAGACTGCAGGAACTGTATCGGAAA

CAACGTGAAGGTGTTATTAAGCCGCAAGTCCCGCCCCCTAATCTTTTAAACCCACATCTG

CGAGAGACGTACACTAAGATGCTCCTGAAACACAAGCTGCAAGAGGCGCAGCCGGTGTAT

CAACCTTCCGGAGAGTCAGATAAAGAAAACAAAAAAATGGAACCACTCAGTCCTTCCACC

AGTGAACTTTCAGAACACACACCTTCTCCGCTGTGCAGGGCTGATTTGGTGCCCCCTGCT

GGTGAGTTCTTCTCCCATCTTCTCAGTGTGGAGCCAGAGTGTGTGAACTCCACCCCACAC

GTGCGAGATACACACACTGAGGACGCACACTGGGTGGGAAAGGGTTGGGTGGATACACAC

ACCATCAGCAGGCTGAACCGTGTGGAGGCTCTAAAGGCGTGCGCTGCTTCACTGTCCAAC

CGAATCGAAAGCGAGGCACGAAAGCTCATCACCTACAGCGACCATGACAATCAGGACGGG

CAGAACCACCAGAGCAAACCGCCAAGCCCGCCGGAGCGCGAACAACCCCATTCCACCTCC

AGAAATCACGACTACCTGAGGGAGCTTCCCGGTGTCGGTAACCTGCACGGCTTCATTACC

CGGGAAGGGTGGGGTGAGATCCCTAAACCCCGCCCACTCACCGCCAGCACCGCCCTTCCA

TTTACTGTGCAGCATGAGGAGCATGACTCAAGCGGCGGCTCCATCAGCGAGGGTGTGCTA

AGTGACGCCAGTCTGTCTGATCACGCCGACCATCCTGACGACCGCATCACCCTGTTCCGG

AAAGAGGCGGAGCTTTACGTGCCCTACCGGCCTGTGGTGACATCACAGCCTCCATGGGAG

GAGCTCAGCAAGGGCAGCCCTCACAGCGTCATCAATATATTCGCCAAAAACCTCAACAAA

TACAACAACGTGATGGACATGGAAGAAAACGGTGCAACGTATGAAGACGACTTTGTTTCC

TCGCGCAGCAGCAGCCAATCAGCATCAAAGAGAACTGCTAGTGGTGTCAGCCGGAGCTCA

GCAGCAGCGGATAGATTTCCTCACTCCTCCGCCTCTGTTCTATTATCCTCACCTTCCTCT

ACACACTCTGTCAGCAAGAGAGGATCTGATAGGAGTTTGGAGCGCTCTCTGGTGGACGGA

CAGAGAAGCATGCCCTTGATTGAGCCTCGGAGGAAAATCGACTCGCCTCGAGGTTCTCAC

AGGAGCGCTGAGGGTAATGGGACACCAGGGGGCGCCTCGGTGCGCTCCTCCCACAGTATG

GTGTCTGAGTTTCCCCACTTTCCCAGAAGTCCAGCTGATTCTCCCAGAATCTCAGGCTCC

TCGTCATCCAGTCCTGCATCTGCAAGAGACTCCTCCCCCAAAGTGGAAACTGGACCCGCC

CCCAACCTGGTCTCATCCCCTGGACCCGCCCACAGCATCACAGAGCGACACTTCCCCCCG

GACGTGCTGCAGCAGCGGTTAAACGCAGAGCTGAACTTCCTGGATGCAGTGGAGGAGTCA

GTGAGACAGCTGAGTGACGTGGAGAGAGTCAGAGCTGTCTCACTCGCTCAGCAGGAAAGC

GTCTCACTCGCACAGATCATCAAGGCTCAGCAGCAGAGGCAGGAGCGAGAGCTGCAACTA

CTGAAATTGAAAGCAGAACAGGAAACGCTGGAGACTCAGAGACACGTCCAGGAGAGCAGA

GAGAGAGCAGCACAGGCCCATGTGGAGCTGTGTATGAATCTGGTTCAGTCTCATCAGAAG

GCTCTCATGGAGTTGCAGGAGAACAGCAGCAAAATAAACCACCAACACACACAGACACCA

CAACACCTCCGAGAGATGACTGAGATGAGTCGTCCTCAGCTGCCCAGTGTTTCTTCTGTC

ACACCTGTAAGGCAGCCACACCCACAAACGGACAGTAGTAGGAGTGAAGTCACAACCGAG

AAAACACCAACAGCACACACTCCACAGAGCATCACAGCTGAGTTGGAGTCCTTCAGTTTC

ACGCAAAGCAACGGCGGTGGGGTGGACAGGAGCGGCTCGGGGGGGGAGGAAGGGAAAACC

CCTGCTGACGACTCTCTCCAAACTGACAAAGACAGCGTCTCGATCAACTACTCTTCAAAG

TTTGATGAGTCTGTGACGGAGGATGAGTTGGAAAAGTCCTTTCGCTCTGTGCTTCCGTCT

GAGTCTCACTGGCGGGGATCCGTGGAAAGGAAACGGCAGTCGGACGAGGAACCTCACGCT

GACAAAATATCTACTAAGGACGGCAGCGTCCCTGTCTTCTCGGCGGATCAGTACAGTTTC

TCGGAGTTCACCATGGCGATGGTGAAGCAGTGCATGCAGGAAGAGGAAGTTCGCTCTCAG

CATCAGAGCTCCCTGCTGCGACTCCGACACCGAGCACTGAAGGAGAAAACCCGAGCTGAG

CTAGAATGGCTCGAGCTCCAAAAAAGGCATCTGCGTGATAAAGGAGAGGATGATAAAATG

CCTCCAATACGCAAGAAACAACGCGGCCTGCTGCTTAAACTACAACAGGAACAGGCAGAG

ATCAGAAGGCTGCAGGAGGTGAACCGTGCAGCACGAAGGGAGAGGAAACTGTTGCTAAAA

CAGCAAGAGGAGATCGAGAGGATCCAACACACCACACAGAAACTGAGAGAGAAACTCCGG

AATGCTGAAGACACAAACCAGAGGTCTCCTGTGTCAGGTGTGAGCGAGGATTTGGCCATT

CCCACTAAGGTGGACATAGAGTTCCGTAGCCCCTCCCCTTTGTCTGTGTCAGGGAGTGAG

ACCAGCAGTGTCATGAAGAAGCTGAAGAAGATGAACTATCACACAGATGAGCGATTTCTT

ACCCAGCGTGAGCTGCGATTGGTACATTGGCGTCAGCAGGTGGAGGCGGAGCTGAGACGT

TCAGATGGGGCGGAGCCTCAGCCGAAGGGACGAGCACTGAACGCCGAACGTAGTACACAG

CAGAAACACGGCACAGACGAGCTGAAGAGAGACAGACAGGAAGAGAGAAAGACAGGACAC

GAAGTGCGCAGCGATTGTGACTCATCATCGGCACTGTCCGTCTCCAGCATACACACAGTG

CAAGAGTGTGACAACACACACAGCCGTGAGTTCTCCTTGGTTCCTGCAAGTTCCAGCAGT

AAAGTGACATCACACAGCAGCACCAGGAAGAGGGACCTCCCTTTATCAGCTCCTACATCT

GAGGCGGCATCGGATCAGAGTGACATTGAGGGGCGTGTCCTGGCTCTGAGGGAGGAGCTT

CGGCGGCGGAAAGCTGAAGTCCAGCTTCTAAAGAGGGAGCAGAGACTGAGACACAAAGAA

CGACTGAAAGCTCAGGAGGCCAGTCTGCTCAAACAGCTAGAGTCTTGCAACAACTTCATC

CAGAAGACTAAAGCCGAGCTGAGTAATGCAGATGTTGCCACCACTTCAACCAAACCTCAG

ATCAAAACTCCAACATCAGACTCGCACAAATCCGGAACCGAAAGCTTCACCACAGACAGC

TCTGGAACAGAGAAGAGTAGAGAAGAGAAGAGTCACGAGTCTGAATCAGAGAGATCGAAC

AGGATACACACACAGTCTCCGGCGCTCAGCGATGAAGATCCTTCCACCGTGACCCCGACT

CCGGTTTTGGGAAGCCCCAACCTCCTGAGTCCTCGTGAACTCCAGCATGTTGAGCAAACC

CCACAACCCGAAAGCCCCGGCAGACCCGGGAAGGACGCCTCGACCGATCTGAGCACAGCC

TCCAGCCCGGGGTCGGATGTCATAGAGGAGCTGGAATCTCTAAGGTCGGATCCAACCCGC

TCTGAACACTCGCATCCGCTGCTTAATTTAAAACTCCAAGACTCCGATGGGAATTCCAGC

AAAAATCAACCGGAAATTGAGGTAAAATTCAGCCATGTAGAAGAATTTACAAATTCCGAA

GCGGTTAAATCCAGTTCCATAATAGTAACTAAACTAGAACCAGAAGAACCCAAACTTCCA

GAGAGGTCCTTAACCTTAGACGTCTCAGACCTTGAAGACAAACCACCTGAGTCGCTGTCT

GAACACAGCTACAATGATGATTTTGAATCAGCAATTAAGGAGGAAACTGGATCTCACTCA

CTGTCTTCTGAAGATCCCGAAGAGGTATCCTACAAATTCTCCTTCAGCAGCGAAGATGAG

ATTGAGGAGAAGATTAGTCTCCAGTCAGGAAATACCAGTAGGACTTTTGAGCAGGAAAGA

CCTCCTGATCTCCACTTCTTGGGGAGGTGTACAGCAGAAAGCATCCATGAAGATGTTCCC

AGTCTTCCGAAGTCTTCGAAGGATGAAATGATGGATGAAATGCCTGATTACGTAATTGGT

GAGCGTGTCCTGGTTAGCAACCTTCAGCCAGGTATTCTAAGGTTCAAGGGACAGACTAGC

TTTGCAGATGGATTCTGGGGCGGAGTGGAGCTGGACGCATCCGAAGGAAGCAACGACGGC

ACTTACAACGGTATTGTTTATTTCCAGTGCAAAGAGAAACACGGGATATTTGCTCCTCCG

GGTAAGATCTCTCACCTACCTGAGACACATGAAGACCATACAAGAAGCTCAGAGGAGAAA

GATTCCTCGTTTAAAGATCAAACATGCAGGAAGACGAACAAGTCTGAAAGCTCACTTCAG

AATAAAAGAGTGGAATCGGATCCGCCTTCTGAGACGGAGGAACTGTCCGGAAAACCTGTG

GTTCTTGATGCATCCGTTACGAAAGACTTGATGATTGATCACGTTGCTCTCAATGGTGAG

ACTTTTGAAAATCTTAATGGAGAAAAAAATCTTCTGGCACTAGAGAGCTTTAAAGACATT

GTCCTGGAATTTAAAAACGTCTCAAAACATGAGGAAGGTAGAGATCAAACTCCAGATATT

CTGGACCTGCTGATCCGTGATGAGTCGTCCACGGCAACCGAGGACCTACAGAAATCTTCA

GTAGTCTCTCCTGAAAAGACAAACGAGGACAACGTGGATGGATCGGTGGTGACTGAACAC

AAACCCCTCACGGCTCTGGCAGATACGCTGATAGAACGCTTCATGAGCGATGCCCTGAAA

CAGTTTCAGCAGATCAAAAAAAACAAGGAGCAAAAAATCTTAGCTGCTAATCAACTCGTC

TTCTTCGGTGAGGACGAGGAACCCAGTGAAAATGATGTGTTCCGGTTCGGAACTTCAGCC

AGCTGCAGAGTGGACTTCGACAAGGACCAGGAAGAGGTGTCTTCTCCTGAGATGTGCAAC

CGATTGGAGAGCCCTGTTTTGGGTCAAAAGGAACTGGTCCAGCGTCTGGAGGAACTGGAG

CTGAACCGCGAGTTCCTACATGTGCTGGGAGACGAGCCGGACTGGTTTGACGAGGACTTT

GGCCTCAGCTCTCGCAGACAAACTCAGAACAAGAACAAACTCCGGCAGGACGAACTGACG

CCCACGTCTGCGGCCAAACTCCCGCACCAGAGGCAGCAGGATGAGGCGGTGGCACCGGCG

GCCATGTTGGTCCCGCACAAAGCCGAGGAAGTGGAGAAGTTGGTGAGTGCCGCCCTGCTG

GAGATCTGGATGAGCTGCGGGATGGGGCGTGGTAAAGCAAGTCTGACGGGCGTGGCCAAA

CCTCAGGCGTCGGAATCGCTGCTGGGGGATCAGAGCGGCGAGGCGTGTGTACGCAGTTAC

AAACAGGCAGTGTTTGACCTGGTATGGGAGCTCATGCAGGAAATCTACGCTGAGGATCCC

AACACCTACAGACCACTGTGGATCAAACCACACCGGATTCATTCACCCTACATCCACAGA

ATCGACAACCCCACTGACCTCCATAAAGTCCAGGCGTTTGTCACCGGTGAGGTTCTAAAG

CTGTACAATTTAAATAAAGAGCAGAACCACAGAACCGACTGGCAGAAGATGCTGAAGTTC

GGCCGTAAGAAGAGAGACAAAGTCGACCACATTCTGGTGCAGGAACTCCATGAAGAAGAG

GCTCTGTGGGTGAACTATGATGAAGACGAGCTGTTGGTGAAGATGCAGTTAGCAGATGGG

ATCCTGGAGGCTCTGCTTAAGGACACTGTGGACGTCCTCAATAAAATCCACCAGAGTAGA

TCCTCACGGGCTCTGACCCCATCCTCATCCACACACCTGCTCTTACAGCCCTGACACACT

GCGCCATCGTTCAGGACAGATCCCGGAATAGCCTAAACCTCGAGACGGTCTGTGTCCCGA

ATTTGCTGCTCAGATCTCCGGTCCCTCCCTTTAAAACCCCACCCTGTGTTTGGGACAGAA

CCGGACCCCCTCCAGCGTCTGTACCACATTTGCCTTCGAGCTGGAGTTTTAATTTATTTG

TAAAGAGTAAACAGTTTTTAAAACTATGTGAAAATGTGTAAAGAGATGATTTAAGCTCAC

ATGAACCCATCTAAGCACACGTGGAACCTACAGAACCCAGTGGAACCTGCAGGAGCCTAA

AGGAACCCAGAAACAGAACGAGACGGTTTAGAGAGGATAAATCAATATAAACTGTGTATT

TTAAAGAGATTTAGAAGAGCAACAATCAGAAACTAAAGTGAACGAGTGCATGATGAGAGA

TTAGTGCTTTATTTACCCTCCATATTAAGTGCCTGACGGTGTAGGCTCCCTCTCTAGGGG

AGAACCGGTTTCAGCTTCCTCTCGTGTGTCGAGCCGAGTCGCTTTTATACTCTGGCGTTT

CTGCTTTCCAGCTGGACCCCTGATTCTGCTGCTTCATTAACTCTTTCCTACACTCAGTAG

GGAACCTCCTCAGAGGGCTGCGTCCTAAATTTTCCACACTTCCTTAAAGACCGCCCCC

>Unigene000045

TCTCTCTCTCTCTCTCTCTCTTCTTTTCTCTCCCCCGATGAACTGCTTCAGTAATGTGGG

AGACGGCGATTCTGTCAGCGGCTCAAGATGGCAGATTTTTCCCTGAGTTCTGAACTGAAG

CATCTCTTCACATTTTTGCCATGAATAGCAGGAACCCTTGCACCGATGGCCCCTGTATAC

GAAGGTATGGCCTCGCAAGTGCAAGTCTTTTCCCCTCACACTCTCCAGTCAAGTGCCTTC

TTTAGCGTGAAGAAACTGAAGGTGGAGCAAAGTTGCAACTGGGATATGACAGGGTACGGC

ACGCACAGCAAGGTCTACGGTCAGAACAGCAAGCAGAGCGTGTCCGCTGCCCCAGTGGGC

CTTAACGCCGCTTCGCTCCAGGTCTCCAACTCCTCCCTGCCATACGAGCAAGCTCTCCTC

TTCCCTGCCAGCTCTGGCCATATCGTAGTTGCCTCAGCGAGTAGCACGTCTGGGGTGGCC

GGACAACTTTTGGGGAGTACGGGCAGCAGTAGTGGCAGTGGCAGCGGAGGGCACAACCTG

ACGCGCCGCAGCACTGTGAGCTTGCTGGATACGTACCAGCGATGCGGGCTTAAGCGCAAG

AGTGAGGAGTTAGATAACAACAACGGCAGTGGAAGCAGTGTGCACGTGGTGGAGGAACTG

CAGCAGCCTCCAGCAGCGCAGATGCTCCAAAACAATGGCCAGAGCGGCCCGACAGGCACA

GTGGCCACCGCATCCACAACTGCCACATCCAAGACCAGTGGCGCCAACAGCGAAGGTGAC

TATCAACTCCTGCAGCATGAAGTTCTGTGCTCCATGACCAACACATATGAGGTGCTGGAG

TTCTTAGGTCGAGGTACCTTCGGGCAGGTGGTGAAGTGCTGGAAGCGTGGCACTAGTGAG

ATTGTGGCCATCAAGATCCTGAAAAACCACCCATCATATGCGCGCCAGGGCCAGATCGAG

GTCAGCATCCTGGCACGCCTTAGCACAGAGAGTGCAGATGACTACAACTTTGTGCGGGCA

TACGAGTGCTTTCAGCACAAGAACCACACGTGCCTGGTCTTTGAGATGTTGGAGCAGAAC

CTGTACGACTTTCTCAAGCAGAACAAGTTCAGCCCACTGCCCCTCAAGTACATTCGGCCT

ATTCTGCAGCAGGTGGCTACAGCGCTGATGAAGCTGAAAAGTCTGGGTCTGATTCATGCC

GACCTGAAGCCTGAGAACATCATGCTGGTGGATCCTTCTCGGCAACCCTACAGAGTCAAA

GTCATTGACTTTGGCTCTGCAAGCCATGTGTCCAAAGCTGTGTGCTCTACTTACCTCCAG

TCCAGATACTACAGAGCTCCAGAGATCATCCTTGGCCTGCCCTTCTGCGAGGCTATAGAC

ATGTGGTCCTTGGGCTGCGTCATCGCTGAGCTCTTCCTGGGCTGGCCGCTTTACCCTGGC

GCGTCCGAGTACGATCAAATTCGGTACATTTCTCAAACCCAGGGGCTGCCTGCCGAGTAT

CTGCTGAGTGCTGGAACAAAAACCACCAGGTTCTTTAACCGAGACCCAGACTCCACCTAT

CCACTGTGGAGATTAAAGACACCAGAGGATCATGAGGGTGAGACAGGGATCAAGTCTAAG

GAAGCCCGCAAGTACATTTTCAACTGCCTGGATGATATGGCACAGGTGAATGTGAGCACA

GAGCTGGAGGGCAGTGACATGCTGGCGGAGAAAGCAGACCGGAGAGAGTTCATCGACCTG

CTGACCAAGATGCTGACCATCGACGCAGATAAAAGGATCACGCCCATTGAGACGCTCAAC

CATCCATTTGTTACTATGGCTCACCTGCTGGATTTCCCCCACAGCACACATGTCAAATCC

TGCTTCCAAAACATGGAGATCTGTAAGCGCCGTGTGAACATGTACGACGCGGTCAACCAG

AGCAAGACCCCGTTCATCACTCACGTAGCCCCCAGCACTTCCACCAACCTCACCATGACC

TTTAACAACCAACTCAACACTGTGCACAGCCAGCCTTCTGCAGCACCCATGGCTGCCGTG

GCACCCAGGACTATGCCCCTGCAGCCTGGCGCCCCTCAGCTCTGCGCCACACGGCCTGAC

CCCTTCCAGCAGGCGCTTATTGTTTGCCCGCCCACCTTTCAAGGGTTGCAGGCGTCCCCT

TCCAAGCACAGCGGGTATTCCGTACGGATGGAGAATGCTGTTCCCATAGTGACTCAGGCC

CCCGGTGGTCAACCCCTGCAGATTCAACCCGGGCTCCTTACACAGCAGGCCTGGCCCTCT

GGTACCCAGCAGATCTTACTTCCCCCAGCATGGCAGCAGCTCACCCACACCTCTGTACAG

CACGCTACCGTCATCCCAGACTCCATGGGCACCTCTCAGCCTTTGCCCAACTGGAGGAAT

CCCCATCCCCATGGCAGCCATTACAATCCCATCATGCAACAGCCAGCCTTGCTTGCCAGT

CACGTGTCATTGCCTTCCCAACAACCATTGAATGTGGGCGTGGCTCATGTCATGAGGCAG

CCAGTAAACAACAGTACAGCTTCTAAAAAGAGTAAGCCACAGCAGATGAGCAACAGGAAC

GTGTCGGCCTACGAGGTGTCCTCCTCCCAGGCGATGTTGTCTCCACAGCGTTCAAAGCGT

GTCAAGGAGAACACACCGCCACGCTGCGCTGTGGTGCAAAACGGACCCTCTGTTCCATGC

GCCCTGTCCCAAGCTTGTGGAGGAGGTGGAGGAGGATGGGGAGAGGAGCAAGCTTCCAGC

ACAACGCGAGAGCACCACGGACCACACAACCAACACCTGCAACACAATCAACACAACCCC

CCAAGACAGACCATCGTCATCCCCGACACCCCGAGTCCAGCAGTCAGCATCATCACCATC

AGCAGCGACACAGACGAGGAAGATGAGCACAAGCAGCCCGGTAACACCAGCACATCATCC

AAGCAGCGGAAGAACGTCATCAGCTGTGTCACTGTCCACGACTCCCCAGAATCCGATTCC

AGCAATATCAGCCCCTACGCTGTCGAGTCCCGAAGCAACTGCCCCAACGCCAACAACTAC

GACTCGAAGAGCACAGTGCTAGACAACTACAACAACGGCAACCCCCGAACCATTATCATC

CCTCCCCTGAAGACCCAGACCAGCGAGAGCCTAAGCGAGTGTGATCGACTGATGCCTGAC

ACAATGAACCACCAGAATCCTGCCTACAAGTTTAATTCCTCCAATGGAGGCCTGTCAACT

AACAATCACTCATCAGTAGGCATACCCGGAGGCGGAGCATATCGCCAACAACGTTCCGGG

CCACATCCTTTCCAGCAGCAGCCGCTTAATCTCAGCCAGGCTCAGCAGCACATGGCAGTG

GACAGAACCAGAGGGCACAGGAGACAGCAAGCCTACATCACACCTACAATGACTGCTCAA

GCTCCCTATTCCTTTCACCACAACAGTCCCTCCCACAGCACTAATGTGCACCCCCACCTA

GCTGCCACCCACTTATCTGGCCAACCCCATCTTTACACATACACCACCCCCGCAGCCCTG

GGATCCACTGGCACTGTGGCACATCTGGTGGCGTCGCAGGGTTCAGCTCATCACGCGGTC

CAACACGCTGGCTACCCACCAAGCATCGTGCACCAGGTACCTGTCAGCATGGGCCACCGT

GTGCTCCCATCCCCCACGCTCCACCACGGCCAGTACCAGCCCCAGTTCGCCCACCAAACC

TACATCAGCGCTTCGCCTGCTTCCACTGTCTACACTGGATATCCCCTGAGCCCCACAAAG

ATGAACCAGTACCCGTATCTGTAGATCAGAAAACACTGGAGCCGAGCCCAACGCTGCTTT

TTGATCTCTTCCTACTAGACACTGAAGAGTCAGTGAGACACGCCCTAGCTTGACAGCCCC

TTAAACCTGTCCTCTCCCTGTCCTCGTCACACTTAAACTTGAAGACAATGAAGACACTGA

TGGAGAAAGAAAGAGGGATGGAATGAATAAGAAAATTTTTTTTTTTCATATGAGTTTCTT

TTTAGAGGGCCAGTTCTGATTCTTTTAGGTTTTCCAATATTTTTATAATGGAAATCATAT

ATATGTGTTTTTTTTTCATTTAATCATTTTTACAGCATGCTAACGCTACTGCTGGCATTT

TGGGAGTGACTGGGAATTTAAATTAGATCGAGTCTTGTTTCTTTCTCTTTTTTTTTTGTT

TCACCGACTGTCGAGCCGAGGTCACGTGGTGGATGAGAACGTTCTATTAGGTGAAATACG

GATATCTGCTGCTCTACAAAGAAAAAAAAAATAACGATGGCCTTGAACTGTCTTAAACAT

TTCCGAATCAGTTGGTGATGGGTGCTGCAGGTACTGCGCTGCGCTGCACTGCACGTACTC

CGCCGTTCACTCCAAACAACAGCCGGAGGAATCTTCTGTTATATTACAAGGAAAAAAAAA

AGACAAAAGACTTTCTAGTGTATTTATATATTTTTTGTGTTTCGTTTGTGTTCACACTTA

GATGACAAATTTGAGTTTCGACCCCCTCTCCTACCCCCCCACACACCTACGGTCCCCTTT

CTGATTTTTTTTTCTAAAATGTAGCAAATTTCACCTGCTTATTTTATGAAGAACCTTTAA

GATGCCAATAGAATTTTTGTTCCTCTTTAAATGTGATTGCACAACGTTGTTGTAATAACA

TAAAGCATGTATAACCTAATAGTCTGTGCACGTGTGTGCTGCTGTCGGCCATGTTTGTGG

TCCACCTTCCTCTGCGCCCTTGTGCGTTGTACTCCTTCACGGTCTTAGGATTATCGTTGT

TCCCTCGTAGTGCCTTACAGTACACATATTACAATTTTTTCCGAAACCAACAGGAACTAG

TTTGCAGTTTGGCATCTAGGTTGGACATCTCTGTCTGAGATGTTTTGCTCCTAACTTCCA

GGTGCAAGCATAGTTCTAAATGTAAGTAGCTAGATTATTATACATGACACCATTTTGTAT

AAAAAAAAAAAAACAACAACACAACAACGCTTCTTGTTTAGCCTGCATGAACTTATTTAG

AGCGAGATTCTTCCTTGAAATAATGAACCAAAGTTGTCATTCAGTAAAGAGGAGTAACAT

TTTATTACTGTTTTATAGGATGTTTAGTAGCTGTGTGTCTTAGAAACCTGTACGGTGGCC

CGGAGCTGTTTGTTTTGTTAAAACTTGTTAGAAATAAAGCGCTACAAGTAATTCATTTCG

AATACCCGCACTAATGCGTCGGATTAACCGTCATAAAACTTTAAGGTGAAGGGCAAAGTG

TTCTAGTATCTGCTAATGAGGTCCAGCTGCAACACATCTGGGAGGGATTTTTGAAGGAAG

GTCGAAAGAGCAGGAGTGTTTGCCGAGCGAGCAGCATTGGTGCAGAAACGATGTTGAAAC

AGTGAACCTGTGAGGGTGAGCGAGCGGGTCGAGGTAGGGACCGATTAAGGTGCTAAAAGA

TGGCACTGGATGGGTTTTTTGGATGGGAATTTTAGAAAGCAGAGAGAGTGAGAAAGAGAG

ACAGAGATATGAAATTCCACATCCTAATGAGAATAGCATGTTGGTTTTGAGCATGTCAGT

AAGATCTGGAGGAATCTGCGCATAAAAAAACAACACCTTTTGATCCAAAGGGTAAGTTTA

AAGAACCAGGCTGGGTTTTGAAAGCTTTGGACACAGTGAAGATTGTTCACCGGGAACATG

TGTTGTTACTCTTTTGTCGGAATTACAGGAAGAACTTGTGCTAGATGATACAGGGAATAA

AATATGGGCCAATGTTTAGCTTTTATTTTATTTTCGTTGCCCGAGTTGATCAAGGCTTGT

TTTTTCTCCGATGATTGACTGTCTTGATGAGGGCACGTGCATGTGAGTTTCTGAACACAT

CCATTTTTCCCCTCTCACCTCACGCCATAGTACTTGCATTTAAAGTGATTTTATTTTTGC

TGGGGTTTTTTTAACATGTCATGTTTAAATATATTTTGTCAGTTCGTGATACCTTTATTG

TAATTGTTTTGCTAGATTGTGTTAATTATCCGTTGTTTATTTGTATCTGTTATGGTCAAT

AAGTTAAGTGTATTCTTAGGGGCATCTAGTAAGTAGGAAGTGTTGATTCTGATTGGGATA

CCTTTAACCCAAACATTGTTTTATTCGTTCGGATCACAACGATGTGAGGATGTGTGTTGA

TTTTTGAGTTGTATTTGGCTTGTTTTGGCTCTGTTATGGACTCACACTGGCTCCAGGACG

TTGAAACATAGAGGTTCCCATGTGGATTCGATTGCATCTAACATTCCACATAAGAAACTG

TTATCCTGTCTCACTGCACTGTCTATGAGCTTTGCTCCTGTCTTACCTCACGTAGACCGG

CTGTGTATGACCGCATGGGGATATGGTGTCATTTTACACTTTTTTTCCACCAAAGAAATT

TGTTGTCGAAGAGGCACAGATTGTCCATGGAAGGTAATTGCAGGCCCAAGGCATAGGCCA

ACTAAGCGGCTGCTTATGGCACCTCATGGCCACCAGGGGGCCCCCAAGAGTTTATGAAAT

GACATCATGTTTTTTGTTGTTGTTTTTTATTTATATTTTAGTGAACAGTAGTAATTATAC

AAAAAAATGCAATATTAAACAAAGAAAAACAACGCAATATTGGGATGTAAGCAACACAGC

GAAAGCAAATAAAATTGATAATGGGTGTGTTAAGATTTATAGTGTGCAAATAATTAAGGA

TTGACTATATTGGCAGCACTGAAGGGCCGCTAAATAAAAATACTGCCTAGGGACCCTAAA

GGTTTATGCGGCCCTGAGTTACTAAAATGACAGATGGTATCATATTAGAAAATACTCCAG

AGCACAACATAAAATAAATTGACATCATTTTTGCTAAATCATCATCAAATCCCATTGATA

CACAAACGTGAGAAAGTACGACCACTTGTACTTTTTACCTCATTACATTTCTGTTACAAG

CTCTTCCAGCTCTCCACATGTGAATCGTGAATTAGTTGCAAACCAATGAAAGAGGCAGGA

CTCCATAGTAACTGCCAGTAATGATCACTAGCACGTACTCCGATATAATTGTATAATGTC

AGACTAAGCAATTCATATCTATACATACATATACAGAGAGTTAAACAGCCATCTTTTTAT

TGTAAGTACATTTTTTCTTTAAAAAAAAAATTAGAATGCAATATGTCTCACAACTCGGTT

ACGCTTAACATCCAGAAAAGAATTATTATTCAAGTAGTATTCAAGTAATGTTGTGGTTTA

TTTTATTTAATTTAGCTTATCAAATTTTGACCTTATTTGTACCTGAATGTCTGAAAACTG

GGCATCTTTTGTATAAGTGACATATTTCGAAGATTTTTTAAGTTTTGATTTTAGTTCTCA

CCACCTGAAAATTCGTCCACATGTCAAATTGTTGCCTCGCAAACTACAAACTCAAAGCTT

TAAGCAAAGGTCATAATTGACCCTTTTCTGTCTTTTAAACATTTTTTTTTTTTTTGCTTT

TTATCTATACACGTCTTCTGTCCCCTTCAGTTTGAAAGGCGTTTCCAGCACCAGATGTCC

TTCTGATGTTCCGAAGCGTTTTTAAAAATTCCAGACAAGTTGTAAATGCTGTTTATTACC

CCAAACAGTCTAATCAGTGTACGAGCGCTTTGTTAAGCACACGCTGTTGAACATCGTCGG

CGTCGATGCTGACAGGTGTTTTATCGCTTCTTTTCGGTGGCAAAATGACAGCACTTCAAC

AATGTTTAAACTTTTTGTGTTTAAGTGTTTGTGAAAATGTGTTTTTTTGTGTGTTTTATT

ACTGTTGGACGAGGGTTTAAGCAGTGCGGTTGCAGGTTTTGCTGGAGTCATTGATCTGTG

CTGAGAGGCCAAACGTTACAAGCGGCTATAGACAACTGTATACTTTTAAAGAACCAAAAA

CAAAATGTCAAAAAGTCTTTAACCCTGATTCCAAAAGACGGTGCAGATCTAAAAGGCCCT

CGCTTTACATCCGTCATGAAGGTGTCTTAGCGACCGTAAATCCTGCTCTCCACATGGACG

GTGCTGCAGTCGCACTCCATCACAAAAAAAACCTGTCAGACGCAACCAGATGAGACATTT

CCTCATCGCTAATCATGCTAATCTGTTGTGTAGCTGTGATGCGCTGTAGCCATCCACGTG

TGCAGCGATTTATGACCAGGACGTTTTTTCTATTTGCACAGTCAAATGTAATCGAAATAT

TTTTCTATAATATCTCAAGACCTAAAAAAAAAAAAAAAAAACATCGCACCCTGACCCTGA

TTTTGTGTCTATCGCTGTATGAATTCTTGATGGCGTGTCTCATTAGCTGTTGTCAAAAGC

TATTGTGTGCTACAGTATTTTCATGTCTTTGTTCATGTCTTCTTTTCTTTTTTTTTTTAG

TCTTTATTGTCATTTTTCTTTATCAGAATTCGAAGAAGAAGAAGAAA

>Unigene000046

ATCGGAAGAGCGTCGTGCTGGTGGAATAGAGGATGCAAAAATTCGGTGGATACAAGACTT

TAAAAAAGAACAGGCGGAGCTTCAGGAAGCCCTGAACAAAGCCGAGGACCGTTTAGCCGA

GACGAAACGCAAACTGGAGGAGCTTCAGAGATCCAAAGAGGAGGAAATCAAACATCTGGA

GTCGGAATTGAGTCAGGCGTGGGCCGACCGAGACTCTGCTGCTCGTGCTGTTGAAGAACT

GGTCGCTAGTCACAGGGTGGTGCTACAGGAGCAGCAGGCTCACACACAAGAGCTGGAGGA

AAAGGAGAAATGGCTGAAGCAAGAGGTGGATCGTCTGCTTGAACAGAAATCCAGCTCCGA

GCAGGAGATCACAAACCTCTGGTCTCAGCTGGAGAGCATGAGGACCAGCAGGCAGGAACT

AGGAGAGTTGAAGGAGCAGTTGCTAGCACGCAAGTCACATGTTAATGACATCGAGCAGTT

GAAGCAGGACTTCTCGCAGCAGCGGCGAGAGCTCCAGGAGCAAAACGAGACCGAGCTGGA

GAACCTGCGTGTGTACTTCGAACAGCGGCTCCGGGCCAGCGAAGAGAACCACCGGGAGGA

AATCGCTCTGCTGCAGCTACGCCTCGTAGAGGGAGCTTTGGAGGACTCGGTTCTCAAGAC

CGGAGAGGAAAGCTTCCTCTCTGATGGAAAGTCTGAGGAGGAGAAGGGAGACTCGTTGGC

AGAGATTACAGAACAACTGGATAAACACAAGGTGGAGTTGGATGCACTGCGGCTGCAGCT

GGAGGAGAAACACAAGCAGGACCTGGAGCAGCTGCGCTGTAACATGGCTCTATCCTATAG

AGAGGAGTTACTGCAAGCTCGTACCGAGCTCACCGACCGCTACTACAGCGACATGGAGCA

GCTCAAAACTAAACACGCCTTGGAGATCGAACAGCTGCGGGCCAAGCTCTCCGACAGCCA

CCTCAAAGAAATAACAAGGCTGCGGCTGCAGTCAGTAGCAGATGTAGCGCGTCAGGTGGA

GACTGAGCTGGAAGAGAAGAGCCGGCTCCAAACCCAGGAGCACCAAGCCCGACTCGCTCA

GCTCGCCTCAGACACAGAGAGCATACTGAGCCTGGAGAAGAAGCTCGCCGCGCTCACCCT

GCAGCACAGCCGAGAGCTGGAGAGAGCAACTGAGCAGCATGCGGAGGAGCTCCGACAGCT

GGAGGAGAGGCTGAAGAAAAACTTTTCAGAAGAACTGCACACACGTCTCGCAGAGGCTCA

ACAGGAAGAAAGGACCCATGTGACCGAAGAGCTGCTCAACAGGAGCAGTGAGGAACGGCT

CCAGCTCCGGGAACAGCTTCAAGCACAGGCTGAGGAGCGCGTGGCTTCCCTAAGAGAGGA

GCTCCAACAAGCAGCCAGTAAGGACAGACAAGAGTTGGAGCAGAAGCTTGTTCAGGCTGA

GGAGCTCCTGGCTGAGGAAAAGCAGAGGCTACAAGCTTTACAGGTGAGTCTGGAGAGGGA

GGAGAGCCCTCAGCTGCTGGCCCTGAAGCAGAGGCTGGAATCTCAATACGCTGGCGAGTT

GCACACAGCAAAGAGCACCATGGCAGCTGAGGTAAAGGAGCTAAACACTTTGCTGCAGGA

GCAGACGGAGAGCAGACTGAAGGAGGCCACGTGCAGACATCAAGAGGAACAGCGGCAGCT

AGCAGAGAAACTCGTGCAACAGAAAGAGGAGGCTCTTCAGAAGCTGAGGCAACAACATGC

TCAGGAGCTGCAGGTCCAGGAAACTCTCCTGGAACAGAACGTTTCAGAACTCCAACACCT

CCAGAAGGAGTACCAGGAACTCAAATCCCGACACCAGGAGGAGCTCGACCTGCTCTCTTC

CAATCACAAGACAGCGCTGGACTCCCTCGCTGAGAAACACAAGGCTGAGCTGGATAAGCT

GCAGGTGGTATTTCAGGAGACCAACTTGGCACAGCTCGAAGCTCAGGAGGCGGAGCTTGA

AGAGCGTCACAAGCAGGAAATGGAAGAACTGGAGACCCGGATGTTGTGCAACATGGACAC

TCTGGAGAGCACATACCTGAAGGAGATCCAGGCTGTGCGCGAGGAGAAGGAGGCGGCGCT

CCGGGATCTCGGGGCCTCCCTGGAGCAACAGCAGGCTGAAGAGATTGAAAGACTGAAGCA

GGAGGAGCTGATTGTCAGAGAAGAGCTGAGAAAAAAACTGGCACAGGTGCACATGGACAA

GTTTAGTGTCATGGCGACTGAACTCAGCCACGCCCATCAGGCAGAGCTGTCTGCGGCGCT

CGCTTCTCAGAAAGCGGCTCTGGAGTCAGAGCACCACTCCGCGTTGGAGACCCTGCGACA

GCACGTGCTCGATCTGGAGGCGCAACACGGCGCCGCCCTCCACGAGGTCACCGACATGAC

TACCAAGGAGAATCAGCAGCTGAAGAAACAGCTGGATGCACTGAATGCTCAGCACCAGCA

ACAACTCCAGGAACTGAGGGCAACATCCTCCAGGGAAATCGAAGCTCTACGCAGGGAGCT

GGAAGAAGAAGCATCAAGACAGCGGCTCCATTTCCTGGAAGAGGCGGAGCTTCTAAAGTG

CCAGTCAGAGGAACAGCTACAGAAAAAGCTAATGGAGTTAAAGGAGGAAAGTGATCGAGA

AAAGGTCTCTGCTCTAGAGGAACTGGAGAGAAGTCTGTGTGAGCAGCACCAGCAGAACGA

GCTCTCCTACACTGAAAAAATGAACCATCTGACCACACAGCTGCAACAGCTGGACACCGT

GGTCTCTCAGCTGCGTGCAGAGGTGAGCGGTTTGCAAGGGGAGCTGGAGGGTAAGCGCTC

GGAGATGGACACGCTGGAGACTCTGCTGCAGCGACGCGAGCGCGAGAGTCAGGAAGGAGC

CAACCTACTCGCCATGCTGCGTGATGACCTCAACACCGCCACCCAGCAGAGACAAGATCT

TCAACTGACTCATGACCGACTCCAGCGGCTTGTCCTGGAGATGCTTCGGATTACAATTGC

GACAGAGGAGGAGATCAGTCGCAGGTTGGGGATATCCATGGAAGGTGATGACATCACTCT

GAACAGGAAGCAAAGTGTCAACCGTGACCCGCAGAAAACGGACGCTGAGGTGGGCGACGG

TGCTGCTGCAGCTGTAGAAGCCAGTGCTAACTGCAGCATGTTCTCGTCACTGATGGATGA

AGGTCTGGAGCTGTCTCAGCGACTGTGTGAAAGTTTGTTTACAGGACCTGATGGAGAGCT

GGACCCTGAAAGAGAGGAGCTGGTACTGGGGGCCTGCACTCATCTACGTGTCGCAGTGGA

CAAATTATTGGAGGTAGTTTCCGACTCCACAGTGCAGCTGGAGCAGCTGCAAGCTCTGCA

GGCCCTGCTGGATGAAAAGTTCAGTGCTGGTGGAGAAGCGAAGAACGCGCTCTTCCTGCA

GCACACTCAGCTTCTGGAGCAACTAGACCAGGAGGCCAGCGTGAAGAGTCAGCTGCAGCT

AGAACTGCACAAGGCTGATGGTCTGATCGAGGGTTATGTGGTGGAGAAAGCTGCTTTGGA

GGATGCACTGCAGCAGAAGGAGTCCCGTGAGCAGAAGCTGGTCAAAGAGCTGGAGAGCAC

TCGGGCTCAGCTGCAGGAACTTGGTGAAGAACACGCACTCCTCCAGCGTCAACGGGATGC

TCTCTCTGCAAGCCTCGGAGAACCTGAGAAAGCACTGCTGGCCGAGGCGGAGCGTTTGGG

GCAGGAGCGTGTCGACGTGCAGCGGCAGGCTGAGAAGGACCGCGTCGGACTTGCAGGTCG

GCTGCGTTTGCTGGAACAGACGCTGGAGGAACACGAGAGCCGAACGCAGCAGCAGGAGGA

GCAGCACCGCACCCAAACCGAGGACCTGCAGCAGCACATCAGCGCGCTGGAGAAACAGCT

CAAACACCACCGCCAGTTTATAGACGAGCAAGCTGTGGAGCGGGAGCACGAGCGAGATGA

GTTCCAGCAGGAGATTAAAAACCTGGAAGCACAGCTGAAAACCACCTCTAAAGGACATGC

TGGAGAGGACACCAAGGGTCAAAGGGTGGAAAGCCTACAAGCTTTAATCAAAGACAAAAC

CGAGGACTACAGCGTTCTGCTCTCGCTGAAGGAACAGTGCCAGCGTGACCTGGAGGAACG

CAACGAAGAGATCGATAAGATGGCATCTCGCATCCGAGAGCTGGAGCAAGCTCTCCTGAG

CAGCGTGGAGGAGCGACGGGCCGCCGCGCAGCTGGAACAGGAGCTGCAGAAAGCGCGCAA

AAACTGCCAGGAACTTTCTCAGGACAAGGAAGCCCTGCAGCAGCAGCTGTACTCTAATAA

ACTCCAGATCTCAGCTCTGCAGTCCAAACTGGATGAAACGAGGCACCGCTACCCCGACCC

AGGTGCTGACCCCAACCTCAGAGAGCGGCTCGAGACGCTACAACAGGACCTGCAGAACAA

AGAGGAGCAGGTGGAGGTGTTGCTGGAGCGAGTGGAGGTGTTGGAGAAAGCTCTGGCAGT

GAAGGAGGAGGACGTCAGGCAGCTCTCTCTGCAGCTGGAGCTCAAAACTACCGAAAGCCG

AGCGAGAGAAGAGGAGCTACGGCTGAGCGTCGCCGAGCTGCAGGATACAGCGAGTCTGTT

ACGCAGACAGCGGCAGGAAGAAGCCGATGCCTCGGCGCTACAGCTTCCTTCTGCTCTGCT

GGAGGAGAAGAACCTGGAGATCGACCACCTGAACCAGCAGCTACTCCATCTGCAGCAGGA

GCTGGACGCATCCAAAGAGAACAAGACAGAGGAGAAGCAAGCAGACGTTGAAGATCTACG

TGCTCAGGTGGAGCGACTCCATGGCGATCAGGAACGTCTGCATCAAGCTAAAGAGGAGGA

GGTGGAACAGCTGCATGAGATCATCCACAAGCTGCAGGAGGAGCTGAGCCAGCTCAATCC

CAATCGGCACGAGGTCAGCGACCCGAACACGGACAGCCCTGAACCTCCTGATTTCCCCTG

GTCGCCCCGGCCTCAGCACGGTGCCGAGGAGAGCCTGTGTCACGAGCTGAGCAGCCAGAC

CCTTCAGAGCTGTCGCACCAAGATCTGTGAGCTCCAGGCGGACTTGGAGCGCAGTGTTGA

GGAGAAGGTGGCTCTCGAGAGACTGCTCCTGATGCAGGAAGAACAGTACGGACAGCAGCT

GGAGGCACTGGGTTGCAGTCTGGGAGAGGGAAAAAGAAAGCTGGCGCTCCTGGAGCAAGA

GGCCGGAGAGCTGAAGCTCCAGTTAGACCAGAAGGAGACGAAGGTGGAGGAGCTGCAAGC

TCGCATTCAAACGCTGGAGGATGGAGAGAGGAGTGTAAATGATTTGGAACTCCGGCTGAT

TAAAGCAGAGGAAACGATGAAGGAGACTCAGCAGGAGATGACCAGGTTGAACGACAAGGC

ACGAAAGCACACAGAAGAGCGAGAGAGGACAGAGAGTCTGATTGCTGAGCTGCAGCAGAG

GTATGAAGACAGCGAGAGAACTGCGACGCAGGCGAGGCTGCAGGTTGCCGAGTTGAAGGA

GACGATTCAGCAGCGGAAATCCGACATCGCCATCTTGGAGACGGGCAAGACGGAGCTGTA

CACGGAGAAACAGGCTCTTCAAAAACGCGAGAGAAAACTTCAAGAGGAAATCGAGAAGCT

GAAACAGGAAGTGTCTGCTAAGAGCCTTCAGATACAGGAAGTGAGTTTGCAGCTGGAGGA

GAGAGCAGCTGACCCGGATCAGAGTCAGGAAGAAGTTCTGACGTGTGCTGAGGAGACCCT

GGCTAAAGCTGCAGCAGCTTTGCGAGAAAAGGAGGAGCAGTTGGCTCATCTGAGGTCGCA

GCACGATGCATTGCGAGCCGAACTCGCTGCGGTGAAGGAAGGCCTGAGCAGCAGTACAGA

GAGAGCTGAGAAACTACAGGAGGAGGGGCAGACTAAGGACCGTGCTCTGGCTGATCTAGA

GGAACATAACCAGCGTCTTCGGGCCGAGCTGCGTGGCTTGCAGGACGATCTGGAGGTGCA

GGAGGAGGAACTGGCCTACCAGCAAAGGGAGTTGGATGAGCTGCGGCAGCGCTGCCGCCT

TCAGGAGCACACCGAGCTCGACTGCACCAAACCCGAACGCCGCTTCCTGGATGGCCTGTC

GCATAACTCCACCCTTTCTTCTCCCGAGGTGCTACGGCGGCTCGACTGCAGCGAAGAGCG

ACCGTCTCATGTACACGCGTCTCACCTGTCAGAACTGAGCGGCCTGCGCAATACCAGCCT

CGAGCTGATGAGCAAACACTCGCTTATGGAGAGAAACGAGTTAAAGCGACCGCTCGTCCT

GCCGCCAGAGACCGACCCGCCCAGCACACACTCGCCATCTGCTTCAACACATTCACTCTC

CATCTCCGAGAACCTCTCTGTGCTCAACTCACTGGACGATGACAAGGTTCAAGTGCTGGA

GAATCTGGATGATGTGACTCCGTCTCGCTCTCCTCTCTCTTCTACCTCCCCGGTGTCTGC

ACCAGAGTGGGCCAGCGATGGATACGGTAGCAACGTGAGCTCCGAGCTGGAGGCTAAACT

AAAGAACGAGCTGGAGCATATGGAGCGGCTTGACGCTCACTTTGTTGAGTACCTACGCAG

CAGGGACATGGCACCCGCTCCATGCTCGGACAGCGCCGCAGGCAGCCTGCATCACACTTA

CGAGCTGCTCTCACCTGAGTTACAGGCCATGCTGAACCGCGTGTACCGCGAAAGCTACAG

AGTGCTGTCTCTGTCTCATCGCCCTCCCCCTGCCGGACAGCCTCATTCAGACGCCGAGGA

GGCTCCACCCGCATCCTGGCAGAGAGAGAGGCGAGCCTTGCAGGAAACGGTGCTGTCACT

CAGGGAGCTCCTGTGCAGGATGGCTGAGCGTGAACCCAAGGTGGAGGGTGAAGTAGACTG

GCGCACAGAGCTGTTGCAGGCCGTGCGGAGTGTGTTTGACAGTGAGAGGAAGTGGCTTCA

TGCACAGCTCCAGGACATCATCACAACCAACACTGGCATAGACTACACGTCTCTCCTGAA

GGATTTAGAGACGCTTCTGCAAAAACAGGAGGATCAGCAGAGGAGGTCTTTGGAGCAGTT

GCTAAGTGCAGACCGAAACAGTCTCCTGGCTGAAATCCGCAGTCTGCATAAACAACTGCA

CACCTGCACGCTGCAGAACCAGGAGCAGCTGCAGCGCTCGCTCGGTGCTGTTCGTGAGGA

AGGTGCGCAGACGCAACAGCAGCTGCACAGACAGGTTGGTGAGCTGGAGAGGCAGCTACA

GCAGGAGCAGTCGTCGTCACATGACCTGCAGCGGTCGCTGGGACAGGAACAGTCCAGATC

AACAAATCTACATCAGCAGCTGGAGATCCAGCAGAAGCTGGCGGTAGAGATGAAGTTGGA

GCTGGAGGAAGGAACCCGGCAGCTTCGTGCGTCTGAGAAAAGCAGAGAGGAGCTGCAGGC

TCAGATTCACAAACTCCGGCTCAAGCTGGAGAGTGAGGAGCAGGAACATCAAGCTTGTGT

GGAGGCTGCAAACGAGATGAGGTCAGGGTTAAGCAGCTTCAGGAGGAACTTCATCACGAG

AGGCTCAGCAGCAAACGCGCGGCGGAGGAGCATGCGCACATGCAAGAGTCTCTCCGGGCC

TCCCTGTGCGAGCATGCGACTCGTCTGAATGAGCTGAACGCTGCACTGGAGCAGGAGCGT

GTAGCAGTCAGTAACCTGCGTGCCGAGCTGCAGATCGAACAATCCCGCTGTGAAGCCCTG

CTGGCGCAGGAACGTGAGCGCACGGAGCTAACCCTCTCGCGCCTGGACGAGGAGCGCTCG

CGCTGTGCCGAACTTTCTCGCTCACTTTCGCACCAGGCTCAGGAACATGCTCGCCGCCTG

GAGGAGGAGGTCCGCTCCCAACAGGCTGCGAGCGCCGACGATAGGAAGTTCATCCAGGAG

CTACGGGCACAGCTGGAGCAAGAGCGGCGCCAGGCCGAGGACCTCGCCGCCACAGTGGAC

AATCTGCAGTCCCAGGTACTGCAAAGCAAACGCAGGCAGGAGGAAGCAGCGCAGCAGGAA

GCGCAAAGAGGACAGGAGGAGGTGAAGAGGCTGCGCTCTGCTCTGGATGGTCTGCAGGCA

CAGCGGGTAGAGGTGAACCGCACCCTGGAGACGGAGAGACACAGAGCTGCTCAGCTTCAG

ACAGAACTGGATGCCGTGAAGGAGAAGATGAGGGAGGTGAAGGAAAAGGAGAGAGCGAAG

GAGGAGCAAAGGGAAAAGCAAAGGTGGTGCGAGAAACAGGAGCAGGAGGACAGGGAGCGG

CGGCAGGAGCAAACCAAAGAAAAACTGGTACTGCAGAGATGAATTTCATACCTGTTAACA

TGAT

>Unigene000047

CACACACACACACACACACGCACGGTTAAAAAAAAATAAGACCTAAAGACGGAGTTTCAC

CGACAGCCAGATAAGATGCCTGCGACGGAGAAAGACCTGGCCGAGGACGCGCCGTGGAAG

AAGATCCAGCAGAACACGTTCACACGCTGGTGCAACGAACACCTGAAGTGCGTGAACAAG

CGGATAGCCGACCTGCAGATGGACCTGAACGATGGGCTGCGACTCATCGCCCTGCTGGAG

GTGCTCAGCCAGAAGAAAATGTACCGGAAGTACCACGCCAGGCCCACCTTTCGCCAAATG

AAGCTCGAGAACGTGTCCGTGGCGCTGGAGTTTCTGGACCGGGAGAACATTCGACTGGTT

TCCATAGACAGTAAAGCGATCGTGGATGGAAACCTGAAGCTGATCCTGGGTCTGGTCTGG

ACTCTTATCCTGCACTACTCCATCTCCATGCCCGTGTGGGAGGAAGACGAGGATGAAGAG

TCCAAAAAGGCCACGCCCAAACAGAGACTCCTGACCTGGGTGCAGAACAAAGTACCCGAC

CTGCCAATCAGTAACTTCAGCCAGGACTGGAGGGATGGCAGGGCTCTGGGGGCACTAGTG

GACAGCTGTGCACCAGGTTTGTGTCCTGATTGGGAGACGTGGGATCCCGTGAAGCCGGTG

GAGAACGCTACAGAGGCCATGCAGCTGGCTGATGACTGGTTGGGAATCCCTCAGGTGATC

GCTCCTGAGGAGATCATCGACCCCAGCGTGGACGAGCAGTCAGTCATGACCTACCTGTCG

CAGTTCCCCAAGGCCAAACTCAAACCCGGAGCACCTCTTAAACCCAAACTCAACCCCAAA

AAGGCCCGTGCTTATGGTCCAGGTATTGAGCCAGTGGGTAACCGTGTGATGCAGCCAGCC

GTGTTCACCGTGGACACATTCAGCGCAGGACAGGGACACGTCACCGTCTACGTGGAGGAT

CCCGAAGGCCGACGGGAAGAAGTGAAGGCCGTTTACAATGAAAGCAAGAAGACCTACAGC

GTAACATACATTCCACAAGTCATGGGACCGCACAAGGTGACAGTGTTGTTTGCCGGACAA

GAAATTCCCAAAAGCCCTTTCGAAGTCAACGTGGACAAGGCTCAGGGTGACCCCACCAAG

GTCACGGCTAAAGGTCCTGGGCTGGAGCCAGTGGGCATAATCGCAAACAAACCCACCTAC

TTCGACATCTACACAGCAGGCGCTGGTGTGGGCGACGTCACGGCGGTAATTAAAGACCCC

CAGGGGAATAAGAACACTGTGGAGGCCATGATGGAGGACAAGGGCGACAGCGTGTTCCGC

TGCACGTACAGACCAGTACAGGCCGGTCCTCACACGGTCAGCATCATGTTCGGAGGCATC

GCTATTCCCAAGAGTCCTTACGCCGTCAGCGTTGGTCCAGCGTCCACCCCTAGTGCTTGC

CGAGCAACGGGTCGGGGCCTGCAGCCCAGAGGAGTGCGTGTGAATCAAGTGGCCGACTTC

AAGGTGGACACCAGAAATGCAGGAAGTGGAGACCTGAAGGTCACAGTGAAGGGACCTAAA

GGCCTGGAGGAGCAGGTGAAGCTGAAGGAAGCGATGGACGGCGTCTACGCCTACGAGTAT

TATCCCAACACGCAGGGCAAATACACAGTGAGCGTCACATGGGGTGGCCAGCACATTCCC

AAGAGTCCTTTTGAGGTCCAGGTCAGCCCTGAAGCAGGACCACAGAAGATCCGAGCTTGG

GGTCCGGGTCTGGAGGGGGGCATTGTGGGCAAGTCGGCTGATTTTGTTGTGGAGTCTGTT

GGCACGGATGTAGGAGTGTTGGGCTTTGCTATCGAGGGTCCATCTCAGGCCCGGATCGAG

TGCGAGGATCAGAATGACGGATCCTGTGACGTCAAGTATTGGCCCACGGAGCCGGGCGAG

TACGCCGTCCATATCATGTGTGATGACGAGGACATTGAGGAGAGTCCGTTCATGGCTTAC

ATCATTCCTGACAACAAATCAAGCAATCCTGACCAGGTTAAAGCCTATGGTCCGGGCCTG

GAGAAGAGCGGTTGCATCATCAACAAGCCTGCAGAGTTCACAGTGGAAGCCAAAGATGCC

GGAAAGGGTCCTCTGAAAATCTCTGCTCAGAACGCGGACGGCGAGCCCATCGACGTGAAG

GTGAAAAGTAAAGGAGACGGCGTGTACGCCTGCTCGTACACCCCCACCTCCGCCGTCAAA

CACACGGTCGTGGTTACCTGGGGAGGGATCAGCGTTCCTAACAGCCCTTTCAGGGTTCAA

GTTGGGAAAGGCAGTCATCCTCACAAGGTCAAAGTGTTTGGTCCAGGAGTGGAGAGAACG

GGTCTGAAAGCTCATGAGCCTACACACTTTACTGTGGATTGCACAGACGCTGGGGAAGGT

GATGTCAGTGTGGGTATCAAGTGCGATGCCAACATCATCAGTGATAAAGAAGAAGACGTG

GATTTTGGTATCATTCCCAATGCCAACGACACCATTACAGTGAAATACATTCCCCCAGGA

GCAGGACGTCTGACCATCAAAGTCCTTTTCACAGATCAGGAAATTCCTGCCAGTCCGTTC

CGGGTTAAAGTCGATCCTTCACACGATGCATCCAAAGTTAAGGCTGAAGGTCCTGGACTC

GCTCAAGCAGGTGTGGAGAGCGGAAAGCCCACCCACTTCACCGTTTACACCAAAGGAGCA

GGGAAAGCGCCGCTGGACGTGCAGTTCTCCGGTCCGTCCAGAACACAACCTGTCCAAGAC

TTCGAGATCATCGACAACTATGATTATTCCTACACGGTCAAATACACCCCAGTCCAACAG

GGTGAAATGGTGATAACTGTGACTTTTGGGGGTGACCCCATCTCTAAAAGCCCCTTTGCA

GTGGGGGTTGCTGCACCGCTTGACCTCAGCAAGATTGAAGTCGATGGACTGGAAAGCAGA

GTGGAGGTTGGACAGAACCAGGAATTTGCAGTGGACACTAAAGAAGCAGGAGGTCAGGGC

AAACTGGAGGCTCGGATTACCAGCCCTACAGGAAAGTCGGTGCCGTGTGTCGTCCAGCCT

CAGCGCGGGAAAGGAGCCAGCCTTGTCAAATACGTCCCGAAAGAGGAAGGTGCCTACAGT

GTAGAGCTGCTCTACGATAGCAACCCGGTGCCCGGAAGCCCCTTCACTGTGGACGCCATG

TTGCCTCCTGATCCCAGCAAGGTCAAAGCTTATGGTCCAGGATTGAAAGGAGGTTTGGTC

GGGAATCCTGCGCAATTCACAATCGATACCAATGGAGCCGGAACAGGCGGACTTGGTCTG

ACCGTCGAGGGTCCGACCGAAGCTAAAATCGAGTGTTCAGACAATGGGGACTGCACATGC

TCTGTGTCCTACCTTCCTACTGAGCCTGGCGAATACCTTGTTAACATTCTCTTCGAAGGC

GTCCACATACCTGGATCTCCTTTCCATGCCGACGTCGAGTACCCTTTCGACCCTACTAAG

GTGGTGGTGTCAGGTACGGGCCTGAAACGCGGTAAGGTCGGCGAGACCAGCGTGCTCAAC

GTAGACTGTGCCAAAGCCGGGCCTGGACAGTTGGTGCTGGAGGCGGTGTCTGATGCAGGA

TCCAAAGCGAAGACTGAAGTGCTGGACAACAAGGATGGCACCTACACAGTGACCTACGTG

CCACTTACAGCAGGCATGTACACACTGCTGCTGAAGTACGGAGGAAAGACCGTCCAAAAT

TTCCCTGCCAAAGTGGTGGTGGATCCAGCTGTTGACACCAGCAAGGTGAAGGTGTTTGGA

CCAGGAGTGGCTGGAGAAGGCGTCTTCAGGGAGGCTACCACCGACTTCACTGTCGATGCT

CGGGCTTTGACCAAAGTGGGTGGGCAGCACATTAAAGCTCAGATTATAAACCCATCCGGT

GCTTCCACAAGCTGCTCCATTTCTGACCAGAACGATGGCACTTATAAAGTGGAGTACACC

CCGTTTGAAAATGGTGTTCATGCTGTGGAGGTGCTGTATGATGACACTCCTCTGCCTAAA

AGCCCCTTCCAGGTAGGAGTAAGTGAGGGTTGTGATCCCACTCGAGTTCTAGCTAAAGGC

CCTGGATTGGACGAAGCGCTCACAGACAAACCTAACAAGTTCTCCATAATCACCAGAGGA

GCTGGTATCGGTGGCCTGGGCATCACAGTCGAGGGTCCGTCCGAGTCCAAGATGTCCTGC

AAAGACAACAAGGACGGTAGTTGTGACGTGGAGTACATTCCCTACGCTCCTGGCCTTTAC

GATGTGAACATTACATACGGAGGGGAGCACATTCCAGGAAGTCCGTTCAGAGTGCCAGTG

AAGGACGTGGTGGACTCCAGTAAAGTGAAGCTGTCTGGACCTGGAGTGAGTTCAGGTGTT

CGAGCCAAGATCCCACAGACTTTTACTGTGGACTGCAGCAAAGCCGGAATAGCACCGCTG

TCTGTAGCTGTAACCGGCCCTAAAGGCAAAGCTGAACCGGTTCAAATAATGGATAACGGA

GACGGAACCCACACCGTAGCCTACACGCCCTCTGTAGAAGGCCCGTACGCTGTAGCAGTC

AAGTACGCTGAAGAGGAAATCCCTCGCAGTCCGTTTAAACTTCGTGTCCTGCCAACACAC

GATGCCAGTAAGGTCCGTGCCAGTGGGCCAGGACTGACTACCAGCCTTCCTGCCAGCCTT

CCTGTGGCGTTTACCATAGATGCCAAGGATGCAGGAGAAGGCCAGCTCTCAGTGGAGATA

ACCGACAAAGACGGGAAGCCGAAGAAGATCAGCATCCACGATAACCAGGACGGGACGTAC

GACGTGTCTTATGTGCCGGATAAAGTGGGCCGGTACACCATCGTCATTAAATACGGCGGT

GACGAGATTCCGACTTCTCCATACAGAGTGCGGGCAACAACGACAGGCGACGCTAGCAAG

TGCACAGTCACCGGTCCTGGAATCGGTCCTACCATCGGCATCGGAGAAGAAGTAGGCTTC

GTGGTGAACACAAAAGGCGCAGGGAAGGGAAAGGTTTCGTGTGTCGTCGTCACACCCGAC

GGAACTGAAGTGGATGCGGAAGTCATCGAGAATGAGGATGGAACCTTCGACATATTCTAC

ACCGCGCCCAAGCCGGGAACCTACATCATCTACGTGCGGTTCGGGGGCGAAAATATCCCT

CACAGCCCTTTCAAAGTTATGGCTACTGATGAACCACCTTTGCTGCAGCAGCAGCAGCAG

TCCGTCCAGCAGAATCGGCTTGCTCCAGGCGCCGGCTTCCAGCCCTGGGTCACAGACGGC

GCATACATTCCGGTGAGCAGCATGAACGGTGCAGGGTTCCGGCCCTTCGACATGGTGATT

CCGTTCACTTTAAGCAAAGGGGAAATTACAGGCGAAGTGCACATGCCATCAGGCAAAACA

GCCCAACCTGAAATTATCGACAATAAGGACGGGACGGTCACGGTGAAGTACGCTCCTACA

GAGTTAGGACTTCATGAGATGCACATCAAATACAACGGCGCTCACATACCAGAGAGTCCT

CTACAGTTCTACGTCAACAGCGCTAACAGTCCCAACGTGACTGCGTACGGCCCCGGACTC

GTGTATGGCGTCGCTAACCAACCAGCTACTTTTACCATCTGCACTGAGGATGCTTCTGAG

GGTGGTCTTGACTTGGCTGTTGAAGGCCCATCAAAGGCAGAGATCGGTTGCGTCGACAAC

AAGGACGGCACTTGTACGGTCACGTATCTTCCTACACTTCCTGGAGATTACAACATTTTG

GTCCGCTATAACGACAAGCACATTGCTGGTAGCCCCTTCACTGCCCGGATCACAGAGGAC

AATAAGAGGAGGTCTCAGGTCAAGCTAGGCTCAGCCGCTGATTTCGCACTGGACATCACG

GAGACGGACCTCAGTCAGCTCACTGCCAGCATCAAAGCTCCTTCAGGCCGCGACGAGCCC

TGTCTACTCAAAAGACAGCCGAACAACCACATCGGCATTTCTTTCATCCCCAGAGAGGTC

GGAGAGCATTTGGTTAGCATTAAAAAGAACGGACACCATGTAGCCAACAGCCCCATCTCC

ATCATGGTGGTGCAGTCTGAGATCGGCGACGCCAGCAGGGTGAAAGTGTATGGACCGGGT

CTGCTGGAGGGACGCACGTTCCAGATGGCTGACTTTATAGTGGACACGAGGGATGCTGGA

TATGGAGGACTGGCTCTGTCCATCGAAGGCCCAAGTAAGGTTGACATTCAGACGGAGGAC

TTGGAGGATGGAACCTGTGGTGTTGCCTACTGTCCCACAGAGCCTGGAACCTATATGGTG

TCCATCAGGTTTGCTGAAGAACATGTTCCCGGGAGTCCATTCAGTGTAAAAGTAACGGGA

GAAGGACGAATCCGCGAGAGTATCAGCCGCCGCCAACGAGCCGCTTCTGTTGCCACAGTC

GGGAGTGCATGTGATCTTAACCTGAGGATTCCAGAGATTGATATCCACGACCTTTCAGCT

CAGGTCACCAGCCCTTCTGGTGTCACCGAACCTGCTGAGATCGGGGCGGTGGGCAACCAC

ACCTACTGCGTGCGATTCGTTCCACACGAGATGGGTGTGCACACGGTAAACGTCAAGTAC

CGTGGCCAACATGTCCCAGGCAGCCCCTTCCAGTTCACCGTAGGGCCACTGGGAGAAGGA

GGGGCCCAGAAAGTACGAGCAGGAGGTCCTGGGCTGGAGAAGGCTGAAATCGGTGTGCCA

GCGGAGTTCAGTGTTTGGACACGTGAGGCTGGTGCCGGGGGCTTGTCCATCGCCGTGGAG

GGCCCCAGCCGCGCCGAACTTTCCTTTGAGGACCGGAAGGATGGCTCTTGTGGTGTCTCT

TATGTGGCACAGGAACCTGGTGACTACGAGGTCTCTGTGAAGTTCAACGAGCAGCACATC

CCCGACAGCCCTTTCCTGGTGCCAGTCTGTGCACCCCCACACGATGCCCGGTGTCTTACT

GTTACCAGTCTTCAGGAATCAGGCCTCAAGCTGAACCAGCCGGCTTCGTTTGCCGTGCGT

CTGAACGGCGCTCAGGGCGTCATCGCCGCCAAGGTCCACAGTCCCTCCGGAGCGCTCGAG

GACTGTGTGGTTTCTGAACTGGAACATGATAAGTACGCCATCCGCTTCATCCCGCGAGAG

AACGGCATCCACACCATCGATGTTAAGTTTAATGACACGCACATCCCGGGCAGCCCGTTC

CAGGTGAGGGTCGGCGAGCCGGGGCAGGGTGGAGATGCCGGGCTGGTCAGTGCTTACGGT

CCTGGACTGGAGAAAGGAACCACAGGCAACCAATCAGAGTTCACCATCAACACCAGTAAA

GCAGGACCGGGCTCCCTGGCAGTAACGGTGGAGGGACCTTCAAAAGTGAAGCTCGAGTGC

CAGGAGTGTCCCGAGGGCTTTAAAGTACACTACACACCAATGGCTCCTGGAAATTACCTG

ATCAGCATCAAATACGGCGGCCCTAATCACATCACAGGAAGTCCCTTCAAGGCCAAAGTC

ACAGGTCAACGGCTTGTTAACGTGACCAACGCCAGCGAGACCTCCACACTACTGCTGGAT

CCCACGGTCCGGGCGGTCAGTGCCGGTTCCACCAACGCCATGCCCAGAGCTGCATCTGAT

GCTAGCAAAGTGACCAGTCGAGGCCCGGGTCTCAGCAAGGCCTACGTGGGCCAGAAAGCC

AGCTTCTCGGTGGACTGCAGCAAAGCGGGTAAAAACATGCTGCTAGTAGGAGTTCTCGGT

CCTCAGACCCCCTGTGAGGAAATCATGGTGAAGCACATGGGCAGCATGCAGTACAACGTC

AGCTACGTTCTAAAGGAGCGCGGAAACTACGTCCTGGCGGTAAAGTGGGGTGAGGATCAC

ATCCCCGGCTCACCGTTTCAAATCACGGTGCCTTAATCTGACACACACACATTATAAGAG

CTACGGAAACTGGCTTTATAAATTACGCCCTGATTGATGACAAACCTGCACTTGAGTTCC

TATTTTGTACATGTGATGTTTTTTTTAAACCACTGCTGAGTTTCTCAACATCTTCCAGGG

GCAGAACAGCAGCTTTGCGTTGGAATCCGGTGTCAGTTTGAGTGTTTGTCTACATTCCTT

TAAAAATATCCTCTGTTACTACTGATGCTGTTTTGTCTATTTCGACTCGTGTTGTATTAT

CCATTATTTTGATTTCTCCTTCCTGCTGCGTCACCTGGACTACTGCGATTGTTTACATGA

TCACGAAAATCCAACGAGTTATCGATCCGACCACTTTAACACACTTCTATCTGTAGACTG

TAGATACATTTATCATAATATCCCAACCGGGTTCAGTTTAATCCGGTATCCGGGTG

>Unigene000048

GTGTGTGTGTGTGTGTGTGTATGCAGAGCTGTCATCTGCATGGAGGAATCTGAACCACAA

CAAAGCAGCTCTGCGTCACATCGAGAATCAGCTGGAGGCGGCGGTTGGTTCAGGAGTTGT

CATTACCTCGTTGAACACTGAGAAGTCTCACAAACACAAACGCAGAGGTAGGCGAAGCTC

TGATGGTGACGATGCAACAGTTAAGCCCCGCCCACAAAGCCGTCGCAGCCCTGATAAGTC

CTCCCGCAGCCCGCTGAGGACCAGCACACAGGACAACACACACACGTCCTCTCCCGCTGA

CGCATCACTGAGTCGCTGCGTTTACGATCGAGACCGTCGTCCTGTCCACTCAGCTGACGC

AGACAGCACGCCATCCTCAGCGCTGGACGCCACGAACGTACGTGTCCTCAACGACTCCTT

CGCTCTGCAGAGGTCAGAGGTCACGATGGGGGCATCGGAGGTCAGCCCAGGGTCAGCGCG

GCTGGAGAAGTTACGTCAGCGCCAGACGGACGAGAAGCTGGAGAAGCTTAAGGAGAGAAT

CCGCAGGCAGAGGGAGCAGCTGGAGGAGACAGCAGGGAGGGGCGGGGCAATAGAGGCGGG

GCATGGAAACTCCGCCCACCAGCACCTGACCAAAGTGCGCAAAGTGGCAGCTGCTCCACC

TGCGCCGAACTATAAAGGGTTTAACAGCAGTGAGGTGAAGATCCAGTCCTCAGACAGCAG

AACGTGGGGAGACACCGAGTCTCGCTTAAAGCCTGAGAATTATCTCACTGACGCTCTCTC

AGAACGAGTGAAACGTAAACCGGTGGAAAAGGATAAGCAGCAAGAGAGGAAGCTGATCAA

ACCCGTGAGGAAGATCCACCGATCTACATCTGCTCCAGAACCCAAACCAGCCATCAGCGC

GTCGTCATGGCGAGAGGGCCAGAAAGTGGTGCAGATGATGCTGGGGCCGCTGCCACGGGA

ACCTTGGGCTCAGACTGCGGGGAGAGAGAGACTGGCAGCTGCTTCATCGCGCTCCAGTTC

TGTTCCTCGGCTGGAGTCGAGGCACCGCTCAAGATCATCAGGTCATCGCTTGAGCAGCAC

AGAGAAGCCTACAGTAACTCGGCCCTCCAGACCACCAGCAGGCACCAGAGAGCGAAAAAC

CGTAACAACTCAGTCATCTAGACCAATAGAGGGCCCCGGCGCTCTTTCTGCCGATCTGGA

GCTTCTTCCTGCAGAGTTTCGCAGCATCCTAGATGATCTGCATCTGGAGGACCGCGGAGT

CAAGTCACATGACCCAGGTCGAGTCGACAGAAAGAGGGCGGGACGAAGCCCCTCTAAACC

CCGCCCAGATAACACAGAGGGAGCGCTGCCACGGAAACGGCACTACGACGCAGACGAGGT

GCGACGCTACATTGCTAAGCAGCAGGAGGAGAGGAAGAGGAAACAGGTGGAGGAGAGGCG

GAGTCAGAGGGAGGAGCAAGAGAGGCGGAGTCACAGACTGCAGGAACTGTATCGGAAACA

ACGTGAAGGTGTTATTAAGCCGCAAGTCCCGCCCCCTAATCTTTTAAACCCACATCTGCG

AGAGACGTACACTAAGATGCTCCTGAAACACAAGCTGCAAGAGGCGCAGCCGGTGTATCA

ACCTTCCGGAGAGTCAGATAAAGAAAACAAAAAAATGGAACCACTCAGTCCTTCCACCAG

TGAACTTTCAGAACACACACCTTCTCCGCTGTGCAGGGCTGATTTGGTGCCCCCTGCTGG

TGAGTTCTTCTCCCATCTTCTCAGTGTGGAGCCAGAGTGTGTGAACTCCACCCCACACGT

GCGAGATACACACACTGAGGACGCACACTGGGTGGGAAAGGGTTGGGTGGATACACACAC

CATCAGCAGGCTGAACCGTGTGGAGGCTCTAAAGGCGTGCGCTGCTTCACTGTCCAACCG

AATCGAAAGCGAGGCACGAAAGCTCATCACCTACAGCGACCATGACAATCAGGACGGGCA

GAACCACCAGAGCAAACCGCCAAGCCCGCCGGAGCGCGAACAACCCCATTCCACCTCCAG

AAATCACGACTACCTGAGGGAGCTTCCCGGTGTCGGTAACCTGCACGGCTTCATTACCCG

GGAAGGGTGGGGTGAGATCCCTAAACCCCGCCCACTCACCGCCAGCACCGCCCTTCCATT

TACTGTGCAGCATGAGGAGCATGACTCAAGCGGCGGCTCCATCAGCGAGGGTGTGCTAAG

TGACGCCAGTCTGTCTGATCACGCCGACCATCCTGACGACCGCATCACCCTGTTCCGGAA

AGAGGCGGAGCTTTACGTGCCCTACCGGCCTGTGGTGACATCACAGCCTCCATGGGAGGA

GCTCAGCAAGGGCAGCCCTCACAGCGTCATCAATATATTCGCCAAAAACCTCAACAAATA

CAACAACGTGATGGACATGGAAGAAAACGGTGCAACGTATGAAGACGACTTTGTTTCCTC

GCGCAGCAGCAGCCAATCAGCATCAAAGAGAACTGCTAGTGGTGTCAGCCGGAGCTCAGC

AGCAGCGGATAGATTTCCTCACTCCTCCGCCTCTGTTCTATTATCCTCACCTTCCTCTAC

ACACTCTGTCAGCAAGAGAGGATCTGATAGGAGTTTGGAGCGCTCTCTGGTGGACGGACA

GAGAAGCATGCCCTTGATTGAGCCTCGGAGGAAAATCGACTCGCCTCGAGGTTCTCACAG

GAGCGCTGAGGGTAATGGGACACCAGGGGGCGCCTCGGTGCGCTCCTCCCACAGTATGGT

GTCTGAGTTTCCCCACTTTCCCAGAAGTCCAGCTGATTCTCCCAGAATCTCAGGCTCCTC

GTCATCCAGTCCTGCATCTGCAAGAGACTCCTCCCCCAAAGTGGAAACTGGACCCGCCCC

CAACCTGGTCTCATCCCCTGGACCCGCCCACAGCATCACAGAGCGACACTTCCCCCCGGA

CGTGCTGCAGCAGCGGTTAAACGCAGAGCTGAACTTCCTGGATGCAGTGGAGGAGTCAGT

GAGACAGCTGAGTGACGTGGAGAGAGTCAGAGCTGTCTCACTCGCTCAGCAGGAAAGCGT

CTCACTCGCACAGATCATCAAGGCTCAGCAGCAGAGGCAGGAGCGAGAGCTGCAACTACT

GAAATTGAAAGCAGAACAGGAAACGCTGGAGACTCAGAGACACGTCCAGGAGAGCAGAGA

GAGAGCAGCACAGGCCCATGTGGAGCTGTGTATGAATCTGGTTCAGTCTCATCAGAAGGC

TCTCATGGAGTTGCAGGAGAACAGCAGCAAAATAAACCACCAACACACACAGACACCACA

ACACCTCCGAGAGATGACTGAGATGAGTCGTCCTCAGCTGCCCAGTGTTTCTTCTGTCAC

ACCTGTAAGGCAGCCACACCCACAAACGGACAGTAGGAGTGAAGTCACAACCGAGAAAAC

ACCAACAGCACACACTCCACAGAGCATCACAGCTGAGTTGGAGTCCTTCAGTTTCACGCA

AAGCAACGGCGGTGGTGTGGACAGGAGCGGCTCGGTGGAGGAGGAAGTGAACACCGCTGC

TGACGACTCTCTCCAAACTGACAAAGACAGCGTCTCGATCAACTACTCTTCAAAGTTTGA

TGAGTCTGTGACGGAGGATGAGTTGGAAAAGTCCTTTCGCTCTGTGCTTCCGTCTGAGTC

TCACTGGCGGGGATCCGTGGAAAGGAAACGGCAGTCGGACGAGGAACCTCACGCTGACAA

AATATCTACTAAGGACGGCAGCGTCCCTGTCTTCTCGGCGGATCAGTACAGTTTCTCGGA

GTTCACCATGGCGATGGTGAAGCAGTGCATGCAGGAAGAGGAAGTTCGCTCTCAGCATCA

GAGCTCCCTGCTGCGACTCCGACACCGAGCACTGAAGGAGAAAACCCGAGCTGAGCTAGA

ATGGCTCGAGCTCCAAAAAAGGCATCTGCGTGATAAAGGAGAGGATGATAAAATGCCTCC

AATACGCAAGAAACAACGCGGCCTGCTGCTTAAACTACAACAGGAACAGGCAGAGATCAG

AAGGCTGCAGGAGGTGAACCGTGCAGCACGAAGGGAGAGGAAACTGTTGCTAAAACAGCA

AGAGGAGATCGAGAGGATCCAACACACCACACAGAAACTGAGAGAGAAACTCCGGAATGC

TGAAGACACAAACCAGAGGTCTCCTGTGTCAGGTGTGAGCGAGGATTTGGCCATTCCCAC

TAAGGTGGACATAGAGTTCCGTAGCCCCTCCCCTTTGTCTGTGTCAGGGAGTGAGACCAG

CAGTGTCATGAAGAAGCTGAAGAAGATGAACTATCACACAGATGAGCGATTTCTTACCCA

GCGTGAGCTGCGATTGGTACATTGGCGTCAGCAGGTGGAGGCGGAGCTGAGACGTTCAGA

TGGGGCGGAGCCTCAGCCGAAGGGACGAGCACTGAACGCCGAACGTAGTACACAGCAGAA

ACACGGCACAGACGAGCTGAAGAGAGACAGACAGGAAGAGAGAAAGACAGGACACGAAGT

GCGCAGCGATTGTGACTCATCATCGGCACTGTCCGTCTCCAGCATACACACAGTGCAAGA

GTGTGACAACACACACAGCCGTGAGTTCTCCTTGGTTCCTGCAAGTTCCAGCAGTAAAGT

GACATCACACAGCAGCACCAGGAAGAGGGACCTCCCTTTATCAGCTCCTACATCTGAGGC

GGCATCGGATCAGAGTGACATTGAGGGGCGTGTCCTGGCTCTGAGGGAGGAGCTTCGGCG

GCGGAAAGCTGAAGTCCAGCTTCTAAAGAGGGAGCAGAGACTGAGACACAAAGAACGACT

GAAAGCTCAGGAGGCCAGTCTGCTCAAACAGCTAGAGTCTTGCAACAACTTCATCCAGAA

GACTAAAGCCGAGCTGAGTAATGCAGATGTTGCCACCACTTCAACCAAACCTCAGATCAA

AACTCCAACATCAGACTCGCACAAATCCGGAACCGAAAGCTTCACCACAGACAGCTCTGG

AACAGAGAAGAGTAGAGAAGAGAAGAGTCACGAGTCTGAATCAGAGAGATCGAACAGGAT

ACACACACAGTCTCCGGCGCTCAGCGATGAAGATCCTTCCACCGTGACCCCGACTCCGGT

TTTGGGAAGCCCCAACCTCCTGAGTCCTCGTGAACTCCAGCATGTTGAGCAAACCCCACA

ACCCGAAAGCCCCGGCAGACCCGGGAAGGACGCCTCGACCGATCTGAGCACAGCCTCCAG

CCCGGGGTCGGATGTCATAGAGGAGCTGGAATCTCTAAGGTCGGATCCAACCCGCTCTGA

ACACTCGCATCCGCTGCTTAATTTAAAACTCCAAGACTCCGATGGGAATTCCAGCAAAAA

TCAACCGGAAATTGAGGTAAAATTCAGCCATGTAGAAGAATTTACAAATTCCGAAGCGGT

TAAATCCAGTTCCATAATAGTAACTAAACTAGAACCAGAAGAACCCAAACTTCCAGAGAG

GTCCTTAACCTTAGACGTCTCAGACCTTGAAGACAAACCACCTGAGTCGCTGTCTGAACA

CAGCTACAATGATGATTTTGAATCAGCAATTAAGGAGGAAACTGGATCTCACTCACTGTC

TTCTGAAGATCCCGAAGAGGTATCCTACAAATTCTCCTTCAGCAGCGAAGATGAGATTGA

GGAGAAGATTAGTCTCCAGTCAGGAAATACCAGTAGGACTTTTGAGCAGGAAAGACCTCC

TGATCTCCACTTCTTGGGGAGGTGTACAGCAGAAAGCATCCATGAAGATGTTCCCAGTCT

TCCGAAGTCTTCGAAGGATGAAATGATGGATGAAATGCCTGATTACGTAATTGGTGAGCG

TGTCCTGGTTAGCAACCTTCAGCCAGGTATTCTAAGGTTCAAGGGACAGACTAGCTTTGC

AGATGGATTCTGGGGCGGAGTGGAGCTGGACGCATCCGAAGGAAGCAACGACGGCACTTA

CAACGGTATTGTTTATTTCCAGTGCAAAGAGAAACACGGGATATTTGCTCCTCCGGGTAA

GATCTCTCACCTACCTGAGACACATGAAGACCATACAAGAAGCTCAGAGGAGAAAGATTC

CTCGTTTAAAGATCAAACATGCAGGAAGACGAACAAGTCTGAAAGCTCACTTCAGAATAA

AAGAGTGGAATCGGATCCGCCTTCTGAGACGGAGGAACTGTCCGGAAAACCTGTGGTTCT

TGATGCATCCGTTACGAAAGACTTGATGATTGATCACGTTGCTCTCAATGGTGAGACTTT

TGAAAATCTTAATGGAGAAAAAAATCTTCTGGCACTAGAGAGCTTTAAAGACATTGTCCT

GGAATTTAAAAACGTCTCAAAACATGAGGAAGGTAGAGATCAAACTCCAGATATTCTGGA

CCTGCTGATCCGTGATGAGTCGTCCACGGCAACCGAGGACCTACAGAAATCTTCAGTAGT

CTCTCCTGAAAAGACAAACGAGGACAACGTGGATGGATCGGTGGTGACTGAACACAAACC

CCTCACGGCTCTGGCAGATACGCTGATAGAACGCTTCATGAGCGATGCCCTGAAACAGTT

TCAGCAGATCAAAAAAAACAAGGAGCAAAAAATCTTAGCTGCTAATCAACTCGTCTTCTT

CGGTGAGGACGAGGAACCCAGTGAAAATGATGTGTTCCGGTTCGGAACTTCAGCCAGCTG

CAGAGTGGACTTCGACAAGGACCAGGAAGAGGTGTCTTCTCCTGAGATGTGCAACCGATT

GGAGAGCCCTGTTTTGGGTCAAAAGGAACTGGTCCAGCGTCTGGAGGAACTGGAGCTGAA

CCGCGAGTTCCTACATGTGCTGGGAGACGAGCCGGACTGGTTTGACGAGGACTTTGGCCT

CAGCTCTCGCAGACAAACTCAGAACAAGAACAAACTCCGGCAGGACGAACTGACGCCCAC

GTCTGCGGCCAAACTCCCGCACCAGAGGCAGCAGGATGAGGCGGTGGCACCGGCGGCCAT

GTTGGTCCCGCACAAAGCCGAGGAAGTGGAGAAGTTGGTGAGTGCCGCCCTGCTGGAGAT

CTGGATGAGCTGCGGGATGGGGCGTGGTAAAGCAAGTCTGACGGGCGTGGCCAAACCTCA

GGCGTCGGAATCGCTGCTGGGGGATCAGAGCGGCGAGGCGTGTGTACGCAGTTACAAACA

GGCAGTGTTTGACCTGGTATGGGAGCTCATGCAGGAAATCTACGCTGAGGATCCCAACAC

CTACAGACCACTGTGGATCAAACCACACCGGATTCATTCACCCTACATCCACAGAATCGA

CAACCCCACTGACCTCCATAAAGTCCAGGCGTTTGTCACCGGTGAGGTTCTAAAGCTGTA

CAATTTAAATAAAGAGCAGAACCACAGAACCGACTGGCAGAAGATGCTGAAGTTCGGCCG

TAAGAAGAGAGACAAAGTCGACCACATTCTGGTGCAGGAACTCCATGAAGAAGAGGCTCT

GTGGGTGAACTATGATGAAGACGAGCTGTTGGTGAAGATGCAGTTAGCAGATGGGATCCT

GGAGGCTCTGCTTAAGGACACTGTGGACGTCCTCAATAAAATCCACCAGAGTAGATCCTC

ACGGGCTCTGACCCCATCCTCATCCACACACCTGCTCTTACAGCCCTGACACACTGCGCC

ATCGTTCAGGACAGATCCCGGAATAGCCTAAACCTCGAGACGGTCTGTGTCCCGAATTTG

CTGCTCAGATCTCCGGTCCCTCCCTTTAAAACCCCACCCTGTGTTTGGGACAGAACCGGA

CCCCCTCCAGCGTCTGTACCACATTTGCCTTCGAGCTGGAGTTTTAATTTATTTGTAAAG

AGTAAACAGTTTTTAAAACTATGTGAAAATGTGTAAAGAGATGATTTAAGCTCACATGAA

CCCATCTAAGCACACGTGGAACCTACAGAACCCAGTGGAACCTGCAGGAGCCTAAAGGAA

CCCAGAAACAGAACGAGACGGTTTAGAGAGGATAAATCAATATAAACTGTGTATTTTAAA

GAGATTTAGAAGAGCAACAATCAGAAACTAAAGTGAACGAGTGCATGATGAGAGATTAGT

GCTTTATTTACCCTCCATATTAAGTGCCTGACGGTGTAGGCTCCCTCTCTAGGGGAGAAC

CGGTTTCAGCTTCCTCTCGTGTGTCGAGCCGAGTCGCTTTTATACTCTGGCGTTTCTGCT

TTCCAGCTGGACCCCTGATTCTGCTGCTTCATTAACTCTTTCCTACACTCAGTAGGGAAC

CTCCTCAGAGGGCTGCGTCCTAAATTTTCCACACTTCCTTAAAGACCGCCCCC

>Unigene000049

ACACACACACACACACACACTTTGGGATTTTTCTTTGCTGGACTTTTATTTTCAGTGAAA

AAAGAAAGCAGCGCGCGGCCACGATGAGCGCGCACGGAACACCAACCTCGCGCTTCCCTT

CCATGTCGCGCGGGCCGAGTATGCGTGTCAACGAGCAGATGTTCATGAGCGGAAATGGCT

ACCAGTCGGACCTGAGTGAGAACATCATGCAACAGCGCTATAGTACCAACACTTACAGCG

TAAAGAGTCAGGGAAGCATGATGCCTCTGCAGCAGAACAGCCAGGTGGTGTATAACCGTT

GCATGCGCAATCTGGAAAAGGCCCAAAGACAACTGGAGCTAGTGTTACAGGGAGCACCCC

GCAGTGAGTTTGATCAATTGATGGCGATGGCTGAGATGGACATCAAACAGCTTCATCAGC

TTGCGATTGAGGGCAAAAACTTTGGCCAGTACCCTGAATTTATAATCAGAGGTCTGGAGG

AGTGTAATGCGACATACGCAGAGCTCTCAGCCGGTGCAGGAAAAATTTCAGTGCGTCAAA

ACCGTAACAGCACGAGCCGAGAGGAATACAGCCTGACCCTAACTGATGCTTATGCCTGGA

TCAAGCAGCAGAAACGTCTGATTGAGACAGCACCGTTTGGTGATGACTGTGAGGCCATTG

AACAGCAGATCGCAAACCACAACAGGTTCCACAGCTCAATCCAACGCAGTGCAGAAGTGC

AGAGAGCCAGGGATGAACTGATGCAGTTAGGAGACAAGCCGAATATGCATGCTCTGGAGC

AGGAGTGGGACTCCTTACAGAAAATGTCACACGATCGCTTGACTCACCTGCGTGACATGC

AGAACATCCTGACTGACATCTCACAGGCCATCATGTGGGTGAACGATAAAGAGGAGGAGG

AGCTGATGTTCGACTGGGGGGACAAAAACATAGACACCTACATCCCCAAAAAACAGGAGA

GCTACTCGAGATTGATGAGTGAACTGGAGATGAAGGAAAAAGAGCTGAACAAGTTAAAAT

CCAGAGTGGATCAACTGTTGAAGAATAATCACCCTGCATCCGATAAAATCGAGGCCTATA

TGGACACACTGCAGACTCAGTGGAGTTGGATGCTTCAGATCACCAACTGCATCGACACAC

ACCTAAAGGAGAATGCTGCTTATAGCCAGTTCTTTAAGGAGGCGAATGAAACGTACACCA

GACTACAAAAAGATGACGAGAACCTTCGCAAGAAATTCACTTGCAATAAATCCGCACCTC

TGGAGAGTCTGACTGAACTCCTGAGAAACATGGAGAAGGAGAAAGAGAGACTGATGGAGA

ATAAGAGACAGGTGCAGACGTTGGTCAACAAGTCGAAGAACATTGTGTGTCTGAAACCTC

GAAACCCAGAACACAAGAGCAACAACAGAGTGATCGTAAAGGCACTGTGTGATTTTAAAC

AGGACCAGAGAGGTATTTATAAAGGGAACGAGGCGATTCTATTGGATAACGCAGAACGCA

GTAAATGGCAGGTGACCGGTCCTGGAGGATTGAACATGTTCATCCCGTCTGTGTGTCTCA

TCATCCCTCCACCAAACCCGCTGTGTATCGACCTGGCCAACAAAAACGAAAAGTACTTTG

AAGCTGTCATGGGTATCTGGAACCAGCTGTTCATCAACAATAAGAGTCTGATTGCCTGGC

ACTACTGTCTGAGAGACATCAATCAGATCAACTCACTCACCATCAGCATGCTGTCTAAGA

TGAAGCCTGAGGAGTACCGCAGCATCATTAAGAGTCTGGAGTATCACTACGAGGAGTTCC

GGCGTCTCAGTCAGCAGTCCAGCCTGTTTGAAGAAGCAGATCAGAGAAAGATGGCGGTGG

ACTATAACAGCGCACAGACGCACTACGACACCATCCTCACCAAAGTGCAGCCTGGCTTCG

ACGCCAGCCAGAGACCAAAAGCACCTCAACACACACATAACCTACTCACACTCACACACA

CACTGCGGCGCAGGCTGGAGGCGGCCGAGAGTGGACTGAGCCAACACCTCCACGTCCCAC

TGAGGGAGAACAGTGTACAAGAGTGTACACAGAGACTGCAGCTGCTGCAGGGTGTTCATC

ATGACCTGGGTTCGATTCAGGATGAGTATCTCCACCTGAGAGAGTTGGTGTTGAAGCAGG

AAGTAAACGCTGATCCAGAACAAGCCAAATTCCTCCGAGCCGAACTCGACCTCATCAACC

AAAAACTCAGCAGCTTAAGGAGTTATTCAAACTCCTACTTAAATAGGCTCACTGCTCTGA

GAGCTCTGATGCAGAATCTCACACGTGCTGAGGACATCATTAAAGTTCATGAAGCTCGAC

TGACTGAGAAAGAGACTAGCTCTCTGGACCAGGGTGAACTGCAGGACTACCATAAAACAC

TCAAGAACATGAAGGTGGATTTGGACCAGAGAAAGGAGGTTCTGAGTGCGATGGAGGCAG

ATCTGGATCAGGCGTATCACTGGAACGGACAAATCGATCAGAACTTCCATCAGTGTGACG

TGGACCTGAGTAAGTTCTCTGATCTGGTGAAACAGATGATCAGCCGCTGGAACCGGATCA

CCAGCCAGATCAACACCAGAATGGGTGATCTGAATAAACAGGAGAAGCAGCTCAGTCAGT

ATAAACACAACAGTTCAAACACCAACCAGTGGATCGACCAAACCAGCAACCGACTGGACA

AACTGCAGAACTACAAACTAAACACACTGCAGGACCTGATGGACCATCTTAACCAACACA

AGACGCTGCACTCTGAGATTAAAGGAAAAAAGGACAAAGTGGATGATGTGCAGAAGGATG

CAAGCACCTGTTCTGCCTCCATAAAGGATTATGAGCTGGAGCTGGCGTCCTACAGTGCTG

GACTGGAGACACTGCTGAACATTCCCATAAAGAGGACCATGCTACAGTCTCCAGCCACCA

TCCTGAGCCAGGAGGCGGCCGATGTTCAGACTCGTTATATAGAGCTGCTGACTCGCTCCA

CCGATTACTACAAGTTCCTCACTGAGATGCAGAAGAACATGGATGAGCTGAAGATACGAA

ACACTAGAATTGACCTTCTGGAGGAAGAACTGAGGCGTTTGAAGGATGATTTGCAGGATC

GCTCACAGAAGAACCGCTCTCTGGAGGAAATGTTGGCCCGTCTCAAAATAGATCTTTCAC

AGTCTAAGGAACAGCTGATTTCTATGGAGCAAGTGAAAAGTACTGAGGCCATGAGATACA

ATGCAGCTAGGGAGAATCTGGACACCACCCAAAATCAGCTAAAAGACCTGAATGACCAGG

TTGCACGGCTTACACTGCTTATTGATGAGGAGAAACGTAAGCGAAGGCTGGCTGAGGAGC

GTTATTCCAGCCAACAACAGGAATACGAGGGGGCCATCCGCAAGAGACAGAAAGAACTGG

ATGAACTTAACTGGACCAAGATTGACTTTGAAAAGGCCGTTAAGGACAAGGAGCGGGAGA

TCGAACGTCTGAAGGTGCAGTTGGAGGACGAGGCCTCTCGGCGCAGAACCGCGGAATCTG

AAACCGCTAAGGTAAGAAACCAGTTCAACCAGGACCTCACTTCTCTAAAACAAACGTATG

AGTCCGAGATCCACGTTACCAAAACCACTGTGCTAAAGGACATGCAACAAAAGGAGGAGG

ACACAGCTGCCCTAAAGATGCAGCTGGAGAAGCTTATGAATGAGAAAAAGGATCTTGAAG

AAGAACTCAGAAGGCTTCAGATGTCTATCAGACAAATGGAGGAAGCCAGAAAGAGAGCCG

AGCAGGAAGTACATCAACAAAGATCCTCAGGAACAGAGGAGACCCGAAGGAGAAAGGAAC

TGGAGATACATGTTCAAACTGTCACACGACAAAGAACTGAACTTGAGAGCAGGTTCAATG

AAGAACTCGTACAAGCCAACAACAAATTGCAAGAAAAGGCCCGTCAGATTTCAGTTCTTA

CTCAGAACCTAGAGGATGAAACCCGAAAGAAAAGAGCTCTGGAACAAGAAAACCACAGAC

TTCGCCAGTCAGAGGCAGACCTGCAGGCCAAGCACTCTTCCTCTTTGGAACTCATCAACA

AGCTAAAAATCACAGAGAAGGAGACAAAACTTATTTGTGTCGAGTTGGAGAAGCAAGCAA

GTGAAAAAGGAAAAGTAGAGCAGGGTGCCGCCAGGATGCAGACTCGCATTATAGACCTGC

AGAACATGTTAGAAAAACTTGAGTTTGAGCTGGAGAAAGAAAAAAAAAGCAACCAGGATG

AACTTACAAGGAGGAAGCGGATAGAAACTGAGCTGGAAAGAGTGAATCAAGTGTGCAGAG

AGTACACAACTACTATTAACTCTCTCCGTGCCCATAAGGAAGAGGAGAGTGCCACCAGCC

GAAGGCAAGAACAAGACCTCCATCGTGTCCAGCAGGACCTGGAAAGATGTCGGAAGGAAT

ACAATGTAACGTCCGAGAACCTTGCCAGACTGACAGCCGAGCTGAAGGCCTTGCAACAGC

AGCTGATTCAAGAGCAGGCTCGTGTTCGTGAGGTGAACCTGCGCAACGAGAGCCTGTACA

AGACCATAGAGGAGAAGAGCAGAGTGTTAAATGAAAGCATGTCTGAAATTGAGAAGCTCA

AGGGTCTCACTCAAAACCTTACAAAGGAAAGGCTGAAGCTTGAGGAGGAGCTAAGGAGTG

TACGCCAGGAACGGGAAGACTTGCGCAACAACAAAAACAACAATGAGAGTGAACATATGG

CCCAACTGTCAGCCATACAGCTCCAGCTTCAGGCCTCAAATAAGAGAGGCCTAGAACTCC

AGGGCCACATTAGTGACCTGACAAAAGAGAGAGAAAATTTAAAGACGGAAATAGCCAAAA

TCCAAAAGCAATATACGGAGACATCTCTTATGGTCCACGAGTCTCAGATGCACTACAAGG

AGATCCTACAAGAGAGAGATAGTCTTCTGGCCAAGCTTAAATTGCTAGAAATGGACAAAG

GGAAGCACCAGCGCTCCGAAGAAGAGCTAAGTCGCATCAAGATGTCACTGGAATCAGAGC

TGCGTCAGAAACAGCGCCTTCAGGAGGAAATTGACAAAATCCGCAAGGATTTCAACTACT

GGAAGAGTCAATATGAGATAAAGGATGGTCAGGTTAAACAATGCCACGCAGATAAGGACA

AGTCAGAGAGGGACAGGTCTTCATTGCAAAGTGAGATAACGAGACTGACTGCTGAACTTA

GAAGTTTGGACGAGCGCTACCGCACTCGGCTTCAGAGCTCTGAGTTTGAAGTGTCTGAAC

TCACCCGTAAACGAGAGGCTCTGGAAAGGGAGCTGAGGAGGCTACAGCAACGTCCAAGCG

CCTTTACTAAGCAAACTCAGACAGATGAAAATGTGCCCATGATTGATCCATCAAAGCTTG

TATTTGATGGCATTCGCAAAAAGGTGACTGCTCATCAGCTGTGTGACTGTGGGATCATCG

ATAAATCTACCCTTGATAAGCTGTTGAAGGGCCAAATCGCAGTGTCAGATATTTCAGCAA

AAATTCAGCCGTATCTTAAGGGTACCGGGGTTGTTGCAGGTGTTCAAAAAGATTCCCACG

GGAGAATGCCAATCTCCGAAGCTAAAAGTAAGAACCTACTGAGTCCACAGAGTGCCATTA

TGTTGCTAGAAGCTCAAGCTGCCACCGGATACATAATTGATCCAAAGTTTAATGAGAGAA

TGCCAGTAGATACAGCTTGTTCCAGAGGTATTGTTGACACAGAGGATAGAGATGTCCTCG

TTACAGCTGAGGCAGCTTGTACTGGGTTCAAGGATCCATTTTCAGGAAAGGTGTTGTCTG

TTGGACAAGCATGCAAGAAAGGCCAACTTGACAAGGAGAAGGCCCTTCGACTGCTTCAGG

CTCAGGAAACAGTGGGAGGGATAATCGACCCAGTACTGAGCGTGTTTCTACCCAAAGACA

TTGCCTTAGACCGTGGCTTGATTGATGAAGAGCTTTATCGAGCGCTGAACAAAAAGCCAG

CTTGTTACATTAATCCAGCAAATTCAGAGAAGATCAGCTACAGTGATTTAAGAAGGAAGT

GTGTCACAGAACCTACAAATGGCCTTCTTCTTTTCCCTGTAAATGAAAACTCATACTATG

TCAAGGGAATCCGTGGCCAAGTCTCACTTACTGAGCTTGTAAATTCCAGCCTTATTACAG

AGGGGGATGTAGACAAAGTGAAACAAGGCCTGCTCACAACCAAAGATATTGAAAATAGGC

TGAAGACGTACCTGTCTGGTTCCGGCTGCATTGCCGGGATTTACGATGAGGCACGGGATC

GAGTCCTGCCTTTCTACCAGGCCATGAAAGAGAATCTTCTCCGTCCAGGAACAACATTAG

AGCTTCTGGAAGCCCAGGCTGCATCAGGCTTCATGATCGATCCAGTCAACAATGTCTACC

TGACAGTGGAGGATGCATGGAGGAGAGGCCTCGTGGGTCAGGAATTTAAAGGAAAACTTT

TGTCTGCTGAGAAAGCTGTGACTGGATACCACGATCCTAGTACTGGGCAGCTCATCTCAC

TCTTCCAAGCTATTGAAAAAGAACTTATTGAAAAGGGGCATGGTATTCGGCTTCTGGAGT

CTCAGATAGCCAGCGGAGGCATCATTGACCCTAAGGAGAGCCATCGAATTGATGTGGAAG

TGGCTTACCGAAGGGGTTACTTTGACCGTGAGATGAATGAAATTCTGTCGTATGAAGGCG

ACGATACAAAGGGCTTTTTTGACCCAAACACTCAAGAAAATCTGACCTATCTGCAGCTCC

AGAAGAGATGTATGAAAGACCCTGTGACCGGTTTGCTTCTGCTGCCACTGAAAGACAAAA

ACAAGCAAAAGCAGCAGTTAAGTCAAAAAAACACACTTCGCAAGAGGAGGGTGGTAATTG

TGGACCCTGACACGGGCAAGGAAATGTCTGTTCGTGAGGCTTATCACAGAGAGCTGATTG

ACTATGACACATTTTTGAATCTTTCGGAACAAGAGTGCGAATGGGAGGAAATCACTATCA

CAGATTCAGCTGGCAAAACACGTCTAGTTATTGTTGACCGTAAAACAGGTACACAGTATG

ATGTCCAGGATTTTCTGGACAAGGGCCTTATTAGGAAAAGTGACTTAGACCAGTACCGCT

CGGGAAGCCTCACACTCACCCAGTTTGCTAATCTGTTCTCAACCAAAGCTACAAGTGCTG

AGCTCTCCATCTGCACCAGCATTCCAGAAGATGTTACGAACTGCGGTAGCCCCACTGAAG

TTAGACCATCTTCTCCAACTGTCCGCAAACGCTTTGCTAGTGTTTCTATCACACTCTCCC

CTCCTTCGGAAATCCTGGATGATCAGAGCCCGGTGGCAGCCATCTTTGACACTGAAACTT

TGGAGAAGATCACAATAACAGAAGCTCTGCGTCGTGGAATAGTGGATGCGATTACAGCAC

AGAGGCTCCTGGAAGCCCAGGCATGCACTGGAGGTATCATAAACCCTGCAAATGGCAAAA

AGCTTTCTCTGCAAGATGCTGTACATCAGAGCATCATCGATGAGGAAATGGCCAACAAGC

TAAAACCAGCTCAAAAGGCTTACATTGGATATGAGGACGTAAAGACCAGAAGAAAAATGT

CTGCTGCAGAGGCCATTAAAGAGAAATGGTTACCATATGAAGCTGGTCAGAGGTTCCTGG

AATTTCAGTACCTGACTGGAGGACTGCTGGAACCAGAAAGTGGAAAGCGAATTAGCATTG

AAGAAGCCATTCGAAGAGGCTGGCTGGATGGTAAAGGAGCCCAGAAGTTGCAGGACACCA

GAAGCTATCTGAAGAGCCTAACTTGCCCTAAAACCAAGCTGAAGATCTCGTATAAAGAGG

CGATGGACAATTGCATGGTGGAAGAGAACAATGGCATGAAGATGCTTCAAGCCACATCTG

TGTCTACCAAGGGTATCAGTAGCCCCTATAACGTGTCCTCCGGTCCTAGCTCCCGCTCTG

GATCTAGAGCCGGATCCAGGGGAAGCTCTAGAAGTGGATCCCGCAGAGGCAGCGTAGATT

ACTCCACTTACACTTACAGCTCCACCACCTCCAGCTCTTACACTCCATTCCCTTAATATT

TCAACCGTGCAGCTGTGTGTGTGTTGGGGGGGCTCCTTTTTTTTATACTACTCATTTTAC

TCAAATATCAAAAGAATTATATTAACGTGGGTTTGGGAA

>Unigene000050

GGCTGTGTTGTGCTGTCCTGTCTGCGGTATAAAGCCGCTAATAAACGGAACAGATGCTCT

TTGGCGATGTGACACGCGCGTTAGGATTAAACAGGAGTGACCGAAAAGAGAGAAAAGTTT

AAACCGAGTTTATAGACGGAAATACAGCGAATGACCGAGATTATATCTTCAGGTGTGCTT

GGGAAAAAGTTAAGAGCTGCAGACAGCAGCATCAGGAGGACCTGAGTGAAGATGTCTGTC

TCCACTCAGCTGGGCCTGCTGCTATGGAAGAATTTCACTTATAGGAGACGTCAAACGTTC

CAGCTGCTAATTGAGATTATCTGGCCCCTTTTTATCTTCTTCATCTTGATTGCTGTGAGA

CTGAACTACCCCCCATATGAACAACACGAGTGTCACTTTCCCAACAAAGCCATGCCCTCA

GCAGGCACCCTGCCATGGATCCAGGGCATTGTGTGTAACGCCAATAACCCATGTTTCCGT

CACCCCACCCCAGGGGAATCCCCAGGAGTAGTTGGCAATTTCAACGATTCCATAATATCA

CGCCTTTTTTCGGATGCCAAGAAAATCCTCCTGTACAGTCAAAACAACAAAAGTGTGGAT

GGGTTTAAAGAGTTTATCAGTGCAGTGAAACACATGCAGAACAATTCTTCAGGTTTCCGG

CTTAAAGACTTTCTAAATGACAACGAGACCCTGTCTGCATTCCTCATGCAAAATGCTTCC

CTCACGGATAATGTTGTGGAGGACATCCTGAGGGCAGAAATAAACCTGGAAAAGGTGCTA

ACTCGAGGCTATGGGGTACATTTGAGGAACATGTGTAACACATCTGTGTTGGAGCAGTTT

GTCAAATTCCCAAACAGAACAGTAGCAGACCTTACCCACAAGATGATCTGCAGCTCACCT

CAGCACTGGCTTAACAAGGCTGAGAAACACTTCCTGGCCAACTTGGACTTCTTCAGACCT

CTGAGGTCTGATGTAAGATCGGAGAGAAATGACACCCACCATGTTGCCAAAGCCACAGAC

AACCTTTTGGAAAGCCTGGGTTCTGTTGCTGTGGAGTTGGCCAGCATGCGAAGCTGGAGA

GACTTGCGCAATGAGATTCTTTTCCTGACCCAAAATGCCACTGGTTCTCCCGGCCAGATG

TACCAGGCTGTGTCAAGAATAGTGTGTGGACACCCCGAGGGTGGTGGCCTCAAAATCAAG

TCTCTTAACTGGTATGAAGACAGCAACTTTAAAGCAATGTTTGGCAACTACAACAGCAGT

GATGAGGAGCCGGTTTCAGTCTATGACAACACAACAACTCCCTATTGTAACAATCTGATG

AAGAATATTGAGACTAGTCCCATCTCCCGGATGATATGGAGAGCAATTAAGCCTCTGCTG

ATGGGGAAAATTCTGTACACACCTCAAACCCCTGCTACACAAAAGATAATCCAAGAGGTC

AACAGGACATTTCAGGAGCTGGGAATTCTCAGGGATCTGGGTGGGATGTGGGAAGAGGCT

AGACCGAAGGTGTGGAACTTTATGGAGAACAGTGAAGAGGTGGACCTTGTGCGGATGTTA

CTACAGAACAACGTCACAGCCTATTTCTTTAGTGCCCAACTGAGTGGTACCCAGTGGAGT

GTTCAGGATCTTAGCAGTTTCCTGACCAAGCATTCAGAGGACACTCGTCCACCAGGAACA

GTGTTCACATGGAGAAATGTTTTTAATGAGACCGATCAAGCTATTATGAGCATATCTCGC

TTCATGGAGTGTGTAAATCTGGATAAACTTGAAGCTGTGAGCACTGAGGAGAAGTTAGTT

AATGAGTCTCTTAGTCTACTGGACAACAGGAAGTTCTGGGCTGGACTTGTTTTCCCCGAT

ATTGACATAAACAGCTCCGAGCTCCCTCCCCATGTCAGCTACAAAATCCGCATGGACATT

GACAATGTGGAGCGCACCAACAAGATCAAGGATGCGTACTGGGACCCTGGCCCCAGAGCT

GACCCATTTGAGGACATGCGGTACGTTTGGGGTGGTTTCTCTTACCTCCAGGATGTTATA

GAACAGAGCATCATCAGAGCAGTAACTGGAACTAAAGAGAAGACTGGAGTTTACATCCAG

CAGATGCCATACCCATGCTATGTTGATGACATTTTCTTGCGCGTGATGAGTCGCTCAATG

CCCCTCTTTATGACTCTAGCATGGATGTACTCTGTGGCTATTATAATAAAGAGTGTTGTC

TACGAGAAGGAGGCCCGCCTCAAAGAGACCATGAGGATTATGGGATTGGACAACGGTATA

CTGTGGTTCAGCTGGTTCATAAGCAGTCTTATTCCACTTCTTATGAGTGCTGGACTACTA

GTGTTTGCGCTCAAGATGGGGAACCTACTTCCCTACAGTGATCCAGGTGTAGTCTTTCTC

TTCTTGGGCTCTTTCGCTGTGGTCACCATCATGCAGTGTTTTCTGATCAGCACAGCATTT

TCTCGGGCAAACCTGGCTGCGGCGTGTGGAGGAATCATCTACTTCACACTCTATCTGCCC

TATGTGCTGTGTGTGGCCTGGGAGGAATACGCAGGTCTTCCTGCTAAAATAATTGCTAGT

ATACTTTCTCCAGTGGCCTTTGGGTTTGGCTGTGAGTACTTTGCGCTATTTGAAGAGCAG

GGAGTGGGGATCCAGTGGAGCAACCTCATCTCTAGCCCAATGGAAGGAGATGACTACAGT

CTCACTACCTCTATTATCTTGATGTACTTTGACGCCTTTCTCTATGGTGTCATGACCTGG

TACATTGAGGCTGTGTTTCCTGGTCAGTATGGTATTCCCAGACCATGGTACTTCCCATTC

ACCAAGTCTTACTGGTTTGGAGAGGAAGAGCAGAAACAAACACCCATTATACAAGGAAAG

AAGGCAAATGCTGGAGCTGTGTGCATTGAGGAAGAACCTGGTCATCTTGAACTTGGGGTG

TATATTAAGAACTTGGTAAAGGTCTATCGTCATGGAAAAAAGCTTGCTGTGGACGATCTT

ACACTAGGCTTCTATGAGGGTCAGATTACCTCTTTTCTCGGGCACAATGGAGCAGGAAAA

ACCACCACTATGTCCATACTGACAGGATTGTTTCCTCCAACATCTGGCACAGCTTACATC

ATGGGCAAAGACATCCAGTCGGAGCTAAGTGCTATCCGGCAGAGCCTGGGGGTTTGCCCT

CAGCACAACGTCCTCTTTAGCATGCTAACAGTGGAGGAGCACATTTGGTTTTATGCACGA

CTGAAAGGCCTCTCCGAAGAGGAGGTAAAAGATGAGATGGATCAGATCTTGTATGACACC

GGTCTGCCACACAAACGCAAATCCAAGACCAGTGAGCTCTCTGGTGGCATGCAAAGGAAG

CTTTCGGTGGCCCTTGCATTTGTTGGAGGGTCAAAAGTGGTCATTCTGGATGAACCAACT

GCCGGAGTAGACCCTTATGCTCGCAGGGGCATCTGGGACCTCTTGCTGAAATATCGTGCA

GGACGAACAATCATCCTTTCCACTCATCATATGGACGAAGCAGACATCTTAGGCGACCGT

ATTGCCATCATCTCGCACGGAAAGCTATGCTGTGTTGGCTCCTCTCTCTTCCTAAAAACA

CACCTGGGAACTGGGTACTACTTAACACTGGTTAAGAAGGATCAAGAGGCTTCTCTGAGC

TCTAGCAGAAACTCCACCACCACAGAGTCTTATATCAAGAAGGATGAAGACGACTCAGAG

AGCAGTTCAGATGCTGGTTTAGGCAGTGACCAAGAAAGTGATGCTGCTACAGCTATAGGC

ACCTCATGGAATGATTCACCTGTTGTTCCTGCAGACGTCTCTCTAATCTTAGGTCTGATC

CTAAAGCACGTCCCTAACGCACGCATGGTGGAGGATCTGGGACATGAAATCACCTTCGTC

CTGCCCTATGAATCGGCTAAAGATGGTGCCTTTGTTGATCTCTTTCATGATTTGGATGAT

CACTTATCTGCACTGGGTATCTCCAGTTATGGGGTTTCAGACACCACACTTGAAGAGATT

TTCCTGAAGGTAGCAGAGGACAGTGGGGTTGATACTGAAATGATGTCGGGAGACGGCACG

CTTCCTGTACGTAGACGCAGACGACATGCTTTTGGAGCTGATCATCAGAGCTGCCTTAAA

CCCTTGACTGAGGATGAAAACTTTGACTGCAATGAATCTGAAGTTGAACCAGATACCAAA

GAGACTGATCATTTAAATAGCACTGATGGTAAAGGTTCCTATCAAGTCCAGGGCTGGAGC

CTGATGAGACAACAGTTTGTAGCCTTGCTGTGGAAAAGGTTCTTGTATGCTCGTCGATCC

AGAAAAGGCTTCTTTGCTCAGATTGTTCTGCCTGCTGTGTTTGTGTGCATAGCTTTGGTC

TTCAGTCTTATTGTTCCCCCCTTCGGAAAATACCCCAGTCTTGCTCTCCAGCCGTGGATG

TATGAGGACCAGGTTACCTTTATCAGCAATGATGCACCTGAAGATGCCAGCACTCAGAAA

TTACTCTCTGCACTATTAGACGATCCCGGCTTCGGAACACGCTGCATGAAAGGAGAACCA

GTACCAGATGTTCCATGTACTATGGGCGATGAGGATTGGTCCATACCAGAGGTCCCAGAT

AATGTGTGGGAGGTGTTCAAGAATGAAAACTGGACCATGGAAAACCCATCGCCAGAATGT

GAGTGCAGCTGTGACGGCAATAAGAAGATGCTTCCAGAATGCCCTGCTAGTGCTGGAGGA

CTTCCACCTCCACAGATAAAGATCAGTGAGAGGGACACCCTTCAGAACCTGACTGGAAGA

AACATCTCAGACTACTTGGTGAAAACATATGCGCAAATCATCGGCAAAAGTTTAAATAAC

AAGGTGTGGGTGAATGAGTTCCGATATGGAGGTTTCTCACTGGGTGCCAGAAGCACTCAG

GTTCTTCCTCCAGCTCAAGAGATTGATGATACCATTTCTCGAGTAAGGGAAATATTCCAT

CTGCATAAGAACTCAGGGACAGCAGCTGACCGTTTTCTCAAAAGTCTGTCTGCTTTCATC

AATGGCCTGGACACTAAAAATAATGTGAAGATCTGGTTCAATAATAAAGGCTGGCACAGC

GTTGTGTCTTTCCTCAATGTGATGAACAACGGTATGTTGCGGGCCAATCTACCTCCAGGA

AAGGACCCAAGTAAATATGGCATTACAGCCTACAATCACCCTTTAAACCTCACCAAGGAG

CAGATCTCCCAGGTGGCACTGGTGACAACTTCAGTGGATGTACTGGTGTCTATCTGCGTT

ATCTTCGCCATGTCATTCGTCCCTGCCAGCTTTGTGGTGTTTCTTATCCAAGAACGTGTG

AGCAAAGCCAAGCATATGCAGTTTATCAGTGGAGTACAGCCCTACCTCTACTGGCTGGCA

AATTTTATCTGGGATATGTGCAATTACATCGTCCCAGCAACCCTGGTCATAATCATCTTT

ATGTGCTTCCAACAAAAAGCCTATGTGTCTTCCACTAATTTGCCAGTACTTGCCCTGTTG

CTGCTTCTGTATGGATGGTCCATCACACCTTTGATGTACCCAGCCTCTTTCTTGTTCAAT

ATCCCCAGCACCGCATATGTTGTTCTGACCAGCGTAAACATACTGATTGGTATCAACGGC

AGTGTGTCTACATTTGTTTTGGAGTTATTCGGTGGTGATGAAGTTGGTGGCATTAATGAC

ATACTGAAGAACGTGCTCTTGATCTTCCCTCACTTCTGTCTGGGACGAGGCTTGATTGAC

ATGGTGAAGAATCAGGCCATGGCTGACGCTCTGGAGAGATTTGGAGAAAACCGTTTCCGC

TCCCCTTTGGAATGGGACATGGTGGGAAAGAACCTTTTTGCCATGGCAGTAGAAGGAGTG

GTCTTCTTCATTATCACTGTCCTGATTCAGTACCGTTTCTTTATCAAACCCAAAGCTGTT

CGCCCTAAGCTGAGTCCGATTGGAGAGGAAGATGAGGATGTGGCCAGGGAGCGACAGAAA

ATAATGAGTGGAGCAGGGCATGGAGACATCCTCGAGCTCAGAGAACTCACAAAGGTATAT

GAAAGGAAGGAGAAGCCTGCTGTGGATAGGCTTTGTGTTGGAATTCCTCCAGGAGAGTGT

TTTGGTCTTCTGGGAGTGAATGGAGCTGGAAAGACAAGTACCTTTAAAATGCTGACCGGA

GACTGTGCAGTCACCAGCGGAGAAGCATTCCTAGCTAGAAAGAGTATACTCAGGGAAATA

GATGAGGTACATCAGAACATGGGCTATTGCCCTCAGTTTGATGCCATCAACGACCTGCTG

ACAGGCAGAGAGCACCTGGAGTTCTATGCCATCCTACGTGGTGTTCCTGAAGGAGAAGTG

TGTGAGGTTGCTGAGTGGGGCATTCGCAAACTGGGCCTTATAAAATATGCTGACAAAGCA

GCTGGAAGCTACAGCGGGGGCAACATGCGCAAACTCTCTACAGCCATGGCCCTCATTGGA

GGCCCACCAGTAGTCTTTCTGGATGAACCCACAACTGGTATGGATCCTAAAGCACGCCGC

GCTTTGTGGAACTGCATCCTCAGCATCATTAAAGAGGGACGATCCGTAGTTCTCACCTCA

CACAGTATGGAAGAGTGTGAGGCACTGTGCACTCGCATGGCCATCATGGTCAATGGCAGG

TTCCGTTGCCTGGGCAGCGTTCAGCATCTAAAGAACAGGTAGGACCTCAGCAGACTTCAG

AAGGGACAGTAAAGTCACAGTTTGACTAACGGTCTTTGTGTAGAACTCTTAGAGTGTTTG

ATGAGTTTTCTCCCCTCAGGTTTGGTGATGGCTACACCATCATCCTGCGCGTGGCAGGTT

CGGCGCCCCAGCTGGAGCCAGTGATGGAGCTGATTGAGCGAGAGTTACCCGGCAGCACGC

TAAAGGAGAAACACAGGAACATGCTGCAGTTCCAGCTTCCGTCATCGCTCTCATCACTCG

CTCGGATCTTCAGCATCCTGTCCAAGAACAAGGAGCGGCTCCACATCGAAGACTACTCTG

TCTCACAGACCACACTGGACCAAGTTTTTGTAAATTTTGCCAAGGACCAAAGTGATGAGG

ACCACATTAAAGACATTTCCATGAACAAGAGCGATGCTGTGGTGGACTTCTCACAGCTGG

ACGCATTCCTCACGGATGCTAAAGCACAGGAGACGGTGGTGTGATCCAAGAGAACACACA

CCAATGGACAGAGGTTCTGAGATCCGTTTCTAAAGACTCTACTTTTATTTGTAATATTTT

TGTAGTATTTTTGTTATGAGGTTAGTGGTGTGTTTGCCCAGTAGAGGGCGCTAGACCTCA

GACACGCCACTGAAACCAAACTCAATGCAAATCAATGCAAAGACTATACTGCACGGCGAT

GTGGGCGGGGTCCAGCCAGGTGCCGCTTCTCCGCATCACAGCGTTTCCGTGCGAGTTAAA

ACTTGAAGTTTAAGCAGTATTTTTACAGATGTTCTTTCTGAGGTTCTTCATGGTTCCACT

TAGACACTGCTAGTGAGACGTGAACTTCATCTCCTCCAACACAGAGACACTTTTGGGTGT

CTGGATCTCAAAGTGACGTCTCGATTTTGTTATCCTGCTGTAACAGGAAGTTAAGACAGG

AGCTGGTCAGTTGGTGTGTGTCGGTGTTGCATGCTTTGTATCCTGAGAGCTGCAGTCTGT

GAGATCTGGTGTTTCATCACTGTCAAGATTTAAAGATAAAAACAGGACAGCAAAAGGTTT

AAGGTAAATATTGTATAAAGATCATGAATCATTTTGTGCCAAACAAAGCATTGGGTTCCG

TTAGTCAGATCTTTTGTCCGGTGTCTTCTCTTGCATGTGCCGTGGACGGTGATGTTCCTG

CAGTGATCTTTACTGTGTGTATGTTCACTTTAACACCATGTTTTTAATCCAGATGTTTTT

GTATCTGGCTGTGGGTCGTGTTTCTGCGGTCTGTACAACAGCCCCGTGCAGAATTTCAAC

TAGAGGATAAAACTCAGGATTTTATTCTGTCAGCATTTCAGAACCTCTTTCGATTTTTAC

TCTTCCTGAACCAGAGATCCAGAAAACATGGTGGATTTTCAGGCCCTTCATTTACAGTGT

GAAGTTTAAGCTGTGACCAAAAGTGCCACTTACACACACACACACACTCGAGTAAAATTC

CCGCCGA

>Unigene000051

CACACACACACACACACACGCACGGTTAAAAAAAAATAAGACCTAAAGACGGAGTTTCAC

CGACAGCCAGATAAGATGCCTGCGACGGAGAAAGACCTGGCCGAGGACGCGCCGTGGAAG

AAGATCCAGCAGAACACGTTCACACGCTGGTGCAACGAACACCTGAAGTGCGTGAACAAG

CGGATAGCCGACCTGCAGATGGACCTGAACGATGGGCTGCGACTCATCGCCCTGCTGGAG

GTGCTCAGCCAGAAGAAAATGTACCGGAAGTACCACGCCAGGCCCACCTTTCGCCAAATG

AAGCTCGAGAACGTGTCCGTGGCGCTGGAGTTTCTGGACCGGGAGAACATTCGACTGGTT

TCCATAGACAGTAAAGCGATCGTGGATGGAAACCTGAAGCTGATCCTGGGTCTGGTCTGG

ACTCTTATCCTGCACTACTCCATCTCCATGCCCGTGTGGGAGGAAGACGAGGATGAAGAG

TCCAAAAAGGCCACGCCCAAACAGAGACTCCTGACCTGGGTGCAGAACAAAGTACCCGAC

CTGCCAATCAGTAACTTCAGCCAGGACTGGAGGGATGGCAGGGCTCTGGGGGCACTAGTG

GACAGCTGTGCACCAGGTTTGTGTCCTGATTGGGAGACGTGGGATCCCGTGAAGCCGGTG

GAGAACGCTACAGAGGCCATGCAGCTGGCTGATGACTGGTTGGGAATCCCTCAGGTATTG

AGCCAGTGGGTAACCGTGTGATGCAGCCAGCCGTGTTCACCGTGGACACATTCAGCGCAG

GACAGGGACACGTCACCGTCTACGTGGAGGATCCCGAAGGCCGACGGGAAGAAGTGAAGG

CCGTTTACAATGAAAGCAAGAAGACCTACAGCGTAACATACATTCCACAAGTCATGGGAC

CGCACAAGGTGACAGTGTTGTTTGCCGGACAAGAAATTCCCAAAAGCCCTTTCGAAGTCA

ACGTGGACAAGGCTCAGGGTGACCCCACCAAGGTCACGGCTAAAGGTCCTGGGCTGGAGC

CAGTGGGCATAATCGCAAACAAACCCACCTACTTCGACATCTACACAGCAGGCGCTGGTG

TGGGCGACGTCACGGCGGTAATTAAAGACCCCCAGGGGAATAAGAACACTGTGGAGGCCA

TGATGGAGGACAAGGGCGACAGCGTGTTCCGCTGCACGTACAGACCAGTACAGGCCGGTC

CTCACACGGTCAGCATCATGTTCGGAGGCATCGCTATTCCCAAGAGTCCTTACGCCGTCA

GCGTTGGTCCAGCGTCCACCCCTAGTGCTTGCCGAGCAACGGGTCGGGGCCTGCAGCCCA

GAGGAGTGCGTGTGAATCAAGTGGCCGACTTCAAGGTGGACACCAGAAATGCAGGAAGTG

GAGACCTGAAGGTCACAGTGAAGGGACCTAAAGGCCTGGAGGAGCAGGTGAAGCTGAAGG

AAGCGATGGACGGCGTCTACGCCTACGAGTATTATCCCAACACGCAGGGCAAATACACAG

TGAGCGTCACATGGGGTGGCCAGCACATTCCCAAGAGTCCTTTTGAGGTCCAGGTCAGCC

CTGAAGCAGGACCACAGAAGATCCGAGCTTGGGGTCCGGGTCTGGAGGGGGGCATTGTGG

GCAAGTCGGCTGATTTTGTTGTGGAGTCTGTTGGCACGGATGTAGGAGTGTTGGGCTTTG

CTATCGAGGGTCCATCTCAGGCCCGGATCGAGTGCGAGGATCAGAATGACGGATCCTGTG

ACGTCAAGTATTGGCCCACGGAGCCGGGCGAGTACGCCGTCCATATCATGTGTGATGACG

AGGACATTGAGGAGAGTCCGTTCATGGCTTACATCATTCCTGACAACAAATCAAGCAATC

CTGACCAGGTTAAAGCCTATGGTCCGGGCCTGGAGAAGAGCGGTTGCATCATCAACAAGC

CTGCAGAGTTCACAGTGGAAGCCAAAGATGCCGGAAAGGGTCCTCTGAAAATCTCTGCTC

AGAACGCGGACGGCGAGCCCATCGACGTGAAGGTGAAAAGTAAAGGAGACGGCGTGTACG

CCTGCTCGTACACCCCCACCTCCGCCGTCAAACACACGGTCGTGGTTACCTGGGGAGGGA

TCAGCGTTCCTAACAGCCCTTTCAGGGTTCAAGTTGGGAAAGGCAGTCATCCTCACAAGG

TCAAAGTGTTTGGTCCAGGAGTGGAGAGAACGGGTCTGAAAGCTCATGAGCCTACACACT

TTACTGTGGATTGCACAGACGCTGGGGAAGGTGATGTCAGTGTGGGTATCAAGTGCGATG

CCAACATCATCAGTGATAAAGAAGAAGACGTGGATTTTGGTATCATTCCCAATGCCAACG

ACACCATTACAGTGAAATACATTCCCCCAGGAGCAGGACGTCTGACCATCAAAGTCCTTT

TCACAGATCAGGAAATTCCTGCCAGTCCGTTCCGGGTTAAAGTCGATCCTTCACACGATG

CATCCAAAGTTAAGGCTGAAGGTCCTGGACTCGCTCAAGCAGGTGTGGAGAGCGGAAAGC

CCACCCACTTCACCGTTTACACCAAAGGAGCAGGGAAAGCGCCGCTGGACGTGCAGTTCT

CCGGTCCGTCCAGAACACAACCTGTCCAAGACTTCGAGATCATCGACAACTATGATTATT

CCTACACGGTCAAATACACCCCAGTCCAACAGGGTGAAATGGTGATAACTGTGACTTTTG

GGGGTGACCCCATCTCTAAAAGCCCCTTTGCAGTGGGGGTTGCTGCACCGCTTGACCTCA

GCAAGATTGAAGTCGATGGACTGGAAAGCAGAGTGGAGGTTGGACAGAACCAGGAATTTG

CAGTGGACACTAAAGAAGCAGGAGGTCAGGGCAAACTGGAGGCTCGGATTACCAGCCCTA

CAGGAAAGTCGGTGCCGTGTGTCGTCCAGCCTCAGCGCGGGAAAGGAGCCAGCCTTGTCA

AATACGTCCCGAAAGAGGAAGGTGCCTACAGTGTAGAGCTGCTCTACGATAGCAACCCGG

TGCCCGGAAGCCCCTTCACTGTGGACGCCATGTTGCCTCCTGATCCCAGCAAGGTCAAAG

CTTATGGTCCAGGATTGAAAGGAGGTTTGGTCGGGAATCCTGCGCAATTCACAATCGATA

CCAATGGAGCCGGAACAGGCGGACTTGGTCTGACCGTCGAGGGTCCGACCGAAGCTAAAA

TCGAGTGTTCAGACAATGGGGACTGCACATGCTCTGTGTCCTACCTTCCTACTGAGCCTG

GCGAATACCTTGTTAACATTCTCTTCGAAGGCGTCCACATACCTGGATCTCCTTTCCATG

CCGACGTCGAGTACCCTTTCGACCCTACTAAGGTGGTGGTGTCAGGTACGGGCCTGAAAC

GCGGTAAGGTCGGCGAGACCAGCGTGCTCAACGTAGACTGTGCCAAAGCCGGGCCTGGAC

AGTTGGTGCTGGAGGCGGTGTCTGATGCAGGATCCAAAGCGAAGACTGAAGTGCTGGACA

ACAAGGATGGCACCTACACAGTGACCTACGTGCCACTTACAGCAGGCATGTACACACTGC

TGCTGAAGTACGGAGGAAAGACCGTCCAAAATTTCCCTGCCAAAGTGGTGGTGGATCCAG

CTGTTGACACCAGCAAGGTGAAGGTGTTTGGACCAGGAGTGGCTGGAGAAGGCGTCTTCA

GGGAGGCTACCACCGACTTCACTGTCGATGCTCGGGCTTTGACCAAAGTGGGTGGGCAGC

ACATTAAAGCTCAGATTATAAACCCATCCGGTGCTTCCACAAGCTGCTCCATTTCTGACC

AGAACGATGGCACTTATAAAGTGGAGTACACCCCGTTTGAAAATGGTGTTCATGCTGTGG

AGGTGCTGTATGATGACACTCCTCTGCCTAAAAGCCCCTTCCAGGTAGGAGTAAGTGAGG

GTTGTGATCCCACTCGAGTTCTAGCTAAAGGCCCTGGATTGGACGAAGCGCTCACAGACA

AACCTAACAAGTTCTCCATAATCACCAGAGGAGCTGGTATCGGTGGCCTGGGCATCACAG

TCGAGGGTCCGTCCGAGTCCAAGATGTCCTGCAAAGACAACAAGGACGGTAGTTGTGACG

TGGAGTACATTCCCTACGCTCCTGGCCTTTACGATGTGAACATTACATACGGAGGGGAGC

ACATTCCAGGAAGTCCGTTCAGAGTGCCAGTGAAGGACGTGGTGGACTCCAGTAAAGTGA

AGCTGTCTGGACCTGGAGTGAGTTCAGGTGTTCGAGCCAAGATCCCACAGACTTTTACTG

TGGACTGCAGCAAAGCCGGAATAGCACCGCTGTCTGTAGCTGTAACCGGCCCTAAAGGCA

AAGCTGAACCGGTTCAAATAATGGATAACGGAGACGGAACCCACACCGTAGCCTACACGC

CCTCTGTAGAAGGCCCGTACGCTGTAGCAGTCAAGTACGCTGAAGAGGAAATCCCTCGCA

GTCCGTTTAAACTTCGTGTCCTGCCAACACACGATGCCAGTAAGGTCCGTGCCAGTGGGC

CAGGACTGACTACCAGCCTTCCTGCCAGCCTTCCTGTGGCGTTTACCATAGATGCCAAGG

ATGCAGGAGAAGGCCAGCTCTCAGTGGAGATAACCGACAAAGACGGGAAGCCGAAGAAGA

TCAGCATCCACGATAACCAGGACGGGACGTACGACGTGTCTTATGTGCCGGATAAAGTGG

GCCGGTACACCATCGTCATTAAATACGGCGGTGACGAGATTCCGACTTCTCCATACAGAG

TGCGGGCAACAACGACAGGCGACGCTAGCAAGTGCACAGTCACCGGTCCTGGAATCGGTC

CTACCATCGGCATCGGAGAAGAAGTAGGCTTCGTGGTGAACACAAAAGGCGCAGGGAAGG

GAAAGGTTTCGTGTGTCGTCGTCACACCCGACGGAACTGAAGTGGATGCGGAAGTCATCG

AGAATGAGGATGGAACCTTCGACATATTCTACACCGCGCCCAAGCCGGGAACCTACATCA

TCTACGTGCGGTTCGGGGGCGAAAATATCCCTCACAGCCCTTTCAAAGTTATGGTCACAG

ACGGCGCATACATTCCGGTGAGCAGCATGAACGGTGCAGGGTTCCGGCCCTTCGACATGG

TGATTCCGTTCACTTTAAGCAAAGGGGAAATTACAGGCGAAGTGCACATGCCATCAGGCA

AAACAGCCCAACCTGAAATTATCGACAATAAGGACGGGACGGTCACGGTGAAGTACGCTC

CTACAGAGTTAGGACTTCATGAGATGCACATCAAATACAACGGCGCTCACATACCAGAGA

GTCCTCTACAGTTCTACGTCAACAGCGCTAACAGTCCCAACGTGACTGCGTACGGCCCCG

GACTCGTGTATGGCGTCGCTAACCAACCAGCTACTTTTACCATCTGCACTGAGGATGCTT

CTGAGGGTGGTCTTGACTTGGCTGTTGAAGGCCCATCAAAGGCAGAGATCGGTTGCGTCG

ACAACAAGGACGGCACTTGTACGGTCACGTATCTTCCTACACTTCCTGGAGATTACAACA

TTTTGGTCCGCTATAACGACAAGCACATTGCTGGTAGCCCCTTCACTGCCCGGATCACAG

AGGACAATAAGAGGAGGTCTCAGGTCAAGCTAGGCTCAGCCGCTGATTTCGCACTGGACA

TCACGGAGACGGACCTCAGTCAGCTCACTGCCAGCATCAAAGCTCCTTCAGGCCGCGACG

AGCCCTGTCTACTCAAAAGACAGCCGAACAACCACATCGGCATTTCTTTCATCCCCAGAG

AGGTCGGAGAGCATTTGGTTAGCATTAAAAAGAACGGACACCATGTAGCCAACAGCCCCA

TCTCCATCATGGTGGTGCAGTCTGAGATCGGCGACGCCAGCAGGGTGAAAGTGTATGGAC

CGGGTCTGCTGGAGGGACGCACGTTCCAGATGGCTGACTTTATAGTGGACACGAGGGATG

CTGGATATGGAGGACTGGCTCTGTCCATCGAAGGCCCAAGTAAGGTTGACATTCAGACGG

AGGACTTGGAGGATGGAACCTGTGGTGTTGCCTACTGTCCCACAGAGCCTGGAACCTATA

TGGTGTCCATCAGGTTTGCTGAAGAACATGTTCCCGGGAGTCCATTCAGTGTAAAAGTAA

CGGGAGAAGGACGAATCCGCGAGAGTATCAGCCGCCGCCAACGAGCCGCTTCTGTTGCCA

CAGTCGGGAGTGCATGTGATCTTAACCTGAGGATTCCAGAGATTGATATCCACGACCTTT

CAGCTCAGGTCACCAGCCCTTCTGGTGTCACCGAACCTGCTGAGATCGGGGCGGTGGGCA

ACCACACCTACTGCGTGCGATTCGTTCCACACGAGATGGGTGTGCACACGGTAAACGTCA

AGTACCGTGGCCAACATGTCCCAGGCAGCCCCTTCCAGTTCACCGTAGGGCCACTGGGAG

AAGGAGGGGCCCAGAAAGTACGAGCAGGAGGTCCTGGGCTGGAGAAGGCTGAAATCGGTG

TGCCAGCGGAGTTCAGTGTTTGGACACGTGAGGCTGGTGCCGGGGGCTTGTCCATCGCCG

TGGAGGGCCCCAGCCGCGCCGAACTTTCCTTTGAGGACCGGAAGGATGGCTCTTGTGGTG

TCTCTTATGTGGCACAGGAACCTGGTGACTACGAGGTCTCTGTGAAGTTCAACGAGCAGC

ACATCCCCGACAGCCCTTTCCTGGTGCCAGTCTGTGCACCCCCACACGATGCCCGGTGTC

TTACTGTTACCAGTCTTCAGGAATCAGGCCTCAAGCTGAACCAGCCGGCTTCGTTTGCCG

TGCGTCTGAACGGCGCTCAGGGCGTCATCGCCGCCAAGGTCCACAGTCCCTCCGGAGCGC

TCGAGGACTGTGTGGTTTCTGAACTGGAACATGATAAGTACGCCATCCGCTTCATCCCGC

GAGAGAACGGCATCCACACCATCGATGTTAAGTTTAATGACACGCACATCCCGGGCAGCC

CGTTCCAGGTGAGGGTCGGCGAGCCGGGGCAGGGTGGAGATGCCGGGCTGGTCAGTGCTT

ACGGTCCTGGACTGGAGAAAGGAACCACAGGCAACCAATCAGAGTTCACCATCAACACCA

GTAAAGCAGGACCGGGCTCCCTGGCAGTAACGGTGGAGGGACCTTCAAAAGTGAAGCTCG

AGTGCCAGGAGTGTCCCGAGGGCTTTAAAGTACACTACACACCAATGGCTCCTGGAAATT

ACCTGATCAGCATCAAATACGGCGGCCCTAATCACATCACAGGAAGTCCCTTCAAGGCCA

AAGTCACAGGTCAACGGCTTGTTAACGTGACCAACGCCAGCGAGACCTCCACACTACTGC

TGGATCCCACGGTCCGGGCGGTCAGTGCCGGTTCCACCAACGCCATGCCCAGAGCTGCAT

CTGATGCTAGCAAAGTGACCAGTCGAGGCCCGGGTCTCAGCAAGGCCTACGTGGGCCAGA

AAGCCAGCTTCTCGGTGGACTGCAGCAAAGCGGGTAAAAACATGCTGCTAGTAGGAGTTC

TCGGTCCTCAGACCCCCTGTGAGGAAATCATGGTGAAGCACATGGGCAGCATGCAGTACA

ACGTCAGCTACGTTCTAAAGGAGCGCGGAAACTACGTCCTGGCGGTAAAGTGGGGTGAGG

ATCACATCCCCGGCTCACCGTTTCAAATCACGGTGCCTTAATCTGACACACACACATTAT

AAGAGCTACGGAAACTGGCTTTATAAATTACGCCCTGATTGATGACAAACCTGCACTTGA

GTTCCTATTTTGTACATGTGATGTTTTTTTTAAACCACTGCTGAGTTTCTCAACATCTTC

CAGGGGCAGAACAGCAGCTTTGCGTTGGAATCCGGTGTCAGTTTGAGTGTTTGTCTACAT

TCCTTTAAAAATATCCTCTGTTACTACTGATGCTGTTTTGTCTATTTCGACTCGTGTTGT

ATTATCCATTATTTTGATTTCTCCTTCCTGCTGCGTCACCTGGACTACTGCGATTGTTTA

CATGATCACGAAAATCCAACGAGTTATCGATCCGACCACTTTAACACACTTCTATCTGTA

GACTGTAGATACATTTATCATAATATCCCAACCGGGTTCAGTTTAATCCGGTATCCGGGT

G

>Unigene000052

GGAGCTCTAACGGCGCCTCAGAGCAGCAGCGAGCGGCGCACAGCAGCGAGTCCACAAACC

AGCTACTGACGTTTAACCCGCATTTACAGGAACATCTTTTTTTTTTTTATCTGCTGGGAA

ACGGGATTGTTTATATTAAAATATTAGTCCGGAAGTGGACAACTAGCTGTGTCGAGTGAG

GAAGAGATCCAGCAGAAGCCCCGAAACTGCCACATCCCTGTGGGAGGTTTTCTCCCATTT

CTAGAAGATTCCATCAGGTGAAACGGTAACAGATACCAGAGCGCAGTCATGGTGGCGCTG

TCGCTAAAGATCGGGGTGGGAAATGTGGTTAAAACAATGCAGTTCGAGCCCTCCACCATG

GTGTACGACGCCTGTCGCATCATCCGGGAGAGAGTTCCTGAAGCGCAGCTTGGCCAGCCC

AACGACTATGGCCTCTTCCTGTCAGATGAGGACCCAAAGAAAGGCATTTGGCTGGAAGCT

GGAAAAGCTCTGGACTACTACATGTTGAGAAATGGGGACACACTCGAATACAAGAAGAAA

CAGAGGCCACTCAAGATCCGCATGTTGGACGGGACAGTAAAAACAGTCATGGTGGATGAC

TCCAAAATCGTTACCGACTTGCTCATGACCATCTGTGCCAGAATAGGCATTACAAATTAT

GATGAGTACTCCCTGGTGCGGGATATCGGCGAGGAGAAGAAGGAGGAAACCACAGGAACG

CTGAAGAGAGACAAAACTCTCCTGAGGGATGACAAAAAGATGGAGAAACTCAAGCAGAAG

CTCCACACTGACGATGAGCTGAACTGGCTGGATCACGGGCGAACGCTGCGTGAGCAGGGA

GTAGAGGAAACCGAGATGCTTCTCCTCAGGCGAAAGTTCTTTTACTCTGACCAGAATGTC

GACTCCAGAGACCCAGTTCAGCTCAACCTGCTCTATGTACAGGCTCGGGATGACATTCTG

AACGGTTCCCATCCTGTCTCTTTCGACAAAGCCTGCGAGTTTGCCGGTTACCAGTGTCAG

ATCCAGTTCGGTGACCACAATGAGTCCAAACACAAACCAGGATTCCTAGACTTGAAAGAG

TTTTTACCCAAAGAGTATGTGAAGAACAAAGGAGAGAAGAAGATTTTTCAGGCACACAAG

AATTGCCAGAATATGACTGAGATTGAGGCCAAAGTCAGTTACGTCAAACTGGCCCGCTCC

CTCAAAACCTACGGCGTGTCTTTCTTCTTAGTAAAGGAAAAAATGAAAGGAAAAAACAAG

CTGGTTCCTCGTCTGCTGGGTATCACCAAGGAGAGTGTGATGAGGGTGGATGAGAAGACC

AAGGAGGTGATGCAGGAGTGGAGCCTGACTAATATTAAGCGCTGGGCTGCCAGTCCTAAA

AGCTTCACTCTGGACTTTGGAGACTATCAGGATGGCTACTACTCAGTCCAGACCACTGAA

GGGGAACAGATTGCACAGTTGATTGCTGGATACATCGACATCATCCTCAAAAAGAAAAAG

AGCAAAGACCACTTTGGCCTGGAGGGAGATGAGGAGTCGACTATGCTGGAGGATTCGGTT

TCACCCAAAAAGTCGACAGTGTTGCAGCAGCAGTTTAATAAAGTGGCGCGAGTGGAGACG

GGCTCTGTGGCCCTGCCTGTCATCATGCGTCCAGGAGCGGGAGGACCGGAGAGCATCTAC

ACAGGCTCCATGCCCAAGGCCAAGCAGCAAATCACCACTGGCCAAATGCACACAGGACAC

ATGCCACCGCTGACTTCAGCTCAGCAGGCACTGACTGGCACAATTAACTCCAGTATGCAG

GCAGTGCAGGCGGCCCAGGCCTCTTTAACCGACTTTGACAGTCTACCACCACTGGGCACA

GATGCGGCATCTCAAGCCTGGAGGAAGAACAAGATGGATGAGTCCAAACATGAAATTCAT

TCTCAGGTGGATGCTATTACTGCAGGAACAGCATCTATGGTTAATCTTACTGCAGGAGAC

CCAGCAGAGACAGACTATACAGCTGTGGGCTGTGCAGTGACCACCATCTCATCCAACTTG

ACCGAAATGTCAAAGGGTGTGAAGTTGCTGGCAGCTCTAATGGAGGATGAGGGTGGAAAC

GGACAGCAGCTTCTGGGTGCCGCCCGCAACCTGGCCTGTGCTGTATCAGACATGCTTAAA

ACGGCTCAGCCTGCCAGCGCAGAGCCTCGTCAGAACCTTCTGCAGGCCGCAGGTAACGTG

GGCCAGGCCAGCGGAGAGCTACTGCAGCAGATAGGAGAGAGTGACGCTGACCAGCAGTTC

CAGGACATGTTGATGAAGCTTGCTAAAGCTGTAGCTACAGCAGCAGCAACTCTGGTCTTA

AAGGCAAAGAACGTGGCCCAGAAGACAGAAGATTCAGCCCAGCAAAACCGGGTCATCGCT

GCTGCCACACAGTGCGCCCTTTCCACCTCTCAGCTGGTGGCATGCACAAGGGTGGTTGCT

CCAACTATCAGTTCCCCAGTATGTCAGGAGCAGCTGATTGAGGCAGGGAAACTAGTGGCT

AAATCAGTTGAAGGCTGTGTGGAGGCATCACAGGGAGCCACCAACGATGAGCAACTGCTG

AAACAAGTGGGTGTGGCCGCCACAGGTGTCACCCAGGCTCTTAACGAGCTGCTGCAGCAT

ATCAAGCAGTACGCCAGCGGAGGCCAGCCAATCGGGCGCCACGGGGAGGCAACTGACCGC

ATTCTGGACGTAACTGAGAACATCTTCAGCTCCATGGGAGATGCAGGTGAAATGGTCCGA

CAGGCCCGGATCCTTGCTCAAGCCACCTCCGATTTGGTCAACGCCATTAAAGCAGATGCA

GAGGGAGAGACAGACCTTGAGAACTCTCGTAAACTGCTCTCAGCTGCGAAGCTTCTAGCC

GATGCAACTGCCAAGATGGTGGAGGCCGCCAAGGGTGCTGCCGCCAACCCAGACAGTGAG

GAGCAGCAGCAGAAGTTGCGTGAGGCCGCTGAGGGCCTGCGCATGGCCACTAATGCAGCA

GCACAGAACGCCATCAAAAAGAGGCTGGTTAATAAGCTAGAGAACGCTGCAAAGCAGGCA

GCTGCAGCCGCAACTCAGATCATAGCTGCCGCCCAGCACGCTGCCTCATCTAATAAGAAC

CCGGCTGCACAACAGCAACTTGTACAGAGCTGCAAGGTGGTGGCAGACCAGATCCCCCAG

CTGGTGCAGGGAGTCAGAGGCAGCCAGGCACAGCCTGACAGCCCCAGTGCTCAGCTTGCC

CTAATTGGAGCCAGCCAGAACTTCCTGCAGCCTGGAGCCAAGATGGTGACTGCAGCTAAG

GCTACGGTGCCCACTATTGGTGACCAGGCCTCTGCCATGCAGCTCAGCCAGTGTGCCAAA

AACCTGGCCAGTGCATTGGCTGAACTCCGCACGGCTGCCCAAAAGGCTCAGGAGGCTTGT

GGTCCTCTGGAGATTGATAATGCCCTGAATGCAGTAAGGGATTTGGAGAAAGACATGCAG

GAGGCTAAAGCTTCAGCAGCTGAAGGAAAACTCAGACCTCTTCCTGGAGAAACGCTGGAG

AAGTGCTCCCAGGACCTGGGCTCCAACACTAAGGCAGTGAGCTCTGCTGTTGCCCAGCTT

CTGAGTGAAGCTACACAGGGCAACGAGAATTACACCGGTATGGCTGCACGGGATGTGGCC

CAGGCTCTCCGTTCTCTTGCCTCCGCAGCCCGTGGAGTGGCCGCAACCACAGACGACCCA

CAGGCGTGCAGCGCCATGCTGGACTGCGCTGGAGATGTCCTCGACAAATCAGGCAACCTC

ATTGAAGAAACCAAAAGAGCCATCGCTAAACCTGGAGACCCAGAGAGCCAGCAGCGACTT

GCCCAGGTGGCCAAGGCTGTCGCTCAGGCACTGAACCGCTGTGTTAACTGCCTTCCTGGT

CAGAGAGATGTGGATAACGCTATCAGGACTGTGGGAGAGGCCAGCAAAAAGCTTCTTTCC

GATCAGTTCCCAGCTAGTGGAAAAACCTTCCAGGAAGCTCAGGCAAACCTGAACCAGGCG

GCCGCCGGGCTTAACCAATCAGCCAACGAGCTGGTGCAGGCTTCTCGCGGCACCACACAG

GACCTGGCCAAAGCGTCGGGCAAATTTGGCCAAGACTTCAGCCACTTCCTGGAGGCTGGC

GTGGACATGGCTGGCCAATCTCAGTCTAAAGAAGACCAGACTCAGGTGGTGACCAACTTG

AAGACCATCTCTATGTCATCCAGCAAGCTGCTGTTGGCTGCTAAGGCTCTGTCTACTGAC

CCCAATTCCCCCAACCTGAAGAACCAGTTGGCTGCTGCTGCACGTGCCGTGACTGACAGC

ATCAACCAGCTGATCACCATGTGTACGCAGCAAGCACCGGGTCAGAAGGAATGTGACAAC

GCTCTGAGGGAGCTCGAGACGGTGCGGGGCATGCTGGAGAACCCAACTGAAGCAGTGAAT

GACATGGGCTACTTTGACTGCATCGACAGTGTAATGGAGAACTCAAAGGTATTGGGTGAA

GCTATGGCCGGCATCTCCCACAATGCAAAGAACAGTAACCTGCCTGAATTCGGAGACGCA

GTCAGCTCGGCCTCCAAAGCTCTGTGCGGCCTCACCGAAGCTGCTGCTCAGGGTGCCTAT

CTGGTGGGTGTCTCTGACCCTAACAGTCATGCTGGACAGAAGGGGTTGGTAGATCCATCT

CAGTTTGCCAAAGCCAAGCAATCGATTCAGATGGCCTGTCAGAACCTGGTGGACCCTGCC

TGCACACAGTCCCAGGTCCTCTCTGCTGCCACTATTGTTGCCAAACACACCTCCGCCCTA

TGCAATGCCTGCCGCCTCGCCTCCTCCAAAACATCAAACCCTGTTGCTAAGCGACAGTTT

GTCCAGAGTGCCAAAGAAGTGGCAAATAGCACAGCCAACCTTGTAAAGTCAATCAAGGCC

CTGGATGGAGCCTTCAACCAAGAGAACAGACAGAAGTGCAAGGCCGCCACAGGGCCATTA

ATCGAGGCTGTGGATAATCTCACTGCCTTTGCTTCCAATCCTGAGTTTGCTAGTGTGCCT

GCACAGATCAGCCCAGAGGGTCTGGCTGCCATGGAGCCCATCGTGGCTGCAGCTAAAACT

ATGCTGGAGAGCTCAACAGGTCTGATACAGACCGCACGCTCTCTAGCCGTGAACCCCAAA

GATCCACCCAAGTGGTCAGTGCTTGCCGGTCACTCCAGGACTGTGTCTGACTCTATCAAG

AAGCTAATCACCAACATGAGAGATAAGGCTCCAGGTCAAAGAGAGTGTGACGAAGCCATT

GAGGTTCTCAATAACTGCATCCGTGAAGTGGACCAGGCTTCTCTCGCTGCCATCAGCCAA

CACCTTACACCCAGAGACGACATTTCCCATGAGGCTCTGCATGAGCAGATGGCAGCCTCG

GTGCAGGAGATTAGCCACTTAATTGACCCAGTGGCTATCGCAGCTCGTTCCGATGCCTCC

CAGCTAGGACATAAGGTTTCTCAGATGGCGAGCTACTTCGAGCCACTCATCATGGCAGCC

ATTGGTACTGCATCAAAAATTCTCAACAGCCAACAGCAAATGAGTGTTCTGGACCAGACA

AAGACTCTGGCAGAGTCTGCCTTACAGATGCTCTATACAGCCAAGGAAGCAGGAGGCAAC

CCTAAGGCGGCTCACACCCAGGAAGCTCTGGAGGAGTCTGTGCAGATGATGAAGGAGGCC

GTTGATGACTTGGGAGGTACAATGGCTGAAACAGCAAGTGCAGCCGGAGCTGTAGGAGGC

ATGGTGGACTCCATCACTCAGGCTCTTAACAAATTGGAGGACCCTGGAGTCGAGCCTGAG

GGAACCTTTGTGGATTATCAGACTACTATGGTGAAAACGGCCAAGGCTATTGCAGTCACT

GTGCAGGAGATGGTCACTAAATCAAACACCAACCCTGATGAGCTCGGAGGTCTAGCCAAT

CAGTTGACCACCGAGTTTGGTGACTTGGCATCAGAGGCTAAATGTGCTGCCATCACTGCA

GAAAATGCTGAGATTGGCTCTCACATTAAGAAGCAGGTAACAGAGCTGGGCTATGGTTGC

ACTGGATTAGTCACCAAGGCAGGAGCTCTACAATGCAGTCCCAACGATTCCATCACTAAG

AAAGAGCTGATTGACGCTGCCCGCAAAGTCTCTGAGAAAGTGTCCCATGTGTTGGCGGCA

CTGCAGGCTGGTAACCGTGGTACCCAGGCTTGCATCACCGCTGCCAGTGCTGTAGCTGGC

ATCATTGCTGACCTTGACACCACTATTATGTTTGCCACTGCTGGTACACTTAACAGAGAG

AATGCAGAGACCTTCGCTGATCACAGAGAAAACATTTTGAAGACTGCTAAGGTTCTGGTG

GAGGACACCAAGCTACTGGTGTCAGGAGCAGGAGCCAGTCAAGAAAAGCTGGCCCAGGCG

GCCCAGTCCTCAGTCAACACTATCACCAAACTTGCCGATGTGGTTAAGCTGGGGGCCGCC

AGCCTGGGCTCTGAGGACCCTGAGACACAGGTGGTCCTGATCAACGCAGTCAAAGACGTG

GCTAAAGCTCTGGGTGATCTTATTAGAACCACCAAAGCAGCCGCTGGCAAGCCTCATGAC

GACCCGGCCATGCTCCAGCTGAAGAGCTCTGCGAAGGTGATGGTTACGAACGTGACGTCC

CTGCTGAAGACGGTGAAGGCTGTGGAGGATGAGGCCACTAAAGGCACACGGGCGCTGGAG

GCCACCATCGAGCACATCAAACAGGAGCTTGCCGTGTTCAGCAGTCCAGATCCTCCTCCA

AAGACAGCTACACCAGAGGAGTTTATCCGCATGACCAAAGGCATCACGCAAGCCACAGCC

AAAGCCGTGGCTGCTGGAAACTCATGTCGCCAGGAGGACATCATTGCTACCGCCAACCTG

AGCAGAAGAGCCATCGCCGAAATGCTGCACTCGTGCAAGCAAGCGGCTTATCACCCAGAG

GTTAATCTGGAGGTGCGGACCAGAGCCCTGCGCTTCGGAAAGGAGTGTGCTAATGGCTAT

CTCGGCCTCCTGGAGCATGTGCTTGTGATCATCCAAAAGCCCACTCACGATCTGAAGCAG

CAGCTGGCAACCTTCTCCAAACGAGTAGCTGGCTCAGTCACTGAGCTCATCCAGGCAGCT

GAAGCCATGAAAGGCACTGAATGGGTAGACCCTGAGGATCCCACAGTCATTGCAGAGAAC

GAGCTGCTGGGTGCAGCGGCCGCCATCGAAGCTGCTGCTAAGAAACTGGAGCAGCTGAAA

CCACGAGCCAAACCCAAGGAGGCTGATGAGAGCCTGAACTTCGAGGAGCAGATTCTGGAG

GCAGCCAAGTCCATCGCTGCAGCTACCAGTGCGCTGGTGAAGGCTGCATCTGCCGCACAA

AGAGAGCTGGTGGCCCAGGGCAAGGTTGGCGCTATTCCAGCGAATGCAGTTGATGATGGA

CAGTGGTCTCAGGGTCTTATTTCAGCCGCCCGCATGGTTGCCGCCGCCACCAATAATCTG

TGTGAAGCCGCGAACTCTGCAGTACAAGGCCACGCTAGTGAGGAGAAGCTCATTTCCTCA

GCCAAGCAGGTGGCGGCATCCACAGCTCAGCTGCTTGTGGCCTGCAAGGTCAAGGCTGAC

CAGGATTCACAAACCATGAAAAGACTGCAGGCTGCGGGCAACGCTGTCAAGAAGGCGTCA

GATAATCTAGTAAAGGCAGCACAGAAGGCTGCTTTCGATGCGCAGGATGATCAGGCTGTA

ATGGTGAAGAGCAGGATGGTGGGCGGCATTGCTCAGATTATTGCTGCTCAGGAGGAGATG

TTGAGGAAGGAGAGGGAGCTGGACGAAGCCAGGAGGAAGCTAGCTCAGATCAGACAGCAG

CAGTATAAATTCTTGCCCTCAGAGCTGAGGGAGGACGGTCATGAGCAGTGAAGAGCTTCT

TTCACACACACATACACACACACACACAGGTCTGCTACTCAGGACTGCACGGCCTGAGCG

CTTTTCTCTCCATGTGTTTAGCTACAATGGCAGGCTTGCTGAAGCAACATTCAGGCTAAA

ATTATAAAAAAAGCGAGGC

>Unigene000053

TGTGTGTGTGTGTGTGTGTTTCAGCTGTACTCCCTGAACGAATACAAGCCCCCGATCTCT

AAGGCGAAGATGACCCAGATCACCAAATCAGCCATTAAAGCGATAAAGTTCTACAAACAT

GTGGTCCAGAGTGTTGAGAAGTTCATTCAGAAGTGCAAACCCGAGTATAAAGTTCCCGGT

CTGTACGTCATCGACTCCATCGTGCGCCAGTCGCGACACCAGTTCGGTCAGGAGAAGGAC

GTGTTCGCACCCCGCTTCAGCAAGAACATCATCAGCACCTTTCAGAACCTCTACAGATGC

CCCTCTGATGATAAGAGCAAAATCGTCCGCGTGCTCAACCTGTGGCAGAAGAACAGCGTG

TTTAAAAGTGACATCATCCAGCCGTTATTGGACATGGCAGCAGGAATCCCGCCCCCAAGT

ATCACACCTGTCTCTGCCAGCACCGCACTTCCTGTTAACAACGCCACTCCTGGAACTCCT

GCCACTCCGGCTACTCCAGCCAACATCATGCCCAGTTTACCAGACTGGGCTTCTCAGATC

TCCAACTCGGACACCGTGGCAGCTGTAGCACAGATCCTACAGAGTCCTCAAGGACAGCAG

TTGCAGCAGTTGGTACAGAGTCTACAGATGCAGCAGCAGAAGCCTCAGCCCTCTCTCCTG

CAGGCTCTGGATGCTGGACTGGTCGTCCAACTGCAGGCCCTAACCGCTCAGCTGACAGCG

GCCGCAGCGGCCAACACACTCCCGCTCGAGCAGAGAGTCTCCTCTTTCAACAAGAAACTC

TTGGGACAGTTTGACTTTGTTGGCGAGTCTGAACCCAGTGAGGATTCTAAAAAGGACACT

GCAGCATCTCAGCTGCCGATGGTTTCGGAGTCCATTAACAACTCCCTCTTCCATCAGTTA

GCTGAGCAGCTCCAGCAGCAGAACCTGGAACAGTTTCAGAAACAAATTCTAGAACATCAG

CAGAAGTCCATGACTCTGGAGAGTCAGGAGAACATTTTCTGTCCCGAGAACTCGGCACCT

CCGTCACAGAGTAGCGTCCAATCACAGCTTCAAGAACAGGACGTCAAACTGGACGATTCT

ATCGACAACCAACAGCAGGACATGGACCTGGATGAGGGACAGGACACTGTGGATGAGGAT

ATGTTTGAATCGGACGAGAAGAAGACCAGCGGTACTCGTTCCAGAACACACTCTCGATCC

CGCTCCAGGTTGGACACATACAGCTGCTCTAACAGACTCCATATAATCCCTCACTGCACA

TCACATCTTATTTCACTTCCTACTGCAGGTCCCCAAAGAGAAGGAGGTCACGGTCACGCT

CGGGGTCTCGAAAGCGCAAACACCGGAAACGGTCCCGCTCACGGTCCAGAGACCGGAAGA

GGAAATCCTCCCGTTCGTACTCGAGCGAGCGCCGAGCGCGGGAAAGAGAAAAGGAGCGGC

AGAAAAAAGGTCTTCCTCCAATCCGCTCCAGATTTCTCAGCGTGTGTAGCACGACATTGT

GGGTGGGCCAAGTTGATAAGAAAGCCACGCAGCAGGACCTGACCAACCTTTTTGAAGAAT

TCGGACAGATTGAATCCATCAATATGATTCCTCCCCGAGGATGTGCTTATATCTGCATGG

TTCATCGGCAGGACGCTTATCGCGCACGGCAGAAATTAAGCACCGGCTCGTATAAAATCG

GGTCAAAGGTTATTAAGATTGCCTGGGCCCTTAATAAAGGTGTGAAACAGGAGTATAAAC

AGTTCTGGGACGTGGATCTCGGAGTGACGTATATCCCCTGGGAGAAGGTCAAACTGGACG

ACCTGGACGGATTTGCCGAAGGAGGAATGATTGACCAGGAAACAGTGAACAGTGAATGGG

AATCTCAGAAGAATGTAGAGGCACCAAAGGATCTCCAGGTTCAGCAGGTGTCTGCTGAGA

GCAGTGCAGCCAGCAACTCACAGAATGAAACCTACACACAGCCGGTGGCCATGATGCCTG

TGCAGATCCCAGTGGCGCAGACTGTCTCTGCTGTGGGCCTGGTCCCGCCCACTTTCCCCG

TCACCATGGCAATGCCTCCACCAGGTTTCGGTCCCCCACCTCCCTTTCTCCGGGCAGGAT

TTAACACTGCACAGCCTCCACCAGGTTTCATGGCTCCGTCCGGTGTGGCACCGTCTGCCG

TTGGAGCTGCTCTCACCTCAGGACCAACAGCTCACAATCAAGACGGAAACAAGGACTCGA

CGTTTGGAGCGATTCTTCCTCCGAGCAGCACTATTCCAGCCAGCTTCATGCCCTCTGGAA

TGCCGGGAGCGGTATTCAACTCTGTTGGAGCACAAGCACAGCAGGCCAGTACTGACAAGA

CGAGTGTGTCTGGAGAGAGCGTGGACACACCGAACGAATTAACACTACAGGGAATGCAGA

ATGCAGTGCGCAGCGGAATGGGTCTACTGGGCATGCACCCTTCTGCAGCTCTCTCTCACC

CCCTCTCTGGTCAGAGGATGCCCGGGCTGCTGCCCATCGACATGCGTCCGAACCTCCTTC

AGGGAGCTGGGGCTCGGTTCTCTATGCTCATGCAGCAAGGCCTGGCGCAGCAAGCTAACA

GCCTCCTGGACGCGTCTCTTCAGACACGCGCTCGAGCCCCTTTCCCTCAGATGGAGCACT

TTAACTGCGCAGACGCTCCGTTTGTCAGAGCCCCCAATCCTGCAGCTCCTGCACCAGACA

GTATAGACAAATCTAGTAACAATATCACAGCAACTGCTACAGACAGCAGGAGCCAACAAG

AAGATCAGGACTATCGCTTTCCACCGCCGGAGAAACAGAGCACGGGTCTGCTGAGGACGC

CTCCTCCTGAGATGAGGACGGAACCGACAGCTGTAAGAGCACCGCTGCTGGACAGACCTC

TCAGAGCAGGCATGCACATGGAGGGCCGAGAGGGACCCGGGAGACGGGAACCCATGCCGG

CCCCTTTCAGAGCAGAGACACGCTGGGGTGCTCCGCGTGGGGACCTGGATGAGCGGGACA

TGAGAGGAATGTCAGCAGGACCGCCGAAAAGCTTTCCGGAGGATCCAGGGAATCCGAACT

TCCAAAACCGGTTTGAGATGCGAGGCGGAGGAGCGATGGGTGGGGCAGGACCCGGTTGGA

CTCGTGGAGGAGGAGGGGGCGGACCCTTTAACATGAACATGCATCAGGACTTTGATGATC

GACGTCGACCCTGGGAAAGGCAGAAAGACCGAGACGACAGAGACTTCCGCAGAGATATAA

ACGACAACCGGCACAGGGATCGAGACAGAGAACGGGAACGAGACCGCGAACGAGGTCGGG

ATCGGAATCGGGAGCGAGAGAGAGACAGAGACCGAGATAACGATAAGGAAAGAGAGAGAG

AGCGCGGAGGCTGGGCACCTGTTCAACCTCTCCTCCCTCTCCCGAGACCGTCTCAACCAC

TGCTCCCTCTACCGACACCACTGCTTAACCTTCCTCCAACTCAGCCCGAATCGCATCCCA

AACCACAACCCGCATCCAGACCGGAGTCGCAGCCCGACGGGAAGCAGCCGCAGCCTGAAA

AACAGAACTCAGGCGAGCGAGAGAAGACGCACGTCTTTAGTGACATCGCTCACGGCCCGG

ACACTGAACACGAAACGGTGACAGAACCTACAGCCGAGTCGTCTCGCTCCTCTTCACCTG

CACCCACCGCCGAACTCCAACACCAAACACAAACCCCAGAAGTGTCCGCTTCCACAGGCG

GCAGACAGACCCCGCTGAAGGAAGAAGCCGAGACTGGGACTGAGCCTGAGCCTGAGCTTG

TGAAAACGGACACTGAGGAAACATAATCGTCTCTCAGTAGACCAATTTGGTGGATTGCTA

GCTGACCAAAAGACGATGATGACTGCGGGGCTCTAACACTGTGGTGCTCTTGATCTGTCC

TGATTACTAAATCTGGTCAGAGATGGGAAACTCAGAGAGCCAGTTCAGAACCCAGGGATC

TCGAAGCAGTAGCTTCACACTCTCAAACAGACAAGAGCCCTATCCAGTTAGGTGTCGTAC

TTTCAAAGAAGACAGTTTGACCCCTCATTGTCGCTGGAGGACTGGATCCAGTTTGAAATC

CAAGGCGGAGAAGCGTTGCGGTTTGTCGCAGCACAGGGCCAATCAGTCGTCCTTGTTAAG

GCATTGTGACTACATTACTAAAGGAGGAGAAGGAAAGAGAAATATCAGCGCCCAGTGGAA

CAACGGACACTTTGAGGAAAACAGGCGGGATTGTAACGGTCATGCATTAAATGGCTACAA

GACCCCTGAGAGGAAGCCGAAATTAGAGGATTTGGATGATCACAGCAGCCCAAAAGTCGT

CATCAAGAGCGACGGCAGCGTTCGGGTCGAGTTCAGTCACATGTCTAAAAACTCAACACT

CCCGGATGAGAACGGAGGTTCCGTCCAGCTGCTGAAGTTTTCCCCCACATCACACTCCGT

GTCCACCCCTGAGTCTGCCCTGGTACAGAACAGCCCTATCAACAGGACCAGCAAGAGAAA

CTCGCTGAGCTCAGAAGGCAGCTGGTACGACTCACCCTGGGGGCCCGGAATCGAGTTGAA

CTACGACGGCGTCTGCGCCCTGCCCAACAGGCTATCAGAAACACTTCCCTCTGTACGCAT

CGAGTACTTCGACGAGCCATGCATTCCGCATCTACTTTACCGGGACCCTGTCATGGCTGC

CACTTTTTCCACTGCTGAAGATCTAAAGCTTGCAAATGAGCTGTCTTTTAAACACAGGAC

TCCGTTTCTGCATGTGATGGAGGAACCTGTTTTAGCAGAGTCCTCTGGGGCAAGGCAGTA

CTCTTCATACACTTTACCGTGCCCCCAAACCAAACCGACCGCCGAGAACTCTGGAAAGAA

GGAAATCATCAAAAACAGGATGAGGCGTTTTAGTGACTGGACCGGGAGCTTAACCAGACG

GAAGAAGAAGTCAAAGGAGTTGAGGTATAAGGATTCCATCGATTGTTTTGACAGCGGTAT

AGATGGACTGACTGCCGACACCAGCTCTCCTTCTCAGGTGTCCAGCCTGTTGGGGTACCA

CAAGGTTCAGTGTCGGGGTCGCTCCCAGTCCCAGGTGCTGCCATCCTGCAGCGGCGGCTC

TTTCAGTGCCCTTCACCCGGCCACAGATGGTCTGCGGCAGAACATCTATGACAACTTCAT

GCGGGAGCTGGAGACAAGCCAGACAACGACAGGAAGCGGCGAGAGGAGAGGCGAGAACTC

TGCAGAGGACACTGAGAGCAGCAGCCAGGAGACGGTGGGCTCGCTGGAACAGCTAGACCT

TCTGTGTGAGAAGGAACAGGACGTGGTCCGAAGAGCCGGCTGGTTGACCTTTAAACCGCT

GCTCACACTGCACAAAGACCGTAAGCTGGAATTGGTTGCCAGGCGCAAGTGGAAACAGTA

CTGGGTGACTCTTAAAGGCTGCACTCTCCTGTTTTACGAGACCTATGGCAAAGGTTCTCC

AGAGCAGGAGGCTTCACCCAGCTATGCTCTGCTCGCTGAAGACGGAATTGTCCAGGCCGT

CCCCGAACACCCGAAGAAGGAGAACGTGTTCTGCCTCAGCAACGCCTTCGGTGATGTCTA

CCTCTTTCAGGCCACCAGCCAAACGGATTTGGAGAATTGGGTGACCGCAATCCATTCGGC

CAGCGCGTCTCTCTTCGCTAAACGCCACGGCAAGGAGGACACGCCGCTCCTGCTCCGGGG

ACAGATCCGCAGCCTACTGCAGAAGATCGACATGGACGGAAAGATGAAGAAGATGGCAGA

GCTCCAGATGTCCATCGTAACTGATTCCAAAAGCAGGAAGGCCATCGAGAACCAGATTCA

GCAGTGGGATCAGAATCTGGAGCGGTTCAACATTGATTTGTTCCGTATGCGCTGTTACCT

GTCCAGCCTGCAGGGGGGGGAGCTGCCCAACCCCAAGAGCCTCCTGGCCACCGCAAGCCG

GCCCTCCAAAGCGGTCCTCGGACGTCTGGGCGTCTTCTCCGTCTCCTCTTTCCACGCACT

GATCTGCTCCCGAGACGAGGCCACACTGAGAAAGCGTTCTCTGTCTTTGCCCCGGAGAAG

CCGCGGTAAAAGAGGCATGTTTTCGTCACACAAAGCTCTGGACAGTTTGACCAAACGCAG

TCGAGACAGAAGACCCTGCACCTCACAGGATTGCATTTCCGTGAGCTCCTGCTCGAACCC

TGAGGTGTTTGATGGCGCTGCAGGCGATCACTCCGGTTTGGTTCCTTCGTACAGCAGAGA

GAGACCGGACGCTCCAGCTGACACTTACTCACAGGCGCCACCCGATGGCAGTCAGTGGGA

GCGCAGCGTGACTGAGAGGCTGGTGTGGGTTTACATGCCTGACCACCAGGTGGTTACAGT

GACACTGAGCGGACAGCACACAGTCGAGGAGCTCCTGAACACCGTCTGCAAGATGCAGAA

TCTGGACCCCAGCGCACACTGCCTGCGTCTGCGCAGATGCGTCAGCGATCGGATGGAGGT

CTGGACTCCAGGACCGCACAAGCTTTTTCAGGACCTGTTGTATGATGAGTTAGAAGTCAG

CGCTCTCAATGTGTACAGGCTCCACATGACCAAGCCCACATACGCAGGGGATTTTGGCTT

TGCTATTTCAGGAAGTGTGGACAGTCTCAACCAAAGCCGCATCTTCGTCACTCAGGTTTT

CTCTGGAGGAATCGCGTTCAGTGAAGGTCTGAGGCCGGGTGATGAGATCCTGGTTCTGAA

CGGATGTCAGGTGTCGCGTCTGGACCTGGAACTCATTCAGACTCTGTTTAGTGAGAACAC

ACTGCACCTGACCATCAGACGAGAGCCGACCGCCATGACACACACACACACCGGCCAGCA

GCCAATGAGGGACCTCCTGTACCGCCATCACAGAGCCAAGTCTGCTACAGACGTGTCGTG

TGTGTCTGAGGGTAAAGCCGGTGTAGAGACGAGGCAACATCATCGTCTGAGTCTCAGAGC

CGTACAGCACAGCAAGAGTGCGGAGACGGTGTTTGCTCTCTATCACTCCTGCCAGGACTC

CTCCGTACGACTGATGGAGGTTCAGACGGAGACGGCGCCCCCGGGCGGGACCCTGCTCAG

AGCCTGTCCACGGCACATGTCCGTCAGCGAGAGACTACGCAAAGTGGTGCAGGAGTTAGT

GGACACGGAGAAGTCCTACGTCAAGGATTTGAGCTGTTTGTTGGAGATCTACCTGAAGCC

CCTGCAGAAGGAGACGTTTCTCACACAGGATGAGATGGAGAGTTTGTTTGGGAGTCTCCC

TGAGATGCTGAACTTCCAGAAGGTGTTTCTGCAGACGTTGGAGGAGAGAATCGCTTCGAC

TCCAGACTTCGGCACGCTGCAGACGCCGGTGCAGTTCAGGAAGTTGCTGTTTTCTCTCGG

TGGCTCCTTCCTCTACTACGCCGATCACTTCAAACTCTACAGTGGATTTTGTGCCAATCA

CATCAAAGTCCAGAAGGTTCTTGAAAGAGCTAAAACGGACCGCGCCTTTAAGGACTTCCT

GGATGCCAGAAATCCCACGGAGCAGCACTCGACTACGCTGGAGTCTTACCTCATTAAACC

GGTGCAGAGAGTGCTGAAGTACCCGCTACTACTGCGAGAACTGGTGTCTCTGACCGACAC

GGACAGCGAGGAGCACTATCACCTCACAGAGGCACTGAAGGCGATGGAGAAGGTGGCCAG

TCATATCAACGAGATGCAGAAGATCCACGAGGATTACGGCGCCGTGTTCGACCAGCTGGT

GGCGGAACAAAGTGGCTCAGAAAGAGAGGTACTGTACCACACCGTCTCGCGCTTCTTCTG

CTAGTACACAGTTCCTACAGTTGGGGGCGCCGCTAGCAAATTTAGGCTCCTTGGAAAAAA

TGAGTTTGGGCCTCCTAGACGACCTTTCCACACCAGACATACTGTTACATCACTGCTGGT

ACTGTTACTTACAATAACACACATCTCTGGAGGGCCCCAAAGTAAGTGGGCCCCTCCTAA

CTCCCCCGCCGTGCATCCCACCACCCGCCACCCTTAATGGCGGCCCCCTTTAACTGTATG

AAGAGCA

>Unigene000054

GTGTCTGCGCACCTCTGTGTCCGGAGTGAGAACGTCGGTGATGTTCTCGAGAGACTCTCG

CTGAGCTGTGTGGAGCTGCTGCTCTCCATTGCTGAGGATTTTCCAGATGGATTGTGGGAA

CAGTTTCAGTCCTCCATACAGATGACTCATGAGCTACTGCAGGCCAACGCCATCCCTCAG

CTGCATCTGCTCTCTGCAGTCACTCAGGAACGAGGGATGTGGAGCAACTGCACACTGCAG

AGCCTTTTGAGAAATGAGATGCCTCCAGAAGCAAAAGTGCATGAGTTCCTGATGCGTGAA

GGTCCAGTCCTGCTGCAGCTCCGGGTAAAGTACCTGCTAAAAGTGAACTGCATGGAGAAG

GCGGCGTTGCTGGCTAAAGCCTGTGCTGATGTCCAGGAATTTGGGGGAAGCAGAGGACAC

TTTACACAGTGCTTTCTTGTGTGTGTGTGCTCTTGTGCACCGCAGGAGACCCTGATGGAG

GAGCTCTCGAAGGTGGACTGTCGTGATGCTCTGGAGATGATCTGTAACCTGGAGGCGGAG

GGAGATGAGAGAGGAGCGTTCACACTTTGTTCAGGCTTCCTGACCCGACAGCTTCTGCAG

GAGGACTCGTACTGCGCCTGGGAACTGACTCTTTTCTGGAGTAAACTCCTAAAGCGATTG

GAGCCTTCAGAGCAGAGCTTTCTGGAGAAGTGCCAGAAGATGTCGCTACTGGCTAAAACT

GTCTTTCACCTGCTCTTCTTCATCAAAGTGATCCAGTCTGAGTTGGATAAGATTGGCTTG

CCAGCCTGCATTGAGATGTGTATACGTGCTTTGAGGATGGAGAACTGCGAGGGAGCTAAC

AAGGCCACCATCTGCAAAACCATCTCGTGTCTACTGCCTTCTGACCTGGAAGTTAAGCGG

GCCTGCCAGCTAACAGAGTTCCTCCTAGATCCGACAGTTGACTCTTATTACGCCGTGGAG

ACCCTGTACAATGAGCCGGACCAGAAGCTTGAGGAGGAAACCCTTCTGATACCCACCTCT

CTTCGCTGCGATCTTTTCCTAGTTTTTAAAACCCAATGGTCTTTTGATCCAGAGTTCTGG

GATTGGAAGACACTAAAGCGTCACTGCTTAGGCCTTATGGGTGAGGAAGCATCTATAGTG

TCCTCTATTGATGAGCTAAACGATGATCGCTTCTTAGAGATTGAGCGAGATGAATTGGAT

TCTGCTCAGGAGGAGTTTAAGGATGTTTTTGAATGCTTTTTGGACACAACAAAAGAACTT

AAGGAGATATCGGATCATCGACAGAAAAACAGAGAAGTTAAAAAGTTGAGGGAAAAAGGG

TTTGTGTCTGCGAGGTTCAGGAACTGGCAAGCGTACATGCAGTACTGTGTGTTGTGTGAT

AAAGAGTTCCTTGGTCATAGAATAGTGCGACATGCACAGACTCACTTCACAGATGGATAT

TATAGCTGTCCGATTTGCACAGAGACGTTTGAAACGAGAGAAACTCTAGATCCGCATGTT

GCATCACACGTAAAGTTGTCTTGCAAAGAGCGCTTGGCTGCGATGAAAACCACTCGGAAA

CTGACTGGTGCAAAAAACTCTGCTTCCAATGATGCACCTCTGGATAACAAAAGTGGGGAA

AATCAGGCACGAAAAGCGAAGGCTAAACACTGCAATGGAGAATCTGATGATGTGTACAAC

ATGGGCTCTTGCATTGAGGATGCAGGAGTCAGGGTCCACAGGAAAGAGTTCAATGTTTGT

CCTGTTTCAAGCTGCAGAAAAGTGTTTAAGTTCTTTCGCAATCTTCTCACCCATGTAAAG

GATCACGGAGACGTGGAGGAGGCTAAGCGCTTCCTTGAATTACAGGCCACCAAGATAGTT

TGTCAGTATTGTCGCCGACACTTTGTGAGTGTAGGTCACCTCAATGACCATCTTCAGGTC

CATTGTGGTGCGAAGCCTTATACCTGCATTCAGTTGAACTGCAAGGCTAGCTTTGACACC

AATGCCGAGCTCCTTGTGCACAGCAAAAGACATCCTTGTTTTAAGGCCAAATGCATGTTT

CCTGGCTGTGGAAAGATTTTTAACGAAGCTTATAAACTCTATGACCATGAGGCGCAGCAT

TATAAAACATTCACTTGCAAAGTGCCCGGTTGTGGGAAGGTATTTCATGCTCAGTCTCAG

TTAGACATGCACAAGGAGAGCCACAATCCTCCTAAATTGGAGACACAGCCCGCTGGGAGT

GTCAGCCCGCTGGGAGAGCCTCAGTCGCACCTCTCCAGTGAACCTGCCCAGCAAGATGCC

CATTCAGGGCCTCCTGCCGACTCCGGCTGCATGATTAGTTCAGAAGATCAGTCAACCGGT

CTAGTAAAAGTAAAGCACTCTATTGAAAACATGCTCAGTGTTGCTGGTCAAGGGCTCTCA

CAAGAGTTTGGCCATGTTAACTGCAAAGCCGAGCCATCTGTTCCAAACCTGGTGCAGCAG

CCACCAATGCCACACATGGTGAATACCGAGCAAATCCAACCTCAATTAACCCCTTCAATG

CCTTCCTTGATTGAACCGAGATCTGAAGAATCCTTGTTAGATGCATTGATGAACGATCCA

ATGCTCACTTCATCTTGCTCACTTCCACCAGCATCTTCGTATCAGAGCCTCTTAGAGGAT

TTCCAGCAAGCTCCATCAGGAGATGTTTTACAAGCTCAAATGGAGTCTGCTGTTTCCCAG

GGTCACACCCAGTCTCTGTACAGTAATGAAAACACTGAATATGGCCAATACAATATCAAA

CCTGAAACCCCAATTTCATACCCACCTCCGTATGATAACACTGTACCCTTGGCGCAGAAT

GGTCATAATGATCCACCGGTACTGCCTGTATCTCAGAGGCTTCCCCCACAGCTCACCATG

TTTTCTTCGAATAGACCAGTGCAGGCACTGCAGGCAAACACACTCAGTTCTGTAGTGGAA

GAAAAAGACAGGTATAAATGTGCATACGAAACATGTTCTCGAGACTACAGCTCGTATAGA

AGTCTAACCAAACACATGAAAGCTGCTCATTGTGAGTTTTATACACAGTGGAAACTGGCA

AAACGGAGCAATAAAGTTTCACCAAACACTTGCAGCGCGTCATCTATAAATGGAAAATGT

ATGTCAGCAGAACCCTCACAGAACCAGCTGAGTCAGAGGACTCCTGCTCCTCAGAACCCA

GTGCAGAAACCACCTGCTCAGACCCACAGCCCAGCAAGCTGTCTAAGCTCCCATTACCCC

TCTGGATCCTCCCATTTAACCGGTCCAAATGGACAGACTTTTCCAAATCAAATGGACAGT

ATTTTGAATCCCATAGTGCTCTCACACTTGGGCAGCATTGCAAATCAGTCTAATGTAGTC

TCTCACTCTTCGTGGAATTCCATCTCAGTGAACACATCACTCCGGCAGCAGACTTGCCAT

TCACAAGCAATGACCTCATCTAGTGGTCAGTTCCCGTCCCAGTTAGACACTGTTCCACAA

ACCCAACTGTCAGATTCCTATAATAGAGTAATGGCCTACCCTTCTTTGCAACATCAAAAC

AGTCCCTCATGTCGATTCATACAGACCCGACCATGTGCCCCCCAAACAATAAACAGTCCT

GCTAAAATAAGCCATCCCGGCGATTCCTCTTTACCTCAGTATGTTCAGCAACCGAAGACC

AGTTCTAAATCCACACTTAAACATACAGACTGTGTCGTAAAACTAGAGAGAAACCCAATT

TTAATTCCATCCCATTCAAACTTATTACAACAAAGCAACCCTCCTACTGCTATGAAACCT

GAAACTTGTCAAAAAGATATCAGTGGTTTTGCACATGATGGGGATGTCAAAAAAAGGAAG

CGAAGCAGCAGAACAAAGTGGCCTGCTATTGTCAAAGACGGCAAGTTTATTTGCTGCCGG

TGCTTTAGAGAGTTTCAGAGTCCTAAATCTCTTGGTGGACATTTGTCTAAACGTACTGTC

TGCAAACCCTACGATGAAGCTGTTTTGTCTGCAGACCTGCCAAGTTCATTTCTGGAGCTT

CTGAACTCCCCTCATCCATCGGAAACACCCCAGTCCTCGCCGTCCACGCAGGTCACTTCT

AGTAAAGCTTGGCCTTCCCTGACCAAAGGCCCCCTTGACCCAAAATTGTTCCCCAATGTC

ACGTTCCTGAACACTAAAAATTCCACATACAACAGTGGAACCATCCATCAAAATTCACCG

AGTCTGGAAAAGGGTGTTACAGGTGAGCAGACTTTTCCTGAGTCATCTGGGTCTTACCAT

CAAAAAAGCAAAAACAGTGTGATTCAACACACCGGAAATATTAGAGATAAGTGCATTTAC

GATCTAATGAATTATACCCAGCCATGCAATACCTTACCAAGTGACACCAGTTCCTCAGAA

CAACTTTTGTCTCACATGTTAAGTGAAAATAAAACTTTAGACTCAAACTTGAGCAGAAGT

CCTAATAACTATGTTGACAGAATCCTCCAAGCTGAAACACTAAACAAGATTAGGGAGATT

AAAGAAGAGCCTACCAGAACAAACATTAGTGGATTATCGAATGATGGGCTTCTTGCTGCA

ATGGCTAGTCTGGCCCAAAATCTGGTGTCAGAAAAGAGCGTCAAGGAAAGGCTTCGTGAA

CAGATATTAGCTGGGGACTTTCAAAAAAGAAGCAGTTTAGGCCAAGGGCAAGGAGTTGAA

AACTCTAATTCACAAAGTCCGATGACTGATGTTCCACTTGGACTTCAAACAGCTGTTCAA

AGTAAGGAGCATATTGTCAGACGTGCTAGTGAACTTGGTGAACTTTACACCTCAGTTGTA

CCTGATGAGACCTTGACAAATATTTCCTTGGCTGGTGCGTCTAACAACAGGGAGACAAAC

AATGCGTTTAGTAACAGCCTAGAACAAGAAGATGATAAAGATCTCTCTGAAATTCTGAAA

TCGTTTGAAAGATTGGATCTAAATAAGGAAATAGAAAAGAATGAGCAGACCACTTCCAAC

CTGCTAGAGGCTGAGAGAGTAACCAAGGCCTCAGAAGCTACATGCACTCTGATGTTACGA

GGCTATTCCTGTGACAACGATGGTTGTGGTTACAGAGCGATGACAAAAGATGCTCTTTTT

AAACATCTTGTAAAACAGCATAACTACACAGAAGACATGATGAATCAGTGTCGCAAGGAA

CAGTCTAACTTGGCACCTTTTAATTGTCAACTATGCCCCAAAACCTTTACAAGAAACTCC

AACCTCAGAACCCATTACCAAGCCGTTCATAATTATTCACATGAGCAAGTGGTCAAGTTG

GGGATTACACGGCAATACAGTCGAAAATCCATTGAGGCCGATCAAGGTCTTTGTACCACG

CTGAACATCTCGGAGTTGGCCAGTCCAGTCTTTGCTGGACAGAATGCCGAGTCTGGTGCC

GGTATCACAGCTTTGGTATCTGGAGTTAGAAGCATAAAATCAGAGTTCAGTGTTCAACCA

TTTGAACAACAGCATCCTGGTGCTTCCTTCGACTCATGTCCTGTTTCCAGTACAATACTG

CGAGCGCAGACAACTCCACATCATCTCCAGAGTCAGACTACTGTGCATTTATCAGTGATT

AAAGCTACTCCTTCCTTGCTAACCTCCACAAGTGTGGAGCAGCAGTTAAATCGACCAAAT

GAATTTGTTCCGGAATCGCTTGTGCCTTCTCAGCACACTCAAGGATTTCTGTCTGGAGGT

CTTTCGTCTTCAGGTGCGCCTCTTCTAGCCCCGATTTCTTTGGCGTCAGCACAGCTAGGT

AGCCTTTCCTCAGATACGAAGCCCAAATTTGGAAAACCTAAAATGGCAAAAGTGAAGGAA

GTGACAAAAAAACCTAAGGAGAAAAAGCTTGACATGGTTGATGTCTTTAGTCCCTATCGG

CCTTACCGCTGCGTCCATCAGGGCTGTGTTGCTGCCTTTACTATACAGCACAATTTGATT

CTTCACTACAAAGCTGTACATCAGTCTGCCCTCCCCAAATTTGAGGTTAATGAAGATGAA

GAACCGATCGAGGAAGAACATAACGAAAACCAGGAGGAGATTGGCATGCCTGACTCTGAG

GTTAATGAGGAGTTCCGCTGTCAAGTCAAAGACTGCTCTCGAATATTTCAGGTCGTTACA

GACCTGCTACAGCATTACCTTCAGCTCCACAAACTCGGCCTTGATAAAGCAGGTATTATG

ATGTCTGGTATGAACTTGGGCAGGTTTCGGTGTGACCAACCAGACTGTCATGTGACATTC

ACTGGATTCTGGAAGTATGTTAACCACATTGATAATGAACACAAGCATGTAAAGCTCTCC

AGGACTGAGCCTGTTAATGGCATGTTCCAGTGTGAGGTTGAAGGATGTAGCTGCGTTTAT

GCAACACGGTCAAACTTGTTGCGACACTTTATGAAAAAACACCCAGAACTTTACAAACTC

CGACTGCTGAACTCGGGAGGTGTCAAACTTGGTCGTCCTCCGAAAAACAGATTTGTCTCC

CTTGAGAAAGAAAATAGAGAAATCAATAAAAAAACTTTTCAGAAGGGAGGTGCGAAAAAG

AAAAGGAGACAAAAGAATCTTTGGACAAAGTATGGAAACCCTATTTTGAAATCGAAAGAG

GAGGCTTCCTCAATGTGCACCAAGCGGATTCAGTTACAGTATCCTTGCATGCTTAAAGGC

TGTGAGACAGTTGCAGAGTCTGAGCGAAAGCTTTTAAAGCACTATGTCCAACATGGCTTG

CCAAAACAATACCTGGAGGAGCAGAGAAGCAACTACATATTCTGCAAAAAAATGCCTCGC

TCAAAGTATAAACGCATTGCGTCCAGGAGTGACGATACTGACAAGTCCGATGGAAGCTCG

ATTGAGGCGTCTGAGAACGAGGACGCAGCAGACACTGGACCCAGCGAATCGGAGTTCTCA

AAGCCGACGTCTGAAAAGGAGAGCACGGAGGACACTGAAGTTTCAGACGCTAAATTGTCT

AGTGACCTGAGCTCCGACATCTCTGTGGTGGTAAAGCGGAAGAGAGGACGACCACGCAAG

GGGGAACAGGTTAAGCGCACCCAGCTCGCACGCAAGAGAATGACGAGGCTACGAGCGGCG

CAGACGCGCTCCATCAACTATGCAGATATGAACTCCGATTCCACTTCTTCCAGCGTTACG

CTTCCACAAGAGCCAAACACATCACTGAGCTCTTTCAAGCCAATGGGCTTCGAGGTGTCC

TTCCTCAAGTTCCTGGAGGAATCGAGTCAGTCTCCCGGGAAGAGAAAGAGTTGTTCTTTA

ATGGATTTACTTCCACACAGGAAATTCCCGACTGTGCGACTGAAGACTGCCTCGGTGGTA

TGCAGCAGGATCAACCCTGACTATCATCATTGCCGAGATGTATTGAAACATGTCGAATTT

AAAAACCCTCAGAAACTAACGTCTCTGAGTAGGGTGACGTTCGAGGTGCACAGAGGGTTC

TCCAATGTCTACGAACTGTTGCTCAAGCAGCTGCATGACATGCGGCCTGCAGTGGTCATT

CAGAAAGAGGGCGTGTCTGAGACGAGGGCGTAGTTTTCAGGGTGGGGGTAGCTAATGTGA

GAGTGGGTCAAGAGCTGTTCAGTGTTGCTGTTTATGTGGAGCATCAGTGTTGAGATGTTT

CCATGCCTTTGTAATGAACCGCTAGTCCCTTTCCAGGACTGTATACTAAAGTGTACTATT

GTAATGAAGTCGGTGAAGGATACGCCTCTCGTGCATGCCCTAAAACACCTGATCTGGCAC

GTGTGCGCAGCTGTGGAGATAAAACCCTATAATGGAAGAGTGAAACTGCTCCGTGTAAGA

ATGTGAGCTCGCCTTTTGTTGGACTGACCGCGTGTCGTGCAGTGGTGAAATGTAGATGAG

TAACATTTTGGTCTTGTTCATCTCGCAGTGTAAAATTTGATAGCGTTGTGTTTATCTGTG

ACTCTCACATACTGACTCTCCAGATAATCTACTGCCACCAGCAGTCTGTACCGCAAACAA

CAGAGACCAGTTTATTACAGCTCGCGTTTTCTACCTCTGTGTGTGTGTGTGTG

>Unigene000055

AGGAAAGTGTCAAAGCCTAATTTAGTTTGTCTTTTGTTGTAGGTATTACATCTAAAAGAT

GCAGTTAAGTTGGATTTGCCAAAATAATTATCGAACAGAGTGTAGAAACTAGTAACCATG

AAGGTCGACAGGAGCAAATTAAAGAAAACCCCCACCGAAGCGCCTGCAGACTGCAGAGCG

CTGATAGAGAAGCTAAAGGGATGCAGCGACGAGCAGCTGCTTGTGGAGTTGCAGCACATC

AAAACATGGAACATCGGAAAGTGTGAGCTCTACCACTGGGTAGATCTTCTGGACCGCTTC

GATGGCATCCTCTGTGATGCAGGCCAGACGGTGGAGAACATGTCCTGGATGCTGGTATGT

GACCGACCAGACAACGGGCAGCTCAAAGCTTTACTGCTGGCAGTGCTCAATTTTACAGCT

CTGCTAATAGAATACAGCTTCTCCCGGCACCTCTACAGCTCCATAGAGCATTTGACCACG

CTTCTAGCATCATGTGACATGCAGGTGGTGCTGTCTGTGCTGAACCTTTTGTATGTGTTC

AGCAAGCGGTCCAACTACATCACCAGGTTAGGCTCGGACAAGAGGAGCCCTCTGCTCGCA

CGCCTGCAACACCTGGCTGAGAGCTGGGGCGGAAAAGAGAATGGCTTCGGCCTAGCAGAG

TGCTGTAGAGATCTGCCTATGACGAAATACCCACCCAGTGCCACCACGCTGCACTTTGAG

TTCTACGCTGAGCCGAGCTCCGAAGTCAAAGTGGAGAAAAAGTCTAGCAGCAACACTCTA

CACTACATCCACATTGAACAGCTTGATAAGATCTCAGAAAGTCCGTCTGAGATCATGGAG

TCGCTGACGGTCATGTACAACATCCCGAAAGACAAGCAGACCCTGCTGTTCACGCACATC

CGCCTAGCGCACGGCTTCTCCAACCACAAGAAGCGACTGCAGGCCGTGCAGGCCAGACTG

CACGCCATCTCTATACTAGTGTACTCCAACGCCCTTCAGGAATCAGCTAACAGTATCCTC

TACAACGGGCTGATCGAGGAGCTGGTGGATGTTCTCCAGATCACAGACAAACAGCTGGTG

GACATAAAGGCTGCCTCTCTGCGTACTTTGACGTCTATTGTGCACTTGGAACGGACTCCT

AAACTTTCAAATATTATTGACTGCACCGGCACAGCCTCCTACCATGGCTTTTTACCTGTG

CTGGTCCGCAACTGTATACAAGCCATGATTGACCCAATAATGGAGCCATACCCGCACCAG

TTTGCCACAGCTCTCTTTTCATTCCTCTACCATTTGGCCAGTTATGATGCTGGTGGAGAG

GCCCTGGTTTCATGTGGAATGATGGAGGCCTTGTTAAAGGTGATTAAGTTCCTGGGCGAC

GAACAGGACCAAATCACTTTTGTGACGCGGGCGGTGCGTGTCGTGGACCTCATTACCAAT

TTGGACATGGCTGCCTTTCAATCACACAGCGGCCTTTCTATCTTCATCTGCAGACTAGAG

CATGAAGTGGACCTGTCCAGGAAAGAGTGCCCTTTTGTTATCAAGCCAAAGATCCAAAGG

CCTAGTACAACTGCTGAAACGGAGGACATGGACACGGATATGGATGTGTCTGAAGTTGCC

ATGGAGAGCAGCCCAGGACCCTCAACTTCCACAGGCTCCAAATCCGACCCTGATCCTCGT

ACACAGACGAGCACAGTGAGCGCACCTAGAACAGGTGTGCAGTGCATCCCACAGAGGGCA

GCGCTGCTTAAATCTATGTTGAACTTTTTGAAGAAAGCCATTCAGGACCCGGCCTTTTCT

GACGGCATACGTCATGTGATGGATGGGTCCCTGCCTACCTCGTTGAAGCACATCATCAGT

AATGCAGAGTATTATGGGCCATCACTTTTCCTTTTGGCAACGGAGGTGGTGACAGTATTC

GTGTTCCAGGAGCCATCTCTTCTCTCCTCCCTGCAGGACAACGGCCTCACCGATGTCATG

CTTTACGCATTGCTCATCAAAGATTTTCTGTCTCTGCAGGTCCCTGCCACCCGAGAGGTG

CTGGGTTCCTTGCCCAATGTCTTCAGTGCCCTGTGCCTAAACGCTCGTGGCCTGCATTCG

TTCGTCCAGTGTCAGCCCTTCGAACGTCTTTTCAAGGTTTTACTGTCGCCCGACTATCTG

CCAGCCATGCGACGTCGCCGCAGTTCTGACCCACTGGGAGACACTGCCTCTAACTTGGGA

AGTGCTGTGGACGAGCTCATGAGACATCAACCCACACTAAAAACCGATGCCACCACAGCA

ATCATCAAGTTGCTTGAGGAGATCTGTAACTTGGGCAGAGCTCCTGAGTACATCTGTCAA

AAGCCCTCCATCCAGAAAGCAGATGGAACTGTTACAGTACCTCCAGCTCGCTCTAACCAT

GCAGCAGAGGAAGCCTCCAGTGAAGATGAGGAAGAAGAAGAGGCTCTGCATACTTTCACC

CAGCAGCAAGGGGAACCTGAAAGCAACAGGCAAGTGGTTGGCACAGAAGAGCGTATTCCC

ATTCCTCTTATGGATTACATACTGAATGTGATGAAGTTTGTGGAGTCCATTCTGAGTAAC

AACACAACAGATGACCACTGTCAAGAGTTTGTAAACCAGAAAGGCCTTCTACCCCTGGTG

TCAATTTTGGGGCTTCCCAACCTTCCTATCGACTTCCCTACGTCTGCTGCATGCCAGGCT

GTGGCTGGCGTATGCAAATCAATTTTGACTTTATCCCACGAGCCAAAAGTTTTACAGGAG

GGACTTTGCCAGTTGGACAGCATTCTGTCAGCTCTGGAGCCGTTACATCGGCCCATTGAG

GAACCGGGTGGTTCGGTTTTATTGAGGGAACTGGCCAGCGCTGGCCATGTCACCGATGCT

ACGTTGTCTGCTCGTGCTACACCACTGTTGCATGCACTTACAGCTGCACACGCATACATC

CTTATGTTTGTCCACACATGCAGAGTTGGGCAGAGTGAGATCCGGGCGATCTCTGTAAAC

CAGTGGGGCTCCCAGCTGGGGCTGAGTGTACTTAATAAGTTAAGTCAGCTCTACTGCTCG

CTGGTGTGGGAGAGCACCGTGCTCTTGTCCCTCTGCACACCCAACAGTCTTCCTCCTGGC

TGTGAATTTGGCCAGGTTGACATGCAGAAGCTGGTGCCCAAAGAAGAAAAACCCTCCGGC

AGCACTGTGACCAGTGGAGGCCGGAGAACCGGTAAAAAAACACTGCTCCATGCTGAGACA

GAGTCCCTTTCTGTGGGTGTGGACCCTTCTGCTCAGGGGTTGCTGGAGGGCATGGGTCTG

GATGGAGACTCGCTCGCTCCAATGGAGACCGACGAACCCACAACTGCTGACCCAAAAGCC

AAGTCTAAACTCACGCCTGCCATGGCAACACGCATTAAGCAGATCAAACCCCTGCTCTCA

GCATCCTCTCGTTTAGGAAGGGCGTTGGCTGAACTGTTTGGTTTGCTGGTTAAGCTTTGT

GTGGGCTCACCTGTCCGTCAGCGCCGCTCCCACCACACCACAAACACAGGCACTGCCCCG

ACTCCAGCTGCTCGTTCAACTGCTTCTTCACTTACCAAACTGCTCACCAAGGGCTTGAGC

TGGCAGCCCCCTCCCTATACCCCCACCCCACGCTTCAGGCTGACCTTTTTCATTTGTTCC

GTTGGATTCACTTCGCCCATGCTATTTGATGAGAGGAAGTATCCCTACCATCTAATGCTG

CAGAAGTTCTTTTGCTCTGGAGGCCATGATGCACTGTTTGAGACATTTAACTGGGCATTG

TCTATGGGAGGCAAAGTGCCAGTGTCTGAGGGGTTGGAGCATCCTGAACTGCCCGATGGA

ACTGGGGAGTTTTTAGATGCCTGGCTAATGCTTGTGGAAAAGATGGTAAACCCCAGCACA

GTGCTGGACTCTCCTCACTCTCTGCCTGCCAAGGTCCCTGGAACAACAACCACCACACCA

CAGTTCAGCGCTCCACGTTTCCTCATCGTCACTCAGAAAGCTGCCTTTAACTGCATCCGC

AACCTGTGGAACCGCAAGCCACTGAAGGTGTACGGCGGTCGTATGGCCGAGTCCATGCTG

GCTATTCTTTGCCACATATTACGAGGTGAGCCAGTCATTCAAGAGCGTTTGTCCAAGGAG

AGGGAAGGCACGGCCCGGGCTGAGGAGGATGGAGCCTCGGTGGTTGGAGGAGCTAGTGGA

GGTGCAGCAGCTGGAGCAGTAGGAGAAAGTGCTTCTGGGTCTGGAGGTAGTGCCAGCACG

GCTGGAGGAACTGCAGATGACGCGAGCGCCACGACTCGCCGCGAACCACAGGTTAACCAG

GCGCAACTTACACAGCTCATAGATATGGGTTTCTCCAGAGAGCATGCTATGGAGGCTTTG

CTTAACACCACCACCATGGAGCAGGCCACAGAGTATCTCCTCACACATCCACCACCGTTA

CTCAGTGCTGCCATTCGGGAATTCACCATGTCTGAAGAGGATCAGATGATGAGGGCCATT

GCCATGTCTCTGGGTCAGGAAGTCAGCATGGAGCAGCGCTCAGACTCTCCTGAGGAGGCT

GCCCGCAGGCGAGAGGAGGAGGAGAGGAGAGCCCGAGAACGAGCTGAGGAAGAGGAGGCT

CGCTGTCTGGAACGTTTCCTGGAGGCTGAGCCTCTGGACAGCAGCGAGCTCCATGCCTTC

ACTGACACGATGCTACCAGGTTGCTTCCACCTGCTGGATGAGCTGCCAGACACCGTCTAT

CGCCTATGCGACCTGCTTATGACTGCCATTAAAAGAAATGGCCCAGAGTACAGAGACCTC

ATCCTGCGCCAGGTTGTCAACCAGGTGTGGGAGGCTGCTGATGTGTTGATAAAGGCTGCT

GTACCACTCACTACCAGCGACACAAAGACAGTGTCTGAATGGACCAAACAGATGGCGACT

CTGCCCCAGGCCTCCAATCTGGCCACTCGTATTCTCCTCCTCACTTTGCTCTTCGAGGAG

CTGAAGCTGTCCTGTGCACAGATTGTGGAAAGTAGTGGAGTTCTGTCAGTGCTGATTAAG

CTGCTGGAGGTGGTTCAGCCCTGCCTGCAGGCGGCCAAGGAGCAGAAAGACATCCAAACA

CCCAAATGGATCACTCCAGTGCTTCTGCTCATCGATTTCTACGAGAAAATGGCCGTTTCC

TCCAAACGAAGAGCGCAAATGAACAAGTACCTTCAGCCCAATGGGAATAATTGGCGGTGG

TTTGATGACCGTTCCGGCCGCTGGTGCAGCTACAGTGCGAGTAACAACAGCACTATAGAC

TCTGCATGGAGAGCAGGAGAGACCAGTGTCCGTTTCACCGCAGGTCGCAGGAGATACACT

GTACAGTTCAACACCATGGTTCAGGTGAACGAAGAGACAGGAAACAGGCGTCCGGTCATG

CTGACGGTGCAGAGAGTGCCCCGCATGCCCAAACCAAACAAATCGGGCAATGTAGCTGAC

CCTGAGAGAGAGGAAGACCAGAGAGCCAAGACTGAGGAGACTCAGGTTCTAGCAGACCCA

GCGTCTGTAGCAGTCGAGATGGTGGCCCCTAAAGAAGAACCTGAGCTAAAAGTGTCCTCA

GCACCTGCTTCCGCTGCCCCCGCTGCATCACAGGACTCCTCATCTGGATCCAATAGCATT

GTGGTGCAGGGCCTAACTGACGAAATGACCACGGTCCTGATCCGTGCCTGCGTCAGCATG

ATCAGTGTACCCGTGGACCCAGACACACTGCACGCCACACTTCGCCTATGCCTGCGCCTC

ACACGTGTCCACCACTATGCTATGATGTTTGCGGAGCTCAAGAGTACACGCATGATCTTA

GGACTGACCCAAAGCTCTGGCTTCAACGGCTTTACACCACTCGTCACACTGCTCTTCCGC

CACATCATCGAGGACCCGGCTACACTGCGCCACACCATGGAAAAGGTGGTTCGTTCTGCA

GTGACCAGTGGAGCAGGAAGCACCACGTCTGGTGTAGTGTCTGGTAGTTTAGGTTCCCGG

GAGATTAATTACATTCTGCGTGTGCTGGGTCCAGCAGCCTGCCGGAACCCGGAAATATTC

ACAGAAACAGCCAACAGCTGTGTCCGAATCGCTCTCCCAGCTCCAAGAGGTGCCGGAACA

GCTTCTGATGACGAATTTGAGAACTTCAGAATTAAGGGACCCAATGCTGTACAATTAGTG

AAGACAACACCGCTAAAGCTTTCTCCACTGCCACAAATACCAGAAACCATTAAAGAGGTC

ATCTACGACATGCTTAATGCCCTGGCAGCTTACCATGCACCTGAGGAAGCAGAGAAAGGA

GAGGAACGTCTAGCGACAGTCCCCGGCAGTCAGGACCTCTGTCAAATCCTACAGGATGTT

GGGGATGATGTTTACCAGCAGTACAGACTTACACGGCAAGGGAGCGACTTTGACTCTCAG

TCCGCTTTCCATATAAATACTCAGGTGTTTGCTGCTGATGGTGCAGTTGCAGAATCCTCC

CAATCTGGAACACCTCAGGGAGAGGCCTCAACTCCAGAGGAGATGCGAGAGGACAAGAAG

GAACAGGAAGGTGACAAAGCAGGCTCTTCGGAAGAAAACAAAGCAGCAATGGCCAAAGCA

AGCAAACCCCTGATGCCCACCTCCACCATTTTGCGGTTACTAGCAGAGCTGGTGCGCTCC

TACGTCGGCATTGCCACACTTATCGCCACTTACAACTACACCGCTGGCCAGTCCGAGCTG

ATTAAAGAGGATTGCAGTGTGTTGGCCTTCGTGTTGGATCATCTGCTCCCACACACTCAG

AATTCTGAGGATAAAGACACTCCTGCCCTTGCACGCCTTTTCCTGGCCAGTCTGGCAGCA

GCAGGCACTGGCACAGAGGCACAGGTGACCCTGGTCAACGAGGTGAAGGCTGCTCTTAGC

CGAGCTTTGGCAATGGCAGAGGGTACAGAAAAGCACGCTCGGCTTCAAGCAGTGATGTGC

ATCATCAGCACTATTATGGAGTCCTGCCCCTCCACCTCCAGTTTTTACAGTACCGCCACT

GCCAAGACACAACACAATGGCATGAACAACATCATTCGTCTGTTCTTGAAGAAAGGCCTG

GTGAATGACCTCGCCCGTGTGCCCCACAGCCTTGACCTGTCTAGTCCTAACATGGCAAAC

ACAGTGAATGCTGCTCTTAAACCATTAGAGACGCTTTCACGCATCGTCAACCAGCCCAGC

AGCCTGTTTGGAGGGAAAGGAGGCTCCAGTAAGAGCAAGACTGAACATGATTCTGTGGGC

ACAGCCAGGGACAGCAACAGCAATACTCAGGATCAAGGGGAGGCAGGCGAGGCCGAGCCA

GTAGAAAACAGTCACAGAGGCCAGGCTACTGATGGTGATCTGATGGATGGAGAGGCAGAA

GGAGACACCGTGGTTATCGCAGGACAGCCAGAGGTGCTCAGCACCCAAGCTATGCAAGTG

GAAAATGAGTTGGTCGATTTGATAGATGAACTTTTAGAAAGGGATGCAGGAACCGTTAAT

AGTACCATCATTGTTGGTCGCTCTGGAGATGAAGAGTCTCAGGAAGACGTGCTGATGGAT

GAAGCTCCTTCAAACATGAGTCAAGCCTCCACTTTGCAAGCCAACAGAGAAGACTCTATG

AACATCCTAGAGCCAGAAGATGAAGAGCACACTCAAGAAGAAGACTCGAGTGGCAGCAAC

GAGGATGAAGATAGTCAAGATGAAGAGGAGGAGGAGGAAGAAGAGGAGGAAGATCCGGAT

GATGAAGAAGGAGATGAGGATGACGACGACGAAGGCTCGGAGATGGAATTGGATGAAGAT

TTTCCAGATATCAACGCAGCCCCACATATACGATTCGAGAGATTCGATCGAGATGATGAT

CTCATTATAGAGTTTGACAACATGTTCTCCAATTCTGCTGACATCCCTCCTTCTCCAGGA

AACATCCCTAGTTCACATCCTCTGATGGTGCGCC

>Unigene000056

GGGATCCCTGTGGAGAAAGAAAGATGGCGGCGCATTGACTAGCGAACGCTCTGTAACGAA

GCTTCTTGTTTGGACCGTTTCTAAAGTATTAGTCACTACAGTAAACTTGTAGAACTCTCA

TTAGTCAGCGAATGCACTACAGCACTAACGGAACATACATTGGGACTTAAAGTAAACACT

GAGGGGATAAAATGGCGACAGGCGGTGCTCCTTTTGATGACGAACAGGAGCTGCACAACT

GGACTATCAGCAACGGCAGCCTGGATGACCGACTCAATAACATGGACTGGGGCGTTCAGC

AGAAAAAGGCTAACCGGTCTTCAGAGAAGAACAGGAAAAAGCTCTCCACCGTGTCCGAGA

GCCGCCTGACCAATGACATTTCTCCCGAATCGACACCGGGGGCGGGGCGCAGGCGTGCGA

GAACACCGCACTCCTTCCCTCACGTTAAGTACACTACGCAGATGTCCGTTCCTGATCAAG

CCGAGCTTGACCGCCTACGGCAGCGCATAAACTTCACCGACCTGGACGAGAGAAGCATCG

GTAGCGACTCCCAGGGACGCGTCACTGCAGCCAACAACCAGCGGCAGCTTTCATCCGAGC

CAAAGAAACCTTTCAACTTTCTGTCGCTTCACGTCAACACTAACAAAAGCAGAGAGTCTT

CTGCTTCTACCCCACCTGCAACCACTGCAGGGGGTAAAGAGACAAAGAAACAGAGTCCAG

GGAAGGAGCTGTTTGTCCCGGTGCCTGTCGTACCTGTTAAAGAGCCACGCAGAGACGAGA

GAACCTCAGGAGGGCCCCCGGTGGACGGGAGACAGGAACCAGCTATCGACAGCAGCCAGG

TGGTCAGTAAGCTGGTGCAGATCCGTGAGTACATCAGTAGAGCCAGCTCTATGAGGGACG

ACTTACTAGAGAAGAACGACGTTCCGGCTAATGTCGAGCGCCTCTCTCACCTCATCACAC

ACCTGAAGGAACAAGAGAAGTCTTACCTGCGTTTTTTGCAAGAGATGCTGGCGAGACAGA

ACATGGAGGAGGAGGCTGAAGGTGCCACGGTGGACTCGGCGGTGTGTTCGGGCTCGATGG

CCAAGAGCACGTCTCTGAACCTAGAGCCTCGGTCAGAGGCCGCAGATTCCACCTGTTACC

GGGTTGGTGGACAGCACAGTGAGGAGTTGGAGAACCTGCGCAAACAGCACGACCTACTGC

AGAAGATGCTGCAGCAGCAGGAGGAGCTGCGGGAGCTGCAGAACCGCCAGGCCACCCTGC

TTAGCATGCAGAACCGTTCTGAACATGCCATGGAAGACCCAGTTGTAACAGAGACCACTG

GAAGCGTGTCAGGTGTCAGCATCACTTCAGAACTCAATGATGAACTCAATGACCTAATAC

GACGCTTCCACAACCAACTGCATGACACTCGGTCTCAGGCAGTTCCTGATAACCGACGTC

AGGCTGAGAGTCTGTCCCTGTCTAGAGAGGTGTCCCGGTCTCGTTCTACCCACAGTCCTC

GGGGGCAGCAATCCCCTGGCACCGATGCTGCTGTTTCTGCTTCCCCTGCTAGCGCCAAGA

TCACCAAACTCCAGGAGCTCCAGGATAAGAAACAGACGATGGACAAAATCCTGCAGGAGC

TTCATTCCCTCCGAGACCAGACGCTAAACAACAGCTCAAGCCGTGGTGCGTCTCAGCGTG

TTTCTGAGCGTCCCTCAGCATCAGGGCGAGACGGGGACAGTGTTTGTGTAGCAAGGCAAG

AATCTTCATCTTATGATGATGGAGGAAATTCAGCTGAAAAACTCAGGAAGCTGAAGGAGG

TACACAAGCGTCTAAATGAGCTGAGAGAGCTGGTACAGTATTATGAGCAGACGTCCGACA

TGATGGTGGACACGGTGAACGAGAATGTGAACGAAGATGAGGAGGACGAGACAGATGACG

GGTCGCTCTTTGAAGCTATGTTTGATTCGGAACAGGAGAATCGCGAACCGATAACTAACA

TCAGAAACCCTGCCCAGCCTCAGTCTACGTCTAACTGGATGGACATGAACACACTGACAA

ATGCCTGTAGCAGCTCTAACAACAGAGTCAGCCAGCTGAACTCTCAGTGTGAGGCCAACA

ATCGCTCAGCCGCTAACCTGCGCAGCTTCAACGGTCCCTCTGCCATCGAATGCCAGTATA

ACCGGGACGGCTCCTATAATGGCGTCAAGCACGGGAATGGAGATGGAGATGAAGACGAAG

AGGACGATGACGATGATGGCGCTGAAGGGGCAGCTCAAGCAAGGCAACGAAGAAGAGAAA

GCTCTGGTTCAGGGTCCAGTCGCCAGAGCAGCATTGCAGATGATGCAGTGTTTGCTCAGA

AAGTCCATCGTCTTCAAACAGCAAAGCAGAAACTTCAGCAGCTCCGTGAGCTTGTTGCCA

TGGTGCAGAGTGATGACACAGACACCACAACAGCCAATGAGGATGAAGGATTGCAACAGC

AACCCAACAACACTAGAGCGACTGCACCAAAGGCCAAGAGGGAACTTGCTCTCTCTGATA

AAGCCAGGGAAAAACTGTACGAGGAGAAGCTGAGGCAGCAGCAGGAGGAGCTGAAACAGC

TTCACGAGGAGAGACAGAGACTGATGGAAATTCAGGGCAAAATCCAGGACTTGCAGTGGG

CCTGTCCTGATCTACAGTCCTCTGTGTCGAGCGCAGCGAGCGGCATGGTAAAGAAGATCC

CTGCTGCAGTTTCCACTCCTGCTGTTGTACCATCCACATCAGCAAAGTGTAACACCACCG

TACTCAAACACACCACCGAACCTGCTCCTGTTACCATCACAGATAATGAGTTGTGGTCAG

AGATGCGCAGACACCAGGTCTTGCGCGAGGAACTGAGGCAGAGACGGAAACATCTGGAAA

GTCTGATGGCTGAGCAGCAAAGGAGAAGTGATGCACAACCTTCACACAGCTTTACCCAGG

ATGACCGGACCATGGCAACCTGGGGCAGCTCCACACAGTGTCCACTGGGTGAGGGCGTTG

ATGATGATGATGAGGATGAGGATGATGATGATTATTTCCTTCTCGGAGTTTGTCTTTTTC

TGCTCTTTTGCGCTGAGTGTCGGTTCTCCTCTGCTGCTCTTTGCTAATCGGCGTGATGTT

CGTCTGGTGGACGTGGACTCGGTGCGCGCCGACTCGGCGGTGTTGGTGGGGGATCTGGAG

GACGCGGCTGCGGTGGACTTCCTGTTCTCAGAGAGTTTGGTGTTTTGGACCGACGTTAGT

GAGGAGGCCATCAAACAAACCTACTACAACCAGAGCAGCAACGCGGTGAAAGAAGCGGTC

CTGGGTTCGGGTCAGAAGGTGGTGGTTTCAGGTCTGGACTCTCCGGACGGTCTGGCGTGT

GATTGGCTTGGACGGAAACTTTACTGGACCGATTCAGAGACCAACCGCATCGAAGTAGCA

AATCTGGACGGAACCTCACGGAAAGTTCTGTTCTGGCAGGATCTCGACCAACCCAGAGCC

ATCGCACTCAATCCAGCACAAAGGTACATGTACTGGACAGATTGGGGGGAGGAGCCACGG

ATCGAGCGCTCTGGGATGGACGGCAGCAGCAGGAAGGTGATTATAGATGCGGATATTTAT

TGGCCCAACGGACTGACCATCGACCTCGACGAGCAGAAACTCTACTGGGCCGATGCCAAA

CTCAGCTTTATACACAGAGCTAACCTGGACGGGACCGCAAGAGAGACGGTGGTGGAGGGG

ACACTCACACACCCGTTTGCTCTCACACTCTTTGATGAGACTCTGTACTGGACAGACTGG

CAGACTCGCTCCATTCACTCCTGCAACAAACACACAGGAAACAACCGCAGAGAAATCCTG

AACGGCATCTACTCCCCCATGGACATCCAGGTGCTGGAGCAGAGGAGACAACCTTACATC

CACACGCCCTGCAGTGATGGAAACGGAGGATGTTCTCACCTGTGTCTGCTCTCGCCTGCA

CCGCCGTATTACAGCTGCTCCTGTCCCACGGGGGTAAAACTGCGCGAGGACGGCAGGATG

TGTCGAGCAGGAGCGGAGGAGGTTCTGCTGCTGGCCCGTCGGACTGATCTGCGCCGGATC

TCTTTGGACCTCCCGGATTTCACTGATGTGGTCCTGCAAGTAGGCGACGTCCGTCACGCC

ATCGCTATCGACTACGACCCGGTGGACGGCTACGTGTACTGGACGGACGATGAGGTGCGG

GCCATTCGCCGATCTAGACTGGATGGAAGTGAAGCCCACACGCTTGTGAGCGCAGAGGTG

GAGCATCCCGACGGCCTCGCCATTGACTGGATCGCACGAAACCTGTACTGGACCGACACC

GGGACCGACCGCATCGAGGTGACACGCCTTAACGGCTCCTCTAGGAGAATCCTCATCTCT

GAGGGCCTGAGCGAGCCCCGGGCCATCGTCCTTGACCCGGTCAACGGCTACATGTACTGG

ACGGATTGGGGCCAAAAGCCAAAGATCGAGCGAGCTAATCTGGACGGCTCCGAGCGCGTG

GTGCTGGTGAACACGTCTCTGGGCTGGCCGAATGGATTAGCGCTGGATCACGCCGCAGGA

AAGCTGTACTGGGGTGACGCTAAAACAGATAAGATCGAGGTGATAAACGTGGACGGCTCG

AACCGAGCGACTCTGCTGGACGATAAACTGCCGCACATTTTTGGCTTCACGCTGTTGGGT

GACTACATCTACTGGAGTGACTGGCAGCGGAGGAGCATCGAACGCGTTCACACCACCAAG

ATCACACGAGAGGTCATCGTTGAACAGCTGCCCGACCTCATGGGACTCAAAGCCACTAAA

GTCACCGAAATATTCGGCACCAATGGCTGCACAGACTATAACGGCGGCTGCAGTCACCTG

TGCTTCAATGGGCCTCGCGGTGTGACGTGTGCATGTCCGACGGGTCTGGAGCTCCTCAGT

GATCTGCGCACCTGCGTTGTTCCTGAAGCGTTTCTGCTTTTCACTAACCGCGCAGACATC

CGCAGCATCTCACTGGGAACCAACAGCAACGACGTGGCCATTCCGCTCAGCGGCGTTAAA

GAGGCTTCAGCGCTCGACTTTGACTTTGCAGAGAACCGAATCTTCTGGACTGACGTAAGC

ACCAAGACGATCAGCCGTGCGTTTATAAATGGCAGCGGTGTGGAGGCGGTGATCGAGTTT

GGTCTGGATTATCCTGAGGGAATGGCTGTGGACTGGATGGGCAGGAACATCTACTGGGCC

GACACTGGAACCAACCGGATCGAGGTGGCCCGTCTGGACGGTCAGTACCGTCAAGTTTTG

GTGTGCAAAGATCTGGACAACCCCCGCTCGTTAGCACTGGATCCAGCTAATGGGTATATG

TACTGGACGGAGTGGGGGGGGCGACCCCGCATCGCCAGCGCTCACATGGACGGCACCAAC

ATCATCACTCTGGTAGATAAAGTGGGCCGAGCCAACGGTCTGACCATCGACTACATGGAT

CAGAGACTGTACTGGACTGACCTGGACACCTGCATGATCGAGTCCACCAACATGCAGGGT

CTGCAGCGTGAGATCGTAGCTGATGATCTTCCACACCCCTTTGGTCTGACTCAGTACCGG

GACTTTATCTACTGGACTGACTGGAACCTGCGCAGTATAGAGCGAGCAGATAAACGGAGT

GGTCAGAACCGGACCTTGGTGCAGGGACAGCTGGAGTACGTCATGGACATCCTGGTGTTC

CACTCATCACGACAGGACGGATCAAACCCGTGTTCACACTATAACGGTTACTGTGCTCAC

CTGTGCCTGGCCTCACCATCAGGGGCTCAGTGTCGCTGCGCGTCCCACTACACACTGCAG

CCCGACGGACACAACTGCAGCTCTCCCGCCAGTTTCCTGTTGTTCAGTCAGAAGAGTTCT

ATCAGTCGGATGGTTTTGGGGGAACAGAGTCCGGATATGATTTTGCCGATTCATGTCTTT

AGGAATTTGCGAGCGATCAGCTTCGATCCGCTGAAGCGCTTCATCTACTGGATCGACGGG

AAGCAGAGCGTCCGCAAGGCCCGGGACGACGGCTCTCAGGCGGCGACTGTGGTGTCCCCA

GGCCCCACACAGCCTCTTAAACAGCCTCACGATCTGAGCGTGGATCCATTTAGCCGCACT

GTTTACTGGACCTGCGAGAACACCAACACCATCAACGTGCAGCGCATGGACGGGCAGAAC

ATCGGAGAGGTGCTGAGAGACGAGGTGGACAAACCCAGAGCCATCGTCCTCAACGCTGAG

AAAGGCTACATGTACTTCACCACACTGCAGGAACGCTCGGCAAAGATCGAGGGGGCGTCT

TTGGACGGGACGGAGCGCGAATCTCTGTTCACGACCGGTTTGATCCGGCCTGTGGCGCTT

GCTCTGGATAACAAACTGGGCAAGCTCTTCTGGGTGGACGCTGATCTGAAACGAATAGAG

AGCAGCGACCTTTCCGGAGCCAATCGGATCGTCCTTCAGGACTCGAACATCCTGCAGCCG

ATTGGTCTGACCGTCCTCGGGGATCACCTGTACTGGATTGATAAGCAGCACCAGATGATC

GAGCGTGTGGATAAGCGCAGCGGGGACAGGAGGACACGGATTCAGGGACGGATTCAGTCT

CTGACCGGAATTCATGCGGTGGAACTGATGGAGACGGATGAGTTCTCGTCTCATCCCTGT

TCCCGTGATAACGGCGGATGCTCACACATCTGCATTGCTAAGGGAGACGGGACACCGCGG

TGCTCGTGTCCTATGCACCTGGTACTGCTGCAGAACCTGCTCAGCTGTGGAGACCCACCC

ACATGCTCGGCGGATCATTTCACATGCGCGACAGGCGAAATCGACTGCATACCAATGACG

TGGCGGTGTGACGGCATTGCTGAGTGCGCCGACCACAGCGATGAGATGAACTGCCCCGTG

TGCTCGGAGCAGCAGTTCCAGTGTCACGCCGGACAGTGTGTCGATGCCAAGCTGCGCTGC

AACGGAGAACAGGACTGCACTGATGGATCAGACGAACTCGACTGCCACACCATCTGCATG

CCCAATCAGTTCCGCTGTAAAAATAATAAATGCATCTCTAAGAAGCAGCAGTGTGACTCG

TATCCCGACTGCTCCGACGACTCGGACGAGCTTTTCTGCGATTTCTCACCACCCAGCAGT

GAGGAGCGACACACCAACACCATCGGCCCGGTGATCGGAATCATCCTGTCTGTCTTCGTC

CTCGGCGGAATCTGCTTTGTATGTCAGCGTGTTGTGTGTCGTCGCTACAAAGGATCAAGC

GGCGCATTTTCTCATGATTACATCACCGGGACCGCCCACGTCCCACTGAACTTTATCAGC

ACCAATAACACACAACATGCAAACTGTACAGGTATATCATGTGGGAAGTCAGTAATGAGT

TCAGTCAGTCTGATGGGTAGTAGCAGCAGTGGAGCTCCTCTATACGACAGGAACCACGTG

ACTGGAGCTTCATCCAGCAGCTCATCCAGCACCAAAGCCGCCTTTTACCCTCAGATTCTG

AATCCTCCTCCGTCACCAGCCACCGATCGCTCACTCTACAACGCAGAGGTGTTTTATTCC

TCCAACAGTCCGTCCACCACCAGGTCCTACAGGCCCTATGTGGTACGTGGTGGTGCCCCG

CCCACCACGCCCTGCAGCACAGACGTGTGTGACAGCGACTACACGCCGAGCCGCTGGAAG

AGCAATAAATACTACATGGAGGTGAACTGGGACTCGGAGCCCTGCCCTCCCCCACCCACG

CCCCGCAGTCACTACATGTCTGCTGAGGAGAGTTGCCCTCCATCGCCCTCCACAGAGCGC

AGCTACTTCCACCTGTGTCCACCTCCACCCTCACCCTGCACAGACTCGTCCTGACCACAC

ACACACACACACACACA

>Unigene000057

GGTCCCAGACTGGACAGGAACCCTCAGAAAGAGGACATGTGTGTAAAAGATAAAGTCATC

GAGTTTAGAATGAACTCTGTGTATGGAAGAGGAATGTTCCTTCTGCTCATCTTTGTAGGA

ACAGAATTACAATCGACTTTCGCACAGAACCGCTGCAACAAGAATACACAAGTAATGACA

AAGTCAGAGTGCCATCTGTGTGCTGTAAGTGATTTAAAAAACTGCCCAGCAGGCTATAGC

AAGAAGACTCCAGGCAAGGGGCAACAGAACTGCAGGTATCAATTAAAAACATCCTTCTCA

TCTGACCTCTACCTTCGTGGCTGCTCGCATGAATGTTTCAAAGAGGTGATAGAAGAACGA

TGCTGTTCAGGATACTGGGGTTACGACTGTCTCGAGTGTCCCGGCTCAGCTTCTACCCCA

TGCAATAATAACGGGGTTTGCACTGATGGCATCGGGGGCTCAGGGAACTGCACCTGCAAA

GAAGGATTTGTGGGAACAGCATGTGAGAAATGTGAAAAGAACCGGTACGGATCCACATGC

AGCAACGTGTGCACCTGTAAGCATGGGCTCTGTAACAGTGGACTTAAAGGGAATGGACAG

TGTACCTGTTTTTCAGGATACACAGGGCCGGATTGTGACCAGGAGCTCCCAGGTTGCGCT

GTTCTGCACTGTGGTCCTGATGCGCGCTGTTTTGAAGATATGAATGCTGGGAAACTAGTG

TGCAAATGTCAGCCTGGTTACTTCGGGGATGGAGTTCAGTGTACCTCAATTAATCCATGC

CAGAAGCGTGTATGTCACGAGCATGCTGTGTGTACACACACTGGGCCGAACCAGCACACA

TGCACCTGTACAGAAGGCTATCAGGGAGACGGGCTGGTGTGTCTTCCCATTGATCCCTGT

CAGACTGATTACGGCGGCTGTCAAAGCAACTCCTCGCGGTGTGTGTATGACGGCCCCGGA

AAGGCTCACTGTGAATGTCTGGAAGGGTTTGAGAAGTTAGTCGTAGGAGCGGGCTGCAGT

CTGAGAGATGTGTGTAAACCAGACTCCTGCCATTTACAAGCCTATTGCATAACGGTGGCA

CCAAACACAGTGGAGTGTAAATGTAAAGATGGCTTTCTTGGCAATGGGAATGTCTGCTTT

GGCAACATAATCCAGCGTCTGCAAGAACTGAATGCAGACCCTAAGAGCCGTTCCTACGGT

CAGCTGTCCAGTGCCATCAGCCTGTTCGAGGGTGTCATGTCCTGGCCATTGACCTCACTT

GGACCATTTACTGTATTTGTGCCAAAGAACAAGGCTTTCAAGTCCACTCAACCCGTGAAA

AGTCTGCTGGCCGATCAGGCAAAGGCTCGTTATCTCGTAAAGATGCACATGATTCCTGGA

GAAGTGAACTATGATATGCTGAAGAACGGAATACTCTATTACACTCTGACTGGAAAATCA

GCTGAGTCAATTACTGAGTCAGGGCAGGTGAAGGTTCGTATTCATGGAAGCCGTAAAAAA

GCAGCGCTGCTGGAGACAGATATTATCTCTTCCAATGGGATGATCCACATAACTGATAAA

CTTTTGGACTCAGTTCCTTCTACTGTAATAAGTGACAGTCAGGAAAACCTGATGAAAATT

CTGTCAGACAACGGCAAGTTCTCCATTTTTAAGTCTTTAATCGAGAAAACCAGCATGGTG

GACATTTTGAACAAGGATGGCCCTTATACACTGTTTGCTCCCACAAATACTGCATTCTAT

CTAATGAAGGACAACCAGCTGAATTACCTCAAATCTGAGGAGGGTAAACCAAAACTACTG

GAATTTCTACGAAACCATGTTGTCTCCTCGGCCCAGATGAAAGCCAGCTACATCGTGTCC

ATCTCACAAATCGTGTCCATGGCCAACCAAGTCCTGACGATTAATGTGACTGCTGCAGGT

CAAATCCTGATCGGTGGGGTAGCAGTAGCTGAGGCAGATGTTGAAGCAAAAAACGGATGT

CTGTATTCACTGCTGGATGTTCTAATCCCGTCATCCATAGAGCCCATTCTTCCACAGCGA

TGTGACTTGACAGAAAGTAACATTTATAAGAGTCCATGTGTAAGCTGTACAGAAGTCTCT

AAGGCTTCCTGTCCTACTGGTGTGTCATTGAACACTACTTCCACAGGCTGTGTGTACAGG

AAGAGATCTTTGGTAATAGATGTCCCTGTTCTAGGCTGCAGTTTGTTCTGCAATGAGACC

CGTACTACACCTCAGTGCTGTAAAGGCTTCTTTGGAGCAGACTGCTCACCTTGTCCTGGT

GGATTCACTACCCCATGCTCCTCTAATGGCAAGTGTTTGGATGGTATTGAAGGCAATGGT

ACGTGTCAGTGTGAACCCAAATTCAGCGGGTGGCGATGCCAGTATTGCTCAGACCCGAAC

AAATATGGTCCTTTTTGCAACCAAACTTGTAGGTGTGTACGCGGTATTTGTGACAATCGG

CCTGAATCAAATGGTGCGTGTAAGCCACAGTCCTGCCAGGAGGGCATCACTGGAGAGCTG

TGTAATAAACATGCACTTCCCTGTGGGCCAAATCAAATATGCCACATCCACGCCAACTGC

ATCCTGGAACAAGCAGGAGAGCAAACCTGTGTATGTAAGCCTGGTTACATGGGCGATGGA

TTCCAGTGTACACACGAAGACCCGTGTACATGGAGTCCGAATGGGGGCTGCAGTGACAAT

GCACGATGTGTACAGCTGGGAAAGGGAAGACACAAATGTGAGTGTCTGACTGGGTGGAAA

GAGGATGGAGACGAGTGTCAGCCTGTTAATAATTGTCTGGAGCCGTCCAGAGGAGGGTGT

CATCCTAATGCCACCTGTGTCTTTTTGGGTCCTGGACAGAATGATTGTGTTTGCAAGAGA

AATTTCCGTGGGACTGGAACAGATTGTGAGCCCGTAAACCAGTGTGTGAATATAATGGGA

GGGTGCCATCAACTGGCCAGTTGTGTGATGATGTATCCTGGAAAGTTCGAGTGTGTGTGT

CCAGCTGACTACATAGGTGATGGAAGAATTTGCTATGGCACATTGGGACAGGAACTAGAA

AGCAATCTTGACCTAAGTGGATTCAATACCTGGGTCACCAAGGCAGATCTCAGGCAGATG

ATGCATGAGAAGGAGAACATTACCCTTTTTGTGCCCTCATTATCAGCCATCATGACCATG

AGTAAAGACGACCAGCATTTCTGGCTGTCCAGCAGCAACATTCAAAGCCTGGTCATGGGT

CATGTAGTTCCTGGACATTATAGATTATCAGATCTGCGTAGCTCCACCTCTCCACAGCTG

GTTTCTTTTTTAAGAGGAACGCTTCCTGTTTCCTGGACCAATGAGACCACAACAATTGGA

GGAGCCACTGTAACATTGGGAGACATAGTCACCAAGAATGGACTCATTCATCTGATTGAC

AAAGTCTTAATTCCTGATCGTAAGCTGAGTACTGGTTTAATCGGAGTGCTGGACAAGACC

CCAGAATTCTCTCTGTTCAAGAACGCTCTTATAACATACAACCTGAGTACAGAGATAGAG

GAGGCCACCGGCTTTACAGTATTCGCACCAACAGACAGCGCCATCAGAGAATACCTGAGG

AATACAGGCCAGGATTCACTGGATCTGAATGTTACACAGTACCACATTGTCCTGGATGAG

ACTTTAAAGCAGAGTGACTTGCAGGATGGTCTTTATAAAGACACTATGTTGGGCTTCTCC

TATCAGCTGGGTATATTTCTCAACAACAGCAATTGGTTTGTAAACAAGGCATGGATGAAT

GCCACAGACATTGAGACTGATAACGGAGTAATACACGGATTGTCTGCTGTCCTGAAGATT

CCTAAAAACCGTTGTTATACAGCTTCTAGCACAGTTTTTTTGGGAAAGTGTATGGACTGC

TTCCATCCTTCTGAAAGTCGCTGTCCTGCTGGAACCAAGCAAGTGAAAGTGTCACTAAAG

AGATGTATGTTCAGTGTGAGGAAATTTGGACGGTTCATCGGCTGTGAGGCTTCTTGTGAA

AAACTCACTATCACCAAAAAATGTTGTGAGGGATTTTATGGCACGGAATGTGAGCCTTGT

CCAGGTCCTGAGGGCCAATCATGTTTCGGTAACGGTGTGTGTATGGACGGAATCAACGGC

ACTGGTGTGTGTCAGTGTAACCCTGGTTTTAATGGGACAGCCTGTGAGAACTGCCAACCT

GGCAAATATGGCATCCATTGTGATCAAGACTGTAAGTGTGTAAATGGCCGCTGTAATGAT

GGTCTGGAGGGAGACGGCAGCTGTCAGTGTGATGTGGGATGGAGAGGAATTTATTGCTCC

ATAGCCATTAAGACAGACTTGTGCGGAGGGAAGTGTCACACAAGTGCGAATTGCCTACTT

AAAATACCAGATAATTCGTATTACTGTAGTTGTGCAGCTGGTTACGAGGGAAACGGTACA

CACTGCACAGTGATGAACGCTTGTGCAAAGAATAACGGAGGCTGTTCTCCAAATGCTGTA

TGTAAGAGGACTCTGCCAGGACGCAGGCAATGTCTGTGTAATCCAGGCTATTCAGGAGAT

GGACAAGTGTGTGTCTCAATAAATCCATGTCTTGATGGCAGTGGAGGCTGTAGTAATAAT

TCAGAGTGTGTACACACAGGACCAAACAAGTCAGCATGTGTGTGTCTGCCAGGATACACT

GGAAATGGAAAAATATGCTCTCCTATTAATTTGTGTAAAAAGAAAAACGGAGGATGCTAC

GTATTTGCCAAGTGTACGATGACAGGCCCAGGAGAAAGGAACTGCACATGTCCAGCAGAT

CAAATTGGAGATGGGATCAAGTGCAAAAGCAAACTCATGCCGGAGATTCGAAAGAAAGGA

CTGACTAACTTTGGCGTCGCTTTGCAGAGAGCTGACATTTATTATCTTCAAAGCCAAGGT

CCATTCACTGTGTTTGCTCCTAATAATAATGCATTTCACAAACTGGGGAATCAAGAGACT

GTAGATAAGGAGAAAAATGCTGCGATTCTGCTGTACCACATAGTACCGTGCCGGCTCTTG

CGCCCAGAAGAGCTGCAACAACCTCGCAACCTCACCACCCTCACCGGTGAAATCCTCACC

ATTTCATATTCGGAGGGTACGATACAAATTAACAACAACGCCAAAGTGGTGTACAGCGAT

GAAGAGAGTAGGAACGGGATTTTCTATGAAATTGACACCGTTTTGATTCCAAACAGTTTT

TATAAAATGGAAGAGACGAGCAAAAAAAATACTTCTCTCAAAGACGTAGTCGATCTCTAT

GGGTTTAAGACGTTCTACAAGCTGCTGGAGGATACAGAGGTGATCAAACAAATCCAGGAA

CCTCTCCACCAGCCAGTCACTCTGTTCATGCCGACAGACACCACCATGGAGGCTTTACCT

CAAGCGCAAAAAGACTTCCTGTATGGTCTGCATAACCGAGCACAACTGGTGGAGTATCTT

GGGTACCATATCATCCAATCCAAAAAGTTGGCAGAGGTGATGCATTTGGATTCCGTCAAG

ACACTTCAGGGATCTGGTATTTCCGTAAGCTGTGTTGGAGAAGATGATATAGGTTCAATC

TATGTCAACGATAAAAGTTGTCGGATTATAAAAAGGAATCTAAATTTCAACGAGGGTCTT

GTTTATGGTATTGATTGCTTGCTGATCCCACCCAGCCTGGGAGGACGTTGTGATAAGAAG

GACACTGTAGACATAACTATGCCTTGTAGATATTGTGGTTCTGTAAGATCATCTGATTGC

CCTGAAGAAAGCAAACCAAAGACTACACAGCCTTGTCACCTTGCAAGACACTCTCTGTAT

GTAAATTCTGGATGCCAGTCTGTGTGCTCCATGATCGTATGGAAACCCAGGTGCTGCCCA

GGCTATTATGGCAGGGACTGTCTGAGCTGTCCTGGAGGGCCTGGAGCTCCATGCAGTAAC

CATGGGCAATGTGATGAGGGTCTTTTAGGAAACGGCACATGCACATGTAATGCAGGGTTT

CAGGGCGTCGCATGTGAATTGTGCACAGATGGCCACTTTGGACCTGAATGCAAAGAATGT

AACTGCACAGAGCACGGAACATGTAACGAAGGACGAGAAGGAACAGGCTCCTGTTTCTGT

GACGAGGGCTGGGCAGGTCTACAGTGTGAACATAAATTAGCCGATGGTCCTGTATGTTTA

CCCGCATGCCACAAGAAGGCAGTGTGCACCGAGAACAACACCTGTGTGTGTAAACCGTTC

TACGAGGGAGATGGAATCACTTGTAAAGTAACAGATATGTGTAAGTTCTGGAATGGTGGC

TGCTCCAAAAATGCCAAATGTACCCAGAAAGGAGAAAAAGTGAACTGCACATGTCTAATA

GACTTTTCAGGAGATGGATATGTCTGTTCTCCGGTGGACCCATGTATCGGGGAAAACAAC

GGAGGCTGCGATGAGCATGCCCTCTGTACCATGACAGGACCTGGAAAAAGAAAGTGTGCA

TGTAAAACAAACTATGTTGGGGACGGGCTGACGTGTGAGCTGAAGGTGCTTCCAGTGAAC

CGTTGTATGTTAGATAACGGTCAATGTCATTTCGACGCACAGTGCACTGACCTGCACTAC

GAAGATAAAACCGTCGGTGTCTTTCACTACCGATCTCCGCTGGGGGCATACAAGCTGAAC

TACACATCAGCTCAAGAAGCATGTAAGATGGCAGGAGGCACTATTGCTACCTATACACAG

CTCTCATACGCTCAGCAGGCTGGATATGCTATGTGTTCAGCCGGATGGCTAGCACAGAAC

CGGGTAGCTTATCCCATGAGCTACTCCAACCCTAACTGTGGTTTTGGTCATGTGGGGATA

GTGGACTATGGCACTCAGACGGATCTAAGTAGGACCTGGGACACATTCTGTTACCAAGTA

AAAGACGTAAAGTGTGAGTGTAAGATCGGCTACATTGGTGACGGATACTCTTGCACTGGT

AACATCCTGCAGGTTCTCACCACCAAACCCGACTTTTCCAACTTTCTTTCACAAATCATG

AATTATTCTAGCGCTGCAGCAAAAGGGCAAGAGTTTATAAGGCGCCTCAGCAATATAACT

ACCAAGTCTACACTATTTGTGCCTGACAACAATGGCCTCTATGAGAACCAGACTTTATCC

TTCAGAGATTTAGAGCACCACCTGCTGGATGGGAGAGCTTTAGTGCTGCAGGACCTCATC

AACATCAGTCATGTTAAATCACATCTGGGAAACACTCTTAACGTAAAACCTGTTCCTAGT

CTCCAAAATCCCCAAACAGAGACTTCTTCTGGATATGTCAATGACCGCTACATCATCGAA

TCTGACATTCTTGCATCCAATGGAGTCATCCATGTGCTACAAGGACCACTGAAAGCCCCA

CCCCCTCCTCCCTCCACTCTTCCTCCAGCACATACAGCAGGATTGGGGATCGGTGTCATA

CTGCTGGTGATTCTTATTGTCACTGCAGGATTCGTAGCCTATTACTTCTACAACCGGCAA

ACCAAACCATTCCAGTTCCACTACTTTAGGGAGGATGAGAAAGACGAAAACAGCTCACCT

GACTCAACACTATATATAAGCAACCCCATGTATGAGAGTTCAGTTGCCCCCGGTACCACA

GACCTCCCTGTGCAAGATAAGGATGACAAGCACCAGGTAGTTGATGGAGGCTCGTATGAC

CTGCTCCAGAATAGCTAAATGGACCACAGATGTTCTTTAAAGTTCAGGATCTGTTCTGGC

AATGAGCATTGAACATAACAGATAAAATCTGTAGTACGTTTGGGGACTAAACACAGCAGC

AACATGGCAAATAATAGTAATAATAATAATGGATGATAATTTTGTGTGTGTGTGTGTGTG

T

>Unigene000058

GGTGATTATGAAAGAGTAAAGTTTCTCCATCCTCCATCATAACTCATAGTTGAAGCGAGA

TTTGGGTGGATTTTTTCATGCAAGCTTATCTTTAAGGATTTTAGGAGTGCCCTAAGTGTA

CTTGACCGAAGTAAGCAGCCATGCGTTATCTATTCCTTTTGGGATTCGTAGTGCTGCATA

GTTTACAAATTAAGGCTACCTCGAATCGCTGTGATGAGCCACGCACCGTCCGCTACTTCA

CCGTGTGCACGTCATGCGCTGCCGCACCTGCAATCAGCTGCCCAAAAGGCTACATAAAGA

CTTCCACTGATGCTGAGTGCCGCTACAATGTGCAAATTGGCGACCGCCTGCTGGAGCTGC

CTGGCTGTTCTCGCTCCTGTGAGAAAGTCACGCTCATCAAGCGATGCTGTCCTGGTTTCT

TCGGGTCTTTGTGTTCACCTTGCCCTAGCTTGTCAGGGAAAGTGTGTAACTGGCATGGTA

CCTGCATGGACGGTGAATCAGGAAACGGCACCTGTGTGTGTGAGAAGGGCTACACTGGAT

TTCTGTGCAATAAATGCCCTAATGGGACCTACGGTGAGCGCTGCAGTTCAGTTTGCGATT

GTGTTCATGGAGAGTGTAATCCAGACCCTGATGGAGATGGACAGTGTTACTGTCAACCAC

CGTATTCAGGACCAAAGTGTGACAAAGTTACATCCTCCTGCGCAGATTGCAGTCCCTACT

CCTATTGTGCAGGAGAGGGTCAGAATGCTAGGTGTGAATGCCTTCCATCTTTTGCAAAAG

TTGGACGATTTTGTGTAGGCGTTTGCACAAAGGATGTTTGTGACATAAATGCAGACTGTT

CCTACAAGGGTGCAGGGCAATTTCAGTGCACTTGCAAAACAGGTTTTGAGGGAGATGGCA

AATTCTGCACCCCGATCAACTCTTGTGCTAAGAATAATGGAGGCTGTCCAACAGACTCCA

CTATTTGTGAAATTACAGGATCTGGCCAATCAATATGTGTATGTAAGAGCGGGATGGAAG

GAGAAGACCCTCGTGCTGGCTGTAGGTTGAAATCTGCCTGTAAAGAGAACACATGCCACA

GAAGTGCACGATGTGAAACGGGCCAGGATGGAACAGCCAGATGTGTTTGCGGTTCTGAAC

AGATCAGTGATGGGTGGCGCTGTTATAGTGATATCATGGAGCGTGTTCTAGAACTGGATC

GGGAAGGGAGCCAGGCAGGAAACCTCACTGGAAGCATCGCGTTGTTTGAGAAGGGATGTA

AGCTCAGTTTGAGCAAGCATGGGCCATTTACAGCATTTATCCCAGTGGATACATCACAAA

TATCTTCAGGCAAGCTCAACCAGGCAAGAACAGCAGAAGCTATCTGCAAACACCACCTCA

TTCTTGGTCAGCATCTTTACAAAGATCTGGAGGGTCACGATTTATGGACATACGGAGGGG

AACTTATGAGATTTAAACCAAACAAAAAGTTTATTTCAAAAAATGATCCTGATACTGTGT

TCACCATAATCAACAGTGACATACCTGCATCCAATGGAATTATACATATAGTGGACAAAG

TGTTTACTTTTGCTACTGTTGACACTTATGACAGTGCTGAGTTTTCTTCAAAAACCATTG

GGGACATTCTTGCAGAAGACACCAGATTCAACAGATTTGTATCTCTAGTAGATAACTGTG

GTGCTCCGATGCCACTCCAGGGTCCAGGGCCGCTCACACTGTTTGTCCCAACCAATAAAG

CTATAGACAGATCCAGGGATGGGAGTATTATATACATGCTAAAGGAGGCCAAACACAAAC

TTCAGGAGCTTTTGAGGCACCATATGTTCTCTCAGGCAGCGGTAACTATGGACCAGGTGG

CTAGCATGACGGAGATTCATACAATGGCTAATCAGGCCATAACAGTCAGTATCTCTGATG

ATGGAAGAGTGTTGCTCACTGAAAAAAAAATATCTTTTGATATGAAGGACATCATTGCAT

CCAATGGAATCATTCACATTGTTGACGGACTTCTCTTGCCTCCGTCTATCATCCCGATCA

TGCCACACCGATGTGATGTAAATGAGACCACCATTATCACGAGTCCATGTGTGATGTGCA

AATTCATTTCTGAGTCAAAATGTCCACCTGGAAGTGAGAAACAGACTGACCGTTTTCAAT

TTTGTGAGCCTCCTCCTGATCCACGTTGGAGCGAATTTACCACCGTACGAGGCTGTGCGA

GATACTGCAAGGCAAAGCGAGCGAGAGCTGAATGCTGCAAAGGTTTTTATGGACCTGACT

GCAAACCCTGTATTGGAGGTTTCCAACACCCATGTTATGGCAAAGGCACTTGCTCTGATG

GCATTTATGGAGATGGCTCATGTAAATGTAAGCCAGACTTTATGGGCATCGGGTGCCACA

TCTGCTCTAACCCAATGAAACATGGAGAAAACTGTGATGAAGATTGCCGCTGTGTCCATG

GCGTGTGTGATAACAGGCCTGACAGTCAGGGTGTGTGTCGCAGGAACTCCTGTTTGTCCG

GATACACCGGAGAGCTTTGTGATCTCACAGCCATGCTATGTGATTCAGATGGTGCCTCTG

AACACTGCCATATTCATGCTTACTGTACACGTGTCAACAACATAAACATGTGTGTGTGTA

ACCCTGGATATGAAGGTGATGGATATTCCTGCACTGAAGCCAACCTGTGCCTCAGAGCTG

ACAGGGGAGGCTGCCATATAGGTGCACAGTGTTTGTATGCAGGCCCTGGAAATATATCGT

GTATATGTAATGAGGGATGGACAGGTGATGGTTTTGTGTGTGTGGAGATCAACAACTGTT

TAACAGCAAATCGTGGTGGGTGTCATGAACAAGCTGAATGCATCTACATTGGACCTGGCC

AGAGTGAATGTGCCTGTAAAAAGGGTTTCATGGGAGATGGCATCATGTGTCAGATTGTTA

ACCCCTGTCTGACAGACAATGGAGGCTGCCATGCCACGGCAACATGTTCATTTAATAATA

GCGGCATACACCAGTGTACATGTCCAGAGGATCATGCAGGAGATGGCTTTAAATGCTATG

GGAATATACTGATGGAGCTAGAGGGAAACTCAGATTTCTACACTTTCAACCGCTATCTAC

TAAGACACCCTGTGATCACTGCTGAGAGTAATGTGACTGCTTTAGTTCCTTCCAAGGATG

CGTTTAAAAATCTGAGTGATGCTGATCAGGTGTTTTGGACTGATTATTACCACCTTCCCT

ACCTCATGCAAGCTCACTTTTTGGATGGCATTTACTCCTATGATGACTTAAGTCAGCAAG

TCAATAAAACAGTCTCAACTAAATCCAAGACAAAATGGAAGATCACATTTAAAAATGGGG

AACTTATGATTGATAATGCAATTGTTCTTGTCTCTGATCTCCAAGCAACCAATGGGTTCA

TTCACATCATCAATACTGTATTAAAACCACCCATTTCGGATATCCCCCCTCCACCCCCTG

ACCTAATGGAGGTCCTTCATAACACTCCCTCATTCAGCCTGTTCAGAGAAGCAGCACTGC

TGTACAATTTAACAGAAAGCATCTCGAAGAAGGACTTCACTATCTTTATTCCATCTGACA

GTGCAGTTAAGGAGTACCTGTTGAAAACAAATTCCACACAACTGGATGAAAACATTGTGA

AATATCACGTTATCACAAAAGAGCAGCTTTTTCCTGAACACTTGACTGATGGAACTCTAA

AAACTACACTGCTTGGAAATGAGTATCAAATCATGATTCACTTAAACAGTGAAAATAAGA

CGCTTATCAACGATGTAGAATTGGATGGAAACATGACTGAGATAAGACACGGTGTCATTA

TCAGCATATCCCGGGTTTTAGTCATTCACAAAAACTACTGCAGCAAAGATATTTATATGA

AAACGTTTGGAAGGTGTGGTGACTGTAACTCATCTCCCAAATGCCTGTTTGGCAGCAAGC

CGATAAAGGATGAATTCCCTTTGAATATGAAGTCCAACTGTAGATACAGGATGCGAGTAG

GGAAAAGAAGAAAGTCTGTGCCAGGTTGCATGATAGACTGTCTGAAAAATGTAAAGGATC

ATTCCTGCTGCCCAGGATACTTTGGACGTGACTGTTTTAAATGTCCTGGAACTGTTGACA

ACTGGTGCTCAAACAACGGCAAATGCCAGGATGGCCTTTTTGGCAATGGGGAATGTCTGT

GTAGCGAAGGGTTTCATGGCACAGCATGTGAAATGTGCGAGCCTGGGAGATATGGTAAAG

ACTGCAAATCAGAATGTCATTGCGATCATGGAAAATGCCTGGATGGCATAGATGGTAATG

GTCAGTGCATCTGCTACAAGGGCTGGAAAGGGGTAAACTGTTCTGTAGAGATCGTTAATG

ATGAATGTGGAGGAATTTGTGATGAAAATGCAAACTGTTTGTCTCCAAGTTCAGGAGTGA

AACCATCATGTGTCTGTTCAGCAGGTTACCATGGAAACGGCACCTTCTGTCAAGAAACTG

AGTTGTGTGCCATTAATAATGGGGGCTGCTCAGAACATGCAACATGCACAAAAATCTCAC

CTGGAGAAAGGAGCTGCACATGTAAGAAAGATTACACTGGAGATGGAACTGTGTGTCTGG

AATTGGACCCCTGCCTAGTTAATAATGGAGGATGTTCTGAAAATGCAGATTGCATCAAAA

CTGGACCAAACACAGCTGCATGTGTTTGTAAATCAGGTTTTGCTACAATAAGAAACTACT

GTATACCAATAAATCTCTGCCTAAAGGATAATAACGGAGGATGCAGCGTCAATGCTATTT

GTCAAAACACTGGCCCGGGTGAGAGGAACTGCACTTGTCGATTTGGCTACAAAGGAAATG

GCATTGAGTGTATGGGAACTGTCAGTAGAGAACTCCTTCACAATTCTGCTGCTAATTGGT

TTAGGAAAAGTCTTTCGCAATCAAGAGTCAGGGATTTGTACAGTAAAGGGCCATTCACTG

TGTTTGTACCTCACACAGATTACATTGGAAACTACTCGATTGATCTCTGGGAGAGTAAAA

ACCGCATCCCAGATCTGTTGCGGTACCACATTGTAAGTTGTGAGGAAATCTCTGGATCAC

AGCTTAAGTCTATGACACATGTTGTCTCTGCTTCTGGGCATGTGTTGAATTTCAGCGTAC

GAGATGGTGCTGTATACATAAACGGAGATACAAAGATAATCACTAGTGACTATGAATGTT

CCAATGGAGTCATCCATTTTATAAATAAAGTTCTAATCCCGTATGAACTGAAAAACGAAA

CTAAAGGGATTGAAGAAATGTTAAATATTACGAATGCGGTAGAAGCGTATGGATATACCG

TGTTTGGGAAGTTGCTACAGAAAGCTAATTTGATGGCTACGCTGCAGCATACAGCATTTC

AACCCTTCACCATGTTCTGGCCTACTGACAAAGCATTTAACAGTCTGCCACAAGAGCAGA

AAAACTGGCTGTATAGTGAAGATCATCTTGATAAGCTACAGGCCTATTTGAAATTTCACA

TTATTCGTGACCAGAGAACAATTGCTATTGCTCTGCCGACAGAAAAAACTGTCAGAACGC

TGTATGGATCATGGCTGACTTTCAGTTGCAGCCAAGACTTAACTGGGGATATTTTAATCA

ATGGAAATGATGCCAAGATTGTGGACCGCAATCTGCAGTTCCTCACAGGAATGGCCCACG

GGATTGACAAGGTTCTAGAACCTCCAAATATAGGAGCACACTGTGATGATTTTTCATCTA

TAGAGGTTAAAGGTCGCTGTGGATCTTGTTTATTTCCACCACCTTGCCCCTACGGAACTG

TGTACACTAACAAAACCAATGTGTGCAGTAGGCCATGGAATTCCTACAGACGGCATTATT

TTTACGACGACTTTGAGTTTCCTAGCCCTTACAGTTTCAAGCCGTTCGGCTGTAACCGAA

TGTGCTCCAAAATGTCATGGGTTTCAAAGTGTTGTAAGAACCACTATGGACGAAACTGTC

AAGTCTGTTCTGGAGGCTTGGAGGCACCATGTGGAGAGCACGGTGATTGTGATGATGGGA

GATATGGGACAGGGACATGTAAATGTCACACTGGATTTAATGGGACTGCCTGCGAATTAT

GTGAGAAGAACCATTACGGTTCTAATTGTACAGCCTGTACATGTACCGCGAATGGGAAAT

GTGATGATGGCATAGATGGTGACGGCAGCTGTTTCTGCCAGGAGGGCTGGACGGGAGTCA

TGTGCGAATCCAAAATCGGTGTGAAACCTGTGTGTTCTCCTGAGTGTCACTCGAAAGCTG

TTTGTCTGCCTGAGAATCAGTGCCAGTGTGAACCAGCATATGTAGGAAATGGCTACAATT

GCACAGCCCCAGATTTGTGCAGTGAATATAATGGAGGCTGCCATGAACAGGCTGATTGCA

CACAGTCTGAGATTAACGTGATTTGCACTTGTAAATCGGGTTACTCTGGAGATGGCAATG

TCTGCTCCCCCATTAACGCATGTGTTGAGGAAACTAATGGTGGCTGCAGTGACTTTGCAA

ACTGCATTTTCATTGGACCTAATGAGCGACGGTGCGAATGTCAACAAGGTTATGTGGGCA

ATGGACTCCAGTGCTTTGAAAAAGTAGTGCCTCCGGTTGATCGCTGTCTGGAGAACAACG

GAGACTGTCATGCTAATGCGATCTGCAAAGACTTGCATTTTCACACCAAAACAGCAGGGG

TCTTTCACCTGCGATCACCGGCTGGGAAATATATGATGAACTACAGCACAGCAGGGGCAG

CCTGCAAAGCTGAGGGGGCCACACTCGCTACTCTGAACCAGCTCTCTGATGCCCAACAGT

TGGGTATGCATTTGTGTGTCGCTGGATGGATTGATGGGAAAAAAGTAGGCTATCCAATCC

GCTTTCCATCAGTTAAATGTGGTGACAATCAAGTAGGCATAATTCTATACAAGGATCCTG

ATGTAAACACTCCCTATGATGCTTACTGCTACAGAATTCGAGAGGTAAAATGTGAATGTG

GACCCGGATATGTTGGGGATGGAGATTTCTGTAATGGCAATCTCGCCTCTGTCATCGCTA

CCCATTCAAACTTCTCTATATTTTACTCTATGTTAGTAAAGACTGCAGAGGCTGCTGAAG

AAGGAAAAAAGCTCTTGGATTTCCTGTCAACAAGTTCAACAAACTTAACACTTTTTGTGC

CACATAACGCAGGATTTTCTCCAAATGAGACTCTGTCATTAAGGGATTTGGAATATCATG

TATCCTCAAACAACAGTCTTCACTACTATGAAGATCTGAAACACAACACAGCAATCCCAT

CACGACTGGGTTTTAATCTTAAAGTCATAATAAAAACAGACAACCAAACACAAGTTGAGG

ATTCTTCACCAGTAAAACTGGTCAATAAACAGACAGTATTAATCTGGGATATTCCAGCTA

CTAACGGCTTGATCCACATTATTGAGGGACCGCTAAAAGCACCTCCTATCAAAGTGACCC

CCTCAACTCCTTCAGCTGTCCGCTCACAATCCAGCGCACCCACTGTTACTGCTGTGTTGC

TTGTTATTTTTATCGGTGGCATTATCGCTGGTGTGGCTTATTATTTCCTAAGAAATAAGA

ATGATGCCTTCAGCTTCAAGTATTTTAAGAATGACGATGAAGATGGCAACTCCACTAAGA

CCAGTGCCAATCCTGCTATCATGTCGATCCCAAACCCTTTATACAGTGGTTACAGGGCAT

TTGTTGAGCCATTTGGGGAGGAAGAACAATGTGAATCTCCTGCTTCAGTTGCAGCAAACA

CACCAAACATCCTAGACTAGCAA

>Unigene000059

TGAAATAAATGTTGGGTGACTCGTCAGCCGGCTAATCAATCGTTTACATAGATTCATTCC

TAGCATAGCTGGTCAAATTTCAGTAAAGAAGAATAACAAACTGTTTTTGACTAAAAACTT

GTTGTTTTTTTGTTTTAGTTTGGGATCCAGTAGTGATTTATGTATTTGCACTAAATAATA

ATCAATATTCAATAAGGCAATCCGGCGGTTAGACTAGCAACTTCACTCGGAATCGATTCC

GAACCGATTCTGCTGGCAGTTCAGTGTGAAAAGTTTGCTTTGACAGTTTGTCTCCGAGTT

TCAGGAGTTCTATGGCAGAGATTCCCAGCTGAGATTTGTGACAGTTTTTTAGGAAGTTAA

CTGTCCTCCTTTCTGACTGACTTTTAGGACTATTTATTATCTTAATAACTGTAAATCATC

ACGAATTACACGCCGAAGGAAAGTATGTGATGCGGGAGAGAGCTGACGTCATCATGGCCT

CTTTTCCTGGGTATGACTTTACTGCTCTGGAGGCTGAAATGTTCCAGAAATAAAGGCCAC

ATTTCTGAGGAGTTTGGGATTTATGGATCTGTACGTGGATAAGAATCAGTCTGGATCCGA

CGTCTAGTTCTGTCTCCGTGTCTCTCTTTACATTGGTCTTCTTGAGGAGATCTGTGTTTG

TGTGCAAACATGGGAGAAACCAGCCTGAACGACTCGAACGTCAAGGTGGCCGTCCGGGTC

CGACCCATGAACCGGAGAGAAAAGGACCTGAACACAAAATGTGTGATCGAAATGGAGGGA

AACCAAACCTTTCTGTATCCAGCCAGTGGGAACCTGGGAAAATCCGACTCCAGGAGTCAG

CCCAAGGTTTTTGCATACGATTACTGCTTCTGGTCCATGGACGAATCCGAAAAGGAAAAA

TTTGCAGGACAAGACGTAGTGTTCCAGTGCCTCGGGGAGAGTCTGCTGCACAACGCATTC

CAGGGCTACAACGCCTGCATCTTCGCTTATGGACAGACCGGTTCAGGAAAGTCGTACACC

ATGATGGGTTCAGCTGACCAGCCTGGTCTGATCCCCCGTCTCTGCAGCTCGCTGTTCGAG

CGCACAGTACAGCACCAGCGAGAGGAGGAGAGCTTCACTGTGGAGGTCTCCTACATGGAA

ATCTATAATGAGAAAGTTCGGGACCTCCTCGACCCCAAGGGGAGTCGCCAGGCTCTAAAA

GTGCGAGAGCACAAAGTCCTGGGGCCGTACGTAGACGGGCTCTCTCGACTCGCCGTGGCC

AGCTATAAGGACATCGAGTCGTTAATGTCGGAGGGGAATAAATCGCGCACAGTGGCCGCC

ACCAACATGAACGAAGAGAGCAGTCGCTCCCACGCCGTCTTCAACATCATCCTCACGCAC

ACACTGAGGGATCTGCAGTCCGGGACCAGTGGGGAGAAAGTGAGTAAGCTGAGTTTGGTG

GATTTGGCTGGAAGTGAGAGAGCAGCTAAGACTGGTGCGGCCGGCGAGCGGCTGAAGGAA

GGCAGCAACATTAATAAGTCTTTAACCACACTGGGGCTGGTGATCTCAGCGCTGGCTGAC

CAGGGGGCAGGAAAGAACAAGAGCAAGTTTGTGCCTTACAGAGACTCTGTGCTCACATGG

CTGCTTAAGGACAGTTTAGGGGGGAACAGTCGGACGGCCATGGTGGCCACGGTGAGCCCG

GCTGCCGATAACTACGACGAGACCCTGTCCACGCTGCGCTATGCCGACCGCGCCAAGAGC

ATCGTCAACCACGCCGTCGTGAACGAGGATCCCAACGCCAGGATCATCCGAGAGCTGAGG

GAGGAGGTGGAGAAGCTCCGGGATCAGCTCACACAAGCAGAGTCTATGAAGGCTCCAGAG

CTGAAGGAGAGGCTGGAGGAGTCTGAAAAGTTGATCCAGGAGATGACCGTCACCTGGGAG

GAGAAACTGAGAAAGACTGAGGAGATTGCTCAGGAAAGGCAAAAACAGCTGGAGAGTCTG

GGCATCTCTCTGCAGTCGTCAGGGATCCGTGTGGGAGAGGATAAATGCTTCCTGGTCAAC

CTGAACGCCGACCCTGCCCTAAACGAACTGCTGGTTTATTATTTAAAGGAACACACAATG

GTGGGATCTGCAGACTCGCAGGACATTCAGCTATGCGGAATGGGCATCCAGGCCGAGCAC

TGCGTCATCAACATTACCGCAGACGGAGCGGTTTTCCTCAATCCCTACAAGAATTCCAGG

ACGTGTGTGAACGGTTCTCCAGTCACCAGCCAGCAGCAGCTTCAACATGGAGACCGCATA

CTGTGGGGCAACAACCACTTTTTCAGAATAAACCTTCCGAAGCGGCGTCTGCGTCCTGCA

GCTGAGGAGGAGGAAGGAGAGGGGGAGAACATGAAAAACAGCAACAGCAGCGAGCAGCTG

GATGCAGACGGAGACACGGCCAGCGAAGTTTCCAGTGAAGTCAGCTTTAGCTATGAGTTT

GCACAGACCGAGGTCATGATGAAGGCACTGGGGAGCAACGACCCCATGCAGGCCGTGCTG

CAGAGCCTTGAGCGACAGCATGAGGAAGAGAAGCGCACGGCTCTGGAGCGTCAGCGGCTG

ATGTACGAACAGGAGCTGCAGCAGCTGCGCAGGCGCCTCACCCCCGAACGCCAGAGCACG

TCGTCTCAGCAACACTACCGCAGCATGGAGAGACTCAGTCTGGGCGGAACCTCGTCGTCT

TCTCGTCTGCGCCAGTGGAGTGAGGAAAGGGAGGCGATGCTGACACGGAGTTTGCGGAAA

CTTCGGGAACAGGTAGTTAAAGCCAACCTGCTGGTGCAGGAGGCAAACTTCATCGCTGAG

GAAATGGATAAGAAAACCGAGTATCGAGTGACCCTGCAGATTCCTGCTGCCAACCTCAAC

GCCAACAGGAAGCGAGATGCTGTGCTGAGTGAACCGGCCATCCAGGTGAGACGGAAAGGT

AAAGGCAAGCAGATCTGGGCTTTGGAGAGAATGGAGAACAGACTGGTGGATATGAGGGAG

CTTTATCAGGAGTGGAAGGACCACGATGAGGACAATCCAGTGATGCGTTCCTACTTTAAA

AGGGCAGACCCTTTCTTTGACGAGCAAGTCAATCACAGTCTGATCGGAGTGGCTAACGTC

TTCCTGTCCTGCCTGTTTTACGACGTAAAGCTGCAGTACGCTGTGCCCATCATTAACCAG

AAAGGAGAGGTGGCAGGACGGCTGCATGTGGAGGTGGTGCGTGTCGCTGGAGGTGTAGAG

GACAGCATGGCTGGGGGAGAGGACGGGGACTACAGCATTGAAGGAGATTCTACTGAGCGC

AAACTCGTCTGTATGATTAAGATCCTGCAGGCTACCGGACTCCCTCAGTACCTGTCCAAC

TTTGTCTTCTGTCAGTATTCGTTTTGGGACCAGGCCGAGCCCATCATTGTGGCCCCTGAG

GTGGACCCGTCTGCCTCGTCACCCAGCAGCAAAGACCCTCACTGCATGGTGGTGTTCGAC

AGCTGTAAGGAATTGGCTGTAACAGTAACAGAGGATTTTATCGAGTATCTAACAGAAGGA

GCTCTGGCTATAGAGGTGTTCGGCCACAGGCAAGCTGATCCGGGCCGGAACCTGGCCCTG

TGGGACCTCAGCATCATTCAGGCCAAAACTCGCACGCTGCGCGACCGGTGGAGTGAGGTG

ACGCGGAAGCTGGAGCTGTGGGTGCAGATGCTGGAGCTGAATGAGAACGGAGAGTACACG

CCTGTCGAGGTGGTGGCAGCTGGAGACGTTCGCACTGGAGGCATCTTTCAGCTGCGACAG

GGTCAGTCACGGCGGCTGCAGGTGGAGGTTCGTTCAGTACAGGATTCTGGCACTATGCCA

CTGATAGCCGAGATCATTCTGGGTGTGTCCATCGGGTGTGTGGAGATCAGACAGACTCGC

CCTGCTAAAAGCAGTGACTCACAACAGCTGGAAGGGGATGAAATGGACAGCTATCAGGAA

CGAGACCTGGAGCGGCTGAGACGCCAGTGGTTGGCAGCCCTCACTAAGAGACAGCAGTAC

CTGGACGAACATCTGCAGAGTCTGGTCGGAAAAACAGATAAGTCAGAAGATGATGTGGAA

CGTGAAGCTCAGCTGCTCGAATGCAGACTTACTCTCACAGAGGAGAGGAATGCAGTGATG

GTGCCCTCTGCTGGCAGCGGCATACCAGGAGCTCCTGCCGAGTGGGTACCGGTGCCTGGA

ATGGAAACTCACATTCCAGTCCTCTTCCTTGACCTGAGCGCTGATGATTTTAGCTCACAG

GAAAACCTGGAGAGCCCGGAACCCGGTGGCTGGGATGCCATGCTGAGTGGAGAGGACGAG

GACGACTTCTTTGATTTGCAGATTGTTAAGCACTATGATGAAGAGGTGAAAGCAGAGGCT

TCCTGGGATTCCACGGTGCATGAGTGTCCTCAGCTGAGTCGAGGCGGAGCTCAAGACCAG

AGGGTCTACCTGACCATACGCGCTGTAGTGCAGCTCAGCCACCCTGCGGAAATGCAGCTC

GTATTGCGCAAACGCATCTGCGTGAATCTCTCCGGGCGGCAGGGTTTTGCCCAGAACTTC

CTAAAGAGAATGTCTACGCGGAGCACCGTGCCTGGCTGCGGGGTGACTTTTGAGGTTGTG

TCCAATATCCCTGGGGATGCTCAGAGTTCTGAGGACAGGGAGATGCTCGCCCGCATGGCC

GCCAACACCGAGAACCCCAAAGCGTCAGGCAACGAGGCGGCCATTGAAAAATACCTGCGC

AGTGTCCTGGCTGTGGAAAACATCCTTACACTGGACCGCCTGAGACAGGAGGTGGCTGTA

AAGGAACACCTGGCTGGTAAAGGAAGGGAGCAGACGGAGTCTCAGCTCGCCTAGTGTACA

CAGGTTATCTGGTAGCAGACAGGATTTGTCTCCAAACTCAAGCCTGGAGGACAACAAGAA

TCGATGGGAAAGTCAGCAGGACATCTTAATGACGGCTCCGCAGTTCAGCCGCACGCTGCC

ACGGCCCGCCAGAGAACCCACACCGCCATCAAGGAATCACAGCCCACCTCAAAACCAGGA

TCCAGAGCAAGTTAAGGCCCTTGTCCCACAGATGCCCAAGCTGCTCAAATCCCTTTTCCC

TGCTCGCGAAGACAAAAAGGACCTGCGTCCCTCTCCACAATCACAACAGCACATGCCTCG

TATTATGATGCAAACTAGCAGCAGCTCCGATGACGGCAGAGTTCGAGGAGAACCGGTGCC

AGCGGTAGGAAGGAGTTCTGGTTCTGAGAAATCATCAGACACCCCTGATGCCTCCATCTC

ATCCATGCAGGATTCACATGAGCTGCCTCTCAGCCCCATCAGTGAAGCTTCAAGTGGCTA

TTTCTCCACTAGTGTCTCCACAGCCACTCTATCCGACGTCTCTGCTGTGTCTGCGGACCT

TACGTCAGGCTCCAACGCTAACGATGCGCCCCGGCAGGCTGCAGACGAGTGCGGGGGTAA

CAGTGTTCCTGCAGATACCGGGCGAAAATCCAGTGAGGAGATACCTCCGCAGAACCTGAA

CCTCAAAGACCTACAGTCCATGCCTCAGAATGGCGTTCCCTGTTCTCGCACCGAAGCCAA

GCTTATAACTGTCCCTTCTTTAGTATCTAAAAGTGAATATGCATTCTCCCTTCAAAGGGT

CAAACCCTCGAACCTCAAATCGTTCAGCCCCATCCTGCCCACGGACGAGAAGGAGAAAGG

AGAAACGGGTATAGTGGAGGGCAGTTACAAGGGAGCGGGCAAAGACGCCGAGCTCCCTCA

CTGGCTCCGAGCTGGAGAGAGCGTCATTGTGGCCAACAGTAAGTGTGGCAGAGTGCGTTA

CGTCGGGCACGTCGACTTCTCAGACGGCATCTGGGTTGGAGTGGAGCTGGACACGCCCGC

AGGTAAAAACGACGGTTCTGTGGGGGGTCGTCAGTATTTTCGCTGTAACCCCAATCATGG

ACTGCTGGTGCGGCCGGACCGGGTGAGTCTGCGCGACACGTCCAGACGGCATATGGAAAA

CCGGCATAGCAAAGAGTTCCTACAGGTTAAGGCGGGTTCAGGCCTGACCAATCAGAAAGC

AGAGAACCGCAAATCCTGGAGTAACTAAACGCAGTCCGAGGACGAAATGGAATTTGTGCA

TTTGTGCTGTTTTACCCTGCTGTTCAGTTCTACACAGAGGAGCTACAGCGTCCCCTCCCT

CTCACCTTTACACCAGCGTACCTGCCCACAACACTGAATAAACTGTGCGAGTCCAGACTC

CAGTAGAACCCTGTTCTCAGGGGTGGAACAGTTGCTTTTATCCTGCCCTGGTTCTCTCGA

GTTCTTTTAATGACTCGTCTTTTAAGTGTGTTTAACGCTTGATTAATCTCCAGAACGTGC

CTTTTTTTTTTTTTGTTCCCCATCAAGTCACCCCTAACCCTAGCGAGAGAGCCTTCCATC

AGCGTGTGGTCTGATCTCAGGGATTGGTCAGAGAGAACGGTTGTGTCTCATGCTAGAGAA

TGTCAAAATTGCCAAACGTAGTATATGCATGTATATGTATAATTCTAGGGTGGTTCAGTT

GAAGAGCCTCACTTTGCTTTCCGAACAATCCGTATCCAATCATGTTCTTTTATCGCTGCC

GTACTGACAGGTTGACCGCTCGCGTTAGAGCTGTATGATCCTTATTATAAAAATAAAATA

TCAGTGCTATAGCAGGTTACAGCTCCGCCATTAGTTTAGCTCCGGCGTTGCTTTATTAGA

TCCTCTAATGATTTATCAGATTAGATCGATCTAATTCTATAGGGAAACGTGCGAGCGAGC

GCTGCTGGGGATTCTACCGCAGAAAAAACATATCAAGCATGTGTGTTAGGAATAAAAGCG

TTCTCTTGCACTTTCTCTACATGTTTAATATGGGATAAGGGCTGAAGACAGGAAGTTGCA

TGTGGGAAAGAAAAAAAAACGATGGAACTGCAGTGGGAGAAAAGTTTGTGGCTTATACAA

AGGGAGGCCACCACATACCTCAGCGCATGTAGCATGTGGATTCGAGCGGTTTTAATAGAA

GGCATATGTTTTACTGGGAGAACAGATTTTAACCTCGCTCTGGAATTCACGAGAATAAAC

CTCGGTCCAGTGCGGAGCGTCGAGCGAAATGTCCCAAGCGTCAGTTAACGACAGCGGTGA

GATCATTTCAGTGAGTCTTCCATGAAGAGCTTTGAATATAGCAAAGACGCAGTGCAAGAA

GACTAAGCGGACCGTCTGTAGTCACGTGAACGAAAGTCATTCCTAGAGAAAGTATTAAAT

GTGCTGTATCATCACGTAGTCGGAGATGCGCGTACTTCAGTGTGATGGAACCTTCTTGCA

CATGGGAGCGGACCAGAACGATGGGTTATACCATTGCGATTTTAATCCTGACCCTGTGTT

CACTCAAGCAGGACATGTCGATCCGACAGCAGTAGCTCTAAACCGCTCCAAAACCAAACA

CACAGCTGGAACATGCCGAGGAACCGTGTGAGCCGATCGTTACTCGGCGTTGGCGTGTAA

TCGTGAGAATCTCGGCGATAATGTGTCGGCGATAATGTGTCGGCGGTTTGTGTGAACACG

GGGTTAAAGCCGGTAACGTGATTCCTGAAATCGAATGCAAATATTGACTTTATTAAAGTA

CATTTTACTGAGTATATTTAGCTGTGGATTTTTTGATATGAAAGTGAATTCCTTTGAATT

AAAATCTTGCACATTTTGATTTAGATTAGATTTAAATCTAATGCTCTGTTTAAGTCTAAC

CGCGGTGCTGAAGTCTAAAGCCATCCTTTAACTCGAGTCTCCCAACAAAACAGGGCA

>Unigene000060

GCCCTTTTATAGTTTCCTTTATTGCAGTTTGCTTTATTGTTCATTATTTTGTACTGTGAT

TTTTTTCTGTTTGTTTGTTTTTGAAAGGGAAGGGTTTTTTTCTTCTCTTTTGTTGGACAT

TCATAAGTGGTTTTTTTTGTGTCATTATATATTTTGTGAACTATGGATCACAGTGAATGT

GGCTCTTCATCATTCAACTCATTGTACCACCATGAAAGCATTGTTCTTTTTTTATATGCT

ACAAGCACCACTGACCAATTTTTTCCCTCTGGTCTCTCTGTTTCCCAATCATTTTTGTTG

ATTTTGTGTTGGATTTTAATTTATCTGTTTAAAGAAACAGTTGAGCGAAGTACAACAAGA

ACACTGCCTGCTGGTTACCCTCACAAACCTGTCTTTTCAACAAGATCTTACCAGGCATGG

TCGGCTGCTCCTGTTACCATCCCTGGGCAAACAAAATGCGGACTGGGTTCCCTCTCCAGC

TCACCTTCCAACACGCCATCTGCTTCGCCATTAAAGTCAGTTTGGTCAATAACCTCAGCT

TCTCCCCTCAAGTCCACTCTGGGAACCACAAATGCATCTCCTGTTAAGTCTGTTAGTGAC

ATCGCATCACCCATCAGAACATACAGATCTATCTCTTCACCTATAAAAACTGTTGTTCAG

CAAGGCCAGTACCCTGCTTCAGTTTCTAGTGGTCTCATGTCTCCATCTTTTAAAACCGGC

ACAGATCCTGTGTCGATCAAAGGACTTGCTGCCTCACTGTCTTCGAGAACTATCTCTGTA

CCACCATCTGGCTCTATACTTGATAAAACTTTCACCACAGTGACCCCTCCTGAATCTCCC

AAGTCAGTTGCTAATTTGTATACTTCAGCTTTGTCACTACGATCAGCTTATACCTCTGCA

CCTACTACAGCAACACCTACGTTTAAAACCTTAACAAGTCTGTCGTCTATGAAGACTTTA

GATCGAGGTACAATGTCCAACGGTTCCATTCTCTCACCTTACACCTCACCAGCTGGGCCT

GCTGTTGCCTCTGCTGAAACAGTTCTGCTTAATGGATCCATGTCACCTGTAAAATACCCT

TCTTATTCTTCCGTATCACCCCGTTTACTCACAGAAGGAGCTGCCAGTTTGCAGGAAAGG

ATACAAGCTACAACACAAGCGGCTGCTTCTAATGTTAGCGCAGCTTTTTGTGAGGCTGAA

AAAACTTTAATTGGCTATTCCACATCAGGCTATGACACAATAAAATCCATGTCATCTCCT

TTGAGATCAAATGTGTCAAACTCACTGTATTCTTCCTATAGAGCCCCTGGTCCTTCCAGC

GCCCAACTATCAACTACTGCTATGACCGTACCGGTTTTCTCTGTAGTGAACGTTCTCCCA

GAGCCCCAAGAAAAGAAACTACCAGACACGGTATCAACTGTACCCAGGACAACCACTCAA

TCAACATCGTTTACCCACAGATCTAAACCTTCAAAACCCCAACTCTATATCCCTTCAGCT

GCATTTAAGTCAGCTACTTCCACACAAATGTCATCGAGTCAAGACATTTTGCGTGATGTG

GCAGACATGAAAGAGGGTCTTATAAAAATGTCTGCAATATTGCAAACTGATGTATCGAAC

ACGAAGAATTATCAGTCCCAGGCTACCAGGGAAGCTAAAATGGAGGATGAGGAACCTTTC

AGTTTAGTTGAAAAAGTTAAGGAGGATCTTGTGAAAGTTAGTGAAATCCTCTCTAAGGAT

GTACTGAGTGATACAAAAGTCTTTACTAAGGACAAAGCATCTGAGGATGAGTGGGAAGAG

TTCTCTAAAGATGAAATAGAGGAGGCTCAACAGAGTTTCCTCAAATCTCTTCCCACTTTT

GAAACCACCCATCCAGTTAGACCACAGTCAGTGCAAGACAAAGACTTAAATTTAGCTAAG

GTGGTTGATTACTTAATGAATGACATTGGTGCTAGTTCGCTTTCAAAAATTGCTGGTGAA

AAAAACAAATATATTGAAATGAAGGAGGGGGAGGAAAAACAGAAACGTTTGTTCAAATCT

GAGCATAAACTAAAAATGCCACCTCCAGGCATGCGCACCTCTCCTTCAGAAAAAGATTTA

TGCAAAATAACTGATTCATACAGTGGGACAGATGCAATTTTGGAATCCCCTGATGACTTT

TCCCATGATCAGGATAAAAGCCCACTTTCAGACAGTGGTTTTGAAACCCGAAGTGAAAAG

ACTCCTTCAGCTCCTCAAAGTGCAGAGAGCACAGGACCCAAACCTCTATTTTCGGATGTC

CCTATCCCCCCAGTCATAACTGAAACCAGAACAGAGGTCGTTCATGTCATTAGAAGCTAT

GAACATCCCGAGGAGGCCTGTGAGCCCCTGCTGGAAGAAGCTGCTGCATTGCCATCACCT

GCTTGCATTGAACCCGAGTCCCATGCTGGCTCAATCAAAGACAAAGTAAAGGCCTTTCAA

ATAAAAGTCAGTTCTGAAGACGATTCTGTAATGAGCAAAAGCATGTGTCTGAAAGAGGAA

ACTCATATAACCACCACAACTAGAATGGTCTACCATAAACCACAGTTAAAAGATGTAGGC

TCTGACAGAATTGAGGAAACCATGTCAGTTCGAGATATAATGAAGGCTTTCCAGTCAGGA

AGAGACCCTTCCAAAGAGTTAGCTGGCCTGTTTGAACACAAGGCTAATAAGGATTCTATC

AAAGGAGATGAATTAACACCACGGTTCCTTGACAAGGACACAAAACCCAAAGTTGAAAGA

ATAATCGAGGTTCATATAGAAAAGGGTAACACTACAGACCCAACAGAGGTAATAATCAGA

GAGACAAAGAAACATCCTGAGTATGTATGCGCAGGCAGCCGTGGTCTGAGGGAACTTTCT

AACAGAGTAGAAGGTGATTTTGGGGCTTTGCAAGAAGATGAACTGTTTACTGCTGAGGAA

ACAGTGCCTTCTTTCCTAGAAACCTCTAGAGTTAATACACCTGTACCTCAAGGGGAGGAG

AGTCGACCCAGTTCTGCTCAACTTATTGCTGATGACACATATAAAGCTTTTAAACTTCTA

AGCCAACAGTCTATTGAATATCATGATGATGAACTGTCCGAGTTAAGGGGGGAGTCATAC

AGATTTGCTGAGAAAATGCTTCTATCTGAACAATTAGACACATCACAGTCTGATACTGAG

GATTTAGTCATTAACCAGGGACATGGCCGCACTCCTGAAGGATTTCATAGCCCTAAAAAA

GAATCAGTTTATAGGTCGGCAAGAGACACATCTGACAATTTTTTCCATAAAGATCAATAT

GATAAACTGACAGTGTTGCACTATACAACAGAACCTGGCAGCCCTAAACATACAGTCAGA

ATGCGCTTCACCCAAGACAGGCAAGACAGAAATAGAGAGAAACTTATATATGAGGATCGC

ATTGACCAAACAGTTAAAGAGGCTGAGGAAAAACTCAGTGAAGTTTCACAGTTTTTTAGA

GACAAGACAGAAAAGCTAAATGATGAACTTCAGTCACCTGAGAAAAAGCTCCCCATGAAA

GACTTCAGGGAGACAAGATCAGGCCCAAGCTCTGTGTGCAGTAGTCCTCAGAAAACAAAG

GTAAAAACTGAAGTGAGCTCTGAGGAATGGGATAAAGGGAAACAGAAATTGTTCGGTAGT

ATGAATGAACGAAAAAGTGCTAGTTTGCCAAGCAGCCCAGAGAGGCATGTGCAGTCACGG

TTAAATGAGGACAAATCTAAACAGGAAAATAACGTTTGGCAGGCAAAGGCAAGTGATATT

TCTTCCCCTGTTCAAACCTCAAGAGTGAGCTCTGTAAGGCTTAAATTTGAAACAGAGGCC

CAAAATCAAGATAAAAATATGCAGAGTATACAATCAAAACCACCTGTAAAAAAATTGCAG

GAAAGCAAGTTACCTGTGTATCAATTTTTTGCTGGTTCAAAGGCTTCAAATGTAGATTCC

ACAGAGGAAGGAAAGCCTCTGAACAAATCAGCAGACAGTGATACAAGTAAAGTAACCTCC

GGGCAAAATTCGCCTTTTAGCAAGTTTAGCAATGCGGCATGTATGTCGGAAACAGACGAC

ACCATTTTTAGCAAGCCACAGTTTGAGGTCGGTAAGCCAAAAGATGACACAGATGAAAAA

CCTGACTTCAGTAGAGATAGAGGTAGTGAAAGCAGTAAAGAAAAAGTCTGTAACTTCTTT

AAAAATGCTAATACTGTTGATTTGGCATCAAAAAAGAACAGCACAGACAGTGACGATACA

CAAATAAAGACTCTTAAAAAAAAGATCACAGAATCACAAATTCCAGTGAGAATATCATCC

ACGCTTCTTGATGCTGATCAAGGAAAGAAAGCAAAACAGGATACAGTTGGTAAACAATTA

GAAAAGAATCTATCACATATACCTACTTTAGCTAAAACAAGAGTTCAAGGCCGCTCGGAA

GTGTCTAGGGAAGAGGCTGGAAAACAATCATTTGAGGGTTCTGTAGAGGGGGGTGGTATT

AGAGATCCAGATTCAGGTAACTTCAAGGAAACAATGGAAAAGGTTACCACAGTTACTACC

AGAGAAAGTTTCAAGGGACTGAAAACATTGCCAGTTTATGTCAGTGTACAAGTAGGAAAA

CAAGGTGAGAAAGACTTAGCAGGAGGGACAAATGGATCGGTCAGAAAAATAGTAGGTTCT

GAGAATCGCACAATTTACACAGTCAAACAGAGACAGCCAACATCTCCTCAGAGAAGCCCA

GACGATGACACTTTAGAACAAATATCTTTTCTAGATAGCTCTGGGAAAAGTCCAATGACC

CCAGAAACACCTAGCTCCGAAGAAGTTAGTTATGATCTCACTGCAAAACACCCTGAATGT

TTTACGACATTCATACCTGGCGGTGCTGGTCCACTAGCTGAGGTCTCCGAAGAATCAGAG

GATTCAGAGCAAGCCAGAGCATTTACATCTAAAGACCCTGCTCCAGAAAAGGTCATAGAC

ACCCCAGCAGTCAACACGTGTCAGGATACTGGACAGAAAAGTAAAGATAAACGAATTGCC

TACATAGAGTTTCCTCCGCCCCCTCCCTTGGACTCTGATTCATCACAAACAGAGAAAAAA

GGGTCTACTCCTTCTTTAGAGATGGACACGGACATGATGGAGGTGAACCTCCAGGAGGAG

CATGATAAGCATCTTCTTGCTGAGCCCATAATTAGAGTCCAGCCCCCATCCCCTGTGCCT

TCAGGAGCTAATGACAGTGACTCAAGTGAAGACGAATCAGTATTTCAGCCCATTGCTGTA

AAGAAATATACCTTTAAAATTGATGAACAGGGCCAAGAAAAAGAATCCAAACCCAAAAAA

CACAACAGAAATGGCAATTATAAAGAACCTGGGATAAACGGTAATGGCAAAGCTGAGGAT

AATGACTATGAACAAAATGGCAACGATCAGTCTATTACAGACTGCTCGATAGCAACAACT

GCTGAGTTCTCTCATGACACAGATGCCACAGAGATTGATTCTCTGGATGGATATGACTTT

CAGGATGAGGATGATGGCCTTCATGAACAGGATGTAAAAACATGTGGATTTTCCAACGAC

AACAGAAAAGACATTTGGGGATCAGAAAGCATTCTCAGACCAAATGACCGTACATTCAGC

CAGAGTAAACTAGAAGTCATTGAAGAGGAATCGCCGACTGAGGAGTACAAAAAAGTACAA

ACTAAGAGTGAACCCTCTGTTAAAAAAACAGAAAATGAAAAAGACAACGAAAATGAAAAA

GACAAAAAGCAATCGAAGGAAGAAGGACTGTCTGATACTTATTTTAGCTACAAACTTGAT

GAAGAGTTTAATACTCCTTTTAAAACTGTCGCAACAAAAGGCTTAGACTTTGATCCCTGG

TCGAGTAAAGGAGGAGATGATGAAGTTGTCGATGCTCGAATTAAAGATGATGAGCCCAAG

CCTTTTGGACTCGCTATTGATGAGAAGTCTCAAGCAACTACCCCTGACACGACTCCTGCA

AGAACTCCAACTGATGAAAGCACACCAACTAGTGAACCTAACCCATTCCCCTTCCATGAA

GGGAAGATGTTTGAGATGACCCGCAGTGGTGCAATTGACATGAGCAAACGGGATTTTGTT

GAAGAGAGGCTGCAGTTTTTCCAGATTGGTGAGCATTTATATGGACGGAGACCTGGGAAT

AAAAGGGAAGGGGGCACAAATCCAAGTGTAGTCACATCACAACCACAGCAAGTAGAGCAT

GCACAGGTTAAGATGGATGACAGCTCAGTAATATCATACAATATCATTCAGATTTGTGCT

GATCGCTCGGACACTCCATCTCATGACAACATTATGGAGCCACCCTCATATTCAGAAGTC

AGTTCTAATTTGTGTTCCTCTTTTAAGATAGGAGTCAAAGACACTTCTGGTGAGAATAAA

GATTTGGTGTGTATGGACTGGGACAGTGTTACACAAAAACCATCACTAGCTTCAGAAGGC

TGTAAATCAGAAACAAGTACAGAAAGCCATAATGAGCAAAGGACAGATTTATCAAAGACA

GAGCAGAAATGTATTTTGATACAGAACTTCAATAATAATAATAATCTACAAGCTTCCAGT

GGTGCAAATAAGTACATGCAATACAGCACAATAGAACAGCATCAGCAGTCACATTACGAG

GCCAGCGGTTTTAACCCTATTTCAGAGGATTTACTCAAAACAAACTCAGACTCAGTGAGA

GTAGATAGCAATTATTCGAACAAAGTGAAGATAATTCTGCAGTGCCCTGAACAAAGTGCT

GTGCACAAAACTAACATAATTCCACAATGCAAGGATGTAAAAAAAAAGCTGCTACAGTCT

AGGTTGCCGATATTGGAGCATCGATCAACAAAACGCTATTGTAGTGCAGGTATACGTTTA

GAGCTTTCAAACAAAGAAATCAAACAAGTGCCGAGGGAAAGATTAGATCCATTTCCGATT

GGGAAACCCAAATCTCGCATCCCGATGTTGAAGGTTAAGAAAGATTCTAATAGTTCCTCG

ATAGGGAAAGGTAGGACAACCACATTAGTAAAAGAGAAAAAGAGGACTCAGGTATTTATA

GGTAAAAATAAGTCAAAGACAGACTCTTCAAGCACATCAAAAAGTAAGACAAGCCAGAAG

CAGGTGCAGGCCGCGGGCAAAATCGCTAACTCAAAGGCTAGTGAAACAGTAAAGCTTGTG

GACTGCCATGCCAATGAAGACAAAAAGATGCAGGGAGATGATAAGGATGAAGAAAGGAGC

ACATCCAGGAACACCTCTTTGTCAGAAGCATCCCAGCCCTCCACTACATCCAGGTCTTCT

AGAGACGTGAGAGCAGAGGTTGAGCAGGCCGGCAGAGACAGGAGGAGGAGAAGGAGGAGT

AAGAGGACGAGTGGAAGTGAAGGCAGTCAGGGTGCTGGGTCTCAAGTGGCCCCCGGCGTG

GAAATCGAGCCTAGTTTGTAAAATTTTCCACTTGTATGATCTTAGTTGCATGAACCATTG

TGATTGCTTGTGGTGTGATTGAACTGTAGATGTCTTTCCTATCATTATTTCATCTTCTTA

TCTTTGTGTACTTGTGTCTCTTGTTATAATTTCAGTTTATATGACGTAGGTAGCTTTACA

GTAGCATGCCAATATGAGAGGACAGATTGCTGGACAGATCGTCAACCTATGCAGAGTGCT

AAGCAGTTTGCAGTAGCTTGTATGAATGATTCGACTTTTGGAAGCTTTAGAAGCATTCGA

AGCATTTGTCCATTGTTCATTTGTGAGTCTTGTTCATATTCAGCCTC

>Unigene000061

CCCGGTTGTTCTTGGTTTGTGCTTCAGTGCATGAGTTCCTGATGCGTGAAGGTCCAGTCC

TGCTGCAGCTCCGGGTAAAGTACCTGCTAAAAGTGAACTGCATGGAGAAGGCGGCGTTGC

TGGCTAAAGCCTGTGCTGATGTCCAGGAATTTGGGGGAAGCAGAGGACACTTTACACAGT

GCTTTCTTGTGTGTGTGTGCTCTTGTGCACCGCAGGAGACCCTGATGGAGGAGCTCTCGA

AGGTGGACTGTCGTGATGCTCTGGAGATGATCTGTAACCTGGAGGCGGAGGGAGATGAGA

GAGGAGCGTTCACACTTTGTTCAGGCTTCCTGACCCGACAGCTTCTGCAGGAGGACTCGT

ACTGCGCCTGGGAACTGACTCTTTTCTGGAGTAAACTCCTAAAGCGATTGGAGCCTTCAG

AGCAGAGCTTTCTGGAGAAGTGCCAGAAGATGTCGCTACTGGCTAAAACTGTCTTTCACC

TGCTCTTCTTCATCAAAGTGATCCAGTCTGAGTTGGATAAGATTGGCTTGCCAGCCTGCA

TTGAGATGTGTATACGTGCTTTGAGGATGGAGAACTGCGAGGGAGCTAACAAGGCCACCA

TCTGCAAAACCATCTCGTGTCTACTGCCTTCTGACCTGGAAGTTAAGCGGGCCTGCCAGC

TAACAGAGTTCCTCCTAGATCCGACAGTTGACTCTTATTACGCCGTGGAGACCCTGTACA

ATGAGCCGGACCAGAAGCTTGAGGAGGAAACCCTTCTGATACCCACCTCTCTTCGCTGCG

ATCTTTTCCTAGTTTTTAAAACCCAATGGTCTTTTGATCCAGAGTTCTGGGATTGGAAGA

CACTAAAGCGTCACTGCTTAGGCCTTATGGGTGAGGAAGCATCTATAGTGTCCTCTATTG

ATGAGCTAAACGATGATCGCTTCTTAGAGATTGAGCGAGATGAATTGGATTCTGCTCAGG

AGGAGTTTAAGGATGTTTTTGAATGCTTTTTGGACACAACAAAAGAACTTAAGGAGATAT

CGGATCATCGACAGAAAAACAGAGAAGTTAAAAAGTTGAGGGAAAAAGGGTTTGTGTCTG

CGAGGTTCAGGAACTGGCAAGCGTACATGCAGTACTGTGTGTTGTGTGATAAAGAGTTCC

TTGGTCATAGAATAGTGCGACATGCACAGACTCACTTCACAGATGGATATTATAGCTGTC

CGATTTGCACAGAGACGTTTGAAACGAGAGAAACTCTAGATCCGCATGTTGCATCACACG

TAAAGTTGTCTTGCAAAGAGCGCTTGGCTGCGATGAAAACCACTCGGAAACTGACTGGTG

CAAAAAACTCTGCTTCCAATGATGCACCTCTGGATAACAAAAGTGGGGAAAATCAGGCAC

GAAAAGCGAAGGCTAAACACTGCAATGGAGAATCTGATGATGTGTACAACATGGGCTCTT

GCATTGAGGATGCAGGAGTCAGGGTCCACAGGAAAGAGTTCAATGTTTGTCCTGTTTCAA

GCTGCAGAAAAGTGTTTAAGTTCTTTCGCAATCTTCTCACCCATGTAAAGGATCACGGAG

ACGTGGAGGAGGCTAAGCGCTTCCTTGAATTACAGGCCACCAAGATAGTTTGTCAGTATT

GTCGCCGACACTTTGTGAGTGTAGGTCACCTCAATGACCATCTTCAGGTCCATTGTGGTG

CGAAGCCTTATACCTGCATTCAGTTGAACTGCAAGGCTAGCTTTGACACCAATGCCGAGC

TCCTTGTGCACAGCAAAAGACATCCTTGTTTTAAGGCCAAATGCATGTTTCCTGGCTGTG

GAAAGATTTTTAACGAAGCTTATAAACTCTATGACCATGAGGCGCAGCATTATAAAACAT

TCACTTGCAAAGTGCCCGGTTGTGGGAAGGTATTTCATGCTCAGTCTCAGTTAGACATGC

ACAAGGAGAGCCACAATCCTCCTAAATTGGAGACACAGCCCGCTGGGAGTGTCAGCCCGC

TGGGAGAGCCTCAGTCGCACCTCTCCAGTGAACCTGCCCAGCAAGATGCCCATTCAGGGC

CTCCTGCCGACTCCGGCTGCATGATTAGTTCAGAAGATCAGTCAACCGGTCTAGTAAAAG

TAAAGCACTCTATTGAAAACATGCTCAGTGTTGCTGGTCAAGGGCTCTCACAAGAGTTTG

GCCATGTTAACTGCAAAGCCGAGCCATCTGTTCCAAACCTGGTGCAGCAGCCACCAATGC

CACACATGGTGAATACCGAGCAAATCCAACCTCAATTAACCCCTTCAATGCCTTCCTTGA

TTGAACCGAGATCTGAAGAATCCTTGTTAGATGCATTGATGAACGATCCAATGCTCACTT

CATCTTGCTCACTTCCACCAGCATCTTCGTATCAGAGCCTCTTAGAGGATTTCCAGCAAG

CTCCATCAGGAGATGTTTTACAAGCTCAAATGGAGTCTGCTGTTTCCCAGGGTCACACCC

AGTCTCTGTACAGTAATGAAAACACTGAATATGGCCAATACAATATCAAACCTGAAACCC

CAATTTCATACCCACCTCCGTATGATAACACTGTACCCTTGGCGCAGAATGGTCATAATG

ATCCACCGGTACTGCCTGTATCTCAGAGGCTTCCCCCACAGCTCACCATGTTTTCTTCGA

ATAGACCAGTGCAGGCACTGCAGGCAAACACACTCAGTTCTGTAGTGGAAGAAAAAGACA

GGTATAAATGTGCATACGAAACATGTTCTCGAGACTACAGCTCGTATAGAAGTCTAACCA

AACACATGAAAGCTGCTCATTGTGAGTTTTATACACAGTGGAAACTGGCAAAACGGAGCA

ATAAAGTTTCACCAAACACTTGCAGCGCGTCATCTATAAATGGAAAATGTATGTCAGCAG

AACCCTCACAGAACCAGCTGAGTCAGAGGACTCCTGCTCCTCAGAACCCAGTGCAGAAAC

CACCTGCTCAGACCCACAGCCCAGCAAGCTGTCTAAGCTCCCATTACCCCTCTGGATCCT

CCCATTTAACCGGTCCAAATGGACAGACTTTTCCAAATCAAATGGACAGTATTTTGAATC

CCATAGTGCTCTCACACTTGGGCAGCATTGCAAATCAGTCTAATGTAGTCTCTCACTCTT

CGTGGAATTCCATCTCAGTGAACACATCACTCCGGCAGCAGACTTGCCATTCACAAGCAA

TGACCTCATCTAGTGGTCAGTTCCCGTCCCAGTTAGACACTGTTCCACAAACCCAACTGT

CAGATTCCTATAATAGAGTAATGGCCTACCCTTCTTTGCAACATCAAAACAGTCCCTCAT

GTCGATTCATACAGACCCGACCATGTGCCCCCCAAACAATAAACAGTCCTGCTAAAATAA

GCCATCCCGGCGATTCCTCTTTACCTCAGTATGTTCAGCAACCGAAGACCAGTTCTAAAT

CCACACTTAAACATACAGACTGTGTCGTAAAACTAGAGAGAAACCCAATTTTAATTCCAT

CCCATTCAAACTTATTACAACAAAGCAACCCTCCTACTGCTATGAAACCTGAAACTTGTC

AAAAAGATATCAGTGGTTTTGCACATGATGGGGATGTCAAAAAAAGGAAGCGAAGCAGCA

GAACAAAGTGGCCTGCTATTGTCAAAGACGGCAAGTTTATTTGCTGCCGGTGCTTTAGAG

AGTTTCAGAGTCCTAAATCTCTTGGTGGACATTTGTCTAAACGTACTGTCTGCAAACCCT

ACGATGAAGCTGTTTTGTCTGCAGACCTGCCAAGTTCATTTCTGGAGCTTCTGAACTCCC

CTCATCCATCGGAAACACCCCAGTCCTCGCCGTCCACGCAGGTCACTTCTAGTAAAGCTT

GGCCTTCCCTGACCAAAGGCCCCCTTGACCCAAAATTGTTCCCCAATGTCACGTTCCTGA

ACACTAAAAATTCCACATACAACAGTGGAACCATCCATCAAAATTCACCGAGTCTGGAAA

AGGGTGTTACAGGTGAGCAGACTTTTCCTGAGTCATCTGGGTCTTACCATCAAAAAAGCA

AAAACAGTGTGATTCAACACACCGGAAATATTAGAGATAAGTGCATTTACGATCTAATGA

ATTATACCCAGCCATGCAATACCTTACCAAGTGACACCAGTTCCTCAGAACAACTTTTGT

CTCACATGTTAAGTGAAAATAAAACTTTAGACTCAAACTTGAGCAGAAGTCCTAATAACT

ATGTTGACAGAATCCTCCAAGCTGAAACACTAAACAAGATTAGGGAGATTAAAGAAGAGC

CTACCAGAACAAACATTAGTGGATTATCGAATGATGGGCTTCTTGCTGCAATGGCTAGTC

TGGCCCAAAATCTGGTGTCAGAAAAGAGCGTCAAGGAAAGGCTTCGTGAACAGATATTAG

CTGGGGACTTTCAAAAAAGAAGCAGTTTAGGCCAAGGGCAAGGAGTTGAAAACTCTAATT

CACAAAGTCCGATGACTGATGTTCCACTTGGACTTCAAACAGCTGTTCAAAGTAAGGAGC

ATATTGTCAGACGTGCTAGTGAACTTGGTGAACTTTACACCTCAGTTGTACCTGATGAGA

CCTTGACAAATATTTCCTTGGCTGGTGCGTCTAACAACAGGGAGACAAACAATGCGTTTA

GTAACAGCCTAGAACAAGAAGATGATAAAGATCTCTCTGAAATTCTGAAATCGTTTGAAA

GATTGGATCTAAATAAGGAAATAGAAAAGAATGAGCAGACCACTTCCAACCTGCTAGAGG

CTGAGAGAGTAACCAAGGCCTCAGAAGCTACATGCACTCTGATGTTACGAGGCTATTCCT

GTGACAACGATGGTTGTGGTTACAGAGCGATGACAAAAGATGCTCTTTTTAAACATCTTG

TAAAACAGCATAACTACACAGAAGACATGATGAATCAGTGTCGCAAGGAACAGTCTAACT

TGGCACCTTTTAATTGTCAACTATGCCCCAAAACCTTTACAAGAAACTCCAACCTCAGAA

CCCATTACCAAGCCGTTCATAATTATTCACATGAGCAAGTGGTCAAGTTGGGGATTACAC

GGCAATACAGTCGAAAATCCATTGAGGCCGATCAAGGTCTTTGTACCACGCTGAACATCT

CGGAGTTGGCCAGTCCAGTCTTTGCTGGACAGAATGCCGAGTCTGGTGCCGGTATCACAG

CTTTGGTATCTGGAGTTAGAAGCATAAAATCAGAGTTCAGTGTTCAACCATTTGAACAAC

AGCATCCTGGTGCTTCCTTCGACTCATGTCCTGTTTCCAGTACAATACTGCGAGCGCAGA

CAACTCCACATCATCTCCAGAGTCAGACTACTGTGCATTTATCAGTGATTAAAGCTACTC

CTTCCTTGCTAACCTCCACAAGTGTGGAGCAGCAGTTAAATCGACCAAATGAATTTGTTC

CGGAATCGCTTGTGCCTTCTCAGCACACTCAAGGATTTCTGTCTGGAGGTCTTTCGTCTT

CAGGTGCGCCTCTTCTAGCCCCGATTTCTTTGGCGTCAGCACAGCTAGGTAGCCTTTCCT

CAGATACGAAGCCCAAATTTGGAAAACCTAAAATGGCAAAAGTGAAGGAAGTGACAAAAA

AACCTAAGGAGAAAAAGCTTGACATGGTTGATGTCTTTAGTCCCTATCGGCCTTACCGCT

GCGTCCATCAGGGCTGTGTTGCTGCCTTTACTATACAGCACAATTTGATTCTTCACTACA

AAGCTGTACATCAGTCTGCCCTCCCCAAATTTGAGGTTAATGAAGATGAAGAACCGATCG

AGGAAGAACATAACGAAAACCAGGAGGAGATTGGCATGCCTGACTCTGAGGTTAATGAGG

AGTTCCGCTGTCAAGTCAAAGACTGCTCTCGAATATTTCAGGTCGTTACAGACCTGCTAC

AGCATTACCTTCAGCTCCACAAACTCGGCCTTGATAAAGCAGGTATTATGATGTCTGGTA

TGAACTTGGGCAGGTTTCGGTGTGACCAACCAGACTGTCATGTGACATTCACTGGATTCT

GGAAGTATGTTAACCACATTGATAATGAACACAAGCATGTAAAGCTCTCCAGGACTGAGC

CTGTTAATGGCATGTTCCAGTGTGAGGTTGAAGGATGTAGCTGCGTTTATGCAACACGGT

CAAACTTGTTGCGACACTTTATGAAAAAACACCCAGAACTTTACAAACTCCGACTGCTGA

ACTCGGGAGGTGTCAAACTTGGTCGTCCTCCGAAAAACAGATTTGTCTCCCTTGAGAAAG

AAAATAGAGAAATCAATAAAAAAACTTTTCAGAAGGGAGGTGCGAAAAAGAAAAGGAGAC

AAAAGAATCTTTGGACAAAGTATGGAAACCCTATTTTGAAATCGAAAGAGGAGGCTTCCT

CAATGTGCACCAAGCGGATTCAGTTACAGTATCCTTGCATGCTTAAAGGCTGTGAGACAG

TTGCAGAGTCTGAGCGAAAGCTTTTAAAGCACTATGTCCAACATGGCTTGCCAAAACAAT

ACCTGGAGGAGCAGAGAAGCAACTACATATTCTGCAAAAAAATGCCTCGCTCAAAGTATA

AACGCATTGCGTCCAGGAGTGACGATACTGACAAGTCCGATGGAAGCTCGATTGAGGCGT

CTGAGAACGAGGACGCAGCAGACACTGGACCCAGCGAATCGGAGTTCTCAAAGCCGACGT

CTGAAAAGGAGAGCACGGAGGACACTGAAGTTTCAGACGCTAAATTGTCTAGTGACCTGA

GCTCCGACATCTCTGTGGTGGTAAAGCGGAAGAGAGGACGACCACGCAAGGGGGAACAGG

TTAAGCGCACCCAGCTCGCACGCAAGAGAATGACGAGGCTACGAGCGGCGCAGACGCGCT

CCATCAACTATGCAGATATGAACTCCGATTCCACTTCTTCCAGCGTTACGCTTCCACAAG

AGCCAAACACATCACTGAGCTCTTTCAAGCCAATGGGCTTCGAGGTGTCCTTCCTCAAGT

TCCTGGAGGAATCGAGTCAGTCTCCCGGGAAGAGAAAGAGTTGTTCTTTAATGGATTTAC

TTCCACACAGGAAATTCCCGACTGTGCGACTGAAGACTGCCTCGGTGGTATGCAGCAGGA

TCAACCCTGACTATCATCATTGCCGAGATGTATTGAAACATGTCGAATTTAAAAACCCTC

AGAAACTAACGTCTCTGAGTAGGGTGACGTTCGAGGTGCACAGAGGGTTCTCCAATGTCT

ACGAACTGTTGCTCAAGCAGCTGCATGACATGCGGCCTGCAGTGGTCATTCAGAAAGAGG

GCGTGTCTGAGACGAGGGCGTAGTTTTCAGGGTGGGGGTAGCTAATGTGAGAGTGGGTCA

AGAGCTGTTCAGTGTTGCTGTTTATGTGGAGCATCAGTGTTGAGATGTTTCCATGCCTTT

GTAATGAACCGCTAGTCCCTTTCCAGGACTGTATACTAAAGTGTACTATTGTAATGAAGT

CGGTGAAGGATACGCCTCTCGTGCATGCCCTAAAACACCTGATCTGGCACGTGTGCGCAG

CTGTGGAGATAAAACCCTATAATGGAAGAGTGAAACTGCTCCGTGTAAGAATGTGAGCTC

GCCTTTTGTTGGACTGACCGCGTGTCGTGCAGTGGTGAAATGTAGATGAGTAACATTTTG

GTCTTGTTCATCTCGCAGTGTAAAATTTGATAGCGTTGTGTTTATCTGTGACTCTCACAT

ACTGACTCTCCAGATAATCTACTGCCACCAGCAGTCTGTACCGCAAACAACAGAGACCAG

TTTATTACAGCTCGCGTTTTCTACCTCTGTGTGTGTGTGTGTG

>Unigene000062

GCCCAGAAAGGGCGCTGATAGTGAGAGAATGATGCGCTCATGATGACCGCCGCAACTTTG

CGCTTTTGCACGGTGGGTTAAAGCCCCGCGAGCTTCGCTCTGCCCCCCGCTCCCTGATGG

CGTCCCTGCCGGAGCGCCGGGCGTTTGCGCTCAAAATCAACAGGCACTCGTCTGCTGAAA

TCCGTAAGCACTTCACTGGGTTCGGCTATGGCACCGCTCAGAGCGGCCTGCACCAGCGCC

ACAGCTCCAGCACCTCTGCGTCCATCTGTCTCAACATTCCTCAGACTTATGACGCAGTGG

ATCCGCTGGATGTGGAGGAATTCCTGATGACGCAGCTGCGCAGTGGAGATGCCGAGCTCA

TGCAGGAGCTGGGAGAATTCCCTGATGATGACCTGGAGGTGGATTTTGTGGACAGGGAAT

GTCGAACTGCTTACCACTCTGTCCCTGAGGAGGGGGTTGAACTGGATCCCCATGTGAGAG

ACTGTGTACTGGGTTACACTCAGCCTTGGTTATTAGTCGGCAGAAGATGCCAAGGGGATG

GATGGAGTGCGTATTCCGAGAGGACAGGCTTTCACAAACTACTTTCGAAGCAGATATTTG

AGTCCGATGTAAAACCTGAAAAACAGGAGCCATCGGTAAAATCTCCATCCTTGGCAGTGC

TATGCGATAATGACAGTGCTCGCAGCACCATCACCTCCTCAGATTTTGATCTCCGGGCAC

TTCAGCCAGACCCTCGACTTGACAGCCTTCTGCAGTTTAGTAACTTTGAGGATCTAGACC

GCTTTAACTTTGAATCCAGAAAGAACAGCCGTCATCCGGAACTTTTCTCACTTTATCCGC

CTCCTGATGAGGAGGATGCAGTGGAAATTCGTCCTATTCCTGACAGTCCAAAGGAGCACA

TGGGCCTCAGGATCCTGGTGCGGTGCCAGGCCATCAAGTTTGAAATTGAGATCGAGCCTA

TATTTGCCACCATGGCCCTGTATGATCTGAAAGAAAAGAAGAAGATTTCAGAGAACTTCC

ACTTTGATCTAAACACAGATCAAATGAGAAGCTTACTACGATCCCACACCCCTAATATTG

ACAGCTCCACACTGGCAAAGGCTGCCATTTTCTCCATTACCTACCCTTCTCCAGACATCT

ACCTGGTCATTAAGTTAGAAAAAGTCCTGCAGCAGGGGGAAATTGGAGACTGTGCAGAAC

CTTACATAGGGATCATTGACAATGCTAAGAACAAAGACAAGTTAGAGAAACTTCGAAGTC

AGACAGAGAACTTCTGTCAGAGGCTGGGTCAGTACAGGATGCCCTTCGCTTTTGGTACCG

TCAACATCATGAATGTGATCAGCGCAGCCACACTGGATCGAGACACCACAGACTCCGACA

GCATCAATGGCAAGAACAATGTGGATAAGAAGGCTTACCTGCCCAGACGGAACTCGGAGA

GGTTCACCACCCTGGAGGACCAGTCCAACCTGTCCAGTTTTAAACCTGCTATCATAAACA

TTACAACTATTTTCAAACAGGAAGGGGATCGTCTCAGCGATGAAGATTTATTCAAGTTCC

TGGCTGACATCAAAAGAACGTCCACTATTCAGCGACGGATCAAGATTATACCAGGCTTTC

TAAGGTTAGAGGTGAACCCAGTGCCAGATACAGTGATGCACTGTCTGTCTCCTGAGCTCA

TCCCCATTAAACCTGTATCCGAAAAGAACGCAAGACCAGTTAAAGAATTGCTTGAGTTTC

CGTCCAACGATGTCTACGTCCCTCACTGCATATACAGAAACCTGCTGTTTGTTTATCCCC

AGAGGTTCAACTTCGGCAACAGGGGTTTAACATCTCTTAGGAACATCACTATAAAGATCC

AGTTTATGAATGGAGACGAGCCACTGAAGGTTATATTTGGGAAATCAAGTGGTCAAGAGT

TTCTATCAGAGATCTACACCCCAGTCACATACCATAACAAATCTCCTGATTTTTACGAAG

AGGTGAAGATCCGGCTACCGGCTCGACTGACTGAGAGACACCACCTGCTCTTCTCTTTCT

TCCACATCAGCTGCCAGCAGAAGCAGAACCAGAGCGGCAGTATGGAGACTCTCATCGGAT

ACTCGTGGTTACCCATTCTGAATAACGAGCGGCTGCAGATGGGAGCTTTCTGTTTGCCCC

TTGTTCTAGAACGTCTTCCTGTGAACTACTCGCTACATTCACCTGAGAAGTTGCCAATCC

AGGTTCCTCCAGTCAAGTGGATTGATGGACATAAAGGAATTTTTAGCCTGGAAATTCATG

CCGTGTCTTCAGTTCACACACAGGAAAGTTACCTGGAGCGCTTCTTCACGCTGTGCCATG

CTCTGGAGGGAAAGGCTACATTCCCGATCCGTGTAGGGGACGAGAAAGTGCCAGAAAACA

AGCTGGAGCATGAGCTTAAACTCAGCATTATCTCCCTGTCCTCCTCCAAGCTGGAACCAC

TTGTACTCTTCCTCCACCTGGTTCTGGACAAGCTGTTCCGCCTCATCATGCAGCCAATGG

TCATCGCTGGCCAGACCGCCAACCTGGCGCAGATCGCATTCGAGTCGGTGGTGTCTGTAG

TAAACATCCTTCATAACAGCCAGGAACTGGCTAAGGACCACCAAGGCAGGAACTGTCTCC

TAGCAACCTACCTCTACTATGTGTTTCGGTTGCCAGACACTCTGCTGGAAGTCATTAACA

CAGGTGCGCCGGTACAGGAGAGCAGATACAGCACTATGGGCCGAGCCACAGCTACAAGCG

TAGGCAACATGCTGCTCTATTCACGCGTCCGCTCCAGCAGCAATCCTGATATTCCTAATC

CACAGAGCCCAGAGGATGCTGAGGTGAACAGCATACTGGTTGAAAAGGGTTTGAATCATC

CAGGCAGCAGGATGTCAACTTTCATTGATGTTACTAACCGCTATCAAAATTCCGCTAGTA

GCACAAGGCCCTCAAACAAAAAGCAATTTCATGAAGAGCTGGCTCTTCAGATGGTGGTGA

GCACCGGCGTGTGCAGAGAGATGGTGTATAAATACGCCTGGTTCTTCTTTGAGTTGCTGG

TGAAAAGCATGGCCCAGCATGTCTCTCAGATGGACAAACAGGAAGTGTCTCGCAAAAATC

GCTTTTCTGACCGCTTTAAGGACGACATCACCACCATCGTCAACGTGGTGACGGCTGAAA

TTGGAACGATATTAGTAAAACAGCAGAAGGAACAAGAACAGGCCGAAAAGATGAACGTCA

GTCTGGCGTTTTTCCTTTATGACTTGATGTCTCTTATGGACAGAGGCTTTGTCTTTAATC

TGGTTAGAAATTACTGCAACCAGATGTCAGCAAAGAGTGTGTCCATGTTCACTCTGATCA

GCATGAGGCTGGAGTTTTTGCGAGTGCTCTGCAGTCATGAGCACTACCTCAACCTGAGCC

TGTACTTCAGCAGCCCTTCATCCGCTCCCACATCCCCTTCCCCATCTGTCACCTCACAGA

CCTCGAGTTCATGTAGTTTGCCGGACAATAAGATTGTGCAAATGTTTGATCTGACTCAAG

AGTTTAAACAGCAGCACTACCTGACTGGGCTGCTGCTGACGGAGCTCAGTGCTGCTCTGG

ATATGGAATCAGAAGGAAGTAAGGTGCAACGCAAGGCTATAAATGCCATCTGCAGCCTAT

TGTGTGCTCATGATTTGGACTCTCGGTGCTCAAGACTAGAAGTCAGGACTAAGATTGCCT

CTCTCTACCTCCCCCTCGTGGGTATCATCATCGACTCCCTCAACTACCTTGACTTCACAG

TTTCTGAGTCACGTGGCAACAAGGCCAAGTCCAGCGGGCCGGAGGATGACTTTGATAATA

TTCCACCTATCAGTCAGTCAGTTGCAATGGCAATTGCAGGAAATCCGTTTAACACCCTGG

CGAGAAACGCACTAGTGTCCATGGCCTCAGTGGCAAACAGAAACTGTAGCACGCTTTCTG

CAGAGACCAGCCGTAATCTGTTAGTGTGCTTCCTGTGGATTATGAAAAATGCAGACCAGA

GCCTGATTCAGCGCTGGATAGTGGACATAGCCCCATCTCAGCTCCATCGCCTTCTCGAGC

TTCTTACAATCTGCATCTCCTGTTTTGACTACAGGGGGAAGAGTTCAGATAAAGTGAGTG

TGCAGGCCTTACAGAAGTCTCAGCAGGCTAAAGCTCGCCTGGAGGAAGCACTACTGGGAG

GCCTGGGAGCAAGAGGGGAGATGATGAAGAGGGTGGGAGCTAATGACCGAACTATGGGCC

AGAGAGAGAACCTGCGCTGGAGGAAAGACCTCACACAGTGGCGCCAGACCAACGACAGAC

AGGACAAATCTAAAGCTGAATTGGACCAGGAGGCAATAATCAGTGGAAATCTGGCCACTG

AAGCCAACGTCATAGTTTTAGACCTACTGGAGATCATAGTGCAGACCGTACCACTAGTGG

ACTATAAGGATAGTGCAGTTGGTGGAGTTCTGAGGGTTCTCCTGCACTCACTAACATGCA

ACCAGAGCACCATGTATCTATCACACTGCTTCAGCACCATCCGGGCTCTGATTGTGAAGT

TTGGAGACATGTTGTTCGAGGAGGAGGCGGAACAGTGTGCAGATCTGTGCCAGAAAGTGC

TTCAGTACTGCAGCAGCTGTGTGGACAGCAACAGGAACCAGGCCTGTGCTACACTCTACC

TCATCATGAGATACAGCTTCAGTTCAGCCAGTAATTTCTCTAGGGTGAAGATGCAGGTGA

CCATGTCATTGGCGTCCCTAGTAGGAAAGTCATCGGACGTGCACGAGGAGTACCTGCGCC

GCTCTTTTCGCACTATTCTAGCCTATGCAGAAGAAGACACAGAGATGCAATCCACACAAC

TTCCTTCGCAGGTGGACGAACTTCTGAGGAATCTGAACAGCATTCTTTCAGATACAGTTA

AAATGAAGGAATTTCAGGAAGATCCTGAAATGCTAATGGACCTTATGTACAGGATTGCAA

AGGGCTACCAGACATCGCCTGATCTGCGTCTGACCTGGCTCCAGAACATGGCTGAGAAGC

ATAACAGCAGGAAGTGCTTCACCGAATCGGCTATGTGCCTGGTACACGCCGCTGCTCTGG

TGGCTGAGTACCTCAGCATGTTGGAGGACCACAAGTACCTGCCTATAGGCAGTGTTACCT

TTCAAAACATTTCCCCTAACGTTCTGGAGGAGTCCGCGGTGTCAGATGATATCCTGTCCC

CAGATGAGGACGGCGTTTGTTCGGGACGTTACTTTACAGAAATTGGTCTGGTGGGGCTTC

TCGAGCAGGCCGCAGAACTCTTTAGCAACGGTGGTTTATATGAGGCAGTGAATGAGGTGT

ATAAGGTCATCGTTCCCATACTGGAGGCCCATCGGGATTTCCGGAAACTGGCCACCACTC

ACGACAATCTGAAGAGAGCGTTTGAGAACGTTGTTCAAAAGGGGAGTAAGCGGATGTTCG

GGACATATTTCCGTGTGGCATTTTTTGGCTCAAAGTTTGGGGACCTTGATGAGCGAGAGT

TCATTTATAAAGAGCCAGGAATCACACATCTCCCTGAAATCTCCCACCGACTGGAGAATT

TCTACAGACAGTGTTTGGGAGACGGTGTCCTGGAAATGATCAAGGATTCCACACCTGTAG

ACAGAAACAAACTGAATCCCAACAAGGCATACATTCAGATCACGTTTGTGGAGCCGTACT

TTGACGACTATGAGATGAAAGATCGTATGACCATTTTCGAGAAGAACTTCAACCTGCGCC

GGTTCATGTACACCACCCCGTTCACCAAGAGCGGACGTCCTCGAGGAGAGCTGAACGAGC

AGTACAAAAGGAAGACCATCCTGACCACACAGCATGCATTTCCTTATGTGAAGACACGGA

TCAATGTCATTCAGAAGGAGGAGTTTGATCTCACTCCGATTGAGGTGGCTATCGAAGACA

TGCAGAACAAAACTCGTGAGCTGGCTATCGCTACGCACAAAGAGAAGCCTGACCCCAAGA

TGCTGCAGATGGTGTTGCAGGGCTCTGTAACTGCTACAGTTAACCAGGGACCATTAGAGG

TAGCCCAAGTCTTTTTGAATGAAATCCCAGCAGACCCCAAACTCTACCGTCATCACAACA

AACTGCGTCTGTGTTTCAAAGAGTTCATCCTACGGTGTGGGGAAGCAGTGGAAAAGAACA

AGCACCTGATCACGCCAGACCAGCGAGAGTATCAGCAGGAGCTAAAGAAAAACTACAATA

AGCTGAGAGAAAACCTGAGGCCCATGCTGGAGAGGAAGATTCCTGAGCTGTACAAGCCCA

TCGTCAAACCCCGTTTGGAGAACAGGGACTCAACAAAACGACACAGCTTCCGGAGGATTC

CAGAGGACAGTTCATGATGTCATAAAAATGGAAGAAAATGAGTAGAGAAGGTTTCATCAT

TTAAACAGCTCCAATGATGTCTTGATGCCTTGAGCAGGGTGTCATGAGCAGTATTATGTT

AAATATTTTAAATAGTTAATGTTGACCTATTTTAGTAGGTTGTGTTGCAGCACTCTAAAT

TTATTTTTAATGCATTTTTTCTTAAACTGGTAGAAAATAAAAGCTGGTAGTTAAGAAAAC

CCCAAAAAACTATCAGACTATGAACAACTAGTTTGAATATTGATTTAAACGCCACACACC

TGTTTATATAATCTGAAGTAATTGAACTGTTTAACATACTTTAAAATAATATATTTGTAA

TAAAAGTATTCCAGAATAAATAGTATCATTCCCTCTTTTCATGAAAAGATTATTTAGAAT

ACAGTAACTCCCTACTTCAATTCTGTATTTTAAGTTTATCAGTAAATAAGAATTTTGTGG

TCTTCACCTTCATCTATAAACAAATTATTTACCGCAGGTAACAATGGCAATAAAATAACA

TGAAAATCTACTTACAGTTTCGGATACCAAAAAAAAAACCAAACAGTACATTATAACTAT

GGTTATATTTTTACAACCAAGCAGTGAGAAACCTTTGTAGGGCCCAAGCACCCAGACTGG

ACACGGCACATATTCTTAAAACAAAGTAATCGTAGGGAAAGCAGAAGTAAACTGTTTTAG

AGGTGATTTGTAGTTTTACCCATGATGCTCAGTTCTGACCACAAACAGTGGTGGAATTCA

CTGAGTGTAGAAAGAAAAGTCGAACCAAATGTTTAGACAGGTGTCAAAATAATGTAAACC

ACATCTGGGGAAATTTGGAGGCGAAGTAGCACAAATCGAATTTGGATAGAAGAATTTTTC

GGTATTTGTAGCAGCTTTACTGTTAAATGTGTCGGCCTGCGGCTCAATGGCAAATCATTG

AAGTATTAAAAAACGTTTGATTTGTGTGACTATAGAAATGGCATTTGACAAATTTGGTAA

TGACCGGATAATGTGGAGGATTATTAAAAGTTAACAGAATATAATGTGTAGAATTTGTCC

TATCTAAAGAATACCAGAATGTTTGGACCTTTGGTAGTCTGGACCTTTTGTAGAACAGGT

TAAAAACCGAATTAGTGCGAATTTGGTTAGAATGTTTCTAAGACTCCTGCGCAGACTTTT

AACAGATAGCACTTGTTGCCAAACTTTTAGACTACCTGCAGGGGTTAAGGTCTTGGACTA

CTAATTAGAAGATCTGGGTTCGATTCCCACCACCAGCAAGTTGCCAATGCTGGGCCCCTG

AGCAAGGCCCTCAACCCCCAGTTGCTCAGCTGTATAAATAGGAGATAAATGTAA

>Unigene000063

GGCACGGTGCGGCTTGGACGCGTCAGGAGCGCAGCGCACACCCAGAATCAGAAACACGCA

CCAAGTCCAGCAAAGGGTTTTTATATAATAAATTAAAGAGTTTAGTTATAAATTAGATTT

GAAAATATTTTTTTCTGCGCTCCGGGATGAGGCGCTTTGTGCTGTTGGCGCTGTGCGCAT

GCAGTGCGGTGGTGTGTTTGCCGAAGTCCGACAGGAATAAGCGGCAGGGACAACAACAAC

AGGTGCTGGACATACCGGTGGACGAGCTGGCGTACGCACGCCAAGCTGGCTGTACGGAGA

ACGGGCAATTTTACAAAGTCAACGACCAATGGGAACGACCCTACATGGACAGCACGTTAC

TGTGCACGTGCAGAGGATCGGCGGGCGTCCAGTGCGAATCCAAACCTGCAGCTGAAGAAA

TCTGCTTTGATAAAATCAACGCGAGGTCCTACCGCGTGGGTGAGACCTACGAGCGGCCGA

AGGACGGGATGATCTGGGACTGCACCTGCATCGGCTCGGGTCGAGGCAAAATCAGCTGCA

CCATCGCAAATCGTTGCCATGAAGGTGGACGCTCGTACAGAATCGGAGAAACCTGGACGC

GTCCCCATGACACGGGCGACTACATGCTAGAATGTATGTGTCTCGGAAACGGAAAGGGCG

AGTGGACTTGCAAACCCATCGCTGAGCGCTGCTACGACACGTCCCTGGGCACATCGTACG

TGGTGGGACAGACGTGGGAGAAACCTTACCAGGACTGGATGATCGTCGACTGCACGTGTC

TGGGTGAAGGAAATGGACGCATCACCTGCACCTCCAGGAATCGTTGTAACGACCAGGACA

TGAGGAAGGCGTACCGTATCGGTGAGACGTGGACCAAAGTGGACTCACAAGGCCGCCAGC

AGCAGTGTGTGTGTACAGGAAACGGACGCGGCGAATGGAAGTGTGAGAGTCACTCATCTG

CTCAAAACGCCCTCGGTTCAGGCTCGGCCTTGTCCACTGAGGTCAGACCCGTCACCCATC

AGCTAGCCATTCTGCCCGAGTTGGTCGAAATCGGCGCATGTCAAACCAGATCTGGAACCA

CCTACTACAACGGCATGCGCTGGGTTCAGACACAGGGCAGCCAGCAGATGATCTGCACCT

GCGTTAATGGTGGCATCAGCTGCGAGGAATGGGAAGGTCAAGGTCACGTGTACGGCGGGA

ACTCCAACGGCCAACCGTGTGTGTTCCCCTTTGTTTTTGGTGGAAAAACACACTATTCCT

GCATCTCTGAGGGCCGAACGGATGGGCAGCTGTGGTGCAGCACCACCTCTGACTACGACA

CGGATCGCCAGTACTCCTTCTGTACTCAGAGGAACTTGATGGTGACGACTCGTGGTGGAA

ACTCCAATGGAGCTCTGTGCTACTTTCCCTTCCTGTACAACGATCGTAATTACACAGACT

GCACAGCTGATGGCCGACGCGATGGGATGAAGTGGTGCGGCACTACAGCAAACTACGATA

ATGAGCGACACTACGGATTCTGTCCCATGGCAGCTCATGAGGAAATCTGCACCGTAAACG

ACGTAATGTACCGCCTGGGGGATGAGTGGGATAAGCGCCATGACACCATGGGTCACATGA

TGCGTTGCAAGTGTTTGGGCAATGGTCGGGGCGAGTGGAGCTGCGTAGCTTACTCGCAGC

TTCGAGATCAGTGTATTGTAGACGGACTCACATATGAAGTTGACCAGAAATTTGACAAGC

GCCACAGAGAGGGCTACATGATGAACTGCACATGCTTTGGTCAGGGCCGCGGCCGCTGGA

AATGCGACCCTATTGATCAGTGCCAGGACGCAGAGACAAAAGTGTTCTATCAAATTGGAG

AGACGTGGGATAAAGTGAACCATGGCGTTCAGTATCGGTGTACCTGTTATGGAAACGGGA

TCGGAGAACAGGCCTGTGAGCCACTCCAGTCCAGGCTTCCAGTGCGCGTGACCATCACAG

AGACAGGAAACCAGCCCAACTCGCACCCTATCCAGTGGAACCCCCCAGCATCATCCCATA

TCACCCAGTACATCCTCAAGTGGAAAGTGAAAAACACTCGCACTCCATGGAAGGAGGTCG

TCATTCCGGGCCACGTTAACTCCTACACTATCTCAGGCCTGAAGCCAGGGCTTACCTATG

AGGGCCAGCTCATCAGCATCCTGAGCTACGGTCACAGAGAGGTCACCAGATTCGACTTCA

CCACATCCTATGGCTCACTGATTCCGACGGAGGGCGTGACCAGAAATAACATTCGAGTTG

TGGATGCTTCTGAATCCATCACCGAGATCACGTCCAGCAGTTTTGTTGTTTCCTGGACAT

CAGCATCAGAAACCATCTCAGGGTTCAGGGTACAGTACGAGCTGTCAGAGGAGGGAGCAG

AGCCCACTGTGATCGATCTTCCTCGCACATCCACATCGGTGAACATCGACAGACTCCTGC

CAGGCCGAACGTATCAGGTTCAGGTTTACGAAGTCGAGCCGGAGGGAAACATGAACCTTA

TCCTCACCACCACTCAGACCACTGCTCCTGACACTACAACTGAACACAGAGTTACAGATG

TTGGAGAAACATCCATCGTAATCTCCTGGACCAAACCACAAGCACCGATCACCGGTTACC

GTGTGGTGTACACACCAAGCCTTGAGGGCAGCAGCACTGAACTCATTCTGCCTGAGACGG

TGACCTCAGTGACCTTGGTTGACCTCCAGCCGGGTCAGTCATACAACGTCAGTATCTTTG

CTGTAGAGGGAAACCTGGAGAGTGAGCCAGTGGTGCTGCAGGTGCACACAGCTGGAGAGG

CGCAGCCCGAGGAAGTCCAGGCACCGACTGAGCTGCAGTTTTACGAGGTGACTGATGTAA

AGATCACCATCACATGGAAAGGACCACCCAGCGAGGTGTCGGGCTACCGTGTGACCTACG

AGCCCGTGGGCTCAGATGGCCGTGCAACACAGAGACCTCTACAGCTTCCTGTCACGCCCA

ACGCCTATGCCGAGATCACACATCTGCAGCCTGGAACCTTGTACCGCATTTACGTCTACT

CTGTCTACGGAGGAACCGAGAGCCAGCCGCTGGTGGGCGAGAAGTTGACAAGACCTGACG

CTCCTACGGAGCTGCGCTTCCCAGACGTCACAGAAGACACTGTTTTGGTTGCTTGGTCTG

CTCCACAAGCCCCAATCACCGGTTATCGCCTTATCATTACGGCTGAGGACACCACCCCCC

CGAAGCAGTGGAGGGTCCGACCCGAAGAGACGCAGTACACCATCCAGGACCTGCGACCAG

ACACGATGTACACTATCACACTGCACTCGGAACAGGGCAGCACTTTGAGTGAGGGTGTCT

CTGGAACGGTTACCACCTCTGTGCCAGTCGGTAACGCCCCACGTTTCTCCACGGACGTAA

CAGACACTTCCATCATCATCTCCTGGACTCCTGTTCCTCGCTTCAGCTACAAGATGTCAG

TAAAGCCCAGTCAGGGTGGCGAGGCTCCCAGGGTCGTGACCTCCGACTCAGGAAGTATCT

ACATCTCAGGCCTAACCCCCGGAGTGGAGTACACCTACAGCCTGCAGCCCATCTTCAGTG

GACGCAGACCGGGCAGCCCCATCACACGCAACGTTGTCACACCTTTGTCCCCACCGAGTC

ATCTAAATCTAGTGTCCAACCCCGACACCGGCAACCTCAATGTATACTGGCAACCGACCA

CCACTCCCGACATCACAGGGTACCGGGTGACATGTACACCAACCACAGGGCAGCGTGGGA

ATTCTCTGGAAGAGTTTGTTCGAGATGGGGAGACCTCCTGTACGCTGGAGAACCTGAGCC

CTGGTGTTGAGTACAACATCAGCGTCTACGCTGTTAAAAACCATATGGAGAGCGAACCCA

TCTCATCGGTTATCACACAAGCCGTTCCCGCTCCCACCAACCTGAAATTCGGCGAGGTTG

GACCAGACTCCATGCGCCTCTCCTGGACCCGCCCCTCTGTCCGCCAGTCAGAGATCAGCC

GCTTTGTGATTCGCTATCATCCGAGCAACGATGACGACAATGTGCAAGAAGTCAATGTGG

GCGGCGCCACCAGCACCTACCTGCTTCAGAATCTGCTTCCCGTCACTGAGTACATGGTGA

GCGTGTCGTGTGTGTATAGTGAGCGAGAGAGCATCCCGATCACAGGCAGACAGACCACGA

TTCTCGACGCTCCCACCGCTCTTATCTTCTCTGATGTCTTAACACACTCTTTCACTGCAA

ACTGGCGAGCCCCACGCGCCCAGATCACGGGCTACCGACTGGTATACGAGGCGACCAGCG

GGGGCCGCCGTCATGAGGAGCGTCTGCCACCAAGCCGCACACATTACGCCCTGAACAACC

TGCAGCCCGACACACTGTACACCCTGCACATCTACGCCATCAGCGGCCGCCAGGAGAGTC

AACCTCTCACCGGCACACAGTCTACCATCTCGGATGCTCCCACTGACCTGGAGGTGACCT

TATCCACCCCAACCAGCATCACCATCTCCTGGGACGCTCCTGCTATTCCAGTGCGTTACT

ACAGAATCAAATATGGACAAAGTGGAGAACGAGGTCCTGGCCAGGAGTTCACTGTGCCTG

GGACAGACTCTACAGCCACCATCAGTGATCTGCAGCCAGAAAGAGATTACACCATCACCA

TTTACGCAGTGACCGGAAGAGGAGACAGTCCAGCATCCAGCACACCGACCATCATTACAC

ACCGCACCGGTAGCCACGGTGCTCCGTCTACCACAGATCTAGATGTGAGTGATGTCCAGG

ACCAGGCCATTATAGTGCGCTGGACTCCTGCCAGAGGCCCGGTCGCAGGCTACAGGGTCA

CTGGGAAACCCAAAAAAGGAGTGGGACCCACCTTCTCCAAGGAGGTGGGACCTGATAAGA

CAGAGTTGCGAATAACAGGTCTGGTACCAACAGTGGAGTACGTAATCAGCGTCTATGCCA

TCAGTCGTGATGGAGAAAGCACACCGGTGGTGCAGAAAGCAACTAAAATCGAAACACCAG

ACCGTCCCACAGACATGAGGTTCCCTGATGTAGACAGTTCTGGGTTGACCGACCGAAGCT

CCACCCCAGAGTCGGAGGGAACACACACCACTGCAGGTGTTCCTTCCCCCACAAACATTC

AGTTCGGAGACGTGGGCCCGTCATCCTTCGTGGTGCTCTGGAGGCCACCAAGTGTCAGAC

TAAATGGATACCGTATTCTTGTGACACCCAAAAATAATTTTGCACATCCAAAAGAGTTGA

ACGTCGCTCCTGATGCCATGCAGGCCACAGTCACTGGACTTCTGGTCTCCACTTTGTATG

AGGTGCATGTGTACGCTTTAAAGGATTCAGCCAGCAGCCCACCACTTATTGGAGAGATCT

CTACAACTGAAGACATCAGCCCACCCCGGCGCTCTCGCATCAACGAAGTGAAGGACACAA

GCATTACGATCAGCTGGCGCTCCAAGATCGAGCCAATGACAGGCTTCCTGATTGAAGCCA

AGCCCCTTTCTGGAGAGAGTCCAACTATCAGGAAGGAGATTCCAGCTGAACAACGCAGTG

TCGTGATAACCGGTCTTCAGCCTGCCACCACTTACTCAATCAACATCTACACACTTAATG

GAGACCGACGCAGTGCACCCTTTACCCTAATCGCCAAAACAATTGGTTCATCCCTCCCGC

CCCCCACTGACCTCCAGTTCCTGGCTCTGACTCCGACTTCAATTTCCTTCAAATGGCAGC

CACCCACCAGCCGCATTACAGGATATTACGTCACTTACGAAGAAGAAGGAAAAAGCCCAA

GGGAGCTTACACCACGGCCACATGCCGGTGCCAACTATGCCTCCATCACAGATCTGAAAC

CAGCCACTGTGTACATCATAAAAATCATCGCTCTGCAGAACACAATGAGAAGTCCAGCAC

TGGTGGGCAGGGCCACTACACAGGAATCAGACTTTACACTGCCTTCACGATCGCACAGCA

GCCTTGGTCCCCTGGACGTACCTGAGACGGAAATTGTGGTGAACGTGATGGGTCCGACAG

TGCAGCCGAGACCAGACAAGCACGGCCAGGGCATGGAGTACACCGAATACAACAATCAGC

CCACTCTGCCTCACAGTGGGCAACGGCCGAACCCCTACGTCCCTGGAACGGGCCAAACTC

TGATCTATGTGCCCGCTCCAGGGTCGGACGGTTCGCGAGTGCCCAAAGTGGTGCAACTGA

GCGAAAGAAATGCTCACAATTTCCTTTTTCCAGAAAATAAGACGGGGACACCGCAGGAGG

CTCAGACTCAGACTACTATCAGCTGGAAACCCTTTAGGCAGAGCAAAGCCTACATTGTGT

CCTGCCAACCTGTCACAAATCGCAACGAGAAGATGTTCCAGATGCAACTTCCTGCCACCT

CCACCAGCGCTACACTGATCGGTCTCACATCTGGCGCTTCATACCGCGTCCTCGTCGAGG

CTCTGAAGGACGCACTTAAGTACAAAATCCTTGACGAGGTCATAACCGCCGGTAACACAG

ATCCAGCAAGGGTTCCAGCCAGCGACGATTCATGCTACGACACCATAACCGCTGCTCACT

ACAATGTGGGAGATGAATGGGAGCGCATGTCTGAGACAGGCTTCAAGTTGTGGTGCAGGT

GCCTGGGACTGGGCAGTGGTCACTTCCGCTGCGACTCCTCCAAATGGTGCCATGACAACG

GCAACAACTATCGCATTGGCGAGCGGTGGGAGAGACAGGCAGAAAACGGTCACATGATGA

GCTGCACCTGCCTCGGCAACGGCAAGGGAGAGTTCAAGTGCGAACCACACGAGTCGACGT

GTTACGATGATGGTAAGACATACCAGGTTGGCAACCAGTGGCAGAAAGAGTACCTCGGTG

CAATCTGTACCTGCACCTGTTACGGAGGACAGCAGGGCTGGCGGTGTGAAAACTGCAAAA

AACCTGGAACTGAGATCAACACTCAAATGCTGAGGCCAGTCCGCTACGGAGACGGCATCG

CCAAAGTGAACATTCACTGCCCAATCGAGTGTCTCCGGCCGGACATCCTTGCTGACGCCG

TGGCGAATCCCAACCCAAGAGAATAAAATGATAAACACATCCCCTATTGAACCTACCCTG

ATACGTCAACTTCTAAACTGAACTACCCCTCCAGTGCCTTAAAAGCATTCCTATTGGTTG

CTGAAAACACAGCTGCTGCTTCCCAGCCAATCATCACAGCCAGCATGACACCTTAGCAAT

TCCATAGCAACACCAATGCTTTTAATTGGTTGGTGCTATTATTCAGTCATTTGATTGGCT

TACTAGTTTTTTTTTTCAGTTTTAAACCATATGAAAAATGTTGCCAGATTTTATAAAAAT

CTATTTTAATTTTAAAAAATCTTTAAAGAAAAATTAAAAAAAAGTTTTTTAATTTTACAA

AAACTGATTTGTGAGAGATTTGTGAGTGTTTTTCCAAAGGCTTTTTAGCACACTTTTTTA

ATTCACATACTTATTTTCCTCTTTGGCATGAGTTTTTTCCCTCAGAGTCAGCACAGTTCT

TTACATTGACATTTTTTTTTTTACCGTCTAATGCTTTAAGATTTATTTTTTCCCCTTAAA

CATTTTCATTAGATGCAGTTACCGTCGGTGTTGAAGTCTTCACTACTGTCAAAATTCCAT

TTTTAATAAAGATTTACGAAGG

>Unigene000064

GGCACGGTGCGGCTTGGACGCGTCAGGAGCGCAGCGCACACCCAGAATCAGAAACACGCA

CCAAGTCCAGCAAAGGGTTTTTATATAATAAATTAAAGAGTTTAGTTATAAATTAGATTT

GAAAATATTTTTTTCTGCGCTCCGGGATGAGGCGCTTTGTGCTGTTGGCGCTGTGCGCAT

GCAGTGCGGTGGTGTGTTTGCCGAAGTCCGACAGGAATAAGCGGCAGGGACAACAACAAC

AGGTGCTGGACATACCGGTGGACGAGCTGGCGTACGCACGCCAAGCTGGCTGTACGGAGA

ACGGGCAATTTTACAAAGTCAACGACCAATGGGAACGACCCTACATGGACAGCACGTTAC

TGTGCACGTGCAGAGGATCGGCGGGCGTCCAGTGCGAATCCAAACCTGCAGCTGAAGAAA

TCTGCTTTGATAAAATCAACGCGAGGTCCTACCGCGTGGGTGAGACCTACGAGCGGCCGA

AGGACGGGATGATCTGGGACTGCACCTGCATCGGCTCGGGTCGAGGCAAAATCAGCTGCA

CCATCGCAAATCGTTGCCATGAAGGTGGACGCTCGTACAGAATCGGAGAAACCTGGACGC

GTCCCCATGACACGGGCGACTACATGCTAGAATGTATGTGTCTCGGAAACGGAAAGGGCG

AGTGGACTTGCAAACCCATCGCTGAGCGCTGCTACGACACGTCCCTGGGCACATCGTACG

TGGTGGGACAGACGTGGGAGAAACCTTACCAGGACTGGATGATCGTCGACTGCACGTGTC

TGGGTGAAGGAAATGGACGCATCACCTGCACCTCCAGGAATCGTTGTAACGACCAGGACA

TGAGGAAGGCGTACCGTATCGGTGAGACGTGGACCAAAGTGGACTCACAAGGCCGCCAGC

AGCAGTGTGTGTGTACAGGAAACGGACGCGGCGAATGGAAGTGTGAGAGTCACTCATCTG

CTCAAAACGCCCTCGGTTCAGGCTCGGCCTTGTCCACTGAGGTCAGACCCGTCACCCATC

AGCTAGCCATTCTGCCCGAGTTGGTCGAAATCGGCGCATGTCAAACCAGATCTGGAACCA

CCTACTACAACGGCATGCGCTGGGTTCAGACACAGGGCAGCCAGCAGATGATCTGCACCT

GCGTTAATGGTGGCATCAGCTGCGAGGAATGGGAAGGTCAAGGTCACGTGTACGGCGGGA

ACTCCAACGGCCAACCGTGTGTGTTCCCCTTTGTTTTTGGTGGAAAAACACACTATTCCT

GCATCTCTGAGGGCCGAACGGATGGGCAGCTGTGGTGCAGCACCACCTCTGACTACGACA

CGGATCGCCAGTACTCCTTCTGTACTCAGAGGAACTTGATGGTGACGACTCGTGGTGGAA

ACTCCAATGGAGCTCTGTGCTACTTTCCCTTCCTGTACAACGATCGTAATTACACAGACT

GCACAGCTGATGGCCGACGCGATGGGATGAAGTGGTGCGGCACTACAGCAAACTACGATA

ATGAGCGACACTACGGATTCTGTCCCATGGCAGCTCATGAGGAAATCTGCACCGTAAACG

ACGTAATGTACCGCCTGGGGGATGAGTGGGATAAGCGCCATGACACCATGGGTCACATGA

TGCGTTGCAAGTGTTTGGGCAATGGTCGGGGCGAGTGGAGCTGCGTAGCTTACTCGCAGC

TTCGAGATCAGTGTATTGTAGACGGACTCACATATGAAGTTGACCAGAAATTTGACAAGC

GCCACAGAGAGGGCTACATGATGAACTGCACATGCTTTGGTCAGGGCCGCGGCCGCTGGA

AATGCGACCCTATTGATCAGTGCCAGGACGCAGAGACAAAAGTGTTCTATCAAATTGGAG

AGACGTGGGATAAAGTGAACCATGGCGTTCAGTATCGGTGTACCTGTTATGGAAACGGGA

TCGGAGAACAGGCCTGTGAGCCACTCCAGTCCAGGCTTCCAGTGCGCGTGACCATCACAG

AGACAGGAAACCAGCCCAACTCGCACCCTATCCAGTGGAACCCCCCAGCATCATCCCATA

TCACCCAGTACATCCTCAAGTGGAAAGTGAAAAACACTCGCACTCCATGGAAGGAGGTCG

TCATTCCGGGCCACGTTAACTCCTACACTATCTCAGGCCTGAAGCCAGGGCTTACCTATG

AGGGCCAGCTCATCAGCATCCTGAGCTACGGTCACAGAGAGGTCACCAGATTCGACTTCA

CCACATCCTATGGCTCACTGATTCCGACGGAGGGCGTGACCAGAAATAACATTCGAGTTG

TGGATGCTTCTGAATCCATCACCGAGATCACGTCCAGCAGTTTTGTTGTTTCCTGGACAT

CAGCATCAGAAACCATCTCAGGGTTCAGGGTACAGTACGAGCTGTCAGAGGAGGGAGCAG

AGCCCACTGTGATCGATCTTCCTCGCACATCCACATCGGTGAACATCGACAGACTCCTGC

CAGGCCGAACGTATCAGGTTCAGGTTTACGAAGTCGAGCCGGAGGGAAACATGAACCTTA

TCCTCACCACCACTCAGACCACTGCTCCTGACACTACAACTGAACACAGAGTTACAGATG

TTGGAGAAACATCCATCGTAATCTCCTGGACCAAACCACAAGCACCGATCACCGGTTACC

GTGTGGTGTACACACCAAGCCTTGAGGGCAGCAGCACTGAACTCATTCTGCCTGAGACGG

TGACCTCAGTGACCTTGGTTGACCTCCAGCCGGGTCAGTCATACAACGTCAGTATCTTTG

CTGTAGAGGGAAACCTGGAGAGTGAGCCAGTGGTGCTGCAGGTGCACACAGCTGGAGAGG

CGCAGCCCGAGGAAGTCCAGGCACCGACTGAGCTGCAGTTTTACGAGGTGACTGATGTAA

AGATCACCATCACATGGAAAGGACCACCCAGCGAGGTGTCGGGCTACCGTGTGACCTACG

AGCCCGTGGGCTCAGATGGCCGTGCAACACAGAGACCTCTACAGCTTCCTGTCACGCCCA

ACGCCTATGCCGAGATCACACATCTGCAGCCTGGAACCTTGTACCGCATTTACGTCTACT

CTGTCTACGGAGGAACCGAGAGCCAGCCGCTGGTGGGCGAGAAGTTGACAAGACCTGACG

CTCCTACGGAGCTGCGCTTCCCAGACGTCACAGAAGACACTGTTTTGGTTGCTTGGTCTG

CTCCACAAGCCCCAATCACCGGTTATCGCCTTATCATTACGGCTGAGGACACCACCCCCC

CGAAGCAGTGGAGGGTCCGACCCGAAGAGACGCAGTACACCATCCAGGACCTGCGACCAG

ACACGATGTACACTATCACACTGCACTCGGAACAGGGCAGCACTTTGAGTGAGGGTGTCT

CTGGAACGGTTACCACCTCTGTGCCAGTCGGTAACGCCCCACGTTTCTCCACGGACGTAA

CAGACACTTCCATCATCATCTCCTGGACTCCTGTTCCTCGCTTCAGCTACAAGATGTCAG

TAAAGCCCAGTCAGGGTGGCGAGGCTCCCAGGGTCGTGACCTCCGACTCAGGAAGTATCT

ACATCTCAGGCCTAACCCCCGGAGTGGAGTACACCTACAGCCTGCAGCCCATCTTCAGTG

GACGCAGACCGGGCAGCCCCATCACACGCAACGTTGTCACACCTTTGTCCCCACCGAGTC

ATCTAAATCTAGTGTCCAACCCCGACACCGGCAACCTCAATGTATACTGGCAACCGACCA

CCACTCCCGACATCACAGGGTACCGGGTGACATGTACACCAACCACAGGGCAGCGTGGGA

ATTCTCTGGAAGAGTTTGTTCGAGATGGGGAGACCTCCTGTACGCTGGAGAACCTGAGCC

CTGGTGTTGAGTACAACATCAGCGTCTACGCTGTTAAAAACCATATGGAGAGCGAACCCA

TCTCATCGGTTATCACACAAGCCGTTCCCGCTCCCACCAACCTGAAATTCGGCGAGGTTG

GACCAGACTCCATGCGCCTCTCCTGGACCCGCCCCTCTGTCCGCCAGTCAGAGATCAGCC

GCTTTGTGATTCGCTATCATCCGAGCAACGATGACGACAATGTGCAAGAAGTCAATGTGG

GCGGCGCCACCAGCACCTACCTGCTTCAGAATCTGCTTCCCGTCACTGAGTACATGGTGA

GCGTGTCGTGTGTGTATAGTGAGCGAGAGAGCATCCCGATCACAGGCAGACAGACCACGA

TTCTCGACGCTCCCACCGCTCTTATCTTCTCTGATGTCTTAACACACTCTTTCACTGCAA

ACTGGCGAGCCCCACGCGCCCAGATCACGGGCTACCGACTGGTATACGAGGCGACCAGCG

GGGGCCGCCGTCATGAGGAGCGTCTGCCACCAAGCCGCACACATTACGCCCTGAACAACC

TGCAGCCCGACACACTGTACACCCTGCACATCTACGCCATCAGCGGCCGCCAGGAGAGTC

AACCTCTCACCGGCACACAGTCTACCATCTCGGATGCTCCCACTGACCTGGAGGTGACCT

TATCCACCCCAACCAGCATCACCATCTCCTGGGACGCTCCTGCTATTCCAGTGCGTTACT

ACAGAATCAAATATGGACAAAGTGGAGAACGAGGTCCTGGCCAGGAGTTCACTGTGCCTG

GGACAGACTCTACAGCCACCATCAGTGATCTGCAGCCAGAAAGAGATTACACCATCACCA

TTTACGCAGTGACCGGAAGAGGAGACAGTCCAGCATCCAGCACACCGACCATCATTACAC

ACCGCACCGGTAGCCACGATCTAGATGTGAGTGATGTCCAGGACCAGGCCATTATAGTGC

GCTGGACTCCTGCCAGAGGCCCGGTCGCAGGCTACAGGGTCACTGGGAAACCCAAAAAAG

GAGTGGGACCCACCTTCTCCAAGGAGGTGGGACCTGATAAGACAGAGTTGCGAATAACAG

GTCTGGTACCAACAGTGGAGTACGTAATCAGCGTCTATGCCATCAGTCGTGATGGAGAAA

GCACACCGGTGGTGCAGAAAGCAACTAAAATCGAAACACCAGACCGTCCCACAGACATGA

GGTTCCCTGATGTAGACAGTTCTGGGTTGACCGACCGAAGCTCCACCCCAGAGTCGGAGG

GAACACACACCACTGCAGGTGTTCCTTCCCCCACAAACATTCAGTTCGGAGACGTGGGCC

CGTCATCCTTCGTGGTGCTCTGGAGGCCACCAAGTGTCAGACTAAATGGATACCGTATTC

TTGTGACACCCAAAAATAATTTTGCACATCCAAAAGAGTTGAACGTCGCTCCTGATGCCA

TGCAGGCCACAGTCACTGGACTTCTGGTCTCCACTTTGTATGAGGTGCATGTGTACGCTT

TAAAGGATTCAGCCAGCAGCCCACCACTTATTGGAGAGATCTCTACAACTGAAGACATCA

GCCCACCCCGGCGCTCTCGCATCAACGAAGTGAAGGACACAAGCATTACGATCAGCTGGC

GCTCCAAGATCGAGCCAATGACAGGCTTCCTGATTGAAGCCAAGCCCCTTTCTGGAGAGA

GTCCAACTATCAGGAAGGAGATTCCAGCTGAACAACGCAGTGTCGTGATAACCGGTCTTC

AGCCTGCCACCACTTACTCAATCAACATCTACACACTTAATGGAGACCGACGCAGTGCAC

CCTTTACCCTAATCGCCAAAACAATTGGTTCATCCCTCCCGCCCCCCACTGACCTCCAGT

TCCTGGCTCTGACTCCGACTTCAATTTCCTTCAAATGGCAGCCACCCACCAGCCGCATTA

CAGGATATTACGTCACTTACGAAGAAGAAGGAAAAAGCCCAAGGGAGCTTACACCACGGC

CACATGCCGGTGCCAACTATGCCTCCATCACAGATCTGAAACCAGCCACTGTGTACATCA

TAAAAATCATCGCTCTGCAGAACACAATGAGAAGTCCAGCACTGGTGGGCAGGGCCACTA

CACAGGAATCAGACTTTACACTGCCTTCACGATCGCACAGCAGCCTTGGTCCCCTGGACG

TACCTGAGACGGAAATTGTGGTGAACGTGATGGGTCCGACAGTGCAGCCGAGACCAGACA

AGCACGGCCAGGGCATGGAGTACACCGAATACAACAATCAGCCCACTCTGCCTCACAGTG

GGCAACGGCCGAACCCCTACGTCCCTGGAACGGGCCAAACTCTGATCTATGTGCCCGCTC

CAGGGTCGGACGGTTCGCGAGTGCCCAAAGTGGTGCAACTGAGCGAAAGAAATGCTCACA

ATTTCCTTTTTCCAGAAAATAAGACGGGGACACCGCAGGAGGCTCAGACTCAGACTACTA

TCAGCTGGAAACCCTTTAGGCAGAGCAAAGCCTACATTGTGTCCTGCCAACCTGTCACAA

ATCGCAACGAGAAGATGTTCCAGATGCAACTTCCTGCCACCTCCACCAGCGCTACACTGA

TCGGTCTCACATCTGGCGCTTCATACCGCGTCCTCGTCGAGGCTCTGAAGGACGCACTTA

AGTACAAAATCCTTGACGAGGTCATAACCGCCGGTAACACAGATCCAGCAAGGGTTCCAG

CCAGCGACGATTCATGCTACGACACCATAACCGCTGCTCACTACAATGTGGGAGATGAAT

GGGAGCGCATGTCTGAGACAGGCTTCAAGTTGTGGTGCAGGTGCCTGGGACTGGGCAGTG

GTCACTTCCGCTGCGACTCCTCCAAATGGTGCCATGACAACGGCAACAACTATCGCATTG

GCGAGCGGTGGGAGAGACAGGCAGAAAACGGTCACATGATGAGCTGCACCTGCCTCGGCA

ACGGCAAGGGAGAGTTCAAGTGCGAACCACACGAGTCGACGTGTTACGATGATGGTAAGA

CATACCAGGTTGGCAACCAGTGGCAGAAAGAGTACCTCGGTGCAATCTGTACCTGCACCT

GTTACGGAGGACAGCAGGGCTGGCGGTGTGAAAACTGCAAAAAACCTGGAACTGAGATCA

ACACTCAAATGCTGAGGCCAGTCCGCTACGGAGACGGCATCGCCAAAGTGAACATTCACT

GCCCAATCGAGTGTCTCCGGCCGGACATCCTTGCTGACGCCGTGGCGAATCCCAACCCAA

GAGAATAAAATGATAAACACATCCCCTATTGAACCTACCCTGATACGTCAACTTCTAAAC

TGAACTACCCCTCCAGTGCCTTAAAAGCATTCCTATTGGTTGCTGAAAACACAGCTGCTG

CTTCCCAGCCAATCATCACAGCCAGCATGACACCTTAGCAATTCCATAGCAACACCAATG

CTTTTAATTGGTTGGTGCTATTATTCAGTCATTTGATTGGCTTACTAGTTTTTTTTTTCA

GTTTTAAACCATATGAAAAATGTTGCCAGATTTTATAAAAATCTATTTTAATTTTAAAAA

ATCTTTAAAGAAAAATTAAAAAAAAGTTTTTTAATTTTACAAAAACTGATTTGTGAGAGA

TTTGTGAGTGTTTTTCCAAAGGCTTTTTAGCACACTTTTTTAATTCACATACTTATTTTC

CTCTTTGGCATGAGTTTTTTCCCTCAGAGTCAGCACAGTTCTTTACATTGACATTTTTTT

TTTTACCGTCTAATGCTTTAAGATTTATTTTTTCCCCTTAAACATTTTCATTAGATGCAG

TTACCGTCGGTGTTGAAGTCTTCACTACTGTCAAAATTCCATTTTTAATAAAGATTTACG

AAGG

>Unigene000065

AGTTCAGATTTCCTGGTTTTGCACAGACCAATGTAGTATATAAACTACCTCCAAAGCACT

GTTTTTAATCACACATGCACACCAAGCCGCATCACAAGTCCATACAGCACAGTATCACTT

AAAGCTAAACCAGAGGTACATTTCTAAAAGTTAATTAGTGCCATTACGGTACATTAATTA

AGCTCATAGAACAGATAGAATCGGTAAAAAAGGCTCTTGTGTGTCTTTGCAGGACACACT

CGAATACAAGAAGAAACAGAGGCCACTCAAGATCCGCATGTTGGACGGGACAGTAAAAAC

AGTCATGGTGGATGACTCCAAAATCGTTACCGACTTGCTCATGACCATCTGTGCCAGAAT

AGGCATTACAAATTATGATGAGTACTCCCTGGTGCGGGATATCGGCGAGGAGAAGAAGGA

GGAAACCACAGGAACGCTGAAGAGAGACAAAACTCTCCTGAGGGATGACAAAAAGATGGA

GAAACTCAAGCAGAAGCTCCACACTGACGATGAGCTGAACTGGCTGGATCACGGGCGAAC

GCTGCGTGAGCAGGGAGTAGAGGAAACCGAGATGCTTCTCCTCAGGCGAAAGTTCTTTTA

CTCTGACCAGAATGTCGACTCCAGAGACCCAGTTCAGCTCAACCTGCTCTATGTACAGGC

TCGGGATGACATTCTGAACGGTTCCCATCCTGTCTCTTTCGACAAAGCCTGCGAGTTTGC

CGGTTACCAGTGTCAGATCCAGTTCGGTGACCACAATGAGTCCAAACACAAACCAGGATT

CCTAGACTTGAAAGAGTTTTTACCCAAAGAGTATGTGAAGAACAAAGGAGAGAAGAAGAT

TTTTCAGGCACACAAGAATTGCCAGAATATGACTGAGATTGAGGCCAAAGTCAGTTACGT

CAAACTGGCCCGCTCCCTCAAAACCTACGGCGTGTCTTTCTTCTTAGTAAAGGAAAAAAT

GAAAGGAAAAAACAAGCTGGTTCCTCGTCTGCTGGGTATCACCAAGGAGAGTGTGATGAG

GGTGGATGAGAAGACCAAGGAGGTGATGCAGGAGTGGAGCCTGACTAATATTAAGCGCTG

GGCTGCCAGTCCTAAAAGCTTCACTCTGGACTTTGGAGACTATCAGGATGGCTACTACTC

AGTCCAGACCACTGAAGGGGAACAGATTGCACAGTTGATTGCTGGATACATCGACATCAT

CCTCAAAAAGAAAAAGAGCAAAGACCACTTTGGCCTGGAGGGAGATGAGGAGTCGACTAT

GCTGGAGGATTCGGTTTCACCCAAAAAGTCGACAGTGTTGCAGCAGCAGTTTAATAAAGT

GGCGCGAGTGGAGACGGGCTCTGTGGCCCTGCCTGTCATCATGCGTCCAGGAGCGGGAGG

ACCGGAGAGCATCTACACAGGCTCCATGCCCAAGGCCAAGCAGCAAATCACCACTGGCCA

AATGCACACAGGACACATGCCACCGCTGACTTCAGCTCAGCAGGCACTGACTGGCACAAT

TAACTCCAGTATGCAGGCAGTGCAGGCGGCCCAGGCCTCTTTAACCGACTTTGACAGTCT

ACCACCACTGGGCACAGATGCGGCATCTCAAGCCTGGAGGAAGAACAAGATGGATGAGTC

CAAACATGAAATTCATTCTCAGGTGGATGCTATTACTGCAGGAACAGCATCTATGGTTAA

TCTTACTGCAGGAGACCCAGCAGAGACAGACTATACAGCTGTGGGCTGTGCAGTGACCAC

CATCTCATCCAACTTGACCGAAATGTCAAAGGGTGTGAAGTTGCTGGCAGCTCTAATGGA

GGATGAGGGTGGAAACGGACAGCAGCTTCTGGGTGCCGCCCGCAACCTGGCCTGTGCTGT

ATCAGACATGCTTAAAACGGCTCAGCCTGCCAGCGCAGAGCCTCGTCAGAACCTTCTGCA

GGCCGCAGGTAACGTGGGCCAGGCCAGCGGAGAGCTACTGCAGCAGATAGGAGAGAGTGA

CGCTGACCAGCAGTTCCAGGACATGTTGATGAAGCTTGCTAAAGCTGTAGCTACAGCAGC

AGCAACTCTGGTCTTAAAGGCAAAGAACGTGGCCCAGAAGACAGAAGATTCAGCCCAGCA

AAACCGGGTCATCGCTGCTGCCACACAGTGCGCCCTTTCCACCTCTCAGCTGGTGGCATG

CACAAGGGTGGTTGCTCCAACTATCAGTTCCCCAGTATGTCAGGAGCAGCTGATTGAGGC

AGGGAAACTAGTGGCTAAATCAGTTGAAGGCTGTGTGGAGGCATCACAGGGAGCCACCAA

CGATGAGCAACTGCTGAAACAAGTGGGTGTGGCCGCCACAGGTGTCACCCAGGCTCTTAA

CGAGCTGCTGCAGCATATCAAGCAGTACGCCAGCGGAGGCCAGCCAATCGGGCGCCACGG

GGAGGCAACTGACCGCATTCTGGACGTAACTGAGAACATCTTCAGCTCCATGGGAGATGC

AGGTGAAATGGTCCGACAGGCCCGGATCCTTGCTCAAGCCACCTCCGATTTGGTCAACGC

CATTAAAGCAGATGCAGAGGGAGAGACAGACCTTGAGAACTCTCGTAAACTGCTCTCAGC

TGCGAAGCTTCTAGCCGATGCAACTGCCAAGATGGTGGAGGCCGCCAAGGGTGCTGCCGC

CAACCCAGACAGTGAGGAGCAGCAGCAGAAGTTGCGTGAGGCCGCTGAGGGCCTGCGCAT

GGCCACTAATGCAGCAGCACAGAACGCCATCAAAAAGAGGCTGGTTAATAAGCTAGAGAA

CGCTGCAAAGCAGGCAGCTGCAGCCGCAACTCAGATCATAGCTGCCGCCCAGCACGCTGC

CTCATCTAATAAGAACCCGGCTGCACAACAGCAACTTGTACAGAGCTGCAAGGTGGTGGC

AGACCAGATCCCCCAGCTGGTGCAGGGAGTCAGAGGCAGCCAGGCACAGCCTGACAGCCC

CAGTGCTCAGCTTGCCCTAATTGGAGCCAGCCAGAACTTCCTGCAGCCTGGAGCCAAGAT

GGTGACTGCAGCTAAGGCTACGGTGCCCACTATTGGTGACCAGGCCTCTGCCATGCAGCT

CAGCCAGTGTGCCAAAAACCTGGCCAGTGCATTGGCTGAACTCCGCACGGCTGCCCAAAA

GGCTCAGGAGGCTTGTGGTCCTCTGGAGATTGATAATGCCCTGAATGCAGTAAGGGATTT

GGAGAAAGACATGCAGGAGGCTAAAGCTTCAGCAGCTGAAGGAAAACTCAGACCTCTTCC

TGGAGAAACGCTGGAGAAGTGCTCCCAGGACCTGGGCTCCAACACTAAGGCAGTGAGCTC

TGCTGTTGCCCAGCTTCTGAGTGAAGCTACACAGGGCAACGAGAATTACACCGGTATGGC

TGCACGGGATGTGGCCCAGGCTCTCCGTTCTCTTGCCTCCGCAGCCCGTGGAGTGGCCGC

AACCACAGACGACCCACAGGCGTGCAGCGCCATGCTGGACTGCGCTGGAGATGTCCTCGA

CAAATCAGGCAACCTCATTGAAGAAACCAAAAGAGCCATCGCTAAACCTGGAGACCCAGA

GAGCCAGCAGCGACTTGCCCAGGTGGCCAAGGCTGTCGCTCAGGCACTGAACCGCTGTGT

TAACTGCCTTCCTGGTCAGAGAGATGTGGATAACGCTATCAGGACTGTGGGAGAGGCCAG

CAAAAAGCTTCTTTCCGATCAGTTCCCAGCTAGTGGAAAAACCTTCCAGGAAGCTCAGGC

AAACCTGAACCAGGCGGCCGCCGGGCTTAACCAATCAGCCAACGAGCTGGTGCAGGCTTC

TCGCGGCACCACACAGGACCTGGCCAAAGCGTCGGGCAAATTTGGCCAAGACTTCAGCCA

CTTCCTGGAGGCTGGCGTGGACATGGCTGGCCAATCTCAGTCTAAAGAAGACCAGACTCA

GGTGGTGACCAACTTGAAGACCATCTCTATGTCATCCAGCAAGCTGCTGTTGGCTGCTAA

GGCTCTGTCTACTGACCCCAATTCCCCCAACCTGAAGAACCAGTTGGCTGCTGCTGCACG

TGCCGTGACTGACAGCATCAACCAGCTGATCACCATGTGTACGCAGCAAGCACCGGGTCA

GAAGGAATGTGACAACGCTCTGAGGGAGCTCGAGACGGTGCGGGGCATGCTGGAGAACCC

AACTGAAGCAGTGAATGACATGGGCTACTTTGACTGCATCGACAGTGTAATGGAGAACTC

AAAGGTATTGGGTGAAGCTATGGCCGGCATCTCCCACAATGCAAAGAACAGTAACCTGCC

TGAATTCGGAGACGCAGTCAGCTCGGCCTCCAAAGCTCTGTGCGGCCTCACCGAAGCTGC

TGCTCAGGGTGCCTATCTGGTGGGTGTCTCTGACCCTAACAGTCATGCTGGACAGAAGGG

GTTGGTAGATCCATCTCAGTTTGCCAAAGCCAAGCAATCGATTCAGATGGCCTGTCAGAA

CCTGGTGGACCCTGCCTGCACACAGTCCCAGGTCCTCTCTGCTGCCACTATTGTTGCCAA

ACACACCTCCGCCCTATGCAATGCCTGCCGCCTCGCCTCCTCCAAAACATCAAACCCTGT

TGCTAAGCGACAGTTTGTCCAGAGTGCCAAAGAAGTGGCAAATAGCACAGCCAACCTTGT

AAAGTCAATCAAGGCCCTGGATGGAGCCTTCAACCAAGAGAACAGACAGAAGTGCAAGGC

CGCCACAGGGCCATTAATCGAGGCTGTGGATAATCTCACTGCCTTTGCTTCCAATCCTGA

GTTTGCTAGTGTGCCTGCACAGATCAGCCCAGAGGGTCTGGCTGCCATGGAGCCCATCGT

GGCTGCAGCTAAAACTATGCTGGAGAGCTCAACAGGTCTGATACAGACCGCACGCTCTCT

AGCCGTGAACCCCAAAGATCCACCCAAGTGGTCAGTGCTTGCCGGTCACTCCAGGACTGT

GTCTGACTCTATCAAGAAGCTAATCACCAACATGAGAGATAAGGCTCCAGGTCAAAGAGA

GTGTGACGAAGCCATTGAGGTTCTCAATAACTGCATCCGTGAAGTGGACCAGGCTTCTCT

CGCTGCCATCAGCCAACACCTTACACCCAGAGACGACATTTCCCATGAGGCTCTGCATGA

GCAGATGGCAGCCTCGGTGCAGGAGATTAGCCACTTAATTGACCCAGTGGCTATCGCAGC

TCGTTCCGATGCCTCCCAGCTAGGACATAAGGTTTCTCAGATGGCGAGCTACTTCGAGCC

ACTCATCATGGCAGCCATTGGTACTGCATCAAAAATTCTCAACAGCCAACAGCAAATGAG

TGTTCTGGACCAGACAAAGACTCTGGCAGAGTCTGCCTTACAGATGCTCTATACAGCCAA

GGAAGCAGGAGGCAACCCTAAGGCGGCTCACACCCAGGAAGCTCTGGAGGAGTCTGTGCA

GATGATGAAGGAGGCCGTTGATGACTTGGGAGGTACAATGGCTGAAACAGCAAGTGCAGC

CGGAGCTGTAGGAGGCATGGTGGACTCCATCACTCAGGCTCTTAACAAATTGGAGGACCC

TGGAGTCGAGCCTGAGGGAACCTTTGTGGATTATCAGACTACTATGGTGAAAACGGCCAA

GGCTATTGCAGTCACTGTGCAGGAGATGGTCACTAAATCAAACACCAACCCTGATGAGCT

CGGAGGTCTAGCCAATCAGTTGACCACCGAGTTTGGTGACTTGGCATCAGAGGCTAAATG

TGCTGCCATCACTGCAGAAAATGCTGAGATTGGCTCTCACATTAAGAAGCAGGTAACAGA

GCTGGGCTATGGTTGCACTGGATTAGTCACCAAGGCAGGAGCTCTACAATGCAGTCCCAA

CGATTCCATCACTAAGAAAGAGCTGATTGACGCTGCCCGCAAAGTCTCTGAGAAAGTGTC

CCATGTGTTGGCGGCACTGCAGGCTGGTAACCGTGGTACCCAGGCTTGCATCACCGCTGC

CAGTGCTGTAGCTGGCATCATTGCTGACCTTGACACCACTATTATGTTTGCCACTGCTGG

TACACTTAACAGAGAGAATGCAGAGACCTTCGCTGATCACAGAGAAAACATTTTGAAGAC

TGCTAAGGTTCTGGTGGAGGACACCAAGCTACTGGTGTCAGGAGCAGGAGCCAGTCAAGA

AAAGCTGGCCCAGGCGGCCCAGTCCTCAGTCAACACTATCACCAAACTTGCCGATGTGGT

TAAGCTGGGGGCCGCCAGCCTGGGCTCTGAGGACCCTGAGACACAGGTGGTCCTGATCAA

CGCAGTCAAAGACGTGGCTAAAGCTCTGGGTGATCTTATTAGAACCACCAAAGCAGCCGC

TGGCAAGCCTCATGACGACCCGGCCATGCTCCAGCTGAAGAGCTCTGCGAAGGTGATGGT

TACGAACGTGACGTCCCTGCTGAAGACGGTGAAGGCTGTGGAGGATGAGGCCACTAAAGG

CACACGGGCGCTGGAGGCCACCATCGAGCACATCAAACAGGAGCTTGCCGTGTTCAGCAG

TCCAGATCCTCCTCCAAAGACAGCTACACCAGAGGAGTTTATCCGCATGACCAAAGGCAT

CACGCAAGCCACAGCCAAAGCCGTGGCTGCTGGAAACTCATGTCGCCAGGAGGACATCAT

TGCTACCGCCAACCTGAGCAGAAGAGCCATCGCCGAAATGCTGCACTCGTGCAAGCAAGC

GGCTTATCACCCAGAGGTTAATCTGGAGGTGCGGACCAGAGCCCTGCGCTTCGGAAAGGA

GTGTGCTAATGGCTATCTCGGCCTCCTGGAGCATGTGCTTGTGATCATCCAAAAGCCCAC

TCACGATCTGAAGCAGCAGCTGGCAACCTTCTCCAAACGAGTAGCTGGCTCAGTCACTGA

GCTCATCCAGGCAGCTGAAGCCATGAAAGGCACTGAATGGGTAGACCCTGAGGATCCCAC

AGTCATTGCAGAGAACGAGCTGCTGGGTGCAGCGGCCGCCATCGAAGCTGCTGCTAAGAA

ACTGGAGCAGCTGAAACCACGAGCCAAACCCAAGGAGGCTGATGAGAGCCTGAACTTCGA

GGAGCAGATTCTGGAGGCAGCCAAGTCCATCGCTGCAGCTACCAGTGCGCTGGTGAAGGC

TGCATCTGCCGCACAAAGAGAGCTGGTGGCCCAGGGCAAGGTTGGCGCTATTCCAGCGAA

TGCAGTTGATGATGGACAGTGGTCTCAGGGTCTTATTTCAGCCGCCCGCATGGTTGCCGC

CGCCACCAATAATCTGTGTGAAGCCGCGAACTCTGCAGTACAAGGCCACGCTAGTGAGGA

GAAGCTCATTTCCTCAGCCAAGCAGGTGGCGGCATCCACAGCTCAGCTGCTTGTGGCCTG

CAAGGTCAAGGCTGACCAGGATTCACAAACCATGAAAAGACTGCAGGCTGCGGGCAACGC

TGTCAAGAAGGCGTCAGATAATCTAGTAAAGGCAGCACAGAAGGCTGCTTTCGATGCGCA

GGATGATCAGGCTGTAATGGTGAAGAGCAGGATGGTGGGCGGCATTGCTCAGATTATTGC

TGCTCAGGAGGAGATGTTGAGGAAGGAGAGGGAGCTGGACGAAGCCAGGAGGAAGCTAGC

TCAGATCAGACAGCAGCAGTATAAATTCTTGCCCTCAGAGCTGAGGGAGGACGGTCATGA

GCAGTGAAGAGCTTCTTTCACACACACATA

>Unigene000066

TGAAGGTGGAGTAAGGTAAAGGGCAGGCTCCAGATCTCTGATTGGTTAAAAGGGGCACTC

GGGGCAGGATATACCTGCCTATTAAATAGAGCTCTCGAAGGTGGACTGTCGTGATGCTCT

GGAGATGATCTGTAACCTGGAGGCGGAGGGAGATGAGAGAGGAGCGTTCACACTTTGTTC

AGGCTTCCTGACCCGACAGCTTCTGCAGGAGGACTCGTACTGCGCCTGGGAACTGACTCT

TTTCTGGAGTAAACTCCTAAAGCGATTGGAGCCTTCAGAGCAGAGCTTTCTGGAGAAGTG

CCAGAAGATGTCGCTACTGGCTAAAACTGTCTTTCACCTGCTCTTCTTCATCAAAGTGAT

CCAGTCTGAGTTGGATAAGATTGGCTTGCCAGCCTGCATTGAGATGTGTATACGTGCTTT

GAGGATGGAGAACTGCGAGGGAGCTAACAAGGCCACCATCTGCAAAACCATCTCGTGTCT

ACTGCCTTCTGACCTGGAAGTTAAGCGGGCCTGCCAGCTAACAGAGTTCCTCCTAGATCC

GACAGTTGACTCTTATTACGCCGTGGAGACCCTGTACAATGAGCCGGACCAGAAGCTTGA

GGAGGAAACCCTTCTGATACCCACCTCTCTTCGCTGCGATCTTTTCCTAGTTTTTAAAAC

CCAATGGTCTTTTGATCCAGAGTTCTGGGATTGGAAGACACTAAAGCGTCACTGCTTAGG

CCTTATGGGTGAGGAAGCATCTATAGTGTCCTCTATTGATGAGCTAAACGATGATCGCTT

CTTAGAGATTGAGCGAGATGAATTGGATTCTGCTCAGGAGGAGTTTAAGGATGTTTTTGA

ATGCTTTTTGGACACAACAAAAGAACTTAAGGAGATATCGGATCATCGACAGAAAAACAG

AGAAGTTAAAAAGTTGAGGGAAAAAGGGTTTGTGTCTGCGAGGTTCAGGAACTGGCAAGC

GTACATGCAGTACTGTGTGTTGTGTGATAAAGAGTTCCTTGGTCATAGAATAGTGCGACA

TGCACAGACTCACTTCACAGATGGATATTATAGCTGTCCGATTTGCACAGAGACGTTTGA

AACGAGAGAAACTCTAGATCCGCATGTTGCATCACACGTAAAGTTGTCTTGCAAAGAGCG

CTTGGCTGCGATGAAAACCACTCGGAAACTGACTGGTGCAAAAAACTCTGCTTCCAATGA

TGCACCTCTGGATAACAAAAGTGGGGAAAATCAGGCACGAAAAGCGAAGGCTAAACACTG

CAATGGAGAATCTGATGATGTGTACAACATGGGCTCTTGCATTGAGGATGCAGGAGTCAG

GGTCCACAGGAAAGAGTTCAATGTTTGTCCTGTTTCAAGCTGCAGAAAAGTGTTTAAGTT

CTTTCGCAATCTTCTCACCCATGTAAAGGATCACGGAGACGTGGAGGAGGCTAAGCGCTT

CCTTGAATTACAGGCCACCAAGATAGTTTGTCAGTATTGTCGCCGACACTTTGTGAGTGT

AGGTCACCTCAATGACCATCTTCAGGTCCATTGTGGTGCGAAGCCTTATACCTGCATTCA

GTTGAACTGCAAGGCTAGCTTTGACACCAATGCCGAGCTCCTTGTGCACAGCAAAAGACA

TCCTTGTTTTAAGGCCAAATGCATGTTTCCTGGCTGTGGAAAGATTTTTAACGAAGCTTA

TAAACTCTATGACCATGAGGCGCAGCATTATAAAACATTCACTTGCAAAGTGCCCGGTTG

TGGGAAGGTATTTCATGCTCAGTCTCAGTTAGACATGCACAAGGAGAGCCACAATCCTCC

TAAATTGGAGACACAGCCCGCTGGGAGTGTCAGCCCGCTGGGAGAGCCTCAGTCGCACCT

CTCCAGTGAACCTGCCCAGCAAGATGCCCATTCAGGGCCTCCTGCCGACTCCGGCTGCAT

GATTAGTTCAGAAGATCAGTCAACCGGTCTAGTAAAAGTAAAGCACTCTATTGAAAACAT

GCTCAGTGTTGCTGGTCAAGGGCTCTCACAAGAGTTTGGCCATGTTAACTGCAAAGCCGA

GCCATCTGTTCCAAACCTGGTGCAGCAGCCACCAATGCCACACATGGTGAATACCGAGCA

AATCCAACCTCAATTAACCCCTTCAATGCCTTCCTTGATTGAACCGAGATCTGAAGAATC

CTTGTTAGATGCATTGATGAACGATCCAATGCTCACTTCATCTTGCTCACTTCCACCAGC

ATCTTCGTATCAGAGCCTCTTAGAGGATTTCCAGCAAGCTCCATCAGGAGATGTTTTACA

AGCTCAAATGGAGTCTGCTGTTTCCCAGGGTCACACCCAGTCTCTGTACAGTAATGAAAA

CACTGAATATGGCCAATACAATATCAAACCTGAAACCCCAATTTCATACCCACCTCCGTA

TGATAACACTGTACCCTTGGCGCAGAATGGTCATAATGATCCACCGGTACTGCCTGTATC

TCAGAGGCTTCCCCCACAGCTCACCATGTTTTCTTCGAATAGACCAGTGCAGGCACTGCA

GGCAAACACACTCAGTTCTGTAGTGGAAGAAAAAGACAGGTATAAATGTGCATACGAAAC

ATGTTCTCGAGACTACAGCTCGTATAGAAGTCTAACCAAACACATGAAAGCTGCTCATTG

TGAGTTTTATACACAGTGGAAACTGGCAAAACGGAGCAATAAAGTTTCACCAAACACTTG

CAGCGCGTCATCTATAAATGGAAAATGTATGTCAGCAGAACCCTCACAGAACCAGCTGAG

TCAGAGGACTCCTGCTCCTCAGAACCCAGTGCAGAAACCACCTGCTCAGACCCACAGCCC

AGCAAGCTGTCTAAGCTCCCATTACCCCTCTGGATCCTCCCATTTAACCGGTCCAAATGG

ACAGACTTTTCCAAATCAAATGGACAGTATTTTGAATCCCATAGTGCTCTCACACTTGGG

CAGCATTGCAAATCAGTCTAATGTAGTCTCTCACTCTTCGTGGAATTCCATCTCAGTGAA

CACATCACTCCGGCAGCAGACTTGCCATTCACAAGCAATGACCTCATCTAGTGGTCAGTT

CCCGTCCCAGTTAGACACTGTTCCACAAACCCAACTGTCAGATTCCTATAATAGAGTAAT

GGCCTACCCTTCTTTGCAACATCAAAACAGTCCCTCATGTCGATTCATACAGACCCGACC

ATGTGCCCCCCAAACAATAAACAGTCCTGCTAAAATAAGCCATCCCGGCGATTCCTCTTT

ACCTCAGTATGTTCAGCAACCGAAGACCAGTTCTAAATCCACACTTAAACATACAGACTG

TGTCGTAAAACTAGAGAGAAACCCAATTTTAATTCCATCCCATTCAAACTTATTACAACA

AAGCAACCCTCCTACTGCTATGAAACCTGAAACTTGTCAAAAAGATATCAGTGGTTTTGC

ACATGATGGGGATGTCAAAAAAAGGAAGCGAAGCAGCAGAACAAAGTGGCCTGCTATTGT

CAAAGACGGCAAGTTTATTTGCTGCCGGTGCTTTAGAGAGTTTCAGAGTCCTAAATCTCT

TGGTGGACATTTGTCTAAACGTACTGTCTGCAAACCCTACGATGAAGCTGTTTTGTCTGC

AGACCTGCCAAGTTCATTTCTGGAGCTTCTGAACTCCCCTCATCCATCGGAAACACCCCA

GTCCTCGCCGTCCACGCAGGTCACTTCTAGTAAAGCTTGGCCTTCCCTGACCAAAGGCCC

CCTTGACCCAAAATTGTTCCCCAATGTCACGTTCCTGAACACTAAAAATTCCACATACAA

CAGTGGAACCATCCATCAAAATTCACCGAGTCTGGAAAAGGGTGTTACAGGTGAGCAGAC

TTTTCCTGAGTCATCTGGGTCTTACCATCAAAAAAGCAAAAACAGTGTGATTCAACACAC

CGGAAATATTAGAGATAAGTGCATTTACGATCTAATGAATTATACCCAGCCATGCAATAC

CTTACCAAGTGACACCAGTTCCTCAGAACAACTTTTGTCTCACATGTTAAGTGAAAATAA

AACTTTAGACTCAAACTTGAGCAGAAGTCCTAATAACTATGTTGACAGAATCCTCCAAGC

TGAAACACTAAACAAGATTAGGGAGATTAAAGAAGAGCCTACCAGAACAAACATTAGTGG

ATTATCGAATGATGGGCTTCTTGCTGCAATGGCTAGTCTGGCCCAAAATCTGGTGTCAGA

AAAGAGCGTCAAGGAAAGGCTTCGTGAACAGATATTAGCTGGGGACTTTCAAAAAAGAAG

CAGTTTAGGCCAAGGGCAAGGAGTTGAAAACTCTAATTCACAAAGTCCGATGACTGATGT

TCCACTTGGACTTCAAACAGCTGTTCAAAGTAAGGAGCATATTGTCAGACGTGCTAGTGA

ACTTGGTGAACTTTACACCTCAGTTGTACCTGATGAGACCTTGACAAATATTTCCTTGGC

TGGTGCGTCTAACAACAGGGAGACAAACAATGCGTTTAGTAACAGCCTAGAACAAGAAGA

TGATAAAGATCTCTCTGAAATTCTGAAATCGTTTGAAAGATTGGATCTAAATAAGGAAAT

AGAAAAGAATGAGCAGACCACTTCCAACCTGCTAGAGGCTGAGAGAGTAACCAAGGCCTC

AGAAGCTACATGCACTCTGATGTTACGAGGCTATTCCTGTGACAACGATGGTTGTGGTTA

CAGAGCGATGACAAAAGATGCTCTTTTTAAACATCTTGTAAAACAGCATAACTACACAGA

AGACATGATGAATCAGTGTCGCAAGGAACAGTCTAACTTGGCACCTTTTAATTGTCAACT

ATGCCCCAAAACCTTTACAAGAAACTCCAACCTCAGAACCCATTACCAAGCCGTTCATAA

TTATTCACATGAGCAAGTGGTCAAGTTGGGGATTACACGGCAATACAGTCGAAAATCCAT

TGAGGCCGATCAAGGTCTTTGTACCACGCTGAACATCTCGGAGTTGGCCAGTCCAGTCTT

TGCTGGACAGAATGCCGAGTCTGGTGCCGGTATCACAGCTTTGGTATCTGGAGTTAGAAG

CATAAAATCAGAGTTCAGTGTTCAACCATTTGAACAACAGCATCCTGGTGCTTCCTTCGA

CTCATGTCCTGTTTCCAGTACAATACTGCGAGCGCAGACAACTCCACATCATCTCCAGAG

TCAGACTACTGTGCATTTATCAGTGATTAAAGCTACTCCTTCCTTGCTAACCTCCACAAG

TGTGGAGCAGCAGTTAAATCGACCAAATGAATTTGTTCCGGAATCGCTTGTGCCTTCTCA

GCACACTCAAGGATTTCTGTCTGGAGGTCTTTCGTCTTCAGGTGCGCCTCTTCTAGCCCC

GATTTCTTTGGCGTCAGCACAGCTAGGTAGCCTTTCCTCAGATACGAAGCCCAAATTTGG

AAAACCTAAAATGGCAAAAGTGAAGGAAGTGACAAAAAAACCTAAGGAGAAAAAGCTTGA

CATGGTTGATGTCTTTAGTCCCTATCGGCCTTACCGCTGCGTCCATCAGGGCTGTGTTGC

TGCCTTTACTATACAGCACAATTTGATTCTTCACTACAAAGCTGTACATCAGTCTGCCCT

CCCCAAATTTGAGGTTAATGAAGATGAAGAACCGATCGAGGAAGAACATAACGAAAACCA

GGAGGAGATTGGCATGCCTGACTCTGAGGTTAATGAGGAGTTCCGCTGTCAAGTCAAAGA

CTGCTCTCGAATATTTCAGGTCGTTACAGACCTGCTACAGCATTACCTTCAGCTCCACAA

ACTCGGCCTTGATAAAGCAGGTATTATGATGTCTGGTATGAACTTGGGCAGGTTTCGGTG

TGACCAACCAGACTGTCATGTGACATTCACTGGATTCTGGAAGTATGTTAACCACATTGA

TAATGAACACAAGCATGTAAAGCTCTCCAGGACTGAGCCTGTTAATGGCATGTTCCAGTG

TGAGGTTGAAGGATGTAGCTGCGTTTATGCAACACGGTCAAACTTGTTGCGACACTTTAT

GAAAAAACACCCAGAACTTTACAAACTCCGACTGCTGAACTCGGGAGGTGTCAAACTTGG

TCGTCCTCCGAAAAACAGATTTGTCTCCCTTGAGAAAGAAAATAGAGAAATCAATAAAAA

AACTTTTCAGAAGGGAGGTGCGAAAAAGAAAAGGAGACAAAAGAATCTTTGGACAAAGTA

TGGAAACCCTATTTTGAAATCGAAAGAGGAGGCTTCCTCAATGTGCACCAAGCGGATTCA

GTTACAGTATCCTTGCATGCTTAAAGGCTGTGAGACAGTTGCAGAGTCTGAGCGAAAGCT

TTTAAAGCACTATGTCCAACATGGCTTGCCAAAACAATACCTGGAGGAGCAGAGAAGCAA

CTACATATTCTGCAAAAAAAATGCCTCGCTCAAAGTATAAACGCATTGCGTCCAGGAGTG

ACGATACTGACAAGTCCGATGGAAGCTCGATTGAGGCGTCTGAGAACGAGGACGCAGCAG

ACACTGGACCCAGCGAATCGGAGTTCTCAAAGCCGACGTCTGAAAAGGAGAGCACGGAGG

ACACTGAAGTTTCAGACGCTAAATTGTCTAGTGACCTGAGCTCCGACATCTCTGTGGTGG

TAAAGCGGAAGAGAGGACGACCACGCAAGGGGGAACAGGTTAAGCGCACCCAGCTCGCAC

GCAAGAGAATGACGAGGCTACGAGCGGCGCAGACGCGCTCCATCAACTATGCAGATATGA

ACTCCGATTCCACTTCTTCCAGCGTTACGCTTCCACAAGAGCCAAACACATCACTGAGCT

CTTTCAAGCCAATGGGCTTCGAGGTGTCCTTCCTCAAGTTCCTGGAGGAATCGAGTCAGT

CTCCCGGGAAGAGAAAGAGTTGTTCTTTAATGGATTTACTTCCACACAGGAAATTCCCGA

CTGTGCGACTGAAGACTGCCTCGGTGGTATGCAGCAGGATCAACCCTGACTATCATCATT

GCCGAGATGTATTGAAACATGTCGAATTTAAAAACCCTCAGAAACTAACGTCTCTGAGTA

GGGTGACGTTCGAGGTGCACAGAGGGTTCTCCAATGTCTACGAACTGTTGCTCAAGCAGC

TGCATGACATGCGGCCTGCAGTGGTCATTCAGAAAGAGGGCGTGTCTGAGACGAGGGCGT

AGTTTTCAGGGTGGGGGTAGCTAATGTGAGAGTGGGTCAAGAGCTGTTCAGTGTTGCTGT

TTATGTGGAGCATCAGTGTTGAGATGTTTCCATGCCTTTGTAATGAACCGCTAGTCCCTT

TCCAGGACTGTATACTAAAGTGTACTATTGTAATGAAGTCGGTGAAGGATACGCCTCTCG

TGCATGCCCTAAAACACCTGATCTGGCACGTGTGCGCAGCTGTGGAGATAAAACCCTATA

ATGGAAGAGTGAAACTGCTCCGTGTAAGAATGTGAGCTCGCCTTTTGTTGGACTGACCGC

GTGTCGTGCAGTGGTGAAATGTAGATGAGTAACATTTTGGTCTTGTTCATCTCGCAGTGT

AAAATTTGATAGCGTTGTGTTTATCTGTGACTCTCACATACTGACTCTCCAGATAATCTA

CTGCCACCAGCAGTCTGTACCGCAAACAACAGAGACCAGTTTATTACAGCTCGCGTTTTC

TACCTCTGTGTGTGTGTGTGTG

>Unigene000067

TCCACATCTCCCCCTCCCCCTGCCATCGCTGTTCCCCTTCTCGAGATGGGGTTCTCCCTC

AGACAGATCATGAAGGCTCTGGAAGCTACAGGTGCACACACGGTCTCTAACACAAATACA

CACAATTTAAAATCCACCGTCTTTACCATCATTGTTTTTCTCAGGTACAAGAGGTGAGGC

TGACGCGCAAAACATCACGGTTCTGGCCATGTGGATGATCGAGCACCCGGGCAACGAGGA

CACAGAGGAGCGGACCCTGTCCTCCGACTGCTCGGGGGCAGCAGGAGGGGGCAAAAGTGC

GGAGCGGAGCTTCCTGCGTTCACCTGGAGATATTCCCAACGCTGATGCCGCAGAGCTTGA

AGAGGGATTTAGCGAGAGCCCTGAAGGAATAGATCAAGACTCCGCCTCTGCATCCAACGG

CCCCGCCCTCCGGGGGCGCTCAGCCGCCAGTAGGAAGCATCGCTTCGATCTGGCAGCACG

TACTCTATTGGCCCGTGCAGCTGGACTGTACCATTCGGTGCAGGCCCACCGCTCTCAGGT

GCGTAGGGAGGGCTCAGGGCTGCAGCAGGACTCCAGCTCACTGGGAGGAGTTTACGACTT

TAACCTGGATGAGGAGCTGGAGCTAGAGCTGGACCAAGAGGCCATGGAGGCCATGTTCAC

TCAGGAGCTTGCATCAGACTCGGATATTTTGGGAATGTGGATTCCGGAGGTACTGGACTG

GCCAACATGGCGTGTGTGTGAGTCAGAAGAGCGCGAGGAGGTGGTGGTCTGTGAGCTCTG

CGACGTCAGCGTGTCCAGTTTTAACCAGCACATGAAGAAAAGTCATCCTGGCTGCGGACG

GAGCGCAAATCGGCAGGGTTACCGCAGTAACGGGTCCTACGTGGACGGGTGGTTTGGGGG

AGAGTGTGGGAGCGGGAACCCCTATTACTTACTGTGCGGAAGCTGCAGGGAAAAGTACCT

GGGCATGAAGAGCAAGCACAAAGGAGCAGTGTCGGAGAGATACAAGGGCCAGGCTCCAGA

CCTGCTGGGAAAACAGGACAGTGTCTATGAAGAAGACTGGGACATGCTGGACGTGGATGA

GGCGGAGAAGCTGACAGGCGAGGAGGAGTTTGAGCTGCTTTCAGCACCGCTGGGACTCAC

GGAACGGAAACCCGTCCCCGAGGCTGTTCAGTTCCCTGACACAGACCCCCTCGGGGCCTC

CGCTGCCATGGTTACCGCCACCAATAGCATGGAGGAGACCCTGCTACAGATAGGGTGTCA

GAGTTCGGTGGATAAAAGCACATCAGGTCGCATGTGTTTGGGTGAGCAAGCAGCCGGTCT

CCAGAGTTCTGCTGATCGCATGGTTGCTTTGAGGAGAGTCACAGCAGCTGCTCAAGTCCT

GGTGGCAAGAACCATGGTGATGAGGGCGCTGTCTTTGCTGTCTGTCAGCGGTTCCAGCTG

CAGTCTCGCTGCTGGTCTGGAAGCTCTGGGTCTGACGGACATTAGGACCCTGGTGCGTTT

GATGTGTCTGGCTGCGGCCGGTCGAGCAGGTCTTTCTACCGGTCTCGCTTCAGGCCCCGG

CGCTTTAGAGCGGCCACGTGGCACGAACAAGCCCACCAAACCCATCTCCTGTCTGGCCTA

CCTCAGCACAGCAGTGGGCTGCCTCGCCTCCAACAGCCCAAACGCCGCCAAGCTTCTGGT

CCAGCTGTGCACTCAGAACCTGATTTCAGCAGCTACAGGAATGAATCTGACCACTGTGGA

TGACCCAATTCAGAGGAAGTTCCTGCCCAGTTTCCTGCGGGGCGTGGCTGAGGAAAACAA

ACTCATCACCTCGCCAAATTTCATAGTGACTCAGGCATTGGTGGCGCTCTTAGCCGATAA

AGGAGCCAGACTGAGACCCGGGTACGACAAGGCCGAAATGGAGAAGAGAGGCCCGTTGGA

GCTGGCCAATGCGCTGGCGGCGTGCTGCCTCTCATCCCGCCTCTCCTCACAGCACCGCCA

GTGGGCTGCGCAGCAGCTGGTGCGGACACTTGCCGCCCATGACCGCGACAACCAGAGTCG

ACCGCAGACCTTCGCCGACATGGCCGGTGACTTACGCAAGTGTTCTACCATCAAGCTGGA

GGCTCATCAGGGCAGAGTGATCAGCTTCGGGTGGTGTAACAAGAAAGGTCTTTTGGCGAC

CAGCGGTAGTGATGGAACAGTTCGAGTCTGGAACGTGAACAAGAACCAATACACTCTGCA

GCAGACCTGCGTCTTTAACAAAACAGACTTTACCTCTGAGGAGTGTTCCACCGGACTGGG

GTCTCCTGGTGAACCGAGCTTGGCGCCCGTGGCCTGGAGTGTGAGCGGGAAATTAGTTGC

AGCTGCCATGGAGAAAGTGGTGAACATCTGGCAGGTCAATGGCGGTAAGGCCTTGTTGGA

CCTGCAGCCTCACTGGGTGTCGTCGCTGGTGTGGCCGGAGAACGAGGCAGGGTGTCTTTG

GGCCGGAGAGTCTCGAGAGCTCCTATTGGTCGGTCGCATAGATGGAAGTTTGGGCTTGCT

GGAAATTTTAGATAGCTCAAACATACAGAGAACCGAACTTCTGCACTGTTACAGGAAAGA

CGCTGTGATGAACATCGCCTGGTACAGCGAGGATCGACCGTTTGCTGTCGGCTACGCTGA

CGGAAAGCTTCTGATTGGCTGCAAAGAAGCGCTGGAGAACGGATCCGTGGTCGTCATCGA

TGCTCACAAGGAGAGCATCAGTTCTCTGCGCTGGGCTCCAGGAGGGCAAATTTTATTAAG

CTGTGCCAAAGAGGAGACTGCCTGCCTTTGGGCGGAGTCTGGGGCGGGGCTTGGGCGGAG

TTGGGTGTGTCTCCAGAGCATCGCTCACCCGAGTGTTGTAAATGCTGTCGCCTGGTGTGG

TCTCACCGGACAGGGACCTAAAGCACTAAACATGCTGGCCACGTGTTGTCAGAATGGCTT

GGTGTCTGTGTGGACCGTGCCTCAGGACCCCACAGCCTTCTCTCAGTCTGGCTCATCCAG

CACTGATGCATGGTGGGAAACGGACTCCAAGTCAAAACCAAGGTTCACTCCACAGTGGCA

CGAGGCCGCAGTGTGCGTCTTCCAGCTAAGAGGTCACATGACTCCGGTTAGGACGATAGC

CTTTAGTCCTGACGGTCTTGCTCTGGTTTCTGGTGGTGTTGGTGGTCTCCTAAACATCTG

GTCTCTCCGAGATGGCTCTGTGCTGCAGACGGTGGTTGTCGGCTCAGGAGCGATTCAGAA

CACTGTGTGGATTCCAGACGTCGGGGTGGCCGTCTGTTCCAACCGCTCAAAGGACGTTCT

GGTTGTGAACTGCTCCTCGGAGTTTATGACCGCTAATCAGGTGTTAGCAACCTGTCGCAC

CGCGCTGAAGAAGCAGGGGGTTGTGGGATTGAACACGGCTCCCTGTATGAGGACTTTTCT

CGAGAGACTTCCCATAATGCTTCAGGAGCAGTACGCCTACGAGAAGCCCCATGTAGTGTG

TGGAGAGCAGCTGATCCACAGTCCCTACATGCAGTGTTTGGCATCTCTGGCCGTCGGGTT

GCAGTTGGACTCGTTACTATGTCGTCCCGCAGTGCCTCTTCACCTGTCTCATTGCCTCGC

CCAACCTGTCTCTTCATCATCGTCATCATCACAATCATCCTCCTCATCCGGTCCTTCAGC

ACTCATGCACTCATGTGGCAGCAGCGAGTGGGCGTGGCTTCACTGCTTCTCCACCACCAT

AAAGACGGCTGAGGCGCTCGCCCGCGCTCACACTTTCCCAGAATCCTTTAACGTGCCTGA

TCTGGAGCCGGTGGCCAAAGACAAGATGGCGGTGCTGATGGACAACAGTAAATGGGTTCC

TGGGATGGATGAGCAGTTGATGGCTTGGGCCACCTCCAGACCCGAGGACTGGCACCTGGG

TGGGAAGTGTGACGTGTATCTGTGGGGCGCCGGTCGCCACGGTCAGCTCGCCGAGGTCGG

CCGGAACATTCTGGTGCCGACGCCTGCTGCGTCTTTCTCTCAGGCTCAACAGGTTGTATG

TGGGCAGAACTGCACATTTGTGATTCAGGCGAACGGCACAGTGTCTGCGTGTGGGGAGGG

CAGCTACGGTCGACTGGGTCAGGGAAACTCTGATGACCTGCACATTCTTACGGTCATCTC

AGCACTTCAAGGGTTTGTTGTTACCCAGCTGGTCACGTCGTGTGGCTCTGATGGACACTC

CATGGCTCTGACTGAGAGCGGTGAGGTGTTCAGCTGGGGAGATGGCGACTACGGCAAACT

GGGCCACGGCAACAGTGACCGCCAACGCCGGCCGCGCCAGATAGAGGCTTTACAGGGAGA

GGAGGTGGTGCAGATGTCCTGTGGGTTTAAACACTCTGCCGTCGTCACAGCAGACGGTAA

ACTCTTCTGCTTCGGCAATGGAGACTACGGGAGACTCGGACTGGGAAACACATCCAATAA

GAAACTTCCAGAACGAGTCACAGCTTTGGAGGGACAACAGATAGGCCAGGTGGCGTGCGG

GCTAAATCACACACTGGTGGTGTCATCTGACGGGTCGACCGTCTGGGCGTTTGGAGACGG

AGATTATGGAAAACTTGGCTTGGGAAACTCGACTGCTAAATCATCACCGCAGAAAGTGGA

TGTTCTGTGTGGAATTGGAATCAAAAAGGTTGCATGTGGAACCCAGTTCTCTGTGGCTTT

AACCAAAGATGGAAATGTTTACACTTTTGGGCAAGATCGCCTGATCGGTCTCCCAGAGGG

GCGTGCCCGCAATCACAACCGCCCGCAGCAGCTCCCCGCTCTGGTGGGCGTGATTATCAA

AGATGTCGCTGTAGGGGCGGAGCATACACTCGCGCTGGCGTCTACAGGAGATGTTTATGC

TTGGGGGAGCAACTCTGAGGGACAGCTCGGTTTGGGCCACACCAACCATGTACGAGAGCC

GACTCTAATCACAGCTCTGCAGGGTAAAAACACCCAGCAGATTTCAGCAGGACGCTGCCA

CAGTGCCGCATGGACCGCGCCCTCAGTACCACCCAGAGCACCAGGCTCGTCCATGCCGCT

GCAGCTGGGTTTGCCCGTGTGTGTTCCTCCGCAGTACGGCAGTCTGAGAGACATCAGCAT

GGAGGCACTAAGAGCTCGGCTACGTCTGCTCTACCACTTCTCTGACCTCATGTACTCCTC

CTGGAGACTGCTTAACCTCAGCCCTAACAACCAGAGCTGCACGTCTCATTATAACCCTGG

GACGTGGGGTATCGTACAGGGGCAGCTGCGCCCTCTACTGGCTCCACGTGTGTATATGCT

CCCGATGGTACGCTCCATCGGCAAGACCATGGTGCAGGGCAAGAATTACGGACCTCAGAT

CACCGTCAAGAGAATCTCCACTCGTGGGAGGAAGTGTAAGCCCATTTTCGTGCAGATCGC

TCGGCAGGTAGTGAAGCTGAATGCGTCTGATCTCCGGCTGCCGTCTCGGGCGTGGAAGGT

GAAGCTGGTGGGGGAGGGAGCGGACGACGCTGGAGGAGTTTTTGATGACACCATTACAGA

AATGTGTCAGGAGCTCGAGAACGGGGTGGTGGATCTGCTCATCCCTTCTCCTAATGCCAC

TGCGGAAGTGGGCTACAACAGAGACAGGTTTCTGCTGAATCCCTCGGCTTGCCTGGACGA

ACATTTGCTGCAGTTCAAGTTCCTGGGCATCCTGATGGGCGTGGCCATCCGCACCAAGAA

ACCTCTGGACCTGCATCTGGCTCCGATGGTGTGGAAACAGCTGTGCTGCATCCCGCTGAC

GCTGGAGGACCTGGAGGAGGTGGACCTGCTGTATGTCCAGACACTCAACAGCATTGTGCA

CCTGGAGGACAGCAGCATCACAGAGCAGAATTTCCACGAGATGATTCCTCTGGATTCATT

TGTGGGTCAGAGCGCTGATGGGAAAATGGTGCCCATCATTCCGGGAGGAAACAGTATTCC

ACTCACCTTCTCCAACAGGAAGGAGTACGTAGAACGCGCCATAGAGTACCGGCTACATGA

ACTGGATCGTCAGGTGGCGGCCGTGCGTGAGGGCATGTCATGGATTGTACCGGTTCCTTT

ACTCTCGCTGCTGACCGCTCGGCAGTTGGAGCAGATGGTGTGTGGTCTGGCGGAGATCAG

TGTGGAGGTGTTGAAGAAGGTGGTTCGTTATCGGGAGGTGGAGGAGCAGCAGCAGTTGGT

GCAGTGGTTCTGGCAGACGCTTGACGAGTTCTCCAACGAGGAACGAATGCTGTTCATGCG

CTTCGTCTCCGGACGCTCGCGTCTCCCTGCTAACACAGCCGATATCTCGCAGCGCTTCCA

GATCATGAAGGTGGACCGGCCGTACGACAGCCTTCCCACGTCTCAGACCTGTTTCTTCCA

GCTCCGCCTCCCGCCCTACTCCAGCCAATCGGTGATGGCAGAGCGGCTACGCTACGCCAT

TAATAACTGTCGTTCTATCGACATGGACAATTACATGCTGTCACGGAATGTGGACAATAC

TGAGGGTTCGGACACGGACTACTGACCGAGAGTTAAAGCACAAAGACACTCGCTGAACTT

TAAACCGTCAACACAAAAATCACCACCTTTAATTCTTCTCTTCTTTTATTAATCATTAAA

GCAGTCAGACGGCGTTAACCAGGTACAGATAAATATAACCATTTCTGTTATTCACAACTC

CCCACACACACACACACACACACCGTTTTTTTTAAAAGGTTTTTTGTATTTCTTAATCCT

GGGGGAAAAAATATCAATAGCAATGGACTGGTCAAGGAAAACCATGTTGTACATTGTGTA

TAATGTTTCATGAGAAATTGTTTTAAGTTCATATTTATTTTGTTTTAATTTCAGATTTGC

CTTCGGTTCGTTTCAGCTCAGTGAGACGATTAAACGCTTTCAACTCTGAGGGTTTTTTTC

TTTTATTGCGGTTAAAATGCTAAAATCGAAGCTTTATGAAGCGCCACGTTTGATCAACTT

TCTTGTCTCACAGAGAAAAATCAAAAAACCATCAATGAAAGTTTAGGACAAAACACGACT

CGTCACTGAATCGGGACGTTGCGGCGGCGATGTTGTTGAAACGTTGTATTAAACATTAAT

AGCTTTTTCGGATGCATTTAGTGTCAACAAAATTTTAATATTTTTAAAGTGTGTGTGTGA

ACCTGATCAGGTAAATTTGCATAATGTTGAAGTTTTAAAACAGTTTTCTTAAAAAGGCAA

AAAAAACCCCAACTTAAACACGTCATGGCTTCTCTGGGCTTCCGTAGCTGATTGGGGGGG

TCTGGATTTGTGTGTGTGTGTGTATTTTTAAACTCAAGCATAAAACAGTTTAGATTAAAA

AATAACAAAGAATCGACACAAAACCGGCAGTAACGTGAGGTTTGTTTTTTAAAAAAAAAA

AACAACAAGCTGTTTTACAATCTTTCCCACTCAAATAATGAGTTGATGTACAAACACACA

CACACACACAAAGTGCTAAACACACACGATCCTCTGTCCCACAGCGGCGATCCAGTCGAG

TCAGAGATTTAAAGATCTGTGGAGTCTTTACAGAGAGAGAGACAGTCTGTTAT

>Unigene000068

TGGCCACCACATTGGAGCCCTGAGCATCCAGGATGACCCACTGCTCATGCTGGCTCTGAA

CTCAGCTAAGATTGCTAGCGATGCCCTGTACAAAAAGGACTTTAACAAGTCTAAGACCAA

ATTCCACCTTCCAGTGGACATGCTGGCTTTTGAACTGGCCAAGAAATGTCAGGTCCAGGT

CAATGATGCCAACTACAGAACTTACCTGCACAACTGGACCTGCCTGCCAGACTCCAATGA

TGTTGTTCAAGCCAGACAGACATATGAACTTCAGAGTGATGCTGTGTACAGGGCCGACCT

GAAATGGTTGAAAGGCCTGGGATGGATCCCTATTGGTTCACTTGATGTTGAGAAAGCAAA

GAAAGCTGGAGAGGCTTTGAGTGAAAGAAAATATCGTCAGCCCCCGAGCAACTTCAAATT

CACAAGCACAACTGACGACATGCCGATAGTGTTGGCGAAAGCCAATAATAATATCATGAA

CAAGCGGTCTTATATCCAAGCTTGGGAGAGTGATAAGACTAAGATCCACATTATGCCAGA

TGCAATGGAGGTTCTTCATGCCAAACAGAACAAGATCAACTACAGTGAGAAACTGTACAA

ACTTGCCAATGAGCTGGCAAAGAAGAAAGGCTATGACATGCGCGGTGATGCTATTTCAAT

TCTAGCAGCCAGGGCATCAACAACGATCGCTAGTGACTACAAGTACAAAACTGGTTACCG

TAAGCAAGTTGGCCACCATATTGGAGCCCTAAGCGTCCGGGATGACCCACTGCTCATGCT

GGCTCTGAACTCAGCTAAGATTGCTAGCGATGTCCTGTACAAAAAGGACTTCAACAAGTC

CAAAACAAAATTCCACCTTCCAGTGGACATGCTGGCTTTTGAACTGGCCAAAAAGAACCA

GATCCAGGTCAATGACGATAACTACAGAACTTACCTGCACAACTGGACCTGCCTGCCCGA

TCAGAATGATGTCATTCAGGCTCGCAAAGCATATGATCTTGCCAGTGATAATATCTACAA

GACAGATTTGGAATGGATCCGTGGCTGTGGATGGGTGGCAGCTGACTCTGTTGATCATGT

CAAAGTCAGAAAAGCTCAGCAGATCCTCAATGATAGAATGTACAAGAAAGATGCCAAGGA

TAATTTTGCCAAGTTCACAAATATTGTGGATCGACCAGAAGTTCTCTTGGCTAAGGTTAA

TGCCTTCAATCTCAGTGATCTGAAATACAAAGAATCTTTCAACCTTGAGAAAGGCCACTA

CATCGGATCTGACGATACACCCCAACTAGCACACTCCAGAGAGGCTGCAAAGATCATCAG

TGAGAAACTGTACAAACTTGGCTGGGATGAAGCTAAAGCCACAGGATATCAGCTGAATCA

TGAGTACCTGCCACTTGTATCAGCTAAGAAGGTCGGAAGCATCGTCAGTGAAGCTAAGTA

CCATGATGCGCATGAAAAAGCTAAGGGTCATTACCTGGCCAACACTCTGGTTGACTTCCC

AGCAGTGGTCCATTCTTCACAAATGGAAAAGATGAAGAATATGAGGACCTATCAAAAGGA

TTACCATACGAGCAAGACTAAGCTTCACATTCCACATGACATGATCAGCCATGTGGTAGC

CAAGAAATGCCAGGAAATCCTAAGCGATGTGCTGTACCGCAGCTACCTGCACCAGTGGAC

GTGCCATCCGGAACAAAACGATGCTATTCGTGCCCGCAAGGCCAATGAGATCCTGAGTGA

TGTGTTCTATAAAGATGATTTGAACTGGATGAAGGGTATTGGCTGCTATGCCTGGGACAC

ACCAGAAATTGTGCGAGCCAAGAAGTCTTATGAGCTTCAGAGTGACATCAAGTACAAGGC

AGAGGGCAAGAAAGAATTTAATAACTACTCTATAGTGACAGATACCCCTGTGTATGTGAC

TGCTATACTGGGCCACACATGGGCAAGCGAGCTGAACTACAGAGAGGCCTACCACAAAGA

GAAGCACCTGTACACTACTGTTTTGGACACCTATGATTATGCAAGGTGCTACAACTTGAA

GCAACTGTACAGCAGTAAAACCTATTCTGCTCTTTGGGATAGAATCAAGGCCAAGAGCTA

TACTATTCCTATTGATGCTAATGCTTTGGTCCACGCCAAACAGCAGAAGGTCCTCCTCAG

CAAAGTGAAATATAAGGAGGATTATGAGAAGTTTAAGTCCCTGTACAGCCTGCCCAAGTG

TCTGGCGGACGACCCAGCCACTGCCAGATGCATCAAAGCTGGAAAACTTAACTTGGATCG

CTTGTACAAAGATAAATGGGAAAAGACAAAAGCCAAAATTCATATACCTCCAGATATGCT

AGATGTGGTGGCTGCCCGCAACACCCAGAAAATGATCAGTGAAGTGGACTACCGTAAATA

TCTGCACCAGTGGATCTGCCTGCCTGACATGCAGGTCTATGTACATGCCCGCAAAGTCAA

CGAGCAGCTGAGTGATATATTTTACAAGGAAGATATGACCTGGCTAAAGGGTGTTGGATG

TTATGCATGGGATACTCCAGAAATTCTGCGTTGCAAACAGGCTATGAATCTGCAGAGTGA

GAACCTATACAGAGCTAAAGGAGTGGAGCATTTTAAGAACTTCTCTGTAGTAACAGACAC

CCCGGTGTATGAGACATGCAAGCAGAGTGCCCACAACCTAAGTGATCTCAATTATCACCA

TGACTATGTCACCAATGTAAGAGGTAAAAACACTGCTCCTGCTACGACTGTGGACACTGA

AAGGGCTCGTTTAGCCAACTACATCCAGAGTGACCACATTTACAAAGAGGCAAGCAAGAG

TAATATGTCCACTGGCTACTCTCTGCCCTTCGACACTCCACTTGCCCTGCAAGCCAAAGC

CAACAACGTCATTACCAGCAATGTAAAGTACAAGGAAGCATATGAAAACACTAAGGCTAG

GAGCTACAAACTGGACCCCAATGGTGTCAATTTCGTCACCATAAGAAAGGCCAACCAGGT

CACCAATCAAAGACTATACCGTGCCCTGTATGAAAAGGAGAAGGACAAAGTTCACTCTGT

ATATGACACCCCTGAGATCAAACAGGTTAAGGCCACTCAGGAGGCCATCAGTGATGTTTG

CTATAAAGAAAAGTACTACAACAGCAGAGGGACACTACTGCCAATGCCATTCACGCCACA

GCTGATGCACTGCGCTCATGTCAATCAGATTAACAGTGATCTGAAATACAAAGAGGATTT

GCAGTGGCTGAGAGGAATTGGTTGCTTCGTGTTTGATTCTCCTGAAATGGTCCGCATCCG

TGAAATCAACAAATTCAGGACTTCCTACGATATTGATGCCAAGAAAAACTTTTCCAACTT

CTCTGTGGTCTTGGACACGCCAGAGTACAAGCGTGTGTCTGAACTCAAGACGCACTTAAG

TGAGCGGGTGTACAAAGCTGCGGGAAATATACAGAGAACTCAGTGCAGCACAACTGGTGA

CGCTATGGAATTTAAGCGAGCCAAGTGGGCCCAGACTCTTACCAATAAGTGGCTATATAC

TGATTTGGCTTCCAAAGAAAGAGCCTTCTACACTCCAGAGCTTCACACTCCTCTTCTGGA

TCATGCTAGATCTATGAAAGTGGTGTACAGTGAGAACAAGTATAAAGAGCAGTATGAGAA

GATGAAGCACAAGTACACTCCCGTCCATGACACCCCCATCCTAATCCGTGCTAAAAAGTC

TTACCTTAATTCCAGTGACCTGCGCTATAAAGAGACCTTTGAGTTAGCCAAAGGACACTA

CCACACCACTAAAGATGCCCTGGACATTATTTGTGCTAAGCGGGTCAGAGATGACATCAG

TGAGGTTAAATACAGAGAGAAATACATCAACTCACTAGGAACATGGAAGTCTATCCCGGA

TCGTCCTGAATTCTTCCACAGCAAGGTTGTGCGAGACTGCATCAGTGATTACAAATACAA

AGAGGACCTGGAATGGATCAAAGGTGTTGGCTGTTTCGTGTGGGACACACCATTGCTGAA

GCAGGCTGAGCATAACAAAGCACTGTACAGTGAGAGATTGTACAAGGCCTCATATGAGAA

GACCAAATGCAACTTTAAGTACACATGCGACACACCATTCTTTCAAGCGGTTAAAAATGC

CTCCGCCCTCTCCAATGATAGATCCTACCGTGCATCCTATGAGAAGACTAAGGATAAGTA

CACCATAGTTGTAGATGACCCTAGAAACCTCTTGGCTAAAGAGGTCAACAATATGAGCCA

GTGGAAGTACAGGTCACAATATGAGCGCCTCAAGTCAAAATACACATCAGTGCTGGAGAC

CCCCGAACACAAAGTCCACAAGCTTAGAAAGCAGATCAGCGATAAAATCTACACGATGGA

GTATAATAAGAATAAGGCTAAGGGCTACACACTCGGACACGACACTCCTCTGAATCTGCA

CATGAAGAAGGTCAAAGAAATTACCAGCAACCTGAAATATAAGGAAGTGTATGAGAAAAA

CAAAGCTCAAATCAACATCGCTCCTGATGCTTTTGACATCCGGGCTGCTAAAGAAGCCTA

CAAGAACATCAGCAATCTTGACTACAAAAAGAAGTATGAGGCCACCAAGAATAAGTGGAT

TTGGACCGTGGATAGACCTGACTTTGTCCACGCTGCCAAGATCAACTACCAGCAGAGTGA

CGTTGAGTACAAGTACGACAAGGAGATGCTGAAAGGTTGCGTGATGCCTGTTACTGATGA

TAAGTACACACTTCTAGCCAAGCAGAACACAGAGCTTTCCAGTGACATTAAATACAAGCA

AAAGTATGAAGAGGCCAGAGGACATTACCACGTTGTACACGATACTCCTCAGATCCTCCA

TGCAAAATCAGTGTCTGGTCTGGTTTCTGAGAGCAAATACAAGCTGGAAAGCAAGAAGGC

TCGACAGTCTGGCTCTTTCACCACCTTGCCTGAGACCCGTGACACAGCCCACAGCAAAGC

AATGACCAAAATCACAAGTTCTAAAGTGTACAAGCAGAAGTTTGAGAAAGACAAAGGAAA

GTCGATATATAACAATATGACCGTACCTCCAGATGTAAAGCATGCCATGGATGTGGCCAA

GAGCCAGAGCAATATTAATTATAAACAGGAAGCTAAAGCCAAGTTGCATTACACACCTGT

TGCTGACCGCCCTGACATCCTGAAGGCTACCCAGGCTGCCAAACTCATCAGTGAGGTGGG

TTATCGTGACAAAGCTCGTCAGGAAGCCAGTCGTGGAGGATCGCTGGTCAACCGTCCTGA

TATCAACCTGGCCACCGAAGTGTCCAAGCTTACTAGCCTGGTGAAATACAAAGAGAAGTT

TGACAAGGAGATGAAAGGAAAGAGACCACAGTATGACTTGAAGAACAGTAAGATTTATCA

GACATTGAAGGATGCCAATACGCTTGCAAGTGAGGTGAAATATAAGGGTGACCTGAAGAA

GATTCATAAGCCTGTGACTGACATGTCTGAGTCTCTGGCCATGCAGCACAACCTGAGTAC

CAGCAAACTGGCCAGTAGTGTAAAATACAAGGAGAAGTACGAGAGGGAGAGAGGCAAGGC

CATGCTGGACTTTGAGACTCCGACTTATGTGAGCGCCAAAGAAGCACAGCATATGCAGAG

CCAGAGAGAGTATAAGAAGGCATTGGAGCAGGAGATAAAGGGCAAAGGAATGTTGGCTTT

GGCTAACGATACTCCAGACTTCATGAGAGCAAGAAATGCTACAGAAATCCTCAGCCAGAG

TAAATACAAGCACAACGCTGAGCAGGACAGGGCCAGCTATACCACAGTGATTGACACTCC

CGACATCATCCACGCTCAGCAGATCAGGAATATTGTGAGCCAGAAAAAGTACAAGGAGGA

GGCAGAGAAGACCATGTCCCACTATAATGCAGTACTGGACACTCCTGAGATGCAGAGAGT

GCGGGAGAACCAGAGGAACTTCAGCACTATAAAGTACAAAGACATGTTGGGGCAAGGAAC

AGCTATATCGGACCTCCCTGAGGTGAAGCGAGTCCGAGAGACTCAGAAGCACATCAGTTC

GGTTTTGTATAAAGAACCTTCTGGCAAAGGAACTTCTGTCATATTTACACCAGAGATGGA

GCGGGTCAAACGGAACCAAGAACATATTAGCTCGGTTCTATACTCCGACAGCTTCCGCAA

GCAGGTGCAGGGTAAAGCAGCCTTCGTACTGGACACTCCTGAAATGAGACGTGTGAAGCA

GACTCAGCGTATCATCTCTGGGGTGCAGTACCACCAGGACTTTGAGAAGAGTAAGGGCAG

CTTCACTCCCACTACCACTGACCTGGTGACTGAACGTGTTAAGAAAAACACTCAGGACTT

CAGTGACATCAGTTATCGTGGCATTCAGAGGCGTGTGGTGGAAATGGAGAGGAGACGTGC

TATAGAACATGACCAGGAAACCATCACTGACCTGCGTGTGTGGCGTACAAACCCAGGCTC

TGTGTTTGACTATGACCCTGCTGAAGATAACATCCAGTCTAGAAGTCTCCACATGATGTC

AGTTCAAGCTCAGCGCCGTAGTAAAGAACACTCTCGCTCGACAAGTGCCATGAGCGGCTT

AGGAGAAGAGAAATCTGAGCCGTCCGAGAGCGTGGACCACCACATGTCCGTCTACAGCAA

CGGATTCCAGACTCATACTACGGGATACCAGCAGGCCAAGACAGTTGAACTTCAGCAAAG

ATCCTCTTCTGTAGTCACCCAGCAGACCACTGTGTCCTCTGTTCCCTCACACCCCTCCAC

TACTGGGAAAACTGTCCGTGCCATGTACGACTACGCTGCTGCCGACAATGATGAGGTGTC

CTTCAAAGATGGAGACGTTATCGTGAACGTGCAGTCCATCGATGAAGGTTGGATGTACGG

CACAGTGCAGCGTACTGGAAAAACCGGCATGCTGCCTGCCAACTACGTAGAGGCGGTCTA

AAGACTAGTGCGAGTACGACATCCACACAGCTGAGCGTCTCCAGCATTTCCCTAGTTTAC

GTGTAGTTGATGGTAGTTTAGAATAAGAATAGCACTGACCCCTGTTCGTCTGCATTAACA

GTATGTGTAATTCGCTTGTGCCAGTTTCAGTGGATGTGACATGACACTATGTACTAAACC

CAGCAGGACATTTAAAAGACCATTATTTTGTATATTAATGACAAAAAAACATTAAGTTGG

TTTCAATGCAGGCTTTCTGCATTGAATGTTTCACACCAGAAGAGATGAGCTTATGAGATG

TAAAAAAAAAAATAAAAAATCCGACATTCGCTACACTAAAAAAAAAAGACGTTGTTAAAC

AGATTAGGAATAAAAATAAAAATCGTGAGGAGAAACCATCAGTTCCACACAAATTAAGAA

TGATACACTAAAGACTCCCGAGTAACACACCTCTTCCCTCCTTTGCTGAAGGCTAGTTAC

AAGTTACATGTGTGTTTTGCTTCTTCTAATTCCCTTGGTTTAGGGTTAGAGTCATATTTT

GACTCGGTAGCTAATAAAGCACACGAAGCTTCTGCAGT

>Unigene000069

GGTCGCGTGGTGCTGGTCGCTGAGGGAGAAGTCAGGCCGAGAGGTTCACTGCAGGATTTA

TTATTATTTTTTTTACAACATTTTACATGTTTTAACGAGAATTTGCCATTCTGCGGCTCA

GATCGAAGCCGGGTAGCGCGGAGAGGAGCAGGAGTGAAGAAAAAGACAATTAAAAGAGAG

ACTTTCTTGTGGTTTAAGTTGTAAAGCACGTTCAGGTTGGGGGAAAGGAGGACCGGAGAC

ATGGCCACTCAGGTGGAGACTCTCCTGCATACCACCATCAACACCACCACCGCCGCGCTC

CTGCAGGACGATGACCTCCAGTCGCCTGTAGGACATGGAGAGCTCTCCCCGGTGCCGAGC

TCGAAGCAGGCCGGGAATGAGAACAGCCCGCAGGAGAACATGGAGGAAAAGAAAGAGTTC

AAAGAAGCGCCTCCGCCCAAAGTCAATCCCTGGACCAAGAAACTCAACTCGGTGACTGTG

AATGGTCAGACCGCGCCCGAGCAAACTGGACCAGCAAAAGTGGTACGAGCAGGAAACCCC

CGACCAAGGAGAGGAGGAAAGGTTGGAGATTTTGGAGATGTGACCAACTGGCCGACACCT

GGAGAGATTGCCACCAAGGAAGTGCAGGGCTGTAAGAAGGCGTCAGTAAAAAAGGACTCG

AGAGAGAAGCGGGACAGTGAGGAATGTAAAGAGAACCAGAAGGCCAAATCAGATGATTCT

GGAGAGGAGAAGAACCGAGAGGAAGACCTGCACAAGAACAACGCTCACCGAAATAAACAT

AAATGGGTTCCTCTGATGATCGAGGTGAAGTCCGAAGGACCTCGGGAGCGTTCGGCTTCC

AGAAACAATGCACGACAGGGGGAAACTCCCAGAATGCACCACAGCAACAGGAATGACCTG

AGAGACTGGCACAGTGGAAAATACGAGCGTGATCAGTTCCGAGACGATCATGACGAGGTG

TCGAGTGTGAAGAGTGAGGGAGCGCCGTTCAGAGGAGGCTTCAGAGGACGAGGAAGAGGA

CGCGGTCGAGGAAGAGGACGGGGACGGGGAGGACGAGGTCAGTATGACTATCCATACAGC

TACAAGTGTGAGGGAAAAGACGGTGTGTATGACCCCAAATACACACAGTCAGTGACCTAT

TACTATGACAGCATGAGCAGCGCTGAGCTGTACAGCGTGGACCAGGACCTGCTTAAAGAG

TATATCAAGCGACAGATTGAGTACTACTTTAGCATGGAGAATCTGCAGCGGGACTTTTTT

CTTCGGCGAAAGATGGATGAGGGTGGATTCCTGCCTGTAGCTCTGATCGCTAGCTTCCAC

CGTGTTCAAGCTCTCACCACTGACATTCAGCTCATCATGGAGGCTCTAAAGGACAGTAAG

GTGGTGGAGATTATTGACATGAAGATTCGATGTAAAGAGGAACCGGCGAAATGGCCTCTT

CCTGGATTAACTCTTTCTGAACACACACACACGGACTTCTCCCAACTCATCAACTGTCCT

GAGTTCATCCCCCGCAACACTTCAGACACGCCTCACACAGGTTCACCTCGTGTGTCGTCT

GCTGCTGAGCAGAAGTTGGAGGAAGTGTGTAACCTCAAGACTATGCCAAAGGGTTTATCA

GCGAGTCTCCCTGATCTTGACTCGGAATGCTGGATAGAAGTGAAGAAACGGCCACGCCCC

TCACCAGCCAGACCCAAAAAGCTGGAGGACCTTCGGAGTGCCCTTACTCCAGGGGATCAG

GACGAACCAGAGGAGCTGGACTTCTTGTTTGATGAGGAGATGGAGGCGATGGATGGCCGG

AGGAACACTTTCACGGAGTGGACGGATGACGAGTCGGACGGAGAGATCGACGACCACGAC

GTCAACAAGATCCTTATTGTGACTCAGACACCACCGTACCTGCGCAAACACCCAGGGGGC

GACCGGACTGGCAACCACGTCTCCCGTTCCAAACTCACTAACGAGCTTGTTAAGGTTATT

AACGACGGCCTGTTCTACTATGAACAGGATCTGTGGCACGACACGTATGAGCCTGAGTAC

GCCACCATCAAGCAAGAGGTAGAAAACTTTAAGAAGGTTCATCTTATTAGCCGTGAGCAG

TTTGACTGCTTGACCCCTGAACCCCCCATCGACCCCAACCAGGAAGTGCCGCCCTGCCCG

CCCCGCCCACAACACATTCCCACGGATGATCTGGCGAATAAACTGTTTGGTGCTCCGGAG

CGTTCTGTGATGGCACGCTCTCTCCCCACCGCTGTGCCTGACTCGCCGGCATACAGAGCA

CCGCACACACCTCACACACCCCGCACACCCCGATGCAAACACAACACACCTCGGTTTTAT

CCTGTCATAAAGGACGGCAGGCCTGTGGATGCCAAGACACCACGGAAAAGAAAAACACGT

CACAGCTCAAACCCCCCAATGGAGTGTCATGTCGGTTGGGTGATGGACTCCAGAGAGCAC

AGGTCACGGACTGCCTCTGTCAGCTCTAACGCAAGTCCATCTGAAGGCACAGCGGCGCTG

GGAAACATCGGCTGCACTCCTCAGTCTTTACCTAAATTCCAGCATCCGTCACATGAGCTG

CTAAAGGAAAACGGCTTCACACAGCACGTCTACCACAAATACAGACGACGCTGTCTCAAT

GAGCGTAAGCGTTTGGGAATCGGTCAATCTCAGGAGATGAACACACTCTTCCGTTTCTGG

TCATTCTTCCTGCGTGATCACTTTAACAAAAAAATGTATGAAGAGTTCAGACAGCTGGGA

GTCGAGGATGCTAAGGAGGGTTACAGGTATGGCTTGGAGTGTCTGTTCCGGTATTACAGT

TACGGTCTGGAGAGAAAGTTCAGAGCTGAAATCTTTAAAGATTTTCAGGAGGAAACAATC

AAAGATTATGAAGCAGGTCAGCTGTATGGTCTGGAGAAGTTCTGGGCTTTTCTTAAATAC

TCTAAAGCGAAGAATCTGGAGATCGACTCCAGACTGCAGGAGTATTTGTGCAGCTTCAGA

CGTCTGGAGGACTTCAGAGTTGATCCTCCTATGGATGAGGGTCGTAAACGTCACTCCTCT

AGTGGTGATGGACGTCGCCGTCACCCGTCTCAGTCCTCTGCTCGCCACACCCATACCAGT

GACCACAACCTAGACCCTAAGCCTGGTCCGGCTCCCACTGCTGACTGTAGCAAGTGACTG

ACTAAGTGAAACCCACACCGCCGTCTTCACCGTTACCCGCCCATTTATCATCCCCCTCTG

GGGTCCAGTCGTTCCCGCACGCATCATGCGCTGTTCGAGTGGCTGTGCGAGGACATCCAG

CTCTTTCCTTCAGACATTAGTGATCTAATTTCACACCTAAGAGGCTGCTGAAGGGAACCT

GTTCCACTCGTCACTTTTATTTTCATCTTTTTTTTTTTTTTTAATTTGAACATCACTCGG

TGTGACCTTTGATATTTACAGCCTGAACCCTTCTGATTGGCTGGGAAATGGGCAATGAGG

CGTTTTTGTTTTGGGTTTTCTGATTTGGGACTCAGTTTGTTGATAAATGATGAGGGAGGA

AGACTGAACATTATAGCCGAACCCTCAGGACATTTCACATCGCTTTTCCCCCCCACAGTT

GCCTTATTTTACGGAGGTTTTTTCTGCTTATCGTGCGCCGGACACCTTAAACCCCCCCCC

CCCTTTCCCCATCCACATCACCTCTTATTTGTAGCTGAAAACACACACAGCTGTTGTAAG

CAGACGCTTCCCCTCGTTTCTGCGTCTTCCATGTGCTCCGTTCTGGCCGTTATCGTCTGT

TGCCGTGTGTTTGTCTCTGTGTCGTCCCCCAGTGTCCAAACTTAATCACAATTACTTGAA

GGAGAGAAGCAGGTAAAAACATCTGATCATGCTGCAGGTGCTCAAGGACTTGAAACTCAT

GCACAAGATGTAGAAATGGGGAAAAAAAAAAAAAAAAACCAAAAAAAAAAAAAAAAAAAA

AATTGTCTTGTGGCTACCTTCTGAGGAACAAAAAAAATGTAAAGGAAAAAACAGAGGAAA

AAAAAGTATTTGACAAAAATAAGTTTTATTTAAACTTCCTCTGTACAACTTAACATCGCC

CATCACTGTCATTAAAAAAAAGAAATATGGATTGGATGCAGTAAGCAAACAAACACAGTA

AGAACCCTCTAACTCTTACTTGTTAATTATTTTTTTCCTGTTGCTTTAGTTTATCATTTT

GTTTTTCCTCTTGATTTGGAGTTGCAGCGTTCTGCTGAGCCGATGATGCGCTGTGAGTCT

GCAGTGACGCATGGGTTGCGCAGCGACAAAAACGCTCTCTATTGCGATTCAACAGCGCCT

GCGTGCGACAAATGCTCCCTTTTCCCTTGCCCTCCCCTTTCGGCCTGCGGACCACAGATC

TGCCCGTCTAGCCCGCTAGTATGCCACCCAGCGGACGCCATGCCCAGTACAGGACACTAT

TGGCAAGCAGTTTAATTGGTTCGTGTTGTGCTTACAATACATTCTAAAAGTCCAAACAGC

AGTCAAACCGAACTTGTGTTCCTGAGGGTTGGTTGGATCCAGAGAGTGTGTCAGTCTGGT

GGGCCCAGGTCTACCTGCGGCTTTAGGTCTGTGGTTTGTTCACTGGCCGACGTAAGCGCA

GCGAAAAGCGACAAGCAAGCACAGTGCGACCGCGGCGTGGCAAAACACAGTGGCCGCCCG

TGTGGCTATTTTGGCGCCTGCATTACAGGGACCTTCACCTACACTCTGCACAGGCATTTA

AGGTTGTGACTATAGTCTAGCTAAATCTGTTGGGGCTTTTGGCTTTGAAATTTCTTTTCA

TGTGTAGCCGCAGCCTGGGTTTCTGTAGATTTTAATGCGTCTCAACGGTTTTCTTCCAAT

AATGAGACCATAACTGAAAAATGTCCGTCAGGGTGAAGGTGCTGGTCAGATTTGCGCAGC

GCAGGAATAAGCATGCAGCTTAGCGCGGCAGTATTCTGCGCATGCGAGCCCACGCTGTAG

ACTCACTTTTTGCTTTATGGAGTACGGGTTCTAACTCTGGTTTTCTCTGCCCTTTTTGTT

TTCTTCCTTCTTTCAACCTCTTATTTTTTTTTTTTTCTTTCAACCCCACATCTCTCTCTC

TCTCTTTCTCACTCTCTTGCTCCTCTATTACTCTTTTTCCTCTCTTCCTTCTGTTGTTTT

CATCGTGAACCTATTAAAGGCTTGCTACCTGAAGGCAATCGAGACTCAGCCAAACTTTGC

GGTGGCATGGAGCAACCTGGGCTGCGTGTTCAATGCTCAGGGCGAGATCTGGCTGGCCAT

TCATCATTTCGAGAAGGCGGTAACATTGGATCCAAACTTTTTGGATGCTTACATAAACTT

GGGCAATGTGCTTAAAGAAGCCCGCATCTTCGACAGAGCTGTTGCTGGCTATCTGCGAGC

CCTCAGTCTGAGCCCCAACCATGCTGTTGTCCATGGCAACTTGGCCTGCGTGTACTATGA

ACAGGGTCTTATCGATTTGGCCATCGACACCTATCGACGTGCTATTGAGCTGCAGCCTCA

CTTCCCTGATGCTTACTGCAACCTTGCTAATGCGCTTAAGGAAAAGGGCAATGTGTCTGA

GGCTGAGGAATGCTACAACACAGCGCTGCGTCTTTGCCCCACACATGCTGACTCGCTTAA

TAACCTGGCTAACATCAAACGGGAGCAAGGCAACATTGAAGAGGCTGTGCAGCTCTACAG

GAAAGCTCTTGAGGTGTTCCCAGAGTTTGCTGCAGCCCACTCCAACCTTGCCAGTGTCCT

TCAACAGCAGGGCAAGCTGCAGGAAGCCCTCATGCATTACAAAGAAGCTATCAGAATCAG

CCCCACCTTTGCTGATGCCTACTCTAACATGGGAAACACACTGAAGGAGATGCAGGATGT

GCAGGGGGCGCTGCAGTGCTACACACGCGCTATTCAGATCAACCCTGCGTTCGCAGATGC

TCACAGCAACCTGGCTTCAATACACAAGGACTCTGGGAATATTCCAGAAGCTATCGCATC

TTACCGCACGGCATTGAAGCTCAAACCTGACTTCCCGGACGCTTACTGCAATCTTGCACA

CTGTCTGCAGATTGTGTGTGACTGGACCGACTATGATGAGCGCATGAAGAAGTTGGTTAG

CATTGTGGCTGACCAGCTGGAAAAGAACCGCCTGCCCTCAGTCCATCCTCATCACAGCAT

GCTGTACCCTCTTTCTCATGGGTTCCGCAAAGCCATTGCTGAAAGGCATGGCAACCTCTG

CTTGGATAAGATCAATGCTCTCCATAAACCTGCATTTGAGCATCCTAAGGACCTGAAAGC

CAGTGGGGGGCGTTTGCGCGTTGGCTATGTCAGCTCAGATTTTGGCAACCACCCCACCTC

CCATCTCATGCAGTCCATACCTGGCATGCATAACTCTGAAAAGTTTGAGGTGTTCTGCTA

TGCCCTCAGTCCTGATGATGGCACAAACTTCAGAGTGAAAGTTATGGCTGAGGCTCATCA

CTTTACTGATCTTTCCCAGATTCCCTGCAATGGAAAAGCTGCAGACAGGATCCATCAAGA

TGGAATTCACATCCTGGTCAACATGAATGGCTATACCAAAGGTGCTCGCAATGAGCTGTT

CGCCTTGCGCCCAGCTCCTATTCAGGCCATGTGGCTTGGCTACCCGGGCACAAGTGGAGC

TCCTTTTATGGACTACATCATTACCGATAAGGAGACGTCTCCAGCTGAGCTGGCTGAGCA

GTATTCTGAGAAGATGGCCTACATGCCTAATACATTCTTCATTGGGGATCATGCTAACAT

GTTCCCACATCTTAAGAAAAAGGCTGTGATTGACTTCAAGTCCAATGGACACATTTTTGA

CAATCGCATCGTGCTGAATGGAATTGATCTCAAAGCCTTTTTAGACAGCCTACCCGACGT

CAAGGTCATCAAGATGGAGGGTGATGGCCAGGAGTCCACAGATATTAACGGAGCCCTCTC

CATGCCAGTCATTCCAATGAACACAGCCGCTGAGGCCATTATAAACATGATCAATCAAGG

CCAGATCCAAGTCACTATCAATAGTTTTACAGTTAGCAATGGCTTGGCTACCACTCAGAT

TAATAACAAAGCTGCCACAGGAGAGGAGGTTCCTAGAACAATAGTTGTGACCACTCGCTC

CCAGTATGGTCTGCCTGAGGATTCCATTGTCTACTGTAACTTTAACCAACTCTACAAGAT

TGACCCTCCGACTCTGCAGATGTGGGCTAATATTCTAAAGCGTGTGCCAAACAGTATACT

CTGGCTGTTGCGCTTCCCAGCTGTGGGTGAACCCAACATCCAGCAGTATGCCCAGAACAT

GGGATTGCCTACTAGCCGCATCGTCTTCTCACCTGTTGCCCCGAAAGAGGAACATGTAAG

GCGTGGCCAGCTGGCTGATGTCTGCCTTGACACACCTCTCTGCAATGGCCACACTACTGG

CATGGATGTGCTCTGGGCTGGCACGCCAATGGTCACAATGCCAGGTCTGGGTAATAGATT

TACAACAAACACAAG

>Unigene000070

AGATCGGAAGAGCGTCGAGAGGGCTAAAGTCCTGTTTACTGCAGGTCAGTCGGTGGCTGC

TATGGAGATGCTGAGCTCATGTGCGCTATCCTACTGCCACTCGGGGAAGTGCGAGCGCGC

CGCCTGCCGCTCTATCCTCACACTGTGTAAGTGGCTGCTGGCCGACTGGAAGGAACTGAC

GCCTCAGCTCAAACAGGTAGTGAAGAGAAACAGCAGCGGAGCACCGTCCAACCTCAGCAA

GAACATCGCCGCCCTGCTGGAGCTGCCCGTGGAGGAGCAGAGCATACTGCGCATTACCAC

GGAGACGACGGTGAGTGTAGGAGTCGGAGAAGCAGACTTTGTCCTGGGTCAGCTCTACCA

GCTTTCTGCTAGCCAGGCACCTGAGGTGGCCAAATCCTGGGCAGCTTTAGCAAGCTGGGC

GTACAGATGGGGCCGGAAAGTTGTGGACAATGCCAGTCAAGGAGAAGGGGTTCCTCTGCT

GCCAGGAGAAAAGAAGGAGATTGAGGAGTTATTGCCATCTGGCACCACCGATGAGGACAA

GGAGGCCATTTTTAGCATTCTGGGCCAGGCCATGTGTCGACCTGCTGGGATTCAGGATGA

GGACATGGCTCTGCAGAATGAAGAGGATGATGATGACATGGTGGATGTGATCTGGAGGCA

GCTGTTAAGCTCGTGCCCGTGGCTGGCAGAAGTGGAGGATGGTGTGACTGAGGGTCTGAT

CCGGGTGTGGAGGCGGGTTGTGGACCGCATCTTCAGTCTCTACCGCGTCTCCTGCAGGGC

TTATTTCACCTTCCTGAAGCTCAACGCTGGACAGACGCCTATCGATGAGGACGACCCCAA

ACTTCTGCTGAACCCTCAGAGCAGCAAGCAAAGCAGCGATGACATGATCGTGATGGCAAC

GCTGCGTCTGCTGAGGCTGCTAGTGAAGCACGCTGGAGAGCTGAGGGAGGGACTGGAGCT

TGGTCTGGCTTCCACACCCACCGCACCCTGGAGAGGCATCATCCCGCAGCTGTTCTCCAG

ACTGAACCACCCCGAAGCCTACATCCGCCAGAGCATCTGCAGTCTGTTGTGTCGCGTAGC

CCAGGACTCGCCCCACCTCATCCTCTACCCTGCCATCGTGGGTTTAATCTCACTTGGAGC

GGAGGCTCAGACCACAGGTATCAAGCTGCCCTCTGCTCTGCCTACTCTGTTGGGCACTAT

GCCGGGCGAACCTCTGTGTGTGGGCGAAAGTGAGGGCGGCAGTCCCCCTGCGTCTCAGGA

GAGCGGCCGAGGAGACGAACTGGGTCTGTGTTCGGTCGAGGACCAGGCCATGATGCAGGA

CTGCTACAGCAAGATTGTGGACACACTTTCTTCAGCTAACCCCACCATGGTCCAACAGGT

GCAGTTGCTGGTTGGGGAACTGCGCAGGGTCACTGTACTTTGGGATGAGCTGTGGTTGGG

TGTCCTGCAGCAGCAACACATGCACGTCCTGCGCAGGATCCAGCAGCTCGAGGACGAAGT

AAAGAGAGTCCAGAACAACAACACGCTGCGCAAGGAGGAGAAGGTGGCCATTATGAGGGA

GAAGCACTCGGCTCTTATGCGGCCGGTAGTCTTTGCTCTGGATCACGCACGTAGCATCAC

CGCCGCCGCAGCGGAGACGCCTCACGAAACATGGTTCCAGGAGACATACGGAGAAGGCAT

CAACAATGCTCTCGAGCGACTGAAGAACCCACAAAACCCAGCCAACCCTGCCAATAGCTG

GGTTCCCTTTAAACAGATCATGTTAAGTCTGCAGCAGCGAGCTCAGAAGCGAGCAAGTTA

TCTCCTGCGTCTGGAGGAAATCAGTCCTCGTCTGGGCTCTATGACACACACAGAGATGGC

TCTTCCGGGAGAGGTTTCCGCCACCGACGCCATTACCATCCACAACGTCGGCAACACTAT

CACTATCCTGCCCACCAAAACCAAGCCCAAAAAACTCTACTTTCTCGGCTCAGACGGCAA

GAACTACCCATACCTCTTTAAAGGTTTGGAGGATTTGCATTTAGATGAGAGGATCATGCA

GTTTTTGTCTATTGTCAACACAATGTTTACTAAAGTCAACCAGCAGGAGAGTCCACGCTT

TCATGCCCGCCACTACTCCGTCACGCCTCTGGGCACTCGCTCGGGTCTCATCCAGTGGGT

AGACGGAGCCACGCCCCTGTTTGGACTCTATAAACGGTGGCAGCAGCGTGAGGCTGTGCT

TCAGGCTCAGAAGGCTCAGGACTCAATCCAGCAGCCACAGAACCCGCCCATGGTTCCGCG

GCCCAGTGAGCTGTACTACAGTAAGATCGGGCCTGCTCTGAAAGCCGTGGGACTCAGCCT

AGACGTGTCACGCCGTGATTGGCCGCTCAGCGTCATGAGAGACGTGCTTCGGGAGCTGAT

GGAGGCCACGCCCCCCAACCTGCTGGCTAAGGAGCTGTGGTGCTCGTGTACCACACCGAG

TGAGTGGTGGAGAGTCACGCAGTCATACGCCAGGTCCACTGCCGTGATGTCGATGGTGGG

TTACATTATTGGTTTAGGTGATCGTCACCTGGACAACGTGTTGATTGACATGACGACCGG

TGAGGTGGTGCACATCGACTACAACGTCTGCTTTGAGAAAGGAAAAAGTCTGAGGGTTCC

TGAGAAGGTTCCTTTCCGTATGACTCACAACATCGAGACAGCGTTAGGAGTCACGGGAGT

GGAGGGAATCTTTAGACTTTCCTGTGAACAGGTGATCCAGATGATGCGTAGAGGCAGAGA

AACTCTTCTGACCCTGCTGGAGGCATTTGTGTATGACCCTCTGGTGGACTGGACAGCAGG

GGGAGAGGTGGGCTTCGCCGGAGCTGTGTACGGAGGAGGAGGACAGCAGGCCGAGAGCAA

GCAAAGCAAGAGGGAGATGGAGCGAGACATCACACGCAGCCTGTTCTCCTCAAGGGTAGC

CGAGATCAAGGTTAACTGGTTTAAGAACCGTGACGAGATGCAGGCGGTGCTGCCGCAGGT

GGAGATGGCTGTGGAGGAGTACCTGAACCTTCAGGAACAGCTCTCTCAGGTGGACAAGGC

GCAGGGCAAGCTGCTGGAGGAGATGGAGTTCCTGGAGGGAGCAGACAGCAGAGTGGACCA

CTCAATTCATACACTGGAGCACAGATATTCCGAGCACACGCAGCTGCAGTCGAGGCAGAG

GACTGTACAGGACGACATCCAGAGCAAGCTGTCTGATCTAGACCAGTGGATCTCTCAGTA

CCAGGCTGCGTTTGGAAGTCTGGAGGCCACGCAGCTTGCTAGCCTACTGCAGGAGATCAG

CAGCCCCATAGATCTCGGTCCTCCCAGTTATGTCCCTGCTACAGCGTTTCTGCAGAACGC

AGGTCAGGCTCATCTCATCAGCCAGTGTGAAGCTCTGGAGGCGGAGGTGAGCGCCCTCTT

GCAGCAGCGCCGCTCTATACTGCGCGCCTGCCTCGAGCACTTGCACAGTTACGCCACCGT

GGCCCTGCTGTACCCGCGTGCTGTCCTGCACCGCCACAGAGTCTACACTTGGAAGCAGTG

GATGGAAGAGCTGATGTGTGATATGACTGTGGAGAACTGCCAGGCCATCTATCACCATTA

TGAAATGCAGTTCGCACCCCAGCCACCGACTGCTACGTGTCAGTTCTTGTCAAGTGTGGA

GCTGGCGCTGCAGCACCACGCTGCGGAGACAAACACACGGCTTCTGCGTCAGATGGAAAG

GCTGAAGGCAGAGGGAGCGAGCGTGCCTGTGTGCGAGGAGCAGCTGCAGGAGATTGAACG

CTGCATCAAGGTCTTCCTGCATGAGGATGCCGAGCTCGCCTCCTTCAGCCTGGCTGGGAT

TATAATCTCCGCTCTCTGCACCCTTACCAGGCGTAACCTGGTGATGGAGGGTGCGGCAGC

CAGTGCAGGAGAGCAGCTGGTGGAGCTGACGTCCCGGGACGGAGCCTGGTTTTTGGAGGA

GCTCTGCAGCATGAGTGGAAACGTCACTTGTCTGGTGCAGCTGTTGAAAGAATGCCAGCT

CCAGTCCCACGAACTGGACATCCTTTGCCTCGAGGACACCTCCCAGGCTGTCTACCTGGC

CAATGGGGTCTACACCTGCCTACAGGAGCTGAACACAAACTTCCGTCAGATTATCTTCCC

AGAGGCTCTGCGCTGCATGCTGAAGGGAGAGAGCACTCTGGAGAGCATGCTGTCGGAGCT

GGACTCTCTGATAGAGCAGTGTGCAGACGGTGTGTCCCTGCAGGGCCTGGCAGAGGCGCT

ACAGACTCACCTCCGCAACGCTTCCATGGGGCTGCAAGAGGATGTGGATGCACACTACCT

CCAGGTCACCAGGGTTCTTCGTGTTCAGTACTCAGAGCTGATTCAGCCTCGGAACATGGA

GGGCTCCATACAGGACACGCCCAAAATGAGCGCTGGACAGATGCTGCTAGTGGCCTTTGA

TGGGATGTTTGCTCAGCTGGAGACTGCTTTTGGACTCTTGATAGAGAAGTTGAACTCCAT

GGATATTCCGACTGCCTGGAGGAAGGTGGATGTGATTTGGGAGGCTCGAGCCACACAGGT

GCATTTTTTTGACACGGTTCAGACGCGGCAGGTCCTGGAGGAGATCTTTTTCCTTAAGAG

GCTCCAAACGATTCGTGACTTCTTCCGGCTGTGTGCCTCATTTGCTCAGACACTGTCAGG

CACCTGTCCTCCGCCCAACGACGAGCCCCCACCGTCTAACGGCCCTGTTTCGTTAGTGAA

GCCCATATACCGTACCTCCACTGTAGTCAATGAGGACCAGATGACGCGGCCCATCAAGGC

TTTTACGGCAGACTTTGTGCGGCAAATGTTGATGGGTCTCCCCACTCAGGCTCTTGGGCT

GGCTCTCTGCAGTGCCCTCAGTGCTCTCGGCTCAGACCTCATCGCTCAGGTTGAGGCTAA

AGACTTTGGGGCAGAGGGGAAAGTGTCTCTGGACGACCTGTGTAAGAAGGCGGTGGAACA

GGGTGTCCAGGCAGGACGTATCTCCCAGCTGCTGCTAAACAGAGCCACACTGTTGGCCAG

CTCGTATGACACGGCCTGGAAAAAGCTGGACCTGGTGCGCCGCCTGGAGCTCAGCATCGA

CGCTTGCAAGGTCAGCCTGCAGAGAGCGCAGCTACACATCGCCATGTTTCAGTGGCAGCA

TGAGGATGTTCTTGGGACTCGCCCTCAGCCCATGACTGTCAGCCCTCCACCTCGCTCTAT

CATACTCAGCAACATGAAGAAGAAGCTGTACAAGCTCAGCCAGGATGACGCAGCTATCGC

CAGTGTTCAGGAGAAACTGGCATCACTTGAGGGCAGCATCGAACAGAGGTTGAAGTGGGC

GGGCGGGGCAAACCCGGCTCTGGCGCCTGTACTGCAGGATTTCGAGAGCACCATAACTGA

GCGGCGCGCCCTAGTGGTGAAGGAGAGTCAGCGCACCACTCAGGTCACCTTCCTGTGCAG

CACTGTTTTGAACTTCGAGGGCCTTCGGACACGTACCTCAGAAGCTCTGAGCATGGACGC

CGCTCTGTTTGATCTGGTGAAGCGTTGCCAGGCCACGTGCTCCTACGCCGCCCAGTTCAG

CTCCACTGTCTCCACTTTAGAGCTTCAGCTTCTCCACAGACTGAGTCCTGTAATGGAGCA

GTCTATCGGCACCCCTGAGTGGCTGGCATGCGCTCAGAAGCATCTGGCTCAGGAGATGGC

GAATCAGAGGGCAGCGCAGGAGGAACGAGAGCAGCAGCTGGACTCAGTTACTGAAACCCT

GCAGCTGCTTGTGGACACCATTAAAGGCACCCTGTCTAATCACAACCGACTCCTGGCTGA

CGTCAAGCACCTGCTACGTGCTATGGCCAAGGATGAGGAAAGCGCTCTTGCAGAAGGAGA

GGAAGTAATGTACGAGGGCAGTGTGCGTCAGTTCCTGTGCGAGTACAAAGGGTGGCAGGA

TAATGTGCAGATCGTGCTGTTCACGGTGGTGCAGGCGACTGGGCAGCCACGCAGCCTGGA

GCAGGTGGAGCTGCTGCAGGAGATTCCCCCCACCCTGAAAGAGCTCAAAGGACAAAGTCA

GAGAGTGTATAACGGGCTGGTTGGATTTGCGTTCCCCCTAGTCACCGACAGAGGAGGTGA

TTGTGCAAGCCCCACAGCAACTGTTCAGACCAGCTTTGCTGCCGCGGTGCGATGCAGTGG

CGTAAAGACACAGCCTGACAGCATGTCTCAGAATGCACGCAAAGCCCTGCCTCGCAACCT

GGGGACCCCGGCAGACACACCACCCAGCACCCTGCTTATCACCAGCAAAAGCCTGATGCC

GAGCCCCAAACGTACCGTCAGGGACCCCAAAACCGGGAGAGCTGTGCAAGAGAGAAACTC

ATATGCTGTGAGTGTTTGGAAAAGAGTGAAGGCAAAGCTGGAGGGAAGGGATGTGGACCC

GAACCGCAGAATGAGCGTTACAGAGCAGGTGGATTATGTCATCAAGGAAGCCACAAATCT

GGACAACCTGGCACAGCTGTACGAGGGCTGGACGGCGTGGGTGTGAACAGGAATTTCCGC

GTCGGTTCACTTCGGTTCCAGCGAGGGTCAAAAATCCACCAGGTCTGCGGCATGGAAGCC

ACAGAGCTGCATGCGGTCAAATAAACGGGGTTTGGGTGGGGCGTCGAGGCCCTCCCGTGC

GCCCACACGGGATCCTCCCTCCCCTCCGTAGCACAGCAGCAGATCCAGCTAACACCCACA

CTAGGATTCTCTGGTCTGCATCCGTCTCCCTGTAAACGTGCAAAGGGGGGAACCCACTGA

GGACAGGGGAGGGGGGTGTGGGGGGTGAGGATGATGATGATTCGGCCCTTCCAGCTTTAA

CATCCCCCTCCCCACAAACCAACCCCTGCTTCATCAGCGATATTAACGCCACGTCTGTCA

ACGATAATGAAGCTTGTGTTTTAGCGCAGGTGCGTTGCACCTCTGGACTTCTCTCAGGAG

ATGGGGGCTGGGCGCCTCCCAACATCCTATGGCCTTCTGTTTTTTTGGATGGCTTTATCA

AGGGTGGGGCAGTTGAGGCTGGCGTTCACCGGGGCAGGTGTGCCAGGCTTTAAAAATGGA

ACTCTGTTCAAAGTCAGACTGCCGCTAGAGGATTTGCGAGTGCTTAAACTTTTTCTCAAA

ATCTTTCCTTTTTCCCCCCTTTCTCTTTAGTCAAACATTTTCCCCTGAATCTTACTTTAC

ACACCTCCGCCACTTTCACACTTGAGCGGACATTTAAAAAAAATCTGTTTTGGGGAAATT

TTCATTTAGTATTGGTCGTGAGGACGTGAGCTTGTGGATTTTTTTTTGATTTTTTTTTTT

TAGCCTCCTTTAGGATTCCACACTTTCTGAAGCATTCCACTCCGATCCTTATGACTGCTG

GCCTGTTTCCAGAATAGTCATTGTTTTTCTGATTCTTTGCCTCCTCCCATTTTTCTCTCT

CTCTCTCTCTCTC

>Unigene000071

TCGGAAGAGCGTCGTGCAGGAGGACTCGTACTGCGCCTGGGAACTGACTCTTTTCTGGAG

TAAACTCCTAAAGCGATTGGAGCCTTCAGAGCAGAGCTTTCTGGAGAAGTGCCAGAAGAT

GTCGCTACTGGCTAAAACTGTCTTTCACCTGCTCTTCTTCATCAAAGTGATCCAGTCTGA

GTTGGATAAGATTGGCTTGCCAGCCTGCATTGAGATGTGTATACGTGCTTTGAGGATGGA

GAACTGCGAGGGAGCTAACAAGGCCACCATCTGCAAAACCATCTCGTGTCTACTGCCTTC

TGACCTGGAAGTTAAGCGGGCCTGCCAGCTAACAGAGTTCCTCCTAGATCCGACAGTTGA

CTCTTATTACGCCGTGGAGACCCTGTACAATGAGCCGGACCAGAAGCTTGAGGAGGAAAC

CCTTCTGATACCCACCTCTCTTCGCTGCGATCTTTTCCTAGTTTTTAAAACCCAATGGTC

TTTTGATCCAGAGTTCTGGGATTGGAAGACACTAAAGCGTCACTGCTTAGGCCTTATGGG

TGAGGAAGCATCTATAGTGTCCTCTATTGATGAGCTAAACGATGATCGCTTCTTAGAGAT

TGAGCGAGATGAATTGGATTCTGCTCAGGAGGAGTTTAAGGATGTTTTTGAATGCTTTTT

GGACACAACAAAAGAACTTAAGGAGATATCGGATCATCGACAGAAAAACAGAGAAGTTAA

AAAGTTGAGGGAAAAAGGGTTTGTGTCTGCGAGGTTCAGGAACTGGCAAGCGTACATGCA

GTACTGTGTGTTGTGTGATAAAGAGTTCCTTGGTCATAGAATAGTGCGACATGCACAGAC

TCACTTCACAGATGGATATTATAGCTGTCCGATTTGCACAGAGACGTTTGAAACGAGAGA

AACTCTAGATCCGCATGTTGCATCACACGTAAAGTTGTCTTGCAAAGAGCGCTTGGCTGC

GATGAAAACCACTCGGAAACTGACTGGTGCAAAAAACTCTGCTTCCAATGATGCACCTCT

GGATAACAAAAGTGGGGAAAATCAGGCACGAAAAGCGAAGGCTAAACACTGCAATGGAGA

ATCTGATGATGTGTACAACATGGGCTCTTGCATTGAGGATGCAGGAGTCAGGGTCCACAG

GAAAGAGTTCAATGTTTGTCCTGTTTCAAGCTGCAGAAAAGTGTTTAAGTTCTTTCGCAA

TCTTCTCACCCATGTAAAGGATCACGGAGACGTGGAGGAGGCTAAGCGCTTCCTTGAATT

ACAGGCCACCAAGATAGTTTGTCAGTATTGTCGCCGACACTTTGTGAGTGTAGGTCACCT

CAATGACCATCTTCAGGTCCATTGTGGTGCGAAGCCTTATACCTGCATTCAGTTGAACTG

CAAGGCTAGCTTTGACACCAATGCCGAGCTCCTTGTGCACAGCAAAAGACATCCTTGTTT

TAAGGCCAAATGCATGTTTCCTGGCTGTGGAAAGATTTTTAACGAAGCTTATAAACTCTA

TGACCATGAGGCGCAGCATTATAAAACATTCACTTGCAAAGTGCCCGGTTGTGGGAAGGT

ATTTCATGCTCAGTCTCAGTTAGACATGCACAAGGAGAGCCACAATCCTCCTAAATTGGA

GACACAGCCCGCTGGGAGTGTCAGCCCGCTGGGAGAGCCTCAGTCGCACCTCTCCAGTGA

ACCTGCCCAGCAAGATGCCCATTCAGGGCCTCCTGCCGACTCCGGCTGCATGATTAGTTC

AGAAGATCAGTCAACCGGTCTAGTAAAAGTAAAGCACTCTATTGAAAACATGCTCAGTGT

TGCTGGTCAAGGGCTCTCACAAGAGTTTGGCCATGTTAACTGCAAAGCCGAGCCATCTGT

TCCAAACCTGGTGCAGCAGCCACCAATGCCACACATGGTGAATACCGAGCAAATCCAACC

TCAATTAACCCCTTCAATGCCTTCCTTGATTGAACCGAGATCTGAAGAATCCTTGTTAGA

TGCATTGATGAACGATCCAATGCTCACTTCATCTTGCTCACTTCCACCAGCATCTTCGTA

TCAGAGCCTCTTAGAGGATTTCCAGCAAGCTCCATCAGGAGATGTTTTACAAGCTCAAAT

GGAGTCTGCTGTTTCCCAGGGTCACACCCAGTCTCTGTACAGTAATGAAAACACTGAATA

TGGCCAATACAATATCAAACCTGAAACCCCAATTTCATACCCACCTCCGTATGATAACAC

TGTACCCTTGGCGCAGAATGGTCATAATGATCCACCGGTACTGCCTGTATCTCAGAGGCT

TCCCCCACAGCTCACCATGTTTTCTTCGAATAGACCAGTGCAGGCACTGCAGGCAAACAC

ACTCAGTTCTGTAGTGGAAGAAAAAGACAGGTATAAATGTGCATACGAAACATGTTCTCG

AGACTACAGCTCGTATAGAAGTCTAACCAAACACATGAAAGCTGCTCATTGTGAGTTTTA

TACACAGTGGAAACTGGCAAAACGGAGCAATAAAGTTTCACCAAACACTTGCAGCGCGTC

ATCTATAAATGGAAAATGTATGTCAGCAGAACCCTCACAGAACCAGCTGAGTCAGAGGAC

TCCTGCTCCTCAGAACCCAGTGCAGAAACCACCTGCTCAGACCCACAGCCCAGCAAGCTG

TCTAAGCTCCCATTACCCCTCTGGATCCTCCCATTTAACCGGTCCAAATGGACAGACTTT

TCCAAATCAAATGGACAGTATTTTGAATCCCATAGTGCTCTCACACTTGGGCAGCATTGC

AAATCAGTCTAATGTAGTCTCTCACTCTTCGTGGAATTCCATCTCAGTGAACACATCACT

CCGGCAGCAGACTTGCCATTCACAAGCAATGACCTCATCTAGTGGTCAGTTCCCGTCCCA

GTTAGACACTGTTCCACAAACCCAACTGTCAGATTCCTATAATAGAGTAATGGCCTACCC

TTCTTTGCAACATCAAAACAGTCCCTCATGTCGATTCATACAGACCCGACCATGTGCCCC

CCAAACAATAAACAGCCCTGCTAAAATAAGCCATCCCAGCGATTCCTCTTTACCTCAGTA

TGTTCAGCAACCGAAGACCAGTTCTAAATCCACACTTAAACATACAGACTGTGTCGTAAA

ACTAGAGAGAAACCCAATTTTAATTCCATCCCATTCAAACTTATTACAACAAAGCAACCC

TCCTACTGCTATGAAACCTGAAACTTGTCAAAAAGATATCAGTGGTTTTGCACATGATGG

GGATGTCAAAAAAAGGAAGCGAAGCAGCAGAACAAAGTGGCCTGCTATTGTCAAAGACGG

CAAGTTTATTTGCTGCCGGTGCTTTAGAGAGTTTCAGAGTCCTAAATCTCTTGGTGGACA

TTTGTCTAAACGTACTGTCTGCAAACCCTACGATGAAGCTGTTTTGTCTGCAGACCTGCC

AAGTTCATTTCTGGAGCTTCTGAACTCCCCTCATCCATCGGAAACACCCCAGTCCTCGCC

GTCCACGCAGGTCACTTCTAGTAAAGCTTGGCCTTCCCTGACCAAAGGCCCCCTTGACCC

AAAATTGTTCCCCAATGTCACGTTCCTGAACACTAAAAATTCCACATACAACAGTGGAAC

CATCCATCAAAATTCACCGAGTCTGGAAAAGGGTGTTACAGGTGAGCAGACTTTTCCTGA

GTCATCTGGGTCTTACCATCAAAAAAGCAAAAACAGTGTGATTCAACACACCGGAAATAT

TAGAGATAAGTGCATTTACGATCTAATGAATTATACCCAGCCATGCAATACCTTACCAAG

TGACACCAGTTCCTCAGAACAACTTTTGTCTCACATGTTAAGTGAAAATAAAACTTTAGA

CTCAAACTTGAGCAGAAGTCCTAATAACTATGTTGACAGAATCCTCCAAGCTGAAACACT

AAACAAGATTAGGGAGATTAAAGAAGAGCCTACCAGAACAAACATTAGTGGATTATCGAA

TGATGGGCTTCTTGCTGCAATGGCTAGTCTGGCCCAAAATCTGGTGTCAGAAAAGAGCGT

CAAGGAAAGGCTTCGTGAACAGATATTAGCTGGGGACTTTCAAAAAAGAAGCAGTTTAGG

CCAAGGGCAAGGAGTTGAAAACTCTAATTCACAAAGTCCGATGACTGATGTTCCACTTGG

ACTTCAAACAGCTGTTCAAAGTAAGGAGCATATTGTCAGACGTGCTAGTGAACTTGGTGA

ACTTTACACCTCAGTTGTACCTGATGAGACCTTGACAAATATTTCCTTGGCTGGTGCGTC

TAACAACAGGGAGACAAACAATGCGTTTAGTAACAGCCTAGAACAAGAAGATGATAAAGA

TCTCTCTGAAATTCTGAAATCGTTTGAAAGATTGGATCTAAATAAGGAAATAGAAAAGAA

TGAGCAGACCACTTCCAACCTGCTAGAGGCTGAGAGAGTAACCAAGGCCTCAGAAGCTAC

ATGCACTCTGATGTTACGAGGCTATTCCTGTGACAACGATGGTTGTGGTTACAGAGCGAT

GACAAAAGATGCTCTTTTTAAACATCTTGTAAAACAGCATAACTACACAGAAGACATGAT

GAATCAGTGTCGCAAGGAACAGTCTAACTTGGCACCTTTTAATTGTCAACTATGCCCCAA

AACCTTTACAAGAAACTCCAACCTCAGAACCCATTACCAAGCCGTTCATAATTATTCACA

TGAGCAAGTGGTCAAGTTGGGGATTACACGGCAATACAGTCGAAAATCCATTGAGGCCGA

TCAAGGTCTTTGTACCACGCTGAACATCTCGGAGTTGGCCAGTCCAGTCTTTGCTGGACA

GAATGCCGAGTCTGGTGCCGGTATCACAGCTTTGGTATCTGGAGTTAGAAGCATAAAATC

AGAGTTCAGTGTTCAACCATTTGAACAACAGCATCCTGGTGCTTCCTTCGACTCATGTCC

TGTTTCCAGTACAATACTGCGAGCGCAGACAACTCCACATCATCTCCAGAGTCAGACTAC

TGTGCATTTATCAGTGATTAAAGCTACTCCTTCCTTGCTAACCTCCACAAGTGTGGAGCA

GCAGTTAAATCGACCAAATGAATTTGTTCCGGAATCGCTTGTGCCTTCTCAGCACACTCA

AGGATTTCTGTCTGGAGGTCTTTCGTCTTCAGGTGCGCCTCTTCTAGCCCCGATTTCTTT

GGCGTCAGCACAGCTAGGTAGCCTTTCCTCAGATACGAAGCCCAAATTTGGAAAACCTAA

AATGGCAAAAGTGAAGGAAGTGACAAAAAAACCTAAGGAGAAAAAGCTTGACATGGTTGA

TGTCTTTAGTCCCTATCGGCCTTACCGCTGCGTCCATCAGGGCTGTGTTGCTGCCTTTAC

TATACAGCACAATTTGATTCTTCACTACAAAGCTGTACATCAGTCTGCCCTCCCCAAATT

TGAGGTTAATGAAGATGAAGAACCGATCGAGGAAGAACATAACGAAAACCAGGAGGAGAT

TGGCATGCCTGACTCTGAGGTTAATGAGGAGTTCCGCTGTCAAGTCAAAGACTGCTCTCG

AATATTTCAGGTCGTTACAGACCTGCTACAGCATTACCTTCAGCTCCACAAACTCGGCCT

TGATAAAGCAGGTATTATGATGTCTGGTATGAACTTGGGCAGGTTTCGGTGTGACCAACC

AGACTGTCATGTGACATTCACTGGATTCTGGAAGTATGTTAACCACATTGATAATGAACA

CAAGCATGTAAAGCTCTCCAGGACTGAGCCTGTTAATGGCATGTTCCAGTGTGAGGTTGA

AGGATGTAGCTGCGTTTATGCAACACGGTCAAACTTGTTGCGACACTTTATGAAAAAACA

CCCAGAACTTTACAAACTCCGACTGCTGAACTCGGGAGGTGTCAAACTTGGTCGTCCTCC

GAAAAACAGATTTGTCTCCCTTGAGAAAGAAAATAGAGAAATCAATAAAAAAACTTTTCA

GAAGGGAGGTGCGAAAAAGAAAAGGAGACAAAAGAATCTTTGGACAAAGTATGGAAACCC

TATTTTGAAATCGAAAGAGGAGGCTTCCTCAATGTGCACCAAGCGGATTCAGTTACAGTA

TCCTTGCATGCTTAAAGGCTGTGAGACAGTTGCAGAGTCTGAGCGAAAGCTTTTAAAGCA

CTATGTCCAACATGGCTTGCCAAAACAATACCTGGAGGAGCAGAGAAGCAACTACATATT

CTGCAAAAAAATGCCTCGCTCAAAGTATAAACGCATTGCGTCCAGGAGTGACGATACTGA

CAAGTCCGATGGAAGCTCGATTGAGGCGTCTGAGAACGAGGACGCAGCAGACACTGGACC

CAGCGAATCGGAGTTCTCAAAGCCGACGTCTGAAAAGGAGAGCACGGAGGACACTGAAGT

TTCAGACGCTAAATTGTCTAGTGACCTGAGCTCCGACATCTCTGTGGTGGTAAAGCGGAA

GAGAGGACGACCACGCAAGGGGGAACAGGTTAAGCGCACCCAGCTCGCACGCAAGAGAAT

GACGAGGCTACGAGCGGCGCAGACGCGCTCCATCAACTATGCAGATATGAACTCCGATTC

CACTTCTTCCAGCGTTACGCTTCCACAAGAGCCAAACACATCACTGAGCTCTTTCAAGCC

AATGGGCTTCGAGGTGTCCTTCCTCAAGTTCCTGGAGGAATCGAGTCAGTCTCCCGGGAA

GAGAAAGAGTTGTTCTTTAATGGATTTACTTCCACACAGGAAATTCCCGACTGTGCGACT

GAAGACTGCCTCGGTGGTATGCAGCAGGATCAACCCTGACTATCATCATTGCCGAGATGT

ATTGAAACATGTCGAATTTAAAAACCCTCAGAAACTAACGTCTCTGAGTAGGGTGACGTT

CGAGGTGCACAGAGGGTTCTCCAATGTCTACGAACTGTTGCTCAAGCAGCTGCATGACAT

GCGGCCTGCAGTGGTCATTCAGAAAGAGGGCGTGTCTGAGACGAGGGCGTAGTTTTCAGG

GTGGGGGTAGCTAATGTGAGAGTGGGTCAAGAGCTGTTCAGTGTTGCTGTTTATGTGGAG

CATCAGTGTTGAGATGTTTCCATGCCTTTGTAATGAACCGCTAGTCCCTTTCCAGGACTG

TATACTAAAGTGTACTATTGTAATGAAGTCGGTGAAGGATACGCCTCTCGTGCATGCCCT

AAAACACCTGATCTGGCACGTGTGCGCAGCTGTGGAGATAAAACCCTATAATGGAAGAGT

GAAACTGCTCCGTGTAAGAATGTGAGCTCGCCTTTTGTTGGACTGACCGCGTGTCGTGCA

GTGGTGAAATGTAGATGAGTAACATTTTGGTCTTGTTCATCTCGCAGTGTAAAATTTGAT

AGCGTTGTGTTTATCTGTGACTCTCACATACTGACTCTCCAGATAATCTACTGCCACCAG

CAGTCTGTACCGCAAACAACAGAGACCAGTTTATTACAGCTCGCGTTTTCTACCTCTGTG

TGTGTGTGTGTG

>Unigene000072

GCCATTTTGTTGACAGTTGGTGTTTTTTTTAATAAGACCGTACAGCCGGATTTATCCGAA

TAGCGCTGGCTTTTACTTCAACTTTCACCGTTTTGAAGGACGCATCAATTTACCGAGGGA

ACCATGCTGGCTTTCCTGAAGGAAGGATTTTCATTGGAATGATATAAAAAGCTGGTTTTG

GATCCTAAAATAAACTGCAGTGACAGGGGTGGACAGCCAGAAAAGGACGGCACTGCTTCA

ATTCAACTCTCACCATATCCCTCATCTTCCACATCCATCTGGATTCTACTGTGCCCGGTT

CTGTACTGCTTGTGAGCACATCCTGCTCATTCTCTCCCTCTTTTTTACATCATCAATGGA

TCAAAGAACACAAGGAGGGCCCACCACACCACCCCCTCTTCCTGCAAATGCCCTACCGGT

TGAGTGTGAAAAAGAAGGAGGAGGTGGAAAGAAAGATGATGAAGATGAAAAAAAGAAGCG

TGAGAAAGAAAGAGATGCTGTGCCGGTTGTTTCCTGCAACTCAGAGAGTGCTGCTGGTGA

CCAACAGCAGCAGCAGTCTCCACAGTTCTCAGTCAAAGAGACCAGCTACTCGGAGGGCAA

TGTCAAACTTAAGATAGGTCTTCAGGCCAAACGAATGAAAAAACCCCCTAAAATCCTCGA

AAACTATGTCTGCCGCCCAGCATTTAGAGCAACAGTTAGACATGGAAGTCGAGGTGGTGG

GCGAGGAAGTCGTCGAGTGGAACCCCCCAATGTCAACACTCCTTGTTTGCCACATATTAA

AGATGCGGAAAAGGACCCTAATGAAAACTTGGGCGTGTCTCAGTCATCAGCTGTTTTACC

CAATAATATACCTACACCTTCTCTTCCCAGTTCTACTTCCACCAACCCTGAAATTCATGT

GAATGCAAATGTACCAGCTAAAAGAGGTCCTCCAAAGCTGGCCTGCAAGTCTGAAGAAAT

TGTAGATGTCAAATCCATTGCTTTCTCAGACAAATCCCTTAAGAGTCACTGCCCTTTGTC

AGACAGCAAATCACACTCCTCTGGAAAAAGGAGCCTTGCACCCCAAACCGGCCCACCAGC

CCCGTTACTACAAACTCCTACCAGCAATGACTCCCCACATGATCCCAAAATTGACAGAGT

CACAAAACCACTTCAGTACACCTACACAGATAGCCAAAGGGAACAAGAAAGAGGGGCTCC

TAATTGGGGCACGCCCACAGTAACTGAGAAACTTGCGCAGCTTATAGCCACCTGCCCACC

CACAAAATGTCCTAAGCCTAAAGCCAGAAAAGTATCTCCAGCTCCATCGCATAGCAGTTC

ACCACCTTTGATCTCTAATTTGCAGCGACCTGAGCGGGCCATGGCTAACAGAAATACTTA

TTCAAGAGTAGCTCATCTTGCCCCTCCTCCACCTGTGTCACGGCCTCCAGGTCGTCCATA

TGGCTCACGAAACAAAGACAGTATCCTGGATAAGCTATCTGGCATTCCAAGAAAGGAGGA

AACAAGTGGACTTTCAAAAGAGATTACAAGCAGCAAAAGTATCTGCAGCAACATTTCTCC

AGTACCTTATAACAGTCGCTTAACCAATAATGATAGCGTTGACTATGATAATAACAATGA

ACTAAAATCACAGTCTCGTCTTGAGTCACATTTATCCACACCTTCCCTTTTCTCCCCTGC

CTTGGAGAGAATAGGAAGTGGAATCAGCCACCAGATTATGCCTGCCTCACTTGGGTTAAG

AATCACTGCAAGCCCCTCCCAAACAGAGGAGCCTGTTGATATTGAGAAACAACGTGAAAG

GGACATTTTTAATGTCAGAGAAATGAATAAAGGCTCTTCTACTGCAGTGTTGGGAAAGCA

AGACAGTAAAGAGAAAGGGTCCATCTTAACTTCCGGGGAAAGAAGGAAAAGCTTAAGTCC

AGCTAAACGAAATCCTGCACAAGAAAGCCATGCTTGTGAGGAACTTGGTGCTGTTGAGTC

TACCAGACGCACTGCATCTCCTTCTATATCTACTGGTACTAGTAGCAGCTTCCCAACACT

TTGTGACGATCATGACCCTGCATCCCCTGGATCACCAGCAGAACAGGAGTCTAAGCCTCT

AAAAAAGCGGCGGGGTCGAAGACCTCGCTGGACTAAGGCAGTCAGTCGCACGCACAGAGC

GATAGATGATGCTTCTCCAAGTCAAACCAAAATCACCACATTGCCTACTGACTCTTCATC

CTCTCAAATAATCCGTCGGCCTGTGGGAAGGCCGCCTAATGCTAATCGTATGCTTCCGGT

TGTCTCCGAGCTCTATTCGCAACCACTCAAGAAAAGGGGCCGACCGAAGTCCAAAATGCC

AACACTGGATGCACCTGCACGTGGTCGCTTGCCTCTTAAGGCGACTCCCCCAAAAGTATA

CTTGATGTCTAAGGAGGAACAAGATCCTCCTGTCCTGCACCCAGAGGTGGATCTGAATCC

CCCTAAACCTATGCCTCGAAAAAGAGGAAGACCAAAACGCTTACCTCCAACCCTGCCACA

GGAGACTCAGCCCCCAACGCTTGCTCCAGAGCCAGGAGATACTGTGGCAGGTGACAAGGG

CTTTCAAAACAAAGGCAATGGCCAGCTTATAATGAAAACCATTATAGGCAAGATCAACAA

GATGAAGACGGTGAAGAGGAAGAGGTTTCTTAGCCAGATCCTGTTAGGGTCAGGATCAGG

AACTGAGCCAGAGGTCACTAAAAGCACAGGCATGGTGGGGCCAGTGGCTTCCACTTCACA

GTCGCTTTCATCGCTTGCTGCAACATTTGGTGGCAAACTTGGACCTCAAATCAATGTAAG

CAAAAAAGGAACAATATACATGGGTAAAAGAAGGGGCCGCAAACCCAAAGCACCAGTCAA

CAATTCACCAGGTGCCCCTACACCACCACCTGAACCTTTTTTAACCCCAAACACTTCCTC

CCCACACCATTCACATCAGTCTTTGGCTTCGCAAAACCAGATTCCCCCCTCTGACATTTT

CCCCTCCCCTTCACTTTCTCAGTCTAGTGGGGGACAAAGCCCCATTAGTGAAGCTAGTTT

TGTGGAGCCTGGGTCAGGCCACTTGATATCCCATTCACACTTTAGCCACCATTCATTTCC

CTTTACCCCTCCAACTCTTACAGCACCCTCCCCACGTACTTTGGGAGGTTCCACTTCTTC

ACACTCCTCTCAGAAGAAGGCTACTTGCAGAGGTCACCACTATCACCACCACCACTACCG

ACAACATTACCATTGCCGCAAGCTCTCACCTCCCAGGCCCCTGTTGCCCACATCCCCTGC

TCCTCTTAGTGAGTTAAAGGAGGCCACACCTTCCCCAGTGAGTGAGTCACACAGTGAGGA

GACTGTGCCCAGTGACAGTGGCATAGGAACTGACAACAACAGTACATCAGATCGTGGGGA

GAAAGCTGGGAATGCTTGTAGCTTAGCTGGGTTAGGTGTTTCACAAAGTTTGACCAATGG

ACTGCTAATGCCTGGACTGATGGGTTCATCAGTGGGTATTGGGCTAGGGCATAATTTTAG

AGGTAGCAGGAGACATTCATCTACTGTACTGCTTGATTGTCCTTCACCCACACCATCCCC

ACTTGGAGAAAGATCAACAGCTAATCCACGCCGACCACACCCTTTGGCTCAGGCTTCAGC

ACTAGTGGGCCACAAAGAAAAGCATAAGCATAAGTGCAAACGGCGCGGGCACGGATGCCC

AGGTTATGATAAGTTAAAAAGGCAAAAACGCAAACGTAAAAAGAAGTACCTTCAGCTGCG

TTCACGGAGACAGGATCCTGATTTCCTGGCTGAGCTTGATGACATATGTGTGCGGCTTTG

TGGAATACGAATATCACATCGCACACCAGCTCTTCGTCTGGGCAGCAGCCTAGGTCTTAG

TGCAGGAACAGCAGGCACAAGTCATGGGGCTGGTTTGACTGGTAGCAGAGCAGGTAGTAG

CATTGGTAGTGGCACCAGCACCACACCACATCATTACCTGCATAGAGACCTACTACCAAC

AATCTTTAGAATCAACTTCAGTGGGTACTACTCACCACACCCAGCCTACCCATGTGATCC

CCTGCACTATGTCCGTAAGCCTGATCTTAAGAAAAAAAGGGGAAGACCTCCCAAGCTTCG

AGAGTCAATGTCAGAGGTGCCTTTTGTTCCTGGGCTAGGGTTCCCCCTGACTAGTGGAGG

GTTCTATCACCCTTCTTATAGTGTTCCGTACTCCTCAGCCCCTCTTGGACTTGGTTATTA

TCGGGGCTATCCCCCAGCCAGTGCATTGTACCCTCACCCTCACCACCAGGCTACGCATTC

TGGTCCATCTCACCATAGCCACCATAGCTCATCCTTCCCTCCACCACCTCCTCCTTCATA

TGTACATCATCATCACCCTCCACATCTGCTTCTCAACCCTTCCAAGTTTCACAAAAAAAA

ACATAAGCTTCTACGACAAGAGTATCTCAGTGGGGGTCGTTCAGCAGTGCTGTACCCTCC

TATGTCTTCTGAACTGTCATTTAGCTGGCACCACAAACACAAGCACAGACATAAACATCG

TGACCGCTGTGGAGAGGAAGAGGGAGATGATGGTGGAGGAGTCAGTGGTGGAGGAGGCAG

AAATGGAGGTGAGGGCATTGGAACAGGAAGAGGGGAACGAGCAAGAAGAGGGGGAAGCTT

GGAATCACTCAAGCACTGTGGGTATGGTCCAGATGGCTCAGCTGATTCAAGTGCTAACAA

GCAAACAGCTGCCACCTCTGGCAACTCTCCCTCTTCCTCTTCGTCATCATCAGCAGAAAG

GTACAAACAGAAAGAAGCCTCTATGTCTTGTCTGGTTCCTTCAAGACTATCCTTGAGCCA

GGGTAGTCGGGGGCAGCATCCTGCAGACTCTTGGTTTAAAATTGGCAGCTATGATGTTGA

TTACTCCAAACTCTCCCATACTCAGGCTCTTGGGCAGGGGGGATTCTCTGATGGGCATCT

GCATAACTCGGTTGTGGGTTGTTCAGACAGCGAGGATGAAGATCACACTTCACCAAATGA

GGAACAGGAGTCAGGTTCCCACCACACTAACCTTTTTGCTTCAGCCCTTACACGGACTTC

CCTCAGATGTAATCAGGGCAGGAAAGGTGGACGCTTAGAAGGCCCAAGTCTTACGCGTTT

GGACAGATCACTGCGGAAGGAACTGTCCACATCTGCTGAGAGAAGGGACTCAGGAAACTG

TGGTATACAGACAAGATATAGTCAGTTTAGCACCCCTGAAGTTGCTTCTCACCGACATCA

CCATACTCCTCTCCCTGCCCCTCACTCTTCTTCCTGCCTCTCCACCTGTGGTCTGGAGCA

GCCAGGGGAAAGTCCATCTGCACGCTCCTCACAGCCTGCATGCTGCTTGGACTCCTCTCC

CTCTTGCCCATCCCAGTGTTCAGCCCCTCTTACTCCCAAAAACAACTTGCATCACGTCAA

CAAAATTCTCCGGGCTAAGAAGCTGCAGAGGCAGGCACGTACAGGAAATAACATGGTTAA

GAAACGAGGACCGGGGCGGCCCCGCAAGAATCCTTTACCCTCGCCGTCACCGGTGCCTCC

AACGCCATCTCCACCACCAGAACTGGAACCAGAAAAGCACATTGAGGAATGGCAGGAAGA

CACTGTTACGGATGCCATTGAGTCTGTTATACAGAGGCAAAAGCGGAAAGGCCGAAAGAG

GAGGTACTGGGAGGCAGACAGGGATGAAAATGCAGAAGATGAAGATGAGACCATGCATGA

GAGGGAGGAGGAAGAAGAGGAGGAAGCAAGGGTAGAGGATAGAGAGGAACAGCTGGTAAC

ACAGGGACCATTAACAGGAGGGAAAGGACGACTGGCACATGAGGAGATGCCAAGCTTTCA

AAGTGCTGTTGAAGGAAAATCTGATGGCCACTCCTCCCTGGAATGCGCAGGTACCACGCC

CACTGTACAAGCTCCACCCACATCAATGACTACCCAAAGGGAGAAAAAAGCTGCTAGGCC

ACCTAAAAAGAAATTTCAGAAGGCTGGCCTTTATTCTGATGTGTACAAGACAGATGATCC

CCGCGGTCAGCTGTTACAGTTAAAGAAGGAGAAGTTAGAGTATATTCCAGGTGAACATGA

ATATGGCCTATTGCCTGCTCCCATTCATGTTGGAAAGTACTTAAGACAGAAGCGTATAGA

CTTCCAGTTGCCTTATGACATCCTGTGGCTTTGGAAACATGACCAGCTGCATAAGAGACC

AGATGTCCCTCTTTACAAGAAAATACGATCCAATGTATACGTTGATGTAAAGCCACTCTC

GGGCTATGAAGCCACCACCTGTAATTGCAGGTGCCCCGAGGACTGCAACGCTAAAGGCTG

CTCAGATGACTGTCTTAACAGGATGATTTTTGCAGAGTGTTCCCCCAACGCTTGTCCATG

TGGAGAGCAGTGTGATAACCAGCGAATTCAGAGGCATGAGTGGGTTCAGTGTCTGGAGCG

TTTCAGGACAGAAGGGAAGGGCTGGGGCATCCGCACTAAGGAGTCACTGCGGGCTGGCCA

GTTCATCATCGAGTACCTCGGAGAGGTGGTCAGCGAGCAGGAGTTCAGGAGTCGAATGAT

GGAGCAGTACTTCTCTCACAGCGGGCAGTACTGTCTAAATCTGGACAGTGGTATGGTGAT

CGACAGCTACCGCATGGGCAATGAGGCACGTTTTATCAACCACAGCTGTGAGCCCAACTG

CGAGATGCAGAAATGGTCAGTAAATGGGGTGTACAGGATTGGACTGTTTGCCCTGAAAGA

CATGGACAGCGGGACAGAGCTTACTTACGACTACAATTTTCACTCCTTCAACACCGAAGA

GCAGCAAGTGTGTATGTGTGGCTCAGAAGGCTGCCGGGGCATCATTGGTGGAAAGAGTCA

GAGGATCAATGGCCTGCCAGGCAAAGCTGGAGGATCTGGAAGTGGAGCTCGAAGGCTTGG

GAGACTGAAGGAGAAACGCAAATCAAAGCACCACCTTAAAAAGCGGGAGGAGGAGTCCGG

GGACAGCAGCAAGTTTTTCCAGCACCTTTTGATGAAGCCGATGTCTAACAGAGAGAGGAA

CTTTGTCCTAAAGCATCGCGTGTTCCTGGTTCGCAACTGGGAGAAAATGAGGGACAAACG

GGAACTGTCGAAGAGGGATGGAGAGAGGGAGCGAGAGAAGGAGAGCAGCCTGTCACAGTA

CGCCCGGTGGGGAGGGGTCATCCGTGATGATGGCAATATCAAATCAGGTATGTGTATGTT

TGCATGGTTGCCTGTCTAAACAAAGTCAGTTTTTTGTTGTATGCTTAATTATCTGTGTGT

GTGTGTGTGTGTGTGTGTGTGTTACAGACGTGTTCCTAACACAGTTCTCAGCATTACAGA

CCTCTCGTTCAGTGCGCACGCGCAGACTCGCCGCTGCGAGG

>Unigene000073

GCATGTTTTCTGATTTCTTCACAGAGCTGGGGCGGAAAAGAGAATGGCTTCGGCCTAGCA

GAGTGCTGTAGAGATCTGCCTATGACGAAATACCCACCCAGTGCCACCACGCTGCACTTT

GAGTTCTACGCTGAGCCGAGCTCCGAAGTCAAAGTGGAGAAAAAGTCTAGCAGCAACACT

CTACACTACATCCACATTGAACAGCTTGATAAGATCTCAGAAAGTCCGTCTGAGATCATG

GAGTCGCTGACGGTCATGTACAACATCCCGAAAGACAAGCAGACCCTGCTGTTCACGCAC

ATCCGCCTAGCGCACGGCTTCTCCAACCACAAGAAGCGACTGCAGGCCGTGCAGGCCAGA

CTGCACGCCATCTCTATACTAGTGTACTCCAACGCCCTTCAGGAATCAGCTAACAGTATC

CTCTACAACGGGCTGATCGAGGAGCTGGTGGATGTTCTCCAGATCACAGACAAACAGCTG

GTGGACATAAAGGCTGCCTCTCTGCGTACTTTGACGTCTATTGTGCACTTGGAACGGACT

CCTAAACTTTCAAATATTATTGACTGCACCGGCACAGCCTCCTACCATGGCTTTTTACCT

GTGCTGGTCCGCAACTGTATACAAGCCATGATTGACCCAATAATGGAGCCATACCCGCAC

CAGTTTGCCACAGCTCTCTTTTCATTCCTCTACCATTTGGCCAGTTATGATGCTGGTGGA

GAGGCCCTGGTTTCATGTGGAATGATGGAGGCCTTGTTAAAGGTGATTAAGTTCCTGGGC

GACGAACAGGACCAAATCACTTTTGTGACGCGGGCGGTGCGTGTCGTGGACCTCATTACC

AATTTGGACATGGCTGCCTTTCAATCACACAGCGGCCTTTCTATCTTCATCTGCAGACTA

GAGCATGAAGTGGACCTGTCCAGGAAAGAGTGCCCTTTTGTTATCAAGCCAAAGATCCAA

AGGCCTAGTACAACTGCTGAAACGGAGGACATGGACACGGATATGGATGTGTCTGAAGTT

GCCATGGAGAGCAGCCCAGGACCCTCAACTTCCACAGGCTCCAAATCCGACCCTGATCCT

CGTACACAGACGAGCACAGTGAGCGCACCTAGAACAGGTGTGCAGTGCATCCCACAGAGG

GCAGCGCTGCTTAAATCTATGTTGAACTTTTTGAAGAAAGCCATTCAGGACCCGGCCTTT

TCTGACGGCATACGTCATGTGATGGATGGGTCCCTGCCTACCTCGTTGAAGCACATCATC

AGTAATGCAGAGTATTATGGGCCATCACTTTTCCTTTTGGCAACGGAGGTGGTGACAGTA

TTCGTGTTCCAGGAGCCATCTCTTCTCTCCTCCCTGCAGGACAACGGCCTCACCGATGTC

ATGCTTTACGCATTGCTCATCAAAGATGTCCCTGCCACCCGAGAGGTGCTGGGTTCCTTG

CCCAATGTCTTCAGTGCCCTGTGCCTAAACGCTCGTGGCCTGCATTCGTTCGTCCAGTGT

CAGCCCTTCGAACGTCTTTTCAAGGTTTTACTGTCGCCCGACTATCTGCCAGCCATGCGA

CGTCGCCGCAGTTCTGACCCACTGGGAGACACTGCCTCTAACTTGGGAAGTGCTGTGGAC

GAGCTCATGAGACATCAACCCACACTAAAAACCGATGCCACCACAGCAATCATCAAGTTG

CTTGAGGAGATCTGTAACTTGGGCAGAGCTCCTGAGTACATCTGTCAAAAGCCCTCCATC

CAGAAAGCAGATGGAACTGTTACAGTACCTCCAGCTCGCTCTAACCATGCAGCAGAGGAA

GCCTCCAGTGAAGATGAGGAAGAAGAAGAGGCTCTGCATACTTTCACCCAGCAGCAAGGG

GAACCTGAAAGCAACAGGCAAGTGGTTGGCACAGAAGAGCGTATTCCCATTCCTCTTATG

GATTACATACTGAATGTGATGAAGTTTGTGGAGTCCATTCTGAGTAACAACACAACAGAT

GACCACTGTCAAGAGTTTGTAAACCAGAAAGGCCTTCTACCCCTGGTGTCAATTTTGGGG

CTTCCCAACCTTCCTATCGACTTCCCTACGTCTGCTGCATGCCAGGCTGTGGCTGGCGTA

TGCAAATCAATTTTGACTTTATCCCACGAGCCAAAAGTTTTACAGGAGGGACTTTGCCAG

TTGGACAGCATTCTGTCAGCTCTGGAGCCGTTACATCGGCCCATTGAGGAACCGGGTGGT

TCGGTTTTATTGAGGGAACTGGCCAGCGCTGGCCATGTCACCGATGCTACGTTGTCTGCT

CGTGCTACACCACTGTTGCATGCACTTACAGCTGCACACGCATACATCCTTATGTTTGTC

CACACATGCAGAGTTGGGCAGAGTGAGATCCGGGCGATCTCTGTAAACCAGTGGGGCTCC

CAGCTGGGGCTGAGTGTACTTAATAAGTTAAGTCAGCTCTACTGCTCGCTGGTGTGGGAG

AGCACCGTGCTCTTGTCCCTCTGCACACCCAACAGTCTTCCTCCTGGCTGTGAATTTGGC

CAGGTTGACATGCAGAAGCTGGTGCCCAAAGAAGAAAAACCCTCCGGCAGCACTGTGACC

AGTGGAGGCCGGAGAACCGCTGAGACAGAGTCCCTTTCTGTGGGTGTGGACCCTTCTGCT

CAGGGGTTGCTGGAGGGCATGGGTCTGGATGGAGACTCGCTCGCTCCAATGGAGACCGAC

GAACCCACAACTGCTGACCCAAAAGCCAAGTCTAAACTCACGCCTGCCATGGCAACACGC

ATTAAGCAGATCAAACCCCTGCTCTCAGCATCCTCTCGTTTAGGAAGGGCGTTGGCTGAA

CTGTTTGGTTTGCTGGTTAAGCTTTGTGTGGGCTCACCTGTCCGTCAGCGCCGCTCCCAC

CACACCACAAACACAGGCACTGCCCCGACTCCAGCTGCTCGTTCAACTGCTTCTTCACTT

ACCAAACTGCTCACCAAGGGCTTGAGCTGGCAGCCCCCTCCCTATACCCCCACCCCACGC

TTCAGGCTGACCTTTTTCATTTGTTCCGTTGGATTCACTTCGCCCATGCTATTTGATGAG

AGGAAGTATCCCTACCATCTAATGCTGCAGAAGTTCTTTTGCTCTGGAGGCCATGATGCA

CTGTTTGAGACATTTAACTGGGCATTGTCTATGGGAGGCAAAGTGCCAGTGTCTGAGGGG

TTGGAGCATCCTGAACTGCCCGATGGAACTGGGGAGTTTTTAGATGCCTGGCTAATGCTT

GTGGAAAAGATGGTAAACCCCAGCACAGTGCTGGACTCTCCTCACTCTCTGCCTGCCAAG

GTCCCTGGAACAACAACCACCACACCACAGTTCAGCGCTCCACGTTTCCTCATCGTCACT

CAGAAAGCTGCCTTTAACTGCATCCGCAACCTGTGGAACCGCAAGCCACTGAAGGTGTAC

GGCGGTCGTATGGCCGAGTCCATGCTGGCTATTCTTTGCCACATATTACGAGGTGAGCCA

GTCATTCAAGAGCGTTTGTCCAAGGAGAGGGAAGGCACGGCCCGGGCTGAGGAGGATGGA

GCCTCGGTGGTTGGAGGAGCTAGTGGAGGTGCAGCAGCTGGAGCAGTAGGAGAAAGTGCT

TCTGGGTCTGGAGGTAGTGCCAGCACGGCTGGAGGAACTGCAGATGACGCGAGCGCCACG

ACTCGCCGCGAACCACAGGTTAACCAGGCGCAACTTACACAGCTCATAGATATGGGTTTC

TCCAGAGAGCATGCTATGGAGGCTTTGCTTAACACCACCACCATGGAGCAGGCCACAGAG

TATCTCCTCACACATCCACCACCGTTACTCAGTGCTGCCATTCGGGAATTCACCATGTCT

GAAGAGGATCAGATGATGAGGGCCATTGCCATGTCTCTGGGTCAGGAAGTCAGCATGGAG

CAGCGCTCAGACTCTCCTGAGGAGGCTGCCCGCAGGCGAGAGGAGGAGGAGAGGAGAGCC

CGAGAACGAGCTGAGGAAGAGGAGGCTCGCTGTCTGGAACGTTTCCTGGAGGCTGAGCCT

CTGGACAGCAGCGAGCTCCATGCCTTCACTGACACGATGCTACCAGGTTGCTTCCACCTG

CTGGATGAGCTGCCAGACACCGTCTATCGCCTATGCGACCTGCTTATGACTGCCATTAAA

AGAAATGGCCCAGAGTACAGAGACCTCATCCTGCGCCAGGTTGTCAACCAGGTGTGGGAG

GCTGCTGATGTGTTGATAAAGGCTGCTGTACCACTCACTACCAGCGACACAAAGACAGTG

TCTGAATGGACCAAACAGATGGCGACTCTGCCCCAGGCCTCCAATCTGGCCACTCGTATT

CTCCTCCTCACTTTGCTCTTCGAGGAGCTGAAGCTGTCCTGTGCACAGATTGTGGAAAGT

AGTGGAGTTCTGTCAGTGCTGATTAAGCTGCTGGAGGTGGTTCAGCCCTGCCTGCAGGCG

GCCAAGGAGCAGAAAGACATCCAAACACCCAAATGGATCACTCCAGTGCTTCTGCTCATC

GATTTCTACGAGAAAATGGCCGTTTCCTCCAAACGAAGAGCGCAAATGAACAAGTACCTT

CAGCCCAATGGGAATAATTGGCGGTGGTTTGATGACCGTTCCGGCCGCTGGTGCAGCTAC

AGTGCGAGTAACAACAGCACTATAGACTCTGCATGGAGAGCAGGAGAGACCAGTGTCCGT

TTCACCGCAGGTCGCAGGAGATACACTGTACAGTTCAACACCATGGTTCAGGTGAACGAA

GAGACAGGAAACAGGCGTCCGGTCATGCTGACGGTGCAGAGAGTGCCCCGCATGCCCAAA

CCAAACAAATCGGGCAATGTAGCTGACCCTGAGAGAGAGGAAGACCAGAGAGCCAAGACT

GAGGAGACTCAGGTTCTAGCAGACCCAGCGTCTGTAGCAGTCGAGATGGTGGCCCCTAAA

GAAGAACCTGAGCTAAAAGTGTCCTCAGCACCTGCTTCCGCTGCCCCCGCTGCATCACAG

GACTCCTCATCTGGATCCAATAGCATTGTGGTGCAGGGCCTAACTGACGAAATGACCACG

GTCCTGATCCGTGCCTGCGTCAGCATGATCAGTGTACCCGTGGACCCAGACACACTGCAC

GCCACACTTCGCCTATGCCTGCGCCTCACACGTGTCCACCACTATGCTATGATGTTTGCG

GAGCTCAAGAGTACACGCATGATCTTAGGACTGACCCAAAGCTCTGGCTTCAACGGCTTT

ACACCACTCGTCACACTGCTCTTCCGCCACATCATCGAGGACCCGGCTACACTGCGCCAC

ACCATGGAAAAGGTGGTTCGTTCTGCAGTGACCAGTGGAGCAGGAAGCACCACGTCTGGT

GTAGTGTCTGGTAGTTTAGGTTCCCGGGAGATTAATTACATTCTGCGTGTGCTGGGTCCA

GCAGCCTGCCGGAACCCGGAAATATTCACAGAAACAGCCAACAGCTGTGTCCGAATCGCT

CTCCCAGCTCCAAGAGGTGCCGGAACAGCTTCTGATGACGAATTTGAGAACTTCAGAATT

AAGGGACCCAATGCTGTACAATTAGTGAAGACAACACCGCTAAAGCTTTCTCCACTGCCA

CAAATACCAGAAACCATTAAAGAGGTCATCTACGACATGCTTAATGCCCTGGCAGCTTAC

CATGCACCTGAGGAAGCAGAGAAAGGAGAGGAACGTCTAGCGACAGTCCCCGGCAGTCAG

GACCTCTGTCAAATCCTACAGGATGTTGGGGATGATGTTTACCAGCAGTACAGACTTACA

CGGCAAGGGAGCGACTTTGACTCTCAGTCCGCTTTCCATATAAATACTCAGGTGTTTGCT

GCTGATGGTGCAGTTGCAGAATCCTCCCAATCTGGAACACCTCAGGGAGAGGCCTCAACT

CCAGAGGAGATGCGAGAGGACAAGAAGGAACAGGAAGGTGACAAAGCAGGCTCTTCGGAA

GAAAACAAAGCAGCAATGGCCAAAGCAAGCAAACCCCTGATGCCCACCTCCACCATTTTG

CGGTTACTAGCAGAGCTGGTGCGCTCCTACGTCGGCATTGCCACACTTATCGCCACTTAC

AACTACACCGCTGGCCAGTCCGAGCTGATTAAAGAGGATTGCAGTGTGTTGGCCTTCGTG

TTGGATCATCTGCTCCCACACACTCAGAATTCTGAGGATAAAGACACTCCTGCCCTTGCA

CGCCTTTTCCTGGCCAGTCTGGCAGCAGCAGGCACTGGCACAGAGGCACAGGTGACCCTG

GTCAACGAGGTGAAGGCTGCTCTTAGCCGAGCTTTGGCAATGGCAGAGGGTACAGAAAAG

CACGCTCGGCTTCAAGCAGTGATGTGCATCATCAGCACTATTATGGAGTCCTGCCCCTCC

ACCTCCAGTTTTTACAGTACCGCCACTGCCAAGACACAACACAATGGCATGAACAACATC

ATTCGTCTGTTCTTGAAGAAAGGCCTGGTGAATGACCTCGCCCGTGTGCCCCACAGCCTT

GACCTGTCTAGTCCTAACATGGCAAACACAGTGAATGCTGCTCTTAAACCATTAGAGACG

CTTTCACGCATCGTCAACCAGCCCAGCAGCCTGTTTGGAGGGAAAGGAGGCTCCAGTAAG

AGCAAGACTGAACATGATTCTGTGGGCACAGCCAGGGACAGCAACAGCAATACTCAGGAT

CAAGGGGAGGCAGGCGAGGCCGAGCCAGTAGAAAACAGTCACAGAGGCCAGGCTACTGAT

GGTGATCTGATGGATGGAGAGGCAGAAGGAGACACCGTGGTTATCGCAGGACAGCCAGAG

GTGCTCAGCACCCAAGCTATGCAAGTGGAAAATGAGTTGGTCGATTTGATAGATGAACTT

TTAGAAAGGGATGCAGGAACCGTTAATAGTACCATCATTGTTGGTCGCTCTGGAGATGAA

GAGTCTCAGGAAGACGTGCTGATGGATGAAGCTCCTTCAAACATGAGTCAAGCCTCCACT

TTGCAAGCCAACAGAGAAGACTCTATGAACATCCTAGAGCCAGAAGATGAAGAGCACACT

CAAGAAGAAGACTCGAGTGGCAGCAACGAGGATGAAGATAGTCAAGATGAAGAGGAGGAG

GAGGAAGAAGAGGAGGAAGATCCGGATGATGAAGAAGGAGATGAGGATGACGACGACGAA

GGCTCGGAGATGGAATTGGATGAAGATTTTCCAGATATCAACGCAGCCCCACATATACGA

TTCGAGAGATTCGATCGAGATGATGATCTCATTATAGAGTTTGACAACATGTTCTCCAAT

TCTGCTGACATCCCTCCTTCTCCAGGAAACATCCCTAGTTCACATCCTCTGATGGTGCGC

C

>Unigene000074

AAAAAGAACCAGATCCAGGTCAATGATGCCAACTACAGAACTTACCTGCACAACTGGACC

TGCCTGCCAGACTCCAATGATGTTGTTCAAGCCAGACAGACATATGACATCCAGAGTGAT

GCTGTGTACAGGGCCGACCTGAAATGGTTGAAAGGCCTGGGATGGATCCCTATTGGTTCA

CTTGATGTTGAGAAAGCAAAGAAAGCTGGAGAGGCTTTGAGTGAAAGAAAATATCGTCAG

CCCCCGAGCAACTTCAAATTCACAAGCACAACTGACGACATGCCGATAGTGTTGGCGAAA

GCCAATAATAATATCATGAACAAGAAAGCTTATGTTCAAGCCTGGGATAATGAAAAGTCC

AATATCCACATCATGCCAGATGCAATGGATGTGGTCCTTGCCAAACAAAATAAAGTGAAT

TACAGTGAGAAACAATACAAACTTGCCAACGAACTGTCAAAGAAAAAAGGCTATGATTTG

CGCGGTGATGCTATTTCTATCATTGCTGCTAAAGCGTCGCGGGATATTGCTAGTGATTAC

AAGTACAAAACTGGTTACCGTAAGCAAGTTGGCCACCATATTGGAGCCCTAAGCGTCCGG

GATGACCCACTGCTCATGCTGGCTCTGAACTCAGCTAAGATTGCTAGCGATGTCCTGTAC

AAAAAGGACTTCAACAAGTCCAAAACAAAATTCCACCTTCCAGTGGACATGCTGTCATTT

GAACTGGCCAAAAAGAACCAGATCCAGGTCAATGACGATAACTACAGAACTTACCTGCAC

AACTGGACCTGCCTGCCCGATCAGAATGATGTCATTCAGGCTCGCAAAGCATATGATCTT

GCCAGTGATAATATCTACAAGACAGATTTGGAATGGATCCGTGGCTGTGGATGGGTGGCA

GCTGACTCTGTTGATCATGTCAAAGTCAGAAAAGCTCAGCAGATCCTCAATGATAGAATG

TACAAGAAAGATGCCAAGGATAATTTTGCCAAGTTCACAAATATTGTGGATCGACCAGAA

GTTCTCTTGGCTAAGGTTAATGCCTTCAATCTCAGTGATCTGAAATACAAAGAATCTTTC

AACCTTGAGAAAGGCCACTACATCGGATCTGACGATACACCCCAACTAGCACACTCCAGA

GAGGCTGCAAAGATCATCAGTGAGAAACTGTACAAACTTGGCTGGGATGAAGCTAAAGCC

ACAGGATATCAGCTGAATCATGAGTACCTGCCACTTGTATCAGCTAAGAAGGTCGGAAGC

ATCGTCAGTGAAGCTAAGTACCATGATGCGCATGAAAAAGCTAAGGGTCATTACCTGGCC

AACACTCTGGTTGACTTCCCAGCAGTGGTCCATTCTTCACAAATGGAAAAGATGAAGAAT

ATGAGGACCTATCAAAAGGATTACCATACGAGCAAGACTAAGCTTCACATTCCACATGAC

ATGATCAGCCATGTGGTAGCCAAGAAATGCCAGGAAATCCTAAGCGATGTGCTGTACCGC

AGCTACCTGCACCAGTGGACGTGCCATCCGGAACAAAACGATGCTATTCGTGCCCGCAAG

GCCAATGAGATCCTGAGTGATGTGTTCTATAAAGATGATTTGAACTGGATGAAGGGTATT

GGCTGCTATGCCTGGGACACACCAGAAATTGTGCGAGCCAAGAAGTCTTATGAGCTTCAG

AGTGACATCAAGTACAAGGCAGAGGGCAAGAAAGAATTTAATAACTACTCTATAGTGACA

GATACCCCTGTGTATGTGACTGCTATACTGGGCCACACATGGGCAAGCGAGCTGAACTAC

AGAGAGGCCTACCACAAAGAGAAGCACCTGTACACTACTGTTTTGGACACCTATGATTAT

GCAAGGTGCTACAACTTGAAGCAACTGTACAGCAGTAAAACCTATTCTGCTCTTTGGGAT

AGAATCAAGGCCAAGAGCTATACTATTCCTATTGATGCTAATGCTTTGGTCCACGCCAAA

CAGCAGAAGGTCCTCCTCAGCAAAGTGAAATATAAGGAGGATTATGAGAAGTTTAAGTCC

CTGTACAGCCTGCCCAAGTGTCTGGCGGACGACCCAGCCACTGCCAGATGCATCAAAGCT

GGAAAACTTAACTTGGATCGCTTGTACAAAGATAAATGGGAAAAGACAAAAGCCAAAATT

CATATACCTCCAGATATGCTAGATGTGGTGGCTGCCCGCAACACCCAGAAAATGATCAGT

GAAGTGGACTACCGTAAATATCTGCACCAGTGGATCTGCCTGCCTGACATGCAGGTCTAT

GTACATGCCCGCAAAGTCAACGAGCAGCTGAGTGATATATTTTACAAGGAAGATATGACC

TGGCTAAAGGGTGTTGGATGTTATGCATGGGATACTCCAGAAATTCTGCGTTGCAAACAG

GCTATGAATCTGCAGAGTGAGAACCTATACAGAGCTAAAGGAGTGGAGCATTTTAAGAAC

TTCTCTGTAGTAACAGACACCCCGGTGTATGAGACATGCAAGCAGAGTGCCCACAACCTA

AGTGATCTCAATTATCACCATGACTATGTCACCAATGTAAGAGGTAAAAACACTGCTCCT

GCTACGACTGTGGACACTGAAAGGGCTCGTTTAGCCAACTACATCCAGAGTGACCACATT

TACAAAGAGGCAAGCAAGAGTAATATGTCCACTGGCTACTCTCTGCCCTTCGACACTCCA

CTTGCCCTGCAAGCCAAAGCCAACAACGTCATTACCAGCAATGTAAAGTACAAGGAAGCA

TATGAAAACACTAAGGCTAGGAGCTACAAACTGGACCCCAATGGTGTCAATTTCGTCACC

ATAAGAAAGGCCAACCAGGTCACCAATCAAAGACTATACCGTGCCCTGTATGAAAAGGAG

AAGGACAAAGTTCACTCTGTATATGACACCCCTGAGATCAAACAGGTTAAGGCCACTCAG

GAGGCCATCAGTGATGTTTGCTATAAAGAAAAGTACTACAACAGCAGAGGGACACTACTG

CCAATGCCATTCACGCCACAGCTGATGCACTGCGCTCATGTCAATCAGATTAACAGTGAT

CTGAAATACAAAGAGGATTTGCAGTGGCTGAGAGGAATTGGTTGCTTCGTGTTTGATTCT

CCTGAAATGGTCCGCATCCGTGAAATCAACAAATTCAGGACTTCCTACGATATTGATGCC

AAGAAAAACTTTTCCAACTTCTCTGTGGTCTTGGACACGCCAGAGTACAAGCGTGTGTCT

GAACTCAAGACGCACTTAAGTGAGCGGGTGTACAAAGCTGCGGGAAATATACAGAGAACT

CAGTGCAGCACAACTGGTGACGCTATGGAATTTAAGCGAGCCAAGTGGGCCCAGACTCTT

ACCAATAAGTGGCTATATACTGATTTGGCTTCCAAAGAAAGAGCCTTCTACACTCCAGAG

CTTCACACTCCTCTTCTGGATCATGCTAGATCTATGAAAGTGGTGTACAGTGAGAACAAG

TATAAAGAGCAGTATGAGAAGATGAAGCACAAGTACACTCCCGTCCATGACACCCCCATC

CTAATCCGTGCTAAAAAGTCTTACCTTAATTCCAGTGACCTGCGCTATAAAGAGACCTTT

GAGTTAGCCAAAGGACACTACCACACCACTAAAGATGCCCTGGACATTATTTGTGCTAAG

CGGGTCAGAGATGACATCAGTGAGGTTAAATACAGAGAGAAATACATCAACTCACTAGGA

ACATGGAAGTCTATCCCGGATCGTCCTGAATTCTTCCACAGCAAGGTTGTGCGAGACTGC

ATCAGTGATTACAAATACAAAGAGGACCTGGAATGGATCAAAGGTGTTGGCTGTTTCGTG

TGGGACACACCATTGCTGAAGCAGGCTGAGCATAACAAAGCACTGTACAGTGAGAGATTG

TACAAGGCCTCATATGAGAAGACCAAATGCAACTTTAAGTACACATGCGACACACCATTC

TTTCAAGCGGTTAAAAATGCCTCCGCCCTCTCCAATGATTTCAAGTATAGAATCAGGGCC

AAAGAGCTGCTCAAGAGTGGCTGCAATGAGCTACGCCGTCCCGATCTTTTGACCGCCATA

TACAACACTGGCATGTGGAGTAAGTGGAAGTACAGGTCACAATATGAGCGCCTCAAGTCA

AAATACACATCAGTGCTGGAGACCCCCGAACACAAAGTCCACAAGCTTAGAAAGCAGATC

AGCGATAAAATCTACACGATGGAGTATAATAAGAATAAGGCTAAGGGCTACACACTCGGA

CACGACACTCCTCTGAATCTGCACATGAAGAAGGTCAAAGAAATTACCAGCAACCTGAAA

TATAAGGAAGTGTATGAGAAAAACAAAGCTCAAATCAACATCGCTCCTGATGCTTTTGAC

ATCCGGGCTGCTAAAGAAGCCTACAAGAACATCAGCAATCTTGACTACAAAAAGAAGTAT

GAGGCCACCAAGAATAAGTGGATTTGGACCGTGGATAGACCTGACTTTGTCCACGCTGCC

AAGATCAACTACCAGCAGAGTGACGTTGAGTACAAGTACGACAAGGAGATGCTGAAAGGT

TGCGTGATGCCTGTTACTGATGATAAGTACACACTTCTAGCCAAGCAGAACACAGAGCTT

TCCAGTGACATTAAATACAAGCAAAAGTATGAAGAGGCCAGAGGACATTACCACGTTGTA

CACGATACTCCTCAGATCCTCCATGCAAAATCAGTGTCTGGTCTGGTTTCTGAGAGCAAA

TACAAGCTGGAAAGCAAGAAGGCTCGACAGTCTGGCTCTTTCACCACCTTGCCTGAGACC

CGTGACACAGCCCACAGCAAAGCAATGACCAAAATCACAAGTTCTAAAGTGTACAAGCAG

AAGTTTGAGAAAGACAAAGGAAAGTCGATATATAACAATATGACCGTACCTCCAGATGTA

AAGCATGCCATGGATGTGGCCAAGAGCCAGAGCAATATTAATTATAAACAGGAAGCTAAA

GCCAAGTTGCATTACACACCTGTTGCTGACCGCCCTGACATCCTGAAGGCTACCCAGGCT

GCCAAACTCATCAGTGAGGTGGGTTATCGTGACAAAGCTCGTCAGGAAGCCAGTCGTGGA

GGATCGCTGGTCAACCGTCCTGATATCAACCTGGCCACCGAAGTGTCCAAGCTTACTAGC

CTGGTGAAATACAAAGAGAAGTTTGACAAGGAGATGAAAGGAAAGAGACCACAGTATGAC

TTGAAGAACAGTAAGATTTATCAGACATTGAAGGATGCCAATACGCTTGCAAGTGAGGTG

AAATATAAGGGTGACCTGAAGAAGATTCATAAGCCTGTGACTGACATGTCTGAGTCTCTG

GCCATGCAGCACAACCTGAGTACCAGCAAACTGGCCAGTAGTGTAAAATACAAGGAGAAG

TACGAGAGGGAGAGAGGCAAGGCCATGCTGGACTTTGAGACTCCGACTTATGTGAGCGCC

AAAGAAGCACAGCATATGCAGAGCCAGAGAGAGTATAAGAAGGCATTGGAGCAGGAGATA

AAGGGCAAAGGAATGTTGGCTTTGGCTAACGATACTCCAGACTTCATGAGAGCAAGAAAT

GCTACAGAAATCCTCAGCCAGAGTAAATACAAGCACAACGCTGAGCAGGACAGGGCCAGC

TATACCACAGTGATTGACACTCCCGACATCATCCACGCTCAGCAGATCAGGAATATTGTG

AGCCAGAAAAAGTACAAGGAGGAGGCAGAGAAGACCATGTCCCACTATAATGCAGTACTG

GACACTCCTGAGATGCAGAGAGTGCGGGAGAACCAGAGGAACTTCAGCACTGTTTTGTAT

AAAGAACCTTCTGGCAAAGGAACTTCTGTCATATTTACACCAGAGATGGAGCGGGTCAAA

CGGAACCAAGAACATATTAGCTCGGTTCTATACTCCGACAGCTTCCGCAAGCAGGTGCAG

GGTAAAGCAGCCTTCGTACTGGACACTCCTGAAATGAGACGTGTGAAGCAGACTCAGCGT

ATCATCTCTGGGGTGCAGTACCACCAGGACTTTGAGAAGAGTAAGGGCAGCTTCACTCCC

ACTACCACTGACCTGGTGACTGAACGTGTTAAGAAAAACACTCAGGACTTCAGTGACATC

AGTTATCGTGGCATTCAGAGGCGTGTGGTGGAAATGGAGAGGAGACGTGCTATAGAACAT

GACCAGGAAACCATCACTGACCTGCGTGTGTGGCGTACAAACCCAGGCTCTGTGTTTGAC

TATGACCCTGCTGAAGATAACATCCAGTCTAGAAGTCTCCACATGATGTCAGTTCAAGCT

CAGCGCCGTAGTAAAGAACACTCTCGCTCGACAAGTGCCATGAGCGGCTTAGGAGAAGAG

AAATCTGAGCCGTCCGAGAGCGTGGACCACCACATGTCCGTCTACAGCAACGGATTCCAG

ACTCATACTACGGGATACCAGCAGGCCAAGACAGTTGAACTTCAGCAAAGATCCTCTTCT

GTAGTCACCCAGCAGACCACTGTGTCCTCTGTTCCCTCACACCCCTCCACTACTGGGAAA

ACTGTCCGTGCCATGTACGACTACGCTGCTGCCGACAATGATGAGGTGTCCTTCAAAGAT

GGAGACGTTATCGTGAACGTGCAGTCCATCGATGAAGGTTGGATGTACGGCACAGTGCAG

CGTACTGGAAAAACCGGCATGCTGCCTGCCAACTACGTAGAGGCGGTCTAAAGACTAGTG

CGAGTACGACATCCACACAGCTGAGCGTCTCCAGCATTTCCCTAGTTTACGTGTAGTTGA

TGGTAGTTTAGAATAAGAATAGCACTGACCCCTGTTCGTCTGCATTAACAGTATGTGTAA

TTCGCTTGTGCCAGTTTCAGTGGATGTGACATGACACTATGTACTAAACCCAGCAGGACA

TTTAAAAGACCATTATTTTGTATATTAATGACAAAAAAACATTAAGTTGGTTTCAATGCA

GGCTTTCTGCATTGAATGTTTCACACCAGAAGAGATGAGCTTATGAGATGTAAAAAAAAA

AATAAAAAATCCGACATTCGCTACACTAAAAAAAAAAAGACGTTGTTAAACAGATTAGGA

ATAAAAATAAAAATCGTGAGGAGAAACCATCAGTTCCACACAAATTAAGAATGATACACT

AAAGACTCCCGAGTAACACACCTCTTCCCTCCTTTGCTGAAGGCTAGTTACAAGTTACAT

GTGTGTTTTGCTTCTTCTAATTCCCTTGGTTTAGGGTTAGAGTCATATTTTGACTCGGTA

GCTAATAAAGCACACGAAGCTTCTGCAGT

>Unigene000075

ACACACACACACACACACACGCATCTTTCACATTGCTCTGAACTTCCAGGAAAGGACGGC

TGGCACAAGAGAGAGGGGGTGGTGTCAGCTGACTGGATCCGTCCAATCAGATAAGTCCTG

ATGTAAGTGTCAAGCAGCAGCTGAGATAGAATCGGCGTGCCCTGAGGACTCGTGCGCCAG

TAGCGCAAAAGAGAGTGCAGCATGGCTTGCTGAGAGACGGAACAAACGTGTGCGAGGGAG

AGAAGTGTGTGTGAGAAAAAGAGAGAGAGGGGTGGGGAGAGGACAAGGTTGGTTCCTCTC

CAGTATGGACGAGTCCAGCATTCTGAGGAGACGAGGGCTACAGAAGGAGCTCAGTTTGCC

CAGAAGAGGGAGCTTCTGTAGGACGGGGAACCGGAAGAGTCTGATAGGAAGCGGACAGTC

ATCAGGACTTCCCCGGCCGCATTCCCCCTTTTCATCACACACCGGCACAAGTCCCCAGGA

CAGCCCTAGAAACTTCTCCCCGAGTACCTCAGCACACTTCTCCTTCGCTCGCAGGACCGA

CGGACGGCGTTGGTCCCTGGCATCGTTGCCGTCGTCCGGATATGGAACCAACACCCCCAG

CTCTACCATTTCTTCGTCCTGTTCATCACAGGAGAAGCTCCATCAGCTGCCCTTCCAGCC

CACTGCTGACGAGCTCCACTTCCTGTCCAAGCACTTCTGCACTGAGAGCATTGCTGGAGA

CGACCATCGCAGGGCCGCGTCCATGCGCCCGCGCTCACGTAGTCTCAGCCCTGGAAGATC

TCCATCCTGCTGTGATCACGAGATTATTATGATGAATCACGTGTACAAAGAACGTTTTCC

TAAGGCCACTGCGCAGATGGAGGAGCGCATTCAAATTATCATCAGCAGCAGCTCCCCGGA

GAACGTGCTTCCTCTGGCAGACGGAGTGCTGAGCTTCGCCCATCACCAAATCATCGAACT

GGCACGCGACTGTCTGGAGAAATCTCGGCTCGGCCTCATCACCTCACGCTACTTCTGCGA

GCTCACTGACAAACTCGAGAGACTTTTTCAAGAGTCCACTGAGAGATCGGAGAGTGCTGA

GGTGACTTTCATTAAGGAACTGGTGAAGAAGATCCTAATCGTCATCGCCAGACCGGCGCG

GCTTCTGGAGTGTCTCGAGTTTAACCCAGAGGAGTTCTACCACCTGCTGGAGGCGGCTGA

GGGTCACGCCAAGGAGGGACAAGGCATCAAGACCGACATTCCGCGCTACATCATAAGCCA

ACTCGGTCTGACACGAGACCCTTTGGAAGAAATAGCCCAGTTAACCAGCTACGACAGTGG

AATCGCGGAAACTCCCGATACCGATGATTCTGTGAGTTCCCAGAGTCTGAGCGCACCGCC

GCAGCCCCGCCGTAAACCCTGCGAGATAGATTTCGAGATGATCAAACTCATCAGTAACGG

AGCTTACGGCGCTGTCTATCTGGTGCGACACAAAGAGACCAATCAGCGTTTCGCCATGAA

GAAGATCAACAAGCAGAACCTGATGCTGAGGAACCAGATCCAGCAGGCGTTTGTAGAGAG

GGACATCCTGACCTTCGCCGAGAACCCGTTTGTGGTCAGCATGTACTGTTCCTTCGAGAC

ACGCAGACACCTGTGCATGGTCATGGAGTACGTCGAAGGTGGGGACTGTGCCACTCTGCT

GAAGTACATGGGTCCTTTGCCGGTGGACATGGCCAGAATGTACTTTGCCGAGACTGTCCT

TGCTTTGGAATACCTCCATAATTATGGCATCGTCCACCGAGACCTAAAACCAGACAATTT

GCTGGTGACTTCGATGGGACACATCAAGCTGACAGACTTTGGCTTGTCCAAGGTGGGCCT

GATGAACATGACCACTAACCTGTATGAAGGTCACATAGAGAAAGACGCCAGAGAGTTCTC

GGATAAACAGGTGTGTGGCACCCCCGAATACATTGCTCCCGAGGTAATTCTGAGGCAGGG

ATATGGCAAGCCTGTAGACTGGTGGGCCATGGGGATCATTCTCTACGAGTTCCTAGTAGG

ATGTGTGCCGTTCTTCGGAGACACGCCGGAGGAGCTGTTTGGTCAGGTTATAAGTGATGA

GATCAACTGGCCTGAGGGAGAGGACGCACCTCCGGCTGACTCTCAGGAGCTCATCACGCT

GCTGCTTAGACAGAACCCACTGGAGCGCCTGGGGACTGCAGGAGGAGCTTACGAGGTCAA

ACACCACCAGTTCTTCCACAGTCTGGACTGGAACAGTCTGCTCAGACAGAAGGCTGAATT

TATCCCCCAGCTTGAGTCCGAGGACGACACCAGCTACTTTGACACCCGATCCGAAAGGTA

CCATCACCTGGAAACGGAGGAAGAGGAAGACACGAACGATGAAGACTTTAACGTGGAACT

GCGTCAGTTTTCCTCGTCCTCACACCGGTTTTCTAAAGTCTACAGCAGTCTGGATCTGTC

ACGTGGGCAACTGGAGGAAAAGGGAGAGCAACCAGAGAAGAAGAGTGAGAGTCCCCTGAC

TGTCGACTCGCTCAGCTGGACTCCAGACTTCACTGAAATTCCATCACTGTCCCACTCCTC

TGACCTGGAGAGCAGCACGAACAGCCTGAGCCGAAACTCAGGACTTCTGCCCAAATTTGC

CATTTCAGCAGAGGCTGATAGCTGCGATAGTTCGCTGTCCCTGGACAAGGCGAGTAAAGT

GGCCTTCTCTGTTGGAGAACTTCCTCAGACGGAGCCTGATGCCATCACACCATCCAGCCC

TGTCAGCAACGCCACCCTGTCAGGCAGTTTCTCTGAACATCTGGACCAGCTGATATCACG

TCCAGAGGGAGCAGACAGTCTGGACCCTCCCAACAAGCCATCCACTGATACCGTCAGCCA

TTTGACCTCTCTAAGACCAGCGACCAGTATACCGAAATCATCATCCACCAGCGCTCTGTC

TCTCATGATTCCTGCAGATGTTTTTGGCACATCCCCACTGGCTAGCCCCATGTCTCCACA

TTCACTGTCGTCAAACCCCTCCTCCAGAGACTCCTCACCCAGCCGAGACTCCTCCCTCTC

TGCCGCCAACTCGCGCCAGCCAATCATCATTCACAGTTCAGGGAAGAAGTTTGGTTTCAC

ACTGCGAGCGATTAGAGTCTACGCCTGCGACAGCGATGTTTATACTGTCTATCACATGGT

CTGGAATGTGGAAGACAGTGGTCCTGCTCAAAAGGCTGGACTCAAAGCTGGAGACTTGAT

TACTCATGTCAATGGGGAAACGGTCCATGGACTGGTGCACACTGAAGTGGTGGAACTGTT

GCTGAAGAGTGGAAGCAAGGTTGCTATATCCACTACTCCTTTTGAGAACACGTCGATCAA

AACGGGTCCGGCTAGAAGGAACAGTTATCGGAGCAAAATGATTAGGAGAACCAAGAAGCC

CAAGAAGGAAAAGACACAAGAAAGGCGACGATCAGTATTCAGGCGCTTCGCCATGCAGCC

CTCTCCACTGCTTCACACCAGTCGCAGCTTTACTTCCTTAAACCGATCCTTGTCCTCAGG

GGAGAGTCTGCCAGGATCCCCCACACACAGTCTGTCTCCTCACTCCCCCACCACTCCCTT

CCGGCCCACGACGGAACTCAATCAATCAGGTGGCACATCATCTCAGAGCAGTTCCCCCAG

CTCTAGTGCACCAAATTCCCCAGCTGGCTCCGGCCATGTCCGTCCTAACACTTTGCACGG

TTTGGGCCCTAAGTTGCCTGGCCAGCGGCTTCGTCAAAGTCGCCGGAAATCAGCGGGAAG

CATTCCTCTCTCCCCGTTGGCTCGAACACCTTCCCCTACCCCTCAGCCCACCTCCCCTCA

GCGATCTCCTTCCCCATTGCTCGCTCACTCGGTGGGAAGCTCCAAAACTACTCAGACTTT

CTCAGCCAAGATGCACTCGCCCCCTACAATCGTAAGGCACATGGTGCGACCTAAAAGTGC

CGAACCTCCTCGGTCTCCGCTCCTTAAAAGGGTTCAGTCGGAGGAAAAGCTGTCGCCATC

CTACACAGGGGATAAAAAGCACCTCTGCTCCCGGAAACAAAGTCTAGAGGTGACCCAAGA

AGAAGTTCAGACTGGGTTTGCATCTGGAGGTGAGCACACACTTCAGTGTGTGGAGGAGAG

TTCTTGTGAACTTCCCACCATCACCCGAGTAAGACCTGCTGAGCAGGGCTGCCTAAAAAG

ACCTGTGACTCGTAAGGTTGGACGACAGGAAATGATGGAGGATCTTGATAAGGATAAATT

GAAGTCCAATGTTGTAGCGAAAAGACAGGACTGGCATGAGAGAAGAGAATCTCTACAAAA

ACAAGATGCCATTCAGGAGGCTGAATCTACCCAAAGCCTGACTGGGTGTTGCTCGGTTGA

TAACAAAGTTGTCACTGCTACAGTAAAAGATGTTCTTTATAAAAAACTAAACTCCAGAGC

ATGTGAAAGCATTGCAGACTCCATGAGCAGCACTGGAGACTCTGCGTCGGTTTTCAATGA

GAGTATAAGATCATCCCCTGCTCAGTCAGACCGACAGCTGGCCAGGCAGATCAAAGAGGG

CAGTAAACCAGACAGGCATGATTTCAAAGCCCCTAATATGGAATTTGCTCGGAAAAGACA

GTCCTTTGAGGAGCGTGAAGACTGTTTGTGTCGAATCACCTCTGGCGTTCACGAAAGTCT

ACATTTTAATGCCACTAGATCCAAAAGTCTACAACTGGATTCAGCTGGAATTGCTACTCC

TGGAATCACCACAGATTCGTTGAGCCCTAAGATATTCAGTGGGCGAGGGGAAAGTGCCGT

AGAGAAGCTTCAGATTATCTCGTCCACAGAGGGCTCCATCAGAAAGACTTCATCTGAGTA

CAAATTAGAAGCCCGACTTGTTTCTTCCCTGAAACCACTAAAGGGCACACTGGACATAGG

GCTGCTCTCTGGGCCTAGAATCTCCAAAACTGATACATGTCTTTCAAAAATAGCCAGTAG

CCAAGCTGATAGTGGTGGTCAGGTCGGAGCCATGCAAAACCAGAGTGAAAAGCAGCCCCT

GATGCTAACACATAAACCGACACAGGCTGCCTTGGAGATAAAAAGTCCCGGTGCTTCGAG

CAGCAAAAATGCAGGAAAAGATGAACCCACTTGCAGTACAGACTATGTTTTTGGCTTGTT

TAGCCATGAAGGCACTCATGACAAAAATAAGCAGGGTGAAGGAATCAAATTCCAGACCGT

AGCCAGTAAAATGGAGCCGAAAGATTTGTCAACAGGTGGAAAAACAGAGAATAAGTTGAA

AACAGCTCAAGAAATGAGGCCTGCTCGACACAGTAATCATTTTTCTTGTGGGAAAACACC

CTCAATAAGGGAGGTCAGCAATGAAGACCAGGATGATGAGATGGAAAATCCGGTGGAAAA

ACCTACCCTGCAAAACAACACAGTAGAAACTAAGACGCCAATGACCCATGACCAGGTCAT

CACACAAAACACATCACTACCCTCAGGCCCGTGTAGTGTTGATAAAGATAAAAATGTGAG

AGAAATCCTTATCAGTGCCCAGAAATCGGAGCAGGCTTTTGCAAACACCAATAAAATAAC

AATGGCAAAGGATAGAGTTCTGAATTCTCTAGGGTTAACTGTGGTTACACATGAAGTTTA

TCAGTGTAAAACTGTCCAAGATGATGCTTCAGGATCTCGAAGCTCAGAGGCATTGGTGCA

GACTTTGCCTCCAAAACCAGACAGAACAGTTATTGTGCAAAAGACAGGCTCAGATGTAAA

AGCTGGGATTGTGGCAATTGATGAGAAAAAAACACTAAGCACAACTGGAAAGAGTACAGA

TAAGAAACATGAACAGATCACAGCAAGTAGCACACCAGTGTTGCATGCGAAAACAGAACC

GTTTCACTCTGAGGGTCTAGTAAAAGAAAAGGTATCGGTTCCACGGAGTTCAACTGTAAC

CACATCATCTCCTTCGGCAGCTTCGATAGTGCCAGATATAAAGTTGCCTCCAAATTTACC

TCTTCAAACCAAACAAGATAAAAACAATGTAGCTCCCAAAGGTACCCCTGTTAAAGCCAC

GTCTGCTGTGGTTCCCCAATTACCCCAGCATGTACATAAAAGCCCACAGTCATTTGCTGA

AATCCAATCAGACAGCACTGCAATAAACCAGTCTCAGCAAACGACTTTGAAAACACCAAC

TGGTGCTGACTCAGGTAACGGGCAAAAACGTTTAAATTCTATCAGCAGAACAGATGACCA

AAATGCTGAAGTGGAGAATGGCAAAAGATCCAGTTCAAAATGTCAGGAGACATGTGGAGT

TAAAACCCAAATCGTAGACAAGGATCATAAGACGTGTATGGTTCCAGAAGGGTCAATCGA

TAAATCCAAGATTACATCATCCAAAGATAAAAAGGCACAAAATATAGCAGTTTCAAAGGA

GGAAGTAAAATGCATTATGACAAACGAGCCTCGCAGTCCTACAGACCTCAAAACTGATGG

GGCAAAGTGTGTCCTAGCTACTGCTCACACGCCCCCTAGTAAGTCAAATCAGTATTCTGG

TGTCAAAGGCAGTGAAAAAAATAGCAATACTGTGCAGGTTAAGTCAGTGAATATACTTGA

AGAAAAGTTGAAAGATAAGAATACTCCAGATGTATCCTGTCAGAAGGTCACCAAGCCGGT

ACTCGACGTTTGCAAGAAGGAGGTTGTTACTGCAACAAAAAAACAACCACAATTGACAGG

TACAAGTTCCAATGGAAAGCAGGGCCTGTCGAGCTCTAAGAGCAAAGCTAAACTAGAACC

ATCTCCTGCTACAAACCAGAGTACAAAAGCCACCGAAGGAAGAGGAACTCAAGAAGGCAA

AGCACCAGTGCAGGTCATCATAGATCATACAAATCTAAAACCAAAGGAGCAAAGAGAGGT

AACACTTCCTACATTAAAAACTCAATCACAGAGTGAGAACCTGCCTCAGACCACCAGCCC

TCAAGTTGGTTCTGTACCTGTACCTACTGTTAAGGTTCACACACCAAGTGGATCTCCTAT

AGGCAAGGGTACAGGGTCCACAGTGGAGACCTCAAAGTCGCCTGACTCCATGCAGAAAGG

AAACCGCAAAGACTGTCAGAAATCGTCAGGATTGGCGAAAGACGGTGCAGCACCAGAAAA

AGATTCACCTCGCACTAAGCAGTCCAAAGATCTGCCCCGAGGTTCAAATAATAAAAAGTG

ACAGTGAGGATGCCGAAGGTTGCCAAATCATTTTTTGTAAGAAAAACTGCAAAGCAATCT

CTTT

>Unigene000076

GGTCATCCCTGTTTGGAGATGCACCGACATTCATTTTCCTACATCTAGAAATGAACGATG

GCCGCTAAAGGAGGAACGAGGGGGTTTTATTTTAACACTGTACTGTCTTTAGCTCGCTCT

TTGGCTGCTCATCGTCCGGCTCCGATTGACAAGGTCCAGAAGCTACAATGCATGTGTCCG

GTGGATTTCCGTGGTGTTTACCAGCTGGACGAGAGAAGAAGAGACGCTGTGATAGCACTC

GGTATCTTCCTAGTAGAGTCTGAGCTGCAGCACAAAGATCCTATCGTCCCGTACCTCCTG

AGTTTGCTGAAAGGGCTGCCGCGAGTCCAATGGATCGAAGAAAGCTCGGAGAGGAAGGGG

AGAGAAACTCTCCCTGTAGCTGAGAACTTCAGCTTTTGCTTGGTGACTATGCTGTCTGAC

GTGGCTCAGAGGGATGAGACCTTACGCATCCAGATTCTGGAGGCTGTGATGGACATCATG

CAGGTTCTTCTGGATTTATGTCGCAACCCTGAGGATCAAGACAAAGAATACCTGTGTCGA

TACACTTTGCCGTGCCTGCTTGGTGTGGTGCGAGCGTCTGGACGCTACAGCAACACAGAC

GAAGCACTGCTGTCTAAACTGTTCCCACGGACATCAACGCAGCCGGTGATCGCCTCGGAG

GAGCCGGAGTCAGTAAGACGGCGCTCGTTTAACGACTTTCGCTCCATCTTACCCAGCTCG

CTGCTTACCGTGTGCCAGAGCGATACACTGCGCCGCAAGACTAGCAGCGTCTCCAGCATC

TCCCAGCAGGCCAGTCCTGAGCGTACAGGATTACCTCCCAGCTCTCCTTCTGACCCGTCT

GCTCCTTATTTTGAAGCAGGATCATATTTACCTGATGGCAGTGCTGTAGACCCGGACTAT

TACTTTTCCACCATTAGCTCCAGTTTCTCAGTGTCTCCGCTCTTCACTGGCTGCAGCACC

AGAGGCTTTGGTGTGCCTTTGGACATGCTGAGGCGTCTCCTTCAGATGATTGAGCAATTT

GTCGCCGAGTCGTTTTTAAAGAAGTTGGATCAAACCATGGCCGAGTTTTTGGAGAGCAAT

CCTGGTCATCATCTGTTCTACAAAACCTTCAGTGACCCTCTGTATGTGGCAATATTTAAA

ATGTTAAGGGACACACTGTACTATATGAAAGACCTGCAGACGTCCTTCGTAAAGGAGGTG

CACGACTTTGTGCTGGAGCAGTTCAGCAGCAGCCAATCAGAGCTGCAGCGCATCCTGCAC

GAAGTGGAGTCTCTGCACAGCGAACTGAGCCCGCTAAAGTTACGGTGTCAGGCCAATGCC

GCTTGTGTGGACCTCATGGTGTGGGCCGTCACCGAGGAACAGGGTGCCGAGAATCTTTGC

ACCAAGCTGTCAGAGAAGCTGCAGTCTAAGACGTCCAGTAAGGTCATCATCGCCCACATG

CCTCTGCTCATCTGCTGCCTGCAGGGTCTTGGCAGGCTGTGTGAGCGCTTCCCGGTGGTG

GCTCACTCAGTCACCATGTCTCTAAGAGACTTTCTGGTGGTGCCGTCACCTGTACTGGTT

AAGCTCTACAAATACCACAGCCAGTATGCCAGTGGAACCGGTGAGATTAAGATCCATGTG

ACCAATGAGCATTCCCAGTCCACTTTCAGTGCCCACGCCAGTAAGAAAAGCCAACCGTCC

ATGTATGAGCAGCTCAGAGACATCTCCATAGACAACATCTGCAGGTGTCTAAAATCAGGA

CTGACCATGGATTCTGTCATTGTCGAGGCGTTTCTTGCCAGCCTGTCCAACCGTCTTTAC

ATCTCACAGGAAAATGATAAGGATGCTCACCTTATTCCGGACCACACCATCCGGGCATTG

GGTCACATTGCCGTGGCATTAAGGGACACGCCACGGGTCATGGAACCTATTCTGCAGATC

CTGCAGCAAAAGTTTTGTCAGCCGCCCTCTCAGCTCGATGTGCTTATTATTGACCAGCTG

GGCTGCATGGTCATAACTGGCAATCAATACATATACCAGGAGGTCTGGAACCTGTTCCAG

CAAATAAGTGTGAAAGCCAGCTCCATGGTGTATTCCACCAAAGACTACAAAGATCATGGA

TACAGGCATTGTTCCCTAGCTGTGATCAATGCTCTAGCTAACATCGCGGCTAATCTGCAG

GCTGAGCAGAAGGTGGACGAGTTGCTTGTAAACCTTCTAGAGCTGTTTGTTCAGCTAGGT

CTGGAGGGCAAGCGTGCCAGCGAGCGAGCCAGTGAAAAAGGGCCAGCGCTTAAGGCTTCA

AGCAGTGCCGGTAATTTAGGAGTGCTCATTCCTGTCATTGCAGTGCTCACCAGACGTTTG

CCACCAATAAAGGAGGCTAAGCCCAGGCTGCAGAAGCTTTTCCGAGATTTTTGGCTCTAT

TCGGTAGTCATGGGCTTTGCGGTGGAGGGCTCAGGTTTATGGCCAGAGGAATGGTATGAA

GGTGTGTGTGAAATTGCCACTAAATCCCCATTGTTGACGTTTCCCAGCGGTGAGCCTCTG

CGCTCAGAACTGCAGTATAACTCCGCTCTGAAGAATGACACCGTCACTGCGCCGGAGTTA

ACTGATTTGCGAAGCACCATCATTAACTTACTGGATCCTTCACCTGAAGTCGCAGCCCTT

ATCAATAAACTGGACTTCGCCATGTCGACCTACCTGCTTTCAGTCTACCGGCTGGAGTAT

ATGAGAATGCTACGCTCTCTGGATTCCGACCGTTTTCAGGTGATGTTCCGTTATTTTGAA

GATCGAGCCATCCAGAAAGACAAGTCGGGGATGATGCAGTGTGTGATTGCGGTTAGTGAC

AAGGTCTTCGATGTCTTCCTTCAAATGATGGCAGATAAGCCCAAGACCAAGGCTCATGAA

GAGGAGCTGGAGCGACACGCGCAATTCCTGCTGGTGAACTTTAATCACATCCACAAACGG

ATCAGGCGCGTGGCTGATAAATACCTCTCGGGATTGGCTGAAACCTTCCCTCACCTGTTG

TGGAGCGGTCGAGTGTTAAAAACAATGCTGGACATTTTGCAGACGCTCTCCCTGTCACTC

AGCGCTGACATCCACAAAGACCAGCCATATTACGACATTCCAGATACTCCGTACCGCATC

ACTGTTCCTGACACTCACGAAGCCAGAGAGAGTATCGTGAAAGACTTTGCTGCACGATGC

GGTGAGATATTAAAGGAGGCCATGAAGTGGGCACCATCCGTCACTAAATCTCATCTACAG

GAATACCTGAACAAGCACCAGAACTGGGTTTCAGGTCTGTCCCAGCACACAGGCCTTGCT

ATGGCTACAGAGAGCATTCTGCACTTCGCAGGCTACAACCGACAGAGCACGGCTTTGGGG

ACCAATCAGCTCACAGAACGGCCGGCGTGCGTTAAAAAAGATTACTCCAACTTCATGGCC

TCGTTGAACCTGAGGAATCGATATGCTGGAGAGGTGGCGGGGATGATCCACTTCCTGGAG

GCGACAAGCAGCACCTCCGATCTGAATAAGCTGATGATCCGGCAGCTGAATGAGGCTCTG

GAAAGGCAGCAGCCTGAAGAGTTCACTGAAGCTATGTTCAAAATGGCTGCCCTCCTGATC

ACCTCCAAAGACTGCGACCCGCAGCTCCTGCATCACCTGTGCTGGTCCCCACTGAAGATG

TTCACTGAGAACGGCATGGAGACAGCCATAGCCTGTTGGGAATGGCTTTTAGCAGCTAGG

ACAAGCTTGGAAGTCCCATTTATGCGTGAGATGGCAGGTGCCTGGCAGATGACGGTGGAG

CAGAAGATAGGGCTTTTCTCTGAGGCAGAAGTGGAAGCTGATCCACTGGCAGCATCTGAG

GAGAGTCAGCCCAAACCCTGTGCCCCCAATGCCATCCCACATTACATCTGGATCGAGTTT

CTTTTCCAGCGCTTTGAGATCGCCAAGTACTGCAGCGCGGACCAGGTGGAGATTTTCAGT

AGCTTGTTGCAAAGGTCGCTTTCGCTTACTGTAGGAGGCTCAAATAGCAGCCTGAACCGA

CACGTGGCAGCTATCGGACCTCGCTTCAGGTTGCTGACGCTTGGTCTCTCGCTGCTTCAT

TCTGACGTGGTTAGTAATGCCACCATCCGCAATGTGCTAAGGGAGAAGATCTACTCTACT

GCCTTCGATTACTTTAGTGTGGCTTCCAAATTCCCCACTCAGGGGGACAAGCGTCTACGG

GAGGACATCAGTGTCATGATCAAGTTCTATGCCAGCATCCTCTCGGACAAGAAGTACCTT

GCTGCTAGCCAGCTCGTGCCACTTGACAATCAGGACCCGTCACTAAACAGCCTGTCGGTG

ATGACTGCTGCCGACCCCAGGAACACCATGGACATGTCGGTGGGTACACGCCAACAGAGT

GCCCAAGGCTGGATCAACACCTACCCACTCTCCAGTGGAATGTCCACCACTTCCAAGAAG

TCGGGAATATCAAAGAAGAGTAACCGAGGAACTCAGCTGCACAAGTACTACATGAAGCGA

AGAACCCTTCTACTGGCTTTGCTGGCAAGCGAGATTGAGCGCCTGACCACGTGGTACAAC

CCACTGTCTGCCCAAGAGCTGGCAATCTCCACAGAGCAGTCTGTAGAGACAAGCATTGCT

AATTGGAGGTCTAAGTATATCAGTCTGAGTGAGAAACAGTGGAAGGACAATGTCAACCTG

GCCTGGAGTATCTCTCCCTATCTTGCCCTGCACCTCCCATCCAGGTTCAAACACACAGAG

GCCATTGTGGCTGAAGTGACCCGGTTGGTTCGCCTGGACCCTGGTGCAGTCAGTGATGTC

CCAGAGGCAGTCAAGTTTCTGGTCACATGGCATACCATTGATGCTGACTCGCCCGAACTG

AGCCACATCCTTTGTTGGGCCCCTGCTGATCCCCCGACTGGTCTGTCCTATTTCTCCAGC

ATGTACCCTCCTCACCCTCTTACTGCCCAGTATGGGGTGAAAGTGCTGCGCTCTTTTCCC

CCAGATGCGATTCTGTTCTACATTCCACAAATTGTCCAAGCACTCCGGTATGACAAGATG

GGGTATGTGCGCGAATACATACTGTGGGCCGCGCAGAAATCCCAGCTGCTCGCACATCAG

TTCATCTGGAACATGAAGACCAACATCTACCTGGATGAAGAGGGTCACCAGAAAGATCCG

GACATTGGCGAGCTGCTGGAACAAATGATGGAGGAAATCACAAGCTCTCTGTCTGGCCCG

GCGAAAGACTTCTACCAGCGCGAATTTGACTTTTTTAACAAGATCACCAACGTCTCTGCC

ATTATTAAGCCGGTTCCTAAAGGCGAGGAGCGGAAAAGAGCTTGTCTCAAAGCCTTATCC

GATATCAGAGTTCAACCAGGCTGCTACTTACCCAGCAATCCTGAAGCCATCGTTCTGGAT

ATTGACTATGAGTCAGGAACTCCCATGCAGAGTGCTGCCAAAGCTCCTTATCTAGCCAAG

TTCAAAGTCAAACGATGTGGAGTGAGTGAGCTTGAGAAAGAAGGTCTGCGCTGCCCCAGC

GATTCTCTAGAAGAGGGCGGTGAAAACTCTGAAGCCGCCCAGAAGGTCTGCTGGCAAGCG

GCCATCTTTAAAGTAGGAGACGACTGCAGGCAGGACATGCTGGCGCTGCAGATCATCGGG

CTGTTTAAAAACATCTTTGAGCTGGTGGGACTCGACCTGTTTGTTTTCCCATACAGAGTT

GTTGCTACAGCACCTGGATGTGGTGTAATCGAGTGTATCCCGGACTGTAAGTCTCGTGAT

CAGCTGGGGAGACAGACCGACTTCGGCATGTACGACTACTTCCGAAACAAGTACGGAGAC

GAGTCCACTCTGGCCTTCCAGAAGGCACGCTATAATTTTATCCGCAGCATGGCAGCCTAC

AGCCTGCTCCTCTTCCTGCTGCAGATCAAAGACCGGCACAACGGCAACATCATGCTGGAC

AGCAAAGGCCATCTGATTCACATCGATTTCGGCTTTATGTTCGAGAGTTCTCCCGGTGGA

AACCTCGGCTGGGAGCCGGACATCAAGCTGACCGATGAGATGGTGATGATCATGGGGGTA

AAATGGAAGCCACTCCGTTTAAGTGGTTCATGGAAATGTGTGTGCGTGGATATCTCGCTG

TAAGACCCTATATGGATGCAGTGGTGTCACTGGTGACCTTGATGCTGGATACTGGTCTTC

CATGCTTCAGAGGACAGACCATAAAACTGCTGAAACAAAGATTCAACCCCAACATGAGTG

AGAAGGAAGCGGCTGCGTTCATGATTAAAGTTATTGAGCGCTGCTTCCTCAGCAGCAGGA

GCAAAACCTACGACATGCTACAGTATTATCAAAACCAGATCCCATACTGATGTCCCAAAC

GTCGTCCCCCGTCTCTCTATAGCTTTCCACACCGTATAACCGTCTGTCTCCCCTGTCCTC

TGATATTAACGGCTATATCAGAAAAAACGTCCTTATACACTGTCAGAGGACCCATTTCGC

AGTGTTACTACTAATAAAATAGATCTTTTAGGGCCCTCCAAAGTGCATCCCTTGGAATTG

TCCTAACCCAAACTTTCACCCCCACCTGTGGGCACTGAATCTGTTTCAATTCCGCTTTAA

GATTTGCATCGTGACAGACTTGTACGCTTGTGAAAGCGCTAACATTTCATTTTGAACGTT

TCTAGCATTATAATCTAATTTTACTAGAAGGAATTCCGGTTTTGTCACCTTGACCAACTT

GTACTGTAATTAGCTTGTGTGCTTTTGCAACGATTCGCTATCGCCTGCAGGGTGGCATGA

TTTTAAACAGGATGTGCCCTGGATGTTAATGGTTATTCAGACGAAAATGTGCAGAAAATA

AGTGCTTTGGCATGCACCGGATTAGTTTTATTGGTAATGTCTATAATTTATTACTCAGAC

TTCACGATATAAAGCATCGGTTTTATTTTTTTTCGAATTTATTTGTAGTTTTGTTGGGTG

GAATCTTCTAGTTGATTTTGTTTTAATAGACCAGAAAAATTGTGTTTAGTATATGAAATG

TAAAGGCAAACCCTATTTTGTGTGTGTGTATAAGGTCTAATCTACATGTGTATGATTACA

ATCAAGGAAATAATGGTCTAGAATTTTATTTGTATTTTTAAGCATCGATATTCCCATTAG

TGCATTAACAGATCTTACCTTCTGTATCTGTGCATCCGAGATTTGTTTCATTGGAGTTGT

ATTATTTATACCGCCGTGCAATAACCTGCGTGCTGAACGTGGAGCCGCCGT

>Unigene000077

ACACACACACACACACACACGCATCTTTCACATTGCTCTGAACTTCCAGGAAAGGACGGC

TGGCACAAGAGAGAGGGGGTGGTGTCAGCTGACTGGATCCGTCCAATCAGATAAGTCCTG

ATGTAAGTGTCAAGCAGCAGCTGAGATAGAATCGGCGTGCCCTGAGGACTCGTGCGCCAG

TAGCGCAAAAGAGAGTGCAGCATGGCTTGCTGAGAGACGGAACAAACGTGTGCGAGGGAG

AGAAGTGTGTGTGAGAAAAAGAGAGAGAGGGGTGGGGAGAGGACAAGGTTGGTTCCTCTC

CAGTATGGACGAGTCCAGCATTCTGAGGAGACGAGGGCTACAGAAGGAGCTCAGTTTGCC

CAGAAGAGGGAGCTTCTGTAGGACGGGGAACCGGAAGAGTCTGATAGGAAGCGGACAGTC

ATCAGGACTTCCCCGGCCGCATTCCCCCTTTTCATCACACACCGGCACAAGTCCCCAGGA

CAGCCCTAGAAACTTCTCCCCGAGTACCTCAGCACACTTCTCCTTCGCTCGCAGGACCGA

CGGACGGCGTTGGTCCCTGGCATCGTTGCCGTCGTCCGGATATGGAACCAACACCCCCAG

CTCTACCATTTCTGAGAAGCTCCATCAGCTGCCCTTCCAGCCCACTGCTGACGAGCTCCA

CTTCCTGTCCAAGCACTTCTGCACTGAGAGCATTGCTGGAGACGACCATCGCAGGGCCGC

GTCCATGCGCCCGCGCTCACGTAGTCTCAGCCCTGGAAGATCTCCATCCTGCTGTGATCA

CGAGATTATTATGATGAATCACGTGTACAAAGAACGTTTTCCTAAGGCCACTGCGCAGAT

GGAGGAGCGCATTCAAATTATCATCAGCAGCAGCTCCCCGGAGAACGTGCTTCCTCTGGC

AGACGGAGTGCTGAGCTTCGCCCATCACCAAATCATCGAACTGGCACGCGACTGTCTGGA

GAAATCTCGGCTCGGCCTCATCACCTCACGCTACTTCTGCGAGCTCACTGACAAACTCGA

GAGACTTTTTCAAGAGTCCACTGAGAGATCGGAGAGTGCTGAGGTGACTTTCATTAAGGA

ACTGGTGAAGAAGATCCTAATCGTCATCGCCAGACCGGCGCGGCTTCTGGAGTGTCTCGA

GTTTAACCCAGAGGAGTTCTACCACCTGCTGGAGGCGGCTGAGGGTCACGCCAAGGAGGG

ACAAGGCATCAAGACCGACATTCCGCGCTACATCATAAGCCAACTCGGTCTGACACGAGA

CCCTTTGGAAGAAATAGCCCAGTTAACCAGCTACGACAGTGGAATCGCGGAAACTCCCGA

TACCGATGATTCTTCCCAGAGTCTGAGCGCACCGCCGCAGCCCCGCCGTAAACCCTGCGA

GATAGATTTCGAGATGATCAAACTCATCAGTAACGGAGCTTACGGCGCTGTCTATCTGGT

GCGACACAAAGAGACCAATCAGCGTTTCGCCATGAAGAAGATCAACAAGCAGAACCTGAT

GCTGAGGAACCAGATCCAGCAGGCGTTTGTAGAGAGGGACATCCTGACCTTCGCCGAGAA

CCCGTTTGTGGTCAGCATGTACTGTTCCTTCGAGACACGCAGACACCTGTGCATGGTCAT

GGAGTACGTCGAAGGTGGGGACTGTGCCACTCTGCTGAAGTACATGGGTCCTTTGCCGGT

GGACATGGCCAGAATGTACTTTGCCGAGACTGTCCTTGCTTTGGAATACCTCCATAATTA

TGGCATCGTCCACCGAGACCTAAAACCAGACAATTTGCTGGTGACTTCGATGGGACACAT

CAAGCTGACAGACTTTGGCTTGTCCAAGGTGGGCCTGATGAACATGACCACTAACCTGTA

TGAAGGTCACATAGAGAAAGACGCCAGAGAGTTCTCGGATAAACAGGTGTGTGGCACCCC

CGAATACATTGCTCCCGAGGTAATTCTGAGGCAGGGATATGGCAAGCCTGTAGACTGGTG

GGCCATGGGGATCATTCTCTACGAGTTCCTAGTAGGATGTGTGCCGTTCTTCGGAGACAC

GCCGGAGGAGCTGTTTGGTCAGGTTATAAGTGATGAGATCAACTGGCCTGAGGGAGAGGA

CGCACCTCCGGCTGACTCTCAGGAGCTCATCACGCTGCTGCTTAGACAGAACCCACTGGA

GCGCCTGGGGACTGGAGGAGCTTACGAGGTCAAACACCACCAGTTCTTCCACAGTCTGGA

CTGGAACAGTCTGCTCAGACAGAAGGCTGAATTTATCCCCCAGCTTGAGTCCGAGGACGA

CACCAGCTACTTTGACACCCGATCCGAAAGGTACCATCACCTGGAAACGGAGGAAGAGGA

AGACACGAACGATGAAGACTTTAACGTGGAACTGCGTCAGTTTTCCTCGTCCTCACACCG

GTTTTCTAAAGTCTACAGCAGTCTGGATCTGTCACGTGGGCAACTGGAGGAAAAGGGAGA

GCAACCAGAGAAGAAGAGTGAGAGTCCCCTGACTGTCGACTCGCTCAGCTGGACTCCAGA

CTTCACTGAAATTCCATCACTGTCCCACTCCTCTGACCTGGAGAGCAGCACGAACAGCCT

GAGCCGAAACTCAGGACTTCTGCCCAAATTTGCCATTTCAGCAGAGGCTGATAGCTGCGA

TAGTTCGCTGTCCCTGGACAAGGCGAGTAAAGTGGCCTTCTCTGTTGGAGAACTTCCTCA

GACGGAGCCTGATGCCATCACACCATCCAGCCCTGTCAGCAACGCCACCCTGTCAGGCAG

TTTCTCTGAACATCTGGACCAGCTGATATCACGTCCAGAGGGAGCAGACAGTCTGGACCC

TCCCAACAAGCCATCCACTGATACCGTCAGCCATTTGACCTCTCTAAGACCAGCGACCAG

TATACCGAAATCATCATCCACCAGCGCTCTGTCTCTCATGATTCCTGCAGATGTTTTTGG

CACATCCCCACTGGCTAGCCCCATGTCTCCACATTCACTGTCGTCAAACCCCTCCTCCAG

AGACTCCTCACCCAGCCGAGACTCCTCCCTCTCTGCCGCCAACTCGCGCCAGCCAATCAT

CATTCACAGTTCAGGGAAGAAGTTTGGTTTCACACTGCGAGCGATTAGAGTCTACGCCTG

CGACAGCGATGTTTATACTGTCTATCACATGGTCTGGAATGTGGAAGACAGTGGTCCTGC

TCAAAAGGCTGGACTCAAAGCTGGAGACTTGATTACTCATGTCAATGGGGAAACGGTCCA

TGGACTGGTGCACACTGAAGTGGTGGAACTGTTGCTGAAGAGTGGAAGCAAGGTTGCTAT

ATCCACTACTCCTTTTGAGAACACGTCGATCAAAACGGGTCCGGCTAGAAGGAACAGTTA

TCGGAGCAAAATGATTAGGAGAACCAAGAAGCCCAAGAAGGAAAAGACACAAGAAAGGCG

ACGATCAGTATTCAGGCGCTTCGCCATGCAGCCCTCTCCACTGCTTCACACCAGTCGCAG

CTTTACTTCCTTAAACCGATCCTTGTCCTCAGGGGAGAGTCTGCCAGGATCCCCCACACA

CAGTCTGTCTCCTCACTCCCCCACCACTCCCTTCCGGCCCACGACGGAACTCAATCAATC

AGGTGGCACATCATCTCAGAGCAGTTCCCCCAGCTCTAGTGCACCAAATTCCCCAGCTGG

CTCCGGCCATGTCCGTCCTAACACTTTGCACGGTTTGGGCCCTAAGTTGCCTGGCCAGCG

GCTTCGTCAAAGTCGCCGGAAATCAGCGGGAAGCATTCCTCTCTCCCCGTTGGCTCGAAC

ACCTTCCCCTACCCCTCAGCCCACCTCCCCTCAGCGATCTCCTTCCCCATTGCTCGCTCA

CTCGGTGGGAAGCTCCAAAACTACTCAGACTTTCTCAGCCAAGATGCACTCGCCCCCTAC

AATCGTAAGGCACATGGTGCGACCTAAAAGTGCCGAACCTCCTCGGTCTCCGCTCCTTAA

AAGGGTTCAGTCGGAGGAAAAGCTGTCGCCATCCTACACAGGGGATAAAAAGCACCTCTG

CTCCCGGAAACAAAGTCTAGAGGTGACCCAAGAAGAAGTTCAGACTGGGTTTGCATCTGG

AGGTGAGCACACACTTCAGTGTGTGGAGGAGAGTTCTTGTGAACTTCCCACCATCACCCG

AGTAAGACCTGCTGAGCAGGGCTGCCTAAAAAGACCTGTGACTCGTAAGGTTGGACGACA

GGAAATGATGGAGGATCTTGATAAGGATAAATTGAAGTCCAATGTTGTAGCGAAAAGACA

GGACTGGCATGAGAGAAGAGAATCTCTACAAAAACAAGATGCCATTCAGGAGGCTGAATC

TACCCAAAGCCTGACTGGGTGTTGCTCGGTTGATAACAAAGTTGTCACTGCTACAGTAAA

AGATGTTCTTTATAAAAAACTAAACTCCAGAGCATGTGAAAGCATTGCAGACTCCATGAG

CAGCACTGGAGACTCTGCGTCGGTTTTCAATGAGAGTATAAGATCATCCCCTGCTCAGTC

AGACCGACAGCTGGCCAGGCAGATCAAAGAGGGCAGTAAACCAGACAGGCATGATTTCAA

AGCCCCTAATATGGAATTTGCTCGGAAAAGACAGTCCTTTGAGGAGCGTGAAGACTGTTT

GTGTCGAATCACCTCTGGCGTTCACGAAAGTCTACATTTTAATGCCACTAGATCCAAAAG

TCTACAACTGGATTCAGCTGGAATTGCTACTCCTGGAATCACCACAGATTCGTTGAGCCC

TAAGATATTCAGTGGGCGAGGGGAAAGTGCCGTAGAGAAGCTTCAGATTATCTCGTCCAC

AGAGGGCTCCATCAGAAAGACTTCATCTGAGTACAAATTAGAAGCCCGACTTGTTTCTTC

CCTGAAACCACTAAAGGGCACACTGGACATAGGGCTGCTCTCTGGGCCTAGAATCTCCAA

AACTGATACATGTCTTTCAAAAATAGCCAGTAGCCAAGCTGATAGTGGTGGTCAGGTCGG

AGCCATGCAAAACCAGAGTGAAAAGCAGCCCCTGATGCTAACACATAAACCGACACAGGC

TGCCTTGGAGATAAAAAGTCCCGGTGCTTCGAGCAGCAAAAATGCAGGAAAAGATGAACC

CACTTGCAGTACAGACTATGTTTTTGGCTTGTTTAGCCATGAAGGCACTCATGACAAAAA

TAAGCAGGGTGAAGGAATCAAATTCCAGACCGTAGCCAGTAAAATGGAGCCGAAAGATTT

GTCAACAGGTGGAAAAACAGAGAATAAGTTGAAAACAGCTCAAGAAATGAGGCCTGCTCG

ACACAGTAATCATTTTTCTTGTGGGAAAACACCCTCAATAAGGGAGGTCAGCAATGAAGA

CCAGGATGATGAGATGGAAAATCCGGTGGAAAAACCTACCCTGCAAAACAACACAGTAGA

AACTAAGACGCCAATGACCCATGACCAGGTCATCACACAAAACACATCACTACCCTCAGG

CCCGTGTAGTGTTGATAAAGATAAAAATGTGAGAGAAATCCTTATCAGTGCCCAGAAATC

GGAGCAGGCTTTTGCAAACACCAATAAAATAACAATGGCAAAGGATAGAGTTCTGAATTC

TCTAGGGTTAACTGTGGTTACACATGAAGTTTATCAGTGTAAAACTGTCCAAGATGATGC

TTCAGGATCTCGAAGCTCAGAGGCATTGGTGCAGACTTTGCCTCCAAAACCAGACAGAAC

AGTTATTGTGCAAAAGACAGGCTCAGATGTAAAAGCTGGGATTGTGGCAATTGATGAGAA

AAAAACACTAAGCACAACTGGAAAGAGTACAGATAAGAAACATGAACAGATCACAGCAAG

TAGCACACCAGTGTTGCATGCGAAAACAGAACCGTTTCACTCTGAGGGTCTAGTAAAAGA

AAAGGTATCGGTTCCACGGAGTTCAACTGTAACCACATCATCTCCTTCGGCAGCTTCGAT

AGTGCCAGATATAAAGTTGCCTCCAAATTTACCTCTTCAAACCAAACAAGATAAAAACAA

TGTAGCTCCCAAAGGTACCCCTGTTAAAGCCACGTCTGCTGTGGTTCCCCAATTACCCCA

GCATGTACATAAAAGCCCACAGTCATTTGCTGAAATCCAATCAGACAGCACTGCAATAAA

CCAGTCTCAGCAAACGACTTTGAAAACACCAACTGGTGCTGACTCAGGTAACGGGCAAAA

ACGTTTAAATTCTATCAGCAGAACAGATGACCAAAATGCTGAAGTGGAGAATGGCAAAAG

ATCCAGTTCAAAATGTCAGGAGACATGTGGAGTTAAAACCCAAATCGTAGACAAGGATCA

TAAGACGTGTATGGTTCCAGAAGGGTCAATCGATAAATCCAAGATTACATCATCCAAAGA

TAAAAAGGCACAAAATATAGCAGTTTCAAAGGAGGAAGTAAAATGCATTATGACAAACGA

GCCTCGCAGTCCTACAGACCTCAAAACTGATGGGGCAAAGTGTGTCCTAGCTACTGCTCA

CACGCCCCCTAGTAAGTCAAATCAGTATTCTGGTGTCAAAGGCAGTGAAAAAAATAGCAA

TACTGTGCAGGTTAAGTCAGTGAATATACTTGAAGAAAAGTTGAAAGATAAGAATACTCC

AGATGTATCCTGTCAGAAGGTCACCAAGCCGGTACTCGACGTTTGCAAGAAGGAGGTTGT

TACTGCAACAAAAAAACAACCACAATTGACAGGTACAAGTTCCAATGGAAAGCAGGGCCT

GTCGAGCTCTAAGAGCAAAGCTAAACTAGAACCATCTCCTGCTACAAACCAGAGTACAAA

AGCCACCGAAGGAAGAGGAACTCAAGAAGGCAAAGCACCAGTGCAGGTCATCATAGATCA

TACAAATCTAAAACCAAAGGAGCAAAGAGAGGTAACACTTCCTACATTAAAAACTCAATC

ACAGAGTGAGAACCTGCCTCAGACCACCAGCCCTCAAGTTGGTTCTGTACCTGTACCTAC

TGTTAAGGTTCACACACCAAGTGGATCTCCTATAGGCAAGGGTACAGGGTCCACAGTGGA

GACCTCAAAGTCGCCTGACTCCATGCAGAAAGGAAACCGCAAAGACTGTCAGAAATCGTC

AGGATTGGCGAAAGACGGTGCAGCACCAGAAAAAGATTCACCTCGCACTAAGCAGTCCAA

AGATCTGCCCCGAGGTTCAAATAATAAAAAGTGACAGTGAGGATGCCGAAGGTTGCCAAA

TCATTTTTTGTAAGAAAAACTGCAAAGCAATCTCTTT

>Unigene000078

TTTACACACACACACACACACACTGAGACTCAATTACTGCCCAATTCCCCCTTCTTCATC

ATCTCCTGCTACGCCGGCCTGTCGGAACAAAAAAAAAAAGGAAAAAGAACCCGAACCGTA

AATCTAAAAAAAAAAAAAAAAAGTATTTCCTTTGAAGCGCGAGGTGGATTTTTAAACGCG

TCGACGGACAAAAGTGTCGCAAAAAACCTGACGGTGGAAGAGCAGTGATGTCACAGAGCT

GAGGACGCTTCCACACAATATGGAGTCTGGAGAGCGGCTGGCTTCGCCTTCCTCCACCCC

GGTCTCCGTACACACCAGCTCATCTTCATCCTCATCTTCAGTGTCTCCAGCCTCCAACGC

CAAGAGCAGCCTCAACCACGGAGCCGCTGCCTCGCTCGCTGCCTGCGGTCCCCTGTTTGG

GGTAACAGGAAGTGAGCAGCCCTTCAGCGTGACCTCTGTGACCTCGGTGCCCAGTGCCTT

CCCCGTGATGGCGCACCCCGCTTTTGGCCTGCTCTCCCCCGGCACGGCTCGGCCCGAGTT

CGGCGGTCTCGGAGCGCTGGGGGTCACGGCGGCCCTCGCGGCGCATCCGCAACTAGGGGC

TTTCACAGAGTGGTGGAGAGCAGCCGAAGCTCACGGACGGAGTCCAGCCGCCTTCTTCCC

TCCTTTCCTCGGCCTGCCCCCGCTGTTTGCCCCTCCCCTTCAGAACCACGAGGCCACGCC

CTACACGTCCAAAACCCTGAGCAAAAGCAGCCAGGGGCCCAAAGGTGTTAACGGAGCTAT

GAACGGCAGCGTGGTCTCTCCGTCCACGACGAAAAGTGGCGGGTCCGTGGCATCCTCGGC

CGCTCTCAACACCTCTGGCGGAAAACCTCGCGCCCGCAAAGCCTCTCAAAACAGCAACAG

TGCGGGAGAGCTGCAGGAGAAACTCTCACAGAAACCGAAAGAGAAGAAACCGCGTAAGAA

ACAGACCGAAGGCTCAGGCATGAGCGACAGTGAATCAGGATCATCTCTGGACAGCGACAT

TGAAGGCGTGAGCAGCAGTGACCTGGATGATCTCGGTGAGGAGGACGACGACGATGACGA

TGACGACCAGAGTGAAGAGTCCGACTCTGAGAAAGAACCCCATAAAAAAAAGAAAGCCAA

GGTCGTAGCCTCAAACCTGCGGCCAGGTAAGAAGGAACATTCCAGAACTGCAGAGGTCTG

GGACCTTCGGGAGGGCAACGAACCACCAGGCGCCTGCGTTTCCAAATCCAGAGACAGACC

CGCGCAACCGACCAGTGTCATCCAGTCCACCGGCCTTGCAGTCAGCACCAACCCGCTGGC

TCTGATTGGCCAATCGCAGCATGACTCCTCCCCTCAGCGCCTGAGCTCCTCCTCTCCCCG

CCCCATCGCCTCTCATCAGCCACTCCCACTATCTCTTTGTTCCTCCCCTAAACCACTCTC

GGTCCCTTCTCCTCCCAAACCGCTCCCTCTGTCGTCCTCTCCCAAACCACCCTCATTAAG

CCCCTCCCCCAGGGCATGGGGCTCCGCCCACAAGTCTCAGAACTCGCTGAGCAGGAGGAA

GCATTTGGAGAGCTCGCTGTCCCACATAGCCGACTACAGACTGAAACAGTCTTTACTTGC

GCACGACCAGGAGTTCCCACTCCAGCTCAAGAAGCAGCAGGACCTTTACAAGACCTCGAA

GAATTCCGGGGTCCGCTCCTCCTCCTCCTCTTCCTCCACCTCCGCTTCTTCCATCCTCCC

TTCCAAATCCACGGCAGGTCGTACCAAGGCCCCGGGTGCTCAGGCCGTGACCGGGTCTCC

AAGCCTCATGCTCACTCAGACGCTCCTGGGGTTGGGCCACACCAACGGCGTCATCCAGAA

CTCCACTCAGGACACCCCGCTCGCTCTTACCACCAAACCTCGTGCCGACCTGCCCGTCAA

CCTCAGCACCGGCGGCAGGAAGGACACATCCTTGCACACCTCTTCACTTGCCCCGCTACC

CGCTCTGGTACCTGCATCCGTCTTGCCAGCACGACCCCGTGCCTCCCGCAAAAACAAGAC

CCCTCGAGCCCTGGAGGTTCCTAAAGATGTTTCCCAAAAGCACTTGGTGAAGTCTCTCGT

GGATCTGTTCCACCATGCAGCGGGAGAGCAGGAGACCCCTAGCAAGAAGGACTCTGATGA

GTCGGGCGAGGATGACGAGGATGACGAAGATGACGATGTGGATGACGACGAGGATGACGA

AGATGAGGATGACAGTCTCTCAGAGTCAGACAGCAACTCGGACAGCGAGCTGAATGGCTC

AACACGGAGGAACATGGACACCACTGAGACGGAGACCGACGGCGAAAGGACACCGCTAAA

GCTCAGCAAAAACCTGCCGCTGCTCGCCTCTGCCTCGGCCAGTTACAGCCTTGCGGACTG

CTCGCCGCTCAACCTTCAAGTCATTAAGCCCTCCGGCATGGCCACGCCCACCATCGTGAG

CGGCTCCGGTGCCTTGACCTACCACAGCTCGCCTTCCTCCTCGTATTCCGTCGGCACGCC

TCCAGGATCTGGAAAGAGGAAGCGTGTGATGAACGAGGAAGATCTAAAGACTCCTTTAGA

AATTGGTTGGCGCAGAGAGACCCGGATCAAATCAGCCGGAGGTCGTCTCCAAGGAGACGT

AGCGTATTACGCGCCCTGTGGCAAGCGCCTGCGCCAGTACCCCGACGTTGTGAAGGGTCT

GCAGTGGTGCCCGCTAAGCGAGGACGAGGTTATACCGCGTATCAGAGCCATGGAGGGCCG

GAGGGGTCGTCCGCCAAACTCCGAACGCCAGCACGGCGCCGGTAACGGCGAGGGCTCCGG

GGCCCGCCGCAGGAAGGGCCGTCCGCCCAACGTGGGCCACACCGAGTTCCCCAGCCCCTC

GGAGGCCAAACTACTCCGCAAACTGGAAGCTCAGGAAATCGCCCGTCAGGCTGCTCAGAT

GAAGCTGATGAGAAAGCTGGAGAAACAGGCGCTCGCTAGAGCAGCTAAAGAAGCTCGCAA

ACAGCAAGCCATCATGGCTGCGGAAGAGAGGAGAAAACAGAAGGAGCAAATCAAGATCCT

CAAACAGCAGGAAAAGATAAAGCGCATTCAGCAAATCCGGATGGAAAAGGAGCTCCGTGC

GCAGCAGATTCTCGAGGCGAAGCGGAAAAAGCGTGAGGAGGCCGCCAACGCCAAAATCAT

GGAGGCAGAAAAGCGTCTAAAGGAGAGAGAGATGAGACGGCAGCAGGCTGTTATACTAAA

GCACCAGGAGTTGGAGAGGCATAGACTAGATATGGAGAGGGAGAGGAGGAGGCAGCATGT

CATGCTAATGAAAGCTGTAGAAGCTCGCAAAAAAGCTGAGGAGCGCGAGCGCCTTAAGCA

GGAGAAGCGTGACGAGAAGCGTCTTAACAAAGAGCGCAAGCTGGAGTTGCGGCGCCTCGA

GCTGGAGATGCTGCGGGAGATGAAAAAACCCAACGAGGACATGTGCTTAACTGATCACAA

GGCCCTGCCGGAGTTCTCCCGGATGGCGGGGTTGGTGCTCCCGGGGCAGGTTTTCTCTGA

CTGTCTGATGGTGGTTCAGTTCCTACGCAGTTTTGGGAAGGTTTTGGGGATGGAGCAGAG

CGAGGTTCCTACACTGGGTGTGCTGCAGGAGGGGCTCCTCAACCTGGGCAACAGCATGGG

GCAGGTGCAGGACCTGCTGGTGCGTCTGCTGTCCTCGGCCGTCTGCGACCCGGGGCTGCC

TCCAGGGCACAGGGCCAAGTCCATTCTGGGTGATCACCTGACCAACGTGGGTCTAAACCG

CGACAACGTTTCAGAGGTTCTGCAGCAGTACATGGAGTCTCACTGTGCTCAGACCGAGCT

GGCCGACGTGGCCCTCAGTCTGAAGACCAAGGCGTTCCAGGCACACACTCCGGCGCAGAA

AGCATCGATTCTCGCCTTCCTGGTGAACGAGCTGGCCTGCAGCAGGAGTGTGGTCAGTGA

GATCGATAAAAGTCTGGACCAGATGAATGTACTGCGGAAAGACGAGTGTATCGTGGAGGG

AAAGCTGAAAAAGTTGAAAAACATTCATGCTAAACGCACAGGGAGGCGCGAAGCCGGCGG

AGGGGACGAGTCTCATGCCACGGGTACACCGAGCGCCGGACAAAAGCGCAAGAGAAAGAC

TAACGACAGCGACGACGACGAGGAAGAAGACGACGACAGCGATGATGCCGTAGACGACGA

TGATGAGGATGATGAAGAAGAGGTAAAGAAATCGCGGAGGGTGGAAACGTGTGACGAGGA

TGAAGGAGATCAAGCCACAAGCGTAGAAGAACTGGAAAAACACATTGACAAAATATCAAA

GCAGCAGAATCTTATCAGACGGAAGCTCTTCGAGTCGTCTCATGCTTTACGCTCCATGTG

CTACGGACAGGACCGCTACCGCCGCCGCTACTGGGTCCTCCCACAGTGTGGAGGAGTCTT

CATCGAAGGGGTGGAGAGCGGAGAGGGTCCCGAGGAGCTCGAGAAAGAACAAGAGCGTCT

ACGCAATTTCAAGCCGGTGAGGATCAAGGAGGAGCCACAGGAAGAGGGGGATGAGGAGGA

GGAGGAGGAGGAGGCGCAGGTGGAGGATGCACAGCAGCAGCAGCCGGAGGGAAAGACGCA

GCCTCAGGTTAAGCAGGAGGAGGAGCAGCGTGCTGAACAGGAAGTGAAAAAGGAAGACGA

GAAGCCGTGCTCGCCGCCGAACAAGGACACGCCGCTATCAGACTCCGCCTCCTGTCCTCA

CAAGGAGCCCTCAAAGCCACTCGGTGACCTCGCGACACTTTGCTGTACTCCTGACGTCAG

CATGCCGAGTGTAACCACGGTGACCGCATCAGCATCGTCTCCGCCTACCTCCAAGCCCCC

TGTACTGTGCAGCGCTGCTACAGACTCTGTAGCTCTGGTGGCTCAGTTTGGTGCTCCTCC

CCCACAATTCGGGATTCCTCTGTCCCTGCAGCATCATCAGCACCTCCTGGCTAACGATCA

ACTCCTGCGTGTTTTAACCGAGCGCAGCGGACATTGGTTTAGCCTCCTGCCTCGTTCTCC

GTGCGACGAGTCTTCTCTCATCCAACCCACCACGTCCAACGTCCAGCCCAGGAGCCCTCC

GGCAAGGTCCTGCTCGCCACACCAGCATCTCCAACCTCCGTCTGCCTCCAGCAGCCCCAG

TAACGCTGCTCACGACGGGCAGGGCAACCCCACCGTCTCCCCTCTGCAGGTAAAACCTGG

TGGTGCTCTGCTGTCTTTGCCCATGTGCGCATGGTCAGGTGGCATGATAAGCCCTAACCT

ACCTATTTGCAGCAGCCCTATGCCCATTTGCCCACTCGCCGAGGGCAACGCCAGCCCCCT

GATGGCACCCAGTGTCTCCACCAGCAAAAGCGGCTCTCCTCCGCCCCCTGGTGACAAACC

ATGTGCACTGTCACCTGCTATAGATCTGCCAAGAAACCATGACCAGCCGCAGCCACAACC

CATTCCAGAAGACATGTTGCTGGGCTGGTGGAAGATGATAGACATGGAGGAACTGCAGGT

TCTAATGAAGACGCTCCACAGCAGAGGCATCAGGGAGCGAGCGCTGCACAAACAGCTCCA

AAAATCCATCGAGCTCGTCTCACACACCTTCAACAAGAACAAGGAGGTAGCTGTGATGGA

GGTGTCGGAGCTGGACGAAGGCCAGGTTTCTGTGGAGACGCTACAGGAGTGGTGCGTGGA

GGAGCAGGCGATGGAGACGGACATCGCTTTACTACAGCAGGTGGAGGAGCTCGAGCGCAG

GGTCACTTCAGCCAGCCTGCAGGTCAAGGGCTGGATGCACCCGGAGCCCCAGTCTGAGAG

GGAGGACCTGCTGTATTACGAGCACAAGCCCCTTCCCAAAGTCTGCCCAGGGGCGGAGTT

ACATGAGGAGCGGGGTTCGGAGAAGGGTCTGAGACGGCAACCCAGCAATCCGCTGGACAT

CGCCGTGATGCGCCTGGCCGAGCTGGAGCGCCACATCGAGAGAAGGTACCTGCGGAGCCC

CTTAGGGACAACCATCCAGATCAGGCTGGATAATGTGGGGACGGTCACTGTGCCCGCCCC

CGCGCCATCCACTAGTGCTGGAGGGGAAGGAGGTGAGGAGGAGATCGCTCCGGGAATGAA

GGTGTGGAGAAAGGCTCTGAGTGAGGTGAGGAACTCGTCACAGCTGGCCATGTGTCTGCA

ACAGCTGCAGAAGTCCATCGCCTGGGAGAGATCCATCATGAAAGTGTACTGCCAGATCTG

CCGTAAAGGGGATAATGAGGACTTACTGCTGCTGTGTGATGGCTGTGATAAAGGCTGTCA

CACCTACTGCCACAAGCCCAAGATCAACGCCATCCCAGAGGGAGACTGGTACTGCCCTGC

CTGCATCTCCAAGGCGAGCAGCCAGTCGCCCAAGAATAAGAAGCCTCAGAGTCGCGTGCA

GTCGGGCGGAGGGGGCAAGAAATCGGCAGAGACGACCAAGAAGAGCAAAAAACAGCAGGA

GGCGTGCGAGGAAGACGAAGCGGTGGGAGGCAGCAGCACCAGCAACAGCAGTCCAAAGAA

AACAGCCACCGCTTCCAGCCAGGCCAAAAAGAGCTCACCCGTTCCTTCAGCCGCCAAACC

CGAGAGCCCGGCCTGCGTAAAGCGAGCCAAGACAGCCAGGGACAACAACCGCGACCTGGG

ACTCTGCAGGATTCTTTTGGCTGAACTGGAGCGTCATCAAGACGCCTGGCCTTTCCTCAC

ACCCGTCAACCTAAAATCAGTCCCTGGATACCGGAAAGTCATTAAAAAGCCCATGGACTT

CTCCACCATCCGCGAGAAACTCGTCAGCAGCCAGTACCAGAACCTCGAGACGTTCATCAT

CGACGTCAACCTGGTGTTCGACAACTGCGAGAAATTTAACGAGGACAACTCGGACATCGG

CCGCGCGGGACACAACATGAGGAAGTTCTTTGAGAAGAGGTGGACCGAGCTCCTGAAGCA

GACTAACTGATTCGCTCAGAGCCGCCATGTGACATGCCACTTTTACCAAATCCCTCATAA

CAGACCTCGGACCAGAACTGGCCAGAAATTTCCTCAAAGCAAAGCGCCGATCTCAAACCT

GAACCTGTCTCACACACACACACACACA

>Unigene000079

AAAGCCCGTGCGAGCTGGGAAGGAGCTTTAGATGGCGGATGAGCCTTGGGAAGGGTAACC

CGTGGAGCTTGTCGACAGCACTTCGCCAACTTTAAACTTTTGCGCTCGCGGTTGGTCTTA

TTTGTTAAAATATTATATTAAATTATATTTAAATGTAAACTAATGGTGCATTGTTCGTGC

GGTTTTATTTTGTCGACAAAATATGTACCGGTTTAACGTAGTCGTTGTTTTCCGTGCCTC

GCGTAGGCAGCCATGGCTGTCCTCCATTTTTAGTGCGCTCCGCGCGGCGCTTTAAAACCA

CCGCTCTACATTCAAAGGCTATTTTGTCTTTACTTTATATATATTTAAACTACATGAAAT

ATTTCATAGTTTATCGTCATCCGGAGTTATGGAGATTTGTGTTTTTTTTTCTTGCGTTTC

TTGAGTAGTAAGTTCGATAGCTTTATTCGGGCTTTGGATGTTACTGCTCCATGCGTGTTT

TTCCCTAACGTTACCTGTCTATGGAATCGGTGTGTGTACTTTCTATTTTTCTGCGATCCG

ATATTTTTTTAAATTTCTTTTAGATATGCTTTCAGTCGTCCTTTGTCCTGGTGACGCAGT

AAGAATTTATGCATGCTGGATCTATTGCATTTAAAAGTCTGCACTCGTCTGTTGCTTGTG

TTATCTACCGGGTGCTGTTTAAGTTCATTTATCTAATATTTTTTATGAAATTTGTTCTTT

TCTTCGACAATTGTCAAAACCGCTCACCTTTTTCCTTACGAGACAACGGGGTATATTCTG

CAGTCCTAAAGCAAAAGCCGGACAGGTTGGACTTGCATGATGGTTTCATCCCATCTCAAC

TTTTGACAGTTTAAAACTCAAAGATCACCCTTTACAGTTAGACAATTTAACCATGTCGAA

GATCAACGACAAGAAAGGAGACGAAACTTCGAATCATAGCAATGCTTCGAGCTCTGGATC

AGAGGAGTCAAATCATTCAGGCTCAGAGTTGGCCAGCCAGTCTGAGAGTGAATGTGGCAG

TGAACATGAACAGTCCCAGCATTCCGAGTCAAACAACACGTCCGAGTCTGAAACCTGTTT

TGAATCTGGCAGCGAATCAGCTGGGTCAAAGTCACGCCAGACTGTTGCAGAGATTAAAGA

GAAACGGGTCAGGAAGAGAGATCTGGCCGATGTAAAAAAGATGTGGGAGGAACATCCAGA

TATTTACGGTGTCAGAAGGTCCAATCGGAGCAGACAGGAGCCTGCTCGTCTGAACGTTGG

GGCAGAGGTCAGCAGTGAGTCTGGGAGTGAAAGCCACAAAAGAAAAAATTCACGACAGAA

AAAAAAAGAAAATACCTGGAAAGATGACTGTCACGATGAAGACGACTGTGAGGAAGAAGT

TAGCAGTTCAGAGATTGAACAGGAAGGGGAAAAAAATAGATCCAGACGACTTCCTGCTAG

AAGGCCACAGGCCAAATTCTACACAACCAAGCAGAATCTGTCTCAAAAGGGGCGGAAACT

AAGGATGCAGGAAACATCAGATGAAGATGATGAGGACGACATTGTTGAGGAAGAGAATAC

CCCAAAAAGACAAACCCGACGAAGAGCAGCTACTAAAGTTAGTTATAAGGAGGACCAGCA

TGATTTTGAAACAGACTCTGATGACCTGATTGAGATGGCAGAGGGAACAGAGGATGTACG

AGATGATGACAGTGAAACCATTGAGAAAGTCTTGGATACCAGGATTGGTAAAAAAGGAGC

TACAGGAGCAGCCACCACAGTCTATGCTGTAGAGGCCAACGGAGACCCTATGGCTGATTT

TTCACCTGAGAAGGAGGGAGGTGAGACTCAGTTTCTTATCAAATGGAAGGGCTGGTCCTA

CATCCACAACACATGGGAAAGTTTGGACTCCCTTTCTCAGCAAAAGGCTAAAGGGATGAA

AAAAATGGAAAATTTTAAGAAGAAAAATGAAGAGCTCAGTGCATGGTTAGAGAAAGCGTC

ACCTGAGGATTTGGAATATTACAACTGCCAGCAGGAGTTAACCAATGATCTAAACAAGCA

GTTTCAGATAATAGAGAGAGTTATTGCAATGAAAACAGGGAAGGCACAGGTACCCTCTGA

CTTTCCATCACATAAAACCATGTCTGATGAACCTGAGTACTTATGCAAGTGGATGGGTCT

GCCCTATGCAGAGTGCACTTGGGAAGATGGTGCCTTAATAGGTAAAAAGTTCCAGCATTG

CATAGATTGTTTTAACAACAGGAATGTTTCCAAGACAATTCCCTCCAAGGACTGTAAGGT

TTTAAAGCAAAGGCCAAGGTTTGTGCCTCTAAAAAAGCAGCCATCTTATATTGGAGATGA

GAACCTGGAGCTGAGAGACTACCAGTTAGATGGGCTAAACTGGCTTGTGCACTCTTGGTG

CAGGTGTAACAGTGTTATCTTAGCTGATGAGATGGGCTTAGGGAAGACCATCCAGACCAT

TTCATTCCTTTCCTACCTGTTCCATCAACACCAGCTATATGGACCCTTTCTCTTGGTTGT

GCCCCTTTCCACCCTCACCTCCTGGCAGCGCGAGTTCAGTACTTGGGCTCCTGACATGAA

CGTTGTGGTCTATCTGGGAGATGTTTTGAGCAGAAAAACGATTCGTGATTATGAATGGAT

TCACCAACAAACCAAAAGAATAAAGTTTAACACACTCTTGACCACATATGAAATTTTACT

GAAGGATAAGGGTGTTTTGGGAAACATAAACTGGGCATTTCTTGGGGTGGATGAGGCTCA

CCGCTTAAAGAATGATGACTCTTTGCTGTATAAAACACTAATAGATTTCAGGTCCAACCA

CAGGCTGCTCATAACAGGAACACCACTGCAAAATTCACTGAAAGAGTTATGGTCACTCCT

GCACTTTCTTATGCCTGACAAGTTTGAATTGTGGGAGGATTTTGAAGAAGAGCATAGTAT

GGGCAGAGACAATGGCTACCAGAGCCTGCACAAAGTGCTTGAGCCCTTTCTTTTACGCCG

TGTAAAAAAAGACGTAGAAAAATCGCTGCCTGCCAAAGTGGAGCAGATCCTCCGAGTGGA

CATGTCCGCACAGCAGAAACAGTTCTACAAGTGGATTCTTACAAGGAATTACAAAGCACT

ATCTAAAGGCACCAAAGGCAGTTCCACAGGCTTCCTCAACATTGTTATGGAGCTAAAGAA

ATGCTGTAATCATGGGTTCCTTATCAAACAGCCAGATGAGGCAGAAAATGAGAATTCTCA

GGAGCACCTGCAGGCAGTGATCCGTGGCAGTGGGAAGCTTGTGCTCTTGGATAAATTGCT

AACAAGACTAAAAGAACGAGGCAGCCGTGTGCTAATCTTCTCTCAGATGGTCCGTATGCT

GGACATCTTGGCTGATTATCTTGCCATGAAACGTTACCCTTTCCAGAGATTGGATGGCTC

AATCAAGGGAGAACTCCGGAAGCAAGCGCTCGACCACTTCAATGCAGAGGGTTCTGAAGT

ATCTACCATAACTATATATCATGGCATCACACAGTTTCTGTAACTGTACTGGTTAAGCAG

TATTTCTGCACTACGTAAACTTTTGATTTATACTACAAAAGTGTAGTATAAATTAGTGAT

GAAATTAATCAAATGACCTTGTAGTTTTATATAATCCGGTTTTTACTTGTTCCCTTTTGT

CTGCCCCAGGATTTCTGTTTCCTTTTGTCCACTCGTGCTGGAGGTCTGGGCATTAACTTG

GCCTCAGCTGACACTGTGGTCATCTTTGACTCTGACTGGAACCCTCAAAATGACCTGCAG

GCCCAGGCCAGGGCACACAGAATAGGCCAAAAGAAACAAGTAAATATTTATCGCCTGGTT

ACAAAAGGCACTGTGGAGGAGGATATTATTGAAAGGGCAAAGAAGAAGATGGTGCTAGAC

CACCTTGTCATACAGAGGATGGATACTACTGGAAGGACAATACTAGACAACAACTCGGGC

AATTCAAATTCAAATCCATTTAACAAAGAGGAGCTGGCAGCTATCTTAAAGTTTGGAGCA

GAAGATCTTTTTAAAGAACCTGAAGGGGAGGAATCAGAGCCGCAGGAAATGGACATTGAT

GAGATTCTGAGGCTTGCTGAAACCAGGGAAAGTGATCAAGGCTCCAGTGCTACAGATGAG

CTTCTGTCCCAATTTAAGGTGGCTAACTTTACCATGGATGAGATCACACCGGAGCTTGAG

GAGAAGCCCATGCGGAACTGGGATGAGATCATTCCAGAAGATCAAAGGCGGCAGTTAGAG

GAGGAAAAGAAACAGAAGGAGATGGAGGACATTTACATGTTGCCCCGAAGCAGAAGCTTG

AATAAAAGAGCACTGGCAAATGACAGTGACAGCGATATTGGCTCAAAGCTCAAACTGAAA

AGCCACTCATCAGGTTCAGAGAGTGAAACGGACGACAGTGAGGATGATAAAAAGCCCAAA

CGGCGAGGCCGGCCACGTGCGCGCAAAAACAATGTTGTTGGCTTTTCAGATGCTGAGATT

CGTAGGTTTATAAAAGCTTATAAAAAATTTGGCGCTCCACTTGAAAGGTTGGAGGCCATT

GCGCGTGACTCTGAACTGGTTGACAAATCCATAGCAGATCTAAAGAGACTAGGGGAGCTG

GTGCATACAACATGCGTAGCTGCAGTACAGGAGCATGAAGAACAAATAAAGGAGAACCCC

ACCGAAGCTAAAGGCCCAGGAAAAAGACGTGGCATTAACATTAAGATCTCTGGGGTTCAG

GTTAATGCTAAATCCATAATCCAGCATGAGGAGGATTTTGAGCCTTTGCACAGGGCTGTG

CCTTCCAGTTCCATTGAGAGAAGCAAATTTCAGCTTGGTTGTCGTGTGAAGATGCCACAC

TTTGATGTAGAATGGGATGTACAGGATGACGCACATCTACTGTTGGGAATATATGAGCAT

GGCTATGGTAACTGGGACCAGATCAAGACTGATTCAGAGCTGAAACTTATAGACAAGATT

CTTCCAGACAACTCTGACAAAAAGCCACAGGCAAAGCAGCTGCAAATGAGAGCTGATTAC

TTGTTGAAGATCCTAAAGAAGAAACAGGAGGGTGAAGATGCATCCAGAGCAAAAGAAGAG

TCTCAGGTAAAGAAGAGAAAACCTAGAGTAAAAAAAGAGAAGTCCCAAAAAGATGAACTG

GCCAGTGAAATCTCCTCACCTCATCTCTCAGAACATACTTCAGAAGAAGGTGAAGTAAAG

GATGATAAAGCTGAAAAATTTCCTACAAAGGTAAAACAAAAAAAGGACAACAAAGAGAAC

AAAGAGAAGCAGGGCACTGCAAAGAAAGACAAGGAAAGGGACAGGGAAAAAAAACGGACA

AAGCTTAAAAAAGAAAAGACAAAATTAGGAAGCAAAGCCAAGAAAACTCAGGGCCCTGTG

CACATTACAGCAGGCAGTGAGCCAGTTCCTATTGGAGAGGAGGATGATGAGCTGGACCAG

GATACATTCAGTATTTGTAAAGAACGGATGAGACCAGTAAAGAAAGCTCTTAAACAGCTG

GATAAGCCTGATGAAGGTCTGTCAGTGCAGGAACAGCTGCAGCACACACGTTCCTGCTTA

TTGAAGATTGGAGACCGAATTACAGAGTGCCTTAAAGCCTACAGTGATCCAGAACATGTC

AAAGCCTGGCGCAGGAATCTATGGATATTTGTGTCGAAATTTACCGAGTTTGGAGCTAAG

AAACTTCACAGGTTATACAAAATGGCTCAAAAAAAGCGCTCCCAAGAAGAGGCGAAAGAG

CACAAAAAGAAAGATGAACCATTCAAAAAGAAGACATTTCGTCCAGAACCTTCTGGATCA

AGTCGAGACTCCACTGGCACACAACCTTCATCGAAAGCACGCTTTCCACATAATCACCCA

ACAGGATTTTCATCTCAATCACAGCACCGGGAGCATTACAACCAACATAAGAGACACTTC

CCTAATGATTATAGGGGAGATTGGCACAGGGAAAGAAAATATACCTACCCAGGAAACAGT

AACCAGCCATGGCAAGGTGACCGATATAACCCTTATGACCAGCACAGTTATAAGGAGCAT

CACTACAGTAACCGACGGGCTCACACAGACTTGTACCACAGTACTGGCTTCCGAAACCCG

GGTTCAGCTCGTAAACGAATGTATGACCAGTACAGCAATGATCGAGACCACCGTGTTCAC

CGTGAATATTATGACAGGCATTCAGAACCAAAGAGGAGACGCTCAGGTGACTATCACTCT

GCAAACTGCAACCAAGGAGGAGGTGGAATATGTTACAACCAGGACTTCCGGAGGATGCCT

GATCATCAGGGTATCCCAGAGACTGATCACTACCGGTCGTTTAACCCAGACAAACCCCTT

CCTCTGCTGGACCCACGTTCTCCTCAGGCCCAGAAATCCCCACTTGAGTCCCTATCACCT

CTGGAGAAAACCACTGAGCCGAAACCCAGTCCAGATCTTAAATGGAACAGAAAGTGATTT

AATCCTTAATGATAGTGGAACATATAGAAAGACACCAGTTTTAGACAAGGCCACCAGTCC

CCACCATTTATTCCTTAATCACGATCCAATAGCTGCAGCTCAAGCTTCCATGTATTTGAG

CCCTCTGGATCTTTTGGGGTTAAATATGAAGCCCTCTTAGTCCTGTGGACTACTTAATGA

ACAATGTGATTTGAAAGCTTGGAAGTGCCTCACTTGTTTGTCCTTTTTTTAGCCATAAGC

ATTGATTAATTTAATTGTTTATTTAATGTTTTTGTTGCTTTGCTTTGAGGAGGTTTATCC

ACAGGAAAGTAGGCCTTGTGGCAAAAACCTAAACCACATTAGTACTTATAAAGCCTGAGT

TGATTTGACCCTGTTTTATGTGGAAGCATCCCTTGTCCTTTACATTTTAGTGTTTTGCAT

CTAATAAACCAGATTCATGTGATTGTCAGACCTGTTTATGTTCCAAGAGCAGGGGAAACA

CTAAAATGTACAGTTCAGGGTTGGGAAGCTATAATGTTTTTGAAATAGGCTTATAATTTT

GTGTTTGGCCCATTATACCAATAAAAGAAATTATGCTTAGAATGTGCCACCAAATGAGAA

TTTGTACTTTTAAATAACAAA

>Unigene000080

GGAAAACAATAAACAACCGTATGAGAAGAAACGCAGGAGGACCTGTCGCTTGTGCCAAAA

TCATATTTGTTTAGGATTAAAGACTGACTTGAAAGAAAAAAAGATCGTCGCTGTTTAAAA

GCTTCACCGGACAAATCGTTGTCCTGACTGGAGGAAAAAAAATCCAGAATCTTTGTGTTA

AAGATAAGGAAGTAAGCTAGCCAGTCTATGCAGCTAGCCCCCTTCAAGCTCCAGCCTAGC

CTAGCCTCCCCTCTCCTCTCCCCTCTGTTTGTGCCTCGTAAGTTAGGGTGGATTGTTATG

CAGTTCCCGGAGGACTGTTGTCGCTCTTGTTTTCTTTCTCCTCCGCCACTACTTCAGTTC

CTCACCCTAGTTTCTCTGGCCCTATGTCTCGGTGGTTGTTCCCCTGGTCGGGCTCGATAA

AGAAGCGGGCCTGTCGCTACCTTTTACAGCATTACCTCGGCCACTTCTTGGAGGAACGTT

TAAGTTTGGACCAGCTGAGTTTGGACTTGTACAATGGCAGCGGTGTTATTAAAGAAATCA

ACCTCGACGTGTGGGCAGTAAACGAGCTTCTGGAGTCACTGGCTGCCCCGTTGGAGATTG

TAGAAGGTTTTGTCCAGAGTATAAAAGTGACAATTCCATGGGCAGCGCTGGTCACCGATC

ACTGCACACTTGAAATAACCGGATTACAGATAACATGCAGACCCAAAGACCGAACCAGTG

GTACCTGGGATTCTCAAGGCTGGTCGTCATGTATGACTTCCAGCATGCAGCTTGCCCAGG

AGTGTCTGAAGGACCCACCCGAGGCATCGGAAGAACCACCCGCTCCTCTGGAAGGGCTGG

AAATGTTTGCACAGACCATTGAAACAGTACTTCGGCGAATAAAAGTCACGTTTCTGGACA

CTATCGTAAGACTTGAGCATCATCCCCTTGACAGTAAAAATGGGGTCGCCTTAGAGATGC

AAATTAAAAGGCTGGATTACTGTGATGAAGCTGTGAGAGACTCTAATCAAACAGTGCCTG

TAGATATCCACCAGCCTCCAGCCTTTCTGCACAAAATTCTTCAGCTCAACTCAGTACAGC

TGCTTTACGAGACTCTCGGAGAGCCACAGAGTTTTGTTCCTGAACATACTGAGGTGGAGG

ATGAAGAAAAGGATTCCGCCCCCAAGCCGTTACGGATGCATCCTGGGCCTCTAGTCATTG

GACGCTGCTCCGGTTTCATGGAGACCACCGTTAAAATCAAGCAGAACGACATGCTCCCAG

GGCCCAAGCTGGAGCTGGACGGTAAGGTGGGCTGTCTACACTTGCTGCTTTCTCCCAATC

AAATTACAAACCTAACGGACCTTCTGACCGCTTTATGCATCGAGACAGAGGGTAGCAGTA

ATAGTCGTGGTGGTGGTGGTGCTCCGAGTAGGCCTCTGGTTTCTGATGATCTTCGGTTGA

TAGAGGAGGACCTCAGTAAGCAACTCAGTGTGGATACAGTAGATAAAGAACTGGAGCTAA

ATGCGGAGCCTTATATCTACAGCCTGGAGAATGGAGAGATGTTTTACTCTATGGGGCCTG

GTGGTATGTCTAGCAGTGTAACATCCATACGTTCAGGAAGTGAGCTTTCAGACAGTGAGA

TGGAGTCCTCAATACACAGCCAAAGCAGCCTTGTTGCTCCAACACACCCTCTGTCCCCAC

AGGGAATTCTTAACTGCCCCAGACGTTATCCTGTACCAGGAACCTTTTCTAGCTTGCCTC

AATGTAGCAGGCCGTCAAGGCGTTCAACACACACTGGCCAGTCCGAATCCGCAAAGCCTG

ATTGCTTGCTTCGGCTCTCACTGGGTGGAATGACTCTAACTCTTCTGGAACAGGAGCCAG

TCGAAGATCGGTCATCCATGGCTAAAGTGTCACAAGTGTTCTTCCATGAGCTAGGCTTTT

TTAAAGACAGTATATTCAGTGAGAGAGACTTTGAGAACCTGAGGGGTAGCTTTGCTAAAG

CATGCCCTCACTCTCATCTCAGGCTAACTGGCGCAGCGGTGCAGATAGCATGTGAGATGC

GAAGTGCCGGACAGAGTGCCCGCTCCATAACCTCTGACCTCTCCTTCAGCAGGCTGGAAC

TGCTGGAGTGTCTTTGGGAGCCAGGAAAGCCCCAGTACACTGAGCTGTTGCAGTTCAAAA

ATGCTGGTCACTTTACCGTGGGAGCGACGGCTAGGCCTTGTGCCCAAATTCATTATTCTC

TGACAGAAAAACAGCAGCGCAAGAGTGGTTCGAAGCAGCGGATAGTGCGCAGGGAGAGCT

CTTTACATATAGAGCTGGGCGAGCTCACCACTGAAGTAGATCTGGACATAGTGGGCAGAC

TGGACAGCATCATACAGGCTCTGAGTCATTGTCCTAGTCAGCCCAAACAGCCAGCAAACA

ACCAGACGCCGCTGTTAGAGCTCCACTCCTCTTTTGTTCTTCTCTCCCCTTTCGCTGTAC

TAAAGCTCCGCTTCCCATCCCCGACCTTCGACCTTTAAATGAGCGAAGACCCGTTAGCGA

GAGAGCGGTGCGTAATGAGATTCTGAGCCTGGAGATGTGCAATCTAGAACTGAAGTCTCA

ACATGGATCAGACCTCCAGCAGCAGATGCCATCCAACTCCACAGCTAAACTAACCAAACT

GCTGGAGGCGAGCTTCAGTGATCTGCACGGAGTGTATGAGGGTTGGGAAGGAGGCTCTTT

CCCCTGTATCCGTGTACAAAAGGACTCGAACACACAGAATCCAGACAGCACACCCAAAAT

TGCGGTGCATGTAAGTGGAGCAGCAGACTCGGATGGTAAAGGGGGCGTAGGTGTGTGTTT

GAACAGGGATTTGGGGTTTGGCTTTGTGTCTATGGAAAATCCCTGTGAGCTAAGAGAGAA

GAACAGCTCCCCCTTCTCCTCTAACCGCACCATGTTTGAGACAGAAGAGATGGTAATCCC

AGCAGATCCCGAGGAAATGAAGGAATTCCAGGCTCAGTGTGTGGCTCAGAGTCAGTGTGT

GGTGGATATCATTCTACCTGTGGCCTACATTCTCTTACCCAGCAAAGAGGCATTTCAAAG

CATCTACAACAGGATCAATAATGATTTGCTGATGTGGGAACCCCCACCTCCTCCTCAACC

ACCTCCCAATATCTCTCCCAGCCCCATGCAGCAGCGCAGATCTGAGGGTGACGAGTTTCA

GCTCTGCAAATCTGCCTTCAGACTTGATTCTGACTCAGAAGAAGATGAACCTCAGTTTCA

TTCAGCCAGTGAACCAAGAAAAAAGGCACAGCCAGAGCCTCAATCAAACCATGCCCTCAG

TCTGCTTAGTTTATCAGTACAGGTTAACAAGGGTCGCCTGCAGTTTCTGACAGACAGTAA

GAATGAGGCGGGACGGAGGATGGAACAATGCCATGGAGAATTGCTGCTAGACTTTGAAGG

CGGGAAGATTTTTAGTGTTACCCAGCACCTGAATAATCCCAATTTAAACTTCTTGACTAT

CGAGGGCAAAAAAGTTGAACTCTATCATAAAGCTGAAGTCCCAGACTCTGCTGTCCCAAC

ACGATTGGAAATGCCCAAGTTTACACCCCCAAACCATTTGGACCCTACTATTTATCCCAC

AGAAGTTGGGGTTAGCAGTGTCGGGCGTAGAGGAACAGGGGGTCAGATGTTGTCAACTGC

CATCAAAATCACACTAGACCTCCAACGCAACGTCAAGGAGTTTTTGGTTGCATTACGGCT

CCAGGGTGCTACCTTAAGGCATCATATGGCACTGACCAATCACAGTTGGCATGAGCAGCT

GGTCGATTTTTTGGATGTGATTGATGATGATATTTTAGGATACACACCCCCTGCTATCAT

CACAGTGTTACATACTCACTTGGCCACATGTGCAGTCGATTACAGGCCACTCTATCTTCC

ATTGCGAGTGCTGTTCACAGCAGAATCCTTTTCCTTGTCCAGTAATATCATAGTAGATAC

TGCCACCTTCCACCTCAGGTTTATTCTGGATGACTCGGCCTTGTACCTTACCGACAAGTG

TGACAGCAACAGCGTTGATTTAAAACGAGATTATGTGTGCGTATTAGACATTGACCTGTT

GGAGTTGGCCATAACTACCTGGAAAGGCAACAATACTGGCAAACTAACTCAACCACTGTT

CGAGCTGCGCTGTTCAAATAACGTGGTTCACGTGCACACCTGTGCAGACTCTTGTGCTGC

TTTAGTGAATATGCTTCAGTACCTGATCTCCCAAGGCGACCTGCACCCGCCTCCTCGTCA

TACTTCCCCTACAGAGATTGCTGGACAAAAGCTCCCACTTTCTGAAGCTCCTACATCTTT

ACCACCCTGCCCACCAGCAGAGACAGCTGAAATTAACCAGTGTGACCTTACAGATGCCCT

GATTGACACCGATCGAACGCTGCATGAAGAGGATAAAGAGTTTGCAGGATCTCCTTCATT

AGTAAAGCGAGCATCACCTGTCTCCGTGTACCTGTTTCCTGGGGAAGGTCCAAAAGCTCA

CTCCTCTTTGCTAGAGGGGGTTGAGTCTGAGCTTGATCAACTGGTGGTCACCGCTCCTGA

AGCTCATGTTGATATGATGTCAGAGGAATGTTCTGAAGGCTCCACAGATAATGAGGACTT

CTGTATTTTAGAGGCGCCTGGTATAGGCATTCCTCCTAAAGATGGCGAGCCGGTGGTCAA

AGTGTTGTGTCAGACTCCTATAAAGGTGCGTGACGGCTATTACTCTCGGCCACGGGGCAG

TTCGGACCTGCTCCGGGCACCTGCACGCTTTGCGATGCCTCAGAGCAGGGTAGTACTCCG

AGAGGTATCAGTAGTCTGGCATCTCTATGGAGGCAAAGACTTTGGAGGAAAACCAATCTC

TGCTCACACACAACACAGCCATCGGGGTCGTCCAGCACCTGCCGGAGCCCGAGGTTCTCC

TTCACGCTCTGCTGGCTCATCCAGACCACAGAACTCATGGCGTTGGGTGGGAGGCAGTGG

CAGACAACACTCACTGCTCATGGAAATACAGTTGACAAAGGTCAGCTTTCAGCATGAGTC

GTACCCTGTGGTCGTGCCCCCTGTAGTCTCAGGGCAGGATATGGAAGGGGCAGGATTGGG

AGAGCAGCCTCTTTCCCGGCAGGTGTTTATTGTGCAAGAGCTGGAGGTGCGTGACAGACT

GGCCTCCTCACAGATCAACAAATTCCTCTACCTGTATACCAGCGAGAGTATGCCACGTAG

AGCCCACTCCAACATGTTAACAGTAAAGGCTCTGCAGGTACTTCCTGAATCTGGATTGGG

CGGTCCAGAATGTTGCCTGAGAATCAGTCTGCTGCCCCTCCGTCTGAACATAGATCAGGA

TGCCCTTTTCTTTTTGAAAGATTTCTTTAGTAACATGGCTGCTGGGATTAACCCATATTT

CCCTGTTGATCCGGCTGCAGAGGTAAAAGCAGACATTACTCAGAAACCGTCAGAGGAAGT

CGAGTCTGGCACTGGATTGGGCCCTGAGCTCACTGCTTCTATAGAGACAACGTATAGTGA

GCAGAGCTCCTCCTCTGCTGGCTCCTCCTCTTCCTCTGATCAGCCAATTTACTTCCGAGA

GTTTCGTTTTACATCAGAAGTTCCGATATGGCTGGACTATCATGGGAAGCATGTGGCTAT

CGAACAGGGGACATTTGCAGGGATTCTTATTGGCCTGGCCCAACTCAACTGCTCAGAGCT

GAAGCTGAAAAGGCTGTGTTGTAGACACGGACTGCTGGGAGTTGATAAAGTCATTCAGTA

CGCTGTAAACGAGTGGCTGATTGACATCAGGAAGAACCAGTTACCAGGAATTCTGGGAGG

TGTTGGTCCAATGCACTCTGTGGTTCAGCTTTTTCACGGGGTGCGGGACCTGTTCTGGAT

GCCCATCGAGCAGTACAGGAAAGATGGACGTATAATTCGGGGTCTTCAAAAGGGGGCAGC

GTCCTTTGGCACCTCTACAGCTTCGGCTGCACTGGAGCTAAGCAACAGACTGGTGCAGGC

CATACAGGCCACAGCAGAGACAGTGTATGACATCCTGTCCCCTACTCCACCCCTGACCCG

ATGCATCACAGAGGGACGTCCTGCCTCCCGGCCTAGACGCACCCCACAGCCAGCTGACCT

CAGAGAAGGAGTGGCCAAGGCCTACGACACCGTCAGAGAGGGGGTGATAGACACTGCTCA

GACACTGTGTGACGTGGCATCACGTGGACACGAGCAGAAAGGTCTTCCAGGAGCAGTGGG

TGGGGTTCTGCGTCAGATTCCTCCCACCGTAGTGCGTCCCTTAATAGTAGCCTCCGAAGC

CACCTCCAACCTGCTTGGAGGTATGCGCAACCAAATCAAACCGGATGCACGAAAAGAGGA

CTTCTTAAAATGGCGCACAGAAGATGGCCAAGAATGACTGCTCGGGTACAAATCAGGCTT

TTTGCTAGTGTCAAGCGTGTAGCGTGGAGGCCCGAGTGTGAAAATGCGGATACCTCCATG

AGTATTAATGCAAAGATGAAGGAAAAAAATGCAGTTCAGCCACTCAGCACGAGCCGACAT

GTTTGAATTGGCTTTCACGTGGTAGTTGCACATGGTCACGTTCATGCACGAGCAGAGCAT

TATTAGGTAAACAAGTGCAGTTTGCAGCTATCCACCTGATCTGAACTTATGCAATCATAT

CGATTAGGAAACGCTGTTTACCATTTAACAGACACACTTGCTTAAAATTGTATTTGTTCT

GTTCTCTTAATCACACACTGGGATGTTTCTAAGTACCTGGGATGTTTCTTTATAGATCTA

TGATCATTAGAATTGTTTAATATGTCCTTATCAAATGCAACAAGTTATAAAAATGATTAA

AATTGTACAAATTGGAAGCTAAAAGGGCATTTTTTTCTTAGTGGGTGTCAGAATGTAAAA

GTGGCCTTTCTATAATAAAGATTAAAAGATTCTGAATTGATTCTTATTCATCCCTTTGAC

CACGTTACGCGACGCCCCCAGTGTTATTGTGCCATAATTACATCAGTTTGAATGTTTCTT

CTCTTCGTGTCAGTATAGATGCTTCTATGACTTTTCCACACTAAACTCC

>Unigene000081

GGAAAACAATAAACAACCGTATGAGAAGAAACGCAGGAGGACCTGTCGCTTGTGCCAAAA

TCATATTTGTTTAGGATTAAAGACTGACTTGAAAGAAAAAAAGATCGTCGCTGTTTAAAA

GCTTCACCGGACAAATCGTTGTCCTGACTGGAGGAAAAAAAATCCAGAATCTTTGTGTTA

AAGATAAGGAAGTAAGCTAGCCAGTCTATGCAGCTAGCCCCCTTCAAGCTCCAGCCTAGC

CTAGCCTCCCCTCTCCTCTCCCCTCTGTTTGTGCCTCGTAAGTTAGGGTGGATTGTTATG

CAGTTCCCGGAGGACTGTTGTCGCTCTTGTTTTCTTTCTCCTCCGCCACTACTTCAGTTC

CTCACCCTAGTTTCTCTGGCCCTATGTCTCGGTGGTTGTTCCCCTGGTCGGGCTCGATAA

AGAAGCGGGCCTGTCGCTACCTTTTACAGCATTACCTCGGCCACTTCTTGGAGGAACGTT

TAAGTTTGGACCAGCTGAGTTTGGACTTGTACAATGGCAGCGGTGTTATTAAAGAAATCA

ACCTCGACGTGTGGGCAGTAAACGAGCTTCTGGAGTCACTGGCTGCCCCGTTGGAGATTG

TAGAAGGTTTTGTCCAGAGTATAAAAGTGACAATTCCATGGGCAGCGCTGGTCACCGATC

ACTGCACACTTGAAATAACCGGATTACAGATAACATGCAGACCCAAAGACCGAACCAGTG

GTACCTGGGATTCTCAAGGCTGGTCGTCATGTATGACTTCCAGCATGCAGCTTGCCCAGG

AGTGTCTGAAGGACCCACCCGAGGCATCGGAAGAACCACCCGCTCCTCTGGAAGGGCTGG

AAATGTTTGCACAGACCATTGAAACAGTACTTCGGCGAATAAAAGTCACGTTTCTGGACA

CTATCGTAAGACTTGAGCATCATCCCCTTGACAGTAAAAATGGGGTCGCCTTAGAGATGC

AAATTAAAAGGCTGGATTACTGTGATGAAGCTGTGAGAGACTCTAATCAAACAGTGCCTG

TAGATATCCACCAGCCTCCAGCCTTTCTGCACAAAATTCTTCAGCTCAACTCAGTACAGC

TGCTTTACGAGACTCTCGGAGAGCCACAGAGTTTTGTTCCTGAACATACTGAGGTGGAGG

ATGAAGAAAAGGATTCCGCCCCCAAGCCGTTACGGATGCATCCTGGGCCTCTAGTCATTG

GACGCTGCTCCGGTTTCATGGAGACCACCGTTAAAATCAAGCAGAACGACATGCTCCCAG

GGCCCAAGCTGGAGCTGGACGAGGGTAGCAGTAATAGTCGTGGTGGTGGTGGTGCTCCGA

GTAGGCCTCTGGTTTCTGATGATCTTCGGTTGATAGAGGAGGACCTCAGTAAGCAACTCA

GTGTGGATACAGTAGATAAAGAACTGGAGCTAAATGCGGAGCCTTATATCTACAGCCTGG

AGAATGGAGAGATGTTTTACTCTATGGGGCCTGGTGGTATGTCTAGCAGTGTAACATCCA

TACGTTCAGGAAGTGAGCTTTCAGACAGTGAGATGGAGTCCTCAATACACAGCCAAAGCA

GCCTTGTTGCTCCAACACACCCTCTGTCCCCACAGGGAATTCTTAACTGCCCCAGACGTT

ATCCTGTACCAGGAACCTTTTCTAGCTTGCCTCAATGTAGCAGGCCGTCAAGGCGTTCAA

CACACACTGGCCAGTCCGAATCCGCAAAGCCTGATTGCTTGCTTCGGCTCTCACTGGGTG

GAATGACTCTAACTCTTCTGGAACAGGAGCCAGTCGAAGATCGGTCATCCATGGCTAAAG

TGTCACAAGTGTTCTTCCATGAGCTAGGCTTTTTTAAAGACAGTATATTCAGTGAGAGAG

ACTTTGAGAACCTGAGGGGTAGCTTTGCTAAAGCATGCCCTCACTCTCATCTCAGGCTAA

CTGGCGCAGCGGTGCAGATAGCATGTGAGATGCGAAGTGCCGGACAGAGTGCCCGCTCCA

TAACCTCTGACCTCTCCTTCAGCAGGCTGGAACTGCTGGAGTGTCTTTGGGAGCCAGGAA

AGCCCCAGTACACTGAGCTGTTGCAGTTCAAAAATGCTGGTCACTTTACCGTGGGAGCGA

CGGCTAGGCCTTGTGCCCAAATTCATTATTCTCTGACAGAAAAACAGCAGCGCAAGAGTG

GTTCGAAGCAGCGGATAGTGCGCAGGGAGAGCTCTTTACATATAGAGCTGGGCGAGCTCA

CCACTGAAGTAGATCTGGACATAGTGGGCAGACTGGACAGCATCATACAGGCTCTGAGTC

ATTGTCCTAGTCAGCCCAAACAGCCAGCAAACAACCAGACGCCGCTGTTAGAGCTCCACT

CCTCTTTTGTTCTTCTCTCCCCTTTCGCTGTACTAAAGCTCCGCTTCCCCATCCCCGACC

TTCGACCTTTAAATGAGCGAAGACCCGTTAGCGAGAGAGCGGTGCGTAATGAGATTCTGA

GCCTGGAGATGTGCAATCTAGAACTGAAGTCTCAACATGGATCAGACCTCCAGCAGCAGA

TGCCATCCAACTCCACAGCTAAACTAACCAAACTGCTGGAGGCGAGCTTCAGTGATCTGC

ACGGAGTGTATGAGGGTTGGGAAGGAGGCTCTTTCCCCTGTATCCGTGTACAAAAGGACT

CGAACACACAGAATCCAGACAGCACACCCAAAATTGCGGTGCATGTAAGTGGAGCAGCAG

ACTCGGATGGTAAAGGGGGCGTAGGTGTGTGTTTGAACAGGGATTTGGGGTTTGGCTTTG

TGTCTATGGAAAATCCCTGTGAGCTAAGAGAGAAGAACAGCTCCCCCTTCTCCTCTAACC

GCACCATGTTTGAGACAGAAGAGATGGTAATCCCAGCAGATCCCGAGGAAATGAAGGAAT

TCCAGGCTCAGTGTGTGGCTCAGAGTCAGTGTGTGGTGGATATCATTCTACCTGTGGCCT

ACATTCTCTTACCCAGCAAAGAGGCATTTCAAAGCATCTACAACAGGATCAATAATGATT

TGCTGATGTGGGAACCCCCACCTCCTCCTCAACCACCTCCCAATATCTCTCCCAGCCCCA

TGCAGCAGCGCAGATCTGAGGGTGACGAGTTTCAGCTCTGCAAATCTGCCTTCAGACTTG

ATTCTGACTCAGAAGAAGATGAACCTCAGTTTCATTCAGCCAGTGAACCAAGAAAAAAGG

CACAGCCAGAGCCTCAATCAAACCATGCCCTCAGTCTGCTTAGTTTATCAGTACAGGTTA

ACAAGGGTCGCCTGCAGTTTCTGACAGACAGTAAGAATGAGGCGGGACGGAGGATGGAAC

AATGCCATGGAGAATTGCTGCTAGACTTTGAAGGCGGGAAGATTTTTAGTGTTACCCAGC

ACCTGAATAATCCCAATTTAAACTTCTTGACTATCGAGGGCAAAAAAGTTGAACTCTATC

ATAAAGCTGAAGTCCCAGACTCTGCTGTCCCAACACGATTGGAAATGCCCAAGTTTACAC

CCCCAAACCATTTGGACCCTACTATTTATCCCACAGAAGTTGGGGTTAGCAGTGTCGGGC

GTAGAGGAACAGGGGGTCAGATGTTGTCAACTGCCATCAAAATCACACTAGACCTCCAAC

GCAACGTCAAGGAGTTTTTGGTTGCATTACGGCTCCAGGGTGCTACCTTAAGGCATCATA

TGGCACTGACCAATCACAGTTGGCATGAGCAGCTGGTCGATTTTTTGGATGTGATTGATG

ATGATATTTTAGGATACACACCCCCTGCTATCATCACAGTGTTACATACTCACTTGGCCA

CATGTGCAGTCGATTACAGGCCACTCTATCTTCCATTGCGAGTGCTGTTCACAGCAGAAT

CCTTTTCCTTGTCCAGTAATATCATAGTAGATACTGCCACCTTCCACCTCAGGTTTATTC

TGGATGACTCGGCCTTGTACCTTACCGACAAGTGTGACAGCAACAGCGTTGATTTAAAAC

GAGATTATGTGTGCGTATTAGACATTGACCTGTTGGAGTTGGCCATAACTACCTGGAAAG

GCAACAATACTGGCAAACTAACTCAACCACTGTTCGAGCTGCGCTGTTCAAATAACGTGG

TTCACGTGCACACCTGTGCAGACTCTTGTGCTGCTTTAGTGAATATGCTTCAGTACCTGA

TCTCCCAAGGCGACCTGCACCCGCCTCCTCGTCATACTTCCCCTACAGAGATTGCTGGAC

AAAAGCTCCCACTTTCTGAAGCTCCTACATCTTTACCACCCTGCCCACCAGCAGAGACAG

CTGAAATTAACCAGTGTGACCTTACAGATGCCCTGATTGACACCGATCGAACGCTGCATG

AAGAGGATAAAGAGTTTGCAGGATCTCCTTCATTAGTAAAGCGAGCATCACCTGTCTCCG

TGTACCTGTTTCCTGGGGAAGGTCCAAAAGCTCACTCCTCTTTGCTAGAGGGGGTTGAGT

CTGAGCTTGATCAACTGGTGGTCACCGCTCCTGAAGCTCATGTTGATATGATGTCAGAGG

AATGTTCTGAAGGCTCCACAGATAATGAGGACTTCTGTATTTTAGAGGCGCCTGGTATAG

GCATTCCTCCTAAAGATGGCGAGCCGGTGGTCAAAGTGTTGTGTCAGACTCCTATAAAGG

TGCGTGACGGCTATTACTCTCGGCCACGGGGCAGTTCGGACCTGCTCCGGGCACCTGCAC

GCTTTGCGATGCCTCAGAGCAGGGTAGTACTCCGAGAGGTATCAGTAGTCTGGCATCTCT

ATGGAGGCAAAGACTTTGGAGGAAAACCAATCTCTGCTCACACACAACACAGCCATCGGG

GTCGTCCAGCACCTGCCGGAGCCCGAGGTTCTCCTTCACGCTCTGCTGGCTCATCCAGAC

CACAGAACTCATGGCGTTGGGTGGGAGGCAGTGGCAGACAACACTCACTGCTCATGGAAA

TACAGTTGACAAAGGTCAGCTTTCAGCATGAGTCGTACCCTGTGGTCGTGCCCCCTGTAG

TCTCAGGGCAGGATATGGAAGGGGCAGGATTGGGAGAGCAGCCTCTTTCCCGGCAGGTGT

TTATTGTGCAAGAGCTGGAGGTGCGTGACAGACTGGCCTCCTCACAGATCAACAAATTCC

TCTACCTGTATACCAGCGAGAGTATGCCACGTAGAGCCCACTCCAACATGTTAACAGTAA

AGGCTCTGCAGGTACTTCCTGAATCTGGATTGGGCGGTCCAGAATGTTGCCTGAGAATCA

GTCTGCTGCCCCTCCGTCTGAACATAGATCAGGATGCCCTTTTCTTTTTGAAAGATTTCT

TTAGTAACATGGCTGCTGGGATTAACCCATATTTCCCTGTTGATCCGGCTGCAGAGGGTA

AACTTAACGGATAAACAAGCATCAGTTACATTTTCCATCTTTTCCATGAATGTATAAAGT

GCATTGTGTCTTTATTTCATAGTAAAAGCAGACATTACTCAGAAACCGTCAGAGGAAGTC

GAGTCTGGCACTGGATTGGGCCCTGAGCTCACTGCTTCTATAGAGACAACGTATAGTGAG

CAGAGCTCCTCCTCTGCTGGCTCCTCCTCTTCCTCTGATCAGCCAATTTACTTCCGAGAG

TTTCGTTTTACATCAGAAGTTCCGATATGGCTGGACTATCATGGGAAGCATGTGGCTATC

GAACAGGGGACATTTGCAGGGATTCTTATTGGCCTGGCCCAACTCAACTGCTCAGAGCTG

AAGCTGAAAAGGCTGTGTTGTAGACACGGACTGCTGGGAGTTGATAAAGTCATTCAGTAC

GCTGTAAACGAGTGGCTGATTGACATCAGGAAGAACCAGTTACCAGGAATTCTGGGAGGT

GTTGGTCCAATGCACTCTGTGGTTCAGCTTTTTCACGGGGTGCGGGACCTGTTCTGGATG

CCCATCGAGCAGTACAGGAAAGATGGACGTATAATTCGGGGTCTTCAAAAGGGGGCAGCG

TCCTTTGGCACCTCTACAGCTTCGGCTGCACTGGAGCTAAGCAACAGACTGGTGCAGGCC

ATACAGGCCACAGCAGAGACAGTGTATGACATCCTGTCCCCTACTCCACCCCTGACCCGA

TGCATCACAGAGGGACGTCCTGCCTCCCGGCCTAGACGCACCCCACAGCCAGCTGACCTC

AGAGAAGGAGTGGCCAAGGCCTACGACACCGTCAGAGAGGGGGTGATAGACACTGCTCAG

ACACTGTGTGACGTGGCATCACGTGGACACGAGCAGAAAGGTCTTCCAGGAGCAGTGGGT

GGGGTTCTGCGTCAGATTCCTCCCACCGTAGTGCGTCCCTTAATAGTAGCCTCCGAAGCC

ACCTCCAACCTGCTTGGAGGTATGCGCAACCAAATCAAACCGGATGCACGAAAAGAGGAC

TTCTTAAAATGGCGCACAGAAGATGGCCAAGAATGACTGCTCGGGTACAAATCAGGCTTT

TTGCTAGTGTCAAGCGTGTAGCGTGGAGGCCCGAGTGTGAAAATGCGGATACCTCCATGA

GTATTAATGCAAAGATGAAGGAAAAAAATGCAGTTCAGCCACTCAGCACGAGCCGACATG

TTTGAATTGGCTTTCACGTGGTAGTTGCACATGGTCACGTTCATGCACGAGCAGAGCATT

ATTAGGTAAACAAGTGCAGTTTGCAGCTATCCACCTGATCTGAACTTATGCAATCATATC

GATTAGGAAACGCTGTTTACCATTTAACAGACACACTTGCTTAAAATTGTATTTGTTCTG

TTCTCTTAATCACACACTGGGATGTTTCTAAGTACCTGGGATGTTTCTTTATAGATCTAT

GATCATTAGAATTGTTTAATATGTCCTTATCAAATGCAACAAGTTATAAAAATGATTAAA

ATTGTACAAATTGGAAGCTAAAAGGGCATTTTTTTCTTAGTGGGTGTCAGAATGTAAAAG

TGGCCTTTCTATAATAAAGATTAAAAGATTCTGAATTGATTCTTATTCATCCCTTTGACC

ACGTTACGCGACGCCCCCAGTGTTATTGTGCCATAATTACATCAGTTTGAATGTTTCTTC

TCTTCGTGTCAGTATAGATGCTTCTATGACTTTTCCACACTAAACTCC

>Unigene000082

CGTGAGACTGAAGAAGTAGCCAATAAAATCGCCTATATGCGGGATAGTGTGAGCCAATCA

CAGTGTAGTAGGCGGGATTTGACGTAATACGGTTAGGAAAAACATTCCGTGTGTCTTTGC

ATGGCTAATACAGTAACATGGGCGTAACCGAAAGATTTACATTATCATTGAACTAAAACA

ATAAAAAACAGATTAAAAATAATCCACAAAATTTTTAGTTAACATTTAACCAACTACGTG

TTTTATAAACCCTAAAGCAAAACCAGTCATTAATTATATATAATACTTTTGATATATAAT

GTGATAGAAAATATTGAGCCATGTTTTATTTATTTTGTAAATCTGACTTTAAATGAGATT

TAAGGTTTTAATCCAAAGCTGATATGAGGGAAAAAGTTAATAAATGATTCTTTCTAGAAT

AAATCTTGTGTTTATAGTTTATACTGAAGAATTACGGCGAGGATCCAGACACCTACAACG

AGTCTCTGAAGAAGCTGGAGCAGCTACGGCAGAGTGCAGTGAACGTGACGCGGGACTTCG

AGGGCTGCAGTACGCTACGCAAATACTTCGGGCAGCTGCACTACCTGCAAAGCCGCGTGC

CTTTGGGTCCTGGGCAGGAAGCTGCGGTTCCGGTGTCCTGGACGGAGATCTTTTCAGGGA

AGACGGTCACACACGACGATATCAGTTACGAGCAAGCGTGCATTCTGTACAATCTGGGTG

AGTGTCTGCACCGGGTGTCACAGAGTCTCACACACACATACACACACTGTGTGTGTGTGT

TAGTGAGGAGGTCACGTTTCCTGATTCCAGAAATGTGTGTGTTTTTGTGTGTGTAGGAGC

GCTGCATTCGATGCTGGGAGCAATGGACAACCGAATCTCAGAGGAGGGGATGAAGGTGTC

GTGCACACATTTCCAGTGCGCCGCTGGAGCCTTCGCCTACCTGCGAGATCACTTCAGCCA

TAACTACAGCGTAGACATGAGCCACCAGATCCTTAATCTCAACATCAATCTCATGCTGGG

TCAGGCTCAGGAATGCCTGCTGGAGAAGTCCATGCTGGACAACAGAAAGAGTTTCCTTGT

TGCACGGATTAGTGCTCAGGTAGTGGAGTATTATAAAGAGGCTTGCCGCGCCCTGGAGAA

CTCCGACACCGCCTCGATGCTTGGAAAGATCCAGAAAGACTGGAAGAAACTTGTGCAGAT

GAAGATTTACTACTTTGCTGCTGTCGCTCATCTTCATATGGGAAAGCAGGCTGAGGAACA

GCAGAAATATGGAGAAAGGCTTGCTTATCTTCAAAGTTCACTAGACAAACTGAACGAAGC

GATAAAGTTGGCTAAAGGCCAGCCCGACAGCGTACAAGAAGCCCTGCGCTTTACAATGGA

CGTCATCGGGGGAAAATTTAACTCTGCTAAAAAGGACAATGACTTTATTTACCACGAGAC

TGTGCCCTGTCTGGAGACGCTGCCTTCAGTTAAAGGTGCACCGCTGGTCAAAGCTTTACC

CGTGAATCCAACAGACCCTAGTGTGACAGGACCAGATATTTTCTCCAAGTTGGTGCCTAT

GGCTGCTCATGAGGCCTCCTCACTCTACAGTGAAGAAAAAGCCAAGCTGCTACGAGATGT

CATGACAAAAATAGACAGCAAGAACGAGACTTTAGAACAATTTATGGACTCTCTGAATCT

GGACCCGGACTCCGTGGACAACATGGACAGATACAACAGCATTCCCTCTGTCCTCATGGA

GAAGTGTGCAGCTCTAAGTGTGAGACCGGACACCGTAAAAAGTCTCATACAGTCTATGCA

GGTACTATCGGGTGTGTTCACGGATGTGGAGGCCTCTCTTAGAGAGATTCGGTTAGTGCT

AGACGACGACGAAGCTAGTGACCGGAAGTTAGAGGAGGTAGCAGGAAAGCAGTGTGTTCC

TGCACAGCCAGCTACCATAGCGGAAATCCGTCGGGACCTTGAGAAATATGTAGAGGCCCA

CGAGAAGGCAAGCTTTACCAACACAGAGCTACACCGGGCCATGAATCTGCACATTAGTAA

CCTGCGACTCCTGGGTGGACCACTGGATACTCTGAGAGAGGCCTTGCCCAGACCCCAGCT

CTCTGAAGATGAAGTAGCGGGTCTCCAGTGTATGAAAAGGATTCTGGGTAAAGTGCAGGA

GATGAAAGACCAGCGCAATTCACTTGAGAAGCAACTAAGGGAACTGATCCAGCAGGATGA

TATAACTGCTGCTTTAGTTACCACTGAGAGGGCTGATATGAAGAGATTATTTGAGGAGCA

GCTGAAGAAGTATGAACAGGTAAAAGTGTACATTGACCAAAATCTAGCTGCACAGGAGAA

CATTTTGAAAGCACTGACGGATGCGAATGTCCAGTATGCAACAGTACGCAAGGGCCTAGC

TGAGACGGAGCACAAATGGAACAGTACCGTGCAAATGCTTGTTGCTTCTTATGAAGCCTA

TGAAGACCTGATGAAAAAATCGCAGGAGGGAAAAGAGTTTTATGAAGACCTGGAGGCTAA

GTCCTCTCGCCTCTTAGAAAGGGCTAAGTCCATCTGCAAAACCAGAGATGAAGAAAGACA

AGCCATTTTGGAAAAAGAGCTTCAAAAAAAGGTACCCCAAAGGCCCACAGCCCCAAAGCC

AGCACAAAAGAAAGCCCCTGACATGGATTTATCTGGAATAGAAGACCCTGAGTTAGCCAA

GATAAATGCTGCCATTTTAGCCTTAGGTGGAGATTTACCAGAAGATCTTCGCAGTTTGCC

TCCCGAGCTTGGTCGTCCATCAATACCAGAGGCTTTTCATCCAGGGGCAGCTGCTCTATA

TGCACGACCATTGTTTCCTCCGAACCTTCCGCCTCCCGAACTTCTTGCACAAATCTCCCG

TTTTACGCTGCCCCCAACCCAGGGCTTTAACCGGAATCCAACAACAGGCCCCTTGCCCCA

CCAACATCAGGTTCCCCAGTTGCCCAACCCTCAAATTGCACAGCTCCCTTCAGTACCTGG

GTATGGTCTTCCATCTTCACAAACCCAGCCTGCTGCATCAGGTCCACCAACTGCCATTGG

CATGTCTACCACAACTGTGGATAGTGTGCATACGCCAATTCCCAGCTATACTTCAGCACC

AGGACATCCAGTAATTTCTACTACACCTGGACACCCTATGGCCACTTCAGTGGGGTATAA

TGCCCCATCTGCAGTAGCTTCTCAGTATTTTCAACAGCCTGGCGTTCCTGTGAGCCAGCC

ACCTCAGGGAGTACAAGTTCCGCAACCAAAGCCACCGCCCTCTCAATCATTTCCCCAAGT

TCGCAGACAGGGCCAGCCACAACCGAGTCCACCGCAGACATTTTATCAAGCAGTGCCCGC

TCAGCAGCATATAGCTGCACCTGTTACTGGACCTCAACTCCCACAACAAACTCAACCTTA

TTCTCTACACCACATGGCACAACCAAGACCACCTAATATTCAGCAACAGGCAAATAATAT

TCACTCTACACCATCTGCTTTGTCCCACCAGGTGTACCCTGCTGTTTCATTTGTTCCAGG

TTGCCAGCCTGCTCCAAATCAGCTGCCCTATCATTCCATGCAACCACAAATGCCCCCAAA

CTCCCAGCCAATGCCTCAAGTTTCTATGTCACACTTACCAAATGTAAAACAAATTCCCCC

TTGTTCCCAGCCCTACATGCCCCCACTCAATCAGCAATTACATACTATTCCACAAACACC

CCTTTCCACTCAGCAGGCAGTACCTGCAAGCCATAGGCAAATGCCACCTACTCCACAACA

AATACCCCCACTCTCACAACCACAAATGCTACCATCTTCAGCCCAAATATCTCCTCCCAC

TCACCTGCAAATGCCTGCAGTTTCTCAACCAGGCTTGCATGCTTCTCATTCACCAGTATC

TGTTTCTCAGTCTTTACCTCGCTCATCTCACCCACCACTGCACAGTGTTTCCCAACCATT

ACCACACCCCTCTCAAGTCCATCCTTCCTATCAGCAAGTATTTCCAGGAACCACACCAAG

AGTTCCAGTCCCTGTCCAGCCCCAGTTACCTCCAGCCTCTCTGCCAGCACAGTTCCCGGT

GCACACCAATGCTCCCCCTTCACAGCCTCACAGTCTTCCCTCACAACTCCCTCATGGATC

TCTTTCGTCAGTGCCCTCACAGATCAGTTTGCCTCAGCATCATCTACCTCCTCAGTCCAC

ACCTATGGGTTATCCTGGTGGTATTCCTTTAGTTCCCCACCAGTCCACAGTTCCTCAGCA

GCCTCAGGCTCTGCCTCCACAGGTACCATCAGGGGCACAAGCATCTTACTCTGGGCCGCA

TGCTGGGATTCATCCTGGACCAATACAGAATTCTGGACCAGTCATTACGCCTGGAATACA

GTCACTCCCTCAGGGTATGATCCCACCATCTTCCGCTGCACCAGGGGCTACACCAAGTAA

TGTCTTGGCTACTCCATCAGCTTCTCCATCACCAGGGCCTTCCTCACTGGGTATCGCGCC

TCAGAGACCTTCTCCTGCTCTCACGCCTGTTCCAGGTGCAGCGTTACCACAGACTGTGGT

GACACCCTTGCCATCATCCACCCATCTGTTTCAGCACCAAAATTCCAGTACAGATGATCT

TCTCTCTTCAAGCCCTGAAAGCCAACATGGTAGTAGCAAAGACACTGCCAATGTCTTATT

GCCCATGAAGGCTGATCCACAAGATGAACAGCTTCGCAAAAAATCTGAGGGGTTAAAAAT

TATTCAAGGTGATCCGTACCAGGCTCCGGAAAGGGTCTCGCAGCTTTGCGCAGAGCTTGA

AAGGTTTCGGTCAACTGTTCAGTCCTTGGAGCGTCCCTCAGGAGAAGGAAGTTTATCGGA

ACTTGATGCTCGATGGAAAGAGCTACAAGATCAGCAAGAGAAAGATTCTCGTCAGCTTTC

TATCGCCATAGCCAGGTGCTACACAATGAAAAACCGCCACCAGGATGTCATGCCTTATGA

CTGTAATAGAGTTGTTTTGGACTCTGGCAAAGATGACTATATCAATGCCAGCTTTATAGA

GGAACTTTCGCCATATTGTCCCCGTCTTATTGCAACACAGGCACCTCTTACTGGAACTGC

TGCTGATTTCTGGCTCATGGTCTACGAGCAGAAAGTGTCTCTCATAGTAATGCTGGTGTC

TGAGCAAGAACTTGAAAAGCAAAAGGTCTTGCGGTACTTTCCATCTGAAAGAGGACAGCA

GATCACACAAGGCTCAATTACACTAAGCCTAACCACACAGAAAAACACAGCCACTCATGT

GGAGCGCATGATTGGACTGCAGTACAGAGATCAGAGTTTAAAGCGCACAGTCATACACCT

ACAGTTCACCTCATGGCCTGAACTTGGCTTGCCTGAGAGTAAAAGTAACCTCATTCGCTT

CATTCAGGAAGTTCACGGACATTACCTTCTCCAACGCCCTTTGCACACACCTGTTGTGGT

GCACTGCAGCTCTGGCGTTGGTCGAACCGGAGCCTTCTCTCTGTTGTATGCTGCATTACA

AGAACTGGAGGCTGGGAATGGTATACCTGACCTACCCCAGTTGGTGAGAAAAATGAGACA

ACAGAGAAAGAACATGCTACAGGAGAAGTTACACCTAAAATTCTGCTATGAAGCAGTCCT

GAAACATGCAGAGCAAGTGCTTCAGCGTCGTGGCATTGTTACTGCCTCGAGCCAAAAGTC

TACAAACAGTGCAGCAGTTAAGCAATACTCACAGCCAGAGGCTCAGGATATTGTTCTTGG

CGGCGATATGCCCATTAGCTCTATTCAGGCTACGGTTGCTAGACTCAGCATCCGTCCACC

CAGTGTCGAGTCTGACCAGGACCCAAATCAGGACCCAGTTGCTTGTGTTGCTGCAGGACT

AGAACCAGAGTTGGCACTAGTCCAGGACCTCCAAGCTTTTTCTCCCAGTGACACTATTCC

TGCCTCGCTAAGCCCTCCAATTTCCTGCCCTACTTCACCAGTTAAAATGCTGTCACCTTC

ACCTCCTAATTACCAAGGTAATGGGTTCGAGGGGTCCACAATAATGCCAGTCTCTAACAA

CCATGCTGCACTTGAAGCAACTCTTCCAGCACCGTCATCCCTGGACCTACTGGCATCGTT

AACGCCTGAAGCTTTCACACTCGACAGTGGTTGCCGTGGGAAACAGCGAATCAGCAAGCA

AAGCTTTTTTCAGGCACAGGAAGGCCAGGGTTTGCAGGGTCCTTCGAATCAGGATGACCC

ACTGAGCACCCTGGATCCACTTTGGACTCTGAACAAGTCCTGACTCCCTTTTGATAAGGC

CACTTCTGTTTATTTCTTAGTGTCCTGTATCCCCAGATCACCTAACAGTTTAGCATTTCC

AGCGCTTATCGGCTAGCTCACAGCCTTTATTAGTTGATTCGGTTGTTTTGGAGCAGGAAA

AGGCCCAGATTCCAGAGTCTGATGTGGATCTCTAGGACCACATCACTGTGTGTTGGTCCA

TGTGTATCTCTTCGATTCCTTGTATTTTTAAGCTGTTTCCTAGCATGCACCCCTCTTTTA

AAGACAAGTCAAGCCTTCTAGATGGACATCTGCATTTTTAACAATAAAAACATCTTTTAA

AGGAGAAACGTTAAGGTTCTCAGCACTTTGAGAAAAGGGAACAATGGATTTGTCATTTGG

TTGGTTTGCGTGTATCTTGGACTGAAATAAAAATGGATTTCTTAGAGGAACACCAGTCAT

TAACAAGTGCTTGACTAATGATTAGTACTATTGCACAGACTCCTCTCATTCCACGCTTCA

TCTCCTTTTTTGTTGTCAACTTTGACCAAGTTGAATAATGGAAAAAGTGCTAACTTACAA

CTGCAAAAGCCAGTAAGGTCTAGTTCACAGAATAAAAGTTTCAATAATGCTAGCTGCCTT

TTAACATAAGATAACCTAGGGTTAGCTGTTTTTAGGGGGACTACTTGCTAACTTTATGTA

CGGCTAAGTGATGCTGGTATCCTGTATACGCACTTTTCCTTTATTACATGTTAAACGACT

TTATAACTTATTTCTGATTCAGCAC

>Unigene000083

TTTACACACACACACACACACACTGAGACTCAATTACTGCCCAATTCCCCCTTCTTCATC

ATCTCCTGCTACGCCGGCCTGTCGGAACAAAAAAAAAAAGGAAAAAGAACCCGAACCGTA

AATCTAAAAAAAAAAAAAAAAAGTATTTCCTTTGAAGCGCGAGGTGGATTTTTAAACGCG

TCGACGGACAAAAGTGTCGCAAAAAACCTGACGGTGGAAGAGCAGTGATGTCACAGAGCT

GAGGACGCTTCCACACAATATGGAGTCTGGAGAGCGGCTGGCTTCGCCTTCCTCCACCCC

GGTCTCCGTACACACCAGCTCATCTTCATCCTCATCTTCAGTGTCTCCAGCCTCCAACGC

CAAGAGCAGCCTCAACCACGGAGCCGCTGCCTCGCTCGCTGCCTGCGGTCCCCTGTTTGG

GGTAACAGGAAGTGAGCAGCCCTTCAGCGTGACCTCTGTGACCTCGGTGCCCAGTGCCTT

CCCCGTGATGGCGCACCCCGCTTTTGGCCTGCTCTCCCCCGGCACGGCTCGGCCCGAGTT

CGGCGGTCTCGGAGCGCTGGGGGTCACGGCGGCCCTCGCGGCGCATCCGCAACTAGGGGC

TTTCACAGAGTGGTGGAGAGCAGCCGAAGCTCACGGACGGAGTCCAGCCGCCTTCTTCCC

TCCTTTCCTCGGCCTGCCCCCGCTGTTTGCCCCTCCCCTTCAGAACCACGAGGCCACGCC

CTACACGTCCAAAACCCTGAGCAAAAGCAGCCAGGGGCCCAAAGGTGTTAACGGAGCTAT

GAACGGCAGCGTGGTCTCTCCGTCCACGACGAAAAGTGGCGGGTCCGTGGCATCCTCGGC

CGCTCTCAACACCTCTGGCGGAAAACCTCGCGCCCGCAAAGCCTCTCAAAACAGCAACAG

TGCGGGAGAGCTGCAGGAGAAACTCTCACAGAAACCGAAAGAGAAGAAACCGCGTAAGAA

ACAGACCGAAGGCTCAGGCATGAGCGACAGTGAATCAGGATCATCTCTGGACAGCGACAT

TGAAGGCGTGAGCAGCAGTGACCTGGATGATCTCGGTGAGGAGGACGACGACGATGACGA

TGACGACCAGAGTGAAGAGTCCGACTCTGAGAAAGAACCCCATAAAAAAAAGAAAGCCAA

GGTCGTAGCCTCAAACCTGCGGCCAGGTAAGAAGGAACATTCCAGAACTGCAGAGGTCTG

GGACCTTCGGGAGGGCAACGAACCACCAGGCGCCTGCGTTTCCAAATCCAGAGACAGACC

CGCGCAACCGACCAGTGTCATCCAGTCCACCGGCCTTGCAGTCAGCACCAACCCGCTGGC

TCTGATTGGCCAATCGCAGCATGACTCCTCCCCTCAGCGCCTGAGCTCCTCCTCTCCCCG

CCCCATCGCCTCTCATCAGCCACTCCCACTATCTCTTTGTTCCTCCCCTAAACCACTCTC

GGTCCCTTCTCCTCCCAAACCGCTCCCTCTGTCGTCCTCTCCCAAACCACCCTCATTAAG

CCCCTCCCCCAGGGCATGGGGCTCCGCCCACAAGTCTCAGAACTCGCTGAGCAGGAGGAA

GCATTTGGAGAGCTCGCTGTCCCACATAGCCGACTACAGACTGAAACAGTCTTTACTTGC

GCACGACCAGGAGTTCCCACTCCAGCTCAAGAAGCAGCAGGACCTTTACAAGACCTCGAA

GAATTCCGGGGTCCGCTCCTCCTCCTCCTCTTCCTCCACCTCCGCTTCTTCCATCCTCCC

TTCCAAATCCACGGCAGGTCGTACCAAGGCCCCGGGTGCTCAGGCCGTGACCGGGTCTCC

AAGCCTCATGCTCACTCAGACGCTCCTGGGGTTGGGCCACACCAACGGCGTCATCCAGAA

CTCCACTCAGGACACCCCGCTCGCTCTTACCACCAAACCTCGTGCCGACCTGCCCGTCAA

CCTCAGCACCGGCGGCAGGAAGGACACATCCTTGCACACCTCTTCACTTGCCCCGCTACC

CGCTCTGGTACCTGCATCCGTCTTGCCAGCACGACCCCGTGCCTCCCGCAAAAACAAGAC

CCCTCGAGCCCTGGAGGTTCCTAAAGATGTTTCCCAAAAGCACTTGGTGAAGTCTCTCGT

GGATCTGTTCCACCATGCAGCGGGAGAGCAGGAGACCCCTAGCAAGAAGGACTCTGATGA

GTCGGGCGAGGATGACGAGGATGACGAAGATGACGATGTGGATGACGACGAGGATGACGA

AGATGAGGATGACAGTCTCTCAGAGTCAGACAGCAACTCGGACAGCGAGCTGAATGGCTC

AACACGGAGGAACATGGACACCACTGAGACGGAGACCGACGGCGAAAGGACACCGCTAAA

GCTCAGCAAAAACCTGCCGCTGCTCGCCTCTGCCTCGGCCAGTTACAGCCTTGCGGACTG

CTCGCCGCTCAACCTTCAAGTCATTAAGCCCTCCGGCATGGCCACGCCCACCATCGTGAG

CGGCTCCGGTGCCTTGACCTACCACAGCTCGCCTTCCTCCTCGTATTCCGTCGGCACGCC

TCCAGGATCTGGAAAGAGGAAGCGTGTGATGAACGAGGAAGATCTAAAGACTCCTTTAGA

AATTGGTTGGCGCAGAGAGACCCGGATCAAATCAGCCGGAGGTCGTCTCCAAGGAGACGT

AGCGTATTACGCGCCCTGTGGCAAGCGCCTGCGCCAGTACCCCGACGTTGTGAAGGGTCT

GCAGTGGTGCCCGCTAAGCGAGGACGAGGTTATACCGCGTATCAGAGCCATGGAGGGCCG

GAGGGGTCGTCCGCCAAACTCCGAACGCCAGCACGGCGCCGGTAACGGCGAGGGCTCCGG

GGCCCGCCGCAGGAAGGGCCGTCCGCCCAACGTGGGCCACACCGAGTTCCCCAGCCCCTC

GGAGGCCAAACTACTCCGCAAACTGGAAGCTCAGGAAATCGCCCGTCAGGCTGCTCAGAT

GAAGCTGATGAGAAAGCTGGAGAAACAGGCGCTCGCTAGAGCAGCTAAAGAAGCTCGCAA

ACAGCAAGCCATCATGGCTGCGGAAGAGAGGAGAAAACAGAAGGAGCAAATCAAGATCCT

CAAACAGCAGGCGAAGCGGAAAAAGCGTGAGGAGGCCGCCAACGCCAAAATCATGGAGGC

AGAAAAGCGTCTAAAGGAGAGAGAGATGAGACGGCAGCAGGCTGTTATACTAAAGCACCA

GGAGAGGGAGAGGAGGAGGCAGCATGTCATGCTAATGAAAGCTGTAGAAGCTCGCAAAAA

AGCTGAGGAGCGCGAGCGCCTTAAGCAGGAGAAGCGTGACGAGAAGCGTCTTAACAAAGA

GCGCAAGCTGGAGTTGCGGCGCCTCGAGCTGGAGATGCTGCGGGAGATGAAAAAACCCAA

CGAGGACATGTGCTTAACTGATCACAAGGCCCTGCCGGAGTTCTCCCGGATGGCGGGGTT

GGTGCTCCCGGGGCAGGTTTTCTCTGACTGTCTGATGGTGGTTCAGTTCCTACGCAGTTT

TGGGAAGGTTTTGGGGATGGAGCAGAGCGAGGTTCCTACACTGGGTGTGCTGCAGGAGGG

GCTCCTCAACCTGGGCAACAGCATGGGGCAGGTGCAGGACCTGCTGGTGCGTCTGCTGTC

CTCGGCCGTCTGCGACCCGGGGCTGCCTCCAGGGCACAGGGCCAAGTCCATTCTGGGTGA

TCACCTGACCAACGTGGGTCTAAACCGCGACAACGTTTCAGAGGTTCTGCAGCAGTACAT

GGAGTCTCACTGTGCTCAGACCGAGCTGGCCGACGTGGCCCTCAGTCTGAAGACCAAGGC

GTTCCAGGCACACACTCCGGCGCAGAAAGCATCGATTCTCGCCTTCCTGGTGAACGAGCT

GGCCTGCAGCAGGAGTGTGGTCAGTGAGATCGATAAAAGTCTGGACCAGATGAATGTACT

GCGGAAAGACGAGTGTATCGTGGAGGGAAAGCTGAAAAAGTTGAAAAACATTCATGCTAA

ACGCACAGGGAGGCGCGAAGCCGGCGGAGGGGACGAGTCTCATGCCACGGGTACACCGAG

CGCCGGACAAAAGCGCAAGAGAAAGACTAACGACAGCGACGACGACGAGGAAGAAGACGA

CGACAGCGATGATGCCGTAGACGACGATGATGAGGATGATGAAGAAGAGGTAAAGAAATC

GCGGAGGGTGGAAACGTGTGACGAGGATGAAGGAGATCAAGCCACAAGCGTAGAAGAACT

GGAAAAACACATTGACAAAATATCAAAGCAGCAGAATCTTATCAGACGGAAGCTCTTCGA

GTCGTCTCATGCTTTACGCTCCATGTGCTACGGACAGGACCGCTACCGCCGCCGCTACTG

GGTCCTCCCACAGTGTGGAGGAGTCTTCATCGAAGGGGTGGAGAGCGGAGAGGGTCCCGA

GGAGCTCGAGAAAGAACAAGAGCGTCTACGCAATTTCAAGCCGGTGAGGATCAAGGAGGA

GCCACAGGAAGAGGGGGATGAGGAGGAGGAGGAGGAGGAGGCGCAGGTGGAGGATGCACA

GCAGCAGCAGCCGGAGGGAAAGACGCAGCCTCAGGTTAAGCAGGAGGAGGAGCAGCGTGC

TGAACAGGAAGTGAAAAAGGAAGACGAGAAGCCGTGCTCGCCGCCGAACAAGGACACGCC

GCTATCAGACTCCGCCTCCTGTCCTCACAAGGAGCCCTCAAAGCCACTCGGTGACCTCGC

GACACTTTGCTGTACTCCTGACGTCAGCATGCCGAGTGTAACCACGGTGACCGCATCAGC

ATCGTCTCCGCCTACCTCCAAGCCCCCTGTACTGTGCAGCGCTGCTACAGACTCTGTAGC

TCTGGTGGCTCAGTTTGGTGCTCCTCCCCCACAATTCGGGATTCCTCTGTCCCTGCAGCA

TCATCAGCACCTCCTGGCTAACGATCAACTCCTGCGTGTTTTAACCGAGCGCAGCGGACA

TTGGTTTAGCCTCCTGCCTCGTTCTCCGTGCGACGAGTCTTCTCTCATCCAACCCACCAC

GTCCAACGTCCAGCCCAGGAGCCCTCCGGCAAGGTCCTGCTCGCCACACCAGCATCTCCA

ACCTCCGTCTGCCTCCAGCAGCCCCAGTAACGCTGCTCACGACGGGCAGGGCAACCCCAC

CGTCTCCCCTCTGCAGGTAAAACCTGGTGGTGCTCTGCTGTCTTTGCCCATGTGCGCATG

GTCAGGTGGCATGATAAGCCCTAACCTACCTATTTGCAGCAGCCCTATGCCCATTTGCCC

ACTCGCCGAGGGCAACGCCAGCCCCCTGATGGCACCCAGTGTCTCCACCAGCAAAAGCGG

CTCTCCTCCGCCCCCTGGTGACAAACCATGTGCACTGTCACCTGCTATAGATCTGCCAAG

AAACCATGACCAGCCGCAGCCACAACCCATTCCAGAAGACATGTTGCTGGGCTGGTGGAA

GATGATAGACATGGAGGAACTGCAGGTTCTAATGAAGACGCTCCACAGCAGAGGCATCAG

GGAGCGAGCGCTGCACAAACAGCTCCAAAAATCCATCGAGCTCGTCTCACACACCTTCAA

CAAGAACAAGGAGGTAGCTGTGATGGAGGTGTCGGAGCTGGACGAAGGCCAGGTTTCTGT

GGAGACGCTACAGGAGTGGTGCGTGGAGGAGCAGGCGATGGAGACGGACATCGCTTTACT

ACAGCAGGTGGAGGAGCTCGAGCGCAGGGTCACTTCAGCCAGCCTGCAGGTCAAGGGCTG

GATGCACCCGGAGCCCCAGTCTGAGAGGGAGGACCTGCTGTATTACGAGCACAAGCCCCT

TCCCAAAGTCTGCCCAGGGGCGGAGTTACATGAGGAGCGGGGTTCGGAGAAGGGTCTGAG

ACGGCAACCCAGCAATCCGCTGGACATCGCCGTGATGCGCCTGGCCGAGCTGGAGCGCCA

CATCGAGAGAAGGTACCTGCGGAGCCCCTTAGGGACAACCATCCAGATCAGGCTGGATAA

TGTGGGGACGGTCACTGTGCCCGCCCCCGCGCCATCCACTAGTGCTGGAGGGGAAGGAGG

TGAGGAGGAGATCGCTCCGGGAATGAAGGTGTGGAGAAAGGCTCTGAGTGAGGTGAGGAA

CTCGTCACAGCTGGCCATGTGTCTGCAACAGCTGCAGAAGTCCATCGCCTGGGAGAGATC

CATCATGAAAGTGTACTGCCAGATCTGCCGTAAAGGGGATAATGAGGACTTACTGCTGCT

GTGTGATGGCTGTGATAAAGGCTGTCACACCTACTGCCACAAGCCCAAGATCAACGCCAT

CCCAGAGGGAGACTGGTACTGCCCTGCCTGCATCTCCAAGGCGAGCAGCCAGTCGCCCAA

GAATAAGAAGCCTCAGAGTCGCGTGCAGTCGGGCGGAGGGGGCAAGAAATCGGCAGAGAC

GACCAAGAAGAGCAAAAAACAGCAGGAGGCGTGCGAGGAAGACGAAGCGGTGGGAGGCAG

CAGCACCAGCAACAGCAGTCCAAAGAAAACAGCCACCGCTTCCAGCCAGGCCAAAAAGAG

CTCACCCGTTCCTTCAGCCGCCAAACCCGAGAGCCCGGCCTGCGTAAAGCGAGCCAAGAC

AGCCAGGGACAACAACCGCGACCTGGGACTCTGCAGGATTCTTTTGGCTGAACTGGAGCG

TCATCAAGACGCCTGGCCTTTCCTCACACCCGTCAACCTAAAATCAGTCCCTGGATACCG

GAAAGTCATTAAAAAGCCCATGGACTTCTCCACCATCCGCGAGAAACTCGTCAGCAGCCA

GTACCAGAACCTCGAGACGTTCATCATCGACGTCAACCTGGTGTTCGACAACTGCGAGAA

ATTTAACGAGGACAACTCGGACATCGGCCGCGCGGGACACAACATGAGGAAGTTCTTTGA

GAAGAGGTGGACCGAGCTCCTGAAGCAGACTAACTGATTCGCTCAGAGCCGCCATGTGAC

ATGCCACTTTTACCAAATCCCTCATAACAGACCTCGGACCAGAACTGGCCAGAAATTTCC

TCAAAGCAAAGCGCCGATCTCAAACCTGAACCTGTCTCACACACACACACACACA

>Unigene000084

CACACACACACACACACACTGTCTCTCTCCCAGACAACAGCACCGCTTTACTCTCTGGGT

CTGACATCTGCTTACTGACGCGTCGCTTTTTTATAATTCTGGAAGAAAGACAGATATAAA

GACTGCTTGGAGCTGCAGTTTAACAGTTTAAAGACAAAAGCCGGGACATAAAGTTATGGA

GTCTGAAGAGAGACTGTCGTCTTCCAGCCACTCGCAGGCTTCAATCAGCTCTTCTGCCAC

ACCTCCAGCTTCATCTGCAAACATAACGAGTGAAAGTGGACATCTGTTTCAGCTGGTTAG

TGATCCAAGTTTTAGAGCCTCTTTTCCCCTGTTGAGCCATCCAGCATTTAGGTTGTACGC

TCCTGTATCTGGATGCTCAGATTTCAGAGGCCTGGGGATTTTGGGATTGCAGGGAGGCTT

AACAGCCCACCCACATCTTGGAGCATTTCCAGACTGGTGGCGGGTATACGAGGCACAACT

TCACGGAGCTGCTGCGCTTTTTCCTCCTTTCCGGGGCCTGCCTCCTCTATTCACACCTCC

ATCACTGAGCCAGTCACACACTCCCAAGAAGAGCATACGTGGCTCTGCCAAAGGGTTGAA

TGGTGCAGTCAAAGCAAAATCGGCTTCCTCTGGAGCAAGCTCTACCTCCTCCTCACCCTC

ACCAGCCTATACAACCTCTGAACAGACCAGCACACTTAAATCAGGTCTAAACCCTCAAAA

CAAAACCACCAAATCTAACCTCAACTACTGTAAAGAGAAACAATCAGCCACCAATCCCTC

AAACTGGAACAGGGTGCAAAAGAGCAAGATGGGTCAAGTCCAGGAGAGGACAGGCAACAA

AGCAAAAGAGAATCCCTGTATGACAGTGGCAGAGATTTCCAGCACCAGTGATAGCCAATC

AAGATCCAGTTCTGACAGCTCCAGTGACACACTCAGCAGTTTGGGCTCGGTCGACCTGGA

GAATGAAGATGATGATGAGGATCTGAGTGTATGCAGCATGGACTCGGACACACAGAGTGA

CTCCTCACACATAGACCAAGCTGCAGGCTGCTTTTCCAGTGGACCAAATATGTGCTCTAT

GGACTCTTTAAAACATTCCCAAGCAGCTCCGTTACTTTTCGGCAGTCCATCAATCAGAGA

AGAACACCGCAAACACACAAGTGTCATCCAGACTACAGGAGCAGCAGGCATTGCCAAGAC

TTTCTCCCTCCCTCTACAACCCAATCAAGATGCCTTGTCGAAACCTTTGTGTCTCACCAT

GTCTCCCAAACCTTCGTCAGTATCATCGTCTCCCAAGCCTCTTTCTGTGTCCTCATCTCC

AAAGCAACACTCCGTCTCTGCCTCTCCAAAACCTCTTTTGTATACCCGCTCTCCAAATCC

AGCATCACTTTCCTTCTCTCCTAACATCACCTCCTCTCCCAAACCCACCTCCATAACAAA

TGCCAGGAATTCGCCTGACGGGTCCCGTCTACACGGCAATGAAAGTAATCAGGACAAGCA

GGCAAAGGAACACCAAGAATCAACTAAATCCCTGAAGAAAAATGTGTCATCTTCCTCACC

ATCGCCTCAGATTAGCACTAGCCTCAATCCCCAGCACTGCAACCTGTTTCTCACCTCTAA

CATCACGAAGAACTCTTCCCATCTTAATGGAGTTGTTCAGGGTGCTGTGCAGGATGCTCC

CTTGGCACTTATTACCAAACCTCGTCTAGACAGCTCTAATACCCCAGATAAGACTTTACT

TGCAACCACCAGTCCCTGCTTTAACACCCCTATCAACTTGAGCACTGGAACCCGACATTC

TTACCCCGGACCTCCCACATCAGGCAAAGCCTTCACACACCAAGAGTCCCACAGGGCAAG

AGAAGGATCACAGCCCCAAAGAAAAACATTTAGGGAAATAGAGTCTGACATTCCCAGCAG

CAGAGACTCTGATGATTCTGGGGAATATGAGGATGAGGAGGATTTAAGTGACAGTCTGTC

AGACTCCGGTAGTAACCTTGATACCGACTCTGATAACGATGAAGAGGACAATAAAGATGA

GGAAATGAATACTGATGCAGAGAGCCACACAGACAGCATGCCACTTAAACTTACTAAAAG

CTCAACCTCTTTGGCAGACCCCTCTACTGGCAGGAGCAACAGCTCTAGTTCACTTAACCT

GCAAATTTATGAGTCACCTAACATTTTACAGACTCTGAATGGATCCTGGGCTCTGACTTA

TCAAAGCCCCCCAAGCTCATCATGTGTGGTTCCGCCCCCAGCAAAGAGACGAAGAGTGAC

TGATGAGCAGGCACTGCGCCGGCCTCTTGAACATGGGTGGAAAAGAGAGACCCGCATCAG

CAATATCGGTGGCCGTGTGCAGGGTGAGGTGGCCTACTATGCTCCCTGCGGAAAAAAACT

CAAACAGTACCCAGACATTATGAAATACCTAGCAAGAAATGGAATAAATGAGATTACACG

TGACCATTTTAGCTTCAGTGCTAAAATAAGAGTTGGCAACTTCTATGAAGCTCAAGAAGG

ACCTGAGGGATTGCAGTGGTGCTTGTTGAAAGAGGATGAGGTTGTCTCCTGCATCTCAGC

AATGGATTCACATAGAGGTCGTCCTAAAAGTCTGGAACTTCAGCCTACTGATGATGCCTT

CCATTCTCGCAAAAGGAAAAGACCACCTAGTGTGAGCGAAAGTGAGATGGCTAATGCTAC

TCTGATTAAACTACAACAGAAACTGGTGGCGCAGGAAATTGCACGGCAGGCTGCTCAGAT

AAAGATGATGCGAAAGCTGGAAAAAAGAGCTCTTGCACAAGCTGCTAAAGATGCCAAACG

ACAAAAAGCACTGCTGGAAGCAGAGGAGAAACGCAAGCAGAAAGAGCAGCTGAAGATTCT

GAAGCAGCAGGAGAAGGTCAGAAGACTCGAACAAATTCGTGCCGAGAAGGAGCTTCGAGC

TCAGCAGATACTTGAGGCAAAAAGGAAAAGAAAAGAAGAGTCCATTAATGCAAAAACTCT

AGAAGCTGAAAAACGATTGAAGGAGAAGGAACTACGCAGGCAACAGGCATTAATTCTGAA

GCAGCAGGAATTGGAGAGGCATAGACTAGAAATGGTATGGGAGCGGGAAAGGCGAAAGCA

GCACATGATTCTGGTCAAAGTCACGGAGGCTCGGAAGAAAGCTGAGGAGCGAGAACGTCT

GAAGCAGGAGAAGAGAGATGAGAAACGGCTAAACAAAGAGCGCAAACTGGAGCTCAGACG

GTTGGAGCTAGCGATGGTTAAAGAGCTGAATAAACCCAATGAAGACCTGTGTCTGCCAGA

TCAAAAAGCTCTGCCTGATCTGTCACGGATACCAGGTCTTCTATTACCAGACAGCTGTTT

TGGGGACTGCCTGATGGTGATGCAGTTCCTGCGCTGCTTTGGAAAGGTGCTAGGTCTGGA

CCGCAATATCAAGCTGCCCACCCTTCACATGCTGCAGGCAGGACTCCTCAACCTAAGCCC

AAGTGCAGCTCAGCTGCAGAATCTGGTCATTGGACTGCTCTCTGCAGCAGTGCGTGATCC

AGGCTTTCCACCAGGATCCATGGCTCAAACAACTCTCGGTCAGCATGTTTCCAGTGTGGA

GATAAACCAGGAGAACATGTCCGAGATTTTGCAGATCTACATGGCCGCTCATGGCAGTCA

GACTGATCTCTCGCCACTGGTAGAAAGTTTGAAGACCAAAGGCTTTATGGCCCACACCCC

CACACAGAAAGCCTCAATCATGGGTTTTTTGGTCAATGAGCTGGCCAGCAGCAGAAGTGT

GATTATGGAAATTGATAAAAGTATAGAACATATGACAAATCTACGAAGAGAAAAATGTGT

CATTGAAAGTAGCCTTCGCAAATTGAGAAGCAGCTACGTTAAGCGGACTGGGAAGCGTGA

TTTTAGTTTAGGAGGTGAAGAGAGCCAGGCTTTGGAAACACCTACCACAGGACACAAACG

TAGAAGAAAAGTCGGAGGCATTGAGGAGGAAGATGATGAGGATGATGACAGTAATGATCA

TGGAGAGGAGGATGAGGATGAAGAGGAAGACGGGGGAAAGAAACAACGGAAAACTGAAAC

ATGTGAGGAAGAGGATGATGAGGATCAATCAGCCAGTGTGGAAGAGCTTGTGAAAAATAT

AGAGAAATTGACCAAACACCAGCTTCTAATCAGGCATAAACTATTCGAGTCCTCATACTC

CCTGCAATCGATGATGCTCGGGCAGGATCGCTATAAGAGGCGCTACTGGGCTCTTCCTCA

GTGTGGGGGTGTGTTTGTTGAGAGTACAGTGAGTAAAGAAGGACCACAAGGGGTTAATCA

AGAGTCTGGGAGGATGCACAGTGCTCAGTTGACCTCGGTTAAAAAGGAACCTGCAGAGGT

ACCAGATGGGGAACCGTATGACGGCCCGCAAACAAAGCTTGAAAATTCCTCTCTGGATCT

ACAGCAGGAGAACGGTTCATTCAATTTGTTTTTGCAGAAACCGAGTTCTTTTTCCAAACT

TAGTAACCTCTTAGAGGTGCCAAAGACGTCCAGCAGTGATTCTCAGAGCAAAAATTCCAC

TGCTGCCCCCTTTTCTACAAATGCTTTAACAGTGTTACCCAACAGCCTTTCTACTAACCT

AAACCAGAACATAAAATATGAGGTCAGCTCTCAATTACTAAACCCCACTCACACAGGAAA

CCAGCAACATCCTCTACATAATGACCAACTCTATACGACTCAGGCAGAGAAACATTCTGC

CTGGTTCAGCCTGCTGCCACGATCGCCATGTGACGAGTTTTCTTTGACAACCAGCTGTAC

AAATCCATCATCTTGCCGTGCCAAAGCATCATCACCTGCACCTGCCACCAGCAACAGCAT

GACGCAATCTACATCCACCTTCTCCAGCAATAGTCAGAACATCACCTCTCCTGTCAGTCC

CTCTGCCACTGCACCTGTCAGCAACTTTTTGGGAAAGGAACTTCAGATGAAACACACGAT

GAATCCGAACATGTCCTTCAAGGACATGTTTACCAAAGCAGCTGACCCGCTCCCATCCAC

CAGTGTGTCCACCAGTAAAATCGGGTCCCCAGTCTTGTTGAGTGACAAATCACCTGTTCT

TGAAATGGCCAAAAGAAAGGACCTGCCCTTCCCACAGCCTGTTCCACAAGAGATGCTTAC

TGGATGGTGGAAGGTGACCAGTTCTGAAGAGTTAAGCCGTATAGTGAGTGCTTGTCACCC

TCGAGGGATCAGAGAGAGAGTCTTACAGAAACAAATCCAGAAACACATGGACTACCTCAC

TCAAGTCTGTGCCAAAAACAAAAATGCTGCTGTGATCGATGTATGTGAGCTGAAGCAGAG

TCAGGTGTGTGAGGAGACAGTGCAGAGCTGGTGTGTTGAGGAGCATGCCATGGATAGGGA

CATTGCTGTGCTGCAACAGGTGGAAGAGCTGGAGCGTAAAGTTATCTCTGCCAGCCTTCA

TGTCAAAAGTTGGATGCCCACAAAGCCACAGTCGGAGCGTGAAGACTTGCTATATTATGA

GCACAAGGTTCTAAGCCATAGTGAAAGCACAGACATTGTACGACAGGCTGATAATCCACT

GGACATTGCAGTGGCACGTCTGTACGAGCTGGAGAGGAACATAGAGAGAAGATACCTAAA

GAGTCCATTAAGCACTACTATCCAGGTGCCTCTGGATAACCGGGGTACGGTTAGCATACT

GGCTCCAGCAACACCAGCTAATACTAAGTGTGATAGTGATGTGGAAGACCTGGTCCCGGG

GTTAAAGCTGTGGAGAAAAGCCCTTAGCAAAGTGCGTAGTGGTGCCCAGCTTTCAGTCTG

CCTGCAGCATCTACACAATTCTATCGCATGGGAGAAATCCATCCTGAAAGTGGTGAGGAA

GATACATATACACTGAAACACTCCACAGGAAGTCGGAGTAAACAGTAATAATCATTTATA

TAAATTGGCAATTTAAAGAGCAATGATAGTCGTAATAAGGGTTTATGAAGGGCAGGAGAA

GCCAAGTTTGGTGCTTAGTGTCAGTACTGTGTTGTGTCACTATCAGTTCTGCCAGTTATG

CCAGACGGGAGACAATGAAGAGCTGCTGCTGTTGTGTGATGGGTGTGATAAAGGATGCCA

CACATACTGCCATAATCCCAAAATTACTGCTATACCTGAGGGAGACTGGTTCTGCCCTGC

CTGCATAGCCAAGACAAGCGAACCGCCTCAGAGGAGTAGGAAACAGGTCAGTCGGAACAG

TGGAGGGGGTGAAAAATTGTCCGAAACCAAACGCAGCAGGAAAGCCTGTGTGTCAGGAGA

GGGTTCGGAAAACAGCACAGTCAGCACCAGCGTCAGCAGCACAGCCAAAAAAGGAGGAAA

GGAAAGCAGAAAGAGAAAACCGGAGGACAACCTTTACAACACCACCACCACTAAGCAGGA

AAGAGACAGATCCAAAACCACTACAGACAACAGTAAAGACCTGGAGCTCTGCAGATTGCT

TTTGGCAGAGCTGCAAACGCATCAGGACGCCTGGCCGTTTATGACTCCTGTCAATCCCAA

GTCAGTCCCTGGATACCGCAAAGTCATTAAGAAACCCATGGACTTTTCCACCATACAGGA

CAAACTTTCCAACAGCCAATACCTGAATCTGGAGACATTTATCATTGATGTTAACTTGGT

TTTTGAGAACTGTGAAAAGTTTAATAAGGATGACTCTGACATTGGACGTGCAGGACACAG

TATGAGGAGATTTTTCCAGCGAAGATGGACTGAGCTCTTAAAACAGGTCAACTGAACTGT

CTGAAAAACCTCCTGCATATAGACTACTGTCGCTTTATAACTGTCCTCAAGCAAGCCCTC

TTCTCCAAATGTTCTTTACCCACTCGCATCACTGGTTTGCATTATGAGCTCTACAGGCAG

TCCGTCCAGCGTGTGGTAGGATGACCGCAGTGTTTCGGATAACATAAGGTGTCTCTTCAA

GTGCAATAAAAAATTTCTTTACTTTACAGAGCTTTCATCATTTTAAAACTGGGAC

>Unigene000085

AAATAAAGGGCGGAGGGAGAAAAACTTTACGACGGGTTTTTTTTTTTTAACTGTTAAACA

GGAAGGAAACTGTCGAAATGCAAACCTGCAATCTCTAAAATAGGCCTCATTTAGGCTGGG

AAATTAACAAGCGTTATTAATATAAGTGGAGGTAATCGCTCGATCTGTACAGTGCGTTTC

ATTTTTAACTTATTATTCTTTGAAGCTGGTTTGAACAAACACAGCTGAAAAGGAAACGCG

CGTAAGCCGTTTACACGCGCGTCTCTTCCGCACCGGAAACTTCGACATTTTTCTTGCACA

CCACCAACTGAATTTGGAAATACTAAAGGACGACCTCTTATCGCTTTAAACTAACTATAC

AGTAAAAAAGGACCGATGTCTTATGGCTTCACACGGGCAAGGACGCAAAAGAACTCCGAA

TAGAAACGGAATAACTCCAGAAGAAGATGCGTTAAATGTAATCGCACGAGAGGCTGAGGC

CCAACTAACAGCTAAGAGGGTTGCCAGGGCAGAGGCTCGTGAGATACGCATGAAGGAGCT

GGAAAGACAACAAAAAGAGAAATACAATGGACTGGAGAACCTGGACAACAAATGCTGGGA

TATTGAGCAGTGGATGGAGGACAGTGAGCAGTATACACACCACCCCCAGCAACATGTTTT

GGTGTTGGGAAATGAAGAAGGTACATCAGCAGGGAGCAAGAGAAGTGTGCAGGGCTCCTC

TCGAGGACCGACTCTATCCACTGAGACTTCCCTGAGTGGGGCGTCCTCAAGTAAAGGAAG

TGGTGGTTCATCTGTCACTGCTGATACAGAAACATCCTTAAGGGATATTAAAGAGATTCA

TGAACTAAAAGACCAGATCCGTGATGTGGAGATCAATTACATGCAGATCCTCAAAGTAAC

AAAGGATTCTTTGTCAGAGATGGAAATGAAATATCGCAAGGCTATGGTGTCCAATGCTCA

GTTGGATAATGAAAAATCTAATCTGGTGTATGAGATGGACAGTTTAAAAGACTCACTAAT

GGAGTTGGAAGAGCTTCTGTCTGAAACTCGTCGCGAGTGTGAGAAAAAGAATAATGATCT

GGAACGAGAGAAGCATGCTCACAGTATACTGCATTTTCAGTTCAGTAATCGAAAAGAAAC

ACTAAAGCAAAGTGAAGAGCTGCTGAGTGAGATCCGACAATTACATCTCAAACAAGAAGG

GTTTACTAGAGAGATTTCTGACCTGCAGGAAACTGTTAAATGGAAGGATAAAAAAATTGG

GGCTTTAGAAAAACAAAAGGAGTTTTCTGATGCCATTCGAAATGAACGGGATGAACTTAG

GGATGAGGTGGTTCAACTCAAAGACACATTAAAGAAACATGGCATTGCTCTCGGATCAGA

ATTACGCACCAATGGGGAAGTGGGAGTAAATGTAAGTGGTGGATCAGAATTATGCACCAA

TAGGGAAGTGGGAGTAAATGTAAGTGGTGGATCAGAATTATGCACCAATAGGGAAGTGGG

AGTAAATGTAAGTGGTGGATCTGTCTGCACTGAATTGAAACATGGAAGCAGTGTTTTAGG

TAAGGTTTTTTTTTCCAATACAAGTAGATTTGCTCTTGCATTGTGCTTGCTTAAGCATCT

TGACTGGTATTTCTAGCCAGCTTGACTGTTATTAACCAGCAGCTTTATTTCTCTTTCACT

ATTTCTCTTTTCACAGAATTCATACTTTAATTTGAAACATTCAAATTATCTCAAAATGTA

GGACTAGGTACTTGAAATACCTGTTCGTCTGATTTATTTTCTACCCCTCTAATATCTTTA

CTGACAAATATTTAAACTTCTATTTTATTATATCTACAGCTGCATATCAGCAAAGATTTT

TTTTCTACACATGATGATAGATCATTGCTCAATAACTTTCATTTTGATCTTCACTGAAAC

AAATGTCGACCTAATTTAGTCTATCCATGTTTTAATTGATTAACTGCGGTGACCCGGAGG

GGACATGTTCGGTTCGCTTGGTTTTGTCGTGGCCACAGCATGTTGGCTTGTGGGAGGTTG

ATGTTGTGACGTGGCCACGGGATATTAATTCAAGGGAATGTCATATTAACTCATGGGAAC

GAGATATTAACTCGTGGGAACATGATATCTGATTGTTTATTTCTTATGTTTATCACAATG

TGAAAGATTATGAAAGTTTCTGACTTTTATATTTATGTAAAAACGTTTACATTATCAGAC

CTGTAAGAACGCTCTTTTTTTTCCTATTCAGTGCCTTTAACAATATGTATGTAAAAGCTG

TGCCACCAGGTTAATAGTTCGGCTACCAAGTTGCTCAAAGCGCAGCTGCTTTAATCGCAG

TTGGACCGGTTAACTGGTTAATTATGAATTAGAAGACCTCATTAAAGCTATAAAAAACTG

TCGATAGTACATCCCGTTAAACTTAACACTGCGCTTTAGGAGCCTTATAAGCCTCACTTA

TAACCTACCTGTGATTTAAATATAACAGACCACAGCATGTCCTTATGATATTACGAGTAA

AGGAAAATGTTATTCAATCAATTATGTCCATATTTACCTCTTATTCCGACTGCCGGTGTC

GCCATATGTGCGGTTACGCTCAGTGTGTTCATTACATTAAAAAGTCATTCTCTCAACATA

TGAAGTCGTGGCCACGAGAAACAGATGTCATTCCCTCAACATAGCATCTCGAGGCCCCGA

CTTAACGTCGACATATCGTGCTTCCACGAGTTGGTGTCTCGTTCCCACCAATTGATGTCT

CGTGGCCACGTCGTGGTGTCGCGTTCCCACGAGTTGGTGTGTTGTGGCCGCGACGGGGCT

GGGTGGGCCGGGCATGTCCCCTCCCGGTCACCGTAGTTAACACCAGCTTAGATGTCTATC

TCTTCAAAATATTCACACCTGTGCTGTGTTGTAGCTATGCTTTGGAGCCTAAACTTTCTC

ATTCTTTCAGCACATATTTATTTTACCCATTACATTGTATATTTTAATGGAATATTTTTT

TCTATAACACTAATTCCTTAATCAACCTCCCAGGCACCCAGAAGTTGCAGCTGGTTAGAG

ATGAAAATTTAGGGGGCTTCAGATGTGACCAATTACAATTCCCTCAGCAATTTGACCATG

GAATTGAAGAAAATCATCTGCCTTCACCTGCATCTGGCAGTTCTTTTAACTCTCGTATAA

AAACACAATTTAAAGATGTGGATCTTGGGAATTATTTAATTAACAGCATAGAGAAAATTC

AAAACAATGTTCAAAAACCTCAGGCTGTATTTGAACCTAATGCAAAGAAACAAATTGTAT

CAGACATTTTGAAAGGTAATGATGTTCCAGAAACTGATGCAAATATTATGAAAGGTATAG

GAAAAGATCAGCAGGAAAGTTATGTTAAAAGAAGTAAGACTGGAAAAGAAGGAAAGCATG

ATTTCACACACATGTTCACCTGTGAGATATCAGAAAAAGATATCAATCCACATTTTGATG

AAACTGTGTTTTGCAACTCTCAAATTAAAATGGCTACTGACAGTTCCCCATTAGCGAAAA

CAGATACAACATTTAATTCCCAAAGTTCCAATGTTTCAGTAAAAAAGAAGAAGAAAAAGA

AACAGAAACAAAAGTAGCGCTGTGCAAAAAATGTTCATTTAAAAATAGGGAGAAAAAACA

GCTCAGAGAAAGCAGTTCAGTTCAGACAGTATTGTCAGAGTGCAAGTTTGTACAACAATA

AATAAGAAAGCAAAAGATAATGTGAATTTCGATGAAAAGATAGACCTAATGCACAAAGGA

ACAGAATCATTGGAACTACAAATTCCCAATACTGGCCCTGAAAATATTGTATTACCAATC

ATTAATCAAGACGATTGCATGAGCAAGATTGCATGGGTGGACAGAAAATTGGCATTTCTA

CTCATGACCATCCTAGAGATTTTCTTCTTTTGGCAAATGCATCATACAAATTAACAATGA

TGATGCTGAGTTAAAAGCTATTGCTGAATTATGTGATGATTTTTAATTATCTGAAAACTC

TTCTGAGGTTGAACCTATGAAAGATAAAACTGGAACTACCAGAGATGTAGTAAAAATTGA

AGCATTTATTCATTACTGTGAAACATACACCCTTGAAAAAAAATCCACAAACACAAAGCT

GGGTTACACTGTAGTTAAATCTGAAGCCCCTGAGGAGAAGCAAGAAACCTTTACTGGCAT

ACAGCTTGATGATACTGCATTTAAAGCTAAAATACATAGAGAAAAGAAAGAAATGTCTAC

TGGCACACCATTGGGTGACACTGTAGTTACAGCTAAAGTCCAACAAGAGAAGCAGGGAAC

CCTTATTAGCACACAGCTTAATGACAATGTAGTTACAGCTAAATCCTTAAGAAGCAAGAC

ACATTCATAAACATGTAGCTGGGTGACACTGCAGATACAGCTGAAAAAACCCAAAAAGCA

GCAAGAAACAATCACTGGCATACAGCTTTATGAAACTGTAGTTACAGCTAAGGTCCTTGA

AGAGAAACAAGAAACATTCATAGACACACAGCTGGATGACACTGTAGTTAAAGCTGAAGT

CCCTAAAAAGAAGAAAAAATCAGAGCCACCAATGCATATGGGTCAGAAATTTAATATAGA

AGAGGATGACAGTGAGGAGGAGTTTAAATCATTTTTTTTTATTAAAATAAAGAGATTTTT

TGGAGGTAATGACTGACAAAAGGAATACACATTACTTGTTGTGCAAAGCAATCTTGTACA

ACCAGTGAAAACCCGGCGCACGCAAATACTAAAGATAAAGACCAAATACTACAGACAGAC

AGACAGAATGTTTTAATGGAGAAAGACAAGCGTGTGTCTCTAGTGCAAATAACCACACCA

GGAGGGACTCAGCAACAGTTTTATCCAGGGTATGAGGAACAAGGCTCCAAAAGCCAAGAA

GAAATAATACATTTAAAACACAGTGAAAAAGTTAAGCAGGAAATGTCCAGCAAATGTTAT

AAAGGCCCTTGATGAGCTGCTCTCTGAGACAGCAGTGTTAAACTTAGTGGCTGATGGTAC

TGTACAGCAGAAAGCATTTCAAAATATGGGAGAGCAGGAATTCAAAGAAGTCAAAGAAGT

GACACATATAAAAGAAACAAGCAGAATGCAAAGAATGGTTCTAAAAAGAAAAGCAAAAAA

AGTAAAAGGGAAGACTAAAGAGGACTGTAAAATGACCTAATACTTTACCCAAGGATGTTA

ATTATAAAGTGTCCAGAGTCTTAAAGCCTAACTAAATCTATGTTAATAAATAATTGAAAG

TTTAAAATAGTTAAAAGCTTCTAATCTTTAATCGAGATTAAAAAAGTCATTAAATTTCAT

TTCAGCATTTAAATTTTTTGTTGTGTGTGGTATGTATGCTTCTACATCTGGATTGTGTGA

GATGACATGACATGCTTAGTGAAATTCATATAGTTATTTATTTTCTCATGAATATATCTG

CAGCAGTTTTCACAATGTTTTATCTCTGACATGCAGCAGTTTGCTACTATTTCACAAATT

ATATAATAAAAGTTCTTTTGTAAGATTTCTTTCAGAAACGTTTGTTTTTGTTTTTTTACC

CAGTGACGTACCGTGGGGTTTCAGCAGGGGCGGGTGGTGCATTTGGTACCTTGGACTTGG

ACTTACCCGACTGATACACCTCTTAATACCACCACTATCACATCGCAATTATAGAAACCA

CCACTACTATACAAAAACGCAATATAACATGGTGTGAAGTTAGAGACAATGAGGCATTTC

GCGTATTTAGGGAAAATACCATGTTTATAAAAGCAAGAAACCCCCGTTAAGACAATTTCA

ATCCTCTACAACAACAACTGTACCACATACATCATCTGGGGCAGTTCAGAATCAATATTA

ATATTTTATTTATATAAAGAAACAGTGTTTAATTAATTAATACTGCTTGTTGGCCCTGAC

TGCACACCACTGGGTTTCAGTCAGGCCTTTAAGTAAGCAGTAACTTTAAAATTACTTGGA

CAATCCTACAAGCTAACACAGATATATGATAATAATGTGCATATGCACAATTGACACCAT

ATGGAACTCTGAGCCAGACAATCAAAGTACTAAAGACTAAACTGAAAAAAATAGTTTTTT

AGTAGCTGTTAGCCACTCATAGCACTTAGAGTTGAAATCTTAAAAAAACTGCTGGCCATT

GTTAGTGAAAATCAGTTTGGGTGTAGGTCTTGCTTAATTCGTTGTCTGCAAAAAGTTGCC

TTGAAAAGGCGTTTTTAATAGGTTGGTGACAGAATTTGGCCGGCAGCTGCAGCAGTCAAT

TTGTTATTGTTGACTTTCACTTTCATACCTTGGAAAATCCTGATAAAATCAATCATTTTA

TTTTTATAGGGCCAGCAGAGAAGGTAGAAACATATTCTATATTAAAATAACTGGTTCGTA

ATAAAACATCTATTATAAAAATAAAATTACAGCTGTTTTACATTCTATTTACTTTGGTCT

GTAAACAATTTCAAAAGTATGGTGGCCTTTTTGGTAATTTTATCATACCGATAATATGAT

TCCTTGGTCTTTTGTGTATTGTTAGCCTAAATAATATAACTAATTCCATAATTAACTATG

AAATGTATGTATGCTGGTTAACTCAATTTCACATAGAGTTGTCACTATTTGGGTCCCCTT

GAATTGTGATCTTTGCTATTTGCAGTGGGTTTTTGCATTTGATAGTGTTTTTGTGTATTT

GGTCGTGAAGATTTGAAGAGCGTTTCAGTATTTGTGTCTGCATTGTGAGGATATGCAGTG

TGTTTTCTTAAGTTGCAGTGCATTGGGCTTTCTCTGTTACCATACAAAAGTCCTAGGAAG

AGGGACTTTACGATAAAGACATTTTAATGGTCTACAAATTTATTCCAAAGTTGGCATGAC

TGTGTTTTTTACAGAATACTTTTACAGAAGTTACTGCATGCTATTCATTCTCATATATAT

ATATATTATATATATATATATATATATATGTGTGTGTGTGTGTG

>Unigene000086

TTTACACACACACACACACACACTGAGACTCAATTACTGCCCAATTCCCCCTTCTTCATC

ATCTCCTGCTACGCCGGCCTGTCGGAACAAAAAAAAAAAGGAAAAAGAACCCGAACCGTA

AATCTAAAAAAAAAAAAAAAAAGTATTTCCTTTGAAGCGCGAGGTGGATTTTTAAACGCG

TCGACGGACAAAAGTGTCGCAAAAAACCTGACGGTGGAAGAGCAGTGATGTCACAGAGCT

GAGGACGCTTCCACACAGTCCCCTGTTTGGGGTAACAGGAAGTGAGCAGCCCTTCAGCGT

GACCTCTGTGACCTCGGTGCCCAGTGCCTTCCCCGTGATGGCGCACCCCGCTTTTGGCCT

GCTCTCCCCCGGCACGGCTCGGCCCGAGTTCGGCGGTCTCGGAGCGCTGGGGGTCACGGC

GGCCCTCGCGGCGCATCCGCAACTAGGGGCTTTCACAGAGTGGTGGAGAGCAGCCGAAGC

TCACGGACGGAGTCCAGCCGCCTTCTTCCCTCCTTTCCTCGGCCTGCCCCCGCTGTTTGC

CCCTCCCCTTCAGAACCACGAGGCCACGCCCTACACGTCCAAAACCCTGAGCAAAAGCAG

CCAGGGGCCCAAAGGTGTTAACGGAGCTATGAACGGCAGCGTGGTCTCTCCGTCCACGAC

GAAAAGTGGCGGGTCCGTGGCATCCTCGGCCGCTCTCAACACCTCTGGCGGAAAACCTCG

CGCCCGCAAAGCCTCTCAAAACAGCAACAGTGCGGGAGAGCTGCAGGAGAAACTCTCACA

GAAACCGAAAGAGAAGAAACCGCGTAAGAAACAGACCGAAGGCTCAGGCATGAGCGACAG

TGAATCAGGATCATCTCTGGACAGCGACATTGAAGGCGTGAGCAGCAGTGACCTGGATGA

TCTCGGTGAGGAGGACGACGACGATGACGATGACGACCAGAGTGAAGAGTCCGACTCTGA

GAAAGAACCCCATAAAAAAAAGAAAGCCAAGGTCGTAGCCTCAAACCTGCGGCCAGGTAA

GAAGGAACATTCCAGAACTGCAGAGGTCTGGGACCTTCGGGAGGGCAACGAACCACCAGG

CGCCTGCGTTTCCAAATCCAGAGACAGACCCGCGCAACCGACCAGTGTCATCCAGTCCAC

CGGCCTTGCAGTCAGCACCAACCCGCTGGCTCTGATTGGCCAATCGCAGCATGACTCCTC

CCCTCAGCGCCTGAGCTCCTCCTCTCCCCGCCCCATCGCCTCTCATCAGCCACTCCCACT

ATCTCTTTGTTCCTCCCCTAAACCACTCTCGGTCCCTTCTCCTCCCAAACCGCTCCCTCT

GTCGTCCTCTCCCAAACCACCCTCATTAAGCCCCTCCCCCAGGGCATGGGGCTCCGCCCA

CAAGTCTCAGAACTCGCTGAGCAGGAGGAAGCATTTGGAGAGCTCGCTGTCCCACATAGC

CGACTACAGACTGAAACAGTCTTTACTTGCGCACGACCAGGAGTTCCCACTCCAGCTCAA

GAAGCAGCAGGACCTTTACAAGACCTCGAAGAATTCCGGGGTCCGCTCCTCCTCCTCCTC

TTCCTCCACCTCCGCTTCTTCCATCCTCCCTTCCAAATCCACGGCAGGTCGTACCAAGGC

CCCGGGTGCTCAGGCCGTGACCGGGTCTCCAAGCCTCATGCTCACTCAGACGCTCCTGGG

GTTGGGCCACACCAACGGCGTCATCCAGAACTCCACTCAGGACACCCCGCTCGCTCTTAC

CACCAAACCTCGTGCCGACCTGCCCGTCAACCTCAGCACCGGCGGCAGGAAGGACACATC

CTTGCACACCTCTTCACTTGCCCCGCTACCCGCTCTGGTACCTGCATCCGTCTTGCCAGC

ACGACCCCGTGCCTCCCGCAAAAACAAGACCCCTCGAGCCCTGGAGGTTCCTAAAGATGT

TTCCCAAAAGCACTTGGTGAAGTCTCTCGTGGATCTGTTCCACCATGCAGCGGGAGAGCA

GGAGACCCCTAGCAAGAAGGACTCTGATGAGTCGGGCGAGGATGACGAGGATGACGAAGA

TGACGATGTGGATGACGACGAGGATGACGAAGATGAGGATGACAGTCTCTCAGAGTCAGA

CAGCAACTCGGACAGCGAGCTGAATGGCTCAACACGGAGGAACATGGACACCACTGAGAC

GGAGACCGACGGCGAAAGGACACCGCTAAAGCTCAGCAAAAACCTGCCGCTGCTCGCCTC

TGCCTCGGCCAGTTACAGCCTTGCGGACTGCTCGCCGCTCAACCTTCAAGTCATTAAGCC

CTCCGGCATGGCCACGCCCACCATCGTGAGCGGCTCCGGTGCCTTGACCTACCACAGCTC

GCCTTCCTCCTCGTATTCCGTCGGCACGCCTCCAGGATCTGGAAAGAGGAAGCGTGTGAT

GAACGAGGAAGATCTAAAGACTCCTTTAGAAATTGGTTGGCGCAGAGAGACCCGGATCAA

ATCAGCCGGAGGTCGTCTCCAAGGAGACGTAGCGTATTACGCGCCCTGTGGCAAGCGCCT

GCGCCAGTACCCCGACGTTGTGAAGGGTCTGCAGTGGTGCCCGCTAAGCGAGGACGAGGT

TATACCGCGTATCAGAGCCATGGAGGGCCGGAGGGGTCGTCCGCCAAACTCCGAACGCCA

GCACGGCGCCGGTAACGGCGAGGGCTCCGGGGCCCGCCGCAGGAAGGGCCGTCCGCCCAA

CGTGGGCCACACCGAGTTCCCCAGCCCCTCGGAGGCCAAACTACTCCGCAAACTGGAAGC

TCAGGAAATCGCCCGTCAGGCTGCTCAGATGAAGCTGATGAGAAAGCTGGAGAAACAGGC

GCTCGCTAGAGCAGCTAAAGAAGCTCGCAAACAGCAAGCCATCATGGCTGCGGAAGAGAG

GAGAAAACAGAAGGAGCAAATCAAGATCCTCAAACAGCAGGAAAAGATAAAGCGCATTCA

GCAAATCCGGATGGAAAAGGAGCTCCGTGCGCAGCAGATTCTCGAGGCGAAGCGGAAAAA

GCGTGAGGAGGCCGCCAACGCCAAAATCATGGAGGCAGAAAAGCGTCTAAAGGAGAGAGA

GATGAGACGGCAGCAGGCTGTTATACTAAAGCACCAGGAGTTGGAGAGGCATAGACTAGA

TATGGTATGGGAGAGGGAGAGGAGGAGGCAGCATGTCATGCTAATGAAAGCTGTAGAAGC

TCGCAAAAAAGCTGAGGAGCGCGAGCGCCTTAAGCAGGAGAAGCGTGACGAGAAGCGTCT

TAACAAAGAGCGCAAGCTGGAGTTGCGGCGCCTCGAGCTGGAGATGCTGCGGGAGATGAA

AAAACCCAACGAGGACATGTGCTTAACTGATCACAAGGCCCTGCCGGAGTTCTCCCGGAT

GGCGGGGTTGGTGCTCCCGGGGCAGGTTTTCTCTGACTGTCTGATGGTGGTTCAGTTCCT

ACGCAGTTTTGGGAAGGTTTTGGGGATGGAGCAGAGCGAGGTTCCTACACTGGGTGTGCT

GCAGGAGGGGCTCCTCAACCTGGGCAACAGCATGGGGCAGGTGCAGGACCTGCTGGTGCG

TCTGCTGTCCTCGGCCGTCTGCGACCCGGGGCTGCCTCCAGGGCACAGGGCCAAGTCCAT

TCTGGGTGATCACCTGACCAACGTGGGTCTAAACCGCGACAACGTTTCAGAGGTTCTGCA

GCAGTACATGGAGTCTCACTGTGCTCAGACCGAGCTGGCCGACGTGGCCCTCAGTCTGAA

GACCAAGGCGTTCCAGGCACACACTCCGGCGCAGAAAGCATCGATTCTCGCCTTCCTGGT

GAACGAGCTGGCCTGCAGCAGGAGTGTGGTCAGTGAGATCGATAAAAGTCTGGACCAGAT

GAATGTACTGCGGAAAGACGAGTGTATCGTGGAGGGAAAGCTGAAAAAGTTGAAAAACAT

TCATGCTAAACGCACAGGGAGGCGCGAAGCCGGCGGAGGGGACGAGTCTCATGCCACGGG

TACACCGAGCGCCGGACAAAAGCGCAAGAGAAAGACTAACGACAGCGACGACGACGAGGA

AGAAGACGACGACAGCGATGATGCCGTAGACGACGATGATGAGGATGATGAAGAAGAGGT

AAAGAAATCGCGGAGGGTGGAAACGTGTGACGAGGATGAAGGAGATCAAGCCACAAGCGT

AGAAGAACTGGAAAAACACATTGACAAAATATCAAAGCAGCAGAATCTTATCAGACGGAA

GCTCTTCGAGTCGTCTCATGCTTTACGCTCCATGTGCTACGGACAGGACCGCTACCGCCG

CCGCTACTGGGTCCTCCCACAGTGTGGAGGAGTCTTCATCGAAGGGGTGGAGAGCGGAGA

GGGTCCCGAGGAGCTCGAGAAAGAACAAGAGCGTCTACGCAATTTCAAGCCGGTGAGGAT

CAAGGAGGAGCCACAGGAAGAGGGGGATGAGGAGGAGGAGGAGGAGGAGGCGCAGGTGGA

GGATGCACAGCAGCAGCAGCCGGAGGGAAAGACGCAGCCTCAGGTTAAGCAGGAGGAGGA

GCAGCGTGCTGAACAGGAAGTGAAAAAGGAAGACGAGAAGCCGTGCTCGCCGCCGAACAA

GGACACGCCGCTATCAGACTCCGCCTCCTGTCCTCACAAGGAGCCCTCAAAGCCACTCGG

TGACCTCGCGACACTTTGCTGTACTCCTGACGTCAGCATGCCGAGTGTAACCACGGTGAC

CGCATCAGCATCGTCTCCGCCTACCTCCAAGCCCCCTGTACTGTGCAGCGCTGCTACAGA

CTCTGTAGCTCTGGTGGCTCAGTTTGGTGCTCCTCCCCCACAATTCGGGATTCCTCTGTC

CCTGCAGCATCATCAGCACCTCCTGGCTAACGATCAACTCCTGCGTGTTTTAACCGAGCG

CAGCGGACATTGGTTTAGCCTCCTGCCTCGTTCTCCGTGCGACGAGTCTTCTCTCATCCA

ACCCACCACGTCCAACGTCCAGCCCAGGAGCCCTCCGGCAAGGTCCTGCTCGCCACACCA

GCATCTCCAACCTCCGTCTGCCTCCAGCAGCCCCAGTAACGCTGCTCACGACGGGCAGGG

CAACCCCACCGTCTCCCCTCTGCAGGTAAAACCTGGTGGTGCTCTGCTGTCTTTGCCCAT

GTGCGCATGGTCAGGTGGCATGATAAGCCCTAACCTACCTATTTGCAGCAGCCCTATGCC

CATTTGCCCACTCGCCGAGGGCAACGCCAGCCCCCTGATGGCACCCAGTGTCTCCACCAG

CAAAAGCGGCTCTCCTCCGCCCCCTGGTGACAAACCATGTGCACTGTCACCTGCTATAGA

TCTGCCAAGAAACCATGACCAGCCGCAGCCACAACCCATTCCAGAAGACATGTTGCTGGG

CTGGTGGAAGATGATAGACATGGAGGAACTGCAGGTTCTAATGAAGACGCTCCACAGCAG

AGGCATCAGGGAGCGAGCGCTGCACAAACAGCTCCAAAAATCCATCGAGCTCGTCTCACA

CACCTTCAACAAGAACAAGGAGGTAGCTGTGATGGAGGTGTCGGAGCTGGACGAAGGCCA

GGTTTCTGTGGAGACGCTACAGGAGTGGTGCGTGGAGGAGCAGGCGATGGAGACGGACAT

CGCTTTACTACAGCAGGTGGAGGAGCTCGAGCGCAGGGTCACTTCAGCCAGCCTGCAGGT

CAAGGGCTGGATGCACCCGGAGCCCCAGTCTGAGAGGGAGGACCTGCTGTATTACGAGCA

CAAGCCCCTTCCCAAAGTCTGCCCAGGGGCGGAGTTACATGAGGAGCGGGGTTCGGAGAA

GGGTCTGAGACGGCAACCCAGCAATCCGCTGGACATCGCCGTGATGCGCCTGGCCGAGCT

GGAGCGCCACATCGAGAGAAGGTACCTGCGGAGCCCCTTAGGGACAACCATCCAGATCAG

GCTGGATAATGTGGGGACGGTCACTGTGCCCGCCCCCGCGCCATCCACTAGTGCTGGAGG

GGAAGGAGGTGAGGAGGAGATCGCTCCGGGAATGAAGGTGTGGAGAAAGGCTCTGAGTGA

GGTGAGGAACTCGTCACAGCTGGCCATGTGTCTGCAACAGCTGCAGAAGTCCATCGCCTG

GGAGAGATCCATCATGAAAGTGTACTGCCAGATCTGCCGTAAAGGGGATAATGAGGACTT

ACTGCTGCTGTGTGATGGCTGTGATAAAGGCTGTCACACCTACTGCCACAAGCCCAAGAT

CAACGCCATCCCAGAGGGAGACTGGTACTGCCCTGCCTGCATCTCCAAGGCGAGCAGCCA

GTCGCCCAAGAATAAGAAGCCTCAGAGTCGCGTGCAGTCGGGCGGAGGGGGCAAGAAATC

GGCAGAGACGACCAAGAAGAGCAAAAAACAGCAGGAGGCGTGCGAGGAAGACGAAGCGGT

GGGAGGCAGCAGCACCAGCAACAGCAGTCCAAAGAAAACAGCCACCGCTTCCAGCCAGGC

CAAAAAGAGCTCACCCGTTCCTTCAGCCGCCAAACCCGAGAGCCCGGCCTGCGTAAAGCG

AGCCAAGACAGCCAGGGACAACAACCGCGACCTGGGACTCTGCAGGATTCTTTTGGCTGA

ACTGGAGCGTCATCAAGACGCCTGGCCTTTCCTCACACCCGTCAACCTAAAATCAGTCCC

TGGATACCGGAAAGTCATTAAAAAGCCCATGGACTTCTCCACCATCCGCGAGAAACTCGT

CAGCAGCCAGTACCAGAACCTCGAGACGTTCATCATCGACGTCAACCTGGTGTTCGACAA

CTGCGAGAAATTTAACGAGGACAACTCGGACATCGGCCGCGCGGGACACAACATGAGGAA

GTTCTTTGAGAAGAGGTGGACCGAGCTCCTGAAGCAGACTAACTGATTCGCTCAGAGCCG

CCATGTGACATGCCACTTTTACCAAATCCCTCATAACAGACCTCGGACCAGAACTGGCCA

GAAATTTCCTCAAAGCAAAGCGCCGATCTCAAACCTGAACCTGTCTCACACACACACACA

CACA

>Unigene000087

AAATAAAGGGCGGAGGGAGAAAAACTTTACGACGGGTTTTTTTTTTTTAACTGTTAAACA

GGAAGGAAACTGTCGAAATGCAAACCTGCAATCTCTAAAATAGGCCTCATTTAGGCTGGG

AAATTAACAAGCGTTATTAATATAAGTGGAGGTAATCGCTCGATCTGTACAGTGCGTTTC

ATTTTTAACTTATTATTCTTTGAAGCTGGTTTGAACAAACACAGCTGAAAAGGAAACGCG

CGTAAGCCGTTTACACGCGCGTCTCTTCCGCACCGGAAACTTCGACATTTTTCTTGCACA

CCACCAACTGAATTTGGAAATACTAAAGGACGACCTCTTATCGCTTTAAACTAACTATAC

AGTAAAAAAGGACCGATGTCTTATGGCTTCACACGGGCAAGGACGCAAAAGAACTCCGAA

TAGAAACGGAATAACTCCAGAAGAAGATGCGTTAAATGTAATCGCACGAGAGGCTGAGGC

CCAACTAACAGCTAAGAGGGTTGCCAGGGCAGAGGCTCGTGAGATACGCATGAAGGAGCT

GGAAAGACAACAAAAAGAGAAATACAATGGACTGGAGAACCTGGACAACAAATGCTGGGA

TATTGAGCAGTGGATGGAGGACAGTGAGCAGTATACACACCACCCCCAGCAACATGTTTT

GGTGTTGGGAAATGAAGAAGGTACATCAGCAGGGAGCAAGAGAAGTGTGCAGGGCTCCTC

TCGAGGACCGACTCTATCCACTGAGACTTCCCTGAGTGGGGCGTCCTCAAGTAAAGGAAG

TGGTGGTTCATCTGTCACTGCTGATACAGAAACATCCTTAAGGGATATTAAAGAGATTCA

TGAACTAAAAGACCAGATCCGTGATGTGGAGATCAATTACATGCAGATCCTCAAAGTAAC

AAAGGATTCTTTGTCAGAGATGGAAATGAAATATCGCAAGGCTATGGTGTCCAATGCTCA

GTTGGATAATGAAAAATCTAATCTGGTGTATGAGATGGACAGTTTAAAAGACTCACTAAT

GGAGTTGGAAGAGCTTCTGTCTGAAACTCGTCGCGAGTGTGAGAAAAAGAATAATGATCT

GGAACGAGAGAAGCATGCTCACAGTATACTGCATTTTCAGTTCAGTAATCGAAAAGAAAC

ACTAAAGCAAAGTGAAGAGCTGCTGAGTGAGATCCGACAATTACATCTCAAACAAGAAGG

GTTTACTAGAGAGATTTCTGACCTGCAGGAAACTGTTAAATGGAAGGATAAAAAAATTGG

GGCTTTAGAAAAACAAAAGGAGTTTTCTGATGCCATTCGAAATGAACGGGATGAACTTAG

GGATGAGGTGGTTCAACTCAAAGACACATTAAAGAAACATGGCATTGCTCTCGGATCAGA

ATTACGCACCAATGGGGAAGTGGGAGTAAATGTAAGTGGTGGATCAGAATTATGCACCAA

TAGGGAAGTGGGAGTAAATGTAAGTGGTGGATCTGTCTGCACTGAATTGAAACATGGAAG

CAGTGTTTTAGGTAAGGTTTTTTTTTCCAATACAAGTAGATTTGCTCTTGCATTGTGCTT

GCTTAAGCATCTTGACTGGTATTTCTAGCCAGCTTGACTGTTATTAACCAGCAGCTTTAT

TTCTCTTTCACTATTTCTCTTTTCACAGAATTCATACTTTAATTTGAAACATTCAAATTA

TCTCAAAATGTAGGACTAGGTACTTGAAATACCTGTTCGTCTGATTTATTTTCTACCCCT

CTAATATCTTTACTGACAAATATTTAAACTTCTATTTTATTATATCTACAGCTGCATATC

AGCAAAGATTTTTTTTCTACACATGATGATAGATCATTGCTCAATAACTTTCATTTTGAT

CTTCACTGAAACAAATGTCGACCTAATTTAGTCTATCCATGTTTTAATTGATTAACTGCG

GTGACCCGGAGGGGACATGTTCGGTTCGCTTGGTTTTGTCGTGGCCACAGCATGTTGGCT

TGTGGGAGGTTGATGTTGTGACGTGGCCACGGGATATTAATTCAAGGGAATGTCATATTA

ACTCATGGGAACGAGATATTAACTCGTGGGAACATGATATCTGATTGTTTATTTCTTATG

TTTATCACAATGTGAAAGATTATGAAAGTTTCTGACTTTTATATTTATGTAAAAACGTTT

ACATTATCAGACCTGTAAGAACGCTCTTTTTTTTCCTATTCAGTGCCTTTAACAATATGT

ATGTAAAAGCTGTGCCACCAGGTTAATAGTTCGGCTACCAAGTTGCTCAAAGCGCAGCTG

CTTTAATCGCAGTTGGACCGGTTAACTGGTTAATTATGAATTAGAAGACCTCATTAAAGC

TATAAAAAACTGTCGATAGTACATCCCGTTAAACTTAACACTGCGCTTTAGGAGCCTTAT

AAGCCTCACTTATAACCTACCTGTGATTTAAATATAACAGACCACAGCATGTCCTTATGA

TATTACGAGTAAAGGAAAATGTTATTCAATCAATTATGTCCATATTTACCTCTTATTCCG

ACTGCCGGTGTCGCCATATGTGCGGTTACGCTCAGTGTGTTCATTACATTAAAAAGTCAT

TCTCTCAACATATGAAGTCGTGGCCACGAGAAACAGATGTCATTCCCTCAACATAGCATC

TCGAGGCCCCGACTTAACGTCGACATATCATGCTTCCACGAGTTAATATCTCGTTCCCAC

CAATTGATATCTCGTGGCCACGTCGTGGTGTCACGTTCCCACGAGTTGGTGTGTTGTGGC

CGCGACGGGGCTGGGTGGGCCGGGCATGTCCCCTCCCGGTCACCGTAATTAACACCAGCT

TAGATGTCTATCTCTTCAAAATATTCACACCTGTGCTGTGTTGTAGCTATGCTTTGGAGC

CTAAACTTTCTCATTCTTTCAGCACATATTTATTTTACCCATTACATTGTATATTTTAAT

GGAATATTTTTTTCTATAACACTAATTCCTTAATCAACCTCCCAGGCACCCAGAAGTTGC

AGCTGGTTAGAGATGAAAATTTAGGGGGCTTCAGATGTGACCAATTACAATTCCCTCAGC

AATTTGACCATGGAATTGAAGAAAATCATCTGCCTTCACCTGCATCTGGCAGTTCTTTTA

ACTCTCGTATAAAAACACAATTTAAAGATGTGGATCTTGGGAATTATTTAATTAACAGCA

TAGAGAAAATTCAAAACAATGTTCAAAAACCTCAGGCTGTATTTGAACCTAATGCAAAGA

AACAAATTGTATCAGACATTTTGAAAGGTAATGATGTTCCAGAAACTGATGCAAATATTA

TGAAAGGTATAGGAAAAGATCAGCAGGAAAGTTATGTTAAAAGAAGTAAGACTGGAAAAG

AAGGAAAGCATGATTTCACACACATGTTCACCTGTGAGATATCAGAAAAAGATATCAATC

CACATTTTGATGAAACTGTGTTTTGCAACTCTCAAATTAAAATGGCTACTGACAGTTCCC

CATTAGCGAAAACAGATACAACATTTAATTCCCAAAGTTCCAATGTTTCAGTAAAAAAGA

AGAAGAAAAAGAAACAGAAACAAAAGTAGCGCTGTGCAAAAAATGTTCATTTAAAAATAG

GGAGAAAAAACAGCTCAGAGAAAGCAGTTCAGTTCAGACAGTATTGTCAGAGTGCAAGTT

TGTACAACAATAAATAAGAAAGCAAAAGATAATGTGAATTTCGATGAAAAGATAGACCTA

ATGCACAAAGGAACAGAATCATTGGAACTACAAATTCCCAATACTGGCCCTGAAAATATT

GTATTACCAATCATTAATCAAGACGATTGCATGAGCAAGATTGCATGGGTGGACAGAAAA

TTGGCATTTCTACTCATGACCATCCTAGAGATTTTCTTCTTTTGGCAAATGCATCATACA

AATTAACAATGATGATGCTGAGTTAAAAGCTATTGCTGAATTATGTGATGATTTTTAATT

ATCTGAAAACTCTTCTGAGGTTGAACCTATGAAAGATAAAACTGGAACTACCAGAGATGT

AGTAAAAATTGAAGCATTTATTCATTACTGTGAAACATACACCCTTGAAAAAAAATCCAC

AAACACAAAGCTGGGTTACACTGTAGTTAAATCTGAAGCCCCTGAGGAGAAGCAAGAAAC

CTTTACTGGCATACAGCTTGATGATACTGCATTTAAAGCTAAAATACATAGAGAAAAGAA

AGAAATGTCTACTGGCACACCATTGGGTGACACTGTAGTTACAGCTAAAGTCCAACAAGA

GAAGCAGGGAACCCTTATTAGCACACAGCTTAATGACAATGTAGTTACAGCTAAATCCTT

AAGAAGCAAGACACATTCATAAACATGTAGCTGGGTGACACTGCAGATACAGCTGAAAAA

ACCCAAAAAGCAGCAAGAAACAATCACTGGCATACAGCTTTATGAAACTGTAGTTACAGC

TAAGGTCCTTGAAGAGAAACAAGAAACATTCATAGACACACAGCTGGATGACACTGTAGT

TAAAGCTGAAGTCCCTAAAAAGAAGAAAAAATCAGAGCCACCAATGCATATGGGTCAGAA

ATTTAATATAGAAGAGGATGACAGTGAGGAGGAGTTTAAATCATTTTTTTTTATTAAAAT

AAAGAGATTTTTTGGAGGTAATGACTGACAAAAGGAATACACATTACTTGTTGTGCAAAG

CAATCTTGTACAACCAGTGAAAACCCGGCGCACGCAAATACTAAAGATAAAGACCAAATA

CTACAGACAGACAGACAGAATGTTTTAATGGAGAAAGACAAGCGTGTGTCTCTAGTGCAA

ATAACCACACCAGGAGGGACTCAGCAACAGTTTTATCCAGGGTATGAGGAACAAGGCTCC

AAAAGCCAAGAAGAAATAATACATTTAAAACACAGTGAAAAAGTTAAGCAGGAAATGTCC

AGCAAATGTTATAAAGGCCCTTGATGAGCTGCTCTCTGAGACAGCAGTGTTAAACTTAGT

GGCTGATGGTACTGTACAGCAGAAAGCATTTCAAAATATGGGAGAGCAGGAATTCAAAGA

AGTCAAAGAAGTGACACATATAAAAGAAACAAGCAGAATGCAAAGAATGGTTCTAAAAAG

AAAAGCAAAAAAAGTAAAAGGGAAGACTAAAGAGGACTGTAAAATGACCTAATACTTTAC

CCAAGGATGTTAATTATAAAGTGTCCAGAGTCTTAAAGCCTAACTAAATCTATGTTAATA

AATAATTGAAAGTTTAAAATAGTTAAAAGCTTCTAATCTTTAATCGAGATTAAAAAAGTC

ATTAAATTTCATTTCAGCATTTAAATTTTTTGTTGTGTGTGGTATGTATGCTTCTACATC

TGGATTGTGTGAGATGACATGACATGCTTAGTGAAATTCATATAGTTATTTATTTTCTCA

TGAATATATCTGCAGCAGTTTTCACAATGTTTTATCTCTGACATGCAGCAGTTTGCTACT

ATTTCACAAATTATATAATAAAAGTTCTTTTGTAAGATTTCTTTCAGAAACGTTTGTTTT

TGTTTTTTTACCCAGTGACGTACCGTGGGGTTTCAGCAGGGGCGGGTGGTGCATTTGGTA

CCTTGGACTTGGACTTACCCGACTGATACACCTCTTAATACCACCACTATCACATCGCAA

TTATAGAAACCACCACTACTATACAAAAACGCAATATAACATGGTGTGAAGTTAGAGACA

ATGAGGCATTTCGCGTATTTAGGGAAAATACCATGTTTATAAAAGCAAGAAACCCCCGTT

AAGACAATTTCAATCCTCTACAACAACAACTGTACCACATACATCATCTGGGGCAGTTCA

GAATCAATATTAATATTTTATTTATATAAAGAAACAGTGTTTAATTAATTAATACTGCTT

GTTGGCCCTGACTGCACACCACTGGGTTTCAGTCAGGCCTTTAAGTAAGCAGTAACTTTA

AAATTACTTGGACAATCCTACAAGCTAACACAGATATATGATAATAATGTGCATATGCAC

AATTGACACCATATGGAACTCTGAGCCAGACAATCAAAGTACTAAAGACTAAACTGAAAA

AAATAGTTTTTTAGTAGCTGTTAGCCACTCATAGCACTTAGAGTTGAAATCTTAAAAAAA

CTGCTGGCCATTGTTAGTGAAAATCAGTTTGGGTGTAGGTCTTGCTTAATTCGTTGTCTG

CAAAAAGTTGCCTTGAAAAGGCGTTTTTAATAGGTTGGTGACAGAATTTGGCCGGCAGCT

GCAGCAGTCAATTTGTTATTGTTGACTTTCACTTTCATACCTTGGAAAATCCTGATAAAA

TCAATCATTTTATTTTTATAGGGCCAGCAGAGAAGGTAGAAACATATTCTATATTAAAAT

AACTGGTTCGTAATAAAACATCTATTATAAAAATAAAATTACAGCTGTTTTACATTCTAT

TTACTTTGGTCTGTAAACAATTTCAAAAGTATGGTGGCCTTTTTGGTAATTTTATCATAC

CGATAATATGATTCCTTGGTCTTTTGTGTATTGTTAGCCTAAATAATATAACTAATTCCA

TAATTAACTATGAAATGTATGTATGCTGGTTAACTCAATTTCACATAGAGTTGTCACTAT

TTGGGTCCCCTTGAATTGTGATCTTTGCTATTTGCAGTGGGTTTTTGCATTTGATAGTGT

TTTTGTGTATTTGGTCGTGAAGATTTGAAGAGCGTTTCAGTATTTGTGTCTGCATTGTGA

GGATATGCAGTGTGTTTTCTTAAGTTGCAGTGCATTGGGCTTTCTCTGTTACCATACAAA

AGTCCTAGGAAGAGGGACTTTACGATAAAGACATTTTAATGGTCTACAAATTTATTCCAA

AGTTGGCATGACTGTGTTTTTTACAGAATACTTTTACAGAAGTTACTGCATGCTATTCAT

TCTCATATATATATATATTATATATATATATATATATATATGTGTGTGTGTGTGTG

>Unigene000088

GGTAAGTCGAGGCCTCGCGCTTCAATGGCGACTGAAAGCAGGCTGAACGGAAAGCGCGAG

CGATCAGGAAGAGGAGCGGAACCGTGCAGTCGGAATTAAATAAATATATAAACAGGATAT

AAAGCACACACCGCTGTAAGTCTGAGGAGTCGCGCGCCAGCTGAGCGAAAGCTGACAGAA

TGGACAAAAGAGGGACTATTTCACTCCTCGAATCGGAATTATATTATCTGATTTCTCGCT

TTTTATCCAGCGGTCCGTGTCAGAGAGCGGCCGAGGTTCTCGTGAGTGAACTGGAGGAAC

ATCAGCTTCTCCCCCGTAGATTGGACTGGGAAGGAAATGTGCACCCCAGATCTTATGAAG

ACTTGATTGCTGCGAACAAGCATATTGCGCCAGACCACTTGCTGCAGATATGTAAGCAGA

TCGGCCCGATCCTTGACAAGCAGGTGCCATCTGGTTTGCCTGGTGTGCATTCTCTGCTGG

GTACGGGAAGACACTCCTTACTTCGTGCAGCTAAAGATTGTGGCCATAGTCGATGGAAAA

GCTCGACGTTTGCTGCACTTCATCGAGGAAGACCACCGGAGAGGCCTTTGAGCTGCAGAG

ATGCTCCAAATCTGGTGGAGGTGTGCAGAGGCAGGGAGTTGACAGGCACTCAGCGTTTCA

GCTTTGTCCATCCTGTCGGTATATATCAAAACATTAAAATGCACAGGAGGATTCTGGGAC

ATCTGTCTGCGGTTTATTGCATCGCCTTCGACCGAACAGGGTCCCGAATATTCACGGGTT

CAGATGATGCCTTGGTAAAAATCTGGTCCTCCTTTGATGGGAGGCTCCACTCTACTCTGC

GAGGACACCATGCTGAGATCTCCGACCTTGCAGTCAACTTTGAGAACACCCTCATCGCAG

CGGGGAGCTGTGATAAAACCATCCGAGTCTGGTGCCTTCGTACCTGCGCCCCGGTGGCAG

TGCTGCAGGGTCACAGCGGATCTATTACCTCTTTACAGTTTTCTCCATTTGCCAAGGGCT

CCAAACGGTACATGGTCTCTACAGGAACTGATGCAACTGTCTGCTTCTGGCAGTGGGATG

TCATGAACACCACATTCAATGATCGTCCCGTAAAGTTCACAGAACGGCCGCGACCCGGTG

TTCAGATGGTGTGTTCCTCATTCAGCCCAGGAGGCGTGTTCCTGGCCACAGGGAGCACAG

ATGATGTCATCAGGATATACTACCTGGGCAGCGGGCTTCCAGAAAAAATAGCAGAGCTCA

ACTCGCATACAGACAAAGTTGACAGCATCCAGTTCTGCAATACAGGAGAGAGGTTTGTAA

GCGGCAGTCGTGATGGCACTGCTCGCATCTGGAAGCTTCATCAGAGACATCAGTGGAGAA

GCATTTTGCTGGAGATGTCTGCTACTTTGCCAGGAACTGTGCCTGCGGAGGAAGAGAGTT

TCTTCAAGCCTAAGGTCACTATGGTGTCGTGGGATCGCCATGACAACACGGTTATCACGG

CCGTTAACAATTATCTTCTCAAAGTGTGGAACTCCTACACAGGGCAGCTGTTGCATATCT

TAAAGGGTCATGAGGCAGAGGTGTTTGTACTGGAGCCACACCCATACGATCCCCGTGTCA

TGCTGTCTGCGGGTCATGATGGCAACATTTTCATATGGGATCTCATCCGAGGCACAAAGA

CTCATCACTACTTTAACATGATTGAAGGGCAAGGGCATGGTGCTGTTTTTGACTGCAAGT

TTACCCCTGATGGTCATCGCTTTGCCTGTACAGACTCTCATGGTCACCTGGTAATTTTTG

GATTTGGAAGCTCCAAGCCTTATGAAAAGCTTCCAGATCAGATGTTTTTCCACACTGACT

ACCGTCCATTGATCCGGGATTCCAATGGGTTCGTGTTGGACGAGCAGACACAACAGGCAC

CTCACCTTATGCCTCCACCCTTCCTCGTGGATGTTGATGGGAATCCTCATCCTCCCAAAT

ATCAGAGACTTGTGCCAGGGAGGGAAAACTTCACAGATGAGCACCTGGTTCCTCAGCTTG

GATACGTTGCTACAAGTGATGGAGAGGTGGTGGAGCAGGTGATCAGCCAGCAGCCTGTGG

AGAGTGAGGATCCTCAGGCACGCCGAAGTCTTTTAGATGATGCAATCCGTCAGCTGCAAG

AGCAACAGGACCAGCAGGCCGGAAGGCAGCAGGGTACCCCCAGAAGAGTGTCTTTAAGCG

AGCGGATGGAGGTACAGTCTCCTCCTAACGTAGGGCTCCGGCGCAGTGGGCAAGTAGAGG

GAGTGCGTCAGATGCATCAAAACGCACCACGTAGCCAGATGGCAACAGAAAGGGACCTGC

AGGCATGGCGGCGCAGAGTGGTGGTGCCTGAACTCACCTCCAGTGGTTACATGGATCAGG

AGTCTTTCCGAATTGCTAAGGGTGAGGAAGAGATGGTTCTGTATAATGCCAAGAAACGCA

GAATTGCACAAAGCAGCTTAAGGGATGACTCTGATGACGAGCCGGTGTGCACCAAGCGGG

AACAGCTACGCAAGCGGCACCGCAATTTGGAAAGCACCAGCAGTTACGCTGAGAGTCTGA

TTGAGTTTTCCTGTGAGGAGGGAGAGGAGACTGCTTCGTCTGAGGAAAATGAAATAGAAG

CTAGCGAGGTCGACTCCTCAAACGAAGAGGAAGAGGAGGAGTGGAAAAGTGACAGCTCAA

GTCGCTCATCCAGCGAGCATTCTGATTGGACAGCTGATGCTGGCATTAGCGCGCAGGCAG

CCATGTCGTCTCGAAGACGTGCTCGTAGGCAAATAAGCAGTGACGAAGAAGAGGAAGACA

ACTCTGCAGAGGAAGAACAAACCGAACAAACGCAGGACGAGGATGTGCAGTCCTCCTCCC

CTCATAAGTCGAAGAAAAAAGCAAAAACACAGAAGAGTCCAAAACTCAGACCGTCTTTAA

ACAGAGAGATTTCCAACGAGTTTCGTCCCTCCGCCTGGATTACTGACGTCATTCCCCGAA

AGTCTCCATTCGTTCCACAGATGGGAGATGAGGTTATCTACTTCCGTCAGGGTCACGAGG

CCTACGTGGAGGCAGTTTGCAGGAGTAATCTGTACCCCATTAATCTGGACAAACAGCCGT

GGAAGAAGATGGAGCTGAGAGACCAAGAGTTTGTTAAAATAACTGGGATCAAATATGAGG

TGTGTCCTCCGACACTTTGTTGCTTAAAGCTGACGCAGATTGACACTGGAACAGGAAAAA

TAACAACCAAGTCTTTTTCTGTGAAGTATCATGATATGGCTGATGTTATTGACTTCCTGG

TGCTCCGCCAAAGCTATGATGAAGCACGTAGCAGGAACTGGAGGCCAAACGATGCTTTCC

GGTCAGTAATTGATGATGCTTGGTGGTTCGGGACCATAGTCTGTCAGGAGCCCTATCAGC

CTGAGTATCCTGACAGCCATTTCCAATGCTTTAAAGTCAAGTGGGACAACGGAGAAATTG

AAAAGCTCAGTCCATGGGATATTGAAGCTATTCCAGAGAATGCTCAGCGTCCGGAGACGG

TGGGTGCAAGTGTTCAAGTCACAGACTCAGAGCGCGCCGACATCCTGTACAAACCACAGG

CGGACGAATGGGGTGGAAAGAGTCGAGAAGAAGAGTGTGCACGCATCGCTGCTGGCATCG

ACCAGCTCATCACCATAGATATCGCTGCACCCTTCTCTGGCCCAGTGGACCTCACTCAAT

ATCCCACTTACTGTACTGTAATCGCCTACCCCACTGACCTCGGCTCCATCCGAATGAGAC

TGAAGCACAACTTCTACAGGAGATTGTCTGCATTAATTTGGGACACGAGATATATTTATT

TTAATGCAAGGACCTTCAACGAACCACGTAGCAAGATTGCACAATCGGCAAAACTAATAA

CAGACGTTCTCTTGAAGTTTATTGAGTAGGTCTATAGGTCATATTTGAGCACTTATTAAT

CAAAGTAGTTATGTAAACGCTTCCTCAAAGCATGTGTGTTTTATTCTGCAGCAAGCCACA

CTGTACGGATATCATGGAGATTTACAATGCAGTTGAAAACATGGAGTACTCTGAGGATGA

GGATATGGAAGATGTTGATGCACCAGGAACATCTACTGGGCGAAGAGCAAATCAGTTGGA

ACAGATCCCAGACAGGGATGCATGGAAGGACGCATGCAAGCAGCTGTTGGATTTCATGTT

TGAGCGCGAGGACTCCGAGCCTTTCCGCCAGCCTGTAGATCAGGATGAGTATCAGAATTA

TTCAGACATCATCGACACTCCCATGGATCTGGAGACGGTTCGACGAAACCTTGAAGAAGA

CCGCTATGAAAACCCCAATGAACTATGCAAAGATACCAGACTTATTTTCACTAATGCTAA

AGTCTACACACCAAACAAGCGCTCAAAGATTTACAGCATGACTCTGCGACTGTCTGCATT

CTTTGAGGAGAGCATCCGAAACATCATATCTGACTATAAAACTGCAGTCAAAAGTAGTTT

GAAGCTGAGACGCAGTCAGAGATTTAAAAGAAGAACCCAGCAGCAGGAACCGCTTTTCCC

TCCTCCCCCGGTTGAATCAACTAGTCAGAAGAGGGCTGCTATTAAAACTCAGGAACAGGT

TGAAGCGGCTACGACCAAGTCTACCTCGGCTAAGACCTCGACAGCGGAGCACCGTCGCAG

TAAGAAGCGAAGACGTCGCAGATCTACCAACACGTCCATGGAGGACTCCAATAAGAGTGC

ATCATCTCTATCAGAATCTGAAAATGACAGCGAATTGTCATCAAACAAATCAAAGGCAGA

AGAATTCTCCTCATCCTCTCAGCCATTCAGACGTGAAACAAGAGCATCGCGAAGAGCCAT

GCAGAACAGAGAGGCCAGAAGAAAGATCGTCAGTCAAAGTGGAAGTGAGATTGACTGCTC

GACTGAGGATGATGATGACGAGGATGATGATTACTCTGGGAAGTCCAGCTCCTTAAAACG

GCTGCAGTCAAGCAGAAACAAAAGCAGGACCAGCCGACTCACTCGGAACTCAGAGAAAAG

AAAGCTGGAGGGTCAGGTGAATGGGCTCAGCAGCAGATCCTCCCGTGCAGAACGCAGGAG

AGTTCAACGCAGCAGTGGATCTGACCCAGAATTTTCTAAAGAAGAGGAAAACACAGGTAA

AGAAGAGGACCCGGAACAGAGTCGCCAGCTCAGGAAAACTGCTATGGTGGCAGTTAGTAA

GATGAAACTGTTGGACGCATCTGAGGAGGAGGATACTTCAGATCATAAAGCTCGTTCCAG

GAGCAGCAAACGTGCCACAGTTATTCAGAGCAGCTCGGAGTCTGAGGAACATGACCAACA

GGAGTCGAATGAAAGTGAGGATTGTGCCAGTGAGGACGAGAAAAGTGAGGCCAGTCCTGA

TAGCGACTTCTCAGGTTCACAACAAAATGGCGAGAGTAAAAGTGGTAACCGTGGAACATC

TAGAAGGAGAGTCGTGAAAGAAGAGGGTTCTCTTCGTGTCAATGGACACAAAAAGCAAAG

CCAGGAAGAGAGTGAGGACAACAGTGAGGACAACGAGGGAGAAAGTGTGAGTGAAAGTGT

GGATGAGGAGGAGGACTCCATCAGCAGGAGAAAGCTCCTCAGGAAAACTGCAAGAGTAGC

AGTATGTAAGATCAAGCAAATGAATGATAGTGACGAGGAGGAAGAGGATCACTGTAGAAC

CAGACGGCAGAAAAATCATCCAAACAATGCGAATAACTTCAAAACATCTGACGAGGAAAC

AGAGACTCCGCAGGCTGAGAAACACCCTCATCAAAACGGAAGAAAATTAAACACACAGGA

CATTAAATACTCTGAGAAAATGAATCACATGACTTCAAAAAGCCAAGAGCTAGTATCTGT

CTCAAAAGCTTGTGCAGCATTAAAATCAGCGGTCAAGGATTCCAGTGCTTTAGGGAGAAA

AATCAGGTCTGAAGATAAAGAAGAGGAAATAAACGAGAGCAATAGTGGAGAAGAAAAAAT

CACGAACAAAAGATGCTCAGTGGCAAGAAAGAACAAATGCAGCAGACCAGCCAAGTCTCG

GAGTTTGGATGAGGACACCAACACTTCTGACTCTGAGAGTGAAACACCTTCATACCAGGG

ACGCAAGAAAAAAGAGTCGGATTCTTCCGAAGAGTGGCGACAAAGCGACGAATCAAAAAA

GGATAAAAAAAGGTTTTCCAGAAGGCCCACTCTGAGAGCCCTTCCCAAAAAGAAATACAT

CACAGACTCTGAGGAAGATCTGGATTCTGATGGAGAAAAGCAGTCAGGGAATGGAAGGAG

AAGCTCTGATTCAGTGGAAGACGGCAACACCCATCGACAGAAATCATCAGAAGGATCAAA

AAGGAAACATGTCAGCACCTCCGAATCTGATAACAAAGAGGAAAACGGTTCTGCCACTGA

TGAAAGTGACTGTGACAAACCTCCAGGTAGGAGAGGAAAGCGGTTTAAAAAGCTTTCAGG

ATCCGAGAGTGATTCTGGAAGTAGAGAGAAAAGAACAAACGGTCCTTCAAAAAGGCACCG

ATCTGAGGACGAACTCCCTGAAGGCGCTAGTTGTCCAGGAAAACGGCAAGCTGGGAAACG

CAAACACAGCAAGAGCTACAGTAGTAATGATGGCACGGACTCCGAGAGCCAGGCAGAGTC

CAGCAATGAGTCACAGGTCTCCAGCTCAGGATCACAAAGCAATTCAGAAAGATACGGGAA

GGGAAGACTGAGAGCCCGAAGACGAGGAACCGCCCATCGCAAACTGTCTACTTCCGATAT

AGACAGTGACACTTCCTACGGAAAGCCTCAACGAGCCACACGCATTCGGACACGGAATCT

CGGTAAACGGACAGTTAACTACAGAGACAGTGAGTGAAGAGTTGTGGCTGTGGAAGAGAC

ACTAACTGGAGAACTTGACGACAATTGATTGTCCTAAATAAATGTAGTAGCACTGG

>Unigene000089

GGTAAGTCGAGGCCTCGCGCTTCAATGGCGACTGAAAGCAGGCTGAACGGAAAGCGCGAG

CGATCAGGAAGAGGAGCGGAACCGTGCAGTCGGAATTAAATAAATATATAAACAGGATAT

AAAGCACACACCGCTGTAAGTCTGAGGAGTCGCGCGCCAGCTGAGCGAAAGCTGACAGAA

TGGACAAAAGAGGGACTATTTCACTCCTCGAATCGGAATTATATTATCTGATTTCTCGCT

TTTTATCCAGCGGTCCGTGTCAGAGAGCGGCCGAGGTTCTCGTGAGTGAACTGGAGGAAC

ATCAGCTTCTCCCCCGTAGATTGGACTGGGAAGGAAATGTGCACCCCAGATCTTATGAAG

ACTTGATTGCTGCGAACAAGCATATTGCGCCAGACCACTTGCTGCAGATATGTAAGCAGA

TCGGCCCGATCCTTGACAAGCAGGTGCCATCTGGTTTGCCTGGTGTGCATTCTCTGCTGG

GTACGGGAAGACACTCCTTACTTCGTGCAGCTAAAGGTACAGTAGAATTATTGGTGAAAT

ATTACTGCAATGGGCAGATGCTTTTTACCCACTATGTCTTGGTGTGGTACAGATTGTGGC

CATAGTCGATGGAAAAGCTCGACGTTTGCTGCACTTCATCGAGGAAGACCACCGGAGAGG

CCTTTGAGCTGCAGAGATGCTCCAAATCTGGTGGAGGTGTGCAGAGGCAGGGAGTTGACA

GGCACTCAGCGTTTCAGCTTTGTCCATCCTGTCGGTATATATCAAAACATTAAAATGCAC

AGGAGGATTCTGGGACATCTGTCTGCGGTTTATTGCATCGCCTTCGACCGAACAGGGTCC

CGAATATTCACGGGTTCAGATGATGCCTTGGTAAAAATCTGGTCCTCCTTTGATGGGAGG

CTCCACTCTACTCTGCGAGGACACCATGCTGAGATCTCCGACCTTGCAGTCAACTTTGAG

AACACCCTCATCGCAGCGGGGAGCTGTGATAAAACCATCCGAGTCTGGTGCCTTCGTACC

TGCGCCCCGGTGGCAGTGCTGCAGGGTCACAGCGGATCTATTACCTCTTTACAGTTTTCT

CCATTTGCCAAGGGCTCCAAACGGTACATGGTCTCTACAGGAACTGATGCAACTGTCTGC

TTCTGGCAGTGGGATGTCATGAACACCACATTCAATGATCGTCCCGTAAAGTTCACAGAA

CGGCCGCGACCCGGTGTTCAGATGGTGTGTTCCTCATTCAGCCCAGGAGGCGTGTTCCTG

GCCACAGGGAGCACAGATGATGTCATCAGGATATACTACCTGGGCAGCGGGCTTCCAGAA

AAAATAGCAGAGCTCAACTCGCATACAGACAAAGTTGACAGCATCCAGTTCTGCAATACA

GGAGAGAGGTTTGTAAGCGGCAGTCGTGATGGCACTGCTCGCATCTGGAAGCTTCATCAG

AGACATCAGTGGAGAAGCATTTTGCTGGAGATGTCTGCTACTTTGCCAGGAACTGTGCCT

GCGGAGGAAGAGAGTTTCTTCAAGCCTAAGGTCACTATGGTGTCGTGGGATCGCCATGAC

AACACGGTTATCACGGCCGTTAACAATTATCTTCTCAAAGTGTGGAACTCCTACACAGGG

CAGCTGTTGCATATCTTAAAGGGTCATGAGGCAGAGGTGTTTGTACTGGAGCCACACCCA

TACGATCCCCGTGTCATGCTGTCTGCGGGTCATGATGGCAACATTTTCATATGGGATCTC

ATCCGAGGCACAAAGACTCATCACTACTTTAACATGATTGAAGGGCAAGGGCATGGTGCT

GTTTTTGACTGCAAGTTTACCCCTGATGGTCATCGCTTTGCCTGTACAGACTCTCATGGT

CACCTGGTAATTTTTGGATTTGGAAGCTCCAAGCCTTATGAAAAGCTTCCAGATCAGATG

TTTTTCCACACTGACTACCGTCCATTGATCCGGGATTCCAATGGGTTCGTGTTGGACGAG

CAGACACAACAGGCACCTCACCTTATGCCTCCACCCTTCCTCGTGGATGTTGATGGGAAT

CCTCATCCTCCCAAATATCAGAGACTTGTGCCAGGGAGGGAAAACTTCACAGATGAGCAC

CTGGTTCCTCAGCTTGGATACGTTGCTACAAGTGATGGAGAGGTGGTGGAGCAGGTGATC

AGCCAGCAGCCTGTGGAGAGTGAGGATCCTCAGGCACGCCGAAGTCTTTTAGATGATGCA

ATCCGTCAGCTGCAAGAGCAACAGGACCAGCAGGCCGGAAGGCAGCAGGGTACCCCCAGA

AGAGTGTCTTTAAGCGAGCGGATGGAGGTACAGTCTCCTCCTAACGTAGGGCTCCGGCGC

AGTGGGCAAGTAGAGGGAGTGCGTCAGATGCATCAAAACGCACCACGTAGCCAGATGGCA

ACAGAAAGGGACCTGCAGGCATGGCGGCGCAGAGTGGTGGTGCCTGAACTCACCTCCAGT

GGTTACATGGATCAGGAGTCTTTCCGAATTGCTAAGGGTGAGGAAGAGATGGTTCTGTAT

AATGCCAAGAAACGCAGAATTGCACAAAGCAGCTTAAGGGATGACTCTGATGACGAGCCG

GTGTGCACCAAGCGGGAACAGCTACGCAAGCGGCACCGCAATTTGGAAAGCACCAGCAGT

TACGCTGAGAGTCTGATTGAGTTTTCCTGTGAGGAGGGAGAGGAGACTGCTTCGTCTGAG

GAAAATGAAATAGAAGCTAGCGAGGTCGACTCCTCAAACGAAGAGGAAGAGGAGGAGTGG

AAAAGTGACAGCTCAAGTCGCTCATCCAGCGAGCATTCTGATTGGACAGCTGATGCTGGC

ATTAGCGCGCAGGCAGCCATGTCGTCTCGAAGACGTGCTCGTAGGCAAATAAGCAGTGAC

GAAGAAGAGGAAGACAACTCTGCAGAGGAAGAACAAACCGAACAAACGCAGGACGAGGAT

GTGCAGTCCTCCTCCCCTCATAAGTCGAAGAAAAAAGCAAAAACACAGAAGAGTCCAAAA

CTCAGACCGTCTTTAAACAGAGAGATTTCCAACGAGTTTCGTCCCTCCGCCTGGATTACT

GACGTCATTCCCCGAAAGTCTCCATTCGTTCCACAGATGGGAGATGAGGTTATCTACTTC

CGTCAGGGTCACGAGGCCTACGTGGAGGCAGTTTGCAGGAGTAATCTGTACCCCATTAAT

CTGGACAAACAGCCGTGGAAGAAGATGGAGCTGAGAGACCAAGAGTTTGTTAAAATAACT

GGGATCAAATATGAGGTGTGTCCTCCGACACTTTGTTGCTTAAAGCTGACGCAGATTGAC

ACTGGAACAGGAAAAATAACAACCAAGTCTTTTTCTGTGAAGTATCATGATATGGCTGAT

GTTATTGACTTCCTGGTGCTCCGCCAAAGCTATGATGAAGCACGTAGCAGGAACTGGAGG

CCAAACGATGCTTTCCGGTCAGTAATTGATGATGCTTGGTGGTTCGGGACCATAGTCTGT

CAGGAGCCCTATCAGCCTGAGTATCCTGACAGCCATTTCCAATGCTTTAAAGTCAAGTGG

GACAACGGAGAAATTGAAAAGCTCAGTCCATGGGATATTGAAGCTATTCCAGAGAATGCT

CAGCGTCCGGAGACGGTGGGTGCAAGTGTTCAAGTCACAGACTCAGAGCGCGCCGACATC

CTGTACAAACCACAGGCGGACGAATGGGGTGGAAAGAGTCGAGAAGAAGAGTGTGCACGC

ATCGCTGCTGGCATCGACCAGCTCATCACCATAGATATCGCTGCACCCTTCTCTGGCCCA

GTGGACCTCACTCAATATCCCACTTACTGTACTGTAATCGCCTACCCCACTGACCTCGGC

TCCATCCGAATGAGACTGAAGCACAACTTCTACAGGAGATTGTCTGCATTAATTTGGGAC

ACGAGATATATTTATTTTAATGCAAGGACCTTCAACGAACCACGTAGCAAGATTGCACAA

TCGGCAAAACTAATAACAGACGTTCTCTTGAAGTTTATTGACAAGCCACACTGTACGGAT

ATCATGGAGATTTACAATGCAGTTGAAAACATGGAGTACTCTGAGGATGAGGATATGGAA

GATGTTGATGCACCAGGAACATCTACTGGGCGAAGAGCAAATCAGTTGGAACAGATCCCA

GACAGGGATGCATGGAAGGACGCATGCAAGCAGCTGTTGGATTTCATGTTTGAGCGCGAG

GACTCCGAGCCTTTCCGCCAGCCTGTAGATCAGGATGAGTATCAGAATTATTCAGACATC

ATCGACACTCCCATGGATCTGGAGACGGTTCGACGAAACCTTGAAGAAGACCGCTATGAA

AACCCCAATGAACTATGCAAAGATACCAGACTTATTTTCACTAATGCTAAAGTCTACACA

CCAAACAAGCGCTCAAAGATTTACAGCATGACTCTGCGACTGTCTGCATTCTTTGAGGAG

AGCATCCGAAACATCATATCTGACTATAAAACTGCAGTCAAAAGTAGTTTGAAGCTGAGA

CGCAGTCAGAGATTTAAAAGAAGAACCCAGCAGCAGGAACCGCTTTTCCCTCCTCCCCCG

GTTGAATCAACTAGTCAGAAGAGGGCTGCTATTAAAACTCAGGAACAGGTTGAAGCGGCT

ACGACCAAGTCTACCTCGGCTAAGACCTCGACAGCGGAGCACCGTCGCAGTAAGAAGCGA

AGACGTCGCAGATCTACCAACACGTCCATGGAGGACTCCAATAAGAGTGCATCATCTCTA

TCAGAATCTGAAAATGACAGCGAATTGTCATCAAACAAATCAAAGGCAGAAGAATTCTCC

TCATCCTCTCAGCCATTCAGACGTGAAACAAGAGCATCGCGAAGAGCCATGCAGAACAGA

GAGGCCAGAAGAAAGATCGTCAGTCAAAGTGGAAGTGAGATTGACTGCTCGACTGAGGAT

GATGATGACGAGGATGATGATTACTCTGGGAAGTCCAGCTCCTTAAAACGGCTGCAGTCA

AGCAGAAACAAAAGCAGGACCAGCCGACTCACTCGGAACTCAGAGAAAAGAAAGCTGGAG

GGTCAGGTGAATGGGCTCAGCAGCAGATCCTCCCGTGCAGAACGCAGGAGAGTTCAACGC

AGCAGTGGATCTGACCCAGAATTTTCTAAAGAAGAGGAAAACACAGGTAAAGAAGAGGAC

CCGGAACAGAGTCGCCAGCTCAGGAAAACTGCTATGGTGGCAGTTAGTAAGATGAAACTG

TTGGACGCATCTGAGGAGGAGGATACTTCAGATCATAAAGCTCGTTCCAGGAGCAGCAAA

CGTGCCACAGTTATTCAGAGCAGCTCGGAGTCTGAGGAACATGACCAACAGGAGTCGAAT

GAAAGTGAGGATTGTGCCAGTGAGGACGAGAAAAGTGAGGCCAGTCCTGATAGCGACTTC

TCAGGTTCACAACAAAATGGCGAGAGTAAAAGTGGTAACCGTGGAACATCTAGAAGGAGA

GTCGTGAAAGAAGAGGGTTCTCTTCGTGTCAATGGACACAAAAAGCAAAGCCAGGAAGAG

AGTGAGGACAACAGTGAGGACAACGAGGGAGAAAGTGTGAGTGAAAGTGTGGATGAGGAG

GAGGACTCCATCAGCAGGAGAAAGCTCCTCAGGAAAACTGCAAGAGTAGCAGTATGTAAG

ATCAAGCAAATGAATGATAGTGACGAGGAGGAAGAGGATCACTGTAGAACCAGACGGCAG

AAAAATCATCCAAACAATGCGAATAACTTCAAAACATCTGACGAGGAAACAGAGACTCCG

CAGGCTGAGAAACACCCTCATCAAAACGGAAGAAAATTAAACACACAGGACATTAAATAC

TCTGAGAAAATGAATCACATGACTTCAAAAAGCCAAGAGCTAGTATCTGTCTCAAAAGCT

TGTGCAGCATTAAAATCAGCGGTCAAGGATTCCAGTGCTTTAGGGAGAAAAATCAGGTCT

GAAGATAAAGAAGAGGAAATAAACGAGAGCAATAGTGGAGAAGAAAAAATCACGAACAAA

AGATGCTCAGTGGCAAGAAAGAACAAATGCAGCAGACCAGCCAAGTCTCGGAGTTTGGAT

GAGGACACCAACACTTCTGACTCTGAGAGTGAAACACCTTCATACCAGGGACGCAAGAAA

AAAGAGTCGGATTCTTCCGAAGAGTGGCGACAAAGCGACGAATCAAAAAAGGATAAAAAA

AGGTTTTCCAGAAGGCCCACTCTGAGAGCCCTTCCCAAAAAGAAATACATCACAGACTCT

GAGGAAGATCTGGATTCTGATGGAGAAAAGCAGTCAGGGAATGGAAGGAGAAGCTCTGAT

TCAGTGGAAGACGGCAACACCCATCGACAGAAATCATCAGAAGGATCAAAAAGGAAACAT

GTCAGCACCTCCGAATCTGATAACAAAGAGGAAAACGGTTCTGCCACTGATGAAAGTGAC

TGTGACAAACCTCCAGGTAGGAGAGGAAAGCGGTTTAAAAAGCTTTCAGGATCCGAGAGT

GATTCTGGAAGTAGAGAGAAAAGAACAAACGGTCCTTCAAAAAGGCACCGATCTGAGGAC

GAACTCCCTGAAGGCGCTAGTTGTCCAGGAAAACGGCAAGCTGGGAAACGCAAACACAGC

AAGAGCTACAGTAGTAATGATGGCACGGACTCCGAGAGCCAGGCAGAGTCCAGCAATGAG

TCACAGGTCTCCAGCTCAGGATCACAAAGCAATTCAGAAAGATACGGGAAGGGAAGACTG

AGAGCCCGAAGACGAGGAACCGCCCATCGCAAACTGTCTACTTCCGATATAGACAGTGAC

ACTTCCTACGGAAAGCCTCAACGAGCCACACGCATTCGGACACGGAATCTCGGTAAACGG

ACAGTTAACTACAGAGACAGTGAGTGAAGAGTTGTGGCTGTGGAAGAGACACTAACTGGA

GAACTTGACGACAATTGATTGTCCTAAATAAATGTAGTAGCACTGG

>Unigene000090

TTTTCTTTTGTTTTTGTCTTTTTTTTTCTGTCCCATCTCTCCCCATCTTTTTCTTATTCA

TCCTTGGGCAATACATCAGGCCCTCCAAGATGAGGTGTTGGCTCGGGAAAAAGAGGTGGA

CCATCTAGAGGCCCTTGGCCAATCTCTCTCCCCTCTCAGCTGTGCGGCAGACCGTGATTG

GCTTAGTGAGCGAGTGGGAGCAGTGCGCAGCGGGCACACAGAGCTCCGGAATTGGTGCTT

TCGGCGAGCAGCTATGTTGGAGCAGGCTCTGGCCAATGCACAGCTGTTTGGGGAGGAGGA

GGTGGAGGTGCTGAACTGGCTGGCTGAGGTGGCCCAGCGCCTGTCTGACGTCTCAGTGCA

GAGCTACCAGCCCGAGCTTTTGGCTGAGCAGCACAAGTACACTCTGTCTCTGAACGAGGA

GATTGTGAGCCGGAAGAAGACAGTAGACCAGGCCATCAAGAATGGACAAGCATTGCTGAA

ACAGACCACTGGTGAAGAAGTGCTGCTTATCCAGGAGAAGCTGGATGGAATAAAGAGCCG

ATATTCCGAGATGACAGCAGGTAGCAGCAAGGCCCTTCGTAACCTGGAGCAGGCACTGCA

GCTCGCTACCCGATTTGCCAGCGCCCACGAGGACCTCAGCCAGTGGCTTGACTCTATGGA

GGCGGAGCTTAATAACATGGAACCAGACACAACTCCAGCCTATCAGGAGAGACAAAAGGA

CCTGAAGTGTGTGTCAGCTGAGAAGCGTCTCATTCTGGATACAGTTAATGAGGTGGGCAG

TGCACTGCTGGACCTGGTGCCCTGGCGAGCACGTGAAGGTCTGGATCGTCTAGTGGCCGA

TGCTAACCAACGCTACAGACAAGCCGATGAGACCATTACGCAAAGAGTGCAGCTAGTGCA

GGCAGCCATCCAGAGGTCACAGCAGTATGAAGAGGCCGTGGATGCGGAACTGGCCTGGGT

GGGGGAGACAGAGAGGAAGTTGACCTCCCTGGGGCCTCTAAGTCTGGAACCTGACGTGAC

AGTGGCACAGCTCCAAGTCCAGAGAGCCTTCAACATTGACATTATCCGCCACAAGGACAC

GGTGGACCAGCTCCTCCACACCCGAGAGGATATCCTGGAGAGCTGCAGTGACCAGCAAAG

AGAAGCACTGAAGGTAAAGACAGATTCATTAAGTATGCGGTATGAGGCAGTGAGTCAGAA

CCATGCTGAAAGATTTTCAGCACTGGAACAAGCACAGGTGTTGGTAGCCCGCTTCTGGGA

GACTTACGAGGAGCTTGACCCATGGCTGGGTGAGACAGAGACCCTGATATCCCAGCTGCC

TCCTCCTGCCATTGACACTGAGGCTCTACGACAACAGCAGGACCAGATGAGGATGCTGAG

AGAGTCTATAGCTGAGCACAAACCCCACATTGACAAGCTGCTGAAGATTGGCCCACAGTT

GGCTGAGTTAAGTTCCCAGGAGGGGGCGACACTGAGACAACGCTACAGTGAGGCTGAAAG

GCGTTACCTCGCCATCAAAGAGGACGTTAAAGGTCGTGCTGCAGTACTGGATGAGGCCTT

CTCTCAGTCTGCACAGTTCCATGATAAGATGGACCCCCTGTTAGAAACTCTGGAGGGAGC

AGTGCAGCGTTTGCGGCAGCCTCCTCCCGTGGCTGCTGAGGTAGAGAAAATCCGTGAACA

GCTGGCGGAGCACCGAGCAGCCGGGCTGGAACTGGACAAATTGTTGCCGTCCTTCAGCAC

TCTTTGTGCCCGTGGGGAGGAGCTCATCACCCGTGCTGCACATGATGACCCTGCGGCTCA

GGCAGTGCGTTCTCGGCTTCTAAGACTCCGTTCCCTGTGGGATGAGATCAGACAGAGAGC

AGAAGAAAGAGAGGGAAAACTGCAGGATGTACTGGACTTGGCTGGAAAGTTCTGGGCTGA

CATGGCAGCTTTGCTTAGCACCCTGCGAGACTCTCAGGAAATTGTAAAAGAACTGGAAGA

CCCTGGATTGGATCCATCACTCATCAAACAGCAAATAGAGGCAGCAGAAGCTATCAAAGC

AGAGACAGACGGTCTGCGTGAAGAACTGGAGATCGTCCGCACACTGGGAGCTGATCTAAT

TTTTGCTTGTGGTGAAACCGAGAAACCAGAGGTCAAGAAAACCATCGATGAGATGAATGC

TGCGTGGGAAGGCTTGAACCGCACATGGAGAGAGAGAATGGAGAAGCTGGAGGAGGCTAT

GACAGCCTCTGTGCAGTATCAGGATGCACTGCAGGGTATGTTTGACTACCTAGACAACGC

TGTCATTAAACTGTGTGACATGCAAGCCGTAGGAACTGACCTCAGCACCGTCAAACAACA

GATAGAAGAGCTCAAGCAATTTAAGGTGGAAGTGTATCAGCAACAGATAGACATGGAGAA

GCTCTGCCACCAGGGGGAGCTACTGCTCAAGAAGGTTTCTGACCAAGCGGACAGAGACAT

GATCCAGGAACCTCTCACTGAGCTCAGGCACCTGTGGGACAATCTGGGAGACAAGATCAC

TGTCAGACAGCACAAGCTGGAAGGAGCTCTTCTAGCCCTGGGTCAGTTTCAGCATGCCCT

CTCTGAACTGCAGTCCTGGCTAAGCCACACTCATGCTACCCTGGACACCCAGAGACCTGT

CAACTCTGACCCCAAAGCCATAGAGATCGAGCTGGCCAAACACCATGTATTGCGCAACGA

CGTCCTGTCCCATCGAGCCACAGTAGAGACTGTGAACAAGGCAGGCTCAGAGCTGCTGGA

GTCCAGTCCCGGAGACGAGGCCAGCCACCTGAGAGACCAGCTGGATGAGCTGAACCGCAG

CTGGGACAGCTTACTTCTGAAAACAGACGAAAGACAGAAGCTACTAGAGACGGCACTGCA

GCAGGCGGAGGGTTTCCATGGTGAGCTGGAGGAGTTCCTGCAGTGGCTGAGACGCACAGA

GAGTCAGCTATCAGCAGCCAAACCCACAGGTGGCCTTCCAGAGACCGCCAGGGAGCAGTT

GCAGCAGCACATGGAGCTGCAGGCTCAGTTGACCCAGCGCGGTGAACAGTACCACCGACT

GCTAGATCAGGGAGAGTCCATGCTGTTGGCTAGAGGGGCAGAGGAAAACAGCCCTGGAAC

CACTCAAACTCAGCAGAACCTGGCCCTGCTCCAAAACAAGTGGGCCAGTTTGAATGCCAA

GATGGATGACAGGAGGGCTAAGCTGGAGGAAGCAGTATCACTGGCAACAGGATTCCAGTC

GTCACTGCAGGACACCATCAACTGGCTGACGCAGGCAGAGCAGACACTAAACATGGCTCA

GTCACCGAGCCTCATCCTCGACACTGTCCTCTTCCAGATAGATGAGCACAAGGTGTTTGT

AAATGAGGTGAACACACACAGGGAGCAGGTGCTGGCTCTAGAGAAGGCCGGGTCTCAGCT

GCGCTTTGCCAGCCTTAAACAGGACGTGGTGCTCATCAAGAACCTTCTGTTGAGCGTGCA

AGCTCGCTGGGACAAGCTGGTGCAGAGGTCTCTGGACAGGGGACGGCACCTGGACGAGGC

CCGCAAAAGAGCCAAACAGTTCCATGAGGCCTGGAGGAAGCTGACAGACTGGCTGGAGGA

GGCTGAGAAACGGCTGGATGCTGAGCTGGAAATCTCCAATGAGCCAGATAAAATTAAAGT

GCAGCTTACTAAGCATAAGGAGTTTCAGAAAACTCTTGGTTCTAAGCAGCCAGTATATGA

CACCACAGTGCGCTCTGGGAAGGCCATGAGAGACAAAGCTACACTGCCAGCCGACACACA

GAAACTGGACAACCTCCTCGGGGAGGTCAGAGACAAATGGGACACTGTGTGTGGAAAGAG

TGTTGAAAGGCAGCACAAGTTGGAGGAGGCCCTGCTGTTCTCGGGTCAGTTTGCTGAGGC

ACTGCAGGCTCTTGTGGACTGGTTGTACCGGGTAGAGCCACAGTTGGCTGAAGACCAGCC

TGTCCATGGTGACCTGGACCTTGTGTCTAATCTCATGGATTCTCATAAGGCCTTTCAAAA

AGAGCTGGGGAAAAGGACCAGTAACATACAGGCACTGAAACGTTCTGCTCGGGAGCTAAT

GGAGACAGGCAGGGATGACACCGCCTGGGTCAAAGTTCAGCTGCAAGAGCTCAGCAACCG

CTGGGAGACGATCTGCGCACTGTCTGTCTCCAAACAGACCCGTCTGCAGCAGGCTCTCAA

ACAGGCTGAGGAGTTCCGCACAGCAGTGCAGATGTTATTGGAGTGGCTCTCAGAAGCAGA

GCAAACTCTACGTTTTCGAGGCATCCTGCCAGAGGAGGTGGAGACCTTGCAGGTTTTATT

ACACACCCATCGAAATTTTATGCAGACCGTAGAAGAGAAGCGAGTGGATGTGAACAAGGC

AGCTGGCATGGGGGAGGCCATACTGGCAGTGTGCCATCCTGACTGTATCACCACCATCAA

ACACTGGATCACCATCATCAGAGCCCGCTTTGAGGAGGTTTTAACCTGGGCGAAGCAGCA

TGAGCAGCGTCTGGAGGCAGCACTTGCCGAACTGCTCAACAATGCAACACTGCTGGAAGA

CCTGTTATCATGGCTACAGTGGGCGGAGACCACATTAGTGCAACGAGACACTGAGCCCCT

CCCCCAGGATATCACTCAGCTTAAAACACTGATCACAGAGCATCAGGTTTTCATGGAGGA

AATGACACGTAAACAACCTGACGTCGATAAAGTGACAAAAACCTACAAGAGAAAACCATC

AGAAACGTCTAGCAGTCTTGCTGAGAGACGGGGTGTCCGTAAGCAGCAGCAACAGCAGCA

GCAGCCTGCTATGCAGGTAAGTGGAGGAAACCCTCGTCTTAACCAGCTCTGCTCACGATG

GCAACAAGTTTGGCTACTAGCACTGGACCGCCAACGCAAGCTCCATGATGGCCTTGACAG

GCTTGAAGAGCTGAAAGAATTTGCCAACTTTGACTTTGATGTGTGGAGGAAGAAGTACAT

GCGCTGGATGAATCATAAGAAGTCCCGCGTCATGGACTTTTTCAGACGCATTGACAAGGA

CCAGGATGGCAAAATCACCCGGCAGGAATTTATCGACGGCATTCTGGCTTCAAAGTTCCC

CACCAGCAGGCTGGAGATGACTGCAGTGGCTGATATCTTTGACCGAGATGGTGACGGTTA

TATTGATTACTATGAGTTTGTGGCAGCTCTGCATCCTAATAAAGATGCCTACAAACCCAC

AACAGATGCAGACAAGATTGAAGACGAGGTCACCAGACAAGTCGCCCAGTGCAAGTGTGC

TAAGAGATTCCAGGTTGAGCAGATTGGCGAGAACAAGTACAGGTTCTTCCTTGGGAACCA

GTTTGGCGACTCTCAGCAGCTGCGTCTGGTGCGGATTTTGCGTAGCACAGTTATGGTACG

AGTGGGAGGGGGATGGATGGCGCTAGACGAGTTCCTGGTCAAAAACGACCCTTGCAGAGT

GCAACATCCAGGACTTAGAATTCTTCGCTCTGACTCCAGCTGCTCCATATCATCTCGTAT

AGCACGCGGACGTACCAACCTGGAGCTACGAGAAAAATTCATTCTTCCTGAAGGGGTTTC

ACAAGGTATGGCGGCCTTCCGTTCCCGTGGTCGACGATCTAAACCTTCATCACGCACAGC

CTCGCCAACACGCTCATCCTCCTCAGCCTCGCAGAGTGCCCAGAGCTGTGCCTCTGGGCC

ATCTGCCCCTGCCACACCCACTGCTTCTGCTAGGGGGGGCTCCTCATCCAAGCTTAAAAG

GCCACCTTTCCATTCCAGCCGTGGATCCCTGACAGGTGAAAATGGCGGAACCACACATGC

TACTAAACCTGTGCGCTCTGACAATAAGCGCACGCCGTCCTCAACCAGCGGCCCGACTAG

CCGCGCAGGCAGCCGTGCAGGAAGCAGAGCCAGCAGCCGAAGGGGCAGTGACGCCTCCGA

TGCATCCGAGCTGATGGAGACGCGCTCTGCCTGCTCTGACACTTCAGACACACCACGCCG

CCCAGGCACTAAACCCTCCAAAATCCCCACCATCTCCAAGAAAACACCCAGTCCCAAAAC

CCCCACCACTAAGAAGTAGCAGGTTTTCAGCATGGACAGATGGAGACGGGGGATTTTTTT

TTCTCTAGTGCATGCCACTCAACCTTCCTGTCCACCAGCACAGGCATGTGGGATTGATGA

GAATAGAGAACTGAAAAATCTCTCTTTTAAAAACACCTGGGCAGTGCTGACCCCACATCT

GCCCCTGCAACAAACCTAATAGTGCTGCCTTATTTGTGTTACAATATGACTGACTTTATA

GTTTCTTGAACATGTGACTGACCTGGAAAGCAACTACCACGTACGCACATAGACACGTGG

ACTGGCACCACCACCGGACCTGGGATTTTATCTCAGCCTTGTTGATATGCAATGGCCAGG

CGCTCTTCAGTGCTGAAGCTGACAGATGGTACAGCCACCATTCCAATACTGACTCTCTTA

GGCACAAAGATTTTTTTGTGAAACAAACAGTGGCAATGATTCTAAAGTTTCATCCACTGT

ATGCAAAGCTACTTGAGTATTCTGCATCCATGTGGTGTGTGTTTTAAGTGCTAGTTTCTT

TTTTTTCGGTGGACAAACGGAAAACATTCTAAGGAAGTGGTGAGCGATTTATCATCTCAG

CCTTGCTAGTGAGTCAGAAGCATGGTCATGTGACCGCAGCTTCACTTATCTTTCTCAAGA

AAGCATGCACCAACACTCACTGATGGCTTCTGATGTGATGTGTGTATGTATCGCCAGTCA

GCAAATGAAGAAAGTCCTAATTCCTGAGAGCACTTAAGTGACTCGTAGAGAAGAATTTCA

CATGTGCACTGACATTTCCTTTCCTTTCAAACCTGATGCTAACATTAGGCACGCTTACCG

CTGTGGTTTAACCATGATTAGTTACACGCATCATGTTACAG

>Unigene000091

CCACCCGGGCGGCGCGGTCCCCCTGCCGTGACTCGGCAAGGTGTATCAGTAAGGCGTGGG

TGTGTGATGGAGACAGCGACTGTGAGGATAACTCTGATGAAGATAATTGTGAAGCGTTGT

TGTGTAAACTTTCGCACCATACATGCGCCCAGAACGATTCCATCTGCCTTCCTGCCGAAA

AGCTGTGCGACGGTAAGGACGACTGTCCCGACGGCTCGGACGAGAAACTCTGCGACCTGT

GCTCGCTAGAGAACGGCGGCTGCAGCCATAACTGCACCATTGCCCCCGGCGAGGGCATCC

TGTGCTCCTGCCCGACTGGCATGGAACTCGGCTCTGATAACAAGACCTGCCAGATCCAGA

GCTTTTGCGCCAAACATCTGAAGTGCAGTCAACGCTGCATCCAGGAGAAAGCCACCGTCA

AGTGTGCCTGCTACGAGGGCTGGGCTCTGGAACCGGACAACGAGAGCTGCAAGAGCACCG

ACCCTTTCAAGCCTTTCATCATTTTCTCCAACCGGCACGAAATTCGACGTATCGACCTGT

ACAAGGGCGAGTTCAGTGTCCTGGTGCCCAGCCTGAGGAACACCATCGCCCTGGACTTCC

ACCTCAATCAGAGCTCCCTCTACTGGACCGACGTGGTGGAGGACAAGATCTATCGTGGCA

AACTGTCTGAAAACGGAGCCCTGACCAGTTTTGAGGTGGTGATCCAGTATGGTCTGGCCA

CTCCAGAGGGTTTGGCTGTGGACTGGATTGCTGGAAACATCTACTGGGTGGAGAGCAACT

TGGACCAGATCGAGGTGGCCAAGCTAGACGGAACCATGCGCACGACACTGCTCGCCGGAG

AGGTGGAGCATCCACGAGCTATAGCCCTCGATCCACGTGACGGTATTTTGTTTTGGACAG

ACTGGGATGCCAGTTCTCCCCGAATCGAGGCTGCCTCCATGAGCGGCGATGGCCGGCGTA

CCATTCACAGAGAAACAGGAAGTGGAGGCTGGCCTAATGGGCTCACCGTTGACTACTTAG

AGCGCCGTATCCTCTGGATCGATGCTCGGTCTGATGCCATCTACTCTGCTGCTTACGACG

GTTCGGGCCTGATCGAGGTGCTGCGTGGACACGAATACCTGTCGCACCCGTTCGCCGTCA

CCATGTACGGCGGCGAGGTTTACTGGACAGACTGGCGCACTAACACACTGGCTAAAGCCA

ACAAATGGACAGGACACAACGTTACAGTTGTACAGAGGACCAACACACAGCCGTTCGACC

TGCAGGTCTATCATCCATCCAGACAGCCTCAGGCTCCAAACCCTTGTGCTGCTAACGATG

GAAGGGGTCCGTGCTCTCACCTCTGCCTCATCAACTACAACCAGACCTTCTCCTGCGCCT

GCCCCCACCTTATGAAGCTGCAGGCAGACAAACACACCTGCTACGAATCTCGTCAGTTCT

TACTCTACGCTCGTCAGATCGAGATCAGAGGAGTGGACATCGACAATCCATATTACAACT

ACATCATCTCCTTCACTGTTCCTGTGATCGATAATGTAACAGCGGTGGATTACGACGCCC

AGGAACAGCGCATCTACTGGTCTGACGTTCGCACGCAAATGATCAAACGAGCCTTCATTA

ACGGCACGGGGGTGGAGACAGTGGTGTCGGCCGATGTACCCAACGCTCAAGGCTTGGCTG

TGGACTGGGTGTCCAGGAACCTGTTCTGGACTAGCTACGATACCAGCAAGAAGCAGATCA

ACGTAGCCCGATTGGACGGCTCCTTTAAGAACTCGGTCGTCCACGGACTGGACAAACCAC

ACTGCCTGGTGGTACATCCCATGCTTGGAAAGCTCTACTGGACAGACGGGGACAACGTCA

GCATGGCCAACACAGATGGCAGCAACCAGTCGTTACTCTTCACCAGTCAGAAAGGCCCCG

TGGGACTGTCTATTGACTTCGATGAGGGGAAGCTGTACTGGATCAGCTCAGGCAACAGCA

CGATAAATCGCTGCAATCTGGACGGCACCGGCCTGGAGGTCATTGAAAGTGTAAAAGGAA

AACTCACCAGAGCCACCGCTCTCGCCATCATGGGGGACAAGCTGTGGTGGGCGGACCAGG

GCACAGACCAGGTAGGCACCTGCGACAAGAAGGATGGCGGCAGCTGGAAGGTTCTTAGGA

ACGGCACGTCTCCGGTGATGCATATGAAGATCTACAACGAGACTGTCCAACAAAAAGGTA

CGAACCTGTGCACAATGAACAACGGGGACTGTTCTCAGCTTTGCTTGCCCACCTCACCCA

CCAGCAGAGCCTGCATGTGTACGGCAGGCTACAACCTGAAGAGCGGCCAGCAGTCCTGTG

AAGGTATGGGCTCGTTCTTGCTGTACTCGGTGCATGAGGGCATCCGCGGAATTCCTCTGG

ACCCGCTGGACAAATCTGACGCTCTGGTGCCAGTTTCTGGAACCTCTTTGGCAGTGGGCA

TCGACTTTCACGCCGAAAACGACACCATTTACTGGGTGGACATGGGTCTTAGCACCATCA

GCCGAGCCAAAAGAGACCAGACATGGAGAGAAGATGTAGTTACTAACGGCATTGGACGTG

TGGAAGGCATTGCTGTCGACTGGATTGCAGGAAACATCTACTGGACTGACCAAGGCTTCG

ACATGATTGAGGTGGCCAGACTCAACGGCTCCTTCCGCTACGTGGTCATCTCTCACGGGC

TGGACAAACCCCGCGCCATCGCCGTCCATCCAGAGAAAGGGTACCTTTTCTGGACCGAGT

GGGGTCAGTACCCACGCATCGAAAGGTCTCGACTGGACGGGTCAGAAAGAGTGGTTCTGG

TTAATGTCAGTATTAGCTGGCCCAATGGCATCTCCATCGATTATGAGGAAGGGCTGCTCT

ACTGGTGTGACGCTCGCACAGACAAAATCGAGCGTATTAATCTGGAGACCGGTGGGAACC

GAGAACTTGTCCTGGCAGTTCATAACATGGATATGTTCGCCGTGTCTGTATTCGAAAACT

ACATCTACTGGAGTGACAGAACCCATGCCAATGGCTCCATTAAGAGGGGTAATAAGAACA

ATGCCACTGATATGGTTTATCTAAGAAAAGGCATTGGTGTGCAGCTCAAGGATATTAAAG

TTTTCAACCGAGCCCGGCAGCAAGGCACCAACATCTGTAAAGACAAAAACGGTGGATGTG

AGCAACTATGTCTATTCCGTGGCAACGGAGCACGCACCTGCGCCTGTGCCCATGGCATGC

TAGCCGAAGACGGGCGCACTTGCCGCGACTACGACGGCTACCTTCTTTACTCCGAGCGTA

CAATACTGAAAAGCATACACTTATCAGACGAAACCAACCTCAACGCCCCGATCAAACCTT

TCGAGGATCCAGATCATATGAGAAATGTCATCGCTCTCACCTTCGACCACCAAGGTGGCG

ACGGCAAAGGAGCTAACCGCATCTTCTTCAGCGATATACACTTCGGCAACATCCAGCAGA

TCAACAGTGATGGCTCAGCGCGCAAGACCGTTGTTGAAAATGTGGGCTCGGTGGAAGGCT

TGGCGTATCACAGGGGTTGGGACACGCTTTACTGGACCAGCTACACCACCTCCACCATTA

CGCGCCACACGGTGGACCAGACACTCAGCGGCGCCTTTAACAGAGAGACGGTGGTCAGCA

TGTCTGGAGATGATCACCCCAGAGCCTTTGCGTTAGATGAGTGCCAAGGGTTAATGTTCT

GGACTAATTGGAATGAGTTGGCTCCCAGCATCATGCGCTCATCTCTAGCAGGCTCTAATG

TCCTTGTGATCGTGGGTAACAATATCCGCACCCCAAATGGCCTCGCTATAGACCATCGCT

CCGAGAAGCTTTATTTCTCTGATGCCACGTTGGATAAGATTGAACGCTGCGAGTATGACG

GCACCAATCGCTTTGTGGTTTTGAAGAACGAGCCGGTCCACCCGTTTGGCTTGGCTGTCT

ACGGCGAGTACATCTTCTGGACGGACTGGGTGCGCAGAGCCGTGCTGAGAGCGGATAAAT

TCGGCAGAGACATGAAAGTACTGCGTGCTGACATCCCCCAGCAGCCCATGGGCATCATAG

CCGTTGCCAAAGACACCAACAGCTGTGAGTTCTCTCCATGTCACACCAATAACGGCGGCT

GTCAGGATCTGTGTCTTCTCACGTCCGAGGGCCGGGTGAACTGCAGCTGCCGCGGAGATC

GCAAACTGGTGGAGGGCAACGTGTGTGTTGCCGAGAACACTACATGCCACAGCGTGGACG

AGTTCGAATGCGGAAATGGTGACTGCATCAACTACAGCCTGACATGCGACGGACGCGCTC

ACTGCAAAGACAAATCAGACGAGAAACCGTCCTACTGCTCTAATCGAGGCTGTAAGAAGG

GATACAGACGCTGCCTTAACGGACGCTGCATCAAACACAGCTCCTGGTGCGACGGCACAG

ACGACTGCGGAGACCGATCAGACGAACGACCCTGCAACATGACCTTGTGTAGTGCATCAG

AGTTCCAATGTAAGGACGGTTCCTGTATCACCAACACGTCCCGCTGTAATCAGGTGGTAG

ACTGTGAGGATGCCAGTGATGAAATGAACTGCAGTCCAACAGATTGCCACAGCTTCTACC

TGTTAGGAGTTAAAGGTGTGATCTTCCAGAAATGTGAATTTACTACGCTGTGCTACACCC

CTATCTGGCAGTGCGACGGCTCGAACGACTGTGGAGACTTTTCGGATGAGAGGAACTGCC

CAGAGAAGAGGCAGCTGAAATGTCCTGTGAACTTTTTCGCCTGTCCCAGCGGTCGCTGTA

TTCCCATGAGCTGGACTTGCGACAAGGAGAACGACTGTGAAAACGGAACAGACGAGACTC

ACTGTGATAAGTTCTGCTCACCAACACAGTTTGAGTGCGGGAATCACCACTGTATCTCCA

GCTCCTGGGTGTGTGACGGCACCGATGACTGTGGAGACGGCACCGATGAGGGCAGCCGAT

GCAGTTCAAAGACATGCAGTCCCGAAGCCTTTCAATGCCCTGGCTCTCACTGCGTGCCCC

AGCGCTGGAAGTGTGATGGGGACAACGACTGTCCAGATGGTGCTGATGAGGGTGTGCAGG

CTGGCTGCACAAACAATAAGACCTGCGACGACACGGAGTTCCAGTGCCAGAATAAGCAGT

GCATCCCCAAGCATTTCGTCTGCGATCACGATTTGGACTGCCGTGACGGCTCTGACGAGT

CTCCCGAATGCGAGTACCCAACGTGTGGTCCCAATGATTTCCGCTGTGAAAACGGGCAGT

GCCTTAAGCAGAAGAGCTGGGAGTGTGACGGCGAATTTGACTGTCGTGACCACTCGGACG

AAGCGCCCAAGAACCTCCACTGTACCGAGCCAGAGAAGCGCTGTAACGACTCGGCATACC

TGTGCAACAACGGGAAGTGTGTGAGCGAGGACTCGCTGTGCGACTGGAAGGACGACTGCG

GCGACGGCTCCGACGAGCACAACTGCTTCGTCAACGAGTGTCTTAACAACAAGCTCAGCG

GCTGCACACAGCACTGCGAGGACCTAAAGATTGGATACAAGTGCCGCTGTGATCCCGGCT

TCCGTCTGAAAAACGACGGCAAGACATGCGTGGATGTGGATGAGTGCACCACCACTTACC

CTTGTTCACAGCGATGTTTCAACAGCTACGGCAGCTTCCACTGCTTCTGCATAGAGGGTT

ACGTGGCCTATGCGAACGACTGGACAAGCTGCAAGTCCTCTTCTGAAGAGGAGGCTTATC

TAATCTTTGCCAACCGTTACTACCTGAGGAAGCTCAATCTTGATGGCAGCAATTACACCC

TCATCAAAACGGATTTGAACAACGCTGTGGCTCTGGATTATCACTACGCAGAGCAGATGA

TCTACTGGACAGACGTGACCACGCAGGGCAGCATGATCCGCCGCATGCTTATGAACGGCA

GTAACGTCGAGGTGCTGCACCGGACGTCTCTGAGCAATCCGGACGGTCTGGCGGTGGACT

GGGTCGGGGGCAACCTGTACTGGTGCGATAAGGGTCGAGACACCATCGAGGTGTCCAAGT

TGAACGGGGCTTACAGGAGCATGCTGGTCAACAGCGGTCTGAGGGAGCCTCGGGCGGTGG

CTGTGGACGTCCGCAATGGTTATCTGTACTGGTCAGACTGGGGAGATGTGCCTCACATCG

GCCGTATTGGAATGGATGGAACAGACCGCAGCATCATCATCAAGGATAAGATCACATGGC

CTAACGGCCTCACGCTGGACTTCATCAACGACCGCATCTACTGGGCCGACGCTCGCGAGG

ACTACATCGCCTTCGCCAGTCTCGATGGCACCAACCGACACATCGTTCTGAATCAGGACA

TCCCACACATCTTCGCCATGTCTCTCTTCGAGGATTACATCTACTGGACCGACTGGGAGA

CCAAGTCTATCAATCGAGCTCACAAAACACTGGGAACCAACAAGACCATGCTGATCAGCA

CGCTGCACAGACCTATGGATATTCACATCTACCATCCATACAGACAGCCTGCAGTTGAAA

ATCATCCCTGTCAGGTAGATAACGGAGGCTGCAGTAACCTGTGCTTGTTGTCCCCTGGAG

GAGGTTACAAGTGCGCCTGCCCCACCAACTTCTACCTGGCTGCAGATGGGAAGCAGTGCC

TGTCTAACTGTACAGCGAGTCAGTTTGTGTGTAAGAATGATAAGTGCATCCCCTTCTGGT

GGAAGTGCGACACTGAAGATGACTGTGGAGATCGCTCCGATGAACCTGCTGACTGCCCGG

AATTTAAATGCCGTCCGGGGCAGTTCCAGTGCGGCACAGGCATCTGCACAAACCCCGCCT

ACATCTGTGATGGCGACAATGACTGCCAGGACAACTCGGATGAAGCTAACTGCGACATCC

ACGTGTGCCTGCCGAGCCAGTTCAA

>Unigene000092

TTGTAGGTGTTCAATACAGAAGGGAGAAAAATACAAAGTGAAATAATCTGAAACAAAAGA

AGCCTTTTCCTGAAAACCTCATCCATCTCTGTATTCTTCCATGAATCACGATAGTGAACA

AGTGTTTTTATTACTGCAACTAAACATTCACGAGGCAGACAACACCAGAAAAAGTAACAC

CCTGAACCACAGTAACTTGTAAGAGGGTTTAGTGGCATTACATGAAAGAATACAGAAACC

AGAACAAGCATTTATTGGCGCTCGAGTAAATCTTTCTAAGACACTATAGGAACCTTCCTG

CAATTGATGAGGATTTACAACCTTAAATTGAGTTAAGATTAGATTAGAATATGTGAGGTT

AAATAAGTTCTGGATGATACTGTGGCATAAATTTAACTGAATACATTCTCTGTGGAATTT

ATAGGGAAGCCTGAAAATATATGTGTAAAGTCAAGTTTATTCACTATAAAAGCAGGTATG

TGCATTCTCTTTGGTCTAGCATTTCATTACCATACAAAAGAAATTTGAGACATATGAAGA

CTAGCAAGAAATAACCATCAAGTGTCTTGAAAAGAAGAGGCAAACAAAATGTTTGAGTCA

CAGACAGATAGCACATTAGTGATGCAGATAGAAAAATATAGAAAAATATCAACTGTGATC

AGTTTAATTGACATGACCTGCATTGTATATCTCTTAAGGCTGCCATTGGTAAGTGCACTT

AGTCACACATACACGTATACATGTTCAGCTTTAGATTAGTGGAAAATACAGAAGTGTATA

AGTCATTCGGTGAAGGGTGTATAAAAATTTGATCTGAAATTGCTTAATTAGTCAGACAAA

ATGGGGAATTTTACTCTCAATATAAAGTGAATGCACACACATATTGCACAGTCTTTGGGG

AAAATGCAGATATTGCTTGTTTGATGTAAACATATTTTCCTCACTGTGCTACCTGGCAAT

AAAAAAATTAATAATCACACGTTTTAAAGATATACATCGATATATATGCCTACTAAAGCA

TATAGTGCTAAATACTACAATTCACCATTTTGTGCCTTAAACGAATTGCAGATTATATCA

TGACTTGGTCAACAGAGTGGAATCGGCCATATACAAAATGTGCCCTTGGTCTCATTATGC

TTTTATAGCCTTCATAAATAACTCAAACAAAATTAAATGTATTTACCTGATAAGATTATG

TTAAGAGATGTTAATTATTAAACAAAGCTATATATTTACAACAGAGCACAAAGTACAGCC

TGGCTGCTGGGCTACATGAAGGACAGACATAACTTATGGAAACCTGAATTTCTAAAGTTA

GTATATGATAATAACAAACTGAGTGGGGGAAATTGAGAACGATAAACAGGCTACTTATTT

TTGGTGTTGTTAGCAAAACAGTACTTAGCATTTAAACTGAAAGAATAGCTGTATTCATTA

AAACATGATTCAGAAGAGATTTTTTAAAACGGCAGCCTGTTTACTAATGAATGTATTCAG

TGCAATTGCAAACAGGTGTCATTTATTCAGGATAGATAATGAGAATATTAATGAAGTGAT

CGATATGAAGCAGAAGCACCAGATTCTGGGGTCCCATCACTCTGGAACTTCTTATGGTTG

GCATTCATAAGTCTAAGAGAGCATTTTCACAAGTCCATTTTAACTCTCTCTCTTTCTCTC

TCTGTGAAGTAGCACCATACTGTTTCTTTTGTAAAACTGGAACATAAAAGCCAAATGCAG

AAGAAGGTGTGTGACCAGGCTAAGTCCTCTTCATGCACACTTGACATCTACTGATGTGAG

TTCGCAGATTCCCAGCCTTAAGCGTGGTGGGGACAGGTTTGCTAAACATCTCATTATCTT

CGATGGTAGCCAACCAGAAGCTGTAGGAGTTAGCATAGTAGTTGCATGTGCCACGGCCAT

GACATTCAATAAATGGAGCAGAGCGGAACTCCTCCAGACAGGAGCCAGGAGAGGCCAGGG

CCTGACCGGAGCCCTCTGCACCTGCACTAGTATGCATGACAAAGGAATAACCAATCCACA

GAGAGATCCAGCCTTGTGGGCAGGATGGGATGGCTATAGTCTGACTGTGCACAGCAATGA

CCATAGCAGGAGCCTCACACACTGCACATCTGCTGATAAAAGGTTTGATGCTCTCGCCAG

TAATTGGAGCCATGCTCATCGGCATGGGCTCAGGAGATGTGAGCCAATAGGAATAGTCAT

TACGAGAGGCAAAGTTACACACGTTGTTGATGTTACAGAAGAGGAATGGCATAGGACTGA

ACTTTCTCAAACAGCTACCAGCCGTACCCAGGTCTTGGCCATGGGATCGCTCATTTCCCT

GCACATACAGGAGGGAGTACCCATCAAAGATCATAGTGGTGCCCTGTGGACATAGAGGCA

CCTCAACTGACTGACTGTGCCGAGTTACTAGGAATCCATGGTCCATAGAGGAAGGTCCAG

GAGGGCCAACCTGACCTGGAACACCATCAGGACCTGGGGGACCTGGATATCCCCTTGATC

CAGGGGGTCCAGTAGGGCCAGGTTCTCCTTTATTTCCAGGAAGCCCATTAAAGCCTGGTG

CCCCATCTTCTCCTTTAATACCCTGTATTCCAATAAGTCCTTGCTGTCCTTTTTGCCCAA

AATACCCTTGAGGTCCAGGTAAACCTTGAGGACCCTGTGGTCCTGGAAAGCCAATGTCTC

CTTTTATAAAGGTATGGGGTCCAGGAGGTCCAGGAAGGCCATCGTTACCTTTAGCCCCTG

GAAATCCTCTGTCACCAATTTCTCCTTTAAATCCAGGTAATCCACCCTCTCCTTTTATTC

CTTGGAAGCCAGGAGGGCCTGGAGGTCCCATTTCACCCTTTAGTCCTTTATAGCCTGGGG

TGCCAGGTTCACCAGGTAGGCCAGTAAAGCCTTTATCGCCTTTTAAACCCTGGGTACCCT

GTATGCCTGGGAAGCCTTTGTCTCCTTTCTCCCCTTTGAGACCAGCACCACCTGGAACAC

CTGCTGGACCTGGAGCACCAGGCTGCCCTGGCTCCCCAGGCTCTCCCTGAACTCCTTTAT

CCCCTTTAGGTCCCTCCATTGATGGCCCCGGTAGTCCTCTTTCACCAGTTCGACCCTGAA

CACCTTGAAATCCAGGCAGGCCTTTTTCTCCTTTACTGCCAGGAACTCCAGGGCTTCCAG

ACATGCCAGGGGGACCTGGGTTGCCTTTTTCACCTTTTTCACCAGCATTCCCAGGAGTTC

CTGGAAAACCTCTACCAGGTAAACCTGGTTCACCCCTTTCTCCAGAGACTCCAGGGAACC

CATCTTTTCCTGCCTCTCCTTTCTTCCCTTGTGGCCCTGGAGAACCCTGTAGACCAGGTT

CACCTGGCCGTCCTTTAAGACCAGCCTCTCCTGTTAATCCAGGTAAACCGGGAGTTCCTT

TTTCACCTGGTACTCCAGGGCTTCCTGGGTAACCAATCTGTCCTATGTCTCCTTTTTGCC

CTGGAGTTCCAGGTATGCCTGGGATTCCCATGGAACCTTTTACACCCTTTTGCCCAGACT

CCCCAGGAAATCCAGGTGGACCCATTTCACCCTTTTCTCCAGGGAGGCCAGGAATTCCTA

TTCCAGGCAGACCTGCTAATCCTTTTTCACCTTTTCCTCCTGGATCACCCTTAAACCCAT

GATGGCCCGGCTGACCAGCAACACCTTTGCTGCCTTTTGGCCCAAAAGGTCCTGGCAATC

CCATTTCCCCAATGCTGCCCTTTACTCCGGGTGCTCCAATTTGTCCTGGTTGTCCAGGAA

GTCCTGAATCACCCTTCTCACCTGGCTTTCCTGGTGAGCCAGGCATTCCATCCTTTCCAG

GCAATCCAGGATCTCCAGATAATCCAATAATCCCCTTTTGTCCAGTAGGGCCTATTTCCC

CTTTATTTCCGATGTCTCCCTTCTCGCCTTTCATATGTTCCATTTCCACTTCAGTCATAT

TACCAGGTGGTCCTTGAAGACCAGGATCACCTCTCTCTCCTTTAGGACCCTGTTCCCCAG

TCACTCCCCTAGGTCCAATTAGACCAGGGTCACCCCTTGGTCCAATAGCTCCAGGACCTC

CTGTTTGACCCGGGTGCCCAGGTGAACCTGGAATTCCCATTATTCCCATATCTCCTTTGA

TACCTCTTTCCCCAGGGAATCCATCTAGTCCTGGATTTCCTGGTTCCCCTGGTAAGCCCT

TTGGGCCTGGTGTTCCAGGAAAACCAGGAGTGCCTTTCTCACCTTGAGGACCAGGCATTG

CAGGACCCGGAAAACCTTGGGGACCTTTCTCCCCCTTTTCACCTATACGACCTGCGGGAC

CAGATTCTCCAGGAGGACCTATAAGTCCAGGAGGTCCAGGGTCACCAAGTCCTGGGGGTC

CAGGTGGTCCTGACAAACCAGGAAATCCAGGAGGGCCAGCGTCACCTTTTACACCCTGAG

GACCAGGTGGGCCAGTTTTTCCTTCAAATCCAGGTACACCTGGCATTCCAACATTACCCT

TTTCACCAGGAAACCCTTGCATTCCTGGCTGTCCCATAGAGCCCTTGGGTCCTCGGAGGC

CAATGCCAGGTTCACCTTTCTGTCCAGATGTTCCTGGGCTTCCAGGAGAACCGGCTTGCC

CTGGATTGCCAGGATCTCCAGGAAATCCTGGTGCACCTGGTAAACCAGAGTGACCTTTTG

GACCTGGTGGACCTGGGAGTCCGATTCCCGGAAGACCAGGGGAACCTTTTTCACCTGGTG

TTCCTGGCCAACCTTGATCTCCAGGAGAACCTCTTTCACCTTGAAGGCCACGCAATCCTG

TCTCACCACGAGGTCCTGGTGGACCTGGAGGCCCTGGAGTACTTATACCTTTTCCAGGTT

CACCTTTAGGTCCTGGTATTCCTGGTTTTCCTGGAACACCAGGACTTCCTGGACTGCCCT

TTTCTCCTGGCTCTCCAGTTTGGCCAAATCCAGGAACACCTGGGGGTCCTCTTTCACCAA

TGGGTCCCATTATTCCAGGATCTCCATCTAGACCTCGGTCACCTTTAATACCCTCCCTAG

CTGGTTCTCCTTTCAGTCCTGGCAGTCCTGGACGTCCATCTTTTCCAGAAAGTCCATCTA

TTCCTGGCAATCCCCTACTACCAGGAAGCCCTGGTAAACCCTTCTCTCCCTTCAGGTCCG

GGGCTATATAGATATCACCAGGTTCACCCTTTGACCCAGGAGAACCCATCAAACCCTGAG

AGCCATCATTCCCCGGAAATCCAGGCAGTCCAGTAGGGCCAGGCAAACCCTTTTCTCCTT

TACCACCTGGTTGTCCAGGGAAGCCGTGATTTCCTTTTGGCCCAGGAGAACCAGGTAAAC

CTGGAGGTCCGCTAGCAGAGCACTGTATACAAGTGTCTCCTTTGTCACCTTTTTGTCCCA

CCTCCCCTTGCAAGCCAGGTGGTCCAGGAGGGCCTGGTTCTCCCCTATCAAAGTCACACT

CATCACTTAATCGTCCTGGTGGTCCAGGTGGACCAGGAGGTCCATCAGCTCCATTGAAGC

CTCTGAGAGACTCGCCATCAACACCTTTGTCTCCTTTCTGTCCAATCTCTCCAGGTAAGC

CTCTCTCTCCCGGAGGACCCACAATGTTCCTACCTGGGGGACCAGGCTCTCCAGGATACC

CAGGGGGGCCTATTTCTCCCAATGGCCCGGGCGAACCTAGAGGACCCGGAGGGCCAGGTG

CACCATCACCATATCCTGGAGGTCCTTTCTCTCCCTTTTGTCCCTTGAAACCTGGAAAGC

CAGGAATACCCAGTGTACCTTTGTCCCCCTTAGCTCCATTCTCTCCGTCCTTTCCTATTT

TTCCTGGTAGGCCTGGAGGTCCTTGTTCACCTTTGATTCCTGCTTGAAACGGTGAACACA

TGTCACCCTTTTCTCCAGTGTCTCCTTTATCACCTTCTGGTCCGGGTATGTAAGTCAGCG

TATTTTCTCCAAACTCTCCAGCAGTTCCAGGGGGTCCTGGTGGACCTCGAAGCCCCTTTT

CACCCTTATCCCCTTTTGGACCAACAAAAGAAAGCTTATCACCTGGAGGGCCAGGAAGGC

CGGGTTCACCAGGAAATCCCCGAATCCCATCTAGTCCTGGTGGGCCGACTGGTCCTGGTA

GTCCAGGATTTCCTGGTGGCCCTAATAATCCTGGTGATCCTGGAAAACCTCTTTCTCCTT

TCTGAGGAATAAACCCGCCAATAATACCACCTGGGTCACCCTTAATTCCTGGTTCACCTG

GAAAACCAGGTGGTCCAGAATTTCCTGGTATGCCTGGGATTCCATCTCTACCTCGATCCC

CTTAGTAACAAAAATAAAAAATTTCAATGTTTGCCTTTGTTCCGTTGCAACCTGGGATAC

CCGGTAATCCCTGTGGGCCATCTTGACCTGGGATACCCGGTTGCCCAGGAATTCCTGGAA

TACCAGGTAAACCAGAAGGACCACGTATTCCTTTCATTCCCTGAGCCCCTGCATCACCAC

GATCACCCTTCGGTCCCATTGGCCCTTGGGGGCCCTCATGACCTTGCATACCTGGGAATC

CCATAGTGCCTTGTAAGCCAGGTAACCCTCTCTCACCCTTCGACCCTTTTACACCACTGC

AGTCACACTTTCCACATGATGAGCCTGAACATCCCTCAGCTCTACTCGGACGCGCGCACA

ACAAAACGGCCGCCACGAGAATCAGCAAGACGCGCATGGCGCCTATTACAACGCTACAGA

GAGTAAAACACGCACAGCTCCTTGAATAGCTGATTTTCCTTCTTAAATATTTACTTTATT

TCACTGGAGCCTAATCTACAGGTTTTGTCCGCATCTTTGCTCTCGACGTTATGCGGTTCT

GTTCAAACCAACAGCTTCTAGATGAATGTAATACACTCCCCTCTCAGACCTCCCACCACC

TTCTGCACC

>Unigene000093

TCCAAGAGGTAAACAAGTTCATAACTGAATGCAACATAGAAAAATAAAGCTATTTACATA

TTGTATGGTTCTAGCTTACCTGTGATAAAAAGTAAATAACATAAACAATTTTGCTCTGCA

TGCATTTTTTTGTGATTTAGGTCAACAGGACATTTCAGGAGCTGGGAATTCTCAGGGATC

TGGGTGGGATGTGGGAAGAGGCTAGACCGAAGGTGTGGAACTTTATGGAGAACAGTGAAG

AGGTGGACCTTGTGCGGATGTTACTACAGAACAACGTCACAGCCTATTTCTTTAGTGCCC

AACTGAGTGGTACCCAGTGGAGTGTTCAGGATCTTAGCAGTTTCCTGACCAAGCATTCAG

AGGACACTCGTCCACCAGGAACAGTGTTCACATGGAGAAATGTTTTTAATGAGACCGATC

AAGCTATTATGAGCATATCTCGCTTCATGGAGTGTGTAAATCTGGATAAACTTGAAGCTG

TGAGCACTGAGGAGAAGTTAGTTAATGAGTCTCTTAGTCTACTGGACAACAGGAAGTTCT

GGGCTGGACTTGTTTTCCCCGATATTGACATAAACAGCTCCGAGCTCCCTCCCCATGTCA

GCTACAAAATCCGCATGGACATTGACAATGTGGAGCGCACCAACAAGATCAAGGATGCGT

ACTGGGACCCTGGCCCCAGAGCTGACCCATTTGAGGACATGCGGTACGTTTGGGGTGGTT

TCTCTTACCTCCAGGATGTTATAGAACAGAGCATCATCAGAGCAGTAACTGGAACTAAAG

AGAAGACTGGAGTTTACATCCAGCAGATGCCATACCCATGCTATGTTGATGACATTTTCT

TGCGCGTGATGAGTCGCTCAATGCCCCTCTTTATGACTCTAGCATGGATGTACTCTGTGG

CTATTATAATAAAGAGTGTTGTCTACGAGAAGGAGGCCCGCCTCAAAGAGACCATGAGGA

TTATGGGATTGGACAACGGTATACTGTGGTTCAGCTGGTTCATAAGCAGTCTTATTCCAC

TTCTTATGAGTGCTGGACTACTAGTGTTTGCGCTCAAGATGGGGAACCTACTTCCCTACA

GTGATCCAGGTGTAGTCTTTCTCTTCTTGGGCTCTTTCGCTGTGGTCACCATCATGCAGT

GTTTTCTGATCAGCACAGCATTTTCTCGGGCAAACCTGGCTGCGGCGTGTGGAGGAATCA

TCTACTTCACACTCTATCTGCCCTATGTGCTGTGTGTGGCCTGGGAGGAATACGCAGGTC

TTCCTGCTAAAATAATTGCTAGTATACTTTCTCCAGTGGCCTTTGGGTTTGGCTGTGAGT

ACTTTGCGCTATTTGAAGAGCAGGGAGTGGGGATCCAGTGGAGCAACCTCATCTCTAGCC

CAATGGAAGGAGATGACTACAGTCTCACTACCTCTATTATCTTGATGTACTTTGACGCCT

TTCTCTATGGTGTCATGACCTGGTACATTGAGGCTGTGTTTCCTGGTCAGTATGGTATTC

CCAGACCATGGTACTTCCCATTCACCAAGTCTTACTGGTTTGGAGAGGAAGAGCAGAAAC

AAACACCCATTATACAAGGAAAGAAGGCAAATGCTGGAGCTGTGTGCATTGAGGAAGAAC

CTGGTCATCTTGAACTTGGGGTGTATATTAAGAACTTGGTAAAGGTCTATCGTCATGGAA

AAAAGCTTGCTGTGGACGATCTTACACTAGGCTTCTATGAGGGTCAGATTACCTCATTTC

TGGGACACAACGGAGCAGGAAAGACCACCACTATGTCTATTCTGACTGGCCTGTTCCCTC

CCACATCTGGCACCGCTTACATAATGGGCAAAGACATTCGTACAGATCTGAGTGCCATCC

GTCAGAACCTGGGCGTGTGTCCACAGCACAATGTCCTGTTCAGCATGTTGACTGTAGAGG

AGCACATCTGGTTCTACGCTCGGCTGAAGGGTCTGTCCGAAGAGAGGGTGAAGTCTGAGA

TCCATCAGATCGTTACAGATTTAGGCCTTCCGCACAAACGCAAATCTCGTACCAGTCAGC

TTTCAGGAGGAATGCAGAGGAAGCTCTCTGTAGCTTTGGCATTTGTTGGAGGTTCAAAGG

TTGTGATCCTGGACGAACCCACAGCTGGTGTTGATCCATACGCTCGCAGGGGTATCTGGG

ATCTGCTGCTAAAATACCGTCAAGGTCGAACCATCATTCTCTCCACTCACCACATGGATG

AGGCTGACATTCTTGGTGACCGCATTGCCATCATCTCTCATGGAAAGCTGTGTTGTGTGG

GTTCTTCTTTGTTCCTGAAGACTCAGCTGGGCACTGGATACTACCTAACGCTGGTCAAGA

GGGATTTTGACCTGTCCCTGACCTCCTGCACAAACTCCACTAACATCGTATCATATTCCA

AGAATAGTCTCAAAAAGGATGACAGCATCTCCGAGAGCAGCTCTGATGGCGGTCTTGGAA

GTGACCAGGAAAGTGAAACTACAACCATCGACATCTCCCTGATCTCCAATGTCATCTTCA

AGCACGTTCCCTCAGCCAGACTGGTGGAGGATCTGGGCCATGAGATCACCTACGTGCTGC

CTTATGAAGCGGCAAAAGACGGCGCTTTTGTGGAACTCTTTCATGAGATTGATGATCGCC

TTATCGACCTCGGAATCTCCAGCTACGGAATTTCAGACACCACACTCGAGGAGATTTTCC

TCAAAGTTGCAGATGAGAATGGAGTTGATGCTGTAACCTCGGATGGCACTGTTCTAGCAA

GAAGACACCGCCGACATGCGTTTGGAGACCATCAGAGCTGTCTAAAGCCCTTCACCGAGG

ATGACAGTTTTGATTTCAACGATTCAGAAGAATCGAGAGAAACTGACTGGTTGGGAGTCT

CTGATGGAAAAGGCTCATACCAGGTTAAAGGTTGGCGTCTGAAGCAGCAACAGTTTGTAG

CGCTACTCTGGAAACGTTTCCTGTACGCTCGACGCTCTCGGAAAGGATTCTTTGCTCAGA

TTGTTCTTCCAGCCGTGTTTGTGTGCATCGCCCTGGTCTTCAGTCTCATCGTGCCGCCAT

TCGGAAAGTACCCGAGTCTAGAGCTGGAGCCCAGCATGTATGAGGAGCAGTACACCTTCA

TCAGTAATGATGTGCCGGAGGATTTGCACACAAACAAGCTGCTAGGATCGCTAACAGACA

GTCTGGCCCACAGGGGGCAGTGTGCGGAGGACAGGACCACCGAAGATGGTCTATGCCCGG

TTCGAGTCAGCGAGTGGACGATTCCCGTGGTTCCCTACAGCGTATCAGACATGTTCCAGC

GGGGAAACTGGAGCATGGAGAACCCATCACCTCTTTGTGAGTGCAGCTGTAAGAAGAAGA

AAAAGATGCTGCCGGATTGTCCCCCAGGAGCAGGAGGTCTTCCTCCACCACAGGTAAACT

AATGCAGAAAAGATTTTCATTCCTCATGTTTGATACCTGTGGGGGGGTGTCGGATTTTAG

AGAACTATTTTGTTGGTTTGTAGATCACAGTCAGCAGCACAGACACACTGCAGGATCTAA

CAAACAGAAACATCTCTGACTACCTGGTTAAGACCTACGCACAGATCATCGGGAAGAGTT

TGAAGAACAAACTCTGGGTGAACGAGTTCAGATATGGTGGATTCTCATTGGGTGGCAGAA

ATTTGCATGGCCTGCCTCCTTCAGATGAGATCTCCAAAGTCATCGCTCACATGCGCGCTC

GATTCAATTTAGAAAGAGGCACAGCAGCAGACAGGTTTCTAAGAGGTTTATCGAGCTTCA

TCCATGGCATGGACACCAAGAACAACGTCAAGATCTGGTTCAATAATAAAGGCTGGCACA

GCGTTGTGTCTTTCCTCAATGTGATGAACAACGGTATGTTGCGGGCCAATCTACCTCCAG

GAAAGGACCCAAGTAAATATGGCATTACAGCCTACAATCACCCTTTAAACCTCACCAAGG

AGCAGATCTCCCAGGTGGCACTGGTGACAACTTCAGTGGATGTACTGGTGTCTATCTGCG

TTATCTTCGCCATGTCATTCGTCCCTGCCAGCTTTGTGGTGTTTCTTATCCAAGAACGTG

TGAGCAAAGCCAAGCATATGCAGTTTATCAGTGGAGTACAGCCCTACCTCTACTGGCTGG

CAAATTTTATCTGGGATATGTGCAATTACATCGTCCCAGCAACCCTGGTCATAATCATCT

TTATGTGCTTCCAACAAAAAGCCTATGTGTCTTCCACTAATTTGCCAGTACTTGCCCTGT

TGCTGCTTCTGTATGGATGGTCCATCACACCTTTGATGTACCCAGCCTCTTTCTTGTTCA

ATATCCCCAGCACCGCATATGTTGTTCTGACCAGCGTAAACATACTGATTGGTATCAACG

GCAGTGTGTCTACATTTGTTTTGGAGTTATTCGGTGGTGATGAAGTTGGTGGCATTAATG

ACATACTGAAGAACGTGCTCTTGATCTTCCCTCACTTCTGTCTGGGACGAGGCTTGATTG

ACATGGTGAAGAATCAGGCCATGGCTGACGCTCTGGAGAGATTTGGAGAAAACCGTTTCC

GCTCCCCTTTGGAATGGGACATGGTGGGAAAGAACCTTTTTGCCATGGCAGTAGAAGGAG

TGGTCTTCTTCATTATCACTGTCCTGATTCAGTACCGTTTCTTTATCAAACCCAAAGCTG

TTCGCCCTAAGCTGAGTCCGATTGGAGAGGAAGATGAGGATGTGGCCAGGGAGCGACAGA

AAATAATGAGTGGAGCAGGGCATGGAGACATCCTCGAGCTCAGAGAACTCACAAAGGTAT

ATGAAAGGAAGGAGAAGCCTGCTGTGGATAGGCTTTGTGTTGGAATTCCTCCAGGAGAGT

GTTTTGGTCTTCTGGGAGTGAATGGAGCTGGAAAGACAAGTACCTTTAAAATGCTGACCG

GAGACTGTGCAGTCACCAGCGGAGAAGCATTCCTAGCTAGAAAGAGTATACTCAGGGAAA

TAGATGAGGTACATCAGAACATGGGCTATTGCCCTCAGTTTGATGCCATCAACGACCTGC

TGACAGGCAGAGAGCACCTGGAGTTCTATGCCATCCTACGTGGTGTTCCTGAAGGAGAAG

TGTGTGAGGTTGCTGAGTGGGGCATTCGCAAACTGGGCCTTATAAAATATGCTGACAAAG

CAGCTGGAAGCTACAGCGGGGGCAACATGCGCAAACTCTCTACAGCCATGGCCCTCATTG

GAGGCCCACCAGTAGTCTTTCTGGATGAACCCACAACTGGTATGGATCCTAAAGCACGCC

GCGCTTTGTGGAACTGCATCCTCAGCATCATTAAAGAGGGACGATCCGTAGTTCTCACCT

CACACAGTATGGAAGAGTGTGAGGCACTGTGCACTCGCATGGCCATCATGGTCAATGGCA

GGTTCCGTTGCCTGGGCAGCGTTCAGCATCTAAAGAACAGGTAGGACCTCAGCAGACTTC

AGAAGGGACAGTAAAGTCACAGTTTGACTAACGGTCTTTGTGTAGAACTCTTAGAGTGTT

TGATGAGTTTTCTCCCCTCAGGTTTGGTGATGGCTACACCATCATCCTGCGCGTGGCAGG

TTCGGCGCCCCAGCTGGAGCCAGTGATGGAGCTGATTGAGCGAGAGTTACCCGGCAGCAC

GCTAAAGGAGAAACACAGGAACATGCTGCAGTTCCAGCTTCCGTCATCGCTCTCATCACT

CGCTCGGATCTTCAGCATCCTGTCCAAGAACAAGGAGCGGCTCCACATCGAAGACTACTC

TGTCTCACAGACCACACTGGACCAAGTTTTTGTAAATTTTGCCAAGGACCAAAGTGATGA

GGACCACATTAAAGACATTTCCATGAACAAGAGCGATGCTGTGGTGGACTTCTCACAGCT

GGACGCATTCCTCACGGATGCTAAAGCACAGGAGACGGTGGTGTGATCCAAGAGAACACA

CACCAATGGACAGAGGTTCTGAGATCCGTTTCTAAAGACTCTACTTTTATTTGTAATATT

TTTGTAGTATTTTTGTTATGAGGTTAGTGGTGTGTTTGCCCAGTAGAGGGCGCTAGACCT

CAGACACGCCACTGAAACCAAACTCAATGCAAATCAATGCAAAGACTATACTGCACGGCG

ATGTGGGCGGGGTCCAGCCAGGTGCCGCTTCTCCGCATCACAGCGTTTCCGTGCGAGTTA

AAACTTGAAGTTTAAGCAGTATTTTTACAGATGTTCTTTCTGAGGTTCTTCATGGTTCCA

CTTAGACACTGCTAGTGAGACGTGAACTTCATCTCCTCCAACACAGAGACACTTTTGGGT

GTCTGGATCTCAAAGTGACGTCTCGATTTTGTTATCCTGCTGTAACAGGAAGTTAAGACA

GGAGCTGGTCAGTTGGTGTGTGTCGGTGTTGCATGCTTTGTATCCTGAGAGCTGCAGTCT

GTGAGATCTGGTGTTTCATCACTGTCAAGATTTAAAGATAAAAACAGGACAGCAAAAGGT

TTAAGGTAAATATTGTATAAAGATCATGAATCATTTTGTGCCAAACAAAGCATTGGGTTC

CGTTAGTCAGATCTTTTGTCCGGTGTCTTCTCTTGCATGTGCCGTGGACGGTGATGTTCC

TGCAGTGATCTTTACTGTGTGTATGTTCACTTTAACACCATGTTTTTAATCCAGATGTTT

TTGTATCTGGCTGTGGGTCGTGTTTCTGCGGTCTGTACAACAGCCCCGTGCAGAATTTCA

ACTAGAGGATAAAACTCAGGATTTTATTCTGTCAGCATTTCAGAACCTCTTTCGATTTTT

ACTCTTCCTGAACCAGAGATCCAGAAAACATGGTGGATTTTCAGGCCCTTCATTTACAGT

GTGAAGTTTAAGCTGTGACCAAAAGTGCCACTTACACACACACACACACTCGAGTAAAAT

TCCCGCCGA

>Unigene000094

TTGTAGGTGTTCAATACAGAAGGGAGAAAAATACAAAGTGAAATAATCTGAAACAAAAGA

AGCCTTTTCCTGAAAACCTCATCCATCTCTGTATTCTTCCATGAATCACGATAGTGAACA

AGTGTTTTTATTACTGCAACTAAACATTCACGAGGCAGACAACACCAGAAAAAGTAACAC

CCTGAACCACAGTAACTTGTAAGAGGGTTTAGTGGCATTACATGAAAGAATACAGAAACC

AGAACAAGCATTTATTGGCGCTCGAGTAAATCTTTCTAAGACACTATAGGAACCTTCCTG

CAATTGATGAGGATTTACAACCTTAAATTGAGTTAAGATTAGATTAGAATATGTGAGGTT

AAATAAGTTCTGGATGATACTGTGGCATAAATTTAACTGAATACATTCTCTGTGGAATTT

ATAGGGAAGCCTGAAAATATATGTGTAAAGTCAAGTTTATTCACTATAAAAGCAGGTATG

TGCATTCTCTTTGGTCTAGCATTTCATTACCATACAAAAGAAATTTGAGACATATGAAGA

CTAGCAAGAAATAACCATCAAGTGTCTTGAAAAGAAGAGGCAAACAAAATGTTTGAGTCA

CAGACAGATAGCACATTAGTGATGCAGATAGAAAAATATAGAAAAATATCAACTGTGATC

AGTTTAATTGACATGACCTGCATTGTATATCTCTTAAGGCTGCCATTGGTAAGTGCACTT

AGTCACACATACACGTATACATGTTCAGCTTTAGATTAGTGGAAAATACAGAAGTGTATA

AGTCATTCGGTGAAGGGTGTATAAAAATTTGATCTGAAATTGCTTAATTAGTCAGACAAA

ATGGGGAATTTTACTCTCAATATAAAGTGAATGCACACACATATTGCACAGTCTTTGGGG

AAAATGCAGATATTGCTTGTTTGATGTAAACATATTTTCCTCACTGTGCTACCTGGCAAT

AAAAAAATTAATAATCACACGTTTTAAAGATATACATCGATATATATGCCTACTAAAGCA

TATAGTGCTAAATACTACAATTCACCATTTTGTGCCTTAAACGAATTGCAGATTATATCA

TGACTTGGTCAACAGAGTGGAATCGGCCATATACAAAATGTGCCCTTGGTCTCATTATGC

TTTTATAGCCTTCATAAATAACTCAAACAAAATTAAATGTATTTACCTGATAAGATTATG

TTAAGAGATGTTAATTATTAAACAAAGCTATATATTTACAACAGAGCACAAAGTACAGCC

TGGCTGCTGGGCTACATGAAGGACAGACATAACTTATGGAAACCTGAATTTCTAAAGTTA

GTATATGATAATAACAAACTGAGTGGGGGAAATTGAGAACGATAAACAGGCTACTTATTT

TTGGTGTTGTTAGCAAAACAGTACTTAGCATTTAAACTGAAAGAATAGCTGTATTCATTA

AAACATGATTCAGAAGAGATTTTTTAAAACGGCAGCCTGTTTACTAATGAATGTATTCAG

TGCAATTGCAAACAGGTGTCATTTATTCAGGATAGATAATGAGAATATTAATGAAGTGAT

CGATATGAAGCAGAAGCACCAGATTCTGGGGTCCCATCACTCTGGAACTTCTTATGGTTG

GCATTCATAAGTCTAAGAGAGCATTTTCACAAGTCCATTTTAACTCTCTCTCTTTCTCTC

TCTGTGAAGTAGCACCATACTGTTTCTTTTGTAAAACTGGAACATAAAAGCCAAATGCAG

AAGAAGGTGTGTGACCAGGCTAAGTCCTCTTCATGCACACTTGACATCTACTGATGTGAG

TTCGCAGATTCCCAGCCTTAAGCGTGGTGGGGACAGGTTTGCTAAACATCTCATTATCTT

CGATGGTAGCCAACCAGAAGCTGTAGGAGTTAGCATAGTAGTTGCATGTGCCACGGCCAT

GACATTCAATAAATGGAGCAGAGCGGAACTCCTCCAGACAGGAGCCAGGAGAGGCCAGGG

CCTGACCGGAGCCCTCTGCACCTGCACTAGTATGCATGACAAAGGAATAACCAATCCACA

GAGAGATCCAGCCTTGTGGGCAGGATGGGATGGCTATAGTCTGACTGTGCACAGCAATGA

CCATAGCAGGAGCCTCACACACTGCACATCTGCTGATAAAAGGTTTGATGCTCTCGCCAG

TAATTGGAGCCATGCTCATCGGCATGGGCTCAGGAGATGTGAGCCAATAGGAATAGTCAT

TACGAGAGGCAAAGTTACACACGTTGTTGATGTTACAGAAGAGGAATGGCATAGGACTGA

ACTTTCTCAAACAGCTACCAGCCGTACCCAGGTCTTGGCCATGGGATCGCTCATTTCCCT

GCACATACAGGAGGGAGTACCCATCAAAGATCATAGTGGTGCCCTGTGGACATAGAGGCA

CCTCAACTGACTGACTGTGCCGAGTTACTAGGAATCCATGGTCCATAGAGGAAGGTCCAG

GAGGGCCAACCTGACCTGGAACACCATCAGGACCTGGGGGACCTGGATATCCCCTTGATC

CAGGGGGTCCAGTAGGGCCAGGTTCTCCTTTATTTCCAGGAAGCCCATTAAAGCCTGGTG

CCCCATCTTCTCCTTTAATACCCTGTATTCCAATAAGTCCTTGCTGTCCTTTTTGCCCAA

AATACCCTTGAGGTCCAGGTAAACCTTGAGGACCCTGTGGTCCTGGAAAGCCAATGTCTC

CTTTTATAAAGGTATGGGGTCCAGGAGGTCCAGGAAGGCCATCGTTACCTTTAGCCCCTG

GAAATCCTCTGTCACCAATTTCTCCTTTAAATCCAGGTAATCCACCCTCTCCTTTTATTC

CTTGGAAGCCAGGAGGGCCTGGAGGTCCCATTTCACCCTTTAGTCCTTTATAGCCTGGGG

TGCCAGGTTCACCAGGTAGGCCAGTAAAGCCTTTATCGCCTTTTAAACCCTGGGTACCCT

GTATGCCTGGGAAGCCTTTGTCTCCTTTCTCCCCTTTGAGACCAGCACCACCTGGAACAC

CTGCTGGACCTGGAGCACCAGGCTGCCCTGGCTCCCCAGGCTCTCCCTGAACTCCTTTAT

CCCCTTTAGGTCCCTCCATTGATGGCCCCGGTAGTCCTCTTTCACCAGTTCGACCCTGAA

CACCTTGAAATCCAGGCAGGCCTTTTTCTCCTTTACTGCCAGGAACTCCAGGGCTTCCAG

ACATGCCAGGGGGACCTGGGTTGCCTTTTTCACCTTTTTCACCAGCATTCCCAGGAGTTC

CTGGAAAACCTCTACCAGGTAAACCTGGTTCACCCCTTTCTCCAGAGACTCCAGGGAACC

CATCTTTTCCTGCCTCTCCTTTCTTCCCTTGTGGCCCTGGAGAACCCTGTAGACCAGGTT

CACCTGGCCGTCCTTTAAGACCAGCCTCTCCTGTTAATCCAGGTAAACCGGGAGTTCCTT

TTTCACCTGGTACTCCAGGGCTTCCTGGGTAACCAATCTGTCCTATGTCTCCTTTTTGCC

CTGGAGTTCCAGGTATGCCTGGGATTCCCATGGAACCTTTTACACCCTTTTGCCCAGACT

CCCCAGGAAATCCAGGTGGACCCATTTCACCCTTTTCTCCAGGGAGGCCAGGAATTCCTA

TTCCAGGCAGACCTGCTAATCCTTTTTCACCTTTTCCTCCTGGATCACCCTTAAACCCAT

GATGGCCCGGCTGACCAGCAACACCTTTGCTGCCTTTTGGCCCAAAAGGTCCTGGCAATC

CCATTTCCCCAATGCTGCCCTTTACTCCGGGTGCTCCAATTTGTCCTGGTTGTCCAGGAA

GTCCTGAATCACCCTTCTCACCTGGCTTTCCTGGTGAGCCAGGCATTCCATCCTTTCCAG

GCAATCCAGGATCTCCAGATAATCCAATAATCCCCTTTTGTCCAGTAGGGCCTATTTCCC

CTTTATTTCCGATGTCTCCCTTCTCGCCTTTCATATGTTCCATTTCCACTTCAGTCATAT

TACCAGGTGGTCCTTGAAGACCAGGATCACCTCTCTCTCCTTTAGGACCCTGTTCCCCAG

TCACTCCCCTAGGTCCAATTAGACCAGGGTCACCCCTTGGTCCAATAGCTCCAGGACCTC

CTGTTTGACCCGGGTGCCCAGGTGAACCTGGAATTCCCATTATTCCCATATCTCCTTTGA

TACCTCTTTCCCCAGGGAATCCATCTAGTCCTGGATTTCCTGGTTCCCCTGGTAAGCCCT

TTGGGCCTGGTGTTCCAGGAAAACCAGGAGTGCCTTTCTCACCTTGAGGACCAGGCATTG

CAGGACCCGGAAAACCTTGGGGACCTTTCTCCCCCTTTTCACCTATACGACCTGCGGGAC

CAGATTCTCCAGGAGGACCTATAAGTCCAGGAGGTCCAGGGTCACCAAGTCCTGGGGGTC

CAGGTGGTCCTGACAAACCAGGAAATCCAGGAGGGCCAGCGTCACCTTTTACACCCTGAG

GACCAGGTGGGCCAGTTTTTCCTTCAAATCCAGGTACACCTGGCATTCCAACATTACCCT

TTTCACCAGGAAACCCTTGCATTCCTGGCTGTCCCATAGAGCCCTTGGGTCCTCGGAGGC

CAATGCCAGGTTCACCTTTCTGTCCAGATGTTCCTGGGCTTCCAGGAGAACCGGCTTGCC

CTGGATTGCCAGGATCTCCAGGAAATCCTGGTGCACCTGGTAAACCAGAGTGACCTTTTG

GACCTGGTGGACCTGGGAGTCCGATTCCCGGAAGACCAGGGGAACCTTTTTCACCTGGTG

TTCCTGGCCAACCTTGATCTCCAGGAGAACCTCTTTCACCTTGAAGGCCACGCAATCCTG

TCTCACCACGAGGTCCTGGTGGACCTGGAGGCCCTGGAGTACTTATACCTTTTCCAGGTT

CACCTTTAGGTCCTGGTATTCCTGGTTTTCCTGGAACACCAGGACTTCCTGGACTGCCCT

TTTCTCCTGGCTCTCCAGTTTGGCCAAATCCAGGAACACCTGGGGGTCCTCTTTCACCAA

TGGGTCCCATTATTCCAGGATCTCCATCTAGACCTCGGTCACCTTTAATACCCTCCCTAG

CTGGTTCTCCTTTCAGTCCTGGCAGTCCTGGACGTCCATCTTTTCCAGAAAGTCCATCTA

TTCCTGGCAATCCCCTACTACCAGGAAGCCCTGGTAAACCCTTCTCTCCCTTCAGGTCCG

GGGCTATATAGATATCACCAGGTTCACCCTTTGACCCAGGAGAACCCATCAAACCCTGAG

AGCCATCATTCCCCGGAAATCCAGGCAGTCCAGTAGGGCCAGGCAAACCCTTTTCTCCTT

TACCACCTGGTTGTCCAGGGAAGCCGTGATTTCCTTTTGGCCCAGGAGAACCAGGTAAAC

CTGGAGGTCCGCTAGCAGAGCACTGTATACAAGTGTCTCCTTTGTCACCTTTTTGTCCCA

CCTCCCCTTGCAAGCCAGGTGGTCCAGGAGGGCCTGGTTCTCCCCTATCAAAGTCACACT

CATCACTTAATCGTCCTGGTGGTCCAGGTGGACCAGGAGGTCCATCAGCTCCATTGAAGC

CTCTGAGAGACTCGCCATCAACACCTTTGTCTCCTTTCTGTCCAATCTCTCCAGGTAAGC

CTCTCTCTCCCGGAGGACCCACAATGTTCCTACCTGGGGGACCAGGCTCTCCAGGATACC

CAGGGGGGCCTATTTCTCCCAATGGCCCGGGCGAACCTAGAGGACCCGGAGGGCCAGGTG

CACCATCACCATATCCTGGAGGTCCTTTCTCTCCCTTTTGTCCCTTGAAACCTGGAAAGC

CAGGAATACCCAGTGTACCTTTGTCCCCCTTAGCTCCATTCTCTCCGTCCTTTCCTATTT

TTCCTGGTAGGCCTGGAGGTCCTTGTTCACCTTTGATTCCTGCTTGAAACGGTGAACACA

TGTCACCCTTTTCTCCAGTGTCTCCTTTATCACCTTCTGGTCCGGGTATGTAAGTCAGCG

TATTTTCTCCAAACTCTCCAGCAGTTCCAGGGGGTCCTGGTGGACCTCGAAGCCCCTTTT

CACCCTTATCCCCTTTTGGACCAACAAAAGAAAGCTTATCACCTGGAGGGCCAGGAAGGC

CGGGTTCACCAGGAAATCCCCGAATCCCATCTAGTCCTGGTGGGCCGACTGGTCCTGGTA

GTCCAGGATTTCCTGGTGGCCCTAATAATCCTGGTGATCCTGGAAAACCTCTTTCTCCTT

TCTGAGGAATAAACCCGCCAATAATACCACCTGGGTCACCCTTAATTCCTGGTTCACCTG

GAAAACCAGGTGGTCCAGAATTTCCTGGTATGCCTGGGATTCCATCTCTACCTCGATCCC

CCTTTGTTCCGTTGCAACCTGGGATACCCGGTAATCCCTGTGGGCCATCTTGACCTGGGA

TACCCGGTTGCCCAGGAATTCCTGGAATACCAGGTAAACCAGAAGGACCACGTATTCCTT

TCATTCCCTGAGCCCCTGCATCACCACGATCACCCTTCGGTCCCATTGGCCCTTGGGGGC

CCTCATGACCTTGCATACCTGGGAATCCCATAGTGCCTTGTAAGCCAGGTAACCCTCTCT

CACCCTTCGACCCTTTTACACCACTGCAGTCACACTTTCCACATGATGAGCCTGAACATC

CCTCAGCTCTACTCGGACGCGCGCACAACAAAACGGCCGCCACGAGAATCAGCAAGACGC

GCATGGCGCCTATTACAACGCTACAGAGAGTAAAACACGCACAGCTCCTTGAATAGCTGA

TTTTCCTTCTTAAATATTTACTTTATTTCACTGGAGCCTAATCTACAGGTTTTGTCCGCA

TCTTTGCTCTCGACGTTATGCGGTTCTGTTCAAACCAACAGCTTCTAGATGAATGTAATA

CACTCCCCTCTCAGACCTCCCACCACCTTCTGCACC

>Unigene000095

GGTATGTTGCAAGGTAACTATGCAGAGTGGGGGGAGGGGTAAGTCTGTGTGTATATGTGT

GTAGGGGGTTATTATGGGATGTCTGCCCCCTGATCTCTTTGTGTGTATGCAAGGGGTGTT

AGTAGGAGAGGGAGAGACGGCGACGTCGTACGGACTATAGACCGCAGAAATCCTATAGAT

TTATATCAACACAAAAGGAGCCACACTGTTGTTGGAAGAGAAGACAAGAGAACGTGGAAA

CTGTACTTGGAGACCGTCTCACGTTTTCTTCTTTATTTCGACCAGCGGCTGTAACGTTAC

TCAAGTTGTCCACAAGACCGAGTACGAAGAGTCAAGTAGCCGTGTATTTAACCAAACCAG

ACGGTGAAAATACACAAAAACTATGTCTGGGAGTGAAGACGACAGAGATGATTTTGGACC

GACAGAAGACCTGTCCATGATGCAAGACGATGACCTGGAGGAGGACTTGTCAGAGAATGA

AGCCCCTAAAGTAAAAAAGAAGAAAAAAGCAAAGAAGAGCAGCCGTGAGAACAAAAGCAG

CAAGAGGCAGCGCTCTCGTCGGGAGGACATCCCAATTAGTTCCCCTGAGCCTGTAGAGGG

GCGTGATGTGGATGAGGTAGAGGATGATGGGCCTGGAGGCCGTTCTGACAGTGAGGGCAG

TGATTACACACCTGGCCGGAAGAAGAAAAAGAGAGCCAGCACCTCCAGAGACAAAAAAAG

GAGCAGTACTGGTGCTGACAGAAGCTCCAGCTTGGCCGTCTCTAAAAGAAAGGAGCCTGA

GGAGGAAGATGATGAAGACGATGACGATAGCTCTGAGCCGAAGACCTCCTCCCAGCTGCT

GGATACCTGGGGCATGGAGGACATTGATCATGTCTTTACTGAAGAGGACTACAGATCTCT

TACCAACTACAAGGCCTTCAGTCAGTTTGTCAGGCCTCTTATTGCAGCAAAGAACCCAAA

GATTGCGGTTTCCAAAATGATGATGGTACTTGGGGCAAAATGGCGTGAATTCAGCACTAA

CAACCCCTTGCGTGGTTCTGCCAGTGCCAATGCTGCCCTGGCAGCTGCTAATGTGGCTGC

GGCTGTGGAGAGTATGGTTGCAGCTGAGACTCCAGCCACTGCTCCTACACCCGCTCCCCT

TGCTGAGCCCCAGCCCCCACCGGCTCCCCCACTCCGAAAGGCTAAAACTAAAGAGGGCAA

AGGTCCTAATGCTCGTAGAAAGTCCAAACCTACCCCAAAACCTCAAGAGAAGAAGGTCAA

GACTAAGAAAGTTGCACCTTTAAAAATTAAGCTGGGTGGCTTTAACAGCAAGAGAAAGCG

CTCGTCTAGTGAAGAGGAAGATATGGATGTAGACAGCGACTTTGATGATGGCAGTATAAA

CAGCGTCTCTGTGTCGGATAGCTCCAATAGCCGCAGCAGCCGCTCAAAAAAGAAGCCTAA

GCCCAAGAAGAAGAAGAAAGTGGATGAAGATGCTGATGGCTATGAGACTGACCACCAAGA

CTATTGTGAGGTGTGTCAGCAGGGAGGAGAGATTATCTTGTGTGATACCTGTCCCAGAGC

TTATCACATGGTGTGTCTGGATCCTGATATGGAAAAAGCCCCTGAAGGCACTTGGAGCTG

TCCACATTGTGAGAAGGAGGGTATTCAGTGGGAAGCACGTGAGGAGGCTTCAGATGGGGA

AGAGGAGAATGATGCAGGTGGCGGTGAGGCAGAGGAAGATGACCACCACATGGAGTTCTG

TAGGGTGTGTAAGGATGGAGGAGAGCTGCTGTGCTGTGATACCTGTCCATCATCCTATCA

CATGCACTGTCTGAACCCGCCTCTACCAGACATCCCCAACGGACAGTGGATTTGTCCCCG

CTGCAGGTGTCCTCCTCTAAAGGGAAAGGTCCAGAAAGTTCTCACATGGCGCTGGGGTGA

AGCTCCGCCTCCCATCCCAGTGCCCCGCCCACCCGATCTTCCAGTCAACGCCCCTGATCC

GCCTCCTCTGGTTGGTCGAAAGGAGCGGGAGTTTTTTGTAAAGTGGTGCAACATGTCATA

CTGGCACTGTTCCTGGGTACAGGAGCTACAGTTGGAGCTGAATTGTCAGGTGATGTTCCG

GAATTTTCAGCGTAAGACGGATATGGAGGAGCCCCCGAGTGTGGATGCGTCTGAGTGTGA

TGATAATAAGAGCATGAGGAGGAAGAGTAAAGACCCGCTGTACGCTCGCATGGACCACCA

GTTCGGACGCTACGGAGTTAAAATGGAGTGGCTGCTCATCCAGCGCATCCTAAATCACAG

GTACACACTAATCACTAAACACAGAGAGATCACACACTCTACTAACTGTTTGTCTGTCTG

TCTGTCTCTCAGTGTGGATAAGAAAGGTAATGTTCATTATCTGCTGAAATGGAGAGATCT

GCCGTATGATCAGTCTGCGTGGGAGAGTGAGGACATGGACATCCCTGATTACGACACCTA

TAAACAGCAGTACTGGAACCACAGAGAGATGATGATTGGGGATGAAGGACGACCAGGGAA

GAAGTTGAAGAAGGTGAAACTGCGAAAGATAGAAAAGCCTCCTGTAAACCCTGTGGTGGA

TCCCACGGTGAAGTTTGACAGACAGCCGGAGTATTTGGACATGACGGGGGGGACGTTACA

CCCCTATCAGTTGGAGGGATTGAACTGGCTGCGGTTCTCCTGGTCTCAGGGTACAGACAC

TATCCTGGCTGATGAAATGGGTCTCGGCAAAACTGTGCAGACCGCCGTCTTCCTCTACTC

TCTCTTCAAAGAGGGTCACTCTAAAGGCCCGTTCCTGGTGAGCGCTCCTCTCTCCACTAT

CATTAACTGGGAGCGGGAGTTTGAGATGTGGGCTCCGGACATGTACGTGGTGACGTATGT

GGGCGATAAGGACAGCCGAGCTGTAATCCGCGAGAACGAGTTCAGCTTCGAGAATCACGC

CATCCGCGGAGGAAAGAAAGCCTTCAAGATGAAGGAGGCGGCGGTGAAGTTCCATGTGTT

GTTGACGTCGTATGAGTTGATCACTATTGATCAGGCGGTGTTGGGATCTATAGACTGGGC

GTGTCTGGTGGTGGATGAAGCTCACCGGCTGAAAAACAACCAGTCCAAGTTTTTCCGGGT

GTTGAATAATTATCCGCTGCAGCATAAACTCTTGTTAACGGGAACTCCACTGCAGAACAA

CCTGGAGGAACTGTTTCACCTGCTCAACTTCCTCACTCCAGAGCGGTTCAATAATCTGGA

AGGATTTCTGGAGGAGTTTGCAGACATAGCTAAGGAGGATCAGATTAAAAAGCTGCACGA

CATGTTGGGACCTCACATGCTGCGGCGTCTCAAAGCAGACGTCTTCAAACACATGCCCTC

AAAAACCGAGCTTATTGTCCGGGTGGAGCTCAGCATTATGCAGAAGAAATACTATAAGTA

CATCCTCACACGTAACTTTGAGGCTCTAAACACACGTGGCGGTGGGAATCAGGTGTCTCT

CCTGAACGTGGTGATGGATCTGAAGAAGTGCTGTAACCACCCCTATCTCTTCCCCACAGC

TGCCATTGAAGCCCCCAAAATGCCTAATGGCATGTATGAAGGTGGAGCTCTCACAAAGTC

TTCTGGGAAATTGATGCTGCTGTGTAAGATGTTAAAGAAGCTAAAGGAGGGGGGGCACAG

GGTGCTCATCTTCTCACAGGTACACACACTCTTACTCACACACTTTCTTGCAACATATAA

TAGAATTATTTTATTATACACAAAGTGTGTTTAATGCAGATGACGAAAATGTTGGACCTG

CTAGAGGATTTCCTGGAGAACGAGGGCTATAAATACGAACGGATTGATGGAGGAGTGACG

GGAGTAATGAGACAGGAAGCTATTGACAGGTTCAATGCTCCCGGAGCTTCTCAGTTTGTC

TTCCTGCTATCGACACGAGCGGGGGGGTTAGGCATCAACCTGGCAACCGCAGACACTGTC

ATCATCTATGACTCAGACTGGAATCCACACAATGACATTCAGGCATTCAGCAGAGCTCAC

CGTATTGGTCAGAACAGGAAGGTGATGATCTACCGGTTCGTCACTAAGGCCTCTGTGGAG

GAGAGAATCACTCAGGTGGCTAAGAAAAAGATGATGCTGACTCATTTAGTTGTGCGACCC

GGTTTGGGTTCGAAGGCGGGCTCCATGTCCAAACAGGAACTGGACGACATTTTGAAATTT

GGCACAGAGGAGCTGTTTAAAGACGACGCCGGAGAAGGTGATAATAAGGAGGAGGACAGC

AGCGTGATTCACTACGACGATAAAGCCATTGATAAACTGTTGGACCGGAACCAGGACGCC

ACCGATGACACCGAGCTGCAGAGCATGAACGAATACCTGAGCTCCTTTAAAGTGGCCCAG

TACGTGGTCAAAGACGAGGACGAGGAGGAGGTGGACAGGGAGATCATTAAGCAGGAAGAG

AATGTGGACCCTGATTACTGGGAGAAGTTATTGCGTCATCATTATGAGCAGCAGCAGGAA

GTGTTGGCCAGCCACCTGGGGAAGGGCAAGAGACCGCGCAAACCCGTCAACTACAACGAC

TGCTCGCAGGAGGAACAAGCAGATTGGCATGACGACCAGTCTGACAACCAATCAGATTAC

TCCGTTGCCTCAGAGGAGGGAGATGAGGACTTTGACGAGCGCTCTGAAGCGAACAATCGC

AGGTCAAGTCGTAGAGGTCTGAGGAACGACAAAGAGAAGCCACTGCCCCCCCTGCTGGCC

AGAGTGGGCGGGAACATTGAGGTGTTGGGTTTTAACTCCCGTCAGAGAAAGGCCTTCTTG

AATGCGGTGATGCGTTATGGGATGCCTCCACAGGATGCGTTCACCACACAGTGGCTCGTC

CGAGACCTTAGGGGCAAATCTGAGAAAGAGTTTAAGGCATATGTCTCTCTGTTCATGCGT

CACCTGTGTGAACCGTGTGCTGACGGCGCTGAAACCTTTGCTGATGGAGTTCCTCGTGAG

GGGTTGTCACGGCAACACGTCCTGACACGCATTGGTGTGATGTCACTCATACGGAAGAAG

GTCCAGGAGTTTGAGCATGTGAACGGTCAGTGGTCCATGCCATGGATGAAGGAACTGGAG

GAGAATAAAAGAGCAGTTGCTGTTGCTATGGGAGACGACCCCAAAACCCCCTCTACTGGA

ACCCCTGGAGACACACAACCCAACACACCTGCACCAGAGGACCTGTCCAGAGGGGAGGAG

TCAGAACGGGAGACGGACGACAAGATGGAGGGAGAGGGAGAGAAAGACAGAAAGAGCACT

CAGCTACAAGATGACGAGGTGATTGAGATTCCAGACGATGGGGAGACCCCCCTGAGTCCA

GAGAAGACAGGACCAGGACAGGAGATGGAAAAAGACAGCACAGCGCCTTCTTTGGAGAAG

GATGGCGAGATAAAGGACATAGCGGGAGAAAAAGACAAAGAAAAGGGAGGGGCAAAAGAG

GAAAGCACTACATGTTCTTTAAAAATTGAAGAGGTGAAAGGGGAAAGGTCAGAGGTCACC

AGTGCTGGGGACTCCTCTCAGCCTAAAGAAGAGTGCAGTGAGGAGAAGAGTGAAAAGACA

GACAGCAACCTAACAGGAGAGGAGAAGAAAGAAGTGAAGGAGGAGAAGGATGGCGTGAGA

GCGGAGGAGTCTCGGATAAATGGAGAGGACGGAGGAGTGGACAATGAGGAGAAGAAAAAA

CATGTCAAACAGAGATTCATGTTCAACATCGCTGACGGAGGATTCACAGAGCTCCACTCT

CTGTGGCAGAACGAGGAGAGAGCCGCCACAGTCACTAAGAAGACATATGACATTTGGCAC

CGTCGTCATGACTACTGGCTCCTGGCGGGAATCATACAACACGGCTACGCCCGCTGGCAG

GATGTTCAGAACGACCCGCGTTTCTCCATCCTTAATCAGCCGTTTAAAGGAGAGATGAGC

CGAGGAAACTTCCTGGAGATCAAAAACAAATTCCTCGCCCGCAGGTTTAAGCTGTTGGAG

CAGGCGTTGGTGATTGAGGAGCAGCTGCGTCGAGCGGCATACCTTAACATGACACAAGAC

CCCACTCATCCGGCCATGGCCCTGAATGCACGCTTCGCCGAGGTGGAGTGTCTGGCAGAA

TCACACCAACACCTCAGCAAAGAATCTCTTGCGGGAAACAAACCGGCCAACGCTGTCCTA

CACAAGGTTCTCAACCAACTGGAGGAACTGCTCAGTGATATGAAAGCTGATGTAACCCGG

CTTCCTGCCACACTGTCCAGAGTCCCGCCAATCGCAGCGCGCCTGCAGATGTCTGAGAGG

AGCATCCTTAGCAGACTGGCCAACAAGGGCAATGAGACCCACACACCACCGCCTATCCCA

CCTGGGCCGTATGCAACTCCACAAAACTACGGTCCCCCGTTTACTCCAGCACCACCAGCC

ATGGCTCTGGCTGCAGCCAACTACAGCCAAATGCCGCCAGGCTCGTTTATATCAGTGCTG

AATGGGCCTCCAATGTCAGTCAAAAAAGAGCGAGAGGCAGAGCTTATGATGGGAAGGAGG

GAGCAACGCAGCGGAGAAGTGATCTGCATCGACGATTGAGGCCAGCCATAATCCCCAGAC

ACACACACAGACATACACACTGTTTTTTCGTTCTCTCAGCAGAACTTAAGTAAAGAAAGT

ATGTTATGGCCAGGATATAATTAGCAAAATGTACAACAAATTGGGGGGGTTGAGGAGGTG

CACCAGATCACACATCATATGACAAAAAAACTATGTAACAGGCAATATAATCCAAACATA

ACACTCTCTCTCTTACAACACACTAATTCTCACTTTTACACAGAGACAAAAATCATTACA

CACACACTCAAAACACACATACACAC

>Unigene000096

GACACACTGTTACGTCATTTCCGCTACATTTCAGTAGGCCGAGCCGCAGCTACAGGCAAC

AGTATAAGAAACGGGAAAGAAAAAAAAATTCCAGAATAATCAAATTTGTCCCGAAGAATC

TTTCTCCATCCTTTACTAATTAAACAGCATTTGGAAATATTACTAAATATCTGATATAAA

CTGAACCCGACACGTTGATTCGAAACAGACGGTACAGAGGAAGAGGCGCAAAGAAAAATT

TCCATACTCGATGACATTTCATCGCAATGTCAGATCGTTTGGGGCAAATAACCAAGTCCA

AGGATGGGAAAAGCAAGTATTCCTCACTCAGCCTATTTGACAAGTACAAAGGAAAGTCAA

TAGAAACTCAGAAACCCACAGTTGTTGCTCGACATGGCTTACAGAGTCTTGGTAAAGTGG

CCGCTGCCCGGCGCATGCCCCCGCCCGCTCACCTGCCGAGCCTGAAGTCCGAGAACAGAG

GAAACGATCCAAACGTGATTATTGTGCCCAAAGACGGAACAGGATGGGCGAACAAGCAGG

AACAACCCGATCAAAAGATTTCCGTTGCGTCGACAGCACAGCCGCCGGAGTCGCAGCCGC

CGCTGCCTTTGCAGAAATCCGTCTCCAATCTTCAGAAGCCTTCAGCAGCAGCCAACCAGG

AGAGCACAAGCAACGGTGGACCAAAGCAATGGGCACAACTAAACGGAAAGGCACCAGATC

AAGATGGTTTAAGGGCCTCAAACCGACTGCAGCCCTTCTCTCACGAGGAATTTCCGACGC

TGAAAGCAGCAGGAGAACAGGACAAGGGTGGCAAGGAAAAAAGCGCCTTCGATCCGTCGT

ATGGGCCAGGACCAAGCCTCCGCCCTCAGAATGTGACAAGTTGGAGGGAGGGTGGAGGGA

GGTACCTACAGCCTCCATCTCTGTCCTCAGCCCTCTCTTCTGATTCAGAGGGCAAAACCA

GTGCCTCTCCTGAGGCGACCACCCCTCTTCCTCCTCCATCCTCATCTTCCACCTCCGCTG

CTTCCTCTTCCGCCAATGCCGGCACCACAGCGTCTCCTGACCCGAAGGATCCTTCTCTCC

GACCGGCTCAACCCACCCGCCGCCTGACGCCTGGGCTGCAGTATCAGCTCCATCACTCCT

CTAACACCACCTACCACGACATGCTGCCCGCCTTTATGTGCCCCAAGGAGACTCGTGATG

GTCCAGCTTCCTCCGAGCCTGGCTCTGCTGCAGCTGTTGCCCCAATGCGCTTCGATGCCC

GACCCACATTCAGACCCACTTACGAAACCATTAATGGAGACCTGCGGAGAGAGAACCGTT

TCACACGAGTCCCACCCCGTCCCTCGCCTCGCCCAATCCGCCGTCCCGGTGATAGACCTT

CTCGTCCTGCTATCATCAACCCTGACGATCTGAAAGACCTGGACGACCTGGACAACGACT

GCGAAGATGGATGGGCAGGTCTTCATGAGGAGGTGGATTACAGCGAGAAGCTAAAATTTA

GCGACGATGAAGAGGATCACTCTCCCAGCGATAAGAGCAAGATCTGCCTTGTCGTATATA

GGGGAGAGTGGGAATCTCGCCGTGAACGCCAGTCATCCATGAGCTCAGACGGCCAGGAAG

ACTCTTACCATCACCAGCACGACCCTCTGCCTTCCAGAAAAACCAACAGCAGATACGGCT

CTACAGACCTGCATCAGAAGAGCAATGGCACAAGTGAGTCTGTGGAAGAACTGGACGAAC

CTCAGCGGTCCGCTGCCGCTCCCCCGCGGGGAAAATTCGTCCCACCAGACGTGTCCGAGG

CAGTGGAAAGGGCACGGCGTAGGCGCGAGGAGGAGGAGAGGCGAGCCCGGGAGGAAAGGT

TGGCCGCCTGCGCTGAAAAACTCAAGAAATTGGACGAGAAGTTTGGCAAAATCGAGAAGC

CGGCGCTTACCGGAGATGGACATGGAGCGGAAAATAAGGAGCTTTTACTTTCACCCGGAC

GAGAGGCGGGCAAAACTCAGCCAGAAACCTGGCAGCATGTTAAAGATGTTTCCGAGCAGA

AATCTGACTCACTTCACAGCCAGGAATATAGAGATGATTTCAGCTACAGGAACGAGGATG

ACTGTTCAGAATACACACAGCGACACCAGAAAGCCGTCGCCCCACGCTTTCAAAAGCAGC

AGCAGGATCAGGTGTATAAGATGCAGCAATGGCAGCAGTCAGGCTCTGGTCCAGCTCAGA

GAGGGTATTACCCCTCCCATGTGCTTGGTTTTGACCCCCGCTGGATGATGATGCCCCCTT

ACATGGACCCACGCATGACTCAGGGACGCTCAACAGTTGATTATTACTCTTCTAATGTCC

ATTCTTCAGTGAAGCCAGCTGGGCAGCAGGATCACCTCCACAGTCCCGGGTCCACCTCAG

ATGAGGGCTGCCATCCCACCATGCACCTGGATAAGAAAGTGCTCTCTGCTGACACTTATT

CAGTTTGGAGCCAAGAAGGCTACCCACCTGCACGCAGTTTCACCCCACCTTACCAGAGAC

AGCACGAGAGCGGTGAGAGGAGCCAAACAGATGACAGAAGTGAGCGGTCTGGCTCTCAGC

AGGACTCTTATGAGGACAGAGGAATAGAACGAGCTGAAAGTCCTCTGGAGGATCATCTTC

ATCAGGGCTACCGGCCAAATCGTGGGCCAGACATTTCTCTCGGTTCGCAGCGGGAAGATG

CCTCTCATCCCCTAGGACATAACGAGTACTTCAAACCCGAGAACCAGAAGGAGGTTATTT

CTAACCGGCACCATAAAGACAGTCTGGATTCTCATGATGAAGTGTGTGAAGGTAAAAAGA

GTTTTGACTCTGGTTTCTGGAGGAAAGAAGATTCTATGAGAAAAGACAAAGATGCCACAC

AGTGGTCGGATCCTGGGTCAGGTGGTAACGGTGGGGTTGGCCAGCCATCTGAACCGATGA

GCAGAACTCTCAGAAGGACAGGTCCCATCAAGAAACCAGTGCTCAAGGCCTTGAAAGTGG

AGGACAAAGAAAATGAGAAGCCCAAATCCGAACCGGAGGAGAAGTCTGTTCCATACAGAC

TTGAGAAGGAAGTCCTTACCAACGTCTATGATTTAAAAAAGGACAACCAGCCTATAATCA

GCAGGCAGCTGCCTCCTCCTGTGACCTGCCAAGAGGAAAAGCAGACTTTCCGCCCAAAAG

TAGAAAAATTAAGCAATCCGACAGAAGAATCCATTAAGGAGAACGGCTGGGATGGCAGTA

ATTCCCAGCTGAGAGACAACTCTGAAAGCAGAGAACCTCCAGCACCTCGTCGCAACAACT

GGATCTTTATTGATGAAGAGCAGGCATTCGCCGGAGTCAGAGGGGCAGGGCGAGGCAGAG

GCAGGGGGTTTAGAGAGTTCAACTCACGTGGTGGAGGCAGAGGAGGACGTGGTGACAACA

ATAGAGGCATGTACAACACCGGGAATATCCAGCGAGGAGGAAGGGGGCGAGGATCTCGGG

AGTACGTCAAAACCGAGGATTTCCAAAGAGGCAAACCACGGAGGCGAAACGTGAGCGAGA

CTTTAAGCGAAGCCTCTGAATATGAAGAGCTGCCCAAACGGCGCCGCCAGAAAAATGGAG

AGAACGGAGAAGCTGCTTATTCTACAGCTGGAGACACTAAAAGGGCAGATCGGGATTCCT

GGCGGTCCAATAAGGTTTACAATAACGACCAGAGCACCAGTGGAGAAGCGAGGGAGAAAC

CCAAGATGAGAAGTTTCAGTCGGTCTCTTCCTCCACGCCTAAACACCGGATATAACCGGG

GGTTCGGATCAAAAGAATTTTCCACTTGGAGAGGAAGAGGTGGTCAGTTTGGAAGCGGAT

CCATGCAGGAGAACGGCTATAATTTTACAAATGACACGTTTTCTAGAAAAAATGCAGAAG

GTGACTCTCTTAAATACCCTTCAAAGTTTGCTTCGTCCTTCACAGAGAACGGAGTTGAAG

ATCGGGAGGGAGACTACCATCTTGACAAAGATAATTTGGACAACCGGCCACTGAGAAGAA

GACGTCCTCCTCGCCAAGACAAACCGCCACGGTTTAGACGTCTACGCCAGGAGCGAGAAA

CCGGAGGGCAGTGGAACAGAGACGAGTACGTTAACGGGGATTTTACAAATCCATGGTCCA

GTCGATCTAAAACCAGTGAAGACCATTGGCCTGGTCAGTATCCTGGAAACAGGCAGCAGG

ATTTTGCACCACAGGCAGAGGACTGGGAGACTGGGTCGGAAAACAGTGACCTAAGTGATT

GGAGAGAGAAACGTGGGGAAGCCCCAGGGGAACCGGGCCACGGCGAGCCAGGCTCTGAAA

AACGGGAGCTCTCCAAAAGGAGTTTCTCCAGTCAGCGCCCAATCGTGGACCGACAGAATC

GCAAGACTGACATTTTGGAGAGCTCAAAGTTGCCCCGATCCACAGATACACCGTCCAGCA

GCAGTTCGTCTAACCGAAACGACAGCTGGCAGAATGGAGTGCGCCGGAGTCCCGAGGACT

CGACTTCAGGATTGAACGCAGGGTCGGTGTATGGAGTCGAACAGTCAGAAGACGGCCTGT

CCACCAGCAATTCTGAAACTTCAGTGAAAAAACTGGATAAAGATCTAAAACCAAGAAGCA

TGAAGGGAGACATGTCTGAGTCTCTATCACAATATGATCTCAACAGCTACCCGATTGAAA

GCGATTCTGGAGTCTCTATTCCAAGTCCCGACGGATTCCAAGACGCCCTTAACAAAAAAC

AGAGGCGTCCACAAGAGGACGAGCGAAGGAGGAAGGAGCAAGGAGCGACGGTAAAGAACA

GAGCCATCACTTCAAAAATGCCTCCTCGTTTCGCAAAGAAGCAGGGTGGGATCTCCATCG

ATCAGTCTGAGGAAACCCTGTCGACCAGTAATCTCGGCACTGAGATCTGGGAGGCCAGCA

GTGCAGCTCTTCCAGTTCAATCGTCTGGTGGAGACTCATGGACGAAGCAGGTTTCGTACA

ATGCAAGTGAACCGAATTCAGAGGATTCGGACGCAGGGCCGGAGCACAGCAAAGAGCACA

AACCCGGGCCAATCGGAAACGAGCGCTCTCTGAAGAACCGTAAGGGTTCCGAGGGCGTGG

AGCGCCTGGAGGGCCCCATCACGCCTGTAAACGGAGTGGATATCCACGTCGAAAACGTCC

TTCCTGTCCCACCCATTGAGTTTGGGGTCAGCGCCAAGGACTCTGACTTCAGCCTTCCTC

CGGGATCAGCACCGGTGGTCGTGTCCAACCCTGTCACCAAGCTGCAGGATGCTCTTGCCA

GCAATGCGGGCCTGGCTCAGGGCATTCCTATGCTGCGCAGAGATCACCTGCAGCCTGCTA

TCAACCTCAACCCCATTTCCTACCCCACCACTGACCTCACTCTCAAGATGGAGTCTGCGC

GTAAAGCCTGGGAGAACTCTCAGTCCCTTCCTGAGCAGGGTTCTCCAGGTGGTGCTGGGT

CCGTCACGCAGCCCACATGCAGTTCGTCCAGTGCCGTCAGCTACAGCTCTTTCGGAGGAG

TCTCCATGCCTCCCATGCCTGTGGCTTCTGTCGCACCCTCCGTCTCATTGCAGGGTAATC

ATATCCCTCCCTTGTACCTGGACGGCCATGTGTTTCCAAGCCAGCCTCGCTTGGTGCCGC

CGACTATGACTCAGCAGCAAAGCTATCAACAGGCTGCAGCCGCTCAGCAGATCCCCATCT

CCCTCCACACATCCATGCAGGCTCAGGCTCAGCTGGGACTCCGGGGTGGTCTGCCCGTCT

CTCAGTCCCAGGAGATGTTCAGTTCAATTCCCACTTTTAGGTCGCAGGTATACATGCAGC

CCAGCCTGTCTCACCCGAGTGCGATGGTGCTTTCTGGAGCAGGCCCTCTCAAGGGGCCGT

ACTCTGCGTTCCCCGGCATGCAGCCGTCCGAGCTGGTGAAGCCGCAGTCCAGCTCGCACT

TTCAGCCCATTAATGGCAGCCAGGCACTTGTTTATGACAACGCAATGAACCAGGCTGCGG

GTTTGGGCACGTCTCAGCTCATGGACTCCCAGCTCATTCAGGTTACGATGCCTTTACCTG

GCTCTCAGTTGCGTTACGGTTCGGCCCAGCAGCACTTGATCCTCCCACAGTCCATCCAGC

TCCAGCAGGGACAGAGCCTCTCTGTCGGCACCCCTCGCCGCATTCTTTCCCATGGTTCCC

AGCCACCAGTCATGACTGCCAGTCGAGAGCCATCTCAGCTGGAGATGAAAGGATTTCAGT

TCCCTGATAAAGCCAGCCATTCACCTGGGATACCTGCCGGTTCATACAGACCCAGTTCTG

CCAGTCCCAGTGGAAAGCCGTCTGGTCCTGGTGCTCCCCCGACTGTCGGCCCGCTCCCTG

GACATTATGCTCAACAGGTGCCCCCTCCACAAGGCAACATGGTGATGCACATGCGCACCC

CCACCAACGGGCCTTTCCCAAACCCCATCCAGAGACCTGTAATGCAGGTAAACAAGGCTG

TAATTATGCGCTCTACCCCCTACACGAGCCCAGGCCGTGATCCTACCCACTGCACCCCCC

CTACCAACCCAGAGTCCCTTAACAAGGGCACAGAGGAGGGCACGAAGGTGAAGCTCCTCC

ATGAAGCTCGGCATGTTGTATCCGATCTGAAGACGTCCTCTTCCTCATCTGTGATCTCTG

GAAAACTGCAGGACGTCCTCACCGGAGCGCCGAAGCCGAACCGCACCGGAGCCATTAAAC

CTCAGAGCGTTAAAATGGAGGAGAGCAAGGCTTGATCACAGAGCTCACACACACACACAC

ACACCGTTCACCTCATTGTACTGAACCTCCCAGCTCAAAACGAGGACTTTCTCTTTTTCT

CACACACACACACACACAC

>Unigene000097

AAGGAGCAGATGGATCTTCAGGAAGAGAAGCACAGGCAGGAGATTGAGCGTCTCAGAAGT

TACTTCAGTGAGCAGATCAGAGAAATGGAGGAGCGTTACACGAAGGAGGTTCTCCATCTA

CAGGACAGACTGAAGGACCTGAAACCAATGGAAACACTGCACAGTATGGCTGGTATTTCT

GCTGAAGAGCAGCATCTTCAGATGCGACAGTATGAGATGGAGGAGGAGATCGCCCGGGTG

ATAGTGCAGATGTCGGTGGAATTTGCCCGTCAGAGTGAGATGAGTCGTGTCAGTGTGCAG

GAAGAGACGCGGGACAGGGCGGAGCCTGAGCGAGGGATTCAGATGACGGATGAGTCGTGG

GGAGGAGGTGTGGAGTGGTGGAATCCCAGCATGCAGTGCAGCAGCACACGGACAGAACAG

CTAAGTAGAAGTGAAAGTGAGGATGAGGAGAGGAAGAGGAAGGATAAGCAGTGTGAAAAG

GAGGAAGTGACTGTGACATCACAGCTGACATCAACAGAGACCCAGGAAAGGGTACATCAG

GTGTTAATGGACTTGTTAAAGATGGCTGCGTTAACGGAGGAGACGGTCAGAGACGAGTCC

ATCCTGAGCGCAGCAGACGAACCTGAGACGGGCAAATTGAGCTTGTTGTCAGGGGCAACA

GACACTGACGAGGGACTGGACGTGTGCTGGGAGGCGGGGCTTGCTGTAGAGAGGGCGGGG

TCTCTATGGGGCGTCTGCTCCAGGCTGCAGAAGACAGTGGAGAAGCTGATCATCACTCTG

ACCAGCACACACACACAGCTGGATCACGCGCGGTTAACTCAGACGGAGTTAATGAAGGAG

AAGTTCACTTACGATCAACAAATCACTGACTTACTGAACAGACAAGAGGAACTGCAGCGG

AGACTGGAGGAGGAGAGAACAGCTCGAGAGAAGCTCACACTGCAGCTCCATCATGCTGAA

GGTTTGATTGACGGGTATTCAGATGAGAGGCGGGCTCTGGAGGAGCAGGTGCGTGAGGGG

GCGGGACGGCAGCAGCAGCTGGAGGCGGAGCTTCAGGTGACAAACAGTCGACTTCAGGAA

CTTCATCAGGAAAGAAAGGAAGTGACACGGCAGAAAGACTTATTACTACGTCAACAAGAT

GCTGTTATTGGTGGAGCCACAGAGGCAGAGCTAAGCTTAGTTGAGGCTGCGGTTGATGAT

GCACCTGCGGCAGGGCTTTTGGAGGAGACTGAGAAACTGTTGCAGGAGAAGGTTGATGTT

CAACGTCAGGCCCAGAAGGATCATACAGAACTTCTGGAACAGGTGAAGCATTTGGAGGCA

GAGCTTAATGAGCAGGTGAGGAGCACAGTCAAGATGGAGGAGACCCACCAGTCTGTGACC

TCTGACCTCCATCAGCAGATTCATGCGCTTGAAAAACAGCTGGAGAACAATCGACGCTTC

CTGGACGAACAGGCTGCAGACAGGGAGCAGGAACGAGACGTTTTCCAGCAGGAGATCCAG

AAACTGGAGCAGCAGCTGAAGAGCAGCAGTAAGACTCAGGCTGAACACAGAGAGGTGGAG

CGGTTGAGTGTGGCCCTGCAGGAGAAAGCTGATTGGTGCAGTGAGCTGTCTTTGCGAGTG

CAGCAGCTGCAGAGGGATGTGCAGGAGCGTAACGAGGAGATCGAGCGGCAGGAGGAGCGA

GTGAGAGTGCTGGAGGAGGTCCTCGTCTCCCACGACAACCACCACATCACCTCAGCCGTG

GAGGACAGTAAGCAGTATGCCTCCATGGAAGGCGGGGCAGATGCCACTCTGGAGTCTTTA

CTCCAGACAGAGAGAGAGGCACTGGACAGGAAAGAAAAAGAGATTGTGAACTTGGAGGAG

CAGTTGGAGCAGTTCAGAGATGAACTCCAGAATAAGAGTGAGGAGGTTCAGCAGCTCCAC

ATGCAGCTGGAGATCCAGAAGAAGGAGATCAGCACACAGCAGCAGGATCTTCAGACTCAC

ACTCGCTTAAAGACGGAGGTGGAAGTAAAGAACCAGCTGGTGTGTGATCTCGAGTCTCAG

GTTGAGTACCTTCGTGGAGAACAGGATCGACTCAAACGAGGCAGCGAGGAAGAAGTGGAG

CAGCTGAACTCTGTAATCGAGAAACTCCAGCAGGAGCTCTCCAACATTGAGCACAAACAG

TCTGTCCAGGAAGAGGATAATGATGAGGAAGATCAGAAAGATGAACTGAAACTGAAGATT

GACCAAATTCAACGAGAGCTCGACACCCTCAAGGAGGAGCACTTTCTGCTCGTCAGCAAA

TATGGATTGCTGCAAGAGGAACGAGAGACTAAAGAAAAGAAGGAGAAGAAGAACGAGATG

TTGGTTCATGAACTTGAAGACATGTTGAGGGATAAAATGGCGGCATTGGTGGTTGCTCAA

GTTCAGATTCAAGCTTTGGGAGAAAGTGCCAATTCCATTGTGAGTGGTTTAACTCAGCGT

GTGGAGCAGTTACAGACCTGCCTGGAGGAGCGTGAGGATGAGCTAAGAGAGTGTAGATCA

AAGGGGGACGAAGCTAAATCTGAAGCAGGAACTCTTCGTCTGAAAGTCTCCCAGCTGGAG

GAGAAACTTAAGCAGAAGGTTGCCATTTTGAGCCAGATGGTCAACCAAAAGCATTGTGAA

ACAGACATCCAAACCAAATGTGAGATCAGAAGTAGGACTTCTCTGGATGATGAATTAGAA

ATAAAGCATGCGGAAACATTAAGCGATTCCATCGGTGGGAAAGAGATGGATTTAACGCAG

CAGTATCAGGAGAATGATGGGAAAGAGTGGAAATTTAAGGTGATTTCTGAAGACAAAAAA

GAAATAAATCTAATAAAACCCGATTCTGCATTAATTGCTTCTGGTTCAGAGATGCAGGTT

AAACAGTGTGCAGTAAATGCGATGGAAAACGTTCTCTTCCTGATAAAAAAGTTGAGAAAG

TTGGAAGTAGATCTTTGCTCCATGCCAAAGGACCAGGAACTGCAGAAACATCTGCTGGCC

AGTTCAGAAGAGGACATTAAGGAGTACGAGAAAATGCTGACAAAGCTGATGAAGATACTT

GACCAAATGAAATCAACACCTTCATCACCTGCAGGGACTGAAAGTGGACACGTTACTGAG

ACAGAGAAGCTTCATCAGAAACTACAGCAGGCCAGGAATCAGTCAGCAGCTGCTAATGAA

CATCTGGACAGCTGCAAAATTCAAAACCTGCAGGAACAAATACAGTTTAAGGATGAACTG

CTGGAGAAGATACGGGAGGATTTACGCAGGGCCTCCGAGCGGAGTAGTGAGGACTCCACA

GTGATTGTAGCACTTCATCAGGAGCTCCATGAGGTTAAATGTCAATCAGAAGCTACAGAA

AAGGAATTAAACAGCTGCAAAGAACTTAGCCAAAGACTTCAGGAGCAGATTGAGGAGCAA

GAGAAGATGATCAGCATGTTGAAGGATCAGATGCACAAGACCACCGATGAAGCTGAGCGA

TCAGAGCTAATTCAGGAACTACAGCACATCAAACTTCAAACAGCTGCTAATGAAGAAGAA

CTGAACAACTACAAAGAGCAGAATCTCAAACTGCAGGAGCAAATACAGGTTTGTGATGCG

TCTGTCGTCCAGCTGATGGAGGAACTTCAGCATCACAGAACAGCTTTAAACAAATCCGGA

GAAGAGAAACACTCGAGATCTCACAGGAACACTGGACAAAATGATGGGAAGATCATGAGT

GACCGGGCAGCCCGACAGCAGGACAGCCTGTCTCCCTCCAGTTCTTTACCTGCAGTTCCT

TCAGCTGACCGCGCTCAGTGCATCTCCACAGAGGAGGTGGGGACACAGGTGGAGGGGGCG

GAGCTGGGGGCAGGGCTGGAGGAGGTGATTGGGGAATACACACAACGGATTGGACAGATT

AGGGAGCTGCACGCGGCTGAGATCATGGACATGGAAAACAGGCACATCACAGAGAGTGAG

AGTCTAAAGAACGAGAACAGGAGACTGGAGCAGGAGTGTGTCACACTTAGAGAGGGAATT

GATCAACTCCACCCACTACAGGTGATGAGACCAGATCACTCAACATTCTCTGTGTACAGA

GACGGATACACCAGTGACAGTAGCTCAGATGTAGGCACAGAGTACAGAAGCACACCCGAG

GGGGCACGACCAGATGATGCACACCTGCCTGACAGGCTTAAGTCTCTCCTCCGGGAGGTT

CATCAGGAGGGGGCGGAGCTGGGGGCAGGGCTGGAGGAGGTGATTGGGGAATACACACAA

CGGATTGGACAGATTAGGGAGCTGCACGCGGCTGAGATCATGGACATGGAAAACAGGCAC

ATCACAGAGAGTGAGAGTCTAAAGAACGAGAACAGGAGACTGGAGCAGGAGTGTGTCACA

CTTAGAGAGGGAATTGATCAACTCCACCCACTACAGGTGATGAGACCAGATCACTCAACA

TTCTCTGTGTACAGAGACGGATACACCAGTGACAGTAGCTCAGATGTAGGCACAGAGTAC

AGAAGCACACCCGAGGGGGCACGACCAGATGATGCACACCTGCCTGACAGGCTTAAGTCT

CTCCTCCGGGAGGTTCATCAGGAGGGAATGCAGGTTCTCTCTCTGTCAGAGTTGAGTGTC

TGTGATGAATCTGCAGACCACAGCAGTCTCTTAGCACAGATCACACACCTCCAAGCTCAG

CTCACACAGCTGCGTCAGGACACACACACCAACCAACAGCTGCACACAACGCCGACAGAG

ACGGACAGACAAGTGGAGCAGTGTGACAGACTGAGGGCGGAGCTGGCACAGGTCAGATTG

GAGCTGAAGACCTCACTAAAAACCCAACACACACACAAGAGAGAGCTAGACACGCTCAGG

GCGGAAGTGTGTGTAAAGGTGTCTGAGCTGGACGCAGTAAGTGAGCGTCTGGCGGAGGAG

CAGCGCCGGGCGAGAGAGCTGCAGTGGGAGGTGGAAAGAGAGAGAAGCAGGACAGACAGG

AGGACAGAGGGACAGAGGGAGGAGCAAGAGGATATGAAACTTGCTCTGGAGGAGGAGAGA

CTGGTTTTAGCACAGAAAGAGACAGAATTGGCAAAATACACAGGCCTGGTCAAACAGCTT

CAGGTCCAGTTAGAATCTCAGAACTCTCGACTGAAGGAGATGCGTTCTGACCTTCAGAAG

GAGAAAGAGCTAAATGCTGAACTGCTTAAACACGCACAGCACCCTCTACAGGTGGGGGAT

GGCGTTCCTTCTGTGGCAGACAGCATGCTCCAGTCTCTGCAGGTCCAGCTGGAGAAGAAG

CAAACCACCCTGATGGAGCTGAGGGCACAGGCAGAGGAGAAGATGCTGCAGGAGCTGGAA

CAGAAGCAGCAGTGGCAGGATGAAAGGAGACACCTCCAGAGACAGGTAGATGAGCTCCAG

AGCCTGTTGGAGGGAGAGAAGGAGCGAGGCAAGGCGACAGAGAGAGAGAGAATGAGGCTG

GAGGGTAGAGTCTCTCAAATGATGGAGAGAAGCAGCACCAGTGAGCAAGTAAGTATGCAG

GATTCGGAGCAGAAATCATACAAATCTGTGTCTCTGGACTCTACCGGTACCCCTGTTCTG

AACATCGCAGGAGTAACATGCAGCAGAGACACGGAAAGTGTTGTTGCAAAACTCCAGAAA

ATGTCCAGCAAAATCAGTCGGTTTGCAGTAGAGGGAGCTGATGATGAGAGCTTCAGCTGG

TTACTGAAAAGCATTCAGGAGGTCATATCATTTCTACAGAAATACTCCTCAGTGCCCCCT

GTTGGACCAGAGAGTGCTGCCCAACTGACTGGAGGTGTCTCCAGCGTGTTATCGGAGCGA

CTGCTGCATCAGAATGCAGAACTAACAGGTTTTGTCAGTCGTCTAACAGAGGAAAAGAAC

GAGCAGAGAAATCAGATTCTGAGACTGGAGGAGGAGCTCCGGAGGCAGCGCCATCTAAAG

GCTCACAATTCTCTCGGGTTTGGCTCCGAGGTCCCAGGGGGTGTGTTGGAGGTACAGAAG

GAGTTGTGGATGAGAGAGAAGAGTAAGATGGAGAAATCCCTTCTTCAGGCAGAGGCAGAG

ATTTCACGTCTTCGGGCAGACATACGGGCCGACGTAGTGCGAGATCTTAACACGTCCGAC

GCCGACAACGCCACGCTAAAGAGGATTTATGGAAAATACTTGCGTTCCGAGAGTTTCCGC

AAAGCTTTGATCTACCAGAAAAAATACCTGCTGCTGCTGCTGGGTGGCTTTCAGGAGTGT

GAGGAGGCGACACTGTCACTAATTGCAGGCATGGCCAGCCCACATGTCCACACACACACC

ACCTTTCTGGAGTGTATAAGTCAGAGGCGGCGTGGATACTCGCGCTTCCGATCCGCCGCC

CGTGCCTGCATCGCCCTCTTCAGGATGCGGTTTCTGGTTCGAAGATGGCAAAAGGTGACT

GGAGGATCAACCACCATCATCAGGAACGGTGTAGGACAGAGTCAAGTGATTGAGATGAGA

ACTGACTCCCCCTACCTGCAGCCAAGAGGAACAACTAGAGGAAGAACAGGACGAGAGTCA

CCTCACACTACACACACTACACACCACAGGTATGGGGGGATAGTTACAGATGGAGGAGTT

GTGTGTTCTCATCTACAGAGTTATGATCCAGATCGAGCTCTGTCTGATTACATCACCAGA

CTGGAAGCACTGCAGCGTAGACTGGGGAGTGTTCAATCGGGATCATCTTCATACACTCAG

ATGCACTTTGGACTGAGAAGATAAAACAATTTTAATGAAAGAGAATAAACACGCACGCAC

ACACAAACACACACAAACACACACACACAC

>Unigene000098

AACACATTTAATTCATAAACACGCGCGCTTTCGGGTCTGATCACATTCCACACGGCCGGT

TCGCTGCTGTGAGCCTATTTTTTGTGGTAACAACTACATTACCCAGAAGTCCTATCAGCC

TACACAATTACGTCATTTCCGAAACACCAGCAGTTTCCTGGGACATGACACAGTCGTTTA

GATGAGAAAACACGGTTCGCCGGCTGAGGAAATTTAGCTGGCTGAAATGGAGGCGGTGCC

GCGAATGCCGATGATTTGGCTGGATTTAAAAGAAGCTGGAGAGTTTCAGTTCAGCTCGAC

TGTTAAACAGTTTATACTGAAGAATTACGGCGAGGATCCAGACACCTACAACGAGTCTCT

GAAGAAGCTGGAGCAGCTACGGCAGAGTGCAGTGAACGTGACGCGGGACTTCGAGGGCTG

CAGTACGCTACGCAAATACTTCGGGCAGCTGCACTACCTGCAAAGCCGCGTGCCTTTGGG

TCCTGGGCAGGAAGCTGCGGTTCCGGTGTCCTGGACGGAGATCTTTTCAGGGAAGACGGT

CACACACGACGATATCAGTTACGAGCAAGCGTGCATTCTGTACAATCTGGGAGCGCTGCA

TTCGATGCTGGGAGCAATGGACAACCGAATCTCAGAGGAGGGGATGAAGGTGTCGTGCAC

ACATTTCCAGTGCGCCGCTGGAGCCTTCGCCTACCTGCGAGATCACTTCAGCCATAACTA

CAGCGTAGACATGAGCCACCAGATCCTTAATCTCAACATCAATCTCATGCTGGGTCAGGC

TCAGGAATGCCTGCTGGAGAAGTCCATGCTGGACAACAGAAAGAGTTTCCTTGTTGCACG

GATTAGTGCTCAGGTAGTGGAGTATTATAAAGAGGCTTGCCGCGCCCTGGAGAACTCCGA

CACCGCCTCGATGCTTGGAAAGATCCAGAAAGACTGGAAGAAACTTGTGCAGATGAAGAT

TTACTACTTTGCTGCTGTCGCTCATCTTCATATGGGAAAGCAGGCTGAGGAACAGCAGAA

ATATGGAGAAAGGCTTGCTTATCTTCAAAGTTCACTAGACAAACTGAACGAAGCGATAAA

GTTGGCTAAAGGCCAGCCCGACAGCGTACAAGAAGCCCTGCGCTTTACAATGGACGTCAT

CGGGGGAAAATTTAACTCTGCTAAAAAGGACAATGACTTTATTTACCACGAGACTGTGCC

CTGTCTGGAGACGCTGCCTTCAGTTAAAGGTGCACCGCTGGTCAAAGCTTTACCCGTGAA

TCCAACAGACCCTAGTGTGACAGGACCAGATATTTTCTCCAAGTTGGTGCCTATGGCTGC

TCATGAGGCCTCCTCACTCTACAGTGAAGAAAAAGCCAAGCTGCTACGAGATGTCATGAC

AAAAATAGACAGCAAGAACGAGACTTTAGAACAATTTATGGACTCTCTGAATCTGGACCC

GGACTCCGTGGACAACATGGACAGATACAACAGCATTCCCTCTGTCCTCATGGAGAAGTG

TGCAGCTCTAAGTGTGAGACCGGACACCGTAAAAAGTCTCATACAGTCTATGCAGGTACT

ATCGGGTGTGTTCACGGATGTGGAGGCCTCTCTTAGAGAGATTCGGTTAGTGCTAGACGA

CGACGAAGCTAGTGACCGGAAGTTAGAGGAGGTAGCAGGAAAGCAGTGTGTTCCTGCACA

GCCAGCTACCATAGCGGAAATCCGTCGGGACCTTGAGAAATATGTAGAGGCCCACGAGAA

GGCAAGCTTTACCAACACAGAGCTACACCGGGCCATGAATCTGCACATTAGTAACCTGCG

ACTCCTGGGTGGACCACTGGATACTCTGAGAGAGGCCTTGCCCAGACCCCAGCTCTCTGA

AGATGAAGTAGCGGGTCTCCAGTGTATGAAAAGGATTCTGGGTAAAGTGCAGGAGATGAA

AGACCAGCGCAATTCACTTGAGAAGCAACTAAGGGAACTGATCCAGCAGGATGATATAAC

TGCTGCTTTAGTTACCACTGAGAGGGCTGATATGAAGAGATTATTTGAGGAGCAGCTGAA

GAAGTATGAACAGGTAAAAGTGTACATTGACCAAAATCTAGCTGCACAGGAGAACATTTT

GAAAGCACTGACGGATGCGAATGTCCAGTATGCAACAGTACGCAAGGGCCTAGCTGAGAC

GGAGCACAAATGGAACAGTACCGTGCAAATGCTTGTTGCTTCTTATGAAGCCTATGAAGA

CCTGATGAAAAAATCGCAGGAGGGAAAAGAGTTTTATGAAGACCTGGAGGCTAAGTCCTC

TCGCCTCTTAGAAAGGGCTAAGTCCATCTGCAAAACCAGAGATGAAGAAAGACAAGCCAT

TTTGGAAAAAGAGCTTCAAAAAAAGGTACCCCAAAGGCCCACAGCCCCAAAGCCAGCACA

AAAGAAAGCCCCTGACATGGATTTATCTGGAATAGAAGACCCTGAGTTAGCCAAGATAAA

TGCTGCCATTTTAGCCTTAGGTGGAGATTTACCAGAAGATCTTCGCAGTTTGCCTCCCGA

GCTTGGTCGTCCATCAATACCAGAGGCTTTTCATCCAGGGGCAGCTGCTCTATATGCACG

ACCATTGTTTCCTCCGAACCTTCCGCCTCCCGAACTTCTTGCACAAATCTCCCGTTTTAC

GCTGCCCCCAACCCAGGGCTTTAACCGGAATCCAACAACAGGCCCCTTGCCCCACCAACA

TCAGGTTCCCCAGTTGCCCAACCCTCAAATTGCACAGCTCCCTTCAGTACCTGGGTATGG

TCTTCCATCTTCACAAACCCAGCCTGCTGCATCAGGTCCACCAACTGCCATTGGCATGTC

TACCACAACTGTGGATAGTGTGCATACGCCAATTCCCAGCTATACTTCAGCACCAGGACA

TCCAGTAATTTCTACTACACCTGGACACCCTATGGCCACTTCAGTGGGGTATAATGCCCC

ATCTGCAGTAGCTTCTCAGTATTTTCAACAGCCTGGCGTTCCTGTGAGCCAGCCACCTCA

GGGAGTACAAGTTCCGCAACCAAAGCCACCGCCCTCTCAATCATTTCCCCAAGTTCGCAG

ACAGGGCCAGCCACAACCGAGTCCACCGCAGACATTTTATCAAGCAGTGCCCGCTCAGCA

GCATATAGCTGCACCTGTTACTGGACCTCAACTCCCACAACAAACTCAACCTTATTCTCT

ACACCACATGGCACAACCAAGACCACCTAATATTCAGCAACAGGCAAATAATATTCACTC

TACACCATCTGCTTTGTCCCACCAGGTGTACCCTGCTGTTTCATTTGTTCCAGGTTGCCA

GCCTGCTCCAAATCAGCTGCCCTATCATTCCATGCAACCACAAATGCCCCCAAACTCCCA

GCCAATGCCTCAAGTTTCTATGTCACACTTACCAAATGTAAAACAAATTCCCCCTTGTTC

CCAGCCCTACATGCCCCCACTCAATCAGCAATTACATACTATTCCACAAACACCCCTTTC

CACTCAGCAGGCAGTACCTGCAAGCCATAGGCAAATGCCACCTACTCCACAACAAATACC

CCCACTCTCACAACCACAAATGCTACCATCTTCAGCCCAAATATCTCCTCCCACTCACCT

GCAAATGCCTGCAGTTTCTCAACCAGGCTTGCATGCTTCTCATTCACCAGTATCTGTTTC

TCAGTCTTTACCTCGCTCATCTCACCCACCACTGCACAGTGTTTCCCAACCATTACCACA

CCCCTCTCAAGTCCATCCTTCCTATCAGCAAGTATTTCCAGGAACCACACCAAGAGTTCC

AGTCCCTGTCCAGCCCCAGTTACCTCCAGCCTCTCTGCCAGCACAGTTCCCGGTGCACAC

CAATGCTCCCCCTTCACAGCCTCACAGTCTTCCCTCACAACTCCCTCATGGATCTCTTTC

GTCAGTGCCCTCACAGATCAGTTTGCCTCAGCATCATCTACCTCCTCAGTCCACACCTAT

GGGTTATCCTGGTGGTATTCCTTTAGTTCCCCACCAGTCCACAGTTCCTCAGCAGCCTCA

GGCTCTGCCTCCACAGGTACCATCAGGGGCACAAGCATCTTACTCTGGGCCGCATGCTGG

GATTCATCCTGGACCAATACAGAATTCTGGACCAGTCATTACGCCTGGAATACAGTCACT

CCCTCAGGGTATGATCCCACCATCTTCCGCTGCACCAGGGGCTACACCAAGTAATGTCTT

GGCTACTCCATCAGCTTCTCCATCACCAGGGCCTTCCTCACTGGGTATCGCGCCTCAGAG

ACCTTCTCCTGCTCTCACGCCTGTTCCAGGTGCAGCGTTACCACAGACTGTGGTGACACC

CTTGCCATCATCCACCCATCTGTTTCAGCACCAAAATTCCAGTACAGATGATCTTCTCTC

TTCAAGCCCTGAAAGCCAACATGGTAGTAGCAAAGACACTGCCAATGTCTTATTGCCCAT

GAAGGCTGATCCACAAGATGAACAGCTTCGCAAAAAATCTGAGGGGTTAAAAATTATTCA

AGGTGATCCGTACCAGGCTCCGGAAAGGGTCTCGCAGCTTTGCGCAGAGCTTGAAAGGTT

TCGGTCAACTGTTCAGTCCTTGGAGCGTCCCTCAGGAGAAGGAAGTTTATCGGAACTTGA

TGCTCGATGGAAAGAGCTACAAGATCAGCAAGAGAAAGATTCTCGTCAGCTTTCTATCGC

CATAGCCAGGTGCTACACAATGAAAAACCGCCACCAGGATGTCATGCCTTATGACTGTAA

TAGAGTTGTTTTGGACTCTGGCAAAGATGACTATATCAATGCCAGCTTTATAGAGGAACT

TTCGCCATATTGTCCCCGTCTTATTGCAACACAGGCACCTCTTACTGGAACTGCTGCTGA

TTTCTGGCTCATGGTCTACGAGCAGAAAGTGTCTCTCATAGTAATGCTGGTGTCTGAGCA

AGAACTTGAAAAGCAAAAGGTCTTGCGGTACTTTCCATCTGAAAGAGGACAGCAGATCAC

ACAAGGCTCAATTACACTAAGCCTAACCACACAGAAAAACACAGCCACTCATGTGGAGCG

CATGATTGGACTGCAGTACAGAGATCAGAGTTTAAAGCGCACAGTCATACACCTACAGTT

CACCTCATGGCCTGAACTTGGCTTGCCTGAGAGTAAAAGTAACCTCATTCGCTTCATTCA

GGAAGTTCACGGACATTACCTTCTCCAACGCCCTTTGCACACACCTGTTGTGGTGCACTG

CAGCTCTGGCGTTGGTCGAACCGGAGCCTTCTCTCTGTTGTATGCTGCATTACAAGAACT

GGAGGCTGGGAATGGTATACCTGACCTACCCCAGTTGGTGAGAAAAATGAGACAACAGAG

AAAGAACATGCTACAGGAGAAGTTACACCTAAAATTCTGCTATGAAGCAGTCCTGAAACA

TGCAGAGCAAGTGCTTCAGCGTCGTGGCATTGTTACTGCCTCGAGCCAAAAGTCTACAAA

CAGTGCAGCAGTTAAGCAATACTCACAGCCAGAGGCTCAGGATATTGTTCTTGGCGGCGA

TATGCCCATTAGCTCTATTCAGGCTACGGTTGCTAGACTCAGCATCCGTCCACCCAGTGT

CGAGTCTGACCAGGACCCAAATCAGGACCCAGTTGCTTGTGTTGCTGCAGGACTAGAACC

AGAGTTGGCACTAGTCCAGGACCTCCAAGCTTTTTCTCCCAGTGACACTATTCCTGCCTC

GCTAAGCCCTCCAATTTCCTGCCCTACTTCACCAGTTAAAATGCTGTCACCTTCACCTCC

TAATTACCAAGGTAATGGGTTCGAGGGGTCCACAATAATGCCAGTCTCTAACAACCATGC

TGCACTTGAAGCAACTCTTCCAGCACCGTCATCCCTGGACCTACTGGCATCGTTAACGCC

TGAAGCTTTCACACTCGACAGTGGTTGCCGTGGGAAACAGCGAATCAGCAAGCAAAGCTT

TTTTCAGGCACAGGAAGGCCAGGGTTTGCAGGGTCCTTCGAATCAGGATGACCCACTGAG

CACCCTGGATCCACTTTGGACTCTGAACAAGTCCTGACTCCCTTTTGATAAGGCCACTTC

TGTTTATTTCTTAGTGTCCTGTATCCCCAGATCACCTAACAGTTTAGCATTTCCAGCGCT

TATCGGCTAGCTCACAGCCTTTATTAGTTGATTCGGTTGTTTTGGAGCAGGAAAAGGCCC

AGATTCCAGAGTCTGATGTGGATCTCTAGGACCACATCACTGTGTGTTGGTCCATGTGTA

TCTCTTCGATTCCTTGTATTTTTAAGCTGTTTCCTAGCATGCACCCCTCTTTTAAAGACA

AGTCAAGCCTTCTAGATGGACATCTGCATTTTTAACAATAAAAACATCTTTTAAAGGAGA

AACGTTAAGGTTCTCAGCACTTTGAGAAAAGGGAACAATGGATTTGTCATTTGGTTGGTT

TGCGTGTATCTTGGACTGAAATAAAAATGGATTTCTTAGAGGAACACCAGTCATTAACAA

GTGCTTGACTAATGATTAGTACTATTGCACAGACTCCTCTCATTCCACGCTTCATCTCCT

TTTTTGTTGTCAACTTTGACCAAGTTGAATAATGGAAAAAGTGCTAACTTACAACTGCAA

AAGCCAGTAAGGTCTAGTTCACAGAATAAAAGTTTCAATAATGCTAGCTGCCTTTTAACA

TAAGATAACCTAGGGTTAGCTGTTTTTAGGGGGACTACTTGCTAACTTTATGTACGGCTA

AGTGATGCTGGTATCCTGTATACGCACTTTTCCTTTATTACATGTTAAACGACTTTATAA

CTTATTTCTGATTCAGCAC

>Unigene000099

AGAGTGAGAGAGAGAGAGAGAGAAAGAGAAAGAGAGAGAGACGCTCGCTAGCTTTGAACG

GAAGAAGTTTGCGACGCTGTATGACAGAGAGGGCGCGCGCGAGAGAGAGAGAATCTGGAT

AAAGCTCTACCCGGCGACTGCGTTTCTCTTTATCCATCTGGCACTAGAAAAAAACAGCGT

CTAACACGCAGCAACCATGTCGGCTGCAGATAGGTACCTGTATGTGGACCGCTCGCTGGT

GAACAACCCGCTGGCGCAGGCCGACTGGGCCTCCAAAAAGCTCGTCTGGATTCCGTCTGA

GAAACTGGGCTTCGAGTCGGGCTCGGTTAAAGAGGAGACGGGGGACGAGTGCACCGTGGA

GCTCACCGACTCTGGAAAAAAGATCAAAGTGAATAAAGACGACATCCAGAAGATGAACCC

ACCCAAATTCAACAAGGTGGAGGACATGGCGGAGCTCACCTGTCTCAACGAGGCGTCTGT

CCTCCACAACCTGAAGGACAGATACTACTCCGGACTCATCTACACATACTCTGGGTTGTT

CTGTGTGGTCATTAACCCATATAAAAACTTGCCCATCTACTCGGAGGAGATCATAGACAT

GTACAAGGGAAAGAAGAGGCACGAGATGCCACCGCACATCTACGCCATCACAGACACTGC

ATACCGGAGCATGATGCAGGATCGTGAGGATCAGTCCATCCTGTGCACAGGAGAATCTGG

AGCTGGAAAAACAGAAAACACTAAGAAGGTCATTCAGTATCTGGCGTCTGTTGCCTCATC

CACTAAAACCAAAAAAGACCCTACCAGCACCATCAGCAGTCTGTCACATGGGGAGCTCGA

GAAGCAGCTGCTGCAGGCTAACCCTATCCTGGAGGCGTTCGGTAACGCCAAGACGGTGAA

GAATGACAACTCCTCACGCTTCGGAAAATTTATCAGGATTAACTTTGATGTGAACGGTTA

CATCGTTGGAGCAAACATCGAGACCTACCTGTTGGAGAAGTCTCGTGCTATCAGACAGGC

TAAAGAAGAACGCGCCTTCCACATCTTCTACTATCTGCTGTCTGGAGCTGGTGATAAACA

GCGCTCTGAGTTGTGCCTGGAGGATTATGGGAAATATCGCTTTCTGTCCAATGGAAAGGT

GACAATTGCTGGACAGCAGGACCGAGATCTTTACACTGAGACGATGGACGCCTTCAGAAT

AATGAGCATCCCTGAGGACGAGCAGACGGGTTTGCTGAAGGTGGTTTCTGCAGTTCTTCA

TTTGGGAAACATCATCTTTAAGAAGGAGCGTAACTCTGATCAGGCCTCCATGCCTGATGA

CACGGCTGCACAGAAGGTCTGCCACCTGCTGGGCATGAACGTGACCGACTTTGTGAGAGC

AATTCTCTCGCCGAGGATTAAAGTGGGCCGTGATTTCGTGCAGAAAGCGCAGACGCAGGA

GCAGGCAGAGTTTGCAGTTGAAGCTTTAGCCAAAGCCACGTATGAAAGATTGTTCCGCTG

GTTAGTGATGCGGATTAATAAAGCTCTGGACAAAACCAAACGCCAGGGAGCTTCGTTCCT

CGGTATCTTGGACATAGCCGGCTTTGAGATCTTTGAGTTGAACTCGTTTGAGCAGCTGTG

TATTAATTACACTAACGAGAAGCTGCAGCAGCTCTTCAATCACACCATGTTCATACTGGA

GCAGGAGGAGTATCAGCGTGAGGGCATCGAGTGGAACTTCATCGACTTTGGGCTGGACCT

TCAGCCCTGCATCGAGCTTATCGAGAGGCCGAACAACCCTCCAGGTATCCTGGCCCTACT

GGATGAAGAATGTTGGTTCCCTAAAGCCACAGACGTGTCGTTTGTAGAGAAACTCACAAA

TCAACATGCCAATCATTTGAAATTCGCCAAACCCAAACAGTTGAAGGACAAAACTGAGTT

CTCAGTGCTACACTACGCCGGCCGGGTGGATTATAACGCTGTTGCATGGCTGACGAAGAA

CATGGATCCCCTGAATGATAACGTTACTGCACTGCTCAACAACTCCTCCAGTGTGTTTGT

GCAGGATCTGTGGAAGGACGCGGATCGTGTGGTGGGACTGGAGACCATCGCCAAGATGTC

GGACAGCTCCATGCCCAGTGCCTCCAAAACGAAGAAGGGAATGTTCCGCACCGTCGGTCA

GCTTTACAAGGAGTCTCTGGCCAAACTGATGACCACGCTACACAACACGCAGCCCAATTT

CGTCCGCTGCATCATCCCCAACCACGAGAAGAGGGCGGGTAAGCTCGATGCTCACCTAGT

TTTAGAGCAGCTCAGGTGTAACGGCGTGTTAGAGGGAATCAGGATCTGCAGACAGGGCTT

CCCTAACCGCATCGTCTTCCAGGAGTTTAGACAAAGATACGAGATTTTGGCAGCCAACGC

CATTCCAAAGGGCTTCATGGACGGAAAACAGGCCTGTATACTGATGATTAAGCACCTGGA

CTTGGACCCCAACCTCTACCGCATCGGTCAGAGTAAGATCTTTTTCAGGACTGGTGTTCT

GGCTCAGCTGGAGGAGGAGCGCGACCTGAAGATCACAGTTATCATTATCGCCTTCCAGGC

ACAGGCCCGAGGCTTTCTCGCCAGGAAGTCATTTGCCAAACGGCAGCAGCAGCTGACAGC

CATGCGAGTGATCCAGAGGAACTGTGCTGCTTATCTGAAGCTCAGGAACTGGCAGTGGTG

GAGACTCTTCACTAAGGTGAAGCCTCTACTCCAGGTGACACGTCAGGAGGAGGAGCTAAG

TGTCAAAGAGGAGGAGCTTCAAAAAGCCAAAGATTCAGCGCAGAAGTACGAAACTGAGCT

GAAAGATATTACGCTGAAACACACACAGCTTGTGGAGGAGAGGAACGCTTTGCAGGAGCA

ACTTCAGGCAGAAACTGAGCTTTATGCTGAGGCAGAGGAGATGCGAGTTCGATTAGTTGC

TAAAAAGCAGGAGCTGGAGGAGATCCTTCATGAAATGGAGGCACGGCTGGAGGAGGAGGA

GGAGCGGAGCGCCACCCTGCAGGTTGACAGGAAGAAGATGCAGCAGCAGATCCAGGAACT

GGAGGAACACCTTGAAGAAGAGGAGGATGCGCGACAAAAGCTGCAGCTGGAGAAAGTCAC

CTGTGAGGGAAAGATCAAGAAACTGGAGGACGACCTGCTGGTGATCGAGGATCAAAACAA

CAAACTGCTGAAAGAGAAAAAGCTGTTAGAGGAGCGTGTTGCTGATTTTAGTGCTAATTT

AGCTGAAGAAGAGGAGAAATCTAAAAACCTGACCAAGCTAAAGAACAAGCACGAGTCCAT

GATCTCAGACCTGGAAGTGCGTTTAAAGAAAGAGGAAAAAACTCGGCAGGAGCTCGACAA

GATAAAGAGGAAGCTGGAGGCGGAGTCTAATGACCTTCAGGAACAGATCGCTGACCTTCA

GGCGCAGATTGCTGAGCTAAAGGCACAGCTGGCTAAAAAGGAGGAGGAGCTTCAGGCTGC

ACTAGCAAGGCTGGAGGACGAGACAGCACAAAAGAACAACGCATTGAAGAAGATTCGTGA

GCTGGAGGGCATCATCTCAGACCTGCAGGATGACCTGGACTCTGAACGTGTCGCCCGAAA

CAAAGCAGAGAAGACCAAGAGAGACTTGGCAGAGGAGCTGGAGGCCCTAAAGTCTGAGCT

GGAGGACACATTGGACACCACAGCCACTCAGCAGGAGCTCAGGGCAAAGCGTGAACAGGA

AGTGACCCTGCTGAAAAAAGCAATAGAGGAAGAGAGCCGAAACCATGAAGCTCAGGTGCA

GGAGATGAGACAGAAACACACACAGGCTGTAGAGGAGCTCACAGAACATTTGGAACAGGC

CAAGAGGGTGAAGACAAACTTGGAGAAGGCAAAGCAGACGCTGGAGAAGGAGACATCAGA

GCTGACAGTTGAGATCCGCACCCTCTCTCAGGCCAAACAGGAAGTGGAACACAAGAGAAA

GAAAGTGGAGGGACAGTTAGCAGATCTGCAGTCCCGCTTCAACGACAGTGAAAAACAGAA

AGCTGAGCTTGGAGACAGGGTGTCCAAAATGACTGTGGAGCTGGAGAGTGTCACCAACCT

GCTAAATGAGGCTGAGGGGAAGAACATTAAACTGAATAAGGATGTGGCCAGTCTGACCTC

TCAGGTCCAAGACACACAGGAGCTGCTGGCTGAAGAGACACGTCAGAAACTGCAGTTCTC

CACAAAACTGCGTCAGATGGAGGATGAGAGAAACAGCCTACAGGATCAGCTGGAGGAAGA

AACAGAGGCCAAGAGGAACGTGGAGAGACATGTCTCCACCCTCAACATCCAGCTTTCAGA

TTTTAAGAAGAAGCTGGATGACGTGTCAGGAAATGTTGAGCTGCTGGAGGAGAGCAAGAA

ACGTCTGCAGAAAGACCTAGAGTCCTCAAACTCCCAGCTGGAGGAGAAGTCTTCTGCCTA

TGAGAAACTGGAGAAGACCAAGAACAGACTGCAGCAGGAATTGGAGGACATTCTTATGGA

CCTGGACAACCAGAGACAGCTTGTCTCCAATCTGGAAAAGAAGCAAAAGAAGTTTGACCA

GATGCTTGCAGAGGAGAAGAGTGTGTCCTGTAAGTACGCCGATGAGCGGGACCGGGCAGA

GGCAGAGGCACGTGAGAAGGAGACAAAGGCGTTGTCTCTGGCGAGGGCACTGGAGGAGGC

GCAGGACACACGTGAGGAACTGGAGAGAGCCAACAAAGCCTTACGGGCTGAGATGGAGGA

CCTGATCAGCTCCAAGGATGACGTAGGAAAGAATGTCCACGAGCTGGAGAAATCAAAGCG

TGGTCTGGAGCAGCAGGTTGAGGAGATGAAGACGCAGCTAGAGGAGCTGGAGGACGAGCT

GCAGGCTGCCGAGGACGCAAAACTACGGCTGGAAGTGAACATGCAGGCCCTCAAAGCACA

ATTTGAACGAGACCTGCAGGCACGAGACGAGCTCGGAGAGGAGAAGAGAAAACAGCTGAT

GAAACAGGTGCATGAGATGGAAGCGGAGCTGGAGAATGAGAGGAAACAGAGATCTCTGGC

TTTAGCAGTGAAAAAGAAGTTGGAGCTCGACCTGTCCGACCTGGAGGCTCAAATAGACCA

GGCCATTAAAGCTCGAGATGAAGCACAGAGACAGCTGAAAAAACTACAGGCCCAGGTAAA

GGATCAGTTACGGGATATGGATGAACTCCGAGCGGCTCGAGATGCGTCACTGAACAGCGC

TAAAGAAAACGAACGCAAACTCAAGAGTCTGGAGGCTGAAAACATGCAGTTACATGAGGA

TCTGTCTGCAGCTGATCGCAGTAGGAAGCAGATTCAGCAGGAGAGAGATGATCTGCATGA

TGAAATGACCAGCTGTAATTCCAAAATGTCTGTGCTTAGTGATGAGAAGCGGAAGTCGGA

GGCTCGTATTGCTCAGCTGGAAGAGGAACTTGATGAGGAACAGTGCAGCACTGAACTTGT

TAATGACCAACTAAAGAAGGCAAATGCACAGGTGGACACTCTGGGTAGTGAGTTAGCGGG

TGAGCGTAGCGCGGTTCAGAAGAGTGAGACGGCTCGACAGCAGCTGGAGAGACAGAATAA

GGATCTGAAGAGTAAACTGCAGGAACTGGAGGGTTCCGTCAAATCCAAGTTCAAATCCAC

TATCGCCGCCCTGGAGGCCAAAATCCTGCAGCTGGAGGAACAACTGGAGCAGGAGGCCAA

GGATAAACAGCAGACTTCAAAGTCAATGCGCCGTACGGAGAAGAGACTGAAGGAGGTCCT

GCTGCAGGTGGAGGACGAGAGACGCAACAGTGAGCAGTTTAAAGATCAGATGGAGAAGGC

GAACTCGCGCATGAAGCAGCTGAAGCGTCAGTTGGAGGAGGCGGAGGAGGAAGCGACCCG

CGCCAACGCCTCCCGCAGGAAGTTACAGCGAGAACTGGAGGACGCCACCGAGGCCAGTGA

GACCATGAACCGTGAAGTCAACACCCTGAAGAGCAAACTCCGACGCGGTGGTGACTTCAC

CCTCAACGTACGGCGTGTGGTCCGTGGTGGTCTGGACAGCGAGGAGGACGCTGACCTCAC

CAGTGAGACATCAGAGGCCACACCTGAGTAACACCTCCAGCTGACAGCCAATCAGATCGC

CTGAGTCCAGGACAGCTGCATCACTTTTACTCCTTTAATCCACATCTTCATCACGAAACG

ATTCAAACTCATTCTGATACCCGAATACTGAGCGCTTTAATAGCTGCCAATCAAAACGGT

TGCCAGTTCATTTAAAATCCTGCCGTCTACCCAATGAAAAACTTTATTGGTGTCCGGCTG

TGGGACTGGTGTCCGGCTGTGGCTTTACATCTTAATATTTTTCAGCTTGTTTTTATTTAC

GTTTTTTACAGCACTTTTTTTTTGCTTTGTTGCCGTGTGCAGGTCAGGTTTTTCCTCACG

GTCACACTCGTCCACTGGATTAATAAAGATCTATAATTTGATTAAATGCTCACTATTATT

AAATTATCAGTCGTGCACAAAAATCAGGTTGGATGGGATCCGCTCCCGAAAATGGATTTT

AGAGAAACTACACTGGGTTGAGTTACTGTGGGTTGCCAGATCAGTGGAACTCGCACTGTT

CCACACTAATGATTTATTAGCAGGTTCTTTTGTTAATTGTTTAAACACTGCGGTGTTTTT

TTCACTTCAGTCTCTTCACATTAAATGGTGTTTAGTTATTCATCACTCTTTTTCTTACTT

TTCTGTGTTTATTTTACATTTTCACACTCGTTTCTCTCCGTAAAGCTCCTGACCCCATCC

CCCCCCCCCCC

>Unigene000100

AGAGTGAGAGAGAGAGAGAGAGAAAGAGAAAGAGAGAGAGACGCTCGCTAGCTTTGAACG

GAAGAAGTTTGCGACGCTGTATGACAGAGAGGGCGCGCGCGAGAGAGAGAGAATCTGGAT

AAAGCTCTACCCGGCGACTGCGTTTCTCTTTATCCATCTGGCACTAGAAAAAAACAGCGT

CTAACACGCAGCAACCATGTCGGCTGCAGATAGGTACCTGTATGTGGACCGCTCGCTGGT

GAACAACCCGCTGGCGCAGGCCGACTGGGCCTCCAAGAAGCTGGTGTGGGTTCCGTCGGA

GCGGCTGGGGTTCGAGGCCGGCTCCCTCAGAGAGGAACATGGAGACGAGGTGGTGGTGGA

ACTCGCCGACTCGGGTAAGAAGATCCGCATCAACAAGGACGATGTCCAGAAGATGAACCC

GCCCAAATTTAACAAGGTGGAGGACATGGCCGAGCTGACATGCCTGAATGAAGCGTCCGT

GCTCCATAACCTGAAGGAGAGATACTACTCCGGCCTCATCTACACATACTCGGGGCTGTT

CTGTGTGGTGATTAACCCCTATAAGAACCTGCCTATCTACTCAGAGGAGATTGTGGAGAT

GTACAAGGGCAAGAAAAGGCACGAGATGCCGCCACACATCTACGCCATCACAGACACGGC

GTACAGGAGCATGATGCAGGATCGTGAGGATCAGTCCATCCTGTGCACGGGTGAGTCTGG

TGCTGGAAAAACGGAGAACACCAAAAAGGTCATTCAGTATTTGGCCCATGTAGCTTCTTC

TCACAAGACCAAGAAAGACCAGAGCAGCTCGGTCCTGTCACATGGGGAGCTGGAGAAACA

GCTGTTGCAGGCTAACCCCATTCTCGAGGCCTTCGGAAACGCCAAAACAGTCAAGAACGA

CAACTCGTCACGATTCGGAAAATTCATCCGAATCAATTTCGACGTTAATGGTTACATCGT

CGGCGCCAACATCGAGACCTACCTGCTGGAGAAATCTCGCGCCATCCGTCAGGCCAGAGA

GGAGAGAACCTTCCACACCTTCTACTACCTGCTGACCGGCGCTGGAGACAAACTGCGCAA

TGAACTATGTCTGGAGGGCTACAACAAGTATCGCTTCCTGTCTAATGGAAATGTGACCAT

CCCTGGACAGCAGGACAAAGACATGTTCGTTGAGACCATGGAGGCCATGCGCATCATGGG

CATCCCAGAGGACGAGCAGATCGGTCTGCTCCGTGTCGTCTCCTCTGTCCTACAGTTGGG

GAACATGAGCTTCAAGAAGGAGCGTCACTCAGACCAAGCCTCCATGCCTGATGACACAGC

GGCTCAGAAGGTGTGTCACCTAATGGGCATGAACGTGACTGACTTCACCCGAGCCATCCT

GAGCCCCCGCATTAAGGTGGGGCGCGACTATGTGCAGAAGGCGCAGACGCAGGAGCAGGC

CGAGTTCGCAGTTGAGGCTCTGGCTAAAGCTACATACGAAAGGATGTTCCGCTGGCTCGT

CATGCGCATCAACAAAGCACTGGACAAGACCAAGCGCCAGGGAGCGTCCTTCATCGGCAT

CCTGGACATCGCCGGCTTCGAGATCTTCGAGCTGAACTCGTTCGAGCAACTGTGCATCAA

CTACACCAACGAGAAACTGCAGCAGCTCTTCAACCACACCATGTTCATCCTGGAGCAGGA

GGAGTACCAGCGCGAGGGCATTGAGTGGAGCTTCATCGACTTCGGTCTCGACCTGCAGCC

CTGCATCGACCTCATAGAGAAGCCTGCGAGTCCTCCAGGTATCCTGGCTCTGCTGGATGA

GGAGTGCTGGTTCCCCAAAGCCACAGATAAGAGTTTTGTGGAGAAGGTTCTGCAGGAGCA

GGGCACACACCCCAAATTCTTCAAACCTAAGAAACTGAAGGATGAGGCAGATTTCTGTAT

CATTCACTACGCCGGAAAGGTGGACTACAAAGCTGACGAGTGGCTGATGAAGAACATGGA

CCCTCTGAATGATAACGTGGCCTCACTGCTCAACCAGTCCACCGATAAATTTGTGTCTGA

ACTCTGGAAGGACGTGGACCGGATTGTGGGTTTGGATAAGGTGGCGGGAATGTCGGAGAT

GCCCGGAGCATTTAAGACCCGTAAAGGAATGTTCCGGACTGTCGGTCAGCTCTATAAGGA

GCAGCTGTCCAAGCTGATGGCCACGCTGAGGAACACCAACCCCAACTTCGTGCGCTGCAT

CATTCCAAACCACGAGAAAAAGGCGGGTAAACTGGACCCTCACCTGGTTCTGGATCAGCT

CAGGTGTAACGGTGTGTTGGAAGGTATCCGTATCTGCAGACAGGGTTTCCCCAATCGCAT

CGTCTTCCAGGAGTTCAGACAGAGGTACGAGATCCTGACTCCTAACGCCATTCCAAAGGG

CTTCATGGATGGCAAGCAGGCCTGTGTGCTGATGATCAAAGCTTTGGAATTGGACCCAAA

CCTGTATCGGATCGGACAGAGCAAGGTGTTCTTCCGTGCCGGAGTTCTGGCCCACCTGGA

GGAGGAGCGTGATATGAAGATCACAGATGTCATCATCAGCTTCCAGGCCTGGTGCCGTGG

CTACGTCGCCCGAAAGGCATTTGCCAAGAGGCAGCAGCAGCTGACCGCCATGAGAGTGAT

CCAGAGGAACTGTGCTGCTTACCTCAAACTCAGGAACTGGCAGTGGTGGAGACTCTTCAC

TAAGGTGAAGCCCTTGCTCCAGGTGAGCCGTCAGGAGGAGGAGATGCAGGCCAAAGATGA

GGAGCTGACCAAAGTCCGTGAGAAACAGGTGGTGGCTGAGAAAATGATCGAGGAGATGGA

GGTCAAACACCAGCAGCTGAATGCAGAGAAGATGGCTCTACAGGAGCAGCTGCAGGCAGA

GATGGAGCTGTGTGCTGAGGCGGAGGAGATGAGATCTCGACTAGCGACTCGGCAGCAGGA

GCTGGAGGAGATACTACACGACCTGGAGAACCGTGTGGAGGAGGAGGAAGAGCGGGTCAC

TCAACTGCAAAACGAGAAGAAGAAGATGCAGCAGAACATCACGGATTTGGAGCAGCAGCT

GGATGAAGAAGAAGCAGCACGGCAGAAACTGCAGTTGGAGAAAGTGACCACAGATGCCAA

GCTGAAGAAGTTAGAAGAAGATGTCATGGTGCTTGATGATCAGAATGCCAAACTCAACAA

GGAGAAAAAACAGCTAGAGGACAGGATCTCCGAGTTTAACATTAACCTGGCTGAGGAAGA

GGAGAAATCCAAGAGTTTACAGAAACTTAAAAACAAACATGAGGCCATGATCACAGACCT

GGAGGATCGTCTGAAGAAGGAGGAAAAGACTCGTCAGGAACTGGAAAAGAACCGTAGAAA

GCTGGAAAGTGACTTCACAGAGCTCAATGATCACATCGCTGATCTTCAATCTCAGATCGC

GGAACTTCGGGCACAGCTTGCCAAGAAAGAGGAGGAGCTACAGGCTGCGCTGGCCAGAGT

AGAGCAGGAAACGTCTCTGAAAAACACGGCACAGAAGAGAGTGAGGGAACTGGAGGCTCA

GGTCACAGAACTTCAGGAGGATCTGGAGCTGGAGAGAGCAGCAAGAAATAAAGCTGAAAA

ACAGAGACGGGATCTGGGTGAGGAACTGGAGGCACTGAAGAGTGAACTGGAAGACACGCT

GGACTCAACCGCTGCTCAGCAGGAGCTCAGAGCTAAACGTGAATCTGAAGTGGCTCAGTT

AAAGAAGACTCTGGAGGATGAAGTTCGGAGTCATGAACAGCTCCTCGCTGAAATGCGGCA

GAAACATACACAGGCTTTTGAAGAGCTCAATGAACAACTGGAACAGGCCAAGAGGAGCAA

GGCATCACTGGAGAAGGTGAAACATGCTGCTGAAAGCGAGCGCAGTGAACTACAGATTGA

ACTTAAATCCATCACTCAGAGCAAAAACGAATCTGAACAGCGGAGGAAAAAAGCTGAAAC

TCAGCTGCAGGAACTCCAACTGAAATACAATGAAAGTGAGAAGCAGAAGAAGGACATGGC

AGATAAAATGAGCAAAATGCAGGTGGAGGTGGAGAATCTGAGCAGTGCTTTGTCTGAAGT

GGAGAGTAAATGCATCAAAGCAACTAAAGACTCTTCATCTGTGGAGTCTCAGCTTCAGGA

TACACAGGGTTTGCTGCAGGAGGAGACTCGTCAGAAACTAGCGCTGAGCTCACGGCTGCG

TCAGGTCGAGGATGAACAGAACCGTCTGAAGGAACTTCTTGAGGAGGAAGAAGAGGGAAA

AAAGAGTGTGGAGAAACAGCTTCATGCCTCTCAAGCCCAGTTAACAGAGTTGAGGAAGAA

GATGGAACAGGAAGCATCAACTCTGGAGACTTTGGAAGAAGGTAAGAGGAAGCTGCACCG

CGAGATTGAGAACCTGACCCAGCGTCTGGAGGAGAAGTCTTCAGCCTATGACAAGCTGGA

GAAGACCAAGGTGCGTCTGCAGCAGGAGCTCGACGACATGCTGGTGGGTCAGGATCACCT

GCGGCAGAACGTCCAGGAGCTCGAGAGGAAACAGAAAAAATTCGACCAGATGCTAGCCGA

AGAGAAGAGTATTTCCACTCGTTATGGTGAAGAGAGGGACCGAGCAGAAGCTGAAGCTCG

TGAAAAGGAGACGAAGGCGCTGACTTTAGCTCGAGAGCTGGAGGCTCTGCAGGAACAGCG

TAATGAACTGGAAAGAGCCAATAAACTCCTCAAAACTGAGATGGAGGACCTGGTGTCTTC

TAAAGACGACGTTGGCAAAAGTGTCCATGAGCTGGAGCGTGCTAAGCGAGCGATGGAGCA

ACAGTTGGAGGAGATGAAGCTTCAGGTGGAGGAACTGGAGGATGAGCTGCAGCTGACCGA

AGACGCGAAGCTGCGATTGGAGGTGAACCTTCAGGCCATGAAAGCACAATTTGAACGAGA

CCTGCAGGCACGAGACGAGCTCGGAGAGGAGAAGAGAAAACAGCTGATGAAACAGGTGCA

TGAGATGGAAGCGGAGCTGGAGAATGAGAGGAAACAGAGATCTCTGGCTTTAGCAGTGAA

AAAGAAGTTGGAGCTCGACCTGTCCGACCTGGAGGCTCAAATAGACCAGGCCATTAAAGC

TCGAGATGAAGCACAGAGACAGCTGAAAAAACTACAGGCCCAGGTAAAGGATCAGTTACG

GGATATGGATGAACTCCGAGCGGCTCGAGATGCGTCACTGAACAGCGCTAAAGAAAACGA

ACGCAAACTCAAGAGTCTGGAGGCTGAAAACATGCAGTTACATGAGGATCTGTCTGCAGC

TGATCGCAGTAGGAAGCAGATTCAGCAGGAGAGAGATGATCTGCATGATGAAATGACCAG

CTGTAATTCCAAAATGTCTGTGCTTAGTGATGAGAAGCGGAAGTCGGAGGCTCGTATTGC

TCAGCTGGAAGAGGAACTTGATGAGGAACAGTGCAGCACTGAACTTGTTAATGACCAACT

AAAGAAGGCAAATGCACAGGTGGACACTCTGGGTAGTGAGTTAGCGGGTGAGCGTAGCGC

GGTTCAGAAGAGTGAGACGGCTCGACAGCAGCTGGAGAGACAGAATAAGGATCTGAAGAG

TAAACTGCAGGAACTGGAGGGTTCCGTCAAATCCAAGTTCAAATCCACTATCGCCGCCCT

GGAGGCCAAAATCCTGCAGCTGGAGGAACAACTGGAGCAGGAGGCCAAGGATAAACAGCA

GACTTCAAAGTCAATGCGCCGTACGGAGAAGAGACTGAAGGAGGTCCTGCTGCAGGTGGA

GGACGAGAGACGCAACAGTGAGCAGTTTAAAGATCAGATGGAGAAGGCGAACTCGCGCAT

GAAGCAGCTGAAGCGTCAGTTGGAGGAGGCGGAGGAGGAAGCGACCCGCGCCAACGCCTC

CCGCAGGAAGTTACAGCGAGAACTGGAGGACGCCACCGAGGCCAGTGAGACCATGAACCG

TGAAGTCAACACCCTGAAGAGCAAACTCCGACGCGGTGGTGACTTCACCCTCAACGTACG

GCGTGTGGTCCGTGGTGGTCTGGACAGCGAGGAGGACGCTGACCTCACCAGTGAGACATC

AGAGGCCACACCTGAGTAACACCTCCAGCTGACAGCCAATCAGATCGCCTGAGTCCAGGA

CAGCTGCATCACTTTTACTCCTTTAATCCACATCTTCATCACGAAACGATTCAAACTCAT

TCTGATACCCGAATACTGAGCGCTTTAATAGCTGCCAATCAAAACGGTTGCCAGTTCATT

TAAAATCCTGCCGTCTACCCAATGAAAAACTTTATTGGTGTCCGGCTGTGGGACTGGTGT

CCGGCTGTGGCTTTACATCTTAATATTTTTCAGCTTGTTTTTATTTACGTTTTTTACAGC

ACTTTTTTTTTGCTTTGTTGCCGTGTGCAGGTCAGGTTTTTCCTCACGGTCACACTCGTC

CACTGGATTAATAAAGATCTATAATTTGATTAAATGCTCACTATTATTAAATTATCAGTC

GTGCACAAAAATCAGGTTGGATGGGATCCGCTCCCGAAAATGGATTTTAGAGAAACTACA

CTGGGTTGAGTTACTGTGGGTTGCCAGATCAGTGGAACTCGCACTGTTCCACACTAATGA

TTTATTAGCAGGTTCTTTTGTTAATTGTTTAAACACTGCGGTGTTTTTTTCACTTCAGTC

TCTTCACATTAAATGGTGTTTAGTTATTCATCACTCTTTTTCTTACTTTTCTGTGTTTAT

TTTACATTTTCACACTCGTTTCTCTCCGTAAAGCTCCTGACCCCATCCCCCCCCCCCCC

>Unigene000101

GGGTTGCGAAATAATTATAGCCTGTCAGTGCGTAGTTGTGGTAATGTAACTCATGTTGTT

TTTAGATGTGTTTGCGGTTCTGAACAGATCAGTGATGGGTGGCGCTGTTATAGTGATATC

ATGGAGCGTGTTCTAGAACTGGATCGGGAAGGGAGCCAGGCAGGAAACCTCACTGGAAGC

ATCGCGTTGTTTGAGAAGGGATGTAAGCTCAGTTTGAGCAAGCATGGGCCATTTACAGCA

TTTATCCCAGTGGATACATCACAAATATCTTCAGGCAAGCTCAACCAGGCAAGAACAGCA

GAAGCTATCTGCAAACACCACCTCATTCTTGGTCAGCATCTTTACAAAGATCTGGAGGGT

CACGATTTATGGACATACGGAGGGGAACTTATGAGATTTAAACCAAACAAAAAGTTTATT

TCAAAAAATGATCCTGATACTGTGTTCACCATAATCAACAGTGACATACCTGCATCCAAT

GGAATTATACATATAGTGGACAAAGTGTTTACTTTTGCTACTGTTGACACTTATGACAGT

GCTGAGTTTTCTTCAAAAACCATTGGGGACATTCTTGCAGAAGACACCAGATTCAACAGA

TTTGTATCTCTAGTAGATAACTGTGGTGCTCCGATGCCACTCCAGGGTCCAGGGCCGCTC

ACACTGTTTGTCCCAACCAATAAAGCTATAGACAGATCCAGGGATGGGAGTATTATATAC

ATGCTAAAGGAGGCCAAACACAAACTTCAGGAGCTTTTGAGGCACCATATGTTCTCTCAG

GCAGCGGTAACTATGGACCAGGTGGCTAGCATGACGGAGATTCATACAATGGCTAATCAG

GCCATAACAGTCAGTATCTCTGATGATGGAAGAGTGTTGCTCACTGAAAAAAAAATATCT

TTTGATATGAAGGACATCATTGCATCCAATGGAATCATTCACATTGTTGACGGACTTCTC

TTGCCTCCGTCTATCATCCCGATCATGCCACACCGATGTGATGTAAATGAGACCACCATT

ATCACGAGTCCATGTGTGATGTGCAAATTCATTTCTGAGTCAAAATGTCCACCTGGAAGT

GAGAAACAGACTGACCGTTTTCAATTTTGTGAGCCTCCTCCTGATCCACGTTGGAGCGAA

TTTACCACCGTACGAGGCTGTGCGAGATACTGCAAGGCAAAGCGAGCGAGAGCTGAATGC

TGCAAAGGTTTTTATGGACCTGACTGCAAACCCTGTATTGGAGGTTTCCAACACCCATGT

TATGGCAAAGGCACTTGCTCTGATGGCATTTATGGAGATGGCTCATGTAAATGTAAGCCA

GACTTTATGGGCATCGGGTGCCACATCTGCTCTAACCCAATGAAACATGGAGAAAACTGT

GATGAAGATTGCCGCTGTGTCCATGGCGTGTGTGATAACAGGCCTGACAGTCAGGGTGTG

TGTCGCAGGAACTCCTGTTTGTCCGGATACACCGGAGAGCTTTGTGATCTCACAGCCATG

CTATGTGATTCAGATGGTGCCTCTGAACACTGCCATATTCATGCTTACTGTACACGTGTC

AACAACATAAACATGTGTGTGTGTAACCCTGGATATGAAGGTGATGGATATTCCTGCACT

GAAGCCAACCTGTGCCTCAGAGCTGACAGGGGAGGCTGCCATATAGGTGCACAGTGTTTG

TATGCAGGCCCTGGAAATATATCGTGTATATGTAATGAGGGATGGACAGGTGATGGTTTT

GTGTGTGTGGAGATCAACAACTGTTTAACAGCAAATCGTGGTGGGTGTCATGAACAAGCT

GAATGCATCTACATTGGACCTGGCCAGAGTGAATGTGCCTGTAAAAAGGGTTTCATGGGA

GATGGCATCATGTGTCAGATTGTTAACCCCTGTCTGACAGACAATGGAGGCTGCCATGCC

ACGGCAACATGTTCATTTAATAATAGCGGCATACACCAGTGTACATGTCCAGAGGATCAT

GCAGGAGATGGCTTTAAATGCTATGGGAATATACTGATGGAGCTAGAGGGAAACTCAGAT

TTCTACACTTTCAACCGCTATCTACTAAGACACCCTGTGATCACTGCTGAGAGTAATGTG

ACTGCTTTAGTTCCTTCCAAGGATGCGTTTAAAAATCTGAGTGATGCTGATCAGGTGTTT

TGGACTGATTATTACCACCTTCCCTACCTCATGCAAGCTCACTTTTTGGATGGCATTTAC

TCCTATGATGACTTAAGTCAGCAAGTCAATAAAACAGTCTCAACTAAATCCAAGACAAAA

TGGAAGATCACATTTAAAAATGGGGAACTTATGATTGATAATGCAATTGTTCTTGTCTCT

GATCTCCAAGCAACCAATGGGTTCATTCACATCATCAATACTGTATTAAAACCACCCATT

TCGGATATCCCCCCTCCACCCCCTGACCTAATGGAGGTCCTTCATAACACTCCCTCATTC

AGCCTGTTCAGAGAAGCAGCACTGCTGTACAATTTAACAGAAAGCATCTCGAAGAAGGAC

TTCACTATCTTTATTCCATCTGACAGTGCAGTTAAGGAGTACCTGTTGAAAACAAATTCC

ACACAACTGGATGAAAACATTGTGAAATATCACGTTATCACAAAAGAGCAGCTTTTTCCT

GAACACTTGACTGATGGAACTCTAAAAACTACACTGCTTGGAAATGAGTATCAAATCATG

ATTCACTTAAACAGTGAAAATAAGACGCTTATCAACGATGTAGAATTGGATGGAAACATG

ACTGAGATAAGACACGGTGTCATTATCAGCATATCCCGGGTTTTAGTCATTCACAAAAAC

TACTGCAGCAAAGATATTTATATGAAAACGTTTGGAAGGTGTGGTGACTGTAACTCATCT

CCCAAATGCCTGTTTGGCAGCAAGCCGATAAAGGATGAATTCCCTTTGAATATGAAGTCC

AACTGTAGATACAGGATGCGAGTAGGGAAAAGAAGAAAGTCTGTGCCAGGTTGCATGATA

GACTGTCTGAAAAATGTAAAGGATCATTCCTGCTGCCCAGGATACTTTGGACGTGACTGT

TTTAAATGTCCTGGAACTGTTGACAACTGGTGCTCAAACAACGGCAAATGCCAGGATGGC

CTTTTTGGCAATGGGGAATGTCTGTGTAGCGAAGGGTTTCATGGCACAGCATGTGAAATG

TGCGAGCCTGGGAGATATGGTAAAGACTGCAAATCAGAATGTCATTGCGATCATGGAAAA

TGCCTGGATGGCATAGATGGTAATGGTCAGTGCATCTGCTACAAGGGCTGGAAAGGGGTA

AACTGTTCTGTAGAGATCGTTAATGATGAATGTGGAGGAATTTGTGATGAAAATGCAAAC

TGTTTGTCTCCAAGTTCAGGAGTGAAACCATCATGTGTCTGTTCAGCAGGTTACCATGGA

AACGGCACCTTCTGTCAAGAAACTGAGTTGTGTGCCATTAATAATGGGGGCTGCTCAGAA

CATGCAACATGCACAAAAATCTCACCTGGAGAAAGGAGCTGCACATGTAAGAAAGATTAC

ACTGGAGATGGAACTGTGTGTCTGGAATTGGACCCCTGCCTAGTTAATAATGGAGGATGT

TCTGAAAATGCAGATTGCATCAAAACTGGACCAAACACAGCTGCATGTGTTTGTAAATCA

GGTTTTGCTACAATAAGAAACTACTGTATACCAATAAATCTCTGCCTAAAGGATAATAAC

GGAGGATGCAGCGTCAATGCTATTTGTCAAAACACTGGCCCGGGTGAGAGGAACTGCACT

TGTCGATTTGGCTACAAAGGAAATGGCATTGAGTGTATGGGAACTGTCAGTAGAGAACTC

CTTCACAATTCTGCTGCTAATTGGTTTAGGAAAAGTCTTTCGCAATCAAGAGTCAGGGAT

TTGTACAGTAAAGGGCCATTCACTGTGTTTGTACCTCACACAGATTACATTGGAAACTAC

TCGATTGATCTCTGGGAGAGTAAAAACCGCATCCCAGATCTGTTGCGGTACCACATTGTA

AGTTGTGAGGAAATCTCTGGATCACAGCTTAAGTCTATGACACATGTTGTCTCTGCTTCT

GGGCATGTGTTGAATTTCAGCGTACGAGATGGTGCTGTATACATAAACGGAGATACAAAG

ATAATCACTAGTGACTATGAATGTTCCAATGGAGTCATCCATTTTATAAATAAAGTTCTA

ATCCCGTATGAACTGAAAAACGAAACTAAAGGGATTGAAGAAATGTTAAATATTACGAAT

GCGGTAGAAGCGTATGGATATACCGTGTTTGGGAAGTTGCTACAGAAAGCTAATTTGATG

GCTACGCTGCAGCATACAGCATTTCAACCCTTCACCATGTTCTGGCCTACTGACAAAGCA

TTTAACAGTCTGCCACAAGAGCAGAAAAACTGGCTGTATAGTGAAGATCATCTTGATAAG

CTACAGGCCTATTTGAAATTTCACATTATTCGTGACCAGAGAACAATTGCTATTGCTCTG

CCGACAGAAAAAACTGTCAGAACGCTGTATGGATCATGGCTGACTTTCAGTTGCAGCCAA

GACTTAACTGGGGATATTTTAATCAATGGAAATGATGCCAAGATTGTGGACCGCAATCTG

CAGTTCCTCACAGGAATGGCCCACGGGATTGACAAGGTTCTAGAACCTCCAAATATAGGA

GCACACTGTGATGATTTTTCATCTATAGAGGTTAAAGGTCGCTGTGGATCTTGTTTATTT

CCACCACCTTGCCCCTACGGAACTGTGTACACTAACAAAACCAATGTGTGCAGTAGGCCA

TGGAATTCCTACAGACGGCATTATTTTTACGACGACTTTGAGTTTCCTAGCCCTTACAGT

TTCAAGCCGTTCGGCTGTAACCGAATGTGCTCCAAAATGTCATGGGTTTCAAAGTGTTGT

AAGAACCACTATGGACGAAACTGTCAAGTCTGTTCTGGAGGCTTGGAGGCACCATGTGGA

GAGCACGGTGATTGTGATGATGGGAGATATGGGACAGGGACATGTAAATGTCACACTGGA

TTTAATGGGACTGCCTGCGAATTATGTGAGAAGAACCATTACGGTTCTAATTGTACAGCC

TGTACATGTACCGCGAATGGGAAATGTGATGATGGCATAGATGGTGACGGCAGCTGTTTC

TGCCAGGAGGGCTGGACGGGAGTCATGTGCGAATCCAAAATCGGTGTGAAACCTGTGTGT

TCTCCTGAGTGTCACTCGAAAGCTGTTTGTCTGCCTGAGAATCAGTGCCAGTGTGAACCA

GCATATGTAGGAAATGGCTACAATTGCACAGCCCCAGATTTGTGCAGTGAATATAATGGA

GGCTGCCATGAACAGGCTGATTGCACACAGTCTGAGATTAACGTGATTTGCACTTGTAAA

TCGGGTTACTCTGGAGATGGCAATGTCTGCTCCCCCATTAACGCATGTGTTGAGGAAACT

AATGGTGGCTGCAGTGACTTTGCAAACTGCATTTTCATTGGACCTAATGAGCGACGGTGC

GAATGTCAACAAGGTTATGTGGGCAATGGACTCCAGTGCTTTGAAAAAGTAGTGCCTCCG

GTTGATCGCTGTCTGGAGAACAACGGAGACTGTCATGCTAATGCGATCTGCAAAGACTTG

CATTTTCACACCAAAACAGCAGGGGTCTTTCACCTGCGATCACCGGCTGGGAAATATATG

ATGAACTACAGCACAGCAGGGGCAGCCTGCAAAGCTGAGGGGGCCACACTCGCTACTCTG

AACCAGCTCTCTGATGCCCAACAGTTGGGTATGCATTTGTGTGTCGCTGGATGGATTGAT

GGGAAAAAAGTAGGCTATCCAATCCGCTTTCCATCAGTTAAATGTGGTGACAATCAAGTA

GGCATAATTCTATACAAGGATCCTGATGTAAACACTCCCTATGATGCTTACTGCTACAGA

ATTCGAGAGGTAAAATGTGAATGTGGACCCGGATATGTTGGGGATGGAGATTTCTGTAAT

GGCAATCTCGCCTCTGTCATCGCTACCCATTCAAACTTCTCTATATTTTACTCTATGTTA

GTAAAGACTGCAGAGGCTGCTGAAGAAGGAAAAAAGCTCTTGGATTTCCTGTCAACAAGT

TCAACAAACTTAACACTTTTTGTGCCACATAACGCAGGATTTTCTCCAAATGAGACTCTG

TCATTAAGGGATTTGGAATATCATGTATCCTCAAACAACAGTCTTCACTACTATGAAGAT

CTGAAACACAACACAGCAATCCCATCACGACTGGGTTTTAATCTTAAAGTCATAATAAAA

ACAGACAACCAAACACAAGTTGAGGATTCTTCACCAGTAAAACTGGTCAATAAACAGACA

GTATTAATCTGGGATATTCCAGCTACTAACGGCTTGATCCACATTATTGAGGGACCGCTA

AAAGCACCTCCTATCAAAGTGACCCCCTCAACTCCTTCAGCTGTCCGCTCACAATCCAGC

GCACCCACTGTTACTGCTGTGTTGCTTGTTATTTTTATCGGTGGCATTATCGCTGGTGTG

GCTTATTATTTCCTAAGAAATAAGAATGATGCCTTCAGCTTCAAGTATTTTAAGAATGAC

GATGAAGATGGCAACTCCACTAAGACCAGTGCCAATCCTGCTATCATGTCGATCCCAAAC

CCTTTATACAGTGGTTACAGGGCATTTGTTGAGCCATTTGGGGAGGAAGAACAATGTGAA

TCTCCTGCTTCAGTTGCAGCAAACACACCAAACATCCTAGACTAGCAA

>Unigene000102

ATGGCAGTGACGAGCTGGAGAGAGTGTGCGTCCCACATCTGGTGGCATTTATTTCGCTCT

CAGCTTTTCACACCTGCGAGCCCACCCTGTATACCTGTGGAAATGGCCGCTGTGTGCCGT

ACCACTACCGCTGTGATCACTATAACGACTGTGGTGATAACAGTGATGAGGAAGGCTGTT

TGTTTCGGCCCTGCGACCCAGACACAGAGTTCACCTGCAACAATGGCCGCTGCATTGCTC

GCGAGTATGTGTGCAATGGCATCAATAACTGCTTCGACAACGGAACCTCGGATGAACGCA

ATTGCGCTGAAAGGACTTGTCAGCCTGGTCACACCAAATGCCAAAGTACAAACATCTGCA

TTCCACGATCATACCTGTGTGATGGAGATAACGACTGCGGAGACATGAGTGATGAGAGCC

CCACACACTGCGCAACATCTACATGTACCCAGAGTGAGTTTCGCTGCACAAGCGGGCGAT

GTATCCCTGCACACTGGTACTGTGATGGTGGTACTGACTGTGCAGATGGGTCAGACGAAC

CCTCCACCTGTACCACCATAGTGAGAACCTGTAGCTCAGACCAGTTCCGCTGTGATGATG

GAAGGTGCATCGCATCCTCCTGGATCTGTGATGGAGACAATGACTGTGGGGACATGAGTG

ATGAGGACCAGAGGCACAACTGTGCTAATCGAACCTGCTCTTCACTGGAGTTCACCTGTG

TGAATAATCGGCCCCCACAGAGGAAGTGCATCCCTCAGGACTGGGTGTGTGATGGTGATG

CAGACTGCTCAGATGCATATGATGAACACCAGAACTGTACACGCAGGTCCTGTTCTGCAA

CTGAGTTTACATGCAGCAACGGCCTCTGTATCCGCAGTGCATACAGGTGTGACCGGCGGA

ATGACTGTGGAGACGGTAGTGATGAGCAAAGCTGTACCTACCAGCCGTGTCAACAGCACC

AGTTCACCTGTCAGAATGGACGTTGTATTTCTCAGGACTTCCAGTGCGATGGCGATAATG

ACTGTGGTGATGAATCAGATGAGCTGCCGCACATGTGCCACACTCCAGCGCCTACCTGCC

CTCCTGGGAAGTTCAGGTGTGACAATGGCAACTGTGTGCCGTCCAGCCAAGTCTGTGACC

ACAGTGATGACTGCAATGATAACAGTGATGAGAAAGGCTGTGGCATTAATGAATGTACAG

ACCCATCCGTGCACCATTGTGACCACGAGTGCACTGATACCCCCACCAGCTTTGTGTGCA

GATGCCGTCCTGGCTACCGCCTTATGTCTGATAAGAAGAGCTGTGATGATGTAGATGAGT

GTTCAGAGACCCCCAGTGTATGCAGTCAGATGTGTGAGAACACACTAGGCTCATATGTGT

GTAAATGTGCACCAGGTTTCCTTCGCGAGCCAGACGGGCGTCGTTGTCGGCAAAACAGCA

ACATCAGTCCTTACTTGATTTTTAGCAACCGCTACTACCTGCGTAACTTATCTGCAGACG

GAGCAGCCTACTCGCTCATCCTTCAGGGCCTTAGCAGTGTTGTGGCTCTAGACTTCGATC

GCGTCGACAAGCGTCTTTACTGGATCGATGTGAGCCGTCGTGTGGTGGAGAGAATGTTTT

TTAATGGCACAGGCAGAGAGGTGGTCCTGAATGGCATTATGCACGGAGAAGGCCTGGCTG

TGGACTGGGTGGGACGGAAGCTTTATTGGGTAGACAGCTTTTTAGACTGTCTGAAGGTGT

CTGAGCTAGATGGACGCTTTATTAGGAAGCTGGCTGAGCACTGTGTGGATGTTAACAACA

CCTACTGCTTTGAGAATCCAAGAGCCATTGTGGTGCATCCAAAGTTTGGATATGTGTACT

GGACTGACTGGGGAAGTAAAGCATTTATAGGTCGTGTTGGGATGGACGGTAACAACAAGT

CTGCCATTATTACCTCTAAGATCGAGTGGCCCAATGGTCTTACCATCGACTACACCAATG

ATATGCTCTATTGGGCTGATGCTCCCCTTAACTACATTAAATCCTCTAACTTAGAGGCCC

CCCACCGCCACACTGTTTATGATGGTGTTCTTCCTCACCCGTTTGCTATAACTGTGTTTG

AGGACACTGTGTACTGGACAGACTGGAACACTCGCACTGTAGAAAAAGGCAATAAGTACG

ACGGCACTGGGCGCCAAGCTCTTGTCAACACTACTCACAGGCCCTTTGATATCCACGTGC

TTCACCCGTATCGTCAGCCCATAGTAACCAATCCCTGTGGAATAAACAACGGCGGCTGTT

CCCACCTGTGTCTCTTAAAAGCTGGAGGACGGGGATACACTTGTCAGTGCCCAGACCACT

TTCTGACTGTGCAGATAGGGGGCGTAGCCCGCTGCCTACCCAGCTGCTCCAGCACACAGT

ATAGATGCGCTGACAACGAGCGTTGCATCCCTATCTGGTGGAAGTGTGATGGTCAATCTG

ATTGCAGGGACGGTTCCGACGAGCCGTCCACATGCCCCCTGCGCCATTGTAGGTTGGGCC

AATTCCAATGTAATGACGGAAACTGTACCAGCTCACATTTTCTGTGTAACTCAAATCGCG

ACTGCCCTGATGGATCTGATGAGGACACTGTGCTGTGTGCAACACACCAGTGTGAGTCCC

ACCAATGGCAGTGCAGTAATAAGCGGTGTATCCCGGAGGCGTGGCAGTGCGACGGGGAGA

ATGACTGTGGTGACGGCTCGGATGAGGAACCCTCACACTGCTCCAGCAGAACGTGTCGTC

CTGGCCAGTTCAAGTGCAGGAATGGCCGCTGCATCCCTCAGAGCTGGAAATGTGATGTAG

ATGATGATTGTGGAGATAACTCGGATGAGCCCTTGGATGAGTGCATGGGACCAGAATATC

GATGTGATAACCACACTGAGTTTGACTGCAGTACAAACTACCGCTGTGTTCCTTTGTGGG

CCGTGTGTAATGGACAAAACGACTGCAGAGACAACAGTGATGAGCAGAACTGTGAGGAGA

TGACCTGTGACCCTGCTGGAGATTTCCGCTGTGATAACCACAAGTGTATCCCCCTGCGCT

GGAAGTGTGATGGAGACAGTGACTGTGGAGATGGATCAGATGAACGCAACTGCAACCCTC

GAGCCTGCACAGAGAGCGAGTATCGCTGTGACAACCTGCGCTGCATTCCTGAACGCTGGG

TGTGTGACCATGACAACGACTGTGAGGATAACTCGGATGAAAGGGACTGTGAGTTGCGGA

CCTGCCATCCTGGATACTTTCAGTGTGATAGTGGTCACTGCATTGCTGAGCGCTTAAAAT

GCGATGGCAGTGCAGATTGTATGGACGTCACTGATGAGACATCCTGCCCAACACGTTATC

CAAATGGCACATACTGTCCTCCCTTCCTGTTCGAGTGCAAAAATCATATATGTGTTCAGC

AGCACTGGAAATGTGATGGAGACAACGACTGTGGAGATGGATCGGATGAAGAGCTGCATC

TCTGCTTGGATATACCGTGTGAAGCCCCTTTCCGCTTCCGCTGTGACAATAACCGCTGCA

TCTACAGCCACGAACTCTGCAACTCAGTAGATGACTGTGGAGACGGAACGGATGAAAAGC

AGGAGCACTGCTTGACTCCCACTCATGGACCCTGTTCCTCTGATGAGTTTAAATGTGGGA

ACAGCCAGTGCATTCCTCTGCAGTATGTCTGTGATGATTATGATGATTGTGGAGACCAGA

CTGATGAGTTGGGCTGTAACAGTGGAAATGGACGCTCTTGCAGTGAGAATTTGTGTGAAC

ATAACTGCACCGAACTGACGGGTGGAGGCTTTATATGCTCCTGCAGACAGGGCTACAAAG

CCCGAGAGGACAACAGAAACTCCTGTGAAGATGTAAATGAGTGTGAGCTCTATGGCACTT

GTCCCCAGCTGTGCAAAAACACTAAGGGGAGTTACGAGTGCTTCTGTGCTGAGGGATTCC

GCTCTGTGGGACCGCAACACAGGACTGAATGCGCTGCTGAAGGCAATCCTCCAGTGCTAC

TGTTGCCTGATAATGTGCGTATCCGTCGCTATAACCTGTCGTCTAAGCAGTATTTAGACT

ATATCGATAATGCTGAACACATTCATGCTGTGGACTACATTTGGGACCCAGAGGACCAGG

GGCTCAGCATTGTATACTGGACCATTTTAGGTCATGGTGCTCAGTTCGGATCCATAAAGA

GGGCATATATGACCACATTTGATGACCATGGAAGTAACCAGATGAAAGATGTAGATCTGA

ACCTTAGATACATATCCAGTCCTGATGGAATTGCTGTGGACTGGGTTGGGCGACACATTT

ATTGGACAGATGCTGGAACCAATCGTATTGAAGTGGCAAAACTGGATGGGCGGTACAGGA

AGTGGTTAATCTATTCCAATCTGGATCAACCAGCTGCAATAGTTGTCAACCCTGCTATTG

GTATGATGTACTGGACTGACTGGGGTAGCCGACCAAAGATTGAAGCAGCATGGATGGATG

GTCAGCACAGACAGGTGCTGCTGGATGAGGATCTGGGCTGGCCTACAGGACTCACTCTAG

ATTACCTTAATGGAAACAGGATTTATTGGTGTGACTCAAAAGAGAACATTATTGAGTCTA

TGAAACCAGATGGAACAGACAGGAAGATCGTCCTGTCAGGAGATATTGGAAACCCCTACA

GTCTGGATGTGTTTGAAGGACATGTGTACTGGACAACAAAGGTCAGGGGTGAAGTATGGA

AGGCTGACAAGTTTGGAAGGGGGGATAAAGTCAAGATGTTGACCATAAACCCTTGGTTGA

CTCAAGTCCGCATCTATCATGAGCACAGATACAATCGCTCAGTGTTAAACCCCTGTAAAG

GTGTGTGTAGTCATCTGTGTCTGCTGCGACCTGGTGGATACACCTGTGCCTGTCCACAAG

GCTCTTCTCTGGTCAGCTTTAATGAAAACGAGTGTGATGCTGCCAGCCTACCCGAGGTTG

CTATGCCGGTTGAATGCGGATGTATTAATGGTGGAACCTGCTCCACTGACAAGCCTGGCC

TGCCAAAATGCAAGTGTCCTTATGGTTATTCTGGAAGGCACTGTGAAATAGGAAAGTCCC

GAGGAGCACCTGCTGGTACAGCAGTGGCTGTGCTGTTGGCAGTGATTATTATTCTCTTAA

GTGGGGCTCTCGCTGTTGGGCTTTTCCTGAACTACAAACGCACAGGCTCACTCATCCCTT

CAATGCCTAAGCTACCCAGCCTGAGCAGCCTGGCTAAGTTAAGTGATGCAGGAAATGGTG

TGTCCTTCCGCTCAGGGGATGGCAGTACTGTGGATCCTCAGACTCTTGGGGTATCGTTTA

TTGATAGAGCCATGCAGCTGAATGAAAACTTTACAGATGCTGGGCGGGAACCTGTCACAT

TCGAGAATCCTCTTTATTCCACTGCAACAGGTGCAGCTGCTGATGCTGCGGTCATACATG

CCACGCAGGTGACTGTGAATATAAGTGGAGATTCAACAGAGAACGTATTCGCAAATCCGA

TGTACAATGAACAGCAGCAGGCAGTGGACATGAAAAATACACCAAATGAACAAGCAGCAA

AGGAGTCCAAGTGGAACTTTTTCAAGAAAAAATTAAAACCTAGTACAACCTTTGAAAACC

CAGCATACTCAGAGATGCGAGACAAACAGTCCGCTTTGACTACGGAGGACATTTCAACTA

GCCATCCACAGCCAGCTCTTCCACCACTAAAGCGAGAGAAATCCAGCATGTACTCACCGA

CAGAAGACTCCTTCCAAGACACGGCCAATCTGGTCAAAGAGGACAGCGACATATAACGCT

TACTTTCTTTTTCAATCTTTCCTACACACAAACGCAGATACAACGGGAAAAAAGACACAC

TCTGTTCCTCTTTGCACAGAATCTATTTTTATATGCAGATTTTGTACAAAAAAAAAAAGA

AATCTTAAGATTATTTTAAGAACACTTAATAAGATGTTTTTATAACAGAAAATTAAAAGG

TCTATGGAGACTATGCCTTCTGCTGTTTCTTATGCCAATTCATGTCTATAATTGTTTTTA

TATTGTGATGTACTATATGTACTGTATATCTTTGCACTGATACTACTGATGGTAAAAATG

TTGTACATTTGTAAAAAATTTTTAGAAGATTTCGATGGTAACTGGAAATGATGTGCAGCA

GATCCTGCGTTCTATTAAAGATAAATAAAATGACCGCTGAACATGGTAGTGTGTAGGTTA

ATGCACGCTGCTCAGTGATTTATTCCTTTTTGTTGCTTCTTTGCATAGAAATTTACACTG

ATAATTTGTAAGTGGACTAAAAAAACACATCTCATCCTGAACATTTATATACCTCATGTG

TGGACTGGCCAGCACAGGAAAAAAAGAGCCAATTTCTGTTTTGGTCCATGATTCACAATA

TGTTCTTAAGCAGTATCTTTGTTCAAATTAATATTACATTACCTTAAATACAGTATATAT

TAGACATTTATAGATTTATGAACTGCTATAGATTATTTGTTGTTACCCAAGTTTTAATTG

TAGATATGGTAATTACCAGAAATCAAAATAACTGATGATATTTTTTGCCTTGATATCTAC

CAGGCTAATTTATACGGCGTGTCTTGTAAAAACATTTACTGTATTGTTGCAGTAGGCAAA

TATTAAAGACTGCTGTTATTTAAAAGAAGGGACTGATTTAAAAAAAAAAAAAAGCTAATA

ACCTATTTTCATTCATAATTTATATTACGAATGTCACTAAT

>Unigene000103

CAGTAAAGATCGGAAGAGCGTCCTGGAGAAGCTAGAGGACAGATTGGGAGGCCACGCCCA

TTTTGTGACTCATAAACTGGCCAATGGTAAGATTCGTAAGGCTGTAGGGAGGGAGGAGTT

TCGCCTGCTTCACTACGCCGGAGAGGTCAACTACAGTGTGAATGGATTCCTGGACAAAAA

TAATGATCTTCTGTACAGAAACCTAAAGGAGGTGATGTGCCAGTCGAAGAATTATATAAT

AAAACAGTGTTTTCATCCAAATGAGCTGACAGACCAGAGAAGACCAGAGACGGCTGCTAC

CCAGTTTAAGCTGAGTTTGGCCAAACTCATGGAGATTCTGATGTCTAAGGAACCGTCATA

CATCCGCTGCATCAAACCTAACGATGCCAAACAACCAGGAAGGTTTGAGGAGGTGTTGGT

CAGACACCAGGTGAAGTACCTGGGCTTAATGGAGAATCTGAGGGTAAGAAGAGCCGGATT

TGCTTACAGACGAAGCTTTGAGGCTTTTCTACAGAGGTATAAGCCTCTATGTCCAGACAC

TTGGCCAAATTTTAATGGAAAACTCTCAGATGGTGTCTCCACCCTAGTCAAACATCTGGA

CTACAAACCAGAGGAGTACAAACTGGGCAGATCGAAAATCTTCATCCGTTTCCCAAAAAC

ACTTTTCCGCACCGAGGACGCTCTGGAAAACGTGAAACCAACCATCGTGATCATCCTACA

GAAATGCTGGAGGGGCTACAGGGAATGGGCCAAATATCAGGGCATCAGACACGCAGCAAT

AACAATCCAGTCATGGTGGAGGGGCGTGAAAGGGCGCAGGAGAGCCAAGCGCCGCAGACA

AGCTGCAGACACCATCCGCACCTTTATAAAAGGTTTTATTTTGAGGCAGGAGCCTCGTTG

TCCTGATAACGAGTACTTCCTGGATCATGTGCGCTTCTCCTATTTGATGACCATCAAGCG

CAACTTACCAAAAAGTGTCCTTGATAGGACCTGGCCAGTGCCGCCTCCATCACTGGAAGA

GGCCTCGGTTTACATCCACCGCTTGTGTATACGCAACCTGGTGAATGACTACTGCAGGAA

AATCCAACCCGAGTGGAAGAACCAGTTGGAGCAGAAGGTTGTAGCCAGTGGTATTTTTAG

GGGCCAGAAGGACAACTACCCTCGAAGTGTTCCTAGACTTTTTGTGGGCACCAGGCTGGA

GAATGAAGAGATCAATCTGAAAGTGAGACAAACTCTAGGAACTGACAACAAACTGAAGTA

CGGTGTGCCAGTCATCAAGTATGATCGGCACGGATTTCGCGCGCGGCCCCGGCAGCTGCT

GATGACCGGCTCTTCTGTTGTTCTTGTTCAGGAGTCCAAAATCAAGCAGCGAATCGACTA

CACTTCACTCCTGGGCATCTCAGTCAGTTCCCTCAGCGATGGCTTTTTCGTCCTGCACGT

CCCCACGGCGGATAGCAAACAGAAGGGCGACCTGGTGCTGCAGAGTGACCATGTGATCGA

GGCCGTCACCAAACTGGCCATCCTGTCCGACAAGATACACAACGTCAATGTCAGTCAGGA

CAGTATTTGTTTTGCAATAACCCGGGGCAAAGAGGGCATCATTGACTTCACCTCGGGGGC

GGAGCTACGAGTGATGAAGGCTAAGAACGGACACCTGGCTGTGACGGCCCCGTATATCAA

CAGCAGCGCATGATCAGGTGTGGACCAATCCCAGCTCACTATGAAGAACCCTTCCTCATC

ATGGATCGAGCACTCACAGAAGAATAATCAGCTTATGCCAATCATCGCAACTGCCACACT

TTCTAACAGACTCTCACGCTAAAGTGAATGTCAAGCTCGTTCCGACAGTTTATAACAAAC

CAGCGCCACTAACCTTGTTTGATTTAGTTTTTCACAATAAAAGCATTTACCAGCCAGATT

AACGGAATGAATTCTGCACCAGTTACCATCGATTTTCTTTTGAAGCGATTCTTTTACGAC

TTTCGCACGGGTTGAAGAATTTACAATATGGCGTCCGAGCCATGTTATTAATTTCTTTGA

TGATGTTGCACGTTCTTCAGCATCTTCAGTGGAGCTCGGAGGATCAGACCCCAGCTGGAA

AAAAACCCCAACTGCTACAGAATCAAAAGCAAAACACACGCCAAGTCGAGCTGTCAATCA

TCCGGGGTTATTACAAGCTATAACACGTTTACGTCAGCCGTTGACTGCAGAGCACGATGA

AAACCCCTCGCTGAAGCTACACACACGTTTGCACTTCCAGAAGATCGGAAGAGCGTCATG

TCAGTGAGACAGGAGGAGGGGGTGTAGAGCAGATCGAGTAAAATGAAGGATTTCTGCAAG

TAGAAAAGCTGGTCAGGTTTGACGGTGTGTACCAGAAGGTGTCTGGGGAAACATTTCGAA

GAGTAAGCCGACTGTAAAGGAGATGCACTCACACACACGCTGAGGATCAAGAGAAAACGA

CGACCGAAAGTAAAAGGAACAGGAAAAAAAGCTGAAACTAGAGAAGAGGATGAGGTACAT

TGGCAGGGAGGTGGAGGTGGATGGAGGTGTGAGGTTGGTGATGGAGAGCGCTCTGTCAGC

GAGGGATAGGGTGGGGGTGCAGGATTTCCTGCTGCTGGACAACTACACCAGCGAAGCAGC

GTTTATCGAGAACCTGCGGCGGCGCTATAAGGAGAACCTCATATACACATATATCGGCTC

TGTGCTGGTGTCGGTGAATCCGTACAAAGAGCTGGGGATCTACTCCAAAACCAGCATGGA

GCGCTACAGAGGAGTCAACTTCTACGAGATTTCACCTCACATTTACGGCTTGGCGGATAA

CTCGTACCGCGCGCTGCGCACATCACGCAAGGATCAGTGCATCCTGATCTCCGGAGAGAG

CGGAGCAGGAAAAACGGAGGCTTCTAAGAAAATCCTGCAGTATTACACCACCACCTGCCC

CACACGCAACAACACGCCATCCATCCGAGAGAGGCTGCTGCAGTCAATTCCCGTGCTGGA

GGCTTTCGGGAACGCCAAAACTCTGCGGAATGACAACTCCAGCCGCTTCGGCAAGTACAT

GGACGTGCAGTTCGACTATAAGGGTGCTCCAATCGGTGGCCACATCCTAAACTACCTTCT

GGAGAAGTCTCGAGTGGTGCACCAAAATCATGGAGAGAGGAACTTCCACATCTTCTACCA

GCTGCTGGAGTCTGGGGACAGCTCACTACTGATGAGGCTGGGGCTGGACACCACCAACCC

ACAGCAATACCGCTACCTGGTTAAGGGCAACTGTCCACGGGTAAGTTCCATCAGCGATAA

AAACAGCTGGAAAGCCGTAAGCACCGGTCTTACCATCATTGGCTTCAATGAGAAGGAGGT

GGAGGAGTTACTGAAGGTCGTGGCAAGTGTCCTGCATTTGGGTAACACCCAGTTTGGGGA

GGATGAGTACGGGGAGACACACTTTACCACCGAGACTCAGCTCACATACCTCACTGAGCT

GCTGGGTGTGGAAGTGTCAGCGCTGAGTGAGGCTCTAACCCATAAAAAGATCGTTGCCAA

AGGAGAGGAGATGATTGGCCCGCTGACCCTCGAACAGGCTCTCTCGGCTCGAGACGCTCT

GGCCAAAGCCATATATGGTCGTACTTTTACTTGGCTTGTCCAGAAGATAAACCAATCACT

GGCTTTCCAGGATGAAGTGTACTACACCAGCAAATGCTCCTCAGTCATTGGTCTGTTGGA

TATCTACGGCTTTGAGGTCTTTCAAAACAACAGTTTTGAACAGTTCTGTATTAATTACTG

CAACGAGAAGCTCCAGCAGCTTTTCATCGAGCTCACGCTGAAGAGCGAGCAGGAGGAGTA

CGAGGCTGAGGGCATTGGGTGGGAGTCGGTGGAATATTTCAACAACAAAATCATCTGTGA

CCTGGTGGAGGAGAAGTTCAAAGGAATCATCGCTATTCTGGATGAGGAGTGTTTAAGGCC

AGGAGATGCCACCGACACCACCTTCCTGGAGAAGCTAGAGGACAGATTGGGAGGCCACGC

CCATTTTGTGACTCATAAACTGGCCAATGGTAAGATTCGTAAGGCTGTAGGGAGGGAGGA

GTTTCGCCTGCTTCACTACGCCGGAGAGGTCAACTACAGTGTGAATGGATTCCTGGACAA

AAATAATGATCTTCTGTACAGAAACCTAAAGGAGGTGATGTGCCAGTCGAAGAATTATAT

AATAAAACAGTGTTTTCATCCAAATGAGCTGACAGACCAGAGAAGACCAGAGACGGCTGC

TACCCAGTTTAAGCTGAGTTTGGCCAAACTCATGGAGATTCTGATGTCTAAGGAACCGTC

ATACATCCGCTGCATCAAACCTAACGATGCCAAACAACCAGGAAGGTTTGAGGAGGTGTT

GGTCAGACACCAGGTGAAGTACCTGGGCTTAATGGAGAATCTGAGGGTAAGAAGAGCCGG

ATTTGCTTACAGACGAAGCTTTGAGGCTTTTCTACAGAGGTATAAGCCTCTATGTCCAGA

CACTTGGCCAAATTTTAATGGAAAACTCTCAGATGGTGTCTCCACCCTAGTCAAACATCT

GGACTACAAACCAGAGGAGTACAAACTGGGCAGATCGAAAATCTTCATCCGTTTCCCAAA

AACACTTTTCCGCACCGAGGACGCTCTGGAAAACGTGAAACCAACCATCGGTGAGGGAGT

GACGAGAGGGGGAGTGTTGTTGAGGATGATAGTAACAGGGTGGTCCATGTGGTGTCAGGG

GGTCCATGATGGTAACAGGGGGTCTATGATGGTAACAGGGAGTCTATGAAGGTAACAGGG

GGTCCATAATGGTAATAGGAGGGCCAATATGGTAACAGAGGGTCTATAATGGTAACGGGG

GGGCATGTGGTAAAAGGGGTGTCGAGAGAGAGAGAGAGAGAGAGGAAGGGGAGCAGCGTA

ATTGGAATCCACTCATTACAGAGGCTTGTGAGCGTTACGGTGTTTTTCCGTCAGAGAGAG

GGAACATGTTCATGTCAGTGAGACAGGAGGAGGGGGTGTAGAGCAGATCGAGTAAAATGA

AGGATTTCTGCAAGTAGAAAAGCTGGTCAGGTTTGACGGTGTGTACCAGAAGGTGTCTGG

GGAAACATTTCGAAGAGTAAGCCGACTGTAAAGGAGATGCACTCACACACACGCTGAGGA

TCAAGAGAAAACGACGACCGAAAGTAAAAGGAACAGGAAAAAAAGCTGAAACTAGAGAAG

AGGATGAGGTACATTGGCAGGGAGGTGGAGGTGGATGGAGGTGTGAGGTTGGTGATGGAG

AGCGCTCTGTCAGCGAGGGATAGGGTGGGGGTGCAGGATTTCCTGCTGCTGGACAACTAC

ACCAGCGAAGCAGCGTTTATCGAGAACCTGCGGCGGCGCTATAAGGAGAACCTCATATAC

ACATATATCGGCTCTGTGCTGGTGTCGGTGAATCCGTACAAAGAGCTGGGGATCTACTCC

AAAACCAGCATGGAGCGCTACAGAGGAGTCAACTTCTACGAGATTTCACCTCACATTTAC

GGCTTGGCGGATAACTCGTACCGCGCGCTGCGCACATCACGCAAGGATCAGTGCATCCTG

ATCTCCGGAGAGAGCGGAGCAGGAAAAACGGAGGCTTCTAAGAAAATCCTGCAGTATTAC

ACCACCACCTGCCCCACACGCAACAACACGCCATCCATCCGAGAGAGGCTGCTGCAGTCA

ATTCCCGTGCTGGAGGCTTTCGGGAACGCCAAAACTCTGCGGAATGACAACTCCAGCCGC

TTCGGCAAGTACATGGACGTGCAGTTCGACTATAAGGGTGCTCCAATCGGTGGCCACATC

CTAAACTACCTTCTGGAGAAGTCTCGAGTGGTGCACCAAAATCATGGAGAGAGGAACTTC

CACATCTTCTACCAGCTGCTGGAGTCTGGGGACAGCTCACTACTGATGAGGCTGGGGCTG

GACACCACCAACCCACAGCAATACCGCTACCTGGTTAAGGGCAACTGTCCACGGGTAAGT

TCCATCAGCGATAAAAACAGCTGGAAAGCCGTAAGCACCGGTCTTACCATCATTGGCTTC

AATGAGAAGGAGGTGGAGGAGTTACTGAAGGTCGTGGCAAGTGTCCTGCATTTGGGTAAC

ACCCAGTTTGGGGAGGATGAGTACGGGGAGACACACTTTACCACCGAGACTCAGCTCACA

TACCTCACTGAGCTGCTGGGTGTGGAAGTGTCAGCGCTGAGTGAGGCTCTAACCCATAAA

AAGATCGTTGCCAAAGGAGAGGAGATGATTGGCCCGCTGACCCTCGAACAGGCTCTCTCG

GCTCGAGACGCTCTGGCCAAAGCCATATATGGTCGTACTTTTACTTGGCTTGTCCAGAAG

ATAAACCAATCACTGGCTTTCCAGGATGAAGTGTACTACACCAGCAAATGCTCCTCAGTC

ATTGGTCTGTTGGATATCTACGGCTTTGAGGTCTTTCAAAACAACAGTTTTGAACAGTTC

TGTATTAATTACTGCAACGAGAAGCTCCAGCAGCTTTTCATCGAGCTCACGCTGAAGAGC

GAGCAGGAGGAGTACGAGGCTGAGGGCATTGGGTGGGAGTCGGTGGAATATTTCAACAAC

AAAATCATCTGTGACCTGGTGGAGGAGAAGTTCAAAGGAATCATCGCTATTCTGGATGAG

GAGTGTTTAAGGCCAGGAGATGCCACCGACACCACCTTCCTGGAGAAGCTAGAGGACAGA

TTGGGAGGCCACGCCCATTTTGTGAC

>Unigene000104

GATGTACCGTGTGGACGTAACAGCGGTGATGTCGTTGAAAGCGCGTTGTGCTTTTTTGTA

ACGAACTTCGGTGTCCTGTTAGATAGTACAATAGATTTAATTAAAACCTCTGTTGAGAAT

TAATCACGATTGAGAAAACAAATCATGGCGGATGTTACAGCGCGTAGTTTACAGTACGAG

TATAAAGCGAACTCGAACTTGGTGTTGCAAGCAGACCGCTCACTGATCGACCGCACGCGC

CGGGATGAACCCACTGGCGAGGTTTTGTCTCTGGTTGGAAAATTGGAAGGAACAAAAATG

GGCGATAAAAGTCAAAGAACAAAACCACAGATGTTGGAAGAAAGACGAGCAAAGAGGCGA

AAGCGGGACGAGGACAGACATGACATTAATAAAATGAAAGGTTTCACGCTCCTGTCTGAG

GGAATTGATGATATGGTCGGTATTGTTTACAAACCCAAAACAAAAGAAACCAGAGAAACA

TATGAAATCCTCCTGAGTTTTATTCAGGCTGCACTCGGAGATCAGCCGAGGGACATCCTC

TGTGGAGCTGCTGATGAAGTCTTGGCTGTATTGAAAAATGATAAGATGAGAGATAAGGAA

AGGCGGAGGGAGGTGGAACAGTTGCTTGGCCCTGCGGACGACACACGGTACCATGTTTTA

GTCAATCTTGGCAAAAAGATCACAGACTACGGAGGAGACAAGGAGCTCCAAAATATGGAT

GATAACATTGATGAAACATATGGAGTAAATGTGCAGTTTGAATCTGATGAGGAGGAAGGT

GATGAAGATCAGTTTGGAGAGGTTCGTGATGAAGGCTCTGATGACAGTGAGGGTGAGGAA

GCAGATGTGGGCTGTACTCTGTCTGCTAATTTGGGAAGCGCCGGAGACGTGATGACAAAT

AAAAAGAAAGACCTTCATCCACGGGATATCGATGCCTTCTGGCTCCAGCGTCAACTTAGT

CGCTTCTACGATGACGCTATTGTGTCTCAGAAAAAAGCAGATGAAGTTCTGGAGATTCTT

AAGACTGCAAGTGATGATCGAGAATGTGAAAATCAGTTGGTGATTTTGTTAGGCTTTAAC

ACCTTCGACTTTATTAAGGTTTTACGGCAGCACAGACGGATGATCCTGTACTGCACCATG

TTGGCGAGTGCTCAGAGTGACTCAGAGAAAGAAAGAATTATGAGTAAAATGGAAGCGGAT

CAGGATTTATCTCAGGTTCTCTATCAGTTACAGGAGACGGAAAAGGAAGACATAATTCGT

GAGGAAAGATCACGAAGAGAAAGAGTTCGAAAGTCTCGTGTGGACAGTGATCTGGACTCA

ATAAACATTGATCATGGACAGTCGATGCCAGCGCGGCATCTGCTTGATCTGGAAGACCTT

GCCTTTACCCAGGGCAGTCACTTCATGGCCAACAAACGCTGCCAGCTGCCTGACGGTTCA

TTCCGCAAACAGAGGAAAGGTTATGAGGAGGTTCATGTACCTGCACTTAAACCCAAACCC

TTTTCTGAGGATGAGGCTCTTGTGGCTATTGAGAAGATGCCAAAATACTCTCAAGCTGCG

TTCGAGGGGTTTAAAACCTTGAACCGCATCCAAAGTAAGCTGTTTAAGACCACAATGGAG

ACCGACGGAAACCTGCTGGTGTGCGCTCCCACTGGAGCTGGTAAAACTAATGTTGCTCTA

ATGGCCATGCTCCGTGAAATTGGCAAGCACATCAACATGGACGGCACAATCAACGTAGAT

GACTTTAAAATGATCTATATCGCTCCTATGCGTTCACTGGTGCAGGAAATGGTTGGCAGC

TTTGGGAAGCGTTTGGCCAGTTATGGTATAACAGTGTCTGAGTTAACTGGAGATCACCAG

CTTTGTAAAGAAGAGATCAACGCTTCTCAAATCATTGTGTGCACGCCAGAGAAGTGGGAT

ATCATCACCCGCAAGGGAGGGGAGCGCACATATACCCAGCTAGTGCGCCTCATAATTATC

GATGAGATTCACTTGCTTCATGATGATCGTGGACCAGTGTTGGAGTCTCTAATAGCTCGA

ACGATCCGAAATGTAGAATTGACTCAGGAGGACGTGCGTCTCATTGGTCTTAGTGCCACA

CTGCCAAATTATGAGGATGTGGCCACATGTCTTCGTGTAGATCCTGCTAAAGGACTTTTC

TACTTCGATAACAGTTTCCGTCCAGTGCCACTGGAACAGACGTACGTAGGAATCACTGAG

AAAAAAGCAATCAAACGATTTCAGATCATGAATGAAATCGTCTACGAGAAGATAATGGAA

CATGCTGGAAAGAATCAGGTCTTAGTATTTGTCCATTCACGGAAGGAGACGGGAAAAACT

GCCCGCGCAATCAGGGACATGTGTTTGGAAAAAGATACATTGGGACTTTTTCTCCGAGAA

GGTTCTGCCTCCACCGAAGTCTTAAGAACGGAAGCGGAGCAATGCAAGAATTTGGAGTTA

AAGGATCTGTTGCCGTATGGCTTTGCTATCCATCATGCTGGCATGACTCGAGTGGACAGA

ACGTTAGTGGAAGATCTTTTTGCCGACAGACATATACAGGTACTGGTGTCAACTGCTACC

CTGGCCTGGGGAGTTAATTTACCAGCACATACTGTCATCATTAAAGGTACACAGGTTTAC

AGCCCAGAGAAAGGCAGGTGGACAGAGCTTGGAGCACTGGACATTCTACAGATGTTGGGC

CGTGCGGGCCGTCCACAGTACGACTCTAAAGGCGAGGGTATCCTGATCACCTCACATGGA

GAGCTGCAGTATTACCTGTCTCTACTTAACCAGCAGTTACCTATAGAAAGTCAAATGTTG

GCCAAACTTCCAGACATGCTTAATGCTGAGATTGTGCTGGGAAACGTTCAGAATGCTAAG

GATGCAGTGAACTGGATGGGTTACACTTACCTGTATGTGCGGATGCTGCGTAACCCGACC

CTTTATGGTGTTTCCCATGATGAGCGCAGTACTGATCCCCTGTTAGAGATGCGTCGAATG

GACCTGGTGCACACGGCCGCCACTGTACTGGAAAAAAGCAACCTGGTCAAATATGATAAA

AGATCCGGGACCTTCCAGGTGACGGATCTGGGCCGTATTGCCAGTCACTTTTACATCACA

CACGATTCTATAATGACGTACAATCAGCTGCTGAAGCCCACACTTAGTGAAATTGAGCTC

TTCAGAGTGTTCTCCCTCTCCTCTGAATTCAGGAACATCACTGTCAGAGAGGAAGAAAAG

CTTGAGTTACAGAAACTTTTGGAAAGGGTACCAATTCCAGTGAAGGAGAGTATTGAGGAG

CCCAGTGCTAAGATAAATGTACTGCTTCAGGCCTACATCTCCCAACTCAAGCTGGAGGGC

TTTGCTCTAATGGCTGACATGGTTTATGTTACTCAGAGTGCAGGTCGGCTGATGAGGGCC

ATCTTTGAAATAGTTTTGAGTCGAGGTTGGGCACAGCTCTCTGACAAAACCATGAACCTC

TGCAAGATGATCGACAAAAGGATGTGGCAGTCGATGTCACCTCTGCGACAGTTTCGCAAA

CTTCCTGAAGAGGTTATTAAGAAAATTGAGAAAAAAAGCTTTCCATTCGAACGATTGTAT

GACCTCAACCACAATGAAATCGGTGAATTGATTCGTATGCCTAAGATTGGGAAAACCATT

CATAAGTATGTGCATCAGTTCCCAAAACTGGACCTGGCAGTGCATCTTCAGCCCATCACT

CGGTCCACGCTTAAAGTGGAGCTCACAATCACACCAGACTTTCAATGGGATGAAAAAATC

CATGGATCATCAGAGGCGTTTTGGATCTTGGTGGAAGATGTTGACAGTGAAGTGATCCTA

CACCATGAATATTTCCTGCTTAAGGCAAAGTATGCTCAGGACGAGCACCTGGTCACCTTT

TTTGTGCCTGTGTTTGAGCCGCTGCCCCCTCAGTACTTCATCAGAGTGGCCTCAGACCGC

TGGCTGTCATGTGAGACTCAACTGCCAGTATCATTTCGCCACCTGATCTTGCCTGAGAAG

TATCCACCTCCGACAGAGCTGCTGGACCTACAACCTCTTCCTGTATCTGCCCTGAGAAAT

TCCGCCTTTGAATGCCTGTACCAAAACATGTTTCCTTTTTTCAACCCTATTCAGACTCAA

GTGTTCAATGCTGTGTACAACAGTGACGATAATGTGTTTGTTGGAGCCCCTACTGGAAGT

GGGAAGACCATTTGTGCAGAATTTGCTATTCTCAGGATGCTGCTGCACAATTCAGAAGGC

CGGTGCATCTACATCACACCCATGGAGTCTCTAGCTGAACAAGTGTTTGTGGATTGGCAC

CAGAAGTTTCAGGGTCTTTTGAATAAAAAGGTTGTTATGCTCACTGGAGAAACCAGCACT

GACCTAAAGCTACTGGGGAAAGGTGACATTATCATCAGCACCCCAGAAAAGTGGGATATT

TTGTCCCGCCGATGGAAACAGAGAAAGAATGTGCAGAATGTCAGTCTTTTTATTGTGGAT

GAGGTTCATCTGATTGGAGGAGAAAACGGGCCTGTCCTAGAAGTAATCTGCTCAAGAATG

AGATACATCTCGTCTCAGATAGAGCGTCCTATCCGCATTGTGGCTCTAAGCTCTTCGCTG

TCTAATGCCAAAGATGTGGCTCATTGGTTGGGTTGTAGCACAACAGCAACTTTCAACTTT

CACCCAAATGTCAGGCCTGTACCGTTGGAGCTTCACATTCAGGGTTTCAACGTGAGTCAC

ACACAAACACGTTTACTGTCGATGGCCAAACCTGTTTACCACACAATCATGAAGCATTCC

CCCTCTAAACCTTCACTGGTGTTTGTACCGTCTCGGAGACAGACTCGTCTGACTGCCATC

GACATACTAACTTTCTGCGCCGCTGATGTGGTTCCACAAAGGTTTCTGCACTGCTCGGAG

AAAGAATTGGCACCATTTTTGGACAAGCTGTCTGATTGTGCTCTGAAAGAGACCCTGTCT

AATGGTGTAGGTTATCTACATGAAGGACTGTCTATGACCGAACGCAGGACAGTTGAACAG

CTTTTTAATTCAGGTGTAATTCAGGTTGTGGTGGCCTCTCGTTCTCTTTGTTGGGGAACT

AACATCTCCTCCCACTTGGTCATTGTAATGGACACACAGTATTACAACGGAAAAATCCAT

GCTTATGTGGATTACCCAATCTATGATGTGCTGCAAATGGTGGGAAAGGCCAATCGTCCA

ATACAGGATGATGAGGGCTGCTGTGTCATTATGTGTCAGGGATCTAAAAAGGACTTCTTT

AAGAAGTTTCTTTATGAGCCTTTGCCAGTTGAATCTCATTTGGACCACCGACTTCATGAT

CACTTTAATGCTGAAATAGTCACGAAGACAGTGGAAAACAAACAGGATGCGGTGGACTAC

CTAACCTGGACTTTCCTTTACCGACGAATGACCCAGAACCCCAATTATTATAATCTTCAG

GGTATGTCTCATCGTCATCTGTCTGACCACCTGTCTGAATTGGTGGAAAACACACTGCAT

GATCTGGAGCAGTCCAAGTGCATCAGCATTGAGGATGAAATGGATGTTGCCCCTCTTAAT

CTGGGGATGATTGCTGCATACTACTATATCAACTACACCACCATTGAGTTATTTAGTATG

TCTTTGAATGCCAAGACTAAAATCCGTGGTCTGATCGAGATCATATCCAATGCAGCCGAG

TACAAAAACATACCAATCAGACACCATGAAGACGCTCTACTTCATCAGCTAGCCCAGAAG

GTGCCTCATAAATTGAACAGTCCCAAATTTAATGACCCTCATGTGAAGACAAACCTGCTG

TTACAGGCCCATCTGTCCCGTATGCAACTGAGTGCTGAGCTTCAGTCTGATACTGAAGAG

ATTCTCAGTAAGGCGGTACGTCTCATCCAGGCGTGTGTAGATGTGCTGTCCAGCAATGGC

TGGCTGAGTCCAGCATTAGCTGCTATGGAGCTAGCTCAGATGGTGACCCAGGCCATGTGG

TCTAAAGACTCTTATCTAAAACAGCTGCCTCACTTTACAACAGAGCACATCAAACGCTGT

ACAGAAAAGGGTGTGGAGAGTATATTTGACATCATGGAGATGGAGGATGAAGACCGTATA

AATCTTCTGCAACTGTCAGATGCTCAGATGGCTGATGTGGCACGTTTCTGTAACCGCTAC

CCTAACATAGAGCTCTCCTATGAAGTGGCTGACAAAGAGGACATCAAAAGTGGTAGTCCA

GTTGTTGTTCAGGTACAGTTGGAGAGAGAGGAAGAAGTTACTGGACCCGTCATCGCTCCA

CTATTTCCACAGAAGGTATGAAACACCTCTCAACATAAATAGGTGTATAATTATATTTAC

ACCAATTAGACCCTGATTTATTAAGGTAAGGACTTCATCTATCTGGCATCAAGATGTTAA

CAGCAGATCCTTTGAGTTGTGAGATGGAACCTCCATTTTGTGGCCCTACTTTACATTTAA

CGATATTATCGTTCTTGGTATTTTCAGTATGGCTGGACTGCGACTCAAATTGGTGGGTAC

TTCCTCCCCTGGGTTCAGTATGCCTGGGTCATTACTATCAGCTGCTGAAGGCGAATCAGT

GGGAGTCGTTCGTTTTTGTTTCAGCTCAGGGATTATAGATAAAATTAGGCGGGCGAGCTT

GCGTTCTTTTCATAGTGCATTCTGGTGCCATCTTTACCCAA

>Unigene000105

ATTAAATTGACTCGGTGTGTTTTATCTTTAGCCGCTAACAGGCTGCTCTAGCTTTTCGCA

TTAAAACCTGGTTGTTTCCGTAGTTCGCTTTCAGCCTTGTTCGTGCCAATCAGGGCTGCG

CTCCAACATGCGCAGTCGCCTTCTCTATAGTATGTGTAATCGTCGACGTGTTCTGCACTA

GTTAGGCGCCGTCGGGAAGGTGCAGTTGTTCATATCGGAACACAGCCCAGGGTTTACACG

CATCCACGTCCACCAGTGTGTGTGTTTAAGATTAGGTTAGCCTGCTTCAACCACATAGGC

CGACCTCTTCAAAACGAACGTTGTCATTCTAGGCTGCCGGTTGGGGTGTTTGCACACGGC

TGTGAATGGTAGTGTCTATAAAGGGTATGTCCCTTCAGGAGAGAGGTGCCAATGTCCATC

AGGATTAATCACACTGCAGGGGGGTCACTGCGCCGATCACAGAGGAACACACTCGCCACC

AGGTTGCAGTCCGAACAAACCAAACAGAAAACAAATTCCCCTCCTGAAAACAAAAGATCG

TATAATAAAGCTTCCAAGGCCCCTCCCACGCCTGCAGCCGGCTCCTCGCGTGGCCACGCC

CCTCGAAGAAACAGCCTTTCTCTCGCTTCATTGAGTCAGTCTGCATCTGAGCCGGATTCG

GAGGCAGCTGGAAGCTCGGGCCAACGCGAGCAGCGTGAAGTCAGCAACAATCGCGCCCTT

AAACGCAGCTCTGCAACTGAGCACAACAACGCGTCCTACTCCCCTAGTCCGGCCAAGCGG

CCCAAAGCTCCTCCCCCTGAGAGCACAGAGCAGCTGGAAGCTGCTGACCCAACTGCTGAC

TCCGCCCCCGAGCTCCCACCCTCATCCAGGTCTAAAAAGCGACGTCAAACAGACGAACAT

CCTACTAGCAGGACAGTAAATAAGAAGAGCAGCGCGTGCCCTGCCTCTAGCTCCTCCCAC

AAACGGAAAAAGGCAGACAACTCATTTTGCTCCTCTTCTTCCAGTTCATTCTCGTCCTCT

TCCTCATTTTTATCCGGCTCGAGCAGCGAGCAGGCCAGCCGCTCATCCAAACCCACCAAA

CTGGCGTCCAAATCGGCCGCCTCGGCCAAATCTGTGACTGAGTCAGCCTCTTCCTCCTCT

GCTTCTTCTTCTTCCTTTACTTCCTCTTCCTCCTCCTCTTCTGTGGTGGTTGCGGGTGCC

AGCGGCTGCCCCCCGCCCTCGCAGGGTGCCCGGCTGAAGCAGGGAAAAGATCAGAGCAAG

TCCCGGCGCTCTCGCTCCGCCTCTTCGCCGTCTCCTCGCCGCAGCAGTCGGGATAAAGAG

CACAGCAAATCCAGCACAGCAGCCTCCAAATTCGATTGGACGAGCCGCTTCAGTCCAAGA

GTGAACTTGCCGAAGCCCAGACTCGCCCTGCCCGGATCCTCCAAACCCGAAACGCCTTCA

GGCTCCAGCAAACCCGGCCCGAGCGGACTGCAGGCCAAACTCGCCAGTCTGAGAAAGTCC

AGTAAGAAGCGTAGCGAGTCTCCCCCTGCAGAACTCCCCAGCTTTAGGAGGAGCACACGA

CAAAAGACCACGGGCTCCTGCGCCAGCACCAGTCGGCGAGGTTCAGGTTTAGGGAAGCGT

GGTGCAGCTGAAACTCGCCGACAGGACAAAATGGCTGAAAACGACAACAGTCAGGATGGA

GCCAATTCATCACGCACAGAGGAGACGCCTCAGGGAGCTTCAGCCTCCAGTTCAGTAGCT

GGCGCTGTCGGAATGACCACATCCGGTGAGAGCGAATCGGATGACTCAGAAATGGGACGA

TTGCAGGCTCTGCTGGAGGCTCGTGGTTTGCCCCCACACCTGTTCGGACCCCGTATGTCC

CAACTTTTCCATCGAACCATCGGCACTGGAGCAAGTTCGAAAGCTCAGCAGCTGCTGCAG

GGTCTGCAGGCGACGGGGGACGAGTCTCAGCAGCTGCAGGCAGCGATAGAAATGTGCCAG

CTGTTGGTGATGGGGAACGAAGAAACACTGGGAGGTTTTCCTGTGAAGAGTGTTGTACCT

GCTTTGATCACGTTGTTACAGATGGAGCACAACTTTGATATTATGAATCATGCATCTCGT

GCGCTAACCTACATGATGGAGGCTCTACCACGGTCTTCTGCTGTAGTTGTAGACGCTATT

CCTGTCTTCCTGGAAAAGCTTCAGGTGATCCAGTTTATAGATGTGGCTGAACAGGCTCTG

ACCGCGCTGGAGATGCTCTCCCGAAGACACAGCAAGGCTATCCTGCAGGCCGGCGGATTG

GCTGACTGTCTGCTGTACCTGGAGTTTTTCAGTATTAACGCACAGAGGAACGCATTAGCC

ATTGCCGCTAACTGCTGCCAGAGCATCACACCGGATGAGTTTCACTTTGTGGCCGACTCG

CTGCCTCTGCTCACTCAGAGACTCACACACCAGGATAAAAAATCAGTGGAGAGTACGTGT

CTTTGTTTTGCCCGGCTGGTGGACAACTTTCAGCATGAGGAGAATTTGTTGCAGCAGGTG

GCATCACGTGATTTGCTCACAAACATTCAGCAGCTTCTGGTGGTGACTCCGCCAGTGCTG

AGTTCAGGCATGTTCATCATGGTGGTGCGCATGTTCTCTCTCATGTGCTCCAACTGTCCC

AACCTCGCTGTGCAGCTGATGAAGCAGAATATTGCAGAGACGCTGCGATTCCTGCTTTGT

GGAGCATCTAATGGAAGCTGCCAGGAGCAAATTGACCTCATTCCCAGAAGCCCCCAGGAG

CTCTATGAGCTCACTTCCCTTATCTGTGAGCTGATGCCCTGCCTCCCCAGAGAAGGTATT

TTCGCTGTAGACTCGATGTTGAAGAAGGGCAGCGCACACACTACAGAGGCCGCAGTGTGG

CAGTGGAGGGACGACCGAGGCCTGTGGCATCCATACAACCGAATAGACAGTCGGATCATT

GAGACGGCCCACCAGAACGGTGAGGACGAGATCAGCCTGTCCACACTAGGACGCGTTTAC

ACCATCGACTTTAACTCTATGCAGCAGATCAACGAGGACACAGGAACGGCTCGGGGAATT

CAAAGGAAACCCAACCCCCTGATCAACCCCAACACCGGTCACGTGGATGTGCGTGGCGAG

GACGCGCGTGCTGAGCTGATGCGGGAGGAAGCCGAGCTAGCCCGCTGCTTTATCAAGACT

TTGTTTGGCGTGCTGTATGAGGTGTACAGCTCGTCAGCCGGCCCTGCAGTCAGACACAAG

TGCCTCCGAGCCATCCTGCGTATCATCTACTTCTCCGACTCCGAGCTGCTCAAGGACGTA

CTGCGCAACCACGCCGTCTCCAGTCACATAGCCTCCATGCTGTCCAGTCAGGACCTGAAG

ATTGTGGTCGGCTCTCTGCAGATGGCTGAGATCCTCATGCAGAAACTTCCTGACGTGTTC

AGCGTGTACTTCAGAAGAGAAGGTGTTATGCACCAGGTGAAGAACCTGGCCGAGAGCGAG

TCGTTCCTCCCAAGTCCACCCAAGGCGTGCACAAGCGGCATCACAGCGGCAACCACCACC

ATCACCACTGCCACCACACCTGCTGCCTCTAGTGTCGCACCGGATTTGGGCTCACCGAGT

TTTCATAACAACATGGAGGATTCACTGGACCTCAGCCCACAGGGTCGCCTAAGTGATGTT

CTGAAAAGGAAGCGACTTCCTCGCAGGGTACCGCGCCGTCCCAAATATTCTCCACCCAGA

GATGATGACAAAGTGGACAATCAGGCTAAGAGTCCCACCACCACTGCGTCCCCTAAATCG

TCCTTCCTGGCAAGTCTTAACCCCAAAACCTGGGGAAAACTTAGTGCTCAGAACAACAGT

ACTAACTCCGAACCACCTCGCACAGCCGGGGTTAGCGGCCTTGCACGCACAGCTAACAAA

GACTCCGTCTCTAATAACAGGGACAAGATTAAGGCATGGATTAAGGAGCAGGCCAGTAAA

TTTGTGGAGCGATACTTCAATTCAGAAAACGTGGATGGCAGCAACCCAGCTCTGAACGTT

CTGCAGAGACTGTGTACTGCTACGGAGCAGCTCAGCCTGCAGGTGGATGGCGGTCTGGAG

TGTTTAGTGGAGATCTGCAGCATTGTGTCTGAGTCAGATGTTTCTTCGTTCGAGATTCAA

CACAGTGGCCTGGTGAAGCAGCTGCTTATCTATTTAACCTCCAACAGTGAACGAGACCTC

ATCAGCAGAGACCAGAGGATCAAACGCTTCCTGCACATTTTCTGTGGCAGTCCTCTTCCT

GGGCAGGAGCCACTGTTCCGGTTAGAACCAACAGCGAATGGAGCGCTGCTAGCACTGGTG

CACAAAATGAACAGTTGCTTAAGTCAGATGGAGCAGTTTCCTGTTAAAGTGCACGACTTT

CCTAGTGGTAATGGCAATGGAAGCAGAGGGTCTCAAGCACTGAAATTCTTCAACACACAT

CAGTTGAAGTGCCAGCTGCAGAGGCACCCAGACTGCACCAACGTGAAGCAGTGGAAGGGA

GGACCTGTTAAAATCGACCCTCTTGCACTAGTACAGGCCATCGAAAGATACCTTGTTGTT

AGAGGGTACGGACGAATCAGAGAGGAGGACGAGGACAGCGACGACGACGGATCAGACGAC

GAAATCGATGAGTCACTTGCGGCTCAGTTCCTGAATTCGGGCAGTGTGCGTCACCGGCTA

CAGTTCTACATTGCTGATCACCTGCTGCCCTACAACATGACTGTGTACCAGGCGGTGCGT

CAGTTCAGCCTTCAGCCGGAGGAGGAGCGGGAGAGCACTGACGACGAGGCCAACCCTCTC

GGTCGTGCCGGCATCTGGACCAAAACTCACACAATATGGTATAAGCCGGTAAGAGAGGAC

GAGGATGGCTCTAAGGACTCAGTAGGAGGGAAAAGAGGTCGAGCACAAACGGCCCCCACT

AAAACCTCACCACGTAACGCTAAGAAACAAGACGAGCTGTGGCACGATGGCGTGTGTCCG

AGTGTCAGTAACCCGTTGGAATCATACCTCATATCTGAACCTCCAGAGGGAATTACATTT

GATGACCCCTCACTGGAGGTCATTCTGCTGTTGAGGGTTCTGCATTCCATCAGCAGATAC

TGGTTCTACCTCTACGAGAATGCTGTGTGTAAGGAGATCATTCCCACTAGTGAGTTCATC

AACAGTAAACTGACAGCAAAAGCCAACAGACAGCTGCAGGATCCGCTTGTGATCATGACT

GGAAACATCCCACCCTGGCTTACCGAGCTGGGCAAAACCTGTCCGTTCTTCTTCCCGTTT

GACACGAGGCAAATGTTGTTCTATGTCACCGCGTTTGACCGTGATCGCGCCATGCAGCGT

CTGCTCGACACCAACCCCGAAATCAACCAGTCAGATTCGCAGGACAGCCGCGTAGCCCCC

CGCCTTGACAGAAAGAAGAGGACGATAAACAGAGATGAGCTGTTAAAGCAGGCGGAGTCT

GTCATGCAGGATCTCGGCAGTTCCAGGGCCATGCTGGAGATTCAGTATGAGAATGAGGTG

GGGACAGGTCTTGGTCCAACCCTGGAGTTCTATGCCCTTGTGTCTCAGGAGTTGCAGAGA

GCTGATCTGGGTTTGTGGCGAGGAGAAGAGGTCACCCTCGCCAATCCTAAAGGGAGTCAA

GAGGGTACCAAGTACATGTTTAGCTCTAGGGGTCTCTTTGCTGTGCCTTTCGGTCGCAGC

ACCAAGCCAGCCCACATCGCCAAGATCAAGATGAAATTCCGCTTTTTGGGGAAACTCATG

GCTAAAGCCATCATGGACTTTAGACTGCTGGATCTGCCCCTCGGCCTGCCTTTTTACAAG

TGGATGTTGAGAAACGAGTCGTCGATAAGCTCTCACGATTTGGTGAACATCGACCCTGGT

GTGGCCAAATCCGTTCAGCACCTGGAGGACATCATTCGGCAGAAGAGGAGACTAGAGCAG

GACAAGTCCTATACTAGGGAGACACTACAACAGGCTCTGGAGAGTTTAAATATGAATGGC

TGTTCAGTGGAGGATTTAGGGCTGGACTTTACATTGCCAGGCTTCCCAAACATTGAGCTG

AAGAAGGGAGGCAAAGATGTCCCAGTCACAATACACAATCTGGAGGAGTACCTAAGGCTG

GTGGTGTACTGGACCTTAAATGAGGGTGTGTCCCGGCAGTTCGAGTCATTTAGAGAAGGG

TTTGAGTCCGTTTTCCCTCTCCACCACCTACAGTACTTCTACCCCGAAGAGCTGGACCAG

CTGTTGTGTGGCAGCAAGTCAGAGACGTGGGACGTTAAAACTCTAATGGAATGTTGCAGA

CCGGACCATGGGTATACACACGACAGTGTGTGTGTGTGTAGTCGGGCAGTGCGCTTCCTC

TTTGAGGTGTTGAGTAGTTTTGATGCAGAGCAACAGAGACTGTTTCTGCAGTTTGTGACC

GGGAGTCCGAGACTGCCTGTAGGAGGTTTTCGGAGTTTAAATCCTCCTCTGACGATTGTA

CGCAAGACATTCGAGTCAACGGAGAACCCAGATGACTTCCTGCCGTCAGTGATGACCTGT

GTAAACTACCTGAAACTTCCGGATTATTCCAGCATTGAGATCATGAGGGAAAAACTGTTA

ATGGCGGCGCGGGAGGGCCAGCAGTCTTTTCACCTGTCCTGATCTTACACATACTCACAC

ATATGCACCGACACACACACACACACACACACA

>Unigene000106

GGTCAAACTGGAGATGGGCCGTGCTTGTGTGACTTGTATAAAAGACCGTCTGACAGCGAA

TAATATCGTTGCTGTGGCTGGAGGAACTACGCTAGCTGCTGTTGCGGACATGATGCAGCT

TGATTGCAAAGATTTACATATGCTATTTGTCCCAGCACGTGGTGGAATTGGAGAAGGCGT

CGAGTTAGAAGCCAATACCATTTGTGCCAAAATGGCGCAAAATACGATGAGTAATTATCG

CTTATTGTATGTTCCAGATCATGTTAGCAGCGAAGCATATGCGTCTATTGTGACAGAGCC

TTCCGTGAAAGAAGTTCTTGAGTTGATTCGATCTTCCAATATCGTCATTCATGGAATAGG

TGATGCGTTAACAATGGCACGTCGCAGAAATACTTCGGAAGCAGATTGGTTAAAGATTCA

GGCAAGCGAAGCAGTTGGCGAAGCTTTCGGTTACTACTTTAATGAACAAGGAAATGTTGT

TCATAAAGTAAGAACAGTTGGTATGCAACTAGAGGATTTACAAAATGTATCTCACGTTGT

TGCAGTCGCTGGAGGTTCTTCAAAGGCAAAGGCAATACAGGCTGTAATTAAACAAGGGCA

CACTTCAATTCTAATTACAGATGAAGGTGCAGCAAAACAGTTAACAAAGGGTATTACCCT

TTAATATAATCCCCCAAGGAGGAAATTCAAATGACTAAAATTGGTATTAATGGATTTGGA

CGTATCGGACGTAACGTATTCCGCGCAGCTCTTAACAACTCTGAGGTAGAAGTAGTAGCA

ATCAACGACTTAACAGATGCTAAAACATTAGCTCACCTTTTAAAATATGACACAGTTCAC

GGAACTTTAAATGCAGAAGTATCTGCTAACGAAAACAGCATCGTTGTTAACGGTAAAGAA

ATTAAAGTTATCGCTGAGCGTGACCCAGCTCAATTACCATGGAGCGACTACGGAGTAGAA

GTAGTAGTAGAATCTACTGGCCGTTTCACTAAAAAATCAGACGCTGAAAAACACTTAGGT

GGATCAGTTAAGAAAGTTATCATCTCAGCTCCAGCTTCTGACGAAGATATCACTGTAGTT

ATGGGTGTTAACCACGAACAATACGATGCAGCTAACCACAACGTAGTATCTAACGCTTCT

TGTACTACAAACTGTCTAGCTCCATTCGCTAAAGTATTAAACGAAAAATTCGGCGTAAAA

CGCGGAATGATGACAACAATTCACTCTTACACTAACGACCAACAAATCTTAGACTTACCA

CACAAAGATTTACGTCGTGCTCGTGCAGCAGCTGAAAACATGATCCCAACATCTACTGGT

GCAGCTAAAGCAGTAGCATTAGTATTACCAGAACTTAAAGGTAAATTAAACGGTGGCGCT

GTACGTGTTCCAACTGCTAACGTTTCTCTAGTTGACCTAGTTGTTGAACTTGACAAAGAA

GTAACAGTTGAAGAAGTAAATGCAGCATTCAAAGCAGCAGCTGAAGGCGAATTAAAAGGT

ATCCTTGGATACAGCGAAGAGCCATTAGTATCTATCGACTATAACGGATGTACAGCTTCT

TCTACAATCGATGCATTATCTACAATGGTAATGGAAGGTAACATGGTTAAAGTACTTTCT

TGGTACGATAACGAAACTGGTTACTCTAACCGCGTAGTAGACCTAGCAGCTTACATGACT

TCAAAAGGTCTTTAATTCTTAATTATATAAGTTATCAATGACAAAACGAGGGAGAGGGTT

TGTTCCCTCTCTCTCTTCTTTCGTTTTAAAAGCAAATGTGTTAAGCTTTTGAGTGGGTTT

TGTCAATCCTAACTAGTAGTCGGAGGGAAATCCAATGAACAAAAAATCAATTCGTGACGT

AGATTTAAAAGGTAAGCGTGTATTTTGCCGCGTTGACTTCAACGTACCTATGAAAGAAGG

CAAAATTACAGATGAAACTCGTATCCGTGCAGCTCTTCCTACAATTCAATATTTAGTAGA

GCAAGGTGCGAAAGTTATTTTAGCAAGCCACTTAGGCCGTCCAAAAGGTCAAGTAGTAGA

AGAATTACGTCTTACTCCAGTAGCAGCACGTTTAGGCGAGCTTCTTGGTAAAGATGTTAA

AAAAGCAGACGAAGCATTCGGACCAGTTGCACAAGAAATGGTTGCAGCAATGAACGAAGG

CGACGTATTAGTTCTTGAAAACGTACGTTTCTATGCGGGCGAAGAAAAGAACGATGCAGA

ACTTGCGAAAGAATTTGCAGCTCTTGCTGACATCTTCGTAAACGATGCATTCGGTGCAGC

TCACCGTGCTCATGCTTCTACAGCAGGAATCGCAGACTACCTACCAGCAGTATCTGGTCT

ATTAATGGAAAAAGAGTTAGAAGTATTAGGTAAAGCACTTTCTAACCCAGAACGTCCATT

CACAGCTATCATCGGTGGTGCGAAAGTAAAAGATAAAATCGGTGTAATTCGTCATCTATT

AGACAAAGTAGATAACTTAATCATCGGTGGCGGACTTGCTTACACATTCGTTAAAGCTTT

AGGTCATGAAATTGGTCTATCTCTATGTGAAGACGACAAGATTGAACTAGCTAAAGAATT

TATGCAACTTGCAAAAGAAAAAGGCGTAAACTTCTACATGCCAGTTGATGTTGTAATCAC

TGAGGAATTCTCTGAAACTGCAACAACTAAAATTGTTGGTATCGACTCTATCCCTTCTAA

CTGGGAAGGCGTGGACATCGGTCCTAAAACACGTGAAATTTATGCAGATGTAATTAAAAA

CTCTAAGCTTGTTGTATGGAATGGACCAATGGGTGTATTCGAAATGACTCCATTCTCACA

AGGTACAAAAGCAGTAGGACAAGCATTAGCAGATGCAGAAGGTACATACTCTGTTATCGG

CGGTGGTGACTCTGCAGCAGCTGTTGAAAAATTCGGTATGGCTGATAAAATGAGCCACAT

TTCTACTGGCGGCGGTGCGTCATTAGAATTCATGGAAGGTAAAGAGCTTCCAGGTGTAGT

TTGTCTTAACGACAAATAAGTAGCAGCCAAAGAAAAAGGACGGTGCAAAGCATGCGTAAA

CCAATTATCGCAGGTAACTGGAAAATGAATAAAACTCTATCTGAAGCAGTTAGCTTCGTA

GAGGAAGTTAAAGGTCAAATCCCAGCAGCTTCAGCTGTTGATGCAGTAGTTTGCTCTCCA

GCTCTATTTTTAGAGCGCCTTGTAGCAGCGACTGAAGGAACTGATTTACAAGTAGGTGCA

CAAAACATGCACTTCGAAAAAAATGGTGCATTCACTGGCGAAATTAGCCCAGTAGCACTT

AGCGACTTAAAAGTAGGCTACGTAGTACTTGGCCACTCTGAGCGTCGTGAAATGTTTGCT

GAAACAGATGAGTCAGTAAACAAAAAGACTATCGCAGCATTTGAACATGGTTTAACACCA

ATCGTATGTTGTGGTGAGACTTTAGAAGAGCGCGGAAGCGTAAAAACATTTGATCTAGTA

GCAGGTCAAGTAACAAAAGCACTTGCAGGTTTAACAGAAGAGCAAGTTAAAGCAACTGTT

ATCGCTTATGAGCCAATCTGGGCTATCGGTACAGGTAAATCTTCTTCTTCTGCAGATGCA

AACGAAGTATGTGCGCACATCCGTAAAGTTGTTGCAGAAGCTGTTTCTCCAGAAGCTGCA

GAAGCTGTTCGTATTCAATACGGCGGTAGCGTAAAACCAGAAAACATTAAAGAGTACATG

GCACAATCTGACATCGACGGCGCTTTAGTTGGCGGTGCTAGCTTAGAGCCTGCTTCGTTC

TTAGGTCTTCTGGGGGCGGTAAAATGAGAAAGCCAACAGCTTTAATCATTCTTGACGGTT

TCGGACTTCGTGAAGAAACTTACGGGAATGCTGTAGCACAAGCTAAGAAACCTAATTTTG

ATGGTTACTGGAACAAATTCCCTCACACAACGCTTACAGCTTGTGGCGAGGCAGTAGGTC

TTCCAGAAGGTCAAATGGGTAACTCTGAGGTTGGTCACTTAAATATCGGTGCTGGCCGCA

TTGTATATCAAAGCTTAACACGCGTAAACGTTGCAATTCGTGAAGGTGAGTTCGATAAGA

ACGAAACGTTCCAAAGTGCAATTAAAAGCGTAAAAGAAAAAGGTACTGCACTTCATTTAT

TTGGTTTACTTTCTGACGGTGGTGTGCACAGTCACATGAACCACATGTTTGCTCTTCTTC

GCTTAGCAGCTAAAGAAGGCGTTGAGAAAGTATACATTCATGCATTCTTAGATGGCCGCG

ATGTTGGACCAAAAACAGCACAAAGCTATATCGATGCAACAAATGAAGTAATTAAAGAAA

CAGGAGTAGGACAATTCGCGACTATCTCTGGTCGTTATTACTCCATGGACCGTGACAAGC

GTTGGGATCGCGTAGAAAAATGTTACCGTGCTATGGTGAATGGTGAAGGCCCTACTTATA

AATCAGCAGAAGAGTGTGTAGAAGACTCTTATGCAAATGGTATCTACGATGAATTCGTAT

TGCCGTCTGTAATTGTTAACGAAGATAACACGCCAGTTGCAACAATCAATGATGATGATG

CAGTTATCTTCTATAACTTCCGTCCAGACCGTGCAATTCAAATTGCTCGTGTATTTACAA

ACGAAGACTTCCGTGAGTTCGATCGTGGTGAAAAAGTACCTCACATTCCAGAATTCGTAT

GTATGACACACTTTAGTGAAACAGTAGATGGTTACGTGGCATTCAAGCCAATGAACCTTG

ATAACACTTTAGGTGAAGTTGTTGCGCAAGCGGGATTAAAGCAACTTCGCATCGCGGAAA

CTGAAAAGTATCCGCACGTTACATTCTTCTTTAGCGGTGGTCGTGAGGCTGAATTCCCAG

GAGAAGAGCGTATCTTAATTAACTCACCGAAGGTTGCAACGTATGACTTGAAACCTGAAA

TGAGCATTTACGAAGTAACGGACGCTTTAGTAAATGAAATCGAAAATGATAAACATGATG

TTATCATTCTTAACTTTGCGAACTGTGATATGGTTGGCCATTCTGGGATGATGGAACCAA

CAATTAAAGCAGTAGAAGCAACTGACGAATGTTTAGGAAAAGTTGTAGAAGCGATTCTTG

CAAAAGATGGTGTAGCACTTATTACTGCTGACCATGGTAATGCGGATGAAGAATTAACTT

CTGAAGGAGAGCCAATGACAGCTCATACAACTAACCCGGTTCCTTTCATTGTTACTAAGA

ACGACGTAGAATTACGTGAAGATGGTATCTTAGGTGATATCGCTCCAACTATGCTTACAC

TTCTTGGTGTTGAGCAACCGAAAGAAATGACAGGTAAAACAATTATTAAATAATTTGCTT

ATATAAAAAGGAGAGAATTTATTATGTCAACAATTATTGATGTTTATGCTCGCGAAGTCC

TTGACTCTCGTGGTAACCCAACTGTAGAAGTAGAAGTTTACACAGAAAGCGGCGCTTTCG

GACGCGCTATCGTACCAAGTGGTGCATCTACTGGTGAGCACGAAGCAGTAGAATTACGTG

ACGGTGACAAATCTCGTTACCTAGGTAAAGGTGTTATGAACGCAGTAAACAACGTTAACG

AAGCAATCGCTCCAGAAATCGTTGGTTTCGACGTAACTGACCAAGCTGGTATCGACCGTG

CTATGATCGAATTAGATGGCACTCCAAACAAAGGTAAACTAGGCGCTAACGCTATCCTTG

GTGTATCTATGGCAGTAGCTCACGCAGCAGCTGACTTCGTAGGTCTTCCATTATACCGTT

ACCTTGGTGGATTCAATGCAAAACAATTACCAACTCCAATGATGAACATCATCAACGGTG

GTTCTCACGCTGATAACAACGTTGACTTCCAAGAGTTCATGATCTTACCAGTTGGTGCTC

CAACATTCAAAGAATCAATCCGTATGGGTGCTGAAGTATTCCATGCACTTAAAGCTGTAT

TACATGACAAAGGTCTTAACACTGCAGTAGGTGACGAAGGTGGATTCGCTCCAAACCTTG

GTTCTAACCGTGAAGCATTAGAAGTAATCATCGAAGCTATCGAAAAAGCTGGTTACAAAG

CTGGCGAGAACGTATTCTTAGGAATGGACGTTGCTTCTTCTGAGTTCTACAACAAAGAAA

CTGGTAAATATGACCTTGCAGGCGAAGGCCGTACTGGCTTAACTTCTGCAGAAATGGTTG

ATTTCTACGAAGAGCTTTGCAAAGACTTCCCAATCATCTCTATCGAAGATGGTTTAGACG

AAAACGACTGGGATGGTCACAAATTATTAACTGAGCGTATCGGTGATAAAGTACAATTAG

TTGGTGACGACTTATTCGTAACTAACACTCAAAAACTTGCTGAAGGTATCGAAAAAGGTA

TCTCTAACTCAATCTTAATTAAAGTTAACCAAATCGGTACTTTAACTGAGACTTTCGAAG

CTATCGAAATGGCTAAACGTGCTGGTTACACAGCAGTTGTATCTCACCGTTCTGGTGAAA

CTGAAGATGCTACAATCGCTGACATCGCAGTTGCAACTAACGCTGGCCAAATCAAAACTG

GTTCTATGAGCCGTACTGACCGTATTGCTAAGTACAACCAATTATTACGCATCGAAGACG

AACTAGGCGAAATCGCTGTTTACGATGGTATCAAATCTTTCTACAACATCAAACGATAAT

TGTAGAAAATAACGACAGACCG

>Unigene000107

AGAGTGAGAGAGAGAGAGAGAGAAAGAGAAAGAGAGAGAGACGCTCGCTAGCTTTGAACG

GAAGAAGTTTGCGACGCTGTATGACAGAGAGGGCGCGCGCGAGAGAGAGAGAATCTGGAT

AAAGCTCTACCCGGCGACTGCGTTTCTCTTTATCCATCTGGCACTAGAAAAAAACAGCGT

CTAACACGCAGCAACCATGTCGGCTGCAGATAGGTACCTGTATGTGGACCGCTCGCTGGT

GAACAACCCGCTGGCGCAGGCCGACTGGGCCTCCAAGAAGCTGGTGTGGGTTCCGTCGGA

GCGGCTGGGGTTCGAGGCCGGCTCCCTCAGAGAGGAACATGGAGACGAGGTGGTGGTGGA

ACTCGCCGACTCGGGTAAGAAGATCCGCATCAACAAGGACGATGTCCAGAAGATGAACCC

GCCCAAATTTAGCAAGGTGGAGGACATGGCGGAGCTCACCTGTCTCAACGAGGCGTCTGT

CCTCCACAACCTGCGTGAAAGATACTACTCCGGACTCATCTACACATACTCTGGTCTCTT

CTGTGTTGTAGTGAATCCTTATAAAATGCTGCCAATTTACTCAGAGAAGATCATTGACAT

GTACAAGGGCAAGAAACGTCATGAAGTTCCACCACACATCTACTCCATCACTGACAACGC

CTACAGAAACATGATGCAGGATCGAGAGGACCAGTCTATTCTCTGCACAGGTGAATCTGG

TGCAGGGAAAACAGAGAACACTAAGAAAGTGATTCAGTATTTAGCCGTTGTGGCATCATC

ACACAAAGGCAAGAAAGACACAAGCGCTGGGGAGCTGGAGAAGCAGCTTTTGCAGGCCAA

TCCTATTTTGGAGGCATTTGGTAACGCCAAGACCATCAAGAACGACAATTCTTCACGATT

CGGTAAATTCATCCGCATTAACTTTGATGTAACAGGCTACATCGTTGGAGCAAACATTGA

AACCTACCTGCTGGAAAAATCTCGCTGTATCCGACAGGCTAAAACTGAGAGAGCTTTCCA

CATCTTCTACTACATGGTGGCAGGAGCCAGGGACAAGCTGCGTGAGGAACTGCTGCTGGA

GAATTTCAATAATTACCGCTTCCTGGTAGCAGGACATGTGCAGGTTCCAGGAAATCAGGA

TGACGAGATGTACGATGAAACAATGGAGGCGATGAACATTATGGGCTTTACTGATGAGGA

GAGGATCGGAATCCAGAAGGTTGTGTCCACTGTGCTGCAGCTTGGCAACATCGAGTTTAA

GAAAGAGAGGAACCAGGAGCAGGCCACCATGCCTGACAATACAGCTGCACAGAAGGTCTG

TCACCTGTCAGGCATCAATGTGACGGATTTCACCCGTGCCATCCTGACCCCACGCATCAA

AGTAGGCAGAGAAGTCGTACAGAAGGCGCAGACCAAAGAGCAGGCTGATTTTGCAGTGGA

GGCTTTAGCAAAGGCAACGTATGAGCGTCTGTTCCGCTGGATTCTTGCTCGTGTCAATAA

AGCTCTGGATAAAACCAAGCGTCAGGGCGCGTCTTTCCTTGGCATCCTGGACATTGCCGG

CTTCGAGATCTTCGAGGACAACTCGTTTGAGCAGCTGTGTATTAATTACACTAACGAGAA

GCTGCAGCAGCTCTTCAATCACACCATGTTCATACTGGAGCAGGAGGAGTATCAGCGTGA

GGGCATCGAGTGGAGCTTCATCGACTTTGGACTGGACCTTCAGCCCTGCATCGACCTGAT

CGAGCGGCCTGCGAATCCTCCAGGTGTTCTGGCTCTGCTGGATGAGGAGTGCTGGTTCCC

CAAAGCCACAGATAAGAGTTTTGTGGAGAAGGTTCTGCAGGAGCAGGGCACACACCCCAA

ATTCTTCAAACCTAAGAAACTGAAGGATGAGGCAGATTTCTGTATCATTCACTACGCCGG

AAAGGTGGACTACAAAGCTGACGAGTGGCTGATGAAGAACATGGACCCTCTGAATGATAA

CGTGGCCTCACTGCTCAACCAGTCCACCGATAAATTTGTGTCTGAACTCTGGAAGGACGT

GGACCGGATTGTGGGTTTGGATAAGGTGGCGGGAATGTCGGAGATGCCCGGAGCATTTAA

GACCCGTAAAGGAATGTTCCGGACTGTCGGTCAGCTCTATAAGGAGCAGCTGTCCAAGCT

GATGGCCACGCTGAGGAACACCAACCCCAACTTCGTGCGCTGCATCATTCCAAACCACGA

GAAAAAGGCGGGTAAACTGGACCCTCACCTGGTTCTGGATCAGCTCAGGTGTAACGGTGT

GTTGGAAGGTATCCGTATCTGCAGACAGGGTTTCCCCAATCGCATCGTCTTCCAGGAGTT

CAGACAGAGGTACGAGATCCTGACTCCTAACGCCATTCCAAAGGGCTTCATGGATGGCAA

GCAGGCCTGTGTGCTGATGATCAAAGCTTTGGAATTGGACCCAAACCTGTATCGGATCGG

ACAGAGCAAGGTGTTCTTCCGTGCCGGAGTTCTGGCCCACCTGGAGGAGGAGCGTGATAT

GAAGATCACAGATGTCATCATCAGCTTCCAGGCCTGGTGCCGTGGCTACGTCGCCCGAAA

GGCATTTGCCAAGAGGCAGCAGCAGCTGACCGCCATGAGAGTGATCCAGAGGAACTGTGC

TGCTTACCTCAAACTCAGGAACTGGCAGTGGTGGAGACTCTTCACTAAGGTGAAGCCCTT

GCTCCAGGTGAGCCGTCAGGAGGAGGAGATGCAGGCCAAAGATGAGGAGCTGACCAAAGT

CCGTGAGAAACAGGTGGTGGCTGAGAAAATGATCGAGGAGATGGAGGTCAAACACCAGCA

GCTGAATGCAGAGAAGATGGCTCTACAGGAGCAGCTGCAGGCCGAGATGGATCTCTGCGC

CGAGGCAGACGAGATGAGGAACAGGCTGGTGGCGAAAAAGCAGGAACTGGAGGAGATCCT

GCATGACCTGGAGGCCCGAGTGGAGGAGGAGGAGGAGAGAGCCAATCAGCTTCAGATGGA

GAAGAAGAAGATGCAGCAGAACATCACGGATCTGGAGCAGCAGCTGGACGAGGAGGAGGC

AGCTCGGCAGAAGCTGCAGCTGGAGCGCGTCACCATGGAGGCCAAGCTAAAGAAAATCGA

GGAGGACGTCATGGTGCTGGAGGACCAGAACACCAAGCTGAACAAGGAGAAGAAGCTGAT

GGAGGAAAGAATCAGCGAGTTTACCACTAACCTGGCTGAGGAGGAGGAGAAATCCAAGAG

TCTGCAGAAGCTGAAGAACAAACACGAGGCCATGATCACAGACCTGGAGGATCGTCTCCG

CAAGGAGGAGAAAATGAGACAGGAGCTGGAAAAGAACCGCAGGAAGTTGGAGGGCGACTC

CACCGAGCTTCACGATCAGATCGCCGAACTCCAGGCTCAGATCGCCGAGCTCCGAGCTCA

ACTCGCCAAAAAGGAGGAAGAGCTGCAGGAAGCACTGGCCAGGATCGAGGAAGAGGCGGC

GCAGAAGAACCTGGCCCACAAGAAGATTCGTGAGTTGGAGGCTCAGCTGAGCGAGCTGCA

GGAGGACCTGGAGCTGGAGCGAGCCGCTCGCACCAAGGCAGAAAAACACCGCCGTGACCT

GGGGGAGGAGCTCGAGGCTTTAAAGACCGAGCTGGAGGACACGCTGGACACCACTGCTGC

TCAGGAGGCACTCAGGACAAAGCGTGAGACAGAGGTGACGCACCTGAAGAAGTCTTTGGA

GGATGAAGCTAAAAATCATGAGCAGATTGTGGCAGAAATGAGACAGAAACACGGCCAGGC

TTTCGATGAACTCAATGAACAGCTGGAGCAGTCAAAGAGGAACAAGGCATCAGTGGAGAA

GATGAAGCAGACTCTGGAGTCGGAGCGTAACGAGCTGCAGATCGAGCTGCAGGGTCTGAT

GCAGAGTAAAGGTGAGTCGGAGCACCGCAGGAAGAAGGCGGAGGCTCAGGTGCAGGAGCT

GCAGATCAAACACTCTGAGAGTGAGAAGCAGAGAGCAGAGCTGATGGAGAAAGTCTCCAA

GTTGCAGGCGGAGCTTGATAGCGTTAACAGCGTGCTAAGTGACGTGGAAGGAAAATCCAT

CAAAGCAGTGAAAGACTGTTCAGCTGTGGAGTCTCAACTGCAGGACGTCCAGGAGCTGCT

TCAGGAAGAGACTCGTCAGAAACTGTCTCTTAACACACGAATGAGGCAGCTGGAGGATGA

ACAGCACAATCTGAGAGAACAGCTGGAGGAAGAGGAGGAAGCCAAGAAGAACATGGAGAA

ACAGTTGATTGCAGCTCAGTCCCAGCTGGCTGAGATGAGGAAGAAGATCGAGCAGGAGGC

GAGTTCTCTGGAGAGCACAGAGGAGGTGAAGAAGCGTGTGCAGCGCGACCTCGAGTCTGT

CAGTCAGCGCCTGGATGAGAGGAACCTGGCCTTCGACAAACTGGACAAGACCAAGACCCG

CTTGCAGCAGGAGCTGGATGACCTGCTGGTGGACCAGGACCATCTGAGGCAGATTGTTTC

CAACCTGGAGAAGAAGCAGAAGAAGTTTGACCAGATGTTGGCGGAGGAGAAGACAATCTC

TGCTCGCTATGCTGAGGAACGTGACCGGGCAGAAGCTGAAGCCCGTGAGAAGGAGACCCG

CGCACTCGCTCTGACACGCGAGCTTGAGACCCTGACAGACATAAAGAACGAAATGGAACG

CGCAAACAAGGCTCTCCGGGCTGAGATGGAGGACCTCGTCTCCTCCAAGGACGACGTCGG

CAAGAGCGTGCACGAGCTGGAGAAGTCGAAGCGCGGCATGGAGCAGCAGCTGGAGGAGAT

GCGCACACAGCTGGAGGAGCTGGAGGATGAGCTGCAAGCTACTGAGGACGCCAAGCTGAG

GCTGGAGGTCAACATGCAGGCCATGAAGGCTCAGTACGAGAGAGACCTGGCTGGACGAGA

CGAGATGGGAGAGGAGAAGAAGAGGCAGCTGGTCAAACAGGTGAGGGAGATGGAGATGGA

GCTGGAGGACGAAAGGAAGCAGCGCTCGGTTGCCATGGCAGCGCGGAAGAAGTTGGAGCT

GGATCTGAAGGAGCTGGAGGCTGCCATCGACCAGGCCAACAAGAACCGAGAGGAAGCTCT

GAAACAGCTCAAGAAACTGCAGGCTCAGATGAAGGACGTCCTGCGGGAGCTGGATGAGAC

GCGTCTCTCCAGAGAGGAGATTCTGGCACTAAGCAAAGAGAATGAGAAGAAGTTTAAGAG

CATGGAGGCAGAGATGATTCAAATTCAGGAGGAACTGGCTGCAGCAGAACGGGCAAAGAG

ACAGGCTCAGCAGGAGAGAGACGAGCTGCTGGACGAGATCAACAACCAGAACAGCAAGAA

TGCTCTGACAGTGGAGGAGAGAAGGCGTCTGGAGGCTCGTATTGCTCAGCTGGAGGAGGA

GCTTGAGGAGGAACAGAACAACAGCGAGTTGACAAACGATCGTCTCAAGAGAACGATGCT

GCAGGTGGACCAGGTAAACGTGGAGCTGACAGCCGAGCGCAGCACCGCCCAGCGCCTGGA

GGGGGCGCGCTCACAGATGGAGCGCCAGAACAAGGAGATGAAGCTGAAACTGACCGAGCT

GGAAGGAGCGGTGAAGAGCAAATACAAGGCCTCTATTGGCGCCCTAGAGGCCAAAATCGC

ACAGCTGGAAGAACAGCTGGACATTGAGACCAGAGAGAGGCAGGCAGCCACCAAATTGGT

GCGTCGTTCAGAGAAGAAGCTGAAGGAAGTCATCCTGCAGGTGGATGATGAGAGACGTAA

TGCTGACCAGTACAAAGATCAGGCGGAGAAGATGAACAGTCGTATGAAGCAGCTGAAGCG

GCAGCTGGAGGAGGCGGAGGAGGAGGCGCAGAGAGCTAACGCTAACCGCAGGAAGCTGCA

GAGAGAGCTGGAGGACGCCACCGAGTCGGCCGACGCCATGAACCGCGAGGTCTCCTCACT

GAAGAGCAAGCTGAGACGTGGGGATCTGCCCTTCACCATGCGCCGTATCGTCAGCCGCGC

CGGCATTGAAAGCGATGAGGAGCCCGAGTCCAAGGGCGAAACTCCTGAACCCAAACCGGA

GTGAAGAAACGAGGACACCTGCTGGCTGTAGTGAAGAACTGCAGCACTTCCCTTTATTAT

TATTCTTTTTTTTACGCATATAATAGACCACTGTCTACTCCCATTCAGATTTAATTTGCT

AGTGCCATAACACACACACACACAAACACACACACACACACACTGTAACTCAGACAATCA

GTTCTCCAGCCCATAGTCACTCACCCGAGGACCAAACCGTCATTCTTTTTATTCTATTGC

GTTTATTCAGCAAGATTCTGAAGACGGGATGATATTTTTGTAAGAAGTCTTTTTCTTTTC

TTCCTCCTCTCCGTCTTCTCCTCCGCTTCCTCAGCAGGCTCGTGTCTCAGTCCAGTTTTT

AAACCAAAATCTAAAGAGAAATGATAATTTTTTTTTTTTTTTTGAAAATAAGAGAAGTGA

AGAAAAAAAAAAAGAAAAGGGACCAAAAAGAAAAGAAAAAAAAATTGCCGTTCTTTATAG

AAAATGTATTTTATCCTAAAGTTTTTGTTTTTTTTATAAAGGTGCAAGGATGAAAGATTA

TACCACTTATTTAGGGAAAAGCTGCACACACACACACACTCTTTCTGTCTCTCTCTCTCT

CTCTCTC

>Unigene000108

GGTGACGTCACTGGGCCCTGTACCACGCGTGCGCGACATTTTCACGCATGCGTTGTTTAG

GCTCGAACTGTGACGGGGGGCAAAGCTGGTCGGCTAGTGACAAGAGCTGAGTGTTAAAAA

CACTAACTAGTGTCTATATAAGGACCTGAGTGAGTGAGGAAGCCCTTATTTTGCGGCCTA

TGTAGTGCACTTATACAGACGGAAGAGTTATCTGAAATTTAGCCCAAATTAATATTAGTG

GAGCTGCTTTGAGTGTCTATAACAGCAACTACACAACACTGCAAAAAAAACAAAAACAAA

CAAAAAAAAACCAACAACTCCACACAGTGAATGGTAGTGTCTATAAAGGGTATGTCCCTT

CAGGAGAGAGGTGCCAATGTCCATCAGGATTAATCACACTGCAGGGGGGTCACTGCGCCG

ATCACAGAGGAACACACTCGCCACCAGGTTGCAGTCCGAACAAACCAAACAGAAAACAAA

TTCCCCTCCTGAAAACAAAAGATCGTATAATAAAGCTTCCAAGGCCCCTCCCACGCCTGC

AGCCGGCTCCTCGCGTGGCCACGCCCCTCGAAGAAACAGCCTTTCTCTCGCTTCATTGAG

TCAGTCTGCATCTGAGCCGGATTCGGAGGCAGCTGGAAGCTCGGGCCAACGCGAGCAGCG

TGAAGTCAGCAACAATCGCGCCCTTAAACGCAGCTCTGCAACTGAGCACAACAACGCGTC

CTACTCCCCTAGTCCGGCCAAGCGGCCCAAAGCTCCTCCCCCTGAGAGCACAGAGCAGCT

GGAAGCTGCTGACCCAACTGCTGACTCCGCCCCCGAGCTCCCACCCTCATCCAGGTCTAA

AAAGCGACGTCAAACAGACGAACATCCTACTAGCAGGACAGTAAATAAGAAGAGCAGCGC

GTGCCCTGCCTCTAGCTCCTCCCACAAACGGAAAAAGGCAGACAACTCATTTTGCTCCTC

TTCTTCCAGTTCATTCTCGTCCTCTTCCTCATTTTTATCCGGCTCGAGCAGCGAGCAGGC

CAGCCGCTCATCCAAACCCACCAAACTGGCGTCCAAATCGGCCGCCTCGGCCAAATCTGT

GACTGAGTCAGCCTCTTCCTCCTCTGCTTCTTCTTCTTCCTTTACTTCCTCTTCCTCCTC

CTCTTCTGTGGTGGTTGCGGGTGCCAGCGGCTGCCCCCCGCCCTCGCAGGGTGCCCGGCT

GAAGCAGGGAAAAGATCAGAGCAAGTCCCGGCGCTCTCGCTCCGCCTCTTCGCCGTCTCC

TCGCCGCAGCAGTCGGGATAAAGAGCACAGCAAATCCAGCACAGCAGCCTCCAAATTCGA

TTGGACGAGCCGCTTCAGTCCAAGAGTGAACTTGCCGAAGCCCAGACTCGCCCTGCCCGG

ATCCTCCAAACCCGAAACGCCTTCAGGCTCCAGCAAACCCGGCCCGAGCGGACTGCAGGC

CAAACTCGCCAGTCTGAGAAAGTCCAGTAAGAAGCGTAGCGAGTCTCCCCCTGCAGAACT

CCCCAGCTTTAGGAGGAGCACACGACAAAAGACCACGGGCTCCTGCGCCAGCACCAGTCG

GCGAGGTTCAGGTTTAGGGAAGCGTGGTGCAGCTGAAACTCGCCGACAGGACAAAATGGC

TGAAAACGACAACAGTCAGGATGGAGCCAATTCATCACGCACAGAGGAGACGCCTCAGGG

AGCTTCAGCCTCCAGTTCAGTAGCTGGCGCTGTCGGAATGACCACATCCGGTGAGAGCGA

ATCGGATGACTCAGAAATGGGACGATTGCAGGCTCTGCTGGAGGCTCGTGGTTTGCCCCC

ACACCTGTTCGGACCCCGTATGTCCCAACTTTTCCATCGAACCATCGGCACTGGAGCAAG

TTCGAAAGCTCAGCAGCTGCTGCAGGGTCTGCAGGCGACGGGGGACGAGTCTCAGCAGCT

GCAGGCAGCGATAGAAATGTGCCAGCTGTTGGTGATGGGGAACGAAGAAACACTGGGAGG

TTTTCCTGTGAAGAGTGTTGTACCTGCTTTGATCACGTTGTTACAGATGGAGCACAACTT

TGATATTATGAATCATGCATCTCGTGCGCTAACCTACATGATGGAGGCTCTACCACGGTC

TTCTGCTGTAGTTGTAGACGCTATTCCTGTCTTCCTGGAAAAGCTTCAGGTGATCCAGTT

TATAGATGTGGCTGAACAGGCTCTGACCGCGCTGGAGATGCTCTCCCGAAGACACAGCAA

GGCTATCCTGCAGGCCGGCGGATTGGCTGACTGTCTGCTGTACCTGGAGTTTTTCAGTAT

TAACGCACAGAGGAACGCATTAGCCATTGCCGCTAACTGCTGCCAGAGCATCACACCGGA

TGAGTTTCACTTTGTGGCCGACTCGCTGCCTCTGCTCACTCAGAGACTCACACACCAGGA

TAAAAAATCAGTGGAGAGTACGTGTCTTTGTTTTGCCCGGCTGGTGGACAACTTTCAGCA

TGAGGAGAATTTGTTGCAGCAGGTGGCATCACGTGATTTGCTCACAAACATTCAGCAGCT

TCTGGTGGTGACTCCGCCAGTGCTGAGTTCAGGCATGTTCATCATGGTGGTGCGCATGTT

CTCTCTCATGTGCTCCAACTGTCCCAACCTCGCTGTGCAGCTGATGAAGCAGAATATTGC

AGAGACGCTGCGATTCCTGCTTTGTGGAGCATCTAATGGAAGCTGCCAGGAGCAAATTGA

CCTCATTCCCAGAAGCCCCCAGGAGCTCTATGAGCTCACTTCCCTTATCTGTGAGCTGAT

GCCCTGCCTCCCCAGAGAAGGTATTTTCGCTGTAGACTCGATGTTGAAGAAGGGCAGCGC

ACACACTACAGAGGCCGCAGTGTGGCAGTGGAGGGACGACCGAGGCCTGTGGCATCCATA

CAACCGAATAGACAGTCGGATCATTGAGACGGCCCACCAGAACGGTGAGGACGAGATCAG

CCTGTCCACACTAGGACGCGTTTACACCATCGACTTTAACTCTATGCAGCAGATCAACGA

GGACACAGGAACGGCTCGGGGAATTCAAAGGAAACCCAACCCCCTGATCAACCCCAACAC

CGGTCACGTGGATGTGCGTGGCGAGGACGCGCGTGCTGAGCTGATGCGGGAGGAAGCCGA

GCTAGCCCGCTGCTTTATCAAGACTTTGTTTGGCGTGCTGTATGAGGTGTACAGCTCGTC

AGCCGGCCCTGCAGTCAGACACAAGTGCCTCCGAGCCATCCTGCGTATCATCTACTTCTC

CGACTCCGAGCTGCTCAAGGACGTACTGCGCAACCACGCCGTCTCCAGTCACATAGCCTC

CATGCTGTCCAGTCAGGACCTGAAGATTGTGGTCGGCTCTCTGCAGATGGCTGAGATCCT

CATGCAGAAACTTCCTGACGTGTTCAGCGTGTACTTCAGAAGAGAAGGTGTTATGCACCA

GGTGAAGAACCTGGCCGAGAGCGAGTCGTTCCTCCCAAGTCCACCCAAGGCGTGCACAAG

CGGCATCACAGCGGCAACCACCACCATCACCACTGCCACCACACCTGCTGCCTCTAGTGT

CGCACCGGATTTGGGCTCACCGAGTTTTCATAACAACATGGAGGATTCACTGGACCTCAG

CCCACAGGGTCGCCTAAGTGATGTTCTGAAAAGGAAGCGACTTCCTCGCAGGGTACCGCG

CCGTCCCAAATATTCTCCACCCAGAGATGATGACAAAGTGGACAATCAGGCTAAGAGTCC

CACCACCACTGCGTCCCCTAAATCGTCCTTCCTGGCAAGTCTTAACCCCAAAACCTGGGG

AAAACTTAGTGCTCAGAACAACAGTACTAACTCCGAACCACCTCGCACAGCCGGGGTTAG

CGGCCTTGCACGCACAGCTAACAAAGACTCCGTCTCTAATAACAGGGACAAGATTAAGGC

ATGGATTAAGGAGCAGGCCAGTAAATTTGTGGAGCGATACTTCAATTCAGAAAACGTGGA

TGGCAGCAACCCAGCTCTGAACGTTCTGCAGAGACTGTGTACTGCTACGGAGCAGCTCAG

CCTGCAGGTGGATGGCGGTCTGGAGTGTTTAGTGGAGATCTGCAGCATTGTGTCTGAGTC

AGATGTTTCTTCGTTCGAGATTCAACACAGTGGCCTGGTGAAGCAGCTGCTTATCTATTT

AACCTCCAACAGTGAACGAGACCTCATCAGCAGAGACCAGAGGATCAAACGCTTCCTGCA

CATTTTCTGTGGCAGTCCTCTTCCTGGGCAGGAGCCACTGTTCCGGTTAGAACCAACAGC

GAATGGAGCGCTGCTAGCACTGGTGCACAAAATGAACAGTTGCTTAAGTCAGATGGAGCA

GTTTCCTGTTAAAGTGCACGACTTTCCTAGTGGTAATGGCAATGGAAGCAGAGGGTCTCA

AGCACTGAAATTCTTCAACACACATCAGTTGAAGTGCCAGCTGCAGAGGCACCCAGACTG

CACCAACGTGAAGCAGTGGAAGGGAGGACCTGTTAAAATCGACCCTCTTGCACTAGTACA

GGCCATCGAAAGATACCTTGTTGTTAGAGGGTACGGACGAATCAGAGAGGAGGACGAGGA

CAGCGACGACGACGGATCAGACGACGAAATCGATGAGTCACTTGCGGCTCAGTTCCTGAA

TTCGGGCAGTGTGCGTCACCGGCTACAGTTCTACATTGCTGATCACCTGCTGCCCTACAA

CATGACTGTGTACCAGGCGGTGCGTCAGTTCAGCCTTCAGCCGGAGGAGGAGCGGGAGAG

CACTGACGACGAGGCCAACCCTCTCGGTCGTGCCGGCATCTGGACCAAAACTCACACAAT

ATGGTATAAGCCGGTAAGAGAGGACGAGGATGGCTCTAAGGACTCAGTAGGAGGGAAAAG

AGGTCGAGCACAAACGGCCCCCACTAAAACCTCACCACGTAACGCTAAGAAACAAGACGA

GCTGTGGCACGATGGCGTGTGTCCGAGTGTCAGTAACCCGTTGGAATCATACCTCATATC

TGAACCTCCAGAGGGAATTACATTTGATGACCCCTCACTGGAGGTCATTCTGCTGTTGAG

GGTTCTGCATTCCATCAGCAGATACTGGTTCTACCTCTACGAGAATGCTGTGTGTAAGGA

GATCATTCCCACTAGTGAGTTCATCAACAGTAAACTGACAGCAAAAGCCAACAGACAGCT

GCAGGATCCGCTTGTGATCATGACTGGAAACATCCCACCCTGGCTTACCGAGCTGGGCAA

AACCTGTCCGTTCTTCTTCCCGTTTGACACGAGGCAAATGTTGTTCTATGTCACCGCGTT

TGACCGTGATCGCGCCATGCAGCGTCTGCTCGACACCAACCCCGAAATCAACCAGTCAGA

TTCGCAGGACAGCCGCGTAGCCCCCCGCCTTGACAGAAAGAAGAGGACGATAAACAGAGA

TGAGCTGTTAAAGCAGGCGGAGTCTGTCATGCAGGATCTCGGCAGTTCCAGGGCCATGCT

GGAGATTCAGTATGAGAATGAGGTGGGGACAGGTCTTGGTCCAACCCTGGAGTTCTATGC

CCTTGTGTCTCAGGAGTTGCAGAGAGCTGATCTGGGTTTGTGGCGAGGAGAAGAGGTCAC

CCTCGCCAATCCTAAAGGGAGTCAAGAGGGTACCAAGTACATGTTTAGCTCTAGGGGTCT

CTTTGCTGTGCCTTTCGGTCGCAGCACCAAGCCAGCCCACATCGCCAAGATCAAGATGAA

ATTCCGCTTTTTGGGGAAACTCATGGCTAAAGCCATCATGGACTTTAGACTGCTGGATCT

GCCCCTCGGCCTGCCTTTTTACAAGTGGATGTTGAGAAACGAGTCGTCGATAAGCTCTCA

CGATTTGGTGAACATCGACCCTGGTGTGGCCAAATCCGTTCAGCACCTGGAGGACATCAT

TCGGCAGAAGAGGAGACTAGAGCAGGACAAGTCCTATACTAGGGAGACACTACAACAGGC

TCTGGAGAGTTTAAATATGAATGGCTGTTCAGTGGAGGATTTAGGGCTGGACTTTACATT

GCCAGGCTTCCCAAACATTGAGCTGAAGAAGGGAGGCAAAGATGTCCCAGTCACAATACA

CAATCTGGAGGAGTACCTAAGGCTGGTGGTGTACTGGACCTTAAATGAGGGTGTGTCCCG

GCAGTTCGAGTCATTTAGAGAAGGGTTTGAGTCCGTTTTCCCTCTCCACCACCTACAGTA

CTTCTACCCCGAAGAGCTGGACCAGCTGTTGTGTGGCAGCAAGTCAGAGACGTGGGACGT

TAAAACTCTAATGGAATGTTGCAGACCGGACCATGGGTATACACACGACAGTGTGTGTGT

GTGTAGTCGGGCAGTGCGCTTCCTCTTTGAGGTGTTGAGTAGTTTTGATGCAGAGCAACA

GAGACTGTTTCTGCAGTTTGTGACCGGGAGTCCGAGACTGCCTGTAGGAGGTTTTCGGAG

TTTAAATCCTCCTCTGACGATTGTACGCAAGACATTCGAGTCAACGGAGAACCCAGATGA

CTTCCTGCCGTCAGTGATGACCTGTGTAAACTACCTGAAACTTCCGGATTATTCCAGCAT

TGAGATCATGAGGGAAAAACTGTTAATGGCGGCGCGGGAGGGCCAGCAGTCTTTTCACCT

GTCCTGATCTTACACATACTCACACATATGCACCGACACACACACACACACACACACA

>Unigene000109

ACACACTCACACACACTCACTCCTCTCAACAGACCCTTACTAAAGCTTGTCTTTCAGGCT

ATGGCTCTCCAGCGGCTCCAGTAAAATCATCTAATAGTCTCACAGGACAGACCCCACCCC

AACAGCAGCCTCAATCATTCTCTCCCAACCAGCCTCCATCACACTTATCTCAGCCATATG

CAGGGTTTAGTTCTCCACAAACACATGATCTTCCTTCAGCCAGGACGGCGGGAGTAGCAA

AAGGATACGGAAATTTGGGAAGCCAGTCATTTTCTGCAGAATCAGTATATGGGACAGATT

CAGGTTATGGCTCACTGCCTTCATCACTAGGGGGAGCTGGAAGCCCCTCCATGGGCTATG

CTGCATCAGGACATTCTCCTGCACTTCTTCGTTCAGGGGCAAGCGGGGGAACAGCTGGTG

GAAGCAGCAATGGAGGAAGTACAAGTACAGGAGGCGGAAATAATGTCACTGGCGGAGGTG

GCTCTTACCACATTCCTGATTCCAGTCCATCACCATCTGGAAACTCTGGAATCATTCGGC

CAGGTCTTCATTCTCCAGCTCCTTCACGACCTGCTCAGTCACCTGTTGGAACAGGATCCA

ACAAATACCTATCATCTGTTCTCTCGCCTTCCTTCTTACCCTCCCCACAAGGCTACCCAG

ACACCCGTGGACCACGATCACAAACGTATCATCCCTCTGCCCCTCCCAAAACCAAGTCTG

ACACAAGCCTTCTTGGTGTGACTGAGTCTCGACCACAACAGGATGATGACGATGACGATT

TCCTCATCCAGCATTTACTTCAGGCTCAAAGTCCTACTCCCCAAGCATCCCATCATCATC

CACCACAGAGTCATCATTCCTCACAGTCTACACAACAGCCACCAGTTCTTCCGAATCAAG

ATGGAAGCAAGGGTTTATCCTATGAGATTGGAAAGAGTTCAGAAGAGCGATATCATCTTC

AGAGTGTTATTCGAACTCACAGTGCAACCACAAATGCTGCAGTATCTGGAACAGGAAATG

GAGCAGGCCTGGACAGTCAGTTGGAATTGTCTTTAAAAAAGCAGCAGCATCAGCAACAAC

ATCAAACACAGCATCAACAACAGCAACAACAACACAAACATCAACAGCAGAAAAGCAGCA

GATCTGTCACAGATGGAGGAGGACGAGGAAACCCAGATCAAGCTCATTCACATTCACATC

ATCACCATGATTCAATGAGCTCAGTGGTACACTATGGTCGAGGTGATCCTTATTCTCAAC

ATTCTATTTCACAGCATCCATCTAGTCACCATCAACACACTCCCCACACACCTACACATT

CTCACTTGCACTCTCACCCCCATATGGATCTTCAGAAGAAGCCCCAAGAGTCAGCAGATA

TGGCCTACATTCGCAAGACACCTGATCTGCAGCAGCACCACCACCAACAGCAATCAGGAC

ATCATCAGTCTCATCAGCAACAACACCAACAGCAGCAGCAGCAATCTCAAACATTAATGG

AATCTCCAACTGACCAGTCACGTCAGCCTCCTCATTTGTTACAATCTGTTCTCTCCCATA

CAACACGAAACAAAATAGAAACTCAGCAACAGCAACACCCTGCAAGCCAGCAGGCTTTAA

TAGAAGCAACAGGTGGGGTTACAGCAGCAGAAACCCATTCCCAGCCTCAGGCATCTCAAC

TACAGCTTCAACTCCAGACTCAAGCTTTGGAAACTGCTAGTCACTATAGCCATGGTTCTC

AGCAGCAGGATCAGAACCATCAAAAATCTAATACTGTCTCTTCTCTGGACATGTTGGAGC

GCTCCCTTTCTCGAACCTCAAGCCAGGAAGGAGGAATGGTGGATGATAGACCAGGAAATG

AGGGCGGCAGAGGAAATTCGGCAGCTGGGACCGGAGGACCTGTTGAGAGGCACAGATCAC

AGGATCAGCAGAGACTTCCATCCCATCATCCTACACAACACCATCCCCCTGAGTTACATA

CTTATCTTTCTGAGCCTGATTTGGGATTATCTACCCCGTCTCATGGGCACCACCTTTCCC

ATCACCACCATCAGTCACAGCAACCACCTCCAGCTCAACAACCTAACTCACACCACCATG

CTCACCATCAGCATCAAAATCCACAGAGCCATCACCATCTTGCTTCACAGTCAGCCTCTG

CAACAGCACAGCAACAAGAGCAGTCTCACTCCTCCCATTCCTCTCAAATCACTCAAGCTC

AGCTTGACCAGCAGCATCAAGGCGATCATCACTTTGATAGTAGTGGCTCAGCAGTAAAGT

CAAATGCAAATCAGAATCAGTCCCATCACAACCAGAGATTTGTTCCTTTAACATCTATTT

GTTTTCCAGACTCTCTTCTTCCAGATGAAGAACGTTCTTTCTTTCCTGGGATGGAGGACA

TGTTCTGCCCCGAAGAGTACAAGTCAAGCTGCTCTGGAGGAACCGGGCAGGGTCAAGATG

GAGTATCCCAAGCTCAGACAGGACAAGAAGGTATGGAGGGCATAAAATCAACTGCTGAGG

GTGGAGAGGATGATACAGGTGCAAGCTATGACATGCTTGGTCACCCTGGCGATCAGGATT

ATGGGCAGTATTGCCATGATCTCACAGAGCATGATAATGGAACCATGCATCTTGACCTTG

ACTCTTTAAAAACCCATGAACTCCCATCAACAGTCAATACAGAGCAACTTGGACTTATTC

AGTCTCAGCCAACTAACCTTGGAATAGGTAGCAGTGCAAATGTCGGGGATGGAGCTGGTG

CTAAGATGGTAGGTTCTGGAGGTTCTGTTTCTGGATCAGGAGGACTTACCTCTCCAATCT

TCTGTTCATCCCGACCCAAAAAGCTTCTGAAATCCAGCTCTTTTCACCTGCTTAAAGAAA

GACCTGATCCAAATTCCATTCCCAAAAAGAGCTATGCACAGGAGTATGAATTTGAAGATG

ATGAAGATAAGGCTGATGTTCCTGCTGACATTCGACTAAATAGCCGAAGACTTCCTGACC

TGATTCCTGACCTTGTATCCAGCTGTCGGAAAGCCGGTGGTGGAACTATGAGTCCTTTAA

TGAGCGACTTGGATTTTGGATATCCCTCTCTTGGTCCGCCACCACAATTACTTCCCAATG

ATGGACCAAAAAAGAGAGGCAGGAAGCCTACCAAGCCCAAACGGGAGGGCCCCCCAAGGC

CTCGTGGCCGTCCACGAATACGCCCTTTGCCGGAGCCCCACCACTCCAGGGCCATGATGG

GAGAGTGTGGAACAGGATATATTCCTGAGAGAGGCAGGGGTAGAGGTAGAGGCAGAGGAA

GAGGCAGGCGAGATGAATCTATGATGGACAGCAAAGATCAGAGTCAGCTGTATCAACAAC

ACATGCAGCAACAGCAAATGCATCACCTTCCACAACAGCACCAAGAGCCAATCAAACCTA

TTAAGATCAAGCTTCCTATCGGTTCCATGTCATCTTCTGATGCCCTCCTGCGGACAGATT

CTTTGTCTGGTACTGACCCAGCCCTCTCAGATGGTTCTGTTGGCTCTGCCCCTTCTTTGG

GGTTAAGTCCAGGGACACCAGGAGTGCCTGATATGACCAGACCATTAGACAAGAATAAGG

CTAAAACTCAAGATATGGAAGAGAAAGATTCTGAAAAGACTGGCTTTGTGGCATCTTTTT

TGGATTTTCTGAAATCCGGGAAAAGACCAGGTAGCAGCTCTGGGAATGGTGATTCATCTC

CAGGAAAAAGTGGAGGTATACATCCCATATCTCCTCAACCACCACCTCCACCAGCACCTG

CAGGATCATCTGGTTATGGGGATAGTGAAGGCGATGGAGGGCTGTCTTTGGGTGGATGCC

CAAGTCCTTGTAAGCGCTTAGACGAAGAACTGAAAAGGAATCTGGAAACACTTCCATCAT

TTTCTTCTGATGAAGAAGACTCCGTGGGAAAAAACCAGGACCTCCAGAAGAGTATTTCCT

CAGCCATCTCTGCTCTGTATGACACTCCACAGCTAAGCACCTCTTCAATGCAGCCTCCCT

CTCTCCCTCCGCCGCCTCCTCCAGTTGCTCCTACACCATTGACACCAACACAACATCCAC

CTTCCCTTAGCCCACAAACCCCAACACACATGCACACACAGCCTTCTTCACATACACATC

CACAGTCTATTGAGTCTACCATATTATCAAGAGATGAGCAAGGGGATGAGGTTGAGGAGG

AAATAATTAATGATGAAGACAAAGAAGAGGAACAGGACAAAGGAGTAGAAGAGAAAGTCA

CAGAAATGGAGTTGCTAAGTGTACCAAAAGTAGGAGACTCTCCTAAAGAAGCTTCTCAAG

CAGTAGCTCCGCATTCACTTCTACCTCTTTTCCCATCATCCCCTGCATCTTCATCTTCAC

CCTCTCCACCACCTCTTCCTCCACTTTCTCTTCCTTCACCCCTTCCTGAGCTGGAAGAGA

ACCCATCTCCACCACAGAAACCTTCAGCTCAGCTGTCTTCACCTCCAGCCAGCGATCTTG

CCACTACATTACCTGTCCTTTCTCCTCCACATCCTCTGACTGTAGCCTCTCCTCCCCCCT

TGGAGACACCCACACCATCAAGCCCTCCTCCAACTCAGCAGACTGTTCCAGCTCCTCCAT

CCCCAGAAGAAACCCCAGCAACTCAAATTTTGCCTTTACATCTGGCACAGAAACAGAGTG

GTGCTGCTATTGCAGGAGAGACTGATGATGATGAGAGTGAAAGTGGAGGAGAGGGTATCT

TTAGGGAAAGGGATGAATTTGTGGTACGGATAGAAGACATCAAAACTCTTAAGCTGGCCC

TGCAGACTGGTCGGGAGCCTCCACCCATTTGGCGTGTACAAAAAGCTCTGCTCCAGAAGT

TTGCGCCAGAAATTAAAGATGGACAGAGGCAGTTCTGTGCCACTAGCAATTATCTGGGTT

ACTTTGGTGACGCTAAAAAACGGTACCAACGACTATATGTGAAATTCCTAGAAAATGTGA

ATAAGAAGGATTATGTAAGAGTTTGCTCTTGCAAGCCCTGGCACAGGCCTTCTGTGACAC

TCAGACGACAGGCACAGTCTCACCCGGTCTCAAAGGTGAACCCATCTCCTAATAATCAGA

CACTGCCTCGTGTTGTAAGAGATGAGAAAGATAAAGACAGAGAAAAAGGACGAAATAAGG

AGCAGCGTGACAGCAGAGATAAGAACAACATCAAGGTGAAAGAGCAACAAGAAAAGGAAA

AGGTGGTAAAAATTAAGGAGCGAGAAGAAAAGAAAGACAAAGAAAAGAAAATGCCACCAC

CTCCTGCACCTCAAAGGGCAGGGAAACAAACAGCTGTGACAGAACCTGGACGAAAGGAGG

AGAAAAGAGGAGTGGAAAGAAAGATAGAACGAACTACAAAACAGCAGCCACTAAAAGTAA

AGGCTGAGCCTCCACCAAAAAAGAGAAGAAAGTGGCTGAAAGAAGTTCCGTCATCATCCG

ATTCCGATTCTTCGGCCAGTGATGATGAAATTTCTGTGAGAGCAGGCCTGAATACACGTG

CCATGCGAGAGATGTACAGAAGCTATGTGGAAATGCTGGTCAGTACAGCTCTGGACCCTG

ACATGATACAAGCACTGGAGGATACAGAAGATGAATTGTACTTGCCTCCAATGCGGAAGA

TTGACAGCATTCTTAGTGAGCATAAAAGAAAGCTGCTAAAACGGATCAACATGAACCCCC

AACACCAGGAGGCCCTACACCTTTTTCCTCAGATCACAGCAGAGCCCTTGGACTCAGGAG

CTGTGAGGGTCAAACTTGGTGGGGAGTGCTATAATAGAAAAACCCTGAACCGTGTCAAGA

AGAGTATCTCCAAACCACAGGACATCAAGTTGTCAACAGAGACTTGTCGCCTCTACAGCT

TTTACCATTCTCTGCATCATTATAAATACCACACCTTCCTAATCTGCAAAAAAGAGACCA

ACACTATAGAACAAGCGTCAGAAGATCCTGGGCAAGAGGAGGTGGTTCAGCAGTGCATGG

CCAATCAGAGCTGGCTGGACACACTCTTTAATGCCTTCTTAGAACTGATGACCCTCAGTG

CCAAAGCCTGACAGAATAAAAGCGTTCAAGAGGGGGGGGGCAGCGAGCGACAAAGGACAA

GCCCTGAGGATGCAGCAGAAGGCTGTTGTGGAACAAAAGAAATGGATTTGTTTTCACAGT

GTCCCCCCTGCAGTCCTGTTCTATTGTTTTTCTTAAGGCATTTCTTAAGATGATTGTGAT

ACATGGCCAGGCAATAGAGCCGCCTCCCAGTAGGTAGCCTTTGCCGAGTAAACTAATCAG

CACTTCATTACTCAAGTCAATTAGAGCAAAGTTTTCATGCTGAATGGATTCAAGGAAAAA

TGGGGACCATATTCACTAATTTTTTGTGTAAGGGGATGAGTGGATGGATTGTCCCCTTTC

GCTGTGCTGAATTTGCTAACAGACAGAAGAACAGAGAATGAATATGACTGAAGAATTCCG

TACAAATTTTAAGGGTAGATTTGGGGGAAAAAACAAACTTTGAACAACTAGAATTTAAAG

ACATTGCAAGAAAAAAGTCTGTTACAATTTGAATTTTACACAGATTATTTTTAAGGATTA

CAAACTTTTATTCTGCTTTTTTTCCTTGAGTGGAAG

>Unigene000110

ATTAAATTGACTCGGTGTGTTTTATCTTTAGCCGCTAACAGGCTGCTCTAGCTTTTCGCA

TTAAAACCTGGTTGTTTCCGTAGTTCGCTTTCAGCCTTGTTCGTGCCAATCAGGGCTGCG

CTCCAACATGCGCAGTCGCCTTCTCTATAGTATGTGTAATCGTCGACGTGTTCTGCACTA

GTTAGGCGCCGTCGGGAAGGTGCAGTTGTTCATATCGGAACACAGCCCAGGGTTTACACG

CATCCACGTCCACCAGTGTGTGTGTTTAAGATTAGGTTAGCCTGCTTCAACCACATAGGC

CGACCTCTTCAAAACGAACGTGAATGGTAGTGTCTATAAAGGGTATGTCCCTTCAGGAGA

GAGGTGCCAATGTCCATCAGGATTAATCACACTGCAGGGGGGTCACTGCGCCGATCACAG

AGGAACACACTCGCCACCAGGTTGCAGTCCGAACAAACCAAACAGAAAACAAATTCCCCT

CCTGAAAACAAAAGATCGTATAATAAAGCTTCCAAGGCCCCTCCCACGCCTGCAGCCGGC

TCCTCGCGTGGCCACGCCCCTCGAAGAAACAGCCTTTCTCTCGCTTCATTGAGTCAGTCT

GCATCTGAGCCGGATTCGGAGGCAGCTGGAAGCTCGGGCCAACGCGAGCAGCGTGAAGTC

AGCAACAATCGCGCCCTTAAACGCAGCTCTGCAACTGAGCACAACAACGCGTCCTACTCC

CCTAGTCCGGCCAAGCGGCCCAAAGCTCCTCCCCCTGAGAGCACAGAGCAGCTGGAAGCT

GCTGACCCAACTGCTGACTCCGCCCCCGAGCTCCCACCCTCATCCAGGTCTAAAAAGCGA

CGTCAAACAGACGAACATCCTACTAGCAGGACAGTAAATAAGAAGAGCAGCGCGTGCCCT

GCCTCTAGCTCCTCCCACAAACGGAAAAAGGCAGACAACTCATTTTGCTCCTCTTCTTCC

AGTTCATTCTCGTCCTCTTCCTCATTTTTATCCGGCTCGAGCAGCGAGCAGGCCAGCCGC

TCATCCAAACCCACCAAACTGGCGTCCAAATCGGCCGCCTCGGCCAAATCTGTGACTGAG

TCAGCCTCTTCCTCCTCTGCTTCTTCTTCTTCCTTTACTTCCTCTTCCTCCTCCTCTTCT

GTGGTGGTTGCGGGTGCCAGCGGCTGCCCCCCGCCCTCGCAGGGTGCCCGGCTGAAGCAG

GGAAAAGATCAGAGCAAGTCCCGGCGCTCTCGCTCCGCCTCTTCGCCGTCTCCTCGCCGC

AGCAGTCGGGATAAAGAGCACAGCAAATCCAGCACAGCAGCCTCCAAATTCGATTGGACG

AGCCGCTTCAGTCCAAGAGTGAACTTGCCGAAGCCCAGACTCGCCCTGCCCGGATCCTCC

AAACCCGAAACGCCTTCAGGCTCCAGCAAACCCGGCCCGAGCGGACTGCAGGCCAAACTC

GCCAGTCTGAGAAAGTCCAGTAAGAAGCGTAGCGAGTCTCCCCCTGCAGAACTCCCCAGC

TTTAGGAGGAGCACACGACAAAAGACCACGGGCTCCTGCGCCAGCACCAGTCGGCGAGGT

TCAGGTTTAGGGAAGCGTGGTGCAGCTGAAACTCGCCGACAGGACAAAATGGCTGAAAAC

GACAACAGTCAGGATGGAGCCAATTCATCACGCACAGAGGAGACGCCTCAGGGAGCTTCA

GCCTCCAGTTCAGTAGCTGGCGCTGTCGGAATGACCACATCCGGTGAGAGCGAATCGGAT

GACTCAGAAATGGGACGATTGCAGGCTCTGCTGGAGGCTCGTGGTTTGCCCCCACACCTG

TTCGGACCCCGTATGTCCCAACTTTTCCATCGAACCATCGGCACTGGAGCAAGTTCGAAA

GCTCAGCAGCTGCTGCAGGGTCTGCAGGCGACGGGGGACGAGTCTCAGCAGCTGCAGGCA

GCGATAGAAATGTGCCAGCTGTTGGTGATGGGGAACGAAGAAACACTGGGAGGTTTTCCT

GTGAAGAGTGTTGTACCTGCTTTGATCACGTTGTTACAGATGGAGCACAACTTTGATATT

ATGAATCATGCATCTCGTGCGCTAACCTACATGATGGAGGCTCTACCACGGTCTTCTGCT

GTAGTTGTAGACGCTATTCCTGTCTTCCTGGAAAAGCTTCAGGTGATCCAGTTTATAGAT

GTGGCTGAACAGGCTCTGACCGCGCTGGAGATGCTCTCCCGAAGACACAGCAAGGCTATC

CTGCAGGCCGGCGGATTGGCTGACTGTCTGCTGTACCTGGAGTTTTTCAGTATTAACGCA

CAGAGGAACGCATTAGCCATTGCCGCTAACTGCTGCCAGAGCATCACACCGGATGAGTTT

CACTTTGTGGCCGACTCGCTGCCTCTGCTCACTCAGAGACTCACACACCAGGATAAAAAA

TCAGTGGAGAGTACGTGTCTTTGTTTTGCCCGGCTGGTGGACAACTTTCAGCATGAGGAG

AATTTGTTGCAGCAGGTGGCATCACGTGATTTGCTCACAAACATTCAGCAGCTTCTGGTG

GTGACTCCGCCAGTGCTGAGTTCAGGCATGTTCATCATGGTGGTGCGCATGTTCTCTCTC

ATGTGCTCCAACTGTCCCAACCTCGCTGTGCAGCTGATGAAGCAGAATATTGCAGAGACG

CTGCGATTCCTGCTTTGTGGAGCATCTAATGGAAGCTGCCAGGAGCAAATTGACCTCATT

CCCAGAAGCCCCCAGGAGCTCTATGAGCTCACTTCCCTTATCTGTGAGCTGATGCCCTGC

CTCCCCAGAGAAGGTATTTTCGCTGTAGACTCGATGTTGAAGAAGGGCAGCGCACACACT

ACAGAGGCCGCAGTGTGGCAGTGGAGGGACGACCGAGGCCTGTGGCATCCATACAACCGA

ATAGACAGTCGGATCATTGAGACGGCCCACCAGAACGGTGAGGACGAGATCAGCCTGTCC

ACACTAGGACGCGTTTACACCATCGACTTTAACTCTATGCAGCAGATCAACGAGGACACA

GGAACGGCTCGGGGAATTCAAAGGAAACCCAACCCCCTGATCAACCCCAACACCGGTCAC

GTGGATGTGCGTGGCGAGGACGCGCGTGCTGAGCTGATGCGGGAGGAAGCCGAGCTAGCC

CGCTGCTTTATCAAGACTTTGTTTGGCGTGCTGTATGAGGTGTACAGCTCGTCAGCCGGC

CCTGCAGTCAGACACAAGTGCCTCCGAGCCATCCTGCGTATCATCTACTTCTCCGACTCC

GAGCTGCTCAAGGACGTACTGCGCAACCACGCCGTCTCCAGTCACATAGCCTCCATGCTG

TCCAGTCAGGACCTGAAGATTGTGGTCGGCTCTCTGCAGATGGCTGAGATCCTCATGCAG

AAACTTCCTGACGTGTTCAGCGTGTACTTCAGAAGAGAAGGTGTTATGCACCAGGTGAAG

AACCTGGCCGAGAGCGAGTCGTTCCTCCCAAGTCCACCCAAGGCGTGCACAAGCGGCATC

ACAGCGGCAACCACCACCATCACCACTGCCACCACACCTGCTGCCTCTAGTGTCGCACCG

GATTTGGGCTCACCGAGTTTTCATAACAACATGGAGGATTCACTGGACCTCAGCCCACAG

GGTCGCCTAAGTGATGTTCTGAAAAGGAAGCGACTTCCTCGCAGGGTACCGCGCCGTCCC

AAATATTCTCCACCCAGAGATGATGACAAAGTGGACAATCAGGCTAAGAGTCCCACCACC

ACTGCGTCCCCTAAATCGTCCTTCCTGGCAAGTCTTAACCCCAAAACCTGGGGAAAACTT

AGTGCTCAGAACAACAGTACTAACTCCGAACCACCTCGCACAGCCGGGGTTAGCGGCCTT

GCACGCACAGCTAACAAAGACTCCGTCTCTAATAACAGGGACAAGATTAAGGCATGGATT

AAGGAGCAGGCCAGTAAATTTGTGGAGCGATACTTCAATTCAGAAAACGTGGATGGCAGC

AACCCAGCTCTGAACGTTCTGCAGAGACTGTGTACTGCTACGGAGCAGCTCAGCCTGCAG

GTGGATGGCGGTCTGGAGTGTTTAGTGGAGATCTGCAGCATTGTGTCTGAGTCAGATGTT

TCTTCGTTCGAGATTCAACACAGTGGCCTGGTGAAGCAGCTGCTTATCTATTTAACCTCC

AACAGTGAACGAGACCTCATCAGCAGAGACCAGAGGATCAAACGCTTCCTGCACATTTTC

TGTGGCAGTCCTCTTCCTGGGCAGGAGCCACTGTTCCGGTTAGAACCAACAGCGAATGGA

GCGCTGCTAGCACTGGTGCACAAAATGAACAGTTGCTTAAGTCAGATGGAGCAGTTTCCT

GTTAAAGTGCACGACTTTCCTAGTGGTAATGGCAATGGAAGCAGAGGGTCTCAAGCACTG

AAATTCTTCAACACACATCAGTTGAAGTGCCAGCTGCAGAGGCACCCAGACTGCACCAAC

GTGAAGCAGTGGAAGGGAGGACCTGTTAAAATCGACCCTCTTGCACTAGTACAGGCCATC

GAAAGATACCTTGTTGTTAGAGGGTACGGACGAATCAGAGAGGAGGACGAGGACAGCGAC

GACGACGGATCAGACGACGAAATCGATGAGTCACTTGCGGCTCAGTTCCTGAATTCGGGC

AGTGTGCGTCACCGGCTACAGTTCTACATTGCTGATCACCTGCTGCCCTACAACATGACT

GTGTACCAGGCGGTGCGTCAGTTCAGCCTTCAGCCGGAGGAGGAGCGGGAGAGCACTGAC

GACGAGGCCAACCCTCTCGGTCGTGCCGGCATCTGGACCAAAACTCACACAATATGGTAT

AAGCCGGTAAGAGAGGACGAGGATGGCTCTAAGGACTCAGTAGGAGGGAAAAGAGGTCGA

GCACAAACGGCCCCCACTAAAACCTCACCACGTAACGCTAAGAAACAAGACGAGCTGTGG

CACGATGGCGTGTGTCCGAGTGTCAGTAACCCGTTGGAATCATACCTCATATCTGAACCT

CCAGAGGGAATTACATTTGATGACCCCTCACTGGAGGTCATTCTGCTGTTGAGGGTTCTG

CATTCCATCAGCAGATACTGGTTCTACCTCTACGAGAATGCTGTGTGTAAGGAGATCATT

CCCACTAGTGAGTTCATCAACAGTAAACTGACAGCAAAAGCCAACAGACAGCTGCAGGAT

CCGCTTGTGATCATGACTGGAAACATCCCACCCTGGCTTACCGAGCTGGGCAAAACCTGT

CCGTTCTTCTTCCCGTTTGACACGAGGCAAATGTTGTTCTATGTCACCGCGTTTGACCGT

GATCGCGCCATGCAGCGTCTGCTCGACACCAACCCCGAAATCAACCAGTCAGATTCGCAG

GACAGCCGCGTAGCCCCCCGCCTTGACAGAAAGAAGAGGACGATAAACAGAGATGAGCTG

TTAAAGCAGGCGGAGTCTGTCATGCAGGATCTCGGCAGTTCCAGGGCCATGCTGGAGATT

CAGTATGAGAATGAGGTGGGGACAGGTCTTGGTCCAACCCTGGAGTTCTATGCCCTTGTG

TCTCAGGAGTTGCAGAGAGCTGATCTGGGTTTGTGGCGAGGAGAAGAGGTCACCCTCGCC

AATCCTAAAGGGAGTCAAGAGGGTACCAAGTACATGTTTAGCTCTAGGGGTCTCTTTGCT

GTGCCTTTCGGTCGCAGCACCAAGCCAGCCCACATCGCCAAGATCAAGATGAAATTCCGC

TTTTTGGGGAAACTCATGGCTAAAGCCATCATGGACTTTAGACTGCTGGATCTGCCCCTC

GGCCTGCCTTTTTACAAGTGGATGTTGAGAAACGAGTCGTCGATAAGCTCTCACGATTTG

GTGAACATCGACCCTGGTGTGGCCAAATCCGTTCAGCACCTGGAGGACATCATTCGGCAG

AAGAGGAGACTAGAGCAGGACAAGTCCTATACTAGGGAGACACTACAACAGGCTCTGGAG

AGTTTAAATATGAATGGCTGTTCAGTGGAGGATTTAGGGCTGGACTTTACATTGCCAGGC

TTCCCAAACATTGAGCTGAAGAAGGGAGGCAAAGATGTCCCAGTCACAATACACAATCTG

GAGGAGTACCTAAGGCTGGTGGTGTACTGGACCTTAAATGAGGGTGTGTCCCGGCAGTTC

GAGTCATTTAGAGAAGGGTTTGAGTCCGTTTTCCCTCTCCACCACCTACAGTACTTCTAC

CCCGAAGAGCTGGACCAGCTGTTGTGTGGCAGCAAGTCAGAGACGTGGGACGTTAAAACT

CTAATGGAATGTTGCAGACCGGACCATGGGTATACACACGACAGTCGGGCAGTGCGCTTC

CTCTTTGAGGTGTTGAGTAGTTTTGATGCAGAGCAACAGAGACTGTTTCTGCAGTTTGTG

ACCGGGAGTCCGAGACTGCCTGTAGGAGGTTTTCGGAGTTTAAATCCTCCTCTGACGATT

GTACGCAAGACATTCGAGTCAACGGAGAACCCAGATGACTTCCTGCCGTCAGTGATGACC

TGTGTAAACTACCTGAAACTTCCGGATTATTCCAGCATTGAGATCATGAGGGAAAAACTG

TTAATGGCGGCGCGGGAGGGCCAGCAGTCTTTTCACCTGTCCTGATCTTACACATACTCA

CACATATGCACCGACACACACACACACACACACACA

>Unigene000111

GTAGCCGCTCGCGCAGGTCCACGTCGGCAGCTGCGCAGTGTTCATCTTACCTCAGAAGTG

AGAGGCGACATGACAGATACACAAATACTGTAATCTGGACACAGAGCCAGAATAAAAACT

GTAATAATAATTATAATAAAAACACTCTGGAGGGTTCAACCTTCTGCCGGCAGTCATGTT

CAAGAAACTGAAGCAAAAAATTAATGAGGAACAGTCACCCCAGAGAAATGTCCTGTCACC

TCAACAGGCCCAGGTGAGTTCAGGTGGGAGGGCAGTTAAATCACCGTTACTGCAGCCGGA

CACCGCTTCCTCCTCCAGTGAGCGAGAGAACTTTAGTAAAGGAGCAGTTAGTCCTCAGAG

TGGGAGTGTGAACGGAGATGGAGCTGTCTCTCCTCTGAGAGAGGAGGCGCCGTCTTTGGC

ACAGAAGCTGCAGCAAAGGGTGTCCTCGGTCGAGTCTCTCTTTCGGGGTTCGGGACGTGC

CGAGGGGCTTTTCCGCTCCGGCTCAAGGGACAGCCTAGTCCGTAGCGCGTCACGTGAATC

TCTTACTCCTCTGGGAGAGAATGAGGCTCCGGGGGTCCCTTCCTTCGATCCTCCATCTGA

CATCGAGAGCGAGGCTGAAGATTCTCCTGGAAACGCAGAGGCTTTGTCCAAAGATCAGCT

CGTATATCGGCTGCACAGAGTGGAGAAGAGTCTGGCGAATTACAGAGGGAAATACTCGGA

GCTGGTTACAGCCTACAGAACAGTGCAGAGAGATAAAGAGAAAACACAGGTTGTCCTCAG

TCAGAGTCAAGACAAAGCACTACGGAGAATAGGGGAACTCAGAGAAGAGCTGCAGATGGA

TCAGCAGGCCAAAAAGCACCTGCAGGAGGAGTTTGATGCTGCTCTGGAGGAAAAAGACCA

AATGATTACCGTCCTGCAGACTCAAGTTGCCCTTCTGAAGAAGCGGCTGCACGGTTCCCC

AGGGTCTTTACTCACATCTGAGGCAGGAGATTCCCAAACCGCTGAGACTACAGAGACGAT

CGCTGACCCTCAGAGCGTCACCCAGGCGGAGATTGAGGAACTAATTCAAGCAGGGGGCAG

TAGTGAGCCAGAAAACACTGCAGACTCTGAGGCTCTGCAGAAGCGAGTACGGAGACAGGA

GACGCTGCTGCAGCGCTGCAATGAGCTGATGAAAACCAACAAAGAGCGCAGTGCTCAACT

CGGCAGCGAGAACGACGTCCTGCAGCAGCAACTGCAGGAGAGACTGCAAGAACTGGAAAA

GATGAAGGAGCTTCACACCAGTGAGAAGACAAAGCTGATCACACAGCTAAGAGATGCCAA

GAACCTCATCGAGCAGCTGGAGCAAGACAAGGGCATGGTTATCGCTGAGACGAAGCGGCA

GATGCACGAGACTCTGGAGATGAAAGAGGAGGAGATCGCTCAGTTACGCACCAGGATCCA

GCAGACCCTGACCCAGAAAGAGGAACTGCAGGAACAGCGGGAAAAAGCTGAGAAAGCAGC

GTTTGAGGAGTTGGAACGCGCCCTAGGCGTGGCACAGAGAGCAGAAGAAGCCAGACGTCA

GCTGCAGGAACGCATGGAGAAGGAGGTAAAGCAGGTGGAGCAGACCAGTGAGGAGGAAAG

GCGGAGCCTACAACAGGAGCTCACCAGAGTCAAACAGGAGGTCATATCCATTATGAAGAA

GTCTACAGAAGAGAGGATAGCGGAGATGGAGCAGCAGCACCTAGAAGATCTGGCCAACAA

GGACCTGCAAATGAGTGTTCAGATACAGGAAGCGGTGGTAGAGTTACTGAGTTGATTCTC

CTCACTTTTATCACTCAGGACAGTACAGAGCTGAAGTGTGTGACGGATTGTTGTTGGTTG

TTTAGGAGAATTGCCGTAAGGAGTTGTTGGAAGCAGCGCAGGAGAAGGAGCAGCAGGCCT

CGCTGGCAGTGGAGGAGGCGGAGCTGCAGAAGTCTGCTGTCCAGGCTGAAGGAGAAGCTA

AAACCAGGGAGCTGCAGCTGGAGCTGGAGAGCGCAAGAACCAGAATTCTTGAGCTGGAAA

GTTCCATGGCCAAGTCATGTCAAGCAGATCAGTTCTCAGTGCAGATTGAAGAACAGAGGA

AGAAGTATGCAGATGAAATAGCTTCTTTAGGAGAAAGACATCAGCAGGAAGTGGAGAAAC

TGAAGATGGAACATGCAGAAGCTCTGAATCAGCAGCACTGCACTGATGTTGAGGCGCTCG

AGCAGAAACATCAGACTGAAACTGACGTCATGATGAAAGACAAAGAAAGAGAGTTTCAAG

AACATGTCAAAGAAATGAACCAAAAGATGCTGGAGAAGCTGGAGATCAAGCAGGCCAAGC

TGGAGGCTTTGTCCTCAGAGCTCAGTGAGATGCAGAAGTGTAAAGAGCAGCTGGAGGAGC

ATAAGGAGTCGGTGAGGATGGAGTTAGAAGTGAGACTAAAGGATGAGCAGGTGAAATTTC

AGAAGGAGGTGGATGATTTGAAGCAAAAGCACGAGGAGATGTTAGAAGGACTTGAGAAGC

ATCAGAAAGAAGAGATAAATCAGCTACAACTGGTGCTGGAGGAGAAGAATAAAGAGCTGG

AACAATATGTTGAAAAGGAAAGAATCCTACAGGAAGATACTAACAAGGCTAAACAGGATG

TGGAGACGATGTTAAAGGAGATAGAAGATCTCCGGTGTGTTGCTCAGTCTGAGAAAGAGA

AGCTTGCTGAAGCAGCTGCTCAGACTACTTTACTCAAAGAGGAACTTACGAAGTCACAGA

GTCGAGTACAAGACTTGGAGAAACTTCTAGAGGAAAGTCGTAATGAGTGCCAGCATAAAG

AAGAACATCTTCAACAGAAGAGTCATGAAAACTTTGAACTTCAGCAGAGGATACAGCAAG

CTTCCAATGACCTGGCTGAAACAACGTCTTCTCACGCTCAGACCCACCAAGCAATGCAGG

AGGAACAGGATGGGTTAAAGAAGCAGTTGGAAGAGGAGAGAAGTTCTTCTGAGAAAAGAG

TCGAGAGCTTTAGAAAAGAAATGGAGAGCAAGTTAAAATCCCAGGAGACCAAGATGGAGA

AACTCAGACAGAAAGCCAAAGAAATGCAGGAAAAGTTCAAGAAAAAACTGCAAGAACAAG

AGGAGAATGCGAAAGCAGAGATGGCAAAGAAGGAGAAAGAACTGCAGCAGAAAGAGCAAC

AGGTGAAAGAGAAGATCCTGGAGATAGCTCAAGCTAACTCTGAGGGTCTCAGCAGCGCTT

TGTCAGACTTGGAGGCGAATCACAAGGAGCAGTTTGAGAAGATTCAGAAGGTTCATAAGC

TGGAGCAGGATGACCTTCTGCGTATTTGGCAGGAGAAGCTGAGCCAGCAGGAGGAGGAGC

TGCAGGAGAAGCATGCTCTGACGATGCAAGAAAAAATGCAAGAATTGGAGGACATCTCTC

AGCAGCTTTTGACCCACAGAGAGGAGAAGCGACAAATGGATCAGGAAATAAAAAACCTTA

AAGAGGAGCTTTCAATGAGGGAAACCACTGTGCAGAAGCTTCAGACTGAGCTCAGGGAAG

CTGCAGGGAAACTGGACAGCCTGTCTAAAGTAGAGGAGCTCCTCAAAACACAAGTGGAGA

CTGTAGAGAAAAACCTGAACCAGGCTTTAAATGAAAGAAACATCTTTCAGGATCAGCTCA

CTGCAGCTGAGGAGACCAGTAAGGAAAAAATTCTGGCTTTGTCTGAAGAGCTCGCAAACG

CATGCAAGAAGCTCACTATTCTTGAAGCTTCCAAAAGTAAAGAAGGAGAAAACCTACAAA

AGAGCCTTGAGGAGAAAACGTTTGAGCTTCAGAATAAAGAAAGCAAGTTTTTGGAACAAA

TCCATAATGTCGGTGAGGAGCTGGAGCAGCACTGTCACACGGCACAGACAATCATCAATG

ATTTCTCAGCTCGCCTGTGCGAAAGAGTCGAGACGAAGGTGAACGAGCTGCAGAGTAGAG

TCATGTTCAACCAGAAGAAAGTGTGTCATTTAAAAAACCTCATTTTAACAAAGAACCACC

GAATCTGTTGTTTAGAGCAAGAGCTTCAGCAAGTCGTAGAGGAGAACCAGAAGCTCAAGA

ACTCACTCGAAGAAACAACTCTTCAGCTCCATGCAAATTCAGAGAATCTCAAAGCCTTAA

ACGTGGAGAAGGAGACCCTGCAAAATGATGCTAAGAATCATTCGCAGTTACTTTCTGAGA

AAGATCTTTCTATAGAAAAAACTCATGAAGAAAACAAAAACTTATCCGAAAACCTTAAAG

CAAATATTTTGCATATCAGTAACTTGGAGAACGTCATAGATGACCTGAAGAAGCAGTTAT

CATGTAGCATAACTGAGAAGGAGGAAGCCATATCTCTGCTGAATCAGCAGCACCAGGAGG

AGAAACAGAGCGTGCAGAACCAAATGGAGCAAGCAGAAAATGAAAAAGGTTTAGCTCTCA

AGCAAGTCGAGACACTCAGGAATAAACTGGCTGATTTTAAAAAGAAAACCGACTTCAAGA

TTTCTCAGAACCAGAACACGGTTAGATCTTTGCAGCGCCAGATAGAGGACATGGAGAAGC

AGATTGCGGAGAAGGACGAGCAGCTACAAAAACTCACTGCCAGCATCGACAATCAGTGTA

TCAGCAAGTCCGAGCTGGACCAGGTGCTGAGCGAGAAAGAGCAGAAGGTAAGTGCCTTAA

ATGTAGAACTGGATGACTGCACGAAGAAGATAAGTGAACTGGAGCAGCGACTCAAATCAC

AGAGCATGGAACAGGAGAAGAAAGAAGCTGAGCTGCAAAATCACGTCACCTCGAGCGAGA

TGGAAAAGACGGAGCTGATTCAGCAGCTGGAGCAAACCAGGCAGCAGTGTTCTCTTCAAC

AGGACCTCGTCTCTAAAACCCAAGAACAGCTCCATGCTTTGGAGAACGAGTTCCAAGCCG

TCAAGCAGAAACTGGAGAGCCAGCAAGAAGAATTTGAGCGGGAGAAGTCGAAAGAAGAGG

CTTTGAAAGCGGCGGAAGGTAAAGTCGCTGACCTGAAAAAGAAAGCTGAGCAAAAGATCA

GCTTGATCCGTAAGCAGCTGACTTCCCAGATCGAGCAGAAGGAGCAGACGATCAAAGAGC

TGCAGGCACAGCTGGAGGACATCATGCAGACGCAGAATGAGAAGGAGCAACAGGTGAGGA

ATTTGGAGGAGAATGGAAGGAAAATGGAGGAGGCCATTAATAATCTAATGGAGGAACACA

AAAAGCACCTGGAGCAGGTACTGAGTGATGGACAAGTAGAAAAAGAGTGCATGGACAAGG

AGGATTTGGTTGCTGCTGCTGCTGCTGCTGCTACTGCTACAGTAAATGACAGAGAGGAGG

AGACCTGGTCAAGGATCAGAGATCTGGAAGCCAAACTTCTTCAGTCTGAAGAACAGAACA

CCAGCTGCCGAGCTGAAATAAACCAATTGAAAGAAGAACTCTGTGAACAGACTAGTCTGG

TTCAGGAGCTTCAGAAGAGTCTGGAGGTCCAGGTTAAAGAGAAAGAGACGAATGTCATTC

AAATGGACAAATGCTTTGTGGAGAAAAGCAACAATCTCTTAGGAATGGAACATGTTCAAT

ATGTAGGAGACCAGAACACTAAATCACACCAAGACATTTGGGAGACGGAGAAGGAGCTCT

TGGTGAAAGACTATGAGATGAAGCTCCAAGACCTCCATAAGAGACTGGAGGAAAAGGAGA

ACGTACAGCTGGAACATAGTTTAGAGCAAAAACTGCTAGACGCTGAAAACGAGAAGCAGA

AGATTCATAAAGACTACACTCGCTTACAGAAAGACCTTCGGTCTTTGAGACGAGAACATG

AAAAGGACCTGGAATATCTGCGGAAAGAAATCGCAGAAGAGAGCGAGAAAAAGCTGAAAA

GTGACGTGGAGGATTTAGAAATGAAGCACAACTCAACCCTAAAGCAGATGATGAGAGAGT

TTAACACTCAGATAGCGCTGAAGGATAAAGAGCTGGAGACGTCAGTACGAGAGGCTGTGG

AAAAAGCGCAGATGGTCGAAGCGGAACTGATGGGAATCCATAAAGAAGAAGCCAATCAAC

TTCAACAGATCATTTCCCAGAAAGACGACGACCTCAAAAAAAACCATCCAACGTTACGAG

CAACTCCTTCAGAGTCGAGAAGAAGAAATGGGCAGTCGAGTGTGGGAGGTGCAGCAGGAG

CTGGAGGAGCTGCAGCAGAGGAGTCAAACCGGTCCTCAGGGTGTTGATGAGCTTCAGGTG

CAGCTCGCCGAGAAGACCACCCTGCTGAGTGAAACCCGGCTTAAAGAGCAGGAGTACCAG

GATCGGATCCACACTCTGGAGGACATGATACGCAAGGCTTATAAGAACTCAGTGGTGACC

CACCTGGGCAAAGATGTCTCACACCACACTGCGGAGTCCCTTAGCGAGCCCACCGAGTTG

GAGTACCTGCGCAAGGTGATGTTCGAGTACATGATGGGCCGCGAAACAAAGACGATGGCT

AAAGTCATCACGTCCGTGCTGAAGTTCCCCGAGGATCAGGCGCAGAAGATTCTGGAGCGA

GAAGATTCCCGACTGATGACTTGGCTGCG

>Unigene000112

GACAGTTTTGAAAAGGAGGGGGAAAGGAAGCAGACTCAAAAAAAAAAAAAAAGATATACT

GGGCTTCAGAAAGACAGAACGATGTGAGGAGAGAACTGTTCCATTTATAGAGAAATTCAG

GAGTAGAATGAACGGATCTGTGTGAGGTCTGAAAGTGAAGTTTTTAATAGTGGTACGGAA

CAGATACACAACAGGACTGAAACCACAACGAGCTGTGCAATGAGGAGGTGCAGGCATGAC

ACTCATCAGCGGGTAGCAATTACTCTAAACTACAAGGCTTAGAAAAGAACTGATGGCAGA

GTGAATCAATGAGGAAGAGAACGCTTCCTCTGCTGCTGAAACTACCCCACAGGACGAGCT

GCCCCAGTGCACAGAAAGCTGCTTATTCAAACTTCCTGACAAGGTCATGGCTTCACCCAG

TTAATGAGATACAATGAAGAGATTGGACGATGAACCCGTACACAGTGGGAACAGGACGAG

CCTTTCTGACAATCTCACCAACGGTGACTCAGTGACTCAGAGAAACCCTGTGGCTTTACT

GCAGGGGTTACATCTGTGTCAAGAGGCCTGGAACCCAGGAAGCCCGTGGGACAGAGAGGG

AGCCCACGCTGCTCTCTGGGGGAGGCCACCAGGGAGTTTCCTGGTGGTGTCAGAATCTTC

TTCTGAGAATAAACTCCTCTGTGTGTCTGTCAGTGATGAGGACAAAAAAGTAGAAGACTT

TCCCATCATACACACTGGCTCAACACAGCGTCTAAACACATCTCACCTGGCCTTTGCTGA

TCTGCTGCAACTGGTGATCTTCTACAACTTCAGCAGGGATGTGTTACCTTTTTGTCTGAG

GATTCCTTCATGGGTATGTGGCCTAACCGAACAGCCAAAGCATCTGGTTTCTGAACTGGG

TCCAAAGGCCTGGCTTTGTCCGCCAACAGATCTGCTGCCCGACACCATGAGTCCAGAAAC

AGCAGATATTCAGGACACTGTAATGTGCACCATACAGTTAACAGCAGACAATGGTGCACT

CTGCTTCATCAACCCTCTGTATCTCCAAGAACATGGAGATGACTGGCTGACACATTCCCA

GGTCAGCGCAAATCTTGCCAATCACCTCTTCACTTCCAAGCGTGACAGGAGGCTGAGCAC

TACAAGACCATGGGCAGGGGCGGGGCTTAAAAATCAAACCAAGTCAGTGGGAGACTCTGC

TGTTATGGAAATCTCAGGCTCTCCTGTTCATGGCTTGACTTCCCCAGTGACTGGGGTGAT

TTTGCGCAGAGCAAGCAGTACCTCAGATCCTCAATGGAGGATGAGTGCAGAATCAATCAG

CAGCCCCATCCCTCAGTCACCGCATCGAGTTTCTTGGATTGAGGACAAAGTCTGGATGAG

CCCTCCTGCTCCTTCCTCCCTGCTCCATCCTCCATGCCTAGAATTTGATTCTTTGTCGAT

GAGCAGCATAGAAGAAGAGCCTGAAGTTGAGCCAGCCGTAAGCTCCTGCCAGGTCCAAAA

CTCACCGCGCCTCCCACTGGCTGACAAGGTTAAGAACAGGCTTTCAGCGGTTGGCCAGGC

TCTGGGTGGCTTTATTAAACCACAGAAACGTCTGAGTAAGCGTGTCCAGGAAATGAGCGA

ACGCAAAGGCAGTCCTTTCGCAGAGGCTCTAAAGGGATTTGTAGAGCAGACGCTGAAACT

GAGAGCTTCTTGTCATGTGACGAGTACAGAGATGCTGCAGGAAGTGCGATCCTCCCTTAC

CGCTTTGAGGGAAATGCTGTATGATAGTGCTGAAATTCAGAGCATTATTGACACTCTGGG

GGATGTGCCTGATTTTGAACTGGATACAATGATGGAGCAAGCCTTACATAAAGTTGCTCT

GAAGCCTCTGTGTTCCCATTTGTACGAGTGCATGAAGACAGCACGACAGCAAGATGGCAG

CCTGCAGCGACTGCAAGCAAACCAAAACACGCTCAAATGTCGGCGCCTAGAGGAACTGGA

GGGGACTGCGGGTGCAGGTGTGCCTGATGCGGCCATGCTGGAGAAAATCCAACAGCGCTG

GGCTACCATGCACCAACAATATTCACCACAAATGAAAGTGGAACTGCTGCTCAAAGTGTG

TAAAAACGTTTACCACAGCATGACTGTTAATGCCAAGTCTGGTGTGGTTTTCGGAGCAGA

TGACTTTCTGCCCTGTCTAACATGGGTAATACTGCGCAGTGATGTTGTTACTCTGCAGAT

AGACACAGATTACATGATGGAGCTGCTGGACCCCTCTCAGTTACAGGGAGAAGGTGGATA

TTACCTTACGTCCCTTTATGCCTCCTTGTACTACATAAGCAGCTTTCAGTCTCGTTTGGC

AGCGCGGCAGCTGAGTGCCGAAGCCCAGAAATCCCTTAGTCAGTGGCATCGCAGACGCAC

ACTCCACTGTAACCAATCACGACGCAGCAGAAACCGGCGGACCATCCGAAGGCACAGGAA

TAACGAAAAAGAGGATGGGGAATCAGGGGAGTTGGATATAAGAAACATGTCTAAAGAGAA

AGACTGGACTCACACTGATGATGTGACAGAGACATTGCCGAGTGTGAATGAGATTTCTGG

AGAGGAAAAGGAAGAGTAAGCGAATGAAGGATCAGAGGGTTAATAGCGTGTGGGTAACAT

TAACATTAAAACTGTCCTGCACTTTTGCTTTAACAGTAGCTGCGTTTACATGGACCCTAA

TGATCAGACTATAAGCACACTCAGATTAAAAAAGTGACATGTAAACATGTCAATCAGAAT

TAATCAGCCAAATCTGATTAAAATTTCACTCGGATTGGGAAGTGGTTTAAACCTTTTTCA

CCCAATTTAGGGGACATGTGGACACCTCAATCCGACTGAAAATAAAACAAATGAAAACAA

ATCACGTGATGACTGTTCAACAGATTAAATCTGATTGTAAGCCCCATGCACATTAACCTA

ATCATTTCATTCAGTGTTTGTGTAAACTCTGCATTTCGGATTCATCAATCTGATTAATTT

AATTTCGATCGAAACAAAAAGTGTCCATGTAAACATAGCTAGTGTGTAAAAAACTTTTCT

TGTCAAAAGCTACACAACTCTTGCCTTTGCATAATTTTTGAGGCCTTAATACACTTTTTG

GGTAATCTATAGCCTTGACTTAGTGTTGTATAATATATTCAGATGTATCTTTAAGAGTTT

TAATTTAATTTGTAGCAGCATTTAATAAGAGCTGATCGAAAGAGCTAAATAAACCAAACA

CAACCAATTACTGATCTCATCTACAGTCCAAATTATACAAATTATAATTTGTTATGCAGC

TGCAGAATAAGACATGATAACTCACTGCATCGACGACAGTACACTATTAACATTGAGCAC

ACTTTCTGGATTAGATGCAATTCGGATTGCTTAATCTTCCTTCCGAGGCTTGCACATTTC

TAGTGGGGGTTATTCGAAACTGGGGACACACTGAAAATCTAACCCTAGTAATATAGATGA

GGACAATGAGGTAGGTAAGACAATTAAAGACTAATTTAGAGCTTTTTATTAATAATTCTA

AGCATTTTATTTAGTAGGCTATTCTGTCTATGCAGCCAGCTATTTTCTGTTTGTCTTATC

CTGCACATGGAGTAATTTGAAATTGAAATATGAAATTTGTTCATGGAGTATGTAAGTGGA

GTGAAGCGGCAAAAATTTCTACTCAAGGCATTTTATAATTGCTCACTCTGACTTCACTCT

GGATTTTCTTTATCCTGGTTTTTGCTCACTCCACGTGTGCCTTATTAATGCCCCAGTGTT

TTTTTTTATTCTAGACAGATAGATAGTATTTAAGTGCCTTTTGTAATCCAGACCTGAGAT

TTAAATCCTAAATGAACTTGAGCTGGTTAAGACCTTGCTACTGTTACTGGTAGTGTTGTG

TCCAGATATTGCAGAATGGTAATAGTACAAATATAAAGGCAATCACATGACCCAACATAA

TTTCTGTTCATTCTATGCTGCAGTTACAATATAAATGCCGTCATTATTAAAACAATGTTT

ACAGGATAAAGACAAAATCTGTGCTTATAACTGAATAACTTAAGCTTCGCTGAGGATCTT

TAACAATATTTTTATTGATTTTAAACAGGGATACAACAAAGCCAACATGTAATATTCCAA

TGCACACATTTACAATCTGTGTTATATACCCCTGATAGATCCAATAACCCCCACCCCACC

CATCCCTAACTCCAAGCCAGCCTTACAGGTCTAAGACAGGCTAAACAACAGGCATATTTA

AAGAAGGAGAAGAAAGAAACAAAAAACAAAACAAAAAAACATGAAGCTGGATGTCCGGGT

TTTGTAATTAAAGTATAAATGTATACGAAATAAGCGAAGGAAGGGCCCCCACACCTGTTG

AAATTTTAATTCTGAATTACGTATTAAATAATGAATCTTCTCCAGGGACAAACAGGACAT

AATATCTCTGAGGCACTGGGTGTGAGTGGGTGACGTGACTTTCACTTAAATAAAAGGGCA

TGTCTAGCCAGGAGGGACGCAAAAGACACTAAACAATGTTTAGCTGGAGTGAGACGATTA

CCTTCCTCTCGAGTAATGCCAAACTGAGCAACCAGGGGATCTGGTTCAAGATTACAATTA

AGAATGTAACACAAGGTTTTAAAGACATCCCTCCAGAATTTTTCCAAATTAGGGCATGTC

AAATACATATGAATAAGAGACGCCTGTCCGCTTTTACATTTATCACAGCAAGGACCGAAT

TCTGGGTAGAAACGAGAGAGCTTAACTTTGGAGAAATGAGTCCTGTGCACAACCTTAAAC

TGTAGTAAAGTATGGCGGGGGCCTAAAGAGGCTGAATTGACAAGCCTGAGAATTGAGTTC

CATGTGTCATCTGACAGTGTCAGATTTAGGTCATCTTCCCAAGCAGTTTTGATTTTATTT

AAAGTGGTGTGACTAAATCCCGTCAGTCCACCATAAATAGTGGATATTAGGCCTTTATGC

AGCGGAGACAAACAGAGGAAATTATCTATAATATTAGCATCCGGAGCCTCAGGGAAATTT

GACATTGAAGAGCTAATGAAGTGTCTTACTTGAGGATTTTTAACAACAGTGTGGAGAATT

CGTTCTTCTTACAACGTATAATCTCATTTTAGTAGATCATAGACATGGCCTATGATTTAA

CTAAACAAAAACAATTATGACTTCTTAATCGGTGGCTAGTTTCCTAGACTATTTTATATT

CTGTCTACACATCCTATGCTAGGCTCTGTGTAAGGATAGCTAATTTCTGATGGCAATTTT

TTTTATATACACTGTACACACTGAATGCATTAATGCAGGCTAAACAATGAACAATGATGC

TTTCTATAACCTCCTCTGCACCGCAAGATGTTTCATGAAAGTGATTTTCTTCATAAAACA

CAAGCCGGTCATTTCTAACGATGGGATGTCCTGTATGCGCAGAATAGAACAACTCGGATC

AGTTTATTCAGACATGGATTGCATTTCTTAGATATTTTTTTATTTTTTAATAAGTAAAAG

TATACTGTCAATAAACTTTGACAATTTAATAGTTTATAGTTTTAGTGGAAGCCAGAGTCG

AGGGGCATAGCGCGGTAGAAAACTGTGGAAAAGTTAAGAAATCGAAACAAGAATATTAAA

GCAAGAAACAGACACTAAACCAGCCACAATAAATAAAAGGCTGAACGTGTTATAATCAGC

ACTTTGTGTCCAGCTGGTTCTCCCCTGGGACAGGGAGTGCATTGTGGGTAAACTTTCTGA

TCTACACAGACGCGAGACAAGATTTAAGGTGCGTTTTCTGCATCACATAGCGGCTGTCAA

GGAGAGATTAAAACTCTGGGCTGGTATGAGGAAGTGCTGGTGTAGTAACATGACAGTGGT

GTTTAACTGTTTAGCAACAGATAAATTTGTTCATGCAGTTGCATTATGGGCTGTTAACAT

GATGTGTGCTCATAATGGAGTGTTAGTAGAGCACGGTCCGAGTGACAGAAAACTACATAA

AAAGCACACAAAATCACGCAAACGCCTGACTCCGCTCCGTTCACATACTCTGGCCAGGGT

GAACCTGGAGCCAAGAGACATAAGTAGGAGAGCACCCTGAAAATGGGCCAACCGGTCACA

GGGCACCATCATGTATAGACTCATATCATTTTCAATTCAGTGGTGATAATCAACATATGT

TATTAGACTAGGAGAGAAACTGGGGTAACTACAGTAAACCCTCAAGATCTAAGAAGAACA

TGCACATTCCACACAAACTAGCATGAATTATGAATGAACTTGTTTCTACTATATCACTAC

TATATTATACAGAGCATTTTACAGGCATGGTGGTAGCTGAGTGGGCAAGTTCAGTTCAGT

TGTACAAATCAGATAACTGTTTTTCCATATAAAAGTGTCTGCCAAATGACAGAAATGTTT

ACCATGTGAAGATTATTAGTGTCTTTACAGAGCTCAAGGTACAAAAGCTTCTAAATCATA

TAGGATGAGATTTTTTGAGAAAGTAAAAATACAGAAAGTTTACGGTTAGGATTAGGGTTA

GTGTTAGGGTTAAGATTAGGGTTAGGT

>Unigene000113

GGCAAACACAGGAACGCTGAGTGGATTGATCTGAATCGTGAAGCTGCTGGGTGGCTGAAG

CGGAAAGGGGACAGGCAGCCTGGGGCAGCTGCTATGCCGCTGGGACCATGGCTGCTTTCG

GGATGTTGAGTTACGAGCACCGGCCTTTGAAGCGGCCCAGACTCGGTCCTCCGGACGTTT

ATCCCCAAGACCCGAAGCAGAAAGAGGATGAATTGACTGCACTGAATGTCAAGCAGGGAT

TCAATAACCAGCCGGCCGTATCAGGGGATGAACACGGAAGTGCTAAAAATGTCAACTTCA

ACCCCTCTAAGATCAGCTCCAACTTCAGCAGCATCATTGCAGAAAAGCTGCGCTGTAACA

CCTTCCCGGACACAGGAAAGAGGAAGCCCCAGGTCAATCAGAAGGACAATTTTTGGCTGG

TCACAGCACGCTCACAGAGCTCAATCAACAACTGGTTTACAGATCTGGCGGGAACAAAAC

CTCTCACTCAGCTGGCTAAAAAGGTCCCTATCTTTAGTAAGAAAGAGGAGGTTTTCGGAT

ATCTGGCCAAATACTCGGTCCCAGTCATGCGTTCCGCCTGGATGATCAAAATGACCTGCG

CTTATCACGCCGCCATTACCGAAACGAAAGTCAAGAAGAGGCACGTCATTGATCCCTGCA

TAGAATGGACTCAGATCATCACGAAGTACCTCAGCGAGCAGCTGCAGAAGGTGGCCGAGT

TTTACCGACAGTCTCCCAGTCAGGGCTGCGGTTCTCCTCTGCCTGCTCCTCCGGCCGAGG

TGGAGACAGCCATGAAGCAGTGGGAGTATAATGAGAAGCTGGCCATGTTTATGTTTCAGG

ACGGCATGTTAGACAGACACGAGTTCTTGACGTGGGTCCTGGAATGCTTTGAAAAGATAC

GTCCAGGAGAAGACGAACTTCTGAAGTTGCTGCTGCCGCTCCTTTTACAGTATTCAGGGG

AGTTTGTGCAGTCAGCTTATTTGTCTAGAAGGCTGGCATATTTCTGCACACGACGCCTAA

ACCTGCTGTTAAGCGATGGGAGTATAGGACCCAGCTCAGGAGGACATCCACCACACAGCA

TCACCAGTCAGCCAGGCAACGCCTTACCCACCCCTCCCACCTCCCAGCCTGCAGGGACCA

CCCAACCCCAGACTCAGTTCACCGACTACTACATCTGCCCTCAGCACCGCCCTGTGGTGT

TTGGCCTTAGCTGCATGCTCCAGAGTATTGTGCTGTGCTGTCCTAGCGCTCTGGTGTGGC

ACTATTCGCTTACAGACAGTCGAAATAAGACCGGATCACCGCTGGACCTTCTGCCTATCG

CCCCCTCAAACCTGCCCATGCCAGGCGCCAACAGCACCTTCAATCAGCAGGTCCGTGCTA

AACTACGGGAAATTGAGGAGCAGATCAAGGAGCGGGGGCAGGCGGTGGAGTTTCGATGGT

CTTTTGATAAATGTCAGGAAACAACAGCGGGATTTACAATCGGTCGGGTTCTGCACACAC

TAGAGGTTCTGGACAACCACAGCTTTGAGAAGTCGGACTTTAGTAATTCTTTGGACTCGC

TTTACAACCGAATATTTGGTTCAGGCCAGAGTAAAGATGGGCATGAGTTGAGTCCAGATG

ATGACGCAGTGGTAACTCTGCTGTGCGAGTGGGCTGTGTGCTGTAAACGCTCAGGGAGGC

ACAGGGCCATGGTGGTGGCCAAGCTGTTGGAGAAGAGACAGACTGAGATCGAAGCAGAGA

GGTGTGGGGAGTCAGAGGTGGTGGATGAAAAGGGCTCTGTGTCTTCTGGCTCTCTCTCAG

CCGCCACTCTTCCCGTCTTCCAGGATGTTCTACTGCAGTTCCTGGACACTCAAGCTCCAG

TCCTTACGGAGCCTGGTAACGAGAGCGAGCGAGTGGAGTTCTCCAACCTGGTGCTGCTCT

TCTGCGAGCTCATTAGGCACGATGTCTTCTCCCACAACATCTACATGTGCACGCTCATTT

CCCGTGGCGACCTGGCCTCTGATTCCCACCTTTCCCGCCCGCGTTCCCCTAGCGACGAGC

CCTCTGATGAATCCGAACGCAAAGAGCAGGACGCTGGCAGCGGGGTTAAAATGGAGGACA

CTGGTCTGTCTGAGCCTATGGAAATTGATAACAACTCCAGTGCTAATTTTGATGAGATGT

TTTCACCCCCGATGCACTGTGAGGCCAAAGGAAGCCCCTCCCCAGAAAAAAATGCCCCGG

AACAGGATGGGAAGAGCACTGCCAAGGAGAAGGGTTTAGACCCGACTTTCCCGCAGGTGT

ACGATCAGCCACGCCACATCCAGTACGCCACACACTTCCCCATCCCGCAGGAGGAAAGTG

CCAGTCACGAATGTAACCAGCGTCTTGTGGTCCTGTACGGCGTGGGCAAGCAGCGGGATG

AGGCTCGACACGCCATCAAGAAGATCACCAAAGACATCCTGAAAGTGCTGAACCGCAAGA

GCACTGCCGAGACTGGAGGAGAGGAAGGACAGAAAAGGAAAAGAAGTAAGCCTGAAGCGT

TTCCTACTGCAGAAGATATTTTCTCTAAGTTTCAGCATCTCTCCCACTTTGACCAGCACC

AGGTCACCTCTCAGGTGTCCAGGAACGTTCTGGAACAAATCACCAGCTTTGCCTTAGGGA

TGTCTTATCACCTTCCCCTGGTCCAGCACATCCAGTTTATCTTTGACCTAATGGAATACT

CCTTAAACATAAGTGGCCTCATCGACTTTGCCATTCAGCTGCTGAACGAGCTGAGTCTGG

TGGAGGCTGAGCTCCTTCTGAAGTCCTCCAGTCTGGCAGGGAGCTACACCACTAGCCTTT

GTCTGTGCATCGTGGCTGTGCTTCGACGCTACCACTCCTGTCTCATCCTCAACCCTGACC

AAACTGCACAGGTTTTCGACGGGCTTCGCATTGTGGTGAAGTCAGGGGTGAATCCTGCAG

ACTGTTCCTCAGCTGAACGCTGTATCCTGGCCTACCTGTATGACCTCTATACTTCCTGCA

GTCACCTGAAAAGCAAGTTTGGAGAGATCTTTAGTGAGTTTTGTTCCAAGGTAAAGAACT

CGATCTACTGGAATATTGACCCATCAGACTCCAACATGCTGTGGGACCAGGTCTTCATGA

TCGACGCCATAGCCAACCCTTCAGCCCACAACATCAACCACTCCATGGTGGGCAAGATTC

TCAACGACAGCCCAGCAAACCGCTACAGCTTTGTGTGTAACGTGCTGATGGATGTGTGTG

TGGACCATCGTGACCCTGACAGGGTTAACGATATTGGTATCCTGTGTGCGGAGCTGACAG

CATACTGCCGTTCTCTGAGCGCAGAATGGCTTGGGGTCCTAAAGGCTCTGTGCTGTTCCT

CCAACAACGGCAACTGTGGCTTCAACGACTTGCTGTGTAACGTAGATGTGAGCGATTTAT

CGTTTCACGACTCTCTGGCTACATTTGTTGCTATCCTCATAGCCCGGCAGTGTCTGCTTC

TGGAGGATCTGGTGCGCTGCGTGGCTATTCCTTCTCTCCTCAATGCTGCCTGTAGTGAGC

AGGACTCCGAGCCAGGTGCCAGACTGACCTGCAGGATTCTGCTGCATCTCTTCAGAACCC

CACAGAGAAATCCATGTCCACAGGACAGCAAGACAGATAAGGCAACGGTGGGCATCAGGT

CATCATGTGATCGTCACTTGTTAGCTGCATCTCAGAATAGCATCGTAGTGGGTGCAGTGT

TTGCTGTCCTAAAGGCTGTCTTTATGCTCGGAGACGCTGAGTTAAAAGGCTCAGGCTTCT

CCCATCCTGCTGGCTTAGATGACATCGGGGAGGATGACATAGGCTCCAAAAAGTCCGGTG

GCCGCAACATCTCCATAGAAACAGCCAGCCTGGACGTTTACGCCAAATATGTGCTGAAAA

GCATCTGTCAGCAGGAGTGGGTAGGAGAACGCTGTCTCAAATCTCTCTCTGAGGACAGCA

GCGCTCTTCAGGACCCAGTGCTAGTCAACATCCAGGCCCAGAGGCTGCTGCAGCTCATCT

GTTACCCACACAGGCAGCTGGACAGCGAAGAAGGAGAAAACCCTCAGAGACAGCGCATCA

AACGGATCCTCCAGAACCTGGATCAGTGGACCATGAGACAGTCATCTCTTGAACTCCAGC

TAATGATCAAGCAAAGTTCTAATAACGAGCTCTATTCCCTTCTGGAAAACATTGCCAAAG

CAACCATCGAGGTTTTCCAGAAGTCTGCAGAGATGAACTCCAGTAACCCTTCCTGGAACG

GCTCGGCTGCCTCCAGCAGCTCGGTCTCCAATAGCAACAACACCAGCAAGCTCAAACCGG

TGCTCAGTTCTTCTGAGCGCTCAGGTGTGTGGTTGGTGGCCCCACTAATTGCCAAACTGC

CCACCTCAGTACAGGGACACGTACTGAAAGCAGCAGGAGAGGAGCTGGAGAAAGGGCAGC

ATCTCGGTTCCTCTTCCCGCAAGGAGAGAGATCGACAGAAACAGAAGAGTATGTCACTGC

TGAGTCAGCAGCCTTTCCTGTCTCTGGTGCTGACATGCTTAAAAGGCCAGGACGAGCAGA

GAGAAGGCCTCCTTACTTCACTCTACAGCCAAGTGCACCAGATCGTCACTAACTGGCGTG

AGGATCAGTATCAGGACGACTGTAAGGCCAAGCAGATGATGCATGAGGCTCTTAAGCTGC

GGCTCAACTTGGTGGGTGGAATGTTTGACACGGTGCAACGCAGCACACAGCAGACCACCG

AGTGGGCCGTGCTTCTTCTTGACATCATCAGCAGCGGCACAGTCGACATGCAGTCCAACA

ACGAGCTGTTCACCACCGTGCTGGACATGCTGAGCGTGCTGATTAATGGAACACTGGCAG

CTGACATGTCCAGCATTTCTCAGGGTGGCATGGAGGAGAACAAGAGGGCTTATATGAACC

TGGTGAAGAAGCTCAGGAAAGAATTGGGGGATCGTCAGTCCGAGAGTTTGGAGAAGGTGC

GCCAGTTACTGCCACTACCCAAGCAGACTCGGGATGTCATTACCTGTGAACCTCAGGGAT

CCCTTATAGACACTAAGGGGAACAAAATTGCTGGCTTTGAGAAAGAGGGTCTACAGGTGT

CGACGAAGCAAAAGATCTCCCCATGGGACGTATTCGAGGGTCTGAAACACTCGGCTCCTC

TGTCCTGGGGCTGGTTCGGCACGGTGCGCGTTGACCGTAAGGTCACCCGCTTCGAGGAAC

AGCAGAGACTCCTGCTTTATCACACGCATCTAAAACCTAAACCACGCAGCTATTACCTGG

AGCCTTTACCCCTTCCTCCAGAAGAGGAGGAGCCTCCGACGCCTGTGCAGCAAGAACCAG

AAAAGAAGATGGTGGAGGCTGTGAAGACAGATAAGAGCGTGCCGAGTGTGGGTCCTGATA

CGGTCAAGAAAAAATCCAAAAAGAAGAAAACTTCGTCCGTGAATAAAGAGGACTATACAG

CACGTAACCCCGGTGTAATGACGTTTCATGGCCCAGGCATGAATCCTGACCTCATGAACA

TGAGCCAGCCCAACCACTCTTACAGAGCAATGCCCTACAACCAGACACCCATCATGTACA

CCCAGAATCAACCTCTTCCTCCAGGCGGACCGGGACTGGAGCCTCCATATCGACCTCGCG

TGCCTATGAACAACCATCAAAAGATCATGCCAATGCGGCCCAACTACCCGAACATGATGT

CCAACCTTCAGGGTGGCATGGGCATGGTGGGCATGGATAACAAGCAGTACCCTATAGGCT

TCAAACCGCAGCCTGCCATACCACAGGGACAGATACGCCAGCAGCTGCAGGTCAGATTGA

ACCAGAGCATGATTGGACAGCAGATGAGACAGATGGCTCCCAATCAGCAATACACTCCTA

TGCAGCAATCACAGGGTTACACCAGCTACAGTTCTCATGTGGGGATGCAGGCACACCCCT

CTCAAAGCGGAGGAATAGTTCCTTCTTCATACACCAACCAGGGCTTCCCAGGAGCCCATC

CTGGTACCAACCCTGCCGTTGTGGATCCTCTCAGGCAGATGCAGCAGAGGCCCAGCGGAT

ACGTCCACCAGCAGGCACCTGGAGCTTACACAGCCAACATGCAAAACACCCCAAGGTTTA

CCCATCAGCCCATGCAGCAGACTCCTATGATGCATGGCTTGGGGCAGGGTCACATGCAGG

CACAGGGCATGCACCCTAACATGCGCACCAATCAGATGCTGGACCAGCAGCAGCAGCAGC

AGCAGGCGCAGCAGCAGCAGTTCTTGAGACAGCAGGCTCTCCGGCAACAAGCCCAGCAAC

AGCAACAGCAACAGCAGCAGGTGCAGGTGCAGCAGCAGCAGGTCCCCACACAGCAGGTTC

AGCAGCAGCAGGTGGGCACGCAACAGCCTCCTGGCCAGAACCAAAGCCTGGGCATGCAAC

CACTTCCTCCACAGCAGCCCATGTTTCCTCGACAGGGGCAGGGCATGCAGCAGACACAAC

AGCAACAACAGACTGCTGCTTTAGTCAGACAACTACAACAGCAGCTGTCCAGCAGCAGAC

GCAGCAGGTCCCCACACAGCAGGT

>Unigene000114

CGCAGCTGCAAGGCTCCCGGGCAAAACAGATACAGTGGCCTGCGCTGACATTCAAGCCTC

TGGTAGGAAAGAGTGTCCTGGGCTCCATCACCGCCGTGGAATTCTTCTCCGACAGACAGC

TGGACTTCCTAACCGATGACGGAGCATGCCAGCCCTATCAGGATGAAGTGGACAGCTTAA

GTCCTGTTCTCAGGGACAATCCACAGCTTCATGAAGAAGTGAAAAGCTGGGTAAAAGACC

AAAAGGTGCAGGAAATTTTTATGCAAGGTCCCTATTCTTTAAATGGATACCGCGTACGAG

TTTACAGGCAGGACTCAGCCACCCAGTGGTTCACGGGCATCATTACGCATCACGACCTCT

TCAGTCGCACTATGGTTGTAATGAATGACCAGGTACTAGAGCCTCAGAATGTTGATCCCT

CAATGGTACAGATGACTTTTCTGGACAATGTGGTTCATTCTTTGCTGAAAGGAGAAAATA

TAGGCATCACATCCAGAAGGAGATCACGCTCTAGCCAGAATAACAACACTGCCCACGGTC

ATTACACACGTGCCCAGGCCAACAGTCCCAGACCGGTAATGAACTCATCAGGCCCTGCCA

CCAAGCAGGGTTCACAAGCCCAACAGCAACAGCCCCAGCATGCTCAACAGCAGGTATCCC

AGCAGCAGCAGCAGCAGCCGCAGGTATCACCAGGGCAACAGCGTGGGTCCCGGTCGACAC

GTAGAAAGGGATCAGACAGCAGCATCCCTGATGATGAGAAAATCAGCGACGAAAAGACGG

ACGCGGGAGGCGGAAAAGGAGATGTTTCTAAAAACAAATCAAAACAGGTGATGAACAAGA

GGAGGAAAGCTGAAGATGAGGAGAAGAAGGCTGGGCTTAAACGGTTAAAGATGGAGGCAT

CGGATCTTTCCGAGAGCAGCGACTCTGAGAACTCTAATAAGAGGCTCCTGGACTCATCCT

CAGAGCCGAGCTCTGAAAATGAATTAAAAAATAAAGGCATTTTAAAAGCCAGTGAGGAAG

AGGAAAAGTCCCAGAGCTGCAAGGCTATGGAGGAGCCTGACGCTAATAGCAGGATGTCTC

CCTGGGAGGAGGCTTCTACGGTGGATCAGATTGTCAAGCCGACAGCCATGGAAGTGATGC

CCTCTACCAAGAGTGAAAATACAGGCGACATGAGGTCCCTAGCGCAACCTTCCCCACTGC

CTCCTCCCAGCTCGTGTGCGCAGCCTCGGAGCTTAGTTCCTGCTGAAGTTCAGGGTTGCA

TTATGGAAATAAAAAGCACTGTGAAAACTTCTCCTAAGGACCATTATAGCACAGGGGCAC

CAAGAACACAAACTCCAAAGTGTGTAATTGACATCACAGAAGACTCCAGCTCCCACCCCA

CAACCCGAGAGAACTCAGAGACTGTGCCTGCGACTTTAGCTTCTCAAACACGTGAAACTT

ATGTCCCTGAAAGCAGACATTTGGTGTTAAACCCCTTAGCCTCAGAATGCAGGAAAGCAG

AGGGTGAACTTCAGCAGCAGCTAGGTCAGAGCCTGGGCAGCAAGATTGAATTTGCTCACT

CTGAGGTGATCCGACCTGTGGTTTCGGTAAGCGAGTTAGCGGCTGTTGCGGAAAGGGAAA

GGGAGAAAGTACAGCAGCAGTACCCTTCAATCATGCCTTGCATCAAAAATGCCTCACTGG

CTGAAGATGTGCGAAAACCACAGAAACTTAGCTCGTCACCAGATGTGGCCAAGTCCAAGT

CTAACCCTTCACCTGACGCATTTAAGCCCAAGTGCAATCCCTCACCTGACGCCATGAAGT

CAAAAACCCACAGTGTCCTGGAGGCCATGAAGCCTAAACCTAACACTTCACCTGAGGTCA

CCAAACACAAAGAGATCATTGAGATCACTTCATCCTCTGTTGCCCGGTCGGCAGTCAAGT

CGAGAAGTCAAGAAATACCACGCTCCAGCTTTAAGCCTGTTCCTGCTCGTAGCACTCCCT

CTGAGTCCATCAAGAGCGCTCTCATTGTCGACAAAAACGAGCACTTTACAGTGTATCGTG

ACCCGGCGCTGGTGCGGCCCGAGGCTGATACCAACCATGTAACTTACCTTCCCCCACACC

TCCATCCACTCCACAGCTCCTCCCATGCTACTTGCCTGACACCTAGCTCACATCACCACT

CCCATCTATTGCCTGCATCTTCCCTTAGCCCGCACCCCTCAGTGCACCACCCCTTGCTCC

CCACCGTGTTGCCTGCCATGCCGCCGTCCTCCCTACTGGGTGGCCACCCGCGCCTCGACT

CAGGGGGACTCAGTCATCTAGCCCTAGCTCACCACCACCCACACCAGCAACAGCAGTTTC

TGCAACAGCAACCACCCCCACCGCTGCTGGCCCAGACACATGGTGGTGCCAGCTACAACC

AGCTCGGCCTTTATCCCATAATCTGGCAATACCATAATGGCACACAGCACTCCTATCCAT

CTGGAATTAGCCTGCCTGGGTCAAAGTGGGTGCATCCAGAAAATGCTGTGAACTCAGATG

GTAGCCTGTCAAGGAACACCTCCAGTCCCTGGCTGCATCAGCCCACTCCTGTGTCCTCAG

CGGATAGTCTAGGAATTCTGAGCCATGTCCCAGGTAGGCCAGCCAGTGCTGATCCCCACA

GACCCCTTAAAATCTCCTCACACTCCAGCCCTCCGCTCTCCAAAACTGTGGATCACCACA

AAGGAGAGCTGGAAAGTAAGGTTTTTGTAGATCCAATGCGTGGCCTGGTAACCGCCCACC

TAAAGCAAGAGCCAGACCAAAGCCGGACACCGAACAGCAGGGATTTACAGCGCCTCTACG

CGGATTCATCTCACGTAAAGCAGCAGGCGCTGCCTCCCCGCCTGCCACTAGACACTCCAG

ACCGGGCTGTTAAGTACAAAGAGGAGAATCGACGCATTCTACAAGAGAGCATTGAGGTGG

CACCCTTTACTGCCAAAATCCGCTCAGGAGAGCCAGAGCGTGAGCCCTATATCCGCATTC

CCTCTCTCCCCATCAGTGGACCGCCCAAGGAGAATGAGCATTCGCCATCTGATCTTTATA

AGTACAAGCACTCGGCTGCTCAGTCACTGCCACAGAGCAACTATTTTACCACCATTTCCA

ACAGTGTGGTGAATGAGCCACCTCGTCTTTACCCCTCCAAAGAGCTTAGCTCCCTTAATG

TTTCAAGTCCCCTGACTCTCGGCTCCTACCCCAACAGCGGAGGCAACACCAAGTCCTTAT

CCAAGCCTCCCCCGCTCATCAAGCACCATCCAGACGGGGAGGGCTTGGTGGGTAAGATCA

GTGAGCAGCTCAGTCATCAGGTGGCCCTGAACCCTCTGAGTACCCCCGTGGCATCTTGCG

AACCCCGCAGTCCGGCTGTGTCACCCTCAAACCAACTCAGGAGCATGCCTGCTCTACACA

GGGCACCTGTTTTTCACCCTCCTACCCAGCAAACACTGGACCGCAAAGAGTCTGGCTACG

GTCGCCTCTCTCCACCAACGCTAACCCCTATTCAGCCGGTTAGTGTGGCTGGGAAGGTAT

CAGAGCAACAAAAGCCGCCCACTCTGCTTCCAGAGCTTAGGGAGGTGAAAGGCACTGTAG

AGCTGAGCTCCAGTGAGCCATGGAGGCCCAGCTCTAACTTACAGGGCTATGATAAAACAG

TTCAGTGGCAGTTAGACAAAAGTCAAGGAAAGCCACAGGTAGCCACAGCTTCTGTTATTG

TTCGCTCCAGAACCTGCATTAAGTATGACAGCTCACCAGGCTCCAAACCTGGTGCCAGGG

AGACGTCTAACAGTAAACCACATACAGGAAAATACCAACTGGACTGCAGCAAACTGGCTG

AAACCAGAGAACCCGGCAGAGTTATACAGCAAAACACAAATAAGGAGGACATTTTTTTGC

AGTACAAAAACAATTTTGTCCGAGTCTCCCAGGGCAGTTTTCCCAGCTCTGCGGTAGCTG

TTGTTAACTCTGTGTGCAATAACAGTACTGCTGTAACCACTTCTGCCTCTGCTGCTTCCA

CTCACAATGTGCTGAGTCGTGGCACAGCTGAACTGCCTTACTCCACCTCAGCAAATCCAA

GCAGCAGTAACATTAACAGACTGGAGGGCGCTGCACCCAAATGCAGGACTCCTACCAGCC

TTGAGCTACAGGAGTGCAATGCCAGGACAGCCTCCCCCATTGGCCCACTGCCTCAGCCTG

GTTCGGCCCTAGCTCCACAGCCTTATTCTGGGAACTTTATCCACCTGAAGAAGCACAAGG

CTGCCCTGGCCGCTGCCCAGTCCCGTGGATCAAGCAGTGCCTCAGAGAGTGAAGGTAGCA

GTCGATCCTCTCAGGAATCACCCACAATCATCACGCAGGATCGTGCATCCCCAGGCAACC

CGTCCAGCAAAGTCAGCCCCCTACCCAATGGTCAGCCATCACTGATGAACCAGCCCAACT

ACCACAAGCTAAAGAAAGCATGGCTGACTCGGCACTCAGAGGAGGATCGCAACACTAACA

AGGTGGAGATGACTTCCAATGCCGTCTCTGAGATCATTAGGCCATGCACAGTAAACCTTA

TAGCATCCACCTCCAGCGACACAGAGATAAGCAAAGATGGCAAATGCTTAGAGGATAAAT

TGAGCCCGGAGGACAGGAAACCACGACGAGTTCCTTCCAAGCGGCCACACGAATCAGGCT

CGGATAGCGGCGATGACTCCGATGGCAGTGACAGCAAGCATGAGCAGAGAGCCAAAAGGC

AGCCTAAGCCCACCTTCAAGAAGAAGCAGAATGACATGCAAAAAAAGAAAGGGGAAAATG

ACAAGGAGGAGGAGGATGTCAAGCCCAATGGCATATTCAGAAGCGCCAAGGAGAAAACCA

AACTAAAATTGGCCAGCAGCAACGGTATTCCTCGCTCGGTGTTGAAAGACTGGAGGAAGG

TGAAGAAGCTGAAGCAGACGGGTGAGTCCTTTCTGCAGGACGATTCGTGCTCGGAGATTG

GACCCAACCTGCAGAAGTGCCGCGAGTGCCGCTCGATCCGCACCAAGAAGGGAGAAGAGC

CCACCCACTCCCCCGTATTCTGCCGCTTCTACTATTTTCGCCGCCTTTCATATAGTAAAA

ACGGGGTTATCCGGATCGACGGCTTCTCATCCCCTGACCAGCACGACGAGGAGGCCTTGA

GTCTCTGGGCTCCAGATGCTTACGAAGAGAATGATCTGGACCTGGAAACCTCCAAATACA

TTCTCAGTTGCATCGGGGATAAGTTTTGCCAGCTGGTCATGAGCGAGAAGACCGCCGCCA

CATGGATAAAGAAAGATGCCAAAATAGCCTGGAAACGGGCAGTGAGGGGGGTGCGTGAGA

GCTGCGACGCATGCGAGGCCACATTGTTCAACATTCACTGGGTGTGCCAGAAATGTGGAT

TTGTGGTGTGCTTGGATTGCTACAAGGCAAAGGAGAAGAAGAGCTCCAAAGACAAAGATC

TGTACACCTGGTTAAAGTGTGTCAAGGGACAGCCACATGATCACAAGCACTTGATGCCAA

CGCAGATTATTCCAGGAACAGTCTTGACGGACCTGGTAAATGCCATGCACATGCTCAGGG

AGAAGTTTGGCATCAAAGCGCACTGCGTCTGTGCCAACAAGCATAATATACTCAACAAGC

TCCCATCCACTAACGGCGTTTCTCAGGTCCTCCAGAACGTGCTGAATCACAGCAACAAAC

TTTCCCTGTGCAAGCCTGAGGCCGGCCAGCACAACTTGGGACAGAAGGTTGAGGCAAACG

GGGGCAGCAGTCCAGCCAGTGACACCAGCACTGATTGCAAGCTCCCCCCACCTGAATCCC

AGTCACCACTGCACTTTCTGGCCGACCTGGCTGAGCAAAAATCCCGGGAAGAGAAGAAAG

AGAATAAGGAGTCGGTGGTAGGGAAGGTGAAGGAAGAGAGCACAGATGCTCTGGAGACTT

TGCACTGTAAAACCTCGTCTCTGGTGGCCAACAGCACAGAGCAGGGCTCCACGCTTCGAG

ACCTGCTCACCACCACGGCCGGAAAACTGCGTTTGGGCTCCACCGACGCTGGCATCGCTT

TCGCACCGGTTTATTCCACCGCCTCACAGACGGGGAAAAGTGGTCGCTCTATGCCCAACA

TCCTGGATGACATCATTGCTTCCGTAGTGGAGAATAAGATACCAGCCGACCGCAGCACTA

AGCAGAGCCCAAAGGCTAAACCCCAGGACGAGGTCAAGGCTGAGAGAAGGAAACAAGCTG

AGGATGTTCCTGAGCAGCATACTGACATTCCCCACTGCTGGCTGTATGATCACAGACTGC

TCTGGCTTAAAGACCACCGCAAGAGCAGTAACTGGAAACTGTTTCGAGAGTGCTGGAAGC

AGGGACAGCCTGTGCTCGTCTCAGGGGTTCATAAGAAGCTCAACGCCAGCCTGTGGAAAG

CTGAAGCTTTCAGCCAGGAGTTCGCCGACCACCAGGGGGATCTGCTGAACTGCAAAGACG

GCGTGGTTTCCAACTCGGGCATCAAAGAATTCTGGGACGGCTTCGAGGACTTGACAAAAC

GGCTCAAATCCAG

>Unigene000115

GCCCTCTTGCTCAGCGCCAGATGAGGTGTGACGTCCTCGCCGTTTGCTGGCAGTCACGTG

GTCGGCGCTAGCAGGTTCTGGTAAGCGCGTGCGCGAGAATCCAGTCCAGCGCATCCTTAA

GCGGCGGACCGAGAGGATGTGGTGACGAATCCTACAGATTTAAATACACGAATCTATGAA

GAATTGATTCATTGTTTGTTTATTAAATTAAACATTAGAGAAACAAACGTGAACGTTTCG

AGCTGAATAATAAACGGGGCTGAGCTTTAACGAACAGATCATCACAGAGCAGCTTTACTG

AAACCTTCCACTGTTCTTTCTCTATGGGGAAGCTAGAAGATGGAGGACAAAAGCCCACGT

GTAGCGGACTACTTAGTGGTGGCGGGCCTCACTGAGTCTCCAGAGCCCTTTGAAGACGAC

ATAACAAGCAATGAAAACTGCCAGAGGAGCTCAGTCGAAGCTGGGGCTCCAATCACAGAT

GTGGCGGTGGTGTTTCGCTCTCAGGGGGAGGAGGTGCCGCAGGGCTACACCTGCATCGAG

TTCACCCCTTCAGGCCTTTCGGCGGAGCTGAACAGCGGCAGCATCATGGGTCCACAGATC

TTCCTGTGCTACAGGCGAGGACTAGACAAGCCGCCTCTCACTGACCTTGGCGTCCTCTAT

GAGTGGAGAGAGACTTTAAAGCCGGGATGTCACATCATCCAAACCACACCTTCCGGTCGC

CCTGCCAATATCAACAGCTCTTCCTCACAGAGAATCTACATCACATACCGCCGTGCACCC

GAGTTTCATTCTCATGCCATGTTGGCTGTCACAGACATTTCTGTTATCATTCCTGGCAAA

GGAGAAGCACCACCATATGCCTTTTGCAAAGTGGACAAGAACCTAAACAGCAGCATGTGG

GGTTCCTCTGTGTACCTTTGCTACAAAAAGTCTTTGGCCAAGACAAACACTCTTGCATTC

AAAGCAGGCTTGCTGTCCCGGTACCCAGAAGAGGACTATGAGTCGTTTCCTTTGCCTGAG

TCAGTTCCCCTCTTCTGTTTGCCAATGGGAGCATCTATAGAGTGCTGGCCCTCTCGAACA

AAATACTCCCTCCCAGTGTTTTCCACCTTTGTTCTTACTGGAGCTTCTGGAGAGAAGGTT

TATGGTGCAGCGATCCAGTTTTATGAGGCGTACCCAGAAGAGCGCCTGACTGAGAGACAG

CGCACACAGCTCGGCCTACAAGCCAGTGGCCTGAAGTCTAAGGAATGTATGACGGTCCAC

ACCAACAAGAGCATCTGCCTTCTTTCTCACTGGCCCTTTTTTGACGCTTTCCGCACTTAC

CTTACCTTCCTTTACCGCTACTCAATTTCTGGGCCACACACATTGCCCATTGAGAAGCAT

ATCTGTCATTTTATGCACAAAGTTCCTTTCCCATCTTCTCAAAGACCACGCATTCTAGTC

CAGTTATCTCCACATGATAGCCTCATGCTAAGCCAGCCTGTCTCCTCTCCTCTGCCTCTG

AGTGGTGGAAGTCTCTCCACACTGTTGCTAAACCTGGGTCCTAAAAATGCAGTGACTCTG

CTGGTTCTGGCTGTGACCGAGCACAAGATTCTTGTGCATTCACTGCGGCCGGCTGTGCTA

ACCAGCGTTACAGAGGCCCTGGTGTCAATGATCTTTCCCTTCCATTGGCCATGCCCATAC

ATTCCCCTGTGCCCGCTGGCATTGGCAGATGTGCTCAGTGCTCCATGCCCCTTTATTGTT

GGTGTGGATTCACGCTATTTCGACTTGTATGAGCCCCCAGCAGACATCAGCTGCATTGAC

CTCGACACCAACACCATCTCAAACAAAGAAGATCGCCGAGCCCTAACATGGAAAATCCTG

CCAAGAAAAGCCTGCAAACGTCTGATCAACACCCTCAGCAATCTCCATCATCAGCTGGTA

CAGGATTATTTGCTGAGTCATGAGGAAGGTCAGATCGATGTGTCCATGAGCGAACGGGAC

CCAGGAAGCGGTGAAAAAAGTCTCCAAACACTCGAGCTAGAGATCCAAGAAGCTTTTTTA

CGCTTTATGGCAGCCATCTTAAAGGGTTACCGCTCCTACTTGCTTCCCATTACTCAGGCG

CCCTCAGAGAAAGCGACTGACGCCAGCTCTCTCTTTGATCTACAAGGCTTCTTAAAGAGT

CGTGATCGCTCCCATCAGAAGTTTTACTCGCTCATGACCAAGACTCAGATGTTCATCCGC

TTCATCGAGGAGTGCTCTTTTGTCAGCGACAAGGATGCGAGCTTGGCCTTCTTTGATGAC

TGTGTAGACAAGGTTGACAGCGAGAGGATAGAGGACACCAGGCTTATTGAACTGGACAAA

TCACATCGCAGTGAGCACACAGTTTTTATCACACCTCCAGAGCTTCCATTCGTTCCTGAA

GGAGAGGAGCCCTCCATACTTTATAGCTACAGTGGTTTTCCTGTGCTGAATGCTGAACTC

TGTGAACCCCAAGAGGGCCCCGGCATCCCTATAGCAACCACAAACTCCAGACACACCAGC

CCTGGAAGCCCAGCTGCCATTTTCCGCCGCTCCAAACAGGAGATTAAATCAGCTCAGCGA

ATGGCCAAGACATACTCATCCATACCTCACATGTGGTCTAAGTGTTTGCTAAGGCACTGC

CATGGCCTATGGTTTATCTGCCTGCCAGCCTATGTAGCAGCATGCCAGTCTAAGGTGCGG

GCACTCCGAGCTGCCTATGATGTTCTGAGAATGATGCAAGACAAAAAGCTGCAACCTCCT

GATGAGGTATGTTACCGGATTCTGATGCAGCTCTGCGGGCAGTATGGGCAGCCCGTCCTG

GCTGTGCGGGTGCTGTTTGAAATGAAGAAAGCTGGAGTTCAGCCTAATGCTATAACGTAT

GGATATTATAATAGAGCGGTGTTGGAGAGCACATGGCCTTCGTCCACCAGAGGGGGCCAT

TTCCTGTGGGGTAAACTGCGCAACGTGGTCCGAGGTGTGGTACAGTTCAAACAAGTGTGG

AGGAGACAGGCTAGTCATGCCAAAGAGCCCCAGCTCTCAGATGCCAGTGATCTGGACAGC

GTGAGCCACGGCAGTCTGGACAGCACTAATGACTCAGCAGAGCGAGCGTCTATCGATACA

GACTTCACCAAAATGGACTCCAGCGATGATGGATCCAGCACAGGTGGACAATCAGATCAA

GGCTATGACTCATTGTCTAAAGAAGAGGTGCAGGTTAGCTCACCTCACATGAAGGAGAAG

AAGGAGAGAGACAGCCTTCCACTGGCCAAAACAGGAGCGTCTATAAAAAATGATTCATCT

GCTGCACTGAAACCATTAAATCCCTCAAATGTAAGAGACAATGCAGCTGTACCCATACGA

CCCAACTCATTGGACATCTTTGGTAAAAATACTGTGAGACCCAAAACATTAGAGCTGCTG

CTCATCTCTGCAAGCTGTAAACAGAAGCAAAGGTATTCAGATAAGGCGATGGTGGAGGAG

GAAACAGGCACCACAAACCCACTCAAACCTGCTGCAGAGCGGAGCACAAGTTGCACAGTT

GCCATGGAGACTAGTACTAGCGATGCAAGTTGTAGTGTGTCAAGGAGTGTGAGCTTCAGT

GGCACTCTAGGCCGGTTGCCACAACGGACAGGGATTGAGTCTGGCTTTGACCCCTTGTCA

CTTTTGGCAGCTGAGAGCAAGGTGCCAGATGAGCAAGGAGAAACAGACGATGCTCCTAGC

ACACGCCGGCATCTGGCTGAGGAAATCCAGCTTCATCTGGAGCACCTGAGCAGTCCAGTG

AGTCAGCGTTTATTTAGCACAGATATTCGCAGCCTGCAGAGTCCTTCCCCAAGCAGCTCA

CCTTGCCAGACTGCTATACCAGGATCTCCAAGCACCCAGTTGCAGCCCCAACCCCGGAGC

AGACTTTTCTCCTCACCATCCTTGCCACTTGGATGCCCACGTAAGAGCAAGGAAGCCCGT

CCCACCTCTTTTGTTTCACCCTCCTCTCCCACCCCTTCTGCCTCATCCTTTTCCATGGAA

TCCCTACTTACACCAACATTAGATGTTTTCAGGAGCAGCTTTATGTCTGCAGGCAAGGGC

GTGGCTGAAAAAGCTAGCCGTCTCTACTCAAGGCTTTCATCACAGACTTCTATTGCACAG

GACCTGAACTCTGATCGCGTCAGCGTGTCTTCATTAGGCTCAGTTGAGCCTGACTGCTCC

TCAGTGTTTGAGGGTGATCTTTGTCTTGATTCAGAAGGTCTCTCCTCACCTCAGAGAGAC

GCCTCTGTCAGCGTCACTCCCTTCAAAAGGAGTCCAAACAGGAATAGCCGCTGCTTAGAG

AGTCCTTCAGCACCTCCCAGGCTTTTCCGCCAGGCTTCTCTCCTTGGTTCGTCACTGTCT

GTTGTGAAAGCCCCACAAACCCCAGACGTGTGTCCCGATATAAGCCTCACACCAGGCCAG

CAGAATTATGACATAGAGGTTCGCATGTCTAGTTGCTCACGCTGTAACACCTGTGAGTGC

CTGGCGTATGATGAGGAGATCATGGCTGGCTGGACAGCTGATGACTCTAATTTAAATAGC

ACTTGTCCTTTCTGTGGCTCAGCCTTTTTGCCCCTGCTTAATGTAAACATCCAAGACCTG

AACAACCAGGACAGAATCCCTTTTCAAGACCTGCCCAGTTCCCAGGCAGAAACAAATGAT

GCCTCAGAGAATGAGACAAAAACATGCATTGAAACTACAGATAACGGCTCCAACAGTTCC

AGCCATAGCGCAGAGCCAATCACCGTACCCTACTTGAGTCCTTTGGTGCTATGGAAAGAG

CTGGAGAGCCTCTTAGAGAATGAAGGCGAGCAGATTATCTCTTCACCAGTGATTGTGGAC

CATCACCCTATTGTCTTCTGGAATCTGGTCTGGTTTTTCCGTAGGCTGGAGCTGCCGAGC

AGCCTGCCTGCTCTCATCCTTAGCTCCAAGCACTGCAGCCAGAACATGCAGCCTTTCCAG

TGCAGTGCATCAGAGGACAGTAAGAATGTGTTAGTCACGATTTTGTGGGACAACCCCAAG

CTGCACCAGGACCCAATCCCACCCTGCTACATTCTGTGGAATGCATACTGTTCAAACACT

AATGTTGGTGTGTCCTCATTGTTGGAGGATAAACAGCCTGTAAGCCTGGAGCTTCTGCAG

AGTGTTGTCAGGAGCATTCAGAAAAATGATGTCTACCAGCCCATGAGCCAAACACTTCAG

CTGCTTGGTACCAGGCTAGGATTTATTAGGCAACGGAGCCTGTATAGAGATATCTTATTC

CTGACATTAGTGGCATTGGGCAGGAACAGTATTAATATTGATGCTTTTGACCGAGAGTAC

AAGTTGGCTTATGATCGACTGACCCCTGGCCAGGTCAAACTTACTCACAACTGTGATCGT

CCACCTGGTGCTGGAGCCATGGAATGTAGAAGAACTTTTGGGCTGCCGAGTCTGTAATTT

CTTTCGTTTTATTCCTCTCCGACATCTCCTTTCCCTTCCTTCATCAAAGGATGTGAAGTC

TGAGCTAGCCAAAGAAGAAGGATTCACTACTACCAAAGCTACCTCTGGCTCGAAACACGG

CCATGTAGACACTTACAGGCATTTCCCAGATCGTTGCTTAAACCAACACGTGCGTGACCC

ATTCTTATTAACTACATGCTACCTTTGAACACTATGTCATATATTGTCCATGTTTCTGTT

TACCTCATGTCATGTCCTTCATTTTAACCTACTACACATTTCACTCTGAAGTCCCAGGAC

ACATTTACACATTTAGTCTCACTCAGGTAAGATTTACTGCCGAGGCCATCGTAAAGTGAG

AGAAGTATCATCTAGTGCTCAGGTCACAGCATCACTCATCACCATTGAATCATATAGACA

GGATTGTAGAGTGGTTTTGGGACCAGTAGCACTATGTATTGTATTGTTTAGAAAAGCACA

ACGAATAAACCCTAACGAGACTAAAGTTTTATCACATTCCTCTCTCAGTATTCTAAAGGC

AGTTGTATATTACCATAAAAAGTCACCTTTCTGTATATAGCCTATTATTCTATTATTTAG

AATATTTGTATTATAAAGGTATATTTTAAAATTTGTATTTGTTATAAATGAGTTGTCTTC

ATATGTAAAGACAGTACCAAGCAGTGTATTGGTTACAATTTCCAAGTGTGTCGTGTAAGA

CTATGGTCCTGGAGACCGTTATTGTGATAACTGTGAAGCGTTTTGTCTTTTGATTAGCTG

AGGTACAGAACTGGACCAAATCAGATGTGAAATCATGAATTATATGACTGTTTGTTAGAG

GTGATTTAAAAGAAGTGTAATAAAATATGAATATTGTAATTCCACTTATCAACTGAAAAT

GCCTTATTAAAGGTCAATCCAGCCACAGAAATGAATGGAGACTAAAACATGCATAGAATA

CAATAAAACTCTATTGGAAATACAGCAGAGACCTAAAACAAATCATTTCCTTGTCTACAC

AAACTGATGCAGATTAGTATTAGTTTTTATTAAATATTACATACGGACTCTCATTTTGTT

ATAAACCCACCA

>Unigene000116

AGGACGCGCCACATGACAGGGGGCAGTGGAGATGGGGGACAGAAGCTGTTGCAACAGGCT

CAGACTACAGAGAAGGAGTTTGGAGAAGTAACTGAAAAGATCCAACAATGCTGCTCGTCT

CTCGAGTCACGGCTGCAGGGTGTAGGAGAAGTCCAATCACACGTCCGTGATATTTTCTCT

CGGCTGGCGGACCTGGACGACGAGCTGGACAGCCTGAGCCCTGTAGGACGCGATGTGGAC

TCCCTTGCCTCACAGGCAGATGCAATAAGGGGATTCCTGGGGCGTCTGGCCTCTCTGCGG

GCTGAACTTGAAGGCCATGGCAGTGCCTGCACCACCATGCTGAAGCGAGAAGGTAGCTCC

CCTGATCTCCTGGCTATTAGAAGGGAGACGGAAGCTCTAAATCGGCAGGCAGCCAAGCTG

GCCGAGCGGGGTCAGGGTCGTCTAGCACTCATAGAAGAAGCCGAGGGGCGAGTAAAGGAG

TTCTATGCCCGCCTCATGGAGCTACAGAGGCTACTTGACCAGGCAGAGGAAGGGCTGAAC

GTACAAAGTGTTGTGGGAACCGAGGTGGATGTCATCAAACGACAGCTCCAGGAATTTAAG

GCTGTGGAGCGAGAGCAGGTAGACAGCATCCAGCCTAAACTGCATCACGTGAACGCTGTT

GGCCAGGGGCTAATCCAAAGTGCAGCTAAACATACCGACACGCAGGCCCTGGAGCATGAC

CTGGAAACCACCAACCTGCAGTGGAACTCCCTCAACAAAAGGGTGGCCGAGAGAATTGCT

CAGCTCCAGGAAGCCCTTTTGCACTGCGGGAAGTTTCAAGACGCTCTGGAACCTCTTTTA

AGCTGGTTGAGTGACACAGAGGAGCTGATCGCTAATCAGAAGCCTCCATCAGCTGAATAC

CGTGTGGTTAAAGCACAAATCCAGGAACAGAAGCTGCTGCAGCGGTTGTTAGATGACCGG

CTTGGGACTGTAGAGATGATCCGGGCTGAAGGTGAGCGCATCGCAGCCACAGCAGAGACG

CAAGACCGAGAAAAGATCCAGAGACAGCTGCAATGTTTGGGAGAACGATGGACAGATCTG

CTTGAAAAGGCCAATGCCAGGCAACGTCAGCTGGAGGAGCTGCAGGTACTTGCGCTGCAG

TTCCATGAGTCAGTGGATCCTTTGGGAGAGTGGCTGAGTGCTACAGAGAGGCGACTAAGC

TCTGCTGAACCCATGGGAACTCAGGCTTCCAAAATTAGCCAGCAAATCAATCGGCACAAG

GCCCTCCAAGATGAGGTGTTGGCTCGGGAAAAAGAGGTGGACCATCTAGAGGCCCTTGGC

CAATCTCTCTCCCCTCTCAGCTGTGCGGCAGACCGTGATTGGCTTAGTGAGCGAGTGGGA

GCAGTGCGCAGCGGGCACACAGAGCTCCGGAATTGGTGCTTTCGGCGAGCAGCTATGTTG

GAGCAGGCTCTGGCCAATGCACAGCTGTTTGGGGAGGAGGAGGTGGAGGTGCTGAACTGG

CTGGCTGAGGTGGCCCAGCGCCTGTCTGACGTCTCAGTGCAGAGCTACCAGCCCGAGCTT

TTGGCTGAGCAGCACAAGTACACTCTGTCTCTGAACGAGGAGATTGTGAGCCGGAAGAAG

ACAGTAGACCAGGCCATCAAGAATGGACAAGCATTGCTGAAACAGACCACTGGTGAAGAA

GTGCTGCTTATCCAGGAGAAGCTGGATGGAATAAAGAGCCGATATTCCGAGATGACAGCA

GGTAGCAGCAAGGCCCTTCGTAACCTGGAGCAGGCACTGCAGCTCGCTACCCGATTTGCC

AGCGCCCACGAGGACCTCAGCCAGTGGCTTGACTCTATGGAGGCGGAGCTTAATAACATG

GAACCAGACACAACTCCAGCCTATCAGGAGAGACAAAAGGACCTGAAGTGTGTGTCAGCT

GAGAAGCGTCTCATTCTGGATACAGTTAATGAGGTGGGCAGTGCACTGCTGGACCTGGTG

CCCTGGCGAGCACGTGAAGGTCTGGATCGTCTAGTGGCCGATGCTAACCAACGCTACAGA

CAAGCCGATGAGACCATTACGCAAAGAGTGCAGCTAGTGCAGGCAGCCATCCAGAGGTCA

CAGCAGTATGAAGAGGCCGTGGATGCGGAACTGGCCTGGGTGGGGGAGACAGAGAGGAAG

TTGACCTCCCTGGGGCCTCTAAGTCTGGAACCTGACGTGACAGTGGCACAGCTCCAAGTC

CAGAGAGCCTTCAACATTGACATTATCCGCCACAAGGACACGGTGGACCAGCTCCTCCAC

ACCCGAGAGGATATCCTGGAGAGCTGCAGTGACCAGCAAAGAGAAGCACTGAAGGTAAAG

ACAGATTCATTAAGTATGCGGTATGAGGCAGTGAGTCAGAACCATGCTGAAAGATTTTCA

GCACTGGAACAAGCACAGGTGTTGGTAGCCCGCTTCTGGGAGACTTACGAGGAGCTTGAC

CCATGGCTGGGTGAGACAGAGACCCTGATATCCCAGCTGCCTCCTCCTGCCATTGACACT

GAGGCTCTACGACAACAGCAGGACCAGATGAGGATGCTGAGAGAGTCTATAGCTGAGCAC

AAACCCCACATTGACAAGCTGCTGAAGATTGGCCCACAGTTGGCTGAGTTAAGTTCCCAG

GAGGGGGCGACACTGAGACAACGCTACAGTGAGGCTGAAAGGCGTTACCTCGCCATCAAA

GAGGACGTTAAAGGTCGTGCTGCAGTACTGGATGAGGCCTTCTCTCAGTCTGCACAGTTC

CATGATAAGATGGACCCCCTGTTAGAAACTCTGGAGGGAGCAGTGCAGCGTTTGCGGCAG

CCTCCTCCCGTGGCTGCTGAGGTAGAGAAAATCCGTGAACAGCTGGCGGAGCACCGAGCA

GCCGGGCTGGAACTGGACAAATTGTTGCCGTCCTTCAGCACTCTTTGTGCCCGTGGGGAG

GAGCTCATCACCCGTGCTGCACATGATGACCCTGCGGCTCAGGCAGTGCGTTCTCGGCTT

CTAAGACTCCGTTCCCTGTGGGATGAGATCAGACAGAGAGCAGAAGAAAGAGAGGGAAAA

CTGCAGGATGTACTGGACTTGGCTGGAAAGTTCTGGGCTGACATGGCAGCTTTGCTTAGC

ACCCTGCGAGACTCTCAGGAAATTGTAAAAGAACTGGAAGACCCTGGATTGGATCCATCA

CTCATCAAACAGCAAATAGAGGCAGCAGAAGCTATCAAAGCAGAGACAGACGGTCTGCGT

GAAGAACTGGAGATCGTCCGCACACTGGGAGCTGATCTAATTTTTGCTTGTGGTGAAACC

GAGAAACCAGAGGTCAAGAAAACCATCGATGAGATGAATGCTGCGTGGGAAGGCTTGAAC

CGCACATGGAGAGAGAGAATGGAGAAGCTGGAGGAGGCTATGACAGCCTCTGTGCAGTAT

CAGGATGCACTGCAGGGTATGTTTGACTACCTAGACAACGCTGTCATTAAACTGTGTGAC

ATGCAAGCCGTAGGAACTGACCTCAGCACCGTCAAACAACAGATAGAAGAGCTCAAGCAA

TTTAAGGTGGAAGTGTATCAGCAACAGATAGACATGGAGAAGCTCTGCCACCAGGGGGAG

CTACTGCTCAAGAAGGTTTCTGACCAAGCGGACAGAGACATGATCCAGGAACCTCTCACT

GAGCTCAGGCACCTGTGGGACAATCTGGGAGACAAGATCACTGTCAGACAGCACAAGCTG

GAAGGAGCTCTTCTAGCCCTGGGTCAGTTTCAGCATGCCCTCTCTGAACTGCAGTCCTGG

CTAAGCCACACTCATGCTACCCTGGACACCCAGAGACCTGTCAACTCTGACCCCAAAGCC

ATAGAGATCGAGCTGGCCAAACACCATGTATTGCGCAACGACGTCCTGTCCCATCGAGCC

ACAGTAGAGACTGTGAACAAGGCAGGCTCAGAGCTGCTGGAGTCCAGTCCCGGAGACGAG

GCCAGCCACCTGAGAGACCAGCTGGATGAGCTGAACCGCAGCTGGGACAGCTTACTTCTG

AAAACAGACGAAAGACAGAAGCTACTAGAGACGGCACTGCAGCAGGCGGAGGGTTTCCAT

GGTGAGCTGGAGGAGTTCCTGCAGTGGCTGAGACGCACAGAGAGTCAGCTATCAGCAGCC

AAACCCACAGGTGGCCTTCCAGAGACCGCCAGGGAGCAGTTGCAGCAGCACATGGAGCTG

CAGGCTCAGTTGACCCAGCGCGGTGAACAGTACCACCGACTGCTAGATCAGGGAGAGTCC

ATGCTGTTGGCTAGAGGGGCAGAGGAAAACAGCCCTGGAACCACTCAAACTCAGCAGAAC

CTGGCCCTGCTCCAAAACAAGTGGGCCAGTTTGAATGCCAAGATGGATGACAGGAGGGCT

AAGCTGGAGGAAGCAGTATCACTGGCAACAGGATTCCAGTCGTCACTGCAGGACACCATC

AACTGGCTGACGCAGGCAGAGCAGACACTAAACATGGCTCAGTCACCGAGCCTCATCCTC

GACACTGTCCTCTTCCAGATAGATGAGCACAAGGTGTTTGTAAATGAGGTGAACACACAC

AGGGAGCAGGTGCTGGCTCTAGAGAAGGCCGGGTCTCAGCTGCGCTTTGCCAGCCTTAAA

CAGGACGTGGTGCTCATCAAGAACCTTCTGTTGAGCGTGCAAGCTCGCTGGGACAAGCTG

GTGCAGAGGTCTCTGGACAGGGGACGGCACCTGGACGAGGCCCGCAAAAGAGCCAAACAG

TTCCATGAGGCCTGGAGGAAGCTGACAGACTGGCTGGAGGAGGCTGAGAAACGGCTGGAT

GCTGAGCTGGAAATCTCCAATGAGCCAGATAAAATTAAAGTGCAGCTTACTAAGCATAAG

GAGTTTCAGAAAACTCTTGGTTCTAAGCAGCCAGTATATGACACCACAGTGCGCTCTGGG

AAGGCCATGAGAGACAAAGCTACACTGCCAGCCGACACACAGAAACTGGACAACCTCCTC

GGGGAGGTCAGAGACAAATGGGACACTGTGTGTGGAAAGAGTGTTGAAAGGCAGCACAAG

TTGGAGGAGGCCCTGCTGTTCTCGGGTCAGTTTGCTGAGGCACTGCAGGCTCTTGTGGAC

TGGTTGTACCGGGTAGAGCCACAGTTGGCTGAAGACCAGCCTGTCCATGGTGACCTGGAC

CTTGTGTCTAATCTCATGGATTCTCATAAGGCCTTTCAAAAAGAGCTGGGGAAAAGGACC

AGTAACATACAGGCACTGAAACGTTCTGCTCGGGAGCTAATGGAGACAGGCAGGGATGAC

ACCGCCTGGGTCAAAGTTCAGCTGCAAGAGCTCAGCAACCGCTGGGAGACGATCTGCGCA

CTGTCTGTCTCCAAACAGACCCGTCTGCAGCAGGCTCTCAAACAGGCTGAGGAGTTCCGC

ACAGCAGTGCAGATGTTATTGGAGTGGCTCTCAGAAGCAGAGCAAACTCTACGTTTTCGA

GGCATCCTGCCAGAGGAGGTGGAGACCTTGCAGGTTTTATTACACACCCATCGAAATTTT

ATGCAGACCGTAGAAGAGAAGCGAGTGGATGTGAACAAGGCAGCTGGCATGGGGGAGGCC

ATACTGGCAGTGTGCCATCCTGACTGTATCACCACCATCAAACACTGGATCACCATCATC

AGAGCCCGCTTTGAGGAGGTTTTAACCTGGGCGAAGCAGCATGAGCAGCGTCTGGAGGCA

GCACTTGCCGAACTGCTCAACAATGCAACACTGCTGGAAGACCTGTTATCATGGCTACAG

TGGGCGGAGACCACATTAGTGCAACGAGACACTGAGCCCCTCCCCCAGGATATCACTCAG

CTTAAAACACTGATCACAGAGCATCAGGTTTTCATGGAGGAAATGACACGTAAACAACCT

GACGTCGATAAAGTGACAAAAACCTACAAGAGAAAACCATCAGAAACGTCTAGCAGTCTT

GCTGAGAGACGGGGTGTCCGTATGTTCGCTTTCTCTTTTACAGGTAAGCAGCAGCAACAG

CAGCAGCAGCCTGCTATGCAGGTAAGTGGAGGAAACCCTCGTCTTAACCAGCTCTGCTCA

CGATGGCAACAAGTTTGGCTACTAGCACTGGACCGCCAACGCAAGCTCCATGATGGCCTT

GACAGGCTTGAAGAGCTGAAAGAATTTGCCAACTTTGACTTTGATGTGTGGAGGAAGAAG

TACATGCGCTGGATGAATCATAAGAAGTCCCGCGTCATGGACTTTTTCAGACGCATTGAC

AAGGACCAGGATGGCAAAATCACCCGGCAGGAATTTATCGACGGCATTCTGGCTTCAAAG

TTCCCCACCAGCAGGCTGGAGATGACTGCAGTGGCTGATATCTTTGACCGAGATGGTGAC

GGTTATATTGATTACTATGAGTTTGTGGCAGCTCTGCATCCTAATAAAGATGCCTACAAA

CCCACAACAGATGCAGACAAGATTGAAGACGAGGTCACCAGACAAGTCGCCCAGTGCAAG

TGTGCTAAGAGATTCCAGGTTGAGCAGATTGGCGAGAACAAGTACAGGGTGAGTTCTGAA

TTAGTACAAC

>Unigene000117

CACACACACACACACACACTGTCTCTCTCCCAGACAACAGCACCGCTTTACTCTCTGGGT

CTGACATCTGCTTACTGACGCGTCGCTTTTTTATAATTCTGGAAGAAAGACAGATATAAA

GACTGCTTGGAGCTGCAGTTTAACAGTTTAAAGACAAAAGCCGGGACATAAAGTTATGGA

GTCTGAAGAGAGACTGTCGTCTTCCAGCCACTCGCAGGCTTCAATCAGCTCTTCTGCCAC

ACCTCCAGCTTCATCTGCAAACATAACGAGTGAAAGTGGACATCTGTTTCAGCTGGTTAG

TGATCCAAGTTTTAGAGCCTCTTTTCCCCTGTTGAGCCATCCAGCATTTAGGTTGTACGC

TCCTGTATCTGGATGCTCAGATTTCAGAGGCCTGGGGATTTTGGGATTGCAGGGAGGCTT

AACAGCCCACCCACATCTTGGAGCATTTCCAGGGTTGAATGGTGCAGTCAAAGCAAAATC

GGCTTCCTCTGGAGCAAGCTCTACCTCCTCCTCACCCTCACCAGCCTATACAACCTCTGA

ACAGACCAGCACACTTAAATCAGGTCTAAACCCTCAAAACAAAACCACCAAATCTAACCT

CAACTACTGTAAAGAGAAACAATCAGCCACCAATCCCTCAAACTGGAACAGGGTGCAAAA

GAGCAAGATGGGTCAAGTCCAGGAGAGGACAGGCAACAAAGCAAAAGAGAATCCCTGTAT

GACAGTGGCAGAGATTTCCAGCACCAGTGATAGCCAATCAAGATCCAGTTCTGACAGCTC

CAGTGACACACTCAGCAGTTTGGGCTCGGTCGACCTGGAGAATGAAGATGATGATGAGGA

TCTGAGTGTATGCAGCATGGACTCGGACACACAGAGTGTAAGCAGGAAGGTTAAGGACTC

CTCACACATAGACCAAGCTGCAGGCTGCTTTTCCAGTGGACCAAATATGTGCTCTATGGA

CTCTTTAAAACATTCCCAAGCAGCTCCGTTACTTTTCGGCAGTCCATCAATCAGAGAAGA

ACACCGCAAACACACAAGTGTCATCCAGACTACAGGAGCAGCAGGCATTGCCAAGACTTT

CTCCCTCCCTCTACAACCCAATCAAGATGCCTTGTCGAAACCTTTGTGTCTCACCATGTC

TCCCAAACCTTCGTCAGTATCATCGTCTCCCAAGCCTCTTTCTGTGTCCTCATCTCCAAA

GCAACACTCCGTCTCTGCCTCTCCAAAACCTCTTTTGTATACCCGCTCTCCAAATCCAGC

ATCACTTTCCTTCTCTCCTAACATCACCTCCTCTCCCAAACCCACCTCCATAACAAATGC

CAGGAATTCGCCTGACGGGTCCCGTCTACACGGCAATGAAAGTAATCAGGACAAGCAGGC

AAAGGAACACCAAGAATCAACTAAATCCCTGAAGAAAAATGTGTCATCTTCCTCACCATC

GCCTCAGATTAGCACTAGCCTCAATCCCCAGCACTGCAACCTGTTTCTCACCTCTAACAT

CACGAAGAACTCTTCCCATCTTAATGGAGTTGTTCAGGGTGCTGTGCAGGATGCTCCCTT

GGCACTTATTACCAAACCTCGTCTAGACAGCTCTAATACCCCAGATAAGACTTTACTTGC

AACCACCAGTCCCTGCTTTAACACCCCTATCAACTTGAGCACTGGAACCCGACATTCTTA

CCCCGGACCTCCCACATCAGGCAAAGCCTTCACACACCAAGAGTCCCACAGGGCAAGAGA

AGGATCACAGCCCCAAAGAAAAACATTTAGGGAAATAGAGTCTGACATTCCCAGCAGCAG

AGACTCTGATGATTCTGGGGAATATGAGGATGAGGAGGATTTAAGTGACAGTCTGTCAGA

CTCCGGTAGTAACCTTGATACCGACTCTGATAACGATGAAGAGGACAATAAAGATGAGGA

AATGAATACTGATGCAGAGAGCCACACAGACAGCATGCCACTTAAACTTACTAAAAGCTC

AACCTCTTTGGCAGACCCCTCTACTGGCAGGAGCAACAGCTCTAGTTCACTTAACCTGCA

AATTTATGAGTCACCTAACATTTTACAGACTCTGAATGGATCCTGGGCTCTGACTTATCA

AAGCCCCCCAAGCTCATCATGTGTGGTTCCGCCCCCAGCAAAGAGACGAAGAGTGACTGA

TGAGCAGGCACTGCGCCGGCCTCTTGAACATGGGTGGAAAAGAGAGACCCGCATCAGCAA

TATCGGTGGCCGTGTGCAGGGTGAGGTGGCCTACTATGCTCCCTGCGGAAAAAAACTCAA

ACAGTACCCAGACATTATGAAATACCTAGCAAGAAATGGAATAAATGAGATTACACGTGA

CCATTTTAGCTTCAGTGCTAAAATAAGAGTTGGCAACTTCTATGAAGCTCAAGAAGGACC

TGAGGGATTGCAGTGGTGCTTGTTGAAAGAGGATGAGGTTGTCTCCTGCATCTCAGCAAT

GGATTCACATAGAGGTCGTCCTAAAAGTCTGGAACTTCAGCCTACTGATGATGCCTTCCA

TTCTCGCAAAAGGAAAAGACCACCTAGTGTGAGCGAAAGTGAGATGGCTAATGCTACTCT

GATTAAACTACAACAGAAACTGGTGGCGCAGGAAATTGCACGGCAGGCTGCTCAGATAAA

GATGATGCGAAAGCTGGAAAAAAGAGCTCTTGCACAAGCTGCTAAAGATGCCAAACGACA

AAAAGCACTGCTGGAAGCAGAGGAGAAACGCAAGCAGAAAGAGCAGCTGAAGATTCTGAA

GCAGCAGGAGAAGGTCAGAAGACTCGAACAAATTCGTGCCGAGAAGGAGCTTCGAGCTCA

GCAGATACTTGAGGCAAAAAGGAAAAGAAAAGAAGAGTCCATTAATGCAAAAACTCTAGA

AGCTGAAAAACGATTGAAGGAGAAGGAACTACGCAGGCAACAGGCATTAATTCTGAAGCA

GCAGGAGCGGGAAAGGCGAAAGCAGCACATGATTCTGGTCAAAGTCACGGAGGCTCGGAA

GAAAGCTGAGGAGCGAGAACGTCTGAAGCAGGAGAAGAGAGATGAGAAACGGCTAAACAA

AGAGCGCAAACTGGAGCTCAGACGGTTGGAGCTAGCGATGGTTAAAGAGCTGAATAAACC

CAATGAAGACCTGTGTCTGCCAGATCAAAAAGCTCTGCCTGATCTGTCACGGATACCAGG

TCTTCTATTACCAGACAGCTGTTTTGGGGACTGCCTGATGGTGATGCAGTTCCTGCGCTG

CTTTGGAAAGGTGCTAGGTCTGGACCGCAATATCAAGCTGCCCACCCTTCACATGCTGCA

GGCAGGACTCCTCAACCTAAGCCCAAGTGCAGCTCAGCTGCAGAATCTGGTCATTGGACT

GCTCTCTGCAGCAGTGCGTGATCCAGGCTTTCCACCAGGATCCATGGCTCAAACAACTCT

CGGTCAGCATGTTTCCAGTGTGGAGATAAACCAGGAGAACATGTCCGAGATTTTGCAGAT

CTACATGGCCGCTCATGGCAGTCAGACTGATCTCTCGCCACTGGTAGAAAGTTTGAAGAC

CAAAGGCTTTATGGCCCACACCCCCACACAGAAAGCCTCAATCATGGGTTTTTTGGTCAA

TGAGCTGGCCAGCAGCAGAAGTGTGATTATGGAAATTGATAAAAGTATAGAACATATGAC

AAATCTACGAAGAGAAAAATGTGTCATTGAAAGTAGCCTTCGCAAATTGAGAAGCAGCTA

CGTTAAGCGGACTGGGAAGCGTGATTTTAGTTTAGGAGGTGAAGAGAGCCAGGCTTTGGA

AACACCTACCACAGGACACAAACGTAGAAGAAAAGTCGGAGGCATTGAGGAGGAAGATGA

TGAGGATGATGACAGTAATGATCATGGAGAGGAGGATGAGGATGAAGAGGAAGACGGGGG

AAAGAAACAACGGAAAACTGAAACATGTGAGGAAGAGGATGATGAGGATCAATCAGCCAG

TGTGGAAGAGCTTGTGAAAAATATAGAGAAATTGACCAAACACCAGCTTCTAATCAGGCA

TAAACTATTCGAGTCCTCATACTCCCTGCAATCGATGATGCTCGGGCAGGATCGCTATAA

GAGGCGCTACTGGGCTCTTCCTCAGTGTGGGGGTGTGTTTGTTGAGAGTACAGTGAGTAA

AGAAGGACCACAAGGGGTTAATCAAGAGTCTGGGAGGATGCACAGTGCTCAGTTGACCTC

GGTTAAAAAGGAACCTGCAGAGGTACCAGATGGGGAACCGTATGACGGCCCGCAAACAAA

GCTTGAAAATTCCTCTCTGGATCTACAGCAGGAGAACGGTTCATTCAATTTGTTTTTGCA

GAAACCGAGTTCTTTTTCCAAACTTAGTAACCTCTTAGAGGTGCCAAAGACGTCCAGCAG

TGATTCTCAGAGCAAAAATTCCACTGCTGCCCCCTTTTCTACAAATGCTTTAACAGTGTT

ACCCAACAGCCTTTCTACTAACCTAAACCAGAACATAAAATATGAGGTCAGCTCTCAATT

ACTAAACCCCACTCACACAGGAAACCAGCAACATCCTCTACATAATGACCAACTCTATAC

GACTCAGGCAGAGAAACATTCTGCCTGGTTCAGCCTGCTGCCACGATCGCCATGTGACGA

GTTTTCTTTGACAACCAGCTGTACAAATCCATCATCTTGCCGTGCCAAAGCATCATCACC

TGCACCTGCCACCAGCAACAGCATGACGCAATCTACATCCACCTTCTCCAGCAATAGTCA

GAACATCACCTCTCCTGTCAGTCCCTCTGCCACTGCACCTGTCAGCAACTTTTTGGGAAA

GGAACTTCAGATGAAACACACGATGAATCCGAACATGTCCTTCAAGGACATGTTTACCAA

AGCAGCTGACCCGCTCCCATCCACCAGTGTGTCCACCAGTAAAATCGGGTCCCCAGTCTT

GTTGAGTGACAAATCACCTGTTCTTGAAATGGCCAAAAGAAAGGACCTGCCCTTCCCACA

GCCTGTTCCACAAGAGATGCTTACTGGATGGTGGAAGGTGACCAGTTCTGAAGAGTTAAG

CCGTATAGTGAGTGCTTGTCACCCTCGAGGGATCAGAGAGAGAGTCTTACAGAAACAAAT

CCAGAAACACATGGACTACCTCACTCAAGTCTGTGCCAAAAACAAAAATGCTGCTGTGAT

CGATGTATGTGAGCTGAAGCAGAGTCAGGTGTGTGAGGAGACAGTGCAGAGCTGGTGTGT

TGAGGAGCATGCCATGGATAGGGACATTGCTGTGCTGCAACAGGTGGAAGAGCTGGAGCG

TAAAGTTATCTCTGCCAGCCTTCATGTCAAAAGTTGGATGCCCACAAAGCCACAGTCGGA

GCGTGAAGACTTGCTATATTATGAGCACAAGGTTCTAAGCCATAGTGAAAGCACAGACAT

TGTACGACAGGCTGATAATCCACTGGACATTGCAGTGGCACGTCTGTACGAGCTGGAGAG

GAACATAGAGAGAAGATACCTAAAGAGTCCATTAAGCACTACTATCCAGGTGCCTCTGGA

TAACCGGGGTACGGTTAGCATACTGGCTCCAGCAACACCAGCTAATACTAAGTGTGATAG

TGATGTGGAAGACCTGGTCCCGGGGTTAAAGCTGTGGAGAAAAGCCCTTAGCAAAGTGCG

TAGTGGTGCCCAGCTTTCAGTCTGCCTGCAGCATCTACACAATTCTATCGCATGGGAGAA

ATCCATCCTGAAAGTGACAAGCGAACCGCCTCAGAGGAGTAGGAAACAGGTCAGTCGGAA

CAGTGGAGGGGGTGAAAAATTGTCCGAAACCAAACGCAGCAGGAAAGCCTGTGTGTCAGG

AGAGGGTTCGGAAAACAGCACAGTCAGCACCAGCGTCAGCAGCACAGCCAAAAAAGGAGG

AAAGGAAAGCAGAAAGAGAAAACCGGAGGACAACCTTTACAACACCACCACCACTAAGCA

GGAAAGAGACAGATCCAAAACCACTACAGACAACAGTAAAGACCTGGAGCTCTGCAGATT

GCTTTTGGCAGAGCTGCAAACGCATCAGGACGCCTGGCCGTTTATGACTCCTGTCAATCC

CAAGTCAGTCCCTGGATACCGCAAAGTCATTAAGAAACCCATGGACTTTTCCACCATACA

GGACAAACTTTCCAACAGCCAATACCTGAATCTGGAGACATTTATCATTGATGTTAACTT

GGTTTTTGAGAACTGTGAAAAGTTTAATAAGGATGACTCTGACATTGGACGTGCAGGACA

CAGTATGAGGAGATTTTTCCAGCGAAGATGGACTGAGCTCTTAAAACAGGTCAACTGAAC

TGTCTGAAAAACCTCCTGCATATAGACTACTGTCGCTTTATAACTGTCCTCAAGCAAGCC

CTCTTCTCCAAATGTTCTTTACCCACTCGCATCACTGGTTTGCATTATGAGCTCTACAGG

CAGTCCGTCCAGCGTGTGGTAGGATGACCGCAGTGTTTCGGATAACATAAGGTGTCTCTT

CAAGTGCAATAAAAAATTTCTTTACTTTACAGAGCTTTCATCATTTTAAAACTGGGAC

>Unigene000118

GAAAGGCGTTTCATAGCCCGGCAGAGAAATGAGAGCACATCCCGCTCCGGCTGAACCTCT

GCGTCTTGTCTTCCTGTCCTAAAGCAGTTGAATAGATAAGACGTAAACAGAGGAGTCGTC

GCCATGGGGGACGACAGCGAGTGGATGAAGCTGCCCATCGACCAGAAGTGCGAACACAAG

GTTTGGAAGGCCAGACTGAACGGATATGAAGAAGCACTGAAGCTGTTCCAGAGGATCGGG

GACGAAAAGAGTCCTGAATGGGGAAAGTACCTGGGACTGATCAAAAAATTTGTTACAGAA

TCCAACGCTGTTGCTCAGCTGAAAGGCTTGGAAGCAGCACTTATCTTTATCGAAAACGCT

CATGTAGCCGGCAAGACAACAGGGGAAGTGGTGTCAGGTGTAGTCAGTAAAGTTTTTAAT

CAGCCAAAAGCCCGAGCCAAAGAGCTGGGAATCGATATCTGCCTCATGTACGTGGAGATC

GAGAAAGCGGAAATTGTCCAGGATGAGCTTATCAAAGGACTGGATAACAAAAACCCGAAG

ATTATAGTGGCCTGCATCGAAACTCTCAGAAAGGCTTTGAGTGAATTCGGTTCCAAAATT

ATAACCCTGAAGCCAGTAGTAAAAGTGTTGCCAAAGCTGTTCGAGTCCCGAGAGAAGGCG

GTGCGTGACGAGGCCAAATTGCTGGCGGTGGAGATTTATAAATGGATTAGAGATGCCTTG

CGAACTCCACTGCAGAACATCAACTCTGTACAGTTAAAAGAGTTGGAGGAGGAGTGGGTG

AAGCTGCCAACCGGTGTGCCAAAGCAGAGTCGCTTTTTGCGCTCTCAGCAGGACCTAAAG

GCAAAGTTTGAGCAGCAGCAGGCAGCCGGTGGGGACGAGGCTGATGGTGATGACGATGAT

GTGGCTGAAACCCAAGTGGATCCATATGAGCTGCTGGAAGCTGTTGACATCCTTTCAAAA

ATTCCAAAAGATTTCTATGAAAAAATCGAAGCAAAGAAATGGCAGGAAAGGAAGGAGGCC

TTAGAGGCGGTGGAGGCCCTTGCTAAAAATCCCAAGCTTGAGGGCGGGGACTATGGGGAC

ATGGTCCGAGCCCTAAAGAAGGTTATTGGAAAAGATGCCAATGTTATGCTTGTGACTTTG

GCAGCCAAATGTCTTGCTGGATTGGCAGCAGGACTGCGCAAAAAGTTTGGAACATATGCG

GGCCATGTGGTGCCAACCATTTTGGAAAAGTTTAAAGAGAAGAAACCTCAGGTGGTCCAG

GCTTTACAGGAGGCAATAGATGCAGTCTTTCTAACTACAAATCTACAGAACATCAGTGAA

GATGTGTTGGCGGTGATGGACAACAAGAACCCATCGATCAAACAGCAGGCATCGCTGTTC

CTGGCCCGAAGCTTTCGCCACTGTACTCCTACCACGCTGCCAAAGAGCGTCCTGAAGCCT

CTCTGTGCCGCTTTACTCAAGCAAGTAAACGATTCCGCTCCAGAGGTGAGAGATGCTGCG

TTCGAGGCTTTAGGAACGGCCATGAAGGTGGTGGGAGAGCGAGCGGTCAACCCCTTCTTG

GCTGATGTGGACAAACTCAAGTTAGACAAGATCAAAGAATGTGCTGATAAAGTGGAGCTT

GTTGGAGGGAAAAAGGGAGAAGGTGGAGGAGGGCAAAAGAAAGAGAAACCTGCAGCTAAA

CCTCCCCCTGTGGAAGAGCCTCCTGCTAAACCTGCTGGTCCTCCTAAAAAAGCTCCTGCA

GCCAAAGCCGCAGGTCCCTCCAAGAAAAGCAAACCTGCTCCTGCTGCCAGCAGCAAGTCG

AAGAAAGCGGCAGAAACAAAAGAAGTTAACGAGACTGAATTATCTCTGGAAGTGTGTGAG

GAGAGGGCCGCAGCTGTGCTCCCAGCTTCCTGTATGCAGCTGCTGGACAGCGCTAACTGG

AAGGAGAGGCTAGCTAGCATGGAGGAATTTCAAAGGGCTGTTGAACAGATGGACAAGACC

GAGATGCCATGCCAGGCTTTAGTGAGGATGTTGGCTAAGAAACCTGGCTGGAAAGAGACA

AATTTTCAGGTAATGCAGATGAAGCTGCACATCGTCCGCCTGATTGCTGAGAAAGGTTCC

TTCTCCAAAACGTCGGCTCTCGTCGTATTGGACGGCCTGGTGGACAAAATCGGAGACGTA

AAGTGCGGTAACAATGCCAAAGAGGCTCTGACCGCTATTGGAGAAGCCTGTTCGCTGCCG

TGGGCCGCTGAACAGGTGGTCTCTTTGGCCTTTGCACAAAAGAACCCCAAAAACCAGGCT

GAGACGCTGAACTGGCTGGCCAACGCCATGAAGGAGTTTGGCTTTGCAGGGATAAACGTG

AAGGCTTTCATCAACAATGTCAAAACCGCTTTGGGCGCCACCAACCCGGCTGTGAGAACG

TCCGCTATCACTCTGCTGGGTGTGATGTTCTTGTATATGGGGGCTCCACTGCGTATGTTC

TTTGAAGATGAAAAGCCAGCCCTGCTTTCTCAAATAGACACAGAGTTCGAGAAGATGCAG

GGCCAGTCTCCTCCTGCTCCCACACGGGGCTTGTCAAAGAAACCGGCGAAGGATGACAGA

GAAGAGGTGGATGAGGATGAAGCAGACGGAGGTGCTGGTGACATTATGGACCTGCTGCCA

AGAACTGATATCAGTGATAAAATCTCATCTGACATGGTGGCCAAGATTAGTGATAAGAAC

TGGAAGATCCGTAAAGAAGGACTGGATGAGGTGACGGCTGTCATTTCAGAGGCCAAGTTC

ATCCAAGCTAACCTCGGAGAGCTGCCCATGGCACTGAAGGGCCGTCTCAGTGACTCCAAC

AAACTACTGGTTCAGCAAACCCTGAATATTCTGCAACAGATTGCTACAGCCATGGGTCCT

GCTCTCAAACAGCATGTGAAGAATTTGGGCTTCACCATCATCACCGTATTGGGAGACAGC

AAGCCCAACGTCCGGGCTGCTGCCTTGACAACACTAAATGCATGGGTGGAGCAAACAGGC

CTGAAGGAGTGGCTGGAGGGTGAAGATTTGGCAGAAGAGCTAAAACGAGAGAATCCTTTT

CTCAGACAGGAGATTCTGGGCTGGTTGGCTGAGCGGCTGCCCAACATGCGCTCGGTGCCA

GCTGATTTGATGCTGTGCGTCCCCCTCCTTTACACCTGCCTGGAGGACCGTAGTGGAGAT

GTGCGAAAGAAAGCCCAGGATGCCCTACCCATGTTTATGATGCACCTGGGTTATGAAAAG

ATGTGCAAAGCCACCAGTAAACTCAAGCCGGCTTCAAAGGACCAGGTGGTTGCCATGTTG

GAGAAGGCCAGGGCTGTAATGCCTGCCAAACCTGCTGCACCTGCAAAAGCTGCCCCATCT

AAAGCAGCTCAAAGTACTCCTCCTGCAAAGTCTTCCCCAGCACCTGCTAAAAGCCAGCCT

GCAGTGGACGACTATAGCCCACCAGAACCCAAACAGGACACCAAGAAACCCAAAACAACA

GGGCCTGCTCAGAAAAAGGGAGTGTTGGGTAAGAAGGCTCCAGTCAAGGCTGCTAATAAA

GATGATGAAGACAAGACTGGTCCTATCTTTATCCTTGTGCCTAATGGCAAGGAGCAGAGG

ATGAAGGAGGAAAAAGCACTGAAGATCCTGAAGTGGAACTTCATCACACCACGGGACGAG

TATGTGGAACAGCTAAAAACTCAAATGGCCACATGCCTGGCAAAGTGGCTACAGGATGAA

CTGTTTCACTACGACTTCCAGCACCATGTGAAGGCCATCAACGCAATGATTGAGCACCTC

GATGGCGAGTCAGACGCCGCGATTGGCTGTCTGGATCTAATTCTAAAGTGGTTCACGCTG

CGGTTCTTCGACACAAACACTAGTGTCCTGATGAAGGCCATGGAATTTCTTAAACTGCTT

TTTACCATGCTGAGCAGAGAGAACCATCACCTGAGCGAGTACGAAGCCTCGTCTTTCATT

CCCTACCTCATCCTCAAGGTCGGGGAATCAAAAGACGTTGTGCGCAAAGACGTCCGAACC

ATTTTGTCCATGCTGTGTAACGTTTATTCTCCCAGCAAAATGTTTCCGTTCCTTATGGAG

GGGACAAAGTCCAAGAATGCCAAGCAGAGATCAGAATGTCTTGAAGAGCTCGGATGTCTG

ATTGAGAATAATGGCATGAATGTGTGCCAGCCAACTCCCGCCAAGGCTCTAAAAGACATC

GCTGTTCATATCGGAGATCGGGACACGTCAGTGCGCAATGCAGCACTAAACACGGTGGTG

GCAGCTTACAACGTTTGTGGGGAACAGGTCTTCAAACTCATCGGAAATCTGTCCGAGAAG

GACATGAGCATGCTGGAGGAGCGGATCAAGCGCTCGGCTAAGAAGGCACCTGCACCTGTT

TCGACCAAACAGGAGAAAGCACAGAGGGAACAGCCCAACAATCCTAACGCTACCTTCCTG

CGCAAGCCTGCACAAGAGGAAGTGCCGAACAAACTAAATCAAGCTCGATCACAGAATGCC

CATGAGCACACTGCCCCCTCCATCCCCAAGGAGTTTCAGCTTGACCTGGACATGATTGAG

AACGACCACACGCGCGTCAGCGACTTCCCTGATCTTGTGCAGCACAAACTGGATGAGCTT

CTCGAGCCAGTCATGATCCCCGAGCCCAAGATTCGCTCAATCTCTCCCCACTTCGACGAC

CTCCACAACAGCACAGCCTCCACCATCAACTTTGTCATCTCGCAGGTTGCCAGTGGAGAT

ATTAACACAAGTATTCAGGCCCTGGCACAGATCGATGAGGTGCTGCGGCAGGAAGACAAG

GCGGAAGCGATGTCAGGCCACATAGACCAGTTCCTGATTGCAACTTTCATGCAGCTCCGC

CTCATCTACAACACGCACATGGCTGACGAGCGGCTGGACAAGAAGGACATCTTCAAACTG

TACAGCTGCATCATCGGCAACATGCTCTCGTTGTTCTCTATGGAGAGTCTGGCCCGGGAG

GCGTCGATGGGTGTGCTGAAGGATCTGATGCACGGTCTGATCACACTGATGCTGGACTCG

CGGGTGGAGGACATTGAGGACGGCCAGCAGCTGATTCGCTCAGTCAACCTGCTGGTGGTG

CGAGTGCTGGAGAAGTCGGATCAAACCAACATACTTAGCGCTTTGCTGGTGCTACTGCAG

GACAGCCTGATCACCACTGCGGGTTCCCCCATGTTCTCAGAGCTTGTCATGAAGTGTCTG

TGGAGAATGATCCGCTTCCTACCCGAGACCATTAACAGTATTAACCTGGACCGCATCCTG

CTGGACGTCCATAACTTCATGAAGGTTTTCCCTAAAGAGAAGCTTAAGCAGCTGAAGAGC

GACGTGCCACATCGCACCCTGAAAACTTTATTGCACACACTCTGCAAGCTCACTGGGGCC

AAGATACTGGACCATCTGTCTATGATTGATAACCGGAATGAGTCAGAACTGGAGGCACAT

CTGAGGCGGGTTGTAAAGCATTCAGGAAATCTCTCAGGCCTCAAGTCGGACCAGCACTCT

GAGAAAATGGCCCTACGATCCGACGATAAAGTAATCAAAGCAAAAGTGAGTGACATCCTC

TCCGAGATCTTCAAAAAGATCGGTTCAAAGGAGAACACTAAAGAGGGCTTGACAGAACTT

TATGAATATAAGCAGAAGTATACAGACGCTGACTTGGAGCCCTTCCTGAGGAACACGTCT

CAGTTTTTCCAGAGCTACGTCGAGCGCGGCCTCCGTATGATCGAGTCTGAGCGGGAGGGG

AAAAGCCGTCTGCAGAGTTCTTCAGTGATTCCACAGCACAGTGTGGATTCTGCGTACCCC

TCCAACAACAGCTCCATGTCAGTCAGCAGTAATGGAGAAGATCTGAAACCTGCTGTTTAC

TACGAGAGACTGAAGATCCTCCGGCAACGTCACGGACTCGAGAACGCTAAGCAACAGCAG

CAGCAGGAAGACGAGCGTCCTCTGTCCAACCTGCTGTCTCGTCCACCCCTGGCCTCCTCC

ACCGACATGCTTCACAGTAAGCTGTCGCAGCTGAAAGAGACACGTGAATCTCAGCTACAG

CTGGAGCAGACGCGCTCACACAGCCCAGCCCGAGCCTCCTCACCCGCCTCCAACCTCGAT

GACCTCAAAAAGAGGCTCGAGCGCATCAAAAGTAACCGCCAGTAGAAGCGAGCTCCTCCC

TGATAGCTAAACTGTTAACCTCCGTTAAGATGCTCTAGTCTTTTTTTCAATCCTGCTCTC

AGCTTTTTTCTTTTTCCTTCAGGTCTGGTATATGTATAGATTAACATAATGACAATAGGG

GGGAAAATTAAACCACTGGTTTGTCTAATTGTATAATATAAAGTAAACTGCATTTTTGCT

TGTTTAGTATCGTGCACAACTGTCTGAGAGGTTGTTGGGTTTTTTTTTGTTGTTGTATAT

ACTGACGGTTGTTAACCACTCCAGGCTTTTCTGTGTCATGTTTAGGAGCTGCAGTAGATG

TGCAAAAGTGCTGTAGTATGTGTTATCTGGAGATTTTCCCAGACGACG

>Unigene000119

GACAGTTTTGAAAAGGAGGGGGAAAGGAAGCAGACTCAAAAAAAAAAAAAAAGATATACT

GGGCTTCAGAAAGACAGAACGATGTGAGGAGAGAACTGTTCCATTTATAGAGAAATTCAG

GAGTAGAATGAACGGATCTGTGTGAGGTCTGAAAGTGAAGTTTTTAATAGTGGTACGGAA

CAGATACACAACAGGACTGAAACCACAACGAGCTGTGCAATGAGGAGGTGCAGGCATGAC

ACTCATCAGCGGGTAGCAATTACTCTAAACTACAAGGCTTAGAAAAGAACTGATGGCAGA

GTGAATCAATGAGGAAGAGAACGCTTCCTCTGCTGCTGAAACTACCCCACAGGACGAGCT

GCCCCAGTGCACAGAAAGCTGCTTATTCAAACTTCCTGACAAGGTCATGGCTTCACCCAG

TTAATGAGATACAATGAAGAGATTGGACGATGAACCCGTACACAGTGGGAACAGGACGAG

CCTTTCTGACAATCTCACCAACGGTGACTCAGTGACTCAGAGAAACCCTGTGGCTTTACT

GCAGGGGTTACATCTGTGTCAAGAGGCCTGGAACCCAGGAAGCCCGTGGGACAGAGAGGG

AGCCCACGCTGCTCTCTGGGGGAGGCCACCAGGGAGTTTCCTGGTGGTGTCAGAATCTTC

TTCTGAGAATAAACTCCTCTGTGTGTCTGTCAGTGATGAGGACAAAAAAGTAGAAGACTT

TCCCATCATACACACTGGCTCAACACAGCGTCTAAACACATCTCACCTGGCCTTTGCTGA

TCTGCTGCAACTGGTGATCTTCTACAACTTCAGCAGGGATGTGTTACCTTTTTGTCTGAG

GATTCCTTCATGGGTATGTGGCCTAACCGAACAGCCAAAGCATCTGGTTTCTGAACTGGG

TCCAAAGGCCTGGCTTTGTCCGCCAACAGATCTGCTGCCCGACACCATGAGTCCAGAAAC

AGCAGATATTCAGGACACTGTAATGTGCACCATACAGTTAACAGCAGACAATGGTGCACT

CTGCTTCATCAACCCTCTGTATCTCCAAGAACATGGAGATGACTGGCTGACACATTCCCA

GGTCAGCGCAAATCTTGCCAATCACCTCTTCACTTCCAAGCGTGACAGGAGGCTGAGCAC

TACAAGACCATGGGCAGGGGCGGGGCTTAAAAATCAAACCAAGTCAGTGGGAGACTCTGC

TGTTATGGAAATCTCAGGCTCTCCTGTTCATGGCTTGACTTCCCCAGTGACTGGGGTGAT

TTTGCGCAGAGCAAGCAGTACCTCAGATCCTCAATGGAGGATGAGTGCAGAATCAATCAG

CAGCCCCATCCCTCAGTCACCGCATCGAGTTTCTTGGATTGAGGACAAAGTCTGGATGAG

CCCTCCTGCTCCTTCCTCCCTGCTCCATCCTCCATGCCTAGAATTTGATTCTTTGTCGAT

GAGCAGCATAGAAGAAGAGCCTGAAGTTGAGCCAGCCGTAAGCTCCTGCCAGGTCCAAAA

CTCACCGCGCCTCCCACTGGCTGACAAGGTTAAGAACAGGCTTTCAGCGGTTGGCCAGGC

TCTGGGTGGCTTTATTAAACCACAGAAACGTCTGAGTAAGCGTGTCCAGGAAATGAGCGA

ACGCAAAGGCAGTCCTTTCGCAGAGGCTCTAAAGGGATTTGTAGAGCAGACGCTGAAACT

GAGAGCTTCTTGTCATGTGACGAGTACAGAGATGCTGCAGGAAGTGCGATCCTCCCTTAC

CGCTTTGAGGGAAATGCTGTATGATAGTGCTGAAATTCAGAGCATTATTGACACTCTGGG

GGATGTGCCTGATTTTGAACTGGATACAATGATGGAGCAAGCCTTACATAAAGTTGCTCT

GAAGCCTCTGTGTTCCCATTTGTACGAGTGCATGAAGACAGCACGACAGCAAGATGGCAG

CCTGCAGCGACTGCAAGCAAACCAAAACACGCTCAAATGTCGGCGCCTAGAGGAACTGGA

GGGGACTGCGGGTGCAGGTGTGCCTGATGCGGCCATGCTGGAGAAAATCCAACAGCGCTG

GGCTACCATGCACCAACAATATTCACCACAAATGAAAGTGGAACTGCTGCTCAAAGTGTG

TAAAAACGTTTACCACAGCATGACTGTTAATGCCAAGTCTGGTGTGGTTTTCGGAGCAGA

TGACTTTCTGCCCTGTCTAACATGGGTAATACTGCGCAGTGATGTTGTTACTCTGCAGAT

AGACACAGATTACATGATGGAGCTGCTGGACCCCTCTCAGTTACAGGGAGAAGCTTTCAG

TCTCGTTTGGCAGCGCGGCAGCTGAGTGCCGAAGCCCAGAAATCCCTTAGTCAGTGGCAT

CGCAGACGCACACTCCACTGTAACCAATCACGACGCAGCAGAAACCGGCGGACCATCCGA

AGGCACAGGAATAACGAAAAAGAGGATGGGGAATCAGGGGAGTTGGATATAAGAAACATG

TCTAAAGAGAAAGACTGGACTCACACTGATGATGTGACAGAGACATTGCCGAGTGTGAAT

GAGATTTCTGGAGAGGAAAAGGAAGAGTAAGCGAATGAAGGATCAGAGGGTTAATAGCGT

GTGGGTAACATTAACATTAAAACTGTCCTGCACTTTTGCTTTAACAGTAGCTGCGTTTAC

ATGGACCCTAATGATCAGACTATAAGCACACTCAGATTAAAAAAGTGACATGTAAACATG

TCAATCAGAATTAATCAGCCAAATCTGATTAAAATTTCACTCGGATTGGGAAGTGGTTTA

AACCTTTTTCACCCAATTTAGGGGACATGTGGACACCTCAATCCGACTGAAAATAAAACA

AATGAAAACAAATCACGTGATGACTGTTCAACAGATTAAATCTGATTGTAAGCCCCATGC

ACATTAACCTAATCATTTCATTCAGTGTTTGTGTAAACTCTGCATTTCGGATTCATCAAT

CTGATTAATTTAATTTCGATCGAAACAAAAAGTGTCCATGTAAACATAGCTAGTGTGTAA

AAAACTTTTCTTGTCAAAAGCTACACAACTCTTGCCTTTGCATAATTTTTGAGGCCTTAA

TACACTTTTTGGGTAATCTATAGCCTTGACTTAGTGTTGTATAATATATTCAGATGTATC

TTTAAGAGTTTTAATTTAATTTGTAGCAGCATTTAATAAGAGCTGATCGAAAGAGCTAAA

TAAACCAAACACAACCAATTACTGATCTCATCTACAGTCCAAATTATACAAATTATAATT

TGTTATGCAGCTGCAGAATAAGACATGATAACTCACTGCATCGACGACAGTACACTATTA

ACATTGAGCACACTTTCTGGATTAGATGCAATTCGGATTGCTTAATCTTCCTTCCGAGGC

TTGCACATTTCTAGTGGGGGTTATTCGAAACTGGGGACACACTGAAAATCTAACCCTAGT

AATATAGATGAGGACAATGAGGTAGGTAAGACAATTAAAGACTAATTTAGAGCTTTTTAT

TAATAATTCTAAGCATTTTATTTAGTAGGCTATTCTGTCTATGCAGCCAGCTATTTTCTG

TTTGTCTTATCCTGCACATGGAGTAATTTGAAATTGAAATATGAAATTTGTTCATGGAGT

ATGTAAGTGGAGTGAAGCGGCAAAAATTTCTACTCAAGGCATTTTATAATTGCTCACTCT

GACTTCACTCTGGATTTTCTTTATCCTGGTTTTTGCTCACTCCACGTGTGCCTTATTAAT

GCCCCAGTGTTTTTTTTTATTCTAGACAGATAGATAGTATTTAAGTGCCTTTTGTAATCC

AGACCTGAGATTTAAATCCTAAATGAACTTGAGCTGGTTAAGACCTTGCTACTGTTACTG

GTAGTGTTGTGTCCAGATATTGCAGAATGGTAATAGTACAAATATAAAGGCAATCACATG

ACCCAACATAATTTCTGTTCATTCTATGCTGCAGTTACAATATAAATGCCGTCATTATTA

AAACAATGTTTACAGGATAAAGACAAAATCTGTGCTTATAACTGAATAACTTAAGCTTCG

CTGAGGATCTTTAACAATATTTTTATTGATTTTAAACAGGGATACAACAAAGCCAACATG

TAATATTCCAATGCACACATTTACAATCTGTGTTATATACCCCTGATAGATCCAATAACC

CCCACCCCACCCATCCCTAACTCCAAGCCAGCCTTACAGGTCTAAGACAGGCTAAACAAC

AGGCATATTTAAAGAAGGAGAAGAAAGAAACAAAAAACAAAACAAAAAAACATGAAGCTG

GATGTCCGGGTTTTGTAATTAAAGTATAAATGTATACGAAATAAGCGAAGGAAGGGCCCC

CACACCTGTTGAAATTTTAATTCTGAATTACGTATTAAATAATGAATCTTCTCCAGGGAC

AAACAGGACATAATATCTCTGAGGCACTGGGTGTGAGTGGGTGACGTGACTTTCACTTAA

ATAAAAGGGCATGTCTAGCCAGGAGGGACGCAAAAGACACTAAACAATGTTTAGCTGGAG

TGAGACGATTACCTTCCTCTCGAGTAATGCCAAACTGAGCAACCAGGGGATCTGGTTCAA

GATTACAATTAAGAATGTAACACAAGGTTTTAAAGACATCCCTCCAGAATTTTTCCAAAT

TAGGGCATGTCAAATACATATGAATAAGAGACGCCTGTCCGCTTTTACATTTATCACAGC

AAGGACCGAATTCTGGGTAGAAACGAGAGAGCTTAACTTTGGAGAAATGAGTCCTGTGCA

CAACCTTAAACTGTAGTAAAGTATGGCGGGCGCATAAAGAGGCTGAATTGACAAGCCTGA

GAATTGAGTTCCATGTGTCATCTGACAGTGTCAGATTTAGGTCATCTTCCCAAGCAGTTT

TGATTTTATTTAAAGTGGTGTGACTAAATCCCGTCAGTCCACCATAAATAGTGGATATTA

GGCCTTTATGCAGCGGAGACAAACAGAGGAAATTATCTATAATATTAGCATCCGGAGCCT

CAGGGAAATTTGACATTGAAGAGCTAATGAAGTGTCTTACTTGAGGATTTTTAACAACAG

TGTGGAGAATTCGTTCTTCTTACAACGTATAATCTCATTTTAGTAGATCATAGACATGGC

CTATGATTTAACTAAACAAAAACAATTATGACTTCTTAATCGGTGGCTAGTTTCCTAGAC

TATTTTATATTCTGTCTACACATCCTATGCTAGGCTCTGTGTAAGGATAGCTAATTTCTG

ATGGCAATTTTTTTTATATACACTGTACACACTGAATGCATTAATGCAGGCTAAACAATG

AACAATGATGCTTTCTATAACCTCCTCTGCACCGCAAGATGTTTCATGAAAGTGATTTTC

TTCATAAAACACAAGCCGGTCATTTCTAACGATGGGATGTCCTGTATGCGCAGAATAGAA

CAACTCGGATCAGTTTATTCAGACATGGATTGCATTTCTTAGATATTTTTTTATTTTTTA

ATAAGTAAAAGTATACTGTCAATAAACTTTGACAATTTAATAGTTTATAGTTTTAGTGGA

AGCCAGAGTCGAGGGGCATAGCGCGGTAGAAAACTGTGGAAAAGTTAAGAAATCGAAACA

AGAATATTAAAGCAAGAAACAGACACTAAACCAGCCACAATAAATAAAAGGCTGAACGTG

TTATAATCAGCACTTTGTGTCCAGCTGGTTCTCCCCTGGGACAGGGAGTGCATTGTGGGT

AAACTTTCTGATCTACACAGACGCGAGACAAGATTTAAGGTGCGTTTTCTGCATCACATA

GCGGCTGTCAAGGAGAGATTAAAACTCTGGGCTGGTATGAGGAAGTGCTGGTGTAGTAAC

ATGACAGTGGTGTTTAACTGTTTAGCAACAGATAAATTTGTTCATGCAGTTGCATTATGG

GCTGTTAACATGATGTGTGCTCATAATGGAGTGTTAGTAGAGCACGGTCCGAGTGACAGA

AAACTACATAAAAAGCACACAAAATCACGCAAACGCCTGACTCCGCTCCGTTCACATACT

CTGGCCAGGGTGAACCTGGAGCCAAGAGACATAAGTAGGAGAGCACCCTGAAAATGGGCC

AACCGGTCACAGGGCACCATCATGTATAGACTCATATCATTTTCAATTCAGTGGTGATAA

TCAACATATGTTATTAGACTAGGAGAGAAACTGGGGTAACTACAGTAAACCCTCAAGATC

TAAGAAGAACATGCACATTCCACACAAACTAGCATGAATTATGAATGAACTTGTTTCTAC

TATATCACTACTATATTATACAGAGCATTTTACAGGCATGGTGGTAGCTGAGTGGGCAAG

TTCAGTTCAGTTGTACAAATCAGATAACTGTTTTTCCATATAAAAGTGTCTGCCAAATGA

CAGAAATGTTTACCATGTGAAGATTATTAGTGTCTTTACAGAGCTCAAGGTACAAAAGCT

TCTAAATCATATAGGATGAGATTTTTTGAGAAAGTAAAAATACAGAAAGTTTACGGTTAG

GATTAGGGTTAGTGTTAGGGTTAAGATTAGGGTTAGGT

>Unigene000120

TATATACATATCTACAGTATATACAGATTTAATTAACATTTACTATTTTCCTCTCCAGCT

GAAAGGACTTGTCAGCCTGGTCACACCAAATGCCAAAGTACAAACATCTGCATTCCACGA

TCATACCTGTGTGATGGAGATAACGACTGCGGAGACATGAGTGATGAGAGCCCCACACAC

TGCGCAACATCTACATGTACCCAGAGTGAGTTTCGCTGCACAAGCGGGCGATGTATCCCT

GCACACTGGTACTGTGATGGTGGTACTGACTGTGCAGATGGGTCAGACGAACCCTCCACC

TGTACCACCATAGTGAGAACCTGTAGCTCAGACCAGTTCCGCTGTGATGATGGAAGGTGC

ATCGCATCCTCCTGGATCTGTGATGGAGACAATGACTGTGGGGACATGAGTGATGAGGAC

CAGAGGCACAACTGTGCTAATCGAACCTGCTCTTCACTGGAGTTCACCTGTGTGAATAAT

CGGCCCCCACAGAGGAAGTGCATCCCTCAGGACTGGGTGTGTGATGGTGATGCAGACTGC

TCAGATGCATATGATGAACACCAGAACTGTACACGCAGGTCCTGTTCTGCAACTGAGTTT

ACATGCAGCAACGGCCTCTGTATCCGCAGTGCATACAGGTGTGACCGGCGGAATGACTGT

GGAGACGGTAGTGATGAGCAAAGCTGTACCTACCAGCCGTGTCAACAGCACCAGTTCACC

TGTCAGAATGGACGTTGTATTTCTCAGGACTTCCAGTGCGATGGCGATAATGACTGTGGT

GATGAATCAGATGAGCTGCCGCACATGTGCCACACTCCAGCGCCTACCTGCCCTCCTGGG

AAGTTCAGGTGTGACAATGGCAACTGTGTGCCGTCCAGCCAAGTCTGTGACCACAGTGAT

GACTGCAATGATAACAGTGATGAGAAAGGCTGTGGCATTAATGAATGTACAGACCCATCC

GTGCACCATTGTGACCACGAGTGCACTGATACCCCCACCAGCTTTGTGTGCAGATGCCGT

CCTGGCTACCGCCTTATGTCTGATAAGAAGAGCTGTGATGATGTAGATGAGTGTTCAGAG

ACCCCCAGTGTATGCAGTCAGATGTGTGAGAACACACTAGGCTCATATGTGTGTAAATGT

GCACCAGGTTTCCTTCGCGAGCCAGACGGGCGTCGTTGTCGGCAAAACAGCAACATCAGT

CCTTACTTGATTTTTAGCAACCGCTACTACCTGCGTAACTTATCTGCAGACGGAGCAGCC

TACTCGCTCATCCTTCAGGGCCTTAGCAGTGTTGTGGCTCTAGACTTCGATCGCGTCGAC

AAGCGTCTTTACTGGATCGATGTGAGCCGTCGTGTGGTGGAGAGAATGTTTTTTAATGGC

ACAGGCAGAGAGGTGGTCCTGAATGGCATTATGCACGGAGAAGGCCTGGCTGTGGACTGG

GTGGGACGGAAGCTTTATTGGGTAGACAGCTTTTTAGACTGTCTGAAGGTGTCTGAGCTA

GATGGACGCTTTATTAGGAAGCTGGCTGAGCACTGTGTGGATGTTAACAACACCTACTGC

TTTGAGAATCCAAGAGCCATTGTGGTGCATCCAAAGTTTGGATATGTGTACTGGACTGAC

TGGGGAAGTAAAGCATTTATAGGTCGTGTTGGGATGGACGGTAACAACAAGTCTGCCATT

ATTACCTCTAAGATCGAGTGGCCCAATGGTCTTACCATCGACTACACCAATGATATGCTC

TATTGGGCTGATGCTCCCCTTAACTACATTGAATCCTCTAACTTAGAGGCCCCCCACCGC

CACACTGTTTATGATGGTGTTCTTCCTCACCCGTTTGCTATAACTGTGTTTGAGGACACT

GTGTACTGGACAGACTGGAACACTCGCACTGTAGAAAAAGGCAATAAGTACGACGGCACT

GGGCGCCAAGCTCTTGTCAACACTACTCACAGGCCCTTTGATATCCACGTGCTTCACCCG

TATCGTCAGCCCATAGTAACCAATCCCTGTGGAATAAACAACGGCGGCTGTTCCCACCTG

TGTCTCTTAAAAGCTGGAGGACGGGGATACACTTGTCAGTGCCCAGACCACTTTCTGACT

GTGCAGATAGGGGGCGTAGCCCGCTGCCTACCCAGCTGCTCCAGCACACAGTATAGATGC

GCTGACAACGAGCGTTGCATCCCTATCTGGTGGAAGTGTGATGGTCAATCTGATTGCAGG

GACGGTTCCGACGAGCCGTCCACATGCCCCCTGCGCCATTGTAGGTTGGGCCAATTCCAA

TGTAATGACGGAAACTGTACCAGCTCACATTTTCTGTGTAACTCAAATCGCGACTGCCCT

GATGGATCTGATGAGGACACTGTGCTGTGTGCAACACACCAGTGTGAGTCCCACCAATGG

CAGTGCAGTAATAAGCGGTGTATCCCGGAGGCGTGGCAGTGCGACGGGGAGAATGACTGT

GGTGACGGCTCGGATGAGGAACCCTCACACTGCTCCAGCAGAACGTGTCGTCCTGGCCAG

TTCAAGTGCAGGAATGGCCGCTGCATCCCTCAGAGCTGGAAATGTGATGTAGATGATGAT

TGTGGAGATAACTCGGATGAGCCCTTGGATGAGTGCATGGGACCAGAATATCGATGTGAT

AACCACACTGAGTTTGACTGCAGTACAAACTACCGCTGTGTTCCTTTGTGGGCCGTGTGT

AATGGACAAAACGACTGCAGAGACAACAGTGATGAGCAGAACTGTGAGGAGATGACCTGT

GACCCTGCTGGAGATTTCCGCTGTGATAACCACAAGTGTATCCCCCTGCGCTGGAAGTGT

GATGGAGACAGTGACTGTGGAGATGGATCAGATGAACGCAACTGCAACCCTCGAGCCTGC

ACAGAGAGCGAGTATCGCTGTGACAACCTGCGCTGCATTCCTGAACGCTGGGTGTGTGAC

CATGACAACGACTGTGAGGATAACTCGGATGAAAGGGACTGTGAGTTGCGGACCTGCCAT

CCTGGATACTTTCAGTGTGATAGTGGTCACTGCATTGCTGAGCGCTTAAAATGCGATGGC

AGTGCAGATTGTATGGACGTCACTGATGAGACATCCTGCCCAACACGTTATCCAAATGGC

ACATACTGTCCTCCCTTCCTGTTCGAGTGCAAAAATCATATATGTGTTCAGCAGCACTGG

AAATGTGATGGAGACAACGACTGTGGAGATGGATCGGATGAAGAGCTGCATCTCTGCTTG

GATATACCGTGTGAAGCCCCTTTCCGCTTCCGCTGTGACAATAACCGCTGCATCTACAGC

CACGAACTCTGCAACTCAGTAGATGACTGTGGAGACGGAACGGATGAAAAGCAGGAGCAC

TGCTTGACTCCCACTCATGGACCCTGTTCCTCTGATGAGTTTAAATGTGGGAACAGCCAG

TGCATTCCTCTGCAGTATGTCTGTGATGATTATGATGATTGTGGAGACCAGACTGATGAG

TTGGGCTGTAACAGTGGAAATGGACGCTCTTGCAGTGAGAATTTGTGTGAACATAACTGC

ACCGAACTGACGGGTGGAGGCTTTATATGCTCCTGCAGACAGGGCTACAAAGCCCGAGAG

GACAACAGAAACTCCTGTGAAGATGTAAATGAGTGTGAGCTCTATGGCACTTGTCCCCAG

CTGTGCAAAAACACTAAGGGGAGTTACGAGTGCTTCTGTGCTGAGGGATTCCGCTCTGTG

GGACCGCAACACAGGACTGAATGCGCTGCTGAAGGCAATCCTCCAGTGCTACTGTTGCCT

GATAATGTGCGTATCCGTCGCTATAACCTGTCGTCTAAGCAGTATTTAGACTATATCGAT

AATGCTGAACACATTCATGCTGTGGACTACATTTGGGACCCAGAGGACCAGGGGCTCAGC

ATTGTATACTGGACCATTTTAGGTCATGGTGCTCAGTTCGGATCCATAAAGAGGGCATAT

ATGACCACATTTGATGACCATGGAAGTAACCAGATGAAAGATGTAGATCTGAACCTTAGA

TACATATCCAGTCCTGATGGAATTGCTGTGGACTGGGTTGGGCGACACATTTATTGGACA

GATGCTGGAACCAATCGTATTGAAGTGGCAAAACTGGATGGGCGGTACAGGAAGTGGTTA

ATCTATTCCAATCTGGATCAACCAGCTGCAATAGTTGTCAACCCTGCTATTGGTATGATG

TACTGGACTGACTGGGGTAGCCGACCAAAGATTGAAGCAGCATGGATGGATGGTCAGCAC

AGACAGGTGCTGCTGGATGAGGATCTGGGCTGGCCTACAGGACTCACTCTAGATTACCTT

AATGGAAACAGGATTTATTGGTGTGACTCAAAAGAGAACATTATTGAGTCTATGAAACCA

GATGGAACAGACAGGAAGATCGTCCTGTCAGGAGATATTGGAAACCCCTACAGTCTGGAT

GTGTTTGAAGGACATGTGTACTGGACAACAAAGGTCAGGGGTGAAGTATGGAAGGCTGAC

AAGTTTGGAAGGGGGGATAAAGTCAAGATGTTGACCATAAACCCTTGGTTGACTCAAGTC

CGCATCTATCATGAGCACAGATACAATCGCTCAGTGTTAAACCCCTGTAAAGGTGTGTGT

AGTCATCTGTGTCTGCTGCGACCTGGTGGATACACCTGTGCCTGTCCACAAGGCTCTTCT

CTGGTCAGCTTTAATGAAAACGAGTGTGATGCTGCCAGCCTACCCGAGGTTGCTATGCCG

GTTGAATGCGGATGTATTAATGGTGGAACCTGCTCCACTGACAAGCCTGGCCTGCCAAAA

TGCAAGTGTCCTTATGGTTATTCTGGAAGGCACTGTGAAATAGGAAAGTCCCGAGGAGCA

CCTGCTGGTACAGCAGTGGCTGTGCTGTTGGCAGTGATTATTATTCTCTTAAGTGGGGCT

CTCGCTGTTGGGCTTTTCCTGAACTACAAACGCACAGGCTCACTCATCCCTTCAATGCCT

AAGCTACCCAGCCTGAGCAGCCTGGCTAAGTTAAGTGATGCAGGAAATGGTGTGTCCTTC

CGCTCAGGGGATGGCAGTACTGTGGATCCTCAGACTCTTGGGGTATCGTTTATTGATAGA

GCCATGCAGCTGAATGAAAACTTTACAGATGCTGGGCGGGAACCTGTCACATTCGAGAAT

CCTCTTTATTCCACTGCAACAGGTGCAGCTGCTGATGCTGCGGTCATACATGCCACGCAG

GTGACTGTGAATATAAGTGGAGATTCAACAGAGAACGTATTCGCAAATCCGATGTACAAT

GAACAGCAGCAGGCAGTGGACATGAAAAATACACCAAATGAACAAGCAGCAAAGGAGTCC

AAGTGGAACTTTTTCAAGAAAAAATTAAAACCTAGTACAACCTTTGAAAACCCAGCATAC

TCAGAGATGCGAGACAAACAGTCCGCTTTGACTACGGAGGACATTTCAACTAGCCATCCA

CAGCCAGCTCTTCCACCACTAAAGCGAGAGAAATCCAGCATGTACTCACCGACAGAAGAC

TCCTTCCAAGACACGGCCAATCTGGTCAAAGAGGACAGCGACATATAACGCTTACTTTCT

TTTTCAATCTTTCCTACACACAAACGCAGATACAACGGGAAAAAAGACACACTCTGTTCC

TCTTTGCACAGAATCTATTTTTATATGCAGATTTTGTACAAAAAAAAAAAGAAATCTTAA

GATTATTTTAAGAACACTTAATAAGATGTTTTTATAACAGAAAATTAAAAGGTCTATGGA

GACTATGCCTTCTGCTGTTTCTTATGCCAATTCATGTCTATAATTGTTTTTATATTGTGA

TGTACTATATGTACTGTATATCTTTGCACTGATACTACTGATGGTAAAAATGTTGTACAT

TTGTAAAAAATTTTTAGAAGATTTCGATGGTAACTGGAAATGATGTGCAGCAGATCCTGC

GTTCTATTAAAGATAAATAAAATGACCGCTGAACATGGTAGTGTGTAGGTTAATGCACGC

TGCTCAGTGATTTATTCCTTTTTGTTGCTTCTTTGCATAGAAATTTACACTGATAATTTG

TAAGTGGACTAAAAAAAACACATCTCATCCTGAACATTTATATACCTCATGTGTGGACTG

GCCAGCACAGGAAAAAAAGAGCCAATTTCTGTTTTGGTCCATGATTCACAATATGTTCTT

AAGCAGTATCTTTGTTCAAATTAATATTACATTACCTTAAATACAGTATATATTAGACAT

TTATAGATTTATGAACTGCTATAGATTATTTGTTGTTACCCAAGTTTTAATTGTAGATAT

GGTAATTACCAGAAATCAAAATAACTGATGATATTTTTTGCCTTGATATCTACCAGGCTA

ATTTATACGGCGTGTCTTGTAAAAACATTTACTGTATTGTTGCAGTAGGCAAATATTAAA

GACTGCTGTTATTTAAAAGAAGGGACTGATTTAAAAAAAAAAAAAAGCTAATAACCTATT

TTCATTCATAATTTATATTACGAATGTCACTAAT

>Unigene000121

GTAGCCGCTCGCGCAGGTCCACGTCGGCAGCTGCGCAGTGTTCATCTTACCTCAGAAGTG

AGAGGCGACATGACAGATACACAAATACTGTAATCTGGACACAGAGCCAGAATAAAAACT

GTAATAATAATTATAATAAAAACACTCTGGAGGGTTCAACCTTCTGCCGGCAGTCATGTT

CAAGAAACTGAAGCAAAAAATTAATGAGGAACAGTCACCCCAGAGAAATGTCCTGTCACC

TCAACAGGCCCAGGTGAGTTCAGGTGGGAGGGCAGTTAAATCACCGTTACTGCAGCCGGA

CACCGCTTCCTCCTCCAGTGAGCGAGAGAACTTTAGTAAAGGAGCAGTTAGTCCTCAGAG

TGGGAGTGTGAACGGAGATGGAGCTGTCTCTCCTCTGAGAGAGGAGGCGCCGTCTTTGGC

ACAGAAGCTGCAGCAAAGGGTGTCCTCGGTCGAGTCTCTCTTTCGGGGTTCGGGACGTGC

CGAGGGGCTTTTCCGCTCCGGCTCAAGGGACAGCCTAGTCCGTAGCGCGTCACGTGAATC

TCTTACTCCTCTGGGAGAGAATGAGGCTCCGGGGGTCCCTTCCTTCGATCCTCCATCTGA

CATCGAGAGCGAGGCTGAAGATTCTCCTGGAAACGCAGAGGCTTTGTCCAAAGATCAGCT

CGTATATCGGCTGCACAGAGTGGAGAAGAGTCTGGCGAATTACAGAGGGAAATACTCGGA

GCTGGTTACAGCCTACAGAACAGTGCAGAGAGATAAAGAGAAAACACAGGTTGTCCTCAG

TCAGAGTCAAGACAAAGCACTACGGAGAATAGGGGAACTCAGAGAAGAGCTGCAGATGGA

TCAGCAGGCCAAAAAGCACCTGCAGGAGGAGTTTGATGCTGCTCTGGAGGAAAAAGACCA

AATGATTACCGTCCTGCAGACTCAAGTTGCCCTTCTGAAGAAGCGGCTGCACGGTTCCCC

AGGGTCTTTACTCACATCTGAGGCAGGAGATTCCCAAACCGCTGAGACTACAGAGACGAT

CGCTGACCCTCAGAGCGTCACCCAGGCGGAGATTGAGGAACTAATTCAAGCAGGGGGCAG

TAGTGAGCCAGAAAACACTGCAGACTCTGAGGCTCTGCAGAAGCGAGTACGGAGACAGGA

GACGCTGCTGCAGCGCTGCAATGAGCTGATGAAAACCAACAAAGAGCGCAGTGCTCAACT

CGGCAGCGAGAACGACGTCCTGCAGCAGCAACTGCAGGAGAGACTGCAAGAACTGGAAAA

GATGAAGGAGCTTCACACCAGTGAGAAGACAAAGCTGATCACACAGCTAAGAGATGCCAA

GAACCTCATCGAGCAGCTGGAGCAAGACAAGGGCATGGTTATCGCTGAGACGAAGCGGCA

GATGCACGAGACTCTGGAGATGAAAGAGGAGGAGATCGCTCAGTTACGCACCAGGATCCA

GCAGACCCTGACCCAGAAAGAGGAACTGCAGGAACAGCGGGAAAAAGCTGAGAAAGCAGC

GTTTGAGGAGTTGGAACGCGCCCTAGGCGTGGCACAGAGAGCAGAAGAAGCCAGACGTCA

GCTGCAGGAACGCATGGAGAAGGAGGTAAAGCAGGTGGAGCAGACCAGTGAGGAGGAAAG

GCGGAGCCTACAACAGGAGCTCACCAGAGTCAAACAGGAGGTCATATCCATTATGAAGAA

GTCTACAGAAGAGAGGATAGCGGAGATGGAGCAGCAGCACCTAGAAGATCTGGCCAACAA

GGACCTGCAAATGAGTGTTCAGATACAGGAAGCGGTGGAGAATTGCCGTAAGGAGTTGTT

GGAAGCAGCGCAGGAGAAGGAGCAGCAGGCCTCGCTGGCAGTGGAGGAGGCGGAGCTGCA

GAAGTCTGCTGTCCAGGCTGAAGGAGAAGCTAAAACCAGGGAGCTGCAGCTGGAGCTGGA

GAGCGCAAGAACCAGAATTCTTGAGCTGGAAAGTTCCATGGCCAAGTCATGTCAAGCAGA

TCAGTTCTCAGTGCAGATTGAAGAACAGAGGAAGAAGTATGCAGATGAAATAGCTTCTTT

AGGAGAAAGACATCAGCAGGAAGTGGAGAAACTGAAGATGGAACATGCAGAAGCTCTGAA

TCAGCAGCACTGCACTGATGTTGAGGCGCTCGAGCAGAAACATCAGACTGAAACTGACGT

CATGATGAAAGACAAAGAAAGAGAGTTTCAAGAACATGTCAAAGAAATGAACCAAAAGAT

GCTGGAGAAGCTGGAGATCAAGCAGGCCAAGCTGGAGGCTTTGTCCTCAGAGCTCAGTGA

GATGCAGAAGTGTAAAGAGCAGCTGGAGGAGCATAAGGAGTCGGTGAGGATGGAGTTAGA

AGTGAGACTAAAGGATGAGCAGGTGAAATTTCAGAAGGAGGTGGATGATTTGAAGCAAAA

GCACGAGGAGATGTTAGAAGGACTTGAGAAGCATCAGAAAGAAGAGATAAATCAGCTACA

ACTGGTGCTGGAGGAGAAGAATAAAGAGCTGGAACAATATGTTGAAAAGGAAAGAATCCT

ACAGGAAGATACTAACAAGGCTAAACAGGATGTGGAGACGATGTTAAAGGAGATAGAAGA

TCTCCGGTGTGTTGCTCAGTCTGAGAAAGAGAAGCTTGCTGAAGCAGCTGCTCAGACTAC

TTTACTCAAAGAGGAACTTACGAAGTCACAGAGTCGAGTACAAGACTTGGAGAAACTTCT

AGAGGAAAGTCGTAATGAGTGCCAGCATAAAGAAGAACATCTTCAACAGAAGAGTCATGA

AAACTTTGAACTTCAGCAGAGGATACAGCAAGCTTCCAATGACCTGGCTGAAACAACGTC

TTCTCACGCTCAGACCCACCAAGCAATGCAGGAGGAACAGGATGGGTTAAAGAAGCAGTT

GGAAGAGGAGAGAAGTTCTTCTGAGAAAAGAGTCGAGAGCTTTAGAAAAGAAATGGAGAG

CAAGTTAAAATCCCAGGAGACCAAGATGGAGAAACTCAGACAGAAAGCCAAAGAAATGCA

GGAAAAGTTCAAGAAAAAACTGCAAGAACAAGAGGAGAATGCGAAAGCAGAGATGGCAAA

GAAGGAGAAAGAACTGCAGCAGAAAGAGCAACAGGTGAAAGAGAAGATCCTGGAGATAGC

TCAAGCTAACTCTGAGGGTCTCAGCAGCGCTTTGTCAGACTTGGAGGCGAATCACAAGGA

GCAGTTTGAGAAGATTCAGAAGGTTCATAAGCTGGAGCAGGATGACCTTCTGCGTATTTG

GCAGGAGAAGCTGAGCCAGCAGGAGGAGGAGCTGCAGGAGAAGCATGCTCTGACGATGCA

AGAAAAAATGCAAGAATTGGAGGACATCTCTCAGCAGCTTTTGACCCACAGAGAGGAGAA

GCGACAAATGGATCAGGAAATAAAAAACCTTAAAGAGGAGCTTTCAATGAGGGAAACCAC

TGTGCAGAAGCTTCAGACTGAGCTCAGGGAAGCTGCAGGGAAACTGGACAGCCTGTCTAA

AGTAGAGGAGCTCCTCAAAACACAAGTGGAGACTGTAGAGAAAAACCTGAACCAGGCTTT

AAATGAAAGAAACATCTTTCAGGATCAGCTCACTGCAGCTGAGGAGACCAGTAAGGAAAA

AATTCTGGCTTTGTCTGAAGAGCTCGCAAACGCATGCAAGAAGCTCACTATTCTTGAAGC

TTCCAAAAGTAAAGAAGGAGAAAACCTACAAAAGAGCCTTGAGGAGAAAACGTTTGAGCT

TCAGAATAAAGAAAGCAAGTTTTTGGAACAAATCCATAATGTCGGTGAGGAGCTGGAGCA

GCACTGTCACACGGCACAGACAATCATCAATGATTTCTCAGCTCGCCTGTGCGAAAGAGT

CGAGACGAAGGTGAACGAGCTGCAGAGTAGAGTCATGTTCAACCAGAAGAAAGTGTGTCA

TTTAAAAAACCTCATTTTAACAAAGAACCACCGAATCTGTTGTTTAGAGCAAGAGCTTCA

GCAAGTCGTAGAGGAGAACCAGAAGCTCAAGAACTCACTCGAAGAAACAACTCTTCAGCT

CCATGCAAATTCAGAGAATCTCAAAGCCTTAAACGTGGAGAAGGAGACCCTGCAAAATGA

TGCTAAGAATCATTCGCAGTTACTTTCTGAGAAAGATCTTTCTATAGAAAAAACTCATGA

AGAAAACAAAAACTTATCCGAAAACCTTAAAGCAAATATTTTGCATATCAGTAACTTGGA

GAACGTCATAGATGACCTGAAGAAGCAGTTATCATGTAGCATAACTGAGAAGGAGGAAGC

CATATCTCTGCTGAATCAGCAGCACCAGGAGGAGAAACAGAGCGTGCAGAACCAAATGGA

GCAAGCAGAAAATGAAAAAGGTTTAGCTCTCAAGCAAGTCGAGACACTCAGGAATAAACT

GGCTGATTTTAAAAAGAAAACCGACTTCAAGATTTCTCAGAACCAGAACACGGTTAGATC

TTTGCAGCGCCAGATAGAGGACATGGAGAAGCAGATTGCGGAGAAGGACGAGCAGCTACA

AAAACTCACTGCCAGCATCGACAATCAGTGTATCAGCAAGTCCGAGCTGGACCAGGTGCT

GAGCGAGAAAGAGCAGAAGGTAAGTGCCTTAAATGTAGAACTGGATGACTGCACGAAGAA

GATAAGTGAACTGGAGCAGCGACTCAAATCACAGAGCATGGAACAGGAGAAGAAAGAAGC

TGAGCTGCAAAATCACGTCACCTCGAGCGAGATGGAAAAGACGGAGCTGATTCAGCAGCT

GGAGCAAACCAGGCAGCAGTGTTCTCTTCAACAGGACCTCGTCTCTAAAACCCAAGAACA

GCTCCATGCTTTGGAGAACGAGTTCCAAGCCGTCAAGCAGAAACTGGAGAGCCAGCAAGA

AGAATTTGAGCGGGAGAAGTCGAAAGAAGAGGCTTTGAAAGCGGCGGAAGGTAAAGTCGC

TGACCTGAAAAAGAAAGCTGAGCAAAAGATCAGCTTGATCCGTAAGCAGCTGACTTCCCA

GATCGAGCAGAAGGAGCAGACGATCAAAGAGCTGCAGGCACAGCTGGAGGACATCATGCA

GACGCAGAATGAGAAGGAGCAACAGGTGAGGAATTTGGAGGAGAATGGAAGGAAAATGGA

GGAGGCCATTAATAATCTAATGGAGGAACACAAAAAGCACCTGGAGCAGGTACTGAGTGA

TGGACAAGTAGAAAAAGAGTGCATGGACAAGGAGGATTTGGTTGCTGCTGCTGCTGCTGC

TGCTACTGCTACAGTAAATGACAGAGAGGAGGAGACCTGGTCAAGGATCAGAGATCTGGA

AGCCAAACTTCTTCAGTCTGAAGAACAGAACACCAGCTGCCGAGCTGAAATAAACCAATT

GAAAGAAGAACTCTGTGAACAGACTAGTCTGGTTCAGGAGCTTCAGAAGAGTCTGGAGGT

CCAGGTTAAAGAGAAAGAGACGAATGTCATTCAAATGGACAAATGCTTTGTGGAGAAAAG

CAACAATCTCTTAGGAATGGAACATGTTCAATATGTAGGAGACCAGAACACTAAATCACA

CCAAGACATTTGGGAGACGGAGAAGGAGCTCTTGGTGAAAGACTATGAGATGAAGCTCCA

AGACCTCCATAAGAGACTGGAGGAAAAGGAGAACGTACAGCTGGAACATAGTTTAGAGCA

AAAACTGCTAGACGCTGAAAACGAGAAGCAGAAGATTCATAAAGACTACACTCGCTTACA

GAAAGACCTTCGGTCTTTGAGACGAGAACATGAAAAGGACCTGGAATATCTGCGGAAAGA

AATCGCAGAAGAGAGCGAGAAAAAGCTGAAAAGTGACGTGGAGGATTTAGAAATGAAGCA

CAACTCAACCCTAAAGCAGATGATGAGAGAGTTTAACACTCAGATAGCGCTGAAGGATAA

AGAGCTGGAGACGTCAGTACGAGAGGCTGTGGAAAAAGCGCAGATGGTCGAAGCGGAACT

GATGGGAATCCATAAAGAAGAAGCCAATCAACTTCAACAGATCATTTCCCAGAAAGACGA

CGACCTCAAAAAAACCATCCAACGTTACGAGCAACTCCTTCAGAGTCGAGAAGAAGAAAT

GGGCAGTCGAGTGTGGGAGGTGCAGCAGGAGCTGGAGGAGCTGCAGCAGAGGAGTCAAAC

CGGTCCTCAGGGTGTTGATGAGCTTCAGGTGCAGCTCGCCGAGAAGACCACCCTGCTGAG

TGAAACCCGGCTTAAAGAGCAGGAGTACCAGGATCGGATCCACACTCTGGAGGACATGAT

ACGCAAGGCTTATAAGAACTCAGTGGTGACCCACCTGGGCAAAGATGTCTCACACCACAC

TGCGGAGTCCCTTAGCGAGCCCACCGAGTTGGAGTACCTGCGCAAGGTGATGTTCGAGTA

CATGATGGGCCGCGAAACAAAGACGATGGCTAAAGTCATCACGTCCGTGCTGAAGTTCCC

CGAGGATCAGGCGCAGAAGATTCTGGAGCGAGAAGATTCCCGACTGATGACTTGGCTGCG

>Unigene000122

CCATGCAGATCGGAAGGAGTAGGAGAGGGAGAGACGGCGACGTCGTACGGACTATAGACC

GCAGAAATCCTATAGATTTATATCAACACAAAAGGAGCCACACTGTTGTTGGAAGAGAAG

ACAAGAGAACGTGGAAACTGTACTTGGAGACCGTCTCACGTTTTCTTCTTTATTTCGACC

AGCGGCTGTAACGTTACTCAAGTTGTCCACAAGACCGAGTACGAAGAGTCAAGTAGCCGT

GTATTTAACCAAACCAGACGGTGAAAATACACAAAAACTATGTCTGGGAGTGAAGACGAC

AGAGATGATTTTGGACCGACAGAAGACCTGTCCATGATGCAAGACGATGACCTGGAGGAG

GACTTGTCAGAGAATGAAGCCCCTAAAGTAAAAAAGAAGAAAAAAGCAAAGAAGAGCAGC

CGTGAGAACAAAAGCAGCAAGAGGCAGCGCTCTCGTCGGGAGGACATCCCAATTAGTTCC

CCTGAGCCTGTAGAGGGGCGTGATGTGGATGAGGTAGAGGATGATGGGCCTGGAGGCCGT

TCTGACAGTGAGGGCAGTGATTACACACCTGGCCGGAAGAAGAAAAAGAGAGCCAGCACC

TCCAGAGACAAAAAAAGGAGCAGTACTGGTGCTGACAGAAGCTCCAGCTTGGCCGTCTCT

AAAAGAAAGGAGCCTGAGGAGGAAGATGATGAAGACGATGACGATAGCTCTGAGCCGAAG

ACCTCCTCCCAGCTGCTGGATACCTGGGGCATGGAGGACATTGATCATGTCTTTACTGAA

GAGGACTACAGATCTCTTACCAACTACAAGGCCTTCAGTCAGTTTGTCAGGCCTCTTATT

GCAGCAAAGAACCCAAAGATTGCGGTTTCCAAAATGATGATGGTACTTGGGGCAAAATGG

CGTGAATTCAGCACTAACAACCCCTTGCGTGGTTCTGCCAGTGCCAATGCTGCCCTGGCA

GCTGCTAATGTGGCTGCGGCTGTGGAGAGTATGGTTGCAGCTGAGACTCCAGCCACTGCT

CCTACACCCGCTCCCCTTGCTGAGCCCCAGCCCCCACCGGCTCCCCCACTCCGAAAGGCT

AAAACTAAAGAGGGCAAAGGTCCTAATGCTCGTAGAAAGTCCAAACCTACCCCAAAACCT

CAAGAGAAGAAGGTCAAGACTAAGAAAGTTGCACCTTTAAAAATTAAGCTGGGTGGCTTT

AACAGCAAGAGAAAGCGCTCGTCTAGTGAAGAGGAAGATATGGATGTAGACAGCGACTTT

GATGATGGCAGTATAAACAGCGTCTCTGTGTCGGATAGCTCCAATAGCCGCAGCAGCCGC

TCAAAAAAGAAGCCTAAGCCCAAGAAGAAGAAGAAAGTGGATGAAGATGCTGATGGCTAT

GAGACTGACCACCAAGACTATTGTGAGGTGTGTCAGCAGGGAGGAGAGATTATCTTGTGT

GATACCTGTCCCAGAGCTTATCACATGGTGTGTCTGGATCCTGATATGGAAAAAGCCCCT

GAAGGCACTTGGAGCTGTCCACATTGTGAGAAGGAGGGTATTCAGTGGGAAGCACGTGAG

GAGGCTTCAGATGGGGAAGAGGAGAATGATGCAGGTGGCGGTGAGGCAGAGGAAGATGAC

CACCACATGGAGTTCTGTAGGGTGTGTAAGGATGGAGGAGAGCTGCTGTGCTGTGATACC

TGTCCATCATCCTATCACATGCACTGTCTGAACCCGCCTCTACCAGACATCCCCAACGGA

CAGTGGATTTGTCCCCGCTGCAGGTGTCCTCCTCTAAAGGGAAAGGTCCAGAAAGTTCTC

ACATGGCGCTGGGGTGAAGCTCCGCCTCCCATCCCAGTGCCCCGCCCACCCGATCTTCCA

GTCAACGCCCCTGATCCGCCTCCTCTGGTTGGTCGAAAGGAGCGGGAGTTTTTTGTAAAG

TGGTGCAACATGTCATACTGGCACTGTTCCTGGGTACAGGAGCTACAGTTGGAGCTGAAT

TGTCAGGTGATGTTCCGGAATTTTCAGCGTAAGACGGATATGGAGGAGCCCCCGAGTGTG

GATGCGTCTGAGTGTGATGATAATAAGAGCATGAGGAGGAAGAGTAAAGACCCGCTGTAC

GCTCGCATGGACCACCAGTTCGGACGCTACGGAGTTAAAATGGAGTGGCTGCTCATCCAG

CGCATCCTAAATCACAGTGTGGATAAGAAAGGTAATGTTCATTATCTGCTGAAATGGAGA

GATCTGCCGTATGATCAGTCTGCGTGGGAGAGTGAGGACATGGACATCCCTGATTACGAC

ACCTATAAACAGCAGTACTGGAACCACAGAGAGATGATGATTGGGGATGAAGGACGACCA

GGGAAGAAGTTGAAGAAGGTGAAACTGCGAAAGATAGAAAAGCCTCCTGTAAACCCTGTG

GTGGATCCCACGGTGAAGTTTGACAGACAGCCGGAGTATTTGGACATGACGGGGGGGACG

TTACACCCCTATCAGTTGGAGGGATTGAACTGGCTGCGGTTCTCCTGGTCTCAGGGTACA

GACACTATCCTGGCTGATGAAATGGGTCTCGGCAAAACTGTGCAGACCGCCGTCTTCCTC

TACTCTCTCTTCAAAGAGGGTCACTCTAAAGGCCCGTTCCTGGTGAGCGCTCCTCTCTCC

ACTATCATTAACTGGGAGCGGGAGTTTGAGATGTGGGCTCCGGACATGTACGTGGTGACG

TATGTGGGCGATAAGGACAGCCGAGCTGTAATCCGCGAGAACGAGTTCAGCTTCGAGAAT

CACGCCATCCGCGGAGGAAAGAAAGCCTTCAAGATGAAGAAGGAGGCGGCGGTGAAGTTC

CATGTGTTGTTGACGTCGTATGAGTTGATCACTATTGATCAGGCGGTGTTGGGATCTATA

GACTGGGCGTGTCTGGTGGTGGATGAAGCTCACCGGCTGAAAAACAACCAGTCCAAGTTT

TTCCGGGTGTTGAATAATTATCCGCTGCAGCATAAACTCTTGTTAACGGGAACTCCACTG

CAGAACAACCTGGAGGAACTGTTTCACCTGCTCAACTTCCTCACTCCAGAGCGGTTCAAT

AATCTGGAAGGATTTCTGGAGGAGTTTGCAGACATAGCTAAGGAGGATCAGATTAAAAAG

CTGCACGACATGTTGGGACCTCACATGCTGCGGCGTCTCAAAGCAGACGTCTTCAAACAC

ATGCCCTCAAAAACCGAGCTTATTGTCCGGGTGGAGCTCAGCATTATGCAGAAGAAATAC

TATAAGTACATCCTCACACGTAACTTTGAGGCTCTAAACACACGTGGCGGTGGGAATCAG

GTGTCTCTCCTGAACGTGGTGATGGATCTGAAGAAGTGCTGTAACCACCCCTATCTCTTC

CCCACAGCTGCCATTGAAGCCCCCAAAATGCCTAATGGCATGTATGAAGGTGGAGCTCTC

ACAAAGTCTTCTGGGAAATTGATGCTGCTGTGTAAGATGTTAAAGAAGCTAAAGGAGGGG

GGGCACAGGGTGCTCATCTTCTCACAGATGACGAAAATGTTGGACCTGCTAGAGGATTTC

CTGGAGAACGAGGGCTATAAATACGAACGGATTGATGGAGGAGTGACGGGAGTAATGAGA

CAGGAAGCTATTGACAGGTTCAATGCTCCCGGAGCTTCTCAGTTTGTCTTCCTGCTATCG

ACACGAGCGGGGGGGTTAGGCATCAACCTGGCAACCGCAGACACTGTCATCATCTATGAC

TCAGACTGGAATCCACACAATGACATTCAGGCATTCAGCAGAGCTCACCGTATTGGTCAG

AACAGGAAGGTGATGATCTACCGGTTCGTCACTAAGGCCTCTGTGGAGGAGAGAATCACT

CAGGTGGCTAAGAAAAAGATGATGCTGACTCATTTAGTTGTGCGACCCGGTTTGGGTTCG

AAGGCGGGCTCCATGTCCAAACAGGAACTGGACGACATTTTGAAATTTGGCACAGAGGAG

CTGTTTAAAGACGACGCCGGAGAAGGTGATAATAAGGAGGAGGACAGCAGCGTGATTCAC

TACGACGATAAAGCCATTGATAAACTGTTGGACCGGAACCAGGACGCCACCGATGACACC

GAGCTGCAGAGCATGAACGAATACCTGAGCTCCTTTAAAGTGGCCCAGTACGTGGTCAAA

GACGAGGACGAGGAGGAGGTGGACAGGGAGATCATTAAGCAGGAAGAGAATGTGGACCCT

GATTACTGGGAGAAGTTATTGCGTCATCATTATGAGCAGCAGCAGGAAGTGTTGGCCAGC

CACCTGGGGAAGGGCAAGAGACCGCGCAAACCCGTCAACTACAACGACTGCTCGCAGGAG

GAACAAGATTGGCATGACGACCAGTCTGACAACCAATCAGATTACTCCGTTGCCTCAGAG

GAGGGAGATGAGGACTTTGACGAGCGCTCTGAAGCGAACAATCGCAGGTCAAGTCGTAGA

GGTCTGAGGAACGACAAAGAGAAGCCACTGCCCCCCCTGCTGGCCAGAGTGGGCGGGAAC

ATTGAGGTGTTGGGTTTTAACTCCCGTCAGAGAAAGGCCTTCTTGAATGCGGTGATGCGT

TATGGGATGCCTCCACAGGATGCGTTCACCACACAGTGGCTCGTCCGAGACCTTAGGGGC

AAATCTGAGAAAGAGTTTAAGGCATATGTCTCTCTGTTCATGCGTCACCTGTGTGAACCG

TGTGCTGACGGCGCTGAAACCTTTGCTGATGGAGTTCCTCGTGAGGGGTTGTCACGGCAA

CACGTCCTGACACGCATTGGTGTGATGTCACTCATACGGAAGAAGGTCCAGGAGTTTGAG

CATGTGAACGGTCAGTGGTCCATGCCATGGATGAAGGAACTGGAGGAGAATAAAAGAGCA

GTTGCTGTTGCTATGGGAGACGACCCCAAAACCCCCTCTACTGGAACCCCTGGAGACACA

CAACCCAACACACCTGCACCAGAGGACCTGTCCAGAGGGGAGGAGTCAGAACGGGAGACG

GACGACAAGATGGAGGGAGAGGGAGAGAAAGACAGAAAGAGCACTCAGCTACAAGATGAC

GAGGTGATTGAGATTCCAGACGATGGGGAGACCCCCCTGAGTCCAGAGAAGACAGGACCA

GGACAGGAGATGGAAAAAGACAGCACAGCGCCTTCTTTGGAGAAGGATGGCGAGATAAAG

GACATAGCGGGAGAAAAAGACAAAGAAAAGGGAGGGGCAAAAGAGGAAAGCACTACATGT

TCTTTAAAAATTGAAGAGGTGAAAGGGGAAAGGTCAGAGGTCACCAGTGCTGGGGACTCC

TCTCAGCCTAAAGAAGAGTGCAGTGAGGAGAAGAGTGAAAAGACAGACAGCAACCTAACA

GGAGAGGAGAAGAAAGAAGTGAAGGAGGAGAAGGATGGCGTGAGAGCGGAGGAGTCTCGG

ATAAATGGAGAGGACGGAGGAGTGGACAATGAGGAGAAGAAAAAACATGTCAAACAGAGA

TTCATGTTCAACATCGCTGACGGAGGATTCACAGAGCTCCACTCTCTGTGGCAGAACGAG

GAGAGAGCCGCCACAGTCACTAAGAAGACATATGACATTTGGCACCGTCGTCATGACTAC

TGGCTCCTGGCGGGAATCATACAACACGGCTACGCCCGCTGGCAGGATGTTCAGAACGAC

CCGCGTTTCTCCATCCTTAATCAGCCGTTTAAAGGAGAGATGAGCCGAGGAAACTTCCTG

GAGATCAAAAACAAATTCCTCGCCCGCAGGTTTAAGCTGTTGGAGCAGGCGTTGGTGATT

GAGGAGCAGTTGCGTAGAGCTGCGTATCTGAACATGACTGAGGACCCGTCTCACCCCTCC

ATGACTCTAAACACACGATTCAGTCAGGTGGAGTGTTTAGCCGAGTCCCACCAACACCTG

AGCAAAGAGAGCATGATGGGTAATAAACCTGCCAATGCTGTACTGCACAAAGTGCTGAAA

CAGTTGGAGGAGTTGCTCAGCGACATGAAGGCTGATGTCACGCGTCTCCCCGCCACCATC

GCTCGAATCCCTCCGGTTGCTGTGCGTCTGCAGATGTCAGAGCGCAGCATTCTCAGCCGT

CTGGCCAGTCGGGGGTCAGAGGTCAGCCAGACTCAAACACCCCACTGACCCCTCAGGGTA

ACCTGCTGACCCCTGAGTGACCTTCCCTGCTGTAGAGGCTGTGTTCTAATCCAGATTCAC

TTCACTAGCTCACTCCCTTTGCGCTGGACTGAGTGACTCAGACTCGATGCTACATTCACA

CCATCGAGTGACAATGTGACCTTTGACCTTGACTCTAGATTAGGCTGAAGGAGATAAAAG

GAGACAGAGGGCGGGAACGAGGTTTTTACTCTGTATCTCTGTACTTTATATCCAACTTCC

TTAGTGGTAAAGCGGCGGTGTGATAGCTTTAGTGAGAGTGACAGACTGAAATAGTGCAGC

GTGTGTGTGTGTGTGTGT

>Unigene000123

GGCACTTGACCACGTATCACTGAAATCTTTTTCGCTTTTGTCTCCTTTCATTTCTGCTCT

CTTCCACCAGTTGTCATTCTAGGCTGCCGGTTGGGGTGTTTGCACACGGCTGTGAATGGT

AGTGTCTATAAAGGGTATGTCCCTTCAGGAGAGAGGTGCCAATGTCCATCAGGATTAATC

ACACTGCAGGGGGGTCACTGCGCCGATCACAGAGGAACACACTCGCCACCAGGTTGCAGT

CCGAACAAACCAAACAGAAAACAAATTCCCCTCCTGAAAACAAAAGATCGTATAATAAAG

CTTCCAAGGCCCCTCCCACGCCTGCAGCCGGCTCCTCGCGTGGCCACGCCCCTCGAAGAA

ACAGCCTTTCTCTCGCTTCATTGAGTCAGTCTGCATCTGAGCCGGATTCGGAGGCAGCTG

GAAGCTCGGGCCAACGCGAGCAGCGTGAAGTCAGCAACAATCGCGCCCTTAAACGCAGCT

CTGCAACTGAGCACAACAACGCGTCCTACTCCCCTAGTCCGGCCAAGCGGCCCAAAGCTC

CTCCCCCTGAGAGCACAGAGCAGCTGGAAGCTGCTGACCCAACTGCTGACTCCGCCCCCG

AGCTCCCACCCTCATCCAGGTCTAAAAAGCGACGTCAAACAGACGAACATCCTACTAGCA

GGACAGTAAATAAGAAGAGCAGCGCGTGCCCTGCCTCTAGCTCCTCCCACAAACGGAAAA

AGGCAGACAACTCATTTTGCTCCTCTTCTTCCAGTTCATTCTCGTCCTCTTCCTCATTTT

TATCCGGCTCGAGCAGCGAGCAGGCCAGCCGCTCATCCAAACCCACCAAACTGGCGTCCA

AATCGGCCGCCTCGGCCAAATCTGTGACTGAGTCAGCCTCTTCCTCCTCTGCTTCTTCTT

CTTCCTTTACTTCCTCTTCCTCCTCCTCTTCTGTGGTGGTTGCGGGTGCCAGCGGCTGCC

CCCCGCCCTCGCAGGGTGCCCGGCTGAAGCAGGGAAAAGATCAGAGCAAGTCCCGGCGCT

CTCGCTCCGCCTCTTCGCCGTCTCCTCGCCGCAGCAGTCGGGATAAAGAGCACAGCAAAT

CCAGCACAGCAGCCTCCAAATTCGATTGGACGAGCCGCTTCAGTCCAAGAGTGAACTTGC

CGAAGCCCAGACTCGCCCTGCCCGGATCCTCCAAACCCGAAACGCCTTCAGGCTCCAGCA

AACCCGGCCCGAGCGGACTGCAGGCCAAACTCGCCAGTCTGAGAAAGTCCAGTAAGAAGC

GTAGCGAGTCTCCCCCTGCAGAACTCCCCAGCTTTAGGAGGAGCACACGACAAAAGACCA

CGGGCTCCTGCGCCAGCACCAGTCGGCGAGGTTCAGGTTTAGGGAAGCGTGGTGCAGCTG

AAACTCGCCGACAGGACAAAATGGCTGAAAACGACAACAGTCAGGATGGAGCCAATTCAT

CACGCACAGAGGAGACGCCTCAGGGAGCTTCAGCAGCCTCCAGTTCAGTAGCTGGCGCTG

TCGGAATGACCACATCCGGTGAGAGCGAATCGGATGACTCAGAAATGGGACGATTGCAGG

CTCTGCTGGAGGCTCGTGGTTTGCCCCCACACCTGTTCGGACCCCGTATGTCCCAACTTT

TCCATCGAACCATCGGCACTGGAGCAAGTTCGAAAGCTCAGCAGCTGCTGCAGGGTCTGC

AGGCGACGGGGGACGAGTCTCAGCAGCTGCAGGCAGCGATAGAAATGTGCCAGCTGTTGG

TGATGGGGAACGAAGAAACACTGGGAGGTTTTCCTGTGAAGAGTGTTGTACCTGCTTTGA

TCACGTTGTTACAGATGGAGCACAACTTTGATATTATGAATCATGCATCTCGTGCGCTAA

CCTACATGATGGAGGCTCTACCACGGTCTTCTGCTGTAGTTGTAGACGCTATTCCTGTCT

TCCTGGAAAAGCTTCAGGTGATCCAGTTTATAGATGTGGCTGAACAGGCTCTGACCGCGC

TGGAGATGCTCTCCCGAAGACACAGCAAGGCTATCCTGCAGGCCGGCGGATTGGCTGACT

GTCTGCTGTACCTGGAGTTTTTCAGTATTAACGCACAGAGGAACGCATTAGCCATTGCCG

CTAACTGCTGCCAGAGCATCACACCGGATGAGTTTCACTTTGTGGCCGACTCGCTGCCTC

TGCTCACTCAGAGACTCACACACCAGGATAAAAAATCAGTGGAGAGTACGTGTCTTTGTT

TTGCCCGGCTGGTGGACAACTTTCAGCATGAGGAGAATTTGTTGCAGCAGGTGGCATCAC

GTGATTTGCTCACAAACATTCAGCAGCTTCTGGTGGTGACTCCGCCAGTGCTGAGTTCAG

GCATGTTCATCATGGTGGTGCGCATGTTCTCTCTCATGTGCTCCAACTGTCCCAACCTCG

CTGTGCAGCTGATGAAGCAGAATATTGCAGAGACGCTGCGATTCCTGCTTTGTGGAGCAT

CTAATGGAAGCTGCCAGGAGCAAATTGACCTCATTCCCAGAAGCCCCCAGGAGCTCTATG

AGCTCACTTCCCTTATCTGTGAGCTGATGCCCTGCCTCCCCAGAGAAGGTATTTTCGCTG

TAGACTCGATGTTGAAGAAGGGCAGCGCACACACTACAGAGGCCGCAGTGTGGCAGTGGA

GGGACGACCGAGGCCTGTGGCATCCATACAACCGAATAGACAGTCGGATCATTGAGACGG

CCCACCAGAACGGTGAGGACGAGATCAGCCTGTCCACACTAGGACGCGTTTACACCATCG

ACTTTAACTCTATGCAGCAGATCAACGAGGACACAGGAACGGCTCGGGGAATTCAAAGGA

AACCCAACCCCCTGATCAACCCCAACACCGGTCACGTGGATGTGCGTGGCGAGGACGCGC

GTGCTGAGCTGATGCGGGAGGAAGCCGAGCTAGCCCGCTGCTTTATCAAGACTTTGTTTG

GCGTGCTGTATGAGGTGTACAGCTCGTCAGCCGGCCCTGCAGTCAGACACAAGTGCCTCC

GAGCCATCCTGCGTATCATCTACTTCTCCGACTCCGAGCTGCTCAAGGACGTACTGCGCA

ACCACGCCGTCTCCAGTCACATAGCCTCCATGCTGTCCAGTCAGGACCTGAAGATTGTGG

TCGGCTCTCTGCAGATGGCTGAGATCCTCATGCAGAAACTTCCTGACGTGTTCAGCGTGT

ACTTCAGAAGAGAAGGTGTTATGCACCAGGTGAAGAACCTGGCCGAGAGCGAGTCGTTCC

TCCCAAGTCCACCCAAGGCGTGCACAAGCGGCATCACAGCGGCAACCACCACCATCACCA

CTGCCACCACACCTGCTGCCTCTAGTGTCGCACCGGATTTGGGCTCACCGAGTTTTCATA

ACAACATGGAGGATTCACTGGACCTCAGCCCACAGGGTCGCCTAAGTGATGTTCTGAAAA

GGAAGCGACTTCCTCGCAGGGTACCGCGCCGTCCCAAATATTCTCCACCCAGAGATGATG

ACAAAGTGGACAATCAGGCTAAGAGTCCCACCACCACTGCGTCCCCTAAATCGTCCTTCC

TGGCAAGTCTTAACCCCAAAACCTGGGGAAAACTTAGTGCTCAGAACAACAGTACTAACT

CCGAACCACCTCGCACAGCCGGGGTTAGCGGCCTTGCACGCACAGCTAACAAAGACTCCG

TCTCTAATAACAGGGACAAGATTAAGGCATGGATTAAGGAGCAGGCCAGTAAATTTGTGG

AGCGATACTTCAATTCAGAAAACGTGGATGGCAGCAACCCAGCTCTGAACGTTCTGCAGA

GACTGTGTACTGCTACGGAGCAGCTCAGCCTGCAGGTGGATGGCGGTCTGGAGTGTTTAG

TGGAGATCTGCAGCATTGTGTCTGAGTCAGATGTTTCTTCGTTCGAGATTCAACACAGTG

GCCTGGTGAAGCAGCTGCTTATCTATTTAACCTCCAACAGTGAACGAGACCTCATCAGCA

GAGACCAGAGGATCAAACGCTTCCTGCACATTTTCTGTGGCAGTCCTCTTCCTGGGCAGG

AGCCACTGTTCCGGTTAGAACCAACAGCGAATGGAGCGCTGCTAGCACTGGTGCACAAAA

TGAACAGTTGCTTAAGTCAGATGGAGCAGTTTCCTGTTAAAGTGCACGACTTTCCTAGTG

GTAATGGCAATGGAAGCAGAGGGTCTCAAGCACTGAAATTCTTCAACACACATCAGTTGA

AGTGCCAGCTGCAGAGGCACCCAGACTGCACCAACGTGAAGCAGTGGAAGGGAGGACCTG

TTAAAATCGACCCTCTTGCACTAGTACAGGCCATCGAAAGATACCTTGTTGTTAGAGGGT

ACGGACGAATCAGAGAGGAGGACGAGGACAGCGACGACGACGGATCAGACGACGAAATCG

ATGAGTCACTTGCGGCTCAGTTCCTGAATTCGGGCAGTGTGCGTCACCGGCTACAGTTCT

ACATTGCTGATCACCTGCTGCCCTACAACATGACTGTGTACCAGGCGGTGCGTCAGTTCA

GCCTTCAGCCGGAGGAGGAGCGGGAGAGCACTGACGACGAGGCCAACCCTCTCGGTCGTG

CCGGCATCTGGACCAAAACTCACACAATATGGTATAAGCCGGTAAGAGAGGACGAGGATG

GCTCTAAGGACTCAGTAGGAGGGAAAAGAGGTCGAGCACAAACGGCCCCCACTAAAACCT

CACCACGTAACGCTAAGAAACAAGACGAGCTGTGGCACGATGGCGTGTGTCCGAGTGTCA

GTAACCCGTTGGAATCATACCTCATATCTGAACCTCCAGAGGGAATTACATTTGATGACC

CCTCACTGGAGGTCATTCTGCTGTTGAGGGTTCTGCATTCCATCAGCAGATACTGGTTCT

ACCTCTACGAGAATGCTGTGTGTAAGGAGATCATTCCCACTAGTGAGTTCATCAACAGTA

AACTGACAGCAAAAGCCAACAGACAGCTGCAGGATCCGCTTGTGATCATGACTGGAAACA

TCCCACCCTGGCTTACCGAGCTGGGCAAAACCTGTCCGTTCTTCTTCCCGTTTGACACGA

GGCAAATGTTGTTCTATGTCACCGCGTTTGACCGTGATCGCGCCATGCAGCGTCTGCTCG

ACACCAACCCCGAAATCAACCAGTCAGATTCGCAGGACAGCCGCGTAGCCCCCCGCCTTG

ACAGAAAGAAGAGGACGATAAACAGAGATGAGCTGTTAAAGCAGGCGGAGTCTGTCATGC

AGGATCTCGGCAGTTCCAGGGCCATGCTGGAGATTCAGTATGAGAATGAGGTGGGGACAG

GTCTTGGTCCAACCCTGGAGTTCTATGCCCTTGTGTCTCAGGAGTTGCAGAGAGCTGATC

TGGGTTTGTGGCGAGGAGAAGAGGTCACCCTCGCCAATCCTAAAGGGAGTCAAGAGGGTA

CCAAGTACATGTTTAGCTCTAGGGGTCTCTTTGCTGTGCCTTTCGGTCGCAGCACCAAGC

CAGCCCACATCGCCAAGATCAAGATGAAATTCCGCTTTTTGGGGAAACTCATGGCTAAAG

CCATCATGGACTTTAGACTGCTGGATCTGCCCCTCGGCCTGCCTTTTTACAAGTGGATGT

TGAGAAACGAGTCGTCGATAAGCTCTCACGATTTGGTGAACATCGACCCTGGTGTGGCCA

AATCCGTTCAGCACCTGGAGGACATCATTCGGCAGAAGAGGAGACTAGAGCAGGACAAGT

CCTATACTAGGGAGACACTACAACAGGCTCTGGAGAGTTTAAATATGAATGGCTGTTCAG

TGGAGGATTTAGGGCTGGACTTTACATTGCCAGGCTTCCCAAACATTGAGCTGAAGAAGG

GAGGCAAAGATGTCCCAGTCACAATACACAATCTGGAGGAGTACCTAAGGCTGGTGGTGT

ACTGGACCTTAAATGAGGGTGTGTCCCGGCAGTTCGAGTCATTTAGAGAAGGGTTTGAGT

CCGTTTTCCCTCTCCACCACCTACAGTACTTCTACCCCGAAGAGCTGGACCAGCTGTTGT

GTGGCAGCAAGTCAGAGACGTGGGACGTTAAAACTCTAATGGAATGTTGCAGACCGGACC

ATGGGTATACACACGACAGTCGGGCAGTGCGCTTCCTCTTTGAGGTGTTGAGTAGTTTTG

ATGCAGAGCAACAGAGACTGTTTCTGCAGTTTGTGACCGGGAGTCCGAGACTGCCTGTAG

GAGGTTTTCGGAGTTTAAATCCTCCTCTGACGATTGTACGCAAGACATTCGAGTCAACGG

AGAACCCAGATGACTTCCTGCCGTCAGTGATGACCTGTGTAAACTACCTGAAACTTCCGG

ATTATTCCAGCATTGAGATCATGAGGGAAAAACTGTTAATGGCGGCGCGGGAGGGCCAGC

AGTCTTTTCACCTGTCCTGATCTTACACATACTCACACATATGCACCGACACACACACAC

ACACACACACA

>Unigene000124

GGTGTTGGAAGAGGTGCTGAGACAGAAACGAGGAGAGAGTGACCGAAAGGCTGGAATGGT

GTGAGAGTAGAAGCAGACTCCTCCAGGGCTTTTCGTTTGCACTAGGCCACACCTCCAGGA

CTGAACCATGGATGACGACTTTGAGTGGCGCAGAGAGCAGAGGAGACAGAAGAGAGAGGA

GATGCGACTGGAGGCGGAGAGAATGGCCTACCGCCGGACTGAGGAAGATGAGGAAGAGGC

AGCTCGTGAGCGTCGGCGAAGAGCTCGCCAAGAGCGCATACGAAACAAGGAGAATGAGGA

GATAGCTAACCCAGCTGAGAGCGAAGGGCTGAACAGCCATAGTGTCACAGCGACAAAGAC

GGTGTCGAGCTCAGAGCTAAACACGGGCCAGGCCGGTGAGGGTGATGAGGAGGAGCAAGC

CCTGCTCGATCGTCTGGCCAAGCGTGAGGAGCGCAGACAGAAGAGGATGAAGGAGGCGCT

GGACAGGCAGAAACAGCTGGACTCTGTCGACGATCCGTCCTACAGTACCTACAGTGTGGA

GAGCACCTCCACCACCTCTTACCAAGCATTCAGCAATCAGGAGGACGAGCCGGCACTCAA

CAGCAAGGAGGAGGTGAAGGAGGAGTTGGTTCCACAGATGGAACTAGTGGAGGAGAAGCC

ACGCCGGTCTTACTTGAGGGAGCAGATGGAGGATGAGGACGCAGGAGGGAAGATCAAGCA

GCCGAACGGAGGAGTGTATGAGGACAGCAAGCACAAGAAGGTGGAGAGAACCCAAAGCCG

TGGCAGCGTGCACTCTTTCGAGGGCCCGGCAGCAGAGGAAGGAGACGACAGCGAGCAAAC

ACGCTTGGAAGCTGAGAGGAAGCTGGAGGAGCTAAAGAGGAGAAGAGACGAGACAGAGAA

CGAGGAGTTTGAGAGGATGAAACAGAGGCAGCAGGAGGCTGAGGTCGAGCTGGAGGAGCT

GAAGAGGAAGAGAGAGGATAGGAGGAAGGTGCTGGAGGAGGAAGAGAGGCTGAAAAAGCA

AGAGGAGGCTGAAAGAAAGGAAGAGAAGAGGAGGATGAAGGAGGAGATAGAGAGGAGGAG

AGCGGAGGCAGCTGAGCGGAGGCAGAAGGTCGGAGAAGAGGTAGACGGAGATGCCGGCAA

GCCTTTTAAGTGTGTGAGCCCACGAGGATCCTCTCTGAAGATTGGTGAAAGGGCAGAATT

CCTCAACAGGTCAGCACAGAAGAGCGCCGTGAAGACATCCCACTCTCCCGTGGTGTCAAA

GATTGACAACAGATTGGAGCAGTACACCACAGCTGTGCAGGGTAATAAAGAGGTGCGCTC

GCCACGCGGAGCTGCAATCGACTTCCCCGGGGGTTCTGATGGCGTACGCAACATTAAGAG

CATGTGGGAGAAGGGCAATGTTTTCAGTTCCACCATCAGCTCACCTTCCAGCAATAAGGA

TGCAGCTGGAATAAAAGTGGGCGTTGCAGGACGCATCAATGATTGGCTGAACAAAACACC

AGAGGCGAGCAAAACGCCAGGAGGAAGACCTGTGGACCTGAAACCGGGCGACGTTACCGG

CAAGCGCAATCTATGGGAAAACAAAAGCTCCTCTGCTACCAAGACCTCTTCCTGCTAGTT

GTCTTCAGATTTGGCTCCTTGAGGAGGTGACAAGCAGAGGAGAGAGCAAATCTGTTGCCA

ATGGAACGGGACCCTAACCTTTCCATGAAGCCACAGCACCAGGGACCAAGTGAAAGCACC

GTATTCCTCTTCACATGCATGCACTCGTACCAAAAAACAATAGTCTCATATGTTTTTATT

AATGGCTCGGACTTCACTATCCTAAAGATGAGGTAGAATCTAAAAAAAAAAAGAAAAAAA

AAAAAGAAAAGGAAACTGCTCCTGTCGTTTCTGTCACCTGACTGTCAGAAGAGGGGGATG

ATGAAAACGGCTGGATTACGGCGCTGCTGAACGAGACAGCGGCTTTGACAGCTAGTGAAC

GTTGCAGACTCCAGCAGCTCTGATTGGACGCTTGTGTCAGAATGACAGCAGAGGACTCTA

CTTCTGCCGTCACCATGAGTAACTCTAGCCACTCCTCCAAATCGAACCCTGCCAACAATC

ATGCAGTTCTTCACGGATCCATTACCGAAGGTGTTGCCGGAGCCCCTAATGAAGCGGCCC

TGATGGCTCTGATGGAGCGCACAGGATACAAGATGGTTCAAGAGAACGGACAGCGTAAAT

ACGGCCCACCGCCCGGCTGGCAAGGAGCGTCCCCACCGCGTGGCTGTGAAATCTTCGTAG

GAAAGATCCCGCGCGACGTTTATGAAGACGAGTTGGTGCCGGTGTTTGAGTCAGTGGGCA

GGATCTATGAGATGCGCCTAATGATGGACTTCGATGGAAAAAATCGTGGCTATGCCTTTG

TCATGTACACTCAGAAACACGAGGCTAAACGTGCCGTGAGGGAGCTGAACAACTACGAGG

TGCGACCTGGACGGTTGCTGGGGGTCTGCTCAAGTGTGGACAACTGCCGCCTGTTTATTG

GTGGAATTCCCAAGACCAAGAAGCGAGAAGAGATTCTGGAGGAAGTCTCCAAAGTGACAG

AGGGAGTGTTGGATGTAATTGTATATGCAAGTGCTGCAGACAAAATGAAGAATCGTGGCT

TTGCCTTCGTAGAGTACGAGTCTCATCGTGCAGCTGCCATGGCCAGGAGGAAACTCATGC

CTGGACGCATTCAGCTCTGGGGTCACCAAATTGCCGTCGATTGGGCCGAACCAGAAATCG

ACGTTGATGAGGACATCATGGAAACGGTGAAGATCTTGTATGTTCGCAACCTAATGATCG

AGACCAGCGAGGAGACCCTCCGCAAAATTTTCAGCCAGTTTAACCCAGGCTGCGTGGAGC

GAGTAAAGAAGATCCGCGACTATGCCTTTGTACACTTCACTAGCCGGGATGATGCGGTGG

TGGCTATGGACAACCTGAACAATACAGAAATTGAGGGCTCGTTCATCGAGGTAACGTTGG

CCAAGCCAGTAGATAAGGAGCAGTACACTCGCTATCAGAAAGCATCCAAGGGAACCATGA

CCAGTAGTGACAGTACTCAGCAAAATTTTGTGTATCAGTGTGATCCCTACACCCTTGCCT

ACTATAGCTACCCTTACAACACTCTCATTGGACCCAACCGAGACTACTTCATCAAAGCAG

GTACTGCACGAGGCCGTGGCCGTGCTGGAGCCACTAACAGAGGCCCAGGGCCTCGCGGCT

CGTACCTCGGTGGCTATTCCGCAGGTCGAGGCATTTACAGCCGCTACCATGAAGGAAAGA

CCAAGGTCCCGGACAAACCATATGAACTCATGTCCAGTCTGGAGCTGGCAGCTGTTAATC

CAGTGGGCATTAAGCCTGGCACAATGACTCTACCAGGCCTCGGTGCACAGTACCCCGCCA

TGTTCTCTGCTGCACCCGCTGCTACTAAACTGGTGGAGGAGGGCAAAATCCACCCAGTGG

AGCACTTGATCAACCCCCTCGGCCTTCCGCATGATCACCCAGCTGCTCCCTCGAGTGCTG

CCGTCATTCCTGCCGTCTCGACACCTCCGCCCTTTCAGGGTCGTCCTATCACTCCTGTTT

ATGCTGTGGCTCACAACGTCCCTCGGATCCCTGCTGCCGCCGCGGCTGCCGGCCTGTATG

GAGCGAGCTTCGTGCCCATTGCTGCTCATACCAACACAGCTGCTCTGGCAGCATTGCAGA

AGAATGCAGCTGTAGCGGCTGCTGCGTACGGAGGTTACGCCGGCTACATGCCCCAAGCCT

TCCCTGCTGCCACTTTCCAAGTGCCTATCCACGAAATGTACCCGACCTACTGACCCGTGT

TATGCCTTGACACAGTCTTGACCCGAGACACAGCATCAGATTTATGAAGGCTGTTGACTG

ATCAAACAAAACCCCCAAAACTTTAAGCGCATATATACACAAACAACAGTAACTTTCTAT

AAATATTATAAACTTTATATATACTCTGTAATGCCTCATAGCGCTCTAATAGTGTATTAC

ATATAATTTTCTGACAACCTAATAACCCATGCAAATGCCCTGCCCTGATTTTTATTGTAA

AAGTCCTTATTTTTGGTTGATGTCTCCTCATCCAAAATTACCATCTGGTGGTGCTTTCTA

TTTTAAGCAAAGTTGCAACTTTTAAGTGCATGAGGTGTTCATTTTACAATTGGGTTAAAT

TAGTACATTATCCTATTATGTGCAAATCTCCCTCCTAATGATGAAAGCACGACTCTACAC

CATGGTGTCAAACGCTGTATAAGTTTGGGATTTGGGGCACAACTAACCTTCAGTTGAAAA

TACGATAGCAGGAGACTTTTCTAATATAACGTTTTAATGTTTTTTAATGTCAACTTTTAT

TTGCAATGCCTCACAAAATTACGTGGACAGTGTTTAAAACTGTATGACAGAAAGCATGGT

GTCGAGTAAGCAGCCAGATGTAAAGCCTGGATTAGCGCATGTGCATTTCCAGATGTTTAA

AAATATCTGGGTTGTACAGTTTACTTCCTGAACTTTACAAATACGTTGCAGCAGGTGGAT

TGGCTAAGATACGTCACCCCAGATGTCAATACGTGTGTGTGTGAGTGAGTGTGTGTGCTT

GCAGGGTGTGTTCTCACCTTGCATAAATAGACTCCGGATCTGCCGTGAATAAAGCGGTTA

CTGAAGATAAATCCAACAAATGTTATTAGAGCGTTAGAGGCGTTGTTGGTGTTTTGTACT

GTTGTGCTGTGTATAGTGTGTTTAAAGATAAAATATGATCCACCAGACCTGCTGACAGGA

GACTTAGACCTAAATTAAGAAAAAGAAAACAGATTTTCACCTACGACTACTGAACTGTTC

CTGGAGATTCAAGCACAGGACACGGGATTAAATCTCTGTGGTCCAACAATTAACCAACTG

ACTACATGAAAAGCACCAGTTGTGGCCTGTGTGTATGCTACTCAATTTCCAAGATAAAAA

CCTCCTTCCTTATTGCAGATCTACTGATGCAGTCTCATCTCAAGCTCCACCCACTTGAAC

AAGATTCCCCTGTCAGCCAGCAGGCGAGGTGACAATTCAGGGGGGTTTAAAAAAAGCCCA

TCATTTGTGCCCATCAAGTGCCTTAAGTGCAAATATGTCACATCGTAAAACCACAACAAT

CATTCATTCATTACAACTATGACGTTTTTATTGCTGTCTACATTTAAAAAAAAAGTGGTG

TTTTTGCACTAATCGATCTCGACTGATTGGTTTTAACCATCCAAAATGAAGGGTGAATGG

AAAACCTGCGCTAAAGTGAAGGATCCGATCCCGCCGAGAGTCTATAAGCACTTAAATCAG

CGTCATATCAAAGCCAAACACCCCTTTAAGGAACACTTTACACTCCAAACATAGACTAGA

ACCTTCTGAAACCTTCGTCCTGTCTCTGCAGCTGTGAAAAATACCGAAGGATTCCTGTAT

GGACGATCACCCCAGCACGCTTGCTATAATAGATTGAAAAAATATAGAAAAACCTAAAGA

AAAAAAATATGTAAACTTGCTAAGCAGAGAAATATATGACCACTGAATATTATTGACATG

TCTGACTAATACTATTACATCGTTTTCTTTAATAGGATATTATAGTGTTAGATTTCCTTG

TGGCCTGTTTTATTTTCACTGTTGTTTAAAGGTTTTTCAGACGTTCTTTAAAAATGTTTT

TGAGAATTTTATAAAGCCTAAACGTTGGAGACAAAGCCAATAGTTTGAAAAAAATTGCAA

CATGGGAACCAAGACAAAACTCCAGACACTGTGTTAAAGCACACATACTGTTCACTATAG

AGCCTTAATATAAATACATTCAGCCTTTATAGTTTAGACATTATAGATTTGCAGAACTGC

TTTTTTGTAAACTCGAGACTGAAGACTGACGTTCCTTATTTTAAAAAATATATACAGCTT

CGTTTAGATCAGTCGTTCCAGGCTCACACTTTTGTTTAATGCTATTGTTAGAGGTTGAAT

TTCCAAAAATGTTATTTCTTGTCAAAAACCCAAATTTGTGACTACGACATAGCTGCTTAA

TTATTTAATGTTGTTATAATAAGATGTAGCCATATAGGCATTGAAAACCGTTTTCTGGAC

ACGCTACTCGTGGTACAGGAGCCTGAGCAGGGAGCTGACTAATGAGTTGACGTTAATTTC

AATTAATTACAAAGAAGCTTTAGACAGATTTCTTCTTTTTTCCCTTTTCTGCTAATTGTT

AAACCTAATATTAAACATGGCATTTCACAACATACTAAATTGTATGCACAAGTTATAGCA

CACCTAACTTGTGGGCACTAAGATGAACTTGTGGCCTCAATTTATTATCGTATGGCTTAG

ATATACTAATAATATCCACCATGGCGCGTCAGAGGATTTGTAGATTCCCCTTTCTTTGTT

TCTGGCAC

>Unigene000125

GATGTACCGTGTGGACGTAACAGCGGTGATGTCGTTGAAAGCGCGTTGTGCTTTTTTGTA

ACGAACTTCGGTGTCCTGTTAGATAGTACAATAGATTTAATTAAAACCGTGAGTTTTTTT

ATTCGAGTTTTCGCAAAGGATTTTTCGGAAAAAATAATTTTACTATTGTAAAATAACAGT

CCGTCTTTTTGTTTGCTTCAGTCTGTTGAGAATTAATCACGATTGAGAAAACAAATCATG

GCGGATGTTACAGCGCGTAGTTTACAGTACGAGTATAAAGCGAACTCGAACTTGGTGTTG

CAAGCAGACCGCTCACTGATCGACCGCACGCGCCGGGATGAACCCACTGGCGAGGTTTTG

TCTCTGGTTGGAAAATTGGAAGGAACAAAAATGGGCGATAAAAGTCAAAGAACAAAACCA

CAGATGTTGGAAGAAAGACGAGCAAAGAGGCGAAAGCGGGACGAGGACAGACATGACATT

AATAAAATGAAAGGTTTCACGCTCCTGTCTGAGGGAATTGATGATATGGTCGGTATTGTT

TACAAACCCAAAACAAAAGAAACCAGAGAAACATATGAAATCCTCCTGAGTTTTATTCAG

GCTGCACTCGGAGATCAGCCGAGGGACATCCTCTGTGGAGCTGCTGATGAAGTCTTGGCT

GTATTGAAAAATGATAAGATGAGAGATAAGGAAAGGCGGAGGGAGGTGGAACAGTTGCTT

GGCCCTGCGGACGACACACGGTACCATGTTTTAGTCAATCTTGGCAAAAAGATCACAGAC

TACGGAGGAGACAAGGAGCTCCAAAATATGGATGATAACATTGATGAAACATATGGAGTA

AATGTGCAGTTTGAATCTGATGAGGAGGAAGGTGATGAAGATCAGTTTGGAGAGGTTCGT

GATGAAGGCTCTGATGACAGTGAGGGTGAGGAAGCAGATGTGGGCTGTACTCTGTCTGCT

AATTTGGGAAGCGCCGGAGACGTGATGACAAATAAAAAGAAAGACCTTCATCCACGGGAT

ATCGATGCCTTCTGGCTCCAGCGTCAACTTAGTCGCTTCTACGATGACGCTATTGTGTCT

CAGAAAAAAGCAGATGAAGTTCTGGAGATTCTTAAGACTGCAAGTGATGATCGAGAATGT

GAAAATCAGTTGGTGATTTTGTTAGGCTTTAACACCTTCGACTTTATTAAGGTTTTACGG

CAGCACAGACGGATGATCCTGTACTGCACCATGTTGGCGAGTGCTCAGAGTGACTCAGAG

AAAGAAAGAATTATGAGTAAAATGGAAGCGGATCAGGATTTATCTCAGGTTCTCTATCAG

TTACAGGAGACGGAAAAGGAAGACATAATTCGTGAGGAAAGATCACGAAGAGAAAGAGTT

CGAAAGTCTCGTGTGGACAGTGATCTGGACTCAATAAACATTGATCATGGACAGTCGATG

CCAGCGCGGCATCTGCTTGATCTGGAAGACCTTGCCTTTACCCAGGGCAGTCACTTCATG

GCCAACAAACGCTGCCAGCTGCCTGACGGTTCATTCCGCAAACAGAGGAAAGGTTATGAG

GAGGTTCATGTACCTGCACTTAAACCCAAACCCTTTTCTGAGGATGAGGCTCTTGTGGCT

ATTGAGAAGATGCCAAAATACTCTCAAGCTGCGTTCGAGGGGTTTAAAACCTTGAACCGC

ATCCAAAGTAAGCTGTTTAAGACCACAATGGAGACCGACGGAAACCTGCTGGTGTGCGCT

CCCACTGGAGCTGGTAAAACTAATGTTGCTCTAATGGCCATGCTCCGTGAAATTGGCAAG

CACATCAACATGGACGGCACAATCAACGTAGATGACTTTAAAATGATCTATATCGCTCCT

ATGCGTTCACTGGTGCAGGAAATGGTTGGCAGCTTTGGGAAGCGTTTGGCCAGTTATGGT

ATAACAGTGTCTGAGTTAACTGGAGATCACCAGCTTTGTAAAGAAGAGATCAACGCTTCT

CAAATCATTGTGTGCACGCCAGAGAAGTGGGATATCATCACCCGCAAGGGAGGGGAGCGC

ACATATACCCAGCTAGTGCGCCTCATAATTATCGATGAGATTCACTTGCTTCATGATGAT

CGTGGACCAGTGTTGGAGTCTCTAATAGCTCGAACGATCCGAAATGTAGAATTGACTCAG

GAGGACGTGCGTCTCATTGGTCTTAGTGCCACACTGCCAAATTATGAGGATGTGGCCACA

TGTCTTCGTGTAGATCCTGCTAAAGGACTTTTCTACTTCGATAACAGTTTCCGTCCAGTG

CCACTGGAACAGACGTACGTAGGAATCACTGAGAAAAAAGCAATCAAACGATTTCAGATC

ATGAATGAAATCGTCTACGAGAAGATAATGGAACATGCTGGAAAGAATCAGGTCTTAGTA

TTTGTCCATTCACGGAAGGAGACGGGAAAAACTGCCCGCGCAATCAGGGACATGTGTTTG

GAAAAAGATACATTGGGACTTTTTCTCCGAGAAGGTTCTGCCTCCACCGAAGTCTTAAGA

ACGGAAGCGGAGCAATGCAAGAATTTGGAGTTAAAGGATCTGTTGCCGTATGGCTTTGCT

ATCCATCATGCTGGCATGACTCGAGTGGACAGAACGTTAGTGGAAGATCTTTTTGCCGAC

AGACATATACAGGTACTGGTGTCAACTGCTACCCTGGCCTGGGGAGTTAATTTACCAGCA

CATACTGTCATCATTAAAGGTACACAGGTTTACAGCCCAGAGAAAGGCAGGTGGACAGAG

CTTGGAGCACTGGACATTCTACAGATGTTGGGCCGTGCGGGCCGTCCACAGTACGACTCT

AAAGGCGAGGGTATCCTGATCACCTCACATGGAGAGCTGCAGTATTACCTGTCTCTACTT

AACCAGCAGTTACCTATAGAAAGTCAAATGTTGGCCAAACTTCCAGACATGCTTAATGCT

GAGATTGTGCTGGGAAACGTTCAGAATGCTAAGGATGCAGTGAACTGGATGGGTTACACT

TACCTGTATGTGCGGATGCTGCGTAACCCGACCCTTTATGGTGTTTCCCATGATGAGCGC

AGTACTGATCCCCTGTTAGAGATGCGTCGAATGGACCTGGTGCACACGGCCGCCACTGTA

CTGGAAAAAAGCAACCTGGTCAAATATGATAAAAGATCCGGGACCTTCCAGGTGACGGAT

CTGGGCCGTATTGCCAGTCACTTTTACATCACACACGATTCTATAATGACGTACAATCAG

CTGCTGAAGCCCACACTTAGTGAAATTGAGCTCTTCAGAGTGTTCTCCCTCTCCTCTGAA

TTCAGGAACATCACTGTCAGAGAGGAAGAAAAGCTTGAGTTACAGAAACTTTTGGAAAGG

GTACCAATTCCAGTGAAGGAGAGTATTGAGGAGCCCAGTGCTAAGATAAATGTACTGCTT

CAGGCCTACATCTCCCAACTCAAGCTGGAGGGCTTTGCTCTAATGGCTGACATGGTTTAT

GTTACTCAGAGTGCAGGTCGGCTGATGAGGGCCATCTTTGAAATAGTTTTGAGTCGAGGT

TGGGCACAGCTCTCTGACAAAACCATGAACCTCTGCAAGATGATCGACAAAAGGATGTGG

CAGTCGATGTCACCTCTGCGACAGTTTCGCAAACTTCCTGAAGAGGTTATTAAGAAAATT

GAGAAAAAAAGCTTTCCATTCGAACGATTGTATGACCTCAACCACAATGAAATCGGTGAA

TTGATTCGTATGCCTAAGATTGGGAAAACCATTCATAAGTATGTGCATCAGTTCCCAAAA

CTGGACCTGGCAGTGCATCTTCAGCCCATCACTCGGTCCACGCTTAAAGTGGAGCTCACA

ATCACACCAGACTTTCAATGGGATGAAAAAATCCATGGATCATCAGAGGCGTTTTGGATC

TTGGTGGAAGATGTTGACAGTGAAGTGATCCTACACCATGAATATTTCCTGCTTAAGGCA

AAGTATGCTCAGGACGAGCACCTGGTCACCTTTTTTGTGCCTGTGTTTGAGCCGCTGCCC

CCTCAGTACTTCATCAGAGTGGCCTCAGACCGCTGGCTGTCATGTGAGACTCAACTGCCA

GTATCATTTCGCCACCTGATCTTGCCTGAGAAGTATCCACCTCCGACAGAGCTGCTGGAC

CTACAACCTCTTCCTGTATCTGCCCTGAGAAATTCCGCCTTTGAATGCCTGTACCAAAAC

ATGTTTCCTTTTTTCAACCCTATTCAGACTCAAGTGTTCAATGCTGTGTACAACAGTGAC

GATAATGTGTTTGTTGGAGCCCCTACTGGAAGTGGGAAGACCATTTGTGCAGAATTTGCT

ATTCTCAGGATGCTGCTGCACAATTCAGAAGGCCGGTGCATCTACATCACACCCATGGAG

TCTCTAGCTGAACAAGTGTTTGTGGATTGGCACCAGAAGTTTCAGGGTCTTTTGAATAAA

AAGGTTGTTATGCTCACTGGAGAAACCAGCACTGACCTAAAGCTACTGGGGAAAGGTGAC

ATTATCATCAGCACCCCAGAAAAGTGGGATATTTTGTCCCGCCGATGGAAACAGAGAAAG

AATGTGCAGAATGTCAGTCTTTTTATTGTGGATGAGGTTCATCTGATTGGAGGAGAAAAC

GGGCCTGTCCTAGAAGTAATCTGCTCAAGAATGAGATACATCTCGTCTCAGATAGAGCGT

CCTATCCGCATTGTGGCTCTAAGCTCTTCGCTGTCTAATGCCAAAGATGTGGCTCATTGG

TTGGGTTGTAGCACAACAGCAACTTTCAACTTTCACCCAAATGTCAGGCCTGTACCGTTG

GAGCTTCACATTCAGGGTTTCAACGTGAGTCACACACAAACACGTTTACTGTCGATGGCC

AAACCTGTTTACCACACAATCATGAAGCATTCCCCCTCTAAACCTTCACTGGTGTTTGTA

CCGTCTCGGAGACAGACTCGTCTGACTGCCATCGACATACTAACTTTCTGCGCCGCTGAT

GTGGTTCCACAAAGGTTTCTGCACTGCTCGGAGAAAGAATTGGCACCATTTTTGGACAAG

CTGTCTGATTGTGCTCTGAAAGAGACCCTGTCTAATGGTGTAGGTTATCTACATGAAGGA

CTGTCTATGACCGAACGCAGGACAGTTGAACAGCTTTTTAATTCAGGTGTAATTCAGGTT

GTGGTGGCCTCTCGTTCTCTTTGTTGGGGAACTAACATCTCCTCCCACTTGGTCATTGTA

ATGGACACACAGTATTACAACGGAAAAATCCATGCTTATGTGGATTACCCAATCTATGAT

GTGCTGCAAATGGTGGGAAAGGCCAATCGTCCAATACAGGATGATGAGGGCTGCTGTGTC

ATTATGTGTCAGGGATCTAAAAAGGACTTCTTTAAGAAGTTTCTTTATGAGCCTTTGCCA

GTTGAATCTCATTTGGACCACCGACTTCATGATCACTTTAATGCTGAAATAGTCACGAAG

ACAGTGGAAAACAAACAGGATGCGGTGGACTACCTAACCTGGACTTTCCTTTACCGACGA

ATGACCCAGAACCCCAATTATTATAATCTTCAGGGTATGTCTCATCGTCATCTGTCTGAC

CACCTGTCTGAATTGGTGGAAAACACACTGCATGATCTGGAGCAGTCCAAGTGCATCAGC

ATTGAGGATGAAATGGATGTTGCCCCTCTTAATCTGGGGATGATTGCTGCATACTACTAT

ATCAACTACACCACCATTGAGTTATTTAGTATGTCTTTGAATGCCAAGACTAAAATCCGT

GGTCTGATCGAGATCATATCCAATGCAGCCGAGTACAAAAACATACCAATCAGACACCAT

GAAGACGCTCTACTTCATCAGCTAGCCCAGAAGGTGCCTCATAAATTGAACAGTCCCAAA

TTTAATGACCCTCATGTGAAGACAAACCTGCTGTTACAGGCCCATCTGTCCCGTATGCAA

CTGAGTGCTGAGCTTCAGTCTGATACTGAAGAGATTCTCAGTAAGGCGGTACGTCTCATC

CAGGCGTGTGTAGATGTGCTGTCCAGCAATGGCTGGCTGAGTCCAGCATTAGCTGCTATG

GAGCTAGCTCAGATGGTGACCCAGGCCATGTGGTCTAAAGACTCTTATCTAAAACAGCTG

CCTCACTTTACAACAGAGCACATCAAACGCTGTACAGAAAAGGGTGTGGAGAGTATATTT

GACATCATGGAGATGGAGGATGAAGACCGTATAAATCTTCTGCAACTGTCAGATGCTCAG

ATGGCTGATGTGGCACGTTTCTGTAACCGCTACCCTAACATAGAGCTCTCCTATGAAGTG

GCTGACAAAGAGGACATCAAAAGATTCGGGTATTCATAACCAGTACCACGTTATGGAGAG

TATTATTGATATTTGCACACAAGAAAGTATCCAGTGGTAGTCCAGTTGTTGTTCAGGTAC

AGCTGGAG

>Unigene000126

GTTAAGGGCCCGACAGTGACTGTTTAAACCAACGCCCGATAAATAGATACTAAATTGGGT

GTGATATTCTCGTAAAGGAGAAAAAATGAATGGTCACAAAGCATTTCAGTAAAATGGCCT

CCAATGACTCAAACATCAATGAAGAACTAATGGAAAAGCCCGGTTGTTCCACTGAAATCC

CCAAAGCCACATCGCGCTCAAAAAGGAAACCCGCAGTTGTAACGAAGCACACAGCCGTAA

ACGAGCCAGAGTCAGCGGATGAATGCGAGCAGGACTGCACCGCTGATGATCCCAATGACA

CAACGATCGAAATGGAGGAGATCCCAGAAGATGTAGTTATAGTAAAACCGGAACCCGTCA

GCAATGAAGGAAAAGATGAATTTAAAGGCCCTGAATTCCGCAACAAAGGAGGGAGCAAAG

TTAAAGAGGAGGGTCAGAGGAGGCGTCCTGATGAGCACCTTCAGGAGATCCGTTGTTCTC

GAGTTAATTGCACTGCGTGTGGGCAACAAGTCAATCATTTCCAGAGAGACTCTGTGTATC

AGCACCCCATACTCAAAGTTCTTCTCTGCAAATCCTGTTTCAAGTATTACATGAGCGACG

ACATCAGTAAGGATGAGGACGGCATGGATGAACAGTGCAGGTGGTGTGCCGAGGGAGGTA

ATCTCCTCTGTTGTGACTATTGTAGCAACGCATTTTGCAAAACGTGTATTCTCCGTAATC

TTGGCCGTAAGGAACTGTCTGGAATAATGAACAACGAAGACGAGAAGTGGCATTGCTATG

TGTGCCATCCCGAGCCGCTGGAAGACCACGTAGCTGCTTGTGAGAAAGTTTTGAAAAACC

TAGAATGTATGAAAAAGCCCAAGGCAGAACAGGACAAACCGAAACGGGACGGCATAAAGC

ACAAACAGAGCAAAGTTAAAAATTCAGCTCTTAACGGCAAAGATGTAAATTCGGATGGAT

CAGGGACGCTAACCTTTTCCTACAAAACGTTAAACGTTCCAAAAGAACTGCTGAAAAAGA

CAAAGAAGCTAATTGAGACGACCAACGGCTTGAACAGCACTTTTGTGCAGTTTATACAGC

AGGAACCGGTCTACCTTGACGAAAACATTGTCCGGCACAGACACTTGAAAGCCTTTAGAT

CTGTTTTGGCCGATTTAAAAAAAGCCCATGCGACTTTAGAACAGGCTCTAGAAGAGGAAT

TTAAAGCCCTAGAGGTTGTGAATGGAGACGGGAACGGGTTAGCTGATAACCCGCCTGTAG

CTGTCACAGTTCCTGACTCTGAGCCTGCTGAACAGAGTAAAGATGATCAGGTGGTTGATC

AGACAGTTCAGGATAGTACCACAGATCAAGTTTCAGCAGCATCAGAAGACGAGGTGGACG

AGGATGAGGTGAACGCAGTTCAAGCTGACGTAGAAATGGCCGAAGATGTCGTCCCTGCAG

CAGAAAATCCGGAGGAAAAACTTACAGAAAACGAAGCAAATGCAGAAGTTGTCCAGAGTA

AACCTGACTGTGAAGACGATGCTTCAGCAGCTGGTGAGGCCTCTCTTGACAAAGATATCG

TGTCTATACCGCCTTCTGTTCCCGACGAACTTTTTGAGATGGTTGAGAGTCTTTCAGACT

GCGTCAAACAAGAGAGTGAGAAGGACTCTGTTGATACAGACAGTATGTCGAGTAAGAACT

CGGAGTCTGATCAAAAGCCAAAATCTGGTAAAAGGTCTGCAAAGGTGAGCAAGAAACTTG

TGGTTAAGCTCACACCGATTCCTCTTAAAATCACCATTAAAAAAGATGAAAGCAAGTCAA

AGTCGCAGGAGAAGGAAGGTGCTTTGTCACCAGATGAAAGCAGACGTTCCCCCAGGATGA

AGACCACTCCGTTGCGCAAATCATCAGAAAGGAGAAGTAAAAGCCAGTCTGAGGATACAA

GAGCGAATGACGACGAATGCGACGCTTCAGAGACTCCAGTCGAAACTGGCGATCCAGACT

CTGATTCGGACGAAGTCCCCGAGGTGCTGCGGCAGGTAGAAGACGAGGAGGCCACTTCAG

ACGAACCGGACGCAGAGCCTAACGGCAAGGAAGCAGGCAATACATCGCCCAAAAAATCAG

CTGAACAATCCTCTAAAAAGACAGCAAAGCGCAAGCTCCTCGCATCTTCTTCGGATTCAG

ACAACACAAAGTCAAGAAAAGCAAAGAACGCCGCCAAAAAGAAGCGTCGAGAATCCGATT

CATCTAATCACGACTCTGATCTCGAGAAAGAGCTCAAGAGTTTAAGCAGGCTCCGCACAA

CAAAGAAATCCAAGCCAGGCAAGAAACAGACCACAGAAGACGAGGAAGAGGAGGAGAAGG

AAGGCAAGAAGACGACGAAAAAGAAGAAGGAGGAAGAGACCAAGAAGGAGGGTAAAAAGG

GGCCAAAAAGGTCGTTCGAGAGGAAAAGGAGGTCACAGAAAGGGAAAGAAAAAGCCACCA

AAGATTCCTCCTCGTCCAGCGATGAGGAGGAGGAGGAGGAGCAGGAAGCAGATTCTGGAG

AGGAGAGCGGTGATCAGCAGAAGATTAAACCTATTCTTGAGTCTGCCTTGTCGCACATGG

ATGCATTCCAGCAGTCATCAGGAGATGAAGCTCAAGTGAAGGCAGAACCCTCTCAGATGC

TTGATGACGACGACGATCCTGAGAATAGAATTGCAAAGAAGATGCTGTTAGCCCAGATTA

AAGCTAATTACTCATCGGGGGATGACAGCTCTTCAGAAGATGACGACAGCAAAACCAACA

AAGAAGAAAGATCTCCGACGAAAGCGAAGAACGATGCCAGCTCTGAGGGTGAAGAGGAGG

AGGACTCTGACTCTGACTCTGGTTCGGAAGTGGACGTGAAAAAGGCCGGGCGGAGACACA

AGCTGCTTCGCAAGAAACTGACACTAAGTGAGGGCGAGTCCGACACTGAGAAACCAGCCA

AGAGCAAGAAAGAGGTTAAGAGAAGAGGGAGACGAAAAGCAGTGAACAGCGATGACTCTG

CCGATTCAGACTTTAAGCGTTCAGGCTCTGGATCCCGGAGCGAGTCGGCGATGAGTGATT

TTGTGAGTGAGGATGATGAAGACAACAAAAGCAACAAGCGGAAAACACGGTCCTCTAAAA

AAGCTGACAAAGACGGCGAGAGGAGCTACCAAAAAGAGAAAAAGAAAAAACGCCGGCGGA

TTAAGGTTCAAGATGATTCCTCCAGCAATAAGAGTGCCGAGGAAGGGGACGAGGACAAAG

AAGAGGAAGATGAAGAAGGGGACGATAAGGGTACGCCGAAGGGCCGCAAAAAGATCCGCA

AGATCCTTAAAGATGACAAGCTGCGTTCCGAGACCCAGAATGCATTAAAAGAGGAGGAAG

AGCGACGAAGACGAATAGCTGAAAAAGAGCGGCTGAGAGAAAAACTGCGAGAGACAATCG

TGGTGGAGGAGTCCTCGCAGATCACCTGTCCAATTACAACCAAACTGGTGCTGGATGAGG

ACGAAGAGACTAAAGAGCCACTCGTACAGGTCCACAGAAACATGGTGACCAAGCTGAAAC

CCCATCAAGTGGACGGTGTGCAGTTCATTTGGGATTGCTGTTGTGAGTCGGTTAAAAAGA

CTAAGAACTCCTCTGGCTCAGGCTGTATCCTCGCTCACTGTATGGGTCTGGGCAAAACCC

TTCAGGTGGTGACGTTTCTCCATACTGTGCTTCTATGCGAGAAGCTCAATTTCTCAACGG

CTTTGGTGGTGTGTCCTCTCAACACCGTTCTCAACTGGCTGAACGAGTTTGAAAAGTGGC

AGGAGGGCTTGAAAGATGAGGAAAGCTTAGAGGTGACCGAGCTGGCTACAGTGAAGAGGC

CTCAAGAGCGAGCATACGCTCTTCAACGCTGGCAGGAGGACGGCGGCATCATGATCATCG

GTTACGAAATGTACCGCAACCTCACGCAAGGCCGAAACATCAAGAGCAAAAAACTGAAAG

AGACATTCCAAAAGACCCTCGTTAACCCAGGTCCAGACTTTGTTATTTGCGACGAGGGCC

ACGTCCTGAAGAATGAAGCCTCGGCCGTTTCCAAAGCCATGAACTCCATCAAGACTCGGC

GAAGAGTAATTCTGACCGGAACGCCGCTTCAAAACAACCTGATCGAGTACCACTGCATGG

TGAACTTCATCAAAGAAAACTTGCTGGGTTCAGTGAAGGAGTTCAGGAACCGTTTCATTA

ACCCAATTCAAAATGGGCAGTGCGCCGACTCCACGATGGTCGACGTGCGGGTAATGAAGA

AGCGAGCGCACATTCTCTATGAAATGCTGGCGGGATGTGTTCAAAGAAGAGACTACACTG

CACTTACTAAGTTCCTGCCACCGAAACATGAGTATGTGCTGGCAGTGCGAATAACTCCTG

TGCAGTGCAAGCTGTACCGATACTATCTGGAGCACTTCACAGGTGTCGGTGCTGCCTTGG

AAGGAGGAAGAGGCCGTGCAGGCACAAAGCTTTTCCAGGATTTCCAGATGCTCAGCAGAA

TCTGGACTCATCCATGGTGTCTGCAGCTGGACTATATCAGTAAAGAAAACAAGGGTTACT

TTGATGAAGACAGCATGGATGATTTCATTGCGTCTGAAACAGAGGAGTCCTCCATGAGCC

TGACCTCTGAAGACGAGAAAGCAAAAAGGAAGAAGAAACGAGGGAAAGGCAAAGGCGACA

GCTCTGACAAGTCTGACAGTGACGACGTGGAGGTGATCAAGGAGTGGAACACGAGCTCCC

GTGGGGGAAACCCAGAGGGGCGGAACAGAGCTGAACCTGTAGAGGAAGTTCGTGCCAGCA

GCTCAGGCCCTGGCAGCCCGTCTCCTGATTGGTACAAAGAGTTTGTGACGGAAGCGGACG

CGGAGATTCTGGAACACTCCGGAAAAATAGTGCTTCTGTTTGAAATTCTGCGCATGGCAG

AAGATGTGGAGGATAAAGTGTTGGTGTTTAGTCAGTCGCTTATTTCACTGGACCTCATTG

AGGATTTTCTGGAGTTAGCAGGCCGAGCCAAAGAGGAGGGAAAACCCTCTCCTTACAAAG

GTGAGGGCAAGTGGTTCAAAAACATTGACTACTACCGCCTTGACGGTTCTACTAATGCCA

TGACTAGGAAGAAATGGGCTGAAGACTTCAACGACACCAGCAACGTCCGGGGTCGTCTTT

TCCTCATTTCTACAAGAGCCGGCTCTCTAGGGATCAACCTAGTGGCAGCAAACCGGGTCA

TTATCTTTGATGCCTCGTGGAACCCGTCTTATGACATTCAGAGCATTTTCAGGGTGTACC

GCTTCGGCCAACTAAAAACAGTATACGTCTACAGGTTTTTGGCTCAGGGCACCATGGAGG

AAAAGATTTACGACAGGCAAGTGACCAAACAGTCCCTGTCCTTCCGCGTGGTGGACCAAC

AGCAGATTGAGCGACACTTTACCATGAACGAACTGACTGAACTCTACACGTTCGAGCCCG

ATCAGCTGGATGACCCATCGGAAAAGAAAAGCAAGAGATCCACACCCATGTTACCCAAGG

ATCCTGTTCTGGCTGAGCTGCTTCACAACTTCAAAGATCACATTGTGGGTTACCACGAGC

ATGATTCCTTACTGGACCACAAAGAGGAGGAGGCTTTGAGCGAGGAGGACCGTAAAGCCG

CCTGGGCTGAATACGAAGCAGAGAAGAAGGGTCTCTCTATGCGCATCAACCAGCCGGCAT

ACGCTTCTGTAGGCATCGGATCAAACTACCTCCCCTTCAATGTGGCAGCTCTGGCCTCAA

TGTCCAACCAGCAGCTGGAGGACCTGATCAATCAGGGACGACAGAAGGTGATGGAGGCCA

CTAATGCTCTGAGGACTTTTCCCAGGGAGCCCCTTGAGGACATTATCAGCCAGGTGTGGA

AGGAGAATCCCAATCTGTCCGAGTCCCAGGTGCAGGGGATGGCTCTGGCCAGACAGGCAG

CCATCGACATGGAGCTGAAACACAGAGAGGCCACGTACAGAGATGCTCTCAATAAGCAGC

AGACGCTGATGATGTACGTTCAGAAGGTGATCAACAATCGTAAGGTTCAGGAGCAGCAGC

TGGCGCTTGCCAGGCATGGGATGCTGATGAACCAGATGGCCATGCAGAATGGCATCGCCG

GGGGAATGAGTCAGATTGACCTGCTGGGCCTGTACCAACAACTGGGTGCCCTCCAGGGGC

TGCCAACTTCCCAGATAGGCAAGAACCCAGGACCCTCAAAGGGTCTGTAGTGCCCTACGT

TTCCTGTGTGGGCATTCTAACCTAACCAGCTCGGACACTTTTTGCCCCTTTTTTGAGCGC

TCGTGTAAAAATCTGGGATGGAAATATCTGGAAGACTTTGGCAGTATGCTTTGTAATTCT

TTGTATATACAGCCTTAGCGTTTAGTTGTTTTGTTTTTTTTTTTCTCTCTCGTTTCCTTC

AACAT

>Unigene000127

AGGTTGGGAGGTGTGAGGTGTGCCGTGTGTAGGCTTGATTTGTGTAGGCTGGAGTTAAAA

AAAACGCTTGTGTGTGACTCGCTTGTCGTTAGACACATCTAAGACTTGAATGCTTGTGCT

ACAAATTGCAGTCAGAACAAGTGCGATGGCCACGTGCTCTCAGCAACAAATCATATTAAA

GTTATGTTTTAAATCTGCTAACTGTTTGAGTGTGCCGTTATTTTTTTTTTTAAAGGTGTT

TACCAGCTTGCACAACAAAGGAACGGATTTGTGACCTAAGCTCTGGATGAAGTTATTGAT

ATTGATTGTTTAGCCTCAGCATCTCTTTTTTTCTCTGTTGTGAAACTCAAAGGAAACTGT

AGAAATACGGGTTTGGCAAACTTTACAGCAGCAAACGTTCTGCTGGAAAACGAAAGATCT

CCGTTTTGTTACATGGTGCATGTGTGTGTAAGGGACCTCTTTGTCCTTTAACCTTTATTT

GTGCTGCTGGAGACACTGTCCTGACTGTCACTTCTCTTTTAGAATGGTTTTACGCCACTT

CACATCGCCTGCAAGAAGAACAGGATCAAAGTGATGGAGCTGCTGCTGAAACATGGAGCG

TCCATACAGGCAGTGACAGAGTCTGGACTCACGCCGATTCATGTGGCCGCCTTTATGGGG

CATGAAAACATAGTGACTCAGTTGATGAATCATGGTGCATCGCCAAACACAACCAACGTG

AGAGGAGAGACTGCGCTCCACATGGCCGCCAGGGCTGGACAGGCGGGTGTAGTGAAATAC

CTGGTGCAGAACGGAACTCACGTCGACGCCAAATCTAAGGATGACCAGACTGCGCTGCAT

ATCTCTAGTCGTCTGGGAAAGCCCGAGATTGTACAGCAGCTGCTGCAGCATGGAGCCGCA

CCCGATTCCACCACCACATCCGGATACACACCATTGCACCTGGCAGCCCGTGAAGGCCAC

AAAGACGTCGCCGCTATACTGCTGGACCAGGGCGCTTCACTGAGCATCACTACCAAGAAG

GGATTCACGCCGCTACATGTGGCAGCTAAGTACGGGAAAATAGAAGTCGCCAACCTCCTG

CTGCAGAAGAGAGCTCCTCCAGATGCGTCGGGAAAGAGCGGACTAACTCCACTGCATGTA

GCTGCGCACTACGATAACCAGAAAGTGGCACTTCTTCTCCTGGACCAGGGGGCTTCTCCC

CATGCAGCTGCCAAGAATGGCTACACTCCTCTACATATAGCTGCCAAGAAGAATCAGATG

GAGATCGCTACCACACTGCTGGAATACGGCGCTGATACCAACGCGGTCACTCGGCAGGGC

ATCAGTCCGCTGCACCTGGCTGCTCAGGAGGGCAACGTGGACATGGTCACCCTACTGCTT

GCACGTGATGCCACCATCAATCTGGGTAACAAGAGCGGACTCACTCCCCTTCACCTGGTG

GCCCAGGAGGATAAAGTAAATGTAGCTGAAGTCTTGGTTAACCAGAGTGCTGCTGTGGAC

CCTGAGACCAAGATGGGCTACACTCCGCTGCATGTCGCCTGTCACTATGGCAACATAAAG

ATGGCGCACTTCCTCCTGAAGAACCAGGCTAAGGTCAACGCCAAAACGAAGAACGGATAC

ACTCCTCTGCACCAGGCTGCACAGCAAGGCCACACACACATCATTAACCTCCTCCTGCAG

CACGGAGCCTCGCCCAACGAGCTGACGCTGAATGGAAACACAGCACTGTCCATTGCCAGG

CGACTGGGTTACATTTCAGTAGTGGACACCCTCAAAGTGGTGACAGAAGAGACCCTGACC

ACCATGACGGTGACTGAGAAGCACAAGATAAACGTCCCAGAAACAATGAACGAGGTTCTG

GACATGTCTGATGATGAAGTTCGTAGAGCCAATGTCCCCGAAATGCTCAATGAGGACTAT

ATTTCAGATGTTGAAGAAGGGGAGGATGTGATGATGGGGGACACGGATAAATACCTGGGT

CCCCAGGATCTGCGTGAGCTAGGGGACGACTCTTTGCCTCAGGAAGGTTACGTAGGATTT

AGCATTGGAGCCCGATCCGGAAGCCTGCGCTCCTTCAGTTCGGATAGGTCCAACACACTG

AACCGGAGCTCTTTCACCAGGGACAGCATGATGATCGAGGAGATCCTGGCACCCAGCAAG

GACACGCATCTGACTGTAGCTAAAGACTATGACACTGACTCTCTGAGGCGATACAGTTGG

ACAGCAGATGCTTTGGACAATGTCAACTTGGTCTCCAGCCCAGTCCATTCAGGGTTCCTA

GTGAGCTTTATGGTGGACGCTCGTGGTGGCTCCATGCGAGGCAGCCGCCACAATGGGATG

CGCATCATCATCCCACCACGCAAGTGCACTGCCCCGACTCGCATTACCTGCCGCCTGGTC

AAGCGACACAAGCTTGCCTCGCCACCACCCATGGTGGAAGGAGAAGGACTGGCAAGCCGT

CTGGTGGAGGTGGGCCCTGCCGGAGCTCAGTTTCTCGGCCCTGTGATAGTGGAGATCCCT

CACTTTGGCTCCATGAGAGGGAAGGAGCGTGAACTGATCATTCTCAGAAGCGATAACGGA

GAGACTTGGAAAGAGCACCAGTATGACTGCAAGACCGAAGCCCTTAACGAGCTGCTTAAC

GGCATGGATGAAGAACTGGACTGCATAGAAGACCTGGAGAAGAAACGCATCTGTCGAATC

ATTACAAAAGACTTCCCACAGTATTTTGCCGTGGTGTCACGGATCAAGCAGGAGACCAAC

CATATGGGTCCTGAGGGAGGGGTTCTATCCAGTACGACTGTTCGCAAAGTCCAGGCTTCT

TTCCCTGAAGGAGCGCTCACAAAGAAAATCAGGGTTGGACTTCAGACCCAGCCGGTTCCA

GACGAAACAGTAAAGAAAATCATAGGCAACAGAGCCACGTTTAGCCCCATAGTGACAGTG

GAACCACGCAGAAGGAAGTTCCACAAACCCATCACCATGACTATTCCTGTACCTCCTCGA

TCAGGGGAAGGCATGAACGGCTACAAAGGAGACACAACACCTTGCTTACGTCTTCTGTGT

AGCATCACAGGTGGCACCTCCCCCGCACAATGGGAAGACATCACTGGGACTACTCCGCTC

ACCTTCACGAATGACTGTGTCTCGTTCACTACCAACGTGTCTGCTAGATTCTGGCTCGCA

GACTGCCATCAGATTCCGGAGACAGTTTCTTTGGCTACTCAGCTCTATAGGGAACTCATC

TGTGTGCCCTACATGGCTAAATTCGTAGTGTTTGCCAAAATGAATGACCCTGTGGAATCC

CATCTGCGATGCTTCTGCATGACGGATGACAAAGTGGACAAAACCCTGGAGCAGCAAGAA

AACTTTGAAGAGGTGGCCAGAAGCAAAGACATTGAGGTTTTGGAAGGAAAGCCTATCTAT

GTGGACTGCTACGGCAATTTAACCCCTTTGATTAAAGCCGGACAGCAGCTGGTTCTTAAC

TTTTATGCATTTAAAGAGAACAGGCTGCCATTCTGTGTAAAGGTGAGGGACAACAGCCAG

GAACCGTGCGGACGGCTGTCCTTCCTCAGAGAGATTAAAAACGCAAAGGGGCTTCCCCAA

GTTGCTGTCTGTAATTTAAACATCACTCTGCCCGCGCTCAAAAAGACTGAAAAGCCAGAA

CGACGTCATACCTTTGCATCTCTAGCCTTACGTAAGCGGTACAGCTATCTGACTGAGCCT

GGATTGAGCCCTCAAAGTCCTTGCGAGAGGACAGACCTGCGAATGGCTATAGTGGCTGAT

CATCTCGGCCTTAGCTGGACTGAGCTGGCCAGGGAAATGGATTTCTCTGTTGATGAGATA

AATCACATCCGCGTGGAGAACCCCAACTCGTTGACCGCTCAGAGTTTCATGCTATTAAAG

AAATGGGTCAACAGAGACGGTAAAAATGCCACAACGGATGCCTTAACTGCAGCACTGACA

AAGATCAATCGGATGGATATTGTGACATTGCTGGAAGGGCCAATATTTGATTATGGTTAC

CACAACATCAGTCTGGAGTTGCAGAGTCCCTCAGAGTTGTCCTATGAACCCCCCACCCCT

CTCCGGCAGGATGATTTCTTTAGCGAGGATGGGGGTATTGTAGTCTCGCCCAGTAGAACC

CCTGTTAGACCAAATGAACTGTCCTTACCAACCACATCTAGGGATGCGCTTTCCCCCAAT

GCAGCACCTCCTACTGTGGTGGCAGAGGATTCCTCTATTGGAGGAAGAGGCTCCTATAGT

CGAGAAGGCGGCTCAACTGAAGACGACCTGGACGCCCATGACAGTGATGAGATGCCTAAA

CATCCTGAAGTGTTCATGCAGAGCACATACCAAGAGCAAGAGGTTCCTTCCAACGGAGCT

CACAGGGCTAGAGAAAGTGGCAGGTCTGGTTTTGACGAGGAAGAGGAGGAAGAGATGACG

CAGGAGAAGTCCTGTTTTAAAAGCATAAACTTGGAGGAAGGTTTAGAGATTGTGGAGATA

CCCCAAGAAAAAGTGCAGGAAATTCGCTCACAAGTGGAACAGGCAAAAAAAGAGATGTGT

TCGCTTGCAGGATTGCAAGAAGGGCCGTCCAGTGTGAGCCAGGAGATCTCCGCTTCGGGT

CTGCACACTGATACCCAACTGCAAGATAGAAGTCAAAAGCCAACTGAAAGGCAAAATGGG

GAACATCCAGAGCTCCAAGGCCGAGCCGGCTCACTGCTTGAGCCACAGGAACCATCTAGT

AAGATGAAGGGCAAGGGCAGCAGAGACTCCTTAGATGACACAGCTGCTGGGCCGAGCACC

ATGGACGAAGACACAGTGGAGCCGAAAGCTAAGGTCGGAGAGGGAGACAAGAAGAAAGAA

GGGAGACAACGAGGGAAAAAGGGTCAATCGTAACAGACGGGTGTCCTAAATGAGATCTGA

ACTTGTTTCATCATTGTTTTCCTGCGAAAAGCATCACTGGAAACCCAACGCAGGAGATCA

GGACAGGGGATTGTGCGTCTGCAGCCTTTACATAAACGCATGGTTTTTAGAACGAGGATT

TTATGATTTCGCATGAGCATGGAAAACCTCCGATTTTCTGTCAACGACACATCCAAGCAT

CTGATCGGTGCCAAGAAATGACACTTAAGCTCAAGACTGTCGGTGGCATAAAGCTTCTAG

TCAGTCCTATACATTGGAGGAGTGCCAGCGACTACCAACATGGCACCGAAGCATTCCATG

ACCAAAGATGGTACAATGAGCATTGTGGAATGATTATTTTTTAAAAGAAAAAAAAAAAAA

AAAACCACCTTCTACACTCGCCTTATTTTCTCTCCCCCCACCCTCCCCCTCGCTTCTCGG

ACTAGCGTCGACGCTGTTTTTAAAAAAGATCCTGTATATAAATCAGAACTGTATGAAAGT

AGCTCACGAAATTGTGAACGCAGGAGCAAAGAGCGAGTCACAAACGGAATGAAAAAAAAA

AAGAAGAAGAAAGGAATGCTGATATAACTATTCCGACTTTAAAGATCATTTTAAAGACGA

ATTGATTACACAAAAAAGCGAAAAGAAACTTTTTACACAAAAAAAAACAAAAAACAAGTT

TGCTTGATTTCAACGTTTTTAAGCCTACCACAAAAACGACAAAACATCGAACGACATAGC

TGCAGTTGTGTTAAAAATTTACTCCGAATACTTATATTTTGATGCATGTGATTCACCTTA

TTTTTTGATTTGTTTCCTATCTACATAACCAGAGGTGTGGTAAATGTTCTACCTGTGTGC

ACACTCATAGCCTTGGTGTTTGTCACTAATACTGTTTTATAATATCACTGTACCACAACA

GACTCACTGGAAGTGAATGGACACACAATTCTCGCAGCTAAAGGTTTCTACGCTATGCAG

TCAAAGACGAGCTTGGAGTGTAAGAGAGACCGAAAGAGAAAGAGCGTCGTGTCAACTGGC

ACAGCGTTCAGTTTTAATGGGTTTTGTGTGTATCGGCACTAAAAGGTCTTTCTGTAAGCC

AACCGTTTGTAACCATTCTAACAGTGTTACTAGGCTGCTGTGGACATAAGGGGCATTTTT

TTATTTGGGTTTGGTGAAATATTTCCTTATATTGAATATATTCAAAAAAAAAAAAAAGAG

AGCGAGAATGAAAGAAAGATCACCTTCAGGTATTCGTTGATGAAATGATCTGGTAACAGA

CTTGTATGAGAAAGCGTACACTGGATGATTGAATTAATTATTTAAGCATAAAATAAATTG

TGTTTAAACTCAAATTGTGATGTGAACTTTGATCTGGATGATGTGGTGCATTTGTGCGAG

AAAACTGTGTCTGCATTTGTCTGTCCATCGATCTATCTGTTCATCCATCTGTCCATCTAT

CTATATACAGTATCTATTTAGCTAGCTACACACACACACACACACACACA

>Unigene000128

ACACACACACACACACACACCCAGGACATCAGGGAGGTTTAGAGGAGGAGGAGGAAAAAA

AGTTTTAGTAAACTGTCGTGTGTAGAAGAAGCGAGGAGGCCACAGGAGTTCCTCCGACAC

TTTAATTTCAGGAACTAGTACGAACCCTCACCGCGGATAGAGACAGAAGCTGTGGATTTG

TGTGAACTTCTGGGCAAACATGTCAGTTTCAGATGAAGCAGACGGGCCGCGACCGCGATA

TGGCTCCGTGCTGGACGGCGTGGAGCGACTCACAGCCGAGGAGATGGACGAGCGGCGACA

GCAGAACATGGCCTACGAGTACCTGTGTCACCTGGAAGAGGCCAAACGCTGGATGGAGGC

TTGCTTGGATGAGGAGCTCCCCCCTACCACCGAGCTGGAGGAAGGTCTGCGCAACGGCGT

CTACCTGGCAAAGCTCGGCAACTTCTTCGCCCCACAAGTCGTCTCCCTGAAGAGGATCTA

CGACCGTGAACAGACCCGCTACAAGGCAACCGGACTCCACTTTAGACACACAGACAATGT

GATCCAGTGGCTGAACGCTATGGCTGATAAAGGCCTTCCCAAGATTTTCTATCCTGAGAC

CACAGACATCTACGACAGGAAGAACATGCCACGCTGCATTTACTGCATCCATGCACTCAG

CCTGTACCTGTTCAAGCTTGGTTTGGCCCCGCAGATCACAGACCTGTACGGCAAAGTGGC

CTTCACAGAGGAGGAAATTAACAACATGAAGATCGAGCTGGAGAAGTACAACATCCAAAT

GCCGGCTTTCAGCAAAATTGGCGGAATCCTGGCTAACGAGTTATCCGTTGACGAGGCAGC

GTTGCACGCTGCAGTGATCGCCATTAACGAAGCCATAGACCATGGAGACCCTGCAGGCAC

TCTAGCGGCCATGAAGAACCCCAATGCCATGCTGCTTAACCTGGACGAAAGCGCCGCCCA

GCACTACCAGGACACGCTGTACCAGGCCAAAGCAGAGAAAGTGTGCAATGCTAGGAAAAG

GATCGGGGAGAATGTGAACGCCGAGCGGGATGTCTACGAGGAGCTGCTCACTCATGCTGA

AATTCAGGGCAATGTCAACAAAGTTAACTCGTCTAATTCCCTGGCTTCAGTGGAGCAGGC

GCTATTCAGCGGTGATGAGGACAAGCTTTACAAGGCGCTTTCATCACCTGCGCTGGGTCT

GCAGTCCCTCAAGCCGAAGAACAAAGGCTGGTACCTCAAACAGCTGGTGGCGGATCGGGA

AGCTAAGGAGCAGAACAACCCAGGAGAGTCGTTGACCAAAGAAGAGCTGCAGAACGGCGT

GGAGGCGGCCAATAGCGTCGCAGAGAGCTATCAGAAAATGCTTCAGGCGGTACAGCGGAT

TAATGCTGCCATCAGAAAAGGTGTAGCTGAGGAAACTGTGACTGAGCTGATGAATCCAGA

AGCCCAGCTTCCAGCCGTTTACCCCAGAGCCGCAGATCTGTACCAGAGGGAACTCTTCAG

TCTGCAGCAGCAAAGCCCAGAGGGTTCGCTCACACACCCGGAGCTGTTGGTTGCAGTGGA

GATGCTGTCGTCGGTGGTGCTGATAAACGAGGCTCTGGATGTAGGAGACCGAGGCGCCGT

GTGGAAACAGCTGAGCAGCTCCGTTACAGGTCTCAGTAATGTGGAGGGAGAATACGCACA

GAGGTACATAGACGAGTTGATGCGACTAAAGGCTGCAGCGCGAGAAGAAGGCAGCGATTA

TCTGACGTGGAATGACATCCAGGCGTGTGTGGATCAGGTCAACACCACTATTCAGGAGGA

GCACGAGAGAATCGCTGCTATCGGTCTGATCAACGAGGCTCTTCATGAGGGAGACCCAAA

GAAGACGCTGGAGGCCTTGCAGCACCCTGCAGCCAAGCTGACAGATGTGGATCCGTCAAT

AGCTCAGCATTATTTCGACAAACTCCTGGAGGCCAGGAGAGAGAAAGCTCACGAGACTCG

GGATCCATCTGCGGTGCTTTGGTTGGATGAAATCCAGGAGGGCATCCTCCAAGCTAACAA

AGACACTGAAGAGGCCCTGCAGTTCTCTCAGGCAATCCAGGCCATTAATGAGGCAGTAGA

CAGCGGGGATGCGAGCCAGACGCTGGCAGCTCTGCGTGCTCCGGGTGCTAGTCTGTATGG

CGTGACCCCAGAGTGCGCTCAGACATATCAGAATGACCTGACCAAGATCAAGAATGACAA

AACGGAACAAGGTGATAACGGTAGTAAGTGGATGAAGCACTGGGTGAAGGGAGGCTATAA

TTACTACTACAACCTGAATACTAAAGCGGGCACATGGGACGAGCCTGCAGGATTCGTGCA

GAACAACACACAGCTAAACAAGGACGACATTCAGTCTGTCGTATCAGGAGTAACTGCAGC

TTACAACCGTGAGCAGTTGTGGCTGGCCAATGAGAGCCTAATAGCTAAACTGCAAGCACG

ATGCCGAGGTTTCCTGGTCAGAAAAAATCTTAGAGAGCGGCTCGACTTCCTGCGGTCTCA

GGATCCAGCCATCAGAAGCATCCAGGCCCACTGGAAAGGTTACAAAGAAAGAAAAACCTT

CAATGAAAGAAAGCAGTTCCTGAAGGATCACACCGAGGAGGCAGTTAAGATCCAGTCGAT

GGTTAGGATGCACCAAGCTCGCAAAAAATACAGAGATCGTCTCAAATATTTCAGAGACCA

TATCAATGAAGTGGTGAAAATTCAGGCGTTCATCAGGGCTAACAAAGCAAGAGATGACTA

CAAGACACTCATCAATGCAGACGATCCTCCCATGGCGGTTGTGCGTAAATTTGTGCACCT

GCTGGACAGCAGTGATCAGGACTTCCAGGAAGAGCTGGAACTGATGCGTCTGAGGGAGGA

AGTTATCACCAACATCCGCTCCAACCAGCAACTGGAGAATGATCTCAACCTGATGGACAT

CAAGATCGGTCTTCTGGTAAAGAACAAGATCACCTTGCAGGAGGTCGTCTCGCACAGCAA

GAAGCTCACTAAGAAGAACAAGGGTGAGCTGTCCAATCTTATGATGATGAACAAGCAAAA

AGGAGGCCTGAAGGCTCTCAGCAAAGAGAAGCGAGACAAGCTGGAAGCCTATCAGTACCT

TTTCTACCTGTTGCAGACAAATCCCACCTACCTGGCCAAGCTCATTTTTCAAATGCCTCA

GAACAAGTCCACCAAATTCATGGATTCTGTAATCTTCACGCTGTACAACTATGCATCCAA

CCAGCGTGAAGAGTATCTGCTGCTCAAACTGTTCAAAACAGCACTGCAAGAGGAAATCAA

ATCAAAAGTGGACCAGATCCAGGAGATCGTAACTGGAAACCCCACCGTCATCAAAATGGT

GGTGAGCTTTAACAGAGGAGCTCGTGGTCAAAACGCTCTGAGGCAGATCCTGGCTCCGGT

CGTAAAAGAAATCATGGAGGACAAGACGCTAAACATCAAAACGGACCCGGTGGACATTTA

CAAGGCCTGGGTGAATCAGATGGAGTCTCAGACTGGAGAAGCCAGCAAATTGCCTTATGA

TGTCACACCAGAGCAAGCCATGAGCCACGAGGAGGTCCGAAACCGCCTGGAAGCCTCCAT

TAAGAACATGAGGACAGTGACGGACAAATTCTTGTCCGCCATCATAGTTTCTGTAGACAA

AATCCCCTATGGAATGCGTTTCACTGCCAAAGTGCTGAAGGATACGCTGAAAGAAAAGTT

CCCCGATGCCACAGAGGATGAGCTTTTAAAGATTGTCGGAAACCTGCTGTATTATCGCTA

TATGAACCCTGCCATTGTTGCCCCCGATGCCTTTGACATTATTGACATGTCGGCTGGAGG

GCAGCTGACCACTGATCAGAGGCGGAACCTGGGCTCCATCGCCAAAATGCTACAACACGC

CGCCTCTAACAAGATGTTTCTTGGAGACAACGCCCATCTCAACCCTATTAACGAGTACCT

TACAAACTCCTACCAGAAATTCAGACGTTTCTTCTTGGCAGCTTGCGACGTCCCGTCACT

CGAGGATAAGTTCAACGTGGATCAGTACTCTGACCTAATCACCGTGTCCAAGCCTGTTAT

TTACATCTCCATCGGCGAGATCATTAACACACACACGCTCTTGCTAGATCACCAGGATGC

CATCGCTCCTGAGCACAATGATCCCATTCATGAGCTTCTGGAGGATTTGGGAGAGGTGCC

AACTATCGAGTCTCTGATAGGGGAAAATCCACTTCCTCCTGATGACCCTAACAAGGAAAT

GATGGGCAAGACGGAAGTCTCTCTCACCCTTACCAACAAATTTGACGTTCCTGGGGAAGC

CAATGCTGAAACGGACGCCAAGACCCTCCTTCTGAACACCAAGAGGCTCATTGTGGACGT

AATCCGATTCCAGCCAGGAGAAACGCTGTCTGAAATCCTGGACTCTTCAGCAACCCCGGA

GCAGGAAATAGAGTACCAGCGGGCCATGCAGAGACGAGCCATCCGAGACGCCAAAACACC

CGAAAAAATGAAGCGGGCCAAACCGGTGGTGGACGACAGTCTCACACTGCAGGGCAAAAA

GGACAAGATTAAAAGCAACCTGCAGAGACTGGCTGAGCTGGGGAAAGTACAGCCCGAGAA

CAGATACCAGGATCTTATCAATGACATTGCTAAGGACATAAGAAACCAAAGGAGGTATCG

ACAGAGAAGAAAGGCCGAGTTGGTGAAGCTTCAACAGACCAACAGCGCCCTTAACACCAA

GACCAGTTTCTACAACGTACAAATCGACTACTACAATCAGTACATCAAGACCTGCATGGA

CAACCTAGCCAGCAAGGGCAAGCTGTCAAAGAAGCCTGGAGACAACAAGGCTAAGAAGAG

CAAGCAGGCATCCCAGAAATACACAGCAGCACGTCTGCATGAGAAGGGAGTGCTGATCGA

AATCGAGGATCTGCAGACCAATCAGTTCAAGAATGTTATTTTCGAGATTTCACCCTCTGA

GAATGTGGGAGTGTTTGAAATAAAAGCCAAGTTTATGGGAGTCCATTTGGAGACGCTGTT

GTTAGAATATCAGGATCTTCTGCAGTTACAGTATGAGGGCGTGGCCGTCATGAAGCTTTT

TGACCGAGCCACCGTCAATGTCAACCTGCTCATCTTTCTGCTGAATAAGAAATTCTACGG

CAAATAAAAGAGAGACTCTCAGAAGGGCTTTAATGGACTGTTAAATACAAAGCTAGGCCT

ATAAAACCACACACAACTAGCCCCGCCCCCTGCGGTCCTGTACTCCACCTGCACTACTCC

CGATAATTCTCTCTCTTCACAAGAAGTTACTAGAAATGTGTTTATCATTGTTAGATTAGA

TTAACTCTGCAGAGACGTCCGCCTGCAACAGCTTTAAATATTATGTACTATGTAAAAAGA

ATGATGGAGGTTTTTTTTGTTACCTTAAAATTTGTAAGATGGAAGACAGAGCATGCTTCT

GAGTATGTTTTAGGATGTCATATAGGAAGTATGCAGCCAAAAGAAAAAATCTATTTATCA

AAGCAATACCCATCATCTGCCTTACTTTGAGTTCTGAAATTAAAACCTGGATTGTATTAA

CATTGTAATTTATTGGTAAAGATATCTAGTTATTCCCTCCCAGCTATGAGATGCTGTAAG

AATTTTAACTTTATGTTCTATTTTTTTTGCTTTCTTAGCTCTTTTTTCCCTAAGTTTGTT

TTTACACTGCCTTAACTTGAATAAACTGTTGGGTGTAAAGCGCTGGTATATTCGCTATCT

GTAAGTATGACCGCCGAAAGCAGAGCATGATGCCAGGGGTTTTTCTCAACACTTCCTCCT

CTAGATTTAAAAATGAACTTCATGTGCTGTAGGAATTAGACCGGACGATAGACATGGACG

TAAATGCTAGCTAATTGTCTTAATGTCTAATGTACCCAAACCTGCGTTTTCTCGCCCAAT

TCAATTCAAATGATTTCCGTCGTTTCGGTTTTCTGGTGGCTGTGCTACAGTATGTTGTGG

CGTCCGACTACGGCATTGTTGTTAGCTGGTGTCAGAATATTTCATTATTGTGTTTTGCTC

TTTCAGATGTTTTACTGCATACAATCGGAATGAACAGCACTTAAATGCCTGATCGAACGT

AAAAAAAAGGGGGGGTTTCAAATGCTAATTTAAAGTATTTACCTACCAGTATAGTGCAGT

TTCGCCAAAAAGAAATGCTGGTGATGAAAAGTATAGAGAGTAAAAGATCGGAATTGGGAC

AATCAAAGTAAAGCTGGAAAATAAAATAAACGAGTAAAATCATTTTTGGGGTTTTGTGAT

CTTTTCTTTTTATCGCTGTTTCTGTTTTGTATGTTCTGATCAAATGTACCGTATAAAGCA

TACAACACACAGTGTCTTTGTGAAATGCATTTAAGGCATAACACCCAC

>Unigene000129

GGTGTTGGAAGAGGTGCTGAGACAGAAACGAGGAGAGAGTGACCGAAAGGCTGGAATGGT

GTGAGAGTAGAAGCAGACTCCTCCAGGGCTTTTCGTTTGCACTAGTAAGGGCCACACCTC

CAGGACTGAACCATGGATGACGACTTTGAGTGGCGCAGAGAGCAGAGGAGACAGAAGAGA

GAGGAGATGCGACTGGAGGCGGAGAGAATGGCCTACCGCCGGACTGAGGAAGATGAGGAA

GAGGCAGCTCGTGAGCGTCGGCGAAGAGCTCGCCAAGAGCGCATACGAAACAAGGAGAAT

GAGGAGATAGCTAACCCAGCTGAGAGCGAAGGGCTGAACAGCCATAGTGTCACAGCGACA

AAGACGGTGTCGAGCTCAGAGCTAAACACGGGCCAGGCCGGTGAGGGTGATGAGGAGGAG

CAAGCCCTGCTCGATCGTCTGGCCAAGCGTGAGGAGCGCAGACAGAAGAGGATGAAGGAG

GCGCTGGACAGGCAGAAACAGCTGGACTCTGTCGACGATCCGTCCTACAGTACCTACAGT

GTGGAGAGCACCTCCACCACCTCTTACCAAGCATTCAGCAATCAGGAGGACGAGCCGGCA

CTCAACAGCAAGGAGGAGGTGAAGGAGGAGTTGGTTCCACAGATGGAACTAGTGGAGGAG

AAGCCACGCCGGTCTTACTTGAGGGAGCAGATGGAGGATGAGGACGCAGGAGGGAAGATC

AAGCAGCCGAACGGAGGAGTGTATGAGGACAGCAAGCACAAGAAGGTGGAGAGAACCCAA

AGCCGTGGCAGCGTGCACTCTTTCGAGGGCCCGGCAGCAGAGGAAGGAGACGACAGCGAG

CAAACACGCTTGGAAGCTGAGAGGAAGCTGGAGGAGCTAAAGAGGAGAAGAGACGAGACA

GAGAACGAGGAGTTTGAGAGGATGAAACAGAGGCAGCAGGAGGCTGAGGTCGAGCTGGAG

GAGCTGAAGAGGAAGAGAGAGGATAGGAGGAAGGTGCTGGAGGAGGAAGAGAGGCTGAAA

AAGCAAGAGGAGGCTGAAAGAAAGGCAAGAGAGGAGGAAGAGAAGAGGAGGATGAAGGAG

GAGATAGAGAGGAGGAGAGCGGAGGCAGCTGAGCGGAGGCAGAAGGTCGGAGAAGAGGTA

GACGGAGATGCCGGCAAGCCTTTTAAGTGTGTGAGCCCACGAGGATCCTCTCTGAAGATT

GGTGAAAGGGCAGAATTCCTCAACAGGTCAGCACAGAAGAGCGCCGTGAAGACATCCCAC

TCTCCCGTGGTGTCAAAGATTGACAACAGATTGGAGCAGTACACCACAGCTGTGCAGGGT

AATAAAGAGGTGCGCTCGCCACGCGGAGCTGCAATCGACTTCCCCGGGGGTTCTGATGGC

GTACGCAACATTAAGAGCATGTGGGAGAAGGGCAATGTTTTCAGTTCCACCATCAGCTCA

CCTTCCAGCAATAAGGATGCAGCTGGAATAAAAGTGGGCGTTGCAGGACGCATCAATGAT

TGGCTGAACAAAACACCAGAGGCGAGCAAAACGCCAGGAGGAAGACCTGTGGACCTGAAA

CCGGGCGACGTTACCGGCAAGCGCAATCTATGGGAAAACAAAAGCTCCTCTGCTACCAAG

GTGACAAGCAGAGGAGAGAGCAAATCTGTTGCCAATGGAACGGGACCCTAACCTTTCCAT

GAAGCCACAGCACCAGGGACCAAGTGAAAGCACCGTATTCCTCTTCACATGCATGCACTC

GTACCAAAAAACAATAGTCTCATATGTTTTTATTAATGGCTCGGACTTCACTATCCTAAA

GATGAGGTAGAATCTAAAAAAAAAAAGAAAAAAAAAAAAGAAAAGGAAACTGCTCCTGTC

GTTTCTGTCACCTGACTGTCAGAAGAGGGGGATGATGAAAACGGCTGGATTACGGCGCTG

CTGAACGAGACAGCGGCTTTGACAGCTAGTGAACGTTGCAGACTCCAGCAGCTCTGATTG

GACGCTTGTGTCAGAATGACAGCAGAGGACTCTACTTCTGCCGTCACCATGAGTAACTCT

AGCCACTCCTCCAAATCGAACCCTGCCAACAATCATGCAGTTCTTCACGGATCCATTACC

GAAGGTGTTGCCGGAGCCCCTAATGAAGCGGCCCTGATGGCTCTGATGGAGCGCACAGGA

TACAAGATGGTTCAAGAGAACGGACAGCGTAAATACGGCCCACCGCCCGGCTGGCAAGGA

GCGTCCCCACCGCGTGGCTGTGAAATCTTCGTAGGAAAGATCCCGCGCGACGTTTATGAA

GACGAGTTGGTGCCGGTGTTTGAGTCAGTGGGCAGGATCTATGAGATGCGCCTAATGATG

GACTTCGATGGAAAAAATCGTGGCTATGCCTTTGTCATGTACACTCAGAAACACGAGGCT

AAACGTGCCGTGAGGGAGCTGAACAACTACGAGGTGCGACCTGGACGGTTGCTGGGGGTC

TGCTCAAGTGTGGACAACTGCCGCCTGTTTATTGGTGGAATTCCCAAGACCAAGAAGCGA

GAAGAGATTCTGGAGGAAGTCTCCAAAGTGACAGAGGGAGTGTTGGATGTAATTGTATAT

GCAAGTGCTGCAGACAAAATGAAGAATCGTGGCTTTGCCTTCGTAGAGTACGAGTCTCAT

CGTGCAGCTGCCATGGCCAGGAGGAAACTCATGCCTGGACGCATTCAGCTCTGGGGTCAC

CAAATTGCCGTCGATTGGGCCGAACCAGAAATCGACGTTGATGAGGACATCATGGAAACG

GTGAAGATCTTGTATGTTCGCAACCTAATGATCGAGACCAGCGAGGAGACCCTCCGCAAA

ATTTTCAGCCAGTTTAACCCAGGCTGCGTGGAGCGAGTAAAGAAGATCCGCGACTATGCC

TTTGTACACTTCACTAGCCGGGATGATGCGGTGGTGGCTATGGACAACCTGAACAATACA

GAAATTGAGGGCTCGTTCATCGAGGTAACGTTGGCCAAGCCAGTAGATAAGGAGCAGTAC

ACTCGCTATCAGAAAGCATCCAAGGGAACCATGACCAGTAGTGACAGTACTCAGCAAAAT

TTTGTGTATCAGTGTGATCCCTACACCCTTGCCTACTATAGCTACCCTTACAACACTCTC

ATTGGACCCAACCGAGACTACTTCATCAAAGCAGGTACTGCACGAGGCCGTGGCCGTGCT

GGAGCCACTAACAGAGGCCCAGGGCCTCGCGGCTCGTACCTCGGTGGCTATTCCGCAGGT

CGAGGCATTTACAGCCGCTACCATGAAGGAAAGACCAAGGTCCCGGACAAACCATATGAA

CTCATGTCCAGTCTGGAGCTGGCAGCTGTTAATCCAGTGGGCATTAAGCCTGGCACAATG

ACTCTACCAGGCCTCGGTGCACAGTACCCCGCCATGTTCTCTGCTGCACCCGCTGCTACT

AAACTGGTGGAGGAGGGCAAAATCCACCCAGTGGAGCACTTGATCAACCCCCTCGGCCTT

CCGCATGATCACCCAGCTGCTCCCTCGAGTGCTGCCGTCATTCCTGCCGTCTCGACACCT

CCGCCCTTTCAGGGTCGTCCTATCACTCCTGTTTATGCTGTGGCTCACAACGTCCCTCGG

ATCCCTGCTGCCGCCGCGGCTGCCGGCCTGTATGGAGCGAGCTTCGTGCCCATTGCTGCT

CATACCAACACAGCTGCTCTGGCAGCATTGCAGAAGAATGCAGCTGTAGCGGCTGCTGCG

TACGGAGGTTACGCCGGCTACATGCCCCAAGCCTTCCCTGCTGCCACTTTCCAAGTGCCT

ATCCACGAAATGTACCCGACCTACTGACCCGTGTTATGCCTTGACACAGTCTTGACCCGA

GACACAGCATCAGATTTATGAAGGCTGTTGACTGATCAAACAAAACCCCCAAAACTTTAA

GCGCATATATACACAAACAACAGTAACTTTCTATAAATATTATAAACTTTATATATACTC

TGTAATGCCTCATAGCGCTCTAATAGTGTATTACATATAATTTTCTGACAACCTAATAAC

CCATGCAAATGCCCTGCCCTGATTTTTATTGTAAAAGTCCTTATTTTTGGTTGATGTCTC

CTCATCCAAAATTACCATCTGGTGGTGCTTTCTATTTTAAGCAAAGTTGCAACTTTTAAG

TGCATGAGGTGTTCATTTTACAATTGGGTTAAATTAGTACATTATCCTATTATGTGCAAA

TCTCCCTCCTAATGATGAAAGCACGACTCTACACCATGGTGTCAAACGCTGTATAAGTTT

GGGATTTGGGGCACAACTAACCTTCAGTTGAAAATACGATAGCAGGAGACTTTTCTAATA

TAACGTTTTAATGTTTTTTAATGTCAACTTTTATTTGCAATGCCTCACAAAATTACGTGG

ACAGTGTTTAAAACTGTATGACAGAAAGCATGGTGTCGAGTAAGCAGCCAGATGTAAAGC

CTGGATTAGCGCATGTGCATTTCCAGATGTTTAAAAATATCTGGGTTGTACAGTTTACTT

CCTGAACTTTACAAATACGTTGCAGCAGGTGGATTGGCTAAGATACGTCACCCCAGATGT

CAATACGTGTGTGTGTGAGTGAGTGTGTGTGCTTGCAGGGTGTGTTCTCACCTTGCATAA

ATAGACTCCGGATCTGCCGTGAATAAAGCGGTTACTGAAGATAAATCCAACAAATGTTAT

TAGAGCGTTAGAGGCGTTGTTGGTGTTTTGTACTGTTGTGCTGTGTATAGTGTGTTTAAA

GATAAAATATGATCCACCAGACCTGCTGACAGGAGACTTAGACCTAAATTAAGAAAAAGA

AAACAGATTTTCACCTACGACTACTGAACTGTTCCTGGAGATTCAAGCACAGGACACGGG

ATTAAATCTCTGTGGTCCAACAATTAACCAACTGACTACATGAAAAGCACCAGTTGTGGC

CTGTGTGTATGCTACTCAATTTCCAAGATAAAAACCTCCTTCCTTATTGCAGATCTACTG

ATGCAGTCTCATCTCAAGCTCCACCCACTTGAACAAGATTCCCCTGTCAGCCAGCAGGCG

AGGTGACAATTCAGGGGGGTTTAAAAAAAGCCCATCATTTGTGCCCATCAAGTGCCTTAA

GTGCAAATATGTCACATCGTAAAACCACAACAATCATTCATTCATTACAACTATGACGTT

TTTATTGCTGTCTACATTTAAAAAAAAAGTGGTGTTTTTGCACTAATCGATCTCGACTGA

TTGGTTTTAACCATCCAAAATGAAGGGTGAATGGAAAACCTGCGCTAAAGTGAAGGATCC

GATCCCGCCGAGAGTCTATAAGCACTTAAATCAGCGTCATATCAAAGCCAAACACCCCTT

TAAGGAACACTTTACACTCCAAACATAGACTAGAACCTTCTGAAACCTTCGTCCTGTCTC

TGCAGCTGTGAAAAATACCGAAGGATTCCTGTATGGACGATCACCCCAGCACGCTTGCTA

TAATAGATTGAAAAAATATAGAAAAACCTAAAGAAAAAAAATATGTAAACTTGCTAAGCA

GAGAAATATATGACCACTGAATATTATTGACATGTCTGACTAATACTATTACATCGTTTT

CTTTAATAGGATATTATAGTGTTAGATTTCCTTGTGGCCTGTTTTATTTTCACTGTTGTT

TAAAGGTTTTTCAGACGTTCTTTAAAAATGTTTTTGAGAATTTTATAAAGCCTAAACGTT

GGAGACAAAGCCAATAGTTTGAAAAAAATTGCAACATGGGAACCAAGACAAAACTCCAGA

CACTGTGTTAAAGCACACATACTGTTCACTATAGAGCCTTAATATAAATACATTCAGCCT

TTATAGTTTAGACATTATAGATTTGCAGAACTGCTTTTTTGTAAACTCGAGACTGAAGAC

TGACGTTCCTTATTTTAAAAAATATATACAGCTTCGTTTAGATCAGTCGTTCCAGGCTCA

CACTTTTGTTTAATGCTATTGTTAGAGGTTGAATTTCCAAAAATGTTATTTCTTGTCAAA

AACCCAAATTTGTGACTACGACATAGCTGCTTAATTATTTAATGTTGTTATAATAAGATG

TAGCCATATAGGCATTGAAAACCGTTTTCTGGACACGCTACTCGTGGTACAGGAGCCTGA

GCAGGGAGCTGACTAATGAGTTGACGTTAATTTCAATTAATTACAAAGAAGCTTTAGACA

GATTTCTTCTTTTTTCCCTTTTCTGCTAATTGTTAAACCTAATATTAAACATGGCATTTC

ACAACATACTAAATTGTATGCACAAGTTATAGCACACCTAACTTGTGGGCACTAAGATGA

ACTTGTGGCCTCAATTTATTATCGTATGGCTTAGATATACTAATAATATCCACCATGGCG

CGTCAGAGGATTTGTAGATTCCCCTTTCTTTGTTTCTGGCAC

>Unigene000130

AAAAAAACTTTGTATTCCAGAAAAGGCTGAAATTTTAGCCGTGAATGTTGACTCCAAAGG

AGTCCATGCTGTTTTGAAAACAGGAAGCTGGGTTCGGTACTGTATATTTGACTTGGCCAC

CGGCAAGGCTGAGCAGGAGAATCATTTCCCCACCAGTAACCTGGCGTTTCTGGGTCAAAG

TGAGCGTAATGTTGCCATCTTCACCGCTGGCCAGGAAAGCCCCATCATCCTACGAGATGG

CAATGGCACCATTTACCCAATGGCCAAAGACTGCATGGGCGGCATTCGAGACCCTGATTG

GCTGGATCTGCCGCCTATCGCCAGTCTGGGAATCGGCGTTCACTCACTCACTAACCTGCC

CAACAACTCGACGATTAAAAAGAAAGCTGCAGTCATCATTATGGCCGTCGAGAAACAGAC

TCTGATGCAGCACGTTTTGCGCTGTGATTACGAAGCCTGTCGGCAGTACCTGGTGGGTTT

AGAGCAGGCCGTGCTGCTGGAACAGAACCCTCACGCTCTCGGCGCTCTGCTCGGCCATCG

ATGCGACGGAAACCGAAACCTTCTGCATGCCTGCGTCTCCGTCTGCTTCCCTGTTAGCAA

CAAGGAGACCAAGGAGGAGGAAGAAGCTGAACGCTCGGAAAGAAACACGTTCGCCGAGCG

GCTGTCTGCAGTTGAAGCTATCGCTAATGCTATTTCGGTGGTGTCCAGCAACAGCTCGGG

AAACAGGACAGGATCGTCCAGCAGTCGCGGTCTCCGTCTCCGAGAGATGATGAGGCGCTC

CCTCCGAGCTGCCACAATGAGCCGCCATGAATCAGGGCCCTCCTCCAGTGACCACCAGGA

CCCTGTGTCACCACCCATTGCCCCGCCGAGCTGGGTGCCGGACCCTCCACCTATGGATCC

TGATGGTGACATTGATTTCATCTTGGCCCCTGCTGTGGGCTCACTTACAACAGCATCCTC

TGGCCCCAGTCAGGGTCCGAGTACATCCACCATTCCAGGTCCATCATCTGAAGCGTCCGT

GGTGGAAAGCAAAGACCGCAAGGCCAACGCTCATCTGATCCTCAAACTGATGTGTGACAG

CATCGTCCTGAGACCTCATCTTCGAGATCTGCTCTCTGCAAAGGATGCACGAGGCATGAC

TCCATTTATGCTGGCCGTCAGCGGGAGGGCGTACCCAGCGGCCATCACTGTCCTGGAGGC

AGCCCAGAAAATAGCCAAAGCAGGAGAGACGAATATCGGAGACAAGGACGACGTGAACTC

TGTGTTTCTGGAAATGATCTGTCCTGCTGGAACAAACCCAGACGACTCGCCGCTCTACGT

GCTGTGCTGTAACGACACCTGCAGCTTTACCTGGACAGGAGCTGAGCACATCAACCAGGA

CATCTTTGAATGCAGGACATGTGGTTTACTGGAGTCACTGTGTTGCTGCACCGAGTGTGC

CCGGGTTTGCCACAAAGGCCATGACTGCAAGCTAAAGAGAACGTCTCCCACCGCCTATTG

TGACTGCTGGGAAAAGTGCAAGTGCAAAACGCTGATCGCCGGGCAAAAAGCAGCACGACT

GGACCTTCTCTATAGGCTGCTGACGACCACCAACCTAGTGACCAGTCCCAATAGCAGAGG

GGAGCACATTTTGCTATTCTTGGTGCAGACAGTTGCCCGTCAAAGTGTGGAACACTGTCA

ATATCGGCCACCCCGAATCAGAGAGGACCGGAACCGCAAGGCTGTTAATGCAGAAGACTC

CGACATGCCGGATCATGATTTGGAGCCCCCACGCTTTGCCCAGCTGGCACTGGAGCGTGT

ACTACAGGACTGGAACGCTCTTAAGTGCATGATCCTGTTCGGTTCGCAGGAGAACAAAGA

TCCGTTAAGTGCGAGCAGTCGGATTGCTCACCTCTTGCCCGAGGAGCAGATGTACCTAAA

CCAGCAGAGCGGTACGATCCGGCTGGACTGTTTCACTCACTGTCTCATTGTAAAGTGTGC

TCCAGATCTCACCTTTATCGACACACTCCTGGGCACGCTGGTAAAGGAACTGCAGAACAA

ATACACCCCAGGCCGCAGAGAAGAGGCCGTCTGCGTCACACGTCGCTTCTTGCGCTCCGT

CGCCAGAGTTTTTGTCATCCTCAGCGTCGAGATGGCTTCATCCAAGAAGAAAAATAACTT

CGTCCCGCAGCCTATAGGAAAGTGCAGGCGTGTGTTCCAGGCGCTGCTTCCGTACGCCGT

CGAGGAGTTGTGTAATGTAGCAGAGTCTCTGATTGTGCCGGTGCGCATGGGCATCGCCAG

GCCGACGGCACCGTTTACGCTAGCCAGCACCAGCATCGATGCCGTGCAGGGCAGCGAGGA

GCTTTTCTCTGTGGAGCCACTGCCCCCACGACCCTCACCTGATCAGTCCAACAATTCTAG

CCAATCAGCTTCCTCTTACATCATCAGGAACCCTCAGCCACGGCGCAGCAGCCAATCACA

GACTACACGAGGGCGAGACGAAGAGCAGGACGACATTGTGTCTGCTGATGTTGAGGAGGT

GGAGGTGGTGGAGGGGGTCGCTGGAGAGGAGGATCACCATGATGACCAGGAGGAGCAGGG

GGAGGAGTCTGCAGAAGCTGAGGGACAGCATGATGAACATGACGAAGACGGAAGTGACAT

GGAGCTCGACCTGTTAGCTGCTGCTGAGACCGAAAGCGACAGTGAAAGTAATCATAGTAA

CCAGGACAACGCAAGTGGGCGCCGCAGTGTCGTTACAGCGGCGACGGCGGGATCCGAAGC

AGGTGCCAGTAGCGTTCCTGCTTTCTTCTCTGAGGACGACTCCCAGTCCAACGACTCGAG

TGACTCCGACAGCAGCAGCAGTCAGAGTGACGACGCCGATCAGGAGACATACCTGCTGGA

CGAACCGCTCGAGAGGACCACCGGCTCTGCTCACGCCAACAGTGCTGCTCAGGCCCCGCG

CTCCATGCAGTGGGCTGTGCGCACCACTCCCAGCCAACGCACCGCTGGAGGGGCTCCTGC

AAGCTCCTCCACTCCTGCAGCGAGCTCTGCAGGGCTCATCTACATCGACCCGTCCAACCT

GCGCCGCAGCAGCGCCATCAGCACCAGTGCAGCAGCGGCAGCCGCGGCCCTGGAGGCCAG

CAATTCCAGCAGCTACTTGACATCTGCCAGCAGCCTGGCACGAGCCTACAGCATCGTCAT

CCGCCAGATTTCTGATCTCATGAGCCTCATCCCAAAATACAACCATCTGGTTTACTCTCA

GTACCCTGCTGCAGTAAAACTCACCTATCAGGATGCAGTCAACCTTCAAAACTACGTAGA

GGAGAAGCTGGTTCCTACGTGGAACTGGATGGTGTCCATCATGGACTCGACAGAGGCTCA

GTTGCGCTACGGCTCAGCTCTGTCCTCTGCGGGAGACCCTGGACACCCAAGCCACCCGCT

GCATGCGTCGCAGCACTCCGGACGCAGGGAGCGCATGACTGCACGTGAGGAAGCCAGCCT

ACGCACACTGGAGGGACGCAGGCGTGCGGCCACTTTGCTGACCGCGCGTCAGGGCATGAT

GTCAGCACGCGGAGACTTCCTGAACTACGCACTCTCCCTGATGCGCGCCCATAATGACGA

GCACTCGGATGTGCTGCCCGTGCTGGACGTGTGCTCGCTGAAACACGTGGCCTACGTTTT

CCAGGCCCTCATTTACTGGATTAAAGCCATGAATCTGCAGACCACGCTGGACACCTCACA

GATGGACAGGAAGAGGAGCCGTGACCCGTTGGAACTCGCGTTGGACAATGAGGACTCTGA

GCATGAGAACGATGATGACACCAATCAGAGTTCGACCCTCCAGGATAAAGAGGAGGAGTC

TGTCTCTGCTGAAACCGGTCAGCACCACCCGTTTTTCCGCCGCTCCGACTCCATGACGTT

CCTGGGCTGCATTCCACCCAATCCTTTTGAAGTCCCTGTGGCTGAGGCCATCCCTCTGGC

TGATCAGCCTCACCTGCTGCAGCCCAATGCTAGAAAGGAGGACCTGTTCGGACGCCCGAG

TCAGGGCCTCTACTCCACCTCCTACATGGCTAGCAAAGGTCTCTCAGACCTTACAGTGGA

CATGAACTGCTTACAGATTCTGCCCACAAAGATGTCGTATTCAGCCAATATGAAAAACGT

TATGAGTATGGAGTCTCGGCAGAGGGGCGGAGAAGAGCAGCAGGCGACGGCTCAAGACAT

GGATAGCTCTAAACCTGGTCCCTCCCCTCATGACCTTGCTGCCCAGTTAAAGAGCAGCCT

TTTAGCAGAGATTGGACTGACAGAGAGTGAAGGCCCACCCCTTCCCACTTTTATTCCTCA

CTGCAGCTTCATGGGCATGGTGATCTCTCACGACATGCTGCTGGGACGATGGCGTCTCTC

CCTTGAGCTTTTTGGCCGTGTCTTTATGGAAGACGTCGGCGCTGAACCCGGATCTATCCT

GACGGAGTTGGGTGGTTTCGAGGTGAAGGAGTCCAAGTTTCGTCGGGAAATGGAGAAATT

GCGCAACCTGCAGTCTCGTGATTTGGCCTTGGAGGTGGATCGGGACCGGGACCAGCTCAT

CCAGCAGACCATGCGCCAGCTCAACACGCATTTCGGTCGGCGTTGCACAAGCACCCCCAT

GGCTGTGCATCGCGTCAAGGTGACGTTCAAGGACGAGCCTGGTGAGGGAAGCGGTGTGGC

ACGGAGTTTTTACACCGCCATCGCTCAGGCCTTTCTGTCCAACGACAAACTGCCCAACCT

GGACTGTGTACAGAGTGTCAGCAAAGGCATGCAGGCCAGCAATTTGATGCAGCGCTTAAG

AAACCGAGACCGAGAGAGGGAAAGAAGGAGTGGTGGCCTGAGGACAGGATCCCGGCGGGA

CCGTGACAGGGACTCTCGGAGACAGCTGTCCATCGACACCAGGCCGTTCCGTCCTGCATC

AGAGGGGAACCCCAGTGACGAGCCGGACCCGTTGCCTGCACACAGACAGGCTTTAGGGGA

GCGTCTGTATCCCCGTGTCCATGCCATGCAGCCGGCGTTTGCCAGTAAAATCACAGGCAT

GCTACTGGAGCTCTCCCCTGCTCAGCTGCTCCTGCTGTTGGCCAGTGAGGATTCTCTCCG

TGCCCGTGTAGAGGAGGCCATGGAGCTCCTTATCACACATGGAAGGGAAAACGGTGCTGA

CAGCATACTGGACCTCGGGCTCCTTGACACTCAAGAGAAAGCCCAGGAGAACAGGAAGAG

ACACGCCTCCACCCGCAGTGTCGTGGACATGGAGCTGGATGATACAGATGACGGCGATGA

CAACGCGCCTCTGTTTTACCAGCCTGGAAAAAGAGGCTTCTATTCACCCCGGCCTGGCAA

AAGCTCCGAAGCCAGACTGAACTGCTTCCGTAACATCGGCAGAATACTCGGCCTGTGTCT

CTTGCAGAACGAACTTTGCCCAATTACCCTTAACAGGCATGTCATCAAAGTGTTACTGGG

TAGAAAGGTGAACTGGCACGACTTTGCCTTCTTCGACCCCGTGATGTACGAGAGTTTACG

CCAGTTAATTCGCCACTCGCAGACGAGCGAGGCAGAGGCCGTGTTTGCTGCCATGGACCT

CGCGTTTGCCATCGATCTATGCAAAGAGGAAGGATCTGGACAGGTGGAGCTCTTGGCTGG

TGGCGGCAGCATTCCTGTCACCCCACAGAACGTGTACGAGTATGTGAGGAAGTATGCCGA

ACACAGGATGCTGGTTGTAGCCGAACAGCCTCTGCACGCAATGAGGAAGGGTCTTCTGGA

CGTGCTTCCTAAGAACGCTCTGGAGGACCTCACCGCCGAAGACTTCCGACTTCTCGTCAA

CGGCTGCGGCGAAGTCAACGTCCAGATGCTCATCAGCTTCACCTCCTTCAACGATGAATC

CGGTGAAAACGCAGAGAAGCTTTTGCAGTTTAAACGCTGGTTTTGGTCCATTGTGGAGAA

AATGAGCATGACCGAGAGGCAGGATCTGGTGTATTTCTGGACGTCCAGCCCGTCTCTGCC

TGCCAGCGAGGAAGGATTTCAGCCCATGCCGTCCATCACCATCAGACCTCCAGACGACCA

ACATCTGCCCACGGCCAACACCTGCATCTCGCGCCTCTATGTACCGCTTTACTCCTCCAA

ACAGATTCTCAAACAGAAGCTGCTCCTCGCCATCAAGACCAAGAATTTCGGCTTCGTGTA

GGGTGACGGGTGGCGACGTGTAATATGAGTAGTTTCATTTTTTTGTAGATTTTTTTTGTC

TATACAGTTTTATGAAGTTAAAAAGAAAGTGCTGTCCCACATCCTTGGCGGTATTAGCAT

TTTTTTTTCCTCCCCCCCTGTCATTTTTTTGAAGTCTGGGTGGTGATTAAGTACGTACAC

ACACACACACACACAC

>Unigene000131

TGGCCACCACATTGGAGCCCTGAGCATCCAGGATGACCCACTGCTCATGCTGGCTCTGAA

CTCAGCTAAGATTGCTAGCGATGCCCTGTACAAAAAGGACTTTAACAAGTCTAAGACCAA

ATTCCACCTTCCAGTGGACATGCTGGCTTTTGAACTGGCCAAGAAATGTCAGGTCCAGGT

CAATGATGCCAACTACAGAACTTACCTGCACAACTGGACCTGCCTGCCAGACTCCAATGA

TGTTGTTCAAGCCAGACAGACATATGAACTTCAGAGTGATGCTGTGTACAGGGCCGACCT

GAAATGGTTGAAAGGCCTGGGATGGATCCCTATTGGTTCACTTGATGTTGAGAAAGCAAA

GAAAGCTGGAGAGGCTTTGAGTGAAAGAAAATATCGTCAGCCCCCGAGCAACTTCAAATT

CACAAGCACAACTGACGACATGCCGATAGTGTTGGCGAAAGCCAATAATAATATCATGAA

CAAGCGGTCTTATATCCAAGCTTGGGAGAGTGATAAGACTAAGATCCACATTATGCCAGA

TGCAATGGAGGTTCTTCATGCCAAACAGAACAAGATAAAGCTTATGTTCAAGCCTGGGAT

AATGAAAAGTCCAATATCCACATCATGCCAGATGCAATGGATGTGGTCCTTGCCAAACAA

AATAAAGTGAATTACAGTGAGAAACAATACAAACTTGCCAACGAACTGTCAAAGAAAAAA

GGCTATGATTTGCGCGGTGATGCTATTTCTATCATTGCTGCTAAAGCGTCGCGGGATATT

GCTAGTGATTACAAGTACAAAACTGGATACCGTAGGCAAGTTGGTCACCACATTGGAGCT

CGCAGTGTCCAGGATGACCCACTGATTATGCTAGCCATAAACTCAGCTAAGATTGCTAGC

GATGTCCTGTACAAAAAGGACTTCAACAAGTCCAAAACAAAATTCCACCTTCCAGTGGAC

ATGCTGTCATTTGAACTGGCCAAGAAATGTCAGATCCAGGTCAATGACGATAACTACAGA

ACTTATGGATACCGCAAACAAGTTTGACACCATATTGGAGCTCGCTATGTCCAGGATGAC

CCATTGATGATGTTGGCCATAAACTCTGCTAGGATTGCCAGCGATGCCCTGTACAAGAAA

GACTTTAACAAGTCTAAAACTAAATTCCACCTCCCAGTCGACATGTTGTCTCTTGAGCTT

GCTAAGAAATGCCAAATCCAGGTCAATGACTTTAACTACAGAACTTCCCTGCACAACTGG

ACCTGCCTGCCCGATCAGAATGATGTCATTCAGGCTCGCAAAGCATATGATCTTGCCAGT

GATAATATCTACAAGACAGATTTGGAATGGATCCGTGGCTGTGGATGGGTGGCAGCTGAC

TCTGTTGATCATGTCAAAGTCAGAAAAGCTCAGCAGATCCTCAATGATAGAATGTACAAG

AAAGATGCCAAGGATAATTTTGCCAAGTTCACAAATATTGTGGATCGACCAGAAGTTCTC

TTGGCTAAGGTTAATGCCTTCAATCTCAGTGATCTGAAATACAAAGAATCTTTCAACCTT

GAGAAAGGCCACTACATCGGATCTGACGATACACCCCAACTAGCACACTCCAGAGAGGCT

GCAAAGATCATCAGTGAGAAACTGTACAAACTTGGCTGGGATGAAGCTAAAGCCACAGGA

TATCAGCTGAATCATGAGTACCTGCCACTTGTATCAGCTAAGAAGGTCGGAAGCATCGTC

AGTGAAGCTAAGTACCATGATGCGCATGAAAAAGCTAAGGGTCATTACCTGGCCAACACT

CTGGTTGACTTCCCAGCAGTGGTCCATTCTTCACAAATGGAAAAGATGAAGAATATGAGG

ACCTATCAAAAGGATTACCATACGAGCAAGACTAAGCTTCACATTCCACATGACATGATC

AGCCATGTGGTAGCCAAGAAATGCCAGGAAATCCTAAGCGATGTGCTGTACCGCAGCTAC

CTGCACCAGTGGACGTGCCATCCGGAACAAAACGATGCTATTCGTGCCCGCAAGGCCAAT

GAGATCCTGAGTGATGTGTTCTATAAAGATGATTTGAACTGGATGAAGGGTATTGGCTGC

TATGCCTGGGACACACCAGAAATTGTGCGAGCCAAGAAGTCTTATGAGCTTCAGAGTGAC

ATCAAGTACAAGGCAGAGGGCAAGAAAGAATTTAATAACTACTCTATAGTGACAGATACC

CCTGTGTATGTGACTGCTATACTGGGCCACACATGGGCAAGCGAGCTGAACTACAGAGAG

GCCTACCACAAAGAGAAGCACCTGTACACTACTGTTTTGGACACCTATGATTATGCAAGG

TGCTACAACTTGAAGCAACTGTACAGCAGTAAAACCTATTCTGCTCTTTGGGATAGAATC

AAGGCCAAGAGCTATACTATTCCTATTGATGCTAATGCTTTGGTCCACGCCAAACAGCAG

AAGGTCCTCCTCAGCAAAGTGAAATATAAGGAGGATTATGAGAAGTTTAAGTCCCTGTAC

AGCCTGCCCAAGTGTCTGGCGGACGACCCAGCCACTGCCAGATGCATCAAAGCTGGAAAA

CTTAACTTGGATCGCTTGTACAAAGATAAATGGGAAAAGACAAAAGCCAAAATTCATATA

CCTCCAGATATGCTAGATGTGGTGGCTGCCCGCAACACCCAGAAAATGATCAGTGAAGTG

GACTACCGTAAATATCTGCACCAGTGGATCTGCCTGCCTGACATGCAGGTCTATGTACAT

GCCCGCAAAGTCAACGAGCAGCTGAGTGATATATTTTACAAGGAAGATATGACCTGGCTA

AAGGGTGTTGGATGTTATGCATGGGATACTCCAGAAATTCTGCGTTGCAAACAGGCTATG

AATCTGCAGAGTGAGAACCTATACAGAGCTAAAGGAGTGGAGCATTTTAAGAACTTCTCT

GTAGTAACAGACACCCCGGTGTATGAGACATGCAAGCAGAGTGCCCACAACCTAAGTGAT

CTCAATTATCACCATGACTATGTCACCAATGTAAGAGGTAAAAACACTGCTCCTGCTACG

ACTGTGGACACTGAAAGGGCTCGTTTAGCCAACTACATCCAGAGTGACCACATTTACAAA

GAGGCAAGCAAGAGTAATATGTCCACTGGCTACTCTCTGCCCTTCGACACTCCACTTGCC

CTGCAAGCCAAAGCCAACAACGTCATTACCAGCAATGTAAAGTACAAGGAAGCATATGAA

AACACTAAGGCTAGGAGCTACAAACTGGACCCCAATGGTGTCAATTTCGTCACCATAAGA

AAGGCCAACCAGGTCACCAATCAAAGACTATACCGTGCCCTGTATGAAAAGGAGAAGGAC

AAAGTTCACTCTGTATATGACACCCCTGAGATCAAACAGGTTAAGGCCACTCAGGAGGCC

ATCAGTGATGTTTGCTATAAAGAAAAGTACTACAACAGCAGAGGGACACTACTGCCAATG

CCATTCACGCCACAGCTGATGCACTGCGCTCATGTCAATCAGATTAACAGTGATCTGAAA

TACAAAGAGGATTTGCAGTGGCTGAGAGGAATTGGTTGCTTCGTGTTTGATTCTCCTGAA

ATGGTCCGCATCCGTGAAATCAACAAATTCAGGACTTCCTACGATATTGATGCCAAGAAA

AACTTTTCCAACTTCTCTGTGGTCTTGGACACGCCAGAGTACAAGCGTGTGTCTGAACTC

AAGACGCACTTAAGTGAGCGGGTGTACAAAGCTGCGGGAAATATACAGAGAACTCAGTGC

AGCACAACTGGTGACGCTATGGAATTTAAGCGAGCCAAGTGGGCCCAGACTCTTACCAAT

AAGTGGCTATATACTGATTTGGCTTCCAAAGAAAGAGCCTTCTACACTCCAGAGCTTCAC

ACTCCTCTTCTGGATCATGCTAGATCTATGAAAGTGGTGTACAGTGAGAACAAGTATAAA

GAGCAGTATGAGAAGATGAAGCACAAGTACACTCCCGTCCATGACACCCCCATCCTAATC

CGTGCTAAAAAGTCTTACCTTAATTCCAGTGACCTGCGCTATAAAGAGACCTTTGAGTTA

GCCAAAGGACACTACCACACCACTAAAGATGCCCTGGACATTATTTGTGCTAAGCGGGTC

AGAGATGACATCAGTGAGGTTAAATACAGAGAGAAATACATCAACTCACTAGGAACATGG

AAGTCTATCCCGGATCGTCCTGAATTCTTCCACAGCAAGGTTGTGCGAGACTGCATCAGT

GATTACAAATACAAAGAGGACCTGGAATGGATCAAAGGTGTTGGCTGTTTCGTGTGGGAC

ACACCATTGCTGAAGCAGGCTGAGCATAACAAAGCACTGTACAGTGAGAGATTGTACAAG

GCCTCATATGAGAAGACCAAATGCAACTTTAAGTACACATGCGACACACCATTCTTTCAA

GCGGTTAAAAATGCCTCCGCCCTCTCCAATGATAGATCCTACCGTGCATCCTATGAGAAG

ACTAAGGATAAGTACACCATAGTTGTAGATGACCCTAGAAACCTCTTGGCTAAAGAGGTC

AACAATATGAGCCAGTGGAAGTACAGGTCACAATATGAGCGCCTCAAGTCAAAATACACA

TCAGTGCTGGAGACCCCCGAACACAAAGTCCACAAGCTTAGAAAGCAGATCAGCGATAAA

ATCTACACGATGGAGTATAATAAGAATAAGGCTAAGGGCTACACACTCGGACACGACACT

CCTCTGAATCTGCACATGAAGAAGGTCAAAGAAATTACCAGCAACCTGAAATATAAGGAA

GTGTATGAGAAAAACAAAGCTCAAATCAACATCGCTCCTGATGCTTTTGACATCCGGGCT

GCTAAAGAAGCCTACAAGAACATCAGCAATCTTGACTACAAAAAGAAGTATGAGGCCACC

AAGAATAAGTGGATTTGGACCGTGGATAGACCTGACTTTGTCCACGCTGCCAAGATCAAC

TACCAGCAGAGTGACGTTGAGTACAAGTACGACAAGGAGATGCTGAAAGGTTGCGTGATG

CCTGTTACTGATGATAAGTACACACTTCTAGCCAAGCAGAACACAGAGCTTTCCAGTGAC

ATTAAATACAAGCAAAAGTATGAAGAGGCCAGAGGACATTACCACGTTGTACACGATACT

CCTCAGATCCTCCATGCAAAATCAGTGTCTGGTCTGGTTTCTGAGAGCAAATACAAGCTG

GAAAGCAAGAAGGCTCGACAGTCTGGCTCTTTCACCACCTTGCCTGAGACCCGTGACACA

GCCCACAGCAAAGCAATGACCAAAATCACAAGTTCTAAAGTGTACAAGCAGAAGTTTGAG

AAAGACAAAGGAAAGTCGATATATAACAATATGACCGTACCTCCAGATGTAAAGCATGCC

ATGGATGTGGCCAAGAGCCAGAGCAATATTAATTATAAACAGGAAGCTAAAGCCAAGTTG

CATTACACACCTGTTGCTGACCGCCCTGACATCCTGAAGGCTACCCAGGCTGCCAAACTC

ATCAGTGAGGTGGGTTATCGTGACAAAGCTCGTCAGGAAGCCAGTCGTGGAGGATCGCTG

GTCAACCGTCCTGATATCAACCTGGCCACCGAAGTGTCCAAGCTTACTAGCCTGGTGAAA

TACAAAGAGAAGTTTGACAAGGAGATGAAAGGAAAGAGACCACAGTATGACTTGAAGAAC

AGTAAGATTTATCAGACATTGAAGGATGCCAATACGCTTGCAAGTGAGGTGAAATATAAG

GGTGACCTGAAGAAGATTCATAAGCCTGTGACTGACATGTCTGAGTCTCTGGCCATGCAG

CACAACCTGAGTACCAGCAAACTGGCCAGTAGTGTAAAATACAAGGAGAAGTACGAGAGG

GAGAGAGGCAAGGCCATGCTGGACTTTGAGACTCCGACTTATGTGAGCGCCAAAGAAGCA

CAGCATATGCAGAGCCAGAGAGAGTATAAGAAGGCATTGGAGCAGGAGATAAAGGGCAAA

GGAATGTTGGCTTTGGCTAACGATACTCCAGACTTCATGAGAGCAAGAAATGCTACAGAA

ATCCTCAGCCAGAGTAAATACAAGCACAACGCTGAGCAGGACAGGGCCAGCTATACCACA

GTGATTGACACTCCCGACATCATCCACGCTCAGCAGATCAGGAATATTGTGAGCCAGAAA

AAGTACAAGGAGGAGGCAGAGAAGACCATGTCCCACTATAATGCAGTACTGGACACTCCT

GAGATGCAGAGAGTGCGGGAGAACCAGAGGAACTTCAGCACTGTTTTGTATAAAGAACCT

TCTGGCAAAGGAACTTCTGTCATATTTACACCAGAGATGGAGCGGGTCAAACGGAACCAA

GAACATATTAGCTCG

>Unigene000132

TAGCCACACCTAAACCTTTTTTTTTTCAGTTTTAATATTGAAAACTTTTCATCATAAAGT

TGACTTTTCCCGTGCGGTGGGCACGGAGGTGGTGTGAGGCACGGCTTTGGTGTCATGGAA

ACTTTAGCGTTATCTGATGTTCCAGCACAGAGAAACACTGAGGGAAGATAATCCGTGAAT

TAAAGAGGATCTGTTCTAGAGACTGCCGTGATGAGCGGGCTAGACCCCTCGCCCCCTCGG

CCCTGCTCCGACGCTCCCATGCTGGGGAACAATGGGGAGCCGTCCTGTCTCCCGGCAGGG

CTGGGACACACTTCTGCAGGGGTTTCATTTGCAAATGAATCCCCACACTGATGAAAATGT

AGAGGGCAAAGCCACACAATGAAAGGGATTCATAGAAAATTGCAATTTGTAAATCAACTT

GTATGTAAAACAGCAAGATTTTTTTGAGGGTACAAAGAACAGGCTGGGTGAGAAAAAAAG

AGAGAGAGACAGAGAGAGAAGGAGAAAATAAGAGAGCAAAATGCTGTCATTGAAGACAAG

GGAAGACTGCAGTGTACAGCAGGATGGAATAAGGACAGGGCAAGCAGACATGTGGAGGAT

GCTAAATTGATTGATGGCAAATCCACGAGTGTCTATAGAAGGAAGCTGACATCGAGGATC

TAACCTGGAATTGCATTCTTCTTACTACATTACAATACGTTTTTACAACCTGGGGTGATT

TGACGTTAAGAAGGATAAATATGAGACAAAAGCCTCCTGTTCAGTGGTTTCAAATGATGA

AAGCAAGCTCTCTTGTTATGACATGCCCAGAATGACTTCCTGAATCTGTCTAGTAGTGAG

GCACTACTGCGAGTCACTGTGTGCTGGAAGAAGACACTGATTAGGACTGTGCTAGGCAGA

TGAAACGTCTGCGAAGTGATTGAGTTATGACTTCTTCTCTGCAGCTCGTACGCTCAAGTC

AACCTTTTAGATTTTCTGAAACTGGTCAACAGTCATCCCAGACCAAGAGAGGAGGATAGT

AAGGGAAGAGAATTTAAAGAAAGACTGAGCCACCAAGGAACAGAGCGAAAAAAAGTGCAC

CATGCAGTCAGACGACCTCTTTATCCGGAAGTTCCGCCGACAGAGCCCACGTCCTACCCC

TGTCTTTGAGCCCAAGCGGACAGCGGTGGCGGAATGGCCGCCCAGAAGGGATGGTGAGGG

AGCAGAGGGTGAGAGTTTGACAACACCCAGCAGGGCAGGTTTGAATCAACGTGCAGTTGC

GCGTGGCCATATTATGCAACGGAGCAACAGCGATGTCACACTGGGAGATTTGGAATCAGC

GGCAAAAGCAGGAGCAAAAGCAGTCAGGTCAGCAAGTGAAAAGACCGGGCAGGTAGGACA

GGGTGACTCTAGTATAGTCCTCCACCGGGAATATGGGAGTCTTTCTTCTTTGGAAAAGCA

GATCCAACCTCCAGAGCTCTGCATGGATGAGCAGAATATGCTCAGCCCAAACGCTCTCCG

CTTTAAAGATCCATTTTTGTTGCTCGGGCTGCAGGAGACACCACCTGAACCAGATGGCTT

TTTCCGAGCCCTGGAGTGCCCAAAACCCGCCAAGCCCCCCAAACCTGATGCTCTAAACAA

GAAATCAAAGCCCTCACCCCTTCAGGTCCCTTCTGTACCTTGTGATCTGACATGTGGAGG

TGCATGGGTGCGCAACTTTGCACATTATGACGTGCAGAGCATCCTGTTCGACTTGAGTGA

AGCGGCCACTAGCCGAGACAGCGTTGGTCGCAAGAAGAACATCACCTCAGGGGCGTCAGC

TGCCTCGCAGCTCCGGCCTCTTTCACAGGCTCCTCCTGCCTCCCCCTCACAGAGTCTGGG

AAGTGGGACTGTTGGTACAGCAAGCACAGAGGAACCAGAGCCATGCCTGCTGGACGAAGG

AGACGGCAATGACAACGATTTGCTGCTCAGCTGCCCCCACTTTAGGAACGAGATGGGAGG

GGAGGAGAGGGCTGGACTGGGAAGAAACCAGTGTTGTAGTGGGCGATGGAGAAGTCTACA

GAACCCCAACGATGCTGTGTCTGTATTAGAGGAACCCAGAGAAAGCCATGTCCAACAGCA

AGGAAAGAGCAACTACTTCATTGAGCATGCAGACCTCGGGGCACGGTACTACCACAAGTA

CTTCTACAATAAAGACCATCAAAATTTCTTTGGCATGGACGAGCGCCTGGGGCCCGTGGC

TATCAGTTTTCGGCGAGAGGAGAAAGAAGGCACTAGTGGGGCGCAGTATATGTACAGGAT

CATATTCAGGACTACGGAGCTTAAGACTCTTCGTGGCTCCATCCTGGAAGAGTCTGTGCC

ATCTGCAGCACGACACACCACCCCGAGAGGGCTTTCCCCAAAGAGACTTTTGGAGTTCAT

TTTCCCGGATCTCAACCTGCAATGTCTGAGGCTTGCTTCTACGTCTCCCAAAGTCCGAGA

CACGCTGCTCAAGCTGGATGAGCAAGGGCTAAATTTCCAGCGTAAGGTAGGTGTGATGTA

CTGCCGGGCGGGGCAGAGTACAGAAGAAGACATGTATAATAACGAGAGTGCCGGGCCGGC

GTTCGAAGAGTTCCTGGACCTGCTGGGAGACAGAGTGAGACTGAAGGGCTGGGAGAAATA

CAGAGCACAGCTCGACACCAAGACGGATTCCACTGGGACCCACTCTCTATACACACGTTA

CCAAGATTACGAGATCATGTTCCATGTTTCCACCATGCTTCCATACACAGCCAACAACAC

GCAACAGCTTTTGAGAAAAAGACACATAGGGAACGACATCGTGACGATAGTCTTCCAGGA

ACCGGGGGCCATGCCGTTCACCCCTAAAGCCATCCGCTCACACTTCCAGCACGTGTTCAT

CATTGTCCGAGTGCACGAGCCCTGCACGGAAAACACCTACTATCGCGTTGCTGTTACACG

CTCCAAAGACATCCCTCTATTTGGTCCACTTTTTCCCAAAAGAGCTCGCTTTCCACGCTC

ACAGGCTTTTCGTGACTTCCTTCTGGCCAAGGCGTTAAACGCCGAAAACGCAGCTGAGAA

ATCGGAAAAGTTCCGCTCGATGGCCACACGCACGCGTCAGGAATATCTAAAGGACCTAGC

AGAGAACTACGTGACCACAACGCCTATCGATTCATCTACCAAGTTTCCCCTGCTTTCCCT

GGGAGGCAAACGTAAAGACAAGTTTAAGGGTGCTAAAGGAGCAGAGCTGCACAGTGCCGG

CGCTCTGGTATGGGCCGTGTGCGCCAGGCAGGATGACGACGGCCACAAACTTGCCTGTCT

TTTGGGCGTTTCAGCTGAATTCGTGGTGCTGATCGAACGCTGCAGCAAACGCGTCGTCTT

TAACTGCTCGTGCCGTGACGTGATTGGGTGGAAGGCAGTAACTGAGACTGGCATTAAGGG

TGGGCCTTGTCTGGATATTTTCTACGAAAGGGGCGAGGCGATCTCGGTTACGATGTCAGA

AAGCCAAATCGAGGATATTAAAGAGGTGGTGCAAAGGCTTGAGCTGGTGACAAGAGGATG

TGAGGCACTTGAGATCACTCCTCTGCGGGACAACACAGGTCAGCCGGGATTCCTAATGAG

CGCAGAGGGCTTCGTGATTGAGATGCAGCGATTCTGTTACGCAGAAAGCGGAGGCCTGCA

GCTGAGCGCACGGGTAGTACGACTCTGTGGACACACCCTCGTCCACCTGAGTCAGGAGGA

GAGGACGAGGCTTCTGCGGACGGCACCCAAAATTCACATCACCGTCATCCCACCCGACGA

GAACGGCAAACCTCGCAGGAGCTTTTCGGAACTCTATCAGAAGTCTATCCAAGATGCAGA

AAGCAAGTCTGGTGAAGGCCACTCGAGCGAGGCCTGGGTACTGAACGAGCATGACGAAGA

CGAGGATGACGAGGAGGAGGATGAGAGAGGGGGTGAGCGAGAGCAGGGCGAAGGGGAGGA

GTCAGACAGCATGAACCAATCAGATACAAGAGAGGAAGAAGAAAGTGGAAGTCCAAGCTT

AAGTGGCTTGCTCTCTCCGCCCAGCTTACCTCTTCTTAGGGCGACTTCACTGCAGGACAA

TCCTCCAACCCAAACGCTGGAAATCACTTCCTCCGCTCTCACACGCAGCTACTCACTCGA

GAGAACCCTGCCATGTCAGGACATCTGTGATGGTCATGTTTATGATAACGTCGGAGCGAA

GGTGAACAGACATCATATCTACGAGAATCTAGGAGAGCTGATCGACGCAACCCCTGACCT

CATACTGGCTGTCAAACCAAAGGTTCCTCTTCAGGAAGAAGGACAGGTTGGACTAGATGT

GCTAGGTGAGGAGAGAGCATCGGGCAGCGAAGCACCTCCTCAGTCAGAACATATCGAGAG

GAATTCCAGAGCGGAGAGAAACTCCAGAGCACTGAGCATTCATAACTCCATCACCAAGAT

TCTTTCAGAAACAACAGATTCATCAGAGGAGGAATGGCAGTCTATTTCTGACCTGGCCAC

TGCATGCAGGAATATTCTAGAAGCCCTATCGCATGAAGATCGAAAGGCCGGTGATGGCAC

TCAGGCAGGAGGGGATACAGGGACGCAGACAGATGCCAGAATCAAAGATATGAAAGACAG

TGATTCATCCAGTCACCTGGAGGAAAAGGTGTCCCAATTGGAGGCCATGCTGAAACGATT

ACAGGATGACCTCCAAAAGGGTGAACAGAGGAGATGAGAGAAGATGACCCGAGGGACAGG

CTCCCTCTCTCAACACTAAGCGACCAATGGCAGTCGAGGAGAAAGAGGACAAAGCCATGC

TGCAGGCAGAGGTGCAGAGTCTGAGACAGAACAACCAGCGGCTGCAGGAGGAGTCGCAGA

GCACCGTGGCCCGCCTCATCAAAGTCACCGAGCTGCTGTGTAACGTTAACAAACCCTGCT

AGTAGAACGGGTCAGCGAGATAGATATACAGTACACACACAAACACACACACACCAAAAC

AGACCAGCACCGAATGCTAGCAAGACACGAGCAAAGCAATCCAGACAGAACTGCAAGACC

CGGAGCCCATGCTTAGGCTCATTAATCACAAAGGAATGACTCAAATGCTAGACAAAGCTG

CAAGACCCAGACCTCAGACACTGAATTACAGACCACTAAAGAACGACACAAATTTCACCA

CACTAGCACAAAAAATTATCTAGTGCTACATGAAAGATGGACATAAATTTAAAGCTTGGA

AGGTGCTGGCGTAGTAGACCATTTTTTGGAACACACCATAAGATCCCTAGCAATGTAAGA

CCTCTAATGTAGACCTCAGGGCCTATATTACGTGTAAAAACAATCAATCAAGACTAGCAT

CCCTTTTAGACAAGCATTGCTTTTTTAGTGTATTTAGCTGGCATCATTGGTGAAGTGCAG

CCCACCAAGATTACCAGGTTATTATTATTAATATTATTGCTCACTTGCATTTTACAAAAC

CAAGAGCAAAACCTTAAGGCACGTTATTATCATCGTGCATGTTTGTCAGGCATAACAGAT

CAAGACATTGATGGAAATTTACTGTTACCCAAAGCAGAGCAAGATCCTAACTGCATGATT

TTTTTATTTTAACGTTGCCAGACCCCATATGGTTAGTACTCAAGGCTGGGTAACCCATAT

GCCTTGAGTTACTTTGTTACACTTGTTTTCCAGCTACCCCAGGTATCAGAGGGTAGGAGA

TACCAAATCCTCAGATCTTCATCCTCTTCTGAAATTTATCTTCCAGCTTCCTATTGACTG

AACTTACCTGAACAAGGCAATCAGCTGCAGAAGGAAGCATGTATGATAAATGAGGCTTAT

GGAAATCCAAAATAGTAAGCAAAAGTCATAACTAGGAATAGTATAATAAAGGAATGGCAA

CGCAACTGGAATAAGGAAGTAACAGGCAGACATTATTAACAAATCCAGGAGAAAGCAGGA

AATAGAAGGGCATGTAAAGGAAATATAGGAATAGAAAGTATAATTACACAATTCAGAATA

GGGCACATGAGTCAATGCCGACTATAATAAAACACGAAGATAAAGAACCATGTAATCGGC

AGTGGGTAGAACGGGGTAGGTGAAAAACAAGAGGAACTTAAGAAACATGGGTGAGGCAGC

CCTGTCAGTACTTTTGCCTAAAATTCTAACGCTTGATAAGAAGTTAATAGTGTTCAGTCA

GCATGATGAGCCAGTAGTAACTTTCATTTATTGTTGGAAAATTTTTTAAAAACTGGACCT

AATTTTTTTGTCCAGATATTGACAAATCTACAGAGTATTCTAGTAAAAACCACAGCTAAC

ATTTGGCAGACGTCCTTATTCAGAACGACTTACATTTATCTATTTTATGTAACTGAGCAA

TTGGGGGTTAAGGG

>Unigene000133

GTCAAAGGTCACAGTGCGTATCGAAAAATACACATGCAAAACATCTGACTGCATGTTTGT

ACAATTTCTGTCTCACACACACATACACACACTGTGTGTGTGTGTTAGTGAGGAGGTCAC

GTTTCCTGATTCCAGAAATGTGTGTGTTTTTGTGTGTGTAGGAGCGCTGCATTCGATGCT

GGGAGCAATGGACAACCGAATCTCAGAGGAGGGGATGAAGGTGTCGTGCACACATTTCCA

GTGCGCCGCTGGAGCCTTCGCCTACCTGCGAGATCACTTCAGCCATAACTACAGCGTAGA

CATGAGCCACCAGATCCTTAATCTCAACATCAATCTCATGCTGGGTCAGGCTCAGGAATG

CCTGCTGGAGAAGTCCATGCTGGACAACAGAAAGAGTTTCCTTGTTGCACGGATTAGTGC

TCAGGTAGTGGAGTATTATAAAGAGGCTTGCCGCGCCCTGGAGAACTCCGACACCGCCTC

GATGCTTGGAAAGATCCAGAAAGACTGGAAGAAACTTGTGCAGATGAAGATTTACTACTT

TGCTGCTGTCGCTCATCTTCATATGGGAAAGCAGGCTGAGGAACAGCAGAAATATGGAGA

AAGGCTTGCTTATCTTCAAAGTTCACTAGACAAACTGAACGAAGCGATAAAGTTGGCTAA

AGGCCAGCCCGACAGCGTACAAGAAGCCCTGCGCTTTACAATGGACGTCATCGGGGGAAA

ATTTAACTCTGCTAAAAAGGACAATGACTTTATTTACCACGAGACTGTGCCCTGTCTGGA

GACGCTGCCTTCAGTTAAAGGTGCACCGCTGGTCAAAGCTTTACCCGTGAATCCAACAGA

CCCTAGTGTGACAGGACCAGATATTTTCTCCAAGTTGGTGCCTATGGCTGCTCATGAGGC

CTCCTCACTCTACAGTGAAGAAAAAGCCAAGCTGCTACGAGATGTCATGACAAAAATAGA

CAGCAAGAACGAGACTTTAGAACAATTTATGGACTCTCTGAATCTGGACCCGGACTCCGT

GGACAACATGGACAGATACAACAGCATTCCCTCTGTCCTCATGGAGAAGTGTGCAGCTCT

AAGTGTGAGACCGGACACCGTAAAAAGTCTCATACAGTCTATGCAGGTACTATCGGGTGT

GTTCACGGATGTGGAGGCCTCTCTTAGAGAGATTCGGTTAGTGCTAGACGACGACGAAGC

TAGTGACCGGAAGTTAGAGGAGGTAGCAGGAAAGCAGTGTGTTCCTGCACAGCCAGCTAC

CATAGCGGAAATCCGTCGGGACCTTGAGAAATATGTAGAGGCCCACGAGAAGGCAAGCTT

TACCAACACAGAGCTACACCGGGCCATGAATCTGCACATTAGTAACCTGCGACTCCTGGG

TGGACCACTGGATACTCTGAGAGAGGCCTTGCCCAGACCCCAGCTCTCTGAAGATGAAGT

AGCGGGTCTCCAGTGTATGAAAAGGATTCTGGGTAAAGTGCAGGAGATGAAAGACCAGCG

CAATTCACTTGAGAAGCAACTAAGGGAACTGATCCAGCAGGATGATATAACTGCTGCTTT

AGTTACCACTGAGAGGGCTGATATGAAGAGATTATTTGAGGAGCAGCTGAAGAAGTATGA

ACAGGTAAAAGTGTACATTGACCAAAATCTAGCTGCACAGGAGAACATTTTGAAAGCACT

GACGGATGCGAATGTCCAGTATGCAACAGTACGCAAGGGCCTAGCTGAGACGGAGCACAA

ATGGAACAGTACCGTGCAAATGCTTGTTGCTTCTTATGAAGCCTATGAAGACCTGATGAA

AAAATCGCAGGAGGGAAAAGAGTTTTATGAAGACCTGGAGGCTAAGTCCTCTCGCCTCTT

AGAAAGGGCTAAGTCCATCTGCAAAACCAGAGATGAAGAAAGACAAGCCATTTTGGAAAA

AGAGCTTCAAAAAAAGGTACCCCAAAGGCCCACAGCCCCAAAGCCAGCACAAAAGAAAGC

CCCTGACATGGATTTATCTGGAATAGAAGACCCTGAGTTAGCCAAGATAAATGCTGCCAT

TTTAGCCTTAGGTGGAGATTTACCAGAAGATCTTCGCAGTTTGCCTCCCGAGCTTGGTCG

TCCATCAATACCAGAGGCTTTTCATCCAGGGGCAGCTGCTCTATATGCACGACCATTGTT

TCCTCCGAACCTTCCGCCTCCCGAACTTCTTGCACAAATCTCCCGTTTTACGCTGCCCCC

AACCCAGGGCTTTAACCGGAATCCAACAACAGGCCCCTTGCCCCACCAACATCAGGTTCC

CCAGTTGCCCAACCCTCAAATTGCACAGCTCCCTTCAGTACCTGGGTATGGTCTTCCATC

TTCACAAACCCAGCCTGCTGCATCAGGTCCACCAACTGCCATTGGCATGTCTACCACAAC

TGTGGATAGTGTGCATACGCCAATTCCCAGCTATACTTCAGCACCAGGACATCCAGTAAT

TTCTACTACACCTGGACACCCTATGGCCACTTCAGTGGGGTATAATGCCCCATCTGCAGT

AGCTTCTCAGTATTTTCAACAGCCTGGCGTTCCTGTGAGCCAGCCACCTCAGGGAGTACA

AGTTCCGCAACCAAAGCCACCGCCCTCTCAATCATTTCCCCAAGTTCGCAGACAGGGCCA

GCCACAACCGAGTCCACCGCAGACATTTTATCAAGCAGTGCCCGCTCAGCAGCATATAGC

TGCACCTGTTACTGGACCTCAACTCCCACAACAAACTCAACCTTATTCTCTACACCACAT

GGCACAACCAAGACCACCTAATATTCAGCAACAGGCAAATAATATTCACTCTACACCATC

TGCTTTGTCCCACCAGGTGTACCCTGCTGTTTCATTTGTTCCAGGTTGCCAGCCTGCTCC

AAATCAGCTGCCCTATCATTCCATGCAACCACAAATGCCCCCAAACTCCCAGCCAATGCC

TCAAGTTTCTATGTCACACTTACCAAATGTAAAACAAATTCCCCCTTGTTCCCAGCCCTA

CATGCCCCCACTCAATCAGCAATTACATACTATTCCACAAACACCCCTTTCCACTCAGCA

GGCAGTACCTGCAAGCCATAGGCAAATGCCACCTACTCCACAACAAATACCCCCACTCTC

ACAACCACAAATGCTACCATCTTCAGCCCAAATATCTCCTCCCACTCACCTGCAAATGCC

TGCAGTTTCTCAACCAGGCTTGCATGCTTCTCATTCACCAGTATCTGTTTCTCAGTCTTT

ACCTCGCTCATCTCACCCACCACTGCACAGTGTTTCCCAACCATTACCACACCCCTCTCA

AGTCCATCCTTCCTATCAGCAAGTATTTCCAGGAACCACACCAAGAGTTCCAGTCCCTGT

CCAGCCCCAGTTACCTCCAGCCTCTCTGCCAGCACAGTTCCCGGTGCACACCAATGCTCC

CCCTTCACAGCCTCACAGTCTTCCCTCACAACTCCCTCATGGATCTCTTTCGTCAGTGCC

CTCACAGATCAGTTTGCCTCAGCATCATCTACCTCCTCAGTCCACACCTATGGGTTATCC

TGGTGGTATTCCTTTAGTTCCCCACCAGTCCACAGTTCCTCAGCAGCCTCAGGCTCTGCC

TCCACAGGTACCATCAGGGGCACAAGCATCTTACTCTGGGCCGCATGCTGGGATTCATCC

TGGACCAATACAGAATTCTGGACCAGTCATTACGCCTGGAATACAGTCACTCCCTCAGGG

TATGATCCCACCATCTTCCGCTGCACCAGGGGCTACACCAAGTAATGTCTTGGCTACTCC

ATCAGCTTCTCCATCACCAGGGCCTTCCTCACTGGGTATCGCGCCTCAGAGACCTTCTCC

TGCTCTCACGCCTGTTCCAGGTGCAGCGTTACCACAGACTGTGGTGACACCCTTGCCATC

ATCCACCCATCTGTTTCAGCACCAAAATTCCAGTACAGATGATCTTCTCTCTTCAAGCCC

TGAAAGCCAACATGGTAGTAGCAAAGACACTGCCAATGTCTTATTGCCCATGAAGGCTGA

TCCACAAGATGAACAGCTTCGCAAAAAATCTGAGGGGTTAAAAATTATTCAAGGTGATCC

GTACCAGGCTCCGGAAAGGGTCTCGCAGCTTTGCGCAGAGCTTGAAAGGTTTCGGTCAAC

TGTTCAGTCCTTGGAGCGTCCCTCAGGAGAAGGAAGTTTATCGGAACTTGATGCTCGATG

GAAAGAGCTACAAGATCAGCAAGAGAAAGATTCTCGTCAGCTTTCTATCGCCATAGCCAG

GTGCTACACAATGAAAAACCGCCACCAGGATGTCATGCCTTATGACTGTAATAGAGTTGT

TTTGGACTCTGGCAAAGATGACTATATCAATGCCAGCTTTATAGAGGAACTTTCGCCATA

TTGTCCCCGTCTTATTGCAACACAGGCACCTCTTACTGGAACTGCTGCTGATTTCTGGCT

CATGGTCTACGAGCAGAAAGTGTCTCTCATAGTAATGCTGGTGTCTGAGCAAGAACTTGA

AAAGCAAAAGGTCTTGCGGTACTTTCCATCTGAAAGAGGACAGCAGATCACACAAGGCTC

AATTACACTAAGCCTAACCACACAGAAAAACACAGCCACTCATGTGGAGCGCATGATTGG

ACTGCAGTACAGAGATCAGAGTTTAAAGCGCACAGTCATACACCTACAGTTCACCTCATG

GCCTGAACTTGGCTTGCCTGAGAGTAAAAGTAACCTCATTCGCTTCATTCAGGAAGTTCA

CGGACATTACCTTCTCCAACGCCCTTTGCACACACCTGTTGTGGTGCACTGCAGCTCTGG

CGTTGGTCGAACCGGAGCCTTCTCTCTGTTGTATGCTGCATTACAAGAACTGGAGGCTGG

GAATGGTATACCTGACCTACCCCAGTTGGTGAGAAAAATGAGACAACAGAGAAAGAACAT

GCTACAGGAGAAGTTACACCTAAAATTCTGCTATGAAGCAGTCCTGAAACATGCAGAGCA

AGTGCTTCAGCGTCGTGGCATTGTTACTGCCTCGAGCCAAAAGTCTACAAACAGTGCAGC

AGTTAAGCAATACTCACAGCCAGAGGCTCAGGATATTGTTCTTGGCGGCGATATGCCCAT

TAGCTCTATTCAGGCTACGGTTGCTAGACTCAGCATCCGTCCACCCAGTGTCGAGTCTGA

CCAGGACCCAAATCAGGACCCAGTTGCTTGTGTTGCTGCAGGACTAGAACCAGAGTTGGC

ACTAGTCCAGGACCTCCAAGCTTTTTCTCCCAGTGACACTATTCCTGCCTCGCTAAGCCC

TCCAATTTCCTGCCCTACTTCACCAGTTAAAATGCTGTCACCTTCACCTCCTAATTACCA

AGGTAATGGGTTCGAGGGGTCCACAATAATGCCAGTCTCTAACAACCATGCTGCACTTGA

AGCAACTCTTCCAGCACCGTCATCCCTGGACCTACTGGCATCGTTAACGCCTGAAGCTTT

CACACTCGACAGTGGTTGCCGTGGGAAACAGCGAATCAGCAAGCAAAGCTTTTTTCAGGC

ACAGGAAGGCCAGGGTTTGCAGGGTCCTTCGAATCAGGATGACCCACTGAGCACCCTGGA

TCCACTTTGGACTCTGAACAAGTCCTGACTCCCTTTTGATAAGGCCACTTCTGTTTATTT

CTTAGTGTCCTGTATCCCCAGATCACCTAACAGTTTAGCATTTCCAGCGCTTATCGGCTA

GCTCACAGCCTTTATTAGTTGATTCGGTTGTTTTGGAGCAGGAAAAGGCCCAGATTCCAG

AGTCTGATGTGGATCTCTAGGACCACATCACTGTGTGTTGGTCCATGTGTATCTCTTCGA

TTCCTTGTATTTTTAAGCTGTTTCCTAGCATGCACCCCTCTTTTAAAGACAAGTCAAGCC

TTCTAGATGGACATCTGCATTTTTAACAATAAAAACATCTTTTAAAGGAGAAACGTTAAG

GTTCTCAGCACTTTGAGAAAAGGGAACAATGGATTTGTCATTTGGTTGGTTTGCGTGTAT

CTTGGACTGAAATAAAAATGGATTTCTTAGAGGAACACCAGTCATTAACAAGTGCTTGAC

TAATGATTAGTACTATTGCACAGACTCCTCTCATTCCACGCTTCATCTCCTTTTTTGTTG

TCAACTTTGACCAAGTTGAATAATGGAAAAAGTGCTAACTTACAACTGCAAAAGCCAGTA

AGGTCTAGTTCACAGAATAAAAGTTTCAATAATGCTAGCTGCCTTTTAACATAAGATAAC

CTAGGGTTAGCTGTTTTTAGGGGGACTACTTGCTAACTTTATGTACGGCTAAGTGATGCT

GGTATCCTGTATACGCACTTTTCCTTTATTACATGTTAAACGACTTTATAACTTATTTCT

GATTCAGCAC

>Unigene000134

AGATCGGAAGAGCGTCAGTGTGTGGGTTTTTTTTTCTTTTTCTCCAACGCAGGAGTCCAT

GCTGTTTTGAAAACAGGAAGCTGGGTTCGGTACTGTATATTTGACTTGGCCACCGGCAAG

GCTGAGCAGGAGAATCATTTCCCCACCAGTAACCTGGCGTTTCTGGGTCAAAGTGAGCGT

AATGTTGCCATCTTCACCGCTGGCCAGGAAAGCCCCATCATCCTACGAGATGGCAATGGC

ACCATTTACCCAATGGCCAAAGACTGCATGGGCGGCATTCGAGACCCTGATTGGCTGGAT

CTGCCGCCTATCGCCAGTCTGGGAATCGGCGTTCACTCACTCACTAACCTGCCCAACAAC

TCGACGATTAAAAAGAAAGCTGCAGTCATCATTATGGCCGTCGAGAAACAGACTCTGATG

CAGCACGTTTTGCGCTGTGATTACGAAGCCTGTCGGCAGTACCTGGTGGGTTTAGAGCAG

GCCGTGCTGCTGGAACAGAACCCTCACGCTCTCGGCGCTCTGCTCGGCCATCGATGCGAC

GGAAACCGAAACCTTCTGCATGCCTGCGTCTCCGTCTGCTTCCCTGTTAGCAACAAGGAG

ACCAAGGAGGAGGAAGAAGCTGAACGCTCGGAAAGAAACACGTTCGCCGAGCGGCTGTCT

GCAGTTGAAGCTATCGCTAATGCTATTTCGGTGGTGTCCAGCAACAGCTCGGGAAACAGG

ACAGGATCGTCCAGCAGTCGCGGTCTCCGTCTCCGAGAGATGATGAGGCGCTCCCTCCGA

GCTGCCACAATGAGCCGCCATGAATCAGGGCCCTCCTCCAGTGACCACCAGGACCCTGTG

TCACCACCCATTGCCCCGCCGAGCTGGGTGCCGGACCCTCCACCTATGGATCCTGATGGT

GACATTGATTTCATCTTGGCCCCTGCTGTGGGCTCACTTACAACAGCATCCTCTGGCCCC

AGTCAGGGTCCGAGTACATCCACCATTCCAGGTCCATCATCTGAAGCGTCCGTGGTGGAA

AGCAAAGACCGCAAGGCCAACGCTCATCTGATCCTCAAACTGATGTGTGACAGCATCGTC

CTGAGACCTCATCTTCGAGATCTGCTCTCTGCAAAGGATGCACGAGGCATGACTCCATTT

ATGCTGGCCGTCAGCGGGAGGGCGTACCCAGCGGCCATCACTGTCCTGGAGGCAGCCCAG

AAAATAGCCAAAGGAGAGACGAATATCGGAGACAAGGACGACGTGAACTCTGTGTTTCTG

GAAATGATCTGTCCTGCTGGAACAAACCCAGACGACTCGCCGCTCTACGTGCTGTGCTGT

AACGACACCTGCAGCTTTACCTGGACAGGAGCTGAGCACATCAACCAGGACATCTTTGAA

TGCAGGACATGTGGTTTACTGGAGTCACTGTGTTGCTGCACCGAGTGTGCCCGGGTTTGC

CACAAAGGCCATGACTGCAAGCTAAAGAGAACGTCTCCCACCGCCTATTGTGACTGCTGG

GAAAAGTGCAAGTGCAAAACGCTGATCGCCGGGCAAAAAGCAGCACGACTGGACCTTCTC

TATAGGCTGCTGACGACCACCAACCTAGTGACCAGTCCCAATAGCAGAGGGGAGCACATT

TTGCTATTCTTGGTGCAGACAGTTGCCCGTCAAAGTGTGGAACACTGTCAATATCGGCCA

CCCCGAATCAGAGAGGACCGGAACCGCAAGGCTGTTAATGCAGAAGACTCCGACATGCCG

GATCATGATTTGGAGCCCCCACGCTTTGCCCAGCTGGCACTGGAGCGTGTACTACAGGAC

TGGAACGCTCTTAAGTGCATGATCCTGTTCGGTTCGCAGGAGAACAAAGATCCGTTAAGT

GCGAGCAGTCGGATTGCTCACCTCTTGCCCGAGGAGCAGATGTACCTAAACCAGCAGAGC

GGTACGATCCGGCTGGACTGTTTCACTCACTGTCTCATTGTAAAGTGTGCTCCAGATCTC

ACCTTTATCGACACACTCCTGGGCACGCTGGTAAAGGAACTGCAGAACAAATACACCCCA

GGCCGCAGAGAAGAGGCCGTCTGCGTCACACGTCGCTTCTTGCGCTCCGTCGCCAGAGTT

TTTGTCATCCTCAGCGTCGAGATGGCTTCATCCAAGAAGAAAAATAACTTCGTCCCGCAG

CCTATAGGAAAGTGCAGGCGTGTGTTCCAGGCGCTGCTTCCGTACGCCGTCGAGGAGTTG

TGTAATGTAGCAGAGTCTCTGATTGTGCCGGTGCGCATGGGCATCGCCAGGCCGACGGCA

CCGTTTACGCTAGCCAGCACCAGCATCGATGCCGTGCAGGGCAGCGAGGAGCTTTTCTCT

GTGGAGCCACTGCCCCCACGACCCTCACCTGATCAGTCCAACAATTCTAGCCAATCAGCT

TCCTCTTACATCATCAGGAACCCTCAGCCACGGCGCAGCAGCCAATCACAGACTACACGA

GGGCGAGACGAAGAGCAGGACGACATTGTGTCTGCTGATGTTGAGGAGGTGGAGGTGGTG

GAGGGGGTCGCTGGAGAGGAGGATCACCATGATGACCAGGAGGAGCAGGGGGAGGAGTCT

GCAGAAGCTGAGGGACAGCATGATGAACATGACGAAGACGGAAGTGACATGGAGCTCGAC

CTGTTAGCTGCTGCTGAGACCGAAAGCGACAGTGAAAGTAATCATAGTAACCAGGACAAC

GCAAGTGGGCGCCGCAGTGTCGTTACAGCGGCGACGGCGGGATCCGAAGCAGGTGCCAGT

AGCGTTCCTGCTTTCTTCTCTGAGGACGACTCCCAGTCCAACGACTCGAGTGACTCCGAC

AGCAGCAGCAGTCAGAGTGACGACGCCGATCAGGAGACATACCTGCTGGACGAACCGCTC

GAGAGGACCACCGGCTCTGCTCACGCCAACAGTGCTGCTCAGGCCCCGCGCTCCATGCAG

TGGGCTGTGCGCACCACTCCCAGCCAACGCACCGCTGGAGGGGCTCCTGCAAGCTCCTCC

ACTCCTGCAGCGAGCTCTGCAGGGCTCATCTACATCGACCCGTCCAACCTGCGCCGCAGC

AGCGCCATCAGCACCAGTGCAGCAGCGGCAGCCGCGGCCCTGGAGGCCAGCAATTCCAGC

AGCTACTTGACATCTGCCAGCAGCCTGGCACGAGCCTACAGCATCGTCATCCGCCAGATT

TCTGATCTCATGAGCCTCATCCCAAAATACAACCATCTGGTTTACTCTCAGTACCCTGCT

GCAGTAAAACTCACCTATCAGGATGCAGTCAACCTTCAAAACTACGTAGAGGAGAAGCTG

GTTCCTACGTGGAACTGGATGGTGTCCATCATGGACTCGACAGAGGCTCAGTTGCGCTAC

GGCTCAGCTCTGTCCTCTGCGGGAGACCCTGGACACCCAAGCCACCCGCTGCATGCGTCG

CAGCACTCCGGACGCAGGGAGCGCATGACTGCACGTGAGGAAGCCAGCCTACGCACACTG

GAGGGACGCAGGCGTGCGGCCACTTTGCTGACCGCGCGTCAGGGCATGATGTCAGCACGC

GGAGACTTCCTGAACTACGCACTCTCCCTGATGCGCGCCCATAATGACGAGCACTCGGAT

GTGCTGCCCGTGCTGGACGTGTGCTCGCTGAAACACGTGGCCTACGTTTTCCAGGCCCTC

ATTTACTGGATTAAAGCCATGAATCTGCAGACCACGCTGGACACCTCACAGATGGACAGG

AAGAGGAGCCGTGACCCGTTGGAACTCGCGTTGGACAATGAGGACTCTGAGCATGAGAAC

GATGATGACACCAATCAGAGTTCGACCCTCCAGGATAAAGAGGAGGAGTCTGTCTCTGCT

GAAACCGGTCAGCACCACCCGTTTTTCCGCCGCTCCGACTCCATGACGTTCCTGGGCTGC

ATTCCACCCAATCCTTTTGAAGTCCCTGTGGCTGAGGCCATCCCTCTGGCTGATCAGCCT

CACCTGCTGCAGCCCAATGCTAGAAAGGAGGACCTGTTCGGACGCCCGAGTCAGGGCCTC

TACTCCACCTCCTACATGGCTAGCAAAGGTCTCTCAGACCTTACAGTGGACATGAACTGC

TTACAGATTCTGCCCACAAAGATGTCGTATTCAGCCAATATGAAAAACGTTATGAGTATG

GAGTCTCGGCAGAGGGGCGGAGAAGAGCAGCAGGCGACGGCTCAAGACATGGATAGCTCT

AAACCTGGTCCCTCCCCTCATGACCTTGCTGCCCAGTTAAAGAGCAGCCTTTTAGCAGAG

ATTGGACTGACAGAGAGTGAAGGCCCACCCCTTCCCACTTTTATTCCTCACTGCAGCTTC

ATGGGCATGGTGATCTCTCACGACATGCTGCTGGGACGATGGCGTCTCTCCCTTGAGCTT

TTTGGCCGTGTCTTTATGGAAGACGTCGGCGCTGAACCCGGATCTATCCTGACGGAGTTG

GGTGGTTTCGAGGTGAAGGAGTCCAAGTTTCGTCGGGAAATGGAGAAATTGCGCAACCTG

CAGTCTCGTGATTTGGCCTTGGAGGTGGATCGGGACCGGGACCAGCTCATCCAGCAGACC

ATGCGCCAGCTCAACACGCATTTCGGTCGGCGTTGCACAAGCACCCCCATGGCTGTGCAT

CGCGTCAAGGTGACGTTCAAGGACGAGCCTGGTGAGGGAAGCGGTGTGGCACGGAGTTTT

TACACCGCCATCGCTCAGGCCTTTCTGTCCAACGACAAACTGCCCAACCTGGACTGTGTA

CAGAGTGTCAGCAAAGGCATGCAGGCCAGCAATTTGATGCAGCGCTTAAGAAACCGAGAC

CGAGAGAGGGAAAGAAGGAGTGGTGGCCTGAGGACAGGATCCCGGCGGGACCGTGACAGG

GACTCTCGGAGACAGCTGTCCATCGACACCAGGCCGTTCCGTCCTGCATCAGAGGGGAAC

CCCAGTGACGAGCCGGACCCGTTGCCTGCACACAGACAGGCTTTAGGGGAGCGTCTGTAT

CCCCGTGTCCATGCCATGCAGCCGGCGTTTGCCAGTAAAATCACAGGCATGCTACTGGAG

CTCTCCCCTGCTCAGCTGCTCCTGCTGTTGGCCAGTGAGGATTCTCTCCGTGCCCGTGTA

GAGGAGGCCATGGAGCTCCTTATCACACATGGAAGGGAAAACGGTGCTGACAGCATACTG

GACCTCGGGCTCCTTGACACTCAAGAGAAAGCCCAGCAGGAGAACAGGAAGAGACACGCC

TCCACCCGCAGTGTCGTGGACATGGAGCTGGATGATACAGATGACGGCGATGACAACGCG

CCTCTGTTTTACCAGCCTGGAAAAAGAGGCTTCTATTCACCCCGGCCTGGCAAAAGCTCC

GAAGCCAGACTGAACTGCTTCCGTAACATCGGCAGAATACTCGGCCTGTGTCTCTTGCAG

AACGAACTTTGCCCAATTACCCTTAACAGGCATGTCATCAAAGTGTTACTGGGTAGAAAG

GTGAACTGGCACGACTTTGCCTTCTTCGACCCCGTGATGTACGAGAGTTTACGCCAGTTA

ATTCGCCACTCGCAGACGAGCGAGGCAGAGGCCGTGTTTGCTGCCATGGACCTCGCGTTT

GCCATCGATCTATGCAAAGAGGAAGGATCTGGACAGGTGGAGCTCTTGGCTGGTGGCGGC

AGCATTCCTGTCACCCCACAGAACGTGTACGAGTATGTGAGGAAGTATGCCGAACACAGG

ATGCTGGTTGTAGCCGAACAGCCTCTGCACGCAATGAGGAAGGGTCTTCTGGACGTGCTT

CCTAAGAACGCTCTGGAGGACCTCACCGCCGAAGACTTCCGACTTCTCGTCAACGGCTGC

GGCGAAGTCAACGTCCAGATGCTCATCAGCTTCACCTCCTTCAACGATGAATCCGGTGAA

AACGCAGAGAAGCTTTTGCAGTTTAAACGCTGGTTTTGGTCCATTGTGGAGAAAATGAGC

ATGACCGAGAGGCAGGATCTGGTGTATTTCTGGACGTCCAGCCCGTCTCTGCCTGCCAGC

GAGGAAGGATTTCAGCCCATGCCGTCCATCACCATCAGACCTCCAGACGACCAACATCTG

CCCACGGCCAACACCTGCATCTCGCGCCTCTATGTACCGCTTTACTCCTCCAAACAGATT

CTCAAACAGAAGCTGCTCCTCGCCATCAAGACCAAGAATTTCGGCTTCGTGTAGGGTGAC

GGGTGGCGACGTGTAATATGAGTAGTTTCATTTTTTTGTAGATTTTTTTTGTCTATACAG

TTTTATGAAGTTAAAAAGAAAGTGCTGTCCCACATCCTTGGCGGTATTAGCATTTTTTTT

TTCCTCCCCCCCTGTCATTTTTTTGAAGTCTGGGTGGTGATTAAGTACGTACACACACAC

ACACACACAC

>Unigene000135

GGAGGAAAAACAAATCGAGCTACAAAATAGTGCTTCATTGTGACATGTTTTTTTACGATA

ATATCTGGACGTCCTCGGTGTCGACCGTTACATAGCTAGTCAGAATTCAAGACTTGGCGA

ATAATAATAATAATAATAATATTAGTAATAATAATAATACGATTCCCAACAAAAAGGTCT

GGTTAGCATTGCTAGTTGCGAGCTAGCGCTTAAAAACCAAGTGAGGATCGAATCTACTGA

AATGCATCGATTATAACATCAAAAAACATCAACACAAAGGTTATTACTGGATTTAATAAA

AAAGGGACAATAATAGAACCTCCACCAATGACCCCGGAGAATAGCTGGCGCGTGCCAACT

GTGAGCAGTTTGCAGCCATTGTTTGATCCGGACGCGATTTTTTTACATTGCTAGATTTTT

TTAGTTTATTTTTATTAATAAACATTTTTGCTTGACTGGCTCTCGTTTGGGTTTAATTGA

AAATGGCCGCCGTTGTGAATTATTCTCCTCCGTGGTGGGTTAACCTGCTTCACCGTCTTC

CTCACTTTAACCTGCGCTTCGAGCAGATCAGCAGCGAGTTTCAGCCGGAGGACCCGTCAT

ACCAGCAGTCTATTCTGTTGCTGGGAGCAGTGGCTTTAGCCTGCCTGGCCCTGGATCTCC

TCTTTCTTCTCTTCTACTCATTCTGGCTCTGTTGCCGCCGCAAGAAGAGCAGCGAGCAGC

CCAACGCCGACTGCTGCTGCACGGCCTGGTGTGTCATCATTGCCACCCTGGTGTGCAGCG

CTGGCATAGCTGTTGGTTTCTATGGGAATGGAGAGACCTGCGACGGAGTTAACCGGCTCA

CCTACTCACTGCGCCACGCCAACCGTACTATCGCCGGCGTGCAAAAGCTGGTTTCAGACA

GCACCACCTCCCTGAATCTGACCGTAGATGACAATTTGCAGCATTTGGAGAGCCAATACA

CGAAGCATGCTGATTACCTGTCGATCGTGCAGAAGGTGCAGGGCCAGCTGGACGAGCTGC

TTAAACAGATGGTGGAAATCCCGTTTTGGAGCAATTCCAGAATTTCCCTGGAAGCACTGG

CTAGCAAGATCGAACTCTACGACTGGTATAGGTGGTTGGGGTATCTGGGGCTGCTCCTGT

TTGACGTCCTCATCTGCCTGCTGGTACTCTTCGGCCTCATTCGCAATTCAAAAAGAACTT

TAATTGGGGTCTGTTTACTGGGCGTGTTGGCACTGGTGATCAGCTGGGGATCGCTGGGTT

TGGAGCTAGCTGTATCCTCGAGCTCTAGTGACTTTTGCGTGGCACCCGATATGTACGTCG

CCGAAGTGGCTGAACGCTACGGCGTCATCAACAGAGACATCCTGCAGTATTACCTTCATT

GCAGTGTCGGCCAAAACAACCCCTTCCAACAGAAACTGTCCGGAAGCCACAAGGCTCTGG

TGGAGATGCAGGACGACGTGTTGGAGCTCCTGCGTGCCGCAGTGCGAGAATACCCCCAGA

CTAGAACTAATTTGGAGCAGATTCAAGGGATTTTAAACACAACTGAAATCAGCTTGCATC

AGCTAACCGCTCTAGTGGACTGCCGCAGTCTGCACATGGATTACGTGCAGGCTGTGACCG

GGCTGTGCTACGACGGTCTGGAGGGACTGATCTACCTGGTGCTGTTTTCCTTCGTCACCG

CCCTCATGTTCAGCTCTATCATCTGTAGCGTGCCTCACACATGGCAAGGGAGGAGATCCA

GTGAAGACACAGAAGAAGACTCTGCCGCATCTGGAGGAAGACAGAACCACGATAACTTGT

ACCGGGTGCACATGCCCAGTCTTTACAGCTGTGGCAGCAGCTATGGCAGCGAGACCAGCA

TTCCCGCAGCAGCACACACCGTCAGCAACGCCCCCGTTACTGAGTACATGACACAGAATG

CAAACTTTCAGAATCCTCGCTGTGAGAACACCCCTCTGATTGGAAGAGAGTCGCCGCCAC

CTTCGTACACTTCCAGCATGAGGGCCAAATACCTGGCCAACGCCCGACCTGATCCTAACA

GCAGCACTGCAGCACACTAGAGATCTCCAAAAGACGTCCCTACGCTGTCCCCTTTCTATC

GCTCTCTCTCTCTCTCTCTCTCACTCTCTCTCTCTCTCTCTCTCTTTATTTCTGAGTACC

TTTTTTCTTCTTGTTTTAGATTTTTTTTTTACATTTCCGGTGCGTGAAACGCAACCTGTC

AAACTTAACACAACATGTAGAAAAGCATGTGAAAAACTCCGGGTTTTGCTGAAGAACCCT

TTATGATGTTTGCAGTGAAGGTTCTGAAATGATCTTCAGTGAAGAGCAAAAAAACCCCTG

AACGATTTCATGTCCAGAGAAACAAACACACAACACGTCTGAGTTTCCTACAACTTCATG

CAACAGGAAGTGGTTATAAAGCTGAGCGCGTGCAGAAACAGTACAGATCAGTGAAGAAAG

AGGTGAGCGTCAGGTTCTGTTGAGACTCCAACATTTTATCACTCTATAAATCCATCACTC

CATCATTTTATCACTCCATCATCACCGTATTCATGGGAACGGATAAAGCGTCTGAGACGG

AAACGCACGCAAAGATGGAGAAAGAAGAAGTGAGGGGTGAGGAGGAGAGCGGAGAGGAGC

GCGCGGAACAGGACGGGAACTTATTAACCTCGTACACCTGGCAGACGGGAGGAGCGCGGG

CCGATCCGTCAGAGAAAAGCGCGGGAAACAAGTGGAGTCGGATGCAGAGCTGGAAAAAAG

CGCTGAGTGAAGACACACCCGAAAAATCAGCCACACCGACACCGGGGCCTCGCGTCGGGA

GAAAGAACCCCTTCCGCAGAGCGCTGTCAGAACCTCCCGGCTCGCTCCTGTCCTCCATCC

TGTCTCCTTCATCGTCATCACCCTCAGCGGCGGAGACCAGCGCAGGCGCGGACACGCAAC

AGCGCGGGAAACTCCGCAAATACCTGCAGCAGGTATCACAGAGACTGAAAAGGCCGCGGG

CGCAGAACCGCAACAACACGAGCACCACACAACAACAACAACAGCGGGAGGATGTGCCAT

GTGTGGAGGGTGCAGATTTAGTTCAGTTCTCCTGGGTTCCTCAGGATGTTCCTGTGTGGG

ACATCAACAACTGCATCCTTCAGGACGGACAGATATTTATCTCTCGAGAAGAGGAGCCTT

TGTTGCGGATGAGGAATCGAGCGAGCAGCTGTCTGTCATCTGTCAGTTTACAGAACCTCA

CAGAGAGTCACTCCAACCTTGAGTGCAGTCCAGATCCCACGTCTGTTACCGCCCCAAAAC

CCAAAACCCAGGATGGTGGAGTGAGAATGAAGATTAAACGGCGTCTGCAGGGTGGAGCTC

ACAGGAAACTGAGTAACACACAACTCAACGTAGTGGGACTGAGTTCTGCAGCATGGTTCG

GTTCTCGAGACTCTCTCTCTGTGCCACTGAGTGCAGCTGAAAGTCTGAATGTGAGTTCGG

ACTGTACCACCGTGATCCGGCCGGTGCACAGTTCCATCCTGGGAGAGAAGTATTGCTTTG

AGGTAATTACCTCAGACAGCAATCACTGCTTCGGGTGCAGCTCCGCGGCTGAGAGAGATC

GCTGGATCGAGAACCTGCGGCGTGCGGCGCGGCCCAATAAGGATAACTGTGAACGTATGG

AGAACTCTCTCAGCCTGTGGGTAAACGAAGCTAAAGGTCTCCCACCAAAAAAGCGCTACT

ACTGTGAGGTTCACCTGGATGGTACACTGTTTGCTCGCACCAGCAGTCGTGCGGTGGGTA

AAGCGTCCTCAGTCCCTGTGAGTGACAGTGGGTCAAGTGCTGTGGGCGTGTCTTCTGGAG

GAAGTGCAAGTACAGGGGGGTGCCAGCTGTTTTGGGGTGAGTATTTTGAGCTGGATAACC

TGCCCCCTGTTACACAGATCACACTGCACCTGTTCCGGGATGATGACCCAAAGAAGAAGC

GCCACTCAAAGGATGAGAACATCTTATACCCTCTGGGCAGCGTAGCCCTGTCTCTGGCCA

ACATTAAAGGCCGAGTGTTTCAGGAGAAATGGTATCCCATCGTTCCTTACAAACCTCCTG

GGACAGGTGGATCGAAGGAGCAGCTCGGCCCCCAGGCGTGTATCCGAGTTAAGGCGCGAT

TCCAGAACCTAAAAGTGCTACCGATCGAGAGATATAAGGAGTTTGCAGAGTCTGTGACTT

TGGGGTACATGAAGATGTGTAGCAACCTGGAGCCTCTGCTGAACGTCAGAGATAAAGAGG

AGCTGGCTGGAGCACTCGTCCATGTTCTACAGAGCATTGGAAAGGCCAAAGACTTCCTGA

TCGAGCTGGGTCACGCTGAAGTACAGCGCTCGGGCGAGAACGAGGCGCTGCTCTTCAGAG

AAAACACTTTGGCCACCAAAGCCATAGACGAGTATATGAAGCTGGTGGGGCAGAAATATC

TCATCGACACTTTGGGAGATTTCATAAATCGACTATACACAACCTCAGAAAGCTGTGAAG

TCGATCCACAGAAATGTTCTGCCTCTGAGCTGCTGGTCAACCAGCGCCACCTGAAGGATG

CGTGTGGAGAAGTTGTGCGACAGATCACTGAGAGGCATAGCTCATTTCCAGTGGAACTTA

ATGAAATTTTCTCCAGCTGGGTGTCTGATTGTGAGGAACGTGGACGAGCGGACATTGGAC

ACCGGCTCATTTCAGCATCATTGTTCCTCCGGTTTCTCTGTCCAGCCATTCTCAGTCCCT

CTCTGTTTGGACTGATCCAACCTTACCCAGAACACAACACACTCCGCACACTGACGCTCA

CGGCTAAGGGGCTGCAGAACCTCGCCAACTTCACATTGTAAGTTACAGAAACTGTCTAAC

TCTGATGGTGTCACTCATATTACCTACTAACTCCACTGATTACTGAAAGAATGGAAGACA

TCCGACAGAACTGTCACGATCACTTTAGTTAAAAAGGCTTGTTTTAGTCTTTAAAATTAA

AACATTCATTCAATAAATATTTTTCTAAAGTCAAACAAACAGGTGAGCACCGAACTGATA

AAATCAGAAAAAAAATCGAACCCAAACTGTAGTGGAGAGACAATGAGCAGAATAATTGAG

ATGATCACACACACACACACACAGACACTGAACTTATAACAACTAAATTCAGCTGGACTC

CAGTTACTCTCAGCAAAGAGGAAACAGAAAACGCTTTGAGTTTAGTGTTCTGAACCTCCA

TTCGTGTAGGTTTGGAGAGAAAGAAGAGTACATGCTTTTCATGAATGAGTTCCTGGCAGA

ACATTGGGAGGATATGAGGACGTTCCTGCAGAAAGTATCAAACCCTGACAGTGAACTAGA

AATGGCTCGGTTTGATGGCTATGTGGATCTTCCTTTACGCCTAGCTGTGCTTCACAACCT

GTTGGTGGACATCATTTCTCCAATGAAACTGGAAGTTATAAACAACCTGCAGCCGCTGCC

TTCCATCCTGAATCAGATCACTGAGCACCTCGGCCCAGACATTCCTCGAATCACCTGCAG

CAGGTCCACTTGTGAAACAGAGAAACCCACCTATGTTCCTCCCAGAGATCTTGGCAAGTA

CAGTCCCCTCAACAAATCCCTCCCGCACCTGCCCACAGACCCACGTCCCCCTCAGGGCAA

GGGCAAACACCGGAAGCAGGTGGCGAGGACGCAGAGTGTTCCTGCCTCTGAGAGACACCA

TCGCCGTCCTCTGAAGAGAACCAGCAGCAACGCTGAGTTGAGTGAAACAGCTCACATCCA

CCATGAGGAGAGTCCAGAAAGACAAATGGAGATCTCTCTTCCACAGCAAGAGTTTTTTAA

CAAGTCTGCGCCTGCTCCTGTTCCCTGGATCAAAGAGCACGTTCAGGATGAGACCAATAT

TCTGGAAAAGCATGCTCAGGAGCTGTATGAGCTGCGTTCAGGGGTTGACCAGGTGACAGA

CCGTGAGCTGGAGATGGCGAAGAGGTTGGAAGATTTTATTGCGGTGAGTCAGGAGCAGCA

GGCGCAGCTGCAGGCCGAACTGCAGGAAGTGCGCAATGTGTTAGCACGAAGGGAGGAGCA

GCTTGCTAGCGCCACCTTCAGGCTGGGTGTGATTGAGGAAGAGCGTGAGGAAGATGAGAG

GAAGTTAAGTGTCGCTCTGGCAGCAGCTGAACGTGTGAATGTGTTGGAGGAGCAGTTTGT

GGGGCTGTTAAAGGACATGCAGCAGTTAAATACTGTTTATAATGACGTCCTGAGCACATC

AGCACTTAACAGAGACACTGAGACACTGAGACAGTGAGACAGTGTGTATGATGTCATAAT

GTGGGGCTAAGAGTTTCTTTTTTTACACATACTCTGTGTTGTTGGAGTGTTTGTTATTAT

GCAGATA

>Unigene000136

ATACAAGTGGGACAAAGTATACGAGTTCTCATCAGTCTCTCCTCCATCCCAATCTTTCCA

GGTCCCTATTCTTTAAATGGATACCGCGTACGAGTTTACAGGCAGGACTCAGCCACCCAG

TGGTTCACGGGCATCATTACGCATCACGACCTCTTCAGTCGCACTATGGTTGTAATGAAT

GACCAGTCTGTAGGTACTAGAGCCTCAGAATGTTGATCCCTCAATGGTACAGATGACTTT

TCTGGACAATGTGGTTCATTCTTTGCTGAAAGGAGAAAATATAGGCATCACATCCAGAAG

GAGATCACGCTCTAGCCAGAATAACAACACTGCCCACACCACAGGAGGGAGACCGAGCGG

CAACACAGGAAGCTCTCAGGGTCATTACACACGTGCCCAGGCCAACAGTCCCAGACCGGT

AATGAACTCATCAGGCCCTGCCACCAAGCAGGGTTCACAAGCCCAACAGCAACAGCCCCA

GCATGCTCAACAGCAGGTATCCCAGCAGCAGCAGCAGCAGCCGCAGGTATCACCAGGGCA

ACAGCGTGGGTCCCGGTCGACACGTAGAAAGGGATCAGACAGCAGCATCCCTGATGATGA

GAAAATCAGCGACGAAAAGACGGACGCGGGAGGCGGAAAAGGAGATGTTTCTAAAAACAA

ATCAAAACAGGTGATGAACAAGAGGAGGAAAGCTGAAGATGAGGAGAAGAAGGCTGGGCT

TAAACGGTTAAAGATGGAGGCATCGGATCTTTCCGAGAGCAGCGACTCTGAGAACTCTAA

TAAGAGGCTCCTGGACTCATCCTCAGAGCCGAGCTCTGAAAATGAATTAAAAAATAAAGG

CATTTTAAAAGCCAGTGAGGAAGAGGAAAAGTCCCAGAGCTGCAAGGCTATGGAGGAGCC

TGACGCTAATAGCAGGATGTCTCCCTGGGAGGAGGCTTCTACGGTGGATCAGATTGTCAA

GCCGACAGCCATGGAAGTGATGCCCTCTACCAAGAGTGAAAATACAGGCGACATGAGGTC

CCTAGCGCAACCTTCCCCACTGCCTCCTCCCAGCTCGTGTGCGCAGCCTCGGAGCTTAGT

TCCTGCTGAAGTTCAGGGTTGCATTATGGAAATAAAAAGCACTGTGAAAACTTCTCCTAA

GGACCATTATAGCACAGGGGCACCAAGAACACAAACTCCAAAGTGTGTAATTGACATCAC

AGAAGACTCCAGCTCCCACCCCACAACCCGAGAGAACTCAGAGACTGTGCCTGCGACTTT

AGCTTCTCAAACACGTGAAACTTATGTCCCTGAAAGCAGACATTTGGTGTTAAACCCCTT

AGCCTCAGAATGCAGGAAAGCAGAGGGTGAACTTCAGCAGCAGCTAGGTCAGAGCCTGGG

CAGCAAGATTGAATTTGCTCACTCTGAGGTGATCCGACCTGTGGTTTCGGTAAGCGAGTT

AGCGGCTGTTGCGGAAAGGGAAAGGGAGAAAGTACAGCAGCAGTACCCTTCAATCATGCC

TTGCATCAAAAATGCCTCACTGGCTGAAGATGTGCGAAAACCACAGAAACTTAGCTCGTC

ACCAGATGTGGCCAAGTCCAAGTCTAACCCTTCACCTGACGCATTTAAGCCCAAGTGCAA

TCCCTCACCTGACGCCATGAAGTCAAAAACCCACAGTGTCCTGGAGGCCATGAAGCCTAA

ACCTAACACTTCACCTGAGGTCACCAAACACAAAGAGATCATTGAGATCACTTCATCCTC

TGTTGCCCGGTCGGCAGTCAAGTCGAGAAGTCAAGAAATACCACGCTCCAGCTTTAAGCC

TGTTCCTGCTCGTAGCACTCCCTCTGAGTCCATCAAGAGCGCTCTCATTGTCGACAAAAA

CGAGCACTTTACAGTGTATCGTGACCCGGCGCTGGTGCGGCCCGAGGCTGATACCAACCA

TGTAACTTACCTTCCCCCACACCTCCATCCACTCCACAGCTCCTCCCATGCTACTTGCCT

GACACCTAGCTCACATCACCACTCCCATCTATTGCCTGCATCTTCCCTTAGCCCGCACCC

CTCAGTGCACCACCCCTTGCTCCCCACCGTGTTGCCTGCCATGCCGCCGTCCTCCCTACT

GGGTGGCCACCCGCGCCTCGACTCAGGGGGACTCAGTCATCTAGCCCTAGCTCACCACCA

CCCACACCAGCAACAGCAGTTTCTGCAACAGCAACCACCCCCACCGCTGCTGGCCCAGAC

ACATGGTGGTGCCAGCTACAACCAGCTCGGCCTTTATCCCATAATCTGGCAATACCATAA

TGGCACACAGCACTCCTATCCATCTGGAATTAGCCTGCCTGGGTCAAAGTGGGTGCATCC

AGAAAATGCTGTGAACTCAGATGGTAGCCTGTCAAGGAACACCTCCAGTCCCTGGCTGCA

TCAGCCCACTCCTGTGTCCTCAGCGGATAGTCTAGGAATTCTGAGCCATGTCCCAGGTAG

GCCAGCCAGTGCTGATCCCCACAGACCCCTTAAAATCTCCTCACACTCCAGCCCTCCGCT

CTCCAAAACTGTGGATCACCACAAAGGAGAGCTGGAAAGTAAGGTTTTTGTAGATCCAAT

GCGTGGCCTGGTAACCGCCCACCTAAAGCAAGAGCCAGACCAAAGCCGGACACCGAACAG

CAGGGATTTACAGCGCCTCTACGCGGATTCATCTCACGTAAAGCAGCAGGCGCTGCCTCC

CCGCCTGCCACTAGACACTCCAGACCGGGCTGTTAAGTACAAAGAGGAGAATCGACGCAT

TCTACAAGAGAGCATTGAGGTGGCACCCTTTACTGCCAAAATCCGCTCAGGAGAGCCAGA

GCGTGAGCCCTATATCCGCATTCCCTCTCTCCCCATCAGTGGACCGCCCAAGGAGAATGA

GCATTCGCCATCTGATCTTTATAAGTACAAGCACTCGGCTGCTCAGTCACTGCCACAGAG

CAACTATTTTACCACCATTTCCAACAGTGTGGTGAATGAGCCACCTCGTCTTTACCCCTC

CAAAGAGCTTAGCTCCCTTAATGTTTCAAGTCCCCTGACTCTCGGCTCCTACCCCAACAG

CGGAGGCAACACCAAGTCCTTATCCAAGCCTCCCCCGCTCATCAAGCACCATCCAGACGG

GGAGGGCTTGGTGGGTAAGATCAGTGAGCAGCTCAGTCATCAGGTGGCCCTGAACCCTCT

GAGTACCCCCGTGGCATCTTGCGAACCCCGCAGTCCGGCTGTGTCACCCTCAAACCAACT

CAGGAGCATGCCTGCTCTACACAGGGCACCTGTTTTTCACCCTCCTACCCAGCAAACACT

GGACCGCAAAGAGTCTGGCTACGGTCGCCTCTCTCCACCAACGCTAACCCCTATTCAGCC

GGTTAGTGTGGCTGGGAAGGTATCAGAGCAACAAAAGCCGCCCACTCTGCTTCCAGAGCT

TAGGGAGGTGAAAGGCACTGTAGAGCTGAGCTCCAGTGAGCCATGGAGGCCCAGCTCTAA

CTTACAGGGCTATGATAAAACAGTTCAGTGGCAGTTAGACAAAAGTCAAGGAAAGCCACA

GGTAGCCACAGCTTCTGTTATTGTTCGCTCCAGAACCTGCATTAAGTATGACAGCTCACC

AGGCTCCAAACCTGGTGCCAGGGAGACGTCTAACAGTAAACCACATACAGGAAAATACCA

ACTGGACTGCAGCAAACTGGCTGAAACCAGAGAACCCGGCAGAGTTATACAGCAAAACAC

AAATAAGGAGGACATTTTTTTGCAGTACAAAAACAATTTTGTCCGAGTCTCCCAGGGCAG

TTTTCCCAGCTCTGCGGTAGCTGTTGTTAACTCTGTGTGCAATAACAGTACTGCTGTAAC

CACTTCTGCCTCTGCTGCTTCCACTCACAATGTGCTGAGTCGTGGCACAGCTGAACTGCC

TTACTCCACCTCAGCAAATCCAAGCAGCAGTAACATTAACAGACTGGAGGGCGCTGCACC

CAAATGCAGGACTCCTACCAGCCTTGAGCTACAGGAGTGCAATGCCAGGACAGCCTCCCC

CATTGGCCCACTGCCTCAGCCTGGTTCGGCCCTAGCTCCACAGCCTTATTCTGGGAACTT

TATCCACCTGAAGAAGCACAAGGCTGCCCTGGCCGCTGCCCAGTCCCGTGGATCAAGCAG

TGCCTCAGAGAGTGAAGGTAGCAGTCGATCCTCTCAGGAATCACCCACAATCATCACGCA

GGATCGTGCATCCCCAGGCAACCCGTCCAGCAAAGTCAGCCCCCTACCCAATGGTCAGCC

ATCACTGATGAACCAGCCCAACTACCACAAGCTAAAGAAAGCATGGCTGACTCGGCACTC

AGAGGAGGATCGCAACACTAACAAGGTGGAGATGACTTCCAATGCCGTCTCTGAGATCAT

TAGGCCATGCACAGTAAACCTTATAGCATCCACCTCCAGCGACACAGAGATAAGCAAAGA

TGGCAAATGCTTAGAGGATAAATTGAGCCCGGAGGACAGGAAACCACGACGAGTTCCTTC

CAAGCGGCCACACGAATCAGGCTCGGATAGCGGCGATGACTCCGATGGCAGTGACAGCAA

GCATGAGCAGAGAGCCAAAAGGCAGCCTAAGCCCACCTTCAAGAAGAAGCAGAATGACAT

GCAAAAAAAGAAAGGGGAAAATGACAAGGAGGAGGAGGATGTCAAGCCCAATGGCATATT

CAGAAGCGCCAAGGAGAAAACCAAACTAAAATTGGCCAGCAGCAACGGTATTCCTCGCTC

GGTGTTGAAAGACTGGAGGAAGGTGAAGAAGCTGAAGCAGACGGGTGAGTCCTTTCTGCA

GGACGATTCGTGCTCGGAGATTGGACCCAACCTGCAGAAGTGCCGCGAGTGCCGCTCGAT

CCGCACCAAGAAGGGAGAAGAGCCCACCCACTCCCCCGTATTCTGCCGCTTCTACTATTT

TCGCCGCCTTTCATATAGTAAAAACGGGGTTATCCGGATCGACGGCTTCTCATCCCCTGA

CCAGCACGACGAGGAGGCCTTGAGTCTCTGGGCTCCAGATGCTTACGAAGAGAATGATCT

GGACCTGGAAACCTCCAAATACATTCTCAGTTGCATCGGGGATAAGTTTTGCCAGCTGGT

CATGAGCGAGAAGACCGCCGCCACATGGATAAAGAAAGATGCCAAAATAGCCTGGAAACG

GGCAGTGAGGGGGGTGCGTGAGAGCTGCGACGCATGCGAGGCCACATTGTTCAACATTCA

CTGGGTGTGCCAGAAATGTGGATTTGTGGTGTGCTTGGATTGCTACAAGGCAAAGGAGAA

GAAGAGCTCCAAAGACAAAGATCTGTACACCTGGTTAAAGTGTGTCAAGGGACAGCCACA

TGATCACAAGCACTTGATGCCAACGCAGATTATTCCAGGAACAGTCTTGACGGACCTGGT

AAATGCCATGCACATGCTCAGGGAGAAGTTTGGCATCAAAGCGCACTGCGTCTGTGCCAA

CAAGCATAATATACTCAACAAGCTCCCATCCACTAACGGCGTTTCTCAGGTCCTCCAGAA

CGTGCTGAATCACAGCAACAAACTTTCCCTGTGCAAGCCTGAGGCCGGCCAGCACAACTT

GGGACAGAAGGTTGAGGCAAACGGGGGCAGCAGTCCAGCCAGTGACACCAGCACTGATTG

CAAGCTCCCCCCACCTGAATCCCAGTCACCACTGCACTTTCTGGCCGACCTGGCTGAGCA

AAAATCCCGGGAAGAGAAGAAAGAGAATAAGGAGTCGGTGGTAGGGAAGGTGAAGGAAGA

GAGCACAGATGCTCTGGAGACTTTGCACTGTAAAACCTCGTCTCTGGTGGCCAACAGCAC

AGAGCAGGGCTCCACGCTTCGAGACCTGCTCACCACCACGGCCGGAAAACTGCGTTTGGG

CTCCACCGACGCTGGCATCGCTTTCGCACCGGTTTATTCCACCGCCTCACAGACGGGGAA

AAGTGGTCGCTCTATGCCCAACATCCTGGATGACATCATTGCTTCCGTAGTGGAGAATAA

GATACCAGCCGACCGCAGCACTAAGCAGAGCCCAAAGGCTAAACCCCAGGACGAGGTCAA

GGCTGAGAGAAGGAAACAAGCTGAGGATGTTCCTGAGCAGCATACTGACATTCCCCACTG

CTGGCTGTATGATCACAGACTGCTCTGGCTTAAAGACCACCGCAAGAGCAGTAACTGGAA

ACTGTTTCGAGAGTGCTGGAAGCAGGGACAGGTTAGTCACTCCACCACAAACCACCCAGC

TCAACCCGATCAAGCATTTTACATGCACACAAAAAAAAAAAAAAGTATGTCTGAGAAATG

AAGAAACTGACAGCAGAGGATGAAGGTTGTTTATGTACTGCAAGCTCTTTTAGTATTGTA

CTGGTGC

>Unigene000137

ATCTACAGATCGGAAGAGCGTCATTCATGGAATAGGTGATGCGTTAACAATGGCACGTCG

CAGAAATACTTCGGAAGCAGATTGGTTAAAGATTCAGGCAAGCGAAGCAGTTGGCGAAGC

TTTCGGTTACTACTTTAATGAACAAGGAAATGTTGTTCATAAAGTAAGAACAGTTGGTAT

GCAACTAGAGGATTTACAAAATGTATCTCACGTTGTTGCAGTCGCTGGAGGTTCTTCAAA

GGCAAAGGCAATACAGGCTGTAATTAAACAAGGGCACACTTCAATTCTAATTACAGATGA

AGGTGCAGCAAAACAGTTAACAAAGGGTATTACCCTTTAATATAATCCCCCAAGGAGGAA

ATTCAAATGACTAAAATTGGTATTAATGGATTTGGACGTATCGGACGTAACGTATTCCGC

GCAGCTCTTAACAACTCTGAGGTAGAAGTAGTAGCAATCAACGACTTAACAGATGCTAAA

ACATTAGCTCACCTTTTAAAATATGACACAGTTCACGGAACTTTAAATGCAGAAGTATCT

GCTAACGAAAACAGCATCGTTGTTAACGGTAAAGAAATTAAAGTTATCGCTGAGCGTGAC

CCAGCTCAATTACCATGGAGCGACTACGGAGTAGAAGTAGTAGTAGAATCTACTGGCCGT

TTCACTAAAAAATCAGACGCTGAAAAACACTTAGGTGGATCAGTTAAGAAAGTTATCATC

TCAGCTCCAGCTTCTGACGAAGATATCACTGTAGTTATGGGTGTTAACCACGAACAATAC

GATGCAGCTAACCACAACGTAGTATCTAACGCTTCTTGTACTACAAACTGTCTAGCTCCA

TTCGCTAAAGTATTAAACGAAAAATTCGGCGTAAAACGCGGAATGATGACAACAATTCAC

TCTTACACTAACGACCAACAAATCTTAGACTTACCACACAAAGATTTACGTCGTGCTCGT

GCAGCAGCTGAAAACATGATCCCAACATCTACTGGTGCAGCTAAAGCAGTAGCATTAGTA

TTACCAGAACTTAAAGGTAAATTAAACGGTGGCGCTGTACGTGTTCCAACTGCTAACGTT

TCTCTAGTTGACCTAGTTGTTGAACTTGACAAAGAAGTAACAGTTGAAGAAGTAAATGCA

GCATTCAAAGCAGCAGCTGAAGGCGAATTAAAAGGTATCCTTGGATACAGCGAAGAGCCA

TTAGTATCTATCGACTATAACGGATGTACAGCTTCTTCTACAATCGATGCATTATCTACA

ATGGTAATGGAAGGTAACATGGTTAAAGTACTTTCTTGGTACGATAACGAAACTGGTTAC

TCTAACCGCGTAGTAGACCTAGCAGCTTACATGACTTCAAAAGGTCTTTAATTCTTAATT

ATATAAGTTATCAATGACAAAACGAGGGAGAGGGTTTGTTCCCTCTCTCTCTTCTTTCGT

TTTAAAAGCAAATGTGTTAAGCTTTTGAGTGGGTTTTGTCAATCCTAACTAGTAGTCGGA

GGGAAATCCAATGAACAAAAAATCAATTCGTGACGTAGATTTAAAAGGTAAGCGTGTATT

TTGCCGCGTTGACTTCAACGTACCTATGAAAGAAGGCAAAATTACAGATGAAACTCGTAT

CCGTGCAGCTCTTCCTACAATTCAATATTTAGTAGAGCAAGGTGCGAAAGTTATTTTAGC

AAGCCACTTAGGCCGTCCAAAAGGTCAAGTAGTAGAAGAATTACGTCTTACTCCAGTAGC

AGCACGTTTAGGCGAGCTTCTTGGTAAAGATGTTAAAAAAGCAGACGAAGCATTCGGACC

AGTTGCACAAGAAATGGTTGCAGCAATGAACGAAGGCGACGTATTAGTTCTTGAAAACGT

ACGTTTCTATGCGGGCGAAGAAAAGAACGATGCAGAACTTGCGAAAGAATTTGCAGCTCT

TGCTGACATCTTCGTAAACGATGCATTCGGTGCAGCTCACCGTGCTCATGCTTCTACAGC

AGGAATCGCAGACTACCTACCAGCAGTATCTGGTCTATTAATGGAAAAAGAGTTAGAAGT

ATTAGGTAAAGCACTTTCTAACCCAGAACGTCCATTCACAGCTATCATCGGTGGTGCGAA

AGTAAAAGATAAAATCGGTGTAATTCGTCATCTATTAGACAAAGTAGATAACTTAATCAT

CGGTGGCGGACTTGCTTACACATTCGTTAAAGCTTTAGGTCATGAAATTGGTCTATCTCT

ATGTGAAGACGACAAGATTGAACTAGCTAAAGAATTTATGCAACTTGCAAAAGAAAAAGG

CGTAAACTTCTACATGCCAGTTGATGTTGTAATCACTGAGGAATTCTCTGAAACTGCAAC

AACTAAAATTGTTGGTATCGACTCTATCCCTTCTAACTGGGAAGGCGTGGACATCGGTCC

TAAAACACGTGAAATTTATGCAGATGTAATTAAAAACTCTAAGCTTGTTGTATGGAATGG

ACCAATGGGTGTATTCGAAATGACTCCATTCTCACAAGGTACAAAAGCAGTAGGACAAGC

ATTAGCAGATGCAGAAGGTACATACTCTGTTATCGGCGGTGGTGACTCTGCAGCAGCTGT

TGAAAAATTCGGTATGGCTGATAAAATGAGCCACATTTCTACTGGCGGCGGTGCGTCATT

AGAATTCATGGAAGGTAAAGAGCTTCCAGGTGTAGTTTGTCTTAACGACAAATAAGTAGC

AGCCAAAGAAAAAGGACGGTGCAAAGCATGCGTAAACCAATTATCGCAGGTAACTGGAAA

ATGAATAAAACTCTATCTGAAGCAGTTAGCTTCGTAGAGGAAGTTAAAGGTCAAATCCCA

GCAGCTTCAGCTGTTGATGCAGTAGTTTGCTCTCCAGCTCTATTTTTAGAGCGCCTTGTA

GCAGCGACTGAAGGAACTGATTTACAAGTAGGTGCACAAAACATGCACTTCGAAAAAAAT

GGTGCATTCACTGGCGAAATTAGCCCAGTAGCACTTAGCGACTTAAAAGTAGGCTACGTA

GTACTTGGCCACTCTGAGCGTCGTGAAATGTTTGCTGAAACAGATGAGTCAGTAAACAAA

AAGACTATCGCAGCATTTGAACATGGTTTAACACCAATCGTATGTTGTGGTGAGACTTTA

GAAGAGCGCGAAAGCGGAAAAACATTTGATCTAGTAGCAGGTCAAGTAACAAAAGCACTT

GCAGGTTTAACAGAAGAGCAAGTTAAAGCAACTGTTATCGCTTATGAGCCAATCTGGGCT

ATCGGTACAGGTAAATCTTCTTCTTCTGCAGATGCAAACGAAGTATGTGCGCACATCCGT

AAAGTTGTTGCAGAAGCTGTTTCTCCAGAAGCTGCAGAAGCTGTTCGTATTCAATACGGC

GGTAGCGTAAAACCAGAAAACATTAAAGAGTACATGGCACAATCTGACATCGACGGCGCT

TTAGTTGGCGGTGCTAGCTTAGAGCCTGCTTCGTTCTTAGGTCTTCTGGGGGCGGTAAAA

TGAGAAAGCCAACAGCTTTAATCATTCTTGACGGTTTCGGACTTCGTGAAGAAACTTACG

GGAATGCTGTAGCACAAGCTAAGAAACCTAATTTTGATGGTTACTGGAACAAATTCCCTC

ACACAACGCTTACAGCTTGTGGCGAGGCAGTAGGTCTTCCAGAAGGTCAAATGGGTAACT

CTGAGGTTGGTCACTTAAATATCGGTGCTGGCCGCATTGTATATCAAAGCTTAACACGCG

TAAACGTTGCAATTCGTGAAGGTGAGTTCGATAAGAACGAAACGTTCCAAAGTGCAATTA

AAAGCGTAAAAGAAAAAGGTACTGCACTTCATTTATTTGGTTTACTTTCTGACGGTGGTG

TGCACAGTCACATGAACCACATGTTTGCTCTTCTTCGCTTAGCAGCTAAAGAAGGCGTTG

AGAAAGTATACATTCATGCATTCTTAGATGGCCGCGATGTTGGACCAAAAACAGCACAAA

GCTATATCGATGCAACAAATGAAGTAATTAAAGAAACAGGAGTAGGACAATTCGCGACTA

TCTCTGGTCGTTATTACTCCATGGACCGTGACAAGCGTTGGGATCGCGTAGAAAAATGTT

ACCGTGCTATGGTGAATGGTGAAGGCCCTACTTATAAATCAGCAGAAGAGTGTGTAGAAG

ACTCTTATGCAAATGGTATCTACGATGAATTCGTATTGCCGTCTGTAATTGTTAACGAAG

ATAACACGCCAGTTGCAACAATCAATGATGATGATGCAGTTATCTTCTATAACTTCCGTC

CAGACCGTGCAATTCAAATTGCTCGTGTATTTACAAACGAAGACTTCCGTGAGTTCGATC

GTGGTGAAAAAGTACCTCACATTCCAGAATTCGTATGTATGACACACTTTAGTGAAACAG

TAGATGGTTACGTGGCATTCAAGCCAATGAACCTTGATAACACTTTAGGTGAAGTTGTTG

CGCAAGCGGGATTAAAGCAACTTCGCATCGCGGAAACTGAAAAGTATCCGCACGTTACAT

TCTTCTTTAGCGGTGGTCGTGAGGCTGAATTCCCAGGAGAAGAGCGTATCTTAATTAACT

CACCGAAGGTTGCAACGTATGACTTGAAACCTGAAATGAGCATTTACGAAGTAACGGACG

CTTTAGTAAATGAAATCGAAAATGATAAACATGATGTTATCATTCTTAACTTTGCGAACT

GTGATATGGTTGGCCATTCTGGGATGATGGAACCAACAATTAAAGCAGTAGAAGCAACTG

ACGAATGTTTAGGAAAAGTTGTAGAAGCGATTCTTGCAAAAGATGGTGTAGCACTTATTA

CTGCTGACCATGGTAATGCGGATGAAGAATTAACTTCTGAAGGAGAGCCAATGACAGCTC

ATACAACTAACCCGGTTCCTTTCATTGTTACTAAGAACGACGTAGAATTACGTGAAGATG

GTATCTTAGGTGATATCGCTCCAACTATGCTTACACTTCTTGGTGTTGAGCAACCGAAAG

AAATGACAGGTAAAACAATTATTAAATAATTTGCTTATATAAAAAGGAGAGAATTTATTA

TGTCAACAATTATTGATGTTTATGCTCGCGAAGTCCTTGACTCTCGTGGTAACCCAACTG

TAGAAGTAGAAGTTTACACAGAAAGCGGCGCTTTCGGACGCGCTATCGTACCAAGTGGTG

CATCTACTGGTGAGCACGAAGCAGTAGAATTACGTGACGGTGACAAATCTCGTTACCTAG

GTAAAGGTGTTATGAACGCAGTAAACAACGTTAACGAAGCAATCGCTCCAGAAATCGTTG

GTTTCGACGTAACTGACCAAGCTGGTATCGACCGTGCTATGATCGAATTAGATGGCACTC

CAAACAAAGGTAAACTAGGCGCTAACGCTATCCTTGGTGTATCTATGGCAGTAGCTCACG

CAGCAGCTGACTTCGTAGGTCTTCCATTATACCGTTACCTTGGTGGATTCAATGCAAAAC

AATTACCAACTCCAATGATGAACATCATCAACGGTGGTTCTCACGCTGATAACAACGTTG

ACTTCCAAGAGTTCATGATCTTACCAGTTGGTGCTCCAACATTCAAAGAATCAATCCGTA

TGGGTGCTGAAGTATTCCATGCACTTAAAGCTGTATTACATGACAAAGGTCTTAACACTG

CAGTAGGTGACGAAGGTGGATTCGCTCCAAACCTTGGTTCTAACCGTGAAGCATTAGAAG

TAATCATCGAAGCTATCGAAAAAGCTGGTTACAAAGCTGGCGAGAACGTATTCTTAGGAA

TGGACGTTGCTTCTTCTGAGTTCTACAACAAAGAAACTGGTAAATATGACCTTGCAGGCG

AAGGCCGTACTGGCTTAACTTCTGCAGAAATGGTTGATTTCTACGAAGAGCTTTGCAAAG

ACTTCCCAATCATCTCTATCGAAGATGGTTTAGACGAAAACGACTGGGATGGTCACAAAT

TATTAACTGAGCGTATCGGTGATAAAGTACAATTAGTTGGTGACGACTTATTCGTAACTA

ACACTCAAAAACTTGCTGAAGGTATCGAAAAAGGTATCTCTAACTCAATCTTAATTAAAG

TTAACCAAATCGGTACTTTAACTGAGACTTTCGAAGCTATCGAAATGGCTAAACGTGCTG

GTTACACAGCAGTTGTATCTCACCGTTCTGGTGAAACTGAAGATGCTACAATCGCTGACA

TCGCAGTTGCAACTAACGCTGGCCAAATCAAAACTGGTTCTATGAGCCGTACTGACCGTA

TTGCTAAGTACAACCAATTATTACGCATCGAAGACGAACTAGGCGAAATCGCTGTTTACG

ATGGTATCAAATCTTTCTACAACATCAAACGATAATTGTAGAAAATAACGACAGACCG

>Unigene000138

GCAGCGATGTTTTCAAAGCGAGCTGTGAGCTGGTGAGAAACGTCACAGAGTCGTAGGTTA

ATGACCGTTTGGAACGTCATATTCAACAAAACCGTCTGTTCTGCTGAAAGAAATAAATAA

TTAGCGTCTTTTTCTTGTGCTGATGTAAAAGTGGAACTCGGGGAGGGAAAAAAAGACTGC

AGTCCCATGATGAACCCCATGAGACGTAGCTGCCATAGCTAGTCAGAAGCCTCACCATGG

CCAAGCCTGGAACCGACAGAGATGGAGCCATGGTGGAAAAGACGACTGGGAAGAAGAGTA

AAGATAAAATCTCCCCATTTACCAAAACTCCGAAGCTGGATCGGAGTGAGATAATGGGGA

AAGAGGGAAAGCCCAAGTCTTCCATGAAACGCAAGCTGTCCTTCAGTGTCAGCCCGCCCC

GTAACGGGGAACGGGATTCAGACACAGATAAAGATGGTCCTGATAAAAAGAGGGTAAAAA

AGGAGTCTGGAGGTAAAAAGTCCACCCCTGTGAACATTTTGTTCGGTTACCCTCTCTCGG

AACGCAAGCAGATGGCCCTGCTAATGCAGATGACAGCAAGAGACAACAGTCCAGACACCA

CACCAAGCCACCCCTCCCAAGCTCCAGCAGTGCAAAAAAAGACTCCCAATAGCTCAACAT

CTCGTCAGAAAGACAAGGTGAACAAGAGGAATGAGCGTGGAGAAACACCGCTGCACATGG

CTGCCATCAGAGGGGATGTGAAACATGTCAAGGAGCTGATTGGATTAGGGGCGGATGTAA

ATGTGAAAGATTTTGCAGGTTGGACACCTCTACATGAAGCTTGCAACCTTGGTTACTATG

ACGTAGCAAAGGTTCTTATTGGTGCAGGAGCAGAGGTGAACACCCAGGGATTGGACGATG

ATACACCCCTACATGATGCCTCCAGCAGTGGACACAAAGATATTGTGAAGTTACTCTTGC

AGCATGGAGGCAACGCATTTCAGGCAAACAAGAGAGGCGAGCGACCGGTGGATGTTGCTG

ATTCTCAGGAGGTTGAGCAGCTACTGAAGGGGGAGATCCCATTGTCTGACCCAGATGACA

GCTCTTCAGACTCTGAGGACCTTCCATCAATAAACCCTTCGAGTGTAGATGACAATATGG

AGTACTCGGACACTGAGAAGGACTCTGATGGCAAATCGACCACAGCCAAGGCCACATCTT

CAGTGTCTGGTGTGGATGAGTACGAGTTCAAGGACGAGGAAGAGGAAGAAGATCTTAGCA

AGGCCCTAAATGACAGACACATCTTGAGACGAGAAGTGCGACAGAAAGAGGAGCAAAACC

ATTACACACCCAGACAGTCCAACAAGAGCGATCAGACAGTGTCATCATGCAAATTAAAAA

AGCCCAAATCATCCCGAGTACTCTACTGCAGTTCAGATAGTTCAGACAATGAATTGGAGG

CTCCTTCAGAACGAAAGTGTTCCCCGACCAGGTCACTCTGCAACGACACACACAAAACAG

AGAGCCGGACAAAAAGAGATAGCTTAACTCAAACATCAACAGAAAAAGGCAAGGCAAACA

AGAGGAACAGGAGTCAAAGCAAAAACAAGGAAAATCAGGAGGTTCAAGAGGACGGAAAAG

AAAACAGCAAATCTCTTTTGTTCTCGACAGCCACAGCGTCTGACAACTCTGATAAAAGTG

TTCGGGAGGAGGACTCTTTTAAAATGTCCTTTAGCCCAAAGGACGATTCATCTGTGCACC

TCTTTCACTTGTCCGCTGTGAAATCCCCCAAGCTCAGTTATGGTCAACCTGACAAACAGA

TAACTCCTCTAAAACAGGAAAATGCTAAGACTTTTGTCTCCATTGGAGATGGGTCCTGCA

CTATGGATGGTGTCAAGTACAACCACTACACAGATTCTGACTACACAGAAGGTTCCAGCA

GTAAAAGCTGTAAGCACAAGGACAAAAGCAAACATCACTACAGGGAACACATCTTGGATG

GAGATGACAGAAGCTCTAGTCCATTTAAAGATGCCAGTTTAAGCAATAGTATGGATAGCT

CGGAAGGAGCCTTTAGAAAATCTGACAAGGATGGTAAGGTTGTCAAAAAGCATAAGCTTA

AACACAAGGAAAAGGACAAGTACAGGAAAGACTATGAGTCTGAGAGAAATCGGCATCGAC

AGAGAGAACTAAGTAAAGATGGACATAGGAATTTGGAGTTTGACCGAGAATTTTGGAAAG

AAAATTTCTTCAAAAGTGATGAGAGTGAAGAACTCAAGGGGAAATGTGAGACACCAGGCA

GTTGTTCTCCTCAAAAGTCTATAGACTCGTCGCCAGTTAAGGAAGATAAAACAACATTAA

AAGACAAGCATTCGGTTTGTAACAGCACTAAGGAGAGAAAACAAAAAGACGAGCGAGAAA

AAGACAAACCTATAAAAAAAGAAAAGAAAGAGGTAGCATACAAGGATGAAAGAGGAGCAA

AGGACAGTAAAGGGGCTGAAAATGACCGGTCAGATTGTCCAAACTTGATACGAAAGCCTG

AAGACTCAAATTCTTGCATAAAAGAGGAGACCGAAGACAAGCCTCTTACTGGAAACTTGT

TTGATCACAATCAGCTAGAGACTACAGAGAAGAGCTCTCGTGAGAAAAATGATAAACGGC

TACCCACAAAGGACAGAGAATCTGAAAAAGGTGAGAAAAAGCAGGCAGAAAAAGAAAAGA

AAACCAAATCAGATCATTTGTCAGATAAACCTGATCTTCATAACTGTACAGATCGATGGA

AAGAGAGGGATAAAATAATGACCTTGTCTAATTCGTCTGGTGAGAGAATCCATAAAGAAA

GTGACAAACTAAAACCTGTGGCGCCTATGAAAAAGCATGAAGAAAACAAAAAGAGCAAGG

ACAAGTCTGAAAGAAGAAGTGAGAAGGAACGTCAAGAGAGAGAGCATTTCTCAAGCGATC

ACAGGGACAGAGATAAAATGGGTTTTGAAAAGAAAGGAAAGGCTCCTGAGCGGAGCAGTG

ATCATAGCAAAATCGATCGTATTAAAGAAAAAGAAAGAGATAAAGACTTCGACAAGAAGA

AAAAAGAAAAATTAAAAGAAACCATGATCTCCTCCTCAGGTTCAAATCTCAAATTTCTTT

TGGAAGAAAAAAAGGGGTACACATCTGAGAGCAATAAAACAGTAACCCTTAAATTAAAAG

AAGAGGTCATAAAAACCCCTGAAAAGGACAGGGACAGGCGGGAGAGGGATAGAGATTTGG

AAAGGCATCGGGATAAAGACAGAGAGCGGCACAAGGAAAAGGACAAGGAAAGACAACATA

GGGACAGCAAACCCAGCAAAACAAGGCCTGTTGAAATTGAGTCGGATAGGTCCAAATCCA

AAGCTTCACCTGCACAAAAAGAAACCCGCCCCAAAGAGAAAAGATTGGTTAATGATGACC

TCATGCAGACGAGCTTTGAACGAATGCTTAGCTTAAAGGATCAGGAGATTGAGCAGTGGC

ACAAGAAACACTTGGAAAAAATCAAGCAAAAAGAAAGAGAAAGGCTAAAACAACGACCTG

GATCCGACTCTGGGAAGCCAAAAAACAAAGATAAAGCGAAAATTTCCACTTCCAGTGAAC

CAAGTGCAAATAAAGAACTCTTGCGCTCCAAAAGCTCAGAAATCCCAGACGCTTCTGGTC

GGGAAAAGATCTTAAAGGATGCGACCAGCGGAAGGACACTTTCTCTAGATGCAAAGAATC

TGTTGTGTTTTGGAAAGTCTGTTCCAGTAATAGAAAACAGCCTTAGTCGATCTCCAAGAC

CTGACAGTGAACGATCTGCTATCATGTCAAGATCAGTGTCTATGATCTCAGTAGCCAGTT

CAGAGGATTCCTGCCAGGCTACCATGCTAACGCCCAGGCCCACGGAGTACGACTCGGACC

TAAATTTAGAAATGTCTGATTCTCAGCCACCTTTCCTCCAATCTTCCCATATAGTTCAGT

CCTCCAGATCACCAATTTTTAACAATAAAGAAATTAACAGTCTTCCTGACGTAGCCTCTG

GCAGTCGAACACCACTGTCAAGCGGACTTGCATCTTCTCAATCAAGAACTGTCCATAATG

AGGATGATAAAGCCAGTCTAGCTGAGCCAGACAGACTGACTGAAGAATCTCAGAAAGTTG

GCGATGTAGCAAACTCTAGTGAGGTACCTGGTAAAATACACCATACTGAAGCCTGTGGAG

CAGTCACACAGAAAAGTCTTACTATTAACAGCTCCCTGTCAGTGCATCTCCACTGTGCTG

AATCTGAAGTTCAACCTCCTGTCGGTGTTGAGGATGCAAATAGGAGTTCAAGCAACCAGC

TTGCATGTGCCCCTAACTCTGAAGATCTCCCAGGTCACCAAACTAGCTTTCCACGTAGTA

TTGGTTTAACAACAACTACAGTAGAGGTAAGCAAGCTAAACACTGATACCTTACCAGAGC

AGAGTGGACAAAATCAGCCTGACAGCTTTGACACAGAAATGGCCGATGGCTCCAACAAAA

ATTCCCTGTTAGCTTCTGTGCCCCAGGTTGCATCCATGGTATCATCAGAGCAGAGCTTCA

CAAAAACACCTACGCAGACTGGTAACCTGAAATGGAATTCTGTCTTTGATCCCAATAATG

ACCACCCAGCAAGGACAGACATTGATTTGACAGAAGAGAAAAGTTTAAGAAGTTTGTCAA

GCGGTCCTGAAAGCCTGATGGAAGACAGATGCATGGAGAAGGTTTCTGCTGGGAGCAGAA

TGGACTTGACAACAAGCCAAGCTTCATCTATCAGCTCTAGTAGGATCAGTTCTGAAGAGT

CTGACAAAATGCTGCAATGTACAGTCAGTGTGTTTAGAGATTCTCATCAGGTTACAGAAC

AGATCTCAGTGGATGACTGTAAAGCAAGTCACTTCGGTGAGAACATAAGTGTGGCTGATG

CACATCTGGGGAAAAAGGAATATGGTTTGGCTCCCCCTTCAGTTTCAAATGTCCTTCACT

GTACTAGTCCCGAACAGACATCTGAAGAGTTAATGGAAGTATCAGGGGAAAAGTCAGACC

ATAATCAGGTCATTGCTGAGACACAAGTAGAGAAATTGCTGTCTTCAGAGGATGGGGCTC

AAACCCAGCCTTTTCCGGACACTAAAGCGAATGTTGATGTGGAGATGCCAGACCAGCTTA

CTGTTGAAGAGCAACAAGCAAAACAAGGTACTCCATCAGATGGAGAGCAGAATCTGGGCT

GCACTGGTGTTCAGCTTGATTTTCAGTCAGAACAGCTCAGTGGAGCAACATCTGGAGGCT

CTTCACCATTGTCAGTGGCAGACAGAGACAGTGATCTAACAGCAGCAAGGACAAAAGTAA

AAAATCCAGATGACGAGGTGGACATCCAGGTCACTCATCCAAGAAAACGAAAGATGCCCA

AAAGTTCTCTTTCAACCCCAGCAGGCCTTACAGCTCAGCAAATAAAGGAAAAGACCCAGC

AGTCATTAGCAGCAATCGTGGACTCCCTAAAGCTTGAAGAAATTCAGCCCTACCAAACCG

AAAGGGCCAATCCTTATTACGAGTTCCTGCACATCCGTAAGAAAATCGAGGAGAAAAGAA

AGGTGCTATGTAGCGTCATTCCTCAAGCACCACAATACTATGATGAATATGTTACATTCA

ACGGCTCTTACTTGCTTGATGGGAATCCACTGAGTAAGCTTTGTATTCCAACGATAACAC

CACCACCCTCACTACCTGAACCACTAAAGGAGATGTTCAAACAGCAGGAGATTGTGCGCA

TGAAACTGCGCCTGCAGCACAGCATTGAAAGAGAAAAGTTGATTGTGTCCAATGAACAAG

AAGTCCTGCGTGTCCATTATCGTGCTGCAAGAACATTGGCCAATCAGACACTACCCTTCA

GCGCCTGCACTGTTTTATTGGATGCAGAAGTATACAACATGCCCCAGGATGTACAGGGAG

ATGACGGAAAAACCTCAGTGAGAGATCGGTTTAATGCAAGACAATTCATGTCTTGGCTTC

AGGATGTGGATGACAAATTCGACAAGTTAAAGACGTGTCTCCTGATGAGACAGCAGCACG

AGGCAGCAGCTCTTAATGCTGTTCAGCGCTTAGAGTGGCAGTTAAAGCTACAGGAGCTGG

AGCCTGCCACCTACAAATCAACCAGTATCTTCGAAATCCCAGAGTTTCACATCCCCCTCG

TGGAGGTCAATGACGATTTCGATCTCACGCCAATATAACATCAGGAAATCCAATGTCCTG

ATAAGGATCGTCTGTCCAGGACTCGGAAAGAGTAGAGGAACACAAAGCACTTGTTTTGGG

CGAGCTGCACTGTGTGTTTGCGTTTGTGTGTGTTTGTGTTTGTGTGTGTGTGTGTGTG

>Unigene000139

TCCTGTGACAATCACACGCTGATTATTCTCAACCAACCATAAAGACATTGGCACCCTTTA

TTTAGTATTTGGTGCTTGAGCCGGAATAGTTGGCACAGCTCTTAGCCTCCTAATTCGGGC

AGAGCTAGCCCAACCTGGCGCTCTTCTAGGCGATGACCAAATTTATAATGTCATTGTTAC

TGCTCACGCCTTTGTTATAATTTTCTTTATAGTGATGCCAATTATGATCGGTGGGTTCGG

CAATTGACTTGTACCCTTAATGATCGGGGCCCCCGACATAGCATTCCCTCGAATAAATAA

CATAAGCTTCTGACTGCTACCCCCATCCCTTCTATTACTACTTACCTCTTCTGGTGTAGA

GGCGGGGGCAGGAACAGGATGAACCGTATACCCCCCACTTGCAGGAAACCTTGCACATGC

GGGAGCATCCGTGGATCTAACTATTTTTTCACTGCACCTCGCAGGAATCTCATCAATTCT

AGGGGCCATCAATTTTATCACAACTATTATTAACATAAAACCCCCAGCAATCTCCCAATA

CCAAACACCTTTATTTGTATGAGCTGTCCTCATTACAGCAGTACTTCTCCTGCTATCCCT

ACCAGTACTTGCCGCAGGCATCACAATACTATTAACAGACCGGAACCTAAATACCACTTT

CTTTGACCCAGCAGGGGGAGGTGATCCAATTCTATATCAACATCTTTTCTGATTCTTTGG

CCACCCAGAAGTATATATTTTAATCCTCCCAGGATTTGGCATAATTTCCCACATCGTAGC

TTACTATGCCGGTAAGAAAGAACCATTTGGATACATAGGAATAGTGTGAGCTATAATGGC

CATCGGCCTACTGGGATTTATTGTCTGAGCTCACCACATGTTTACTGTAGGAATAGATGT

AGACACCCGAGCATACTTCACATCTGCAACAATAATTATTGCAATCCCCACTGGAGTAAA

AGTATTTAGTTGACTCGCAACACTCCATGGAGGATCCATTAAATGAGAAACACCCATATT

ATGGGCCCTGGGGTTTATTTTCCTTTTCACAGTTGGAGGACTAACTGGCATCGTACTAGC

TAATTCATCCCTAGACATTGTACTACACGATACCTATTATGTGGTGGCCCACTTCCACTA

TGTCCTGTCAATAGGAGCAGTATTTGCCATTATGGGGGCCTTCGTCCACTGATTCCCGCT

ATTTACAGGGTATTCAATGCATGATACATGAACAAAAATTCACTTCGGCACAATATTCGT

AGGAGTTAATCTTACCTTCTTCCCACAGCACTTTTTAGGATTAGCAGGCATGCCCCGACG

ATACTCGGACTACCCAGATGCCTATGCACTATGAAACATTATTTCATCAATTGGCTCCCT

AATCTCTTTAGTGGCAGTAATTATATTCCTCTATATCTTATGGGAAGCTTTCACCGCCAA

ACGAGAAGTACTTTCCGTAGAACTTGCCCACACAAATGTTGAATGACTACACGGATGCCC

ACCCCCCTATCATACATTTGAAGAGCCGGCCTTCGTGCAAGTTCAATCAAATTAACGAGA

AAGGAGGGAATTGAACCCCCATAAACTAGTTTCAAGCCAGCTACATAACCACTCTGTCAC

TTTCTTCAATAAGATACTAGTAAAATGAGTATTACACTGCCTTGTCAAGGCAAAATTGTA

GGTTTAACTCCTGCGCATCTTAAGCCCTCTGGGCCTAATGGCACACCCATCTCAACTAGG

ATTTCAAGACGCAGCCTCACCCGTAATAGAAGAACTTCTGCACTTCCACGACCATGCCTT

AATAATCGTTTTCTTAATTAGCACCTTAGTACTATATATTATTGTTGTAATAGTCACCAC

TAAACTCACTAATAAATATATTCTAGACTCACAAGAAATTGAAATCGTCTGAACTATTCT

ACCAGCAGTAATCCTTGTTTTAATTGCTCTTCCCTCCCTACGAATTCTCTATCTAATAGA

TGAAGTCAACGACCCCCACCTAACTGTAAAAGCCATAGGCCATCAATGATACTGAAGCTA

TGAATACACTGACTACGAAAACCTGGCCTTCGACTCCTACATGGTCCCAACACAAGACCT

ACTACCAGGCCAGTTTCGACTACTAGAAACAGACCACCGAATAGTAATTCCGATAGAATC

ACCTGTTCGAGTGTTAGTTTCAGCTGAAGACGTACTTCACTCCTGAGCTGTACCAGCATT

AGGAGTTAAAATAGACGCGGTCCCAGGACGATTAAATCAAACATCCTTCATCGCCTCCCG

GCCCGGAGTATTTTACGGACAATGCTCCGAAATCTGCGGAGCAAACCACAGCTTCATACC

TATCGTTGTAGAATCCGTCCCCTTAGAACACTTTGAAAACTGGTCTTCCCTAATACTTGA

AGACGCCTCACTAGAAAGCTAAATATGGGCCTAGCGTTAGCCTTTTAAGCTAAAGATTGG

TGACCCCCGACCACCTCTAGTGACATGCCACAATTAAACCCAGCCCCATGATTTGCAATC

TTACTATTCTCGTGATTAATCTTCTTAACACTAATCCCGAACAAAGTACTAAACCACACA

TTTACAAATGAAATTACAGCCCTAAGCGCTAAAAACCTTAAATCAAAAACCTGAAACTGA

CCATGGCACTAAACCTATTTGACCAATTTATAAGCCCCACACACTTAGGAGTTCCCTTAA

TTGCCATTGCACTTACCCTGCCCTGAATTCTAATCCCCACCCCAACTAATCGGTATCAAA

ACAACCGCCTTATTTCCCTCCAAAGTTGATTCATTAAGTCCTTTACACAACAATTACTGC

TGCCACTAAATCAAAGCGGACATAAGTGAGCCATAATCCTGGCCTCCCTAATAATCTTTA

TCCTCACACTTAATATACTAGGACTTCTCCCTTATACATTTACCCCGACTACCCAGCTAT

CTCTAAATATAGGCCTAGCCGTCCCACTATGATTAGCAACCGTAATCATTGGTTTACGAA

ACCAACCTACAGCAGCCCTAGGACATCTACTACCAGAAGGCACCCCGGCCCCTCTAATCC

CAGTCTTAATTGTTATCGAAACAATTAGCCTATTTATTCGACCTTTAGCCCTCGGAGTAC

GACTAACTGCTAACTTAACAGCCGGCCACCTATTAATTCAGCTAATCTCAACCGCAACCA

TTACACTGCTACCAATAATAACTACAGTTGCAACCCTCACTGCTATCCTACTAGTAATAC

TTACTCTACTAGAAATTGCAGTAGCTATTATTCAAGCTTATGTATTTGTTCTACTACTAA

GCTTATATTTACAAGAAAACGTATAATGGCCCACCAAGCACATGCATATCATATGGTAGA

TCCAAGCCCATGGCCCCTGACCGGCGCAGCAGCTGCTCTTCTAATAACTTCAGGACTAGC

AATCTGATTTCATTTCCACTCAGTAACACTTATATCTCTAGGTCTAATATTATTAATTTT

AACAATAGCTCAATGATGACGAGACATTGTACGAGAAGGCACATTTCAAGGCCACCATAC

TCCTCAAGTACAAAAAGGGTTACGATACGGAATAATTCTTTTCATTACCTCAGAAGTATT

TTTCTTCCTGGGGTTCTTCTGAGCCTTTTACCACTCCAGCCTTGCCCCCACCCCTGAATT

AGGAGGATGCTGACCCCCCACAGGAATCACAACTCTAGATCCATTCGAAGTGCCACTCCT

TAACACCGCAGTACTACTAGCCTCAGGTGTAACAGTAACTTGAGCGCACCATAGCCTAAT

GGAAGGAGAACGAAAACAAGCAATTCAATCCCTGGCCCTAACAATTCTACTAGGCTTCTA

TTTTACTGCCCTCCAGGCCATAGAATACTATGAGGCCCCCTTCACTATTGCAGACGGAGT

GTACGGCTCAACATTCTTTGTAGCAACAGGCTTTCACGGCCTACACGTTATCATTGGCTC

TACCTTCCTGGCCGTCTGCCTACTCCGACAAATCAAACATCATTTTACCTCAGAACACCA

CTTCGGATTCGAAGCAGCTGCCTGATATTGACACTTTGTAGATGTTGTATGACTATTCCT

TTATGTCTCTATTTACTGATGAGGCTCATATCTTTCTAGTATTTAATGTTAGTACGAGTG

ACTTCCAATTACCTGGTCTTGGTTAAAATCCAAGGAAAGATAATGAACTTAGTCTTAATA

ATAATCATCTCAGCCATCCTATCAATTGCCCTGGCCCTCGTATCATTTTGACTCCCACAA

ATAAGCCCCGACACAGAAAAACTCTCCCCTTACGAGTGTGGTTTTGATCCTCTAGGATCA

GCACGTCTCCCCTTCTCTCTCCGATTTTTTTTAGTTGCCATCCTATTTCTATTATTTGAT

CTAGAAATCGCGCTACTACTACCACTACCATGAGGTAATCAACTACCAGACCCAGCCCAC

ACTCTAATATGAGCTGCAGCTGTCCTTGTACTACTCACGCTAGGCCTCATTTATGAGTGA

CTACAAGGAGGCCTAGAATGAGCTGAATAGGGGGCTAGTCCAAATACAAGACCTCTGATT

TCGGCTCAGACAATCCCGGTTTAAATCCGTGGCCCCCTTATGACGCCAGTATACCTTAGC

TTCACCTCAGCATTTACACTAGGCCTGACAGGACTGGCCCTCCACCGCTCCCATCTGATG

TCTGCCTTACTATGTTTAGAAGGTATAATACTCTCCCTATTTATTGCCCTTGCCATTTGA

ACCCTCCAACTAGAATCAACCACCTTTTCCGCAGCCCCCATCTTCCTGCTAGCCTTCTCA

GCCTGCGAAGCCAGTGCAGGATTAGCTTTACTAGTTGCTACAGCCCGGACCCACGGAACA

GATCACCTACAAGCCTTAAACCTACTACAATGCTAAAAATCCTAATTCCAACCATTATGC

TATTCCCAACAATCTGGCTTTCTACCCCTAAGTGGCTATGAACGTCTACAACCACACAAA

GCTTAATTATTGCATTACTAAGCCTCACTTGAATTAAACTGCCCCTAGAAATCAGCTGAT

CAACCTCCAACCCATACATGGCTACAGATCCCTTATCAACCCCCTTATTAATCTTAACCT

GCTGACTCCTCCCACTCATAATTTTAGCCAGCCAAAATCACATTAAAACAGAGCCCATTA

ACCGCCAACGAAGTTACATTACTCTAATAGCCTTACTACAAACTTTTCTAATCCTAGCGT

TTAGTGCTACCGAAATTATTATATTCTACGTAATATTTGAAGCAACTCTAATCCCAACCC

TTATTATCATTACCCGATGAGGAAACCAAGCTGAACGGCTAAGCGCCGGAACATATTTCC

TATTTTATACACTAGCCGGATCGCTACCACTCTTAGTAGCGCTCCTATTACTTCACCGTG

ACACCGGGACCCTATCAATATTTATTATTCAATATACACAGCCGCTAACCCTTAATTCTT

GAGGAGATAAAATCTGATGGGCGGCCTGCCTAATCGCTTTCTTAGTAAAAATGCCCTTAT

ATGGTGTACATCTCTGACTGCCAAAAGCCCATGTAGAAGCCCCTGTAGCTGGGTCTATAG

TACTAGCAGCAGTCCTACTAAAGCTAGGAGGCTATGGTATAATACGAATAATAGTAATCC

TCGACCCCCTATCAAAAGATCTAGTATATCCTATTCTTGCTCTGGCCCTATGAGGGGTAA

TCATAACCGGTTCTATTTGCCTACGACAAACAGACCTAAAATCACTAATCGCCTACTCAT

CCGTCAGCCACATAGGCCTGGTAGCAGGGGGAATCCTAATCCAGACCCCATGAGGCTTTA

CTGGAGCATTAGTACTCATAATTGCCCACGGCCTAGTCTCCTCCGCCCTATTCTGCCTAG

CCAACACCACATATGAACGAACCCACAGCCGAACCATACTCCTAGCTCGAGGAATACAAA

TTATCTTTCCACTAACCGCTGTTTGATGATTTATCTCTAACCTAGCAAACTTGGCATTAC

CCCCTATACCTAACCTAATAGGAGAATTAATAATCATCACAGCCATATTTAACTGATCCC

CATGAACCCTTGTAATAACAGGAACTGGGACCCTAATCACAGCTGCCTATTCACTATACC

TATTTCTAATAACACAACGAGGCCCGCTTCCACAACATATTATTAACCTACAACCCTTCC

ACACACGAGAACATCTATTAATAGCACTGCACCTCATTCCAGTTATCCTCTTAATCATCA

AACCAGAGATTATATGGGGATGATGATATTGTAGATATAGTTTAACATAAAATATTAGAT

TGTGGTTCTAAAGATAGAGGTTAAACTCCCCTTATCCACCGAAAGAGGCCC

>Unigene000140

GAGAGAGAGAGAGAGAGAGTGTAAATGATACACCGCGCGCGGCCGCGTTCAGAATTCTGA

AGAGAAAGGTACTGGCGTGCTGAACCGAGTTGCGACGTTCCTCTGGAGAAGACGGCAGAG

GACATCATCGGATGGGACGGAGGAAAGTGTGTCGCCTATTTTAAGCCCGAACGATCCTGC

AGGTTTAAAGCAGGCGTCCCCGGGGGGAGGAGAGGTCGAGCGGGGTTTCTTCTCCGATAA

AGGGAGCCCAAGCGTGCAGAGCGTGGCCTCTCTCGTCACAGACAGCGGCGACCTTCCGTT

TGCGGATAGCGACAGCAGTGGCAGCGTGAGGAACTTGGTGGTGGCAACGAAGCGCGAAGA

GAAAGACGGGACGGAGCTGCTGGTGGCCGAGGCCAAGAAACTCCGGGTTTTTCTGGAGGA

GATCAGCTTTACGGACGACGGACCTGACAGACACGTGACGCAGAAGACGATTGCACAATG

CACGGACATTCCGGTAAAGAACAGCGCCGAACCTAAATCACCAGAGGTCAAAAAGACCGT

TCTGAAACCGGTCGTAGGAGGTAAAGGGATTTACAGCGCTCTCGCCGGGGTGACGCTGAG

TCCCCAGTCCAGGGTCGAGTCCAGGGTCGAGTCCAGCTCTACGGATTTATTTAGCACTGA

AGACATGGGAAAAAAAACAACAAGCCGGAGAAGATCTCGCAAGCTGAGCAGCGGCTCTCA

GGAGCCCACAAGCCCTTCAAAGCCATTGTCCCCTGAGGCAGAGAACGAGTCCGCTCTGGC

CTTACCCTCACCTGTGCAGATTCACAAAGCTGTGTGGGTGGAAACGCACCTGGAAGAGGA

AGGGAGCGAGAGCAGCTCACTTGGTCAAGTCACACCCGCTCAACAAAGCCCCGTGCTGGG

ATTCAGAGCCTCACTTATCGCTCAGGAAGCGCAGGACTCCGGCTCGGATTCTTCTATTTA

TCAGGACGCGGTGGAGGGAAAGACAGACGAGGAAGCGGACACAAAGACTGAGAAACGGAG

GAGCGTCAAGCTTTCCACCAGCGAGAAGTTCTTTGCTAAAAGAGTCTGGCTGAACTCGCA

GTCGAGTTTAGACGGAGAACAAGAAGACACCAGCGTGAGCTCCAATGAAAAGCAGAAATC

CGAAGTCAGAATTCTGCCAAGCCTCAAGAACGTCTGCGTGGAAATTAAAAAGCCTGACCA

GGCGTCATATGACTCTATAAGCGAAAGTAATGATGCATTCGTAAAAGAGGAAGCGCAAAC

CAAACTGCGGCTTGATAATGATGTAGAGAGTCCCTCAGGGCTTCCTAATAACATCACAGA

TCCTGGAGAGGACTTGCCAGATATGTCTGGATACAAGAGAGTTACGAGTTCAGGGGTTAG

AAATCAGCAGACCGCCGGCACATCGTCCGAGGACAAAGCAAGCAGTGGAACAAATGGAGT

TTCTGCTGGAAAGGGTCCAGCCCCACCAGTGCCACTGAAGACAAAAGCCAGTATGGCTCG

AATTACCAGCCACACAGAGGCCAGGAAAGAGGAAAGCACTAAAAAACTTGCACAAAATGG

CACAGAGAAAAAGCTAAGTAAATCTCCAACAAAAGACATGCCTCATGTGTTCACCAAACC

TGCAGACAAGTCAAAAATTCCAAAAAAGCCTGCACCTGAACTTCTGCCAAAACCAATCAA

AAAGCCAAACACAAGCATGTTACCTGACACGCCAGTTTCTTCTCCAGTTCAGACACGTGA

GCCTGCGCTGTCTGAGAGAAGTAAACCCAGCTCAAGTAACAGTGTTACAAAACCCCAGTC

ACCAACATCACAGCCTGATCAGAGCCAGACTAAGTCACCCATTAGGAAAGAAGCAGCTGT

GGCTTCTGTGGATGTAGATCTGAGTCGTGAACCCAAACCTTCTCAAAACAAGAACCCAAA

GAAAGGAAAGGATTCAAAATATATTGTGGAAAAAACAGAGCCTCCATCATCTCCTAGTGG

CTTAGAGGAAACTAAGGAAACAAGTGACCCTAAAGAAGTTACTAAGTCAAAACCACAAAC

TCCTACTGAAAAAACACCCAAGAGTAAAACAGGAAAACTGTTAGTTAGTAAAAGCTCTAA

AGTTAACAAAGCTGAGGTTGATAACAAGCCTCACACTCTTACAGAGGCATCGCAGCCAGA

AAACATTAGCACAAAGGACAAGACTACAAATGAAATGCGGGTCATCGGATCAAAGAATCC

TAAAAAACCCAAGGCTGAGGGATCGCCAACAGGAAGCAGGCTGCCTCGTCTGACTCCACC

TAGCACTCCAAAACAACCCACAGAGGATGAATTCGATTATAGGGAAGATTACTCACCAAA

GCAACCCTCGTTTTCTGATCCAAGGCCAACGACCGATGATTCATCTTTAGTCAACGGTCC

TCTTAGTCCTGGCAAATCTGCAGAATGTGACCAAGCTGCTGAGAATGCTGTGGTGAAGGA

TTCATCAGAAAACAAACCCTTAACACCTTTAAAACATTCGACTAAACTTAAATCCAAAGA

AGAGGAGGAAACATTGAAAGTTAAACCCCCTGTAGACCTTCAGAATGAAGTGTCAGACCA

GAACCTGGAGAAAGCAAAAAAGAAGCAAAATGTGAATGTGGAGACCAAAAAAAATATTTC

AGAGCTGGAAACATCAGCTCAAGTGCCTGAAGAGCACAAGGACAAGGTGCGTGTTGCTAA

AGTCAAGGACCAGACAGTAAACCTGGAAAAAGAGACCCAGCCTGTAACTCTAACGACCAC

AGAACCTACAGCAGACTTGAAAACCTCACCTCGAGATTTAAAAGAGAGACTTAATGTATC

CAAAGAGGCCCAAAACATGTCCTTACAGACCGATGAACATACTGTAGATTTAAAGAGTTT

ATCTAAAGTATCTCAGAAGGAACAGGACCAGACTGAAGAGCACTCAAAAACTTCAGTGTC

TGAGCAGAAAATTGACAGGCCAGAATCTGTGCCATGTGTGGATGATAAGATTAAGGAGAA

TAAAACCAATTTAAAATTAACTCATAAGGTTGAACAAGAAATGGTCAAGGGTGAACCGGA

TGCAAGTGTAGATGATAAGACCAAGGACCTTAAAACGAAATTCAAATTAACTCAGGAGGC

TGAACAGAAACCTCCAAAGGCCAGTGAGAAGTCTACTCATTTGAAAAATCCACCTGAAGT

ATTTAAGAAAGAAACTCAAGCCCAGGTTGTACAGAATGAAAAGGATACAACAGAGATAAC

ATCTTTATCCCAAGTGTCCGAAAGCAGTAAAATAAAGACTGACAAAATGCAAAGCATAGA

TAAGAAAAGGGTTCCTAACACAGATCAGGTGTCTGAACAGAAATCAGACAAAGCTGAAAA

ATCGCCTAAAGCGACAGTGGAGAATAAAGCAGACTTAAAAGCTCAACCTGAATTATCAAA

GGAAGCAAAGAAGGGAGAAAATGTGAGCGGTATTTTAGATTTGCCTCCAAGTCAAGCATC

TTCTCTCGAAGGGGACAAATCTGATAAGTTGAGGGAGGGGCATTGTAAACCTAAAGACCA

TACACAAGACCTGACTGCTCTACATCAAGTATTAAACCAAGCAAGTAGTGAAAAGACTGC

TGTTGAAATAAAACCAGAAACAAAGGCAAACAAGACTCAAGTTACAGAAACATTAAATGT

TGGTACGTCTCAAGTAAAACCAGAGCTTAGTAATAAAGGAGCTCCCCCAGACACAGACAA

GGAGAAGGTAACACTTGACACAGTGGACAAAATATCAGTCAAACCAACAGCGAGCGTCCA

TGAGACCAAAGAAATTAACCAGCAATCCAACACAGCACAAGTCACACCGGATGCTACAAA

ATTCATAGGTAAAGAACAAAACAATGAGATAAATCGTCTTAAATTAAACAAATTGTCTAA

AACCACAGAGGAAGCAGGTCCTAGGGAGGCTGCTAAAGACACAAAAGCCTTTAAGTCAAT

TGACACAATAGTGCCGGCACTTAAAGTCTCAGAGTTTGCCAAGAAGGACATCAAAACTAA

ATCTGGTAACAAGATGGAACTAGAACCTCTTGCATATGGAAACCTCTGTACTAATCAGCA

CCCTGTAGTTAAAAAAGCTGAGCAACCAAAAGTTACAGGGCAAGTGAGCTCCCACCTGGA

GATGACGACTGAGGTCAAAATACCCAGCAATGCCATCCAGAAACATAAAACCGACCCTAC

GAAATCTCAGAAATTACCCATTATTGAGAAGCCGCCGCCATGCAATACTACTGCAAAAAT

GACAGCTCAAGACTTTCTACTGGCCTCAAAAAAACTTTCAAAAAATGAATCTCCATCTAG

CTGGCTGGATGTGGATCAAGGTTTTGAGAAGAAGCAAAAGAAAATGGAAAGAAAAATGGA

CTGTTCAGCAAGCAACAGTAACCTCCAAGACACATCTGATGAACCTGAAGACTTTATACG

CAAAATCAAAGAACTTTGCTCTCCTTTCTCGTTTCCTCCCAAAAAGCATGCTAAGTCTAG

GACGTTTGTGCCTCCGTTTGCAATGCCTGCTATAATAGAAGATCACTTTGAGAAAACCTT

TGATCCTGAGGAGTTCAAGTTTGGAATAAGAAAAAGCACAAGGCCAAAAGATCCATCTCC

AGCTATGTTGATCAAGAAGAAAAGTGAAGATGTGAGAAATAAACAGCTTCCAAAACGCAA

GGCAGCTGAAGATAGTATGGTCTTCAAAGCACTGGTTTCCAGACAGGGACAAGACAAAAC

AGACGAGGAGAAGACCACAGAGAGTAAGGAAAATGGGGAAGACCAGTGTAATTCAGAAGG

TCCCGGAAAGGTGGCTTCTCGGTTAGAAAGAATGTCCATCCTATCAAACCTCTTGAACTC

GAGGAAACCTAAAACCCAGCCGGAAGGGGTATCAAATGGAGTTACATCTCCTACAGTATC

AACACTAGGCGACACGACCAACCTGACGACGCTTAAATCTGAAATAGCGCCTCCAGGCCA

GATAGATGTGAAATTGCAACATCTTGTGATAGATCCTGGTGTTTCTGGGGATTCACTAAA

GAGCCCACTCACTCCTCCTCCTCTTCCGAACTTTTCAGAAATCAAGCTTCCTGATTTCCT

TGGAAAGTTCCTGAAAATGGATCAAGAATCCCCTGCTTTAGGTGTCTCACAAAACCTAGA

AGCACCGTCCAAGTTGCCTGCAATACAGACAGAAGTCAGCGCCGGAGTAGTTCCAGACAT

AAATACTGGTCTGAAAACATTTCCAGAGCCTTTAAAACCAATCTTTCCACCCAAACCACC

AGAACAACAAACCAAATCTCCGTCACCTACACACACTCAGATAACGACAGCAAGAGGCTT

TCACAAGCGGCCCGGAAAGATGGTCATCTTCCAGCAAGCCCAGTTTGGCGGCGAGGCGTA

CGAGGTGTTTAGGGATGTAGACGATGCCACGTCGTTACAGCTGTCCCCTGTTATCTCTCT

AAAAGTTGTCAGAGGATGCTGGCTGCTTTATGAGAAACCAGGTTTCCAGGGACGGACCAT

CGCGCTTGAGGAAGGGCCAACGGAATTGGTGAACATGTGGGCGGAGCCGGACCCTGACCA

GGAAGTGGGACCAGACGAAATTCCCATTCCTAGCAAACCGATGGTGATAGGTTCTATTCG

TCTGGCTGTGAGGGACTACAGCCTGCCCAAGATCGAGATCTACTCCGAGCCGAACGGAAT

GGGCCGCCTGTCCACGTTCTGCGATGACATCATCGAGTTGGGCACGTTCGGACGTCCTCA

CAGCGCCGGATCCATCAGGGTCCATTCCGGAGTTTGGCTCGTCTTCAGTGATCCAGATTT

CCAGGGTTTGTTATCTGTCCTGCCTGAGGGAGAGTATCCGTGTCCGGAATCCTGGGGTTT

TCCCAGTCCCTTCGTCGGATCTTTACGTCCCCTTAAGATGGGCGGCATTAAAGTAGAAAA

CCCTCACGAGGTCCGGGCCGTGCTGTATGAGGCTCCTCTGTTCCAGGGTGCCTGGGTGGA

GATCGACTCGGATGTGTATGATGTCTTGGAGGCAGACGAGGAAGAGGAGAACGATGAGGA

AGAACAGAACTTGGTCCGGAGGAAGAAACCGACTATAATCGGCTCTATAAAGATCCTCAG

CGGCCTGTGGGTGGGATATGCAGAGCCGGGGTTCGAGGGTCGTCAGTATGTGTTGGAGGA

GGGGGAATATGCCGACTGCTCTGATTGGGGAGGTTTGGAAGACGAGCTATGCTCACTCCG

CCCCTTACAGTCGACCTTTGTGTCTCCTCACCTGAAGATGTTCAGTGAGCTTGAGTTCAA

TGATAGAGGTCTCAGTGTTGACCTGCTCGTCCCCGTCATGGCCATGGGGA

>Unigene000141

CAGCTAAGATTGCTAGCGATGCCCTGTACAAAAAGGACTTTAACAAGTCTAAGACCAAAT

TCCACCTTCCAGTGGACATGCTGGCTTTTGAACTGGCCAAGAAATGTCAGGTCCAGGTCA

ATGATGCCAACTACAGAACTTACCTGCACAACTGGACCTGCCTGCCAGACTCCAATGATG

TTGTTCAAGCCAGACAGACATATGAACTTCAGAGTGATGCTGTGTACAGGGCCGACCTGA

AATGGTTGAAAGGCCTGGGATGGATCCCTATTGGTTCACTTGATGTTGAGAAAGCAAAGA

AAGCTGGAGAGGCTTTGAGTGAAAGAAAATATCGTCAACCCCCGAGCAACTTCAAATTCA

CAAGCACGACTGAAGACATGCCGATAGTGTTGGCGAAAGCCAATAATAATATCATGAACA

AGAAAGCTTATGTTCAAGCCTGGGATAATGAAAAGTCCAATATCCACATCATGCCAGATG

CAATGGATGTGGTCCTTGCCAAACAAAATAAAGTGAATTACAGTGAGAAACAATACAAAC

TTGCCAACGAACTGTCAAAGAAAAAAGGCTATGATTTGCGCGGTGATGCTATTTCTATCA

TTGCTGCTAAAGCGTCGCGGGATAAAAGGTACAAGCTTGACAATGAAGAATCCAAGAAGA

AGGGTTATGACCTGCGAAGTGATGCTATTGCTATCAAGGCTGCCAAAGCATCAAGAGACA

TCATCAGTGAGTACAAGTACAAAACTGGATACCGCAAACAAGTTGGACACCATATTGGAG

CTCGCTGGCAAGTTGGCCACCACATTGGAGCCCTGAGTATACAGGATGACCCACTGCTCA

TGCTGGCTCTGAACTCAGCTAAGATTGCTAGCGATGTCCTGTACAAAAAGGACTTCAACA

AGTCCAAAACAAAATTCCACCTTCCAGTGGACATGCTGGCTTTTGAACTGGCCAAGAAAT

GTCAGATCCAGGTCAATGATGCCAACTACAGAACTTATGGATACCGCAAACAAGTTTGAC

ACCATATTGGAGCTCGCTATGTCCAGGATGACCCATTGATGATGTTGGCCATAAACTCTG

CTAGGATTGCCAGCGATGCCCTGTACAAGAAAGACTTTAACAAGTCTAAAACTAAATTCC

ACCTCCCAGTCGACATGTTGTCTCTTGAGCTTGCTAAGAAATGCCAAATCCAGGTCAATG

ACTTTAACTACAGAACTTCCCTGCACAACTGGACCTGCCTGCCCGATCAGAATGATGTCA

TTCAGGCTCGCAAAGCATATGATCTTGCCAGTGATAATATCTACAAGACAGATTTGGAAT

GGATCCGTGGCTGTGGATGGGTGGCAGCTGACTCTGTTGATCATGTCAAAGTCAGAAAAG

CTCAGCAGATCCTCAATGATAGAATGTACAAGAAAGATGCCAAGGATAATTTTGCCAAGT

TCACAAATATTGTGGATCGACCAGAAGTTCTCTTGGCTAAGGTTAATGCCTTCAATCTCA

GTGATCTGAAATACAAAGAATCTTTCAACCTTGAGAAAGGCCACTACATCGGATCTGACG

ATACACCCCAACTAGCACACTCCAGAGAGGCTGCAAAGATCATCAGTGAGAAACTGTACA

AACTTGGCTGGGATGAAGCTAAAGCCACAGGATATCAGCTGAATCATGAGTACCTGCCAC

TTGTATCAGCTAAGAAGGTCGGAAGCATCGTCAGTGAAGCTAAGTACCATGATGCGCATG

AAAAAGCTAAGGGTCATTACCTGGCCAACACTCTGGTTGACTTCCCAGCAGTGGTCCATT

CTTCACAAATGGAAAAGATGAAGAATATGAGGACCTATCAAAAGGATTACCATACGAGCA

AGACTAAGCTTCACATTCCACATGACATGATCAGCCATGTGGTAGCCAAGAAATGCCAGG

AAATCCTAAGCGATGTGCTGTACCGCAGCTACCTGCACCAGTGGACGTGCCATCCGGAAC

AAAACGATGCTATTCGTGCCCGCAAGGCCAATGAGATCCTGAGTGATGTGTTCTATAAAG

ATGATTTGAACTGGATGAAGGGTATTGGCTGCTATGCCTGGGACACACCAGAAATTGTGC

GAGCCAAGAAGTCTTATGAGCTTCAGAGTGACATCAAGTACAAGGCAGAGGGCAAGAAAG

AATTTAATAACTACTCTATAGTGACAGATACCCCTGTGTATGTGACTGCTATACTGGGCC

ACACATGGGCAAGCGAGCTGAACTACAGAGAGGCCTACCACAAAGAGAAGCACCTGTACA

CTACTGTTTTGGACACCTATGATTATGCAAGGTGCTACAACTTGAAGCAACTGTACAGCA

GTAAAACCTATTCTGCTCTTTGGGATAGAATCAAGGCCAAGAGCTATACTATTCCTATTG

ATGCTAATGCTTTGGTCCACGCCAAACAGCAGAAGGTCCTCCTCAGCAAAGTGAAATATA

AGGAGGATTATGAGAAGTTTAAGTCCCTGTACAGCCTGCCCAAGTGTCTGGCGGACGACC

CAGCCACTGCCAGATGCATCAAAGCTGGAAAACTTAACTTGGATCGCTTGTACAAAGATA

AATGGGAAAAGACAAAAGCCAAAATTCATATACCTCCAGATATGCTAGATGTGGTGGCTG

CCCGCAACACCCAGAAAATGATCAGTGAAGTGGACTACCGTAAATATCTGCACCAGTGGA

TCTGCCTGCCTGACATGCAGGTCTATGTACATGCCCGCAAAGTCAACGAGCAGCTGAGTG

ATATATTTTACAAGGAAGATATGACCTGGCTAAAGGGTGTTGGATGTTATGCATGGGATA

CTCCAGAAATTCTGCGTTGCAAACAGGCTATGAATCTGCAGAGTGAGAACCTATACAGAG

CTAAAGGAGTGGAGCATTTTAAGAACTTCTCTGTAGTAACAGACACCCCGGTGTATGAGA

CATGCAAGCAGAGTGCCCACAACCTAAGTGATCTCAATTATCACCATGACTATGTCACCA

ATGTAAGAGGTAAAAACACTGCTCCTGCTACGACTGTGGACACTGAAAGGGCTCGTTTAG

CCAACTACATCCAGAGTGACCACATTTACAAAGAGGCAAGCAAGAGTAATATGTCCACTG

GCTACTCTCTGCCCTTCGACACTCCACTTGCCCTGCAAGCCAAAGCCAACAACGTCATTA

CCAGCAATGTAAAGTACAAGGAAGCATATGAAAACACTAAGGCTAGGAGCTACAAACTGG

ACCCCAATGGTGTCAATTTCGTCACCATAAGAAAGGCCAACCAGGTCACCAATCAAAGAC

TATACCGTGCCCTGTATGAAAAGGAGAAGGACAAAGTTCACTCTGTATATGACACCCCTG

AGATCAAACAGGTTAAGGCCACTCAGGAGGCCATCAGTGATGTTTGCTATAAAGAAAAGT

ACTACAACAGCAGAGGGACACTACTGCCAATGCCATTCACGCCACAGCTGATGCACTGCG

CTCATGTCAATCAGATTAACAGTGATCTGAAATACAAAGAGGATTTGCAGTGGCTGAGAG

GAATTGGTTGCTTCGTGTTTGATTCTCCTGAAATGGTCCGCATCCGTGAAATCAACAAAT

TCAGGACTTCCTACGATATTGATGCCAAGAAAAACTTTTCCAACTTCTCTGTGGTCTTGG

ACACGCCAGAGTACAAGCGTGTGTCTGAACTCAAGACGCACTTAAGTGAGCGGGTGTACA

AAGCTGCGGGAAATATACAGAGAACTCAGTGCAGCACAACTGGTGACGCTATGGAATTTA

AGCGAGCCAAGTGGGCCCAGACTCTTACCAATAAGTGGCTATATACTGATTTGGCTTCCA

AAGAAAGAGCCTTCTACACTCCAGAGCTTCACACTCCTCTTCTGGATCATGCTAGATCTA

TGAAAGTGGTGTACAGTGAGAACAAGTATAAAGAGCAGTATGAGAAGATGAAGCACAAGT

ACACTCCCGTCCATGACACCCCCATCCTAATCCGTGCTAAAAAGTCTTACCTTAATTCCA

GTGACCTGCGCTATAAAGAGACCTTTGAGTTAGCCAAAGGACACTACCACACCACTAAAG

ATGCCCTGGACATTATTTGTGCTAAGCGGGTCAGAGATGACATCAGTGAGGTTAAATACA

GAGAGAAATACATCAACTCACTAGGAACATGGAAGTCTATCCCGGATCGTCCTGAATTCT

TCCACAGCAAGGTTGTGCGAGACTGCATCAGTGATTACAAATACAAAGAGGACCTGGAAT

GGATCAAAGGTGTTGGCTGTTTCGTGTGGGACACACCATTGCTGAAGCAGGCTGAGCATA

ACAAAGCACTGTACAGTGAGAGATTGTACAAGGCCTCATATGAGAAGACCAAATGCAACT

TTAAGTACACATGCGACACACCATTCTTTCAAGCGGTTAAAAATGCCTCCGCCCTCTCCA

ATGATAGATCCTACCGTGCATCCTATGAGAAGACTAAGGATAAGTACACCATAGTTGTAG

ATGACCCTAGAAACCTCTTGGCTAAAGAGGTCAACAATATGAGCCAGTGGAAGTACAGGT

CACAATATGAGCGCCTCAAGTCAAAATACACATCAGTGCTGGAGACCCCCGAACACAAAG

TCCACAAGCTTAGAAAGCAGATCAGCGATAAAATCTACACGATGGAGTATAATAAGAATA

AGGCTAAGGGCTACACACTCGGACACGACACTCCTCTGAATCTGCACATGAAGAAGGTCA

AAGAAATTACCAGCAACCTGAAATATAAGGAAGTGTATGAGAAAAACAAAGCTCAAATCA

ACATCGCTCCTGATGCTTTTGACATCCGGGCTGCTAAAGAAGCCTACAAGAACATCAGCA

ATCTTGACTACAAAAAGAAGTATGAGGCCACCAAGAATAAGTGGATTTGGACCGTGGATA

GACCTGACTTTGTCCACGCTGCCAAGATCAACTACCAGCAGAGTGACGTTGAGTACAAGT

ACGACAAGGAGATGCTGAAAGGTTGCGTGATGCCTGTTACTGATGATAAGTACACACTTC

TAGCCAAGCAGAACACAGAGCTTTCCAGTGACATTAAATACAAGCAAAAGTATGAAGAGG

CCAGAGGACATTACCACGTTGTACACGATACTCCTCAGATCCTCCATGCAAAATCAGTGT

CTGGTCTGGTTTCTGAGAGCAAATACAAGCTGGAAAGCAAGAAGGCTCGACAGTCTGGCT

CTTTCACCACCTTGCCTGAGACCCGTGACACAGCCCACAGCAAAGCAATGACCAAAATCA

CAAGTTCTAAAGTGTACAAGCAGAAGTTTGAGAAAGACAAAGGAAAGTCGATATATAACA

ATATGACCGTACCTCCAGATGTAAAGCATGCCATGGATGTGGCCAAGAGCCAGAGCAATA

TTAATTATAAACAGGAAGCTAAAGCCAAGTTGCATTACACACCTGTTGCTGACCGCCCTG

ACATCCTGAAGGCTACCCAGGCTGCCAAACTCATCAGTGAGGTGGGTTATCGTGACAAAG

CTCGTCAGGAAGCCAGTCGTGGAGGATCGCTGGTCAACCGTCCTGATATCAACCTGGCCA

CCGAAGTGTCCAAGCTTACTAGCCTGGTGAAATACAAAGAGAAGTTTGACAAGGAGATGA

AAGGAAAGAGACCACAGTATGACTTGAAGAACAGTAAGATTTATCAGACATTGAAGGATG

CCAATACGCTTGCAAGTGAGGTGAAATATAAGGGTGACCTGAAGAAGATTCATAAGCCTG

TGACTGACATGTCTGAGTCTCTGGCCATGCAGCACAACCTGAGTACCAGCAAACTGGCCA

GTAGTGTAAAATACAAGGAGAAGTACGAGAGGGAGAGAGGCAAGGCCATGCTGGACTTTG

AGACTCCGACTTATGTGAGCGCCAAAGAAGCACAGCATATGCAGAGCCAGAGAGAGTATA

AGAAGGCATTGGAGCAGGAGATAAAGGGCAAAGGAATGTTGGCTTTGGCTAACGATACTC

CAGACTTCATGAGAGCAAGAAATGCTACAGAAATCCTCAGCCAGAGTAAATACAAGCACA

ACGCTGAGCAGGACAGGGCCAGCTATACCACAGTGATTGACACTCCCGACATCATCCACG

CTCAGCAGATCAGGAATATTGTGAGCCAGAAAAAGTACAAGGAGGAGGCAGAGAAGACCA

TGTCCCACTATAATGCAGTACTGGACACTCCTGAGATGCAGAGAGTGCGGGAGAACCAGA

GGAACTTCAGCACTGTTTTGTATAAAGAACCTTCTGGCAAAGGAACTTCTGTCATATTTA

CACCAGAGATGGAGCGGGTCAAACGGAACCAAGAACATATTAGCTCG

>Unigene000142

GGGGTTTTTATAACTAGTGACGCATTTGGCGTTACGTTTTTTCCCTCCACGCAAGTTCTT

TAGCATGTTCTTCAAATTCTCCATTTCAAAACTTAAATCTTCTAAAACTATTTAGAACGA

GACGAGTTTCTGAGTGTTTGGACGCAGTTTTCGATTTTTAAGATCAGCCACACATCCGAC

TTATGCAGCGTCTATAGCAGCACAAGATGTTCAACAAGTCGTTCGGTACTCCCTTCGGGG

GAGGAACAGGAGGATTTGGGACTTCATCTACATTTGGACAGCAGAACACAGGATTTGGAG

CAGCAGGTGGTTTTGGTGCTTCTGCTTTCGGCACTGCAAACAACACCGGTGGTCTTTTTG

GAGCAACACAGAACAAACCTGGTGGGCTTTTTGGCTCCAACACATTCAGTCAGCCAGTCA

CTTCATCCACTAGCAGTGGATTCGGTTTCGGTGCAACAAGCGGCGGAACCTCGAACAGCC

TCTTCGGAAGTACTAATACGGGAGGTGGCGGTCTTTTCTCCCAGCAAGGCAATGCATTCG

GTGCCAATAAACCCGCATCTTTTGGTACATTTGGTACCAGTACCAGCAGTGGAGGGTTGT

TTGGGACAACTAACACCACTTCTAATCCATTCGGCGGAACTCCGTCTATGTTCGGATCTT

CGGGCTTTACTGCGACTCAACCTGGCACAACCATTAAATTTAACCCACCAACAGGAAGTG

ACACCATGGTGAAGAGCGGCGTGACGACCAGCATCAACACCAAGCACCAGTGCATCACAG

CCATGAAGGAGTATGAAAACAAATCTCTAGAGGAGTTACGGTTAGAGGATTACCAAGCAG

GAAGAAAGGGACCCTCTAACACAGGCGGTCTGTTCGGGGCTACAGCCACAGCAACTCCAA

GCACCGCTACCGGCCTGTTCGGCCCCTCGGGTACCGGCTTCAGCTTCAACCAGCCCAAAC

CCTCCTTCAGCGCTGGTACTAGCGGCTTTGGGGCGACCCCAGGTGGTTTATTTGGCCAGC

CGCCACAGACTAGTAGTCTTTTCAAGTCTTTCGGTCAGCCAACCACCACCCAAAACACAA

GCTTCTCTTTCGGTGCTACCAACACCATGGGCCCGACTAATACTAGCAGCATGGGTCTGT

TTGGAAACACCGCACCCGCGCAGGCTGGAGGTCTGTTTGGAAACACGTCCAACACCAGCA

CGGCCACTGGCTTCGGCACAGCCACCGGACTCTTTGGGCAGCCCAATGCTGGCTTTGGGA

ATAATTTGTTTGGTAACAAGCCTGCTGGTTTTGGCACCACCACCACGAGCGCTCCTTCCT

TTGGCACAGGAACGGGTCTGTTTGGAAACAAACCCACACTCACTTTGGGCACCAACACAA

ATACATTTGGGTTTGGTGCCAATCCTGCTGGGGGCAGTCTGTTTGGAAACAAACCCGCCA

CTGGTACACTCGGAACAGGACTCGGAACAGGATTTGGAGCAGGTGTGGGTACAGGTGGGA

CGTCTCTGTTTGGAAATAACCAGAATAAGATCGGCTCCACTTTGGGGACAGTAGGGGCCT

TTGGGGGAACAAGCTTCAATACAGGAGCCAACGCTCTAAATTTTGGAGCACCGCAGCAAC

CTGTCGCTCTGACGGATCCCAACGCTGCAGCCCAGCAAGCAATCCTACAACAGCAGCTCA

GTGCGCTCAACTATTCACCTTTTGGAGATTCACCTCTGTTTAGAAACCCACTCTCTGACC

CCAAGAAAAAAGAGGAGCGCTTAAAGCCTACAAATCCAGCTGCTCAGAAAGCCCTGACGA

CCCCTACGCACTACAAGCTTACCCCTCGTCCTGCCACACGCGTACGCCCCAAAGCCCTCA

CCTCGTCAGGTTCTGGGAAATCTCAGCTTTTTGATGGGCTAGACGATGACGAACCTTCAC

TTACCAACGGAGCTTTTATGCCACGGAGAAGCATAAAGAAACTTGTGCTAAAGAACCTGA

ATGGAAGCAGTCTATACAGCAGCCCACTAAACAGGGATGCTGACGATTTGAATTCGCCAT

CCGAATACCCGGAGAATGGCCTTAGTCTAAGATCAGATGCAGAGCAAACCCAAGATGTAG

AAGCTGAAGGAGGAGCAGAGGATGATCTGCAAGTCACCAAGTTCTACACCAACCCCATTG

CAAAGCCCATCCCACAAGCCCAACCCAGTCCCTCACTTCAAGACACAATCAGTGAGTTCA

CTATGAGGAGAGTCAGCAGTGTCCGCAACGGCCTGGACCTCAGCAGTGAAGAGATCTCTT

TGGGAGATGACAGCGTTCAGGAAGAGAGAGACGAAGAGCTGGAGGTGCAGAAACCTCCGC

ACCCTGCCGGTATTGTTCTTACGAGGGTGGGCTACTACACCATCCCGTCCATGGAGGAGC

TCGGGAAGATGCTGAATGAAAACGGAGAGTGTATTGTCGAGAACTTTACAGTCGGAAGGA

GAGGATACGGTTCTATTTTCTTCCGCGGTGAAGTAAATTTGACCAACCTGAATCTCGACG

AAATCGTGCACTTCAGACGAAAGGAAGTAATCGTCTACCCTGATGACGAGAATAAACCTT

GTATAGGAGAAGGCCTCAACAGGCGAGGAGAAGTGACTCTTGATGGTGTTTGGCCCATCG

ACAAAACCACTCGCAGCCAGATTAAAAGTCCTCAACGCTTGAGCGAGATGAACTACGAGG

AACGTCTGGAGAACGCCTCACGCAAACAAGGTGCACGCTTCCTGGAGTACAGACCTGAAA

CCGGATCCTGGGTTTTCGAGGTAGCACATTTTTCCAAGTACGGCCTGCAGGACTCCGACG

AGGAAGACGACGTCCCTCCGGCTAAAACCGAGGTGAAAAAGATGAAAACGGCCATCCCGG

CTGGACTGCAACAAATTCCACTGTCCCAACAGCATCAGCAGATGGCGCCACAAGCTCAGA

GCACGGCCGTTCTGGAGCTTCTGAGCCGTGTGCCGGAGCTGGACAGCGACATGGCGGATA

TTACGCAGGAACATCTAGCAGATGGTGTGTTGTTTAAGGAGGATGATAGCATGCTGGAGG

ATGACACCAGAATGGCTCGAAGACCAGAAACTCCTGCTGAGCCGGAGCCCATCTCCGCCT

CCAGTCAGATCGCCTCCTCGTTAGGAATCAACCCGCACGCTCTTCAGATTATGAAGGCCT

CCCTGTTTGCTGCTGATGACGACTGCGACCTCTTTTACGAACAGTCCCCTCCGAAACTCC

TGGAGGAGCTATCGTCTCCCCGGGTTTTACTTGGGAAAACTCCGGCCAGGCTATCAGTGG

GTGGTCTTCTCCAGAGCAAATTCTCCTCTGCTGGAGGGCTGTACTCCCAGCTGCCAGAGG

TTTCTTTTGGGGCGATCTCTCAAAAGCCGAGTAAACCAGCAGTACCAGAAGCTCCATGGC

CCTCACTTGGGACCTCCGTTATTTTGCCTCCTCCTGTCCCGGAGGTCACACTGAGGACAG

TAGGGGCACGCAGACTTGGGGGACCTGTGCCTCTGGATAACTCTGTGACTCTGGGAAAAG

GGCACCTGCTGATGGACGCTGCGCTGTACATGGGCCGCTCGTTCCGGGTGGGATGGGGAC

CCAACTGGACACTAGTGCATTGTGGGAGTCCTCTCAGCACGATTGACGAGTCAAAGGAAC

AGACGAAAGACATGATGGGTTATGGCTTTTTACCCCGACCTACCAAGAACCGGCAGATCA

CTGACAGCCCCTTTAAGGTACACGTTGAGCATGTGGTGGGCATGGAGCCCAAAGAGACCA

CCCAGAGCCAATCTGTGTACCACAAGCCGCTTGAGATCGGCCTCAAGAACAGCACAATCA

GCACAGATGATCTTTGCCCATTCATCAAGCCAGATAAAGGTGTAAATGCCCTGCACGATT

ACGCCGAGTGGATTGTCGAGGTCCTTAAAGAAGCAGCATCTGAAGACGTTGTACTCAATC

ACTGGCAGCAAGTGTGGACGCTGTGTGAGGCTCTGTGGGGACGACTTGGAGAGCGGAACT

TGGATCCTGAGACTGGTGGAGGTTATCGAGAACAACATGCAAGAAGGCACAGTTTCTCTC

GCTGGCTATCTGAAAGTGCTGCCCAACGCATTCAGGACGAGGTGTGCCAAAGTCAGTCGC

ACGCTGACGCCATCTTCAGCTACCTGACTGGTAATTGCATCAGTAAGGCGTGCAGACTGG

CTCAGAAAAATGGAGATCATCGTCTGTCCCTGTTGCTGTCCCAGGCCGTCGGTTCTCAGC

TCACCAGAGATCTTTCGGCTCTGCAGTTAGCAGACTGGAACCGAATGCAGACAGACTCCT

TCATCCAGGATGAGAGACTGCACATTTTTGCCCTGCTTGCAGGAAAACCTGTTTGGGAGG

CAACAGACTGCTGTATAAATGTGTGCGCTAGGCTGGACTGGAAGCGCTGTTTGGCTGTGC

ATCTGTGGTACATGCTGCCCCCTACAGCCTCTGTAGCAGACGCTCTCGCCAAATACGAAG

CTGCCTTTCAGGGTTTAGAGGAAGGAAGAAAATACGCCTGCGCTCCTCTTCCTCCGTATA

TGGATGATGATTATGAACAGGAAGCTTTCGAGGAGATGGAGGATACGGAATCTAAAAAAC

CCCTCTACGACATCTGTTTCCATCTGCTGAAACTTTACAGTGACAAGCACTACAGCCTGC

AGCAGCTGCTCGACCCCACTACAGTGACTGCAGAGCAGCTCGACTACAGGCTAAGTTGGC

ACCTGTGGAACATGCTACAATCCCTAAATTACACTCATCTGGCAGTGCCAAGACAGAATC

TTCTACACATTAGCTACGCTGCCCAGCTGGAGAGCGCAGGCCTCTGGGAGATGGCGGTTT

TTGTTCTGCTGCACATTCCTGACTCAGTGCATCGAGAGCATGCCGTGCGGGAAATACTGA

ACCAACATTGCACTCTGGAGGAGACTGAGGAGTCGGCAGAGAAGGAACACTTCCTCACCC

ACAAGCTGCTCATCCCCATGCAATGGCTCCATGAAGCTAAGGCGGTTCGGGCACGCAGGG

ACGGAGACCGACACGGCGAAGCGCTGCACCTCTTCAGGGCCGGACACTGGAACCGCTGCC

ACCAACTCGTCATCCAGGACCTTGCTTCAGACTGCATCATTAACGATAATCATGAGTATC

TGAAGGAGTTTCTGGAAGGATTTGAAGCGCCTGAGCGCAGCGTGCAGATACAGGGCTGGG

ACACATCAGGACGTGTCTACCTGAACTACATCCGTGTTATTCAGACCCTGAAAGCCATTC

AGAGGATGGACAGTCCAGGATACCAGCTGGAACGACTACACACCGATGTGATGTCCCTCT

GCAGCAGGATTGAGCATCTTCCCTGTTTTAAAGCCAAAGACAGACTGGCTCAGTCAGAAA

TGGCTAAACGTCTGGCCAACATCCTGCGGGCTGTACTAAGTCTTCAGCAGGGTGGGGAGG

GCACGCCGGATCCTCGTCACATCCCCCTGTGCCATCTTGCCCCCCACATAGGACGCCTAC

CCATGCCGGAGGACTACGCTCTGGAGGAACTTCGCAGCCTCACCCAATCGTATTTGCACA

AATACGTCGTTATTCACTGACGACTTGCAGAACTTCAGGGCTTCGTCGGCCCTTTCATGA

GCGCCCTAGTTTTACCTCTTCTTAAATCATAAAGTCTCATAACACACAAACATTTATTCA

GTCATTTTTTGTGTCGTATTTGTACGCTTAGATGTGAGTGAGGTGCGTTTTACTGATTTA

GTGTTGATTTTTTTTCTTTTGTGAACTCGAGTGCATGTTAGTGTTGCGTTTATTTCATGG

TGGACTGCTTGCTAAACTTTTTTTGCCTGGTGATGATTAGAGAGAATACATGCTGCAGTG

GCTGACATCATCAGCTTTTCTGCAATATCATTCGCTAATAAACATCATTTGAGCTTTAGT

TTAGATTTAAGATCAGCGCTGGATTTAGGGATTAATTCGTTTTAAGGATGTGACTCAAGC

AAATGTTTTTCCATCAGCTGATTTTTTTTTTTGTCACTCAACGATGGTGACGGTAAAACC

TGTATAATGGCCATGAATATCTTTATTTTTGTAATATTTTGTACATAATCCAAACAGCAA

AACTGCATTGTTAAAAAGTTTTCAACAAACATGATCTAAATTAATGGATAGAGCTGCAAA

GCGGGTGGATGCGAGGATGTATTTAACGAGTGCAAAACCGAGAATCCTTTACTGCCGTTT

GTTCCTCAGTAACGTTCAGTCGCCGCGAACGTTGGCGTCTATTTTACAATTGCTTAACCA

TCGGTCCTGCTTCCCCAACCCTATTTTTATTCCCTTCGCCAT

>Unigene000143

AGGTTGGGAGGTGTGAGGTGTGCCGTGTGTAGGCTTGATTTGTGTAGGCTGGAGTTAAAA

AAAACGCTTGTGTGTGACTCGCTTGTCGTTAGACACATCTAAGACTTGAATGCTTGTGCT

ACAAATTGCAGTCAGAACAAGTGCGATGGCCACGTGCTCTCAGCAACAAATCATATTAAA

GTTATGTTTTAAATCTGCTAACTGTTTGAGTGTGCCGTTATTTTTTTTTTTAAAGGTGTT

TACCAGCTTGCACAACAAAGGAACGGATTTGTGACCTAAGCTCTGGATGAAGTTATTGAT

ATTGATTGTTTAGCCTCAGCATCTCTTTTTTTCTCTGTTGTGAAACTCAAAGGAAACTGT

AGAAATACGGGTTTGGCAAACTTTACAGCAGCAAACGTTCTGCTGGAAAACGAAAGATCT

CCGTTTTGTTACATGGTGCATGTGTGTAATGGTTTTACGCCACTTCACATCGCCTGCAAG

AAGAACAGGATCAAAGTGATGGAGCTGCTGCTGAAACATGGAGCGTCCATACAGGCAGTG

ACAGAGTCTGGACTCACGCCGATTCATGTGGCCGCCTTTATGGGGCATGAAAACATAGTG

ACTCAGTTGATGAATCATGGTGCATCGCCAAACACAACCAACGTGAGAGGAGAGACTGCG

CTCCACATGGCCGCCAGGGCTGGACAGGCGGGTGTAGTGAAATACCTGGTGCAGAACGGA

ACTCACGTCGACGCCAAATCTAAGGATGACCAGACTGCGCTGCATATCTCTAGTCGTCTG

GGAAAGCCCGAGATTGTACAGCAGCTGCTGCAGCATGGAGCCGCACCCGATTCCACCACC

ACATCCGGATACACACCATTGCACCTGGCAGCCCGTGAAGGCCACAAAGACGTCGCCGCT

ATACTGCTGGACCAGGGCGCTTCACTGAGCATCACTACCAAGAAGGGATTCACGCCGCTA

CATGTGGCAGCTAAGTACGGGAAAATAGAAGTCGCCAACCTCCTGCTGCAGAAGAGAGCT

CCTCCAGATGCGTCGGGAAAGAGCGGACTAACTCCACTGCATGTAGCTGCGCACTACGAT

AACCAGAAAGTGGCACTTCTTCTCCTGGACCAGGGGGCTTCTCCCCATGCAGCTGCCAAG

AATGGCTACACTCCTCTACATATAGCTGCCAAGAAGAATCAGATGGAGATCGCTACCACA

CTGCTGGAATACGGCGCTGATACCAACGCGGTCACTCGGCAGGGCATCAGTCCGCTGCAC

CTGGCTGCTCAGGAGGGCAACGTGGACATGGTCACCCTACTGCTTGCACGTGATGCCACC

ATCAATCTGGGTAACAAGAGCGGACTCACTCCCCTTCACCTGGTGGCCCAGGAGGATAAA

GTAAATGTAGCTGAAGTCTTGGTTAACCAGAGTGCTGCTGTGGACCCTGAGACCAAGATG

GGCTACACTCCGCTGCATGTCGCCTGTCACTATGGCAACATAAAGATGGCGCACTTCCTC

CTGAAGAACCAGGCTAAGGTCAACGCCAAAACGAAGAACGGATACACTCCTCTGCACCAG

GCTGCACAGCAAGGCCACACACACATCATTAACCTCCTCCTGCAGCACGGAGCCTCGCCC

AACGAGCTGACGCTGAATGGAAACACAGCACTGTCCATTGCCAGGCGACTGGGTTACATT

TCAGTAGTGGACACCCTCAAAGTGGTGACAGAAGAGACCCTGACCACCATGACGGTGACT

GAGAAGCACAAGATAAACGTCCCAGAAACAATGAACGAGGTTCTGGACATGTCTGATGAT

GAAGTTCGTAGAGCCAATGTCCCCGAAATGCTCAATGAGGACTATATTTCAGATGTTGAA

GAAGGGGAGGATGTGATGATGGGGGACACGGATAAATACCTGGGTCCCCAGGATCTGCGT

GAGCTAGGGGACGACTCTTTGCCTCAGGAAGGTTACGTAGGATTTAGCATTGGAGCCCGA

TCCGGAAGCCTGCGCTCCTTCAGTTCGGATAGGTCCAACACACTGAACCGGAGCTCTTTC

ACCAGGGACAGCATGATGATCGAGGAGATCCTGGCACCCAGCAAGGACACGCATCTGACT

GTAGCTAAAGACTATGACACTGACTCTCTGAGGCGATACAGTTGGACAGCAGATGCTTTG

GACAATGTCAACTTGGTCTCCAGCCCAGTCCATTCAGGGTTCCTAGTGAGCTTTATGGTG

GACGCTCGTGGTGGCTCCATGCGAGGCAGCCGCCACAATGGGATGCGCATCATCATCCCA

CCACGCAAGTGCACTGCCCCGACTCGCATTACCTGCCGCCTGGTCAAGCGACACAAGCTT

GCCTCGCCACCACCCATGGTGGAAGGAGAAGGACTGGCAAGCCGTCTGGTGGAGGTGGGC

CCTGCCGGAGCTCAGTTTCTCGGCCCTGTGATAGTGGAGATCCCTCACTTTGGCTCCATG

AGAGGGAAGGAGCGTGAACTGATCATTCTCAGAAGCGATAACGGAGAGACTTGGAAAGAG

CACCAGTATGACTGCAAGACCGAAGCCCTTAACGAGCTGCTTAACGGCATGGATGAAGAA

CTGGACTGCATAGAAGACCTGGAGAAGAAACGCATCTGTCGAATCATTACAAAAGACTTC

CCACAGTATTTTGCCGTGGTGTCACGGATCAAGCAGGAGACCAACCATATGGGTCCTGAG

GGAGGGGTTCTATCCAGTACGACTGTTCGCAAAGTCCAGGCTTCTTTCCCTGAAGGAGCG

CTCACAAAGAAAATCAGGGTTGGACTTCAGACCCAGCCGGTTCCAGACGAAACAGTAAAG

AAAATCATAGGCAACAGAGCCACGTTTAGCCCCATAGTGACAGTGGAACCACGCAGAAGG

AAGTTCCACAAACCCATCACCATGACTATTCCTGTACCTCCTCGATCAGGGGAAGGCATG

AACGGCTACAAAGGAGACACAACACCTTGCTTACGTCTTCTGTGTAGCATCACAGGTGGC

ACCTCCCCCGCACAATGGGAAGACATCACTGGGACTACTCCGCTCACCTTCACGAATGAC

TGTGTCTCGTTCACTACCAACGTGTCTGCTAGATTCTGGCTCGCAGACTGCCATCAGATT

CCGGAGACAGTTTCTTTGGCTACTCAGCTCTATAGGGAACTCATCTGTGTGCCCTACATG

GCTAAATTCGTAGTGTTTGCCAAAATGAATGACCCTGTGGAATCCCATCTGCGATGCTTC

TGCATGACGGATGACAAAGTGGACAAAACCCTGGAGCAGCAAGAAAACTTTGAAGAGGTG

GCCAGAAGCAAAGACATTGAGGTTTTGGAAGGAAAGCCTATCTATGTGGACTGCTACGGC

AATTTAACCCCTTTGATTAAAGCCGGACAGCAGCTGGTTCTTAACTTTTATGCATTTAAA

GAGAACAGGCTGCCATTCTGTGTAAAGGTGAGGGACAACAGCCAGGAACCGTGCGGACGG

CTGTCCTTCCTCAGAGAGATTAAAAACGCAAAGGGGCTTCCCCAAGTTGCTGTCTGTAAT

TTAAACATCACTCTGCCCGCGCTCAAAAAGACTGAAAAGCCAGAACGACGTCATACCTTT

GCATCTCTAGCCTTACGTAAGCGGTACAGCTATCTGACTGAGCCTGGATTGAGCCCTCAA

AGTCCTTGCGAGAGGACAGACCTGCGAATGGCTATAGTGGCTGATCATCTCGGCCTTAGC

TGGACTGAGCTGGCCAGGGAAATGGATTTCTCTGTTGATGAGATAAATCACATCCGCGTG

GAGAACCCCAACTCGTTGACCGCTCAGAGTTTCATGCTATTAAAGAAATGGGTCAACAGA

GACGGTAAAAATGCCACAACGGATGCCTTAACTGCAGCACTGACAAAGATCAATCGGATG

GATATTGTGACATTGCTGGAAGGGCCAATATTTGATTATGGTTACCACAACATCAGTCTG

GAGTTGCAGAGTCCCTCAGAGTTGTCCTATGAACCCCCCACCCCTCTCCGGCAGGATGAT

TTCTTTAGCGAGGATGGGGGTATTGTAGTCTCGCCCAGTAGAACCCCTGTTAGACCAAAT

GAACTGTCCTTACCAACCACATCTAGGGATGCGCTTTCCCCCAATGCAGCACCTCCTACT

GTGGTGGCAGAGGATTCCTCTATTGGAGGAAGAGGCTCCTATAGTCGAGAAGGCGGCTCA

ACTGAAGACGACCTGGACGCCCATGACAGTGATGAGATGCCTAAACATCCTGAAGTGTTC

ATGCAGAGCACATACCAAGAGCAAGAGGTTCCTTCCAACGGAGCTCACAGGGCTAGAGAA

AGTGGCAGGTCTGGTTTTGACGAGGAAGAGGAGGAAGAGATGACGCAGGAGAAGTCCTGT

TTTAAAAGCATAAACTTGGAGGAAGGTTTAGAGATTGTGGAGATACCCCAAGAAAAAGTG

CAGGAAATTCGCTCACAAGTGGAACAGGCAAAAAAAGAGATGTGTTCGCTTGCAGGATTG

CAAGAAGGGCCGTCCAGTGTGAGCCAGGAGATCTCCGCTTCGGGTCTGCACACTGATACC

CAACTGCAAGATAGAAGTCAAAAGCCAACTGAAAGGCAAAATGGGGAACATCCAGAGCTC

CAAGGCCGAGCCGGCTCACTGCTTGAGCCACAGGAACCATCTAGTAAGATGAAGGGCAAG

GGCAGCAGAGACTCCTTAGATGACACAGCTGCTGGGCCGAGCACCATGGACGAAGACACA

GTGGAGCCGAAAGCTAAGGTCGGAGAGGGAGACAAGAAGAAAGAAGGGAGACAACGAGGG

AAAAAGGGTCAATCGTAACAGACGGGTGTCCTAAATGAGATCTGAACTTGTTTCATCATT

GTTTTCCTGCGAAAAGCATCACTGGAAACCCAACGCAGGAGATCAGGACAGGGGATTGTG

CGTCTGCAGCCTTTACATAAACGCATGGTTTTTAGAACGAGGATTTTATGATTTCGCATG

AGCATGGAAAACCTCCGATTTTCTGTCAACGACACATCCAAGCATCTGATCGGTGCCAAG

AAATGACACTTAAGCTCAAGACTGTCGGTGGCATAAAGCTTCTAGTCAGTCCTATACATT

GGAGGAGTGCCAGCGACTACCAACATGGCACCGAAGCATTCCATGACCAAAGATGGTACA

ATGAGCATTGTGGAATGATTATTTTTTAAAAGAAAAAAAAAAAAAAAAACCACCTTCTAC

ACTCGCCTTATTTTCTCTCCCCCCACCCTCCCCCTCGCTTCTCGGACTAGCGTCGACGCT

GTTTTTAAAAAAGATCCTGTATATAAATCAGAACTGTATGAAAGTAGCTCACGAAATTGT

GAACGCAGGAGCAAAGAGCGAGTCACAAACGGAATGAAAAAAAAAAAGAAGAAGAAAGGA

ATGCTGATATAACTATTCCGACTTTAAAGATCATTTTAAAGACGAATTGATTACACAAAA

AAGCGAAAAGAAACTTTTTACACAAAAAAAAACAAAAAACAAGTTTGCTTGATTTCAACG

TTTTTAAGCCTACCACAAAAACGACAAAACATCGAACGACATAGCTGCAGTTGTGTTAAA

AATTTACTCCGAATACTTATATTTTGATGCATGTGATTCACCTTATTTTTTGATTTGTTT

CCTATCTACATAACCAGAGGTGTGGTAAATGTTCTACCTGTGTGCACACTCATAGCCTTG

GTGTTTGTCACTAATACTGTTTTATAATATCACTGTACCACAACAGACTCACTGGAAGTG

AATGGACACACAATTCTCGCAGCTAAAGGTTTCTACGCTATGCAGTCAAAGACGAGCTTG

GAGTGTAAGAGAGACCGAAAGAGAAAGAGCGTCGTGTCAACTGGCACAGCGTTCAGTTTT

AATGGGTTTTGTGTGTATCGGCACTAAAAGGTCTTTCTGTAAGCCAACCGTTTGTAACCA

TTCTAACAGTGTTACTAGGCTGCTGTGGACATAAGGGGCATTTTTTTATTTGGGTTTGGT

GAAATATTTCCTTATATTGAATATATTCAAAAAAAAAAAAAAGAGAGCGAGAATGAAAGA

AAGATCACCTTCAGGTATTCGTTGATGAAATGATCTGGTAACAGACTTGTATGAGAAAGC

GTACACTGGATGATTGAATTAATTATTTAAGCATAAAATAAATTGTGTTTAAACTCAAAT

TGTGATGTGAACTTTGATCTGGATGATGTGGTGCATTTGTGCGAGAAAACTGTGTCTGCA

TTTGTCTGTCCATCGATCTATCTGTTCATCCATCTGTCCATCTATCTATATACAGTATCT

ATTTAGCTAGCTACACACACACACACACACACACA

>Unigene000144

GGAGCACAGCCCTGTTTGCCAAAGAAGCACTCACTTTTCAACATAGTTGAGAGAAAGTGT

CTCCAAAGTTCGACAGACCAAGCAGAGATTAACATATTCTTCTAAAAACCGCTTCTAGAG

TCGGGTTTCAGGACGGGTTTTATTTTCTTTCTCCTTCAACGTGTGCGCGCAACTCCTTTC

CCTCTTCCAGGAGCGCAAGCGCTAAAGGTTGTGTGTGGGCTGCCATGTGGTAACGGCGTT

ATTCAGTTGGCGTGTTGGCGAGAGAGAATGAAATGCACATTGCGGATGGAGGGAGTGATG

CAGTCGCAGCATCGGGGACTAGTGACAATCCCAAGGTTTCTCTTGATTTGTCTTGTCGTC

CTAGTCCTGACTTCAGAAGTTAACGCTGGAGGTAAGAAGCACAGTGGGATATGTGGGGGC

AGAGACTGCAGTGGTGGTTGTCAGTGCTATCCTGAAAAAGGTGCAAGGGGCCAGCCTGGT

CCTCTTGGCCCTCAGGGTCCCACAGGGCCTCCAGGACGAGCGGGTGAACCTGGCCTGCAA

GGTCTAAAGGGGGAGAAAGGTAACTCGGGCGAGGCAGGCATCATAGGACCAAAAGGGGCT

CAGGGAATGATAGGAAGTCCGGGGTTTCCAGGAGCAGATGGTATTCCAGGTCACCCAGGT

CAACAAGGGCCTCGAGGAAAACCTGGTGCTGATGGATGTAATGGAACCCGGGGAGATGCT

GGAATACCTGGCCTGGAAGGATATAGTGGTACTATGGGACCAAAAGGTCTGCCAGGCAAA

AAGGGTGAAAAGGGGGACCCTCTGGAGATTAGCGTTTACATGGAGAGATTTCGGGGTGAA

CCAGGAATTCCAGGATATACAGGACCTATAGGCCCTCCTGGAAAAAGAGGGTACCAAGGA

CCACCAGGGCTCTCAGGTGTACATGGTGAAAAGGGTTTTAGAGGACCTCCTGGACTCAAG

GGCAGTGCAACTGCAATACATCCAGGAGAAAGAGGGGAACAGGGGGAAGTTGGAGATCCA

GGTCCACCTGGGACTGATCGAGTTGTGTTCATTGGCTCATTTACGGGCCCTAAAGGGGAG

AAAGGTATGAAAGGTTTACCAGGAGATTTGGGGTTTAATAATAAAGGGGAGACAGGAATT

AAAGGATTTCCTGGGGACCGGGGCTTTCCAGGAAATGCTGGCTTGCCTGGTAAAAAGGGT

GAAGCTGGGCTTGCAGGTGAACATGGGCATCCAGGAAGCAAGGGGGAACCAGGAGAGGCA

GGAGAGCTTATCTATACAGATGGATACAGTGGCGAAGGCCCACCAGGTCCTCCAGGAGAA

CGTGGGCCACCAGGGCCTGTTGGTAGCATTGGAATTAAAGGTTTACCGGGTCCACCTGGG

CCACATGCTCAGGAACAGCAGATAGAGCTGTTTGGTTCTTTTGGACCAAAAGGGGCAAAA

GGTGTAAAAGGAGATGCTGGGGAACCTGGTATCCCAGCCTCAGAGCCTGGGCCGCCAGGA

TTTGATGGTTTACCAGGACCACCTGGACCACCTGGACCAAAAGGATCATGGAGTGTGTTT

TTCAGAGGGTATGAAGGGCCACATGGACGGCCAGGAAATGCTGGAGCAAAAGGACAAAAA

GGTGACCAAGGTCATTGTGAATGCACTTTGCAAACCTCTCCTCCTGGAACCCCTGGCCCA

GCTGGTGATCAGGGTGACCCTGGAGTACTAGGCGAGTGGGGTCATCGAGGTGATCAGGGA

GACCCAGGAGACAGAGGCCGCACTGGTTTGCATGGCTTTCCTGGGCTACAGGGGCTGCCA

GGACCTAAAGGTTTAAAAGGAGATACAGTAGAGGTTAAAGACAAAGGAACAAGTGGAGAC

CCAGGAGACCCAGGGAGACCAGGAGAACAAGGAACTCAGGGCAGACCTGGGACTAGTGGA

ATTATCGGTGCTCCTGGACCTCATGGTCTTCCTGGTGAAGGGCCAGTTGGCGAGTCTGGA

GAGAAAGGCTACCCAGGAAGGCCAGGTCCATCGGGGTTACCAGGACCAGCAGGGGTACCA

GGTCAAGTAATCGGAGGAACTAGGGGCATCAAGGGTATAGCTGGTGATGCAGGATTAGAT

GGTTATGATGGACCATCAGGCCTACCGGGTACTTCAGGAGACTGTATTCTACAAGGAGAA

GGAGGCGAATGGATTACAGCAGGGGATTGTGCAGCTATATCTGGACCCCCAGGAGAGATG

GGACCTCCAGGACTTACTGGATTAGAGGGAATGCCAGGACAACCTGGTCCAAAAGGATTT

GAAGGAGATAATGGCAAGATAGGACTTAAAGGGGAAATAGGAGACCAGGGCATCGGAGGC

CCACCTGGAAAAACAGGTTTCACTGGAACTCGCGGGGACATAGGCACTCCTGGACTAAAA

GGTCACAAAGGTTCTCCTGGCATCCCGGGTAGGCCAGGGCTTGATGGTGATTTTGGACAG

AAGGGTCCTAATGGCGAAGCTTTAGGAGCATCGTCTGGGCCCCCAGGGGAACGAGGACCA

CCAGGGTTTCAAGGTATAAAAGGTGCCTCTGGAAATGCTGGAACAAGTGGATACCCAGGC

ATGGAGGGAATGCCTGGGATGATTGGGGACAAAGGTGAATCAGGTCCATTAGGATTACAG

GGGGAAACAGGAAGACCTGGACCAGCAGGAATTTTTGGCTTCCCTGGAAAGCCTGGTTAC

GCAGGACCTCCTGGACCTTTAGGCCAAACCGGGCCACCAGGGCCTAGGGGAGAAACGGGC

TATCGGGGAGACAGTGGTCCCCCAGGACATGCAGGAGTAAAAGGTGTTAGAGGAGAACCA

GGAAGTGATGCTGGGCCAGGAGATTATGGACAACATGGGGATCCAGGAGCCCCTGGACCT

GTTGGGCAGACTGGTCTGCCTGGACTTAAAGGTGAAAAAGGCTCTCAAGGCATGACGGGT

TTCCCAGGTATGCTAGGTAATTCTGGTGTAAACGGCTTTCAAGGACCAAAAGGACTGGTA

GGAATAACAGGTCTAGAAGGAGTGAAAGGAGGCATGGGCCAACAGGGGCATAAAGGTGAA

AGAGGAGTTATGGGCCCTCCAGGTAAAATGTCTGTTGTAGAAATGACACATATGAAAGAG

GTTATGAAAGGTGAAAAAGGTAGCTGTGGATTTGAAGGAGATTCTGGTTTCACTGGGCCC

CGAGGCACCAAAGGACTCCCAGGAATTCCAGGTGAAGAGGGATATCATGGATTACCTGGG

GAACCCAGCAATATAAAAGGTCTACAAGGACAGACTGGAGCTCAGGGTCTTCCTGGTCAG

AAAGGAATGCCAGGTCCTACAGGACTACCAGGAATTCAAGGATTTTTTGGACCAACTGGC

ACTAGGGGGGAGAAAGGTTCATCTGGTGCTTATGGAGCAAAAGGATCTTTTGGAATTAAG

GGACTCAAAGGTGACCAGGGTGACGTTATCAATTTCCCAGGACCCACAGGAACCAGAGGG

GAAACTGGGCTGCCTGGACCTTTAGGTCACAAAGGTTTCCTGGGTGTGTCAGGGGAAAGA

GGAAGTCCTGGTTTTGATGGTATTAAAGGAAATAAAGGAGTTCAAGGTGAACTCGGAGAA

GAAGGGCTATCAGGTGCTGACGGCCTGCAGGGTGCTCCAGGAAACCAGGGCTCTAAGGGA

TGGCCAGGAGTTCCAGGAGCGGCAGGAACCCCGGGTAAACCAGGACATCCAGGACAAATA

GGTTTTCCTGGCCTTGATGGAATTTCAGGGTTGCATGGTCTTAAAGGTCAAAAGGGCCTT

CAAGGTGTTGCAGGTCTTGACAACTGGGGACTATCAGGTGAAGATGGAGAAAAGGGAGAA

ATAGGAGAACCAGGTATTCCAAACACAGAGATTGGGATCCCCGGAACTCATGGCCTTAAA

GGTTCAACAGGAAATCCAGGTGATCAGGGTGAACCAGGTCCTCCAGGAGATCAAGGCCCT

CCAGGAGCACATGGAATTCCAGGCAGAAGGGGTCTTTCAGGGGACACAGGGCTTCCTGGT

CAGAAAGGTCTTCAAGGCTTTCCAGGAGCCACAGGTCTTCCAGGTTTTCCAGCCCCATCT

GGAGCTAAAGGAATCTTTGGCAGTGAAGGGGATTTCGGAACCCCGGGACCAAAAGGATTA

CTGGGGGATATAGGAGACAAGGGATTTCCGGGACAATTTGGTGTACCAGGAGAAAAAGGT

ATAAAAGGATCCATGGGTGGGATGGGTGTCCCAGGACCTTATGGTTTACAAGGTGATAGA

GGACTTCCTGGTGCTAAAGGAGGGACTGGTCCACCAGGCCCACCTGGCTCCCCTGGTGCT

AGAGGTTTACCTCCTGTTCCTGTAAAGATGCCAGGTGAGAGAGGACCCCCAGGGCCACCG

GGTGATCAAGGACTACAGGGATTGAGAGGGAACTTTGGCTCTATGGGACTCCCTGGCGAT

GCAGGTTATATTGGACCAAAGGGACAAAAGGGGATGCCAGGGCTTTCTGGAATACATGGA

ACACCAGGCATCCGAGGAGATCTCGGACAAACAGGACACCCTGGCTTACAGGGCTTAGAA

GGAAACCGTGGCCGTCCTGGTACTCCTGGTCCCACTGGTATGCCTGGACGCAGTGTCAGT

GTTGGGTACCTCCTGGTAAAACACAGTCAGTCTGATCAGACTCCGATGTGCCCTGTGGGT

ATGTCTAAACTGTGGGATGGCTACAGCTTGCTTTACTTTGAAGGACAGGAGAAAGCTCAT

AATCAGGACCTTGGTCTGGCAGGCTCGTGTCTCCCTCGTTTCAACACCATGCCATTCTTG

TATTGCACCCCTGGTGACGTATGCTACTATGCTAGTCGCAATGACAAGTCCTTTTGGCTT

TCTACAACCGCCCCTATTCCTATGATGCCTGTGGAAGAGTCAGAGATAAAGCCGTACATC

AGCCGCTGTTCAGTATGTGAGGCTCCATCTGTGGCTATAGCTGTGCACAGTCAGGATATT

ACTATCCCACAATGTCCTGCAGGCTGGAGAAGCTTATGGATTGGCTACTCTTTCCTTATG

CATACGGCAGCTGGAGATGAAGGCGGTGGTCAGTCTCTGGTGTCTCCAGGCTCGTGTCTG

GAGGATTTCCGCACCACCCCGTTCATTGAGTGTAATGGAGCTAAGGGTACCTGTCACTAC

TTCTCTAACAAGCACAGTTTTTGGCTTACATCCATTGAGCAAACTTTCCACAGTTCCCCT

GAGTCAGAGACTCTCAAGGCGGGACAGCTTCTCTCACGCATCAGTCGCTGCCAAGTCTGT

ATGAAGAACCTGTGACCTGCATGAACTTTGACATGAGGGTATGATAATTTTGACATCTCT

GTGCACATTCATGACAATAATTCAAGGCTAGGCTCTTAATGTCACTCAGAGATCATATTA

AGTCTATAAACCAAACAGGATTGTTGAAATATATTTTGTTAACAGCTCCCGCTTATATAA

ATAATATACTGCACACTTTTATATCATGGATGACCCTCAGGTTTTCTTTATTACTATGTA

TCCTTCCCTGTGTGCCTAAGGACTGAAAAATATGTAAATGTATTGAGAAAAGTGGTCAAG

CAAGCATCCATTTACTCATGTTTCATTTGAAAAGGTGCTTTTTCTAACTTATTACCTCAC

TTATCAACATGATTTGTATATTACCAAGATCTTCATACAGCCAAACCAAAGACACTCATG

TTAATGCACTGCTTCTCTTATATTCATCTGTGTGTATAACAATTAGTATGACCCGTTATT

ATTATTGTCTCATTGTGACCTATACAACCAGCCAAATGATTTACACTTTAAATGATATGT

TAGAACCGTCCGAGTGACACCTTTGAATCAAGTATTTCATTACTTACGTTCCTAATCATG

ATTTTATCATCAGGTTATGAACTGTGTTTTACTAACACTGTTTTGCAACTTTACATTGTG

ATTTGTAGAAAATTATTTGCACTAATAATACAGTATGTTGTCATCATCATTATGCTAATG

CTTATGCAAAGCTTCCTCCACGTCCGCTGAGCGTAAGCATGCACCTTTTTACAGATATGC

AGACTGTCACAGCTTAGCAAAGAGTTCAGATCAGCTTTTTTAATTGAACATGGATTATAA

CACAAATAATTTTTTCTACTCTGTTACACAGACTGCACTGATTTTTCAATGTTAATTGTC

CAAATATTTGATTTTTTTTCCCCCTTGCACAGCCAACACATAAATAAATGGTATGTTTTT

ACATTTATTCCTTCTTAAAATCTGATGTTTAATAATTAACTGATCAATCTTTTTTAAATA

AATGACCCC

>Unigene000145

TGTGTGTGTGTGTGTGTGTGTAGACATACTCTGGGTTGTTCTGTGTGGTCATTAACCCAT

ATAAAAACTTGCCCATCTACTCGGAGGAGATCATAGACATGTACAAGGGAAAGAAGAGGC

ACGAGATGCCACCGCACATCTACGCCATCACAGACACTGCATACCGGAGCATGATGCAGG

ATCGTGAGGATCAGTCCATCCTGTGCACAGGAGAATCTGGAGCTGGAAAAACAGAAAACA

CTAAGAAGGTCATTCAGTATCTGGCGTCTGTTGCCTCATCCACTAAAACCAAAAAAGACC

CTACCAGCACCATCAGCAGTCTGTCACATGGGGAGCTCGAGAAGCAGCTGCTGCAGGCTA

ACCCTATCCTGGAGGCGTTCGGTAACGCCAAGACGGTGAAGAATGACAACTCCTCACGCT

TCGGAAAATTTATCAGGATTAACTTTGATGTGAACGGTTACATCGTTGGAGCAAACATCG

AGACCTACCTGTTGGAGAAGTCTCGTGCTATCAGACAGGCTAAAGAAGAACGCGCCTTCC

ACATCTTCTACTATCTGCTGTCTGGAGCTGGTGATAAACAGCGCTCTGAGTTGTGCCTGG

AGGATTATGGGAAATATCGCTTTCTGTCCAATGGAAAGGTGACAATTGCTGGACAGCAGG

ACCGAGATCTTTACACTGAGACGATGGACGCCTTCAGAATAATGAGCATCCCTGAGGACG

AGCAGACGGGTTTGCTGAAGGTGGTTTCTGCAGTTCTTCATTTGGGAAACATCATCTTTA

AGAAGGAGCGTAACTCTGATCAGGCCTCCATGCCTGATGACACGGCTGCACAGAAGGTCT

GCCACCTGCTGGGCATGAACGTGACCGACTTTGTGAGAGCAATTCTCTCGCCGAGGATTA

AAGTGGGCCGTGATTTCGTGCAGAAAGCGCAGACGCAGGAGCAGGCAGAGTTTGCAGTTG

AAGCTTTAGCCAAAGCCACGTATGAAAGATTGTTCCGCTGGTTAGTGATGCGGATTAATA

AAGCTCTGGACAAAACCAAACGCCAGGGAGCTTCGTTCCTCGGTATCTTGGACATAGCCG

GCTTTGAGATCTTTGAGTTGAACTCGTTTGAGCAGCTGTGTATTAATTACACTAACGAGA

AGCTGCAGCAGCTCTTCAATCACACCATGTTCATACTGGAGCAGGAGGAGTATCAGCGTG

AGGGCATCGAGTGGAGCTTCATCGACTTTGGACTGGACCTTCAGCCCTGCATCGACCTGA

TCGAGCGGCCTGCGAATCCTCCAGGTGTTCTGGCTCTGCTGGATGAGGAGTGCTGGTTCC

CCAAAGCCACTGATAAAACCTTCGTGGACAAGCTGGTGCAGGAGCAGGGGAACCATCCCA

AATTCCAAAAACCAAAAAAGCTCAAAGATGATGCTGATTTCTGCATCCTTCACTACGCAG

GCAAGGTGGATTATAAAGCAGTGGAGTGGCTGATGAAGAACATGGACCCTCTGAACGATA

ACGTAACAACAATGCTTAATCAGTCATCAGACAGATTTGTATCTGAACTCTGGAAAGATG

TGGACAGAATTGTGGGACTGGATAAAGTTGCAGGAATGGCCGAGTCAGCACACGGGGCGT

TTAAAACCCGTAAAGGAATGTTCCGGACGGTCGGACAGCTTTACAAAGAACAGCTCGCTA

ATCTGATGACCACGCTGAGGAACACCAACCCCAACTTCGTGCGCTGCATCATCCCCAATC

ATGAGAAGAAGGCAGGTAAACTGGATCCTCACCTGGTCCTGGATCAGTTAAAGTGTAACG

GTGTGTTAGAGGGAATCCGAATCTGTAGACAGGGCTTTCCTAACCGCATCATCTTCCAGG

AGTTCAGACAGAGATATGAGCTGCTCACTCCGAACGCGATCCCTAAAGGTTTTATGGATG

GTAAACAGGCCTGTGTGCTGATGATCAAAGCTTTGGAATTGGACCCAAACCTGTATCGGA

TCGGACAGAGCAAGGTGTTCTTCCGTGCCGGAGTTCTGGCCCACCTGGAGGAGGAGCGTG

ATATGAAGATCACAGATGTCATCATCAGCTTCCAGGCCTGGTGCCGTGGCTACGTCGCCC

GAAAGGCATTTGCCAAGAGGCAGCAGCAGCTGACCGCCATGAGAGTGATCCAGAGGAACT

GTGCTGCTTACCTCAAACTCAGGAACTGGCAGTGGTGGAGACTCTTCACTAAGGTGAAGC

CCTTGCTCCAGGTGAGCCGTCAGGAGGAGGAGATGCAGGCCAAAGATGAGGAGCTGACCA

AAGTCCGTGAGAAACAGGTGGTGGCTGAGAAAATGATCGAGGAGATGGAGGTCAAACACC

AGCAGCTGAATGCAGAGAAGATGGCTCTACAGGAGCAGCTGCAGGCCGAGATGGATCTCT

GCGCCGAGGCAGACGAGATGAGGAACAGGCTGGTGGCGAAAAAGCAGGAACTGGAGGAGA

TCCTGCATGACCTGGAGGCCCGAGTGGAGGAGGAGGAGGAGAGAGCCAATCAGCTTCAGA

TGGAGAAGAAGAAGATGCAGCAGAACATCACGGATCTGGAGCAGCAGCTGGACGAGGAGG

AGGCAGCTCGGCAGAAGCTGCAGCTGGAGCGCGTCACCATGGAGGCCAAGCTAAAGAAAA

TCGAGGAGGACGTCATGGTGCTGGAGGACCAGAACACCAAGCTGAACAAGGAGAAGAAGC

TGATGGAGGAAAGAATCAGCGAGTTTACCACTAACCTGGCTGAGGAGGAGGAGAAATCCA

AGAGTCTGCAGAAGCTGAAGAACAAACACGAGGCCATGATCACAGACCTGGAGGATCGTC

TCCGCAAGGAGGAGAAAATGAGACAGGAGCTGGAAAAGAACCGCAGGAAGTTGGAGGGCG

ACTCCACCGAGCTTCACGATCAGATCGCCGAACTCCAGGCTCAGATCGCCGAGCTCCGAG

CTCAACTCGCCAAAAAGGAGGAAGAGCTGCAGGAAGCACTGGCCAGGATCGAGGAAGAGG

CGGCGCAGAAGAACCTGGCCCACAAGAAGATTCGTGAGTTGGAGGCTCAGCTGAGCGAGC

TGCAGGAGGACCTGGAGCTGGAGCGAGCCGCTCGCACCAAGGCAGAAAAACACCGCCGTG

ACCTGGGGGAGGAGCTCGAGGCTTTAAAGACCGAGCTGGAGGACACGCTGGACACCACTG

CTGCTCAGGAGGCACTCAGGACAAAGCGTGAGACAGAGGTGACGCACCTGAAGAAGTCTT

TGGAGGATGAAGCTAAAAATCATGAGCAGATTGTGGCAGAAATGAGACAGAAACACGGCC

AGGCTTTCGATGAACTCAATGAACAGCTGGAGCAGTCAAAGAGGAACAAGGCATCAGTGG

AGAAGATGAAGCAGACTCTGGAGTCGGAGCGTAACGAGCTGCAGATCGAGCTGCAGGGTC

TGATGCAGAGTAAAGGTGAGTCGGAGCACCGCAGGAAGAAGGCGGAGGCTCAGGTGCAGG

AGCTGCAGATCAAACACTCTGAGAGTGAGAAGCAGAGAGCAGAGCTGATGGAGAAAGTCT

CCAAGTTGCAGGCGGAGCTTGATAGCGTTAACAGCGTGCTAAGTGACGTGGAAGGAAAAT

CCATCAAAGCAGTGAAAGACTGTTCAGCTGTGGAGTCTCAACTGCAGGACGTCCAGGAGC

TGCTTCAGGAAGAGACTCGTCAGAAACTGTCTCTTAACACACGAATGAGGCAGCTGGAGG

ATGAACAGCACAATCTGAGAGAACAGCTGGAGGAAGAGGAGGAAGCCAAGAAGAACATGG

AGAAACAGTTGATTGCAGCTCAGTCCCAGCTGGCTGAGATGAGGAAGAAGATCGAGCAGG

AGGCGAGTTCTCTGGAGAGCACAGAGGAGGTGAAGAAGCGTGTGCAGCGCGACCTCGAGT

CTGTCAGTCAGCGCCTGGATGAGAGGAACCTGGCCTTCGACAAACTGGACAAGACCAAGA

CCCGCTTGCAGCAGGAGCTGGATGACCTGCTGGTGGACCAGGACCATCTGAGGCAGATTG

TTTCCAACCTGGAGAAGAAGCAGAAGAAGTTTGACCAGATGTTGGCGGAGGAGAAGACAA

TCTCTGCTCGCTATGCTGAGGAACGTGACCGGGCAGAAGCTGAAGCCCGTGAGAAGGAGA

CCCGCGCACTCGCTCTGACACGCGAGCTTGAGACCCTGACAGACATAAAGAACGAAATGG

AACGCGCAAACAAGGCTCTCCGGGCTGAGATGGAGGACCTCGTCTCCTCCAAGGACGACG

TCGGCAAGAGCGTGCACGAGCTGGAGAAGTCGAAGCGCGGCATGGAGCAGCAGCTGGAGG

AGATGCGCACACAGCTGGAGGAGCTGGAGGATGAGCTGCAAGCTACTGAGGACGCCAAGC

TGAGGCTGGAGGTCAACATGCAGGCCATGAAGGCTCAGTACGAGAGAGACCTGGCTGGAC

GAGACGAGATGGGAGAGGAGAAGAAGAGGCAGCTGGTCAAACAGGTGAGGGAGATGGAGA

TGGAGCTGGAGGACGAAAGGAAGCAGCGCTCGGTTGCCATGGCAGCGCGGAAGAAGTTGG

AGCTGGATCTGAAGGAGCTGGAGGCTGCCATCGACCAGGCCAACAAGAACCGAGAGGAAG

CTCTGAAACAGCTCAAGAAACTGCAGGCTCAGATGAAGGACGTCCTGCGGGAGCTGGATG

AGACGCGTCTCTCCAGAGAGGAGATTCTGGCACTAAGCAAAGAGAATGAGAAGAAGTTTA

AGAGCATGGAGGCAGAGATGATTCAAATTCAGGAGGAACTGGCTGCAGCAGAACGGGCAA

AGAGACAGGCTCAGCAGGAGAGAGACGAGCTGCTGGACGAGATCAACAACCAGAACAGCA

AGAATGCTCTGACAGTGGAGGAGAGAAGGCGTCTGGAGGCTCGTATTGCTCAGCTGGAAG

AGGAACTTGATGAGGAACAGTGCAGCACTGAACTTGTTAATGACCAACTAAAGAAGGCAA

ATGCACAGGTGGACACTCTGGGTAGTGAGTTAGCGGGTGAGCGTAGCGCGGTTCAGAAGA

GTGAGACGGCTCGACAGCAGCTGGAGAGACAGAATAAGGATCTGAAGAGTAAACTGCAGG

AACTGGAGGGTTCCGTCAAATCCAAGTTCAAATCCACTATCGCCGCCCTGGAGGCCAAAA

TCCTGCAGCTGGAGGAACAACTGGAGCAGGAGGCCAAGGATAAACAGCAGACTTCAAAGT

CAATGCGCCGTACGGAGAAGAGACTGAAGGAGGTCCTGCTGCAGGTGGAGGACGAGAGAC

GCAACAGTGAGCAGTTTAAAGATCAGATGGAGAAGGCGAACTCGCGCATGAAGCAGCTGA

AGCGTCAGTTGGAGGAGGCGGAGGAGGAAGCGACCCGCGCCAACGCCTCCCGCAGGAAGT

TACAGCGAGAACTGGAGGACGCCACCGAGGCCAGTGAGACCATGAACCGTGAAGTCAACA

CCCTGAAGAGCAAACTCCGACGCGGTGGTGACTTCACCCTCAACGTACGGCGTGTGGTCC

GTGGTGGTCTGGACAGCGAGGAGGACGCTGACCTCACCAGTGAGACATCAGAGGCCACAC

CTGAGTAACACCTCCAGCTGACAGCCAATCAGATCGCCTGAGTCCAGGACAGCTGCATCA

CTTTTACTCCTTTAATCCACATCTTCATCACGAAACGATTCAAACTCATTCTGATACCCG

AATACTGAGCGCTTTAATAGCTGCCAATCAAAACGGTTGCCAGTTCATTTAAAATCCTGC

CGTCTACCCAATGAAAAACTTTATTGGTGTCCGGCTGTGGGACTGGTGTCCGGCTGTGGC

TTTACATCTTAATATTTTTCAGCTTGTTTTTATTTACGTTTTTTACAGCACTTTTTTTTT

GCTTTGTTGCCGTGTGCAGGTCAGGTTTTTCCTCACGGTCACACTCGTCCACTGGATTAA

TAAAGATCTATAATTTGATTAAATGCTCACTATTATTAAATTATCAGTCGTGCACAAAAA

TCAGGTTGGATGGGATCCGCTCCCGAAAATGGATTTTAGAGAAACTACACTGGGTTGAGT

TACTGTGGGTTGCCAGATCAGTGGAACTCGCACTGTTCCACACTAATGATTTATTAGCAG

GTTCTTTTGTTAATTGTTTAAACACTGCGGTGTTTTTTTCACTTCAGTCTCTTCACATTA

AATGGTGTTTAGTTATTCATCACTCTTTTTCTTACTTTTCTGTGTTTATTTTACATTTTC

ACACTCGTTTCTCTCCGTAAAGCTCCTGACCCCATCCCCCCCCCCCCC

>Unigene000146

GGAGAAGCGCTTACCTTCTCTCCGGATGGTGGTGGTGGCATCACGGTGCACACTGGTGAC

TCAAGCGCTTTTGCCTGAATGGGGTTTCTTTGATCATTCATAATTCAATAATTTCCCGAT

CGTCGCCTACATTTGAGTCATTCAACAATCATCAAAAAATCCTTACCGGAACCTAAAACC

CCGTGGAAAGTAAACAGTTCTTCGTTGTTAAACCGAGTCAAGCTTTGTGTACCGCGTACT

GTCGGGCTTCACAATATTTAAAGTCGTATAGGACAAGTTTACAGCAAAGCTCCCCAAACA

GTTTGTGAGTGTTGGTGTGTGACCCTCTCTCTGCTGCTGCTGTGGAGGGCTTTACATACT

CAGCTAGTGTGGCTGGATGCATCACACCGTGGAAAAATTTAGTGCGCGCAGCTCACGGGA

CCCTTTCCCTCTGGATGGATTCAACCGGGGACCATGGGCTCCTATGGGTGGCCGTGCCTG

GCCTCCTCCTCCCAGGTGCTCACCTGGTGGTCAACCTCAGTTCCTCCCTCACCTTCCACC

CAATCATATGACTGGACTAAATCATCCAAATAAATATTACAACAGTGGGGCTCTGACACG

AGGAGGGGAGCCCTTGCCAGTTTTACACAGGGACATCAGGGTGCCCCCAGCCCCACTCCG

ACCATGGGATCCACTTGGTCAGCATTATGAGCCACTGCCCAGTGATGATCATGTCCGTTT

GCACAACAGATACAATGCTGGACCGTCTGCACACCTCACAGCCCGAGCTAACCAGTTGTT

AAAGTACGGACCACCTCCTCTGCAACATGGATCTAGGCCCATGCCTCCAATTGCAGACAT

GTGGGAACAGTCTCATCAACAGCAGCAACAACCTCCCAGGGCCCCTTTTTCTCGTGTTGG

ACAGTTGAAACGGACAGCACCACTTCTGGGAGAGCAATCGGTCATCCAGCAGACCTCTCT

TGCCCTAAACAGACCTAATGAGGATTGTCCAAGCCCAAGCAAAAGGAAGAAGAGTTCTGA

CTCAGAACAGCTCATAATAGGCACCCACCAATACTCAGGGTCAATCGGGTCATCGCTGCC

TCTGCAGCAGCCCCAGTCCCATTATCCCCCTCTACAAAAGGATACAGCAACTTGGACCCC

AGTAGAACACAAGGCAGACAGAAATGAGTTTCAGGAGTTGCGCAGACAAGACATGGGTGA

CTGCAATTACAAACAGTTTTCATCATCGAAACCTTCACCCATCTCTCCACCCCCCATCAC

TTCTGCCAGCAGCTATGAACATGGCCATGGTCCTCCACTCAAATCCCTTAAGCCGCAATC

ACCACACTCTCTTGGGTCTTTCTCGCAAAATGTCCAACTCCACCACACAACATTTCCCTT

TCCTAAAAACAAGGCTTCCGCTCAAAATTCAATCGAGCACCAGGTCATTTCCATCCATAA

AATGCATTCTTCTGGGGTGAGGACTACTGGCAGCCAAGTGCCCCTGCCCCAACCCTTATC

TTTGGTTGATAGAGAACACCCTGTTCCCACACCAGCACATCAAACCTCCGTGCCTTACAG

TCACCCAAAATTCCAGCCTCATCCAGGACTGGTTTCCACCACCAGCCCCTCTTCCAACAG

TGGGCCACATGCACCAGAATCTCAATGCAACCCTCCTAAGTCATGGAGAAATGAGATCAA

ACATGATAACCAGGCTTTAATACTTGGTCCTTATCACAGATCCGTGGATCCTCAGCTTTC

CACTTCATCTCTGCAGCAACAGCAGAAGAAAACTGGGCAAAATCAGGATGAAGGCACCTC

TTTGGCTCGGGCACCAGTCATCACCTTCACAATGCAACCTGCAGCGCCTCCTTGTTCTGC

GGGATGCTACAGTGGTGTGAATACTATGTCTACCTTAAGCTCTGTGCCCTCTTCTCTCAG

TTCCACTGTCAGCACCATTAAAAACTGGAAAACTGCCCATCCCACAACGCTTAACTCGAA

CCCCAGTTCGATCCCATCAAGTTTCCCACACCAGGACTATCAGAGAGCCCAATCTGGCCC

AGAAAGCCATTTAGACCACAAACAAAAGGTACAAATTCAGCATCAGGAACTTCATCTACC

CCAGCAAGGCCAAGACACACCTCTTTACACCCCCAAAGTGTCCTCAGCTCCTTCCTCTCT

CAGCTCAGGGTTTCAGAGATCAGGAAATAGTGTCATCACCAGCAAGGCAATTGACAACCT

CTTGCAGCCTGCACATTCACCGCTTCGTCAAATGCAGCCTGCAGTGCCCATCACTGTGAA

CACAACCCCTCAACAGTCTAACTCTTTGCATCATAAATTGCCGCACCAGGTAACCCCAGT

CACTTCTCAGGTTCCACCTGCTGAAACATATGAAAGGCCATGTCCCCAACCTGCCATCTC

TAACCCCCAGTCTATTGAGGAAGCGCTCAAGAAGCTTGATGCCGAGCTTCAAAGTCATAT

GCAAGCTGAAGAAAGAAGGAAGGAAAAGGCAGAAGAGGAGAGAAAACACAACATCGGGAA

TAACAAAGTAAAAAAGCATAAGGTCAGTGAGGACTCTGCAACTGAGGACATGGAAAGCTT

GCTTTCAAATAGTCCTACTGATCCGCCTCCTCCATGCCTGTCACCAGCCATTACACCTTC

CACAGTTTTACCTCCATCCCAGACATCCCCACCTTTTCCCTGGCTTAGCAGGGGAGGCGT

ACCTACCCGTCAGCTGGCACCTGGTGCAAATTCTGTAGAAAGATCTCGACCACCACCTCT

CACCCCTCAAACAGACTATGCCAGAGAAAAGCAGAGACAGAGAGACCAGTGGAATGCTTC

AGTCCCTCCTACTTTACAGAATACCTCAGGGATTCCTATATATTCATCCAAACATATCCC

AACAGAGTTTTCGAATAAACCAATTCAGACCTGTTCAACTGAAGTATCAACAACGGAGAA

GAGCTCTCCTAACATCAAAGCTATGGGTCCTGTCCTAAACACTTCTAACCTCAGAGAACC

CCCCAAACTCTACCAGGCCTTTCCCAGGGACACTCAATCATCTTTACAAAAAGAAACAAG

TGGTGGGCTAAGTAGTTTGGGCAGCAGTGCCAGTAGTTCCAGTATGGATTCAGATAGTGC

CCAGTTTGAGGAGGAACCATCTGAGCTTTTACCAGATGGTTTGGCAAACATTATGAAGAT

GTTGGATGAGTCCATTAAGAAGGAGGAGGAACTACAATCTGTCCAGAGTGGTGGGCAGTC

TGACCCAGACATATCATTTTCTCTTACAGTGGCACCCATAAAGACTTACACGTTTGCCCC

TGATCTCATGCCTGCCCTAAAACAGCCATTAACAGTGGATTATTCAACTGAGACCCATGC

TAGCCCACCTGTCCTGAGTCGTCAAGGGTCCTTGGCATCCCCTTGTAGTCGCACTTCTTC

ACTTGAAGAAGAGGAGGATACACTAAAAATCATTGATACATATGACACTAGTGTCGATAA

AGATGGTGGTGTTGGTAGTTATCGCCATAGTGACTTGGCCAAGCTTTATGGATTATCAGA

GGGAGCAAAGAGTGAGTGTGATGAGGAGGACGAAGAGGCAGAACAAGACGAGGATACTCG

TTCATGTTCTCCACCACCTACTAGGCCACACCTGCATCAGACAGGTGTGAACAGCATGTT

TCAAAACTTAGCCTCCATGCTGGAGAGCCAGAAGTATGCCTACCGCGGCGGCCCATTTGG

TCGACCACCTCCTTCAGCTCTGGTTGGAGTGAAGTACTCTTCATCACTTTCATTAGGCCC

AGATATCTGCAGACAACAAAGCACTTCTCCCACATCAGGCTCAACCAAACACCCAGGCTT

CAACCACAAAGATCAAGCCTCAGTGAATTCTAGATCTGAACAAACCCGTTCCCTCTCACC

ATCGAGATCAACACCAGAAACAAAAGATCTTGTAGGCTGCCAGTCGGATCTCTCAGGATG

TGAGGAGGAAGAAGGAGTAAGGAAGGGGCTGATGAATGAAGATGCTTCTTGTATTGTAAA

TAACTTCACAGAGGTAGAGAGGAAGCCCAAGCTCACAACAATCTCTGAATCCTCACTTGC

AGAACTGGGTCGTAGTTGTGAGGTGATGCTAAGCCGGCATAATCTCCCATCAATAGCATC

AGACCATAATAAGAAACAGATGCCACACATAACTGAGAAAGACAGGAAGAGAGATCGTGA

CTATTACCAGGAGAAGAAACACAAACGCAGCAGCAGTAGCAAAAAAAATGAGGAAAGAAA

AGAGAAAAAGAAAAAACACAGAGAAAAACAAGAGAATATGTTGTCAAACTCAAGCCGAAG

ACATAAGGACAGCAAACCGCACAAAGAGAAGAGGGGCCACATAGACAGTCACAGAGAGAA

AGAGAAAGAAAGAGATGGAGAAAAAAAGAAAGGGAAAGAAAAAGAGGAATGGGTATGCCG

GAACAAGGAAAAGAAGTCAGGAAGTTCTTCAGTGGATCATTCGTCATCTTCACCCTCATT

GGGTTCTGCAGACTTCCAGAAGTTAAAGGCTTTGACAGACGGCCCACCAAAAGAGCTAAA

GATTCGTCTAATTAAAGTGGAAAGTGGAGATCGAGAAACCTTTATTGCCTCTGAAGTTGA

GGAGAAAAGAATTCCACTCGGGGAGATTACTATCAAAAACACAGCCAGTGAAATCATCAG

ATCGTGCAAGGGTGCCCGTGTTAAGGGGAAATTCAAGGAGTCCTATCTACTTCCATCATT

TTCTCTGAAACCTGTTGTGACTATGGAGCAACCGATCCCACGCGAAAAACTCAACCCTCC

TACACCCAGTATCTATCTTGAGAGCAAGAGGGATGCTTTCTCTCCTGTGCTGCTTCAGTT

CTGCACAGACCCCAAGAACCCCATTACTGTCATTAGAGGACTAGCAGGCTCACTCAGACT

AAATTTGGGTCTCTTCTCCACAAAGTCACTTGTGGAGGCGAATGCAGAACATGCAGTGGA

AGTGAGAACGCAGGTGCAACAGCCAGCCGATGAGAACTGGGATCCCAGTGGCACTGGGCA

AATTTGGCTATGCGAAAGCAGCCGTTCACATACCACTATAGCCAAATATGCCCAATATCA

AGCCTCCAGCTTCCAGGAAAGCCTACAGGAAGAGAAAGGCAGTGATGATGAGGAGGAGGA

TGAAGATGAAAAGAAACCCGTCACAAACTTGGACAGCACAGGCAGTAACTCCTTAAGCTC

CAGTTTGGAGCAAAAACCGGTGGGAAAAATCATCAAATTTGGTACCAACATTGATCTCTC

TGATCCCAAAAGGTGGAAGCCTCAGCTACAGGAGCTGCAGAAGTTGCCAGCTTTCATGCG

TGTGTCCTCCAGTGGCAATATGCTTAGTCATGTGGGCCACACCATCTTAGGCATGAACAC

TGTACAGCTTTACATGAAGGTCCCTGGCAGTCGCACACCAGGCCACCAGGAGAATAATAA

TTTCTGTTCTGTGAACATTAACATTGGCCCTGGGGACTGTGAATGGTTTGCTGTCCATGA

TAACTACTGGGAATCCATCAGCAATATCTGTGAGAAGCATGGGGTGGATTATTTGACCGG

TTCATGGTGGCCAGTGTTAGAGGATCTGTACAATGCCAATATTCCTGTCTACCGCTTCAT

CCAGAGGCCTGGAGATTTGGTGTGGATCAATGCAGGCACCGTGCACTGGGTCCAGGCTGT

GGGTTGGTGCAATAACATTGCATGGAATGTGGGTCCACTGAATGCATACCAGTACCAGTT

GGCTCTGGAAAGGTTCGAGTGGAACGAGGTGAAGAAGGTCAAGTCCATTGTGCCTATGAT

TCATGTTTCCTGGAATGTGGCCCGCACAGTTAAAGTTACAGACCCCGACACCTACAAGAT

GATTAAACACTGTCTCCTGCAGTCCCTGAAGCACATTCAAATTCTCAGAGACCAGCTTGT

AGCTGCAGGGAAGAAGATCTCTTACCAGAGCAGAGTAAAAGATGAACCAGCCTACTACTG

CAATGAGTGTGATGTGGAGGTTTTTAACTTGTTGTTTGTGACGAGTGAAAACGGGAGTAA

GAAGTCATATGTGGTGCACTGTGAGGACTGTGCCAGGCAACGCAGCCCCAATCTCTCTAA

TGTAGTGGTGCTTGAACAATATCGCATTGAAGACCTGATGAACACTTACGACACCTTCAG

TTTGGCGTCCAGCTCTCGGTGAAATCGACTCTGCCATCCACCCGAGCCATAACCAAAAGT

CTTGCTTACCAGGAC

>Unigene000147

TAAAAGGGGAAAAAAAGCGTGACGGACGACGACGACGGGGGGAAAAAGACTGCAGTCCCA

TGATGAACCCCATGAGACGTAGCTGCCATAGCTAGTCAGAAGCCTCACCATGGCCAAGCC

TGGAACCGACAGAGATGGAGCCATGGTGGAAAAGACGACTGGGAAGAAGAGTAAAGATAA

AATCTCCCCATTTACCAAAACTCCGAAGCTGGATCGGAGTGAGATAATGGGGAAAGAGGG

AAAGCCCAAGTCTTCCATGAAACGCAAGCTGTCCTTCAGTGTCAGCCCGCCCCGTAACGG

GGAACGGGATTCAGACACAGATAAAGATGGTCCTGATAAAAAGAGGGTAAAAAAGGAGTC

TGGAGGTAAAAAGTCCACCCCTGTGAACATTTTGTTCGGTTACCCTCTCTCGGAACGCAA

GCAGATGGCCCTGCTAATGCAGATGACAGCAAGAGACAACAGTCCAGACACCACACCAAG

CCACCCCTCCCAAGCTCCAGCAGTGCAAAAAAAGACTCCCAATAGCTCAACATCTCGTCA

GAAAGACAAGGTGAACAAGAGGAATGAGCGTGGAGAAACACCGCTGCACATGGCTGCCAT

CAGAGGGGATGTGAAACATGTCAAGGAGCTGATTGGATTAGGGGCGGATGTAAATGTGAA

AGATTTTGCAGGTTGGACACCTCTACATGAAGCTTGCAACCTTGGTTACTATGACGTAGC

AAAGGTTCTTATTGGTGCAGGAGCAGAGGTGAACACCCAGGGATTGGACGATGATACACC

CCTACATGATGCCTCCAGCAGTGGACACAAAGATATTGTGAAGTTACTCTTGCAGCATGG

AGGCAACGCATTTCAGGCAAACAAGAGAGGCGAGCGACCGGTGGATGTTGCTGATTCTCA

GGAGGTTGAGCAGCTACTGAAGGGGGAGATCCCATTGTCTGACCCAGATGACAGCTCTTC

AGACTCTGAGGACCTTCCATCAATAAACCCTTCGAGTGTAGATGACAATATGGAGTACTC

GGACACTGAGAAGGACTCTGATGGCAAATCGACCACAGCCAAGGCCACATCTTCAGTGTC

TGGTGTGGATGAGTACGAGTTCAAGGACGAGGAAGAGGAAGAAGATCTTAGCAAGGCCCT

AAATGACAGACACATCTTGAGACGAGAAGTGCGACAGAAAGAGGAGCAAAACCATTACAC

ACCCAGACAGTCCAACAAGAGCGATCAGACAGTGTCATCATGCAAATTAAAAAAGCCCAA

ATCATCCCGAGTACTCTACTGCAGTTCAGATAGTTCAGACAATGAATTGGAGGCTCCTTC

AGAACGAAAGTGTTCCCCGACCAGGTCACTCTGCAACGACACACACAAAACAGAGAGCCG

GACAAAAAGAGATAGCTTAACTCAAACATCAACAGAAAAAGGCAAGGCAAACAAGAGGAA

CAGGAGTCAAAGCAAAAACAAGGAAAATCAGGAGGTTCAAGAGGACGGAAAAGAAAACAG

CAAATCTCTTTTGTTCTCGACAGCCACAGCGTCTGACAACTCTGATAAAAGTGTTCGGGA

GGAGGACTCTTTTAAAATGTCCTTTAGCCCAAAGGACGATTCATCTGTGCACCTCTTTCA

CTTGTCCGCTGTGAAATCCCCCAAGCTCAGTTATGGTCAACCTGACAAACAGATAACTCC

TCTAAAACAGGAAAATGCTAAGACTTTTGTCTCCATTGGAGATGGGTCCTGCACTATGGA

TGGTGTCAAGTACAACCACTACACAGATTCTGACTACACAGAAGGTTCCAGCAGTAAAAG

CTGTAAGCACAAGGACAAAAGCAAACATCACTACAGGGAACACATCTTGGATGGAGATGA

CAGAAGCTCTAGTCCATTTAAAGATGCCAGTTTAAGCAATAGTATGGATAGCTCGGAAGG

AGCCTTTAGAAAATCTGACAAGGATGGTAAGGTTGTCAAAAAGCATAAGCTTAAACACAA

GGAAAAGGACAAGTACAGGAAAGACTATGAGTCTGAGAGAAATCGGCATCGACAGAGAGA

ACTAAGTAAAGATGGACATAGGAATTTGGAGTTTGACCGAGAATTTTGGAAAGAAAATTT

CTTCAAAAGTGATGAGAGTGAAGAACTCAAGGGGAAATGTGAGACACCAGGCAGTTGTTC

TCCTCAAAAGTCTATAGACTCGTCGCCAGTTAAGGAAGATAAAACAACATTAAAAGACAA

GCATTCGGTTTGTAACAGCACTAAGGAGAGAAAACAAAAAGACGAGCGAGAAAAAGACAA

ACCTATAAAAAAAGAAAAGAAAGAGGTAGCATACAAGGATGAAAGAGGAGCAAAGGACAG

TAAAGGGGCTGAAAATGACCGGTCAGATTGTCCAAACTTGATACGAAAGCCTGAAGACTC

AAATTCTTGCATAAAAGAGGAGACCGAAGACAAGCCTCTTACTGGAAACTTGTTTGATCA

CAATCAGCTAGAGACTACAGAGAAGAGCTCTCGTGAGAAAAATGATAAACGGCTACCCAC

AAAGGACAGAGAATCTGAAAAAGGTGAGAAAAAGCAGGCAGAAAAAGAAAAGAAAACCAA

ATCAGATCATTTGTCAGATAAACCTGATCTTCATAACTGTACAGATCGATGGAAAGAGAG

GGATAAAATAATGACCTTGTCTAATTCGTCTGGTGAGAGAATCCATAAAGAAAGTGACAA

ACTAAAACCTGTGGCGCCTATGAAAAAGCATGAAGAAAACAAAAAGAGCAAGGACAAGTC

TGAAAGAAGAAGTGAGAAGGAACGTCAAGAGAGAGAGCATTTCTCAAGCGATCACAGGGA

CAGAGATAAAATGGGTTTTGAAAAGAAAGGAAAGGCTCCTGAGCGGAGCAGTGATCATAG

CAAAATCGATCGTATTAAAGAAAAAGAAAGAGATAAAGACTTCGACAAGAAGAAAAAAGA

AAAATTAAAAGAAACCATGATCTCCTCCTCAGGTTCAAATCTCAAATTTCTTTTGGAAGA

AAAAAAGGGGTACACATCTGAGAGCAATAAAACAGTAACCCTTAAATTAAAAGAAGAGGT

CATAAAAACCCCTGAAAAGGACAGGGACAGGCGGGAGAGGGATAGAGATTTGGAAAGGCA

TCGGGATAAAGACAGAGAGCGGCACAAGGAAAAGGACAAGGAAAGACAACATAGGGACAG

CAAACCCAGCAAAACAAGGCCTGTTGAAATTGAGTCGGATAGGTCCAAATCCAAAGCTTC

ACCTGCACAAAAAGAAACCCGCCCCAAAGAGAAAAGATTGGTTAATGATGACCTCATGCA

GACGAGCTTTGAACGAATGCTTAGCTTAAAGGATCAGGAGATTGAGCAGTGGCACAAGAA

ACACTTGGAAAAAATCAAGCAAAAAGAAAGAGAAAGGCTAAAACAACGACCTGGATCCGA

CTCTGGGAAGCCAAAAAACAAAGATAAAGCGAAAATTTCCACTTCCAGTGAACCAAGTGC

AAATAAAGAACTCTTGCGCTCCAAAAGCTCAGAAATCCCAGACGCTTCTGGTCGGGAAAA

GATCTTAAAGGATGCGACCAGCGGAAGGACACTTTCTCTAGATGCAAAGAATCTGTTGTG

TTTTGGAAAGTCTGTTCCAGTAATAGAAAACAGCCTTAGTCGATCTCCAAGACCTGACAG

TGAACGATCTGCTATCATGTCAAGATCAGTGTCTATGATCTCAGTAGCCAGTTCAGAGGA

TTCCTGCCAGGCTACCATGCTAACGCCCAGGCCCACGGAGTACGACTCGGACCTAAATTT

AGAAATGTCTGATTCTCAGCCACCTTTCCTCCAATCTTCCCATATAGTTCAGTCCTCCAG

ATCACCAATTTTTAACAATAAAGAAATTAACAGTCTTCCTGACGTAGCCTCTGGCAGTCG

AACACCACTGTCAAGCGGACTTGCATCTTCTCAATCAAGAACTGTCCATAATGAGGATGA

TAAAGCCAGTCTAGCTGAGCCAGACAGACTGACTGAAGAATCTCAGAAAGTTGGCGATGT

AGCAAACTCTAGTGAGGTACCTGGTAAAATACACCATACTGAAGCCTGTGGAGCAGTCAC

ACAGAAAAGTCTTACTATTAACAGCTCCCTGTCAGTGCATCTCCACTGTGCTGAATCTGA

AGTTCAACCTCCTGTCGGTGTTGAGGATGCAAATAGGAGTTCAAGCAACCAGCTTGCATG

TGCCCCTAACTCTGAAGATCTCCCAGGTCACCAAACTAGCTTTCCACGTAGTATTGGTTT

AACAACAACTACAGTAGAGGTAAGCAAGCTAAACACTGATACCTTACCAGAGCAGAGTGG

ACAAAATCAGCCTGACAGCTTTGACACAGAAATGGCCGATGGCTCCAACAAAAATTCCCT

GTTAGCTTCTGTGCCCCAGGTTGCATCCATGGTATCATCAGAGCAGAGCTTCACAAAAAC

ACCTACGCAGACTGGTAACCTGAAATGGAATTCTGTCTTTGATCCCAATAATGACCACCC

AGCAAGGACAGACATTGATTTGACAGAAGAGAAAAGTTTAAGAAGTTTGTCAAGCGGTCC

TGAAAGCCTGATGGAAGACAGATGCATGGAGAAGGTTTCTGCTGGGAGCAGAATGGACTT

GACAACAAGCCAAGCTTCATCTATCAGCTCTAGTAGGATCAGTTCTGAAGAGTCTGACAA

AATGCTGCAATGTACAGTCAGTGTGTTTAGAGATTCTCATCAGGTTACAGAACAGATCTC

AGTGGATGACTGTAAAGCAAGTCACTTCGGTGAGAACATAAGTGTGGCTGATGCACATCT

GGGGAAAAAGGAATATGGTTTGGCTCCCCCTTCAGTTTCAAATGTCCTTCACTGTACTAG

TCCCGAACAGACATCTGAAGAGTTAATGGAAGTATCAGGGGAAAAGTCAGACCATAATCA

GGTCATTGCTGAGACACAAGTAGAGAAATTGCTGTCTTCAGAGGATGGGGCTCAAACCCA

GCCTTTTCCGGACACTAAAGCGAATGTTGATGTGGAGATGCCAGACCAGCTTACTGTTGA

AGAGCAACAAGCAAAACAAGGTACTCCATCAGATGGAGAGCAGAATCTGGGCTGCACTGG

TGTTCAGCTTGATTTTCAGTCAGAACAGCTCAGTGGAGCAACATCTGGAGGCTCTTCACC

ATTGTCAGTGGCAGACAGAGACAGTGATCTAACAGCAGCAAGGACAAAAGTAAAAAATCC

AGATGACGAGGTGGACATCCAGGTCACTCATCCAAGAAAACGAAAGATGCCCAAAAGTTC

TCTTTCAACCCCAGCAGGCCTTACAGCTCAGCAAATAAAGGAAAAGACCCAGCAGTCATT

AGCAGCAATCGTGGACTCCCTAAAGCTTGAAGAAATTCAGCCCTACCAAACCGAAAGGGC

CAATCCTTATTACGAGTTCCTGCACATCCGTAAGAAAATCGAGGAGAAAAGAAAGGTGCT

ATGTAGCGTCATTCCTCAAGCACCACAATACTATGATGAATATGTTACATTCAACGGCTC

TTACTTGCTTGATGGGAATCCACTGAGTAAGCTTTGTATTCCAACGATAACACCACCACC

CTCACTACCTGAACCACTAAAGGAGATGTTCAAACAGCAGGAGATTGTGCGCATGAAACT

GCGCCTGCAGCACAGCATTGAAAGAGAAAAGTTGATTGTGTCCAATGAACAAGAAGTCCT

GCGTGTCCATTATCGTGCTGCAAGAACATTGGCCAATCAGACACTACCCTTCAGCGCCTG

CACTGTTTTATTGGATGCAGAAGTATACAACATGCCCCAGGATGTACAGGGAGATGACGG

AAAAACCTCAGTGAGAGATCGGTTTAATGCAAGACAATTCATGTCTTGGCTTCAGGATGT

GGATGACAAATTCGACAAGTTAAAGACGTGTCTCCTGATGAGACAGCAGCACGAGGCAGC

AGCTCTTAATGCTGTTCAGCGCTTAGAGTGGCAGTTAAAGCTACAGGAGCTGGAGCCTGC

CACCTACAAATCAACCAGTATCTTCGAAATCCCAGAGTTTCACATCCCCCTCGTGGAGGT

CAATGACGATTTCGATCTCACGCCAATATAACATCAGGAAATCCAATGTCCTGATAAGGA

TCGTCTGTCCAGGACTCGGAAAGAGTAGAGGAACACAAAGCACTTGTTTTGGGCGAGCTG

CACTGTGTGTTTGCGTTTGTGTGTGTTTGTGTTTGTGTGTGTGTGTGTGTG

>Unigene000148

GGGGGGAGCTTAATGAGAAAGTCGCATCTCTTACAGAAGAGGAGTGAGCAGGACAGCCCG

AGAGGAAACCTCATCAGCTCTTCGTTTCACAGTCTTAAAGCTCAGCTAGACTCGCACTCA

CTGGCAGGGCCACACCTCCAGGACTGAACCATGGATGACGACTTTGAGTGGCGCAGAGAG

CAGAGGAGACAGAAGAGAGAGGAGATGCGACTGGAGGCGGAGAGAATGGCCTACCGCCGG

ACTGAGGAAGATGAGGAAGAGGCAGCTCGTGAGCGTCGGCGAAGAGCTCGCCAAGAGCGC

ATACGAAACAAGGAGAATGAGGAGATAGCTAACCCAGCTGAGAGCGAAGGGCTGAACAGC

CATAGTGTCACAGCGACAAAGACGGTGTCGAGCTCAGAGCTAAACACGGGCCAGGCCGGT

GAGGGTGATGAGGAGGAGCAAGCCCTGCTCGATCGTCTGGCCAAGCGTGAGGAGCGCAGA

CAGAAGAGGATGAAGGAGGCGCTGGACAGGCAGAAACAGCTGGACTCTGTCGACGATCCG

TCCTACAGTACCTACAGTGTGGAGAGCACCTCCACCACCTCTTACCAAGCATTCAGCAAT

CAGGAGGACGAGCCGGCACTCAACAGCAAGGAGGAGGTGAAGGAGGAGTTGGTTCCACAG

ATGGAACTAGTGGAGGAGAAGCCACGCCGGTCTTACTTGAGGGAGCAGATGGAGGATGAG

GACGCAGGAGGGAAGATCAAGCAGCCGAACGGAGGAGTGTATGAGGACAGCAAGCACAAG

AAGGTGGAGAGAACCCAAAGCCGTGGCAGCGTGCACTCTTTCGAGGGCCCGGCAGCAGAG

GAAGGAGACGACAGCGAGCAAACACGCTTGGAAGCTGAGAGGAAGCTGGAGGAGCTAAAG

AGGAGAAGAGACGAGACAGAGAACGAGGAGTTTGAGAGGATGAAACAGAGGCAGCAGGAG

GCTGAGGTCGAGCTGGAGGAGCTGAAGAGGAAGAGAGAGGATAGGAGGAAGGTGCTGGAG

GAGGAAGAGAGGCTGAAAAAGCAAGAGGAGGCTGAAAGAAAGGCAAGAGAGGAGGAAGAG

AAGAGGAGGATGAAGGAGGAGATAGAGAGGAGGAGAGCGGAGGCAGCTGAGCGGAGGCAG

AAGGTCGGAGAAGAGGTAGACGGAGATGCCGGCAAGCCTTTTAAGTGTGTGAGCCCACGA

GGATCCTCTCTGAAGATTGGTGAAAGGGCAGAATTCCTCAACAGGTCAGCACAGAAGAGC

GCCGTGAAGACATCCCACTCTCCCGTGGTGTCAAAGATTGACAACAGATTGGAGCAGTAC

ACCACAGCTGTGCAGGGTAATAAAGAGGTGCGCTCGCCACGCGGAGCTGCAATCGACTTC

CCCGGGGGTTCTGATGGCGTACGCAACATTAAGAGCATGTGGGAGAAGGGCAATGTTTTC

AGTTCCACCATCAGCTCACCTTCCAGCAATAAGGATGCAGCTGGAATAAAAGTGGGCGTT

GCAGGACGCATCAATGATTGGCTGAACAAAACACCAGAGGCGAGCAAAACGCCAGGAGGA

AGACCTGTGGACCTGAAACCGGGCGACGTTACCGGCAAGCGCAATCTATGGGAAAACAAA

AGCTCCTCTGCTACCAAGGTGACAAGCAGAGGAGAGAGCAAATCTGTTGCCAATGGAACG

GGACCCTAACCTTTCCATGAAGCCACAGCACCAGGGACCAAGTGAAAGCACCGTATTCCT

CTTCACATGCATGCACTCGTACCAAAAAACAATAGTCTCATATGTTTTTATTAATGGCTC

GGACTTCACTATCCTAAAGATGAGGTAGAATCTAAAAAAAAAAAGAAAAAAAAAAAAGAA

AAGGAAACTGCTCCTGTCGTTTCTGTCACCTGACTGTCAGAAGAGGGGGATGATGAAAAC

GGCTGGATTACGGCGCTGCTGAACGAGACAGCGGCTTTGACAGCTAGTGAACGTTGCAGA

CTCCAGCAGCTCTGATTGGACGCTTGTGTCAGAATGACAGCAGAGGACTCTACTTCTGCC

GTCACCATGAGTAACTCTAGCCACTCCTCCAAATCGAACCCTGCCAACAATCATGCAGTT

CTTCACGGATCCATTACCGAAGGTGTTGCCGGAGCCCCTAATGAAGCGGCCCTGATGGCT

CTGATGGAGCGCACAGGATACAAGATGGTTCAAGAGAACGGACAGCGTAAATACGGCCCA

CCGCCCGGCTGGCAAGGAGCGTCCCCACCGCGTGGCTGTGAAATCTTCGTAGGAAAGATC

CCGCGCGACGTTTATGAAGACGAGTTGGTGCCGGTGTTTGAGTCAGTGGGCAGGATCTAT

GAGATGCGCCTAATGATGGACTTCGATGGAAAAAATCGTGGCTATGCCTTTGTCATGTAC

ACTCAGAAACACGAGGCTAAACGTGCCGTGAGGGAGCTGAACAACTACGAGGTGCGACCT

GGACGGTTGCTGGGGGTCTGCTCAAGTGTGGACAACTGCCGCCTGTTTATTGGTGGAATT

CCCAAGACCAAGAAGCGAGAAGAGATTCTGGAGGAAGTCTCCAAAGTGACAGAGGGAGTG

TTGGATGTAATTGTATATGCAAGTGCTGCAGACAAAATGAAGAATCGTGGCTTTGCCTTC

GTAGAGTACGAGTCTCATCGTGCAGCTGCCATGGCCAGGAGGAAACTCATGCCTGGACGC

ATTCAGCTCTGGGGTCACCAAATTGCCGTCGATTGGGCCGAACCAGAAATCGACGTTGAT

GAGGACATCATGGAAACGGTGAAGATCTTGTATGTTCGCAACCTAATGATCGAGACCAGC

GAGGAGACCCTCCGCAAAATTTTCAGCCAGTTTAACCCAGGCTGCGTGGAGCGAGTAAAG

AAGATCCGCGACTATGCCTTTGTACACTTCACTAGCCGGGATGATGCGGTGGTGGCTATG

GACAACCTGAACAATACAGAAATTGAGGGCTCGTTCATCGAGGTAACGTTGGCCAAGCCA

GTAGATAAGGAGCAGTACACTCGCTATCAGAAAGCATCCAAGGGAACCATGACCAGTAGT

GACAGTACTCAGCAAAATTTTGTGTATCAGTGTGATCCCTACACCCTTGCCTACTATAGC

TACCCTTACAACACTCTCATTGGACCCAACCGAGACTACTTCATCAAAGTGACTCTACCA

GGCCTCGGTGCACAGTACCCCGCCATGTTCTCTGCTGCACCCGCTGCTACTAAACTGGTG

GAGGAGGGCAAAATCCACCCAGTGGAGCACTTGATCAACCCCCTCGGCCTTCCGCATGAT

CACCCAGCTGCTCCCTCGAGTGCTGCCGTCATTCCTGCCGTCTCGACACCTCCGCCCTTT

CAGGGTCGTCCTATCACTCCTGTTTATGCTGTGGCTCACAACGTCCCTCGGATCCCTGCT

GCCGCCGCGGCTGCCGGCCTGTATGGAGCGAGCTTCGTGCCCATTGCTGCTCATACCAAC

ACAGCTGCTCTGGCAGCATTGCAGAAGAATGCAGCTGTAGCGGCTGCTGCGTACGGAGGT

TACGCCGGCTACATGCCCCAAGCCTTCCCTGCTGCCACTTTCCAAGTGCCTATCCACGAA

ATGTACCCGACCTACTGACCCGTGTTATGCCTTGACACAGTCTTGACCCGAGACACAGCA

TCAGATTTATGAAGGCTGTTGACTGATCAAACAAAACCCCCAAAACTTTAAGCGCATATA

TACACAAACAACAGTAACTTTCTATAAATATTATAAACTTTATATATACTCTGTAATGCC

TCATAGCGCTCTAATAGTGTATTACATATAATTTTCTGACAACCTAATAACCCATGCAAA

TGCCCTGCCCTGATTTTTATTGTAAAAGTCCTTATTTTTGGTTGATGTCTCCTCATCCAA

AATTACCATCTGGTGGTGCTTTCTATTTTAAGCAAAGTTGCAACTTTTAAGTGCATGAGG

TGTTCATTTTACAATTGGGTTAAATTAGTACATTATCCTATTATGTGCAAATCTCCCTCC

TAATGATGAAAGCACGACTCTACACCATGGTGTCAAACGCTGTATAAGTTTGGGATTTGG

GGCACAACTAACCTTCAGTTGAAAATACGATAGCAGGAGACTTTTCTAATATAACGTTTT

AATGTTTTTTAATGTCAACTTTTATTTGCAATGCCTCACAAAATTACGTGGACAGTGTTT

AAAACTGTATGACAGAAAGCATGGTGTCGAGTAAGCAGCCAGATGTAAAGCCTGGATTAG

CGCATGTGCATTTCCAGATGTTTAAAAATATCTGGGTTGTACAGTTTACTTCCTGAACTT

TACAAATACGTTGCAGCAGGTGGATTGGCTAAGATACGTCACCCCAGATGTCAATACGTG

TGTGTGTGAGTGAGTGTGTGTGCTTGCAGGGTGTGTTCTCACCTTGCATAAATAGACTCC

GGATCTGCCGTGAATAAAGCGGTTACTGAAGATAAATCCAACAAATGTTATTAGAGCGTT

AGAGGCGTTGTTGGTGTTTTGTACTGTTGTGCTGTGTATAGTGTGTTTAAAGATAAAATA

TGATCCACCAGACCTGCTGACAGGAGACTTAGACCTAAATTAAGAAAAAGAAAACAGATT

TTCACCTACGACTACTGAACTGTTCCTGGAGATTCAAGCACAGGACACGGGATTAAATCT

CTGTGGTCCAACAATTAACCAACTGACTACATGAAAAGCACCAGTTGTGGCCTGTGTGTA

TGCTACTCAATTTCCAAGATAAAAACCTCCTTCCTTATTGCAGATCTACTGATGCAGTCT

CATCTCAAGCTCCACCCACTTGAACAAGATTCCCCTGTCAGCCAGCAGGCGAGGTGACAA

TTCAGGGGGGTTTAAAAAAAGCCCATCATTTGTGCCCATCAAGTGCCTTAAGTGCAAATA

TGTCACATCGTAAAACCACAACAATCATTCATTCATTACAACTATGACGTTTTTATTGCT

GTCTACATTTAAAAAAAAAGTGGTGTTTTTGCACTAATCGATCTCGACTGATTGGTTTTA

ACCATCCAAAATGAAGGGTGAATGGAAAACCTGCGCTAAAGTGAAGGATCCGATCCCGCC

GAGAGTCTATAAGCACTTAAATCAGCGTCATATCAAAGCCAAACACCCCTTTAAGGAACA

CTTTACACTCCAAACATAGACTAGAACCTTCTGAAACCTTCGTCCTGTCTCTGCAGCTGT

GAAAAATACCGAAGGATTCCTGTATGGACGATCACCCCAGCACGCTTGCTATAATAGATT

GAAAAAATATAGAAAAACCTAAAGAAAAAAAATATGTAAACTTGCTAAGCAGAGAAATAT

ATGACCACTGAATATTATTGACATGTCTGACTAATACTATTACATCGTTTTCTTTAATAG

GATATTATAGTGTTAGATTTCCTTGTGGCCTGTTTTATTTTCACTGTTGTTTAAAGGTTT

TTCAGACGTTCTTTAAAAATGTTTTTGAGAATTTTATAAAGCCTAAACGTTGGAGACAAA

GCCAATAGTTTGAAAAAAATTGCAACATGGGAACCAAGACAAAACTCCAGACACTGTGTT

AAAGCACACATACTGTTCACTATAGAGCCTTAATATAAATACATTCAGCCTTTATAGTTT

AGACATTATAGATTTGCAGAACTGCTTTTTTGTAAACTCGAGACTGAAGACTGACGTTCC

TTATTTTAAAAAATATATACAGCTTCGTTTAGATCAGTCGTTCCAGGCTCACACTTTTGT

TTAATGCTATTGTTAGAGGTTGAATTTCCAAAAATGTTATTTCTTGTCAAAAACCCAAAT

TTGTGACTACGACATAGCTGCTTAATTATTTAATGTTGTTATAATAAGATGTAGCCATAT

AGGCATTGAAAACCGTTTTCTGGACACGCTACTCGTGGTACAGGAGCCTGAGCAGGGAGC

TGACTAATGAGTTGACGTTAATTTCAATTAATTACAAAGAAGCTTTAGACAGATTTCTTC

TTTTTTCCCTTTTCTGCTAATTGTTAAACCTAATATTAAACATGGCATTTCACAACATAC

TAAATTGTATGCACAAGTTATAGCACACCTAACTTGTGGGCACTAAGATGAACTTGTGGC

CTCAATTTATTATCGTATGGCTTAGATATACTAATAATATCCACCATGGCGCGTCAGAGG

ATTTGTAGATTCCCCTTTCTTTGTTTCTGGCAC

>Unigene000149

TTCAGGGAGAGACTGGGTGTAAAGACTGTTAAAGCACTGAAAAGAAACAATAATGGCCTG

ACACATGCTGCTATTGATATGCTCTGTGCTCTTATGTGTCCAATGCATGATGACTATGAC

TTAAGGCAGGAGCAGCTAAACAAGGCCTCTCTTTTATCATCTAAAAAGTTCCTGGAGAGC

CTTTTGGACAAATTCATCATTAATGTGGAACATGGTACAGGTGCTTTAGTGGTCAGTGCT

CTGCTAGATTTCCTGACTTTTGCACTCTGTGCTCCTTACAGCGAGACGAGTGAAAGTCAG

CAGTTCGACATGCTGCTGGAGATGGTGGCTTCCAGTGGTCGCACACTTTTTAAACTTTTT

CAGCATCCCTCTATGGCTATAGTAAAGGGTGCAGGTCTGGTTATGAAGGCCATTATTGAG

GAGGGGGATAAGGAAATAGCCACAAAGATGCAGGAACTGGCGTTGAGTGAAGGTGCTCTA

CTTAGACATCTACACACATCTATGTTCACAGTGAGCACTGATCAGAGAATGCTTACGAAC

AGACAGCTGAGTCGTCACCTAGTAGGTCTGTGGATAGCTGAGAATCCTGTCGCTATGACC

CTTCTCAAGAGAATGTTGCCAACTGGACTTCTTGCATATCTGGACAGCTCAGACCCTGTG

CCAGAAAGGGACATGGACAGAATTCACATTCGTGACAATCTTAAGATTGCATCTGACCAG

TTTGGGCACACTAAAGTTCCAGAGTGGCAACGAATTGCAGGCAAGGCTGCTAAAGAGGTT

GAAAGGTTTGCTAAAGAAAAGTCTGATCTGATGCTTATGCACTGGAGGGATAAAATGGGT

ATTGCACAGAAAGAGCAGGACAGGAATAACTTGAACACAAACCAAAAACCTGTTGTTTTG

AGAAAAAGACGTCAGCGGATCAAGATTGAGGCCAATTGGGATCTCTTTTACCACAAATTT

TCTATAGATCAAGCACGCTCAAATCTTATTTGGAACATGAAGACACGTGAGGAGCTGCGT

GATGCTTTGGAGGGAGAAATGCGCTCCTTCAGCATAGACAGGGAGCTGGGGAGTAACAGT

GTGATTTCCTGGAATCATCAAGAGTTTGAGGTAAGGTATGAGTGCCTTTATGATGAAATC

AGAATTGGAGATTATTATCTGCGCCTCCTTCTGGAGGAGAATGAGAATGAGGAGTCCGGA

GTCATCAAGAGATCGTATGAGTTTTTTAATGAACTATATCATCGTTTTCTCTTAACGCCC

AAGGTGGCAATGAAGTGTCTATGCCTGCAGGCTCTGACCATAGTATATGACAAGTGTTGT

AAGGAAATTGGTCCTTTCACTGACACCAAATACATTGTGGGCATGTTGGATAAGAGTACT

GATAAACTAGAGAGAGACAGACTAATTCTCTTTCTCAACAAGCTCATTCTGAACAAGAAA

AATGTGAAGGACATAATGGATGCAAATGGTGTGCGTCTTCTAGTAGACTTGCTAACCTTA

GCTCACCTCCATACCAGTAGAGCTACTGTTCCACTGCAGAGTAATGTATTGGAAGCAGCT

CCAAATATGAAAAGAGAGAGTGAAAAGGAGTGGTATTTTGGGAATGCTGATAAGGAACGG

AGAGGACCTTTTAGTTTTGAGGAGATGCAAGAGTTTTGGAAATCAGGTACACTAACTGCA

AAGACACGATGCTGGGCACAGGGCATGGATGGCTGGCGCCCTCTTCAGGCTATTCCACAG

CTGAAGTGGTGCACACTAGCCAGTGGACAAGCAGTAATGAATGAAACCGATTTGGCCACA

CTAATCCTCAATATGCTCATTACTATGTGCTCCTACTTTCCTAGCAGAGATCAGGACAAT

GCTGTTATTAGACCTTTACCAAAAGTGAAGCGGCTAATAAGTGACAATGCTTGCCTTCCA

CATATTGTTCAGCTGCTGCTGACCTTTGATCCCATCCTCGTAGAAAAGGTGGCCATCTTG

TTATACCTGGTTATGCAGGACAACCCCAACTTGCCACGCCTCTACCTCACTGGTGTTTTC

TTCTTTATCATGATGTACACAGGCTCTAATGTGCTTCCTGTGGCACGATTCCTGAAGTAC

ACTCACTTGAAGCAGGCCTTCAGATCAGAAGAGGCTAAAAGCCAGGATATTGTGCAGAGG

AGTGTGTTAGGACCAGTTTTACCTGAGGCAATGGTGTGTTACCTAGAGAATTATGAAGCT

GAACTCTTCTCAGAGATCTTTTTGGGAGATTTTGACACTCCTGAAGCAATCTGGAACAGT

GAGATGAGGCGTATGATGATCGAGAAGATTGCTGCCCACCTGGCTGATTTTACTCCTCGG

CTTGAGAGCAACACTCGTGCTTTGTATCAGTACTGCCCGATTCCTGTTCTTAACTTTCCT

CAGTTGGAAAATGAGCTGTTTTGCAACATCTACTACCTCCGTCACCTTTGTGACTTACAG

CGCTTCCCTGAATGGCCTATACGGGACCCGGTAAAGATGTTAAGGGACACCTTGGAAGCC

TGGAAAAAGGAAGTGGGAAAAAAACCCCCCTCTATGTCTGTTGATGATGCCTATGACGTG

CTAAACCTTCCTGCAGGACAGGGCCAGTACGAGGAAAGCAAAATCAGGAAAGCATATTTC

CGATTAGCTCAAAAATACCATCCAGACAAAAATCCAGAAGGCAGGGAAACATTTGAAAAG

GTGAACAAGGCATATGAGTTTCTGTGCACTAAATCGGCAAGATTCGTAGATGGACCTGAC

CCTGAAAACATCATCCTAATACTTAAGGCCCAGAGCATCCTCTTTAGCCAACACAAACAA

GAACTGGAGCCATATAAATATGCAGGTTACCCTATGCTTATTAAAACTATTACCATGGAG

ACCAGTGATAGGCAGCTTTTTTCCAAAACATCCCCACTCTTGCCAGCAGCGTCTGAGCTT

ACTTTCCACACAGTCAACTGCTCAGCACTCAATGCTGAGGAACTGCGACGAGAAAATGGA

TTTGAGGTTCTGAATGAGGCTCTGTCTCGCTGTGTGGCTGTGCTTACTGGTTCCAGCAAA

ATTGGAGACATGTCAGTGCAGGTATGCGGTCACATCTGTAGGTGCTTCAGTGTTGCAGCT

CAGTTTGAGGAGTGCAGAGAGAAAATCATCAAGCTTCCAAACATCATAAGAAATATTTGT

CACATACTTTACTATAGCAATGCTCTTTCTCGTCTAGCCTCGCTGGCTGTAGAATGTGTG

AGCTCGTTCTCCGTGGACTTTTTTCTTCAGACACAATTATATCAAGCTGGTGCACTTTGG

CCACTGCTTCTCAACCTCTTTAATTTTGACTTCACACTGGAAGAAAGTGGCGTCCAAGCT

AGCCAGGAAACCAACCAGCAACATGTAATCAACAGCCTGGCCAAAAACAGTCTGTTGGCT

ATAGCCCGTCTTGGAGGCTACAGAACCAGAACAGAGGCTGACGGAAATCATCAGATTGGG

GGTGAACCAACTCCAGAGAACCCTGCAGTGAGGAAGAGTGTGGCAGCCATGCTTACACCA

TACATTGCATATAAACTGAGCACGAATTCACCTGCCGAGGTCCTAAAGTTGGTTAATTGC

AATTCGGAGACCCCTTATCTTATATGGAATAATGCAACCAGAGCCGAGCTTCTTGAGTTT

CTTGAGGCGCAGCAAGAAGGAAACATGAAGAGGGGTGAATGTGACGAAAGCTTTGGAGCA

GAGTTTGCCTTTAGTGAGCACAATAAAGAACTCATTGTGGGAGAGATCTTTGTTCGCGTG

TATAATGAGCAACCAACATTTCCTTTGGAGTATCCGAAAGTGTTTGCAGGAAGTCTTTTG

GATTATGTGGGTTCTCAGGCACAGTATCTCCACACTCTGCTTGCGCTGACACAGACAAAT

AAAGTGGAGTCACAGCAGCACGCTCAAAGACTTCTTTGGGTTGAAATGGCATTAGAAGCA

CTGTGCAACGTTATCAAGAACAACCCAGGATCTGAAACAGAATGTATTGGCCATTTTAAA

CTGCTCTTTTCCCTGCTCAGAGTCCATGGGGCTGGGAAAGTTCAGCAGCTAGTCCTAGAG

GTTGTGAACACAGTTACTTCAAACCAGGAATGTATCAGCAGTATAGCTGAGTCTTTGGTG

TTGGCCAATTTGCTATTGTTGCTGCACTCCTTACCATCAAGCAGGCAGCTTGTCCTGGAG

ACTCTGTATGCCCTCACGTCTAACACCAAAATTGTCAAAGAGGCAATGAACAAAGGTGCA

TTGATCTTCCTGTTGGATTTGTTCTGTAATTCCACTCACCCTCAGGTGCGCACACAGACA

GCTGAGCTCCTGTCCAAGATGACCTCCGACAAACTTGTGGGCCCCAAGGTTCGTCTGATA

CTGATGCGTTTTCTGCCAGGAGTCTTCATAGATGCCATGCGAGACAATGCTGAAGCTGCT

GTGCATATTTTTGAGGGTACTCATGAAAACCCAGAGCTGATTTGGAATGATGCATCACGA

GAGATCGTCTCGACCACTGTCAGGGAATTAATGCTTGAACACTTTAAGCAGCAGAAGGAT

AACCCTGATGTCAATTGGAAGCTTTCTGAAGAGTTTATGGTCCCATATGCAGCAGGACAA

GGTGAGCTGGAAGTGGGTGGAGTGTTCCTTCGCATCTTTATTGCTCAGCCAGGCTGGGTG

CTGCGCAAGCCTAGAGAATTCCTTGTCTCCTTACTTGATGCCCTCACGGAGCTGCTGGAA

AGGAACCACCCTAATGATGAGGCATTAGAGACAGTCACAACAGCAGCTGTGTGTTTGTTC

AGTACTCAGACTCAGTTGGCTGATCAGATTCCGCCTCTTGGTCACCTGCCGAGGATCCTT

GCAGCTCTCAACCACAAAAATAGTGCTGTTCTTAAAAGCTCCATCCGCCTCATCCATGTC

CTCTCAGAGAATGAGTTGTGTGTGCGCTCCATGGCAGCTTTGGAGACTATTGGACCATTG

ATGTCTGGGATGAAGGTCAGGGCTGATATGGCAGGACTAGCATGTGAAGCCCTCAATCGC

ATGTTTCAGCGAGAGCAAACAGACCTAGTTGCACAGGCTCTTCGAGTAGAACTGGTGCCA

TATTTACTAAAGCTTTTAGAGGGAATAGGATTAGAGACTCTGGACAACCCATCAGCAACC

AAAGCCCAAATAGTCAAAGCACTTAAGTCCATGACTGGCAGTTTACATTATGGAGAACAG

GTGAATGAAATCTTGTCCCGATCCACTGTGTGGAGTGCCTTCAAAGATCAGAAACATGAT

CTTTTTATCTCTGAATCACAACTTGCAGGCTTTCTGTCAGGTCCAGCAGTAGCAGGCTAT

ATAACTGCTGGCACGGGTGCTACAGTTATGTCCACTATCCCGCCACCTGTAGACAATAAC

ATTGGAGACCAGTGCTAAAAATCAAACAAGCACCAACAGTTTAGTACCTGCACCATACAG

AAAGACCAACTAAAGGACTTAAATAGAAAAGTGTTTGGTCGATTTCAGCCCTCTCAATTT

GTGATGCACTGTACTCTGTGTCCATGTTATTTTTTTACAATCATTAATTATTTCCTTTTT

TTCTTTTTGCTCTTATTTCTAGTATATTAAGCTATAATTTTTCAAAAAAGGAAATTTGTT

TACTTTTATTACTTTGTATAAGAATAAATCATTATTTGCTGGAAGGAAAAAATAAATACA

TCTCAAAAGTCAACACATTCTTTTTTTCAGTTCACCTTATTTTCTATGCTATGCTTTCCA

TTGTGTTTTTCTCTATATTATTATGGTAAGCTTGTCTTATGTCATAGATTTGTGTGCCAT

AAAGCTGTCATGCCATGTCATGTTCTTCACCTAGTTGTGTTTTCTATTAACCCGAAAATA

AATAAATAATTTTGAAAATTGAAACCTGTCACTGTCAGTTACAATAACACCCCATAGAGT

GACTTGTATTTAAACTTACCTTATGTCTCTGCAGCCAACCTACCCTTACCTACCAGGAAT

GCAAAAATATCACATGGTAAAAGTAAGTGATAGCTAAGAGTAACCATGATGAATAATGAT

GAGCTTCTTAAATAGACTTATCAAATGTATGTGATTAATTAAATTGTCACTATCTATGTA

AGGAGGCCTATATAAACTGTTTAATTTGAATGTACTATTAAGAAATGTCACACTTGTATA

TAAAATTTTAAGAATAAAAAGGAAGCCAA

>Unigene000150

AGAGCGCACCACAGTTTACATCAAGGGGAGGAAGGGGGCTAGCAGAGTAGTTCAAGCAGT

TAGATAATAAACATAAATAAATAGGCACATTGAGTGGTAAGGAGCGTGGTTTGTTTGGAA

ACCGCCATCGGACACGTTCCGGTCACCTCTTGGTGCCAGGAAGAGGGTCTTGGTGGCGTT

TCAGAGCCGTTCCCTCTGTGAAAAGCAGCGTGCCGAGGGGAGGACGAGTCTCTTTGAATA

GACAGGATGCACGGACCGCCTTCCAGCGACAGCGCATGCCCACTGCGCCTCATCAAGAGA

GTTCAGTTCGGCGTCCTCAGCCCAGATGAGCTTAAACGGATGTCCGTCACCGAGGGGGGT

ATCAAGTACCCCGAGACCACAGAGGGTGGACGGCCCAAACTGGGTGGCCTCATGGACCCT

CGGCAAGGCGTCATCGAGAGGTCTGGGCGGTGCCAGACGTGTGCAGGTAACATGACTGAA

TGTCCGGGTCACTTCGGTCACATCGAGCTGGCCAAGCCTGTCTTTCACGTCGGCTTCATC

ACAAAGATCATGAAAGTCCTCCGCTGTGTGTGTTTCTTCTGCTCCAAGCTGCTGGTTGAC

GCAAACAACCCCAAAATCAAAGACATCCTGACCAAGTCGAAAGGTCAACCGCGGAAGCGC

CTGATGCACGTCTACGACCTGTGCAAAGGTAAAAACATCTGCGAGGGCGGTGAGGAGATG

GACAACAAATTTGGAGTGGAGCAGCAGGAGAATGATGAGGACCTCACTAAAGAAAAGGGT

CACGGAGGTTGCGGGCGCTACCAGCCACGTATCCGGCGCACGGGTCTGGAGCTGTACGCA

GAGTGGAAGCACGTCAACGAAGATTCGCAAGAGAAAAAGATTCTCCTCAGTCCTGAACGT

GTGCACGAGATCTTCAAACGCATCTCGGACGAGGAAGATATGATCCTGGGAATGGACCCC

AAGTTCGCACGACCCGAGTGGATGATCGTCACGGTGCTGCCGGTGCCACCCCTCGCCGTC

AGACCGGCCGTGGTCATGCAGGGCTCGGCCAGGAACCAGGACGATTTGACGCACAAGTTA

GCCGACATCGTAAAAATCAATAACCAGCTACGACGGAACGAGCAGAGCGGCGCTGCGGCA

CACGTTATCGCAGAGGATGTCAAGCTTCTGCAGTTCCATGTGGCCACCATGGTGGACAAC

GAGCTCCCTGGTCTGCCTCGAGCCATGCAAAAATCGGGTCGTCCCCTGAAATCCATCAAA

CAGAGACTGAAGGGAAAAGAAGGTCGAGTGCGAGGAAACCTGATGGGGAAGCGTGTGGAC

TTCTCCGCTCGAACCGTCATCACACCTGACCCCAACCTACAGATCGATCAGGTGGGGGTG

CCCCGTTCCATCGCCGCTAACATGACCTTCCCCGAGATCGTCACACCCTTTAACATCGAC

AGACTCCAGGAGCTGGTGAGGAGAGGAAACAGTCAGTATCCTGGAGCCAAATACATCATC

CGCGATAACGGAGACCGAATTGATCTTCGGTTTCACCCAAAACCGAGCGACCTTCACCTT

CAGATTGGCTATAAGGTGGAGCGGCACATGTGCGACGGTGACATCGTCATTTTCAACAGA

CAGCCCACCTTGCACAAAATGTCTATGATGGGTCACAGGGTTCGAATCCTGCCTTGGTCT

ACGTTTCGACTCAACCTCAGTGTAACGACTCCCTACAATGCTGATTTTGATGGAGACGAG

ATGAACCTGCACCTGCCCCAGTCCCTCGAGACCCGTGCCGAGATCCAGGAGCTGGCCATG

GTGCCCCGTATGATCGTCACCCCCCAGTCCAACAGACCCGTCATGGGTATCGTGCAGGAT

ACGCTCACCGCTGTTCGCAAGTTCACCAAGCGGGACGTCTTCTTAGAGAGGGGTGAAGTT

ATGAATCTGCTGATGTTTTTGTCCTCCTGGGATGGCAAAGTGCCCCAGCCTGCTATCCTA

AAGCCACGCCCACTGTGGACCGGTAAACAGATCTTCAGTTTGATCATTCCAGGTCACATT

AATGTGATCCGTACACACAGCACGCATCCGGACGATGAAGACAGCGGCCCGTATAAACAC

ATCTCTCCTGGAGACACCAAGGTGATTGTGGAGAACGGTGAGCTGATCATGGGAATTCTG

TGTAAGAAGTCCCTCGGTACATCCGCAGGTTCTCTCGTCCACATCTCCTACCTGGAAATG

GGTCATGACATCACCCGCCTCTTTTACTCCAACATTCAGACGGTCGTCAACAACTGGCTG

CTTATTGAAGGTCACTCCATCGGTATCGGTGACTCTATCGCTGACGCAAAGACGTATCTT

GACATTCAGAACACCATCAAGAAGGCCAAGCAAGATGTGATAGAGGTCATCGAGAAAGCA

CACAACAACGAGCTGGAGCCCACGCCAGGTAACACTCTGAGACAGACCTTCGAAAATCAG

GTCAACCGTATTCTGAACGACGCTCGAGACAAAACAGGATCGTCTGCTCAGAAGTCACTG

TCCGAGTACAACAACTTCAAATCCATGGTGGTGGCTGGATCCAAGGGCTCCAAAATTAAC

ATCTCACAGGTTATTGCTGTGGTGGGTCAGCAGAACGTTGAGGGTAAACGAATCCCTTTC

GGCTTTAAGCACCGCACACTTCCTCACTTCATCAAGGACGACTACGGTCCCGAGAGTAGG

GGGTTCGTAGAGAATTCGTACCTCGCCGGTCTGACACCCACAGAGTTCTTTTTCCACGCC

ATGGGAGGCAGAGAGGGTCTGATTGATACGGCTGTGAAAACTGCCGAGACGGGTTACATC

CAGCGTCGTCTGATCAAGTCTATGGAGTCTGTTATGGTGAAATATGACGCCACCGTGAGG

AACTCGATCAACCAGGTGGTACAGCTGAGATACGGAGAGGACGGTCTGGCTGGAGAGGCT

GTGGAGTTCCAAAACATGGCCACGCTCAAACCGTCTCACAAAGCTTTCGAGAAGAAGTTC

AAGTTTGAGTACACTAATGAGCGAGCCCTGCGGCGTACGCTGCAGGAGGACGTGTTGAAG

GAAGTGATGACCAACGCACACGTGCAGAGCGCTTTGGAGAGAGAGTTCGAGAAAATGAAG

GAGGATCGAGAGATCTTAAGAGCCATTTTCCCCAATGGAGACAGCAAGGTGGTGCTGCCG

TGTAATCTAGCCAGAATGATCTGGAACGCGCAGAAGATTTTCCGCATCAACCCGAGAACT

CCGACCGATCTTAACCCCTTAAGAGTGGTGGAAGGTGTGCACGAGCTGAGCAGGAAGCTG

GTGATCGTAAACGGAGACGACCCGTTAAGCAGGCAGGCTCAGCAGAACGCGACGCTGCTT

TTTAACATCCACCTGCGCTCCACCCTCTGCTCCAGACGCATGACCGAAGAGTTTCGTCTC

AGCTCCGAGGCCTTCGAGTGGCTCCTGGGAGAGATCGAGACCAAGTTCAACCAGTCGATC

GCTCACCCAGGTGAGATGGTGGGTGCCCTGGCTGCTCAGTCTCTGGGTGAGCCTGCCACT

CAGATGACCCTGAACACCTTCCACTACGCCGGTGTGTCGGCTAAGAACGTCACCTTGGGT

GTGCCTCGACTCAAAGAGCTGATCAACATTTCCAAACGTCCCAAAACTCCGTCCCTGACC

GTGTTCCTGCTGGGGCAGGCGGCCCGCGACGCAGAGCGTGCCAAGGACATCCTGTGTCGA

CTGGAGCACACGACCCTGCGCAAGGTCACCGCCAACACCGCCATCTACTACGACCCCAAC

CCCCAGAGCACAGTCGTGTCCGAGGATCAGGAGTGGGTGAACGTTTATTATGAGATGCCT

GACTTTGATGTGACGCGTATCTCTCCCTGGCTGCTGCGCATCGAGCTGGACCGCAAACAC

ATGACTGACCGCAAGCTCTCCATGGAGCAGATCGCCGAGAAGATTAACGCAGGTTTTGGT

GACGATCTGAACTGCATCTTTAATGACGATAACGCCGAGAAGCTGGTGCTGCGTATCCGA

ATCATGAACAGTGACGAAAACAAGTTCCAGGAGGATGAGGAGGTGGTGGATAAGATGGAC

GACGATGTGTTTCTGCGCTGCATCGAGTCCAACATGCTAACGGACATGACCCTGCAGGGC

ATTGAACAAATCAGCAAGGTGTACATGCATCTTCCTCAAACCGAGAACAAGAAAAAGACC

ATCATTACTGAAGAGGGAGAGTTCAAAGCTCTACAGGAATGGATTCTCGAGACGGACGGC

GTCAGTCTTATGAGAGTCCTCAGCGAGAAGGACGTCGATCCTGTCCGAACAACCTCCAAC

GACATCGTAGAGATCTTCACTGTGCTCGGTATCGAGGCGGTTCGCAAGGCTCTGGAGAGG

GAGTTGTACCACGTCATCTCCTTTGACGGTTCCTATGTGAACTACCGCCATCTTGCCCTT

CTGTGTGACACCATGACCTGCAGAGGTCACCTGATGGCCATCACGCGTCACGGTATTAAC

AGGCAGGACACCGGGCCGCTCATGAAGTGCTCTTTTGAGGAGACGGTTGACGTCCTGATG

GAAGCGTCGTCTCACGGCGAATGCGACCCAATGAAGGGCGTGTCCGAAAACATTATGCTC

GGACAGCTGGCTCCTGCAGGCACTGGCTGCTTCGACCTGCTTCTGGATGCCGAGAAATGC

AAATACGGCATGGAGATTCCCACCAACATTCCTGGCATCAGCGTGGCTGGACCAACTGGA

ATGTTCTTTGGCTCCGCCCCTAGCCCTATGAGTGGTATGTCTCCAGCCATGACGCCCTGG

AACACTGGGGCCACCCCGGCTTATGGTGCATGGTCTCCCAGCGTGGGAAGCGGAATGACT

CCAGGAGCCGCGGGATTTTCACCCAGTGCTGCATCTGATGCCAGCGGCTTCTCTCCAGGT

TACTCGCCCGCATGGTCCCCAACACCAGGCTCACCTGGGTCTCCTGGTCCAGCCAGTCCT

TACATTCCCTCTCCAGGTGGAGCCATGTCTCCAAATTATTCCCCAACGTCTCCGGCATAC

GAGCCGCGTTCCCCTGGAGGCTACACCCCACAGAGCCCTGGCTACTCTCCTACATCTCCG

TCGTACTCGCCCACCTCTCCGTCCTACTCTCCTACCAGCCCAAACTACAGCCCAACATCC

CCATCTTATTCCCCCACTTCTCCGTCTTACTCTCCCACATCCCCATCCTATTCCCCGACC

TCCCCGAGCTACTCGCCCACGTCGCCCTCTTACTCGCCAACGTCTCCGTCTTATTCCCCC

ACCTCCCCATCGTACTCTCCGACGTCACCATCATATTCTCCAACTTCACCGTCTTATTCT

CCGACGTCTCCCAGCTACAGCCCAACGTCCCCTTCATATTCACCCACTTCCCCGTCGTAC

TCGCCCACCTCCCCATCATACTCGCCAACCTCTCCTTCGTATTCCCCGACTTCACCATCT

TATTCCCCTACGTCTCCCAGTTACAGCCCGACCTCCCCAAATTACACGCCAACTTCGCCC

AGCTACTCGCCCACCTCTCCGTCTTACAGCCCCACCTCGCCGTCGTACTCGCCCACGTCT

CCGAATTACACGCCCACCAGCCCGAATTATTCTCCGACGTCTCCTTCGTACTCCCCCACC

TCTCCGTCTTACTCCCCATCCAGCCCACGGTACACGCCCCAATCGCCCACATACACCCCG

AGTTCGCCTTCTTACAGCCCGAGCTCCCCATCTTACTCCCCAACGTCTCCGAAATACACC

CCCACCTCTCCGTCCTACAGCCCCAGCTCTCCGGAATACACCCCGACTTCACCCAAATAC

TCTCCGACCTCACCCAAGTACTCACCCACGTCTCCCAAATACAGTCCGACGTCTCCAACT

TACTCGCCCACTACACCCAAATACAGCCCCACTTCGCCGACATACTCCCCAACGTCTCCT

ACCTACACCCCAACCAGCCCCAAGTACTCCCCCACTTCCCCCACGTATTCTCCGACATCT

CCAAAATACTCCCCGACTTCACCCACTTACTCGCCTACTAGTCCTAAAGGCTCGACTTAC

AGCCCAACCTCACCGGGGTACAGCCCAACGTCCCCGACCTACAGTCCAGCCATCAGCCCC

GACGACAGCGATGAGGAAAACAACTAAAGCGGGCGACCCCTGGATTAGCTCCGCCGTGTC

GTCTCATCCTTCTCGAAAAACACAGC

>Unigene000151

CTTGTAGTATCACTGGTCCTTTTTTTTTAATTAACAAGACGTCTCTGTTCCATTTTGTTT

TATTTTATTTTCTGTTTTGTTCTCTTCTCTTTTGTTTCTTTGTGAAAAACATAAATTGGG

GATATGCAAGCAGACCACAGGAGGGAGACCGAGCGGCAACACAGGAAGCTCTCAGGGTCA

TTACACACGTGCCCAGGCCAACAGTCCCAGACCGGTAATGAACTCATCAGGCCCTGCCAC

CAAGCAGGGTTCACAAGCCCAACAGCAACAGCCCCAGCATGCTCAACAGCAGGTATCCCA

GCAGCAGCAGCAGCAGCCGCAGGTATCACCAGGGCAACAGCGTGGGTCCCGGTCGACACG

TAGAAAGGGATCAGACAGCAGCATCCCTGATGATGAGAAAATCAGCGACGAAAAGACGGA

CGCGGGAGGCGGAAAAGGAGATGTTTCTAAAAACAAATCAAAACAGGTGATGAACAAGAG

GAGGAAAGCTGAAGATGAGGAGAAGAAGGCTGGGCTTAAACGGTTAAAGATGGAGGCATC

GGATCTTTCCGAGAGCAGCGACTCTGAGAACTCTAATAAGAGGCTCCTGGACTCATCCTC

AGAGCCGAGCTCTGAAAATGAATTAAAAAATAAAGGCATTTTAAAAGCCAGTGAGGAAGA

GGAAAAGTCCCAGAGCTGCAAGGCTATGGAGGAGCCTGACGCTAATAGCAGGATGTCTCC

CTGGGAGGAGGCTTCTACGGTGGATCAGATTGTCAAGCCGACAGCCATGGAAGTGATGCC

CTCTACCAAGAGTGAAAATACAGGCGACATGAGGTCCCTAGCGCAACCTTCCCCACTGCC

TCCTCCCAGCTCGTGTGCGCAGCCTCGGAGCTTAGTTCCTGCTGAAGTTCAGGGTTGCAT

TATGGAAATAAAAAGCACTGTGAAAACTTCTCCTAAGGACCATTATAGCACAGGGGCACC

AAGAACACAAACTCCAAAGTGTGTAATTGACATCACAGAAGACTCCAGCTCCCACCCCAC

AACCCGAGAGAACTCAGAGACTGTGCCTGCGACTTTAGCTTCTCAAACACGTGAAACTTA

TGTCCCTGAAAGCAGACATTTGGTGTTAAACCCCTTAGCCTCAGAATGCAGGAAAGCAGA

GGGTGAACTTCAGCAGCAGCTAGGTCAGAGCCTGGGCAGCAAGATTGAATTTGCTCACTC

TGAGGTGATCCGACCTGTGGTTTCGGTAAGCGAGTTAGCGGCTGTTGCGGAAAGGGAAAG

GGAGAAAGTACAGCAGCAGTACCCTTCAATCATGCCTTGCATCAAAAATGCCTCACTGGC

TGAAGATGTGCGAAAACCACAGAAACTTAGCTCGTCACCAGATGTGGCCAAGTCCAAGTC

TAACCCTTCACCTGACGCATTTAAGCCCAAGTGCAATCCCTCACCTGACGCCATGAAGTC

AAAAACCCACAGTGTCCTGGAGGCCATGAAGCCTAAACCTAACACTTCACCTGAGGTCAC

CAAACACAAAGAGATCATTGAGATCACTTCATCCTCTGTTGCCCGGTCGGCAGTCAAGTC

GAGAAGTCAAGAAATACCACGCTCCAGCTTTAAGCCTGTTCCTGCTCGTAGCACTCCCTC

TGAGTCCATCAAGAGCGCTCTCATTGTCGACAAAAACGAGCACTTTACAGTGTATCGTGA

CCCGGCGCTGGTGCGGCCCGAGGCTGATACCAACCATGTAACTTACCTTCCCCCACACCT

CCATCCACTCCACAGCTCCTCCCATGCTACTTGCCTGACACCTAGCTCACATCACCACTC

CCATCTATTGCCTGCATCTTCCCTTAGCCCGCACCCCTCAGTGCACCACCCCTTGCTCCC

CACCGTGTTGCCTGCCATGCCGCCGTCCTCCCTACTGGGTGGCCACCCGCGCCTCGACTC

AGGGGGACTCAGTCATCTAGCCCTAGCTCACCACCACCCACACCAGCAACAGCAGTTTCT

GCAACAGCAACCACCCCCACCGCTGCTGGCCCAGACACATGGTGGTGCCAGCTACAACCA

GCTCGGCCTTTATCCCATAATCTGGCAATACCATAATGGCACACAGCACTCCTATCCATC

TGGAATTAGCCTGCCTGGGTCAAAGTGGGTGCATCCAGAAAATGCTGTGAACTCAGATGG

TAGCCTGTCAAGGAACACCTCCAGTCCCTGGCTGCATCAGCCCACTCCTGTGTCCTCAGC

GGATAGTCTAGGAATTCTGAGCCATGTCCCAGGTAGGCCAGCCAGTGCTGATCCCCACAG

ACCCCTTAAAATCTCCTCACACTCCAGCCCTCCGCTCTCCAAAACTGTGGATCACCACAA

AGGAGAGCTGGAAAGTAAGGTTTTTGTAGATCCAATGCGTGGCCTGGTAACCGCCCACCT

AAAGCAAGAGCCAGACCAAAGCCGGACACCGAACAGCAGGGATTTACAGCGCCTCTACGC

GGATTCATCTCACGTAAAGCAGCAGGCGCTGCCTCCCCGCCTGCCACTAGACACTCCAGA

CCGGGCTGTTAAGTACAAAGAGGAGAATCGACGCATTCTACAAGAGAGCATTGAGGTGGC

ACCCTTTACTGCCAAAATCCGCTCAGGAGAGCCAGAGCGTGAGCCCTATATCCGCATTCC

CTCTCTCCCCATCAGTGGACCGCCCAAGGAGAATGAGCATTCGCCATCTGATCTTTATAA

GTACAAGCACTCGGCTGCTCAGTCACTGCCACAGAGCAACTATTTTACCACCATTTCCAA

CAGTGTGGTGAATGAGCCACCTCGTCTTTACCCCTCCAAAGAGCTTAGCTCCCTTAATGT

TTCAAGTCCCCTGACTCTCGGCTCCTACCCCAACAGCGGAGGCAACACCAAGTCCTTATC

CAAGCCTCCCCCGCTCATCAAGCACCATCCAGACGGGGAGGGCTTGGTGGGTAAGATCAG

TGAGCAGCTCAGTCATCAGGTGGCCCTGAACCCTCTGAGTACCCCCGTGGCATCTTGCGA

ACCCCGCAGTCCGGCTGTGTCACCCTCAAACCAACTCAGGAGCATGCCTGCTCTACACAG

GGCACCTGTTTTTCACCCTCCTACCCAGCAAACACTGGACCGCAAAGAGTCTGGCTACGG

TCGCCTCTCTCCACCAACGCTAACCCCTATTCAGCCGGTTAGTGTGGCTGGGAAGGTATC

AGAGCAACAAAAGCCGCCCACTCTGCTTCCAGAGCTTAGGGAGGTGAAAGGCACTGTAGA

GCTGAGCTCCAGTGAGCCATGGAGGCCCAGCTCTAACTTACAGGGCTATGATAAAACAGT

TCAGTGGCAGTTAGACAAAAGTCAAGGAAAGCCACAGGTAGCCACAGCTTCTGTTATTGT

TCGCTCCAGAACCTGCATTAAGTATGACAGCTCACCAGGCTCCAAACCTGGTGCCAGGGA

GACGTCTAACAGTAAACCACATACAGGAAAATACCAACTGGACTGCAGCAAACTGGCTGA

AACCAGAGAACCCGGCAGAGTTATACAGCAAAACACAAATAAGGAGGACATTTTTTTGCA

GTACAAAAACAATTTTGTCCGAGTCTCCCAGGGCAGTTTTCCCAGCTCTGCGGTAGCTGT

TGTTAACTCTGTGTGCAATAACAGTACTGCTGTAACCACTTCTGCCTCTGCTGCTTCCAC

TCACAATGTGCTGAGTCGTGGCACAGCTGAACTGCCTTACTCCACCTCAGCAAATCCAAG

CAGCAGTAACATTAACAGACTGGAGGGCGCTGCACCCAAATGCAGGACTCCTACCAGCCT

TGAGCTACAGGAGTGCAATGCCAGGACAGCCTCCCCCATTGGCCCACTGCCTCAGCCTGG

TTCGGCCCTAGCTCCACAGCCTTATTCTGGGAACTTTATCCACCTGAAGAAGCACAAGGC

TGCCCTGGCCGCTGCCCAGTCCCGTGGATCAAGCAGTGCCTCAGAGAGTGAAGGTAGCAG

TCGATCCTCTCAGGAATCACCCACAATCATCACGCAGGATCGTGCATCCCCAGGCAACCC

GTCCAGCAAAGTCAGCCCCCTACCCAATGGTCAGCCATCACTGATGAACCAGCCCAACTA

CCACAAGCTAAAGAAAGCATGGCTGACTCGGCACTCAGAGGAGGATCGCAACACTAACAA

GGTGGAGATGACTTCCAATGCCGTCTCTGAGATCATTAGGCCATGCACAGTAAACCTTAT

AGCATCCACCTCCAGCGACACAGAGATAAGCAAAGATGGCAAATGCTTAGAGGATAAATT

GAGCCCGGAGGACAGGAAACCACGACGAGTTCCTTCCAAGCGGCCACACGAATCAGGCTC

GGATAGCGGCGATGACTCCGATGGCAGTGACAGCAAGCATGAGCAGAGAGCCAAAAGGCA

GCCTAAGCCCACCTTCAAGAAGAAGCAGAATGACATGCAAAAAAAGAAAGGGGAAAATGA

CAAGGAGGAGGAGGATGTCAAGCCCAATGGCATATTCAGAAGCGCCAAGGAGAAAACCAA

ACTAAAATTGGCCAGCAGCAACGGTATTCCTCGCTCGGTGTTGAAAGACTGGAGGAAGGT

GAAGAAGCTGAAGCAGACGGGTGAGTCCTTTCTGCAGGACGATTCGTGCTCGGAGATTGG

ACCCAACCTGCAGAAGTGCCGCGAGTGCCGCTCGATCCGCACCAAGAAGGGAGAAGAGCC

CACCCACTCCCCCGTATTCTGCCGCTTCTACTATTTTCGCCGCCTTTCATATAGTAAAAA

CGGGGTTATCCGGATCGACGGCTTCTCATCCCCTGACCAGCACGACGAGGAGGCCTTGAG

TCTCTGGGCTCCAGATGCTTACGAAGAGAATGATCTGGACCTGGAAACCTCCAAATACAT

TCTCAGTTGCATCGGGGATAAGTTTTGCCAGCTGGTCATGAGCGAGAAGACCGCCGCCAC

ATGGATAAAGAAAGATGCCAAAATAGCCTGGAAACGGGCAGTGAGGGGGGTGCGTGAGAG

CTGCGACGCATGCGAGGCCACATTGTTCAACATTCACTGGGTGTGCCAGAAATGTGGATT

TGTGGTGTGCTTGGATTGCTACAAGGCAAAGGAGAAGAAGAGCTCCAAAGACAAAGATCT

GTACACCTGGTTAAAGTGTGTCAAGGGACAGCCACATGATCACAAGCACTTGATGCCAAC

GCAGATTATTCCAGGAACAGTCTTGACGGACCTGGTAAATGCCATGCACATGCTCAGGGA

GAAGTTTGGCATCAAAGCGCACTGCGTCTGTGCCAACAAGCATAATATACTCAACAAGCT

CCCATCCACTAACGGCGTTTCTCAGGTCCTCCAGAACGTGCTGAATCACAGCAACAAACT

TTCCCTGTGCAAGCCTGAGGCCGGCCAGCACAACTTGGGACAGAAGGTTGAGGCAAACGG

GGGCAGCAGTCCAGCCAGTGACACCAGCACTGATTGCAAGCTCCCCCCACCTGAATCCCA

GTCACCACTGCACTTTCTGGCCGACCTGGCTGAGCAAAAATCCCGGGAAGAGAAGAAAGA

GAATAAGGAGTCGGTGGTAGGGAAGGTGAAGGAAGAGAGCACAGATGCTCTGGAGACTTT

GCACTGTAAAACCTCGTCTCTGGTGGCCAACAGCACAGAGCAGGGCTCCACGCTTCGAGA

CCTGCTCACCACCACGGCCGGAAAACTGCGTTTGGGCTCCACCGACGCTGGCATCGCTTT

CGCACCGGTTTATTCCACCGCCTCACAGACGGGGAAAAGTGGTCGCTCTATGCCCAACAT

CCTGGATGACATCATTGCTTCCGTAGTGGAGAATAAGATACCAGCCGACCGCAGCACTAA

GCAGAGCCCAAAGGCTAAACCCCAGGACGAGGTCAAGGCTGAGAGAAGGAAACAAGCTGA

GGATGTTCCTGAGCAGCATACTGACATTCCCCACTGCTGGCTGTATGATCACAGACTGCT

CTGGCTTAAAGACCACCGCAAGAGCAGTAACTGGAAACTGTTTCGAGAGTGCTGGAAGCA

GGGACAGCCTGTGCTCGTCTCAGGGGTTCATAAGAAGCTCAACGCCAGCCTGTGGAAAGC

TGAAGCTTTCAGCCAGGAGTTCGCCGACCACCAGGGGGATCTGCTGAACTGCAAAGACGG

CGTGGTTTCCAACTCGGGCATCAAAGAATTCTGGGACGGCTTCGAGGACTTGACAAAACG

GCTCAAATCCAG

>Unigene000152

CCCAAAATTTTGCTGGTGTACCCAGGGCTCTGACACGAGGAGGGGAGCCCTTGCCAGTTT

TACACAGGGACATCAGGGTGCCCCCAGCCCCACTCCGACCATGGGATCCACTTGGTCAGC

ATTATGAGCCACTGCCCAGTGATGATCATGTCCGTTTGCACAACAGATACAATGCTGGAC

CGTCTGCACACCTCACAGCCCGAGCTAACCAGTTGTTAAAGTACGGACCACCTCCTCTGC

AACATGGATCTAGGCCCATGCCTCCAATTGCAGACATGTGGGAACAGTCTCATCAACAGC

AGCAACAACCTCCCAGGGCCCCTTTTTCTCGTGTTGGACAGTTGAAACGGACAGCACCAC

TTCTGGGAGAGCAATCGGTCATCCAGCAGACCTCTCTTGCCCTAAACAGACCTAATGAGG

ATTGTCCAAGCCCAAGCAAAAGGAAGAAGAGTTCTGACTCAGAACAGCTCATAATAGGCA

CCCACCAATACTCAGGGTCAATCGGGTCATCGCTGCCTCTGCAGCAGCCCCAGTCCCATT

ATCCCCCTCTACAAAAGGATACAGCAACTTGGACCCCAGTAGAACACAAGGCAGACAGAA

ATGAGTTTCAGGAGTTGCGCAGACAAGACATGGGTGACTGCAATTACAAACAGTTTTCAT

CATCGAAACCTTCACCCATCTCTCCACCCCCCATCACTTCTGCCAGCAGCTATGAACATG

GCCATGGTCCTCCACTCAAATCCCTTAAGCCGCAATCACCACACTCTCTTGGGTCTTTCT

CGCAAAATGTCCAACTCCACCACACAACATTTCCCTTTCCTAAAAACAAGGCTTCCGCTC

AAAATTCAATCGAGCACCAGGTCATTTCCATCCATAAAATGCATTCTTCTGGGGTGAGGA

CTACTGGCAGCCAAGTGCCCCTGCCCCAACCCTTATCTTTGGTTGATAGAGAACACCCTG

TTCCCACACCAGCACATCAAACCTCCGTGCCTTACAGTCACCCAAAATTCCAGCCTCATC

CAGGACTGGTTTCCACCACCAGCCCCTCTTCCAACAGTGGGCCACATGCACCAGAATCTC

AATGCAACCCTCCTAAGTCATGGAGAAATGAGATCAAACATGATAACCAGGCTTTAATAC

TTGGTCCTTATCACAGATCCGTGGATCCTCAGCTTTCCACTTCATCTCTGCAGCAACAGC

AGAAGAAAACTGGGCAAAATCAGGATGAAGGCACCTCTTTGGCTCGGGCACCAGTCATCA

CCTTCACAATGCAACCTGCAGCGCCTCCTTGTTCTGCGGGATGCTACAGTGGTGTGAATA

CTATGTCTACCTTAAGCTCTGTGCCCTCTTCTCTCAGTTCCACTGTCAGCACCATTAAAA

ACTGGAAAACTGCCCATCCCACAACGCTTAACTCGAACCCCAGTTCGATCCCATCAAGTT

TCCCACACCAGGACTATCAGAGAGCCCAATCTGGCCCAGAAAGCCATTTAGACCACAAAC

AAAAGGTACAAATTCAGCATCAGGAACTTCATCTACCCCAGCAAGGCCAAGACACACCTC

TTTACACCCCCAAAGTGTCCTCAGCTCCTTCCTCTCTCAGCTCAGGGTTTCAGAGATCAG

GAAATAGTGTCATCACCAGCAAGGCAATTGACAACCTCTTGCAGCCTGCACATTCACCGC

TTCGTCAAATGCAGCCTGCAGTGCCCATCACTGTGAACACAACCCCTCAACAGTCTAACT

CTTTGCATCATAAATTGCCGCACCAGGTAACCCCAGTCACTTCTCAGGTTCCACCTGCTG

AAACATATGAAAGGCCATGTCCCCAACCTGCCATCTCTAACCCCCAGTCTATTGAGGAAG

CGCTCAAGAAGCTTGATGCCGAGCTTCAAAGTCATATGCAAGCTGAAGAAAGAAGGAAGG

AAAAGGCAGAAGAGGAGAGAAAACACAACATCGGGAATAACAAAGTAAAAAAGCATAAGG

TCAGTGAGGACTCTGCAACTGAGGACATGGAAAGCTTGCTTTCAAATAGTCCTACTGATC

CGCCTCCTCCATGCCTGTCACCAGCCATTACACCTTCCACAGTTTTACCTCCATCCCAGA

CATCCCCACCTTTTCCCTGGCTTAGCAGGGGAGGCGTACCTACCCGTCAGCTGGCACCTG

GTGCAAATTCTGTAGAAAGATCTCGACCACCACCTCTCACCCCTCAAACAGACTATGCCA

GAGAAAAGCAGAGACAGAGAGACCAGTGGAATGCTTCAGTCCCTCCTACTTTACAGAATA

CCTCAGGGATTCCTATATATTCATCCAAACATATCCCAACAGAGTTTTCGAATAAACCAA

TTCAGACCTGTTCAACTGAAGTATCAACAACGGAGAAGAGCTCTCCTAACATCAAAGCTA

TGGGTCCTGTCCTAAACACTTCTAACCTCAGAGAACCCCCCAAACTCTACCAGGCCTTTC

CCAGGGACACTCAATCATCTTTACAAAAAGAAACAAGTGGTGGGCTAAGTAGTTTGGGCA

GCAGTGCCAGTAGTTCCAGTATGGATTCAGATAGTGCCCAGTTTGAGGAGGAACCATCTG

AGCTTTTACCAGATGGTTTGGCAAACATTATGAAGATGTTGGATGAGTCCATTAAGAAGG

AGGAGGAACTACAATCTGTCCAGAGTGGTGGGCAGTCTGACCCAGACATATCATTTTCTC

TTACAGTGGCACCCATAAAGACTTACACGTTTGCCCCTGATCTCATGCCTGCCCTAAAAC

AGCCATTAACAGTGGATTATTCAACTGAGACCCATGCTAGCCCACCTGTCCTGAGTCGTC

AAGGGTCCTTGGCATCCCCTTGTAGTCGCACTTCTTCACTTGAAGAAGAGGAGGATACAC

TAAAAATCATTGATACATATGACACTAGTGTCGATAAAGATGGTGGTGTTGGTAGTTATC

GCCATAGTGACTTGGCCAAGCTTTATGGATTATCAGAGGGAGCAAAGAGTGAGTGTGATG

AGGAGGACGAAGAGGCAGAACAAGACGAGGATACTCGTTCATGTTCTCCACCACCTACTA

GGCCACACCTGCATCAGACAGGTGTGAACAGCATGTTTCAAAACTTAGCCTCCATGCTGG

AGAGCCAGAAGTATGCCTACCGCGGCGGCCCATTTGGTCGACCACCTCCTTCAGCTCTGG

TTGGAGTGAAGTACTCTTCATCACTTTCATTAGGCCCAGATATCTGCAGACAACAAAGCA

CTTCTCCCACATCAGGCTCAACCAAACACCCAGGCTTCAACCACAAAGATCAAGCCTCAG

TGAATTCTAGATCTGAACAAACCCGTTCCCTCTCACCATCGAGATCAACACCAGAAACAA

AAGATCTTGTAGGCTGCCAGTCGGATCTCTCAGGATGTGAGGAGGAAGAAGGAGTAAGGA

AGGGGCTGATGAATGAAGATGCTTCTTGTATTGTAAATAACTTCACAGAGGTAGAGAGGA

AGCCCAAGCTCACAACAATCTCTGAATCCTCACTTGCAGAACTGGGTCGTAGTTGTGAGG

TGATGCTAAGCCGGCATAATCTCCCATCAATAGCATCAGACCATAATAAGAAACAGATGC

CACACATAACTGAGAAAGACAGGAAGAGAGATCGTGACTATTACCAGGAGAAGAAACACA

AACGCAGCAGCAGTAGCAAAAAAAATGAGGAAAGAAAAGAGAAAAAGAAAAAACACAGAG

AAAAACAAGAGAATATGTTGTCAAACTCAAGCCGAAGACATAAGGACAGCAAACCGCACA

AAGAGAAGAGGGGCCACATAGACAGTCACAGAGAGAAAGAGAAAGAAAGAGATGGAGAAA

AAAAGAAAGGGAAAGAAAAAGAGGAATGGGTATGCCGGAACAAGGAAAAGAAGTCAGGAA

GTTCTTCAGTGGATCATTCGTCATCTTCACCCTCATTGGGTTCTGCAGACTTCCAGAAGT

TAAAGGCTTTGACAGACGGCCCACCAAAAGAGCTAAAGATTCGTCTAATTAAAGTGGAAA

GTGGAGATCGAGAAACCTTTATTGCCTCTGAAGTTGAGGAGAAAAGAATTCCACTCGGGG

AGATTACTATCAAAAACACAGCCAGTGAAATCATCAGATCGTGCAAGGGTGCCCGTGTTA

AGGGGAAATTCAAGGAGTCCTATCTACTTCCATCATTTTCTCTGAAACCTGTTGTGACTA

TGGAGCAACCGATCCCACGCGAAAAACTCAACCCTCCTACACCCAGTATCTATCTTGAGA

GCAAGAGGGATGCTTTCTCTCCTGTGCTGCTTCAGTTCTGCACAGACCCCAAGAACCCCA

TTACTGTCATTAGAGGACTAGCAGGCTCACTCAGACTAAATTTGGGTCTCTTCTCCACAA

AGTCACTTGTGGAGGCGAATGCAGAACATGCAGTGGAAGTGAGAACGCAGGTGCAACAGC

CAGCCGATGAGAACTGGGATCCCAGTGGCACTGGGCAAATTTGGCTATGCGAAAGCAGCC

GTTCACATACCACTATAGCCAAATATGCCCAATATCAAGCCTCCAGCTTCCAGGAAAGCC

TACAGGAAGAGAAGGACAGTGAGGATGAAGAGGATGAAGGAGAAAAAAAAACAGAATTGA

TATCAGAGACCTTGTCTAGCAACCTGCCCTCCACACTGAGCTCGGAGCAGAAGACTGGCG

GTAAAATCATTAAGTTTGGTACCAACATCGATCTTTCTGACCCAAAGAGATGGAAGTTGC

AGCTACAGGAGTTGTTGAAGTTGCCGGTGTTTATGCGCGTTTCCTCCACGGAGAACATGC

TGAGCCACGTGGGACACACCATCCTCGGCATGAACACTGTCCAGCTGTACATGAAGGTCC

CGGGCAGCCGCACGCCGGGACACCAAGAGAACAACAACTTCTGCTCGGTAAACATCAACA

TCGGTCCCGGAGACTGTGAGTGGTTCGCTGTTCATGAGCACTACTGGGAGGCCATCAGTC

ACTTCTGTGAGAAAAATGGAGTGGACTACCTGACGGGCTCGTGGTGGCCAGTTTTGGAGG

ATCTGTACCGCTCCAACATCCCTGTGTATCGTTTCATCCAGCGGCCCGGAGACTTGGTGT

GGATTAATGCAGGAACCGTTCACTGGGTTCAGGCCGTCGGCTGGTGCAACAACATCGCCT

GGAATGTGGGGCCGCTGAACTCCTATCAGTATCAGTTGGCTCTGGAGCGCTTTGAATGGA

ACGAGGTCAAAAAGGTCAAATCAATCGTTCCAATGATTCATGTCTCCTGGAACGTCGCCC

GTACAATCAAGATCACCGACCCGAGCACTTACAAAATGATCAGATATTGCCTCCTACAGT

CCATAAAGCACATCCAGATTCTAAGAGAGCAGCTGGTAGCAGAAGGGAAGAAGATCTGCT

ACCAAAGCCGAGTGAAGGACGAGCCGGCCTACTACTGTAACGAGTGTGACGTGGAGGTGT

TTAACCTGCTCTTTGTCACCAGCGAGAGCAGCAATAGGAAGACCTACGTGGTTCACTGCG

AGGACTGCGCTCGCCGGCGGAACTCCAACCTCAGCGGCGTTGTCGTCCTTGAACAGTACA

GAGTGGAGGAGCTGATGAACACCTACGATAACTTCAATCTGGCTCCTGTGCCATGTGTGA

AATGAGCCCGAGGTGTTGATGTCACATAGCCATGACCAAATCTCTTGCAGCAGTGCCAAC

GTTCAGTTCTCGGCTTTATTATTATTATTATTATTTTTAACATTACTCAAAATGACATCC

TGCATGGGATCGTCTTAAGATACGCATAACGAATCAAATGAAAACTGTACAAGTGCCAAA

TAAAATAGAATAACAGAGACTCGTTAACCATCTAAAACACATTAAACTGAACAAGATTTC

AACACATTTCTTCACCCTATTACGGAAAACTTTTACACCTACTCATCGATGCAAAGATCA

TATTAATTATCAATGTGGCTCAAACTGGGCAGATAATAATGAGGATAAAAAGCTAATCTG

ATCCCAAGCCCACATTTGTGTTCTTGGCTGATAGGAGTGTAAACTGATGTGGTCTGCTAT

TGTTTAATATGCTTTTTTGTGTAAAAAAAAACAAACACATTTATGGCAGATGACTAACAT

TTATCTCATTCGTATATCTGCGCAGTCGAAGGTTAAGAGCCTTCGTTCGAGTGCCCAGC

>Unigene000153

TTACTATATATTTCTTGTGAAGTTTTAATATTTCACATTAGTGGTGCTAGTGGTTGTGGT

GGATTTTGTACCCCAGTGCTGGAAAAAGAAACACATGCCAAGTCAGTCCTCCACCATCAT

TAGTGTGTTTGTGTGTGTGTCTTCTTCGGCTTGCTCACAGGGTCATTACACACGTGCCCA

GGCCAACAGTCCCAGACCGGTAATGAACTCATCAGGCCCTGCCACCAAGCAGGGTTCACA

AGCCCAACAGCAACAGCCCCAGCATGCTCAACAGCAGGTATCCCAGCAGCAGCAGCAGCA

GCCGCAGGTATCACCAGGGCAACAGCGTGGGTCCCGGTCGACACGTAGAAAGGGATCAGA

CAGCAGCATCCCTGATGATGAGAAAATCAGCGACGAAAAGACGGACGCGGGAGGCGGAAA

AGGAGATGTTTCTAAAAACAAATCAAAACAGGTGATGAACAAGAGGAGGAAAGCTGAAGA

TGAGGAGAAGAAGGCTGGGCTTAAACGGTTAAAGATGGAGGCATCGGATCTTTCCGAGAG

CAGCGACTCTGAGAACTCTAATAAGAGGCTCCTGGACTCATCCTCAGAGCCGAGCTCTGA

AAATGAATTAAAAAATAAAGGCATTTTAAAAGCCAGTGAGGAAGAGGAAAAGTCCCAGAG

CTGCAAGGCTATGGAGGAGCCTGACGCTAATAGCAGGATGTCTCCCTGGGAGGAGGCTTC

TACGGTGGATCAGATTGTCAAGCCGACAGCCATGGAAGTGATGCCCTCTACCAAGAGTGA

AAATACAGGCGACATGAGGTCCCTAGCGCAACCTTCCCCACTGCCTCCTCCCAGCTCGTG

TGCGCAGCCTCGGAGCTTAGTTCCTGCTGAAGTTCAGGGTTGCATTATGGAAATAAAAAG

CACTGTGAAAACTTCTCCTAAGGACCATTATAGCACAGGGGCACCAAGAACACAAACTCC

AAAGTGTGTAATTGACATCACAGAAGACTCCAGCTCCCACCCCACAACCCGAGAGAACTC

AGAGACTGTGCCTGCGACTTTAGCTTCTCAAACACGTGAAACTTATGTCCCTGAAAGCAG

ACATTTGGTGTTAAACCCCTTAGCCTCAGAATGCAGGAAAGCAGAGGGTGAACTTCAGCA

GCAGCTAGGTCAGAGCCTGGGCAGCAAGATTGAATTTGCTCACTCTGAGGTGATCCGACC

TGTGGTTTCGGTAAGCGAGTTAGCGGCTGTTGCGGAAAGGGAAAGGGAGAAAGTACAGCA

GCAGTACCCTTCAATCATGCCTTGCATCAAAAATGCCTCACTGGCTGAAGATGTGCGAAA

ACCACAGAAACTTAGCTCGTCACCAGATGTGGCCAAGTCCAAGTCTAACCCTTCACCTGA

CGCATTTAAGCCCAAGTGCAATCCCTCACCTGACGCCATGAAGTCAAAAACCCACAGTGT

CCTGGAGGCCATGAAGCCTAAACCTAACACTTCACCTGAGGTCACCAAACACAAAGAGAT

CATTGAGATCACTTCATCCTCTGTTGCCCGGTCGGCAGTCAAGTCGAGAAGTCAAGAAAT

ACCACGCTCCAGCTTTAAGCCTGTTCCTGCTCGTAGCACTCCCTCTGAGTCCATCAAGAG

CGCTCTCATTGTCGACAAAAACGAGCACTTTACAGTGTATCGTGACCCGGCGCTGGTGCG

GCCCGAGGCTGATACCAACCATGTAACTTACCTTCCCCCACACCTCCATCCACTCCACAG

CTCCTCCCATGCTACTTGCCTGACACCTAGCTCACATCACCACTCCCATCTATTGCCTGC

ATCTTCCCTTAGCCCGCACCCCTCAGTGCACCACCCCTTGCTCCCCACCGTGTTGCCTGC

CATGCCGCCGTCCTCCCTACTGGGTGGCCACCCGCGCCTCGACTCAGGGGGACTCAGTCA

TCTAGCCCTAGCTCACCACCACCCACACCAGCAACAGCAGTTTCTGCAACAGCAACCACC

CCCACCGCTGCTGGCCCAGACACATGGTGGTGCCAGCTACAACCAGCTCGGCCTTTATCC

CATAATCTGGCAATACCATAATGGCACACAGCACTCCTATCCATCTGGAATTAGCCTGCC

TGGGTCAAAGTGGGTGCATCCAGAAAATGCTGTGAACTCAGATGGTAGCCTGTCAAGGAA

CACCTCCAGTCCCTGGCTGCATCAGCCCACTCCTGTGTCCTCAGCGGATAGTCTAGGAAT

TCTGAGCCATGTCCCAGGTAGGCCAGCCAGTGCTGATCCCCACAGACCCCTTAAAATCTC

CTCACACTCCAGCCCTCCGCTCTCCAAAACTGTGGATCACCACAAAGGAGAGCTGGAAAG

TAAGGTTTTTGTAGATCCAATGCGTGGCCTGGTAACCGCCCACCTAAAGCAAGAGCCAGA

CCAAAGCCGGACACCGAACAGCAGGGATTTACAGCGCCTCTACGCGGATTCATCTCACGT

AAAGCAGCAGGCGCTGCCTCCCCGCCTGCCACTAGACACTCCAGACCGGGCTGTTAAGTA

CAAAGAGGAGAATCGACGCATTCTACAAGAGAGCATTGAGGTGGCACCCTTTACTGCCAA

AATCCGCTCAGGAGAGCCAGAGCGTGAGCCCTATATCCGCATTCCCTCTCTCCCCATCAG

TGGACCGCCCAAGGAGAATGAGCATTCGCCATCTGATCTTTATAAGTACAAGCACTCGGC

TGCTCAGTCACTGCCACAGAGCAACTATTTTACCACCATTTCCAACAGTGTGGTGAATGA

GCCACCTCGTCTTTACCCCTCCAAAGAGCTTAGCTCCCTTAATGTTTCAAGTCCCCTGAC

TCTCGGCTCCTACCCCAACAGCGGAGGCAACACCAAGTCCTTATCCAAGCCTCCCCCGCT

CATCAAGCACCATCCAGACGGGGAGGGCTTGGTGGGTAAGATCAGTGAGCAGCTCAGTCA

TCAGGTGGCCCTGAACCCTCTGAGTACCCCCGTGGCATCTTGCGAACCCCGCAGTCCGGC

TGTGTCACCCTCAAACCAACTCAGGAGCATGCCTGCTCTACACAGGGCACCTGTTTTTCA

CCCTCCTACCCAGCAAACACTGGACCGCAAAGAGTCTGGCTACGGTCGCCTCTCTCCACC

AACGCTAACCCCTATTCAGCCGGTTAGTGTGGCTGGGAAGGTATCAGAGCAACAAAAGCC

GCCCACTCTGCTTCCAGAGCTTAGGGAGGTGAAAGGCACTGTAGAGCTGAGCTCCAGTGA

GCCATGGAGGCCCAGCTCTAACTTACAGGGCTATGATAAAACAGTTCAGTGGCAGTTAGA

CAAAAGTCAAGGAAAGCCACAGGTAGCCACAGCTTCTGTTATTGTTCGCTCCAGAACCTG

CATTAAGTATGACAGCTCACCAGGCTCCAAACCTGGTGCCAGGGAGACGTCTAACAGTAA

ACCACATACAGGAAAATACCAACTGGACTGCAGCAAACTGGCTGAAACCAGAGAACCCGG

CAGAGTTATACAGCAAAACACAAATAAGGAGGACATTTTTTTGCAGTACAAAAACAATTT

TGTCCGAGTCTCCCAGGGCAGTTTTCCCAGCTCTGCGGTAGCTGTTGTTAACTCTGTGTG

CAATAACAGTACTGCTGTAACCACTTCTGCCTCTGCTGCTTCCACTCACAATGTGCTGAG

TCGTGGCACAGCTGAACTGCCTTACTCCACCTCAGCAAATCCAAGCAGCAGTAACATTAA

CAGACTGGAGGGCGCTGCACCCAAATGCAGGACTCCTACCAGCCTTGAGCTACAGGAGTG

CAATGCCAGGACAGCCTCCCCCATTGGCCCACTGCCTCAGCCTGGTTCGGCCCTAGCTCC

ACAGCCTTATTCTGGGAACTTTATCCACCTGAAGAAGCACAAGGCTGCCCTGGCCGCTGC

CCAGTCCCGTGGATCAAGCAGTGCCTCAGAGAGTGAAGGTAGCAGTCGATCCTCTCAGGA

ATCACCCACAATCATCACGCAGGATCGTGCATCCCCAGGCAACCCGTCCAGCAAAGTCAG

CCCCCTACCCAATGGTCAGCCATCACTGATGAACCAGCCCAACTACCACAAGCTAAAGAA

AGCATGGCTGACTCGGCACTCAGAGGAGGATCGCAACACTAACAAGGTGGAGATGACTTC

CAATGCCGTCTCTGAGATCATTAGGCCATGCACAGTAAACCTTATAGCATCCACCTCCAG

CGACACAGAGATAAGCAAAGATGGCAAATGCTTAGAGGATAAATTGAGCCCGGAGGACAG

GAAACCACGACGAGTTCCTTCCAAGCGGCCACACGAATCAGGCTCGGATAGCGGCGATGA

CTCCGATGGCAGTGACAGCAAGCATGAGCAGAGAGCCAAAAGGCAGCCTAAGCCCACCTT

CAAGAAGAAGCAGAATGACATGCAAAAAAAGAAAGGGGAAAATGACAAGGAGGAGGAGGA

TGTCAAGCCCAATGGCATATTCAGAAGCGCCAAGGAGAAAACCAAACTAAAATTGGCCAG

CAGCAACGGTATTCCTCGCTCGGTGTTGAAAGACTGGAGGAAGGTGAAGAAGCTGAAGCA

GACGGGTGAGTCCTTTCTGCAGGACGATTCGTGCTCGGAGATTGGACCCAACCTGCAGAA

GTGCCGCGAGTGCCGCTCGATCCGCACCAAGAAGGGAGAAGAGCCCACCCACTCCCCCGT

ATTCTGCCGCTTCTACTATTTTCGCCGCCTTTCATATAGTAAAAACGGGGTTATCCGGAT

CGACGGCTTCTCATCCCCTGACCAGCACGACGAGGAGGCCTTGAGTCTCTGGGCTCCAGA

TGCTTACGAAGAGAATGATCTGGACCTGGAAACCTCCAAATACATTCTCAGTTGCATCGG

GGATAAGTTTTGCCAGCTGGTCATGAGCGAGAAGACCGCCGCCACATGGATAAAGAAAGA

TGCCAAAATAGCCTGGAAACGGGCAGTGAGGGGGGTGCGTGAGAGCTGCGACGCATGCGA

GGCCACATTGTTCAACATTCACTGGGTGTGCCAGAAATGTGGATTTGTGGTGTGCTTGGA

TTGCTACAAGGCAAAGGAGAAGAAGAGCTCCAAAGACAAAGATCTGTACACCTGGTTAAA

GTGTGTCAAGGGACAGCCACATGATCACAAGCACTTGATGCCAACGCAGATTATTCCAGG

AACAGTCTTGACGGACCTGGTAAATGCCATGCACATGCTCAGGGAGAAGTTTGGCATCAA

AGCGCACTGCGTCTGTGCCAACAAGCATAATATACTCAACAAGCTCCCATCCACTAACGG

CGTTTCTCAGGTCCTCCAGAACGTGCTGAATCACAGCAACAAACTTTCCCTGTGCAAGCC

TGAGGCCGGCCAGCACAACTTGGGACAGAAGGTTGAGGCAAACGGGGGCAGCAGTCCAGC

CAGTGACACCAGCACTGATTGCAAGCTCCCCCCACCTGAATCCCAGTCACCACTGCACTT

TCTGGCCGACCTGGCTGAGCAAAAATCCCGGGAAGAGAAGAAAGAGAATAAGGAGTCGGT

GGTAGGGAAGGTGAAGGAAGAGAGCACAGATGCTCTGGAGACTTTGCACTGTAAAACCTC

GTCTCTGGTGGCCAACAGCACAGAGCAGGGCTCCACGCTTCGAGACCTGCTCACCACCAC

GGCCGGAAAACTGCGTTTGGGCTCCACCGACGCTGGCATCGCTTTCGCACCGGTTTATTC

CACCGCCTCACAGACGGGGAAAAGTGGTCGCTCTATGCCCAACATCCTGGATGACATCAT

TGCTTCCGTAGTGGAGAATAAGATACCAGCCGACCGCAGCACTAAGCAGAGCCCAAAGGC

TAAACCCCAGGACGAGGTCAAGGCTGAGAGAAGGAAACAAGCTGAGGATGTTCCTGAGCA

GCATACTGACATTCCCCACTGCTGGCTGTATGATCACAGACTGCTCTGGCTTAAAGACCA

CCGCAAGAGCAGTAACTGGAAACTGTTTCGAGAGTGCTGGAAGCAGGGACAGCCTGTGCT

CGTCTCAGGGGTTCATAAGAAGCTCAACGCCAGCCTGTGGAAAGCTGAAGCTTTCAGCCA

GGAGTTCGCCGACCACCAGGGGGATCTGCTGAACTGCAAAGACGGCGTGGTTTCCAACTC

GGGCATCAAAGAATTCTGGGACGGCTTCGAGGACTTGACAAAACGGCTCAAATCCAG

>Unigene000154

TGTACGGCGACCTGCTGCCGTTTAATCGGCTCGTCTTTCTTGATATTTTCGCTGTAATTT

ATTTAATCGGGTTTAAGGGACGTTTGTCCCTCTCGTCCCCTGTTTATTTTATAATCCAGG

GCTACGTTTATCCAATTACAGCCGCTGTTTTTAAAGGGGACACAGAAGCGGAGTGTGGAC

ACTGTGGAAATGGACGCTGTGGATCATCATCCGGGCTTCAGTGTCCGGGATCACACACTG

CTCTCGGACCCGGACAGGACTTTAACCCCGAATGGACACGGGGCTGCTGATGGACATGAA

GCTGCTGAAGTCTGGAGATTAGTGTGTGTGTCTCTGTATGGAGGAGCTCCGTGGGGATTC

ACGCTGAGGGGAGGACAAGAACACCGAGAACCGCTCATCATCACCAAGGTTGAAGACGGC

AGCAAGGCGGCAGCCGTGAGGCTCCAAGTAGGAGACGAGCTTGTAAACATCAACGATGTT

TCCCTGAGCGGCTACAGACAGGAGGCCATCTGCTTAGTCAAGGGCTCCCACAAGACTCTT

ACTCTGGTGGTGAAAAGGAAAAATGAACCCATGAGCAGGCCTCATTCCTGGCATTCCACC

AAATTCAATGAGAACCAATCAGACACTGCCAAAACACAGTCCACACCCTCACCAGTCTGG

CAAACAAGATATGATGCAAGTGCATTCACATCCGACTTTACTACTGGTTGGGACCAGCCT

AATTTGCGCAGAGTATCAGACCAGTTTAGCTCACTGGGCAGTATGGATAGTTTAGAACAC

AGCTCTTGTGCTTATCCACCTGGACGCCTATCCCCAAGCAAATCTAATGGTAACAGTGTT

GAACATTTAGTGTGTGGAAAAAGAGATTCTGCCTATAGCTCCTTTTCGACCAGCTCAGGG

ACACCTGATTATACGCTGTCGAGAAGCAACGCAGCTTCTACAGAGAACATGTTGTGTAAA

GTTAACCAGTGGGATGCAGGAAGTCGAGCTAGCAACAGCAGACATAGTCAATGCCTAATC

GAAGGAGTCCGGCAAGATGACAAAATTGGTTATCTGCAGCCACCTTCAGTAAGCAGTGGC

CGAGAAAGCCCGAGAACAGAGGAGCAACCAGGCTACCGGCATTCTGCTTCAGGAAGGTCT

AGCATTGCACCTGTCTGGCATGTCCCTGATATGAAAAAGGCCGTTGCCCCATCACCTCCG

CCACCTGCACCACCAACACGCAGCGACAGCTTTGCTGCGACTAAAATTCATGAAAAAACT

CTGGTCCCCAGCAACACTGAAAATCCTAGTACTCATACCCAGTCAAAGATTCAGGGCAAA

GGATTGAAGGCAGCAACAGAGGTGTTTGAAGGCAATCAAAGGATTTATCTTGCAGCTGAG

TCAGGCAGAGAAACAAGTCAAAACTACAGCTTGCAACCGAAAAGTGGCTTGATTAATCCA

TATGTGGCAGGCGACGGTCAACAAATTTCTCTTTCAAATTCAAACAAGAAATACTCTCCA

TCCGGCATGGAGCCGTCCTACACTCATTTTGCCTACCATAAACGTCAGTACAGTGATGAG

ACAAATTTTTATGGGCCTTCCAGAACCCCGTCTTCAGGCAAGTCTCAGAATTGCTACAGC

AGCATGCAAGAGTTACCCACCAACAGCTATGCTCCACTCTGTAGCCAACATCAGATCAGA

AGGCATATTGCATCACAGTCCACCACTGCTATTGACCAAAATGCAGATAGTCAAAACTCC

AACAGGTATTACTTTGTCAAATCTCAACCAGCCTCTCAAGGAGGGTCCCAGACCTCTCTA

TTCCGGGTGGAGGACAGGAATGCTAATTCTGTAATGGAAATAACCCAAAGTGGAGGTGAT

AAAACTTCCGTAAGCTCTCAAGGACAGCTCAAGGACAGGTTCACCACATCTCAGGTACTG

CTGCACCCAAATAGCAAAGACAGTAATGGATATTACAGACAGGTTGACAGCCAACATAGG

GGGTACACAGCAATTAATAGATCAACCTTTGGGGATGCAACAACGAAAAATTTGGAGGTC

AGGGCGCCCCAGAAACAAAACACTGTGACCAACATAAACCAGTTTATCACACACTATGAG

CAGGGAAAGATCTCTGAATCACACCGGTTAGGAGAATCCTCCAAAGAAAATTTCCCATCT

AAAACTGAGACCATGTTATGCCCCCAGAAAACCCCTTTGCTGCACTCTTTATCACATGAT

GGCAATAGACTGATTGATGTACAAAGTGAAGTTGTTAGTGGAGCTGGACAACAGGATATT

GTGGATCCCCAAAATGGCAGACAGTTTCATCGTACTGATCGTTTTGCCACCACATTACGA

AATGAAATCCAGATAAGGAGAGCGCAGCTTCAGAAAAGCCGAAGTACAGCGGGCTTGAGC

GGTGTAGCTGATCCTCAAGAAGAGCCTTCAGTTGGGAGGTCCACAGATATCGCTTCACCT

TCCTCTGATGGTTCTTTTACCAGTGCCTACAAAGACCATCTGAAGGAAGCTCAAGCTCGA

GTCCTCAAAGCTACTTCTTTCAGACGAAGGGACTTGGAGCCAGTGCTGTTAGAGCACCCA

GGAGCTGAATTCTCTGAGGTTCTGGCAAATAAGCCTGCTTCAGGTACGAACCACGTGCAC

CGTATATGTGGTCGCAAACGGTTTCCTAATGACAAGAAAGTACGATCTTTCTCAGAGCCT

GAAAAAATTCATGAAGTTGGTGTTGAAGAAAGAGCTCCACAAGATAACGACTTAACTTTA

GTCAGTAGGCGTAAAGTCTCTGAGGCAACAGGAAAACCCACTTATCCCAAGCCGATGTTA

AAGCCAAATTCGCAGATATCTCTAGACTGTAGATCAGACAAGCCTGGAGAATTGTCTCAG

TCTGGACAAAATGATGTCACTAAAAGGAATACCAAGAGTAAGGCAATAGACAGTGGCCCA

AATTCCTTACAGAGACTTGGCACGTTTGCAGAGTATGAGGCCACATGGAGCACGCAGAAA

AAAACTGATCCCAGAACATCTGGAAGGTACCATTCGGCCGATAACATCCTTGATTCAGCA

GTGGATGAGCGACATAAACCCTCCTATTTCCATGAAAGATCACGCTCTTCTCCATCAGAG

GACTTCTATGGTCAGAATATCCCAGCGCAAGGAAGAAAGTCAGCCGAGTCCCACCTGCCA

GAGAGAAAAGTCTCTGATCATTGCAACTCTACATCTCGGTTAAGTGAAGAAGGACACACG

ACTTTATTGCCCAATCAAAATCCAGCATCACTTTTGACAGGAGAAAAGTGGGAGAGCAAC

TTCTCAGATCCCCCATATACTGGCAAATCTCCCTCTAGCCCAGCGGATCCTTTCCAGCAT

TTCCTCACCGATCCCTCTTGCCATGAAAAAAACGAAGGCTCAAATTCTCTGAGACCTCCT

TCAACATGCCCAGACAAGTACAAATGCCCAGAAGCTGCTACTTCTATCAACACCTCACAG

GGTGGAACCGGTGTCTCTCAACCTCCCATTAAAGACACAGTTGCTAAAGACATCCCAGTC

CACACCCCAGTATCCTGGAAGGACCTAGAGCATATCAAAAGTTTAAAAAGAGAGGAGGAG

ACTCAATCTGAGACACTGTCCTTTCCTCCTCCCCCTCCTGAGTCTCCTTCCCACCTGACT

CCACCCAGCATGGAGGAGCAGCGTTCGCCATCACCTCACTTTGCTCCCCAGAGACTGACT

GACAAGCCCCCTGTCTCTGTCTCCCAGCAGGATGAAGGCACAGGAAGGATGGAACAGGTG

ATCGATGATAGCAGCACTGTGAAAAAAGTGCCCATCAAGATTGTCCACTCTGAAAGCAGC

ACAGAGAAAGAAAATCGACAGTACCTTTACCCAAGCACTGAGACACCTCGTAACTCACAG

CCAGCAGTCTTGTCTTTGTCCAGTCTAGGAGCACAAGAGCAATCCTCCTCTCCGTTTTGC

ACATACACACGTCAAAGAGAGCAGGAAGATGAAGCTATTAGACCTCAGAAGGAGCCAGAG

CCTAGCGAAGATGTCTCAGGGCCTCCAAAACCACAAAAAAAACCCTGTGTGGATCAGAGC

ACTAACGGAGACTCCTCAGGTATCTACAGCGCTTCCTTTTACTCCGAGGAAGATCAGAAG

AGAGACATTTTGGCCAGGGACATCATAGACAAAGATAAGTCTTTGGCGGAGATTTTTGAC

CAGAGCAAACGGAGAACTACTATGGACCTAATGGAGGGGATTTTTCCAGAAGGAGAGCAG

CTGGTCGAAGAGGCCCACCAACGCAGGAGAGCTGCACCTAAACTGCCCTCACGCAATACT

GAAGAAAGAAGGGAGGAGGACAGCATGACGACGGCTGCCACCGCCGCAGCAGCAACTCTG

GTGACCAGCTCGGCTTACTACAGCACGTCAGCTCCCAAAGCAGAGCTGCTTATTAAAATG

AAGGACATGCAGGAGAAGAGTGTAGAGCTCGAATCAGAAGAGGAGCTGGAGGAAGACAAC

GACGCTGAGCTTGCCAATAAGAAGCATGAGCTAATCAAGAGCCTGAGCAAGAAACTGAAG

GTGCTTCGAGAGGCACGAGAGAGTCTTCAGGAAGACATGCAGGACAACAACGCTCTGGGT

GAGGAGGTAGAGACCACCGTACAGGCCATCTGCAAACCCAACGAGCTGGAGAAATTCCGC

ATGTTCGTCGGGGACCTGGACAAAGTAGTCAGTCTGCTGCTGTCTCTTTCTGGGCGACTG

GCTCGTGTAGAGAATGCCCTTAACAGTCTGGAGGAGGACGCACCCCTCGAAGAGAGGCGC

ACGTTAATGGAAAAGCGCAAGCTGTTGATCCGGCAGCACGAGGACGCCAAGGAGCTGAAG

GACAACCTGGACCGGCGGGAGAAAGTAGTCTACGACATCCTGACCAGCTACCTGAAGGAG

GAGAACCTGGCCGACTATGAGCACTTTGTCAAGATGAAGTCAGCCCTCATCATTGAACAG

CGCAAACTGGAGGATAAGATCAAACTGGGCGAGGAGCAGCTCAAGTGTTTGACGGACAGT

TTGCCTCCAGAACAAAGGTCACTCTTCTGAGAGATTACAAAAAGACTTTTAAGCTATAGT

TTACCCAAAAACCACTCGCCCTTGTGTTGGTACAAACCCGTAAGACCTTTTTTAATCTTC

GGAACACAATTTATTGCATTTTAATAGCTTTTATGTTGCGCCATCACCACAGATCAGGTG

TCATGACGTACACATACTGTTGTATGTGTATGTCATCAAGTATATGTTATCAAGTACGTC

CATAAATGTCACAGTAGAGATGAAAATGTGGAATAAAGTAGTTATTTTTTGTTTTCTTTC

TGCAAGAAAGGACGCTCGTAGCCTGTATTACAGGTAAACCACCGATGTCACATGAACTAT

TATGCCGATCCTCTTGGTACTTTTCTGGGCCTTGAATATGGTAGTCACCCTGCTGTCTAT

GAAAGGGTCAGAGAGCCTTTGGATTCAATCAAAAATACTTAAAATCGTAGTTCAAACATG

GGGAGAAGAGGTCTCACGGGTTTGTAACAACATGAGAACGAGTAATTGAAGAATTTTCAT

TTTTGGGAGAATTATCCCTTAAACTCTAAAGACACACGGCATCTGGATTTCAATGTCGGG

ACTTATTTCGTCAGACTCTGCTATTGTAACACCATGAAGTTCTTGCTGAGCTTTTGTGAA

CAGAGACAAACCACTTTTTGACTGTTTTAGAACCACGCCCAACTTTGGGCTGCATCATTA

CCGTAAAATACTGTGTGATTATTGTGATTATTAATTACGCTGAACTGGATAAAAAGGCAA

ATTTCAAAGCGCTTACTGTATCAAGTTATGACCATACCACTGTTAATCTGACCTTGTCTC

AATTATTAGATTATCATTTATTGTTTCTGTAATATTGTATTTTGACCACTAGTGGGCAGT

GAAAGATGGTTGCTTTGACCTTATGGTACAAACCACTTTTCATGCATCTCTTACACTAGC

ATACACATAGACTCTAACATAAGCATTTACACCTTATATTCATAACTATTATCTTATATT

TAACTTTTAGCCCAGTTTCAATGACACACGTAGACCTGGAGATTTTCATGATTTTTACAA

TGTGCGTTATATCATACAGCATCATTCCCGGTC

>Unigene000155

GAACACAAGTCAAACGTTCAGCAGCTGCCAGAGCGGCGGTAGATAGGACACCGACTGAAT

GGTAAAAAAGCCTCAAAATTGACTTTTACAGCGGAATGTTTACTCGAAGGGGTTATGGAG

ACGTCAAGAAATCTACACAGAAAGTTCTGGACCCAAAGAAGGATGTTTTAACCCGCTTGA

AGCATCTCCGGTCACTCTTAGATTTCACCGAAAGACAAGAACTCAAGATTTTTTTCGAAG

CGAACTGCTCGCAGATTTATTTTATCTTCTATGAAAATTTTGTTACACTGGAAAGTAACT

TGAAACAAAAAGGGAACAAATCTCAGAGGGAGGAGCTGGACTCCATCCTGTTTATTTTTG

AAAAAATCCTTCAACTGCTACCAGAGAAAATCAACAGTCGATGGCAGTTTCACAGTATAG

GCTCCATTCTAAAGAAACTTTTACACACTGCCAACTCTTTCAAGATCCGCTGTGAGGGTA

TGCGGCTGTTCCTGCTGTGGTTACAGGCTCTGCAGACAAATTGTTCTGAAGAGCAGTTGC

TGATTTTTGCTTGCCTGGTGCCTGGTTTTCCTGCAGTTCCCTCATCTAGAGGTCCCTGCA

CACTAGACACTATCATCTACAGCCCCTTCTCCAATCCTCCTGATGCCAAGATTGTACCAG

AGGAGATTACACCTTTGGTGCCTGCTGTTCCTGGGGAGAAGGCTATAGATGACCAGACAT

GCTACATTCTTCAGACTGTGCTGAAGTTCATGGTGATCCAGGCATCCAGCTTGGAATGGT

ACAGGAAAGAGAAACAGGACTCGGGCTTCAGGTTTCTCTTTGCCCTCTTTAAGAAGTACT

ACCTGCCCCACATCTTTCCTTCCTTCACCAAACTCACAAACCTCTATAAGCCATTGTTAG

ACCTCCCCCACCACAGACCGAAACCTCTATATGTGCCGGTGTCGCGCAACAACGAGAGCA

CCTTCTGTACTCGCGATCAGTACCTGGCCCCCCGTGTAGCTTTTATCACCTGGTTGGTGA

ATTTTTTCCTGGAGAAGAAGTACGTGAGCTCAGCTTCAACTAGCACTAAGAATGGAGGAG

AAGTTTTCCCCAAAATCATCCAGACTGTAACTGCGGGCAGTACCAGTCAGGAGAAGAGTA

ACGAGTTGGAGCCTAACGGGCCGGCAGAGCAGGAGAAAAACCACTCGAACAGCAGCACGC

TGTCCGACCGCAAAGGCAGCAACTCCAGTCTGTGTAGCATCGAGGAAGAGCACCGCTCCG

TGTACGACATGGTGCACAGCATTCTGCTCTCCACACGAGACAACGTCAACTTTGTCAACG

AGGTCTTCCACCAGGCCTTCCTGTTGCCATCCTGTGAGGCGTCTGCCACCCGTAAGGTGG

TTAAAGTGTACAGGAAATGGATTCTGCAGGAAAGGCCCACCTTTATGACTGAGCCTGAAA

AGACAGATCATGACGAGGAGGCAGAAGAGCCCCAGCAACCCGTAGAGATTGACAGTACAC

AACACACACAGGAGGTCCATGGACACAAAAGGTCATCCAGCTGGGGAAGAACTTACTCCT

TTAGCAGTGCCATCAGTAGAGGCTGTCTGACAGAGCAACAGAACCATGATGTAAAAGCAG

GGATTCAGCCTACACTACAGGTCTTTCTGACAAACTCTTCGAACGTGTTCCTGTTGGAGC

CTTGCCATGATGTGCCCAAACTCCTCGAGAACCAAGTGGAGGTGTGCAGAGCCGTGCTAA

GCATCTACCGTCACATGATCATGGAGCACAACATGAACCGACAGACCTGGGAACAGATGC

TGCAGGTACTGCTGCGAATCACTGAAGCAGTCATGAAAAGGTCACAGGATGACCAAAGAA

AAGACATGTTTTCCCAAAGCCTAGCTAGTGTCCTGTTCAGAACCATCATTGTGGCATGGG

TGCGAGCCAATCTCAGTGTGTTCATCTCTCGGGAGCTGTGGGACGACCTGCTGGCAGTGC

TGTCTTCTCTTAGCCACTGGGAGGAGCTGGTGATCGAGTGGGGCAGCATCATGGACTCCC

TGACTGTCGTGCTGGCCCGTTGCGTCTATGGCCTGGACCTCAACAACCTTCCACTTGACA

AGCTGTCTGAGCAAAAAGAAAAGAAGCAGCGGGGCAGAGGAGTGATGCAGGACTCTCAGA

AATCCTCAGTGATATCGAGGACATTCTCTCTGAGTTGGAGAAACGCAGGAGGACAACCTG

GCACTCAGGAGCCCATGCGTATCCGCAGTGCCACCACCACCGGCGCTCCTGCTGTTGAGA

AGGCTCGCAATAACGTCAGACAGAAAGCATCAGATGTGGAAGAATGTCAGTTATCTGAAT

GTGGGGGTGAAGGAGAGGCAGGAGATGGAGACAGTCCTCTCCCTCGCAGCAGCAGCACCT

CAGATATTACCCAACTAACAGAATCTATCCCAGGTCAAAACAGAGAAGATTCTCCTAGTG

CAAGCTTTTCTGATGGCAAATCCAGTGTATGTGACAACAGCTCAGTAGGCAAGAGGGAAA

CACCAACGATTCTGGTTCGGCGGAGCAGCACGCCGGGAGATATTGACTACCCGGGAGAAG

CAGGGACACTGACTGCAAGGACAACGATCCGGGAAAAGAGTGAGAGCTTGAGTAGCGAGA

CCTCAAACTCCTACCTCACTGACACTGATGTAAATCTCCTGACATGGCAAGCCGTGGAGG

AGGAGCAAGACTCTTCCTCTGCCAATGATGTCAGTGTGAGCGTTAACTTGGGACTGACCA

CTGATACAGAAAGCCAGAGAAGTCTATTGCTGAGTCAGAGTGAAGCACTTGCAGGTTCTG

AGTGTGTCCACTCGCTCCACCTTGCCCCTCTGTCCCCATGCCCTTCCGTTTCTCCCACCC

TGCTCCTGCCCCAGGGCTACGGTGATTGCCCTCAGCTCCTGGAGGACGCCATGCACATGC

CTCATGATCTGGACAGCAGCGAGTTTCTGGCTGATGACATCAGCATCATTGCCGGCGGCT

GTTTGACCGGCTGGCATGCCGACTCTGCCTTTGTAATTTGGAGGAGGATTCTGGGTATCC

TCGGGGATGTTAACAACATCCGGTGCCCTCGAATCCATGCCAAGGTCTTCAGCTACCTGT

ATGAACTCTGGCACAAGTTGGCCAAGATAAGAGATAACCTGGGGATCAATATGGACAACC

AGTCAGTTCTACCCAAAACTCTGTTTATTCCTCCTCTTCGAATGTTAGCTTCCTGGCTAT

TTAAGGCCACTATGCTGCCTGCCGAGTTTAAAAGCGGCAAACTGCAGGCTTATAGGCTGA

TCTGTGAAATGATGACTAAACATCAGGATGTGCCACCCAACAGCGACTTCTTGGTACATC

TTTATCACGTCATGCACAAGGGCTTCACCAGTGATGACCAGGATGTGCTGAACACAATGA

TCAGGTGGTGCTCGCCACGTTTCTTCTTCCTTGGCATGCCTGGCTTTACAATGTTGGTCA

GGGACTGTATCAGTGCTGCCGCTCGTGTTCTCAACTCTGACAACTCTGAGTCTCCAAGGA

TTGAAGCTCAAACAGTGCTTGGCTCTCTGGTCTGCTTTCCCAACCTGTACCAGCAGATGC

CTTCACTGCAGTCTGCGGCGAAATCTCAGGACATTACCGTCGGAAGAGAAGACCTGAAGG

ACCACCTTGTGAACATTCTGCTAAGGACGGCACGTAATGAGCCGAGTGAAGGAGCCAGGT

GTGTTGCTATCTGCAGTCTGGGTCTATGGGTGTGTGAGGAGTTGGGGCAGCAAAACATAC

ACCCTCAAGTAAATGATGCCATTAATGTGCTCGGGGTCACTTTAAAGTTTGGCAATAAGG

TGGTGGCCCATGTAGCCTGCGATGTCTTCCAGCTGCTGATCTGTCACTGGCAGCACCTGC

AAAAACTGGAGCAGTCCCTACCCAAAAGAATAATTGAAATCTTTGTAGCAACGATAGCAT

TCCTTTTACCCAGCGCTGAGCACTCGACTGTAGAGGCGGATAAAAAGTTAATAGTGTCCT

TGCTGCTGTGCCTGCTTGACTGGTGTATGACTGTCCCTTTATCCATCTTACTGGAGCCCA

TCACTATGCCTATGCTGGATGACCCTGCCTCGCACAAAACCCCTCTGTTGGACTACATAT

ACCGGGTGCTTCACTGTTGTGTATCCGGCTCTAACCTGCACACACAGCAGAGCCACTACC

TCCTGAGTCTGGCAGATCTTTCCTCAGACTATGACCCCTTCCTGACACTAGGCCAGGTCA

AGAACTCTGAGCCCCCACCTATGCACAACAGCACGGGCGAATTTGGCAACCTTCTCACGG

TGGCTGAGGAAAAAAAGCGACGTAACATGGAGCTGATTCCTCTGACAGCTAGAATGATAA

TGACGCACCTGGTCAATCATTTGGGGCACCAGCCCCTCAGCGGTGGCCCTGCACTATTGC

ACAGCATGGTTAACGAAAACCATGACAACCCCTATGTGGAGTCTTCTGAACTCTCCTCAG

AGGTCTTTAAGAGCCCCAACCTGCAGCTCTTTGTGTTCAATGACAGCACCCTCATATCTT

ACCTGCAGATTCCCTCCGATCCTACATCCCCCTCAAGCCCAGACTCTAAAGAGAACTCGT

CCGAGGTGCGAGTGATTGTGAGGGACATCTCTGGGAAGTACTCATGGGACGGAGGTGTCC

TTTACAAGACTTTTGACAGGACAAACTTAGACTGCAAAGGTGCTGGAGATGAGCCTTCCC

ACCACTACCATTCATCCACTTCCTCCAAGCACTCCTCATCTCAGGCCAGTAGATGCCCAT

CAGAGCTGGAGGTTGAGGATGGGGTAGATGTGCTGGACCAGCTTCTGGAAGAGTTGGGCA

ACAGCAGTCCTGAGTGTCTGCCTCAGCCTCAGCAGAGACTTACGCAACCAGCTCCAGCAC

CTGTGGGCATGAACCCAGAGATGGAGGGTTCCATCATGGAGGCTATATACAGTCAAGCAC

AGCTGGAGGAGGAGGAGCTTACCTGCAGACGAAATGAGGACCCTAGCATCCTAGCATCCG

TCAGTCAGAGGAGACCCATTTACCAGGAACCCAAAGCACCATTCTATTTCTGCCGCCTTC

TGCTCAATGAGCTGGGCATGAACTCCTGGGACCGCAGAAAAAGCTTTCATTTGCTGAAGA

AGAACTCCAAGCTTTTAAGAGAACTGAAGAATTTGGATTCGAGGCAGTGTCGGGAGACGC

ACAAAATTGCCGTGTTCTATATTGGTGAAGGCCAGGAGGACAAGTGCTCAATTTTGTCCA

ATACTGAGGGCAGCCAAGCTTATGAGGACTTTGTCTCTGGACTTGGATGGGAGGTGGATC

TGGCTACTCACTGTGGCTTTATGGGAGGTTTGCAGAGAAATGGCAGCACGGGGAACACCG

CACCATACTACGCTACCTCGAACGTGGAGGTCATCTTTCATGTGTCCACTCGCATGCCGT

CTGACTCGGATGACTCCATCACTAAAAAGCTTCGTCATCTGGGAAACGATGAAGTGCACA

TTGTGTGGTCAGAGCACACTCGGGACTACCGGCGGGGCATCATCCCTACTGACTTTGGAG

ATGTTCTTATCATCATCTACCCCATGAAGAACCACATGTTCTTTATTCAGGTCATGAAGA

AACCACAGGTTCCTTTCTTTGGCCCGCTGTTCGATGGTGCAATAGTTACAGGAACGCTGC

TGCCGAGTCTCGTACGGTCCACGTGCATTAATGCTAGCAGGGCAGTGAAGTCTCGACTGA

CACTCTACCAGAGCTTTTATGAGGAGCGGGCTCTCTATCTGGAGGCCATCATTCTAAACC

ACAAGGAAAGTCTGACATTCGAAGATTTTGCTGCCCAGGTCTTCTCCCCTTTCCCCACTT

ACTACCCAGTGAGTGGGACAGGCTCCCTCACTGGCAGCACGAGCGCTGAGCTAAACAATA

CTGTGACGACCAGCGAATCTACTGAGCAAGCTTCACCCACAATGCCACGTGCCACCAAGA

GCCGGGTTTCAGGAAAACTCCGGCGTTCGGCCAGCGCCATCAGCAAGTCCTCCAACTGAG

CGTCTCTTTTACAAATAAGCACTTCATCAACCCTTTTCAGACTGCAGAGAACTTAAAACT

GTGCTGTGTGTAAGTGTGTGTGTGTGTGTG

>Unigene000156

GAAACACAACGCCAGCGGCTAGCTAACAGTATTAATCACACTGGGAAGATCTCTCACAGA

CATTCTGCAGAACTTATTTAATTATGTGAAATAAACAGCACTTTAAATAAAATTAAGGTT

CATTAAGACGCTAATGAATGATTGGAAGTGGCTGAAGGGACGGCACGGAGAACTCTTTCC

TGAGGAAGTGCTACTTTCCCGGGGAGGACCGTAAACTCGAGCCCGGGGATTTGGTCGCGG

CCTGTAGTAAAGCCAGCCCAGGCAGAGAGGATGTCCGACTCGGACAGTGATGAGGATCAG

GACCGTCCCTTCCATTTGACAGGCTTCCTGTTTGGAAACATCAATGAAGATGGACAGCTG

GAGGACGACACTGTCCTCGACACGGAGAGTAAGAAGCACCTGGCGGGACTGGGCTCTCTC

GGCCTGGGCGCGCTCATTACAGAGATCACAGCCACTGAGGAGGACGCCGAGCAGAGCACT

GACGCAGACGGCTGGGTGAAGAGCACTGATGATGCAGTGGATTATTCAGACATCAGTGAA

GTCGCCGAGGATGAAACCCGAAAATACAAACAAGCCATGAGTAGCATGCAGCTGTCCCAT

AAATCAGATGATGATGATGATGATTATGACGCTGACTCTGAGGACATTGATGCTAAGCTA

ATGCCCCCACCCCCGCCGCCGACCCAGTCAATCACAGCTAAGAAAGAAGACTCACTTAAC

CAGAGCTCCAGTGTGACTGATGAAGGAGATGGTATTATTCTTCCCTCCATCATTGCGCCC

TCCTCTGCGGGGGAGAAGGCGGAGTTTAGCAGCTCCTCAGACAGTGAGAGTGAGTGTGAT

AGGCCGACACCTGCCGGAGGGGGTGGGCTTCAGTCCAGCCTCACGCTCCCATTGGCTGGA

ATCATGCAGAAAGACACGGCTAAAACTCTGCCTCAAGTAACTGAACTGTTTCCTGAATTC

AGACCGGGCCAGGTTCTGCGGTTTCTGCGTCTGTTTGGACCTGGTAAAAATATGCCGTCA

GTTTGGCGCAGCGCTCGAAGGAAAAGAAAGAAGAAACACAAGGACACACACTCGGACACG

CCCAATGCTGACAGCGAATCAAACGCTACAGAGGGCGGAGACAAGAAAAAATCTGGATGG

GATTATGAATATGCCCCACCCCCTCCGCCAGAGCAGTGTTTGTCTGATGATGAGATCTCC

ATGATGGCTCCGGTGGAGTGTAAGAGCGTGTCAGTGGTGTGTGAGGGTGAGGGTGTGTGT

GACTCGAGGCCGCGGGTAGCCGAATGGCGCTATGGTCCAGCTCAGCTGTGGTACGACATG

TTGGGCGTCCCTGAGGACGGGACAGGGTTTCACTACGGCCTCAAGCTGCAGCAGGACAAC

ACCGACGCAGACACACAGACTGACACACACAAAGAAACACACACCGACACAGACACGAAC

ACTGAAACACACACTGGTACGCACACTGATACACATGCTGATACACACACTGACACAAAC

ACACCTGACGCAGAGCAGAGCAGTGAAGAAGGCAAGCAGCAGTCTGAGCTGGAGGACGAG

CTCTTCCTTATGGTCACACAGCTGCAGTGGGAGGACGACATTATCTGGAATGGAGAAGAT

GTCAAACACAAAGGCACTAAGACCCAGAGAGCCAGTCTAGCAGGGTGGCTGCCCTCCAGC

ATGACCCGCAATGCTAATGCTTACAATGCACAGCAGGTTTCCCATGAGGGCGATACTCCC

TGGTACTCTATTTTCCCAATCGATAACGAGGAGCTCGTGTACGGCCGCTGGGAGGACAAC

ATCATCTGGGACGATCAGAACATGAACTGTCTGCTTTGTCCTCCTGTCCTGACCCTCGAC

CCCAATGATGAGAACCTCATACTGGAGATCCCTAGTGAGAAGGAGGAGAGGACTTCAAAC

TCTCCATCTAAAGAGAACAAGAAGGAGTCATCTCTGAAAAAAAGCCGCATCCTGCTCGGA

AAAACGGGCGTCATTAAAGAGGAACCGCAACAGAACATGTCCCAGCCAGAGGTTAAAGAC

CCGTGGAATCTGTCGAATGATGAGTTCTACTACCCCAAACAGCAGGGGCTGAGGGGCACG

TTCGGGGGTAACATCATTCAGCACTCTATCCCGGCGTTGGAATTAAGGCAGCCGTTTTTC

CCGACTCACATGGGCCCGATGAAGTTGCGTCAGTTCCACCGCCCCCCCCTCAAGAAATAC

TCCTTTGGTGCTCTTTCTCAGCCTGGACCTCATGCAGTGCAGCCCCTCCTCAAACACATC

AAGAAGAAAGCCAAGATGAGGGAGCAGGAGCGACAGGCTTCAGGGGGCGGAGACATGTTC

TTCATGCGCACCAATCAGGATCTGACTGGTAAAGATGGAGACCTGATCCTGGCGGAGTAC

AGTGAGGAGTACCCGCCACTCATACATCAGGTTGGCATGGCTACCAAAATCAAAAACTAC

TACAAAAGGAAACCCGGTAAAGATTCTGGCCCTCCAGACTATAAGTATGGTGAGACTGTG

TACTGTCACACATCTCCTTTCCTCGGATCACTTCATCCTGGACAACTACTGCAGGCATTC

GAGAACAATCTATTCCGAGCTCCCATCTACCTGCACAAGGTACCAGAGACGGATTTCCTG

ATTATCCGCACACGGCAGGGTTATTTTATCCGTGAGGTGTTGGACATCTTTGCGGTGGGA

CAGGAGTGTCCTCTGTATGAAGTTCCGGGTCCCAACTCTAAACGGGCCAACACACACATC

CGGGACTTTCTGCAGGTGTTCATCTATCGTTTGTTCTGGAAGAGTAAAGATCGACCCAGA

CGCATCCGGATGGAAGACATAAAGAAGGCGTTTCCCTCTCACTCTGAGAGCAGCATTCGC

AAACGCCTTAAACTCTGCGCAGACTTCAAACGCACTGGAATGGACTCTAACTGGTGGGTG

TTGAAGCCTGATTTCCGTCTGCCCACTGAGGAGGAGATCAGAGCTGTGGTGTCTCCAGAG

CAGTGCTGCGCATATTATAGCATGCTGGTGGCAGAGCAGAGGCTCAAGGATGCGGGTTAC

GGTGAGAAATCCTTCTTTGCTCCAGAGGAAGAAAACGAGGAAGACTTTCAGATGAAGATT

GACGATGAGGTGCGCACAGCTCCGTGGAACACCACGCGTGCGTTTATCGCAGCGATGAAG

GGAAAGTGTTTGCTGGAGGTTTCTGGAGTCGCAGATCCAACCGGCTGTGGAGAAGGATTC

AGCTACGTCAAAGTGCCCAACAAACCCACACAGCAGAAGGACGAACGCGAGCCGCAGCCT

GCGAAGAAAACCGTCACAGGAACAGACGCTGATCTCAGACGCCTCTCACTCAAAAACGCC

AAGCAGCTCCTGCGCAAATTCGGTGTGCCAGAGGAGGAGATTAAGAAGCTGTCCCGTTGG

GAGGTGATTGATGTCGTGCGCACCATGTCTACGGAACAGGCGCGTTCAGGAGAGACACCC

ATGAGTAAATTCGCTCGAGGGTCACGCTTTTCTGTCGCTGAACATCAAGAGCGATACAAA

GAGGAATGTCAGAGAATATTCGACCTGCAGAACAAGGTGCTGGAGAGTACGGAAGTGTTG

TCCACTGACACAGACAGCAGTTCTGCTGAGGACAGTGACTTTGAGGAGATGGGGAAGAAC

ATTGAGAACATGCTGCAGAACAAGAAGACGAGTTCTCAGCTGAGCAGGGAGCGGGAGGAG

GAGGAGAGGAAGGAGCTGCAGCGCATGCTGATGGGGGACGAGGGGGGGGAGCGGGACAAG

GGCAAGAAGGATCGACGCAAAGGCCTGTCCAGCGCCCTGTCCACCGGCTCTCATAAAGAC

GATGACGCGTCCTCAGTGACCAGCCTAAACTCCTCGGCAACGGGGCGGCGGCTTAAGATC

TACCGCACATTTCGGGACGAGGACGGGAAAGAGTATGTGCGCTGTGAGACAGTTCGCAAA

CCAGCCGTCATCGACGCTTACCTACGCATCCGCTCCACCAAGGATGATGACTTCATTCGT

AAGTTTGCCCTGTTTGATGAGCAGCACCGGGAGGAGATGCGTAAAGAACGCAGGCGCATT

CAGGAGCAGCTGCGCAGATTAAAGCGCAATCAGGAGAAGGACAAGTTCAGAGGGCCGCCT

GAGAAACGACCTAAAAAGGCCAAAGAGAGACCTGACCTCAAGCTGAAATGTGGAGCGTGT

GGTGCGATTGGTCACATGAGGACCAATAAGTTTTGCCCATTGTACTATCAGACGAATGCC

CCGCCCAGTAATCCTGTTGCCATGACGGAGGAACAGGAGGAGGAGCTTGAGAAGACGGTG

ATCCACAATGACAATGAAGAGCTGATTAAAGTGGAGGGAACAAAGATCGTCCTGGGCAAA

CAGCTCATCGAGAGTGCGGATGAGGTGAGGAGGAAGTCTCTGGTCTTGAAGTTTCCTAAA

CAGCAGCTTCCTCCGAAGAAGAAGAGACGCGTTGGCAGCACGGTCCACTGCGACTACCTC

AACCGTCCTCACAAGTCGATCCACCGCAGGAGGACGGACCCGATGGTCACACTGTCCTCC

ATCCTGGAGAGCATCATCAACGACATGAGAGACCACCCCAATACATACCCGTTTCACACA

CCGGTGAACAGTAAACTGGTGAAAGATTATTATAAGATCATCACTCGGCCGATGGACCTG

CAGACGCTACGTGAGAACGTGAGGAAGCGACTTTATCCATCCAGAGAGGAGTTCAGAGAG

AGTGTGGAGCTCATCGTCAAAAACAGTGCCACGTATAATGGTGCAAAACATCCTTTGACG

CAAGTGGCACAGTCCATTCTGGATCTCTGTGATGAGAAACTAAAAGAGAAGGAGGAGCGT

CTGGTACGTCTGGAGAAAGCGATTAATCCACTGCTGGATGATGATGATCAGGTGGCCTTC

TCATTTATTCTTGACAACATCGTCACTCAGAAGATGATGGCTGTACCTGAGTCGTGGCCG

TTTCATCATCCTGTAAATAAGAAGTTTGTTCCTGATTATTATAAAGTCATTACTAATCCC

ATGGACCTGGAGAACCTCCGCAAGAACATCTCCAAACACAAGTATCAGAATCGGGAGGTT

TTCCTGGCAGATGTTAACCTGATCCACACCAACAGTGTCAAGTACAATGGTCCTGACAGC

TCCTACACTAAGACAGCTTTGGAGATTGTCAATGTGTGCAAACAGACTTTAGCTGAGTAT

GATGAACACCTGACTCAGTTAGAGAAGGATATCTGTACAGCTAAAGAAGCTGCTTTGGAT

GCTGCTGACCTCGAGAGTCTGGATCCACTCACACCTGGACCATACACACCTCAGGGCCGA

GGGAGAGGAAGATTGGGATTGGGAGAGGAAGAGTCTGATGTTGATATTGAAGGGTTTGAA

GATGATGATGATGGCAAACCTAAAACACCTGCGCCGGCTGAGGAAGGAGACCTGGACGAT

GAAGATGAGGATGATGATGAGATGCTCTTGCCTGGGCGTGGTCATCTGGAGGATGAGGAG

GATGATGAAGGCTCAAGCAGACCAGTGCAATCTAGTGTCCTCTACCAGGACCTGCTGATG

TCCGAGGGAGAGGACGAGTGCAGTGACGAGGAGGGAGACAACCCCTTCTCCTCCATCCAC

CTGTCAGAAAGTGGGAGTGACAGTGATCGAGAGGTGGGACATCAGGACTCCACTCGCATG

GGCATGGAGCACGAGGAGAGCATGATGTCATATGATGGGGAGGGGCCAGATGAGGATACA

CACCTGGAGGACAGCAATGTCAGTTATGGCAGCTATGATGATGGAGACAGCCGACAACAG

GGGCGGGGCTTCAGTCCAGAGGGGGAGGGGCATGAGCAAGGGATCGGGCCTGGGGATGGA

GTGAGTGATGAGGAGGAAGAGGAGGAGGAAGACGAAAGTGAGGAGCAGAGGAGAGGACCA

AGTGTGCTGACCCGAGTCCAACTCAGTGAGGATGAGGAGGACAGTGAGGACTTTCAGAGT

ATTGGGGGAGACAGTGACATGGACTCAGATAACTAACACACAGACACACACAAACACACT

GTTCTGTGTGTATATTCTGATTGGTTGTTATGAAACTGTTGTACTTCCTATTTTTATTGT

TAATATTTTTTTAGATTTGCATTTTGCTAG

>Unigene000157

GATCATTCAGTTTGTGTATCTAAAACAGTGGGATGATGGACCTTTTTTTTCTGCTCTGTG

TTATACATGTGCTGTCAGTGACTGAAGCTCAGCAGAGAACGTACCTCATCACGGCACCCA

AAGTTCTGCGAGTTGATGCGTCAGAGACTGTGCTGGTGCATTTGTATGGCTATGACCAAG

AAACTATAGTAAATCTTTACCTTAAAGACAACCTGGCCCATGAAGGCAGAATTTATGCAT

CTTCAACTCTCAAGCTGAATGACAGAAATAATTACCAAGCTGAAACTACTCTACGGGTTT

TCCCAAACATTGAACAAAAGGTCACACATCTCCACCTGCAGGCCGTTTCTAGTTCCTTTA

CGAATGTCAAAAAAATCCCAGTCATTCATGCCAATGGTTTCCTTTTTATTCAGACGGATA

AACCACTTTATACACCCGAACAGCAAGTTGAGGTGCGAGTGTACTCTTTGAATGAGGAGC

TGAGAAAGACTAGTAGACCGGTTCTCCTCACGTTCAAGGACCCAGATGGCATTAAAGTGG

AAATTATTGAGATGAATGACATTTTCAATGGTGTGAAACCATTGCTTCCACCATTTAAAA

TACCCTTGAAACCTAAGTTTGGGATCTGGAAAATTGAGGCTGCTTATACTATTAACTTTA

CTACAACTGCTGTGGCTGAATTTGAGGTCAAAGAATATGTATTACCCAGCATCTCTCTCC

GTATCCAGCCTGAAGCAAACTATGTCAGCATAGCAAACTATGAGGCTTTTAAACTGAAGA

TATTTGCCAAATACATCAGTGGGAAAGCAGTCAGATCAGCAGATGTCTTCACAAGATTTG

GATACATCAACAATCATGATGTGGTGATGATACCCCAAACTTTAAGCCAATATAAGATGG

ATGATGGTGAAATGGAGGTTGTGTTAAACATCAAACATGACCTGTTCAGTGTAAATTCAG

GACCTCAACAGCTATGGGACATGAACAACCATTTCCTTCGTGTGGTTGTTTTGTTAAAGG

AGTCTACCGGTGGTCTCTCCCAGGAGGCCGTTCTGTCCAATGTGAAGTTTGTTCATGCTC

CGTTCACCCTGAGTCTGATTGCTACTCCTCCTTTCATCAAGCCAGCTTTACCATATCAAA

TCAGAGTCTTAGTGAAAGACCCACTGGGTGAAATAGCGAGAGGTGTTCCTGTCAAGGCAA

GGGTAACAATAACTTATAATAATAATGAACAGGAAGAACTCCATGGATATCGTAATGAAA

TGAGACAGACATCCAGGAATGATGGGGTTGCATATTTTGTTTGCAATATTCCTGATAACG

TTGCAAGAGCTGAACTTTTTTTTGAAACGGACGATCCTAAATATCCACCAGAAAGCCAGA

CTCAGCTTAAACTCAGTACTTTGGCTTATAAGTCTGTCAATCAGCGTTACCTGTATATCA

GTTTACCTGCACATGCCTCAGCTTTTGAGGTAGACGACCATGCCTCTATAAACATCAACT

TTCACTATCGTGACTTTCTTCCTCTAAAGACCTTTAGTTACCAGATTCTTTCCAAGGGTA

AAGTTGTACAGTTTGCCACAGAACAACGAATAAGTCATAGTGTCCAAAGTATCAACTTTA

AAGTGACCTCAAACATGGTGCCCTCTGCTCGCCTGGTGGTCTATTATATTCTTTTTGGAG

AGCAAACAGCTGAATTGGTTGCAGATTCTGTGTGGTTCGATGTCAAAGCCAAATGTGTCA

ACAATTTAGATGTGAACCTATCAGCTCCAAGTAAGAGCTACAAACCTAAAGACAGGCTTA

TGTTATCAGTAAAATCTAGAAGTGCGAATGAAAAGTTCCTGGTGACCCTGTCAGCTGTAG

ACACGGCTCTGTACAACCTCAGAGGTAGCAATAAAGACCCACTAAACAAGGTACTGCAGC

GATTTGAGCACAGTGACTTGGGCTGTGGAGGAGGAGGAGGCCGGAATAACGCTGACGTTT

TTAGCCGTGCTGGACTGACTATTCTTACAAATGCAAATGTTGAGGCCTCTGATGCAGATG

AGACCTGCACAGCCGTGGTCCGACCCAAACGCTCCACTCTTTCAGACACAGAAAAGCAAA

AATATGAGTTCCAAGCTAATTCTGTTCGAAGGAAGCGTGCTGATCTCTCGCTGAATGAAA

AACTGGAGGCAAAAGCTAAAGAATACAGAAATTTTCATCAATGTTGCACAGCAGGAACTG

ACAGTAGCCCAACTCTGGAGACTTGCAGGGAACGCACTCGGAAATTAAAGACTGAACATG

TACAGTGCAAGTTTGCGTTTCGGGACTGTTGCTTCTATGCTGAAAAACTTCGAGCAGAAA

GTGGCGGTGTCAGTCTGGCTCGTTCAGAAATAGAATTTTTGCTGGAGGTGAAGAGTGAGC

AGATTCGCAGCTACTTCCCAGAGAGCTGGTTGTGGGAGGAATATGAAAATGAGAGGTCTG

GACTGCTACAGGTCACTAGAGGACTTCCCCATTCTCTCACTATATGGGAGATCAGAGCTG

TGGGCGTGTTCAACAATGGAATTTGTATGGCAGATCCCCTGCAAGTGTCAGTTCAGCAGG

CAGTGAGTGTAGATGTTCCTCTGCCGTACTCTATAGTGCGTGGGGAGCAAATTGAGCTCA

AAGGCTCAGTCTACAATGAATATGACACAGTTACAACATATAGTGTGACTTTGTCAGCAT

CAGAGGGTGTATGTGTGTTTCGTGGAACTCTTCTGACAGAGGATGGAGACCAACAATCTA

ACAAAGGCACAATTAAAGGCCATTCTGTGGCACTTGTACAGTTTTTCATCATGGCATTAG

AAGTGGGCAATCATAAACTCAGCTTCACTCTTACAACCAGATGGGGACGTGAGACAGTGG

TGAAGACACTCAGAGTAGTGCCAGAAGGCATCAGAAAGGAAATATATGTTGGACGGAGAA

TTGACCCAAGTGGTATCTACGGTACATCAGTGACACGTATTGAGCTCAGAAATTCTCTTC

CTCCAAAGATTGTGCCCAAGTCCACTGTGGAACGTCTGCTGACAGTAAATGGGGAGGTGC

TAGGAGAATTGCTGTCTATCATTATAAACCCAAAGGGGATTATGCAGTTGACTAACCTGC

CCAGAGGCTCTGGAGAGGCGGAGCTTGTGGGCCTTCTTCCCATCTTCTATGTGTATGATT

ATATTGAGCAGTCTGAGCAGTGGGGGAAAATAAGCAAATTTGGTAACACTATTCTGCTAA

AGAGAAAGCTGAAACAGGGTATCACCAGTGTTATGTCATTTAAATCTAAGTGGGAGGACA

GTTTCAGTTTATGGAAAAACAAAGAACCCAGCACCTGGCTGACTGCTCTGGTGGTGAAGA

CTCTGGCTTCTGTGAACAAATATGTCACTGTGGATCATAATCAGCTGTCCAGCACGGTCT

ACTGGCTGATAACAAAGTGTCAGACTGCAGATGGCGCCTTTAAAGAGATATCCAGCTACA

AGCCTATGAGGCTAATGGGTGCTGGAGGAGATGTCAATGAACAGTCTGTGTTTCTTACAT

CATTTGTAGTAATCGCAATTAAGAATGCATTGACAGTTCCCAAATCCAACTTAGAGATGT

ATCATCATGCTGTGGAGAAGGCGACACACTACCTGACCTTGCATTTGACAAAAGTATCAA

GTTTATATGTGAAAGCCATTGCAGCCTACGCACTGACCGTTGTTGACTTCAACAACCATC

ATGCAGTGACACTCTATGAAAACCTAAAAAAAAAGGCAAAGATTAAAGGAAACCCGGTAA

CTGTCCGGTTTTGGGAGGAGAATGAAGCCCCTCAGGACCCACTAAAGCCAAACAGGGCCA

CTGCACAGTCAGTGGAGACCACAGTCTATATGCTGCTCAACACTTTATTTAGAGGAGACA

CAGCATATAGCAAGCCAATCATCCAATGGCTCACGGATGATCAGCGATATGGAGGAGGAT

TTCACTCTACTCAGGATTCTATTCTTACACTGGAGGCTTTAACCAAATATAGTAATTTAG

TTCGTAGAGCAAATTTACAAATGGAGGTTGATGTGTCATATCGAAGGAAAGGCAGTATTG

ATCGTGTCACTCTCACCCAGGACAAACCTGTGGGGAAACCTATTGAGGTAAAATATGATG

ATGATGTCATACTGAAAACTGGATACAGCACAGGCGTGTCGTTTGCGAATTTGAGAACAA

TTTATTATGAAACATCAGATGACAATAATAATTGCAACTTTGACATAACAGTTGATGTTC

ATCCCCGAGACCCAAATTCACAAGATCCTATTACTCTTTCACCGAGGATCGTTGCCTGTG

CCAGATATAAACCACGTAAAAATGAGGTGGAGAATGAAGCGGGTCACACAGTAATGGAAA

TTAATCTTCCCACTGGTATGAGCCCAATTCAGGAGGACCTCAAACGGTTTAGGGATGGAC

TCGAAAGTCGCATTTCTGACTATGAAATAATAGACAACCAAGTGGTTCTCCAGATAGATT

CAATCCCCTCAGAGGAGTTCTACTGTGTAGGCTTTCGTATTCAGGAGGTATTTAGAACCG

GCATGAACAGTGCGTCTGTGTTTAAAGTGTATGAGTATCATGATCCAGACAGTCAGTGTA

CAAAATTCTACTACAGTCAAAGCCACAAGCTTCTTCGACTCTGTGATGAAGACCAGTGTC

AGTGCATGGCAGCTGAATGTTGCAACTTCAGAGCCAACATTGACCCAACTCTTACTGTCG

AAAAGAAATTACATGATGTGTGCAAAGCCAACATCAAATATGCTTTAAAAGTAAAAATCA

CAGCAGTGGATACAGAAGGTGACTTTTTGACATACAGAGCAAATGTGGAAGGAGTCATTG

AAAAAGGGTTATTAGATATTACGAGGTCAGGTACATCTGAGGTCAGCTTTGTAAAAAAGG

CAACCTGCACCTCAGCCAATCTGGAAGTTGGAAAGCAGTACCTGATTATGGGCACTGAAG

TCATGCAGCTTAGAATAAACCGTAGTTACAAGTACAAGTTTCCACTGGATTTACAGGCTT

CAGTGGACTGGTGGCCCTCAGAATGCCAAACAAGCGCTTGCCAACAATACACTGAGGTTT

TAAATAATTTTGAATTCGATTATCTTAGTAGCAGCTGCTCGTGATGCTCGGTGTTTTGCA

TTAGAAGCTTATATAGCATTTGGCTTATAAGCCAGCGTTTGGCGTTATAAACCTTTGACA

TTAATGAAGCTTTTTAAGTGTTATTGTGTAATGTCATGTAGATAAAGGAAAAGGCAAGCG

CAGAATTTTTTTATTGCTGCAATTATGGCAAAAAAACAATGTAGCAAATTAGAGACTAGA

GCGAAACTACTAACAACAAACATTTTTGTAAACAGAAGCTAAACAAAGACAATAAAGAAC

AGTATAAAAAGCTGTGGTCATTATTATACTCTTCTAGGCAATAGGACCAACTAGAACACA

AAATTAGTGCTAGTAAAACCTTAAATGTATAATTTCAATAATTAAATAATAACTTTTGAC

CATGACTAAAGAGTTAGACTTTGCTGCTTTGCTGGCAGATAGAAAGCATCAGGTTGTTTA

ACACTATTTTTTATGCCAAAGCCATCAAATGTCCAATAATGGAGATCTGATCATAAGATC

AACCATAGCAAAAGCATACTTGTCCATTTAGCAATTAAATCGAAACCCTATTAAAAAACC

CTGAAATGTCATTAAGAGTGAGGTGGATGGTCAAAAGCCAAGCTGCTTAAATGTCTGAAC

AGCAATGTAAAAACCTCTGAACGTACAGCATCCTTCCTGTTCTGGGTTATTTTCACCTGT

TGTTATTTTCTGCAAATAAATGCTCAAAATCACACCCCCCTCCTGAGCCTTAAGCCACAT

CACCTTCACATCCTAACACAAAGAACACATCTTTGTAAAGTAATGCTATCATGCCTGCAT

ACCAAACATTTATGATTTACAGAATATTTATGCTACCGATACTTGTATCATTTCATTTCG

ATTCAAGTTTATGCGATTTGTTTTTGCGATCTACTGTTTATTATTTGTGTACCAACCTTT

GCCTCCTTTTCTGATTATGATTCAGCCTTACTATTCTGATTTTTGTTTAAACACCATTCT

GCAACATGGATCCCTCCTAGCCC

>Unigene000158

GGAAGAAGAAGGAGAAAGGACTGCGCGCAACAGACGAAAAATGGAGGTTGTGCTGCAACA

AATCTTGGACGAAGACGAGTTGTCTAAAGTCCCCAGGGAGCTGCAGGAAAAACTGGAGAA

ATACCTGGCGGAGCAGCAGAGTGAGGCTGATTACTATAAAGCGCAATATGAGCAGCTTCG

AGTGGACAGCGAGCAACAGATTTCTAACCTGGAGAAGAGGTACACCGAAAGCCAGGATCA

GTGTGTGTCTATTACAAAAGACCATCAAAAACTCCAGGAAGAGTTTACATCACTTGATGA

AGAGCTGAAATCACTTCGTGTAAAGACAAAGGAACAGGAGTCCTCATATGTGAAGCTGGC

AACAGAGCAGAAGGAGCTTTCTAAATTAAGGGATGAGCTGGAGGCTGAGAAGCAGGAACT

GGTGCGCAGGCTGGAAAGAACATCGCAGGAGGTCGAGGGACAGAGTGAGGATTTGAAACG

GATTAGTAACCGACTGGTGGATGTCAATGCAACAAATATGAAACTGCGCTTAAAGGTGGA

TGAGCTAGAGACTTTGGAAGTCTCTATTAAGTACAAGGAGAAGCGTCTGGAGCAGGAGAA

GGATCTGCTTCAGGGGCAGGTTACCTGGCTGAGTGAGGAACTGAAGACTAAAAGTGAAGA

GTTTCTGTCTCTGTTACGGGAAAAAAGCAGTGAGATCCTGGAGCTCAAGTGCAGGATCAA

CAACAAAGAGGATGAGTATAGCAGAGTACAGGATCAGCTGACCAGTCTAAAATCCTCCAA

TGAGAACCTTCAGAAGCAAGCCGAGGACTTGATTATCAAACTAAAAGAGGCTGAAGAACA

GCAAGCTGTTTCAGAGGAGAAGTTTCGCAATGAGCTGAATGCAAACATCAAGCTGTCTAA

CTTGTACAAGGCAGCAGCGATGGATGCAGAGGCGAAAAGTGAGGAGCTGACCAGAGCCTT

GGAAGAGCTGCATAAGCTACTAAAAGAAGCTGGAGAGGCTCACAAAGCTATGGAGATAAA

GCTGTCTGAGGTTGAGAGCTGCAAACACAAGGACGTGGCTGAACTAAAAGGCCGTATTAG

CGTCCTGGAGAAGGAACTGGACAACGCCAATGACCTTCTGTCCGACTCCACACGGAGAGT

TGGCACCATTTTTGCACCTGCTCTCCCCGATGAACAACTTGGCACAATGTCTTCCACTGC

AGCAGCTGTTAGCAAGATCAAACCCAGTATGAAGCTCACAGAGATCTACACAGCTTATGT

AGAGTCCCAGGAGCAGCTGCAGCTGGAGAAACTGGAGAATAAACGTGTGAACAAGTACCT

GGATGACATTGTGCAGGAGGTTGAGGCTAAAGCACCGATACTGAAAAAGCAGCGGGAGGA

ACTGGAGCGTGCACAGAAATCTGTAGCAAGTCTGTCTGCCAAGCTGGAGCATGCTGTGCA

GGAGGTACAGTGCTTGCAGAAAGAGGCAGATGAAGCCAAGAAACACTCCTCCATGCTGAA

GAGAGACAATCAGAGGTTCGGGGTACAGCTGAGTGACATGGCGCACCAGGTTCGTGTGTT

ATTGATTGAGCTGGAAGAGGCGCGTGGCAATCATGTGGTCCGTGACGAGGATGATCTATG

CTCGGCTGACGTGAGCAGTACATCCGAGGTAATATCTCAGCATCTAGTCTCATTCCGCAG

CGTGGAGGAACTGCAGCAACAGAACCAGCGTCTGCTAGTGGCCCTGCGTGACCTCGGAGA

GGAACAGGAGAAAGGAGAGATGGAGGCTGAGAGCACCAAACTGGCCGAGATGCAGAATAG

TTTGGAGAATGCCCAAATCGAGTTGGAGCAGCTTCGAGAACAGCGAGCCCACCAGATGCA

GCTGGTTGAATCCATTGTGCGTCAGCGAGACATGTACCGTGTCCTGCTTGCCCAGGCTAC

AGGGGTCAGCTTTCCTCAGCAAGACGAGTTCTCGCTCACCTCCACACCCTGTCGTTCTCC

TGCAGCCACACCCACTGCAGCCACGCCCACTGGCCTTGTTGCTACAGCGATAGAGTCTGT

GGAGACAGTCGAGGCCAAGGCTGCCCTGAAACAGCTCCAAGAGGTGTTTGGAGTCTATAA

AAAAGAGAAAGCTGAAAGTGAGAAGACTCTGACTGAGCAGTGTGAGAAGCTGCAGGAGCA

GGTGTCAGAAATGAGATCAAAGAACACCAAGATTTCCACTCAGCTGGAGTTTACATCCAA

ACGATATGAGATGCTGCAGGATAATGTGGAAGGTTACCGTAAGGAGATTGCCTCTCTGAG

AGAAAAGACTCAGAAACAGACCACCACGCTGCAGCAGAGTGAGCAGACTGTCAACACACT

CGCACAAGACCTGCGTGCAGCTAATGAGAAACTGTCCATGATTGAAGCACAAGCAGAGAG

CCTGCGCAAAGAGAAGGACATGCTCAAGATGGTAGAGTTAAGTCTGAGTCAAGAGAAAGA

GTCCCTCCTCACTCAGCAGAGAAACCAGAACCTCCTACTTAACAATCTTAAATCCATTCA

GGCCATGTTGGAGCGCTCGGAGACAGAAACCCGGCAACGTCTCACAGCGCAGGTGGAGAA

ACAGGAGCGGGAGATTTCTCTGCTGCAGAAGAAACTGGAGCACGAGGTGGAGCAGAGACA

CGCACTGGGACGCATTAATGATAAGCAGCTTTTGGATGCCAAGAAGCAGCTGGAGGTGCA

GTGCGCATTGCATCAAAGGACCAGAGAGCAGCTCAGCAGCACACAGCAGGAACTCAGCAG

TCTCCAGCAGAAGCCAAACACCAGAGAGGGCAGAGTTTCCTCACCTGCCACCCACACAGG

CACAGGACTACCGGGTGATGAAGAGAACATGGAGGAACTGCGTAAGCATCTAAACGAGGC

TAATAGCAGAGCTGAGGAACTGACTGAGAGACTGGACAGCACTAACGCCTCTCTTGAGCA

ATACAGGTTCATGTGCCTGAGTCTAGAGGAGGCCCTGCAAAAGGAGAAACAGGTAACCGA

ACAAGTCAAAGTGTCAGTCCAAAGCCAGGTTTCAGAGGCTCATGAGCAGCTTCAGCAAAT

GGAAAATAATCTTTTAGAAGCTGAAAAGGAGAAAAATAAAATACAGCAAGAGAAACAGAA

AACCGCGGCAGCCATCGAGCAACAGGTCAGCGAGCTCCAACGGAACTTGAGACGTCTACA

GACTGAGCATCAGGAAACGGTGCAAAAGGCAGCTCTGGCGTCTGCTCAAATGCAACAGGC

CTTACTGGACAGCCAGGAGCAGGCACGTCTCGCCACAGAGGCTCAGGAGAAGTATGAGCA

GGAGATGCTGTTACATGCAGCAGACATGGAGGCCCTACAGGCAGCCAAAGTCAAGGAACA

ACACGCCTCACAGCTCCGCCAGCAGCTTGAGGAAAAGATCCAAACTGTCAGTGCACAGCT

TTTTGAGGCCCGAGTGTCCTGGGAGGAGCAGGAGAAGATCCTCAAAGAGGAGCAGACTAG

GCTCGAGTCGCACTGTCAAGACCTGCAGAGGCAGAATGAGCTTTTGCATGAGCAGATTCA

GGCTATGAGTGGAAAGATGTTAACCCAGATGCAGCAGAAAGCCGGTGACGGTTCACTAAA

CGTTTCTTTTACTGAAGAGGGGAAGTCTCAGGAGCAGCTCCTTGAGATCCTCAGTTTTGT

TCGTCGGGAGAAAGAGATAGCGGAGTCCCGTTACGAGGTAGCTCAGGGAGAGAGTTTGAG

GTACAAACTGCAAGTGGTACACCTGGAGCGAGAGCTGAAGGAGGTGCAGGACTCCCTCAG

TGCAGAGAGAGAAAAGCTGCAGGTGACTGCTAAAACCTTGTTACAACATGATGAGCTAAT

GAAGAAGACTGAGACCATGAATGTGCTAATGGAGACAAATAAGATGTTGCGAGAGGAGAA

AGAAAAACTGGAGCAGGAGCTGCAGCAGACTCAAGCCAAGGTGAGTAAGCTGCAGTCAGA

CATCTCGCCCATGCAGGAGTCCAATACTGAGCTGAGTGAGAAGAGCGGCATGCTTCAGGC

TGAAAAAAATCTTCTGGAAGAGGAAATCAAGCGCTGGAAAGCACGCACACAGGTCCTGGT

GAGCCAGCAGAAGGACACAGACCCAGAAGAGTACAAACGGCTGCACTTAGAGAAAGAAAC

CCATCTTAAACGTATCCAGCAACTCACAGAAGAAACCAGTCGACTTAAAGCAGAAGCAAC

CAGGTACAAAGACCTTCTCTTTACAATTAAGCTATGAACCAGAAATTAAATTCTGATTCT

TTGAAATCGGTACAAAATAAATTAAACGAAATTCACAATGCTTACAAAAACGGAAAAAAG

GACTTAAAGGGTTATGTTTAACTTTCTTCCTGCAGAAGCAGTAGTTCCATCACTATGCTG

CAGTCTCAGATGCAGAACCTGCGGGAGAATCTTGGCAAGGTGACGACTGAAAGGGACAAT

CTTAAAAAAGAGCTTGAGGCGAGAACTCTGGACATCCAGGAAAAGCTTAGGACCATTACA

CAGGTCAAGAAGGTTGGGCGCCGCTATAAATCTCAATATGAGGAGCTCAAAGTTGAACAT

GACAAGGTGGTGGCAGCTGCTGCCTCTGCTCCAGTCCAAGACCAGGAGGCTCAGCAGACC

TCAGTTCAGGAGCTACAGAGCCTAAAAGATTCCCTAAGCCTATCAGAAGCCAGGACTCAG

GAGCTAGAAGGACAACTAGAAACCTTAAACAAAACGATAGGCGAGCACGAAACAGGTACG

CGCACTGCACAGGACCAGGTATCCCATCTTCAGAGTGAGCTAATGCGTCTGCGCCAAGAG

CTGGTAGAAAAGTCAGCCCAGGAAGACCGGCTCCGGCAGCAGTTTGCCGAGAAGGAGGAT

CGAACCAGGAAGGCCATTTTGGGGGCAAAACAGAAAATCAACCATCTTGTGGATGTAAAG

GGTCAGTTGGTAAAGGAGAACGAAGAGCTGAAACAGCAGCGTGAGGAGCTGGAGGTGCGC

ATAAATGCACTACGCTCACAGTACGAGGGACGTCTGAGCCGGCAGGAAAAGGAGCTTCGG

GAACTGCGGGAGCAGGAGAGACATGTTGAGCAGAGGGATGAGCCACTAGAGCAAGGTCCC

AGCAAGACTCAGGAGCAGCAGAGGACTACAGAGCAGAGGCAAATTTCTCTGAAATCCACA

CCAGTTGCCGAGAGGGGCAGTGCAAGCAACTCTGAGCCTCCTACAGCTAATATAAAACCC

ACCCCCCTAGTTGCGACCCCTAGCAAACCTCCAGTCATTCCAGGAAACAAACCCACTCCA

AGAGCCAGTATTAAACCTATGATTACCCCTGCTGCAGTGCCCACACCCACTCCTACTGCA

ACAGTGATGCCTACCACCCAGCAGGAGAGCCAGGAAGCAATGTCATCAGAGTCACCTTTG

GACCATGTGACTGTGTTTGGCAGCACCACCGGTTCTGTGCGTTCCACCAGCCCCAACATT

CAGACTACGCTGGCTCAGCCCATGCTAAGTGTACAGCAGAGCCAGACCACTGCATTCGTC

CAGCCCACTCAACAACAAAGTTTACCACTCACCGAGCCCGTGAGTCAGGAGCCTGCCTCT

GCCACCATGATGATGGAGGCTACACCCAGCAACTCCCAGATGGAGAGACCCTCCACATCC

ACAACTGTGTTTGGAACTGTATCTGCAACACCTGGAGGCACCCTAACCAAACGGCCCCGA

GAGGAGGAGTTGGATAGCATTACAGTGGAGCTGGAGCCTCAGGAGGAGCCCACCGAACCT

CCCATCTCCAAGAAGCTCCGTATGCAGAGAGTGGAACTAGAGATGGAGATGCTGACTGAG

AACAGCACTGAAGCCGAAAGTGTTGTACCAGAGGGAAGCCAGGAAGCAGCTGTCCATGAG

CTGGAAAATTATCCCACCCTGGAGGTAGATGAAGAGTCTGGAGTGTCTCAGTCTGTACCT

GTAGAATTTCTCCTCTCCCAGGTATCTAATATACAAAGTCAAGAAGAATCAGATGAACCA

GTCATCGTTATTGTCAGTGACTCTGAGAGTGAGGAAGCACCAGAGGAAGAGACCGAAGAA

GAGGAGCAGGGATTTGAGGAAGATGAAGATGAGGCGGAGGACGATGATGATGATGATGAT

GAT

>Unigene000159

CCACTAAGCGACATTGGGGTGATGTATGAAGGCAAGGACAGGCTGATTCAGGGCTGTGAG

GTGATCCAGGCCACGCCGTACGGCCGTTGCGCCAACGTCAACAACAGCTCGGCTACATCT

CAGCGCATCTTCATCACGTTCCGTCGCGCACCCCTCGTCCAGCCTCGCAACTCTCTGGCC

GTTACTGACATCTGTGTGATAGTCACAAGCAAAGGCGAGACTCCACCACACACCTTCCTC

AAATTAGACAAGAACCTCAACTGTGGCATGTGGGGCTCCAGTGTATACCTATGCTATAAG

AAGTCAGTCTCCTCGACCAACTCTATCGCCTATAAAGCAGGTCTGATCTTTCGCTACCCA

GAGGAAGATTATGAGTCCTTCCCCCTTTCCGAGTCAGTGCCTCTGTTCTGCCTTCCAATG

GGGGCCAAGATTGAGTGCTGGGCTCCCAACACACAGTACCCTTTACCGGTCTTCTCTACA

TTCGTCCTAACCAACTCCTCTGGGGAAAAGGTCTACGGTGCAGCCATCCAGTTCTACGAG

CTGTACCCATCAGATAACCTGACTGAAAAGCAAAAGATCCAACTTGGACTCTTTACAACT

GTGGACAAGAAGCCTATTCCCAATCGCACGGTTAATACAAACAAGTGCATCTGCCTGCTG

TCTCGCTGGCCTTTCTTCCAAGCCTTCAAAGACTTCTTACGCTTCCTCTACAAGCTCTCG

GTCAATAGCGTCAACCCTCTGCCTATTGAGAAACACATTTCCCATTTTATGCACAATGTA

CCCTTTCCCTCTCCTCAGAGACCCAGGATACTGGTACAGCTCTCAGCACATGACAGCTTG

ATGCTGTCCCAGCCTGTATGTACACCTTTACCACTTAGTGGTGCAGACTATAGTACTCTA

CTAACTAACCTGGGACCGGAAAACTGTGCCACGCTGCTACATTTCATCCTGCTGGAGAGC

AAGATACTGCTGCACTCCCTCAGACCGGCTGTACTGACTGGTGTCGCTGAGGCTGTGGTG

GCGATGATCTTTCCTTTCCAGTGGCAGTGTCCCTACATCCCTCTGTGTCCTCTTTCTCTG

GCCGGGGTCCTAAATGCACCCTGTCCTTTTATTGTCGGAGTGGACTCTAGATATTTTGAT

CTTTATGATCCCCCACCGGATGTGGTTTGTGTGGACCTGGACACCAACACTATCTACCTG

TCTGATGAGAAAAGGAACTCTAACTGGAAGAATCTCCCAAAGAAGCCTTGCAAGAGTCTG

ATTAACATGTTGGTCAACCTGCACCACCAGCTTAACATTGTCAGACGGACATCCCCAGGA

CTATCGGCGGTAGAGATGACCCCAATGGAGGCTGACTTCACATGGCATAAAAAGATGACT

GCTCTCGAGATGGAGATTCAGGAAGCCTTTCTACGCTTCATGGCCTCGATATTAAAGGGC

TATCGATACTACCTTAAACCCATCACTGAGGCACCCTCTGAGAAAGCCACCGCCGCTGAC

TCACTCTACGATCTGCAGGGGTTTTTAAAAAGTCGGGATCGTGCTCATCAGAAGTTCTAC

TCCCAGCTCACCAAGACTCAGTTATTTATTCGTTTTATAGAGGAATGCACCTTCGTCAGC

GACAAAGATACTGGTCTAGCCTTTTTTGACTACTGCATTGAAAAGCTTTTTCCGTCTGAT

AAAGGAACAGACAAAAGCACGAAGGTTGAAGGGGAGCTGTCAGAAGACACCAGACTGGTA

GAGCTGGATGAGTCTCAGAAGAGCGAGCACACCGTCTTTATTTTGCCGCCAGAGCCCCCC

ACTGATGACAGCCCAGATCCTCCTCCAAAATACAGCTATAAGAGTTTCCCACGGTTAAAG

CTGGAGTTATTCGATCGTCCGGCGCAGTTAAAGCCAGCACTCAGCACCAGAGCTGCAGGT

GCCAGTGTGTCCAGCAGTCCTGCTCTCCTGGCTAAAAGAACCAAGCAGGAAGTCAAGCTG

GCCTACAAGATGGCAAAGCGTTTCTATTCAAACCCTCAGCTGTGGGCTAAGTGTCTATTA

AGTCACTGCTACAGTCTGTGGTTCATTTGCCTGCCTGCAGGCATGCGTTTGGCTCACTCC

AAACCCCGTGCCATGCAGCAGGCGCTGGATGTCCTGCTGAAGATGAGGAGTTCTGAGGTG

GAAGTGCTGGATGAGGTGTGTTACCGTGTCGTGATGCAGCTGTGCGGGCTCTGGGAAATG

CCCGTCATGGCCGTGCTGGTTCTAATGGAGATGAAGAAGGCCGGCGTTGAGGCTAATGCC

ATCACTTATGGATACTACAACAAGGCTGTGTTGGAGAGCCCTTGGCCTAGTCGAAATCGT

AGTGGTCGATTCATGTGGACTAAAATCCGGAATGTGGTGCGAGGGGTGGCCCAGTTTAAG

CAGGCTCTTCGGGGAGAAGCACAGTCCAAAAGAGCGCCTCCTATTACAGCAGTAGTGTCT

CTTGACGCAATAGCAGATGGTGACGGGGACAGACTCAGCCACTGCAGTGCCGACAGCTCC

AGCGAAGCTAACGGAGAAGAACACACGCTGTTCTCACACAGACTGATTGTAGGAGACAAA

AATGGGAGTCAGTTTAGTACAGGAGGGCAGTCGGATCAAGGTTATGGCTCTAAAGATGAG

CTCCATCAGGACTCTACAGATGCTTGTAAAGCTACAGCTTTTCCACATTCTGATAATGAC

CTAGCCAACAAGAGCAAAACCATGAAGACTCAACAGAACGCCCTAGTGGACATTGCTGGC

TCTGTGGATAGTGCGATGGTGGTGTCAGAGCCCCCCAGTCCTGCTGAAACCACCCCCCCT

CCAGCCAGCATCGTCAAACAGTCCAGTGGGAGCTTCGACAAAGCCATGTGTAGAAAAGAT

TCAGGCAGCAAACTCTTCACTGCTCACAGTAAGACTGAGGACATTGTCCTACCAGAGGAT

GAAGGTGCTACACACCCAGACGCCACCCAAAGCAGCCAGCAGCAGAGGGCCAAAGCCTTC

ACTGAGCGCAGCTGCAGTTTCAGCACAGAGAGTCGAGCTGGCATGTTGCGGAAAAAGAGC

AGTCTGGAGTTAAGTGTGGATCATATGGGCGCAGACGCCAAAATTCTTGCTGCAGCTTTT

TCTGGTGGCAGGACTCCTCCTCCTGCTACCGCATCAGCCTCCATCTTTAGTGACCTTGGG

GTGGCACCTATAGCAAGCTCTGGCCTTGAGGAAGATACTGATGAGAAGATGGAGTTAATC

ACCTTTGAGGAGAAAACAGATGAAGGTAAGGGCCAAAAGAAAGAGACATCTGAGGAAGTA

ATGTCTAAGGAAAGCACAGAAGGAATGGGAAGAAGCATCAGCACCTCCTCATCGTCCTCT

AAGGCAGCAGAAAGAGAGGACGTAGAAGTAGGGGCAGATCCTCTTTCTTTGCTGGCGTCG

GAAAGCGATGAAGCAGCGTCGATTCAGAGCCAAGAATCAGCACGCCTCGTCCCACCAGTG

GTCTCGAGAAACCTTGCTGATGAGATTGAGATGTACATGAACCTGAAAAGCCCACTAGGA

GCCAAGGCTTCTAGCATGGAGCTGCACCAGGTGGTTCAGAGTAAAGTTGATCAGGCTGAC

TCTCCTGCTTGTAAGCAGTCTTTGGGACGGAGGTCTAGCCTCCCTGCGGGTCCAGTAAAA

GCCCCTGATACACTGGAAACCCCTAAACGCAGCCCTGCTGTCACACGCTCCAAGACTTTT

GCAATCAAGTCCCTAAAGACTCCTAGCAGTGAGGGTCAAAGGTCATCGTCCCTGACTGCA

CTAGTGAAGACTTCACAGAGTGGATCTTTGGGCTCTGTCATCAACTCCATCTCAGGCATC

AAGATGGACGCCTTGCTTTCAGGACCTAAAGTTGACATGCTGAAGTCTGGCATGAAGCAA

GCGGCTAACGTAGCCAGTAAAGTGTGGGGGGCTGTGGCTTCTGCCTACTCCTATTCAGAT

GATGAGGAGGAGCAGAGCCAGCCTGGAGACAGCTTCCCAGCTCGACTAGAGGAACAGCTG

ATGGGTGGAGATGGAACAGGGGACACTGTGCCTAGAGGACTAATGCCCAACCTGGCCTCC

AATGGCCTCACACACAGCAACACCAGTCTGGGCAGCAGCAGTGGGAGTAGTGAAACTGGC

CGGGGACAGCACATTGCATCTATGACTCCTGGGAGGTCTGTGAGAGGTGTGGACTCCGAG

CAGGGATCTTCTCATCATGCCAGCACTTCCAGTATCTATCAGAACTGCGCTTTAGAGGTG

CTAATGTCGAGCTGCTCTCAGTGTCGCTCCTGTGAGGTTCTGGTGTACGATGAGGAGATC

ATGGCAGGTTGGACAGCAGATGACTCCAACTTGAACACAACATGTCCTTTCTGTAACAAT

TCCTTCTTGCCTCTGCTGCACGTAGAGTTCCATGACCTGCGCCCTCCGTCTGGGTTTTAT

GTGCCCAGCAACTCAGGAGACAGTATCCACAGTGGCAGCACCCATCCTCCAGCCTCCAGC

TCAAAGGTAAAGGTGGGCAGCCCCAGTGACCCTGCAGACCTTATCAGCTTCTTCGAATCC

CCTGACAGCAAGATGGAGCCTGAAGGAAATCTAACCTACCAGACTGGCACACAGACAGCA

CTCGTTCCAGAACCAGTGATTTCGGACCCTCTGGGTCTGCTGGAGAAGCGCAGCACATCC

CTAACTCGCAGTAACAGTGTGGGCGGACCTCTGCAGAGCCTGGATTCCATCCAGCGGCCC

AATCATGGGGTGTCCACCACCAGCCTGCCTAATAGCCTGCAGGAGGTGGTGGATACATTG

GGTCAGAAGCGTCCTAACCCAATGCCAGTCTCGGTGCCTTATCTGAGCCCGCTGGTGTTG

AGAAAGGAGCTGGAATCCTTGCTAGAAAATGAGGGAGATCAGGTTATTTACACTCAAAAG

TTTCTCACCCAGCATCCTATCATCTTCTGGAACATGGTGTGGTATTTCCGCCGACTGGAC

CTGCCCAGCATTCTCCCTGGACTCATTCTGACCTCTGAGCACTGCAACAATGGAGTACAG

CTGCCTCAGACATCTCTGCCTCAAGACAGTAAACAAGTATACGTGCAGTTGCTCTGGGAC

AACGTAAACCTCCACCAGGAGCCCACTGAGCCTCTCTATCAGCTCTGGAGAACCTTTTTA

AAAAAGAAGGGCTCATTGGCTCCCACTGACCACCAGGAGATACGGTTCCTACTCAACAGC

ATTGTCAGGAACATCCAAACCAATGATGTCTACGGCCCCATAAACCTTCTTCTTAGGGAG

ATCAAGCGACACCCGGAGGTCAAACGACAAAGGAGTATCTACAGACAGATTTTGTTCCTT

TCACTTGTGGCCTTGGGAAGGGAGAACATTGATGTTGAGGCCTTTGACCGGGAATATCAA

CTAGCCTTTGAAAAGCTGAGTCCAGAACAGCTGAAAGTGCTCTCTTCTCTTGACCGTCCT

CCATCCAGCAGCGTGCAGTGGTGCCTCAAGTGCTTTGGAACTTCATTCATCTGACCTGGG

ATATTAGGGAATTTTTAAATCCTACCAGACATTTAAGAGGTGTAGAAGTGCACACGGTGA

CAGAGATGTACTGACTGTTTCTTGTACTGTCAGTCCTCTTTCTTTCACCTTTACCTAAAC

TTTATCTGCCTCCATATATCTTATTGCAGTGTTTGGCAACACCAGTATGTTGCACATGGA

TACTTATTGGTGTAAATCTTCACATCTTAATATCAGCACCTGTTTATTTCCTTGGACCAG

TGGAAGTAAAGAAGATGGAAAATTTCTCTGTACAAGGTCATTGCCTTATATCCTTATAAC

ATCCATCCAAACCTACCTGTGGAAAACGTCCAGTGGAAGTAAAGATGTTTTTTGAGACTT

TTGGCAAAGGTCTTAGATAATGTGCACTTTCGTGTTTTTTTTATTTCTATTGTTGTATAT

TTTTTTTTCTATGATGGGATATTCAAACTAGACTTCCTTTTAGAAAGGGATCCTTACTCG

TTTCTCTAAAGTCGGTCTTAAATGCCAAGCCTATGTTATCAATGTGGGTCTTGAGTGTTG

CAGTGGAAGGATTAAAGTGTGTGTGTGTGCATGTTTGTACATAGCTCTGTGTGTGT

>Unigene000160

TATCTGCATAATAACAAACACTCCAACAACACAGAGTATGTGTAAAAAAAGAAACTCTTA

GCCCCACATTATGACATCATACACACTGTCTCACTGTCTCAGTGTCTCAGTGTCTCTGTT

AAGTGCTGATGTGCTCAGGACGTCATTATAAACAGTATTTAACTGCTGCATGTCCTTTAA

CAGCCCCACAAACTGCTCCTCCAACACATTCACACGTTCAGCTGCTGCCAGAGCGACACT

TAACTTCCTCTCATCTTCCTCACGCTCTTCCTCAATCACACCCAGCCTGAAGGTGGCGCT

AGCAAGCTGCTCCTCCCTTCGTGCTAACACATTGCGCACTTCCTGCAGTTCGGCCTGCAG

CTGCGCCTGCTGCTCCTGACTCACCGCAATAAAATCTTCCAACCTCTTCGCCATCTCCAG

CTCACGGTCTGTCACCTGGTCAACCCCTGAACGCAGCTCATACAGCTCCTGAGCATGCTT

TTCCAGAATATTGGTCTCATCCTGAACGTGCTCTTTGATCCAGGGAACAGGAGCAGGCGC

AGACTTGTTAAAAAACTCTTGCTGTGGAAGAGAGATCTCCATTTGTCTTTCTGGACTCTC

CTCATGGTGGATGTGAGCTGTTTCACTCAACTCAGCGTTGCTGCTGGTTCTCTTCAGAGG

ACGGCGATGGTGTCTCTCAGAGGCAGGAACACTCTGCGTCCTCGCCACCTGCTTCCGGTG

TTTGCCCTTGCCCTGAGGGGGACGTGGGTCTGTGGGCAGGTGCGGGAGGGATTTGTTGAG

GGGACTGTACTTGCCAAGATCTCTGGGAGGAACATAGGTGGGTTTCTCTGTTTCACAAGT

GGACCTGCTGCAGGTGATTCGAGGAATGTCTGGGCCGAGGTGCTCAGTGATCTGATTCAG

GATGGAAGGCAGCGGCTGCAGGTTGTTTATAACTTCCAGTTTCATTGGAGAAATGATGTC

CACCAACAGGTTGTGAAGCACAGCTAGGCGTAAAGGAAGATCCACATAGCCATCAAACCG

AGCCATTTCTAGTTCACTGTCAGGGTTTGATACTTTCTGCAGGAACGTCCTCATATCCTC

CCAATGTTCTGCCAGGAACTCATTCATGAAAAGCATGTACTCTTCTTTCTCTCCAAACAA

TGTGAAGTTGGCGAGGTTCTGCAGCCCCTTAGCCGTGAGCGTCAGTGTGCGGAGTGTGTT

GTGTTCTGGGTAAGGTTGGATCAGTCCAAACAGAGAGGGACTGAGAATGGCTGGACAGAG

AAACCGGAGGAACAATGATGCTGAAATGAGCCGGTGTCCAATGTCCGCTCGTCCACGTTC

CTCACAATCAGACACCCAGCTGGAGAAAATTTCATTAAGTTCCACTGGAAATGAGCTATG

CCTCTCAGTGATCTGTCGCACAACTTCTCCACACGCATCCTTCAGGTGGCGCTGGTTGAC

CAGCAGCTCAGAGGCAGAACATTTCTGTGGATCGACTTCACAGCTTTCTGAGGTTGTGTA

TAGTCGATTTATGAAATCTCCCAAAGTGTCGATGAGATATTTCTGCCCCACCAGCTTCAT

ATACTCGTCTATGGCTTTGGTGGCCAAAGTGTTTTCTCTGAAGAGCAGCGCCTCGTTCTC

GCCCGAGCGCTGTACTTCAGCGTGACCCAGCTCGATCAGGAAGTCTTTGGCCTTTCCAAT

GCTCTGTAGAACATGGACGAGTGCTCCAGCCAGCTCCTCTTTATCTCTGACGTTCAGCAG

AGGCTCCAGGTTGCTACACATCTTCATGTACCCCAAAGTCACAGACTCTGCAAACTCCTT

ATATCTCTCGATCGGTAGCACTTTTAGGTTCTGGAATCGCGCCTTAACTCGGATACACGC

CTGGGGGCCGAGCTGCTCCTTCGATCCACCTGTCCCAGGAGGTTTGTAAGGAACGATGGG

ATACCATTTCTCCTGAAACACTCGGCCTTTAATGTTGGCCAGAGACAGGGCTACGCTGCC

CAGAGGGTATAAGATGTTCTCATCCTTTGAGTGGCGCTTCTTCTTTGGGTCATCATCCCG

GAACAGGTGCAGTGTGATCTGTGTAACAGGGGGCAGGTTATCCAGCTCAAAATACTCACC

CCAAAACAGCTGGCACCCCCCTGTACTTGCACTTCCTCCAGAAGACACGCCCACAGCACT

TGACCCACTGTCACTCACAGGGACTGAGGACGCTTTACCCACCGCACGACTGCTGGTGCG

AGCAAACAGTGTACCATCCAGGTGAACCTCACAGTAGTAGCGCTTTTTTGGTGGGAGACC

TTTAGCTTCGTTTACCCACAGGCTGAGAGAGTTCTCCATACGTTCACAGTTATCCTTATT

GGGCCGCGCCGCACGCCGCAGGTTCTCGATCCAGCGATCTCTCTCAGCCGCGGAGCTGCA

CCCGAAGCAGTGATTGCTGTCTGAGGTAATTACCTCAAAGCAATACTTCTCTCCCAGGAT

GGAACTGTGCACCGGCCGGATCACGGTGGTACAGTCCGAACTCACATTCAGACTTTCAGC

TGCACTCAGTGGCACAGAGAGAGAGTCTCGAGAACCGAACCTGCTACACACACCAAAAAA

CAAATACACAGGATTTTAGAAAGCTATAATAAAATAACAGACAGACAAAAAAACACAGAG

AACAGACAGACAGACAGACAGCACCATGCTGCAGAACTCAGTCCCACTACGTTGAGTTGT

GTGTTACTCAGTTTCCTGTGAGCTCCACCCTGCAGACGCCGTTTAATCTTCATCTAAACA

CAAGAGAGAGACACATACTCTGGGGTCATCTCACTCCACCATCCTGGGTTTTGGGTTTTG

GGGCGGTAACAGACGTGGGATCTGGACTGCACTCAAGGTTGGAGTGACTCTCTGTGAGGT

TCTGTAAACTGACAGATGACAGACAGCTGCTCGCTCGATTCCTCATCCGCAACAAAGGCT

CCTCTTCTCGAGAGATAAATATCTGTCCGTCCTGAAGGATGCAGTTGTTGATGTCCCACA

CAGGAACATCCTGAGGAACCCAGGAGAACTGAACTAAATCTGCACCCTCCACACATGGCA

CATCCTCCCGCTGTTGTTGTTGTTGTGTGGTGCTCGTGTTGTTGCGGTTCTGCGCCCGCG

GCCTTTTCAGTCTCTGTGATACCTGCTGCAGGTATTTGCGGAGTTTCCCGCGCTGTTGCG

TGTCCGCGCCTGCGCTGGTCTCCGCCGCTGAGGGTGATGACGATGAAGGAGACAGGATGG

AGGACAGGAGCGAGCCGGGAGGTTCTGACAGCGCTCTGCGGAAGGGGTTCTTTCTCCCGA

CGCGAGGCCCCGGTGTCGGTGTGGCTGATTTTTCGGGTGTGTCTTCACTCAGCGCTTTTT

TCCAGCTCTGCATCCGACTCCACTTGTTTCCCGCGCTTTTCTCTGACGGATCGGCCCGCG

CTCCTCCCGTCTGCCAGGTGTACGAGGTTAATAAGTTCCCGTCCTGTTCCGCGCGCTCCT

CTCCGCTCTCCTCCTCACCCCTCACTTCTTCTTTCTCCATCTTTGCGTGCGTTTCCGTCT

CAGACGCTTTATCCGTTCCCATGAATACGGTGATGATGGAGTGATAAAATGATGGAGTGA

TGGATTTATAGAGTGATAAAATGTTGGAGTCTCAACAGAACCTGACGCTCACCTCTTTCT

TCACTGATCTGTACTGTTTCTGCACGCGCTCAGCTTTATAACCACTTCCTGTTGCATGAA

GTTGTAGGAAACTCAGACGTGTTGTGTGTTTGTTTCTCTGGACATGAAATCGTTCAGGGG

TTTTTTTGCTCTTCACTGAAGATCATTTCAGAACCTTCACTGCAAACATCATAAAGGGTT

CTTCAGCAAAACCCGGAGTTTTTCACATGCTTTTCTACATGTTGTGTTAAGTTTGACAGG

TTGCGTTTCACGCACCGGAAATGTAAAAAAAAAATCTAAAACAAGAAGAAAAAAGGTACT

CAGAAATAAAGAGAGAGAGAGAGAGAGAGAGTGAGAGAGAGAGAGAGAGAGAGCGATAGA

AAGGGGACAGCGTAGGGACGTCTTTTGGAGATCTCTAGTGTGCTGCAGTGCTGCTGTTAG

GATCAGGTCGGGCGTTGGCCAGGTATTTGGCCCTCATGCTGGAAGTGTACGAAGGTGGCG

GCGACTCTCTTCCAATCAGAGGGGTGTTCTCACAGCGAGGATTCTGAAAGTTTGCATTCT

GTGTCATGTACTCAGTAACGGGGGCGTTGCTGACGGTGTGTGCTGCTGCGGGAATGCTGG

TCTCGCTGCCATAGCTGCTGCCACAGCTGTAAAGACTGGGCATGTGCACCCGGTACAAGT

TATCGTGGTTCTGTCTTCCTCCAGATGCGGCAGAGTCTTCTTCTGTGTCTTCACTGGATC

TCCTCCCTTGCCATGTGTGAGGCACGCTACAGATGATAGAGCTGAACATGAGGGCGGTGA

CGAAGGAAAACAGCACCAGGTAGATCAGTCCCTCCAGACCGTCGTAGCACAGCCCGGTCA

CAGCCTGCACGTAATCCATGTGCAGACTGCGGCAGTCCACTAGAGCGGTTAGCTGATGCA

AGCTGATTTCAGTTGTGTTTAAAATCCCTTGAATCTGCTCCAAATTAGTTCTAGTCTGGG

GGTATTCTCGCACTGCGGCACGCAGGAGCTCCAACACGTCGTCCTGCATCTCCACCAGAG

CCTTGTGGCTTCCGGACAGTTTCTGTTGGAAGGGGTTGTTTTGGCCGACACTGCAATGAA

GGTAATACTGCAGGATGTCTCTGTTGATGACGCCGTAGCGTTCAGCCACTTCGGCGACGT

ACATATCGGGTGCCACGCAAAAGTCACTAGAGCTCGAGGATACAGCTAGCTCCAAACCCA

GCGATCCCCAGCTGATCACCAGTGCCAACACGCCCAGTAAACAGACCCCAATTAAAGTTC

TTTTTGAATTGCGAATGAGGCCGAAGAGTACCAGCAGGCAGATGAGGACGTCAAACAGGA

GCAGCCCCAGATACCCCAACCACCTATACCAGTCGTAGAGTTCGATCTTGCTAGCCAGTG

CTTCCAGGGAAATTCTGGAATTGCTCCAAAACGGGATTTCCACCATCTGTTTAAGCAGCT

CGTCCAGCTGGCCCTGCACCTTCTGCACGATCGACAGGTAATCAGCATGCTTCGTGTATT

GGCTCTCCAAATGCTGCAAATTGTCATCTACGGTCAGATTCAGGGAGGTGGTGCTGTCTG

AAACCAGCTTTTGCACGCCGGCGATAGTACGGTTGGCGTGGCGCAGTGAGTAGGTGAGCC

GGTTAACTCCGTCGCAGGTCTCTCCATTCCCATAGAAACCAACAGCTATGCCAGCGCTGC

ACACCAGGGTGGCAATGATGACACACCAGGCCGTGCAGCAGCAGTCGGCGTTGGGCTGCT

CGCTGCTCTTCTTGCGGCGGCAACAGAGCCAGAATGAGTAGAAGAGAAGAAAGAGGAGAT

CCAGGGCCAGGCAGGCTAAAGCCACTGCTCCCAGCAACAGAATAGACTGCTGGTATGACG

GGTCCTCCGGCTGAAACTCGCTGCTGATCTGCTCGAAGCGCAGGTTAAAGTGAGGAAGAC

GGTGAAGCAGGTTAACCCACCACGGAGGAGAATAATTCACAACGGCGGCCATTTTCAATT

AAACCCAAACGAGAGCCAGTCAAGCAAAAATGTTTATTAATAAAAATAAACTAAAAAAAT

CTAGCAATGTAAAAAAATCGCGTCCGGATCAAACAATGGCTGCAAACTGCTCACAGTTGG

CACGCGCCAGCTATTCTCCGGGGTCATTGGTGGAGGTTCTATTATTGTCCCTTTTTTATT

AAATCCAGTAATAACCTTTGTGTTGATGTTTTTTGATGTTATAATCGATGCATTTCAGTA

GATTCGATCCTCACTTGGTTTTTAAGCGCTAGCTCGCAACTAGCAATGCTAACCAGACCT

TTTTGTTGGGAATCGTATTATTATTATTACTAATATTATTATTATTATTATTATTCGCCA

AGTCTTGAATTCTGACTAGCTATGTAACGGTCGACACCGAGGACGTCCAGATATTATCGT

AAAAAAACATGTCACAATGAAGCACTATTTTGTAGCTCGATTTGTTTTTCCTCC

>Unigene000161

ACACTCTTTCCCTATAAGACTCTCCTCTGTAAAAACAATGAGGGACTTATTGTGAAAGAT

GTAGATGACAATGAGTGTGGTGAAGAGCCTGAACATGATCAAGATGCATTTGAGGAAATT

TCCAAGCCTATGGAAATAGATCCGCATGACCTTGAAGAGACAACTCTAAATGTACCGAAA

GAACCCAAAGATGACTACGAGGAAGAGGTCTACGAATCTGCCAAATACTTCCTGTTAAAC

TGTGCCACACCTGATTCTGTGTTGAGGCTAAAAAATTCAGGGCTCGGAAACCATGAAGTA

GAAAGACTTCAGAGATTGTATTTTCATCAGCAAGACCACCATTCACTTAGAACATTTATG

GACAGCCATTTAAAGAGGGCTGACAAGGAAAGCAAGAGATTTATAGAGGTAACAACCTTC

TCTAGTCTGCTGACGAAAGTTGATGTGAGAAATCTGTCCAAAGCTTTGGGTGTGTCCATG

GACGGTCTCCTTTTGCTCTCTTTGCACCAGTTTGACACTGAAGTTTCTTTTTGCAGCAAA

ATACGAGGCTTCCTAAAGACTGCCCAATTGTCTTTACGCATCCTTTTGGTTCAGATAGAT

ATGGAGGAATCACTCTGCAAAAATGAGCTTATTGCTTCTGCAAAGTACTGCACCATGAAT

GAACTTTTAGCCTCTCAGTCAGAAGATCCCAGCTGCTATATAGTGTTTATCACAAAACTT

TCAAGGATTGCAAGTGGCAATAAGTACATTGGATTCCAGGGGGGCGCATGGATTTCGATA

CATATTGATGACCTCAGAGAAACAGAAGACATGAGTTTGGATCTCTCTGTTTTTTGTGGA

ACACCAATTAGCAAATTGCTCTCTATGCCAGCAGTACCTGCTGCTATGGAAGTTGGTGAT

CAAGAAAAAAACACAATCATAAATGCTCAGGATGAATCCGCATACCTGCACAGTTTGTCT

TTGGTAAAATCATGCACCCAGAAAGCTGTTTCACTTCTTCGAGACTCTAATAAAAAAGCT

CCTAGGAGTATGGAGCGCATGAATATCCTTCTTAGCCTTTTGCAAAATGACTTGGGGCCT

GATGGAGCTCATTTTCAGGAAGTGTTGCTGAAGCGTTTAGTGGTTGCACTAACGCAAAAA

GAGGAGAATATACATAATTCAGGAGACTGGGTGAACAGAGAAATAATGAAGCGGGAGGCA

TTACAAGAAGGCGGCACTCTCAGGCACACTCTGTGGCGATATCTACAGGGAGTTCTGACA

CCAATTCTGGCCCGGATATTAGAGGTCCTGGACAGAGACTGTAACCTGGACTTGTTGTAT

AGTGCAGGCCTCGGTGAGGGGCTTGTTCAATTCTGGCTGGACATCTTTGACGATGGACAG

ATTTTAAACTTATCAGTGCCACAAAACTTGAGCATTTCAGACCAAGAGATTGAGGTTCAG

TGCAATCTGATGGTGGGGGAAGAGGCATGCCCCTGCAGTGCTCCTTTTAGTTGGCTTATC

AGACAGTATTGTTATAACCTATGGGAGGAGGCTCAGTTTGTGCAGGGTATTGAACAAAGC

AGCCATCAGAGAATAAAGCAGTTTGTAAGTGCTGTGACTAGCAGTCGACTGTGTGGCTAC

ATGGAAAAACTTTCAGAAAGGGCGCGCATAGAACTGGGCCAGAGATATCTCAATGATTTT

GTGATACTCTCTTTTAAAATCAAGACTGAGGATGGAGTGAGGGTGTTTAGTGCAGCAGTG

TCAGGTTGTGTTGCAGCACTGCAAAAGGACATAGGCGTGACCCCTGATCTTTCACCAGCT

TGGATTATGGCAGCAACTCAACATTTCAGTCCACGACTTGACACACTCTCTCACATTCTG

CAGATCCAGCCTTACTTAGCTCCTCTCATTATTCTAAAACAGTCCACACGTAAAGACCAA

CCAGACATGTGTGAAGATATTCTAGCCCTTGGGATATGTGTGGAGGAGATGAAACTTCAG

TCTTTTAGCTCTTTATCGCAGTGCTCAGACTTTTTGGGTAGAGTTGAATTGCTCCAGCCC

TGCTTGCAGAGAGTGTTCAGTCCCACATACTGCTCCCTCTGCAGCCCTGGCTATCTCAGA

CATCTGTATGCTATAAAGTCTGCATGGCAGGGTATGCTGGTGGTAGCAGCTTTTATCGAG

CAGGTTGTAGTGAAAGTCAATTCGTGTGATAAGAAGCTGGTGGCACTGACACTTAAGCAC

TGCAGCCAGCTACAGCGGCTGATGGAGGAGTGTTCTAACCTGCAGAGTAAAGCCTGTCTG

CAGCAGCTCATCCGCATCCTCAACGAGTACTATGAGGAATCTATCAGCGAGGAGTTATGT

TTTGGTCTGAAATGTCCTGTGTGTCTGGAGGAGCTGGCTGAGCCCTGTGTTCTACAGTGT

AAGCATGTGTTCTGCCTGTCCTGTATGAGGAGATGTATGCAAAAAGAGGAAGTAAAATGC

CCTAAGTGCAGGGCACAGATTCCTCCCAACTATCAGCCAGTTATCTCAACCAGCATAGAT

TTGGCCCTAAAACAACATAAAGAATTGAGAAGGCTCTGGAACTCCTTCTTTTTGGAGGTG

GTTTCTCATTTCTGCTTTTCCAAGGAGCAGAAACCCGAAGACGACATGGTGGAGCTGCTC

TTTTCCCTGCTTATCTCAGCACAAGGGGACGTGTATAAGACTAGAGAGCTAACCCCATTC

CTGGAATGTGTTGACCAGTGTCCTGTGGTACGGTCTGTGTTGCCCAAACTCTTAATGCAA

TATAGTTTTGACCAGGTAAAAGGCCACATTCAAAGATATTTGCAAAACCTGGAGGACAAA

CTTCTGGATTCTAAGGACAGAATGGAACTTTACAGACTGTTTGTCAACTGTTTTCAGGAT

TCTTTGCTTTGTGTGGATTTGAGTGAAGCTCTGGAGGCCAAAGAACTACAGCGACGTCAG

CAAGAAGACATTACATTTCTTGGCCGACTGGCTCGCAAGCAGACTCCATCCAGAGAAGAG

CATCCTGCTGAGTTTCTTTTGTCCATGGCCCGTCTCAGGATGTGTCTGGATTCTGCTGCT

CGAATACTTTCCAGGGCTGTCAGTCAACAAAGCGGAGGTTGTGTTCAATGGGAACAGAGG

TTGTTAGAGGAGGTGGAAGTGGTCTTCAAGTATGCTAAGGATGACTGGTACAAAATTTAC

CTGCTAAGAACGCTGAACAGGCAGGCAGGCACAGACTGTGTACAGGCTGCCATGAACAGC

GCTTCATATGAATGGGTCTTCCCTGCTGAGCTCTTGCGACAACAAAGGTTGATTCCTGTG

GAGGTGGATCGATTTTTGTGCTGCGGTGGGCAGTATCGCGCAATAAGAAATGGAGTGGGC

CAGGTGCTGCTGGAAGGCAATGCAAACGCACTTAAAGCTGCCCTGCAGAATATAAGCAGT

TCAGAGTCTGCAAAGTGCGTTCTTCTGGCTTTGGCTTTGTTCAAACAGGTGACATGCCGC

ATAGATTTGCCTGACTCAGCCATGCACCCTGGAGAACAGAAGTTAGACATGCTGAAACAC

ATTGTAAGGGAGAACACAGCCTGGTCAGGGTTTTTACGTGAACTCCTTACCAGTCTTATA

TCAAATCCAGCTGAAGGTCTTATGTCTCACCTTCATGTTCGTGCAAACAATTCCACACAA

AGACGTCTTCTCTTGGAGCTCCTGGTTCATGCCGTCGCTGTGTTCCATTGTGGAAGCCAC

ATTTTCAATCCCTTCCACCTCATTGCCTCTAGGCCACAATCAATGAGGGATTCATTTCTA

CCCACCATGCAAGATGATGACACCAGTATGGTCATTCAGGGCTTAAGAGAACAAAACCTG

AAGCGATATTACTGTAAAAATGGTCACCTGTCTTTAGTTGGAGAGTGTGGAAAGCCAACA

GCTTTGGGAAAGTGTGACACATGTGGAGTGCCTATTGGTGGACTAAACCACAAGGCAGTA

GCTGGATTCAATCCAGGCCAAAGTAGTGTGGGTGATCAGACCCAACCAGGCCATATTCTC

AATGATGCTTCTCGCAGATCTGAAGCTCCAAACAGAGGTCTGTCTATGGCCCAGTCCTGT

GTACTACGTCTCTTTCTTCATCTGGCCATGCTGCATGGATCTTTTCTCAATCAGCAGGGT

ATCAGTGCAATGATTCACCCCACTGTATATAATGTCAAAGAGTTTTTATGGCGCCATCTT

GAAAAAGACATCGAGGTCCTAGGAAAAACTCTAAATCTGAACTGGGACAATGCAGCCATC

ACGGTTCATCTGATACTCAACACCTCAGCACAGCTTCCTACAGTTCGTGTCCAGCAGGGG

GCAACAGGATGGGCTTCTCGAAATGCTCGAGAGCAGTGGGAGAAGCAGGTTTGCGAAACA

GTCATCAATCCTATTCTAGAGAATTTGAACAGACATCTAAGTAATGCTCAGAAGCAGGTT

GAAGCTGATGATAAGCTGTCCAACAGTGCTCTGATGAAGATTTTAAAAGGGGACCCTCGT

TCTCTCATTCCTCTTCCCTCTGACTGTCCCAGCCATCATGCACTTTTCTGGACTCCTCCA

GACACCCTAAGTGTGGAACATCTTTCTGTGCTTATTGGCCAAAAAAGGGCACAGAAACAA

GTTCCTCTCCTCTCATTGTTTCTCAACAAGGTGCAGTACATACGACACCTGACTTGCCTG

CCTGAACTGGCAGCCCTTTTGTCGGATTTGATACGAATAATGCCGCTAGACTCTGACACA

AACTCCACACCCATTGCTACACTGCTGCAAAACATTTCTGGAGGCCTCCAGAGGAATACA

TTAAAAAAAAAGTTGCAGACCTTTTTCAGGGTGTGGAACCATCTCAGAATGGAGCTGGCT

AATAACACTTCTGTAGTTGTGGATCCTGAGCTGTGTGCAAAGGACATAACTCTGGACAGT

CCTGGTCAGTTTCTCTGTCTCAGTCGTCATGGACCAGGCTCGTGTCTTCACGCTCTCATC

AAAAAGCTGTCAGAAACTCACAACAGCTTGGTGCGAGAAGCTGAAAAACTCAGTCATCAG

GAAAACTGTGACTACAGTGTTCCTGTAGGGGAGCTGTCTGAGAGTCAACTTGCAGTATGC

CACCCAGAGAGGGAGCTGCTGCCCCTGGTGTTAGCTCACTGCCAGTACACACTGGTAAAG

AGTCAACAAATTGCCAGTGACTATAACTTGCTAGCTATTGAGAAACAACTTTACAGACGC

TTCCTAGCAGGAAAGCCACGCATCCAAACGGACACTGAAAAGTATCTAAAGAGACATCAT

CAAGATTTTTCAGTAGTTTTAAAAGACGTCAGAAGCAAGATACCTCAGAAACCACTAAAG

GGCTCAGTATGCAGCTCAATAAGAACCGTGCTGCGCTCCTTCACTGATGTGTGTGATGCT

GTGTATGCTCTGGAGATTGGCCTGCGCTTTCTGGGGAAAACTGGAGGAGACCCAGAATCC

CAACTACTGTCCTATCTTCTTGACTCTCTCAAGATGAAGCAACATATTTCCAGCAGTGTA

GCTAAGTCTCTAGCAGATATCAGGCTAGATCAGTGTACAGCCACATGGCAGCTGATGTCC

TGCTGGAAGAGTGAGCTAAGACTAAGAAGAGGACAGGACCCTATCCCAAGACTTTCACAA

GACTATAGGGAGGCAGTTACTGCCGATGTGCAGAAAGAATTAAACAAATTCCTGGACACC

ACTGATGTTGAGCTTTTTACTCTGGAGCTTCATGAAATCCTGCTTTTAAAGACGGACAAT

AACTCAGAGGACAGATATGATCCGCAATGGGACATCCAAAACACTCTGGAGTCACACTTG

GACGGAAAAGGAGCCCCTGCTTTGCCTGGACTAGACAATCTTTCAGAGGAGATCTGTCTG

AGTAACGGTGCAGAGGTCTGGAGGCTTGCAGTAGACTTTAAGCGATAATTGTGTCTCTTA

GAGCTATTGCGTTGTGTTGCCTAACTCTACTGTATGCGACAAACCTTAAAATTTTTCTTT

CTTTACTCGCTCCCTATGTTATGTAAATTCTGAATGGACCATCTGGAAATTTTGTTTCAC

TGTTTTATTCCAAGCACCTTATTGATTACCTAC

>Unigene000162

GCTGGCAGCTCTAATGGAGGATGAGGGTGGAAACTGACAGCAGCTTCTGGGTGCCGCCCG

CAACCTGGCCTGTGCTGTATCAGACATGCTTAAAACGGCTCAGCCTGCCAGCGCAGAGGT

ATTACACCAATAATCTCAGTGCTCACACACACCCCGACACCGACACAGTATCACCGGGTG

TATCGTAAACACGAGTTTGTCTTTCACAGCCTCGTCAGAACCTTCTGCAGGCCGCAGGTA

ACGTGGGCCAGGCCAGCGGAGAGCTACTGCAGCAGATAGGAGAGAGTGACGCTGACCAGC

AGTTCCAGGACATGTTGATGAAGCTTGCTAAAGCTGTAGCTACAGCAGCAGCAACTCTGG

TCTTAAAGGCAAAGAACGTGGCCCAGAAGACAGAAGATTCAGCCCAGCAAAACCGGGTCA

TCGCTGCTGCCACACAGTGCGCCCTTTCCACCTCTCAGCTGGTGGCATGCACAAGGGTGG

TTGCTCCAACTATCAGTTCCCCAGTATGTCAGGAGCAGCTGATTGAGGCAGGGAAACTAG

TGGCTAAATCAGTTGAAGGCTGTGTGGAGGCATCACAGGGAGCCACCAACGATGAGCAAC

TGCTGAAACAAGTGGGTGTGGCCGCCACAGGTGTCACCCAGGCTCTTAACGAGCTGCTGC

AGCATATCAAGCAGTACGCCAGCGGAGGCCAGCCAATCGGGCGCCACGGGGAGGCAACTG

ACCGCATTCTGGACGTAACTGAGAACATCTTCAGCTCCATGGGAGATGCAGGTGAAATGG

TCCGACAGGCCCGGATCCTTGCTCAAGCCACCTCCGATTTGGTCAACGCCATTAAAGCAG

ATGCAGAGGGAGAGACAGACCTTGAGAACTCTCGTAAACTGCTCTCAGCTGCGAAGCTTC

TAGCCGATGCAACTGCCAAGATGGTGGAGGCCGCCAAGGGTGCTGCCGCCAACCCAGACA

GTGAGGAGCAGCAGCAGAAGTTGCGTGAGGCCGCTGAGGGCCTGCGCATGGCCACTAATG

CAGCAGCACAGAACGCCATCAAAAAGAGGCTGGTTAATAAGCTAGAGAACGCTGCAAAGC

AGGCAGCTGCAGCCGCAACTCAGATCATAGCTGCCGCCCAGCACGCTGCCTCATCTAATA

AGAACCCGGCTGCACAACAGCAACTTGTACAGAGCTGCAAGGTGGTGGCAGACCAGATCC

CCCAGCTGGTGCAGGGAGTCAGAGGCAGCCAGGCACAGCCTGACAGCCCCAGTGCTCAGC

TTGCCCTAATTGGAGCCAGCCAGAACTTCCTGCAGCCTGGAGCCAAGATGGTGACTGCAG

CTAAGGCTACGGTGCCCACTATTGGTGACCAGGCCTCTGCCATGCAGCTCAGCCAGTGTG

CCAAAAACCTGGCCAGTGCATTGGCTGAACTCCGCACGGCTGCCCAAAAGGCTCAGGAGG

CTTGTGGTCCTCTGGAGATTGATAATGCCCTGAATGCAGTAAGGGATTTGGAGAAAGACA

TGCAGGAGGCTAAAGCTTCAGCAGCTGAAGGAAAACTCAGACCTCTTCCTGGAGAAACGC

TGGAGAAGTGCTCCCAGGACCTGGGCTCCAACACTAAGGCAGTGAGCTCTGCTGTTGCCC

AGCTTCTGAGTGAAGCTACACAGGGCAACGAGAATTACACCGGTATGGCTGCACGGGATG

TGGCCCAGGCTCTCCGTTCTCTTGCCTCCGCAGCCCGTGGAGTGGCCGCAACCACAGACG

ACCCACAGGCGTGCAGCGCCATGCTGGACTGCGCTGGAGATGTCCTCGACAAATCAGGCA

ACCTCATTGAAGAAACCAAAAGAGCCATCGCTAAACCTGGAGACCCAGAGAGCCAGCAGC

GACTTGCCCAGGTGGCCAAGGCTGTCGCTCAGGCACTGAACCGCTGTGTTAACTGCCTTC

CTGGTCAGAGAGATGTGGATAACGCTATCAGGACTGTGGGAGAGGCCAGCAAAAAGCTTC

TTTCCGATCAGTTCCCAGCTAGTGGAAAAACCTTCCAGGAAGCTCAGGCAAACCTGAACC

AGGCGGCCGCCGGGCTTAACCAATCAGCCAACGAGCTGGTGCAGGCTTCTCGCGGCACCA

CACAGGACCTGGCCAAAGCGTCGGGCAAATTTGGCCAAGACTTCAGCCACTTCCTGGAGG

CTGGCGTGGACATGGCTGGCCAATCTCAGTCTAAAGAAGACCAGACTCAGGTGGTGACCA

ACTTGAAGACCATCTCTATGTCATCCAGCAAGCTGCTGTTGGCTGCTAAGGCTCTGTCTA

CTGACCCCAATTCCCCCAACCTGAAGAACCAGTTGGCTGCTGCTGCACGTGCCGTGACTG

ACAGCATCAACCAGCTGATCACCATGTGTACGCAGCAAGCACCGGGTCAGAAGGAATGTG

ACAACGCTCTGAGGGAGCTCGAGACGGTGCGGGGCATGCTGGAGAACCCAACTGAAGCAG

TGAATGACATGGGCTACTTTGACTGCATCGACAGTGTAATGGAGAACTCAAAGGTATTGG

GTGAAGCTATGGCCGGCATCTCCCACAATGCAAAGAACAGTAACCTGCCTGAATTCGGAG

ACGCAGTCAGCTCGGCCTCCAAAGCTCTGTGCGGCCTCACCGAAGCTGCTGCTCAGGGTG

CCTATCTGGTGGGTGTCTCTGACCCTAACAGTCATGCTGGACAGAAGGGGTTGGTAGATC

CATCTCAGTTTGCCAAAGCCAAGCAATCGATTCAGATGGCCTGTCAGAACCTGGTGGACC

CTGCCTGCACACAGTCCCAGGTCCTCTCTGCTGCCACTATTGTTGCCAAACACACCTCCG

CCCTATGCAATGCCTGCCGCCTCGCCTCCTCCAAAACATCAAACCCTGTTGCTAAGCGAC

AGTTTGTCCAGAGTGCCAAAGAAGTGGCAAATAGCACAGCCAACCTTGTAAAGTCAATCA

AGGCCCTGGATGGAGCCTTCAACCAAGAGAACAGACAGAAGTGCAAGGCCGCCACAGGGC

CATTAATCGAGGCTGTGGATAATCTCACTGCCTTTGCTTCCAATCCTGAGTTTGCTAGTG

TGCCTGCACAGATCAGCCCAGAGGGTCTGGCTGCCATGGAGCCCATCGTGGCTGCAGCTA

AAACTATGCTGGAGAGCTCAACAGGTCTGATACAGACCGCACGCTCTCTAGCCGTGAACC

CCAAAGATCCACCCAAGTGGTCAGTGCTTGCCGGTCACTCCAGGACTGTGTCTGACTCTA

TCAAGAAGCTAATCACCAACATGAGAGATAAGGCTCCAGGTCAAAGAGAGTGTGACGAAG

CCATTGAGGTTCTCAATAACTGCATCCGTGAAGTGGACCAGGCTTCTCTCGCTGCCATCA

GCCAACACCTTACACCCAGAGACGACATTTCCCATGAGGCTCTGCATGAGCAGATGGCAG

CCTCGGTGCAGGAGATTAGCCACTTAATTGACCCAGTGGCTATCGCAGCTCGTTCCGATG

CCTCCCAGCTAGGACATAAGGTTTCTCAGATGGCGAGCTACTTCGAGCCACTCATCATGG

CAGCCATTGGTACTGCATCAAAAATTCTCAACAGCCAACAGCAAATGAGTGTTCTGGACC

AGACAAAGACTCTGGCAGAGTCTGCCTTACAGATGCTCTATACAGCCAAGGAAGCAGGAG

GCAACCCTAAGGCGGCTCACACCCAGGAAGCTCTGGAGGAGTCTGTGCAGATGATGAAGG

AGGCCGTTGATGACTTGGGAGGTACAATGGCTGAAACAGCAAGTGCAGCCGGAGCTGTAG

GAGGCATGGTGGACTCCATCACTCAGGCTCTTAACAAATTGGAGGACCCTGGAGTCGAGC

CTGAGGGAACCTTTGTGGATTATCAGACTACTATGGTGAAAACGGCCAAGGCTATTGCAG

TCACTGTGCAGGAGATGGTCACTAAATCAAACACCAACCCTGATGAGCTCGGAGGTCTAG

CCAATCAGTTGACCACCGAGTTTGGTGACTTGGCATCAGAGGCTAAATGTGCTGCCATCA

CTGCAGAAAATGCTGAGATTGGCTCTCACATTAAGAAGCAGGTAACAGAGCTGGGCTATG

GTTGCACTGGATTAGTCACCAAGGCAGGAGCTCTACAATGCAGTCCCAACGATTCCATCA

CTAAGAAAGAGCTGATTGACGCTGCCCGCAAAGTCTCTGAGAAAGTGTCCCATGTGTTGG

CGGCACTGCAGGCTGGTAACCGTGGTACCCAGGCTTGCATCACCGCTGCCAGTGCTGTAG

CTGGCATCATTGCTGACCTTGACACCACTATTATGTTTGCCACTGCTGGTACACTTAACA

GAGAGAATGCAGAGACCTTCGCTGATCACAGAGAAAACATTTTGAAGACTGCTAAGGTTC

TGGTGGAGGACACCAAGCTACTGGTGTCAGGAGCAGGAGCCAGTCAAGAAAAGCTGGCCC

AGGCGGCCCAGTCCTCAGTCAACACTATCACCAAACTTGCCGATGTGGTTAAGCTGGGGG

CCGCCAGCCTGGGCTCTGAGGACCCTGAGACACAGGTGGTCCTGATCAACGCAGTCAAAG

ACGTGGCTAAAGCTCTGGGTGATCTTATTAGAACCACCAAAGCAGCCGCTGGCAAGCCTC

ATGACGACCCGGCCATGCTCCAGCTGAAGAGCTCTGCGAAGGTGATGGTTACGAACGTGA

CGTCCCTGCTGAAGACGGTGAAGGCTGTGGAGGATGAGGCCACTAAAGGCACACGGGCGC

TGGAGGCCACCATCGAGCACATCAAACAGGAGCTTGCCGTGTTCAGCAGTCCAGATCCTC

CTCCAAAGACAGCTACACCAGAGGAGTTTATCCGCATGACCAAAGGCATCACGCAAGCCA

CAGCCAAAGCCGTGGCTGCTGGAAACTCATGTCGCCAGGAGGACATCATTGCTACCGCCA

ACCTGAGCAGAAGAGCCATCGCCGAAATGCTGCACTCGTGCAAGCAAGCGGCTTATCACC

CAGAGGTTAATCTGGAGGTGCGGACCAGAGCCCTGCGCTTCGGAAAGGAGTGTGCTAATG

GCTATCTCGGCCTCCTGGAGCATGTGCTTGTGATCATCCAAAAGCCCACTCACGATCTGA

AGCAGCAGCTGGCAACCTTCTCCAAACGAGTAGCTGGCTCAGTCACTGAGCTCATCCAGG

CAGCTGAAGCCATGAAAGGCACTGAATGGGTAGACCCTGAGGATCCCACAGTCATTGCAG

AGAACGAGCTGCTGGGTGCAGCGGCCGCCATCGAAGCTGCTGCTAAGAAACTGGAGCAGC

TGAAACCACGAGCCAAACCCAAGGAGGCTGATGAGAGCCTGAACTTCGAGGAGCAGATTC

TGGAGGCAGCCAAGTCCATCGCTGCAGCTACCAGTGCGCTGGTGAAGGCTGCATCTGCCG

CACAAAGAGAGCTGGTGGCCCAGGGCAAGGTTGGCGCTATTCCAGCGAATGCAGTTGATG

ATGGACAGTGGTCTCAGGGTCTTATTTCAGCCGCCCGCATGGTTGCCGCCGCCACCAATA

ATCTGTGTGAAGCCGCGAACTCTGCAGTACAAGGCCACGCTAGTGAGGAGAAGCTCATTT

CCTCAGCCAAGCAGGTGGCGGCATCCACAGCTCAGCTGCTTGTGGCCTGCAAGGTCAAGG

CTGACCAGGATTCACAAACCATGAAAAGACTGCAGGCTGCGGGCAACGCTGTCAAGAAGG

CGTCAGATAATCTAGTAAAGGCAGCACAGAAGGCTGCTTTCGATGCGCAGGATGATCAGG

CTGTAATGGTGAAGAGCAGGATGGTGGGCGGCATTGCTCAGATTATTGCTGCTCAGGAGG

AGATGTTGAGGAAGGAGAGGGAGCTGGACGAAGCCAGGAGGAAGCTAGCTCAGATCAGAC

AGCAGCAGTATAAATTCTTGCCCTCAGAGCTGAGGGAGGACGGTCATGAGCAGTGAAGAG

CTTCTTTCACACACACATACACACACACACACAGGTCTGCTACTCAGGACTGCACGGCCT

GAGCGCTTTTCTCTCCATGTGTTTAGCTACAATGGCAGGCTTGCTGAAGCAACATTCAGG

CTAAAATTATAAAAAAAGCGAGGC

>Unigene000163

AAGAAGAAGAAGAAGAAGAAGAATCGCCTGTGCAGAATATAAAAGGGGATGACGTAGGGG

CTCAGGCCAGTCACAGAAAACAGAGATGAGCCTGACATCCTGGTTCCTGGTAAGCAGTGG

GGGCACACGCCACCGGCTGCCCCGTGAGATGATTTTTGTGGGTCGTGATGACTGCGAGCT

CATGCTACAGTCTCGCAGTGTGGACAAGCAGCACGCCGTGATCAACTACGAACCGGCAAG

TGATGAGCACAAAGTGAAGGATCTGGGAAGCCTTAATGGGACATTTGTGAACGATGTTCG

AATCCAGGAGCAGATGTACATCACCCTGAAGATTGACGATAAGCTGAGGTTTGGATATGA

TACTAACTTGTTCACTGTGGTCCGAGGAGAGCTTCATGTTCCAGAGGAGGCTTTAAAGCA

TGAGAAATTCACCAGTCAGCTTCAGCTAGGGAAGAAGCCAGTTTGCACTGAGTCTGCCAA

AACACCCAAATCTCCTACAGCCAGCGGCGCAGAGAGCAAAGTATCCGTACCCACTGCGGA

ACCTTCTACCAAAGTGGTGGAACCCTGCAAGACCGAGGACAAAATGCAAGGTGATACTGC

AGCACTGCACCGAGGAACACCGTTGTATGGCCAGCCTTCGTGGTGGGGGGATGGTGACGC

CGATGACGAGAACTCGTTTAAACAGGAGAATAAAGCAGCAGGGAAAAAACAGGAGCCTCA

TGGGACAGACACCAAGGACACCAGGCGGACTGATAAAGCCAAGGAGAATGGTGTGAGCAC

TGCAGGCCCTGAGCCCAGCTACTTTGAGATCCCGAGTAAAGACGGCCAGATGGCAGAGGA

CAGCATCCATGAGGTCCCGACCAAGGACACAGAGAGCAGCGCAGCTGCAGCAGCAGCAGC

AGCAGCAGCGAAAGCCGCCCAAGCGAACACCTCCTTTACTATTGAGTTTGACAACACCTC

TCCAGGAAAAGTAACCATTAAGGATCACGTGTCGCGTCCAAGATCGAAAAAATCTCAGCC

AGGGAGTAAAGAGCTCAGCAGCCTGCAGGCAGCCATCATGGCCTCAGAGAGCAAAGTGGC

CGACTGGCTTGCGCACAACAATCCCCCCATGGTGAGACGGGAGTCCACGGAAGAGGACAG

CAAAAGCATTAAAAGTGATGTGCCTGTTCTTCTCAAAAGGCTAAAAGGCAGCAAGCACGA

GGACGGGACCCAAAGTGACTCTGAGAACGGCCTTGGCTTGCGTTTCGGTAGCCGGCGCCA

TGCTGCTCTGGAGGAACGCTTGAAGGCAGCTGGCCTTACGCATGCCAAAGTCACCGGAGC

AGAGGGCTCGGGCTCCAGCAGCCGCAGCGCCTTCACCATCGAGTTCTTCGACGAGGAAAA

CCCGCGCAAACGCCGCTCCTACTCCTTCTCCCAGACTGCACCACTTCTGGGAGGAGAGGG

ACTGTGTCCAGCACCACCATCCCATCCGAAGGGAACTTCATTAACACTGGCAACAGTAGC

GCCTACAGCAGCACGGCTGCTGCAGAAAAAAAGGTCAGAGGAGAAGACCACGACGGTCGG

AGATGAGACTCAGAAACAAGACGACGATCAGAGCGACAAAGGCACTTATACCATTGAGCT

GGAGAACCACAACCCTGAGGAGGAGGAGGCCCGTCGCATGATCGATAAGGTGTTTGGCGT

GGAGAACCCTCAGAATCCCAGTCGCTCAGTGCAACAGACAACTAAAGACAGTAAGAAGGT

CAGCAGCACTAAAGCGGAGGGTCTTTTGGATGAATCGGTTGCGGTTGGAAGTCCACACTG

GGTCTCTCAATGGGCCACTTTGGCCGCTAATCACACAAGAACTGATCCTGAAGGTTCCGG

GGCAGAACCCACAAACTTTGTTCATCAAGAGCGAGAAGCTGATGCATTTGAGTCAGGAGT

GTCTTTGAGAAGCACCAGCTCTGCCACCTCTAGTCAGGCTGAACGTAAAAGAAGGACATT

GCCTCAGCTCCCCTGTGAGGAGAAGAGCAGGCTGGGGAAGGTCTCCTCCACCTCAGGACT

CCGATCCGAGATTGGCGAGAAACAGGACACCGAACTCCAGGAAAAACAACACAAAGGAGA

TGGCGAATGTGCATCCACTCTCATTAAGAAGGACACTACAAGTGGAATTGCAAACCAAGT

GCAAACAGCCACTACCAAGCCTCCTGTGCCCCCTGTGGGAGGGGTGGAGAAGCAAAGTGA

GGAGTTTCGCAGGAGGCGTGCTGAGGACAGGAACAAAGGTAGCGATAGTGAGCGAAGCGG

GAAGCCCCTGGTGCGTCAAGGCAGCTTCACCATTGACAAGCCAAGCACCAATGTTCCGCT

TGAGCTTATCCCACGCATCAACCAGCGGCAGTCCGGAAACAGAGAGCGTAGTGATTCAGC

TGGAAGCATGGACACGACCACCTTGCTCAAGGACACTGAGGCCGTTATGGCCTTTCTAGA

GGCTAAACTGCGTGATGAGAAAAAGCTGGATCCATCACCGCCCGCTGGCTACCCTTTGGG

ACGCTCTTTTTCCCCAGAGTTGGATACTGATGCAATTAAAGCAGCAGGCCGCGCCGAAGT

GTCACAGAAGCGTCGTTCTCTCAGCAGTATACACAGAGAGAAAAGTAACATGAGCTCCGT

TGGAAAGAGCACAACCAATGCACGGGAACGTTTGGAAAGGAAAGTCAAGCCGAAGACCTC

GGATGGGCGTCTGGATACCCGTCGTTCTACCCATACAAGTCAGCGGACCCGCCAGCCGTC

CATGGACCTCACAGATGATGAGCAGACATCTTCCTTGCCCGTTTCTGACATACTTTCAGA

TGACCAGGAAAACGGACGTAGTCCCTTCACCTTTCCTGATGACTTGCTACTTTCCAAACT

GGACTCTTCTAAAGGTGCTAAAGTTAATAAAGCCAGCAGGACACCACAGGCCACCACTGC

CTCTATAGGCAAGCATCAGTTGCCTCAGCCTCGGCCCACAAGAGCATCTCTTCTGAGACG

TGCTCGACTTGGAGACACATCTGACACAGACCTGGCAGATGCAGACCGAGTGTCAGTTGC

ATCCGAAGTGTCTACTACCAGCTCCACATCCAGGCCACCATCGGGCAGGAAGGCACCATC

CCGCATCGATATGCTTGCACAGCCCAGACGCACCAGGGTCGGGACCATGTCAGCACGCAG

CGACTCAGAAGCCCCAATGTCACGCAGCACTACATCCAGAATATCGGCCGAGACAGCTTT

AAGACTGGGCTTGCGCCCAACTAACCCTGCAGACAGCAAGCTGAACCCACGTCTTCGGGC

AAACAGCGTCTCCAAGTTGACAGACACCAAAACCAAGACTACAGCATCCATCTCCAGTGC

TCCAGCAGCCAGCAGCAGGTGGCGGCGACTTAATACAGAATACGGCTCCACCTCTGAGGA

CGAATTTGGCTCAAACAGGAACTCGCCAAAAGCCGGGCGTCTGAGGCCAGGCACTGCTCT

GCGTACCAGTAAACTGAGCAGCTCAGGCATGGTGACAACAAGCCCTGGCAGCATCACCAT

CAAACACAGGATGAGAGAACAGGAGGAGTACATCAGAGACTGGACAGCCCACAGCGAAGA

GATCGCCAGGATCAGTCAGGACCTAGCCAAGGATCTGGCTATTCTGGCGAGGGAGATCCA

CGATGTGGCCGGCGAGATCGACTCGGTCAGCTCCTCAGGCACAGCACCAAGCACTACGGT

GAGCACAGCGGCCACCACGCCCGGGTCTGCCATCGACACCAAGGAGGAGGTACAGCCCGC

TTCCCTAAAAAAACCTTAGAATAAAGGTTTCGGTAATACTCTGCTAGCCACAGAGACTTT

TTAATCTATCCCAGCTACAGCCAGGATAGCAATCAGATGGGTTTTAAAATATTTCACAGA

GCAGCGGTAACACACTGACTTCCATTCTTCATCAATTCACCCTCTCATTTCATCACTTGT

AGTTGGTAGAACGAGTTTTTGATGAAAGTTTGAACTTTCGGAAGATCCCCCCTGTGGTGC

AGACCAAGACCCCAGAGATGAACGGCCGGCCTGTAGAGCTCCGACCTCGAGCCCCAGACA

GTCTGGATCCCCAGAATGCACTGCGTAGACGCACATGGAACCGAGATGAGGTCAGACCGG

CGGTCCTAGAAAGCTTGCTGCTGAGCTCAGTCTCTCAGCTGTCCTCTAAAATCCGCCAAT

CTGTGGACAAAACAGCAAGCAAAATCAAGATTTTGTTCAAAGACAAAGACAGGAACTGGG

ATGACATTGAGAGCAAACTGAGCTCAGAGAGTGATATGCCACTTCTCAAAACCACTAACA

AGGAAATCTCAACAATTCTAACAGAGCTAAAAAGAGTGGAAAAGCAGATCCAAGCTATAA

ATATCATGGTAGACCCAGATGGAACCCTGGATGCTCTTACCAGTCTTGCCCTAGCAAGTC

CCACCACACCCAAACCTAAGACCAGCAACGGAGCCTCCAAAGCCTCCGTGATCATCTCCC

CGAACCGAGAGCTCCTCAGCGCTGGACTGAGTCGTCCTCAAACCAGCGGAGAAGCGAGCA

GCATTACTAACAAGTAAAGACAGAAAACCCTCAGAGTTGAGTAAACTGAGAAACCCTTTT

TGTCCATGTCTGTACCCCTCCCTCTCCTGCACTCCATTGGAATGTCCATCTTTATGTAAA

TGAAGTCACACCTGTGGATTTGGACAGTGATATACAGTGCAGCTCTACAAGTGCACGCAG

CTGCTGGTGAAGAAGAGCTGAAGAGGAGCATTAGTCTAAAGGCTTAAAATGTTGGAACCA

GCCACCTGTATGCTGCTGCAAAGGTGTACACACTCATCCAGGCATGAAGCAGGTGGACTT

AAAATAATTTATCTTCAACAATAAATGACTCAGAGTGCCTGTCTTCTCTCTTTTGTGAGC

ATTCTTTCTTGCAAATAGCAACTTCTTATTACTACGTGACTTTTGTGTCAAAAATAAAAC

AGTGTTGGTCAAAAAGACTTCAAGATTTCAAAGCTGAGCATAAACCAGCTTAGCAAAGTA

AGTCTGATTCTATGCGGGCACTCAAACATTGTTCTTTTTTTTCCATTTCTTATTTCTTGA

TTACAAAAAGTGCATTTAGGCTATTAAAATAAGCAGGGGTCAACTTGGTAAGTTAAACTG

ACCGTTCATTGCATTATCTTATGCACTGATTTTTTATTTTTATTGGCTAATGTATACAGT

AGGTAGCAAACTTTGTACAAGGTTCAGACATTAATTAAAACCCAGCAAACTGGCGTTTGT

GTCATTTTCCCCCTGACATTAGAAGGAAGATCATATGCAGTATTAGAAAAACGTCTTAGT

GGAATGCACCATTGTAGTGTTACACATCACAGTTGCTTAGATCTCTGTGCTTTGTGCTGA

AATGTTAAATCAGCCTTAAATTAACCAGCCCTTAATGTACGGTTCAATCACATTCTGAAC

ATGTTATGGCCAGTTGCTTAAATGCTAACGTTCTTTAATCAGAGTATGCAATTACAAGAA

CCAAATAAAGTCTGTCAGCATTTTTTTTTTTAATTATCACAACCTCATAGATCATGTTTT

TTGTTCATGCAGGTATTCCGGCATATTATGTATGTACTGTATTGTCTTACTCTTCCGTCG

GCGCTTGCTGCTTGGCTGGAACTCATGCGCCTGAGAAAAAGCGTCCAGCGCCCAGCCTTT

CATCGTGAATCTGCGTGTTGTAGAAAGACTAGTGCAAAATAAAACAATAGTGAAGATAGG

TACAGGACATTAGTTGTTGTTGTTTGTTAATAAATGTCAATGCCAAGTGGCTGCTGTGGA

AACGCTAGTTCCTGGACAGGTTTAAGAAAGAAAATCTTAGTTTAGTGACCGCATTTGTGG

TTTTAAAGGTTCCTGGATTAACTGGGTGAGTTAATGTTAAAGGTGATACAGGACAACAAA

CACCAAACTCTATACATTATACCGCTCGCAGAACAGGATAGATTTTCAGGCAGATGATCC

TCCTGCCCCTAAAACACTTAAAAAAAACAGTAGTTATCATTTATAAAACTATTGTACATG

TTTTACATATAAGGGG

>Unigene000164

ACGACCCACCATGACCCACTAAAACACCACACCCCACCCCAGCCGCTACCGCTAACCCCA

ACGCTGCAAAATAGGGGGCAGGATTAGAGGCAACTCCAACTAAACCGACCACCAAACTTA

ACAAAAGCAAGTCAAAAAAATACATAATTCTCACCAGGATTTTAACCAGGACTAATGACT

TGAAAAACCACCGTTGTAATTCAACTATAAGAACTAATGATTACCCGAAAAACGCATCCT

TTATTAAAAATTATTAACGATGCTATAATTGACCTACCTGCCCCCTCCAATATCTCTGCA

TGATGAAACTTTGGCTCCCTCTTATTACTCTGCTTAATAACACAAATCCTCACCGGCCTA

TTTTTAGCTATACATTACACCTCAGACATCTCAACTGCCTTTTCATCAGTAACTCACATC

TGCCGAGATGTAAACTATGGATGAGTTATTCGAAACCTACATGCAAACGGTGCATCCTTC

TTCTTCATTTGTATCTATTTCCACATTGGACGAGGACTATACTATGGGTCATACCTTTAT

AAAGAAACCTGAAACATTGGAGTAGTATTGTTACTTTTAGTAATAATAACTGCATTCGTA

GGATATGTCCTACCATGGGGTCAAATATCATTCTGAGGCGCCACAGTAATCACAAACCTA

CTATCTGCCGTGCCCTACATAGGAGATGCCCTAGTACAATGAATCTGAGGTGGATTCTCC

GTAGACAACGCAACCCTCACCCGATTCTTCGCATTCCACTTCCTTCTCCCATTTATAATT

ATTGCAGCCACAGTACTACATGCCTTATTCCTACATGAAACAGGCTCCAACAACCCTGCC

GGCCTAAATTCCGACACAGACAAAATTCCATTCCACCCATACTTTTCCTATAAAGACATT

CTAGGATTCATCGTGCTAATAACAACTCTAGCATCTCTAGCATTATTCTCACCCAACCTC

CTGGGTGATCCAGAAAATTTTACCCCGGCCAACCCCCTTGTCACTCCCCCTCACATTAAA

CCAGAATGATACTTTCTATTTGCCTATGCCATTCTACGATCTATCCCAAACAAACTAGGA

GGCGTCCTAGCATTGCTATTCTCAATCCTAGTACTAATACTAGTACCAATGCTACACACA

TCAAAACAACAAGGAATAACCTTCCGACCTCTAACCCAACTCCTATTTTGAACCCTAGTT

GCAGACGTCCTCATCCTGACCTGAATCGGAGGCATACCAGTTGAACATCCATTCGTTATT

ATTGGCCAAATCGCCTCAATTCTATACTTCACATTGTTCCTAATCCTGTGCCCAATAGCA

GGTTGACTAGAAAACAAATTACTCAATCTAAACTGCCCTAGTAGCTTAGACATAAAGCGC

CGGTCTTGTAATCCGGAGATCGAAGGTTAGACTCCCTCCTAGTGCCAGAAAAGAGAGATT

TTAACTCCCACCCCTAACTCCCAAAGCTAGGATTCTAAAACTAAACTATTTCCTGAAAAC

AGTAATTGCTCGACTTCATTAAGTGCTCTATGCTCTAGTACATAATATGCATAACTCAAC

ACCATGTTATAGTACATAATATGTATAATTTTACATGATGGTGTAATACATTAAACCAAG

CCCAACATCAAGATTATTCAAAACATTGCTGCCAACAACATAAAACTAATTACGCAAAAA

CATATAATGGTTTTATAAATGTCAAATTGTTTAAAACAAACATATTAAATCAAGATTAAC

CACGTATGGCTTACATTAATCAGACATAAAGGTATTAATCAAATTAAACTGAGTTAAATA

AAGTAATTGGGGTAGTGAGAGACCATCATCAGTTGATACCTTAAGGTACAACGTCCTTGA

TAGGGTCAAGGGCAAGAATTGTGGGGGTCGCACAAAATGCACTATTACTGGCATCTGGTT

CCTATTTCAGGGCCATACATTTATATTCCCCCAAAACTTGAATTGTCGCCGGCATAAGTT

AATGGTGTCGTACTTTATTAGCAAAAACCCCCCATGTAAACATCCATCCTAAAGGGCATG

GGTATCTTTTTTTTTTTCGGTGGATTTCATATACATCTCCAGTGGTTTGGCCCAATGATT

TATCAAGGTGGTCCTACCAAATGTATCTATCCAAAGGTTAATGCATGATTTAATGACATA

TCCTTAAGATTTACATACTCTTAACCTGGTACATAACTTTATCCTTGTTCCACATACTAC

TCTAAGATTCCCCCCCGGCTCGCGCGCGTCAAACCCCCCTACCCCCCTAAGCCGAAGAGT

CCTTAATTAATCCTGCCAAACCCCTAAAACAGGTAAGGCTCGATCGGCGTCATAAGCAAG

TTCAGGTATGTGCTCATATATAGTGTTACAAATTCGCATCATATTATATAGCTAGTGTAG

CTTAAATTAAAGCATAACACTGAAGATGTTAAGATGAACCCTAGAAAGTTCTACAGGCAC

AAAGGCTTGGTCCTGACTTTACTATCAGCTCTGACCCAATTTATACATGCAAGTATCCGC

ATCCCCGTGAGAATGCCCTCAATCCTCTGCCCGAGAACGAGGAGCAGGCATCAGGCACAA

TCTAACTAGCCCAAAACGCCTTGCTACGCCACGCCCTCAAGGGACTTCAGCAGTAATAGA

CATTAAGCCATAAGTGAAAGCTCGACTTAGTTAGGGCTAAAAGGGCCGGTAAAATTCGTG

CCAGCCACCGCGGTTATACGAAAGGCCCAAGTTGATAGATACGGCGTAAAGGGTGGTTAA

GGGAACTAAAAATAGAGCTAAAGACCCTCTGAGCCGTTATACGCACCCCGAGGGCACGAA

ACCCAAACACGAAAGTAGCTTTAAACAAAATTCACCTGATCCCACGAAAGCTAAGAAACA

AACTGGGATTAGATACCCCACTATGCTTAGCCTTAAACCTAGATGTTAAACTACAAACAC

ATCCGCCAGGGCACTACGAGCACAGCTTAAAACCCAAAGGACTTGGCGGTGTCCCAGACC

CACCTAGAGGAGCCTGTTCTATAACCGATAACACCCGATAAACCTCACCACTTCTTGTTT

ATCCCGCCTATATACCGCCGTCGTCAGCTTACCCTGTGAGGGCCTAATAGTAAGCAGAAA

GGGACCACCCAAAAACGTCAGGTCAAGGTGTAGCGTACGAAGTGGGCAGAAATGGGCTAC

ATTTTCTACAACAGAACAATACGGACAGCACCCTGAAATATGTGCTTAAAGGTGGATTTA

GTAGTAAAAAGAAAATAGAGAGTTCTTTTGAATTAGGCTCTGAGACGCGCACACACCGCC

CGTCACTCTCCCCTACTCCTATTATTAAAAATCCATTCATAATAATTTAATTATCAACGC

GGGGAGGCAAGTCGTAACATGGTAAGTGTACTGGAAAGTGCACTTGGACGAACCAGAGTG

TAGCTTAACACAAAGCACCCAACTTACACTTAGGAGATTTCAACTTAACTTGACCGCTCT

GAGCTAAACCTAGCCCCAAACCCACTCCACCTTACTACCAGACAACCTTAGCCAAACCAT

TTACCCAAATAAAGTATAGGCGATAGAAATTGAAACCTGGCGCAATAGATATAGTACCGC

AAGGGAAAGATGAAAAATTATAACCAAGCATAATATAGCAAGGACTAACCCCTATACCTT

CTGCATAATGAATTAACTAGAAATAACTTTGCAAGGAGAGCCAAAGCTAAGACCCCCGAA

ACCAGACGAGCTACCTAAGAACAGCTAAAAGAGCACACCCGTCTATGTAGCAAAATAGTG

GGAAGATTTATAGGTAGAGGCGACAAACCTACCGAGCCTGGTGATAGCTGGTTGTCCAAG

ATAGAATCTTAGTTCAACTTTAAATTTGCCCACAGAACCCTCTAAATCCCCTTGTAAATT

TAACTGTTAGTCCAAAGAGGAACAGCTCTTTGGACACTAGGAAAAAACCTTGTAGAGAGA

GTAAAAAATTTAACACCCATAGTAGGCCTAAAAGCAGCCACCAATTAAGAAAGCGTTCAA

GCTCAACACCCACTACCTAAAAAATCCCAAACATATAACTGAACTCCTCACACCCAATTG

GACCAATCTATCACCCTATAGAAGAACTAATGTTAGTATAAGTAACATGAAAACATTCTC

CTCCGCATAAGCCTGCGTCAGATTAAAACACTGAACTGACAATTAACAGCCCAATATCTA

CAATCAACCAACAAGTCATTATTACCCTCACTGTCAACCCAACACAGGCATGCTCATAAG

GAAAGGTTAAAAAAAGTAAAAGGAACTCGGCAAATCTTACCCCGCCTGTTTACCAAAAAC

ATCACCTCTAGCATCACCAGTATTAGAGGCACCGCCTGCCCAGTGACACATGTTTAACGG

CCGCGGTACCCTAACCGTGCAAAGGTAGCATAATCACTTGTTCCTTAAATAGGGACCTGT

ATGAATGGCTCCACGAGGGTTCAGCTGTCTCTTACTTTTAACCAGTGAAATTGACCTGCC

CGTGAAGAGGCGGGCATAACACAGCAAGACGAGAAGACCCTATGGAGCTTTAATTTATTA

ATGCAAACAGTACCTAACAAACCCACAGGTCCTAAACTACCAAACCTGCATTAAAAATTT

CGGTTGGGGCGACCTCGGAGCAGAACCCAACCTCCGAGCAGTACATGCTAAGACTTCACC

AGTCAAAGCGAACTACTATACTCAATTGATCCAATAACTTGACCAACGGAACAAGTTACC

CTAGGGATAACAGCGCAATCCTATTCTAGAGTCCATATCAACAATAGGGTTTACGACCTC

GATGTTGGATCAGGACATCCCGATGGTGCAGCCGCTATTAAAGGTTCGTTTGTTCAACGA

TTAAAGTCCTACGTGATCTGAGTTCAGACCGGAGTAATCCAGGTCGGTTTCTATCTACTT

CAAATTCCTCCCTGTACGAAAGGACAAGAGAAATAAGGCCTACTTCACAAAGCGCCTTCC

CCCGTAAATGATATCATCTCAACTTAGTATTATACCCACACCCACCCAAGAACAGGGTTT

GTTAAGATGGCAGAGCCCGGTAATCGCATAAAACTTAAAACTTTACAGTCAGAGGTTCAA

TTCCTCTTCTTAACAACATACCCATGGCCAACCTCCTACTCCTCATTGTACCCATTCTAA

TCGCAATGGCATTCCTAATGCTTACCGAACGAAAAATTCTAGGCTATATACAACTACGCA

AAGGCCCCAACGTTGTAGGCCCCTACGGGCTACTACAACCCTTCGCTGACGCCATAAAAC

TCTTCACCAAAGAGCCCCTAAAACCCGCCACATCTACCATCACCCTCTACATCACCGCCC

CGACCTTAGCTCTCACCATCGCTCTTCTACTATGAACCCCCCTCCCCATACCCAACCCCC

TGGTCAACCTCAACCTAGGCCTCCTATTTATTCTAGCCACCTCTAGCCTAGCCGTTTACT

CAATCCTCTGATCAGGGTGAGCATCAAACTCAAACTACGCCCTGATCGGCGCACTGCGAG

CAGTAGCCCAAACAATCTCATATGAAGTCACCCTAGCCATCATTCTACTATCAACATTAC

TAATAAGTGGCTCCTTTAACCTCTCCACCCTTATCACAACACAAGAACACCTCTGATTAC

TCCTGCCATCATGACCCTTGGCCATAATATGATTTATCTCCACACTAGCAGAGACCAACC

GAACCCCCTTCGACCTTGCCGAAGGGGAGTCCGAACTAGTCTCAGGCTTCAACATCGAAT

ACGCCGCAGGCCCCTTCGCCCTATTCTTCATAGCCGAATACACAAACATTATTATAATAA

ACACCCTCACCACTACAATCTTCCTAGGAACAACATATGACGCACTCTCCCCTGAACTCT

ACACAACATATTTTGTCACCAAGACCCTACTTCTAACCTCCCTGTTCTTATGAATTCGAA

CAGCATACCCCCGATTCCGCTACGACCAACTCATACACCTCCTATGAAAAAACTTCCTAC

CACTCACCCTAGCATTACTTATATGATATGTCTCCATACCCATTACAATCTCCAGCATTC

CCCCCC

>Unigene000165

AAGAGAAAGACCTAAAAGACAAGAGGTTGGTGAGCATTCCAGATCTCCTGTCCGCCATTA

AACTTCTGTGCATGAGGTTCCAGCGCGAGCTGGTCACCGTGGTGGATGACCTGAGGCTGG

ACATCCTACTGCGGATGCTTAAGACCCCTCATTTCAGCGCTAAGATGAACTCCCTCAAAG

AGGTCACCAAGCTGATAGAGGAAAGCACAGTGTCTAAAACAGTGAAAAACGCCATCGACA

CAGACCGGCTCCTCGACTGGCTGGTGGAGAACTCTGTCCTCTCGATAGCACTGGAAGGAA

ACATTGACCAGGCGCAGTACTGTGATAGGATAAAGGGAATCATTGAACTGTTGGGCAGTA

AACTCTCACTGGATGAACTTTCAAAGATCTGGAAAATACAGTCTGGTCAGTCCTCCACCG

TCATCGAGAACATCCACACCATCATCGCCGCAGCAGCAGTCAAGTTCAACAGCGAGCAGC

TCAATCATCTGTTTGTCCTCATACAAAAAAGCTGGGAGGTCGAGTCCGATCGAGTCAGAC

AGAAGCTTTTAAGTCTGATCGGGAAGATTGGCAGAGAGGCACGCTCCGAGACAACAACAG

GAAAGGTGTTGGAGGTGCTTTGGGAGTTGGCCCATCTCCCTACTCTTCCCACCACTTTGG

TCCAGCAGGCCCTGGAGGAACACCTGACTATCCTGAGCGATGCCTACGCTGTCAAAGAAA

CTGTTAAGAAAAGCTACATCATCAAGTGTATCGAGGACATTAAGAAGATACAAATTCAGG

AGGACTCAAAGTCTACCAGCGAGATACAGGTGCCTTTTTTTACTGGCTCCTGGGGCAAGA

AGAAACCAGCCTGGGTTAAATCATCCCAACAGAGTAACCCCCAATCAGTATGGGTTGTTC

CAGCCCTACGGCAGCTCCATGAGATTACTCGCTCCTTCATCAAGCAGACATACCAGAAAC

AGGATAAGAGTATAATTCAAGACCTTAAGAAGAATTTCGAGATTGTAAAGCTGATAACTG

GTTCTCTAGTGGCATGTCATCGACTGGCTGTGACAGCCGCTGGCAACAGTGGTCTCTCAG

GGTCCACTCTGGTGGATGGACGATACACTTATCAAGAGTATTTGGAAGGACATCTGAAGT

TTTTGGCCTTCTTTCTACAAGAAGCCAGTCTGTATTTGGTGTGGAACAGAGCTAAAGAGA

TCTGGGAGTGCTTAGTGTCTGGCATTGACGTGTGTGAGCTGGACCGGGAGATGTGTTTTG

AATGGTTCGCTAAAGGGCAACATGATCTTGAAAGTGATGTTCAGCAACAACTTTTCAAAG

AGAAGATCCTTAAACTTGAGCCTTATGAGATTACTATGAACGGGTTTAATCTGTTCAAAA

CCTTCTTTGAGAATGTGAATCTATGTGATCATCGTCTCAAACGGCAGGGAAATCAGCTGT

GTGTGGAGCGTTTGGATCTAATCGGGATGGATTTCATCTGGAGGATTGCCATGGAGTCTC

CTGATGAGGACATTGCCAATGAGGCCATTCATCTTATCATCACCTACAGTTATATAAATA

TCAATCCCAAAATGAAGAAGGATTCAGTGTCCTTACATAAGAAGTTCATAGCTGATTGTT

ATAAGAGGTTGGAGGCTGCCAGCTCTGCTCTGGGTGGCCCAACCTTAACCCATGCTGTTA

CGCGAGCAACCAAAATGTTGACAGCTACTGCCATGCCAACTGTTGCCACATCAGTGCAAT

CACCCTCTAGGTCTACTAAGCTGGTAATCATTGAGAGGCTGCTGTTACTGGCTGAGAGAT

ATGTCATAACTATCGAGGATTTGTACTCTGTTCCTCGGACTATTCTACCTCATGGAGCTT

CTTTCAATGGCCATCCTGTAACTCTCCACATAACCTACGAATCTACCAAAGATACTTTTG

TCCTGGAGGCACACAGTAATGAGACAGTTGGGAGCATCAGGTGGAAAATTGCAGAGCAGC

TTAGCTGCCCAGTGGACAACGTTCAGATATTCGCCAATGACAGCATGCTGACCATGAACA

GGGATCAGAAGCTGTTGAATCAGCTGGGTTTCTGTGATGAGCAGAGTCTGACAGTGAAGA

GTTCGGGAACCGGCACTCCATCTGGCAGCTCTGCAGACAGCTCGGCCTCCGCCAGCTCCA

GCAGCAGTGGAGTCTTTAACTCAGCATATGCACTGGAGCAGGAGAAATCTCTGCCTGGCG

TCGTGATGGCGTTAGTATGTAACGTGTTTGAGATGTTGTATCAGCTGGCTAACCTAGAGG

AACCTAGAATAACTTTGCGTGTGCGGAAGTTGTTGCTGCTTATCCCGACTGATCCCGAGG

TGCAGGATGCCTTGGATAACTTTGTACCAAAGGAGTCCAGTGTGTGGAGCCACCAGAAAA

CTCTGTTCACTTTGGGTGCTGGATCTCAGGCCTCCAAGTCTCCATCTTTATCCAGCAAGC

AGCAGCATCAGCAGAGTGCAGCATCCATCCTGGAGTCGTTGTTTCGCTCCTCTGCCCCGG

GCATGTCCACTTTCCGAGTGCTTTATAATCTTGAGGTGCTAAGCTCTAAATTGATGCCTA

CCTCAGATGATGAGATGGCAAAGACTAGTGTTAAGTCTTTCTGTGAGAATTTTCTGAAAG

CCGGAGGTCTCAGCCTGGTGGTTAATGTCATGCAGAGGGACTCCATTCCCTCTGAAGTAG

ACTATGAGACCAGACAGGGAGTCTACTCTATCTGCCTGCAACTTGCCAGATTCTTACTAG

TAGGACAAACAATGCCAAGCATCCTGGATGAAGAGCTTAGTAAAGAGGGCATAGACACAC

TGTCCTCACGACCCTTTCGTAACGCCAGCAGGCAGAGTGGACGTCAGCTGTCGCTCTGTG

GGACACCCGAGAAATCCTCCTACAGACAGATGTCTATGTCTGATAGATCTTCTATTCGGG

TGGAGGAGATTATACCCGCAGCTCGGGTTGCCATCCAGACAATGGAGGTGGGGGACTTTA

CTTCTACAGTGGCCTGCTTTATGCGCCTTACCTGGGCTGCAGCTGCTGGCCGATTGGACT

TGGTGGGCAGCAGCCAGCCAATCCGAGAAAGCACCAATTCCCTGCTGCCTCTGGGAGTCC

GTAGCAGGGTTAGCAGCACAGGCAGTAACTGCAGTTCCAGCAGTGAAGGAGAAGCCACCA

CATTGCAGGCTGGCATCTGTGTTAAACAACAGTGTGTTTCCATCAAAGACACGATCATCG

CCCGGGAAGCACTTTCACTATTGGTCACCTGTCTGCAGCTTCGCTGTCAACAGCTTTCTT

CTTTCTATAACCTGCCCTGTGTAAATGATTTTATCATCGACATTCTGCTGGGCTCATCCA

GTGGAGAGATCCGGCGGGTGGCATGTGATCAGCTGTACACCTTGAGTCAGAGTGATACAT

CAGCGTATCCTGAGCTGCAGAAGCCCAATCTGTTCCTGTTAAAGGTGGTTTTGGCTGCTC

AGCTCCCGCTCTGGTCTCCCACCAGTGTCATGAGGGGCATCAATCAGAGGTTGCTTTCAC

AGTGCACTGAGTATTTTGACCTGCGATGCCAGCTATTGGATGATCTGACCACTACGGAAA

TGGAGCAGCTGCAGATCAGCCCAGCAGCCATGTTGGAGGATGAGATCAGCTGGCTGGATA

ACTTTGAGCCAACGTGGACCAGCGAGCTGGAGACCAGCGGGGCTGACAACATCCTGCAGG

CCGGACATCTGCGCCTAATTAAGACTCTGCTCTCGCTCTGCGGCAACGAGAAGGAGCAAT

TTGGTGCAGTACAGTCAGCAGTCGATTAGCAGCATATGAGGTGTTGGTGATGTTGGCTGA

CAGCTCTATCACCAACTTACAGCTTATCACTCGAGAACTTCTCTCTATGCACCACCAATC

TGACCCGTCTCTCTCTAAAGAATTTGATTACCTCCCACCAGTGGATGGCCGCTCCATATC

AGGGTTTGTGGGGCTAAAGAACGGTGGAGCGACCTGCTACATGAACGCTGTCTTTCAGCA

ACTCTATATGCAGCCTGGCTTACCCGAGGCCATCCTGTCGATAGAAGATGACGCAGAGAA

CCCGGAGGAAAGTGTATTCTGTCAAGTGCAGTCCCTGTTTGGCCATCTGATGGAGAGCAA

GTTGCAGTACTATGTGCCTGAGAACTTCTGGAAGATTTTTAAGATGTGGAACAAGGAGCT

GTATGTGCGAGAGCAGCAGGATGCCTATGAGTTCTTCACCAGTCTGGTGGACCAGTTAGA

TGAACACCTAAAGAAAATTGGAAGAGAGCAGATCTTTAAAAATACATTTCAGGGCATTTT

CTCTGACCAGAAAATATGCAAAGACTGTCCACACAGATACGAGCGTGAGGAAGCCTTTAT

GGCACTGAACCTTGGGGTGACCTCATGTCAGAGTTTGGAGATTTCCCTGGATCAGTTTGT

TCGTGGAGAGGTGCTGGAGGGAAGCAATGCTTACTATTGTGAAAAATGCAAAGAGAAGAG

AACTACAGTAAAGCGCACTTGTATTAAGACGTTACCCAGTGTCCTGGTTATCCATCTTAT

GCGCTTTGGCTTCGACTGGGAGAGCGGACGCTCCATTAAATATGACGAACAGATAAGATT

CCCCTGGGTGCTAAACATGGAGCCGTACACAGTATCTGGCATGGCTCGACAGGACTCCAG

TTCAGATCATGGCGAGAACAGTCGGGGGGGAGAGGCTGGACCTGGAGGCTCTCCGCGCAA

AAAAGTTACTATTTCTGAGAACTATGAACTGGTGGGAGTGGTGGTACACAGCGGTCAGGC

GCACGCAGGACACTACTACTCCTTTATCAAAGACCGCAGGGGCGGTTCGCGAAGCAGATG

GTACAAGTTTAACGATAATGTTGTGGAAGAGTTCGACATGAACGATGAAACCCTGGAGTA

CGAGTGTTTTGGTGGAGAATACAGACCCAAAGTCTACGATCAGTCGAACCCATATCCAGA

TGTGCGGCGACGTTATTGGAATGCCTACATGCTATTCTACCAGCGCATCAGTGAGCAGAA

CTCTCCTGTGCTGCCGAAAAAGAGCCGTGTCAGCGTCATGAGACAGGAAGCCGAGGACCT

TACTCTGTCTGCACCTTCCTCCCCTGACATTTCACCCCAGTCATCTCCACGACCCCCCAG

AGCCAATAACGACAGACTGACTCTGCTAACCCGGCTGGTCAGGAAAGGGGAGAAGAAGGG

CTTGTTTGTGGAGAAGATGCCTGCCAGGATCTTTCAGATGGTGAGGGATGAAAACCTAAA

GTTTATGAAGAACAGAGATGTGTACAATAGTGATTACTTTAACTTCACCTTGTCACTAGC

TTCTGTCAACTCGACGAAATTGAAGCATCAATCCTATCAGGCCATGGCGAAGACCAGCCT

GCAGCTGGCTGTGCAGTTCCTGTTCCACACTTACCTGCACACCAAAAAGAAGCTCCGGGT

TGATACAGAGGAGTGGATAGCTACAGTGGAGATGCTGCTGTCTAAGAGCAATGAGGCGTG

TCGCTGGATGGTGCAGTACCTGGTGGGAACTGAAGGACGGGAGATTATCAAGAACTGTCT

GTTGGAGTGTAATGTACGGGAGGTGCGTGTAGTGGTGGCCTCCATCCTCGAGAAAACTCT

AGAGAGTGCACTCTGTTTCCAGGACGCAGGACTGGACTGCCTTTTAGACATGCTGCTCTC

TTTGCTTGACAAGGACGTACCGGAGAACTGCAAGAACTGCTCACAGTACTTCAGCCTGTT

CAGTAACTTTTCCCAGAGGGGGTTTGGGCCTTGTCAACTGCTGTTAAAGCATGCTGCTTA

TCGAAGGATGCTCATCTTCCTGCTGGGTCCGAATAGACAGAACAATCAGAATCGTCGCTG

GAGTTCAGCTCAGGCTCGAGAGTTCCTTCACTTGCATAACACGCTTGCCTTGATAGTGCT

ACACATGGACCTCAAATCTCAAAGAACAGAGACTCCTGGGGTGTTTAAGCATAACTCGTC

TGTCCTGGTGTCATCAACCACTCTTCTGCCACTGCACCCAGATGTCAACACACTGCTGTT

CAA

>Unigene000166

GGGGGATCATCTCAACTGTGTACAGCTTCTACTGCACCATGACGTACCTGTAGATGACGT

CACAAACGATTACCTGACAGCGCTGCATGTGGCCGCCCACTGTGGTCACTATAAAGTGGC

GAAGGTCATCGTGGATAAGAAAGCCAACCCCAATGCCAAGGCACTGAATGGTTTTACGCC

ACTTCACATCGCCTGCAAGAAGAACAGGATCAAAGTGATGGAGCTGCTGCTGAAACATGG

AGCGTCCATACAGGCAGTGACAGAGTCTGGACTCACGCCGATTCATGTGGCCGCCTTTAT

GGGGCATGAAAACATAGTGACTCAGTTGATGAATCATGGTGCATCGCCAAACACAACCAA

CGTGAGAGGAGAGACTGCGCTCCACATGGCCGCCAGGGCTGGACAGGCGGGTGTAGTGAA

ATACCTGGTGCAGAACGGAACTCACGTCGACGCCAAATCTAAGGATGACCAGACTGCGCT

GCATATCTCTAGTCGTCTGGGAAAGCCCGAGATTGTACAGCAGCTGCTGCAGCATGGAGC

CGCACCCGATTCCACCACCACATCCGGATACACACCATTGCACCTGGCAGCCCGTGAAGG

CCACAAAGACGTCGCCGCTATACTGCTGGACCAGGGCGCTTCACTGAGCATCACTACCAA

GAAGGGATTCACGCCGCTACATGTGGCAGCTAAGTACGGGAAAATAGAAGTCGCCAACCT

CCTGCTGCAGAAGAGAGCTCCTCCAGATGCGTCGGGAAAGAGCGGACTAACTCCACTGCA

TGTAGCTGCGCACTACGATAACCAGAAAGTGGCACTTCTTCTCCTGGACCAGGGGGCTTC

TCCCCATGCAGCTGCCAAGAATGGCTACACTCCTCTACATATAGCTGCCAAGAAGAATCA

GATGGAGATCGCTACCACACTGCTGGAATACGGCGCTGATACCAACGCGGTCACTCGGCA

GGGCATCAGTCCGCTGCACCTGGCTGCTCAGGAGGGCAACGTGGACATGGTCACCCTACT

GCTTGCACGTGATGCCACCATCAATCTGGGTAACAAGAGCGGACTCACTCCCCTTCACCT

GGTGGCCCAGGAGGATAAAGTAAATGTAGCTGAAGTCTTGGTTAACCAGAGTGCTGCTGT

GGACCCTGAGACCAAGATGGGCTACACTCCGCTGCATGTCGCCTGTCACTATGGCAACAT

AAAGATGGCGCACTTCCTCCTGAAGAACCAGGCTAAGGTCAACGCCAAAACGAAGAACGG

ATACACTCCTCTGCACCAGGCTGCACAGCAAGGCCACACACACATCATTAACCTCCTCCT

GCAGCACGGAGCCTCGCCCAACGAGCTGACGCTGAATGGAAACACAGCACTGTCCATTGC

CAGGCGACTGGGTTACATTTCAGTAGTGGACACCCTCAAAGTGGTGACAGAAGAGACCCT

GACCACCATGACGGTGACTGAGAAGCACAAGATAAACGTCCCAGAAACAATGAACGAGGT

TCTGGACATGTCTGATGATGAAGTTCGTAGAGCCAATGTCCCCGAAATGCTCAATGAGGA

CTATATTTCAGATGTTGAAGAAGGGGAGGATGTGATGATGGGGGACACGGATAAATACCT

GGGTCCCCAGGATCTGCGTGAGCTAGGGGACGACTCTTTGCCTCAGGAAGGTTACGTAGG

ATTTAGCATTGGAGCCCGATCCGGAAGCCTGCGCTCCTTCAGTTCGGATAGGTCCAACAC

ACTGAACCGGAGCTCTTTCACCAGGGACAGCATGATGATCGAGGAGATCCTGGCACCCAG

CAAGGACACGCATCTGACTGTAGCTAAAGACTATGACACTGACTCTCTGAGGCGATACAG

TTGGACAGCAGATGCTTTGGACAATGTCAACTTGGTCTCCAGCCCAGTCCATTCAGGGTT

CCTAGTGAGCTTTATGGTGGACGCTCGTGGTGGCTCCATGCGAGGCAGCCGCCACAATGG

GATGCGCATCATCATCCCACCACGCAAGTGCACTGCCCCGACTCGCATTACCTGCCGCCT

GGTCAAGCGACACAAGCTTGCCTCGCCACCACCCATGGTGGAAGGAGAAGGACTGGCAAG

CCGTCTGGTGGAGGTGGGCCCTGCCGGAGCTCAGTTTCTCGGCCCTGTGATAGTGGAGAT

CCCTCACTTTGGCTCCATGAGAGGGAAGGAGCGTGAACTGATCATTCTCAGAAGCGATAA

CGGAGAGACTTGGAAAGAGCACCAGTATGACTGCAAGACCGAAGCCCTTAACGAGCTGCT

TAACGGCATGGATGAAGAACTGGACTGCATAGAAGACCTGGAGAAGAAACGCATCTGTCG

AATCATTACAAAAGACTTCCCACAGTATTTTGCCGTGGTGTCACGGATCAAGCAGGAGAC

CAACCATATGGGTCCTGAGGGAGGGGTTCTATCCAGTACGACTGTTCGCAAAGTCCAGGC

TTCTTTCCCTGAAGGAGCGCTCACAAAGAAAATCAGGGTTGGACTTCAGACCCAGCCGGT

TCCAGACGAAACAGTAAAGAAAATCATAGGCAACAGAGCCACGTTTAGCCCCATAGTGAC

AGTGGAACCACGCAGAAGGAAGTTCCACAAACCCATCACCATGACTATTCCTGTACCTCC

TCGATCAGGGGAAGGCATGAACGGCTACAAAGGAGACACAACACCTTGCTTACGTCTTCT

GTGTAGCATCACAGGTGGCACCTCCCCCGCACAATGGGAAGACATCACTGGGACTACTCC

GCTCACCTTCACGAATGACTGTGTCTCGTTCACTACCAACGTGTCTGCTAGATTCTGGCT

CGCAGACTGCCATCAGATTCCGGAGACAGTTTCTTTGGCTACTCAGCTCTATAGGGAACT

CATCTGTGTGCCCTACATGGCTAAATTCGTAGTGTTTGCCAAAATGAATGACCCTGTGGA

ATCCCATCTGCGATGCTTCTGCATGACGGATGACAAAGTGGACAAAACCCTGGAGCAGCA

AGAAAACTTTGAAGAGGTGGCCAGAAGCAAAGACATTGAGGTTTTGGAAGGAAAGCCTAT

CTATGTGGACTGCTACGGCAATTTAACCCCTTTGATTAAAGCCGGACAGCAGCTGGTTCT

TAACTTTTATGCATTTAAAGAGAACAGGCTGCCATTCTGTGTAAAGGTGAGGGACAACAG

CCAGGAACCGTGCGGACGGCTGTCCTTCCTCAGAGAGATTAAAAACGCAAAGGGGCTTCC

CCAAGTTGCTGTCTGTAATTTAAACATCACTCTGCCCGCGCTCAAAAAGACTGAAAAGCC

AGAACGACGTCATACCTTTGCATCTCTAGCCTTACGTAAGCGGTACAGCTATCTGACTGA

GCCTGGATTGAGCCCTCAAAGTCCTTGCGAGAGGACAGACCTGCGAATGGCTATAGTGGC

TGATCATCTCGGCCTTAGCTGGACTGAGCTGGCCAGGGAAATGGATTTCTCTGTTGATGA

GATAAATCACATCCGCGTGGAGAACCCCAACTCGTTGACCGCTCAGAGTTTCATGCTATT

AAAGAAATGGGTCAACAGAGACGGTAAAAATGCCACAACGGATGCCTTAACTGCAGCACT

GACAAAGATCAATCGGATGGATATTGTGACATTGCTGGAAGGGCCAATATTTGATTATGG

TTACCACAACATCAGTCTGGAGTTGCAGAGTCCCTCAGAGTTGTCCTATGAACCCCCCAC

CCCTCTCCGGCAGGATGATTTCTTTAGCGAGGATGGGGGTATTGTAGTCTCGCCCAGTAG

AACCCCTGTTAGACCAAATGAACTGTCCTTACCAACCACATCTAGGGATGCGCTTTCCCC

CAATGCAGCACCTCCTACTGTGGTGGCAGAGGATTCCTCTATTGGAGGAAGAGGCTCCTA

TAGTCGAGAAGGCGGCTCAACTGAAGACGACCTGGACGCCCATGACAGTGATGAGATGCC

TAAACATCCTGAAGTGTTCATGCAGAGCACATACCAAGAGCAAGAGGTTCCTTCCAACGG

AGCTCACAGGGCTAGAGAAAGTGGCAGGTCTGGTTTTGACGAGGAAGAGGAGGAAGAGAT

GACGCAGGAGAAGTCCTGTTTTAAAAGCATAAACTTGGAGGAAGGTTTAGAGATTGTGGA

GATACCCCAAGAAAAAGTGCAGGAAATTCGCTCACAAGTGGAACAGGCAAAAAAAGAGAT

GTGTTCGCTTGCAGGATTGCAAGAAGGGCCGTCCAGTGTGAGCCAGGAGATCTCCGCTTC

GGGTCTGCACACTGATACCCAACTGCAAGATAGAAGTCAAAAGCCAACTGAAAGGCAAAA

TGGGGAACATCCAGAGCTCCAAGGCCGAGCCGGCTCACTGCTTGAGCCACAGGAACCATC

TAGTAAGATGAAGGGCAAGGGCAGCAGAGACTCCTTAGATGACACAGCTGCTGGGCCGAG

CACCATGGACGAAGACACAGTGGAGCCGAAAGCTAAGGTCGGAGAGGGAGACAAGAAGAA

AGAAGGGAGACAACGAGGGAAAAAGGGTCAATCGTAACAGACGGGTGTCCTAAATGAGAT

CTGAACTTGTTTCATCATTGTTTTCCTGCGAAAAGCATCACTGGAAACCCAACGCAGGAG

ATCAGGACAGGGGATTGTGCGTCTGCAGCCTTTACATAAACGCATGGTTTTTAGAACGAG

GATTTTATGATTTCGCATGAGCATGGAAAACCTCCGATTTTCTGTCAACGACACATCCAA

GCATCTGATCGGTGCCAAGAAATGACACTTAAGCTCAAGACTGTCGGTGGCATAAAGCTT

CTAGTCAGTCCTATACATTGGAGGAGTGCCAGCGACTACCAACATGGCACCGAAGCATTC

CATGACCAAAGATGGTACAATGAGCATTGTGGAATGATTATTTTTTAAAAGAAAAAAAAA

AAAAAAAACCACCTTCTACACTCGCCTTATTTTCTCTCCCCCCACCCTCCCCCTCGCTTC

TCGGACTAGCGTCGACGCTGTTTTTAAAAAAGATCCTGTATATAAATCAGAACTGTATGA

AAGTAGCTCACGAAATTGTGAACGCAGGAGCAAAGAGCGAGTCACAAACGGAATGAAAAA

AAAAAAGAAGAAGAAAGGAATGCTGATATAACTATTCCGACTTTAAAGATCATTTTAAAG

ACGAATTGATTACACAAAAAAGCGAAAAGAAACTTTTTACACAAAAAAAAACAAAAAACA

AGTTTGCTTGATTTCAACGTTTTTAAGCCTACCACAAAAACGACAAAACATCGAACGACA

TAGCTGCAGTTGTGTTAAAAATTTACTCCGAATACTTATATTTTGATGCATGTGATTCAC

CTTATTTTTTGATTTGTTTCCTATCTACATAACCAGAGGTGTGGTAAATGTTCTACCTGT

GTGCACACTCATAGCCTTGGTGTTTGTCACTAATACTGTTTTATAATATCACTGTACCAC

AACAGACTCACTGGAAGTGAATGGACACACAATTCTCGCAGCTAAAGGTTTCTACGCTAT

GCAGTCAAAGACGAGCTTGGAGTGTAAGAGAGACCGAAAGAGAAAGAGCGTCGTGTCAAC

TGGCACAGCGTTCAGTTTTAATGGGTTTTGTGTGTATCGGCACTAAAAGGTCTTTCTGTA

AGCCAACCGTTTGTAACCATTCTAACAGTGTTACTAGGCTGCTGTGGACATAAGGGGCAT

TTTTTTATTTGGGTTTGGTGAAATATTTCCTTATATTGAATATATTCAAAAAAAAAAAAA

AGAGAGCGAGAATGAAAGAAAGATCACCTTCAGGTATTCGTTGATGAAATGATCTGGTAA

CAGACTTGTATGAGAAAGCGTACACTGGATGATTGAATTAATTATTTAAGCATAAAATAA

ATTGTGTTTAAACTCAAATTGTGATGTGAACTTTGATCTGGATGATGTGGTGCATTTGTG

CGAGAAAACTGTGTCTGCATTTGTCTGTCCATCGATCTATCTGTTCATCCATCTGTCCAT

CTATCTATATACAGTATCTATTTAGCTAGCTACACACACACACACACACACACA

>Unigene000167

GAGGGAGCAAAGTTAAAGAGGAGGGTCAGAGGAGGCGTCCTGATGAGCACCTTCAGGAGA

TCCGTTGTTCTCGAGTTAATTGCACTGCGTGTGGGCAACAAGTCAATCATTTCAAGAGAG

ACTCTGTGTATCAGCACCCCATACTCAAAGTTCTTCTCTGCAAATCCTGTTTCAAGTATT

ACATGAGCGACGACATCAGTAAGGATGAGGACGGCATGGATGAACAGTGCAGGTGGTGTG

CCGAGGGAGGTAATCTCCTCTGTTGTGACTATTGTAGCAACGCATTTTGCAAAACGTGTA

TTCTCCGTAATCTTGGCCGTAAGGAACTGTCTGGAATAATGAACAACGAAGACGAGAAGT

GGCATTGCTATGTGTGCCATCCCGAGCCGCTGGAAGACCACGTAGCTGCTTGTGAGAAAG

TTTTGAAAAACCTAGAATGTATGAAAAAGCCCAAGGCAGAACAGGACAAACCGAAACGGG

ACGGCATAAAGCACAAACAGAGCAAAGTTAAAAATTCAGCTCTTAACGGCAAAGATGTAA

ATTCGGATGGATCAGGGACGCTAACCTTTTCCTACAAAACGTTAAACGTTCCAAAAGAAC

TGCTGAAAAAGACAAAGAAGCTAATTGAGACGACCAACGGCTTGAACAGCACTTTTGTGC

AGTTTATACAGCAGGAACCGGTCTACCTTGACGAAAACATTGTCCGGCACAGACACTTGA

AAGCCTTTAGATCTGTTTTGGCCGATTTAAAAAAAGCCCATGCGACTTTAGAACAGGCTC

TAGAAGAGGAATTTAAAGCCCTAGAGGTTGTGAATGGAGACGGGAACGGGTTAGCTGATA

ACCCGCCTGTAGCTGTCACAGTTCCTGACTCTGAGCCTGCTGAACAGAGTAAAGATGATC

AGGTGGTTGATCAGACAGTTCAGGATAGTACCACAGATCAAGTTTCAGCAGCATCAGAAG

ACGAGGTGGACGAGGATGAGGTGAACGCAGTTCAAGCTGACGTAGAAATGGCCGAAGATG

TCGTCCCTGCAGCAGAAAATCCGGAGGAAAAACTTACAGAAAACGAAGCAAATGCAGAAG

TTGTCCAGAGTAAACCTGACTGTGAAGACGATGCTTCAGCAGCTGGTGAGGCCTCTCTTG

ACAAAGATATCGTGTCTATACCGCCTTCTGTTCCCGACGAACTTTTTGAGATGGTTGAGA

GTCTTTCAGACTGCGTCAAACAAGAGAGTGAGAAGGACTCTGTTGATACAGACAGTATGT

CGAGTAAGAACTCGGAGTCTGATCAAAAGCCAAAATCTGGTAAAAGGTCTGCAAAGGTGA

GCAAGAAACTTGTGGTTAAGCTCACACCGATTCCTCTTAAAATCACCATTAAAAAAGATG

AAAGCAAGTCAAAGTCGCAGGAGAAGGAAGGTGCTTTGTCACCAGATGAAAGCAGACGTT

CCCCCAGGATGAAGACCACTCCGTTGCGCAAATCATCAGAAAGGAGAAGTAAAAGCCAGT

CTGAGGATACAAGAGCGAATGACGACGAATGCGACGCTTCAGAGACTCCAGTCGAAACTG

GCGATCCAGACTCTGATTCGGACGAAGTCCCCGAGGTGCTGCGGCAGGTAGAAGACGAGG

AGGCCACTTCAGACGAACCGGACGCAGAGCCTAACGGCAAGGAAGCAGGCAATACATCGC

CCAAAAAATCAGCTGAACAATCCTCTAAAAAGACAGCAAAGCGCAAGCTCCTCGCATCTT

CTTCGGATTCAGACAACACAAAGTCAAGAAAAGCAAAGAACGCCGCCAAAAAGAAGCGTC

GAGAATCCGATTCATCTAATCACGACTCTGATCTCGAGAAAGAGCTCAAGAGTTTAAGCA

GGCTCCGCACAACAAAGAAATCCAAGCCAGGCAAGAAACAGACCACAGAAGACGAGGAAG

AGGAGGAGAAGGAAGGCAAGAAGACGACGAAAAAGAAGAAGGAGGAAGAGACCAAGAAGG

AGGGTAAAAAGGGGCCAAAAAGGTCGTTCGAGAGGAAAAGGAGGTCACAGAAAGGGAAAG

AAAAAGCCACCAAAGATTCCTCCTCGTCCAGCGATGAGGAGGAGGAGGAGGAGCAGGAAG

CAGATTCTGGAGAGGAGAGCGGTGATCAGCAGAAGATTAAACCTATTCTTGAGTCTGCCT

TGTCGCACATGGATGCATTCCAGCAGTCATCAGGAGATGAAGCTCAAGTGAAGGCAGAAC

CCTCTCAGATGCTTGATGACGACGACGATCCTGAGAATAGAATTGCAAAGAAGATGCTGT

TAGCCCAGATTAAAGCTAATTACTCATCGGGGGATGACAGCTCTTCAGAAGATGACGACA

GCAAAACCAACAAAGAAGAAAGATCTCCGACGAAAGCGAAGAACGATGCCAGCTCTGAGG

GTGAAGAGGAGGAGGACTCTGACTCTGACTCTGGTTCGGAAGTGGACGTGAAAAAGGCCG

GGCGGAGACACAAGCTGCTTCGCAAGAAACTGACACTAAGTGAGGGCGAGTCCGACACTG

AGAAACCAGCCAAGAGCAAGAAAGAGGTTAAGAGAAGAGGGAGACGAAAAGTGAACAGCG

ATGACTCTGCCGATTCAGACTTTAAGCGTTCAGGCTCTGGATCCCGGAGCGAGTCGGCGA

TGAGTGATTTTGTGAGTGAGGATGATGAAGACAACAAAAGCAACAAGCGGAAAACACGGT

CCTCTAAAAAAGCTGACAAAGACGGCGAGAGGAGCTACCAAAAAGAGAAAAAGAAAAAAC

GCCGGCGGATTAAGGTTCAAGATGATTCCTCCAGCAATAAGAGTGCCGAGGAAGGGGACG

AGGACAAAGAAGAGGAAGATGAAGAAGGGGACGATAAGGGTACGCCGAAGGGCCGCAAAA

AGATCCGCAAGATCCTTAAAGATGACAAGCTGCGTTCCGAGACCCAGAATGCATTAAAAG

AGGAGGAAGAGCGACGAAGACGAATAGCTGAAAAAGAGCGGCTGAGAGAAAAACTGCGAG

AGGTAAACAGAGAGACTGCGAATGCTGTAATTGCATCTCAGACAATCGTGGTGGAGGAGT

CCTCGCAGATCACCTGTCCAATTACAACCAAACTGGTGCTGGATGAGGACGAAGAGACTA

AAGAGCCACTCGTACAGGTCCACAGAAACATGGTGACCAAGCTGAAACCCCATCAAGTGG

ACGGTGTGCAGTTCATTTGGGATTGCTGTTGTGAGTCGGTTAAAAAGACTAAGAACTCCT

CTGGCTCAGGCTGTATCCTCGCTCACTGTATGGGTCTGGGCAAAACCCTTCAGGTGGTGA

CGTTTCTCCATACTGTGCTTCTATGCGAGAAGCTCAATTTCTCAACGGCTTTGGTGGTGT

GTCCTCTCAACACCGTTCTCAACTGGCTGAACGAGTTTGAAAAGTGGCAGGAGGGCTTGA

AAGATGAGGAAAGCTTAGAGGTGACCGAGCTGGCTACAGTGAAGAGGCCTCAAGAGCGAG

CATACGCTCTTCAACGCTGGCAGGAGGACGGCGGCATCATGATCATCGGTTACGAAATGT

ACCGCAACCTCACGCAAGGCCGAAACATCAAGAGCAAAAAACTGAAAGAGACATTCCAAA

AGACCCTCGTTAACCCAGGTCCAGACTTTGTTATTTGCGACGAGGGCCACGTCCTGAAGA

ATGAAGCCTCGGCCGTTTCCAAAGCCATGAACTCCATCAAGACTCGGCGAAGAGTAATTC

TGACCGGAACGCCGCTTCAAAACAACCTGATCGAGTACCACTGCATGGTGAACTTCATCA

AAGAAAACTTGCTGGGTTCAGTGAAGGAGTTCAGGAACCGTTTCATTAACCCAATTCAAA

ATGGGCAGTGCGCCGACTCCACGATGGTCGACGTGCGGGTAATGAAGAAGCGAGCGCACA

TTCTCTATGAAATGCTGGCGGGATGTGTTCAAAGAAGAGACTACACTGCACTTACTAAGT

TCCTGCCACCGAAACATGAGTATGTGCTGGCAGTGCGAATAACTCCTGTGCAGTGCAAGC

TGTACCGATACTATCTGGAGCACTTCACAGGTGTCGGTGCTGCCTTGGAAGGAGGAAGAG

GCCGTGCAGGCACAAAGCTTTTCCAGGATTTCCAGATGCTCAGCAGAATCTGGACTCATC

CATGGTGTCTGCAGCTGGACTATATCAGTAAAGAAAACAAGGGTTACTTTGATGAAGACA

GCATGGATGATTTCATTGCGTCTGAAACAGAGGAGTCCTCCATGAGCCTGACCTCTGAAG

ACGAGAAAGCAAAAAGGAAGAAGAAACGAGGGAAAGGCAAAGGCGACAGCTCTGACAAGT

CTGACAGTGACGACGTGGAGGTGATCAAGGAGTGGAACACGAGCTCCCGTGGGGGAAACC

CAGAGGGGCGGAACAGAGCTGAACCTGTAGAGGAAGTTCGTGCCAGCAGCTCAGGCCCTG

GCAGCCCGTCTCCTGATTGGTACAAAGAGTTTGTGACGGAAGCGGACGCGGAGATTCTGG

AACACTCCGGAAAAATAGTGCTTCTGTTTGAAATTCTGCGCATGGCAGAAGATGTGGAGG

ATAAAGTGTTGGTGTTTAGTCAGTCGCTTATTTCACTGGACCTCATTGAGGATTTTCTGG

AGTTAGCAGGCCGAGCCAAAGAGGAGGGAAAACCCTCTCCTTACAAAGGTGAGGGCAAGT

GGTTCAAAAACATTGACTACTACCGCCTTGACGGTTCTACTAATGCCATGACTAGGAAGA

AATGGGCTGAAGACTTCAACGACACCAGCAACGTCCGGGGTCGTCTTTTCCTCATTTCTA

CAAGAGCCGGCTCTCTAGGGATCAACCTAGTGGCAGCAAACCGGGTCATTATCTTTGATG

CCTCGTGGAACCCGTCTTATGACATTCAGAGCATTTTCAGGGTGTACCGCTTCGGCCAAC

TAAAAACAGTATACGTCTACAGGTTTTTGGCTCAGGGCACCATGGAGGAAAAGATTTACG

ACAGGCAAGTGACCAAACAGTCCCTGTCCTTCCGCGTGGTGGACCAACAGCAGATTGAGC

GACACTTTACCATGAACGAACTGACTGAACTCTACACGTTCGAGCCCGATCAGCTGGATG

ACCCATCGGAAAAGAAAAGCAAGAGATCCACACCCATGTTACCCAAGGATCCTGTTCTGG

CTGAGCTGCTTCACAACTTCAAAGATCACATTGTGGGTTACCACGAGCATGATTCCTTAC

TGGACCACAAAGAGGAGGAGGCTTTGAGCGAGGAGGACCGTAAAGCCGCCTGGGCTGAAT

ACGAAGCAGAGAAGAAGGGTCTCTCTATGCGCATCAACCAGCCGGCATACGCTTCTGTAG

GCATCGGATCAAACTACCTCCCCTTCAATGTGGCAGCTCTGGCCTCAATGTCCAACCAGC

AGCTGGAGGACCTGATCAATCAGGGACGACAGAAGGTGATGGAGGCCACTAATGCTCTGA

GGACTTTTCCCAGGGAGCCCCTTGAGGACATTATCAGCCAGGTGTGGAAGGAGAATCCCA

ATCTGTCCGAGTCCCAGGTGCAGGGGATGGCTCTGGCCAGACAGGCAGCCATCGACATGG

AGCTGAAACACAGAGAGGCCACGTACAGAGATGCTCTCAATAAGCAGCAGACGCTGATGA

TGTACGTTCAGAAGGTGATCAACAATCGTAAGGTTCAGGAGCAGCAGCTGGCGCTTGCCA

GGCATGGGATGCTGATGAACCAGATGGCCATGCAGAATGGCATCGCCGGGGGAATGAGTC

AGATTGACCTGCTGGGCCTGTACCAACAACTGGGTGCCCTCCAGGGGCTGCCAACTTCCC

AGATAGGCAAGAACCCAGGACCCTCAAAGGGTCTGTAGTGCCCTACGTTTCCTGTGTGGG

CATTCTAACCTAACCAGCTCGGACACTTTTTGCCCCTTTTTTGAGCGCTCGTGTAAAAAT

CTGGGATGGAAATATCTGGAAGACTTTGGCAGTATGCTTTGTAATTCTTTGTATATACAG

CCTTAGCGTTTAGTTGTTTTGTTTTTTTTTTTCTCTCTCGTTTCCTTCAACAT

>Unigene000168

AAGCGGCGGCTCCTCTGATGTTTCTCCGGATAAAAATGCTTCCATGAAGATGTCAGGAGA

TGTCATGACAGATGTTGTTCAGGTGATGGAGCTTCCTCCTGTTCTCTTCTCTCTGAAAAA

TCACCTGACGGAAGTGGAACATGGCTGGGTGGAGCTGCATGCTGACATCAGTGCAGTACA

ACAGAAGCTACAGCAGCATGTTCTGGAGAGTGTGTCTCCTCTTTCCCTTCTCTCAGAGGT

GTGTGAATGGCTCGGCGGAGCTGAAAGCTGTCTGCATGACGCGGAGAAAAACGATCCGGG

TTCAAATACAGTAACGCAGCTCACGAGTCGACTCAATACATACAAGGAGCTGAAGCGAGC

ACAGATGTGTGTACAGATGTCAGTAGACTTCCTGAACCGGATGTACCTGAAAGAAGTCGA

GCCGACACTCAGATACAAACACACAGCATTTGCAGAACAGCTTGGGGACCTAAACATGCG

CTGGACTACACTGCAAACACACCTTTACGCACAGACGCAGTACGCTGAGCAGCAGCTGAG

CATTTGCGTAGAGCAGGACGGAAAACTGCGCCTCCTGCTGGCCTGGATCAGAAATCATGA

GGCATTGATGCGACTGGCTGAGAGACCTGTTTGTTATAGTGCAGCGGAAGAGCAGCTGAA

TGAGTGTGAGATATTAAAGGAGGAACTGAAGGTTAAGTCTGCTCAGCTGGAGGAGCTGAA

GAAAAGCCTCAGTCTAAGCGATGGTGCAGATGAAGGCGGAATCAGTCACATCAACACCAT

CACACAGGCGTTAACCGCTTTAAATGAACAGAACTGCTCTCTGAAACAGACGCTTCAGCA

CCTGTGCGAGCAGTGGTCTGGGTTCGAACGTGGGAGGTGTCACGTTGCTCAGAGCACCCA

AAGGATATGTCAATCACTCAGACGATGCCACACCCCTCACATCTCCTTTACAGCGCTCCA

GAAGAGCATCAGTAAACTACAGTCATTACACACTGAATCCGAGGAAGTTGACAAAAAATG

GGCGGAGCTTGACCATACATTCTCCAGTCTTACTGGTTGTATCCATTCAGGCACCTCCGA

CTTATTATCTGAAGAGCTTCAGAAGGAGAGATCACGGTGGACTGAGGTGAAGCAGGATGT

TACACACACCTCGTTGAGGTTACACACTCTGCTGCAGGCGTGGAAACAATACTCAGATCT

GTCCTCCTCCTGCTTAGAGAAGCTTCGGCAGCAGAAGGAGCAGTTCTGTGACGTATTAAA

GATGGCGTCCGAGTCTGACAGCGACACGGAAACACTTGCGACTTGTATGCAATGTTTACA

AACACTCTGATGTTCAGGTTCTGCTGCAGAACGTTCAGTCTCTTCAGACGGACACGGATC

AAACGCTCGAGGCTTCAAAAGAGTCGATCAGACAGATGGAGCCGCCGGCGGCCGCCTCTG

TTCGTTCGGAGTGCCGTTCGCTGACACGGCGGCTGTGTCGATTCGAGCAGGTCGTAAAGA

GGAAGCTGGAGGAGATGAAGGAAGATGTCAAGCAACTTCAGGAATTTAAGCAGCTACTCG

AATCACTAGAGACGCACTTAAAGGAGTGGACGAGTCGGCAAAAACACATCGATGCTCAAG

ACACTGACGTATCAAAGTCTGATCTGTTGGAGTTAAGTGGTCACTCAGCTGACTTGGAGA

TGCTGAACGAACTCAGTTACAATCTAACCCTGAGTGACCATGTGACCCAAAGACTACAGA

GCCTGAACAGGAAGTGGGCTCGGGAGTCTGCACATGCTCAGGAGAGATGCAGCGAGCTGC

AGGCCGTGTCTCTCGAGCGACAAGTGTTTGAGGAGAGGTGTGAGACATGGATGTCGTTCC

TGCAGAAGATGGAGGACAGTCTGGCTGGAGAGATTGCGTCCACATACCCTGGTCTTAGAG

AGCAGCTACGCACTCATCAGAGGTTCCAGGCTGAACTGTCTCTGGGCCATCAGATTCTTC

ACTCGGTCATCGCTGAGGCTCTGCGTGTGCTACAGAGGGGAGAGGTGGAGGACCGGAGTG

ACTTCCTGTTAAAGCTGGCTCAGCTCAGAGAGCAATGGCAGGGCGCGGTACAGAGAGCAG

CTCAGCGGCGCACCCTGGTGGAGGGGCTAATACGCCACTGGCACCGTTACACACATACAC

TTCGCAAACTCAACAGACTGCTCTCGCTTGCACACACACTCCTGCCCCCGGCTGGCCTCA

CACACTGCAGTGTGTCACAGCTACGCCATTCGCTCAAACAGCTGGAGAGAACCGAACTGC

TTTTCCAAAGATCCCTCTCTTCCTACCTGCTGCTGTTGGATGTGGGTCGTCAGCTCTTCT

CTGTGAGCGATGAGGAGACACAGACACTCTTGCAGACGGACCTCGCCACCCTGCAGGAAG

ACTTCGAGCGCTCACAGTGCATGCTGGGAAAATGGAAGACTCTTACTGCTGATGTCATCA

AGACATGGGAGAGCTGTACGACTCGGCTAGACGGTCTGGCACAGCGTCTGGAAGAACTTA

AGGCCAGGCTGGAAAAACATCCCATCCAGGAGATGGAGAAAGAGACGATTACGGAGCTGC

AGGACTCTCTAGAGGACTGGGCCGCGAGTCTGTCCGAAACGAGCAGCATGAAGACGGACT

TGTCTCAGTACATCATCACAGAGGACGTGCTGATGCTGCAGGAGCAAGAGGAGCATCTGC

ACTGCCAGTGGGAGGAACTGTGCTTTAAGGTGTCGATGCGTAAACAGGAGATTGCTGAGC

AGCTGAATGCGTGGATCATCTTCAGTGATAAGAACAGAGAGCTGTGTGAGTGGCTCACAC

ACATGGAGAATAAGATGACACACAGCTCGGAGATCAGCATTCAGGAGATGGTGGAGAAAC

TGAAGAAGGACTGCATGGAGGAGATCAACCTCTTTAGTGAGCACAAGACTCACCTGAAGC

ACCTGGGAGAGCAGCTGATCACAGCCGGCACCAAGAACAAGGAGAGCGAAATCAAGGACA

AAATCGAGGGAATAAATGAGCGCTGGCAGCACCTGTTCAACCACATCGAGGCCAGGGTGC

GGAAGCTGAAGGAGACGCTGGTGATGGTGCAGCAGCTGGATAAGAACATGAGCAACCTGC

GCCTGTGGCTGTCGCATATCGAGGGGGAGCTCACCAAACCCATCGTCTTCACTGCCTGTC

ACGGAGATGAAATCCAGTGCAAACTTGCTGAACAACAGGAGTTGCAGAAGGACATCGAGC

AGCACACAGAGGGTGTGGCGTCGGTGCTCACACTGTGTGACATGTTGTTGCACGACACCG

ATGCCTGCAACAGCAATGTCGAAAATGACTCCATCCAGCAGACCACGCGTAGCCTGGACC

GCCGCTGGAGAAACATCTGCGCCTTGTCTATGGAGAGGAGAATGAGGATCGAGGAGACGT

GGCGTCTCTGGCGCAAATTTCTGGACGATTACTCTCGTTTCGAGGACTGGCTGAGCTCTG

CGGAGCGCACGGCAGCAAACCCAGCCAGCACAGACGTCCTCTACACGTGTGCCAAGGAAG

AGCTTAAAAAGTTTGAGGCTTTTCAGCGGCAGGTCCATGAGCGTCTGACTCAGCTGGAGT

TGGTGAATAAACAGTATCGGCGTCTGGCCCGGGAAAACCGCACAGATGCAGCCTGCAAAC

TGAAGACCATGGTGAATGAAGGGAACCGGCGCTGGGACCAGCTGCACAGGAGAGTCAGCG

CCATCATTCGCAGACTCAAGCACTTCACATCTCAGAGGGAGGAGTTTGAGGGGACGAGGG

ACGCTGTTCTGGTATGGCTGACTGAGATGGACCTACAACTCACTAATGTGGAGCATTTTT

CTGAAAGTGACGTCCATGAAAAGATGAGGCAACTCAAAGCGTTCCAGCAGGAGATCACGC

TGCACACGAATAAGATCGATAGTTTGATCGTGTTTGGAGAGAAACTGATCCAGAAGAGTT

CTGCGCTGGACGCTGTTCTGATCGAAGACGAGTTGGAGGAGCTGCACTCCTACTGCCAGG

AAGTGTTCAGCAGAGTCGCCCGTTTCCACCATCGCCTCATCCATACGATACCGGTTGTAG

ATGATGAGCAGGATTTCTTGGACAGAGACGCGAGTATGGACACTGAGGAGCTGATTGGCG

TGTGGAAGAGAGAGCCCACAGAGGAGGATGTGATAACACCCGTGTCGGGTCATCCTTCGG

GTCGAGAGACACCAGTCAGTGTGGAATCCTTAGCGCTGGAATGGGACCACACCGTGGATG

TGGGCGGATCTTCATCGCCCGAGGACGAAGATGAGGTCACGTACTACAGCGCGCTCTCAG

TGACTGAGTCCAGCTGGCAGAACAGGAAGCGCATCAACAGCCTGACACACACCGATGCCT

CTTACCGTCCTGAGGGATATGCGAAGCTGATGTCCGAATGCACAGACAGCATTGACGACG

TAACGCGAGTCAAACACATCCTGATTGACGACATGCCGCTCAACCACCAGGGACTGACCG

TACCGTCGCCTGAAGACCAACAGACAGGAGTGATTGAGAGATGGGAGATTCTCCAGGCTA

ACAGCAATGATGACAATGAGCTCGGTGTTCAGCAAGATCAATGCAAACTGACCTCTGACC

TCAGGGAACTGACATCCTGTCTGGACTGTGTGCTTCCAGAACTGGAGAGGCTTCAGAGAG

GGAAGGAGTCCCCAAGTGTTCAGGACATGGAGAACAGCATCTGCAAGCTCAAAGAGAGTC

AGAGGAAATTCAGTGGCTACAAGGCTTTGCTGATTAACGTGAATCTAAGTGGGCGGCGTC

TGCTGCGAGGGGAAAGTGCAGAGCTGCAGGAGCTGAAAGCAGAGCTTCGGGCTACCAATA

AGAAATGGACGCAGGCATGTGCGGCTCTCGATGCCTGGGAGAACCAACTGCATCTCGAGC

TTTTGCAGTGTCAGGAGTTTCACAAGACTTTGCACTCGCTTTTGTTGTGGCTGGCTCGAG

CTGAGAGTCAGCGCTACATGGTGAACATCCATGACCCAAATGTAGACGTCGATGTTCTAC

AGGAACACCGAGATACACTTAAGGCAGTGCAGTTTGAGCTCCTGGCCCAGGAGCAGAAAG

TGCAGTCACTGCAGCAGATCTCTTCACACATCCTCCGTGTGGAGCAGGGAGAGGAGAGCA

TGGAGGCCAAGGAGAAGGTGCATGTCGTTAACAACAAGCTTCGTCTCCTGCTTAGACAGA

TCGGTCATGACCTAAACACACTGCAGTCCAGACTGAAAAATGAGGGATCATCAGTTGACA

GTGAGCCAGTACAACGTTCAGCGCTAACGGACAGGTCGAGGGCTGTTGAAGAAAAACCTT

CTCTGAAAGCAAAAGAGGTGTCCAGAGGCAGACCCTTCCTGTACCGGGTTCTGCGCGCCG

CATTTCCACTTCACCTGCTCTTCCTGCTGCTGCTGGTGTTTGCATGCCTGGTGCCAGCAT

CTGAAGAGGATTACAGTTGCGCCCTGTCCAATAACTTTGCCCGCTCCTTCTATCCTATGC

TGCGTTACACCAACGGCCCGCCTCCCACATGATCAACTGAGCTGCGTAATCATATTGTGA

CAGTCAGTAAAAATAAAAACAGAATTTAAGTTAAATGACTATTTTTAAGAAGGCACATAA

TAAACAGAACAAAATATTAACATAATGAAATCTCTTATTTACTACCTGTAGAACGTTGTC

TGGGTGTCTCTCCAGCATGAAATCAGAGTACAGCCCTGTGGGTGAAAACACACAACCTTG

CAGTCGGGAGCGTCTGGCTCCCTCTGCTGGCCTCTATTAATTAACAGAAATATCAAGCGA

CAGTTGATTGATTAGTAAACATTAGTGCATCAACAGTTTGCATTTTATTTTCTTCACATT

AATTTATTAGCAAAAACACTGAGTGACATTAGTGTTCTAGCTTTAAGACCGTATTTGGAC

ACAAAAACCAAAAAGTAGGTTGTAGGTTATACACACACACCACTTACAACAGTTAATAGT

CAACACAATAAAAATAAATATATATCTTATTTATTCCCACCAGCC

>Unigene000169

TCGTTTTTTACGCATGCCTTTAACATCATGCACTGAAATTTGAGATGACCTATTTTAATT

AAACATGCATGTATTTAGACAAGCCATCATGCCTTTTTGGCGATTTCTGAGTTTTCACAT

GTGTTTCATGCAGATAAAGATGGTCCTGATAAAAAGAGGGTAAAAAAGGAGTCTGGAGGT

AAAAAGTCCACCCCTGTGAACATTTTGTTCGGTTACCCTCTCTCGGAACGCAAGCAGATG

GCCCTGCTAATGCAGATGACAGCAAGAGACAACAGTCCAGACACCACACCAAGCCACCCC

TCCCAAGCTCCAGCAGTGCAAAAAAAGACTCCCAATAGCTCAACATCTCGTCAGAAAGAC

AAGGTGAACAAGAGGAATGAGCGTGGAGAAACACCGCTGCACATGGCTGCCATCAGAGGG

GATGTGAAACATGTCAAGGAGCTGATTGGATTAGGGGCGGATGTAAATGTGAAAGATTTT

GCAGGTTGGACACCTCTACATGAAGCTTGCAACCTTGGTTACTATGACGTAGCAAAGGTT

CTTATTGGTGCAGGAGCAGAGGTGAACACCCAGGGATTGGACGATGATACACCCCTACAT

GATGCCTCCAGCAGTGGACACAAAGATATTGTGAAGTTACTCTTGCAGCATGGAGGCAAC

GCATTTCAGGCAAACAAGAGAGGCGAGCGACCGGTGGATGTTGCTGATTCTCAGGAGGTT

GAGCAGCTACTGAAGGGGGAGATCCCATTGTCTGACCCAGATGACAGCTCTTCAGACTCT

GAGGACCTTCCATCAATAAACCCTTCGAGTGTAGATGACAATATGGAGTACTCGGACACT

GAGAAGGACTCTGATGGCAAATCGACCACAGCCAAGGCCACATCTTCAGTGTCTGGTGTG

GATGAGTACGAGTTCAAGGACGAGGAAGAGGAAGAAGATCTTAGCAAGGCCCTAAATGAC

AGACACATCTTGAGACGAGAAGTGCGACAGAAAGAGGAGCAAAACCATTACACACCCAGA

CAGTCCAACAAGAGCGATCAGACAGTGTCATCATGCAAATTAAAAAAGCCCAAATCATCC

CGAGTACTCTACTGCAGTTCAGATAGTTCAGACAATGAATTGGAGGCTCCTTCAGAACGA

AAGTGTTCCCCGACCAGGTCACTCTGCAACGACACACACAAAACAGAGAGCCGGACAAAA

AGAGATAGCTTAACTCAAACATCAACAGAAAAAGGCAAGGCAAACAAGAGGAACAGGAGT

CAAAGCAAAAACAAGGAAAATCAGGAGGTTCAAGAGGACGGAAAAGAAAACAGCAAATCT

CTTTTGTTCTCGACAGCCACAGCGTCTGACAACTCTGATAAAAGTGTTCGGGAGGAGGAC

TCTTTTAAAATGTCCTTTAGCCCAAAGGACGATTCATCTGTGCACCTCTTTCACTTGTCC

GCTGTGAAATCCCCCAAGCTCAGTTATGGTCAACCTGACAAACAGATAACTCCTCTAAAA

CAGGAAAATGCTAAGACTTTTGTCTCCATTGGAGATGGGTCCTGCACTATGGATGGTGTC

AAGTACAACCACTACACAGATTCTGACTACACAGAAGGTTCCAGCAGTAAAAGCTGTAAG

CACAAGGACAAAAGCAAACATCACTACAGGGAACACATCTTGGATGGAGATGACAGAAGC

TCTAGTCCATTTAAAGATGCCAGTTTAAGCAATAGTATGGATAGCTCGGAAGGAGCCTTT

AGAAAATCTGACAAGGATGGTAAGGTTGTCAAAAAGCATAAGCTTAAACACAAGGAAAAG

GACAAGTACAGGAAAGACTATGAGTCTGAGAGAAATCGGCATCGACAGAGAGAACTAAGT

AAAGATGGACATAGGAATTTGGAGTTTGACCGAGAATTTTGGAAAGAAAATTTCTTCAAA

AGTGATGAGAGTGAAGAACTCAAGGGGAAATGTGAGACACCAGGCAGTTGTTCTCCTCAA

AAGTCTATAGACTCGTCGCCAGTTAAGGAAGATAAAACAACATTAAAAGACAAGCATTCG

GTTTGTAACAGCACTAAGGAGAGAAAACAAAAAGACGAGCGAGAAAAAGACAAACCTATA

AAAAAAGAAAAGAAAGAGGTAGCATACAAGGATGAAAGAGGAGCAAAGGACAGTAAAGGG

GCTGAAAATGACCGGTCAGATTGTCCAAACTTGATACGAAAGCCTGAAGACTCAAATTCT

TGCATAAAAGAGGAGACCGAAGACAAGCCTCTTACTGGAAACTTGTTTGATCACAATCAG

CTAGAGACTACAGAGAAGAGCTCTCGTGAGAAAAATGATAAACGGCTACCCACAAAGGAC

AGAGAATCTGAAAAAGGTGAGAAAAAGCAGGCAGAAAAAGAAAAGAAAACCAAATCAGAT

CATTTGTCAGATAAACCTGATCTTCATAACTGTACAGATCGATGGAAAGAGAGGGATAAA

ATAATGACCTTGTCTAATTCGTCTGGTGAGAGAATCCATAAAGAAAGTGACAAACTAAAA

CCTGTGGCGCCTATGAAAAAGCATGAAGAAAACAAAAAGAGCAAGGACAAGTCTGAAAGA

AGAAGTGAGAAGGAACGTCAAGAGAGAGAGCATTTCTCAAGCGATCACAGGGACAGAGAT

AAAATGGGTTTTGAAAAGAAAGGAAAGGCTCCTGAGCGGAGCAGTGATCATAGCAAAATC

GATCGTATTAAAGAAAAAGAAAGAGATAAAGACTTCGACAAGAAGAAAAAAGAAAAATTA

AAAGAAACCATGATCTCCTCCTCAGGTTCAAATCTCAAATTTCTTTTGGAAGAAAAAAAG

GGGTACACATCTGAGAGCAATAAAACAGTAACCCTTAAATTAAAAGAAGAGGTCATAAAA

ACCCCTGAAAAGGACAGGGACAGGCGGGAGAGGGATAGAGATTTGGAAAGGCATCGGGAT

AAAGACAGAGAGCGGCACAAGGAAAAGGACAAGGAAAGACAACATAGGGACAGCAAACCC

AGCAAAACAAGGCCTGTTGAAATTGAGTCGGATAGGTCCAAATCCAAAGCTTCACCTGCA

CAAAAAGAAACCCGCCCCAAAGAGAAAAGATTGGTTAATGATGACCTCATGCAGACGAGC

TTTGAACGAATGCTTAGCTTAAAGGATCAGGAGATTGAGCAGTGGCACAAGAAACACTTG

GAAAAAATCAAGCAAAAAGAAAGAGAAAGGCTAAAACAACGACCTGGATCCGACTCTGGG

AAGCCAAAAAACAAAGATAAAGCGAAAATTTCCACTTCCAGTGAACCAAGTGCAAATAAA

GAACTCTTGCGCTCCAAAAGCTCAGAAATCCCAGACGCTTCTGGTCGGGAAAAGATCTTA

AAGGATGCGACCAGCGGAAGGACACTTTCTCTAGATGCAAAGAATCTGTTGTGTTTTGGA

AAGTCTGTTCCAGTAATAGAAAACAGCCTTAGTCGATCTCCAAGACCTGACAGTGAACGA

TCTGCTATCATGTCAAGATCAGTGTCTATGATCTCAGTAGCCAGTTCAGAGGATTCCTGC

CAGGCTACCATGCTAACGCCCAGGCCCACGGAGTACGACTCGGACCTAAATTTAGAAATG

TCTGATTCTCAGCCACCTTTCCTCCAATCTTCCCATATAGTTCAGTCCTCCAGATCACCA

ATTTTTAACAATAAAGAAATTAACAGTCTTCCTGACGTAGCCTCTGGCAGTCGAACACCA

CTGTCAAGCGGACTTGCATCTTCTCAATCAAGAACTGTCCATAATGAGGATGATAAAGCC

AGTCTAGCTGAGCCAGACAGACTGACTGAAGAATCTCAGAAAGTTGGCGATGTAGCAAAC

TCTAGTGAGGTACCTGGTAAAATACACCATACTGAAGCCTGTGGAGCAGTCACACAGAAA

AGTCTTACTATTAACAGCTCCCTGTCAGTGCATCTCCACTGTGCTGAATCTGAAGTTCAA

CCTCCTGTCGGTGTTGAGGATGCAAATAGGAGTTCAAGCAACCAGCTTGCATGTGCCCCT

AACTCTGAAGATCTCCCAGGTCACCAAACTAGCTTTCCACGTAGTATTGGTTTAACAACA

ACTACAGTAGAGGTAAGCAAGCTAAACACTGATACCTTACCAGAGCAGAGTGGACAAAAT

CAGCCTGACAGCTTTGACACAGAAATGGCCGATGGCTCCAACAAAAATTCCCTGTTAGCT

TCTGTGCCCCAGGTTGCATCCATGGTATCATCAGAGCAGAGCTTCACAAAAACACCTACG

CAGACTGGTAACCTGAAATGGAATTCTGTCTTTGATCCCAATAATGACCACCCAGCAAGG

ACAGACATTGATTTGACAGAAGAGAAAAGTTTAAGAAGTTTGTCAAGCGGTCCTGAAAGC

CTGATGGAAGACAGATGCATGGAGAAGGTTTCTGCTGGGAGCAGAATGGACTTGACAACA

AGCCAAGCTTCATCTATCAGCTCTAGTAGGATCAGTTCTGAAGAGTCTGACAAAATGCTG

CAATGTACAGTCAGTGTGTTTAGAGATTCTCATCAGGTTACAGAACAGATCTCAGTGGAT

GACTGTAAAGCAAGTCACTTCGGTGAGAACATAAGTGTGGCTGATGCACATCTGGGGAAA

AAGGAATATGGTTTGGCTCCCCCTTCAGTTTCAAATGTCCTTCACTGTACTAGTCCCGAA

CAGACATCTGAAGAGTTAATGGAAGTATCAGGGGAAAAGTCAGACCATAATCAGGTCATT

GCTGAGACACAAGTAGAGAAATTGCTGTCTTCAGAGGATGGGGCTCAAACCCAGCCTTTT

CCGGACACTAAAGCGAATGTTGATGTGGAGATGCCAGACCAGCTTACTGTTGAAGAGCAA

CAAGCAAAACAAGGTACTCCATCAGATGGAGAGCAGAATCTGGGCTGCACTGGTGTTCAG

CTTGATTTTCAGTCAGAACAGCTCAGTGGAGCAACATCTGGAGGCTCTTCACCATTGTCA

GTGGCAGACAGAGACAGTGATCTAACAGCAGCAAGGACAAAAGTAAAAAATCCAGATGAC

GAGGTGGACATCCAGGTCACTCATCCAAGAAAACGAAAGATGCCCAAAAGTTCTCTTTCA

ACCCCAGCAGGCCTTACAGCTCAGCAAATAAAGGAAAAGACCCAGCAGTCATTAGCAGCA

ATCGTGGACTCCCTAAAGCTTGAAGAAATTCAGCCCTACCAAACCGAAAGGGCCAATCCT

TATTACGAGTTCCTGCACATCCGTAAGAAAATCGAGGAGAAAAGAAAGGTGCTATGTAGC

GTCATTCCTCAAGCACCACAATACTATGATGAATATGTTACATTCAACGGCTCTTACTTG

CTTGATGGGAATCCACTGAGTAAGCTTTGTATTCCAACGATAACACCACCACCCTCACTA

CCTGAACCACTAAAGGAGATGTTCAAACAGCAGGAGATTGTGCGCATGAAACTGCGCCTG

CAGCACAGCATTGAAAGAGAAAAGTTGATTGTGTCCAATGAACAAGAAGTCCTGCGTGTC

CATTATCGTGCTGCAAGAACATTGGCCAATCAGACACTACCCTTCAGCGCCTGCACTGTT

TTATTGGATGCAGAAGTATACAACATGCCCCAGGATGTACAGGGAGATGACGGAAAAACC

TCAGTGAGAGATCGGTTTAATGCAAGACAATTCATGTCTTGGCTTCAGGATGTGGATGAC

AAATTCGACAAGTTAAAGACGTGTCTCCTGATGAGACAGCAGCACGAGGCAGCAGCTCTT

AATGCTGTTCAGCGCTTAGAGTGGCAGTTAAAGCTACAGGAGCTGGAGCCTGCCACCTAC

AAATCAACCAGTATCTTCGAAATCCCAGAGTTTCACATCCCCCTCGTGGAGGTCAATGAC

GATTTCGATCTCACGCCAATATAACATCAGGAAATCCAATGTCCTGATAAGGATCGTCTG

TCCAGGACTCGGAAAGAGTAGAGGAACACAAAGCACTTGTTTTGGGCGAGCTGCACTGTG

TGTTTGCGTTTGTGTGTGTTTGTGTTTGTGTGTGTGTGTGTGTG

>Unigene000170

TGTCTGTGTGTGTGTGTGTCTGTGTGTGTGTGTGTTGTATTTCAGAAGTCTCCCGACCCG

ACGGCAGGATGAGTAAGACCCTGAGTGAGGATGAGAAGTTCCTTTTTGCTGATAAGGATT

TCAAAAGTCCGGTGGCGCAGGCCGACTGGGCCGCCAAGAAGTTGGTGTGGGTTCCATCTG

AGAAACACGGGTTTGAGGCGGCGAGTATGAAGGAGGAGCGAGGAGACGAGGTGCTAGTGG

AGCTGATGGACAACGGCAAGAAGATCACTGTCAACAAAGACGACATCCAGAAGATGAACC

CACCCAAATTCAACAAGGTGGAGGACATGGCGGAGCTCACCTGTCTCAACGAGGCGTCTG

TCCTCCACAACCTGCGTGAAAGATACTACTCCGGACTCATCTACACATACTCTGGTCTCT

TCTGTGTTGTAGTGAATCCTTATAAAATGCTGCCAATTTACTCAGAGAAGATCATTGACA

TGTACAAGGGCAAGAAACGTCATGAAGTTCCACCACACATCTACTCCATCACTGACAACG

CCTACAGAAACATGATGCAGGATCGAGAGGACCAGTCTATTCTCTGCACAGGTGAATCTG

GTGCAGGGAAAACAGAGAACACTAAGAAAGTGATTCAGTATTTAGCCGTTGTGGCATCAT

CACACAAAGGCAAGAAAGACACAAGCGCTGGGGAGCTGGAGAAGCAGCTTTTGCAGGCCA

ATCCTATTTTGGAGGCATTTGGTAACGCCAAGACCATCAAGAACGACAATTCTTCACGAT

TCGGTAAATTCATCCGCATTAACTTTGATGTAACAGGCTACATCGTTGGAGCAAACATTG

AAACCTACCTGCTGGAAAAATCTCGCTGTATCCGACAGGCTAAAACTGAGAGAGCTTTCC

ACATCTTCTACTACATGGTGGCAGGAGCCAGGGACAAGCTGCGTGAGGAACTGCTGCTGG

AGAATTTCAATAATTACCGCTTCCTGGTAGCAGGACATGTGCAGGTTCCAGGAAATCAGG

ATGACGAGATGTACGATGAAACAATGGAGGCGATGAACATTATGGGCTTTACTGATGAGG

AGAGGATCGGAATCCAGAAGGTTGTGTCCACTGTGCTGCAGCTTGGCAACATCGAGTTTA

AGAAAGAGAGGAACCAGGAGCAGGCCACCATGCCTGACAATACAGCTGCACAGAAGGTCT

GTCACCTGTCAGGCATCAATGTGACGGATTTCACCCGTGCCATCCTGACCCCACGCATCA

AAGTAGGCAGAGAAGTCGTACAGAAGGCGCAGACCAAAGAGCAGGCTGATTTTGCAGTGG

AGGCTTTAGCAAAGGCAACGTATGAGCGTCTGTTCCGCTGGATTCTTGCTCGTGTCAATA

AAGCTCTGGATAAAACCAAGCGTCAGGGCGCGTCTTTCCTTGGCATCCTGGACATTGCCG

GCTTCGAGATCTTCGAGGACAACTCGTTTGAGCAGCTGTGTATTAATTACACTAACGAGA

AGCTGCAGCAGCTCTTCAATCACACCATGTTCATACTGGAGCAGGAGGAGTATCAGCGTG

AGGGCATCGAGTGGAACTTCATCGACTTTGGGCTGGACCTTCAGCCCTGCATCGAGCTTA

TCGAGAGGCCGAACAACCCTCCAGGTATCCTGGCCCTACTGGATGAAGAATGTTGGTTCC

CTAAAGCCACAGACGTGTCGTTTGTAGAGAAACTCACAAATCAACATGCCAATCATTTGA

AATTCGCCAAACCCAAACAGTTGAAGGACAAAACTGAGTTCTCAGTGCTACACTACGCCG

GCCGGGTGGATTATAACGCTGTTGCATGGCTGACGAAGAACATGGATCCCCTGAATGATA

ACGTTACTGCACTGCTCAACAACTCCTCCAGTGTGTTTGTGCAGGATCTGTGGAAGGACG

CGGATCGTGTGGTGGGACTGGAGACCATCGCCAAGATGTCGGACAGCTCCATGCCCAGTG

CCTCCAAAACGAAGAAGGGAATGTTCCGCACCGTCGGTCAGCTTTACAAGGAGTCTCTGG

CCAAACTGATGACCACGCTACACAACACGCAGCCCAATTTCGTCCGCTGCATCATCCCCA

ACCACGAGAAGAGGGCGGGTAAGCTCGATGCTCACCTAGTTTTAGAGCAGCTCAGGTGTA

ACGGCGTGTTAGAGGGAATCAGGATCTGCAGACAGGGCTTCCCTAACCGCATCGTCTTCC

AGGAGTTTAGACAAAGATACGAGATTTTGGCAGCCAACGCCATTCCAAAGGGCTTCATGG

ACGGAAAACAGGCCTGTATACTGATGATTAAGCACCTGGACTTGGACCCCAACCTCTACC

GCATCGGTCAGAGTAAGATCTTTTTCAGGACTGGTGTTCTGGCTCAGCTGGAGGAGGAGC

GCGACCTGAAGATCACAGTTATCATTATCGCCTTCCAGGCACAGGCCCGAGGCTTTCTCG

CCAGGAAGTCATTTGCCAAACGGCAGCAGCAGCTGACAGCCATGCGAGTGATCCAGAGGA

ACTGTGCTGCTTATCTGAAGCTCAGGAACTGGCAGTGGTGGAGACTCTTCACTAAGGTGA

AGCCTCTACTCCAGGTGACACGTCAGGAGGAGGAGCTAAGTGTCAAAGAGGAGGAGCTTC

AAAAAGCCAAAGATTCAGCGCAGAAGTACGAAACTGAGCTGAAAGATATTACGCTGAAAC

ACACACAGCTTGTGGAGGAGAGGAACGCTTTGCAGGAGCAACTTCAGGCAGAAACTGAGC

TTTATGCTGAGGCAGAGGAGATGCGAGTTCGATTAGTTGCTAAAAAGCAGGAGCTGGAGG

AGATCCTTCATGAAATGGAGGCACGGCTGGAGGAGGAGGAGGAGCGGAGCGCCACCCTGC

AGGTTGACAGGAAGAAGATGCAGCAGCAGATCCAGGAACTGGAGGAACACCTTGAAGAAG

AGGAGGATGCGCGACAAAAGCTGCAGCTGGAGAAAGTCACCTGTGAGGGAAAGATCAAGA

AACTGGAGGACGACCTGCTGGTGATCGAGGATCAAAACAACAAACTGCTGAAAGAGAAAA

AGCTGTTAGAGGAGCGTGTTGCTGATTTTAGTGCTAATTTAGCTGAAGAAGAGGAGAAAT

CTAAAAACCTGACCAAGCTAAAGAACAAGCACGAGTCCATGATCTCAGACCTGGAAGTGC

GTTTAAAGAAAGAGGAAAAAACTCGGCAGGAGCTCGACAAGATAAAGAGGAAGCTGGAGG

CGGAGTCTAATGACCTTCAGGAACAGATCGCTGACCTTCAGGCGCAGATTGCTGAGCTAA

AGGCACAGCTGGCTAAAAAGGAGGAGGAGCTTCAGGCTGCACTAGCAAGGCTGGAGGACG

AGACAGCACAAAAGAACAACGCATTGAAGAAGATTCGTGAGCTGGAGGGCATCATCTCAG

ACCTGCAGGATGACCTGGACTCTGAACGTGTCGCCCGAAACAAAGCAGAGAAGACCAAGA

GAGACTTGGCAGAGGAGCTGGAGGCCCTAAAGTCTGAGCTGGAGGACACATTGGACACCA

CAGCCACTCAGCAGGAGCTCAGGGCAAAGCGTGAACAGGAAGTGACCCTGCTGAAAAAAG

CAATAGAGGAAGAGAGCCGAAACCATGAAGCTCAGGTGCAGGAGATGAGACAGAAACACA

CACAGGCTGTAGAGGAGCTCACAGAACATTTGGAACAGGCCAAGAGGGTGAAGACAAACT

TGGAGAAGGCAAAGCAGACGCTGGAGAAGGAGACATCAGAGCTGACAGTTGAGATCCGCA

CCCTCTCTCAGGCCAAACAGGAAGTGGAACACAAGAGAAAGAAAGTGGAGGGACAGTTAG

CAGATCTGCAGTCCCGCTTCAACGACAGTGAAAAACAGAAAGCTGAGCTTGGAGACAGGG

TGTCCAAAATGACTGTGGAGCTGGAGAGTGTCACCAACCTGCTAAATGAGGCTGAGGGGA

AGAACATTAAACTGAATAAGGATGTGGCCAGTCTGACCTCTCAGGTCCAAGACACACAGG

AGCTGCTGGCTGAAGAGACACGTCAGAAACTGCAGTTCTCCACAAAACTGCGTCAGATGG

AGGATGAGAGAAACAGCCTACAGGATCAGCTGGAGGAAGAAACAGAGGCCAAGAGGAACG

TGGAGAGACATGTCTCCACCCTCAACATCCAGCTTTCAGATTTTAAGAAGAAGCTGGATG

ACGTGTCAGGAAATGTTGAGCTGCTGGAGGAGAGCAAGAAACGTCTGCAGAAAGACCTAG

AGTCCTCAAACTCCCAGCTGGAGGAGAAGTCTTCTGCCTATGAGAAACTGGAGAAGACCA

AGAACAGACTGCAGCAGGAATTGGAGGACATTCTTATGGACCTGGACAACCAGAGACAGC

TTGTCTCCAATCTGGAAAAGAAGCAAAAGAAGTTTGACCAGATGCTTGCAGAGGAGAAGA

GTGTGTCCTGTAAGTACGCCGATGAGCGGGACCGGGCAGAGGCAGAGGCACGTGAGAAGG

AGACAAAGGCGTTGTCTCTGGCGAGGGCACTGGAGGAGGCGCAGGACACACGTGAGGAAC

TGGAGAGAGCCAACAAAGCCTTACGGGCTGAGATGGAGGACCTGATCAGCTCCAAGGATG

ACGTAGGAAAGAATGTCCACGAGCTGGAGAAATCAAAGCGTGGTCTGGAGCAGCAGGTTG

AGGAGATGAAGACGCAGCTAGAGGAGCTGGAGGACGAGCTGCAGGCTGCCGAGGACGCAA

AACTACGGCTGGAAGTGAACATGCAGGCCCTCAAAGCACAATTTGAACGAGACCTGCAGG

GACGAGACGAGCAGGGAGAGGAGAAAAGAAAACAGCTGATAAAACAGGTCCGCGAGCTGG

AGACGGAGCTGGAGGACGAGCGAAAACAGAGAGCCGCCATGGCTGCGTCCAAGAAGAAGA

TGGAGGTAGACATAAAAGACCTGGAGGGACAGATAGACACAGCCAACAAGAACAGAGACG

AGGCCATCAAACAACTGCGCAAACTACAGGCTCAGATGAAGGACTTTCAGAGAGAGCTGG

ACGACACGCGAGCTGCCAGAGAAGAAGTTCTGGCCACCGCTAAAGAAAACGAAAAGAAGG

CCAAGAACCTGGAGGCGGAGCTTATGCAACTTCAGGAGGACTTGGCAGCTGCAGAACGTG

CAAAGAAGCAGGCTGAAGCAGAGCGTGATGAACTTGCTGATGAACTCTCCAGCAACGCAT

CTGGAAAGTCTGCACTGAGTGATGAAAAACGCCGTCTTGAGGCTAAGATTGCTCAGCTAG

AGGAGGAACTGGAGGAGGAACAGAGCAACATGGAGATAATGAACGACAGGCTGAGAAAGA

GCGCTCAGCAGGTGGACCAACTGACAAGTGAGCTTCAGACAGAAAGAACAACTTCACAGA

AGAATGAAAGTGCTCGTCAGCAGCTGGAGAGACAGAACAAAGAACTGAAAGCCAAACTGC

AGGAGATGGAAAACCAGGTGAAGTCCAAGTTCAAGTCCTCAATTACTGCTCTAGAGGCTA

AAGTGGCACAACTGGAGGAACAGCTGGAGCAGGAGAATAGGGAGAAACAGGCCACAGGTA

AAACAATGCGTCAGAAGGAGAAGAAGTTGAAGGACCTCACGATCCAGTTGGAGGATGAAA

GAAAGCAGGCTGAGCAATACAAAGACCAGGCAGAGAAGGCAAATGCACGGGTAAAGCAGC

TGAAGCGACAAGGGGAGGAGGCGGAGGAAGAACCCCAGCGCATCACCGCCGCTCGCCGTA

AACTACAGCGAGAACTGGAGGAAGCAACCGAGTCGAATGATACACTGAGCCGAGAGATTA

CATCACTGAAAAACAAGCTGAGGCGCGGTAATGAGGCTCCATCATTTGCTAGCGGTGCGC

GGCGTACCGGTGGAAGGCGGGGCCTGATCGACGCTGATGCTAATGACGAAGATGCCGAAT

CTCAGGGCGACTACAATGGCACCAAATCAACTGAATAAACACACACACACACACACA

>Unigene000171

AAGAAGAAGAAGAAGAAGAAGAATCGCCTGTGCAGAATATAAAAGGGGATGACGTAGGGG

CTCAGGCCAGTCACAGAAAACAGAGATGAGCCTGACATCCTGGTTCCTGGTAAGCAGTGG

GGGCACACGCCACCGGCTGCCCCGTGAGATGATTTTTGTGGGTCGTGATGACTGCGAGCT

CATGCTACAGTCTCGCAGTGTGGACAAGCAGCACGCCGTGATCAACTACGAACCGGCAAG

TGATGAGCACAAAGTGAAGGATCTGGGAAGCCTTAATGGGACATTTGTGAACGATGTTCG

AATCCAGGAGCAGATGTACATCACCCTGAAGATTGACGATAAGCTGAGGTTTGGATATGA

TACTAACTTGTTCACTGTGGTCCGAGGAGAGCTTCATGTTCCAGAGGAGGCTTTAAAGGT

AGCCTGATGAAATTGTGTGTGTAAATTTGTGAATTGATTGAATTGTTGAGTGATAAGCGA

TGTCATAAAAATGTTCCTTTTTTTTTTGTTTAAAGCATGAGAAATTCACCAGTCAGCTTC

AGCTAGGGAAGAAGCCAGTTTGCACTGAGTCTGCCAAAACACCCAAATCTCCTACAGCCA

GCGGCGCAGAGAGCAAAGTATCCGTACCCACTGCGGAACCTTCTACCAAAGTGGTGGAAC

CCTGCAAGACCGAGGACAAAATGCAAGGTGATACTGCAGCACTGCACCGAGGAACACCGT

TGTATGGCCAGCCTTCGTGGTGGGGGGATGGTGACGCCGATGACGAGAACTCGTTTAAAC

AGGAGAATAAAGCAGCAGGGAAAAAACAGGAGCCTCATGGGACAGACACCAAGGACACCA

GGCGGACTGATAAAGCCAAGGAGAATGGTGTGAGCACTGCAGGCCCTGAGCCCAGCTACT

TTGAGATCCCGAGTAAAGACGGCCAGATGGCAGAGGACAGCATCCATGAGGTCCCGACCA

AGGACACAGAGAGCAGCGCAGCTGCAGCAGCAGCAGCAGCAGCAGCGAAAGCCGCCCAAG

CGAACACCTCCTTTACTATTGAGTTTGACAACACCTCTCCAGGAAAAGTAACCATTAAGG

ATCACGTGTCGCGTCCAAGATCGAAAAAATCTCAGCCAGGGAGTAAAGAGCTCAGCAGCC

TGCAGGCAGCCATCATGGCCTCAGAGAGCAAAGTGGCCGACTGGCTTGCGCACAACAATC

CCCCCATGGTGAGACGGGAGTCCACGGAAGAGGACAGCAAAAGCATTAAAAGTGATGTGC

CTGTTCTTCTCAAAAGGCTAAAAGGCAGCAAGCACGAGGACGGGACCCAAAGTGACTCTG

AGAACGGCCTTGGCTTGCGTTTCGGTAGCCGGCGCCATGCTGCTCTGGAGGAACGCTTGA

AGGCAGCTGGCCTTACGCATGCCAAAGTCACCGGAGCAGAGGGCTCGGGCTCCAGCAGCC

GCAGCGCCTTCACCATCGAGTTCTTCGACGAGGAAAACCCGCGCAAACGCCGCTCCTACT

CCTTCTCCCAGACTGCACCACTTCTGGGAGGAGAGGGACTGTGTCCAGCACCACCATCCC

ATCCGAAGGGAACTTCATTAACACTGGCAACAGTAGCGCCTACAGCAGCACGGCTGCTGC

AGAAAAAAAGGTCAGAGGAGAAGACCACGACGGTCGGAGATGAGACTCAGAAACAAGACG

ACGATCAGAGCGACAAAGGCACTTATACCATTGAGCTGGAGAACCACAACCCTGAGGAGG

AGGAGGCCCGTCGCATGATCGATAAGGTGTTTGGCGTGGAGAACCCTCAGAATCCCAGTC

GCTCAGTGCAACAGACAACTAAAGACAGTAAGAAGGTCAGCAGCACTAAAGCGGAGGGTC

TTTTGGATGAATCGGTTGCGGTTGGAAGTCCACACTGGGTCTCTCAATGGGCCACTTTGG

CCGCTAATCACACAAGAACTGATCCTGAAGGTTCCGGGGCAGAACCCACAAACTTTGTTC

ATCAAGAGCGAGAAGCTGATGCATTTGAGTCAGGAGTGTCTTTGAGAAGCACCAGCTCTG

CCACCTCTAGTCAGGCTGAACGTAAAAGAAGGACATTGCCTCAGCTCCCCTGTGAGGAGA

AGAGCAGGCTGGGGAAGGTCTCCTCCACCTCAGGACTCCGATCCGAGATTGGCGAGAAAC

AGGACACCGAACTCCAGGAAAAACAACACAAAGGAGATGGCGAATGTGCATCCACTCTCA

TTAAGAAGGACACTACAAGTGGAATTGCAAACCAAGTGCAAACAGCCACTACCAAGCCTC

CTGTGCCCCCTGTGGGAGGGGTGGAGAAGCAAAGTGAGGAGTTTCGCAGGAGGCGTGCTG

AGGACAGGAACAAAGGTAGCGATAGTGAGCGAAGCGGGAAGCCCCTGGTGCGTCAAGGCA

GCTTCACCATTGACAAGCCAAGCACCAATGTTCCGCTTGAGCTTATCCCACGCATCAACC

AGCGGCAGTCCGGAAACAGAGAGCGTAGTGATTCAGCTGGAAGCATGGACACGACCACCT

TGCTCAAGGACACTGAGGCCGTTATGGCCTTTCTAGAGGCTAAACTGCGTGATGAGAAAA

AGCTGGATCCATCACCGCCCGCTGGCTACCCTTTGGGACGCTCTTTTTCCCCAGAGTTGG

ATACTGATGCAATTAAAGCAGCAGGCCGCGCCGAAGTGTCACAGAAGCGTCGTTCTCTCA

GCAGTATACACAGAGAGAAAAGTAACATGAGCTCCGTTGGAAAGAGCACAACCAATGCAC

GGGAACGTTTGGAAAGGAAAGTCAAGCCGAAGACCTCGGATGGGCGTCTGGATACCCGTC

GTTCTACCCATACAAGTCAGCGGACCCGCCAGCCGTCCATGGACCTCACAGATGATGAGC

AGACATCTTCCTTGCCCGTTTCTGACATACTTTCAGATGACCAGGAAAACGGACGTAGTC

CCTTCACCTTTCCTGATGACTTGCTACTTTCCAAACTGGACTCTTCTAAAGGTGCTAAAG

TTAATAAAGCCAGCAGGACACCACAGGCCACCACTGCCTCTATAGGCAAGCATCAGTTGC

CTCAGCCTCGGCCCACAAGAGCATCTCTTCTGAGACGTGCTCGACTTGGAGACACATCTG

ACACAGACCTGGCAGATGCAGACCGAGTGTCAGTTGCATCCGAAGTGTCTACTACCAGCT

CCACATCCAGGCCACCATCGGGCAGGAAGGCACCATCCCGCATCGATATGCTTGCACAGC

CCAGACGCACCAGGGTCGGGACCATGTCAGCACGCAGCGACTCAGAAGCCCCAATGTCAC

GCAGCACTACATCCAGAATATCGGCCGAGACAGCTTTAAGACTGGGCTTGCGCCCAACTA

ACCCTGCAGACAGCAAGCTGAACCCACGTCTTCGGGCAAACAGCGTCTCCAAGTTGACAG

ACACCAAAACCAAGACTACAGCATCCATCTCCAGTGCTCCAGCAGAGTACGTTTACACAG

AGATGTCTCAAGCAGAGCTGGAGGATGACCCAGCAGACCAGGACCCCATGGCCAGCAGCA

GGTGGCGGCGACTTAATACAGAATACGGCTCCACCTCTGAGGACGAATTTGGCTCAAACA

GGAACTCGCCAAAAGCCGGGCGTCTGAGGCCAGGCACTGCTCTGCGTACCAGTAAACTGA

GCAGCTCAGGCATGGTGACAACAAGCCCTGGCAGCATCACCATCAAACACAGGATGAGAG

AACAGGAGGAGTACATCAGAGACTGGACAGCCCACAGCGAAGAGATCGCCAGGATCAGTC

AGGACCTAGCCAAGGATCTGGCTATTCTGGCGAGGGAGATCCACGATGTGGCCGGCGAGA

TCGACTCGGTCAGCTCCTCAGGCACAGCACCAAGCACTACGTTGGTAGAACGAGTTTTTG

ATGAAAGTTTGAACTTTCGGAAGATCCCCCCTGTGGTGCAGACCAAGACCCCAGAGATGA

ACGGCCGGCCTGTAGAGCTCCGACCTCGAGCCCCAGACAGTCTGGATCCCCAGAATGCAC

TGCGTAGACGCACATGGAACCGAGATGAGGTCAGACCGGCGGTCCTAGAAAGCTTGCTGC

TGAGCTCAGTCTCTCAGCTGTCCTCTAAAATCCGCCAATCTGTGGACAAAACAGCAAGCA

AAATCAAGATTTTGTTCAAAGACAAAGACAGGAACTGGGATGACATTGAGAGCAAACTGA

GCTCAGAGAGTGATATGCCACTTCTCAAAACCACTAACAAGGAAATCTCAACAATTCTAA

CAGAGCTAAAAAGAGTGGAAAAGCAGATCCAAGCTATAAATATCATGGTAGACCCAGATG

GAACCCTGGATGCTCTTACCAGTCTTGCCCTAGCAAGTCCCACCACACCCAAACCTAAGA

CCAGCAACGGAGCCTCCAAAGCCTCCGTGATCATCTCCCCGAACCGAGAGCTCCTCAGCG

CTGGACTGAGTCGTCCTCAAACCAGCGGAGAAGCGAGCAGCATTACTAACAAGTAAAGAC

AGAAAACCCTCAGAGTTGAGTAAACTGAGAAACCCTTTTTGTCCATGTCTGTACCCCTCC

CTCTCCTGCACTCCATTGGAATGTCCATCTTTATGTAAATGAAGTCACACCTGTGGATTT

GGACAGTGATATACAGTGCAGCTCTACAAGTGCACGCAGCTGCTGGTGAAGAAGAGCTGA

AGAGGAGCATTAGTCTAAAGGCTTAAAATGTTGGAACCAGCCACCTGTATGCTGCTGCAA

AGGTGTACACACTCATCCAGGCATGAAGCAGGTGGACTTAAAATAATTTATCTTCAACAA

TAAATGACTCAGAGTGCCTGTCTTCTCTCTTTTGTGAGCATTCTTTCTTGCAAATAGCAA

CTTCTTATTACTACGTGACTTTTGTGTCAAAAATAAAACAGTGTTGGTCAAAAAGACTTC

AAGATTTCAAAGCTGAGCATAAACCAGCTTAGCAAAGTAAGTCTGATTCTATGCGGGCAC

TCAAACATTGTTCTTTTTTTTCCATTTCTTATTTCTTGATTACAAAAAGTGCATTTAGGC

TATTAAAATAAGCAGGGGTCAACTTGGTAAGTTAAACTGACCGTTCATTGCATTATCTTA

TGCACTGATTTTTTATTTTTATTGGCTAATGTATACAGTAGGTAGCAAACTTTGTACAAG

GTTCAGACATTAATTAAAACCCAGCAAACTGGCGTTTGTGTCATTTTCCCCCTGACATTA

GAAGGAAGATCATATGCAGTATTAGAAAAACGTCTTAGTGGAATGCACCATTGTAGTGTT

ACACATCACAGTTGCTTAGATCTCTGTGCTTTGTGCTGAAATGTTAAATCAGCCTTAAAT

TAACCAGCCCTTAATGTACGGTTCAATCACATTCTGAACATGTTATGGCCAGTTGCTTAA

ATGCTAACGTTCTTTAATCAGAGTATGCAATTACAAGAACCAAATAAAGTCTGTCAGCAT

TTTTTTTTTTAATTATCACAACCTCATAGATCATGTTTTTTGTTCATGCAGGTATTCCGG

CATATTATGTATGTACTGTATTGTCTTACTCTTCCGTCGGCGCTTGCTGCTTGGCTGGAA

CTCATGCGCCTGAGAAAAAGCGTCCAGCGCCCAGCCTTTCATCGTGAATCTGCGTGTTGT

AGAAAGACTAGTGCAAAATAAAACAATAGTGAAGATAGGTACAGGACATTAGTTGTTGTT

GTTTGTTAATAAATGTCAATGCCAAGTGGCTGCTGTGGAAACGCTAGTTCCTGGACAGGT

TTAAGAAAGAAAATCTTAGTTTAGTGACCGCATTTGTGGTTTTAAAGGTTCCTGGATTAA

CTGGGTGAGTTAATGTTAAAGGTGATACAGGACAACAAACACCAAACTCTATACATTATA

CCGCTCGCAGAACAGGATAGATTTTCAGGCAGATGATCCTCCTGCCCCTAAAACACTTAA

AAAAAACAGTAGTTATCATTTATAAAACTATTGTACATGTTTTACATATAAGGGG

>Unigene000172

AGAGAGAGAGAGAGAGAGAGACTGTGTTCTGAGACAGAGGAAGAGACCAGAAGGCTTTGT

TGACTTATGCAGCCCACCATGCCGAGCTCAAACAAGGTGTGAGTGTGTACGCTCAACACA

CACCCACCTACACACACTCATTTTGGAGGGAAGGCTGAAGACCGAGAGCGTTGAACTTTG

CTCATCAGAGAAGAAAGGAAAGTCGGCAGGTCCGTACAGGAGGAAGGAGCAAAAGAAGTG

GAACAAGCTGTTCTTTCTTTATCCTTTTTGTTCTTAATTCATCCACTGGGTTTCTACATT

GAAGAAAGGAGGGGGTTTTTAGGGACGGCATGGCGTGACAAACTCTGCTACCTGTGGACG

TGTCGCTTTGCACGCAACTTGCTTCGAAGCCGGACTCTTGCACCTGGACCGACCTGCTGA

TTCCACTCTTCTTCTTCTGCTACTGCTTCTTTCTTCTTCTTTTTTTTTTACATCTTTTAA

CGCTGGTTGGACGAGTCCTAGCATGCCCTGAACCCTCGCTGTCTTTTTAACGGAGCTGGT

CATTCGTATCGGGAGCAGGTTATTGTGTTGGCTGGTGTAAAAGCAAAAGGAAAAAATAAA

CTCGAGAGGTAGAGATGGAGTACGGGCGGTTGGAGAGGGAGCCATGTCTCTCTCCCGCTG

CATTCGTCAACCAGGTGCAGTATTCCAACATCCTGGAGGGCCGTTTCAAACAGCTGCAAG

ATGAGCGAGAAGCCGTACAGAAGAAGACCTTCACTAAGTGGGTGAACTCTCACCTGGGCA

GGGTGACATGCAGGATCAACGATCTGTACACGGACCTGCGGGATGGACGCATGCTCATCC

GCCTGCTGGAGGTTCTTTCCTCCGAACAGCTGCCGAAGCCGACAAAAGGACGGATGAGAA

TCCACTGTCTGGAGAACGTGGATAAAGCGCTGCAGTTCCTCAAGGAGCAGAAGGTCCATT

TGGAAAACATGGGTTCTCACGACATCGTCGACGGCAACCACCGCCTCACGCTCGGCCTCA

TCTGGACCATCATCCTGCGCTTCCAGATTCAGGATATCAGCGTGGAGACGGAGGACAACA

AGGAGAAAAAATCCGCCAAAGATGCTTTACTGCTGTGGTGCCAGATGAAGACTGCTGGGT

ACCCGAATGTCAATGTTCACAATTTCACCACTAGCTGGAGAGACGGCCTGGCGTTCAACG

CTATCGTGCACAAACACAGACCGGATCTAATTGAGTTTGACACTCTGAAGCGATCCAACG

CTCACTACAACCTACAGAACGCCTTCAACATTGCCGAGAAGGAGATGGGGCTCACCAAAC

TGCTGGATCCTGAGGATGTAAACGTGGATCAGCCAGACGAGAAGTCGATCATCACCTATG

TGGCCACGTACTACCACTACTTCAGCAAGATGAAGGCGCTCGCCGTGGAGGGCAAAAGAA

TCGGCAAGGTGCTGGACTATGCCATCGAGGCAGACCAGCTGATCGAGAAGTATGAGACGT

TGGCGTCGGAGCTGCTGCAGTGGATCGAACAGACCATCGAGACGCTCAACGACCGGCAGC

TCGCCAACTCACTGAGCGGCGTGCAGAACCAGCTGCAGGCTTTCAACACCTATCGCACAG

TGGAGAAACCACCCAAGTTTACAGAGAAAGGCAATTTGGAGGTTCTTCTCTTCACCATCC

AGAGCAAAATGAGAGCCAACAACCAGAAAGTCTACATGCCTCGAGAGGGAAAGCTCATTT

CCGACATAAACAAGGCGTGGGAGCGTCTGGAGAAGGCGGAGCATGAACGTGAACTGGCTC

TGAGAAATGAGTTGATTCGTCAAGAGAAGCTAGAGATGCTGGCAGCGCGATTTGACCGCA

AAGCTGCCATGAGGGAGACGTGGCTCAGTGAGAACCAGCGTCTCGTCTCGCAGGACAACT

TCGGTTTTGATCTCAGCGCCGTGGAGGCTGCGACACGCAAGCATGAGGCCATCGAGACGG

ACATTGTCGCGTATGGAGAGCGTGTGGCTGCTGTAGAAGCCGTTGCCCGTGAGCTTGATG

CGGAGCGATACCATGACGTCCGGCGCATCCTGGCACGCCGGGATAACGTGCTACGCCTCT

GGGAGTACCTAAAGGAGCTGCTTGCAGCCAGGAGGGAAAGACTTGTGGCACACCGCGACC

TACAAAGACTTCTGCAGGAGATGAGCTACATCATGGACTGGATGGAAGACATGAAGGGTC

GTCTCCAGTCCCAGGACAGTGGGAAACATCTTCACGACGTGGAGGACTTACTTCAGAAAC

ACACTCTAGTAGAGGCGGACATATCGGCGCAGGCAGAGAGGATCCGAGCGGTGCAGGCAG

CAGCCAATCGCTTTACCTCGGAAGAAATAACCTATAAGCCCTGCGAGCCAGCGCTGGTGG

AGGAGAAGGTGGCGCTTCTGGGCCAGACGTATGAAGAGCTGGGTCAGCTGGTAGTCGAGC

GACGGGCACAGCTTGAGGACTCGCGGCGTCTCTGGCAGCTGCTGTGGGAGCTCGGGGAGG

AGGCAGCGTGGATGCGGGAACAGGAGCAGATCATGGCAACAGAGGAGTGCGGGAAGGATC

TGTCCTCTGCACTGCGTCTCCTTTCTAAACATGAGGCATTTCGAGAAGAGATGGCAGCAC

GCTATGGCCCGCTGGGAAACAGCATCGCTGCTGGGGAGCAACTGGTGAAGGAGGGGCACT

GCGGAGCACCCGAGATTACAGAGAGGATCAAAGAGATCAAAGCTCAGTGGGGCAGACTAG

AGGAGGCATCTCGTTTGCGAGAGGAGCGTCTAAAGGAGTCTGTGACTTTCCACCAGTTCC

AGACTGACGCCTGCGATATGGAGGCCTGGCTTCAGGAAGCTCTTAGACAGGTGTCCAGTC

AGGAGGTCGGCCATGATGAGTATTCTACACAGACGCTGGCACGGAAACACAAAGAGGTAG

AGGAGGAGATCCAGAGCCACCGCTCTCTCATAGACTCGCTGCATGAGCAGGCTCAATCAC

TGCCCGAAGAATACGCCCAGTCTCCACAGGTGGAGGGTCGTCTTCCTGCTATAGAGCAGC

GCTATGAAGAGTTGAAAGCTCTGTCCTCTGCACGACGCGACGCCCTGGAGGGCGCACTAG

CCCTTTACCGGATGTACAGTGAAGCCGACGCCTGCAAGCTGTGGATTGGAGAAAAGGAGC

AGTGGCTCCTCACTATGGAAATCCCTTCCAAACTCGAGGACCTGGAGGTGGTCCAGCAGA

GGTTCGAGACACTGGAGCCGGAAATGAATAATATTGGCTCTCGCGTCTCTGACGTCAACC

AGGTCGCGGAACAGCTGCTTAAATCCGACAACTGCAACAAGGAGCAAATTCACCAGACTC

AAGACCAGCTCAATGACCGGTGGCATGAGTTCCAGCGGCTTGCTAATGAGAGGAAACAGG

CTATGGAATCAGCTCTAAATCTCCAGAACTACCATCTAGAGTGCAACGAGCTGCAGAGCT

GGATGAAGGAAAAAACCAAAGTGATCGAGTCTACACAAGGGCTCGGCAATGATCTGGCTG

GGGTCATGGCACTGCAGCGCAAGCTCACGGGCATGGAGCGAGACCTAGAGGCCATACAGG

GGAAGCTGGGCGGCCAGCGTGCCGAGGCCGAAAAGTTGGCGTCCGAGCATCCGGAGCAGG

CTGAGGAGATCCAGGCACGGTTAACTGACATGGAGGATGTGTGGGAGGAGCTGTGTGGCA

CCATGAAGAAGCGTGAGGAGAGTCTGGGCGAGGCCTCCAAACTCCAGGGCTTCCTGCGTC

ACCTCGACGACTTCCAAGCCTGGCTCTCGCGCACGCAGACCGCCGTGGCTTCTGAGGACA

CACCCACCTCACTTGCTGAGGCGGAGCAACTCCTGGCTCAGCATGAAGCCATCAAAAATG

AGGTGGACAACTATCGCGACGACTACGAAAGGATCAAGGCCACCGGTGCCGAGGTGACCC

AGGGCCAAACGGATGCCCAGTACATGTTCCTGGCTCAGCGGCTGCAGGCACTGGACACGG

GCTGGCAAGATTTGCGCAGGATGTGGGAGAGCAGGCAGTGCCTCCTGGCCCAGGCATTCG

ACTTCCAGACGTTCCTTAGAGACGCCAAGCAGGCCGAGAGCTTCCTCAACAGCCAGGAGT

ATGTTCTCTCGCACACCGAGATGCCCTCGAGCCTGGAGGGGGCGCTGGAGGCTATCAAAA

AGCACGAGGACTTCCTCACCACCATGGAGGCCAGTGAGGAGAAGATCAACGGTGTGGTGG

AGTCAGGGCGACGCCTTGTTTCTGACGGCAACGCCAACGCCGACAAGATCCAGGAGAAAG

CCGACTCCATTGAGGAGCGGCACCAAAAGAACAAGCAGGCTGCTAATGAGTTGCTGGGTA

AACTGAAGGATAACAGAGAGTTGCAGCACTTCCTCCAGGATGGACAGGAGCTGAGTCTGT

GGATTAATGAGAAGATGTTGACAGCTCAGGACATGACATACGACGAGGCCAGAAACTTGC

ACAGTAAGTGGCAGAAACACCAAGCTTTCATGGCCGAGCTGGCCTCCAACAAGGACTGGC

TGGACAAAATCGCTAAGGAGGGTCAGGCTCTGGTGAAGGAGAAACCCGAGCTGGAGGAGA

CAGTGTCCGAGACCCTGCGTGAGCTGCAGAAGCAGTGGGATTTGTTGGAGACCACCACAC

AGACCAAAGCGCAGTGTCTGTTTGACGCCAACCGCGCTGAGCTTTTCACCCAGAGCTGCT

CGGCTCTCGACACATGGCTGCAGAACCTCTCCTCACAGCTGCAGAGCGACGACTTCGGCA

AGGACCTGACCAGCGTCAACATCCTGCTCAAGAAACACCAGATGCTGGAGCATCAGATGG

AAGTGCGAGAGAAGGAGGTGCAGTCTCTGCAGTCACAGGCTCTGGCCCTGTCTCAGGAGG

ACGCCAGGATCGTCGAGGTAGACGGTCAGCAGAGGAGAGTGATAGACACCTTCGCTCAGC

TCCAGGATCCTCTTAGAGACAGACTACAACACTTACTAGCATCGAAAGAGGCCCACCAGT

TCAACAGAGACCTGGAGGATGAGATACTGTGGGTAAAGGAAAGAATGCCACTGGCTACAT

CGGTAGATCACGGCAAAGACCTGCCCAGTGTTCAGCTGCTCATAAAAAAGAACCAGACGC

TTCAAAAAGAAATCCAGGGCCACCAGCCTCGCATCGATGACCTGCAAGCCCACGGGCGCA

ACATGGCCCCCGGGGGCGGGGCAGAGATGAAGGGGCAGCGGGACGAGCCTCTGAACGAGC

TTGGGGATCTGTGGACGTGGCTCATCGCAGAGACGGAGCAGAGACACAAACGCCTAGTTG

AGGCCCATCGCTCTCAGCAGTTCTATGCAGACGCGGCCGAGGCTGAGGCCTGGATGGGAG

AGCAGGAGCTGCACATGATGTCTGAAGAGAAGGCGAAGGACGAGCAGAGCGCTTTGGTGA

TGGTAAAGAAGCACCAGATCCTAGAGCAGGCGTTGGAGGATTATGCTCAGACCATCCACC

AGCTGGCCAATAGCAGCAGACTTATGGTGGACAGTGAACATCCTGAGAGTGAGAGGATCG

CGCTGAGGCAGGCTCAGGTGGATAAGCTGTACGCCGGGCTGAAGGACCTGGCTGAGGAGA

GGCGGGGCAAACTCCAGGAGCGACTGCGTCTCACACAGCTTAAGCGAGAGGTGGACGATC

TGGAGCAGTGGATCGCTGAGAGGGAGGTGGTCGCTGGATCGCACGAGCTCGGCCAGGACT

ACGAGCACGTCACTGTAAGCGCAACATTAGGCAGAAATCTCTGATACATGAACTGGAATA

GATACAGGAAATACAGTATGGTATCGGTTACTGGCAAAGACAAACTATCTTTTATGTGCT

TTAAATTTAATATTCCAGCAGAGATCAGTTTACGACACAGTAGACTATAATATAATTATT

CCATCCTATGAATAGTTCTGTCTCTGTGTCTGTCTCCTCGTCTCAG

>Unigene000173

GGACATCCGGTTGGAGCAGCGTCTGAGCTGGTGATTATTTGGGGGGGGGCACTGTGGCAG

ATTTGCACGGCTTGTAAAAACATGAATGCATAACATTCATGCGGTTTCTGTTTTAAAAAG

CTCAATCGGACATCTCGATGCACAACCTCCTTCACTGTAAAGCAGCGCGGTAAGAAACCT

TTAACGCAAGTACACCTGAGTGCTGGGATCATGAATAAGCAGCCCAATAAGGAGACTCTG

AAGGACAAGTTGAGGGGGGTGTTTGGATTCGGGCAGCGAATCCCCACCAAACAGACTGAG

AGCACACCTTCTGAGTTTATCATCACCCATGACATCCTAAGGGAGCTAGCTCCAGAGTGT

GGACTGAGCAACAGAATCAGAGTCATCAACCATGTATGTGACCTCGCCAGGTCCAAAAAG

TTTGAGGAGAATGCTGTGGAGGCGTTATGGAAAGCGGTGGAGGACATGATGCAGCCCGAA

CAACCATCTGAAGCTCGACATGCTGTGCTACTGCTGCTCAGAGCCATAATACAGGGACAG

GGGGAGAGACTGGGTCCTCTGAGAGCATATTTCTTTAAGATCATTTGGGATTACCAGCCG

TGTAACGAGGACCTGCCGGAGAGACTCGGTGTGTTCAAGGCTCTCACAGAGAACGGAAAA

GATGTCACCTACTTGGAGGATGAGATAGCTCGTTTCGTGGTGGTTTGGATGGAAACTGGT

TTGACAGCTGATTTCTTGCATGTGTTGGTAAACCTGGTCAAGTTCAACTCCTGCTACCTG

GATCAGAACGTCTCACTTATGGTGCAGAAGATCTGCTTGCTCTGTAACCGAACTACATCC

TCTACAGACATCGAGGTAGCTCTGCAGGTGCTGGACGCGGTCGTGTGTTATAACTGCCTG

CCGTCTGACTCTCTGACTATCTTTATCATCACACTCTGCAGAACAGTTAACGTCAAGGAG

TATTGTGAGTCCTGCTGGAAGCTGATGAGGAAGGTGTTGGGGACTCACCTGGGCCACAGT

GCAATCTACACTATGTGTCGCATCATGGAGGAAAGGGTGTATACGGAGGATGCTGCTCTG

CTCAGAGGAGCCGTGTTTTTTGTCGGCATGGCGCTGTGGGGTGCTCACAGACTCCCTGCA

CTAAAAAACACACCCACTTTGGTGCTGCCCTCATTCTACAAGGCGATGAACTGCGCTAAT

GAGGTGGTGTCCTATGAGATCGTCCTCTCGATCACGCGGCTGATAAAGAAATATGGCAAA

GAACTGCAGGTGGTCACCTGGGACATCCTGCTGGCCATCATAGAAAGGCTCCTGCAGCAG

ATACAAAGTGCAGGCAGTCCCGAGCTGAAGGCGATTGTGTTTGAATTGTTGAGCACAGTG

GAGGAGCTGTACGAACAGAACGACTACCACGGATCCACAGAGCGCTTCTTTAGCCTGGTG

GAGAAATGCGCTGAAAAACGACCGGATGCGTCAGTGCTGACTCTAATCTCGTATCGAGCT

CAGTCCATCCATCCTGCTAAAGACGGCTGGATCCAAAGCCTCCATCGCCTTATGGAGAAA

TTCTTCAGGAATGACACTCGTAGCGTGATCAGAATCAAAGTGCTGCACATCCTCTCTTTT

GTCCTCAGCACCAACAGGCAGCTGTATGAGGAGGAGCTGATCGAGGTGGTGGTGCTGCCC

CAGCTGGGTCAGATCGCAGAGGACCGGGACCTGTCCGTGCGCAAACAGGCTACGCAGCTG

CTGGTGGATCTCGCCGAGGGCTGCAACACACACCACTTTAACAGTCTGCTTGACATCATC

GAGAAGGTGGCAAGTCGTCCTCTATCATGCTCTCAAGATGGAGAGAGAGAACTCTCAGCT

GACTCGCCTATGGAGGATGTCAAAACGGCTATATTGGGGCTGCTGGAGATCCTGCAGAGT

AAGCTGTACAGTCTGCCAGCCAGTCATGCAAGTCGAGTGTATGAGCTGCTGATCAGTCAC

CTTCAGCTTCACTACAAAAACAAATACTGCTCTGCCCTCGCCAGTTCTGTACGCCTGCAG

GTGTTTGATTTCTTGTTGTTAATGAGGGCTGACTCACTGCACCGTTTGGGCGTGCCCAAT

AAAGACGGAGTCCTGCGCTTCAGCCCCTACTGCTATTGTGATGTGGGTGGAACAGAGAAG

AGAGAAGCAGAAAAAAAAGCAACCGGAGCTGTGTCTTCGCCGACAGGAAGTCCTGCCCCT

GTGGCTCCGCCCACTTCTATTCGCACCGCCTGCCTGCCATACAGCCTTGCATTTAGTGTG

CTGCTACAGTGTCTAAAGATGGAAACTGACTGGAAGGTTCTGAAATTGGTTCTAGATAAG

ATGCCGTGCATGTTGCAGTATAAAGTTTTGCTCCTCACATCACTGTGCAGTGTAGATCAG

CTGTGCTCCACTCTCTGCTTAATGGTGACTGATCGTCTGATCTCAGAGAGATTAAAACGA

ACTCCAGATGGTTTCTCCCGCACTGATGTCCAGCTGTCTGTGGTTCCTGTTCTGACTGCA

CTCACATCCTATCACAGTTACTTAGAACAGTCCAGACAGAGGGAGCTGGTGCAGTGTTTG

GATACAGGGCTAATCTACCGCTGTGCTAAGCAGTGTGTTGTGGCTCTGACCATGTGTACT

GTGGAAATGCCTGACATCATGATCAAGCTGATGCCTGCTCTCATCGTCAAGCTCACACAC

ATCTCGGCAACCGTCACCATGGCGTCACCCATGCTCGAGTTCCTGTCAACACTGGTCAGG

TTGCCTCATCTGTACGCTAACTTCGTAGCCGAGCACTACGTCAGTGTGTTTGCCATCTCA

CTGCCCTACACTAATCCCTCGAAGTTCAATCAATACATTGTGTCTCTCGCTCATCATGTG

ATCGCAATGTGGTTTATCCGCTGCAGGATGCCTTTCAGAAAAGACTTTGTGCAGTACATC

ACCAAGGGTCTGCGGTCAAACGCTCTGCTTCCGTTTGATGACACACACGAGCAAAGCAGC

TTTCGGGCGCGCAGCACCAGTCTAAACGAGAGGCCAAAGAGGATGCAGACGTCCACTACC

ACGTGCAGTCTGGGCTCCGCAGACGAAAATGCTGTGATGCAGGCGGATGACGCGCTGAAG

ACTGTTCACCTCGAGCTCACTGAAACCTGCCTAGACATGATGGCCCGATACGTCTTCTCT

AACTTCTCTGCTCTGCCCAAGAGGTAGAAGTGTCTGTGTGGGTGTTGGGGGGGGTACATA

GCTCTATTGTGTTATTGTTTCGTCATATGTCTGCAGGTCCCCCATAGCCGAGTTCCTGTT

GGCTGGCGGCCCAAGCATGACCTGGCTGGTTGGCAACAAGCTGGTTACAATAACTACCAG

CGGTGGCTCACGTACTCAGACTTTACTGGGCCTCGACCCGGCAGAGAGACCAGGGGGTGG

AGAGATGACCCGGTCCGACCCGACTCTACACACCAGACAAACAAAGGAAGCTCCAGCCAA

ATTGGAGTCTCAGTCGAGCCAACAACTGAGCCGAGCCACTCGCATCAGAGTTCGCTCTAT

GTCTGGCGGTCATGCTCTGCGTGCTGGAGCAGTGCAAAGTCTTAGCCCCCTGGTTTCTCC

CTCTGAGGGGGAATTCGGCTCTCCCCTTGAAAGCCTAAACTGTCCTCGAGTAGATGATGA

GATCCCACAGTGCTCCTCCTCTTCGTCCTCGCAAATGGCTCCACCCATTCGGGACAAGAG

CGGCCTGGCTGAGTTTGTGCCCATGCTTACCCAGGGCTGGGCGCAGATCTATATTAGGAG

ACCGTCAGGAAACACTAGCTGGCTGATGTGTCTGGAGAACCCTCCAAGTCCGTTCTCCTC

AGAGCTGGGAAACATGCCCCTACAGGAGCTTTCCAGTGTCCTCATGGCTATGGACGGGGT

AAGAGAACCCCAAACTGACGCTCCGTCTGCCTCCTCTGCCCCATCTGATGCCCCTCAGCG

TCATAACAAACCCAGCCTTATCCAGCGCTCCAACACTGACTCTGTAGTAGTCCTGGAGGA

GGGTGGGCATGGCAAGGCACCAGAGTCCGACCCAGAGTCGCCACAGATGGACTTGGAGGC

TACGTCCTCGGAACCTATTTATATGAGCGCTGATATGTTCAAACCTTCCAACGCCATGCT

AAGCAGGTCCTCCAGCACCTCCAGTCAGGATGACAAGTCTACTCTTGAGGAAGTGAGCGA

GGGTGGCATTCCAATCGACCAGCCTGCGTTGACCTTCTCCACTCCTGGGGCTCAATACTC

AGACTTGCCCTTCTCACACTCGCAGACTCTGAACAAGTCCAGCTCATCCCCAGAGCTTCA

AACCCTGCCTGAGGCTTTTGCAAGGGCATCCTGTTCTGGGGCTGGAGCAGGAGATCCGCC

GAGGTCTAAAAGCCAAATTGAGGAAAAGAACATACCAGGTCAGGGTTCATCTGAGAGAGA

AAACTTAGGAGGCGGAGAAAGTGTTGCATCAACAGGACCACAGAGCCTGGGTGCCAGTGT

ATCCACATCTTCTTCAATCTCTAAAATGAAGCTGGAGTTCCCGAGCACCCAGCCAGGCTC

CTTGTCACCTAGTGGGCATAGACCTCGTGGGCACACTATCTCTGTTTCTGCTCCCTCATC

AAGAAGAGAGAGGAAGTCTGACCGAGATTCCTACCACAACCGAGGAGGCGTGTTTAATGC

TGAGAAGAGCTCTGGACTGAGTCCAAGTTTTGTCTTTCTTCAGCTTTACCATTCGCCCTT

TTTTGGCAACGAGGCCAACAAGCCCCTTCTCCTTCCCAAATCAGAGCTGATCGATCGAGC

AGTTAAAGTGCTGGATCAGATGCCGCCGTATGACACGCATAAGATAGGAGTGGTGTTTGT

GGGACCTGGACAGGCGAACAACGAGGTGGCGATCCTGTCGAATGAATACGGGTCGAACCG

TTACGCTCGGTTTCTGACTGGTCTCGGGAAACTCATCCACCTGAAAGACTGTGATCCTGA

CCAGATCTTCCTGGGAGGACTGGACCACGGGCGCTACGCAGACGACGGCGAGTTCACCTA

CTGCTGGCACGATGACATCATGCAAGCAATCTTCCACATCGCCACCCTGATGCCAAACAG

AGAGAGCGATAAAAGCTGCTGCAACAAGAAACGCCACATTGGGAACGACTTTGTGGTGGT

GGTTTACAACGACTCAGGAGAGGAATACAAACTCGGTACCATTAAGGGCCAGTTTAACTT

TGTGGAGGTGCTTATAAAGCCCCTGGACTACGAGAGTAACCTGGTGACACTGCAGTGTCG

CAGAGACCTCGAGGGTTTGGTTGACACCACAGTAGCGAAGATTGTCTCAGACCGGAACCT

TCCTCTGCTGGTGAGACAGATGGCTCTGCACGCTAATATGGCATCACTGGTGCACCAGTA

CAGAGCAAATCCGTCTGACGCCTATGCATCCAAATGGCTCGCTCGACTCCGACACATTAA

GAGGATCAGAATTCGGGCTCAAGAGGAAATCCAGTCTCGCCCGACTCCTGGAATCTCGCT

CACTCAAAGCCACACCCCCATGCAGAACAAAGCTGCCCACCAGTCCTCAGGCTCCGCCTC

CAACACCGATTGCGGCCAGAGGAAGCGGCTGGTTTCTACAGTCGATGATTTCACTGATTT

CGTTTAGTGTTGATCTTTTACTTTAACCCAAAACAGTTAATTAGTATTAAATGTATTTTA

TTATTTCTACTGATATGAAAATTGTACGAAAATACACCTTTTTTAATTAATGTTGGGGGA

AAAATCAGTTTTCTCTCAGCACCTGCACATCCCAACACTCAAGATTTGGCTTCGTTCTGT

TTACTCGCTGCTCGATAAAACTTTTCTTAGAAACACAATTGAAATATTTTTATCACATGA

TCGTTTTTTCGGGAACACAATTATATTTTGTTTGATGGTTATTTTGTGATTTTTTTTTTT

TAGTTGCACGTTTTTGCTTTTTTTCAGCTTTAATGAACCA

>Unigene000174

AAGAAGAAGAAGAAGAAGAAGAATCGCCTGTGCAGAATATAAAAGGGGATGACGTAGGGG

CTCAGGCCAGTCACAGAAAACAGAGATGAGCCTGACATCCTGGTTCCTGGTAAGCAGTGG

GGGCACACGCCACCGGCTGCCCCGTGAGATGATTTTTGTGGGTCGTGATGACTGCGAGCT

CATGCTACAGTCTCGCAGTGTGGACAAGCAGCACGCCGTGATCAACTACGAACCGGCAAG

TGATGAGCACAAAGTGAAGGATCTGGGAAGCCTTAATGGGACATTTGTGAACGATGTTCG

AATCCAGGAGCAGATGTACATCACCCTGAAGATTGACGATAAGCTGAGGTTTGGATATGA

TATCCTTTTCAAAGCACCCGCCGTCACTCCCTGCGTAACCTCTGAAGAGCTGATTTGATG

TGGGTCAATGTTCTTGCTGTCTTTTGTTGTCTCAGTTCCTTTTGTGTGGGTTTGGATTCA

CATACACTCTCTTTTTTCTTAACCTTGTGCACATACTAACTTGTTCACTGTGGTCCGAGG

AGAGCTTCATGTTCCAGAGGAGGCTTTAAAGCATGAGAAATTCACCAGTCAGCTTCAGCT

AGGGAAGAAGCCAGTTTGCACTGAGTCTGCCAAAACACCCAAATCTCCTACAGCCAGCGG

CGCAGAGAGCAAAGTATCCGTACCCACTGCGGAACCTTCTACCAAAGTGGTGGAACCCTG

CAAGACCGAGGACAAAATGCAAGGTGATACTGCAGCACTGCACCGAGGAACACCGTTGTA

TGGCCAGCCTTCGTGGTGGGGGGATGGTGACGCCGATGACGAGAACTCGTTTAAACAGGA

GAATAAAGCAGCAGGGAAAAAACAGGAGCCTCATGGGACAGACACCAAGGACACCAGGCG

GACTGATAAAGCCAAGGAGAATGGTGTGAGCACTGCAGGCCCTGAGCCCAGCTACTTTGA

GATCCCGAGTAAAGACGGCCAGATGGCAGAGGACAGCATCCATGAGGTCCCGACCAAGGA

CACAGAGAGCAGCGCAGCTGCAGCAGCAGCAGCAGCAGCAGCGAAAGCCGCCCAAGCGAA

CACCTCCTTTACTATTGAGTTTGACAACACCTCTCCAGGAAAAGTAACCATTAAGGATCA

CGTGTCGCGTCCAAGATCGAAAAAATCTCAGCCAGGGAGTAAAGAGCTCAGCAGCCTGCA

GGCAGCCATCATGGCCTCAGAGAGCAAAGTGGCCGACTGGCTTGCGCACAACAATCCCCC

CATGGTGAGACGGGAGTCCACGGAAGAGGACAGCAAAAGCATTAAAAGTGATGTGCCTGT

TCTTCTCAAAAGGCTAAAAGGCAGCAAGCACGAGGACGGGACCCAAAGTGACTCTGAGAA

CGGCCTTGGCTTGCGTTTCGGTAGCCGGCGCCATGCTGCTCTGGAGGAACGCTTGAAGGC

AGCTGGCCTTACGCATGCCAAAGTCACCGGAGCAGAGGGCTCGGGCTCCAGCAGCCGCAG

CGCCTTCACCATCGAGTTCTTCGACGAGGAAAACCCGCGCAAACGCCGCTCCTACTCCTT

CTCCCAGACTGCACCACTTCTGGGAGGAGAGGGACTGTGTCCAGCACCACCATCCCATCC

GAAGGGAACTTCATTAACACTGGCAACAGTAGCGCCTACAGCAGCACGGCTGCTGCAGAA

AAAAAGGTCAGAGGAGAAGACCACGACGGTCGGAGATGAGACTCAGAAACAAGACGACGA

TCAGAGCGACAAAGGCACTTATACCATTGAGCTGGAGAACCACAACCCTGAGGAGGAGGA

GGCCCGTCGCATGATCGATAAGGTGTTTGGCGTGGAGAACCCTCAGAATCCCAGTCGCTC

AGTGCAACAGACAACTAAAGACAGTAAGAAGGTCAGCAGCACTAAAGCGGAGGGTCTTTT

GGATGAATCGGTTGCGGTTGGAAGTCCACACTGGGTCTCTCAATGGGCCACTTTGGCCGC

TAATCACACAAGAACTGATCCTGAAGGTTCCGGGGCAGAACCCACAAACTTTGTTCATCA

AGAGCGAGAAGCTGATGCATTTGAGTCAGGAGTGTCTTTGAGAAGCACCAGCTCTGCCAC

CTCTAGTCAGGCTGAACGTAAAAGAAGGACATTGCCTCAGCTCCCCTGTGAGGAGAAGAG

CAGGCTGGGGAAGGTCTCCTCCACCTCAGGACTCCGATCCGAGATTGGCGAGAAACAGGA

CACCGAACTCCAGGAAAAACAACACAAAGGAGATGGCGAATGTGCATCCACTCTCATTAA

GAAGGACACTACAAGTGGAATTGCAAACCAAGTGCAAACAGCCACTACCAAGCCTCCTGT

GCCCCCTGTGGGAGGGGTGGAGAAGCAAAGTGAGGAGTTTCGCAGGAGGCGTGCTGAGGA

CAGGAACAAAGGTAGCGATAGTGAGCGAAGCGGGAAGCCCCTGGTGCGTCAAGGCAGCTT

CACCATTGACAAGCCAAGCACCAATGTTCCGCTTGAGCTTATCCCACGCATCAACCAGCG

GCAGTCCGGAAACAGAGAGCGTAGTGATTCAGCTGGAAGCATGGACACGACCACCTTGCT

CAAGGACACTGAGGCCGTTATGGCCTTTCTAGAGGCTAAACTGCGTGATGAGAAAAAGCT

GGATCCATCACCGCCCGCTGGCTACCCTTTGGGACGCTCTTTTTCCCCAGAGTTGGATAC

TGATGCAATTAAAGCAGCAGGCCGCGCCGAAGTGTCACAGAAGCGTCGTTCTCTCAGCAG

TATACACAGAGAGAAAAGTAACATGAGCTCCGTTGGAAAGAGCACAACCAATGCACGGGA

ACGTTTGGAAAGGAAAGTCAAGCCGAAGACCTCGGATGGGCGTCTGGATACCCGTCGTTC

TACCCATACAAGTCAGCGGACCCGCCAGCCGTCCATGGACCTCACAGATGATGAGCAGAC

ATCTTCCTTGCCCGTTTCTGACATACTTTCAGATGACCAGGAAAACGGACGTAGTCCCTT

CACCTTTCCTGATGACTTGCTACTTTCCAAACTGGACTCTTCTAAAGGTGCTAAAGTTAA

TAAAGCCAGCAGGACACCACAGGCCACCACTGCCTCTATAGGCAAGCATCAGTTGCCTCA

GCCTCGGCCCACAAGAGCATCTCTTCTGAGACGTGCTCGACTTGGAGACACATCTGACAC

AGACCTGGCAGATGCAGACCGAGTGTCAGTTGCATCCGAAGTGTCTACTACCAGCTCCAC

ATCCAGGCCACCATCGGGCAGGAAGGCACCATCCCGCATCGATATGCTTGCACAGCCCAG

ACGCACCAGGGTCGGGACCATGTCAGCACGCAGCGACTCAGAAGCCCCAATGTCACGCAG

CACTACATCCAGAATATCGGCCGAGACAGCTTTAAGACTGGGCTTGCGCCCAACTAACCC

TGCAGACAGCAAGCTGAACCCACGTCTTCGGGCAAACAGCGTCTCCAAGTTGACAGACAC

CAAAACCAAGACTACAGCATCCATCTCCAGTGCTCCAGCAGCCAGCAGCAGGTGGCGGCG

ACTTAATACAGAATACGGCTCCACCTCTGAGGACGAATTTGGCTCAAACAGGAACTCGCC

AAAAGCCGGGCGTCTGAGGCCAGGCACTGCTCTGCGTACCAGTAAACTGAGCAGCTCAGG

CATGGTGACAACAAGCCCTGGCAGCATCACCATCAAACACAGGATGAGAGAACAGGAGGA

GTACATCAGAGACTGGACAGCCCACAGCGAAGAGATCGCCAGGATCAGTCAGGACCTAGC

CAAGGATCTGGCTATTCTGGCGAGGGAGATCCACGATGTGGCCGGCGAGATCGACTCGGT

CAGCTCCTCAGGCACAGCACCAAGCACTACGTTGGTAGAACGAGTTTTTGATGAAAGTTT

GAACTTTCGGAAGATCCCCCCTGTGGTGCAGACCAAGACCCCAGAGATGAACGGCCGGCC

TGTAGAGCTCCGACCTCGAGCCCCAGACAGTCTGGATCCCCAGAATGCACTGCGTAGACG

CACATGGAACCGAGATGAGGTCAGACCGGCGGTCCTAGAAAGCTTGCTGCTGAGCTCAGT

CTCTCAGCTGTCCTCTAAAATCCGCCAATCTGTGGACAAAACAGCAAGCAAAATCAAGAT

TTTGTTCAAAGACAAAGACAGGAACTGGGATGACATTGAGAGCAAACTGAGCTCAGAGAG

TGATATGCCACTTCTCAAAACCACTAACAAGGAAATCTCAACAATTCTAACAGAGCTAAA

AAGAGTGGAAAAGCAGATCCAAGCTATAAATATCATGGTAGACCCAGATGGAACCCTGGA

TGCTCTTACCAGTCTTGCCCTAGCAAGTCCCACCACACCCAAACCTAAGACCAGCAACGG

AGCCTCCAAAGCCTCCGTGATCATCTCCCCGAACCGAGAGCTCCTCAGCGCTGGACTGAG

TCGTCCTCAAACCAGCGGAGAAGCGAGCAGCATTACTAACAAGTAAAGACAGAAAACCCT

CAGAGTTGAGTAAACTGAGAAACCCTTTTTGTCCATGTCTGTACCCCTCCCTCTCCTGCA

CTCCATTGGAATGTCCATCTTTATGTAAATGAAGTCACACCTGTGGATTTGGACAGTGAT

ATACAGTGCAGCTCTACAAGTGCACGCAGCTGCTGGTGAAGAAGAGCTGAAGAGGAGCAT

TAGTCTAAAGGCTTAAAATGTTGGAACCAGCCACCTGTATGCTGCTGCAAAGGTGTACAC

ACTCATCCAGGCATGAAGCAGGTGGACTTAAAATAATTTATCTTCAACAATAAATGACTC

AGAGTGCCTGTCTTCTCTCTTTTGTGAGCATTCTTTCTTGCAAATAGCAACTTCTTATTA

CTACGTGACTTTTGTGTCAAAAATAAAACAGTGTTGGTCAAAAAGACTTCAAGATTTCAA

AGCTGAGCATAAACCAGCTTAGCAAAGTAAGTCTGATTCTATGCGGGCACTCAAACATTG

TTCTTTTTTTTCCATTTCTTATTTCTTGATTACAAAAAGTGCATTTAGGCTATTAAAATA

AGCAGGGGTCAACTTGGTAAGTTAAACTGACCGTTCATTGCATTATCTTATGCACTGATT

TTTTATTTTTATTGGCTAATGTATACAGTAGGTAGCAAACTTTGTACAAGGTTCAGACAT

TAATTAAAACCCAGCAAACTGGCGTTTGTGTCATTTTCCCCCTGACATTAGAAGGAAGAT

CATATGCAGTATTAGAAAAACGTCTTAGTGGAATGCACCATTGTAGTGTTACACATCACA

GTTGCTTAGATCTCTGTGCTTTGTGCTGAAATGTTAAATCAGCCTTAAATTAACCAGCCC

TTAATGTACGGTTCAATCACATTCTGAACATGTTATGGCCAGTTGCTTAAATGCTAACGT

TCTTTAATCAGAGTATGCAATTACAAGAACCAAATAAAGTCTGTCAGCATTTTTTTTTTT

AATTATCACAACCTCATAGATCATGTTTTTTGTTCATGCAGGTATTCCGGCATATTATGT

ATGTACTGTATTGTCTTACTCTTCCGTCGGCGCTTGCTGCTTGGCTGGAACTCCTGAGAA

AAAGCGTCCAGCGCCCAGCCTTTCATCGTGAATCTGCGTGTTGTAGAAAGACTAGTGCAA

AATAAAACAATAGTGAAGATAGGTACAGGACATTAGTTGTTGTTGTTTGTTAATAAATGT

CAATGCCAAGTGGCTGCTGTGGAAACGCTAGTTCCTGGACAGGTTTAAGAAAGAAAATCT

TAGTTTAGTGACCGCATTTGTGGTTTTAAAGGTTCCTGGATTAACTGGGTGAGTTAATGT

TAAAGGTGATACAGGACAACAAACACCAAACTCTATACATTATACCGCTCGCAGAACAGG

ATAGATTTTCAGGCAGATGATCCTCCTGCCCCTAAAACACTTAAAAAAAACAGTAGTTAT

CATTTATAAAACTATTGTACATGTTTTACATATAAGGGG

>Unigene000175

GGAGGAAAAACAAATCGAGCTACAAAATAGTGCTTCATTGTGACATGTTTTTTTACGATA

ATATCTGGACGTCCTCGGTGTCGACCGTTACATAGCTAGTCAGAATTCAAGACTTGGCGA

ATAATAATAATAATAATAATATTAGTAATAATAATAATACGATTCCCAACAAAAAGGTCT

GGTTAGCATTGCTAGTTGCGAGCTAGCGCTTAAAAACCAAGTGAGGATCGAATCTACTGA

AATGCATCGATTATAACATCAAAAAACATCAACACAAAGGTTATTACTGGATTTAATAAA

AAAGGGACAATAATAGAACCTCCACCAATGACCCCGGAGAATAGCTGGCGCGTGCCAACT

GTGAGCAGTTTGCAGCCATTGTTTGATCCGGACGCGATTTTTTTACATTGCTAGATTTTT

TTAGTTTATTTTTATTAATAAACATTTTTGCTTGACTGGCTCTCGTTTGGGTTTAATTGA

AAATGGCCGCCGTTGTGAATTATTCTCCTCCGTGGTGGGTTAACCTGCTTCACCGTCTTC

CTCACTTTAACCTGCGCTTCGAGCAGATCAGCAGCGAGTTTCAGCCGGAGGACCCGTCAT

ACCAGCAGTCTATTCTGTTGCTGGGAGCAGTGGCTTTAGCCTGCCTGGCCCTGGATCTCC

TCTTTCTTCTCTTCTACTCATTCTGGCTCTGTTGCCGCCGCAAGAAGAGCAGCGAGCAGC

CCAACGCCGACTGCTGCTGCACGGCCTGGTGTGTCATCATTGCCACCCTGGTGTGCAGCG

CTGGCATAGCTGTTGGTTTCTATGGGAATGGAGAGACCTGCGACGGAGTTAACCGGCTCA

CCTACTCACTGCGCCACGCCAACCGTACTATCGCCGGCGTGCAAAAGCTGGTTTCAGACA

GCACCACCTCCCTGAATCTGACCGTAGATGACAATTTGCAGCATTTGGAGAGCCAATACA

CGAAGCATGCTGATTACCTGTCGATCGTGCAGAAGGTGCAGGGCCAGCTGGACGAGCTGC

TTAAACAGATGGTGGAAATCCCGTTTTGGAGCAATTCCAGAATTTCCCTGGAAGCACTGG

CTAGCAAGATCGAACTCTACGACTGGTATAGGTGGTTGGGGTATCTGGGGCTGCTCCTGT

TTGACGTCCTCATCTGCCTGCTGGTACTCTTCGGCCTCATTCGCAATTCAAAAAGAACTT

TAATTGGGGTCTGTTTACTGGGCGTGTTGGCACTGGTGATCAGCTGGGGATCGCTGGGTT

TGGAGCTAGCTGTATCCTCGAGCTCTAGTGACTTTTGCGTGGCACCCGATATGTACGTCG

CCGAAGTGGCTGAACGCTACGGCGTCATCAACAGAGACATCCTGCAGTATTACCTTCATT

GCAGTGTCGGCCAAAACAACCCCTTCCAACAGAAACTGTCCGGAAGCCACAAGGCTCTGG

TGGAGATGCAGGACGACGTGTTGGAGCTCCTGCGTGCCGCAGTGCGAGAATACCCCCAGA

CTAGAACTAATTTGGAGCAGATTCAAGGGATTTTAAACACAACTGAAATCAGCTTGCATC

AGCTAACCGCTCTAGTGGACTGCCGCAGTCTGCACATGGATTACGTGCAGGCTGTGACCG

GGCTGTGCTACGACGGTCTGGAGGGACTGATCTACCTGGTGCTGTTTTCCTTCGTCACCG

CCCTCATGTTCAGCTCTATCATCTGTAGCGTGCCTCACACATGGCAAGGGAGGAGATCCA

GTGAAGACACAGAAGAAGACTCTGCCGCATCTGGAGGAAGACAGAACCACGATAACTTGT

ACCGGGTGCACATGCCCAGTCTTTACAGCTGTGGCAGCAGCTATGGCAGCGAGACCAGCA

TTCCCGCAGCAGCACACACCGTCAGCAACGCCCCCGTTACTGAGTACATGACACAGAATG

CAAACTTTCAGAATCCTCGCTGTGAGAACACCCCTCTGATTGGAAGAGAGTCGCCGCCAC

CTTCGTACACTTCCAGCATGAGGGCCAAATACCTGGCCAACGCCCGACCTGATCCTAACA

GCAGCACTGCAGCACACTAGAGATCTCCAAAAGACGTCCCTACGCTGTCCCCTTTCTATC

GCTCTCTCTCTCTCTCTCTCTCACTCTCTCTCTCTCTCTCTCTCTTTATTTCTGAGTACC

TTTTTTCTTCTTGTTTTAGATTTTTTTTTTACATTTCCGGTGCGTGAAACGCAACCTGTC

AAACTTAACACAACATGTAGAAAAGCATGTGAAAAACTCCGGGTTTTGCTGAAGAACCCT

TTATGATGTTTGCAGTGAAGGTTCTGAAATGATCTTCAGTGAAGAGCAAAAAAACCCCTG

AACGATTTCATGTCCAGAGAAACAAACACACAACACGTCTGAGTTTCCTACAACTTCATG

CAACAGGAAGTGGTTATAAAGCTGAGCGCGTGCAGAAACAGTACAGATCAGTGAAGAAAG

AGGTGAGCGTCAGGTTCTGTTGAGACTCCAACATTTTATCACTCTATAAATCCATCACTC

CATCATTTTATCACTCCATCATCACCGTATTCATGGGAACGGATAAAGCGTCTGAGACGG

AAACGCACGCAAAGATGGAGAAAGAAGAAGTGAGGGGTGAGGAGGAGAGCGGAGAGGAGC

GCGCGGAACAGGACGGGAACTTATTAACCTCGTACACCTGGCAGACGGGAGGAGCGCGGG

CCGATCCGTCAGAGAAAAGCGCGGGAAACAAGTGGAGTCGGATGCAGAGCTGGAAAAAAG

CGCTGAGTGAAGACACACCCGAAAAATCAGCCACACCGACACCGGGGCCTCGCGTCGGGA

GAAAGAACCCCTTCCGCAGAGCGCTGTCAGAACCTCCCGGCTCGCTCCTGTCCTCCATCC

TGTCTCCTTCATCGTCATCACCCTCAGCGGCGGAGACCAGCGCAGGCGCGGACACGCAAC

AGCGCGGGAAACTCCGCAAATACCTGCAGCAGGTATCACAGAGACTGAAAAGGCCGCGGG

CGCAGAACCGCAACAACACGAGCACCACACAACAACAACAACAGCGGGAGGATGTGCCAT

GTGTGGAGGGTGCAGATTTAGTTCAGTTCTCCTGGGTTCCTCAGGATGTTCCTGTGTGGG

ACATCAACAACTGCATCCTTCAGGACGGACAGATATTTATCTCTCGAGAAGAGGAGCCTT

TGTTGCGGATGAGGAATCGAGCGAGCAGCTGTCTGTCATCTGTCAGTTTACAGAACCTCA

CAGAGAGTCACTCCAACCTTGAGTGCAGTCCAGATCCCACGTCTGTTACCGCCCCAAAAC

CCAAAACCCAGGATGGTGGAGTGAGAATGAAGATTAAACGGCGTCTGCAGGGTGGAGCTC

ACAGGAAACTGAGTAACACACAACTCAACGTAGTGGGACTGAGTTCTGCAGCATGGTGCT

GTCTGTCTGTCTGTCTGTTCTCTGTGTTTTTTTGTCTGTCTGTTATTTTATTATAGCTTT

CTAAAATCCTGTGTATTTGTTTTTTGGTGTGTGTAGCAGGTTCGGTTCTCGAGACTCTCT

CTCTGTGCCACTGAGTGCAGCTGAAAGTCTGAATGTGAGTTCGGACTGTACCACCGTGAT

CCGGCCGGTGCACAGTTCCATCCTGGGAGAGAAGTATTGCTTTGAGGTAATTACCTCAGA

CAGCAATCACTGCTTCGGGTGCAGCTCCGCGGCTGAGAGAGATCGCTGGATCGAGAACCT

GCGGCGTGCGGCGCGGCCCAATAAGGATAACTGTGAACGTATGGAGAACTCTCTCAGCCT

GTGGGTAAACGAAGCTAAAGGTCTCCCACCAAAAAAGCGCTACTACTGTGAGGTTCACCT

GGATGGTACACTGTTTGCTCGCACCAGCAGTCGTGCGGTGGGTAAAGCGTCCTCAGTCCC

TGTGAGTGACAGTGGGTCAAGTGCTGTGGGCGTGTCTTCTGGAGGAAGTGCAAGTACAGG

GGGGTGCCAGCTGTTTTGGGGTGAGTATTTTGAGCTGGATAACCTGCCCCCTGTTACACA

GATCACACTGCACCTGTTCCGGGATGATGACCCAAAGAAGAAGCGCCACTCAAAGGATGA

GAACATCTTATACCCTCTGGGCAGCGTAGCCCTGTCTCTGGCCAACATTAAAGGCCGAGT

GTTTCAGGAGAAATGGTATCCCATCGTTCCTTACAAACCTCCTGGGACAGGTGGATCGAA

GGAGCAGCTCGGCCCCCAGGCGTGTATCCGAGTTAAGGCGCGATTCCAGAACCTAAAAGT

GCTACCGATCGAGAGATATAAGGAGTTTGCAGAGTCTGTGACTTTGGGGTACATGAAGAT

GTGTAGCAACCTGGAGCCTCTGCTGAACGTCAGAGATAAAGAGGAGCTGGCTGGAGCACT

CGTCCATGTTCTACAGAGCATTGGAAAGGCCAAAGACTTCCTGATCGAGCTGGGTCACGC

TGAAGTACAGCGCTCGGGCGAGAACGAGGCGCTGCTCTTCAGAGAAAACACTTTGGCCAC

CAAAGCCATAGACGAGTATATGAAGCTGGTGGGGCAGAAATATCTCATCGACACTTTGGG

AGATTTCATAAATCGACTATACACAACCTCAGAAAGCTGTGAAGTCGATCCACAGAAATG

TTCTGCCTCTGAGCTGCTGGTCAACCAGCGCCACCTGAAGGATGCGTGTGGAGAAGTTGT

GCGACAGATCACTGAGAGGCATAGCTCATTTCCAGTGGAACTTAATGAAATTTTCTCCAG

CTGGGTGTCTGATTGTGAGGAACGTGGACGAGCGGACATTGGACACCGGCTCATTTCAGC

ATCATTGTTCCTCCGGTTTCTCTGTCCAGCCATTCTCAGTCCCTCTCTGTTTGGACTGAT

CCAACCTTACCCAGAACACAACACACTCCGCACACTGACGCTCACGGCTAAGGGGCTGCA

GAACCTCGCCAACTTCACATTGTTTGGAGAGAAAGAAGAGTACATGCTTTTCATGAATGA

GTTCCTGGCAGAACATTGGGAGGATATGAGGACGTTCCTGCAGAAAGTATCAAACCCTGA

CAGTGAACTAGAAATGGCTCGGTTTGATGGCTATGTGGATCTTCCTTTACGCCTAGCTGT

GCTTCACAACCTGTTGGTGGACATCATTTCTCCAATGAAACTGGAAGTTATAAACAACCT

GCAGCCGCTGCCTTCCATCCTGAATCAGATCACTGAGCACCTCGGCCCAGACATTCCTCG

AATCACCTGCAGCAGGTCCACTTGTGAAACAGAGAAACCCACCTATGTTCCTCCCAGAGA

TCTTGGCAAGTACAGTCCCCTCAACAAATCCCTCCCGCACCTGCCCACAGACCCACGTCC

CCCTCAGGGCAAGGGCAAACACCGGAAGCAGGTGGCGAGGACGCAGAGTGTTCCTGCCTC

TGAGAGACACCATCGCCGTCCTCTGAAGAGAACCAGCAGCAACGCTGAGTTGAGTGAAAC

AGCTCACATCCACCATGAGGAGAGTCCAGAAAGACAAATGGAGATCTCTCTTCCACAGCA

AGAGTTTTTTAACAAGTCTGCGCCTGCTCCTGTTCCCTGGATCAAAGAGCACGTTCAGGA

TGAGACCAATATTCTGGAAAAGCATGCTCAGGAGCTGTATGAGCTGCGTTCAGGGGTTGA

CCAGGTGACAGACCGTGAGCTGGAGATGGCGAAGAGGTTGGAAGATTTTATTGCGGTGAG

TCAGGAGCAGCAGGCGCAGCTGCAGGCCGAACTGCAGGAAGTGCGCAATGTGTTAGCACG

AAGGGAGGAGCAGCTTGCTAGCGCCACCTTCAGGTAACTCTCTCACACACACAGATGATC

ATTCACACACGTGCACTTTTACAGTGGTGTGTGTGTGTGTGTGTGTAGGCTGGGTGTGAT

TGAGGAAGAGCGTGAGGAAGATGAGAGGAAGTTAAGTGTCGCTCTGGCAGCAGCTGAACG

TGTGAATGTGTTGGAGGAGCAGCTTGCTAGC

>Unigene000176

GAAAAACAAACACAGCTCGCGCGGAAGATGGCGGAGCATTTAGAAGTGGCTTAAAGGAAA

CTGGTGTGTTTGAAGTGGACACATATTAACCCGTGGTGGCCTTGGGGAAGCTTTAGATGC

GACTGGGTCTTTAGTCAGATCTTCCGCATTGATACCCAAAAGGATGTGAGGTGAAACGGA

GTGGATGTCCTCATTCTCCACTGCTGCAAAGGTGCTGATATGTGTTGAAGATGAGTGGAC

AAGGGGAGAACTCTCTGGACTCATTGGGCTACGACCGGAAAAGGAAACTCTCCACTTGTG

ACACACCAGGACTAGGGTGTGATAAGCGTCGGAGAGAACAGGAGTGTAAATACATCGAGG

AGCTTGCTGAGCTGATCTCAGCCAACCTCAGCGACATCGATAACTTCAACGTCAAACCGG

ACAAATGCGCCATTCTTAAAGAGACAGTACGCCAGATACGGCAGATTAAAGAGCAAGGAA

AGGGGTCGTGTGGCGATGATGATGTCCAAAAGGCAGATGTGTCCTCTACGGGTCAGGGGG

TCATCGACAAGGACCATCTGGGCCCTCTGCTATTGCAGGCATTGGATGGTTTCCTGTTCG

TTGTCAATCGAGAGGGAAGCATCGTCTTTGTGTCCGACAATGTGACTCAATATCTGCAGT

ACAAGCAGGAGGAGCTGATTAACACCAGCGTGTATAACATCCTGCATCAAGAGGACCGTG

AAGAGTTGCAGAAAAACCTGCCCAAAGCCAGTGGAGCGAATGGAGTTTCGTGGACTGGAG

ATGCCGCTCGTCAGAAAAGCCACACCTTTAACTGTCGGATGCTGGTAAAGTTCGGTCATG

GAGGCCATGGACCGGAGGACGGATCAGGAAGGCCACGTTATGAGACCATGCAATGTTTTG

CCCTTACCCAACCTAAAGCCATGATGGAGGAGGGCGAAGATCTGCAGTCTTGTATGATCT

GTGTGGCGCGGCGAGTGACCGCGGTGGAGTGCATGGAAAGGTTCAGCACCAGGCACGAGC

TCTCAGGTAAGCTGATCGAGATCGAGCATCAAAGCACCTTACACACCACAATGCGTCCCG

GTTGGGAGGATCTGGTACGCAGATGTTTGCAGATGTTCCTGCATCACAGTGAAGGCCAGC

CCTGGTCCTGTAAACGGCACTACCAGGACGCCTGCATACGTGGTCGAGCAGAGACTCCAC

TCTACCACTTCTCCCTATCGGACGGAACGCCAGTAACGGCACAGACACGCAGCGAGCTCT

GTCGAAACCCAAATACCAACGAACCTCACAGCTACCTCTCCACCCACCTATTGCAGAGAG

AGCCGAATGGTTATAGGTCAAACCACGGAGGTGTAATGCGTTCCCAGGGAATGGCTGGTG

CCAATCATAATTCCCAAATGAACATGACTCCTAGTGGGCAGATGGGCATGGGCAATGCCA

GAGGATACGTCATGAATGACTCAGGGCATATGGTTCCCAGAGGAGGCCCCATGTATGGCC

CTGGGAACAGAATGAATCAGATGAACTCAATGCACCAGATGAGTCAAATGAACCAGATGA

ACTCAATGAATGCAATGAACCAGATGAATCAAATGAACCAAATGAATCCTGTTAATCAGA

TGAATCAAATGGGTTCGATGAACCAGATGAACCCCATGAATCAGATGAATCAGCATAACC

CTATGAGTCAAATGAACCAGATGAATCAGATGGGGCATCCCGGAATGAACCAGCACCCAC

AGCACTTCCATAATGATGGGTATGGGTTGGGCATGACCAGTCCGTCCCACGGTAGCCCAA

GTATGAACCCATCACAACAGATCATCATGGGATCTCCACGAATACGAGGAAGCCCCAAGA

TAGGAGCCAGCCCTTTCTCGCCAGGAGGTATGCACTCTCCTATGGGGCCTGTTGGAATGA

CTGGAAGTAGTGGCTCCTCTGGTGGTAGCACTTTCTCAAGCAGCTCTCTTAATGCCCTAC

AGGCCATCAGTGAGGGAGTTGGTTCCTCCCTTCCCTCTACTCTCACCTCACCTTCTCATA

AGTCAGACAGCTCCCCTAGTATCAACTCCACTCAGTCTGGTCAACCAAACCTGTCTGGGA

CTAATAAAGGACCTAATGCAGGCGAAACGAAAAGCTCTGCTAATACGCCTAACACTGGGA

CTGACCAGCAACATGCAAACATGTCCGAACCCTCAACAGATAAACCAGATAGTCACACTA

ACAAAGATGGCCAGACTGGTGCTGATGTGCAGCGGAAGGGCCCAGAAGGAAAGGGCCATA

AGAAGTTACTCCAGCTGCTTAGATCTCCACCGGAGGAGCTTGCTTTGGGCGGAGGAAGTG

GAGCAGCCCCTCCTGGACGGGCTTCCACTCCCATTGGTGCTGACCCAAAAGAGGCTGGAG

GTAGTATGACAAGCCCGTCTTCCACAGGTGTGTCTTCATCATCATCTGCTTCATCGGGAG

GCGCCACATCGCAGCATGGATCGGGATGCGTGTCATCTACAGGCTGCATGGGTAGCCATC

TGTCTTCTCACTCGCTGCAGGAGAAACACAAAATTCTTCACAAGCTCTTGCAAAACGGCA

ACACACCGGACGAGGTGGCGCGCATCACCGCCGAGGCCACGGGGAAGATCACTCTTGGTT

CTGAACACCAAGGCTCAGACGGCATAGGGTTGGAGGTCAAGCAGGAGCAGCACAGTCCAA

AAAAGACTAATGCTTTGCTCCACTATCTTCTAAACAAGGATGATAGCAAGGAGACTAGAA

TTGACGGCAAACCAGGACTGGATGAGCTGGAGGGTCGTGGGGTTGGTGGAGTAGGTGTCA

CAGACTCCAGCGGCCAGGAAAGCAAGGTCAAAATCGAGCCGCCTGATGAGTTGCAAAGCC

TGGAGAGCATTCTGGGAGGCTTGAAACCCAGTTCTGGGATGTTTGGTGATTCTGGAAACA

CAGATGGAGAAAATAAGAAAGGAAATGGACCCAACAACAGTGCACATGATCATGAGGGTG

CAGTTATGAACTCAGGGCCTCGAGTGCCGTTCCAGAGAGCAATGTCTGTGGACAGTAAAC

CCTTTAGCGGGGCGAGAGGGACAGGCAGAAGAAGTGCCCCCTCCTCTCTTATTAAACAGG

AGAGCTTGGACAACCACATAGGCATGGGTGGTCCTAACAGGCAAATGGGAATGAACCAGA

TGCCCCCTATGATGGGCCCAGGTGATTGGAGGATGCCAGGTTCTACAGGCAGTCCAGGGG

GATCTAACGGACATCCTGCTATGGGTTGTCCAGGCATGGATCCAAATCCTAAAGGAATGA

TGGGAGGTCCCATGATCAACAGGTCAAACAGTGTACCAACACCAAGATCCATGTTACAGC

AGCAATTAATGGATATGGGTCAAAATGAAATGGGGATGAACACATTTAGAGGGCAGCCAC

ATCCTCCTCAGTCTCCATCGTGGCCAGACTGTGGCATGGGCATGGAGGGACCCCCTAATG

CAAACAGCTTGCCATTTGGCAACCCCCTGGACGACCTACTTGTTCCTCCATCCACCACTG

ATGGGCAAAGTGACGAGCGAGCTCTGCTTGACCAGCTCGACTCCCTCCTCAACAACACAG

ACAACCTCGGCCTGGAGGAGATCGACAGAGCGCTTGGGATCCCAGATCTGGTTGGCCAGA

GCCCAAGTACTGAACAACCAGCAACTCCTACAGATGCTTTCCCAGGACCATCTCCCGATT

CATCAATGAGTATGGATCCTAAGGTCATGTATGGAGCAGGATATCCTGGTCCACCATCTA

TGGGCATGCAGGGAGGCTATGGCTCTGGACCTAATCCTGGTACAGGTCCTGGTCCTGGCA

ACATGCAGGGACATCCGGGACAGGGATTTAATCCTATGATGCAGCAGATGGGTGGAATGG

TGGGCATGAACCCCCGTGCTGCCATGATGCGTCCACGCATGGTCAACGCTAAGCAGCCGC

TCCGCCTGCATCTTCAGCAGAGACTTCAGGGACAGCAGTTTATGAACCAGAGCCGACAGG

GCATGCCAATAAAAATGGACAATGCACCAGTGGCAAATCATGGAGTGAGGCCTGGCATGC

AGCCTGGAATGCAACCAGGCATGGGGGGACAGCCTAGTTTTCTGAACGCTCAGATGTTGG

CTCAGCGAAACCGAGAAATGATAACACAGATGAGAAGGCAGAGGATGATGATTATGCAGC

AGCAGGCTGCAGCTGGAGGCTTCAGTCCCCCTCCTAATGTCACAGCTCCCACTGGCATGG

AGAACCCCATGGGAGGAACAGCTATGAACCAGCCTGGCCACCAGCCTGGCCAACAGCCAT

TTAACTATGGAGGAAACTATGCTATGGGCCAGCAGGGTGACCCCTCTTTCATGGGCACTG

GCAACAGCCCACCAAATATGATGGCTGGACGCATGGGAGGCCCACAGAATCCCATGATGC

AACAGCACCCTCAGAGTGGGCCCATGTACCAGTCAGCAGATATGAAGGGATGGGGGCAGG

GTGGCATGCCTATGAACAACTCCTACCCTCAACAACAACAGCAGCAGCAGTTCCCACAGC

AGGGGGCGCCAGCGCAATACGGAGGAATGATGATGAACGGAGCCATGCAGGGCGCCGTGA

ACGGGCCAGGGCAGATGATGCCAATGCAAGGGCAGATGGGCATGAACCCCATGGGCATGG

GTCGCATGCCACTGGGTCCTGATCAGAAATACTGAAGCAGAGCATCGTTTAAGCAAGAGA

AACAGCATACAGTGAGAGAAGTGAGACACACGCCACCGCCAGCTCCTGCTACACATGGAA

TGGGACAAGCTGGACATGAGACCAAGGACTCGCCCGCATCACTGTGTTTTAATAGACACT

CGAGCATTTTTGTGTGCCCCAGAATACCCTCTGTTGATCTGCAGCCAAACTCGAGCTCTT

ATGTGAAGCTGGGTGTGAGCGTAATGCTGTACGTCTCCACTTTTCTCTCGCTGTGGAACT

GCCCCGGTGCTGGGTGGGGATGCACTTTATCACACGGCTAATAAGGAAAAGAATACCTGC

TCGTTCTAGCCTTATTTAACAACTTGACCCGTTCAGAAACCAGTTTGTTGATGACGTGTT

TTAGCAGAAACTTTGCTTTCAGAACAGACCGCTTCAGTTCCAGCTATATGCAGCTACGAG

CAATAATTCTTCTCTCTATGCCATAAATATGTTAAAACGGTGCACCAGTGGAAAAAAACA

GCAAAAGGTACTGCAGTATTAGCTGGGCTTTTTGTTTAACGTGTGAATTAGGACAGATCT

GTACATGCTGTACGCTCCAAATGTATCATATGTGCCCTTATTATATACTGTACATTTTTG

CACAGATTTGGCACATGTGATGTCCAGCATATTATATTTTAGGTCAATACTGCTTTGGGT

GTTAATTCTATTTTAACTTTCTCAAAAGGGAAAAATATTTTAAAATGTTATATATATATA

TATTTTTTTTTATCTTTTCTGATCTAGCCTCATTTTCTTTACAGTATTGCAGTTATAACA

GCCATTTGTTTTCGGAGGGCACTTTTTTCCTGTTGAAACGCAACGGAGATCTGATCGAGC

AGTTACATCAAGGATACATAATGGTTTAACGTGCGTCTTTTTATCTGTATATAATATGGA

CATGGTGGTGAGTTTAAGTAAGACGGTTATTTGCTCAGCCTTTTTCTGTGAGTTATCTGT

TACTATCGTTGACGATTTCACTTTTAAGCGCTGGGTCTTTTATAAATCTACCTTTAAAAA

TTGCACAATCCAAAGATATAGATATTTCAATTCTTTGCTAAAATTTTAACCTTACCACTA

GTCTTTTATTGTGTAAGGTACATATCATATAAGCTTATTGTTATGATTTTGTACTACATG

CTGTACCCGATCGCTATATTTCTTTAATTTTAAGGCCAGTTCATAAAGCAAAGGGTTTTT

TTTTTTTTTTTTGGTTTTGT

>Unigene000177

TGTGTGTGTGTGTGCATGCACAGGCTCACTGCTCTGAGAGCTCTGATGCAGAATCTCACA

CGTGCTGAGGACATCATTAAAGTTCATGAAGCTCGACTGACTGAGAAAGAGACTAGCTCT

CTGGACCAGGGTGAACTGCAGGACTACCATAAAACACTCAAGAACATGAAGGTGGATTTG

GACCAGAGAAAGGAGGTTCTGAGTGCGATGGAGGCAGATCTGGATCAGGCGTATCACTGG

AACGGACAAATCGATCAGAACTTCCATCAGTGTGACGTGGACCTGAGTAAGTTCTCTGAT

CTGGTGAAACAGATGATCAGCCGCTGGAACCGGATCACCAGCCAGATCAACACCAGAATG

GGTGATCTGAATAAACAGGAGAAGCAGCTCAGTCAGTATAAACACAACAGTTCAAACACC

AACCAGTGGATCGACCAAACCAGCAACCGACTGGACAAACTGCAGAACTACAAACTAAAC

ACACTGCAGGACCTGATGGACCATCTTAACCAACACAAGACGCTGCACTCTGAGATTAAA

GGAAAAAAGGACAAAGTGGATGATGTGCAGAAGGATGCAAGCACCTGTTCTGCCTCCATA

AAGGATTATGAGCTGGAGCTGGCGTCCTACAGTGCTGGACTGGAGACACTGCTGAACATT

CCCATAAAGAGGACCATGCTACAGTCTCCAGCCACCATCCTGAGCCAGGAGGCGGCCGAT

GTTCAGACTCGTTATATAGAGCTGCTGACTCGCTCCACCGATTACTACAAGTTCCTCACT

GAGATGCAGAAGAACATGGATGAGCTGAAGATACGAAACACTAGAATTGACCTTCTGGAG

GAAGAACTGAGGCGTTTGAAGGATGATTTGCAGGATCGCTCACAGAAGAACCGCTCTCTG

GAGGAAATGTTGGCCCGTCTCAAAATAGATCTTTCACAGTCTAAGGAACAGCTGATTTCT

ATGGAGCAAGTGAAAAGTACTGAGGCCATGAGATACAATGCAGCTAGGGAGAATCTGGAC

ACCACCCAAAATCAGCTAAAAGACCTGAATGACCAGGTTGCACGGCTTACACTGCTTATT

GATGAGGAGAAACGTAAGCGAAGGCTGGCTGAGGAGCGTTATTCCAGCCAACAACAGGAA

TACGAGGGGGCCATCCGCAAGAGACAGAAAGAACTGGATGAACTTAACTGGACCAAGATT

GACTTTGAAAAGGCCGTTAAGGACAAGGAGCGGGAGATCGAACGTCTGAAGGTGCAGTTG

GAGGACGAGGCCTCTCGGCGCAGAACCGCGGAATCTGAAACCGCTAAGGTAAGAAACCAG

TTCAACCAGGACCTCACTTCTCTAAAACAAACGTATGAGTCCGAGATCCACGTTACCAAA

ACCACTGTGCTAAAGGACATGCAACAAAAGGAGGAGGACACAGCTGCCCTAAAGATGCAG

CTGGAGAAGCTTATGAATGAGAAAAAGGATCTTGAAGAAGAACTCAGAAGGCTTCAGATG

TCTATCAGACAAATGGAGGAAGCCAGAAAGAGAGCCGAGCAGGAAGTACATCAACAAAGA

TCCTCAGGAACAGAGGAGACCCGAAGGAGAAAGGAACTGGAGATACATGTTCAAACTGTC

ACACGACAAAGAACTGAACTTGAGAGCAGGTTCAATGAAGAACTCGTACAAGCCAACAAC

AAATTGCAAGAAAAGGCCCGTCAGATTTCAGTTCTTACTCAGAACCTAGAGGATGAAACC

CGAAAGAAAAGAGCTCTGGAACAAGAAAACCACAGACTTCGCCAGTCAGAGGCAGACCTG

CAGGCCAAGCACTCTTCCTCTTTGGAACTCATCAACAAGCTAAAAATCACAGAGAAGGAG

ACAAAACTTATTTGTGTCGAGTTGGAGAAGCAAGCAAGTGAAAAAGGAAAAGTAGAGCAG

GGTGCCGCCAGGATGCAGACTCGCATTATAGACCTGCAGAACATGTTAGAAAAACTTGAG

TTTGAGCTGGAGAAAGAAAAAAAAAGCAACCAGGATGAACTTACAAGGAGGAAGCGGATA

GAAACTGAGCTGGAAAGAGTGAATCAAGTGTGCAGAGAGTACACAACTACTATTAACTCT

CTCCGTGCCCATAAGGAAGAGGAGAGTGCCACCAGCCGAAGGCAAGAACAAGACCTCCAT

CGTGTCCAGCAGGACCTGGAAAGATGTCGGAAGGAATACAATGTAACGTCCGAGAACCTT

GCCAGACTGACAGCCGAGCTGAAGGCCTTGCAACAGCAGCTGATTCAAGAGCAGGCTCGT

GTTCGTGAGGTGAACCTGCGCAACGAGAGCCTGTACAAGACCATAGAGGAGAAGAGCAGA

GTGTTAAATGAAAGCATGTCTGAAATTGAGAAGCTCAAGGGTCTCACTCAAAACCTTACA

AAGGAAAGGCTGAAGCTTGAGGAGGAGCTAAGGAGTGTACGCCAGGAACGGGAAGACTTG

CGCAACAACAAAAACAACAATGAGAGTGAACATATGGCCCAACTGTCAGCCATACAGCTC

CAGCTTCAGGCCTCAAATAAGAGAGGCCTAGAACTCCAGGGCCACATTAGTGACCTGACA

AAAGAGAGAGAAAATTTAAAGACGGAAATAGCCAAAATCCAAAAGCAATATACGGAGACA

TCTCTTATGGTCCACGAGTCTCAGATGCACTACAAGGAGATCCTACAAGAGAGAGATAGT

CTTCTGGCCAAGCTTAAATTGCTAGAAATGGACAAAGGGAAGCACCAGCGCTCCGAAGAA

GAGCTAAGTCGCATCAAGATGTCACTGGAATCAGAGCTGCGTCAGAAACAGCGCCTTCAG

GAGGAAATTGACAAAATCCGCAAGGATTTCAACTACTGGAAGAGTCAATATGAGATAAAG

GATGGTCAGGTTAAACAATGCCACGCAGATAAGGACAAGTCAGAGAGGGACAGGTCTTCA

TTGCAAAGTGAGATAACGAGACTGACTGCTGAACTTAGAAGTTTGGACGAGCGCTACCGC

ACTCGGCTTCAGAGCTCTGAGTTTGAAGTGTCTGAACTCACCCGTAAACGAGAGGCTCTG

GAAAGGGAGCTGAGGAGGCTACAGCAACGTCCAAGCGCCTTTACTAAGCAAACTCAGACA

GATGAAAATGTGCCCATGATTGATCCATCAAAGCTTGTATTTGATGGCATTCGCAAAAAG

GTGACTGCTCATCAGCTGTGTGACTGTGGGATCATCGATAAATCTACCCTTGATAAGCTG

TTGAAGGGCCAAATCGCAGTGTCAGATATTTCAGCAAAAATTCAGCCGTATCTTAAGGGT

ACCGGGGTTGTTGCAGGTGTTCAAAAAGATTCCCACGGGAGAATGCCAATCTCCGAAGCT

AAAAGTAAGAACCTACTGAGTCCACAGAGTGCCATTATGTTGCTAGAAGCTCAAGCTGCC

ACCGGATACATAATTGATCCAAAGTTTAATGAGAGAATGCCAGTAGATACAGCTTGTTCC

AGAGGTATTGTTGACACAGAGGATAGAGATGTCCTCGTTACAGCTGAGGCAGCTTGTACT

GGGTTCAAGGATCCATTTTCAGGAAAGGTGTTGTCTGTTGGACAAGCATGCAAGAAAGGC

CAACTTGACAAGGAGAAGGCCCTTCGACTGCTTCAGGCTCAGGAAACAGTGGGAGGGATA

ATCGACCCAGTACTGAGCGTGTTTCTACCCAAAGACATTGCCTTAGACCGTGGCTTGATT

GATGAAGAGCTTTATCGAGCGCTGAACAAAAAGCCAGCTTGTTACATTAATCCAGCAAAT

TCAGAGAAGATCAGCTACAGTGATTTAAGAAGGAAGTGTGTCACAGAACCTACAAATGGC

CTTCTTCTTTTCCCTGTAAATGAAAACTCATACTATGTCAAGGGAATCCGTGGCCAAGTC

TCACTTACTGAGCTTGTAAATTCCAGCCTTATTACAGAGGGGGATGTAGACAAAGTGAAA

CAAGGCCTGCTCACAACCAAAGATATTGAAAATAGGCTGAAGACGTACCTGTCTGGTTCC

GGCTGCATTGCCGGGATTTACGATGAGGCACGGGATCGAGTCCTGCCTTTCTACCAGGCC

ATGAAAGAGAATCTTCTCCGTCCAGGAACAACATTAGAGCTTCTGGAAGCCCAGGCTGCA

TCAGGCTTCATGATCGATCCAGTCAACAATGTCTACCTGACAGTGGAGGATGCATGGAGG

AGAGGCCTCGTGGGTCAGGAATTTAAAGGAAAACTTTTGTCTGCTGAGAAAGCTGTGACT

GGATACCACGATCCTAGTACTGGGCAGCTCATCTCACTCTTCCAAGCTATTGAAAAAGAA

CTTATTGAAAAGGGGCATGGTATTCGGCTTCTGGAGTCTCAGATAGCCAGCGGAGGCATC

ATTGACCCTAAGGAGAGCCATCGAATTGATGTGGAAGTGGCTTACCGAAGGGGTTACTTT

GACCGTGAGATGAATGAAATTCTGTCGTATGAAGGCGACGATACAAAGGGCTTTTTTGAC

CCAAACACTCAAGAAAATCTGACCTATCTGCAGCTCCAGAAGAGATGTATGAAAGACCCT

GTGACCGGTTTGCTTCTGCTGCCACTGAAAGACAAAAACAAGCAAAAGCAGCAGTTAAGT

CAAAAAAACACACTTCGCAAGAGGAGGGTGGTAATTGTGGACCCTGACACGGGCAAGGAA

ATGTCTGTTCGTGAGGCTTATCACAGAGAGCTGATTGACTATGACACATTTTTGAATCTT

TCGGAACAAGAGTGCGAATGGGAGGAAATCACTATCACAGATTCAGCTGGCAAAACACGT

CTAGTTATTGTTGACCGTAAAACAGGTACACAGTATGATGTCCAGGATTTTCTGGACAAG

GGCCTTATTAGGAAAAGTGACTTAGACCAGTACCGCTCGGGAAGCCTCACACTCACCCAG

TTTGCTAATCTGTTCTCAACCAAAGCTACAAGTGCTGAGCTCTCCATCTGCACCAGCATT

CCAGAAGATGTTACGAACTGCGGTAGCCCCACTGAAGTTAGACCATCTTCTCCAACTGTC

CGCAAACGCTTTGCTAGTGTTTCTATCACACTCTCCCCTCCTTCGGAAATCCTGGATGAT

CAGAGCCCGGTGGCAGCCATCTTTGACACTGAAACTTTGGAGAAGATCACAATAACAGAA

GCTCTGCGTCGTGGAATAGTGGATGCGATTACAGCACAGAGGCTCCTGGAAGCCCAGGCA

TGCACTGGAGGTATCATAAACCCTGCAAATGGCAAAAAGCTTTCTCTGCAAGATGCTGTA

CATCAGAGCATCATCGATGAGGAAATGGCCAACAAGCTAAAACCAGCTCAAAAGGCTTAC

ATTGGATATGAGGACGTAAAGACCAGAAGAAAAATGTCTGCTGCAGAGGCCATTAAAGAG

AAATGGTTACCATATGAAGCTGGTCAGAGGTTCCTGGAATTTCAGTACCTGACTGGAGGA

CTGCTGGAACCAGAAAGTGGAAAGCGAATTAGCATTGAAGAAGCCATTCGAAGAGGCTGG

CTGGATGGTAAAGGAGCCCAGAAGTTGCAGGACACCAGAAGCTATCTGAAGAGCCTAACT

TGCCCTAAAACCAAGCTGAAGATCTCGTATAAAGAGGCGATGGACAATTGCATGGTGGAA

GAGAACAATGGCATGAAGATGCTTCAAGCCACATCTGTGTCTACCAAGGGTATCAGTAGC

CCCTATAACGTGTCCTCCGGTCCTAGCTCCCGCTCTGGATCTAGAGCCGGATCCAGGGGA

AGCTCTAGAAGTGGATCCCGCAGAGGCAGCGTAGATTACTCCACTTACACTTACAGCTCC

ACCACCTCCAGCTCTTACACTCCATTCCCTTAATATTTCAACCGTGCAGCTGTGTGTGTG

TTGGGGGGGCTCCTTTTTTTTATACTACTCATTTTACTCAAATATCAAAAGAATTATATT

AACGTGGGTTTGGGAA

>Unigene000178

TGACGCTGACCAGCAGTTCCAGGTGGGATAACTGTTTACATACACACACATTAGATTCTG

GAGCGTCAAAATAACTACATTTATCCTAATTCTTTTTGGTCATGGCCTTTAACCAGTCTT

TAGGCAAAGGATCGTTCAGACACACAAGATGGAGTCATCATTATAGTCAAATGCTGGACA

TGTTGATGAAGCTTGCTAAAGCTGTAGCTACAGCAGCAGCAACTCTGGTCTTAAAGGCAA

AGAACGTGGCCCAGAAGACAGAAGATTCAGCCCAGCAAAACCGGGTCATCGCTGCTGCCA

CACAGTGCGCCCTTTCCACCTCTCAGCTGGTGGCATGCACAAGGGTGGTTGCTCCAACTA

TCAGTTCCCCAGTATGTCAGGAGCAGCTGATTGAGGCAGGGAAACTAGTGGCTAAATCAG

TTGAAGGCTGTGTGGAGGCATCACAGGGAGCCACCAACGATGAGCAACTGCTGAAACAAG

TGGGTGTGGCCGCCACAGGTGTCACCCAGGCTCTTAACGAGCTGCTGCAGCATATCAAGC

AGTACGCCAGCGGAGGCCAGCCAATCGGGCGCCACGGGGAGGCAACTGACCGCATTCTGG

ACGTAACTGAGAACATCTTCAGCTCCATGGGAGATGCAGGTGAAATGGTCCGACAGGCCC

GGATCCTTGCTCAAGCCACCTCCGATTTGGTCAACGCCATTAAAGCAGATGCAGAGGGAG

AGACAGACCTTGAGAACTCTCGTAAACTGCTCTCAGCTGCGAAGCTTCTAGCCGATGCAA

CTGCCAAGATGGTGGAGGCCGCCAAGGGTGCTGCCGCCAACCCAGACAGTGAGGAGCAGC

AGCAGAAGTTGCGTGAGGCCGCTGAGGGCCTGCGCATGGCCACTAATGCAGCAGCACAGA

ACGCCATCAAAAAGAGGCTGGTTAATAAGCTAGAGAACGCTGCAAAGCAGGCAGCTGCAG

CCGCAACTCAGATCATAGCTGCCGCCCAGCACGCTGCCTCATCTAATAAGAACCCGGCTG

CACAACAGCAACTTGTACAGAGCTGCAAGGTGGTGGCAGACCAGATCCCCCAGCTGGTGC

AGGGAGTCAGAGGCAGCCAGGCACAGCCTGACAGCCCCAGTGCTCAGCTTGCCCTAATTG

GAGCCAGCCAGAACTTCCTGCAGCCTGGAGCCAAGATGGTGACTGCAGCTAAGGCTACGG

TGCCCACTATTGGTGACCAGGCCTCTGCCATGCAGCTCAGCCAGTGTGCCAAAAACCTGG

CCAGTGCATTGGCTGAACTCCGCACGGCTGCCCAAAAGGCTCAGGAGGCTTGTGGTCCTC

TGGAGATTGATAATGCCCTGAATGCAGTAAGGGATTTGGAGAAAGACATGCAGGAGGCTA

AAGCTTCAGCAGCTGAAGGAAAACTCAGACCTCTTCCTGGAGAAACGCTGGAGAAGTGCT

CCCAGGACCTGGGCTCCAACACTAAGGCAGTGAGCTCTGCTGTTGCCCAGCTTCTGAGTG

AAGCTACACAGGGCAACGAGAATTACACCGGTATGGCTGCACGGGATGTGGCCCAGGCTC

TCCGTTCTCTTGCCTCCGCAGCCCGTGGAGTGGCCGCAACCACAGACGACCCACAGGCGT

GCAGCGCCATGCTGGACTGCGCTGGAGATGTCCTCGACAAATCAGGCAACCTCATTGAAG

AAACCAAAAGAGCCATCGCTAAACCTGGAGACCCAGAGAGCCAGCAGCGACTTGCCCAGG

TGGCCAAGGCTGTCGCTCAGGCACTGAACCGCTGTGTTAACTGCCTTCCTGGTCAGAGAG

ATGTGGATAACGCTATCAGGACTGTGGGAGAGGCCAGCAAAAAGCTTCTTTCCGATCAGT

TCCCAGCTAGTGGAAAAACCTTCCAGGAAGCTCAGGCAAACCTGAACCAGGCGGCCGCCG

GGCTTAACCAATCAGCCAACGAGCTGGTGCAGGCTTCTCGCGGCACCACACAGGACCTGG

CCAAAGCGTCGGGCAAATTTGGCCAAGACTTCAGCCACTTCCTGGAGGCTGGCGTGGACA

TGGCTGGCCAATCTCAGTCTAAAGAAGACCAGACTCAGGTGGTGACCAACTTGAAGACCA

TCTCTATGTCATCCAGCAAGCTGCTGTTGGCTGCTAAGGCTCTGTCTACTGACCCCAATT

CCCCCAACCTGAAGAACCAGTTGGCTGCTGCTGCACGTGCCGTGACTGACAGCATCAACC

AGCTGATCACCATGTGTACGCAGCAAGCACCGGGTCAGAAGGAATGTGACAACGCTCTGA

GGGAGCTCGAGACGGTGCGGGGCATGCTGGAGAACCCAACTGAAGCAGTGAATGACATGG

GCTACTTTGACTGCATCGACAGTGTAATGGAGAACTCAAAGGTATTGGGTGAAGCTATGG

CCGGCATCTCCCACAATGCAAAGAACAGTAACCTGCCTGAATTCGGAGACGCAGTCAGCT

CGGCCTCCAAAGCTCTGTGCGGCCTCACCGAAGCTGCTGCTCAGGGTGCCTATCTGGTGG

GTGTCTCTGACCCTAACAGTCATGCTGGACAGAAGGGGTTGGTAGATCCATCTCAGTTTG

CCAAAGCCAAGCAATCGATTCAGATGGCCTGTCAGAACCTGGTGGACCCTGCCTGCACAC

AGTCCCAGGTCCTCTCTGCTGCCACTATTGTTGCCAAACACACCTCCGCCCTATGCAATG

CCTGCCGCCTCGCCTCCTCCAAAACATCAAACCCTGTTGCTAAGCGACAGTTTGTCCAGA

GTGCCAAAGAAGTGGCAAATAGCACAGCCAACCTTGTAAAGTCAATCAAGGCCCTGGATG

GAGCCTTCAACCAAGAGAACAGACAGAAGTGCAAGGCCGCCACAGGGCCATTAATCGAGG

CTGTGGATAATCTCACTGCCTTTGCTTCCAATCCTGAGTTTGCTAGTGTGCCTGCACAGA

TCAGCCCAGAGGGTCTGGCTGCCATGGAGCCCATCGTGGCTGCAGCTAAAACTATGCTGG

AGAGCTCAACAGGTCTGATACAGACCGCACGCTCTCTAGCCGTGAACCCCAAAGATCCAC

CCAAGTGGTCAGTGCTTGCCGGTCACTCCAGGACTGTGTCTGACTCTATCAAGAAGCTAA

TCACCAACATGAGAGATAAGGCTCCAGGTCAAAGAGAGTGTGACGAAGCCATTGAGGTTC

TCAATAACTGCATCCGTGAAGTGGACCAGGCTTCTCTCGCTGCCATCAGCCAACACCTTA

CACCCAGAGACGACATTTCCCATGAGGCTCTGCATGAGCAGATGGCAGCCTCGGTGCAGG

AGATTAGCCACTTAATTGACCCAGTGGCTATCGCAGCTCGTTCCGATGCCTCCCAGCTAG

GACATAAGGTTTCTCAGATGGCGAGCTACTTCGAGCCACTCATCATGGCAGCCATTGGTA

CTGCATCAAAAATTCTCAACAGCCAACAGCAAATGAGTGTTCTGGACCAGACAAAGACTC

TGGCAGAGTCTGCCTTACAGATGCTCTATACAGCCAAGGAAGCAGGAGGCAACCCTAAGG

CGGCTCACACCCAGGAAGCTCTGGAGGAGTCTGTGCAGATGATGAAGGAGGCCGTTGATG

ACTTGGGAGGTACAATGGCTGAAACAGCAAGTGCAGCCGGAGCTGTAGGAGGCATGGTGG

ACTCCATCACTCAGGCTCTTAACAAATTGGAGGACCCTGGAGTCGAGCCTGAGGGAACCT

TTGTGGATTATCAGACTACTATGGTGAAAACGGCCAAGGCTATTGCAGTCACTGTGCAGG

AGATGGTCACTAAATCAAACACCAACCCTGATGAGCTCGGAGGTCTAGCCAATCAGTTGA

CCACCGAGTTTGGTGACTTGGCATCAGAGGCTAAATGTGCTGCCATCACTGCAGAAAATG

CTGAGATTGGCTCTCACATTAAGAAGCAGGTAACAGAGCTGGGCTATGGTTGCACTGGAT

TAGTCACCAAGGCAGGAGCTCTACAATGCAGTCCCAACGATTCCATCACTAAGAAAGAGC

TGATTGACGCTGCCCGCAAAGTCTCTGAGAAAGTGTCCCATGTGTTGGCGGCACTGCAGG

CTGGTAACCGTGGTACCCAGGCTTGCATCACCGCTGCCAGTGCTGTAGCTGGCATCATTG

CTGACCTTGACACCACTATTATGTTTGCCACTGCTGGTACACTTAACAGAGAGAATGCAG

AGACCTTCGCTGATCACAGAGAAAACATTTTGAAGACTGCTAAGGTTCTGGTGGAGGACA

CCAAGCTACTGGTGTCAGGAGCAGGAGCCAGTCAAGAAAAGCTGGCCCAGGCGGCCCAGT

CCTCAGTCAACACTATCACCAAACTTGCCGATGTGGTTAAGCTGGGGGCCGCCAGCCTGG

GCTCTGAGGACCCTGAGACACAGGTGGTCCTGATCAACGCAGTCAAAGACGTGGCTAAAG

CTCTGGGTGATCTTATTAGAACCACCAAAGCAGCCGCTGGCAAGCCTCATGACGACCCGG

CCATGCTCCAGCTGAAGAGCTCTGCGAAGGTGATGGTTACGAACGTGACGTCCCTGCTGA

AGACGGTGAAGGCTGTGGAGGATGAGGCCACTAAAGGCACACGGGCGCTGGAGGCCACCA

TCGAGCACATCAAACAGGAGCTTGCCGTGTTCAGCAGTCCAGATCCTCCTCCAAAGACAG

CTACACCAGAGGAGTTTATCCGCATGACCAAAGGCATCACGCAAGCCACAGCCAAAGCCG

TGGCTGCTGGAAACTCATGTCGCCAGGAGGACATCATTGCTACCGCCAACCTGAGCAGAA

GAGCCATCGCCGAAATGCTGCACTCGTGCAAGCAAGCGGCTTATCACCCAGAGGTTAATC

TGGAGGTGCGGACCAGAGCCCTGCGCTTCGGAAAGGAGTGTGCTAATGGCTATCTCGGCC

TCCTGGAGCATGTGCTTGTGATCATCCAAAAGCCCACTCACGATCTGAAGCAGCAGCTGG

CAACCTTCTCCAAACGAGTAGCTGGCTCAGTCACTGAGCTCATCCAGGCAGCTGAAGCCA

TGAAAGGCACTGAATGGGTAGACCCTGAGGATCCCACAGTCATTGCAGAGAACGAGCTGC

TGGGTGCAGCGGCCGCCATCGAAGCTGCTGCTAAGAAACTGGAGCAGCTGAAACCACGAG

CCAAACCCAAGGAGGCTGATGAGAGCCTGAACTTCGAGGAGCAGATTCTGGAGGCAGCCA

AGTCCATCGCTGCAGCTACCAGTGCGCTGGTGAAGGCTGCATCTGCCGCACAAAGAGAGC

TGGTGGCCCAGGGCAAGGTTGGCGCTATTCCAGCGAATGCAGTTGATGATGGACAGTGGT

CTCAGGGTCTTATTTCAGCCGCCCGCATGGTTGCCGCCGCCACCAATAATCTGTGTGAAG

CCGCGAACTCTGCAGTACAAGGCCACGCTAGTGAGGAGAAGCTCATTTCCTCAGCCAAGC

AGGTGGCGGCATCCACAGCTCAGCTGCTTGTGGCCTGCAAGGTCAAGGCTGACCAGGATT

CACAAACCATGAAAAGACTGCAGGCTGCGGGCAACGCTGTCAAGAAGGCGTCAGATAATC

TAGTAAAGGCAGCACAGAAGGCTGCTTTCGATGCGCAGGATGATCAGGCTGTAATGGTGA

AGAGCAGGATGGTGGGCGGCATTGCTCAGATTATTGCTGCTCAGGAGGAGATGTTGAGGA

AGGAGAGGGAGCTGGACGAAGCCAGGAGGAAGCTAGCTCAGATCAGACAGCAGCAGTATA

AATTCTTGCCCTCAGAGCTGAGGGAGGACGGTCATGAGCAGTGAAGAGCTTCTTTCACAC

ACACATACACACACACACACAGGTCTGCTACTCAGGACTGCACGGCCTGAGCGCTTTTCT

CTCCATGTGTTTAGCTACAATGGCAGGCTTGCTGAAGCAACATTCAGGCTAAAATTATAA

AAAAAGCGAGGC

>Unigene000179

CGACCAAGATCGGAAGATGGCGGAGCATTTAGAAGTGGCTTAAAGGAAACTGGTGTGTTT

GAAGTGGACACATATTAACCCGTGGTGGCCTTGGGGAAGCTTTAGATGCGACTGGGTCTT

TAGTCAGATCTTCCGCATTGATACCCAAAAGGATGTGAGGTGAAACGGAGTGGATGTCCT

CATTCTCCACTGCTGCAAAGGTGCTGATATGTGTTGAAGATGAGTGGACAAGGGGAGAAC

TCTCTGGACTCATTGGGCTACGACCGGAAAAGGAAACTCTCCACTTGTGACACACCAGGA

CTAGGGTGTGATAAGCGTCGGAGAGAACAGGAGTGTAAATACATCGAGGAGCTTGCTGAG

CTGATCTCAGCCAACCTCAGCGACATCGATAACTTCAACGTCAAACCGGACAAATGCGCC

ATTCTTAAAGAGACAGTACGCCAGATACGGCAGATTAAAGAGCAAGGAAAGGGGTCGTGT

GGCGATGATGATGTCCAAAAGGCAGATGTGTCCTCTACGGGTCAGGGGGTCATCGACAAG

GACCATCTGGGCCCTCTGCTATTGCAGGCATTGGATGGTTTCCTGTTCGTTGTCAATCGA

GAGGGAAGCATCGTCTTTGTGTCCGACAATGTGACTCAATATCTGCAGTACAAGCAGGAG

GAGCTGATTAACACCAGCGTGTATAACATCCTGCATCAAGAGGACCGTGAAGAGTTGCAG

AAAAACCTGCCCAAAGCCAGTGGAGCGAATGGAGTTTCGTGGACTGGAGATGCCGCTCGT

CAGAAAAGCCACACCTTTAACTGTCGGATGCTGGTAAAGTTCGGTCATGGAGGCCATGGA

CCGGAGGACGGATCAGGAAGGCCACGTTATGAGACCATGCAATGTTTTGCCCTTACCCAA

CCTAAAGCCATGATGGAGGAGGGCGAAGATCTGCAGTCTTGTATGATCTGTGTGGCGCGG

CGAGTGACCGCGGTGGAGTGCATGGAAAGGTTCAGCACCAGGCACGAGCTCTCAGGTAAG

CTGATCGAGATCGAGCATCAAAGCACCTTACACACCACAATGCGTCCCGGTTGGGAGGAT

CTGGTACGCAGATGTTTGCAGATGTTCCTGCATCACAGTGAAGGCCAGCCCTGGTCCTGT

AAACGGCACTACCAGGACGCCTGCATACGTGGTCGAGCAGAGACTCCACTCTACCACTTC

TCCCTATCGGACGGAACGCCAGTAACGGCACAGACACGCAGCGAGCTCTGTCGAAACCCA

AATACCAACGAACCTCACAGCTACCTCTCCACCCACCTATTGCAGAGAGAGCCGAATGGT

TATAGGTCAAACCACGGAGGTGTAATGCGTTCCCAGGGAATGGCTGGTGCCAATCATAAT

TCCCAAATGAACATGACTCCTAGTGGGCAGATGGGCATGGGCAATGCCAGAGGATACGTC

ATGAATGACTCAGGGCATATGGTTCCCAGAGGAGGCCCCATGTATGGCCCTGGGAACAGA

ATGAATCAGATGAACTCAATGCACCAGATGAGTCAAATGAACCAGATGAACTCAATGAAT

GCAATGAACCAGATGAATCAAATGAACCAAATGAATCCTGTTAATCAGATGAATCAAATG

GGTTCGATGAACCAGATGAACCCCATGAATCAGATGAATCAGCATAACCCTATGAGTCAA

ATGAACCAGATGAATCAGATGGGGCATCCCGGAATGAACCAGCACCCACAGCACTTCCAT

AATGATGGGTATGGGTTGGGCATGACCAGTCCGTCCCACGGTAGCCCAAGTATGAACCCA

TCACAACAGATCATCATGGGATCTCCACGAATACGAGGAAGCCCCAAGATAGGAGCCAGC

CCTTTCTCGCCAGGAGGTATGCACTCTCCTATGGGGCCTGTTGGAATGACTGGAAGTAGT

GGCTCCTCTGGTGGTAGCACTTTCTCAAGCAGCTCTCTTAATGCCCTACAGGCCATCAGT

GAGGGAGTTGGTTCCTCCCTTCCCTCTACTCTCACCTCACCTTCTCATAAGTCAGACAGC

TCCCCTAGTATCAACTCCACTCAGTCTGGTCAACCAAACCTGTCTGGGACTAATAAAGGA

CCTAATGCAGGCGAAACGAAAAGCTCTGCTAATACGCCTAACACTGGGACTGACCAGCAA

CATGCAAACATGTCCGAACCCTCAACAGATAAACCAGATAGTCACACTAACAAAGATGGC

CAGACTGGTGCTGATGTGCAGCGGAAGGGCCCAGAAGGAAAGGGCCATAAGAAGTTACTC

CAGCTGCTTAGATCTCCACCGGAGGAGCTTGCTTTGGGCGGAGGAAGTGGAGCAGCCCCT

CCTGGACGGGCTTCCACTCCCATTGGTGCTGACCCAAAAGAGGCTGGAGGTAGTATGACA

AGCCCGTCTTCCACAGGTGTGTCTTCATCATCATCTGCTTCATCGGGAGGCGCCACATCG

CAGCATGGATCGGGATGCGTGTCATCTACAGGCTGCATGGGTAGCCATCTGTCTTCTCAC

TCGCTGCAGGAGAAACACAAAATTCTTCACAAGCTCTTGCAAAACGGCAACACACCGGAC

GAGGTGGCGCGCATCACCGCCGAGGCCACGGGGAAGATCACTCTTGGTTCTGAACACCAA

GGCTCAGACGGCATAGGGTTGGAGGTCAAGCAGGAGCAGCACAGTCCAAAAAAGACTAAT

GCTTTGCTCCACTATCTTCTAAACAAGGATGATAGCAAGGAGACTAGAATTGACGGCAAA

CCAGGACTGGATGAGCTGGAGGGTCGTGGGGTTGGTGGAGTAGGTGTCACAGACTCCAGC

GGCCAGGAAAGCAAGGTCAAAATCGAGCCGCCTGATGAGTTGCAAAGCCTGGAGAGCATT

CTGGGAGGCTTGAAACCCAGTTCTGGGATGTTTGGTGATTCTGGAAACACAGATGGAGAA

AATAAGAAAGGAAATGGACCCAACAACAGTGCACATGATCATGAGGGTGCAGTTATGAAC

TCAGGGCCTCGAGTGCCGTTCCAGAGAGCAATGTCTGTGGACAGTAAACCCTTTAGCGGG

GCGAGAGGGACAGGCAGAAGAAGTGCCCCCTCCTCTCTTATTAAACAGGAGAGCTTGGAC

AACCACATAGGCATGGGTGGTCCTAACAGGCAAATGGGAATGAACCAGATGCCCCCTATG

ATGGGCCCAGGTGATTGGAGGATGCCAGGTTCTACAGGCAGTCCAGGGGGATCTAACGGA

CATCCTGCTATGGGTTGTCCAGGCATGGATCCAAATCCTAAAGGAATGATGGGAGGTCCC

ATGATCAACAGGTCAAACAGTGTACCAACACCAAGATCCATGTTACAGCAGCAATTAATG

GATATGGGTCAAAATGAAATGGGGATGAACACATTTAGAGGGCAGCCACATCCTCCTCAG

TCTCCATCGTGGCCAGACTGTGGCATGGGCATGGAGGGACCCCCTAATGCAAACAGCTTG

CCATTTGGCAACCCCCTGGACGACCTACTTGTTCCTCCATCCACCACTGATGGGCAAAGT

GACGAGCGAGCTCTGCTTGACCAGCTCGACTCCCTCCTCAACAACACAGACAACCTCGGC

CTGGAGGAGATCGACAGAGCGCTTGGGATCCCAGATCTGGTTGGCCAGAGCCCAAGTACT

GAACAACCAGCAACTCCTACAGATGCTTTCCCAGGACCATCTCCCGATTCATCAATGAGT

ATGGATCCTAAGGTCATGTATGGAGCAGGATATCCTGGTCCACCATCTATGGGCATGCAG

GGAGGCTATGGCTCTGGACCTAATCCTGGTACAGGTCCTGGTCCTGGCAACATGCAGGGA

CATCCGGGACAGGGATTTAATCCTATGATGCAGCAGATGGGTGGAATGGTGGGCATGAAC

CCCCGTGCTGCCATGATGCGTCCACGCATGGTCAACGCTAAGCAGCCGCTCCGCCTGCAT

CTTCAGCAGAGACTTCAGGGACAGCAGTTTATGAACCAGAGCCGACAGGGCATGCCAATA

AAAATGGACAATGCACCAGTGGCAAATCATGGAGTGAGGCCTGGCATGCAGCCTGGAATG

CAACCAGGCATGGGGGGACAGCCTAGTTTTCTGAACGCTCAGATGTTGGCTCAGCGAAAC

CGAGAAATGATAACACAGATGAGAAGGCAGAGGATGATGATTATGCAGCAGCAGGCTGCA

GCTGGAGGCTTCAGTCCCCCTCCTAATGTCACAGCTCCCACTGGCATGGAGAACCCCATG

GGAGGAACAGCTATGAACCAGCCTGGCCACCAGCCTGGCCAACAGCCATTTAACTATGGA

GGAAACTATGCTATGGGCCAGCAGGGTGACCCCTCTTTCATGGGCACTGGCAACAGCCCA

CCAAATATGATGGCTGGACGCATGGGAGGCCCACAGAATCCCATGATGCAACAGCACCCT

CAGAGTGGGCCCATGTACCAGTCAGCAGATATGAAGGGATGGGGGCAGGGTGGCATGCCT

ATGAACAACTCCTACCCTCAACAACAACAGCAGCAGCAGTTCCCACAGCAGGGGGCGCCA

GCGCAATACGGAGGAATGATGATGAACGGAGCCATGCAGGGCGCCGTGAACGGGCCAGGG

CAGATGATGCCAATGCAAGGGCAGATGGGCATGAACCCCATGGGCATGGGTCGCATGCCA

CTGGGTCCTGATCAGAAATACTGAAGCAGAGCATCGTTTAAGCAAGAGAAACAGCATACA

GTGAGAGAAGTGAGACACACGCCACCGCCAGCTCCTGCTACACATGGAATGGGACAAGCT

GGACATGAGACCAAGGACTCGCCCGCATCACTGTGTTTTAATAGACACTCGAGCATTTTT

GTGTGCCCCAGAATACCCTCTGTTGATCTGCAGCCAAACTCGAGCTCTTATGTGAAGCTG

GGTGTGAGCGTAATGCTGTACGTCTCCACTTTTCTCTCGCTGTGGAACTGCCCCGGTGCT

GGGTGGGGATGCACTTTATCACACGGCTAATAAGGAAAAGAATACCTGCTCGTTCTAGCC

TTATTTAACAACTTGACCCGTTCAGAAACCAGTTTGTTGATGACGTGTTTTAGCAGAAAC

TTTGCTTTCAGAACAGACCGCTTCAGTTCCAGCTATATGCAGCTACGAGCAATAATTCTT

CTCTCTATGCCATAAATATGTTAAAACGGTGCACCAGTGGAAAAAAACAGCAAAAGGTAC

TGCAGTATTAGCTGGGCTTTTTGTTTAACGTGTGAATTAGGACAGATCTGTACATGCTGT

ACGCTCCAAATGTATCATATGTGCCCTTATTATATACTGTACATTTTTGCACAGATTTGG

CACATGTGATGTCCAGCATATTATATTTTAGGTCAATACTGCTTTGGGTGTTAATTCTAT

TTTAACTTTCTCAAAAGGGAAAAATATTTTAAAATGTTATATATATATATATTTTTTTTT

ATCTTTTCTGATCTAGCCTCATTTTCTTTACAGTATTGCAGTTATAACAGCCATTTGTTT

TCGGAGGGCACTTTTTTCCTGTTGAAACGCAACGGAGATCTGATCGAGCAGTTACATCAA

GGATACATAATGGTTTAACGTGCGTCTTTTTATCTGTATATAATATGGACATGGTGGTGA

GTTTAAGTAAGACGGTTATTTGCTCAGCCTTTTTCTGTGAGTTATCTGTTACTATCGTTG

ACGATTTCACTTTTAAGCGCTGGGTCTTTTATAAATCTACCTTTAAAAATTGCACAATCC

AAAGATATAGATATTTCAATTCTTTGCTAAAATTTTAACCTTACCACTAGTCTTTTATTG

TGTAAGGTACATATCATATAAGCTTATTGTTATGATTTTGTACTACATGCTGTACCCGAT

CGCTATATTTCTTTAATTTTAAGGCCAGTTCATAAAGCAAAGGGTTTTTTTTTTTTTTTT

TGGTTTTGT

>Unigene000180

GGATTGTTTTATCAGCTTGCTGTGATGTGTCGTTATTAAGGATTAAGATTTTGCTCTTCT

GATTTTTTCGTTGTAAAGGATGCTAAATTGATTGATGGCAAATCCACGAGTGTCTATAGA

AGGAAGCTGACATCGAGGATCTAACCTGGAATTGCATTCTTCTTACTACATTACAATACG

TTTTTACAACCTGGGGTGATTTGACGTTAAGAAGGATAAATATGAGACAAAAGCCTCCTG

TTCAGTGGTTTCAAATGATGAAAGCAAGCTCTCTTGTTATGACATGCCCAGAATGACTTC

CTGAATCTGTCTAGTAGTGAGGCACTACTGCGAGTCACTGTGTGCTGGAAGAAGACACTG

ATTAGGACTGTGCTAGGCAGATGAAACGTCTGCGAAGTGATTGAGTTATGACTTCTTCTC

TGCAGCTCGTACGCTCAAGTCAACCTTTTAGATTTTCTGAAACTGGTCAACAGTCATCCC

AGACCAAGAGAGGAGGATAGTAAGGGAAGAGAATTTAAAGAAAGACTGAGCCACCAAGGA

ACAGAGCGAAAAAAAGTGCACCATGCAGTCAGACGACCTCTTTATCCGGAAGTTCCGCCG

ACAGAGCCCACGTCCTACCCCTGTCTTTGAGCCCAAGCGGACAGCGGTGGCGGAATGGCC

GCCCAGAAGGGATGGTGAGGGAGCAGAGGGTGAGAGTTTGACAACACCCAGCAGGGCAGG

TTTGAATCAACGTGCAGTTGCGCGTGGCCATATTATGCAACGGAGCAACAGCGATGTCAC

ACTGGGAGATTTGGAATCAGCGGCAAAAGCAGGAGCAAAAGCAGTCAGGTCAGCAAGTGA

AAAGACCGGGCAGGTAGGACAGGGTGACTCTAGTATAGTCCTCCACCGGGAATATGGGAG

TCTTTCTTCTTTGGAAAAGCAGATCCAACCTCCAGAGCTCTGCATGGATGAGCAGAATAT

GCTCAGCCCAAACGCTCTCCGCTTTAAAGATCCATTTTTGTTGCTCGGGCTGCAGGAGAC

ACCACCTGAACCAGATGGCTTTTTCCGAGCCCTGGAGTGCCCAAAACCCGCCAAGCCCCC

CAAACCTGATGCTCTAAACAAGAAATCAAAGCCCTCACCCCTTCAGGTCCCTTCTGTACC

TTGTGATCTGACATGTGGAGGTGCATGGGTGCGCAACTTTGCACATTATGACGTGCAGAG

CATCCTGTTCGACTTGAGTGAAGCGGCCACTAGCCGAGACAGCGTTGGTCGCAAGAAGAA

CATCACCTCAGGGGCGTCAGCTGCCTCGCAGCTCCGGCCTCTTTCACAGGCTCCTCCTGC

CTCCCCCTCACAGAGTCTGGGAAGTGGGACTGTTGGTACAGCAAGCACAGAGGAACCAGA

GCCATGCCTGCTGGACGAAGGAGACGGCAATGACAACGATTTGCTGCTCAGCTGCCCCCA

CTTTAGGAACGAGATGGGAGGGGAGGAGAGGGCTGGACTGGGAAGAAACCAGTGTTGTAG

TGGGCGATGGAGAAGTCTACAGAACCCCAACGATGCTGTGTCTGTATTAGAGGAACCCAG

AGAAAGCCATGTCCAACAGCAAGGAAAGAGCAACTACTTCATTGAGCATGCAGACCTCGG

GGCACGGTACTACCACAAGTACTTCTACAATAAAGACCATCAAAATTTCTTTGGCATGGA

CGAGCGCCTGGGGCCCGTGGCTATCAGTTTTCGGCGAGAGGAGAAAGAAGGCACTAGTGG

GGCGCAGTATATGTACAGGATCATATTCAGGACTACGGAGCTTAAGACTCTTCGTGGCTC

CATCCTGGAAGAGTCTGTGCCATCTGCAGCACGACACACCACCCCGAGAGGGCTTTCCCC

AAAGAGACTTTTGGAGTTCATTTTCCCGGATCTCAACCTGCAATGTCTGAGGCTTGCTTC

TACGTCTCCCAAAGTCCGAGACACGCTGCTCAAGCTGGATGAGCAAGGGCTAAATTTCCA

GCGTAAGGTAGGTGTGATGTACTGCCGGGCGGGGCAGAGTACAGAAGAAGACATGTATAA

TAACGAGAGTGCCGGGCCGGCGTTCGAAGAGTTCCTGGACCTGCTGGGAGACAGAGTGAG

ACTGAAGGGCTGGGAGAAATACAGAGCACAGCTCGACACCAAGACGGATTCCACTGGGAC

CCACTCTCTATACACACGTTACCAAGATTACGAGATCATGTTCCATGTTTCCACCATGCT

TCCATACACAGCCAACAACACGCAACAGCTTTTGAGAAAAAGACACATAGGGAACGACAT

CGTGACGATAGTCTTCCAGGAACCGGGGGCCATGCCGTTCACCCCTAAAGCCATCCGCTC

ACACTTCCAGCACGTGTTCATCATTGTCCGAGTGCACGAGCCCTGCACGGAAAACACCTA

CTATCGCGTTGCTGTTACACGCTCCAAAGACATCCCTCTATTTGGTCCACTTTTTCCCAA

AAGAGCTCGCTTTCCACGCTCACAGGCTTTTCGTGACTTCCTTCTGGCCAAGGCGTTAAA

CGCCGAAAACGCAGCTGAGAAATCGGAAAAGTTCCGCTCGATGGCCACACGCACGCGTCA

GGAATATCTAAAGGACCTAGCAGAGAACTACGTGACCACAACGCCTATCGATTCATCTAC

CAAGTTTCCCCTGCTTTCCCTGGGAGGCAAACGTAAAGACAAGTTTAAGGGTGCTAAAGG

AGCAGAGCTGCACAGTGCCGGCGCTCTGGTATGGGCCGTGTGCGCCAGGCAGGATGACGA

CGGCCACAAACTTGCCTGTCTTTTGGGCGTTTCAGCTGAATTCGTGGTGCTGATCGAACG

CTGCAGCAAACGCGTCGTCTTTAACTGCTCGTGCCGTGACGTGATTGGGTGGAAGGCAGT

AACTGAGACTGGCATTAAGGGTGGGCCTTGTCTGGATATTTTCTACGAAAGGGGCGAGGC

GATCTCGGTTACGATGTCAGAAAGCCAAATCGAGGATATTAAAGAGGTGGTGCAAAGGCT

TGAGCTGGTGACAAGAGGATGTGAGGCACTTGAGATCACTCCTCTGCGGGACAACACAGG

TCAGCCGGGATTCCTAATGAGCGCAGAGGGCTTCGTGATTGAGATGCAGCGATTCTGTTA

CGCAGAAAGCGGAGGCCTGCAGCTGAGCGCACGGGTAGTACGACTCTGTGGACACACCCT

CGTCCACCTGAGTCAGGAGGAGAGGACGAGGCTTCTGCGGACGGCACCCAAAATTCACAT

CACCGTCATCCCACCCGACGAGAACGGCAAACCTCGCAGGAGCTTTTCGGAACTCTATCA

GAAGTCTATCCAAGATGCAGAAAGCAAGTCTGGTGAAGGCCACTCGAGCGAGGCCTGGGT

ACTGAACGAGCATGACGAAGACGAGGATGACGAGGAGGAGGATGAGAGAGGGGGTGAGCG

AGAGCAGGGCGAAGGGGAGGAGTCAGACAGCATGAACCAATCAGATACAAGAGAGGAAGA

AGAAAGTGGAAGTCCAAGCTTAAGTGGCTTGCTCTCTCCGCCCAGCTTACCTCTTCTTAG

GGCGACTTCACTGCAGGACAATCCTCCAACCCAAACGCTGGAAATCACTTCCTCCGCTCT

CACACGCAGCTACTCACTCGAGAGAACCCTGCCATGTCAGGACATCTGTGATGGTCATGT

TTATGATAACGTCGGAGCGAAGGTGAACAGACATCATATCTACGAGAATCTAGGAGAGCT

GATCGACGCAACCCCTGACCTCATACTGGCTGTCAAACCAAAGGTTCCTCTTCAGGAAGA

AGGACAGGTTGGACTAGATGTGCTAGGTGAGGAGAGAGCATCGGGCAGCGAAGCACCTCC

TCAGTCAGAACATATCGAGAGGAATTCCAGAGCGGAGAGAAACTCCAGAGCACTGAGCAT

TCATAACTCCATCACCAAGATTCTTTCAGAAACAACAGATTCATCAGAGGAGGAATGGCA

GTCTATTTCTGACCTGGCCACTGCATGCAGGAATATTCTAGAAGCCCTATCGCATGAAGA

TCGAAAGGCCGGTGATGGCACTCAGGCAGGAGGGGATACAGGGACGCAGACAGATGCCAG

AATCAAAGATATGAAAGACAGTGATTCATCCAGTCACCTGGAGGAAAAGGTGTCCCAATT

GGAGGCCATGCTGAAACGATTACAGGATGACCTCCAAAAGAGCTGTTTCACAGAGCCAGT

CAGATTGAGGGGTGATAGAGGTAGGGCGGGACAAGCCAAAGAAACCTGTCTTGTCATTGG

GCAGGGTGAACAGAGGAGATGAGAGAAGATGACCCGAGGGACAGGCTCCCTCTCTCAACA

CTAAGCGACCAATGGCAGTCGAGGAGAAAGAGGACAAAGCCATGCTGCAGGCAGAGGTGC

AGAGTCTGAGACAGAACAACCAGCGGCTGCAGGAGGAGTCGCAGAGCACCGTGGCCCGCC

TCATCAAAGTCACCGAGCTGCTGTGTAACGTTAACAAACCCTGCTAGTAGAACGGGTCAG

CGAGATAGATATACAGTACACACACAAACACACACACACCAAAACAGACCAGCACCGAAT

GCTAGCAAGACACGAGCAAAGCAATCCAGACAGAACTGCAAGACCCGGAGCCCATGCTTA

GGCTCATTAATCACAAAGGAATGACTCAAATGCTAGACAAAGCTGCAAGACCCAGACCTC

AGACACTGAATTACAGACCACTAAAGAACGACACAAATTTCACCACACTAGCACAAAAAA

TTATCTAGTGCTACATGAAAGATGGACATAAATTTAAAGCTTGGAAGGTGCTGGCGTAGT

AGACCATTTTTTGGAACACACCATAAGATCCCTAGCAATGTAAGACCTCTAATGTAGACC

TCAGGGCCTATATTACGTGTAAAAACAATCAATCAAGACTAGCATCCCTTTTAGACAAGC

ATTGCTTTTTTAGTGTATTTAGCTGGCATCATTGGTGAAGTGCAGCCCACCAAGATTACC

AGGTTATTATTATTAATATTATTGCTCACTTGCATTTTACAAAACCAAGAGCAAAACCTT

AAGGCACGTTATTATCATCGTGCATGTTTGTCAGGCATAACAGATCAAGACATTGATGGA

AATTTACTGTTACCCAAAGCAGAGCAAGATCCTAACTGCATGATTTTTTATTTTAACGTT

GCCAGACCCCATATGGTTAGTACTCAAGGCTGGGTAACCCATATGCCTTGAGTTACTTTG

TTACACTTGTTTTCCAGCTACCCCAGGTATCAGAGGGTAGGAGATACCAAATCCTCAGAT

CTTCATCCTCTTCTGAAATTTATCTTCCAGCTTCCTATTGACTGAACTTACCTGAACAAG

GCAATCAGCTGCAGAAGGAAGCATGTATGATAAATGAGGCTTATGGAAATCCAAAATAGT

AAGCAAAAGTCATAACTAGGAATAGTATAATAAAGGAATGGCAACGCAACTGGAATAAGG

AAGTAACAGGCAGACATTATTAACAAATCCAGGAGAAAGCAGGAAATAGAAGGGCATGTA

AAGGAAATATAGGAATAGAAAGTATAATTACACAATTCAGAATAGGGCACATGAGTCAAT

GCCGACTATAATAAAACACGAAGATAAAGAACCATGTAATCGGCAGTGGGTAGAACGGGG

TAGGTGAAAAACAAGAGGAACTTAAGAAACATGGGTGAGGCAGCCCTGTCAGTACTTTTG

CCTAAAATTCTAACGCTTGATAAGAAGTTAATAGTGTTCAGTCAGCATGATGAGCCAGTA

GTAACTTTCATTTATTGTTGGAAAATTTTTTAAAAACTGGACCTAATTTTTTTGTCCAGA

TATTGACAAATCTACAGAGTATTCTAGTAAAAACCACAGCTAACATTTGGCAGACGTCCT

TATTCAGAACGACTTACATTTATCTATTTTATGTAACTGAGCAATTGGGGGTTAAGGG

>Unigene000181

GAACCTCAAGCACCCTGCCAAGTATTCAAAATCTGCCAGTCAACCTACCATCGGCCAGTT

TCTCCCTGACATCAAAAAAGTAACCTGCCACCATGGGGGATGGAGAAATGGAGTGTTTCG

GCCCGGCGGCTGTCTTTCTCCGGAAGCCGGAAAGAGAGAGAATTGAGGCTCAGAACAAAC

CTTTTGATGCCAAAACAGCAGTCTTTGTGACCGATGCTGCTGAGATGTACCTAAAGGGAA

CACTGAAGAGTAAAGAGGGTGGCAAAGCCACTGTCGAAACTCTGGATGGAAAAAGTGTCA

CAGTTAAGGAAGATGAAGTCTGTCCAATGAATCCTCCAAAGTATGATAAAATTGAGGACA

TGGCCATGATGACCCACCTTAATGAACCTGCTGTGCTGTATAACCTTAAAGAGCGTTACG

CAGCATGGATGATCTACACCTACTCTGGGCTGTTCTGCGTCACTGTCAACCCCTACAAGT

GGCTTCCAGTGTACGACCAAAAAGTTGTGTCTGGATACAGAGGCAAAAAGAGGATTGAAG

CCCCACCTCACATTTTCTCCATCTCTGATAACGCCTATCAGTTCATGCATACTGATCGTG

AAAACCAGTCAGTCCTGATTACTGGAGAATCTGGTGCAGGAAAGACTGTGAACACTAAAC

GTGTCATTCAGTACTTTGCAATGGTTGGCATGACTGGAAAGAAGGTAGAGGCTACTCCTG

GAAAAATGCAGGGGTCACTGGAAGACCAAATCGTTGCAGCCAACCCCCTGCTGGAGGCTT

ATGGTAATGCAAAAACTGTGAGAAATGACAACTCCTCTCGTTTTGGTAAATTCATCAGAA

TTCACTTTGGCCAAACTGGCAAGCTGGCCTCAGCCGATATAGAAACCTATCTGCTGGAAA

AGTCTAGAGTTACTTTCCAGTTGTCTGCTGAGAGAAGTTACCACATTTTCTATCAACTTA

TGACTGGACACAAACCAGAGCTGCTTGAGGCACTGCTCATCACCACCAACCCCTACGACT

ACCCGATGATCAGCCAGGGTGAAATTACTGTCAAGAGCATCAATGATGTGGAGGAGTTTA

TAGCCACAGATACGGCTATTGATATCCTTGGCTTTACCGCTGATGAGAAAATTAACATCT

ACAAGTTGACTGGTGCTGTGATGCATCACGGCAACATGAAGTTCAAGCAGAAGCAGAGGG

AGGAGCAGGCTGAACCTGATGGCACTGAAGTTGCCGATAAAATCGCATACCTCCTGGGCC

TAAACTCAGCAGACATGCTGAAAGCTTTGTGCTACCCCAGAGTCAAAGTCGGAAATGAGT

TTGTGACCAAAGGCCAGACTGTACCTCAGGTGAACAATGCAGTTTCTGCTCTCTGCAAGT

CCATCTATGAGAAAATGTTCTTGTGGATGGTCGTGCGGATCAATGAGATGTTGGACACAA

AACAACAAAGACAGTTCTTCATTGGTGTGCTGGACATCGCTGGATTTGAGATCTTTGATT

TCAACAGCTTGGAGCAGCTCTGCATTAACTTCACAAATGAGAAACTGCAACAGTTTTTCA

ACCACCACATGTTTGTTCTGGAACAAGAAGAGTACAAGAAAGAAGGTATTGAGTGGGAGT

TCATCGACTTCGGTATGGACTTGGCTGCCTGCATTGAGCTTATTGAGAAGCCAATGGGCA

TCTTCTCCATCCTTGAAGAGGAGTGCATGTTCCCTAAAGCCACAGATATGTCATTTAAAA

ACAAGCTTCATGACCAGCACCTTGGCAAAAACAATGCCTTCCAAAAGCCAAAACCTGCCA

AAGGCAAAGCTGAGGCCCACTTCTCACTGGTGCACTACGCCGGCACTGTGGACTACAACA

TTTCTGGCTGGTTGGACAAGAACAAGGATCCACTGAATGATTCTGTTGTGCAGCTGTACC

AGAAGTCAGGAAACAAACTGCTGTGCTTGCTGTATGCTGGTCATGCTGCTGGAGATGCTG

ATAGTGGTGGTGGAAAGAAAGCTGGCAAGAAGAAGGGTGGTTCATTCCAGACAGTGTCTG

CTCTCTTTAGAGAAAATTTGGGCAAGTTGATGACCAACTTAAGAAGCACTCATCCTCACT

TTGTCCGTTGTTTGATTCCTAATGAGTCCAAGACTCCTGGTCTGATGGAGAACTTCCTCG

TCATCCACCAGTTGAGATGTAATGGTGTACTAGAAGGTATCAGAATTTGCAGAAAAGGTT

TCCCCAGCAGAATCCTCTATGGTGACTTCAAGCAGAGATACAAAGTGTTGAATGCCAGTG

TTATTCCTGAGGGACAGTTCATTGACAACAAGAAGGCTTCAGAGAAACTCTTAGGATCTA

TTGATGTGGATCATACCCAGTACAAGTTTGGACACACCAAGGTGTTTTTCAAAGCTGGTC

TGCTGGGTACCCTTGAGGAGATGCGAGATGAGAAATTGGCATCACTGGTAACCATGACTC

AGGCTTTGTGCCGTGGCTACCTCATGCGTAGAGAGTTTGTCAAAATGATGGAAAGGAGGG

AATCCATCTATTCCATTCAATACAACATCCGCTCATTCATGAATGTGAAACACTGGCCAT

GGATGAAGCTGTACTTCAAGATCAAGCCTCTTCTCAAGAGTGCAGAAACTGAGAAGGAAA

TGGCTGCTATGAAGGAGAACTTTGAGAAAATGAAGGAGGACTTGGCAAAGGCGCTGGCTA

GGAAAAAGGAACTTGAGGAGAAAATGGTATCTCTGCTACAGGAGAAAAATGACCTGCAGC

TGCAAGTGGCATCTGAATCAGAAGGCCTTGCTGATGCTGAGGAAAGGTGTGAGGGACTCA

TCAAGAGCAAGATCCAGCTTGAAGCTAAACTCAAAGAGACAAATGAGAGACTGGAGGATG

AGGAGGAAATCAATGCTGAGCTGACAGCAAAGAAAAGGAAACTGGAGGATGAGTGCTCTG

AGCTGAAGAAGGACATTGATGACCTGGAGCTCACCTTGGCCAAAGTGGAAAAGGAGAAAC

ACGCTACTGAGAACAAGGTCAAGAATCTCACTGAGGAAATGGCCTCTCAGGATGAGAGCA

TTGCCAAGCTCACAAAGGAGAAGAAAGCCCTTCAAGAGTCACACCAGCAGACTCTAGATG

ATCTCCAGGCAGAGGAAGACAAAGTCAACACTTTGACCAAAGCAAAGACCAAGCTTGAGC

AACAAGTTGATGATCTTGAGGGTTCTCTGGAACAAGAGAAGAAGCTCCGTATGGACCTTG

AGAGAGCCAAGAGAAAGCTTGAAGGTGACTTGAAACTTGCTCAAGAGTCTATAATGGATC

TGGAGAATGACAAGCAGCAGTCTGAGGAGAAGATCAAGAAGAAGGACTTTGAAATGAGTC

AACTCCTGAGCAAGATTGAAGATGAACAGACCATGGGTGCACAACTTCAGAAGAAGATCA

AAGAGCTCCAGGCTCGCATTGAAGAGCTTGAGGAGGAAATTGAGGCTGAGCGTGCAGCTC

GTGCTAAAGTTGAGAAGCAGAGAGCTGATCTCTCCAGGGAACTTGAGGAGATCAGTGAAA

GGCTTGAGGAAGCTGGAGGTGCCACTTCTGCTCAGATTGAAATGAACAAAAAGCGTGAGG

CTGAGTTCCAGAAGCTGCGCCGTGATCTGGAGGAGTCCACCCTGCAGCATGAAGCCACTG

CTGCGGCCCTTCGCAAGAAACAAGCTGACAGTGTTGCTGAGCTTGGAGAGCAAATTGACA

ACCTCCAACGTGTGAAACAGAAGCTGGAGAAGGAAAAGAGTGAATATAAAATGGAGATCG

ATGACCTCTCCAGTAACATGGAGGCTGTTGCTAAAGCAAAGGCTAACTTAGAAAAGATGT

GCCGCACACTTGAGGACCAACTGGGTGAACTTAAGGCCAAGAACGATGAGAATGTTCGCC

AATTAAATGACATTAACACCCAGAAAGCAAGACTTCAGACTGAGAATGGTGAATTTGGTC

GGCAGCTGGAGGAGAAAGATGCACTTGTTTCCCAACTCACAAGAGGAAAACAAGCCTACA

CTCAGCAGATTGAAGAACTCAAGAGAGTCATTGAGGAGGAAGTGAAGGCCAAGAACGCCC

TGGCCCATGCTGTCCAATCAGCACGTCATGACTGTGACCTGCTAAGAGAGCAGTTTGAGG

AAGAGCAGGAGGCCAAAGCTGAGCTTCAGCGTGGAATGTCCAAGGCCAACAGCGAGGTGG

CTCAGTGGAGAACCAAATATGAGACTGATGCCATTCAGCGTACCGAGGAACTTGAGGAAG

CCAAGAAAAAGCTTGCTCAGCGTCTACAAGAGGCAGAGGAATCCATTGAAGCTGTAAATT

CCAAATGTGCTTCTCTGGAAAAAACCAAGCAAAGACTGCAGGGTGAAGTGGAGGACCTCA

TGATCGACGTTGAGAGAGCTAATGCCCTGGCTGCCAATCTTGACAAGAAGCAGAGAAACT

TTGATAAGGTTTTGGCAGAATGGAAGCAGAAGTATGAGGAAGGTCAGGCTGAACTAGAGG

GAGCTCAAAAAGAGGCTCGCTCGCTCAGCACTGAACTCTTTAAAATGAAGAATTCTTATG

AGGAAGCTCTGGACCATCTGGAGACACTCAAAAGGGAGAACAAGAATCTCCAGCAGGAGA

TCTCTGACCTGACAGAACAGATTGGTGAGACTGGAAAAACTATTCATGAGCTGGAGAAAG

CAAAGAAGACGGTGGAAACTGAGAAATCGGAAATCCAGACTGCTCTCGAGGAAGCTGAGG

GCACGCTCGAGCACGAGGAATCCAAGATCCTTCGTATTCAGCTTGAGCTTAACCAGGTTA

AGGGCGAGATTGACAGGAAACTGGCTGAGAAGGATGAGGAGATCGAACAGATCAAGAGAA

ACAGCCAGAGGGTGATTGAGTCCATGCAAACCACCCTAGACTCTGAAGTTAGAAGCAGAA

ATGATGCTTTGAGAGTCAAAAAGAAGATGGAGGGAGATCTCAATGAGATGGAGATTCAGC

TGAGCCATGCCAATCGCCAGGCTGCTGAGGCCCAGAAACAACTCAGGAATGTCCAAGGAC

AGCTAAAGGATGCCCAACTGCACCTTGATGAGGCTATCAGAGGACAGGAAGACATGAAGG

AGCAGGTGGCCATGGTTGAGCGCAGGAATAACCTGATGCTGGCAGAGATTGAGGAACTAA

GATCTGGATTGGAGCAGACAGAGAGAGGACGCAAAGTGGCTGAACAGGAGCTGGTTGATG

CCAGTGAGCGTGTGACACTGCTGCACTCTCAGAATACCAGTCTAATCAACACCAAGAAGA

AGCTTGAGTCTGACCTGGTTCAAATACAAGGTGAGGTGGATGACACTGTCCAGGAGGCCA

GAAATGCTGAAGAGAAGGCCAAGAAAGCCATCACAGATGCTGCCATGATGGCTGAAGAGC

TTAAGAAAGAACAGGACACCAGTGCTCACTTGGAGAGGATGAAGAAAAACCTTGAGATTA

CAGTCAAAGATCTGCAGCATCGTCTAGATGAGGCTGAAAACCTTGCAATGAAGGGTGGAA

AGAAGCAGCTCCAGAAACTGGAATCTAGGGTGCGTGAACTGGAGAGTGAGGTGGAATCTG

AGCAGAGACGTAGTGCTGAATCTATTAAAGGAGTTCGTAAATATGAGAGAAGAGTAAAGG

AGCTCACCTACCAGACTGAAGAGGACAAGAAGAATGTGATAAGACTGCAGGACCTGGTGG

ACAAACTACAGATGAAAGTAAAGTCCTACAAGAGGCAGGCTGAGGAAGCTGAAGAGCAGG

CCAACACTCACATGTCAAGGCTCAGGAAAGTGCAGCATGAGCTAGAGGAGTCTATAGAGC

GTGCTGATATCGCCGAGTCCCAGGTCAACAAGCTGAGAGTCAAGAGCAGGGAGGCTGGAA

AGGGCAAAGAGGTAGCTGAATAAAGGGTGAAGAGAAGACAAAGACAGCCAT

>Unigene000182

CCCCCCCCCCCCCCCCCCCCCGGAGGGGGGCGCCGGGCCCCCCCCCCGCCCCCCCGCCGT

AAACCCTGCGAGATAGATTTCGAGATGATCAAACTCATCAGTAACGGAGCTTACGGCGCT

GTCTATCTGGTGCGACACAAAGAGACCAATCAGCGTTTCGCCATGAAGAAGATCAACAAG

CAGAACCTGATGCTGAGGAACCAGATCCAGCAGGCGTTTGTAGAGAGGGACATCCTGACC

TTCGCCGAGAACCCGTTTGTGGTCAGCATGTACTGTTCCTTCGAGACACGCAGACACCTG

TGCATGGTCATGGAGTACGTCGAAGGTGGGGACTGTGCCACTCTGCTGAAGTACATGGGT

CCTTTGCCGGTGGACATGGCCAGAATGTACTTTGCCGAGACTGTCCTTGCTTTGGAATAC

CTCCATAATTATGGCATCGTCCACCGAGACCTAAAACCAGACAATTTGCTGGTGACTTCG

ATGGGACACATCAAGCTGACAGACTTTGGCTTGTCCAAGGTGGGCCTGATGAACATGACC

ACTAACCTGTATGAAGGTCACATAGAGAAAGACGCCAGAGAGTTCTCGGATAAACAGGTG

TGTGGCACCCCCGAATACATTGCTCCCGAGGTAATTCTGAGGCAGGGATATGGCAAGCCT

GTAGACTGGTGGGCCATGGGGATCATTCTCTACGAGTTCCTAGTAGGATGTGTGCCGTTC

TTCGGAGACACGCCGGAGGAGCTGTTTGGTCAGGTTATAAGTGATGAGATCAACTGGCCT

GAGGGAGAGGACGCACCTCCGGCTGACTCTCAGGAGCTCATCACGCTGCTGCTTAGACAG

AACCCACTGGAGCGCCTGGGGACTGGAGGAGCTTACGAGGTCAAACACCACCAGTTCTTC

CACAGTCTGGACTGGAACAGTCTGCTCAGACAGAAGGCTGAATTTATCCCCCAGCTTGAG

TCCGAGGACGACACCAGCTACTTTGACACCCGATCCGAAAGGTACCATCACCTGGAAACG

GAGGAAGAGGAAGACACGAACGATGAAGACTTTAACGTGGAACTGCGTCAGTTTTCCTCG

TCCTCACACCGGTTTTCTAAAGTCTACAGCAGTCTGGATCTGTCACGTGGGCAACTGGAG

GAAAAGGGAGAGCAACCAGAGAAGAAGAGTGAGAGTCCCCTGACTGTCGACTCGCTCAGC

TGGACTCCAGACTTCACTGAAATTCCATCACTGTCCCACTCCTCTGACCTGGAGAGCAGC

ACGAACAGCCTGAGCCGAAACTCAGGACTTCTGCCCAAATTTGCCATTTCAGCAGAGGCT

GATAGCTGCGATAGTTCGCTGTCCCTGGACAAGGCGAGTAAAGTGGCCTTCTCTGTTGGA

GAACTTCCTCAGACGGAGCCTGATGCCATCACACCATCCAGCCCTGTCAGCAACGCCACC

CTGTCAGGCAGTTTCTCTGAACATCTGGACCAGCTGATATCACGTCCAGAGGGAGCAGAC

AGTCTGGACCCTCCCAACAAGCCATCCACTGATACCGTCAGCCATTTGACCTCTCTAAGA

CCAGCGACCAGTATACCGAAATCATCATCCACCAGCGCTCTGTCTCTCATGATTCCTGCA

GATGTTTTTGGCACATCCCCACTGGCTAGCCCCATGTCTCCACATTCACTGTCGTCAAAC

CCCTCCTCCAGAGACTCCTCACCCAGCCGAGACTCCTCCCTCTCTGCCGCCAACTCGCGC

CAGCCAATCATCATTCACAGTTCAGGGAAGAAGTTTGGTTTCACACTGCGAGCGATTAGA

GTCTACGCCTGCGACAGCGATGTTTATACTGTCTATCACATGGTCTGGAATGTGGAAGAC

AGTGGTCCTGCTCAAAAGGCTGGACTCAAAGCTGGAGACTTGATTACTCATGTCAATGGG

GAAACGGTCCATGGACTGGTGCACACTGAAGTGGTGGAACTGTTGCTGAAGAGTGGAAGC

AAGGTTGCTATATCCACTACTCCTTTTGAGAACACGTCGATCAAAACGGGTCCGGCTAGA

AGGAACAGTTATCGGAGCAAAATGATTAGGAGAACCAAGAAGCCCAAGAAGGAAAAGACA

CAAGAAAGGCGACGATCAGTATTCAGGCGCTTCGCCATGCAGCCCTCTCCACTGCTTCAC

ACCAGTCGCAGCTTTACTTCCTTAAACCGATCCTTGTCCTCAGGGGAGAGTCTGCCAGGA

TCCCCCACACACAGTCTGTCTCCTCACTCCCCCACCACTCCCTTCCGGCCCACGACGGAA

CTCAATCAATCAGGTGGCACATCATCTCAGAGCAGTTCCCCCAGCTCTAGTGCACCAAAT

TCCCCAGCTGGCTCCGGCCATGTCCGTCCTAACACTTTGCACGGTTTGGGCCCTAAGTTG

CCTGGCCAGCGGCTTCGTCAAAGTCGCCGGAAATCAGCGGGAAGCATTCCTCTCTCCCCG

TTGGCTCGAACACCTTCCCCTACCCCTCAGCCCACCTCCCCTCAGCGATCTCCTTCCCCA

TTGCTCGCTCACTCGGTGGGAAGCTCCAAAACTACTCAGACTTTCTCAGCCAAGATGCAC

TCGCCCCCTACAATCGTAAGGCACATGGTGCGACCTAAAAGTGCCGAACCTCCTCGGTCT

CCGCTCCTTAAAAGGGTTCAGTCGGAGGAAAAGCTGTCGCCATCCTACACAGGGGATAAA

AAGCACCTCTGCTCCCGGAAACAAAGTCTAGAGGTGACCCAAGAAGAAGTTCAGACTGGG

TTTGCATCTGGAGGTGAGCACACACTTCAGTGTGTGGAGGAGAGTTCTTGTGAACTTCCC

ACCATCACCCGAGTAAGACCTGCTGAGCAGGGCTGCCTAAAAAGACCTGTGACTCGTAAG

GTTGGACGACAGGAAATGATGGAGGATCTTGATAAGGATAAATTGAAGTCCAATGTTGTA

GCGAAAAGACAGGACTGGCATGAGAGAAGAGAATCTCTACAAAAACAAGATGCCATTCAG

GAGGCTGAATCTACCCAAAGCCTGACTGGGTGTTGCTCGGTTGATAACAAAGTTGTCACT

GCTACAGTAAAAGATGTTCTTTATAAAAAACTAAACTCCAGAGCATGTGAAAGCATTGCA

GACTCCATGAGCAGCACTGGAGACTCTGCGTCGGTTTTCAATGAGAGTATAAGATCATCC

CCTGCTCAGTCAGACCGACAGCTGGCCAGGCAGATCAAAGAGGGCAGTAAACCAGACAGG

CATGATTTCAAAGCCCCTAATATGGAATTTGCTCGGAAAAGACAGTCCTTTGAGGAGCGT

GAAGACTGTTTGTGTCGAATCACCTCTGGCGTTCACGAAAGTCTACATTTTAATGCCACT

AGATCCAAAAGTCTACAACTGGATTCAGCTGGAATTGCTACTCCTGGAATCACCACAGAT

TCGTTGAGCCCTAAGATATTCAGTGGGCGAGGGGAAAGTGCCGTAGAGAAGCTTCAGATT

ATCTCGTCCACAGAGGGCTCCATCAGAAAGACTTCATCTGAGTACAAATTAGAAGCCCGA

CTTGTTTCTTCCCTGAAACCACTAAAGGGCACACTGGACATAGGGCTGCTCTCTGGGCCT

AGAATCTCCAAAACTGATACATGTCTTTCAAAAATAGCCAGTAGCCAAGCTGATAGTGGT

GGTCAGGTCGGAGCCATGCAAAACCAGAGTGAAAAGCAGCCCCTGATGCTAACACATAAA

CCGACACAGGCTGCCTTGGAGATAAAAAGTCCCGGTGCTTCGAGCAGCAAAAATGCAGGA

AAAGATGAACCCACTTGCAGTACAGACTATGTTTTTGGCTTGTTTAGCCATGAAGGCACT

CATGACAAAAATAAGCAGGGTGAAGGAATCAAATTCCAGACCGTAGCCAGTAAAATGGAG

CCGAAAGATTTGTCAACAGGTGGAAAAACAGAGAATAAGTTGAAAACAGCTCAAGAAATG

AGGCCTGCTCGACACAGTAATCATTTTTCTTGTGGGAAAACACCCTCAATAAGGGAGGTC

AGCAATGAAGACCAGGATGATGAGATGGAAAATCCGGTGGAAAAACCTACCCTGCAAAAC

AACACAGTAGAAACTAAGACGCCAATGACCCATGACCAGGTCATCACACAAAACACATCA

CTACCCTCAGGCCCGTGTAGTGTTGATAAAGATAAAAATGTGAGAGAAATCCTTATCAGT

GCCCAGAAATCGGAGCAGGCTTTTGCAAACACCAATAAAATAACAATGGCAAAGGATAGA

GTTCTGAATTCTCTAGGGTTAACTGTGGTTACACATGAAGTTTATCAGTGTAAAACTGTC

CAAGATGATGCTTCAGGATCTCGAAGCTCAGAGGCATTGGTGCAGACTTTGCCTCCAAAA

CCAGACAGAACAGTTATTGTGCAAAAGACAGGCTCAGATGTAAAAGCTGGGATTGTGGCA

ATTGATGAGAAAAAAACACTAAGCACAACTGGAAAGAGTACAGATAAGAAACATGAACAG

ATCACAGCAAGTAGCACACCAGTGTTGCATGCGAAAACAGAACCGTTTCACTCTGAGGGT

CTAGTAAAAGAAAAGGTATCGGTTCCACGGAGTTCAACTGTAACCACATCATCTCCTTCG

GCAGCTTCGATAGTGCCAGATATAAAGTTGCCTCCAAATTTACCTCTTCAAACCAAACAA

GATAAAAACAATGTAGCTCCCAAAGGTACCCCTGTTAAAGCCACGTCTGCTGTGGTTCCC

CAATTACCCCAGCATGTACATAAAAGCCCACAGTCATTTGCTGAAATCCAATCAGACAGC

ACTGCAATAAACCAGTCTCAGCAAACGACTTTGAAAACACCAACTGGTGCTGACTCAGGT

AACGGGCAAAAACGTTTAAATTCTATCAGCAGAACAGATGACCAAAATGCTGAAGTGGAG

AATGGCAAAAGATCCAGTTCAAAATGTCAGGAGACATGTGGAGTTAAAACCCAAATCGTA

GACAAGGATCATAAGACGTGTATGGTTCCAGAAGGGTCAATCGATAAATCCAAGATTACA

TCATCCAAAGATAAAAAGGCACAAAATATAGCAGTTTCAAAGGAGGAAGTAAAATGCATT

ATGACAAACGAGCCTCGCAGTCCTACAGACCTCAAAACTGATGGGGCAAAGTGTGTCCTA

GCTACTGCTCACACGCCCCCTAGTAAGTCAAATCAGTATTCTGGTGTCAAAGGCAGTGAA

AAAAATAGCAATACTGTGCAGGTTAAGTCAGTGAATATACTTGAAGAAAAGTTGAAAGAT

AAGAATACTCCAGATGTATCCTGTCAGAAGGTCACCAAGCCGGTACTCGACGTTTGCAAG

AAGGAGGTTGTTACTGCAACAAAAAAACAACCACAATTGACAGGTACAAGTTCCAATGGA

AAGCAGGGCCTGTCGAGCTCTAAGAGCAAAGCTAAACTAGAACCATCTCCTGCTACAAAC

CAGAGTACAAAAGCCACCGAAGGAAGAGGAACTCAAGAAGGCAAAGCACCAGTGCAGGTC

ATCATAGATCATACAAATCTAAAACCAAAGGAGCAAAGAGAGGTAACACTTCCTACATTA

AAAACTCAATCACAGAGTGAGAACCTGCCTCAGACCACCAGCCCTCAAGTTGGTTCTGTA

CCTGTACCTACTGTTAAGGTTCACACACCAAGTGGATCTCCTATAGGCAAGGGTACAGGG

TCCACAGTGGAGACCTCAAAGTCGCCTGACTCCATGCAGAAAGGAAACCGCAAAGACTGT

CAGAAATCGTCAGGATTGGCGAAAGACGGTGCAGCACCAGAAAAAGATTCACCTCGCACT

AAGCAGTCCAAAGATCTGCCCCGAGGTTCAAATAATAAAAAGTGACAGTGAGGATGCCGA

AGGTTGCCAAATCATTTTTTGTAAGAAAAACTGCAAAGCAATCTCTTT

>Unigene000183

TTCCTCCTCCATCCTCATCTTCCCCCTCCGCTGCTTCCTCTTCCGCCAATGCCGGCACCA

CAGCGTCTCCTGACCCGAAGGATCCTTCTCTCCGACCGGCTCAACCCACCCGCCGCCTGA

CGCCTGGGCTGCAGTATCAGCTCCATCACTCCTCTAACACCACCTACCACGACATGCTGC

CCGCCTTTATGTGCCCCAAGGAGACTCGTGATGGTCCAGCTTCCTCCGAGCCTGGCTCTG

CTGCAGCTGTTGCCCCAATGCGCTTCGATGCCCGACCCACATTCAGACCCACTTACGAAA

CCATTAATGGAGACCTGCGGAGAGAGAACCGTTTCACACGAGTCCCACCCCGTCCCTCGC

CTCGCCCAATCCGCCGTCCCGGTGATAGACCTTCTCGTCCTGCTATCATCAACCCTGACG

ATCTGAAAGACCTGGACGACCTGGACAACGACTGCGAAGATGGATGGGCAGGTCTTCATG

AGGAGGTGGATTACAGCGAGAAGCTAAAATTTAGCGACGATGAAGAGGATCACTCTCCCA

GCGATAAGAGCAAGATCTGCCTTGTCGTATATAGGGGAGAGTGGGAATCTCGCCGTGAAC

GCCAGTCATCCATGAGCTCAGACGGCCAGGAAGACTCTTACCATCACCAGCACGACCCTC

TGCCTTCCAGAAAAACCAACAGCAGATACGGCTCTACAGACCTGCATAAGAGCAATGGCA

CAAGTGAGTCTGTGGAAGAACTGGACGAACCTCAGCGGTCCGCTGCCGCTCCCCCGCGGG

GAAAATTCGTCCCACCAGACGTGTCCGAGGCAGTGGAAAGGGCACGGCGTAGGCGCGAGG

AGGAGGAGAGGCGAGCCCGGGAGGAAAGGTTGGCCGCCTGCGCTGAAAAACTCAAGAAAT

TGGACGAGAAGTTTGGCAAAATCGAGAAGCCGGCGCTTACCGGAGATGGACATGGAGCGG

AAAATAAGGAGCTTTTACTTTCACCCGGACGAGAGGCGGGCAAAACTCAGCCAGAAACCT

GGCAGCATGTTAAAGATGTTTCCGAGCAGAAATCTGACTCACTTCACAGCCAGGAATATA

GAGATGATTTCAGCTACAGGAACGAGGATGACTGTTCAGAATACACACAGCGACACCAGA

AAGCCGTCGCCCCACGCTTTCAAAAGCAGCAGCAGGATCAGGTGTATAAGATGCAGCAAT

GGCAGCAGTCAGGCTCTGGTCCAGCTCAGAGAGGGTATTACCCCTCCCATGTGCTTGGTT

TTGACCCCCGCTGGATGATGATGCCCCCTTACATGGACCCACGCATGACTCAGGGACGCT

CAACAGTTGATTATTACTCTTCTAATGTCCATTCTTCAGTGAAGCCAGCTGGGCAGCAGG

ATCACCTCCACAGTCCCGGGTCCACCTCAGATGAGGGCTGCCATCCCACCATGCACCTGG

ATAAGAAAGTGCTCTCTGCTGACACTTATTCAGTTTGGAGCCAAGAAGGCTACCCACCTG

CACGCAGTTTCACCCCACCTTACCAGAGACAGCACGAGAGCGGTGAGAGGAGCCAAACAG

ATGACAGAAGTGAGCGGTCTGGCTCTCAGCAGGACTCTTATGAGGACAGAGGAATAGAAC

GAGCTGAAAGTCCTCTGGAGGATCATCTTCATCAGGGCTACCGGCCAAATCGTGGGCCAG

ACATTTCTCTCGGTTCGCAGCGGGAAGATGCCTCTCATCCCCTAGGACATAACGAGTACT

TCAAACCCGAGAACCAGAAGGAGGTTATTTCTAACCGGCACCATAAAGACAGTCTGGATT

CTCATGATGAAGTGTGTGAAGGTAAAAAGAGTTTTGACTCTGGTTTCTGGAGGAAAGAAG

ATTCTATGAGAAAAGACAAAGATGCCACACAGTGGTCGGATCCTGGGTCAGGTGGTAACG

GTGGGGTTGGCCAGCCATCTGAACCGATGAGCAGAACTCTCAGAAGGACAGGTCCCATCA

AGAAACCAGTGCTCAAGGCCTTGAAAGTGGAGGACAAAGAAAATGAGAAGCCCAAATCCG

AACCGGAGGAGAAGTCTGTTCCATACAGACTTGAGAAGGAAGTCCTTACCAACGTCTATG

ATTTAAAAAAGGACAACCAGCCTATAATCAGCAGGCAGCTGCCTCCTCCTGTGACCTGCC

AAGAGGAAAAGCAGACTTTCCGCCCAAAAGTAGAAAAATTAAGCAATCCGACAGAAGAAT

CCATTAAGGAGAACGGCTGGGATGGCAGTAATTCCCAGCTGAGAGACAACTCTGAAAGCA

GAGAACCTCCAGCACCTCGTCGCAACAACTGGATCTTTATTGATGAAGAGCAGGCATTCG

CCGGAGTCAGAGGGGCAGGGCGAGGCAGAGGCAGGGGGTTTAGAGAGTTCAACTCACGTG

GTGGAGGCAGAGGAGGACGTGGTGACAACAATAGAGGCATGTACAACACCGGGAATATCC

AGCGAGGAGGAAGGGGGCGAGGATCTCGGGAGTACGTCAAAACCGAGGATTTCCAAAGAG

GCAAACCACGGAGGCGAAACGTGAGCGAGACTTTAAGCGAAGCCTCTGAATATGAAGAGC

TGCCCAAACGGCGCCGCCAGAAAAATGGAGAGAACGGAGAAGCTGCTTATTCTACAGCTG

GAGACACTAAAAGGGCAGATCGGGATTCCTGGCGGTCCAATAAGGTTTACAATAACGACC

AGAGCACCAGTGGAGAAGCGAGGGAGAAACCCAAGATGAGAAGTTTCAGTCGGTCTCTTC

CTCCACGCCTAAACACCGGATATAACCGGGGGTTCGGATCAAAAGAATTTTCCACTTGGA

GAGGAAGAGGTGGTCAGTTTGGAAGCGGATCCATGCAGGAGAACGGCTATAATTTTACAA

ATGACACGTTTTCTAGAAAAAATGCAGAAGGTGACTCTCTTAAATACCCTTCAAAGTTTG

CTTCGTCCTTCACAGAGAACGGAGTTGAAGATCGGGAGGGAGACTACCATCTTGACAAAG

ATAATTTGGACAACCGGCCACTGAGAAGAAGACGTCCTCCTCGCCAAGACAAACCGCCAC

GGTTTAGACGTCTACGCCAGGAGCGAGAAACCGGAGGGCAGTGGAACAGAGACGAGTACG

TTAACGGGGATTTTACAAATCCATGGTCCAGTCGATCTAAAACCAGTGAAGACCATTGGC

CTGGTCAGTATCCTGGAAACAGGCAGCAGGATTTTGCACCACAGGCAGAGGACTGGGAGA

CTGGGTCGGAAAACAGTGACCTAAGTGATTGGAGAGAGAAACGTGGGGAAGCCCCAGGGG

AACCGGGCCACGGCGAGCCAGGCTCTGAAAAACGGGAGCTCTCCAAAAGGAGTTTCTCCA

GTCAGCGCCCAATCGTGGACCGACAGAATCGCAAGACTGACATTTTGGAGAGCTCAAAGT

TGCCCCGATCCACAGATACACCGTCCAGCAGCAGTTCGTCTAACCGAAACGACAGCTGGC

AGAATGGAGTGCGCCGGAGTCCCGAGGACTCGACTTCAGGATTGAACGCAGGGTCGGTGT

ATGGAGTCGAACAGTCAGAAGACGGCCTGTCCACCAGCAATTCTGAAACTTCAGTGAAAA

AACTGGATAAAGATCTAAAACCAAGAAGCATGAAGGGAGACATGTCTGAGTCTCTATCAC

AATATGATCTCAACAGCTACCCGATTGAAAGCGATTCTGGAGTCTCTATTCCAAGTCCCG

ACGGATTCCAAGACGCCCTTAACAAAAAACAGAGGCGTCCACAAGAGGACGAGCGAAGGA

GGAAGGAGCAAGGAGCGACGGTAAAGAACAGAGCCATCACTTCAAAAATGCCTCCTCGTT

TCGCAAAGAAGCAGGGTGGGATCTCCATCGATCAGTCTGAGGAAACCCTGTCGACCAGTA

ATCTCGGCACTGAGATCTGGGAGGCCAGCAGTGCAGCTCTTCCAGTTCAATCGTCTGGTG

GAGACTCATGGACGAAGCAGGTTTCGTACAATGATTCGGACGCAGGGCCGGAGCACAGCA

AAGAGCACAAACCCGGGCCAATCGGAAACGAGCGCTCTCTGAAGAACCGTAAGGGTTCCG

AGGGCGTGGAGCGCCTGGAGGGCCCCATCACGCCTGTAAACGGAGTGGATATCCACGTCG

AAAACGTCCTTCCTGTCCCACCCATTGAGTTTGGGGTCAGCGCCAAGGACTCTGACTTCA

GCCTTCCTCCGGGATCAGCACCGGTGGTCGTGTCCAACCCTGTCACCAAGCTGCAGGATG

CTCTTGCCAGCAATGCGGGCCTGGCTCAGGGCATTCCTATGCTGCGCAGAGATCACCTGC

AGCCTGCTATCAACCTCAACCCCATTTCCTACCCCACCACTGACCTCACTCTCAAGATGG

AGTCTGCGCGTAAAGCCTGGGAGAACTCTCAGTCCCTTCCTGAGCAGGGTTCTCCAGGTG

GTGCTGGGTCCGTCACGCAGCCCACATGCAGTTCGTCCAGTGCCGTCAGCTACAGCTCTT

TCGGAGGAGTCTCCATGCCTCCCATGCCTGTGGCTTCTGTCGCACCCTCCGTCTCATTGC

AGGGTAATCATATCCCTCCCTTGTACCTGGACGGCCATGTGTTTCCAAGCCAGCCTCGCT

TGGTGCCGCCGACTATGACTCAGCAGCAAAGCTATCAACAGGCTGCAGCCGCTCAGCAGA

TCCCCATCTCCCTCCACACATCCATGCAGGCTCAGGCTCAGCTGGGACTCCGGGGTGGTC

TGCCCGTCTCTCAGTCCCAGGAGATGTTCAGTTCAATTCCCACTTTTAGGTCGCAGGTAT

ACATGCAGCCCAGCCTGTCTCACCCGAGTGCGATGGTGCTTTCTGGAGCAGGCCCTCTCA

AGGGGCCGTACTCTGCGTTCCCCGGCATGCAGCCGTCCGAGCTGGTGAAGCCGCAGTCCA

GCTCGCACTTTCAGCCCATTAATGGCAGCCAGGCACTTGTTTATGACAACGCAATGAACC

AGGCTGCGGGTTTGGGCACGTCTCAGCTCATGGACTCCCAGCTCATTCAGGTTACGATGC

CTTTACCTGGCTCTCAGTTGCGTTACGGTTCGGCCCAGCAGCACTTGATCCTCCCACAGT

CCATCCAGCTCCAGCAGGGACAGAGCCTCTCTGTCGGCACCCCTCGCCGCATTCTTTCCC

ATGGTTCCCAGCCACCAGTCATGACTGCCAGTCGAGAGCCATCTCAGCTGGAGATGAAAG

GATTTCAGTTCCCTGATAAAGCCAGCCATTCACCTGGGATACCTGCCGGTTCATACAGAC

CCAGTTCTGCCAGTCCCAGTGGAAAGCCGTCTGGTCCTGGTGCTCCCCCGACTGTCGGCC

CGCTCCCTGGACATTATGCTCAACAGGTGCCCCCTCCACAAGGCAACATGGTGATGCACA

TGCGCACCCCCACCAACGGGCCTTTCCCAAACCCCATCCAGAGACCTGTAATGCAGGTAA

ACAAGGCTGTAATTATGCGCTCTACCCCCTACACGAGCCCAGGCCGTGATCCTACCCACT

GCACCCCCCCTACCAACCCAGAGTCCCTTAACAAGGGCACAGAGGAGGGCACGAAGGTGA

GCCACCCACTGTAACAAAAAATATAGGTGAAGCTCCTCCATGAAGCTCGGCATGTTGTAT

CCGATCTGAAGACGTCCTCTTCCTCATCTGTGATCTCTGGAAAACTGCAGGACGTCCTCA

CCGGAGCGCCGAAGCCGAACCGCACCGGAGCCATTAAACCTCAGAGCGTTAAAATGGAGG

AGAGCAAGGCTTGATCACAGAGCTCACACACACACACACACACCGTTCACCTCATTGTAC

TGAACCTCCCAGCTCAAAACGAGGACTTTCTCTTTTTCTCACACACACACACACACACCG

TTCACCTCATTGTACTGAACCTCCCAGCTCAAAAC

>Unigene000184

GAACCTCAAGCACCCACAAAAGTACTCAACAAGTGCCATTCCTGTTTTGCCCTTAATTTA

AGGTAACCTGCCACCATGGGGGATGGAGAAATGGAGTGTTTCGGCCCGGCGGCTGTCTTT

CTCCGGAAGCCGGAAAGAGAGAGAATTGAGGCTCAGAACAAACCTTTTGATGCCAAAACA

GCAGTCTTTGTGACCGATGCTGCTGAGATGTACCTAAAGGGAACACTGAAGAGTAAAGAG

GGTGGCAAAGCCACTGTCGAAACTCTGGATGGAAAAAGTGTCACAGTTAAGGAAGATGAA

GTCTGTCCAATGAATCCTCCAAAGTATGATAAAATTGAGGACATGGCCATGATGACCCAC

CTTAATGAACCTGCTGTGCTGTATAACCTTAAAGAGCGTTACGCAGCATGGATGATCTAC

ACCTACTCTGGGCTGTTCTGCGTCACTGTCAACCCCTACAAGTGGCTTCCAGTGTACGAC

CAAAAAGTTGTGTCTGGATACAGAGGCAAAAAGAGGATTGAAGCCCCACCTCACATTTTC

TCCATCTCTGATAACGCCTATCAGTTCATGCATACTGATCGTGAAAACCAGTCAGTCCTG

ATTACTGGAGAATCTGGTGCAGGAAAGACTGTGAACACTAAACGTGTCATTCAGTACTTT

GCAATGGTTGGCATGACTGGAAAGAAGGTAGAGGCTACTCCTGGAAAAATGCAGGGGTCA

CTGGAAGACCAAATCGTTGCAGCCAACCCCCTGCTGGAGGCTTATGGTAATGCAAAAACT

GTGAGAAATGACAACTCCTCTCGTTTTGGTAAATTCATCAGAATTCATTTTGGACAAACT

GGCAAGCTGGCCTCAGCTGATATTGAAACCTATCTGCTGGAAAAGTCAAGAGTCACTTTT

CAGTTGTCTGCTGAAAGAAGCTACCACATTTTCTATCAGCTCATGACTGGACACAAACCA

GAGCTGCTTGAGGCACTGCTCATCACCACAAACCCCTATGACTACCCAATGATCAGCCAG

GGTGAAATTACAGTCAAGAGCATCAATGATGTAGAGGAGTTTATAGCCACAGATACGGCT

ATTGATATCCTTGGCTTTACCGCTGATGAGAAAATTAACATCTACAAGTTGACTGGTGCT

GCGATGCATCACGGAAACATGAAGTTTAAGCAGAAGCAGAGGGAGGAGCAGGCTGAACCT

GATGGCACTGAAGTTGCCGATAAAATCGCATACCTCCTGGGCCTAAACTCAGCAGACATG

CTGAAAGCTTTGTGCTACCCCAGAGTCAAAGTCGGAAATGAGTTTGTGACCAAAGGCCAG

ACTGTACCTCAGGTGAACAATGCAGTTTCTGCCCTTTGCAAGTCCATCTATGAGAAAATG

TTCTTGTGGATGGTCTTTCGTATCAATGAGATGTTGGACACAAAGCAGCCAAGACAGTTC

TTTATTGGTGTGCTGGACATCGCTGGATTTGAGATCTTTGATTTCAACAGCTTGGAGCAG

CTCTGCATTAACTTCACAAATGAGAAACTGCAACAGTTTTTCAACCACCACATGTTTGTT

CTGGAACAAGAAGAGTACAAGAAAGAAGGTATTGAGTGGGAGTTCATCGACTTCGGTATG

GACTTGGCTGCCTGCATTGAGCTTATTGAGAAGCCAATGGGCATCTTCTCCATCCTTGAA

GAGGAGTGCATGTTCCCTAAAGCCACAGATATGTCATTTAAAAACAAGCTTCATGACCAG

CACCTTGGCAAAAACAATGCCTTCCAAAAGCCAAAACCTGCCAAAGGCAAAGCTGAGGCC

CACTTCTCACTGGTGCACTACGCCGGCACTGTGGACTACAACATTTCTGGCTGGTTGGAC

AAGAACAAGGATCCACTGAATGATTCTGTTGTGCAGCTGTACCAGAAGTCAGGAAACAAA

CTGCTGTGCTTGCTGTATGCTGGTCATGCTGCTGGAGATGCTGATAGTGGTGGTGGAAAG

AAAGCTGGCAAGAAGAAGGGTGGTTCATTCCAGACAGTGTCTGCTCTCTTTAGAGAAAAT

TTGGGCAAGTTGATGACCAACTTAAGAAGCACTCATCCTCACTTTGTCCGTTGTTTGATT

CCTAATGAGTCCAAGACTCCTGGTCTGATGGAGAACTTCCTCGTCATCCACCAGTTGAGA

TGTAATGGTGTACTAGAAGGTATCAGAATTTGCAGAAAAGGTTTCCCCAGCAGAATCCTC

TATGGTGACTTCAAGCAGAGATACAAAGTGTTGAATGCCAGTGTTATTCCTGAGGGACAG

TTCATTGACAACAAGAAGGCTTCAGAGAAACTCTTAGGATCTATTGATGTGGATCATACC

CAGTACAAGTTTGGACACACCAAGGTGTTTTTCAAAGCTGGTCTGCTGGGTACCCTTGAG

GAGATGCGAGATGAGAAATTGGCATCACTGGTAACCATGACTCAGGCTTTGTGCCGTGGC

TACCTCATGCGTAGAGAGTTTGTCAAAATGATGGAAAGGAGGGAATCCATCTATTCCATT

CAATACAACATCCGCTCATTCATGAATGTGAAACACTGGCCATGGATGAAGCTGTACTTC

AAGATCAAGCCTCTTCTCAAGAGTGCAGAAACTGAGAAGGAAATGGCTGCTATGAAGGAG

AACTTTGAGAAAATGAAGGAGGACTTGGCAAAGGCGCTGGCTAGGAAAAAGGAACTTGAG

GAGAAAATGGTATCTCTGCTACAGGAGAAAAATGACCTGCAGCTGCAAGTGGCATCTGAA

TCAGAAGGCCTTGCTGATGCTGAGGAAAGGTGTGAGGGACTCATCAAGAGCAAGATCCAG

CTTGAAGCTAAACTCAAAGAGACAAATGAGAGACTGGAGGATGAGGAGGAAATCAATGCT

GAGCTGACAGCAAAGAAAAGGAAACTGGAGGATGAGTGCTCTGAGCTGAAGAAGGACATT

GATGACCTGGAGCTCACCTTGGCCAAAGTGGAAAAGGAGAAACACGCTACTGAGAACAAG

GTCAAGAATCTCACTGAGGAAATGGCCTCTCAGGATGAGAGCATTGCCAAGCTCACAAAG

GAGAAGAAAGCCCTTCAAGAGTCACACCAGCAGACTCTAGATGATCTCCAGGCAGAGGAA

GACAAAGTCAACACTTTGACCAAAGCAAAGACCAAGCTTGAGCAACAAGTTGATGATCTT

GAGGGTTCTCTGGAACAAGAGAAGAAGCTCCGTATGGACCTTGAGAGAGCCAAGAGAAAG

CTTGAAGGTGACTTGAAACTTGCTCAAGAGTCTATAATGGATCTGGAGAATGACAAGCAG

CAGTCTGAGGAGAAGATCAAGAAGAAGGACTTTGAAATGAGTCAACTCCTGAGCAAGATT

GAAGATGAACAGACCATGGGTGCACAACTTCAGAAGAAGATCAAAGAGCTCCAGGCTCGC

ATTGAAGAGCTTGAGGAGGAAATTGAGGCTGAGCGTGCAGCTCGTGCTAAAGTTGAGAAG

CAGAGAGCTGATCTCTCCAGGGAACTTGAGGAGATCAGTGAAAGGCTTGAGGAAGCTGGA

GGTGCCACTGCTGCTCAGATTGAGATGAACAAAAAGCGTGAGGCCGAGTTCCAGAAGCTG

CGCCGTGATCTGGAGGAGTCCACCCTGCAGCATGAAGCCACTGCTGCGGCCCTTCGCAAG

AAACAAGCTGACAGTGTTGCTGAGCTTGGAGAGCAAATTGACAACCTCCAACGTGTGAAA

CAGAAGCTGGAGAAGGAAAAGAGTGAATATAAAATGGAGATCGATGACCTCTCCAGTAAC

ATGGAGGCTGTTGCTAAAGCAAAGGCTAACTTAGAAAAGATGTGCCGCACACTTGAGGAC

CAACTGGGTGAACTTAAGGCCAAGAACGATGAGAATGTTCGCCAATTAAATGACATTAAC

ACCCAGAAAGCAAGACTTCAGACTGAGAATGGTGAATTTGGTCGGCAGCTGGAGGAGAAA

GATGCACTTGTTTCCCAACTCACAAGAGGAAAACAAGCCTACACTCAGCAGATTGAAGAA

CTCAAGAGAGTCATTGAGGAGGAAGTGAAGGCCAAGAACGCCCTGGCCCATGCTGTCCAA

TCAGCACGTCATGACTGTGATATGCTAAGAGAGCAGTTTGAGGAAGAGCAGGAGGCCAAA

GCTGAGCTTCAGCGTGGAATGTCCAAGGCCAACAGCGAGGTGGCTCAGTGGAGAACCAAA

TATGAGACTGATGCCATTCAGCGTACCGAGGAACTTGAGGAAGCCAAGAAAAAGCTTGCT

CAGCGTCTACAAGAGGCAGAGGAATCCATTGAAGCTGTAAATTCCAAATGTGCTTCTCTG

GAAAAAACCAAGCAAAGACTGCAGGGTGAAGTGGAGGACCTCATGATCGACGTTGAGAGA

GCTAATGCCCTGGCTGCCAATCTTGACAAGAAGCAGAGAAACTTTGATAAGGTTTTGGCA

GAATGGAAGCAGAAGTATGAGGAAGGTCAGGCTGAACTAGAGGGAGCTCAAAAAGAGGCT

CGCTCGCTCAGCACTGAACTCTTTAAAATGAAGAATTCTTATGAGGAAGCTCTGGACCAT

CTGGAGACACTCAAAAGGGAGAACAAGAATCTCCAGCAGGAGATCTCTGACCTGACAGAA

CAGATTGGTGAGACTGGAAAAACTATTCATGAGCTGGAGAAAGCAAAGAAGACGGTGGAA

ACTGAGAAATCGGAAATCCAGACTGCTCTCGAGGAAGCCGAGGGCACCCTTGAACATGAA

GAGTCCAAGATTCTTCGTGTTCAGCTTGAGCTCAACCAGGTTAAGGGTGAGATTGACAGG

AAACTGTCTGAGAAGGATGAGGAGATGGAGCAGATCAAGAGAAACAGCCAGAGGGTGATT

GAGTCCATGCAAACCACCCTAGACTCTGAAGTTAGAAGCAGAAATGATGCTTTGAGAGTC

AAAAAGAAGATGGAGGGAGATCTCAATGAGATGGAGATTCAGCTGAGCCATGCCAATCGC

CAGGCTGCTGAGGCCCAGAAACAACTCAGGAATGTCCAAGGACAGCTAAAGGATGCCCAA

CTGCACCTTGATGAGGCTATCAGAGGACAGGAAGACATGAAGGAGCAGGTGGCCATGGTT

GAGCGCAGGAATAACCTGATGCTGGCAGAGATTGAGGAACTAAGATCTGGATTGGAGCAG

ACAGAGAGAGGACGCAAAGTGGCTGAACAGGAGCTGGTTGATGCCAGTGAGCGTGTGACA

CTGCTGCACTCTCAGAATACCAGTCTAATCAACACCAAGAAGAAGCTTGAGTCTGACCTG

GTTCAAATACAAGGTGAGGTGGATGACACTGTCCAGGAGGCCAGAAATGCTGAAGAGAAG

GCCAAGAAAGCCATCACAGATGCTGCCATGATGGCTGAAGAGCTTAAGAAAGAACAGGAC

ACCAGTGCTCACTTGGAGAGGATGAAGAAAAACCTTGAGATTACAGTCAAAGATCTGCAG

CATCGTCTAGATGAGGCTGAAAACCTTGCAATGAAGGGTGGAAAGAAGCAGCTCCAGAAA

CTGGAATCTAGGGTGCGTGAACTGGAGAGTGAGGTGGAATCTGAGCAGAGACGTAGTGCT

GAATCTATTAAAGGAGTTCGTAAATATGAGAGAAGAGTAAAGGAGCTCACCTACCAGACT

GAAGAGGACAAGAAGAATGTGATAAGACTGCAGGACCTGGTGGACAAACTACAGATGAAA

GTAAAGTCCTACAAGAGGCAGGCTGAGGAAGCTGAAGAGCAGGCCAACACTCACCTGTCC

AGGTACAGGAAAGTGCAGCATGAGCTAGAGGAGTCTATAGAGCGTGCTGATATCGCCGAG

TCCCAGGTCAACAAGCTGAGAGTCAAGAGCAGGGAGGCTGGAAAGGGCAAAGAGGTAGCT

GAATAAAGGGTGAAGAGAAGACAAAGAC

>Unigene000185

GGAGGAAAAACAAATCGAGCTACAAAATAGTGCTTCATTGTGACATGTTTTTTTACGATA

ATATCTGGACGTCCTCGGTGTCGACCGTTACATAGCTAGTCAGAATTCAAGACTTGGCGA

ATAATAATAATAATAATAATATTAGTAATAATAATAATACGATTCCCAACAAAAAGGTCT

GGTTAGCATTGCTAGTTGCGAGCTAGCGCTTAAAAACCAAGTGAGGATCGAATCTACTGA

AATGCATCGATTATAACATCAAAAAACATCAACACAAAGGTTATTACTGGATTTAATAAA

AAAGGGACAATAATAGAACCTCCACCAATGACCCCGGAGAATAGCTGGCGCGTGCCAACT

GTGAGCAGTTTGCAGCCATTGTTTGATCCGGACGCGATTTTTTTACATTGCTAGATTTTT

TTAGTTTATTTTTATTAATAAACATTTTTGCTTGACTGGCTCTCGTTTGGGTTTAATTGA

AAATGGCCGCCGTTGTGAATTATTCTCCTCCGTGGTGGGTTAACCTGCTTCACCGTCTTC

CTCACTTTAACCTGCGCTTCGAGCAGATCAGCAGCGAGTTTCAGCCGGAGGACCCGTCAT

ACCAGCAGTCTATTCTGTTGCTGGGAGCAGTGGCTTTAGCCTGCCTGGCCCTGGATCTCC

TCTTTCTTCTCTTCTACTCATTCTGGCTCTGTTGCCGCCGCAAGAAGAGCAGCGAGCAGC

CCAACGCCGACTGCTGCTGCACGGCCTGGTGTGTCATCATTGCCACCCTGGTGTGCAGCG

CTGGCATAGCTGTTGGTTTCTATGGGAATGGAGAGACCTGCGACGGAGTTAACCGGCTCA

CCTACTCACTGCGCCACGCCAACCGTACTATCGCCGGCGTGCAAAAGCTGGTTTCAGACA

GCACCACCTCCCTGAATCTGACCGTAGATGACAATTTGCAGCATTTGGAGAGCCAATACA

CGAAGCATGCTGATTACCTGTCGATCGTGCAGAAGGTGCAGGGCCAGCTGGACGAGCTGC

TTAAACAGATGGTGGAAATCCCGTTTTGGAGCAATTCCAGAATTTCCCTGGAAGCACTGG

CTAGCAAGATCGAACTCTACGACTGGTATAGGTGGTTGGGGTATCTGGGGCTGCTCCTGT

TTGACGTCCTCATCTGCCTGCTGGTACTCTTCGGCCTCATTCGCAATTCAAAAAGAACTT

TAATTGGGGTCTGTTTACTGGGCGTGTTGGCACTGGTGATCAGCTGGGGATCGCTGGGTT

TGGAGCTAGCTGTATCCTCGAGCTCTAGTGACTTTTGCGTGGCACCCGATATGTACGTCG

CCGAAGTGGCTGAACGCTACGGCGTCATCAACAGAGACATCCTGCAGTATTACCTTCATT

GCAGTGTCGGCCAAAACAACCCCTTCCAACAGAAACTGTCCGGAAGCCACAAGGCTCTGG

TGGAGATGCAGGACGACGTGTTGGAGCTCCTGCGTGCCGCAGTGCGAGAATACCCCCAGA

CTAGAACTAATTTGGAGCAGATTCAAGGGATTTTAAACACAACTGAAATCAGCTTGCATC

AGCTAACCGCTCTAGTGGACTGCCGCAGTCTGCACATGGATTACGTGCAGGCTGTGACCG

GGCTGTGCTACGACGGTCTGGAGGGACTGATCTACCTGGTGCTGTTTTCCTTCGTCACCG

CCCTCATGTTCAGCTCTATCATCTGTAGCGTGCCTCACACATGGCAAGGGAGGAGATCCA

GTGAAGACACAGAAGAAGACTCTGCCGCATCTGGAGGAAGACAGAACCACGATAACTTGT

ACCGGGTGCACATGCCCAGTCTTTACAGCTGTGGCAGCAGCTATGGCAGCGAGACCAGCA

TTCCCGCAGCAGCACACACCGTCAGCAACGCCCCCGTTACTGAGTACATGACACAGAATG

CAAACTTTCAGAATCCTCGCTGTGAGAACACCCCTCTGATTGGAAGAGAGTCGCCGCCAC

CTTCGTACACTTCCAGCATGAGGGCCAAATACCTGGCCAACGCCCGACCTGATCCTAACA

GCAGCACTGCAGCACACTAGAGATCTCCAAAAGACGTCCCTACGCTGTCCCCTTTCTATC

GCTCTCTCTCTCTCTCTCTCTCACTCTCTCTCTCTCTCTCTCTCTTTATTTCTGAGTACC

TTTTTTCTTCTTGTTTTAGATTTTTTTTTTACATTTCCGGTGCGTGAAACGCAACCTGTC

AAACTTAACACAACATGTAGAAAAGCATGTGAAAAACTCCGGGTTTTGCTGAAGAACCCT

TTATGATGTTTGCAGTGAAGGTTCTGAAATGATCTTCAGTGAAGAGCAAAAAAACCCCTG

AACGATTTCATGTCCAGAGAAACAAACACACAACACGTCTGAGTTTCCTACAACTTCATG

CAACAGGAAGTGGTTATAAAGCTGAGCGCGTGCAGAAACAGTACAGATCAGTGAAGAAAG

AGGTGAGCGTCAGGTTCTGTTGAGACTCCAACATTTTATCACTCTATAAATCCATCACTC

CATCATTTTATCACTCCATCATCACCGTATTCATGGGAACGGATAAAGCGTCTGAGACGG

AAACGCACGCAAAGATGGAGAAAGAAGAAGTGAGGGGTGAGGAGGAGAGCGGAGAGGAGC

GCGCGGAACAGGACGGGAACTTATTAACCTCGTACACCTGGCAGACGGGAGGAGCGCGGG

CCGATCCGTCAGAGAAAAGCGCGGGAAACAAGTGGAGTCGGATGCAGAGCTGGAAAAAAG

CGCTGAGTGAAGACACACCCGAAAAATCAGCCACACCGACACCGGGGCCTCGCGTCGGGA

GAAAGAACCCCTTCCGCAGAGCGCTGTCAGAACCTCCCGGCTCGCTCCTGTCCTCCATCC

TGTCTCCTTCATCGTCATCACCCTCAGCGGCGGAGACCAGCGCAGGCGCGGACACGCAAC

AGCGCGGGAAACTCCGCAAATACCTGCAGCAGGTATCACAGAGACTGAAAAGGCCGCGGG

CGCAGAACCGCAACAACACGAGCACCACACAACAACAACAACAGCGGGAGGATGTGCCAT

GTGTGGAGGGTGCAGATTTAGTTCAGTTCTCCTGGGTTCCTCAGGATGTTCCTGTGTGGG

ACATCAACAACTGCATCCTTCAGGACGGACAGATATTTATCTCTCGAGAAGAGGAGCCTT

TGTTGCGGATGAGGAATCGAGCGAGCAGCTGTCTGTCATCTGTCAGTTTACAGAACCTCA

CAGAGAGTCACTCCAACCTTGAGTGCAGTCCAGATCCCACGTCTGTTACCGCCCCAAAAC

CCAAAACCCAGGATGGTGGAGTGAGAATGAAGATTAAACGGCGTCTGCAGGGTGGAGCTC

ACAGGAAACTGAGTAACACACAACTCAACGTAGTGGGACTGAGTTCTGCAGCATGCAGGT

TCGGTTCTCGAGACTCTCTCTCTGTGCCACTGAGTGCAGCTGAAAGTCTGAATGTGAGTT

CGGACTGTACCACCGTGATCCGGCCGGTGCACAGTTCCATCCTGGGAGAGAAGTATTGCT

TTGAGGTAATTACCTCAGACAGCAATCACTGCTTCGGGTGCAGCTCCGCGGCTGAGAGAG

ATCGCTGGATCGAGAACCTGCGGCGTGCGGCGCGGCCCAATAAGGATAACTGTGAACGTA

TGGAGAACTCTCTCAGCCTGTGGGTAAACGAAGCTAAAGGTCTCCCACCAAAAAAGCGCT

ACTACTGTGAGGTTCACCTGGATGGTACACTGTTTGCTCGCACCAGCAGTCGTGCGGTGG

GTAAAGCGTCCTCAGTCCCTGTGAGTGACAGTGGGTCAAGTGCTGTGGGCGTGTCTTCTG

GAGGAAGTGCAAGTACAGGGGGGTGCCAGCTGTTTTGGGGTGAGTATTTTGAGCTGGATA

ACCTGCCCCCTGTTACACAGATCACACTGCACCTGTTCCGGGATGATGACCCAAAGAAGA

AGCGCCACTCAAAGGATGAGAACATCTTATACCCTCTGGGCAGCGTAGCCCTGTCTCTGG

CCAACATTAAAGGCCGAGTGTTTCAGGAGAAATGGTATCCCATCGTTCCTTACAAACCTC

CTGGGACAGGTGGATCGAAGGAGCAGCTCGGCCCCCAGGCGTGTATCCGAGTTAAGGCGC

GATTCCAGAACCTAAAAGTGCTACCGATCGAGAGATATAAGGAGTTTGCAGAGTCTGTGA

CTTTGGGGTACATGAAGATGTGTAGCAACCTGGAGCCTCTGCTGAACGTCAGAGATAAAG

AGGAGCTGGCTGGAGCACTCGTCCATGTTCTACAGAGCATTGGAAAGGCCAAAGACTTCC

TGATCGAGCTGGGTCACGCTGAAGTACAGCGCTCGGGCGAGAACGAGGCGCTGCTCTTCA

GAGAAAACACTTTGGCCACCAAAGCCATAGACGAGTATATGAAGCTGGTGGGGCAGAAAT

ATCTCATCGACACTTTGGGAGATTTCATAAATCGACTATACACAACCTCAGAAAGCTGTG

AAGTCGATCCACAGAAATGTTCTGCCTCTGAGCTGCTGGTCAACCAGCGCCACCTGAAGG

ATGCGTGTGGAGAAGTTGTGCGACAGATCACTGAGAGGCATAGCTCATTTCCAGTGGAAC

TTAATGAAATTTTCTCCAGCTGGGTGTCTGATTGTGAGGAACGTGGACGAGCGGACATTG

GACACCGGCTCATTTCAGCATCATTGTTCCTCCGGTTTCTCTGTCCAGCCATTCTCAGTC

CCTCTCTGTTTGGACTGATCCAACCTTACCCAGAACACAACACACTCCGCACACTGACGC

TCACGGCTAAGGGGCTGCAGAACCTCGCCAACTTCACATTGTTTGGAGAGAAAGAAGAGT

ACATGCTTTTCATGAATGAGTTCCTGGCAGAACATTGGGAGGATATGAGGACGTTCCTGC

AGAAAGTATCAAACCCTGACAGTGAACTAGAAATGGCTCGGTTTGATGGCTATGTGGATC

TTCCTTTACGCCTAGCTGTGCTTCACAACCTGTTGGTGGACATCATTTCTCCAATGAAAC

TGGAAGTTATAAACAACCTGCAGCCGCTGCCTTCCATCCTGAATCAGATCACTGAGCACC

TCGGCCCAGACATTCCTCGAATCACCTGCAGCAGGTCCACTTGTGAAACAGAGAAACCCA

CCTATGTTCCTCCCAGAGATCTTGGCAAGTACAGTCCCCTCAACAAATCCCTCCCGCACC

TGCCCACAGACCCACGTCCCCCTCAGGGCAAGGGCAAACACCGGAAGCAGGTGGCGAGGA

CGCAGAGTGTTCCTGCCTCTGAGAGACACCATCGCCGTCCTCTGAAGAGAACCAGCAGCA

ACGCTGAGTTGAGTGAAACAGCTCACATCCACCATGAGGAGAGTCCAGAAAGACAAATGG

AGATCTCTCTTCCACAGCAAGAGTTTTTTAACAAGTCTGCGCCTGCTCCTGTTCCCTGGA

TCAAAGAGCACGTTCAGGATGAGACCAATATTCTGGAAAAGCATGCTCAGGAGCTGTATG

AGCTGCGTTCAGGGGTTGACCAGGTGACAGACCGTGAGCTGGAGATGGCGAAGAGGTTGG

AAGATTTTATTGCGGTGAGTCAGGAGCAGCAGGCGCAGCTGCAGGCCGAACTGCAGGAAG

TGCGCAATGTGTTAGCACGAAGGGAGGAGCAGCTTGCTAGCGCCACCTTCAGGTAACTCT

CTCACACACACAGATGATCATTCACACACGTGCACTTTTACAGTGGTGTGTGTGTGTGTG

TGTGTAGGCTGGGTGTGATTGAGGAAGAGCGTGAGGAAGATGAGAGGAAGTTAAGTGTCG

CTCTGGCAGCAGCTGAACGTGTGAATGTGTTGGAGGAGCAGCTTGCTAGC

>Unigene000186

ACCAGCAGTTCCAGTCTTTAGGCAAAGGATCGTTCAGACACACAAGATGGAGTCATCATT

ATAGTCAAATGCTGGACATGTTGATGAAGCTTGCTAAAGCTGTAGCTACAGCAGCAGCAA

CTCTGGTCTTAAAGGCAAAGAACGTGGCCCAGAAGACAGAAGATTCAGCCCAGCAAAACC

GGGTCATCGCTGCTGCCACACAGTGCGCCCTTTCCACCTCTCAGCTGGTGGCATGCACAA

GGGTGGTTGCTCCAACTATCAGTTCCCCAGTATGTCAGGAGCAGCTGATTGAGGCAGGGA

AACTAGTGGCTAAATCAGTTGAAGGCTGTGTGGAGGCATCACAGGGAGCCACCAACGATG

AGCAACTGCTGAAACAAGTGGGTGTGGCCGCCACAGGTGTCACCCAGGCTCTTAACGAGC

TGCTGCAGCATATCAAGCAGTACGCCAGCGGAGGCCAGCCAATCGGGCGCCACGGGGAGG

CAACTGACCGCATTCTGGACGTAACTGAGAACATCTTCAGCTCCATGGGAGATGCAGGTG

AAATGGTCCGACAGGCCCGGATCCTTGCTCAAGCCACCTCCGATTTGGTCAACGCCATTA

AAGCAGATGCAGAGGGAGAGACAGACCTTGAGAACTCTCGTAAACTGCTCTCAGCTGCGA

AGCTTCTAGCCGATGCAACTGCCAAGATGGTGGAGGCCGCCAAGGGTGCTGCCGCCAACC

CAGACAGTGAGGAGCAGCAGCAGAAGTTGCGTGAGGCCGCTGAGGGCCTGCGCATGGCCA

CTAATGCAGCAGCACAGAACGCCATCAAAAAGAGGCTGGTTAATAAGCTAGAGAACGCTG

CAAAGCAGGCAGCTGCAGCCGCAACTCAGATCATAGCTGCCGCCCAGCACGCTGCCTCAT

CTAATAAGAACCCGGCTGCACAACAGCAACTTGTACAGAGCTGCAAGGTGGTGGCAGACC

AGATCCCCCAGCTGGTGCAGGGAGTCAGAGGCAGCCAGGCACAGCCTGACAGCCCCAGTG

CTCAGCTTGCCCTAATTGGAGCCAGCCAGAACTTCCTGCAGCCTGGAGCCAAGATGGTGA

CTGCAGCTAAGGCTACGGTGCCCACTATTGGTGACCAGGCCTCTGCCATGCAGCTCAGCC

AGTGTGCCAAAAACCTGGCCAGTGCATTGGCTGAACTCCGCACGGCTGCCCAAAAGGCTC

AGGAGGCTTGTGGTCCTCTGGAGATTGATAATGCCCTGAATGCAGTAAGGGATTTGGAGA

AAGACATGCAGGAGGCTAAAGCTTCAGCAGCTGAAGGAAAACTCAGACCTCTTCCTGGAG

AAACGCTGGAGAAGTGCTCCCAGGACCTGGGCTCCAACACTAAGGCAGTGAGCTCTGCTG

TTGCCCAGCTTCTGAGTGAAGCTACACAGGGCAACGAGAATTACACCGGTATGGCTGCAC

GGGATGTGGCCCAGGCTCTCCGTTCTCTTGCCTCCGCAGCCCGTGGAGTGGCCGCAACCA

CAGACGACCCACAGGCGTGCAGCGCCATGCTGGACTGCGCTGGAGATGTCCTCGACAAAT

CAGGCAACCTCATTGAAGAAACCAAAAGAGCCATCGCTAAACCTGGAGACCCAGAGAGCC

AGCAGCGACTTGCCCAGGTGGCCAAGGCTGTCGCTCAGGCACTGAACCGCTGTGTTAACT

GCCTTCCTGGTCAGAGAGATGTGGATAACGCTATCAGGACTGTGGGAGAGGCCAGCAAAA

AGCTTCTTTCCGATCAGTTCCCAGCTAGTGGAAAAACCTTCCAGGAAGCTCAGGCAAACC

TGAACCAGGCGGCCGCCGGGCTTAACCAATCAGCCAACGAGCTGGTGCAGGCTTCTCGCG

GCACCACACAGGACCTGGCCAAAGCGTCGGGCAAATTTGGCCAAGACTTCAGCCACTTCC

TGGAGGCTGGCGTGGACATGGCTGGCCAATCTCAGTCTAAAGAAGACCAGACTCAGGTGG

TGACCAACTTGAAGACCATCTCTATGTCATCCAGCAAGCTGCTGTTGGCTGCTAAGGCTC

TGTCTACTGACCCCAATTCCCCCAACCTGAAGAACCAGTTGGCTGCTGCTGCACGTGCCG

TGACTGACAGCATCAACCAGCTGATCACCATGTGTACGCAGCAAGCACCGGGTCAGAAGG

AATGTGACAACGCTCTGAGGGAGCTCGAGACGGTGCGGGGCATGCTGGAGAACCCAACTG

AAGCAGTGAATGACATGGGCTACTTTGACTGCATCGACAGTGTAATGGAGAACTCAAAGG

TATTGGGTGAAGCTATGGCCGGCATCTCCCACAATGCAAAGAACAGTAACCTGCCTGAAT

TCGGAGACGCAGTCAGCTCGGCCTCCAAAGCTCTGTGCGGCCTCACCGAAGCTGCTGCTC

AGGGTGCCTATCTGGTGGGTGTCTCTGACCCTAACAGTCATGCTGGACAGAAGGGGTTGG

TAGATCCATCTCAGTTTGCCAAAGCCAAGCAATCGATTCAGATGGCCTGTCAGAACCTGG

TGGACCCTGCCTGCACACAGTCCCAGGTCCTCTCTGCTGCCACTATTGTTGCCAAACACA

CCTCCGCCCTATGCAATGCCTGCCGCCTCGCCTCCTCCAAAACATCAAACCCTGTTGCTA

AGCGACAGTTTGTCCAGAGTGCCAAAGAAGTGGCAAATAGCACAGCCAACCTTGTAAAGT

CAATCAAGGCCCTGGATGGAGCCTTCAACCAAGAGAACAGACAGAAGTGCAAGGCCGCCA

CAGGGCCATTAATCGAGGCTGTGGATAATCTCACTGCCTTTGCTTCCAATCCTGAGTTTG

CTAGTGTGCCTGCACAGATCAGCCCAGAGGGTCTGGCTGCCATGGAGCCCATCGTGGCTG

CAGCTAAAACTATGCTGGAGAGCTCAACAGGTCTGATACAGACCGCACGCTCTCTAGCCG

TGAACCCCAAAGATCCACCCAAGTGGTCAGTGCTTGCCGGTCACTCCAGGACTGTGTCTG

ACTCTATCAAGAAGCTAATCACCAACATGAGAGATAAGGCTCCAGGTCAAAGAGAGTGTG

ACGAAGCCATTGAGGTTCTCAATAACTGCATCCGTGAAGTGGACCAGGCTTCTCTCGCTG

CCATCAGCCAACACCTTACACCCAGAGACGACATTTCCCATGAGGCTCTGCATGAGCAGA

TGGCAGCCTCGGTGCAGGAGATTAGCCACTTAATTGACCCAGTGGCTATCGCAGCTCGTT

CCGATGCCTCCCAGCTAGGACATAAGGTTTCTCAGATGGCGAGCTACTTCGAGCCACTCA

TCATGGCAGCCATTGGTACTGCATCAAAAATTCTCAACAGCCAACAGCAAATGAGTGTTC

TGGACCAGACAAAGACTCTGGCAGAGTCTGCCTTACAGATGCTCTATACAGCCAAGGAAG

CAGGAGGCAACCCTAAGGCGGCTCACACCCAGGAAGCTCTGGAGGAGTCTGTGCAGATGA

TGAAGGAGGCCGTTGATGACTTGGGAGGTACAATGGCTGAAACAGCAAGTGCAGCCGGAG

CTGTAGGAGGCATGGTGGACTCCATCACTCAGGCTCTTAACAAATTGGAGGACCCTGGAG

TCGAGCCTGAGGGAACCTTTGTGGATTATCAGACTACTATGGTGAAAACGGCCAAGGCTA

TTGCAGTCACTGTGCAGGAGATGGTCACTAAATCAAACACCAACCCTGATGAGCTCGGAG

GTCTAGCCAATCAGTTGACCACCGAGTTTGGTGACTTGGCATCAGAGGCTAAATGTGCTG

CCATCACTGCAGAAAATGCTGAGATTGGCTCTCACATTAAGAAGCAGGTAACAGAGCTGG

GCTATGGTTGCACTGGATTAGTCACCAAGGCAGGAGCTCTACAATGCAGTCCCAACGATT

CCATCACTAAGAAAGAGCTGATTGACGCTGCCCGCAAAGTCTCTGAGAAAGTGTCCCATG

TGTTGGCGGCACTGCAGGCTGGTAACCGTGGTACCCAGGCTTGCATCACCGCTGCCAGTG

CTGTAGCTGGCATCATTGCTGACCTTGACACCACTATTATGTTTGCCACTGCTGGTACAC

TTAACAGAGAGAATGCAGAGACCTTCGCTGATCACAGAGAAAACATTTTGAAGACTGCTA

AGGTTCTGGTGGAGGACACCAAGCTACTGGTGTCAGGAGCAGGAGCCAGTCAAGAAAAGC

TGGCCCAGGCGGCCCAGTCCTCAGTCAACACTATCACCAAACTTGCCGATGTGGTTAAGC

TGGGGGCCGCCAGCCTGGGCTCTGAGGACCCTGAGACACAGGTGGTCCTGATCAACGCAG

TCAAAGACGTGGCTAAAGCTCTGGGTGATCTTATTAGAACCACCAAAGCAGCCGCTGGCA

AGCCTCATGACGACCCGGCCATGCTCCAGCTGAAGAGCTCTGCGAAGGTGATGGTTACGA

ACGTGACGTCCCTGCTGAAGACGGTGAAGGCTGTGGAGGATGAGGCCACTAAAGGCACAC

GGGCGCTGGAGGCCACCATCGAGCACATCAAACAGGAGCTTGCCGTGTTCAGCAGTCCAG

ATCCTCCTCCAAAGACAGCTACACCAGAGGAGTTTATCCGCATGACCAAAGGCATCACGC

AAGCCACAGCCAAAGCCGTGGCTGCTGGAAACTCATGTCGCCAGGAGGACATCATTGCTA

CCGCCAACCTGAGCAGAAGAGCCATCGCCGAAATGCTGCACTCGTGCAAGCAAGCGGCTT

ATCACCCAGAGGTTAATCTGGAGGTGCGGACCAGAGCCCTGCGCTTCGGAAAGGAGTGTG

CTAATGGCTATCTCGGCCTCCTGGAGCATGTGCTTGTGATCATCCAAAAGCCCACTCACG

ATCTGAAGCAGCAGCTGGCAACCTTCTCCAAACGAGTAGCTGGCTCAGTCACTGAGCTCA

TCCAGGCAGCTGAAGCCATGAAAGGCACTGAATGGGTAGACCCTGAGGATCCCACAGTCA

TTGCAGAGAACGAGCTGCTGGGTGCAGCGGCCGCCATCGAAGCTGCTGCTAAGAAACTGG

AGCAGCTGAAACCACGAGCCAAACCCAAGGAGGCTGATGAGAGCCTGAACTTCGAGGAGC

AGATTCTGGAGGCAGCCAAGTCCATCGCTGCAGCTACCAGTGCGCTGGTGAAGGCTGCAT

CTGCCGCACAAAGAGAGCTGGTGGCCCAGGGCAAGGTTGGCGCTATTCCAGCGAATGCAG

TTGATGATGGACAGTGGTCTCAGGGTCTTATTTCAGCCGCCCGCATGGTTGCCGCCGCCA

CCAATAATCTGTGTGAAGCCGCGAACTCTGCAGTACAAGGCCACGCTAGTGAGGAGAAGC

TCATTTCCTCAGCCAAGCAGGTGGCGGCATCCACAGCTCAGCTGCTTGTGGCCTGCAAGG

TCAAGGCTGACCAGGATTCACAAACCATGAAAAGACTGCAGGCTGCGGGCAACGCTGTCA

AGAAGGCGTCAGATAATCTAGTAAAGGCAGCACAGAAGGCTGCTTTCGATGCGCAGGATG

ATCAGGCTGTAATGGTGAAGAGCAGGATGGTGGGCGGCATTGCTCAGATTATTGCTGCTC

AGGAGGAGATGTTGAGGAAGGAGAGGGAGCTGGACGAAGCCAGGAGGAAGCTAGCTCAGA

TCAGACAGCAGCAGTATAAATTCTTGCCCTCAGAGCTGAGGGAGGACGGTCATGAGCAGT

GAAGAGCTTCTTTCACACACACATACACACACACACACAGGTCTGCTACTCAGGACTGCA

CGGCCTGAGCGCTTTTCTCTCCATGTGTTTAGCTACAATGGCAGGCTTGCTGAAGCAACA

TTCAGGCTAAAATTATAAAAAAAGCGAGGC

>Unigene000187

GTAACAGCAGTGATTTAAGGGTGTTATGGGTGTTACAGCAGTGATGTAAGAGTGTTATGG

GTGTTACAGCAGGGTGTTATGGGCATTTAAACAGTAGTTTGAGGTTGTTAAGTGTGATAT

ACCTGGTGTTAATTATTATTATCTGCAGAACCTGGCAGCTGAATCTGTAAGTGACTTATC

ATCTGTGTTGAGTCTTGCTGACAGTGAAGATTTGTTGGAAACCTTTCAGCAAGATATTAA

TGACTGCATTAAGGACAATGTTGAGGATGATTCAGAGGATGATGCAGAGGATGAGGATGA

GGATCTCTTTGAACTGGCATCAGAGAAGAACGAGAAAGTGAACGGCAGTGTTGAGAGTAA

GGAAGAGGATGAAGAGACTAATGACGTAGACAGTATTCCTAAAGGCCATGCAGAAGAAGA

TGAAACTTTCAGTGAGCAGCTCAGCCAGCAGATGGCTATCATCGAGGACATCAGGAAAAA

TGAATTCGGTGTGGGAGTGGAGCTGAACGAAGCAGGCCAGAACCTCATGAAGAAGCAACA

GGCTCGTCTTGGCCGCAGTCTCGAACGCCTCTCCACTGAACTCTACAGCAAAGACACACA

CTTTGTTCTGGAGCTCATCCAGAATGCTGACGATAACAGCTACCCGCTACATAGAAAGGA

GGAGCCTGCTTTGGCCTTTATAGTGCAGAAAGACTGCATCACCATTCTAAACAACGAGTT

CGGCTTCGAAGAGAAGAACGTGCGAGCTATCTGTGACGTGGGAAAAAGCACCAAAGGAAA

ACATACTTGTGGCTACATCGGTCAGAAAGGCATTGGATTTAAGTCAGTTTTTAAAGTCAC

AGACTGTCCTGAGATCCACTCCAATGACTTCCATATTCAGTTTGACAAGGCCAGCGGCCC

TATGGGATACATCCTGCCTCACTGGGTGGAAGAAGAGAGGCATGTGCCCCCTGTTGCCAA

GGAGATAGCAGAAAAAAGCTGGACAACAAAGATTTTCCTGCCTCTTCGATCAGAGAGCTA

CCAGACCAAAAACCTTTTTCACGACGTACACCCATCTCTGCTTCTCTTCCTTCACCGTCT

CCGCTCCATCACTATCTACAGCGAGGCCGAGAGAAAAGTGGTGTCAATGATGAGACGAGA

TCTAAATCACAACGTGCTGGAGGTAGAGCACACCGGCGGTGTGGAGCGCTGGTTGGTTGT

CAAGAAGATACTCTACCCTGAGAAAGTTAAAGTGGATGTGGAATCTACCGAACTAGCGCT

TGCCTTCCAGCTCAATAATGGCTCAGAGGGCAAAGTAAAAGTTCAGCCTCGGCAGCAGCC

CGTCTTCGCCTTCCTTCCCCTGCGAAGCTTTGGCTTCCGCTTCGTCATCCAAGGAGATTT

TGACATTCCTTCTTCTAGAGAGGATGTTGATAGAGACAGTTCCTGGAATCAGTGGCTCCG

CTCTGAAATCCCCCAACTCTTCTTGCAAGCCATGGATGTTTTCACTACTCACCCTGAATT

CAGTGGACTGCAAGGACTCTGCCACTTCCTGCAGTATATCCCTCAGCCTAGTGAAATCCT

GGACTTTTTCAACCCGGTGGCCAAACAGATTATCCAGCTGCTGAAGGGGAAACCCTGCCT

TCCTGCTAAGATGGACAGCAATGGAGGCGTGCAGTTCAAGCTGCCTTCGCAGGTGGCTGT

GTGCCAGGACCCCTTAATCCAGGATGTGATCGGAGGAGAAGAGCTGTGTAAGCACCTGAA

CCTGTCCTACCTTCACCCTGAATTACAGTCATCACTGTCATCTTCTTTGCTCTCTGCTTT

GGGTGTCCAGCGTCTGAAGGCTGCCAACATCACCACCGTAATCTGTGCAATGGCCAAGAG

TCTTGTCCAGGAGGGTGATATTTACACAGATTCCAACCTGAAAAAACTGGCCAAGCTGCT

GGCTTGTAATTTCCGTGCTCTGGAATCTGAGTACAATGAGGTGGATAGCCTCCTGCAGGC

TCTCCGGGACATTCCCATGATTCCTCTGGCGGATGGCCGTGTGGTGTCTCTCAGCGCAGA

GGGTGTCTTTTTTCCTCTCAGTGAGGAGACTCACAAAGGCCTGAACGCTTTGTACAAGGA

CCTGAGCATCGTAGAGCCGCGACTGCTAGAGTGTCTGGATAAATTGGGTAACTCTCAGGT

GCGTGAGCTGCTCTGTAGGCTGGAGGTTCATGAACTGGAGCCCAAAGAGGTGCTGCAGAA

TCACATCTACCCCATACTGAAGCAAGCTGCTTGGAAGGAAAAGCCGGAGAAAATCACTGT

CAGTTACCTGGTGTTTATCAAGGAGCATTCACAGGAGCAAGACTACAGGGCGCTGAGGGA

AGCTATCCCCGTACTCACCAACAAGGGTTTCCTCTGCCCCGGCCAGTGCAAAGTGCAGTT

CTCCAAAGAGTACGGCAACATTGATCTGCCCAACAAGCTGCCTGGTGTCGACTGGGTTCT

TTTGGATTCTTGCTACCTGTGTGATGGCGATTTATCAGGATGGAGGGATTTTTTGAGTGA

TCTCGGAGTAAGAGATCTCCTAATTTTCAAGAAGGAGCGACGCAACCTCAAAGACACAGA

GCTGGTATCGAGCCCATGGGCAGCGGAGGCAGAGCTGTGGCCCAAACCTACAGATAAACT

GTATGTGATAGAGGATCAGCAGTGTGAAGAGCTTCACTCACTAATAACGGCAGATCAACT

TCCCCCTACCATCAAACTTCAGCAAAGACAAGCTCTAATCAACCTTCTACAGAACAACTG

GGACACTGGAGAGAAATATTCTCAGTACCTCAGTGCTCAGGTACTGGACAACCAGGGCCG

CACTCTAAAAGACACCAGGTCCTCGTTTTACTACCATCTCACCCAGTTGGCATGGATGCC

TTCCTACAAGCCCACGCAGGACGAGAAAAACTCGGTGGAGTACTTGATTCCAAACAGGGT

GTATCTCTTTTCTAACAAAGTGCACAGTCTATTGGGATCCCACGTCTTTTATGTCAATCT

GGATCCCAGCGAATTCTCCAGAGCTGTAGGAATGAGACACACTGTTGTGGTAGACGAGAT

GATCAGCTACCTGAAACGCTGGTGTACGAAGGAGTCCGATAGCGGCGAGGCAGAAGGAGC

TGATTTTATCACCACCGCGCAGCACATCCACACCGTGTACCTCTACTTGCAAGCCGAGTG

CTCGCACACACAGCTTAGGGACCTCTTCCAGCACTCACCTGCTGTTTACATAGAGTATGA

CAGAAAGGATGAGTGGTCTTCAGGACGCTTCTATCATCTCAAAGACGTGTGCTGGAGAGA

TCCAACAGACATGTTTGTCAGATACAAGGAGCTGATCCGCAAACCAGACAGCAGGGTCCA

GGAACCCAAAGTACTGGCTCCATTCTACAGCAAATTGCCCCAAATGGAAGAACTGTTTAA

GTCTCTAAACGTTTACCCCAGTCCATCCATGAAGCAGTACGTGGACCTGTTGGAGACTGT

ATGTGAGTCGTCACCACTTCCCACAGGAGAGGTGCTACAGGATGTCTCCATCATTTTTGC

CAGACTGGCGGACAAATGTAAAAACCCTACCAACGTAGGGCAAGATCAAGACATCGAAAT

GAACCAGTGCTACTGCATGACCCTAAAAGAGATGGTGTCTCGTCTGAAGGTGTTTCCTAC

CAAGACACAGGGTTGGGTCACACTGGCACGGAAACCTATGATAGCAGACAGCCCAAGCCT

GGAGAAGATCTTCAAATCTTACAAAGAAGTCTGCCTCCTCAACCTGCCCTCGGCTCAGAA

GAAAGTACAAAGACACAAACCGAACCAAAAAGCCAAGCTGCATGATGAGAAGCTGGCCTT

CAATGAAGGAGACCGAGAGCTCTTTCTGGAGATCTGCGGCGTAAAGAAACTCGCCCAGTG

TGTCACCACCGAACCTACAACGGAGCTCTACAGGCCGTGTCCAGCTATGCAAAACCTGGT

CCGTAATCTCATTCCTTACATCCAGAGGTTTATCTATCACCACGATGAGCTTGGAGAAAT

ATACGATGACCTAAAAGAAAATGGAATTGCCCAGGAGATCAAATCTCTCAGCTTCGGTCA

GGTGGGAATGCTGTACATTAATTACCGACTGGAACAGCCAGATGAGAGCCCCATTGTAGA

AAGTGAGGACATCATTTGTCTTCTGAAAGACAGAAAAGAGTTGTACATTCAGAAAGACCA

TCTTTCATCCAAAATGGCCATCTGCAGAGAACTGGTGAAACTGTTCACCACAGAGAAGAG

CTTTGTAGAGGAACTGAAACACTTTCTCGAGGAGCTTGTTATCCGTATCAGTGATCCGGA

TTCTTTAAAGAGATTACTGGATGGTAAAAACATACAGGAGCTTCCTCAGGATGAAGAGCG

GTGGGAGGTTCCGGCACCGCTGGAGACATTCGAACCCAGGACCTTTACTATCAGCTCATC

AGGGATTGTGAGTGCAGAAGAAGAGGAAATAAACACAGAAGAAGATACACTGCACAGCTG

GCCTCCTAAAGCATCCATCAGCAAAACAGGAGGCAGAAGCACAGGAAAAGGAAGCCAGGC

AGTAGAGGCAGTAATGAAGATGTGGCCTCCTCCAGCACCTCTGACCTCTTCAGCTCAGTC

TGTTTCTCCTGCACCTTCAGCTCACTGGAGTTCAGGAGCTGAGAGGAGTCCTTCCAATCT

GAACTCTCAGGAACGCACGACTGAACTTCAGATGCACCGAGAGCCTGGCAGTTTAAATAT

TCCAGGGAATCAGGAGCCCAGGGAACGTGCTGGCATCCCACGTGCGGAAGCACAAACTAG

TAACGCCGATGTGCCGGTGGTCCAGGGTGTCGAAGCTCCTAATGAAGCCCATGCTGGGTC

CAATGGAGCTCCTGATGAGCACACTGAGGTGTGTGTGGACAAGCCAGAGAACAGCACTCT

ATCTCCAGCAGAGGAACAACAACCCACACTACAGCCCACGGAACAGGTTAATAAACCAGT

TCACCCTCCAGAAGTCATGGAAAGCCAGTTCCAGGGAAGTGTTATTTACCGTCCACCCAT

GCCACTGGACAGCGCAGTATGGACCAAACCCACAGTCGCTGGAGTGCTGGAGGACCTTGC

GCTGGACTTCAGTTTACCCAACACCCTGCAGGTGCCGTGTGCTTACGATGACAGCGATGA

TATCGGGCGATGGGGCGAGCAGCTGGTCTACTCCTTCCTCACCCGCTGGCGAGAAAGTGG

AGAAGGACCGCGGGAGATCAGCTGGTCCAACGAAAAGGGTGAGAGCGGCCAGCCGTACGA

TTTTAAACTGATCTTCCCGACTGGCTGTAACACCACGCGGGAAGTGTTTGTGGAAGTCAA

GACAACAGTGAAGCAAGAGAGGCATTTTATTCACATGTCAGCCAACGAGATCGATTTTGC

GCTCAAGGAGAAGGAAAAGTACCACATATACAGAGTGTACGGCGCTGGAGATTCCCAACA

TACCCGTCTCTGTCGCATCAAGAACCTTGCCCAACATCTGCATTCCAAAACACTGGAACT

GTTTTTATTCGTGTAGGGAAAAAAAAATATTTTAATCTTTTTAGCAAGTGTAACTTTTGA

ACTTTATAATTTTATTTCTGCACTAGTAACGTCATGCACAAGTTTAGTTATTTTATACCT

CTCTGTCATCTGTTTAAATCCTTTATATGTTTAAAATTAAATACACTAAATTATTACTTA

TTTTTTTATTTTAGATGTTTTAAAGTTAAAATGATGTATATTAACTTTTATTTGAAAAAA

AAAAAAACATTAAACCAGAAATAACATGTCGGTCATTTAATCATTATTCATTCATCCTTA

TAAAGCC

>Unigene000188

AAGAAGAAGAAGAAGAAGAAGAATCGCCTGTGCAGAATATAAAAGGGGATGACGTAGGGG

CTCAGGCCAGTCACAGAAAACAGAGATGAGCCTGACATCCTGGTTCCTGGTAAGCAGTGG

GGGCACACGCCACCGGCTGCCCCGTGAGATGATTTTTGTGGGTCGTGATGACTGCGAGCT

CATGCTACAGTCTCGCAGTGTGGACAAGCAGCACGCCGTGATCAACTACGAACCGGCAAG

TGATGAGCACAAAGTGAAGGATCTGGGAAGCCTTAATGGGACATTTGTGAACGATGTTCG

AATCCAGGAGCAGATGTACATCACCCTGAAGATTGACGATAAGCTGAGGTTTGGATATGA

TACTAACTTGTTCACTGTGGTCCGAGGAGAGCTTCATGTTCCAGAGGAGGCTTTAAAGGT

AGCCTGATGAAATTGTGTGTGTAAATTTGTGAATTGATTGAATTGTTGAGTGATAAGCGA

TGTCATAAAAATGTTCCTTTTTTTTTTTGTTTAAAGCATGAGAAATTCACCAGTCAGCTT

CAGCTAGGGAAGAAGCCAGTTTGCACTGAGTCTGCCAAAACACCCAAATCTCCTACAGCC

AGCGGCGCAGAGAGCAAAGTATCCGTACCCACTGCGGAACCTTCTACCAAAGTGGTGGAA

CCCTGCAAGACCGAGGACAAAATGCAAGGTGATACTGCAGCACTGCACCGAGGAACACCG

TTGTATGGCCAGCCTTCGTGGTGGGGGGATGGTGACGCCGATGACGAGAACTCGTTTAAA

CAGGAGAATAAAGCAGCAGGGAAAAAACAGGAGCCTCATGGGACAGACACCAAGGACACC

AGGCGGACTGATAAAGCCAAGGAGAATGGTGTGAGCACTGCAGGCCCTGAGCCCAGCTAC

TTTGAGATCCCGAGTAAAGACGGCCAGATGGCAGAGGACAGCATCCATGAGGTCCCGACC

AAGGACACAGAGAGCAGCGCAGCTGCAGCAGCAGCAGCAGCAGCAGCGAAAGCCGCCCAA

GCGAACACCTCCTTTACTATTGAGTTTGACAACACCTCTCCAGGAAAAGTAACCATTAAG

GATCACGTGTCGCGTCCAAGATCGAAAAAATCTCAGCCAGGGAGTAAAGAGCTCAGCAGC

CTGCAGGCAGCCATCATGGCCTCAGAGAGCAAAGTGGCCGACTGGCTTGCGCACAACAAT

CCCCCCATGGTGAGACGGGAGTCCACGGAAGAGGACAGCAAAAGCATTAAAAGTGATGTG

CCTGTTCTTCTCAAAAGGCTAAAAGGCAGCAAGCACGAGGACGGGACCCAAAGTGACTCT

GAGAACGGCCTTGGCTTGCGTTTCGGTAGCCGGCGCCATGCTGCTCTGGAGGAACGCTTG

AAGGCAGCTGGCCTTACGCATGCCAAAGTCACCGGAGCAGAGGGCTCGGGCTCCAGCAGC

CGCAGCGCCTTCACCATCGAGTTCTTCGACGAGGAAAACCCGCGCAAACGCCGCTCCTAC

TCCTTCTCCCAGACTGCACCACTTCTGGGAGGAGAGGGACTGTGTCCAGCACCACCATCC

CATCCGAAGGGAACTTCATTAACACTGGCAACAGTAGCGCCTACAGCAGCACGGCTGCTG

CAGAAAAAAAGGTCAGAGGAGAAGACCACGACGGTCGGAGATGAGACTCAGAAACAAGAC

GACGATCAGAGCGACAAAGGCACTTATACCATTGAGCTGGAGAACCACAACCCTGAGGAG

GAGGAGGCCCGTCGCATGATCGATAAGGTGTTTGGCGTGGAGAACCCTCAGAATCCCAGT

CGCTCAGTGCAACAGACAACTAAAGACAGTAAGAAGGTCAGCAGCACTAAAGCGGAGGGT

CTTTTGGATGAATCGGTTGCGGTTGGAAGTCCACACTGGGTCTCTCAATGGGCCACTTTG

GCCGCTAATCACACAAGAACTGATCCTGAAGGTTCCGGGGCAGAACCCACAAACTTTGTT

CATCAAGAGCGAGAAGCTGATGCATTTGAGTCAGGAGTGTCTTTGAGAAGCACCAGCTCT

GCCACCTCTAGTCAGGCTGAACGTAAAAGAAGGACATTGCCTCAGCTCCCCTGTGAGGAG

AAGAGCAGGCTGGGGAAGGTCTCCTCCACCTCAGGACTCCGATCCGAGATTGGCGAGAAA

CAGGACACCGAACTCCAGGAAAAACAACACAAAGGAGATGGCGAATGTGCATCCACTCTC

ATTAAGAAGGACACTACAAGTGGAATTGCAAACCAAGTGCAAACAGCCACTACCAAGCCT

CCTGTGCCCCCTGTGGGAGGGGTGGAGAAGCAAAGTGAGGAGTTTCGCAGGAGGCGTGCT

GAGGACAGGAACAAAGGTAGCGATAGTGAGCGAAGCGGGAAGCCCCTGGTGCGTCAAGGC

AGCTTCACCATTGACAAGCCAAGCACCAATGTTCCGCTTGAGCTTATCCCACGCATCAAC

CAGCGGCAGTCCGGAAACAGAGAGCGTAGTGATTCAGCTGGAAGCATGGACACGACCACC

TTGCTCAAGGACACTGAGGCCGTTATGGCCTTTCTAGAGGCTAAACTGCGTGATGAGAAA

AAGCTGGATCCATCACCGCCCGCTGGCTACCCTTTGGGACGCTCTTTTTCCCCAGAGTTG

GATACTGATGCAATTAAAGCAGCAGGCCGCGCCGAAGTGTCACAGAAGCGTCGTTCTCTC

AGCAGTATACACAGAGAGAAAAGTAACATGAGCTCCGTTGGAAAGAGCACAACCAATGCA

CGGGAACGTTTGGAAAGGAAAGTCAAGCCGAAGACCTCGGATGGGCGTCTGGATACCCGT

CGTTCTACCCATACAAGTCAGCGGACCCGCCAGCCGTCCATGGACCTCACAGATGATGAG

CAGACATCTTCCTTGCCCGTTTCTGACATACTTTCAGATGACCAGGAAAACGGACGTAGT

CCCTTCACCTTTCCTGATGACTTGCTACTTTCCAAACTGGACTCTTCTAAAGGTGCTAAA

GTTAATAAAGCCAGCAGGACACCACAGGCCACCACTGCCTCTATAGGCAAGCATCAGTTG

CCTCAGCCTCGGCCCACAAGAGCATCTCTTCTGAGACGTGCTCGACTTGGAGACACATCT

GACACAGACCTGGCAGATGCAGACCGAGTGTCAGTTGCATCCGAAGTGTCTACTACCAGC

TCCACATCCAGGCCACCATCGGGCAGGAAGGCACCATCCCGCATCGATATGCTTGCACAG

CCCAGACGCACCAGGGTCGGGACCATGTCAGCACGCAGCGACTCAGAAGCCCCAATGTCA

CGCAGCACTACATCCAGAATATCGGCCGAGACAGCTTTAAGACTGGGCTTGCGCCCAACT

AACCCTGCAGACAGCAAGCTGAACCCACGTCTTCGGGCAAACAGCGTCTCCAAGTTGACA

GACACCAAAACCAAGACTACAGCATCCATCTCCAGTGCTCCAGCAGCCAGCAGCAGGTGG

CGGCGACTTAATACAGAATACGGCTCCACCTCTGAGGACGAATTTGGCTCAAACAGGAAC

TCGCCAAAAGCCGGGCGTCTGAGGCCAGGCACTGCTCTGCGTACCAGTAAACTGAGCAGC

TCAGGCATGGTGACAACAAGCCCTGGCAGCATCACCATCAAACACAGGATGAGAGAACAG

GAGGAGTACATCAGAGACTGGACAGCCCACAGCGAAGAGATCGCCAGGATCAGTCAGGAC

CTAGCCAAGGATCTGGCTATTCTGGCGAGGGAGATCCACGATGTGGCCGGCGAGATCGAC

TCGGTCAGCTCCTCAGGCACAGCACCAAGCACTACGGTGAGCACAGCGGCCACCACGCCC

GGGTCTGCCATCGACACCAAGGAGGAGGCGGTCCTAGATAGCTTGCTGCTGAGCTCAGTC

TCTCAGCTGTCCTCTAAAATCCGCCAATCTGTGGACAAAACAGCAAGCAAAATCAAGATT

TTGTTCAAAGACAAAGACAGGAACTGGGATGACATTGAGAGCAAACTGAGCTCAGAGAGT

GATATGCCACTTCTCAAAACCACTAACAAGGAAATCTCAACAATTCTAACAGAGCTAAAA

AGAGTGGAAAAGCAGATCCAAGCTATAAATATCATGGTAGACCCAGATGGAACCCTGGAT

GCTCTTACCAGTCTTGCCCTAGCAAGTCCCACCACACCCAAACCTAAGACCAGCAACGGA

GCCTCCAAAGCCTCCGTGATCATCTCCCCGAACCGAGAGCTCCTCAGCGCTGGACTGAGT

CGTCCTCAAACCAGCGGAGAAGCGAGCAGCATTACTAACAAGTAAAGACAGAAAACCCTC

AGAGTTGAGTAAACTGAGAAACCCTTTTTGTCCATGTCTGTACCCCTCCCTCTCCTGCAC

TCCATTGGAATGTCCATCTTTATGTAAATGAAGTCACACCTGTGGATTTGGACAGTGATA

TACAGTGCAGCTCTACAAGTGCACGCAGCTGCTGGTGAAGAAGAGCTGAAGAGGAGCATT

AGTCTAAAGGCTTAAAATGTTGGAACCAGCCACCTGTATGCTGCTGCAAAGGTGTACACA

CTCATCCAGGCATGAAGCAGGTGGACTTAAAATAATTTATCTTCAACAATAAATGACTCA

GAGTGCCTGTCTTCTCTCTTTTGTGAGCATTCTTTCTTGCAAATAGCAACTTCTTATTAC

TACGTGACTTTTGTGTCAAAAATAAAACAGTGTTGGTCAAAAAGACTTCAAGATTTCAAA

GCTGAGCATAAACCAGCTTAGCAAAGTAAGTCTGATTCTATGCGGGCACTCAAACATTGT

TCTTTTTTTTCCATTTCTTATTTCTTGATTACAAAAAGTGCATTTAGGCTATTAAAATAA

GCAGGGGTCAACTTGGTAAGTTAAACTGACCGTTCATTGCATTATCTTATGCACTGATTT

TTTATTTTTATTGGCTAATGTATACAGTAGGTAGCAAACTTTGTACAAGGTTCAGACATT

AATTAAAACCCAGCAAACTGGCGTTTGTGTCATTTTCCCCCTGACATTAGAAGGAAGATC

ATATGCAGTATTAGAAAAACGTCTTAGTGGAATGCACCATTGTAGTGTTACACATCACAG

TTGCTTAGATCTCTGTGCTTTGTGCTGAAATGTTAAATCAGCCTTAAATTAACCAGCCCT

TAATGTACGGTTCAATCACATTCTGAACATGTTATGGCCAGTTGCTTAAATGCTAACGTT

CTTTAATCAGAGTATGCAATTACAAGAACCAAATAAAGTCTGTCAGCATTTTTTTTTTTA

ATTATCACAACCTCATAGATCATGTTTTTTGTTCATGCAGGTATTCCGGCATATTATGTA

TGTACTGTATTGTCTTACTCTTCCGTCGGCGCTTGCTGCTTGGCTGGAACTCATGCGCCT

GAGAAAAAGCGTCCAGCGCCCAGCCTTTCATCGTGAATCTGCGTGTTGTAGAAAGACTAG

TGCAAAATAAAACAATAGTGAAGATAGGTACAGGACATTAGTTGTTGTTGTTTGTTAATA

AATGTCAATGCCAAGTGGCTGCTGTGGAAACGCTAGTTCCTGGACAGGTTTAAGAAAGAA

AATCTTAGTTTAGTGACCGCATTTGTGGTTTTAAAGGTTCCTGGATTAACTGGGTGAGTT

AATGTTAAAGGTGATACAGGACAACAAACACCAAACTCTATACATTATACCGCTCGCAGA

ACAGGATAGATTTTCAGGCAGATGATCCTCCTGCCCCTAAAACACTTAAAAAAAACAGTA

GTTATCATTTATAAAACTATTGTACATGTTTTACATATAAGGGG

>Unigene000189

CCATCACTCCTCTAACACCACCTCCCACGACATGCTGCCCGCCTTTATGTGCCCCAAGGA

GACTCGTGATGGTCCAGCTTCCTCCGAGCCTGGCTCTGCTGCAGCTGTTGCCCCAATGCG

CTTCGATGCCCGACCCACATTCAGACCCACTTACGAAACCATTAATGGAGACCTGCGGAG

AGAGAACCGTTTCACACGAGTCCCACCCCGTCCCTCGCCTCGCCCAATCCGCCGTCCCGG

TGATAGACCTTCTCGTCCTGCTATCATCAACCCTGACGATCTGAAAGACCTGGACGACCT

GGACAACGACTGCGAAGATGGATGGGCAGGTCTTCATGAGGAGGTGGATTACAGCGAGAA

GCTAAAATTTAGCGACGATGAAGAGGATCACTCTCCCAGCGATAAGAGCAAGATCTGCCT

TGTCGTATATAGGGGAGAGTGGGAATCTCGCCGTGAACGCCAGTCATCCATGAGCTCAGA

CGGCCAGGAAGACTCTTACCATCACCAGCACGACCCTCTGCCTTCCAGAAAAACCAACAG

CAGATACGGCTCTACAGACCTGCATCAGAAGAGCAATGGCACAAGTGAGTCTGTGGAAGA

ACTGGACGAACCTCAGCGGTCCGCTGCCGCTCCCCCGCGGGGAAAATTCGTCCCACCAGA

CGTGTCCGAGGCAGTGGAAAGGGCACGGCGTAGGCGCGAGGAGGAGGAGAGGCGAGCCCG

GGAGGAAAGGTTGGCCGCCTGCGCTGAAAAACTCAAGAAATTGGACGAGAAGTTTGGCAA

AATCGAGAAGCCGGCGCTTACCGGAGATGGACATGGAGCGGAAAATAAGGAGCTTTTACT

TTCACCCGGACGAGAGGCGGGCAAAACTCAGCCAGAAACCTGGCAGCATGTTAAAGATGT

TTCCGAGCAGAAATCTGACTCACTTCACAGCCAGGAATATAGAGATGATTTCAGCTACAG

GAACGAGGATGACTGTTCAGAATACACACAGCGACACCAGAAAGCCGTCGCCCCACGCTT

TCAAAAGCAGCAGCAGGATCAGGTGTATAAGATGCAGCAATGGCAGCAGTCAGGCTCTGG

TCCAGCTCAGAGAGGGTATTACCCCTCCCATGTGCTTGGTTTTGACCCCCGCTGGATGAT

GATGCCCCCTTACATGGACCCACGCATGACTCAGGGACGCTCAACAGTTGATTATTACTC

TTCTAATGTCCATTCTTCAGTGAAGCCAGCTGGGCAGCAGGATCACCTCCACAGTCCCGG

GTCCACCTCAGATGAGGGCTGCCATCCCACCATGCACCTGGATAAGAAAGTGCTCTCTGC

TGACACTTATTCAGTTTGGAGCCAAGAAGGCTACCCACCTGCACGCAGTTTCACCCCACC

TTACCAGAGACAGCACGAGAGCGGTGAGAGGAGCCAAACAGATGACAGAAGTGAGCGGTC

TGGCTCTCAGCAGGACTCTTATGAGGACAGAGGAATAGAACGAGCTGAAAGTCCTCTGGA

GGATCATCTTCATCAGGGCTACCGGCCAAATCGTGGGCCAGACATTTCTCTCGGTTCGCA

GCGGGAAGATGCCTCTCATCCCCTAGGACATAACGAGTACTTCAAACCCGAGAACCAGAA

GGAGGTTATTTCTAACCGGCACCATAAAGACAGTCTGGATTCTCATGATGAAGTGTGTGA

AGGTAAAAAGAGTTTTGACTCTGGTTTCTGGAGGAAAGAAGATTCTATGAGAAAAGACAA

AGATGCCACACAGTGGTCGGATCCTGGGTCAGGTGGTAACGGTGGGGTTGGCCAGCCATC

TGAACCGATGAGCAGAACTCTCAGAAGGACAGGTCCCATCAAGAAACCAGTGCTCAAGGC

CTTGAAAGTGGAGGACAAAGAAAATGAGAAGCCCAAATCCGAACCGGAGGAGAAGTCTGT

TCCATACAGACTTGAGAAGGAAGTCCTTACCAACGTCTATGATTTAAAAAAGGACAACCA

GCCTATAATCAGCAGGCAGCTGCCTCCTCCTGTGACCTGCCAAGAGGAAAAGCAGACTTT

CCGCCCAAAAGTAGAAAAATTAAGCAATCCGACAGAAGAATCCATTAAGGAGAACGGCTG

GGATGGCAGTAATTCCCAGCTGAGAGACAACTCTGAAAGCAGAGAACCTCCAGCACCTCG

TCGCAACAACTGGATCTTTATTGATGAAGAGCAGGCATTCGCCGGAGTCAGAGGGGCAGG

GCGAGGCAGAGGCAGGGGGTTTAGAGAGTTCAACTCACGTGGTGGAGGCAGAGGAGGACG

TGGTGACAACAATAGAGGCATGTACAACACCGGGAATATCCAGCGAGTAGGAAGGGGGCG

AGGATCTCGGGAGTACGTCAAAACCGAGGATTTCCAAAGAGGCAAACCACGGAGGCGAAA

CGTGAGCGAGACTTTAAGCGAAGCCTCTGAATATGAAGAGCTGCCCAAACGGCGCCGCCA

GAAAAATGGAGAGAACGGAGAAGCTGCTTATTCTACAGCTGGAGACACTAAAAGGGCAGA

TCGGGATTCCTGGCGGTCCAATAAGGTTTACAATAACGACCAGAGCACCAGTGGAGAAGC

GAGGGAGAAACCCAAGATGAGAAGTTTCAGTCGGTCTCTTCCTCCACGCCTAAACACCGG

ATATAACCGGGGGTTCGGATCAAAAGAATTTTCCACTTGGAGAGGAAGAGGTGGTCAGTT

TGGAAGCGGATCCATGCAGGAGAACGGCTATAATTTTACAAATGACACGTTTTCTAGAAA

AAATGCAGAAGGTGACTCTCTTAAATACCCTTCAAAGTTTGCTTCGTCCTTCACAGAGAA

CGGAGTTGAAGATCGGGAGGGAGACTACCATCTTGACAAAGATAATTTGGACAACCGGCC

ACTGAGAAGAAGACGTCCTCCTCGCCAAGACAAACCGCCACGGTTTAGACGTCTACGCCA

GGAGCGAGAAACCGGAGGGCAGTGGAACAGAGACGAGTACGTTAACGGGGATTTTACAAA

TCCATGGTCCAGTCGATCTAAAACCAGTGAAGACCATTGGCCTGGTCAGTATCCTGGAAA

CAGGCAGCAGGATTTTGCACCACAGGCAGAGGACTGGGAGACTGGGTCGGAAAACAGTGA

CCTAAGTGATTGGAGAGAGAAACGTGGGGAAGCCCCAGGGGAACCGGGCCACGGCGAGCC

AGGCTCTGAAAAACGGGAGCTCTCCAAAAGGAGTTTCTCCAGTCAGCGCCCAATCGTGGA

CCGACAGAATCGCAAGACTGACATTTTGGAGAGCTCAAAGTTGCCCCGATCCACAGATAC

ACCGTCCAGCAGCAGTTCGTCTAACCGAAACGACAGCTGGCAGAATGGAGTGCGCCGGAG

TCCCGAGGACTCGACTTCAGGATTGAACGCAGGGTCGGTGTATGGAGTCGAACAGTCAGA

AGACGGCCTGTCCACCAGCAATTCTGAAACTTCAGTGAAAAAACTGGATAAAGATCTAAA

ACCAAGAAGCATGAAGGGAGACATGTCTGAGTCTCTATCACAATATGATCTCAACAGCTA

CCCGATTGAAAGCGATTCTGGAGTCTCTATTCCAAGTCCCGACGGATTCCAAGACGCCCT

TAACAAAAAACAGAGGCGTCCACAAGAGGACGAGCGAAGGAGGAAGGAGCAAGGAGCGAC

GGTAAAGAACAGAGCCATCACTTCAAAAATGCCTCCTCGTTTCGCAAAGAAGCAGGGTGG

GATCTCCATCGATCAGTCTGAGGAAACCCTGTCGACCAGTAATCTCGGCACTGAGATCTG

GGAGGCCAGCAGTGCAGCTCTTCCAGTTCAATCGTCTGGTGGAGACTCATGGACGAAGCA

GGTTTCGTACAATGCAAGTGAACCGAATTCAGAGGATTCGGACGCAGGGCCGGAGCACAG

CAAAGAGCACAAACCCGGGCCAATCGGAAACGAGCGCTCTCTGAAGAACCGTAAGGGTTC

CGAGGGCGTGGAGCGCCTGGAGGGCCCCATCACGCCTGTAAACGGAGTGGATATCCACGT

CGAAAACGTCCTTCCTGTCCCACCCATTGAGTTTGGGGTCAGCGCCAAGGACTCTGACTT

CAGCCTTCCTCCGGGATCAGCACCGGTGGTCGTGTCCAACCCTGTCACCAAGCTGCAGGA

TGCTCTTGCCAGCAATGCGGGCCTGGCTCAGGGCATTCCTATGCTGCGCAGAGATCACCT

GCAGCCTGCTATCAACCTCAACCCCATTTCCTACCCCACCACTGACCTCACTCTCAAGAT

GGAGTCTGCGCGTAAAGCCTGGGAGAACTCTCAGTCCCTTCCTGAGCAGGGTTCTCCAGG

TGGTGCTGGGTCCGTCACGCAGCCCACATGCAGTTCGTCCAGTGCCGTCAGCTACAGCTC

TTTCGGAGGAGTCTCCATGCCTCCCATGCCTGTGGCTTCTGTCGCACCCTCCGTCTCATT

GCAGGGTAATCATATCCCTCCCTTGTACCTGGACGGCCATGTGTTTCCAAGCCAGCCTCG

CTTGGTGCCGCCGACTATGACTCAGCAGCAAAGCTATCAACAGGCTGCAGCCGCTCAGCA

GATCCCCATCTCCCTCCACACATCCATGCAGGCTCAGGCTCAGCTGGGACTCCGGGGTGG

TCTGCCCGTCTCTCAGTCCCAGGAGATGTTCAGTTCAATTCCCACTTTTAGGTCGCAGGT

ATACATGCAGCCCAGCCTGTCTCACCCGAGTGCGATGGTGCTTTCTGGAGCAGGCCCTCT

CAAGGGGCCGTACTCTGCGTTCCCCGGCATGCAGCCGTCCGAGCTGGTGAAGCCGCAGTC

CAGCTCGCACTTTCAGCCCATTAATGGCAGCCAGGCACTTGTTTATGACAACGCAATGAA

CCAGGCTGCGGGTTTGGGCACGTCTCAGCTCATGGACTCCCAGCTCATTCAGGTTACGAT

GCCTTTACCTGGCTCTCAGTTGCGTTACGGTTCGGCCCAGCAGCACTTGATCCTCCCACA

GTCCATCCAGCTCCAGCAGGGACAGAGCCTCTCTGTCGGCACCCCTCGCCGCATTCTTTC

CCATGGTTCCCAGCCACCAGTCATGACTGCCAGTCGAGAGCCATCTCAGCTGGAGATGAA

AGGATTTCAGTTCCCTGATAAAGCCAGCCATTCACCTGGGATACCTGCCGGTTCATACAG

ACCCAGTTCTGCCAGTCCCAGTGGAAAGCCGTCTGGTCCTGGTGCTCCCCCGACTGTCGG

CCCGCTCCCTGGACATTATGCTCAACAGGTGCCCCCTCCACAAGGCAACATGGTGATGCA

CATGCGCACCCCCACCAACGGGCCTTTCCCAAACCCCATCCAGAGACCTGTAATGCAGGT

AAACAAGGCTGTAATTATGCGCTCTACCCCCTACACGAGCCCAGGCCGTGATCCTACCCA

CTGCACCCCCCCTACCAACCCAGAGTCCCTTAACAAGGGCACAGAGGAGGGCACGAAGGT

GAGCCACCCACTGTAACAAAAAATATAGGTGAAGCTCCTCCATGAAGCTCGGCATGTTGT

ATCCGATCTGAAGACGTCCTCTTCCTCATCTGTGATCTCTGGAAAACTGCAGGACGTCCT

CACCGGAGCGCCGAAGCCGAACCGCACCGGAGCCATTAAACCTCAGAGCGTTAAAATGGA

GGAGAGCAAGGCTTGATCACAGAGCTCACACACACACACACACACCGTTCACCTCATTGT

ACTGAACCTCCCAGCTCAAAACGAGGACTTTCTCTTTTTCTCACACACACACACACACAC

CGTTCACCTCATTGTACTGAACCTCCCAGCTCAAAAC

>Unigene000190

GGATTGTTTTATCAGCTTGCTGTGATGTGTCGTTATTAAGGATTAAGATTTTGCTCTTCT

GATTTTTTCGTTGTAAAGGATGCTAAATTGATTGATGGCAAATCCACGAGTGTCTATAGA

AGGAAGCTGACATCGAGGATCTAACCTGGAATTGCATTCTTCTTACTACATTACAATACG

TTTTTACAACCTGGGGTGATTTGACGTTAAGAAGGATAAATATGAGACAAAAGCCTCCTG

TTCAGTGGTTTCAAATGATGAAAGCAAGCTCTCTTGTTATGACATGCCCAGAATGACTTC

CTGAATCTGTCTAGTAGTGAGGCACTACTGCGAGTCACTGTGTGCTGGAAGAAGACACTG

ATTAGGACTGTGCTAGGCAGATGAAACGTCTGCGAAGTGATTGAGTTATGACTTCTTCTC

TGCAGCTCGTACGCTCAAGTCAACCTTTTAGATTTTCTGAAACTGGTCAACAGTCATCCC

AGACCAAGAGAGGAGGATAGTAAGGGAAGAGAATTTAAAGAAAGACTGAGCCACCAAGGA

ACAGAGCGAAAAAAAGTGCACCATGCAGTCAGACGACCTCTTTATCCGGAAGTTCCGCCG

ACAGAGCCCACGTCCTACCCCTGTCTTTGAGCCCAAGCGGACAGCGGTGGCGGAATGGCC

GCCCAGAAGGGATGGTGAGGGAGCAGAGGGTGAGAGTTTGACAACACCCAGCAGGGCAGG

TTTGAATCAACGTGCAGTTGCGCGTGGCCATATTATGCAACGGAGCAACAGCGATGTCAC

ACTGGGAGATTTGGAATCAGCGGCAAAAGCAGGAGCAAAAGCAGTCAGGTCAGCAAGTGA

AAAGACCGGGCAGGTAGGACAGGGTGACTCTAGTATAGTCCTCCACCGGGAATATGGGAG

TCTTTCTTCTTTGGAAAAGCAGATCCAACCTCCAGAGCTCTGCATGGATGAGCAGAATAT

GCTCAGCCCAAACGCTCTCCGCTTTAAAGATCCATTTTTGTTGCTCGGGCTGCAGGAGAC

ACCACCTGAACCAGATGGCTTTTTCCGAGCCCTGGAGTGCCCAAAACCCGCCAAGCCCCC

CAAACCTGATGCTCTAAACAAGAAATCAAAGCCCTCACCCCTTCAGGTCCCTTCTGTACC

TTGTGATCTGACATGTGGAGGTGCATGGGTGCGCAACTTTGCACATTATGACGTGCAGAG

CATCCTGTTCGACTTGAGTGAAGCGGCCACTAGCCGAGACAGCGTTGGTCGCAAGAAGAA

CATCACCTCAGGGGCGTCAGCTGCCTCGCAGCTCCGGCCTCTTTCACAGGCTCCTCCTGC

CTCCCCCTCACAGAGTCTGGGAAGTGGGACTGTTGGTACAGCAAGCACAGAGGAACCAGA

GCCATGCCTGCTGGACGAAGGAGACGGCAATGACAACGATTTGCTGCTCAGCTGCCCCCA

CTTTAGGAACGAGATGGGAGGGGAGGAGAGGGCTGGACTGGGAAGAAACCAGTGTTGTAG

TGGGCGATGGAGAAGTCTACAGAACCCCAACGATGCTGTGTCTGTATTAGAGGAACCCAG

AGAAAGCCATGTCCAACAGCAAGGAAAGAGCAACTACTTCATTGAGCATGCAGACCTCGG

GGCACGGTACTACCACAAGTACTTCTACAATAAAGACCATCAAAATTTCTTTGGCATGGA

CGAGCGCCTGGGGCCCGTGGCTATCAGTTTTCGGCGAGAGGAGAAAGAAGGCACTAGTGG

GGCGCAGTATATGTACAGGATCATATTCAGGACTACGGAGCTTAAGACTCTTCGTGGCTC

CATCCTGGAAGAGTCTGTGCCATCTGCAGCACGACACACCACCCCGAGAGGGCTTTCCCC

AAAGAGACTTTTGGAGTTCATTTTCCCGGATCTCAACCTGCAATGTCTGAGGCTTGCTTC

TACGTCTCCCAAAGTCCGAGACACGCTGCTCAAGCTGGATGAGCAAGGGCTAAATTTCCA

GCGTAAGGTAGGTGTGATGTACTGCCGGGCGGGGCAGAGTACAGAAGAAGACATGTATAA

TAACGAGAGTGCCGGGCCGGCGTTCGAAGAGTTCCTGGACCTGCTGGGAGACAGAGTGAG

ACTGAAGGGCTGGGAGAAATACAGAGCACAGCTCGACACCAAGACGGATTCCACTGGGAC

CCACTCTCTATACACACGTTACCAAGATTACGAGATCATGTTCCATGTTTCCACCATGCT

TCCATACACAGCCAACAACACGCAACAGCTTTTGAGAAAAAGACACATAGGGAACGACAT

CGTGACGATAGTCTTCCAGGAACCGGGGGCCATGCCGTTCACCCCTAAAGCCATCCGCTC

ACACTTCCAGCACGTGTTCATCATTGTCCGAGTGCACGAGCCCTGCACGGAAAACACCTA

CTATCGCGTTGCTGTTACACGCTCCAAAGACATCCCTCTATTTGGTCCACTTTTTCCCAA

AAGAGCTCGCTTTCCACGCTCACAGGCTTTTCGTGACTTCCTTCTGGCCAAGGCGTTAAA

CGCCGAAAACGCAGCTGAGAAATCGGAAAAGTTCCGCTCGATGGCCACACGCACGCGTCA

GGAATATCTAAAGGACCTAGCAGAGAACTACGTGACCACAACGCCTATCGATTCATCTAC

CAAGTTTCCCCTGCTTTCCCTGGGAGGCAAACGTAAAGACAAGTTTAAGGGTGCTAAAGG

AGCAGAGCTGCACAGTGCCGGCGCTCTGGTATGGGCCGTGTGCGCCAGGCAGGATGACGA

CGGCCACAAACTTGCCTGTCTTTTGGGCGTTTCAGCTGAATTCGTGGTGCTGATCGAACG

CTGCAGCAAACGCGTCGTCTTTAACTGCTCGTGCCGTGACGTGATTGGGTGGAAGGCAGT

AACTGAGACTGGCATTAAGGGTGGGCCTTGTCTGGATATTTTCTACGAAAGGGGCGAGGC

GATCTCGGTTACGATGTCAGAAAGCCAAATCGAGGATATTAAAGAGGTGGTGCAAAGGCT

TGAGCTGGTGACAAGAGGATGTGAGGCACTTGAGATCACTCCTCTGCGGGACAACACAGG

TCAGCCGGGATTCCTAATGAGCGCAGAGGGCTTCGTGATTGAGATGCAGCGATTCTGTTA

CGCAGAAAGCGGAGGCCTGCAGCTGAGCGCACGGGTAGTACGACTCTGTGGACACACCCT

CGTCCACCTGAGTCAGGAGGAGAGGACGAGGCTTCTGCGGACGGCACCCAAAATTCACAT

CACCGTCATCCCACCCGACGAGAACGGCAAACCTCGCAGGAGCTTTTCGGAACTCTATCA

GAAGTCTATCCAAGATGCAGAAAGCAAGTCTGGTGAAGGCCACTCGAGCGAGGCCTGGGT

ACTGAACGAGCATGACGAAGACGAGGATGACGAGGAGGAGGATGAGAGAGGGGGTGAGCG

AGAGCAGGGCGAAGGGGAGGAGTCAGACAGCATGAACCAATCAGATACAAGAGAGGAAGA

AGAAAGTGGAAGTCCAAGCTTAAGTGGCTTGCTCTCTCCGCCCAGCTTACCTCTTCTTAG

GGCGACTTCACTGCAGGACAATCCTCCAACCCAAACGCTGGAAATCACTTCCTCCGCTCT

CACACGCAGCTACTCACTCGAGAGAACCCTGCCATGTCAGGACATCTGTGATGGTCATGT

TTATGATAACGTCGGAGCGAAGGTGAACAGACATCATATCTACGAGAATCTAGGAGAGCT

GATCGACGCAACCCCTGACCTCATACTGGCTGTCAAACCAAAGGTTCCTCTTCAGGAAGA

AGGACAGGTTGGACTAGATGTGCTAGGTGAGGAGAGAGCATCGGGCAGCGAAGCACCTCC

TCAGTCAGAACATATCGAGAGGAATTCCAGAGCGGAGAGAAACTCCAGAGCACTGAGCAT

TCATAACTCCATCACCAAGATTCTTTCAGAAACAACAGATTCATCAGAGGAGGAATGGCA

GTCTATTTCTGACCTGGCCACTGCATGCAGGAATATTCTAGAAGCCCTATCGCATGAAGA

TCGAAAGGCCGGTGATGGCACTCAGGCAGGAGGGGATACAGGGACGCAGACAGATGCCAG

AATCAAAGATATGAAAGACAGTGATTCATCCAGTCACCTGGAGGAAAAGGTGTCCCAATT

GGAGGCCATGCTGAAACGATTACAGGATGACCTCCAAAAGGAGAAAGAGGACAAAGCCAT

GCTGCAGGCAGAGGTGCAGAGTCTGAGACAGAACAACCAGCGGCTGCAGGAGGAGTCGCA

GAGCACCGTGGCCCGCCTCATCAAAGTCACCGAGCTGCTGTGTAACGTTAACAAACCCTG

CTAGTAGAACGGGTCAGCGAGATAGATATACAGTACACACACAAACACACACACACCAAA

ACAGACCAGCACCGAATGCTAGCAAGACACGAGCAAAGCAATCCAGACAGAACTGCAAGA

CCCGGAGCCCATGCTTAGGCTCATTAATCACAAAGGAATGACTCAAATGCTAGACAAAGC

TGCAAGACCCAGACCTCAGACACTGAATTACAGACCACTAAAGAACGACACAAATTTCAC

CACACTAGCACAAAAAATTATCTAGTGCTACATGAAAGATGGACATAAATTTAAAGCTTG

GAAGGTGCTGGCGTAGTAGACCATTTTTTGGAACACACCATAAGATCCCTAGCAATGTAA

GACCTCTAATGTAGACCTCAGGGCCTATATTACGTGTAAAAACAATCAATCAAGACTAGC

ATCCCTTTTAGACAAGCATTGCTTTTTTAGTGTATTTAGCTGGCATCATTGGTGAAGTGC

AGCCCACCAAGATTACCAGGTTATTATTATTAATATTATTGCTCACTTGCATTTTACAAA

ACCAAGAGCAAAACCTTAAGGCACGTTATTATCATCGTGCATGTTTGTCAGGCATAACAG

ATCAAGACATTGATGGAAATTTACTGTTACCCAAAGCAGAGCAAGATCCTAACTGCATGA

TTTTTTATTTTAACGTTGCCAGACCCCATATGGTTAGTACTCAAGGCTGGGTAACCCATA

TGCCTTGAGTTACTTTGTTACACTTGTTTTCCAGCTACCCCAGGTATCAGAGGGTAGGAG

ATACCAAATCCTCAGATCTTCATCCTCTTCTGAAATTTATCTTCCAGCTTCCTATTGACT

GAACTTACCTGAACAAGGCAATCAGCTGCAGAAGGAAGCATGTATGATAAATGAGGCTTA

TGGAAATCCAAAATAGTAAGCAAAAGTCATAACTAGGAATAGTATAATAAAGGAATGGCA

ACGCAACTGGAATAAGGAAGTAACAGGCAGACATTATTAACAAATCCAGGAGAAAGCAGG

AAATAGAAGGGCATGTAAAGGAAATATAGGAATAGAAAGTATAATTACACAATTCAGAAT

AGGGCACATGAGTCAATGCCGACTATAATAAAACACGAAGATAAAGAACCATGTAATCGG

CAGTGGGTAGAACGGGGTAGGTGAAAAACAAGAGGAACTTAAGAAACATGGGTGAGGCAG

CCCTGTCAGTACTTTTGCCTAAAATTCTAACGCTTGATAAGAAGTTAATAGTGTTCAGTC

AGCATGATGAGCCAGTAGTAACTTTCATTTATTGTTGGAAAATTTTTTAAAAACTGGACC

TAATTTTTTTGTCCAGATATTGACAAATCTACAGAGTATTCTAGTAAAAACCACAGCTAA

CATTTGGCAGACGTCCTTATTCAGAACGACTTACATTTATCTATTTTATGTAACTGAGCA

ATTGGGGGTTAAGGG

>Unigene000191

GATTCCTTTTAAAGACTGTACTTAACTTAATTTATGTTTTTTTTTTTTTGTTTGTTTGAG

GGCGGGGGGAGTTGTTCAATTAAAAAAAAATGCTTCTGTGTTGCTTGTAGACAGCTGAGT

CGTCACCTAGTAGGTCTGTGGATAGCTGAGAATCCTGTCGCTATGACCCTTCTCAAGAGA

ATGTTGCCAACTGGACTTCTTGCATATCTGGACAGCTCAGACCCTGTGCCAGAAAGGGAC

ATGGACAGAATTCACATTCGTGACAATCTTAAGATTGCATCTGACCAGTTTGGGCACACT

AAAGTTCCAGAGTGGCAACGAATTGCAGGCAAGGCTGCTAAAGAGGTTGAAAGGTTTGCT

AAAGAAAAGTCTGATCTGATGCTTATGCACTGGAGGGATAAAATGGGTATTGCACAGAAA

GAGGACAGGAATAACTTGAACACAAACCAAAAACCTGTTGTTTTGAGAAAAAGACGTCAG

CGGATCAAGATTGAGGCCAATTGGGATCTCTTTTACCACAAATTTTCTATAGATCAAGCA

CGCTCAAATCTTATTTGGAACATGAAGACACGTGAGGAGCTGCGTGATGCTTTGGAGGGA

GAAATGCGCTCCTTCAGCATAGACAGGGAGCTGGGGAGTAACAGTGTGATTTCCTGGAAT

CATCAAGAGTTTGAGGTAAGGTATGAGTGCCTTTATGATGAAATCAGAATTGGAGATTAT

TATCTGCGCCTCCTTCTGGAGGAGAATGAGAATGAGGAGTCCGGAGTCATCAAGAGATCG

TATGAGTTTTTTAATGAACTATATCATCGTTTTCTCTTAACGCCCAAGGTGGCAATGAAG

TGTCTATGCCTGCAGGCTCTGACCATAGTATATGACAAGTGTTGTAAGGAAATTGGTCCT

TTCACTGACACCAAATACATTGTGGGCATGTTGGATAAGAGTACTGATAAACTAGAGAGA

GACAGACTAATTCTCTTTCTCAACAAGCTCATTCTGAACAAGAAAAATGTGAAGGACATA

ATGGATGCAAATGGTGTGCGTCTTCTAGTAGACTTGCTAACCTTAGCTCACCTCCATACC

AGTAGAGCTACTGTTCCACTGCAGAGTAATGTATTGGAAGCAGCTCCAAATATGAAAAGA

GAGAGTGAAAAGGAGTGGTATTTTGGGAATGCTGATAAGGAACGGAGAGGACCTTTTAGT

TTTGAGGAGATGCAAGAGTTTTGGAAATCAGGTACACTAACTGCAAAGACACGATGCTGG

GCACAGGGCATGGATGGCTGGCGCCCTCTTCAGGCTATTCCACAGCTGAAGTGGTGCACA

CTAGCCAGTGGACAAGCAGTAATGAATGAAACCGATTTGGCCACACTAATCCTCAATATG

CTCATTACTATGTGCTCCTACTTTCCTAGCAGAGATCAGGACAATGCTGTTATTAGACCT

TTACCAAAAGTGAAGCGGCTAATAAGTGACAATGCTTGCCTTCCACATATTGTTCAGCTG

CTGCTGACCTTTGATCCCATCCTCGTAGAAAAGGTGGCCATCTTGTTATACCTGGTTATG

CAGGACAACCCCAACTTGCCACGCCTCTACCTCACTGGTGTTTTCTTCTTTATCATGATG

TACACAGGCTCTAATGTGCTTCCTGTGGCACGATTCCTGAAGTACACTCACTTGAAGCAG

GCCTTCAGATCAGAAGAGGCTAAAAGCCAGGATATTGTGCAGAGGAGTGTGTTAGGACCA

GTTTTACCTGAGGCAATGGTGTGTTACCTAGAGAATTATGAAGCTGAACTCTTCTCAGAG

ATCTTTTTGGGAGATTTTGACACTCCTGAAGCAATCTGGAACAGTGAGATGAGGCGTATG

ATGATCGAGAAGATTGCTGCCCACCTGGCTGATTTTACTCCTCGGCTTGAGAGCAACACT

CGTGCTTTGTATCAGTACTGCCCGATTCCTGTTCTTAACTTTCCTCAGTTGGAAAATGAG

CTGTTTTGCAACATCTACTACCTCCGTCACCTTTGTGACTTACAGCGCTTCCCTGAATGG

CCTATACGGGACCCGGTAAAGATGTTAAGGGACACCTTGGAAGCCTGGAAAAAGGAAGTG

GGAAAAAAACCCCCCTCTATGTCTGTTGATGATGCCTATGACGTGCTAAACCTTCCTGCA

GGACAGGGCCAGTACGAGGAAAGCAAAATCAGGAAAGCATATTTCCGATTAGCTCAAAAA

TACCATCCAGACAAAAATCCAGAAGGCAGGGAAACATTTGAAAAGGTGAACAAGGCATAT

GAGTTTCTGTGCACTAAATCGGCAAGATTCGTAGATGGACCTGACCCTGAAAACATCATC

CTAATACTTAAGGCCCAGAGCATCCTCTTTAGCCAACACAAACAAGAACTGGAGCCATAT

AAATATGCAGGTTACCCTATGCTTATTAAAACTATTACCATGGAGACCAGTGATAGGCAG

CTTTTTTCCAAAACATCCCCACTCTTGCCAGCAGCGTCTGAGCTTACTTTCCACACAGTC

AACTGCTCAGCACTCAATGCTGAGGAACTGCGACGAGAAAATGGATTTGAGGTTCTGAAT

GAGGCTCTGTCTCGCTGTGTGGCTGTGCTTACTGGTTCCAGCAAAATTGGAGACATGTCA

GTGCAGGTATGCGGTCACATCTGTAGGTGCTTCAGTGTTGCAGCTCAGTTTGAGGAGTGC

AGAGAGAAAATCATCAAGCTTCCAAACATCATAAGAAATATTTGTCACATACTTTACTAT

AGCAATGCTCTTTCTCGTCTAGCCTCGCTGGCTGTAGAATGTGTGAGCTCGTTCTCCGTG

GACTTTTTTCTTCAGACACAATTATATCAAGCTGGTGCACTTTGGCCACTGCTTCTCAAC

CTCTTTAATTTTGACTTCACACTGGAAGAAAGTGGCGTCCAAGCTAGCCAGGAAACCAAC

CAGCAACATGTAATCAACAGCCTGGCCAAAAACAGTCTGTTGGCTATAGCCCGTCTTGGA

GGCTACAGAACCAGAACAGAGGCTGACGGAAATCATCAGATTGGGGGTGAACCAACTCCA

GAGAACCCTGCAGTGAGGAAGAGTGTGGCAGCCATGCTTACACCATACATTGCATATAAA

CTGAGCACGAATTCACCTGCCGAGGTCCTAAAGTTGGTTAATTGCAATTCGGAGACCCCT

TATCTTATATGGAATAATGCAACCAGAGCCGAGCTTCTTGAGTTTCTTGAGGCGCAGCAA

GAAGGAAACATGAAGAGGGGTGAATGTGACGAAAGCTTTGGAGCAGAGTTTGCCTTTAGT

GAGCACAATAAAGAACTCATTGTGGGAGAGATCTTTGTTCGCGTGTATAATGAGCAACCA

ACATTTCCTTTGGAGTATCCGAAAGTGTTTGCAGGAAGTCTTTTGGATTATGTGGGTTCT

CAGGCACAGTATCTCCACACTCTGCTTGCGCTGACACAGACAAATAAAGTGGAGTCACAG

CAGCACGCTCAAAGACTTCTTTGGGTTGAAATGGCATTAGAAGCACTGTGCAACGTTATC

AAGAACAACCCAGGATCTGAAACAGAATGTATTGGCCATTTTAAACTGCTCTTTTCCCTG

CTCAGAGTCCATGGGGCTGGGAAAGTTCAGCAGCTAGTCCTAGAGGTTGTGAACACAGTT

ACTTCAAACCAGGAATGTATCAGCAGTATAGCTGAGTCTTTGGTGTTGGCCAATTTGCTA

TTGTTGCTGCACTCCTTACCATCAAGCAGGCAGCTTGTCCTGGAGACTCTGTATGCCCTC

ACGTCTAACACCAAAATTGTCAAAGAGGCAATGAACAAAGGTGCATTGATCTTCCTGTTG

GATTTGTTCTGTAATTCCACTCACCCTCAGGTGCGCACACAGACAGCTGAGCTCCTGTCC

AAGATGACCTCCGACAAACTTGTGGGCCCCAAGGTTCGTCTGATACTGATGCGTTTTCTG

CCAGGAGTCTTCATAGATGCCATGCGAGACAATGCTGAAGCTGCTGTGCATATTTTTGAG

GGTACTCATGAAAACCCAGAGCTGATTTGGAATGATGCATCACGAGAGATCGTCTCGACC

ACTGTCAGGGAATTAATGCTTGAACACTTTAAGCAGCAGAAGGATAACCCTGATGTCAAT

TGGAAGCTTTCTGAAGAGTTTATGGTCCCATATGCAGCAGGACAAGGTGAGCTGGAAGTG

GGTGGAGTGTTCCTTCGCATCTTTATTGCTCAGCCAGGCTGGGTGCTGCGCAAGCCTAGA

GAATTCCTTGTCTCCTTACTTGATGCCCTCACGGAGCTGCTGGAAAGGAACCACCCTAAT

GATGAGGCATTAGAGACAGTCACAACAGCAGCTGTGTGTTTGTTCAGTACTCAGACTCAG

TTGGCTGATCAGATTCCGCCTCTTGGTCACCTGCCGAGGATCCTTGCAGCTCTCAACCAC

AAAAATAGTGCTGTTCTTAAAAGCTCCATCCGCCTCATCCATGTCCTCTCAGAGAATGAG

TTGTGTGTGCGCTCCATGGCAGCTTTGGAGACTATTGGACCATTGATGTCTGGGATGAAG

GTCAGGGCTGATATGGCAGGACTAGCATGTGAAGCCCTCAATCGCATGTTTCAGCGAGAG

CAAACAGACCTAGTTGCACAGGCTCTTCGAGTAGAACTGGTGCCATATTTACTAAAGCTT

TTAGAGGGAATAGGATTAGAGACTCTGGACAACCCATCAGCAACCAAAGCCCAAATAGTC

AAAGCACTTAAGTCCATGACTGGCAGTTTACATTATGGAGAACAGGTGAATGAAATCTTG

TCCCGATCCACTGTGTGGAGTGCCTTCAAAGATCAGAAACATGATCTTTTTATCTCTGAA

TCACAACTTGCAGGCTTTCTGTCAGGTCCAGCAGTAGCAGGCTATATAACTGCTGGCACG

GGTGCTACAGTTATGTCCACTATCCCGCCACCTGTAGACAATAACATTGGAGACCAGTGC

TAAAAATCAAACAAGCACCAACAGTTTAGTACCTGCACCATACAGAAAGACCAACTAAAG

GACTTAAATAGAAAAGTGTTTGGTCGATTTCAGCCCTCTCAATTTGTGATGCACTGTACT

CTGTGTCCATGTTATTTTTTTACAATCATTAATTATTTCCTTTTTTTCTTTTTGCTCTTA

TTTCTAGTATATTAAGCTATAATTTTTCAAAAAAGGAAATTTGTTTACTTTTATTACTTT

GTATAAGAATAAATCATTATTTGCTGGAAGGAAAAAATAAATACATCTCAAAAGTCAACA

CATTCTTTTTTTCAGTTCACCTTATTTTCTATGCTATGCTTTCCATTGTGTTTTTCTCTA

TATTATTATGGTAAGCTTGTCTTATGTCATAGATTTGTGTGCCATAAAGCTGTCATGCCA

TGTCATGTTCTTCACCTAGTTGTGTTTTCTATTAACCCGAAAATAAATAAATAATTTTGA

AAATTGAAACCTGTCACTGTCAGTTACAATAACACCCCATAGAGTGACTTGTATTTAAAC

TTACCTTATGTCTCTGCAGCCAACCTACCCTTACCTACCAGGAATGCAAAAATATCACAT

GGTAAAAGTAAGTGATAGCTAAGAGTAACCATGATGAATAATGATGAGCTTCTTAAATAG

ACTTATCAAATGTATGTGATTAATTAAATTGTCACTATCTATGTAAGGAGGCCTATATAA

ACTGTTTAATTTGAATGTACTATTAAGAAATGTCACACTTGTATATAAAATTTTAAGAAT

AAAAAGGAAGCCAA

>Unigene000192

GGCAGAACGAGACACACAACACAACAACACTAAACACAACTCAACTCAACAAAACTAAAC

ACAACACACGGTTACCGTGTGTGTGTTTGTGTGTGTGTGCAGGCAGTAATGTGATGCGCT

CCATCCATGGTGTGGGGGAACTGATGACTCAGGTGGTCCTCAGAGGAAGTGGCTTCCTGC

CCACAACAAGCCGGAATTCTCCCGATCGAGACTCTGTAAAAGCTCAAGCAGAACCTCAGA

AACAGGACATCCTGGTGATGGACACCAAGCTGAAGATCATAGAGATACTACAGTTTATTC

TAAACGTGAGGTTGGATTATCGTATCTCATGCCTACTGAGTATCTTTAAGAGTGAGTTTG

ATGAGAGTAATCCACAGACAGAGCCATCCATCAACCTGGAATCACAGATCAGTGTTCAGG

GAGCACTTGACTTTGAGCACATTGAGGAGCAGGCAGAGGGAATATTTGGAGGAAGTGAGG

AGAACACTCCACTGGATCTGGATGATCATGGTGGTCGAACATTTCTTAGGGTTCTCCTTC

ACCTCACCATGCATGACTATCCTCCTCTAGTGTCCAGAGCTCTGCACCTCCTTTTCAGAC

ATTTCAGTCAGAGACAAGAGGTTCTGCAGGCCTTCAAACAGGTGCAGCTCTTAGTCACCA

GTCAGGATGTGGAAAACTATAAACAGATTAAATCTGATCTGGACCAGTTGAGGTCTATTG

TGGAGAAGTCTGAGCTGTGGGTGTATAAAAGACAGGGGTCCGATTCAGGACTGCACACGG

GAGCGGTCATTACCACGGAAATACATCATAAGAGCAACTCGGTTTCTGATGGATCATACA

AACCCAAAGTGGAGAACACCAGCAGCTCCAACTACCGACTGGTCAAAGAGATCCTCCTGA

GACTCAGTAGATTGTGTGTGATGGAGTGTCTATCCGGCAGAAAGAACAAAAAGCAGCAGC

AGCGCCTCCTTAGGAATATGGGGGCTCATTCAGTGGTGTTGGAACTTCTACAGATCCCTT

ATGAAAAGGGTGAGGATGTGTGGATGCAGGAGATCATGACTCTCGCTCATCAGTTCCTCC

AGAACTTTTGTGCTGGAAACCAGCAGAACCAGACTCTGCTGCACAAACACATCAACCTGT

TCCTCAACCCTGGAATCCTGGAGGCAGTGACCATGCAGCACATTTTTATGAATAATTTTC

AGCTGTGCAGTGAGATTAATGAGCGTGTGGTGCAGCACTTTGTCCACTGCATTGAGACAC

ATGGTCGTAGTGTCCACTACCTCAAATTCCTGCAGACCATCGTCAAAGCCGAAAACAAGT

TCATCAAGAAGTGTCAGGACATCGTTATGGCCGAGATGATGCGTCTGGAGAGGGAGCGTC

TAGACGAGAACAGTGCTCTCAGGTATCATATCCACCTGGTGGAGCTGTTAGCTGTGTGCA

CAGAGGGTAAAAACGTCTACACTGAGATAAAGTGTAATTCACTTCTACCGCTGGATGACA

TTGTGCGAGTGGTCACACATGAGGACTGCATCCCTGAGGTGAAGATGGCATATGTGAACT

TCCTTAATCACTGTTATGTGGATACAGAAGTGGAGATGAAAGAGATTTACACCAGCAACC

ACATGTGGAGATTATTTGATGATTTCCTGGTGGATATCTGCAGAGTGTGTAACAGTACGA

GTGATCGGAAGCATGCAGACACTGTTCTGGAGCGCTATGTGACTGACACTATCATGAGCA

TCGTCACCACCTTCTTTAGCTCTCCCTTCTCCGACCAGAGCACCAGCCTGCAGACTCGTC

AGCCGGTGTTTGTGCAGCTGCTGCAGGGTGTGTTCCGTGTGTATCACTGTAGCTGGCTGG

TTACGAGTCAGAAGGGCAGCGTGGAGGCGTGTCTAAAGGTCCTGAGTGACGTGGCTAAAG

GGCGAGCCATTGCTATCCCGGTGGATCTGGACTGTCAGGTTAATAACCTCTTCATCAAGT

CTAATAACATTGTGCAGAAAACAGCTCTGAGCTGGAGACTGTCTGCTCGTAACGCTGCTC

GCAGGGACTCGGTCCTTACTGCCTCCAGAGACTACCGCAACATTATCGAGAGGTTGCAGG

ACATAGTCTCTGCGTTAGAGGAGCGTTTGAGACCTCTGGTGCAGGCGGAGCTGTCCGTGT

TGGTGGATGTACTACATCGTCCAGAGCTGCTGTTTCCTGAACACACCGAAGCTCATCGCA

AGTGTGAGAGCGGAGGATTCATCTGCAAGCTGATTAAACACACAAAGCAGCTGCTGGAGG

AGAACGAAGAGCGTCTGTGCATCAAAGTGCTGCAGACGCTCCGAGAGATGATGACCAAAG

ACCGAGGATATGGAGAGAAGCTCATGGCTTATGATGATGAGATGGATGTTACTAAGGTAG

ATATGAATCTGCCACCAAAACAGCAGGAGGATCACAGAAGGGGCGAGGCTCTTAGGCAGT

TACTGGTGAACCGTTACTATGGTAACTTCCGGACTGGAAGCAGGAGAGAGAGTCTGACCA

CCTTCACTAACTCAGCACTCACACCTGTCACTCCCAGCAAGAACCAATCAGGTCGAGCTG

AGATGTCTTTAACAGAAGTACAGTGTCATTTGGATCGCGAGGGAGCTTCAGATCTGGTCA

TTGACCTCATCATGAACACAAACAGTGACCGTGTCTTTCATGAGAGCATCCTATTGGCTA

TCGCTCTGCTGGAGGGCGGGAACACCACCATACAGCACTCGTTTTTTAAACGTCTCACTG

AAGATAAAAAGTCCGAAAAGTTTTTCCGGGTGTTTTTTGATCGAATGAAAGCTGCACAGC

TGGAGATCAAAGCTACAGTAACTGTAAACACCAGTGACCTCAGCAACAAACGCCGTGACG

ACCAAGCAGAACGAGAGACACATCAAAATCTTCGAGGTAAGGACTCTGTTGTTATGGTGA

CAGAAGATGTTCGGGAGCAGCTCCTGGAGGCGTCTGCAGTCACTAAGAAGGCGTTCAGCT

CATACCGACGTGACGCCGATCCCGAAGAGTCGTACGCTCATGCTGACTGTGATAAAGGGG

GCGGGGACAAAGGGGCGGAGCATGGGGAGATGAGCCCGGTCATCCTCATCATGCAGCCAA

TCCTGCGCTTCCTTCAGCTGCTTTGTGAAAACCACAACAGAGACCTTCAGAACTTTCTGC

GTTGCCAGAACAATAAGAACAATTACAATCTGGTGTGTGAGACGCTGCAGTTTCTAGACT

GTATCTGCGGAAGCACCACAGGAGGTCTGGGACTGCTGGGACTTTACATTAACCAGCACA

ACGTCACCCTCATCAACCAAACTGTAGAGAGTCTCACAGAGTACTGCCAAGGACCCTGCC

ATGATAACCAGAACTGCATTGCCACTCATGAGTCTAATGGCATTGACATCATCATCGCTC

TAATCCTGAACGACATCAATCCCCTCGGGAGGAAAAGGATAGAACTAGTGCTAGAGCTGA

AGAATAACGCCTCCAAACTGCTGCTGGCCATCATGGAGAGTCGCCATGACAATGAGAACG

CAGAGCGAATCCTGTATAACATGAGACCCAAAGAGCTGGTGGAGGTGATAAAGAAAGCAT

ATCAGCAGGGAGAGACAGACTTTGATGAGGAGGAGGAGAATGCAGAAGAACATGCTGCTT

CACCTCGCAATGTCGGCCACAACATCTACATCCTCGCACATCAGCTGTCTCGTCACAATA

AGGAGCTTCAGGTGCTGCTAAAGCCCACAGGAGAAGATCAGGCTCTGGAGTTTTACACTG

AACACACTGCACAAATCGAGATCGTCCGTCAGGATCGCACCATGGAGCAGATTGTGTTTC

CAGTTCCTCATATCTGCTCATTCCTGACAAATGAGTCGAAGCTGCGTGTGTATTACAGCA

CAGAGAGGGACGAGCAGGGCAGCAAAATCAACGACTTCTTCCTCCGAGCTGATGACCTGT

ACAGCGAAATGAGGTGGCAGAAAAAACTAAGAGCCCAGCCTGTGTTGTACTGGTGCAGTA

GAAACATGTCTTTCTGGAGTAACGTCTCATTTAACTTGGCTGTGCTGATTAACATGCTAG

TGGCATTTTTCTATCCATTGGAAAGTTTAACTGATAGTCACTTGGATCCGTCAGTGTCTC

TGTTACTGTGGGGGTGTGTGTTGGGCTCATTGGTGTTTGTGTTATTATGGCCCAGTCCCA

ATGCAGTAAGGGTTCTAGTTATCGCCTCAGTTCTGCGCCTTGGCTTTTCCTTAGGCTTAC

ATCACATGCTGTCTCTGCTGGGAGCCTTTAATGTTGGTAATAAGATTGTGTTCCTGTTGA

GTTTTGTGGGTAATAGAGGGACGTTCACTCGCGGTTACAGAGCAATGGTGATGGACAGAG

AGTTCCTCTTTCACCTGCTTTATCTTCTCATCTGTTCACTGGGTCTCTGTGGACATGTCT

TCTTCTACAGCTTACTGTTGTTTGACCTGGTGAACAGAGAGGAAACGTTGTTGAACGTTA

TTAAAAGTGTGACGAGAAATGGACGCTCCATTGTCCTCACTGCTGTACTGGGACTCATAC

TGGTTTATCTCTTTTCCATCGTGGGCTACATCTTCTTCAAAGATGATTTTATACTGGAGG

TGGACAGTATCTCTAACGCCACACTGGAGGAAGGGGTGAGTCAGGTGAGCGGTTTTCTGT

CTGAGGGTTCGTGTGTATTGGAGAACGGGACGTGTCTGTCTGTGGGTTCAGGAGAAGCTG

AGTTTGAGAGAGTGTGTGATTCTCTGTGGATGTGTATGATTACTGTTTTGAGTCATGGTC

TGAGAAGTGGAGGAGGAGTTGGAGACGTCCTAAGAAAACCTTCTAAAGAGGAGCCCCTGT

TTGCTGCAAGAGTTATTTATGACCTCTTGTTCTTCTTCCTGGTCATCATCATTGTCTTAA

ATCTGATCTTTGGCGTCATCATCGACACATTTGCTGACCTGAGAAGTGAAAAACAGAGAA

AAGAAGAAGTCCTGAAGACCACCTGCTTTATATGCGGGTTGGAGAGAGACAAGTTTGATA

ACAAAACCGTGACATTTGAGGAGCACATTAAGGAGGAACACAACCTGTGGCATTACCTGT

ACTTCATAGTGCTGGTGCGAGTGAAGGACTCGACGGAGTACACGGGTCCCGAGAGCTACG

TGGCTCAGATGATCAAGGAACACAATCTGGACTGGTTTCCCCGAATGAGAGCCATGTCTC

TGGTGAGCAGCGACTCAGAGGGAGAACAGAATGAACTTCGCTTGCTGCAGGACAAACTTG

AGTCCACCATGAGACTGGTCACCAACCTGACCAATCAGCTCACAGAGCTTAAAGAGCAGA

TGACTGAGCAAAGAAAGCACAAGCAGCGGATTGGTCTACTGGGAAACCCTGCCCATCTTA

ATATCAACCCCCAGCAACCTGCCTGAGGAGTGACACACACAAGGGACACACACAAAACGG

GGTGTGTTTACTATAATGATGAAGACAGTGATGATGATAATGGCAATAATGATGATGAAG

ATGATGGTGATAAATACACTGTTTTTTAATTAAAGCTAAATTTTTATAATTAAACTTTCA

CACAGCGTTTTGGATAATCGAGTGTTTTATTTCATTCTAATTGTGTATGTGATGTAATAG

AACACAGACCCAGAATAGAACGTTTCACCCAGACACTGAGTTTTGCACTGTTTTTGTCAC

TGTGTTAATAATGCAGGCATGCAAATAAATTTATTCTAATTAAAGTTTATTGAATAGTTT

ATTGATCAACAC

>Unigene000193

GGGCTTTTAATAGATGCATTGCATTGAAACGGGTTATGGCTGAACCGCAGAGGAACAGCG

GGGCTGTGGCGTAGTGGCATCAGCGGGAAATCCCTGCGGTTGGTTTTTTTTCCTAACTGC

GTGGAAAATAGAGTGAGAGTGTTGGTGTTGAGCTCCGTCCGGACTGCAGCGCTGGATTGC

GGCTTGTGTTGCTCGGTGTTCAGGAGCGGCCGAATGGACGAGCCGGACTACAGCATGACC

TAAAGCACCTCCCCGGAGATGTCACTTAACACCGCGCAGTGTCCGATCCCAGCACAGATC

CAGTGTGGTGGGTAGATAGGCGGTATGACTGAGGAGAAATGTCCTCAGTTGGTGGATTAT

TTCGTGGTGGCGGGGCTGGCTGCCGACTCTGCTCCACCAGACGAGGAATGTCAGCAGAGA

GGAGGCCGTGGCATGGAGCCTATCAGCGACCTGGCTGTGATAGCTCGTGGCCTTGGGGAA

GAGGTGCCTGAGGGCTTCACATGCATCGAGAAGACGTTGGGGGGCCACCCAGCTGAGCTC

AGCGCTGGCCTCATCAACAATCCACACATGTTTCTCTGCTACCGACGTAGCCATGACAAG

CCACCCATTGTGGAGCTTGGTGTGCTGTATGATGGGAAAGAGGCGGTGAAACAGGGTTGG

TATGTCATCGAGACGACGCCATACAGCCGCTGTGCCAGTCTAAGCTCAGGAGCTCCCGCC

ACCCATAAGACGTTCCTCATGTATCGACGTGCCCCTGACTCGCAGGCCCTGAATACACTG

GGAGTGACTGATATCTCATTACTCATGCCCAGCAAAGGAGAAGTGGCACCACACACCTTC

TGCAAAGTGGACAAGAACCTAAACACTGGCATGTGGGGCCCTGCACTCTTCCTTTGCTAT

AAAAGAGCTGTTGCAAAAGCCAACGCCCTTGTGTACGAGGCAGGGCTGATTAGCCGCTAC

CCTGAGGAAGATCTTGAGTCGTTCGCATTGCCGGACTCTGTCCCAGTCTTTTGTCTACCC

ATGGGCGTCACGGTAGAGAGCTGGCCCCTCAATACCAAGTACCAACTTCCTGTCTTCTCC

ACTTTCGTCCTCACCACGGCATCTGGAGATAAGGTGTATGGAGCTGCTATCCAGTTCTAT

GAAGCATACGCACGGGAGGCGCTGTCTGAAAGGCAGCGAGTTCGTCTTGGACTGGTCAGT

GTTGTGGACCGCCAACCCATCACCAACCGTACGGTGCAAGTAAGGAAGAGTGTGTGTGTT

CTCTCTCACTGGCCATTCTTTACAGTCTTCCAGAAGTTTCTCACCTTCATCTACCGCTAC

TCCATATCTGGACCTCATGTGCTGCCCATTGAGAAGCATATCTCCAGTTTTATGCACAAC

GTGCCCTTCCCTTCACCACAGCGACCGCGCATTCTGGTACAGCTGTCTCCATATGACAAC

CTCCTACTGTGTCAGCCAGTGTCTTCTCCCTTACCACTGAGTGGTGCCAGCTTTGTAAAA

CTTCTGCAGAATCTGGGGCCAGAAAATGCATGTACCCTTCTGTTGGCTGTTCTCACTGAG

CACAAGCTGCTGCTACATTCCCTTCGGCCAGATGTACTGACCTCCGTCAGTGAAGCCCTG

GTGTCGATGACGTTTCCGTTGCGTTGGCCCTGTCCGTACATCCCACTTTGCCCGCTTCGC

TGCGCAGATGTGCTATGTGCTCCAATGCCCTTCATCGTGGGTGTTCACTCCAGCTATTTC

GACCTGTACGACCCTCCTTCAGATGTGGTGTGTGTGGACCTTGACACCAACACAATTTTC

CAATCAGAGGACAAGAAACCATTGTCATGGAAATCGTTACCCAGGAAACATGGAAAAATT

CTGGTTAGCTCGCTCACCCACTTGCACAAAACTTTGGAAAAAATCTGTACCCCTGGCCAG

GAGGAGGCGACCTTAGAGTTCCTGCTGACAGATTACGATCTGATCTATGGCCGACAAAAA

CAGCTTGAGCTGGAGATTCAGGAGGCTTTCCTGCGCTTTATGAGCTGTCTCCTTAAAGGC

TATCGCTCCTATCTCCTGCCCATCACACAGGCTCCTTCTGACAGAACCACCGACTGTAGC

TCCCTCTTTAATTTGCAAGGCTTTCTGAAGTCTCGAGATCGGACACAGCAAAAGTTCTAT

TTTCACCTAACCAAGACTCAGATGTTCACGCAGTTCATAGAGGAGTGCTCCTTCGTCAGT

GACAGACACGCATGCCTTGAATTCTTTGACGAGTGTGTTCAAAAGGTAGATGTGGAGAAG

CCTGAGGAAGTGAAGTTGATAGACCTTGATGAGACACACAGTGGAGAGCACACAGTGTTT

ATAATGCCACCAGAGGAACCCCAAGAACCGGATGGCTCGGAATGCCCTGCACTGTACAGC

TATGAGACGTTTCCTATTCTAAATCCTGAGTTGTTTGATCAGCCTCAGGATCAACTCCGT

ACCACCGCAAAGGGAAGTGCCCCCAGTAGTCCTGCTCCTCGTCGTACCAAACAGGAAATC

AAGCTGGCACAAAAATGTGCACAAAAGTATTCTGCAGTACCTGACATGTGGTCCAAGTGT

CTGCTGGGCCACTGTTATGGTCTGTGGTTTATCTACCTTCCCACATTTGTTCGGGCTGAG

AGCACCAAGGTGCGGGCACTGCACACCGCTTATGAGGTGCTCAAGCACATGGAGACACGG

AAGGTGGTGCTGCCTGATGAAGTGTGCTACAGGATCCTAATGCAGCTGTGTGGGCAGTAC

GGGCAGCCTGTGCTTGCCGTCAGAGTCCTGCTGGAGATGAAAAAAGCAGGAATTACTCCT

AACACTATCACGTACGGGTACTACAATAAGGCTGTATTAGAGAGTAAGTGGCCATCCACC

AATCAGGGAGGTCGCCTTCGATGGGCTAAGCTTCGCAACGTTCTTTTAGCCGTGTCACAT

TTCAGGCAACCAATCAAACACAGGCAAAAGGGTGACTCTGTTAGCTCAAAACAAGGTAAC

TCGAACTGTAATCTTCACCCGCACTCCACTCTTGTGCGTCAGACCAGCTGGAGTGGTTTG

AGTGAAAGCTCGAGTCATGAGTCCTTGACTGGTCCCATCACTAAGAGCAACAGTCTAAGC

AGCATGCGACCTGACAAATCTAAGTTAGCTAGAAAAGCAATGCTCCAAAACAAAAGCAGC

GATGACCCCGCCTCCCGCAAACCTCCCATTAGTCCTCGTGATATGGTCACACCCTCCCCT

GTTCCACCTGATGAGCATATTAGAGTGCGACGGAGTGATGTCTGCTTGTCCAACTTTTAC

AAGGAATGTGCAGAGCCAGATCCAGAGTGCATGTGCCAGCCCACTCAAAGAGACAGCAGA

TCTACAGAGCAGCGGGAAACGCTGAATCGAAAAACAGTAGATGAGAACTACAATAACGTG

TTGTCCCCTAGTCGTGGACTGGCCGGTAAACTGCAGCAGCTTCTCACACCCACACGTCAC

CGGAACTCATCACGCCGTGCAGCCAGCATGGATAACCGGCGGCCTGCCATCAACGGGCCG

TCACGCAAATCCTCCGAACAGAGGCAGTCCCGCAAATCACAGGTGGCTGAGACTCTGCTG

AGAGCCAAAGATCGTCTCTACAACGCCACGTCAGAGAGCTCATTGTCTGTAGGAAGTGAC

ATTGACCTGACAGAACCTCCAAATTCAGTTTTTTCACTCAGGAAGTCATGGGATTCGACA

CAGGAAGGGGCGGGGCTTGAGGTGTTGATGTCGAGTTGTACACTTTGCCGGAGCTGTAAC

TCGTTGGTGTATGATGAAGAAATCATGGCGGGCTGGACATCTGATGACTCTAATCTTAAT

TCTACCTGCCCGTTCTGCTCAGCCACATTTGTACCACTGCTTAACGCAGAGATCTGTGAC

CTTGGGCCTGTCAGCAGTCAGAATCGCAGTAACTGGAACATGGAGGATGAGGTTGAAAGT

GCAGTGAGGCCCCCAACTGGCCACGATGGATTTTTGCAACATCTGTACAATGGTGTAAAT

GAGCATGACTCCAGTTCGGAGACCAGCACATACTCTGAGAACAGCAGTAATACTCTAAGC

TCCTCTCTGGGTGGCACCCAGGTAACAGTGGCATACCTGAGCCCTCTGGTCTTACGTAAA

GAACTGGAAAGTCTTTTGGAGAATGAGGGAGAGAGTGTTCTTTCTCAGGTCCAGTTACTG

GACAGCCACTCCATACTCTTTTGGAATCTGGTGTGGTACTTCACTCGCCTTGGTCTTCCC

AGCAATCTGCTGCAGCTGGTGCAGGCCTCCCCTCTCGCTTCACAGATTTCACAGACTCCT

GAGAATACTTCAGTCAGAGTAAGATTAATGTGGGATACCCTGACTCCTGATACGGATTAC

TGGCCTCCTCTTTATGTGCTCTGGAGAATACACAGTGGAGTGCCAATGCGTAACTATTCG

TGGCGCAGACACAACCACCCCTTTACCCTAAACTTTCTAGAGGAAGTCCTGCGCTGTGTT

GGCATGAATGAGATACACAAAGGCATCACGTTCTTCCTGGACACCACCAGCAAGCAATTA

GACAATCCGCGGATACAGAGGAGTTTGTACAGGGAATTTCTGTTTCTTACTTTGGCTGCT

ATGGGCAAAGACCATGTCGCTGCTTTTGACAAGAAATACAAGGCTGCCTACGCCAGACTG

AGCGGATCCCTGGGTCGAGAGGAGCTGAGGAGAAAAAGGGCGCAGCCACCTAGCCCTAAA

GCTGTGGACTGCAGACGATGTTTCCTAGTGCCCCTGGACTGCTGAGTGTCAAGACACACA

CCTGTGCTCCACAGCTGGAACGTTCCACTCTCTGTCTCTCTTACTCTCTCTTTCTCTCTG

TCAGTGGATGTCTCCTGCAGTTACCCCTTAAAGGCAGTTCTTATTAGACTTTTGACTCAT

TGAGTCTCACACATGCTCACATAACAGAGACTGTTGTTCTAAAGTTCTTTTTCATCTGTT

GCTCTACACGGTACTTCTTACGGAACTTGTACGACAAGATAACAGTTCTCTCTGCCCTAT

AAATGTTCTGCCGTTGCTTTTACTTTTGTTTCCCATTGCCTTCAGGTGATTTAATAGGGT

GTGTTTTACCAGATCTTTCTATTTTGTAGTACAGCAAATAGTCACCTTTAGGCTGAGCCT

CTTCTCTTTGCCAAGTAACTAATCCAGACACTGCAGTCATGCTTCAGCGGCGTTCTGATC

TATGCATCGAGCAGATCATCCTGATACCAGCATCATATGTTTTACAGACAAGGCCTTATC

ATCACATCACTCCTTCAGAGTATACATTCTGTAATTCTGCCTTTGTAATGTATATTTTGT

GTGCGTGTGTGATTACCAGACAGCATCTATTTAAGACCCCTTTACGTTCCTTTCTCCTAC

CGCCTCTCTCTCTGTTTGGCGTTAGTGTTTTCGGCTGTTTGTGGTTAGCTGTGCCTCTAG

CTGATTGGCCGTGAAAGATCCACCACAACACTTCAGACCGATCACCACTGAAGAGGAGAA

AAATACTCCAGACAGGAGGCTACAGTGTGGCACGGACTTAGTCTGAGATTTAGAGGGCAG

CTTTAATAACAGGAGCACAGTGATGTGTGTTAGATCCCCCCCAAAAAAAGCACTGATTTT

TGCACCGCAATTAAGTGGAATATATGAATGGCATTGGAATTTACCTCCATTTATTTTTTT

GGTTATTTTATGTGTTGAAAGACTGATTTTATTGTTTTTAAAAACATTGGACGCGTGTAT

GAGCCCCAC

>Unigene000194

TGTGTGTGTGTGTGTGTGTTGTAGGAGCTGCAGATGGATCAGCAGGCCAAAAAGCACCTG

CAGGAGGAGTTTGATGCTGCTCTGGAGGAAAAAGACCAAATGATTACCGTCCTGCAGACT

CAAGTTGCCCTTCTGAAGAAGCGGCTGCACGGTTCCCCAGGGTCTTTACTCACATCTGAG

GCAGGAGATTCCCAAACCGCTGAGACTACAGAGACGATCGCTGACCCTCAGAGCGTCACC

CAGGCGGAGATTGAGGAACTAATTCAAGCAGGGGGCAGTAGTGAGCCAGAAAACACTGCA

GACTCTGAGGCTCTGCAGAAGCGAGTACGGAGACAGGAGACGCTGCTGCAGCGCTGCAAT

GAGCTGATGAAAACCAACAAAGAGCGCAGTGCTCAACTCGGCAGCGAGAACGACGTCCTG

CAGCAGCAACTGCAGGAGAGACTGCAAGAACTGGAAAAGATGAAGGAGCTTCACACCAGT

GAGAAGACAAAGCTGATCACACAGCTAAGAGATGCCAAGAACCTCATCGAGCAGCTGGAG

CAAGACAAGGGCATGGTTATCGCTGAGACGAAGCGGCAGATGCACGAGACTCTGGAGATG

AAAGAGGAGGAGATCGCTCAGTTACGCACCAGGATCCAGCAGACCCTGACCCAGAAAGAG

GAACTGCAGGAACAGCGGGAAAAAGCTGAGAAAGCAGCGTTTGAGGAGTTGGAACGCGCC

CTAGGCGTGGCACAGAGAGCAGAAGAAGCCAGACGTCAGCTGCAGGAACGCATGGAGAAG

GAGGTAAAGCAGGTGGAGCAGACCAGTGAGGAGGAAAGGCGGAGCCTACAACAGGAGCTC

ACCAGAGTCAAACAGGAGGTCATATCCATTATGAAGAAGTCTACAGAAGAGAGGATAGCG

GAGATGGAGCAGCAGCACCTAGAAGATCTGGCCAACAAGGACCTGCAAATGAGTGTTCAG

ATACAGGAAGCGGTGGTAGAGTTACTGAGTTGATTCTCCTCACTTTATCACTCAGGACAG

TACAGAGCTGAAGTGTGTGACGGATTGTTGTTGGTTGTTTAGGAGAATTGCCGTAAGGAG

TTGTTGGAAGCAGCGCAGGAGAAGGAGCAGCAGGCCTCGCTGGCAGTGGAGGAGGCGGAG

CTGCAGAAGTCTGCTGTCCAGGCTGAAGGAGAAGCTAAAACCAGGGAGCTGCAGCTGGAG

CTGGAGAGCGCAAGAACCAGAATTCTTGAGCTGGAAAGTTCCATGGCCAAGTCATGTCAA

GCAGATCAGTTCTCAGTGCAGATTGAAGAACAGAGGAAGAAGTATGCAGATGAAATAGCT

TCTTTAGGAGAAAGACATCAGCAGGAAGTGGAGAAACTGAAGATGGAACATGCAGAAGCT

CTGAATCAGCAGCACTGCACTGATGTTGAGGCGCTCGAGCAGAAACATCAGACTGAAACT

GACGTCATGATGAAAGACAAAGAAAGAGAGTTTCAAGAACATGTCAAAGAAATGAACCAA

AAGATGCTGGAGAAGCTGGAGATCAAGCAGGCCAAGCTGGAGGCTTTGTCCTCAGAGCTC

AGTGAGATGCAGAAGTGTAAAGAGCAGCTGGAGGAGCATAAGGAGTCGGTGAGGATGGAG

TTAGAAGTGAGACTAAAGGATGAGCAGGTGAAATTTCAGAAGGAGGTGGATGATTTGAAG

CAAAAGCACGAGGAGATGTTAGAAGGACTTGAGAAGCATCAGAAAGAAGAGATAAATCAG

CTACAACTGGTGCTGGAGGAGAAGAATAAAGAGCTGGAACAATATGTTGAAAAGGAAAGA

ATCCTACAGGAAGATACTAACAAGGCTAAACAGGATGTGGAGACGATGTTAAAGGAGATA

GAAGATCTCCGGTGTGTTGCTCAGTCTGAGAAAGAGAAGCTTGCTGAAGCAGCTGCTCAG

ACTACTTTACTCAAAGAGGAACTTACGAAGTCACAGAGTCGAGTACAAGACTTGGAGAAA

CTTCTAGAGGAAAGTCGTAATGAGTGCCAGCATAAAGAAGAACATCTTCAACAGAAGAGT

CATGAAAACTTTGAACTTCAGCAGAGGATACAGCAAGCTTCCAATGACCTGGCTGAAACA

ACGTCTTCTCACGCTCAGACCCACCAAGCAATGCAGGAGGAACAGGATGGGTTAAAGAAG

CAGTTGGAAGAGGAGAGAAGTTCTTCTGAGAAAAGAGTCGAGAGCTTTAGAAAAGAAATG

GAGAGCAAGTTAAAATCCCAGGAGACCAAGATGGAGAAACTCAGACAGAAAGCCAAAGAA

ATGCAGGAAAAGTTCAAGAAAAAACTGCAAGAACAAGAGGAGAATGCGAAAGCAGAGATG

GCAAAGAAGGAGAAAGAACTGCAGCAGAAAGAGCAACAGGTGAAAGAGAAGATCCTGGAG

ATAGCTCAAGCTAACTCTGAGGGTCTCAGCAGCGCTTTGTCAGACTTGGAGGCGAATCAC

AAGGAGCAGTTTGAGAAGATTCAGAAGGTTCATAAGCTGGAGCAGGATGACCTTCTGCGT

ATTTGGCAGGAGAAGCTGAGCCAGCAGGAGGAGGAGCTGCAGGAGAAGCATGCTCTGACG

ATGCAAGAAAAAATGCAAGAATTGGAGGACATCTCTCAGCAGCTTTTGACCCACAGAGAG

GAGAAGCGACAAATGGATCAGGAAATAAAAAACCTTAAAGAGGAGCTTTCAATGAGGGAA

ACCACTGTGCAGAAGCTTCAGACTGAGCTCAGGGAAGCTGCAGGGAAACTGGACAGCCTG

TCTAAAGTAGAGGAGCTCCTCAAAACACAAGTGGAGACTGTAGAGAAAAACCTGAACCAG

GCTTTAAATGAAAGAAACATCTTTCAGGATCAGCTCACTGCAGCTGAGGAGACCAGTAAG

GAAAAAATTCTGGCTTTGTCTGAAGAGCTCGCAAACGCATGCAAGAAGCTCACTATTCTT

GAAGCTTCCAAAAGTAAAGAAGGAGAAAACCTACAAAAGAGCCTTGAGGAGAAAACGTTT

GAGCTTCAGAATAAAGAAAGCAAGTTTTTGGAACAAATCCATAATGTCGGTGAGGAGCTG

GAGCAGCACTGTCACACGGCACAGACAATCATCAATGATTTCTCAGCTCGCCTGTGCGAA

AGAGTCGAGACGAAGGTGAACGAGCTGCAGAGTAGAGTCATGTTCAACCAGAAGAAAGTG

TGTCATTTAAAAAACCTCATTTTAACAAAGAACCACCGAATCTGTTGTTTAGAGCAAGAG

CTTCAGCAAGTCGTAGAGGAGAACCAGAAGCTCAAGAACTCACTCGAAGAAACAACTCTT

CAGCTCCATGCAAATTCAGAGAATCTCAAAGCCTTAAACGTGGAGAAGGAGACCCTGCAA

AATGATGCTAAGAATCATTCGCAGTTACTTTCTGAGAAAGATCTTTCTATAGAAAAAACT

CATGAAGAAAACAAAAACTTATCCGAAAACCTTAAAGCAAATATTTTGCATATCAGTAAC

TTGGAGAACGTCATAGATGACCTGAAGAAGCAGTTATCATGTAGCATAACTGAGAAGGAG

GAAGCCATATCTCTGCTGAATCAGCAGCACCAGGAGGAGAAACAGAGCGTGCAGAACCAA

ATGGAGCAAGCAGAAAATGAAAAAGGTTTAGCTCTCAAGCAAGTCGAGACACTCAGGAAT

AAACTGGCTGATTTTAAAAAGAAAACCGACTTCAAGATTTCTCAGAACCAGAACACGGTT

AGATCTTTGCAGCGCCAGATAGAGGACATGGAGAAGCAGATTGCGGAGAAGGACGAGCAG

CTACAAAAACTCACTGCCAGCATCGACAATCAGTGTATCAGCAAGTCCGAGCTGGACCAG

GTGCTGAGCGAGAAAGAGCAGAAGGTAAGTGCCTTAAATGTAGAACTGGATGACTGCACG

AAGAAGATAAGTGAACTGGAGCAGCGACTCAAATCACAGAGCATGGAACAGGAGAAGAAA

GAAGCTGAGCTGCAAAATCACGTCACCTCGAGCGAGATGGAAAAGACGGAGCTGATTCAG

CAGCTGGAGCAAACCAGGCAGCAGTGTTCTCTTCAACAGGACCTCGTCTCTAAAACCCAA

GAACAGCTCCATGCTTTGGAGAACGAGTTCCAAGCCGTCAAGCAGAAACTGGAGAGCCAG

CAAGAAGAATTTGAGCGGGAGAAGTCGAAAGAAGAGGCTTTGAAAGCGGCGGAAGGTAAA

GTCGCTGACCTGAAAAAGAAAGCTGAGCAAAAGATCAGCTTGATCCGTAAGCAGCTGACT

TCCCAGATCGAGCAGAAGGAGCAGACGATCAAAGAGCTGCAGGCACAGCTGGAGGACATC

ATGCAGACGCAGAATGAGAAGGAGCAACAGGTGAGGAATTTGGAGGAGAATGGAAGGAAA

ATGGAGGAGGCCATTAATAATCTAATGGAGGAACACAAAAAGCACCTGGAGCAGGTACTG

AGTGATGGACAAGTAGAAAAAGAGTGCATGGACAAGGAGGATTTGGTTGCTGCTGCTGCT

GCTGCTGCTACTGCTACAGTAAATGACAGAGAGGAGGAGACCTGGTCAAGGATCAGAGAT

CTGGAAGCCAAACTTCTTCAGTCTGAAGAACAGAACACCAGCTGCCGAGCTGAAATAAAC

CAATTGAAAGAAGAACTCTGTGAACAGACTAGTCTGGTTCAGGAGCTTCAGAAGAGTCTG

GAGGTCCAGGTTAAAGAGAAAGAGACGAATGTCATTCAAATGGACAAATGCTTTGTGGAG

AAAAGCAACAATCTCTTAGGAATGGAACATGTTCAATATGTAGGAGACCAGAACACTAAA

TCACACCAAGACATTTGGGAGACGGAGAAGGAGCTCTTGGTGAAAGACTATGAGATGAAG

CTCCAAGACCTCCATAAGAGACTGGAGGAAAAGGAGAACGTACAGCTGGAACATAGTTTA

GAGCAAAAACTGCTAGACGCTGAAAACGAGAAGCAGAAGATTCATAAAGACTACACTCGC

TTACAGAAAGACCTTCGGTCTTTGAGACGAGAACATGAAAAGGACCTGGAATATCTGCGG

AAAGAAATCGCAGAAGAGAGCGAGAAAAAGCTGAAAAGTGACGTGGAGGATTTAGAAATG

AAGCACAACTCAACCCTAAAGCAGATGATGAGAGAGTTTAACACTCAGATAGCGCTGAAG

GATAAAGAGCTGGAGACGTCAGTACGAGAGGCTGTGGAAAAAGCGCAGATGGTCGAAGCG

GAACTGATGGGAATCCATAAAGAAGAAGCCAATCAACTTCAACAGATCATTTCCCAGAAA

GACGACGACCTCAAAAAAACCATCCAACGTTACGAGCAACTCCTTCAGAGTCGAGAAGAA

GAAATGGGCAGTCGAGTGTGGGAGGTGCAGCAGGAGCTGGAGGAGCTGCAGCAGAGGAGT

CAAACCGGTCCTCAGGGTGTTGATGAGCTTCAGGTGCAGCTCGCCGAGAAGACCACCCTG

CTGAGTGAAACCCGGCTTAAAGAGCAGGAGTACCAGGATCGGATCCACACTCTGGAGGAC

ATGATACGCAAGGCTTATAAGAACTCAGTGGTGACCCACCTGGGCAAAGATGTCTCACAC

CACACTGCGGAGTCCCTTAGCGAGCCCACCGAGTTGGAGTACCTGCGCAAGGTGATGTTC

GAGTACATGATGGGCCGCGAAACAAAGACGATGGCTAAAGTCATCACGTCCGTGCTGAAG

TTCCCCGAGGATCAGGCGCAGAAGATTCTGGAGCGAGAAGATTCCCGACTGATGACTTGG

CTGCG

>Unigene000195

TACTACATTAACGTGTGTAATAATATCATCAAGCAGGAGGAGACGGGTTCGTGTCCTGAG

GATGCAGCTGTGTGTGCTGTGGGGAATGGAAAGTGGAGCAGCCTGGGAAAATTCCTCTCC

TCACCACAGAAAGACCTGAGCGGGAAAGACCTCCGGCTCATCTACACCGACGGAGACGAA

TGCCGGAAGAACACGAGGATTAAAACTATCATCAAGTTGAAGTGCAAACCCGGTGACATG

GACAGTCCCCCCGTCATGAGGAGCGTCTCGAGCGACGGCTGCGTTTATGAGTTCGAGTGG

CAGACCGCCGCCGCCTGCGTGCTGACCAGAACAAAGGGGGACGAGTGTAAAGTGGAAAAC

TCCACCGCAGGAATTTCTTTTGACTTGACCCCGCTGAGGAAAACTGATGGCGGTTATTAC

AACATATCCAACGATAAATATGACTATTTGATTAACGTGTGTGGGAACGTTAAGGCTGCA

AACTGCCCTGAGAAATCAGGAGCCTGTCAGGTTGACAAAAGTACTAAAAGTAATTCCTGG

AACTTGGGCGAGTTTAATTCCAGTCTGTCGTATTATGATGGAATGATCGAGCTGGACTAC

CTGAACGGCTCTCGGTACAACAACGAGGAACACACACAGCGCGCCACACACATCTCCTTC

CTGTGTGACCGAGAGGCCGGGCCGGGCAAACCTGAGTTTCAGGCTGAGGATGAATATACC

TACAACTTCAGGTGGTACACTTCTTACGCCTGTCCAGAGAGACCACAGGAGTGTGTGGTT

ACTGACCCCGTCACCTTGCAGCAGTTTGACTTGTCTAGTTTGTCGAAGTTGAGCGGATCC

ACTAACTGGGAGGCCGTGGATGTGGATACCATGAAGGTTTATTATATCAACGTGTGCCGG

CCGCTTAGACCCGTGCCAGGCTGCGACCGACTCGCCTCAGTGTGCCAGATCAATTACGTG

AGCGATCAGGGGACACCGTCTGCGAAAGTGACCGTAAGTAACATGGGCGTGGCTAAAAAA

GGCCCCATGATCTTCGAGGACAATCAGTTGATGTTGGAGTACACGGACGGCTCGGCGTGT

GAGGCAGACGGGAAAATGAGCACTTACACCACCCGCATTCACTTCACCTGCTTGACAGGA

TTTGTGTCATCCGGTCCACGCTTCTTATCAGTTCAGGACTGCGTAGTGAACTTTGTGTGG

GACACCGATGTAGCCTGCGCCATTTCAACTGCTGTGGACACCAATCAGACTTGTTCTTTA

AAGCATCCCAACACCGGCTTTGAGTTCAACCTGCTTCCTCTGGCTTCCGAAAACTCATAC

AAGACAACTGCAAACGGAAAAGAGTTCATCGTGAACATCTGCTCGGCCGCACGCCAATGC

GGATCGGACAAACCGGACGCTGCGGGCTGCGTGGTGGAAGACAACAAGCCGCACAGTTTT

GTAGGAGTGGAGAGGTCCCTGCAGCTGTCCACTGATGGCTTAATTACTCTCACCTATAGA

GGAGAGCTGGATAAAAAGCTGGGCACGAGGGCCACGTTCACTATCAATTTCGTGTGCGAT

CAAAGCGCCACTAACGGCTCCCTAAAGGTCATTCGTGAGGAAATGGCCACGTCCACACAC

GTGCAGCATGACGTTCTTTTCGAGTTCTCCACAGCTCTGGCGTGCGAGCCTGCACCAGTG

GATTGTCGCGTTACTGACTTTCGTGGAAATGAGTACGACCTGAATGACCTCAGTCTAAAT

GATAAGCCATATGTCCCCTTGGACACTTCAGACCAGGCCAAATCTCAGAAGTTCTTCATC

AGTGTGTGTAAACCTTTGCCATTTGTCCAGGGCTGCCCAGTTGGAGTAATTGGCGCATGT

GGTCAGTTAAATGGCCGAGCAGTGAACCTGGGCTTTGTGCAGTCCAGCCTGCAGGCTGGC

TCAGACGGCTCCATCAGCATCGTTTATCAGAACGGCGATAAGTGTGTCTCAGGAGGGCGT

TACTCAACACGCGTTATCTTCCAGTGCGATGACAGCCCAGGCTCACCGATATTCGACCGT

AAAGACGGCTGCGAATATGTCTTCATCTGGAGGACCTCTGAGGCTTGCCCGGTTAAGAGA

GTACAAGGTACAAATTGTAAGGTAAAGGATCCCAGGAGCGGCTATGAGTTTGACCTGACT

CCACTGACTGGGAAGGATTACGAGGTGAAGAACCCACAGTATGAATATCACTTTGCTGTA

TGCGGCCCGATCAAGACGTCAGTGTGCCCCCACGGAGATGATAACATGGTCTCGTCCTGC

CAAGTGGACGGGTCCAATCACAGAATTTCTGGACTGGCGAATCAAAACTTGACCTTAGAT

GATGGCATCATCATGATCAACTACACACACGGGGAAACCTGCCACACCATTTACGAGCGT

TCGACAGCCATTATGTTTTCCTGCGACCACAGCAAGAATCCGGGGAAGCCGGTGTTTATT

AGGGAGACAGCAGACTGCACCTACTTGTTTGAATGGCACACGGCGTTGGCTTGTCCTCCT

TTTAAAACCAGCAGCTGCTCCTACAATGACGGCAGCGGGAACTCGTACGATCTGTCCTCT

TTGGCTCTGCACAGTAGTAACTGGGTGGTTCTGCCTCAGACTGGAAACAAAGACCGGCGC

TACTACATCAATGTGTGCAAGTCTCTCGTACCTCAGAGCGGTTCATGGGGCTGCCCGAGC

AGCGCAGCAGCATGTCAGAAGACCGGTGATGAGTACATGAGTCTGGGAGAAGCTCAGTCC

GCACCACAGTGGGAGGAAAAGATGCTGATACTGAAGTACATGGACGGAACGACCTGCCCG

GGAGGAGACCGCAATCGAACCACCATTATTCGCTTCAAATGCGATCGCAGCAAAGTGGAT

TCGAAGCCCACTCTGACCAGCGTACTAGAGGACTGCATGTACACCTTTGTGTGGTTTACG

GCAGCAGCGTGTCCTCTTAACAGCACAGAACACGGAGATTGCAAAGTCACTAATCCACGC

ACAGGGCATCAATTTGACCTCAACTCCCTCACCAAGGAAGCTGGATATACCGTGTACCAC

AGCGTCGATATAAGAAAGATGTATCGCCTCAACGTCTGTGGCCCTGTAAAGAACTCGGGC

TGTTCTTCAGAAGCAGGAGTGTGTATTAAAGACACTAACACTGCTGTGAGCGGAGGAACG

TCTAACAAGAAGCTGGTCTACAAGGACCAAGTGGTGGAGCTGACGTACGAGGGCGGAGAC

GTCTGCAAGGCCAATCCGGCGCTGAAACACAAGAGCATCATCAGCTTCGTCTGCAAGTCT

GAGGGAGGGGAAGAAGAAAGCTATAAACCCGTGCTGGTGGTCTCAGACGAGGACACGTGC

ACTCACTTTTTTTCCTGGCACACGCCGCTGGTCTGCGAAGAACAGTTGAGGTGTTCAGTT

TGGAACAAAACCAGCCTGATCGATCTGAGTCCTCTAATTCACCTCAGTGGATTCTACACA

GCCACGGACGAGGACACGGGTGGGACCGGAGAGGGGAACAAGGACACGTCTCGTGTCTTC

TACATAAACATCTGCCAACCACTTAATCCCATTCCAGGAGTGAAGTGTCCTCCTGGAGCA

GCAGTGTGTATGGACCCTCCTGAAGGAGACCCCATTGACATTGGACATGCCATCTCTCCT

CCCAAGTATAACTCTGCTACTGATGAGGTGGAGATTGATTTCTCCAGTACGACACCATGT

GTGGCGGATCCATCTGTCAACTACACCTCCAAAATCATCTTCACCTGCCAGAAAGACACA

GACCTGGGATCTCCCGAAATGATGCACCAGCTGGACTGCATGTACGTGTTCGAGTGGCCG

ACGCCTCTGGTGTGTCCGGAGACGGTCAGCGCGCAGGGATGCAAGCTCCGCGTCTCCCAG

CTACAGTTCACCTTCGACTTATCGGTTTTGTCAAAGGATGTTCAGGTTCCTGTTTCCTCT

GGGACGTATAAACTGAACGTGTGCGGCTCCGTGTGGGACAAAGCATGTAAAAACAGTTCG

GTGTGTTTGGTCTCCGGGTCGTCTGCGTCCTCGTTCGGCCTCAGCCGGGCCATGACTCTG

AACTACAGCCGTGAAGATCAGGCTGTCATCATGCAGTACGGAGGTGGAGACCCGTGTCCT

TCAGTGACTACAAAAGGAGATGTGTGTGTGTTTCCCTTCATCTTCTTGGGCAAATCCTAT

TTCAAGTGCACCCAAGATGGCAGGACTGATGGCAGGAAGTGGTGCTCCACCACAGCCAAC

TATGATAAAGACAAGCAGTGGGGCTTCTGTTCTAACGTGACTTCTAAGAGGCAGTCGACA

ATCCTCTTCACGTGTGACCCCTCGGTCGGCCAAGGTAAACCCCAGCTGCTCAGCGAGACG

CAGGCATGTTCGAGTAATTTCCTGTGGCCCACCGATGCAGTCTGTCCTCCAAAAAAGATG

GAGTGCAAGCTGGTGAGCCACCACCAAACCTACGACCTGCGAGCGCTCTCGTCTCTCACC

GAGCCGTGGAAGTTCACCGATAAGAACAACGCTTACTATCTAAACTTGTGCCAAGAAATC

CACGGAGGTCTAACGGGCTGTCCAGACGGAGCCGCCGTGTGTCTCAGACGCTCCAGTGGC

AAGATCGAGACCCTGGGACGAGTGTTTACTCAGAGCATGGGCTTAACAGATGGAAAAATC

CAGCTGAATTACAGCATGGGTGATGCAGTGTGTGGGAATTCCAAAGCAGCCAAAACCATT

GTGCAGCTGTCGTGCGGCAGTACCGTGGGGCGACCCAAACTTCTGAAGGAAGATACGGCA

GCCTGTGAGTTCTGGGTTGACTGGGAGACTCGAGCAGCCTGTGCAGTGAAGCAGCAAGGA

GAGCAGGTGGAGATGGTGAACGGTATCATTAAAGTTCCCCAGACAGGAGCAGAGTTCAGC

CTGGGAGATCTTTACTACAGCCTCCACAAAGCCACAGGAGACATCAGACCCAATGGAGAT

ATCTACATTTATCACATCCAGCTGTCAGGCATTACAAACAGCTCCTTATCCCAGTGCTTG

GGTGCCAACATCTGCCAGGTGAAGAAAGACGCCACACATTGCAGGAAGATCGGTTCATCC

AGCAAGGCCAAGTACTACATTAAAGGGGACAGTCTGGACGTCCTGGTTCCTTCAGAGTCG

GTGTGCGGACGCGACAAATCGAAGACCATGTCGTCCACCATCTTGTTTCATTGCAAGCCA

GAGGCTGGAGTGGGCATCCCAGAGTTCCTGCTGGAGACCGACGGGTGTCAGTACCTGTTC

GTCTGGCACACCGATAAAATCTGTGAACTTTTCACTATGACTCAGCAGAGTGACACCGCT

GAGGTTGGGTTATCAGGGCGGAGTCAGGCTGTGGGCATGGTGCTTACCCTTCTGCTGCTG

GTGCTGTTCATTTGTCTGCTCATTCTGCTGCTTCACAAGCGAGAAAGGAGGGGGCAATTT

ATGCAGAAACTCACCAGCTGCTGCAAACGCAAAACTCCGGTGTCTTATAAATACTCCAAG

GTACTGATAATTATTTCTTTTGGTTCATTCATCTATTTTTTAGATAAAGATAAAGATCAC

AAGCAAGTATTTATGGGATACGGTAAGAGAAGCGGTGGTTCAAATGGTCAAGGTGCTAAG

TTGTTAATTGGAAGACCAGGAAGTCCGGGAAGGTTCACTGTTGGGCTCTTAGGTATGACG

ATAAACCCTTCCTACACCATGGGGGCGCTATCATACCTGACCCTGCACTCTGACCCCC

>Unigene000196

AAAAGGAGGGCAAACAGAGAACACGCAGAAAGTATGGGGTTCAGTATATCCTGGACTCTA

TTCGTACACACTACAGTGTGGAGAAGAATGGCAGTGCTCTATCGGATGAGAAGAAAACAG

TACAGATATCACTCTTAGAGCTACTGAAAGACTTCCTGAAGTCTCCCACTACCAAGGATC

TGCACAGTATTCTGGCTTATACTGTCGTGGTGCAGGACCAGCAGCAGGCGATCATGCTGC

TGGATATTTTGTATACAGTGCTAAAAAACAATCCTCAGGCTCTGTCTGTGCCGCTGGAAT

GGGGGGTGGAGCAGTTTCTCTGTCTGCTGCTGAAGCCCAGCTATAACGATGAAGCCCGTG

AAAAAGTCTTTAGGATCATGTACAAACTGCTAAAGAGCGAGCGAGTCCCAGAACGTAGCA

AACAGCGGGTCAAGCTGCGAGAGACAACCTACATGGGCCTGGTCTGCTTCTTGGAGGAGG

TCCCAGTCACAATGACCATCATCCGCTGTCTGTGTGAGCAGGTGCTGGCTACAGATCCCT

CACCTAACTTCAGAGACCTCTTGTCAGTTGTGTATTTGTCCCATCGGGCTGATCTCTCTG

TCCGACTGGACATATGTCGCAAGCTTTTCAGTCTGATTGACTCCAATGAAGACTGCGTTA

AGCAGCTGGCCCGGCAGCCAGGCTGGCAGGACATTCTCATAAAACTCTATGTCAAAGAGT

CCTTTGAGTCTCATAAGACCAGTGTATCCAGCCCTTATCCCTCCCTGGAGCCTATTCCCA

CCAGAACTCTGCTCCGAAAGGATGACAGCATAGATGGTCCTCGCTCTGACCCCTTCAATC

CCTATAGCTCCCAGCGTGAGGAAGCAGAAGAGGATGAATGTGATGAAGAGGAAGAAGCAT

CTCGTGACATTTCAGAGCGTTTTATTGGATTGTCTCAGTCGCCGCCTGCAAGAGGTCAGC

TGAAGGGCTTTAGTGACTCGATGAACTTCAAGTCTTTCGACTCGGTGGAGCAAAGCAGCC

GCTCGTCATCCCTGTCCAACGCCGTGGACGTGCCATCCACACAGCTGTTGGAGGAAGAAG

AAGAGAGTCTATATAATCCACTCTCACCCTTAGGCATGTCACCTGATCTAGAGCTGGGAG

GGCAGAGAGCATCTCTGACTCCAGACACGCCGCCTTCTACAGAACATAACAAACTTTTTC

TTGGCCCACGAGCTCGCAAGAGTTCCAGCTTGTCTAATGTGTTGGATGAAACCAGTTACT

GCACAGAGCCTCCAACCGCCGACACCATCTCTAACAACTCCAACCCACAGCAGGCCCCAG

AGGAGGAGTTGTGCAACTTGCTGACTTACATTGTGTTCCGAGTGCTGTGGTCTGGCACAG

AAGGAGCAGAAGATGCAGTGTGGCGGGAGAGAGGGCAAGTTTTCTCGGCCCTCACCAAAC

TGGGATCTTCCTGGCAGCTTGTGCGTCACCCGGATGATATCAAACGCAGTCTTCTGGAGA

TGATGCTGGAGTCGTCTCTTTCGGATCTGCGGGACTCTCAGGGCTTGTCATTGCCTCATA

TTCCCTCCTTACTTCGTCTCCTCAGACTGCTACACGATTTCCTGTTTGCAGAAGGCACTG

ACAATCAAACACTGTGGAGTGAAAAGATCTTTGAAGGAGTGGTGAATTTGCTCGACAGGC

TGCAGGCGTGGCACACCACCTCTGCAACAGCCGGCGCAACAGAGCTCAAACGGATGGCTC

AGATTGGCTTGTGGATCATAACAGGATACATACAGCAGCAGAACTCGCAGGTGTGTGAGA

TGGCGTGTGTAAAGCTGCACAGTCTGCTGCAGACGGTCTTGTGTCTCTCATGGGAGGAGG

TATACTTTCTGTTGGGCCGCCTGGGAGCCACACTGTGGCCTGCAGAAGGAATGTCTTTGA

CGAGCCCAGCTGGCACAGAGGCAGTCTTGAGGCCTATGGTACCAGTAGTGCGCACACTCC

TAGACCAGCACGCTGACCCTACCAAACTGCAGCAGTCTCTACCCAACCTGCCCCCCACCA

ATGGCAGTGCCACCTTTGCCCAGGACCTACTGTCGTACTGCAACACAGTCGAGTGGCAGC

TTTTCTACTGCAATCATGTGAGACCCACCATGCAGCAGTATGAGCAGGACACTTTTGGGA

AAAGCCACGATTTACTCTCCAACTTTTGGAACTCGTGTTTCGATGATCTGATGAGCACCG

CTGAAAAGAAGAACAAGGACAAAGCAGAATTCAAAGCCAAGTTTCAGAGTTGATTGTGGA

CCCATATTTGAAGCGTGTACGCGTTGAGAACAGTCGCTATGTGAGTGGACAGAAGCTGAA

TAACAGCCAGCAGGAGGTGGTGTGGAAGCACTGGAAATGCCAGCGCAGACTGATGACCAG

TGAACGAGCAACTTGGGCAGACAGGGATCAGCCAGAAATGAAGTGGAAGCTGTCCAGCGC

AGAAACATACTCCAAGATGCGTTTGAAGCTCGTGCCCAACTACAACTTCGACTCCCACAG

CGAGGCCAGTGCACTCCGGGATAACATGGAGAGCCCTCGCAATTCCACCGAGCCCCTCCC

ACTGGCTGTAGCTAAGGAAGCTAAAGTGAGTGACATGGAAGATGACCAACTGGAGGAAGA

AGACCTTCTCGCCATGGACAGCCAAGCAGAGGAAGTGGAGGACGAGAGCCAAAAGGAGAA

GCTGGTACTCTCTGAGGACTGTGAGCTCATCACTATAGAAGCTGTGGTCATGGGCCGGCT

AGAAGTTACTACACATCACATTTACTTCTATGATGCCAGCAGTGAGAAGGAGGAGACAGA

GGAAGGGATCGGCTTCGATTTCAAGCGGCCACTGAGTCAGCTGAGAGAAGTTCATCTCAG

ACGCTATAACCTGCGCCGCTCGGCCCTGGAGCTCTTCTTCATTGATCAGGCTCACTACTT

TATCAACTTTAAGAAGAAGGTGCGGAACAAAGTCTACTCCAGGATTCTCGGTCTTCGACC

ACCTAATTTGTTTTATTTTGGTTCCCGCTCTCCTCAAGAACTCCTCAAAGCCTCCAATCT

TACACAGAAATGGGTGTGCAGAGAGATCTCAAACTTTGAGTATCTTATGCAGCTCAACAC

CATCTCAGGTCGCACTTACAACGATCTGTCGCAGTACCCTGTGTTCCCTTGGGTGCTGTG

TGACTACACATCTAATGTATTGGATTTGGAAGACCCGTCTGTTTTCAGAGATCTGTCTAA

GCCTATCGGAGTGGTAACCCCCCGCCATGCCCAGGATGTCCGGGAAAAGTACGAGAGCTT

TGAAGACCCCACAGGCACCGTTGATAAGTTCCACTACGGCACACACTACTCCAACGCGGC

AGGCGTCATGCACTACATGATCCGCATGGAGCCTTTCACCAGGCTGCACATCCAGCTGCA

GAGCGGCAAGTTCGACTGTGCAGACAGACAGTTCCACTCAGTAGCAGCAGCATGGCAGGC

TCGTATGGAGAGCCCAGCTGATGTGAAAGAGCTCATCCCTGAGTTCTTTTACTTCCCAGA

ATTCCTGCAGAATATCAACGAATTTAATTTAGGCTGTCTTCAGGTCAATCAGGAGCAGGT

GAATAATGTGGTGCTGCCACGCTGGGCTTCTTCCAGAGAAGATTTTATTAGAAAACACCG

CCAGGCACTGGAGAGTGAGTATGTGTCTGCTCACCTACATGAATGGATCGATCTAATATT

TGGCTATAAACAGCGCGGGCCAGAGGCTGTGCAGGCACTCAATGTGTTTTATTACTGCAC

ATATGAGGGTGCTGTGGATCTGGATGCCATAGCCAATGAGACTGAACGTAAGGCTCTGGA

GGGCATCATCAGTAACTTTGGCCAAACACCCTGTCAGCTTCTCAAGGTAAAACCTCAGAA

ACACATTATATCAACCACTTTATCCGTTTTAGCGCATAAATCATGCCTAGGAATGTTTTT

AAACTATGTCCAAGCTTGTTTATGCTGTTTATGGTTGTGCCCCAGGAGCCGCATCCTCCC

CGTATGTTAGCACAGAGCGCGTGCAGGCGGCAGGCACGTATCAATACTCTGCCTCTCAAC

CTCTTCGACCAGCTGGACAAACTGAGACCCTTCGTTGAGGTGGTAAATGATGGTGTGCCT

CTAGTGCAGGCTGTGGTGCCCAGAAATCAGACACACTCCTTCATCATTCCAGGGTCTGAT

GTGCTGGTGACTGTGAGTGCTAAAGGACAGATAGGGACACACAGCTGGCTGCCCTATGAC

AAGAACATTGCCAATTATTTTACTTTTACTAAAGACCCCACTATCAACAATCCAAAAACA

CAACGGTTCCTGAGCGGCCCCTTCTCCCCAGGGGTGGACATGGGGGCCCAGGTGCTGGTG

GTGTCCAATGACGCTCGCTTGCTGTTTAGTGGGGGACACTGGGACTGCAGCCTACGCGTC

ACCACGCTGGCCAAAGCTAAACTAGTGGGCAGAATCTGCCGTCACATAGATGTGGTAACG

TGTCTGGCTTTGGATCTATGTGGTATCTACCTCATCTCAGGCTCTCGTGACACCACTTGC

ATGGTGTGGCAGGTTCTCCAGCAGGGTGGTTTCTCCAGTGGTCTGTCCCCCCGACCAGTA

CAGGTGCTATGTGGACATGATGCAGAGGTTACCTGTGTCTCCATTAGCACTGAGTTGGAC

ATGGCTGTTTCAGGATCAAAAGACGGGACAGTGATTGTACACAGCATCCGGCGAGGTCAG

TATCTCCGTACGCTGCGTCCTCCCAGTGAGGGCTGCGTTTCTGTACGGGTGAACCAGCTG

CAGGTGGGAATGGAGGGTCACATCGTTGCACAAAGCGTCCTAGAGGGCTGTTCTGCCAGA

AAGGAAAAGTGTGTGCTGCATGTGTACTCTGTGAACGGCACACTGTTGGCGTCCACGGTG

ATGGAGGAGCCTGTGTCAGCACTCTATATGGTTTCAGACTACCTCATTCTGGGCACACTA

CAGGGCAACCTGCACATCAAAGACCTGTTCAGCTTAGAGCAGGCTGCTAAGCCCTTGGCG

CTGATGCTCCCTATTCGCATTGTATCAGTCACCAAGGAGAACAGTCACATTCTGGTGGGA

TTGGAAGACGGGAAACTCATTGTTGTGGGAGCTGGAAAACCAGAGGAGTTGCGTTCAGGT

CAGTTCCCACGGCGTCTCTGGGGATCAACACGCCGAATCTCTCAGGTGTCTGCAGGGGAG

ACTGAGTACAACCCTACAGAGAATGCTGGGAAATAAGCATCATACACACTCCACATGCCT

TTATATAACAGGCCTCTTCACATACACATACATACATGTACACGAACATGACCTGAGATG

TTGACCCGGAACACAGGGTGACTTAATGTACTGTGGACTGTTTCTCTGGTTTAATGGAAA

AACACAGAGTCGTCCAAAGCTGCACTTTTTTTACTTCTTCTTTTTTAAAGTTTCTATTTT

AGACCGTTTTTAAGCTGAAATCATTCCAAAAATTCCAAACATGAACTGGGATGTGAGAGT

GGAAGTCAATCTCAGAGTCAGAACTAAATGTGGATAACACACACGCCTGATGCTGAGAGC

TGCTGGCTGAAGTGTGTCGTCATGGTCGACGCTCGCTTTTGATCTGCCGTGCACAACACA

ACTTGACTAAAAAACACACACTGGAACAACAAGAGTTCTATTTTTAAAGTGTTTGGGTGA

ACTTGTAGACGTGAAGAAAAAACATGGGAAAAAAAAGAGAG

>Unigene000197

AAAGCCCGTGCGAGCTGGGAAGGAGCTTTAGATGGCGGATGAGCCTTGGGAAGGGTAACC

CGTGGAGCTTGTCGACAGCACTTCGCCAACTTTAAACTTTTGCGCTCGCGCTCTGGATCA

GAGGAGTCAAATCATTCAGGCTCAGAGTTGGCCAGCCAGTCTGAGAGTGAATGTGGCAGT

GAACATGAACAGTCCCAGCATTCCGAGTCAAACAACACGTCCGAGTCTGAAACCTGTTTT

GAATCTGGCAGCGAATCAGCTGGGTCAAAGTCACGCCAGACTGTTGCAGAGATTAAAGAG

AAACGGGTCAGGAAGAGAGATCTGGCCGATGTAAAAAAGATGTGGGAGGAACATCCAGAT

ATTTACGGTGTCAGAAGGTCCAATCGGAGCAGACAGGAGCCTGCTCGTCTGAACGTTGGG

GCAGAGGTCAGCAGTGAGTCTGGGAGTGAAAGCCACAAAAGAAAAAATTCACGACAGAAA

AAAAAAGAAAATACCTGGAAAGATGACTGTCACGATGAAGACGACTGTGAGGAAGAAGTT

AGCAGTTCAGAGATTGAACAGGAAGGGGAAAAAAATAGATCCAGACGACTTCCTGCTAGA

AGGCCACAGGCCAAATTCTACACAACCAAGCAGAATCTGTCTCAAAAGGGGCGGAAACTA

AGGATGCAGGAAACATCAGATGAAGATGATGAGGACGACATTGTTGAGGAAGAGAATACC

CCAAAAAGACAAACCCGACGAAGAGCAGCTACTAAAGTTAGTTATAAGGAGGACCAGCAT

GATTTTGAAACAGACTCTGATGACCTGATTGAGATGGCAGAGGGAACAGAGGATGTACGA

GATGATGACAGTGAAACCATTGAGAAAGTCTTGGATACCAGGATTGGTAAAAAAGGAGCT

ACAGGAGCAGCCACCACAGTCTATGCTGTAGAGGCCAACGGAGACCCTATGGCTGATTTT

TCACCTGAGAAGGAGGGAGGTGAGACTCAGTTTCTTATCAAATGGAAGGGCTGGTCCTAC

ATCCACAACACATGGGAAAGTTTGGACTCCCTTTCTCAGCAAAAGGCTAAAGGGATGAAA

AAAATGGAAAATTTTAAGAAGAAAAATGAAGAGCTCAGTGCATGGTTAGAGAAAGCGTCA

CCTGAGGATTTGGAATATTACAACTGCCAGCAGGAGTTAACCAATGATCTAAACAAGCAG

TTTCAGATAATAGAGAGAGTTATTGCAATGAAAACAGGGAAGGCACAGGTACCCTCTGAC

TTTCCATCACATAAAACCATGTCTGATGAACCTGAGTACTTATGCAAGTGGATGGGTCTG

CCCTATGCAGAGTGCACTTGGGAAGATGGTGCCTTAATAGGTAAAAAGTTCCAGCATTGC

ATAGATTGTTTTAACAACAGGAATGTTTCCAAGACAATTCCCTCCAAGGACTGTAAGGTT

TTAAAGCAAAGGCCAAGGTTTGTGCCTCTAAAAAAGCAGCCATCTTATATTGGAGATGAG

AACCTGGAGCTGAGAGACTACCAGTTAGATGGGCTAAACTGGCTTGTGCACTCTTGGTGC

AGGTGTAACAGTGTTATCTTAGCTGATGAGATGGGCTTAGGGAAGACCATCCAGACCATT

TCATTCCTTTCCTACCTGTTCCATCAACACCAGCTATATGGACCCTTTCTCTTGGTTGTG

CCCCTTTCCACCCTCACCTCCTGGCAGCGCGAGTTCAGTACTTGGGCTCCTGACATGAAC

GTTGTGGTCTATCTGGGAGATGTTTTGAGCAGAAAAACGATTCGTGATTATGAATGGATT

CACCAACAAACCAAAAGAATAAAGTTTAACACACTCTTGACCACATATGAAATTTTACTG

AAGGATAAGGGTGTTTTGGGAAACATAAACTGGGCATTTCTTGGGGTGGATGAGGCTCAC

CGCTTAAAGAATGATGACTCTTTGCTGTATAAAACACTAATAGATTTCAGGTCCAACCAC

AGGCTGCTCATAACAGGAACACCACTGCAAAATTCACTGAAAGAGTTATGGTCACTCCTG

CACTTTCTTATGCCTGACAAGTTTGAATTGTGGGAGGATTTTGAAGAAGAGCATAGTATG

GGCAGAGACAATGGCTACCAGAGCCTGCACAAAGTGCTTGAGCCCTTTCTTTTACGCCGT

GTAAAAAAAGACGTAGAAAAATCGCTGCCTGCCAAAGTGGAGCAGATCCTCCGAGTGGAC

ATGTCCGCACAGCAGAAACAGTTCTACAAGTGGATTCTTACAAGGAATTACAAAGCACTA

TCTAAAGGCACCAAAGGCAGTTCCACAGGCTTCCTCAACATTGTTATGGAGCTAAAGAAA

TGCTGTAATCATGGGTTCCTTATCAAACAGCCAGATGAGGCAGAAAATGAGAATTCTCAG

GAGCACCTGCAGGCAGTGATCCGTGGCAGTGGGAAGCTTGTGCTCTTGGATAAATTGCTA

ACAAGACTAAAAGAACGAGGCAGCCGTGTGCTAATCTTCTCTCAGATGGTCCGTATGCTG

GACATCTTGGCTGATTATCTTGCCATGAAACGTTACCCTTTCCAGAGATTGGATGGCTCA

ATCAAGGGAGAACTCCGGAAGCAAGCGCTCGACCACTTCAATGCAGAGGGTTCTGAAGTA

TCTACCATAACTATATATCATGGCATCACACAGTTTCTGTAACTGTACTGGTTAAGCAGT

ATTTCTGCACTACGTAAACTTTTGATTTATACTACAAAAGTGTAGTATAAATTAGTGATG

AAATTAATCAAATGACCTTGTAGTTTTATATAATCCGGTTTTTACTTGTTCCCTTTTGTC

TGCCCCAGGATTTCTGTTTCCTTTTGTCCACTCGTGCTGGAGGTCTGGGCATTAACTTGG

CCTCAGCTGACACTGTGGTCATCTTTGACTCTGACTGGAACCCTCATAATGACATTCAGG

CCTTCAGTCGAGCCCATAGAATTGGTCAGGCAAATAAGGTGATGATCTATCGATTTGTCA

CCCGTGCTTCTGTTGAGGAACGGATTACCCAGGTAGCCAAACGTAAGATGATGCTGACTC

ACCTGGTGGTGCGACCAGGACTGGGCTCCAAAGCTGGCTCCATGTCCAAACAGGAGCTGG

ATGATATCCTAAAGTTTGGCACAGAGGAGCTATTTAAAGATGAAACTGAAGCTGGAGAAA

ATAAGGATGAGGACAGCAGCGTAATTCACTATGATAATGCTGCTATTGAACGTTTGCTGG

ATCGGAGTCAGGATGCCACTGATGACACGGACATGCAGAACATGAACGAGTACCTTAGCT

CTTTTAAAGTAGCCCAGTACACAGTTCGAGAGGATGACAAGATTGAGGAGATTGAGCGAG

AGATCATTAAGCAGGAGGAGAACGTAGACCCAGATTACTGGGAGAAGCTTCTCCGGCACC

ATTATGAGCAGCAGCAGGAAGATCTGGCACGCAACCTGGGCAAAGGCAAGAGAGTCCGCA

AACAGGTTAACTACAACGACGCTGCACAGGAGGATCAAGAATGGCATGCCGATATCTCCG

ACAACCAGTCTGAATACTCTGTGGGATCTGAGGAGGAGGATGAGGACTTTGATGAGAGGC

CAGAAGGTCGAAGGCAGTCTCGGAGGCAGATCAGAAATGAAAAGGATAAACCTCTTCCTC

CCTTGTTGGCTCGAGTGGGTGGCAACCTTGAGGTATTGGGTTTTAATACACGTCAGAGGA

AAGCCTTCCTAAACGCAGTGATGAGATGGGGAATGCCTTCTCAGGATGCTTTCACCTGTC

AGTGGCTGGTACGAGACCTGCGGGGCAAAACCGAGAAAGAGTTCAAGGCATATGTTTCTC

TCTTCATGAGGCACTTGTGTGAGCCTGTAGCTGATGGCTCGGAGACATTTGCTGATGGCG

TGCCGAGAGAGGGTCTGTGCAGACAACCTGTGCTGACCCGCATCGGAGTCATGAGCCTGG

TGAAAAAAAAGGTTCAGGAATTTGAACACATTAATGGAAAATGGAGTATGCCTGAGCTAA

AGCCTGAGGTCACTGGGGACCCCAAAAAACCCTCAAGAGCTTCTTCCCCTGCCATTAAAA

CAGCTACACCCACTCCAGATCAGAGCTGCAACAACACACCCTGCACCTCAAAGCCAGCCA

CACCCTCTCCACCTGAGAAACCAGAAAAGATTAAAAAGGAGGGAGAACGAGAAGAGGGAG

AGAGCTTAACTGATGTAGAAAAGGAGAAAGGGAGAGAGGAGAAAGAAGGTGAAAACACAG

ACAGTAAAGAAAATAAAGAGGAGCCTGAAAAAGATACAACATCCAATGAAAAGCCCAGAC

CATCTGAAGGGCTCACAGGAGGAAGTGCAGGAGACTCCAAATCAAAAGAAGCTTCTGACA

CCTTTCCACCCTCACCAAAGGCAAGGTGTTTAAAAGAAAAGGGTGGAGAGGAGGAGAGCC

TGAAGAAAGAAGCTAGTACTGATAAAGTAAAGGAAGAATGTTCATCGGAGCTTTCTACAG

TCAAAGAAGAGAAGCAAGCAACAGAGAGAGAGGAAAAAAGTGAAAATGGAAGAACAGGTG

AAAAAGGCATGGAAGAGATAGAAAAAAACGAAGAGGCACCTGTTCCTGCAGAATCAGTGG

AGAAGAAGGAGAAAGCAGAAGGCCAAGTGCAGGACGTTAAGAACGATGAAGGCAAGATGG

AAAAGGATGCTGGGAAAGAGGTCAAAAGCCAGAAGGAGGAGGCGACCAAGGGGAATGGCA

AACCAGCAGAGCGTCCGCGGTTCATGTTCAACATAGCAGATGGAGGATTTACAGAGTTGC

ACACACTCTGGCAGAACGAAGAGCGTGCTGCAATCTCATCTGGCAAGATGAATGAGATCT

GGCATCGGCGGCATGACTTCTGGCTGCTGGCTGGCATTGTACTTCATGGCTATGCTAGAT

GGCAGGACATTCAGAACGACCCCCAGTTTGCTATCGTCAATGAGCCGTTCAAGTCTCAAG

CCAACAAGGGCAACTTCCTGGAGATGAAAAACAAGTTCCTGGCCAGGAGGTTTAAGCTGT

TGGAGCAGGCGTTGGTGATTGAGGAGCAGTTGCGTAGAGCTGCGTATCTGAACATGACTG

AGGACCCGTCTCACCCCTCCATGACTCTAAACACACGATTCAGTCAGGTGGAGTGTTTAG

CCGAGTCCCACCAACACCTGAGCAAAGAGAGCATGATGGGTAATAAACCTGCCAATGCTG

TACTGCACAAAGTGCTGAAACAGTTGGAGGAGTTGCTCAGCGACATGAAGGCTGATGTCA

CGCGTCTCCCCGCCACCATCGCTCGAATCCCTCCGGTTGCTGTGCGTCTGCAGATGTCAG

AGCGCAGCATTCTCAGCCGTCTGGCCAGTCGGGGGTCAGAGGTCAGCCAGACTCAAACAC

CCCACTGACCCCTCAGGGTAACCTGCTGACCCCTGAGTGACCTTCCCTGCTGTAGAGGCT

GTGTTCTAATCCAGATTCACTTCACTAGCTCACTCCCTTTGCGCTGGACTGAGTGACTCA

GACTCGATGCTACATTCACACCATCGAGTGACAATGTGACCTTTGACCTTGACTCTAGAT

TAGGCTGAAGGAGATAAAAGGAGACAGAGGGCGGGAACGAGGTTTTTACTCTGTATCTCT

GTACTTTATATCCAACTTCCTTAGTGGTAAAGCGGCGGTGTGATAGCTTTAGTGAGAGTG

ACAGACTGAAATAGTGCAGCGTGTGTGTGTGTGTGTGT

>Unigene000198

GGCGGTTCCAGGCTGCGCTTAAAGCATACACGGGATCGGTGTGGGGGAGAGCGAGGGGAA

AACGGAACAGGCTGAGCGGCTCTTTGTTTCTAGCTAAGATAGCTAACTGAAGAAAAACTG

GGGGACTTACCGGCGAAAACTGACTTCACAATAACTATACATTTTTATCTTTAATTCCTC

CCGGCTTTGCTGAGAGATGTCCACTCCAGACCCCCCCATGGGAGGGACCCCTCGGCCCGG

CCCTTCTCCTGGCCCTGGTCCATCTCCAGGAGCCATGCTAGGTCCAAGCCCAGGACCTTC

ACCTGGCTCTGCTCATGGAATGATGGGTCCCAGCCCCGGACCACCACCTACAGGACACAA

CCTACCTCCACAGGGGCCCTCAGGATACTCGCAGGAAAATATGCATCAGATGCACAAGCC

TATTGAGGGCATGCATGAGAAGGGCATGCCAGATGACCCTCGCTATGGCCAGATGAAGAG

CATGGGTATGAGACAGGGTGGACATAGTGGCATGGGACCACCACCCAGCCCCATGGACCA

ACATTCTCAAGGTTATCCCTCTCCTCTTGGTGGCTCTGAACATGCTCCCAGTCCTGTGCC

TGCTAATGGACCGCCATCTGGCCCGATGATGCCGTCTGGCCCTGTTGGTGTGCCAATGGA

GGGCGGTGACCCACAAGTTATGGGCCAGCAAAATCGAGGAGGGGGAGCTCCACCAGGTCC

AGGCACAGGTCCCAATGGTGGCCCAGCAGGTCCTAGTGGCCCAGGTGGCACTGGAGGACC

GACCCCATTCAACCAGAACCAGTTGCACCAGCTCCGGGCACAGATCATGGCATACAAGAT

GCTGGCTCGTGGACAGCCACTGCCTGACCACCTGCAGATTGCTGTGCAGGGCAAACGGCC

AATGCCAGGAATGCAGCCAGGAATGCCCAATATGCCCCCTGCTTCAGGACCTGCACCTGG

CCCTGCTGGACCTGGAGGCCCTGGCCCCGCAGGACCTAATTACAACAGACCACATGGAAT

GGTAGGACCGAATATGCCTCCTTCAGGACCATCTGGAGTTCCCCCAGGTATGCCGGGACA

GCCCCCTAATGGACCACCCAAACCATGGCCTGAAGGGCCCATGGTAAATGCTGCTGCTCC

AGCTAGCGCACCTCAGAAGTTAATCCCACCTCAACCTACCGGTCGCCCCTCTCCTGCTCC

ACCTTCTGTGCCCCCAGCTGCATCACCAGTCATCCCCCCTCAGACCCAGTCTCCGGGGCA

ACCAGCCCAACCACCTCCCATGATGCTACATCCAAAACAGAACCGCATCACACCTATTCA

GAAACCACGTGGCCTTGATCCTGTAGAGATTCTACAGGAACGGGAATACAGATTGCAAGC

TCGTATAACCCACCGTATCCAAGAACTGGAAAGTCTTCCTGGCTCTCTAGCTGGAGACCT

GCGGACCAAAGCCACGATTGAGCTAAAGGCTCTGAGGCTTCTTAACTTTCAGAGACAGCT

GCGTCAGGAGGTGGTGGTGTGTATGAGGCGTGACACAGCACTCGAGACAGCACTCAATGG

CAAAGCCTACAAGCGCAGCAAGAGACAGTCCCTGCGCGAGGCGCGTATAACAGAGAAACT

TGAGAAGCAGCAGAAGATCGAGCAAGAGCGCAAACGTCGTCAGAAGCACCAGGAATATCT

CAACAGTATTCTCCAGCATGCTAAAGATTTCAAGGAGTACCATCGCTCCATTACAGGAAA

AAACCAGAAAATTACCAAAGCTGTGGCCACATACCATGCCAACACCGAGCGTGAGCAGAA

AAAAGAGAATGAACGCATTGAGAAGGAGAGAATGCGGAGGCTGATGGCCGAAGATGAGGA

AGGTTACAGGAAGCTGATTGATCAGAAGAAAGACAAACGTCTGGCCTACCTGCTACAGCA

GACAGACGAGTATGTGGCCAACCTCACAGAGCTCGTGCGTGCTCATAAAGCTGTTCAGGC

CCTAAAGGAGAAAAAGAAGAAGAAGAAAAAGAAGACTGCAGACAACGGTGAGGCTGGAGC

TCCAGCACTGGGCCCAGATGGAGAGCCTTTGGATGAGACCAGTCAGATGAGTGACCTGCC

TGTAAAAGTTATTCATGTGGACAGTGGCAAGATTCTAACTGGTGTGGATGCTCCTAAAGC

AAACCAGCTAGAGGCCTGGCTGGAGATGAACCCAGGATATGAAGTGGCACCACGGTCAGA

TAGTGAAGACAGTGGCTCAGATGATGATGAGGAGGAGGATGATGATCAGCCCCAACCATC

CCAGTCCTCTTCAGAAGAGAAGAAGAAAATTCCAGATCCTGACAGTGAGGATGTATCTGA

GGTTGATGCCCGTCACATTATTGAGCATGCCAGGCAGGATGTAGATGATGAGTATGGTCA

TGCTCAGTTTGCTGTGGGTTTGCAGTCCTATTACGCTGTGGCACATGCTGTCAATGAGAA

AGTGGAAAGACAGTCATCTCTCATGGTTAATGGGACACTTAAACAGTACCAGATTAAAGG

ATTGGAGTGGCTTGTGTCCCTGTATAATAATAATCTTAATGGCATTCTTGCGGACGAGAT

GGGCTTAGGAAAGACTATCCAGACCATTGCACTTATTACCTACCTCATGGAGTACAAACG

TCTCAATGGACCTTTTCTCATTATCGTACCTCTCTCAACTTTGTCCAACTGGGTATACGA

GTTTGATAAATGGGCTCCATCTGTGGTGAAAGTCTCCTATAAGGGCTCTCCTGCTGCTCG

CCGGGCTTTTCTGCCCATTCTACGCAGTGGCAAGTTTAATGTGCTTGTAACTACATACGA

GTATATTATCAAAGATAAGCAAGTGCTGGCCAAGCTTCGCTGGAAGTACATGATTGTTGA

TGAGGGTCACCGTATGAAGAATCATCACTGTAAGCTGACTCAGGTTCTAAACACGCACTA

CCTGGCCCCCAGGCGCGTTCTACTCACCGGGACACCCCTGCAGAATAAACTGCCTGAGCT

ATGGGCCCTGCTTAACTTCCTGCTGCCCACAATTTTCAAAAGCTGCAGCACCTTTGAGCA

GTGGTTCAATGCACCTTTTGCTATGACTGGTGAAAAGGTTGACCTCAATGAGGAAGAGAC

CATTCTAATCATTCGGCGTCTGCACAAAGTGCTCCGCCCCTTTTTGCTCAGGAGGCTCAA

GAAAGAAGTGGAGGCCCAGCTGCCCGAGAAGGTTGAGTATGTGATAAAGTGTGATATGTC

AGCACTGCAGCGAGTACTGTACAGGCACATGCAAGCTAAAGGAGTTCTGCTTACTGATGG

CTCAGAGAAAGACAAGAAGGGTAAAGGTGGAACTAAGACACTGATGAACACAATTATGCA

GCTGCGGAAGATCTGCAACCACCCATACATGTTTCAGCATATTGAAGAGTCCTTCTCTGA

GCACCTGGGCTTTACTGGAGGCATTGTTCAAGGGCTGGACTTGTATCGAGCGTCTGGAAA

GTTTGAGCTTCTGGACCGCATTCTTCCTAAGCTACGTGCAACTAACCACAAAGTGCTGCT

CTTCTGTCAAATGACTTCACTCATGACCATTATGGAGGATTATTTTGCCTACCGTAATTT

TAAGTACTTGCGTCTGGATGGAACCACTAAAGCTGAGGACCGTGGCATGCTGCTTAAGAA

CTTTAATGACCCTGCCTCACAATACTTTGTGTTCCTGCTGAGTACCAGAGCTGGTGGCCT

GGGGCTCAACCTGCAATCTGCTGACACCGTCATCATTTTTGATAGTGACTGGAATCCTCA

TCAGGACTTGCAAGCTCAGGATCGTGCTCATCGCATTGGGCAGCAAAACGAGGTTCGTGT

TCTGCGTCTGTGCACTGTAAACAGCGTGGAAGAAAAGATCTTGGCCGCCGCCAAGTACAA

ATTAAACGTGGACCAAAAGGTGATCCAGGCTGGCATGTTTGACCAGAAGTCTTCCAGCCA

TGAGCGCAGAGCCTTTTTGCAGGCCATCCTGGAGCATGAGGAACAGGATGAGGAGGAGGA

TGAGGTCCCCGATGATGAGACGGTCAATCAAATGATTGCCAGAAGCGAGGAGGAGTTTGA

TCAGTTTATGCGCATGGATCTAGACCGGCGCCGTGAGGAGGCCCGGAACCCCAAGCGCAG

ACCTCGTCTGATGGAGGAAGATGAGCTCCCCACCTGGATTATGAAGGATGATGCAGAGGT

AGAAAGACTTACTTGTGAGGAAGAAGAGGAGAAGATGTTTGGCAGAGGATCGAGGCAGAG

AAAAGAGGTGGACTACAGTGACTCGCTTACTGAGAAACAGTGGCTCAAGGCTATAGAGGA

AGGTACCCTGGAGGAAATTGAAGAGGAAGTACGGCACAAGAAGACAACCCGAAAAAGGAA

GCGAGACCGTGATCTAGACCTCCCTGGCCCAGCCATGCCCAGCTCCAGTGGCCGAAGCAG

AGAGAAAGACGATGACAGTAAAAAGCAAAAGAAGAGAGGCCGCCCCCCTGCTGAGAAACT

CTCTCCCAATCCACCATCCCTCACCAAGAAGATGAAGAAAATTGTTGATGCAGTCATCAA

GTATAAGGACAACAGTGGCCGGCAGCTGAGTGAGGTCTTCATCCAACTTCCTTCTCGTAA

GGAGCTGCCTGAATATTACGAGCTTATTCGCAAACCTGTGGACTTCCGCAAGATCAAGGA

ACGCATAAGAAGCCATAAATACCGAAGTCTAAATGACTTGGAGAAAGATGTTATGCTGCT

TTGCCAGAACGCTCAGACCTTCAACTTGGAGGGGTCGCTGATCTACGAGGACTCCATCGT

GCTTCAGTCTGTGTTCACAAGTGTTCGGCAGAAGATTGAAAAGGAGGAGGACACTGAGGG

AGAGGAGAGTGAGGAAGAGGAAGAGGAGGCAGATGAAGGCTCAGAGTCTGAATCACGCTC

TGTAAAAGTGAAGATTAAGATTAGCCGTAAAGAGAAAGCACTCGAGAAAGGGAAAGGCAG

GCGGCGCACTGGACGAGGATCACGTCCTAAACCGGTAGTAAGCGATGATGACACTGAAGA

GGAGCAGGAAGAGGAGCACTCTGCCAGTGGAAGTGAGGAAGATTAAGTTCTCAACTGGAC

CACACGATTTTATTTTTTCTGATCTAGACATCATTTTTCACTTAAGCTTTACACGAATCC

ATGTAAAAGGCCTGGTTAGACCTTAAATGAATTTTCAAATGAATTAAAACCTAAACAATT

AACTAAAACATTGTGCAGGTTTGTGCCGATGTTTTAATGGAGGGTATACATCATGCAGTG

CAGCAGTGAACGTTCTGTCTCAGGAGATTTTAATCCAGTTTCAAAAACACTTTCATATTT

AAACAGGCATTCCCGGTTTATGTATAGTTGGAATGTATTTGTCTCTTTGCTTTATATATA

TATATATTTTCCTTTATGAAAGTGATGTGGACAGTAGCATGGCTAGCTAACAGACTATTG

GGAAAATATTACTTGACCATTTTATAGGTGAAATTCTTTTCCTGTAATTTCATGTCATTG

TGAGCAGTGGACAGTAGTCATTAATGGCTTTTGTGTGGTTTTTTAAGTCTTTCCAGAGTG

CATGTTGCAAGTCTGTTAAACACGGTGTATGGGTTGTTCTGTCATTATTCTCTTTTACTT

TGACACTTCATGTCTTTCAATTTCCTATGGTTAATTTATGGTTAGCAGGCTCATGGTACT

TCCTACTTTTTTAAGCTCCAGTTGGCTTAAAGGAGGA

>Unigene000199

TGTGTGTGTGTGTGTGTGTGTAGACATACTCTGGTCTCTTCTGTGTTGTAGTGAATCCTT

ATAAAATGCTGCCAATTTACTCAGAGAAGATCATTGACATGTACAAGGGCAAGAAACGTC

ATGAAGTTCCACCACACATCTACTCCATCACTGACAACGCCTACAGAAACATGATGCAGG

ATCGAGAGGACCAGTCTATTCTCTGCACAGGTGAATCTGGTGCAGGGAAAACAGAGAACA

CTAAGAAAGTGATTCAGTATTTAGCCGTTGTGGCATCATCACACAAAGGCAAGAAAGACA

CAAGCGCTGGGGAGCTGGAGAAGCAGCTTTTGCAGGCCAATCCTATTTTGGAGGCATTTG

GTAACGCCAAGACCATCAAGAACGACAATTCTTCACGATTCGGTAAATTCATCCGCATTA

ACTTTGATGTAACAGGCTACATCGTTGGAGCAAACATTGAAACCTACCTGCTGGAAAAAT

CTCGCTGTATCCGACAGGCTAAAACTGAGAGAGCTTTCCACATCTTCTACTACATGGTGG

CAGGAGCCAGGGACAAGCTGCGTGAGGAACTGCTGCTGGAGAATTTCAATAATTACCGCT

TCCTGGTAGCAGGACATGTGCAGGTTCCAGGAAATCAGGATGACGAGATGTACGATGAAA

CAATGGAGGCGATGAACATTATGGGCTTTACTGATGAGGAGAGGATCGGAATCCAGAAGG

TTGTGTCCACTGTGCTGCAGCTTGGCAACATCGAGTTTAAGAAAGAGAGGAACCAGGAGC

AGGCCACCATGCCTGACAATACAGCTGCACAGAAGGTCTGTCACCTGTCAGGCATCAATG

TGACGGATTTCACCCGTGCCATCCTGACCCCACGCATCAAAGTAGGCAGAGAAGTCGTAC

AGAAGGCGCAGACCAAAGAGCAGGCTGATTTTGCAGTGGAGGCTTTAGCAAAGGCAACGT

ATGAGCGTCTGTTCCGCTGGATTCTTGCTCGTGTCAATAAAGCTCTGGATAAAACCAAGC

GTCAGGGCGCGTCTTTCCTTGGCATCCTGGACATTGCCGGCTTCGAGATCTTCGAGGACA

ACTCGTTTGAGCAGCTGTGTATTAATTACACTAACGAGAAGCTGCAGCAGCTCTTCAATC

ACACCATGTTCATACTGGAGCAGGAGGAGTATCAGCGTGAGGGCATCGAGTGGAACTTCA

TCGACTTTGGGCTGGACCTTCAGCCCTGCATCGAGCTTATCGAGAGGCCGAACAACCCTC

CAGGTATCCTGGCCCTACTGGATGAAGAATGTTGGTTCCCTAAAGCCACAGACGTGTCGT

TTGTAGAGAAACTCACAAATCAACATGCCAATCATTTGAAATTCGCCAAACCCAAACAGT

TGAAGGACAAAACTGAGTTCTCAGTGCTACACTACGCCGGCCGGGTGGATTATAACGCTG

TTGCATGGCTGACGAAGAACATGGATCCCCTGAATGATAACGTTACTGCACTGCTCAACA

ACTCCTCCAGTGTGTTTGTGCAGGATCTGTGGAAGGACGCGGATCGTGTGGTGGGACTGG

AGACCATCGCCAAGATGTCGGACAGCTCCATGCCCAGTGCCTCCAAAACGAAGAAGGGAA

TGTTCCGCACCGTCGGTCAGCTTTACAAGGAGTCTCTGGCCAAACTGATGACCACGCTAC

ACAACACGCAGCCCAATTTCGTCCGCTGCATCATCCCCAACCACGAGAAGAGGGCGGGTA

AGCTCGATGCTCACCTAGTTTTAGAGCAGCTCAGGTGTAACGGCGTGTTAGAGGGAATCA

GGATCTGCAGACAGGGCTTCCCTAACCGCATCGTCTTCCAGGAGTTTAGACAAAGATACG

AGATTTTGGCAGCCAACGCCATTCCAAAGGGCTTCATGGACGGAAAACAGGCCTGTATAC

TGATGATTAAGCACCTGGACTTGGACCCCAACCTCTACCGCATCGGTCAGAGTAAGATCT

TTTTCAGGACTGGTGTTCTGGCTCAGCTGGAGGAGGAGCGCGACCTGAAGATCACAGTTA

TCATTATCGCCTTCCAGGCACAGGCCCGAGGCTTTCTCGCCAGGAAGTCATTTGCCAAAC

GGCAGCAGCAGCTGACAGCCATGCGAGTGATCCAGAGGAACTGTGCTGCTTACCTCAAAC

TCAGGAACTGGCAGTGGTGGAGACTCTTCACTAAGGTGAAGCCCTTGCTCCAGGTGAGCC

GTCAGGAGGAGGAGATGCAGGCCAAAGATGAGGAGCTGACCAAAGTCCGTGAGAAACAGG

TGGTGGCTGAGAAAATGATCGAGGAGATGGAGGTCAAACACCAGCAGCTGAATGCAGAGA

AGATGGCTCTACAGGAGCAGCTGCAGGCCGAGATGGATCTCTGCGCCGAGGCAGACGAGA

TGAGGAACAGGCTGGTGGCGAAAAAGCAGGAACTGGAGGAGATCCTGCATGACCTGGAGG

CCCGAGTGGAGGAGGAGGAGGAGAGAGCCAATCAGCTTCAGATGGAGAAGAAGAAGATGC

AGCAGAACATCACGGATCTGGAGCAGCAGCTGGACGAGGAGGAGGCAGCTCGGCAGAAGC

TGCAGCTGGAGCGCGTCACCATGGAGGCCAAGCTAAAGAAAATCGAGGAGGACGTCATGG

TGCTGGAGGACCAGAACACCAAGCTGAACAAGGAGAAGAAGCTGATGGAGGAAAGAATCA

GCGAGTTTACCACTAACCTGGCTGAGGAGGAGGAGAAATCCAAGAGTCTGCAGAAGCTGA

AGAACAAACACGAGGCCATGATCACAGACCTGGAGGATCGTCTGAAGAAGGAGGAAAAGA

CTCGTCAGGAACTGGAAAAGAACCGTAGAAAGCTGGAAAGTGACTTCACAGAGCTCAATG

ATCACATCGCTGATCTTCAATCTCAGATCGCGGAACTTCGGGCACAGCTTGCCAAGAAAG

AGGAGGAGCTACAGGCTGCGCTGGCCAGAGTAGAGCAGGAAACGTCTCTGAAAAACACGG

CACAGAAGAGAGTGAGGGAACTGGAGGCTCAGGTCACAGAACTTCAGGAGGATCTGGAGC

TGGAGAGAGCAGCAAGAAATAAAGCTGAAAAACAGAGACGGGATCTGGGTGAGGAACTGG

AGGCACTGAAGAGTGAACTGGAAGACACGCTGGACTCAACCGCTGCTCAGCAGGAGCTCA

GAGCTAAACGTGAATCTGAAGTGGCTCAGTTAAAGAAGACTCTGGAGGATGAAGTTCGGA

GTCATGAACAGCTCCTCGCTGAAATGCGGCAGAAACATACACAGGCTTTTGAAGAGCTCA

ATGAACAACTGGAACAGGCCAAGAGGAGCAAGGCATCACTGGAGAAGGTGAAACATGCTG

CTGAAAGCGAGCGCAGTGAACTACAGATTGAACTTAAATCCATCACTCAGAGCAAAAACG

AATCTGAACAGCGGAGGAAAAAAGCTGAAACTCAGCTGCAGGAACTCCAACTGAAATACA

ATGAAAGTGAGAAGCAGAAGAAGGACATGGCAGATAAAATGAGCAAAATGCAGGTGGAGG

TGGAGAATCTGAGCAGTGCTTTGTCTGAAGTGGAGAGTAAATGCATCAAAGCAACTAAAG

ACTCTTCATCTGTGGAGTCTCAGCTTCAGGATACACAGGGTTTGCTGCAGGAGGAGACTC

GTCAGAAACTAGCGCTGAGCTCACGGCTGCGTCAGGTCGAGGATGAACAGAACCGTCTGA

AGGAACTTCTTGAGGAGGAAGAAGAGGGAAAAAAGAGTGTGGAGAAACAGCTTCATGCCT

CTCAAGCCCAGTTAACAGAGTTGAGGAAGAAGATGGAACAGGAAGCATCAACTCTGGAGA

CTTTGGAAGAAGGTAAGAGGAAGCTGCACCGCGAGATTGAGAACCTGACCCAGCGTCTGG

AGGAGAAGTCTTCAGCCTATGACAAGCTGGAGAAGACCAAGGTGCGTCTGCAGCAGGAGC

TCGACGACATGCTGGTGGGTCAGGATCACCTGCGGCAGAACGTCCAGGAGCTCGAGAGGA

AACAGAAAAAATTCGACCAGATGCTAGCCGAAGAGAAGAGTATTTCCACTCGTTATGGTG

AAGAGAGGGACCGAGCAGAAGCTGAAGCTCGTGAAAAGGAGACGAAGGCGCTGACTTTAG

CTCGAGAGCTGGAGGCTCTGCAGGAACAGCGTAATGAACTGGAAAGAGCCAATAAACTCC

TCAAAACTGAGATGGAGGACCTGGTGTCTTCTAAAGACGACGTTGGCAAAAGTGTCCATG

AGCTGGAGCGTGCTAAGCGAGCGATGGAGCAACAGTTGGAGGAGATGAAGCTTCAGGTGG

AGGAACTGGAGGATGAGCTGCAGCTGACCGAAGACGCGAAGCTGCGATTGGAGGTGAACC

TTCAGGCCATGAAAGCACAATTTGAACGAGACCTGCAGGCACGAGACGAGCTCGGAGAGG

AGAAGAGAAAACAGCTGATGAAACAGGTGAGGGGACGACAGTCACATGATCACACGTCTG

CTATGGGTGTAGGAAAGTGTATGAAACTGTGTGATATCTGATGAGAATGTAGACTTTTAA

GGTGTGTATGTAGGTGCATGAGATGGAAGCGGAGCTGGAGAATGAGAGGAAACAGAGATC

TCTGGCTTTAGCAGTGAAAAAGAAGTTGGAGCTCGACCTGTCCGACCTGGAGGCTCAAAT

AGACCAGGCCATTAAAGCTCGAGATGAAGCACAGAGACAGCTGAAAAAACTACAGGCCCA

GGTAAAGGATCAGTTACGGGATATGGATGAACTCCGAGCGGCTCGAGATGCGTCACTGAA

CAGCGCTAAAGAAAACGAACGCAAACTCAAGAGTCTGGAGGCTGAAAACATGCAGTTACA

TGAGGATCTGTCTGCAGCTGATCGCAGTAGGAAGCAGATTCAGCAGGAGAGAGATGATCT

GCATGATGAAATGACCAGCTGTAATTCCAAAATGTCTGTGCTTAGTGATGAGAAGCGGAA

GTCGGAGGCTCGTATTGCTCAGCTGGAAGAGGAACTTGATGAGGAACAGTGCAGCACTGA

ACTTGTTAATGACCAACTAAAGAAGGCAAATGCACAGGTGGACACTCTGGGTAGTGAGTT

AGCGGGTGAGCGTAGCGCGGTTCAGAAGAGTGAGACGGCTCGACAGCAGCTGGAGAGACA

GAATAAGGATCTGAAGAGTAAACTGCAGGAACTGGAGGGTTCCGTCAAATCCAAGTTCAA

ATCCACTATCGCCGCCCTGGAGGCCAAAATCCTGCAGCTGGAGGAACAACTGGAGCAGGA

GGCCAAGGATAAACAGCAGACTTCAAAGTCAATGCGCCGTACGGAGAAGAGACTGAAGGA

GGTCCTGCTGCAGGTGGAGGACGAGAGACGCAACAGTGAGCAGTTTAAAGATCAGATGGA

GAAGGCGAACTCGCGCATGAAGCAGCTGAAGCGTCAGTTGGAGGAGGCGGAGGAGGAAGC

GACCCGCGCCAACGCCTCCCGCAGGAAGTTACAGCGAGAACTGGAGGACGCCACCGAGGC

CAGTGAGGGTCTCAGCAGAGAGGTTAACACACTCAAGAACCGTCTGAGGCGTGGGGGTCC

GGTCAGTTTCCCATCAGGCCGTTCAGGTCGCAGGCTGCAGATGGAGGGTGAATTCTCAGA

TGATGAAGCTGACAGTAAAGTGAATGACACAAATGAAGTACAACCTCCACCGAGCGACTT

TTCTTAGTGCTAAAACTCCTG

>Unigene000200

TGTGTGTGTGTGTGTGTGTGTTCAAGAGAGGAGAGACTGCGCTCCACATGGCCGCCAGGG

CTGGACAGGCGGGTGTAGTGAAATACCTGGTGCAGAACGGAACTCACGTCGACGCCAAAT

CTAAGGATGACCAGACTGCGCTGCATATCTCTAGTCGTCTGGGAAAGCCCGAGATTGTAC

AGCAGCTGCTGCAGCATGGAGCCGCACCCGATTCCACCACCACATCCGGATACACACCAT

TGCACCTGGCAGCCCGTGAAGGCCACAAAGACGTCGCCGCTATACTGCTGGACCAGGGCG

CTTCACTGAGCATCACTACCAAGAAGGGATTCACGCCGCTACATGTGGCAGCTAAGTACG

GGAAAATAGAAGTCGCCAACCTCCTGCTGCAGAAGAGAGCTCCTCCAGATGCGTCGGGAA

AGAGCGGACTAACTCCACTGCATGTAGCTGCGCACTACGATAACCAGAAAGTGGCACTTC

TTCTCCTGGACCAGGGGGCTTCTCCCCATGCAGCTGCCAAGAATGGCTACACTCCTCTAC

ATATAGCTGCCAAGAAGAATCAGATGGAGATCGCTACCACACTGCTGGAATACGGCGCTG

ATACCAACGCGGTCACTCGGCAGGGCATCAGTCCGCTGCACCTGGCTGCTCAGGAGGGCA

ACGTGGACATGGTCACCCTACTGCTTGCACGTGATGCCACCATCAATCTGGGTAACAAGA

GCGGACTCACTCCCCTTCACCTGGTGGCCCAGGAGGATAAAGTAAATGTAGCTGAAGTCT

TGGTTAACCAGAGTGCTGCTGTGGACCCTGAGACCAAGATGGGCTACACTCCGCTGCATG

TCGCCTGTCACTATGGCAACATAAAGATGGCGCACTTCCTCCTGAAGAACCAGGCTAAGG

TCAACGCCAAAACGAAGAACGGATACACTCCTCTGCACCAGGCTGCACAGCAAGGCCACA

CACACATCATTAACCTCCTCCTGCAGCACGGAGCCTCGCCCAACGAGCTGACGCTGAATG

GAAACACAGCACTGTCCATTGCCAGGCGACTGGGTTACATTTCAGTAGTGGACACCCTCA

AAGTGGTGACAGAAGAGACCCTGACCACCATGACGGTGACTGAGAAGCACAAGATAAACG

TCCCAGAAACAATGAACGAGGTTCTGGACATGTCTGATGATGAAGTTCGTAGAGCCAATG

TCCCCGAAATGCTCAATGAGGACTATATTTCAGATGTTGAAGAAGGGGAGGATGTGATGA

TGGGGGACACGGATAAATACCTGGGTCCCCAGGATCTGCGTGAGCTAGGGGACGACTCTT

TGCCTCAGGAAGGTTACGTAGGATTTAGCATTGGAGCCCGATCCGGAAGCCTGCGCTCCT

TCAGTTCGGATAGGTCCAACACACTGAACCGGAGCTCTTTCACCAGGGACAGCATGATGA

TCGAGGAGATCCTGGCACCCAGCAAGGACACGCATCTGACTGTAGCTAAAGACTATGACA

CTGACTCTCTGAGGCGATACAGTTGGACAGCAGATGCTTTGGACAATGTCAACTTGGTCT

CCAGCCCAGTCCATTCAGGGTTCCTAGTGAGCTTTATGGTGGACGCTCGTGGTGGCTCCA

TGCGAGGCAGCCGCCACAATGGGATGCGCATCATCATCCCACCACGCAAGTGCACTGCCC

CGACTCGCATTACCTGCCGCCTGGTCAAGCGACACAAGCTTGCCTCGCCACCACCCATGG

TGGAAGGAGAAGGACTGGCAAGCCGTCTGGTGGAGGTGGGCCCTGCCGGAGCTCAGTTTC

TCGGCCCTGTGATAGTGGAGATCCCTCACTTTGGCTCCATGAGAGGGAAGGAGCGTGAAC

TGATCATTCTCAGAAGCGATAACGGAGAGACTTGGAAAGAGCACCAGTATGACTGCAAGA

CCGAAGCCCTTAACGAGCTGCTTAACGGCATGGATGAAGAACTGGACTGCATAGAAGACC

TGGAGAAGAAACGCATCTGTCGAATCATTACAAAAGACTTCCCACAGTATTTTGCCGTGG

TGTCACGGATCAAGCAGGAGACCAACCATATGGGTCCTGAGGGAGGGGTTCTATCCAGTA

CGACTGTTCGCAAAGTCCAGGCTTCTTTCCCTGAAGGAGCGCTCACAAAGAAAATCAGGG

TTGGACTTCAGACCCAGCCGGTTCCAGACGAAACAGTAAAGAAAATCATAGGCAACAGAG

CCACGTTTAGCCCCATAGTGACAGTGGAACCACGCAGAAGGAAGTTCCACAAACCCATCA

CCATGACTATTCCTGTACCTCCTCGATCAGGGGAAGGCATGAACGGCTACAAAGGAGACA

CAACACCTTGCTTACGTCTTCTGTGTAGCATCACAGGTGGCACCTCCCCCGCACAATGGG

AAGACATCACTGGGACTACTCCGCTCACCTTCACGAATGACTGTGTCTCGTTCACTACCA

ACGTGTCTGCTAGATTCTGGCTCGCAGACTGCCATCAGATTCCGGAGACAGTTTCTTTGG

CTACTCAGCTCTATAGGGAACTCATCTGTGTGCCCTACATGGCTAAATTCGTAGTGTTTG

CCAAAATGAATGACCCTGTGGAATCCCATCTGCGATGCTTCTGCATGACGGATGACAAAG

TGGACAAAACCCTGGAGCAGCAAGAAAACTTTGAAGAGGTGGCCAGAAGCAAAGACATTG

AGGTTTTGGAAGGAAAGCCTATCTATGTGGACTGCTACGGCAATTTAACCCCTTTGATTA

AAGCCGGACAGCAGCTGGTTCTTAACTTTTATGCATTTAAAGAGAACAGGCTGCCATTCT

GTGTAAAGGTGAGGGACAACAGCCAGGAACCGTGCGGACGGCTGTCCTTCCTCAGAGAGA

TTAAAAACGCAAAGGGGCTTCCCCAAGTTGCTGTCTGTAATTTAAACATCACTCTGCCCG

CGCTCAAAAAGACTGAAAAGCCAGAACGACGTCATACCTTTGCATCTCTAGCCTTACGTA

AGCGGTACAGCTATCTGACTGAGCCTGGATTGAGCCCTCAAAGTCCTTGCGAGAGGACAG

ACCTGCGAATGGCTATAGTGGCTGATCATCTCGGCCTTAGCTGGACTGAGCTGGCCAGGG

AAATGGATTTCTCTGTTGATGAGATAAATCACATCCGCGTGGAGAACCCCAACTCGTTGA

CCGCTCAGAGTTTCATGCTATTAAAGAAATGGGTCAACAGAGACGGTAAAAATGCCACAA

CGGATGCCTTAACTGCAGCACTGACAAAGATCAATCGGATGGATATTGTGACATTGCTGG

AAGGGCCAATATTTGATTATGGTTACCACAACATCAGTCTGGAGTTGCAGAGTCCCTCAG

AGTTGTCCTATGAACCCCCCACCCCTCTCCGGCAGGATGATTTCTTTAGCGAGGATGGGG

GTATTGTAGTCTCGCCCAGTAGAACCCCTGTTAGACCAAATGAACTGTCCTTACCAACCA

CATCTAGGGATGCGCTTTCCCCCAATGCAGCACCTCCTACTGTGGTGGCAGAGGATTCCT

CTATTGGAGGAAGAGGCTCCTATAGTCGAGAAGGCGGCTCAACTGAAGACGACCTGGACG

CCCATGACAGTGATGAGATGCCTAAACATCCTGAAGTGTTCATGCAGAGCACATACCAAG

AGCAAGAGGTTCCTTCCAACGGAGCTCACAGGGCTAGAGAAAGTGGCAGGTCTGGTTTTG

ACGAGGAAGAGGAGGAAGAGATGACGCAGGAGAAGTCCTGTTTTAAAAGCATAAACTTGG

AGGAAGGTTTAGAGATTGTGGAGATACCCCAAGAAAAAGTGCAGGAAATTCGCTCACAAG

TGGAACAGGCAAAAAAAGAGATGTGTTCGCTTGCAGGATTGCAAGAAGGGCCGTCCAGTG

TGAGCCAGGAGATCTCCGCTTCGGGTCTGCACACTGATACCCAACTGCAAGATAGAAGTC

AAAAGCCAACTGAAAGGCAAAATGGGGAACATCCAGAGCTCCAAGGCCGAGCCGGCTCAC

TGCTTGAGCCACAGGAACCATCTAGTAAGATGAAGGGCAAGGGCAGCAGAGACTCCTTAG

ATGACACAGCTGCTGGGCCGAGCACCATGGACGAAGACACAGTGGAGCCGAAAGCTAAGG

TCGGAGAGGGAGACAAGAAGAAAGAAGGGAGACAACGAGGGAAAAAGGGTCAATCGTAAC

AGACGGGTGTCCTAAATGAGATCTGAACTTGTTTCATCATTGTTTTCCTGCGAAAAGCAT

CACTGGAAACCCAACGCAGGAGATCAGGACAGGGGATTGTGCGTCTGCAGCCTTTACATA

AACGCATGGTTTTTAGAACGAGGATTTTATGATTTCGCATGAGCATGGAAAACCTCCGAT

TTTCTGTCAACGACACATCCAAGCATCTGATCGGTGCCAAGAAATGACACTTAAGCTCAA

GACTGTCGGTGGCATAAAGCTTCTAGTCAGTCCTATACATTGGAGGAGTGCCAGCGACTA

CCAACATGGCACCGAAGCATTCCATGACCAAAGATGGTACAATGAGCATTGTGGAATGAT

TATTTTTTAAAAGAAAAAAAAAAAAAAAAACCACCTTCTACACTCGCCTTATTTTCTCTC

CCCCCACCCTCCCCCTCGCTTCTCGGACTAGCGTCGACGCTGTTTTTAAAAAAGATCCTG

TATATAAATCAGAACTGTATGAAAGTAGCTCACGAAATTGTGAACGCAGGAGCAAAGAGC

GAGTCACAAACGGAATGAAAAAAAAAAAGAAGAAGAAAGGAATGCTGATATAACTATTCC

GACTTTAAAGATCATTTTAAAGACGAATTGATTACACAAAAAAGCGAAAAGAAACTTTTT

ACACAAAAAAAAACAAAAAACAAGTTTGCTTGATTTCAACGTTTTTAAGCCTACCACAAA

AACGACAAAACATCGAACGACATAGCTGCAGTTGTGTTAAAAATTTACTCCGAATACTTA

TATTTTGATGCATGTGATTCACCTTATTTTTTGATTTGTTTCCTATCTACATAACCAGAG

GTGTGGTAAATGTTCTACCTGTGTGCACACTCATAGCCTTGGTGTTTGTCACTAATACTG

TTTTATAATATCACTGTACCACAACAGACTCACTGGAAGTGAATGGACACACAATTCTCG

CAGCTAAAGGTTTCTACGCTATGCAGTCAAAGACGAGCTTGGAGTGTAAGAGAGACCGAA

AGAGAAAGAGCGTCGTGTCAACTGGCACAGCGTTCAGTTTTAATGGGTTTTGTGTGTATC

GGCACTAAAAGGTCTTTCTGTAAGCCAACCGTTTGTAACCATTCTAACAGTGTTACTAGG

CTGCTGTGGACATAAGGGGCATTTTTTTATTTGGGTTTGGTGAAATATTTCCTTATATTG

AATATATTCAAAAAAAAAAAAAAGAGAGCGAGAATGAAAGAAAGATCACCTTCAGGTATT

CGTTGATGAAATGATCTGGTAACAGACTTGTATGAGAAAGCGTACACTGGATGATTGAAT

TAATTATTTAAGCATAAAATAAATTGTGTTTAAACTCAAATTGTGATGTGAACTTTGATC

TGGATGATGTGGTGCATTTGTGCGAGAAAACTGTGTCTGCATTTGTCTGTCCATCGATCT

ATCTGTTCATCCATCTGTCCATCTATCTATATACAGTATCTATTTAGCTAGCTACACACA

CACACACACACACACA

>Unigene000201

AATTCCTGGTCAAAAACATGCAGGTCTTTCCAGAGCATTATTCGGGATATAAATAATTTG

CTTTTGGAGATACAAGATGTCCTGCTGTTTCCTGTAATAATGAAGCTCGATCTGTATTTG

TGTACAGATTCTACAAGATGCTGCAGGAGGCGCTGTGGCTATCGCCAACCAGGAAGTTCG

AGCCAACGGCTTCAGACCCAGCAAGCAAGATGTGTATGAGTTTATCAAGCAATGTGGAGA

AACAGATCCTTCAAAGCTGCCATTTATCGAGGCTGCCATCAGGAGTCACTACAAGGTTCA

GGACAGTCGAGAGCTGGGCTATGGAAAACTTCATACACTCGCAGATTTCGCCAGGCACCA

GAAGGAGCTGTGTGATGGGGTCGCGTCCACTTTTGTGTTCTATGAATGCCCTCTGTTCCA

TAAAGAGCCAGAGGACATGCTGGTGAATTCAGAGGGCCTGCTGGGGGACGTTAGCCGGGA

ACAGGCAGTGGCGAGTCTTCTTTCTGCTCCATTGCTGGAGGATTTGGCTGAATGGAGTCA

GTGGGAGCTGGTCTTTCAGTCTAATCATGGACCTCTCAAACACTTTATCGACAAATACTG

TGGTTTGTTTGATGCAGGTAACACAGACCTGCTGGCTTTGGAAGTGTCTCCTGGAGTGCT

GTTGAGGATCACCTCTGTCACTGGTGATAAATTCTTCTCTCAGGCCAGCCAGGCCCTTGA

CCCTGCAGGCACCGCCGGTCACCTGGTGTCCATCGTAGTGCGTGATGGGATTTTAAACAC

CCCCACAGCACTGCTCGCCAATCTCATGGAAAGTTCGCTAGCAGCTGCAGCTGCTAAAGA

AGATCCATCCCTGGGTGAAGACAGATGCTCCTTTAGCATAGTGGCCAAGTTTGTGCTCGA

TTGTGTAAGCCGGATGCCTACACGAATTTGTAGAGAGCTGCTGCAACAGGTGTTCCTGGA

GCCTCTTTCAAAAGTGCTTGGTCAGGCCCGGTCTAAAATTGTCCTTTTGGACACAGCCAG

GTCAGAGGCAAGATACCTCAACAAGCTTCATCAGCTGGGTATCATGCTGGGCATCACAGA

GTGGGTGAAGGACTTCCACAACAAACTCATCCTGCCTCCCACACCTGAACTGCCTCAACA

GAACCTGGCAGCTGAATCTGTAAGTGACTTATCATCTGTGTTGAGTCTTGCTGACAGTGA

AGATTTGTTGGAAACCTTTCAGCAAGATATTAATGACTGCATTAAGGACAATGTTGAGGA

TGATTCAGAGGATGATGCAGAGGATGAGGATGAGGATCTCTTTGAACTGGCATCAGAGAA

GAACGAGAAAGTGAACGGCAGTGTTGAGAGTAAGGAAGAGGATGAAGAGACTAATGACGT

AGACAGTATTCCTAAAGGCCATGCAGAAGAAGATGAAACTTTCAGTGAGCAGCTCAGCCA

GCAGATGGCTATCATCGAGGACATCAGGAAAAATGAATTCGGTGTGGGAGTGGAGCTGAA

CGAAGCAGGCCAGAACCTCATGAAGAAGCAACAGGCTCGTCTTGGCCGCAGTCTCGAACG

CCTCTCCACTGAACTCTACAGCAAAGACACACACTTTGTTCTGGAGCTCATCCAGAATGC

TGACGATAACAGCTACCCGCTACATAGAAAGGAGGAGCCTGCTTTGGCCTTTATAGTGCA

GAAAGACTGCATCACCATTCTAAACAACGAGTTCGGCTTCGAAGAGAAGAACGTGCGAGC

TATCTGTGACGTGGGAAAAAGCACCAAAGGAAAACATACTTGTGGCTACATCGGTCAGAA

AGGCATTGGATTTAAGTCAGTTTTTAAAGTCACAGACTGTCCTGAGATCCACTCCAATGA

CTTCCATATTCAGTTTGACAAGGCCAGCGGCCCTATGGGATACATCCTGCCTCACTGGGT

GGAAGAAGAGAGGCATGTGCCCCCTGTTGCCAAGGAGATAGCAGAAAAAAGCTGGACAAC

AAAGATTTTCCTGCCTCTTCGATCAGAGAGCTACCAGACCAAAAACCTTTTTCACGACGT

ACACCCATCTCTGCTTCTCTTCCTTCACCGTCTCCGCTCCATCACTATCTACAGCGAGGC

CGAGAGAAAAGTGGTGTCAATGATGAGACGAGATCTAAATCACAACGTGCTGGAGGTAGA

GCACACCGGCGGTGTGGAGCGCTGGTTGGTTGTCAAGAAGATACTCTACCCTGAGAAAGT

TAAAGTGGATGTGGAATCTACCGAACTAGCGCTTGCCTTCCAGCTCAATAATGGCTCAGA

GGGCAAAGTAAAAGTTCAGCCTCGGCAGCAGCCCGTCTTCGCCTTCCTTCCCCTGCGAAG

CTTTGGCTTCCGCTTCGTCATCCAAGGAGATTTTGACATTCCTTCTTCTAGAGAGGATGT

TGATAGAGACAGTTCCTGGAATCAGTGGCTCCGCTCTGAAATCCCCCAACTCTTCTTGCA

AGCCATGGATGTTTTCACTACTCACCCTGAATTCAGTGGACTGCAAGGACTCTGCCACTT

CCTGCAGTATATCCCTCAGCCTAGTGAAATCCTGGACTTTTTCAACCCGGTGGCCAAACA

GATTATCCAGCTGCTGAAGGGGAAACCCTGCCTTCCTGCTAAGATGGACAGCAATGGAGG

CGTGCAGTTCAAGCTGCCTTCGCAGGTGGCTGTGTGCCAGGACCCCTTAATCCAGGATGT

GATCGGAGGAGAAGAGCTGTGTAAGCACCTGAACCTGTCCTACCTTCACCCTGAATTACA

GTCATCACTGTCATCTTCTTTGCTCTCTGCTTTGGGTGTCCAGCGTCTGAAGGCTGCCAA

CATCACCACCGTAATCTGTGCAATGGCCAAGAGTCTTGTCCAGGAGGGTGATATTTACAC

AGATTCCAACCTGAAAAAACTGGCCAAGCTGCTGGCTTGTAATTTCCGTGCTCTGGAATC

TGAGTACAATGAGGTGGATAGCCTCCTGCAGGCTCTCCGGGACATTCCCATGATTCCTCT

GGCGGATGGCCGTGTGGTGTCTCTCAGCGCAGAGGGTGTCTTTTTTCCTCTCAGTGAGGA

GACTCACAAAGGCCTGAACGCTTTGTACAAGGACCTGAGCATCGTAGAGCCGCGACTGCT

AGAGTGTCTGGATAAATTGGGTAACTCTCAGGTGCGTGAGCTGCTCTGTAGGCTGGAGGT

TCATGAACTGGAGCCCAAAGAGGTGCTGCAGAATCACATCTACCCCATACTGAAGCAAGC

TGCTTGGAAGGAAAAGCCGGAGAAAATCACTGTCAGTTACCTGGTGTTTATCAAGGAGCA

TTCACAGGAGCAAGACTACAGGGCGCTGAGGGAAGCTATCCCCGTACTCACCAACAAGGG

TTTCCTCTGCCCCGGCCAGTGCAAAGTGCAGTTCTCCAAAGAGTACGGCAACATTGATCT

GCCCAACAAGCTGCCTGGTGTCGACTGGGTTCTTTTGGATTCTTGCTACCTGTGTGATGG

CGATTTATCAGGATGGAGGGATTTTTTGAGTGATCTCGGAGTAAGAGATCTCCTAATTTT

CAAGAAGGAGCGACGCAACCTCAAAGACACAGAGCTGGTATCGAGCCCATGGGCAGCGGA

GGCAGAGCTGTGGCCCAAACCTACAGATAAACTGTATGTGATAGAGGATCAGCAGTGTGA

AGAGCTTCACTCACTAATAACGGCAGATCAACTTCCCCCTACCATCAAACTTCAGCAAAG

ACAAGCTCTAATCAACCTTCTACAGAACAACTGGGACACTGGAGAGAAATATTCTCAGTA

CCTCAGTGCTCAGGTACTGGACAACCAGGGCCGCACTCTAAAAGACACCAGGTCCTCGTT

TTACTACCATCTCACCCAGTTGGCATGGATGCCTTCCTACAAGCCCACGCAGGACGAGAA

AAACTCGGTGGAGTACTTGATTCCAAACAGGGTGTATCTCTTTTCTAACAAAGTGCACAG

TCTATTGGGATCCCACGTCTTTTATGTCAATCTGGATCCCAGCGAATTCTCCAGAGCTGT

AGGAATGAGACACACTGTTGTGGTAGACGAGATGATCAGCTACCTGAAACGCTGGTGTAC

GAAGGAGTCCGATAGCGGCGAGGCAGAAGGAGCTGATTTTATCACCACCGCGCAGCACAT

CCACACCGTGTACCTCTACTTGCAAGCCGAGTGCTCGCACACACAGCTTAGGGACCTCTT

CCAGCACTCACCTGCTGTTTACATAGAGTATGACAGAAAGGATGAGTGGTCTTCAGGACG

CTTCTATCATCTCAAAGACGTGTGCTGGAGAGATCCAACAGACATGTTTGTCAGATACAA

GGAGCTGATCCGCAAACCAGACAGCAGGGTCCAGGAACCCAAAGTACTGGCTCCATTCTA

CAGCAAATTGCCCCAAATGGAAGAACTGTTTAAGTCTCTAAACGTTTACCCCAGTCCATC

CATGAAGCAGTACGTGGACCTGTTGGAGACTGTATGTGAGTCGTCACCACTTCCCACAGG

AGAGGTGCTACAGGATGTCTCCATCATTTTTGCCAGACTGGCGGACAAATGTAAAAACCC

TACCAACGTAGGGCAAGATCAAGACATCGAAATGAACCAGTGCTACTGCATGACCCTAAA

AGAGATGGTGTCTCGTCTGAAGGTGTTTCCTACCAAGACACAGGGTTGGGTCACACTGGC

ACGGAAACCTATGATAGCAGACAGCCCAAGCCTGGAGAAGATCTTCAAATCTTACAAAGA

AGTCTGCCTCCTCAACCTGCCCTCGGCTCAGAAGAAAGTACAAAGACACAAACCGAACCA

AAAAGCCAAGCTGCATGATGAGAAGCTGGCCTTCAATGAAGGAGACCGAGAGCTCTTTCT

GGAGATCTGCGGCGTAAAGAAACTCGCCCAGTGTGTCACCACCGAACCTACAACGGAGCT

CTACAGGCCGTGTCCAGCTATGCAAAACCTGGTCCGTAATCTCATTCCTTACATCCAGAG

GTTTATCTATCACCACGATGAGCTTGGAGAAATATACGATGACCTAAAAGAAAATGGAAT

TGCCCAGGAGATCAAATCTCTCAGCTTCGGTCAGGTGGGAATGCTGTACATTAATTACCG

ACTGGAACAGCCAGATGAGAGCCCCATTGTAGAAAGTGAGGACATCATTTGTCTTCTGAA

AGACAGAAAAGAGTTGTACATTCAGAAAGACCATCTTTCATCCAAAATGGCCATCTGCAG

AGAACTGGTGAAACTGTTCACCACAGAGAAGAGCTTTGTAGAGGAACTGAAACACTTTCT

CGAGGAGCTTGTTATCCGTATCAGTGATCCGGATTCTTTAAAGAGATTACTGGATGGTAA

AAACATACAGGAGCTTCCTCAGGATGAAGAGCGGTGGGAGGTTCCGGCACCGCTGGAGAC

ATTCGAACCCAGGACCTTTACTATCAGCTCATCAGGGATTGTGAGTGCAGAAGAAGAGGA

AATAAACACAGAAGAAGATACACTGCACAGCTGGCCTCCTAAAGCATCCATCAGCAAAAC

AGGAGGCAGAAGCACAGGTGCGTGCAGAGTCTCTGCCTCGATTTGCCGAATCATGTGACC

ATGCTAAGAGGAAGCAGGCTGTTCCTGTTCTTTATATACGATTTATGGCATTCTTTGCAG

TGCTACCTCAGTAATAAAAGTGTTTGGATTACTGATCAAAAGGTTGCGAGTTTAAATCCA

AAGACCCCCA

>Unigene000202

GGATTCCAAGCATCATTGATCCATCTCAGCATGACTAGTTCAAGGGGCAACTCACTTTCT

CTGGACTCTGCACTCAGAAGTGTGTAAATACCCACAGACGCCCGGACGCTCCACAGCGTA

CCTGCCGGAGCCATGACCACCTTCGACGACATCCTGGAAGAAGCCGGGAAATTCGGCTTG

TTCCAGAAACGCATCTTCTCCCTGCTGAGTCTCGTCTCTGTGCCTTTCGCCGGAGTTTAC

GTAGGCATCGTGTTTCTGGGCTTCTCCCCCGAGCACTGGTGTCGTCACCCTGCAGTGAAG

CAGGTCCAGATGCAATGCGACTGGAAAGTAGAAAAGGTGTTGAACTTCACTGTGCCCTTT

CAGAACACTTCTGCTGGACCGCTGCGCAGTCGGTGCGAGAGGTTTGACGTGGAATGGAAT

GTTTCCGATTTCAGCTGCGATGACCTGGACGGTGAGATCGCTCGGGCAAAGCTTGCGGTT

TTGCCCACCAGTTCCTGCACAGATGGCTGGGAGTACGACTATGAGGGTAGAGAGTCTTTT

GTCACTGAGTTTAATATGGTGTGTGAGGACGCCTGGTTGTTAGACATGTGCCAGGCGGCG

ATCTCCGCCGGCTTTCTAGTCGGCAGCATTGCCATCGGATACTTCGCCGACAGGTTCGGC

CGGAAATCCACTTTCCTGATGTCCTGTGTTCTGAATACAGTCACAGGAATTCTGGTTGCG

GTGTCTCCTAATTACATCTCCCTGCTGGTGTTCCGAGCGCTGTACGGGTTTGGCACTAAG

GGCGGGTGGATGGTCGCATTTGTTCTGATCACAGAGCTGGTAGGAGTGGAGTACCGGAGA

CCAGTCGCTGTGTTCTTTCAGATGTTCTTCAGCCTCGGGGTTCTTATCCTGCCCCTCATA

GCCTACCTCATCACCAACTGGCGCTGGCTGCAGGTGGCCATCACAGCTCCATATCTCGGC

TTCCTGGTCTACTACTGGATTATTCCAGAATCTCCTAGGTGGCTTCTCTCTCAGAACAAC

AACAAAAAAGCTGTAGAGATCACAAAGAAAATGGCCAAAGTGAACAAGAAGACGCTGTCC

AAGAACATTGAGACACTTAAAGACGATTGCACAGACACGGCTGTTGCCTCATTCATGGAC

TTGATCCGGACGCCCAATATGAGAAAGCACACCTTCATCCTCAGCTACAACTGGTTCACC

AGTGCTGTGGTGTATCAGGGTTTGGTCATGCGTCTGGGGATCATGGGTGGAAACGTCTAC

ATTGACTTTCTCATCTCGGGCATTGTGGAGTTTCCCGCTGCCATCCTCATCCTCTTTACC

ATCGACCGTATCGGCCGCCGGCTACCATTCGCAACCGCCAATATTGTCTCTGGAGTCGCC

TGCCTAATAACGGCTTTCATTCCTGAGAGTTTCTTCTGGCTGAAAACAGCTGTGGCGTGT

GTCGGGAGGCTGGGAATCACCATGGCGTTTGAAATGGTGGTGTTCGTAAACACAGAGTTG

TACCCGACCTTCATCAGGAACCTGGGTTTTTCCGTCTGCTCCACTTTATGTGATATCGGA

GGGATCGTAGCGCCGTTCCTGCTTTACAGACTTGCGGTCGTCTGGCTGGAACTACCGCTT

ATTATTTTTGGAATCCTGGCAGTGATTGCTGGTGGTTCAGTGTTGCTGCTTCCGGAGACC

AGCGGCGTCCCTCTACCTGAGACTATTGATGACATCGAACACCCACACAATGCCTCTTCT

CACCTCAGAAACAAAAAGGGAAACGCAGGGAAAATCCATCAGCTGGAGAATCTGCTGCAC

CCGGATGTGACCAATAATAAAGAAACCACGACGGTCTGAGTGGAAGTGGAGGTTTTCTGC

TCTGACGGCGGTAACGTGGTAATATTTTATGACACTTGGACAGCAGTTGAAGTGATGGAC

GGTCTTCTGTTCATGAACTGGAAACCAGGACTGGTCGTCACAGTATTTATGTCCAAAAAT

TTAATTTATGTCCAAAAATTTTTGATGGACAGGTCACCAGTCCATCACAGGACCTCACAC

ATACTCCTATGGGCAATTTAGAATCACTAATTAATCTAACATGTTTTTGGACTGTGGGAG

GAAACTGGAGAACCCGGAGTAAACCCACACAAGCACGTGGAGAACATGCAAACTCCACAC

AGCAAGGCCCCTGCAGGGGAATCGAACCCGGGACCTCCTTGCTGTAAGGCAACAGTGCAA

AACCACTAAGCCACCGTGCTGCCCCTGATTTTTCTGATTGTTTTCCTAATTTCTTTTTAA

GTGCCGCGTCGCCGCTTACACCATTTTTTATATAAGAAGTAATCTCTCTTGTGGAATCAT

GGGATTGTGTCTATAGTTGGCATACATTGTAGATCATCCAGGAATTTTTGGACACATTTT

TACTTGCTATGGTTTCGGACATACTACTCGGCCTGTATTTCTTTTTAGTATTAAAGTATG

TACGATTTTGGATTTACACAGTTTTTTTTTTTGATTAAAATGATTTTTCTGATCACAACA

TTAGTGTCTAATCTCACTAATACTATTGGAACTGAATGATCAAATCCACAAGGTCACATG

ACCCAATCTTGTAAAAACATCTTACCAGAAGTTTGGAGATCATTATAAGAGGGAGAGTGG

GAAGATGGGGAGGTGTCCTAATACTTTTAAACAAACAACACATATAAAGTCATTTACTTT

TTTCTGAAAGGTTTTTTTAATTGATAGGACAGACAGAGAAGAAACAGAGAGGGGAGGGTG

GGGGGTAGGACCGGAAAGGACCCAAGCCGGAATCAAACCAGGGTCACCGGAGCACAACTG

CTCAAGTTGTTGCCAGCACAATGGTGTAATGTGTTTTGTTCTTTGGTGACATGGGTTCAG

ACGGGTCTCTGGGCGTCTCTGCAATTTTATTGTTTTAAGTTTTTTATGTTTACTGTGAAT

CAATACTCTAGAATTCAAATATCTCTTTTATTATTTGTGAAAATTTCCAGCTGTTAAAAT

AAAAGGCAGACTGAATCAACGGCGCTTTTTTTTTTTTAAATAAAGCTTTAAAACAAGTTC

CAAAGTTTCGGTTTGCATTTCAGGCTTTGATGCCTGTCTGGCTTTCGATCCAGTCGACGA

ACTTGGAGACGCGAGCGTACACACCAGGCTTCATGGCATTGGCACAGCCGAGACCCCACG

ATGTCACACCCTGAAGAACAAAGGTGTTCTGACCATAGCATACAAGAGGACCGCCACTGT

CACCCTGACAGCTGTCAGTTCCTCCCTCGATGTTTCCTGCACACATCTCATTGTCCTTCA

CTCGGTGGTTCAGGTATTCGGGACGGTTACACACTTTGTTTTCGATCACTGGGAATCCCG

TCTCCTTCAGAATGCCTTCTCCACCCGTTCCTTGGGTTTCTCCCCATCCTGTAACATAGC

ATTCTGTCCCACTGGGAACGATGTGATCTTTCTGAGGCAAACACACTTTAGACACTTTCT

CATTCAGGATCGCCGGTGTTTCCAACTTGAGTAAAGCGATATCCGTTCCTGCCGGCCCTT

TTATAAGCTTTTCCAGGTTGCGGATCTGTCTGGACGACTCTACCGCCCGTTCTGTATGAA

TTCCCATGTACACTTTGTAGGCAGACGGTCTGTTGGATCGTTCAAGGCAGTGAGCGGCTG

TAAGGACCCACTGGGCATCGATCAAAGTTCCTCCACAGAAGTGCATGCCAGTGTTGGTTC

TGAGGCTAATCTGCCAGGGCCAAGAGTGAGGTTTGGATATGCATCCTCCAACGATTCGTC

CGAAACAGCGTCTGGGTTTTACTGCCGGCTGTCCGCACTTCCCCTCCGCACAGTCTTGAA

TGTTGCAGTAATCCCATTTCTTTGAGGGATCCATCGTGTAGCACCAAGGGCCGTTTGTAT

CCCCATCAGGGTTTCTGCATTTATTAGACTCCAGACCCTTCTCAGGGTGTGTCAGTGGAC

TAAAGTTGGTCTTGTGAGGGGTGGACGCACTCCATGCCTGGCAGGTCACTCCGGCTGCTG

TCATTGCTACTGAACCTCGATAATTTTCCCCAGTACTAATTTTGCAATCTTCATTTTCTT

TTTCTGCGCCTTGTGTAGTAGGACGGTTCTGTTGTGTGACGACCGAAGGAGGAGGAGGAG

GAGCCACGCCCTGATCGGGCTGCGTGGGGCGACTGATTCCACAGCGCTCTATCCTGCAGT

ACTCCCAGCGCACAGAGGGGTCAGTGGTGAAGCACCATGGAGCTCTGTCCTCATCAGGGT

TCCTGCACAGGTTTCTTCTCAAGTCTGCATTGGGGAATTCCTGTGGTGTCTTTGAATGTT

TATGTGGGACCATCGAGCTCCAGGACTGACACTTCTTTCCAGTGATGGTCTCCGACATGA

CTCCACGGTACGAGCTGCCATCTCCTGTGTAACAGTCCTCGGCTGGTGCGATGACCGGCT

CATCTGGGTGTGGCTCGGCACCACAGCTCGATATATTGCAGTACTCCCAGCGGGTTTCTG

GGTCATTGGTGTAACACCAGGGACTCCTCTCGTTATCAGGGTTTCTGCAGTAGTTGTTAT

CCAGACCTTTGCATGGGTAGTTCTCCGGGGTTCTTGAATGCTTCTGAGGTAACTGAGATA

ACCACGCTTGACAAGTTTTCCCTGATTTGGTCACAGAAACTGTCCCTCTGTAGGAGCTTC

CATCTCCTGTGATACAGGTAAGCTCTGGTGCAATAGTTGGAGGCTGGGTTGCGCAGCGTG

GGATGGGGCAGTAATCCCAGCGTTTGGATGGACTTGTGGTGAAACACCACGGTCGGGGCT

CTCCGTCCGGATTTCTGCAGTAGTTCTCCTCTAGGTACTTCTCAGGAAAAGATTTGGGGT

TGTATCCATGGTTATGGGGTTTCTGGGAGTCCCAGCGTTGACAAGTGAATCCTCCCTCGG

TGCTGGCGATCTTTCCTCTGTAGTTTTCTCCACTACAGTACATGCATTCCTCGCTGCAGT

TGGGGATGGAACAGTGCTCCCATCTCTTATTCACGTCTGTGGTGTAGCACCAGGGTCCCT

GGCTGTCTCCATCTGGGTTTCGACAGTAATTAGACTCCAGGTCTGCTCTCGGATGCGTCT

TCGGGGTGATGTTTGGCTTGTGAGGGAACATTGCTTCCCAGCGCTGACACAGGATTCCTG

ATTTTGACCTGCTCTGTGTTCCTCTGTAGTCGCTCCCGGCACCGTTCATACATTCAAGTA

AATAACGTTTCTTCTCGAACAGAGCTGTACTCATCCTCCGGTACACTGGCTCGATCTTTG

AGTTGGCTGGTATTGTTATACATTCCTGATCTTTTTCAATGAATACAAACGACCTGCACA

CAAACTGAGTTTCTGCATCACACTTCAGTGCACATTCTTCCACCTTTGCTGTGACGTATG

TTCTTTTTTGCATCGTGAAAATCCAGGCGCCGTCGGTTTTAACATACTCGTCTAAATCTG

TGCTCTGGGATTGAACTTGAAAGACACCTGCATAAAGAAAAAGCCCAAAAAGAAGTAGAG

GGACTTTGTAGACATCCATACTGGAGCACTAACACACACTGATTTCAGAGCAAGTTTTGT

CTCATAGGCCGCGTCAATAAGACAAATAATCATCGGTCAATCATTTTCTTAGCAAGGACA

TAGAACAGAGTTCATCGTGTGTTTACTGGACCTACATTTACTGTTTGGAATCCATGTTTT

AAC

>Unigene000203

GGCATACACAGACCGAGTGACTACTGCGCAGTGACAGTTATTTTAGTTCCCGCGCGGGTG

ACCGCGTCCTCCGCTGTCATAACAAATAACACGCAGTTCACGGAAACCGGAAGTTAGTGT

TCGGTAAAGCGGAAAAGATGTCCTGTCAGCTCATATAACGGTCTTTTTTTCTCTCTCTTC

CTAATATAAAATTGCAGCATATCCAGACTACCAGACTCTCTAAACACAGACATCAGCATG

GACAATGTGACAGCCTCCATGTTCCCCGGGCAGAAGATGTCTCCATCCAAAGCGATGACA

ATGAAAGACTACGAGAATCAAATTACAGCTCTGAAAAAAGAGAACTTTAACCTCAAGCTG

AGGATCTACTTCCTGGAGGAGCGCGTGCAGCAAAAATGCGACGATTCTACCGAAGAAATT

TACAAAACGAACATTGAGTTAAAGGTGGAGGTGGAATCTATGAAGAGGGAACTTGCTGAA

AAACAGGAGCTGCTTGTGTCGGCATCGAAAGCGCTAGAGAGTTTAGCTAACAGAGATTCC

GAGGCGGGTCACCGGGAACGGGCTCAGAGGGACATGGACGCACTCCGAGAAGCATTTAAT

GCCAGAATCAGAGACCTGGAAGAGTCTTTGCGCTGTGCAGAAGACGAAGTAGAAAAAATG

GCTTCCATTGCCGAGCAGGAGAAGCTGAGGAGTATAGAGTTGGAGAAAGAACTTGAAGCT

GTCTGCCAGTCTGGACCATTGCACGATGCTGACTCTGCCCGGGGACCGGACCACAAACTC

CAGCAGGCTCTGCAGGAAAAAGAGCGTTTAATCAATCAACTTCGGGAGTCCATCAAAAAA

CAAGATGCCCTGATAGGAGAACTTCAGGAGCGAGATCAGGATCAGCCAATCAGAGAGCAG

ATGCTCCAGCTCAACAAACTGATTGGTGAAAAGGATGCTGAAGTACAGGCTTTAAAAGAA

GAGTTAGACCGGGAGAGAGGAAAAGCCGAGAAGGAAAGTCAGGTGTGTATGATAACACAG

GACGAGCTGAAGACGCTACATCAGAAAACCCGACAGCTGACTGAAGAACTGAACTCCACC

AGGAGCAGCGGTCAAAAACTGAAACACAGACTGGAGGAGATGGAAGGAGAGAACAAGATT

CTCTCTGGACAACTGGAGGAGAGGGAGAGTGAACTGGCGGGAGAAAAGAAAAACGCCCTG

AAGCGAGACAAGACCATTCAGGGACTGAGCATCCTGCTTAAAGAGAAAGAACGACAGGTG

GATGAGTTGTATGGAAATCTGGAGGAGAAAGAACGGACCTTAACCAAAGCCAGAGAAGCT

CTTCACAAAGCTCAGCTACAGAAATATCAGGGTGGTGAGGAACAACAGGCCCTGCTACAG

TCGCAGAATGCTGAGCTGTCTCGTCTACAGGCAGAGTGTCATTCGTCTCTGCTGGAGCTT

CAGAGGTTACAGCGTGCACTCAGCAGCAGAGATGCACAACTGGAGCTACTGCAGCAAGAT

AAAGTGCAGCTGGAGAACGAGCTGGAGCTGCTTCAGCAACACAAAAGAAAAGGAGACAAG

ACCATTAATGATCTGCAGAACCAGTTAAAAAAGCTGAGTGGAGAGCTGGCCGAGCGTGAG

AACCTTCTGGAACATCAGCGTTTAGCTCAGCAGGAGGAAACCCGCACAACAGAACAGAAA

CTGCAAAGCACAATACAACAGTTAACAACCAACATCACAAAAAAAGAACAACAGCTACAG

GAATACATGAGCATGATGCAACATCTGGAACAGAATCAAGCTTCAGCAGGAAGTGATGTA

ATGCTGGCCAAACTCAGAGAGAAGCTGAGGGAAAAGGAAAAGGCTTTAGAGAAGGCACTG

GATGAAAAGTTTGTGGCAGTGGAAGAGAAGGAGAATGAAATTCATCTGCTGCAGCTGAGC

ATGAGAGAAAAGGACAGAGATCTGGAGCGTCTCAATCACCTGCTCACACACAACGAGGAG

ACCGTTAACAGCTTTGATGCGTTACTGAAGGAGAAAGACATGGAATTGCAACAGCTGCTG

AATTCCTTTAAAAACCTGCAGCGCTCCAAACAGGAGAGTGAAGAGAACCTGCAAAGAGCT

CTAAGGGAGAAAGATTCCATCATCCAGCAGCTCCAATATGCACTGCAACTCAAAACTAAA

GACACCGAGGACGGCGCTACTACGGGTCTCACACAGTCGGATTCTCAAGAGTATAATTCT

GCCGAGCAAATGAGTCAAAGGTTAAAGGTCACAGAGGCTATGCTAATCGAGGAAGTGAAG

CACAGGGAAAGGCTGGTGTGTGAAAACCAGACCGTTGTGGACAACCTGCTTGCCATTATC

AGTAGTAAAGACCAGCTACTTAAGGAAAGTGCAGAGCGACATTCGCAGGCACTCGGTGAT

CAGACAGCAGAGGTGCAGGATCTGCGCAGACAGCTCGCAGACACAGAGCGGCGAGTTAGC

AACGGCCAGAATCTCAGTGCTGGTCTGAATGAGAAGGATGTTCTAATAAATCTGTTGGAT

CACACTCCAGAAAGAGATCTGGAGGTTTCGCCCCCTCAAGTGGTAGAGCTTAGACAGACC

ATTAATATACTGCAACAGAGACTGGAGGAGAAAGAGGATGAACTGTCTCAGCGGCGCAGC

GAGGAAAACAGCGAGAAATTGCGTGCAGCCAATAAAAAAACCCGTAGTAATACTGAAGAA

AGAACTCGCCCAGAAGACAGAGGCACTCAACAGTGCTCTGAAGAGAGAAAATCAGCTCCA

GATGTCTCTTGCGGAACTTCAGTCCTCTGTGTCCGAGCTGGAAGGTCGACTAGAGGGCCA

GACAGCCAACATCGAGTCCCTCAAATCCATCATAAGCACTAAAGATGAAATTATTACTGA

GCTACAGCAACATTTGTCAGAAATGGGATTAAGACGGTCGACTGTTGATCACCACTCCCA

AGCCGCAGAGGAGCTCAGCTTCCGCTCTCTTCCTCAGAGAGAGAGAACCGTCATCGGGGG

AGACAGACAGCAGCAGCAGGAGACGTTGTCTCTGGGTGACCTTTGCTCTGAACAGGCTGA

ACTAAACCACATTCTTAGGGAAGAGCAGCAGCTTTACACCAAGCTTATTCATGCTGTAAA

AGAGCAAGACAGTGTTCAGCAGCTGCAGTCCTTACAGCTGGAGCTGAGCTCCGTAACACT

CCTCAGGCAGCAGTTGGAAGATGGCGTGCGTAACAACAATAAACTCCGTGAGCAGCTGCA

CCAAGAGATCCAGAGAGTTAATCAGAGAGAAGGTGTGGACCCTGCAGAGCTGAAAAGTAT

GCGAGATGCTCTAGAAGATGCTCAGCGCTGGAACGCATCACTGCAAGCACGACTTGGGCA

GATCCAAAGCAGAGGAGGGGGAGTAGGACAGGCTAATGACACAGATGATTCGCTGAGTCT

GATTGGCGAGCAGACCTCCTATATGAGTATATGTCTAGGGGAGGGGCAGGAGGAGGAGCT

ACAGCAGCTCTCTATAGAACAACTCAGACTGAAGGTCTCGGAGCTACAGGCCAAAAATGC

AGAGCTGCAGAAACAGTTGGCGCAGCCAGAGAGAGACGTTATGAGCAATTCTGAAAGAGA

GCCACAGGGACAACGGGACCTTATTGGGTTTTCACCTCCATGCACGCAGCCACAGAGACC

GGTCACGCCACTAGCTGTTAATGCTAACGAGAGACAGACCGCTTCCAGTGTCAAGAAGAC

AGCTGATGATAGTCTTGAAGTCCAAAGACGGATGGCAAGTTCCCCTTCACTTTTGCCTAA

GAAAGATAATGGAAGTGTCTCATATTCTCCCGGACAGCAGAGTGAGAATGAACAGGAGGA

ACTCGCAGCCTTGCTTAGAGACTGTGGAGCGGAATCCATTTCCCAACTCAGGGAGCAGGT

AAATAAACTTCGCTCAGAAATGTCAAAGCTCGGTGTGCTTTTGAAGGAAGAAACTGTGGC

CGAGTCCAAGGAAAGCACCGAAAGTTCCGGAGAAAACGACAGCCATACAAACCTGCATCA

GACGGTGCAGACGCTCCGCTCCGAGGCTCGAAGTCACCGCAAAATTGTTCGACTGTTGAA

GGAACAGCTGCTGCGCGATAGAAAACCAAAAACAGGCGGCGAGCCAGGACTCGACTCGGA

ACGGGTTGACAGCATGGCTCGCAAGATGGAGCGTCTGAGAAAGGAGCATGAAGCCACTCA

GAGACATGCTGCCATCCTGGAGGAAAGGCTAAAGGAGGTACAAAGCAGAGAAAGAAATGA

GGACCGAGAACAAGAAGAGAAAGACAGTAAAAAGAAAGGCAGACAAGAACATAGCAAAAA

GTTGAGACATGCGGCATACAGCAAATCACGGCTGCCAGTAGCAATAAGACCTGCAAAGCC

GAAAGACAGACATAGAATGACTCATCATGATCCAGAGAAAAGTCCTGAGCCGCTTAGTAC

ATCAGATTCTGAGCAGCTCAGCCCAAAAAGCGAAGGTTCTTGTGGGGCGGTTCTTCAAAG

GTCCTCTGAAAACAGCTTTAAAACCCTTCGGGGTTCTGAGTCTAGACAAAACAGAGAAGT

CAGAAAAAAATCCAAAAATGTGTCTGACATTCCCCAGTCGGAGCTTTTATCTCAGCTGGA

GTTACTGAACCAGGAATGCCAGGAGAAAACGGAGCTTATCTCACAACTTCAGCTTCAGAT

GCAGAACTGGGAGAAGCTCCAAGCCGAGCTCCAAGAGAAGGAGCGGTTAAATAGTCAGTA

TCTGGAAGCCTTGCATGCTGCAGAGTCTACAATTGCATACCTTACAGCCTGTAACCTGGA

TTCGGAAAGTGGCCTGGGTCAGCCCAAACCGGGATCTGATTCATCACTGCAACAGCAGTG

CAGCGAGCTTCAACGAGCCGTGCAGGAAAAAGATCAAGTCAGCATTCAGCTGCTTGAGTG

CTTAAACAAAGCCGAAGCTGCCATGGCATCACTGACTTCGGCTAATCTGCCTGAAAGCTT

TTTCTCTGCCCAGAAAGACCCACATGCCTTAAGTGAGAGGTTTGACTTCCTTCTCAGCCA

AATAAAGACCTTACAGGAGCAAAAAAATGCTTGCACTTCTAAGGAGTTGCAGCAGAACAG

GCTGAATGCTGAGTTACAGGAAAAACTCCGGGCCGCAGAAGAAACTATTACCAAACTATC

TGCTCAAGACGATACTACCACTGGTCAGAAGCTGATTTACAGCCACAGTTGCACAAGTCC

ACAAAAGGAGATGGTGGATGAGTATGAAGAGGGACAGCAGAGACTGGCTGCAAGTTTTTC

AGAGTGTATTTTTGCAGTAGAACAAGCAGTAGGGTCTCTTGCTGAATATGGCTTAAGGTC

TGATCGGCTTTTGCATGGTGTTGAAACCAGCACCAACGCAGAGCTTGAACAGAACTTGGA

CAGGCTTCAAAAGGTGCTCCTGGAAAGAGACAGGTTTTGGAACTCCATCAGTACAGATAT

AAGCCACAGCACTATTGTGGGTCCGTTCCACCAACCAGTACATCACAATCTACAGATTTT

AGACGCTGCACAGCAGAAGACAAGCACACCAATTTCTCAAGAACAGCAGGGGCAACAGAA

AATATCCGAAACATGCGATCCTGATCGATCCGGAAGTCAGCATCTAAACCTTTACAAAAA

CCTAACTCTGCTTGTCCAGATCTTTAAACAACATGCTCTGAAGGTCCGGGAGCTAGAGGA

G

>Unigene000204

GGATTCCAAGCATCATTGATCCATCTCAGCATGACTAGTTCAAGGGGCAACTCACTTTCT

CTGGACTCTGCACTCAGAAGTGTGTAAATACCCACAGACGCCCGGACGCTCCACAGCGTA

CCTGCCGGAGCCATGACCACCTTCGACGACATCCTGGAAGAAGCCGGGAAATTCGGCTTG

TTCCAGAAACGCATCTTCTCCCTGCTGAGTCTCGTCTCTGTGCCTTTCGCCGGAGTTTAC

GTAGGCATCGTGTTTCTGGGCTTCTCCCCCGAGCACTGGTGTCGTCACCCTGCAGTGAAG

CAGGTCCAGATGCAATGCGACTGGAAAGTAGAAAAGGTGTTGAACTTCACTGTGCCCTTT

CAGAACACTTCTGCTGGACCGCTGCGCAGTCGGTGCGAGAGGTTTGACGTGGAATGGAAT

GTTTCCGATTTCAGCTGCGATGACCTGGACGGTGAGATCGCTCGGGCAAAGCTTGCGGTT

TTGCCCACCAGTTCCTGCACAGATGGCTGGGAGTACGACTATGAGGGTAGAGAGTCTTTT

GTCACTGAGTTTAATATGGTGTGTGAGGACGCCTGGTTGTTAGACATGTGCCAGGCGGCG

ATCTCCGCCGGCTTTCTAGTCGGCAGCATTGCCATCGGATACTTCGCCGACAGGTTCGGC

CGGAAATCCACTTTCCTGATGTCCTGTGTTCTGAATACAGTCACAGGAATTCTGGTTGCG

GTGTCTCCTAATTACATCTCCCTGCTGGTGTTCCGAGCGCTGTACGGGTTTGGCACTAAG

GGCGGGTGGATGGTCGCATTTGTTCTGATCACAGAGCTGGTAGGAGTGGAGTACCGGAGA

CCAGTCGCTGTGTTCTTTCAGATGTTCTTCAGCCTCGGGGTTCTTATCCTGCCCCTCATA

GCCTACCTCATCACCAACTGGCGCTGGCTGCAGGTGGCCATCACAGCTCCATATCTCGGC

TTCCTGGTCTACTACTGGATTATTCCAGAATCTCCTAGGTGGCTTCTCTCTCAGAACAAC

AACAAAAAAGCTGTAGAGATCACAAAGAAAATGGCCAAAGTGAACAAGAAGACGCTGTCC

AAGAACATTGAGACACTTAAAGACGATTGCACAGACACGGCTGTTGCCTCATTCATGGAC

TTGATCCGGACGCCCAATATGAGAAAGCACACCTTCATCCTCAGCTACAACTGGTTCACC

AGTGCTGTGGTGTATCAGGGTTTGGTCATGCGTCTGGGGATCATGGGTGGAAACGTCTAC

ATTGACTTTCTCATCTCGGGCATTGTGGAGTTTCCCGCTGCCATCCTCATCCTCTTTACC

ATCGACCGTATCGGCCGCCGGCTACCATTCGCAACCGCCAATATTGTCTCTGGAGTCGCC

TGCCTAATAACGGCTTTCATTCCTGAGAGTTTCTTCTGGCTGAAAACAGCTGTGGCGTGT

GTCGGGAGGCTGGGAATCACCATGGCGTTTGAAATGGTGGTGTTCGTAAACACAGAGTTG

TACCCGACCTTCATCAGGAACCTGGGTTTTTCCGTCTGCTCCACTTTATGTGATATCGGA

GGGATCGTAGCGCCGTTCCTGCTTTACAGACTTGCGGTCGTCTGGCTGGAACTACCGCTT

ATTATTTTTGGAATCCTGGCAGTGATTGCTGGTGGTTCAGTGTTGCTGCTTCCGGAGACC

AGCGGCGTCCCTCTACCTGAGACTATTGATGACATCGAACACCCACACAAAAACAAAAAG

GGAAACGCAGGGAAAATCCATCAGCTGGAGAATCTGCTGCACCCGGATGTGACCAATAAT

AAAGAAACCACGACGGTCTGAGTGGAAGTGGAGGTTTTCTGCTCTGACGGCGGTAACGTG

GTAATATTTTATGACACTTGGACAGCAGTTGAAGTGATGGACGGTCTTCTGTTCATGAAC

TGGAAACCAGGACTGGTCGTCACAGTATTTATGTCCAAAAATTTAATTTATGTCCAAAAA

TTTTTGATGGACAGGTCACCAGTCCATCACAGGACCTCACACATACTCCTATGGGCAATT

TAGAATCACTAATTAATCTAACATGTTTTTGGACTGTGGGAGGAAACTGGAGAACCCGGA

GTAAACCCACACAAGCACGTGGAGAACATGCAAACTCCACACAGCAAGGCCCCTGCAGGG

GAATCGAACCCGGGACCTCCTTGCTGTAAGGCAACAGTGCAAAACCACTAAGCCACCGTG

CTGCCCCTGATTTTTCTGATTGTTTTCCTAATTTCTTTTTAAGTGCCGCGTCGCCGCTTA

CACCATTTTTTATATAAGAAGTAATCTCTCTTGTGGAATCATGGGATTGTGTCTATAGTT

GGCATACATTGTAGATCATCCAGGAATTTTTGGACACATTTTTACTTGCTATGGTTTCGG

ACATACTACTCGGCCTGTATTTCTTTTTAGTATTAAAGTATGTACGATTTTGGATTTACA

CAGTTTTTTTTTTTGATTAAAATGATTTTTCTGATCACAACATTAGTGTCTAATCTCACT

AATACTATTGGAACTGAATGATCAAATCCACAAGGTCACATGACCCAATCTTGTAAAAAC

ATCTTACCAGAAGTTTGGAGATCATTATAAGAGGGAGAGTGGGAAGATGGGGAGGTGTCC

TAATACTTTTAAACAAACAACACATATAAAGTCATTTACTTTTTTCTGAAAGGTTTTTTT

AATTGATAGGACAGACAGAGAAGAAACAGAGAGGGGAGGGTGGGGGGTAGGACCGGAAAG

GACCCAAGCCGGAATCAAACCAGGGTCACCGGAGCACAACTGCTCAAGTTGTTGCCAGCA

CAATGGTGTAATGTGTTTTGTTCTTTGGTGACATGGGTTCAGACGGGTCTCTGGGCGTCT

CTGCAATTTTATTGTTTTAAGTTTTTTATGTTTACTGTGAATCAATACTCTAGAATTCAA

ATATCTCTTTTATTATTTGTGAAAATTTCCAGCTGTTAAAATAAAAGGCAGACTGAATCA

ACGGCGCTTTTTTTTTTTTAAATAAAGCTTTAAAACAAGTTCCAAAGTTTCGGTTTGCAT

TTCAGGCTTTGATGCCTGTCTGGCTTTCGATCCAGTCGACGAACTTGGAGACGCGAGCGT

ACACACCAGGCTTCATGGCATTGGCACAGCCGAGACCCCACGATGTCACACCCTGAAGAA

CAAAGGTGTTCTGACCATAGCATACAAGAGGACCGCCACTGTCACCCTGACAGCTGTCAG

TTCCTCCCTCGATGTTTCCTGCACACATCTCATTGTCCTTCACTCGGTGGTTCAGGTATT

CGGGACGGTTACACACTTTGTTTTCGATCACTGGGAATCCCGTCTCCTTCAGAATGCCTT

CTCCACCCGTTCCTTGGGTTTCTCCCCATCCTGTAACATAGCATTCTGTCCCACTGGGAA

CGATGTGATCTTTCTGAGGCAAACACACTTTAGACACTTTCTCATTCAGGATCGCCGGTG

TTTCCAACTTGAGTAAAGCGATATCCGTTCCTGCCGGCCCTTTTATAAGCTTTTCCAGGT

TGCGGATCTGTCTGGACGACTCTACCGCCCGTTCTGTATGAATTCCCATGTACACTTTGT

AGGCAGACGGTCTGTTGGATCGTTCAAGGCAGTGAGCGGCTGTAAGGACCCACTGGGCAT

CGATCAAAGTTCCTCCACAGAAGTGCATGCCAGTGTTGGTTCTGAGGCTAATCTGCCAGG

GCCAAGAGTGAGGTTTGGATATGCATCCTCCAACGATTCGTCCGAAACAGCGTCTGGGTT

TTACTGCCGGCTGTCCGCACTTCCCCTCCGCACAGTCTTGAATGTTGCAGTAATCCCATT

TCTTTGAGGGATCCATCGTGTAGCACCAAGGGCCGTTTGTATCCCCATCAGGGTTTCTGC

ATTTATTAGACTCCAGACCCTTCTCAGGGTGTGTCAGTGGACTAAAGTTGGTCTTGTGAG

GGGTGGACGCACTCCATGCCTGGCAGGTCACTCCGGCTGCTGTCATTGCTACTGAACCTC

GATAATTTTCCCCAGTACTAATTTTGCAATCTTCATTTTCTTTTTCTGCGCCTTGTGTAG

TAGGACGGTTCTGTTGTGTGACGACCGAAGGAGGAGGAGGAGGAGCCACGCCCTGATCGG

GCTGCGTGGGGCGACTGATTCCACAGCGCTCTATCCTGCAGTACTCCCAGCGCACAGAGG

GGTCAGTGGTGAAGCACCATGGAGCTCTGTCCTCATCAGGGTTCCTGCACAGGTTTCTTC

TCAAGTCTGCATTGGGGAATTCCTGTGGTGTCTTTGAATGTTTATGTGGGACCATCGAGC

TCCAGGACTGACACTTCTTTCCAGTGATGGTCTCCGACATGACTCCACGGTACGAGCTGC

CATCTCCTGTGTAACAGTCCTCGGCTGGTGCGATGACCGGCTCATCTGGGTGTGGCTCGG

CACCACAGCTCGATATATTGCAGTACTCCCAGCGGGTTTCTGGGTCATTGGTGTAACACC

AGGGACTCCTCTCGTTATCAGGGTTTCTGCAGTAGTTGTTATCCAGACCTTTGCATGGGT

AGTTCTCCGGGGTTCTTGAATGCTTCTGAGGTAACTGAGATAACCACGCTTGACAAGTTT

TCCCTGATTTGGTCACAGAAACTGTCCCTCTGTAGGAGCTTCCATCTCCTGTGATACAGG

TAAGCTCTGGTGCAATAGTTGGAGGCTGGGTTGCGCAGCGTGGGATGGGGCAGTAATCCC

AGCGTTTGGATGGACTTGTGGTGAAACACCACGGTCGGGGCTCTCCGTCCGGATTTCTGC

AGTAGTTCTCCTCTAGGTACTTCTCAGGAAAAGATTTGGGGTTGTATCCATGGTTATGGG

GTTTCTGGGAGTCCCAGCGTTGACAAGTGAATCCTCCCTCGGTGCTGGCGATCTTTCCTC

TGTAGTTTTCTCCACTACAGTACATGCATTCCTCGCTGCAGTTGGGGATGGAACAGTGCT

CCCATCTCTTATTCACGTCTGTGGTGTAGCACCAGGGTCCCTGGCTGTCTCCATCTGGGT

TTCGACAGTAATTAGACTCCAGGTCTGCTCTCGGATGCGTCTTCGGGGTGATGTTTGGCT

TGTGAGGGAACATTGCTTCCCAGCGCTGACACAGGATTCCTGATTTTGACCTGCTCTGTG

TTCCTCTGTAGTCGCTCCCGGCACCGTTCATACATTCAAGTAAATAACGTTTCTTCTCGA

ACAGAGCTGTACTCATCCTCCGGTACACTGGCTCGATCTTTGAGTTGGCTGGTATTGTTA

TACATTCCTGATCTTTTTCAATGAATACAAACGACCTGCACACAAACTGAGTTTCTGCAT

CACACTTCAGTGCACATTCTTCCACCTTTGCTGTGACGTATGTTCTTTTTTGCATCGTGA

AAATCCAGGCGCCGTCGGTTTTAACATACTCGTCTAAATCTGTGCTCTGGGATTGAACTT

GAAAGACACCTGCATAAAGAAAAAGCCCAAAAAGAAGTAGAGGGACTTTGTAGACATCCA

TACTGGAGCACTAACACACACTGATTTCAGAGCAAGTTTTGTCTCATAGGCCGCGTCAAT

AAGACAAATAATCATCGGTCAATCATTTTCTTAGCAAGGACATAGAACAGAGTTCATCGT

GTGTTTACTGGACCTACATTTACTGTTTGGAATCCATGTTTTAAC

>Unigene000205

GGGATTCAAGATGGCGTAGAAAGTGAAGCTAGATACTCTGTCCCAGAGTCATGGAGGATA

GGAGGCCCCAGAAGTCATGTGAAGAGGCCATTGGATCAATAGTAAGGCAGGAGTATGAGG

CAGCAGTGGCCCACAGCACGGAGGCCCTATTGGCCCTGGGATCAACCCCACTCACCCCCA

ACGCAACCCTTCTCCGCAAGTGTGCATTGCTCTACCGGATGGCAGCCCTGCTCCAGTTGA

AGAAGTACGATCAAGCTGATGAAGACTGTAAGCATGTGCTGGCTGAGGAGCTGGGTATAG

GTGAGGGAAGTTTCAGAGCTGGTTTGCAGAAAATGATGCTGGATGGAAGCCTACAGGAAG

TCTATACCATTCTTTCTAAAGCTCTGTATGGAGAACCGCTGAATGGTATCATGGCGAAGG

ATATGACGAGGTTAAAGATACTCCTTTCAGAAATGGAGGTTGTAAGCAGTAAAATAGTAA

CCGAACCGCAGGGAGAAACAGAAGAGGACGTTCAAGAAGGTTGGCAGTTTCGGCCTCCGC

CCAGAGGGGTCACTAGCAGCGAGGAGTACACGTTGTGTAAAAGGTTTTTAGAGCAGGGTC

TGTGTCGGTACGGTGCACAGTGTACCTCAGCCCACTCTCAAGAGGAACTGACCGAATGGC

AGCAGCGCTATGCCTCCAGACTCATCCGCCTCAAGCAGCAACAAGATGGGAAGCATTACA

GCGAGAACTACACGGAGGCCCTTATCGAAAAGTGGATCAACTCTCTCACACCAGAAAAAG

TGCTTAATGACTGTGTAGATGGAGTTACGGTGGAACATAACTCAGATCTTTCAGTCACCG

TCAACACCAAGAAGTCATCCCATTCCTGGATATTTTTACTTTACTGTGAGCCTGCCCGCA

TGTTGCACCGCATCGCACTGCTCTATGATGCCAACCGTCCCCACTTCTCCATAACAGCCG

TGTCAGCAGGTGACCCCTCCACCCAGGTACCTCAGCAGGTATGTGAAGGTGGCTGTCAAG

AGTGGACTGGAGTAGACCTGCTGCAGAATGGCATGGATCACTGCCTCTACACTGTGGAGG

TGGCTTTTAAAACCGAGATCTTTGGCACTTTCCGCCAGACAGTAGTGTTTGATTTCGGCT

CTGAGCCTGTGCTGATGCAGAGAGTGATGGTGGACGCAGCGTCAATTGAAGACCTGGAAC

ATTTGATGCAGGCCCGGCAGCAGCTTCTTATGACAGCGAAACGCTGGGACTCCACCTGCA

AGACCATCGTGGATTTTGTACCCAACGAGAACATGGAGCTGGAGCGCAGTCTGCTTAGCC

GCTATCAGATCCCACTGTCAGCTGACCAGCTCTTTACTCAGTCTGTATTAGACAAGACTC

TAACCCGTGAGAACTACCAGCCCCGGCTGCATGACCTGCTCTACATCGAGGAGATTGCAC

AGTACAAAGAAGTTAGCAAGTTCAATATCAAGGTGAACCTGCAGCTGGTTTCCAGCTTCA

TGCTGACTGGAATATCTGGGGGTGCTAAATATGCCCAAAACGGTGAGCTGTATGCCCGCT

TCAAGCTAACAGAGACACTGTCTGAGGACACACTGGCAGGACGGCTGGTAATGACAAAGG

TGAACTCGGTGCTGCTACTACCTCTGAGCCGGGATCGTCAAGGCCAGCCAGCCAGCGCCA

AGGAGCGCGTCTATGAGGCCGGGATTGAGGAGAAGACTAAGGACTACATCTTCCTGCGCA

TCTGCAAGGACTGCTGCGAGGAGCTCCATCTGATGCCTGACAGGGAGCTGCAGGTGGAGC

TACAGTTTCAGCTCAACAGGCTTCCGTTGTGCGAGATGCACTATGCTTTAGACCGCCTAC

GTGACAACGGCATCCTTTTTCCTGACATCAGCCTAACGCCAATAATCCCTTGGAGCCCTA

ACAGACAGCACAAGGAGGACTGAAGTTGAAATGGGATGTCCAAAAAACCTCATACAAATG

CAGTGGGATGAGCAGTTGGACCCTCGTTTGAATGCTAAGCAAAAGGAGGCCATTTTGGCC

ATCACCACTCCACTGTCAATCAGTCTCCCTCCAGTACTAATCATCGGACCCTACGGTACA

GGAAAAACATTCACACTGGCACAGGCAGTGAAACACATCCTCAAACAGCCTGACACACGG

GTCCTCATCTGCACCCATTCAAACAGCGCAGCAGATCTCTACATAAAGGACTACCTGCAT

CCATATGTGGAGGCGGGCAATTCGCATGCAAGACCTCTCAGGGTGTACTTCAGGAACCGC

TGGGTAAAAACAGTGCATCCAGTGGTGCAGCAGTATTGCCTGATTTCCGGAGCTTACCTC

ACCTTCCAGATGCCGACTCGGCAGGACATAGAGCGCCACCGAGTTGTGGTAGTGACTCTT

AGTACCTCCCAGTACCTCTGTCAGCTTGATCTGGAACCTGGGCTCTTCACACACATACTA

TTGGACGAGGCGGCTCAGGCAATGGAGTGTGAGACTATTATGCCCTTGGCACTGGCTAAT

AAGAGTACACGCATCGTCCTTGCTGGAGACCACATGCAGCTCAGTCCATTTGTGTACAGT

GAATTTGCACGGGAGAGGAACCTCCATGTGTCTCTGCTGGACCGGCTGTATGAGCACTAT

CCCCCTGACTACCCCTGCAGGATCCTGCTGTGTGAAAACTATCGCTCCCATGAGGCAATC

ATCAACTACACATCGGAGCTGTTCTATGAGGGCAAACTGATGGCCAGCGGCAAGCAGCCC

CCACATAAGGACTTCTACCCTCTGACCTTCTTCACGGCTCGCGGGGAGGACGTGCAGGAG

AAGAACAGCACGGCGTACTACAACAATGCTGAGGTGTTTGAGATTGTCGAGCGTGTGGAG

GAAATAAGGAAGAAGTGGCCAGTAGCGTGGGGGAAACTGGACGAAGGCAGCATCGGCGTT

GTGTCCCCATACGCAGACCAGGTGTTTCGCATTCGAGCCGAACTCCGCAAAAAGCGCATG

CCTGAGGTCAGCGTGGAGCGAGTGCTCAACGTCCAGGGTAAGCAGTTCCGGGTGCTATTT

CTCAGCACGGTTCGCACTCGCCACACCTGTAAGCACAAGCAGACAGCCATCAAAAGGAAG

GAACAGCTGGTAGAAGACTCCACCGAGGACCTGGACTATGGCTTCCTATCCAACTACAAA

CTCCTGAACACTGCCATTACACGAGCTCAGTCCCTGGTGGCCGTGGTGGGAGACCCCATA

GCGCTCTGCTCGGTGGGCCGCTGCAGAAAATTCTGGGAAACGTTCATCTCCATTTGCCAT

TATAACAACAGTCTGCATGGAATTACATTCGAGCAAATTAAAGCCCAGTTGGAGGCTCTG

GAGCTCAAGAAGACCTATGTGCTAAATCCACTGGCTCCTGAGTTTATTCCCCGAGCCCTG

AGAGCGCAGCATCCACAGGGACCCGGCAAACACCAGCACTCCCCTCCCAAGGTAAAAGGA

CCGCTTGCCAATCATGCAGACCATTTTCCTCCAGAAGGATTCGTTCAGCCCAGCGCTGCT

GTGTTAATGGGGAATCCCATCCAGGCTTTCACTCCAACAGCTGGAGGAGCACCATCCTCC

GGCAAATCCCCCAGTCCTGTACAGCGCATTGACCCTCACACTGGAGCCAGCATCCTGTAT

GTCCCTGCTGTGTATGGGGGAAACATGGTCATGCCTATGTCTCTGCCGTTGCCATGGCCA

GGCTATCAGAACCGCTTTGCTATGGACCCTCGCATCATGAGTCACCAGGCAGCCATGGCA

GCCTATAATCTGAATCTGCGACCCGCAGTCAGTCGGGCATCACCCGTGCTATATGGTCTG

AACCAACCATCCTCACTTGGCATTGGCCAACTTCCGTCCACCGACAAGGACTTGCTGCAG

GAACCTACTGCCAATGGAAAAGTTGACATGAATGGAAAAGTGGAACATAGCAGGCTATTG

ACACCTGAAAGAAAAGCTCCTGAGCTGAAGGACAAACAGGGAGAGCCTGAACCAGTTCAG

AACAAAAGCCCTGAAGCCCATGCCGGCCTAATGTTCCCCCCAAGCCGTCTGCTGCGTAAA

GACCCACCCGCTCAGCGTCCCCCACTCAGCTTTCCTTTACCCCCTCCTCATCCTGCATTT

GCCCCTACTGGCCCCCTGGCACATTATGGCCTTCCTAGACCTCCCCTTCGCGTGCAGACC

CAACAGCCACAGCCGGCTGCCTTCCCTCCCTCATTTTTTGGTGGCCATCCTCTGGCCCAG

CGCACCTTGCACTCTCCAGTGCTGGATGGCCTTGATGGAGGGGAGAGCCTGATGGGCACT

GTGCCTGATGAACTGAAGAACGTTGTGAGAGGAATACCTCCTCAACACCAGCCCCATCAG

CAGCCAATGCGCCTCAACAGTTTCGGGGAGGAAAGCCTCGACTCGCTGCTTGGTGACTCT

CTGGATGGTCAGAGCTGTTCGGGCATGGACACACTGCCTCCCCAACCGCAGCAGCAGCCC

ATGCGTATAGGTCAGTGGAACGAGCCTGGACCCTTCCTGCAAGGTGCTGTGCCTCCTTTC

ACCTTGGCTCAGCAGTTAGCACACCTGAGTCAGCCAGGCATGCGGATCCCCCCGCAGCTG

CTTCGTGCCAGCTGGGGCCTGGGCACCACTGCTGACAACGAGCCTCGAGCACCAATGCCC

AGGTATCCTGGTCTCCCACGTGAGGCACTCCCTCATGAGTCGTATGATCCACGTGAAGCT

GCCGAAATGCCCCCTCCTCAGTCACGCCTGCTACAGTACCGGCAGGTGCAGCAGCCCCAC

GTTCCCGGAGAGCTGCCATCACCTTCCGCTGCTTCCTCTGTCCTCCCTTCATCCACCCCC

AGCAGTTCCAACAACCACAACCATACTCCATCATCGGGTCCGTTCCCTTTCCCGGAGACC

AACCGTGATCTCCCTTTTCACCAGCAACAGGCCTTCAGCCAGGCCGCTCTCCCTCTACCC

CCAGAGCATGTGCCCGTCAAATACCTTCTGCCTGAGGCCCACTGGCATCATCCCGGGACG

CAGCAGTCCCAGGCGCTGCCGTTCAACCTGCAGCAGGGCCCTTTTACACAGCTTCAGCTT

CAACAGCACAAACAGCAACAGCAGCAGCTCCAGGCCTCACACAATGCCCTGCACAGTCTA

GAAGAGTATGAACCGCGAGGGCCCGGGCGGCCCCTGTACCAGAGGCGGATATCCTCCAGC

TCTCCTCAGCCATACGGGGAGAGTGTGGTGCAGTGTAAGGAGTCTCAGGATCCCATAGCC

TGTTTCACATACCCCCCTGCAGACCTGTGGCCCAGCCCAGGCCCCGCTCCATTCCACAAC

ATTCCATGCAATGGAGCGAGTCACCTCGTCCCTCAGAGGGATATTTTAGTTTCTAAGGCG

GTGAATGCTACAGAGGAACACACCCAGGCTGAGTGTGTGCAGCAGCCTCCTCACCCCGCA

GGCCTTGCAGCGCTTCAGCAGCATGGACAGTTTCCAGCTCTCCTGCCCAGCAAGCAGACC

CCCCCAGACCCAGGAGGAGTGGGCTCCATGCCAGCCTCGAGTCACAGCAAACCCCCTGCC

ATGTCCTATGCCAGCGCTCTTCGAGCTCCCCCCAAACCCCGCTGCCCGCTACCTGAGCAG

ACCAAAAAGAACACCGACCCCATCTCTCTGTTACA

>Unigene000206

CCGCGCAGATCGGAAGAGCGTTGGCTATGAGACTGACCACCAAGACTATTGTGAGGTGTG

TCAGCAGGGAGGAGAGATTATCTTGTGTGATACCTGTCCCAGAGCTTATCACATGGTGTG

TCTGGATCCTGATATGGAAAAAGCCCCTGAAGGCACTTGGAGCTGTCCACATTGTGAGAA

GGAGGGTATTCAGTGGGAAGCACGTGAGGAGGCTTCAGATGGGGAAGAGGAGAATGATGC

AGGTGGCGGTGAGGCAGAGGAAGATGACCACCACATGGAGTTCTGTAGGGTGTGTAAGGA

TGGAGGAGAGCTGTTATGCTGTGACACCTGTCCTTCTTCATACCACATCCACTGCCTTAA

CCCACCTCTACCTGAAATTCCCAATGGAGAGTGGATCTGCCCACGTTGCACTTGTCCTTC

ATTAAAGGGGAAAGTGCAGAAGATTCTTACCTGGCGTTGGGGAGACCCCCCACCCCCCAC

TCCTGTTCCACGCCCTCCTGACCTTCCAGCAGATGCCCCAGACCCTGCTCCGCTGGCTGG

CCGGCCAGAGAGGGAATTTTTTGCAAAGTGGCAGAACATGTCCTATTGGCATTGTTCCTG

GGTGTCTGAGCTGCAGCTGGAGCTGCATTGCCAAGTGATGTTCAGAAATTACCAGCGCAA

AAATGACATGGACGAGCCCCCACCCATCGACTTTGGAGGTGAGGGTGATGAAGATAAAAG

TGAAAAGAGGAAGAACAAAGACCCCATCTATGCCCAGATGGAAGAGAAGTATTACCGTTT

TGGCATTAAGATGGAGTGGATGATGATTCACCGCATCCTCAACCACAGTCTGGATAAAAA

AAATAACTGTCACTACCTCATCAAATGGAGAGATCTACCTTATGACCAAGCCACCTGGGA

GTCGGAGGACATGGAGATCCCAGAATATGATGCTTACAAACTGCAGTACTGGAACCACAG

GGAGCTTATGATGGGAGATGAAGGCAAACCAAGTAAAAAATTGAAGATTAAAGGAAAAAT

GAAGAGACTGGACAGGCCACCAGACAACCCAGTTGTTGATCCCACTATTAAATTTGAACG

CCAGCCGGAATATCTCGACTCAACAGGAGGAACTTTGCATCCATACCAGCTGGAAGGGCT

GAACTGGCTGCGCTTTTCCTGGGCCCAGGGTACTGACACCATCCTGGCTGATGAGATGGG

CTTAGGGAAGACCATCCAGACCATTTCATTCCTTTCCTACCTGTTCCATCAACACCAGCT

ATATGGACCCTTTCTCTTGGTTGTGCCCCTTTCCACCCTCACCTCCTGGCAGCGCGAGTT

CAGTACTTGGGCTCCTGACATGAACGTTGTGGTCTATCTGGGAGATGTTTTGAGCAGAAA

AACGATTCGTGATTATGAATGGATTCACCAACAAACCAAAAGAATAAAGTTTAACACACT

CTTGACCACATATGAAATTTTACTGAAGGATAAGGGTGTTTTGGGAAACATAAACTGGGC

ATTTCTTGGGGTGGATGAGGCTCACCGCTTAAAGAATGATGACTCTTTGCTGTATAAAAC

ACTAATAGATTTCAGGTCCAACCACAGGCTGCTCATAACAGGAACACCACTGCAAAATTC

ACTGAAAGAGTTATGGTCACTCCTGCACTTTCTTATGCCTGACAAGTTTGAATTGTGGGA

GGATTTTGAAGAAGAGCATAGTATGGGCAGAGACAATGGCTACCAGAGCCTGCACAAAGT

GCTTGAGCCCTTTCTTTTACGCCGTGTAAAAAAAGACGTAGAAAAATCGCTGCCTGCCAA

AGTGGAGCAGATCCTCCGAGTGGACATGTCCGCACAGCAGAAACAGTTCTACAAGTGGAT

TCTTACAAGGAATTACAAAGCACTATCTAAAGGCACCAAAGGCAGTTCCACAGGCTTCCT

CAACATTGTTATGGAGCTAAAGAAATGCTGTAATCATGGGTTCCTTATCAAACAGCCAGA

TGAGGCAGAAAATGAGAATTCTCAGGAGCACCTGCAGGCAGTGATCCGTGGCAGTGGGAA

GCTTGTGCTCTTGGATAAATTGCTAACAAGACTAAAAGAACGAGGCAGCCGTGTGCTAAT

CTTCTCTCAGATGGTCCGTATGCTGGACATCTTGGCTGATTATCTTGCCATGAAACGTTA

CCCTTTCCAGAGATTGGATGGCTCAATCAAGGGAGAACTCCGGAAGCAAGCGCTCGACCA

CTTCAATGCAGAGGGTTCTGAAGATTTCTGTTTCCTTTTGTCCACTCGTGCTGGAGGTCT

GGGCATTAACTTGGCCTCAGCTGACACTGTGGTCATCTTTGACTCTGACTGGAACCCTCA

AAATGACCTGCAGGCCCAGGCCAGGGCACACAGAATAGGCCAAAAGAAACAAGTAAATAT

TTATCGCCTGGTTACAAAAGGCACTGTGGAGGAGGATATTATTGAAAGGGCAAAGAAGAA

GATGGTGCTAGACCACCTTGTCATACAGAGGATGGATACTACTGGAAGGACAATACTAGA

CAACAACTCGGGCAATTCAAATTCAAATCCATTTAACAAAGAGGAGCTGGCAGCTATCTT

AAAGTTTGGAGCAGAAGATCTTTTTAAAGAACCTGAAGGGGAGGAATCAGAGCCGCAGGA

AATGGACATTGATGAGATTCTGAGGCTTGCTGAAACCAGGGAAAGTGATCAAGGCTCCAG

TGCTACAGATGAGCTTCTGTCCCAATTTAAGGTGGCTAACTTTACCATGGATGAGATCAC

ACCGGAGCTTGAGGAGAAGCCCATGCGGAACTGGGATGAGATCATTCCAGAAGATCAAAG

GCGGCAGTTAGAGGAGGAAAAGAAACAGAAGGAGATGGAGGACATTTACATGTTGCCCCG

AAGCAGAAGCTTGAATAAAAGAGCACTGGCAAATGACAGTGACAGCGATATTGGCTCAAA

GCTCAAACTGAAAAGCCACTCATCAGGTTCAGAGAGTGAAACGGACGACAGTGAGGATGA

TAAAAAGCCCAAACGGCGAGGCCGGCCACGTGCGCGCAAAAACAATGTTGTTGGCTTTTC

AGATGCTGAGATTCGTAGGTTTATAAAAGCTTATAAAAAATTTGGCGCTCCACTTGAAAG

GTTGGAGGCCATTGCGCGTGACTCTGAACTGGTTGACAAATCCATAGCAGATCTAAAGAG

ACTAGGGGAGCTGGTGCATACAACATGCGTAGCTGCAGTACAGGAGCATGAAGAACAAAT

AAAGGAGAACCCCACCGAAGCTAAAGGCCCAGGAAAAAGACGTGGCATTAACATTAAGAT

CTCTGGGGTTCAGGTTAATGCTAAATCCATAATCCAGCATGAGGAGGATTTTGAGCCTTT

GCACAGGGCTGTGCCTTCCAGTTCCATTGAGAGAAGCAAATTTCAGCTTGGTTGTCGTGT

GAAGATGCCACACTTTGATGTAGAATGGGATGTACAGGATGACGCACATCTACTGTTGGG

AATATATGAGCATGGCTATGGTAACTGGGACCAGATCAAGACTGATTCAGAGCTGAAACT

TATAGACAAGATTCTTCCAGACAACTCTGACAAAAAGCCACAGGCAAAGCAGCTGCAAAT

GAGAGCTGATTACTTGTTGAAGATCCTAAAGAAGAAACAGGAGGGTGAAGATGCATCCAG

AGCAAAAGAAGAGTCTCAGGTAAAGAAGAGAAAACCTAGAGTAAAAAAAGAGAAGTCCCA

AAAAGATGAACTGGCCAGTGAAATCTCCTCACCTCATCTCTCAGAACATACTTCAGAAGA

AGGTGAAGTAAAGGATGATAAAGCTGAAAAATTTCCTACAAAGGTAAAACAAAAAAAGGA

CAACAAAGAGAACAAAGAGAAGCAGGGCACTGCAAAGAAAGACAAGGAAAGGGACAGGGA

AAAAAAACGGACAAAGCTTAAAAAAGAAAAGACAAAATTAGGAAGCAAAGCCAAGAAAAC

TCAGGGCCCTGTGCACATTACAGCAGGCAGTGAGCCAGTTCCTATTGGAGAGGAGGATGA

TGAGCTGGACCAGGATACATTCAGTATTTGTAAAGAACGGATGAGACCAGTAAAGAAAGC

TCTTAAACAGCTGGATAAGCCTGATGAAGGTCTGTCAGTGCAGGAACAGCTGCAGCACAC

ACGTTCCTGCTTATTGAAGATTGGAGACCGAATTACAGAGTGCCTTAAAGCCTACAGTGA

TCCAGAACATGTCAAAGCCTGGCGCAGGAATCTATGGATATTTGTGTCGAAATTTACCGA

GTTTGGAGCTAAGAAACTTCACAGGTTATACAAAATGGCTCAAAAAAAGCGCTCCCAAGA

AGAGGCGAAAGAGCACAAAAAGAAAGATGAACCATTCAAAAAGAAGACATTTCGTCCAGA

ACCTTCTGGATCAAGTCGAGACTCCACTGGCACACAACCTTCATCGAAAGCACGCTTTCC

ACATAATCACCCAACAGGATTTTCATCTCAATCACAGCACCGGGAGCATTACAACCAACA

TAAGAGACACTTCCCTAATGATTATAGGGGAGATTGGCACAGGGAAAGAAAATATACCTA

CCCAGGAAACAGTAACCAGCCATGGCAAGGTGACCGATATAACCCTTATGACCAGCACAG

TTATAAGGAGCATCACTACAGTAACCGACGGGCTCACACAGACTTGTACCACAGTACTGG

CTTCCGAAACCCGGGTTCAGCTCGTAAACGAATGTATGACCAGTACAGCAATGATCGAGA

CCACCGTGTTCACCGTGAATATTATGACAGGCATTCAGAACCAAAGAGGAGACGCTCAGG

TGACTATCACTCTGCAAACTGCAACCAAGGAGGAGGTGGAATATGTTACAACCAGGACTT

CCGGAGGATGCCTGATCATCAGGGTATCCCAGAGACTGATCACTACCGGTCGTTTAACCC

AGACAAACCCCTTCCTCTGCTGGACCCACGTTCTCCTCAGGCCCAGAAATCCCCACTTGA

GTCCCTATCACCTCTGGAGAAAACCACTGAGCCGAAACCCAGTCCAGATCTTAAATGGAA

CAGAAAGTGATTTAATCCTTAATGATAGTGGAACATATAGAAAGACACCAGTTTTAGACA

AGGCCACCAGTCCCCACCATTTATTCCTTAATCACGATCCAATAGCTGCAGCTCAAGCTT

CCATGTATTTGAGCCCTCTGGATCTTTTGGGGTTAAATATGAAGCCCTCTTAGTCCTGTG

GACTACTTAATGAACAATGTGATTTGAAAGCTTGGAAGTGCCTCACTTGTTTGTCCTTTT

TTTAGCCATAAGCATTGATTAATTTAATTGTTTATTTAATGTTTTTGTTGCTTTGCTTTG

AGGAGGTTTATCCACAGGAAAGTAGGCCTTGTGGCAAAAACCTAAACCACATTAGTACTT

ATAAAGCCTGAGTTGATTTGACCCTGTTTTATGTGGAAGCATCCCTTGTCCTTTACATTT

TAGTGTTTTGCATCTAATAAACCAGATTCATGTGATTGTCAGACCTGTTTATGTTCCAAG

AGCAGGGGAAACACTAAAATGTACAGTTCAGGGTTGGGAAGCTATAATGTTTTTGAAATA

GGCTTATAATTTTGTGTTTGGCCCATTATACCAATAAAAGAAATTATGCTTAGAATGTGC

CACCAAATGAGAATTTGTACTTTTAAATAACAAA

>Unigene000207

GCAGTGGAAGCAGCAGTGGGGGAGGAGGAGGTGGGGGTGGTGGTGGTGGTGGTGTGTGGC

ACATTACAGTAGAGACTGACTCACCATCTACTACTTCTGGGAGCACGCAAAGCAACCTTT

CCGCGAAGCTCGGTCACTTGATCAGCCCACAGGATTCGTCAATGCTGCGCAATATTCAAA

ATACACTAAAGAATAAAATACAGAGCAAGGGCAAAGACAATCGCTTCCTATCCCCAGACG

GCTATTTGGGCTCACCGCGCAAGGGCATGCGTCGCATTCGGCAGCGCAGTAATAGCGATA

TAACTATAAGCGAGTTAGACGGAGATGGCAATGAGGGTTGGTCCTTTTCCGGCTGGACTC

CAATGCACCGTGAGTATGGCAGTACTTCCTCCATCGACAAGCATGGCGTTTCCGGAGAGA

GCTTCTTTGACATGCTGAAAGCTTATCAGACTGATGACCCTGATCAGCGTAGCCCTGCTC

CTGAGAAATTAAGCGAACTGCTTACTGTGGCACATAAACAGACCGCCCTCGACCTGCCAG

ATGGGCCCCTGGGACCCCCACGAGGTAGTCCTGCAAGTCGCCTTAAGGAACGGGAAAAGC

ACTTGAAACGGCGTTCCAAGTCAGAAACCGGAGGTGAGTCCATATTCCGCAAGTTACGAA

GCGTAAAGGTAGAGGGTGACTCCTCTCGCGCTGGTTCGGATGCAGAAGATGGGAAGTCTG

AGGATGCAGGTCCTCCCCCAAAGCCCTGGGTTTGTCAAAAAGGTTTCGCCCATTATGATG

TCCAGAGCGTTCTGTTTGACCTGAACGAAGCTGTACAAAACCGACAGAGTGCCTCAAAAC

GGAAGAACACCACTACCGGCGCCTCTGCCGCAGCTGCTGCTTCGGCCTCCACCTCCTCCT

CCTTATCGTCCACCCAAAGCGGAGCCTATGGCTCCCCCTGTGGCAGTCAAGAAGAGCTCA

GTGCCAAGGATGGACCAGCATTGGACCCTGGTGACGATAAAAGCAACGATCTGGTCCTCA

GCTGTCCCTGCTTTCGTAATGAGATAGGTGGTGAAGGTGAGAGAAACATCAGCCCTTCTA

AACAGGGCAGCATCGGAAGTGGATCGACCAATTCCTCCAGGGACGAGGATGGAAACCGTG

AGGCGGCTCCCAACACCCATTTCAGTAACGCAGGGGTAGCTGTGCTGGAAGCTCCCAAAG

ATGGAAAGAGCTTACATCATGAGAGAGGAAAGACAAACATTTTGGAACATGTGGACCTGG

GGGCCTATTACTACAGAAAGTACTTCTATTTAAGAGAACACTGGAACTATTTGGGAGTGG

ACGAGACACTCGGGCCGGTCGCCGTGAGTCTTCGGCGAGAAAAGCTGGACGATCATAAAG

AGCACGGCCCACAGTACAATTACAGGGTCATCTTTAGAGCCAGTGAGCTGATGACACTGC

GAGGCTCCATTTTGGAGGATGCTGTTCCCTCCACATCCAAGCACGGTTTACCTCGAGGCC

TGCCTCTGAAGGAGGTTCTGGAGTACCTCGTCCCTGAACTGAACACTCACTGCCTCCGCC

TCGCCCTGAACACGCCCAAAGTCACGGAGCAGCTGATGAAACTGGATGAACAAGGGCTAA

GCTTCCAGCTGAAGGTAGGAGTGATGTACTGTCTGGCTGGCCAGAGCACCGAGGAGGAGA

TGTACAATAACGAGGCAGCCGGTCCTGCGCTCGAGGAATTCCTGCAGCTGCTTGGAGAGC

GAGTGCGTCTCAAAGGATTCAGCAAGTACCGCGCTCAGCTCGACACCAAGACCGACTCGA

CTGGCACTCACTCTTTATACACCGCTTACAAAGACTACGAGATCATGTTCCACGTGTCCA

CCATGCTTCCTTACACACCCAACAACAAGCAGCAGGTAGGTCTGGATCCTGGAGTTAAAA

AAAACCCCAACCAATCGCTACAGTTCCCCCGCATGCTTTAACAAACATCTGTGCTTTATC

ACAGCTACTCAGGAAACGCCACATCGGGAACGACATCGTCACCATCGTGTTCCAGGAGCC

CGGAGCCAAAGCCTTCAGCCCCAAGAACATTCGCTCTCACTTCCAGCATGTGTTCGTGGT

GGTGCAGGTGCACAACCCGTGCTCGGAGAACACCTGCTACAGCGTTGCTGTGGCCCGTTC

AAGAGACGTCCCTGCTTTCGGGCCGCTCATCCCAGACGACGCAAAGTTCCCCAAATCCTC

AGTCTTCCGGGACTTCCTGTTGACCAAGTTGATCAACGCGGAGAACGCCGCACACAAGTC

GGACAAGTTCCGCGCCATGGCGACCCGCACGCGCCACGAGTACCTGAGGGATCTGGCCGA

GCGCCACGTCACCAGCACCCCTATCGACCCCTCGGGCAAATTTCCCTTCATCTCTCTGGC

GCACAAGCGCAAGGAGAAAAGCCGACCCTACCTGGGGGCGGAGCTACGCAGCCCGGGGTC

CATCACTTGGGTTGTGCACGCAGAAGATCACAGCACTGGAGGAGAACTGGAAGCCCTGCT

CGGTATCTCTAACGACTTCCTCATCCTGGTGGATCCAGAGGCCAAGGCTGTTGTGTTTAA

CTGTGCTGTACGTGATGTCATCAGCTGGACTCTGGGAAGCCCCGCCTCCATGAAGATCTT

CTATGAGCGTGGAGAGTATATCTCACTGCGCTCGCTCAACAACAACACTGAGGACTTCAG

GGAGGTCGTGAAACGTCTGGAGTTTCTAACGAAAGGCTGTGAGACGTCGGAGATGACGCT

GCGCAGAAACGGACTCGGCCAGCTGGGCTTCCACGTTAACTACGAGGGCATCGTGGCTGA

AGTGGAGCCGTACGGTTACGCCTGGCAGGCCGGGCTGCGGCAGGGCAGTCGCTTGGTGGA

GATCTGCAAAGTCGCAGTGGCGACGCTTTCGCACGAGCAGATGATCGATCTACTGCGCAC

CTCTGTCACTGTAAAGGTGGTCATCATCCCGCCGCACGAGGATGGCACTCCCCGCAGGGG

CTGTTCGGAGCTGTACCGCATGCCGGTGGCAGAGTATAAGGGAAGTAATGAGAGCGGTTC

CTACGAATACAAGTTCCCGTTCCGCACCAACAACAATAAGTGGCAGCGCACCTCCTCCAG

CCCACAGCAGTCCCTCTCTGCCTCGCCTCAGAGCCACACCGCGACCCCCAACCGCGCCGT

CAGTCTGGGAGCCAGCATGGGCAAGACACTGTCGGCCGAGAGACCCGAGCGAGCCACCGC

TATCCCACGCAGTGTGTCCAGCGACGGCCGGCCTCTCGACTCCAAAAGAATGTCTCCCGG

GTCGGAAAACTACGCCCTAGCGTCCTCCATGGTGATGGCGCGACCCACTCACACCCGCAG

CTCCCCCAGCAACCTGTCCTGCGTCAGTGACACCGGCTCAGGCGGCTCAGCCCACTGGAG

GCAGAAGTCTATGCCTGAGGGATTCGCCCCGAACAGACACTCTCCTCTTCCGCCAGAGAG

ACCAATGATGGGAGACGGCAGCGGGAAGTCCACCCCCAGCTGGCCCCGTGTGGACGACTC

TGACAGAGCAGCAGACGCCCCGCAGAGTAAATCCGGACCGGCGTACTCGGGCGGCCCGCG

CATCCAGAGGCAGGAGCAGGTCATCCATCTATCACCCAAAAAGAGCAGCCAGTCCGAGGG

CCCGTACTCCCAGTCGAGCAGTAACACCCTTTCCAGTACCGCCTCCAGCGGTGGTCACAG

CGATGACAAGTGGTACGATATGGGAAGCGGAGGTCAAGGAGACGGTGCTGAACCCGAGCC

CAACGGTCTGGGTGGAGGCGGGTACCTGCAGGGCTCCTCCGCTGACAGTGGCATCGACGC

CACCTCCTATGTTCCCCATCATGGCAGCGCCAGCTCCCTTCTCGCCGTGGGCACTTCGGC

ATCCCGGGAGAGGGTAGCCTCGCCTTGGCACGGGCCTTCTGAGGGAGGCCGGCGTGTGCT

GGAGAGGTCACCTCCGGCCGCGGAGTCGTCTAACGCTCCTGCAGAGGCTCCCACAACACG

CAGTCCTCCTACACATCTGCTAATGCGAGACAGCAGCACCTACAGCCTCAGTGACATGAC

CTCCCACTCCAGTTCGCGCCACTCTGGCAGCTCCGGTGTGCTCAGCTCCAGTCACAACTC

CCCTAGAGACGAGTCTCCCTCCGCCACCTCACCCTCCTCCTCCTCCCAGAGCTCAGTCTC

TCCAGGACCTAAAAGCTTTTATCCCCGCCAGGGAGCCACCAGCAAGTACCTGATCGGTTG

GAGGAAACCGGGGAGCACTATCAACTCGGTGGACTTTGGGGACACTCGGAAGAGGCCTCA

GGGCGAAGGGGCAGAAAGTGGGCCGTCTCAGACCCGACCCTCTCTCAGAGACCTGCACTC

TCCTCAGCCTCTGGGCAAATCCACTGTGGAGGAGGATCTAAAGAAACTCATCACACTGGA

CAGCCCACCAGCTATCCATCATGATGACAAGTCTCTGCAGCCTTCCTGTTCGGGTGCGAG

TGGTTCGGGCCGGCGGTCGCTACAGCGCACCCTTTCTGATGAGAGCATCTATCGAGGCCA

GCGCTTGCCATCGCTTGGAGATGCGGTGTTGGAGCAGGCGCTCAGCAGCGACGTGCTGTT

CAGCTGCTCTACCCTGCCGCGCTCACCCACCACCCGCGGGGCCCCACTGCGCAGACCTTC

CTACAAACTGGGCATCAAGATGCATGGTGACCTCTACGCGTCTGACACCTCTCTAGCAGA

CCTGCACGAGCGCCAGCGGCTGCCATCCCCCGAGCTGGGCCTTATGCCCCTGCCTGACAC

TGACATGGACAGCGGTCTGGACTGGAGCCACCTGGTGGACGCTGCCAACGCTTTCGAGGG

TGAAGGGAGACCCCCACGCATTCAGAGAAGTTTTATGTTCAGCTCTCAGGACATCAGTCA

CCGTAGTGAGAGTGCAGTGAGCTCTCAGCAGCTGGAGCTGCAGCCTGTACCGCACTCCCG

ACTCTCTCCCAGTGACATTCCTGTTGGTTTTGCTGGGAAAGTGTCTCAGCTCGAGGCTTT

GGTGAAGATGCTGCAGGACAGTTTGAAGAAGGAGCGAGAGGAGAAGCTGAAGCTTCAGGC

TCAGATCAAGCGGCTGTGGGAGGACAACCAGAGACTCCAGGAAGAGTCTCAAAACTCTGC

AGCCAAGCTGAAGAAGTTCACAGAGTGGGTCTTCAACACCATCGATTTGAACTGAAAGCA

CATCTGGAACAGCTGCTTTGTCCTCCAGCAGTCAGTAATCAAACACCAGTTCTGTCCAGC

ATTCCTGTACAGGGAACCATCAGTCCTCCAGTCCAGAGTTATCCCTTTCACTAATGTTTT

TCTTAGATCCAAACTGCAGGGATGAGCAGATTTCTGCAGCTACGTCATTCCTCAGATCTT

GGAGGAACGGCTTTTTTTTTTTTAACACTGCTTGTGATATTTTGCTGGATCACTGTGCTA

ATCAGAGTAGGTGTGTGTGTAGTCTTCTTTCCGTTTCTCTGTAATTAGCAAAGATCGATG

GTGTGACTCGAAAAACGCAGCCATACAATCAAGAAAACAACAGGAAAAAAAGGTTCCTGA

CGTTCTAAAGAAGCCCAGAACTCATCCTGGCTTTTTTTTTCTTAAACTCATAAGCAATAG

ACCGACGTTCTCAACAC

>Unigene000208

ATAGATCGGAAGAGCGTCATCAGGTCAACGGGCACAAGTTCATGTCCACATTTCTGAAGC

AGCCAACTTTCTGTTTTCACTGCAAAGACTTTATATGGGGTGTTTTTGGAAAACAGGGGT

ACCAGTGTCAAGTGTGTACTTGCGTCGTCCACAAACGTTGTCATCAGTTGGTTGTCACTG

TGTGTCCACGCATGAAAAAACCCGCCAAAGAACAGTCGGCGAATCAGGGTTTCAGCATCA

ACGTTCCCCACAGGTTCAATGTGCACAATTATAAGGTTCCTACTTTCTGTGATCACTGCG

GTTCTTTACTCTGGGGCCTCGTCAAGCAGGGACTTCACTGTAGCATCTGTAAGATGAACA

TTCATATCCGCTGTAAGGGAAACGTCGCTCCAAATTGTGGTGTGAACAACGTGGAGCTTG

CTAACAAATTAGCAGAGATGGGACTTCAGCCAGGAGGGACGCTCAAGCGCAGTGCATCGA

ACCAGTCTACTAGCGAGTCCTCATCTCATCGTGTCAGTGTGAAGAAGAGTTTGTCTCAAG

AAGAGAATAAAGGTCAAATGCTGCACATCGAAAACTTTACTTTCCTCCAGGTTCTTGGCA

AAGGCAGCTTCGGCAAGGTGATGCTGGCCCGCTTGAACGGCTCGGATCGCGCTTTCGCCG

TGAAAATTTTAAAGAAAGACGTGATCCTGCAAGATGACGATGTTGAATGCACGCTGACTG

AGAAGCGAGTACTGACGCTCGCACAGTCGCACCCCTACCTCACACACCTGTACTGCTGCT

TCCAGAACCAGGAGCGCCTGTTCTTCGTAATGGAGTTTGTAAACGGTGGCGATCTCATGT

TCCACATTCAGAAGTCAAGGAAGTTCGAGGAGAACCGAGCTCGATTTTACACAGCAGAGA

TCACCTGTGCGCTCATCTTCCTGCATGGCAAGGGCATCATATACAGAGATCTGAAACTGG

ATAATGTGCTGCTGGATCGGGACGGTCACTGTAAGCTGGCGGATTTCGGAATGTGTAAAG

AGGGGATGTTCGAAGGAGCCTCTACAGGAACGTTTTGCGGGACTCCCGATTACATCGCTC

CTGAGATCCTGCAGGAGATGCTGTACGGGCCGTCAGTCGATTGGTGGGCTCTGGGCGTGC

TCCTTTACGAGATGCTGTCAGGCCACGCCCCTTTTGAGGCAGAGAATGAGGACGATTTGT

TTGAGTGCATTCTAAATGAGGAGATCGTTTATGCGTCGTGGCTGAGCGCCGAAGCCGTGG

ACATCCTCAAAGCTCTCTTGATGAAGAATCCGGCTCGCCGTCTGGGCTGCGTGGCTGCAA

GTGGTGGAGAAAACGCTGTCCCGGCGCACAGGTTCTTCAGCACTATCGACTGGGACAAAC

TGAACCGGCGGGAACTCGAACCTCCGTTTAAACCTCAAATCAAAACCAGTGAGGACGTGA

ACAACTTCGACCCCGACTTTACAAACGAAGACGTCGTCCTCACTCCCATCGAGGACGCCT

TGCTTCCGCAGATCCACCAGGATGAGTTCCAAGATTTCTCTTTTACCTCGGAGAAAATGC

TGGAGATCTGCACATTTTAAAAAAAAACAGGGATTAAAAACAAAAAACAAACCGATGACA

CAGGACCAGGATGACGTCTGGCTTGTTGAGTCATGACATCCAATCATAGCTATGTATTTC

AGCGTTTTGTAAAGCTGTGACGCGAGACATCAGAGTTCATCGTGATTGTTTGAGTTTTGA

GGACTTATTCTTTTCGTACACAAAGGGTAATGATGCATTTTTTTTACTTAAAATGTGTTA

TTTTGTTAAGCAAAACCAAAGTATTTAAACTCTTATTGAATTTAGCGAAGACTGTTCTGA

TCTTTTTAATGGAATTAGAGGAAATGTTTAAGTCTCAGTAAAACTAATTTGTATAAAAAA

ATGTTTTATTGCAGCCAAGGTTAGTTTTTATTCTAGGGCAAAAATAATGAACTCAATTTC

GATTCAGAGGACAACGGAAAAAAATGGGTTGCAGTATTAAGTCTGAATTTTTATAATGTG

CCGGACCCATGAGAGGCGGAGCTTCTGTAACTTTAGTACACTTTTAGTTGATAGTTTAAA

AAAAAATTATTCAGTTAGCAGAATTATTTGCCAGATTTATTAGAGGATGAAAAAGGCTTT

TTGCTGCCATCTGTTGGTGTGTCTGGAAACATTTTGATGCCTCCTTGTAATAATAGTTAT

ACATTTTGAATTGGACTATCTAAGATTAGCTATGTAACACTAGCTAAACAAGTATTAAGT

GGTTAAAGTGATGTAACAAAAGCTCCGCCCAATCAATGTTTTGTGGTCTGATTGCCTTTT

CTCTACAGTCACAGCTGGATTGTCTGACCTTTTAACATCATAACCTGTTGCTTTTGGTTA

AAAATTTACTAAAGATAAATTCATCTTTTTTTGTCCTTAGAACTGCAGTCAGAATCTTAT

TGCATTATAGTTTTTGGAGTTTTTTTTTTTTTTTGGTGTCTACGATGAAGTGACAGCGCG

TCCTTGCGTGTCTGCGATCTCGGGATTTCTGCCTAACTACATTTCCGACACTTGGATAAT

TCAGGAACAGAGTGAGGCTGGCCGGTGTGTGGAGAGACAGACAGCATGGCTGAGCCCCGG

CGAGACAGTACCAGTAGTCTACAGAGGAAGAAGCCTCCATGGCTGAAACTGACCATTCCC

ACTGCTCAGATGTCTTTGGACGAACCCCCCACTTTCATCCAGCCTGTGAAGAGACAGGGG

TTCCTGCGCAGTGTCAGCATGCCTGTCGAAACATCTCACTTCGCATCACCGACACGACCT

GTTTGATCCGCGCCGACCTCCGCTGCAGCACCAGACCTCCATCACACAGTCCATCAAAAG

GGGCACAGCAGACTGGTTCGGGGTCAGCAAGGATGGTGACGCCACACAGAAATGGCAGAG

AAAGAGTCTGCGCCATTGCAGCCTGCGCTATGGTAAACTGAAGCCACAGGTGATCCGGGA

GATGGAGCTTCCAAGCCAGGACAACATCTCAATGACCAGTACAGAGACTCCACCTCCTCT

CTATGTACCAACATCTCAGCATGGCATACCAAAGATCGTGGACCCCTTGGCGCGTGGTAG

GGCTTTCCGCATGGTGGAGGAAGTGGACGGATACAGTGTGCCTCATACACCCATCACACC

CGGTGCCACCTCTCTGTGTTCCTTCACCAGCTCCCGATCCGGCCTAAACCGGATACCTCG

ACGACGCAAGCGGGAGTCTGTGGCTAAGATGAGCTTCAGGGCAGCAGCTGCGCTGGTCAA

GGGACGCTCGTTTAAAGAAAGCACCCTGCGGCGGGTTCACAGACGCAGCTTCACCCCAGC

CAGCTTTATCGAGGAGGACGTTGTGGACTTTCCTGACGAGCTGGACACGTCCTTCTTCGC

ACGAGATGGATTGATGCAGGAAGAGATGTCAACCTTTCATGACGAGGTGTTTGAGTCTCC

CTCTGACTCCACCACCAAGGACGTGGACAGTAAGCAGCTGGACGAATCGGAGCTGACGGG

AAGTGCACTAGACAAGAACGAACTCGAGAGAAGTCATCTGATGCTACCTCTAGAGCGAGG

CTGGAGAAAAGCCAAGGAGGGAACCCCTGCTCCGCCCAAGGTCCGGCTGAAACAGGAAGT

GGTCAGCGTAAGCGGGCAGAGACGGGGGCAGAGGATCGCTGTACCGGTCAAAAAGCTCTT

TGCGCGAGAGAAACGTCCATACGGGCTTGGCATGGTGGGCAAGCTGACCAATCGGACGTA

CCGCAAGCGCATAGACAGCTATGTGAAAAGGCAGATAGAAGATATGGACGATCACAGGCC

GTTCTTTACCTACTGGATCACGTTTGTCCACCTACTCATCACTATTCTGGCGGTGTGTAT

TTATGGCATCGCACCCGTGGGTTTCTCCCAGCATGAGACGGTGGATTCTGTTTTAAGAAA

CAAAGGTGTCTATGAGAATGTCAAGTTTGTGCAGCAAGAGAACTTCTGGATTGGGCCGAG

TTCCGAGGCTCTAATCCATCTGGGAGCCAAATTCTCTCCCTGCATGCGACAGGATCAGGA

GGTACACGATCAAATCCAGAGGAAACGTGAGATTGAGCGCAACTCCGCCTGCTGTGTGCG

AAACGACCGCTCGGGCTGCCTGCAGACGTCTGAGGAGGAGTGTTCGAGCACACTGGCTGA

GTGGGTAAAGTGGCCCTATCATCCCAGCACTCCACAACTGGACGGAAAACCTCGTAACTA

CGGCTCTGTTTGCCATCAGGACCCCAGGATTTGTTTGGAGCCAGCCTCTGGACCGCCTCA

CGAGTGGCCTGATGACATCACCAAGTGGCCGGTTTGCACCAAGTACAATACTGGCAATCA

CACTAACCTGCCCCACATCGACTGTACCATCACCGGGCGGCCGTGCTGTATCGGAACTAA

AGGAAGGTGTGAGATCACATCACGGGAATACTGTGACTTCATGAAAGGCTCTTTCCACGA

AGAGGCAACACTGTGTTCACAGGTTCACTGTATGGATGATGTCTGTGGCCTGCTGCCGTT

CCTAAACCCGGAGAAACCCGACCAGTTCTACCGCTTGTGGCTGTCTCTCTTCCTACACGC

TGGGATTTTGCACTGCCTGGTTTCTGTGATATTTCAGATGACAATCCTGAGAGATTTGGA

GAAGTTGGCTGGTTGGCTGCGAATCTCAATCATCTACATCCTGAGTGGCATCACAGGGAA

CCTGGCCAGTGCCATCTTCCTGCCCTACAGAGCAGAGGTGGGTCCTGCCGGTTCACAGTT

TGGCATTCTGGCATGTCTTTTCGTGGAACTCTTTCAGAGCTGGCAGATTCTCGCTCAGCC

ATGGCGGGCATTTGCTAAACTGCTGTGTGTGGTGCTCTTCCTCTTCGCCTTCGGTTTGCT

GCCCTGGATCGACAACTTTGCCCACATCTGCGGCTTCGTGTCAGGTTTCTTCCTGTCCTT

TGCGTTCCTTCCCTACATCAGCTTCGGCCGTATGGACATGTACCGTAAGCGCTGCCAGAT

CCTGGTCTCACTTACACTCTTCCTGGGCATCTTCTCTGGATTTGTGGTGCTCTTTTATGT

CCATCCCATTAAGTGTGAGTGGTGCGAGCTGCTCACCTGCATTCCCTTGACAGACAAGTT

TTGTGAGAAGTATGACCTAAACGCGCATCTCCACTGACGCAGTCATGAGGAAGCTCAGGT

GAGGCTTTTGGACGGGGCAGGATCCCATCACCTCCAGTTAGGAGACCTGCCATGTTTCTC

TGCTGACCCTGAGGCAAGGACAATAGCAACATGGATGATACACATCACATTTTTTTCTCT

CTTACTGTAAGTTCAAACTCCTCTTTTGACCATTAACGACCTGAACGTTATTTTTCCTCA

TCATTAGTTTGTTATCGGTCGTCTGTGAAACAGGGACAGTTGCCCCACCCGCCTCTCCTT

CAGATACTCCACCTGGAGATTGTGAGCGAGAATAAGCACTGGTACAAACAAACAGAAGAA

TAGATGCTAATAGATGTAATATTTTATACTTTTCATATTTTATGATGCCACGAAATCAGG

TGAGGGTTTTAGAGTAGTTCAGATTCAAATCACCTCCGACTTCCAGGGACGAAAGAGAAA

TTTAATCAGAGCCACC

>Unigene000209

GTGTGTGTGTGTGTGTGTAGTGTGGAGAAGAATGGCAGTGCTCTATCGGATGAGAAGAAA

ACAGTACAGATATCACTCTTAGAGCTACTGAAAGACTTCCTGAAGTCTCCCACTACCAAG

GATCTGCACAGTATTCTGGCTTATACTGTCGTGGTGCAGGACCAGCAGCAGGCGATCATG

CTGCTGGATATTTTGTATACAGTGCTAAAAAACAATCCTCAGGCTCTGTCTGTGCCGCTG

GAATGGGGGGTGGAGCAGTTTCTCTGTCTGCTGCTGAAGCCCAGCTATAACGATGAAGCC

CGTGAAAAAGTCTTTAGGGTGAGCACACGCAACCCTCTGCCACTGCTACCTATTGTGTTC

GAATATGCAGTCTTTGTATAGGCCAAATGACATACTGCTTCTCTGCAGATCATGTACAAA

CTGCTAAAGAGCGAGCGAGTCCCAGAACGTAGCAAACAGCGGGTCAAGCTGCGAGAGACA

ACCTACATGGGCCTGGTCTGCTTCTTGGAGGAGGTCCCAGTCACAATGACCATCATCCGC

TGTCTGTGTGAGCAGGTGCTGGCTACAGATCCCTCACCTAACTTCAGAGACCTCTTGTCA

GTTGTGTATTTGTCCCATCGGGCTGATCTCTCTGTCCGACTGGACATATGTCGCAAGCTT

TTCAGTCTGATTGACTCCAATGAAGACTGCGTTAAGCAGCTGGCCCGGCAGCCAGGCTGG

CAGGACATTCTCATAAAACTCTATGTCAAAGAGTCCTTTGAGTCTCATAAGACCAGTGTA

TCCAGCCCTTATCCCTCCCTGGAGCCTATTCCCACCAGAACTCTGCTCCGAAAGGATGAC

AGCATAGATGGTCCTCGCTCTGACCCCTTCAATCCCTATAGCTCCCAGCGTGAGGAAGCA

GAAGAGGATGAATGTGATGAAGAGGAAGAAGCATCTCGTGACATTTCAGAGCGTTTTATT

GGATTGTCTCAGTCGCCGCCTGCAAGAGGTCAGCTGAAGGGCTTTAGTGACTCGATGAAC

TTCAAGTCTTTCGACTCGGTGGAGCAAAGCAGCCGCTCGTCATCCCTGTCCAACGCCGTG

GACGTGCCATCCACACAGCTGTTGGAGGAAGAAGAAGAGAGTCTATATAATCCACTCTCA

CCCTTAGGCATGTCACCTGATCTAGAGCTGGGAGGGCAGAGAGCATCTCTGACTCCAGAC

ACGCCGCCTTCTACAGAACATAACAAACTTTTTCTTGGCCCACGAGCTCGCAAGAGTTCC

AGCTTGTCTAATGTGTTGGATGAAACCAGTTACTGCACAGAGCCTCCAACCGCCGACACC

ATCTCTAACAACTCCAACCCACAGCAGGCCCCAGAGGAGGAGTTGTGCAACTTGCTGACT

TACATTGTGTTCCGAGTGCTGTGGTCTGGCACAGAAGGAGCAGAAGATGCAGTGTGGCGG

GAGAGAGGGCAAGTTTTCTCGGCCCTCACCAAACTGGGATCTTCCTGGCAGCTTGTGCGT

CACCCGGATGATATCAAACGCAGTCTTCTGGAGATGATGCTGGAGTCGTCTCTTTCGGAT

CTGCGGGACTCTCAGGGCTTGTCATTGCCTCATATTCCCTCCTTACTTCGTCTCCTCAGA

CTGCTACACGATTTCCTGTTTGCAGAAGGCACTGACAATCAAACACTGTGGAGTGAAAAG

ATCTTTGAAGGAGTGGTGAATTTGCTCGACAGGCTGCAGGCGTGGCACACCACCTCTGCA

ACAGCCGGCGCAACAGAGCTCAAACGGATGGCTCAGATTGGCTTGTGGATCATAACAGGA

TACATACAGCAGCAGAACTCGCAGGTGTGTGAGATGGCGTGTGTAAAGCTGCACAGTCTG

CTGCAGACGGTCTTGTGTCTCTCATGGGAGGAGGTATACTTTCTGTTGGGCCGCCTGGGA

GCCACACTGTGGCCTGCAGAAGGAATGTCTTTGACGAGCCCAGCTGGCACAGAGGCAGTC

TTGAGGCCTATGGTACCAGTAGTGCGCACACTCCTAGACCAGCACGCTGACCCTACCAAA

CTGCAGCAGTCTCTACCCAACCTGCCCCCCACCAATGGCAGTGCCACCTTTGCCCAGGAC

CTACTGTCGTACTGCAACACAGTCGAGTGGCAGCTTTTCTACTGCAATCATGTGAGACCC

ACCATGCAGCAGTATGAGCAGGACACTTTTGGGAAAAGCCACGATTTACTCTCCAACTTT

TGGAACTCGTGTTTCGATGATCTGATGAGCACCGCTGAAAAGAAGAACAAGGACAAAGCA

GAATTCAAAGCCAAGTTTCAGAGTTGATTGTGGACCCATATTTGAAGCGTGTACGCGTTG

AGAACAGTCGCTATGTGAGTGGACAGAAGCTGAATAACAGCCAGCAGGAGGTGGTGTGGA

AGCACTGGAAATGCCAGCGCAGACTGATGACCAGTGAACGAGCAACTTGGGCAGACAGGG

ATCAGCCAGAAATGAAGTGGAAGCTGTCCAGCGCAGAAACATACTCCAAGATGCGTTTGA

AGCTCGTGCCCAACTACAACTTCGACTCCCACAGCGAGGCCAGTGCACTCCGGGATAACA

TGGAGAGCCCTCGCAATTCCACCGAGCCCCTCCCACTGGCTGTAGCTAAGGAAGCTAAAG

TGAGTGACATGGAAGATGACCAACTGGAGGAAGAAGACCTTCTCGCCATGGACAGCCAAG

CAGAGGAAGTGGAGGACGAGAGCCAAAAGGAGAAGCTGGTACTCTCTGAGGACTGTGAGC

TCATCACTATAGAAGCTGTGGTCATGGGCCGGCTAGAAGTTACTACACATCACATTTACT

TCTATGATGCCAGCAGTGAGAAGGAGGAGACAGAGGAAGGGATCGGCTTCGATTTCAAGC

GGCCACTGAGTCAGCTGAGAGAAGTTCATCTCAGACGCTATAACCTGCGCCGCTCGGCCC

TGGAGCTCTTCTTCATTGATCAGGCTCACTACTTTATCAACTTTAAGAAGAAGGTGCGGA

ACAAAGTCTACTCCAGGATTCTCGGTCTTCGACCACCTAATTTGTTTTATTTTGGTTCCC

GCTCTCCTCAAGAACTCCTCAAAGCCTCCAATCTTACACAGAAATGGGTGTGCAGAGAGA

TCTCAAACTTTGAGTATCTTATGCAGCTCAACACCATCTCAGGTCGCACTTACAACGATC

TGTCGCAGTACCCTGTGTTCCCTTGGGTGCTGTGTGACTACACATCTAATGTATTGGATT

TGGAAGACCCGTCTGTTTTCAGAGATCTGTCTAAGCCTATCGGAGTGGTAACCCCCCGCC

ATGCCCAGGATGTCCGGGAAAAGTACGAGAGCTTTGAAGACCCCACAGGCACCGTTGATA

AGTTCCACTACGGCACACACTACTCCAACGCGGCAGGCGTCATGCACTACATGATCCGCA

TGGAGCCTTTCACCAGGCTGCACATCCAGCTGCAGAGCGGCAAGTTCGACTGTGCAGACA

GACAGTTCCACTCAGTAGCAGCAGCATGGCAGGCTCGTATGGAGAGCCCAGCTGATGTGA

AAGAGCTCATCCCTGAGTTCTTTTACTTCCCAGAATTCCTGCAGAATATCAACGGTCAAT

CAGGAGCAGGTGAATAATGTGGTGCTGCCACGCTGGGCTTCTTCCAGAGAAGATTTTATT

AGAAAACACCGCCAGGCACTGGAGAGTGAGTATGTGTCTGCTCACCTACATGAATGGATC

GATCTAATATTTGGCTATAAACAGCGCGGGCCAGAGGCTGTGCAGGCACTCAATGTGTTT

TATTACTGCACATATGAGGGTGCTGTGGATCTGGATGCCATAGCCAATGAGACTGAACGT

AAGGCTCTGGAGGGCATCATCAGTAACTTTGGCCAAACACCCTGTCAGCTTCTCAAGGAG

CCGCATCCTCCCCGTATGTTAGCACAGAGCGCGTGCAGGCGGCAGGCACGTATCAATACT

CTGCCTCTCAACCTCTTCGACCAGCTGGACAAACTGAGACCCTTCGTTGAGGTGGTAAAT

GATGGTGTGCCTCTAGTGCAGGCTGTGGTGCCCAGAAATCAGACACACTCCTTCATCATT

CCAGGGTCTGATGTGCTGGTGACTGTGAGTGCTAAAGGACAGATAGGGACACACAGCTGG

CTGCCCTATGACAAGAACATTGCCAATTATTTTACTTTTACTAAAGACCCCACTATCAAC

AATCCAAAAACACAACGGTTCCTGAGCGGCCCCTTCTCCCCAGGGGTGGACATGGGGGCC

CAGGTGCTGGTGGTGTCCAATGACGCTCGCTTGCTGTTTAGTGGGGGACACTGGGACTGC

AGCCTACGCGTCACCACGCTGGCCAAAGCTAAACTAGTGGGCAGAATCTGCCGTCACATA

GATGTGGTAACGTGTCTGGCTTTGGATCTATGTGGTATCTACCTCATCTCAGGCTCTCGT

GACACCACTTGCATGGTGTGGCAGGTTCTCCAGCAGGGTGGTTTCTCCAGTGGTCTGTCC

CCCCGACCAGTACAGGTGCTATGTGGACATGATGCAGAGGTTACCTGTGTCTCCATTAGC

ACTGAGTTGGACATGGCTGTTTCAGGATCAAAAGACGGGACAGTGATTGTACACAGCATC

CGGCGAGGTCAGTATCTCCGTACGCTGCGTCCTCCCAGTGAGGGCTGCGTTTCTGTACGG

GTGAACCAGCTGCAGGTGGGAATGGAGGGTCACATCGTTGCACAAAGCGTCCTAGAGGGC

TGTTCTGCCAGAAAGGAAAAGTGTGTGCTGCATGTGTACTCTGTGAACGGCACACTGTTG

GCGTCCACGGTGATGGAGGAGCCTGTGTCAGCACTCTATATGGTTTCAGACTACCTCATT

CTGGGCACACTACAGGGCAACCTGCACATCAAAGACCTGTTCAGCTTAGAGCAGGCTGCT

AAGCCCTTGGCGCTGATGCTCCCTATTCGCATTGTATCAGTCACCAAGGAGAACAGTCAC

ATTCTGGTGGGATTGGAAGACGGGAAACTCATTGTTGTGGGAGCTGGAAAACCAGAGGAG

TTGCGTTCAGGTCAGTTCCCACGGCGTCTCTGGGGATCAACACGCCGAATCTCTCAGGTG

TCTGCAGGGGAGACTGAGTACAACCCTACAGAGAATGCTGGGAAATAAGCATCATACACA

CTCCACATGCCTTTATATAACAGGCCTCTTCACATACACATACATACATGTACACGAACA

TGACCTGAGATGTTGACCCGGAACACAGGGTGACTTAATGTACTGTGGACTGTTTCTCTG

GTTTAATGGAAAAACACAGAGTCGTCCAAAGCTGCACTTTTTTTACTTCTTCTTTTTTAA

AGTTTCTATTTTAGACCGTTTTTAAGCTGAAATCATTCCAAAAATTCCAAACATGAACTG

GGATGTGAGAGTGGAAGTCAATCTCAGAGTCAGAACTAAATGTGGATAACACACACGCCT

GATGCTGAGAGCTGCTGGCTGAAGTGTGTCGTCATGGTCGACGCTCGCTTTTGATCTGCC

GTGCACAACACAACTTGACTAAAAAACACACACTGGAACAACAAGAGTTCTATTTTTAAA

GTGTTTGGGTGAACTTGTAGACGTGAAGAAAAAACATGGGAAAAAAAAGAGAG

>Unigene000210

TGTGTGTGTGTGTGTGTGTTCCATTTGTCCGGTTTATCTGCTTTAGATTGAATTCTATAG

TCTGTGTTTGTGGATGACATCAGAACCATGTGTTGTCTGTTCCCCTTAAGCTGAGTTGTG

CCTGGAGGATTATGGGAAATATCGCTTTCTGTCCAATGGAAAGGTGACAATTGCTGGACA

GCAGGACCGAGATCTTTACACTGAGACGATGGACGCCTTCAGAATAATGAGCATCCCTGA

GGACGAGCAGACGGGTTTGCTGAAGGTGGTTTCTGCAGTTCTTCATTTGGGAAACATCAT

CTTTAAGAAGGAGCGTAACTCTGATCAGGCCTCCATGCCTGATGACACGGCTGCACAGAA

GGTCTGCCACCTGCTGGGCATGAACGTGACCGACTTTGTGAGAGCAATTCTCTCGCCGAG

GATTAAAGTGGGCCGTGATTTCGTGCAGAAAGCGCAGACGCAGGAGCAGGCAGAGTTTGC

AGTTGAAGCTTTAGCCAAAGCCACGTATGAAAGATTGTTCCGCTGGTTAGTGATGCGGAT

TAATAAAGCTCTGGACAAAACCAAACGCCAGGGAGCTTCGTTCCTCGGTATCTTGGACAT

AGCCGGCTTTGAGATCTTTGAGTTGAACTCGTTTGAGCAGCTGTGTATTAATTACACTAA

CGAGAAGCTGCAGCAGCTCTTCAATCACACCATGTTCATACTGGAGCAGGAGGAGTATCA

GCGTGAGGGCATCGAGTGGAGCTTCATCGACTTTGGACTGGACCTTCAGCCCTGCATCGA

CCTGATCGAGCGGCCTGCGAATCCTCCAGGTGTTCTGGCTCTGCTGGATGAGGAGTGCTG

GTTCCCCAAAGCCACTGATAAAACCTTCGTGGACAAGCTGGTGCAGGAGCAGGGGAACCA

TCCCAAATTCCAAAAACCAAAAAAGCTCAAAGATGATGCTGATTTCTGCATCCTTCACTA

CGCAGGCAAGGTGGATTATAAAGCAGTGGAGTGGCTGATGAAGAACATGGACCCTCTGAA

CGATAACGTAACAACAATGCTTAATCAGTCATCAGACAGATTTGTATCTGAACTCTGGAA

AGATGTGGACAGAATTGTGGGACTGGATAAAGTTGCAGGAATGGCCGAGTCAGCACACGG

GGCGTTTAAAACCCGTAAAGGAATGTTCCGGACGGTCGGACAGCTTTACAAAGAACAGCT

CGCTAATCTGATGACCACGCTGAGGAACACCAACCCCAACTTCGTGCGCTGCATCATCCC

CAATCATGAGAAGAAGGCAGGTAAACTGGATCCTCACCTGGTCCTGGATCAGTTAAAGTG

TAACGGTGTGTTAGAGGGAATCCGAATCTGTAGACAGGGCTTTCCTAACCGCATCATCTT

CCAGGAGTTCAGACAGAGATATGAGCTGCTCACTCCGAACGCGATCCCTAAAGGTTTTAT

GGATGGTAAACAGGCCTGTGTGCTGATGATCAAAGCTCTGGAATTGGACTCTAATCTCTA

CCGGATCGGACAGAGCAAAGTGTTTTTCCGTGCTGGAGTTCTGGCTCACCTGGAGGAGGA

GCGGGACATCAAAATTACGGATGTTATCCTCAAGTTCCAGGCTTGGTGTCGTGGTTACGT

CGCTCGCAAGTCATTTGCCAAACGGCAGCAGCAGCTGACAGCCATGCGAGTGATCCAGAG

GAACTGTGCTGCTTATCTGAAGCTCAGGAACTGGCAGTGGTGGAGACTCTTCACTAAGGT

GAAGCCTCTGCTCCAGGTGACCCGTAAGGAGGAGGAAATGCAGGCGAAAGAGGAGGAGCT

GGAGAAAGTGAAGGAGAAACAGCAGCAGTTTGAACAACAGTTGCAAGACTATGAGACCAA

ACAGCAGCAGCTAAGCACAGAGAAGCAGGCTCTACAGGAGCAGCTGCAGGCAGAGATGGA

GCTGTGTGCTGAGGCGGAGGAGATGAGATCTCGACTAGCGACTCGGCAGCAGGAGCTGGA

GGAGATACTACACGACCTGGAGAACCGTGTGGAGGAGGAGGAAGAGCGGGTCACTCAACT

GCAAAACGAGAAGAAGAAGATGCAGCAGAACATCACGGATTTGGAGCAGCAGCTGGATGA

AGAAGAAGCAGCACGGCAGAAACTGCAGTTGGAGAAAGTGACCACAGATGCCAAGCTGAA

GAAGTTAGAAGAAGATGTCATGGTGCTTGATGATCAGAATGCCAAACTCAACAAGGAGAA

AAAACAGCTAGAGGACAGGATCTCCGAGTTTAACATTAACCTGGCTGAGGAAGAGGAGAA

ATCCAAGAGTTTACAGAAACTTAAAAACAAACATGAGGCCATGATCACAGACCTGGAGGA

TCGTCTGAAGAAGGAGGAAAAGACTCGTCAGGAACTGGAAAAGAACCGTAGAAAGCTGGA

AAGTGACTTCACAGAGCTCAATGATCACATCGCTGATCTTCAATCTCAGATCGCGGAACT

TCGGGCACAGCTTGCCAAGAAAGAGGAGGAGCTACAGGCTGCGCTGGCCAGAGTAGAGCA

GGAAACGTCTCTGAAAAACACGGCACAGAAGAGAGTGAGGGAACTGGAGGCTCAGGTCAC

AGAACTTCAGGAGGATCTGGAGCTGGAGAGAGCAGCAAGAAATAAAGCTGAAAAACAGAG

ACGGGATCTGGGTGAGGAACTGGAGGCACTGAAGAGTGAACTGGAAGACACGCTGGACTC

AACCGCTGCTCAGCAGGAGCTCAGAGCTAAACGTGAATCTGAAGTGGCTCAGTTAAAGAA

GACTCTGGAGGATGAAGTTCGGAGTCATGAACAGCTCCTCGCTGAAATGCGGCAGAAACA

TACACAGGCTTTTGAAGAGCTCAATGAACAACTGGAACAGGCCAAGAGATTTAAACTGAA

CCTGGAGAAGAGTAAGCAGACTCTGGAGGGTGATAATAAGGAGTTGAGTAGTGAGGTGAA

GTTACTGCAACAGGCAAAAACCGAATCTGAACACAAACGCAAGAAACTGGAGACGCAACT

GCAGGAAGTGCAGAACCGCATTAATGAAGGAGATAAACACAAAGGAGAACTGACTGAACG

CACACACAAACTACAGACGGAGTTAGAGAGTGTGTCTGCACTGTTGGAGGAAACTGAGAA

AAAAGGGCTCAAACTGGCTAAAGATGCATCAAGTCTTGAGAGCCAACTACAGGACACACA

GGAGCTGCTTCAGGAGGAGACACGTCAGAAGCTGAATTTGAGCAGTCGGATCAGACAGCT

GGAGGAGGAGAAGACCAACCTGCAGGAGCAGCAGGAGGAAGAGGAGGAGTCACGCAAAAA

CCTGGAGAAACAACTGGCAACCCTGCAGGCACAGCTGTGTGAGACAAAGAAGAAGCTGGA

GGATGATGTGGGTGTGTTGGAGGCTCTTGAAGAGGTGAAGCGGAAGCTGCAGAAGGACCT

TGAAGCCAGTAACACTCGTTTGGAGGAGAAGATTCTGGCCTTCGATAAATTGGAGAAGAC

AAAGACTCGTCTTCAGCAGGAGCTGGAGGACCTGACAGTCGACCTGGACCACCAGAGACA

GATCGTCTCCAACCTGGAGAAAAAACAGAAAAAATTTGACCAGATGCTGGCAGAGGAGAA

GAGTATTTCAGCGCGTTATGCTGAGGAGCGAGACCGCGCGGAAGCGGAGGCGAGGGAGAA

GGAGACGAAGGCGCTGTCATTGGCACGCGCTCTGGATGAAGCTCTGGAGGCCAAAGAGGA

GTTTGAGCGACTGAACAAACAGCTGAGAGCTGAAATGGAGGACCTGATGAGCTCCAAGGA

CGACGTGGGCAAAAACGTGCACGAACTGGAGAAGTCGAAACGCACCCTGGAGCAGCAGGT

GGAGGAGATGCGCACTCAGCTGGAGGAGCTGGAGGACGAGCTGCAGGCTACTGAAGATGC

TAAACTGCGCTTAGAAGTGAACATGCAGGCCATGAAGGCGCAATTCGAGCGAGATCTTCA

GGCCAGAGATGAGCAGAACGACGAGAAGAAGAGACTCCTAATCAAACAGGTGCGTGAGAT

GGAGGCAGAGCTGGAGGATGAGAGGAAACAGAGAGCTCTGGCCGTCGCTGCAAAGAAGAA

GCTGGAAATGGACCTGAAGGATGTGGAAGCTCAGATTGAAGCTGCTAATAAAGCTCGAGA

TGAAGCTATCAAACAACTCCGCAAACTCCAGGCTCAGATGAAGGATTATCAAAGAGAGCT

GGAAGAAGCTCGAACATCACGAGATGAAATCTTTGCTCAGTCCAAAGAGAACGAGAAGAA

GCTCAAAACCCTTGAGGCCGAGATCCTGCAGCTGCAAGAGGACCTGGCTGCATCAGAGCG

AGCACGGCGTCATGCTGAACAGGAGAGAGATGAACTGGCTGATGAAATCTCCAACAGCAC

CTCGGGAAAATCTGCGCTGATGGATGAGAAGAGGAGGCTGGAGGCTCGTATCACTCAGCT

GGAGGAAGAACTGGAAGAAGAGCAGAGCAACATGGAGCTGCTGAATGATCGCTTCCGCAA

AACCGCCATGCAGGTGGACACTCTGGGTAGTGAGTTAGCGGGTGAGCGTAGCGCGGTTCA

GAAGAGTGAGACGGCTCGACAGCAGCTGGAGAGACAGAATAAGGATCTGAAGAGTAAACT

GCAGGAACTGGAGGGTTCCGTCAAATCCAAGTTCAAATCCACTATCGCCGCCCTGGAGGC

CAAAATCCTGCAGCTGGAGGAACAACTGGAGCAGGAGGCCAAGGATAAACAGCAGACTTC

AAAGTCAATGCGCCGTACGGAGAAGAGACTGAAGGAGGTCCTGCTGCAGGTGGAGGACGA

GAGACGCAACAGTGAGCAGTTTAAAGATCAGATGGAGAAGGCGAACTCGCGCATGAAGCA

GCTGAAGCGTCAGTTGGAGGAGGCGGAGGAGGAAGCGACCCGCGCCAACGCCTCCCGCAG

GAAGTTACAGCGAGAACTGGAGGACGCCACCGAGGCCAGTGAGGGTCTCAGCAGAGAGGT

TAACACACTCAAGAACCGTCTGAGGCGTGGGGGTCCGGTCAGTTTCCCATCAGGCCGTTC

AGGTCGCAGGCTGCAGATGGAGGGTGAATTCTCAGATGATGAAGCTGACAGTAAAGTGAA

TGACACAAATGAAGTACAACCTCCACCGAGCGAGTAAACACCAGAACACACACACACACA

CACTTAATTCATTCAGATTTTTTCCCCTTTTGTGTGTGTGTGTGTGTTCATTGTTGTGAG

CGAATAGGAAAGCAACGGAGTTTAAACTGAACTCGTGAGAAACGTCAGCATTGACAGGAA

GTGCCGTCTGTTCTAAAACTTTTAAAACTTTTTCCATTTATTATTAAAAATAATTATATA

AAAAAAAACAATGTTAAAGTTGAAATCAATATTTTATAGCTTTTTTTAAAAAAAAAATTT

TTGTGGTATTTTACATTTATTTCGCCATTTTTATTTATTTTTAACAAATATATATTCAGA

AGTCCTTTTTGTAGCACTAATGCTTTTTTTCTGTGACCAGGGCGACCCATGGTGTAGGCG

AAATAGGCCTAGGAAAGTTTTTTTTTTCTTTTTCTGGAAGTGCGCCCTCTTGTGACCATT

ATGCGCTTTTACAATTTATTTACTACTTATGCTTAATAGGGGGG

>Unigene000211

TGAGCTGGCTTTGTGTTCGGGCTGAACGCGGAACAGAGTGAAAGCGGTGCCCGGGACGGA

GGGCGGGATTTGAACTTTTCGAAACGACAGGGACGTTTAGTTTATTCTGCTCTCAGTAGC

CGGACTGTCATTTATGTTTTCTAGGCTTGTGCTTGGCAGGCGTTTGGTTCTGAAGTCGGA

AAACCAGTGCAGGCATCTTGTTTAATCCAGACAGTCCTGTCCTGTGCGCCAAGTCTTCTC

CAAAAAAAAAACAAAGACTTGGCATTCAAATGAGAAGTGCACTTTTAGTTCGGCTTAAAA

GACAGGAAAAACAGCACATACTGCAGTCAGAAGGAAATGCATTAACGATCTAGGGTTAAT

GCTGCAAACTTTGCCTGAAGAGCTGACTTGTGTAATGGAGTAGGTCTGATTGGGAATAAA

CACTAAACACAGGTCTCCTGCCGTGTGAACTATTAAATGCAAGCTCCCAGATGTGGCCCA

TTAACCCCTGCTCTGCACGGCAGTGATCCGGCAGAGTCAAGCTTACTTCGCTCTCTCCTA

CAACTTAATTGAGATGCTTTGAGCAAGACGGGATCATTCCTTGGAAAATAACCTGCGAGC

CTAACTTCTCCAGTCAGCTGATACTTGAATTCTGAGCGTTTTTACAAACCCAGGGCCCAA

GAGTTGTTCTGTAATAGGATGGACACGTTAGATCCTCTGTCGGGCTCTGCGTTGGAGTCC

AAAGCAGACCGCATTGCTCGCTACAAGGCTGAGAGGCGGCGCGAGTTGGCAGAACGATAT

GCCAACCTGGAGGACATCAACTCCAAATACACCAGAAAGGAGAGACCTAGAGACAATTCA

CAGGCTCCAGACATAAGTCCAAGCACAGAGCAAAAGCCTAAAGAGGTCTCTGTCTATACA

CAAGGTCTGCCTACCGAAGCTAACAACACGGAGGAAGAAGAGAGAGAGACAAAAGGTCAA

AGTGAAACTAATAAGAAACAGGGAACTGCAACAAGATCCCGGCTCGGTCTGCTGGGCACA

GAGGAAGTCAAACTGCCAACGGCTGATGAGAAAGAAGATGACAGTTTTAAGACCAGTGCG

TCTGCCAGGATGTCTCGCTCTAAAGATTCCTATACACCTGAAATCACGTCCAGCTCAAAG

ACCATCTCAGAGAAAACCATACAGGCGAGTCAGTGTGCCAACAATCAGAGAGAGCCAATC

AGCACTCAGGACAAACCAGCTAAAGACCGGCAGTCTTTAATGACTTCATCATCAGAGGAA

GAGGAGCAAGATGAAGAGACGCACAGTGAGAGGTTGGAAGTGCAGGATGAAGGGGAAGAG

GACTTGGTCAGTGAATCCGGGGTTGGCGAAAGACACCGTACACCAGTAATAAATGATCTC

TACTCAATCTCCTCGCCATCTGTGAGTGTTGAAGAGCGGGAGCCCAAGGTGATCTCAGCA

GGCTTGCAAAGGCAGGACTCAGGTTCCAATGGCCTCAGGGGCATCCTAAAGAAGAGTCGC

TCTGCCAGCCTGGAGGCTGAGCCTCCAGCAGAGAGAAGCAAAGCTAACGGCAAAGACAAT

GAGGAGAATCAGCAGAAAGACAGAACAGAAGAAATAGAAGAGGAGACAAAAGTTGATGGT

GGGATAAAAGTTAGGCAGAGGGAAAACAGCAGCTCCAGCGATGCTCCTTGTTCATCATCA

CTGTGTGCATCTGAGGACAGCGAGGAGCTGGAGAGCAGTCTGGATGGAAGCATGAGCTCC

TCGTTTAACCAGAGGCTGGCTGAGCTGACGAGTAACGATGATGATTGGCGTAGCAAGCTA

AAGAAAACCAAAACCAGCGATGTGGTCCAGACGTCCCTGGCTGACCGCTTTAGCCAGCTG

CAGGATGCCGAGAACTCCTGGAAGAAGAAGAAATCTTCAGCTGACGTGGAGACAAAAATG

TCCCTGGCACAGCGGATGAAGATTCTGCAGGAGAAGGAAGAGCAGTGGAAAGCCAAAGGA

AAAGGAGCTGCCAATGATTCCACACAGTTCTCCGTGGCTGGACGTATGGCCAAGAGAGGT

CTTGTGTCCCCCAGTAGAGACGATGTGCCGGTTATCCTCAGCAAGAAAGCAAGCCAAAGC

ACACCGGTTAAGCCTGTAGAAGAGATCGCCTCCCGACCTGACGCTGAGGGCGACAAAAGG

CTCGACAAACTGGAGTCCTTTTTAGACAAACTTCACAATAAAGGTGTGAGCAGTAAAGCT

TCTACCATCGAGGTGACGGAGGAGAAGGAGAAGGAGGTGATGACCCTGGATGACGATGAG

ACGTTTCAGCGCTTTTTCAGACCCGTGTCCCAGACAAATCTGACCTTAGATTTGGTTTTG

AATGAAGAGGAGGATTTCAGTGCGCTCCAGTTCGACTCGCCAAAGTTAACAACATCAGTA

GCAGATCACCGGAGGTCAGTGAGGCCCTCTCGCAACACAAAAGGCTCAAAGAACCCTCTA

CGTGCCCTGGCAGCTCGAGAGGACCTGAAACAGGCTTACACTGAGCAGAGACTCAACATA

GCAACCTTAGAGGCTAATAGGATTCAAGTAGAGAGGATGTCCAAACACTCACGCCTGGCT

GGTTGTGCCCTGGCCGGATTGGCGAGTAAAGAGAACTTCAGGAACGTAAACCTTCGAAGT

GTAAAGTCCACTGAAGTGGTGACCAACAACAGTGCAGTGCCTTTTCACAAGCTGATGCTC

ATCCACATCAAAGGGCGCAGGCACGTTCAGGTGCGTCTGGTAGAGCCCACAGCCCGCTCT

CTAAACAGCGGTGACTGCTTTCTGCTAATCACCCCCAATCACTGCTTCCTGTGGTGTGGG

GAGTTTTCCAACGTTATTGAGAAGGCCAAGGGCTCAGAGATGGCACAATATGTCCAGGCT

AAGCGAGACCTTGGCTGTAAAGCCCCTCAAGTCACTGTGCTGGAAGAGGGTATCAACACA

AACAGCCGCTCAGCCAAAGACTTCTGGAACCTTCTTGGAGGACAAACCGAATACAGAGCT

GCCGGAGAACCCGATGAGGACGAGCTGTATGAAAGCGGCATTGTGGAGTCGAATGGTATC

TACAAAGTGGTGGAAGACAAATTAGTGCCTTACGAGGAAGCCTGGGCCTCCATCCCCAGT

GTGACTCTGCTCAACTCCAAAGAGGCCTTGGTGTTTGATTTTGGCAGCGAGGTATATGTA

TGGACGGGGAAGGATGTTGCACTGAGTGACAGGAAAGTGGCAGTGCAGCTGGGCAAACAG

CTCTGGAGCGGCGCCTACGATTACACCACCTGCAGGGTCAACCCCCTGGACTCCTCCTCC

GCCAATAAGGAGCTTCCTCAACAAGGTGATGGCAGACCAGGTTGGGCTTTGTTTGGAAGG

CTTTCAGAACATAACGAGACAGCGCTGTTCAGAGAGAAGTTTCTGGATTGGGCCGAACGT

AAACCCGCCAAAGACGAGCCGGCCACGGAGGAAGTAAAGAGTCCAGTACAGCAACACTTT

ACTGCCCCTCTGGAGTCAGAGCTGAAGCCGTGTGATGTTAAGGCTTTGCTGGATGGTGGA

GACGTTCCTGTAAAAATGGCTTTAGAGGGTGTAGACGTGCACCGAGGTTATGGATTAGTC

AGTTCAGAAGACGGACGACAGGCTCAGTTAGCCACGGTGGCAGTAGACGCCTGGCACATT

TGCGAGTTCGACGACTTCGAGATCCCGTCCGAGAGCGTGGGTCAGCTGCACGAGGGTGAC

ACATACGTCATTCGCTGGAAGTATTCCATCACCAGTGTGGTGGGTAAAAGGCAGAAGCCT

GGTGAGTTAAGTGCCACAGGGCCCGGCAGGGAGCGAACTGCATGCTTCTTCTGGCAGGGC

CGTCAATCAAGCGTGAGTGGGCGGGGCACATCAGCTCTAATGACGGTGGAGCTGGGAAAC

CAGCGCGGGGCACAAGTGCTTGTCACACAGGGCAAAGAGCCACCCTGCTTCCTACAGCTG

TTCAACGGAGGACTAATCATCCACCGGGGCTCCAGAGAAGACAGTGACAAAAACACAGGT

GGCTGGAGAATGTTCTGTGTCCGTGGTGAGGCGGTAGAGGAGGCATCTCTTCTAGAAGTA

GAGTGCTGCAATGCTAGCCTGCGATCCCGCGCCTGTGTCCTTGTCCTCGGCGTACAACAG

AGGCTGCTGTACTTGTGGCGTGGCTGCAAGGCTCAGCCAACAGCCCACCATGTGGCCAAG

AGGACTGTGGAGCGGCTCACACACACCTGTCCTCCTGAGCTCGGGTTAAACAGCAACACT

AATATGAAAGTGCAGGAAATTGAAGAAGGCTCCGAGCCTCGTGAGTTCTGGGATGCACTT

AGCCATAAGGACAGGAAATCCTATGACTGTATGCTGGAAGATCCAGGAAAATACAACTTC

ACACCTCGTCTTTACCGCCTCAGTGCCAGTTCCGGAAGCTTTGAGGGGGAGGAACAACTG

AGTCCTTCCCGAGTGACAGGTTCTGTGATGGCAATGCCGTTCCTGCAGGAGAACCTTTAT

TCTGCCCCTCAACCAGCTCTGTTTCTGCTTGATAACAGGATGGAGGTGTATCTGTGGCAA

GGCCAGCAGCCTGAGGATGCAGAGTGCACAGGTTCTGCAAAGATTCGCTGGGACAACGAA

AGGAAGTGCGCCATGGAGACTACCCTGCAGTACTGCAAAGCAAAGAATTCCAGGCGACCC

CCGCCGGCTTATCTAATCATAGCTGGAGCTGAGCCTCTCACCTTTACTAACATCTTCCCC

TACTGGGAAAAAGATCCCAGCATCAGAGTGCAGGGTATGAGGAATAAACTAATCCTGGTG

GAGGATGCTTTGTCCAAACTGAGTAAGAATCAGTACTCTGTGGAGGAGTTGACCACCAGA

CCGCTGCCTGAGGGTGTGGACCCTCTGCACCTTGAGCTCTACCTGTCTGACACAGACTTC

CAGACGGCTCTGGAGATGACGCGAGAGGAGTTCAGTTCTCTCCCCAACTGGAAGCAGCTC

AACATAAAGAAGAGCAAAGGCCTCTTTTAGATATTCAAATGAATTATTTTCAAAAAGGAA

GAACAAATCCTACGGTGTCCTTTATGTATATTACTGAATGTACAGCGTAAACTTTTTTAA

GCAATAAACTGACCTAGCAGTTTTGTAGAGATATTTTCTAATGGCCAATTATTGTAGCAA

ACAAAACTTATACATGATATAAATAGATATCAATTGGTGATTTATTATCAGTTAGACAAT

ATGGACAAAAGTATTGGGACACCTGACTTGCTTTAAAAAACATCCCAGTCAGTATGTAGT

CTTACTCTTACTCCTATAATAACCTTCACACTTTTAATAAGACTTTCCAGTAGATTTTGG

ACTGGATTTTCTCATTCAGCTACAAAATGATTAGTGTGATAAGACGCTCGTGTCGGGTAA

GGAGGTCTGAAGTGTCGTTAGCGTTCCATTTCATCCTGATGATATTCAGTGTGTTTGAGG

TCAGGGCTCTGTTCCACACTAACCAAAATACACCACTTCTTCATGGGATTTGTGCACAGG

AGCATTTACACTCTGGAACAATGTTTGTGTCTCGAAATCTACAGCATACAGAGACCTCCT

ATAAAATATGTGGCTTACATTGTGGCAGCAGTTTGGATGTGTTG

>Unigene000212

CGCAGCTGCAAGGCTCCCGGGCAAAACAGATACAGTGGCCTGCGCTGACATTCAAGCCTC

TGGTAGGAAAGAGTGTCCTGGGCTCCATCACCGCCGTGGAATTCTTCTCCGACAGACAGC

TGGACTTCCTAACCGATGACGGAGCATGCCAGCCCTATCAGGATGAAGTGGACAGCTTAA

GTCCTGTTCTCAGGGACAATCCACAGCTTCATGAAGAAGTGAAAAGCTGGGTAAAAGACC

AAAAGGTGCAGGAAATTTTTATGCAAGGTCCCTATTCTTTAAATGGATACCGCGTACGAG

TTTACAGGCAGGACTCAGCCACCCAGTGGTTCACGGGCATCATTACGCATCACGACCTCT

TCAGTCGCACTATGGTTGTAATGAATGACCAGGTACTAGAGCCTCAGAATGTTGATCCCT

CAATGGTACAGATGACTTTTCTGGACAATGTGGTTCATTCTTTGCTGAAAGGAGAAAATA

TAGGCATCACATCCAGAAGGAGATCACGCTCTAGCCAGAATAACAACACTGCCCACACCA

CAGGAGGGAGACCGAGCGGCAACACAGGAAGCTCTCAGGGTCATTACACACGTGCCCAGG

CCAACAGTCCCAGACCGGTAATGAACTCATCAGGCCCTGCCACCAAGCAGGGTTCACAAG

CCCAACAGCAACAGCCCCAGCATGCTCAACAGCAGGTATCCCAGCAGCAGCAGCAGCAGC

CGCAGGTATCACCAGGGCAACAGCGTGGGTCCCGGTCGACACGTAGAAAGGGATCAGACA

GCAGCATCCCTGATGATGAGAAAATCAGCGACGAAAAGACGGACGCGGGAGGCGGAAAAG

GAGATGTTTCTAAAAACAAATCAAAACAGGTGATGAACAAGAGGAGGAAAGCTGAAGATG

AGGAGAAGAAGGCTGGGCTTAAACGGTTAAAGATGGAGGCATCGGATCTTTCCGAGAGCA

GCGACTCTGAGAACTCTAATAAGAGGCTCCTGGACTCATCCTCAGAGCCGAGCTCTGAAA

ATGAATTAAAAAATAAAGGCATTTTAAAAGCCAGTGAGGAAGAGGAAAAGTCCCAGAGCT

GCAAGGCTATGGAGGAGCCTGACGCTAATAGCAGGATGTCTCCCTGGGAGGAGGCTTCTA

CGGTGGATCAGATTGTCAAGCCGACAGCCATGGAAGTGATGCCCTCTACCAAGAGTGAAA

ATACAGGCGACATGAGGTCCCTAGCGCAACCTTCCCCACTGCCTCCTCCCAGCTCGTGTG

CGCAGCCTCGGAGCTTAGTTCCTGCTGAAGTTCAGGGTTGCATTATGGAAATAAAAAGCA

CTGTGAAAACTTCTCCTAAGGACCATTATAGCACAGGGGCACCAAGAACACAAACTCCAA

AGTGTGTAATTGACATCACAGAAGACTCCAGCTCCCACCCCACAACCCGAGAGAACTCAG

AGACTGTGCCTGCGACTTTAGCTTCTCAAACACGTGAAACTTATGTCCCTGAAAGCAGAC

ATTTGGTGTTAAACCCCTTAGCCTCAGAATGCAGGAAAGCAGAGGGTGAACTTCAGCAGC

AGCTAGGTCAGAGCCTGGGCAGCAAGATTGAATTTGCTCACTCTGAGGTGATCCGACCTG

TGGTTTCGGTAAGCGAGTTAGCGGCTGTTGCGGAAAGGGAAAGGGAGAAAGTACAGCAGC

AGTACCCTTCAATCATGCCTTGCATCAAAAATGCCTCACTGGCTGAAGATGTGCGAAAAC

CACAGAAACTTAGCTCGTCACCAGATGTGGCCAAGTCCAAGTCTAACCCTTCACCTGACG

CATTTAAGCCCAAGTGCAATCCCTCACCTGACGCCATGAAGTCAAAAACCCACAGTGTCC

TGGAGGCCATGAAGCCTAAACCTAACACTTCACCTGAGGTCACCAAACACAAAGAGATCA

TTGAGATCACTTCATCCTCTGTTGCCCGGTCGGCAGTCAAGTCGAGAAGTCAAGAAATAC

CACGCTCCAGCTTTAAGCCTGTTCCTGCTCGTAGCACTCCCTCTGAGTCCATCAAGAGCG

CTCTCATTGTCGACAAAAACGAGCACTTTACAGTGTATCGTGACCCGGCGCTGGTGCGGC

CCGAGGCTGATACCAACCATGTAACTTACCTTCCCCCACACCTCCATCCACTCCACAGCT

CCTCCCATGCTACTTGCCTGACACCTAGCTCACATCACCACTCCCATCTATTGCCTGCAT

CTTCCCTTAGCCCGCACCCCTCAGTGCACCACCCCTTGCTCCCCACCGTGTTGCCTGCCA

TGCCGCCGTCCTCCCTACTGGGTGGCCACCCGCGCCTCGACTCAGGGGGACTCAGTCATC

TAGCCCTAGCTCACCACCACCCACACCAGCAACAGCAGTTTCTGCAACAGCAACCACCCC

CACCGCTGCTGGCCCAGACACATGGTGGTGCCAGCTACAACCAGCTCGGCCTTTATCCCA

TAATCTGGCAATACCATAATGGCACACAGCACTCCTATCCATCTGGAATTAGCCTGCCTG

GGTCAAAGTGGGTGCATCCAGAAAATGCTGTGAACTCAGATGGTAGCCTGTCAAGGAACA

CCTCCAGTCCCTGGCTGCATCAGCCCACTCCTGTGTCCTCAGCGGATAGTCTAGGAATTC

TGAGCCATGTCCCAGGTAGGCCAGCCAGTGCTGATCCCCACAGACCCCTTAAAATCTCCT

CACACTCCAGCCCTCCGCTCTCCAAAACTGTGGATCACCACAAAGGAGAGCTGGAAAGTA

AGGTTTTTGTAGATCCAATGCGTGGCCTGGTAACCGCCCACCTAAAGCAAGAGCCAGACC

AAAGCCGGACACCGAACAGCAGGGATTTACAGCGCCTCTACGCGGATTCATCTCACGTAA

AGCAGCAGGCGCTGCCTCCCCGCCTGCCACTAGACACTCCAGACCGGGCTGTTAAGTACA

AAGAGGAGAATCGACGCATTCTACAAGAGAGCATTGAGGTGGCACCCTTTACTGCCAAAA

TCCGCTCAGGAGAGCCAGAGCGTGAGCCCTATATCCGCATTCCCTCTCTCCCCATCAGTG

GACCGCCCAAGGAGAATGAGCATTCGCCATCTGATCTTTATAAGTACAAGCACTCGGCTG

CTCAGTCACTGCCACAGAGCAACTATTTTACCACCATTTCCAACAGTGTGGTGAATGAGC

CACCTCGTCTTTACCCCTCCAAAGAGCTTAGCTCCCTTAATGTTTCAAGTCCCCTGACTC

TCGGCTCCTACCCCAACAGCGGAGGCAACACCAAGTCCTTATCCAAGCCTCCCCCGCTCA

TCAAGCACCATCCAGACGGGGAGGGCTTGGTGGGTAAGATCAGTGAGCAGCTCAGTCATC

AGGTGGCCCTGAACCCTCTGAGTACCCCCGTGGCATCTTGCGAACCCCGCAGTCCGGCTG

TGTCACCCTCAAACCAACTCAGGAGCATGCCTGCTCTACACAGGGCACCTGTTTTTCACC

CTCCTACCCAGCAAACACTGGACCGCAAAGAGTCTGGCTACGGTCGCCTCTCTCCACCAA

CGCTAACCCCTATTCAGCCGGTTAGTGTGGCTGGGAAGGTATCAGAGCAACAAAAGCCGC

CCACTCTGCTTCCAGAGCTTAGGGAGGTGAAAGGCACTGTAGAGCTGAGCTCCAGTGAGC

CATGGAGGCCCAGCTCTAACTTACAGGGCTATGATAAAACAGTTCAGTGGCAGTTAGACA

AAAGTCAAGGAAAGCCACAGGTAGCCACAGCTTCTGTTATTGTTCGCTCCAGAACCTGCA

TTAAGTATGACAGCTCACCAGGCTCCAAACCTGGTGCCAGGGAGACGTCTAACAGTAAAC

CACATACAGGAAAATACCAACTGGACTGCAGCAAACTGGCTGAAACCAGAGAACCCGGCA

GAGTTATACAGCAAAACACAAATAAGGAGGACATTTTTTTGCAGTACAAAAACAATTTTG

TCCGAGTCTCCCAGGGCAGTTTTCCCAGCTCTGCGGTAGCTGTTGTTAACTCTGTGTGCA

ATAACAGTACTGCTGTAACCACTTCTGCCTCTGCTGCTTCCACTCACAATGTGCTGAGTC

GTGGCACAGCTGAACTGCCTTACTCCACCTCAGCAAATCCAAGCAGCAGTAACATTAACA

GACTGGAGGGCGCTGCACCCAAATGCAGGACTCCTACCAGCCTTGAGCTACAGGAGTGCA

ATGCCAGGACAGCCTCCCCCATTGGCCCACTGCCTCAGCCTGGTTCGGCCCTAGCTCCAC

AGCCTTATTCTGGGAACTTTATCCACCTGAAGAAGCACAAGGCTGCCCTGGCCGCTGCCC

AGTCCCGTGGATCAAGCAGTGCCTCAGAGAGTGAAGGTAGCAGTCGATCCTCTCAGGAAT

CACCCACAATCATCACGCAGGATCGTGCATCCCCAGGCAACCCGTCCAGCAAAGTCAGCC

CCCTACCCAATGGTCAGCCATCACTGATGAACCAGCCCAACTACCACAAGCTAAAGAAAG

CATGGCTGACTCGGCACTCAGAGGAGGATCGCAACACTAACAAGGTGGAGATGACTTCCA

ATGCCGTCTCTGAGATCATTAGGCCATGCACAGTAAACCTTATAGCATCCACCTCCAGCG

ACACAGAGATAAGCAAAGATGGCAAATGCTTAGAGGATAAATTGAGCCCGGAGGACAGGA

AACCACGACGAGTTCCTTCCAAGCGGCCACACGAATCAGGCTCGGATAGCGGCGATGACT

CCGATGGCAGTGACAGCAAGCATGAGCAGAGAGCCAAAAGGCAGCCTAAGCCCACCTTCA

AGAAGAAGCAGAATGACATGCAAAAAAAGAAAGGGGAAAATGACAAGGAGGAGGAGGATG

TCAAGCCCAATGGCATATTCAGAAGCGCCAAGGAGAAAACCAAACTAAAATTGGCCAGCA

GCAACGGTATTCCTCGCTCGGTGTTGAAAGACTGGAGGAAGGTGAAGAAGCTGAAGCAGA

CGGGTGAGTCCTTTCTGCAGGACGATTCGTGCTCGGAGATTGGACCCAACCTGCAGAAGT

GCCGCGAGTGCCGCTCGATCCGCACCAAGAAGGGAGAAGAGCCCACCCACTCCCCCGTAT

TCTGCCGCTTCTACTATTTTCGCCGCCTTTCATATAGTAAAAACGGGGTTATCCGGATCG

ACGGCTTCTCATCCCCTGACCAGCACGACGAGGAGGCCTTGAGTCTCTGGGCTCCAGATG

CTTACGAAGAGAATGATCTGGACCTGGAAACCTCCAAATACATTCTCAGTTGCATCGGGG

ATAAGTTTTGCCAGCTGGTCATGAGCGAGAAGACCGCCGCCACATGGATAAAGAAAGATG

CCAAAATAGCCTGGAAACGGGCAGTGAGGGGGGTGCGTGAGAGCTGCGACGCATGCGAGG

CCACATTGTTCAACATTCACTGGGTGTGCCAGAAATGTGGATTTGTGGTGTGCTTGGATT

GCTACAAGGCAAAGGAGAAGAAGAGCTCCAAAGACAAAGATCTGTACACCTGGTTAAAGT

GTGTCAAGGGACAGCCACATGATCACAAGCACTTGATGCCAACGCAGATTATTCCAGGAA

CAGAGAATAAGGAGTCGGTGGTAGCGGA

>Unigene000213

GGAGGTTTCTTTTTCTCGTCTCTCAGTACTAAAAGTAATTCCTGGAACTTGGGCGAGTTT

AATTCCAGTCTGTCGTATTATGATGGAATGATCGAGCTGGACTACCTGAACGGCTCTCGG

TACAACAACGAGGAACACACACAGCGCGCCACACACATCTCCTTCCTGTGTGACCGAGAG

GCCGGGCCGGGCAAACCTGAGTTTCAGGCTGAGGATGAATATACCTACAACTTCAGGTGG

TACACTTCTTACGCCTGTCCAGAGAGACCACAGGAGTGTGTGGTTACTGACCCCGTCACC

TTGCAGCAGTTTGACTTGTCTAGTTTGTCGAAGTTGAGCGGATCCACTAACTGGGAGGCC

GTGGATGTGGATACCATGAAGGTTTATTATATCAACGTGTGCCGGCCGCTTAGACCCGTG

CCAGGCTGCGACCGACTCGCCTCAGTGTGCCAGATCAATTACGTGAGCGATCAGGGGACA

CCGTCTGCGAAAGTGACCGTAAGTAACATGGGCGTGGCTAAAAAAGGCCCCATGATCTTC

GAGGACAATCAGTTGATGTTGGAGTACACGGACGGCTCGGCGTGTGAGGCAGACGGGAAA

ATGAGCACTTACACCACCCGCATTCACTTCACCTGCTTGACAGGATTTGTGTCATCCGGT

CCACGCTTCTTATCAGTTCAGGACTGCGTAGTGAACTTTGTGTGGGACACCGATGTAGCC

TGCGCCATTTCAACTGCTGTGGACACCAATCAGACTTGTTCTTTAAAGCATCCCAACACC

GGCTTTGAGTTCAACCTGCTTCCTCTGGCTTCCGAAAACTCATACAAGACAACTGCAAAC

GGAAAAGAGTTCATCGTGAACATCTGCTCGGCCGCACGCCAATGCGGATCGGACAAACCG

GACGCTGCGGGCTGCGTGGTGGAAGACAACAAGCCGCACAGTTTTGTAGGAGTGGAGAGG

TCCCTGCAGCTGTCCACTGATGGCTTAATTACTCTCACCTATAGAGGAGAGCTGGATAAA

AAGCTGGGCACGAGGGCCACGTTCACTATCAATTTCGTGTGCGATCAAAGCGCCACTAAC

GGCTCCCTAAAGGTCATTCGTGAGGAAATGGCCACGTCCACACACGTGCAGCATGACGTT

CTTTTCGAGTTCTCCACAGCTCTGGCGTGCGAGCCTGCACCAGTGGATTGTCGCGTTACT

GACTTTCGTGGAAATGAGTACGACCTGAATGACCTCAGTCTAAATGATAAGCCATATGTC

CCCTTGGACACTTCAGACCAGGCCAAATCTCAGAAGTTCTTCATCAGTGTGTGTAAACCT

TTGCCATTTGTCCAGGGCTGCCCAGTTGGAGTAATTGGCGCATGTGGTCAGTTAAATGGC

CGAGCAGTGAACCTGGGCTTTGTGCAGTCCAGCCTGCAGGCTGGCTCAGACGGCTCCATC

AGCATCGTTTATCAGAACGGCGATAAGTGTGTCTCAGGAGGGCGTTACTCAACACGCGTT

ATCTTCCAGTGCGATGACAGCCCAGGCTCACCGATATTCGACCGTAAAGACGGCTGCGAA

TATGTCTTCATCTGGAGGACCTCTGAGGCTTGCCCGGTTAAGAGAGTACAAGGTACAAAT

TGTAAGGTAAAGGATCCCAGGAGCGGCTATGAGTTTGACCTGACTCCACTGACTGGGAAG

GATTACGAGGTGAAGAACCCACAGTATGAATATCACTTTGCTGTATGCGGCCCGATCAAG

ACGTCAGTGTGCCCCCACGGAGATGATAACATGGTCTCGTCCTGCCAAGTGGACGGGTCC

AATCACAGAATTTCTGGACTGGCGAATCAAAACTTGACCTTAGATGATGGCATCATCATG

ATCAACTACACACACGGGGAAACCTGCCACACCATTTACGAGCGTTCGACAGCCATTATG

TTTTCCTGCGACCACAGCAAGAATCCGGGGAAGCCGGTGTTTATTAGGGAGACAGCAGAC

TGCACCTACTTGTTTGAATGGCACACGGCGTTGGCTTGTCCTCCTTTTAAAACCAGCAGC

TGCTCCTACAATGACGGCAGCGGGAACTCGTACGATCTGTCCTCTTTGGCTCTGCACAGT

AGTAACTGGGTGGTTCTGCCTCAGACTGGAAACAAAGACCGGCGCTACTACATCAATGTG

TGCAAGTCTCTCGTACCTCAGAGCGGTTCATGGGGCTGCCCGAGCAGCGCAGCAGCATGT

CAGAAGACCGGTGATGAGTACATGAGTCTGGGAGAAGCTCAGTCCGCACCACAGTGGGAG

GAAAAGATGCTGATACTGAAGTACATGGACGGAACGACCTGCCCGGGAGGAGACCGCAAT

CGAACCACCATTATTCGCTTCAAATGCGATCGCAGCAAAGTGGATTCGAAGCCCACTCTG

ACCAGCGTACTAGAGGACTGCATGTACACCTTTGTGTGGTTTACGGCAGCAGCGTGTCCT

CTTAACAGCACAGAACACGGAGATTGCAAAGTCACTAATCCACGCACAGGGCATCAATTT

GACCTCAACTCCCTCACCAAGGAAGCTGGATATACCGTGTACCACAGCGTCGATATAAGA

AAGATGTATCGCCTCAACGTCTGTGGCCCTGTAAAGAACTCGGGCTGTTCTTCAGAAGCA

GGAGTGTGTATTAAAGACACTAACACTGCTGTGAGCGGAGGAACGTCTAACAAGAAGCTG

GTCTACAAGGACCAAGTGGTGGAGCTGACGTACGAGGGCGGAGACGTCTGCAAGGCCAAT

CCGGCGCTGAAACACAAGAGCATCATCAGCTTCGTCTGCAAGTCTGAGGGAGGGGAAGAA

GAAAGCTATAAACCCGTGCTGGTGGTCTCAGACGAGGACACGTGCACTCACTTTTTTTCC

TGGCACACGCCGCTGGTCTGCGAAGAACAGTTGAGGTGTTCAGTTTGGAACAAAACCAGC

CTGATCGATCTGAGTCCTCTAATTCACCTCAGTGGATTCTACACAGCCACGGACGAGGAC

ACGGGTGGGACCGGAGAGGGGAACAAGGACACGTCTCGTGTCTTCTACATAAACATCTGC

CAACCACTTAATCCCATTCCAGGAGTGAAGTGTCCTCCTGGAGCAGCAGTGTGTATGGAC

CCTCCTGAAGGAGACCCCATTGACATTGGACATGCCATCTCTCCTCCCAAGTATAACTCT

GCTACTGATGAGGTGGAGATTGATTTCTCCAGTACGACACCATGTGTGGCGGATCCATCT

GTCAACTACACCTCCAAAATCATCTTCACCTGCCAGAAAGACACAGACCTGGGATCTCCC

GAAATGATGCACCAGCTGGACTGCATGTACGTGTTCGAGTGGCCGACGCCTCTGGTGTGT

CCGGAGACGGTCAGCGCGCAGGGATGCAAGCTCCGCGTCTCCCAGCTACAGTTCACCTTC

GACTTATCGGTTTTGTCAAAGGATGTTCAGGTTCCTGTTTCCTCTGGGACGTATAAACTG

AACGTGTGCGGCTCCGTGTGGGACAAAGCATGTAAAAACAGTTCGGTGTGTTTGGTCTCC

GGGTCGTCTGCGTCCTCGTTCGGCCTCAGCCGGGCCATGACTCTGAACTACAGCCGTGAA

GATCAGGCTGTCATCATGCAGTACGGAGGTGGAGACCCGTGTCCTTCAGTGACTACAAAA

GGAGATGTGTGTGTGTTTCCCTTCATCTTCTTGGGCAAATCCTATTTCAAGTGCACCCAA

GATGGCAGGACTGATGGCAGGAAGTGGTGCTCCACCACAGCCAACTATGATAAAGACAAG

CAGTGGGGCTTCTGTTCTAACGTGACTTCTAAGAGGCAGTCGACAATCCTCTTCACGTGT

GACCCCTCGGTCGGCCAAGGTAAACCCCAGCTGCTCAGCGAGACGCAGGCATGTTCGAGT

AATTTCCTGTGGCCCACCGATGCAGTCTGTCCTCCAAAAAAGATGGAGTGCAAGCTGGTG

AGCCACCACCAAACCTACGACCTGCGAGCGCTCTCGTCTCTCACCGAGCCGTGGAAGTTC

ACCGATAAGAACAACGCTTACTATCTAAACTTGTGCCAAGAAATCCACGGAGGTCTAACG

GGCTGTCCAGACGGAGCCGCCGTGTGTCTCAGACGCTCCAGTGGCAAGATCGAGACCCTG

GGACGAGTGTTTACTCAGAGCATGGGCTTAACAGATGGAAAAATCCAGCTGAATTACAGC

ATGGGTGATGCAGTGTGTGGGAATTCCAAAGCAGCCAAAACCATTGTGCAGCTGTCGTGC

GGCAGTACCGTGGGGCGACCCAAACTTCTGAAGGAAGATACGGCAGCCTGTGAGTTCTGG

GTTGACTGGGAGACTCGAGCAGCCTGTGCAGTGAAGCAGCAAGGAGAGCAGGTGGAGATG

GTGAACGGTATCATTAAAGTTCCCCAGACAGGAGCAGAGTTCAGCCTGGGAGATCTTTAC

TACAGCCTCCACAAAGCCACAGGAGACATCAGACCCAATGGAGATATCTACATTTATCAC

ATCCAGCTGTCAGGCATTACAAACAGCTCCTTATCCCAGTGCTTGGGTGCCAACATCTGC

CAGGTGAAGAAAGACGCCACACATTGCAGGAAGATCGGTTCATCCAGCAAGGCCAAGTAC

TACATTAAAGGGGACAGTCTGGACGTCCTGGTTCCTTCAGAGTCGGTGTGCGGACGCGAC

AAATCGAAGACCATGTCGTCCACCATCTTGTTTCATTGCAAGCCAGAGGCTGGAGTGGGC

ATCCCAGAGTTCCTGCTGGAGACCGACGGGTGTCAGTACCTGTTCGTCTGGCACACCGAT

AAAATCTGTGAACTTTTCACTATGACTCAGCAGAGTGACACCGCTGAGGTTGGGTTATCA

GGGCGGAGTCAGGCTGTGGGCATGGTGCTTACCCTTCTGCTGCTGGTGCTGTTCATTTGT

CTGCTCATTCTGCTGCTTCACAAGCGAGAAAGGAGGGGGCAATTTATGCAGAAACTCACC

AGCTGCTGCAAACGCAAAACTCCGGTGTCTTATAAATACTCCAAGGTAAGCACGGACGAG

GACGGCTGCGAGGACGAGATGGAGTGGCTGATGGAGGAGGTGGAGGCTCCGGCACAAACA

CCTCACGAGAACGGCCACATCACCACCAAACCGGTGAGCGCCGACGCCTTGCGCACGTTC

TCCCTCGACGAGCAGGACAGCGAGGAAGAAGTTCTCACCGTCCCCGGAGTTCGAGTCCAC

TCGTCTCGCTTTTTGTTGAAAAACGAGAGCGACGAGGATCTGGTGGGGCTGCTGGACGAC

ACAGGGAGAAAGAACACGTCCAAATCGAGCTCCAGACATCTGAGAAAAGCGCAGGAAGAC

CCAGACGACAGCGACGAGGACCTCCTTAAAGTCTGAGGTGTCGAGTCACAAACTTCTAAA

CGTCCCTCCAAACTCACGCCGGTGTTTTTGGGTTTTTTTTTTTATTTTATTGTGGTGTTT

TCTAAAGGGAAGTCTCTTTCTGTCTCTTCAGTGCTCATCCATAGACCATCAGTGTGTTGA

CGATAAAAATATTTTGTCGTTTAATCGTATGATTTAGCTTTTTTTTTTCCCCCAAAAATC

AGTTTTGCAGAGCTGTTAATTTATGT

>Unigene000214

TGTACGGCGACCTGCTGCCGTTTAATCGGCTCGTCTTTCTTGATATTTTCGCTGTAATTT

ATTTAATCGGGTTTAAGGGACGTTTGTCCCTCTCGTCCCCTGTTTATTTTATAATCCAGG

GCTACGTTTATCCAATTACAGCCGCTGTTTTTAAAGGGGACACAGAAGCGGAGTGTGGAC

ACTGTGGAAATGGACGCTGTGGATCATCATCCGGGCTTCAGTGTCCGGGATCACACACTG

CTCTCGGACCCGGACAGGACTTTAACCCCGAATGGACACGGGGCTGCTGATGGACATGAA

GCTGCTGAAGTCTGGAGATTAGTGTGTGTGTCTCTGTATGGAGGAGCTCCGTGGGGATTC

ACGCTGAGGGGAGGACAAGAACACCGAGAACCGCTCATCATCACCAAGGTTGAAGACGGC

AGCAAGGCGGCAGCCGTGAGGCTCCAAGTAGGAGACGAGCTTGTAAACATCAACGATGTT

TCCCTGAGCGGCTACAGACAGGAGGCCATCTGCTTAGTCAAGGGCTCCCACAAGACTCTT

ACTCTGGTGGTGAAAAGGAAAAATGAACCCATGAGCAGGCCTCATTCCTGGCATTCCACC

AAATTCAATGAGAACCAATCAGACACTGCCAAAACACAGTCCACACCCTCACCAGTCTGG

CAAACAAGATATGATGCAAGTGCATTCACATCCGACTTTACTACTGGTTGGGACCAGCCT

AATTTGCGCAGAGTATCAGACCAGTTTAGCTCACTGGGCAGTATGGATAGTTTAGAACAC

AGCTCTTGTGCTTATCCACCTGGACGCCTATCCCCAAGCAAATCTAATGGTAACAGTGTT

GAACATTTAGTGTGTGGAAAAAGAGATTCTGCCTATAGCTCCTTTTCGACCAGCTCAGGG

ACACCTGATTATACGCTGTCGAGAAGCAACGCAGCTTCTACAGAGAACATGTTGTGTAAA

GTTAACCAGTGGGATGCAGGAAGTCGAGCTAGCAACAGCAGACATAGTCAATGCCTAATC

GAAGGAGTCCGGCAAGATGACAAAATTGGTTATCTGCAGCCACCTTCAGTAAGCAGTGGC

CGAGAAAGCCCGAGAACAGAGGAGCAACCAGGCTACCGGCATTCTGCTTCAGGAAGGTCT

AGCATTGCACCTGTCTGGCATGTCCCTGATATGAAAAAGGCCGTTGCCCCATCACCTCCG

CCACCTGCACCACCAACACGCAGCGACAGCTTTGCTGCGACTAAAATTCATGAAAAAACT

CTGGTCCCCAGCAACACTGAAAATCCTAGTACTCATACCCAGTCAAAGATTCAGGGCAAA

GGATTGAAGGCAGCAACAGAGGTGTTTGAAGGCAATCAAAGGATTTATCTTGCAGCTGAG

TCAGGCAGAGAAACAAGTCAAAACTACAGCTTGCAACCGAAAAGTGGCTTGATTAATCCA

TATGTGGCAGGCGACGGTCAACAAATTTCTCTTTCAAATTCAAACAAGAAATACTCTCCA

TCCGGCATGGAGCCGTCCTACACTCATTTTGCCTACCATAAACGTCAGTACAGTGATGAG

ACAAATTTTTATGGGCCTTCCAGAACCCCGTCTTCAGGCAAGTCTCAGAATTGCTACAGC

AGCATGCAAGAGTTACCCACCAACAGCTATGCTCCACTCTGTAGCCAACATCAGATCAGA

AGGCATATTGCATCACAGTCCACCACTGCTATTGACCAAAATGCAGATAGTCAAAACTCC

AACAGGTATTACTTTGTCAAATCTCAACCAGCCTCTCAAGGAGGGTCCCAGACCTCTCTA

TTCCGGGTGGAGGACAGGAATGCTAATTCTGTAATGGAAATAACCCAAAGTGGAGGTGAT

AAAACTTCCGTAAGCTCTCAAGGACAGCTCAAGGACAGGTTCACCACATCTCAGGTACTG

CTGCACCCAAATAGCAAAGACAGTAATGGATATTACAGACAGGTTGACAGCCAACATAGG

GGGTACACAGCAATTAATAGATCAACCTTTGGGGATGCAACAACGAAAAATTTGGAGGTC

AGGGCGCCCCAGAAACAAAACACTGTGACCAACATAAACCAGTTTATCACACACTATGAG

CAGGGAAAGATCTCTGAATCACACCGGTTAGGAGAATCCTCCAAAGAAAATTTCCCATCT

AAAACTGAGACCATGTTATGCCCCCAGAAAACCCCTTTGCTGCACTCTTTATCACATGAT

GGCAATAGACTGATTGATGTACAAAGTGAAGTTGTTAGTGGAGCTGGACAACAGGATATT

GTGGATCCCCAAAATGGCAGACAGTTTCATCGTACTGATCGTTTTGCCACCACATTACGA

AATGAAATCCAGATAAGGAGAGCGCAGCTTCAGAAAAGCCGAAGTACAGCGGGCTTGAGC

GGTGTAGCTGATCCTCAAGAAGAGCCTTCAGTTGGGAGGTCCACAGATATCGCTTCACCT

TCCTCTGATGGTTCTTTTACCAGTGCCTACAAAGACCATCTGAAGGAAGCTCAAGCTCGA

GTCCTCAAAGCTACTTCTTTCAGACGAAGGGACTTGGAGCCAGTGCTGTTAGAGCACCCA

GGAGCTGAATTCTCTGAGGTTCTGGCAAATAAGCCTGCTTCAGGTACGAACCACGTGCAC

CGTATATGTGGTCGCAAACGGTTTCCTAATGACAAGAAAGTACGATCTTTCTCAGAGCCT

GAAAAAATTCATGAAGTTGGTGTTGAAGAAAGAGCTCCACAAGATAACGACTTAACTTTA

GTCAGTAGGCGTAAAGTCTCTGAGGCAACAGGAAAACCCACTTATCCCAAGCCGATGTTA

AAGCCAAATTCGCAGATATCTCTAGACTGTAGATCAGACAAGCCTGGAGAATTGTCTCAG

TCTGGACAAAATGATGTCACTAAAAGGAATACCAAGAGTAAGGCAATAGACAGTGGCCCA

AATTCCTTACAGAGACTTGGCACGTTTGCAGAGTATGAGGCCACATGGAGCACGCAGAAA

AAAACTGATCCCAGAACATCTGGAAGGTACCATTCGGCCGATAACATCCTTGATTCAGCA

GTGGATGAGCGACATAAACCCTCCTATTTCCATGAAAGATCACGCTCTTCTCCATCAGAG

GACTTCTATGGTCAGAATATCCCAGCGCAAGGAAGAAAGTCAGCCGAGTCCCACCTGCCA

GAGAGAAAAGTCTCTGATCATTGCAACTCTACATCTCGGATGGAACAGGTGATCGATGAT

AGCAGCACTGTGAAAAAAGTGCCCATCAAGATTGTCCACTCTGAAAGCAGCACAGAGAAA

GAAAATCGACAGTACCTTTACCCAAGCACTGAGACACCTCGTAACTCACAGCCAGCAGTC

TTGTCTTTGTCCAGTCTAGGAGCACAAGAGCAATCCTCCTCTCCGTTTTGCACATACACA

CGTCAAAGAGAGCAGGAAGATGAAGCTATTAGACCTCAGAAGGAGCCAGAGCCTAGCGAA

GATGTCTCAGGGCCTCCAAAACCACAAAAAAAACCCTGTGTGGATCAGAGCACTAACGGA

GACTCCTCAGGTATCTACAGCGCTTCCTTTTACTCCGAGGAAGATCAGAAGAGAGACATT

TTGGCCAGGGACATCATAGACAAAGATAAGTCTTTGGCGGAGATTTTTGACCAGAGCAAA

CGGAGAACTACTATGGACCTAATGGAGGGGATTTTTCCAGAAGGAGAGCAGCTGGTCGAA

GAGGCCCACCAACGCAGGAGAGCTGCACCTAAACTGCCCTCACGCAATACTGAAGAAAGA

AGGGAGGAGGACAGCATGACGACGGCTGCCACCGCCGCAGCAGCAACTCTGGTGACCAGC

TCGGCTTACTACAGCACGTCAGCTCCCAAAGCAGAGCTGCTTATTAAAATGAAGGACATG

CAGGAGAAGAGTGTAGAGCTCGAATCAGAAGAGGAGCTGGAGGAAGACAACGACGCTGAG

CTTGCCAATAAGAAGCATGAGCTAATCAAGAGCCTGAGCAAGAAACTGAAGGTGCTTCGA

GAGGCACGAGAGAGTCTTCAGGAAGACATGCAGGACAACAACGCTCTGGGTGAGGAGGTA

GAGACCACCGTACAGGCCATCTGCAAACCCAACGAGCTGGAGAAATTCCGCATGTTCGTC

GGGGACCTGGACAAAGTAGTCAGTCTGCTGCTGTCTCTTTCTGGGCGACTGGCTCGTGTA

GAGAATGCCCTTAACAGTCTGGAGGAGGACGCACCCCTCGAAGAGAGGCGCACGTTAATG

GAAAAGCGCAAGCTGTTGATCCGGCAGCACGAGGACGCCAAGGAGCTGAAGGACAACCTG

GACCGGCGGGAGAAAGTAGTCTACGACATCCTGACCAGCTACCTGAAGGAGGAGAACCTG

GCCGACTATGAGCACTTTGTCAAGATGAAGTCAGCCCTCATCATTGAACAGCGCAAACTG

GAGGATAAGATCAAACTGGGCGAGGAGCAGCTCAAGTGTTTGACGGACAGTTTGCCTCCA

GAACAAAGGTCACTCTTCTGAGAGATTACAAAAAGACTTTTAAGCTATAGTTTACCCAAA

AACCACTCGCCCTTGTGTTGGTGCAGACCCGTAAGACCTTTTTTAATCTTCGGAACACAA

TTTATTGCATTTTAATAGCTTTTATGTTGCGCCATCACCACAGATCAGGTGTCATGACGT

ACACATACTGTTGTATGTGTATGTCATCAAGTATATGTTATCAAGTACGTCCATAAATGT

CACAGTAGAGATGAAAATGTGGAATAAAGTAGTTATTTTTTGTTTTCTTTCTGCAAGAAA

GGACGCTCGTAGCCTGTATTACAGGTAAACCACCGATGTCACATGAACTATTATGCCGAT

CCTCTTGGTACTTTTCTGGGCCTTGAATATGGTAGTCACCCTGCTGTCTATGAAAGGGTC

AGAGAGCCTTTGGATTCAATCAAAAATACTTAAAATCGTAGTTCAAACATGGGGAAAAAA

GGTCTCACGGGTTTGTAACAACATGAGAACGAGTAATTGAAGAATTTTCATTTTTGGGAG

AATTATCCCTTAAACTCTAAAGACACACGGCATCTGGATTTCAATGTCGGGACTTATTTC

GTCAGACTCTGCTATTGTAACACCATGAAGTTCTTGCTGAGCTTTTGTGAACAGAGACAA

ACCACTTTTTGACTGTTTTAGAACCACGCCCAACTTTGGGCTGCATCATTACCGTAAAAT

ACTGTGTGATTATTGTGATTATTAATTACGCTGAACTGGATAAAAAGGCAAATTTCAAAG

CGCTTACTGTATCAAGTTATGACCATACCACTGTTAATCTGACCTTGTCTCAATTATTAG

ATTATCATTTATTGTTTCTGTAATATTGTATTTTGACCACTAGTGGGCAGTGAAAGATGG

TTGCTTTGACCTTATGGTACAAACCACTTTTCATGCATCTCTTACACTAGCATACACATA

GACTCTAACATAAGCATTTACACCTTATATTCATAACTATTATCTTATATTTAACTTTTA

GCCCAGTTTCAATGACACACGTAGACCTGGAGATTTTCATGATTTTTACAATGTGCGTTA

TATCATACAGCATCATTCCCGGTC

>Unigene000215

ACAGTTTTTTTTTATGCCCTAAAATTGTTTTTGGAGTCAGTCTAGAAAATGGATTAAAAT

ACCTCATCCCCTGAGCTTAATCAATTTGATGAGACATGCAGCCCAGTAGGGACATCTCTC

TGGTGTTTACATTTGGATGATCACACTTTGGTAGAATAAGTTGATTAGACGATGTCTTTG

CATCTTTAGGTTTATGGTGCAGCGATCCAGTTTTATGAGGCGTACCCAGAAGAGCGCCTG

ACTGAGAGACAGCGCACACAGCTCGGCCTACAAGCCAGTGGCCTGAAGTCTAAGGAATGT

ATGACGGTCCACACCAACAAGAGCATCTGCCTTCTTTCTCACTGGCCCTTTTTTGACGCT

TTCCGCACTTACCTTACCTTCCTTTACCGCTACTCAATTTCTGGGCCACACACATTGCCC

ATTGAGAAGCATATCTGTCATTTTATGCACAAAGTTCCTTTCCCATCTTCTCAAAGACCA

CGCATTCTAGTCCAGTTATCTCCACATGATAGCCTCATGCTAAGCCAGCCTGTCTCCTCT

CCTCTGCCTCTGAGTGGTGGAAGTCTCTCCACACTGTTGCTAAACCTGGGTCCTAAAAAT

GCAGTGACTCTGCTGGTTCTGGCTGTGACCGAGCACAAGATTCTTGTGCATTCACTGCGG

CCGGCTGTGCTAACCAGCGTTACAGAGGCCCTGGTGTCAATGATCTTTCCCTTCCATTGG

CCATGCCCATACATTCCCCTGTGCCCGCTGGCATTGGCAGATGTGCTCAGTGCTCCATGC

CCCTTTATTGTTGGTGTGGATTCACGCTATTTCGACTTGTATGAGCCCCCAGCAGACATC

AGCTGCATTGACCTCGACACCAACACCATCTCAAACAAAGAAGATCGCCGAGCCCTAACA

TGGAAAATCCTGCCAAGAAAAGCCTGCAAACGTCTGATCAACACCCTCAGCAATCTCCAT

CATCAGCTGGTACAGGATTATTTGCTGAGTCATGAGGAAGGTCAGATCGATGTGTCCATG

AGCGAACGGGACCCAGGAAGCGGTGAAAAAAGTCTCCAAACACTCGAGCTAGAGATCCAA

GAAGCTTTTTTACGCTTTATGGCAGCCATCTTAAAGGGTTACCGCTCCTACTTGCTTCCC

ATTACTCAGGCGCCCTCAGAGAAAGCGACTGACGCCAGCTCTCTCTTTGATCTACAAGGC

TTCTTAAAGAGTCGTGATCGCTCCCATCAGAAGTTTTACTCGCTCATGACCAAGACTCAG

ATGTTCATCCGCTTCATCGAGGAGTGCTCTTTTGTCAGCGACAAGGATGCGAGCTTGGCC

TTCTTTGATGACTGTGTAGACAAGGTTGACAGCGAGAGGATAGAGGACACCAGGCTTATT

GAACTGGACAAATCACATCGCAGTGAGCACACAGTTTTTATCACACCTCCAGAGCTTCCA

TTCGTTCCTGAAGGAGAGGAGCCCTCCATACTTTATAGCTACAGTGGTTTTCCTGTGCTG

AATGCTGAACTCTGTGAACCCCAAGAGGGCCCCGGCATCCCTATAGCAACCACAAACTCC

AGACACACCAGCCCTGGAAGCCCAGCTGCCATTTTCCGCCGCTCCAAACAGGAGATTAAA

TCAGCTCAGCGAATGGCCAAGACATACTCATCCATACCTCACATGTGGTCTAAGTGTTTG

CTAAGGCACTGCCATGGCCTATGGTTTATCTGCCTGCCAGCCTATGTAGCAGCATGCCAG

TCTAAGGTGCGGGCACTCCGAGCTGCCTATGATGTTCTGAGAATGATGCAAGACAAAAAG

CTGCAACCTCCTGATGAGGTATGTTACCGGATTCTGATGCAGCTCTGCGGGCAGTATGGG

CAGCCCGTCCTGGCTGTGCGGGTGCTGTTTGAAATGAAGAAAGCTGGAGTTCAGCCTAAT

GCTATAACGTATGGATATTATAATAGAGCGGTGTTGGAGAGCACATGGCCTTCGTCCACC

AGAGGGGGCCATTTCCTGTGGGGTAAACTGCGCAACGTGGTCCGAGGTGTGGTACAGTTC

AAACAAGTGTGGAGGAGACAGGCTAGTCATGCCAAAGAGCCCCAGCTCTCAGATGCCAGT

GATCTGGACAGCGTGAGCCACGGCAGTCTGGACAGCACTAATGACTCAGCAGAGCGAGCG

TCTATCGATACAGACTTCACCAAAATGGACTCCAGCGATGATGGATCCAGCACAGGTGGA

CAATCAGATCAAGGCTATGACTCATTGTCTAAAGAAGAGGTGCAGGTTAGCTCACCTCAC

ATGAAGGAGAAGAAGGAGAGAGACAGCCTTCCACTGGCCAAAACAGGAGCGTCTATAAAA

AATGATTCATCTGCTGCACTGAAACCATTAAATCCCTCAAATGTAAGAGACAATGCAGCT

GTACCCATACGACCCAACTCATTGGACATCTTTGGTAAAAATACTGTGAGACCCAAAACA

TTAGAGCTGCTGCTCATCTCTGCAAGCTGTAAACAGAAGCAAAGGTATTCAGATAAGGCG

ATGGTGGAGGAGGAAACAGGCACCACAAACCCACTCAAACCTGCTGCAGAGCGGAGCACA

AGTTGCACAGTTGCCATGGAGACTAGTACTAGCGATGCAAGTTGTAGTGTGTCAAGGAGT

GTGAGCTTCAGTGGCACTCTAGGCCGGTTGCCACAACGGACAGGGATTGAGTCTGGCTTT

GACCCCTTGTCACTTTTGGCAGCTGAGAGCAAGGTGCCAGATGAGCAAGGAGAAACAGAC

GATGCTCCTAGCACACGCCGGCATCTGGCTGAGGAAATCCAGCTTCATCTGGAGCACCTG

AGCAGTCCAGTGAGTCAGCGTTTATTTAGCACAGATATTCGCAGCCTGCAGAGTCCTTCC

CCAAGCAGCTCACCTTGCCAGACTGCTATACCAGGATCTCCAAGCACCCAGTTGCAGCCC

CAACCCCGGAGCAGACTTTTCTCCTCACCATCCTTGCCACTTGGATGCCCACGTAAGAGC

AAGGAAGCCCGTCCCACCTCTTTTGTTTCACCCTCCTCTCCCACCCCTTCTGCCTCATCC

TTTTCCATGGAATCCCTACTTACACCAACATTAGATGTTTTCAGGAGCAGCTTTATGTCT

GCAGGCAAGGGCGTGGCTGAAAAAGCTAGCCGTCTCTACTCAAGGCTTTCATCACAGACT

TCTATTGCACAGGACCTGAACTCTGATCGCGTCAGCGTGTCTTCATTAGGCTCAGTTGAG

CCTGACTGCTCCTCAGTGTTTGAGGGTGATCTTTGTCTTGATTCAGAAGGTCTCTCCTCA

CCTCAGAGAGACGCCTCTGTCAGCGTCACTCCCTTCAAAAGGAGTCCAAACAGGAATAGC

CGCTGCTTAGAGAGTCCTTCAGCACCTCCCAGGCTTTTCCGCCAGGCTTCTCTCCTTGGT

TCGTCACTGTCTGTTGTGAAAGCCCCACAAACCCCAGACGTGTGTCCCGATATAAGCCTC

ACACCAGGCCAGCAGAATTATGACATAGAGGTTCGCATGTCTAGTTGCTCACGCTGTAAC

ACCTGTGAGTGCCTGGCGTATGATGAGGAGATCATGGCTGGCTGGACAGCTGATGACTCT

AATTTAAATAGCACTTGTCCTTTCTGTGGCTCAGCCTTTTTGCCCCTGCTTAATGTAAAC

ATCCAAGACCTGAACAACCAGGACAGAATCCCTTTTCAAGACCTGCCCAGTTCCCAGGCA

GAAACAAATGATGCCTCAGAGAATGAGACAAAAACATGCATTGAAACTACAGATAACGGC

TCCAACAGTTCCAGCCATAGCGCAGAGCCAATCACCGTACCCTACTTGAGTCCTTTGGTG

CTATGGAAAGAGCTGGAGAGCCTCTTAGAGAATGAAGGCGAGCAGATTATCTCTTCACCA

GTGATTGTGGACCATCACCCTATTGTCTTCTGGAATCTGGTCTGGTTTTTCCGTAGGCTG

GAGCTGCCGAGCAGCCTGCCTGCTCTCATCCTTAGCTCCAAGCACTGCAGCCAGAACATG

CAGCCTTTCCAGTGCAGTGCATCAGAGGACAGTAAGAATGTGTTAGTCACGATTTTGTGG

GACAACCCCAAGCTGCACCAGGACCCAATCCCACCCTGCTACATTCTGTGGAATGCATAC

TGTTCAAACACTAATGTTGGTGTGTCCTCATTGTTGGAGGATAAACAGCCTGTAAGCCTG

GAGCTTCTGCAGAGTGTTGTCAGGAGCATTCAGAAAAATGATGTCTACCAGCCCATGAGC

CAAACACTTCAGCTGCTTGGTACCAGGCTAGGATTTATTAGGCAACGGAGCCTGTATAGA

GATATCTTATTCCTGACATTAGTGGCATTGGGCAGGAACAGTATTAATATTGATGCTTTT

GACCGAGAGTACAAGTTGGCTTATGATCGACTGACCCCTGGCCAGGTCAAACTTACTCAC

AACTGTGATCGTCCACCTGGTGCTGGAGCCATGGAATGTAGAAGAACTTTTGGGCTGCCG

AGTCTGTAATTTCTTTCGTTTTATTCCTCTCCGACATCTCCTTTCCCTTCCTTCATCAAA

GGATGTGAAGTCTGAGCTAGCCAAAGAAGAAGGATTCACTACTACCAAAGCTACCTCTGG

CTCGAAACACGGCCATGTAGACACTTACAGGCATTTCCCAGATCGTTGCTTAAACCAACA

CGTGCGTGACCCATTCTTATTAACTACATGCTACCTTTGAACACTATGTCATATATTGTC

CATGTTTCTGTTTACCTCATGTCATGTCCTTCATTTTAACCTACTACACATTTCACTCTG

AAGTCCCAGGACACATTTACACATTTAGTCTCACTCAGGTAAGATTTACTGCCGAGGCCA

TCGTAAAGTGAGAGAAGTATCATCTAGTGCTCAGGTCACAGCATCACTCATCACCATTGA

ATCATATAGACAGGATTGTAGAGTGGTTTTGGGACCAGTAGCACTATGTATTGTATTGTT

TAGAAAAGCACAACGAATAAACCCTAACGAGACTAAAGTTTTATCACATTCCTCTCTCAG

TATTCTAAAGGCAGTTGTATATTACCATAAAAAGTCACCTTTCTGTATATAGCCTATTAT

TCTATTATTTAGAATATTTGTATTATAAAGGTATATTTTAAAATTTGTATTTGTTATAAA

TGAGTTGTCTTCATATGTAAAGACAGTACCAAGCAGTGTATTGGTTACAATTTCCAAGTG

TGTCGTGTAAGACTATGGTCCTGGAGACCGTTATTGTGATAACTGTGAAGCGTTTTGTCT

TTTGATTAGCTGAGGTACAGAACTGGACCAAATCAGATGTGAAATCATGAATTATATGAC

TGTTTGTTAGAGGTGATTTAAAAGAAGTGTAATAAAATATGAATATTGTAATTCCACTTA

TCAACTGAAAATGCCTTATTAAAGGTCAATCCAGCCACAGAAATGAATGGAGACTAAAAC

ATGCATAGAATACAATAAAACTCTATTGGAAATACAGCAGAGACCTAAAACAAATCATTT

CCTTGTCTACACAAACTGATGCAGATTAGTATTAGTTTTTATTAAATATTACATACGGAC

TCTCATTTTGTTATAAACCCACCA

>Unigene000216

GCACACAGGCTTCGTTATTGTCCCCTACAGCAGGGCAGCTTTGGCTCCACACGCCTCGCT

GCTGCTTTTTACCCCAGTGTCTGAAACGGTTCTGTTCCCCTCCTAATTACAGATTAATAA

TCATGGCATTCCAGCGCTAGTGGGATTATGACTCAATGTACCACAGAAGTGTCCCTCTCC

TGCGCTAGTTACCACACTCCATGCTGCAGCCACTATCGGAAGCTTTGCTGTTGGTAGCCT

TTTGGCTTGGAAGGAAAAGAGAGCTGAACCAGCTGTTGTCAGAACACATATGGTGCATTG

TTGCCATTCCCAGGCCTACATCCTCAAGGCTCAGCCTTTCGCTGCCCTGCTGTGGAAGCT

GAATTTGGCGTAAGACGAGATATTAATATATTGCTGATTTGAAATTTGGGTCAAGACACT

AAAAGTCTTGTTTAGTGTTTTCTTCCACTGCCCTTGAGGTGGTGGTCGAGCAGCTTAGGC

CATGGAGCCCGGGATGGAGTACTGTCTGGCTCAAGTGCTACAAAAGGATGTGGGCCGACG

GCTGCAGGTTGGGCAGGAGCTCATCGACTACATCGTGGATAAAGAGAAGTCTTATGACTT

GGAGCAGGACCAGACAGCGCTGGACAAAATGGTCGATGGCCTTGCATCCTCTTGGGTCAA

CTCCAGCAACTTCAAGTTGGCTTTGCTGGGCATTGACATTTTGTCTGCCCTAGTGACCAG

ATTACAAGACCGCTTTCGGGCCCAGATTGGAACAGTTTTACCTAGCTTGATAGATCGATT

AGGGGACGCCAAAGACCAAGTAAGAGACCAAGACCAGACATTGCTGCTGAAGATCATGGA

CCAAGCTGCTAACCCTCAGTATGTGTGGGACAGAATGCTAGGGGGATTCAAACACAAAAA

CAACAGGACCAGAGAAGGAGTGTGTCTTTGTCTCATTGCAACCCTAAATGTGTATGGTGC

CCAGGGTTTAACTCTAAACAAAATTGTACCACATATATGTAATCTGTTGGGAGACCCTAC

AAGTCAGGTGAGAGACAGTGCCATGAACTGCCTCGTGGAAATATATCGACATGTTGGAGA

AAGGGTTAGAGTAGACCTCAGCAAAAAAGGATTGCCACAGTCACGGTTGAATGTAATTTT

TGCCAGATTTGATGAGGTCCAAAGGTCTGGAAACATGATCCTGTCTACTGGATCAGATAA

AAACTTTGATGATGAGGACTCTGTTGATGGAGGCAGGTCCTCCTCTTCCACTTCATCCAA

AGGTTTTTCCAGCAGCAGGCGAGGAGGAAACATGGGTTCCATGAGACGACCCAGTTCTGC

CTCAGCACCCAGAGCTGCAGCGAAAGAGGGAGCCAGTGCAGGAGCTGTTGACGAGGAAGA

CTTTATTAAGGCATTTGAAGACGTCCCTACAGTTCAAATCTATTCTAGTAGAGAACTGGA

AGACGCCGTAACTAAGATACGAGAGGTGTTGTCAGATGATAAACATGACTGGGAGCATAG

AGTGACTGCTCTAAAGAAAGTTCGCTCATTGATTTTGTCTGGAGCTGCAGAACACGAGGG

TTTCCTTCAACAGCTCCGCCTTTTGGAAGGTGCCTTTAAACTGTCTGCGAAAGATTTACG

CTCACAAGTGGTTCGGGAGGCCTGTATCACTCTTGGATACGTGTCCTCAGTGTTGAGGAA

CAAATTTGACCATGGAGCAGAAGCCATTATGCCCACGCTGCTCAATCTCGTGCCCAACAG

CGCCAAAATCATGGCCACATCAGGCGCGGCTGCTATCCGTCTTATTTTACGGCATACACA

CTATCCACGTCTAATCCCAATCATCACCAGCAACTGCACATCTAAATCAGTGGGTGTTCG

AAGGCGGTGTTATGAATTTCTAGACCTGTTGTTGCAGGAATGGCAAACCCATACACTAGA

GAGACATGTTGCAGTTCTTACCGAGACCATCAAGAAAGGAATTCATGACGCCGACTCCGA

AGCCAGATCGATAGCAAGAAAATGTTACTGGGGATTTCACGGTCATTACAACAGAGAGGC

AGAGCACCTGTTCCAGTCTTTGGAGTCATCTTATCAGAAGGCGATCCAATCTTTTCTGAA

AAGTTCAGATAGCATAGTGTCTCTTCCTCAGTCGGATCGTTCTTCTTCCAGCTCACAGGA

GAGCCTCAATCGGCCACTGTCTGTGAGGAGTGTGATTGGAGGAAGCATGAATAGAAGTAA

GGTAGTGAGCTCCAGAGCACCATCCACCCCTGGCTCGCTCCAGCGGTCACGCAGTGACAT

TGACGTGAATGCTGCTGCCAGTGCCAAGTCCCGGATGCCTGCTGTGACCTCTGTTGCCTC

CCCTTCCCCTTTCAGCTCAGCTGCAGCCCTTCCACCTGGATCCTATGCCTCTCTGGGTCG

TGTGCGCACCAGAAGGCAGAGCTCTGGAAGTACAGTTGGTGCAAACGCTTCGGTAACAGA

CAGCCGAGGTCGAAGCAGAGCTAAAGTGGTGTCTCAGTCCCAGCCTGGCAGCCGTTCTAG

TTCCCCTGGAAAGCTTCTGGGGCACACGTATGGCAGAGTTCCGCGTACCATAGCTATGGC

TTCTACACCTGCAGACAAACGAGCAAAGGTCCCAAGAAGTCAGGGCTGCAGCCGAGAGAC

CAGCCCCAGCCGACTGGGCATAGCCGAGCTGTGTCATAAAGCTCTTCTTCCTGCTGCTTC

TCGTTACCGCACGCTAGCACGGAGCAGTCGTATCCCACGCCCCAGCATGAGTCAGGGCTG

CAGCCGTGACACAAGTCGCGAGAGCAGCCGCGATACCAGCCCTGCTCGTGGTTTCACTCC

TTTGGATCGATTTGGGCTGATTCACCAAGCCCGTATTTCTGCATCTGTTAATGCCATGAG

AGTTCTAAACACCGGGACAGAGGTTGAGGCAGCTGTGGCTGATGCTTTGCTGTTAGGAGA

CTCCAGGAATAAGCGAAAACCAATGAGAAGGCGATACGAGTCTCCAGGGATGTACTCTGA

TGACGATGCTAACAGCGACGCGTCCAGTGCCTGCTCTGAGCGCTCGTATGGATCCCGGAA

TGGAGGAATTCCACATTATTTGCGTCAAACGGAGGATGTTGCGGAGGTGCTAAACCACTG

TGCTAGCTCCAATTGGTCTGAGAGGAAGGAAGGGCTTCTGGGCTTGCAGAATTTGCTTAA

GAGTCAGCGTGTCTTGAGCCGAGTGGAGATGAAAAGATTATGCGAGATCTTCACAAGGAT

GTTTGCAGACCCCCATAGCAAGGTGTTCAGCATGTTCCTGGAGACTCTGGTAGACTTCAT

CACTGTTCATAGAGAAGATCTTCAGGACTGGCTTTTTGTCCTGCTCACTCAACTGCTTAA

GAAGATGGGAGCTGATCTCCTTGGTTCAGTTCAGGCCAAAGTTCAAAAGGCTCTGGATGT

CACTCGCGAGTCCTTTCCATTCGATCAACAATTTAACATCCTTATGAGGTTTATTGTTGA

TCAAACCCAAACTCCAAACTTAAAGGTTAAGGTGGCCATCCTAAAATACATTGAGTCTCT

GGCAAAACAAATGGACCCGGCAGACTTTGTGAATTCCAGTGAAACAAGATTAGCCGTGTC

CCGTATCATAACCTGGACCACAGAGCCCAAAAGCTCTGATGTGAGGAAGGCTGCTCAGGT

GGTACTGATCTCACTGTTTGAGTTGAATACTCCGGAGTTTACCATGCTTCTTGGAGCGCT

ACCCAAAACATTCCAGGATGGAGCCACTAAGCTCCTCCATACCCATTTAAAGAACTCTAG

CAGCACCAGTGGAGTGAGTTCTCCCAGCAACACAATTGGCCGTACACCACCCAGGCATCC

ATCTAGCAGGACGAGCCCTCTCACCTCTCCCACTAACTCCTCTCATGCTGGCCTTTCACC

AAGTATGCTGGAGTATGACACAGAGAATATGAACTCTGATGAAATCTACAGCTCACTGCG

CGGTGTTACAGAGGCCATCCAGAGCTTTAGATATCGCAGTGAGGAAGACCTTAACGAGCC

AATCAGACGTGAATTTAAGAAGGATGATGTGGTTGGCCGAGAGACTGCTTCACCTGGTTC

AGATTCTCGATTGGCTTTGGATATAGTCGAAGGAGGACGTACTGCCTTGGATAACAAGAC

CTCTCTACTCAACACCCCCTCTCCTCGCTCTTTTGCTGGGCCAAGGACGCGAGAATTTGC

ACCTTATGGATATGGGGACACCATTGGTGGTTATGATAAAAGTGCTTTGAAAGAAGCTGT

CTTTGATGATGACGTGGAGCAGTTCCGTGATTGCCGTCGGCAGGAGTGCAGTGAAAACAA

GATGGTCCTCTCCAAGGGCTTTTCTCCTGACCTGGTGGCAGACCTGCTGAAAGAACTGTC

TAATCATAACGAGCGAGTGGAGGAGAGAAAAGGTGCACTGGTGGAACTGCTAAAGATCAC

CCGAGAAGATAGCCTAGCTGTGTGGGACGAGCATTTCAAAACGATCCTGCTGCTTCTTTT

GGAAACACTTGGAGACAAAGATCACACTATTCGAGCTATGGCTTTGCGTGTCTTAAAAGA

AATCTTAAGGATTCAACCAGCCCGCTTCAAGAACTATGCAGAACTTACAATCATGAAGAC

ATTAGAGGCCCACAAAGACTCTCACAAAGAGGTGGTAAGAGCTGCTGAAGAGTCTGCATC

CACACTTGCTGGCTCCATCCACCCCGAGCAGTGCATCAAAGTTCTGTGTCCCATTGTGCA

GACGGCGGATTACCCCATCAACCTGGCAGCTATTAAAATGCAGACTAAAGTTGTTGAACG

CATTTCAAAAGATTCGCTTCACCAGCTTCTTCCAGATATTATTCCTGGACTCTTACAAGG

CTATGACAACACAGAAAGCAGTGTTCGCAAGGCCAGTGTCTTCTGCCTGGTTGCTATCTA

CTCTGTTATCGGTGAGGAGCTCAAACCCCACCTGGCGCAGCTCACAGGCAGTAAGATGAA

GTTGCTAAACCTGTACATCAAGAGAGCTCAGACAACCAACAGTAACAGCAGCAGCTCATC

TGACGTTTCCTCTCACAGCTAGTGCAGAAGGCATGCTCATCAATGCCCCGCCCTCAATAT

TGCAATTCTACCTTTGCCTCCTTCAGATGATCCACTTTGGGGTTTTTTTGTTTGTTTTAA

GTATTCCAATACATTTTCATCAGTATTCCATTTTATTTTCTGTTCATACATTTTCTGAAT

GTAAAAATAGTTGGAAAGTTTCTTTAAAGTGGTTTATAGGGATCACATCATCTTTTTAGA

TTGGCACCAGTTACTTTAAAAAAAAAAAAAGAAATTACTTGATATTTAATATTCATAATG

TAATGATGTGGTCTTTTTTATATAGTGTTTGAGATTTTGACATTGTTTGTTTTAAAATGG

GGATCAGTTGTGTAGGTTGAATGTCAATTGAATCGGAGTTTCATTTGGCTGGCCGAGTGA

TTTAAATGTAATCGTGAGAAAAGATTGAGTGTCGTTTTAAACACAAATGTTGACAAAACA

TGTAGGTTTAAATGTATAAATGTATTTAATTTTGTATTTAAGTTCAATTACCAGCCACCA

GATCTGCACCCCT

>Unigene000217

GGGCTTTTAATAGATGCATTGCATTGAAACGGGTTATGGCTGAACCGCAGAGGAACAGCG

GGGCTGTGGCGTAGTGGCATCAGCGGGAAATCCCTGCGGTTGGTTTTTTTTCCTAACTGC

GTGGAAAATAGAGTGAGAGTGTTGGTGTTGAGCTCCGTCCGGACTGCAGCGCTGGATTGC

GGCTTGTGTTGCTCGGTGTTCAGGAGCGGCCGAATGGACGAGCCGGACTACAGCATGACC

TAAAGCACCTCCCCGGAGATGTCACTTAACACCGCGCAGTGTCCGATCCCAGCACAGATC

CAGTGTGGTGGGTAGATAGGCGGTATGACTGAGGAGAAATGTCCTCAGTTGGTGGATTAT

TTCGTGGTGGCGGGGCTGGCTGCCGACTCTGCTCCACCAGACGAGGAATGTCAGCAGAGA

GGAGGCCGTGGCATGGAGCCTATCAGCGACCTGGCTGTGATAGCTCGTGGCCTTGGGGAA

GAGGTGCCTGAGGGCTTCACATGCATCGAGAAGACGTTGGGGGGCCACCCAGCTGAGCTC

AGCGCTGGCCTCATCAACAATCCACACATGTTTCTCTGCTACCGACGTAGCCATGACAAG

CCACCCATTGTGGAGCTTGGTGTGCTGTATGATGGGAAAGAGGCGGTGAAACAGGGTTGG

TATGTCATCGAGACGACGCCATACAGCCGCTGTGCCAGTCTAAGCTCAGGAGCTCCCGCC

ACCCATAAGACGTTCCTCATGTATCGACGTGCCCCTGACTCGCAGGCCCTGAATACACTG

GGAGTGACTGATATCTCATTACTCATGCCCAGCAAAGGAGAAGTGGCACCACACACCTTC

TGCAAAGTGGACAAGAACCTAAACACTGGCATGTGGGGCCCTGCACTCTTCCTTTGCTAT

AAAAGAGCTGTTGCAAAAGCCAACGCCCTTGTGTACGAGGCAGGGCTGATTAGCCGCTAC

CCTGAGGAAGATCTTGAGTCGTTCGCATTGCCGGACTCTGTCCCAGTCTTTTGTCTACCC

ATGGGCGTCACGGTAGAGAGCTGGCCCCTCAATACCAAGTACCAACTTCCTGTCTTCTCC

ACTTTCGTCCTCACCACGGCATCTGGAGATAAGGTGTATGGAGCTGCTATCCAGTTCTAT

GAAGCATACGCACGGGAGGCGCTGTCTGAAAGGCAGCGAGTTCGTCTTGGACTGGTCAGT

GTTGTGGACCGCCAACCCATCACCAACCGTACGGTGCAAGTAAGGAAGAGTGTGTGTGTT

CTCTCTCACTGGCCATTCTTTACAGTCTTCCAGAAGTTTCTCACCTTCATCTACCGCTAC

TCCATATCTGGACCTCATGTGCTGCCCATTGAGAAGCATATCTCCAGTTTTATGCACAAC

GTGCCCTTCCCTTCACCACAGCGACCGCGCATTCTGGTACAGCTGTCTCCATATGACAAC

CTCCTACTGTGTCAGCCAGTGTCTTCTCCCTTACCACTGAGTGGTGCCAGCTTTGTAAAA

CTTCTGCAGAATCTGGGGCCAGAAAATGCATGTACCCTTCTGTTGGCTGTTCTCACTGAG

CACAAGCTGCTGCTACATTCCCTTCGGCCAGATGTACTGACCTCCGTCAGTGAAGCCCTG

GTGTCGATGACGTTTCCGTTGCGTTGGCCCTGTCCGTACATCCCACTTTGCCCGCTTCGC

TGCGCAGATGTGCTATGTGCTCCAATGCCCTTCATCGTGGGTGTTCACTCCAGCTATTTC

GACCTGTACGACCCTCCTTCAGATGTGGTGTGTGTGGACCTTGACACCAACACAATTTTC

CAATCAGAGGACAAGAAACCATTGTCATGGAAATCGTTACCCAGGAAACATGGAAAAATT

CTGGTTAGCTCGCTCACCCACTTGCACAAAACTTTGGAAAAAATCTGTACCCCTGGCCAG

GAGGAGGCGACCTTAGAGTTCCTGCTGACAGATTACGATCTGATCTATGGCCGACAAAAA

CAGCTTGAGCTGGAGATTCAGGAGGCTTTCCTGCGCTTTATGAGCTGTCTCCTTAAAGGC

TATCGCTCCTATCTCCTGCCCATCACACAGGCTCCTTCTGACAGAACCACCGACTGTAGC

TCCCTCTTTAATTTGCAAGGCTTTCTGAAGTCTCGAGATCGGACACAGCAAAAGTTCTAT

TTTCACCTAACCAAGACTCAGATGTTCACGCAGTTCATAGAGGAGTGCTCCTTCGTCAGT

GACAGACACGCATGCCTTGAATTCTTTGACGAGTGTGTTCAAAAGGTAGATGTGGAGAAG

CCTGAGGAAGTGAAGTTGATAGACCTTGATGAGACACACAGTGGAGAGCACACAGTGTTT

ATAATGCCACCAGAGGAACCCCAAGAACCGGATGGCTCGGAATGCCCTGCACTGTACAGC

TATGAGACGTTTCCTATTCTAAATCCTGAGTTGTTTGATCAGCCTCAGGATCAACTCCGT

ACCACCGCAAAGGGAAGTGCCCCCAGTAGTCCTGCTCCTCGTCGTACCAAACAGGAAATC

AAGCTGGCACAAAAATGTGCACAAAAGTATTCTGCAGTACCTGACATGTGGTCCAAGTGT

CTGCTGGGCCACTGTTATGGTCTGTGGTTTATCTACCTTCCCACATTTGTTCGGGCTGAG

AGCACCAAGGTGCGGGCACTGCACACCGCTTATGAGGTGCTCAAGCACATGGAGACACGG

AAGGTGGTGCTGCCTGATGAAGTGTGCTACAGGATCCTAATGCAGCTGTGTGGGCAGTAC

GGGCAGCCTGTGCTTGCCGTCAGAGTCCTGCTGGAGATGAAAAAAGCAGGAATTACTCCT

AACACTATCACGTACGGGTACTACAATAAGGCTGTATTAGAGAGTAAGTGGCCATCCACC

AATCAGGGAGGTCGCCTTCGATGGGCTAAGCTTCGCAACGTTCTTTTAGCCGTGTCACAT

TTCAGGCAACCAATCAAACACAGGCAAAAGGGTGACTCTGTTAGCTCAAAACAAGGTAAC

TCGAACTGTAATCTTCACCCGCACTCCACTCTTGTGCGTCAGACCAGCTGGAGTGGTTTG

AGTGAAAGCTCGAGTCATGAGTCCTTGACTGGTCCCATCACTAAGAGCAACAGTCTAAGC

AGCATGCGACCTGACAAATCTAAGTTAGCTAGAAAAGCAATGCTCCAAAACAAAAGCAGC

GATGACCCCGCCTCCCGCAAACCTCCCATTAGTCCTCGTGATATGGTCACACCCTCCCCT

GTTCCACCTGATGAGCATATTAGAGTGCGACGGAGTGATGTCTGCTTGTCCAACTTTTAC

AAGGAATGTGCAGAGCCAGATCCAGAGTGCATGTGCCAGCCCACTCAAAGAGACAGCAGA

TCTACAGAGCAGCGGGAAACGCTGAATCGAAAAACAGTAGATGAGAACTACAATAACGTG

TTGTCCCCTAGTCGTGGACTGGCCGGTAAACTGCAGCAGCTTCTCACACCCACACGTCAC

CGGAACTCATCACGCCGTGCAGCCAGCATGGATAACCGGCGGCCTGCCATCAACGGGCCG

TCACGCAAATCCTCCGAACAGAGGCAGTCCCGCAAATCACAGGTGGCTGAGACTCTGCTG

AGAGCCAAAGATCGTCTCTACAACGCCACGTCAGAGAGCTCATTGTCTGTAGGAAGTGAC

ATTGACCTGACAGAACCTCCAAATTCAGTTTTTTCACTCAGGAAGTCATGGGATTCGACA

CAGGAAGGGGCGGGGCTTGAGTCAGAATCGCAGTAACTGGAACATGGAGGATGAGGTTGA

AAGTGCAGTGAGGCCCCCAACTGGCCACGATGGATTTTTGCAACATCTGTACAATGGTGT

AAATGAGCATGACTCCAGTTCGGAGACCAGCACATACTCTGAGAACAGCAGTAATACTCT

AAGCTCCTCTCTGGGTGGCACCCAGGTAACAGTGGCATACCTGAGCCCTCTGGTCTTACG

TAAAGAACTGGAAAGTCTTTTGGAGAATGAGGGAGAGAGTGTTCTTTCTCAGGTCCAGTT

ACTGGACAGCCACTCCATACTCTTTTGGAATCTGGTGTGGTACTTCACTCGCCTTGGTCT

TCCCAGCAATCTGCTGCAGCTGGTGCAGGCCTCCCCTCTCGCTTCACAGATTTCACAGAC

TCCTGAGAATACTTCAGTCAGAGTAAGATTAATGTGGGATACCCTGACTCCTGATACGGA

TTACTGGCCTCCTCTTTATGTGCTCTGGAGAATACACAGTGGAGTGCCAATGCGTAACTA

TTCGTGGCGCAGACACAACCACCCCTTTACCCTAAACTTTCTAGAGGAAGTCCTGCGCTG

TGTTGGCATGAATGAGATACACAAAGGCATCACGTTCTTCCTGGACACCACCAGCAAGCA

ATTAGACAATCCGCGGATACAGAGGAGTTTGTACAGGGAATTTCTGTTTCTTACTTTGGC

TGCTATGGGCAAAGACCATGTCGCTGCTTTTGACAAGAAATACAAGGCTGCCTACGCCAG

ACTGAGCGGATCCCTGGGTCGAGAGGAGCTGAGGAGAAAAAGGGCGCAGCCACCTAGCCC

TAAAGCTGTGGACTGCAGACGATGTTTCCTAGTGCCCCTGGACTGCTGAGTGTCAAGACA

CACACCTGTGCTCCACAGCTGGAACGTTCCACTCTCTGTCTCTCTTACTCTCTCTTTCTC

TCTGTCAGTGGATGTCTCCTGCAGTTACCCCTTAAAGGCAGTTCTTATTAGACTTTTGAC

TCATTGAGTCTCACACATGCTCACATAACAGAGACTGTTGTTCTAAAGTTCTTTTTCATC

TGTTGCTCTACACGGTACTTCTTACGGAACTTGTACGACAAGATAACAGTTCTCTCTGCC

CTATAAATGTTCTGCCGTTGCTTTTACTTTTGTTTCCCATTGCCTTCAGGTGATTTAATA

GGGTGTGTTTTACCAGATCTTTCTATTTTGTAGTACAGCAAATAGTCACCTTTAGGCTGA

GCCTCTTCTCTTTGCCAAGTAACTAATCCAGACACTGCAGTCATGCTTCAGCGGCGTTCT

GATCTATGCATCGAGCAGATCATCCTGATACCAGCATCATATGTTTTACAGACAAGGCCT

TATCATCACATCACTCCTTCAGAGTATACATTCTGTAATTCTGCCTTTGTAATGTATATT

TTGTGTGCGTGTGTGATTACCAGACAGCATCTATTTAAGACCCCTTTACGTTCCTTTCTC

CTACCGCCTCTCTCTCTGTTTGGCGTTAGTGTTTTCGGCTGTTTGTGGTTAGCTGTGCCT

CTAGCTGATTGGCCGTGAAAGATCCACCACAACACTTCAGACCGATCACCACTGAAGAGG

AGAAAAATACTCCAGACAGGAGGCTACAGTGTGGCACGGACTTAGTCTGAGATTTAGAGG

GCAGCTTTAATAACAGGAGCACAGTGATGTGTGTTAGATCCCCCCCAAAAAAAGCACTGA

TTTTTGCACCGCAATTAAGTGGAATATATGAATGGCATTGGAATTTACCTCCATTTATTT

TTTTGGTTATTTTATGTGTTGAAAGACTGATTTTATTGTTTTTAAAAACATTGGACGCGT

GTATGAGCCCCAC

>Unigene000218

CATCATCATCATCATCATGAAGGCGGCTTGGGGACCCCCGTTTAACGACCTGCGATATTT

TTACTGTGTGTAAAGTCTAAAAAAAAATCACAACAGTTTATGAAACTGGACGTGGTGCTT

AACTAAACATTTCGCCCTGCAGAATGTCCAAGATCAGGCGGAAGGTGACAGTGGAAAACT

CGAAGACTATCTCAGAGAGCAGCAGCAGCAGTACCACCACTCCATCCCGCAGACCCAGCG

TCTTCGAGAGACTCGGACCCAGCACGGGCAGTAATGCCACAGAGACTCAGTGTAGAAATT

GGTTAAAGACTGGAAATTGCAGTTACGGCAACACTTGTCGCTACACGCATGGAACTCCAG

CAAGAGGCAAGAATTTTTCTGGAGCGTTCAGCAGGTCATCCGAGAGGCTTACTGGTGATC

TCCGGGAGAGGATGAAAAATAAGAGACAGGATGTAGATACAGACACTCAGAAAAGAGATC

AAGACGAACCTACTTCACCCACAACTAGAAAAGATTCATCTCGTAGCAGACATAGAGAGA

AACAGGATATCAAGATTACTAAAGAGCGAACGCCAGCCAGCGAAGAAGAAGCAGCAGACT

GGGAAACCACTCGCGAAGACTCTGATAACGGTGACTACGATTACGAGTTATCCCTGGAAA

TGAAACGGCAGAAAATCCAAAACGAGCTGATGAAGCTCGAGCAGGAGAACATGGACAAAA

AAGAGGAGCTCCTTATTAAGAAGGAGGAATCGGTGGTAACCAAGACCAGAACCAGTAGCA

GCACGTCTAAGGATAAGGGGTCTCCTGATCACTCCAGCTCCAAGAATTCACCGCTGTCCC

GTAAGTCCAGCGGCTCCCCCAAAAGAAAATCCAGCGGATCTCCCAAAAGAAAATCCCTCG

GTAAAGGCAGTGGCTCTGGCAAGAAGGAGAAGAAATCCTCCTCTTCCTCAGTCATCTCCT

CCCCGGTGGCAGAGCAGAGCAAAACTGAGGAGGCTGACAGCAGATCATCTAAGAGTGGAC

ACAGCAAGAAGAAAGGACCACGCACGCCCAGCCCCCCACCGCCTGTGCCCTTGGACCTGC

CCGTAGGGGGGAAGAAGCACAAGGGCAAACACAAAAACAAAGAGAAAAGTGAGGACAAGC

AAAAAGAGGCTAAGGAGCGAGGCAGGGATACTGATAAGCACAAGGAAAAGAAAGAGAAGC

GCAGGGATCGGTCTGACAGTTCCCACAAGGCTAAGCGATCCGTGACGTCAGAGGAACGTT

CTGAGAGCGTGTCGTCTCCTGGGCGAGAGGTGTCTCCCCAGACCAGAAGGAAGTCCTTGT

CTCCCAAAGTGGCCACCCAAAAACAAAGCTCGCCACAAAGGTCTCGCACCCCACCACGTC

ACCAACGGAGCCCCTCTCCTATGACCCGCCATCACCATTCTCCCTCATCTCACTCTGGGT

CTTCTCAACAACGTCACTCTCTATCTCCTCGCCGACGTCGTGGTTCCACCTCCCCGTCCT

ACCAGCGGGACCTGCTTCCCCACTTGGCATCCTCTCCCTCAGCTGCTCAGACCTCACGTC

GCTCACGTTCCCGTTCGTCCCCTACCCCGCAAAGGTCAAGCCCTGCCTCACGTCACCACT

CACAGGGCCGGGATCGGGAAAAAGAGAGAGGACGGGGAGAGAGGGAGAGAAGCCCCGCCC

CCGACAAACGTCATGAGCGTAGAGATGAAAGCCGCGGGAAGCGTGAGAAAGACAGCGGAC

GGGATGATCGCAACTTTGAGGCTGATCAAAGCTCCTCAAGAGAGGAGCGAGAAAGCCGTG

ACCGAGACCGTCGCTTAGATCGGGACCGCCGGGACACTCGTGATGACCGACAACACCGCA

GCATTGATGCAAAGGATGTTAGGGACACCAGAGACCGTTGCGGCCGTGAGTCAGTGGAAC

GCAGAGAGAGAGACCGGGATAGGGAGCGGGACCGTGAGAGAGAGAGGGACAGAGACAAAG

AAAGAAACGATGCCCACAGGAAAGATGACACGTCCGTGCAGGAAGAGAAAAGTTACGGAA

GAGGACATGGCAGAGAAGAGGGTCGGAGCGAGAACAGGGCAGAAAGCAGGAACGAATCGC

GGGCCGACAGAATGGGACGAGGAAGAGGACGTGCAGCAGACAACCCTGAGAAAGGATCAG

CTCGTGGCTCTCGAACATCACAGGCAGAAAGCGGGGGTCAAGACGGGTGGGAATCTCGTA

GCAGTGGCCTAAGAGAAAGAAGCTCTGAAAGGAACACAGATAGGGGTTCCGACCGTGATC

GATACGACAACGACAGGCCACGTGGAGAACAGGCCCGAGATTCGTCTTACGACCGCCGTG

GAAGCCAGGCAGAACGAGATCGCCGTGACCACAGGGACAGAGATCAGAGAGCATCCTCTC

TAGGTCGCCACCAAGGACGCAATGAGGACACCGATCGCGAGGAGAGGAGAGATGAGAGAA

GGAGCGACCGGGGAGAAGACAGACGCGAGGACAGAGGGCGTGAGCGAGAGGGCCGTGAGC

GAGATAGAGAAAGGGACAGGGAGAGAGAGCGGGAGCGGGAGCGAGAAAGAGAGAAAGAGA

GGGAGAGAGAGGCTGAGAGGGAGAGAGAAAGAGCCAGAGAGAGAGAACGTGAGAGAGAAC

GAGAAAGGGAGAGGGAGCAAAGGGAAAGAGAGCGGGAACGTGAAAGGGAAGAACGAGAGC

GAGAGCGTATGGAGAGAGAGCGTGAACGGGAGAGAGAGGAGCGTGAACGAGAAAGAGAAC

GGGAAAGGAAAGAAAGAGAACGGGAGAGGGAGAGGCAGAGAGAATGGGAGGAGAGAGAGA

AGAGCAGGGACGAAAGAAGGGATAGAAGAGAGGATGCACGAGACGAACGCTCGACTAGAG

ATACTCATGAAGAGCGAAAGAATACTGGGCGCAAGAGGCACCGTGTGGAGAACACTCCCA

GCCCCCGGCCCTCTCCTAAAAAAGTGCGGGAAGGAAGTCCTGCAGACAGTGATGGCTACA

ACAGTGCAGAGGAAAAAGGTGATAAACACAGGCTGCTGAGTCAGGTTGTCCGTCCTCAGG

AGCCAAGCCGCTCCCCTCCCCGTGTATCTACCGACGACAAGCCCACGCGCTGGAGAGACA

ATGAACGTCGCGGTGACAAGAAGGACGGACGAAGCCGGCATGAAGAAGCAGAGCCTCGCA

ATGAGCGAGCTGCAGGTCGAGTGGCGGAGCGACGTGGGGATCTTCCTTCTGAGAGTTCAC

TAAATACGCCAGCAACTGAGTCTCGAAGAGGTAAAGAACAGAGGGATGCCATCGTGCCAC

CTGCCTCCTCACCTAGTGGCCCTGACGGACGAGATTCTGCATTGGCCGGTCATGAAGAAG

GCAAAAAGAAAACTAAGGCTCAGAAAAAGAACCTGAAGAAAAACCGGAAAGAGGATGAGG

GCCCTAGCACTGGTGGGGACCGTTTTAACCCTGAGCCCCTGCCGTTTTCTTCCTCCTCAG

TACCTTCTGAAGCTCCACGCCCACTTCTCTCACCTCCTAAAATGGCTAAAAAGAAATTAC

TGGAGCGGAGGCAGAAGCGATCCCGGGGCGCTGAATCTGACCCTTCAGAAGATGATCAAC

CCATCAGCAAAAGACGTCGTGGCCCTCAGACACCTCCACCGATCAACAAGGAGGACTTGC

CTTCCCAGCGAGCGCTGATGTCTGGGCATGCTGTAGCCGCACCACTCAAAATGGACACTA

ATTTTAGTGACTGGTCTGATGAGGAGGTTCCAGAACGCAGCGAGGCTGTTTCTGAGCGAC

CACCAGAGAGACTGCTGCCTCGCAGAAATCCACCTCTAGCACCACTGCTTCCTGACCCAC

CCATGATGCATGCACTCCCACTACAACCACTACTTCCACAGCACATGTTGCGAAAATCAC

AGCAGGCCGAACAAAGGCAGCAACGCAGTAGCAGCATGGGTAGCAACCCAAGTCGAGCAA

CCTCACGCCGACTACGTTCACCCTCCAATGACTCAACTCAACGCACAGATGATACCCAGC

AGGGGCCACGATCACGCAGGGGACGCGGACAGGGTGCCAACTCCCGTGACAGAGAGAGGG

ACAGAGAAAGGGAGAGGGATAGGGCAGCAGCCCCTGAGCCAATCGGAGAAAGGGAGGAGA

GAAAGTCCAGGATTGACCAGCTGAGAAGAGGAGAGCCGAGTCGCAGTACTTCATCAGACC

GTCAGGACTCTCGTAGTCACAGTTCTCGTCGCAGCTCTCCAGAGTCAGAGCGGCAAGGTC

GCTCTCGGGCCGGCTCGCATGACAGCCGGGACCGTGACCGTGACCAATTTGACCGGGACA

GAAAGGACTACCGCCAACCACAGAACCAGCCACTATCACAGCAACGTGATTGGGAGCCAG

AACCCCGTGAGTGGGGAGTCCGAGGTCGTGAACCCCTCCTTCTCCGCCCTAATCGTGAGC

CCATCAGAGAAAGAGACTTGCGTGAAATGCGCGAGAGAGAGAGACTGCTGCCCGAAGGAC

TGCTTTCACAGGAACGCCTTGAACGTGATCGTGTTGATAGGGAGAGAGAGCGGGACAGAG

ACAGGGGGGAACGAGAGCGTGTGATTCCCTTTGACCCCCCGTCATTAGGGGAACTCAAGA

GTCGAGCAGAAATGCGAAAGGAGCGAGGAGACTATGAGCCCCTGCTGCCTCGAGAAGCTC

TGGTTGACATGGACAAACCAAGTGCTACCAACGAGCCACATGTCCAAATGGAACCTTGTG

AGACCGAGAAGATAGACAGCTTTGATGAAGATGAAGGAAAGGGTGAGGATGCCCAGTCTG

CAGTTTCGGCTGGGGAGGAATACGAGCCTATCAGTGATGACGAGCTGGATGAGATTTTGG

CTGACAGCCAAAAAAGAGATGATCAGCAAGATGAAGAAAAAATTCCAGGTCCCCTGGATG

TGATTGATGTAGACTGGTCCAGTCTTATGCCAAAGCAGAAACAAGAACCCCGAGCACCTG

GGGCCGCACTTCTTAGATTTACGCCCGGAGCCATGCTGCTGCGATCTGGCGTCTCTCGTC

GGCTGGCCGGTCCGCACCTCCTCACCCGTGTTAAAGACATCTGTGCTAAAGAGGTGGAGG

ATGCTAAAGATGCAGAAAAGCTGTTTGAGCATGACCTTGGTGCTCTGAACATGGCTGCCT

ACAACCGGAGGACAGAAAGAGCAAACCTGCTGAGAAACCTGGGGCCATGTGGAAAGGCTC

TCTGTGCACGTCAGGACATCGCCATCCGCAGGCAACTTCTCAAAAACGACAAGGGGACAA

CTAAGCAGATCTACACTAGCGCTCCAGTGGTGGACCAGGAACTGCTGCAACTCAGCATAC

GTCTGTTCAAGAAGAAAATGGCCGCCGGGCAAGAGAAATCGGATGCGCTCGGGACTAACA

CCCCAGCTGATCCAACCGAGCCCAGCATGTGGAAACTGGACACCCGGACTCGGCCATCTT

GCGCTGTGAAAAACTGAAACTGTTTGTAAGTCAGTGCTAAAGGAGAATTAGCCACACTGG

CTTAAGAGATCAGGATCATAGAGACTGGAAAACTGCTAGCCTATGAAGACACCACCAGTG

>Unigene000219

GCAGTGGAAGCAGCAGTGGGGGAGGAGGAGGTGGGGGTGGTGGTGGTGGTGGTGTGTGGC

ACATTACAGTAGAGACTGACTCACCATCTACTACTTCTGGGAGCACGCAAAGCAACCTTT

CCGCGAAGCTCGGTCACTTGATCAGCCCACAGGATTCGTCAATGCTGCGCAATATTCAAA

ATACACTAAAGAATAAAATACAGAGCAAGGGCAAAGACAATCGCTTCCTATCCCCAGACG

GCTATTTGGGCTCACCGCGCAAGGGCATGCGTCGCATTCGGCAGCGCAGTAATAGCGATA

TAACTATAAGCGAGTTAGACGGAGATGGCAATGAGGGTTGGTCCTTTTCCGGCTGGACTC

CAATGCACCGTGAGTATGGCAGTACTTCCTCCATCGACAAGCATGGCGTTTCCGGAGAGA

GCTTCTTTGACATGCTGAAAGCTTATCAGACTGATGACCCTGATCAGCGTAGCCCTGCTC

CTGAGAAATTAAGCGAACTGCTTACTGTGGCACATAAACAGACCGCCCTCGACCTGCCAG

ATGGGCCCCTGGGACCCCCACGAGGTAGTCCTGCAAGTCGCCTTAAGGAACGGGAAAAGC

ACTTGAAACGGCGTTCCAAGTCAGAAACCGGAGGTGAGTCCATATTCCGCAAGTTACGAA

GCGTAAAGGTAGAGGGTGACTCCTCTCGCGCTGGTTCGGATGCAGAAGATGGGAAGTCTG

AGGATGCAGGTCCTCCCCCAAAGCCCTGGGTTTGTCAAAAAGGTTTCGCCCATTATGATG

TCCAGAGCGTTCTGTTTGACCTGAACGAAGCTGTACAAAACCGACAGAGTGCCTCAAAAC

GGAAGAACACCACTACCGGCGCCTCTGCCGCAGCTGCTGCTTCGGCCTCCACCTCCTCCT

CCTTATCGTCCACCCAAAGCGGAGCCTATGGCTCCCCCTGTGGCAGTCAAGAAGAGCTCA

GTGCCAAGGATGGACCAGCATTGGACCCTGGTGACGATAAAAGCAACGATCTGGTCCTCA

GCTGTCCCTGCTTTCGTAATGAGATAGGTGGTGAAGGTGAGAGAAACATCAGCCCTTCTA

AACAGGGCAGCATCGGAAGTGGATCGACCAATTCCTCCAGGGACGAGGATGGAAACCGTG

AGGCGGCTCCCAACACCCATTTCAGTAACGCAGGGGTAGCTGTGCTGGAAGCTCCCAAAG

ATGGAAAGAGCTTACATCATGAGAGAGGAAAGACAAACATTTTGGAACATGTGGACCTGG

GGGCCTATTACTACAGAAAGTACTTCTATTTAAGAGAACACTGGAACTATTTGGGAGTGG

ACGAGACACTCGGGCCGGTCGCCGTGAGTCTTCGGCGAGAAAAGCTGGACGATCATAAAG

AGCACGGCCCACAGTACAATTACAGGGTCATCTTTAGAGCCAGTGAGCTGATGACACTGC

GAGGCTCCATTTTGGAGGATGCTGTTCCCTCCACATCCAAGCACGGTTTACCTCGAGGCC

TGCCTCTGAAGGAGGTTCTGGAGTACCTCGTCCCTGAACTGAACACTCACTGCCTCCGCC

TCGCCCTGAACACGCCCAAAGTCACGGAGCAGCTGATGAAACTGGATGAACAAGGGCTAA

GCTTCCAGCTGAAGGTAGGAGTGATGTACTGTCTGGCTGGCCAGAGCACCGAGGAGGAGA

TGTACAATAACGAGGCAGCCGGTCCTGCGCTCGAGGAATTCCTGCAGCTGCTTGGAGAGC

GAGTGCGTCTCAAAGGATTCAGCAAGTACCGCGCTCAGCTCGACACCAAGACCGACTCGA

CTGGCACTCACTCTTTATACACCGCTTACAAAGACTACGAGATCATGTTCCACGTGTCCA

CCATGCTTCCTTACACACCCAACAACAAGCAGCAGCTACTCAGGAAACGCCACATCGGGA

ACGACATCGTCACCATCGTGTTCCAGGAGCCCGGAGCCAAAGCCTTCAGCCCCAAGAACA

TTCGCTCTCACTTCCAGCATGTGTTCGTGGTGGTGCAGGTGCACAACCCGTGCTCGGAGA

ACACCTGCTACAGCGTTGCTGTGGCCCGTTCAAGAGACGTCCCTGCTTTCGGGCCGCTCA

TCCCAGACGACGCAAAGTTCCCCAAATCCTCAGTCTTCCGGGACTTCCTGTTGACCAAGT

TGATCAACGCGGAGAACGCCGCACACAAGTCGGACAAGTTCCGCGCCATGGCGACCCGCA

CGCGCCACGAGTACCTGAGGGATCTGGCCGAGCGCCACGTCACCAGCACCCCTATCGACC

CCTCGGGCAAATTTCCCTTCATCTCTCTGGCGCACAAGCGCAAGGAGAAAAGCCGACCCT

ACCTGGGGGCGGAGCTACGCAGCCCGGGGTCCATCACTTGGGTTGTGCACGCAGAAGATC

ACAGCACTGGAGGAGAACTGGAAGCCCTGCTCGGTATCTCTAACGACTTCCTCATCCTGG

TGGATCCAGAGGCCAAGGCTGTTGTGTTTAACTGTGCTGTACGTGATGTCATCAGCTGGA

CTCTGGGAAGCCCCGCCTCCATGAAGATCTTCTATGAGCGTGGAGAGTATATCTCACTGC

GCTCGCTCAACAACAACACTGAGGACTTCAGGGAGGTCGTGAAACGTCTGGAGTTTCTAA

CGAAAGGCTGTGAGACGTCGGAGATGACGCTGCGCAGAAACGGACTCGGCCAGCTGGGCT

TCCACGTTAACTACGAGGGCATCGTGGCTGAAGTGGAGCCGTACGGTTACGCCTGGCAGG

CCGGGCTGCGGCAGGGCAGTCGCTTGGTGGAGATCTGCAAAGTCGCAGTGGCGACGCTTT

CGCACGAGCAGATGATCGATCTACTGCGCACCTCTGTCACTGTAAAGGTGGTCATCATCC

CGCCGCACGAGGATGGCACTCCCCGCAGGGGCTGTTCGGAGCTGTACCGCATGCCGGTGG

CAGAGTATAAGGGAAGTAATGAGAGCGGTTCCTACGAATACAAGTTCCCGTTCCGCACCA

ACAACAATAAGTGGCAGCGCACCTCCTCCAGCCCACAGCAGTCCCTCTCTGCCTCGCCTC

AGAGCCACACCGCGACCCCCAACCGCGCCGTCAGTCTGGGAGCCAGCATGGGCAAGACAC

TGTCGGCCGAGAGACCCGAGCGAGCCACCGCTATCCCACGCAGTGTGTCCAGCGACGGCC

GGCCTCTCGACTCCAAAAGAATGTCTCCCGGGTCGGAAAACTACGCCCTAGCGTCCTCCA

TGGTGATGGCGCGACCCACTCACACCCGCAGCTCCCCCAGCAACCTGTCCTGCGTCAGTG

ACACCGGCTCAGGCGGCTCAGCCCACTGGAGGCAGAAGTCTATGCCTGAGGGATTCGCCC

CGAACAGACACTCTCCTCTTCCGCCAGAGAGACCAATGATGGGAGACGGCAGCGGGAAGT

CCACCCCCAGCTGGCCCCGTGTGGACGACTCTGACAGAGCAGCAGACGCCCCGCAGAGTA

AATCCGGACCGGCGTACTCGGGCGGCCCGCGCATCCAGAGGCAGGAGCAGGTCATCCATC

TATCACCCAAAAAGAGCAGCCAGTCCGAGGGCCCGTACTCCCAGTCGAGCAGTAACACCC

TTTCCAGTACCGCCTCCAGCGGTGGTCACAGCGATGACAAGTGGTACGATATGGGAAGCG

GAGGTCAAGGAGACGGTGCTGAACCCGAGCCCAACGGTCTGGGTGGAGGCGGGTACCTGC

AGGGCTCCTCCGCTGACAGTGGCATCGACGCCACCTCCTATGTTCCCCATCATGGCAGCG

CCAGCTCCCTTCTCGCCGTGGGCACTTCGGCATCCCGGGAGAGGGTAGCCTCGCCTTGGC

ACGGGCCTTCTGAGGGAGGCCGGCGTGTGCTGGAGAGGTCACCTCCGGCCGCGGAGTCGT

CTAACGCTCCTGCAGAGGCTCCCACAACACGCAGTCCTCCTACACATCTGCTAATGCGAG

ACAGCAGCACCTACAGCCTCAGTGACATGACCTCCCACTCCAGTTCGCGCCACTCTGGCA

GCTCCGGTGTGCTCAGCTCCAGTCACAACTCCCCTAGAGACGAGTCTCCCTCCGCCACCT

CACCCTCCTCCTCCTCCCAGAGCTCAGTCTCTCCAGGACCTAAAAGCTTTTATCCCCGCC

AGGGAGCCACCAGCAAGTACCTGATCGGTTGGAGGAAACCGGGGAGCACTATCAACTCGG

TGGACTTTGGGGACACTCGGAAGAGGCCTCAGGGCGAAGGGGCAGAAAGTGGGCCGTCTC

AGACCCGACCCTCTCTCAGAGACCTGCACTCTCCTCAGCCTCTGGGCAAATCCACTGTGG

AGGAGGATCTAAAGAAACTCATCACACTGGACAGCCCACCAGCTATCCATCATGATGACA

AGTCTCTGCAGCCTTCCTGTTCGGGTGCGAGTGGTTCGGGCCGGCGGTCGCTACAGCGCA

CCCTTTCTGATGAGAGCATCTATCGAGGCCAGCGCTTGCCATCGCTTGGAGATGCGGTGT

TGGAGCAGGCGCTCAGCAGCGACGTGCTGTTCAGCTGCTCTACCCTGCCGCGCTCACCCA

CCACCCGCGGGGCCCCACTGCGCAGACCTTCCTACAAACTGGGCATCAAGATGCATGGTG

ACCTCTACGCGTCTGACACCTCTCTAGCAGACCTGCACGAGCGCCAGCGGCTGCCATCCC

CCGAGCTGGGCCTTATGCCCCTGCCTGACACTGACATGGACAGCGGTCTGGACTGGAGCC

ACCTGGTGGACGCTGCCAACGCTTTCGAGGGTGAAGGGAGACCCCCACGCATTCAGAGAA

GTTTTATGTTCAGCTCTCAGGACATCAGTCACCGTAGTGAGAGTGCAGTGAGCTCTCAGC

AGCTGGAGCTGCAGCCTGTACCGCACTCCCGACTCTCTCCCAGTGACATTCCTGTTGGTT

TTGCTGGGAAAGTGTCTCAGCTCGAGGCTTTGGTGAAGATGCTGCAGGACAGTTTGAAGA

AGGAGCGAGAGGAGAAGCTGAAGCTTCAGGCTCAGATCAAGCGGCTGTGGGAGGACAACC

AGAGACTCCAGGAAGAGTCTCAAAACTCTGCAGCCAAGCTGAAGAAGTTCACAGAGTGGG

TCTTCAACACCATCGATTTGAACTGAAAGCACATCTGGAACAGCTGCTTTGTCCTCCAGC

AGTCAGTAATCAAACACCAGTTCTGTCCAGCATTCCTGTACAGGGAACCATCAGTCCTCC

AGTCCAGAGTTATCCCTTTCACTAATGTTTTTCTTAGATCCAAACTGCAGGGATGAGCAG

ATTTCTGCAGCTACGTCATTCCTCAGATCTTGGAGGAACGGCTTTTTTTTTTTTAACACT

GCTTGTGATATTTTGCTGGATCACTGTGCTAATCAGAGTAGGTGTGTGTGTAGTCTTCTT

TCCGTTTCTCTGTAATTAGCAAAGATCGATGGTGTGACTCGAAAAACGCAGCCATACAAT

CAAGAAAACAACAGGAAAAAAAGGTTCCTGACGTTCTAAAGAAGCCCAGAACTCATCCTG

GCTTTTTTTTTCTTAAACTCATAAGCAATAGACCGACGTTCTCAACAC

>Unigene000220

GACTTTCCAGTGGTTCCTCTCCTAAAAGCATGTACTGTATCAGTGTACACGCTGGTAAAG

TGCCATCTTGTTCTTTTCTTTTATATACAGAAGGGATTCACGCCGCTACATGTGGCAGCT

AAGTACGGGAAAATAGAAGTCGCCAACCTCCTGCTGCAGAAGAGAGCTCCTCCAGATGCG

TCGGGAAAGAGCGGACTAACTCCACTGCATGTAGCTGCGCACTACGATAACCAGAAAGTG

GCACTTCTTCTCCTGGACCAGGGGGCTTCTCCCCATGCAGCTGCCAAGAATGGCTACACT

CCTCTACATATAGCTGCCAAGAAGAATCAGATGGAGATCGCTACCACACTGCTGGAATAC

GGCGCTGATACCAACGCGGTCACTCGGCAGGGCATCAGTCCGCTGCACCTGGCTGCTCAG

GAGGGCAACGTGGACATGGTCACCCTACTGCTTGCACGTGATGCCACCATCAATCTGGGT

AACAAGAGCGGACTCACTCCCCTTCACCTGGTGGCCCAGGAGGATAAAGTAAATGTAGCT

GAAGTCTTGGTTAACCAGAGTGCTGCTGTGGACCCTGAGACCAAGATGGGCTACACTCCG

CTGCATGTCGCCTGTCACTATGGCAACATAAAGATGGCGCACTTCCTCCTGAAGAACCAG

GCTAAGGTCAACGCCAAAACGAAGAACGGATACACTCCTCTGCACCAGGCTGCACAGCAA

GGCCACACACACATCATTAACCTCCTCCTGCAGCACGGAGCCTCGCCCAACGAGCTGACG

CTGAATGGAAACACAGCACTGTCCATTGCCAGGCGACTGGGTTACATTTCAGTAGTGGAC

ACCCTCAAAGTGGTGACAGAAGAGACCCTGACCACCATGACGGTGACTGAGAAGCACAAG

ATAAACGTCCCAGAAACAATGAACGAGGTTCTGGACATGTCTGATGATGAAGTTCGTAGA

GCCAATGTCCCCGAAATGCTCAATGAGGACTATATTTCAGATGTTGAAGAAGGGGAGGAT

GTGATGATGGGGGACACGGATAAATACCTGGGTCCCCAGGATCTGCGTGAGCTAGGGGAC

GACTCTTTGCCTCAGGAAGGTTACGTAGGATTTAGCATTGGAGCCCGATCCGGAAGCCTG

CGCTCCTTCAGTTCGGATAGGTCCAACACACTGAACCGGAGCTCTTTCACCAGGGACAGC

ATGATGATCGAGGAGATCCTGGCACCCAGCAAGGACACGCATCTGACTGTAGCTAAAGAC

TATGACACTGACTCTCTGAGGCGATACAGTTGGACAGCAGATGCTTTGGACAATGTCAAC

TTGGTCTCCAGCCCAGTCCATTCAGGGTTCCTAGTGAGCTTTATGGTGGACGCTCGTGGT

GGCTCCATGCGAGGCAGCCGCCACAATGGGATGCGCATCATCATCCCACCACGCAAGTGC

ACTGCCCCGACTCGCATTACCTGCCGCCTGGTCAAGCGACACAAGCTTGCCTCGCCACCA

CCCATGGTGGAAGGAGAAGGACTGGCAAGCCGTCTGGTGGAGGTGGGCCCTGCCGGAGCT

CAGTTTCTCGGCCCTGTGATAGTGGAGATCCCTCACTTTGGCTCCATGAGAGGGAAGGAG

CGTGAACTGATCATTCTCAGAAGCGATAACGGAGAGACTTGGAAAGAGCACCAGTATGAC

TGCAAGACCGAAGCCCTTAACGAGCTGCTTAACGGCATGGATGAAGAACTGGACTGCATA

GAAGACCTGGAGAAGAAACGCATCTGTCGAATCATTACAAAAGACTTCCCACAGTATTTT

GCCGTGGTGTCACGGATCAAGCAGGAGACCAACCATATGGGTCCTGAGGGAGGGGTTCTA

TCCAGTACGACTGTTCGCAAAGTCCAGGCTTCTTTCCCTGAAGGAGCGCTCACAAAGAAA

ATCAGGGTTGGACTTCAGACCCAGCCGGTTCCAGACGAAACAGTAAAGAAAATCATAGGC

AACAGAGCCACGTTTAGCCCCATAGTGACAGTGGAACCACGCAGAAGGAAGTTCCACAAA

CCCATCACCATGACTATTCCTGTACCTCCTCGATCAGGGGAAGGCATGAACGGCTACAAA

GGAGACACAACACCTTGCTTACGTCTTCTGTGTAGCATCACAGGTGGCACCTCCCCCGCA

CAATGGGAAGACATCACTGGGACTACTCCGCTCACCTTCACGAATGACTGTGTCTCGTTC

ACTACCAACGTGTCTGCTAGATTCTGGCTCGCAGACTGCCATCAGATTCCGGAGACAGTT

TCTTTGGCTACTCAGCTCTATAGGGAACTCATCTGTGTGCCCTACATGGCTAAATTCGTA

GTGTTTGCCAAAATGAATGACCCTGTGGAATCCCATCTGCGATGCTTCTGCATGACGGAT

GACAAAGTGGACAAAACCCTGGAGCAGCAAGAAAACTTTGAAGAGGTGGCCAGAAGCAAA

GACATTGAGGTTTTGGAAGGAAAGCCTATCTATGTGGACTGCTACGGCAATTTAACCCCT

TTGATTAAAGCCGGACAGCAGCTGGTTCTTAACTTTTATGCATTTAAAGAGAACAGGCTG

CCATTCTGTGTAAAGGTGAGGGACAACAGCCAGGAACCGTGCGGACGGCTGTCCTTCCTC

AGAGAGATTAAAAACGCAAAGGGGCTTCCCCAAGTTGCTGTCTGTAATTTAAACATCACT

CTGCCCGCGCTCAAAAAGACTGAAAAGCCAGAACGACGTCATACCTTTGCATCTCTAGCC

TTACGTAAGCGGTACAGCTATCTGACTGAGCCTGGATTGAGCCCTCAAAGTCCTTGCGAG

AGGACAGACCTGCGAATGGCTATAGTGGCTGATCATCTCGGCCTTAGCTGGACTGAGCTG

GCCAGGGAAATGGATTTCTCTGTTGATGAGATAAATCACATCCGCGTGGAGAACCCCAAC

TCGTTGACCGCTCAGAGTTTCATGCTATTAAAGAAATGGGTCAACAGAGACGGTAAAAAT

GCCACAACGGATGCCTTAACTGCAGCACTGACAAAGATCAATCGGATGGATATTGTGACA

TTGCTGGAAGGGCCAATATTTGATTATGGTTACCACAACATCAGTCTGGAGTTGCAGAGT

CCCTCAGAGTTGTCCTATGAACCCCCCACCCCTCTCCGGCAGGATGATTTCTTTAGCGAG

GATGGGGGTATTGTAGTCTCGCCCAGTAGAACCCCTGTTAGACCAAATGAACTGTCCTTA

CCAACCACATCTAGGGATGCGCTTTCCCCCAATGCAGCACCTCCTACTGTGGTGGCAGAG

GATTCCTCTATTGGAGGAAGAGGCTCCTATAGTCGAGAAGGCGGCTCAACTGAAGACGAC

CTGGACGCCCATGACAGTGATGAGATGCCTAAACATCCTGAAGTGTTCATGCAGAGCACA

TACCAAGAGCAAGAGGTTCCTTCCAACGGAGCTCACAGGGCTAGAGAAAGTGGCAGGTCT

GGTTTTGACGAGGAAGAGGAGGAAGAGATGACGCAGGAGAAGTCCTGTTTTAAAAGCATA

AACTTGGAGGAAGGTTTAGAGATTGTGGAGATACCCCAAGAAAAAGTGCAGGAAATTCGC

TCACAAGTGGAACAGGCAAAAAAAGAGATGTGTTCGCTTGCAGGATTGCAAGAAGGGCCG

TCCAGTGTGAGCCAGGAGATCTCCGCTTCGGGTCTGCACACTGATACCCAACTGCAAGAT

AGAAGTCAAAAGCCAACTGAAAGGCAAAATGGGGAACATCCAGAGCTCCAAGGCCGAGCC

GGCTCACTGCTTGAGCCACAGGAACCATCTAGTAAGATGAAGGGCAAGGGCAGCAGAGAC

TCCTTAGATGACACAGCTGCTGGGCCGAGCACCATGGACGAAGACACAGTGGAGCCGAAA

GCTAAGGGTGATCAGGCCAAAAACATTCCTGGCGAGTCGGTCACTGAGGAGCAGTTTACA

GATGAAGAGGGAAACGTTGTCACCAGAAAAGTCGGAGAGGGAGACAAGAAGAAAGAAGGG

AGACAACGAGGGAAAAAGGGTCAATCGTAACAGACGGGTGTCCTAAATGAGATCTGAACT

TGTTTCATCATTGTTTTCCTGCGAAAAGCATCACTGGAAACCCAACGCAGGAGATCAGGA

CAGGGGATTGTGCGTCTGCAGCCTTTACATAAACGCATGGTTTTTAGAACGAGGATTTTA

TGATTTCGCATGAGCATGGAAAACCTCCGATTTTCTGTCAACGACACATCCAAGCATCTG

ATCGGTGCCAAGAAATGACACTTAAGCTCAAGACTGTCGGTGGCATAAAGCTTCTAGTCA

GTCCTATACATTGGAGGAGTGCCAGCGACTACCAACATGGCACCGAAGCATTCCATGACC

AAAGATGGTACAATGAGCATTGTGGAATGATTATTTTTTAAAAGAAAAAAAAAAAAAAAA

ACCACCTTCTACACTCGCCTTATTTTCTCTCCCCCCACCCTCCCCCTCGCTTCTCGGACT

AGCGTCGACGCTGTTTTTAAAAAAGATCCTGTATATAAATCAGAACTGTATGAAAGTAGC

TCACGAAATTGTGAACGCAGGAGCAAAGAGCGAGTCACAAACGGAATGAAAAAAAAAAAG

AAGAAGAAAGGAATGCTGATATAACTATTCCGACTTTAAAGATCATTTTAAAGACGAATT

GATTACACAAAAAAGCGAAAAGAAACTTTTTACACAAAAAAAAACAAAAAACAAGTTTGC

TTGATTTCAACGTTTTTAAGCCTACCACAAAAACGACAAAACATCGAACGACATAGCTGC

AGTTGTGTTAAAAATTTACTCCGAATACTTATATTTTGATGCATGTGATTCACCTTATTT

TTTGATTTGTTTCCTATCTACATAACCAGAGGTGTGGTAAATGTTCTACCTGTGTGCACA

CTCATAGCCTTGGTGTTTGTCACTAATACTGTTTTATAATATCACTGTACCACAACAGAC

TCACTGGAAGTGAATGGACACACAATTCTCGCAGCTAAAGGTTTCTACGCTATGCAGTCA

AAGACGAGCTTGGAGTGTAAGAGAGACCGAAAGAGAAAGAGCGTCGTGTCAACTGGCACA

GCGTTCAGTTTTAATGGGTTTTGTGTGTATCGGCACTAAAAGGTCTTTCTGTAAGCCAAC

CGTTTGTAACCATTCTAACAGTGTTACTAGGCTGCTGTGGACATAAGGGGCATTTTTTTA

TTTGGGTTTGGTGAAATATTTCCTTATATTGAATATATTCAAAAAAAAAAAAAAGAGAGC

GAGAATGAAAGAAAGATCACCTTCAGGTATTCGTTGATGAAATGATCTGGTAACAGACTT

GTATGAGAAAGCGTACACTGGATGATTGAATTAATTATTTAAGCATAAAATAAATTGTGT

TTAAACTCAAATTGTGATGTGAACTTTGATCTGGATGATGTGGTGCATTTGTGCGAGAAA

ACTGTGTCTGCATTTGTCTGTCCATCGATCTATCTGTTCATCCATCTGTCCATCTATCTA

TATACAGTATCTATTTAGCTAGCTACACACACACACACACACACACA

>Unigene000221

CCCGCCTACATCTGTGATGGCGAAAATGACTGCCAGGACAACCCGGATGAAGCTAACGGC

GCCACCCCCGTGTGCCTGCCGAGCCAGTTCAAGTGCTCGCACCCGAGCCGCTGCATTCCT

GGTATTTTCCGCTGTAACGGCCAAAACAACTGCGGACAAGGAGAAGATGAGAAAGATTGT

CCTGAGGTGACCTGTGCACCCAACCAGTTCCAGTGCGTCATCACTAAGCGCTGCATTCCA

CGGGTCTGGATGTGTGACCGGGACAATGATTGTGTGGACGGCTCTGATGAACCAGCCAAC

TGCACTCAGATGACGTGCGGTGTGGACGAGTTCCGCTGTAAGGATTCGGGTCGCTGCATC

CCTGCACGTTGGAAGTGTGATGGTGAGGATGACTGTGGCGACGGCTCGGATGAGCCCAAG

GCAGAGTGCGACGAGAGAACGTGTGAGCCTTATCAGTTCCGCTGCAAGAACAACCGCTGC

GTCCCGGGCCGCTGGCAATGCGACTACGACAACGACTGCGGCGACAACTCCGACGAGGAG

AAGTGCGTGCCGCGTCAGTGCTCAGAAAGCGAGTTCGCCTGCACCAATGGCCGCTGTATC

GCCGGGCGCTGGAAATGTGACGGGGACCATGACTGTGCCGACGGCTCTGATGAGCATGGC

TGTGCTCTGAAGTGTGATGCGGACCAGTTTCAATGTAAAAATGGCCACTGCATTCCGCTG

CGCTGGCGCTGCGACGCAGACGCTGACTGCATTGATGGCAGTGACGAAGAGAGGTGTCAT

GAGACAGTGGAACGTCTTTGCCCCAATGATGAGTTCCAGTGTAACAATACTCTGTGCAAG

CCGCTGGCCTGGAAGTGTGACGGCGAAGACGACTGTGGTGATAACTCCGACGAAAACCCA

GAAGAGTGTCGGAAGTTCCTGTGTCCGCCTACCAGAAACTTCCGCTGCTTGAACGATCGT

GTGTGTCTGTGGAACGGAAAACGCTGCGACGGCATTAACCACTGTGGAGACAACTTAGAC

GAGTTAAACTGCGATCAGACGGTGAGCAAGTGCGGCAAGGATAAGTTCCAGTGCAGCAAC

GGCAAGTGCATCAGCTCCACCCTGCGCTGCAACTACTTCAACGACTGTGACGACTACGGC

TCTGACGAGATCGGCTGCAAGACAGAAATCATGCTAAACGACTGTAAGAGTAACAGGTCT

CTGTGTGGAGAGACAGAAGAGGTCCACTGTACTACGAACGGCACCGACTCTTTCTGCTCC

TGCAAACCCGGCTACGAGAGCAACGGCTTCGGCAACTGCCAAGATAAAAACGAGTGCAGA

CAGTTTGGAGTGTGCTCGCAGATCTGCAACAACAGCAAGGGATCGTACAAGTGCGGCTGC

CACAAGAACTTTATTAAGATCAACGACACGTGCAAGGCTGACGGTTCAAATATCAATAGG

CAGGTGCTGTACATCGCCGACGATAACGAAATCCGTAGTCTGGACCCCGGCATGCCCAAC

TGGGTGTACGAGCAGACCTTCCAAGGCGACGCTAATGTGCGTATCGACGCCATGGACCTG

CACGTCAAGACCAACCGTATCTTCTGGACCAACTGGCACACCAGCGGCATCTCTTCCTTC

CAGCTGCCCTCCACATCCTCGTCTTCCTCATCCGGCACCTCATCCAACTCCCACCGCAAC

ACACGGCAGAGTGAGGGGACCGTCACCAACCTCGCGATCCCAGGTCTAAAGACGCCCCGA

GGCATTGCTGTGGACTGGGTGGCCGGGAACCTGTACTGGACAGACTCGGGCCGGGACGTC

ATTGAGGTGGCCCAGATCACCGGCCAGAACAGGAAGACTTTGATCTCAGGCATGATCGAT

GAGCCCTATGCGATAGTTGTGGATCCACCAAGAGGAACCATGTACTGGGCTGACTGGGGA

AATCACCCCAAAATCGAGACGGCAGCCATGGACGGCACTCTGAGGCAGACGCTGGTGCAC

GAGAACCTGCAGTGGCCCACAGGCCTGGCAGTGGACTACTTAAACGAGCGCCTCTACTGG

GCCGATGCCAAACTGTCTGTAATCGGCAGCGTGCGTCTGGACGGCACAGACCCTGTAGTG

GCCGTATCCAACATCAAAAACAATCTGCTCCACCCGTTCAGCATTGATATCTTTGAGGAC

TACATATATGGCGTGACGTACGTCAACAACTTCGTGTTCCGCGTCAACAAATTCGGCAAA

GGCACAACGGAGAATCTGACAACGGGCATCAATCACGCCACTGACATAGTGCTTTACCAC

CGCTATAAACAGCCTGAGATGGCGAATCCATGCGACAGGAAGAAGTGTGAATGGCTGTGT

TTGCTCAGCCCCAGTGGTCCGGTGTGTACCTGCCCTAACGACCGTGTGGCTGATAACGGC

ACGTGTGTGGAGGTTCCTCCTGCCACTCCGCCTCCTGTCTCCCCTACGTGTAACGTTCAA

TGTCTGAATGGGGGCAGTTGTTTCCTGAACGCTCGCAGTCAGCCCAAGTGCCGCTGCCCG

ACCAGCTACAGCGGCGAGAGGTGCGAGATAGACCAGTGCAGAGACTACTGCAAGAACGGA

GGAACCTGTTCTCCATCACGCACAGGAGCACCCACGTGCCGCTGCCCAACAGGCTTTACA

GGACCTAACTGTAACCAGAACACGTGCAAAGACTACTGCCAGAACGGAGGCAGCTGCACT

GTGAGCCTCGGAAACCAGCCCATGTGTCAGTGCCCTGACGGCTTTCAGGGAGATCAGTGC

CAGTACAGCCACTGTGAGGGCTTCTGTTCCAACGGTGGAACCTGCCTTCAGACAGCAAAT

GGAACCAAGCAATGCCGCTGCCCGTTGAAGTTTCTCGGCCCCACCTGCAGCTTGGACAAG

TGCATGTACTGCGGCAAGGGCAAATGTGTCCCACATCCCACTGACGGAGTGGCATGCAGC

TGTGCTAATGACCGGATACAGCCCAGCTGCTACAGCTGCAAAGACTTCTGCATTACTGGC

CAGTGTCTGTTAGACCAGCGCACCATGCGCCCATATTGCAAGTGTCCTTCAGGTTGGATT

GGGTATCGCTGTGAGGACGCTGCCTCTTCTGCAGGCTCGGTAGACTCCTCCAGCAACGCC

ATCTCTGTGATCATCCCTGTACTGTTGCTGCTCTTACTGGCTCTGCTGGTGGTTGTCGCC

ATCTTGTGGTATAAGAAGAGAATGCGTGGTGCTAAGGGCTTCCAGCACCAGAGGATGACC

AATGGAGCAATGAATGTAGAAATCGGGAACCCCGCATACAAGATATACGAGGGAGAACCC

GACGACGACGCCGGAGAGCTGCTAGACTCGGACTTCACTCTGGATCCGGACAAGCCCACA

AACTTCACCAACCCTGTTTACGCTACGCTCTACATGGGCGCCCACAACAGCCGCAACTCT

CTGGCCAGCACGGACGAAAAGAAGGAGCTGCTGTCCGCAGGGGACGAGGACATGGGAGAC

CCGCTGGCATAGAGCCGGCGACAAAGCACCAAGCTGTGCCCATCTGTCTGAACACCCACC

CTTCCCACCACCAACACCTACCCACCCACCCACCAAACCCCCCACTCATTCACTTGACCC

CCATATGCCTTTACAACATTCGGAAGAAAAGCAACAAGGGAGCAAGAACACTGTACAAAA

TGTAAAAAAGGGGGAGAAAAATAGAAAAAAATATGAAGGACTACTTTTGTATGTGATTGC

AGTATTATATTTTATTCTTTCAGTCAGAACAAAAAAAAACCCACGGAAAACATTTTTCTA

TACTTTTTTTTAATCATTACTCACTTAGCGCCCCTTTTCCTCCCGTTAGCTCTGTCTGTC

TCGACCCCTCCCCTCTTCCATGTAGCCTCTACCTCTGTGCTAAATGAGAACGCAAACAAC

CATCACAGCTAGGGAATTATTATTTCTATTTTATTTTTTGTATGAATTGTATAGACGTCA

AGGAAATAGCCAGGATTTTATTTTATTTTTTTGTCGTCGTCAGTAAATAATAAAGGGAGA

GGAGAGAGCAGCAGGGGGCGCGATCCCCCAATCCCGACGATGGTACCTGATCTGTGCGTA

CGTGTATCGGTACTGCTGGAGGAAAAAGAGGTGTGTGTGTATGTGTGTGTGTGTATGTGT

GTGTGAGTGTGTCGCAGATTTTAAAACTCCGGGAAAGGGAAGGGGAGCTCGCGCGCTCCA

CGGAAAATGCTAGAAAACGCAGGAGTGTCCGGATAACGGGACAGAACCGGGACAGCGCGA

GCTTCCGCACTGATCGCAGAGCCAAAAATAAAGTGGAATCGCACGCGCGAGAAACTGAAA

GCGAATCGATTGGTAAATTAATCGCGGCGGAAATGGAGCTGTCATCCATCGGGGAGCGCG

TGTTCGCGGTGGAGTCCATCACCAAGAAGAGAGTCAGAAAGGGGAATGTGGAGTATCTGT

TGAAGTGGCAGGGTTGGCCTCCTAAGTACAGCACGTGGGAACCAGAGGATAATATCCTGG

ACCCTCGTCTGGTTTTGGCCTTTGAGGAGAAGGAGGAGCGGGACAGAGTGCAGGCTCACA

GGAGGAAAGGCTTGAGGCCCCGCAGACTCGTCTTACGGAACATCTACACGATGGAGCTGC

GCAGTGCTCAGAAACCGCCGGAGAAACCTGCTCCCCGATTCCGCCTGTCACTCACCCGCT

CCATGGGGGCGGGGCTACCACATAACGGGCGGCGCTGCAGGAACGGTGAGGGAGGAGTCT

ACCAACACCCGGACCGCCTTCTAAAGCGCAGGAGCAGGCCCTATGTGTCCAAAACACCAG

AAAGAACTGAATCGCCACGGCAACCGACACCAACCTCTGAGAGAGACTCAGCGGAGGAGG

ATGAAGAGGAGAACGAGTGGGAGAAAGAGGAGGAGAAAAAGAGGAGAAAGATGGAGAATG

AGGAGCGTCATGCCATTCCTCACGGTACGTGTAATGCAGCATTGGCATGGCAACAGTTGC

TAAGGTCCAACGCATGCACGATGCACTGAGGGGGTCAGAGAGACCACGGTGAGACTCTCG

AGCAGTCACGACTCACGCCCGGAGAAACTAAAGGCAGGAACGTGACAAGGAAGTCAGTGC

GCAAATCTGAGCAAAGATAAACCACACACACACACTGTAGAAGCTCTCATCTCCTCTGCG

AGATCTTCTCTGATGACTCACTGAGAAAACGCTCAGAGACATGAGGGATGAAGCGTCAGT

GTGTGATTGGTCGGTGATCTCATGTTGTTCAACATTCCTGAAAAACAAGCGGCTTCAGAG

TGTTTTAGCTGCAGTTACATCATCCTCACTTCCAGCAGCAGAGGTGACAAACTGCCACCA

TCAAACCGCACCGCAGATAAGCGTTCTCCCCAAAACACTCAGAGAACGCATCACAAACAT

CCCTTACTTTACTTAACATTAACATTTCAGTGGTTACGGTGTTGGACTGCTGATCAGAAG

ATCATAAGTTCAATTTCCAGCACCACCAAGCTGCTACTGCTTCCTCAAATAAATGAGATA

AAGAAATGGATAAAAGTGCGTGCAGCTAATGTCAG

>Unigene000222

GGCACTAAGAGGCTGGCCGGTGTTTTTCCACTCATGACGATGTCTATTGGTCACCCATTT

TAAGAGACAAAGGTCGGAAAGAAAAGGCTTATAAATTAAATTTACCGCGACACATCCCAA

TTCAGCGTCGCACGAGACCAGAGACGAGAGACACTTCTAAAGGAATAAAAAAAGAGAATT

CAGAGGAGCATGGCACAAAAAGAGAAGCTTCAATGTCTGAAGGACTTCCACAAAGACACT

TTAAAGCCATCGCCGGGAAAAAGTCCTGGCACCCGACCTGAAGATGAAGCTGAGGGCAAA

CATCCTCAGCGAGAGAAGTGGGCCAGCAAACTTGACTTTCTTCTGTCTGTAGCTGGAGGA

TTCGTTGGTTTAGGAAATGTCTGGCGTTTCCCATACCTCTGCTATAAGAACGGTGGAGGT

GCATTTCTCATCCCATACTTCATTTTCCTGTTTGGCGGAGGCCTGCCTGTGTTTTTCCTG

GAGGTGGCTCTTGGACAGTTTACCTCGCAGGGAGGAATCACATGTTGGGAAAAAATCTGC

CCTATTTTTACTGGTATCGGCTATGCTTCCATTGTGATCGTTTCTTTACTGAACATCTAC

TACATTGTGATTTTGGCTTGGGGCTTATATTACCTGTTCCAGTCCTTCCGTCCTGACCTT

CCTTGGGCCAGCTGCAATAATTTATGGAACACGGACAGCTGTATAGAAGACACACTCCGC

AAGAACATGAGCCTTACTAATGTCACCAATTTCACGTCACCAGTCACCGAGTTCTGGGAG

CGTAATGTCCTCAGTATTTCGGATGGCATCGAGGACATAGGCCCTGTTAAATGGGACCTG

GCTCTGTGTCTTCTTGCTATGTGGGTCATCTGTTTCTTCTGCATATGGAAGGGTGTGAAG

TCCACAGGAAAAGTCGTGTACATCACAGCAACTTTTCCGTTTGTCATGCTGGCTATTCTC

TTGATCCGGGGTGTTACACTTCCTGGGGCGGCCGAGGGCATCAAATTCTATCTGTATCCA

AATGTCACCCGTCTGCAAGACCCAGAGGTTTGGATTGATGCAGGGACGCAGATTTTCTTT

TCCTATGCCATTTGCCTGGGAGCAATGACGTCACTGGGGAGCTACAACAAGTACAAATAC

AACTGCTACAGGGACTGTTTGCTGCTGGGAGGCCTGAACAGCGCTACCAGTTTTGTGTCT

GGCTTCGCAATATTTTCCATCCTGGGCTTCATGGCACAAGAGCAAGGGGTGGACATTGCC

GATGTGGCAGAGTCAGGTCCTGGCCTGGCTTTCATTGCTTACCCTAAAGCTGTGACAATG

ATGCCACTGCCTACATTCTGGGCCATTCTCTTCTTTATCATGCTTCTGCTACTAGGTCTC

GACAGTCAGTTTGTTGAAGTGGAAGGACAGATCACCTCACTGGTGGATTTGTATCCATCC

TTCCTACGGAAGGGTTACCGTCGGGAAATCTTCATAGCTATAATATGCTTTTTGAGCTAC

CTGCTGGGACTTACCATGGTCACTGAAGGTGGCATGTATGTGTTTCAACTTTTTGACTAC

TATGCAGCCAGTGGTGTGTGCCTTTTGTGGGTTGCATTCTTTGAATGCATTGCAGTAGCC

TGGATTTATGGTGCTGATAATTTCTATGATGCAATTGAGGACATGATTGGATACAGACCA

AATCCTTGGATGAAGTGGTGCTGGACTGTAATTACACCTTTTCTTTGTGTGAGAATTAAA

GCAATGGCAGCTCCAGTGCGAGGCAGTACAAATTCGTGTCCTCAGGAGTATCGAACGAAG

GGCTCAGAACTGGCACATCCCCTTGACCCCAACGGGAACAACGGCTTGATCAAGCCCAGC

CACACTATTGTAGAAACCATGATGTGAGCGCTCTCTGTGAGAGAATCCAATCATCTGCTT

TCCATTATATATAAATACATATATATTAAATAAAATGTAAAATAGTCGTAGGACCAGGTT

TACATATCTACGTATCTGCACTAGAAGTTCTTTCCTTTTTCTTTGCTTTCTTCTTTTTTT

TTTTTTTTACACAAAGGAAGTTGTTTTTGACAGTGTCTGCTTTTAATATTTTATCATAAC

ATTGTTATATTACTATTATTACTGTTGCTGTAATTCAAGATGGAAGATTAATTTCCAGAC

ATAAACTCCAGTATCTCAAAACATTTCTTTTTTTTTTTTTTAATCTGTAAATAAGAAATA

ACGTTTTAAAGGGTTTATTGTTGAAGTGGACAAATCAGACCAATTAAGTGCTGCAAATAA

TAGACGTTTATAGTGTTTTTAAAAAAAGTAACATTAGTGTACCTAGACAAAAGCGTCTTT

CCGGTAAGACAGTTGTGCATATTTATTCTGACAACGAGCGTAAATTTCCAAACTAATACG

ATTTTTAAACTCATGCATTATTTCGTTATGGACCAGAGACACTACGTTATTTTCTGGTGC

GAGTGTAACACACTGAAATCATCACCGCACATGCGGGATGAGTTACGACATCAAAGTTAC

TTTGAGCATCTTTCAAAGCACAAATGGTCACACTAATTCTTACCCCATACCACATAAGTG

TATTCAGAGTTGACAACTTCAGTCTAAAAAAAGCTTCAATATTTTTGGAAGTGGAAAGTT

TAGAGATGGGCTACACATGTGATTAAATTAGGGCTCCACATGTAGGTTACGCAATTTACC

TGTAAAGTTGCCAGATACTTTTGAAGCAGAAGTGTGTGAGACATGTTTTTCCGTAGTGTC

TGGACCAACGACAAAAAAAAATTGAAAACAAGAAAGAATAAAAAAAGCCGGAATAGGAAA

ATGTTAGTCATTGAATAAATCTAATAGGCGTTTAGTTTGATTCGATTGGTGGTAATTAAC

ACATTACTCATTGCAATTTGTATTATTGCTAAGCTTTTTCTGACTTAGCGATTTAAGCTA

ATATCACAAGCTAATTGTTCACACAGTTAAATGAAAAGTGAAGAAGATTAATAAGCCGAA

GAGCGTTTTAGTGTTTTTATTTGTTTATTCATTAATGACTATAATGTTCACATAATGCCA

CAATAAATACTAGCCAATTAATAGCTTTGCAATTTGAGCTCAATTAAAATTTTAATGTAA

GTCAAGTCATTTTTATTTATATAGATCAAATAAGTCAATAGTAAACCCTCATTAAAAAAA

ATCACAGCGCAGTGGTTAAGGTGTTGATCTGCTAATTGGAAGATTGATGTTCGATTCCCA

GCACCACCAAGTTACCACTGTTGGGCCCCTGAGCAAGGTCCAAGACCCCTCAATTGCCCA

GTTGTATAAAAAAAGAGATCAATGTAAGTCACTCTGAATAAGGGCGTCTGCCAAATGCCG

TAGATATAAATGTAAAACACTCAGTTCAATTTAACTCGTATCTGTAAATTAAGAGTCCCC

AAGTAAGCAAGCCAGAGGCAACAGCAGCAAGGAACCAAAAAAAAATATTTCTTGGGAGAA

ACCAGGCTCAGTCGGGGGTCAGTTCTCCTCTGACCAGACCAACCAAGTTTCCATTATCTT

GTCTGGAGGGCCGTCGATTGTAATATCAATCTATCTCACCATGGCAGTGACAGTAGACCA

CTATAGTAGAGGTTGAGAGATCTCTGCAACCAAGACAGACCTCCAGTTTTAATAAACTCA

AACCTAAATTAGCAACTCTTACCTGAGCGTCTGAATATGTCAGCAGGACGCACCAGTTCG

CTAATGTCAATCCTTATTAAGCCTGCATACGTAAGTCAACTCTGAATACAGCCCAGCACC

TTTTTCCATGATTTATATATCGATATAATTAAAGGAGGTGCTAAAACAAATTTACTTCTA

CCCACCTTTTTACACTCCCTTAAAATCATATTTTTATTAGCCATCCTTATAAGAAGGTAT

CAGGATTTAGACACCAACTGAACGGTAGTATTTTATAGTCGAATCGAAGCTGTTTAAACA

TTTCATTCTTTTCTTTTCCATTGCCTTTTGTATTTTCTTCACATTAAGATTGTACTTTTC

TTCTGTCAGCATATTTTTTTTTATAGGAAAACATTTTATATAGAGCTTTTAATCAGTGCA

ATTGGTGGAATTTGTAATGTGCAATATTTTTTTTTGTAGGATAATATGCATTGTTCCTGT

CTTTTAAAAAGATGGTACTTGCAGTGAAATTTCTTTATGACAAAATAAGAGGAAACATCA

GGCACGTCGGGGGCCAATTAACGATTACTTATAACGATAAGTAACATCGAACGAAAAAAG

AAAAGAAAAAACGTTATTAGTTACTGTGACGCTTACATAAATTAGTATTTTTTAACGAGG

AACGCTCAGAGTTTTCTATCACTTCTGTCAGATAATAGACATTAATCTTATTATATGATT

TTTAACAGCCTGATATCTGATTTATCTGATATATCATGTAGCCAAACAGCTTTTTCTATT

GTTATTATTATTTTTTTTTTTAACATTCTCTAAATGACGTTTATATGTTGCTTTTAAAAA

ATTGGCTCAAATAATAAGATCCCGTGCAAATAGTTATAAGATCAGAGATTAATGGAATAT

TCCTGTTCGTTACTAAACTAATCTTATTTACGACCTTTTTTTACTATTAACGAGGAAAAA

AAGTCTAATTGTGATAAAACTAAATGCTCATATATATATAAAAAAAATAGTTTGGACTAT

TCCATTTAGTCTCAGAATCTGATCCCAATCCTAATGCAGAGATTTTGTACACCAACAATA

TTTGGCACAATGTCACAATTATTTTTTTTTTTTTTATTCTTGAAAACAACATGGAAGTAA

AAATGTATTTCAAACGATTCTGGAAGGCCTAAAACCTTTTCTATTGCTTAATAAACATTT

ATTTTTGAAAAAAAAATTTAAAAAACCCAGAAAAAAGCCTGCTTTTGCTGAATGTTGCCA

GATTTAAATGAATCCCAAAATTTGTAATAAACTTAATCAATCCGATTGTTTAATTATAAC

CCAGTCCTTCCCTTGTGTTCAGAAATGGCACAGTGCCTCTTTAAGGTGACCCAACAGCAA

TGTTATAGCCCAGCCAGGGTATTTTAACACACGCCTGGCTTCCTAAAATATGGCTTGTGA

GGACACAATCAGGAGCGCCATGTTAGGGAATGGAAAATTTGTCCTGTATATAACAGAACA

TTGTTTAAATGTGCATCCTCGTTCTGTTCCCTACAGTTTTAGCAGTTACTCAGAGTTGCA

CCGAGCCAAAAGATCTTTTTCCCCCCTCAGCGACTGCTGGTAGTAGTGATCTGATCTGAA

AAAGATTAATAGCACCCAAGGGATGACAATTCTGGAAACTTGCACAAAAACTTCCCAAGC

TTAACTTTTTAAAGGATGAATAGTTCCTTTCTCAGGGTACAAGTGATTAATGAGTCCAGT

TCCCATTGGACATGGAAATATCTGCTTTGTTTATTATCCGGCCATGGAAATTATAATTAT

CCTATTATGGCTTAAATATAAATCTTTAAAAATAAAGAAATCATTTTGCACTACAAATCA

GTGGGGTTTGTGTGTGTGTGTGTGTGTG

>Unigene000223

AAATAAAGGGCGGAGGGAGAAAAACTTTACGACGGGTTTTTTTTTTTTAACTGTTAAACA

GGAAGGAAACTGTCGAAATGCAAACCTGCAATCTCTAAAATAGGCCTCATTTAGGCTGGG

AAATTAACAAGCGTTATTAATATAAGTGGAGGTAATCGCTCGATCTGTACAGTGCGTTTC

ATTTTTAACTTATTATTCTTTGAAGCTGGTTTGAACAAACACAGCTGAAAAGGAAACGCG

CGTAAGCCGTTTACACGCGCGTCTCTTCCGCACCGGAAACTTCGACATTTTTCTTGCACA

CCACCAACTGAATTTGGAAATACTAAAGGACGACCTCTTATCGCTTTAAACTAACTATAC

AGTAAAAAAGGACCGATGTCTTATGGCTTCACACGGGCAAGGACGCAAAAGAACTCCGAA

TAGAAACGGAATAACTCCAGAAGAAGATGCGTTAAATGTAATCGCACGAGAGGCTGAGGC

CCAACTAACAGCTAAGAGGGTTGCCAGGGCAGAGGCTCGTGAGATACGCATGAAGGAGCT

GGAAAGACAACAAAAAGAGATTTATCAGGTTCAGAAGAAATACAATGGACTGGAGAACCT

GGACAACAAATGCTGGGATATTGAGCAGTGGATGGAGGACAGTGAGCAGTATACACACCA

CCCCCAGCAACATGTTTTGGTGTTGGGAAATGAAGAAGGTACATCAGCAGGGAGCAAGAG

AAGTGTGCAGGGCTCCTCTCGAGGACCGACTCTATCCACTGAGACTTCCCTGAGTGGGGC

GTCCTCAAGTAAAGGAAGTGGTGGTTCATCTGTCACTGCTGATACAGAAACATCCTTAAG

GGATATTAAAGAGATTCATGAACTAAAAGACCAGATCCGTGATGTGGAGATCAATTACAT

GCAGATCCTCAAAGTAACAAAGGATTCTTTGTCAGAGATGGAAATGAAATATCGCAAGGC

TATGGTGTCCAATGCTCAGTTGGATAATGAAAAATCTAATCTGGTGTATGAGATGGACAG

TTTAAAAGACTCACTAATGGAGTTGGAAGAGCTTCTGTCTGAAACTCGTCGCGAGTGTGA

GAAAAAGAATAATGATCTGGAACGAGAGAAGCATGCTCACAGTATACTGCATTTTCAGTT

CAGTAATCGAAAAGAAACACTAAAGCAAAGTGAAGAGCTGCTGAGTGAGATCCGACAATT

ACATCTCAAACAAGAAGGGTTTACTAGAGAGATTTCTGACCTGCAGGAAACTGTTAAATG

GAAGGATAAAAAAATTGGGGCTTTAGAAAAACAAAAGGAGTTTTCTGATGCCATTCGAAA

TGAACGGGATGAACTTAGGGATGAGGTGGTTCAACTCAAAGACACATTAAAGAAACATGG

CATTGCTCTCGGATCAGAATTACGCACCAATGGGGAAGTGGGAGTAAATGTAAGTGGTGG

ATCAGAATTATGCACCAATAGGGAAGTGGGAGTAAATGTAAGTGGTGGATCAGAATTATG

CACCAATAGGGAAGTGGGAGTAAATGTAAGTGGTGGATCTGTCTGCACTGAATTGAAACA

TGGAAGCAGTGTTTTAGGCACCCAGAAGTTGCAGCTGGTTAGAGATGAAAATTTAGGGGG

CTTCAGATGTGACCAATTACAATTCCCTCAGCAATTTGACCATGGAATTGAAGAAAATCA

TCTGCCTTCACCTGCATCTGGCAGTTCTTTTAACTCTCGTATAAAAACACAATTTAAAGA

TGTGGATCTTGGGAATTATTTAATTAACAGCATAGAGAAAATTCAAAACAATGTTCAAAA

ACCTCAGGCTGTATTTGAACCTAATGCAAAGAAACAAATTGTATCAGACATTTTGAAAGG

TAATGATGTTCCAGAAACTGATGCAAATATTATGAAAGGTATAGGAAAAGATCAGCAGGA

AAGTTATGTTAAAAGAAGTAAGACTGGAAAAGAAGGAAAGCATGATTTCACACACATGTT

CACCTGTGAGATATCAGAAAAAGATATCAATCCACATTTTGATGAAACTGTGTTTTGCAA

CTCTCAAATTAAAATGGCTACTGACAGTTCCCCATTAGCGAAAACAGATACAACATTTAA

TTCCCAAAGTTCCAATGTTTCAGTAAAAAAGAAGAAGAAAAAGAAACAGAAACAAAAGTA

GCGCTGTGCAAAAAATGTTCATTTAAAAATAGGGAGAAAAAACAGCTCAGAGAAAGCAGT

TCAGTTCAGACAGTATTGTCAGAGTGCAAGTTTGTACAACAATAAATAAGAAAGCAAAAG

ATAATGTGAATTTCGATGAAAAGATAGACCTAATGCACAAAGGAACAGAATCATTGGAAC

TACAAATTCCCAATACTGGCCCTGAAAATATTGTATTACCAATCATTAATCAAGACGATT

GCATGAGCAAGATTGCATGGGTGGACAGAAAATTGGCATTTCTACTCATGACCATCCTAG

AGATTTTCTTCTTTTGGCAAATGCATCATACAAATTAACAATGATGATGCTGAGTTAAAA

GCTATTGCTGAATTATGTGATGATTTTTAATTATCTGAAAACTCTTCTGAGGTTGAACCT

ATGAAAGATAAAACTGGAACTACCAGAGATGTAGTAAAAATTGAAGCATTTATTCATTAC

TGTGAAACATACACCCTTGAAAAAAAATCCACAAACACAAAGCTGGGTTACACTGTAGTT

AAATCTGAAGCCCCTGAGGAGAAGCAAGAAACCTTTACTGGCATACAGCTTGATGATACT

GCATTTAAAGCTAAAATACATAGAGAAAAGAAAGAAATGTCTACTGGCACACCATTGGGT

GACACTGTAGTTACAGCTAAAGTCCAACAAGAGAAGCAGGGAACCCTTATTAGCACACAG

CTTAATGACAATGTAGTTACAGCTAAATCCTTAAGAAGCAAGACACATTCATAAACATGT

AGCTGGGTGACACTGCAGATACAGCTGAAAAAACCCAAAAAGCAGCAAGAAACAATCACT

GGCATACAGCTTTATGAAACTGTAGTTACAGCTAAGGTCCTTGAAGAGAAACAAGAAACA

TTCATAGACACACAGCTGGATGACACTGTAGTTAAAGCTGAAGTCCCTAAAAAGAAGAAA

AAATCAGAGCCACCAATGCATATGGGTCAGAAATTTAATATAGAAGAGGATGACAGTGAG

GAGGAGTTTAAATCATTTTTTTTTATTAAAATAAAGAGATTTTTTGGAGGTAATGACTGA

CAAAAGGAATACACATTACTTGTTGTGCAAAGCAATCTTGTACAACCAGTGAAAACCCGG

CGCACGCAAATACTAAAGATAAAGACCAAATACTACAGACAGACAGACAGAATGTTTTAA

TGGAGAAAGACAAGCGTGTGTCTCTAGTGCAAATAACCACACCAGGAGGGACTCAGCAAC

AGTTTTATCCAGGGTATGAGGAACAAGGCTCCAAAAGCCAAGAAGAAATAATACATTTAA

AACACAGTGAAAAAGTTAAGCAGGAAATGTCCAGCAAATGTTATAAAGGCCCTTGATGAG

CTGCTCTCTGAGACAGCAGTGTTAAACTTAGTGGCTGATGGTACTGTACAGCAGAAAGCA

TTTCAAAATATGGGAGAGCAGGAATTCAAAGAAGTCAAAGAAGTGACACATATAAAAGAA

ACAAGCAGAATGCAAAGAATGGTTCTAAAAAGAAAAGCAAAAAAAGTAAAAGGGAAGACT

AAAGAGGACTGTAAAATGACCTAATACTTTACCCAAGGATGTTAATTATAAAGTGTCCAG

AGTCTTAAAGCCTAACTAAATCTATGTTAATAAATAATTGAAAGTTTAAAATAGTTAAAA

GCTTCTAATCTTTAATCGAGATTAAAAAAGTCATTAAATTTCATTTCAGCATTTAAATTT

TTTGTTGTGTGTGGTATGTATGCTTCTACATCTGGATTGTGTGAGATGACATGACATGCT

TAGTGAAATTCATATAGTTATTTATTTTCTCATGAATATATCTGCAGCAGTTTTCACAAT

GTTTTATCTCTGACATGCAGCAGTTTGCTACTATTTCACAAATTATATAATAAAAGTTCT

TTTGTAAGATTTCTTTCAGAAACGTTTGTTTTTGTTTTTTTACCCAGTGACGTACCGTGG

GGTTTCAGCAGGGGCGGGTGGTGCATTTGGTACCTTGGACTTGGACTTACCCGACTGATA

CACCTCTTAATACCACCACTATCACATCGCAATTATAGAAACCACCACTACTATACAAAA

ACGCAATATAACATGGTGTGAAGTTAGAGACAATGAGGCATTTCGCGTATTTAGGGAAAA

TACCATGTTTATAAAAGCAAGAAACCCCCGTTAAGACAATTTCAATCCTCTACAACAACA

ACTGTACCACATACATCATCTGGGGCAGTTCAGAATCAATATTAATATTTTATTTATATA

AAGAAACAGTGTTTAATTAATTAATACTGCTTGTTGGCCCTGACTGCACACCACTGGGTT

TCAGTCAGGCCTTTAAGTAAGCAGTAACTTTAAAATTACTTGGACAATCCTACAAGCTAA

CACAGATATATGATAATAATGTGCATATGCACAATTGACACCATATGGAACTCTGAGCCA

GACAATCAAAGTACTAAAGACTAAACTGAAAAAAATAGTTTTTTAGTAGCTGTTAGCCAC

TCATAGCACTTAGAGTTGAAATCTTAAAAAAACTGCTGGCCATTGTTAGTGAAAATCAGT

TTGGGTGTAGGTCTTGCTTAATTCGTTGTCTGCAAAAAGTTGCCTTGAAAAGGCGTTTTT

AATAGGTTGGTGACAGAATTTGGCCGGCAGCTGCAGCAGTCAATTTGTTATTGTTGACTT

TCACTTTCATACCTTGGAAAATCCTGATAAAATCAATCATTTTATTTTTATAGGGCCAGC

AGAGAAGGTAGAAACATATTCTATATTAAAATAACTGGTTCGTAATAAAACATCTATTAT

AAAAATAAAATTACAGCTGTTTTACATTCTATTTACTTTGGTCTGTAAACAATTTCAAAA

GTATGGTGGCCTTTTTGGTAATTTTATCATACCGATAATATGATTCCTTGGTCTTTTGTG

TATTGTTAGCCTAAATAATATAACTAATTCCATAATTAACTATGAAATGTATGTATGCTG

GTTAACTCAATTTCACATAGAGTTGTCACTATTTGGGTCCCCTTGAATTGTGATCTTTGC

TATTTGCAGTGGGTTTTTGCATTTGATAGTGTTTTTGTGTATTTGGTCGTGAAGATTTGA

AGAGCGTTTCAGTATTTGTGTCTGCATTGTGAGGATATGCAGTGTGTTTTCTTAAGTTGC

AGTGCATTGGGCTTTCTCTGTTACCATACAAAAGTCCTAGGAAGAGGGACTTTACGATAA

AGACATTTTAATGGTCTACAAATTTATTCCAAAGTTGGCATGACTGTGTTTTTTACAGAA

TACTTTTACAGAAGTTACTGCATGCTATTCATTCTCATATATATATATATTATATATATA

TATATATATATATGTGTGTGTGTGTGTG

>Unigene000224

GGCAGGTTTCTCAGCTTCAAGAAAGGCGACATGATCGATGTTTACCACAAACTGTCGAGA

CTGAGGAGCGAGCTGTGGGCTGGAAGTAAAGATAAAGATTTCGGTTATTTTTCCAGAGAT

GCTGTAAAACTGGATGAAGTGTTTGTTGATGACAAAAAGGAAATAGAGATGCAAACTCAG

AAAACAGATTTCCTGTGTATGGATGCGTTTGGGACTATAATCTCAAGTGAGGAAGACTTC

GAAGATCACGAGTTTGGAGATCAAACTAGCAAGTCTGAAGATTTAGGTAACAGTGATTTT

GATGATCGAGACTCCAAATCATCTAAACCGAATGAGAATGTCAAAGTGAAGCTCATTGAT

AATAATAAAGACAGAGGAAAGAAAGCAGAACAAAGCCGAGCTTCCTGGATCAGAGCCGCT

GTTAGCGGATGGTTTGCTAACGATGCTGGAGATTCGAAGGATAAAGAGGAGAACTCTAAG

AGCAGGAAACTGGCTTTAAACATGAAGGAACTAAGCGAAAAGTGTAGACGAACGAAAGCT

TCGGCCCAGACTGAAGATGAATTGAGCAGCGCTCTGGAATTAAAGGAGGAGGAAACAGAG

AATGATGAGGAATCAACCAGTGAGCTAGGAAATAGAATAGAAAGTGTTAGGGGTTTTAAA

GCAGGAGAAGACACATCATCTTCTGAAACCATAAAACAGAGTCAGAATGAAAATATGCAA

ATTCTAAGCACAGAACAGGAAGTAGACAGTCTTAAAGAAAAAGCCTCGAAAGTGATGATC

ACTGAAGATGAGAAGAGAAACGCAGGATGGTACGGCAATGTCTTCAACACCATCACTAAA

CTCTATGGAGATCCTTCTTATAGAAGCGAAGCCAGTAAAAAGGAGGGAAAAGTGAGATCC

AGCAACGAAGACACTGATGTGCTAACAGTTCAAACAATTTCCAAAAATGACAAAAATCCT

GTTGACGATGAGCAGAAACAGGAAGTAAGCAGTCGGGATGACCGGAGCAAAAACAGGAAG

CTCGGAGAAGGATGGTACGGAAGCGTCTACAACAGATTAACAGAATTTTATGGAGATACT

CCTGATGAAAGCACTGCTGATGGTGATGAAGGTGGAGAAACATCTAAGAGTGAGGATGTA

GATCCAAACCTGGAAAATAAACTGGAAACTAGTAGGGAGAACATGAATGAGGATAATAAT

GCAGAATGGTACAGCAGTATCTACAGCAGAATCAGCAATGTATATGGAGATCCATCTGAC

AAAAGAGATGTTCCTGATGTCATGGGAGAGCGCCAGAGCCAAACACAGAGTGAAAGCACC

AAAGAAACATCACTATCAACCACATTGTTGATGAATAGCCTGTCGACTGGTAGGAACGGC

CTGGCAGAAGCAGGAAGTAATGATGAAACAAAAACAAGCGAGTCTGTAAGATCAAAGCCA

AACTTTTCGCCTGATCAGAGCGAAACCAAAAAAACCCAAAAAAACACTGAGAACCTGAGA

GACTCGTCACATGTGTTTTCTATCAAACAGTTTTTGATGTTGATTACTGGCCCGAAAACA

CCTTCTAAACCTTTTAAAGAAGAGAAGACACCTGATAAAGGTGAGGAGGATCCGATCAAT

GAGGATGATGAGCTTAGTGTAAGGCTGACCGAGATTAAAGTAGAAGAGGAGGAAAAGAAT

GAGAAAGATGTTTGTGAGGTAAAGGTATCCGAGGCTCAAACACATGCTGTGGAAGGTTCT

TTAGACTGTAATAAACAGGATACTTGTGGGAATGTTGTAAATAATCATGACGTCCTTGTG

GAGAGAGATGAGGTCAGTGGTCAGTCTGATTCCACCAGCCAGATGACACAGGAACCAGGC

TGGGCCAGGTTATCTGAACACATCTCAGGCGAGCTGCAGTCTGAGAGTCAGACCAGGCCT

GAGCCATGGCTGCAGCTCACAGAGAGCAACCCTGAGACTGTAGGTGAATCTGAAGAAACT

CAGGATGAATATGGAGGAGCAGAAACAGAGGGAGAAAAGACAGAGAGGAGAAAAGACAGG

GAGCAGAGCAACCATACAGGTTCCAACACTAAGAAACACGTGGTAGATGATTTTCAGGCA

GAGTATAGTTTAGAGAACCAAAGTGTGAACGTTTCTTTAATGAAGGATGATGAAGAACAA

GATGGTGCTGATGTTACCGAAACAGAGCTTTATGGAAATAAAGTAGGTGTTGATGTTACT

CCAGTGGAGTCTGCTGGCGAAATTAAGGACACTGATGCCACTAGCATGGAAACTAAAGAA

GAGAAGAAAAATTTAGACGTTCCTCAGATGGAGTCCGCTGGAAGAAACACAAGCGGTGAG

GTTATCCGAGTAAAGAACGCTCGAGGACAAGACAGAGTAAAAGATCCTCAAAATAAAACT

GACAAACAAAAAGACAGTGTTGATGTTATTCAGAAAAAGAATGAAGGAGAAGCAGGAGGT

GATGTCACTCAAGTAAAATGTAATGGAGAAAAAGAAGGTGAAGATATTATTCAGAAAGAA

GAGTTAAGTTTACATAATGGTCAAACTCAAAACTCTCACAGCATCTTCTTAACCAAACCC

AATGAGAAGATCGATCCAGACAAATCCAGAGAGTCTGAGACACCCAAAAGTGCTGTGTTA

CAGTGGTCTCAATCAACACACGCCTCTGGAGCAGATGGTCCCATTGCTAATACGTCTGAT

GGAGCTCAAACTCATCATGATGGCAAAAAAACCAAAGATAAAATGATGGATGTTAATCAC

CACAAACGTTTGGCTCAGATGCAGAAACCTACATATCCTAACCTCCAAACTCATCTCAAT

GAGAAGGACATACAGGTTCTTTCAAGGTTTTTCAGTAAAGAAACGTTGACACGAATGGAT

TTCATCATCGAACACTCTGAAAATATTCACAGTAAAGAGCTTGCTGAGGTTCATGACTTT

AAACAAATGCTGGAACATCTTTTGAAAACCAAAAATGAAGAAATCAAGCCGTCTCTACAA

TTCATACACACTGTAGTAGCCATGCTTCAAAAGACGTTTAGACCCGTGATAGCTGATGGC

AGTGTAGATAAACACCCAGATCCAGGTCAGACGGAGTGTTTCACTGAATTGTGTGTGAGT

ACAACTGAGAACACACCCATCTCTGAGAAAGACACCAGTAAAAACTACCACATGGAGACC

AGCTCCAATACAGACGTCTCGTTCTCTGATCACGAAGAGGTTGTCTCTGACAGGATGAGA

ACATCACACACCCTCCAGACTGAACGGAAACCTGATCCTCGTCTGGACACAGAGATGATC

ACTGAAGAGTCTCTGAAACCCGAGGAATCCAGAAACGGAGCTTCTCTTTCGTACACAGCT

CTCGTTGCTGAATTAGTGGGTGGTGTGTTCATGTACACACTGAAGGAGAGTTTACACACG

ACTCACACCATCCGTCAGGTTGTGATGTCACTTCCTGATGATATCCGCCCGGGACCGGAT

CTTTACGGTGTGCCATGGGAAGCCGTGCTCTTCACCGCCACGTTGGGTTTCTTTACCTTC

CTGTTGTTCACCTGCAGATTCTTCCAGGCAATTAGGAGCCGGTTATACTGCAGTAAAGAA

CAGAGGATGGGGCAAAAAGTGGCAGAGCTTCTGGAGGAGAAGTGTAAAGTTCTGCAGACA

CTCAGCGAGTGTGAACACAAGTTTAAGAAGCTGCAGGCTGCGCTACAGAATGGTGGACTA

TCGGCTCAGGCTTCAGAGAGAAAGGATTTAGAGGTTTTGTCGAAGCAGCTGGAGAAGGTC

AATGCGCTGATGCAGAGTGATCTGGAGAAAATGCAGAAGGAACTGGAGACTCAGGATCAT

CTCAGGAAACAACAGGAGGAGCAGATCGTAAATCTGGCGGAGATATTAAAGAAGCTTGAG

CAGGAGGCCATGGAGCGCAAATCTCAACTAGAGCAGGATAAAACGACTCTGAAAATCCAT

CAGATGAACACTGAGAGACTGCAGAAGAAACTCCAGCAGGATAAAGAGGAGAACAGCATG

CTACAGGAGAGCAAAGCACAGCTGGAGCAGGAGGCAGAGGGATGGAACGAGCGACTAAGT

GAACTGGAGGAAGAGAAGAAGATGTGTGAGACGTCCCACAACAGAATGATGGAAAACTGC

GTCAATAAAGACGAGAGCATAAAGTCTCTTACAGAGTGTCTGTTAAAGGTGAGAGACTGG

GACTCGGAGGAGGAGAGCACTGTGAATGATGCGAGTGGATCAGAAAAAAATTCTCTAGAC

TTCAGCCAGAAACAGAAAGTACAGAAACTCATTAATGCAGCCAAGATGAGTGCAGATTTG

AAGGCAATGGAGGAGGACAAAAACCGAATGTTTGCCAAACTGTCAGATGAGATTAAAGCC

AAAGAAGATCTACAAGGAGGTTTGGATCAGCTGCAGCGTGAGAAAGAGTCGGTCCAGTCC

GAGAGCTCAGTGTATTCCAGTGAAATCCAGAAACTCCAGCACAAACTGCAGATCATGTCT

GAGATGTACCAGGAGAAAGAACTCAAACTGCACAGGATGTTAACAGTGGAGGAGAAGGAG

CGCTCGCAGAAAGAGGAACAGCTCGACAAAGCTGGGAAGAAAATCAGTCTGGCTACAGAC

GAGCTCAACTCCTACAGACAACGAGCCAAAGAGCTGGAGGAGGAGCTGGAAAAAACTAAT

CAGGCCTACAGGAACCAGATTGCGTCACATGAGAAAAAGGCTCATGATAACTGGTTGGCT

GCTCGAGCTGCTGATCGTGACCTGGCTGATGTAAAGAGAGAAAACTCAGTGCTGCGTCAG

AGGTTAACAGACTGGCAGTTTAAACTGGAGCTGATGGAGAAAAATCCTCAGGCTGGTGTG

CTGCACTTCAGAGGTGAAAGATCTCCGTTTGGTCCGTCTCCACTAGGTCGTCCCCCCTCT

GAAACCCGTCCCTTCCTGTCTCCACCCACGCTGATGGACGGCCCTCCCAGAGTCTCTCCA

CAGTTTCCCATGATGCCCGGCGGCAGAGCGCCCACTGGTGGTGTGGAAGGTGACAGGAGT

GTTTCGTATTCTGAAGGGGGCTCCCCTACGTGGGAGAGAGAGCGAGAGCGCAGGGGTTTC

CCCCCTCCAGGTCCAGATCCAGGGTTGGTGTACAGAAGACCTCTATCGGCACCGTTCCTG

CGAGCGCCACTTCCTCCTGAAGCTTACTTCAACGATAAATCAGACTCGGCGATCGGAGAA

GAAGCTGGATTTAATGAAAACGGGATCCGAGATAACGGACGCTTAATGCCTGGTCCTGAT

ACAAGGGCAGGGCCTTTTCCTCTTCCATTTCCTCCACTGGATCCTCGAGACCCTCATTTT

CCTCGTCGATCTTATCGTCCCGACTTCTTCCCTTCTCCTCATCTGTCCATGCGAGGACCT

CCTCCTCTTCCTCATGGAGTTTTTCCCAGAATTCCGCTGCCTCCTCACTTGGCATTTCCA

CCAACAAGATCTCAGTCTGATCACGTTTCACCTGAACCTCCATCCAGACCTTCACCTCCA

GGCAGTGAGCAGCCTTCATCTGCAA

>Unigene000225

TATGTGTGTGTGTGTGTGTGTAGTATGGTGTCTGAGTTTCCCCACTTTCCCAGAAGTCCA

GCTGATTCTCCCAGAATCTCAGGCTCCTCGTCATCCAGTCCTGCATCTGCAAGAGACTCC

TCCCCCAAAGTGGAAACTGGACCCGCCCCCAACCTGGTCTCATCCCCTGGACCCGCCCAC

AGCATCACAGAGCGACACTTCCCCCCGGACGTGCTGCAGCAGCGGTTAAACGCAGAGCTG

AACTTCCTGGATGCAGTGGAGGAGTCAGTGAGACAGCTGAGTGACGTGGAGAGAGTCAGA

GCTGTCTCACTCGCTCAGCAGGAAAGCGTCTCACTCGCACAGATCATCAAGGCTCAGCAG

CAGAGGCAGGAGCGAGAGCTGCAACTACTGAAATTGAAAGCAGAACAGGAAACGCTGGAG

ACTCAGAGACACGTCCAGGAGAGCAGAGAGAGAGCAGCACAGGCCCATGTGGAGCTGTGT

ATGAATCTGGTTCAGTCTCATCAGAAGGCTCTCATGGAGTTGCAGGAGAACAGCAGCAAA

ATAAACCACCAACACACACAGACACCACAACACCTCCGAGAGATGACTGAGATGAGTCGT

CCTCAGCTGCCCAGTGTTTCTTCTGTCACACCTGTAAGGCAGCCACACCCACAAACGGAC

AGTAGTAGGAGTGAAGTCACAACCGAGAAAACACCAACAGCACACACTCCACAGAGCATC

ACAGCTGAGTTGGAGTCCTTCAGTTTCACGCAAAGCAACGGCGGTGGTGTGGACAGGAGC

GGCTCGGTGGAGGAGGAAGTGAACACCGCTGCTGACGACTCTCTCCAAACTGACAAAGAC

AGCGTCTCGATCAACTACTCTTCAAAGTTTGATGAGTCTGTGACGGAGGATGAGTTGGAA

AAGTCCTTTCGCTCTGTGCTTCCGTCTGAGTCTCACTGGCGGGGATCCGTGGAAAGGAAA

CGGCAGTCGGACGAGGAACCTCACGCTGACAAAATATCTACTAAGGACGGCAGCGTCCCT

GTCTTCTCGGCGGATCAGTACAGTTTCTCGGAGTTCACCATGGCGATGGTGAAGCAGTGC

ATGCAGGAAGAGGAAGTTCGCTCTCAGCATCAGAGCTCCCTGCTGCGACTCCGACACCGA

GCACTGAAGGAGAAAACCCGAGCTGAGCTAGAATGGCTCGAGCTCCAAAAAAGGCATCTG

CGTGATAAAGGAGAGGATGATAAAATGCCTCCAATACGCAAGAAACAACGCGGCCTGCTG

CTTAAACTACAACAGGAACAGGCAGAGATCAGAAGGCTGCAGGAGGTGAACCGTGCAGCA

CGAAGGGAGAGGAAACTGTTGCTAAAACAGCAAGAGGAGATCGAGAGGATCCAACACACC

ACACAGAAACTGAGAGAGAAACTCCGGAATGCTGAAGACACAAACCAGAGGTCTCCTGTG

TCAGGTGTGAGCGAGGATTTGGCCATTCCCACTAAGGTGGACATAGAGTTCCGTAGCCCC

TCCCCTTTGTCTGTGTCAGGGAGTGAGACCAGCAGTGTCATGAAGAAGCTGAAGAAGATG

AACTATCACACAGATGAGCGATTTCTTACCCAGCGTGAGCTGCGATTGGTACATTGGCGT

CAGCAGGTGGAGGCGGAGCTGAGACGTTCAGATGGGGCGGAGCCTCAGCCGAAGGGACGA

GCACTGAACGCCGAACGTAGTACACAGCAGAAACACGGCACAGACGAGCTGAAGAGAGAC

AGACAGGAAGAGAGAAAGACAGGACACGAAGTGCGCAGCGATTGTGACTCATCATCGGCA

CTGTCCGTCTCCAGCATACACACAGTGCAAGAGTGTGACAACACACACAGCCGTGAGTTC

TCCTTGGTTCCTGCAAGTTCCAGCAGTAAAGTGACATCACACAGCAGCACCAGGAAGAGG

GACCTCCCTTTATCAGCTCCTACATCTGAGGCGGCATCGGATCAGAGTGACATTGAGGGG

CGTGTCCTGGCTCTGAGGGAGGAGCTTCGGCGGCGGAAAGCTGAAGTCCAGCTTCTAAAG

AGGGAGCAGAGACTGAGACACAAAGAACGACTGAAAGCTCAGGAGGCCAGTCTGCTCAAA

CAGCTAGAGTCTTGCAACAACTTCATCCAGAAGACTAAAGCCGAGCTGAGTAATGCAGAT

GTTGCCACCACTTCAACCAAACCTCAGATCAAAACTCCAACATCAGACTCGCACAAATCC

GGAACCGAAAGCTTCACCACAGACAGCTCTGGAACAGAGAAGAGTAGAGAAGAGAAGAGT

CACGAGTCTGAATCAGAGAGATCGAACAGGATACACACACAGTCTCCGGCGCTCAGCGAT

GAAGATCCTTCCACCGTGACCCCGACTCCGGTTTTGGGAAGCCCCAACCTCCTGAGTCCT

CGTGAACTCCAGCATGTTGAGCAAACCCCACAACCCGAAAGCCCCGGCAGACCCGGGAAG

GACGCCTCGACCGATCTGAGCACAGCCTCCAGCCCGGGGTCGGATGTCATAGAGGAGCTG

GAATCTCTAAGGTCGGATCCAACCCGCTCTGAACACTCGCATCCGCTGCTTAATTTAAAA

CTCCAAGACTCCGATGGGAATTCCAGCAAAAATCAACCGGAAATTGAGGTAAAATTCAGC

CATGTAGAAGAATTTACAAATTCCGAAGCGGTTAAATCCAGTTCCATAATAGTAACTAAA

CTAGAACCAGAAGAACCCAAACTTCCAGAGAGGTCCTTAACCTTAGACGTCTCAGACCTT

GAAGACAAACCACCTGAGTCGCTGTCTGAACACAGCTACAATGATGATTTTGAATCAGCA

ATTAAGGAGGAAACTGGATCTCACTCACTGTCTTCTGAAGATCCCGAAGAGGTATCCTAC

AAATTCTCCTTCAGCAGCGAAGATGAGATTGAGGAGAAGATTAGTCTCCAGTCAGGAAAT

ACCAGTAGGACTTTTGAGCAGGAAAGACCTCCTGATCTCCACTTCTTGGGGAGGTGTACA

GCAGAAAGCATCCATGAAGATGTTCCCAGTCTTCCGAAGTCTTCGAAGGATGAAATGATG

GATGAAATGCCTGATTACGTAATTGGTGAGCGTGTCCTGGTTAGCAACCTTCAGCCAGGT

ATTCTAAGGTTCAAGGGACAGACTAGCTTTGCAGATGGATTCTGGGGCGGAGTGGAGCTG

GACGCATCCGAAGGAAGCAACGACGGCACTTACAACGGTATTGTTTATTTCCAGTGCAAA

GAGAAACACGGGATATTTGCTCCTCCGGGTAAGATCTCTCACCTACCTGAGACACATGAA

GACCATACAAGAAGCTCAGAGGAGAAAGATTCCTCGTTTAAAGATCAAACATGCAGGAAG

ACGAACAAGTCTGAAAGCTCACTTCAGAATAAAAGAGTGGAATCGGATCCGCCTTCTGAG

ACGGAGGAACTGTCCGGAAAACCTGTGGTTCTTGATGCATCCGTTACGAAAGACTTGATG

ATTGATCACGTTGCTCTCAATGGTGAGACTTTTGAAAATCTTAATGGAGAAAAAAATCTT

CTGGCACTAGAGAGCTTTAAAGACATTGTCCTGGAATTTAAAAACGTCTCAAAACATGAG

GAAGGTAGAGATCAAACTCCAGATATTCTGGACCTGCTGATCCGTGATGAGTCGTCCACG

GCAACCGAGGACCTACAGAAATCTTCAGTAGTCTCTCCTGAAAAGACAAACGAGGACAAC

GTGGATGGATCGGTGGTGACTGAACACAAACCCCTCACGGCTCTGGCAGATACGCTGATA

GAACGCTTCATGAGCGATGCCCTGAAACAGTTTCAGCAGATCAAAAAAAACAAGGAGCAA

AAAATCTTAGCTGCTAATCAACTCGTCTTCTTCGGTGAGGACGAGGAACCCAGTGAAAAT

GATGTGTTCCGGTTCGGAACTTCAGCCAGCTGCAGAGTGGACTTCGACAAGGACCAGGAA

GAGGTGTCTTCTCCTGAGATGTGCAACCGATTGGAGAGCCCTGTTTTGGGTCAAAAGGAA

CTGGTCCAGCGTCTGGAGGAACTGGAGCTGAACCGCGAGTTCCTACATGTGCTGGGAGAC

GAGCCGGACTGGTTTGACGAGGACTTTGGCCTCAGCTCTCGCAGACAAACTCAGAACAAG

AACAAACTCCGGCAGGACGAACTGACGCCCACGTCTGCGGCCAAACTCCCGCACCAGAGG

CAGCAGGATGAGGCGGTGGCACCGGCGGCCATGTTGGTCCCGCACAAAGCCGAGGAAGTG

GAGAAGTTGGTGAGTGCCGCCCTGCTGGAGATCTGGATGAGCTGCGGGATGGGGCGTGGT

AAAGCAAGTCTGACGGGCGTGGCCAAACCTCAGGCGTCGGAATCGCTGCTGGGGGATCAG

AGCGGCGAGGCGTGTGTACGCAGTTACAAACAGGCAGTGTTTGACCTGGTATGGGAGCTC

ATGCAGGAAATCTACGCTGAGGATCCCAACACCTACAGACCACTGTGGATCAAACCACAC

CGGATTCATTCACCCTACATCCACAGAATCGACAACCCCACTGACCTCCATAAAGTCCAG

GCGTTTGTCACCGGTGAGGTTCTAAAGCTGTACAATTTAAATAAAGAGCAGAACCACAGA

ACCGACTGGCAGAAGATGCTGAAGTTCGGCCGTAAGAAGAGAGACAAAGTCGACCACATT

CTGGTGCAGGAACTCCATGAAGAAGAGGCTCTGTGGGTGAACTATGATGAAGACGAGCTG

TTGGTGAAGATGCAGTTAGCAGATGGGATCCTGGAGGCTCTGCTTAAGGACACTGTGGAC

GTCCTCAATAAAATCCACCAGAGTAGATCCTCACGGGCTCTGACCCCATCCTCATCCACA

CACCTGCTCTTACAGCCCTGACACACTGCGCCATCGTTCAGGACAGATCCCGGAATAGCC

TAAACCTCGAGACGGTCTGTGTCCCGAATTTGCTGCTCAGATCTCCGGTCCCTCCCTTTA

AAACCCCACCCTGTGTTTGGGACAGAACCGGACCCCCTCCAGCGTCTGTACCACATTTGC

CTTCGAGCTGGAGTTTTAATTTATTTGTAAAGAGTAAACAGTTTTTAAAACTATGTGAAA

ATGTGTAAAGAGATGATTTAAGCTCACATGAACCCATCTAAGCACACGTGGAACCTACAG

AACCCAGTGGAACCTGCAGGAGCCTAAAGGAACCCAGAAACAGAACGAGACGGTTTAGAG

AGGATAAATCAATATAAACTGTGTATTTTAAAGAGATTTAGAAGAGCAACAATCAGAAAC

TAAAGTGAACGAGTGCATGATGAGAGATTAGTGCTTTATTTACCCTCCATATTAAGTGCC

TGACGGTGTAGGCTCCCTCTCTAGGGGAGAACCGGTTTCAGCTTCCTCTCGTGTGTCGAG

CCGAGTCGCTTTTATACTCTGGCGTTTCTGCTTTCCAGCTGGACCCCTGATTCTGCTGCT

TCATTAACTCTTTCCTACACTCAGTAGGGAACCTCCTCAGAGGGCTGCGTCCTAAATTTT

CCACACTTCCTTAAAGACCGCCCCC

>Unigene000226

GGCAGGTTTCTCAGCTTCAAGAAAGGCGACATGATCGATGTTTACCACAAACTGTCGAGA

CTGAGGAGCGAGCTGTGGGCTGGAAGTAAAGATAAAGATTTCGGTTATTTTTCCAGAGAT

GCTGTAAAACTGGATGAAGTGTTTGTTGATGACAAAAAGGAAATAGAGATGCAAACTCAG

AAAACAGATTTCCTGTGTATGGATGCGTTTGGGACTATAATCTCAAGTGAGGAAGACTTC

GAAGATCACGAGTTTGGAGATCAAACTAGCAAGTCTGAAGATTTAGGTAACAGTGATTTT

GATGATCGAGACTCCAAATCATCTAAACCGAATGAGAATGTCAAAGTGAAGCTCATTGAT

AATAATAAAGACAGAGGAAAGAAAGCAGAACAAAGCCGAGCTTCCTGGATCAGAGCCGCT

GTTAGCGGATGGTTTGCTAACGATGCTGGAGATTCGAAGGATAAAGAGGAGAACTCTAAG

AGCAGGAAACTGGCTTTAAACATGAAGGAACTAAGCGAAAAGTGTAGACGAACGAAAGCT

TCGGCCCAGACTGAAGATGAATTGAGCAGCGCTCTGGAATTAAAGGAGGAGGAAACAGAG

AATGATGAGGAATCAACCAGTGAGCTAGGAAATAGAATAGAAAGTGTTAGGGGTTTTAAA

GCAGGAGAAGACACATCATCTTCTGAAACCATAAAACAGAGTCAGAATGAAAATATGCAA

ATTCTAAGCACAGAACAGGAAGTAGACAGTCTTAAAGAAAAAGCCTCGAAAGTGATGATC

ACTGAAGATGAGAAGAGAAACGCAGGATGGTACGGCAATGTCTTCAACACCATCACTAAA

CTCTATGGAGATCCTTCTTATAGAAGCGAAGCCAGTAAAAAGGAGGGAAAAGTGAGATCC

AGCAACGAAGACACTGATGTGCTAACAGTTCAAACAATTTCCAAAAATGACAAAAATCCT

GTTGACGATGAGCAGAAACAGGAAGTAAGCAGTCGGGATGACCGGAGCAAAAACAGGAAG

CTCGGAGAAGGATGGTACGGAAGCGTCTACAACAGATTAACAGAATTTTATGGAGATACT

CCTGATGAAAGCACTGCTGATGGTGATGAAGGTGGAGAAACATCTAAGAGTGAGGATGTA

GATCCAAACCTGGAAAATAAACTGGAAACTAGTAGGGAGAACATGAATGAGGATAATAAT

GCAGAATGGTACAGCAGTATCTACAGCAGAATCAGCAATGTATATGGAGATCCATCTGAC

AAAAGAGATGTTCCTGATGTCATGGGAGAGCGCCAGAGCCAAACACAGAGTGAAAGCACC

AAAGAAACATCACTATCAACCACATTGTTGATGAATAGCCTGTCGACTGGTAGGAACGGC

CTGGCAGAAGCAGGAAGTAATGATGAAACAAAAACAAGCGAGTCTGTAAGATCAAAGCCA

AACTTTTCGCCTGATCAGAGCGAAACCAAAAAAACCCAAAAAAACACTGAGAACCTGAGA

GACTCGTCACATGTGTTTTCTATCAAACAGTTTTTGATGTTGATTACTGGCCCGAAAACA

CCTTCTAAACCTTTTAAAGAAGAGAAGACACCTGATAAAGGTGAGGAGGATCCGATCAAT

GAGGATGATGAGCTTAGTGTAAGGCTGACCGAGATTAAAGTAGAAGAGGAGGAAAAGAAT

GAGAAAGATGTTTGTGAGGTAAAGGTATCCGAGGCTCAAACACATGCTGTGGAAGGTTCT

TTAGACTGTAATAAACAGGATACTTGTGGGAATGTTGTAAATAATCATGACGTCCTTGTG

GAGAGAGATGAGGTCAGTGGTCAGTCTGATTCCACCAGCCAGATGACACAGGAACCAGGC

TGGGCCAGGTTATCTGAACACATCTCAGGCGAGCTGCAGTCTGAGAGTCAGACCAGGCCT

GAGCCATGGCTGCAGCTCACAGAGAGCAACCCTGAGACTGTAGGTGAATCTGAAGAAACT

CAGGATGAATATGGAGGAGCAGAAACAGAGGGAGAAAAGACAGAGAGGAGAAAAGACAGG

GAGCAGAGCAACCATACAGGTTCCAACACTAAGAAACACGTGGTAGATGATTTTCAGGCA

GAGTATAGTTTAGAGAACCAAAGTGTGAACGTTTCTTTAATGAAGGATGATGAAGAACAA

GATGGTGCTGATGTTACCGAAACAGAGCTTTATGGAAATAAAGTAGGTGTTGATGTTACT

CCAGTGGAGTCTGCTGGCGAAATTAAGGACACTGATGCCACTAGCATGGAAACTAAAGAA

GAGAAGAAAAATTTAGACGTTCCTCAGATGGAGTCCGCTGGAAGAAACACAAGCGGTGAG

GTTATCCGAGTAAAGAACGCTCGAGGACAAGACAGAGTAAAAGATCCTCAAAATAAAACT

GACAAACAAAAAGACAGTGTTGATGTTATTCAGAAAAAGAATGAAGGAGAAGCAGGAGGT

GATGTCACTCAAGTAAAATGTAATGGAGAAAAAGAAGGTGAAGATATTATTCAGAAAGAA

GAGTTAAGTTTACATAATGGTCAAACTCAAAACTCTCACAGCATCTTCTTAACCAAACCC

AATGAGAAGATCGATCCAGACAAATCCAGAGAGTCTGAGACACCCAAAAGTGCTGTGTTA

CAGTGGTCTCAATCAACACACGCCTCTGGAGCAGATGGTCCCATTGCTAATACGTCTGAT

GGAGCTCAAACTCATCATGATGGCAAAAAAACCAAAGATAAAATGATGGATGTTAATCAC

CACAAACGTTTGGCTCAGATGCAGAAACCTACATATCCTAACCTCCAAACTCATCTCAAT

GAGAAGGACATACAGGTTCTTTCAAGGTTTTTCAGTAAAGAAACGTTGACACGAATGGAT

TTCATCATCGAACACTCTGAAAATATTCACAGTAAAGAGCTTGCTGAGGTTCATGACTTT

AAACAAATGCTGGAACATCTTTTGAAAACCAAAAATGAAGAAATCAAGCCGTCTCTACAA

TTCATACACACTGTAGTAGCCATGCTTCAAAAGACGTTTAGACCCGTGATAGCTGATGGC

AGTGTAGATAAACACCCAGATCCAGGTCAGACGGAGTGTTTCACTGAATTGTGTGTGAGT

ACAACTGAGAACACACCCATCTCTGAGAAAGACACCAGTAAAAACTACCACATGGAGACC

AGCTCCAATACAGACGTCTCGTTCTCTGATCACGAAGAGGTTGTCTCTGACAGGATGAGA

ACATCACACACCCTCCAGACTGAACGGAAACCTGATCCTCGTCTGGACACAGAGATGATC

ACTGAAGAGTCTCTGAAACCCGAGGAATCCAGAAACGGAGCTTCTCTTTCGTACACAGCT

CTCGTTGCTGAATTAGTGGGTGGTGTGTTCATGTACACACTGAAGGAGAGTTTACACACG

ACTCACACCATCCGTCAGGTTGTGATGTCACTTCCTGATGATATCCGCCCGGGACCGGAT

CTTTACGGTGTGCCATGGGAAGCCGTGCTCTTCACCGCCACGTTGGGTTTCTTTACCTTC

CTGTTGTTCACCTGCAGATTCTTCCAGGCAATTAGGAGCCGGTTATACTGCAGTAAAGAA

CAGAGGATGGGGCAAAAAGTGGCAGAGCTTCTGGAGGAGAAGTGTAAAGTTCTGCAGACA

CTCAGCGAGTGTGAACACAAGTTTAAGAAGCTGCAGGCTGCGCTACAGAATGGTGGACTA

TCGGCTCAGGCTTCAGAGAGAAAGGATTTAGAGGTTTTGTCGAAGCAGCTGGAGAAGGTC

AATGCGCTGATGCAGAGTGATCTGGAGAAAATGCAGAAGGAACTGGAGACTCAGGATCAT

CTCAGGAAACAACAGGAGGAGCAGATCGTAAATCTGGCGGAGATATTAAAGAAGCTTGAG

CAGGAGGCCATGGAGCGCAAATCTCAACTAGAGCAGGATAAAACGACTCTGAAAATCCAT

CAGATGAACACTGAGAGACTGCAGAAGAAACTCCAGCAGGATAAAGAGGAGAACAGCATG

CTACAGGAGAGCAAAGCACAGCTGGAGCAGGAGGCAGAGGGATGGAACGAGCGACTAAGT

GAACTGGAGGAAGAGAAGAAGATGTGTGAGACGTCCCACAACAGAATGATGGAAAACTGC

GTCAATAAAGACGAGAGCATAAAGTCTCTTACAGAGTGTCTGTTAAAGGTGAGAGACTGG

GACTCGGAGGAGGAGAGCACTGTGAATGATGCGAGTGGATCAGAAAAAAATTCTCTAGAC

TTCAGCCAGAAACAGAAAGTACAGAAACTCATTAATGCAGCCAAGATGAGTGCAGATTTG

AAGGCAATGGAGGAGGACAAAAACCGAATGTTTGCCAAACTGTCAGATGAGATTAAAGCC

AAAGAAGATCTACAAGGAGGTTTGGATCAGCTGCAGCGTGAGAAAGAGTCGGTCCAGTCC

GAGAGCTCAGTGTATTCCAGTGAAATCCAGAAACTCCAGCACAAACTGCAGATCATGTCT

GAGATGTACCAGGAGAAAGAACTCAAACTGCACAGGATGTTAACAGTGGAGGAGAAGGAG

CGCTCGCAGAAAGAGGAACAGCTCGACAAAGCTGGGAAGAAAATCAGTCTGGCTACAGAC

GAGCTCAACTCCTACAGACAACGAGCCAAAGAGCTGGAGGAGGAGCTGGAAAAAACTAAT

CAGGCCTACAGGAACCAGATTGCGTCACATGAGAAAAAGGCTCATGATAACTGGTTGGCT

GCTCGAGCTGCTGATCGTGACCTGGCTGATGTAAAGAGAGAAAACTCAGTGCTGCGTCAG

AGGTTAACAGACTGGCAGTTTAAACTGGAGCTGATGGAGAAAAATCCTCAGGCTGGTGTG

CTGCACTTCAGAGGTGAACACCAGAAAAATCTCTGCAGTCGTCCCCCCTCTGAAACCCGT

CCCTTCCTGTCTCCACCCACGCTGATGGACGGCCCTCCCAGAGTCTCTCCACAGTTTCCC

ATGATGCCCGGCGGCAGAGCGCCCACTGGTGGTGTGGAAGGTGACAGGAGTGTTTCGTAT

TCTGAAGGGGGCTCCCCTACGTGGGAGAGAGAGCGAGAGCGCAGGGGTTTCCCCCCTCCA

GGTCCAGATCCAGGGTTGGTGTACAGAAGACCTCTATCGGCACCGTTCCTGCGAGCGCCA

CTTCCTCCTGAAGCTTACTTCAACGATAAATCAGACTCGGCGATCGGAGAAGAAGCTGGA

TTTAATGAAAACGGGATCCGAGATAACGGACGCTTAATGCCTGGTCCTGATACAAGGGCA

GGGCCTTTTCCTCTTCCATTTCCTCCACTGGATCCTCGAGACCCTCATTTTCCTCGTCGA

TCTTATCGTCCCGACTTCTTCCCTTCTCCTCATCTGTCCATGCGAGGACCTCCTCCTCTT

CCTCATGGAGTTTTTCCCAGAATTCCGCTGCCTCCTCACTTGGCATTTCCACCAACAAGA

TCTCAGTCTGATCACGTTTCACCTGAACCTCCATCCAGACCTTCACCTCCAGGCAGTGAG

CAGCCTTCATCTGCAA

>Unigene000227

AACCTGAGATCGGAAGAGCGTCAGGCTACCAAAATACTTTTTTATTGCAATCCACTTTGT

ATGGGGAGCATCAGTGATTTTACAAAATATTTGTACAAGATTTGTACTGTATTTTTTCTT

TAACTGACAAATTCTAGCCAGAACTAGAACTAGACTGGGAAGGAATAGAAGTCATTGTTC

TAGGATTAGGCGGAACAGGATTTGCCCCTAGTTTTTTTCTTGGGACGGGATTTATTTTGT

GGGTGTGGGACGGGAGAGGATCGAAAATTCACACCCATGTGACTCTCTGTTGAGTGTGTA

TGTGTGTATGTGTGTGTGTGTGTGTGTGTGTGTGTGTATACACTGAAGATTTTTTCAACA

GCAACTTGACTTTTTATTTTGTGGCTGTGGCTGTAATGTTGTATGTTTGTCCTCTTTCCT

CATTCTCTGTTTCATCAGCAACCATGCTAAAGTCTTTAGAGATTCTGACAGATTTTCACC

AGTTGCAGCACTGCACTCCATGAAATGAATGTTATACTCGTTTGATAGATGCTCCCCATC

TTGTAGCAGGACTTCTCGATGAGCACAGTCATTTTTATTGCCAAGTAACATCAAGATGAC

ATCAGGAGCAGCATTTTGCTGAACCTGGGATATCCAGTCACGAACAGCATGAAAACTCTG

AGCTGATGTTATATCATACATCAACAGAAGCCCATGGGCTTTGTGGAAGACCTGCCTGGT

GATGCTGTGGTACCTCTCCTGACCTGCAGTGTCCCACACATACAGTTTAACTGTTCTATT

TCCAAATGTAACAGTATGAACGAAGGTGTCAACACCAATGGTAGCACTGTAATCTTGGGT

GAAATGTCCACTCTGTAATCGTTTTATAAAAGACGTCTTGCCCACATTGCTGTTTCCCAC

CATCACAACATTATATAGTGCTTGTCCAAAATCTGGAAATTGCTCAAAGTCGATTATCTG

TCTTTCTCCAGTGCTGCCAGCAGATGACATAGTTTTTCTTATGCTATCCAGAGAAACATT

GCTACCTTCAGTAAAATTCTTAACCTCTTCAGTTCCTGGTCTTGGCTCAGAGACTTCAGG

AGAGATTAGTGTGCTTTTTGGTTCTAAGATGTAACCTGTGTCAGAGACCTTTAATTCACC

CTCTGTCACTTGAATATCCTTTTCCCTCTTATATTCATCCAAACCATCTTTATTTGCAAC

TGACCCACTGCTTTCCTTGGTTGTGCATCTGGACCCAAACTTTTGCCTACCTTCTGAAAG

TTGTTCAGTGTCTTGTGCACTGCTCTCATTTATTTGAGACAGTTCAACACATTCTTCCAT

TTCTTTTGTGCTTTCTGCTATAACAGTATCTTCAACTGTCCTGGCATCTATTGTAGGTTC

ATCATTTGTAACATTTGCATTCTTGTTTTTAGACATCTTGCGACTTGACCCCATTTTTCT

CCTTCGTGCCTGGGAGCTTGGCTCTATATGAATTTGAGAGTTTTGGGATGATAGGTGCTG

AAGAGACCCCTCAGAAATTGACTCTGGCATTTCTAAGATGTCAGGGTTAACAGCTGGTGT

ATTTACCATACTTTTATTCGGTTGAAGCTCTATTTCATTATGAAGGAAGTCCACTGACTG

ATCAAGTTTATGGTGAATATGTGGTTCATTCTCTTGTCTGCTTGTTGTTGTGGGGCAAAA

GTTTGATTCTGGGTTACAAATGTCTTCTTTCGCTATTAATTGTAAGCATTCTAAATTATT

TTCTCCAGCAAAATCTGTAGTTTTTTGCCATGCTTCCACTTCATTCTGTTTTAAAGAAGT

TGGACGCATTTTTTCATCTTCATCTTCTATATTTAGTGTCTCAAAATGCATTTGTTTAGA

TTGATGAGAAATGTCGGCACTTAAGTTTAATGTCTCTTTTTCTGCACCGAATGTCTCTGA

GTTTGGTGTACTTTCTTCTGTTGTTTGTTCCTGGATGTTTTCTTTTTCTTTCATCACTCT

TAGACTTTTTCTAGTTGAACCTATCTTTTTCCTCTTCCCATGAGTACTATCCCCCAAATT

CTTTGTTGCCAAATCACCTGGGGCTATTTTTAATTCCTGTATGTGAACAACTGGACATGT

TATTTCACATTGATCTGGTTTCGATTCTGTATTTTTTGGATTTTCTTGCAGTTTCTCAGT

CTTTGTATGATACTGTTCCAATACAACTGGCATGTCAAATTTAGATTCATCTATGCTATG

TGTTTTTTCTGTATTTTCCAGATCCTTCCATTCTTCTTCCACGATTTCAGCAGTCGTTTC

GTGTCTACCTTGGAGTCTCCTTGTTGATCCAAATTTCCTTCCTTTCTTAGTTTGAGTTTG

CGTATGAATTTCTGCATTCCGATTGTCTACATTTTCATATGTGGAATGAATATTAAAATC

ATTGACACATTCATTATTCAAACTCTTGTCACTGGGCATTTTGACATGTTCTTTAAATGT

ATCATCTTGTGTTTTTGTGATGACTGGTGCCATTTTTAACTTATTTAAGTGTAAGGGCAT

GTCATTCTTCTCCTCATTCATTTCAGCTTTTTCATTTTGCTCCATAATTTTTGTCAAACC

TTCTGCATTAATTGCTGTGTCCAAAACCACTTTGCGAGACTGGTCACTTTCGACTGATAA

TATTCTAGGTGATGAATCATTTTCTGTTCCTGTTTCAACTTCTGTGGTAATAATTTGTTG

TTTTGTTATTGTCACTGATGAGTTTTGGATGTCAGGTGTCACCTCACTTGTAGTTATCTG

TTTTTCAGTGTGAAGGTTTAAAGGAGTAGCTTGGGGTATGATTTGATGTTTATTTTCTTC

GATATTTTCCATATCTTTTAATTCTTCTTCCTCTGCTTCAGCAGTTAACTGGTGTTTTCC

ATAGGGTCTGCGTGTTGATCCAAATTTCTTCTTTTTTTTAGATTGATTTTTTGGTTGAAT

ATCTGCATTTATATTGTCCACATGCTCTGTTGCAGGTGAAGGATGCTCAATTGTAGTTAA

ACCATGATCAGTATTTTGAGAAGATAAGTCACAAAGTACATGTTGTTGTGATTGTACAGT

TTTGATGTCCTTTTGGGTAAAGCTTTCTGTAAGACCAGCATTTATCTCTTCCTTTATGTC

CTTTATGTCTTCTTTGATAACCCCAATCATGGTACTATTTTGTTCCGTGTTTTGTTCCTG

TATGTTTTCATTGTCTTTAATCACTTCAAATTTGTTTTTCTCGTCACTACCAGGAATCTT

CCCTTTAAGTCTCTTTCGACTTAAATCTGTGTTCCTCCTTTTCTCAGAGCTGCCAAGTTC

CACATTCTCTTTTATCGAATCAACCTGAGAGGATTTTAATTCTGCTATCTGAATACATGT

GCTTGTGCTTGCAAATTGATCTGGTTCGGATTCCCTGTTAGTTACATTCTCTAAATGTTC

TTCAGTTATTTTTTGGTTTTGATTTGATAAAACTGTATGGAAATTTTCTGTGTTTTTTTG

CATGACTAATGTGACCTGTGTTTCTGGAATGAGCCCCACTAGACCACATGTAGAATAAAC

ATTCACTTTATTTGCACTTTCATTTTCAGTAATGACTGGCACCGTTACTTGTTTATTTAA

ATGCATGAACTCAGAATCCTTTTCCGTCTGTACCTTTGGTCTTTCATTCTGCTCTATAAT

GTCAGTCAGTCCTTTTGCATTGCTCACCTCATAAGAGACCACAGGGTAAATCGGATCAGC

TTTAACTGGTGATATAGCAGGTGGTATATCAATCTCTCCAGTCCTGGTTTGTTTTTGTTC

ACTCGTTACTGGTAAGCCATAGATGTCAAGTGCCACTTCACTCATTTTACTCTGTGGTTT

ATTGAGCAGGTTTGACGTAGCAGATTGAGTTATGATTCCATGTCTGAGCTCGTCTGTATT

TTCCAGATCTTTCCATCTAATTTCCTGATTTTCTCCTTTCTCTCTTTCAGCAAGTGGCAG

AGTACTTCTTTTTACGTTCTCTGTACTTTTTTCATCATCTTCCTTGAAATCAGGATGATC

TGTTGCAAGTAATAAATGTGATGAAAACTTTTGATTGGACAGTTCAGAATCTGTTAAATT

TTCAGCAGAAGTGATGGGGTGATCACTATTTATTGTACAACTAAATTCCAACGATTTTGT

ATGTTCTAAGTTAAGATCTGAGTAAATGTTTTGATCTTCATTTCGTACTGCACTATCGCT

CAATTTTGTTTCGCCTAGCATTTTGAGGTTTTCAGTATCAACCAGATGTGCAGCTACATC

TGAAAATGCTACATCTGTAGTATTTAATACTGCACCAATATTTTCTACCAATAATGTTTC

CTCGATCTGTCTGCCAAGTACACGACATTCTTGATTTTTCTTCACAAGTTCTGTTGAGTT

GTACCAAGCTGTTTCATGTTGCCCTTGTGAAGTGCTTATGTTGTCGTTTGTAACCAAAGC

TTTACTCACATTTTCTTCATTTTTTTGCTTCAGGTTCCATGATATTTAGGGATCCATCAG

CCGTGGTTTGCTCCTGATCAGGTATAGTTATTGGACAATACACCTGCATGTCTTGAGTTT

CAGGAATTTGCTCCACTAAATCACTTGTAGGATAAGCATGAACTATACTAGCACTGGTGG

GACTGTCAATATAGTGGTCATTGACTACTATGGATGCCTGGACTGTACATGCATAATCTT

GTGTATTTGATGCCATTTCAAGCTCATCTATAAACGTGGACATGCCAGCCTTATTATCAT

CCACGTCTATAAAATCCCCAATAGTAAAACTTTCTGTGGAACCTGCATTAATGTCTTCCT

TTATGTCATTTTTCTCCTTAATAACCTCAGTTTTTATATCTGGAGACTCTGATGTACCTT

TGATTGCTTTGTCAAGCATCTGATCTTCTGCTTCTAATTCAGTTGGAAATTGTTGAAGGT

TGCATGTCTCAGTTTTAGCTTCTGTCTCTTTATGCAGTGTTTCATCAGGTATTAATTGAG

ATTCTGCAATAAAATTTTCAGATTGCATGTCATTGTTTTCTACCTCATTCTCTGTCTCTA

TGACTCTACATTTTTTTCCTTCATCATAGTGCATTACCACTTCCTTCTGAAATATTTCCT

CATCTGCAGTGCCTTTACCTGTCAAACATATAGCCTCAGGAAGATCATTTTTTTCTTTTC

TGTAACAGTTAATTTCACTGTCATCCTGAAATTTCAATTCATTTGAAATAACTTGTTCTG

ATGTTTCTTCCTCCAGTTTTAGAGGTGAAATGATGTTTGTATCCGACTCTATTGGTAGGT

TTAGGTTTGTTGCAGTCACCTCAGCTAAATCATTGTCTGTTCTATCCACAGCTACACAAA

ACTTTTTACGCTTGTCATTATCCTCTTCTTCATACGTATAAATGTTGTCTTCAGTTAATT

GTACAGTTAGCTCCTCGTCCATATGCTCAATGCTGTGGTCATATTTTGTATTTGCTTTGG

TACCAACATTTTCCCATATAACTGATGCTACTTCACTGTCTTTTGTTCCTTGCACGACGC

TCTTCCGATCTA

>Unigene000228

AAATAAAGGGCGGAGGGAGAAAAACTTTACGACGGGTTTTTTTTTTTTAACTGTTAAACA

GGAAGGAAACTGTCGAAATGCAAACCTGCAATCTCTAAAATAGGCCTCATTTAGGCTGGG

AAATTAACAAGCGTTATTAATATAAGTGGAGGTAATCGCTCGATCTGTACAGTGCGTTTC

ATTTTTAACTTATTATTCTTTGAAGCTGGTTTGAACAAACACAGCTGAAAAGGAAACGCG

CGTAAGCCGTTTACACGCGCGTCTCTTCCGCACCGGAAACTTCGACATTTTTCTTGCACA

CCACCAACTGAATTTGGAAATACTAAAGGACGACCTCTTATCGCTTTAAACTAACTATAC

AGTAAAAAAGGACCGATGTCTTATGGCTTCACACGGGCAAGGACGCAAAAGAACTCCGAA

TAGAAACGGAATAACTCCAGAAGAAGATGCGTTAAATGTAATCGCACGAGAGGCTGAGGC

CCAACTAACAGCTAAGAGGGTTGCCAGGGCAGAGGCTCGTGAGATACGCATGAAGGAGCT

GGAAAGACAACAAAAAGAGAAATACAATGGACTGGAGAACCTGGACAACAAATGCTGGGA

TATTGAGCAGTGGATGGAGGACAGTGAGCAGTATACACACCACCCCCAGCAACATGTTTT

GGTGTTGGGAAATGAAGAAGGTACATCAGCAGGGAGCAAGAGAAGTGTGCAGGGCTCCTC

TCGAGGACCGACTCTATCCACTGAGACTTCCCTGAGTGGGGCGTCCTCAAGTAAAGGAAG

TGGTGGTTCATCTGTCACTGCTGATACAGAAACATCCTTAAGGGATATTAAAGAGATTCA

TGAACTAAAAGACCAGATCCGTGATGTGGAGATCAATTACATGCAGATCCTCAAAGTAAC

AAAGGATTCTTTGTCAGAGATGGAAATGAAATATCGCAAGGCTATGGTGTCCAATGCTCA

GTTGGATAATGAAAAATCTAATCTGGTGTATGAGATGGACAGTTTAAAAGACTCACTAAT

GGAGTTGGAAGAGCTTCTGTCTGAAACTCGTCGCGAGTGTGAGAAAAAGAATAATGATCT

GGAACGAGAGAAGCATGCTCACAGTATACTGCATTTTCAGTTCAGTAATCGAAAAGAAAC

ACTAAAGCAAAGTGAAGAGCTGCTGAGTGAGATCCGACAATTACATCTCAAACAAGAAGG

GTTTACTAGAGAGATTTCTGACCTGCAGGAAACTGTTAAATGGAAGGATAAAAAAATTGG

GGCTTTAGAAAAACAAAAGGAGTTTTCTGATGCCATTCGAAATGAACGGGATGAACTTAG

GGATGAGGTGGTTCAACTCAAAGACACATTAAAGAAACATGGCATTGCTCTCGGATCAGA

ATTACGCACCAATGGGGAAGTGGGAGTAAATGTAAGTGGTGGATCAGAATTATGCACCAA

TAGGGAAGTGGGAGTAAATGTAAGTGGTGGATCAGAATTATGCACCAATAGGGAAGTGGG

AGTAAATGTAAGTGGTGGATCTGTCTGCACTGAATTGAAACATGGAAGCAGTGTTTTAGG

CACCCAGAAGTTGCAGCTGGTTAGAGATGAAAATTTAGGGGGCTTCAGATGTGACCAATT

ACAATTCCCTCAGCAATTTGACCATGGAATTGAAGAAAATCATCTGCCTTCACCTGCATC

TGGCAGTTCTTTTAACTCTCGTATAAAAACACAATTTAAAGATGTGGATCTTGGGAATTA

TTTAATTAACAGCATAGAGAAAATTCAAAACAATGTTCAAAAACCTCAGGCTGTATTTGA

ACCTAATGCAAAGAAACAAATTGTATCAGACATTTTGAAAGGTAATGATGTTCCAGAAAC

TGATGCAAATATTATGAAAGGTATAGGAAAAGATCAGCAGGAAAGTTATGTTAAAAGAAG

TAAGACTGGAAAAGAAGGAAAGCATGATTTCACACACATGTTCACCTGTGAGATATCAGA

AAAAGATATCAATCCACATTTTGATGAAACTGTGTTTTGCAACTCTCAAATTAAAATGGC

TACTGACAGTTCCCCATTAGCGAAAACAGATACAACATTTAATTCCCAAAGTTCCAATGT

TTCAGTAAAAAAGAAGAAGAAAAAGAAACAGAAACAAAAGTAGCGCTGTGCAAAAAATGT

TCATTTAAAAATAGGGAGAAAAAACAGCTCAGAGAAAGCAGTTCAGTTCAGACAGTATTG

TCAGAGTGCAAGTTTGTACAACAATAAATAAGAAAGCAAAAGATAATGTGAATTTCGATG

AAAAGATAGACCTAATGCACAAAGGAACAGAATCATTGGAACTACAAATTCCCAATACTG

GCCCTGAAAATATTGTATTACCAATCATTAATCAAGACGATTGCATGAGCAAGATTGCAT

GGGTGGACAGAAAATTGGCATTTCTACTCATGACCATCCTAGAGATTTTCTTCTTTTGGC

AAATGCATCATACAAATTAACAATGATGATGCTGAGTTAAAAGCTATTGCTGAATTATGT

GATGATTTTTAATTATCTGAAAACTCTTCTGAGGTTGAACCTATGAAAGATAAAACTGGA

ACTACCAGAGATGTAGTAAAAATTGAAGCATTTATTCATTACTGTGAAACATACACCCTT

GAAAAAAAATCCACAAACACAAAGCTGGGTTACACTGTAGTTAAATCTGAAGCCCCTGAG

GAGAAGCAAGAAACCTTTACTGGCATACAGCTTGATGATACTGCATTTAAAGCTAAAATA

CATAGAGAAAAGAAAGAAATGTCTACTGGCACACCATTGGGTGACACTGTAGTTACAGCT

AAAGTCCAACAAGAGAAGCAGGGAACCCTTATTAGCACACAGCTTAATGACAATGTAGTT

ACAGCTAAATCCTTAAGAAGCAAGACACATTCATAAACATGTAGCTGGGTGACACTGCAG

ATACAGCTGAAAAAACCCAAAAAGCAGCAAGAAACAATCACTGGCATACAGCTTTATGAA

ACTGTAGTTACAGCTAAGGTCCTTGAAGAGAAACAAGAAACATTCATAGACACACAGCTG

GATGACACTGTAGTTAAAGCTGAAGTCCCTAAAAAGAAGAAAAAATCAGAGCCACCAATG

CATATGGGTCAGAAATTTAATATAGAAGAGGATGACAGTGAGGAGGAGTTTAAATCATTT

TTTTTTATTAAAATAAAGAGATTTTTTGGAGGTAATGACTGACAAAAGGAATACACATTA

CTTGTTGTGCAAAGCAATCTTGTACAACCAGTGAAAACCCGGCGCACGCAAATACTAAAG

ATAAAGACCAAATACTACAGACAGACAGACAGAATGTTTTAATGGAGAAAGACAAGCGTG

TGTCTCTAGTGCAAATAACCACACCAGGAGGGACTCAGCAACAGTTTTATCCAGGGTATG

AGGAACAAGGCTCCAAAAGCCAAGAAGAAATAATACATTTAAAACACAGTGAAAAAGTTA

AGCAGGAAATGTCCAGCAAATGTTATAAAGGCCCTTGATGAGCTGCTCTCTGAGACAGCA

GTGTTAAACTTAGTGGCTGATGGTACTGTACAGCAGAAAGCATTTCAAAATATGGGAGAG

CAGGAATTCAAAGAAGTCAAAGAAGTGACACATATAAAAGAAACAAGCAGAATGCAAAGA

ATGGTTCTAAAAAGAAAAGCAAAAAAAGTAAAAGGGAAGACTAAAGAGGACTGTAAAATG

ACCTAATACTTTACCCAAGGATGTTAATTATAAAGTGTCCAGAGTCTTAAAGCCTAACTA

AATCTATGTTAATAAATAATTGAAAGTTTAAAATAGTTAAAAGCTTCTAATCTTTAATCG

AGATTAAAAAAGTCATTAAATTTCATTTCAGCATTTAAATTTTTTGTTGTGTGTGGTATG

TATGCTTCTACATCTGGATTGTGTGAGATGACATGACATGCTTAGTGAAATTCATATAGT

TATTTATTTTCTCATGAATATATCTGCAGCAGTTTTCACAATGTTTTATCTCTGACATGC

AGCAGTTTGCTACTATTTCACAAATTATATAATAAAAGTTCTTTTGTAAGATTTCTTTCA

GAAACGTTTGTTTTTGTTTTTTTACCCAGTGACGTACCGTGGGGTTTCAGCAGGGGCGGG

TGGTGCATTTGGTACCTTGGACTTGGACTTACCCGACTGATACACCTCTTAATACCACCA

CTATCACATCGCAATTATAGAAACCACCACTACTATACAAAAACGCAATATAACATGGTG

TGAAGTTAGAGACAATGAGGCATTTCGCGTATTTAGGGAAAATACCATGTTTATAAAAGC

AAGAAACCCCCGTTAAGACAATTTCAATCCTCTACAACAACAACTGTACCACATACATCA

TCTGGGGCAGTTCAGAATCAATATTAATATTTTATTTATATAAAGAAACAGTGTTTAATT

AATTAATACTGCTTGTTGGCCCTGACTGCACACCACTGGGTTTCAGTCAGGCCTTTAAGT

AAGCAGTAACTTTAAAATTACTTGGACAATCCTACAAGCTAACACAGATATATGATAATA

ATGTGCATATGCACAATTGACACCATATGGAACTCTGAGCCAGACAATCAAAGTACTAAA

GACTAAACTGAAAAAAATAGTTTTTTAGTAGCTGTTAGCCACTCATAGCACTTAGAGTTG

AAATCTTAAAAAAACTGCTGGCCATTGTTAGTGAAAATCAGTTTGGGTGTAGGTCTTGCT

TAATTCGTTGTCTGCAAAAAGTTGCCTTGAAAAGGCGTTTTTAATAGGTTGGTGACAGAA

TTTGGCCGGCAGCTGCAGCAGTCAATTTGTTATTGTTGACTTTCACTTTCATACCTTGGA

AAATCCTGATAAAATCAATCATTTTATTTTTATAGGGCCAGCAGAGAAGGTAGAAACATA

TTCTATATTAAAATAACTGGTTCGTAATAAAACATCTATTATAAAAATAAAATTACAGCT

GTTTTACATTCTATTTACTTTGGTCTGTAAACAATTTCAAAAGTATGGTGGCCTTTTTGG

TAATTTTATCATACCGATAATATGATTCCTTGGTCTTTTGTGTATTGTTAGCCTAAATAA

TATAACTAATTCCATAATTAACTATGAAATGTATGTATGCTGGTTAACTCAATTTCACAT

AGAGTTGTCACTATTTGGGTCCCCTTGAATTGTGATCTTTGCTATTTGCAGTGGGTTTTT

GCATTTGATAGTGTTTTTGTGTATTTGGTCGTGAAGATTTGAAGAGCGTTTCAGTATTTG

TGTCTGCATTGTGAGGATATGCAGTGTGTTTTCTTAAGTTGCAGTGCATTGGGCTTTCTC

TGTTACCATACAAAAGTCCTAGGAAGAGGGACTTTACGATAAAGACATTTTAATGGTCTA

CAAATTTATTCCAAAGTTGGCATGACTGTGTTTTTTACAGAATACTTTTACAGAAGTTAC

TGCATGCTATTCATTCTCATATATATATATATTATATATATATATATATATATATGTGTG

TGTGTGTGTG

>Unigene000229

ACTACGCAGATGTCCGTTCCTGACCAAGCCGAGCTTGACCGCCTACGGCAGCGCATAAAC

TTCACCGACCTGGACGAGAGAAGCATCGGTAGCGACTCCCAGGGACGCGTCACTGCAGCC

AACAACCAGCGGCAGCTTTCATCCGAGCCAAAGAAACCTTTCAACTTTCTGTCGCTTCAC

GTCAACACTAACAAAAGCAGAGAGTCTTCTGCTTCTACCCCACCTGCAACCACTGCAGGG

GGTAAAGAGACAAAGAAACAGAGTCCAGGGAAGGAGCTGTTTGTCCCGGTGCCTGTCGTA

CCTGTTAAAGAGCCACGCAGAGACGAGAGAACCTCAGGAGGGCCCCCGGTGGACGGGAGA

CAGGAACCAGCTATCGACAGCAGCCAGGTGGTCAGTAAGCTGGTGCAGATCCGTGAGTAC

ATCAGTAGAGCCAGCTCTATGAGGGACGACTTACTAGAGAAGAACGACGTTCCGGCTAAT

GTCGAGCGCCTCTCTCACCTCATCACACACCTGAAGGAACAAGAGAAGTCTTACCTGCGT

TTTTTGCAAGAGATGCTGGCGAGACAGAACATGGAGGAGGAGGCTGAAGGTGCCACGGTG

GACTCGGCGGTGTGTTCGGGCTCGATGGCCAAGAGCACGTCTCTGAACCTAGAGCCTCGG

TCAGAGGCCGCAGATTCCACCTGTTACCGGGTTGGTGGACAGCACAGTGAGGAGTTGGAG

AACCTGCGCAAACAGCACGACCTACTGCAGAAGATGCTGCAGCAGCAGGAGGAGCTGCGG

GAGCTGCAGAACCGCCAGGCCACCCTGCTTAGCATGCAGAACCGTTCTGAACATGCCATG

GAAGACCCAGTTGTAACAGAGACCACTGGAAGCGTGTCAGGTGTCAGCATCACTTCAGAA

CTCAATGATGAACTCAATGACCTAATACGACGCTTCCACAACCAACTGCATGACACTCGG

TCTCAGGCAGTTCCTGATAACCGACGTCAGGCTGAGAGTCTGTCCCTGTCTAGAGAGGTG

TCCCGGTCTCGTTCTACCCACAGTCCTCGGGGGCAGCAATCCCCTGGCACCGATGCTGCT

GTTTCTGCTTCCCCTGCTAGCGCCAAGATCACCAAACTCCAGGAGCTCCAGGATAAGAAA

CAGACGATGGACAAAATCCTGCAGGAGCTTCATTCCCTCCGAGACCAGACGCTAAACAAC

AGCTCAAGCCGTGGTGCGTCTCAGCGTGTTTCTGAGCGTCCCTCAGCATCAGGGCGAGAC

GGGGACAGTGTTTGTGTAGCAAGGCAAGAATCTTCATCTTATGATGATGGAGGAAATTCA

GCTGAAAAACTCAGGAAGCTGAAGGAGGTACACAAGCGTCTAAATGAGCTGAGAGAGCTG

GTACAGTATTATGAGCAGACGTCCGACATGATGGTGGACACGGTGAACGAGAATGTGAAC

GAAGATGAGGAGGACGAGACAGATGACGGGTCGCTCTTTGAAGCTATGTTTGATTCGGAA

CAGGAGAATCGCGAACCGATAACTAACATCAGAAACCCTGCCCAGCCTCAGTCTACGTCT

AACTGGATGGACATGAACACACTGACAAATGCCTGTAGCAGCTCTAACAACAGAGTCAGC

CAGCTGAACTCTCAGTGTGAGGCCAACAATCGCTCAGCCGCTAACCTGCGCAGCTTCAAC

GGTCCCTCTGCCATCGAATGCCAGTATAACCGGGACGGCTCCTATAATGGCGTCAAGCAC

GGGAATGGAGATGGAGATGAAGACGAAGAGGACGATGACGATGATGGCGCTGAAGGGGCA

GCTCAAGCAAGGCAACGAAGAAGAGAAAGCTCTGGTTCAGGGTCCAGTCGCCAGAGCAGC

ATTGCAGATGATGCAGTGTTTGCTCAGAAAGTCCATCGTCTTCAAACAGCAAAGCAGAAA

CTTCAGCAGCTCCGTGAGCTTGTTGCCATGGTGCAGAGTGATGACACAGACACCACAACA

GCCAATGAGGATGAAGGATTGCAACAGCAACCCAACAACACTAGAGCGACTGCACCAAAG

GCCAAGAGGGAACTTGCTCTCTCTGATAAAGCCAGGGAAAAACTGTACGAGGAGAAGCTG

AGGCAGCAGCAGGAGGAGCTGAAACAGCTTCACGAGGAGAGACAGAGACTGATGGAAATT

CAGGGCAAAATCCAGGACTTGCAGTGGGCCTGTCCTGATCTACAGTCCTCTGTGTCGAGC

GCAGCGAGCGGCATGGTAAAGAAGATCCCTGCTGCAGTTTCCACTCCTGCTGTTGTACCA

TCCACATCAGCAAAGTGTAACACCACCGTACTCAAACACACCACCGAACCTGCTCCTGTT

ACCATCACAGATAATGAGTTGTGGTCAGAGATGCGCAGACACCAGGTCTTGCGCGAGGAA

CTGAGGCAGAGACGGAAACATCTGGAAAGTCTGATGGCTGAGCAGCAAAGGAGAAGTGAT

GCACAACCTTCACACAGCTTTACCCAGGATGACCGGACCATGGCAACCTGGGGCAGCTCC

ACACAGTGTCCACTGGGTGAGGGCGTTGATGATGATGATGAGGATGAGGATGATTGCCCA

TCTGATCTTTGTGCACGTGAAGACGAAGATGAGGATGAGGAAGACGAGTCAGAGTCAAGC

TCCACTGATGAACCACACTCCTACTCTAACAGAAAGACCAACAGCAATAGCAAAAACAGT

CTCAAGGGTTCATCTCCACACTGCACCCGATCACGACCTAATCCTAATCCCAGAGGAGCA

AAGAGGCAGGAAAACCTGCGCTGGGCTGCTGACGTGTCTGTCACAGAGAACACCACTCAG

ACTCATGCACACTGGCAGGAACAGACCACACAGCTGCAGAGACAGCTGGATTTCAGCACA

GCCATGTGTCAGATGCTGCTGCAGGACCAGCAGACTCTTTCATGTATGCTGCAGTCGATG

TTGACACCATATAACATCCTGCCAAATAACCTTGCCTCACCGCAGGTGCCCCTTATTATG

CACCAACTCAACAACTGTTACACCCAACTTGCCTGGCAACAAAACAACGTCAATAGGTTG

AAGCAAACTCTCAGCGACCTCCTTCAACAACAACAGCAGCATCAGAACGAAGCACAGGAT

TCAGTCTCTGCTTCACCATTTCTACCCTTTCCTCCCATCAACACACTTGCCATGAATAGT

TTCCCCCCTTTTGCCACTGGTTTTAATATCTCTTCCTTGTTCCCATCGGCGGGTTCAGAG

ATTCAGCAAGTGTCAGGAGCTCAGCCCAGCTCCGATCAGCCTCCTGACCCCAATATGTCC

CTCAAAACAGAGTACATGAGCTTCCCACCACCGCTGCAGAGGACTCCCCTTAACAACACT

TACACTCGCTCTCAGGATGATCCTAAAAACGCCAGTTGGCTAAATACCTCCATGAACCGG

TCATCTGATCAGCGAGGCAGTGAACAAGGGCAGCTGGACTCCCATGATTCTTTAAGCAGC

TTGCCTGACTGTGTAGACCCCTCCACCGTCACTAAGAGCTTCAGACCCGGACGCAAAGCC

TCGGCACAGGCCAGCCTTGCCTCCAGAGACAAAACTCCAAAGAACCGACGCAGAAGGACC

AAGAACCACAGCAACGCTGCATGTTTGGACAGTGACAGCATCTCGAGTGCCTCAGAGCAT

AATCACGAGTGCAAAAAAGGGAGGGAGAGAGAACGAGAGCGCTCAGCTCAACCAAGAGCA

CGAGATCCTAACCACAGCCTCCTGGAAAAGCTAACCCAGGAGAAACTGGATAGCAAAGCC

AAAAAGCATGACATCTCCTCAGCAGCATATGCATGGAGAACCCCCTTCCTCTCTAACCAA

ATTGCATGCACAGAAGCTCCAGACGGCAGCAGTGACTTTTCTCTGTTCGAGGCTCTGCGG

GAGACTATCTACTCCGAGGTCGCCACTCTGATCTCACAGAATGAGTCTCGCCCACACTTC

CTTATCGAGCTCTTCCACGAGTTGCAGCTCCTCAACACAGACTACCTTCGCCAGAGGGCG

CTTTACTCACTACAGGATATTGTAACCAGGCACCTGACAGAGAAAAGCGTTGCAGAGGAG

CCGGGGTCGTCGCTAGGGCTGGGGTCACAGTCTGAACTCACTCCCAGTGAGAGTGTCGCC

ACTACTGATAACGATGTCTCTGAGAAAGATGTAAGGAAGAAATTTAACTTCGGAAGGAAA

AGAGCGGTGGAGCGAGATTCGGTGGACAGCGAGAGCTTGTTGTCTACGTCCTCGAATCTG

GAGCCTTTTGCCAGAGATGACCTGGGTCAGGACGCAAATTGCCCTCAGATTGACACACAG

CAGTTGGATAGACAAATCAAAGCCATTATGACTGAAGTTATTCCCTTCCTAAAGGAGCAT

ATGGATGAGGTGTGTTCTCCGCATCTCCTGACATCAGTGAGGCGTATGGTTCTGACGCTG

ACTCAGCAAAATGATGACAGCCGGGAGTTTATGCGCTTCTTCCATAAGCAGCTCGGGCGT

ATCCTACAGGAGTCCCTGAGTAAGTTTGTGGGTCGTACGCTGCAGGACTGTGGTGAGGAT

CTGTTGGTGGAGATTTCGGAGATCCTCTTCAATGAGCTTGCCTTTTTCAGACTCATGCAG

GACCTCGACCAGAACACCCACCTCTCCAAGAGATACACACAGACCACGCCCACTAAACAC

ACACCCTCCACTGAGGGAAAGAAGTCTGATGGAGAAAAGACCTTCTCACCCTCTTATTTC

GATGAAGATAAAGACAGGGATGAGACCGAGCAGAGACGGACGAGTCTCTGCCTCGAGGGG

GAAAGACAGCAAACAGCTGAGAAACCAAACAGGGATACCTTGGAGGCAGAGGAGGAAGAT

GAAGAGGAAGAACACAAACTTCCTCTGTCTATAAGCTTGTCCAAAGCCGAGACTCAGCCG

TTAAACAACTATGGCAGCGGAGAGGATGAGAACGAGGTGGAGGAGCAGGAGGAGTTTGAG

GCCGGATCTGTGGAGGTACAGACTTCCTTGCAAGCCTCAGACAACAACACAGAACACTCA

CAGTGTAACGGAATTTCACTAAACGCTGCTGCACAGGAGAACAAGCCTGAACAACAGAGC

ACAGAAAGTACTGAAGTGAGAAGCTCTCAGTCTGCTGATGTGATGGAAGATGAACAAGAT

GCTGAAGCTGAGCAAATACACACAACCACCAACCACAAGGACGAGACTGCTGCCCCGCCT

TCACAAGAAAGTCCTCACGACTCCACCACAACCAGCCCTCAGACAGAATCACCTGTTCTG

GTTAACGCAGATGAGGTTGGGTCTGGAAACACAAGTCAGAAATCTGATGAAGAGGATTTT

GTTAAGGTCGAAGATCTCCCGATGCAGCTGTCAGTGATGTGCGAGGAGGAGCTGTGTAAT

CAGATCTTAGAGGAACAGAGGAATAACAATCTGACCAGTGAGATCCTGAATGGAATCACA

GAGGGGTTGAGTGGAATTGTGGGAAATGCACATGCACTCAAAGAGCCTGGTAACTATACA

CAACCCCAC

>Unigene000230

GCCCATAAGCGAGAAGACTAGCAAACTCTGCAGAGCTGTGTAACATTAGCTTTAGTAAAC

CCCTGTGCTGTCCAGACTGTTAAGGTGCCATAAGTGAGTGAGTTAACACCGTTTCTGGCA

TAAGAAAAGCACGACTTCCCCCGTCACATGCCTCATACCGGCCGGGTCTTCATCTTTTAT

GCATTCGAGAGAGTCGCAGGTGGCGGAGTTTAGTTTCAGGCAGACGGTCCGCGATACCAA

AGATGCTTTATTAGACGGATTTCGGTGACACTCGTAGAGACGTGGAATATGACTCCGGCC

AGACGAAACAAATATCCGAAATTGAAATTAAATAGATTGCAGCCTGAAGTATTTATAAAC

TGACAAATCGGTCGGCGGTTTCTTCGAGAGAGCCGTGTGGCACAATTCGAACACACTTGT

CAAGCATGTGGGCAAATGTGGTGTTCATTTTTTTATGTACGACTCTTCTTTGAGGGGAAA

AGCAGCATCATTTTCACACACTTGCTGAAGAAAATTGTGCGTTTCCTTTCTGGTTAAGCA

GTGCTGTACACGTGTCCAACTCGGTATTTTGTTGCTGTCTTGCTGTGTGTACCTACAATC

ATCCATAGCCAGCAGGCAGTGAGCTCATTTGTCCTCCACCCCTGCGCTATGAGCAGAGGT

AGGGGAGGGCACTCCAAAGAGTACCAGAGGTTTTACCTTTCACCCCACCAGAGCAAATCC

AACCAAGGATTCTACTCCCAACCTTTTTCAGCTCACCCTAGAACTCCTCACGCCCCAAGC

CCGAACACTAACTCGGGCAGATACCAGTATCCTCCCCCTTACTCATCCGCTGGCCCTCCT

GGTGCTTCTTCAAGTCCCTCTCTGCCTTCAAATTCTTATACGTCTCGGGTTCCTGGACCT

TTCCAAGTGCAAGATCAGAGGCCGTCATCGAATAGAGGAGCTTGCGCTGACGAAGCAGCA

GACTTTCGGCAGAAGCAAACTCTGTTCCTTCAGGGGCGGACTGCTGAGGCGCCCAAATTT

GTGCCAGGCCAGTCCAAACACCACAGCAGTCCTCCCCAGCGTCACACAGAATATTATAAT

TATATAAATAATAGCAGGTATATAAGCAGAGGGGGTTATAGTCAAGGCAGGCCAAGCTGG

GAAAAGGAGTTTAACACACAGTACTACCCTGGATCTAAGTCTGTCTGGGAGCAACAGCAG

TTCAGAAGACAGAATAATCAGTATCATTGTGACACTGCCTCACTATCTTCAGCATTTCAC

GGTCTATCAGTAGAGAACTGTAGTTCACATTCGTCCTTCGGTAGTACTGGAAGTAAGGCG

TCTCATACATTTTCCTCGGGTAGATATTCCTTGCAGGTGACAGAGGATATTCAGGAGAAG

GTCCACAATTCTTTGGCCTCATTGCAGCAGGGTGAAACCCTTCAGGCCAGGGTCTTGGCT

AAAAAAACTTCGTTTGCCTAAGAAGATTGTTAACCAAGCACTGTATGCTTTATCCAGATC

AGGCCAGGCAGTGAGGTTAGAAGGGACTCCTCCTCTCTGGAGACTCTGGAAAGAAGGTGT

CCCTGAATACTCCCAACGAACCTACTTTAGCGGAAATACTGAAAAATACCTGGTCCAGGA

TACATTTATAAAGAAAAACCAGTGTATTGGTGAGGTAAAACCAGACAGCAGCTTTACCAC

TGGAACATCACCCGCTGCAGAGGCCAGTAGTGATTCCAGTGACGAATCTGAAGACTCTGA

AAAAGAAAGTTTCTCTGACCAGGCCTGTGAAAAGGACTCGAGTCTTTCCATGACAATGGA

CGCAAAAGACTGCAAACTGCAAATTATGCAGTACTTGCATGAAACTGGCCAGGCCAGTGC

ATTGATGATTGCCAAAAACCTGGGCTTGAGAAATGCCATGCAGGTTAATCCTACATTGTA

TGCCATGGAAAAGCATGGCGATCTCAGTCGCAACGCTGGCGTTATGCCACCCGTTTGGGA

GCTGACTGCTCACCGGCGTGATAGGATGGCCCGACAGCGAAAAGCAGCTGTTGCTGCCGC

TATAATACACAATGAGTCAAAGCTTTGTGACACATCAGGCCCAGCAAAAACTGCACAAAC

AGCCCCAAACACGGTGTATGGATCTGGGTTGTCTGAAGAAGTGATGGCGTGGGAGAACCA

CCCAGCAAGTCAGAAAAGTGCTAGCATGGACAGCACATTTGGGAACGGAATGCTTGATAA

GAAGTCACATTTAGATAGAGACTTTCTTCCTGCTCTTGAAAAAATTTCCTCAAATGATAA

AGACCATACATCTGTCCCCTACTCCACACATGATTCTTCATACTACCTGACACAAGACAA

GAATGGAGGACACTCGGAGTGGGCATCCGATGATATCCCAGAATTCTTAAATGCAATTCG

ATCAGAAGTGGCCGTGTCCCTAGCAGCACCTATTCCTCCAGCCCAGAATACAGAGGCCGT

CAGGTTGCAGAAGCTAAGACTGGCACTCAGCAAGAACCCTGTTAGTGGACTGATGGAGTA

CGCACAGTACCTTGGCTACAGCTGTGAATTCCTCCTGCTTGAACAGTCCGGACCCTCACA

TAACCCCAGGTTTCAAATGCAAGTGATGCTCGATGGACGCAGGTTCCCACCAGCTGAAGC

CTCAAGTAAGAAAGTGGCAAAAAAGGATGCAGCTGCAGAGACTCTTCGGGCCCTCCAAAG

AGAGATAGAAGGAACTACTACTGTAGTAAATAAAGCAGAGCTGCCTGCTGTGCCATTAGC

AGAACTGAATGATTTTGCTGAGGATTGTTTTGACAATATGGATGAACCTCCAAAGGCCCT

GGCTCGCTCCCTTCCAGGCGGCAAAAACCCTGTTTCGGTTCTAATGGAGCACAGCCAACG

TAGTGGCCATGCCATTGAGTTTATTCATACTGGGCAGGAGGGGCCAGCACATGACCCACG

CTTCATGTTCCAAGTAAAGGTCGGGGACAGTCTTTACCCGGAGGCATCAGCACCCAGTAA

GAAGGCAGCACGGCAACTGGCGGCAGAGGAAGCTGTGAAGGCTCTTCTGGCAGAAGGACA

ACTACAGCTCAACAAGCCACATCAGGCGTTTTGTCTACTCGGCGAGGGCGATTTGTCCCA

GGCGATGCCCCCATGCCCGTCATTGCCCCCGCTGACAGCAGCTGAGCTTCAGGCAGCACA

CGAGGCCGGAGTAGGCGACCTGATCAACCATCTAAACAACAACGCTGTGTCAGGCTTGCT

GGAGTACGCACGCTCACGAGGCTTTGCTGCAGAGATTCGCCTGGTGGGCCAGTCAGGACT

CCAGCATGAGCCACGATTTACATATCAGGCAAAACTGGGGGGTCGCTGGTTTCCTGCGGT

GTGTGCCAGCACCAAAAAGCAGGGCAAGCAGGAAGCAGCAGACGCAGCTCTCCGAGTGTT

GATTGGAGAAGCGGAAAAGGCAGCACGCACCGGGGAGCTCACAGCAGAGTTGCCGGTGAC

GGGTAGCACGGTGCACGATCAGTTTGCCATGCTAAGCCACCAGCGCTTCAATGCGCTTAC

TGCACGCATCCAGCACAGCCTTTTGGGTAGGAAAATCCTTGCCACGATCATCATGAAGAG

AGGTGCTGACAGTCTGGGCACAGTCGTCAGCCTTGGGACAGGAAATCGATGCGTTAAAGG

AGAAGAACTAAGTCTTAAAGGTGATACCGTTAACGACTGCCATGCTGAAATCATTTCCAG

AAGAGGCTTTATCCGCTTTTTGTACGGTGAGTTGATGAAGTACTGGGAAAGACCAGAAGA

GGACAACATATTTGAGCTTGCGGAGGATGGTTTGCTCAAGATTAAAGACGGCATCACTTT

CCATCTGTACATCAGCACCGCTCCATGTGGGGACGGCGCACTGTTCGATAAATCGTGCAG

CGAGGTAGCGGAGGTCATCGGCTCGGCTCACGTGCCCGTGTTTGAAAACGCTAAACAGGG

CAAACTCAGGACCAAAGTGGAAAATGGTGAGGGGACCATCCCAGTGGAGTCGAGTACAAT

TGTGCCTACTTGGGACGGCATCCAGCATGGCGAGCGCCTCAGAACTATGAGCTGTAGTGA

CAAAATTCTGCGCTGGAACGTGCTGGGCCTGCAGGGGGCTCTGCTCAGCCATTTTATGCA

CCCCGTCTACCTTCAGTCTATCACGCTTGGTTACCTGTACAACCACGGCCATTTGACTCG

AGCCGTGTGCTGCCGATTGGCCAGAGAGGGCAGCGAGTTGATGAAAAGCTTGCCTACCCA

CTTTAAACTTTGCCACCCGGAGGTGGGCAGAGTGAGTGTGTACGACTCAACACGTCACAC

AGGAAAGACCAAAGAATCCAGTGTGAACTGGAGCTTGCCAGACCAGCTAAAAGTAGAGAT

ACTGGATGGAACCAAGGGCAAAGTAGACAGTCCAAAGCTAGAGGTGTCCCGCGTCTCCAA

GTCCAACCTGTTTCGTCATTTCCGGGACGTGTGCAGGAAAGCCGGTCGGCGCGACTTGCT

GTCTCTGACTTCCTACGCCCATGCTAAGATGGCCGCCCGTTCTTTCCAGCAGGCCAAGGA

ACAGTTCTACAAAGCTCTGAGTCAGCTGGGTTACGGAACCTGGATCGGCAGGCCGCAAGA

GGAGAAGAGCTTTGAAGCGTCAGAAAGTGAAGCGCAGAACATTGTTTCAGCTCAGTAAAA

ACAAAAGGGAGCAGAAAGAGGAGGGACGGAGGAGTATGGATGAAAAGGACAGTCAGTGTT

TTGAATCTTCTAGGTTGTAGAAGAATGATGGACACAACTTGAAACAGAGAAAGAGAGCGG

GAAGGAAAAAAGCGCAGTAGATCTCTTGGATTACTTCAGCCTGGGTTTGAGGGAGAGAAA

AAAAATAAAATAAATCGATAAAATACCAGCTCATGTATAAGAATGATGTATGGCGCTTTC

TTTGGAAGCCTCTCTAAACTCTGTTATGCACTAAGAGCTCGGGATCTGTCGATCTGTCTC

AGGCTGGTGGGTGCATATAAGCAGAGATTTAAAGAAGGGATGTTGTAAATTTTTATTTTA

CTTGAGATCAACCATGTATTCGTTTCAGTTGGGGAATCAATGGGCTTCAGCACAGATCTT

CCTGTCGCTTTTTTCGATCTCTTTCTTCTATAGAAAACGTTTCCTATTAAGGTTAAACTA

GTGGACTGAGGTGCTTTTTTTTGTCCCATTTCATTCACATTCCTGGTACTAAAATATAAA

ACTGTTTTAAATAACAGGATAACTGCAGGTTAGGACTGTTCATTCTTCCCCAGACATCCA

CCCCTTCCTCATATATCTATCTATATTAGCTTGATCTCTTTATCTTAACCAGTCAAAACT

AATTTGAATTGCTTCTACACAAATGTATACATGATGTACATACAGATACATACGCTCACA

AATGTATATAAGGTCTCATCACACCGTGTGTGAGTTTATATACGTGTGTGTGTTTATGTA

TATGTATATATTTTGTGTATATATTTTATAAGGTTTCATAAATCTATGTTGCTTTTGGCC

AG

>Unigene000231

GGCCTGTGCACTTATTTTTGCGTATAAATTGATAAGGTTCCATCTTTATCAGCTTTGTTA

GGTGTAAGCTTTAATAAAATGGCTCTGGTCATGTGACTTGTCCCTAACACAGTGTAAAAG

ATGTTTAAATGCATATACGTGTTCAATGATTCACATCAAACTTTACAGATTAAGGAAAAA

GTACTACCAATGCCATGATTTAGGATGTGAGTGGTGAAAAAAATGATTTATTCAGTGCCA

TGTAAGAATGATCGAGCACATAATAGTTACAAAAAGAATGTGAGTTCTGAAGGGCCGTAC

TTACATCATCACCTCTACAGCAGAGTTAAGCTTTTACACAGACGTATTATAAAGCATGTC

TTTATATAAATTACTCTCAGTACTGAATTATCAGAAAGCAGCACCTGAATGAGTTTTCCA

TTCAGCGGGGAATGACTGCAGTTTCAAATAGTACTGATTTTTAATTTTTTTTTTTTATAA

AGAGCACCTTTAACATGTTTAATGTTTTCTGAAATATACAGGAAAGCAGTGGTCTGGAAT

GAACAGAAACAATAACAGAAATTATGGTCTATATTAATGATCATATAATTTGATCTTATA

GTCTACATGCAAGTGATACTCGTTTGTTTAAATCAGGGATCACCAAACTCGGTTCTGCGG

GGTTGGTTTAGCTCCAACCCTAACCAAACACACCTAAACTAGCTAATTAAGGTCTTGCTA

GCTATACGCGAGACCTACACGCAGGTGTGTTGGGGAAAGTTGAAGTTTAACCCTGGAGTC

CCTCAAGGATCGAGTCTGGGGACCCCTGATTTAAAGGATTATCAGGGGGCTAAAATACTG

ACATAATTAAAGAATCACAAAACTAGTGTTCTGTGGTCAAAGTTTAGCTTCACACTTTCT

CCTTCCTATAGAGAAAACAAATGCAGCAAAAAGTGCCAGCTAAAATAGAATAAAACTATA

TTCTAGGATTCAAATCCATCAGTTGGGGTTGTACTTTTGTGTTCTGATTTCAGCTCTAAA

TAAATATTTACTCTAAATTGGTTTTCAAAATATGATTTCTTTTCAATTTTCAAAATCCTC

GAATACAAATAAAAAAGCTGAAAGTAAAAAGTTACATAAAAAATATGTAGCTAAAACTGG

CTAAATATAACACTTTCAAAAAAAACCCAACAGGAATGACAAACGTTTCATATACATATT

GATATGGAAAATACAGTTAAAAAATAAAAAACCTCTGCCTCTCAGCATTTCCTGTCCTCT

CTGGTTCGAACCCATCCTGAACAATATGTGCCAATGCCGGGAGACAGCACCAACAGCCTG

TCTTGTTGGCATTTCGAAAGGAACCAGAGGACAGTAGGCGACCCCTCTGCGATGCAGACC

CGGTCCCTGTCATTTTAATTTTGCTTTATTTTTTACACTTTATAAACATGGTGGATCATT

GAAAAAAAAAAAACAATCTGCCCAGACCGTTAATCTAATGTGGCCTGCAGTGTGTAGGAC

TGACGGATCCTTTACTTTTTGGACTGTGTGATATAGCTGTTCTCGATACACAGCACTATA

AAGTTGTTAGAACAGCTGCTGGGTTTTTGCTGTAGGAGCTGACCAGCCTGTAGAGATGAG

GCCAGACCCATCACGGCGTGATCAGACGTCCTCCACGTCTCGCAGTAGTTATCCATCTGA

CGGTGACCCTTCCCACTGGAGCCATGCCAGATCATCTTCTCTGGCCATGTACTATCGCTG

AGAATATCTCTGCCATCGAACGAATACAAGGGTACGTTGGTCTTTATCCTGCCCTCTGAA

TCACCAAACAGCGATTCCCAGCTGCTGAAGAGCACTTGGTCCTTGAGGTTGACAATAGGC

AATGTGTTTCTATCGGATTTGCGAACGATGCTGTAGAGATCCTGCAGTTTGGAGGACAGG

AATGCCCTAAATGTTCCCTGTAGGCCTGCAGCACGTGCCTGCTGGAAGCACAGGAAATCT

GCGCCGCGGATGCCCCTCATGTTCCCTACCTGAGGGCTGTTCAGGGCGATCAGATGCAGC

CCTGGTCCTGATGTGTGCACATGACTCTCAGGCTGATGGACAGGTATGCTGGGTCTGTGG

ATTGAGGTTATGGGGTACCTCTCTGGTGGTACAGGAATGTTTTGATCAGTGCGGGGTGGG

TATCTGTTGTCCGGCTGATGCGGGTATCTGATGTCTGGATGAGAGGAGTAGTGTGTGTTG

TAGTGTGAATTCGAGCCTGGCTTAGGATGCTCGCTGTCAGGGCTTTCCGGCTGATGGATT

GCAGTGTCAACAGGGGAGTAAACCTCCGCTCCGTTATTTGTCGAGTGGTTTGGAGGGTAG

TGAACCACTGGAGGAGGCTGTACCGCTGCCACCTCGTTGTTCGAATCTCTGTAAAAGGAG

TTATGAGTGCCGAGCAAGACTTTTCTGACGCCTTCACGCACTCTGACGTAAAGTTCACTT

CTGTCGATTACGTAAAAGAGAGCGCCCTCTGGTTGTCTCTGTGCTGTTGCGAGCATCAAC

TCGAATGATCTCATTACCGTCACTCCAGAGCTCTGGCCAGGTGGACCAGGTGGACCAGGA

GGGCCAGGAATGCTGACTGCAGGCCTGTAAGCACCTGGTAATGAGGGAATACCAGGTGGC

CCAGGAGGTCCAGGTGGTCCTGGGGTTCCAGGTCGACCTTCATAACCAGCACCAGGAGGT

CCTGGTGGACCCTGTGGACCGGGTGGCCCTTCAATAGAGTCTCCTTTGGGACCAGGGGGT

CCTGGTGGTCCCTGTACACCTCCATAGTGAGAACCGTAGTGACCTCCTGGCTCGCCCTTC

TCTCCTTTCACTCCAGGGTCTCCACGTTCACCCCTCAGCTCGTTCTTTAGTGTATAAATG

TCAAAGTTGCCTGGTGCTCCAGGAATACCAGGGGTCCCACGGTCTCCCTTCTCTCCTTTC

ACACCAGGATACACTCTTCCAGAATCTTCATATCTACCAAATCTTTCCACAGCTGTTGAT

GGTCCTGGTGGACCCGGAGGACCTGGAGGGCCAGGTGGGCCTGGATAGCCGTATCCGGAG

CCAGAACCAGCCTCTCCTTTTTCACCTTTACTTCCAAGAATACCTGGACGTCCAGGCCTG

CCTGGCATTCCGATCTCACCCTTCATGCCGGGAGGACCATATGCTCCAGGAGGTCCAATA

GGTCCAGTAATTCCTCTTTCCCCCTTCTGACCAACCGTCGAGCCAAGAAATAGAGGGCTT

CCATCAGGTCCAATAACCACACCTGGTTCTCCTTTCTCACCCTTAATGCCAATCCCTGGA

GCACCTGGTTCTCCTCTCTCTCCTTTAGGTCCTATCGGGCCAGGAAATCCCGCTTGACCA

GGAAGGCCAGACGGCTGTCGATCAACCCCAGTATCACGCCCATCTGCAGACCGATAAATG

ATTTCTCCTGGAGGTCCCGGTGGTCCTGGAAGGCCAATTCCATCACGACCTGGTTCACCT

TGGTCTCCTTTATAACCGTGGTCTCCTTTGGGTCCCTGTGGTCCAGGAAAGCCGTCAAGT

CCAGGACGGCCAACCGCTCCTGGTGGTCCCTGATCTCCTTTCTCCCCAGGTACACCAGGT

TGACCCGGCAGACCAGGTGGACCCCGAATGCCTGCTGGTCCTCTGACTCCTGGCAACCCA

CTGTAAACGTTTACACCTGAACCCTCCAAGTCGTCAAATCCAACTTGATAGTTCGGCCCT

GGAGGCCCAGGTGGTCCCACAGGTCCCATCGGTCCTGGAAGACCGGCAAGGCCAGGCTGC

CCAGTTGCTCCTGGAGGACCAGGTGGCCCCAGTGCACCCTTTTCACCAACAGCTCCAGGT

AAACCCAGCTCACCGGTATCACCCTTCTCGCCTTTAGGGCCAGGCAGGCCGGGCTTTCCA

TCAGAACCGGGAGCACCTGGAAGTCCTGGATTTCCTGAAGCACCATCCTTTCCAGGTGGA

CCAGAAGGTCCGGGGACACCCGAAACAGAACCATGTAAAGCAGTAGAAGTATTTAAAAGA

CCTGGGGGTCCAGGTGGGCCGGGTGGGCCAGGAGGACCAGGTAAACCAGGCATTCCCCGC

AAACCCTCAAAATCACCAAAGCCAGAGCCCTCCATGTCTACAAACGTAGGCCTGTCTGAG

CGAGAGAGGGGTCCAGGGGGTCCTGGAGGTCCCGGTGGTCCCCGTGGGCCAGGATGACCT

TCACCCTTGTCACCTTTTTCTCCTTTAGCTCCAGGATCTCCAGGGGTTCCTGGGAAACCA

GGTGGTCCGGCTGGTCCAGCTTTTCCATCTTCACCAGGGTCACCAGCTTCTCCATCAGCC

CCGGGTTGTCCTGGTGGCCCTCGAGGTCCTGGAGACCCCCTCGGTCCTGTTACACGCTGC

ACCACTGAGCCGTCTCCTTTTTCCACTATGTTCACACTGGACCCAGGGGGACCAGGAGGG

CCCTGAGGACCAGGAGGTCCTTGATCTCCCTTTGGTCCAGGATAGCCAAATCCTGCAGCA

CCCTTCATCCCTTTATCTCCTTGGCTACCTTTCTCTCCCTTAGCACCACCTCCAGATCCA

ATGCTGGATCCTGCATCCCCCTTGGGTCCCTCGAGACCACGGTCTCCTTTAGGTCCTCTG

TCTCCTTTGTCCCCTTTTTCTCCCCGAACTCCAGCACCGGTTAGCACTGTGTGCACGTCT

TTGCTCACTGTAACCACAGTCGGGTTCACTGGCGGCTGCTGGAGAGGTTTGGAAGGTTGA

CTTGTGGTCGAAACTGTGTGCCCGGGGCTGTAAAAACCGCTTCCGTATTCACCTGATGCT

CCATCAGAATCATCCTCTTCTTCCTCACAATATTGTGCAGCTACACGAGGGTCTCCAACC

ACCCTCAGATCTCCAATAACACCCACAAACTTATCATTATCGGCTCCTCCAGCTTGACCC

ACAAAGATCCCGGCTCCTGGTTCCAACTCCATTTCATCTGGAGAACGCTCAAAAGGAAGC

ACCTTGGGTTCCGAGTCACAACTGGGGTACAGGGTGACTTTATCATCCAAAACGCTAATA

GAGAAACGATTCCAGTAGTTGACAAGAGAGGGAACAGTGAAGCTGGCAGCCTCATAAGAT

GCCTGGGAGTCTGGCTCGGTGTAGAACAGGACAATATTCTGCTTTCCATCCTTCACGGGT

GTAAGCTTCACCCCTATGTACATAATTTTCTGGTTGGTGTCGGTGATGGAGAAGATGACT

CCAGCTTTGGTGGAAGTTGGTTTCAGGTTGAACATGAGCGAGAAGTCACGGTAAAATGAT

GTGGGCAGGTACACGCGGGCCAGCTGACCAGTCTTGGAGTTGTGATCGAGAACAAAGCCA

GGGTCTGGTTCATAACTCTTGGTGACGCCTTCAGGAAGAGGGTAGCCAATCAGCTGTGAT

AGCGTAACACCTCTCTCTGTCGAAATGGGGTCACCGGGTGCTCCGACAGCTTTGCCAATG

CGTTCGTCTGTTTCAGAGAGATTCACTTTGGTCAGATTCTTC

>Unigene000232

TAGATCGGAAGAGCGTCGTGCAAGGAACAAAAGACAGTGAAGTAGCATCAGTTATATGGG

AAAATGTTGGTACCAAAGCAAATACAAAATATGACCACAGCATTGAGCATATGGACGAGG

AGCTAACTGTACAATTAACTGAAGACAACATTTATACGTATGAAGAAGAGGATAATGACA

AGCGTAAAAAGTTTTGTGTAGCTGTGGATAGAACAGACAATGATTTAGCTGAGGTGACTG

CAACAAACCTAAACCTACCAATAGAGTCGGATACAAACATCATTTCACCTCTAAAACTGG

AGGAAGAAACATCAGAACAAGTTATTTCAAATGAATTGAAATTTCAGGATGACAGTGAAA

TTAACTGTTACAGAAAAGAAAAAAATGATCTTCCTGAGGCTATATGTTTGACAGGTAAAG

GCACTGCAGATGAGGAAATATTTCAGAAGGAAGTGGTAATGCACTATGATGAAGGAAAAA

AATGTAGAGTCATAGAGACAGAGAATGAGGTAGAAAACAATGACATGCAATCTGAAAATT

TTATTGCAGAATCTCAATTAATACCTGATGAAACACTGCATAAAGAGACAGAAGCTAAAA

CTGAGACATGCAACCTTCAACAATTTCCAACTGAATTAGAAGCAGAAGATCAGATGCTTG

ACAAAGCAATCAAAGGTACATCAGAGTCTCCAGATATAAAAACTGAGGTTATTAAGGAGA

AAAATGACATAAAGGAAGACATTAATGCAGGTTCCACAGAAAGTTTTACTATTGGGGATT

TTATAGACGTGGATGATAATAAGGCTGGCATGTCCACGTTTATAGATGAGCTTGAAATGG

CATCAAATACACAAGATTATGCATGTACAGTCCAGGCATCCATAGTAGTCAATGACCACT

ATATTGACAGTCCCACCAGTGCTAGTATAGTTCATGCTTATCCTACAAGTGATTTAGTGG

AGCAAATTCCTGAAACTCAAGACATGCAGGTGTATTGTCCAATAACTATACCTGATCAGG

AGCAAACCACGGCTGATGGATCCCTAAATATCATGGAACCTGAAGCAAAAAAATGAAGAA

AATGTGAGTAAAGCTTTGGTTACAAACGACAACATAAGCACTTCACAAGGGCAACATGAA

ACAGCTTGGTACAACTCAACAGAACTTGTGAAGAAAAATCAAGAATGTCGTGTACTTGGC

AGACAGATCGAGGAAACATTATTGGTAGAAAATATTGGTGCAGTATTAAATACTACAGAT

GTAGCATTTTCAGATGTAGCTGCACATCTGGTTGATACTGAAAACCTCAAAATGCTAGGC

GAAACAAAATTGAGCGATAGTGCAGTACGAAATGAAGATCAAAACATTTACTCAGATCTT

AACTTAGAACATACAAAATCGTTGGAATTTAGTTGTACAATAAATAGTGATCACCCCATC

ACTTCTGCTGAAAATTTAACAGATTCTGAACTGTCCAATCAAAAGTTTTCATCACATTTA

TTACTTGCAACAGATCATCCTGATTTCAAGGAAGATGATGAAAAAAGTACAGAGAACGTA

AAAAGAAGTACTCTGCCACTTGCTGAAAGAGAGAAAGGAGAAAATCAGGAAATTAGATGG

AAAGATCTGGAAAATACAGACGAGCTCAGACATGGAATCATAACTCAATCTGCTACGTCA

AACCTGCTCAATAAACCACAGAGTAAAATGAGTGAAGTGGCACTTGACATCTATGGCTTA

CCAGTAACGAGTGAACAAAAACAAACCAGGACTGGAGAGATTGATATACCACCTGCTATA

TCACCAGTTAAAGCTGATCCGATTTACCCTGTGGTCTCTTATGAGGTGAGCAATGCAAAA

GGACTGACTGACATTATAGAGCAGAATGAAAGACCAAAGGTACAGACGGAAAAGGATTCT

GAGTTCATGCATTTAAATAAACAAGTAACGGTGCCAGTCATTACTGAAAATGAAAGTGCA

AATAAAGTGAATGTTTATTCTACATGTGGTCTAGTGGGGCTCATTCCAGAAACACAGGTC

ACATTAGTCATGCAAAAAAACACAGAAAATTTCCATACAGTTTTATCAAATCAAAACCAA

AAAATAACTGAAGAACATTTAGAGAATGTAACTAACAGGGAATCCGAACCAGATCAATTT

GCAAGCACAAGCACATGTATTCAGATAGCAGAATTAAAATCCTCTCAGGTTGATTCGATA

AAAGAGAATGTGGAACTTGGCAGCTCTGAGAAAAGGAGGAACACAGATTTAAGTCGAAAG

AGACTTAAAGGGAAGATTCCTGGTAGTGACGAGAAAAACAAATTTGAAGTGATTAAAGAC

AATGAAAACATACAGGAACAAAACACGGAACAAAATAGTACCATGATTGGGGTTATCAAA

GAAGACATAAAGGACATAAAGGAAGAGATAAATGCTGGTCTTACAGAAAGCTTTACCCAA

AAGGACATCAAAACTGTACAATCACAACAACATGTACTTTGTGACTTATCTTCTCAAAAT

ACTGATCATGGTTTAACTACAATTGAGCATCCTTCACCTGCAACAGAGCATGTGGACAAT

ATAAATGCAGATATTCAACCAAAAAATCAATCTAAAAAAAAGAAGAAATTTGGATCAACA

CGCAGACCCTATGGAAAACACCAGTTAACTGCTGAAGCAGAGGAAGAAGAATTAAAAGAT

ATGGAAAATATCGAAGAAAATAAACATCAAATCATACCCCAAGCTACTCCTTTAAACCTT

CACACTGAAAAACAGATAACTACAAGTGAGGTGACACCTGACATCCAAAACTCATCAGTG

ACAATAACAAAACAACAAATTATTACCACAGAAGTTGAAACAGGAACAGAAAATGATTCA

TCACCTAGAATATTATCAGTCGAAAGTGACCAGTCTCGCAAAGTGGTTTTGGACACAGCA

ATTAATGCAGAAGGTTTGACAAAAATTATGGAGCAAAATGAAAAAGCTGAAATGAATGAG

GAGAAGAATGACATGCCCTTACACTTAAATAAGTTAAAAATGGCACCAGTCATCACAAAA

ACACAAGATGATACATTTAAAGAACATGTCAAAATGCCCAGTGACAAGAGTTTGAATAAT

GAATGTGTCAATGATTTTAATATTCATTCCACATATGAAAATGTAGACAATCGGAATGCA

GAAATTCATACGCAAACTCAAACTAAGAAAGGAAGGAAATTTGGATCAACAAGGAGACTC

CAAGGTAGACACGAAACGACTGCTGAAATCGTGGAAGAAGAATGGAAGGATCTGGAAAAT

ACAGAAAAAACACATAGCATAGATGAATCTAAATTTGACATGCCAGTTGTATTGGAACAG

TATCATACAAAGACTGAGAAACTGCAAGAAAATCCAAAAAATACAGAATCGAAACCAGAT

CAATGTGAAATAACATGTCCAGTTGTTCACATACAGGAATTAAAAATAGCCCCAGGTGAT

TTGGCAACAAAGAATTTGGGGGATAGTACTCATGGGAAGAGGAAAAAGATAGGTTCAACT

AGAAAAAGTCTAAGAGTGATGAAAGAAAAAGAAAACATCCAGGAACAAACAACAGAAGAA

AGTACACCAAACTCAGAGACATTCGGTGCAGAAAAAGAGACATTAAACTTAAGTGCCGAC

ATTTCTCATCAATCTAAACAAATGCATTTTGAGACACTAAATATAGAAGATGAAGATGAA

AAAATGCGTCCAACTTCTTTAAAACAGAATGAAGTGGAAGCATGGCAAAAAACTACAGAT

TTTGCTGGAGAAAATAATTTAGAATGCTTACAATTAATAGCGAAAGAAGACATTTGTAAC

CCAGAATCAAACTTTTGCCCCACAACAACAAGCAGACAAGAGAATGAACCACATATTCAC

CATAAACTTGATCAGTCAGTGGACTTCCTTCATAATGAAATAGAGCTTCAACCGAATAAA

AGTATGGTAAATACACCAGCTGTTAACCCTGACATCTTAGAAATGCCAGAGTCAATTTCT

GAGGGGTCTCTTCAGCACCTATCATCCCAAAACTCTCAAATTCATATAGAGCCAAGCTCC

CAGGCACGAAGGAGAAAAATGGGGTCAAGTCGCAAGATGTCTAAAAACAAGAATGCAAAT

GTTACAAATGATGAACCTACAATAGATGCCAGGACAGTTGAAGATACTGTTATAGCAGAA

AGCACAAAAGAAATGGAAGAATGTGTTGAACTGTCTCAAATAAATGAGAGCAGTGCACAA

GACACTGAACAACTTTCAGAAGGTAGGCAAAAGTTTGGGTCCAGATGCACAACCAAGGAA

AGCAGTGGGTCAGTTGCAAATAAAGATGGTTTGGATGAATATAAGAGGGAAAAGGATATT

CAAGTGACAGAGGGTGAATTAAAGGTCTCTGACACAGGTTACATCTTAGAACCAAAAAGC

ACACTAATCTCTCCTGAAGTCTCTGAGCCAAGACCAGGAACTGAAGAGGTTAAGAATTTT

ACTGAAGGTAGCAATGTTTCTCTGGATAGCATAAGAAAAACTATGTCATCTGCTGGCAGC

ACTGGAGAAAGACAGATAATCGACTTTGAGCAATTTCCAGATTTTGGACAAGCACTATAT

AATGTTGTGATGGTGGGAAACAGCAATGTGGGCAAGACGTCTTTTATAAAACGATTACAG

AGTGGACATTTCACCCAAGATTACAGTGCTACCATTGGTGTTGACACCTTCGTTCATACT

GTTACATTTGGAAATAGAACAGTTAAACTGTATGTGTGGGACACTGCAGGTCAGGAGAGG

TACCACAGCATCACCAGGCAGGTCTTCCACAAAGCCCATGGGCTTCTGTTGATGTATGAT

ATAACATCAGCTCAGAGTTTTCATGCTGTTCGTGACTGGATATCCCAGGTTCAGCAAAAT

GCTGCTCCTGATGTCATCTTGATGTTACTTGGCAATAAAAATGACTGTGCTCATCGAGAA

GTCCTGCTACAAGATGGGGAGCATCTATCAAACGAGTATAACATTCATTTCATGGAGTGC

AGTGCTGCAACTGGTGAAAATCTGTCAGAATCTCTAAAGACTTTAGCATGGTTGCTGATG

AAACAGAGAATGAGGAAAGAGGACAAACATACAACATTACAGCCACAGCCACAAAATAAA

AAGTCAAGTTGCTGTTGAAAAAATCTTCAGTGTATACACACACACACACACACACACACA

CACATACACACATACACACTCAACAGAGAGTCACATGGGTGTGAATTTTCGATCCTCTCC

CGTCCCACACCCACAAAATAAATCCCGTCCCAAGAAAAAAACTAGGGGCAAATCCTGTTC

CGCCTAATCCTAGAACAATGACTTCTATTCCTTCCCAGTCTAGTTCTAGTTCTGGCTAGA

ATTTGTCAGTTAAAGAAAAAATACAGTACAAATCTTGTACAAATATTTTGTAAAATCACT

GATGCTCCCCATACACGACGCTCTTCCGA

>Unigene000233

TCACGACGTACTGGTTGGACGTTGCGCTCGTCTGCAGGTGTCAACATCAAACATACACAC

ACACAGCTAACATCTGCGACAGGTCGCCACATCTGGTGTTCTACAGCTAAACACACCCCA

GGAAGATTATACAATGCTCAGGCGAATTTTACAAATGACTCCTGGGAAAGGAGGTTCCCA

GGCACCTGATTCTGAGCAGTCATCTGCTGAAATTAATAATGAGACTTCAGAGGGCTTCAT

TTGCCCACAGTGTATGAAGTCTCATAATTCTGCTGAGGAGCTTTTTAAGCATTATGAAGT

TTTTCATGAGCCTCAGGAGCACCCGTCTCATCTTAGCCCCGGAAGGGAGGATTTGGTTCT

TCTGCGACAAGAAGTGCAGGATTTACAAGCTTCCCTGAAGGAAGAACGATGGTTTTCAGG

AGAACTGAAAAAAGAACTAGACAAAGTGCAAGGACAACTGAAGCAAGGACCTCAGAATGA

TGGCCAGGTGGGCGTAGAGGAATCAGAGCTAGAAATGAAACTCCATGAGGCTGAGACTGA

AAAGTTCAACATCAAACAGATGAAAGACCTGTTTGAACAAAAGGCAGCGCAGCTGGCCAC

CGAGATTGTGGAAATCAAGTCACGCTACGATGAGGAGAAGAGCCTGAGAGAGGTGGCTGA

GCAGAAAGTGAACAGCCTGCAGCAGGACCTGCAGAAAGAGAGACAAGAAAAAGACAAACT

ACAGACAGAGCTGCTGCAGAGACCGGGACTAGAGGATGTGGAGGTGTTAAAGAAGGAGCT

GGTGCAGGTGCAGACGCTGATGGACAACATGACTCGCGAAAGGGAGGAGGAATCCGGACG

CCTCAAAAACGAGTATGAACAGCTGCAGAGGAATTTCACTACCTCAGAGAAAACTATCTC

TAAACTGAAGGCGGAACTGGAGAAGGGCCCCCAGGAAGCTGCCGTGTACACGCAACAAAT

CCACCAACTACAGAGTAACCTGGACAACCTTCAGCAACAGAGCCAGATGCTGTCAACAAA

ACTGAAACAACGGGAAAAGGAGAACCAAGAGTTGGATGAGCGCCTGGGGCAGGAGCAAGT

GTCCAAAAAGAATCTGCAGGCCAGTCTGCACCAACGAGAACTAGAGCTTCAGGAGAGTCA

AGCACGTGCTACAACAGCAGAGGCCTCCCTCAGTCGGGCACAGACCGAGTTAACAGAGCG

TATGGAAGACGTTGGGCGACTCAGAAAGGAGCTGGCTGAGCTCGAGAAGAGCCACCAGGA

TCTGAAAGCTGAGCGCAAGCAGCTCCAGCAGCAGAAGGAGGACAGAGAGAGCCAGGGCCT

GCAGCAGCAGAGTGAACTCAGCCAGCTGCATGGGAAGCTGCTGGAGACAGAGCGTCAGCT

GGGCGAGGTGCAGGGCCGGCTAAAGGAGCAGAGACAGCTCGCAGGAGAAAAACTCAAAGA

CCGGGAGCAGCAGGTTGCTGACTACCAGCTCAAACTTTCACGATTAGAAGAACAGCTAAA

AGAGAACAATACCAAGTCCACTGACCTGCAGCACCAGCTGGACAAATCTAAACAGCAACA

TCAAGAGTTGCAGGCGCTCCAGCAGAGCACCAACGGCAAACTACGTGAAGCACAGAATGA

CTTGGAGCAGGTGCTGCGGCAGATTGGCGATAAGGACCAGAAGATTCAGAATCTGGAGGC

CCTACTAGAGAAAAGCAAAGCCAGCCTGAGTCAACTGGAAGCTGAAAGAGAAGACCTGTG

TGCCAAGATTCAGGCAGGAGAAGGGGAAGCTGCTTTACTCAACCAACTGCAGGAAAAGAA

CCACACACTGCAGGAGCAGATAAATCAGCTGACCGAAAAACTGAAGAACCAGTCAGAGAG

TCATAAACAAGCCCAAGAGAACCTCCATGAGCAGTTACAGGAGCAAAAGAGCCAGCTGCG

CTCAGCACAGGATCGCTGCCAGTCCTTTGAGTGTAATGTAACAGATCTCACTGCCCAGCT

TACCCAGAGCAAGGAGAGGCTTGCCCAACTTGACACCCAGCTAAAGGCTAAGACAGAGAT

GCTGCTGTCGGCTGAAGCAGCAAAAACTGCCCAAAGAGCAGATCTGGAGAGTCATCTAGA

AACAGCCCAGCATGCATTGCAAGACAAGCAACAGGAGGTGAGTAAAATTCAGGCTCAGCT

AGAGGAGCAAAGCTGTCGGCTGCAGGAGAAACAGGAACAGTGCTTACAGATGGAGAGCAG

TCTGAAAGAGATCAGAGAGAAGCTATTGGCTGCTGAGCAGAAGACTGACTCCCTGAATAT

CCAGGCTAAGAAAGCAGATGCTGAGTTGGTGGAGCTGCGCTCAGGCAGAGAACAAGCGCA

GATGACACTGCAAACACAGCAGCAGCAAATTACTGAGCTGGAGAAGAAGAACAAGGAGCT

CAGTCAGAAACTGAGCAGTGAAAAAGAAACTGCTATAGCTCTGCAGGAGGAGCGGCAGAA

AATGAGCTCAGCTTTGGGAGATGCTCAGAAGCATGTGGAGAGGTTGGAACAGGAGCAGAG

TGACCTTAAGACAAGACTGGAGAAAGTCGCTGAGGAAGCACACAAGCAAAAGGCAGAGCT

AGAGAAGAAGCTACAGGGGATAAACACAGAGCACCAGAACACTAAACAGGAGAAAGACGC

ACAGGCTAAAGAGCTGCAGAAGGTGAAAGAGGCTCTAAGCAAGACTTCCACAGCCCTAAA

GGAGAGTCAGACCCAGATGGAGAAAGAGAAAAAGAACAGCGCAGCAGTCCTAGCAGAGAA

GGAGAACGCTCAGAAGGCCCAGCAGGAGCTTCATAGGAATGCAGAAGCAACGGCTAAAGA

ACTGAGCACGGCCAAAGCTCAGCTGGCAAAAATGGCAGAGGCAGAGAAAGGGCTTCAGGC

ACAACTGAAAGAGCTGAGTAAGCAGTTAAAGCAGTTTCAGGATGCGCTGAAGGACAAGGA

GAAGGACATCCAGCAGGTCCAGACTCTGCTTAAGACAACTCAAGGATCCTTTGATGAGGA

GAGGAAAAAAATGCAAAGTCAAGCGGTAGAGCTGCAGAAGACTCAAAAAGAAAAGGTGGA

GGAGTTGTCTAGGCTAAAGGAACAGATGAGTGTGATGTCAGAACAGCTGGCTTTAGAAAA

AGGCCGCAGTGCTGAGCTGCAGAAAATCTGTGACAGCACCAAGGAGAGCTTGGACAAACT

ACAGTCTGACTATTACGGCAAAGAATCAGAGGTCTCTGCTCTGCGCCAGGACCTCAAGGC

ACGTGAGGAGAAACTGACTCTGACTCAGGAGGAACTGGTGGCCAGCACCAACCAGCTGAA

CAATTTTGAGGCCCGAATTCAGGAGCTGAAAACAGGAAATGCAGCACTGGAGAAAGACTT

AAAGAAGAGAGATGAGAAGCTCAAACAGCAGGAGGAGGCGCTCCGTGAACTGCAGAAGCA

GCAGGAGCTAATGCAGGAGGAGTTAAAAAAGGAGCGTTCTCAAGTAGAGGAGCTGAGGGA

GGCTCGGAGCACTCTAGAGAAAGAGCGAAGCAGACTAAGTGCTGAACTCAAAGATCTGGC

AGGCAAGCGTGAGAAGGAACTAAAGGACCTGCAGGCAGCGAAGCAGCTATTACTTCAGCA

GAAAGTAGACTTACAGGGCCGAGTGGAGGCAAACCAGGCAGCACTGGAGCAGGAAAAAAA

AGAGCATCAGAACACCCGGGAGTCCATCATGCAAACACAGCAGCAACACAAAGCAGAGAC

AGACAAACTACATGCACAACTGGTGTTGGAGGCAAAGGCCAAAGAGGAAGTGGAGAAGAG

GAAAGAGGAGGCTGAGACAAAGCTTACAATGCAGGTTTCTGCCCTGAATGAGAATGTTGC

CACACTGAAGCGCGAGTGGCAGAGCAGTCAGCGGCGTGTGAGCGAGCTGGAGAAACAGAC

CGATGAATTGCGCGGAGAGATCGCTGTGCTCGAGGCTACTGTGCAGAACAACCAAGACGA

GAGACGTGCTCTGCTTGAGAGGCGAGGACCTGCCTCTGATTGCAGTGTTTATCTTTCTTT

TTTTTCCTCCTCTGCTTCATATGCTTATACACATACACACTCTCTGTGTTTTCTCTGCAC

TGTAGATGTGTAAAGGATGAGGGCGAGATAGAAAAGTTACAGGCCAAAGTGGTGGAGATG

AGAAGAAAACTTGACGACACTACAGCAGCTATGCAGGAGCTGGGCCGAGAGAACCAGTCC

TTACAGATAAAGCAGTCGCAGACCCTCACACGGAAGTGGGCAGAGGACCATGAGGTGCAG

AACTGCATGTCTTGTGGAAAAGGTTTCTCCGTTACCATTCGGAAGCACCACTGTCGTCAC

TGCGGAAACATCTTTTGTGCCGACTGCTCATCCAAGAATGCCCTGACGCCATCCTCTAAA

AAGCCTGTGCGGGTGTGTGAGACCTGCTACGAAGAGCTGCAGGGTTAACAAGCCTCTCTC

CGCCTGCTGCTCTGCCACACGCTCACCACAGACTCTTAGCCTCATCCTAAAAGCAGGAGC

AGGAGTGGTGGTGTGACAAAGCTGCAGGATTACATGAGGACTGGCCTGCTTCAATCTGCT

TACCGGGCATGAGGTGACCTCAGGGACAGAGAGAGAGAGAAAGACCCGGAGGATCACAGC

AGTCCCTCAACTGAGCAGTGCCTTGGTCACCTCTAGGTCCATTTGGGGTTCCAAAGAATC

AAATAAGGGAAAGTTGTTTAACATTAAAAAATTATATAAATATAGAACGTCTTTTGGATG

AGGTTTCTCTCACATGTTTTGTGTTTCGGGTCCCAGGCTGAAAGATTTATACCTCAAGCT

TTTTGAACAAAGGCAGTAGTGAATCTGCCAGAATGGCATCCAGACTGAGAGCGGTTTCTA

CAGCCTCCATGGGCAGGTCAGGTTAGTGCAGTCAGAAAAGAAAGCACAGAGCTTTAAAAA

AAATATAGAAAAATAACACCCCTTTTCCAGCTGACAGGATGGGGCGGCGTAATTCGATCA

CTAAGCTGAATCTGGAACTACTATTATCCACTGCTTATGTGTACCTGTGGATGTAAGAGT

TAACTGGCGGCATCGAGGTGCTGGCCGTTTCTTTCAGCAATGCGAGTGATAATGATATAA

ACGATCGACAGCAGCAAGAATGCAACATTTTTGTGTTTGAGTAACAGTGTACATTAACGT

TAACTTGAATTTACTCGGGATTTGGTTTTACGTGATAGACAGAGACACTAAAACTGACGC

TTGCTGCATCATACAGCCTGGTAAAGACGCAGTTATAGCCAGATGTTTTTTCAAACAACT

ACATCTGACCAGTATTCTGTCCCATTTCTTGCTTTTCCCACAATGCATTGCTAAATACCC

TGCCAAAATTCAGGTATTCTTGCAGAAGTATAACTTTTCCATATAACTTTTTCACATATA

ATCCTTTTAGTTAATTCCAAGCTGTAACCTACAAAATAGATCATTTTAAGAGTACGGAAG

TGTGAATATTTACGCAAAGCTGGCA

>Unigene000234

TGTGTGTGTGTGTGTGTGTGTGAACATATACAGAGATAAACTGGAAAACCTGTTTTGTGG

TTTATTTCTCAGCGCTGCTTTCTGTATGTGAGAGGTTGCGCTCAGTGTTCTGCTCATGTG

GTAGGTTTATTGCCCCTCCACCATGTTTTAGTTTAAAGCCCTGGGTAGCGTCATTTCTGC

CCGATTGAGGCGCCGTGTTCAGATATGAGGCCGCGGTGGGCTGAGGTCGCATTCAGTGGG

TTTCTGTTTTACACACACCTCTGAAAGAACCGGACTCGTCATGCCCACTCTGAACTCTAA

GTGGCGCTATTTGTTCATGCTGCTCGGCCTGCAACTCATCATCATGGCCCTTCTGTCCAG

AGAGGTTTATCAGAAGAGGGTCACCTACTTCTTCAGAATCTTCCGCAAACTGGACCTGCT

GAGCGGCTCGTGGACGGGACATGGAACCAACCAAACCGGTGGTGCTGATATCTACGCCAA

CCTGTCCCAACTGGTGCTGCTACCCAAACAACAGGACATGCACTACTGTCCTAAAAAATC

ACCACTGCTCGGTGGGCCGATCCACATCAGCTTCCCCTCAGGACTCACACTTGCTCAGGT

GAGGAAGAAGAATCCGTTGGTGGTACGCGGGGGGCGCTACAGACCTCCGGACTGCGAGCC

TCATCATCGCACCGCTATAATCATCCCTCACAGGAACAGAGAACAACACCTGAAGTACCT

CCTGTACTACCTGCACCCTTTCCTACAGAGGCAACAGCTGAGCTACGGCATCTACGTCAT

CCACCAGGCCGGTGACTACACGTTTAACCGGGCAAAGTTGATGAACATCGGATTCCGGGA

GGCGATGAGAGAGGAGGACTGGGACTGTCTGTTCTTCCATGACGTGGATCTGATCCCTGA

GGACGACCGCAACATCTACACATGTGACAAGAACCCCAAACACGCTGCCGTCGCCATGGA

CAAGTTCGGCTACAAACTGCCCTATAAGTCGTATTTTGGCGGCGTGTCTGCGTTGACTCC

GCTGCACTATCTGAAGATGAACGGTTTTCCCAACAACTACTGGGGCTGGGGGGGTGAGGA

CGACGACATAGGCATCCGTGTGTCTCTGGCCAAGATGTCCATTAGCCGTCCGTCTTTGAG

GGTCGGACGCTACAAAATGATAAAGCACAAACATGACAAGGGCAATGAGGTCAACCCTAA

AAGGTTCAGTATGTTGGCGAAGACGAAGAAGACGTGGAAGTCGGATGGAATGAATTCTGT

GGTGTATAAACTGGTGTCTCGGGAGTATCTGCCTCTTTACACCAACATCACCGTCAACAT

TGGCACCGTCCACGGTCTGCACCCCACCACCACCACCACCGCCACCACCATCAAATCCTG

AACACAAAGAACCTGGACACACACACACAGAGACAGTAAACACAGGGTCGACGTCTGAAT

GGAGTGTGTGATTCAGGGGCGGAGCCTTTAGTGCATTAATCAGGTTAATAACTGTAGTGT

TCTTTCTGACAGCAGTCTGTCTCTCTGACTGTCTCTCTCTGTCTTAAGCTCGCTCTCGCT

TTTATACTTGCCATTGTGTGTTCAGTTCACAAACATGGACCTACACACTCTTCTGCACAT

GCTCAGGTATGTGTGTGTGTGTGTGTGTGTATGGAATCAGGGCTGGAGATGTGTTAATAG

TCAGCGTGTTTTTGGACTAAAGGCGGAGGAAGGAATGTCCGGATGAACAAACTCTCCGAC

AAAAAAGACTCAACTTCTCTAATGAGCCGTTTGATGTGATCGTAATGGCAACAAAGGCGC

AGGTTTTATACGACTTCACAGCAGAGCCGGGCAACAACGAGCTGTCGGTGAAAGAGGGCG

AGACTATCACGATCACCAATCAGGGCATAGGCGGTGGATGGATCGAGGCGCGGAACAGCA

GAGGCGAGGTCGGTCTGGTGCCCGAGGACTATGTGGAGGTCAGTCAGCCTCAGACGTTTG

CAGCTGGAGGAAGTGTACCTCCGCTTGACTTGTCATTCTTTGAAAGTCAGGCAGCAGCAA

GCAACACACAAGCCCAGGTGGATGTAGGTCAGCCGGAATCCCCACTCACCCCCATCTCCC

CCATGACCCCCAACATCCCCGTGTTCCCGGCCCTGTCATCCCCCGATGAGACGAGTAATG

GCAGTGACCCGTGGGCTGTGTTTGACACTAATGCCTCTGGTGGCTTCTCTAATAACTGGG

CCGCGCAGCCTGAAGGCACAGAGACGGGGCAGAGTGGCACCAACGCGTGGGGTTCTGGAG

GACATGGCCACCCTCACGCCTACCCAGGCCCAGGTGCCGAGGATGACGAGTGGGACGACG

ACTGGGACGAAAGGAAGTCCTCGGGCGGCTTTGGCGAGAGTGAAACGAGCGAGGGCGGAG

CCATGTCCAGAGGAGGAGCTCATGGATCCTCCATGAAAATCACCCTTAACAAATTCCCAG

GATTCTCCAAATCAGGTCCTGAGCTGTACCTCCTGTCCAAACAGGGCCCCAAAGGACCCA

AGCTGTCTGTCTATAGTGGTGAGGTCGGCCCGGTGTGGGAGTACACTGAGCCTCAGCTGG

ACTGCGTTGTAGCAGACCCCAAAAAAGGCTCCAAAATGTACGGGCTGAAGAGCTACATCG

AGTACCAGGTCACCCCTAATACCACCAACAAACCTGTCAATCATCGCTACAAGCACTTTG

ATTGGCTGTATGAGAGACTGCTGGAGAAGTTTGGCTCGGCCATCCCCATCCCATCCCTAC

CTGACAAACAGGTCACAGGCCGTTTTGAGGAGGAGTTCATTAAGATGCGAATGGAGAGGC

TGCAGGCCTGGATGTCTAGAATGTGCCGGCATCCTGTCGTCTCTACCAGCGAGGTCTTCC

AGCTCTTCCTCACTTACAAGGACGAGAAGGACTGGAAAATGGGGAAGAGGAAAGCAGAGA

AGGACGAGACGGTCGGGGTGATGATCTTCTCCACCATCGAGCCGGAGGGACTGCCTGACT

TGGAGCTCCTCGATGTTGAGCAGAAGTGTGAGCAGTTCAGTCGTTTTACCAAAGCGATGG

ACGACGGCGTGAAGGACCTGCTGACTGTGGGACAGGAGCACTGGAAGCGCTGTACAGGCC

CGCTGCCTAAAGAGTACCAGAGGATCGGGAAAGCCTTCCAGAACCTGTCGTCTGTCTTTA

ACAGCAGCGGATATGAAGGAGAAGCTACACTCACGGACGCTCTCACGGCAGCGGGGAAGA

CCTACGAGGAGATCGCTCAGATGGTGGCCGAGCAGCCTAAAAAAGATCTTCACTTCCTCA

TGGAGACCAACAACGAGTACAAAGGGCTTCTGGGCTGTTTTCCCGACACCATCGGAGTCC

ATAAGGTGAACTGTGGACTCTAAAGTCGATTAGATACCACCGGTGTAGGAACTGCTGCTC

AGACGTCCCGTTGCGTGTTATGTTTCTATTCCCGCAGGCCGCCATCGAGAAAGTGAAAGA

GGCAGACAAACTGGTGGTGACCAATAAAATCACACCTGCAGAAAAGGTCACTATGGGCAA

GAGAACCAGCACCATGTCTTATGCATTACAAGCGGAGATGAATCATTTCCATAGTAACCG

CATCTACGACTATAACCGAGTGATGCAGCTGTACCTGGAGCAGCAAGCCAAATTCTACGA

GACGATTGCTGAGAAGCTGAGACACGCTCACAGTCAGTTTACTACCCTGTAAAGAAGACT

CCGTCTCTCTCTCTCTCTCTCTTTCAACTTCTCCTTTCTCTCTCTCCCTTTATGTCTCTC

TCTCTTTCTCTCCCTTCAAACACTGTTACTGTTCAGGCGGTGTTTTTTTTTAACTAAACC

GGGAGAGCAATAACTTGACAGAACACAGTTTTGGCCCACCGGAACTCTGCAGCAGCGAAA

GCCTCTACAGCCAAAGTTTTAAGTTAAAGATTTTACTCTAGCCCAAGGTACTTCAGAAGT

TGGTGAAAATGCTGGAGACGGTCAGAGACAGAGTGCTGGAAGTGTATGACAAACTCTCAG

CGGCGAGAGACCCACGGCTGAGGGGCTACCTGCTGATGGATAGTCCGGTCAGCATGACAG

CGATTTTGCTTGGCTACCTCTTCTTCGTACTGTACGCCGGCCCTCGACTGATGGCCAACC

GCAAACCCTTCGAGTTAAAAGGGCCCATGATTTTTTACAACTTCTGCTTGGTGTTTTTGT

CTACTTTTATTGTATATGAGTTTTTGATGTCAGGATGGGCTACAGGATATACGTGGAGAT

GTGACCCGTGTGATTACTCCGACAGCCCTCAAGGACTTCGAATGGTCAGAGTGGCCTGGC

TGTTCCTCTTCTCAAAGTTCATTGAACTTTTAGACACGGTGTTTTTTGTGCTACGTAAAA

AACACAGTCAGATTACCTTCCTGCATATCTTCCACCATTCTTTCATGCCCTGGACCTGGT

GGTGGGGCGTAAGCATCGCTCCTGGTGGAATGGGTTCATTTCACGCCATGGTCAACTCTG

TGGTCCACGTGATCATGTACTTTTACTACGGCCTCTCTGCTGCAGGACCTCGCTTCCAGA

AGTACCTCTGGTGGAAGAAATACATGACTGCCATCCAGCTGGTGCAGTTCGTGCTGGTAT

CCCTTCACGCTACCCAGTATTACTTCATGTCCAGTTGCGATTACCAGATGCCAGTGATTC

TCCACCTCATCTGGATGTACGGCACTTTCTTCTTCATCCTCTTCTCCAACTTCTGGTACC

AGGCCTACGTTAAGGGCAAGAGGCTGCCCAAGAACGTCGAACAGCAGACTCAGAAAGGCA

AAGCCGGCACTTTCAATGGAGCCACGACCGTTGCTAACGGAGACTCGCTCCTGTCCAATG

GCTTACATCAAAAGAACGGCCTGGCCAATGGGAAGAAGCACTACGAGAACGGAAGCACTC

ACAACGGGAAGATGAAGAAAGCCTAACCACTGAGGGAACGTCCAAAAACAGTGCCTGCAT

ACAACGCCATGGATTCCAAAGAGTGTTTTGGGATTTTTTTTTTCCTGTCAGCCTCATTTC

GTTTATTATTATTATTATTATTTTTTTCTTTTTGTAATTTTAAGATGCACAAATACATAG

GGAGATGAATATTTCTGGGATCCCTGTTTGTATTTTTGTACTCATCATTAATTTTAATTG

TTAATCTGTAAAAAAAAAATAAATAACGAGCACAATATATGAAGAGAAGAGATAATTTAA

TGACTGGCTGTGTTCTTCACTGATGGTGCTCACCTTGTCAGTGAACGCACTCCTCCTTCC

GTCCCTTCCCTCTTTCCCAAACTCCCCCCACCCCACCTCCTTCCTCCGACTTTTCCAGTG

ACCGTCAGCACTGCACATTTCTCCTAAGACAACTTGGGCTCCAACTGTAAATACGGCAGG

TTTACTGTCCGAACTAATGAGATTGTTAGCGCTCTACATGTTTTGTAACTTAAGCATATT

GACTTGAAAATATCCAAAA

>Unigene000235

GGAAGGGTTTTTTACATGCATCTCTGTCACACCACTTCTTTGCCTCTTACTGTTGAAACC

TCCTATGCTTATTTCACACATCCTCTGCATGTGACACTTCAAGAGTTCGAAAGAATGACT

CACCTTAACTTCCACACCAAGGACACCAGGCGGACTGATAAAGCCAAGGAGAATGGTGTG

AGCACTGCAGGCCCTGAGCCCAGCTACTTTGAGATCCCGAGTAAAGACGGCCAGATGGCA

GAGGACAGCATCCATGAGGTCCCGACCAAGGACACAGAGAGCAGCGCAGCTGCAGCAGCA

GCAGCAGCAGCAGCGAAAGCCGCCCAAGCGAACACCTCCTTTACTATTGAGTTTGACAAC

ACCTCTCCAGGAAAAGTAACCATTAAGGATCACGTGTCGCGTCCAAGATCGAAAAAATCT

CAGCCAGGGAGTAAAGAGCTCAGCAGCCTGCAGGCAGCCATCATGGCCTCAGAGAGCAAA

GTGGCCGACTGGCTTGCGCACAACAATCCCCCCATGGTGAGACGGGAGTCCACGGAAGAG

GACAGCAAAAGCATTAAAAGTGATGTGCCTGTTCTTCTCAAAAGGCTAAAAGGCAGCAAG

CACGAGGACGGGACCCAAAGTGACTCTGAGAACGGCCTTGGCTTGCGTTTCGGTAGCCGG

CGCCATGCTGCTCTGGAGGAACGCTTGAAGGCAGCTGGCCTTACGCATGCCAAAGTCACC

GGAGCAGAGGGCTCGGGCTCCAGCAGCCGCAGCGCCTTCACCATCGAGTTCTTCGACGAG

GAAAACCCGCGCAAACGCCGCTCCTACTCCTTCTCCCAGACTGCACCACTTCTGGGAGGA

GAGGGACTGTGTCCAGCACCACCATCCCATCCGAAGGGAACTTCATTAACACTGGCAACA

GTAGCGCCTACAGCAGCACGGCTGCTGCAGAAAAAAAGGTCAGAGGAGAAGACCACGACG

GTCGGAGATGAGACTCAGAAACAAGACGACGATCAGAGCGACAAAGGCACTTATACCATT

GAGCTGGAGAACCACAACCCTGAGGAGGAGGAGGCCCGTCGCATGATCGATAAGGTGTTT

GGCGTGGAGAACCCTCAGAATCCCAGTCGCTCAGTGCAACAGACAACTAAAGACAGTAAG

AAGGTCAGCAGCACTAAAGCGGAGGGTCTTTTGGATGAATCGGTTGCGGTTGGAAGTCCA

CACTGGGTCTCTCAATGGGCCACTTTGGCCGCTAATCACACAAGAACTGATCCTGAAGGT

TCCGGGGCAGAACCCACAAACTTTGTTCATCAAGAGCGAGAAGCTGATGCATTTGAGTCA

GGAGTGTCTTTGAGAAGCACCAGCTCTGCCACCTCTAGTCAGGCTGAACGTAAAAGAAGG

ACATTGCCTCAGCTCCCCTGTGAGGAGAAGAGCAGGCTGGGGAAGGTCTCCTCCACCTCA

GGACTCCGATCCGAGATTGGCGAGAAACAGGACACCGAACTCCAGGAAAAACAACACAAA

GGAGATGGCGAATGTGCATCCACTCTCATTAAGAAGGACACTACAAGTGGAATTGCAAAC

CAAGTGCAAACAGCCACTACCAAGCCTCCTGTGCCCCCTGTGGGAGGGGTGGAGAAGCAA

AGTGAGGAGTTTCGCAGGAGGCGTGCTGAGGACAGGAACAAAGGTAGCGATAGTGAGCGA

AGCGGGAAGCCCCTGGTGCGTCAAGGCAGCTTCACCATTGACAAGCCAAGCACCAATGTT

CCGCTTGAGCTTATCCCACGCATCAACCAGCGGCAGTCCGGAAACAGAGAGCGTAGTGAT

TCAGCTGGAAGCATGGACACGACCACCTTGCTCAAGGACACTGAGGCCGTTATGGCCTTT

CTAGAGGCTAAACTGCGTGATGAGAAAAAGCTGGATCCATCACCGCCCGCTGGCTACCCT

TTGGGACGCTCTTTTTCCCCAGAGTTGGATACTGATGCAATTAAAGCAGCAGGCCGCGCC

GAAGTGTCACAGAAGCGTCGTTCTCTCAGCAGTATACACAGAGAGAAAAGTAACATGAGC

TCCGTTGGAAAGAGCACAACCAATGCACGGGAACGTTTGGAAAGGAAAGTCAAGCCGAAG

ACCTCGGATGGGCGTCTGGATACCCGTCGTTCTACCCATACAAGTCAGCGGACCCGCCAG

CCGTCCATGGACCTCACAGATGATGAGCAGACATCTTCCTTGCCCGTTTCTGACATACTT

TCAGATGACCAGGAAAACGGACGTAGTCCCTTCACCTTTCCTGATGACTTGCTACTTTCC

AAACTGGACTCTTCTAAAGGTGCTAAAGTTAATAAAGCCAGCAGGACACCACAGGCCACC

ACTGCCTCTATAGGCAAGCATCAGTTGCCTCAGCCTCGGCCCACAAGAGCATCTCTTCTG

AGACGTGCTCGACTTGGAGACACATCTGACACAGACCTGGCAGATGCAGACCGAGTGTCA

GTTGCATCCGAAGTGTCTACTACCAGCTCCACATCCAGGCCACCATCGGGCAGGAAGGCA

CCATCCCGCATCGATATGCTTGCACAGCCCAGACGCACCAGGGTCGGGACCATGTCAGCA

CGCAGCGACTCAGAAGCCCCAATGTCACGCAGCACTACATCCAGAATATCGGCCGAGACA

GCTTTAAGACTGGGCTTGCGCCCAACTAACCCTGCAGACAGCAAGCTGAACCCACGTCTT

CGGGCAAACAGCGTCTCCAAGTTGACAGACACCAAAACCAAGACTACAGCATCCATCTCC

AGTGCTCCAGCAGCCAGCAGCAGGTGGCGGCGACTTAATACAGAATACGGCTCCACCTCT

GAGGACGAATTTGGCTCAAACAGGAACTCGCCAAAAGCCGGGCGTCTGAGGCCAGGCACT

GCTCTGCGTACCAGTAAACTGAGCAGCTCAGGCATGGTGACAACAAGCCCTGGCAGCATC

ACCATCAAACACAGGATGAGAGAACAGGAGGAGTACATCAGAGACTGGACAGCCCACAGC

GAAGAGATCGCCAGGATCAGTCAGGACCTAGCCAAGGATCTGGCTATTCTGGCGAGGGAG

ATCCACGATGTGGCCGGCGAGATCGACTCGGTCAGCTCCTCAGGCACAGCACCAAGCACT

ACGGTGAGCACAGCGGCCACCACGCCCGGGTCTGCCATCGACACCAAGGAGGAGGTACAG

CCCGCTTCCCTAAAAAAACCTTAGAATAAAGGTTTCGGTAATACTCTGCTAGCCACAGAG

ACTTTTTAATCTATCCCAGCTACAGCCAGGATAGCAATCAGATGGGTTTTAAAATATTTC

ACAGAGCAGCGGTAACACACTGACTTCCATTCTTCATCAATTCACCCTCTCATTTCATCA

CTTGTAGTTGGTAGAACGAGTTTTTGATGAAAGTTTGAACTTTCGGAAGATCCCCCCTGT

GGTGCAGACCAAGACCCCAGAGATGAACGGCCGGCCTGTAGAGCTCCGACCTCGAGCCCC

AGACAGTCTGGATCCCCAGAATGCACTGCGTAGACGCACATGGAACCGAGATGAGGTCAG

ACCGGCGGTCCTAGAAAGCTTGCTGCTGAGCTCAGTCTCTCAGCTGTCCTCTAAAATCCG

CCAATCTGTGGACAAAACAGCAAGCAAAATCAAGATTTTGTTCAAAGACAAAGACAGGAA

CTGGGATGACATTGAGAGCAAACTGAGCTCAGAGAGTGATATGCCACTTCTCAAAACCAC

TAACAAGGAAATCTCAACAATTCTAACAGAGCTAAAAAGAGTGGAAAAGCAGATCCAAGC

TATAAATATCATGGTAGACCCAGATGGAACCCTGGATGCTCTTACCAGTCTTGCCCTAGC

AAGTCCCACCACACCCAAACCTAAGACCAGCAACGGAGCCTCCAAAGCCTCCGTGATCAT

CTCCCCGAACCGAGAGCTCCTCAGCGCTGGACTGAGTCGTCCTCAAACCAGCGGAGAAGC

GAGCAGCATTACTAACAAGTAAAGACAGAAAACCCTCAGAGTTGAGTAAACTGAGAAACC

CTTTTTGTCCATGTCTGTACCCCTCCCTCTCCTGCACTCCATTGGAATGTCCATCTTTAT

GTAAATGAAGTCACACCTGTGGATTTGGACAGTGATATACAGTGCAGCTCTACAAGTGCA

CGCAGCTGCTGGTGAAGAAGAGCTGAAGAGGAGCATTAGTCTAAAGGCTTAAAATGTTGG

AACCAGCCACCTGTATGCTGCTGCAAAGGTGTACACACTCATCCAGGCATGAAGCAGGTG

GACTTAAAATAATTTATCTTCAACAATAAATGACTCAGAGTGCCTGTCTTCTCTCTTTTG

TGAGCATTCTTTCTTGCAAATAGCAACTTCTTATTACTACGTGACTTTTGTGTCAAAAAT

AAAACAGTGTTGGTCAAAAAGACTTCAAGATTTCAAAGCTGAGCATAAACCAGCTTAGCA

AAGTAAGTCTGATTCTATGCGGGCACTCAAACATTGTTCTTTTTTTTCCATTTCTTATTT

CTTGATTACAAAAAGTGCATTTAGGCTATTAAAATAAGCAGGGGTCAACTTGGTAAGTTA

AACTGACCGTTCATTGCATTATCTTATGCACTGATTTTTTATTTTTATTGGCTAATGTAT

ACAGTAGGTAGCAAACTTTGTACAAGGTTCAGACATTAATTAAAACCCAGCAAACTGGCG

TTTGTGTCATTTTCCCCCTGACATTAGAAGGAAGATCATATGCAGTATTAGAAAAACGTC

TTAGTGGAATGCACCATTGTAGTGTTACACATCACAGTTGCTTAGATCTCTGTGCTTTGT

GCTGAAATGTTAAATCAGCCTTAAATTAACCAGCCCTTAATGTACGGTTCAATCACATTC

TGAACATGTTATGGCCAGTTGCTTAAATGCTAACGTTCTTTAATCAGAGTATGCAATTAC

AAGAACCAAATAAAGTCTGTCAGCATTTTTTTTTTTAATTATCACAACCTCATAGATCAT

GTTTTTTGTTCATGCAGGTATTCCGGCATATTATGTATGTACTGTATTGTCTTACTCTTC

CGTCGGCGCTTGCTGCTTGGCTGGAACTCCTGAGAAAAAGCGTCCAGCGCCCAGCCTTTC

ATCGTGAATCTGCGTGTTGTAGAAAGACTAGTGCAAAATAAAACAATAGTGAAGATAGGT

ACAGGACATTAGTTGTTGTTGTTTGTTAATAAATGTCAATGCCAAGTGGCTGCTGTGGAA

ACGCTAGTTCCTGGACAGGTTTAAGAAAGAAAATCTTAGTTTAGTGACCGCATTTGTGGT

TTTAAAGGTTCCTGGATTAACTGGGTGAGTTAATGTTAAAGGTGATACAGGACAACAAAC

ACCAAACTCTATACATTATACCGCTCGCAGAACAGGATAGATTTTCAGGCAGATGATCCT

CCTGCCCCTAAAACACTTAAAAAAAACAGTAGTTATCATTTATAAAACTATTGTACATGT

TTTACATATAAGGGG

>Unigene000236

GACAGGATGAGAGACGCGTGGCTGTAGAGAGATTAACTGAATAACTCGCTTTAAAGACAG

TGGAGCCAGCGCCGTGGAAAAATGATGATGTTTTCTTTCATCCTAGGCATGTCTTTGCCT

CCTTATAGGTTGATCTATCATTGAACTAACAAGAACTGCAGAATCTCGACGCATAAAGAA

AAAAAAAATATATAAGAAAGAACAGGAAAGCCAGATTTAAAATTTGTTTTTACACTGTAC

TGGTGGTCATACTAAAGTGACCATGCCCCACCTGCTCAACAATGACAGCTCCTTCAGCTC

CAGGCAAGAGCTTCTGGATCTGCAAGATGGACCTATTGGCCCCAGTGTGGACTTGCCAAG

CCCAGTGTCACAGATCAGCACACCTACATGCTCCACCACTCACCGCCCATCTCTAGATGC

CCCTATCAAGACAACAGAGAATTCTGTCACTCGTTCTGGGACAGCGGTTGGGGTTGATTT

TTTGTCCACAGACACAGAGAACCTGTGTGGATGGGGGGCTGTGACACCCCGTTTTATCCA

GAGCTTTAACACAGTCCGCTGGGTGCTCTTTTTCCTGTGTGTGGCCTCATTTCTCCAGGG

CATGATAATCAACGGATTTATCAACACTGTCATCACGTCAATTGAGAGACGCTTTGACTT

GCGCAGCTATCAGGCTGGACTAATTGCTAGCTCCTATGACATTGCTGCCTGCATGTGCCT

GACATTTGTCAGCTATTTTGGTGGCACAGGGCACAAGCCCCGCTGGCTGGGCTGGGGAGT

TTTCATAATGGCTCTCGGCTCACTTGTGTTTGCACTACCGCACTTCGCCACAACAGCTTA

CCAGCCTAGAGTACTGGATCACACTGGGCTGTGCTCAGCTAATCAGACAAATGCATGCGG

GGACAGTCAAGGGAGTGGACTGTCATTGTATCGATACATCTTCATGTTGGGCCAGTTTCT

ACATGGTGTTGGTGCTACACCTCTCTACACGCTTGGCGTTACTTACCTGGATGAAAACGT

CAAGTCCAGTTTTGCACCAGTCTACATAGGTATCTTTTACACGGCAGCTATTATAGGCCC

AGCAGCTGGATACCTGCTGGGTGGATTCTTCCTCAACATGTACACGGAAATTGGCCAAAT

AACTCTACTTACCCCAGAAAGTCCACTGTGGGTGGGGGCATGGTGGATTGGGTTCCTGGT

AGGAGGAGCCGCTGCCCTCATCACTGCTTTACCCATTTTGGGCTATCCTCGCGAACTACC

AGGTTCTCAGCGCTTCATTGCCATGCGTGTGTCTGAGGCTCATCAGCTAAAGGATAAAAG

TCAAGCGACTGCTTCTGATCCTCAGTTTGGCAAGACAGTGAAAGACATGCCAAGGTCAGT

GTTGCTCCTGCTTAAGAACCCTACGTTTATTTTTCTGTGTCTGGCTGGTGCTACAGAGGC

CACTCTTATCGCTGGCATGTCTACCTTTGGGCCCAAGTTTCTGGAATCTCAGTTCAGTCC

TAGTGCGTCTGAGGCAGCTACCTGGTTTGGGTACATGGTGGTACCTGCTGGAGGAGGCGG

CACATTCCTAGGTGGCTACATCGTGAAAAAGCTGAACCTACGTTGCCGTGGCATCATCCG

TTTTTGCATGCTTTGTGCCCTGGTGAGTCTTTTGGCTGTCTTTGTCTTCCTCCTGCACTG

CCCCAACATGCCTATGGCTGGAGTGACTCAACCATACATGAGCAGCAATCAAAATATCAG

CTTCCCCTACACACATCTTCAGAACAGTTTTGTAGCAGGAAGTAATTTGACTGCAGAATG

TAACAAAGAATGTCACTGTGTGGAGGCGCAATATAACCCAGTGTGTGGAGCTAATGGTGT

AATGTACTACTCTGCCTGCCATGCTGGTTGCAGGGCTAATAACAACACGCACATGGGCAC

AGGAAAACAGACTTTCACAGGCTGTAGCTGCATTCTGGGTAATGTTTTGAGAGGGGAAAA

AGGCCTGGCCGCAGCAGGAAAGTGTAGTTCCTCCTGTAACCACATGCCAGTTTTTCTGAC

CTTCTTATTCGTCACCATTTTCTTCACCTTTCTGTCTAGTATCCCTGCTCTCACTGCTAC

GCTGAGGTGTGTCCCTGACAGTCAGAGATCGTTCGCACTGGGCATTCAGTGGATCTTGGT

ACGAACTCTAGGTAGCATCCCAGGTCCTATCGCGTTTGGGTCAGTGATTGATATCTCATG

TCTGCTGTGGCAAGAGCAGTGTGGAGAACGTGGGTCCTGTTACCTTTACCAGAATTCTGC

CATGAGCCAATACACACTCATTGCTGGCATCGTCTACAAGTTTTTAGGCACTGTATTCTT

CATGCTGGCAAGTGTACTGTATAAACCTCCTTCACAATCTCCTCAAAATAGTTGTCAGAA

CACAGACCACAATGCTGCAGATCTAGGTGTCGACATTGGACATCTGCACCTGCCACCTGA

GATCATAGTCAATGTTCATGCCAAGTTGTGACGTCCAACTCCAACTGCACACCCTGCATG

AGAGTGTGTGAATAAGTGTGCCCATCAACTTTTTTCTTAACCTCAGAGAAATGAACCTGC

ACTGTGGTCTTTTCCTTTTGGGCCAGATTTGCCTTGCACAAATATTTATCTGCTACAGAA

GGATCCAACATTGCAAATATCTTGCAGATGCATCAAATTTGTTCATAATGGACTGTTAAT

AAAATGCTGAAGGATACAAAAAAAGGGACATGGTCATAGATTAACTGTTTGTGTTTTAAA

AAAAAAAAATCACTATAGTAGTTTTTAGGCGTAATACTGTAGGCCCACCACTTGTCCAAC

TTCTGCTAGGATAAGTTATTCATCAGATGGATAGGGTATGTTTGGAACAGAAAAAGAAAA

GAAAGCCAAAGAATTTTTTTGTGTTTTTGTTTAGAAATTTCAATTTTAAGCCAGTGAAAT

CAGTACTTGTAAACATACACATAAGCACGCAAACATAATTGCAAAAAAATAATAGGCTGC

AGGGATAAAGTATTTTTCTAGACCTACCCTCAAGTAAGATTCACACTAGTCTCAGCCTTG

GCTAGTCCCACCCCTACAAACCGCTAGCTAAACATGTACTACTCAGGATTTCCAAAATAC

ATGTTTATCATATTGTTTAGGTTTTAACTGTAAACAGAGCCATCATAAAGCTCTGCTTTC

CTCATGGAAGTCAGAAGCTGTTGGGTTCACAAATAAAGGATGATTTTTTTTCAATAGTTT

GCACCAGAGTGGATTAAAACTTGATGTCAAATGGTAAATACAGAATAACTCTGAGATATT

TATTGTCCCAGACTTCCTTTGTAGTCTCCATTTGACACCAGTTTTAGCACCTCTTTATTC

TATATACAGTAAAGCTCAAAAACAACACTAGTAAAGGGATAGAATAGAATATTAAAATAT

TTTGAGCTTGTGTTAACCATAAACATTATTTCAAGCATTTCATCGAAAGTCTTTTTGAAA

ACGATGCATTGTATGCAAATTTTGGGGGGTTTTTGAACTTGGGACATTAGTTAGAAAGAC

AGTCAATTCTATCTGTTGTCCTTCACAAAGTCACTTCGGCCGCATTTACACCGCATGTAA

CAAGTAACTCAATTCCGATTTCTAATTTCCAATTCAGATTAGATATGACCAACAACTGTG

TTTGCAAAAAAAGGACTTTAATGGATTTTAATATTCTTTCAGGCATCATTAATATGTGAC

CAAAATTGAATTTAAATGATAAAAAGATATAACTTGGCATTCAAATGCTCTCTTTTATTT

TCCATCCAAAGAGAGCTTTTTTTGGTGACCTTTAAATATGACGCGGAAGCAACATACTGA

ATAATCATTTCGTCAATCATTTCTTGGTCCATTACGTCCACGGCTTTACGCGCTTACTTA

CTTTTTACTTACTTTTTTGATCAGCACATCTACCTTGGACTATTTTGCTGTGTATAGTGT

AAATGCAAAATTTGGATATGGGTCAGTTTTTAAAAGATGATGTAAGCGGTTTGTCAAAAA

ATCGCAAATAATCAGAAAACTGGAATTGGGCATCAAGATCTGCAATGTAAATGCGGCCTC

TGTTATCCTTAACAGGGTCATAGTTGATCCAGTATTGGGTATGAAGAAGGCATATACCCT

GGTTTAAATCTATCGCAAGGCACCATACTCACATATTCACACACAAATTCTGCTCACACA

CAATTCCACATTAGCAGCTGCAAGGAAACGAGGAACGCAAAAGAACTATAATAAGAATCC

ATCCTGCTACAGTGAGGCAGCAATGATACCTGCTGCTCTATTGTGGCTTTCATAATTTTT

TAATTATAAAATAAACAGTATGTTTATTTGTTTTTTTGTCTATTTACAAAGCACTTATTA

AAATTGGTACTAATACATAATAAATAATGCAAAAAACTGTTATTAAATAATTAAAACCAC

AGTAATTAAATCAACCATTTATTTAAAAAGCTTTTATGCACATGGCAAGAATCTATGTAT

CATGCTACACTGCTTCACCCACAACCATGTTTGCACTTCTTGAAACTAATTCACTGAACA

CTATATATAGGAGCGTATATGATACAATATTAATGCAGTATAGAACTATGTCACACAATT

TGAGTTATTTACCTAAAATAACTAAAAAGTATAATAGAGTTTATAGCTTGATACTTGATG

AAAAACGATTTCAAGTGCCAAAATGTCCTTTAGGACTGCGCAGGTTAGAATGGTCTCATA

AATATGGTTTATGTTACAGCTTACAAGGAAAGGTGATGGTTGTATAAAACAAAGATAATC

TGTACAAAAGTATTTGGAAACCTGATCTATATGTGTTTTTTTTTTAACATTTTAATGCAG

ATTAAGGGTGTGTTAAACAAGTACACAGCATTTCCGACAACTTTTTCAAACAATAAGCAA

CGTAAGTTCATTTGATGAACCGGGAACCGGGTAGTTATGCTTGGAAATTCTGTCCTTTAA

AGAATCACGTTTAGAATTGCGATTGTGTTGCACCTTGTGACATCATGTCTTTTCTGTTTT

AGTTAATCGAACAGCACCAGAGTTAATTTGGTAGCAGACTGAGACCCATCTTAGTCTGCT

TGTTTGGTGTACACCAGGGTTCATATGTCAGCATTTATACTTACTTAAATAAACCTCATT

ATTCGAGCAATCGCATCGAGTTCACTGTAGTCAAACCAAATGCGAGTCTCACACTTATTC

CTAAAAAACCTTTATAGATTTTGAAGTGTGAATGTGGGGATTGTTTTATTTAGATAAGAA

GGTCTGGGGTTGCAGTCAGTATTCCAGTTAATCTGTACAGGACACTAGAGTTCTGTCACA

CAAACTTTAAAGTTTCATTGCTCAATAATGGGATGCTACAATGTACTAAGGCATTTTAAA

AAAAACTGTTCGGTTCCCACCTTTTGCAACAGTTTGGGGAAGACAGGCTCTTGTAGGTTT

GCTGGTA

>Unigene000237

GGGGATGAAAACACAGCAGCGAACCGGTGCGGTCTATGTGCGGGAGTGAAGGGGAGCGAA

TGGCTCGTGCGGTGCTCAGTTAATCTGACTCGTGTTTCAGTTTTCCGGTAATCCGACATC

ATCCGCGCCTTAAACATGGACCTCGCGTCCAGAGGCTCGCGCCGGAGACATCCCACAAGT

TTGTAGTGACTTTTGAAAACTGACTCGAGCCATTTTTAAAAACACACAGTCACTTTTTTT

GGCCTTTTTCCTGCGCGCGCTTGTTGGGATTTAAACGGAAAGTGACGCGGAGCTGCAGGG

ACGTGTTTCTGCTCATCATTCCGGCGGTTTTTCTGTGCTCGGGGTTTATTCTGCGCGCCG

CGTGCTGTGTGTTTTAGAACCTAAAGATTAAATACTGAGTGGGTTTAGCGCGCGCTCCGA

GCTGCCAGGACACACCGGCTCCCCTACTAAGTGTTTTGAAGAGCTGAACATCATTGATTC

ACAGTTTGGCAGTAGGTTTGAGACCCTCCTCATGCAGAACCACGGGAAATGAATAAACCG

CCTCAGCCTATCACAGGACCGCCCTCTGTCCCTCACCCTGCCCCCTCACCTGGATTGACG

TCGGCTGTTTATCCCCCCGGTCAGGCTGCTTCTGTTGTTTTCGCCACCCCACCTCCACCA

ATGAACTCTGCACCACAACCCAGACAGTCGTATTATCCCCGGGGAAGTTTGCAGAGTAAC

GCTCCACGAGTGGCAGCAAGTAACCCGCCACGCCCTGTCCCGCCCCCTCACGTCTACCAA

TCGTCTTCACAGATGGTGATGATCCCGCAGCAGCAGCTCCCGTTTCCTACCAACTCTCAA

GGTCACGCCTATTTCATTCAGTCGGGACAGTACCGCGCGCCTTACGTTCACCAGACGCAG

CAGTATTCAGTGGCTAGCGCACCAGCAGGCTTTTACCCTGGAACCAACCCAGACTACAGC

TCCACATACGCTGGAGCATATTATCCTGCTCAAGCTCAGTACCAACCCTCCGTGCCCACC

GCACCCGTTATGATGAACCCTACCCAGCAGCAGCAGGCTCCGCCCCCTCCACAGCAGCCC

CTGCCCCAACAGCACAAAAGGGAGCGCAAACAGATTAGAATACGAGACCCTAATCAGGGA

GGACGGGACATCACAGAAGAGATCATGTCTGGAGGGAGGACCATGTCCACACCTACGCCT

CCACATTCTGTAGGTACTGAAGTTCAGACAAATGGCGAAAGTGTGACCCCTGCAGCTCCT

GTGGTCCAACTCGATGATCATGTAAAGTCTTCAAGTCCTGCCTTGACCCCGCCGCCTTGT

TCAAATCCTTCTCCTGAGCCTCAACATGTCCCGGATGTCCTCGCTGATCCGGTGGTTCCT

GTGCCTCCAGGGCAAAGTGAAGACTCCATTTCTAACTCGTCTCCAGCTCTCCCCACAGAG

CCTGTGCCTAGTGATGTTGCAGATGCTCCTGAGACCACACCCCAAAAGCAGGAAAAGGAG

GAGCTTAGCACCACTGTTACTGAACAGTTGTGTGAGAATACAGAGCCTGAAAAGGAGCTG

GAGCCCAGCTCAAAAAAGTTGTTTGCTGACTCTAACTCTGACATCATGCCTGCTGAGGAG

GACACAAGACCTGCAGAAGAAGCTAATGGGGAGACTGAATTGTACGAGAAAACACCTGCC

TTACCACTTACAGCCATGCCTGTACCTGAGACCAAGATGGTCCCGGTGCTCAATGGTCTG

CCTCGGGAGCATGAAGATCTGCAAGACGAGAAAATTGAGGCAGAAGCTGAACTAATTCCT

GAGCTTTCAGAGTTTCAGGAAGTTACTGTAAAAACCTCTGCAGAAAAGGAGGAGAGAGCG

AAGGAAGAGCTCACTCCTGCTGATGAGATGACTATGCAAGCTGTTCTGTCTGTGCCAAAG

AAGAAAAAGCTGAAAAAAAGCGATCTGAATAATAAAGTAGTTGGAGACGTGTTGGATGCC

TATAAAGAGCCGGAAGAAAAGGACCTTATTCCTGAGCCTGCTCCCATCGAAGTCCAGACC

TCTGAGCCTCTCCCTGCCACTTCCCCCTTACCTGAGGACACTGAGGAGACGTGGGAGGAA

AAAGAGGACAAAATGGACACTGAGAACATTGAGCCGGACATGGCAAAATCTGTGGAGCAG

AAATACCAGTATAAAGAGGAGCACTGGAAACCAATTAATCCGGAAGAGAAGAAGAGGTAC

GATCGGGAATTCCTGCTGGGCTTCCAGTTTATCAGTGCCAGCATGCACAAACCCGAGGGC

CTGCCGCACATCAGCGATGTCGTTCTGGACAAGGCTAATAAGACCCCACTGCGTCCTTTG

GACCCAAGTCGTCTGATTAATTGTGGTCCTGATTTCACACCTTCATTTGCTAATCTTGGC

AGAGCGCCCCCTGGAGCTGGAAGAGGGCCTCCTCCAGGGCCACGTCGATCTCAGCAGGGT

CAGCGTAAAGAGCCTCGAAAGATTATTGCCAGCGTTTCTCTGAACGACGACGTGCAACTG

AACAAAGCTGAGAAAGCCTGGAAGCCAGCAGTGAAGAAGAACCGTGGCGATGAGGACGAC

CCTGAATCGCTCAAGACCCAGGATCTGTTCCGGCGAGTGCGCAGCATCCTGAACAAACTG

ACACCACAGATGTTTCAGCAGCTCATGAAGCAGGTCACAGAGCTGACCATTGACACAGAG

GAAAGACTTAAAGGAGTCATTGACCTCATCTTCGAAAAAGCCATTTCCGAACCCAACTTT

TCTGTTGCTTATGCCAACATGTGCCGCTGCCTTATGGGGCTGAAAGTTCCCACTTCAGAC

AAACCGGGAGCCACTGTGAATTTCCGCAAACTGCTGCTGAATCGCTGCCAGAAAGAGTTT

GAGAAGGACAAAGATGACGACGAGATCTTTGAGCAGAAGCAGAAGGAGCTGGACGCTGCT

ACAGAGGAGGAAGTCCAGCAGCGGTTGAGGGAAGAGCTGGAGGAAGCAAAGGATCAGGCC

CGCCGCCGTTCCCTTGGCAACATCAAGTTTATCGGGGAGCTGTTCAAGCTGAAGATGCTG

ACTGAGCCAATTATGCATGACTGCATTGTGAAGCTGCTAAAGAACCACGATGAGGAGAGT

CTCGAATGTCTCTGCAGGCTTCTGTCTACTATCGGCAAGGACCTGGACTTCGAAAAGGCC

AAGCCCCGTATGGACCAGTACTTCCACCAAATGGAGAAAATAATAAAGGAGAGAAAAACT

TCTTCAAGGATCCGCTTTATGCTGCAGGACGTGTTGGACCTGAGAAGAAATAACTGGGTT

CCCCGGCGAGGTGACCAGGGACCTAAAACCATTGACCAGATCCATAAAGAGGCAGAACTG

GAGGAGCACCGTGAGCAGATTAAAGTGCAGCAGCAGCTTCTTTCCAAGAAAGACTCGACT

CGAGGAAGAGGAAGCAGGATGAATCCGCCACAGGACGAGGGTTGGAACACCGTACCCATT

AGCACCAAGAACCGGCCCATTGACACGTCCCGCCTCAGCAAAATCACCAAGCCTGGTGCT

TTGGACTTCAACAATCAGCTTCTTGCCCCTGGTGGAAAGGGCTCATGGGGCAGTTGGGGT

AAAGGAAGCAGTGGTGGAACTGGAGCCAAAACCAGCACTGAACAAGATGCAGGAAGGCCG

GCTACCAGCACCCTAAACCGATTCTCAGCTCTTCAGCAGTCAGGACCTTCATCCACATCT

TCCTCATCCTCACTGGACTCAGATCGCAGAGTACCTCAAAGGAGTAGCTCAAGTCGAGAC

CGTGGTGAGCGAGACCGCGACAGGTTCGATCGCTTTGATCGCCGGGACAGCCGTGAGGAT

CGAGACAGAGGGCTGGACAGAGCTCGTGTGCCTGTTACTAAACGCAGCTTCAGCAGGGAG

AGCGAGGACCGGGCTCGAGGTGGAGATGTGCGGCGTGTCTCCAGCATGACGGATGAGAGG

GAACGTGGGAGCAGAGATCGAACCGCCAGTCGAGAGGCAGCAGTGAAGCGTGAAGCAGCT

CCAACACCTGCCCTGATTAAACCATCCCTGAGCGAGGAGGAAGTGGAGAAGAAATCCACC

GCCATTGTGGAGGAGTACCTGCACATCAATGACATGAAGGAGGCAGTGCAGTGCGTGAAG

GAGCTGAACAGCTCGTCTTTGCTCTTCGTGTTCGTTCGTTGTGCCGTGGAGTCGACGCTC

GAGCGCAGCACCATTGCCAGAGAGCACATGGGCCTTCTACTGCAGAAATTAATTAGCGCT

GCCATTTTACCCCCCGAGCAGTACTTCAAGGGGCTTCAAGAGATTCTGGAGGTGGCTGAT

GACATGGCCATAGACATTCCCCACATCTGGCTGTATCTGGCAGAGATCACCACCCCAATG

CTGCAGGAGGGAGGAATCCCCATGGGGCTGCTCTTCAGAGAGGTGGCAAAGCCATTGCTG

CCAATAGGAAAAGCTGCTGTGCTGCTGGTTCACATCCTCAACCTGCTCTCTAAAGGACTG

AGTCACAAAAAGGTTGGTGCCCTGTGGATTGATGCCGGACTCAACTGGAAGGACTTTCTA

CCTGCTGATGAGGATGTCAACAAGTTTGTGACTGAACAGAAAGTGGAGTTTACGCTGGGA

GAGGAGTCTGAAAAGCCAAGTAAAAAGGAGCTAAGTGCAGAAGAGCTAAGCAGAAACCTG

GACCGTCTGATTCAAGACAAGGCCAGTAACCAGAGGATCCATGACTGGTTGGAGGCCAAT

CTTGACGAGCTTCAGATGACCTCTAGCTTGTTTATCCGAGCACTAATGACGTCCGTGTGC

CAGGCGGCGATCATCTGTGAGAACCCATACAAGGTGGATGCAGAGCAGGTTACCCAGAGG

GCCAAGCTGCTTCAGAAGTATTTAATGGATGAGCAGAAGGAGCTGCAGGCGCTTTATGCT

CTCCAGGCTCTCATGGTGCGACTGGAGCAACCAGCAAATTTGCTGCGGATGTTCTTCGAT

ACCTTATACGACGAAGACGTTATCAAGGAGGAAGCGTTTTACAAGTGGGAGTCAAGTAAA

GACCCGGCTGAGCAAATGGGCAAAGGTGTGGCTCTTAAATCAGTCACGGCATTCTTCGTC

TGGCTCCGGGAGGCCGAGGACGAGTCTGATAACAGCTAAAAAATAATAATAATCTTAAAA

AAAAAAAAAACTTGCAGCACAGGAGTGGTACTGTGCTGTGTGGAAAGACTATAAACTGAA

TTTTGAAACTCCATTAAAAACTCAAGTCAATGGACATGACTACAATCACTTCAAATTATT

TTCCTCTCACTTCTGAAAAAAAATTATTAAAATAAAGGGTTTTTTTGTTTCTTCCAAGGG

AGGAAACGCAAATGATATCAATTGCTTAATGGCCGTGTGCCTCGGCTGTAGTGGACGAGA

ATTTTTTTTTTTTGTTTGTTTTTTTTTCTCTTTTCTTCCCCATTAAAAGTACATGACCCC

GCCCC

>Unigene000238

CGTCCGTACTGCTTATACTGTTGTAAATGTTGATGGTGTATTAATCTACCTACAGTATGT

TTGATAATAATGCTAATGATGATGATGATTGCTTCAAGGTGATGAAGATGATGGGCTTAA

ATAATGCTGTTCACTGGGTGGCCTGGTTCATCACAGGCTTCGTGCAGCTCTCCATCTCAG

TGACAGCGCTGACGGCCATATTAAAGTACGGACGAGTGCTTCTCCACAGCGACCCCTTCA

TCATCTGGCTTTTCCTCACTATATATGCCATCGCCACCATCATGTTCTGTTTTTTAGTGT

CTGTAATCTACTCCAAAGCCAAACTGGCCTCCGCTTGCGGTGGGATTATCTATTTTTTGA

GTTACGTTCCCTACATGTACGTGGCCATCAGAGAAGAAGTGGCCCATGACAAGATCACCG

CCTTTGAGAAGTGTATTGCGTCTCTGATGTCCACCACTGCTTTCGGACTCGGTTCAAAGT

ATTTTGCTTTGTACGAGGTGGCCGGTGTGGGCATTCAGTGGCGTACCATCAATCAGTCAC

CGGTGGAAGGTGATGATTTCAACCTGCTGTTGTCTATATTTATGCTGATCATCGATGCCA

CTGTGTACGGCGTGCTCACCTGGTACATCGAGGCCGTGCATCCAGGGATGTATGGTCTGC

CCCGGCCTTGGTACTTCCCTCTGCAAAAGTCATATTGGCTTGGCAGTGGACGGATTGAAA

CCTGGGATTGGCCGTGGGGTGGTAGCGCTAGGCTAAGCGTAATGGAGGAGGATCAGGCCT

GTGCCATGGAGCACAGACGCACAGAGGAAACCCGTGGAATAGAAGAGGAACCCAGTCACT

TACCTCTGGTGGTGTGCATAGACAAACTCACCAAAGTGTACAAGACGGGCAGTAAGCTGG

CGCTCAACAAGCTTAGCTTAAATCTGCATGAAAACCAGGTTGTTTCCTTCCTGGGACACA

ACGGAGCCGGCAAGACCACCACTATGTCGATCCTTACAGGACTGTTCCCCCCTACCTCGG

GTTCGGCCACCATATATGGTCACGACATTCGCACTGATATGGAGCGTATCCGGCAGAACT

TGGGTATGTGCCCGCAGCATAACGTCCTCTTTGACAAGCTGACGGTTGAGGAACACCTGT

GGTTTTACTCACGCCTGAAGGGAATGGCTGAAGAGGACATTCGAAAAGAGATGGATAAGA

TGATTGAAGACCTGGAATTGTCTAATAAACGCCACTGCCTGGTCCAGACGCTTTCAGGAG

GTATGAAGAGAAAGCTGTCAGTGGCTATAGCGTTTGTGGGCGGTTCTAAGGCAGTGATCC

TGGACGAGCCAACAGCTGGAGTGGACCCATACGCCCGCCGTGCCATATGGGACCTCATCC

TCAAATACAAACAAGGCCGGACCATCCTACTCTCCACACACCACATGGACGAGGCTGACC

TTTTAGGTGACCGCATCGCAATCATCTCCCACGGAAAGCTCAAGTGCTGCGGCTCACCTC

TCTTCCTCAAGAGCACCTACGGAGATGGATATAAGCTCACGCTTGTGAAGAAACAAAGCG

ACTCCAATCCTGCAGATCAGTCGAGTGCACCTCCTTCATCCCTGTCCCTGTGCTCAGAGA

CCAGGGTAACTCAGTTTATCCGCCAGTATCTCGCCTCCTGCCTGCTGGTGTCAGACTCCA

ACACTGAGCTGTCCTACATCCTGCCATCTGAAGCTGTCAGGAAGGGCTGCTTCGAGAGGC

TGTTTCAGGCTCTGGAGCAGAGTTTGGACAGTCTGGCTCTCACTAGTTTTGGTGTAATGG

ACACCACACTGGAGGAGGTCTTTCTAAAGGTGTCAGAGGAAGATCTGTCGCTTGAGAACA

GTGACGCAGATATGAAGAGCTCTCCAGGAGGGGCATCAGTGGGAAAGACTTCCTCCCTTT

TAACAGAACAGCAGTGTGATGGAGACTCGCATGGTGCCGTGACCCGGCCTGAAGTGGAGA

TGAGCAATATAGTGATGAGCTCGAGACTCAGCTCCAGTCAGGCCTCGCTCCACTCAAACT

CCTCGGTGGGCTCAGTGAGAGGTGATGAGGGAGGACCGTACTCTGACTTCTATGGTGAAT

ACTACCCACTCTTTATTAATCCCCAGGACCTCGACTCAGCCAGCCTCAGAGATGAGGAGG

AGCCGACTTCCCTTGAGCCGCACGTGCTAGAGGGCCAGGGCAGTTTTAAGCTGGAGGGCT

GGTGGCTCCGATTTCGCCAATTCCATGGCCTAATTGTCAAGAGGTTCCACTGTGCCAAGA

GGAACACCAAGGTCCTGTTCTCGCAGATCCTACTGCCTGCGTTCTTCGTTTGTGTGGCAA

TGACCGTCGCTCTCTCTGTGCCTGAGATAGGTGATCTGCCTCCGCTCATTCTGTCACCGT

CTCAGTATCATAACTACACTCAGCCCAGAGGAAACTTCATCCCCTTCGCCAACGAGGACC

GGCTTCAGTACAGGAGCAAACTGTCCCCAGACGCCAGCCCTCAAAAGATTATCAACACCC

TGCGTTTACCATCAGGAGTGGGTGCAACCTGCGTGCTTAAAACCCCCTTTAATAGTACAC

TGGACCAGCTAGCGCAGAGCCTTAACCCCTACGCCAACAACGCTAAAACACTGGCTGCAC

GCTACTTTGACCCTATGTGCCTGGACTCGTTTACGCAGGGCGTGCCACTGTCTAACTTCG

TCCCACCTCCACCTTCGCCAGCACCCTCTGATTACCCCGAACCACGCTTTGAAGATACTC

TGTGGAACTACACAGCAGCTCCGCCCACCACTGCCCATGAGATGGTGACGTCTCCTCCTC

CACCACTGCCGCTGTCTATTCGAGAACCGGTGCGCTGCATCTGCTCTATGCAGGGAACAG

GCTTTTCGTGCCCCAGCGGAGTCGGTGGGCGGCCACCTCTCACTAAAGTGGTCACCGGCG

ATATTCTGGTGGACATCACAGGCCGCAACGTGTCCGAGTACCTTCTCTATACCTCCGACC

GCATGAGACTGCACAGGTATGGAGGCATCACGGTAGGAAACATCCAGAAATCCGTCCCAC

CATCCTTTGGAAGAAAGATGTCGCTCATGGCACGAAAGATAGCAGTGCGACGATCGGCTC

AGGTACTCTACAACAATAAAGGCTACCACAGCATGCCCACATACCTGAACGTGCTAAACA

ATGCCATTCTACGTGCTAACCTTCCCATTAACAGAGGCAACCCTGCAGCTTATGGTATTA

CATTGACTAATCACCCCATGAACAGGACCAGTGCCAGTCTTTCTCTGGATTACCTACTCC

AAGGTACTGATGTGGTCATTGCCATCTTCATTATCGTGGCCATGTCCTTTGTCCCAGCCA

GCTTTGTGGTCTTTCTGGTAGCTGAGAAATCCACCAAGGCTAAACACCTGCAGTTCGTGA

GCGGCTGTGACCCTGTCATCTACTGGCTGGCCAATTACATCTGGGACATGCTGAATTACC

TGGTTCCAGCTACATGTTGCGTTCTCATTCTCTTTGTGTTTGACTTGCCTGCGTACACAT

CCCCCAAAAACTTCCCGGCAGTGGTGGCGCTCTTCCTGCTCTATGGTTGGTCCATAACCC

CTATTATGTATCCTGCCTCGTTCTGGTTCGAGGTTCCCAGCACAGCCTACGTTTTCCTCA

TCGTCATCAACCTTTTTATAGGAATCACAGCCACCGTGGCCACTTTCCTCCTTCAGCTGT

TTGAACATGACAAGGATCTAAAGTTGGTGAACAGCTACCTGAAGTCATGTTTCCTCATCT

TTCCTAACTACAACTTGGGTCATGGGCTGATGGAAATGGCCTACAATGAATACATAAATG

AATACTATGCCAAGATAGGTCAGTTCGATAAGGTAAAATCTCCGTTTGAGTGGGATATCG

TGACACGTGGACTGGTGGCCATGACCATCGAGGGGTTTGTCGGCTTTCTAATCACCATCC

TGTGCCAGTACAATTTCCTTCGGAAACCTCAGAGATTGCCCGTGAACAGTCAGCCTATCG

AAGACGATGACGTCGACGTAGCACGAGAGAGACGACGAGTGATCCGAGGAGACGCTGTGA

ATGATATGCTGAAGATTGAAAATCTCACCAAGGTGTATAAGTCTCGTAAGATGGGGCGCA

TCTTGGCGGTGGACCGCTTGTGTCTGGGCGTCCGTCCCGGAGAGTGTTTTGGGCTGCTGG

GGGTGAACGGTGCAGGAAAGACGACTACGTTTAAAATGTTGACTGGAGATGAAAGCACAA

CAGGAGGAGAGGCGTTTATTGAAGGACACAGCATACTGAAGGATCTTTTACATGTGCAGC

AGAGTATTGGTTACTGTCCTCAGTTTGACGCTCTGTTCGAGGACCTAACCGCCCGAGAGC

ATCTGGAGCTGTACACACGTCTCCGAGGTATACCATGGAAAGACGAGGACCGGGTGGTAA

AATGGGCTCTGGAGAAGCTCCAGCTCTCAAAGTACGCCGACAAGCCGGCCGGCACGTACA

GCGGAGGAAACAAACGCAAGCTGTCCACAGCCATCGCGCTCATCGGATACCCGTCTTTAA

TTTTCTTGGTTGGAGATTATTATGACTAAATTGTCTAATCTTCATTCTGTAAAACAGACT

TTGTTAACGATCACGTCCTCTATTGTCTTACAGGATGAACCCACTACCGGCATGGACCCG

AAGGCCCGCCGTTTCCTCTGGAATCTTATCCTCGACATCATCAAGACAGGACGATCGGTG

GTGCTGACATCACACAGTATGGAGGAGTGTGAGGCATTGTGCACTCGCTTAGGAATCATG

GTTAATGGACGGTTCAAGTGTCTGGGCAGCATTCAGCATCTCAAGAACAGGTTTGGGGAC

GGCTACATGATCACCGTGCGCACCAAGACCACCGCTAGTGTGAAGGAAGTGATCCGCTTC

TTTAACAGGAATTTCCCAGAGGCCATCCTAAAGGAGAGACACCACACTAAGATCCAGTAC

CAGCTAAAGTCGGAGAATATCTCTCTGGCTCAGGTGTTCAGTAAAATGGAGCAGGTGGTG

GAGGTGCTGAGCATCGAGGATTACTCTGTCAGCCAGACCACCCTGGATAATGTCTTTGTG

AACTTTGCTAAAAAGCAAAGTGACAACCTGGAGCAGCAGGAGAGCTCGCCGAAGAAAAGC

AACGAGTCGCCCCTGCAGCGTCTGCTGAGAATCCTGCTGCCCCGGACGGCCAACACGGAG

CTGAGCGCTCTGGTCATTGACGAGCCCGAGGAACTGGAGAGCGATGACGATGAAGGACTC

ATCAGCTTTGAGGAAGAGAGGGTGCAGTTGTCCTTTAACACGGATACTCTTTGCTGAAAC

CGCGACCTCCGTCATATAAAAAGTGGAAAACCGCAGCCTCAAATTAAAGACTTCTCGTGA

TCATGGAAAGATGTTCCGCAGCTCCTTTTTCTCTGCGGCTTTGCTCAGTGTGTGTATGTG

GCCC

>Unigene000239

TGATCACATGAGAGTTTAGTTCTTGGTGGAGGAGGGGGATTCCATAAAATCATAAATATG

ATTTTTTTTTTTATTGACTTGCAGGATGTCTGGCTACCCCAACATGTCCCCTGGAATAAT

GAGCTCAGGACCTCCATATGGGCCCTCCATGAATAATATGCCAGGAATGATGAACACCCA

GGGACCACCTTTTCCTATGGGTGGAAACATGCCAAACAACAACAGTGGTATGTCTCCTGG

TCCAGAATTTGCTATGGATGGAAAACTTAACCAGACCCAAAAGATGAATAACAAGGCTGA

AGGGACACCCAAGACGGAGTCCAAATCCAAGAAGTCAAACTCTTCCACAACAACCAACGA

GAAGATCACTCGTCTGTATGAGCTTGGTTCTGAGCCTGAACGCAAGATGTGGGTGGACCG

TTACTTGGCATTCACTGAGGAAAAGGCAATGGGCATGAGCAACTTACCCGCTGTTGGCCG

CAGGCCACTTGATCTCTTTCGTCTCTACATGTCTGTTAAGGAAATTGGAGGTCTTACACA

GGTTAACAAGAATAAAAAGTGGCGAGAACTGGCCACCAGTCTAAATGTTGGCACATCTAG

CAGTGCAGCTAGCTCACTAAAGAAGCAGTACATCCAGTGTCTGTATGCTTTTGAATGCAA

GATTGAGCGTGGAGAGGATCCACCACCTGACATCATGACTGCAGAGGGCAAGAAAAACCA

AGCTAAGGTCCAGCCACCCTCCCCAGCTGGATCTGGTTCCCTGCAGGGACCTCAGACCCC

CCAGTCCACTAGCAGCTCAATGGCTGAAAGTGGAGATTTAAAACCTCCTACTCCTGCTTC

TACCCCTCATAGCCAGATGCCCTCAATGCAAAGTGGAAGTGTGAACCTTCAGGATCCATT

TGCTGATAACAATGACCCAGCTTTCTCTAGGAGAAACACCATGACTCCCAACTCTGGCTA

CCAGTCTGGTATGAGCACTCCAGAGATGCCTAGCCGCATGGGCCCTTATGAGCCCAACAA

GGACCCATTCAGTGGCATGCGCAAGGTTGGGGAGCCATATATGACTGGTGGACAGGGTCC

CAACAGCAACATTGGAGAGCAGTTTAACCGTGGGTCAGGCGGTCCAATTGGAAATATGCC

TATGGGGCAGAGGCAGCAGTATCCATACGGAACAGGCTACGAGAGAAGGCCAGAATCAGG

GATGGGCCCAGAAAACAATATGGGACCTGGAGTACCACAGCCAAACATGATGCCTTCCAA

CACTGACACTGGGATGTACTCGCCAAATCGCTATCCATCGCAGCAACAGCGGCATGATTC

CTATGGTAATCAGTATCCTGGCCAGGGTGCGCCTCCTGGTGGCGCTTACCCAAATCAACA

ACCAGGAATGTTTCCACAGCAACCGACTAACTACAAAAGACCAGGGGAGGGAGGTTACCC

TCCAGCTAAGCGGCACCATGATGGTGAGATGTATGGCGGGCCATTTGGTGGTCAACAGCC

ACAGCAGCAACCTCAGGCTCCTCCTAATGCACCCTCATCAGCTCAGCCAGAAATGTACAA

CCAGTTTGGCAGCTCTTACCCTGGACCAGAGCGACGACCTCCTGGACCACAAAACCAGTT

TCCTTTCCCTTTTGGAAGGGACCGTATGCAGCCTGGGTCAGGCCCCAACTCTCAAGGGTC

CATGCCACCGCAGATGATGGGTAGCTCCATGCAGTCAGGTCCTGATGGCCCTCAAGCTGG

TATGTGGCAGGGCAGAGGCGAAATGGGCTACCCCAATTATCCTAACAGACAAGGACCTCT

TAGTGGACCTCCTCAGGCCCCAGGATACCACGGTATGAACCGTTCTGAAGAAATTATGTC

ATCTGAGCAGCGTATGAACCATGAAGGCCCATGGCCAGGACAGAGGCAGCCTGCGTACAG

CCCAGCGGGGGCTGGGCCGCCCATGAGCCGCCCCCTGCAGTCCAACTACCAGTCTCCCCA

AGCCATGCAGAATCACATTCCACAGGTGTCCAGCCCCACTCCCATGCCCCGACCAATGGA

GAGCAGGACATCCCCAAGCAAATCTCCCTACATGCATAGCAACATCAAGATGCAGAAGGC

AGGTCCTCCAGTGCCTGCATCCCATATCATCCCTTCACCTGTACAGCCTCCAATGATGAG

GAGAGATCCTCCATTTCCTCCAGGATCTGTGGAAGCCACACAGCCTGTTCTCAAGCACCG

TAGGCGACTTACCATGAAAGATATTGGTACACCAGAGGCATGGCGAGTCATGATGTCACT

AAAGTCGGGTTTGTTAGCAGAGAGCACATGGGCTTTAGACACCATTAACATCTTGCTGTA

TGATGATAACAGTATCTCCAATTTTAACCTCATTCAGCTTCCTGGTTTCTTGGAACTAAT

AGTGGAGTATTTCAGGCGTTGCCTTATTGAGATCTTTGGTATTCTGAGGGAATATGAAGT

TGGTGACCCAGGACAGAGAACCTTGCTGGACCCCAATGCCCTTAAAGAAGAAGAGAATGG

TGAAGATGAAGAAGAGCCAGTTTTAATGGGCATGGAGGAAGATGGGGAAACCGAGGAGGA

GGGTGAAGATGAAATATCACCACAGCCACATGTGGAACATACGTCAGCATTGCAGCCACA

GATTAAAACAGAGAAAAAGGCAGGTGAATCTAGGAAAGATGAAGAGGGCAAAGTGGCGGA

AAACATTTTGCCTGGAGAGGTAAAAACAGAGGCACCTCCTCTAGATCTTTGTGTGACTCA

GGAGAGGCCAAAACAGGCCAGCAAGTTTGACAAATTACCCTTGAAAGTGGTAAAAAAGAA

GGATCCATTTGTTGTAGACTGCTCCGGCAAGCTTGGGCGGCTTCAAGAGTTTGAAAGTGG

TCTGGTGCACTGGAGTATTGGTGGGGGTGATACTACAGAGCACATTCAGACCCATTTTGA

GAGCAAGATTGAATTATTGCAGTCAGGGAACCAGACTCTGTCTACCCAAACATGTAGAAG

GAAAAAAGCATTAGAGAAGACTAAGACTGAGGCATCCATAGTCCCCTCATGTAGTGCAGA

GCCAAGGAAAGAGAAGGTGCAAACACAACCACAGCAGTCTTCTACAAGACAATTAACTGA

CGGTGTTTTAAAAGAAAATGAGGATAATAATGCCAATGTTCCTGAGTCTTCACTGGAATC

AGTATCGCAAAAAGAGAGAGAGGATGTTGGAGCAGGGGGATTGAGCCAAGAGGCCAGCAA

GTTGGCTCTCCCGATAATCAGTTCTCCCTCACATAATCAGCGTTCTGTTACTGTCCTAGA

GGATGAACCTCACAGCAAAGATGAAGCACCTCTTATTACACTTTCAGACTGGCAGGACTC

CCTTGCCCGCCGCTGTGTGTGCGTCTCAAACATAGTCCGAAGTCTTTCGTTTGTGCCTGG

CAATGACTTGGAGATGTCCAAGCACCCAGGGCTGCTACTTCTATTGGGCCGCCTTGTGCT

GCTTCATCATCATCATCCTGAGCGTAAACAGGCCCCTTTAACGTATGAGAAAGAAGAAGA

GGCAGACAAGGAAATTGGGCAGAAGGATGAATGGTGGTGGGACTGTCTGGAGCTGCTGCG

TGAGAACTGCTTGGTAACACTGGCTAACATTTCAGGTCAGCTTGACTTTTCTGTCTACTC

TGAGAGCATCTGCTTGCCTCTGCTTGATGGCCTGCTCCACTGGGCTGTATGTCCCTCTGC

AGAAGCCCAGGATCCCTTCCCTACCCTTGGGTCTAATGCAGCTTTATCTCCTCAGAGACT

GGTTTTGGAAACTCTTAGCAAACTCAGTATCCAAGACAATAACGTTGACCTTATTTTGGC

AACCCCTCCCTTCAGTCGACTAGAGAAGCTTTTCGGAAACCTTGTACGTCTGGTTGGGGA

CCGAAAGGTGGCTGTATGCCGGGAGATGGCAGTTGTGCTTTTGGCCAACCTTGCCCAGGG

CGACAGCCTTGCAGCCCGTGCCATTGCGGTACAGAAAGGCAGTATAGGAAATCTTTTGGG

CTTCCTTGAGGACAGTTTAGCAGCCACACAGTTCCAACAGAACCAAGGCTCCCTGCTTCA

CATGCAGGGGGCGCACTTTGAACCTACAAGTGTGGACATGATGCGTCGGGCTGCTCGGGC

ACTTCATGCCCTTGCAAAAGTCGAAGAGAACCACTCAGAGTTTACACTCTATGAGTCACG

GCTGCTGGATCTCTCAGTCTCCCCCCTTATGAACTCTTCAGTTTCCCATGTCATCTGTGA

TGTACTGTTTCTGATTGGCCAGTCATGACAGCTGTGGGGAGCGGTGGCTCCACCTTCTCT

GTTTTTTGTTCTCCCCTTTCCTCCTGTGTCCTTGGTGTGCAGGGAATGAAAAGCAGAGAA

AATGACTTTACCCCCTTTTTTATTTATGCAAGGATATTACCCCATTGGTCCCTTTTTTGC

TGTCTCACTTTGGGGAGATTATCACAAATAAGCTGTGGCTCATGAACCAAGAAACAAAAC

AATAACAAACTGTAAATTCTGCCAACTGATATGGCCAGGGACCCCAGGTTTTATGCTCTG

CCCAATGGGGAAGGGTGGGTTACTTTTTTATATCAAAATAAATAAATGACTAAAATAAAT

AGCAGAGAACCTTTTGACCAAAGTTGCTGTCTCGTTTACAGTGAGTTTGGGGTTAAAATG

CCCTTGCTCTACACAGGGGCATCTCTGGTATTTGAGTCTGATTAGACGAGTGAACACTTT

GTTGGTGTTGTAGGTGTTGTGATGCTGCACGTTCAACCCCGAAGGAGCCACTTATCAGCC

TTGAGCAGGATACTCTTACATTGGAAGCCTGTGCAGTAGATTGTAGACGTTTTCTGTACA

GATCTGTAACCCTGTTCCTTATGACTGTAAACACTGCAGCACTGTTTCATGTGGGGAAAT

CTTGGCTTAGCAGCTCCACTCTTGTTTTTAAACTGTTTTAATTTTGGTTTGATGTTAAAG

AGAAAGGCTTTCTCCCCAAAGTATTGAGAACCATGGACACCCCTTACCGCCTCTCTTTAA

CCCCTTGATTGTATGACTTGGCCCTTATAAAGAAATCACCTCTGAGGGACTGATTTTGAA

ATAGGATACAGCCCATGCTATGATGTATATAAGATGTTGTACATTACACTTTTTCCCCCT

CTGTCTCTTTCTCACCCTGTCTTACACTCTTATGCTTTTCACTTGTCTATGAATAATAAA

AGCGGACACTTTGATTCATAGCATAAAATCTTTAGATATATAAACGGAACACCTTTTGGC

CTCCCCACAGTTTCTCCATAGTTCAGCAAACAAATAATTTTTTTTTCTCCTGGTACAAAA

TGAGCTCTAGATGAAGAAAGTTGGGATATTTTTTTCTCCAGCGACATGAACTCTTCATAA

TTTGTATTTCAGACTATGTGGCTAAAACGTAATGTAAATTCCTCCTATTCCCCCC

>Unigene000240

GGCCTGTGCACTTATTTTTGCGTATAAATTGATAAGGTTCCATCTTTATCAGCTTTGTTA

GGTGTAAGCTTTAATAAAATGGCTCTGGTCATGTGACTTGTCCCTAACACAGTGTAAAAG

ATGTTTAAATGCATATACGTGTTCAATGATTCACATCAAACTTTACAGATTAAGGAAAAA

GTACTACCAATGCCATGATTTAGGATGTGAGTGGTGAAAAAAATGATTTATTCAGTGCCA

TGTAAGAATGATCGAGCACATAATAGTTACAAAAAGAATGTGAGTTCTGAAGGGCCGTAC

TTACATCATCACCTCTACAGCAGAGTTAAGCTTTTACACAGACGTATTATAAAGCATGTC

TTTATATAAATTACTCTCAGTACTGAATTATCAGAAAGCAGCACCTGAATGAGTTTTCCA

TTCAGCGGGGAATGACTGCAGTTTCAAATAGTACTGATTTTTAATTTTTTTTTTTTATAA

AGAGCACCTTTAACATGTTTAATGTTTTCTGAAATATACAGGAAAGCAGTGGTCTGGAAT

GAACAGAAACAATAACAGAAATTATGGTCTATATTAATGATCATATAATTTGATCTTATA

GTCTACATGCAAGTGATACTCGTTTGTTTAAATCAGGGATCACCAAACTCGGTTCTGCGG

GGTTGGTTTAGCTCCAACCCTAACCAAACACACCTAAACTAGCTAATTAAGGTCTTGCTA

GCTATACGCGAGACCTACACGCAGGTGTGTTGGGGAAAGTTGAAGTTTAACCCTGGAGTC

CCTCAAGGATCGAGTCTGGGGACCCCTGATTTAAAGGATTATCAGGGGGCTAAAATACTG

ACATAATTAAAGAATCACAAAACTAGTGTTCTGTGGTCAAAGTTTAGCTTCACACTTTCT

CCTTCCTATAGAGAAAACAAATGCAGCAAAAAGTGCCAGCTAAAATAGAATAAAACTATA

TTCTAGGATTCAAATCCATCAGTTGGGGTTGTACTTTTGTGTTCTGATTTCAGCTCTAAA

TAAATATTTACTCTAAATTGGTTTTCAAAATATGATTTCTTTTCAATTTTCAAAATCCTC

GAATACAAATAAAAAAGCTGAAAGTAAAAAGTTACATAAAAAATATGTAGCTAAAACTGG

CTAAATATAACACTTTCAAAAAAAACCCAACAGGAATGACAAACGTTTCATATACATATT

GATATGGAAAATACAGTTAAAAAATAAAAAACCTCTGCCTCTCAGCATTTCCTGTCCTCT

CTGGTTCGAACCCATCCTGAACAATATGTGCCAATGCCGGGAGACAGCACCAACAGCCTG

TCTTGTTGGCATTTCGAAAGGAACCAGAGGACAGTAGGCGACCCCTCTGCGATGCAGACC

CGGTCCCTGTCATTTTAATTTTGCTTTATTTTTTACACTTTATAAACATGGTGGATCATT

GAAAAAAAAAAACAATCTGCCCAGACCGTTAATCTAATGTGGCCTGCAGTGTGTAGGACT

GACGGATCCTTTACTTTTTGGACTGTGTGATATAGCTGTTCTCGATACACAGCACTATAA

AGTTGTTAGAACAGCTGCTGGGTTTTTGCTGTAGGAGCTGACCAGCCTGTAGAGATGAGG

CCAGACCCATCACGGCGTGATCAGACGTCCTCCACGTCTCGCAGTAGTTATCCATCTGAC

GGTGACCCTTCCCACTGGAGCCATGCCAGATCATCTTCTCTGGCCATGTACTATCGCTGA

GAATATCTCTGCCATCGAACGAATACAAGGGTACGTTGGTCTTTATCCTGCCCTCTGAAT

CACCAAACAGCGATTCCCAGCTGCTGAAGAGCACTTGGTCCTTGAGGTTGACAATAGGCA

ATGTGTTTCTATCGGATTTGCGAACGATGCTGTAGAGATCCTGCAGTTTGGAGGACAGGA

ATGCCCTAAATGTTCCCTGTAGGCCTGCAGCACGTGCCTGCTGGAAGCACAGGAAATCTG

CGCCGCGGATGCCCCTCATGTTCCCTACCTGAGGGCTGTTCAGGGCGATCAGATGCAGCC

CTGGTCCTGATGTGTGCACATGACTCTCAGGCTGATGGACAGGTATGCTGGGTCTGTGGA

TTGAGGTTATGGGGTACCTCTCTGGTGGTACAGGAATGTTTTGATCAGTGCGGGGTGGGT

ATCTGTTGTCCGGCTGATGCGGGTATCTGATGTCTGGATGAGAGGAGTAGTGTGTGTTGT

AGTGTGAATTCGAGCCTGGCTTAGGATGCTCGCTGTCAGGGCTTTCCGGCTGATGGATTG

CAGTGTCAACAGGGGAGTAAACCTCCGCTCCGTTATTTGTCGAGTGGTTTGGAGGGTAGT

GAACCACTGGAGGAGGCTGTACCGCTGCCACCTCGTTGTTCGAATCTCTGTAAAAGGAGT

TATGAGTGCCGAGCAAGACTTTTCTGACGCCTTCACGCACTCTGACGTAAAGTTCACTTC

TGTCGATTACGTAAAAGAGAGCGCCCTCTGGTTGTCTCTGTGCTGTTGCGAGCATCAACT

CGAATGATCTCATTACCGTCACTCCAGAGCTCTGGCCAGGTGGACCAGGTGGACCAGGAG

GGCCAGGAATGCTGACTGCAGGCCTGTAAGCACCTGGTAATGAGGGAATACCAGGTGGCC

CAGGAGGTCCAGGTGGTCCTGGGGTTCCAGGTCGACCTTCATAACCAGCACCAGGAGGTC

CTGGTGGACCCTGTGGACCGGGTGGCCCTTCAATAGAGTCTCCTTTGGGACCAGGGGGTC

CTGGTGGTCCCTGTACACCTCCATAGTGAGAACCGTAGTGACCTCCTGGCTCGCCCTTCT

CTCCTTTCACTCCAGGGTCTCCACGTTCACCCCTCAGCTCGTTCTTTAGTGTATAAATGT

CAAAGTTGCCTGGTGCTCCAGGAATACCAGGGGTCCCACGGTCTCCCTTCTCTCCTTTCA

CACCAGGATACACTCTTCCAGAATCTTCATATCTACCAAATCTTTCCACAGCTGTTGATG

GTCCTGGTGGACCCGGAGGACCTGGAGGGCCAGGTGGGCCTGGATAGCCGTATCCGGAGC

CAGAACCAGCCTCTCCTTTTTCACCTTTACTTCCAAGAATACCTGGACGTCCAGGCCTGC

CTGGCATTCCGATCTCACCCTTCATGCCGGGAGGACCATATGCTCCAGGAGGTCCAATAG

GTCCAGTAATTCCTCTTTCCCCCTTCTGACCAACCGTCGAGCCAAGAAATAGAGGGCTTC

CATCAGGTCCAATAACCACACCTGGTTCTCCTTTCTCACCCTTAATGCCAATCCCTGGAG

CACCTGGTTCTCCTCTCTCTCCTTTAGGTCCTATCGGGCCAGGAAATCCCGCTTGACCAG

GAAGGCCAGACGGCTGTCGATCAACCCCAGTATCACGCCCATCTGCAGACCGATAAATGA

TTTCTCCTGGAGGTCCCGGTGGTCCTGGAAGGCCAATTCCATCACGACCTGGTTCACCTT

GGTCTCCTTTATAACCGTGGTCTCCTTTGGGTCCCTGTGGTCCAGGAAAGCCGTCAAGTC

CAGGACGGCCAACCGCTCCTGGTGGTCCCTGATCTCCTTTCTCCCCAGGTACACCAGGTT

GACCCGGCAGACCAGGTGGACCCCGAATGCCTGCTGGTCCTCTGACTCCTGGCAACCCAC

TGTAAACGTTTACACCTGAACCCTCCAAGTCGTCAAATCCAACTTGATAGTTCGGCCCTG

GAGGCCCAGGTGGTCCCACAGGTCCCATCGGTCCTGGAAGACCGGCAAGGCCAGGCTGCC

CAGTTGCTCCTGGAGGACCAGGTGGCCCCAGTGCACCCTTTTCACCAACAGCTCCAGGTA

AACCCAGCTCACCGGTATCACCCTTCTCGCCTTTAGGGCCAGGCAGGCCGGGCTTTCCAT

CAGAACCGGGAGCACCTGGAAGTCCTGGATTTCCTGAAGCACCATCCTTTCCAGGTGGAC

CAGAAGGTCCGGGGACACCCGAAACAGAACCATGTAAAGCAGTAGAAGTATTTAAAAGAC

CTGGGGGTCCAGGTGGGCCGGGTGGGCCAGGAGGACCAGGTAAACCAGGCATTCCCCGCA

AACCCTCAAAATCACCAAAGCCAGAGCCCTCCATGTCTACAAACGTAGGCCTGTCTGAGC

GAGAGAGGGGTCCAGGGGGTCCTGGAGGTCCCGGTGGTCCCCGTGGGCCAGGATGACCTT

CACCCTTGTCACCTTTTTCTCCTTTAGCTCCAGGATCTCCAGGGGTTCCTGGGAAACCAG

GTGGTCCGGCTGGTCCAGCTTTTCCATCTTCACCAGGGTCACCAGCTTCTCCATCAGCCC

CGGGTTGTCCTGGTGGCCCTCGAGGTCCTGGAGACCCCCTCGGTCCTGTTACACGCTGCA

CCACTGAGCCGTCTCCTTTTTCCACTATGTTCACACTGGACCCAGGGGGACCAGGAGGGC

CCTGAGGACCAGGAGGTCCTTGATCTCCCTTTGGTCCAGGATAGCCAAATCCTGCAGCAC

CCTTCATCCCTTTATCTCCTTGGCTACCTTTCTCTCCCTTAGCACCACCTCCAGATCCAA

TGCTGGATCCTGCATCCCCCTTGGGTCCCTCGAGACCACGGTCTCCTTTAGGTCCTCTGT

CTCCTTTGTCCCCTTTTTCTCCCCGAACTCCAGCACCGGTTAGCACTGTGTGCACGTCTT

TGCTCACTGTAACCACAGTCGGGTTCACTGGCGGCTGCTGGAGAGGTTTGGAAGGTTGAC

TTGTGGTCGAAACTGTGTGCCCGGGGCTGTAAAAACCGCTTCCGTATTCACCTGATGCTC

CATCAGAATCATCCTCTTCTTCCTCACAATATTGTGCAGCTACACGAGGGTCTCCAACCA

CCCTCAGATCTCCAATAACACCCACAAACTTATCATTATCGGCTCCTCCAGCTTGACCCA

CAAAGATCCCGGCTCCTGGTTCCAACTCCATTTCATCTGGAGAACGCTCAAAAGGAAGCA

CCTTGGGTTCCGAGTCACAACTGGGGTACAGGGTGACTTTATCATCCAAAACGCTAATAG

AGAAACGATTCCAGTAGTTGACAAGAGAGGGAACAGTGAAGCTGGCAGCCTCATAAGATG

CCTGGGAGTCTGGCTCGGTGTAGAACAGGACAATATTCTGCTTTCCATCCTTCACGGGTG

TAAGCTTCACCCCTATGTACATAATTTTCTGGTTGGTGTCGGTGATGGAGAAGATGACTC

CAGCTTTGGTGGAAGTTGGTTTCAGGTTGAACATGAGCGAGAAGTCACGGTAAAATGATG

TGGGCAGGTACACGCGGGCCAGCTGACCAGTCTTGGAGTTGTGATCGAGAACAAAGCCAG

GGTCTGGTTCATAACTCTTGGTGACGCCTTCAGGAAGAGGGTAGCCAATCAGCTGTGATA

GCGTAACACCTCTCTCTGCTGGCCACTGCGACGCTCTTCCGATCTCCACCC

>Unigene000241

TCTCTCTCTCTCTCTCTCTCTATCTCTATTTTTGTCTTTGTCTCAATCACTTCGTGGTTT

CTCCATCATCTACTCGCATTTGCATGAACGATGTCCCATATTTCCTCCATCACTAAAACA

AACAACTGAGACAAAAGAGGAAGAGGGCCTCACAGAAAGAAGTGGCAGGCTCATATCACA

CACTCGCAGGTCAGTTAACGGATCGGATTCCTTCAGCGTACAGACCGAAGCTGGAGCGGC

TTCTCAAATAAGTTTGGGTAATCAACGGGCTCAGTCTCGGAACATTAAGAGAGTGCTGTT

ACCATGGCAACTGGCAAGCAAGGCTTTGTAAGAGCTACCGAGCCTCAGCATTTGGTCTCC

ATACAGAGAGAGAAACAGACGCATGTTCAGGCCCCAGTACCGGCCATGTATATCCAAGCG

GTGTCCGGTCTGCCATCCTACCCTAAGAGCCAGCCAGCAGTCGTGCAGGATGCCGCAGTC

ATTCCGTTTTCCGTGCCAATGATGTATGCCAACCAGCCTCTGCCTCTTCTGACTCTACAC

ATAGCCAGTGGGGCAGGTCTCCAGCAGCAGAGGCCTGAACTTTCAGCATCTTCTTCAAGA

CCCAAGTCTGCAGGAAAACATGTTTGCTTACATTGTGGAAGGGATTGTCTGAAACCCAGT

GTTCTGGAAAAGCATCTGCGCTGCCACACCGGTGAGAGGCCATACCCTTGCCCCACCTGT

GGCATCTCTTTTAAGACACAGAGTAACCTTTACAAACACAAGCGCACCCAGGCCCATGCT

CGTCTATCCAGCGAATCTGGAAAGGGAACTTTCAGCAGCCAGGAGAGTACAGACAGCTCA

GGAGAGAAATGTTGCAGTCCCTTACTGGAATTACAAAACGAAGATTCAGAAGACATAAAC

AAGAATAATGACATTTTTCCTGCTGTGACAACGAGCACCTGTCCTTCAAAAGAGGTTAAG

ACAGGCTCTGAGACAGAGTGGGCTTTATGCAAAGCTGCAGTGGTTGGAGTCATCGCTTCA

CCAGCACATGGACAACAAGTAATTTCGCATGCTATAACCCAGAAGGCACCCACGTTATCA

TCTGATGCTGCACCAATACAGGTAAAAAGCACAAATGGAGATGGCCGTGGTCCATTGACT

CCAAATCGCACTCCTCTACAGAGGCAGGAGGCACTCTTTCCCAAGTCTCAAAGTCATGAC

AGCACAGACTCTGGCTTCAGTGAAAGCAGTGAACAACAATCAACCTCTAGTCCTGCTGCT

ACTGTGAGTGAGCCCAGTATGGATCTGGAAGAAGCTGAAACTTCTGATAAACCGTCATAT

GTTGGTAACGATGACACCAAAAGCAAAGTGTCACTTCAGGAGAAACAACAACTAGAGAAA

CACATCTTAAAGCTTATCTCTGAGAACAGCGTAGTAATGGACGACAAACACCTGGTGAAT

GTCCGGCCGAGGAAGACGGTGTTGTCGAAACAAGGCAGTATTGATCTTCCTATGCCATAC

ACTTACAAAGACTCATTCCATTTCGAAATACGAAGCAGCAAACACCAAGTATCTGGTTTA

CAACTGCAAGACAGACATGGCAGGGCTTTTTACAGCTCTATGCCTACACAAAATTCTACC

AGCTTGGAACACGGACCGTTAACCCGCAGCAGTTCTTTACCATTTAGCATGGGCATTCGG

AAAAGTGACAGCACAGTTTCACCCCATAAAGTAGACAGCGTTCCATTAAACAGGAGGTGT

AGCGCAGGACATATTAACCCATTTAAGTCGGTAGATCACAACACACCCAGTCATCGTTCT

CTGGTCAGACAAGTAGCAGTGGACGGTTTATCTGCAGTGGAAGGATCTTTAATAAACAAA

AGCATTGTTAGTAGCCTTAGTTCTGATGGAGACTGCAACGACACAATGTCAGAGTCGGCT

GTTAAAAAATGCCGTAAAAAGAAAGCAGAGAAGTTTGCCTATAATAAATGGTACATGTAT

GGAGGCGGTACATTCACAAAGCTCTACAACACGGATAAGGATTCTGGATTGAAGAACAAA

AAGTCCACAGTTAGTCCTGAGCAAACTGATAGACAGGGTATTCAAATAAGCCAGGAAGAA

GATAGCAATTCGTCTGCATCTATAACTGGAAGCCATACTTCCTGTCCTACTTCAAGTTTG

TCTCCTGTTTTCACAGCGCAGACATCAAGTCAGGTTCTTTCCTCTTATGTACTTCAAAAT

ACACCAATAAAACAATCAACTGGGCAACCCCAGCAAAGTCAGACTAATCAGAATGTTTCA

ACTTCAGACCTGAGTACTGTGATAAATGCAAGGAAAGAAACAGAACAAAAAGACAAAGCT

CCTGAAACACAATACACAACCCCAATACCTTCAGAGAGGAAAAAGCAAAAGACAGACAAT

CATCTTTCTGTACTTGCTAAAAACACAGAAGCCTTCGTAAGAATGCCGCAAATTGTGAGC

CGACAGGTAAACCCCGACCTACGTCAAACGTTACATACAGAATGTGCTGACCTGCCCCAG

AATTCAGGTCTATCTTTACTGCAGGGAGACAGTGGGTTATGTCTTAACAGACAAGGCAGT

TTTACTAATATGTCTTTGTCCTCAAATCAGAGACAGCTACTTTTTAAGAGGCACAGCTCT

CCACTTTTTGTGGCCAATATGACTGGGACATCTGCGTCTCCTTCTGTTTCCTCCAGCGCC

TCCTCGGTTTCTGAAGCCAAGACCAGCTTTCTTCCCAAGTACCAGCTGAAGATTCCTGTT

TTGACAGATGGCATGTCAGGTCCTTGTCTTGGCAAAGGTTCCTTACAGAATCAGTCTTCA

CCCAGACAGAATGTTAGTCTTAATCAGCAATCTGCAACAACTTTGCCATCCTCACCACAA

AAGCAGGCAACTATGACTGTCAAGTCTGATGAACAAGGACAAGGTACATTATGTATATCA

GCTGTAACAAAGGCAATATCTGTGACATCTTCTGTCTACAGTTGCCAGAATTCTAATGCC

TACACGTCGACCCTCACCTCAACAACGTCAGCCCCGGTAGCATTTCCGTTAGCATTTACA

GAGATTAGTGTCAATCCCACCACAGTACAGAGCTCAATCTCTCCAATATGCCATTCATCT

CAATCTACAAACAAAATTCATGTTCACACTGAAAATGTGCCATCTGTACAACACAATCTT

GTAAAGATTCCAACGCAATCATCTGAAGTAACACCATCACCTCTACTAATGAGCACTAAA

GATGGCATACCTTTGGAGATATGCAGTACTGAGATTCAGGGATCACAAGACACATTTTAC

GTGCAAACAGCTGACCTCCAAATAGTCATGCAACTCATTTCAGATGAGCAGCTTGCACTG

ATAGAACCACAGATTGAAACACAGGACTATAAAACTAATGAAGGTCACTGTTTTCTCAGC

AAAGATCCGGAAACCTCTGGGACTGCCACAATACAAACAGGTGTTTCACAGGAAATACAT

AAAAAATGCTCTGGTAATACCGAAAAAAAGCTAGCTATTGATCAGAGAAACGTGAACAGG

AGTTTGCTCAGCAATATCCCAGATATAACGTCGATGTCGACTGAATTAACAGTTGAAGAC

CACAAGGCAAAGTATACAAAAAGTGCTCAAGATGTTTATTTAACAAGCACCAGCAGTAAA

GGAGTTATTTCAAGGTCACCTTCCAAGTTAAAGCATGATGTTTCAGCTTTACTGGATAAC

AAGACATTGGGACATCAGTGTAAATTGCTGGGTTCAAAACACAAGGACAGTATGGTGCCA

AAAGACCCAGAGACTCTAGAGTGTAGTGTTAAACAAATTCAGAGCGAGGAGTTAGGTAAG

ATTTTAGCAGACAAATCCGCAGCAAAAAATGTGACAGATAGAAGCAGACCTTGTGTGCAT

AACCAGATCCTGGGAAACTTTTCAACGTCCATAATATGCAGCAACCAGCAAAGTTCTCAA

GACGTTACACACAGTCCACCAAATGTGGCTTCTACCAGCAATCCAGCAGCATCTTATGAG

GGCAAATTGGTGAAAGTTGCAGAAATATCAGAAATAAAAAAAAAAAGCCCAAAACCTCAG

CCTTTGCAAATGACTGTTCAGAAGGACAAACCACTTGTCATCACAAGATCAACAGAAAAA

CTGACAGCTGAAATAAACGCATCATGTATTACTGAGATATCTGAATTCTGTATTCTGAAT

CCACCACCGACCCCCGTTTCATATACAAACCGCTACGAACCAATCCAAGCTAAATCGTGT

CAGACATCAACCATGGCAAATAAGATCAGCTCACAGAATGTAACGGCATGCCGTGTCTGT

CCACAACAACCTTTAAACTGGACAGATACATGCAGTGGTGACAAGCCAACGTCCGAAGAG

AATCAAAGCTCCACTTTAGGTCAATCTGGTCAACATTCAACCCGACAAGACTGGTCTAAA

ATTGAGGAGGTCAAGACAATCAAACCCATTACTGCTCATGTGCCCGAGTCACATGGACCG

GTCTTAAATTGTCTTAATAAGGAAAATATGAAAAGTGCACATGGTCCTGGTGGAATACTT

CACATCATTCAGGATTCCTTTACAGGGCCTCCGGTGAGCTGCTCTCTTCAAAGTTTAAAG

TTCCACTGGGAGGCTGGGACAACGAGACCGAAGTGGATCCCGCTGGAAGCTAAGAACAGG

GGAGAAGATGGAGCAGAGCGGGTGGCGATGTTAGATGTGAAGAGTACATGGACGAAACAA

GAGGAGGAGGTAGAGGGGGCCATGGGAAACAAAAGAGAGCAGGAAGCAAAATGCACTGAA

GTCCACCACAAAGCACTTAATACACATTTGGAACTTTCCACATCCCAGCACGTGTCTAAC

ATCAATGCAGCATCACCCGAGATTGTTTCCAACAGCACAACTGCACCGGTGTATGGACTC

TCTAACCCTTCATCTGTTAGCGAGTGTCTCACCAATAAAGATCTTAAAGGACACACTCCA

AAACACATTGACCAAAATAACACAAAAGAGAAGAAGTACAGTTCTTCTGAATGCTGTGAA

AGGTTTCCAGCCTTATCCAAAGGACAAGGTGACGTGAAGCATCTCTTGGCCATTCAGGAC

AAAGTCCAAAATGAGACACAGTCCAAAAACTCTATACCTCACCTGAATCATTCGACAAGC

GCAAGAGTACAGACTCCAGAGCGAGAAACGGATGGCGAATCAAACAAACCTGCCATGATG

TCTCCTTGCAACATAGAACACTGTCAAATTAAAAAAAACACAACAACATACACACATTCT

TCATTTCCAATCCAGACCAGCAGTTCTTGCATACATTTAAATCCATCATGTACATCCAGC

AATCACAGCAATCCTCCAGAACCTAGTGCTCCACACAGCTACATGGATGAGAAGAAGGAG

GAGGAGGAGGAGAAAGAGGAGGAAGAGGAGAGGTCCAGCAGCAGCGACGA

>Unigene000242

GGTGCACCGGCGGAAGTCAACACAATTGTAAAGATATTCGTAGGGGCGAGCACAAGAGGG

AAAAAAGTGATAATTGGTCACATTGGAACGTTTCAGCCCCTTTTAGACGGTATGGCTTCC

AGTTCCAGTATAGATATAGAAGGCGCGACTCAGCACCTCAGAGATATCTTAAAACTGGAT

CGACCCGCTAACTTAACCGAGTGAAGCAAAGATTGGGAAAGCGTGCTTTTGCCCGGAGTT

TGAAGCAGGTATGAAATTCTGAACGTTATGGTTTTCTGTGACTCCGACCACGCTCCACTA

TCGCGCCATCAGGAACTATCAGCCTATAATACAACTACATAGAACAGCACTGCAGGTGAG

AGCCAGCGGAAGACCTCGTGTAATGGAGAACTAAATGGCCTGCTGGGGACCGACCTTATT

GGAGCAGGAGTTCGAAGTGCTGTAATGGAACCAACAGCTCACGACTCCACTAGCAGAAAT

ATGGGGGATACCCAGACAATAGCCCTGACAGGCGACGACGGCTCGACCTGTATTCCCATC

ACAGCTTGCAACGTGGTTATTGTCGCCAGTCGAGACTCTAGTATTGACACTAAAGCTCGT

GGAAGTAACAAGGTAAAAATTCAACCAGTGGCAAAATATGATTGGGAGCACAAGTATTAC

TATGGCAACCTAATAGCAGTGTCTAATGCATACTTGGCATATGCGATTAGAGGTGCCAAT

AATCAATATATGATACGGGTCCTGCGATTGGGCTCGCCGGAGCGCACCCTGCTAAAGGGA

TTTACTGGAGCAGTCACAGACCTGGCCTTCGCCCATCTAGACTCCACGCTTCTGGGCTGT

GTGGATGAAGCTGGCAACATGTTTATTTGGCAGCTCACCAGCCAAAACAGCAAAATCCAA

GATGAAGTGATTGTGCACATCCGAAGACCTGATGACACACCGCTGAACTCAAGCCGCAGA

CTGATCTGGTGTCCCTTTATTCCTGAGGACAATGAGGAGAACCCAGAGGATGGCTGCCAG

ACAGTGGCATTGCTGCATGAAGACAGGGCTGAGGTTTGGGACCTTGACATTTTGCGCTCC

AACAACAGCACATGGCCTGTGGATGCTACAGAGATTAAAGAGGGCTTTATTGCAATAAAG

GGACACACAGAACGTATCAGTGAGGGAGCTCTGTCTCCAGACGGCACTGTTCTCGCCACA

GCCAGTCATGATGGCTATGTCAAATTCTGGCAGATCTACATTGAAGGACAGGACCAACCA

AGATGTCTTCATGAGTGGCAGCCACATAACGGACGTCCTCTTTCCTGTCTTTTGTTTTGT

GACAACCATAAAAAGCAGGATCCAGAAGTTCCGTTCTGGCGATTCCTAATTACAGGCGCA

GATCAAAACCAGGAACTGAAGATGTGGTGCACAGTATCATGGACCTGTTTACAGACTATT

AAGTTCAGCCCTGATCCAATTAACTCGAGTGTTCTGCCCAGTTTAAAAGCCAGTCTGGAC

CTTTCTGCTGAGTGCCTCATCCTAACAGATGTGCAGAGGAAGGTTCTTTATGTAATGGAG

CTCCTTCAAGATCAGGAGAAGGGACATGCCAGCTTTATGGCTGTCTCGGAGTTCTTGCTC

ACACACCCTGTTCTGAGCTTCGGTGTACAGGATGTCAGTCGTGCTCGCCTTAGACACACC

GAAGTCCTTCCTCCAGACGAGGAGAGCGAAAGCATAACTACAGAGGGAAGCCAGGGACCT

TTAGAGTCGAAATCAGGAATCCAGATCAAACTTTACTGTGTGCATACAAAGTCTCTTCAG

GATGTGCAGATCTGGTTCCAGCCCAACGAGGGTTCTGCCAGCTCCCTGTTTTTGCCTACA

AATTCTGGCTCTCAGGATGGCTTTGGTTTTGCTGACCCTTTGACTGATTTAAGCATGGAG

GTGTTGAGCTCAGATAAGGAATCTGGTGCAAGTGGGTCACAGACTGACCTGTGTAAGATC

CCCTCTCTCCCGGCTCCAGCTGACTTTCTGAGCCCCTCTTGCTCAGCTATGCCAAAACTC

ATGACCCCGGATGCATTTATGACCCCCAGTGCATCCGTACCACCCTCACCTGGAAGCAGT

GCCAGCAGTTTGACTATAGTAACTGCCATGAGCAGCTCAGACAGTGGGCCAAGGGTTGCA

GAGGACCTCACCCAGAGCCCTAAGCTGTGTACAGATGCAGCCAGCAGCAGCTTGAACCTC

AGTGCCAGTACTAGCAGCCCAAGAGCCAACTCCATCCTCATTTCAGGCCTGAGTGAAAAC

ATCCAGGTGCTGCCCTCTCCAAATCCCTCCATGTCCCTGGATCTCCAGGCCACAGAACCT

GTGCAGCTGCCGCAGGCGTCACCTACTAGAGCTCGCTCACCCGATGTCATTTCTTCTGCC

TCTACGGCCATGTCACAGGACATTATTCCTGAGATTGCCTCTGAGACATTGCCAAGGGGG

TTGAACGAGGCACGTGATCCACTTCAGGGCCTGCAAGACAGCATGGCATCAGCAGTTTCA

GTCCTGCACCTCCTTTCACCTCTGGAACTAAGTGGAGGGGCATCTGGGGAATGTGAGCAA

AGGCTAAGCAGCACACCCTCGCTGCTGGAGACGGCTTTGTCACAGGATAACACTGTAAAT

GTGGTCGCATCATCTTTTTCTGAAAATGGTGCAAGCCGCAGCTGGCCTGCAGCCCCAGAT

ATCACGCGCGAAACAAGGAATAGTCTGCGAGATAACGGCCTGGGAGATTGCTCAAGAGAG

GACTTGAAGGACTTGCACATGTCATCCCCCTTTCACAGACGCACCTACCACCTAACACAA

AATGACAGCCAGGATGCCAGTGCAGAGCAGAGTGATCATGATGATGAGGTGGCCAGTCTG

GCATCATCATCAGGAAACTGTGGCCCTCGGGCCTCACACAGGCTGCCAGTGAAGGACTGG

AAGACCTCACCACGTGGATCACCTAAACTTAAGCGCAAAAGCAAAAAAGATGAGGGTGAG

TCCCCGCAGTCCAGGCATTTGGATAATCAGATAAGCATAGAAGTACAGGAGGAGCTGCTG

TTGCTGTTGAGAAGCCAACAGAGGGAGCTAGACGAGCTGCGGCAGAGCCAGCAGGAGCTG

TTGCAAAGGCTCACAGGCCACATGGATGCTGTTCAGAGCTCCATAATGGCACATGTAGAG

CATGTCCTGCTCACACAACAGGAACAGGGACAAAGAACAATGGAGCGGGTAATGGCAGAG

GGACACACACGTAACCAGCAGCTTCAGGAACACATGGCACAGCAGCTCGCTCACACCTTG

GGCACCACCGTATCAAATCGCCTGGACAAAGTTTTGCGTGAGGAGCTGAAGAAATCTGTT

CCACAGACCATATCAAAGAGTCTGGAACCCATGACTGGACAGATGAGTAGTGTTATTGCA

GCCAAGCTTACTGCAGTGGAGGCCACGCTGAAGGACAATGTCACCAAAGTTGTTAAGTCA

AAAAACACTGCTGATGCCATTGGCCGAGCTGCAGCTGAAGCTATGCAGGGTCCAATTCAG

GCAGCCTACAAAGACGCCTTCCAAAGCATTGTGCTGCCTGTGTTTGAGCGGGGGTGCCAG

TCCATGTTTCAGCAGATAAATGACAGCTTTAAACAAGGAACCAATGAGTACATTCAGCAG

CTGGAGACGCATGTGAAAAACAGGAAGCAGCGTGAGCAAGATGCTCGAGACCCTGTAATT

GGTCAGCTGCAGCAGATGATTGATTCATTACAGACTTCCCAAGACCAGCTGGCCACTACG

GTAGCAGCAAGTGTGCGCACTGAGGTTCAGCACCAGCTCCACATGATTGTGGGAAATCTG

CAGGATTCAATCCTGAGTCAGGTGCAGCGTGTAGTGAAGGGAGAAGTGAGCCATGCCATG

AAGGAGCAGCAAGCAGTCGTCACTTCTAGTATCATGCAGGCCATGCGCTCAGCGGCCAGC

ACGCCTGTCCCCACTGCCCACCTCGACTTCCAGGCACAGCAAGCCAGCATCCTGCAGCTG

CTTCAGCAGGGCCACCTTAACGAGGCTTTTCAACAGGCCCTGTCTGCAGCCGATCTAAAC

CTGGTACTGTATGTGTGCGAGACTGTCGACTCTCAGCAGGTGTTCGGCCAGAGTCCCTGC

CCCCTGCACCAGGCCGTGCTGCTCTCGCTCATCCAGCAGCTCTCCACCAACCTGGTTACA

CGCACCGAACTCAAGATCAGCTATCTGGAGGACGCAGTGATGAACCTAGATCATGGTGAC

CCAGTCACTAGAGACCATATGAGTGCCGTGCTGTCACAAGTGCGTCAAAAGCTGTTCCAG

TTCCTGCAGCAGGATCCTCACAGCTCCCTGAGCAAGCGTGCTCGCCGCCTCCAGATGATG

CTGCAGGGTCTTGTTAGCCACTAGCACACTACAGTTCAGCTCTCCGCTCACCCATCTGCA

GTCTTATCGACTTTCATGCGCTCCTACCCTGCCCCCTAGCCTCCTCCCTCGCCTTGCCTG

TACAGCTCACTTCACTTTGCTCAGTACCACAAATGACCCTTAGAGGGTTGTGTGATGGAA

GCTGTAGTCCAGACACACTCTGTAATTTGCTTAACAATAGCACTACATTTTCTATCAATC

CTTCTGAGATAGGGTAGTTATAGTATGTGACATTCTTAAGCCCATTTTCGCTGTTTGGTC

TTTCATCGTTACAGTCCTATAGTCGTAATACGGATAAAAAAGTTTGTATAAAAGGGTTTT

TTATTGTTCATTCCTGCAGTGTGATTACCCCCGATTTTTTTTGTTCCAGAACTAATCATT

ACATAAATTTTTAAGAGAAGCTTCTCTATCCAAATATATACTCTAAATTAAACTGCTGGT

TTCGTAGCTCCTGCAACCCCTGCATTAAGCATCTGTTTAGTCCTAATGAAAGTAACATCT

ATAACATTAAAGAACTTTATTTACCAAAAATGATTGCTTAGTTTGGTTTTATATATAGGC

TCTCCATTGTCACACTGAGAGTTAACATTATAAAAGTGTAGTGCTTATGTTTAGTCTCCA

GAGGGGCAGGAATTTATATGGCCTCCATCTGCTTTATACCCGTCTATATTTTTCTACTCA

TTCTTATTGAGAAGCACTGTTTACCTTGCTTAGATTTAGTTTTGCTAATTTTTTTTTGTT

CTGATTTTGTTGGACAGACTGTCTAAATCCAGACGTTTGGTTTGGTTCTACACAATTTAA

AGCAAACAAAAAATGCTTGTGATACAACTGGAAAGAAAAAAAGGAAACGGTGCAATGTGA

TGGTACCCACATTTATTTGGTCATATTCTTGTTAGAGCAAGAAAACATTTACAGAAAGTG

CCTTATTAGACATTTGGTAATCTGCTACTGGATCCCTACCAGATGGGCCATATAACTTTC

ACATGTGCTAAATGTCCACTCTGGAAACATTAATTTTTTTGGCTAGTAA

>Unigene000243

GGACAATGCCATGGCGGTTGCGTGATGTAACAATGCGGGAGAAGTGTGTGTAGGTGTGCA

GCACGTAAACACGCTGGCGGGTGCGTATGGTAGGTTTGTTTAAGCCGCAGATGACCGCAG

ACTGAAATGGAGTGTCCCGGTGGCAGAGCCGCAGACAGAGCGCAGCCGCGCGCCGCGAAC

GCGCAGAACCAAGATGGCGATCCGAGATCCGACTGTGAGGAAGAAGAATCAGCGGCGGAA

TTTCGGGACGTACCGAGAGTCAAAGCCTGTGACACGAAACGCGCGTCCTCTTTGGTGGCT

AACAGATTAGCGGAAGATCCGCCGAGGCTCAACTTTCAAATACCGAGGAGGAACAAAGAA

AAGAGAGCTCTGTTTCAGTACTTGTCGTCGGAGTCCAGAGAGTTTGCAGAAATCCTCAAC

ATAATCACCTCCAGCTACAAGGATCCTGCCTCCAAAGCGAATTTCGTGTATTCTCAACCT

CGCCTCGTTCATAACGAACCCCTGGAGAAAGAATTCATCGAGAAGAGAAAAGAGCTGAAG

CAGGACGGGCGCACCGAGAAAGAGCTGGCGGAGTCTTTTTGTTTCCTGCTCTCTGAAAAG

GTCCCTTTAGTTTGTGAAAAAGGTTTATCAGTTGGCAACAGCTGGATGAGCCTTTTGGGA

AATTCCACTAAAGGTGTTTACTTGTGTCAGTTTTCTGACTTGCTACAGAGCAGTCCCTTT

GAGCCTGGCTTTACTGGAGAGATAATTATCTTTAAAGTAATAAAGGGAAAAGTTAAGAGT

GTGCACGATAACATACGAGGTTTGGATCCTACACCCAAGTTTGACAGCCATTTTTCTAAG

AATGCCAGCAAGATGACGTCGCTGGCGTCATACAAGGCATTCGAGAATACACAGCAATAT

TTCTATGAGTATGTGGATTATGAACTTGCATCCAGACCAAGGCACGTGTGTCCATATGCC

GTAGTTTCTTTTCAGTTCAAATCCAAAGAGGCAATAACAGCGGGCCCCAAAACCTCACCT

TTACAAAGGTCTAACAGTCTGCCCTCAGGATCAGAAACTCATGGCTACCTTGTCTGGAAA

GGACATTTAGTGAACAGCGGAAAGGAAGTGTATCAAGCAAGCCTGCATTCGATTTCTCAA

CCTTTTCTACCTTTTAAGATACCCGACAGACTGGAGATGGATAAAGTCATGAGGTTGGAC

CAAGTAAAGAAGTTAATTCCAGCTACACTTTTCTCCTGGGAGCTTTACTCCGGCAGTCAT

GAAGTGTTTAGGAGCGGAATGTACTGCAGTCTGTTCGAGGTTAGGATGGAAAAAAACAAA

TCAGGAGAGGGCCTGACCGAGTTATTTCATGAGCTCGAAAAAAACGGATTGGTGTTGATG

AAGTTGATGAACGACAAGGGTTTTCTTTTCCTTTTATGTTCAGGCCAAATGTCCGGCTCA

AACGGTGTGTTGCCTTTTCTCACTGAGTGCTTGTGTGTTACACTAAATATTACATGTGGG

GGGAAAAAATGTTACGCTATTGTTTGAATGTATTTAGAGCGCAGAGCAGCCTGGAAATCA

ACATGTCAAGCTCTTTTCGTCTACCGGCATGCACGGGATGTGTCCAAATTCTCACCCGAA

CCCCACACACCGTTGTCCCCGGTACCCCAGGACCCTATAATGCCACGGCTGAATTGCTTC

ATCCCGGCTTACCACTACGCTCTTAGCAAAGTGCGCTGTAACCCCACAGCCAACTTAAGT

GTCCGGGTAGAGCAGCAGGCCCACGAATACCTCAGCAGCCTTCACGAATCCAAACCCATC

CAGCAAGTCCGACTCGACTATGACATCAAACTGGACAAGAGGGAGAAGCTCTTTCCGGCT

CCAAGGCAGAGATTTAACTGGGAAGGAAACATTCATTCTTACTTTTACAACCCCAACCTG

TACACATTGCCTGTGGTAAAGGCCAAGAAAATAGTGGAGATGTTCAGGTTGGTTCCCGAA

TCTACAATCGACCCAGAGACCACAGATGTTGCCACGGCTCACGGTGACCCCAAAGGAATT

TTAAAAAATCTCATTCAGGCGAATAAAAGGAAAGAAGCAAAACAAAAAGAAGTGCCTGTG

TATGCCCACGGGTTGAAAAGGAAGTCTGAGGAAGACGCACAGGATGTGAGCGTGAAGTCT

TCAAGGATGGCCACGCCCAGCAACGGAGACGCTGAATGTCAAGCTGAAAAGTCCTGCCAG

TCATTGGACGACATGCTGACCAGTATTGGTCTTCATGACACAGACCTCAGGAAGGACAAA

ACTCAAGAGGCCCTGAAGGTTGTGGAGATACTGGACAAGCTCACCAAAACCACTCAGGAC

ACAGACTTGAGGAAAGACAACACTCAGGTAGAGCTGGTTAACAAGGTGCTGGATAATCTA

ACCAAATCTCTACGCAGCTCACTTTTGTCTAACGACAATGGAGAATTTGAAAATAACAAT

GAAGCTACTGAAAAGATTCTAAAGGACAGCATGACCAGACTGGGCCTCCCTACTAATCGA

GACACTGACCTTCGGAATCGTTTTGTGGACGACGGGGATCAGGAAGCACAAAGAAGAAAT

GATCTCGAGGAGGAAACTGTTGGCAGCTTAAGTAGCCTGGAGGCTTTCAGTCCATGCTCG

GATAGCAACGTACAGCAACGGAGTGAAAGCGTGCCTGGAGAAAGATCCATCCCCTGGGTG

CTCATTCCCATAACAGGCTATAAGACTGAGAGATACAGCCACAGACGGGACATCAATTTC

GATGATCCACGTTTCATCCAAAGCCCCACCGTGTCGACTCACTCGGGTCCAGAGAAACAG

GATGTCTCGCCGCCTGACCAGCCTGACAGCAGTAACGCGGAGCCTGAACCCGAGACTGCC

ACGATGGAGGATCAGGATATGGATGTCACCGACAGGCTGAACGTTTCTGATGGTCAGGAG

CAGAAAGATACTCAGTACAGGGGCGTGGACGGCATAGTAGACGAGCAAATCTTCGGCTTC

TCCGCTGAGGTAGAGGATCTTCTACGAGAGGAACGTGTGTACTATATTCCCTGTTCTAGC

TCTCAGACCCAGAGAAATCCACCACAGATTCCTATAAGCCCTTTCTCCGAGTATGTATCA

CATTTTAACACGCCGTTTCCTGTGCAGAGTTACGTCAGCTCGTTCAGAGATACCGTAAGA

GCTTTCTTAGATCCCCGGAGGCAAAGACGTGATAGTGCGACTCTCGTTTCTTCCTCAGTT

ACTCAAGTTCCTCCCGTCTCATCTTGCCCTCTCCTTAATGTACCCGCTCACTCCCCCAGC

GCTGCCACATTACCAAAACCTGCGTACGGCTACAATTCTCCGACCCCCAATGGCGACCAC

CAGCAAGTGATAAACGGGTTAATGCAGGCAGAACACAGCACAGGGGTGTGTGAAAGGCTT

GAATCGCATCAAGACAACACTTCGAGCTGTTGGCGGAGCTCGGATGACGGAGATGGCGCA

GCTCGCGTCGCGGCTACAAACGACAACGCGGCTAGGACTGTGGAACTGGCTCCAGCTTCT

ATCAGCAATTTTATTAGTCAGCTAAAACCTGAGGTGATCAACAACCTTGAGAAGATCATA

CGGGATGTGCAGAAGAACACCGTGCACTTCTACATCCATTGTGTGGATGAAGAAAGTGAT

GTTTGCTGGGAAATAAAGGACTATTTAATAAGACTCGGCAATCCGGAGTGCGATCCTCAA

AAATTTCTTGAGAAAAAAGACAGTCAAGACAAGCTCCTGATAGTCATTCAAAATATAGAC

ATTGCTGCCCACATTCATAAGATTCCTGCGTTAGTGTCTTTAAAAAAGCTGCGATCCGTC

AGTTTTGCTGGAGTCGACAGTCTCGACGACATCAAGAATCACACCTACAACGAGCTGTTC

GTTTCTGGTGGCTTCATCGTCTCTGACGAGTTTGTTCTTAATCCTGACTTCATCACACAG

GAGAGACTTCAGGCTCTTCTTCAATATCTCGAGGAGCTTAACACACCTGAGAGTCCGTGG

CGATGGCGGGTGCATTGTAAAACTCACAAGAAGGTCAAAGAGTTAAGCAGGAGTAAAAGC

GAAGCCCTGAACGTGTTGAATCTCCTAACGACCTACCACAAGAAACAAATAGTCGAGTTT

CTGCCGTATCACGAGTGTGATGGACCGTTACACCGAGCACCTGACCTGGACTGTCTGGTG

AAGCTTCAGGCTCAAAACACCCGGCAGCGTCATGTCATTTATCTCACAGAGCGTCGCTTC

GAGATGTTTCCGCAATATACTAACAGCGGCATCGTTATAGCGAACATCGATGACCTCCTG

TATAGTATGGCCAGTCTGATCGGTGAAGCCGGTGATAAACCAGACTCCTCGGATCTGCAA

TCCTGTCCCACACCTCCAATAGTGAGGGAAGAGGACATGGGACTACACAGGACATCTGCC

CTTGATGAAAGACCCTCTGTGTGTACTGACCTACATCCTTGTCCTCTAACTGATCCAGAC

CCTAGTGTTCCTGACCAAACACAAAGAGAAAAAGACACGCTAGACACTTTAACCTCACCC

GCTACTGGGATACCAAGGGAATCCGATTTTAAAGCTCTGAGAGACGTGATTACCCATTTC

AGAAACGCCCGACTGAAAGCAAGTTCCTGTGCAGGCCAGAAGTCACCGGGATCTTTCGCT

AACAATCCTCTTCAAAGTTTACTAGGACAAAACAACGGCTCTTCATCTGCTTCTCCAAGT

CTCTTAGAGTCTGCCTTAAATGACTGGCATCATCCAAGTCAGATAGAGGAAGTCGGGTCA

GGAGAGCAAACTCAGACAGAAACTAGTTTAGAATGTGTCACAGTTCCAGATCAGGTTGGC

TCTGGAACTCAAGATGAAATGGCTAAGCTGGTTTCTGTAGCCATGGAGACCACCAGTAAT

ACATGTGATCCAGAACCAAGCTGGAATCGCACAACAGACACAGACAACGCCGCAGAATCC

CTAATTAATCCGACCGTCAGTGGTGCAGAGAGTGGCACGAATCAGAAGAAAGCCTCAGAA

GAGGGACCTGCAAAAAATCCGGTGCCCACTACGAGCTCATCATGCGGCAAGTCTGCAGAC

AATACCGCGACAAGCAAGAACTACGGTAGTCGAGGCGGTAAACGTCCAGTGCCTGTACGG

ACGTCTTACAGGATGAATTCTTTAGTTACTATGGACAGGATGCTACAGAGCAGCAATTTG

TGGCCCAGTGCTCAAGTTTCACAGAACAATGCTATGGGATTCCGATCACGGGTATTGAAC

AGTTCGGTGGCTAACACATTTACACATTCAGGCCTGAGAGGACTTTTACCCAGCAGTAAC

ATTGCCTGGAACAATTTTGCACAGGGGACAGCCACAAACATGTGGGGAATTCAACCAGGT

GTAGGACTAGGACAAGTTCACCAGAGTCCGTTTATCCAGACTTATGCCT

>Unigene000244

GGGGATGAAAACACAGCAGCGAACCGGTGCGGTCTATGTGCGGGAGTGAAGGGGAGCGAA

TGGCTCGTGCGGTGCTCAGTTAATCTGACTCGTGTTTCAGTTTTCCGGTAATCCGACATC

ATCCGCGCCTTAAACATGGACCTCGCGTCCAGAGGCTCGCGCCGGAGACATCCCACAAGT

TTGTAGTGACTTTTGAAAACTGACTCGAGCCATTTTTAAAAACACACAGTCACTTTTTTT

GGCCTTTTTCCTGCGCGCGCTTGTTGGGATTTAAACGGAAAGTGACGCGGAGCTGCAGGG

ACGTGTTTCTGCTCATCATTCCGGCGGTTTTTCTGTGCTCGGGGTTTATTCTGCGCGCCG

CGTGCTGTGTGTTTTAGAACCTAAAGATTAAATACTGAGTGGGTTTAGCGCGCGCTCCGA

GCTGCCAGGACACACCGGCTCCCCTACTAAGTGTTTTGAAGAGCTGAACATCATTGATTC

ACAGTTTGGCAGTAGGTTTGAGACCCTCCTCATGCAGAACCACGGGAAATGAATAAACCG

CCTCAGCCTATCACAGGACCGCCCTCTGTCCCTCACCCTGCCCCCTCACCTGGATTGACG

TCGGCTGTTTATCCCCCCGGTCAGGCTGCTTCTGTTGTTTTCGCCACCCCACCTCCACCA

ATGAACTCTGCACCACAACCCAGACAGTCGTATTATCCCCGGGGAAGTTTGCAGAGTAAC

GCCCCACGAGTGGCAGCAAGTACCCCGCCACGCCCTGTCCCGCCCCCTCACGTCTACCAA

TCGTCTTCACAGATGGTGATGATCCCGCAGCAGCAGCTCCCGTTTCCTACCAACTCTCAA

GGTCACGCCTATTTCATTCAGTCGGGACAGACGCAGCAGTATTCAGTGGCTAGCGCACCA

GCAGGCTTTTACCCTGGAACCAACCCAGACTACAGCTCCACATACGCTGGAGCATATTAT

CCTGCTCAAGCTCAGTACCAACCCTCCGTGCCCACCGCACCCGTTATGATGAACCCTACC

CAGCAGCAGCAGGCTCCGCCCCCTCCACAGCAGCCCCTGCCCCAACAGCACAAAAGGGAG

CGCAAACAGATTAGAATACGAGACCCTAATCAGGGAGGACGGGACATCACAGAAGAGATC

ATGTCTGGAGGGAGGACCATGTCCACACCTACGCCTCCACATTCTGTAGGTACTGAAGTT

CAGACAAATGGCGAAAGTGTGACCCCTGCAGCTCCTGTGGTCCAACTCGATGATCATGTA

AAGTCTTCAAGTCCTGCCTTGACCCCGCCGCCTTGTTCAAATCCTTCTCCTGAGCCTCAA

CATGTCCCGGATGTCCTCGCTGATCCGGTGGTTCCTGTGCCTCCAGGGCAAAGTGAAGAC

TCCATTTCTAACTCGTCTCCAGCTCTCCCCACAGAGCCTGTGCCTAGTGATGTTGCAGAT

GCTCCTGAGACCACACCCCAAAAGCAGGAAAAGGAGGAGCTTAGCACCACTGTTACTGAA

CAGTTGTGTGAGAATACAGAGCCTGAAAAGGAGCTGGAGCCCAGCTCAAAAAAGTTGTTT

GCTGACTCTAACTCTGACATCATGCCTGCTGAGGAGGACACAAGACCTGCAGAAGAAGCT

AATGGGGAGACTGAATTGTACGAGAAAACACCTGCCTTACCACTTACAGCCATGCCTGTA

CCTGAGACCAAGATGGTCCCGGTGCTCAATGGTCTGCCTCGGGAGCATGAAGATCTGCAA

GACGAGAAAATTGAGGCAGAAGCTGAACTAATTCCTGAGCTTTCAGAGTTTCAGGAAGTT

ACTGTAAAAACCTCTGCAGAAAAGGAGGAGAGAGCGAAGGAAGAGCTCACTCCTGCTGAT

GAGATGACTATGCAAGCTGTTCTGTCTGTGCCAAAGAAGAAAAAGCTGAAAAAAAGCGAT

CTGAATAATAAAGTAGTTGGAGACGTGTTGGATGCCTATAAAGAGCCGGAAGAAAAGGAC

CTTATTCCTGAGCCTGCTCCCATCGAAGTCCAGACCTCTGAGCCTCTCCCTGCCACTTCC

CCCTTACCTGAGGACACTGAGGAGACGTGGGAGGAAAAAGAGGACAAAATGGACACTGAG

AACATTGAGCCGGACATGGCAAAATCTGTGGAGCAGAAATACCAGTATAAAGAGGAGCAC

TGGAAACCAATTAATCCGGAAGAGAAGAAGAGGTACGATCGGGAATTCCTGCTGGGCTTC

CAGTTTATCAGTGCCAGCATGCACAAACCCGAGGGCCTGCCGCACATCAGCGATGTCGTT

CTGGACAAGGCTAATAAGACCCCACTGCGTCCTTTGGACCCAAGTCGTCTGATTAATTGT

GGTCCTGATTTCACACCTTCATTTGCTAATCTTGGCAGAGCGCCCCCTGGAGCTGGAAGA

GGGCCTCCTCCAGGGCCACGTCGATCTCAGCAGGGTCAGCGTAAAGAGCCTCGAAAGATT

ATTGCCAGCGTTTCTCTGAACGACGACGTGCAACTGAACAAAGCTGAGAAAGCCTGGAAG

CCAGCAGTGAAGAAGAACCGTGGCGATGAGGACGACCCTGAATCGCTCAAGACCCAGGAT

CTGTTCCGGCGAGTGCGCAGCATCCTGAACAAACTGACACCACAGATGTTTCAGCAGCTC

ATGAAGCAGGTCACAGAGCTGACCATTGACACAGAGGAAAGACTTAAAGGAGTCATTGAC

CTCATCTTCGAAAAAGCCATTTCCGAACCCAACTTTTCTGTTGCTTATGCCAACATGTGC

CGCTGCCTTATGGGGCTGAAAGTTCCCACTTCAGACAAACCGGGAGCCACTGTGAATTTC

CGCAAACTGCTGCTGAATCGCTGCCAGAAAGAGTTTGAGAAGGACAAAGATGACGACGAG

ATCTTTGAGCAGAAGCAGAAGGAGCTGGACGCTGCTACAGAGGAGGAAGTCCAGCAGCGG

TTGAGGGAAGAGCTGGAGGAAGCAAAGGATCAGGCCCGCCGCCGTTCCCTTGGCAACATC

AAGTTTATCGGGGAGCTGTTCAAGCTGAAGATGCTGACTGAGCCAATTATGCATGACTGC

ATTGTGAAGCTGCTAAAGAACCACGATGAGGAGAGTCTCGAATGTCTCTGCAGGCTTCTG

TCTACTATCGGCAAGGACCTGGACTTCGAAAAGGCCAAGCCCCGTATGGACCAGTACTTC

CACCAAATGGAGAAAATAATAAAGGAGAGAAAAACTTCTTCAAGGATCCGCTTTATGCTG

CAGGACGTGTTGGACCTGAGAAGAAATAACTGGGTTCCCCGGCGAGGTGACCAGGGACCT

AAAACCATTGACCAGATCCATAAAGAGGCAGAACTGGAGGAGCACCGTGAGCAGATTAAA

GTGCAGCAGCAGCTTCTTTCCAAGAAAGACTCGACTCGAGGAAGAGGAAGCAGGATGAAT

CCGCCACAGGACGAGGGTTGGAACACCGTACCCATTAGCACCAAGAACCGGCCCATTGAC

ACGTCCCGCCTCAGCAAAATCACCAAGCCTGGTGCTTTGGACTTCAACAATCAGCTTCTT

GCCCCTGGTGGAAAGGGCTCATGGGGCAGTTGGGGTAAAGGAAGCAGTGGTGGAACTGGA

GCCAAAACCAGCACTGAACAAGATGCAGGAAGGCCGGCTACCAGCACCCTAAACCGATTC

TCAGCTCTTCAGCAGTCAGGACCTTCATCCACATCTTCCTCATCCTCACTGGACTCAGAT

CGCAGAGTACCTCAAAGGAGTAGCTCAAGTCGAGACCGTGGTGAGCGAGACCGCGACAGG

TTCGATCGCTTTGATCGCCGGGACAGCCGTGAGGATCGAGACAGAGGGCTGGACAGAGCT

CGTGTGCCTGTTACTAAACGCAGCTTCAGCAGGGAGAGCGAGGACCGGGCTCGAGGTGGA

GATGTGCGGCGTGTCTCCAGCATGACGGATGAGAGGGAACGTGGGAGCAGAGATCGAACC

GCCAGTCGAGAGGCAGCAGTGAAGCGTGAAGCAGCTCCAACACCTGCCCTGATTAAACCA

TCCCTGAGCGAGGAGGAAGTGGAGAAGAAATCCACCGCCATTGTGGAGGAGTACCTGCAC

ATCAATGACATGAAGGAGGCAGTGCAGTGCGTGAAGGAGCTGAACAGCTCGTCTTTGCTC

TTCGTGTTCGTTCGTTGTGCCGTGGAGTCGACGCTCGAGCGCAGCACCATTGCCAGAGAG

CACATGGGCCTTCTACTGCAGAAATTAATTAGCGCTGCCATTTTACCCCCCGAGCAGTAC

TTCAAGGGGCTTCAAGAGATTCTGGAGGTGGCTGATGACATGGCCATAGACATTCCCCAC

ATCTGGCTGTATCTGGCAGAGATCACCACCCCAATGCTGCAGGAGGGAGGAATCCCCATG

GGGCTGCTCTTCAGAGAGGTGGCAAAGCCATTGCTGCCAATAGGAAAAGCTGCTGTGCTG

CTGGTTCACATCCTCAACCTGCTCTCTAAAGGACTGAGTCACAAAAAGGTTGGTGCCCTG

TGGATTGATGCCGGACTCAACTGGAAGGACTTTCTACCTGCTGATGAGGATGTCAACAAG

TTTGTGACTGAACAGAAAGTGGAGTTTACGCTGGGAGAGGAGTCTGAAAAGCCAAGTAAA

AAGGAGCTAAGTGCAGAAGAGCTAAGCAGAAACCTGGACCGTCTGATTCAAGACAAGGCC

AGTAACCAGAGGATCCATGACTGGTTGGAGGCCAATCTTGACGAGCTTCAGATGACCTCT

AGCTTGTTTATCCGAGCACTAATGACGTCCGTGTGCCAGGCGGCGATCATCTGTGAGAAC

CCATACAAGGTGGATGCAGAGCAGGTTACCCAGAGGGCCAAGCTGCTTCAGAAGTATTTA

ATGGATGAGCAGAAGGAGCTGCAGGCGCTTTATGCTCTCCAGGCTCTCATGGTGCGACTG

GAGCAACCAGCAAATTTGCTGCGGATGTTCTTCGATACCTTATACGACGAAGACGTTATC

AAGGAGGAAGCGTTTTACAAGTGGGAGTCAAGTAAAGACCCGGCTGAGCAAATGGGCAAA

GGTGTGGCTCTTAAATCAGTCACGGCATTCTTCGTCTGGCTCCGGGAGGCCGAGGACGAG

TCTGATAACAGCTAAAAAATAATAATAATCTTAAAAAAAAAAAAAACTTGCAGCACAGGA

GTGGTACTGTGCTGTGTGGAAAGACTATAAACTGAATTTTGAAACTCCATTAAAAACTCA

AGTCAATGGACATGACTACAATCACTTCAAATTATTTTCCTCTCACTTCTGAAAAAAAAT

TATTAAAATAAAGGGTTTTTTTGTTTCTTCCAAGGGAGGAAACGCAAATGATATCAATTG

CTTAATGGCCGTGTGCCTCGGCTGTAGTGGACGAGAATTTTTTTTTTTTGTTTGTTTTTT

TTTCTCTTTTCTTCCCCATTAAAAGTACATGACCCCGCCCC

>Unigene000245

GCACAGCAGCCTCCAAATTCGATGGGACGAGCCGCTTCAGTCCAAGAGTGAACTTGCCGA

AGCCCAGACTCGCCCTGCCCGGATCCTCCAAACCCGAAACGCCTTCAGGCTCCAGCAAAC

CCGGCCCGAGCGGACTGCAGGCCAAACTCGCCAGTCTGAGAAAGTCCAGTAAGAAGCGTA

GCGAGTCTCCCCCTGCAGAACTCCCCAGCTTTAGGAGGAGCACACGACAAAAGACCACGG

GCTCCTGCGCCAGCACCAGTCGGCGAGGTTCAGGTTTAGGGAAGCGTGGTGCAGCTGAAA

CTCGCCGACAGGACAAAATGGCTGAAAACGACAACAGTCAGGATGGAGCCAATTCATCAC

GCACAGAGGAGACGCCTCAGGGAGCTTCAGCAGCCTCCAGTTCAGTAGCTGGCGCTGTCG

GAATGACCACATCCGGTGAGAGCGAATCGGATGACTCAGAAATGGGACGATTGCAGGCTC

TGCTGGAGGCTCGTGGTTTGCCCCCACACCTGTTCGGACCCCGTATGTCCCAACTTTTCC

ATCGAACCATCGGCACTGGAGCAAGTTCGAAAGCTCAGCAGCTGCTGCAGGGTCTGCAGG

CGACGGGGGACGAGTCTCAGCAGCTGCAGGCAGCGATAGAAATGTGCCAGCTGTTGGTGA

TGGGGAACGAAGAAACACTGGGAGGTTTTCCTGTGAAGAGTGTTGTACCTGCTTTGATCA

CGTTGTTACAGATGGAGCACAACTTTGATATTATGAATCATGCATCTCGTGCGCTAACCT

ACATGATGGAGGCTCTACCACGGTCTTCTGCTGTAGTTGTAGACGCTATTCCTGTCTTCC

TGGAAAAGCTTCAGGTGATCCAGTTTATAGATGTGGCTGAACAGGCTCTGACCGCGCTGG

AGATGCTCTCCCGAAGACACAGCAAGGCTATCCTGCAGGCCGGCGGATTGGCTGACTGTC

TGCTGTACCTGGAGTTTTTCAGTATTAACGCACAGAGGAACGCATTAGCCATTGCCGCTA

ACTGCTGCCAGAGCATCACACCGGATGAGTTTCACTTTGTGGCCGACTCGCTGCCTCTGC

TCACTCAGAGACTCACACACCAGGATAAAAAATCAGTGGAGAGTACGTGTCTTTGTTTTG

CCCGGCTGGTGGACAACTTTCAGCATGAGGAGAATTTGTTGCAGCAGGTGGCATCACGTG

ATTTGCTCACAAACATTCAGCAGCTTCTGGTGGTGACTCCGCCAGTGCTGAGTTCAGGCA

TGTTCATCATGGTGGTGCGCATGTTCTCTCTCATGTGCTCCAACTGTCCCAACCTCGCTG

TGCAGCTGATGAAGCAGAATATTGCAGAGACGCTGCGATTCCTGCTTTGTGGAGCATCTA

ATGGAAGCTGCCAGGAGCAAATTGACCTCATTCCCAGAAGCCCCCAGGAGCTCTATGAGC

TCACTTCCCTTATCTGTGAGCTGATGCCCTGCCTCCCCAGAGAAGGTATTTTCGCTGTAG

ACTCGATGTTGAAGAAGGGCAGCGCACACACTACAGAGGCCGCAGTGTGGCAGTGGAGGG

ACGACCGAGGCCTGTGGCATCCATACAACCGAATAGACAGTCGGATCATTGAGACGGCCC

ACCAGAACGGTGAGGACGAGATCAGCCTGTCCACACTAGGACGCGTTTACACCATCGACT

TTAACTCTATGCAGCAGATCAACGAGGACACAGGAACGGCTCGGGGAATTCAAAGGAAAC

CCAACCCCCTGATCAACCCCAACACCGGTCACGTGGATGTGCGTGGCGAGGACGCGCGTG

CTGAGCTGATGCGGGAGGAAGCCGAGCTAGCCCGCTGCTTTATCAAGACTTTGTTTGGCG

TGCTGTATGAGGTGTACAGCTCGTCAGCCGGCCCTGCAGTCAGACACAAGTGCCTCCGAG

CCATCCTGCGTATCATCTACTTCTCCGACTCCGAGCTGCTCAAGGACGTACTGCGCAACC

ACGCCGTCTCCAGTCACATAGCCTCCATGCTGTCCAGTCAGGACCTGAAGATTGTGGTCG

GCTCTCTGCAGATGGCTGAGATCCTCATGCAGAAACTTCCTGACGTGTTCAGCGTGTACT

TCAGAAGAGAAGGTGTTATGCACCAGGTGAAGAACCTGGCCGAGAGCGAGTCGTTCCTCC

CAAGTCCACCCAAGGCGTGCACAAGCGGCATCACAGCGGCAACCACCACCATCACCACTG

CCACCACACCTGCTGCCTCTAGTGTCGCACCGGATTTGGGCTCACCGAGTTTTCATAACA

ACATGGAGGATTCACTGGACCTCAGCCCACAGGGTCGCCTAAGTGATGTTCTGAAAAGGA

AGCGACTTCCTCGCAGGGTACCGCGCCGTCCCAAATATTCTCCACCCAGAGATGATGACA

AAGTGGACAATCAGGCTAAGAGTCCCACCACCACTGCGTCCCCTAAATCGTCCTTCCTGG

CAAGTCTTAACCCCAAAACCTGGGGAAAACTTAGTGCTCAGAACAACAGTACTAACTCCG

AACCACCTCGCACAGCCGGGGTTAGCGGCCTTGCACGCACAGCTAACAAAGACTCCGTCT

CTAATAACAGGGACAAGATTAAGGCATGGATTAAGGAGCAGGCCAGTAAATTTGTGGAGC

GATACTTCAATTCAGAAAACGTGGATGGCAGCAACCCAGCTCTGAACGTTCTGCAGAGAC

TGTGTACTGCTACGGAGCAGCTCAGCCTGCAGGTGGATGGCGGTCTGGAGTGTTTAGTGG

AGATCTGCAGCATTGTGTCTGAGTCAGATGTTTCTTCGTTCGAGATTCAACACAGTGGCC

TGGTGAAGCAGCTGCTTATCTATTTAACCTCCAACAGTGAACGAGACCTCATCAGCAGAG

ACCAGAGGATCAAACGCTTCCTGCACATTTTCTGTGGCAGTCCTCTTCCTGGGCAGGAGC

CACTGTTCCGGTTAGAACCAACAGCGAATGGAGCGCTGCTAGCACTGGTGCACAAAATGA

ACAGTTGCTTAAGTCAGATGGAGCAGTTTCCTGTTAAAGTGCACGACTTTCCTAGTGGTA

ATGGCAATGGAAGCAGAGGGTCTCAAGCACTGAAATTCTTCAACACACATCAGTTGAAGT

GCCAGCTGCAGAGGCACCCAGACTGCACCAACGTGAAGCAGTGGAAGGGAGGACCTGTTA

AAATCGACCCTCTTGCACTAGTACAGGCCATCGAAAGATACCTTGTTGTTAGAGGGTACG

GACGAATCAGAGAGGAGGACGAGGACAGCGACGACGACGGATCAGACGACGAAATCGATG

AGTCACTTGCGGCTCAGTTCCTGAATTCGGGCAGTGTGCGTCACCGGCTACAGTTCTACA

TTGCTGATCACCTGCTGCCCTACAACATGACTGTGTACCAGGCGGTGCGTCAGTTCAGCC

TTCAGCCGGAGGAGGAGCGGGAGAGCACTGACGACGAGGCCAACCCTCTCGGTCGTGCCG

GCATCTGGACCAAAACTCACACAATATGGTATAAGCCGGTAAGAGAGGACGAGGATGGCT

CTAAGGACTCAGTAGGAGGGAAAAGAGGTCGAGCACAAACGGCCCCCACTAAAACCTCAC

CACGTAACGCTAAGAAACAAGACGAGCTGTGGCACGATGGCGTGTGTCCGAGTGTCAGTA

ACCCGTTGGAATCATACCTCATATCTGAACCTCCAGAGGGAATTACATTTGATGACCCCT

CACTGGAGGTCATTCTGCTGTTGAGGGTTCTGCATTCCATCAGCAGATACTGGTTCTACC

TCTACGAGAATGCTGTGTGTAAGGAGATCATTCCCACTAGTGAGTTCATCAACAGTAAAC

TGACAGCAAAAGCCAACAGACAGCTGCAGGATCCGCTTGTGATCATGACTGGAAACATCC

CACCCTGGCTTACCGAGCTGGGCAAAACCTGTCCGTTCTTCTTCCCGTTTGACACGAGGC

AAATGTTGTTCTATGTCACCGCGTTTGACCGTGATCGCGCCATGCAGCGTCTGCTCGACA

CCAACCCCGAAATCAACCAGTCAGATTCGCAGGACAGCCGCGTAGCCCCCCGCCTTGACA

GAAAGAAGAGGACGATAAACAGAGATGAGCTGTTAAAGCAGGCGGAGTCTGTCATGCAGG

ATCTCGGCAGTTCCAGGGCCATGCTGGAGATTCAGTATGAGAATGAGGTGGGGACAGGTC

TTGGTCCAACCCTGGAGTTCTATGCCCTTGTGTCTCAGGAGTTGCAGAGAGCTGATCTGG

GTTTGTGGCGAGGAGAAGAGGTCACCCTCGCCAATCCTAAAGGGAGTCAAGAGGGTACCA

AGTACATGTTTAGCTCTAGGGGTCTCTTTGCTGTGCCTTTCGGTCGCAGCACCAAGCCAG

CCCACATCGCCAAGATCAAGATGAAATTCCGCTTTTTGGGGAAACTCATGGCTAAAGCCA

TCATGGACTTTAGACTGCTGGATCTGCCCCTCGGCCTGCCTTTTTACAAGTGGATGTTGA

GAAACGAGTCGTCGATAAGCTCTCACGATTTGGTGAACATCGACCCTGGTGTGGCCAAAT

CCGTTCAGCACCTGGAGGACATCATTCGGCAGAAGAGGAGACTAGAGCAGGACAAGTCCT

ATACTAGGGAGACACTACAACAGGCTCTGGAGAGTTTAAATATGAATGGCTGTTCAGTGG

AGGATTTAGGGCTGGACTTTACATTGCCAGGCTTCCCAAACATTGAGCTGAAGAAGGGAG

GCAAAGATGTCCCAGTCACAATACACAATCTGGAGGAGTACCTAAGGCTGGTGGTGTACT

GGACCTTAAATGAGGGTGTGTCCCGGCAGTTCGAGTCATTTAGAGAAGGGTTTGAGTCCG

TTTTCCCTCTCCACCACCTACAGTACTTCTACCCCGAAGAGCTGGACCAGCTGTTGTGTG

GCAGCAAGTCAGAGACGTGGGACGTTAAAACTCTAATGGAATGTTGCAGACCGGACCATG

GGTATACACACGACAGGTACAAACACCTACATTCATTTTTCTTGAAGCTCAGTCTGGTAT

GTGTGGTATGTTTAATTACATTTTAGTGTGTGTGTGTGTAGTCGGGCAGTGCGCTTCCTC

TTTGAGGTGTTGAGTAGTTTTGATGCAGAGCAACAGAGACTGTTTCTGCAGTTTGTGACC

GGGAGTCCGAGACTGCCTGTAGGAGGTTTTCGGAGTTTAAATCCTCCTCTGACGATTGTA

CGCAAGACATTCGAGTCAACGGAGAACCCAGATGACTTCCTGCCGTCAGTGATGACCTGT

GTAAACTACCTGAAACTTCCGGATTATTCCAGCATTGAGATCATGAGGGAAAAACTGTTA

ATGGCGGCGCGGGAGGGCCAGCAGTCTTTTCACCTGTCCTGATCTTACACATACTCACAC

ATATGCACCGACACACACACACACACACACACA

>Unigene000246

CTCTCTCTCTCTCTCTCTCCAGTCGGCGAATCAGGGTTTCAGCATCAACGTTCCCCACAG

GTTCAATGTGCACAATTATAAGGTTCCTACTTTCTGTGATCACTGCGGTTCTTTACTCTG

GGGCCTCGTCAAGCAGGGACTTCACTGTAGCAGGAAACGTCGCTCCAAATTGTGGTGTGA

ACAACGTGGAGCTTGCTAACAAATTAGCAGAGATGGGACTTCAGCCAGGAGGGACGCTCA

AGCGCAGTGCATCGAACCAGTCTACTAGCGAGTCCTCATCTCATCGTGTCAGTGTGAAGA

AGAGTTTGTCTCAAGAAGAGAATAAAGGTCAAATGCTGCACATCGAAAACTTTACTTTCC

TCCAGGTTCTTGGCAAAGGCAGCTTCGGCAAGGTGATGCTGGCCCGCTTGAACGGCTCGG

ATCGCGCTTTCGCCGTGAAAATTTTAAAGAAAGACGTGATCCTGCAAGATGACGATGTTG

AATGCACGCTGACTGAGAAGCGAGTACTGACGCTCGCACAGTCGCACCCCTACCTCACAC

ACCTGTACTGCTGCTTCCAGAACCAGGAGCGCCTGTTCTTCGTAATGGAGTTTGTAAACG

GTGGCGATCTCATGTTCCACATTCAGAAGTCAAGGAAGTTCGAGGAGAACCGAGCTCGAT

TTTACACAGCAGAGATCACCTGTGCGCTCATCTTCCTGCATGGCAAGGGCATCATATACA

GAGATCTGAAACTGGATAATGTGCTGCTGGATCGGGACGGTCACTGTAAGCTGGCGGATT

TCGGAATGTGTAAAGAGGGGATGTTCGAAGGAGCCTCTACAGGAACGTTTTGCGGGACTC

CCGATTACATCGCTCCTGAGATCCTGCAGGAGATGCTGTACGGGCCGTCAGTCGATTGGT

GGGCTCTGGGCGTGCTCCTTTACGAGATGCTGTCAGGCCACGCCCCTTTTGAGGCAGAGA

ATGAGGACGATTTGTTTGAGTGCATTCTAAATGAGGAGATCGTTTATGCGTCGTGGCTGA

GCGCCGAAGCCGTGGACATCCTCAAAGCTCTCTTGATGAAGAATCCGGCTCGCCGTCTGG

GCTGCGTGGCTGCAAGTGGTGGAGAAAACGCTGTCCCGGCGCACAGGTTCTTCAGCACTA

TCGACTGGGACAAACTGAACCGGCGGGAACTCGAACCTCCGTTTAAACCTCAAATCAAAA

CCAGTGAGGACGTGAACAACTTCGACCCCGACTTTACAAACGAAGACGTCGTCCTCACTC

CCATCGAGGACGCCTTGCTTCCGCAGATCCACCAGGATGAGTTCCAAGATTTCTCTTTTA

CCTCGGAGAAAATGCTGGAGATCTGCACATTTTAAAAAAAAACAGGGATTAAAAACAAAA

AACAAACCGATGACACAGGACCAGGATGACGTCTGGCTTGTTGAGTCATGACATCCAATC

ATAGCTATGTATTTCAGCGTTTTGTAAAGCTGTGACGCGAGACATCAGAGTTCATCGTGA

TTGTTTGAGTTTTGAGGACTTATTCTTTTCGTACACAAAGGGTAATGATGCATTTTTTTT

ACTTAAAATGTGTTATTTTGTTAAGCAAAACCAAAGTATTTAAACTCTTATTGAATTTAG

CGAAGACTGTTCTGATCTTTTTAATGGAATTAGAGGAAATGTTTAAGTCTCAGTAAAACT

AATTTGTATAAAAAAATGTTTTATTGCAGCCAAGGTTAGTTTTTATTCTAGGGCAAAAAT

AATGAACTCAATTTCGATTCAGAGGACAACGGAAAAAAATGGGTTGCAGTATTAAGTCTG

AATTTTTATAATGTGCCGGACCCATGAGAGGCGGAGCTTCTGTAACTTTAGTACACTTTT

AGTTGATAGTTTAAAAAAAAATTATTCAGTTAGCAGAATTATTTGCCAGATTTATTAGAG

GATGAAAAAGGCTTTTTGCTGCCATCTGTTGGTGTGTCTGGAAACATTTTGATGCCTCCT

TGTAATAATAGTTATACATTTTGAATTGGACTATCTAAGATTAGCTATGTAACACTAGCT

AAACAAGTATTAAGTGGTTAAAGTGATGTAACAAAAGCTCCGCCCAATCAATGTTTTGTG

GTCTGATTGCCTTTTCTCTACAGTCACAGCTGGATTGTCTGACCTTTTAACATCATAACC

TGTTGCTTTTGGTTAAAAATTTACTAAAGATAAATTCATCTTTTTTTGTCCTTAGAACTG

CAGTCAGAATCTTATTGCATTATAGTTTTTGGAGTTTTTTTTTTTTTTTGGTGTCTACGA

TGAAGTGACAGCGCGTCCTTGCGTGTCTGCGATCTCGGGATTTCTGCCTAACTACATTTC

CGACACTTGGATAATTCAGGAACAGAGTGAGGCTGGCCGGTGTGTGGAGAGACAGACAGC

ATGGCTGAGCCCCGGCGAGACAGTACCAGTAGTCTACAGAGGAAGAAGCCTCCATGGCTG

AAACTGACCATTCCCACTGCTCAGATGTCTTTGGACGAACCCCCCACTTTCATCCAGCCT

GTGAAGAGACAGGGGTTCCTGCGCAGTGTCAGCATGCCTGTCGAAACATCTCACTTCGCA

TCACCGACACGACCTGTTTGATCCGCGCCGACCTCCGCTGCAGCACCAGACCTCCATCAC

ACAGTCCATCAAAAGGGGCACAGCAGACTGGTTCGGGGTCAGCAAGGATGGTGACGCCAC

ACAGAAATGGCAGAGAAAGAGTCTGCGCCATTGCAGCCTGCGCTATGGTAAACTGAAGCC

ACAGGTGATCCGGGAGATGGAGCTTCCAAGCCAGGACAACATCTCAATGACCAGTACAGA

GACTCCACCTCCTCTCTATGTACCAACATCTCAGCATGGCATACCAAAGATCGTGGACCC

CTTGGCGCGTGGTAGGGCTTTCCGCATGGTGGAGGAAGTGGACGGATACAGTGTGCCTCA

TACACCCATCACACCCGGTGCCACCTCTCTGTGTTCCTTCACCAGCTCCCGATCCGGCCT

AAACCGGATACCTCGACGACGCAAGCGGGAGTCTGTGGCTAAGATGAGCTTCAGGGCAGC

AGCTGCGCTGGTCAAGGGACGCTCGTTTAAAGAAAGCACCCTGCGGCGGGTTCACAGACG

CAGCTTCACCCCAGCCAGCTTTATCGAGGAGGACGTTGTGGACTTTCCTGACGAGCTGGA

CACGTCCTTCTTCGCACGAGATGGATTGATGCAGGAAGAGATGTCAACCTTTCATGACGA

GGTGTTTGAGTCTCCCTCTGACTCCACCACCAAGGACGTGGACAGTAAGCAGCTGGACGA

ATCGGAGCTGACGGGAAGTGCACTAGACAAGAACGAACTCGAGAGAAGTCATCTGATGCT

ACCTCTAGAGCGAGGCTGGAGAAAAGCCAAGGAGGGAACCCCTGCTCCGCCCAAGGTCCG

GCTGAAACAGGAAGTGGTCAGCGTAAGCGGGCAGAGACGGGGGCAGAGGATCGCTGTACC

GGTCAAAAAGCTCTTTGCGCGAGAGAAACGTCCATACGGGCTTGGCATGGTGGGCAAGCT

GACCAATCGGACGTACCGCAAGCGCATAGACAGCTATGTGAAAAGGCAGATAGAAGATAT

GGACGATCACAGGCCGTTCTTTACCTACTGGATCACGTTTGTCCACCTACTCATCACTAT

TCTGGCGGTGTGTATTTATGGCATCGCACCCGTGGGTTTCTCCCAGCATGAGACGGTGGA

TTCTGTTTTAAGAAACAAAGGTGTCTATGAGAATGTCAAGTTTGTGCAGCAAGAGAACTT

CTGGATTGGGCCGAGTTCCGAGGCTCTAATCCATCTGGGAGCCAAATTCTCTCCCTGCAT

GCGACAGGATCAGGAGGTACACGATCAAATCCAGAGGAAACGTGAGATTGAGCGCAACTC

CGCCTGCTGTGTGCGAAACGACCGCTCGGGCTGCCTGCAGACGTCTGAGGAGGAGTGTTC

GAGCACACTGGCTGAGTGGGTAAAGTGGCCCTATCATCCCAGCACTCCACAACTGGACGG

AAAACCTCGTAACTACGGCTCTGTTTGCCATCAGGACCCCAGGATTTGTTTGGAGCCAGC

CTCTGGACCGCCTCACGAGTGGCCTGATGACATCACCAAGTGGCCGGTTTGCACCAAGTA

CAATACTGGCAATCACACTAACCTGCCCCACATCGACTGTACCATCACCGGGCGGCCGTG

CTGTATCGGAACTAAAGGAAGGTGTGAGATCACATCACGGGAATACTGTGACTTCATGAA

AGGCTCTTTCCACGAAGAGGCAACACTGTGTTCACAGGTTCACTGTATGGATGATGTCTG

TGGCCTGCTGCCGTTCCTAAACCCGGAGAAACCCGACCAGTTCTACCGCTTGTGGCTGTC

TCTCTTCCTACACGCTGGGATTTTGCACTGCCTGGTTTCTGTGATATTTCAGATGACAAT

CCTGAGAGATTTGGAGAAGTTGGCTGGTTGGCTGCGAATCTCAATCATCTACATCCTGAG

TGGCATCACAGGGAACCTGGCCAGTGCCATCTTCCTGCCCTACAGAGCAGAGGTGGGTCC

TGCCGGTTCACAGTTTGGCATTCTGGCATGTCTTTTCGTGGAACTCTTTCAGAGCTGGCA

GATTCTCGCTCAGCCATGGCGGGCATTTGCTAAACTGCTGTGTGTGGTGCTCTTCCTCTT

CGCCTTCGGTTTGCTGCCCTGGATCGACAACTTTGCCCACATCTGCGGCTTCGTGTCAGG

TTTCTTCCTGTCCTTTGCGTTCCTTCCCTACATCAGCTTCGGCCGTATGGACATGTACCG

TAAGCGCTGCCAGATCCTGGTCTCACTTACACTCTTCCTGGGCATCTTCTCTGGATTTGT

GGTGCTCTTTTATGTCCATCCCATTAAGTGTGAGTGGTGCGAGCTGCTCACCTGCATTCC

CTTGACAGACAAGTTTTGTGAGAAGTATGACCTAAACGCGCATCTCCACTGACGCAGTCA

TGAGGAAGCTCAGGTGAGGCTTTTGGACGGGGCAGGATCCCATCACCTCCAGTTAGGAGA

CCTGCCATGTTTCTCTGCTGACCCTGAGGCAAGGACAATAGCAACATGGATGATACACAT

CACATTTTTTTCTCTCTTACTGTAAGTTCAAACTCCTCTTTTGACCATTAACGACCTGAA

CGTTATTTTTCCTCATCATTAGTTTGTTATCGGTCGTCTGTGAAACAGGGACAGTTGCCC

CACCCGCCTCTCCTTCAGATACTCCACCTGGAGATTGTGAGCGAGAATAAGCACTGGTAC

AAACAAACAGAAGAATAGATGCTAATAGATGTAATATTTTATACTTTTCATATTTTATGA

TGCCACGAAATCAGGTGAGGGTTTTAGAGTAGTTCAGATTCAAATCACCTCCGACTTCCA

GGGACGAAAGAGAAATTTAATCAGAGCCACC

>Unigene000247

AATTAGAGATCGGAAGAGGCGATGAACATTATGGGCTTTACTGATGAGGAGAGGATCGGA

ATCCAGAAGGTTGTGTCCACTGTGCTGCAGCTTGGCAACATCGAGTTTAAGAAAGAGAGG

AACCAGGAGCAGGCCACCATGCCTGACAATACAGCTGCACAGAAGGTCTGTCACCTGTCA

GGCATCAATGTGACGGATTTCACCCGTGCCATCCTGACCCCACGCATCAAAGTAGGCAGA

GAAGTCGTACAGAAGGCGCAGACCAAAGAGCAGGCTGATTTTGCAGTGGAGGCTTTAGCA

AAGGCAACGTATGAGCGTCTGTTCCGCTGGATTCTTGCTCGTGTCAATAAAGCTCTGGAT

AAAACCAAGCGTCAGGGCGCGTCTTTCCTTGGCATCCTGGACATTGCCGGCTTCGAGATC

TTCGAGGACAACTCGTTTGAGCAGCTGTGTATTAATTACACTAACGAGAAGCTGCAGCAG

CTCTTCAATCACACCATGTTCATACTGGAGCAGGAGGAGTATCAGCGTGAGGGCATCGAG

TGGAGCTTCATCGACTTTGGACTGGACCTTCAGCCCTGCATCGACCTGATCGAGCGGCCT

GCGAATCCTCCAGGTGTTCTGGCTCTGCTGGATGAGGAGTGCTGGTTCCCCAAAGCCACT

GATAAAACCTTCGTGGACAAGCTGGTGCAGGAGCAGGGGAACCATCCCAAATTCCAAAAA

CCAAAAAAGCTCAAAGATGATGCTGATTTCTGCATCCTTCACTACGCAGGCAAGGTGGAT

TATAAAGCAGTGGAGTGGCTGATGAAGAACATGGACCCTCTGAACGATAACGTAACAACA

ATGCTTAATCAGTCATCAGACAGATTTGTATCTGAACTCTGGAAAGATGTGGACAGAATT

GTGGGACTGGATAAAGTTGCAGGAATGGCCGAGTCAGCACACGGGGCGTTTAAAACCCGT

AAAGGAATGTTCCGGACGGTCGGACAGCTTTACAAAGAACAGCTCGCTAATCTGATGACC

ACGCTGAGGAACACCAACCCCAACTTCGTGCGCTGCATCATCCCCAATCATGAGAAGAAG

GCAGGTAAACTGGATCCTCACCTGGTCCTGGATCAGTTAAAGTGTAACGGTGTGTTAGAG

GGAATCCGAATCTGTAGACAGGGCTTTCCTAACCGCATCATCTTCCAGGAGTTCAGACAG

AGATATGAGCTGCTCACTCCGAACGCGATCCCTAAAGGTTTTATGGATGGTAAACAGGCC

TGTGTGCTGATGATCAAAGCTCTGGAATTGGACTCTAATCTCTACCGGATCGGACAGAGC

AAAGTGTTTTTCCGTGCTGGAGTTCTGGCTCACCTGGAGGAGGAGCGGGACATCAAAATT

ACGGATGTTATCCTCAAGTTCCAGGCTTGGTGTCGTGGTTACGTCGCTCGCAAGTCATTT

GCCAAACGGCAGCAGCAGCTGACAGCCATGCGAGTGATCCAGAGGAACTGTGCTGCTTAT

CTGAAGCTCAGGAACTGGCAGTGGTGGAGACTCTTCACTAAGGTGAAGCCTCTGCTCCAG

GTGACCCGTCAGGAGGAGGAAATGCAGGCGAAGGATGAGGAGCTGGTGAAGGTGAAGGAG

AAACAGGTTAAGGTGGAGAATGAGCTGGTGGAGATGGAGAGGAAGCACCAACAGCTGATG

GAGGAGAAGAACATCCTGGCAGAGCAGCTGCAGGCGGAGACGGAGCTGTTTGCTGAGGCG

GAGGAGATGAGAGCTCGATTGGTGGCAAAGAAGCAGGAGCTGGAGGAGATCCTACACGAC

CTGGAGTCTCGCGTGGAGGAGGAGGAGGAGCGCAACCAGACCCTGCAGAACGAGAAGAAG

AAGATGCAGTCGCACATACAGGACCTAGAGGAGCAATTGGATGAAGAAGAAGCTGCTCGA

CAGAAGCTACAGCTGGAGAAAGTAACTGCAGAGGCCAAAATTAAGAAAATGGAGGAGGAT

TTACTGTTACTGGAGGACCAAAACTCCAAATTTATAAAGGAGAAGAAGCTGTTGGAGGAG

AGGGTGGGTGAAATAACGTCTCTGTTAACTGAGGAAGAAGAAAAGGCGAAGAACCTCGGC

AAGGTGAAGAACAAACAGGAGATGATGATGGTTGACCTTGAAGAGCGTCTGAAGAAAGAG

GAGAAAACTCGTCAGGAGTTGGAGAAGGCCAAGAGGAAGCTGGATGGAGAGATGACTGAC

CTGCAGGAACAAATCGCTGAACTTCAGGCTCAGATCGACGAGCTCAAACTGCAGCTGGCC

AAAAAAGAGGAGGAGCTACAAGCTGTGCTGACTAGAGGAGATGAGGAAGTGGCTCAGAAG

AACAACGCTCTGAAGCAGGTGAGGGAGCTCCAGGCTCAGTTAGCGGAGCTTCAGGAGGAT

CTGGAGTCGGAGAAATCTGCTCGGAATAAAGCCGAGAAGCTGAAGAGGGATCTGAGTGAG

GAACTGGAGGCTCTGAAGACTGAACTGGAGGACACGCTGGACACCACAGCCGCTCAGCAG

GAGCTCAGATCTAAGAGAGAACAGGAAGTGGCTGAACTGAAGAAGGCGATTGATGAAGAA

GCTCGAAATCACGAGTCTCAAATTCAGGAGATGAGACAGAGACATGGAACTGCACTGGAG

GAACTCTCTGAACAACTGGAACAGGCCAAGAGATTTAAACTGAACCTGGAGAAGAGTAAG

CAGACTCTGGAGGGTGATAATAAGGAGTTGAGTAGTGAGGTGAAGTTACTGCAACAGGCA

AAAACCGAATCTGAACACAAACGCAAGAAACTGGAGACGCAACTGCAGGAAGTGCAGAAC

CGCATTAATGAAGGAGATAAACACAAAGGAGAACTGACTGAACGCACACACAAACTACAG

ACGGAGTTAGAGAGTGTGTCTGCACTGTTGGAGGAAACTGAGAAAAAAGGGCTCAAACTG

GCTAAAGATGCATCAAGTCTTGAGAGCCAACTACAGGACACACAGGAGCTGCTTCAGGAG

GAGACACGTCAGAAGCTGAATTTGAGCAGTCGGATCAGACAGCTGGAGGAGGAGAAGACC

AACCTGCAGGAGCAGCAGGAGGAAGAGGAGGAGTCACGCAAAAACCTGGAGAAACAACTG

GCAACCCTGCAGGCACAGCTGTGTGAGACAAAGAAGAAGCTGGAGGATGATGTGGGTGTG

TTGGAGGCTCTTGAAGAGGTGAAGCGGAAGCTGCAGAAGGACCTTGAAGCCAGTAACACT

CGTTTGGAGGAGAAGATTCTGGCCTTCGATAAATTGGAGAAGACAAAGACTCGTCTTCAG

CAGGAGCTGGAGGACCTGACAGTCGACCTGGACCACCAGAGACAGATCGTCTCCAACCTG

GAGAAAAAACAGAAAAAATTTGACCAGATGCTGGCAGAGGAGAAGAGTATTTCAGCGCGT

TATGCTGAGGAGCGAGACCGCGCGGAAGCGGAGGCGAGGGAGAAGGAGACGAAGGCGCTG

TCATTGGCACGCGCTCTGGATGAAGCTCTGGAGGCCAAAGAGGAGTTTGAGCGACTGAAC

AAACAGCTGAGAGCTGAAATGGAGGACCTGATGAGCTCCAAGGACGACGTGGGCAAAAAC

GTGCACGAACTGGAGAAGTCGAAACGCACCCTGGAGCAGCAGGTGGAGGAGATGCGCACT

CAGCTGGAGGAGCTGGAGGACGAGCTGCAGGCTACTGAAGATGCTAAACTGCGCTTAGAA

GTGAACATGCAGGCCATGAAGGCGCAATTCGAGCGAGATCTTCAGGCCAGAGATGAGCAG

AACGACGAGAAGAAGAGACTCCTAATCAAACAGGTGCGTGAGATGGAGGCAGAGCTGGAG

GATGAGAGGAAACAGAGAGCTCTGGCCGTCGCTGCAAAGAAGAAGCTGGAAATGGACCTG

AAGGATGTGGAAGCTCAGATTGAAGCTGCTAATAAAGCTCGAGATGAAGCTATCAAACAA

CTCCGCAAACTCCAGGCTCAGATGAAGGATTATCAAAGAGAGCTGGAAGAAGCTCGAACA

TCACGAGATGAAATCTTTGCTCAGTCCAAAGAGAACGAGAAGAAGCTCAAAACCCTTGAG

GCCGAGATCCTGCAGCTGCAAGAGGACCTGGCTGCATCAGAGCGAGCACGGCGTCATGCT

GAACAGGAGAGAGATGAACTGGCTGATGAAATCTCCAACAGCACCTCGGGAAAATCTGCG

CTGATGGATGAGAAGAGGAGGCTGGAGGCTCGTATCACTCAGCTGGAGGAAGAACTGGAA

GAAGAGCAGAGCAACATGGAGCTGCTGAATGATCGCTTCCGCAAAACCGCCATGCAGGTG

GACACTCTGGGTAGTGAGTTAGCGGGTGAGCGTAGCGCGGTTCAGAAGAGTGAGACGGCT

CGACAGCAGCTGGAGAGACAGAATAAGGATCTGAAGAGTAAACTGCAGGAACTGGAGGGT

TCCGTCAAATCCAAGTTCAAATCCACTATCGCCGCCCTGGAGGCCAAAATCCTGCAGCTG

GAGGAACAACTGGAGCAGGAGGCCAAGGAACGGGCAGCAGCCAATAAGATTGTGAGACGT

ACAGAAAAGAAACTGAAGGAAGTGTTTATGCAGGTGGAGGACGAGCGTCGCCATGCCGAC

CAGTACAAAGAGCAGATGGAGAAGGCGAACTCGCGCATGAAGCAGCTGAAGCGTCAGTTG

GAGGAGGCGGAGGAGGAAGCGACCCGCGCCAACGCCTCCCGCAGGAAGTTACAGCGAGAA

CTGGAGGACGCCACCGAGGCCAGTGAGGGTCTCAGCAGAGAGGTTAACACACTCAAGAAC

CGTCTGAGGCGTGGGGGTCCGGTCAGTTTCCCATCAGGCCGTTCAGGTCGCAGGCTGCAG

ATGGAGGGTGAATTCTCAGATGATGAAGCTGACAGTAAAGTGAATGACACAAATGAAGTA

CAACCTCCACCGAGCGAGTAAACACCAGAACACACACACACACACACTTAATTCATTCAG

ATTTTTTCCCCTTTTGTGTGTGTGTGTGTGTTCATTGTTGTGAGCGAATAGGAAAGCAAC

GGAGTTTAAACTGAACTCGTGAGAAACGTCAGCATTGACAGGAAGTGCCGTCTGTTCTAA

AACTTTTAAAACTTTTTCCATTTATTATTAAAAATAATTATATAAAAAAAAACAATGTTA

AAGTTGAAATCAATATTTTATAGCTTTTTTTAAAAAAAAAATTTTTGTGGTATTTTACAT

TTATTTCGCCATTTTTATTTATTTTTAACAAATATATATTCAGAAGTCCTTTTTGTAGCA

CTAATGCTTTTTTTCTGTGACCAGGGCGACCCATGGTGTAGGCGAAATAGGCCTAGGAAA

GTTTTTTTTTTCTTTTTCTGGAAGTGCGCCCTCTTGTGACCATTATGCGCTTTTACAATT

TATTTACTACTTATGCTTAATAGGGGGG

>Unigene000248

AGCAACAACAACAGCAGCAGCAGCAGCAGCAAGTAAACCGCAACAACCAGGAGGAGAAAG

AGGACAAGGAGACCGAGAAGGACGGAGAGCGAGACGACGACAAGGATGGTGGAGACGACA

AGGACGATGGCATGAAAGACGACACCTCTGGAGAGGACGGCGAAGACAAGGATCCACCCG

TAGCGGTTAAAGGACGTCGCACGGCCAACAGCCGCGGCCACCGGAAAGGCCGCATCACTC

GCTCCATGGCCAACGAGCAGGAGGAAAGCAGCTCTCCTCCTCTCAGCGGGGAACTGGGAA

ACCTGGAGATGAACGAAAGCTCTCGCTGGACAGAGGAGGAGATGGAGACCGCCAAAAAAG

GACTCCTTCAATATGGCCGCAACTGGTCTGCCATTGCCAAGCTGGTGGGATCCAAAACCG

TCTCGCAGTGCAAGAACTTCTACTTCAACTACAAGAAGAGGCAGAAACTGGACGAGATTC

TGCAGCAGCATAAAATAAAATCGGAGCAGGAGCGTAAGGCCAGGCGTAAAGGTAAAACTC

TGCAGAACGAAGAGACTAGTGCGCCCTCTGCTGCTGATGAAGAGGAGATGGAGGGATCCG

GGGGCAGCGGCAATGAGGAGGAAGTACAGGAGGATGGAGAGGGAGGCCCGAACCAGAGCT

CGGACACGGAGAGCCTCCCGTCCCCTCATTCATCGGAGAGCAGCAAAGTCAAAGACTCCA

ACACGAGCCGCTTCAAATCAGCGCCGAACACCACCTCCACCTCCGCATCTTCCTCAGACA

AAGACACACCTGAGGACGAGAAACCGCAAGTCAAGAGTGAACAGGACATCGTGAAGCAGG

AAGAGCAACCCGAGGGAGTGGACCGGGTGAAGGAGGTGCAGGGGGAGATGGAGGACAAAA

AACCTTCCATCCTGGAGGTGGAAGAAGGGAAGAGAGACGTAAGGAAAAGAGAGAAAGAGC

ACGGAGGGCAGGACAGTGACTCCAGCGCCACCTGCAGCGCAGACGAGGTGGAAGAGACGG

ACAACACAGAGAAGAACAGAATAACGTCTCCCAGGCCGAGTCTGCTGAGTTTTGCACATG

ATGGCGTGATCTCCACCGCACTGCAAAAACCACTGGACCTCAAACAGCTTAAACAGAGAG

CAGCCGCTATACCTCCTATAATGCCAGATATTTCTGTAGCCGGAGGTGGTGTACTGGGGA

AACAGCAGGTGGTACCTGACGCTCAGGCACTCTACGAGCAGCAAATCATCATCATGGCAC

ATGAGTCATCCCAAGGGGCCAAACCGCAACCGGCACAACCAATCAAATCGCAGCATTACC

CCAGCGCCATCCCCGAGACCAGCAGCCCACGAGTCCGAACCCGAACGCCCACGCCCTCAG

AGAGAGAGAGAGAAGAACTGGAAGAGGGGAGGGAAACATGGCTACACACTCTGCTTCACG

GACTACATAAACCGCGGGCGTTCTCTCCTCTGGCTGAGGTGAAAAAGGATCCGGGTTCCG

AGTACGTCAGAGCCGTGAGTCTCGGTCGTCCTGTGATGTCACCATACCAGCTTTCACCCA

GAGACGTAGCCAAACTAAACCACGAACAACTGCTGCACTACAACTCCTCTCTTCCTCAGC

AGGATAGGCTGCCTGGTGTCCGGCCTCTGCACATCCCCGATCCCCCTCCTCTCATCTCCT

CAGCCAAACCAGGCGGCTCCATCACACAGGGAACTCCAGTGCAGATGCACAGCCCCGCCC

ACAGCGCAGAAAACACCAAAATGACTCCTGGAGGTCTGCCTCTGTCCTGGATGGAGCAGC

GGAAACTTGCTGGGTTTCCTGTGGTTAAGCAGGAGCAGCTCTCCCCACGTGGCCCCGCCT

CTTCTCAGCCTGAGAATTTGTCCACGCACGCTATCGTTCATGAGAGTCTTTCAGCTCGCG

GACCCATGCCAGCTGTACAGGGTGGAAGCATCACAAAGGGCCTTCCTGGCACACGCATTC

ATCAAGAATCCCCCATAACATACTGTGGTGGTTCCATCACTCAGGGCACCCCAGCAGACG

TGCTTTATAAAGGTACCATCACTCGTCTTATCGCCGAGGACAGCCCCAGCCGGGACAGGT

CCAGAGAAGAAACCCAAGCCAAAGGACACGTCCTGTACGAAGGCATCAGCGGGCAAATCC

TATCCTACGAGCGACCTCCCAACCCCAAGGAGGACGGCAGGGGGGAGGTACTAGGACTGA

AACGCCCCTACGACATCATGGAAGGAGGCGGGATCTCCCGAGCGCTGCCGTTACGTGACT

CTCTGTCCGCTGCTGAAGGTTTGATGGGTAGAGCAATGCCTCAGGAAAGAGAGAGTCCTC

GTCCTCATCATATTCGGGGCTCCATCTCGCAGGGTATCCCACGCCATGTCGAGTCCCAGG

ACGATTATTCGCGGCGAGAGCCGAAGCAGCTCAAGCGCGAGAGCTCCCCGTCTCCCCGCG

GCCCTGACGGTATTAAGGGCCGCCCCCACGACAACGTAGTGCCTACAGTTAAAGAGGCGG

GGCGCTCCATCCACCTGATCCCGCCGGAGGGGGTGCTGATGAGCAAAGCACCTAAAGAGG

GTTCCATTACACAGGGAACGCCTCTAAAACTGGAGCCGGCCGGTTCGTCCAAACGCCATG

ACGTGCGCTCCATCATATCCAGCTCCCCTCGGCCGTACTCGGGGCTCACGCCTCATTTAG

AAAGAGGACACTACGAAGACCAGGTCCCCAAGTCGCGTCCCAGCGCGGTAGTTAGCACCT

CCAGCTCTTTGGTGCTGGGGGCAGAACAGGCAGCCAAAAGCCACCACAGCCCGGTGAGCT

ACGACGAGCACAAGAGAGCAGGATATGCAGCAGTTTCACACAGAGGATCACCGCTGACCA

GCAGAGACGCCACGCAGCGGCCGCACGACGGTTCTTCGAAGATTCCTCAGCAGGAGCGCA

AGTCCACTCCGACTCCTCGGGAAATGAGTTCCACCAAGTCTCCGCTGCCCGTAGTAGGAG

ACCACGCTGCTTCTCTGGCCTCCTACGAAAGGCTGCTGCAGGGGCTGGGAGCCGATATGT

ACCAAAGGCAGATCCCGCTCCCGTTCGACCCCGCAGCCCTGCCCAGGGGCATCCCTATCG

ATCCTGCTGCTTACTACCTGCCCCGACACCTGGCTCCCGCTCCCGGTTACCCCCACCCCT

ATCCTCCTTATCTAATCCGAGGCTTCCCTGACACGGCGGCGCTCGAGAACAGACACACAC

TCTTTACCGACTACATCACCTCACAGCAGATGCACCAGTGGCCTGCAGCAGCTATGACAC

AAAGATCAGACCTATTGAGAGGTCCCCGGGACCCACCGCTCACTTTATCCTACTCACCTA

CAACACGGGGGGCGTCTGCTTCCCCGATGGACCGCATTAACTACCTCCCCGGAACCCCAA

CCACTTTCCCCAGCCGAGCGTACACCACCTCACCAATCTCACCAGTGGGAAGCTCACACA

TGAGCAAACTCCAGGGCACGCCGACGTCATCCGAACGAGAGCGCGAGAGAGAGCGCGAAC

AAGAGCGTGAGAGAGACCGGGAAAGAGAGAGAGACCGAGAGAGGGAGCGTGAGAGAGAAC

GAGAGCGGGATAGAGAGAGGGAAAGGGAGCGAGAGAGGGAGAGAGAGAGGGATCGGGACA

GGGAGAGGGAGAAGTCTCTCAGTCATGGCAACGTGGAGCACGCCCCTATCTGGAGACCCG

GAGCAGCAGAACAGACCGGGGGCAGCAGCTCCAGCTCGCGTCCTCCTTCCCTGTCTTACA

GCCACCAGCCGTCCCCCATGAGCCCTCGGCCCCAGGAGAGCCTCCAACACAGACCCACCG

TCCTGCACAACACCAGCTCCAAAAACCTGAGTGCAGAGCACAGCAGTCAGTCTGTACTGA

GGTCTGTGCCACAGAGTTCTTCACGGTACCCAAACTACAGCGGACACCTCCAAAGAGTAA

GCGAGGTCTATCCCGACTCTAGTGCCGTCAAAGAGCTGAGCCGTGAGCCGGGCAAATCCC

AGAGTCGTGGCGTTCTAACCAAACAACCGGAACCCAAGTCCGAGTCCAAGATGTCCAGCC

CGAGCGGGGGTGGAGTTTACACATCAGCTTTCGCTCAGACCCCCAGCGGTCAAGTAGGGC

TCACAGCGGCCCGGGGTCTTCCCATGATGACTTCTGAGAGCAGTATTGTGAGCAGAGATA

AGACTCCGAACAAGGACCATGAGGTCCGCGCGCTCGCTCAGCAGAAGTCTTTGGAGCAAC

ACGCTGTGTTTTTTATTCCAGAGGAAAGACTGTCCCCCAGCCAGCAACCTCCGTCCCATG

GGGTGAAGAGCTCCCAGAGAGTGGTGACCCTCGCCCAGCACATTAGCGAGGTGATTAAGA

AAGATTACATGCGCCAGTCACAGCAGGGACAGACAGGAGGACAGTCCGTGTTCGGCTACC

ACAATTCACCTGTGTTGGACCTCACCCGACCCCCAAGTGCCCCCCAGACGACCTCAGAGC

CTGGAGTCAGCGGCCGTTTTACCTCTGAGGGCTCCAACAGTGACCGAGACAGGCTGAGAG

ACAGATCTCCTCCTCAGAGTAAAGTTTCCCCAGTGAGTCTCTCCGCCGAGTGCATCGAGC

CCATCTCTCCACCAGGAGGCGTGTCCGAGCCCGAATCCCAGACAGCGGCGTGTCCCAGCG

AACAAGCCGAGCAAGGGACGGGGTCTCGCTCTCCCTCCGGCACGTCACAGCCACCGGCGT

TCTTCAGCAAACTCACCGAGAACAACTCCGCCATCGTCAAGAGCAAGAAGGAGATGATGA

AGAAGATGGTGGTGGCCAACGAGAATGACTTTAACTCCGGACAGCCCGGGACAGAAATCT

TCAACATGCCCGCCTCTGCTACCGGAGGGCCGGTAAACATACGTAGTCAGCCTCCTCCAG

ACTCCAGCGGAAACACCATCGGTCTGGAGGCCATCATCCGCAAAGCTCTAATGGGGAAAT

ATGACGAACAGGCGGATGAGCGTTTGCCGTCCAGCTCCGGACCTCCTGCTGCTGCTGATC

CACGCGCTGAAGAGCCCTATTCCCTGCCTGGTAAATCAAAGAGCAGCGGTCGCTCCAACG

GCCGCAAGACAAAGTCCCCCGCTCCAGGACTGTCTGGAGGGGAGAGACCGTCGTCTGTGT

CGTCTGTTCACTCTGAAGGAGACTGTAACCGTCGCACACCTCTCACCAACCGAGTGTGGG

AGGACAGACCATCATCTACTGGCTCCACCCCGTACCCCTGTAACCTCTTCACGATGATGC

GTTTGCCCGGCTCAGTGATGCCCGGACCTTCACCTTCACCCACCCCGACCGCCACCGGAC

CACCACAGCCTCAGAGCCGAACCTGGGAGGAGGAACCCAAACCACTGCTGTGCTCTCAGT

ACGAGACACTGTCAGACAGCGAG

>Unigene000249

GAACAGGCCACTGCAGATCAGTGGTATGGGCCAGGACCAAGCCTCCGCCCCCAGAATGTG

ACGAGTTGGCGGGATGGTGGGGGCCGCGCACTGGCACCCACCATAGCTGGGGAGGGGGCA

GTGGAGGGTGGTGTTGGGGTAGCCATGGGAATGGAGGGTGCAGCTGGGGCCCCTCTTCTT

CAGAATCCCCCCCATGGGCCACCTAGAAACCCGCCTGCTGGTAGCCCCACTCTTCCTCTC

CCTCAGCCAGTTGGCCCGCAGTTCCCTCCTTACAGAGGAATTATGCCTCCCTTTATGTAC

CCACCGTACTTGCCCTTCCCAGCACCATATGGTCCTCAAGGGCCGTACAGGTATCCACCA

CCCAATGAGGCCCCTCGATTCTCTCGTTCTCAGGGTGGGCCTCCTCTAGAGGGAAGGCCA

CCTGGTGAAGTGGTCAAACGGCCCTCTATCCTAAAACAGGATGACCTGAAGGAGCTGGAC

GAGCTAGACCATGATGGAGATGAAGGATGGGCCGGGGCACAAGAGGAGATTGACTACTCA

GCCAAGTTGAAGTTCAGTGATGATGAAGGTGAAGATGAGGGGAATGAAGAGGACACTGAA

AGCAAGAACGAAATTCTGGAACAAAGGGAGCAGCAGAAATCCCAAGACGGACCTGCCCCA

AGTTCACGTTCTCGGACGTCTGAGAGTGGAGGCAACTCAAGGCATACACCTCCTGTCCCA

ACTGATGAAGCTGCCCCAGCACCCTCCAGCAAGCCCAACTGGGCCGAGGAGGGGGGAAGC

AGTGGACCCTACCAGCAGGAACGATCCTCAGCTCAGTCGAAGCCTTCTGGTGCTCAGGTC

CAGCCACAGAAAGGACCCACACCTCCCCCATCCAATGCCCAGACGCAGGGCGAGGATGAA

GATGAGACATGGCGTCAGCGCAGGAAGCAGTCATCGACAGAAATATCTGCAGCTGTAGAG

CGTGCCCGGCGTCGTCGTGAGGAAGAGGAGCGCAGGATGGAGGAAGAGCGTCGTGCTGCA

TGTGCTGAGAAATTGAAAAGGCTGGATGAGAAACAGCAGCAACAACAAAGGCAACAGCAG

CAACCGCAGCAGAAGCCTAATGCCGTCGTAGATGTTCCTGCAAGCAGCCCCACGCCCTCC

ATTACCGCTTCAGTCTCTTCATCCAGCACCAGTCAGCCCCCATCACCCTGCATAGATACA

GAAGAACCACCACTTGCATCTGCTCCGATGGGTGGCGCTACATTAACACTGACTGCTAAT

AACAGACAGAGAGCGGGCAGCAACAGCAGCTATGACTCAAACACTGAGTTGCAGCAGGTT

GCTCAGCCTGCTGCCCCCATGCATCAGCAGCCCCATGATGTCCCTGTACCAGGGGAAGTC

AAAGAGGAACCTGTGTCAAGCAACACACACGTTCGTAGCAGCAGAAGCGGAGATGAGACG

GTGAAGGTAGAAGCAATTATGGGGGGAACAGGAAGGTCAGGGGGTGGAATGCCTAACCAG

GGTTTTTCCAAGTATCAGAAGTCCCTCCCACCGCGGTTTCAGAGGCAACAACAGGAACAG

CTAATGAAACAGCAGCAGCAGTGGCAGCAGCAACACAGCCAGGCTGCCCAAGTCCAGCTC

CAGTCTCAAGCACCCCAGCAGGCCCCACCTCCCAGTGCAACACCACAGGCTGGCCCTAAA

CAGCCAGCTCTATACCCGCCTAGCTCCATGGGACGCCCTCCGCCTCTGCCTATGAGCTTT

GACCCTCGGTGGATGATGATGCCCTATATGGACCCCCGCATAATGCAAGGTCGCCCCCCT

CCCTTGGATTATTATCCAGCCAGCATGCATCCCACTGGGCTGATAAACCGGGAGCGCTCA

GATTCTGGTGGCTCGGGCTCAGAGCAATTTGATAGGCAGCAGCATGGCGGTCCACCTAAT

CCTCCTCGTGGCACACCACCCATGGATCCTAAACTGGCTTGGGGACCTGAGGTTTTTCCA

GGGAGTGGGGAGGGTAGAGCTTTGAGTTCACCTTTAAGGCAGAAACAAGCTCTGGAGGAG

GAGGACATGGGCAAAGGTGCCAGGAGTGACACTCCTCCAGTGCGCAATCGAGATGGGGGT

ACAGGTACCATTCAACCACCTGTCGTGTCTGCCAACTCTCCATCTGGCTCCACTGATCAG

GCAATATCTCCTGTGGGAACTCAAACTGGCCATCACCAGCCCTTTATGGGAGGGCGTGGA

AATTACAGCAGCTTTCCAGATAATGGATCCAGAGTGGTTTCTCACCCGCAGCAGAGGGTT

AATAGTACTGGAGAACTTCGTGGCTTTGGCCATCAAGATGAAGCCCCACAGGGTGGCCAG

CAGAGTCAAATCTGGGGTGCTCCACATACCCACTTTGACCGCAATGGCCGTGCTGATCTC

CCTCTTTTGGAGAACAACCCTCATTTGCAACACCACCATGCTCATCCCTCACATTACACT

CTCCATCCACACAAACAGGAGAATGTTAGAGAGCGAGAAAGAGTAGTAGAGGCCAAGAAG

ACTGAAACATCTCCTCCACTTCCACAGCCATGCATTTCTTCCTCCTGCTCCTCTTCTTCC

TCCTCCTCTTCAGCCAGAGAGGATGGCAGCAGAAAGCAACCAATGCATGATCCACCACCC

CAGCAGGATCATGACTCTGGACACAGGGGTGTTGCTGGACGTAAACAGGACAAACCAGGG

AGTACCCACCATCACACACAGTCCTCCCAGCATTCCTTGCAACAGCACCACCATCCCAAA

TCTAACTCGCGTGGTCGGGAGCAAAAGACTGAAACTCAGTGGGGCCCGAGGCCTGGAAGC

AGTAGTGCAAGTGGTGGACATAACAGAAAGGGAGGCCCTGGAATGGGTGGAAACATGGGA

GCAGAACACTTGACTAGTCATGTCGAAAAGCCTGTTGCCCAATCAGGAGGTGGTAACCCA

AACAAGCGAGTGGGCCCTATTAAGCGTCCCGTGCTAAAGGAAATAAAGAGGGAGGTTGGT

GAAGGTGATGGAGGAGAGAAAAAGACTGTAGCCTCTGCAAAAGATAAAGACCTGGATGCC

AATCATGCATCAGTTAAGCAAGGGTCAGTGTCCCATTTTCAAGCTTCATCTGGACCCTCT

AGTAAGGATGAAACTGCTCCTCTAGGAAAGCTAAGAAACAGTGGCAAGGAGCGAGGTGGT

GGTGGTGGTGGTGGTAAAGCATTCAAAGAAGGTGAAAACCCAGTCCAAGCCACTGGTTCT

TTAGCTTCACATTCAAGAAAGGACAGAGAGCATTTGCATGAGACCAGCGGCAGTGCAGGG

GGTGGTACATACCATGCCGGCTCTTCCCGAGGAAGCCGAACAAGTCGCGGAAGGGGCGAG

TTCTACGGCCGAGGTAGAGGCTACAGGGGCAGCTATGCAGGCAGTGCACGTGGTAGGGCA

GGAGGCAGAAGCACCCGAGACTACAGAGCCACTGCAAGTAGTGGTTATCATCATGGCTCC

TCTAACCAAGATCATAAGCGGGAGGGATCATCTGGGAGACACGGCCTAGCAGGCTCTCAG

CCCAACCCTGGACGTGCCAGAAACCACAGCGAAACTCGAAGCGAGGGCTCAGAGTATGAA

GAAGTGCCGAAAAGAAGGCGGCAAAGAGGTTCTGAAACGGGCAGCGAGAGTGCTGCTAGT

GACCTTGCACACTCTGACAGGGAGGATCGTAAGCCAGCTACTAGGAAAGCAACAGGGGGA

GAGAATCAAGGTGTGGGCAATGCAACATCTTCAGCACCACCAAGAAATACTCAAACCAGA

GTGTTTACCCCAAGAGGAGTGCCATCCAGGCGCGGCAGGGGTGGAGGTGCCACTGGAGGT

GGTTTCTATAGAAATTTAAACAGTGGCAGCGATATGTCTGGCGGACACAGATCTGGGCCT

GGTACCTCTCAAGGCATGTCGGCAAAAACAATTGCATCAGTCCGAAAGCAGCACCCACCC

CAACAATCTTCCTTAATCAAAGAACCAAGCCATGGATCTAATGGGGAAAAGAAGGAGAAA

GCAACTGAACAGGGTCAATCTCAAAATCAGGGTGTAAACTCTTCAGTAATCATTGATCCT

ACAGTACCCTCAACTGCCCATTTGGGCACTGAAAATGGAGGTGTGGCCCAGTCATTAAAC

AGCACCAATTCCTCTGGACAAAAACCGGTGTCCTTGCCTAGTTCAGATGGATGTAACATT

TTGCATAGAGGATTCGAGCGCCCTCCTCGTCGTAGGCGCCATGGCCGTTCCCAGCATCAG

CAAGACAAGCCCCCCAGATTCCGTCGGCTTAAACAGGAGCGGGAAAATGCAGCCCGTATG

AATGGAAGTAGTGGAATAATAGAAACTGTGGGTGGACAACCACAGCGATCTGCATCACCA

CTTCAGAATTCGATTCAAGATGGAACACCTAACGTGTCTGCCACTTCTGGTGTTGCAAAT

ATAAACCACAGCATCTCTGCAACCAACAATAACAATAGCAGCTATCTTGGGGGTGGGAAC

CCGAATTTAAATAGTCACCACCATCACCACCACCACCACCACTACCATGGCAATTCTGGT

AGTCATTCTCAGCACCACAATAACCACCACCAGACTGGTGGTGCCAAATCTCCAGATGTC

TCCAACCAGAACTCTGATCAGGCCAATGAGGAATGGGAAACTGCCTCTGAAAGCAGTGAC

TTTGCAGAGTTTCGAGAGAGAGAAGGTGGCGGAGGTGGAAAACCCTATTCCTCCTATCAT

CATTTCCATCCTTCAGGAAGAGGAAGCGGAGGAGGGGGCGGTATTGACCGAGATGTAACT

ACTAAAGAATTGGCTGCCAATAAGAGGAGCTTCTCCAGCCAGCGGCCTGGGATGGAAAGG

CAGAATCGGAGGGTCGGCAACGGTGGCGGGCGAGGTTCCAGAGGTCCACCTTCTGGTGGC

GGACCTGGTAGTGTATCAGCTAATGGAGGGGCCAATCGTGGTGAGAGGCGTGGCAACTGG

CCATCTCCCAAGAACAGAAAATAATCCCCTTTCTTCCCTCCTTTTACTTTGGCAACATTT

CAAATTTATCTCTCTTTTTCTCCTCCCATTCATCTATTAATTCTCAACTCCTAACATATT

ACAATATTATTATTATTACTGTACAAGATATAGTTATGAAGATGCTGTACACACTCATTC

TAGAACCCTTGGTTTAATTCTTTGTGTCTTGTCCTTTTTATTTGTCCATGCACACCTTTT

TTTCCTTACATACAGGCCCCATATTCTACTTAGATGTCCTGTTATATCGATTGGCAACCC

TTTAAATAAAAAAACGTTTGCATATACAAGCACAATATGACCTCAAACTGACACGCAGAT

ACAAACTCGACATGTGACTGCAGAGTGTGTAGAAGGCCTAGTAGCATGGAGTAGTGTCTG

GAAGTATTTATGGAAGGCC

>Unigene000250

CCTGGGTTGTCACGCGCTTGCGCTCCCTATCGGCACAGCACAGCACACTGAGCACAGTGA

GAGACGCGCGCGGGAAGACTCGCTACACAAGCTCGCGCTTCTCGTCGCTTCACACCGGGT

CTTTATCAATCAGGCAGCCGCCATGACGTCCCGGAGGTGGTTCCACCCCAACATCACCGG

CATCGAGGCAGAGCAGCTTCTCCTGACACGAGGCGTCCATGGCAGCTTCCTGGCTCGACC

AAGCAAAAGCAACCCAGGAGACTTCACGCTTTCAGTCAGGCGGTACGATGAGGTCACTCA

CATCAAGATCCAGAATTCTGGCGATTATTATGACTTGTATGGAGGCGAAAAGTTCGCCAC

TCTGGCTGAGTTGGTGCAGTATTACATGGAGCAGCACGACCTTCTGCGAGAAAGAAACGG

AGACGTCATCGAGCTCAAATATCCGCTCAACTGCAAGGATCCCACATCAGAGAGGTGGTA

TCATGGGCATCTATCTGGCCGAGATGCTGAAAAACTCCTCATGGAGAAAGGCAAGCCTGG

CAGCTTTTTGGTGAGAGAAAGTCAGAGCAAACCTGGAGATTTTGTGCTGTCTGTTCTTAC

CAACGAGGAAAAACATGAAAATGTGGACCGCAAGACAAAGGTCACCCACGTCATGATCCG

CTACCAGGATGTGAAGTATGACGTGGGTGGTGGGGAGAGGTTTGACACCCTAACAGACTT

GGTGGAGCACTATAAAAAAAACCCTATGGTGGAGAAGAGTGGGATCGTAGTGCACCTCAA

GCAGCCCTTCAATGCCACTAGGATAAACGCAGCCAACATTGAGAATCGAGTGCAAGAGTT

GAACAAAGTGGCTGATAATTCAGAGAAACCCAAGCAGGGCTTTTGGGAAGAATTTGAGAT

GTTACAGCAGCAGGAATGTAAACTTCTGTACCCGAGAAAAGAAGGCCAAAGACCTGAGAA

TAAGAGCAAAAACAGATACAAGAACATTCTACCCTTTGACACCACCCGGGTGACAATCAA

AGAAGCAGATCCTGACGTGTCTGGCTCGGACTACATTAATGCCAACTATATTAGAAGTAT

GGAGGAAGAGGGCCGTCATGTGGACGAAGGCAAAGTGTTTATTGCTACTCAGGGCTGCCT

TCAAAACACAGTTCTGGACTTCTGGAAAATGGTCTATCAGGAAAACACTCACGTTATTGT

CATGACGACGAAGGAGATGGAGCGAGGCAGGAATAAATGTGTACGCTACTGGCCAGACAC

AAACACGACAAAAGAGTTTGGAAATGTGTGTGTGAGGAACATGGAGGAGCAGGCTGCTCA

GGACTACATCCGCAGAAAGCTCGAGGTCACACGTACAGACAGAAAGGAATCTTCCCGGTA

TATCTGGCACTATCAGTACCTTAGCTGGCCTGACCATGGCGTGCCCAATGAACCAGGAGG

AGTTCTCAACTTTCTTGAACAGGTCAACCGAACTCAAAACAGCATTCCAAATACCGGACC

TGTCATAGTGCACTGCAGTGCGGGGATTGGAAGAACAGGCACGATAATCGTAATAGACAT

CCTTATCGATATCATAAATAGACAAGGGCTGGACTGTGACATCGATATCCCTAAAACCAT

CCAGCGAGTGCGTCAGCAGCGATCCGGCATGGTCCAGACCGAAGCCCAGTATAAGTTCAT

CTACATGGCCGTACAGCAGCATATCGACACAGCACAGAAGAGACTGGAGGAAGAGCAGAG

GAATAAAACAAAAGAAAGAGAGTACTCCAATATTAAATACCCTCAAATGACAAATACCCG

GGCCGTACCCAACATGAGCTCCTCCCGCTCATCGCCTGTAATGACTGATGAAACATCCAG

CGTATACGAGAACGTACACATCAAAACCCCCAAAAGCCCCGTCTGCACAAACTCCAGGAG

ATAGAGCTCAGGACTACTGTTTCACACTCTCTCTGTTGGAGTTGCTATGTGCAGGCACTT

GACTGTAAAGTGATTCTGGAGCTGGTCGCTGAGCTGAACCCTGTGTGTTGGAGGTGTGAT

TGGTGGACCCTGTCAGCTGCACAGCCCCTTCACTTCTTCCTCATTGACTGACGTGCTGTC

TAAGTGCCTAATCCGCCCCAAACAGCCAGAGCAGAGCTGCCTCAGTGCTTCTCCCTCCGG

TTTGATAAGCCTACCTAGCTCTCATCTACTCGATTCCTTCTAAACAGCTGAAGACTGTTC

CACAGGAGTAACTCCAGGTTTCCCCCGACTTTGTCCTGTTCACACTCTGTACGTAATGCC

AGAATTCTGTGCTGTCCTGAGGAGCTCTGTCTCTGACAGTGCTTTGCTGTGAGATGGAAA

CAGTAAGGCATTCCGACTGTCTGTGGCAGAAGATCTTACCGAGCTCGCACGGCAAAATTA

TAAAAATCGTAACACGTTCCAGCTCTTAAGCTACACAGAATGTTGGCTAACAGCAAAAAC

GGGTGATTTTTTTATTAATTATTGTGCTGATAAGCAGGCTGCATTTTCTGTTTTTGGGTA

AAAGTATTTGCTGTGATGCTTTGCAAAGCGCTATCCTTTACTGATTATATATGACGTCGA

ATGTTAATTAATAATGGGCTCATAGACCAATCAGCTTCTGTGTTTGAGCTTTTTTTGAGG

CATGTTTTTTTTTCTATCTGGTTACATTAACACGGATGATTTCTTTATGGTATATATATA

TATATCATGATGGATTCCCTATGCCAGGTTGTACTATTTGATGGAAATGCTATTTTTTGA

CAGATGTCAGCAGCAATGATTTAAAAATGTCATTTAATTGTTATTTAACATTCTCAGAGG

TAGACCGTAAAACATTTAGGGTTTGTGTATTATAATTCTGGTGATAAATAGGCATTCAGG

GATTGTACTCTGGTCAGGGATTGCACTCGATTGTGTGGGGGTTATGACAGGTTTGTGAAA

GTAGGGGGTTTCCAGACAGAGGACAGGGTTTCGCAGTGCAGAATTAAACAGTCCAGATGG

TTCGAGACCTAAAATAGATCTGCCTGTGAGAAAGCAATTTGATTAAATTGTCAGTAATCC

AGCCAGGATGATTGAAAAAAAACAGCATGATGTCAATAAAAGCCTGATCTTATAGGAGAC

CGTATATGATGGCATGAGGAGGATTAGACATGTGCAACAGATTTCGAATGCTTAAAATAA

CTCAGGTTAGAATAGGACAGATTATTTAATGTCTACATCTATTAAGTGAAGAGTGTAGAA

GAAGGAAGATGATGTGATGAGTAGGGTTTCATAGGGGTGGAAAATGTCTGGTGAATTTTC

GGAAACTTTTAATAGGAACGTCATCTTGAAAATGTTGGAAATATTCCAGGTTAAGAATTT

AACAGGAATTTATGGCGATTTATGGGAATTTATTCAAGTTATTTAGTAATCTGGGGAAAT

TTGTATGTATATGAGCAATTATGCATTTCGTTTGATCATAAACTGGCATGCAAGCGAAAT

AATACACATTTAAATAATTACAATTAAATGAGGGGGGGTGGGGGGACTAACAGTAAATGG

GGATTGTGTATAGTAAGGGGGAAAGGGTCATTTTTAGCCAGTAACATCACCTATCACATC

AACTATTTTTAATAGTTAAATTATTTTGTTGCACGACAGCTTACACAGTACAAGAGGGTT

TAACATATAAATTTATGTCATATTTATTGAATTTACATAAATACTCACCAAGATGGACAT

TTTTGCATTATTTCTTGTGCAGGATTGAAGAAATGATGGGTATATGTTGTGGGGGAGTTC

ACAGTGCGTGTGGAAGTGTGGCGTCTGTAGCGCTGCATTGCTGTGTGCATGTGATTGAGG

AACATACAGGCTAGAAATTCAACTCCAGTTTCATTCAGTCTGGTTGTTATATCGAAAAAT

ATTCTGCAAATATTTTTTAGCTACATTAAGCATCCCTGTTATAGGCTAACCCAGTGCTTT

CCAAACTTTCAGCTCATATTTTGGCCATTTTACTTGTTGAAAATTCCTTATAAAATCTGC

AGCCTTTCCGGTTCCCCCTTTATTATAACTCTTCTTTTCCCAGTTTGGAAACCACTGGGC

TAACCCGAAATTTTACAAACTGCCAGTTTATTTCCAATTATTACTATTAAATTCCATATA

TCCCTGTTAAATTTCCATAAAATCCTGTTAATTCCCATGGAAAGTGATAAGTGGAAGAAC

TGAGACTTTCATAAAGAATTATTCTTGATGCACCTGGTCGTGGTTTAAATTATATATATT

CTCTCAAGAATTATAGAGACTTTTTATAAAAAGTGATTGTATTTACAGTTGGAGAAACTG

TTTCTCATTTAAACTCCAACGTCGATCATAACATTGCCCAACATTCACTTTTAGTCTCCA

GAAACATCTCCTGCTTCCTTTAACCATGTCCTGTTTCCCTGGGGTACGAATTTACAGTTC

TCATCATGGGAAAAGCCAAAGAATTGTCAGGCATCGTTCTGCACAAATCATGAATATACA

AAGATCTACCGCACTTACTAATACCATTAAAAGCACAATTTAGCCAAAATGTAAGTTTCC

CACAACTGAAAAGAATGTTGAATATTTGACAGGTGAACAAGGAACTGCAAAAAATGTTTA

AAGATTTCGCAGTTTAGTCGATTCCTGTGGTTTTAGACATTAAAATCAAACATAACTGCA

AGCTCTTTGTTAAAGCTCAGCATTTCAGAGGTAAAAACATCTTACCGAATGCAGCACCTA

ATCTAAAGCATGTGTCATTAGAACTCCAGCGTGTCAAGCTTAGATGAGACCAAAACCAAT

TGTTCAATCAAGAACACAGGCAGCATTTTTTGGTAATTTAAGGCTACATACAGGAAAAAG

CAGCCGATACGACACTCTGGAATATGGAGGCGGAGATCTGATGATTTTCTGAATTGTCTT

TTAAGTCAAATAGCATCATGAATTATTCCAAGTTGTAAGATATTTTAGTTTATGGCCCTG

CCCCAATGCCCACACTACATTCTAAAGTCTTCCACAAAGTGGGCAATTTTGGAAGTGCAC

ACTTCAGTTGAGAAGTGCGCAAGGAAACGAAGGACGGTTTAGGGCAGGCCACCTATCTGC

GTTAAAAGTCATTGCTACGTCATCGCAACACCCCTCAAACAAAAAACGACAGTGTCTGTA

GTTGCGCTGTAATGATAATTACATGCAAAAACCCACATTTCTTGGCTGCTCTTGGAGGGC

ACAAGTGGAAGGATTTGTAGAAGCTCACCAGCAAATTGGGACACCCTACGCACTTGCACC

TGTCAGCGGGAACACGCCAAATGAGGGGCACAAGGGTGCAAGTACGGGTATTGGGACAGG

GCCCCCAAATTTAAACACTGAGTTTCTGGAACACATAGTGTAATACACTATATATATATA

TATATATATATA

>Unigene000251

CTCAGCCCAGCAGCTCCAGCGCTCAGAGCAGGAGCACAGCATGGAAACGCAGAGCAAACT

TACCCTGCTGGAGGAGGAGAGGCAGAGGTGCTCCAGTCTGACAAAGGTGAACTTCCTGCT

CCGTGAACAACTGGAACAGGCCAACGAAGCCAACAAGGCCCTTTTAGAGAGCTTCCATAA

AGCCAGAGAAGAGGCTGAGCAGAGAGAAACACAACTGGCAAGGGAACATGAGGTGACTGC

CTCCCGGCTGAGCCGTGAGCAGGCACGTGTTAGAGCTCTTTGGGGCCAGGCTGCTTCACT

GAGGAGCACCTTTACCCAGCTCAGGGCTTTTGCTGACAGGAGTCTTTCTGACATGCGTGG

GGAGTATGCATCAGTGAGCCGGACACTGCATGCGGCCTGCATAAATCTGAAGGCATCTGA

GAGTGAGAAGAATACCTCTACTGGCATGGAGGTCTCGGCCCTGGAGAGGCAGCTGAGAGA

TAAACTGAGGGAAGAAATGCAGCTTCAGGCCCGCTTTAATGTAGAGAAAATGGAGTTCAG

CTCCAGGATCCAGGGACTAGAGGAAACAGTGAAGCATCTAAAAGTTCAAAACAGTGAGAA

AGAAAGCAGTCTGGCAGCATTACAAAGCAGTCTTGACAGAATGGAGTTGAGTCATGCCGA

GGTTGCAGCCGAGAGAGACACCTTTAGCAATGAAGTTGTCAGTCTACAGCAGATCCTCTA

CAGCATCCAGCAGGCTGTATGTGAGGACAGGGACAGTAGCAGCTCAGACAGTCCAGAAGA

AGAGATTCTTTCCAACTCTTCTACCCATCTCTCCTCTCCTCAGCGGGACAGCACTCTGAA

AGCTGTACGAAGGGCTTTGTCCAAACGGCAGACACAAACCCAGAAATTACAGGAACGCCT

GGATGCTGCACTGGAGCAGGCTGCTAGCCTGCGTGATCGGCTTCAGGATAGAGAGGCTGA

GCGTAGGCAGCTGGAGCAGAGAATCCAGCAGTTGGAGCAGGAGAACCATCGTGCTGAGAG

GGCTGGAGAGGAATGCAACAGGGATGCCCAAAGATATCGCAACTCACTGGGGATTATTTC

CAGTGAAAGGGATGACTTGGAGAAGCAGCTCTCGACCTTGCAGCAGCAGTTAAACTCTGA

TCAGGCAGAGCTGGAGCGTTTGAGGAGCTCTTCTGTAGATCACCAGCGGCAGAGAGAGCT

CCTCCGGCAGCAGAGGGAGGACCTTGAGAGGCAGCTGGCTCGTGAGCATTCCGAAGCCGA

GAGGGGTCAGCGAACTATTGAACAGCTGGAGGCCAAGCACTCGGATGTGCGTAAGGAGCT

GGTGACTGTAAAAGAAGTTCTGAGTCAGCTCACATTACAGAAAGAAGTACTGGAAGATGA

GAACGGGAGCCTTGTTCAGGCTCTGTCTAAGGCAGAGGCCCAGAATGCAGAGCAGGAGTT

GAGGCTAACCAAACTGCAAAGTGAAGCAGCAGCACTAAAAGATGCCTTAGCTAAGATGGG

TTCCCTGAGTGAAGGCCTGGCCAAAGACAAGGTGGAGCTGAACCGAGTGCTGCTACAGGT

GGAGGCTGATAAAGCAGAACAAAGTGAACGGAAAAGAGAGCTGGAGTTAGAGCGGGCGGC

GGCTAGGGAGGAGCTGGCACATGTGCAGCAGGAGCTGCTGGACATGACAGCTGAAAAGCG

AGCGCTGGAGGCCACACAAATTCACCTCCAAGACGCACGAGGATGTCTGGAAGCAGAGCT

AAGTGGGCTGCAGAGGGAGAGGACCTTAATTCTGGAGCAGCTCACCCAGGCTAATAAGCA

GAAGCAGGCAGCGTTGGAGCAGCTGAGTGTGTGTCGAAAAGAAGCTGAACTTCAGACTGC

CTCGCTGCATAGGATGGGCAAAGAGAGAGAGGAGCTGACAAAGGACAAGGCAGCGCTGAC

TGTCCAGCTCACTACAGAACAGCGAAAAACCAAGGCGCTCACCCAAGAGCTGGCTGCTCT

TCGAGCTGAAAGGGAGTCTCTGGAGTCAGCTCTGTTTGATGGACAGGAGCTGGTCTCATC

TCTGGAGGCTGAACAGACTCAGTTAGAGGGGGAGAGAAGCAGTCTCCAGCAGGCTAATGA

GGCTCTGACATGTGAGATGTCTCGTGTCCAAAGTGAACTGGATCAGCAGGTGTCCCAGTC

TCTTCAGGAGAGACAGGCAATGGAGCTAAAGCTAGCTCAGACTGAGAGAAATGCCCAGCG

AAGCCTAGCCACTGCTCAACACACGCATCAGGAACAACTGGAAGCCGAGCGCAAGGACAA

GGAATGTCTACGCTTGACTCTGACTGCACAGAAGGAGCAGGCTGAGGAGCAGCTGCGTGC

GGAGTGTGAAGAACTTCGTCTGCGCAGTTGCAAAGAACTGCAGCAGCTGCAGGAGCAGCT

GGCTAAACTGCAGCAGCACTGCACAGACAGCTTGTTACAAGCCGAATGCCACAAACAACA

GGCGCTGTCTGAGAAAGAAGCAGAAAAAGCCGCTCTGGCTGAACGCATTCTTATGCTGCA

GCAGGACCAGGAAGCTGGTGCTCTGAAGGTGGAGCGCATGCATCGAGACTTCCTAAGCAA

ACAGGAACAGGATAAGGACACAATTACTGGGCTGCAGAGTGAGCTGCAGCAGCTTCGTTC

GTGTTTTGAGGAATCACTCAGCTCTCGAGAGACCTCGGAGAAGAGACTAAACGAGCAGAT

CAGAGAAGTGTCTTTGCACAGGCATGAGGCTCAGCAGGAGGTGGAGCGATTGCGGAGAGA

ACTGGCCGAGGTGGAAGAAAGCCGAGATGCAGGAAGGAGGGAGCTGATCGAGGCTCATCG

GCAGCTCCGCGAGTGTGTTCAGGAGAGAGATACTCAGCGTAGAGAGAGTCTGGAGCTGAA

GAGAGCTCTGGAAGATGAGAGTCGAGAGCGGGAGGCCATTCAGACGTCCAACCAGGAACT

CCGGACTGCTGTTAAAAGAGCTGAGAGTGACAATAACAGTCTGAAGAGATCTCTTGAGGA

AAGGGAGCAGAGACTGCTTGTTCTGGAGGAGTGTAAGTCCTCGCTTCAGCAGGAGGCGCT

GAAACTGAGGTCTAACCTGCGTGATCTGGAGAAATCGCACCTACAGGCCCGCAGGGAACT

GCAAGAATTGCGCCGCAAGGTTAAAACTCTAGAATCAGAATGCACCCAGAAAATTCAGGA

GCTGAGTGAAGTTCAGGCTCGTCTAGCTCAGGAGGAGCAGAAAGAGGAGGAATGCAGAAG

AGACAACTTTGCCCTCAAACAGAGAATCTTGGAGAGTGAAGCAGGAAGAGAGGCAGCTCT

GAAAGAGATGGCTGGTCTTCAAAGACATGTGTCTGAGCTTGAGGAAGTGCAGCGTGTCGA

GCGGGATACCCAGCGTCAGCGTGAAGTCAGCATGCAGCAGAGCCAACAGTGCCAGCAGGA

AAAGATCACCCGTCTAGAGGGGGCGCTGCAGGAGGAGCAGGTGCAGCTTAGAGAGCTGAA

TGTGGAGTTGAGCCTAGCACATGGTCGGGTTCAGGGTCTGGAGGAGCAGCTAGCTAAGAC

TGATGTTGCACGCACAGATCTGGAGCACAAGCTGAACGGCCTGATATCTGCCCTTAGACG

CACTCTGGGCATCGGGCGCAGAGCAAGTTCACCCACACCAAGACCGAGAGGGAGGAACCC

ATCACCATGGAGAGCTCATTCTCCTTTTAAAGGCGATCGTGTATCTAGCTCTTCTCCAAT

TCGATCTGTTGGAGAAGATGGAGAGCTAGATCTGGACTCTGTGCAAGCAGCATTGTGCAA

CTTTCAGCAGGAGCTCAGAGACACCCAGAGAGAGAGAGATGAGGCCCAGGCTCAGGTGCT

GAATTTAAGCCGGCAACTGTCGGATCTACAGGCTAGTCAGGAGAAGAGCAATGCACGACT

TCAGCAGATGAAGAGGAGCTTAAAAGAGCACGAGCAGGGGAAACGTGATTTGGCAGAGCG

TTTAGAGTTAGCTCAGGCTTCACTCACACAACAGGAAGATGCAAAGCAGTGTGGAGACAG

AGAGAGGAGGATTATGGGAGAGGAGCTGTCCAGGCTGAAGAGTAGTCTGCAATTAGCCGA

GGTGGCAACCAAAGCTTTACAGGAGAAGCTGGAGCGAGCTCAGGTGTCTAGGTCCAGTCT

GGATGAGGAGTACCGGAAGCTGAAGGAGGCATGTAAGACGGCAGAAGAACGAGCGTCCAG

GTGTGAGATCAACCATCGTGCTCTAGAAGGAGAACTGCAACGCTCACGGCTCCAAACTGG

AGAGCTGGAGGCTGAGCGTGCCGCTCTTATGGAGAGAGTGACCAGTCTACGGAGAGAACT

GGGGGAGAGTGAGCAGCACACCGCTCTGCTCAAGGTAGAGCGTGAGAGAGCAGCCACAGC

TCTTGCCCGTGCTGAGCTTCAGGAGGCTCAGCTGCATGAGAAGCTGCAGAACCTGACAGC

GGCTCTGAGTGAAAGTAACAGCAGCACAGGAGCTGCACAGGAGCAGGTCGCTCAGCTTCA

GAGCGCTCTCTCTGCTGGCGAGCATGACCGCAGGACTCTACAAATGGAAAAATACAAATG

ATATAAAAAGTAGAGGGAAGACAAAAAGGACAATATTTTGTGTAAGAACAGGAGCAGATG

GACAAAGCACGAGACACTCTTTTAGATACTAAGAGACAGAACCACATCCTGAACGACAAG

CTGCAGAACCTGCTTAAGAATCAGGATGAGTCTGAGCTCAGAATCACACAACTGGACAAA

CACATACGCAGCCTGCAGGAGACTCTGAGGCAGAGGGAACAGGACATGAAGGAGGCTCAG

GATGGCTGTGAATATCTGAAGAGGAAGAAAGGAGAGCTAGAGGAGCGGGTAACCAACCTT

CAGAGCTCGCTTCAGGGTCTGCAGGAGGACAAGACGCAGCTAGACAAGACGCTCGCTCGG

CTGGGCAAAGACAAGTCCTCCCTGAAACGCACGCTTGAGAAGGTGGAGCAGGAGAGACGG

AGGCGGGAAGAGCAGGCGGTGTGTGTTGGACATCAGAAGGAGCAGCTGGAGCAGACAGTG

TGCAGCCTGGAACGGGAACTGAGCGAGACTCAAGCTGAAACCAACACACTTAAGTCCCAA

CTTTCCCATATGGAGCAGGCTCATGCTCAGCGCCTCCTAGAGGTAACAACTCGCCACAGA

CAGGAACTAGAGCTGGAGACAGGACGTCTGCGTGGCGCACAGCAGCAGGCAGAGCGGGCA

CTGGAAGCTAGAGAGAAAGCACACCGTCAAAGAGTCCGCTGTCTTGAGGAACAGGTGTCC

ACACTGAAGGAGCAGCTGGAACAGGAGATGCGCAGAAGACAGACCTACATAAGCAAAATG

CTGAAGCCTGTGTCCTAAAGAAAGTGTGTGCGTGCGTGAGAGGGAGACAGAGAAAGAAAG

AAAGAAAGAAAG

>Unigene000252

TCTCCTCTAATTTTACTGATCCTCAAACGCTGCAGCTTCTCCAAAACAGTCAGCGATTAC

TGAGCAACCTTTGTGCCATCTTTCACTGCCTGCTGAATGAGGCACAGGAGCTCACACAGA

AAGGTTTGTTAGGTTTGGTGAATAAGTGCATGGTGTCTAGTCTGATCTCTCAGTATGCTC

AGGTGGTCCTGTGGTTCTGCCGCACTGGTCTTCTTCCCGAAAGCGCTGACGAAGATGTCC

TTCAGATTTCAAGGCCTTTCTACAGTTTTTCTGTGATTAAGAATTATTACATAGGTCAGA

GGGAGGAGCTGCAGAGACTAGCCAAGGATAAATGGAACCCTGACTGCCTGATGATTGATG

GATTGGTGGCCCAGTGTGGTGAGCGTCTTTCAGAGCTTTGGAGAAGAGACGAGGGCGGGA

CAGGGCAATACCCACCTCCTTCACTACATGCCCTGCTGGATCTGTACCTCCTGGAAAATG

TTGAAGAACTGACCAAACACGCTATTGTGATTTATCTGCTTCTAGATGTGATGTACTCTT

TTCCTAACAAGAGTGGTGCATCCGTGGAATCTTTCCCCACCGCTTTCTCGATTCCCATCG

GATTAGTAAAACTGGTTCAGGGCCTGTGGTTACTCGATCATCACGACCATGAGAACTCTC

TGGATCTGCTCCTTCACCCGTCTGTCTCTCCGTGTGTGTGGGCGTGGCAGCACTCTCGGG

TTCTGCAGGCTCTGATGATTCAGAATAAACACAGCACAGCACTGCATTACTTTCACATGA

TGAAGCCCTCCATCACCTCCACCCCCCTCACCAAGCTGTGTGTTTCTGTACTGCTGCACA

ACAGGTGTTTAGTGGAGGCGTGGGCGCTGTTACGGCAGCAGGTTAATAAAGTCAGTATGG

AGGAGCTGCTCAGATTTTTCTATGAGATGTGTCAGGAGCTGGGATTAATGAAGGAGCTCC

TCAAACTCCCCCTTCAACCCTCTGAACAGGAGTGCTTAGAGCGCTTCCTGCAGGAAACCG

GAGGGTTTCAGAACAGAGAGCTCCTCATGGTACACCATCTTCAGCAGGCCAACTACGTTC

CTGCGCTGCAGATCAACCACATGCTGAAGATGAACCTGGCCGGTGATCGAGATCCAAAAA

TGAAGGAGAGATGGAACACCAGGAACTCCATCATGAATCAGTATGGAAAAGTTTTACCGC

GCGCTCAGAGGAAACTGGCTATCGACCGTGCAAAACCATATCAGAACCCGTCCACTGTAC

TCCGAGAGGTTCCGAGGCCTCAGCCGTTATCCACCGTGGCTAAACGCTCCGCTAACAAGA

ATGTCCTTAGCAGGGCGGCGTTCATTAAAAACGTTCTCACCAAGATTGAGGAAGTGTGGA

TCGGGAAGGACACTACACCTGAACACTCCCCTCTCAAGAGCCCAAAGGCTCCTGATGCTT

TGATGTCCAGTCCAAATCCCCCAACTCTACCAGTCCCCGAGGCTTTTGTTGGAACTCCGA

TTAATATGCTGACTAAGAGGATGTCCAGATTGTTCGATCTAGCAGTTCAGCCGTCTCCCC

ATGCTCAGACTACTCCGCCCAAATCCCTCAGTTCCTGGAGTGCACCTAAACACATAAGCA

GGGCTCCTGAGCTCAGCCTGTTACATACTCCTCAGGTGGTAAAGAGAGCTCGAGCTTTAG

CAGCATCCGGTCCGGTCTTCTCCTCATTCACCCCTCAGTCCATCTTAAGGAGCAGTCTCC

GCCCCTCTCCCATAGCCACACCCACTGCATCACCCACACGTTCAGCAACACCTCCTTTAC

GCACCAAAGAGAGTCGCATCACCTTTTTGGAGGAAATTGAATCACCTGATATGCCAAAGA

GCTTTTCCCTGCACTGGAGCAAGCAGCTAACTGCATTTAAAGTCACCGATTTGGAACCTC

CATCCCTGCCACCAGGTGGCGGAGTAGAGAAATGGAGTGAACATTCTGCTGAAGAAGAGG

AGGATATAAAAGAACCCAGTGGTATACTCATGTCATCATTAAAACACACAGATGCCCTTG

AGTCGGAGAAAATGAACCAGGTTTTACCCACTCTAGAGCGCAGACCCAGTCTGGGACACG

AGACAAGCACTCAGTCTGCCCAATCAGATTCCACGTTGGAATTCCATGATGCTCCGTCTT

CAGATGATATAATCAAAACAACATGGCAATTACATGACCGCCTTGATGAAGACGATGAAA

TCATTGTTAAGCGGCAGGAGTCAGAAACTGCCACCAGAACACTTTCTGTTGAAGAGCCGA

ATGCGAATCAGGAAACGGAACATAAAAAGGAAGTTCCAGAAATTGAGATGCAGCCGGAAG

CAGAGGAAGAGCATCCAAACAAAGACTCAGAGGTGCAGAACAAGGAATCTTTAGGAAAAA

GAGAGACACACAATTTCCCAGTTGTTCCAGACACTTCACTGGAGGAGTCAAATCAGGATG

ATTTAGCGAATCATGAGATGACAGGTTCGGTACCATGTCAGCCAATCAGAAACACTGAGA

AGATGATTAGAGAAAAGGACACCAAAGGCACGGAGTCAACTCAACCACCAATAATTAACG

ATGCTGTCCCTGAAGAGGAGCCAATCAGAGAGAGTCCACGTAAATCTGTTGAACACTCGG

TGGTTAGTAAAGAGCCAATCAGTGAGCTTCCTGCTGTTTCTGAAGAGCTGAAAGATAAAC

CTGAGGCACTGGAGACATCAGATCTGGACGAGTATGTGGAGCGTGAGTTATTCGGTGATC

TGTCACCCCCGCATTCCCACAGTGACAGTAAAGAGGTTTTGGAATCAAACAGTAAGCCAT

GTAGAGATGGGACAGATCTCGGTGTGGAGGATGAAGCTCACAAATCTGTCACTTCATCTG

AAGCTGCATTGTCTGAGTCGCAATCTGTTGTCAGCGTAAACGACACTGAGGAATTGTCCA

GCAGTGAGGAGGAGGAATATGAAGGAGAGGAGGAGGATGAGAAGGAGGACTCAGGCAGTG

AGGTGGAGATTATAGAAGAAGTTCAAGGAAACGGGACACAGCAGCAGCTTTACCTGCCGG

ACCTACAGCCTGAAGATCAGTTCCTCCGGGAGCAGGCGGCGGCTGTGCTGAGCCTCGTCA

CTGATCCACAGCTGGAGCAAGTGATTGACAGATGTGTGGAAGAGGAAGGTCAGGTGGTAG

TAGTTGGCCTGAGACCTGCGAGCCTGGGTGATGTGTGCTACACTGAGCTGAAACCCTCCA

CCACACTCCTCGTTCCTATTGAGCTGGCTGCTGAACATCAGGAGCTTCTGCACAGCGCAG

AGATGCCAGAAATCGCACTTCCTGTTGACTCCAATAACTTCTCCCTCGTGCTAGAAGGAG

ACGATGAAGAACCAGCAGCTTTAGAAATGGATCCTGAGAGCCTCCTCACTGTACAGATGA

GCACCAAGCATGCTGGAGATCTGGAGGAAGAACCAAGCAGTAACAGTGACAACATTATCA

TTGAGCATGCAGTTCTGGAACCTGAGGAAAGGTTTCAAATTCAAGAGCTACCTTCAGAAA

TGATTGTACCAGAAGCTGTGGAACCTGATAAACCAGTAAATGAAGTGCAAATGGAAACAG

AAGTTTGTGAATCTAAATCAAACACAACAGATCAGCCTGAAAAAACAGAAGAAGTTCAAG

AAGAGCAAACAGGAGAGAAACGGTTGCTAAGGTCCAATTCTGACCCTGCAGATGAAGCTC

AAACAGTGGAGGAACCTGAAGAAAAACCAGTGAAACCTCTTGACCAGCCTATTGGGAATC

CTTTGCAGGAAGCTGTGATTGGAGAAACTTTTGATGTGACTGATGTTTGTTTCAGCAGTC

AGATTCATGATTCTGGCTTGTCTGCAGTAACAGAGGATGAGAAGATGCCGGATGAAGAGG

TTGTTCTAGAATCTAGTGAGATAGTTCCTGCAGCTGACGGCAGAAATGCTAAGCTAAAGC

AAGCTACAGAGGCCACAGCTGACTTAAAGGAAGTCCAGAGGAGCCCAGGGAGAAGAGGAA

GAAAGACGGTTACATTTCCAATAACCATAATGGAATCTCAGAATGATGCTTCAGACGGAG

AGCGAGCTGAATCGAAAGTACCTTCCACACCCAGACGCGTAACACGGAGCAGCAGGCAAA

TGCTGGAGTCTGACATTTTCCTCACACCAAGGAGGAGCACAAGAAGAACCAATGCTGAGG

TTGTAGTGGAGGAGGTGACCTCAACAAAAGTAACTCCTCAGCGCAAGACTCCACTGAAAT

CTACACCCAGAAGAGGATCGCGCAAAACGGACAATATGGGTGAAAATGATGTAGAAGCAC

AGAAAATGGAGGACAATTCAACAGTTGAACATCAGAGTGCACGAAAAACTAGAAAAGTGG

CAACAGTCGGGGTCCCAGAGCCGATCCCCGAGGAGAAGTCACTGGAAAACCAAGCTAACA

CTAACGCTAGTCCCAGCAGGGTGACCCGCCAGTCGAGCAGGAGGCTGAGCTTAACTCTGG

AGAGCTTTCATGCAGTTTCAGATGGTCAGAACATGTCTTTAGTCACACCACCTAGAAGCA

GAAGAAAAAACAAAAGCACGACGGAGGAAAAAACGTACAATGAGACTTACACAGTTGACA

GTGTGCAGCTTCAGAATGTTTCCAGACGATTAACCAGAAGCCACCACTGGAACGACAGGG

AGGAACTTGAAAAACCAGAGACCTTGGAATCTACCAATCTGTTGGAGAGCACTTTGATTG

AGAGATTAAACAACGAACAAGCCCAAGAGAGTGAAGTGATGACAGAGATAGTGCGGGCCA

AGCGAAGGACTAAACCTGTAGTTCCAACCATGGAACACGAAGAGCAGAGCGCTGAAGATA

TGGAGTCCACAGTTTCTACTCAACAGCAGGAGCAAAAGAAGAAGAAATCTGCCCCTTCTG

TTAGAAGAACAAGAGCCAGCAAAGCACCAGAAGCCGAAACTACTTCAGTCAGTGACAACA

CATCTAAAGCTCAGGGAGCGAGAGGAAAGAAAAAAGATGTGTCTGTAAACGACACTGGTT

CAGCTCCATCTTCCACTCGAACCAGACGAGCAACCACAGTCACTGACGACTGCCTCCAGG

AGAATGTTTTAGCGGATGTTGTTGTAGAGGAACATAAAGCAGAGGTTAAAACGAAAAAGA

AGAGAACGATAAGAGCGACTGCAGAGCCTGAACCGGTGATGGTGGCTCTCCTGTCTCCGT

TAGGGAGTCCTGCAGAAGCGGCTCAGCAGAAGAAGCCGGAGGATCGAGAAACCGCTGCTC

CAAGGATGAACCTGCGACGGAGACGCATGATGGAAGCCGTTTTTCCAAAACCAGTTACAC

GCAGAAAGAAACTGTAATCATGACCATACCGCTACTACCACCACCATCATCATCATCATC

ATCACCAC

>Unigene000253

GAGGTAGGTTTGCTGGCAGTGGGTGAGTTTGCAGTTACAGGAGGAGGACGGACCAACTGG

CCTCGCCTTCTCCGGGCTATAGGATTAACAGACTATAGTGTTCTGCTGTGAGGACGATAC

TCGGGTGGATGAAAGACTGTCTAGCGGGTCTGTCAGTTCAGAAAAACACCGCTTGGTCTC

CTGAAACCGGAGCGGCTGGAGGCTCGGCTTTGCAGTAGCAGCAACCATGGCTTTGAAGAT

CGTTAAAGGGAGCATCGATCGGATGTTCGATAAAAACCTGCAGGACTTAGTACGTGGCAT

TCGGAACCACAAGGAAGATGAGGCCAAATACATCTCTACATGCATCGACGAGATCAAACA

GGAACTCAAACAGGACAACATTGCTGTCAAAGCCAATGCAGTGTGCAAGCTCACCTACTT

GCAGATGCTTGGTTATGATGTCAGCTGGGCTGCATTCAACATTGTTGAGGTGATGAGCTC

TTCCAAATTCACTTACAAGAGAATCGGTTACCTCGCCGCCTCACAATGCTTTCACGAAGG

CACCGATGTCATCATGCTGACAACCAATCAGATTCGCAAGGATCTCAGCAGTCCAAATCA

ATATGACACTGGTGTGGCTCTGACGGGCCTCTCTTGCTTTGTGACTCCTGACCTGGCCCG

TGACCTGGCCAATGACATTATGACCTTGATGTCCCACACTAAGCCATACATTAGAAAGAA

AGCTGTGCTTATCATGTATAAAGTGTTCCTAAAGTACCCCGAGTCTCTGCGACCTGCCTT

TCCACGACTGAAGGAGAAACTGGAGGACCCAGACCCAGGTGTGCAATCAGCAGCAGTTAA

TGTCATCTGTGAGCTGGCCAGGAGAAACCCTAAGAACTACCTGTCTTTGGCCCCTCTTTT

CTTCAAGCTGATGACCTCCTCCACCAACAACTGGGTCCTTATTAAGATCATCAAACTGTT

TGGCGCCCTCACCCCTCTGGAGCCTCGTTTAGGGAAGAAGCTGATTGAGCCCTTAACTAA

CCTCATCCACAGTACCTCGGCCATGTCTCTGCTGTATGAGTGTGTGAACACAGTGATTGC

AGTGTTGATTTCTCTCTCCTCTGGAATGCCTAATCACAGTGCTAGCATTCAGCTCTGTGT

GCAGAAACTGCGAATCCTAATCGAAGACTCTGACCAGAACTTGAAGTACTTGGGTCTGCT

GGCTATGTCCAAGATCCTTAAAACGCACCCGAAGTCTGTGCAGTCACACAAGGATCTGAT

CCTGCAGTGTCTGGATGACAAGGATGAGTCCATCCGACTGCGGGCTCTGGATCTTCTCTA

TGGGATGGTCTCTAAGAAGAACCTGATGGAGATTGTGAAGAAGTTGATGCTGCATGTGGA

TAAGGCTGAAGGCACTACATATAGGGATGAGCTGCTAACCAAGATAATTGACATTTGCAG

CCAGAGCAACTACCAGTACATCACTAACTTTGAGTGGTACATTAGCATTCTGGTGGAGCT

AACACGGCTTGAGGGCACTCGGCATGGTCATCTCATTGCCTCTCAGATGTTGGATGTTGC

CATCCGTGTAAAGGCCATCCGAGCGTTCGCGGTGGCTCAAATGGCCATGCTGTTGGATAA

TGCACACCTCCTGACGGGCAACATGCAGCGCAATGGCATTTGTGAGGTCCTCTATGCTGC

TGCATGGATCTGTGGGGAATTCTCAGAAAACCTTGAGGAGCCCATGCAGACACTGGAGGC

AATGTTACGACCCAAGGTGGCATCCCTTCCGGGCCACATCCAGGCTGTGTATGTGCAGAA

TGCTGCAAAGCTGTTCGCAACCTTGCTTCAAAAGCATGAAGGGACCGAAGACAGCCAGGT

TGCTCAGGAAGCTAGCCAACTGCTCATAGAGAGACTGCCTCTGTTTGTCCAGAGTGCTAA

CCTTGAAGTGCAGGAAAGGGCATCCTGTATCCTTCAGCTAGTAAAATACATTCAGAAGCT

GCAGCAGAAGGATGTGGAAGTAGCTGAGGAGGTAACTGCACTCTTTGCTGGAGAACTTAA

CCCGGTGGCTCCTAAAGCGCAGAAAAAAGTGCCTGTGCCTGAAGGACTGGATCTGGATGC

CTGGATCAATGAACCTCCATCTGAGAGCGAGTCTGAAGATGAGAAGCCCAAGACCTTATT

TGCTAAAGAGGAGCCTAAGCACTCTCGACCTCGTCACACTGAAGTGGATGAGAAAGAGCT

GGCGAGGAGAAGAGAAGCCAGGAAGCAGGAACAAGCTAACAATCCTTTCTACATTAAATC

GTCCCCATCCACTCAGAAGGTGTATCAGGATACGCCAGGTGTAGAGCACATTCCAGTGGT

ACAGATTGACCTCAGTGTTCCACTCAAAGTCCCAGGCATGCCCATGTCTGACCAGTACCT

GAAGCTGGAGGAGGAGCGTCGGCAGAAAGAAAAGGCGGAAAAGAAGAAAAAAGAGAAAAA

GAAGAAGAAAGAGAAGCGGGGCAAGGGGAAGAGAGGTGACTCTGGCCCTGAAAGTGAGGA

AGACATCACACCCGCTCACCAGGTGGACATCGTCACTGAGGAGATGCCGGAGAATGCCTT

ACCAAGTGATGACGATGACAAAGATCCTAATGATCCACACAAAGCCCTGGATATTGATTT

AGACAAGCCCCTGGCAGACACTGAGAAGCTGCCGGTGAGAACACACCGCCCTGAGGTAGT

AAAAAGCCCTGAGACAGATGAAGATGGAGGGAGTATGCGACTGGAGCCTAAAAAGAGGAG

CAGTAAAGAAAAGAAAGAGAAGAAGAAGGACAAAGATAAGGACAGAAAGAAGAGCAAAGA

AGAAGAGAAGAAGAAAAAGAAAAAAAAACACAAAAACGATTTGGAGGAAGAGCTAATGGA

GACTCCCTCAGAAACCCAAATGCAATCAGAAGACACCAGCGTAATGGCGGCCCCACCTTC

CTCCACATCTGCCGAGGCCTCTGATTTGGATTTCTGGCTCTCTAGTGCTCCTGTTCCCCC

ACCTGCCAAGGAGGCAGCGGTTCAACCACCAGCCCCAGTCCCTGATACACACCCAGCTGC

AGCTCCAGATTCCGAATCAGATGAGCCCAAAGATGAAGAACCTGAAGAAGTGAAATCTAA

ACTTAAGAAGAAAAAGCAGAAGAAAGAAAAGGACAAGGAGGAAAAGGAGAAGAAGAAGAA

AAAGCGGCATCATCACCACCACCACAGTGAGGCAGAGAGGGGCAATTCAGTACAGAATGG

GACCGTGGAAGAGGAAGAGCCTCTACCGCCCATGTCAAACTACTGTCTTCTGGCTGAAAA

CTCCTACATTAAAATGGTGTACGATATTCAGGGAAATTTGCAGGATGGAAGTCAGGTTGT

GGTGTCTGTCATCTTTGAGAACAAGAGCAACAGTTTTCTTAAGTCCATGGAGTTCAATGT

TCTGGATTCCCTCAACTCCAAACTGCAGCGGCCAGATGGGGCTGGACCACACGATGGGCT

TGTGGTTCCTTTTCAGCTGCCACCAGGGGTGTCAAATGAGGCCCGCTTCGTGTTCTCAGT

ACAGAGCATCGTCATGCCTCAGAAACTGAAGGGAACCCTCACCTTTATAGTAAAGTCTGA

AGACTCTTCAACTCATGAGAAACTGGACTTCAAACTGAACTTTACCTGCACATCATACCT

AATCACCACTCCATGCTACAGTGATGCGTACGCCAAGCTGCTAGAGTCTGGGGACTTGAA

GAGCAGCTCTCTGAAGCTAGAGGGAGTCAGTATGCCTTTTCACCACTTGTTGGCTAGAAT

TTGTTTCCATCACCATTTCTCAGTTGTAGAGAGGATCGACTCCTGTGCATCAATGTATAG

CAGGTCAATTCAGGGTCATCATGTCTGCTTGCTGGTTAAAACCGCTGATCAGACAGTCTC

CATTGACGCTAAATGCGATGAGCCGTCACTGCTGGGGAATGTGCTGGAAGAGATAAAGCA

GACCTTCTCGCAATGCTGATTGTGTCTGAGAGACTCGGTGCCCTTGACCTTCCTGTCCCG

TGCCCTTGTGCTGAATCTGACACTCTGAATCATATCACGAACACTGAGGCTGGTTTCCCT

ACGTCTCTACCTACCTTATATTTATTGGAACTCTGGAATCGAGTTAGCTTTCTCTTTTAC

TCCTCTCTTTAATTTTTCTCATCCTCTTGTGTGTCAGACTCAGTCTGGTGTTTGAGCTCA

CTGCCCTTTTGCCACCTCATTATTCTCCTTTCCTGCCGTCTTTATAGTCAAACTGGATTT

ATTTGTTGTTAAATGTAGGTTCTTTTGTCTTTTTTTGTTTTGTATTGTTGCTTTTTGTTA

GTTGGTTTACTTTTATTTCTGTGTTCACAGCGTCTTCATTAATCTCGAATTAAACTTTAG

TTTACTGTGTGCTCCTTTCCCATCAGGCTGAAGTGTGCTTGTGTCTCTTTCTGTTGAAAT

TCTTTTTCGGAGAGCATTTGAAACACAACTTTTTTTCTTCTTCTTTTTTTTAATTTCATT

TAAATACGTCACTATCCTGCCCAACATTATAACATGATCCTGAATGCAACCTAATGATCG

AGAGGTTCTGTAAACAAATTAATAATCAGGTTTTTTTGTTTTACGTAATGTATTACACGT

ACAAAAAAAAAATCTATTTCGTTTTTGACAATAAATCAAACACAAAACTGTTACTTGTGT

AACTTTATGTTTATTTTTTACAAATATTGTAAACTACCCAAAAGTTTATTACACGCTAAC

AGCCGCTGTGGAGGTTTGCAGTTAATAAACAGTATAACGAACATAAGCGAGATTTCTTTA

CTAAGGTCATTACTCTTGCTCGCTGCTGGCTTAACATCATACCAGAAAAGCTTACAAGGC

AACCAAACCAAAAGTATCAAATGAATCTGTTTTCTTGGTCAGTATTGTAGAAAGTGATTA

CTTGTATATCTCTCTATTTATGGCAAGCTGCACATTTTCTAATATGTGCTCATTTCACCT

CAGTCTGCATTACTTTAACTTCCTGTAAACTGGAAGAAAGCCAGAATTGTTTCAGTCTTT

CTGTTCTCCTGCTGCTTTCCATTTATTCCATGACCACCCCCCCCTTTTTTTCTTTTTTTA

ATGTACTGACTAATAGGAAAACATGCTCAAGTAGCATTCCTAGTTTGGTTCGCCTGGTCC

AGCATTTTGTAGCACACAATTCAAACGTTTCATGCTGAAAAAATTTGGTTGCGCAATTGT

TTAAAGCATTTAACAACTAACTAGCCATTGGTTTAAGGCTAAAACAGGCTGTGTTTATAC

AGACGACTACCCAATATGATACGTTTTCCCCTCTAAAGTGTGCACATACAGTATGTACTC

CTCAGCATGTGAATGCAACTTTTGGTGCATATGTCTAAATGGACAATGCAAACTGTATCA

GCGC

>Unigene000254

GAGAAAGAGAGAGAGAGAGAGAGCGAGTGAGTGAGTGACCCAGTGGCGAACAATAGATCC

TGTGATCCCGCAGACAGGACGGTGCTCCTCACCACAGCCTGGGCTGAAATATTCACCCGA

AGCAGAGCGGTATTTGTGGGGAAGCACAGACACACACCGAGTGGCGGAATGGCAATGGAG

GCCGGGGATGAGTTTATTCCTCCTCCAGAGTGTCCCGTGTTCGAGCCGTCGTGGGAGGAG

TTTGCCGATCCCCTAGGATACATTGCCAAGATCCGTCCAATCGCAGAGAAGTCTGGAATA

TGCAAGATTCGCCCTCCACCAGACTGGCAGCCCCCGTTTGCCGTTGAAGTGGACAATTTT

CGTTTCACACCCCGTATCCAGAGGTTGAACGAGTTGGAGGCTGAGACAAGAGTGAAATTG

AATTATCTGGACCGAATAGCCAAATTTTGGGAGATCCAAGGATCCTCTCTCAAAATCCCC

AACATAGAACGACGCATTCTTGACCTGTTCAGCTTGGCCAAGATTGTCACAGAAGAGGGG

GGTTTCGAGACGGTCAGCAAAGAGAGGCGATGGGCTCGAGTAGCACAGAAGCTCGGCTAC

CCACCGGGCAAAAACATCGGCTCGCTCCTGCGGTCGCACTATGAGAGGATCGTCTATCCC

TTCGAGATGTTTCACTCTGGTGCTAGTTTACTGCAGTGTAAGCCCCGGCCCTATGAAAGC

GAGGACGTGGATAAGGAATACAAACCCCACTCCATCCCCCTCCGCCAGTCAGTCCAGCCT

TCTAAGACCAGCAGTTACGGACGACGAGCCAACCGACTGCAGCCTGAGCCGGAGCCCACC

GAGGAAGACATCGAGAAGAATCCCGAGCTAAAGAAGCTGCAGATTTATGGAGCCGGACCC

AAAATGATGGGACTCGGTCTGGTGGCTCGGGACAAGGCAAGGAAGAAAGACGACCTCCCT

CAGACCGTGATAGTTAAGGACAACAGCAGCATTAAAGAGGAACCCGGCGAGACGTTAATT

ACAAAGTCGGAACCGGACATTCCTCCACCGCCTCCGAACCTCATCATTAAAGATGAAGTG

AAGAAAAAGGAAGAGGACGAAGGCCATTCAGTCAACACACACGATTCTATTAAAGACGAA

CCCTGCACCAAGATGACCATGAGACTGAGGCGCAACCTCAGCAGCTCACAGTTTGTGGAC

TCATTTGTGTGTCGAATGTGTGGCCGTGGGGACGAGGACGAGAAGCTGCTGCTTTGTGAT

GGTTGCGATGATAATTACCACACTTTCTGCCTTCTTCCTCCCCTTGCAGACCCTCCCAAG

GGCAACTGGCGCTGCCCTAAATGTGTAGCAGAGGAATGTAAGAAACCCACAGAAGCGTTT

GGTTTCGAGCAGGCTACACGAGAATACACCTTACAGAGTTTCGGCGAGATGGCAGACACG

TTCAAGGCGGATTACTTCAACATGCCTGTCCATATGGTCCCCACAGAGCTGGTTGAGAAG

GAGTTCTGGCGATTAGTCAGCAGTATTGAAGAGGACGTGACTGTCGAATACGGTGCTGAC

ATTCACTCCAAAGAGTTTGGAAGCGGTTTTCCTGTCAATAATGGCAAGAAACATTTAACT

GAAGAAGAGGAGGCGTATGCACGCAGTGGCTGGAACCTGAATGTGATGCCGGTGTTGGAG

CAGTCAGTATTGTGCCATATCAATGCCGACATCTCGGGCATGAAGGTGCCGTGGTTGTAT

GTTGGCATGGTGTTCTCTGCCTTCTGCTGGCACATTGAGGACCACTGGAGTTACTCGATT

AACTACTTACACTGGGGAGAGCCCAAAACCTGGTATGGTGTTCCTGCAGTAGCAGCAGAG

AAGCTGGAAGAAGTAATGAAGAAGCTCACTCCTGAACTCTTCGAGTACCAGCCAGATCTG

CTCCATCAACTCGTCACAATTATGAACCCCAACATACTCATGGCTCATGGCGTGCCGGTT

GTGCGAACGAATCAGTGTGCTGGAGAGTTTGTCATCACTTTCCCTCGAGCCTATCACAGT

GGCTTCAATCAGGGATATAATTTTGCTGAAGCTGTCAATTTCTGCACTGCAGATTGGCTG

CCCACAGGGCGGTCATGTATTGAGCATTACAGACACCTTAGACGCTACTGCGTGTTTTCT

CATGAGGAGCTCACCTGCAAAATGGCTGCCTGCCCGGAAAAGCTGGACCTGAACCTGGCT

GCAGCCACACACAGGGAAATGTTTATTATTGTCCAAGAAGAGAGGAAACTCCGCAAGGCC

CTGCTGGAGAAGGGTATAACTGAGGCGGAGAGAGAGGCATTTGAGTTGTTGCCTGATGAT

GAGAGACAATGTGATAAGTGTAAGACCACCTGCTTCCTATCTGCCCTGGCTTGCACTAAC

TGCCCTGAGCGCCTTGTTTGCCTCCACCACACACAAGACCTCTGTTCCTGCTCCACAGAA

AAACTCTACCTCAGGTATCGTTACACTCTTGACGAGCTCCTGGCCATGTTGTACCGTTTG

AAGGTTCGAGCCGAGTCTTTTGACTCTTGGGCTAACCGAGTGAAAGAAGCTCTTGAGCAG

GAGGAGGGCAACAAAATAGATATTAAAAATCTTACGGCACTGAAAGAGGAGGCGACGGAA

CGTAAATTTCCAGACAACGAGTTGCTCCGAAAGCTCACAGTTGTATTAGAGGAGATCCAG

ATGTGCCAGAGCAGGAGCGGCGAAATACTTAATGGCACGCAGAGCAGTAAAATGAGCCTC

CAGGAGCTCCAAACGCTGGTGGACACCATGCAAAACCTTCCCTGTGTAATGGAGCAGCTA

GGAGATGTGCAGGTTGTGCTAGAGAAGGTGGAGGAGTTTGAGTCACGTGCCCAGGCTGTA

ATAAGCAGCAGCGGTGAGGAGTGGAAGCAGCCGCCTGCTCCCTCGGAAGAGGACGTGCAG

GCTCTGTTAGAGGAAGGAGCTGCTCTGCCAGCTGTTGCCCCAGCCTGTGGGCTTCTGTCA

GGCCTTCAGGAACAGAGGCGCTGGCTGGAACAGGTGCGCCAGACCATTGGGCCCGGAGGT

GGAGAGGTGACTCTGCCCGTATTGAGAAATTTGATGGAGGCTGGCTGCAACGTGCTGCAG

AGTGTTCGTGTCGAGACCGCCATGGCGGAACTGCAGGAGCTGCTGACCATCGCAGAACGC

TGGGAAGAGAAAGCCCAGATCTGCCTGGAAGACCGGCAGAAGCATCCTTTGTCCACATTG

GAGGCCATAGTGAATGAAGCCCAGCTAATTCCTGTGCAGTTACCCAACATCTTGGCCCTA

CAAGCCTGCCTGAGTCGAGCGCGGGCCTGGGTCACTGACCTAGAGGAAATCCAGCAGAAT

GGAGAGCACTATCCATGTCTGGATGACCTGGAGGGTCTGGTGGCAATCGGTCGTGACCTT

CCTGTACGGATGGAGGAGCTACGACAGCTCGAGCTGCAGGTAGCTGGCGCTCACTCCTGG

AGGGAAAAAGCCAGCAAGACCTTCCTGAAAAAGAACAGTCAGCACAGCCTGCTGGAGAAA

GAAGAAGGCAGAGGAGTCTCCGAGCTCGGAAGCAGACGTTGACGTGTTGGGGCTCACAGC

TCAGGACCTCAGGGACCCAGGAGCCATTGTAATGGCATTCAAGGAGGGTGAACAGCAGGA

GAAAGACGCTCTGCTGCGTACGCGACAGCTGAATCTGGCCAAGGAGGGACAGACTATAAA

CATCAAGCAAGAGACACTGTTACCTGAACCGATGGACGCGGACACACCTCAGTCCTCGGA

AAATGGCACACAACACCACAGCGACTCTACTCACACTCACACGGTCAGCCAGTCCGTGTG

TGTGTGCGGTGGCCAACCCCACTCTCCGCTTCACCGCTGCCATCTGTGCAAAGACTGGTT

TCACGGAGGTTGTGTGCCCTTCCCTTCTGTACTGGGCCCAACTGACGTGCCACTTGTAAA

CCCACTCTGTTGGTGGGACTGGGACACACGCTTTCTCTGCCCGCGGTGCCAGAGAACACG

GCGTCCGCGGCTAGAAACCATTCTGGCTTTGTTGGTGGCATTACAGAAGCTGCCTGTAAG

ACTACCAGAAGGCGAGGCCCTCCAGTGTCTTACAGAGAGAGCTATTAACTGGCAGGGCCG

AGCCAAACAAGCCCTTGATTCCCCCGAGCTGCAGGAGGCGCTGCAAAGACTGCAGGAACT

AAAAGAAACACAGAGCAGGGAGAAAGAGGAGCAGAAGGTTGAAACAAAAAGGAAATCGGA

GGAGGTGATTGTACTGTCGGACTCTGAGGAGCCGGAGGACAGTGTGATCGATCTGACGGA

GGAGCTGAAAAACAAATCGGAGAAGCCTGCTAATGGAATCCAGCCTGGCTGTGAAAATGG

CATCAAGAAGCCAGACACTGAAAATGTGACGGGGGCGGAGTCTCTGGTATCACTGGTCCC

GTGTCTTAAAGGGCCGGTAATCGAACTGGCCACTTCAACTAAAGCTCAGTTAGAGGAGCT

GCAGCTAGAGGGCGACCTGCTGGAGGTAACTCTGGATCAGACACACACCATCTACCGCCT

TCTACAAGCAGCTTCTCCTCCACCCCACGAGGCTCTGCACACACTTCTGCAGATCGAGTT

ACTGGAGCAGAAGCGTTCGGGTCGTGGCAGCAGGACGAGGGACTCAAAGAGAAAAAGGAA

AAGTCAGAGATTCGAGGGAGGAGCAAAGTCCACAGAGGCCTCGGATTCCAAAAAGACACG

ACCGGTTCTTCACACACCTGCACAGCAACGAGAAGAGGTGTTGTGATGCATTGTGCTGGG

AACATGTAAAGGAGAAGATGGCGTGAGTGAGTGACCCACACAGTAACAGGTTTCAGTGGC

TCTGGGCCCAAAAATCACCAAGGCGGACGGTAGAGCAGGTTTCATGGAGGTTCTGTTCAA

TAAGCTCACACATTGATCTTTTTCTTCACACACGCATACATACACACACACACACACTCC

CTCCCGCCAGCTGTTGAGCACTTCACTCCCTCTGTTGTGGTGTTTCCGGATGGCTTTAGC

ACAGAGTTTGCCCTCTCCATTTTATTTTTTTCTTCGACGACTTTGTTCTCCATCCTTGCG

CCGTGCGTCAATCTGTTGCCATTGACTCCGCCCACCTTCGTTCTGCTCCTCCAACCCTGT

TCCATTAAATCCTGTTCCACACTGTGCTCAACTCCCCCAAACAAATGTGATCATTCAGTT

ACACAAACGCAAACAAATCGAGCGGTGGCGCCGTATGTGGGGATGGGATTGTGGCCCAGA

TGCCGTTAAAATAACTTGTATTTTTTACGTACTGAGTCTGGTGTTTCGTTGAGGCTGTGG

ACGGACTCTCTGTTTCCAATAAACTAGGAAAATAATTTGGGTGAGTCGATGTGGGGAGAG

GC

>Unigene000255

CTAAGCAGATCGGAAGAGCGTCCCTAGCGCAACCTTCCCCACTGCCTCCTCCCAGCTCGT

GTGCGCAGCCTCGGAGCTTAGTTCCTGCTGAAGTTCAGGGTTGCATTATGGAAATAAAAA

GCACTGTGAAAACTTCTCCTAAGGACCATTATAGCACAGGGGCACCAAGAACACAAACTC

CAAAGTGTGTAATTGACATCACAGAAGACTCCAGCTCCCACCCCACAACCCGAGAGAACT

CAGAGACTGTGCCTGCGACTTTAGCTTCTCAAACACGTGAAACTTATGTCCCTGAAAGCA

GACATTTGGTGTTAAACCCCTTAGCCTCAGAATGCAGGAAAGCAGAGGGTGAACTTCAGC

AGCAGCTAGGTCAGAGCCTGGGCAGCAAGATTGAATTTGCTCACTCTGAGGTGATCCGAC

CTGTGGTTTCGGTAAGCGAGTTAGCGGCTGTTGCGGAAAGGGAAAGGGAGAAAGTACAGC

AGCAGTACCCTTCAATCATGCCTTGCATCAAAAATGCCTCACTGGCTGAAGATGTGCGAA

AACCACAGAAACTTAGCTCGTCACCAGATGTGGCCAAGTCCAAGTCTAACCCTTCACCTG

ACGCATTTAAGCCCAAGTGCAATCCCTCACCTGACGCCATGAAGTCAAAAACCCACAGTG

TCCTGGAGGCCATGAAGCCTAAACCTAACACTTCACCTGAGGTCACCAAACACAAAGAGA

TCATTGAGATCACTTCATCCTCTGTTGCCCGGTCGGCAGTCAAGTCGAGAAGTCAAGAAA

TACCACGCTCCAGCTTTAAGCCTGTTCCTGCTCGTAGCACTCCCTCTGAGTCCATCAAGA

GCGCTCTCATTGTCGACAAAAACGAGCACTTTACAGTGTATCGTGACCCGGCGCTGGTGC

GGCCCGAGGCTGATACCAACCATGTAACTTACCTTCCCCCACACCTCCATCCACTCCACA

GCTCCTCCCATGCTACTTGCCTGACACCTAGCTCACATCACCACTCCCATCTATTGCCTG

CATCTTCCCTTAGCCCGCACCCCTCAGTGCACCACCCCTTGCTCCCCACCGTGTTGCCTG

CCATGCCGCCGTCCTCCCTACTGGGTGGCCACCCGCGCCTCGACTCAGGGGGACTCAGTC

ATCTAGCCCTAGCTCACCACCACCCACACCAGCAACAGCAGTTTCTGCAACAGCAACCAC

CCCCACCGCTGCTGGCCCAGACACATGGTGGTGCCAGCTACAACCAGCTCGGCCTTTATC

CCATAATCTGGCAATACCATAATGGCACACAGCACTCCTATCCATCTGGAATTAGCCTGC

CTGGGTCAAAGTGGGTGCATCCAGAAAATGCTGTGAACTCAGATGGTAGCCTGTCAAGGA

ACACCTCCAGTCCCTGGCTGCATCAGCCCACTCCTGTGTCCTCAGCGGATAGTCTAGGAA

TTCTGAGCCATGTCCCAGGTAGGCCAGCCAGTGCTGATCCCCACAGACCCCTTAAAATCT

CCTCACACTCCAGCCCTCCGCTCTCCAAAACTGTGGATCACCACAAAGGAGAGCTGGAAA

GTAAGGTTTTTGTAGATCCAATGCGTGGCCTGGTAACCGCCCACCTAAAGCAAGAGCCAG

ACCAAAGCCGGACACCGAACAGCAGGGATTTACAGCGCCTCTACGCGGATTCATCTCACG

TAAAGCAGCAGGCGCTGCCTCCCCGCCTGCCACTAGACACTCCAGACCGGGCTGTTAAGT

ACAAAGAGGAGAATCGACGCATTCTACAAGAGAGCATTGAGGTGGCACCCTTTACTGCCA

AAATCCGCTCAGGAGAGCCAGAGCGTGAGCCCTATATCCGCATTCCCTCTCTCCCCATCA

GTGGACCGCCCAAGGAGAATGAGCATTCGCCATCTGATCTTTATAAGTACAAGCACTCGG

CTGCTCAGTCACTGCCACAGAGCAACTATTTTACCACCATTTCCAACAGTGTGGTGAATG

AGCCACCTCGTCTTTACCCCTCCAAAGAGCTTAGCTCCCTTAATGTTTCAAGTCCCCTGA

CTCTCGGCTCCTACCCCAACAGCGGAGGCAACACCAAGTCCTTATCCAAGCCTCCCCCGC

TCATCAAGCACCATCCAGACGGGGAGGGCTTGGTGGGTAAGATCAGTGAGCAGCTCAGTC

ATCAGGTGGCCCTGAACCCTCTGAGTACCCCCGTGGCATCTTGCGAACCCCGCAGTCCGG

CTGTGTCACCCTCAAACCAACTCAGGAGCATGCCTGCTCTACACAGGGCACCTGTTTTTC

ACCCTCCTACCCAGCAAACACTGGACCGCAAAGAGTCTGGCTACGGTCGCCTCTCTCCAC

CAACGCTAACCCCTATTCAGCCGGTTAGTGTGGCTGGGAAGGTATCAGAGCAACAAAAGC

CGCCCACTCTGCTTCCAGAGCTTAGGGAGGTGAAAGGCACTGTAGAGCTGAGCTCCAGTG

AGCCATGGAGGCCCAGCTCTAACTTACAGGGCTATGATAAAACAGTTCAGTGGCAGTTAG

ACAAAAGTCAAGGAAAGCCACAGGTAGCCACAGCTTCTGTTATTGTTCGCTCCAGAACCT

GCATTAAGTATGACAGCTCACCAGGCTCCAAACCTGGTGCCAGGGAGACGTCTAACAGTA

AACCACATACAGGAAAATACCAACTGGACTGCAGCAAACTGGCTGAAACCAGAGAACCCG

GCAGAGTTATACAGCAAAACACAAATAAGGAGGACATTTTTTTGCAGTACAAAAACAATT

TTGTCCGAGTCTCCCAGGGCAGTTTTCCCAGCTCTGCGGTAGCTGTTGTTAACTCTGTGT

GCAATAACAGTACTGCTGTAACCACTTCTGCCTCTGCTGCTTCCACTCACAATGTGCTGA

GTCGTGGCACAGCTGAACTGCCTTACTCCACCTCAGCAAATCCAAGCAGCAGTAACATTA

ACAGACTGGAGGGCGCTGCACCCAAATGCAGGACTCCTACCAGCCTTGAGCTACAGGAGT

GCAATGCCAGGACAGCCTCCCCCATTGGCCCACTGCCTCAGCCTGGTTCGGCCCTAGCTC

CACAGCCTTATTCTGGGAACTTTATCCACCTGAAGAAGCACAAGGCTGCCCTGGCCGCTG

CCCAGTCCCGTGGATCAAGCAGTGCCTCAGAGAGTGAAGGTAGCAGTCGATCCTCTCAGG

AATCACCCACAATCATCACGCAGGATCGTGCATCCCCAGGCAACCCGTCCAGCAAAGTCA

GCCCCCTACCCAATGGTCAGCCATCACTGATGAACCAGCCCAACTACCACAAGCTAAAGA

AAGCATGGCTGACTCGGCACTCAGAGGAGGATCGCAACACTAACAAGGTGGAGATGACTT

CCAATGCCGTCTCTGAGATCATTAGGCCATGCACAGTAAACCTTATAGCATCCACCTCCA

GCGACACAGAGATAAGCAAAGATGGCAAATGCTTAGAGGATAAATTGAGCCCGGAGGACA

GGAAACCACGACGAGTTCCTTCCAAGCGGCCACACGAATCAGGCTCGGATAGCGGCGATG

ACTCCGATGGCAGTGACAGCAAGCATGAGCAGAGAGCCAAAAGGCAGCCTAAGCCCACCT

TCAAGAAGAAGCAGAATGACATGCAAAAAAAGAAAGGGGAAAATGACAAGGAGGAGGAGG

ATGTCAAGCCCAATGGCATATTCAGAAGCGCCAAGGAGAAAACCAAACTAAAATTGGCCA

GCAGCAACGGTATTCCTCGCTCGGTGTTGAAAGACTGGAGGAAGGTGAAGAAGCTGAAGC

AGACGGGTGAGTCCTTTCTGCAGGACGATTCGTGCTCGGAGATTGGACCCAACCTGCAGA

AGTGCCGCGAGTGCCGCTCGATCCGCACCAAGAAGGGAGAAGAGCCCACCCACTCCCCCG

TATTCTGCCGCTTCTACTATTTTCGCCGCCTTTCATATAGTAAAAACGGGGTTATCCGGA

TCGACGGCTTCTCATCCCCTGACCAGCACGACGAGGAGGCCTTGAGTCTCTGGGCTCCAG

ATGCTTACGAAGAGAATGATCTGGACCTGGAAACCTCCAAATACATTCTCAGTTGCATCG

GGGATAAGTTTTGCCAGCTGGTCATGAGCGAGAAGACCGCCGCCACATGGATAAAGAAAG

ATGCCAAAATAGCCTGGAAACGGGCAGTGAGGGGGGTGCGTGAGAGCTGCGACGCATGCG

AGGCCACATTGTTCAACATTCACTGGGTGTGCCAGAAATGTGGATTTGTGGTGTGCTTGG

ATTGCTACAAGGCAAAGGAGAAGAAGAGCTCCAAAGACAAAGATCTGTACACCTGGTTAA

AGTGTGTCAAGGGACAGCCACATGATCACAAGCACTTGATGCCAACGCAGATTATTCCAG

GAACAGTCTTGACGGACCTGGTAAATGCCATGCACATGCTCAGGGAGAAGTTTGGCATCA

AAGCGCACTGCGTCTGTGCCAACAAGCATAATATACTCAACAAGCTCCCATCCACTAACG

GCGTTTCTCAGGTCCTCCAGAACGTGCTGAATCACAGCAACAAACTTTCCCTGTGCAAGC

CTGAGGCCGGCCAGCACAACTTGGGACAGAAGGTTGAGGCAAACGGGGGCAGCAGTCCAG

CCAGTGACACCAGCACTGATTGCAAGCTCCCCCCACCTGAATCCCAGTCACCACTGCACT

TTCTGGCCGACCTGGCTGAGCAAAAATCCCGGGAAGAGAAGAAAGAGAATAAGGAGTCGG

TGGTAGGGAAGGTGAAGGAAGAGAGCACAGATGCTCTGGAGACTTTGCACTGTAAAACCT

CGTCTCTGGTGGCCAACAGCACAGAGCAGGGCTCCACGCTTCGAGACCTGCTCACCACCA

CGGCCGGAAAACTGCGTTTGGGCTCCACCGACGCTGGCATCGCTTTCGCACCGGTTTATT

CCACCGCCTCACAGACGGGGAAAAGTGGTCGCTCTATGCCCAACATCCTGGATGACATCA

TTGCTTCCGTAGTGGAGAATAAGATACCAGCCGACCGCAGCACTAAGCAGAGCCCAAAGG

CTAAACCCCAGGACGAGGTCAAGGCTGAGAGAAGGAAACAAGCTGAGGATGTTCCTGAGC

AGCATACTGACATTCCCCACTGCTGGCTGTATGATCACAGACTGCTCTGGCTTAAAGACC

ACCGCAAGAGCAGTAACTGGAAACTGTTTCGAGAGTGCTGGAAGCAGGGACAGCCTGTGC

TCGTCTCAGGGGTTCATAAGAAGCTCAACGCCAGCCTGTGGAAAGCTGAAGCTTTCAGCC

AGGAGTTCGCCGACCACCAGGGGGATCTGCTGAACTGCAAAGACGGCGTGGTTTCCAACT

CGGGCATCAAAGAATTCTGGGACGGCTTCGAGGACTTGACAAAACGGCTCAAATCCAG

>Unigene000256

AATAAGATCACCCTGCTGTCCAAAATGGCTGTGCTTCTGTCGACAAACACGCTGAAGACT

GCAAATCAACAAGTCTTCCAGTGAGTGGAGGTGAAAGTGGGGTTTATTACACATCTGGAT

AAAAACCTGAAACAGGCTCAGGGATTAGCAGCATAAACATGGACACCACAAGCTCTCTGA

AGATGACTGCTGTGGCCTCCCAGAGCATCTTCAGTTACGTGGACGAGGAGAACCTGCCTG

CAGTAAAAGCTTACCTGGACAAACACCGTGACGTGGACAGCAGGAGTGATAATGGACAGA

CTCCTCTCATGTTGGCAGCTGAGCAGGGCAGCGTTGAGATTGTGCAGGAGCTTATAAGAA

GAGGAGCCAATGTTAACCTGGATGATGTGGACTGCTGGACTGCGCTGATTTCCGCCGCCA

AGGAAGGCCATGTCGAAGTGGTAAAGGAGCTGCTGGCCAACAATGCGAACCTGGAGCATA

GAGATATGGGCGGCTGGACCGCTCTTATGTGGGCAGCCTATAAGGGCCGTGTGGAAGTGG

CTCGCCTTCTGCTGGAGAAAGGAGCAAACCCGAACATCACTGGACAGTACGGCGTATACC

CCATTATTTGGGCAGCAGGCCGAGGTCATGCCGAAATCGTTCGTCTTCTACTGGAACACG

GTGCTAAAGTCAACTGCTCTGACAAGTACGGCACCACAGCATTGATCTGGGCAGCGAGGA

AAGGTCATTACGACTGCGTGATGCACTTGCTGGAGAATGGCGCAAACGTAGACCAGGAAG

GCGCGAACTCAATGACAGCTCTAATAGTGGCGGTACGTGGAGGGTACACAGAGGGGGTGA

AAAAGCTCCTTAAGAGAAACCCCAACGTCAACATGACCGATAAAGACGGAAACACAGCGC

TGATGATCGCTGCCATGGAGGGTCACACAGAGATAGTGCAGGATCTACTGGATGCAGACA

CCTACGTTAACATTCCCGACAGGAGTGGTGACACAGTACTAATTGCAGCAGTAAGAGGAG

GTCATGTGGAGATTGTCCGGGCTTTGCTGAATAAATATGCTGACATTGATGTTAAGGGCC

AGGACAGCAAGACTGCCCTCTACTGGTCAGTAGAAAAAGGCAATGCCACCATGGTACGAG

ATATCCTGCAGTGTAACCCTGACACAGAGGCTTACACTAAGGAGGGGGATACACCACTTA

TTAAAGCTACCAAAATGAGAAACATTGAAATTGTCGAGCTGCTTCTTGACAAAGGAGCAA

AGGTGTCTGCTGTGGACAAGAAAGGAGACACTGCGCTGCACATTGCTATCAGGGGTCGAA

GCAGAAAATTGGCCGAGCTGCTCTTAAGGAACCCTAAAGATGGCCGCCTTCTGTACAAGC

CCAACAAACAGGGAGAGACGCCCTACAACATCGACTGCACACACCAGAAGAGCATTCTCA

CCCAGATTTTTGGTGCCAAGCACTTGTCCCCTACGGAGTGTGACGGTGACATGCTGGGTT

ACGACCTGTACAGCAGTGCTCTGGCTGACATCCTTAGCGAGCCCACCATGCAGCCGCCCA

TCTGTGTAGGTCTATATGCTCAGTGGGGCAGTGGCAAATCTTTCCTGCTCAAGAAGCTGG

AGGATGAAATGAAGACGTTTGCAGGCCAGCAGGTGGAGCCATTGTTTCAGTTCTCATGGC

TGGTGGTGGTTCTGACTCTGCTGATTTGCGGCTCTTTAGCTGTGATCCTCGGCTTCTCTA

TCGATCCCAGACTGGCCATCGCTGTGGCCCTCAGCCTGCTTGCCCTGCTCTACATATTCT

TTGTGCTGGTGTATTTTGGTGGCCGCAAAGAGGGCGAGAGCTGGAGCTGGGCATGGCTCA

TCAGCACCAGGCTTGCACGCCACATCGGCTACCTTGAGCTGCTGCTTAAACTTATGTTTG

TGAACCCGCCAGAGCTTCCTGAGCAGACGACCAAGGCCCTACCTGTCAGATTTCTGTTTA

CAGACTATAACCGCCTGTCGAGTGTGGGTGGGGAGACCTCTATGGCGGAGATGATTGCCA

CACTGTCAGATGCATGTGAAAGAGAATTCGGTTTCTTGGCCACTAGACTCTTCCGAGTCT

TTAAAAACGATGAGACACAAGGAAAGAAGTGGAAAAAGACGTGCTGTGTTCCATCCTTCG

TTCTGTTTGCACTGACTCTGGCATGCCTGGTGACGGGTATGGCCCTCTTGGCTATATTTA

AAGTAGATTCCGATAACGTGACTGTAAAAGCAGTGCTAGTTGCCGTGGCCAGTGTGGTGG

GCCTCGCTCTGCTGCTCAACTGCAGGACCTGGTGGCAGGTGGCCGACTCTGTGCTGCGCT

CGCAGAGAAGGAGGCTTCACAGCGCCGCCAACAGCATGCACAAGCTGAAGAGCGAAGGAT

TCATGAAGGTGCTCAAATGTGAAGTGGAGCTTATGGCAAAAATGGCAAAGACTATCGATG

GCTTTACTCAGAATCAGACTCGTCTGGTCGTCATCATCGATGGCCTGGACTCCTGTGAAC

AAGATAAAGTGCTGCAGATGCTGGATACGGTGAGAATTCTCTTCTCCAAGGGCCCCTTCA

TTTCCATCTTTGCCAGCGACCCCCACATCATTATAAAAGCTATAAACCAGAACCTGAACA

GCGTGCTGCGTGATTCCAACATCAATGGCCACGACTACATGCGCAACATAGTGCACCTGC

CCGTCTTTCTAAACAGCCGGGGCCTCAGCACGGCCAAGAAGCTCTCCATGGCCGCGCCAA

CCAATGGAGATCTGATGGCGGCTGAGGCATGGCATGAGGAGGCAGACAGAAAGTGTCAGT

CGCAGAACAGTCTTGGAGAACAGGCCAAGCAGGGCAGTAAATTGACACTGAACCGGAGGG

ATACATACCGGAGGCGTCAGATGCAGCGAACAGTCACCCGTCAGATGTCTTTTGACCTGA

CCAAGCTGCTGCTTACTGAGGACTGGTTTAGTGACATCAGCCCTCAGACCATGAGGAGAC

TCCTGAACATCGTATCAGTAACAGGGCGCCTCCTAAGAGCCCATCAGATCATTTTTAACT

GGGACCGACTGGCATCATGGATCAACTTAACGGAGGAGTGGCCATATCGGACGTCCTGGC

TCATCCTCTTCCTGGAGGAGAACGACGTGTTCTCGGACCAGATCACACTTAAAACCATCT

ACGAGAGGATTTCTAAAAATATCCCCACCACCAAAGACGTGGAACCCCTTCTGGAAATCG

ACGGAGACATACGCAGCTTCGAAGTGTTCCTGTCCTCCCGAACCCCCGTGCTTACAGTTC

ATGATGTGGAGATGTTCCTACCCTGCACCGTCAACCTGGACCCTAAACTTCGAGAGATTA

TTGCAGATGTGCGCGATGCCCGGGAGCAGATGCATCTCGGAGGAGTGAGTTATCCCACAA

TGCACCTACAGGATGCTCCGGCGAGAGCACAGTCAGTGTTTGGTCAGGGTCCCGTAGCCT

GTTCACCCACCGGCTCCTTTCCTGCACAAACACACAGCAGCTATTTCAGCGGTCTTACTG

GCCCACAGCACCCCTTCTACAACCGGCCATATTTCCCTCCTCATGTCTATCACGTGCCCC

GTCATTATCCCGGCAACCCCCACCATGCCCCCTCCCGCCCTTCCTCCAAACTGGCCTTCT

ATGAGGACCACAATGCTGGACTAACAAAGTCTCAGCCTCCCAGACTGAAGCAGGGCTCTG

ATTCTGCAGCACCCCCCGGGTTCCTTAGTGCTCTTAGCACCGAAGGTGTATGCGAAAGAC

TGCGACAAATTGAAGGCCTGGACTCTAGCATGATAGAGCAGTACATCAGCACCATCAAGA

AGGCGAATGTGAACGGTCGTGTCCTGTGCCAGTGTCAGCTCGAAGAACTGAAGAAGGAGA

TGGACATGAATTTTGGAGACTGGCATCTCTTTAAAGCTATGGTGCTGGAGCAGCGGCAGG

TGGAAAGCCAGCTGCTGCAGGAGGAGTGTGTAAAGGTTAGTGAACAGGGCAGCAGTGCAT

CGGTTCAACCTGAAGTGCCTGCTGTCTTTTCTACAACAGCCCCCCACAGCAATCTACAGT

ACAGCCTGAACCTCAGCTTCGAAGAGCTCAGCACTATTGGACTGGAGGAGCCCATTAGAC

ACCCCATCAACCACTGGATGGCCGTGACTCACAGGACTTCCAGCATGTCCAGTCTAAATT

CTCAGGAGTCATCCAATGACATTAGTAAGTTGACTGACAGGCAGCAAGCTGAGTACCGCA

GTGCTTACCAGGACTACATCGCTCACATGGCCCAGCTGGAGGCAGGCAGCTCTGCAGCTG

AAAGGTCAGAGGACCGTCCCAAGGATGGCGAGCAGGAAGGACGCAAACCCTTCAAGAGAA

ACTCTAAAGCAGGAGAAAGTGTGATGGACTACTCCACTACCCAGGACTCCTGTCTTCTTC

ATCCAATCATGGAGGAGGACGAGAAACAAGAGCGTAAACCCACCGTGAGCCGCACTAAGG

GACCAGGACCTCGCTACCACAGCGTACCCAATGAAGAGGATGAGGAAGAGGATTTGCCCG

TGGAGACCGATGCCACACCCCTTCTCTGGGAAGAGCAGGAACACGGGGTGCAGAGAAGGA

GCTTTGTTTCTGCAGGGATAGACAAAAAAGAGTCGTCTGACTCAGGCATGAGGTCAAGCG

GCAGCTCCTCTGATCATTCACTACAGGACGAGGAGAGCGAAGAGGCTCAGGAAGAAGATG

AAAAGTCAAAAGAGGAGGATCTTGTGACCACGATACGCATGTCCATCTGCTCAGAAGCTC

TGTCTGATGCCAGCAGCCCTGAAGAGAACTGGGACTCCCCACACATACACACTCAGGCTG

TCACCAACCTGAACCAGGCAGCCAGCAACAACTCGGCCCTTAGCAACAACAATAACGGAG

ACGAGCGCAGCCTCATCATCTCTCCAGGATCGAGCACCGGCACCAACACTCTGCTCAATC

AAAACCTGCTTACAGTGAGAAGGAAGAAAGGAGCAAATACTACACATGATGAGGGCAGTG

AGGAACGAGAGAGTGTGCTGTAAAAAGCAAACTTTCCTTTAAATATACTACATTTAATAT

GGTACAAAGAAAGAACTGGTCTTTCAACTTTTAGTTTATGGTAGATGTGAAACTGTGCAG

AGTGGATGTGTAGAGTGATTAGGTTTATGCATTTCATGGAAAATTTATATATAAAAAAAA

AGCCTTTTAAAGCCAATCGTGTGCATTACAATGCAATCATCAAATCATTTGGGATCGTCG

CTGAAGTAGCCGGGATCGGGATCGGGATCTGTTACATGTGGAGTTGTGTGTAATTTACAT

TAGGTCAGGAACAAAACTCTTCCTATTAGAGGAAAATAGTTTTTAAACTGGCAA

>Unigene000257

TAGGCATGCAACCGAGGGTATTACGAAGTGGCCAAGCAGCTGCTGGCAGCCGGGGCCGAG

GTCAATACCAAGGGCCTGGACGATGATACGCCTTTACACGATGCGTCAAATAACGGCCAC

TTCAAAGTGGTGAAGTTACTTTTGCGGTATGGAGGGGATCCTTGTCAAAGCAACAGGAGA

GGGGAAACTCCGTTAAAGGTTGCAAACTCTCCAACCATGCTGAATTTGTTGCTTGGGAAA

GGCACTTACACCTCTAGTGAGAGCTCATCAGAATCGTCAGAGGAGGAGGATGCTCCATCA

TTTGCCCCATCTAGTTCTGTTGATGGCAATAACACGGATTCAGAGTTCGAGAAGGGCTTA

AAGCTGAAAGGGAAGCCCCTGGACCCGCCCAAATCCACTACCACACCGGTCAAGGACGAA

TACGAGTTCGATGAGGATGACGAGGAGGAGCGCATCCCACCGGTGGATGACAAGCATTTG

CTCAAAAAAGATTTCCGCAAGGATCCTGTCAGCAAGACCAACAACTTCATCTCCATACCC

AAGATGGAGGTTAAAACCTATTCCAAAAGTAACTCGCTCACACCAAAGAAGCCTGTGCGT

CGGATACTGTCGGACAGCAACAGCTCAGATGAGGACGACCGGACGTTGTGCTTTACACCC

ACACCCACGCCAAGGCAATCTGCTGCTCAGAGCAACACCAAGAGTAGAGACTCTAGCACT

CTGTGCTCCAAGCAGCTGAAAGACAAAAACAAAGTCAAAAAGAAGAGGAAGAAGGAGACA

AAAAATAACACCAGTAAAGAGGTGCGCTTTGGCAAAGTGAACGACAAGTTCTGCACCTCA

GACTCTGAGACGGGGGACATGGAGAGCGAAGACGATAAAGGCTCAATGCCAGTTTCAAAC

TGTGTGAAAGACTCTTCAACTTTAAACCTTAAAGAATCTAGTGTATTCAGTTCACTGTCT

GTGTCTTCATCTTCCTCATCTCATGGGAGTTTGGGCTCTCAGAAGCTTACACAGTCTCTG

GCAGAGCAGCACCCAAAACAGTGGCGGACAGATGGATGGAAGACAGTTTCCTCCCCGGCT

TGGTCTGATGTCAGCTCCCTGTCCGACTCGGTCAGAACGAGGCTTTCCAGCGAATCTGAC

TATTCCTCTGCAGACTCAAGTGTGGAGTCTATAAAACAAGTGAAGAAGAAATTGCCAGAG

AAAAAGAAAAACAACACTCATGCAAGTTTGTTAGACAAAAAGAGCTCGGATTTTTATAAA

AACTCAAACACAGATGGAACTGTGTCTAAAACAGACAAAGATGGCAAGATATTAAAAAAA

CACAAAGTAAAACACAAACACAAAAACAAAGAAAAGGAGAAGGTTACAACTTTAGTGTTG

AACCAAGATATGAACGAAAAATTTGTCAAAAGCATCTCCTTTGACTTTGATGACTCGCGC

CAAGACTCACCTTCCGAAAGTAAAGTTAAACTGTCCAAACACGAGAAAGATCACTTTAAG

AAAGAGGACAGATTGTCGAAAGTTAAAACCGAGGATAAAGACTGGACAGGAAAGGAAATT

CAGAGAGTATCTAAAGAAGAAAAGTCCAAGAAAACAAAGGAATCCAACAAAGATAGAGCA

AGTAAAGATGAGAAGGACAAATCTTCAAAAACAGAAAAAGAAAAAGGTGTGAAGGACAAA

GAAAAGCCAAAGGAGGACAAACAAAAATCTCATAAAGAGGATAAAAAGAAAAAGTCTAAG

GACAAATCTCTCAAAACAGAGAAGAAGAGTGAGCAGAGAGAAGAGAAGCATTTTAAATCA

GAAAAAGACAAAGCTTCAAAAGAGGAAAAGTCAAAAAAGGATAAGACCCTCAGAGAAGAA

CCAGAATATGAGGACTATGACATGAAAAATCCTTTTTTAGAAGACACCAAGATGAGTGCA

TCAGATCACGATCGATGGCCATCAGACCTTTCCTCTGACAGCTCTCTCTATGAAGAGGAT

AGTTGGGATTCCCCCATAAAAGAGTACAAAGCAAATAACAACTCGGTAAAACTGATTGTG

GAAACAATGAAAGACGAAAACCGTGAGCGGAAAAAGGACAACAAATTTAAAGACAAGAAG

TCAGAACATGGAGACAAGCGCTTTGAGAAAGACGTTACTTCCAAGAGAAAAGAGAAGGAC

TCCACTGACAAAGCAAGCGACAGAGGAAATGAAAAGAAAAAAGACTGGACTGACAAACAG

AAGCTGAACTCCAGCCACTCGGAGAAGGAGAAGAAACGAAAGGAATCGGCTGATGGGGTC

ATCAAAGAGAAAAAAGAAAAGGAGTCTGTTGACTGCGGGAGAGACAGGAAAGATTCCTAT

GAGTTTACTAAGGAGCGCAAAGACTCCAAAACCAAAACAGAATGTTTGAGAGAGGAGTAT

GGCAATGAGACTTTCTTCAAAGATAAATCAGAGTGCGAAACTACCCTCAAATCAGACCCC

AGGGAGAGGAACCACTCTGGAAAAGAAAAGGAGAAAAAAGGCGATGGCATGGAGAAGAAA

GACAAGCTAAAAGGCGAAAAACACAAAGATAAGCCTAAAGATCGAAATTTGGACCAGGAG

AAACCTGAGAAAACCTCTGCTGACAGGTCTTTGAAAGATAAAGACATTGAAAGGAGCTCC

AAAGACAAAAAAGATGGCGCTAAAGATAAACACAAAGATTTTCACAGCAAGGACAAGGAG

AGAAAGATGTCATCTGAGCAAAGCAAAGACAGGAAAGAAAAAATATCCCAGGACAAACAT

AGTGAAAGAGAAAAGGATTTTTCTGAATTTAAAAAGGAAGAGAAGAAACCTGAAAAGCTG

AGGGAGAAGACCTGGTATAAAATAGAGGACATATTTACAGATGAGAGTGAGGATGAGAAT

GACAATTACAATGGAGGGGTAGCCAAATTGAGTGATTCCTTCGGACTGTTGGACTCGCAC

CGTAAGGATTCCACACCTGATATAGATGACCTAGATCTCCTGATGGATAAACACCGGAAG

TATTCAGGTGAGGGCAAACAACACTGTACAGAAAACAAGAAAGAAAAGGAGCATAAAGAT

AAAAAGAAGGAGAAGATATTTGATGGTGGAAAAGAGAGAAAAGGCTCCATTGAGAAACAC

AAGGATAAAAAAGACAAGGATTCAACAGATATAAAACACAAGGACCGCAAAGACAGAGCA

TCTGTGGACTCGATTCAGGAAAAGAAAATCAGACCGAAAAGCACAGACAAACGGGAGCTC

ACTGAGGAGAAGAGCAAAAGCAAATACAAAGATAAGCCAGACCATCTGAAAGATAGAAAA

CCATCTAAAGGGAGTGGGGAGAATGAGAAATCTTTGCTCGAAAAGCTAGAGGAGGAGGCT

ATGAACGATTACAAAGACGATTCCAATGATAAGAACAGCGAGATCTCATCAGACAGTTTC

ACAGACCGGGGCCACGAGCCAGTTTCAAGCAGCTTCTACGACTCGTCGAATATCACCTTG

CCAGATATCTCAGATGATCGAAGAGAGTCGCTGTTAGTTTCCAACCCACAGGATAAATTT

AGGGAGAAAGAAAGGCATAGGCATTCCTCTTCCTCTTCATCCAAAAAAAGTCATGACAAG

GATAAGGAGAAAGCCAAAAAAGAGAAGAACGATAAGAGAGACAAGTCAGAGGAGATTAGA

GAGTCCTTTGGCCGCAGAGAGAGCCTACCTATCGAGAAAGAGCCCATGCCCCTGGAAGCT

GATCCATACACTTTTCCATATGGCTCCAAGGGTGATGGGGAAGATGACCTGGACAAAACT

TTGGAGTTTGAAAAGGAAATGTCCAAAAAGGACAAGACGAATAGCATCCTTACTAGTGAA

AAGATTAAAGACAAAAAGAAAAAAGAGAAGCATAAAGAAAAGGTAAAAGAGGAGAAACAT

AAATATTCAGATGGTGTCGGGTCTTTCCGACACACTAAAGATGAGCAAAAGTCTGGACTG

AAGGAAAGTCCTCAATTCATAAACTTAAAGGAAAAGTCTAAAGAAGATAGTCCAAAATTT

GATGGTAAAACCAGAGACAGAAATAGGGACATTGCAGACAAAGATTATAACAAGACCAAG

GATAAGCTCAGTGATGGCGACAAGCTGAGCCAGTTAAAAGATACTACCAGAAAAGACACT

CGTCCACGTGAGAAGCTTCTGGTTGATGGTGATCTCAGACTAACAAGCTTTGGTAAAATG

CTTAGTTTAAAAGATCAGGAAAATGAAGAGCGCCATAAGAGACACAAGGAAAAAATGAAA

CAAATGGAAAAATTTAGGCATAAATCAGGAGATCCAAAGCTTAAAGAGAAACTCAAGTCC

AATGAGGATCTAAAAAAGAATCGAAGTGATCTCTCATCCAAGAAATCTGGAACATTAGAC

TCTGCTTTGAAAGACAAGAAACTTAAAGAGATGAGCCTTACATCCCAGATGATGTCTCCT

GATAGAAAATCACAGCCACTGGACAGCCAGAACTCAAAAGACTGGCTAGCAGGTCACCAA

ATGAAGGAAAATCTTCCTGCTTCCCCACGACCCGATCAGAACAGACCAACAGGTGTCCCT

ACTCCTGCATCTGTAATTTCATGTCCAAGCTATGAAGAAGTCATGCAGACTCCTCGTACA

CCATCTTGCAGTACTGAGGACTACCAAGAAATCATTTTTGATGGCCTAGACTGTCAGAAC

TCTTCTGCAATGACCATGTCGATGAATGCCTGCTCCCCAACATTCTTTGATAGGTACACA

TCTCAGAACTCATCCCATAGTTTCCCAGAAGGAACCTGCATTACACCAGCAAAGAATATG

CAGTTACCCCTCGAGAGCCGATCAGTCACCTCTGAAGTTCGAAGACCACTAGAGGAGGAG

TTCAAAGCAGAGGATGAGAAGTTTCTTAAACAGCAGAGCACATCTCAAGAATTTGAGACT

TCTGCCTCACATCTGTCTGAAGACAAATTGCAAATGGAGAAACTGGATAGTCTTTCAGCC

TCATACCCCTCACCTCACATTAACATTCTGTCTCCAAGGCATGATTTGACTCAGCATGTA

CCCGACAGAGCGTCTTCTGCCAATTGTAGCAATGATGCCAATGAACTCCCTCCAGAGAGC

ATGTACAGTTATCTTATGAAACCCTCGACTCCAGTCCATAGACCGGACCCTCAGTGTCTG

GACATAGCTGCTCCTCTAACACCAGCCCCTGCAGCTTTGCCTCCGATGGACATTGATGAC

TTGTCCGAACCACATCAGAGTGAGCCCGGTTTGTTGCCCGCTGATCCTATGAGTGGCAGT

GAGTATTTACCTCCTCTTGTGGAAGAGAATGATGAAGAGGAGGAGGATGAAGATAATGAG

GAGGAGGAGGAGGAAAATGATGATGATGATGAGGAGGA

>Unigene000258

TGTGTGTGTGTGTGTGTTTCAGAGCGACACTTCCCCCCGGACGTGCTGCAGCAGCGGTTA

AACGCAGAGCTGAACTTCCTGGATGCAGTGGAGGAGTCAGTGAGACAGCTGAGTGACGTG

GAGAGAGTCAGAGCTGTCTCACTCGCTCAGCAGGAAAGCGTCTCACTCGCACAGATCATC

AAGGCTCAGCAGCAGAGGCAGGAGCGAGAGCTGCAACTACTGAAATTGAAAGCAGAACAG

GAAACGCTGGAGACTCAGAGACACGTCCAGGAGAGCAGAGAGAGAGCAGCACAGGCCCAT

GTGGAGCTGTGTATGAATCTGGTTCAGTCTCATCAGAAGGCTCTCATGGAGTTGCAGGAG

AACAGCAGCAAAATAAACCACCAACACACACAGACACCACAACACCTCCGAGAGATGACT

GAGATGAGTCGTCCTCAGCTGCCCAGTGTTTCTTCTGTCACACCTGTAAGGCAGCCACAC

CCACAAACGGACAGTAGTAGGAGTGAAGTCACAACCGAGAAAACACCAACAGCACACACT

CCACAGAGCATCACAGCTGAGTTGGAGTCCTTCAGTTTCACGCAAAGCAACGGCGGTGGT

GTGGACAGGAGCGGCTCGGTGGAGGAGGAAGTGAACACCGCTGCTGACGACTCTCTCCAA

ACTGACAAAGACAGCGTCTCGATCAACTACTCTTCAAAGTTTGATGAGTCTGTGACGGAG

GATGAGTTGGAAAAGTCCTTTCGCTCTGTGCTTCCGTCTGAGTCTCACTGGCGGGGATCC

GTGGAAAGGAAACGGCAGTCGGACGAGGAACCTCACGCTGACAAAATATCTACTAAGGAC

GGCAGCGTCCCTGTCTTCTCGGCGGATCAGTACAGTTTCTCGGAGTTCACCATGGCGATG

GTGAAGCAGTGCATGCAGGAAGAGGAAGTTCGCTCTCAGCATCAGAGCTCCCTGCTGCGA

CTCCGACACCGAGCACTGAAGGAGAAAACCCGAGCTGAGCTAGAATGGCTCGAGCTCCAA

AAAAGGCATCTGCGTGATAAAGGAGAGGATGATAAAATGCCTCCAATACGCAAGAAACAA

CGCGGCCTGCTGCTTAAACTACAACAGGAACAGGCAGAGATCAGAAGGCTGCAGGAGGTG

AACCGTGCAGCACGAAGGGAGAGGAAACTGTTGCTAAAACAGCAAGAGGAGATCGAGAGG

ATCCAACACACCACACAGAAACTGAGAGAGAAACTCCGGAATGCTGAAGACACAAACCAG

AGGTCTCCTGTGTCAGGTGTGAGCGAGGATTTGGCCATTCCCACTAAGGTGGACATAGAG

TTCCGTAGCCCCTCCCCTTTGTCTGTGTCAGGGAGTGAGACCAGCAGTGTCATGAAGAAG

CTGAAGAAGATGAACTATCACACAGATGAGCGATTTCTTACCCAGCGTGAGCTGCGATTG

GTACATTGGCGTCAGCAGGTGGAGGCGGAGCTGAGACGTTCAGATGGGGCGGAGCCTCAG

CCGAAGGGACGAGCACTGAACGCCGAACGTAGTACACAGCAGAAACACGGCACAGACGAG

CTGAAGAGAGACAGACAGGAAGAGAGAAAGACAGGACACGAAGTGCGCAGCGATTGTGAC

TCATCATCGGCACTGTCCGTCTCCAGCATACACACAGTGCAAGAGTGTGACAACACACAC

AGCCGTGAGTTCTCCTTGGTTCCTGCAAGTTCCAGCAGTAAAGTGACATCACACAGCAGC

ACCAGGAAGAGGGACCTCCCTTTATCAGCTCCTACATCTGAGGCGGCATCGGATCAGAGT

GACATTGAGGGGCGTGTCCTGGCTCTGAGGGAGGAGCTTCGGCGGCGGAAAGCTGAAGTC

CAGCTTCTAAAGAGGGAGCAGAGACTGAGACACAAAGAACGACTGAAAGCTCAGGAGGCC

AGTCTGCTCAAACAGCTAGAGTCTTGCAACAACTTCATCCAGAAGACTAAAGCCGAGCTG

AGTAATGCAGATGTTGCCACCACTTCAACCAAACCTCAGATCAAAACTCCAACATCAGAC

TCGCACAAATCCGGAACCGAAAGCTTCACCACAGACAGCTCTGGAACAGAGAAGAGTAGA

GAAGAGAAGAGTCACGAGTCTGAATCAGAGAGATCGAACAGGATACACACACAGTCTCCG

GCGCTCAGCGATGAAGATCCTTCCACCGTGACCCCGACTCCGGTTTTGGGAAGCCCCAAC

CTCCTGAGTCCTCGTGAACTCCAGCATGTTGAGCAAACCCCACAACCCGAAAGCCCCGGC

AGACCCGGGAAGGACGCCTCGACCGATCTGAGCACAGCCTCCAGCCCGGGGTCGGATGTC

ATAGAGGAGCTGGAATCTCTAAGGTCGGATCCAACCCGCTCTGAACACTCGCATCCGCTG

CTTAATTTAAAACTCCAAGACTCCGATGGGAATTCCAGCAAAAATCAACCGGAAATTGAG

GTAAAATTCAGCCATGTAGAAGAATTTACAAATTCCGAAGCGGTTAAATCCAGTTCCATA

ATAGTAACTAAACTAGAACCAGAAGAACCCAAACTTCCAGAGAGGTCCTTAACCTTAGAC

GTCTCAGACCTTGAAGACAAACCACCTGAGTCGCTGTCTGAACACAGCTACAATGATGAT

TTTGAATCAGCAATTAAGGAGGAAACTGGATCTCACTCACTGTCTTCTGAAGATCCCGAA

GAGGTATCCTACAAATTCTCCTTCAGCAGCGAAGATGAGATTGAGGAGAAGATTAGTCTC

CAGTCAGGAAATACCAGTAGGACTTTTGAGCAGGAAAGACCTCCTGATCTCCACTTCTTG

GGGAGGTGTACAGCAGAAAGCATCCATGAAGATGTTCCCAGTCTTCCGAAGTCTTCGAAG

GATGAAATGATGGATGAAATGCCTGATTACGTAATTGGTGAGCGTGTCCTGGTTAGCAAC

CTTCAGCCAGGTATTCTAAGGTTCAAGGGACAGACTAGCTTTGCAGATGGATTCTGGGGC

GGAGTGGAGCTGGACGCATCCGAAGGAAGCAACGACGGCACTTACAACGGTATTGTTTAT

TTCCAGTGCAAAGAGAAACACGGGATATTTGCTCCTCCGGGTAAGATCTCTCACCTACCT

GAGACACATGAAGACCATACAAGAAGCTCAGAGGAGAAAGATTCCTCGTTTAAAGATCAA

ACATGCAGGAAGACGAACAAGTCTGAAAGCTCACTTCAGAATAAAAGAGTGGAATCGGAT

CCGCCTTCTGAGACGGAGGAACTGTCCGGAAAACCTGTGGTTCTTGATGCATCCGTTACG

AAAGACTTGATGATTGATCACGTTGCTCTCAATGGTGAGACTTTTGAAAATCTTAATGGA

GAAAAAAATCTTCTGGCACTAGAGAGCTTTAAAGACATTGTCCTGGAATTTAAAAACGTC

TCAAAACATGAGGAAGGTAGAGATCAAACTCCAGATATTCTGGACCTGCTGATCCGTGAT

GAGTCGTCCACGGCAACCGAGGACCTACAGAAATCTTCAGTAGTCTCTCCTGAAAAGACA

AACGAGGACAACGTGGATGGATCGGTGGTGACTGAACACAAACCCCTCACGGCTCTGGCA

GATACGCTGATAGAACGCTTCATGAGCGATGCCCTGAAACAGTTTCAGCAGATCAAAAAA

AACAAGGAGCAAAAAATCTTAGCTGCTAATCAACTCGTCTTCTTCGGTGAGGACGAGGAA

CCCAGTGAAAATGATGTGTTCCGGTTCGGAACTTCAGCCAGCTGCAGAGTGGACTTCGAC

AAGGACCAGGAAGAGGTGTCTTCTCCTGAGATGTGCAACCGATTGGAGAGCCCTGTTTTG

GGTCAAAAGGAACTGGTCCAGCGTCTGGAGGAACTGGAGCTGAACCGCGAGTTCCTACAT

GTGCTGGGAGACGAGCCGGACTGGTTTGACGAGGACTTTGGCCTCAGCTCTCGCAGACAA

ACTCAGAACAAGAACAAACTCCGGCAGGACGAACTGACGCCCACGTCTGCGGCCAAACTC

CCGCACCAGAGGCAGCAGGATGAGGCGGTGGCACCGGCGGCCATGTTGGTCCCGCACAAA

GCCGAGGAAGTGGAGAAGTTGGTGAGTGCCGCCCTGCTGGAGATCTGGATGAGCTGCGGG

ATGGGGCGTGGTAAAGCAAGTCTGACGGGCGTGGCCAAACCTCAGGCGTCGGAATCGCTG

CTGGGGGATCAGAGCGGCGAGGCGTGTGTACGCAGTTACAAACAGGCAGTGTTTGACCTG

GTATGGGAGCTCATGCAGGAAATCTACGCTGAGGATCCCAACACCTACAGACCACTGTGG

ATCAAACCACACCGGATTCATTCACCCTACATCCACAGAATCGACAACCCCACTGACCTC

CATAAAGTCCAGGCGTTTGTCACCGGTGAGGTTCTAAAGCTGTACAATTTAAATAAAGAG

CAGAACCACAGAACCGACTGGCAGAAGATGCTGAAGTTCGGCCGTAAGAAGAGAGACAAA

GTCGACCACATTCTGGTGCAGGAACTCCATGAAGAAGAGGCTCTGTGGGTGAACTATGAT

GAAGACGAGCTGTTGGTGAAGATGCAGTTAGCAGATGGGATCCTGGAGGCTCTGCTTAAG

GACACTGTGGACGTCCTCAATAAAATCCACCAGAGTAGATCCTCACGGGCTCTGACCCCA

TCCTCATCCACACACCTGCTCTTACAGCCCTGACACACTGCGCCATCGTTCAGGACAGAT

CCCGGAATAGCCTAAACCTCGAGACGGTCTGTGTCCCGAATTTGCTGCTCAGATCTCCGG

TCCCTCCCTTTAAAACCCCACCCTGTGTTTGGGACAGAACCGGACCCCCTCCAGCGTCTG

TACCACATTTGCCTTCGAGCTGGAGTTTTAATTTATTTGTAAAGAGTAAACAGTTTTTAA

AACTATGTGAAAATGTGTAAAGAGATGATTTAAGCTCACATGAACCCATCTAAGCACACG

TGGAACCTACAGAACCCAGTGGAACCTGCAGGAGCCTAAAGGAACCCAGAAACAGAACGA

GACGGTTTAGAGAGGATAAATCAATATAAACTGTGTATTTTAAAGAGATTTAGAAGAGCA

ACAATCAGAAACTAAAGTGAACGAGTGCATGATGAGAGATTAGTGCTTTATTTACCCTCC

ATATTAAGTGCCTGACGGTGTAGGCTCCCTCTCTAGGGGAGAACCGGTTTCAGCTTCCTC

TCGTGTGTCGAGCCGAGTCGCTTTTATACTCTGGCGTTTCTGCTTTCCAGCTGGACCCCT

GATTCTGCTGCTTCATTAACTCTTTCCTACACTCAGTAGGGAACCTCCTCAGAGGGCTGC

GTCCTAAATTTTCCACACTTCCTTAAAGACCGCCCCC

>Unigene000259

GACGCGGTGACGTCACGAAGACGACGACGACGACAACAACAAAACAGAGCAGCAGTAACA

GAGTGAAAAAAGCCTGTTTAGTTCCTGATAATAACGTTAATAACATACAGACATTACATT

AATGTCACGCGGGAGTCACGTGATCAGAGATTAGAGTGATGATTGATGAAGTCGGATTTT

AAAGAACAGTGTCAGGTTTTAGCTGCAGACGAGAAGCGAAGCCTGTGTGTAATCACATAT

TAGCTTTTTAGCCCGGAGGCTAATCCAGCTAGCATAACATTCTCTATGTGCAGCTAGCAT

TAGCTGCGGAGTGTCAGCCTCGGAGCGGAGCTTTTTGCTACTGCTCGCTTCCGTGCGAAG

GATGGACACCGACAACTAACAGGTGAGCATCACAGACTAGATGAAGCATCCAGGCAGTGA

AGCTGAGCGGAGAATTCACCATGAAGTTGTACGTGTTTCAGGTCAATAACGGGAGCATGT

TGACCTTCGACACCGAGTTGGCGGTGCAAACTGTTCTGGACCTCAAACATGCAATCCAGA

CCAAGTACAAGATCGCCACTCAGCATCAGGTGCTGGTGGTTAACGGAGGCGAGTGTATGG

TGGCCGAGAGGAGAGTGTGTAGCTACAGCGCAGGGATGGATACAAACCCCATCTTTCTGT

TTAACAAGGAAATGATCCTGTGCGACCGTGCACCGACCATCCCCAAGACCACCTTCTCCA

TTGAGAACGAGATGGAGATGAAGGTGGAGGAGTCGCTCATGATGCCGGCCGTGTTTCACA

CTGTTGCCTCGCGGACGCAGCTGGCAGTGGAAATGTTTGAAATTGCCAAGAAGCTGTGCT

CTTTTTGTGAACGCCTGGTGCACGACGAACACCTTCAGCACCAGGGCTGGGCCGCCATCA

TGGCCAACCTGGACGACTGCACCACCTCCTATCAGAAGTTGCTCTCCAAGTTCGACATGG

CCTACACTAATTACCAACAGGATTTTGAAGACATCAAACTAAAGCTAACAAAGCTCGGCA

CGGCTGTCTCGGTCATGGCTAAGATCCCCCTTCTGGAATGTCTAACTCGCCATAGCTACA

GAGAAAGCCTGGAGAAATCCTCCTCCTCCACACACCCCAAAACAGAAAACAAACCAAACA

ATGAAGCCCAAACGTCCCCCCAGTCGGCTGTCTTGGCCCCTACGGAGGTCCTGCAGGACC

AGAAATCTCCCTCTTCAGGAGAATCCTCATCTCCTGGCAGAGGCAGCAGTGCCAGCGACC

TGCAGGAGGCGCTGGAGGGAGAAAAGGTGCAGGAGGAAACAGAAAAAACGGACATGCCGT

CATTTAACGTCACGCTGCTGGACTGGATCAACGTACAGGACCGGCCCAATGATGTGGAGG

CTGTAGTGAGGAAGTGCTTCGACTCCATCAACAGACTCGATCCTAGGATTATTCAACCGT

TTCTCCTCGAGTGCCGGGACACTATAACCAAACTGGATAATCAAAACATGAAGGCTATAA

AGGGGCTGGAAGACCGATTGTACGCGCTCGACCAAATGATCGCCAGCTGCAAAAAGCTAG

TGAACGAACAGAAGGAGCTCGCTCAGGGATTTTTAGCCAATCAGATGAGGGCTGAAAACC

TGAAAGACAACTCAGTGCTGCCTGACCTGTGTCTGAGTCACGCCAATCAACTGATGATCA

TGCTGACCAACCACAGGAAGCTGCTGGACATTAAGCAGAAGTGCACGACTGCCAAACAGG

AATTGGCCAACAACCTGCAAGTCCGTCTCAAATGGTGCTGCTACGTGATGCTGCATGCGG

ATCAGAACGGCGAGAAGCTTCAAGCCCTTCTGCGCCTCCTGACCGAGCTGCTGGAGCGAG

TGCGTGTAGTGGAGGCTCTTAGTACCGTCCCGCAGATATACTGCCTCGCCGTGGTCGAAG

TGGTGCGCCGGAAAATGTTCATTAAACACTACAGAGAGTGGGCCAGTGCTCTGGTAAAGG

ATGGGAAAAACCTTTATGAAGCAGAGAAGGCCAAAAGGGAGACGTTTGGCAAGCTGTTCA

GAAAGTCATTCCTCCGAAATCGCCTGTTCCGAGGACTTGACTCCTGGCCTCCCTCGTCAT

TTTGCACAAGAAAGCCTCGCAAGTTTGACTTTGAGCTTCCGGACATTTCCCTGAACGACC

TACAGTATCTGAAGAGCTGCTGTCCTGCTGAAGTGCAGCCTTTTCTCAGGGTTCCATCAC

TATGCGACTTTGAGCCGTTAAACCACCACACAGAAGCACTTCATCAGCTGGTACAAGCAG

CTCAGAGTGTAGACGAGATGTCTCAGACCATTACCGACTTACTGAGTGAACTGAAGGTGT

CTGGAAGCCAGAGTCCACAGAGACCCAGTTCGGAACACAGAAAGGAGACCACGCCCACCC

CATCTAAAACTCCGCCTCCCCTCAGCATACAGTCTCTGCCCACTCAGCCGCTTTCCCTTC

CGGCTCCATTAGAAGATTTATCCCCTGACAGTATAGACGCTCATACGTTTGACTTTGAAA

CCATAGGACATCCCAACATGGATCCCGTGCTCCAGCAGGGCTCTCTGGACCTGGACTCTT

TAGCCGAGAGCCCGGAGTCAGACTTTATGTCAGCAGTCAATGAGTTTGTCATTGAAGGGA

ACGTAACGTCACCCAACCCCATCAGTGACCCGACCAGTCCTGAGATGATGGTCGAGTCTC

TTTACTCCTCTGTCATTAATGCTATAGATAACAAAAGAATCCAGGACACGACCATGCTCG

AAAGAGAGAACTCCAGAATCATGGCTCTCAAGCTGACCATAGACAAATACCGATCGGCTG

CTGAAGAGTCGCAGGGTAACATGAGGAAAGTTAAGGACGACCTCTGTCATTTTCAGGGAC

TTGTTTTGAAAGAGCAGAGGGACTTTGGCTTTGCCTTGAAGATGATGAGTGCAGAAATTC

ACAGCATAATCAACAACATAAGACGATGTCAGGAAGAGGAACTTGGAGAGGAACACCAGA

AAGAGCTTGAAAAGCTAAAAGATGATCACGAAAAGCACATCCTTCGGCTTACCGATGAGT

TGGAAGGAAACCACAAGATCGTGCGGGATGTCCAAAGAGCCATGCTCGAACTGGAAGGGC

TTGTGGAGAGGAAGGAGAAGGAGCTGGCACAGATGGAAGGCGAGAGGGAGCGCTTGTTGC

AGGAGATACGTAACAGTCATCAACGGGCTGTCCAGGAGTTGGAGAGGAACCTTGCCGAGC

GCAGCCAGGCACTTCAAGATGCCCTGCATTCTAAAGACGCTCTTGCCAGTCAGCTGGAAG

GCCTGCAGGTCGAGATCGAGCAAAAGGTGCGTCGAGAAATGGAGAATGTGGAGAAGAGAC

GTCTCCAAGACTTGGAATCTCGCCTGAAAACAGAGCACGAGGCACAAACTGAGATCTTAA

AACGCGGCCACCAGACTGTTCTAGACACTTTAACCCTCGAAAACCAAGCCAAACTTAGGG

AACTCACAGAGGCACACGTAATTGAGCGGGCGGAGCATGAGTGTCGCTTTAAAGACTACG

AGGCACGCATCGGTGAGCTCGCCGATGCCCGATGCAAACTTGAAGTGGAAATGGCACTTA

AAGAAACTGAAACAGATGATCTGCGCTTGCAATATGAGGATGCCAAGACCCAGCAGGAGG

AGGCACTTAAGGCAGAGATGGAGGTACAGACGAAGGCCCTCCAGGAGCAGATCGAGGAAC

TGATGAAGCAGCTGCAGAAGAAGAACGAGGAGCACGAGCAAGGCCTTGCCGAGCTGCGCA

CTCTAATGTGTCTGGAGAAGGACCACTGCATCTCTGAACTGGTAGAGCGGCACGACGAGG

AGAGGACGCTGCTGCGTCAGGAGTTCTCCACACTCAAGCAGAAGTCTCAGGATGCTGAGA

AGAGCTGCGAGGAACGCCTGCTGGAGATCCAGCGGCAGCTAGAGGAGCAGCTGAAAACGT

TACGCAGTGAACGGGCTGAAGAGCAGGCGGCCTTCAGGGATAAAGAGCAAGACTTGCAAA

CCACCATCAGTGACCTGCAGGCCGTGAACAACCTTCTGTCTGGGCAGCTGGAAAAAGAGC

GTGAGGAGGCCCGAAAAACGGAGCAGGAGGGAAAAGAGGAGCTAAAACAAGCCTTATTGC

AGAAGGAGGAGCTGGAGGCGAGACTGTTGGGAAAAATTACGTTACTTGAAACTCAACTGG

AGAGACATTTATCTGACAAAGCCGAGGCTGGACATGGTGATGTTGGGGATGCCTCGTTGC

AGCAGAGGTTAGATGAGGAAAGAGCATCACTCCTGGGCAAGCTGGAGCTGCTGGAGAAGA

GAAAAAACGAGGAGCTGCAGAACCTGAAGACTTCACTGATCGCCGAGCAGCAGACTAATT

TCAACACTGTACTGATGCGGGAGAAGTTAAAGAAGGAGCAGATCATCCATGAGCTTAGTG

AGAAGCTAAAGATGGTGACTCTACAGCAGGAGAAGGATAAGAGCCTGATTCAGACTCTCT

CTGAGGATCGTGCCACCATCTTGCAGGAGAAAAAACACCTAGAGGAGGAGCTCAACCGGC

TTCGGAGCTCCGCCCTTGTCTCCTCAACTTTCTTCATGTCTAATCCTGTTGCCGTTGAGA

CACCCGTTGCTTGTGGGCCCATTCCTGGCGAGGTCCCCGTGCTGCCTGTAATGGATTCGG

AGAGGCTGGCGTCTGTGGCCGCGGTGCGAGACGATGATAGGGTGGACTCGGCTGTCGACG

CAAGCATGATGACCGTCCATGATCACCCATCAATGCTGTCAGAAGAGAAGCAGCGGATAC

TGATGTTGGAAAGAACTCTGCACATGAAAGAAGAGGAGAACAAGAGGCTCAGCCAAAGAC

TGATGTCTCAGAGCATGTCCTCGGTGTCCTCAAGACACTCAGAGAAAATCGCCATTCGGG

ATTTTCAGGTTGGTGATTTGGTCCTCATCATCCTGGACGAGCGACACGATAATTACGTGC

TCTTCACCGTTGGCCCCACTCTCTATTTCCTGCACTCGGAATCTCTTGCTGCGCTGGACC

TCAAGCCCGCAGCCGGAGTTACGAGGCGACCCTGGGTCCTGGGGAAAGTCATGGAAAAAG

AATACTGTCAGGCAAAAAAGGCTCAAAATCGCTTCAAAGTTCCTCTGGGCACCAAATTCT

ACCGAGTAAAAGCCGTGCCATGGAATAAGAAGGTGTAAAATTGTAAACGAAAGCGAAAAT

AAGCTGGAATGTTTATATTCCTCCTTTTTTTTTCATCTTATGAGCATCAGAATTCTTTTA

TCACAGAAAAAACTATTATTATTATTATTGTTATTATTATTATTATTATTTTCCTCTGTT

TATTCAGAGGTTTGTAAATGTGGACCA

>Unigene000260

GAGTCCACCACAGCTCACCTCATCACCTTCTTAAACACAGCTGCAGCTGTAGATCAGTGC

AGAATTACAAAGTGAAAATTCTGTCCTAAACACAGCTGCACAGAAGGTCTGTCACCTGTC

AGGCATCAATGTGACGGATTTCACCCGTGCCATCCTGACCCCACGCATCAAAGTAGGCAG

AGAAGTCGTACAGAAGGCGCAGACCAAAGAGCAGGCTGATTTTGCAGTGGAGGCTTTAGC

AAAGGCAACGTATGAGCGTCTGTTCCGCTGGATTCTTGCTCGTGTCAATAAAGCTCTGGA

TAAAACCAAGCGTCAGGGCGCGTCTTTCCTTGGCATCCTGGACATTGCCGGCTTCGAGAT

CTTCGAGGACAACTCGTTTGAGCAGCTGTGTATTAATTACACTAACGAGAAGCTGCAGCA

GCTCTTCAATCACACCATGTTCATACTGGAGCAGGAGGAGTATCAGCGTGAGGGCATCGA

GTGGAGCTTCATCGACTTTGGACTGGACCTTCAGCCCTGCATCGACCTGATCGAGCGGCC

TGCGAATCCTCCAGGTGTTCTGGCTCTGCTGGATGAGGAGTGCTGGTTCCCCAAAGCCAC

TGATAAAACCTTCGTGGACAAGCTGGTGCAGGAGCAGGGGAACCATCCCAAATTCCAAAA

ACCAAAAAAGCTCAAAGATGATGCTGATTTCTGCATCCTTCACTACGCAGGCAAGGTGGA

TTATAAAGCAGTGGAGTGGCTGATGAAGAACATGGACCCTCTGAATGATAACGTGGCCTC

ACTGCTCAACCAGTCCACCGATAAATTTGTGTCTGAACTCTGGAAGGACGTGGACCGGAT

TGTGGGTTTGGATAAGGTGGCGGGAATGTCGGAGATGCCCGGAGCATTTAAGACCCGTAA

AGGAATGTTCCGGACTGTCGGTCAGCTCTATAAGGAGCAGCTGTCCAAGCTGATGGCCAC

GCTGAGGAACACCAACCCCAACTTCGTGCGCTGCATCATTCCAAACCACGAGAAAAAGGC

GGGTAAACTGGACCCTCACCTGGTTCTGGATCAGCTCAGGTGTAACGGTGTGTTGGAAGG

TATCCGTATCTGCAGACAGGGTTTCCCCAATCGCATCGTCTTCCAGGAGTTCAGACAGAG

GTACGAGATCCTGACTCCTAACGCCATTCCAAAGGGCTTCATGGATGGCAAGCAGGCCTG

TGTGCTGATGATCAAAGCTTTGGAATTGGACCCAAACCTGTATCGGATCGGACAGAGCAA

GGTGTTCTTCCGTGCCGGAGTTCTGGCCCACCTGGAGGAGGAGCGTGATATGAAGATCAC

AGATGTCATCATCAGCTTCCAGGCCTGGTGCCGTGGCTACGTCGCCCGAAAGGCATTTGC

CAAGAGGCAGCAGCAGCTGACCGCCATGAGAGTGATCCAGAGGAACTGTGCTGCTTACCT

CAAACTCAGGAACTGGCAGTGGTGGAGACTCTTCACTAAGGTGAAGCCCTTGCTCCAGGT

GAGCCGTCAGGAGGAGGAGATGCAGGCCAAAGATGAGGAGCTGACCAAAGTCCGTGAGAA

ACAGGTGGTGGCTGAGAAAATGATCGAGGAGATGGAGGTCAAACACCAGCAGCTGAATGC

AGAGAAGATGGCTCTACAGGAGCAGCTGCAGGCCGAGATGGATCTCTGCGCCGAGGCAGA

CGAGATGAGGAACAGGCTGGTGGCGAAAAAGCAGGAACTGGAGGAGATCCTGCATGACCT

GGAGGCCCGAGTGGAGGAGGAGGAGGAGAGAGCCAATCAGCTTCAGATGGAGAAGAAGAA

GATGCAGCAGAACATCACGGATTTGGAGCAGCAGCTGGATGAAGAAGAAGCAGCACGGCA

GAAACTGCAGTTGGAGAAAGTGACCACAGATGCCAAGCTGAAGAAGTTAGAAGAAGATGT

CATGGTGCTTGATGATCAGAATGCCAAACTCAACAAGGAGAAAAAACAGCTAGAGGACAG

GATCTCCGAGTTTAACATTAACCTGGCTGAGGAAGAGGAGAAATCCAAGAGTTTACAGAA

ACTTAAAAACAAACATGAGGCCATGATCACAGACCTGGAGGATCGTCTCCGCAAGGAGGA

GAAAATGAGACAGGAGCTGGAAAAGAACCGCAGGAAGTTGGAGGGCGACTCCACCGAGCT

TCACGATCAGATCGCCGAACTCCAGGCTCAGATCGCCGAGCTCCGAGCTCAACTCGCCAA

AAAGGAGGAAGAGCTGCAGGAAGCACTGGCCAGGATCGAGGAAGAGGCGGCGCAGAAGAA

CCTGGCCCACAAGAAGATTCGTGAGTTGGAGGCTCAGCTGAGCGAGCTGCAGGAGGACCT

GGAGCTGGAGCGAGCCGCTCGCACCAAGGCAGAAAAACACCGCCGTGACCTGGGGGAGGA

GCTCGAGGCTTTAAAGACCGAGCTGGAGGACACGCTGGACACCACTGCTGCTCAGGAGGC

ACTCAGGACAAAGCGTGAGACAGAGGTGACGCACCTGAAGAAGTCTTTGGAGGATGAAGC

TAAAAATCATGAGCAGATTGTGGCAGAAATGAGACAGAAACACGGCCAGGCTTTCGATGA

ACTCAATGAACAGCTGGAGCAGTCAAAGAGGAACAAGGCATCAGTGGAGAAGATGAAGCA

GACTCTGGAGTCGGAGCGTAACGAGCTGCAGATCGAGCTGCAGGGTCTGATGCAGAGTAA

AGGTGAGTCGGAGCACCGCAGGAAGAAGGCGGAGGCTCAGGTGCAGGAGCTGCAGATCAA

ACACTCTGAGAGTGAGAAGCAGAGAGCAGAGCTGATGGAGAAAGTCTCCAAGTTGCAGGC

GGAGCTTGATAGCGTTAACAGCGTGCTAAGTGACGTGGAAGGAAAATCCATCAAAGCAGT

GAAAGACTGTTCAGCTGTGGAGTCTCAACTGCAGGACGTCCAGGAGCTGCTTCAGGAAGA

GACTCGTCAGAAACTGTCTCTTAACACACGAATGAGGCAGCTGGAGGATGAACAGCACAA

TCTGAGAGAACAGCTGGAGGAAGAGGAGGAAGCCAAGAAGAACATGGAGAAACAGTTGAT

TGCAGCTCAGTCCCAGCTGGCTGAGATGAGGAAGAAGATCGAGCAGGAGGCGAGTTCTCT

GGAGAGCACAGAGGAGGTGAAGAAGCGTGTGCAGCGCGACCTCGAGTCTGTCAGTCAGCG

CCTGGATGAGAGGAACCTGGCCTTCGACAAACTGGACAAGACCAAGACCCGCTTGCAGCA

GGAGCTGGATGACCTGCTGGTGGACCAGGACCATCTGAGGCAGATTGTTTCCAACCTGGA

GAAGAAGCAGAAGAAGTTTGACCAGATGTTGGCGGAGGAGAAGACAATCTCTGCTCGCTA

TGCTGAGGAACGTGACCGGGCAGAAGCTGAAGCCCGTGAGAAGGAGACCCGCGCACTCGC

TCTGACACGCGAGCTTGAGACCCTGACAGACATAAAGAACGAAATGGAACGCGCAAACAA

GGCTCTCCGGGCTGAGATGGAGGACCTCGTCTCCTCCAAGGACGACGTCGGCAAGAGCGT

GCACGAGCTGGAGAAGTCGAAGCGCGGCATGGAGCAGCAGCTGGAGGAGATGCGCACACA

GCTGGAGGAGCTGGAGGATGAGCTGCAAGCTACTGAGGACGCCAAGCTGAGGCTGGAGGT

CAACATGCAGGCCATGAAGGCTCAGTACGAGAGAGACCTGGCTGGACGAGACGAGATGGG

AGAGGAGAAGAAGAGGCAGCTGGTCAAACAGGTGAGGGAGATGGAGATGGAGCTGGAGGA

CGAAAGGAAGCAGCGCTCGGTTGCCATGGCAGCGCGGAAGAAGTTGGAGCTGGATCTGAA

GGAGCTGGAGGCTGCCATCGACCAGGCCAACAAGAACCGAGAGGAAGCTCTGAAACAGCT

CAAGAAACTGCAGGCTCAGATGAAGGACGTCCTGCGGGAGCTGGATGAGACGCGTCTCTC

CAGAGAGGAGATTCTGGCACTAAGCAAAGAGAATGAGAAGAAGTTTAAGAGCATGGAGGC

AGAGATGATTCAAATTCAGGAGGAACTGGCTGCAGCAGAACGGGCAAAGAGACAGGCTCA

GCAGGAGAGAGACGAGCTGCTGGACGAGATCAACAACCAGAACAGCAAGAATGCTCTGAC

AGTGGAGGAGAGAAGGCGTCTGGAGGCTCGTATTGCTCAGCTGGAAGAGGAACTTGATGA

GGAACAGTGCAGCACTGAACTTGTTAATGACCAACTAAAGAAGGCAAATGCACAGGTGGA

CACTCTGGGTAGTGAGTTAGCGGGTGAGCGTAGCGCGGTTCAGAAGAGTGAGACGGCTCG

ACAGCAGCTGGAGAGACAGAATAAGGATCTGAAGAGTAAACTGCAGGAACTGGAGGGTTC

CGTCAAATCCAAGTTCAAATCCACTATCGCCGCCCTGGAGGCCAAAATCCTGCAGCTGGA

GGAACAACTGGAGCAGGAGGCCAAGGATAAACAGCAGACTTCAAAGTCAATGCGCCGTAC

GGAGAAGAGACTGAAGGAGGTCCTGCTGCAGGTGGAGGACGAGAGACGCAACAGTGAGCA

GTTTAAAGATCAGATGGAGAAGGCGAACTCGCGCATGAAGCAGCTGAAGCGTCAGTTGGA

GGAGGCGGAGGAGGAAGCGACCCGCGCCAACGCCTCCCGCAGGAAGTTACAGCGAGAACT

GGAGGACGCCACCGAGGCCAGTGAGGGTCTCAGCAGAGAGGTTAACACACTCAAGAACCG

TCTGAGGCGTGGGGGTCCGGTCAGTTTCCCATCAGGCCGTTCAGGTCGCAGGCTGCAGAT

GGAGGGTGAATTCTCAGATGATGAAGCTGACAGTAAAGTGAATGACACAAATGAAGTACA

ACCTCCACCGAGCGAGTAAACACCAGAACACACACACACACACACTTAATTCATTCAGAT

TTTTTCCCCTTTTGTGTGTGTGTGTGTGTTCATTGTTGTGAGCGAATAGGAAAGCAACGG

AGTTTAAACTGAACTCGTGAGAAACGTCAGCATTGACAGGAAGTGCCGTCTGTTCTAAAA

CTTTTAAAACTTTTTCCATTTATTATTAAAAATAATTATATAAAAAAAAACAATGTTAAA

GTTGAAATCAATATTTTATAGCTTTTTTTAAAAAAAAAATTTTTGTGGTATTTTACATTT

ATTTCGCCATTTTTATTTATTTTTAACAAATATATATTCAGAAGTCCTTTTTGTAGCACT

AATGCTTTTTTTCTGTGACCAGGGCGACCCATGGTGTAGGCGAAATAGGCCTAGGAAAGT

TTTTTTTTTCTTTTTCTGGAAGTGCGCCCTCTTGTGACCATTATGCGCTTTTACAATTTA

TTTACTACTTATGCTTAATAGGGGGG

>Unigene000261

TTTATTGTTTCGTGTCTGTGCTCTCGTAGGTGTGTGGCACCCCCGAATACATTGCTCCCG

AGGTAATTCTGAGGCAGGGATATGGCAAGCCTGTAGACTGGTGGGCCATGGGGATCATTC

TCTACGAGTTCCTAGTAGGATGTGTGCCGTTCTTCGGAGACACGCCGGAGGAGCTGTTTG

GTCAGGTTATAAGTGATGAGATCAACTGGCCTGAGGGAGAGGACGCACCTCCGGCTGACT

CTCAGGAGCTCATCACGCTGCTGCTTAGACAGAACCCACTGGAGCGCCTGGGGACTGGAG

GAGCTTACGAGGTCAAACACCACCAGTTCTTCCACAGTCTGGACTGGAACAGTCTGCTCA

GACAGAAGGCTGAATTTATCCCCCAGCTTGAGTCCGAGGACGACACCAGCTACTTTGACA

CCCGATCCGAAAGGTACCATCACCTGGAAACGGAGGAAGAGGAAGACACGAACGATGAAG

ACTTTAACGTGGAACTGCGTCAGTTTTCCTCGTCCTCACACCGGTTTTCTAAAGTCTACA

GCAGTCTGGATCTGTCACGTGGGCAACTGGAGGAAAAGGGAGAGCAACCAGAGAAGAAGA

GTGAGAGTCCCCTGACTGTCGACTCGCTCAGCTGGACTCCAGACTTCACTGAAATTCCAT

CACTGTCCCACTCCTCTGACCTGGAGAGCAGCACGAACAGCCTGAGCCGAAACTCAGGAC

TTCTGCCCAAATTTGCCATTTCAGCAGAGGCTGATAGCTGCGATAGTTCGCTGTCCCTGG

ACAAGGCGAGTAAAGTGGCCTTCTCTGTTGGAGAACTTCCTCAGACGGAGCCTGATGCCA

TCACACCATCCAGCCCTGTCAGCAACGCCACCCTGTCAGGCAGTTTCTCTGAACATCTGG

ACCAGCTGATATCACGTCCAGAGGGAGCAGACAGTCTGGACCCTCCCAACAAGCCATCCA

CTGATACCGTCAGCCATTTGACCTCTCTAAGACCAGCGACCAGTATACCGAAATCATCAT

CCACCAGCGCTCTGTCTCTCATGATTCCTGCAGATGTTTTTGGCACATCCCCACTGGCTA

GCCCCATGTCTCCACATTCACTGTCGTCAAACCCCTCCTCCAGAGACTCCTCACCCAGCC

GAGACTCCTCCCTCTCTGCCGCCAACTCGCGCCAGCCAATCATCATTCACAGTTCAGGGA

AGAAGTTTGGTTTCACACTGCGAGCGATTAGAGTCTACGCCTGCGACAGCGATGTTTATA

CTGTCTATCACATGGTCTGGAATGTGGAAGACAGTGGTCCTGCTCAAAAGGCTGGACTCA

AAGCTGGAGACTTGATTACTCATGTCAATGGGGAAACGGTCCATGGACTGGTGCACACTG

AAGTGGTGGAACTGTTGCTGAAGAGTGGAAGCAAGGTTGCTATATCCACTACTCCTTTTG

AGAACACGTCGATCAAAACGGGTCCGGCTAGAAGGAACAGTTATCGGAGCAAAATGATTA

GGAGAACCAAGAAGCCCAAGAAGGAAAAGACACAAGAAAGGCGACGATCAGTATTCAGGC

GCTTCGCCATGCAGCCCTCTCCACTGCTTCACACCAGTCGCAGCTTTACTTCCTTAAACC

GATCCTTGTCCTCAGGGGAGAGTCTGCCAGGATCCCCCACACACAGTCTGTCTCCTCACT

CCCCCACCACTCCCTTCCGGCCCACGACGGAACTCAATCAATCAGGTGGCACATCATCTC

AGAGCAGTTCCCCCAGCTCTAGTGCACCAAATTCCCCAGCTGGCTCCGGCCATGTCCGTC

CTAACACTTTGCACGGTTTGGGCCCTAAGTTGCCTGGCCAGCGGCTTCGTCAAAGTCGCC

GGAAATCAGCGGGAAGCATTCCTCTCTCCCCGTTGGCTCGAACACCTTCCCCTACCCCTC

AGCCCACCTCCCCTCAGCGATCTCCTTCCCCATTGCTCGCTCACTCGGTGGGAAGCTCCA

AAACTACTCAGACTTTCTCAGCCAAGATGCACTCGCCCCCTACAATCGTAAGGCACATGG

TGCGACCTAAAAGTGCCGAACCTCCTCGGTCTCCGCTCCTTAAAAGGGTTCAGTCGGAGG

AAAAGCTGTCGCCATCCTACACAGGGGATAAAAAGCACCTCTGCTCCCGGAAACAAAGTC

TAGAGGTGACCCAAGAAGAAGTTCAGACTGGGTTTGCATCTGGAGGTGAGCACACACTTC

AGTGTGTGGAGGAGAGTTCTTGTGAACTTCCCACCATCACCCGAGTAAGACCTGCTGAGC

AGGGCTGCCTAAAAAGACCTGTGACTCGTAAGGTTGGACGACAGGAAATGATGGAGGATC

TTGATAAGGATAAATTGAAGTCCAATGTTGTAGCGAAAAGACAGGACTGGCATGAGAGAA

GAGAATCTCTACAAAAACAAGATGCCATTCAGGAGGCTGAATCTACCCAAAGCCTGACTG

GGTGTTGCTCGGTTGATAACAAAGTTGTCACTGCTACAGTAAAAGATGTTCTTTATAAAA

AACTAAACTCCAGAGCATGTGAAAGCATTGCAGACTCCATGAGCAGCACTGGAGACTCTG

CGTCGGTTTTCAATGAGAGTATAAGATCATCCCCTGCTCAGTCAGACCGACAGCTGGCCA

GGCAGATCAAAGAGGGCAGTAAACCAGACAGGCATGATTTCAAAGCCCCTAATATGGAAT

TTGCTCGGAAAAGACAGTCCTTTGAGGAGCGTGAAGACTGTTTGTGTCGAATCACCTCTG

GCGTTCACGAAAGTCTACATTTTAATGCCACTAGATCCAAAAGTCTACAACTGGATTCAG

CTGGAATTGCTACTCCTGGAATCACCACAGATTCGTTGAGCCCTAAGATATTCAGTGGGC

GAGGGGAAAGTGCCGTAGAGAAGCTTCAGATTATCTCGTCCACAGAGGGCTCCATCAGAA

AGACTTCATCTGAGTACAAATTAGAAGCCCGACTTGTTTCTTCCCTGAAACCACTAAAGG

GCACACTGGACATAGGGCTGCTCTCTGGGCCTAGAATCTCCAAAACTGATACATGTCTTT

CAAAAATAGCCAGTAGCCAAGCTGATAGTGGTGGTCAGGTCGGAGCCATGCAAAACCAGA

GTGAAAAGCAGCCCCTGATGCTAACACATAAACCGACACAGGCTGCCTTGGAGATAAAAA

GTCCCGGTGCTTCGAGCAGCAAAAATGCAGGAAAAGATGAACCCACTTGCAGTACAGACT

ATGTTTTTGGCTTGTTTAGCCATGAAGGCACTCATGACAAAAATAAGCAGGGTGAAGGAA

TCAAATTCCAGACCGTAGCCAGTAAAATGGAGCCGAAAGATTTGTCAACAGGTGGAAAAA

CAGAGAATAAGTTGAAAACAGCTCAAGAAATGAGGCCTGCTCGACACAGTAATCATTTTT

CTTGTGGGAAAACACCCTCAATAAGGGAGGTCAGCAATGAAGACCAGGATGATGAGATGG

AAAATCCGGTGGAAAAACCTACCCTGCAAAACAACACAGTAGAAACTAAGACGCCAATGA

CCCATGACCAGGTCATCACACAAAACACATCACTACCCTCAGGCCCGTGTAGTGTTGATA

AAGATAAAAATGTGAGAGAAATCCTTATCAGTGCCCAGAAATCGGAGCAGGCTTTTGCAA

ACACCAATAAAATAACAATGGCAAAGGATAGAGTTCTGAATTCTCTAGGGTTAACTGTGG

TTACACATGAAGTTTATCAGTGTAAAACTGTCCAAGATGATGCTTCAGGATCTCGAAGCT

CAGAGGCATTGGTGCAGACTTTGCCTCCAAAACCAGACAGAACAGTTATTGTGCAAAAGA

CAGGCTCAGATGTAAAAGCTGGGATTGTGGCAATTGATGAGAAAAAAACACTAAGCACAA

CTGGAAAGAGTACAGATAAGAAACATGAACAGATCACAGCAAGTAGCACACCAGTGTTGC

ATGCGAAAACAGAACCGTTTCACTCTGAGGGTCTAGTAAAAGAAAAGGTATCGGTTCCAC

GGAGTTCAACTGTAACCACATCATCTCCTTCGGCAGCTTCGATAGTGCCAGATATAAAGT

TGCCTCCAAATTTACCTCTTCAAACCAAACAAGATAAAAACAATGTAGCTCCCAAAGGTA

CCCCTGTTAAAGCCACGTCTGCTGTGGTTCCCCAATTACCCCAGCATGTACATAAAAGCC

CACAGTCATTTGCTGAAATCCAATCAGACAGCACTGCAATAAACCAGTCTCAGCAAACGA

CTTTGAAAACACCAACTGGTGCTGACTCAGGTAACGGGCAAAAACGTTTAAATTCTATCA

GCAGAACAGATGACCAAAATGCTGAAGTGGAGAATGGCAAAAGATCCAGTTCAAAATGTC

AGGAGACATGTGGAGTTAAAACCCAAATCGTAGACAAGGATCATAAGACGTGTATGGTTC

CAGAAGGGTCAATCGATAAATCCAAGATTACATCATCCAAAGATAAAAAGGCACAAAATA

TAGCAGTTTCAAAGGAGGAAGTAAAATGCATTATGACAAACGAGCCTCGCAGTCCTACAG

ACCTCAAAACTGATGGGGCAAAGTGTGTCCTAGCTACTGCTCACACGCCCCCTAGTAAGT

CAAATCAGTATTCTGGTGTCAAAGGCAGTGAAAAAAATAGCAATACTGTGCAGGTTAAGT

CAGTGAATATACTTGAAGAAAAGTTGAAAGATAAGAATACTCCAGATGTATCCTGTCAGA

AGGTCACCAAGCCGGTACTCGACGTTTGCAAGAAGGAGGTTGTTACTGCAACAAAAAAAC

AACCACAATTGACAGGTACAAGTTCCAATGGAAAGCAGGGCCTGTCGAGCTCTAAGAGCA

AAGCTAAACTAGAACCATCTCCTGCTACAAACCAGAGTACAAAAGCCACCGAAGGAAGAG

GAACTCAAGAAGGCAAAGCACCAGTGCAGGTCATCATAGATCATACAAATCTAAAACCAA

AGGAGCAAAGAGAGGTAACACTTCCTACATTAAAAACTCAATCACAGAGTGAGAACCTGC

CTCAGACCACCAGCCCTCAAGTTGGTTCTGTACCTGTACCTACTGTTAAGGTTCACACAC

CAAGTGGATCTCCTATAGGCAAGGGTACAGGGTCCACAGTGGAGACCTCAAAGTCGCCTG

ACTCCATGCAGAAAGGAAACCGCAAAGACTGTCAGAAATCGTCAGGATTGGCGAAAGACG

GTGCAGCACCAGAAAAAGATTCACCTCGCACTAAGCAGTCCAAAGATCTGCCCCGAGGTT

CAAATAATAAAAAGTGACAGTGAGGATGCCGAAGGTTGCCAAATCATTTTTTGTAAGAAA

AACTGCAAAGCAATCTCTTT

>Unigene000262

GGGGATGAAAACACAGCAGCGAACCGGTGCGGTCTATGTGCGGGAGTGAAGGGGAGCGAA

TGGCTCGTGCGGTGCTCAGTTAATCTGACTCGTGTTTCAGTTTTCCGGTAATCCGACATC

ATCCGCGCCTTAAACATGGACCTCGCGTCCAGAGGCTCGCGCCGGAGACATCCCACAAGT

TTGTAGTGACTTTTGAAAACTGACTCGAGCCATTTTTAAAAACACACAGTCACTTTTTTT

GGCCTTTTTCCTGCGCGCGCTTGTTGGGATTTAAACGGAAAGTGACGCGGAGCTGCAGGG

ACGTGTTTCTGCTCATCATTCCGGCGGTTTTTCTGTGCTCGGGGTTTATTCTGCGCGCCG

CGTGCTGTGTGTTTTAGAACCTAAAGATTAAATACTGAGTGGGTTTAGCGCGCGCTCCGA

GCTGCCAGGACACACCGGCTCCCCTACTAAGTGTTTTGAAGAGCTGAACATCATTGATTC

ACAGTTTGGCAGTAGGTTTGAGACCCTCCTCATGCAGAACCACGGGAAATGAATAAACCG

CCTCAGCCTATCACAGGACCGCCCTCTGTCCCTCACCCTGCCCCCTCACCTGGATTGACG

TCGTCGTATTATCCCCGGGGAAGTTTGCAGAGTAACGCTCCACGAGTGGCAGCAAGTAAC

CCGCCACGCCCTGTCCCGCCCCCTCACGTCTACCAATCGTCTTCACAGATGGTGATGATC

CCGCAGCAGCAGCTCCCGTTTCCTACCAACTCTCAAGGTCACGCCTATTTCATTCAGTCG

GGACAGACGCAGCAGTATTCAGTGGCTAGCGCACCAGCAGGCTTTTACCCTGGAACCAAC

CCAGACTACAGCTCCACATACGCTGGAGCATATTATCCTGCTCAAGCTCAGTACCAACCC

TCCGTGCCCACCGCACCCGTTATGATGAACCCTACCCAGCAGCAGCAGGCTCCGCCCCCT

CCACAGCAGCCCCTGCCCCAACAGCACAAAAGGGAGCGCAAACAGATTAGAATACGAGAC

CCTAATCAGGGAGGACGGGACATCACAGAAGAGATCATGTCTGGAGGGAGGACCATGTCC

ACACCTACGCCTCCACATTCTGTAGGTACTGAAGTTCAGACAAATGGCGAAAGTGTGACC

CCTGCAGCTCCTGTGGTCCAACTCGATGATCATGTAAAGTCTTCAAGTCCTGCCTTGACC

CCGCCGCCTTGTTCAAATCCTTCTCCTGAGCCTCAACATGTCCCGGATGTCCTCGCTGAT

CCGGTGGTTCCTGTGCCTCCAGGGCAAAGTGAAGACTCCATTTCTAACTCGTCTCCAGCT

CTCCCCACAGAGCCTGTGCCTAGTGATGTTGCAGATGCTCCTGAGACCACACCCCAAAAG

CAGGAAAAGGAGGAGCTTAGCACCACTGTTACTGAACAGTTGTGTGAGAATACAGAGCCT

GAAAAGGAGCTGGAGCCCAGCTCAAAAAAGTTGTTTGCTGACTCTAACTCTGACATCATG

CCTGCTGAGGAGGACACAAGACCTGCAGAAGAAGCTAATGGGGAGACTGAATTGTACGAG

AAAACACCTGCCTTACCACTTACAGCCATGCCTGTACCTGAGACCAAGATGGTCCCGGTG

CTCAATGGTCTGCCTCGGGAGCATGAAGATCTGCAAGACGAGAAAATTGAGGCAGAAGCT

GAACTAATTCCTGAGCTTTCAGAGTTTCAGGAAGTTACTGTAAAAACCTCTGCAGAAAAG

GAGGAGAGAGCGAAGGAAGAGCTCACTCCTGCTGATGAGATGACTATGCAAGCTGTTCTG

TCTGTGCCAAAGAAGAAAAAGCTGAAAAAAAGCGATCTGAATAATAAAGTAGTTGGAGAC

GTGTTGGATGCCTATAAAGAGCCGGAAGAAAAGGACCTTATTCCTGAGCCTGCTCCCATC

GAAGTCCAGACCTCTGAGCCTCTCCCTGCCACTTCCCCCTTACCTGAGGACACTGAGGAG

ACGTGGGAGGAAAAAGAGGACAAAATGGACACTGAGAACATTGAGCCGGACATGGCAAAA

TCTGTGGAGCAGAAATACCAGTATAAAGAGGAGCACTGGAAACCAATTAATCCGGAAGAG

AAGAAGAGGTACGATCGGGAATTCCTGCTGGGCTTCCAGTTTATCAGTGCCAGCATGCAC

AAACCCGAGGGCCTGCCGCACATCAGCGATGTCGTTCTGGACAAGGCTAATAAGACCCCA

CTGCGTCCTTTGGACCCAAGTCGTCTGATTAATTGTGGTCCTGATTTCACACCTTCATTT

GCTAATCTTGGCAGAGCGCCCCCTGGAGCTGGAAGAGGGCCTCCTCCAGGGCCACGTCGA

TCTCAGCAGGGTCAGCGTAAAGAGCCTCGAAAGATTATTGCCAGCGTTTCTCTGAACGAC

GACGTGCAACTGAACAAAGCTGAGAAAGCCTGGAAGCCAGCAGTGAAGAAGAACCGTGGC

GATGAGGACGACCCTGAATCGCTCAAGACCCAGGATCTGTTCCGGCGAGTGCGCAGCATC

CTGAACAAACTGACACCACAGATGTTTCAGCAGCTCATGAAGCAGGTCACAGAGCTGACC

ATTGACACAGAGGAAAGACTTAAAGGAGTCATTGACCTCATCTTCGAAAAAGCCATTTCC

GAACCCAACTTTTCTGTTGCTTATGCCAACATGTGCCGCTGCCTTATGGGGCTGAAAGTT

CCCACTTCAGACAAACCGGGAGCCACTGTGAATTTCCGCAAACTGCTGCTGAATCGCTGC

CAGAAAGAGTTTGAGAAGGACAAAGATGACGACGAGATCTTTGAGCAGAAGCAGAAGGAG

CTGGACGCTGCTACAGAGGAGGAAGTCCAGCAGCGGTTGAGGGAAGAGCTGGAGGAAGCA

AAGGATCAGGCCCGCCGCCGTTCCCTTGGCAACATCAAGTTTATCGGGGAGCTGTTCAAG

CTGAAGATGCTGACTGAGCCAATTATGCATGACTGCATTGTGAAGCTGCTAAAGAACCAC

GATGAGGAGAGTCTCGAATGTCTCTGCAGGCTTCTGTCTACTATCGGCAAGGACCTGGAC

TTCGAAAAGGCCAAGCCCCGTATGGACCAGTACTTCCACCAAATGGAGAAAATAATAAAG

GAGAGAAAAACTTCTTCAAGGATCCGCTTTATGCTGCAGGACGTGTTGGACCTGAGAAGA

AATAACTGGGTTCCCCGGCGAGGTGACCAGGGACCTAAAACCATTGACCAGATCCATAAA

GAGGCAGAACTGGAGGAGCACCGTGAGCAGATTAAAGTGCAGCAGCAGCTTCTTTCCAAG

AAAGACTCGACTCGAGGAAGAGGAAGCAGGATGAATCCGCCACAGGACGAGGGTTGGAAC

ACCGTACCCATTAGCACCAAGAACCGGCCCATTGACACGTCCCGCCTCAGCAAAATCACC

AAGCCTGGTGCTTTGGACTTCAACAATCAGCTTCTTGCCCCTGGTGGAAAGGGCTCATGG

GGCAGTTGGGGTAAAGGAAGCAGTGGTGGAACTGGAGCCAAAACCAGCACTGAACAAGAT

GCAGGAAGGCCGGCTACCAGCACCCTAAACCGATTCTCAGCTCTTCAGCAGTCAGGACCT

TCATCCACATCTTCCTCATCCTCACTGGACTCAGATCGCAGAGTACCTCAAAGGAGTAGC

TCAAGTCGAGACCGTGGTGAGCGAGACCGCGACAGGTTCGATCGCTTTGATCGCCGGGAC

AGCCGTGAGGATCGAGACAGAGGGCTGGACAGAGCTCGTGTGCCTGTTACTAAACGCAGC

TTCAGCAGGGAGAGCGAGGACCGGGCTCGAGGTGGAGATGTGCGGCGTGTCTCCAGCATG

ACGGATGAGAGGGAACGTGGGAGCAGAGATCGAACCGCCAGTCGAGAGGCAGCAGTGAAG

CGTGAAGCAGCTCCAACACCTGCCCTGATTAAACCATCCCTGAGCGAGGAGGAAGTGGAG

AAGAAATCCACCGCCATTGTGGAGGAGTACCTGCACATCAATGACATGAAGGAGGCAGTG

CAGTGCGTGAAGGAGCTGAACAGCTCGTCTTTGCTCTTCGTGTTCGTTCGTTGTGCCGTG

GAGTCGACGCTCGAGCGCAGCACCATTGCCAGAGAGCACATGGGCCTTCTACTGCAGAAA

TTAATTAGCGCTGCCATTTTACCCCCCGAGCAGTACTTCAAGGGGCTTCAAGAGATTCTG

GAGGTGGCTGATGACATGGCCATAGACATTCCCCACATCTGGCTGTATCTGGCAGAGATC

ACCACCCCAATGCTGCAGGAGGGAGGAATCCCCATGGGGCTGCTCTTCAGAGAGGTGGCA

AAGCCATTGCTGCCAATAGGAAAAGCTGCTGTGCTGCTGGTTCACATCCTCAACCTGCTC

TCTAAAGGACTGAGTCACAAAAAGGTTGGTGCCCTGTGGATTGATGCCGGACTCAACTGG

AAGGACTTTCTACCTGCTGATGAGGATGTCAACAAGTTTGTGACTGAACAGAAAGTGGAG

TTTACGCTGGGAGAGGAGTCTGAAAAGCCAAGTAAAAAGGAGCTAAGTGCAGAAGAGCTA

AGCAGAAACCTGGACCGTCTGATTCAAGACAAGGCCAGTAACCAGAGGATCCATGACTGG

TTGGAGGCCAATCTTGACGAGCTTCAGATGACCTCTAGCTTGTTTATCCGAGCACTAATG

ACGTCCGTGTGCCAGGCGGCGATCATCTGTGAGAACCCATACAAGGTGGATGCAGAGCAG

GTTACCCAGAGGGCCAAGCTGCTTCAGAAGTATTTAATGGATGAGCAGAAGGAGCTGCAG

GCGCTTTATGCTCTCCAGGCTCTCATGGTGCGACTGGAGCAACCAGCAAATTTGCTGCGG

ATGTTCTTCGATACCTTATACGACGAAGACGTTATCAAGGAGGAAGCGTTTTACAAGTGG

GAGTCAAGTAAAGACCCGGCTGAGCAAATGGGCAAAGGTGTGGCTCTTAAATCAGTCACG

GCATTCTTCGTCTGGCTCCGGGAGGCCGAGGACGAGTCTGATAACAGCTAAAAAATAATA

ATAATCTTAAAAAAAAAAAAAACTTGCAGCACAGGAGTGGTACTGTGCTGTGTGGAAAGA

CTATAAACTGAATTTTGAAACTCCATTAAAAACTCAAGTCAATGGACATGACTACAATCA

CTTCAAATTATTTTCCTCTCACTTCTGAAAAAAAATTATTAAAATAAAGGGTTTTTTTGT

TTCTTCCAAGGGAGGAAACGCAAATGATATCAATTGCTTAATGGCCGTGTGCCTCGGCTG

TAGTGGACGAGAATTTTTTTTTTTTGTTTGTTTTTTTTTCTCTTTTCTTCCCCATTAAAA

GTACATGACCCCGCCCC

>Unigene000263

TCATCATCATCATCATCATGAAGGCGGCTTGGGGACCCCCGTTTAACGACCTGCGATATT

TTTACTGTGTGTAAAGTCTAAAAAAAATCACAACAGTTTATGAAACTGGACGTGGTGCTT

AACTAAACATTTCGCCCTGCAGAATGTCCAAGATCAGGCGGAAGGTGACAGTGGAAAACT

CGAAGACTATCTCAGAGAGCAGCAGCAGCAGTACCACCACTCCATCCCGCAGACCCAGCG

TCTTCGAGAGACTCGGACCCAGCACGGGCAGTAATGCCACAGAGACTCAGTGTAGAAATT

GGTTAAAGACTGGAAATTGCAGTTACGGCAACACTTGTCGCTACACGCATGGAACTCCAG

CAAGAGGCAAGAATTTTTCTGGAGCGTTCAGCAGGTCATCCGAGAGGCTTACTGGTGATC

TCCGGGAGAGGATGAAAAATAAGAGACAGGATGTAGATACAGACACTCAGAAAAGAGATC

AAGACGAACCTACTTCACCCACAACTAGACAGAAAGATTCATCTCGTAGCAGACATAGAG

AGAAACAGGATATCAAGATTACTAAAGAGCGAACGCCAGCCAGCGAAGAAGAAGCAGCAG

ACTGGGAAACCACTCGCGAAGACTCTGATAACGGTGACTACGATTACGAGTTATCCCTGG

AAATGAAACGGCAGAAAATCCAAAACGAGCTGATGAAGCTCGAGCAGGAGAACATGGACA

AAAAAGAGGAGCTCCTTATTAAGAAGGAGGAATCGGTGGTAACCAAGACCAGAACCAGTA

GCAGCACGTCTAAGGATAAGGGGTCTCCTGATCACTCCAGCTCCAAGAATTCACCGCTGT

CCCGTAAGTCCAGCGGCTCCCCCAAAAGAAAATCCAGCGGATCTCCCAAAAGAAAATCCC

TCGGTAAAGGCAGTGGCTCTGGCAAGAAGGAGAAGAAATCCTCCTCTTCCTCAGTCATCT

CCTCCCCGGTGGCAGAGCAGAGCAAAACTGAGGAGGCTGACAGCAGATCATCTAAGAGTG

GACACAGCAAGAAGAAAGGACCACGCACGCCCAGCCCCCCACCGCCTGTGCCCTTGGACC

TGCCCGTAGGGGGGAAGAAGCACAAGGGCAAACACAAAAACAAAGAGAAAAGTGAGGACA

AGCAAAAAGAGGCTAAGGAGCGAGGCAGGGATACTGATAAGCACAAGGAAAAGAAAGAGA

AGCGCAGGGATCGGTCTGACAGTTCCCACAAGGCTAAGCGATCCGTGACGTCAGAGGAAC

GTTCTGAGAGCGTGTCGTCTCCTGGGCGAGAGGTGTCTCCCCAGACCAGAAGGAAGTCCT

TGTCTCCCAAAGTGGCCACCCAAAAACAAAGCTCGCCACAAAGGTCTCGCACCCCACCAC

GTCACCAACGGAGCCCCTCTCCTATGACCCGCCATCACCATTCTCCCTCATCTCACTCTG

GGTCTTCTCAACAACGTCACTCTCTATCTCCTCGCCGACGTCGTGGTTCCACCTCCCCGT

CCTACCAGCGGGACCTGCTTCCCCACTTGGCATCCTCTCCCTCAGCTGCTCAGACCTCAC

GTCGCTCACGTTCCCGTTCGTCCCCTACCCCGCAAAGGTCAAGCCCTGCCTCACGTCACC

ACTCACAGGGCCGGGATCGGGAAAAAGAGAGAGGACGGGGAGAGAGGGAGAGAAGCCCCG

CCCCCGACAAACGTCATGAGCGTAGAGATGAAAGCCGCGGGAAGCGTGAGAAAGACAGCG

GACGGGATGATCGCAACTTTGAGGCTGATCAAAGCTCCTCAAGAGAGGAGCGAGAAAGCC

GTGACCGAGACCGTCGCTTAGATCGGGACCGCCGGGACACTCGTGATGACCGACAACACC

GCAGCATTGATGCAAAGGATGTTAGGGACACCAGAGACCGTTGCGGCCGTGAGTCAGTGG

AACGCAGAGAGAGAGACCGGGATAGGGAGCGGGACCGTGAGAGAGAGAGGGACAGAGACA

AAGAAAGAAACGATGCCCACAGGAAAGATGACACGTCCGTGCAGGAAGAGAAAAGTTACG

GAAGAGGACATGGCAGAGAAGAGGGTCGGAGCGAGAACAGGGCAGAAAGCAGGAACGAAT

CGCGGGCCGACAGAATGGGACGAGGAAGAGGACGTGCAGCAGACAACCCTGAGAAAGATC

AGAGAGCATCCTCTCTAGGTCGCCACCAAGGACGCAATGAGGACACCGATCGCGAGGAGA

GGAGAGATGAGAGAAGGAGCGACCGGGGAGAAGACAGACGCGAGGACAGAGGGCGTGAGC

GAGAGGGCCGTGAGCGAGATAGAGAAAGGGACAGGGAGAGAGAGCGGGAGCGGGAGCGAG

AAAGAGAGAAAGAGAGGGAGAGAGAGGCTGAGAGGGAGAGAGAAAGAGCCAGAGAGAGAG

AACGTGAGAGAGAACGAGAAAGGGAGAGGGAGCAAAGGGAAAGAGAGCGGGAACGTGAAA

GGGAAGAACGAGAGCGAGAGCGTATGGAGAGAGAGCGTGAACGGGAGAGAGAGGAGCGTG

AACGAGAAAGAGAACGGGAAAGGAAAGAAAGAGAACGGGAGAGGGAGAGGCAGAGAGAAT

GGGAGGAGAGAGAGAAGAGCAGGGACGAAAGAAGGGATAGAAGAGAGGATGCACGAGACG

AACGCTCGACTAGAGATACTCATGAAGAGCGAAAGAATACTGGGCGCAAGAGGCACCGTG

TGGAGAACACTCCCAGCCCCCGGCCCTCTCCTAAAAAAGTGCGGGAAGGAAGTCCTGCAG

ACAGTGATGGCTACAACAGTGCAGAGGAAAAAGGTGATAAACACAGGCTGCTGAGTCAGG

TTGTCCGTCCTCAGGAGCCAAGCCGCTCCCCTCCCCGTGTATCTACCGACGACAAGCCCA

CGCGCTGGAGAGACAATGAACGTCGCGGTGACAAGAAGGACGGACGAAGCCGGCATGAAG

AAGCAGAGCCTCGCAATGAGCGAGCTGCAGGTCGAGTGGCGGAGCGACGTGGGGATCTTC

CTTCTGAGAGTTCACTAAATACGCCAGCAACTGAGTCTCGAAGAGGTAAAGAACAGAGGG

ATGCCATCGTGCCACCTGCCTCCTCACCTAGTGGCCCTGACGGACGAGATTCTGCATTGG

CCGGTCATGAAGAAGGCAAAAAGAAAACTAAGGCTCAGAAAAAGAACCTGAAGAAAAACC

GGAAAGAGGATGAGGGCCCTAGCACTGGTGGGGACCGTTTTAACCCTGAGCCCCTGCCGT

TTTCTTCCTCCTCAGTACCTTCTGAAGCTCCACGCCCACTTCTCTCACCTCCTAAAATGG

CTAAAAAGAAATTACTGGAGCGGAGGCAGAAGCGATCCCGGGGCGCTGAATCTGACCCTT

CAGAAGATGATCAACCCATCAGCAAAAGACGTCGTGGCCCTCAGACACCTCCACCGATCA

ACAAGGAGGACTTGCCTTCCCAGCGAGCGCTGATGTCTGGGCATGCTGTAGCCGCACCAC

TCAAAATGGACACTAATTTTAGTGACTGGTCTGATGAGGAGGTTCCAGAACGCAGCGAGG

CTGTTTCTGAGCGACCACCAGAGAGACTGCTGCCTCGCAGAAATCCACCTCTAGCACCAC

TGCTTCCTGACCCACCCATGATGCATGCACTCCCACTACAACCACTACTTCCACAGCACA

TGTTGCGAAAATCACAGCAGGCCGAACAAAGGCAGCAACGCAGTAGCAGCATGGGTAGCA

ACCCAAGTCGAGCAACCTCACGCCGACTACGTTCACCCTCCAATGACTCAACTCAACGCA

CAGATGATACCCAGCAGGGGCCACGATCACGCAGGGGACGCGGACAGGGTGCCAACTCCC

GTGACAGAGAGAGGGACAGAGAAAGGGAGAGGGATAGGGCAGCAGCCCCTGAGCCAATCG

GAGAAAGGGAGGAGAGAAAGTCCAGGATTGACCAGCTGAGAAGAGGAGAGCCGAGTCGCA

GTACTTCATCAGACCGTCAGGACTCTCGTAGTCACAGTTCTCGTCGCAGCTCTCCAGAGT

CAGAGCGGCAAGGTCGCTCTCGGGCCGGCTCGCATGACAGCCGGGACCGTGACCGTGACC

AATTTGACCGGGACAGAAAGGACTACCGCCAACCACAGAACCAGCCACTATCACAGCAAC

GTGATTGGGAGCCAGAACCCCGTGAGTGGGGAGTCCGAGGTCGTGAACCCCTCCTTCTCC

GCCCTAATCGTGAGCCCATCAGAGAAAGAGACTTGCGTGAAATGCGCGAGAGAGAGAGAC

TGCTGCCCGAAGGACTGCTTTCACAGGAACGCCTTGAACGTGATCGTGTTGATAGGGAGA

GAGAGCGGGACAGAGACAGGGGGGAACGAGAGCGTGTGATTCCCTTTGACCCCCCGTCAT

TAGGGGAACTCAAGAGTCGAGCAGAAATGCGAAAGGAGCGAGGAGACTATGAGCCCCTGC

TGCCTCGAGAAGCTCTGGTTGACATGGACAAACCAAGTGCTACCAACGAGCCACATGTCC

AAATGGAACCTTGTGAGACCGAGAAGATAGACAGCTTTGATGAAGATGAAGGAAAGGGTG

AGGATGCCCAGTCTGCAGTTTCGGCTGGGGAGGAATACGAGCCTATCAGTGATGACGAGC

TGGATGAGATTTTGGCTGACAGCCAAAAAAGAGATGATCAGCAAGATGAAGAAAAAATTC

CAGGTCCCCTGGATGTGATTGATGTAGACTGGTCCAGTCTTATGCCAAAGCAGAAACAAG

AACCCCGAGCACCTGGGGCCGCACTTCTTAGATTTACGCCCGGAGCCATGCTGCTGCGAT

CTGGCGTCTCTCGTCGGCTGGCCGGTCCGCACCTCCTCACCCGTGTTAAAGACATCTGTG

CTAAAGAGGTGGAGGATGCTAAAGATGCAGAAAAGCTGTTTGAGCATGACCTTGGTGCTC

TGAACATGGCTGCCTACAACCGGAGGACAGAAAGAGCAAACCTGCTGAGAAACCTGGGGC

CATGTGGAAAGGCTCTCTGTGCACGTCAGGACATCGCCATCCGCAGGCAACTTCTCAAAA

ACGACAAGGGGACAACTAAGCAGATCTACACTAGCGCTCCAGTGGTGGACCAGGAACTGC

TGCAACTCAGCATACGTCTGTTCAAGAAGAAAATGGCCGCCGGGCAAGAGAAATCGGATG

CGCTCGGGACTAACACCCCAGCTGATCCAACCGAGCCCAGCATGTGGAAACTGGACACCC

GGACTCGGCCATCTTGCGCTGTGAAAAACTGAAACTGTTTGTAAGTCAGTGCTAAAGGAG

AATTAGCCACACTGGCTTAAGAGATCAGGATCATAGAGACTGGAAAACTGCTAGCCTATG

AAGACACCACCAGTG

>Unigene000264

GAAAAAGAAGAAAGGCTTGAATGCTACGGTACTTCTTTTCCAGATGGCAGAAGGGTAAAA

ACCTGAAACAGGCTCAGGGATTAGCAGCATAAACATGGACACCACAAGCTCTCTGAAGAT

GACTGCTGTGGCCTCCCAGAGCATCTTCAGTTACGTGGACGAGGAGAACCTGCCTGCAGT

AAAAGCTTACCTGGACAAACACCGTGACGTGGACAGCAGGAGTGATAATGGACAGACTCC

TCTCATGTTGGCAGCTGAGCAGGGCAGCGTTGAGATTGTGCAGGAGCTTATAAGAAGAGG

AGCCAATGTTAACCTGGATGATGTGGACTGCTGGACTGCGCTGATTTCCGCCGCCAAGGA

AGGCCATGTCGAAGTGGTAAAGGAGCTGCTGGCCAACAATGCGAACCTGGAGCATAGAGA

TATGGGCGGCTGGACCGCTCTTATGTGGGCAGCCTATAAGGGCCGTGTGGAAGTGGCTCG

CCTTCTGCTGGAGAAAGGAGCAAACCCGAACATCACTGGACAGTACGGCGTATACCCCAT

TATTTGGGCAGCAGGCCGAGGTCATGCCGAAATCGTTCGTCTTCTACTGGAACACGGTGC

TAAAGTCAACTGCTCTGACAAGTACGGCACCACAGCATTGATCTGGGCAGCGAGGAAAGG

TCATTACGACTGCGTGATGCACTTGCTGGAGAATGGCGCAAACGTAGACCAGGAAGGCGC

GAACTCAATGACAGCTCTAATAGTGGCGGTACGTGGAGGTTACACAGAGGTGGTGAAAGA

GCTCCTTAAGAGAAACCCCAACGTCAACATGACCGATAAAGACGGAAACACAGCGCTGAT

GATCGCTGCCATGGAGGGTCACACAGAGATAGTGCAGGATCTACTGGATGCAGACACCTA

CGTTAACATTCCCGACAGGAGTGGTGACACAGTACTAATTGCAGCAGTAAGAGGAGGTCA

TGTGGAGATTGTCCGGGCTTTGCTGAATAAATATGCTGACATTGATGTTAAGGGCCAGGA

CAGCAAGACTGCCCTCTACTGGTCAGTAGAAAAAGGCAATGCCACCATGGTACGAGATAT

CCTGCAGTGTAACCCTGACACAGAGGCTTACACTAAGGAGGGGGATACACCACTTATTAA

AGCTACCAAAATGAGAAACATTGAAATTGTCGAGCTGCTTCTTGACAAAGGAGCAAAGGT

GTCTGCTGTGGACAAGAAAGGAGACACTGCGCTGCACATTGCTATCAGGGGTCGAAGCAG

AAAATTGGCCGAGCTGCTCTTAAGGAACCCTAAAGATGGCCGCCTTCTGTACAAGCCCAA

CAAACAGGGAGAGACGCCCTACAACATCGACTGCACACACCAGAAGAGCATTCTCACCCA

GATTTTTGGTGCCAAGCACTTGTCCCCTACGGAGTGTGACGGTGACATGCTGGGTTACGA

CCTGTACAGCAGTGCTCTGGCTGACATCCTTAGCGAGCCCACCATGCAGCCGCCCATCTG

TGTAGGTCTATATGCTCAGTGGGGCAGTGGCAAATCTTTCCTGCTCAAGAAGCTGGAGGA

TGAAATGAAGACGTTTGCAGGCCAGCAGGTGGAGCCATTGTTTCAGTTCTCATGGCTGGT

GGTGGTTCTGACTCTGCTGATTTGCGGCTCTTTAGCTGTGATCCTCGGCTTCTCTATCGA

TCCCAGACTGGCCATCGCTGTGGCCCTCAGCCTGCTTGCCCTGCTCTACATATTCTTTGT

GCTGGTGTATTTTGGTGGCCGCAAAGAGGGCGAGAGCTGGAGCTGGGCATGGCTCATCAG

CACCAGGCTTGCACGCCACATCGGCTACCTTGAGCTGCTGCTTAAACTTATGTTTGTGAA

CCCGCCAGAGCTTCCTGAGCAGACGACCAAGGCCCTACCTGTCAGATTTCTGTTTACAGA

CTATAACCGCCTGTCGAGTGTGGGTGGGGAGACCTCTATGGCGGAGATGATTGCCACACT

GTCAGATGCATGTGAAAGAGAATTCGGTTTCTTGGCCACTAGACTCTTCCGAGTCTTTAA

AAACGATGAGACACAAGGAAAGAAGTGGAAAAAGACGTGCTGTGTTCCATCCTTCGTTCT

GTTTGCACTGACTCTGGCATGCCTGGTGACGGGTATGGCCCTCTTGGCTATATTTAAAGT

AGATTCCGATAACGTGACTGTAAAAGCAGTGCTAGTTGCCGTGGCCAGTGTGGTGGGCCT

CGCTCTGCTGCTCAACTGCAGGACCTGGTGGCAGGTGGCCGACTCTGTGCTGCGCTCGCA

GAGAAGGAGGCTTCACAGCGCCGCCAACAGCATGCACAAGCTGAAGAGCGAAGGATTCAT

GAAGGTGCTCAAATGTGAAGTGGAGCTTATGGCAAAAATGGCAAAGACTATCGATGGCTT

TACTCAGAATCAGACTCGTCTGGTCGTCATCATCGATGGCCTGGACTCCTGTGAACAAGA

TAAAGTGCTGCAGATGCTGGATACGGTGAGAATTCTCTTCTCCAAGGGCCCCTTCATTTC

CATCTTTGCCAGCGACCCCCACATCATTATAAAAGCTATAAACCAGAACCTGAACAGCGT

GCTGCGTGATTCCAACATCAATGGCCACGACTACATGCGCAACATAGTGCACCTGCCCGT

CTTTCTAAACAGCCGGGGCCTCAGCACGGCCAAGAAGCTCTCCATGGCCGCGCCAACCAA

TGGAGATCTGATGGCGGCTGAGGCATGGCATGAGGAGGCAGACAGAAAGTGTCAGTCGCA

GAACAGTCTTGGAGAACAGGCCAAGCAGGGCAGTAAATTGACACTGAACCGGAGGGATAC

ATACCGGAGGCGTCAGATGCAGCGAACAGTCACCCGTCAGATGTCTTTTGACCTGACCAA

GCTGCTGCTTACTGAGGACTGGTTTAGTGACATCAGCCCTCAGACCATGAGGAGACTCCT

GAACATCGTATCAGTAACAGGGCGCCTCCTAAGAGCCCATCAGATCATTTTTAACTGGGA

CCGACTGGCATCATGGATCAACTTAACGGAGGAGTGGCCATATCGGACGTCCTGGCTCAT

CCTCTTCCTGGAGGAGAACGACGTGTTCTCGGACCAGATCACACTTAAAACCATCTACGA

GAGGATTTCTAAAAATATCCCCACCACCAAAGACGTGGAACCCCTTCTGGAAATCGACGG

AGACATACGCAGCTTCGAAGTGTTCCTGTCCTCCCGAACCCCCGTGCTTACAGTTCATGA

TGTGGAGATGTTCCTACCCTGCACCGTCAACCTGGACCCTAAACTTCGAGAGATTATTGC

AGATGTGCGCGATGCCCGGGAGCAGATGCATCTCGGAGGAGTGAGTTATCCCACAATGCA

CCTACAGGATGCTCCGGCGAGAGCACAGTCAGTGTTTGGTCAGGGTCCCGTAGCCTGTTC

ACCCACCGGCTCCTTTCCTGCACAAACACACAGCAGCTATTTCAGCGGTCTTACTGGCCC

ACAGCACCCCTTCTACAACCGGCCATATTTCCCTCCTCATGTCTATCACGTGCCCCGTCA

TTATCCCGGCAACCCCCACCATGCCCCCTCCCGCCCTTCCTCCAAACTGGCCTTCTATGA

GGACCACAATGCTGGACTAGGTGTTATCAGAGAAGATGCCTTCGAAGGTTCCTACCCCAA

TATGGGCTCTGATTCTGCAGCACCCCCCGGGTTCCTTAGTGCTCTTAGCACCGAAGGTGT

ATGCGAAAGACTGCGACAAATTGAAGGCCTGGACTCTAGCATGATAGAGCAGTACATCAG

CACCATCAAGAAGGCGAATGTGAACGGTCGTGTCCTGTGCCAGTGTCAGCTCGAAGAACT

GAAGAAGGAGATGGACATGAATTTTGGAGACTGGCATCTCTTTAAAGCTATGGTGCTGGA

GCAGCGGCAGGTGGAAAGCCAGCTGCTGCAGGAGGAGTGTGTAAAGGTTAGTGAACAGGG

CAGCAGTGCATCGGTTCAACCTGAAGTGCCTGCTGTCTTTTCTACAACAGCCCCCCACAG

CAATCTACAGTACAGCCTGAACCTCAGCTTCGAAGAGCTCAGCACTATTGGACTGGAGGA

GCCCATTAGACACCCCATCAACCACTGGATGGCCGTGACTCACAGGACTTCCAGCATGTC

CAGTCTAAATTCTCAGGAGTCATCCAATGACATTAGTAAGTTGACTGACAGGCAGCAAGC

TGAGTACCGCAGTGCTTACCAGGACTACATCGCTCACATGGCCCAGCTGGAGGCAGGCAG

CTCTGCAGCTGAAAGGTCAGAGGACCGTCCCAAGGATGGCGAGCAGGAAGGACGCAAACC

CTTCAAGAGAAACTCTAAAGCAGGAGAAAGTGTGATGGACTACTCCACTACCCAGGACTC

CTGTCTTCTTCATCCAATCATGGAGGAGGACGAGAAACAAGAGCGTAAACCCACCGTGAG

CCGCACTAAGGGACCAGGACCTCGCTACCACAGCGTACCCAATGAAGAGGATGAGGAAGA

GGATTTGCCCGTGGAGACCGATGCCACACCCCTTCTCTGGGAAGAGCAGGAACACGGGGT

GCAGAGAAGGAGCTTTGTTTCTGCAGGGATAGACAAAAAAGAGTCGTCTGACTCAGGCAT

GAGGTCAAGCGGCAGCTCCTCTGATCATTCACTACAGGACGAGGAGAGCGAAGAGGCTCA

GGAAGAAGATGAAAAGTCAAAAGAGGAGGATCTTGTGACCACGATACGCATGTCCATCTG

CTCAGAAGCTCTGTCTGATGCCAGCAGCCCTGAAGAGAACTGGGACTCCCCACACATACA

CACTCAGGCTGTCACCAACCTGAACCAGGCAGCCAGCAACAACTCGGCCCTTAGCAACAA

CAATAACGGAGACGAGCGCAGCCTCATCATCTCTCCAGGATCGAGCACCGGCACCAACAC

TCTGCTCAATCAAAACCTGCTTACAGTGAGAAGGAAGAAAGGAGCAAATACTACACATGA

TGAGGGCAGTGAGGAACGAGAGAGTGTGCTGTAAAAAGCAAACTTTCCTTTAAATATACT

ACATTTAATATGGTACAAAGAAAGAACTGGTCTTTCAACTTTTAGTTTATGGTAGATGTG

AAACTGTGCAGAGTGGATGTGTAGAGTGATTAGGTTTATGCATTTCATGGAAAATTTATA

TATAAAAAAAAAGCCTTTTAAAGCCAATCGTGTGCATTACAATGCAATCATCAAATCATT

TGGGATCGTCGCTGAAGTAGCCGGGATCGGGATCGGGATCTGTTACATGTGGAGTTGTGT

GTAATTTACATTAGGTCAGGAACAAAACTCTTCCTATTAGAGGAAAATAGTTTTTAAACT

GGCAA

>Unigene000265

GGACAGGAATGAAAAGTGAAACTCCCAGGCGCCACTTTTGCTGCTTATTACACCAGCTGC

GGCTTTCGTTTGTCAAGAACAGTATAAATGCATTTAATAGTCCTAGCTGGACAGTATTTA

ATAGTCCCAGCTGGACAGTATTTAATAGTCCTAGCTGGACATTTTGGGTTGTATTAGCTG

TATTTTCTGCTTGTTTTTGTATTGTTTGGCCGCCGTTTGGATTAAACTAGCTGGGCGTTT

CAGCTCATAGTTATTGAGTACTTTTTTTAATTTGTGCCGATTTTCAGCCCGCTTGAGGAG

ACGGTAACGTTTGTGTGACTTTTTTTTGCTTATTATTTTTAACTCCAGCATTTCCGGTTA

AGCAGTGCTGTACACGTGTCCAACTCGGTATTTTGTTGCTGTCTTGCTGTGTGTACCTAC

AATCATCCATAGCCAGCAGGCAGTGAGCTCATTTGTCCTCCACCCCTGCGCTATGAGCAG

AGGTAGGGGAGGGCACTCCAAAGAGTACCAGAGGTTTTACCTTTCACCCCACCAGAGCAA

ATCCAACCAAGGATTCTACTCCCAACCTTTTTCAGCTCACCCTAGAACTCCTCACGCCCC

AAGCCCGAACACTAACTCGGGCAGATACCAGTATCCTCCCCCTTACTCATCCGCTGGCCC

TCCTGGTGCTTCTTCAAGTCCCTCTCTGCCTTCAAATTCTTATACGTCTCGGGTTCCTGG

ACCTTTCCAAGTGCAAGATCAGAGGCCGTCATCGAATAGAGGAGCTTGCGCTGACGAAGC

AGCAGACTTTCGGCAGAAGCAAACTCTGTTCCTTCAGGGGCGGACTGCTGAGGCGCCCAA

ATTTGTGCCAGGCCAGTCCAAACACCACAGCAGTCCTCCCCAGCGTCACACAGAATATTA

TAATTATATAAATAATAGCAGGTATATAAGCAGAGGGGGTTATAGTCAAGGCAGGCCAAG

CTGGGAAAAGGAGTTTAACACACAGTACTACCCTGGATCTAAGTCTGTCTGGGAGCAACA

GCAGTTCAGAAGACAGAATAATCAGTATCATTGTGACACTGCCTCACTATCTTCAGCATT

TCACGGTCTATCAGTAGAGAACTGTAGTTCACATTCGTCCTTCGGTAGTACTGGAAGTAA

GGCGTCTCATACATTTTCCTCGGGTAGATATTCCTTGCAGGTGACAGAGGATATTCAGGA

GAAGGTCCACAATTCTTTGGCCTCATTAGCAGGGTGAAACCCTTCAGGCCAGGGTCTTGG

CTAAAAAACTTCGTTTGCCTAAGAAGATTGTTAACCAAGCACTGTATGCTTTATCCAGAT

CAGGCCAGGCAGTGAGGTTAGAAGGGACTCCTCCTCTCTGGAGACTCTGGAAAGAAGGTG

TCCCTGAATACTCCCAACGAACCTACTTTAGCGGAAATACTGAAAAATACCTGGTCCAGG

ATACATTTATAAAGAAAAACCAGTGTATTGGTGAGGTAAAACCAGACAGCAGCTTTACCA

CTGGAACATCACCCGCTGCAGAGGCCAGTAGTGATTCCAGTGACGAATCTGAAGACTCTG

AAAAAGAAAGTTTCTCTGACCAGGCCTGTGAAAAGGACTCGAGTCTTTCCATGACAATGG

ACGCAAAAGACTGCAAACTGCAAATTATGCAGTACTTGCATGAAACTGGCCAGGCCAGTG

CATTGATGATTGCCAAAAACCTGGGCTTGAGAAATGCCATGCAGGTTAATCCTACATTGT

ATGCCATGGAAAAGCATGGCGATCTCAGTCGCAACGCTGGCGTTATGCCACCCGTTTGGG

AGCTGACTGCTCACCGGCGTGATAGGATGGCCCGACAGCGAAAAGCAGCTGTTGCTGCCG

CTATAATACACAATGAGTCAAAGCTTTGTGACACATCAGGCCCAGCAAAAACTGCACAAA

CAGCCCCAAACACGGTGTATGGATCTGGGTTGTCTGAAGAAGTGATGGCGTGGGAGAACC

ACCCAGCAAGTCAGAAAAGTGCTAGCATGGACAGCACATTTGGGAACGGAATGCTTGATA

AGAAGTCACATTTAGATAGAGACTTTCTTCCTGCTCTTGAAAAAATTTCCTCAAATGATA

AAGACCATACATCTGTCCCCTACTCCACACATGATTCTTCATACTACCTGACACAAGACA

AGAATGGAGGACACTCGGAGTGGGCATCCGATGATATCCCAGAATTCTTAAATGCAATTC

GATCAGAAGTGGCCGTGTCCCTAGCAGCACCTATTCCTCCAGCCCAGAATACAGAGGCCG

TCAGGTTGCAGAAGCTAAGACTGGCACTCAGCAAGAACCCTGTTAGTGGACTGATGGAGT

ACGCACAGTACCTTGGCTACAGCTGTGAATTCCTCCTGCTTGAACAGTCCGGACCCTCAC

ATAACCCCAGGTTTCAAATGCAAGTGATGCTCGATGGACGCAGGTTCCCACCAGCTGAAG

CCTCAAGTAAGAAAGTGGCAAAAAAGGATGCAGCTGCAGAGACTCTTCGGGCCCTCCAAA

GAGAGATAGAAGGAACTACTACTGTAGTAAATAAAGCAGAGCTGCCTGCTGTGCCATTAG

CAGAACTGAATGATTTTGCTGAGGATTGTTTTGACAATATGGATGAACCTCCAAAGGCCC

TGGCTCGCTCCCTTCCAGGCGGCAAAAACCCTGTTTCGGTTCTAATGGAGCACAGCCAAC

GTAGTGGCCATGCCATTGAGTTTATTCATACTGGGCAGGAGGGGCCAGCACATGACCCAC

GCTTCATGTTCCAAGTAAAGGTCGGGGACAGTCTTTACCCGGAGGCATCAGCACCCAGTA

AGAAGGCAGCACGGCAACTGGCGGCAGAGGAAGCTGTGAAGGCTCTTCTGGCAGAAGGAC

AACTACAGCTCAACAAGCCACATCAGGCGTTTTGTCTACTCGGCGAGGGCGATTTGTCCC

AGGCGATGCCCCCATGCCCGTCATTGCCCCCGCTGACAGCAGCTGAGCTTCAGGCAGCAC

ACGAGGCCGGAGTAGGCGACCTGATCAACCATCTAAACAACAACGCTGTGTCAGGCTTGC

TGGAGTACGCACGCTCACGAGGCTTTGCTGCAGAGATTCGCCTGGTGGGCCAGTCAGGAC

TCCAGCATGAGCCACGATTTACATATCAGGCAAAACTGGGGGGTCGCTGGTTTCCTGCGG

TGTGTGCCAGCACCAAAAAGCAGGGCAAGCAGGAAGCAGCAGACGCAGCTCTCCGAGTGT

TGATTGGAGAAGCGGAAAAGGCAGCACGCACCGGGGAGCTCACAGCAGAGTTGCCGGTGA

CGGGTAGCACGGTGCACGATCAGTTTGCCATGCTAAGCCACCAGCGCTTCAATGCGCTTA

CTGCACGCATCCAGCACAGCCTTTTGGGTAGGAAAATCCTTGCCACGATCATCATGAAGA

GAGGTGCTGACAGTCTGGGCACAGTCGTCAGCCTTGGGACAGGAAATCGATGCGTTAAAG

GAGAAGAACTAAGTCTTAAAGGTGATACCGTTAACGACTGCCATGCTGAAATCATTTCCA

GAAGAGGCTTTATCCGCTTTTTGTACGGTGAGTTGATGAAGTACTGGGAAAGACCAGAAG

AGGACAACATATTTGAGCTTGCGGAGGATGGTTTGCTCAAGATTAAAGACGGCATCACTT

TCCATCTGTACATCAGCACCGCTCCATGTGGGGACGGCGCACTGTTCGATAAATCGTGCA

GCGAGGTAGCGGAGGTCATCGGCTCGGCTCACGTGCCCGTGTTTGAAAACGCTAAACAGG

GCAAACTCAGGACCAAAGTGGAAAATGGTGAGGGGACCATCCCAGTGGAGTCGAGTACAA

TTGTGCCTACTTGGGACGGCATCCAGCATGGCGAGCGCCTCAGAACTATGAGCTGTAGTG

ACAAAATTCTGCGCTGGAACGTGCTGGGCCTGCAGGGGGCTCTGCTCAGCCATTTTATGC

ACCCCGTCTACCTTCAGTCTATCACGCTTGGTTACCTGTACAACCACGGCCATTTGACTC

GAGCCGTGTGCTGCCGATTGGCCAGAGAGGGCAGCGAGTTGATGAAAAGCTTGCCTACCC

ACTTTAAACTTTGCCACCCGGAGGTGGGCAGAGTGAGTGTGTACGACTCAACACGTCACA

CAGGAAAGACCAAAGAATCCAGTGTGAACTGGAGCTTGCCAGACCAGCTAAAAGTAGAGA

TACTGGATGGAACCAAGGGCAAAGTAGACAGTCCAAAGCTAGAGGTGTCCCGCGTCTCCA

AGTCCAACCTGTTTCGTCATTTCCGGGACGTGTGCAGGAAAGCCGGTCGGCGCGACTTGC

TGTCTCTGACTTCCTACGCCCATGCTAAGATGGCCGCCCGTTCTTTCCAGCAGGCCAAGG

AACAGTTCTACAAAGCTCTGAGTCAGCTGGGTTACGGAACCTGGATCGGCAGGCCGCAAG

AGGAGAAGAGCTTTGAAGCGTCAGAAAGTGAAGCGCAGAACATTGTTTCAGCTCAGTAAA

AACAAAAGGGAGCAGAAAGAGGAGGGACGGAGGAGTATGGATGAAAAGGACAGTCAGTGT

TTTGAATCTTCTAGGTTGTAGAAGAATGATGGACACAACTTGAAACAGAGAAAGAGAGCG

GGAAGGAAAAAAGCGCAGTAGATCTCTTGGATTACTTCAGCCTGGGTTTGAGGGAGAGAA

AAAAAATAAAATAAATCGATAAAATACCAGCTCATGTATAAGAATGATGTATGGCGCTTT

CTTTGGAAGCCTCTCTAAACTCTGTTATGCACTAAGAGCTCGGGATCTGTCGATCTGTCT

CAGGCTGGTGGGTGCATATAAGCAGAGATTTAAAGAAGGGATGTTGTAAATTTTTATTTT

ACTTGAGATCAACCATGTATTCGTTTCAGTTGGGGAATCAATGGGCTTCAGCACAGATCT

TCCTGTCGCTTTTTTCGATCTCTTTCTTCTATAGAAAACGTTTCCTATTAAGGTTAAACT

AGTGGACTGAGGTGCTTTTTTTTGTCCCATTTCATTCACATTCCTGGTACTAAAATATAA

AACTGTTTTAAATAACAGGATAACTGCAGGTTAGGACTGTTCATTCTTCCCCAGACATCC

ACCCCTTCCTCATATATCTATCTATATTAGCTTGATCTCTTTATCTTAACCAGTCAAAAC

TAATTTGAATTGCTTCTACACAAATGTATACATGATGTACATACAGATACATACGCTCAC

AAATGTATATAAGGTCTCATCACACCGTGTGTGAGTTTATATACGTGTGTGTGTTTATGT

ATATGTATATATTTTGTGTATATATTTTATAAGGTTTCATAAATCTATGTTGCTTTTGGC

CAG

>Unigene000266

GAAACACGGCTCATTACTTCACTTCTGCCCATCAAACCGTCGCGTCGCGTAAATTTATTA

ATGTAAAACAGTTTTTGGTGAAATTAATCGAGTCAAAAGTGACAGAAACTTGAGAGAGTT

TCGAGCGAGTAACAACTGGATTTCATCTCAGCACTGAGTGCCTCTCCATCTTTCCTGCAG

TGAGACGAACTTCAGTGAAAACGCTGCAAATAGAAAATGCTGCAGAAATGATAGCGGCTC

ACGAGTTACACACCGAGTACAACTACCCTCATGAAACAGATACTCAGTTCTCTTCAGATA

CAGACTTGGAGGATCCTGAGGGGCGGAGCCTCAGACCCACTAAAGGAAAGAAGGGTAAGA

AGACAGTGGTGCGTCCTCAGGGTCGTGTAAATGGTCATCATCAGGAGAATGGAACAGAAA

ACCTCACACTGTTTGAGATAGTCAAACTGGGAAAGAGTGCCACACAGTCAGTTGTAGATG

ACTGGATTGAAGCATACAAGACTGATCGAGATGCTGCTCTACTGGACCTCATCAACTTCT

TCATACACTGCTCTGGTTGTAAAGGTGCTGTGAGTTCAGAGATGTTTCGTAACATGCAGA

ACTCTGAGATCATCAGAAGAATGACTGAAGAATTTGATGAGGACAGCGGGGACTATCCCC

TGACAATGGCAGGTCCGGGATGGAAGAAGTTTCGGGGGGGTTTCTGTGAGTTTATCGGGG

TTTTGGTGCGTCAGTGTCAGTACAGCATCATCTATGACGAGTACATGATGGACACAGTTA

TCTCACTTCTCACCGGCCTGTCCGACTCACAGGTCCGAGCCTTCAGACACACCTCCACAC

TCGCAGCGATGAAGCTGATGACAGCATTGGTGAACGTTGCTCTGAATCTGAGCATTAACA

TGGACAACACACAGAGACAATATGAGACTGAGCGCAACAAGAGTCTGGGGAAGAGAGCCA

ACGACCGGCTGGAGTTTTTACTGCAGAAACGCAAGGAGCTCCAGGAGAATCAGGACGAGA

TTGAGAACATGATGAATGCCATTTTTAAAGGCGTGTTCATCCATCGATACAGGGATGCGA

TAGCGGAGATCAGGGCCATCTGTATCGAGGAAATCGGTGTGTGGATGAAGATGTACAGTG

ACGCATTTCTGAACGACAGTTACCTGAAATACGTGGGCTGGACCATGCACGATAAGCAGG

GGGAGGTGCGGCTGAAGTGTCTTACTGCTCTGCAGGGTTTGTACTATAACCGAGAGCTGA

ACTCCAGACTGGAACTCTTTACCAGTCGCTTTAAGGACCGGATTGTGTCCATGGCTCTGG

ATAAAGAGTACGACGTTGCAGTCCAGGCCATTAAACTCCTAACTCTTGTCCTGCAGAGCA

GTGATGAGGTGCTGACGGCGGAGGACTGTGAAAGTGTGTATCACCTGGTTTACTCTGCCC

ACCGCCCCATTGCCGTGGCAGCAGGAGAGTTCCTCTATAAGAAGTTATTCAGTCAGCGGG

GTCCGGAGGAGGAGGGGCTGCCGAGGAGAGGGCGACAGTGTCTTAATGCAAACCTCATCA

AAACCACCGTCTTCTTCTTCCTAGAGAGTGAGCTGCATGAACATGGTGCGTACCTTGTGG

ACAGTCTGTGGGAGTGTGGATCAGAACTGCTGAAGGACTGGGAGAGTTACATCAGCCTCC

TACTGGACGAACCCTTTCCTGGAGAGGAGGCTCTAACAGACAGACAGGAGACGGCGCTGA

TTGAGATCATGCTTTGTGCTGTCAGACAGTCGTGCGAGTGTCACCCACCTATCGGCCGTG

GGACAGGAAAGAGAGTTATGACTGCAAAAGAAAAAAAAACACAGCTGGATGATCGGACCA

AAATTACTGAGATCTTTGCAGTGGCTCTGCCCCTGCTTCTTGCCAAATATTGTGTGGATT

CTGAGAAGGTGACAAACCTTCTGCAGTTACCGCAGTATTTTGATCTGGAGATTTACACCA

CGGGTCGACTGGAAAAGCACCTGGACGCACTTCTCCGTCAGATCCGTGATGTGGTGGAGA

AGCACACGGACACAGGTGTGTTGGAGGCGTGTTCAATGACGTTCCACGCTCTGTGTAATG

AGGAGTTCACCATCTACAATCGGGTGGACATCATGCTGAGTCAGCTGATGGACGAGCAGA

TTGACAAGTTCAACCGCCTGCTGGACGACTTCCTTCGCGAGGGGGAGGAGCCTGATGAAG

ATGATGCCTTTCAAGTTTTATCAACACTCAAGAGAATTACAGCATTTCACAATGCCCATG

ACTTGACTAAGTGGGATCTGTTCAGCTACAACTACAGACTACTTAACACAGGCCTGCAGA

ACGGAGACATGCCAGAACAGATAGTGGTCCATGCTATGCAGTGCACTCATTATGTCGTCC

TGTGGAACTTGGCAAAGGTCTCAGAGGGCAGCTCCACACAGGCTGAACTGGTCACCCTGA

GGAAGCAGACCCGAGCGTTCTGTCTGATGTGTCAGCGGTACCTAAGCAGCCTCAGCATGG

CTGTAAAAGAGCAGGCCTTTACTATCCTGTGCGATGCATTAATGATCTTCAGTCATCAGA

TCATGGCGTCGGGTCGAGACGCTCTGGAGCCGCTGGTGTTCGGTCCTGATTCGTCCCTGC

AAAGTGACCTGCTCAACTTTGTCCTGGATCACGTCTTCATTGAGCAGGACGATGATGGTA

GCACGGATGGACAGCAGGATGATGAAGCAGGGAAGATTGAAGCTCTACATAAACGCCGGA

ACCTACTAGCAGCTTACTGTAAACTAATCATCTACAACGTAGTGGAAATGAGCACCGGAG

CCGACATCTTCAAACAGTACATGAGGTACTACAATGATTACGGTGATATCATAAAGGAGA

CGATGAGTAAGACGAGACAGATCGATAAGATTCAGTGTGCCAAAACTCTCGTCCTCAGTC

TGCAGCAGCTGTTTAATGAGATGCTCGGTGAGCTCGGCTGTAACTTTGATCGCTCATCTG

CGGCGTTCTGCGGGATTAAAGAACTCGCCCGTCGCTTCTCTCTTACGTTCGGTTTAGATC

AGATGAAAACCCGCGAGGCCATCGCTATGCTGCATAAGGATGGTATAGAGTTTGCATTTA

AAGAGCCAAGTCCTCAGGGTGAGGGCAGTCCCCCTCTTAACCTCGCCTTTCTCGACATTC

TCAGTGAGTTCTCCTCCAAACTGCTTCGTCAAGACCGCAGGACTGTACACATGTACCTGG

AGCGCTTCATGACGTTTCAGATGTCCCTGCAGAGGGAGGAGTGTTGGCTGCCTCTCATAT

CCTACAGGAACTCTCTGCAGGCCGGAGCAGACGACGACACGCTGTCTGTCCTCAGCGGCG

TCAGTGGTCGAGGCTCCGCCCGCAGCAGAAAGCCGAGAGTAACCCCCACCCCCTCCAAGA

GGAAGCTGCCTGAGGAGGAGAGTAGCTGTAGCAGCACTGATGCGAGTGTGTGGATGGCTC

GAGAGCAGCAGCACCAGCCACAGACTCCTATGATGATGTCACCACCTTCTATGATGATGT

CATCACCCCATATGGCCTCCACTGTGCTGCGGGACGCCAAGAAGCTCCGCCCAGAAGAGG

GATATGTGGGCGTCTACACCATGAGCAACCAAACACAGCACAATGCAATACAGCAGCCAC

ACACACCAATCGACTACAATACACAGGTTGCATGGATGTTAGCACAGCGGCAACAGCAAG

AAAGAGTGAACATCCAGAACGCTAAGATGAGGAGTCACATGCAGCACACCATTCGGCGAA

GTGCAGGTCTGATGGAAGACGATGAAGAGCCGATTGTTGAAGATGTGATGATGTCATCAG

AGCAGCGGTTAGAGGACCTGAATGAGGGCATGGACTTCGACACCATGGACATCGATCTGC

CAGCGTCTAAAAATCGACGTGAGCGGACCGAGCTTAAACCTGATTACTTTGACCCCGCCT

CCATTATGGACGACTCTGTCCTAAATGTGTCGATGTTCTGATGCCATCACCACGGGAGCA

AGCAGCAGGGTCGGTGCGATTTGCTCACTTCCTGTTGTACATCGACGTGGCGAAGCTACA

CTTCCTATTTCATAACTTTGTGTTGTTGCCATGGGGGCGGAGTCACAGATCTGCCCCACT

TTACATGTTATTTTGTTTTTATAACTTATAGAGAAACTTTTAGATGATTTTATAAAGACA

ATCAGCTGTAAATCAGACGTTTATAAAGCGAGTGAGGATTTAAATCGCCTTTTTACTACA

CTTCTCACACACACTTCACTTTATGGTCCTTTAGCTTTCCAGCCACCGCGCTAAAAACAC

TCCAGGACAAAAGCTGATTGGACATCCGATTCACATTATTATTACAATCAGATTCATCAG

CAACGTTTCCGACACGACCACAACATCTGCAACATCAGCTGTGACGTGTCTGCTGTCTAA

CGCTAATGTAGCTAGCATGTAAGTGGAGTGGATGCCCACCGGTTTAGCAAAAGCTAGTTA

CATTTTTTAGCATCAGACTGAGTGTTGAACTGAGACTAGTTTTTTTAAATATTTATACAT

TTTAATACAGACTTTAAAAAAAGATCATCGCTAATTTAGTGGCTTTTAGTTGTACTTACA

TGGTAGCTACCATATCATTGGATCTCCTCCCAGCTGATGGACTGCGTTTGTTGTTTTGTT

TGTTTGGCATGTGTGTGTGTGTTTTTTTCTTTGATCAATTATGGCCTCTCAGATTGAACT

CTTTTATTATTCATTATAATATTAGAATATATTCTGTATCTATTAAATATACAATTCTAT

CTTATTCTACACAAACTGTGTGAAAATGTGAATTGGTGCTGATTTGTTTTGTATTTAAGT

TTTGTTTTGTTTATTAGAATGTGACACTTCAACAGAAGGAAAAAAATGAATCTCCAATCT

TTTCGTTCGGCTTTTCTTTGTGCTCGGCTTTCGCTTTCTTTATTTCTTAAATCATGGAAT

GCGGTTTAATGAGGGGATGTGCTGTTTCTATGGTAACTCGGGTAAGGGTGTAATCCTGGT

GGAGGTTGCTAGGGCAGATAGGGGTGGTGGGGCCTTTTCACAAGTTTGTCACAGTTTGTT

TTAGACTCTTCCCCTCATTATCCATGTGTAGAACTGCTGAAAGACTGTGTGTGTGTGTGT

AATATATAAAACTGTATATAAGTGTTTTTTATTATATTTACAAAAGAAAAATGTCCGCTG

TTGCATTTCCTGTTTGTATTTATGTCTGGAGAAACAAACTTTTGGAACAAAATAAACCCT

CGGCTGTGATTTCACCTTCTTCCTGCAGTGTCAGTGTGTGAACACCAGAGGGCGC

>Unigene000267

GGCACGCACTGGTGTCCACTCTGTAGGGGGAGCTGGAGAAGCAGCTTTTGCAGGCCAATC

CTATTTTGGAGGCATTTGGTAACGCCAAGACCATCAAGAACGACAATTCTTCACGATTCG

GTAAATTCATCCGCATTAACTTTGATGTAACAGGCTACATCGTTGGAGCAAACATTGAAA

CCTACCTGCTGGAAAAATCTCGCTGTATCCGACAGGCTAAAACTGAGAGAGCTTTCCACA

TCTTCTACTACATGGTGGCAGGAGCCAGGGACAAGCTGCGTGAGGAACTGCTGCTGGAGA

ATTTCAATAATTACCGCTTCCTGGTAGCAGGACATGTGCAGGTTCCAGGAAATCAGGATG

ACGAGATGTACGATGAAACAATGGAGGCGATGAACATTATGGGCTTTACTGATGAGGAGA

GGATCGGAATCCAGAAGGTTGTGTCCACTGTGCTGCAGCTTGGCAACATCGAGTTTAAGA

AAGAGAGGAACCAGGAGCAGGCCACCATGCCTGACAATACAGCTGCACAGAAGGTCTGTC

ACCTGTCAGGCATCAATGTGACGGATTTCACCCGTGCCATCCTGACCCCACGCATCAAAG

TAGGCAGAGAAGTCGTACAGAAGGCGCAGACCAAAGAGCAGGCTGATTTTGCAGTGGAGG

CTTTAGCAAAGGCAACGTATGAGCGTCTGTTCCGCTGGATTCTTGCTCGTGTCAATAAAG

CTCTGGATAAAACCAAGCGTCAGGGCGCGTCTTTCCTTGGCATCCTGGACATTGCCGGCT

TCGAGATCTTCGAGGACAACTCGTTTGAGCAGCTGTGTATTAATTACACTAACGAGAAGC

TGCAGCAGCTCTTCAATCACACCATGTTCATACTGGAGCAGGAGGAGTATCAGCGTGAGG

GCATCGAGTGGAACTTCATCGACTTTGGGCTGGACCTTCAGCCCTGCATCGAGCTTATCG

AGAGGCCGAACAACCCTCCAGGTATCCTGGCCCTACTGGATGAAGAATGTTGGTTCCCTA

AAGCCACAGACGTGTCGTTTGTAGAGAAACTCACAAATCAACATGCCAATCATTTGAAAT

TCGCCAAACCCAAACAGTTGAAGGACAAAACTGAGTTCTCAGTGCTACACTACGCCGGCC

GGGTGGATTATAACGCTGTTGCATGGCTGACGAAGAACATGGATCCCCTGAATGATAACG

TTACTGCACTGCTCAACAACTCCTCCAGTGTGTTTGTGCAGGATCTGTGGAAGGACGCGG

ATCGTGTGGTGGGACTGGAGACCATCGCCAAGATGTCGGACAGCTCCATGCCCAGTGCCT

CCAAAACGAAGAAGGGAATGTTCCGCACCGTCGGTCAGCTTTACAAGGAGTCTCTGGCCA

AACTGATGACCACGCTACACAACACGCAGCCCAATTTCGTCCGCTGCATCATCCCCAACC

ACGAGAAGAGGGCGGGTAAGCTCGATGCTCACCTAGTTTTAGAGCAGCTCAGGTGTAACG

GCGTGTTAGAGGGAATCAGGATCTGCAGACAGGGCTTCCCTAACCGCATCGTCTTCCAGG

AGTTTAGACAAAGATACGAGATTTTGGCAGCCAACGCCATTCCAAAGGGCTTCATGGACG

GAAAACAGGCCTGTATACTGATGATTAAGCACCTGGACTTGGACCCCAACCTCTACCGCA

TCGGTCAGAGTAAGATCTTTTTCAGGACTGGTGTTCTGGCTCAGCTGGAGGAGGAGCGCG

ACCTGAAGATCACAGTTATCATTATCGCCTTCCAGGCACAGGCCCGAGGCTTTCTCGCCA

GGAAGTCATTTGCCAAACGGCAGCAGCAGCTGACAGCCATGCGAGTGATCCAGAGGAACT

GTGCTGCTTATCTGAAGCTCAGGAACTGGCAGTGGTGGAGACTCTTCACTAAGGTGAAGC

CTCTACTCCAGGTGACACGTCAGGAGGAGGAGCTAAGTGTCAAAGAGGAGGAGCTTCAAA

AAGCCAAAGATTCAGCGCAGAAGTACGAAACTGAGCTGAAAGATATTACGCTGAAACACA

CACAGCTTGTGGAGGAGAGGAACGCTTTGCAGGAGCAACTTCAGGCAGAAACTGAGCTTT

ATGCTGAGGCAGAGGAGATGCGAGTTCGATTAGTTGCTAAAAAGCAGGAGCTGGAGGAGA

TCCTTCATGAAATGGAGGCACGGCTGGAGGAGGAGGAGGAGCGGAGCGCCACCCTGCAGG

TTGACAGGAAGAAGATGCAGCAGCAGATCCAGGAACTGGAGGAACACCTTGAAGAAGAGG

AGGATGCGCGACAAAAGCTGCAGCTGGAGAAAGTCACCTGTGAGGGAAAGATCAAGAAAC

TGGAGGACGACCTGCTGGTGATCGAGGATCAAAACAACAAACTGCTGAAAGAGAAAAAGC

TGTTAGAGGAGCGTGTTGCTGATTTTAGTGCTAATTTAGCTGAAGAAGAGGAGAAATCTA

AAAACCTGACCAAGCTAAAGAACAAGCACGAGTCCATGATCTCAGACCTGGAAGTGCGTT

TAAAGAAAGAGGAAAAAACTCGGCAGGAGCTCGACAAGATAAAGAGGAAGCTGGAGGCGG

AGTCTAATGACCTTCAGGAACAGATCGCTGACCTTCAGGCGCAGATTGCTGAGCTAAAGG

CACAGCTGGCTAAAAAGGAGGAGGAGCTTCAGGCTGCACTAGCAAGGCTGGAGGACGAGA

CAGCACAAAAGAACAACGCATTGAAGAAGATTCGTGAGCTGGAGGGCATCATCTCAGACC

TGCAGGATGACCTGGACTCTGAACGTGTCGCCCGAAACAAAGCAGAGAAGACCAAGAGAG

ACTTGGCAGAGGAGCTGGAGGCCCTAAAGTCTGAGCTGGAGGACACATTGGACACCACAG

CCACTCAGCAGGAGCTCAGGGCAAAGCGTGAACAGGAAGTGACCCTGCTGAAAAAAGCAA

TAGAGGAAGAGAGCCGAAACCATGAAGCTCAGGTGCAGGAGATGAGACAGAAACACACAC

AGGCTGTAGAGGAGCTCACAGAACATTTGGAACAGGCCAAGAGGGTGAAGACAAACTTGG

AGAAGGCAAAGCAGACGCTGGAGAAGGAGACATCAGAGCTGACAGTTGAGATCCGCACCC

TCTCTCAGGCCAAACAGGAAGTGGAACACAAGAGAAAGAAAGTGGAGGGACAGTTAGCAG

ATCTGCAGTCCCGCTTCAACGACAGTGAAAAACAGAAAGCTGAGCTTGGAGACAGGGTGT

CCAAAATGACTGTGGAGCTGGAGAGTGTCACCAACCTGCTAAATGAGGCTGAGGGGAAGA

ACATTAAACTGAATAAGGATGTGGCCAGTCTGACCTCTCAGGTCCAAGACACACAGGAGC

TGCTGGCTGAAGAGACACGTCAGAAACTGCAGTTCTCCACAAAACTGCGTCAGATGGAGG

ATGAGAGAAACAGCCTACAGGATCAGCTGGAGGAAGAAACAGAGGCCAAGAGGAACGTGG

AGAGACATGTCTCCACCCTCAACATCCAGCTTTCAGATTTTAAGAAGAAGCTGGATGACG

TGTCAGGAAATGTTGAGCTGCTGGAGGAGAGCAAGAAACGTCTGCAGAAAGACCTAGAGT

CCTCAAACTCCCAGCTGGAGGAGAAGTCTTCTGCCTATGAGAAACTGGAGAAGACCAAGA

ACAGACTGCAGCAGGAATTGGAGGACATTCTTATGGACCTGGACAACCAGAGACAGCTTG

TCTCCAATCTGGAAAAGAAGCAAAAGAAGTTTGACCAGATGCTTGCAGAGGAGAAGAGTG

TGTCCTGTAAGTACGCCGATGAGCGGGACCGGGCAGAGGCAGAGGCACGTGAGAAGGAGA

CAAAGGCGTTGTCTCTGGCGAGGGCACTGGAGGAGGCGCAGGACACACGTGAGGAACTGG

AGAGAGCCAACAAAGCCTTACGGGCTGAGATGGAGGACCTGATCAGCTCCAAGGATGACG

TAGGAAAGAATGTCCACGAGCTGGAGAAATCAAAGCGTGGTCTGGAGCAGCAGGTTGAGG

AGATGAAGACGCAGCTAGAGGAGCTGGAGGACGAGCTGCAGGCTGCCGAGGACGCAAAAC

TACGGCTGGAAGTGAACATGCAGGCCCTCAAAGCACAATTTGAACGAGACCTGCAGGGAC

GAGACGAGCAGGGAGAGGAGAAAAGAAAACAGCTGATAAAACAGGTCCGCGAGCTGGAGA

CGGAGCTGGAGGACGAGCGAAAACAGAGAGCCGCCATGGCTGCGTCCAAGAAGAAGATGG

AGGTAGACATAAAAGACCTGGAGGGACAGATAGACACAGCCAACAAGAACAGAGACGAGG

CCATCAAACAACTGCGCAAACTACAGGCTCAGATGAAGGACTTTCAGAGAGAGCTGGACG

ACACGCGAGCTGCCAGAGAAGAAGTTCTGGCCACCGCTAAAGAAAACGAAAAGAAGGCCA

AGAACCTGGAGGCGGAGCTTATGCAACTTCAGGAGGACTTGGCAGCTGCAGAACGTGCAA

AGAAGCAGGCTGAAGCAGAGCGTGATGAACTTGCTGATGAACTCTCCAGCAACGCATCTG

GAAAGTCTGCACTGAGTGATGAAAAACGCCGTCTTGAGGCTAAGATTGCTCAGCTAGAGG

AGGAACTGGAGGAGGAACAGAGCAACATGGAGATAATGAACGACAGGCTGAGAAAGAGCG

CTCAGCAGGTGGACCAACTGACAAGTGAGCTTCAGACAGAAAGAACAACTTCACAGAAGA

ATGAAAGTGCTCGTCAGCAGCTGGAGAGACAGAACAAAGAACTGAAAGCCAAACTGCAGG

AGATGGAAAACCAGGTGAAGTCCAAGTTCAAGTCCTCAATTACTGCTCTAGAGGCTAAAG

TGGCACAACTGGAGGAACAGCTGGAGCAGGAGAATAGGGAGAAACAGGCCACAGGTAAAA

CAATGCGTCAGAAGGAGAAGAAGTTGAAGGACCTCACGATCCAGTTGGAGGATGAAAGAA

AGCAGGCTGAGCAATACAAAGACCAGGCAGAGAAGGCAAATGCACGGGTAAAGCAGCTGA

AGCGACAAGGGGAGGAGGCGGAGGAAGAACCCCAGCGCATCCCCGCCGCTCGCCGTAAAC

TACAGCGAGAACTGGAGGAAGCAACCGAGTCGAATGATACACTGAGCCGAGAGATTACAT

CACTGAAAAACAAGCTGAGGCGCGGTAATGAGGCTCCATCATTTGCTAGCGGTGCGCGGC

GTACCGGTGGAAGGCGGGGCCTGATCGACGCTGATGCTAATGACGAAGATGCCGAATCTC

AGGGCGACTACAATGGCACCAAATCAACTGAATAAACACACACACACACACACA

>Unigene000268

GAGGCGGTTCAAAGAGGAGCAAAGATGGCGGACAACAAAGGCCAAGGGGATACATCGAGC

CCAGCTAGCATGGAGGTAGACCCTCCGACAGCGCTTAACGGAGACGCCACCAACGAACAT

TCTGACACGGCTAACGACACAGACGCCGCCTCGAAGGACGGAGACGACAGACAGTCCCAG

GAAACCGTTCCGGAGATGCCGACACCCAGCGCCGAGAGCAGCGAGCCTGCCGCTGCCGCC

GATGGAGGAGGAGGAGGAGGAGGATCACCACATGGAGTACTGTCGAGTGTGTAAGGATGG

AGGAGAGCTGCTGTGCTGTGATACCTGTCCATCATCCTATCACATGCACTGTCTGAACCC

GCCTCTACCAGACATCCCCAACGGACAGTGGATTTGTCCCCGCTGCAGGTGTCCTCCTCT

AAAGGGAAAGGTCCAGAAAGTTCTCACATGGCGCTGGGGTGAAGCTCCGCCTCCCATCCC

AGTGCCCCGCCCACCCGATCTTCCAGTCAACGCCCCTGATCCGCCTCCTCTGGTTGGTCG

AAAGGAGCGGGAGTTTTTTGTAAAGTGGTGCAACATGTCATACTGGCACTGTTCCTGGGT

ACAGGAGCTACAGTTGGAGCTGAATTGTCAGGTGATGTTCCGGAATTTTCAGCGTAAGAC

GGATATGGAGGAGCCCCCGAGTGTGGATGCGTCTGAGTGTGATGATAATAAGAGCATGAG

GAGGAAGAGTAAAGACCCGCTGTACGCTCGCATGGACCACCAGTTCGGACGCTACGGAGT

TAAAATGGAGTGGCTGCTCATCCAGCGCATCCTAAATCACAGGTACACACTAATCACTAA

ACACAGAGAGATCACACACTCTACTAACTGTTTGTCTGTCTGTCTGTCTCTCAGTGTGGA

TAAGAAAGGTAATGTTCATTATCTGCTGAAATGGAGAGATCTGCCGTATGATCAGTCTGC

GTGGGAGAGTGAGGACATGGACATCCCTGATTACGACACCTATAAACAGCAGTACTGGAA

CCACAGAGAGATGATGATTGGGGATGAAGGACGACCAGGGAAGAAGTTGAAGAAGGTGAA

ACTGCGAAAGATAGAAAAGCCTCCTGTAAACCCTGTGGTGGATCCCACGGTGAAGTTTGA

CAGACAGCCGGAGTATTTGGACATGACGGGGGGGACGTTACACCCCTATCAGTTGGAGGG

ATTGAACTGGCTGCGGTTCTCCTGGTCTCAGGGTACAGACACTATCCTGGCTGATGAAAT

GGGTCTCGGCAAAACTGTGCAGACCGCCGTCTTCCTCTACTCTCTCTTCAAAGAGGGTCA

CTCTAAAGGCCCGTTCCTGGTGAGCGCTCCTCTCTCCACTATCATTAACTGGGAGCGGGA

GTTTGAGATGTGGGCTCCGGACATGTACGTGGTGACGTATGTGGGCGATAAGGACAGCCG

AGCTGTAATCCGCGAGAACGAGTTCAGCTTCGAGAATCACGCCATCCGCGGAGGAAAGAA

AGCCTTCAAGATGAAGGAGGCGGCGGTGAAGTTCCATGTGTTGTTGACGTCGTATGAGTT

GATCACTATTGATCAGGCGGTGTTGGGATCTATAGACTGGGCGTGTCTGGTGGTGGATGA

AGCTCACCGGCTGAAAAACAACCAGTCCAAGTTTTTCCGGGTGTTGAATAATTATCCGCT

GCAGCATAAACTCTTGTTAACGGGAACTCCACTGCAGAACAACCTGGAGGAACTGTTTCA

CCTGCTCAACTTCCTCACTCCAGAGCGGTTCAATAATCTGGAAGGATTTCTGGAGGAGTT

TGCAGACATAGCTAAGGAGGATCAGATTAAAAAGCTGCACGACATGTTGGGACCTCACAT

GCTGCGGCGTCTCAAAGCAGACGTCTTCAAACACATGCCCTCAAAAACCGAGCTTATTGT

CCGGGTGGAGCTCAGCATTATGCAGAAGAAATACTATAAGTACATCCTCACACGTAACTT

TGAGGCTCTAAACACACGTGGCGGTGGGAATCAGGTGTCTCTCCTGAACGTGGTGATGGA

TCTGAAGAAGTGCTGTAACCACCCCTATCTCTTCCCCACAGCTGCCATTGAAGCCCCCAA

AATGCCTAATGGCATGTATGAAGGTGGAGCTCTCACAAAGTCTTCTGGGAAATTGATGCT

GCTGTGTAAGATGTTAAAGAAGCTAAAGGAGGGGGGGCACAGGGTGCTCATCTTCTCACA

GATGACGAAAATGTTGGACCTGCTAGAGGATTTCCTGGAGAACGAGGGCTATAAATACGA

ACGGATTGATGGAGGAGTGACGGGAGTAATGAGACAGGAAGCTATTGACAGGTTCAATGC

TCCCGGAGCTTCTCAGTTTGTCTTCCTGCTATCGACACGAGCGGGGGGGTTAGGCATCAA

CCTGGCAACCGCAGACACTGTCATCATCTATGACTCAGACTGGAATCCACACAATGACAT

TCAGGCATTCAGCAGAGCTCACCGTATTGGTCAGAACAGGAAGGTGATGATCTACCGGTT

CGTCACTAAGGCCTCTGTGGAGGAGAGAATCACTCAGGTGGCTAAGAAAAAGATGATGCT

GACTCATTTAGTTGTGCGACCCGGTTTGGGTTCGAAGGCGGGCTCCATGTCCAAACAGGA

ACTGGACGACATTTTGAAATTTGGCACAGAGGAGCTGTTTAAAGACGACGCCGGAGAAGG

TGATAATAAGGAGGAGGACAGCAGCGTGATTCACTACGACGATAAAGCCATTGATAAACT

GTTGGACCGGAACCAGGACGCCACCGATGACACCGAGCTGCAGAGCATGAACGAATACCT

GAGCTCCTTTAAAGTGGCCCAGTACGTGGTCAAAGACGAGGACGAGGAGGAGGTGGACAG

GGAGATCATTAAGCAGGAAGAGAATGTGGACCCTGATTACTGGGAGAAGTTATTGCGTCA

TCATTATGAGCAGCAGCAGGAAGTGTTGGCCAGCCACCTGGGGAAGGGCAAGAGACCGCG

CAAACCCGTCAACTACAACGACTGCTCGCAGGAGGAACAAGCAGATTGGCATGACGACCA

GTCTGACAACCAATCAGATTACTCCGTTGCCTCAGAGGAGGGAGATGAGGACTTTGACGA

GCGCTCTGAAGCGAACAATCGCAGGTCAAGTCGTAGAGGTCTGAGGAACGACAAAGAGAA

GCCACTGCCCCCCCTGCTGGCCAGAGTGGGCGGGAACATTGAGGTGTTGGGTTTTAACTC

CCGTCAGAGAAAGGCCTTCTTGAATGCGGTGATGCGTTATGGGATGCCTCCACAGGATGC

GTTCACCACACAGTGGCTCGTCCGAGACCTTAGGGGCAAATCTGAGAAAGAGTTTAAGGC

ATATGTCTCTCTGTTCATGCGTCACCTGTGTGAACCGTGTGCTGACGGCGCTGAAACCTT

TGCTGATGGAGTTCCTCGTGAGGGGTTGTCACGGCAACACGTCCTGACACGCATTGGTGT

GATGTCACTCATACGGAAGAAGGTCCAGGAGTTTGAGCATGTGAACGGTCAGTGGTCCAT

GCCATGGATGAAGGAACTGGAGGAGAATAAAAGAGCAGTTGCTGTTGCTATGGGAGACGA

CCCCAAAACCCCCTCTACTGGAACCCCTGGAGACACACAACCCAACACACCTGCACCAGA

GGACCTGTCCAGAGGGGAGGAGTCAGAACGGGAGACGGACGACAAGATGGAGGGAGAGGG

AGAGAAAGACAGAAAGAGCACTCAGCTACAAGATGACGAGGTGATTGAGATTCCAGACGA

TGGGGAGACCCCCCTGAGTCCAGAGAAGACAGGACCAGGACAGGAGATGGAAAAAGACAG

CACAGCGCCTTCTTTGGAGAAGGATGGCGAGATAAAGGACATAGCGGGAGAAAAAGACAA

AGAAAAGGGAGGGGCAAAAGAGGAAAGCACTACATGTTCTTTAAAAATTGAAGAGGTGAA

AGGGGAAAGGTCAGAGGTCACCAGTGCTGGGGACTCCTCTCAGCCTAAAGAAGAGTGCAG

TGAGGAGAAGAGTGAAAAGACAGACAGCAACCTAACAGGAGAGGAGAAGAAAGAAGTGAA

GGAGGAGAAGGATGGCGTGAGAGCGGAGGAGTCTCGGATAAATGGAGAGGACGGAGGAGT

GGACAATGAGGAGAAGAAAAAACATGTCAAACAGAGATTCATGTTCAACATCGCTGACGG

AGGATTCACAGAGCTCCACTCTCTGTGGCAGAACGAGGAGAGAGCCGCCACAGTCACTAA

GAAGACATATGACATTTGGCACCGTCGTCATGACTACTGGCTCCTGGCGGGAATCATACA

ACACGGCTACGCCCGCTGGCAGGATGTTCAGAACGACCCGCGTTTCTCCATCCTTAATCA

GCCGTTTAAAGGAGAGATGAGCCGAGGAAACTTCCTGGAGATCAAAAACAAATTCCTCGC

CCGCAGGTTTAAGCTGTTGGAGCAGGCGTTGGTGATTGAGGAGCAGCTGCGTCGAGCGGC

ATACCTTAACATGACACAAGACCCCACTCATCCGGCCATGGCCCTGAATGCACGCTTCGC

CGAGGTGGAGTGTCTGGCAGAATCACACCAACACCTCAGCAAAGAATCTCTTGCGGGAAA

CAAACCGGCCAACGCTGTCCTACACAAGGTTCTCAACCAACTGGAGGAACTGCTCAGTGA

TATGAAAGCTGATGTAACCCGGCTTCCTGCCACACTGTCCAGAGTCCCGCCAATCGCAGC

GCGCCTGCAGATGTCTGAGAGGAGCATCCTTAGCAGACTGGCCAACAAGGGCAATGAGAC

CCACACACCACCGCCTATCCCACCTGGGCCGTATGCAACTCCACAAAACTACGGTCCCCC

GTTTACTCCAGCACCACCAGCCATGGCTCTGGCTGCAGCCAACTACAGCCAAATGCCGCC

AGGCTCGTTTATATCAGTGCTGAATGGGCCTCCAATGTCAGTCAAAAAAGAGCGAGAGGC

AGAGCTTATGATGGGAAGGAGGGAGCAACGCAGCGGAGAAGTGATCTGCATCGACGATTG

AGGCCAGCCATAATCCCCAGACACACACACAGACATACACACTGTTTTTTCGTTCTCTCA

GCAGAACTTAAGTAAAGAAAGTATGTTATGGCCAGGATATAATTAGCAAAATGTACAACA

AATTGGGGGGGTTGAGGAGGTGCACCAGATCACACATCATATGACAAAAAAACTATGTAA

CAGGCAATATAATCCAAACATAACACTCTCTCTCTTACAACACACTAATTCTCACTTTTA

CACAGAGACAAAAATCATTACACACACACTCAAAACACACATACACAC

>Unigene000269

GGGGTTATTTTATGACAGGATGTACAGTAAAAAAAGTGTGTTATTCTGCTTGTTCATCAG

CAATTTGCTGTAATATATTGATGGCCATTAATAAATGATACTGTGTGTAAACTAATTGCT

TATTATAGTAGATTTCATTATGTGTCCCTGTGTAAGAGATGATACTATAAAAAGAATAAT

TAACAAGGGCATTAATATTAAAACTGTCGTTCATGTTACAGCCGGGGTTAGTGTCCAAGT

GGTTATGCAAAAATAATATAGGCCTTTTGACAGATTGATGCCAATTTCAGTATTTTTTCC

TTCTTGGTGCTTACATCTACATGTTTTTTTTTTCTTACTAGGTTAAGCAGTGCTGTACAC

GTGTCCAACTCGGTATTTTGTTGCTGTCTTGCTGTGTGTACCTACAATCATCCATAGCCA

GCAGGCAGTGAGCTCATTTGTCCTCCACCCCTGCGCTATGAGCAGAGGTAGGGGAGGGCA

CTCCAAAGAGTACCAGAGGTTTTACCTTTCACCCCACCAGAGCAAATCCAACCAAGGATT

CTACTCCCAACCTTTTTCAGCTCACCCTAGAACTCCTCACGCCCCAAGCCCGAACACTAA

CTCGGGCAGATACCAGTATCCTCCCCCTTACTCATCCGCTGGCCCTCCTGGTGCTTCTTC

AAGTCCCTCTCTGCCTTCAAATTCTTATACGTCTCGGGTTCCTGGACCTTTCCAAGTGCA

AGATCAGAGGCCGTCATCGAATAGAGGAGCTTGCGCTGACGAAGCAGCAGACTTTCGGCA

GAAGCAAACTCTGTTCCTTCAGGGGCGGACTGCTGAGGCGCCCAAATTTGTGCCAGGCCA

GTCCAAACACCACAGCAGTCCTCCCCAGCGTCACACAGAATATTATAATTATATAAATAA

TAGCAGGTATATAAGCAGAGGGGGTTATAGTCAAGGCAGGCCAAGCTGGGAAAAGGAGTT

TAACACACAGTACTACCCTGGATCTAAGTCTGTCTGGGAGCAACAGCAGTTCAGAAGACA

GAATAATCAGTATCATTGTGACACTGCCTCACTATCTTCAGCATTTCACGGTCTATCAGT

AGAGAACTGTAGTTCACATTCGTCCTTCGGTAGTACTGGAAGTAAGGCGTCTCATACATT

TTCCTCGGGTAGATATTCCTTGCAGGTGACAGAGGATATTCAGGAGAAGGTCCACAATTC

TTTGGCCTCATTAGCAGGGTGAAACCCTTCAGGCCAGGGTCTTGGCTAAAAAACTTCGTT

TGCCTAAGAAGATTGTTAACCAAGCACTGTATGCTTTATCCAGATCAGGCCAGGCAGTGA

GGTTAGAAGGGACTCCTCCTCTCTGGAGACTCTGGAAAGAAGGTGTCCCTGAATACTCCC

AACGAACCTACTTTAGCGGAAATACTGAAAAATACCTGGTCCAGGATACATTTATAAAGA

AAAACCAGTGTATTGGTGAGGTAAAACCAGACAGCAGCTTTACCACTGGAACATCACCCG

CTGCAGAGGCCAGTAGTGATTCCAGTGACGAATCTGAAGACTCTGAAAAAGAAAGTTTCT

CTGACCAGGCCTGTGAAAAGGACTCGAGTCTTTCCATGACAATGGACGCAAAAGACTGCA

AACTGCAAATTATGCAGTACTTGCATGAAACTGGCCAGGCCAGTGCATTGATGATTGCCA

AAAACCTGGGCTTGAGAAATGCCATGCAGGTTAATCCTACATTGTATGCCATGGAAAAGC

ATGGCGATCTCAGTCGCAACGCTGGCGTTATGCCACCCGTTTGGGAGCTGACTGCTCACC

GGCGTGATAGGATGGCCCGACAGCGAAAAGCAGCTGTTGCTGCCGCTATAATACACAATG

AGTCAAAGCTTTGTGACACATCAGGCCCAGCAAAAACTGCACAAACAGCCCCAAACACGG

TGTATGGATCTGGGTTGTCTGAAGAAGTGATGGCGTGGGAGAACCACCCAGCAAGTCAGA

AAAGTGCTAGCATGGACAGCACATTTGGGAACGGAATGCTTGATAAGAAGTCACATTTAG

ATAGAGACTTTCTTCCTGCTCTTGAAAAAATTTCCTCAAATGATAAAGACCATACATCTG

TCCCCTACTCCACACATGATTCTTCATACTACCTGACACAAGACAAGAATGGAGGACACT

CGGAGTGGGCATCCGATGATATCCCAGAATTCTTAAATGCAATTCGATCAGAAGTGGCCG

TGTCCCTAGCAGCACCTATTCCTCCAGCCCAGAATACAGAGGCCGTCAGGTTGCAGAAGC

TAAGACTGGCACTCAGCAAGAACCCTGTTAGTGGACTGATGGAGTACGCACAGTACCTTG

GCTACAGCTGTGAATTCCTCCTGCTTGAACAGTCCGGACCCTCACATAACCCCAGGTTTC

AAATGCAAGTGATGCTCGATGGACGCAGGTTCCCACCAGCTGAAGCCTCAAGTAAGAAAG

TGGCAAAAAAGGATGCAGCTGCAGAGACTCTTCGGGCCCTCCAAAGAGAGATAGAAGGAA

CTACTACTGTAGTAAATAAAGCAGAGCTGCCTGCTGTGCCATTAGCAGAACTGAATGATT

TTGCTGAGGATTGTTTTGACAATATGGATGAACCTCCAAAGGCCCTGGCTCGCTCCCTTC

CAGGCGGCAAAAACCCTGTTTCGGTTCTAATGGAGCACAGCCAACGTAGTGGCCATGCCA

TTGAGTTTATTCATACTGGGCAGGAGGGGCCAGCACATGACCCACGCTTCATGTTCCAAG

TAAAGGTCGGGGACAGTCTTTACCCGGAGGCATCAGCACCCAGTAAGAAGGCAGCACGGC

AACTGGCGGCAGAGGAAGCTGTGAAGGCTCTTCTGGCAGAAGGACAACTACAGCTCAACA

AGCCACATCAGGCGTTTTGTCTACTCGGCGAGGGCGATTTGTCCCAGGCGATGCCCCCAT

GCCCGTCATTGCCCCCGCTGACAGCAGCTGAGCTTCAGGCAGCACACGAGGCCGGAGTAG

GCGACCTGATCAACCATCTAAACAACAACGCTGTGTCAGGCTTGCTGGAGTACGCACGCT

CACGAGGCTTTGCTGCAGAGATTCGCCTGGTGGGCCAGTCAGGACTCCAGCATGAGCCAC

GATTTACATATCAGGCAAAACTGGGGGGTCGCTGGTTTCCTGCGGTGTGTGCCAGCACCA

AAAAGCAGGGCAAGCAGGAAGCAGCAGACGCAGCTCTCCGAGTGTTGATTGGAGAAGCGG

AAAAGGCAGCACGCACCGGGGAGCTCACAGCAGAGTTGCCGGTGACGGGTAGCACGGTGC

ACGATCAGTTTGCCATGCTAAGCCACCAGCGCTTCAATGCGCTTACTGCACGCATCCAGC

ACAGCCTTTTGGGTAGGAAAATCCTTGCCACGATCATCATGAAGAGAGGTGCTGACAGTC

TGGGCACAGTCGTCAGCCTTGGGACAGGAAATCGATGCGTTAAAGGAGAAGAACTAAGTC

TTAAAGGTGATACCGTTAACGACTGCCATGCTGAAATCATTTCCAGAAGAGGCTTTATCC

GCTTTTTGTACGGTGAGTTGATGAAGTACTGGGAAAGACCAGAAGAGGACAACATATTTG

AGCTTGCGGAGGATGGTTTGCTCAAGATTAAAGACGGCATCACTTTCCATCTGTACATCA

GCACCGCTCCATGTGGGGACGGCGCACTGTTCGATAAATCGTGCAGCGAGGTAGCGGAGG

TCATCGGCTCGGCTCACGTGCCCGTGTTTGAAAACGCTAAACAGGGCAAACTCAGGACCA

AAGTGGAAAATGGTGAGGGGACCATCCCAGTGGAGTCGAGTACAATTGTGCCTACTTGGG

ACGGCATCCAGCATGGCGAGCGCCTCAGAACTATGAGCTGTAGTGACAAAATTCTGCGCT

GGAACGTGCTGGGCCTGCAGGGGGCTCTGCTCAGCCATTTTATGCACCCCGTCTACCTTC

AGTCTATCACGCTTGGTTACCTGTACAACCACGGCCATTTGACTCGAGCCGTGTGCTGCC

GATTGGCCAGAGAGGGCAGCGAGTTGATGAAAAGCTTGCCTACCCACTTTAAACTTTGCC

ACCCGGAGGTGGGCAGAGTGAGTGTGTACGACTCAACACGTCACACAGGAAAGACCAAAG

AATCCAGTGTGAACTGGAGCTTGCCAGACCAGCTAAAAGTAGAGATACTGGATGGAACCA

AGGGCAAAGTAGACAGTCCAAAGCTAGAGGTGTCCCGCGTCTCCAAGTCCAACCTGTTTC

GTCATTTCCGGGACGTGTGCAGGAAAGCCGGTCGGCGCGACTTGCTGTCTCTGACTTCCT

ACGCCCATGCTAAGATGGCCGCCCGTTCTTTCCAGCAGGCCAAGGAACAGTTCTACAAAG

CTCTGAGTCAGCTGGGTTACGGAACCTGGATCGGCAGGCCGCAAGAGGAGAAGAGCTTTG

AAGCGTCAGAAAGTGAAGCGCAGAACATTGTTTCAGCTCAGTAAAAACAAAAGGGAGCAG

AAAGAGGAGGGACGGAGGAGTATGGATGAAAAGGACAGTCAGTGTTTTGAATCTTCTAGG

TTGTAGAAGAATGATGGACACAACTTGAAACAGAGAAAGAGAGCGGGAAGGAAAAAAGCG

CAGTAGATCTCTTGGATTACTTCAGCCTGGGTTTGAGGGAGAGAAAAAAAATAAAATAAA

TCGATAAAATACCAGCTCATGTATAAGAATGATGTATGGCGCTTTCTTTGGAAGCCTCTC

TAAACTCTGTTATGCACTAAGAGCTCGGGATCTGTCGATCTGTCTCAGGCTGGTGGGTGC

ATATAAGCAGAGATTTAAAGAAGGGATGTTGTAAATTTTTATTTTACTTGAGATCAACCA

TGTATTCGTTTCAGTTGGGGAATCAATGGGCTTCAGCACAGATCTTCCTGTCGCTTTTTT

CGATCTCTTTCTTCTATAGAAAACGTTTCCTATTAAGGTTAAACTAGTGGACTGAGGTGC

TTTTTTTTGTCCCATTTCATTCACATTCCTGGTACTAAAATATAAAACTGTTTTAAATAA

CAGGATAACTGCAGGTTAGGACTGTTCATTCTTCCCCAGACATCCACCCCTTCCTCATAT

ATCTATCTATATTAGCTTGATCTCTTTATCTTAACCAGTCAAAACTAATTTGAATTGCTT

CTACACAAATGTATACATGATGTACATACAGATACATACGCTCACAAATGTATATAAGGT

CTCATCACACCGTGTGTGAGTTTATATACGTGTGTGTGTTTATGTATATGTATATATTTT

GTGTATATATTTTATAAGGTTTCATAAATCTATGTTGCTTTTGGCCAG

>Unigene000270

GGACAGGAATGAAAAGTGAAACTCCCAGGCGCCACTTTTGCTGCTTATTACACCAGCTGC

GGCTTTCGTTTGTCAAGAACAGTATAAATGCATTTAATAGTCCTAGCTGGACAGTATTTA

ATAGTCCTAGCTGGACATTTTGGGTTGTATTAGCTGTATTTTCTGCTTGTTTTTGTATTG

TTTGGCCGCCGTTTGGATTAAACTAGCTGGGCGTTTCAGCTCATAGTTATTGAGTACTTT

TTTTAATTTGTGCCGATTTTCAGCCCGCTTGAGGAGACGGTAACGTTTGTGTGACTTTTT

TTTGCTTATTATTTTTAACTCCAGCATTTCCGGTTAAGCAGTGCTGTACACGTGTCCAAC

TCGGTATTTTGTTGCTGTCTTGCTGTGTGTACCTACAATCATCCATAGCCAGCAGGCAGT

GAGCTCATTTGTCCTCCACCCCTGCGCTATGAGCAGAGGTAGGGGAGGGCACTCCAAAGA

GTACCAGAGGTTTTACCTTTCACCCCACCAGAGCAAATCCAACCAAGGATTCTACTCCCA

ACCTTTTTCAGCTCACCCTAGAACTCCTCACGCCCCAAGCCCGAACACTAACTCGGGCAG

ATACCAGTATCCTCCCCCTTACTCATCCGCTGGCCCTCCTGGTGCTTCTTCAAGTCCCTC

TCTGCCTTCAAATTCTTATACGTCTCGGGTTCCTGGACCTTTCCAAGTGCAAGATCAGAG

GCCGTCATCGAATAGAGGAGCTTGCGCTGACGAAGCAGCAGACTTTCGGCAGAAGCAAAC

TCTGTTCCTTCAGGGGCGGACTGCTGAGGCGCCCAAATTTGTGCCAGGCCAGTCCAAACA

CCACAGCAGTCCTCCCCAGCGTCACACAGAATATTATAATTATATAAATAATAGCAGGTA

TATAAGCAGAGGGGGTTATAGTCAAGGCAGGCCAAGCTGGGAAAAGGAGTTTAACACACA

GTACTACCCTGGATCTAAGTCTGTCTGGGAGCAACAGCAGTTCAGAAGACAGAATAATCA

GTATCATTGTGACACTGCCTCACTATCTTCAGCATTTCACGGTCTATCAGTAGAGAACTG

TAGTTCACATTCGTCCTTCGGTAGTACTGGAAGTAAGGCGTCTCATACATTTTCCTCGGG

TAGATATTCCTTGCAGGTGACAGAGGATATTCAGGAGAAGGTCCACAATTCTTTGGCCTC

ATTGCAGCAGGGTGAAACCCTTCAGGCCAGGGTCTTGGCTAAAAAACTTCGTTTGCCTAA

GAAGATTGTTAACCAAGCACTGTATGCTTTATCCAGATCAGGCCAGGCAGTGAGGTTAGA

AGGGACTCCTCCTCTCTGGAGACTCTGGAAAGAAGGTGTCCCTGAATACTCCCAACGAAC

CTACTTTAGCGGAAATACTGAAAAATACCTGGTCCAGGATACATTTATAAAGAAAAACCA

GTGTATTGGTGAGGTAAAACCAGACAGCAGCTTTACCACTGGAACATCACCCGCTGCAGA

GGCCAGTAGTGATTCCAGTGACGAATCTGAAGACTCTGAAAAAGAAAGTTTCTCTGACCA

GGCCTGTGAAAAGGACTCGAGTCTTTCCATGACAATGGACGCAAAAGACTGCAAACTGCA

AATTATGCAGTACTTGCATGAAACTGGCCAGGCCAGTGCATTGATGATTGCCAAAAACCT

GGGCTTGAGAAATGCCATGCAGGTTAATCCTACATTGTATGCCATGGAAAAGCATGGCGA

TCTCAGTCGCAACGCTGGCGTTATGCCACCCGTTTGGGAGCTGACTGCTCACCGGCGTGA

TAGGATGGCCCGACAGCGAAAAGCAGCTGTTGCTGCCGCTATAATACACAATGAGTCAAA

GCTTTGTGACACATCAGGCCCAGCAAAAACTGCACAAACAGCCCCAAACACGGTGTATGG

ATCTGGGTTGTCTGAAGAAGTGATGGCGTGGGAGAACCACCCAGCAAGTCAGAAAAGTGC

TAGCATGGACAGCACATTTGGGAACGGAATGCTTGATAAGAAGTCACATTTAGATAGAGA

CTTTCTTCCTGCTCTTGAAAAAATTTCCTCAAATGATAAAGACCATACATCTGTCCCCTA

CTCCACACATGATTCTTCATACTACCTGACACAAGACAAGAATGGAGGACACTCGGAGTG

GGCATCCGATGATATCCCAGAATTCTTAAATGCAATTCGATCAGAAGTGGCCGTGTCCCT

AGCAGCACCTATTCCTCCAGCCCAGAATACAGAGGCCGTCAGGTTGCAGAAGCTAAGACT

GGCACTCAGCAAGAACCCTGTTAGTGGACTGATGGAGTACGCACAGTACCTTGGCTACAG

CTGTGAATTCCTCCTGCTTGAACAGTCCGGACCCTCACATAACCCCAGGTTTCAAATGCA

AGTGATGCTCGATGGACGCAGGTTCCCACCAGCTGAAGCCTCAAGTAAGAAAGTGGCAAA

AAAGGATGCAGCTGCAGAGACTCTTCGGGCCCTCCAAAGAGAGATAGAAGGAACTACTAC

TGTAGTAAATAAAGCAGAGCTGCCTGCTGTGCCATTAGCAGAACTGAATGATTTTGCTGA

GGATTGTTTTGACAATATGGATGAACCTCCAAAGGCCCTGGCTCGCTCCCTTCCAGGCGG

CAAAAACCCTGTTTCGGTTCTAATGGAGCACAGCCAACGTAGTGGCCATGCCATTGAGTT

TATTCATACTGGGCAGGAGGGGCCAGCACATGACCCACGCTTCATGTTCCAAGTAAAGGT

CGGGGACAGTCTTTACCCGGAGGCATCAGCACCCAGTAAGAAGGCAGCACGGCAACTGGC

GGCAGAGGAAGCTGTGAAGGCTCTTCTGGCAGAAGGACAACTACAGCTCAACAAGCCACA

TCAGGCGTTTTGTCTACTCGGCGAGGGCGATTTGTCCCAGGCGATGCCCCCATGCCCGTC

ATTGCCCCCGCTGACAGCAGCTGAGCTTCAGGCAGCACACGAGGCCGGAGTAGGCGACCT

GATCAACCATCTAAACAACAACGCTGTGTCAGGCTTGCTGGAGTACGCACGCTCACGAGG

CTTTGCTGCAGAGATTCGCCTGGTGGGCCAGTCAGGACTCCAGCATGAGCCACGATTTAC

ATATCAGGCAAAACTGGGGGGTCGCTGGTTTCCTGCGGTGTGTGCCAGCACCAAAAAGCA

GGGCAAGCAGGAAGCAGCAGACGCAGCTCTCCGAGTGTTGATTGGAGAAGCGGAAAAGGC

AGCACGCACCGGGGAGCTCACAGCAGAGTTGCCGGTGACGGGTAGCACGGTGCACGATCA

GTTTGCCATGCTAAGCCACCAGCGCTTCAATGCGCTTACTGCACGCATCCAGCACAGCCT

TTTGGGTAGGAAAATCCTTGCCACGATCATCATGAAGAGAGGTGCTGACAGTCTGGGCAC

AGTCGTCAGCCTTGGGACAGGAAATCGATGCGTTAAAGGAGAAGAACTAAGTCTTAAAGG

TGATACCGTTAACGACTGCCATGCTGAAATCATTTCCAGAAGAGGCTTTATCCGCTTTTT

GTACGGTGAGTTGATGAAGTACTGGGAAAGACCAGAAGAGGACAACATATTTGAGCTTGC

GGAGGATGGTTTGCTCAAGATTAAAGACGGCATCACTTTCCATCTGTACATCAGCACCGC

TCCATGTGGGGACGGCGCACTGTTCGATAAATCGTGCAGCGAGGTAGCGGAGGTCATCGG

CTCGGCTCACGTGCCCGTGTTTGAAAACGCTAAACAGGGCAAACTCAGGACCAAAGTGGA

AAATGGTGAGGGGACCATCCCAGTGGAGTCGAGTACAATTGTGCCTACTTGGGACGGCAT

CCAGCATGGCGAGCGCCTCAGAACTATGAGCTGTAGTGACAAAATTCTGCGCTGGAACGT

GCTGGGCCTGCAGGGGGCTCTGCTCAGCCATTTTATGCACCCCGTCTACCTTCAGTCTAT

CACGCTTGGTTACCTGTACAACCACGGCCATTTGACTCGAGCCGTGTGCTGCCGATTGGC

CAGAGAGGGCAGCGAGTTGATGAAAAGCTTGCCTACCCACTTTAAACTTTGCCACCCGGA

GGTGGGCAGAGTGAGTGTGTACGACTCAACACGTCACACAGGAAAGACCAAAGAATCCAG

TGTGAACTGGAGCTTGCCAGACCAGCTAAAAGTAGAGATACTGGATGGAACCAAGGGCAA

AGTAGACAGTCCAAAGCTAGAGGTGTCCCGCGTCTCCAAGTCCAACCTGTTTCGTCATTT

CCGGGACGTGTGCAGGAAAGCCGGTCGGCGCGACTTGCTGTCTCTGACTTCCTACGCCCA

TGCTAAGATGGCCGCCCGTTCTTTCCAGCAGGCCAAGGAACAGTTCTACAAAGCTCTGAG

TCAGCTGGGTTACGGAACCTGGATCGGCAGGCCGCAAGAGGAGAAGAGCTTTGAAGCGTC

AGAAAGTGAAGCGCAGAACATTGTTTCAGCTCAGTAAAAACAAAAGGGAGCAGAAAGAGG

AGGGACGGAGGAGTATGGATGAAAAGGACAGTCAGTGTTTTGAATCTTCTAGGTTGTAGA

AGAATGATGGACACAACTTGAAACAGAGAAAGAGAGCGGGAAGGAAAAAAGCGCAGTAGA

TCTCTTGGATTACTTCAGCCTGGGTTTGAGGGAGAGAAAAAAAATAAAATAAATCGATAA

AATACCAGCTCATGTATAAGAATGATGTATGGCGCTTTCTTTGGAAGCCTCTCTAAACTC

TGTTATGCACTAAGAGCTCGGGATCTGTCGATCTGTCTCAGGCTGGTGGGTGCATATAAG

CAGAGATTTAAAGAAGGGATGTTGTAAATTTTTATTTTACTTGAGATCAACCATGTATTC

GTTTCAGTTGGGGAATCAATGGGCTTCAGCACAGATCTTCCTGTCGCTTTTTTCGATCTC

TTTCTTCTATAGAAAACGTTTCCTATTAAGGTTAAACTAGTGGACTGAGGTGCTTTTTTT

TGTCCCATTTCATTCACATTCCTGGTACTAAAATATAAAACTGTTTTAAATAACAGGATA

ACTGCAGGTTAGGACTGTTCATTCTTCCCCAGACATCCACCCCTTCCTCATATATCTATC

TATATTAGCTTGATCTCTTTATCTTAACCAGTCAAAACTAATTTGAATTGCTTCTACACA

AATGTATACATGATGTACATACAGATACATACGCTCACAAATGTATATAAGGTCTCATCA

CACCGTGTGTGAGTTTATATACGTGTGTGTGTTTATGTATATGTATATATTTTGTGTATA

TATTTTATAAGGTTTCATAAATCTATGTTGCTTTTGGCCAG

>Unigene000271

GAAAGGTACTGAAGTTGGAGCAGCTAAGAGGGAGCTAAGAGGGCAGCAGCATGCAAAAGG

AACTGTGAATAGGCTTGTTTGTGGCCAAGCCCAGCCCCCAAGCTGGAGCTTGTCACCGAA

GGGTTTTTTACATGCATCTCTGTCACACCACTTCTTTGCCTCTTACTGTTGAAACCTCCT

ATGCTTATTTCACACCAAGGACACCAGGCGGACTGATAAAGCCAAGGAGAATGGTGTGAG

CACTGCAGGCCCTGAGCCCAGCTACTTTGAGATCCCGAGTAAAGACGGCCAGATGGCAGA

GGACAGCATCCATGAGGTCCCGACCAAGGACACAGAGAGCAGCGCAGCTGCAGCAGCAGC

AGCAGCAGCAGCGAAAGCCGCCCAAGCGAACACCTCCTTTACTATTGAGTTTGACAACAC

CTCTCCAGGAAAAGTAACCATTAAGGATCACGTGTCGCGTCCAAGATCGAAAAAATCTCA

GCCAGGGAGTAAAGAGCTCAGCAGCCTGCAGGCAGCCATCATGGCCTCAGAGAGCAAAGT

GGCCGACTGGCTTGCGCACAACAATCCCCCCATGGTGAGACGGGAGTCCACGGAAGAGGA

CAGCAAAAGCATTAAAAGTGATGTGCCTGTTCTTCTCAAAAGGCTAAAAGGCAGCAAGCA

CGAGGACGGGACCCAAAGTGACTCTGAGAACGGCCTTGGCTTGCGTTTCGGTAGCCGGCG

CCATGCTGCTCTGGAGGAACGCTTGAAGGCAGCTGGCCTTACGCATGCCAAAGTCACCGG

AGCAGAGGGCTCGGGCTCCAGCAGCCGCAGCGCCTTCACCATCGAGTTCTTCGACGAGGA

AAACCCGCGCAAACGCCGCTCCTACTCCTTCTCCCAGACTGCACCACTTCTGGGAGGAGA

GGGACTGTGTCCAGCACCACCATCCCATCCGAAGGGAACTTCATTAACACTGGCAACAGT

AGCGCCTACAGCAGCACGGCTGCTGCAGAAAAAAAGGTCAGAGGAGAAGACCACGACGGT

CGGAGATGAGACTCAGAAACAAGACGACGATCAGAGCGACAAAGGCACTTATACCATTGA

GCTGGAGAACCACAACCCTGAGGAGGAGGAGGCCCGTCGCATGATCGATAAGGTGTTTGG

CGTGGAGAACCCTCAGAATCCCAGTCGCTCAGTGCAACAGACAACTAAAGACAGTAAGAA

GGTCAGCAGCACTAAAGCGGAGGGTCTTTTGGATGAATCGGTTGCGGTTGGAAGTCCACA

CTGGGTCTCTCAATGGGCCACTTTGGCCGCTAATCACACAAGAACTGATCCTGAAGGTTC

CGGGGCAGAACCCACAAACTTTGTTCATCAAGAGCGAGAAGCTGATGCATTTGAGTCAGG

AGTGTCTTTGAGAAGCACCAGCTCTGCCACCTCTAGTCAGGCTGAACGTAAAAGAAGGAC

ATTGCCTCAGCTCCCCTGTGAGGAGAAGAGCAGGCTGGGGAAGGTCTCCTCCACCTCAGG

ACTCCGATCCGAGATTGGCGAGAAACAGGACACCGAACTCCAGGAAAAACAACACAAAGG

AGATGGCGAATGTGCATCCACTCTCATTAAGAAGGACACTACAAGTGGAATTGCAAACCA

AGTGCAAACAGCCACTACCAAGCCTCCTGTGCCCCCTGTGGGAGGGGTGGAGAAGCAAAG

TGAGGAGTTTCGCAGGAGGCGTGCTGAGGACAGGAACAAAGGTAGCGATAGTGAGCGAAG

CGGGAAGCCCCTGGTGCGTCAAGGCAGCTTCACCATTGACAAGCCAAGCACCAATGTTCC

GCTTGAGCTTATCCCACGCATCAACCAGCGGCAGTCCGGAAACAGAGAGCGTAGTGATTC

AGCTGGAAGCATGGACACGACCACCTTGCTCAAGGACACTGAGGCCGTTATGGCCTTTCT

AGAGGCTAAACTGCGTGATGAGAAAAAGCTGGATCCATCACCGCCCGCTGGCTACCCTTT

GGGACGCTCTTTTTCCCCAGAGTTGGATACTGATGCAATTAAAGCAGCAGGCCGCGCCGA

AGTGTCACAGAAGCGTCGTTCTCTCAGCAGTATACACAGAGAGAAAAGTAACATGAGCTC

CGTTGGAAAGAGCACAACCAATGCACGGGAACGTTTGGAAAGGAAAGTCAAGCCGAAGAC

CTCGGATGGGCGTCTGGATACCCGTCGTTCTACCCATACAAGTCAGCGGACCCGCCAGCC

GTCCATGGACCTCACAGATGATGAGCAGACATCTTCCTTGCCCGTTTCTGACATACTTTC

AGATGACCAGGAAAACGGACGTAGTCCCTTCACCTTTCCTGATGACTTGCTACTTTCCAA

ACTGGACTCTTCTAAAGGTGCTAAAGTTAATAAAGCCAGCAGGACACCACAGGCCACCAC

TGCCTCTATAGGCAAGCATCAGTTGCCTCAGCCTCGGCCCACAAGAGCATCTCTTCTGAG

ACGTGCTCGACTTGGAGACACATCTGACACAGACCTGGCAGATGCAGACCGAGTGTCAGT

TGCATCCGAAGTGTCTACTACCAGCTCCACATCCAGGCCACCATCGGGCAGGAAGGCACC

ATCCCGCATCGATATGCTTGCACAGCCCAGACGCACCAGGGTCGGGACCATGTCAGCACG

CAGCGACTCAGAAGCCCCAATGTCACGCAGCACTACATCCAGAATATCGGCCGAGACAGC

TTTAAGACTGGGCTTGCGCCCAACTAACCCTGCAGACAGCAAGCTGAACCCACGTCTTCG

GGCAAACAGCGTCTCCAAGTTGACAGACACCAAAACCAAGACTACAGCATCCATCTCCAG

TGCTCCAGCAGCCAGCAGCAGGTGGCGGCGACTTAATACAGAATACGGCTCCACCTCTGA

GGACGAATTTGGCTCAAACAGGAACTCGCCAAAAGCCGGGCGTCTGAGGCCAGGCACTGC

TCTGCGTACCAGTAAACTGAGCAGCTCAGGCATGGTGACAACAAGCCCTGGCAGCATCAC

CATCAAACACAGGATGAGAGAACAGGAGGAGTACATCAGAGACTGGACAGCCCACAGCGA

AGAGATCGCCAGGTTATTCCCCTGTGTGCGCAGGATCAGTCAGGACCTAGCCAAGGATCT

GGCTATTCTGGCGAGGGAGATCCACGATGTGGCCGGCGAGATCGACTCGGTCAGCTCCTC

AGGCACAGCACCAAGCACTACGTTGGTAGAACGAGTTTTTGATGAAAGTTTGAACTTTCG

GAAGATCCCCCCTGTGGTGCAGACCAAGACCCCAGAGATGAACGGCCGGCCTGTAGAGCT

CCGACCTCGAGCCCCAGACAGTCTGGATCCCCAGAATGCACTGCGTAGACGCACATGGAA

CCGAGATGAGGTCAGACCGGCGGTCCTAGAAAGCTTGCTGCTGAGCTCAGTCTCTCAGCT

GTCCTCTAAAATCCGCCAATCTGTGGACAAAACAGCAAGCAAAATCAAGATTTTGTTCAA

AGACAAAGACAGGAACTGGGATGACATTGAGAGCAAACTGAGCTCAGAGAGTGATATGCC

ACTTCTCAAAACCACTAACAAGGAAATCTCAACAATTCTAACAGAGCTAAAAAGAGTGGA

AAAGCAGATCCAAGCTATAAATATCATGGTAGACCCAGATGGAACCCTGGATGCTCTTAC

CAGTCTTGCCCTAGCAAGTCCCACCACACCCAAACCTAAGACCAGCAACGGAGCCTCCAA

AGCCTCCGTGATCATCTCCCCGAACCGAGAGCTCCTCAGCGCTGGACTGAGTCGTCCTCA

AACCAGCGGAGAAGCGAGCAGCATTACTAACAAGTAAAGACAGAAAACCCTCAGAGTTGA

GTAAACTGAGAAACCCTTTTTGTCCATGTCTGTACCCCTCCCTCTCCTGCACTCCATTGG

AATGTCCATCTTTATGTAAATGAAGTCACACCTGTGGATTTGGACAGTGATATACAGTGC

AGCTCTACAAGTGCACGCAGCTGCTGGTGAAGAAGAGCTGAAGAGGAGCATTAGTCTAAA

GGCTTAAAATGTTGGAACCAGCCACCTGTATGCTGCTGCAAAGGTGTACACACTCATCCA

GGCATGAAGCAGGTGGACTTAAAATAATTTATCTTCAACAATAAATGACTCAGAGTGCCT

GTCTTCTCTCTTTTGTGAGCATTCTTTCTTGCAAATAGCAACTTCTTATTACTACGTGAC

TTTTGTGTCAAAAATAAAACAGTGTTGGTCAAAAAGACTTCAAGATTTCAAAGCTGAGCA

TAAACCAGCTTAGCAAAGTAAGTCTGATTCTATGCGGGCACTCAAACATTGTTCTTTTTT

TTCCATTTCTTATTTCTTGATTACAAAAAGTGCATTTAGGCTATTAAAATAAGCAGGGGT

CAACTTGGTAAGTTAAACTGACCGTTCATTGCATTATCTTATGCACTGATTTTTTATTTT

TATTGGCTAATGTATACAGTAGGTAGCAAACTTTGTACAAGGTTCAGACATTAATTAAAA

CCCAGCAAACTGGCGTTTGTGTCATTTTCCCCCTGACATTAGAAGGAAGATCATATGCAG

TATTAGAAAAACGTCTTAGTGGAATGCACCATTGTAGTGTTACACATCACAGTTGCTTAG

ATCTCTGTGCTTTGTGCTGAAATGTTAAATCAGCCTTAAATTAACCAGCCCTTAATGTAC

GGTTCAATCACATTCTGAACATGTTATGGCCAGTTGCTTAAATGCTAACGTTCTTTAATC

AGAGTATGCAATTACAAGAACCAAATAAAGTCTGTCAGCATTTTTTTTTTTAATTATCAC

AACCTCATAGATCATGTTTTTTGTTCATGCAGGTATTCCGGCATATTATGTATGTACTGT

ATTGTCTTACTCTTCCGTCGGCGCTTGCTGCTTGGCTGGAACTCATGCGCCTGAGAAAAA

GCGTCCAGCGCCCAGCCTTTCATCGTGAATCTGCGTGTTGTAGAAAGACTAGTGCAAAAT

AAAACAATAGTGAAGATAGGTACAGGACATTAGTTGTTGTTGTTTGTTAATAAATGTCAA

TGCCAAGTGGCTGCTGTGGAAACGCTAGTTCCTGGACAGGTTTAAGAAAGAAAATCTTAG

TTTAGTGACCGCATTTGTGGTTTTAAAGGTTCCTGGATTAACTGGGTGAGTTAATGTTAA

AGGTGATACAGGACAACAAACACCAAACTCTATACATTATACCGCTCGCAGAACAGGATA

GATTTTCAGGCAGATGATCCTCCTGCCCCTAAAACACTTAAAAAAAACAGTAGTTATCAT

TTATAAAACTATTGTACATGTTTTACATATAAGGGG

>Unigene000272

GACAGACCGAGCAAGAAGAGCACTGGTCAGAGAAGCATTCAAAAGGTCCATGGTCACTCT

GGAGGAGCTGCAGAAATCCACAGCTCAGGTGGGAGAATCTGTCCACAGGATAACTATGAG

TCATCTACTTCACAAATCTATCCTTTATGGAAAAGTGACAAGAAAAAAAATACAAATGCA

CACCACATTTTTATTAGATTTAGATAAAAGCTCCCAATGAGAGGGCAGACAATCAAACAA

GAACAACCAGCAAAACAACACATAACACAACCAATACAGTGCAAGCACACACTCATACAG

CCAGTATAGACTGTCAGCTTATTTCCTGTAGTGAACAAGAAAATCACAGACTAATCAATT

CAACAAAACAGATAAAAAATTATATAACATTCTAATAGTTAAGAGTCACATTTAAACTTA

GTTAAACTAAAGAAACTCATACCTTACCCTATACAAGAGCAGAAGGCCTTCATGTCAATA

TGCTTAAGTCATAATAATACATGAGATGCAACCCAACCAAGGAAATCAGAGAACCAGGTT

TATTTTCATTAAAAAAAATATACAAACACATCAACTGTAGTTTCACATTTTTATTACATT

AAAAACGTGGAACGTTCAGTGTTTAATATATTTTAATGTTTATATCTTAAATCAATTAAA

AGGAGGGCAGAACAATTTACAGCCCCCAAGTCGAGATGTGATCGACTCTTAAAAAAAACG

GGAGTCTTATTTAATAACCCTTTCCCCTAAAATCTTTATTTTATTAATCATTATTATTTT

ATTGTCAGATTAAAGATGAACAGCATGACATGATTTATTTCGATCTTGTTTTGATTGTTT

TACATCACACAAACAGGCTATTTTAACAGGAATGTGTCAACGTTTTATACATGTTTTAAA

CATGTTGACGTTAATATACTTATGCTCTCTGCTTAATTTAGCAACAGACATACCAACTTT

TCCTCCAAGCACCTCCTTACAACAACACCCCTCAAACACAACCTCAGTATTTCTGGCATT

AGACAATCACACAACAGGCTTTCCTTCAATCACCATTTCTCCTCAACAAGAAACACACAC

AAGTTTAAAAGAGCCAGTTAAAGACAGTAACACATCAGGCTTGCCCTCAAACACCATGCA

ACAAGACGCACATTTAACCACGCCCACACCTTCCGCAAGAATTCTAAATGAAAGCACCTC

TTACACCCCACGCACCACCAACACAACCTCACCCACACTCACGCCCAGCACCATACCAGA

GAAATCCTTCAAGTACAACTGCAGCATCGAAATGACACATGATTCGGTGTTGGTGAAAAT

TGAACCACATACAGAAATCTATAACATTACATTCACCGACATGGGCAACATAACTGTACC

AGTGACGAATTACCTCATACCAAAGAATTCATTAAAGCCCTGCCACAATTATATCGTCAA

TTTTCCTCTGAACTGTGTAGATAAATTCCAAAATTTTACAAGTGATTTGGAAAAGCCTGT

TATTTCATTTAAAAACGAGTACCCCACGGAATTGATCTGGGAAAATAAAGGAAACTGCTC

GGGGAACTTAACATTCGACTGCAATGGGATTCGAAATGTGAAAGATTTGGTTCCTTTTCG

TAATTATACGTGCAGTGGGTCATTACATTATAAAGAAGATTTAAAAATTGAAAGCGACCC

CATTACATTTAGGATTGAATGTGATCTGACAAATGCCACTAAGATTAATAAAGGTAGTTC

CAGTATAAATGTAAACTGGGAATTATCCAGCTCAAACTGCATGAACATTTCCACACAAAC

CATCTGGAGCACAACCTGCACCTCTGTGACTAAGAATCCAACTTCCCACTCAGGGGTTTG

TCAAAGGAACCACTGTGATATCCCGAATTTGACAGCCTACACAATTTATAGCTGTGATTT

TAAAGCAAGTTTTAAAGGCCAACAGTTCCGAAACTTCATCATAAGTGTAACGACCTTGTC

TACACATCCCATTTGTGGAGAGGCAAAATCACAAGTAATTGATCATAATTCCGTGATAAT

TAATTGTACTGTTACGGAATGGAACGGGAAACAGGGAAATTTCACTGCAGAGTTGAGGCA

AGATAATAGGGTGGTCTCCATTCAAGAAAACAGATCAAAATGTTTCTTTACATTTACAGA

CCTCAGTTACTTAACCAAATATAATTTACAGATTATTACAAAGAATGGTCAAGGTTTTTC

ATGCCGGACACTGGAAGTGTCTTTTTCTACTAAATACAATGACAAAGCTCTTCTTGGATT

TTTGGGGTTCCTCATTGTTCTTACGTCTATCGCACTGCTGTTTGTCCTGTACAAAATCTA

CCTTCTAAAGAGGGAGAAGTCCAGGAATGAGCAAGAACTGGATGACCTTCTTCCATCAAA

TGCACTGCTTCCAGTGGAACCCATAAGTGTTGACGCTTTGCTGGAGGTATACAAGAAGAA

GAGAGCTGATGAGGGACGTCTGTTTATGGAAGAATTTCAGAGTGTCCCACGCATTTACTC

CAACTACTCAGTCAAAGAGGCCAAAAAAACAGAAAACCAGCCAAAAAATCGCTATGTGGA

CATTCTTCCCTACGACTATAATCGAGTCGAACTTGCTCATGGAGAAACTGACTACATCAA

CGCCAGTTTTATTGAAGGTTACAAGGAAACCAACAAATACATTGCAGCCCAAGGACCCAA

AGAAGAGACAGTGGGAGATTTCTGGACAATGATCTGGGAACAAAAGACTTCCATTATTGT

CATGGTAACCCGTTGTGAAGAGGGAAACAAGAACAAATGTTCTCAGTACTGGCCATCACT

GGAACGAGAAACTGAGATTTACAATGATATGGTTGTGAAAATCAAAGGAGAGGAAAAATG

CCCCGATTACATCATTCGCCACTTGACCCTAATAAATCGTAAAGAGAAGGCGGCAGAACG

TGAAGTCACTCACATCCAGTTTACCAGCTGGCCTGACCATGGCGTCCCCTCTGACCCCGG

ACTGCTCCTTAAACTCCGCCGCAGAGTCAACTCCTTCAAAAACTTCTTTAGTGGTCCTAT

TGTTGTTCACTGCAGTGCTGGAGTTGGACGCACAGGGACCTACATTTGTATCGACGCCAT

GATTGAGAGTCTGGAGGCAGAAGGGCGGGTAGATATTTATGGATATGTGGTCAAACTTCG

CCGTCAAAGATGCCTCATGGTCCAAGTGGAGGCCCAGTATGTTCTTATCCACACGGCACT

GATTGAGTACAGTCAGTTTGGCGAGACGGAAATGGCTCTCTCGGATTTTCACTCAGAGTT

GAACACACTCAGGCAGAAGGAAGGCAATGACTTGTCTTTGATGGAGCTGGAGTTTCAGAA

ACTTCCCAAATTCAAAAACTACAGGTCGTCCACTACGGCACACACCGGAGAGAATAAAGG

AAAAAACCGTTCTTCTATTGTGCCATATGACTTCAACAGAGTCCTGATTAAACTAAATGA

TGAAGCCAGCCATGACAGTGATGATGAGGATGAGGAGGAATACTCCTCCGACGAAGAAGA

TGAAATTCCATCCAAATATATCAATGCTTCCTACATGGATGGATACTGGTTGCCGAGTAG

CTTTATTGTCACGCAGGGACCTATGGCTAATACAGTTGCGGATTTTCTGCACATGCTGTA

CCAGAAGAAGGTTAAAACTGTGTTCATGCTCACTAATTGCACTGAGAATAACAAGGAGTT

CTGCAGCCAGTATTGGGATGATGAGAAGAAAACCTTTGGTGAGATGGTGGTGAAGGTCAA

TGAGACTGAACACACGCCAACACACATTAGACGCTGCCTAGAAATACAGCACACAAAGAG

GAAAGACAGGCACACGCTGCAGCAGTACCACTTCCTGAAATGGGCAGGGCAGGAACTTCC

ATCCAACCCCCTTGACCTGATTGACTTGATAAGAAGTGTCAGACAGAGCAGTGACAACAG

CAACAAAAACAAAAATTTGCCTATTCTGGTGCACTGCAGCGATGGCTCTACACGTTCTGG

AATCTTCTGTGCCTTGTGGAAGCTTCTGGACAGTGCAGACATCGAGAAACTGGTGGACAT

CTTTCAGGTGGCCAAAGACCTGCGCAAGGCTCGTATGGAGATATTCAACACCTATGCGCA

GTACGAGTTTCTGTATGATGCACTGGAGGCTGCGTACCCGCTGCAAAACGGCGAGCTGAA

GAAGCCCAGCGGAGCTCCTGCTGACCTGATACAGCTTATTAACGAATCCACAGCACTAAT

CAGTCCCAGCACAGACACTGGCACAGAGGAGCGCACCAAGGAAACGACTGACTCGATACA

GCCTGAAGATGGAGCCACTGAGGCCAACCACGAACCAGAGAAACCTCTTGGTGAAAAAAC

CTCTAATGGTCCCACATCTGTTTTAGAGGTTCAAAGTGTTTAATCAGACCTGCTTATAGC

GCATCAACTGCAGCTTGCTTCATATTTAACTGTTTGAATTAAAAATAAAGAAAAAAACAC

ACTTTTTAAATGAAATGCAGCTAAACACTCTAAAGCCCAGTGAAAACATTTTAATGTACA

TTTTAATATTAAAGTTTTGTAGTATTAAATAAGGATTAATTTTAATTAACCTGAAAAGAA

ACCCAAAATCATTTGTTCAGAATTTCCAGTCAGATCAGTAAAAAAAACCCCACTATTTTG

TTTTACTGTTAGAGTTATTATTATTTTTTAACATTTCAGAGCTTGACTTGACTTTGTAGT

GGTGTATGTTTGCTAAAGCGCAACATGCTACGATTTCAGAAGAACAGTTAAACCCAGTCT

GTATTTAAGTGTGTATTACAGTCTGTAATTTAACATGCTGTCTCATTATGAATCTCTGTG

CCAGAATCTGCTTCTATTCTGTTTATTCATCTTTAAACTGTGAGTGTGAATGTAATGTAA

ATGGAAAGTTAATTACTGTATATCTTCATGTATCTAAAGATATTTTGTTAACAAAGCTAA

GTTATAAAGTGGCGAATGTATTGTTATGGAATACAAAGATTACTTTGCCCTACAATCTTG

TATTCTACCAAATTAGAATAATTTTCATTTATATATAAATGTTATGTTCTTATAGCCATG

TTGTATTTTAATTGGGAATAAACTGAATAAAAGCTTTAAATATGTTGGTATAGAATGGAC

CTGCATTAAAGCATCGGTTGACTTTAAAACCACATCAGGACCGTTGGCATGTCAATGCCG

CTATGTGGGTTTGTAGGACACAGCAGATGCAGAAAGAAACAAAACAGAAACATTAATTTT

GGGCTCTGACGACATCCTGTGC

>Unigene000273

CTAGGTCCTTTTTTAGAGGTCGTGAAAATCCTTTAAAACTAGTTTTTCTTTCGTATTTTA

TGGAGAAAATGGACAGCGAGGTCTTCACGCCTCAGCTGGAGCAATTTCTGCTCACACCTC

TGGTGGCTTGGGTGAAAACGGTGCTCCAGGCCAGTCCTAGCGACCAGAGCAGTCTTTCTC

TGTACATGGAATTAGTGGATGGTTTCTACCTCAACGAGGTCATGCTGAGGATAAATCCCA

AGGCATCCATTCAGAGAGTGAACAGGAAAGTGAACAATGATGCGGTCTTACGTGTACAAA

ATCTCTCCATCCTCACACGCCACATCAAAACCTACTATCAGGAGACCCTGCAGCAGCTGG

TGATGATGCCTCTGCCCAATGTGCTGGTGCTCGGCAGAAATCCGCTCACCGAACAAGGCC

TGGCCGAGGTGAAGAAGCTGCTTTTGCTGTTGCTAGGCTGTGCCGTTCAGTGTGAGGAGA

AAGAGGAGTATATTGAGAGGATCCAGACGCTGGACTTTGACACAAAGGCAGCCATCGCAT

CTCACATTCAGGAGGTCACACACAACCAGGAAAATGTTCTGGACCTGCAGTGGGTGGAGG

GGGGCGAATCTCCACCTGAGGACTTGGACTCACTCTCCAGAAACCTGGTCTTTCACCTCC

GACGCCTGGTGGACGAGAGAGACGAGCAGCTAGAGACAATCGTAGAGCTGACCCAGGAGA

GAGACTGCAGCCCATGTGCTCCTGCTATTCATCCTCCGCCCTGCACCCAGTCTCCCTGCG

ACTCGCCCAGCATGCGGCGCACTGAGAGCAGGCAGCACCTCAGCGTCGAGCTGGCAGACG

CCAAGGCCAAAATTCGACGCCTCCGCCAGGAGCTAGAAGAGAAAAGCGAACAGTTGTTGG

ACTGTAGACAGGAACTGGAGAATCTGGAGAAAGAGATAAAGAGGATCCAACAGGAGAACC

TTCTGCTGTTGTCTGACGCGCGTTCTGCGAGGGCCTACCGTGACGAGCTGGACGCTCTGA

GGGAGAAGGCCATGCGTGTGGACAAGCTGGAGAGCGAAGTGGGCCGTTATAAAGAAAGAC

TCCACGACATTGAGTTCTACAAAGCCAGGGTGGAGGAGCTCAAAGAGGACAATCAGATTC

TGCTGGAGACCAAGAGCATGTTGGAGGACCAGCTGGAAGGCTCCAGAGCTCGTTCAGACA

AACTGCATGAACTCGAGAAGGAGAATCTTCAGTTCAAGGCTAAAATCCATGACATGGAGA

TGGCACGAGACATGGACAGGAAACGCATCGAGGAGCTGCTGGAGGAGAACCTGGCATTGG

AGATGGCTCAGAAGCAAAGCATGGACGAGTCTCTTCACCTCGGATGGGAGCTGGAGCAGC

TTTCCAAAACCACACCTGAGGTCACTGAAGTTCCTCAGAAGTCTCTGGGCCATGAGGTTA

ATGAGCTGACCTCCAGCCGCCTGCTGAAGCTGGAGATGGAAAACCAGGCACTGCTGAAGA

CGGTGGAGGAGCTAAAGGGTCGCGGTGAGACTGAAACACGCCTCCTCAAGGCTGAAAAGG

AGAAGTTGAAACTCAGCCACAAGCTAGAGCAGCTAAAGAACGAAGCGCAGTCGGACAGAG

AGAGCCTGCGTTGCGCAGAGAACCTGAGCTCAGACCTGATGAAGGAGAAAGCACAGCTGC

AGAAAACCCTGGAGACTCTGAGGGAAAATTCAGAGAGACAGGTGAAAGGGCTGGAGCAGG

AGAACGAGCACTTGAATCAGACCATCACATCCCTCAGGCAACGATGCCAGGTCAGCGCTG

AGGCCAGGGTAAAAGACATTGAAAAGGAAAACCGGGTCCTCCATGAGTCCATCAAAGAAA

CCAGCAGCAAGCTTAACAAGCTGGAGTTTGACCGCAAGCAGCTCCGTAAAGATCTGGAGC

ACTATCAGGAGAAAGGCGAGAAGGCAGAGGAGCTGGAGATAGAGGTCCAGCGCTTGGAAA

GGGAAAACGAAGGTTTGCAGAAACGTGTGGCCAGCCTCGGGATCATCTGTGAGAAGGTGG

ACACTTTGGAAAAGGAAAATGCTGAGCTAGAGGCGGAGAGCAGGAGGTTAAAGAAGAAGA

TTGATGGCTTGAGGAATGCGTCCATGCAGCTGGAATCATTGGAAAAAGAGAACGCCCAGC

TGGACCAAGAGAACCTGGAACTGCGGCGTACAGCCGAAAGTCTGAGATCCGCAGGAGCCA

AAGCTGCTCGTCTGGAACAGGAGAACAGAGAGCTGGAAAGCGAGAAGAATCAAATGCAAA

GAAACCTGGAGCTGCTAAAAGCTTCATCCAAGAAAACTGAACGTCTGGAACTCAGCTACC

AAGGACTGGACTCGGAGAACCAACGTCTCCAGAAAGCCCTGGAAAACAGTGGTCGTAAGA

TCCAACAGCTGGAAGGAGAGCTTCGGGACCTGGAAAGCGAAAACCAGAGTCTGCAGAGAA

ATCTGGAGGAGCTAAAGATCTCCAGCAAGCGTCTGGAGCAGCTGGAAAAGGAAAACAAAG

TGCTGGAGCAGGAGAGTTCACAGCTAGAGAAAGACAAGAAGCAGCTGGAAAAGGAGAACA

AGCGGTTGAGGCAGCAGGCGGAGATCAGAGACTCCAAACTGGATGAGGACAGTCAAAGGA

TCTCGCAGCTGGAGAAGGAAAACCGCACGCTAAGCAAGGAGGTGAGCTCCCTGCAGGATT

CTTATAACCGTGTCAAAGATCTGGAGAGGGACAACAAGGAGCTGGTAAAGCAGGCCACCA

TTGACAAGAAGACGCTGGCCACGCTTAGAGAGGAGCTTGTTAGCGAGAAACTAAAGAGTC

AGCAGACGAATAATGATCTGGAGAAGCTGACTCACGAGCTGGAGAAGATAGGGCTGAATA

AAGAAGGACTGCTACACGATGAACAGAGCTCTGCTGCAAGGTTCAAGCTGTTGGAGTCGA

AGCTGGAGTCCACTCTGAAGTCCTCTCTGGAGATTAAAGAGGAGAAGATCGCCGCCCTGG

AGGCCAGACTGCAAGAATCCTCCAACCTGAACCAGCAGCTGAGACAGGAACTCAAAACAG

TGAAGAAGAACTACGAGGCTCTTCGTCAGAGAGAAGAGGAGGAGCACATGGTGCACAGCT

CACCATCTAGAGGAGGCGAGGACTCGCAGAGTGTCAGGAAGTGGGAGAAGGAGAGTCACG

AGGCCACTCGAGAGCTGCTGAAGGTCAAAGATCGACTCATTGAGGTGGAGAGGAACAATG

CCACTCTTCAGGCGGAAAAGCAGGCGCTACGTACCCAGCTGAAACAGCTGGAGACTCAGA

GCAGTAACCTGCAGGCTCAGATCCTCGCTCTTCAGAGACAGGCCGCCTCACTACAGGAGA

ACAACACTACGCTACAGACTCAAAACGCTAAACTCCAGGTGGAGAACTCGACCCTGAACT

CTCAGAGTGCAGCACTCATAGGCCAGAACTCCCAGCTGCAGAGTCAACAGTGCAGCGCCG

AGAGCGAGCGAGACGGCGCTGTGCGAGAGCGTGAGGAACTTCGCTCCACCTATGAGATGT

TGCTGAGGGACCATGAGAAACTTGCAGCTTTACATGAGCGCCAGGCAGCCGAGTATGAGG

CCCTGATCGGGAAACACGGGGGTTTGAAGAGCGTACACAAGAGCCTGGAGGTTCAGCACC

GAGACCTAGAGGACAGGTACAACCAACTGCTGAAGCAGAAGGCACAGCTGGAGGAGCTGG

AGAAGGTTCTGAAAGCTGAGCAGGAGAAGATGCTGTCGGAAAACCAGAGGCACCAGGCCA

CAACAGCAGAGTATCACAAACTGAAGGAAGAAAATGACAAGTTAAACTTCACTTACCAAC

AGCTGCTGAAAGATAATGAGGGCCTGCAGACCGACCACAAGAACCTGAAGAGCCAGCTGA

ATGCTGCTAAACTGGAGCAGACACGCCTGGAGGCGGAGTTCTCCAAGCTGAAGGAGCAGT

ACCAGCAGCTGGATATCACCTCCACCAAACTCACCAACCAGTGTGAGCTGCTGAGTCAGC

TGAAGGGAAATCTGGAGGAGGAGAACCGACACCTGCTGGACCAGATACAGACGCTGATGT

TGCAGAACCGTACCCTGCTGGAGCAGACCATGGAGAGCAAAGACCTCTTCCATGTGGAGC

AGAGACAGTACATAGACAAACTAAACGAGTTGAGGAGACAGAAGGAGAAGTTGGAGGAGA

AGATCATGGATCAGTATAAATTCTACGACCCCTCACCCCCCAGGAGAAGAGGAAACTGGA

TCACTCTGAAGATGAAGAAGCTAATTAAGCCGAAGAGCCGGGAACGAATGCGTTCCCTCA

CTCTGACTCCGTGCCGCTCCGAGTCCAGCGACGGCTTCCTAGCTGCTCCTCAGGACAGTC

AGGACAGCTCATCCATGGGTTCCGGGTCTCTCGATGACCCTCTTAGTCAGAAGAGGAGTG

GCACAATGAACGATCTGCTGCAGTCCATGGAACTGGCTGGTGCTGGTCAGTGGAGCGGCA

GTACAGATCACCTGGAGGCCCCTGATGACCCAGTGTGCGCGGGTGGGAGCCAGAGAATGA

AGGAGCTTGCGTACTCCACCACCGCCATCAACTACGCTACACTCAGCAACAAGCACAAAC

TCAAGAGCAAAGATAAAGTGAAAGATAATGTCTCCTGTGAGGACATCACTCCCAACTCTT

CAGATGAGCAGAGTGCAGCTCGAAGTCAAGGTGTGATAAACGGCAGTATGAGCAGACCGC

AGAGCGAGAGCAGTGGTGAGTTCAGCCTCAGTCTGGATGCGGAAGTTTGGTCCAGTGAGA

GCAGCCCAGTGCAGAGATCATCTCAATACGGCCAGAGGAGGAGAAGCGGCTGTCACACTA

ATGAGTCTGGCAGTCAGGACAACCTGAGTGTAGAATCTCCGCGTCTCCTCAGAGCGTCTG

ATCCTGCGCCGAAGGAACGCACCAAGGGACGAGGTATCCTGCGATCTGCCAGCGGAAAGG

CCACGCCTACCGAACCCAAAACCGGTCGCCCAAGTCTGCGTAAGGCAGAGAGCACTCGAG

TGAAGGGACCGAATCCACCCAGGTTAGGTCTGAGTGCTCAGGCGAAAGCAACATCCGTGT

GTGAGCGCCTGGACACCCAGTCCTCTTCTGCCGGTCTTCCTCGCGCCAGCAGCGTCATCT

CCACCGCTGAGGGCAGCACACGCCGCTCCAGTGTCCACGACATGCTGGCCAAGGACGCCC

GTGAACCTGTCTCTGTGGATCCGTCTCCCTCAAAAAGTCAGGCGGCATCCAATGTTGAAA

CCTCACGTCAACGCA

>Unigene000274

TTTCCGGATCTCACATCTCTACCCACAAATCATTTGCGTCCGGCTGCCGTAGGCGAGCTA

CGCAAAGGTGGTTTACCGAAGATGGCGGCTTGTAGACGTGTGCAGCTTCTTTTATTGCTT

TTAGTTGTTACCGCTTGCTTTCGTTCTACCGAGGGAGCGAAATCACAAAATATTCTCGTT

GATGGAAATGCAGGAGAAAATATCAACGATAATAAAATTGCCATGGGTGTTCCGGCTGAT

CCGAAAGCTGGAGCGGCGGCCTCTCAGTCCCGCCGCAGCTCCACCGGCTGGAAGCTGGCT

GAAGAGGAGGCCTGCCGCGAGGACGTGATCAGACTATGTTCTAAACATTCATGGAACAAC

AACCTGGCCGTGCTCGAGTGTCTGCAGGATCGGAAAGAGGAAACTGAGATTGCAGCCAAT

TGTAATCATCTATTATGGAATTACAAGCTGAATCTGACGACAGACCCAAAGTTCGAGTCT

GTGGCTGTGGAGGTGTGCAAAAGCACCATTTCTGACATTAAAGAGTGTGCAACAGAGGAG

CGAGGAAAGGGCTATTTGGTGTCATGTTTGGTAGATCATCGAACCAACATCACTGAATAC

CAGTGCAATCAGTACATCACAAAGATGGCTACCATCATCTTCAGTGACTACCGGCTCATA

TGCGGCTTCATGGACAGCTGCAAAGAGGATATCAACTCACTGCACTGTGGGAGTGTAAAC

ACTGGTGAGAAGGATTTACACTCCCAGGGTGAAGTTATAGCATGTCTGGAGAAAGGGCTG

GTGAGTGAGGCAGAGGATCAGCCAGGGCATTATTCTATCAAAGAAGATTGTAAGAAGTCC

ATCATGCGTGTGGCTGAGCTGTCGTCGGATGACTTTCACCTTGACAGGCACCTCTACTTT

GCATGCCGCGACGATCGTGAGCGCTTCTGTGAAAACACTCCAGCAGGTGAAGGCAGGGTC

TACAAATGTTTGTTCAACCACAAATTTGATGATTCCATGTCTTTAAAGTGCAGAGAGGCT

CTGTCCACCAGACAGAAACTGATAGCACAAGATTACAAGGTGAGCTACTCCCTGGCCAAG

GCCTGCAAACAGGACCTGCGCAAATACCGCTGCAATGTGGATACCACGTTGCCCCGTGGC

AGAGAGGCCAAGCTGTCGTACCTGCTGCTGTGTCTCGAGTCTGCCGTACACCGAGGGCGC

ACTGTGAGTGGTGAGTGTCAGGGGGAGATGCTGGACTACAGGCGCATGCTGATGGAAGAT

TATTCGCTGAGTCCTGAGATTGTTTTGCACTGCCGCGGAGAGATCGACACACACTGCTCC

GGTCTGCACCAAAAGGGCCGAACCCTTCACTGCCTCATGAGAGTGTCTCGCGACAAGGGC

ATTGTAGACAACCTTTGTCAGAAATCTCTCCAGACTTTGATCCAGGAGACGGATCCTGGA

GCGGATTACCGGATTGACCGGGCCCTCAATGAGGCCTGTGAGTCAGTAATCCAAACTGCC

TGTAAACACATCAGGAATGGTGACCCCATGATCCTGTCTTGTTTAATGGAGCATCTTTAC

ACTGATAAGATGGTGGAGGACTGTGAGCACAGGTTGCTAGAGCTGCAGTACTTCATCTCC

AGAGACTGGAAGCTGGACCCGATCCTCTACAGGAAGTGTCAGAATGATGCTTCACGTGTT

TGCCACACTCATGGCTGGAATGAGACAAGTGAATTGATGCCCCCAGGAGCGGTGTTTTCC

TGTCTGTACCGCCATGCTTATCGCACTGAGGAGCAGGGCCGTCGGTTATCTCGTGACTGT

AAGACAGAAGTGCAGCGCATCCTCCACCAAAGGGCACTGGATGTTAAGCTGGACCCTGAC

CTGCAGAGACGCTGTATGACTGATCTGGGCAAGTGGTGCAGTGAGAAAACAGAGCCTGGA

CAGGAATTGGAGTGTTTGCAGGATCATTTAGATGATTTAGGTTCAGATTGTAGAGATGTG

GTGGGGAATCTAACTGAGCTGGAGTCTGAAGATATTCAGATTGAAGCCCTTTTAATCCGA

GCCTGTGAACCGGTCATCCAGTCGCACTGCCATGAGATGGCAGATAACCAGATAGATACT

GGAGATCTGATGGAATGCCTTGTGCTCAATAAACATCAGAAGGAAATGAATGAAAAGTGT

GCAGTCGGAGTCACTCACTTTCAGCTGGTTCAAATGAAAGACTTCCGCTTTTCATACAAA

TTCAAGATGGCCTGCAAGGAAGATGTTCTCAAGCTCTGCCCTAACATTAAAAAGAAAGTG

GACGTGGTCATCTGTCTGAGCACCATGGTCAGGAATGATACGTTGCAGGAGGTGAAAGAC

CAGCGTGTGTCCATGAAGTGTCGGCGGCAGCTCCGAGTGGAAGAACTGGAGATGTCCGAG

GATATCCGTCTGGACCCTGATCTGTACGAGAGCTGTAAGCAGGACATTAAGGATCACTGT

CAGAACGTGGTTTACGGCAATGCGCAGGTGATCGAGTGTCTGAAGGACAGTAAAAAGCAT

CTGACCCAACGCTGTCACCAGAAAATCTTTAAACTGCAGGAGACTGAGATGGTCGACCCT

GAACTTGACTTTCAGCTCATGAGAGTGTGTAAGCAGATGATCAGGCGCTTCTGCTCTGAA

GCCGATGCTAAAAGCATGCTCCAGTGCCTGAAACAAAACAAGAATAGTGAACTTATGGAC

CCTAAATGCAAACAGATGATCACCAAAAGACAGATCACTCAAAACACAGATTACAGACTG

AATCCAGTTCTGAGGAAAGCCTGTAAGGCTGACATTCCCAAGTTTTGCCAGCACATCCTG

AACAAAGCAAAAGATGACATTGAATTAGAAGGCCAGGTCATCTCCTGCCTCAAACTTAAA

TATGCTGACCAGCGTCTGTCCTCAGACTGTGAGGATCAGATCCGGGTAATTCTGCAGGAG

TCTGCTTTGGACTACAGACTGGACCCTCAGTTACAGATGCAGTGCAGTGATGAGATCCCG

CGGCTGTGTGCAGAGGAGGTAGCGGCGCAAGAACAGACAGGTCAGGTGGAGGAATGTCTC

AAAGTGAATCTGCTCAAGCTTAAAAATGAAGGCTGTAAAAAGGAGGTGCTGAACATCCTC

AAGGAAAGTAAGGCAGACATTTTTGTGGATCCAGTGTTGCACACGGCATGTGCCCTTGAC

CTTAAGCATCAGTGCGCTGCTATCCCACCCGGCAGAGGAAGACAGATGTCATGTCTGATG

GAGGCTCTTCAGGATAAGAGAGTAAGACTTCAGCCAGAGTGCAAGAAGAGACTGCAAGAT

CGCATAGACATGTGGAGCTACGCTGCCAAGGTGGCACCAGCAGAAGGCTTTTCAGACTTG

GCTGGTCAGGTCCTCTCCTCTCCTGCAAAGAACTATATTCTGAGCATGCTGGCGATGTCT

GTAGTGCTGCTGTTCTTTATGGGGCTGCTATGTGGCCGCATTACCAAGCGTGTCACGCAG

GAGCTAAAGGACAGGTAAAGAGTGTTGCCAGGGGCAGGAGGAGGGGGAGATGCAATGCTC

TTCCTTCCCCATTTCACACAGGGAGGGGGATGGAGAAGATAGCAGTGCTCCTCTCTGTCC

CTCACTCACCCCAGGCCCTGGCAATGGCATAGTGAAGAGGATAAGCAAGACTAAAACTAC

AGATTTTCTTGCTACTGGCAGCAGACATGTTTGGACCTGATGTTACCCTCTTCACACTGT

CCATGTTTTCTGACTGCGGTACTCCTGCATCCTCATCTGCTTCTCTGTGACCAGACCTGC

CTGTTGTGGACCATTGTGTGCGTGTGTGTGTTCTGTGCATTTCTGGTACGGTGTGCCAGC

ATTTTTGAATCATTGTTTTGTTTTTGTTCCCTCTCCCCCCCCTCCCCCAGCTTTTTCTAT

GTTCATGGAGTTCCTGTTGAATCAACAGTTCATGTATTTATTCTTTATGCTTATGTTGTA

CTGGATGTTGTGTATGCTTGGGCTTTGTTGTGCTCATGTGCTCCCCTGTGGTCTACTAAA

GCGATGAGTCACAGGTTGCAGGTTCTCACTACTAAATTAAAGAAATAGTTTTTTTGTGTG

GTTGTGGGTGGTTGCCATACATTTCAGTTACACATGGTGACTAGTATATTTGTGGTATTA

GGTTTGTGTGTGCTAGGGTGGCACAGATCTGAAAACATGTTTCGTCTATTATTTTTAACT

GATTATAACTTTCTTACCATCGACAATAGGTTGTTTGTTTTATTCGGGTCATGGCTGACA

TGTTCACTAACTTGCAGAACATCTAATGGGATTATAACGCCATCATTTGCTTTAAAATAG

TTTATAAATGACTTTATTGACAATGATGTACTAGACATACTGCTAAATAGAGGCTTTGTG

CTAAACACAGGGGTTCAAAAACAGGATTGAACTGAATAATTGTGCTTTGTGTTTGTGTGC

ACATGTGTATATGTGTGTATACTTTCCTGTGAGGGCGAATGAGAAGTTACAGATTAAATA

TCCTGTCTATGGGTTCACTTATTTTCATAATGAAAGTTTGGTTTTTTTTTTGTTTTTTTT

AAACCTCTTTACTTTTGTGACTGCAGGTTTGAGTTTATGACAAACAAAAGCATTCAATCT

AATATTTATCAGACTCTCTGTGCTCCCAGATACTACCAGATTACTGATCAGTGACGGAAC

AGCTACTCATATCTGAGCAAAAGAAACTCAGGATATAGGGGTGATAAATAGACCTTTTTA

AAACCTATCCATGGTTAGAATACACACACACCCTTCAGATATTTTTCAATAATGAAATAA

TAGATCCACAGCCCTGTTCTGACTGGGATGCTTGGTTAATTTTCGATAATGGCCTCAGAC

AGGATGTCAAGGTCCTCATGGTGCTCAGGCAACTTGACCGGGTCTTTCCACTAAACTGCT

ACCGAAGAGTGATTTTTACACAAGTTAAGTCATTTCCTGTGCTCAGATTTTTTTCTCAGT

TTAGCTTGTTAGATGTTTAACACCAGTAGAGGTGTTCTGGTGGTTATTACAGTGCCATAC

TAATAGCATTAGTCCTTGCTTAGTCAGGGTGGCTCTCTGTGCAAGGCACATTTAGTTTTT

GCTTACTTAGCTGTGTGTTAATCAATTTGTTTGTTTTGATGGCCTTAATTTATTAAAGGT

TTTAAGTTTGTGATTTTTTTTTTTTTGATGCTTCAGTGTTTTCGTTGGGGCTAGTTATCT

ACTCTGCCATCCAGGGTAAGAGGATTTTAGATTTTTTTTTTTTTTAAACTTGC

>Unigene000275

GGTAAGTGGCATAAGGTTCATTACCTGCTGAAGGTGCTGAACATCGAAGAACAACACGGA

CGGTTGAGCAGTATAACCTCTGAAAGGTGACGGTGCCTTACAGATGTGCGTTTAACAGCT

GTAATGTCTTTAGCTACAGTGATAGGACGTGTGAAGAGCACGTGACACCGCTTTCACTAA

CTTTAAGTGAGGAGACACCAGCTTCGGACTTCCACAGCAGCACCAACTCACTGCTGAATG

CTTCAGGATTCGGGTTTCACAGGTACATCCTACACCATGTGGACGCTATTACTCGGAGTA

TTAATTCTCTTAAAAGGAGCGATTTGCACGTCTGAATATAACTTTAGCGGAGTTATCAGA

AACTTTGTTGTGGGAAACGATAAACTGTTTGTTGCTACTGACTCTAAGCTCTACCAGATG

CGGTATGATCTCGTAGAGGAAAAGGTAAAAGTCATATCCAACAGTACGTACCAGAACACA

CTGGGCATCCTATTGCCTTTCGAGTCGAACAGAACTCTGATCATCTGCGGCACTTCGGAT

TGCGGCTACTGCGAGTTGCTGGATATAAGCGATATAAGCCGCACTATCTACACGGAGACC

ACGCTGCCGTTTGGACCGCGTCTGAACGAATCATCTGTCTCCTTCCTCGTCGATTATACT

TCTAACAGTAATGGTACATACATGTTGGTGGGGAGAGAACATGATGAGCTCGAGGCTGAC

GGGTGTATAGTGGATGTTGGTGTGACACTGCGCAACACTCTCCAAACTCAGCCAGGAGAC

ATTTTTTCTAAAACAGCAGATGCAATTGAAGCTTACATCAAACTGACTGGTGTTAAGTGG

GTAGATGGTTTCCAGGTATCTTCCCAGTTTGAATCCTACCTCTTTGCTAATAATATTTTA

CGCAGGGAAGTAGTTTTACTAAAGATGGTAAACAGAGGCAATAAACCAGATATAACCAAG

TCTCTGAAAGTTGCAACTTTACACTGCTGTAAGGACCTGTATCGCCACAAACTTGTGTCT

TCAACCCATGTTCTGTCAGAATCTTCTAATTTATGGGTGGGAATATTCACTGCAGAACTT

CCACAGCACCCCGAGAACACTGTTTTGGCTATTTACAACGTTACAGCGCTCAGGCCAGGG

AAACCTACTCAAGACTTCCAGTGTCAGCCATCATGTGTCTCACAGGAAAATATGGCTACT

GAAAATCCTCTTGCTGTGGTGTTTAAACACAGTTCGATGACATCTGTGGCAGTAAAAAGA

AAGGGATCCTGGACGGTGCTGTACATTGGTACTGCAAACGGCCAATTGCTAAAGCTTGTA

CTGGACACAGAATTTAGATCTTCCTGTGCAAAAGTGCTGTACAGATCTGATGATGACACA

ATGGTGTTTCCCAAAATGTATTTTGATCCAGTGGATCCCAATTACATCTACATGGCTTTG

AGAAATCAGATTAAACGTGTTGCAGTGTCTCAGTGTGGTATATACGACACACTAAGAGCA

TGCCTTGGCAGCATGGATCCTTTCTGTGGCTTTTGTGGCATCACAGACAGTTGCTCTAGG

CAGGAGGAGTGTTTAATCTCACCCTGGATCTCCAGTTCAAAGAATTCTTTTCAGAAAGAG

CTGATGTTGTTTCAGGTGGTTTCTGTGTTAACTGGAGAGATTACTCTGAACCTACACCTG

CACCTGGAGGCCACAAAGGAGCTTCCTTTAACATGTGATTTTAAAGCAGGAACTTTACAT

CTGTGTAATAGATTTAATACTACAGTTCCAATTGCACAAATTCCTAAATGCTCCTGCAGT

TTCCCTGAAAAGCAACTGTCTAATAAAGGTCTCAAGACGACAGTGACAGTTACTATCGGT

AATCAGACCTTGACAGAGAGCCTGAACCTGAGGAGCTGCCCAAACATTACACAGAATACT

CCATCAGATGATCAATGTGCAGCCTGTGTCTCTGCTGGGTGTTACTGGCACAATGATAAC

CAGACCTGTAGCTGGACTTCCATACCTGCTCCGTATCTACACATACAGGATGTATGCAAG

GACACTTCATCTGATAGAAATGTCAGGCCAGAGATCCTGTCTGTGTGGCCTAATGAAGTC

TCCATCCATGGCAAAAACAATGCGGTGATTAAAGGAAAGAATCTGGAACTGGTTGTGAAA

ATTCGCTTTAAGGATTATATGAACTGCATTTTAAATGAGACACCAGTGTTGGAGCGCTCT

AACACCACACTTAAGTTCTTTATACCTAGTGGAAACAAAGAAGTCGCGAGGGTATGTGTG

GTAACAGCGGATGGACGTTGTCATAGTAATGCCACTATAAGATATAACTCCCAGCCCACT

TGTACGGAACTGCAGCCTAGAGTTACCTGGTCCAGTGGTGAAAGAAAAATTAAAATGCTT

GGAAGCAATTTAGAGTTTGTGGATGCAGTCAGAATTCATACATCTTCCAAAGAGACCATC

CTGAACTCAGATAACAAGACCTTTTGGTTTTACACTCCCAATCAGAAAGAATTCAATGAT

GCTGGGCCGTTTACTGTCTCTCTCAGGGTGGCCAACTCAACAGTGGTCTGTGCAGGTAAA

CTTGCCTACCATCGGGATCCGGAATTCACCAGCTTTACTACCTCTAAGGTGGTTCATGAT

GTGCTGGTCACTATACAGAAAAAAGAAGATCATTTGAATATAAGTGAAGAGGATCTAAAG

GTGTGGGGTTTACAGGAAGAGAAACATTTTGAGTGTAGATTAGAGAAAGTTAAATCCAGT

GCTGTGATCTGTAAGATTTCTGGAGAAAAGGACAGTGAAATCACGGTGGACTCACTCAGG

ATTGAGTTTGGCAATGTTAGCAAAATTTTGACACCTGATCCTCCGAATTTTATATATATA

CTGGTCACATTGATAGTTTTGATTGTTCTTGGTGCTGTTGCTGGAGTATTAATTCTCCGC

AAGAGCAGACGGCAGATGAATGCACAAATGAATGAGCATCTGGAACTGCTGGAGAATGAA

ATCAGAAATGAGATCAGACAAGGATTTGTGGATTTGCAGACAGAGAGATCAGAGCTGGTT

GAAAATGTTGGCGCCATTCCGTACCTAGATTATAAACATTTTGCTTTAAAAATATTCTTT

CCTGAGGGAGGGCCTTTAGCAAACCAGATGGTCAAAGAAATTGGCCAGGATGCAGTGAAG

ACAGAGATGGATGAGAGATGCCAAGCATTCTTCTCACTCATTCGAAATCAGAGATTCCTC

ACATGCTTTCTTTATGCTCTGGAGGAGCAAAAGAGCTTCAGCATAAAGGACAAATGTACA

GTGGCATCGTTGTTGACTGTAACCCTGCATAGTGACCTGCCCTACCTCACCCAGCTGATG

GAGGAGTTACTCCAGAATCTAATGGATCAGCCCAGCAATGCCCAGCCCAAACTCTTGCTA

CGGCGTACGGCGTCTATTGTAGAAAAACTTCTTACCAATTGGATGTCTGTCTGTCTGTAT

GGCTTTCTTAGGGAGTCGGTTGGTCAGCCACTCTTTCTAATGATATCAGCTTTAAACCAG

CAGATTTCTAAAGGACCAGTGGATGCAGTGACTGAGAAAGCACTGTACACACTGAATGAA

GACTGGCTCCTATGGCAGGCTCAGGGTTTCAACTTCAGCCATCTGAAGCTGAAGGTGCTG

TTTGCTGTAGGGACGGAGGGAGAAGTGAGTGAGCCGCTGGAAGTAAGTCTCCTGACATGT

GATACAATAGATCAGGTGAAGGACAAAATCCTCCAGACCTTCCAGTGCAAGTTCGGCTTT

AGATTTACTCAACAACTCCGAGATCTTGGCATTGAATATATGAAGGAAGGAAGTTATATG

CCTTTGGAGGAAGTAGATGGAAGCTCAGAGGTGCAGGGGGAGGTGACCATGCTGAACACT

CTGAAGCATTATGAAATCCCAGATGGAGCTTGCTTAAAAGTAATCACCAAAAAGGTTCAT

GCTCCTCTCAGTCCACAGATTAGCTTGAAAGATGATAAGAACTTCACTACAAAGTATTTT

CACTTGATTGACCCAGACATCGACACGGACCAGAGCAAACACCCAGAGAGGAAAAAACTG

AAGCTCAAAGAGATTTATCTCACCAAATTACTTTCTACTAAGGTGGCAGTGCACACCTCT

GTGGAGATGCTCTTTCGCAGTATTTGGGGAATGCCCAACAATAAAGCTCCACTAGCAGTA

AAATACTTCTTTGATTTCCTCGATTCACAAGCCGAGAAGAAAAAGATCATGGATCCAGAT

GTACTTCACATCTGGAAAACAAACAGCCTACCACTGCGCTTCTGGGTGAACATTTTGAAG

AACCCCAACTTTGTGTTTAGTGACCTTGAGAAGACGCCTCATCTGGACTCATGTCTGTCT

GTCATAGCTCAAGCCTTCATGGACTCCTTCTCTCTCACTGACCATCAACTGGGCAAGCAT

GCACCGACCAACAAGCTTCTGTATGCAAAAGACATTCCTCAATATAAACAGGAAGTGAAA

ACATACTACAAGCTGGTGAGGGATCAGCAACCTGTCAGCACTCAGGACTTTGAAGTGTTT

CTGCAAGAACAGTCAAAAAAACATGAGGAGGAATTTAATGAACCTGCAGCACTGCGAGAG

CTTTACAAGTACATGGAGCGCTATTTCAATGAGATTGAACAACATTTAGAACAAAAGGGC

GTCTCCTGTGGACTGAAAGAGGAAATGCATCGTGTGAAGGAGCTCTTTGAAAGTGTTAAA

AGGAGCGCATGGAACTGATCAAAAGTTAAAGATCTGCGAGGACATTTTATTTGATATTTT

CCTAATTATATAATTATATAAATGATTTTAATTTCATCTTGTCATGTTTTAAGCAATAAT

CTGAACAGTGGCTCTGGTGACTTATGCCGGTACAGTGGTTGTATATATTATTATCTCACA

TTTTCATTTCCGCGGTGTGCATGTGTGTGTCTAGCTCTAGCTAGTGTGTGTGTGTGTGTG

TGTGAGATATTTAATTATTTATTGAGCGCCATTAGCCATCCGAAGCAGATCAGTACCGAA

TTCTAATCAAATCTAAAATCCCTTTTTTTCTTCCGCTGTTCCCTTTTTAGGGGTCGCCAC

AGCGACTCATCTGTCTCCCCCTACCCCTATTCTCTACATCCTCTTTACTCACACACACTA

ACTTCATGTCCTCGTTTATCACATCCCTATACCTCCTCTTTGGTCTTCCTCTTTACCTTC

TGCCTGGTAGCTTCATTTCAAACATTCTCCTAGCAATATATTCACTATCC

>Unigene000276

TTACCGAGATCGGAGAGAGAGAGAGAGAGAGAGAGAGACTGGCAGTGTGAGCGCTGGGCT

CGTGCACTTGCACTTTCTAATGAAGTTTCAGGGTGGTGAAAGGCGCGGATTTTCTCATTG

TTGTTTTATTTAGCAACACAAATGAATGTTCACAGGAGAACTGAACTGAACCAGCAGTGA

GATGAGATTTTACCGGATACCAACGGGAATTAATTAAAAGAAATCCGTTAGAGAAGTGGG

CGGAGCCTCTGCACGTGAAAGGGCCCATGGAGGGTATCTGCTTTGAGGTGAAGCCCCAAG

TTGACCCCGTCTCTTTCTCCAAGCTGCTCCCGGATGGATCCGGAGATAAAAACAAGGAAG

AGGACACGAATGAGAACGAGATCCAATTTAGGCTGACGCTGGTCGGCTCATTAGCAGTCC

ACCCACAGACCACCATGGCAATGCTGCCGTGGGTGGTGGCTGAAATCCGCAAGCCTCCGC

TGAGGGAAAGGAGCCTCGACGCAATTGTCACTGGATCTCAAGTGCTCTTGCGGGTCTCGT

GTTCTTGGGTGCAGTGTGTGTTGAGCGAGGCATGTGCGGGAGACCCGTATGACCCACTGC

GACATACCGTCCTCTTTCAGCAGCGGCCGCATCACCTCACCAAACTCCTCCATAACAGCA

AAGAACCCAGTTACTTCGCCTGTCTGATTCGGGACAAGACCAGTGCCACATGCTATGTGT

TCCGCTGCCATGACAAATACAAGGTCCCAGAGATCATCAGCACCTTGAGGCAGGCCAGTA

AAAGCTCAGCCCGCCAGGAGGACACAGTCACCGCTGGCCAGATCGGTGACCCCCATCAAC

ATAGCGGCCACACGGACCACCCGGAATCGGCCACAGCCTTTGCCAAAAAGTTTGAGGTGC

TGTTTTGCGGCCGGGCAGTGGTGGCACAGAAGAACGCTCCGCCAGCACTGATAGACGAGT

GCATTGAGAAGTTCAGCCGAGCGAACGCCACCAACTCTCTGACTGCTGGGATCCGGAGAG

CTTTCTCACTCACGCAGGTGATAAATGGTGTGAGCGAGGGAGCAGATGGAGGAAGGAGAA

GGACGCTGTTTTTCCGAAAAGATCCCAGCTTCCCGTCCCTGCAGGCATTGGACGAGAACG

GTCTCTCGCCAGAGCTGCACCGCCGAACCGCCAACGAAGCCACCGGGGTTCAGCCAACCA

GCCTGCAGGAGAACAGGACGATGTTATTTATGATTGGACACTCTCAGATCTTTTTGGTCA

GTCCTGACAGTAAAAAAGTGGCCATCGAGAAGAGCTTCAGGGAAATCTCTTTCTGCTCAC

AGGGTATCACGCACGTGGATCATTTCGGGTTTATCTGCAGAGAGTCGGACGAGGGTGGCA

GCTGCCAGTTTGTGTGCTACGTGTTCCAGTGTACAGATGAAGCCCTGGTGGATGAGATCA

TGCTCACCTTGAAGCAGGCCTTCTCAGTAGCCGCTTTGCAGAAAAGCACTACCACCCAAA

ACCTGCAGTGTGACTCCTGCCCCATGCAGCAGCTGCACTGGCTCTGTGACCGACTCGAGG

GTCTCACTGCATCCAAGGCTAAGATGGAGCTGCAAAAGCATGTGGCATTTCTTAATAATC

AGGAGCAGGCATGTGTGTTTGAGACCGCCCTGAAAGCCAGGCCAAAGTCAGAACAGGAAG

AAAACGAGCTGGTGGTATCATGTTTAAGGCGACTCTATGAAGAGAGACAGAAGACACACA

CGCACACACACATTGCACAAACAAAACAGGAAGCTCCGGTGGCTTGCGATGAGGCTTGTC

CAAGTCCTGAAGGCAGCACGAGCAGCAGCCGACTGCGTCTGGAGCAGCTAAAGAGTCGAG

CTAAAAGGTCGCTTACAGAGTCACTGGAAGGAATATGGAAGAGCAATAGCAGGTCCAGGT

TACAGAGAGAGAGCAGCGAAAGTTCCTCAAACTCCCCCATCAACAGCAGCCAACAGGACA

TCATCTCGGTGGAGCCGCCATCTCCGCCCCTGCTCCTGCAGCCGTCCAGCCCGCTCCGTG

CCCACAATTCCACAGGCGACCTAAAGAACCTGGAGACCCCAGACACGGATCAGGAAGTTC

ATGACCCCCAGGCTGGGAGCTTCAGGCGGCGAGCGAGTACGATCAGTCACTCTCCATCTA

TGTGCCTCAGCAGCCTGAGTGTCCCGGACGAGCAGACAACAGCCGGCAGCAAGCCCAAAC

TCGTACGCCATTACTCCGTCAGCACTGACTCGCCACACCACAGCAAAGATGTAGCGGCCC

TGTCCAGCCCGGGAGCTGTTTGTGTGGTGGGAGCGGACAGTCCACTCCGTCAGCGCAGAC

ACTCCTGGAGACAGCAGATTTTTCTCAGAGTGGCAACACCACAAAAAGGCTCAGACACGC

CGGAACGAGGAGAAGTGTCCCTAGAGGTCGGAGGCAGATTGACTGCAGGGGGCGGAGCTG

TGGGCGGGGACAGCATGCTTGGGCCACTGCTTGAGGAAAGGAGGAAAAAGAGTCGTGAAG

AACTGCGAGAGTTATGGAAAAAAGCCATCCTGCAGCAGATCCTCCTCCTGAGAATGGAGC

GAGAGAACCAGAAACTACAGGCCTCTGAGAGCGACCTTCAAAGCAGACGGTTAAAGCTGG

ACTACGAGGAGATCACGCCCTGTCTTAAGGACGTCACGCTCGTCTGGGAGAAAATGCTCA

GCACTGCTGGGAGGTCAAAGGTCAAGTTTGACACTGAGAAGATCCACACAGCGGTCGGGC

AGGGTGTGCCCCGGCAGCACAGGGGTGAAATCTGGAAGTTTCTATCAGAGCAATATTTAC

TCAGACAGCAAGTTCCCAGCGCCAAACCGCCCAAAAACCACCTGCCTTATAAAGAGCTAC

TGAAACAACTCACCTCCCAGCAACATGCCATCCTCATCGATCTGGGCCGTACATTCCCCA

CTCACCCGTATTTCAGCGCTCAGCTGGGGGCAGGGCAGCTGTCACTCTATAACCTGCTGA

AGGCCTACTCACTGCTGGATCCGGAGGTGGGCTACTGTCAGGGCCTGAGCTTCGTGGCTG

GAGTTCTGCTGCTACACATGAGCGAAGAGGAAGCTTTCAACATGCTCAAGTTCCTCATGT

ATGACTTGGGATTGCGCAAACAGTACAGGCCTGACATGATCACCCTTCAGATTCAGATGT

ATCAGCTTTCGCGGCTGCTACATGATTATCATCGGGAGCTATACAGTCACCTGGAGCAGC

AGGAGATCGGGCCCAGTCTGTACGCCGCACCGTGGTTCCTCACCGCTTTCGCATCCCACT

TCCCTCTGGGTTTTGTGGCCAGAGTCTTTGACATGCTGTTCCTGCAAGGCTCAGAGGTCA

TCTTCAAGGTAGCGTTAAGCCTTCTTGGAAGCCACAAGCCGCTGATTCTTCAACACGACA

ATCTGGAGTCCATCGTGGACTTTATTAAGAGCACGCTGCCTAACCTGGGGCTGGTGCAGA

TGGAGAAAACCATCAACCAGGTCTTTGAGATGGACATCAGTAAGCAGCTGCAGGCTTACG

AGGTGGAATACCACGTTCTCCAGGACGAGCTCCTTGATGGTCCAACCACTCTCAGCCAAT

CACAGCGTGCCGCTCAGCTGGAGAAAACCAACGGAAGCCTGCGGCAACAAAACCTGGATC

TGCTGGAGGAAGTGCAGGTGGCTCATGCAAGAATCAATTTCCTGGAGTCGCAGGTGGAAA

GCTTGGTGAAAAGCGAGACGATGCTGCGGTTTGAGCTCTCATCCCTGCAGCAGGAGCACT

CAGACCTGCAGCAGACTGTCACCCGGCTGCAGACTTTGCTTGCCAGCCATGGAATCCAGT

ACACCTCAACTCCGTCCTAATACACTCCCTCATCCATTCTCTAACTTTCTGTCTTCTCAG

AAGGAGAAATATCGAGAAGAGACCTTCGGGGCAGAAAAGGAGCAAAGACTCCTATTTCGG

CGTAACTATTTTTTCCTGGACGACATTACAGGGTGACAGAATCATAAGTAGGTGCTTTCT

TTGTCTCTCTCTTAATTTAGAGGGACATTCAAGTGCCTAAAGGTTAAAGGTCACTGATTT

TGGATTTGAGGACCCTTGTTATGGTATCCCGATGACACACCAGCACTGATAGACTCTGCG

CCATGCTCCCAAACAAGTGCATACTCTCGTCTAGTGTGAGTGTGTAAAGAATTTGCACCT

GTTGTCTTGTGAATTTTATGTAAAAGCAAAGCTGTATGCTGTTTTACATCACTAAACTAA

AACAAACAAAATGTGCACAATCACCATTTTTCTTTTCATCTGCAGCGTTTCCCAGACATG

AAAGTGGTTTTTTTGTTGTTGTGTTGTTTTGTTAACAATAATTACTCTGCTTATCATGCG

TCCCTGTCTAGACACAGAATCATTGTGTGCATTCAGTCAAAGTTGCCCAGCCCTTCAGAT

ACAAGTCTGAATAACAAATACCCACAATGCACCTCTGACAGACACCAAACTCGTCCAGTC

GTGTTTCCACAATGACTCGCAGCGATCTTTGCTCTACATTCCAACAATCAGCTAAACGAT

ATCTTGCATGTGACCGCTCCAGCTTTCGCACAGAAATTGCAGGTTTCCATAGCGGCCATT

CGTTTAAAGGTCCTCCTTATTTACCGTGTATAAGTGAACGACAGAAAAGGGAAAATTTAT

TTAAAGGAGTAATTTGTTTAAAAATTAATGGAAGAAGCATTTTATGGCGAGGGACAGGCA

CTTTTTAGACATTATTTAAAAAGTCTATCATGAAACGTTTCATAATTAAATCCTGGAAAA

AATTTTTATTATGATCCTTCTTTACTAGATAAACAAAATAATAGACAATACCCAGTTTTT

AACCTGTGAGGTTTTCACAAACCAGAGCTAATATCCATCTGGAAAAATGCTACATTTCTG

TCTCACAGACTGAAAATCCCCACCCACTTATCCTGTAATTTTCACAGACAGGAGGCAGCT

GTGTGGGATCTGTGTTCAGACTAGGGGACGGGTTATTTTTCACACATGCTAATTAGAGTG

AAGATACTTTGTAAGTTCATCCTTTGCCGGTTTAGGCGGGACAAGAGGGCCGTTCATTTT

GTAATGAAATTGTATGTTTTTTTTACAAATGTAAATATTTTCATCAATTTACTACCTGTC

CTGAAAATTCAGATCAAGAATAAATAGGCACAAAGTAAACGCTAGGTTCTTCCCTTCAGC

TTAAAAGTAAGCATACGTCACCTACACACACACACACACACAAACAC

>Unigene000277

CTCAAGATCGGAAGAGCGTCTATAGATCAAGCACGCTCAAATCTTATTTGGAACATGAAG

ACACGTGAGGAGCTGCGTGATGCTTTGGAGGGAGAAATGCGCTCCTTCAGCATAGACAGG

GAGCTGGGGAGTAACAGTGTGATTTCCTGGAATCATCAAGAGTTTGAGGTAAGGTATGAG

TGCCTTTATGATGAAATCAGAATTGGAGATTATTATCTGCGCCTCCTTCTGGAGGAGAAT

GAGAATGAGGAGTCCGGAGTCATCAAGAGATCGTATGAGTTTTTTAATGAACTATATCAT

CGTTTTCTCTTAACGCCCAAGGTGGCAATGAAGTGTCTATGCCTGCAGGCTCTGACCATA

GTATATGACAAGTGTTGTAAGGAAATTGGTCCTTTCACTGACACCAAATACATTGTGGGC

ATGTTGGATAAGAGTACTGATAAACTAGAGAGAGACAGACTAATTCTCTTTCTCAACAAG

CTCATTCTGAACAAGAAAAATGTGAAGGACATAATGGATGCAAATGGTGTGCGTCTTCTA

GTAGACTTGCTAACCTTAGCTCACCTCCATACCAGTAGAGCTACTGTTCCACTGCAGAGT

AATGTATTGGAAGCAGCTCCAAATATGAAAAGAGAGAGTGAAAAGGAGTGGTATTTTGGG

AATGCTGATAAGGAACGGAGAGGACCTTTTAGTTTTGAGGAGATGCAAGAGTTTTGGAAA

TCAGGTACACTAACTGCAAAGACACGATGCTGGGCACAGGGCATGGATGGCTGGCGCCCT

CTTCAGGCTATTCCACAGCTGAAGTGGTGCACACTAGCCAGTGGACAAGCAGTAATGAAT

GAAACCGATTTGGCCACACTAATCCTCAATATGCTCATTACTATGTGCTCCTACTTTCCT

AGCAGAGATCAGGACAATGCTGTTATTAGACCTTTACCAAAAGTGAAGCGGCTAATAAGT

GACAATGCTTGCCTTCCACATATTGTTCAGCTGCTGCTGACCTTTGATCCCATCCTCGTA

GAAAAGGTGGCCATCTTGTTATACCTGGTTATGCAGGACAACCCCAACTTGCCACGCCTC

TACCTCACTGGTGTTTTCTTCTTTATCATGATGTACACAGGCTCTAATGTGCTTCCTGTG

GCACGATTCCTGAAGTACACTCACTTGAAGCAGGCCTTCAGATCAGAAGAGGCTAAAAGC

CAGGATATTGTGCAGAGGAGTGTGTTAGGACCAGTTTTACCTGAGGCAATGGTGTGTTAC

CTAGAGAATTATGAAGCTGAACTCTTCTCAGAGATCTTTTTGGGAGATTTTGACACTCCT

GAAGCAATCTGGAACAGTGAGATGAGGCGTATGATGATCGAGAAGATTGCTGCCCACCTG

GCTGATTTTACTCCTCGGCTTGAGAGCAACACTCGTGCTTTGTATCAGTACTGCCCGATT

CCTGTTCTTAACTTTCCTCAGTTGGAAAATGAGCTGTTTTGCAACATCTACTACCTCCGT

CACCTTTGTGACTTACAGCGCTTCCCTGAATGGCCTATACGGGACCCGGTAAAGATGTTA

AGGGACACCTTGGAAGCCTGGAAAAAGGAAGTGGGAAAAAAACCCCCCTCTATGTCTGTT

GATGATGCCTATGACGTGCTAAACCTTCCTGCAGGACAGGGCCAGTACGAGGAAAGCAAA

ATCAGGAAAGCATATTTCCGATTAGCTCAAAAATACCATCCAGACAAAAATCCAGAAGGC

AGGGAAACATTTGAAAAGGTGAACAAGGCATATGAGTTTCTGTGCACTAAATCGGCAAGA

TTCGTAGATGGACCTGACCCTGAAAACATCATCCTAATACTTAAGGCCCAGAGCATCCTC

TTTAGCCAACACAAACAAGAACTGGAGCCATATAAATATGCAGGTTACCCTATGCTTATT

AAAACTATTACCATGGAGACCAGTGATAGGCAGCTTTTTTCCAAAACATCCCCACTCTTG

CCAGCAGCGTCTGAGCTTACTTTCCACACAGTCAACTGCTCAGCACTCAATGCTGAGGAA

CTGCGACGAGAAAATGGATTTGAGGTTCTGAATGAGGCTCTGTCTCGCTGTGTGGCTGTG

CTTACTGGTTCCAGCAAAATTGGAGACATGTCAGTGCAGGTATGCGGTCACATCTGTAGG

TGCTTCAGTGTTGCAGCTCAGTTTGAGGAGTGCAGAGAGAAAATCATCAAGCTTCCAAAC

ATCATAAGAAATATTTGTCACATACTTTACTATAGCAATGCTCTTTCTCGTCTAGCCTCG

CTGGCTGTAGAATGTGTGAGCTCGTTCTCCGTGGACTTTTTTCTTCAGACACAATTATAT

CAAGCTGGTGCACTTTGGCCACTGCTTCTCAACCTCTTTAATTTTGACTTCACACTGGAA

GAAAGTGGCGTCCAAGCTAGCCAGGAAACCAACCAGCAACATGTAATCAACAGCCTGGCC

AAAAACAGTCTGTTGGCTATAGCCCGTCTTGGAGGCTACAGAACCAGAACAGAGGCTGAC

GGAAATCATCAGATTGGGGGTGAACCAACTCCAGAGAACCCTGCAGTGAGGAAGAGTGTG

GCAGCCATGCTTACACCATACATTGCATATAAACTGAGCACGAATTCACCTGCCGAGGTC

CTAAAGTTGGTTAATTGCAATTCGGAGACCCCTTATCTTATATGGAATAATGCAACCAGA

GCCGAGCTTCTTGAGTTTCTTGAGGCGCAGCAAGAAGGAAACATGAAGAGGGGTGAATGT

GACGAAAGCTTTGGAGCAGAGTTTGCCTTTAGTGAGCACAATAAAGAACTCATTGTGGGA

GAGATCTTTGTTCGCGTGTATAATGAGCAACCAACATTTCCTTTGGAGTATCCGAAAGTG

TTTGCAGGAAGTCTTTTGGATTATGTGGGTTCTCAGGCACAGTATCTCCACACTCTGCTT

GCGCTGACACAGACAAATAAAGTGGAGTCACAGCAGCACGCTCAAAGACTTCTTTGGGTT

GAAATGGCATTAGAAGCACTGTGCAACGTTATCAAGAACAACCCAGGATCTGAAACAGAA

TGTATTGGCCATTTTAAACTGCTCTTTTCCCTGCTCAGAGTCCATGGGGCTGGGAAAGTT

CAGCAGCTAGTCCTAGAGGTTGTGAACACAGTTACTTCAAACCAGGAATGTATCAGCAGT

ATAGCTGAGTCTTTGGTGTTGGCCAATTTGCTATTGTTGCTGCACTCCTTACCATCAAGC

AGGCAGCTTGTCCTGGAGACTCTGTATGCCCTCACGTCTAACACCAAAATTGTCAAAGAG

GCAATGAACAAAGGTGCATTGATCTTCCTGTTGGATTTGTTCTGTAATTCCACTCACCCT

CAGGTGCGCACACAGACAGCTGAGCTCCTGTCCAAGATGACCTCCGACAAACTTGTGGGC

CCCAAGGTTCGTCTGATACTGATGCGTTTTCTGCCAGGAGTCTTCATAGATGCCATGCGA

GACAATGCTGAAGCTGCTGTGCATATTTTTGAGGGTACTCATGAAAACCCAGAGCTGATT

TGGAATGATGCATCACGAGAGATCGTCTCGACCACTGTCAGGGAATTAATGCTTGAACAC

TTTAAGCAGCAGAAGGATAACCCTGATGTCAATTGGAAGCTTTCTGAAGAGTTTATGGTC

CCATATGCAGCAGGACAAGGTGAGCTGGAAGTGGGTGGAGTGTTCCTTCGCATCTTTATT

GCTCAGCCAGGCTGGGTGCTGCGCAAGCCTAGAGAATTCCTTGTCTCCTTACTTGATGCC

CTCACGGAGCTGCTGGAAAGGAACCACCCTAATGATGAGGCATTAGAGACAGTCACAACA

GCAGCTGTGTGTTTGTTCAGTACTCAGACTCAGTTGGCTGATCAGATTCCGCCTCTTGGT

CACCTGCCGAGGATCCTTGCAGCTCTCAACCACAAAAATAGTGCTGTTCTTAAAAGCTCC

ATCCGCCTCATCCATGTCCTCTCAGAGAATGAGTTGTGTGTGCGCTCCATGGCAGCTTTG

GAGACTATTGGACCATTGATGTCTGGGATGAAGGTCAGGGCTGATATGGCAGGACTAGCA

TGTGAAGCCCTCAATCGCATGTTTCAGCGAGAGCAAACAGACCTAGTTGCACAGGCTCTT

CGAGTAGAACTGGTGCCATATTTACTAAAGCTTTTAGAGGGAATAGGATTAGAGACTCTG

GACAACCCATCAGCAACCAAAGCCCAAATAGTCAAAGCACTTAAGTCCATGACTGGCAGT

TTACATTATGGAGAACAGGTGAATGAAATCTTGTCCCGATCCACTGTGTGGAGTGCCTTC

AAAGATCAGAAACATGATCTTTTTATCTCTGAATCACAACTTGCAGGCTTTCTGTCAGGT

CCAGCAGTAGCAGGCTATATAACTGCTGGCACGGGTGCTACAGTTATGTCCACTATCCCG

CCACCTGTAGACAATAACATTGGAGACCAGTGCTAAAAATCAAACAAGCACCAACAGTTT

AGTACCTGCACCATACAGAAAGACCAACTAAAGGACTTAAATAGAAAAGTGTTTGGTCGA

TTTCAGCCCTCTCAATTTGTGATGCACTGTACTCTGTGTCCATGTTATTTTTTTACAATC

ATTAATTATTTCCTTTTTTTCTTTTTGCTCTTATTTCTAGTATATTAAGCTATAATTTTT

CAAAAAAGGAAATTTGTTTACTTTTATTACTTTGTATAAGAATAAATCATTATTTGCTGG

AAGGAAAAAATAAATACATCTCAAAAGTCAACACATTCTTTTTTTCAGTTCACCTTATTT

TCTATGCTATGCTTTCCATTGTGTTTTTCTCTATATTATTATGGTAAGCTTGTCTTATGT

CATAGATTTGTGTGCCATAAAGCTGTCATGCCATGTCATGTTCTTCACCTAGTTGTGTTT

TCTATTAACCCGAAAATAAATAAATAATTTTGAAAATTGAAACCTGTCACTGTCAGTTAC

AATAACACCCCATAGAGTGACTTGTATTTAAACTTACCTTATGTCTCTGCAGCCAACCTA

CCCTTACCTACCAGGAATGCAAAAATATCACATGGTAAAAGTAAGTGATAGCTAAGAGTA

ACCATGATGAATAATGATGAGCTTCTTAAATAGACTTATCAAATGTATGTGATTAATTAA

ATTGTCACTATCTATGTAAGGAGGCCTATATAAACTGTTTAATTTGAATGTACTATTAAG

AAATGTCACACTTGTATATAAAATTTTAAGAATAAAAAGGAAGCCAA

>Unigene000278

AAAAATATTGGCATACAAAGTGTGATTTCAGAACCTCAACTTTGCTCCATTGCTGAGAAT

ACAGATAATGTTTTAGATTCAGCTCTAATCGGTAGTTCCTCTACTGTGCAAGGTGAACAA

ATGAAGTTCATAACCAGTATTTCAAATTCATCCCAGGTTTCTCTTTCCACGAGTATTATT

GATCCAAAGTTGCTTATACTGAAGAGAGGTGAGGCTCTTCTCAAACAACCTCTAACGTTG

ACTCCAGTTTCCAGGAACCAGAGCCCATCTGGTCCATCAAGTGATTGCGTAGACCAAGTG

TCTGTAGAGTGCCTTTCGGGATCTGACTTAACTACAGATGTTACAGATGGAAAGGCAGCC

TTATCACCTCTCAGCTTTCATGTACTAAATGCTCCCTGTAATGCTACTGCTTCATTTTTA

AAGGATGAGTCTAAAAAACAGGCCTTAATCAAGTCTGAACATTTGATAGCATTTGAAAAG

CTGATTCCTCAAAATGAGGCCAACTTAATCTCTGAGTCATATATCCAATCTCAGACTCAT

GACCCACCAAGACTGCCTTCCAGGTCAGACAGTATGTCTACTACGATCACTGAACAGCCT

GAGGATCCTGCTCACCTGCCATGCCAAGATTCTATGCTATTGGAATTGGAGGAGCACCAC

TTATCTGTTCAGCATTTGGAAGAAATAAAAGATGCACAGGATAGCGCGGTGGAACACCTA

GATGCTTGTATAGAAGAGTGCAGTGAGGGAGAAGCTGATGGTTCATTACAAGCCAAGAAC

AACAAATCTATTGCTATTCCCTGTACAGCACACAACACGGTTTGTCAATGGATAGCAGGC

AGAGGTACTACAAGCTCCACTCCCTCCACCTCTCGTACACCATCACCACCTCCTGCTTCC

TTGTCTGCAGACTGGAAACACAGTCTGCCTTCTCCCCAGAACTCTGCTGGAACTTCTCAG

TCAGATCACTCACTCAGCCAGCGGCCTCGCTCAGAAAAGCACACTGATATGGCAGAACTG

GCCAAACACGAAGCAGAAATTGAACATCGGACACTAGCGTTGAAACGTGAGGGTTTCTGG

TCCTTAAAGCGTTTGTCTCGTGTGGCTGAGCCACTTCGGCCCAAGGTGCACTGGGACTAC

CTTTGTGAGGAGATGCAGTGGCTTTCTGCTGACTTTGCACAGGAAAGGCGCTGGAAAAGA

GGCGTGGCCCGAAAAGTAGTACGGATGGTGATGCGCCACCACGAGGAGCTGCGGCAGAAA

GAGGAGAGAGCAAAGCGTGAAGAGCATGCCAAACTAAGAAGGATCGCTGCTTCCATTGCA

AAAGAAGTCCGTGCTTTTTGGAGCAGTGTCGAAAAGGTTGTGCAGTACAAGCAACAATCT

CGGCTGGAAGAGAAACGTAAAAAGGCCCTGGATCTGCAGCTGGATTTTATTGTTGGCCAG

ACAGAGCGTTACTCTGACTTGCTTAGTCAGTCTTTGAAAGCCACACCTGCAGCCCCTGCC

AGTGTTTCTCCCCAACAGCGTCCGCTGAAAGCACCAGAGGATGAAGATATTGACTTTGAG

CCTCCATGCGAGGAGGACGATGATGAAGAGACAATTGAAGTGGAGGAGCAGCAGGAGGGG

AATGATGCAGAGTCTCATAGAAAGGAGATTGAGTTATTGAAACAGGAGAGTACACTGCCA

CTGGATCAGTTACTTGGTGCACTCACTCTTCCCCAGGGATCTGCATCTGAAAATGAAGTG

TCAGACGATTCATCCTCTACAGTTGAAGATGAGGACAAAGAATTTTCAGCTAACGAGGAA

GATTCGGAGGATGTTGAGGACACCATTGAAGCCCAAGAGGTGATTGAGGGAGATGGAGAC

CACGAGGAGGAACTTGATGACTTGGCTAAAGAGGGTGAGCTAAGTATGGAGGAGCTGCTA

GAAAAGTACAAGGGTGCTTATGCCTCCGACTATGAAGAACCTTTAGCAACATCAGAGTCC

TCCGAGGCAACCGAAGATGAAGAAGAGGAAGAGACTGAGGGAGATGAAAGTGATAATGCC

TCTTTAGATTCAACAGAAAACAGTGATGAGGAGGATGATACTGAAGCTGAGGAGAGCGAA

AAGGAGGAAGATAATGGGGGAGAAGGGATGGAGGTTTTACTAAGGGAGGGAGCTCATAGC

CCAACTGTCCCCACATCTCCACGTCCCAAAAAAAGAGATCAGCCATATAGCGGCTACTGC

TGAAAGCCTTCAGCCTAAAGGATATACCCTGGCTACGACAAGTGTGAAAACGCCTATTCC

GTTTTTACTTTATGGCAACCTACGCGAGTACCAGCACATAGGATTAGACTGGTTGGTTAC

AATGTTTGAAAAGAAGCTTAATGGTATCTTGGCAGACGAGATGGGACTTGGTAAAACTAT

TCAGACTATTGCCCTGTTGGCACATCTGGCTTGTGAAAAAGGTAACTGGGGCCCTCACTT

AATTATTGTACCCACCAGTGTAATGCTGAACTGGGAGATGGAGCTGAAACGCTGGTGCCC

TGGCTTTAAAATTCTTACTTACTATGGCAGCCAGAAAGAACGCAAGCTTAAGAGACAGGG

CTGGACTAAACCCAATGCTTTCCATGTGTGCATCACGTCATATAAGCTGGTTTTACAAGA

CCATCAGGCATTCAGACGCAAGTCTTGGAAATACCTCATTCTGGATGAGGCACAGAACAT

CAAAAACTTCAAGTCCCAACGGTGGCAAAGTCTGCTAAACTTCAACAGTCACCGGCGGCT

GCTGCTTACTGGTACTCCGCTGCAGAACAGTCTGATGGAGCTGTGGTCGCTCATGCACTT

TCTCATGCCGCATGTCTTCCAGTCCCATCGTGAGTTCAAGGAGTGGTTCTCCAACCCACT

TACTGGGATGATTGAAGGAAGCCAAGAATATAATGAGGGTCTTGTAAAACGACTACACAA

GGTGTTAAGACCCTTTTTGCTACGACGTATCAAAGCAGATGTAGAGAAACAAATGCCCAA

GAAGTATGAGCATGTTGTGCGCTGTCGCCTCTCCAAGAGACAGCGCTTTCTCTATGATGA

CTTTATGGCACAGGCCTCAACGAAGGAAACGCTAGCCAGTGGACACTTCATGTCTGTAAT

CAACATCTTGATGCAGCTGCGTAAAGTGTGTAACCACCCTAACCTGTTTGATCCACGTCC

AATCCAGTCACCCTTTATCGCTGAGACTATAACCTACTCCACTGCCTCCCTGGTTCTGAC

GGCCTTAGACACATCCCCTTTTCAGCGCTGTGACTTTGCAATGTTTGACCTGGTGGGTCT

AGAGGGCCGTGTCTCTCGGTACCAAGCAGATGTTTACCTACCACAAAGGAAAGTAACACG

CCAATTGATCCAGGAGATCATGGAGTCCCCCGAGCCTTTACCCAGACCCAAACCTATTCG

CATGAAGGTCAACAGGATGTTTCAGCCACTCCCCAAGTCTGATGGACGTTCAACAACAAC

AGCAAATAACCCACGGCCCACATGTTCTGACTCTCCTGCAGTTCAGTCTCCACGGCTACC

TCTAAATACAGAGGTTACTCAGGCCCCTGTACAGCCGACACCCCAAGTGTGCCCTGTCAC

GCCTGTTGCGGCATCACAAGCTTCAGTGTGTCAAAGTGTTGCGTCATTGGGGTCTACAAA

TCCAGTTATGAGCATTCGGCATGCTCCACTTTCCACTGTTCCAACAGTGGCAGTCACCCC

TCAACCTTCCAGCAATGTCATGACGCAGAGGGTGCTTCTTAGCCCTGATATGCAGGCCAG

ATTACCATCTGGGGAGGTGGTGAGTATTGCCCAGCTGGCTTCTTTGGCTGGTCGTCCAGT

GTCATCCAGTCAAGGCAGTAAGCCTGTCACATTTCAACTGCAGGGTAACAAACTTACCCT

ATCTGGTGCTCCCCTCCAAGTGCCCGTTGCTCAGCCACGGTCCATTCAAGGAAGTGTAAT

GCATCTGGTATCCAGCGGTGGGCAGCACCACCTTATTAGTCAGCCAGCTCAGGTTGCAGT

TCTACATACAATTAGTCAGTCAGGTAGCATTTCAGCAAATTCCAACCCAACCAGCACAAT

CCCATCCTGCTCAGGACTAGCGGTACCACTCACTGCTGCTCAAGTTCAATCACCCAGAGT

AAGTGGACCAGGAATTGTAAAGATTGTTGTTCGCCAGGCAGCAGGCAAAGAAGGTGGCCA

GGTCCCCACTCTTGCTGTACCAGCATCTCCTCGTGCTGCCCCTCCTCAGTCTGTTTTGTT

GCATCCACATGGAACAAGTTCTGCTCTCAGACCCACTACCCCACAACAGCCTTCCCAACG

ACTTCCTGTTCACCTTACTCCTCCTCAAGCTCCTACCTCATGCCTTGTTCAAACTCCATC

TTCAAGTTCTGGGCTAAAGGTGGTGGAGAGAACATTACCGAGCACAGGCTTTGCTAAAGC

AAATGCTTCATTAGAGGTTGAAGACAGCAGTAGTGATCCTACTCATAAACCTATGACCAC

ATCTCAAAGCCGTGCATTTGGATTTCAGCGTCCGTTAATCAAACCCCAGATTTCACCACG

AACCCCATTTCACATGTCATGGCTTGCAGATCGTAACAAGGCCCAGAGAGATGACCACAT

CTCTCGTATCTTTCGTGTCAATGAACTGCACTGCAATGTAAAACCTGTGTTTGGTCAAGA

AGTATTGGATTTTCTCACCTTCCTTCCAGGCCCTAGTCCTACCCCGGCTAAACCTGTCTT

TAACAGGTGGAACCACTCAGGATATAGTTCTTGTCTCATTGCTCAGTCCCAACACAAGCG

TGACTACCTGGACCAGAGTAAAATACTGCAAAAAGCTATCAAGAATTCTGAGGAAAGGCT

TTACCTGATGTCAGGGGTCATAGAGAGGTTCATATTTGTCATTCCCCCAGTGGAGGCCAA

GCCCATCACTATGCATAGTTGCCATCCACCTCCTTCCCTTAATCACCAACAGGCTCTGCT

TTCCTCCACACTGTCTGCTCATCTTTCTCCCCTTATGCAAATTCTGCACAGCATTCGCTG

TAATATGCGGACACTGTTTCCTGACCTGCGACTTATCCAGTATGACTGTGGCAAGTTGCA

GACTCTTCATCTTCTGCTCCGGAAGCTGCGGTCTGAAGGTCACAGAGTGCTAATATTTAC

TCAGATGACCCGAATGCTCGATATACTGGAGCAATTCCTTAACTACCATGGACACATTTA

CTTAAGATTGGATGGCAGCACACAAGTGGAACAGAGGCAAGCCCT

>Unigene000279

GCTCTTTGTCTGTTAGTGTCAGTAAATAAGGTGTGGCTTCACATTTCCCAAACGGTGGAT

CGTCTTTAACTGTTATAATCGTTTGTATCTCAGTTTAACGCGAAATGATTGAGGAAAAGC

TTTAAATTGTACAACATATTTTGTGTTTCTGTGCGTGTATTTTTAAGCGGCGACCACAAC

AAACACTGACCCGCCGCTTCATCATCACACTCCATTATTCCTCCATTCACCTAATTTCCT

GGTTCATCAAAGAACGTTATAAGTTTAGGGAGGTGAACACATCAGTAGTCCGTTCAGAGA

AGTGATGATCAGCAGAGCGACGTGTGTTATATCGCTTTCTTATCTAACGTTTACACTGTA

ATGTGTTTGTCATCGAGCAAATCTGATCTATGTTGGTCCTCCCTGTAAACCAACTTTTCG

ACCTCAGGAGCTCAAGACTGAAGACCTGCATGCCGCTCACAGCCTCGTCTAAACGGTTCA

TAGTTTTTGAGTGTCTAACTGAAGATTTATATTTCAAGTTGAAGGATGGAAGGTCCTGAA

GGCGCACTTAACCGAGTTGGTCGTGCAGTTTGGACTGTCTGGGGCTACATCACAGGTGCT

GTAGAAAGATACCTCAGACCAGAGGTCAGAAATGAAGCGGCCCAAATTGTGCATCATAAA

CAAGAAGACACTGTGCCCATCGGTCAGAGTTATAAAGAGTTAAAATTAAAGGACGAGGGG

AAAAAAAGTCCTGAAATAAAAACAAGGGAGAGAAAAACGAGAGATGATTTGCAGTTTTCT

GGGGTAAGTGTGCGGACCGCAGCTGTTCAGTGGGAGAAGGAGAACATTGCTCATAGTATT

CATGAAGTCAAAAGCAAGCAAGCCTACACCAGCTCAGAAGGCATATACACGAAATCACAG

TTGAAGAAAGATGGAGAAAAAACGTTGGATGATAAAGGGTGGTTAAGCAAAACCAGCGAT

AACCAATTTACTGAACAAAACGATTCAGCTGCTTACAGTAATGCAGATGAAAAGAACAAA

GACAAAACATTTTCTGGGAGTGAGGAAACTGAACTAAATCTTGAGAGTAAGGTAACCAAT

CAGAAGCAGACAAGGAAACCAGAAGATAATGAAAAAATCGTGAGCATAAGGAATGATCAA

AGTGAAGCAGACTTGATGAAAAGTGAGTTTATTGGACAAGACACTGATCTAAGTGTTAAG

AGGAGTAAACATGGTGAGAAGCAGAGAAGAAACGAGGAAGATGAACAGGGAAGTGTTGAT

CAAACCTTCAGGAAAGTGATTGAAGAAAATATAAAAGAAGAAATGAAATGCAGTTTGACT

GAGGAAAGCCTGCTGGCCAAGGAAGCTGGTGTAAAATTTCATAGGGCTGAAAGCTTTCAA

GAGCAAAAATGGCAATCCAAAGAGGAAGATGTTAATAAAACATTTGGGAAAAGGTATAAT

GAAACTGAAGAAGAGTCAATTAAAAGTGAGTATGTTGACCAAAACCTACTAAGCAAAGAA

GCTTATCTAAATTCTGAGACAACGGAAAACATCCAAGAGCAGAGAAGAGAACAGAAAGAG

GAAGAGGATAAAGGTGGTGATAAAATTATAAAAGGAGAGAATGATAAAACTGGACAGGAA

CCAATTGAAGTTGAGTTGATTGACCATGACTTAATAACAAAGAAAGATTATCAAAATGTT

GATGTGAGTAAAAACAAACAGGAACAAAAAATAAAACAGGAAGAGAGAGGTGCTGATGAA

ACAATGCAAAGATCTAATGACAAATGCATGACAAAGATACTTGATCAAAATGTTGAATGC

AGAAAAAACAACGAGGAGCAGGGTAAGAAAGTGGAAAAGGAAGGTGTTGAGATGATGCAA

AAAAAGACTGATGAAAACCTGATTCCAGACAAGTTCATTAAACAAAGCTTACTGGCCGAG

GAAGCAGAACTAAATGTAAAGGATGAAAACAGTCAGGAGCAAAAAAGGAAATCCCAAAAA

GAAGAGGAAGATGTTGCTGAAACCTTGAAAGAGCTTGATGAAAAGGAGCTGATTAAAAGT

AAATTTCCTGATCAGGACAAAGTCACTGAGGAAGCTGAGCTAAATGTGGAAATAAGTAAA

AGCTGCCAGGAGCAGAAAAGATATAAGGAAGAGAAATGTTTAGAAATAATGAGAAAGAAA

GAGGAAGCTGGAGAAGAACTGACCAAGGACACTGATCTGAATGTGGAAAGGAGTGAAGCC

AACCAGGAACCGGCAGAAAGGGCAGAAGAAGGTGTTGAACTTATTAAAAGAGGTGATGAT

GAAATTGTAAAAGAACCAATTATAAGTGAGTTTACTGATCCTGACTTATCAATAAAGGAA

GATGATATAAGGAGCAAACACGAAAAAGAGAGAACTGTGGAAAAACCAATTTCAAGCGAA

TCTATCGAGCAAGGCTTATTTACAAAGAAATTAGTTTTATATGTTGAGCACATAAAGACA

GACAAACATGAAGATGTTACAATAGGAGAACATGATGAAAAACTGATTAAAAGGGGACTG

ATAAAGGAAGCTGATCTGAGAATTATAAAAAATCAGGAACATTCAGAGAAAACAGCAGAG

GAAGAAGAAAATGTTGCTGGAATTACTAAAAAGGAGAACGATAATGATGCTGAAGAAGAA

CCAGTTAAACGAGACTTTACTACCGAAAGTTATCTAAATGTTAAAAGCAAAGAAGAGCAA

AGAAGGAAACAGGATCAGAAAGCAGAATGTACTGAAGTTATGACAAAAGAGGATAATGAA

TCTAAAGAAGAACCAATTAAAAGTGAGTTTATAGATCAATGCTTACTAGCCAAAGAAGCT

ACTCTAAGTGTTGAGAAAAGTAAAATCGCTCAGGAACAGACAAAGAAAGTCAAAGAGCAG

AAAGAAGTTGATAAAATAATAAGAACAGAAAATGATGACATGTTTGAAAGGGAAATTATT

GACCTTAGATTATTGACAGAGGAAACTGATCCACATGAGAAGAGGAGTACAAACAATCAG

GAGGAGTTAAGAAAATGTGACGAGAAGGAAAAAAGTGTTAAAATAACGAGAGCGAATGAA

ACAAATGGAGAGAAACCACCTAAAAGTGAGTTTGGAAACCAGGGCTTATTAAAAGAGGAA

GATAATCAGAAACAGAAAAGCAAACAGCAAGAGGAAGTAGATGGTGATGAAATAATGAGT

AGAGAAAATGATAAGTGTGAAGACCAAGGCTTACTGTCCATCAGACATTCTTCTCTAGAA

GAACAACCAATTTCTGCAACAACTGAGAATGAAAATAAAGGAAGTAAGCTACAAACGGTG

GACAAGACTTTATTTATTCAGTTGGCCTCAACATGGGACGAGAGGGACCCTTTCCACCAA

AGCCCTTTAAACCAAAGCCTACAGCCTTTAATAGAAATATCTGAAAATTCCGCACTGCAA

AACATCACATCTGATGTGGCCCTGAATAAAGATTCCAGTCCGTTAGTTGATGCAGATAAT

AAGAGTTCACATAAAACAGCACTAATGACTAAACAAGAGCCAGAGGTTGTCAATACTCGA

CCAGAAACAGGGACAGGAAACCAGACCGAGGCTGTTGAAACCACATCACAATTCAGAGCA

GAAGTTGATGCAGTTGATATGAAGAAATCAGCTCAGAGCAATAAAAGTTTTCATGAACAG

CCTGTAGATAGAGAAGATGTTGCTGTACAGGAAATAACAGGCCAGTGCCTCCCTAAACAG

GAATCTTTGCTAAAACATTCAGCTGATACGAGAACAACCGAACTGACTTCACACAAAGCA

CCATTTAGTGAAATAAAGTTTGAGCAAATAGAAGAAACAGCTGTTGTGTCTCTTGATGCA

ATGCAAGTGAAGAACTTGGATAAGAGCTCTAATGAAGCTGAAGTGGAAGCAACGTTAAGC

TCTTCTAAGCAGACAGCTAAAGTATTTGAAATGCTCAGCAAAGTAAGTGGAATAAGCAAG

CAACCACAACCCAAATGTAAAGATTCTCATGTAGAGAACACTTATAACAGGTTAATTGTG

GATGAGACCCCATTAGAGGAGTTCACAGTGCAATATTCAACTGCTAATGCTTTATCACTT

TTCAAGGTATCAAACTTACTAGAGAATGAATCAGAACAAAAAACACTGGACTTCCAAGCA

GATTCACAGAAAGATACGGAGGAACAACAGGGGACTGGAATTAAAGATGTCACACCCTGT

TTAGGGGTTAAAGAGCAAATAACATGGATAAGTGGATTGACAAAGGATTCAGAGGATAGT

GATTGGGAATTGTTGAGAGAAAGGGGGGCTGCTACAAGAAACCAGGAGAATACAAACAAA

CTTGAAATCCATAGGGAAACAACAGAGACCAACAAAGATGTGGAGTTACAGGATACTGCA

AGGGAATGTGAAAGAGAATTAGAAACAGAAATGACAGATTTTAAAACTATTGTAGTTTCA

ACATGGTTGGAGATGGAGTCAGTGAAAAAAAATGAAAAACCAATTCATCTGGAGGAAGAA

AACTTTGGACATGGAGAATCTGTGATGGACAGTAAGGTAGATATGGAGGAAAGAGGAAGA

TCCCTAAAGAGAGAGCTTGACAAAATATCAGAAGACAGTGACAAGAATCAACCATCTGTA

TTAACAGAGCTTTTCCTTCCACCACAGGCTTCATTTTTGGACTTTACAGTCCAGAAGTCG

AAGATTGCTGTTAAAAACCCTCTTATTCGACCCCCAAAAGACCCTCGTTCACTTATAAAC

ATAGCATCCGTAGAACCCCTGACCCCTCATCCTCCGCCTCCCTCACAGCCCAGCTTTGTA

AAAAAGAGTCACATAGGTGCATCAGTACCGAGCAAAGGTGTGACTGGATTTAAATTTCCA

GGTCTGGATTCTGGTTTTCCTGCACTGAGAAAAACTGATGCTGGAAAAAGAATGAGGGAA

GAGGAAGGTTCAGAGAGTGTAACATCACAGAAATCCGACTCAGGCTCACAGGCAGCAGAT

CATCATGTCAAGCAAGAGACACTGCCTCCCAAACCAAAATGGATGCCACCAAGACAACCT

GGGATGGGCGGGCCGGTGATGATGGCAGAGCTGAAGAGCAAACTTAAAAAACCTGCAGAA

GAGTGAAAGAAATTTTAGCAAACAATAAATAATAATAATTTCTTATCTGTTCAATGTAAA

TAATTAATGTTTAAGAAGATTTAAAGTATGTTTGCCATTAAAATATGTGTTGGCATTTGG

ATATCTCGCTGTGGTGTTTTTTAAGGTCAGACCCAAGAC

>Unigene000280

GTGGTTCAGCAAATGACTGGGTGATGACATTCAACATACAACACACACCCTGAAAAACTC

ATACACCTCTGGTATCATACAGTACCTCACAGTTCACTGAGCGTTGTTAGCCAGAGCGTT

TAGGCACTCATGACTACTTGGTATGACCAACTGGTACGTTGCTAATCTCTCAAAGCTTGT

CAAGTTGCAAGAAAGGGATTTTCCAGTAATTTATGACAGTTTTTGAGCAGATTTTTGTCT

TGTCTGTATAGGACTGCTGGACTGCGCTGATTTCCGCCGCCAAGGAAGGCCATGTCGAAG

TGGTAAAGGAGCTGCTGGCCAACAATGCGAACCTGGAGCATAGAGATATGGGCGGCTGGA

CCGCTCTTATGTGGGCAGCCTATAAGGGCCGTGTGGAAGTGGCTCGCCTTCTGCTGGAGA

AAGGAGCAAACCCGAACATCACTGGACAGTACGGCGTATACCCCATTATTTGGGCAGCAG

GCCGAGGTCATGCCGAAATCGTTCGTCTTCTACTGGAACACGGTGCTAAAGTCAACTGCT

CTGACAAGTACGGCACCACAGCATTGATCTGGGCAGCGAGGAAAGGTCATTACGACTGCG

TGATGCACTTGCTGGAGAATGGCGCAAACGTAGACCAGGAAGGCGCGAACTCAATGACAG

CTCTAATAGTGGCGGTACGTGGAGGTTACACAGAGGTGGTGAAAGAGCTCCTTAAGAGAA

ACCCCAACGTCAACATGACCGATAAAGACGGAAACACAGCGCTGATGATCGCTGCCATGG

AGGGTCACACAGAGATAGTGCAGGATCTACTGGATGCAGACACCTACGTTAACATTCCCG

ACAGGAGTGGTGACACAGTACTAATTGCAGCAGTAAGAGGAGGTCATGTGGAGATTGTCC

GGGCTTTGCTGAATAAATATGCTGACATTGATGTTAAGGGCCAGGACAGCAAGACTGCCC

TCTACTGGTCAGTAGAAAAAGGCAATGCCACCATGGTACGAGATATCCTGCAGTGTAACC

CTGACACAGAGGCTTACACTAAGGAGGGGGATACACCACTTATTAAAGCTACCAAAATGA

GAAACATTGAAATTGTCGAGCTGCTTCTTGACAAAGGAGCAAAGGTGTCTGCTGTGGACA

AGAAAGGAGACACTGCGCTGCACATTGCTATCAGGGGTCGAAGCAGAAAATTGGCCGAGC

TGCTCTTAAGGAACCCTAAAGATGGCCGCCTTCTGTACAAGCCCAACAAACAGGGAGAGA

CGCCCTACAACATCGACTGCACACACCAGAAGAGCATTCTCACCCAGATTTTTGGTGCCA

AGCACTTGTCCCCTACGGAGTGTGACGGTGACATGCTGGGTTACGACCTGTACAGCAGTG

CTCTGGCTGACATCCTTAGCGAGCCCACCATGCAGCCGCCCATCTGTGTAGGTCTATATG

CTCAGTGGGGCAGTGGCAAATCTTTCCTGCTCAAGAAGCTGGAGGATGAAATGAAGACGT

TTGCAGGCCAGCAGGTGGAGCCATTGTTTCAGTTCTCATGGCTGGTGGTGGTTCTGACTC

TGCTGATTTGCGGCTCTTTAGCTGTGATCCTCGGCTTCTCTATCGATCCCAGACTGGCCA

TCGCTGTGGCCCTCAGCCTGCTTGCCCTGCTCTACATATTCTTTGTGCTGGTGTATTTTG

GTGGCCGCAAAGAGGGCGAGAGCTGGAGCTGGGCATGGCTCATCAGCACCAGGCTTGCAC

GCCACATCGGCTACCTTGAGCTGCTGCTTAAACTTATGTTTGTGAACCCGCCAGAGCTTC

CTGAGCAGACGACCAAGGCCCTACCTGTCAGATTTCTGTTTACAGACTATAACCGCCTGT

CGAGTGTGGGTGGGGAGACCTCTATGGCGGAGATGATTGCCACACTGTCAGATGCATGTG

AAAGAGAATTCGGTTTCTTGGCCACTAGACTCTTCCGAGTCTTTAAAAACGATGAGACAC

AAGGAAAGAAGTGGAAAAAGACGTGCTGTGTTCCATCCTTCGTTCTGTTTGCACTGACTC

TGGCATGCCTGGTGACGGGTATGGCCCTCTTGGCTATATTTAAAGTAGATTCCGATAACG

TGACTGTAAAAGCAGTGCTAGTTGCCGTGGCCAGTGTGGTGGGCCTCGCTCTGCTGCTCA

ACTGCAGGACCTGGTGGCAGGTGGCCGACTCTGTGCTGCGCTCGCAGAGAAGGAGGCTTC

ACAGCGCCGCCAACAGCATGCACAAGCTGAAGAGCGAAGGATTCATGAAGGTGCTCAAAT

GTGAAGTGGAGCTTATGGCAAAAATGGCAAAGACTATCGATGGCTTTACTCAGAATCAGA

CTCGTCTGGTCGTCATCATCGATGGCCTGGACTCCTGTGAACAAGATAAAGTGCTGCAGA

TGCTGGATACGGTGAGAATTCTCTTCTCCAAGGGCCCCTTCATTTCCATCTTTGCCAGCG

ACCCCCACATCATTATAAAAGCTATAAACCAGAACCTGAACAGCGTGCTGCGTGATTCCA

ACATCAATGGCCACGACTACATGCGCAACATAGTGCACCTGCCCGTCTTTCTAAACAGCC

GGGGCCTCAGCACGGCCAAGAAGCTCTCCATGGCCGCGCCAACCAATGGAGATCTGATGG

CGGCTGAGGCATGGCATGAGGAGGCAGACAGAAAGTGTCAGTCGCAGAACAGTCTTGGAG

AACAGGCCAAGCAGGGCAGTAAATTGACACTGAACCGGAGGGATACATACCGGAGGCGTC

AGATGCAGCGAACAGTCACCCGTCAGATGTCTTTTGACCTGACCAAGCTGCTGCTTACTG

AGGACTGGTTTAGTGACATCAGCCCTCAGACCATGAGGAGACTCCTGAACATCGTATCAG

TAACAGGGCGCCTCCTAAGAGCCCATCAGATCATTTTTAACTGGGACCGACTGGCATCAT

GGATCAACTTAACGGAGGAGTGGCCATATCGGACGTCCTGGCTCATCCTCTTCCTGGAGG

AGAACGACGTGTTCTCGGACCAGATCACACTTAAAACCATCTACGAGAGGATTTCTAAAA

ATATCCCCACCACCAAAGACGTGGAACCCCTTCTGGAAATCGACGGAGACATACGCAGCT

TCGAAGTGTTCCTGTCCTCCCGAACCCCCGTGCTTACAGTTCATGATGTGGAGATGTTCC

TACCCTGCACCGTCAACCTGGACCCTAAACTTCGAGAGATTATTGCAGATGTGCGCGATG

CCCGGGAGCAGATGCATCTCGGAGGAGTGAGTTATCCCACAATGCACCTACAGGATGCTC

CGGCGAGAGCACAGTCAGTGTTTGGTCAGGGTCCCGTAGCCTGTTCACCCACCGGCTCCT

TTCCTGCACAAACACACAGCAGCTATTTCAGCGGTCTTACTGGCCCACAGCACCCCTTCT

ACAACCGGCCATATTTCCCTCCTCATGTCTATCACGTGCCCCGTCATTATCCCGGCAACC

CCCACCATGCCCCCTCCCGCCCTTCCTCCAAACTGGCCTTCTATGAGGACCACAATGCTG

GACTAACAAAGTCTCAGCCTCCCAGACTGAAGCAGGGCTCTGATTCTGCAGCACCCCCCG

GGTTCCTTAGTGCTCTTAGCACCGAAGGTGTATGCGAAAGACTGCGACAAATTGAAGGCC

TGGACTCTAGCATGATAGAGCAGTACATCAGCACCATCAAGAAGGCGAATGTGAACGGTC

GTGTCCTGTGCCAGTGTCAGCTCGAAGAACTGAAGAAGGAGATGGACATGAATTTTGGAG

ACTGGCATCTCTTTAAAGCTATGGTGCTGGAGCAGCGGCAGGTGGAAAGCCAGCTGCTGC

AGGAGGAGTGTGTAAAGGTTAGTGAACAGGGCAGCAGTGCATCGGTTCAACCTGAAGTGC

CTGCTGTCTTTTCTACAACAGCCCCCCACAGCAATCTACAGTACAGCCTGAACCTCAGCT

TCGAAGAGCTCAGCACTATTGGACTGGAGGAGCCCATTAGACACCCCATCAACCACTGGA

TGGCCGTGACTCACAGGACTTCCAGCATGTCCAGTCTAAATTCTCAGGAGTCATCCAATG

ACATTAGTAAGTTGACTGACAGGCAGCAAGCTGAGTACCGCAGTGCTTACCAGGACTACA

TCGCTCACATGGCCCAGCTGGAGGCAGGCAGCTCTGCAGCTGAAAGGTCAGAGGACCGTC

CCAAGGATGGCGAGCAGGAAGGACGCAAACCCTTCAAGAGAAACTCTAAAGCAGGAGAAA

GTGTGATGGACTACTCCACTACCCAGGACTCCTGTCTTCTTCATCCAATCATGGAGGAGG

ACGAGAAACAAGAGCGTAAACCCACCGTGAGCCGCACTAAGGGACCAGGACCTCGCTACC

ACAGCGTACCCAATGAAGAGGATGAGGAAGAGGATTTGCCCGTGGAGACCGATGCCACAC

CCCTTCTCTGGGAAGAGCAGGAACACGGGGTGCAGAGAAGGAGCTTTGTTTCTGCAGGGA

TAGACAAAAAAGAGTCGTCTGACTCAGGCATGAGGTCAAGCGGCAGCTCCTCTGATCATT

CACTACAGGACGAGGAGAGCGAAGAGGCTCAGGAAGAAGATGAAAAGTCAAAAGAGGAGG

ATCTTGTGACCACGATACGCATGTCCATCTGCTCAGAAGCTCTGTCTGATGCCAGCAGCC

CTGAAGAGAACTGGGACTCCCCACACATACACACTCAGGCTGTCACCAACCTGAACCAGG

CAGCCAGCAACAACTCGGCCCTTAGCAACAACAATAACGGAGACGAGCGCAGCCTCATCA

TCTCTCCAGGATCGAGCACCGGCACCAACACTCTGCTCAATCAAAACCTGCTTACAGTGA

GAAGGAAGAAAGGAGCAAATACTACACATGATGAGGGCAGTGAGGAACGAGAGAGTGTGC

TGTAAAAAGCAAACTTTCCTTTAAATATACTACATTTAATATGGTACAAAGAAAGAACTG

GTCTTTCAACTTTTAGTTTATGGTAGATGTGAAACTGTGCAGAGTGGATGTGTAGAGTGA

TTAGGTTTATGCATTTCATGGAAAATTTATATATAAAAAAAAAGCCTTTTAAAGCCAATC

GTGTGCATTACAATGCAATCATCAAATCATTTGGGATCGTCGCTGAAGTAGCCGGGATCG

GGATCGGGATCTGTTACATGTGGAGTTGTGTGTAATTTACATTAGGTCAGGAACAAAACT

CTTCCTATTAGAGGAAAATAGTTTTTAAACTGGCAA

>Unigene000281

GCTGTCTTTTTTCTGTTTGATTTCTATCTGTCCTTTTGTCCTCTCTCTCACTCTCACTGT

CTCTGTGTCTTCAGGCAGGATCTCGGCAGCAGCTCTGGTCCGGCCCGCTGATTCTGAGCC

GGGATTTCGGTGGTGAGTCTCAGCCGGCTGTCCTGACCCCCTCTGACGACGCCTCCCACC

ACGTACCGCCGGCGCGAGAACACCAGGAAGTGGAGGAAGAAAGCGGCCATCGATCCGAGC

GCCTGAATGGAGATGACCTGTTGGAGGAGGGTGACGTTCCAGCGGCTGAAGTTTCCTCAG

ATCCTGGAAACGAGACTGTGGGACTAGAGGTTGAAGGAGTGTGTGTTGAAAAGCGGAAGA

ACTCTGCAGCTGTCAGCACTGTTTCCTGTAGAGACCAGCAGCAGGAGGAAGTAGATAGAG

AGCTGAGGGTAATCAGGGAGGCACCGAGGAGCAACACAGCGTCTGAGACAAAGACACACA

ATAAAATCCAATCTGGCAACCTGCAGGAGGGAACGATGGGTCAGACTCAGGGACTCCTCC

CAGAAACAGAAGAAAGGAAAGAGGCGAGAGGGGACTTACCAGAGGAAAGGGAACCAGAGC

ATGACTCTGAGCAGGAAACCTTCTGGTTGAGCGAGAAGACGCAGCTGCTCCATGGACAGA

AGGACAACGATAAACGCTTGTGGTTTTACCAACATTTAAACGTTACCACACCAGAAAACG

CCAAGGAGCTTCAGATCCAGGAAGCAAGAAAAGACATCTCACAGATACACCAGTCTGCCG

GCTGCAGCACACAGGATCCTCACGTTGGACAATCATTGGATTCTCTTAGTTTCAGACCCA

ACATGGCTCCAGGACCTGTCCACAACGTACGTCTCGAACCGTCAACTGGAGTCCAACAGA

TGCTTAATGTTACTACAGAAGACCAAATTGACGCTCCTCCTGAAACACCGTCTGATTGCT

TGGAGCAGATGAGCTCAGATTCCGGGAAAGAATCTCTACCTTGTGATGATGTCACTGATG

CACCAGACAGATTACTGCCAGATGTTTCTCTCACAGCTGAAGAAAATTCTGGAAAAATAC

GAATCGATCAGATTTTACATTCTGAGATAAAAGAACATTCGTTACCAGAATCTGAAAGTG

ACGTCCAACCAGTTGTATCTCCAGAAACATCTGAGGAACATTTTCAAATACTTAACAAGA

AACCATCTCAGGAGCATAGTGAGTCTCAGATCCTGGATGCCACACACCAAAATCCCGACA

GATCACAGGGTTTACTTCTGGTTCCAAACCCAGCAGCTCCTTTAGGAATACTAGTTTCGG

GTTCTAGCTCTGGAGAAGCAGAAAATGTTTCCAATCCAAGATGGAAAAGGAGCCGCAAGC

CTTCCAGACACCTCCAGAGTCGCCCTGTGAGTTCTTCTCTGTTGATGGATTTATTAATAT

GACATCTCTTGAACTTCTTTCTGAAGACACTATGGAGAAAGTTGGTAAGGTAGAAACAGA

GCTGCTTGAGAGGTGTGAAAGCTCTAAAAAGTCACCTGTAACTATTTTGTGTGGTTCTTT

CTCCAAATTGACCCTCAGAGGAAGTCCGCTGGATCCTCAGCTTCACAGCATCACCTCAGA

GACCATTGAGAGGACAGCTGATGATTTGGAAACCTCAACAACAATTTCTTTACAGGTTGA

GAAGACCAATGAGGAGGTGATAAAGTTCGAACCAGAGCTCATCAAAATGTGTGAGAGATC

AGGAATGTCTGCAGAACCATCAAGTGATCCTGTAGCTGAGGAGAACCTTGAAGGAACGGC

AGCCATTACACAAAAAGACCAACCTCCAGAATCAAAAGATCAGGAGGTGTTAATGAAGTT

GAAGATGTCTACACAGCTTGGAGAGAAACCAAAAAGTCCATCATTGCCATGTGAGTCATT

ATCGCCAACCATTTCTGTCTTAAAGAACATCACTGACTCTCAGGCTGAGCTCATGACCTC

AGCAGAGAGAACATTAGAAACATCTAATCCAAATCTGGAGGTCAGCGGCTTTAAAGAAAA

CATCCTAGAACATCCTGAGGTCATGACTTTAGAAGAAAGTAATACTGGAAAATCTCTTGA

TCTCATCATATCCTCCAAAGAGACTACTGGAGAAACATCTCCTGAGCCACTTCCTGAGGT

TGAAATCCTTGAGGGGAGTGTTCAAGACCAACAACATAAGGTCATGATCTCTGAGGAGAG

CATTACAGGAACCGTTTTGGACTGTGATGTTGATTTTACGTCTGCTGAGGAATTGATTAG

AGATATGTCTCTTAGTCCAGTTCTTGAAGTTCTTAGCAGCCCTGAAGAAATCCACCAACA

TGAGAAGAGCAGAAATGAACTAGAAAGTAATCTAGAAACTCAAGTATTTCATCATGATCT

CATCAAACCTTTTAAAGAGACAGTCAGAGAAGTTTCTGGTGGCCCGCTTTCTGAGGAAAA

TGCTCCAGACCAGCAACCTGAGGTTCTAATCTTAGAAGAAAGCATTGAAGGAACATCTTT

TGGAATCATTCATGATCTTCCTATGTCCCTTGAGGAAACAGTCAGCACCTGTTCAGAGGT

AAATGTTTTGGACCAACAACATGAAGCAACAACCTGTGAGAACATCATTGATGGAACATT

TCAAGATCCTGAGGATCCTCTTGAAGTCAGAACCTTGGAAAATAGTTTTCAAAACCCACA

TCTTGAGGTGACGATTGGAGAAACAGCTCCTGTGATCACAGCATTTGAGAAGATCATCGG

ATTTCCATCTGATAACACCCAGCCTGGAGAGGCAACCTCTGAGGAGAACCGCAGAGCATC

TCTGGAACCTTTAGCTGTCATAGATGCATGTTTTTCTGATGCTCCAGTTCAACGTGAGAA

TGTAATTCTGGACCCCCCTGAGTTCTGTAGTGTCTCATCTTTGGAGAACATTTCCAACTC

AGGATTACACAAGATCTCGGCGCAGATGCTAGATCAGAATGTTGGTACTGGATTAGATAT

GGACAGCAGCCCCCCTGTAGAAGTGGATAGTGGTTCAATCCAGGATTTACCATCTTCAGA

CAGTGTGTCTATCCATGAAAGTGAAAATCTGGAGGAGGAGAAAAGTTCCACAAAGAAGGA

ACGTGAAGAGGAACCGACAGAAGAACATACAGCAGATAAACAACAAACAGAAAGACAAAA

CATCAAACACAACAATCAGATGTACGAGAGACACCGGCGCCGATACACACTGTGGACGAG

GGGAGAGCACACAGACAACGTGCTGCTGATCAGGATCCACAAGGAGGAGAAGGAGGAGTT

GTATTCTCTCCATTCTGAGAATCTTGAGGAAAACTGCAGTAAAGACAACACAAAAACGGA

TGTTGCAGATGTGAGTGTGTGTCGCTCTGTATCGGCGAGCTCGCTGCCCTGGTCCGAGTC

GGTCAGCGATGGAGACTCACTGTTGGGCCCAGACATGTCGGACGACGCCATCAGACAGAG

CAAGGACACTCTCACCAGTGGAATTTCCATGACCGGCTCCGTTTCCACAGACGACTCGTC

CATCACAAACGCTGTTGGAGAAACGGAGGAAGAGGAGAAGAAAGATCGGCTGGCAGAGGT

TCTAGAGCGTTCAAATGTTCTTCGCTCTGCCAGCCGTTCTCGCTCTCCGTCACGGAGACA

CAGCTGGGAACCAAACAAGAATCAGAGCAGAGAGACGGATATGGACCAGTGCAGTGTGGT

TGATGTTGAGGACGAGGGGAGGGCAGCAGGTCACAGGAGGAGTATGAGCTGGTGTCCATC

ACATGTTCCTCACTCTGAGATGAAGGAGATGAACTCCAGAAGTTACAGTCTGGAGGGTTT

GGCAGATGAGGGTGAGGTAAAAATGCCAAATATTCAACACTCCACACACTCGTCTGTGAC

TGAAAGAGAGGAAAGAGGATCACTCGCATCCCTTACTGAGGAGAACGAGAACACCACAGG

AGATAACAGCAGCTTGGACAGTCAGAGATCGCTGCAGAACGCAGGATGTGGTTTCGTTCC

TCCTCCTCACACACTCACCAAATCCATGTCCATGTCAGCGATCAGTCCCAGAGACCTGGA

CGATTTGGCGTGTGCAGGAGCAATAGAGCACAGTATCTCTGAGGAAGACCCTGCCCCACT

GAGGAGTGACTCTGAGGTCAAAGTGACGAAAGTGAGCCGCACCTTCAGCTATCTCAAGAG

CAAAATGACTAAGAAGAGCAAGGAAAAAGACCGTGAGCGGAATAAAGATGGTAAAGAGAA

GGAGAAGAAGAGTTCTAATGGTCATTTTTTTAATCCCTCTGTAACTCCGCCCCCCAGCCC

CTGCCTGCAATGCAACCGACCAATCAGTATGAAGGATGCGCTCACCTGCACAAACTGCAG

TGTTTACGTCCATAAAGGCTGCAGAGATGCTGTTCCACTGTGCGCTAAAGTCAAGATAAA

GCAGCAGACCGTCACTGATTTCGCTCCTCCAGCTGCGACTTTTGCACGAGGAAAAACTGC

TTCAGCGCGAGAGCGTCCCTGGTCCACGTTTCTGCTTCCTGAAGATCAGACGCCACCTCC

TGTCCCACCACCCAGAAAAAACACCACCATCATGTCCTTTAACAGCGGTCCTCTGTCCAA

AAGTGTCTCCATCAGCAACATCACAGGTCAGTCTGATGAAGCTCCTCTGAAAGCTCTACA

GATTCTCTCTCAGTCCTCAGATTCTCTCAATAAAACCGGCAAAGTCAACGAATCCACCGA

GTCTCTGACTGATGAAGGGGCGGAGCTGATGGACTCTCATCCGATGGTGGAGTTTGAGAC

GGAGGTGAAGGAGCTGGAATTCGATTCCTGGAGCGTCACTGTGGACAAGAAATATTTAAA

ACAACTGAAGAAAGACATCATCAAGAGACAGGACGTCATCTACGGTGAGAGAGAGAGTAA

GGGTGTGAGTAAGTTAAGGAGTGAGTGAAGCGAGTGAGGGAGTGAGGGAGTGAGGATGAG

AAAGAAAAGGCACGTCGGTAAAGATGAGGTGGCGGAAGATACAGAACGGCACTATAGGAT

TTAGGACATCAGTAACGTCCTGCAAACGTCTGATAACTTAAACTGAATTTATTTAAAACT

GGTGGAAGAAATGAAGGGCTGAAGAAAAATTAGAGTTTAGTGGTGGGGTGTGAGGGTAAG

GTGTGTGTGTGTGTGTGGTAAGGTGTGTGTGTGTGAGGGTTAGGTGTGTTTGGAAGGGTT

AGGGCAGGCTGTGAGAGTGGCCAGTGTGTGTG

>Unigene000282

GGAATCAGATCGGAAGAGCGGCTGCCGTAGGCGAGCTACGCAAAGGTGGTTTACCGAAGA

TGGCGGCTTGTAGACGTGTGCAGCTTCTTTTATTGCTTTTAGTTGTTACCGCTTGCTTTC

GTTCTACCGAGGGAGCGAAATCACAAAATATTCTCGTTGATGGAAATGCAGGAGAAAATA

TCAACGATAATAAAATTGCCATGGGTGTTCCGGCTGATCCGAAAGCTGGAGCGGCGGCCT

CTCAGTCCCGCCGCAGCTCCACCGGCTGGAAGCTGGCTGAAGAGGAGGCCTGCCGCGAGG

ACGTGATCAGACTATGTTCTAAACATTCATGGAACAACAACCTGGCCGTGCTCGAGTGTC

TGCAGGATCGGAAAGAGGAAACTGAGATTGCAGCCAATTGTAATCATCTATTATGGAATT

ACAAGCTGAATCTGACGACAGACCCAAAGTTCGAGTCTGTGGCTGTGGAGGTGTGCAAAA

GCACCATTTCTGACATTAAAGAGTGTGCAACAGAGGAGCGAGGAAAGGGCTATTTGGTGT

CATGTTTGGTAGATCATCGAACCAACATCACTGAATACCAGTGCAATCAGTACATCACAA

AGATGGCTACCATCATCTTCAGTGACTACCGGCTCATATGCGGCTTCATGGACAGCTGCA

AAGAGGATATCAACTCACTGCACTGTGGGAGTGTAAACACTGGTGAGAAGGATTTACACT

CCCAGGGTGAAGTTATAGCATGTCTGGAGAAAGGGCTGGTGAGTGAGGCAGAGGATCAGC

CAGGGCATTATTCTATCAAAGAAGATTGTAAGAAGTCCATCATGCGTGTGGCTGAGCTGT

CGTCGGATGACTTTCACCTTGACAGGCACCTCTACTTTGCATGCCGCGACGATCGTGAGC

GCTTCTGTGAAAACACTCCAGCAGGTGAAGGCAGGGTCTACAAATGTTTGTTCAACCACA

AATTTGATGATTCCATGTCTTTAAAGTGCAGAGAGGCTCTGTCCACCAGACAGAAACTGA

TAGCACAAGATTACAAGGTGAGCTACTCCCTGGCCAAGGCCTGCAAACAGGACCTGCGCA

AATACCGCTGCAATGTGGATACCACGTTGCCCCGTGGCAGAGAGGCCAAGCTGTCGTACC

TGCTGCTGTGTCTCGAGTCTGCCGTACACCGAGGGCGCACTGTGAGTGGTGAGTGTCAGG

GGGAGATGCTGGACTACAGGCGCATGCTGATGGAAGATTATTCGCTGAGTCCTGAGATTG

TTTTGCACTGCCGCGGAGAGATCGACACACACTGCTCCGGTCTGCACCAAAAGGGCCGAA

CCCTTCACTGCCTCATGAGAGTGTCTCGCGACAAGGGCATTGTAGACAACCTTTGTCAGA

AATCTCTCCAGACTTTGATCCAGGAGACGGATCCTGGAGCGGATTACCGGATTGACCGGG

CCCTCAATGAGGCCTGTGAGTCAGTAATCCAAACTGCCTGTAAACACATCAGGAATGGTG

ACCCCATGATCCTGTCTTGTTTAATGGAGCATCTTTACACTGATAAGATGGTGGAGGACT

GTGAGCACAGGTTGCTAGAGCTGCAGTACTTCATCTCCAGAGACTGGAAGCTGGACCCGA

TCCTCTACAGGAAGTGTCAGAATGATGCTTCACGTGTTTGCCACACTCATGGCTGGAATG

AGACAAGTGAATTGATGCCCCCAGGAGCGGTGTTTTCCTGTCTGTACCGCCATGCTTATC

GCACTGAGGAGCAGGGCCGTCGGTTATCTCGTGACTGTAAGACAGAAGTGCAGCGCATCC

TCCACCAAAGGGCACTGGATGTTAAGCTGGACCCTGACCTGCAGAGACGCTGTATGACTG

ATCTGGGCAAGTGGTGCAGTGAGAAAACAGAGCCTGGACAGGAATTGGAGTGTTTGCAGG

ATCATTTAGATGATTTAGGTTCAGATTGTAGAGATGTGGTGGGGAATCTAACTGAGCTGG

AGTCTGAAGATATTCAGATTGAAGCCCTTTTAATCCGAGCCTGTGAACCGGTCATCCAGT

CGCACTGCCATGAGATGGCAGATAACCAGATAGATACTGGAGATCTGATGGAATGCCTTG

TGCTCAATAAACATCAGAAGGAAATGAATGAAAAGTGTGCAGTCGGAGTCACTCACTTTC

AGCTGGTTCAAATGAAAGACTTCCGCTTTTCATACAAATTCAAGATGGCCTGCAAGGAAG

ATGTTCTCAAGCTCTGCCCTAACATTAAAAAGAAAGTGGACGTGGTCATCTGTCTGAGCA

CCATGGTCAGGAATGATACGTTGCAGGAGGTGAAAGACCAGCGTGTGTCCATGAAGTGTC

GGCGGCAGCTCCGAGTGGAAGAACTGGAGATGTCCGAGGATATCCGTCTGGACCCTGATC

TGTACGAGAGCTGTAAGCAGGACATTAAGGATCACTGTCAGAACGTGGTTTACGGCAATG

CGCAGGTGATCGAGTGTCTGAAGGACAGTAAAAAGCATCTGACCCAACGCTGTCACCAGA

AAATCTTTAAACTGCAGGAGACTGAGATGGTCGACCCTGAACTTGACTTTCAGCTCATGA

GAGTGTGTAAGCAGATGATCAGGCGCTTCTGCTCTGAAGCCGATGCTAAAAGCATGCTCC

AGTGCCTGAAACAAAACAAGAATAGTGAACTTATGGACCCTAAATGCAAACAGATGATCA

CCAAAAGACAGATCACTCAAAACACAGATTACAGACTGAATCCAGTTCTGAGGAAAGCCT

GTAAGGCTGACATTCCCAAGTTTTGCCAGCACATCCTGAACAAAGCAAAAGATGACATTG

AATTAGAAGGCCAGGTCATCTCCTGCCTCAAACTTAAATATGCTGACCAGCGTCTGTCCT

CAGACTGTGAGGATCAGATCCGGGTAATTCTGCAGGAGTCTGCTTTGGACTACAGACTGG

ACCCTCAGTTACAGATGCAGTGCAGTGATGAGATCCCGCGGCTGTGTGCAGAGGAGGTAG

CGGCGCAAGAACAGACAGGTCAGGTGGAGGAATGTCTCAAAGTGAATCTGCTCAAGCTTA

AAAATGAAGGCTGTAAAAAGGAGGTGCTGAACATCCTCAAGGAAAGTAAGGCAGACATTT

TTGTGGATCCAGTGTTGCACACGGCATGTGCCCTTGACCTTAAGCATCAGTGCGCTGCTA

TCCCACCCGGCAGAGGAAGACAGATGTCATGTCTGATGGAGGCTCTTCAGGATAAGAGAG

TAAGACTTCAGCCAGAGTGCAAGAAGAGACTGCAAGATCGCATAGACATGTGGAGCTACG

CTGCCAAGGTGGCACCAGCAGAAGGCTTTTCAGACTTGGCTGGTCAGGTCCTCTCCTCTC

CTGCAAAGAACTATATTCTGAGCATGCTGGCGATGTCTGTAGTGCTGCTGTTCTTTATGG

GGCTGCTATGTGGCCGCATTACCAAGCGTGTCACGCAGGAGCTAAAGGACAGGTAAAGAG

TGTTGCCAGGGGCAGGAGGAGGGGGAGATGCAATGCTCTTCCTTCCCCATTTCACACAGG

GAGGGGGATGGAGAAGATAGCAGTGCTCCTCTCTGTCCCTCACTCACCCCAGGCCCTGGC

AATGGCATAGTGAAGAGGATAAGCAAGACTAAAACTACAGATTTTCTTGCTACTGGCAGC

AGACATGTTTGGACCTGATGTTACCCTCTTCACACTGTCCATGTTTTCTGACTGCGGTAC

TCCTGCATCCTCATCTGCTTCTCTGTGACCAGACCTGCCTGTTGTGGACCATTGTGTGCG

TGTGTGTGTTCTGTGCATTTCTGGTACGGTGTGCCAGCATTTTTGAATCATTGTTTTGTT

TTTGTTCCCTCTCCCCCCCCTCCCCCAGCTTTTTCTATGTTCATGGAGTTCCTGTTGAAT

CAACAGTTCATGTATTTATTCTTTATGCTTATGTTGTACTGGATGTTGTGTATGCTTGGG

CTTTGTTGTGCTCATGTGCTCCCCTGTGGTCTACTAAAGCGATGAGTCACAGGTTGCAGG

TTCTCACTACTAAATTAAAGAAATAGTTTTTTTGTGTGGTTGTGGGTGGTTGCCATACAT

TTCAGTTACACATGGTGACTAGTATATTTGTGGTATTAGGTTTGTGTGTGCTAGGGTGGC

ACAGATCTGAAAACATGTTTCGTCTATTATTTTTAACTGATTATAACTTTCTTACCATCG

ACAATAGGTTGTTTGTTTTATTCGGGTCATGGCTGACATGTTCACTAACTTGCAGAACAT

CTAATGGGATTATAACGCCATCATTTGCTTTAAAATAGTTTATAAATGACTTTATTGACA

ATGATGTACTAGACATACTGCTAAATAGAGGCTTTGTGCTAAACACAGGGGTTCAAAAAC

AGGATTGAACTGAATAATTGTGCTTTGTGTTTGTGTGCACATGTGTATATGTGTGTATAC

TTTCCTGTGAGGGCGAATGAGAAGTTACAGATTAAATATCCTGTCTATGGGTTCACTTAT

TTTCATAATGAAAGTTTGGTTTTTTTTTTGTTTTTTTTAAACCTCTTTACTTTTGTGACT

GCAGGTTTGAGTTTATGACAAACAAAAGCATTCAATCTAATATTTATCAGACTCTCTGTG

CTCCCAGATACTACCAGATTACTGATCAGTGACGGAACAGCTACTCATATCTGAGCAAAA

GAAACTCAGGATATAGGGGTGATAAATAGACCTTTTTAAAACCTATCCATGGTTAGAATA

CACACACACCCTTCAGATATTTTTCAATAATGAAATAATAGATCCACAGCCCTGTTCTGA

CTGGGATGCTTGGTTAATTTTCGATAATGGCCTCAGACAGGATGTCAAGGTCCTCATGGT

GCTCAGGCAACTTGACCGGGTCTTTCCACTAAACTGCTACCGAAGAGTGATTTTTACACA

AGTTAAGTCATTTCCTGTGCTCAGATTTTTTTCTCAGTTTAGCTTGTTAGATGTTTAACA

CCAGTAGAGGTGTTCTGGTGGTTATTACAGTGCCATACTAATAGCATTAGTCCTTGCTTA

GTCAGGGTGGCTCTCTGTGCAAGGCACATTTAGTTTTTGCTTACTTAGCTGTGTGTTAAT

CAATTTGTTTGTTTTGATGGCCTTAATTTATTAAAGGTTTTAAGTTTGTGATTTTTTTTT

TTTTGATGCTTCAGTGTTTTCGTTGGGGCTAGTTATCTACTCTGCCATCCAGGGTAAGAG

GATTTTAGATTTTTTTTTTTTTTAAACTTGC

>Unigene000283

GGGGGGACAGCACCGCACTCGACTTAAGCAGATATTGGACTGGTGTAAACCTGGCTTTGA

CGGAGTCATTGTTTTTGATGAATGCCACAAAGCCAAAAATGCGACGTCCACAAAAATGGG

CAAAGCCGTCCTGGACCTGCAGAATAAGCTGCCTCGGGCCAGGGTGGTGTACGCTAGTGC

CACAGGTGCCTCTGAGCCAAAGAACATGATATATATGAGCCGGTTAGGGATTTGGGGAGA

AGGGACGCCCTTTAAGACATTTGAGGACTTCCTACATGCTATTGAGAAGAGAGGTGTTGG

CGCCATGGAGATAGTTGCCATGGATATGAAAGTCAGTGGCATGTACATAGCCCGGCAGCT

CAGTTTTTCTGGTGTGTCGTTTCGGATTGAGGAAATCCCACTGGATGAAGAATTTAAGGT

GGTCTACAACAAGGCAGCTCGACTGTGGGCTGAAGCCCTGGAAGTGTTTACCAGAGCAGC

AGATATGCTGGGACTAGTGTCTCGTAAGTCCCTCTGGGGTCAGTTTTGGTCCTCCCATCA

GCGCTTCTTTAAATACCTCTGCATCGCCGCCAAAGTGCGCCGCCTGGTGGAGCTTGCAAA

ATCTGAGATGAATCAGGGCAAGTGCATTGTGATTGGGCTGCAGTCAACTGGTGAAGCCCG

AACGCGTGAAGTTCTGGATGAAAATGATGGACATCTAGACCGATTCGTCTCTGCAGCTGA

GGGTGTGTTCCAGTCTCTGGTTCAAAAGCATTTTCCATTAGAGAAACCTAAAAGAGAAAA

GACTGCTGGGAATAAAAGAAAACGAAAACCAAGAGGACGTCCACCCAAATTTCCAAAACA

GTGTGTAGAGGTAGGCGGAGTTATTAAGATCAGTGACGGTTCCGATTCCGACTCTGATGA

CATGGATAGTGACTCGAACTCGTCACCTGACTCGCTGCAAGACAATGATGATGTCATCTT

TGTTAACCACATTAATGGACCTACTGCAAAACTTGAGGAGTTGAAACATGGCCTGTTGGC

TAAAATAGCAGATCTGGGAAAGGAGCTTCCTCTTAACACACTGGATGAGCTGATTGATCA

ATTCGGAGGGCCAGAGAATGTGTCTGAGATGACCGGTCGTAAAGGTCGAGTAGTGCGGCG

GCTGGACGGCAGGGTGCAGTATGAATCGCGTGCTGAACAAGGCTTTACCATCGACCATGT

GAACATCAAAGAGAAGGAACGCTTTATGGACGGTGAAAAGTGGGTGGCGATCATCTCCGA

GGCTGCCAGTTCAGGGATTTCTTTACAGGCAGATAAACGTGTGCGGAATAGAAGGAGAAG

GGTGCACATGACTCTGGAGCTGCCATGGAGTGCAGACCGCGCCATCCAGCAATTTGGTCG

GACTCATCGCTCTAACCAGGTAACAGCTCCGGAGTACATCTTCCTGATCTCAGAGTTAGC

CGGAGAGAGACGCTTTGCCTCCATTGTAGCTAAACGCCTGGAGAGTTTGGGGGCACTAAC

ACACGGTGATAGACGAGCCACGGAGTCCAGAGATCTGAGCAAATACAATTTCGAAAACAA

GTATGGCACAAAAGCTTTGGATAAAATCAGTAAAGCCATTTTGGGATATATCGACAACAA

AGTCCCACCACCGAAAGGTTATCCCGGGGGTGAAGCCAGATTCTTTGCAGAGATGAAGAA

AGGGCTAATTGACGTGGGAATTTTTTGTAAGGAGCCGCGCTTCGGCCTCAACACGGAGAA

AGATTGCACCATCACTAAATTCCTGAATCGAATCCTGGGCCTGGAGGTGCACCAGCAGAA

CTTTCTCTTCCAGTACTTCATTGATAACTTTGAATACCTGATTGACAAGGACAAAAAGGA

AGGCAAATACGACATGGGCATATTGGATCTGGCTCCAGGTAATGATGAAATCCATGAAGA

AAAACAGGAAGTGTTCCTGACTCCCGGGAACCCCCAGGAAGGCCAGGTCATCCTTTATAA

GATCAGTGTGGATCGAGGGATGCCTTGGTTCGAGGCTTGTAACAAGGTGAACACGCTGAC

TGGGGAACATGAGGGCTTTTATATTTCTCACAAGCTGCGAGGAAGCCAGCCTTGTGTTTT

GCTGGCAGAGCAAGGCAGAGGCCGTAATCTCATTGTTTATAAGCCTAACATCGGCAGACA

GGCACACCCTGAGAATCTGGAGAATCTGCTGCAGCGGTACAGAAAGGTGACCCCAGAGGA

GGCCAAAGACTGCTGGGAGAATCAGTTCGTCTTCTCGTTCCAGAAGTGCAGCCATGCCAA

CTGGAAAGGTAAGTGTAAGCAGGTGGAGCTGGGTCAGGAATGCTGGCTGGGCATGCGTCT

GCGACAGTACCACATGCTGTGTGGCGCTTTGCTAAGAGTGTGGAAGCGAGTCGCAGATGT

CGTCTCTGACATCACCAACTCCAGCATCCTGCAGATTGTGCGCCTCAAAACCAAACAGAA

CAGCAAGCAAGTCGGTATAAAGATACCAGAGAACTGTGTGGAGCGTGTGCGCAGCGAGCT

TTCTCTGATGGACGCCGAGGTGAAGCGAGCTCGGCAGCAGAAGGAGCAGAAGGCACAGGA

GCAGCGTGCTAAGGAACAGCGCATCCGCCAGGAGCTTTTTATTCGCGAACAGCAGCAAAA

ACTTCTAAACAGCCGCAAACCCTTCAGCCACTCTTCTATGCACCTGTCGAGCCCCCCACC

TTACCCCGGTGTACCGTCTGGCCGTGTCACCCTACCCAAGAGCCAGCCTGTGGACGAAGT

GCTGGACCTTACGCTTAGCCCTTGCCCCTCCCCTGAGCCCAAACCGCCTCATGCAAACGG

CGCACTGAACCCGAACGGATTAACGGGCATGAATGGCATCTTCGGTATGCGGAGAGCGGC

AGTAGAAGGAGGGCTGAAGATCCCCGAGACGTTTCACCTGGAAGACTTGATCTCCCAGGA

GCAGCTGCGGGAGAGACGACCGACCGTCCAGAACATAACCCGAAGCATCCAAACACTGAA

GCATCATGCGCCGCTGGGCGTGTTGCAGCAAAACCAGACAAACCTAGGACACTCGCTGAC

ACCCCAGCAGAACTCTGCTCAGTTCAACACGCAGCTTCTGAGACACAACGGACGCAAACC

AGACATTAACACGCTAACCAACCATGGCTTACGTGTGCAGCAGTTTCAACTAAACTCCAT

GTCACAGCCACCTCACTTGCTTCCGCCATCTTCTGTCCAGCAGGACAGTCATCAGCACGC

ACTGATGCGGGGGCACACACACAGTCTGCATGCGCACATTCGGGAAAACAAAATGCCAGT

CAGCGAACCTGCTCCACTTTCACTGAAACTCTGTCAGCAGTCGCAGCAGGGGTCAGTCAG

TCAGTCGGTAACAGAAAATTCTGCAACTCCACGGGACGACTTCAGTTTCAACCTTGACCT

GCTTGGTCATCCTCCATACTCATTAACCCCTCAAGCCCCTCCCTCCCCTTCATTTTCTTG

TTCCTCTCCTGGTACATCGTCTCTTTTCTCCTCAGACCCCCAGTCGTCTTCGCTCTCGGA

CTCCATCTCCCCTTCTTCCCTGTTTGCTCCTTCGTCCGAGCGACTGCCTCTTCAATTAGC

CAACGGACACAGCAGCATGGACGCCCAGAATGCCCACAAGGCTCACGGGCGAATGGTGCA

CGGAGGTTCTGCAGACCAGCGCAACTCAGTCATTCAGTTTAGTCCTCTAGACTGGAACAC

CACCTAGTGGCCACTAAGGCTTACATGCTCACATCACCACTGTTTGTGCACAATGCAGTA

CTGTCGCTCGTTCTTTACTGTGATCTCAGCATGTCTGGCTGAGGTATTTTGCAGTAGGTG

TCATTCTTTATTCTGTTCTTCTGCCCCTAGATCATTCGTTCAGTCTCTAGTGTAGTTCTT

CATCACCAGTGTTTCTTCACTGAAATTAAGGTCCATTTATTAGAGATACTCGACAATGAT

CGCTTTATGGCTTGGTTGTGATCGACGTGTCTCGATAGGTTGGGGTTTTTTTTTCCCCTA

CAAAACTTAAACATTTTATAGCGGGAAACTAGATCCGATTTTAATCAAAGTTTAGAATAT

AATTTCAACGATCTCCAATCTTTTGCAAAGCATTAACAAATCTATTTATCACCATGGCTT

CGGTTGCTGCTTTCGGAATAATGCAGATCACTTTTTTCCATATATGTCGATTTCCATACA

TGTCGATTGTTTTCGTACCGACCAAATGCAAGTGTCACTTAAACGCTAATCAGTTTAGAT

TATTCAGAAGATCCATAGGCGTCCCGGAAAAACCCTCCCTCATCTGTGCACCTTTTTTAA

ACATCAGCTCCATAAAGTCTATTTATTTCTACAGTCCACTCCTGTTTCCTTGTTCAAAAA

TTAAATCAAACCTGGAAGAAAATGCCTTTAGCTCAGTCAGTGCCTTTGTATCATTAAGAT

GCAGTCAACAATATTGTGTGTGTGTGTGTGTGTGTGTGTGTGTGTGTGTGTGTGTGTTCG

TGTGTTCTACAGAAAAGCAGATTAATTTTAGCATATTATTTATTATCAAGATCATCATCC

TGCTCTACTACACCAGAGAAACTCGTACATTTAGAATTTTGTAATACTGCTGTTTAACAT

ATTGACCCCATGAATATATTTTTCCCCTAATTCCATAGGCTGGAGCCTGACACATAGCTG

GAGCTATCTAGGGTATGAAAAAAGCTAACAAATGTCATCCCTAGGTCAATGAAGTACTCG

ATTTGCTTTCCTGCTTGTTTTGTACTTTCTCAGGTATAATGTATACTTATTACTGTCCAG

ACCAATTATTATTTATCTAAGATATCTAAACTAAAGGACCTTCTTTTTAACTTTGGGGGG

GTTGGACACCAAAATATTTATTAGGCAACTAGGTTATCTTTAAAGCTTTCTTTTTCTTTT

CTTTCCTTTTTTTTTCTTTTTACCTAAACCCAATTACAAAGACTTTTAGCAGTTAAACTA

CTGGACCTCACAGAAACCTCAGTCTCCTTCCTGAGGGAGCAAAACATTAAGCAATAAAGT

TACATTTGCCCTTAGAACAATTTTTTTTCAAAAAATAATAATAATTTAAAAATCACAGCC

TAAAACTCGATAGGAATTCTTGTTAGGCTCTCATTACCTTTTATTATTTTTCATATGTAA

GAAATAGACAAATATTATATTTAAAAAAAGGAAAAAAAACAAACTACGGATACGTTTATG

AGGTCAATATATTCTTGTACAGGTGG

>Unigene000284

AAGAGTGTCGGAAGTTCCTGTGTCCGCCTACCAGAAACTTCCGCTGCTTGAACGATCGTG

TGTGTCTGTGGAACGGAAAACGCTGCGACGGCATTAACCACTGTGGAGACAACTTAGACG

AGTTAAACTGCGCAGATCAGACGGTGAGCAAGTGCGGCAAGGATAAGTTCCAGTGCAGCA

ACGGCAAGTGCATCAGCTCCACCCTGCGCTGCAACTACTTCAACGACTGTGACGACTACG

GCTCTGACGAGATCGGCTGCAAGACAGAAATCATGCTAAACGACTGTAAGAGTAACAGGT

CTCTGTGTGGAGAGACAGAAGAGGTCCACTGTACTACGAACGGCACCGACTCTTTCTGCT

CCTGCAAACCCGGCTACGAGAGCAACGGCTTCGGCAACTGCCAAGATAAAAACGAGTGCA

GACAGTTTGGAGTGTGCTCGCAGATCTGCAACAACAGCAAGGGATCGTACAAGTGCGGCT

GCCACAAGAACTTTATTAAGATCAACGACACGTGCAAGGCTGACGGTTCAAATATCAATA

GGCAGGTGCTGTACATCGCCGACGATAACGAAATCCGTAGTCTGGACCCCGGCATGCCCA

ACTGGGTGTACGAGCAGACCTTCCAAGGCGACGCTAATGTGCGTATCGACGCCATGGACC

TGCACGTCAAGACCAACCGTATCTTCTGGACCAACTGGCACACCAGCGGCATCTCTTCCT

TCCAGCTGCCCTCCACATCCTCGTCTTCCTCATCCGGCACCTCATCCAACTCCCACCGCA

ACACACGGCAGAGTGAGGGGACCGTCACCAACCTCGCGATCCCAGGTCTAAAGACGCCCC

GAGGCATTGCTGTGGACTGGGTGGCCGGGAACCTGTACTGGACAGACTCGGGCCGGGACG

TCATTGAGGTGGCCCAGATCACCGGCCAGAACAGGAAGACTTTGATCTCAGGCATGATCG

ATGAGCCCTATGCGATAGTTGTGGATCCACCAAGAGGAACCATGTACTGGGCTGACTGGG

GAAATCACCCCAAAATCGAGACGGCAGCCATGGACGGCACTCTGAGGCAGACGCTGGTGC

ACGAGAACCTGCAGTGGCCCACAGGCCTGGCAGTGGACTACTTAAACGAGCGCCTCTACT

GGGCCGATGCCAAACTGTCTGTAATCGGCAGCGTGCGTCTGGACGGCACAGACCCTGTAG

TGGCCGTATCCAACATCAAAAACAATCTGCTCCACCCGTTCAGCATTGATATCTTTGAGG

ACTACATATATGGCGTGACGTACGTCAACAACTTCGTGTTCCGCGTCAACAAATTCGGCA

AAGGCACAACGGAGAATCTGACAACGGGCATCAATCACGCCACTGACATAGTGCTTTACC

ACCGCTATAAACAGCCTGAGATGGCGAATCCATGCGACAGGAAGAAGTGTGAATGGCTGT

GTTTGCTCAGCCCCAGTGGTCCGGTGTGTACCTGCCCTAACGACCGTGTGGCTGATAACG

GCACGTGTGTGGAGGTTCCTCCTGCCACTCCGCCTCCTGTCTCCCCTACGTGTAACGTTC

AATGTCTGAATGGGGGCAGTTGTTTCCTGAACGCTCGCAGTCAGCCCAAGTGCCGCTGCC

CGACCAGCTACAGCGGCGAGAGGTGCGAGATAGACCAGTGCAGAGACTACTGCAAGAACG

GAGGAACCTGTTCTCCATCACGCACAGGAGCACCCACGTGCCGCTGCCCAACAGGCTTTA

CAGGACCTAACTGTAACCAGAACACGTGCAAAGACTACTGCCAGAACGGAGGCAGCTGCA

CTGTGAGCCTCGGAAACCAGCCCATGTGTCAGTGCCCTGACGGCTTTCAGGGAGATCAGT

GCCAGTACAGCCACTGTGAGGGCTTCTGTTCCAACGGTGGAACCTGCCTTCAGACAGCAA

ATGGAACCAAGCAATGCCGCTGCCCGTTGAAGTTTCTCGGCCCCACCTGCAGCTTGGACA

AGTGCATGTACTGCGGCAAGGGCAAATGTGTCCCACATCCCACTGACGGAGTGGCATGCA

GCTGTGCTAATGACCGGATACAGCCCAGCTGCTACAGCTGCAAAGACTTCTGCATTACTG

GCCAGTGTCTGTTAGACCAGCGCACCATGCGCCCATATTGCAAGTGTCCTTCAGGTTGGA

TTGGGTATCGCTGTGAGGACGCTGCCTCTTCTGCAGGCTCGGTAGACTCCTCCAGCAACG

CCATCTCTGTGATCATCCCTGTACTGTTGCTGCTCTTACTGGCTCTGCTGGTGGTTGTCG

CCATCTTGTGGTATAAGAAGAGAATGCGTGGTGCTAAGGGCTTCCAGCACCAGAGGATGA

CCAATGGAGCAATGAATGTAGAAATCGGGAACCCCGCATACAAGATATACGAGGGAGAAC

CCGACGACGACGCCGGAGAGCTGCTAGACTCGGACTTCACTCTGGATCCGGACAAGCCCA

CAAACTTCACCAACCCTGTTTACGCTACGCTCTACATGGGCGCCCACAACAGCCGCAACT

CTCTGGCCAGCACGGACGAAAAGAAGGAGCTGCTGTCCGCAGGGGACGAGGACATGGGAG

ACCCGCTGGCATAGAGCCGGCGACAAAGCACCAAGCTGTGCCCATCTGTCTGAACACCCA

CCCTTCCCACCACCAACACCTACCCACCCACCCACCAAACCCCCCACTCATTCACTTGAC

CCCCATATGCCTTTACAACATTCGGAAGAAAAGCAACAAGGGAGCAAGAACACTGTACAA

AATGTAAAAAAGGGGGAGAAAAATAGAAAAAAATATGAAGGACTACTTTTGTATGTGATT

GCAGTATTATATTTTATTCTTTCAGTCAGAACAAAAAAAAACCCACGGAAAACATTTTTC

TATACTTTTTTTTAATCATTACTCACTTAGCGCCCCTTTTCCTCCCGTTAGCTCTGTCTG

TCTCGACCCCTCCCCTCTTCCATGTAGCCTCTACCTCTGTGCTAAATGAGAACGCAAACA

ACCATCACAGCTAGGGAATTATTATTTCTATTTTATTTTTTGTATGAATTGTATAGACGT

CAAGGAAATAGCCAGGATTTTATTTTATTTTTTTGTCGTCGTCAGTAAATAATAAAGGGA

GAGGAGAGAGCAGCAGGGGGCGCGATCCCCCAATCCCGACGATGGTACCTGATCTGTGCG

TACGTGTATCGGTACTGCTGGAGGAAAAAGAGGTGTGTGTGTATGTGTGTGTGTGTATGT

GTGTGTGAGTGTGTCGCAGATTTTAAAACTCCGGGAAAGGGAAGGGGAGCTCGCGCGCTC

CACGGAAAATGCTAGAAAACGCAGGAGTGTCCGGATAACGGGACAGAACCGGGACAGCGC

GAGCTTCCGCACTGATCGCAGAGCCAAAAATAAAGTGGAATCGCACGCGCGAGAAACTGA

AAGCGAATCGATTGGTAAATTAATCGCGGCGGAAATGGAGCTGTCATCCATCGGGGAGCG

CGTGTTCGCGGTGGAGTCCATCACCAAGAAGAGAGTCAGAAAGGGGAATGTGGAGTATCT

GTTGAAGTGGCAGGGTTGGCCTCCTAAGTACAGCACGTGGGAACCAGAGGATAATATCCT

GGACCCTCGTCTGGTTTTGGCCTTTGAGGAGAAGGAGGAGCGGGACAGAGTGCAGGCTCA

CAGGAGGAAAGGCTTGAGGCCCCGCAGACTCGTCTTACGGAACATCTACACGATGGAGCT

GCGCAGTGCTCAGAAACCGCCGGAGAAACCTGCTCCCCGATTCCGCCTGTCACTCACCCG

CTCCATGGGGGCGGGGCTACCACATAACGGGCGGCGCTGCAGGAACGGTGAGGGAGGAGT

CTACCAACACCCGGACCGCCTTCTAAAGCGCAGGAGCAGGCCCTATGTGTCCAAAACACC

AGAAAGAACTGAATCGCCACGGCAACCGACACCAACCTCTGAGAGAGACTCAGCGGAGGA

GGATGAAGAGGAGAACGAGTGGGAGAAAGAGGAGGAGAAAAAGAGGAGAAAGATGGAGAA

TGAGGAGCGTCATGCCATTCCTCACGCCCAGGACATGACAGAAGAACACGCCCCCTTCAC

ACAGGAAGCTGTCATCACAGAGAACACTAAACCAGAACATTGTGCTTCCTCCCCTGACTG

TAGGGTGGCGCCACTCACTTCAGAGACCGGTTCAAGTACAGCGGCGCCGGAGCAGCAGCT

AAACACCGTTGAGCACCAGCCAATCACAGCCGCTTCAGGGGGCGGAGTCTCCCAAGATAC

ACTCACGACTGACGAATTACAGAAAAGCAGCTCGGAAGCAGGCGTGCTCACAGCCAGGGT

GCAGGAGCGAGCCTCGGTTATAACGGTGCGTCATTTAATCGACAGGTTTGTGCAGGACGA

GCAGAGACAGACAGGTGAGACAGACAGCAAAGAGAAGAGAGAGACAGACGATGACGAGGC

GACAGCCGAGTGCACCACCACCTTACAGCTGAGCACGGAGATCAGATCAGACGAGCGCCA

AACGTCAGAGCGCACGCGGCATCGCGGGAAGGTAGTCGTGACGAAGGTGACGGTAAATTC

GGTGACAGTAACGTTTAAAGAAGCGACGGCGGCCGAAGGGTTTTTCAGCAGCTCTGGGCT

GCAGGTCTGACTCAGCCGGATGAGCAGGAAATGCAACCAGACGTACTTCCTGTCAAACTG

CTAATGTGGCTAATGACCGAACAGAGAGAGGAGTTCGCTAATCCATCCGAGTAACACGTG

CTGTCAGGAATTTGTTAGAAATGTTAGCGGTTGAGGCAAAGTACGTCTTTTTCCTGTGAG

AGGGAGGGATGACAGGAGCAGCTGACATGTCGATCTGTGGATGTAGTGAAGTATTTCGCA

GCTTTGCTTTACAGATACTCGTCGGTCGTTAAAAAGGGTTACGTTCCCAACTCCGTCCTA

TCGTTTTCGTGAAAATGTGAATAATCGTAAAAGGATTTTCAAACGCACGCAAAAACGAAG

ATGTTCATGAATTTAGTGCGAGTTCCCACTTTAGGGGTGGTCATGACTTGATGACATCAT

CAGTTATTAGTCTCCGCAGCACCGAGCTGGCGGTTTCGGCCGGCTCGGGTTTGTATTCTG

CGCCTCGTGCTCGGAAGCCTCGCTGCATTTTTAACGAGTCCGTGTCAGTTCAGATTACAG

AAGCCGCTGCATGGGCTGTACGGACGCGTCGTTAACACCACCGCAGAAAACAACCCCACG

CTTTAGATGTGTCTCTAGCATGCGAC

>Unigene000285

CAGGAGCCTCATGGGACAGACACATCCTCTGCATGTGACACTTCAAGAGTTCGAAAGAAT
[truncated: 16,623,569 more chars]
